# Digital Genome-Wide ncRNA Expression, Including SnoRNAs, across 11 Human Tissues Using PolyA-Neutral Amplification

**DOI:** 10.1371/journal.pone.0011779

**Published:** 2010-07-26

**Authors:** John C. Castle, Christopher D. Armour, Martin Löwer, David Haynor, Matthew Biery, Heather Bouzek, Ronghua Chen, Stuart Jackson, Jason M. Johnson, Carol A. Rohl, Christopher K. Raymond

**Affiliations:** 1 Institute for Translational Oncology and Immunology, Mainz, Germany; 2 Nugen Inc., Seattle, Washington, United States of America; 3 University of Washington, Seattle, Washington, United States of America; 4 Merck Research Laboratories, Boston, Massachusetts, United States of America; 5 Rosetta Inpharmatics, Seattle, Washington, United States of America; Baylor College of Medicine, United States of America

## Abstract

Non-coding RNAs (ncRNAs) are an essential class of molecular species that have been difficult to monitor on high throughput platforms due to frequent lack of polyadenylation. Using a polyadenylation-neutral amplification protocol and next-generation sequencing, we explore ncRNA expression in eleven human tissues. ncRNAs 7SL, U2, 7SK, and HBII-52 are expressed at levels far exceeding mRNAs. C/D and H/ACA box snoRNAs are associated with rRNA methylation and pseudouridylation, respectively: spleen expresses both, hypothalamus expresses mainly C/D box snoRNAs, and testes show enriched expression of both H/ACA box snoRNAs and RNA telomerase TERC. Within the snoRNA 14q cluster, 14q(I-6) is expressed at much higher levels than other cluster members. More reads align to mitochondrial than nuclear tRNAs. Many lincRNAs are actively transcribed, particularly those overlapping known ncRNAs. Within the Prader-Willi syndrome loci, the snoRNA HBII-85 (group I) cluster is highly expressed in hypothalamus, greater than in other tissues and greater than group II or III. Additionally, within the disease locus we find novel transcription across a 400,000 nt span in ovaries. This genome-wide polyA-neutral expression compendium demonstrates the richness of ncRNA expression, their high expression patterns, their function-specific expression patterns, and is publicly available.

## Introduction

Non-coding RNAs (ncRNAs) are a class of molecular species that, for example, play regulatory roles, have been implicated in human diseases, and are essential for stem cells [1], [2], [3], [4], [5]. ncRNAs are frequently monitored using qPCR and northern assays (e.g., [6], [7]). While successful, neither is typically run as a genome-wide high-throughput platform. High throughput microarray and next-generation sequencing platforms commonly employ RNA amplification protocols with either oligo-dT amplification or random priming of polyA+ purified RNA, with the intended goal of amplifying polyadenylated mRNA transcripts (e.g., [8]). As the majority of ncRNA transcripts are not polyadenylated, ncRNAs transcripts are ineffectively amplified and thus not monitored by these platforms.

## Results and Discussion

To examine ncRNAs in human samples, we amplified eleven tissue pools using a novel amplification protocol that effectively amplifies non-ribosomal RNA molecules with lengths greater than 50 nucleotides (nt), including both polyadenylated and non- polyadenylated transcripts, that additionally preserves the strand of the RNA molecules [9]. We generated an average of 50 million sequence reads per tissue, deposited at EMBL (ENA ERP000257; ArrayExpress E-MTAB-305), and aligned reads to the human genome using the program BWA [10]. The percentage of sequence reads aligning to the genome varied from 79% to 90% per tissue (Table S1).

We first measured the expression of protein-coding mRNAs by counting and normalizing the reads overlapping each transcript, generating measurements, for each transcript in each tissue, of the number overlapped reads per 1000 nt of RNA transcript length per million alignable reads (RPKM [11]), along with a normalized uncertainty derived using Poisson statistics [12]. In this compendium (Table S2), we find highly tissue-enriched genes, such as PRM2 (protamine 2) with 151 RPKM in testes and, amazingly, zero RPKM in all other tissues. The non-polyadenylated histone HIST1H2BA has 1,338 RPKM in testes and less than two in other tissues, demonstrating not only the high tissue-enrichment of this histone but the platform's ability to monitor non-polyadenylated transcripts.

Given the ability to monitor polyA- transcripts, we examined the expression of snoRNAs, scaRNAs, scRNAs, and miscellaneous ncRNAs. Many ncRNAs are duplicated throughout the genome; for example, there are hundreds of 7SK-associated transcripts. Grouping similar transcripts into single ncRNA clusters, such as all the 7SK and 7SK-related loci, we identified 336 ncRNA clusters (Supplementary files contain genomic coordinates). For each cluster, we counted the number of unique reads that map to any of the associated genomic locations and normalized the counts and uncertainties to generate RPKM values and error estimates. We find that 2.9% (in hypothalamus) to 1.1% (in heart) of the aligned reads map to one of these 336 ncRNA clusters.

**Figure 1:**
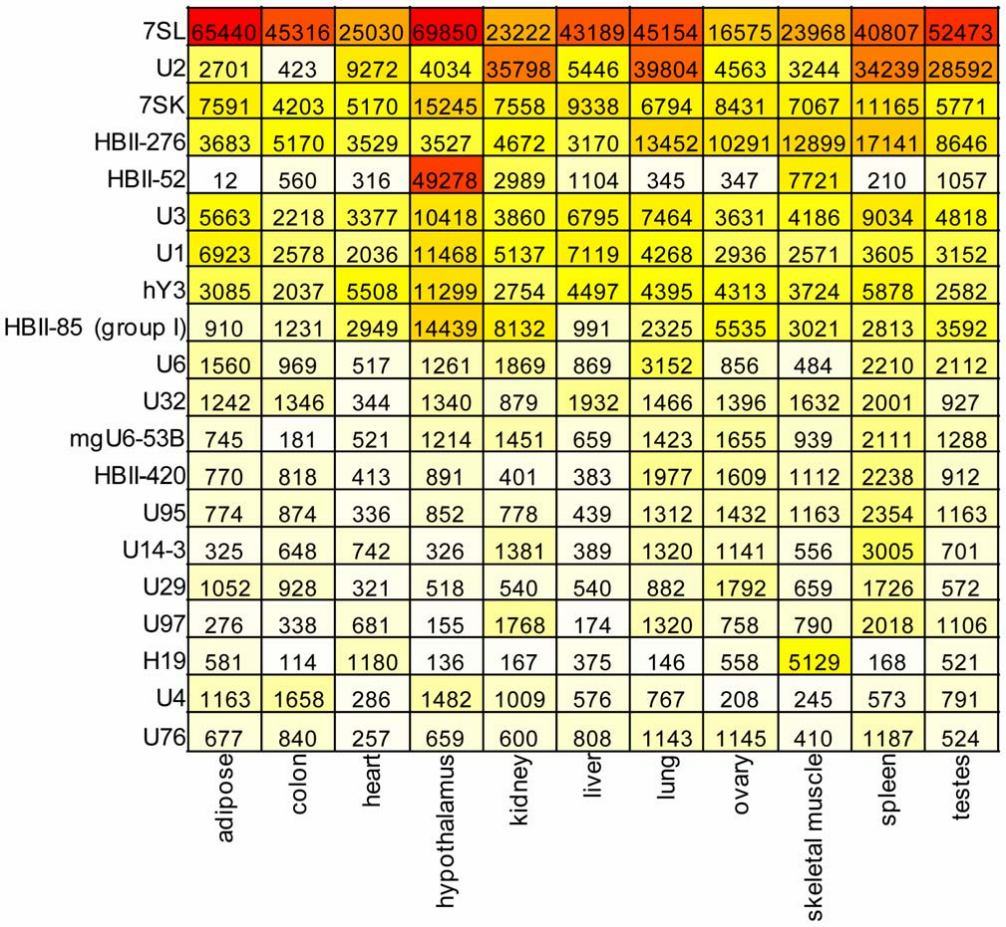
ncRNA expression. Expression (RPKM units) of the 20 monitored ncRNA with highest mean values across eleven tissues.

Figure 1 shows the 20 clusters with highest RPKM values and Table S3 lists expression of all ncRNA clusters in each tissue. The four with highest average RPKM are signal recognition particle scRNA 7SL (at an incredible 70,000 RPKM in hypothalamus), spliceosomal snRNA U2 (40,000 RPKM in lung), snRNA 7SK (15,000 RPKM in hypothalamus), and snoRNA HBII-276 (17,000 RPKM in spleen). In hypothalamus, 104,744 reads align to the snoRNA HBII-52 cluster, 0.4% of all aligned reads, representing an amazing 49,000 RPKM. For comparison, the five mRNAs in hypothalamus with highest RPKM are TTR (transthyretin; 3,300 RPKM), STMN1 (stathmin 1; 1,000 RPKM), MBP (myelin basic protein; 910 RPKM), HSPA8 (heat shock 70 kDa protein 8; 700 RPKM), and GFAP (glial fibrillary acidic protein; 600 RPKM). In hypothalamus, the snoRNA HBII-52 cluster shows RPKM 15 times higher than the highest mRNA.

**Figure 2:**
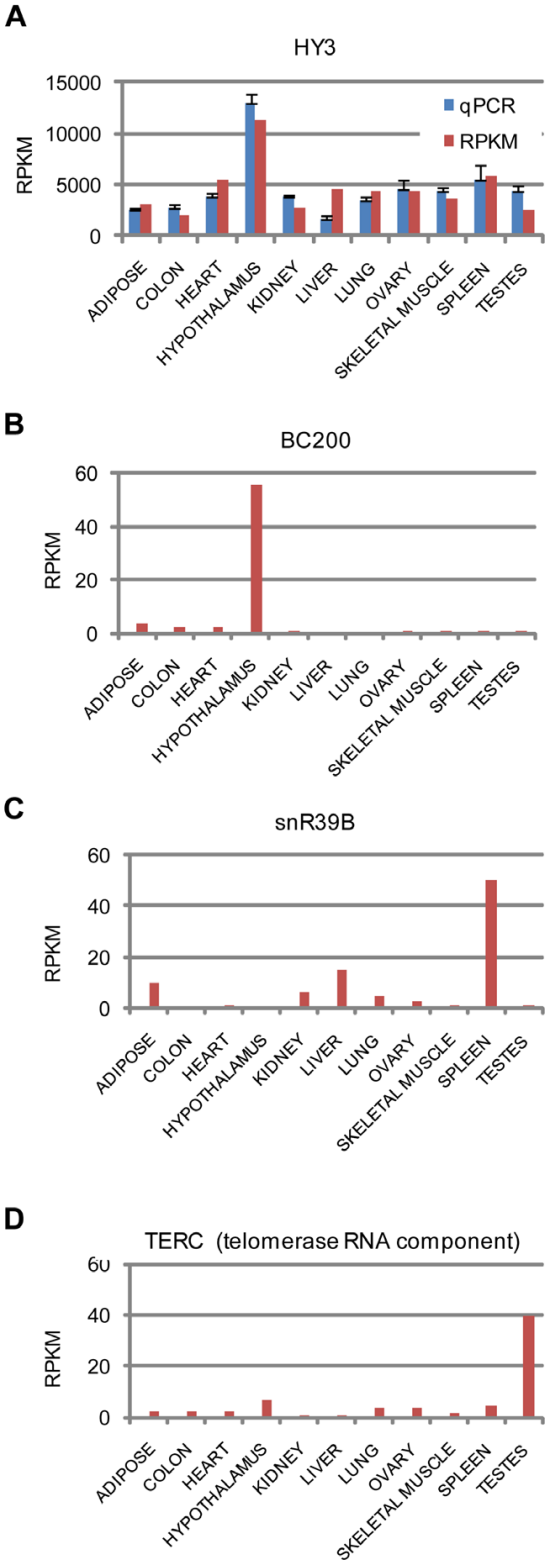
Individual ncRNA expression. A: HY3 sequencing (RPKM) and qPCR expression. qPCR values are normalized to 18S and the mean sequencing value. B, C, and D: expression of BC200, snR39B, and TERC (telomerase RNA component).

Other examples include HY3 RNA (Ro-associated Y3), which is expressed at over 1,000 RPKM in all tissues and over 6,000 in hypothalamus (Figure 2). This expression correlates well with our qPCR validation, and we find HY3 has much higher overall RPKM than HY1, HY4, and HY5, as per [13]. BC200 (BCYRN1) is highly enriched in hypothalamus [14], snoRNA snR39B is enriched in spleen (Figure 2), and tumor suppressor H19 is enriched in skeletal muscle [15] (Figure S1).

Functionally, snoRNAs can be divided into C/D box and H/ACA box based on sequence and structure, and are associated with methylation and pseudouridylation, respectively [16]. As examples, HBII-438 (C/D box) has 107 RPKM in hypothalamus and 25 in testes while HBI-6 (H/ACA) is at 166 in testes and 13 in hypothalamus (Figure S1). 47 H/ACA box snoRNAs have expression above 10 RPKM: 18 have highest RPKM in testes, 11 in spleen, and two in hypothalamus. Conversely, of the 99 C/D box snoRNAs with expression above 10 RPKM, 38 are highest in spleen, 15 in hypothalamus, but none in testes.

Thus, we observe a major shift from C/D box snoRNAs (methylation) in brain to H/ACA box snoRNAs (pseudouridylation) in testes. H/ACA box snoRNAs specify uridines for pseudouridylation, including targets in the ribosome and spliceosome [17], and mutations impacting pseudouridylation slow translation and growth rates [18], suggesting a role for pseudouridine in cellular proliferation, ribosome biogenesis, and pre-mRNA splicing. Human TERC, the RNA component of telomerase, also encodes an H/ACA box and is implicated in dyskeratosis congenital [4]. It is a limiting component of telomerase, and thus essential for tumor, germ, and stem/progenitor cell proliferation [17], [19], [20], [21]. Indeed, induced pluripotent stem cells upregulate TERC to restore telomere elongation[5]. Our data show that TERC is clearly enriched in testes (Figure 2). Together, the higher expression in testes of TERC and H/ACA box snoRNAs suggests that these factors are essential for germ cell proliferation and maintenance.

The sequencing data also enable investigation into snoRNA gene clusters: the 14q, HBII-85, and HBII-52 snoRNA clusters are all groups of adjacently located snoRNAs. While they fall within imprinted regions and have been implicated in disease [22], measuring expression of individual snoRNAs within each cluster has nevertheless been complicated by intra-cluster sequence similarity[23].

**Figure 3:**
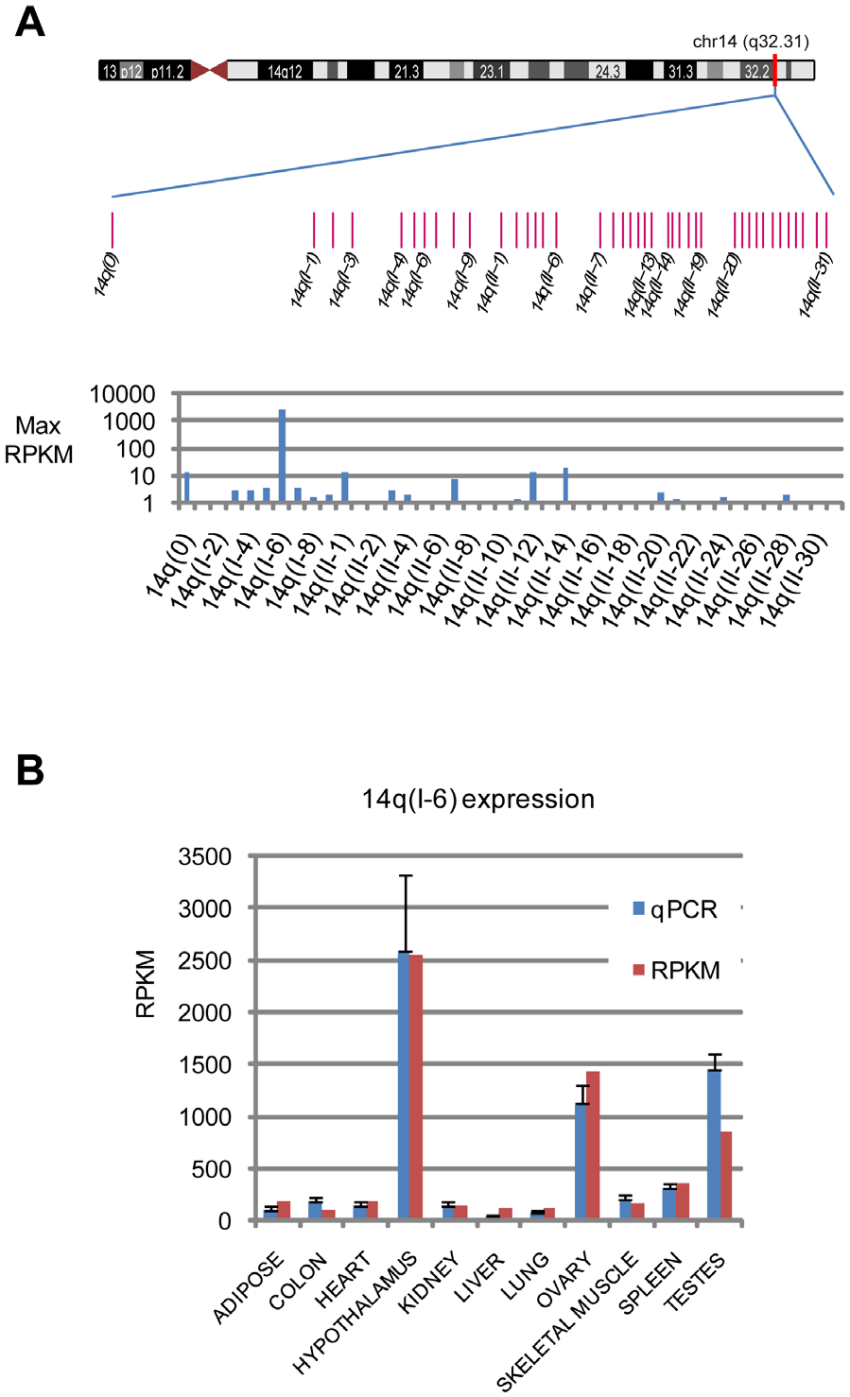
Expression of the 14q snoRNA cluster. A: genomic layout of the 41 members of the 14q snoRNA cluster (upper) and maximum RPKM values for each member, across all tissues (lower). Every second snoRNA is labeled. Y-axis is log10 scale. B: expression of the highly expressed 14q(I-6) snoRNA. qPCR values are normalized to 18S and the mean sequencing value. Y-axis is linear scale.

On chromosome 14, the 41 member 14q snoRNA cluster can be broken into three sub-groups: 14q(0), 1 member; 14q(I), 9 members; and 14q(II), 31 members [24] (Figure 3). Despite the sequence similarity of snoRNAs, the sequencing reads can discriminate individual members (Table S4) and thus enable determination of expression of individual members. 14q(I-6) shows by far the highest expression, at 2,545 RPKM in hypothalamus (Figure 3). 14q(II-14), 14q(II-12), 14q(II-1) are expressed at highest levels in hypothalamus at 19, 14, and 13 RPKM, respectively, and 14q(0) is highest in ovaries, at 13 RPKM. Thus, from the 14q cluster, 14q(I-6) is specifically expressed at significantly higher levels than all other members.

**Figure 4:**
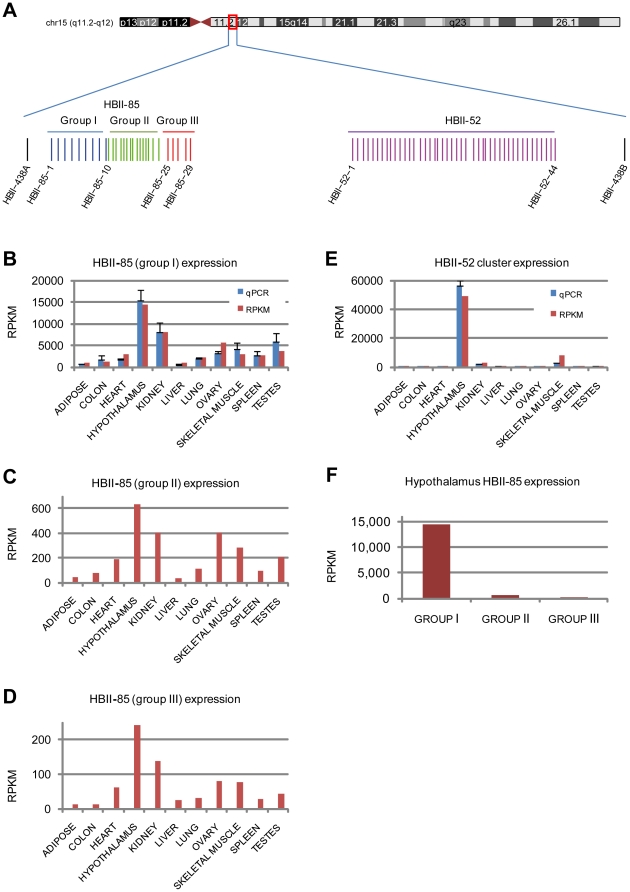
Expression of the HBII-52 and HBII-85 snoRNA clusters. A: genomic layout of the HBII-52 and HBII-85 snoRNA clusters. B, C, and D: expression of the HBII-85 group I, II, and III clusters. E: expression of the HBII-52 cluster. F: expression of HBII-85 group I, II, and III in hypothalamus.

Clusters HBII-85 and HBII-52 fall within the Prader-Willi syndrome (PWS) and Angelman syndrome locus on chromosome 15 [22] (Figure 4). For the HBII-85 29 member cluster, sequencing and qPCR show highest expression in hypothalamus, followed by kidney, as per [6], and expression above 900 RPKM in all tissues. Based on sequence similarity, members of the HBII-85 cluster can be divided into group I (9 members), group II (15 members), and group III (5 members). While sequence reads can map to multiple members within a cluster, they do not align across groups (Table S5). Thus for HBII-85 the cluster, we are able to determine group-specific RPKM values by counting the number of unique sequence reads that align only to any member of an individual group. In hypothalamus, we find group I at 14,439 RPKM, group II at 635, and group III at 242. Consequently, HBII-85 is expressed at significant levels in all tissues examined, HBII-85 is enriched in hypothalamus, and HBII-85 group I expression is significantly higher than group II or III. Thus, while the HBII-85 cluster is frequently referred to as a brain-specific snoRNA, we find, as per original studies of Cavaille et al., [6], [24], that it is expressed in all human tissues examined, albeit at much higher levels in brain.

Finally, the sequence similarity among the 42-member HBII-52 cluster is high enough that sequence reads can align to multiple members, prohibiting determination of individual expression (Table S6). Instead of attempting to monitor individual members, we thus counted the number of unique reads that align to any cluster member. The sequencing and qPCR data show HBII-52 expression in every tissue, ranging from 12 RPKM in adipose to an outstanding 49,278 RPKM in hypothalamus (Figure 4). The high HBII-52 brain enrichment also agrees with northern blots from Cavaille et al., [6], [24]. One discrepancy, however, is that the northern blots in their studies suggest higher HBII-85 than HBII-52 levels in muscle whereas the sequencing data suggest the opposite. This may be due to differences in assays, northerns versus sequencing, the tissue type (skeletal muscle was examined here), or biological variation.

**Figure 5:**
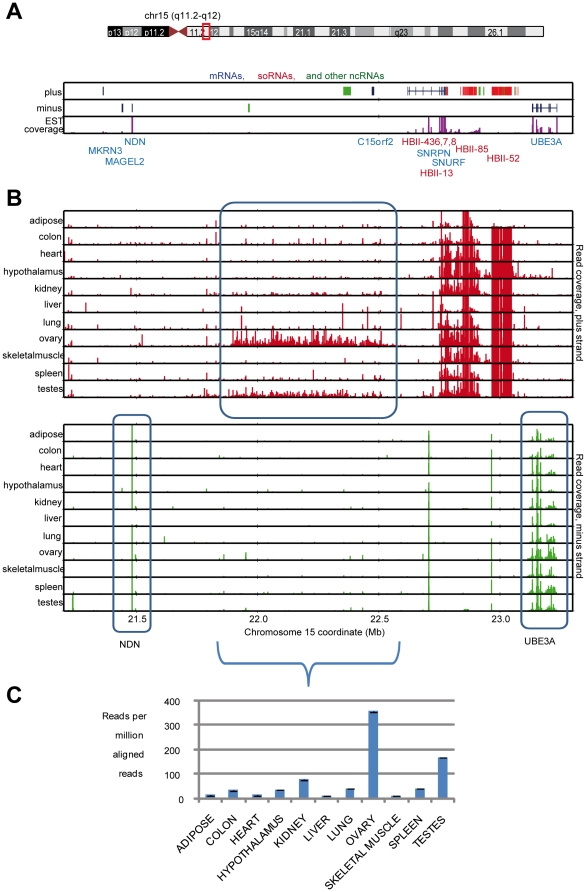
Expression in the PWS/Angelman locus, chromosome 15, 22.0–22.4 Mb. A: gene location and strand and coverage by ESTs (Expressed Sequence Tags). B: sequence read coverage showing positive (red) and minus (green) strand transcription for each tissue. Transcription for genes NDN and UBE3A along with the novel region are circled in blue. C: summed positive strand expression between chromosome 15; 22.0 to 22.4 Mb.

The sequencing of polyA-neutral RNA enables a less biased transcriptional exploration of the genome. We have seen that within the chromosome 15 PWS locus, snoRNA clusters HBII-85 and HBII-52 are expressed at extremely high RPKM. Within the locus, most genes are highest in hypothalamus, including snoRNAs HBII-13 (12 RPKM in hypothalamus), HBII-436 (35 RPKM), HBII-437 (34 RPKM), and HBII-438 (107 RPKM), and mRNAs NDN (33 RPKM) and UBE3A (9 RPKM). Furthermore, between genes NDN and HBII-436, transcription occurs continuously across a 400,000 kb span from 22.0-22.4 Mb (Figure 5). 18,503 reads from ovaries map across this span, resulting in 355 reads per million aligned reads. Testes show 166 reads per million aligned reads. Conversely, other tissues show significantly lower expression, such as 11 reads per million aligned in skeletal muscle. Thus, this identifies a novel, broad span of transcription that falls within a disease locus that is enriched in ovaries and testes.

The amplification protocol does not effectively amplify RNAs less than 50 nt length, such as mature miRNAs, and specifically avoids amplification of long rRNAs 28S, 18S, 16S, and 12S [9]. Nevertheless, reads do align to rRNA 5.8S and 5S (Figure S2) and we find both expressed in all tissues and enrichment in the lung sample. The data also shed light on the signal recognition particle (SRP) ribonucleoprotein protein-RNA complex, including ncRNA 7SL and several protein-coding genes. Across the 11 samples, 7SL is universally highly expressed (above), SRP mRNAs are expressed at less than 100 RPKM, and, among SRP mRNAs, SRP9 is expressed at highest levels in each tissue and highest in hypothalamus (Figure S3, Table S2).

tRNAs are yet another molecular species that can be detected (Table S7), and the sequencing data allow a more absolute measure, expanding on previous ratio measurements [25]. More reads align to mitochondrial than nuclear tRNAs, averaging over 400 RPKM versus 36 across all tissues and tRNAs, respectively. However, more reads align to nuclear glycine tRNAs than mitochondrial glycine tRNAs. Among nuclear glycine tRNAs, the tRNA recognizing the GGG codon (tRNA-Gly-CCC) is more abundant than tRNA-Gly-GCC and tRNA-Gly-TCC, which is different from the codon usage showing GCC is the most commonly used codon for glycine [26].

Large intergenic noncoding RNAs (lincRNAs) are large genomic spans of mammalian-conserved sequence that contain chromatin structure associated with transcription but lacking known protein-coding genes [27]. Using the previously defined lincRNA coordinates [27], we find that the sequence reads here support transcription in many lincRNAs (Table S8). Many reads overlap lincRNAs that contain rRNA transcripts, such as the 28S rRNA element in lincRNA chr11:84867425-84942100. Many reads map to lincRNAs that overlap ncRNAs, such as lincRNA chr20:36470500-36515575 which overlaps ncRNAs ACA60, U71a, U71b, U71c, and U71d. Transcription is active in lincRNA chr11:64946775-64971250, containing the ncRNA NEAT1 (nuclear paraspeckle assembly transcript 1) [28], [29] which is expressed at 350 RPKM in lung. Similarly, lincRNA chr11:65022925-65031750 overlaps many reads, most of which are associated with the ncRNA MALAT1 (metastasis associated lung adenocarcinoma transcript 1) [8], [30] which is expressed at over 2,000 RPKM in both kidney and lung. lincRNA chrX:72949400-72989313 contains ncRNAs XIST[31] and TSIX [32] and shows highest expression in ovaries; chr1:172098475-172103825 contains ncRNA GAS5, associated with growth arrest and the glucocorticoid receptor [33], and C/D box snoRNAs U44, U47, and U74-81 and also shows highest expression in ovaries. Thus, these sequencing data corroborate transcription in many lincRNAs, particularly in those containing known rRNAs and ncRNAs, and provides additional tissue profiles.

In summary, through high-throughput sequencing of a polyA-neutral library, we were able to assemble a genome-wide RNA expression compendium. Rather than acting as housekeeping genes with uniform and ubiquitous expression, ncRNAs have distinctive, tissue-specific, expression patterns. Some ncRNAs are expressed at levels far exceeding mRNA expression. There are novel, broad regions of rumbling transcription that show tissue-variable expression.

## Materials and Methods

### Tissues

We purchased total RNA from Ambion (Austin, USA). Each tissue samples was pooled from multiple donors. Libraries were prepared as per Armour et al, 2009[9].

### Sequencing

We generated an average of 50 million sequence reads per tissue using an Illumina GA-II sequencer, with sequence lengths of 36 nt (adipose, hypothalamus, liver) and 50 nt (colon, heart, kidney, lung, ovary, skeletal muscle, spleen, testes), deposited at EMBL (ENA ERP000257; ArrayExpress E-MTAB-305). We trimmed reads to a common length of 28 nt to avoid aligning sequenced amplification primers.

### Expression profiling

For mRNAs, we downloaded RefSeq transcript coordinates and associated gene symbols from the UCSC genome browser [34], assembly hg18. Using only reads mapping to a single gene, we counted the reads overlapping each transcript in the correct genomic orientation. We modeled the uncertainty of each measurement (error) using Poisson statistics, assigning the square root of the counts as the uncertainty of each measurement [12]. To compare across tissues and transcripts, we normalized both the counts and uncertainties by the number of alignable reads in each tissue and by the transcript length, and similarly normalized the associated normalized uncertainty [11].

For ncRNAs, ncRNA genomic coordinates were downloaded from the two tracks in the UCSC genome browser[34], assembly hg18, tracks RNA Genes [35] and sno/miRNAs [23]. For this analysis, we removed pseudogenes, miRNAs, tRNAs, and rRNAs. We combined genes labeled as “related”, such as 7SK and 7SK-related, into a single cluster while preserving all genomic locations. Preference was given to annotation in the RNA Genes file. Coordinates and names can be found in Supplementary File XXX. For each ncRNA cluster, we counted the number of unique reads that mapped to any of the associated genomic locations and normalized counts and uncertainties to generate RPKM values and error estimates.

### qPCR

Tissue-specific values were normalized to the level of 18S rRNA. Errors are the standard deviations from triplicate measurements. To compare to sequencing RPKM values, the mean for each qPCR was normalized to the corresponding RPKM mean.

## Supporting Information

Figure S1 Expression (RPKM) of H19, HBII-438, and HBI-6.
(0.12 MB PPT)

Figure S2 Expression (RPKM) of 5.8S and 5S rRNA.
(0.09 MB PPT)

Figure S3 Expression (RPKM) of the signal recognition particle (SRP) ribonucleoprotein protein-RNA complex, including 7SL (top) and the protein coding components (bottom).
(0.14 MB PPT)

Table S1 Number of reads and number aligned.
(0.02 MB XLS)

Table S2 Expression of RefSeq transcripts.
(1.28 MB PDF)

Table S3 Expression of 336 ncRNAs.
(0.11 MB PDF)

Table S4 Similarity among 14q cluster snoRNAs, based on the number of identical 28 nt sequences.
(0.09 MB DOC)

Table S5 Similarity among HBII-85 cluster snoRNAs, based on the number of identical 28 nt sequences.
(0.09 MB DOC)

Table S6 Similarity among HBII-52 cluster snoRNAs, based on the number of identical 28 nt sequences.
(0.14 MB DOC)

Table S7 Reads per million aligned per 1000 nt (RPKM) overlapped by each tRNA.
(0.04 MB XLS)

Table S8 Reads per million aligned overlapped by each lincRNA.
(0.15 MB PDF)
