# Supplementary figures and images for: Digital Genome-Wide ncRNA Expression, Including SnoRNAs, across 11 Human Tissues Using PolyA-Neutral Amplification
Source: PLoS One. 2010 Jul 26;5(7):e11779. doi: 10.1371/journal.pone.0011779 (PMC2909899; doi:10.1371/journal.pone.0011779)

## Slide 1
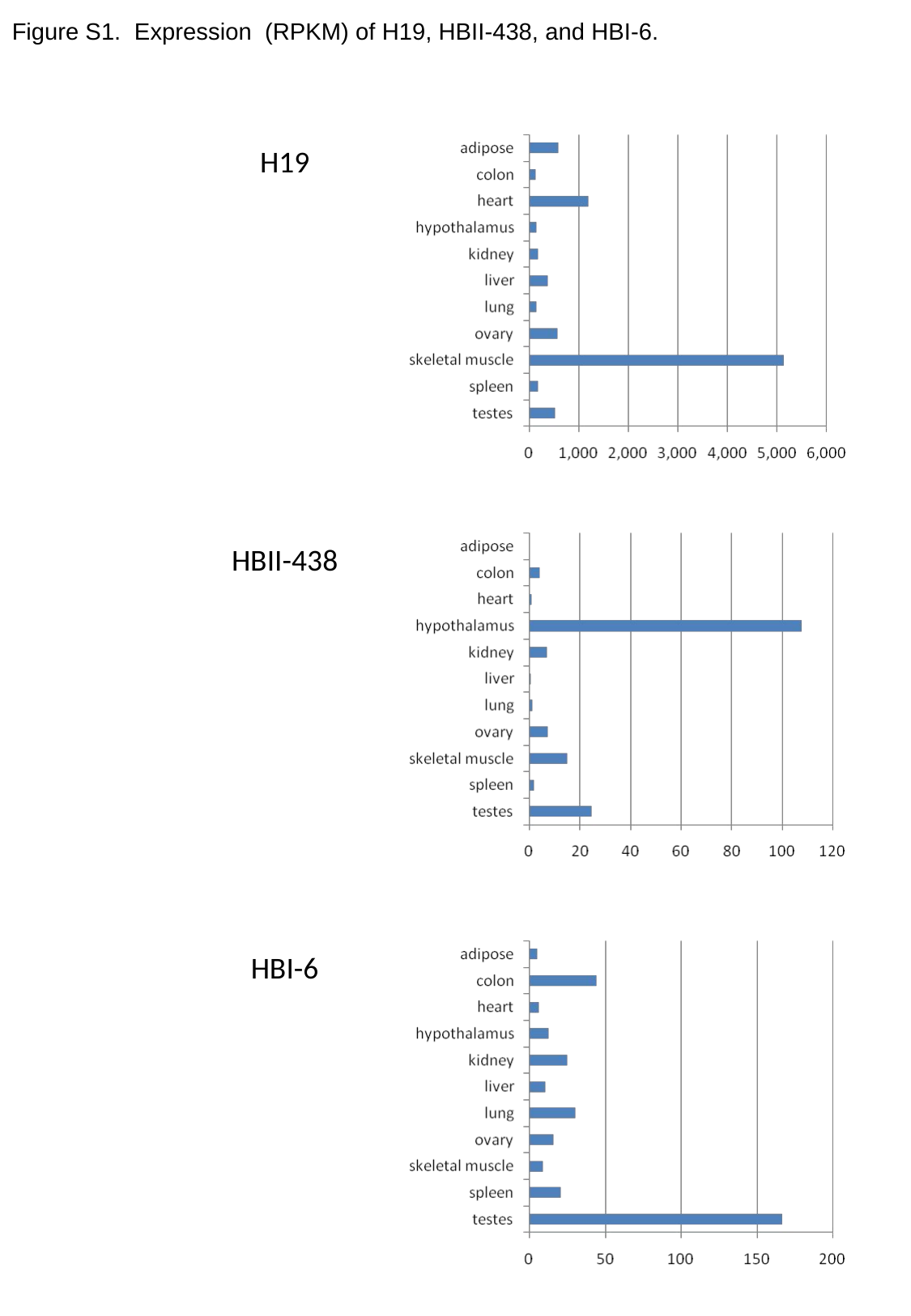

Figure S1. Expression (RPKM) of H19, HBII-438, and HBI-6.
H19
HBII-438
HBI-6

Supplement: Figure S1 — Expression (RPKM) of H19, HBII-438, and HBI-6. (0.12 MB PPT) [file pone.0011779.s001.ppt]

## Slide 1
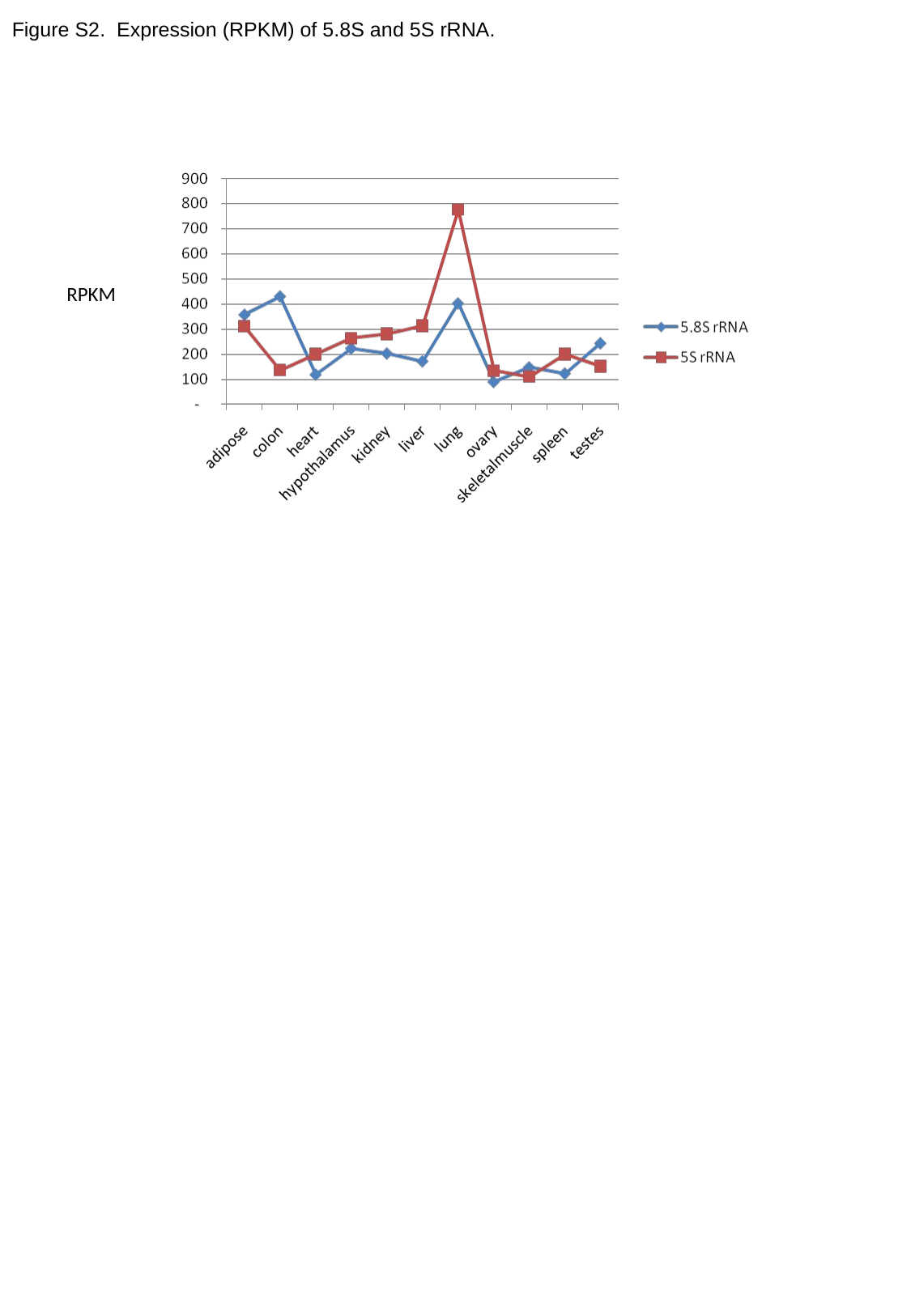

Figure S2. Expression (RPKM) of 5.8S and 5S rRNA.
RPKM

Supplement: Figure S2 — Expression (RPKM) of 5.8S and 5S rRNA. (0.09 MB PPT) [file pone.0011779.s002.ppt]
