# Supplementary material for: Digital Genome-Wide ncRNA Expression, Including SnoRNAs, across 11 Human Tissues Using PolyA-Neutral Amplification
Source: PLoS One. 2010 Jul 26;5(7):e11779. doi: 10.1371/journal.pone.0011779 (PMC2909899; doi:10.1371/journal.pone.0011779)
Supplement: Figure S3 — Expression (RPKM) of the signal recognition particle (SRP) ribonucleoprotein protein-RNA complex, including 7SL (top) and the protein coding components (bottom). (0.14 MB PPT) [file pone.0011779.s003.ppt]

## Slide 1
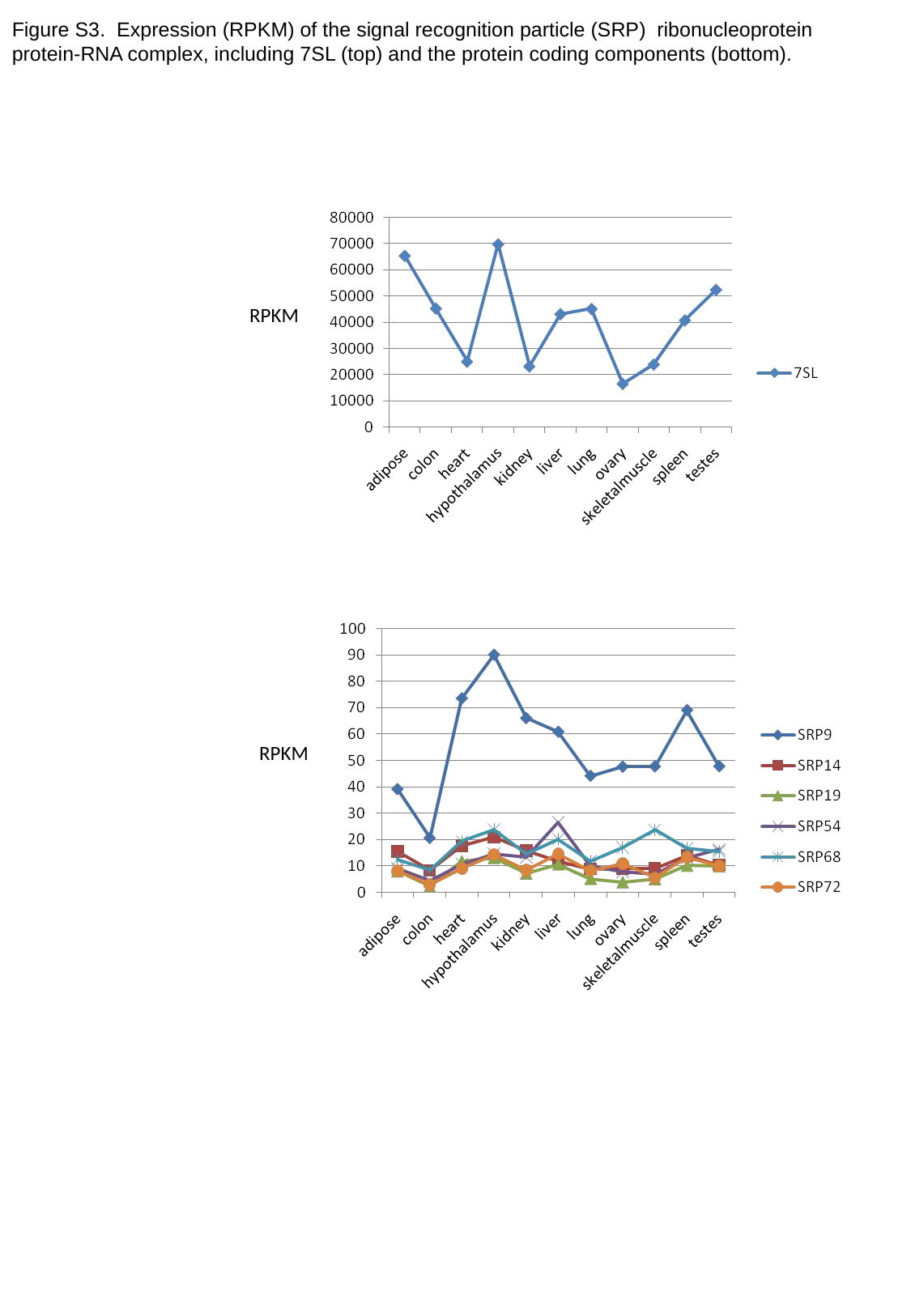

Figure S3. Expression (RPKM) of the signal recognition particle (SRP) ribonucleoprotein protein-RNA complex, including 7SL (top) and the protein coding components (bottom).
RPKM
RPKM
