# Supplementary material for: Digital Genome-Wide ncRNA Expression, Including SnoRNAs, across 11 Human Tissues Using PolyA-Neutral Amplification
Source: PLoS One. 2010 Jul 26;5(7):e11779. doi: 10.1371/journal.pone.0011779 (PMC2909899; doi:10.1371/journal.pone.0011779)
Supplement: Table S3 — Expression of 336 ncRNAs. (0.11 MB PDF) [file pone.0011779.s006.pdf]

ncRNA	transcript length (nt)	type	max	mean	adipose	colon	heart	hypothalamus	kidney	liver	lung	ovary	skeletal muscle	spleen	testes
7SL	160	RNA, 7SL, cytoplasmic 1	69,850	41,002	65440	45316	25030	69850	23222	43189	45154	16575	23968	40807	52473
U2	188	RNA, U2 small nuclear 1;RNA, U2 small nucle	39,804	15,283	2701	423	9272	4034	35798	5446	39804	4563	3244	34239	28592
7SK	310	RNA, 7SK small nuclear	15,245	8,030	7591	4203	5170	15245	7558	9338	6794	8431	7067	11165	5771
HBII-276	77	small nucleolar RNA, C/D box 87	17,141	7,834	3683	5170	3529	3527	4672	3170	13452	10291	12899	17141	8646
HBII-52	76	small nucleolar RNA, C/D box 115	52,220	6,167	13	620	335	52220	3232	1189	368	375	8108	238	1139
U3	75	small nucleolar RNA, C/D box 3C;small nucle	10,418	5,588	5663	2218	3377	10418	3860	6795	7464	3631	4186	9034	4818
U1	159	RNA, U1 small nuclear 1;RNA, U1 small nucle	11,468	4,708	6923	2578	2036	11468	5137	7119	4268	2936	2571	3605	3152
hY3	101	RNA, Ro-associated Y3	11,299	4,552	3085	2037	5508	11299	2754	4497	4395	4313	3724	5878	2582
HBII-85_groupI	95	small nucleolar RNA, C/D box 115	14,439	4,176	910	1231	2949	14439	8132	991	2325	5535	3021	2813	3592
U6	104	RNA, U6 small nuclear 2;RNA, U6 small nucle	3,152	1,442	1560	969	517	1261	1869	869	3152	856	484	2210	2112
U32	73	small nucleolar RNA, C/D box 32A	2,001	1,319	1242	1346	344	1340	879	1932	1466	1396	1632	2001	927
mgU6-53B	104	small nucleolar RNA, C/D box 9	2,111	1,108	745	181	521	1214	1451	659	1423	1655	939	2111	1288
HBII-420	81	small nucleolar RNA, C/D box 99	2,238	1,048	770	818	413	891	401	383	1977	1609	1112	2238	912
U95	63	small nucleolar RNA, C/D box 95	2,354	1,043	774	874	336	852	778	439	1312	1432	1163	2354	1163
U14-3	91	small nucleolar RNA, C/D box 14A	3,005	958	325	648	742	326	1381	389	1320	1141	556	3005	701
U29	65	small nucleolar RNA, C/D box 29	1,792	866	1052	928	321	518	540	540	882	1792	659	1726	572
U97	142	small nucleolar RNA, C/D box 97	2,018	853	276	338	681	155	1768	174	1320	758	790	2018	1106
H19	183	H19, imprinted maternally expressed transcr	5,129	825	581	114	1180	136	167	375	146	558	5129	168	521
U4	91	RNA, U4 small nuclear 1;small Cajal body-spe	1,658	796	1163	1658	286	1482	1009	576	767	208	245	573	791
U59	75	small nucleolar RNA, C/D box 59A	1,352	773	633	817	469	617	1183	900	835	537	1352	818	338
U76	81	small nucleolar RNA, C/D box 76	1,187	750	677	840	257	659	600	808	1143	1145	410	1187	524
ACA3	78	small nucleolar RNA, H/ACA box 3;small nucl	946	653	844	132	328	740	495	875	939	709	430	946	743
hY1	110	RNA, Ro-associated Y1	1,873	646	341	333	664	1873	601	188	897	492	442	838	431
ACA14b	91	small nucleolar RNA, H/ACA box 14B	2,084	629	657	572	179	795	401	306	684	421	258	563	2084
U28	71	small nucleolar RNA, C/D box 28	1,262	612	412	681	250	443	586	435	782	904	329	1262	651
14q(I-6)	75	small nucleolar RNA, C/D box 113-6	2,545	563	179	92	180	2545	142	126	124	1428	166	363	848
U13	98	small nucleolar RNA, C/D box 13	871	556	531	594	287	491	356	750	618	385	710	871	525
ACA42	132	small nucleolar RNA, H/ACA box 42	1,164	489	634	139	478	385	379	136	753	375	128	1164	808
ACA37	127	small nucleolar RNA, H/ACA box 37	996	456	561	176	266	364	480	270	437	397	213	855	996
ACA20	132	small nucleolar RNA, H/ACA box 20	1,149	413	184	101	183	206	408	366	610	633	175	1149	529
U11	133	RNA, U11 small nuclear	987	400	987	410	137	609	415	151	664	67	88	210	662
U104	80	small nucleolar RNA, C/D box 104	790	395	249	333	136	189	279	499	597	546	485	790	237
hY4	91	RNA, Ro-associated Y4	824	369	284	153	229	550	293	431	824	263	104	720	211
U53	75	small nucleolar RNA, C/D box 53	887	310	125	104	104	252	395	238	302	344	887	437	223
U71a	138	small nucleolar RNA, H/ACA box 71A	848	305	190	612	102	156	168	233	431	216	67	334	848
U54	63	small nucleolar RNA, C/D box 54	704	304	138	62	211	69	431	86	522	339	359	704	418
U91	80	small Cajal body-specific RNA 17	965	302	168	261	132	157	244	172	321	965	235	345	322
R38c	80	small nucleolar RNA, C/D box 1C	395	296	303	374	114	206	348	394	352	395	304	295	175
mgU6-53	109	small nucleolar RNA, C/D box 8	484	284	151	149	151	286	472	188	307	328	279	484	332
U34	62	small nucleolar RNA, C/D box 34	596	270	241	187	98	191	182	180	485	376	220	596	214
RNaseMRP	265	ribonuclease MRP RNA	427	264	243	427	153	332	180	249	289	247	201	331	249
U81	72	small nucleolar RNA, C/D box 81	388	234	380	327	86	227	208	196	388	295	102	250	113
HBII-85_groupII	92	small nucleolar RNA, C/D box 115	635	227	47	80	189	635	406	36	116	404	281	98	202
U108	148	small nucleolar RNA, H/ACA box 12	792	225	74	218	151	147	119	62	361	103	76	792	369
RNaseP	328	RNA component of RNaseP	443	224	193	90	120	443	259	437	223	161	128	230	186
U85	330	small Cajal body-specific RNA 10	486	209	163	126	101	183	186	151	482	105	103	486	211
U82	68	small nucleolar RNA, C/D box 82	353	198	136	25	87	117	353	185	331	299	193	310	146
U56	71	small nucleolar RNA, C/D box 56	288	190	288	110	80	283	125	190	232	265	115	286	111
U60	83	small nucleolar RNA, C/D box 60	265	182	129	208	80	104	165	243	216	221	239	265	137
U50	75	small nucleolar RNA, C/D box 50A	403	182	137	191	114	122	149	3	272	256	258	403	99
ACA40	108	small nucleolar RNA, H/ACA box 40	322	157	140	27	91	116	155	220	212	134	110	322	201
U71b	135	small nucleolar RNA, H/ACA box 71B	350	143	162	350	36	133	70	136	157	93	63	150	227
U38a	71	small nucleolar RNA, C/D box 38A	202	116	92	178	48	81	70	68	170	153	111	202	109

ncRNA	transcript length (nt)	type	max	mean	adipose	colon	heart	hypothalam us	kidney	liver	lung	ovary	skeletal muscle	spleen	testes
E2	90	small nucleolar RNA, H/ACA box 62	224	114	110	220	65	112	126	73	184	23	35	76	224
U87	198	small Cajal body-specific RNA 5;small nucleol	247	111	123	51	50	74	192	60	218	58	52	247	100
HBII-239	87	small nucleolar RNA, C/D box 71	253	105	62	72	36	38	117	35	181	166	54	253	136
mgU6-77	149	small nucleolar RNA, C/D box 10	187	101	187	69	73	107	77	145	83	130	63	140	38
U22	126	small nucleolar RNA, C/D box 22	278	98	68	144	49	31	129	17	278	67	44	161	85
U26	75	small nucleolar RNA, C/D box 26	200	97	57	161	43	87	63	48	175	114	58	200	59
HBI-43	238	small nucleolar RNA, C/D box 17	275	93	66	275	41	48	190	21	162	18	24	57	122
HBII-202	73	small nucleolar RNA, C/D box 68	204	90	93	204	24	103	42	46	67	134	65	155	56
U90	330	small Cajal body-specific RNA 7	181	90	33	96	60	32	142	26	181	84	128	133	70
U12	156	RNA, U12 small nuclear	264	87	245	9	58	264	69	49	65	25	22	46	101
ACA55	137	small nucleolar RNA, H/ACA box 55	370	83	54	134	33	25	50	23	141	9	27	49	370
U62	81	small nucleolar RNA, C/D box 62B;small nucl	183	83	75	21	23	57	71	63	143	102	78	183	96
U14-5	92	small nucleolar RNA, C/D box 14B	241	79	69	106	12	35	85	49	29	42	175	241	22
U58b	66	small nucleolar RNA, C/D box 58B	203	76	17	27	25	29	103	42	148	110	67	203	62
ACA21	133	small nucleolar RNA, H/ACA box 21	172	74	19	21	51	23	95	22	131	93	62	124	172
ACA27	133	small nucleolar RNA, H/ACA box 27	198	73	11	4	53	17	70	12	100	100	84	154	198
ACA44	132	small nucleolar RNA, H/ACA box 44	226	72	116	34	28	42	49	39	226	57	35	104	62
HBII-85_groupIII	83	small nucleolar RNA, C/D box 115	242	69	12	14	61	242	138	25	32	79	77	29	45
SNORD119	83	small nucleolar RNA, C/D box 119	101	64	71	48	45	74	68	37	101	86	44	77	49
ACA58	137	small nucleolar RNA, H/ACA box 58	363	63	52	83	8	33	42	5	73	10	8	13	363
U33	78	small nucleolar RNA, C/D box 33	111	61	51	80	28	34	61	49	80	58	80	111	33
E3	77	small nucleolar RNA, H/ACA box 63	121	56	34	57	24	72	73	38	75	43	26	56	121
ACA31	126	small nucleolar RNA, H/ACA box 31	93	54	47	93	30	20	40	81	65	38	43	57	78
HBII-289	115	small nucleolar RNA, C/D box 89	101	53	48	71	15	62	54	54	81	33	29	101	33
U55	75	small nucleolar RNA, C/D box 55	105	51	28	34	21	34	44	4	100	105	56	96	42
SNORD121B	80	small nucleolar RNA, C/D box 121B	103	49	22	3	15	37	73	16	71	36	103	69	98
ACA36	132	small nucleolar RNA, H/ACA box 36A	154	49	13	134	19	13	45	7	154	2	3	72	78
U47	65	small nucleolar RNA, C/D box 47	89	46	75	23	16	62	50	44	54	89	25	56	15
mgU2-19/30	354	small Cajal body-specific RNA 9	76	44	41	71	25	76	39	16	44	63	22	51	40
ACA6	149	small nucleolar RNA, H/ACA box 6	108	44	49	51	19	40	37	20	104	10	20	26	108
U61	73	small nucleolar RNA, C/D box 61	70	40	36	31	27	30	51	30	44	70	64	39	13
U21	95	small nucleolar RNA, C/D box 21	79	38	20	15	20	13	58	16	37	55	73	79	30
U27	72	small nucleolar RNA, C/D box 27	76	37	34	46	16	26	26	31	51	76	9	68	27
ACA60	136	small nucleolar RNA, H/ACA box 60	58	37	50	33	7	55	58	51	58	12	7	42	31
U93	275	small Cajal body-specific RNA 13	72	36	25	45	13	25	25	23	37	71	22	72	34
ACA23	189	small nucleolar RNA, H/ACA box 23	164	34	35	6	9	26	26	32	33	9	13	24	164
ACA61	130	small nucleolar RNA, H/ACA box 61	85	34	40	35	19	22	28	12	85	31	15	51	35
mgU2-25/61	421	small Cajal body-specific RNA 2	78	33	16	31	19	28	46	12	78	39	16	45	38
U45b	71	small nucleolar RNA, C/D box 45B	88	33	38	18	2	88	29	69	19	24	34	28	9
HBI-61	179	small nucleolar RNA, H/ACA box 81	108	32	40	24	6	11	54	7	57	4	10	33	108
ACA7	138	small nucleolar RNA, H/ACA box 7A	51	32	26	31	14	25	42	21	31	40	25	51	45
HBI-6	123	small nucleolar RNA, H/ACA box 26	166	31	5	44	6	13	25	10	30	16	9	21	166
U57	72	small nucleolar RNA, C/D box 57	56	31	52	47	11	56	24	15	41	39	9	35	13
HBII-316	90	small nucleolar RNA, C/D box 92	51	30	16	19	13	22	39	48	51	22	27	45	26
HBII-234	89	small nucleolar RNA, C/D box 70	57	30	10	19	16	14	36	26	34	38	57	54	23
HBII-336	75	small nucleolar RNA, C/D box 93	92	29	24	23	24	3	11	23	49	21	92	31	15
U92	131	small Cajal body-specific RNA 8	87	27	18	18	6	8	18	12	40	30	13	47	87
U25	67	small nucleolar RNA, C/D box 25	55	27	19	45	4	24	20	42	32	27	14	55	12
U24	75	small nucleolar RNA, C/D box 24	69	26	18	39	9	24	18	26	18	28	16	69	17
ACA25	89	small nucleolar RNA, H/ACA box 25	42	25	17	20	42	17	25	15	39	26	11	32	37
ACA26	123	small Cajal body-specific RNA 4	66	25	22	3	9	18	34	24	66	11	2	33	59
U45a	84	small nucleolar RNA, C/D box 45A	45	25	41	17	14	40	22	24	28	16	23	45	10
U74	69	small nucleolar RNA, C/D box 74	53	25	23	20	6	18	21	53	24	26	19	39	21

ncRNA	transcript length (nt)	type	max	mean	adipose	colon	heart	hypothalam us	kidney	liver	lung	ovary	skeletal muscle	spleen	testes
U6atac	122	snRNA U6atac (U12-dependent splicing)	43	23	20	4	18	16	20	14	43	32	21	34	32
U41	70	small nucleolar RNA, C/D box 41	64	23	34	45	13	64	15	22	18	12	5	19	4
U23	126	small nucleolar RNA, H/ACA box 75	64	23	36	11	13	21	19	21	20	8	13	21	64
ACA51	172	small nucleolar RNA, H/ACA box 51	48	21	9	7	22	19	7	5	30	28	14	48	41
U42	62	small nucleolar RNA, C/D box 42A	33	20	19	20	24	26	20	16	33	20	8	23	16
hY5	76	RNA, Ro-associated Y5	32	20	7	6	18	31	27	12	32	31	6	31	17
ACA1	132	small nucleolar RNA, H/ACA box 1	47	19	8	6	11	3	31	25	47	2	4	32	44
ACA50	135	small nucleolar RNA, H/ACA box 50	34	19	11	1	9	34	13	26	13	31	21	21	30
U17b	207	small nucleolar RNA, H/ACA box 73B	33	19	18	31	8	11	17	10	33	21	8	30	18
HBII-55	76	small nucleolar RNA, C/D box 110	46	18	16	31	5	46	13	11	19	21	10	22	8
ACA34	137	small nucleolar RNA, H/ACA box 34	27	18	17	24	13	27	14	10	21	19	21	16	14
U4atac	129	snRNA U4atac (U12-dependent splicing)	32	18	32	10	13	20	19	9	25	15	8	17	28
U96a	72	small nucleolar RNA, C/D box 96A	59	18	8	17	7	9	24	11	26	17	5	59	12
U58a	65	small nucleolar RNA, C/D box 58A	23	18	23	19	10	23	16	22	13	23	16	19	8
U30	70	small nucleolar RNA, C/D box 30	34	17	20	16	6	11	10	34	19	18	13	29	11
ACA24	131	small nucleolar RNA, H/ACA box 24	39	16	8	6	6	5	21	39	15	12	16	36	15
U36c	68	small nucleolar RNA, C/D box 36C	32	16	25	1	7	8	25	32	12	22	11	22	12
U98a	129	small nucleolar RNA, H/ACA box 16A	46	16	11	13	7	3	8	2	46	23	13	42	10
U49B	49	small nucleolar RNA, C/D box 49B	25	16	10	25	18	4	13	19	16	17	21	20	12
ACA68	139	small Cajal body-specific RNA 21	43	15	12	25	5	15	9	3	21	26	3	43	8
HBII-438	67	small nucleolar RNA, C/D box 109	107	15	0	4	1	107	7	1	1	7	15	2	25
U89	270	small Cajal body-specific RNA 12	45	15	8	45	5	13	9	31	11	10	10	16	6
U77	69	small nucleolar RNA, C/D box 77	30	14	14	4	7	9	18	15	28	30	3	24	8
HBII-82	95	small nucleolar RNA, C/D box 111	38	14	2	3	7	4	11	18	16	38	14	29	11
U84	78	small nucleolar RNA, C/D box 84	20	12	8	5	14	8	15	3	11	15	15	20	16
HBII-295	108	small nucleolar RNA, C/D box 90	27	12	11	1	8	13	14	12	20	9	5	27	10
ACA8	139	small nucleolar RNA, H/ACA box 8	28	12	5	4	8	6	16	3	23	17	6	28	10
U83	76	small nucleolar RNA, C/D box 117	29	11	8	0	3	6	13	6	29	6	12	27	17
U17a	87	small nucleolar RNA, H/ACA box 73A	29	11	7	8	9	3	7	5	24	18	5	29	9
U103	88	small nucleolar RNA, C/D box 103A	18	11	7	8	7	11	11	11	15	14	9	18	6
HBII-142	77	small nucleolar RNA, C/D box 66	27	11	9	11	3	4	9	27	16	13	6	13	4
U3b2	216	small nucleolar RNA, C/D box 3B-2;small nuc	19	10	19	5	5	7	9	7	17	11	14	17	3
HBII-437	72	small nucleolar RNA, C/D box 108	34	10	2	1	13	34	15	1	2	18	19	6	2
HBII-296B	87	small nucleolar RNA, C/D box 91B	17	10	7	16	4	7	11	3	10	13	17	11	8
HBII-251	76	small nucleolar RNA, C/D box 85	17	9	3	1	5	10	15	6	16	17	5	13	8
U83a	95	small nucleolar RNA, C/D box 83A	19	9	7	10	4	4	6	14	15	8	9	19	4
U50B	72	small nucleolar RNA, C/D box 50B	18	9	8	6	2	9	5	7	5	13	14	18	7
snR39B	71	small nucleolar RNA, C/D box 2	50	9	10	0	1	0	7	15	5	3	2	50	2
U19	199	small nucleolar RNA, H/ACA box 74A;small n	20	8	12	9	1	9	6	2	20	0	4	4	20
U15b	146	small nucleolar RNA, C/D box 15B	14	8	4	14	4	8	5	8	12	8	8	9	3
HBII-99	91	small nucleolar RNA, C/D box 12	17	7	6	5	2	6	2	16	7	9	17	10	2
U15a	148	small nucleolar RNA, C/D box 15A	29	7	5	6	6	2	9	6	29	0	3	8	8
ACA17	133	small nucleolar RNA, H/ACA box 17	19	7	14	7	2	4	4	6	19	6	1	7	7
U38b	69	small nucleolar RNA, C/D box 38B	15	7	3	4	2	2	6	9	15	5	8	14	8
U49	71	small nucleolar RNA, C/D box 49A	15	7	7	3	3	9	6	5	13	6	4	15	6
U78	65	small nucleolar RNA, C/D box 78	25	7	1	2	3	0	4	9	20	7	0	25	3
ACA5	131	small nucleolar RNA, H/ACA box 5A	18	7	4	18	3	3	9	7	6	4	3	9	8
ACA54	123	small nucleolar RNA, H/ACA box 54	12	6	9	5	0	12	6	11	3	6	3	12	2
U8	136	small nucleolar RNA, C/D box 118	14	6	7	3	3	5	4	14	11	4	2	11	5
hTR_TERC	451	telomerase RNA component	40	6	3	2	3	7	1	1	4	4	2	5	40
U36a	72	small nucleolar RNA, C/D box 36A	12	6	9	10	2	6	5	12	7	3	3	9	4
U58C	66	small nucleolar RNA, C/D box 58C	12	6	3	0	3	2	6	6	11	12	8	12	6
HBII-436	75	small nucleolar RNA, C/D box 107	35	6	4	0	1	35	3	6	3	2	6	4	0

ncRNA	transcript length (nt)	type	max	mean	adipose	colon	heart	hypothalamus	kidney	liver	lung	ovary	skeletal muscle	spleen	testes
HBII-95B	91	small nucleolar RNA, C/D box 11B	21	6	2	2	6	2	5	5	7	21	3	13	2
BC200	200	brain cytoplasmic RNA 1 (non-protein coding	55	6	4	2	2	55	1	0	0	0	0	0	0
HBII-135	74	small nucleolar RNA, C/D box 65	25	6	13	2	0	4	3	25	2	7	3	8	0
HBII-419	68	small nucleolar RNA, C/D box 98	14	6	1	0	2	2	8	1	14	13	3	10	9
HBII-210	78	small nucleolar RNA, C/D box 69	9	6	7	7	4	6	5	5	9	8	5	5	3
U105	85	small nucleolar RNA, C/D box 105	11	6	5	7	5	2	4	8	6	5	1	11	7
U70	105	small nucleolar RNA, H/ACA box 70	13	5	7	8	2	4	3	2	7	13	4	6	6
U35A	87	small nucleolar RNA, C/D box 35A	13	5	1	11	4	1	7	4	4	8	5	13	1
ACA48	133	small nucleolar RNA, H/ACA box 48	11	5	4	3	4	3	6	0	9	6	1	11	9
ACA19	127	small nucleolar RNA, H/ACA box 19	13	5	8	1	3	4	7	1	8	0	2	6	13
ACA39	136	small nucleolar RNA, H/ACA box 39	17	5	2	7	1	3	7	1	5	1	1	4	17
U99	111	small nucleolar RNA, H/ACA box 57	12	4	1	3	3	4	3	1	2	12	5	9	5
14q(II-1)	72	small nucleolar RNA, C/D box 114-1	13	4	4	11	2	13	1	1	0	8	1	1	6
HBII-95	85	small nucleolar RNA, C/D box 11	10	4	1	1	2	1	6	3	6	2	9	10	2
HBII-99B	92	small nucleolar RNA, C/D box 12B	9	4	2	1	1	9	6	4	6	5	2	5	2
ACA10	133	small nucleolar RNA, H/ACA box 10	15	4	2	3	1	1	1	2	3	2	1	11	15
U94	137	small nucleolar RNA, C/D box 94	7	4	1	4	2	5	7	1	3	7	1	6	4
ACA53	251	small nucleolar RNA, H/ACA box 53	7	4	6	5	2	3	7	3	6	0	1	3	5
Z17a	72	small nucleolar RNA, C/D box 4A	7	4	4	3	0	1	4	5	7	5	2	6	4
U5E	51	RNA, U5E small nuclear	5	3	5	4	1	3	3	2	3	5	3	5	2
U44	61	small nucleolar RNA, C/D box 44	8	3	8	3	1	2	4	1	5	4	1	6	1
ACA32	122	small nucleolar RNA, H/ACA box 32	10	3	10	2	1	4	1	3	3	1	2	1	8
U66	133	small nucleolar RNA, H/ACA box 66	13	3	4	13	0	1	4	0	4	0	1	3	6
U40_U46	99	small nucleolar RNA, C/D box 46	5	3	2	2	1	5	5	5	5	4	2	4	1
R38a	74	small nucleolar RNA, C/D box 1A	5	3	2	4	1	2	4	5	3	4	3	5	2
14q(II-14)	75	small nucleolar RNA, C/D box 114-14	19	3	2	2	1	19	2	1	0	6	0	2	0
14q(II-12)	75	small nucleolar RNA, C/D box 114-12	14	3	2	4	1	14	0	0	1	5	0	0	6
14q(0)	78	small nucleolar RNA, C/D box 112	13	3	0	5	4	4	0	0	0	13	2	4	0
Xist	28130	X (inactive)-specific transcript (non-protein c	11	3	0	7	2	0	5	0	3	11	4	0	0
U64	129	small nucleolar RNA, H/ACA box 64	5	3	4	1	3	4	1	1	2	4	5	2	4
HBII-166	112	small nucleolar RNA, C/D box 67	6	3	4	0	0	6	4	1	2	4	3	3	2
ACA35	166	small Cajal body-specific RNA 1	7	3	1	0	1	2	3	0	1	6	2	6	7
ACA11	125	small Cajal body-specific RNA 22	9	3	1	5	1	2	2	0	4	0	0	4	9
mgh28S-2410	74	small nucleolar RNA, C/D box 5	11	3	1	0	2	0	2	2	4	3	1	11	1
U16	102	small nucleolar RNA, C/D box 16	6	2	2	0	1	1	3	4	3	3	2	2	6
ACA46	135	small nucleolar RNA, H/ACA box 46	8	2	0	3	1	1	7	0	8	2	2	3	0
HBII-429	77	small nucleolar RNA, C/D box 100	7	2	1	4	1	0	1	1	3	7	1	6	2
U101	73	small nucleolar RNA, C/D box 101	7	2	6	0	1	1	1	1	7	2	2	3	1
ACA49	137	small nucleolar RNA, H/ACA box 49	4	2	1	3	2	1	1	0	3	4	3	2	4
ACA38	132	small nucleolar RNA, H/ACA box 38	15	2	1	0	2	1	1	0	3	0	1	0	15
14q(II-7)	77	small nucleolar RNA, C/D box 114-7	7	2	0	0	2	4	0	0	1	7	1	4	3
U68	130	small nucleolar RNA, H/ACA box 68	4	2	0	0	2	1	1	1	4	4	4	4	1
HBII-13	67	small nucleolar RNA, C/D box 64	12	2	1	0	2	12	2	0	0	3	2	0	0
U106	79	small nucleolar RNA, C/D box 12C	5	2	0	1	0	1	2	2	2	5	4	2	1
ACA18	105	small nucleolar RNA, H/ACA box 18	4	2	3	0	1	1	3	2	3	1	0	4	0
U75	60	small nucleolar RNA, C/D box 75	5	2	2	0	0	1	0	3	0	1	3	5	4
ACA14a	134	small nucleolar RNA, H/ACA box 14A	5	2	2	0	0	2	1	5	3	1	0	0	2
ACA43	134	small nucleolar RNA, H/ACA box 43	3	2	2	3	1	2	2	2	2	1	2	1	1
HBII-82B	88	small nucleolar RNA, C/D box 111B	4	2	1	1	2	0	3	0	4	2	0	3	1
U88	266	small Cajal body-specific RNA 6	4	1	1	0	1	3	1	1	4	1	2	1	1
U83b	93	small nucleolar RNA, C/D box 83B	5	1	0	1	2	0	1	0	5	2	1	4	1
HBII-108B	85	small nucleolar RNA, C/D box 19B	6	1	2	0	1	0	0	4	0	6	0	2	0
U79	81	small nucleolar RNA, C/D box 79	3	1	2	0	0	2	3	1	2	3	2	0	1

ncRNA	transcript length (nt)	type	max	mean	adipose	colon	heart	hypothalamus	kidney	liver	lung	ovary	skeletal muscle	spleen	testes
U105B	80	small nucleolar RNA, C/D box 105B	5	1	1	1	1	5	1	0	2	0	0	3	2
U43	62	small nucleolar RNA, C/D box 43	5	1	1	0	1	1	0	0	5	2	3	3	0
ACA30	127	small nucleolar RNA, H/ACA box 30	3	1	1	1	2	1	2	1	1	3	0	3	0
ACA57	130	small Cajal body-specific RNA 11	4	1	0	1	2	4	2	0	1	1	1	0	2
U5F	112	RNA, U5F small nuclear	4	1	2	1	0	0	0	0	3	2	1	4	1
ACA2a	134	small nucleolar RNA, H/ACA box 2A	3	1	1	3	0	1	2	1	1	1	1	2	2
U67	59	small nucleolar RNA, H/ACA box 67	5	1	2	1	0	0	0	0	5	0	0	3	1
U107	132	small nucleolar RNA, H/ACA box 11	4	1	1	4	1	1	1	0	4	0	1	0	0
ACA22	134	small nucleolar RNA, H/ACA box 22	5	1	1	0	1	0	1	0	3	1	0	1	5
U80	71	small nucleolar RNA, C/D box 80	3	1	2	1	0	3	1	1	0	2	1	1	0
ACA4	145	small nucleolar RNA, H/ACA box 4	3	1	1	1	0	0	1	3	2	2	1	1	1
ACA47	183	small Cajal body-specific RNA 16	3	1	2	1	1	0	0	0	2	1	0	3	2
SNORD125	97	small nucleolar RNA, C/D box 125	3	1	1	0	2	3	1	1	0	0	3	0	1
U63	68	small nucleolar RNA, C/D box 63	4	1	1	0	0	1	2	2	0	1	0	4	1
R38b	86	small nucleolar RNA, C/D box 1B	3	1	0	0	0	3	1	2	0	2	1	3	0
HBI-115	139	small nucleolar RNA, H/ACA box 47	3	1	2	0	1	1	1	1	3	1	2	0	0
ACA65	141	small nucleolar RNA, H/ACA box 79	7	1	0	1	0	0	1	0	2	0	0	0	7
14q(I-5)	78	small nucleolar RNA, C/D box 113-5	3	1	0	0	0	3	0	1	0	3	1	3	0
SNORA84	134	small nucleolar RNA, H/ACA box 84	4	1	0	2	0	0	0	1	4	1	0	1	1
U7	62	RNA, U7 small nuclear	3	1	0	1	2	3	0	0	1	2	0	1	0
U65	136	small nucleolar RNA, H/ACA box 65	2	1	2	0	0	1	0	1	1	0	0	1	2
U102	72	small nucleolar RNA, C/D box 102	3	1	0	0	1	1	0	1	1	1	1	1	3
U36b	71	small nucleolar RNA, C/D box 36B	2	1	0	0	1	0	2	0	1	1	2	1	1
mgh28S-2412	71	small nucleolar RNA, C/D box 6	3	1	0	3	0	1	2	0	3	1	0	0	0
ACA15	135	small nucleolar RNA, H/ACA box 15	3	1	0	1	0	0	0	0	2	1	1	3	0
ACA33	133	small nucleolar RNA, H/ACA box 33	1	1	1	1	1	1	1	0	1	1	0	1	1
ACA41	132	small nucleolar RNA, H/ACA box 41	2	1	0	2	0	1	1	2	0	1	0	1	2
U18	70	small nucleolar RNA, C/D box 18A	3	1	1	2	0	0	0	1	3	1	0	0	0
U18C	68	small nucleolar RNA, C/D box 18C	3	1	0	0	0	1	1	1	3	1	0	2	0
14q(II-3)	75	small nucleolar RNA, C/D box 114-3	3	1	0	3	0	3	0	0	0	1	0	0	0
PWRN1	1436	Prader-Willi region non-protein coding RNA :	3	1	0	0	0	1	1	0	0	2	0	0	3
U48	65	small nucleolar RNA, C/D box 48	2	1	1	0	0	2	0	0	1	0	1	2	0
U35B	89	small nucleolar RNA, C/D box 35B	2	1	0	1	0	1	1	0	0	2	0	0	1
U5B	88	RNA, U5B small nuclear 1	2	1	2	0	0	0	0	1	1	0	1	1	1
ACA56	129	small nucleolar RNA, H/ACA box 56	4	1	0	1	0	0	0	0	1	0	0	0	4
ACA59	152	small nucleolar RNA, H/ACA box 59B;small nu	1	1	0	0	0	1	1	0	1	0	1	0	1
14q(I-7)	77	small nucleolar RNA, C/D box 113-7	3	1	0	0	0	3	1	0	0	1	0	0	0
14q(I-4)	75	small nucleolar RNA, C/D box 113-4	3	1	0	0	0	3	0	0	0	2	0	0	0
U72	132	small nucleolar RNA, H/ACA box 72	1	1	0	0	0	1	0	1	0	1	0	1	0
SNORA11	129	small nucleolar RNA, H/ACA box 11	5	1	0	0	0	5	0	0	0	0	0	1	0
U42B	68	small nucleolar RNA, C/D box 42B	2	1	1	0	0	0	1	0	0	1	0	2	0
IPW	4498	imprinted in Prader-Willi syndrome (non-pro	1	1	0	0	1	1	1	0	0	1	1	0	1
14q(I-9)	72	small nucleolar RNA, C/D box 113-9	2	1	0	0	0	2	2	0	0	2	0	0	0
U37	66	small nucleolar RNA, C/D box 37	3	0	0	3	0	0	1	1	0	0	0	0	1
U69	132	small nucleolar RNA, H/ACA box 69	3	0	1	0	0	0	0	0	0	1	0	0	3
CDKN2BAS	3832	CDKN2B antisense RNA (non-protein coding)	2	0	0	1	0	0	0	0	1	0	0	1	2
SNORD123	71	small nucleolar RNA, C/D box 123	2	0	0	1	0	1	0	0	0	2	0	0	0
14q(II-28)	72	small nucleolar RNA, C/D box 114-28	2	0	1	1	0	2	0	1	0	0	0	0	0
HVG-1	101	vault RNA 1-1	2	0	0	0	1	0	0	0	0	0	0	2	1
U73a	65	small nucleolar RNA, C/D box 73A	1	0	1	0	0	0	1	0	0	1	1	0	0
U31	66	small nucleolar RNA, C/D box 31	3	0	1	1	0	0	0	0	3	0	0	0	0
HBI-100	145	small Cajal body-specific RNA 3	1	0	1	1	0	0	0	0	1	1	0	0	0
U5A	116	RNA, U5A small nuclear	1	0	0	1	0	0	0	0	0	1	1	0	1

ncRNA	transcript length (nt)	type	max	mean	adipose	colon	heart	hypothalamus	kidney	liver	lung	ovary	skeletal muscle	spleen	testes
ACA63	126	small nucleolar RNA, H/ACA box 77	1	0	1	0	0	0	0	1	0	1	1	0	0
14q(I-3)	72	small nucleolar RNA, C/D box 113-3	3	0	0	1	0	3	0	0	0	0	0	0	0
HBII-296A	93	small nucleolar RNA, C/D box 91A	1	0	0	0	1	0	1	1	1	0	0	0	0
ACA62	134	small nucleolar RNA, H/ACA box 76	2	0	0	1	0	0	0	2	0	0	0	0	0
HBI-36	128	small nucleolar RNA, H/ACA box 35	4	0	0	0	0	4	0	0	0	0	0	0	0
14q(II-26)	72	small nucleolar RNA, C/D box 114-26	1	0	0	1	0	1	0	0	0	0	0	1	1
ACA9	132	small nucleolar RNA, H/ACA box 9	1	0	0	0	0	0	0	0	1	0	0	1	0
ACA29	140	small nucleolar RNA, H/ACA box 29	2	0	0	1	0	0	0	0	2	0	0	1	0
14q(II-20)	72	small nucleolar RNA, C/D box 114-20	3	0	0	0	0	3	0	0	0	1	0	0	0
ACA2b	137	small nucleolar RNA, H/ACA box 2B	1	0	0	0	0	1	0	0	0	0	0	0	1
ACA28	126	small nucleolar RNA, H/ACA box 28	1	0	0	1	0	0	1	0	1	0	0	0	0
HBII-115	111	small nucleolar RNA, C/D box 23	1	0	0	0	0	0	0	0	0	1	1	1	0
14q(II-31)	75	small nucleolar RNA, C/D box 114-31	1	0	0	1	0	0	0	0	0	0	0	0	1
14q(II-23)	72	small nucleolar RNA, C/D box 114-23	1	0	0	0	1	1	0	0	0	1	0	0	0
SNORD127	87	small nucleolar RNA, C/D box 127	2	0	0	0	0	0	0	0	0	0	0	0	2
14q(II-4)	75	small nucleolar RNA, C/D box 114-4	2	0	0	0	0	2	0	0	0	0	0	0	0
14q(II-11)	75	small nucleolar RNA, C/D box 114-11	1	0	0	0	1	0	0	0	0	0	0	0	0
Z17b	74	small nucleolar RNA, C/D box 4B	1	0	0	0	0	0	1	0	1	0	0	0	0
U98b	133	small nucleolar RNA, H/ACA box 16B	1	0	0	0	0	0	0	0	1	0	1	0	0
14q(II-24)	72	small nucleolar RNA, C/D box 114-24	2	0	0	0	0	0	0	0	0	0	0	2	0
U20	80	small nucleolar RNA, C/D box 20	1	0	0	0	1	0	0	0	0	0	0	0	0
U51	70	small nucleolar RNA, C/D box 51	2	0	0	0	0	0	0	0	2	0	0	0	0
14q(II-21)	72	small nucleolar RNA, C/D box 114-21	1	0	0	0	0	0	0	0	0	1	0	0	0
14q(I-8)	74	small nucleolar RNA, C/D box 113-8	2	0	0	0	0	2	0	0	0	0	0	0	0
ACA66	131	small Cajal body-specific RNA 20	1	0	0	0	0	0	0	0	0	0	0	0	1
14q(II-15)	72	small nucleolar RNA, C/D box 114-15	1	0	0	0	0	1	0	0	0	0	0	1	0
PWRN2	5129	Prader-Willi region non-protein coding RNA [illegible]	1	0	0	0	0	0	0	0	0	1	0	0	0
U52	64	small nucleolar RNA, C/D box 52	1	0	0	0	0	0	0	0	0	0	0	1	0
14q(II-18)	72	small nucleolar RNA, C/D box 114-18	1	0	0	0	0	1	0	0	0	1	0	0	0
Z30	97	small nucleolar RNA, C/D box 7	0	0	0	0	0	0	0	0	0	0	0	0	0
14q(II-17)	75	small nucleolar RNA, C/D box 114-17	1	0	0	0	0	1	0	0	0	0	0	0	0
ACA52	134	small nucleolar RNA, H/ACA box 52	0	0	0	0	0	0	0	0	0	0	0	0	0
HBII-108	77	small nucleolar RNA, C/D box 19	1	0	0	0	0	0	1	0	0	0	0	0	0
ACA45	127	small Cajal body-specific RNA 15	1	0	0	0	0	0	0	0	1	0	0	0	0
14q(II-8)	72	small nucleolar RNA, C/D box 114-8	1	0	1	0	0	0	0	0	0	0	0	0	0
14q(I-1)	71	small nucleolar RNA, C/D box 113-1	1	0	0	0	0	0	0	0	0	0	0	1	0
U109	135	small Cajal body-specific RNA 18	1	0	0	0	0	0	1	0	0	0	0	0	0
U100	137	small Cajal body-specific RNA 14	1	0	1	0	0	0	0	0	0	0	0	0	0
HBII-240	81	small nucleolar RNA, C/D box 72	0	0	0	0	0	0	0	0	0	0	0	0	0
HBII-180C	98	small nucleolar RNA, C/D box 88C	0	0	0	0	0	0	0	0	0	0	0	0	0
ACA13	133	small nucleolar RNA, H/ACA box 13	0	0	0	0	0	0	0	0	0	0	0	0	0
SNORD124	105	small nucleolar RNA, C/D box 124	0	0	0	0	0	0	0	0	0	0	0	0	0
14q(II-6)	72	small nucleolar RNA, C/D box 114-6	0	0	0	0	0	0	0	0	0	0	0	0	0
14q(II-9)	72	small nucleolar RNA, C/D box 114-9	0	0	0	0	0	0	0	0	0	0	0	0	0
14q(I-2)	72	small nucleolar RNA, C/D box 113-2	0	0	0	0	0	0	0	0	0	0	0	0	0
14q(II-10)	72	small nucleolar RNA, C/D box 114-10	0	0	0	0	0	0	0	0	0	0	0	0	0
14q(II-13)	74	small nucleolar RNA, C/D box 114-13	0	0	0	0	0	0	0	0	0	0	0	0	0
14q(II-16)	70	small nucleolar RNA, C/D box 114-16	0	0	0	0	0	0	0	0	0	0	0	0	0
14q(II-19)	75	small nucleolar RNA, C/D box 114-19	0	0	0	0	0	0	0	0	0	0	0	0	0
14q(II-2)	78	small nucleolar RNA, C/D box 114-2	0	0	0	0	0	0	0	0	0	0	0	0	0
14q(II-22)	72	small nucleolar RNA, C/D box 114-22	0	0	0	0	0	0	0	0	0	0	0	0	0
14q(II-25)	72	small nucleolar RNA, C/D box 114-25	0	0	0	0	0	0	0	0	0	0	0	0	0
14q(II-27)	70	small nucleolar RNA, C/D box 114-27	0	0	0	0	0	0	0	0	0	0	0	0	0

ncRNA	transcript length (nt)	type	max	mean	adipose	colon	heart	hypothalamus	kidney	liver	lung	ovary	skeletal muscle	spleen	testes
14q(II-29)	70	small nucleolar RNA, C/D box 114-29	0	0	0	0	0	0	0	0	0	0	0	0	0
14q(II-30)	72	small nucleolar RNA, C/D box 114-30	0	0	0	0	0	0	0	0	0	0	0	0	0
14q(II-5)	70	small nucleolar RNA, C/D box 114-5	0	0	0	0	0	0	0	0	0	0	0	0	0
ACA12	130	small Cajal body-specific RNA 23	0	0	0	0	0	0	0	0	0	0	0	0	0
ACA64	128	small nucleolar RNA, H/ACA box 78	0	0	0	0	0	0	0	0	0	0	0	0	0
ACA67	137	small nucleolar RNA, H/ACA box 80	0	0	0	0	0	0	0	0	0	0	0	0	0
HBII-180A	98	small nucleolar RNA, C/D box 88A	0	0	0	0	0	0	0	0	0	0	0	0	0
HBII-180B	98	small nucleolar RNA, C/D box 88B	0	0	0	0	0	0	0	0	0	0	0	0	0
HBII-382	83	small Cajal body-specific RNA 2	0	0	0	0	0	0	0	0	0	0	0	0	0
HVG-2	88	vault RNA 1-2	0	0	0	0	0	0	0	0	0	0	0	0	0
HVG-3	88	vault RNA 1-3	0	0	0	0	0	0	0	0	0	0	0	0	0
SNORA38B	132	small nucleolar RNA, H/ACA box 38B (retrotr	0	0	0	0	0	0	0	0	0	0	0	0	0
SNORD121A	91	small nucleolar RNA, C/D box 121A	0	0	0	0	0	0	0	0	0	0	0	0	0
SNORD126	78	small nucleolar RNA, C/D box 126	0	0	0	0	0	0	0	0	0	0	0	0	0
U18B	71	small nucleolar RNA, C/D box 18B	0	0	0	0	0	0	0	0	0	0	0	0	0
U5Ds	61	RNA, U5D small nuclear	0	0	0	0	0	0	0	0	0	0	0	0	0
U86	87	small nucleolar RNA, C/D box 86	0	0	0	0	0	0	0	0	0	0	0	0	0
U96b	72	small nucleolar RNA, C/D box 96B	0	0	0	0	0	0	0	0	0	0	0	0	0
