# Supplementary material for: Digital Genome-Wide ncRNA Expression, Including SnoRNAs, across 11 Human Tissues Using PolyA-Neutral Amplification
Source: PLoS One. 2010 Jul 26;5(7):e11779. doi: 10.1371/journal.pone.0011779 (PMC2909899; doi:10.1371/journal.pone.0011779)

Table S6. Sequence similarity of HBII-52 snoRNAs, as the number of 28mer sequences in common. Values greater than 0 are filled yellow, values greater than 25 are bright yellow, and values greater than 50 are red.


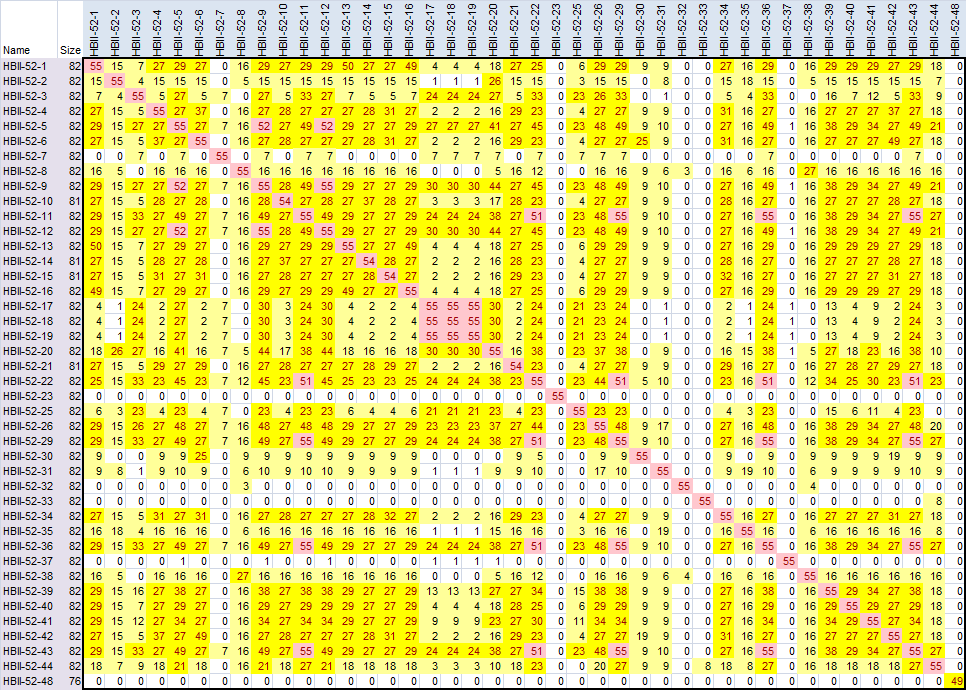

Supplement: Table S6 — Similarity among HBII-52 cluster snoRNAs, based on the number of identical 28 nt sequences. (0.14 MB DOC) [file pone.0011779.s009.doc]
