# Supplementary material for: Digital Genome-Wide ncRNA Expression, Including SnoRNAs, across 11 Human Tissues Using PolyA-Neutral Amplification
Source: PLoS One. 2010 Jul 26;5(7):e11779. doi: 10.1371/journal.pone.0011779 (PMC2909899; doi:10.1371/journal.pone.0011779)
Supplement: Table S2 — Expression of RefSeq transcripts. (1.28 MB PDF) [file pone.0011779.s005.pdf]

| transcript | gene | transcript length (nt) | max | mean | adipose | colon | heart | hypothala mus | kidney | liver | lung | ovary | skeletalm uscle | spleen | testes |
|---|---|---|---|---|---|---|---|---|---|---|---|---|---|---|---|
| NM_000014 | A2M | 4653 | 697 | 165 | 139 | 45 | 180 | 53 | 70 | 697 | 339 | 48 | 27 | 144 | 73 |
| NM_000015 | NAT2 | 1317 | 7 | 1 | 0 | 0 | 0 | 0 | 0 | 7 | 0 | 0 | 0 | 0 | 0 |
| NM_000016 | ACADM | 2603 | 32 | 14 | 12 | 4 | 32 | 10 | 29 | 17 | 4 | 7 | 15 | 11 | 10 |
| NM_000017 | ACADS | 1917 | 2 | 1 | 1 | 2 | 1 | 0 | 1 | 2 | 0 | 0 | 1 | 1 | 0 |
| NM_000018 | ACADVL | 2288 | 8 | 4 | 7 | 5 | 8 | 2 | 7 | 6 | 2 | 2 | 4 | 2 | 2 |
| NM_000019 | ACAT1 | 2132 | 59 | 14 | 6 | 2 | 17 | 4 | 32 | 59 | 3 | 5 | 17 | 3 | 5 |
| NM_000020 | ACVRL1 | 4263 | 5 | 2 | 2 | 3 | 2 | 1 | 1 | 1 | 5 | 0 | 0 | 4 | 0 |
| NM_000021 | PSEN1 | 6089 | 6 | 4 | 3 | 3 | 3 | 6 | 5 | 3 | 5 | 5 | 2 | 6 | 6 |
| NM_000022 | ADA | 1532 | 1 | 0 | 1 | 0 | 1 | 0 | 0 | 0 | 1 | 0 | 0 | 1 | 0 |
| NM_000023 | SGCA | 1432 | 12 | 1 | 0 | 0 | 3 | 0 | 0 | 0 | 0 | 0 | 12 | 0 | 0 |
| NM_000024 | ADRB2 | 2033 | 3 | 1 | 3 | 0 | 1 | 0 | 0 | 1 | 2 | 0 | 1 | 1 | 0 |
| NM_000025 | ADRB3 | 2645 | 0 | 0 | 0 | 0 | 0 | 0 | 0 | 0 | 0 | 0 | 0 | 0 | 0 |
| NM_000026 | ADSL | 1563 | 35 | 11 | 7 | 7 | 12 | 7 | 9 | 14 | 5 | 9 | 35 | 11 | 10 |
| NM_000027 | AGA | 2041 | 2 | 1 | 1 | 1 | 1 | 1 | 2 | 1 | 1 | 1 | 0 | 1 | 1 |
| NM_000028 | AGL | 7449 | 16 | 5 | 3 | 2 | 5 | 3 | 4 | 12 | 2 | 1 | 16 | 3 | 3 |
| NM_000029 | AGT | 2584 | 218 | 24 | 0 | 1 | 6 | 21 | 10 | 218 | 1 | 1 | 2 | 1 | 1 |
| NM_000030 | AGXT | 1598 | 20 | 2 | 0 | 0 | 0 | 0 | 0 | 20 | 0 | 0 | 0 | 0 | 0 |
| NM_000031 | ALAD | 3183 | 30 | 13 | 23 | 5 | 14 | 22 | 18 | 30 | 5 | 8 | 3 | 5 | 10 |
| NM_000032 | ALAS2 | 2026 | 0 | 0 | 0 | 0 | 0 | 0 | 0 | 0 | 0 | 0 | 0 | 0 | 0 |
| NM_000033 | ABCD1 | 3664 | 0 | 0 | 0 | 0 | 0 | 0 | 0 | 0 | 0 | 0 | 0 | 0 | 0 |
| NM_000034 | ALDOA | 2304 | 201 | 46 | 34 | 25 | 73 | 58 | 34 | 9 | 20 | 15 | 201 | 27 | 14 |
| NM_000035 | ALDOB | 2426 | 853 | 151 | 0 | 8 | 1 | 0 | 789 | 853 | 1 | 2 | 1 | 3 | 2 |
| NM_000036 | AMPD1 | 2341 | 95 | 9 | 0 | 0 | 0 | 0 | 0 | 0 | 0 | 0 | 95 | 0 | 0 |
| NM_000037 | ANK1 | 8292 | 2 | 0 | 0 | 0 | 1 | 1 | 0 | 0 | 0 | 0 | 2 | 0 | 0 |
| NM_000038 | APC | 10730 | 28 | 6 | 3 | 2 | 5 | 28 | 4 | 2 | 3 | 5 | 5 | 5 | 4 |
| NM_000039 | APOA1 | 897 | 1,271 | 121 | 0 | 6 | 5 | 0 | 1 | 1,271 | 1 | 4 | 3 | 4 | 40 |
| NM_000040 | APOC3 | 533 | 27 | 3 | 0 | 0 | 0 | 0 | 0 | 27 | 0 | 0 | 1 | 0 | 0 |
| NM_000041 | APOE | 1180 | 99 | 18 | 10 | 2 | 3 | 22 | 32 | 99 | 8 | 5 | 1 | 18 | 3 |
| NM_000042 | APOH | 1201 | 2,188 | 203 | 0 | 6 | 6 | 0 | 4 | 2,188 | 3 | 9 | 4 | 10 | 4 |
| NM_000043 | FAS | 2689 | 2 | 1 | 1 | 0 | 1 | 1 | 1 | 2 | 2 | 2 | 0 | 2 | 1 |
| NM_000044 | AR | 4314 | 5 | 1 | 2 | 0 | 1 | 0 | 1 | 5 | 0 | 3 | 1 | 0 | 2 |
| NM_000045 | ARG1 | 1447 | 181 | 18 | 0 | 1 | 1 | 0 | 0 | 181 | 5 | 1 | 0 | 4 | 1 |
| NM_000046 | ARSB | 6071 | 3 | 1 | 1 | 1 | 2 | 1 | 3 | 1 | 1 | 1 | 0 | 1 | 1 |
| NM_000047 | ARSE | 2220 | 5 | 1 | 0 | 2 | 0 | 0 | 4 | 5 | 0 | 0 | 0 | 0 | 0 |
| NM_000048 | ASL | 1936 | 6 | 1 | 0 | 2 | 0 | 1 | 2 | 6 | 1 | 0 | 0 | 1 | 0 |
| NM_000049 | ASPA | 1418 | 5 | 1 | 1 | 0 | 0 | 2 | 5 | 0 | 1 | 1 | 0 | 1 | 1 |
| NM_000050 | ASS1 | 1848 | 31 | 7 | 5 | 2 | 2 | 2 | 31 | 28 | 1 | 2 | 1 | 1 | 2 |
| NM_000051 | ATM | 13147 | 17 | 5 | 4 | 3 | 4 | 4 | 4 | 3 | 5 | 8 | 2 | 17 | 5 |
| NM_000052 | ATP7A | 8488 | 2 | 1 | 1 | 1 | 1 | 1 | 2 | 0 | 2 | 2 | 1 | 2 | 2 |
| NM_000053 | ATP7B | 6642 | 2 | 0 | 0 | 1 | 0 | 0 | 1 | 2 | 0 | 0 | 0 | 0 | 1 |
| NM_000054 | AVPR2 | 1624 | 0 | 0 | 0 | 0 | 0 | 0 | 0 | 0 | 0 | 0 | 0 | 0 | 0 |
| NM_000055 | BCHE | 2447 | 1 | 0 | 0 | 0 | 0 | 0 | 0 | 1 | 0 | 0 | 0 | 0 | 0 |
| NM_000056 | BCKDHB | 1550 | 4 | 2 | 2 | 1 | 3 | 4 | 3 | 4 | 1 | 2 | 1 | 1 | 2 |
| NM_000057 | BLM | 4528 | 1 | 0 | 0 | 0 | 1 | 0 | 0 | 0 | 0 | 0 | 0 | 1 | 1 |
| NM_000059 | BRCA2 | 11386 | 2 | 0 | 0 | 1 | 0 | 0 | 0 | 0 | 0 | 0 | 0 | 1 | 2 |
| NM_000060 | BTD | 2065 | 2 | 1 | 2 | 0 | 0 | 1 | 1 | 2 | 1 | 1 | 0 | 1 | 1 |
| NM_000061 | BTK | 2611 | 20 | 3 | 2 | 1 | 2 | 1 | 0 | 0 | 4 | 1 | 0 | 20 | 1 |
| NM_000062 | SERPING1 | 1966 | 527 | 85 | 56 | 18 | 50 | 11 | 21 | 527 | 59 | 83 | 25 | 42 | 37 |
| NM_000063 | C2 | 2837 | 48 | 7 | 5 | 2 | 3 | 2 | 2 | 48 | 5 | 1 | 1 | 9 | 1 |
| NM_000064 | C3 | 5101 | 638 | 75 | 35 | 7 | 40 | 20 | 10 | 638 | 26 | 17 | 10 | 6 | 20 |
| NM_000065 | C6 | 3606 | 103 | 12 | 1 | 0 | 21 | 0 | 3 | 103 | 1 | 1 | 1 | 1 | 4 |
| NM_000066 | C8B | 2037 | 116 | 11 | 0 | 0 | 0 | 0 | 0 | 116 | 0 | 0 | 0 | 1 | 0 |
| NM_000067 | CA2 | 1666 | 19 | 4 | 1 | 10 | 1 | 7 | 19 | 5 | 2 | 0 | 2 | 0 | 0 |
| NM_000069 | CACNA1S | 6168 | 18 | 2 | 0 | 0 | 0 | 0 | 0 | 0 | 0 | 0 | 18 | 0 | 0 |
| NM_000070 | CAPN3 | 3316 | 42 | 6 | 1 | 2 | 1 | 3 | 8 | 0 | 1 | 1 | 42 | 1 | 1 |
| NM_000071 | CBS | 2588 | 42 | 6 | 0 | 1 | 3 | 7 | 1 | 42 | 3 | 5 | 0 | 2 | 1 |
| NM_000072 | CD36 | 2104 | 702 | 105 | 702 | 17 | 190 | 2 | 4 | 13 | 37 | 5 | 39 | 135 | 7 |
| NM_000073 | CD3G | 1311 | 3 | 1 | 0 | 1 | 0 | 0 | 0 | 0 | 1 | 0 | 0 | 3 | 0 |
| NM_000074 | CD40LG | 1833 | 1 | 0 | 0 | 0 | 0 | 0 | 0 | 0 | 0 | 0 | 0 | 1 | 0 |
| NM_000075 | CDK4 | 1443 | 3 | 2 | 3 | 3 | 2 | 2 | 2 | 3 | 2 | 2 | 1 | 3 | 2 |
| NM_000076 | CDKN1C | 1930 | 1 | 0 | 0 | 0 | 0 | 0 | 1 | 0 | 0 | 1 | 0 | 0 | 0 |
| NM_000077 | CDKN2A | 1160 | 1 | 0 | 0 | 1 | 0 | 0 | 0 | 0 | 0 | 0 | 0 | 0 | 0 |
| NM_000078 | CETP | 1717 | 2 | 0 | 0 | 0 | 0 | 0 | 0 | 0 | 0 | 0 | 0 | 2 | 0 |
| NM_000079 | CHRNA1 | 1981 | 21 | 2 | 0 | 0 | 0 | 0 | 0 | 0 | 0 | 0 | 21 | 0 | 0 |
| NM_000080 | CHRNE | 2463 | 0 | 0 | 0 | 0 | 0 | 0 | 0 | 0 | 0 | 0 | 0 | 0 | 0 |
| NM_000081 | LYST | 13475 | 9 | 3 | 4 | 1 | 2 | 3 | 2 | 2 | 6 | 3 | 1 | 9 | 5 |
| NM_000082 | ERCC8 | 2044 | 3 | 2 | 3 | 2 | 1 | 3 | 2 | 2 | 1 | 2 | 1 | 3 | 2 |
| NM_000083 | CLCN1 | 3093 | 1 | 0 | 0 | 0 | 0 | 0 | 0 | 0 | 0 | 0 | 1 | 0 | 0 |
| NM_000084 | CLCN5 | 9505 | 9 | 1 | 0 | 0 | 0 | 0 | 9 | 2 | 1 | 1 | 0 | 1 | 1 |
| NM_000085 | CLCNKB | 2591 | 2 | 0 | 0 | 0 | 0 | 0 | 2 | 0 | 0 | 0 | 0 | 0 | 0 |
| NM_000086 | CLN3 | 1877 | 1 | 1 | 1 | 1 | 0 | 0 | 1 | 1 | 0 | 0 | 0 | 1 | 0 |
| NM_000087 | CNGA1 | 2783 | 3 | 1 | 0 | 1 | 0 | 0 | 1 | 1 | 0 | 0 | 0 | 3 | 0 |
| NM_000088 | COL1A1 | 5927 | 18 | 5 | 12 | 8 | 4 | 1 | 1 | 0 | 3 | 18 | 1 | 3 | 2 |
| NM_000089 | COL1A2 | 5411 | 31 | 9 | 16 | 8 | 11 | 1 | 2 | 0 | 6 | 31 | 1 | 6 | 14 |
| NM_000090 | COL3A1 | 5490 | 113 | 29 | 113 | 40 | 42 | 2 | 8 | 5 | 27 | 41 | 2 | 28 | 13 |
| NM_000091 | COL4A3 | 8096 | 2 | 0 | 0 | 0 | 0 | 0 | 2 | 0 | 1 | 0 | 1 | 1 | 0 |
| NM_000092 | COL4A4 | 10341 | 2 | 1 | 0 | 0 | 1 | 0 | 2 | 0 | 1 | 0 | 1 | 1 | 1 |

| transcript | gene | transcript length (nt) | max | mean | adipose | colon | heart | hypothalamus | kidney | liver | lung | ovary | skeletalmuscle | spleen | testes |
|---|---|---|---|---|---|---|---|---|---|---|---|---|---|---|---|
| NM_000093 | COL5A1 | 8438 | 3 | 1 | 2 | 3 | 2 | 0 | 0 | 0 | 1 | 3 | 0 | 1 | 1 |
| NM_000094 | COL7A1 | 9169 | 0 | 0 | 0 | 0 | 0 | 0 | 0 | 0 | 0 | 0 | 0 | 0 | 0 |
| NM_000095 | COMP | 2452 | 16 | 5 | 8 | 0 | 16 | 0 | 0 | 0 | 3 | 0 | 16 | 0 | 7 |
| NM_000096 | CP | 4662 | 404 | 41 | 1 | 2 | 10 | 6 | 1 | 404 | 5 | 3 | 2 | 15 | 3 |
| NM_000097 | CPOX | 2707 | 6 | 2 | 2 | 2 | 1 | 5 | 1 | 6 | 2 | 2 | 1 | 2 | 1 |
| NM_000098 | CPT2 | 3094 | 5 | 2 | 1 | 1 | 2 | 1 | 3 | 5 | 1 | 1 | 1 | 2 | 3 |
| NM_000099 | CST3 | 775 | 46 | 27 | 31 | 14 | 39 | 43 | 22 | 18 | 25 | 17 | 16 | 46 | 31 |
| NM_000100 | CSTB | 658 | 29 | 11 | 29 | 9 | 8 | 8 | 9 | 20 | 12 | 6 | 5 | 13 | 6 |
| NM_000101 | CYBA | 688 | 1 | 0 | 1 | 1 | 0 | 0 | 0 | 0 | 0 | 0 | 0 | 1 | 0 |
| NM_000102 | CYP17A1 | 1870 | 7 | 2 | 0 | 0 | 0 | 0 | 4 | 2 | 0 | 7 | 0 | 0 | 6 |
| NM_000103 | CYP19A1 | 4405 | 3 | 0 | 0 | 0 | 0 | 1 | 0 | 0 | 0 | 3 | 0 | 0 | 0 |
| NM_000104 | CYP1B1 | 5153 | 12 | 5 | 1 | 0 | 12 | 5 | 7 | 1 | 5 | 2 | 1 | 12 | 4 |
| NM_000106 | CYP2D6 | 1659 | 1 | 0 | 0 | 0 | 0 | 0 | 0 | 1 | 0 | 0 | 0 | 0 | 0 |
| NM_000107 | DDB2 | 1848 | 6 | 2 | 2 | 3 | 2 | 1 | 1 | 6 | 2 | 2 | 2 | 2 | 3 |
| NM_000108 | DLD | 3579 | 19 | 10 | 10 | 5 | 19 | 9 | 18 | 12 | 5 | 4 | 9 | 7 | 8 |
| NM_000109 | DMD | 14069 | 67 | 12 | 8 | 3 | 29 | 6 | 1 | 3 | 3 | 9 | 67 | 0 | 5 |
| NM_000110 | DPYD | 4447 | 8 | 3 | 3 | 1 | 3 | 2 | 2 | 4 | 7 | 4 | 1 | 8 | 1 |
| NM_000111 | SLC26A3 | 2894 | 23 | 2 | 0 | 23 | 0 | 0 | 0 | 1 | 0 | 0 | 0 | 0 | 0 |
| NM_000112 | SLC26A2 | 8075 | 29 | 4 | 1 | 29 | 2 | 2 | 1 | 0 | 2 | 2 | 1 | 2 | 2 |
| NM_000113 | TOR1A | 2106 | 5 | 2 | 2 | 2 | 2 | 4 | 2 | 5 | 2 | 2 | 1 | 3 | 2 |
| NM_000114 | EDN3 | 2398 | 2 | 0 | 0 | 2 | 0 | 1 | 0 | 0 | 0 | 0 | 0 | 1 | 0 |
| NM_000115 | EDNRB | 4282 | 12 | 6 | 4 | 1 | 8 | 9 | 7 | 6 | 10 | 1 | 1 | 12 | 2 |
| NM_000116 | TAZ | 1901 | 1 | 0 | 0 | 0 | 1 | 0 | 0 | 0 | 0 | 0 | 0 | 1 | 0 |
| NM_000117 | EMD | 1337 | 12 | 6 | 3 | 3 | 8 | 5 | 6 | 4 | 6 | 8 | 12 | 6 | 4 |
| NM_000118 | ENG | 3196 | 5 | 2 | 2 | 1 | 5 | 1 | 2 | 1 | 2 | 2 | 1 | 4 | 3 |
| NM_000119 | EPB42 | 2540 | 2 | 0 | 2 | 0 | 0 | 0 | 0 | 0 | 1 | 0 | 0 | 0 | 0 |
| NM_000120 | EPHX1 | 1829 | 24 | 6 | 8 | 1 | 2 | 3 | 6 | 24 | 4 | 7 | 1 | 2 | 4 |
| NM_000121 | EPOR | 1848 | 1 | 0 | 0 | 0 | 0 | 0 | 0 | 0 | 0 | 0 | 0 | 1 | 0 |
| NM_000122 | ERCC3 | 2750 | 7 | 4 | 4 | 4 | 3 | 6 | 4 | 3 | 2 | 4 | 3 | 6 | 7 |
| NM_000123 | ERCC5 | 4096 | 16 | 9 | 11 | 6 | 9 | 10 | 10 | 8 | 9 | 11 | 7 | 16 | 9 |
| NM_000124 | ERCC6 | 7006 | 2 | 2 | 1 | 1 | 2 | 2 | 2 | 1 | 1 | 2 | 2 | 2 | 2 |
| NM_000125 | ESR1 | 6329 | 3 | 1 | 1 | 1 | 0 | 0 | 1 | 1 | 0 | 3 | 0 | 0 | 1 |
| NM_000126 | ETFA | 1354 | 73 | 25 | 19 | 10 | 73 | 15 | 33 | 51 | 11 | 10 | 24 | 20 | 12 |
| NM_000127 | EXT1 | 3363 | 13 | 5 | 4 | 5 | 4 | 2 | 3 | 13 | 4 | 6 | 1 | 9 | 3 |
| NM_000128 | F11 | 3278 | 33 | 4 | 0 | 0 | 0 | 0 | 6 | 33 | 0 | 0 | 0 | 0 | 0 |
| NM_000129 | F13A1 | 3863 | 48 | 11 | 19 | 5 | 48 | 5 | 8 | 1 | 14 | 3 | 5 | 3 | 10 |
| NM_000130 | F5 | 9179 | 48 | 5 | 0 | 0 | 2 | 2 | 1 | 48 | 1 | 0 | 0 | 2 | 1 |
| NM_000131 | F7 | 3125 | 62 | 16 | 6 | 8 | 13 | 8 | 9 | 62 | 16 | 12 | 11 | 11 | 15 |
| NM_000132 | F8 | 9035 | 11 | 4 | 2 | 0 | 11 | 1 | 1 | 3 | 4 | 3 | 3 | 8 | 4 |
| NM_000133 | F9 | 2802 | 132 | 12 | 0 | 0 | 0 | 0 | 0 | 132 | 0 | 0 | 0 | 1 | 0 |
| NM_000134 | FABP2 | 2249 | 1 | 0 | 0 | 1 | 0 | 0 | 0 | 0 | 0 | 0 | 0 | 0 | 0 |
| NM_000135 | FANCA | 5460 | 1 | 0 | 0 | 1 | 0 | 0 | 0 | 0 | 0 | 0 | 0 | 0 | 0 |
| NM_000136 | FANCC | 4592 | 1 | 0 | 0 | 1 | 0 | 0 | 1 | 1 | 0 | 1 | 0 | 0 | 0 |
| NM_000137 | FAH | 1447 | 6 | 2 | 5 | 0 | 1 | 1 | 4 | 6 | 1 | 1 | 1 | 2 | 2 |
| NM_000138 | FBN1 | 11694 | 18 | 7 | 18 | 5 | 13 | 1 | 2 | 1 | 4 | 11 | 3 | 8 | 7 |
| NM_000139 | MS4A2 | 3722 | 1 | 0 | 1 | 0 | 0 | 0 | 0 | 0 | 1 | 0 | 0 | 0 | 1 |
| NM_000140 | FECH | 7260 | 5 | 2 | 1 | 1 | 2 | 2 | 5 | 3 | 1 | 1 | 2 | 1 | 1 |
| NM_000141 | FGFR2 | 4640 | 8 | 3 | 1 | 5 | 0 | 8 | 7 | 2 | 2 | 3 | 0 | 0 | 1 |
| NM_000142 | FGFR3 | 4287 | 4 | 1 | 0 | 1 | 0 | 4 | 2 | 1 | 0 | 0 | 0 | 0 | 1 |
| NM_000143 | FH | 1789 | 37 | 13 | 8 | 7 | 24 | 11 | 17 | 37 | 5 | 4 | 14 | 8 | 7 |
| NM_000144 | FXN | 7168 | 1 | 1 | 0 | 0 | 1 | 1 | 0 | 1 | 0 | 1 | 1 | 1 | 1 |
| NM_000145 | FSHR | 2382 | 1 | 0 | 0 | 0 | 0 | 0 | 0 | 0 | 0 | 1 | 0 | 0 | 1 |
| NM_000146 | FTL | 871 | 53 | 25 | 50 | 5 | 17 | 8 | 41 | 30 | 53 | 10 | 6 | 51 | 6 |
| NM_000147 | FUCA1 | 2115 | 29 | 10 | 10 | 8 | 6 | 4 | 13 | 10 | 9 | 8 | 3 | 29 | 7 |
| NM_000148 | FUT1 | 4246 | 0 | 0 | 0 | 0 | 0 | 0 | 0 | 0 | 0 | 0 | 0 | 0 | 0 |
| NM_000149 | FUT3 | 2590 | 2 | 0 | 0 | 2 | 0 | 0 | 0 | 0 | 0 | 0 | 0 | 0 | 0 |
| NM_000150 | FUT6 | 3137 | 5 | 1 | 0 | 1 | 0 | 0 | 5 | 0 | 0 | 0 | 0 | 0 | 0 |
| NM_000151 | G6PC | 3096 | 59 | 7 | 0 | 0 | 0 | 0 | 15 | 59 | 0 | 0 | 0 | 0 | 0 |
| NM_000152 | GAA | 3775 | 2 | 1 | 1 | 0 | 1 | 0 | 1 | 1 | 1 | 1 | 1 | 2 | 1 |
| NM_000153 | GALC | 4175 | 11 | 7 | 5 | 3 | 6 | 11 | 6 | 5 | 10 | 6 | 3 | 8 | 11 |
| NM_000154 | GALK1 | 1361 | 1 | 0 | 0 | 0 | 0 | 0 | 0 | 1 | 0 | 0 | 0 | 0 | 0 |
| NM_000155 | GALT | 1334 | 1 | 1 | 0 | 1 | 1 | 1 | 1 | 1 | 0 | 0 | 1 | 1 | 0 |
| NM_000156 | GAMT | 1058 | 3 | 0 | 0 | 0 | 0 | 0 | 0 | 1 | 0 | 0 | 3 | 0 | 0 |
| NM_000157 | GBA | 2304 | 1 | 1 | 1 | 1 | 1 | 1 | 1 | 1 | 0 | 0 | 0 | 1 | 1 |
| NM_000158 | GBE1 | 3099 | 8 | 4 | 7 | 1 | 8 | 2 | 4 | 4 | 2 | 3 | 6 | 2 | 2 |
| NM_000159 | GCDH | 1822 | 5 | 1 | 1 | 1 | 2 | 1 | 2 | 5 | 0 | 1 | 1 | 1 | 0 |
| NM_000160 | GCGR | 2060 | 0 | 0 | 0 | 0 | 0 | 0 | 0 | 0 | 0 | 0 | 0 | 0 | 0 |
| NM_000161 | GCH1 | 2925 | 3 | 1 | 0 | 0 | 0 | 0 | 1 | 3 | 1 | 0 | 0 | 1 | 0 |
| NM_000162 | GCK | 2733 | 0 | 0 | 0 | 0 | 0 | 0 | 0 | 0 | 0 | 0 | 0 | 0 | 0 |
| NM_000163 | GHR | 4360 | 22 | 6 | 19 | 2 | 2 | 1 | 3 | 22 | 1 | 2 | 8 | 1 | 1 |
| NM_000164 | GIPR | 2024 | 0 | 0 | 0 | 0 | 0 | 0 | 0 | 0 | 0 | 0 | 0 | 0 | 0 |
| NM_000165 | GJA1 | 3130 | 59 | 15 | 9 | 3 | 25 | 59 | 3 | 0 | 18 | 21 | 3 | 2 | 18 |
| NM_000166 | GJB1 | 1654 | 18 | 3 | 0 | 2 | 0 | 1 | 6 | 18 | 0 | 0 | 0 | 0 | 2 |
| NM_000167 | GK | 3620 | 3 | 1 | 0 | 0 | 0 | 0 | 3 | 3 | 1 | 0 | 0 | 1 | 0 |
| NM_000168 | GLI3 | 8212 | 4 | 1 | 1 | 1 | 0 | 1 | 0 | 0 | 0 | 4 | 0 | 0 | 1 |
| NM_000169 | GLA | 1418 | 1 | 1 | 1 | 0 | 1 | 1 | 1 | 1 | 1 | 1 | 0 | 1 | 1 |
| NM_000170 | GLDC | 3809 | 28 | 4 | 0 | 0 | 0 | 2 | 12 | 28 | 0 | 0 | 0 | 0 | 1 |

| transcript | gene | transcript length (nt) | max | mean | adipose | colon | heart | hypothalamus | kidney | liver | lung | ovary | skeletalmuscle | spleen | testes |
|---|---|---|---|---|---|---|---|---|---|---|---|---|---|---|---|
| NM_000171 | GLRA1 | 1796 | 0 | 0 | 0 | 0 | 0 | 0 | 0 | 0 | 0 | 0 | 0 | 0 | 0 |
| NM_000172 | GNAT1 | 2465 | 0 | 0 | 0 | 0 | 0 | 0 | 0 | 0 | 0 | 0 | 0 | 0 | 0 |
| NM_000173 | GP1BA | 2463 | 2 | 0 | 0 | 0 | 0 | 0 | 1 | 0 | 0 | 0 | 0 | 2 | 0 |
| NM_000174 | GP9 | 858 | 0 | 0 | 0 | 0 | 0 | 0 | 0 | 0 | 0 | 0 | 0 | 0 | 0 |
| NM_000175 | GPI | 2075 | 31 | 18 | 20 | 13 | 31 | 30 | 25 | 28 | 8 | 9 | 15 | 15 | 9 |
| NM_000176 | NR3C1 | 6784 | 11 | 6 | 8 | 2 | 5 | 4 | 5 | 5 | 7 | 6 | 11 | 8 | 4 |
| NM_000177 | GSN | 2649 | 31 | 7 | 31 | 8 | 16 | 4 | 2 | 2 | 3 | 3 | 5 | 2 | 4 |
| NM_000178 | GSS | 1900 | 2 | 1 | 1 | 2 | 1 | 1 | 2 | 2 | 0 | 1 | 0 | 1 | 1 |
| NM_000179 | MSH6 | 4328 | 34 | 11 | 6 | 4 | 7 | 10 | 6 | 9 | 7 | 34 | 5 | 10 | 24 |
| NM_000180 | GUCY2D | 3623 | 0 | 0 | 0 | 0 | 0 | 0 | 0 | 0 | 0 | 0 | 0 | 0 | 0 |
| NM_000181 | GUSB | 2296 | 37 | 15 | 25 | 14 | 10 | 7 | 12 | 37 | 13 | 12 | 5 | 23 | 9 |
| NM_000182 | HADHA | 3037 | 55 | 25 | 25 | 15 | 55 | 18 | 26 | 36 | 11 | 19 | 40 | 19 | 13 |
| NM_000183 | HADHB | 2188 | 48 | 14 | 12 | 5 | 48 | 13 | 14 | 16 | 5 | 5 | 25 | 8 | 7 |
| NM_000184 | HBG2 | 583 | 2 | 0 | 0 | 1 | 0 | 0 | 0 | 0 | 2 | 1 | 0 | 1 | 0 |
| NM_000185 | SERPIND1 | 2221 | 19 | 2 | 0 | 1 | 0 | 1 | 0 | 19 | 0 | 1 | 0 | 1 | 0 |
| NM_000186 | CFH | 4127 | 230 | 35 | 13 | 4 | 47 | 5 | 7 | 230 | 15 | 35 | 7 | 7 | 11 |
| NM_000187 | HGD | 1920 | 142 | 15 | 0 | 2 | 1 | 0 | 18 | 142 | 0 | 0 | 1 | 1 | 0 |
| NM_000188 | HK1 | 3601 | 34 | 12 | 8 | 8 | 28 | 34 | 6 | 1 | 9 | 4 | 14 | 14 | 8 |
| NM_000189 | HK2 | 7093 | 14 | 4 | 7 | 4 | 3 | 2 | 0 | 0 | 4 | 2 | 1 | 3 | 14 |
| NM_000190 | HMBS | 1509 | 2 | 1 | 1 | 1 | 1 | 1 | 1 | 2 | 1 | 0 | 1 | 1 | 1 |
| NM_000191 | HMGCL | 1608 | 5 | 2 | 1 | 1 | 1 | 1 | 4 | 5 | 1 | 1 | 1 | 1 | 1 |
| NM_000192 | TBX5 | 3826 | 4 | 1 | 0 | 0 | 4 | 0 | 0 | 0 | 2 | 0 | 0 | 0 | 0 |
| NM_000193 | SHH | 1576 | 0 | 0 | 0 | 0 | 0 | 0 | 0 | 0 | 0 | 0 | 0 | 0 | 0 |
| NM_000194 | HPRT1 | 1415 | 41 | 13 | 6 | 4 | 9 | 40 | 10 | 6 | 5 | 7 | 6 | 10 | 41 |
| NM_000195 | HPS1 | 3714 | 1 | 0 | 1 | 0 | 0 | 1 | 0 | 1 | 0 | 0 | 0 | 1 | 0 |
| NM_000196 | HSD11B2 | 1882 | 6 | 1 | 0 | 5 | 0 | 0 | 6 | 0 | 0 | 0 | 0 | 0 | 0 |
| NM_000197 | HSD17B3 | 1134 | 39 | 4 | 0 | 0 | 0 | 1 | 1 | 0 | 0 | 0 | 0 | 0 | 39 |
| NM_000198 | HSD3B2 | 1708 | 7 | 1 | 0 | 0 | 0 | 0 | 0 | 0 | 0 | 7 | 0 | 0 | 1 |
| NM_000199 | SGSH | 2768 | 1 | 0 | 0 | 0 | 0 | 0 | 1 | 0 | 0 | 0 | 0 | 1 | 0 |
| NM_000200 | HTN3 | 578 | 0 | 0 | 0 | 0 | 0 | 0 | 0 | 0 | 0 | 0 | 0 | 0 | 0 |
| NM_000201 | ICAM1 | 3246 | 13 | 3 | 2 | 1 | 2 | 2 | 2 | 1 | 13 | 1 | 1 | 6 | 1 |
| NM_000202 | IDS | 5852 | 38 | 6 | 2 | 2 | 4 | 38 | 3 | 1 | 5 | 5 | 2 | 6 | 3 |
| NM_000203 | IDUA | 2186 | 0 | 0 | 0 | 0 | 0 | 0 | 0 | 0 | 0 | 0 | 0 | 0 | 0 |
| NM_000204 | CFI | 2161 | 92 | 14 | 2 | 1 | 3 | 1 | 35 | 92 | 5 | 7 | 1 | 8 | 4 |
| NM_000206 | IL2RG | 1451 | 22 | 4 | 3 | 6 | 1 | 1 | 1 | 1 | 7 | 1 | 1 | 22 | 0 |
| NM_000207 | INS | 465 | 12 | 1 | 1 | 12 | 0 | 0 | 0 | 0 | 0 | 0 | 0 | 0 | 0 |
| NM_000208 | INSR | 9059 | 27 | 7 | 3 | 3 | 7 | 3 | 7 | 9 | 2 | 8 | 8 | 27 | 5 |
| NM_000209 | PDX1 | 2573 | 0 | 0 | 0 | 0 | 0 | 0 | 0 | 0 | 0 | 0 | 0 | 0 | 0 |
| NM_000210 | ITGA6 | 5810 | 28 | 14 | 13 | 11 | 13 | 10 | 25 | 5 | 9 | 7 | 7 | 20 | 28 |
| NM_000211 | ITGB2 | 2957 | 9 | 3 | 5 | 1 | 3 | 1 | 0 | 1 | 6 | 1 | 1 | 9 | 0 |
| NM_000212 | ITGB3 | 4894 | 5 | 2 | 2 | 4 | 3 | 1 | 5 | 0 | 2 | 1 | 0 | 3 | 1 |
| NM_000213 | ITGB4 | 5919 | 4 | 1 | 1 | 3 | 0 | 2 | 1 | 0 | 1 | 0 | 0 | 4 | 1 |
| NM_000214 | JAG1 | 5987 | 9 | 4 | 5 | 3 | 6 | 2 | 6 | 0 | 5 | 6 | 3 | 9 | 4 |
| NM_000215 | JAK3 | 5434 | 3 | 1 | 0 | 1 | 0 | 1 | 0 | 0 | 1 | 1 | 0 | 3 | 0 |
| NM_000216 | KAL1 | 6314 | 4 | 1 | 1 | 0 | 1 | 4 | 0 | 0 | 2 | 0 | 3 | 0 | 2 |
| NM_000217 | KCNA1 | 7983 | 1 | 0 | 0 | 0 | 0 | 1 | 0 | 0 | 0 | 0 | 0 | 0 | 0 |
| NM_000218 | KCNQ1 | 3246 | 1 | 0 | 0 | 0 | 0 | 0 | 1 | 0 | 0 | 0 | 0 | 0 | 0 |
| NM_000219 | KCNE1 | 3321 | 1 | 0 | 0 | 0 | 1 | 0 | 1 | 1 | 1 | 0 | 0 | 1 | 1 |
| NM_000220 | KCNJ1 | 2332 | 30 | 3 | 0 | 0 | 0 | 0 | 30 | 0 | 0 | 0 | 0 | 0 | 0 |
| NM_000221 | KHK | 2415 | 7 | 1 | 0 | 0 | 0 | 0 | 7 | 4 | 0 | 0 | 0 | 0 | 1 |
| NM_000222 | KIT | 5176 | 7 | 2 | 1 | 1 | 0 | 3 | 2 | 0 | 3 | 7 | 0 | 1 | 1 |
| NM_000223 | KRT12 | 1992 | 0 | 0 | 0 | 0 | 0 | 0 | 0 | 0 | 0 | 0 | 0 | 0 | 0 |
| NM_000224 | KRT18 | 1467 | 177 | 41 | 1 | 131 | 10 | 4 | 52 | 177 | 48 | 8 | 2 | 10 | 11 |
| NM_000226 | KRT9 | 2289 | 0 | 0 | 0 | 0 | 0 | 0 | 0 | 0 | 0 | 0 | 0 | 0 | 0 |
| NM_000227 | LAMA3 | 5617 | 6 | 1 | 1 | 3 | 1 | 0 | 1 | 0 | 6 | 1 | 0 | 1 | 2 |
| NM_000228 | LAMB3 | 4061 | 9 | 2 | 9 | 3 | 0 | 0 | 1 | 1 | 4 | 0 | 0 | 0 | 0 |
| NM_000229 | LCAT | 1354 | 2 | 0 | 0 | 0 | 0 | 0 | 0 | 2 | 0 | 0 | 0 | 0 | 0 |
| NM_000230 | LEP | 3427 | 420 | 39 | 420 | 8 | 2 | 0 | 0 | 0 | 1 | 0 | 0 | 0 | 0 |
| NM_000231 | SGCG | 1655 | 4 | 1 | 1 | 0 | 4 | 0 | 0 | 0 | 0 | 0 | 4 | 0 | 0 |
| NM_000232 | SGCB | 4279 | 25 | 12 | 12 | 5 | 20 | 18 | 14 | 4 | 7 | 9 | 25 | 7 | 17 |
| NM_000233 | LHCGR | 3093 | 1 | 0 | 1 | 0 | 0 | 0 | 0 | 0 | 0 | 1 | 0 | 0 | 0 |
| NM_000234 | LIG1 | 3083 | 6 | 3 | 2 | 6 | 1 | 3 | 1 | 1 | 2 | 4 | 1 | 4 | 4 |
| NM_000235 | LIPA | 2623 | 85 | 17 | 34 | 2 | 7 | 8 | 6 | 10 | 19 | 7 | 2 | 85 | 4 |
| NM_000236 | LIPC | 1604 | 35 | 4 | 0 | 0 | 0 | 0 | 2 | 35 | 0 | 1 | 0 | 0 | 0 |
| NM_000237 | LPL | 3747 | 312 | 61 | 296 | 7 | 312 | 4 | 7 | 0 | 23 | 3 | 16 | 2 | 2 |
| NM_000238 | KCNH2 | 3897 | 1 | 0 | 0 | 0 | 1 | 0 | 0 | 0 | 0 | 0 | 0 | 0 | 1 |
| NM_000239 | LYZ | 1516 | 133 | 24 | 31 | 24 | 8 | 1 | 2 | 3 | 133 | 4 | 2 | 54 | 3 |
| NM_000240 | MAOA | 4073 | 23 | 11 | 23 | 11 | 12 | 4 | 19 | 20 | 16 | 4 | 6 | 2 | 2 |
| NM_000242 | MBL2 | 3569 | 8 | 1 | 0 | 0 | 0 | 0 | 0 | 8 | 0 | 0 | 0 | 0 | 0 |
| NM_000243 | MEFV | 3499 | 2 | 0 | 0 | 0 | 0 | 0 | 0 | 0 | 1 | 0 | 0 | 2 | 0 |
| NM_000244 | MEN1 | 2768 | 0 | 0 | 0 | 0 | 0 | 0 | 0 | 0 | 0 | 0 | 0 | 0 | 0 |
| NM_000245 | MET | 6622 | 5 | 2 | 1 | 3 | 1 | 1 | 5 | 4 | 3 | 0 | 2 | 1 | 1 |
| NM_000246 | CIITA | 4654 | 1 | 0 | 0 | 0 | 0 | 0 | 0 | 0 | 1 | 0 | 0 | 1 | 0 |
| NM_000247 | MICA | 1365 | 5 | 2 | 2 | 5 | 2 | 2 | 2 | 1 | 3 | 2 | 1 | 3 | 4 |
| NM_000247 | MICA | 1369 | 5 | 2 | 2 | 5 | 2 | 2 | 2 | 1 | 3 | 2 | 1 | 3 | 4 |
| NM_000248 | MITF | 4469 | 15 | 5 | 5 | 1 | 9 | 2 | 6 | 1 | 3 | 9 | 15 | 1 | 4 |
| NM_000249 | MLH1 | 2660 | 7 | 5 | 5 | 4 | 6 | 6 | 4 | 6 | 3 | 4 | 5 | 7 | 7 |

| transcript | gene | transcript length (nt) | max | mean | adipose | colon | heart | hypothalamus | kidney | liver | lung | ovary | skeletalmuscle | spleen | testes |
|---|---|---|---|---|---|---|---|---|---|---|---|---|---|---|---|
| NM_000250 | MPO | 3215 | 5 | 1 | 0 | 0 | 0 | 0 | 0 | 0 | 5 | 0 | 0 | 0 | 0 |
| NM_000251 | MSH2 | 3145 | 7 | 3 | 2 | 2 | 2 | 7 | 2 | 0 | 2 | 3 | 2 | 4 | 4 |
| NM_000252 | MTM1 | 3436 | 6 | 3 | 1 | 1 | 2 | 3 | 3 | 3 | 2 | 3 | 2 | 4 | 6 |
| NM_000253 | MTTP | 4090 | 26 | 3 | 0 | 1 | 0 | 0 | 2 | 26 | 0 | 0 | 0 | 0 | 1 |
| NM_000254 | MTR | 10558 | 8 | 5 | 4 | 2 | 7 | 4 | 8 | 2 | 3 | 6 | 6 | 6 | 7 |
| NM_000255 | MUT | 2958 | 20 | 8 | 4 | 2 | 9 | 7 | 11 | 20 | 5 | 7 | 5 | 5 | 8 |
| NM_000256 | MYBPC3 | 4218 | 40 | 4 | 0 | 0 | 40 | 0 | 0 | 0 | 0 | 0 | 0 | 0 | 0 |
| NM_000257 | MYH7 | 6030 | 2,301 | 404 | 1 | 0 | 2,138 | 1 | 0 | 0 | 0 | 0 | 2,301 | 0 | 8 |
| NM_000258 | MYL3 | 929 | 81 | 9 | 0 | 0 | 81 | 0 | 3 | 0 | 0 | 0 | 18 | 0 | 0 |
| NM_000259 | MYO5A | 12222 | 22 | 4 | 6 | 1 | 3 | 22 | 2 | 0 | 3 | 2 | 1 | 4 | 2 |
| NM_000260 | MYO7A | 7465 | 6 | 1 | 1 | 0 | 0 | 1 | 2 | 2 | 0 | 0 | 0 | 1 | 6 |
| NM_000261 | MYOC | 2045 | 6 | 1 | 6 | 1 | 1 | 0 | 0 | 0 | 1 | 0 | 4 | 0 | 1 |
| NM_000262 | NAGA | 3726 | 4 | 3 | 3 | 3 | 3 | 2 | 4 | 3 | 4 | 2 | 1 | 4 | 2 |
| NM_000263 | NAGLU | 2782 | 1 | 0 | 0 | 0 | 0 | 0 | 1 | 0 | 0 | 0 | 0 | 1 | 0 |
| NM_000264 | PTCH1 | 7943 | 4 | 1 | 0 | 3 | 0 | 1 | 1 | 0 | 1 | 2 | 0 | 1 | 4 |
| NM_000265 | NCF1 | 1398 | 1 | 0 | 0 | 0 | 0 | 0 | 0 | 0 | 0 | 0 | 0 | 1 | 0 |
| NM_000266 | NDP | 2001 | 1 | 0 | 0 | 0 | 0 | 1 | 0 | 0 | 0 | 1 | 0 | 0 | 0 |
| NM_000267 | NF1 | 12361 | 7 | 3 | 2 | 2 | 4 | 7 | 4 | 3 | 3 | 4 | 2 | 3 | 4 |
| NM_000268 | NF2 | 6029 | 3 | 2 | 1 | 1 | 2 | 3 | 3 | 1 | 1 | 1 | 1 | 2 | 1 |
| NM_000269 | NME1 | 811 | 72 | 28 | 20 | 15 | 24 | 72 | 21 | 72 | 9 | 11 | 10 | 34 | 15 |
| NM_000270 | PNP | 2413 | 6 | 2 | 2 | 1 | 2 | 2 | 6 | 2 | 2 | 0 | 0 | 3 | 1 |
| NM_000271 | NPC1 | 4812 | 5 | 2 | 2 | 1 | 2 | 5 | 2 | 2 | 3 | 2 | 1 | 3 | 2 |
| NM_000272 | NPHP1 | 2750 | 6 | 2 | 1 | 1 | 2 | 3 | 3 | 0 | 2 | 2 | 3 | 1 | 6 |
| NM_000273 | GPR143 | 1695 | 1 | 0 | 0 | 0 | 0 | 1 | 0 | 0 | 0 | 0 | 0 | 0 | 0 |
| NM_000274 | OAT | 2066 | 17 | 7 | 5 | 4 | 5 | 17 | 8 | 11 | 5 | 7 | 3 | 6 | 10 |
| NM_000275 | OCA2 | 3140 | 0 | 0 | 0 | 0 | 0 | 0 | 0 | 0 | 0 | 0 | 0 | 0 | 0 |
| NM_000276 | OCRL | 5154 | 14 | 5 | 3 | 3 | 3 | 8 | 3 | 2 | 2 | 7 | 3 | 3 | 14 |
| NM_000277 | PAH | 2681 | 41 | 5 | 0 | 0 | 0 | 0 | 11 | 41 | 0 | 0 | 0 | 0 | 0 |
| NM_000278 | PAX2 | 4057 | 2 | 0 | 0 | 0 | 0 | 0 | 2 | 0 | 0 | 0 | 0 | 0 | 0 |
| NM_000280 | PAX6 | 6944 | 1 | 0 | 0 | 0 | 0 | 1 | 0 | 0 | 0 | 0 | 0 | 0 | 0 |
| NM_000281 | PCBD1 | 1006 | 22 | 6 | 2 | 2 | 7 | 4 | 13 | 22 | 3 | 3 | 3 | 3 | 2 |
| NM_000282 | PCCA | 2493 | 23 | 8 | 5 | 3 | 7 | 7 | 23 | 17 | 3 | 8 | 3 | 6 | 7 |
| NM_000283 | PDE6B | 3402 | 2 | 1 | 0 | 0 | 0 | 2 | 1 | 0 | 0 | 0 | 0 | 1 | 0 |
| NM_000284 | PDHA1 | 3349 | 62 | 19 | 16 | 11 | 62 | 15 | 27 | 18 | 6 | 7 | 25 | 10 | 9 |
| NM_000285 | PEPD | 2010 | 20 | 5 | 4 | 2 | 2 | 3 | 20 | 17 | 2 | 2 | 2 | 4 | 2 |
| NM_000286 | PEX12 | 2682 | 2 | 1 | 0 | 0 | 1 | 1 | 1 | 1 | 1 | 1 | 1 | 1 | 2 |
| NM_000287 | PEX6 | 3498 | 1 | 0 | 0 | 1 | 0 | 1 | 1 | 1 | 0 | 1 | 0 | 1 | 0 |
| NM_000288 | PEX7 | 1481 | 3 | 1 | 1 | 1 | 2 | 2 | 2 | 1 | 1 | 1 | 1 | 1 | 3 |
| NM_000289 | PFKM | 3224 | 26 | 4 | 0 | 1 | 8 | 6 | 2 | 0 | 1 | 2 | 26 | 1 | 2 |
| NM_000290 | PGAM2 | 856 | 5 | 1 | 0 | 0 | 5 | 0 | 0 | 0 | 0 | 0 | 5 | 0 | 0 |
| NM_000291 | PGK1 | 2423 | 16 | 10 | 10 | 5 | 16 | 14 | 16 | 12 | 9 | 4 | 8 | 13 | 5 |
| NM_000292 | PHKA2 | 5325 | 2 | 1 | 1 | 1 | 1 | 0 | 1 | 2 | 1 | 1 | 0 | 1 | 1 |
| NM_000293 | PHKB | 5491 | 6 | 4 | 4 | 2 | 5 | 4 | 5 | 3 | 3 | 3 | 6 | 5 | 5 |
| NM_000294 | PHKG2 | 1578 | 7 | 3 | 2 | 3 | 2 | 4 | 2 | 4 | 2 | 1 | 2 | 3 | 7 |
| NM_000295 | SERPINA1 | 3220 | 4,222 | 397 | 0 | 7 | 9 | 4 | 54 | 4,222 | 19 | 10 | 6 | 26 | 9 |
| NM_000296 | PKD1 | 14135 | 0 | 0 | 0 | 0 | 0 | 0 | 0 | 0 | 0 | 0 | 0 | 0 | 0 |
| NM_000297 | PKD2 | 5056 | 7 | 4 | 5 | 2 | 5 | 3 | 7 | 1 | 4 | 7 | 1 | 3 | 4 |
| NM_000298 | PKLR | 3043 | 8 | 1 | 0 | 0 | 0 | 0 | 8 | 5 | 0 | 0 | 0 | 0 | 0 |
| NM_000299 | PKP1 | 5443 | 1 | 0 | 1 | 0 | 0 | 0 | 0 | 0 | 0 | 0 | 0 | 0 | 0 |
| NM_000300 | PLA2G2A | 999 | 621 | 118 | 19 | 26 | 554 | 1 | 6 | 621 | 9 | 12 | 24 | 3 | 21 |
| NM_000301 | PLG | 3535 | 99 | 11 | 0 | 0 | 0 | 0 | 18 | 99 | 0 | 1 | 0 | 1 | 0 |
| NM_000302 | PLOD1 | 3004 | 7 | 3 | 4 | 2 | 4 | 2 | 4 | 7 | 2 | 3 | 1 | 4 | 2 |
| NM_000303 | PMM2 | 2323 | 16 | 5 | 5 | 10 | 5 | 3 | 5 | 16 | 3 | 3 | 2 | 4 | 4 |
| NM_000304 | PMP22 | 1828 | 8 | 4 | 5 | 2 | 8 | 5 | 1 | 0 | 5 | 4 | 7 | 3 | 3 |
| NM_000305 | PON2 | 1654 | 9 | 3 | 2 | 1 | 2 | 5 | 2 | 9 | 5 | 1 | 1 | 1 | 2 |
| NM_000306 | POU1F1 | 1262 | 0 | 0 | 0 | 0 | 0 | 0 | 0 | 0 | 0 | 0 | 0 | 0 | 0 |
| NM_000307 | POU3F4 | 1507 | 0 | 0 | 0 | 0 | 0 | 0 | 0 | 0 | 0 | 0 | 0 | 0 | 0 |
| NM_000308 | CTSA | 2240 | 15 | 5 | 6 | 4 | 4 | 3 | 15 | 5 | 4 | 3 | 2 | 8 | 3 |
| NM_000309 | PPOX | 1707 | 0 | 0 | 0 | 0 | 0 | 0 | 0 | 0 | 0 | 0 | 0 | 0 | 0 |
| NM_000310 | PPT1 | 2504 | 11 | 3 | 3 | 1 | 2 | 5 | 2 | 1 | 4 | 2 | 1 | 11 | 3 |
| NM_000311 | PRNP | 2740 | 56 | 16 | 6 | 7 | 18 | 56 | 13 | 7 | 12 | 13 | 9 | 15 | 19 |
| NM_000312 | PROC | 1779 | 14 | 1 | 0 | 0 | 0 | 0 | 1 | 14 | 0 | 0 | 0 | 0 | 0 |
| NM_000313 | PROS1 | 3579 | 40 | 9 | 3 | 1 | 17 | 4 | 8 | 40 | 7 | 9 | 1 | 6 | 3 |
| NM_000314 | PTEN | 5547 | 15 | 5 | 4 | 2 | 6 | 4 | 4 | 3 | 6 | 7 | 3 | 15 | 5 |
| NM_000315 | PTH | 814 | 0 | 0 | 0 | 0 | 0 | 0 | 0 | 0 | 0 | 0 | 0 | 0 | 0 |
| NM_000316 | PTH1R | 2128 | 15 | 2 | 1 | 0 | 0 | 0 | 15 | 2 | 0 | 0 | 0 | 1 | 0 |
| NM_000317 | PTS | 934 | 2 | 1 | 1 | 1 | 1 | 2 | 1 | 2 | 0 | 1 | 1 | 1 | 1 |
| NM_000318 | PXMP3 | 4379 | 2 | 1 | 1 | 1 | 2 | 2 | 2 | 1 | 1 | 1 | 1 | 2 | 1 |
| NM_000319 | PEX5 | 3171 | 5 | 3 | 2 | 1 | 2 | 5 | 4 | 4 | 2 | 2 | 3 | 2 | 4 |
| NM_000320 | QDPR | 1649 | 25 | 6 | 3 | 1 | 3 | 25 | 11 | 11 | 2 | 2 | 2 | 2 | 2 |
| NM_000321 | RB1 | 4772 | 31 | 14 | 9 | 6 | 16 | 13 | 14 | 7 | 15 | 12 | 16 | 31 | 21 |
| NM_000322 | PRPH2 | 3027 | 0 | 0 | 0 | 0 | 0 | 0 | 0 | 0 | 0 | 0 | 0 | 0 | 0 |
| NM_000324 | RHAG | 1928 | 0 | 0 | 0 | 0 | 0 | 0 | 0 | 0 | 0 | 0 | 0 | 0 | 0 |
| NM_000325 | PITX2 | 2314 | 3 | 0 | 0 | 1 | 0 | 0 | 0 | 0 | 0 | 0 | 3 | 0 | 0 |
| NM_000326 | RLBP1 | 1752 | 0 | 0 | 0 | 0 | 0 | 0 | 0 | 0 | 0 | 0 | 0 | 0 | 0 |
| NM_000327 | ROM1 | 1876 | 2 | 1 | 1 | 0 | 1 | 2 | 0 | 1 | 1 | 1 | 0 | 1 | 1 |
| NM_000328 | RPGR | 3072 | 6 | 2 | 2 | 1 | 3 | 2 | 2 | 1 | 6 | 2 | 1 | 3 | 3 |

| | | transcript | | | | | | hypothala | | | | | skeletalm | | |
|---|---|---|---|---|---|---|---|---|---|---|---|---|---|---|---|
| transcript | gene | length (nt) | max | mean | adipose | colon | heart | mus | kidney | liver | lung | ovary | uscle | spleen | testes |
| NM_000329 | RPE65 | 2608 | 1 | 0 | 0 | 0 | 0 | 1 | 0 | 0 | 0 | 0 | 0 | 0 | 0 |
| NM_000330 | RS1 | 3024 | 0 | 0 | 0 | 0 | 0 | 0 | 0 | 0 | 0 | 0 | 0 | 0 | 0 |
| NM_000331 | SAA1 | 716 | 7,507 | 747 | 377 | 17 | 49 | 0 | 16 | 7,507 | 22 | 59 | 44 | 72 | 58 |
| NM_000332 | ATXN1 | 10636 | 6 | 3 | 2 | 2 | 2 | 6 | 2 | 1 | 3 | 3 | 4 | 3 | 3 |
| NM_000333 | ATXN7 | 7225 | 3 | 2 | 1 | 1 | 2 | 1 | 2 | 1 | 2 | 2 | 2 | 3 | 2 |
| NM_000334 | SCN4A | 7805 | 4 | 0 | 0 | 0 | 0 | 0 | 0 | 0 | 0 | 0 | 4 | 0 | 0 |
| NM_000335 | SCN5A | 8501 | 3 | 0 | 0 | 0 | 3 | 0 | 0 | 0 | 0 | 0 | 0 | 0 | 0 |
| NM_000336 | SCNN1B | 2597 | 2 | 1 | 0 | 2 | 0 | 0 | 2 | 0 | 2 | 0 | 0 | 0 | 0 |
| NM_000337 | SGCD | 9789 | 9 | 2 | 1 | 0 | 9 | 1 | 0 | 0 | 0 | 4 | 5 | 0 | 1 |
| NM_000338 | SLC12A1 | 4710 | 96 | 9 | 0 | 0 | 0 | 0 | 96 | 0 | 0 | 0 | 0 | 0 | 0 |
| NM_000339 | SLC12A3 | 5567 | 20 | 2 | 0 | 0 | 0 | 0 | 20 | 0 | 0 | 0 | 0 | 0 | 0 |
| NM_000340 | SLC2A2 | 3439 | 9 | 1 | 0 | 0 | 0 | 0 | 2 | 9 | 0 | 0 | 0 | 0 | 0 |
| NM_000341 | SLC3A1 | 2320 | 33 | 3 | 0 | 1 | 0 | 0 | 33 | 0 | 0 | 0 | 0 | 0 | 0 |
| NM_000342 | SLC4A1 | 4953 | 5 | 1 | 0 | 0 | 0 | 0 | 5 | 0 | 1 | 0 | 0 | 0 | 0 |
| NM_000343 | SLC5A1 | 5036 | 4 | 1 | 0 | 1 | 4 | 0 | 1 | 0 | 0 | 0 | 0 | 0 | 0 |
| NM_000344 | SMN1 | 1622 | 0 | 0 | 0 | 0 | 0 | 0 | 0 | 0 | 0 | 0 | 0 | 0 | 0 |
| NM_000345 | SNCA | 3214 | 26 | 3 | 1 | 1 | 1 | 26 | 0 | 0 | 1 | 1 | 0 | 1 | 0 |
| NM_000346 | SOX9 | 3934 | 31 | 7 | 1 | 4 | 6 | 17 | 9 | 1 | 2 | 0 | 1 | 0 | 31 |
| NM_000347 | SPTB | 6725 | 18 | 2 | 0 | 0 | 5 | 2 | 0 | 0 | 0 | 0 | 18 | 0 | 0 |
| NM_000348 | SRD5A2 | 2445 | 2 | 0 | 0 | 0 | 0 | 0 | 0 | 2 | 0 | 0 | 0 | 0 | 0 |
| NM_000349 | STAR | 2695 | 40 | 6 | 0 | 0 | 0 | 1 | 0 | 0 | 0 | 40 | 0 | 0 | 21 |
| NM_000350 | ABCA4 | 7325 | 1 | 0 | 0 | 0 | 0 | 1 | 0 | 0 | 0 | 0 | 0 | 0 | 0 |
| NM_000351 | STS | 6377 | 4 | 2 | 4 | 1 | 3 | 4 | 2 | 1 | 2 | 2 | 3 | 2 | 2 |
| NM_000352 | ABCC8 | 4978 | 1 | 0 | 0 | 0 | 0 | 1 | 0 | 0 | 0 | 0 | 0 | 0 | 0 |
| NM_000353 | TAT | 2757 | 305 | 28 | 0 | 1 | 1 | 0 | 0 | 305 | 0 | 1 | 1 | 1 | 1 |
| NM_000354 | SERPINA7 | 1558 | 30 | 3 | 0 | 0 | 0 | 0 | 0 | 30 | 0 | 0 | 0 | 0 | 0 |
| NM_000355 | TCN2 | 1981 | 24 | 7 | 7 | 2 | 7 | 5 | 24 | 4 | 11 | 4 | 2 | 9 | 2 |
| NM_000356 | TCOF1 | 4864 | 4 | 2 | 2 | 2 | 2 | 3 | 1 | 1 | 1 | 2 | 2 | 4 | 3 |
| NM_000358 | TGFBI | 2805 | 15 | 6 | 10 | 4 | 13 | 2 | 3 | 3 | 10 | 3 | 3 | 15 | 4 |
| NM_000359 | TGM1 | 2777 | 0 | 0 | 0 | 0 | 0 | 0 | 0 | 0 | 0 | 0 | 0 | 0 | 0 |
| NM_000360 | TH | 1817 | 0 | 0 | 0 | 0 | 0 | 0 | 0 | 0 | 0 | 0 | 0 | 0 | 0 |
| NM_000361 | THBD | 4032 | 3 | 1 | 0 | 0 | 2 | 0 | 0 | 0 | 2 | 0 | 0 | 3 | 1 |
| NM_000362 | TIMP3 | 5486 | 136 | 54 | 105 | 19 | 71 | 22 | 51 | 13 | 136 | 16 | 19 | 88 | 58 |
| NM_000363 | TNNI3 | 842 | 1,000 | 91 | 0 | 0 | 1,000 | 1 | 0 | 0 | 0 | 1 | 2 | 0 | 2 |
| NM_000364 | TNNT2 | 1153 | 2,679 | 246 | 0 | 1 | 2,679 | 2 | 11 | 0 | 3 | 1 | 4 | 1 | 1 |
| NM_000365 | TPI1 | 1349 | 71 | 41 | 25 | 29 | 66 | 71 | 55 | 52 | 23 | 21 | 47 | 34 | 25 |
| NM_000366 | TPM1 | 1288 | 660 | 85 | 8 | 57 | 660 | 12 | 22 | 4 | 12 | 28 | 93 | 20 | 16 |
| NM_000367 | TPMT | 3247 | 15 | 5 | 2 | 3 | 4 | 4 | 15 | 11 | 2 | 1 | 2 | 5 | 5 |
| NM_000368 | TSC1 | 8616 | 4 | 3 | 2 | 3 | 3 | 4 | 2 | 1 | 1 | 4 | 2 | 2 | 3 |
| NM_000369 | TSHR | 4400 | 1 | 0 | 0 | 0 | 0 | 0 | 0 | 1 | 0 | 0 | 0 | 0 | 0 |
| NM_000370 | TTPA | 2630 | 24 | 2 | 0 | 0 | 0 | 1 | 1 | 24 | 0 | 0 | 0 | 0 | 0 |
| NM_000371 | TTR | 926 | 3,267 | 508 | 0 | 26 | 4 | 3,267 | 2 | 2,264 | 2 | 7 | 4 | 8 | 5 |
| NM_000372 | TYR | 2065 | 0 | 0 | 0 | 0 | 0 | 0 | 0 | 0 | 0 | 0 | 0 | 0 | 0 |
| NM_000373 | UMPS | 2658 | 7 | 2 | 2 | 2 | 1 | 2 | 3 | 7 | 1 | 3 | 1 | 3 | 2 |
| NM_000374 | UROD | 1383 | 32 | 15 | 20 | 6 | 13 | 15 | 22 | 32 | 9 | 8 | 7 | 11 | 17 |
| NM_000375 | UROS | 1354 | 6 | 3 | 3 | 1 | 4 | 6 | 4 | 3 | 1 | 2 | 3 | 2 | 2 |
| NM_000376 | VDR | 4653 | 4 | 1 | 0 | 4 | 0 | 0 | 4 | 0 | 1 | 0 | 0 | 1 | 0 |
| NM_000377 | WAS | 1830 | 20 | 3 | 1 | 1 | 2 | 1 | 0 | 1 | 4 | 0 | 0 | 20 | 1 |
| NM_000378 | WT1 | 2969 | 8 | 2 | 0 | 0 | 0 | 0 | 1 | 0 | 0 | 7 | 0 | 3 | 8 |
| NM_000379 | XDH | 5717 | 4 | 0 | 0 | 1 | 0 | 0 | 0 | 4 | 0 | 0 | 0 | 0 | 0 |
| NM_000380 | XPA | 1469 | 9 | 6 | 7 | 2 | 5 | 7 | 9 | 6 | 4 | 7 | 4 | 6 | 5 |
| NM_000381 | MID1 | 6217 | 2 | 1 | 2 | 1 | 2 | 1 | 1 | 0 | 2 | 1 | 0 | 0 | 2 |
| NM_000382 | ALDH3A2 | 3702 | 8 | 4 | 8 | 3 | 3 | 4 | 6 | 8 | 4 | 2 | 4 | 2 | 3 |
| NM_000383 | AIRE | 2257 | 0 | 0 | 0 | 0 | 0 | 0 | 0 | 0 | 0 | 0 | 0 | 0 | 0 |
| NM_000384 | APOB | 14121 | 766 | 72 | 1 | 3 | 6 | 0 | 1 | 766 | 1 | 3 | 2 | 3 | 2 |
| NM_000386 | BLMH | 2417 | 6 | 4 | 4 | 3 | 4 | 5 | 6 | 3 | 4 | 5 | 3 | 6 | 6 |
| NM_000387 | SLC25A20 | 1868 | 8 | 2 | 3 | 2 | 2 | 1 | 3 | 8 | 1 | 0 | 2 | 2 | 1 |
| NM_000388 | CASR | 4915 | 4 | 0 | 0 | 0 | 0 | 0 | 4 | 0 | 0 | 0 | 0 | 0 | 0 |
| NM_000389 | CDKN1A | 2149 | 4 | 2 | 2 | 1 | 2 | 2 | 1 | 2 | 2 | 2 | 4 | 1 | 2 |
| NM_000390 | CHM | 5442 | 5 | 3 | 2 | 1 | 3 | 5 | 4 | 2 | 2 | 4 | 2 | 5 | 3 |
| NM_000391 | TPP1 | 3533 | 44 | 14 | 9 | 4 | 16 | 11 | 17 | 9 | 16 | 10 | 5 | 44 | 8 |
| NM_000392 | ABCC2 | 5051 | 21 | 3 | 0 | 0 | 0 | 0 | 6 | 21 | 0 | 0 | 0 | 0 | 0 |
| NM_000393 | COL5A2 | 6930 | 13 | 3 | 13 | 4 | 4 | 1 | 1 | 1 | 3 | 4 | 0 | 3 | 1 |
| NM_000394 | CRYAA | 1114 | 3 | 0 | 0 | 0 | 0 | 0 | 3 | 0 | 0 | 0 | 0 | 0 | 0 |
| NM_000395 | CSF2RB | 4846 | 2 | 0 | 1 | 0 | 0 | 0 | 0 | 0 | 1 | 0 | 0 | 2 | 0 |
| NM_000396 | CTSK | 1702 | 40 | 13 | 40 | 11 | 8 | 3 | 3 | 1 | 13 | 25 | 10 | 6 | 22 |
| NM_000397 | CYBB | 4318 | 129 | 28 | 29 | 4 | 35 | 16 | 8 | 13 | 50 | 12 | 5 | 129 | 12 |
| NM_000398 | CYB5R3 | 1955 | 21 | 10 | 17 | 6 | 15 | 10 | 8 | 21 | 5 | 6 | 5 | 7 | 8 |
| NM_000399 | EGR2 | 2975 | 3 | 1 | 0 | 0 | 0 | 0 | 0 | 0 | 0 | 3 | 0 | 0 | 1 |
| NM_000400 | ERCC2 | 2568 | 1 | 0 | 0 | 0 | 0 | 1 | 1 | 1 | 0 | 0 | 0 | 0 | 1 |
| NM_000401 | EXT2 | 3787 | 2 | 1 | 2 | 1 | 2 | 1 | 1 | 2 | 1 | 1 | 1 | 2 | 1 |
| NM_000402 | G6PD | 2395 | 2 | 1 | 1 | 1 | 0 | 1 | 0 | 0 | 1 | 0 | 0 | 1 | 2 |
| NM_000403 | GALE | 1631 | 2 | 0 | 0 | 1 | 0 | 1 | 2 | 1 | 0 | 0 | 0 | 0 | 0 |
| NM_000404 | GLB1 | 2588 | 5 | 2 | 3 | 2 | 2 | 1 | 5 | 3 | 2 | 2 | 1 | 3 | 2 |
| NM_000405 | GM2A | 3688 | 6 | 3 | 6 | 1 | 3 | 4 | 1 | 1 | 4 | 3 | 1 | 6 | 2 |
| NM_000406 | GNRHR | 5837 | 1 | 0 | 0 | 0 | 0 | 0 | 1 | 0 | 0 | 1 | 0 | 1 | 1 |
| NM_000407 | GP1BB | 958 | 0 | 0 | 0 | 0 | 0 | 0 | 0 | 0 | 0 | 0 | 0 | 0 | 0 |

| transcript | gene | transcript length (nt) | max | mean | adipose | colon | heart | hypothalamus | kidney | liver | lung | ovary | skeletalmuscle | spleen | testes |
|---|---|---|---|---|---|---|---|---|---|---|---|---|---|---|---|
| NM_000408 | GPD2 | 5803 | 9 | 4 | 3 | 2 | 3 | 7 | 3 | 0 | 4 | 3 | 9 | 4 | 2 |
| NM_000409 | GUCA1A | 1902 | 1 | 0 | 0 | 0 | 0 | 1 | 0 | 0 | 0 | 0 | 0 | 0 | 0 |
| NM_000410 | HFE | 2222 | 3 | 1 | 3 | 1 | 2 | 1 | 1 | 2 | 1 | 1 | 0 | 3 | 1 |
| NM_000411 | HLCS | 6010 | 2 | 1 | 1 | 1 | 1 | 2 | 1 | 1 | 1 | 1 | 1 | 1 | 1 |
| NM_000412 | HRG | 1952 | 479 | 45 | 0 | 2 | 2 | 0 | 10 | 479 | 0 | 2 | 1 | 2 | 1 |
| NM_000413 | HSD17B1 | 2223 | 0 | 0 | 0 | 0 | 0 | 0 | 0 | 0 | 0 | 0 | 0 | 0 | 0 |
| NM_000414 | HSD17B4 | 2672 | 55 | 21 | 20 | 8 | 26 | 17 | 26 | 55 | 31 | 8 | 6 | 19 | 16 |
| NM_000415 | IAPP | 1975 | 0 | 0 | 0 | 0 | 0 | 0 | 0 | 0 | 0 | 0 | 0 | 0 | 0 |
| NM_000416 | IFNGR1 | 2120 | 57 | 19 | 17 | 4 | 25 | 11 | 7 | 26 | 31 | 9 | 6 | 57 | 11 |
| NM_000417 | IL2RA | 3216 | 19 | 2 | 1 | 0 | 1 | 0 | 0 | 0 | 2 | 0 | 0 | 19 | 0 |
| NM_000418 | IL4R | 3668 | 13 | 5 | 2 | 6 | 4 | 3 | 2 | 6 | 9 | 2 | 1 | 13 | 3 |
| NM_000419 | ITGA2B | 3334 | 0 | 0 | 0 | 0 | 0 | 0 | 0 | 0 | 0 | 0 | 0 | 0 | 0 |
| NM_000420 | KEL | 2547 | 1 | 0 | 0 | 0 | 0 | 0 | 0 | 0 | 0 | 0 | 0 | 0 | 1 |
| NM_000421 | KRT10 | 2139 | 5 | 3 | 1 | 1 | 5 | 1 | 3 | 2 | 2 | 2 | 1 | 5 | 4 |
| NM_000422 | KRT17 | 1574 | 4 | 1 | 0 | 0 | 0 | 2 | 0 | 0 | 4 | 0 | 0 | 0 | 1 |
| NM_000423 | KRT2 | 2414 | 0 | 0 | 0 | 0 | 0 | 0 | 0 | 0 | 0 | 0 | 0 | 0 | 0 |
| NM_000424 | KRT5 | 2303 | 3 | 0 | 0 | 0 | 0 | 0 | 0 | 0 | 3 | 0 | 0 | 0 | 1 |
| NM_000425 | L1CAM | 5026 | 4 | 1 | 0 | 1 | 0 | 4 | 1 | 0 | 0 | 0 | 0 | 0 | 0 |
| NM_000426 | LAMA2 | 9692 | 32 | 10 | 7 | 2 | 32 | 2 | 3 | 2 | 10 | 22 | 7 | 8 | 12 |
| NM_000427 | LOR | 1229 | 0 | 0 | 0 | 0 | 0 | 0 | 0 | 0 | 0 | 0 | 0 | 0 | 0 |
| NM_000428 | LTBP2 | 8554 | 7 | 3 | 3 | 0 | 6 | 1 | 1 | 0 | 6 | 7 | 3 | 1 | 2 |
| NM_000429 | MAT1A | 3404 | 148 | 14 | 0 | 1 | 0 | 0 | 0 | 148 | 0 | 1 | 0 | 0 | 1 |
| NM_000430 | PAFAH1B1 | 5614 | 39 | 14 | 11 | 6 | 17 | 39 | 11 | 9 | 10 | 13 | 14 | 13 | 16 |
| NM_000431 | MVK | 2073 | 5 | 1 | 1 | 1 | 0 | 1 | 2 | 5 | 0 | 1 | 0 | 1 | 2 |
| NM_000432 | MYL2 | 829 | 1,532 | 237 | 1 | 0 | 1,065 | 0 | 0 | 0 | 0 | 0 | 1,532 | 0 | 4 |
| NM_000433 | NCF2 | 2412 | 15 | 5 | 8 | 1 | 4 | 2 | 0 | 1 | 15 | 1 | 1 | 15 | 1 |
| NM_000434 | NEU1 | 2071 | 7 | 2 | 2 | 1 | 2 | 2 | 7 | 3 | 2 | 1 | 1 | 3 | 1 |
| NM_000435 | NOTCH3 | 8071 | 2 | 1 | 2 | 0 | 1 | 1 | 1 | 0 | 1 | 1 | 1 | 2 | 1 |
| NM_000436 | OXCT1 | 3558 | 34 | 10 | 14 | 4 | 34 | 25 | 10 | 0 | 3 | 4 | 5 | 6 | 4 |
| NM_000437 | PAFAH2 | 3581 | 4 | 2 | 1 | 2 | 1 | 1 | 4 | 3 | 1 | 1 | 1 | 2 | 1 |
| NM_000438 | PAX3 | 1489 | 0 | 0 | 0 | 0 | 0 | 0 | 0 | 0 | 0 | 0 | 0 | 0 | 0 |
| NM_000439 | PCSK1 | 5054 | 3 | 0 | 0 | 0 | 0 | 3 | 0 | 0 | 0 | 0 | 0 | 0 | 0 |
| NM_000440 | PDE6A | 5642 | 0 | 0 | 0 | 0 | 0 | 0 | 0 | 0 | 0 | 0 | 0 | 0 | 0 |
| NM_000441 | SLC26A4 | 4928 | 1 | 0 | 0 | 0 | 0 | 0 | 1 | 0 | 0 | 0 | 0 | 0 | 0 |
| NM_000442 | PECAM1 | 3470 | 77 | 25 | 46 | 7 | 35 | 8 | 13 | 11 | 77 | 7 | 14 | 43 | 11 |
| NM_000443 | ABCB4 | 3967 | 23 | 3 | 0 | 0 | 1 | 0 | 0 | 23 | 0 | 0 | 1 | 3 | 0 |
| NM_000444 | PHEX | 2861 | 1 | 0 | 0 | 0 | 0 | 0 | 0 | 0 | 1 | 1 | 0 | 0 | 0 |
| NM_000445 | PLEC1 | 14798 | 7 | 3 | 3 | 4 | 4 | 4 | 2 | 2 | 3 | 2 | 7 | 4 | 2 |
| NM_000446 | PON1 | 1750 | 20 | 2 | 0 | 0 | 0 | 0 | 0 | 20 | 0 | 0 | 0 | 0 | 0 |
| NM_000447 | PSEN2 | 2298 | 2 | 1 | 2 | 1 | 1 | 2 | 2 | 1 | 1 | 1 | 1 | 2 | 2 |
| NM_000448 | RAG1 | 6580 | 1 | 0 | 0 | 0 | 0 | 0 | 0 | 0 | 0 | 0 | 0 | 1 | 0 |
| NM_000449 | RFX5 | 3618 | 3 | 1 | 1 | 1 | 1 | 1 | 1 | 1 | 1 | 1 | 0 | 3 | 1 |
| NM_000450 | SELE | 3875 | 3 | 1 | 1 | 0 | 2 | 3 | 1 | 1 | 2 | 0 | 0 | 0 | 1 |
| NM_000451 | SHOX | 3757 | 3 | 1 | 1 | 0 | 0 | 0 | 0 | 1 | 3 | 0 | 0 | 0 | 1 |
| NM_000452 | SLC10A2 | 3779 | 2 | 0 | 0 | 0 | 0 | 0 | 2 | 0 | 0 | 0 | 0 | 0 | 0 |
| NM_000453 | SLC5A5 | 3576 | 1 | 0 | 0 | 0 | 0 | 1 | 0 | 0 | 0 | 0 | 0 | 0 | 0 |
| NM_000454 | SOD1 | 965 | 357 | 102 | 88 | 44 | 91 | 223 | 94 | 357 | 28 | 66 | 45 | 42 | 44 |
| NM_000455 | STK11 | 3276 | 14 | 8 | 7 | 8 | 7 | 9 | 5 | 5 | 4 | 6 | 12 | 7 | 14 |
| NM_000456 | SUOX | 2462 | 5 | 2 | 2 | 1 | 2 | 2 | 5 | 3 | 1 | 1 | 1 | 1 | 2 |
| NM_000457 | HNF4A | 3239 | 12 | 3 | 0 | 6 | 0 | 0 | 12 | 10 | 0 | 0 | 0 | 0 | 0 |
| NM_000458 | HNF1B | 2836 | 18 | 2 | 0 | 4 | 0 | 0 | 18 | 2 | 1 | 0 | 0 | 0 | 1 |
| NM_000459 | TEK | 4759 | 6 | 3 | 2 | 2 | 4 | 2 | 4 | 2 | 6 | 1 | 1 | 6 | 1 |
| NM_000460 | THPO | 1805 | 2 | 0 | 0 | 0 | 0 | 0 | 0 | 2 | 0 | 0 | 0 | 0 | 0 |
| NM_000461 | THRB | 7401 | 4 | 1 | 2 | 1 | 1 | 4 | 1 | 2 | 1 | 0 | 2 | 1 | 0 |
| NM_000462 | UBE3A | 4516 | 12 | 8 | 4 | 3 | 11 | 9 | 8 | 6 | 6 | 8 | 12 | 9 | 10 |
| NM_000463 | UGT1A1 | 2357 | 20 | 2 | 0 | 0 | 0 | 0 | 1 | 20 | 0 | 0 | 0 | 0 | 0 |
| NM_000465 | BARD1 | 2594 | 4 | 2 | 0 | 1 | 1 | 2 | 1 | 0 | 1 | 2 | 4 | 1 | 3 |
| NM_000466 | PEX1 | 4382 | 7 | 5 | 4 | 2 | 4 | 5 | 7 | 6 | 3 | 5 | 4 | 4 | 6 |
| NM_000474 | TWIST1 | 1666 | 1 | 0 | 0 | 0 | 0 | 0 | 0 | 0 | 1 | 0 | 0 | 0 | 0 |
| NM_000475 | NR0B1 | 1585 | 3 | 0 | 0 | 0 | 0 | 0 | 0 | 0 | 0 | 0 | 0 | 0 | 3 |
| NM_000476 | AK1 | 2266 | 15 | 4 | 1 | 1 | 10 | 4 | 1 | 2 | 3 | 1 | 15 | 2 | 3 |
| NM_000477 | ALB | 2245 | 8,473 | 796 | 0 | 15 | 37 | 0 | 24 | 8,473 | 17 | 54 | 30 | 62 | 40 |
| NM_000478 | ALPL | 2596 | 8 | 3 | 1 | 0 | 3 | 3 | 6 | 8 | 6 | 0 | 0 | 7 | 0 |
| NM_000479 | AMH | 2018 | 0 | 0 | 0 | 0 | 0 | 0 | 0 | 0 | 0 | 0 | 0 | 0 | 0 |
| NM_000480 | AMPD3 | 4371 | 4 | 1 | 1 | 0 | 0 | 1 | 1 | 0 | 1 | 0 | 4 | 1 | 0 |
| NM_000481 | AMT | 2201 | 2 | 1 | 0 | 2 | 1 | 1 | 2 | 2 | 1 | 1 | 0 | 0 | 1 |
| NM_000482 | APOA4 | 1460 | 73 | 7 | 0 | 6 | 0 | 0 | 0 | 73 | 0 | 0 | 0 | 0 | 0 |
| NM_000483 | APOC2 | 721 | 98 | 10 | 1 | 0 | 1 | 2 | 1 | 98 | 0 | 1 | 0 | 2 | 1 |
| NM_000484 | APP | 3632 | 195 | 72 | 30 | 45 | 64 | 195 | 120 | 43 | 58 | 67 | 14 | 71 | 83 |
| NM_000485 | APRT | 807 | 1 | 1 | 1 | 1 | 0 | 1 | 1 | 1 | 1 | 0 | 0 | 1 | 0 |
| NM_000486 | AQP2 | 4177 | 59 | 5 | 0 | 0 | 0 | 0 | 59 | 0 | 0 | 0 | 0 | 0 | 1 |
| NM_000487 | ARSA | 2054 | 1 | 0 | 0 | 0 | 0 | 0 | 1 | 0 | 0 | 0 | 0 | 0 | 0 |
| NM_000488 | SERPINC1 | 1598 | 92 | 9 | 0 | 0 | 0 | 0 | 0 | 92 | 0 | 0 | 0 | 1 | 1 |
| NM_000489 | ATRX | 11184 | 31 | 19 | 19 | 4 | 24 | 29 | 19 | 7 | 17 | 31 | 15 | 27 | 17 |
| NM_000490 | AVP | 619 | 3 | 0 | 0 | 0 | 0 | 3 | 0 | 0 | 0 | 0 | 0 | 0 | 0 |
| NM_000491 | C1QB | 1044 | 225 | 38 | 24 | 4 | 45 | 25 | 5 | 20 | 53 | 8 | 5 | 225 | 7 |
| NM_000492 | CFTR | 6131 | 5 | 1 | 0 | 5 | 0 | 0 | 0 | 0 | 2 | 0 | 0 | 0 | 1 |

| transcript | gene | transcript length (nt) | max | mean | adipose | colon | heart | hypothalamus | kidney | liver | lung | ovary | skeletalmuscle | spleen | testes |
|---|---|---|---|---|---|---|---|---|---|---|---|---|---|---|---|
| NM_000493 | COL10A1 | 3290 | 1 | 0 | 0 | 0 | 0 | 0 | 0 | 0 | 0 | 1 | 0 | 1 | 0 |
| NM_000494 | COL17A1 | 5610 | 4 | 0 | 0 | 4 | 0 | 0 | 0 | 0 | 0 | 0 | 0 | 0 | 0 |
| NM_000495 | COL4A5 | 6427 | 1 | 1 | 0 | 1 | 1 | 1 | 1 | 0 | 1 | 1 | 0 | 0 | 1 |
| NM_000496 | CRYBB2 | 763 | 1 | 0 | 0 | 0 | 0 | 1 | 0 | 0 | 0 | 0 | 0 | 1 | 1 |
| NM_000497 | CYP11B1 | 3535 | 0 | 0 | 0 | 0 | 0 | 0 | 0 | 0 | 0 | 0 | 0 | 0 | 0 |
| NM_000498 | CYP11B2 | 2936 | 0 | 0 | 0 | 0 | 0 | 0 | 0 | 0 | 0 | 0 | 0 | 0 | 0 |
| NM_000499 | CYP1A1 | 2608 | 1 | 0 | 0 | 1 | 0 | 0 | 0 | 1 | 1 | 0 | 0 | 0 | 0 |
| NM_000500 | CYP21A2 | 2114 | 0 | 0 | 0 | 0 | 0 | 0 | 0 | 0 | 0 | 0 | 0 | 0 | 0 |
| NM_000501 | ELN | 3472 | 3 | 1 | 2 | 1 | 3 | 0 | 1 | 0 | 2 | 0 | 1 | 1 | 1 |
| NM_000502 | EPX | 2716 | 0 | 0 | 0 | 0 | 0 | 0 | 0 | 0 | 0 | 0 | 0 | 0 | 0 |
| NM_000503 | EYA1 | 4326 | 2 | 1 | 0 | 0 | 2 | 2 | 0 | 0 | 0 | 0 | 1 | 0 | 1 |
| NM_000504 | F10 | 1536 | 10 | 2 | 1 | 0 | 1 | 0 | 0 | 10 | 1 | 1 | 0 | 0 | 2 |
| NM_000505 | F12 | 2051 | 9 | 1 | 0 | 0 | 0 | 0 | 1 | 9 | 0 | 0 | 0 | 0 | 0 |
| NM_000506 | F2 | 2010 | 71 | 7 | 0 | 0 | 0 | 0 | 0 | 71 | 0 | 0 | 0 | 0 | 0 |
| NM_000507 | FBP1 | 1594 | 53 | 8 | 5 | 2 | 0 | 0 | 15 | 53 | 5 | 1 | 0 | 2 | 0 |
| NM_000508 | FGA | 3655 | 9,012 | 835 | 0 | 19 | 24 | 0 | 10 | 9,012 | 11 | 32 | 18 | 38 | 25 |
| NM_000509 | FGG | 1661 | 7,715 | 716 | 0 | 10 | 16 | 0 | 6 | 7,715 | 31 | 29 | 15 | 36 | 22 |
| NM_000510 | FSHB | 1936 | 0 | 0 | 0 | 0 | 0 | 0 | 0 | 0 | 0 | 0 | 0 | 0 | 0 |
| NM_000511 | FUT2 | 3099 | 1 | 0 | 0 | 1 | 0 | 0 | 0 | 0 | 0 | 0 | 0 | 0 | 0 |
| NM_000512 | GALNS | 2363 | 1 | 0 | 0 | 0 | 0 | 0 | 1 | 0 | 0 | 0 | 0 | 1 | 1 |
| NM_000513 | OPN1MW | 1997 | 0 | 0 | 0 | 0 | 0 | 0 | 0 | 0 | 0 | 0 | 0 | 0 | 0 |
| NM_000514 | GDNF | 836 | 7 | 1 | 0 | 1 | 0 | 0 | 0 | 0 | 0 | 1 | 7 | 0 | 1 |
| NM_000515 | GH1 | 822 | 0 | 0 | 0 | 0 | 0 | 0 | 0 | 0 | 0 | 0 | 0 | 0 | 0 |
| NM_000516 | GNAS | 1907 | 39 | 15 | 17 | 12 | 19 | 39 | 12 | 10 | 10 | 9 | 21 | 16 | 6 |
| NM_000517 | HBA2 | 605 | 1 | 0 | 0 | 0 | 0 | 0 | 0 | 0 | 1 | 0 | 0 | 0 | 0 |
| NM_000518 | HBB | 626 | 28 | 4 | 6 | 0 | 0 | 4 | 0 | 0 | 28 | 1 | 1 | 5 | 2 |
| NM_000519 | HBD | 774 | 3 | 0 | 0 | 0 | 0 | 0 | 0 | 0 | 3 | 0 | 0 | 0 | 0 |
| NM_000520 | HEXA | 2437 | 8 | 4 | 5 | 3 | 4 | 3 | 8 | 2 | 6 | 3 | 1 | 7 | 5 |
| NM_000521 | HEXB | 1901 | 24 | 13 | 21 | 7 | 17 | 7 | 15 | 16 | 24 | 7 | 5 | 14 | 11 |
| NM_000522 | HOXA13 | 2514 | 2 | 0 | 0 | 2 | 0 | 0 | 0 | 0 | 0 | 0 | 1 | 0 | 0 |
| NM_000523 | HOXD13 | 2327 | 0 | 0 | 0 | 0 | 0 | 0 | 0 | 0 | 0 | 0 | 0 | 0 | 0 |
| NM_000524 | HTR1A | 1269 | 1 | 0 | 0 | 0 | 0 | 1 | 0 | 0 | 0 | 0 | 0 | 0 | 0 |
| NM_000525 | KCNJ11 | 3411 | 2 | 0 | 0 | 0 | 0 | 0 | 0 | 0 | 0 | 0 | 2 | 0 | 0 |
| NM_000526 | KRT14 | 1634 | 0 | 0 | 0 | 0 | 0 | 0 | 0 | 0 | 0 | 0 | 0 | 0 | 0 |
| NM_000527 | LDLR | 5264 | 3 | 1 | 0 | 1 | 1 | 1 | 1 | 2 | 3 | 2 | 0 | 2 | 1 |
| NM_000528 | MAN2B1 | 3224 | 3 | 1 | 2 | 1 | 1 | 0 | 1 | 1 | 1 | 0 | 0 | 3 | 0 |
| NM_000529 | MC2R | 3652 | 0 | 0 | 0 | 0 | 0 | 0 | 0 | 0 | 0 | 0 | 0 | 0 | 0 |
| NM_000530 | MPZ | 1955 | 11 | 2 | 3 | 2 | 2 | 11 | 1 | 0 | 0 | 0 | 2 | 0 | 1 |
| NM_000531 | OTC | 1646 | 8 | 1 | 0 | 0 | 0 | 0 | 0 | 8 | 0 | 0 | 0 | 0 | 0 |
| NM_000532 | PCCB | 1814 | 11 | 4 | 3 | 2 | 6 | 7 | 8 | 11 | 2 | 1 | 2 | 2 | 4 |
| NM_000533 | PLP1 | 3025 | 260 | 25 | 0 | 3 | 2 | 260 | 0 | 0 | 0 | 1 | 0 | 0 | 10 |
| NM_000534 | PMS1 | 3521 | 4 | 2 | 2 | 0 | 2 | 3 | 2 | 1 | 1 | 3 | 2 | 3 | 4 |
| NM_000535 | PMS2 | 2836 | 2 | 1 | 1 | 1 | 0 | 2 | 1 | 0 | 0 | 1 | 0 | 1 | 0 |
| NM_000536 | RAG2 | 2414 | 0 | 0 | 0 | 0 | 0 | 0 | 0 | 0 | 0 | 0 | 0 | 0 | 0 |
| NM_000537 | REN | 1465 | 11 | 1 | 0 | 0 | 0 | 0 | 11 | 0 | 0 | 2 | 0 | 0 | 0 |
| NM_000538 | RFXAP | 2825 | 1 | 0 | 0 | 0 | 0 | 1 | 0 | 0 | 0 | 0 | 0 | 0 | 0 |
| NM_000539 | RHO | 2768 | 0 | 0 | 0 | 0 | 0 | 0 | 0 | 0 | 0 | 0 | 0 | 0 | 0 |
| NM_000540 | RYR1 | 15391 | 78 | 7 | 1 | 0 | 0 | 1 | 0 | 0 | 0 | 0 | 78 | 0 | 1 |
| NM_000541 | SAG | 1749 | 0 | 0 | 0 | 0 | 0 | 0 | 0 | 0 | 0 | 0 | 0 | 0 | 0 |
| NM_000542 | SFTPB | 1990 | 43 | 4 | 0 | 0 | 0 | 0 | 0 | 0 | 43 | 0 | 0 | 0 | 0 |
| NM_000543 | SMPD1 | 2459 | 3 | 1 | 1 | 1 | 1 | 1 | 3 | 2 | 1 | 0 | 0 | 0 | 0 |
| NM_000544 | TAP2 | 5679 | 5 | 2 | 2 | 1 | 1 | 2 | 1 | 1 | 3 | 1 | 2 | 5 | 1 |
| NM_000545 | HNF1A | 3236 | 0 | 0 | 0 | 0 | 0 | 0 | 0 | 0 | 0 | 0 | 0 | 0 | 0 |
| NM_000546 | TP53 | 2586 | 4 | 2 | 1 | 4 | 1 | 1 | 1 | 1 | 1 | 2 | 1 | 4 | 2 |
| NM_000547 | TPO | 3145 | 4 | 0 | 0 | 0 | 0 | 0 | 0 | 0 | 0 | 0 | 0 | 4 | 0 |
| NM_000548 | TSC2 | 5632 | 2 | 1 | 1 | 2 | 1 | 2 | 1 | 1 | 1 | 1 | 2 | 2 | 2 |
| NM_000549 | TSHB | 578 | 0 | 0 | 0 | 0 | 0 | 0 | 0 | 0 | 0 | 0 | 0 | 0 | 0 |
| NM_000550 | TYRP1 | 2876 | 2 | 0 | 0 | 0 | 2 | 1 | 1 | 0 | 0 | 0 | 0 | 0 | 0 |
| NM_000551 | VHL | 2952 | 8 | 4 | 4 | 5 | 3 | 6 | 4 | 4 | 3 | 4 | 2 | 8 | 4 |
| NM_000552 | VWF | 8833 | 13 | 6 | 9 | 2 | 12 | 3 | 1 | 0 | 13 | 4 | 3 | 11 | 4 |
| NM_000553 | WRN | 5746 | 5 | 3 | 2 | 2 | 2 | 2 | 2 | 2 | 3 | 5 | 2 | 5 | 5 |
| NM_000554 | CRX | 4466 | 0 | 0 | 0 | 0 | 0 | 0 | 0 | 0 | 0 | 0 | 0 | 0 | 0 |
| NM_000555 | DCX | 9406 | 1 | 0 | 0 | 0 | 0 | 1 | 0 | 0 | 0 | 0 | 0 | 0 | 0 |
| NM_000557 | GDF5 | 2383 | 0 | 0 | 0 | 0 | 0 | 0 | 0 | 0 | 0 | 0 | 0 | 0 | 0 |
| NM_000558 | HBA1 | 576 | 19 | 3 | 1 | 0 | 1 | 3 | 0 | 0 | 19 | 2 | 1 | 4 | 2 |
| NM_000559 | HBG1 | 584 | 16 | 3 | 4 | 0 | 1 | 6 | 0 | 0 | 16 | 1 | 0 | 4 | 1 |
| NM_000560 | CD53 | 1567 | 35 | 6 | 5 | 1 | 5 | 3 | 1 | 2 | 13 | 1 | 0 | 35 | 2 |
| NM_000561 | GSTM1 | 1184 | 6 | 1 | 1 | 0 | 0 | 0 | 0 | 6 | 0 | 1 | 0 | 1 | 0 |
| NM_000562 | C8A | 2392 | 45 | 4 | 0 | 0 | 0 | 0 | 0 | 45 | 0 | 0 | 0 | 0 | 0 |
| NM_000564 | IL5RA | 2415 | 0 | 0 | 0 | 0 | 0 | 0 | 0 | 0 | 0 | 0 | 0 | 0 | 0 |
| NM_000565 | IL6R | 4176 | 6 | 2 | 1 | 1 | 1 | 0 | 1 | 4 | 1 | 1 | 6 | 2 | 1 |
| NM_000566 | FCGR1A | 2176 | 3 | 1 | 0 | 0 | 1 | 3 | 0 | 0 | 0 | 0 | 0 | 1 | 0 |
| NM_000567 | CRP | 2015 | 2,726 | 254 | 0 | 10 | 9 | 0 | 3 | 2,726 | 5 | 12 | 7 | 14 | 9 |
| NM_000569 | FCGR3A | 2308 | 38 | 7 | 3 | 0 | 9 | 8 | 1 | 6 | 9 | 0 | 0 | 38 | 1 |
| NM_000570 | FCGR3B | 2293 | 5 | 1 | 0 | 0 | 0 | 0 | 0 | 0 | 2 | 0 | 0 | 5 | 0 |
| NM_000572 | IL10 | 1629 | 0 | 0 | 0 | 0 | 0 | 0 | 0 | 0 | 0 | 0 | 0 | 0 | 0 |
| NM_000573 | CR1 | 8616 | 23 | 3 | 1 | 0 | 2 | 0 | 1 | 0 | 2 | 0 | 0 | 23 | 1 |

| | | transcript | | | | | | hypothala | | | | | skeletalm | | |
|---|---|---|---|---|---|---|---|---|---|---|---|---|---|---|---|
| transcript | gene | length (nt) | max | mean | adipose | colon | heart | mus | kidney | liver | lung | ovary | uscle | spleen | testes |
| NM_000574 | CD55 | 2796 | 33 | 10 | 3 | 3 | 14 | 3 | 2 | 4 | 33 | 16 | 13 | 18 | 5 |
| NM_000575 | IL1A | 2928 | 2 | 0 | 0 | 0 | 0 | 0 | 0 | 0 | 0 | 0 | 0 | 0 | 2 |
| NM_000576 | IL1B | 1498 | 2 | 0 | 0 | 0 | 0 | 2 | 0 | 0 | 1 | 0 | 0 | 1 | 0 |
| NM_000577 | IL1RN | 1751 | 6 | 1 | 6 | 0 | 0 | 0 | 0 | 2 | 1 | 0 | 0 | 0 | 0 |
| NM_000578 | SLC11A1 | 3852 | 3 | 1 | 0 | 0 | 1 | 0 | 0 | 0 | 3 | 0 | 0 | 1 | 0 |
| NM_000579 | CCR5 | 3661 | 4 | 1 | 1 | 1 | 1 | 1 | 0 | 0 | 2 | 1 | 0 | 4 | 0 |
| NM_000581 | GPX1 | 904 | 6 | 3 | 6 | 1 | 2 | 2 | 2 | 6 | 3 | 1 | 1 | 4 | 1 |
| NM_000582 | SPP1 | 1583 | 232 | 28 | 33 | 0 | 5 | 31 | 232 | 1 | 1 | 1 | 0 | 0 | 1 |
| NM_000583 | GC | 1780 | 446 | 42 | 0 | 1 | 2 | 0 | 2 | 446 | 1 | 4 | 2 | 5 | 3 |
| NM_000584 | IL8 | 1653 | 19 | 2 | 1 | 0 | 1 | 2 | 0 | 0 | 19 | 0 | 0 | 0 | 0 |
| NM_000585 | IL15 | 1468 | 1 | 0 | 0 | 0 | 0 | 0 | 0 | 0 | 0 | 0 | 0 | 1 | 0 |
| NM_000586 | IL2 | 798 | 0 | 0 | 0 | 0 | 0 | 0 | 0 | 0 | 0 | 0 | 0 | 0 | 0 |
| NM_000587 | C7 | 4012 | 81 | 33 | 7 | 3 | 81 | 2 | 64 | 19 | 28 | 71 | 2 | 32 | 53 |
| NM_000588 | IL3 | 924 | 0 | 0 | 0 | 0 | 0 | 0 | 0 | 0 | 0 | 0 | 0 | 0 | 0 |
| NM_000589 | IL4 | 921 | 0 | 0 | 0 | 0 | 0 | 0 | 0 | 0 | 0 | 0 | 0 | 0 | 0 |
| NM_000590 | IL9 | 591 | 0 | 0 | 0 | 0 | 0 | 0 | 0 | 0 | 0 | 0 | 0 | 0 | 0 |
| NM_000591 | CD14 | 1606 | 5 | 2 | 3 | 1 | 3 | 3 | 1 | 5 | 2 | 1 | 1 | 3 | 1 |
| NM_000593 | TAP1 | 2952 | 7 | 2 | 1 | 2 | 1 | 2 | 1 | 1 | 3 | 1 | 1 | 7 | 2 |
| NM_000594 | TNF | 1669 | 2 | 0 | 0 | 0 | 0 | 0 | 0 | 0 | 1 | 0 | 0 | 2 | 0 |
| NM_000595 | LTA | 1386 | 1 | 0 | 0 | 0 | 0 | 0 | 0 | 0 | 0 | 0 | 0 | 1 | 0 |
| NM_000596 | IGFBP1 | 1650 | 42 | 4 | 0 | 0 | 0 | 0 | 0 | 42 | 0 | 1 | 0 | 0 | 0 |
| NM_000597 | IGFBP2 | 1429 | 8 | 2 | 0 | 1 | 1 | 1 | 1 | 8 | 0 | 1 | 0 | 4 | 1 |
| NM_000598 | IGFBP3 | 2613 | 57 | 32 | 57 | 20 | 50 | 10 | 29 | 56 | 20 | 56 | 16 | 17 | 24 |
| NM_000599 | IGFBP5 | 6239 | 243 | 64 | 125 | 28 | 42 | 22 | 86 | 17 | 22 | 243 | 35 | 18 | 65 |
| NM_000600 | IL6 | 1182 | 8 | 1 | 1 | 8 | 2 | 1 | 0 | 0 | 2 | 0 | 1 | 1 | 0 |
| NM_000601 | HGF | 2805 | 7 | 2 | 2 | 7 | 1 | 1 | 2 | 5 | 4 | 0 | 0 | 2 | 2 |
| NM_000602 | SERPINE1 | 3180 | 11 | 4 | 1 | 0 | 8 | 1 | 1 | 11 | 9 | 5 | 1 | 4 | 1 |
| NM_000603 | NOS3 | 4337 | 5 | 1 | 0 | 0 | 1 | 0 | 0 | 0 | 0 | 0 | 0 | 5 | 0 |
| NM_000605 | IFNA2 | 1142 | 0 | 0 | 0 | 0 | 0 | 0 | 0 | 0 | 0 | 0 | 0 | 0 | 0 |
| NM_000606 | C8G | 877 | 5 | 0 | 0 | 0 | 0 | 0 | 0 | 5 | 0 | 0 | 0 | 0 | 0 |
| NM_000607 | ORM1 | 839 | 3,786 | 351 | 0 | 8 | 10 | 0 | 3 | 3,786 | 5 | 14 | 9 | 19 | 12 |
| NM_000608 | ORM2 | 844 | 395 | 37 | 0 | 1 | 1 | 0 | 1 | 395 | 1 | 2 | 1 | 2 | 1 |
| NM_000609 | CXCL12 | 3542 | 14 | 3 | 2 | 1 | 7 | 1 | 5 | 1 | 1 | 3 | 1 | 14 | 1 |
| NM_000610 | CD44 | 5732 | 29 | 12 | 22 | 10 | 10 | 15 | 3 | 4 | 24 | 4 | 5 | 29 | 6 |
| NM_000611 | CD59 | 7619 | 24 | 12 | 11 | 4 | 24 | 13 | 19 | 9 | 22 | 7 | 10 | 10 | 9 |
| NM_000612 | IGF2 | 5165 | 0 | 0 | 0 | 0 | 0 | 0 | 0 | 0 | 0 | 0 | 0 | 0 | 0 |
| NM_000613 | HPX | 1623 | 249 | 23 | 0 | 0 | 1 | 0 | 0 | 249 | 0 | 1 | 1 | 2 | 1 |
| NM_000614 | CNTF | 1891 | 0 | 0 | 0 | 0 | 0 | 0 | 0 | 0 | 0 | 0 | 0 | 0 | 0 |
| NM_000615 | NCAM1 | 5936 | 37 | 6 | 0 | 4 | 11 | 37 | 0 | 0 | 0 | 8 | 1 | 1 | 3 |
| NM_000616 | CD4 | 3103 | 8 | 2 | 2 | 1 | 1 | 1 | 0 | 1 | 2 | 1 | 0 | 8 | 1 |
| NM_000617 | SLC11A2 | 4162 | 5 | 2 | 1 | 1 | 2 | 4 | 5 | 2 | 3 | 1 | 1 | 1 | 4 |
| NM_000618 | IGF1 | 7321 | 8 | 3 | 8 | 1 | 6 | 0 | 1 | 3 | 1 | 4 | 1 | 2 | 6 |
| NM_000619 | IFNG | 1210 | 1 | 0 | 0 | 0 | 0 | 0 | 0 | 0 | 1 | 0 | 0 | 0 | 0 |
| NM_000620 | NOS1 | 7126 | 2 | 0 | 0 | 0 | 0 | 2 | 1 | 0 | 0 | 0 | 1 | 0 | 0 |
| NM_000621 | HTR2A | 3564 | 2 | 0 | 0 | 0 | 0 | 2 | 0 | 0 | 0 | 0 | 0 | 0 | 0 |
| NM_000623 | BDKRB2 | 4197 | 1 | 0 | 0 | 1 | 0 | 0 | 1 | 0 | 0 | 0 | 0 | 0 | 0 |
| NM_000624 | SERPINA5 | 2312 | 47 | 7 | 0 | 0 | 1 | 0 | 8 | 6 | 0 | 1 | 0 | 10 | 47 |
| NM_000625 | NOS2 | 4205 | 1 | 0 | 0 | 1 | 0 | 0 | 0 | 0 | 0 | 0 | 0 | 0 | 0 |
| NM_000626 | CD79B | 1261 | 5 | 1 | 0 | 0 | 0 | 0 | 0 | 0 | 0 | 0 | 0 | 5 | 0 |
| NM_000627 | LTBP1 | 5290 | 23 | 6 | 2 | 2 | 23 | 2 | 4 | 2 | 6 | 7 | 3 | 2 | 11 |
| NM_000628 | IL10RB | 1935 | 5 | 2 | 2 | 1 | 1 | 1 | 1 | 2 | 2 | 2 | 0 | 5 | 1 |
| NM_000629 | IFNAR1 | 6094 | 18 | 9 | 9 | 2 | 8 | 10 | 8 | 17 | 9 | 8 | 3 | 18 | 7 |
| NM_000631 | NCF4 | 1398 | 5 | 1 | 2 | 1 | 1 | 1 | 0 | 0 | 1 | 0 | 0 | 5 | 0 |
| NM_000632 | ITGAM | 4742 | 3 | 1 | 3 | 0 | 2 | 1 | 0 | 0 | 2 | 0 | 0 | 3 | 0 |
| NM_000633 | BCL2 | 6492 | 5 | 2 | 3 | 2 | 1 | 2 | 2 | 0 | 1 | 4 | 2 | 5 | 1 |
| NM_000634 | CXCR1 | 2486 | 2 | 0 | 0 | 0 | 0 | 0 | 0 | 0 | 1 | 0 | 0 | 2 | 0 |
| NM_000635 | RFX2 | 4112 | 0 | 0 | 0 | 0 | 0 | 0 | 0 | 0 | 0 | 0 | 0 | 0 | 0 |
| NM_000636 | SOD2 | 1593 | 1,594 | 338 | 122 | 22 | 492 | 98 | 287 | 501 | 229 | 68 | 1,594 | 195 | 105 |
| NM_000637 | GSR | 2262 | 14 | 7 | 4 | 6 | 6 | 7 | 13 | 14 | 6 | 6 | 3 | 8 | 7 |
| NM_000638 | VTN | 1677 | 80 | 8 | 0 | 0 | 3 | 0 | 0 | 80 | 0 | 1 | 0 | 1 | 0 |
| NM_000639 | FASLG | 1859 | 1 | 0 | 0 | 1 | 0 | 0 | 0 | 0 | 1 | 0 | 0 | 1 | 0 |
| NM_000640 | IL13RA2 | 1373 | 9 | 1 | 0 | 0 | 1 | 4 | 1 | 0 | 0 | 0 | 0 | 0 | 9 |
| NM_000641 | IL11 | 2354 | 0 | 0 | 0 | 0 | 0 | 0 | 0 | 0 | 0 | 0 | 0 | 0 | 0 |
| NM_000642 | AGL | 7371 | 16 | 5 | 3 | 2 | 5 | 3 | 4 | 12 | 2 | 1 | 16 | 3 | 3 |
| NM_000643 | AGL | 7169 | 17 | 5 | 3 | 2 | 6 | 4 | 4 | 13 | 2 | 2 | 17 | 3 | 3 |
| NM_000644 | AGL | 7109 | 16 | 5 | 3 | 2 | 6 | 4 | 4 | 13 | 2 | 2 | 16 | 3 | 3 |
| NM_000645 | AGL | 7182 | 16 | 5 | 3 | 2 | 6 | 4 | 4 | 12 | 2 | 2 | 16 | 3 | 3 |
| NM_000646 | AGL | 7182 | 16 | 5 | 3 | 2 | 6 | 4 | 4 | 13 | 2 | 2 | 16 | 3 | 3 |
| NM_000651 | CR1 | 9966 | 20 | 2 | 1 | 0 | 2 | 0 | 1 | 0 | 2 | 0 | 0 | 20 | 0 |
| NM_000655 | SELL | 2442 | 22 | 3 | 1 | 0 | 1 | 1 | 0 | 1 | 5 | 0 | 0 | 22 | 1 |
| NM_000657 | BCL2 | 1206 | 8 | 3 | 5 | 4 | 2 | 2 | 3 | 0 | 2 | 4 | 2 | 8 | 3 |
| NM_000658 | AIRE | 1963 | 0 | 0 | 0 | 0 | 0 | 0 | 0 | 0 | 0 | 0 | 0 | 0 | 0 |
| NM_000660 | TGFB1 | 2199 | 19 | 6 | 6 | 4 | 6 | 5 | 3 | 3 | 11 | 3 | 2 | 19 | 3 |
| NM_000661 | RPL9 | 749 | 97 | 45 | 47 | 24 | 46 | 37 | 36 | 48 | 39 | 97 | 28 | 62 | 26 |
| NM_000662 | NAT1 | 1798 | 1 | 0 | 0 | 1 | 0 | 0 | 1 | 1 | 1 | 0 | 0 | 0 | 0 |
| NM_000663 | ABAT | 5586 | 40 | 8 | 0 | 1 | 2 | 19 | 17 | 40 | 1 | 1 | 0 | 1 | 4 |
| NM_000665 | ACHE | 2204 | 3 | 1 | 0 | 1 | 0 | 3 | 0 | 0 | 0 | 0 | 2 | 0 | 0 |

| transcript | gene | transcript length (nt) | max | mean | adipose | colon | heart | hypothalamus | kidney | liver | lung | ovary | skeletalmuscle | spleen | testes |
|---|---|---|---|---|---|---|---|---|---|---|---|---|---|---|---|
| NM_000666 | ACY1 | 1415 | 7 | 1 | 0 | 0 | 0 | 0 | 7 | 2 | 0 | 0 | 0 | 0 | 0 |
| NM_000667 | ADH1A | 1456 | 6 | 1 | 1 | 0 | 0 | 0 | 0 | 6 | 0 | 0 | 0 | 0 | 0 |
| NM_000668 | ADH1B | 2682 | 150 | 27 | 73 | 6 | 11 | 0 | 5 | 150 | 26 | 2 | 8 | 4 | 8 |
| NM_000669 | ADH1C | 1473 | 26 | 4 | 1 | 15 | 1 | 0 | 1 | 26 | 1 | 0 | 2 | 0 | 1 |
| NM_000670 | ADH4 | 1979 | 77 | 7 | 0 | 0 | 0 | 0 | 0 | 77 | 0 | 0 | 0 | 0 | 0 |
| NM_000671 | ADH5 | 2643 | 18 | 11 | 13 | 6 | 13 | 8 | 18 | 15 | 8 | 9 | 5 | 8 | 14 |
| NM_000672 | ADH6 | 1400 | 22 | 3 | 0 | 2 | 0 | 0 | 4 | 22 | 0 | 0 | 0 | 0 | 0 |
| NM_000673 | ADH7 | 2288 | 0 | 0 | 0 | 0 | 0 | 0 | 0 | 0 | 0 | 0 | 0 | 0 | 0 |
| NM_000674 | ADORA1 | 2919 | 2 | 1 | 0 | 0 | 0 | 2 | 0 | 0 | 0 | 0 | 0 | 0 | 2 |
| NM_000675 | ADORA2A | 2566 | 1 | 0 | 0 | 0 | 0 | 0 | 1 | 0 | 1 | 0 | 0 | 1 | 0 |
| NM_000676 | ADORA2B | 1885 | 0 | 0 | 0 | 0 | 0 | 0 | 0 | 0 | 0 | 0 | 0 | 0 | 0 |
| NM_000677 | ADORA3 | 2245 | 1 | 0 | 0 | 0 | 1 | 0 | 0 | 0 | 1 | 0 | 0 | 0 | 0 |
| NM_000678 | ADRA1D | 2666 | 0 | 0 | 0 | 0 | 0 | 0 | 0 | 0 | 0 | 0 | 0 | 0 | 0 |
| NM_000679 | ADRA1B | 2254 | 0 | 0 | 0 | 0 | 0 | 0 | 0 | 0 | 0 | 0 | 0 | 0 | 0 |
| NM_000680 | ADRA1A | 2281 | 4 | 1 | 1 | 0 | 1 | 1 | 0 | 4 | 0 | 0 | 0 | 1 | 0 |
| NM_000681 | ADRA2A | 3873 | 2 | 1 | 2 | 0 | 0 | 0 | 0 | 0 | 0 | 2 | 0 | 2 | 0 |
| NM_000682 | ADRA2B | 3266 | 0 | 0 | 0 | 0 | 0 | 0 | 0 | 0 | 0 | 0 | 0 | 0 | 0 |
| NM_000683 | ADRA2C | 1958 | 1 | 0 | 0 | 0 | 0 | 0 | 0 | 0 | 0 | 0 | 0 | 1 | 0 |
| NM_000684 | ADRB1 | 2862 | 7 | 2 | 2 | 1 | 7 | 3 | 0 | 0 | 3 | 0 | 0 | 1 | 0 |
| NM_000685 | AGTR1 | 2356 | 5 | 1 | 2 | 0 | 1 | 0 | 1 | 5 | 1 | 0 | 1 | 1 | 0 |
| NM_000686 | AGTR2 | 2899 | 2 | 0 | 0 | 0 | 0 | 0 | 0 | 0 | 2 | 0 | 0 | 0 | 0 |
| NM_000687 | AHCY | 2207 | 48 | 17 | 11 | 10 | 12 | 13 | 25 | 48 | 8 | 17 | 11 | 12 | 16 |
| NM_000688 | ALAS1 | 2407 | 25 | 5 | 4 | 2 | 6 | 5 | 5 | 25 | 3 | 2 | 3 | 3 | 3 |
| NM_000689 | ALDH1A1 | 2096 | 63 | 25 | 14 | 11 | 4 | 10 | 43 | 63 | 22 | 27 | 8 | 14 | 60 |
| NM_000690 | ALDH2 | 2400 | 78 | 19 | 32 | 9 | 12 | 16 | 21 | 78 | 12 | 6 | 7 | 5 | 6 |
| NM_000691 | ALDH3A1 | 1776 | 2 | 0 | 0 | 1 | 0 | 0 | 0 | 0 | 2 | 0 | 0 | 0 | 0 |
| NM_000692 | ALDH1B1 | 3024 | 8 | 2 | 1 | 2 | 2 | 1 | 8 | 6 | 0 | 1 | 1 | 1 | 2 |
| NM_000693 | ALDH1A3 | 3495 | 7 | 2 | 7 | 2 | 2 | 1 | 2 | 0 | 2 | 1 | 1 | 1 | 7 |
| NM_000694 | ALDH3B1 | 2815 | 2 | 1 | 1 | 0 | 1 | 0 | 1 | 0 | 2 | 0 | 0 | 2 | 0 |
| NM_000695 | ALDH3B2 | 2650 | 0 | 0 | 0 | 0 | 0 | 0 | 0 | 0 | 0 | 0 | 0 | 0 | 0 |
| NM_000696 | ALDH9A1 | 2500 | 49 | 26 | 38 | 9 | 21 | 19 | 44 | 29 | 18 | 19 | 15 | 25 | 49 |
| NM_000697 | ALOX12 | 2358 | 1 | 0 | 0 | 0 | 0 | 0 | 0 | 0 | 0 | 0 | 0 | 0 | 1 |
| NM_000698 | ALOX5 | 2552 | 15 | 4 | 2 | 1 | 5 | 3 | 1 | 1 | 9 | 0 | 0 | 15 | 1 |
| NM_000699 | AMY2A | 1632 | 1 | 0 | 1 | 0 | 0 | 0 | 0 | 0 | 0 | 0 | 0 | 0 | 0 |
| NM_000700 | ANXA1 | 1399 | 553 | 88 | 553 | 21 | 80 | 25 | 18 | 5 | 136 | 35 | 19 | 48 | 27 |
| NM_000701 | ATP1A1 | 3739 | 109 | 30 | 8 | 36 | 31 | 43 | 109 | 24 | 25 | 11 | 3 | 16 | 22 |
| NM_000702 | ATP1A2 | 5455 | 101 | 20 | 9 | 7 | 16 | 77 | 0 | 0 | 1 | 2 | 101 | 0 | 3 |
| NM_000704 | ATP4A | 3559 | 0 | 0 | 0 | 0 | 0 | 0 | 0 | 0 | 0 | 0 | 0 | 0 | 0 |
| NM_000705 | ATP4B | 1485 | 0 | 0 | 0 | 0 | 0 | 0 | 0 | 0 | 0 | 0 | 0 | 0 | 0 |
| NM_000706 | AVPR1A | 4154 | 3 | 1 | 2 | 0 | 0 | 0 | 1 | 3 | 0 | 0 | 0 | 0 | 0 |
| NM_000707 | AVPR1B | 1773 | 0 | 0 | 0 | 0 | 0 | 0 | 0 | 0 | 0 | 0 | 0 | 0 | 0 |
| NM_000709 | BCKDHA | 1773 | 1 | 1 | 1 | 1 | 1 | 1 | 1 | 1 | 0 | 0 | 1 | 1 | 1 |
| NM_000710 | BDKRB1 | 1289 | 0 | 0 | 0 | 0 | 0 | 0 | 0 | 0 | 0 | 0 | 0 | 0 | 0 |
| NM_000712 | BLVRA | 1081 | 12 | 4 | 4 | 1 | 5 | 3 | 5 | 1 | 7 | 4 | 3 | 12 | 4 |
| NM_000713 | BLVRB | 856 | 5 | 2 | 4 | 1 | 2 | 1 | 2 | 5 | 3 | 1 | 3 | 4 | 1 |
| NM_000714 | TSPO | 866 | 0 | 0 | 0 | 0 | 0 | 0 | 0 | 0 | 0 | 0 | 0 | 0 | 0 |
| NM_000715 | C4BPA | 2243 | 106 | 11 | 0 | 0 | 0 | 0 | 0 | 106 | 10 | 1 | 1 | 1 | 0 |
| NM_000716 | C4BPB | 1115 | 51 | 5 | 0 | 0 | 0 | 0 | 0 | 51 | 0 | 3 | 0 | 0 | 0 |
| NM_000717 | CA4 | 1154 | 1 | 0 | 1 | 1 | 0 | 0 | 1 | 0 | 1 | 0 | 0 | 0 | 0 |
| NM_000718 | CACNA1B | 9789 | 2 | 0 | 0 | 0 | 0 | 2 | 0 | 0 | 0 | 0 | 0 | 0 | 0 |
| NM_000719 | CACNA1C | 13480 | 3 | 1 | 0 | 2 | 3 | 2 | 0 | 0 | 1 | 0 | 0 | 1 | 0 |
| NM_000720 | CACNA1D | 7768 | 1 | 1 | 0 | 0 | 0 | 1 | 1 | 0 | 1 | 0 | 0 | 1 | 1 |
| NM_000721 | CACNA1E | 9723 | 5 | 1 | 0 | 0 | 0 | 5 | 0 | 0 | 0 | 0 | 0 | 0 | 0 |
| NM_000722 | CACNA2D1 | 3822 | 24 | 9 | 17 | 2 | 13 | 22 | 4 | 0 | 2 | 6 | 24 | 4 | 5 |
| NM_000723 | CACNB1 | 3687 | 1 | 0 | 0 | 0 | 0 | 1 | 0 | 0 | 0 | 0 | 1 | 0 | 0 |
| NM_000724 | CACNB2 | 4468 | 5 | 2 | 0 | 2 | 4 | 5 | 1 | 0 | 1 | 3 | 0 | 1 | 1 |
| NM_000725 | CACNB3 | 2698 | 2 | 0 | 0 | 1 | 0 | 2 | 0 | 0 | 0 | 1 | 0 | 0 | 0 |
| NM_000726 | CACNB4 | 7976 | 4 | 1 | 0 | 0 | 0 | 4 | 0 | 0 | 0 | 0 | 0 | 0 | 1 |
| NM_000727 | CACNG1 | 1264 | 1 | 0 | 0 | 0 | 0 | 0 | 0 | 0 | 0 | 0 | 1 | 0 | 0 |
| NM_000728 | CALCB | 1031 | 0 | 0 | 0 | 0 | 0 | 0 | 0 | 0 | 0 | 0 | 0 | 0 | 0 |
| NM_000729 | CCK | 829 | 4 | 0 | 0 | 0 | 0 | 4 | 0 | 0 | 0 | 0 | 0 | 0 | 0 |
| NM_000730 | CCKAR | 1682 | 0 | 0 | 0 | 0 | 0 | 0 | 0 | 0 | 0 | 0 | 0 | 0 | 0 |
| NM_000732 | CD3D | 741 | 16 | 3 | 1 | 4 | 1 | 0 | 0 | 0 | 3 | 0 | 1 | 16 | 0 |
| NM_000733 | CD3E | 1513 | 8 | 1 | 0 | 2 | 0 | 0 | 0 | 0 | 3 | 0 | 0 | 8 | 0 |
| NM_000734 | CD247 | 1678 | 2 | 0 | 0 | 0 | 0 | 0 | 0 | 0 | 1 | 0 | 0 | 2 | 0 |
| NM_000735 | CGA | 704 | 0 | 0 | 0 | 0 | 0 | 0 | 0 | 0 | 0 | 0 | 0 | 0 | 0 |
| NM_000737 | CGB | 879 | 0 | 0 | 0 | 0 | 0 | 0 | 0 | 0 | 0 | 0 | 0 | 0 | 0 |
| NM_000738 | CHRM1 | 2823 | 2 | 0 | 0 | 0 | 0 | 2 | 0 | 0 | 0 | 0 | 0 | 0 | 0 |
| NM_000739 | CHRM2 | 2530 | 6 | 1 | 0 | 1 | 6 | 0 | 0 | 0 | 0 | 0 | 0 | 0 | 0 |
| NM_000740 | CHRM3 | 2745 | 3 | 1 | 0 | 1 | 0 | 3 | 0 | 0 | 0 | 1 | 0 | 0 | 0 |
| NM_000741 | CHRM4 | 1468 | 1 | 0 | 0 | 0 | 0 | 1 | 0 | 0 | 0 | 0 | 0 | 0 | 0 |
| NM_000742 | CHRNA2 | 4067 | 0 | 0 | 0 | 0 | 0 | 0 | 0 | 0 | 0 | 0 | 0 | 0 | 0 |
| NM_000743 | CHRNA3 | 3318 | 1 | 0 | 0 | 1 | 0 | 0 | 0 | 0 | 0 | 0 | 0 | 0 | 0 |
| NM_000744 | CHRNA4 | 5487 | 0 | 0 | 0 | 0 | 0 | 0 | 0 | 0 | 0 | 0 | 0 | 0 | 0 |
| NM_000745 | CHRNA5 | 2428 | 0 | 0 | 0 | 0 | 0 | 0 | 0 | 0 | 0 | 0 | 0 | 0 | 0 |
| NM_000746 | CHRNA7 | 2157 | 0 | 0 | 0 | 0 | 0 | 0 | 0 | 0 | 0 | 0 | 0 | 0 | 0 |
| NM_000747 | CHRNB1 | 2437 | 9 | 2 | 1 | 0 | 1 | 1 | 1 | 1 | 0 | 1 | 9 | 1 | 1 |

| transcript | gene | transcript length (nt) | max | mean | adipose | colon | heart | hypothalamus | kidney | liver | lung | ovary | skeletalmuscle | spleen | testes |
|---|---|---|---|---|---|---|---|---|---|---|---|---|---|---|---|
| NM_000748 | CHRNB2 | 5718 | 3 | 0 | 0 | 0 | 0 | 3 | 0 | 0 | 0 | 0 | 0 | 0 | 0 |
| NM_000749 | CHRNB3 | 1953 | 0 | 0 | 0 | 0 | 0 | 0 | 0 | 0 | 0 | 0 | 0 | 0 | 0 |
| NM_000750 | CHRNB4 | 2448 | 0 | 0 | 0 | 0 | 0 | 0 | 0 | 0 | 0 | 0 | 0 | 0 | 0 |
| NM_000751 | CHRND | 1741 | 3 | 0 | 0 | 0 | 0 | 0 | 0 | 0 | 0 | 0 | 3 | 0 | 0 |
| NM_000754 | COMT | 2302 | 13 | 6 | 4 | 2 | 7 | 8 | 6 | 13 | 5 | 5 | 5 | 6 | 4 |
| NM_000755 | CRAT | 2764 | 6 | 2 | 1 | 1 | 1 | 1 | 2 | 6 | 0 | 1 | 2 | 1 | 2 |
| NM_000756 | CRH | 1279 | 1 | 0 | 0 | 0 | 0 | 1 | 0 | 0 | 0 | 0 | 0 | 0 | 0 |
| NM_000757 | CSF1 | 2973 | 3 | 2 | 3 | 1 | 2 | 2 | 1 | 1 | 3 | 1 | 1 | 3 | 1 |
| NM_000758 | CSF2 | 781 | 0 | 0 | 0 | 0 | 0 | 0 | 0 | 0 | 0 | 0 | 0 | 0 | 0 |
| NM_000759 | CSF3 | 1518 | 1 | 0 | 0 | 0 | 0 | 0 | 0 | 0 | 1 | 0 | 0 | 0 | 0 |
| NM_000760 | CSF3R | 3003 | 3 | 1 | 0 | 0 | 0 | 0 | 0 | 0 | 3 | 0 | 0 | 3 | 0 |
| NM_000761 | CYP1A2 | 3127 | 2 | 0 | 0 | 0 | 0 | 0 | 0 | 2 | 0 | 0 | 0 | 0 | 0 |
| NM_000762 | CYP2A6 | 1764 | 16 | 2 | 0 | 0 | 0 | 0 | 0 | 16 | 0 | 0 | 0 | 0 | 0 |
| NM_000764 | CYP2A7 | 2281 | 6 | 1 | 0 | 0 | 0 | 0 | 0 | 6 | 0 | 0 | 0 | 0 | 0 |
| NM_000765 | CYP3A7 | 2076 | 2 | 0 | 0 | 0 | 0 | 0 | 0 | 2 | 0 | 0 | 0 | 0 | 0 |
| NM_000766 | CYP2A13 | 1747 | 0 | 0 | 0 | 0 | 0 | 0 | 0 | 0 | 0 | 0 | 0 | 0 | 0 |
| NM_000767 | CYP2B6 | 3052 | 28 | 3 | 0 | 0 | 0 | 0 | 1 | 28 | 0 | 0 | 0 | 0 | 0 |
| NM_000769 | CYP2C19 | 1473 | 6 | 1 | 0 | 1 | 0 | 0 | 0 | 6 | 0 | 0 | 0 | 0 | 0 |
| NM_000770 | CYP2C8 | 1924 | 122 | 11 | 0 | 0 | 0 | 0 | 0 | 122 | 0 | 0 | 1 | 1 | 0 |
| NM_000771 | CYP2C9 | 1861 | 116 | 11 | 0 | 0 | 0 | 0 | 1 | 116 | 0 | 0 | 0 | 0 | 1 |
| NM_000772 | CYP2C18 | 2545 | 23 | 2 | 0 | 1 | 0 | 0 | 0 | 23 | 0 | 0 | 0 | 0 | 0 |
| NM_000773 | CYP2E1 | 1667 | 671 | 63 | 0 | 1 | 2 | 0 | 1 | 671 | 1 | 3 | 3 | 4 | 3 |
| NM_000774 | CYP2F1 | 1826 | 0 | 0 | 0 | 0 | 0 | 0 | 0 | 0 | 0 | 0 | 0 | 0 | 0 |
| NM_000775 | CYP2J2 | 1857 | 8 | 2 | 0 | 1 | 6 | 2 | 0 | 8 | 0 | 0 | 1 | 0 | 0 |
| NM_000777 | CYP3A5 | 1707 | 11 | 2 | 0 | 3 | 0 | 0 | 1 | 11 | 1 | 1 | 0 | 0 | 0 |
| NM_000778 | CYP4A11 | 2552 | 35 | 4 | 0 | 0 | 0 | 0 | 35 | 10 | 1 | 0 | 0 | 0 | 0 |
| NM_000779 | CYP4B1 | 2155 | 4 | 1 | 1 | 0 | 0 | 0 | 0 | 0 | 4 | 0 | 0 | 0 | 0 |
| NM_000780 | CYP7A1 | 2875 | 5 | 0 | 0 | 0 | 0 | 0 | 0 | 5 | 0 | 0 | 0 | 0 | 0 |
| NM_000781 | CYP11A1 | 1931 | 12 | 2 | 0 | 0 | 0 | 0 | 0 | 0 | 0 | 12 | 0 | 3 | 5 |
| NM_000782 | CYP24A1 | 3266 | 7 | 1 | 0 | 0 | 0 | 0 | 7 | 0 | 0 | 0 | 0 | 0 | 0 |
| NM_000783 | CYP26A1 | 2119 | 1 | 0 | 0 | 0 | 0 | 1 | 0 | 0 | 0 | 0 | 0 | 0 | 0 |
| NM_000784 | CYP27A1 | 2293 | 3 | 1 | 1 | 0 | 0 | 0 | 1 | 3 | 1 | 0 | 0 | 0 | 0 |
| NM_000785 | CYP27B1 | 2487 | 2 | 0 | 0 | 0 | 0 | 0 | 2 | 0 | 0 | 0 | 0 | 0 | 0 |
| NM_000786 | CYP51A1 | 3208 | 38 | 16 | 6 | 6 | 11 | 26 | 17 | 28 | 16 | 17 | 4 | 9 | 38 |
| NM_000787 | DBH | 2760 | 0 | 0 | 0 | 0 | 0 | 0 | 0 | 0 | 0 | 0 | 0 | 0 | 0 |
| NM_000788 | DCK | 2605 | 8 | 2 | 2 | 2 | 2 | 5 | 1 | 1 | 3 | 2 | 1 | 8 | 2 |
| NM_000789 | ACE | 4195 | 2 | 1 | 1 | 1 | 0 | 0 | 2 | 0 | 1 | 0 | 0 | 0 | 2 |
| NM_000790 | DDC | 1955 | 17 | 2 | 0 | 2 | 0 | 0 | 17 | 2 | 0 | 0 | 0 | 0 | 0 |
| NM_000791 | DHFR | 3917 | 5 | 2 | 5 | 2 | 1 | 2 | 3 | 2 | 2 | 1 | 1 | 3 | 2 |
| NM_000792 | DIO1 | 1860 | 12 | 2 | 0 | 0 | 0 | 0 | 12 | 10 | 0 | 1 | 0 | 0 | 0 |
| NM_000793 | DIO2 | 6387 | 8 | 1 | 0 | 0 | 3 | 8 | 1 | 0 | 0 | 1 | 1 | 0 | 1 |
| NM_000794 | DRD1 | 3373 | 2 | 0 | 0 | 0 | 0 | 2 | 0 | 0 | 0 | 0 | 0 | 0 | 0 |
| NM_000795 | DRD2 | 2699 | 0 | 0 | 0 | 0 | 0 | 0 | 0 | 0 | 0 | 0 | 0 | 0 | 0 |
| NM_000796 | DRD3 | 1640 | 0 | 0 | 0 | 0 | 0 | 0 | 0 | 0 | 0 | 0 | 0 | 0 | 0 |
| NM_000797 | DRD4 | 1360 | 0 | 0 | 0 | 0 | 0 | 0 | 0 | 0 | 0 | 0 | 0 | 0 | 0 |
| NM_000798 | DRD5 | 2376 | 0 | 0 | 0 | 0 | 0 | 0 | 0 | 0 | 0 | 0 | 0 | 0 | 0 |
| NM_000799 | EPO | 1330 | 0 | 0 | 0 | 0 | 0 | 0 | 0 | 0 | 0 | 0 | 0 | 0 | 0 |
| NM_000800 | FGF1 | 3746 | 44 | 7 | 6 | 1 | 7 | 44 | 16 | 0 | 1 | 0 | 1 | 1 | 1 |
| NM_000801 | FKBP1A | 1631 | 35 | 14 | 12 | 7 | 21 | 16 | 13 | 7 | 18 | 13 | 7 | 35 | 10 |
| NM_000802 | FOLR1 | 944 | 23 | 4 | 0 | 0 | 0 | 8 | 23 | 0 | 15 | 0 | 0 | 0 | 0 |
| NM_000803 | FOLR2 | 1131 | 3 | 1 | 3 | 0 | 2 | 0 | 0 | 0 | 1 | 0 | 0 | 1 | 1 |
| NM_000804 | FOLR3 | 845 | 1 | 0 | 0 | 0 | 0 | 0 | 1 | 0 | 0 | 0 | 0 | 0 | 0 |
| NM_000805 | GAST | 435 | 0 | 0 | 0 | 0 | 0 | 0 | 0 | 0 | 0 | 0 | 0 | 0 | 0 |
| NM_000806 | GABRA1 | 4376 | 10 | 1 | 0 | 0 | 0 | 10 | 0 | 0 | 0 | 0 | 0 | 0 | 0 |
| NM_000807 | GABRA2 | 2414 | 9 | 1 | 0 | 1 | 0 | 9 | 1 | 0 | 0 | 0 | 0 | 0 | 0 |
| NM_000808 | GABRA3 | 2785 | 5 | 0 | 0 | 0 | 0 | 5 | 0 | 0 | 0 | 0 | 0 | 0 | 0 |
| NM_000809 | GABRA4 | 11129 | 4 | 0 | 0 | 0 | 1 | 4 | 0 | 0 | 0 | 0 | 0 | 0 | 0 |
| NM_000810 | GABRA5 | 2897 | 14 | 1 | 0 | 0 | 0 | 14 | 0 | 0 | 0 | 0 | 0 | 0 | 0 |
| NM_000811 | GABRA6 | 2519 | 0 | 0 | 0 | 0 | 0 | 0 | 0 | 0 | 0 | 0 | 0 | 0 | 0 |
| NM_000812 | GABRB1 | 2211 | 11 | 1 | 0 | 0 | 1 | 11 | 0 | 0 | 0 | 0 | 0 | 0 | 0 |
| NM_000813 | GABRB2 | 7295 | 20 | 2 | 1 | 0 | 0 | 20 | 1 | 0 | 0 | 0 | 0 | 3 | 0 |
| NM_000814 | GABRB3 | 5810 | 9 | 1 | 0 | 0 | 0 | 9 | 1 | 0 | 0 | 0 | 0 | 0 | 0 |
| NM_000815 | GABRD | 1925 | 1 | 0 | 0 | 0 | 0 | 1 | 0 | 0 | 0 | 0 | 0 | 0 | 0 |
| NM_000816 | GABRG2 | 3933 | 19 | 2 | 0 | 0 | 0 | 19 | 0 | 0 | 0 | 0 | 0 | 0 | 0 |
| NM_000817 | GAD1 | 3474 | 15 | 1 | 0 | 0 | 0 | 15 | 1 | 0 | 0 | 0 | 0 | 0 | 0 |
| NM_000818 | GAD2 | 2824 | 5 | 0 | 0 | 0 | 0 | 5 | 0 | 0 | 0 | 0 | 0 | 0 | 0 |
| NM_000819 | GART | 3360 | 7 | 4 | 3 | 2 | 4 | 4 | 3 | 7 | 2 | 2 | 3 | 5 | 4 |
| NM_000820 | GAS6 | 2503 | 6 | 3 | 1 | 2 | 6 | 2 | 2 | 2 | 3 | 2 | 3 | 1 | 5 |
| NM_000821 | GGCX | 3247 | 7 | 2 | 1 | 1 | 1 | 1 | 2 | 7 | 1 | 1 | 1 | 1 | 1 |
| NM_000823 | GHRHR | 1604 | 0 | 0 | 0 | 0 | 0 | 0 | 0 | 0 | 0 | 0 | 0 | 0 | 0 |
| NM_000824 | GLRB | 3058 | 10 | 1 | 1 | 0 | 1 | 10 | 0 | 0 | 0 | 0 | 0 | 0 | 1 |
| NM_000825 | GNRH1 | 2150 | 1 | 0 | 0 | 1 | 0 | 0 | 0 | 0 | 1 | 1 | 0 | 1 | 1 |
| NM_000826 | GRIA2 | 5754 | 43 | 4 | 0 | 0 | 0 | 43 | 0 | 0 | 0 | 0 | 0 | 0 | 0 |
| NM_000827 | GRIA1 | 5730 | 12 | 1 | 0 | 0 | 0 | 12 | 0 | 0 | 1 | 0 | 0 | 0 | 0 |
| NM_000828 | GRIA3 | 5182 | 17 | 2 | 0 | 0 | 0 | 17 | 1 | 0 | 0 | 1 | 0 | 0 | 1 |
| NM_000829 | GRIA4 | 5508 | 6 | 1 | 0 | 0 | 0 | 6 | 0 | 0 | 0 | 0 | 0 | 0 | 0 |
| NM_000830 | GRIK1 | 3231 | 2 | 0 | 0 | 0 | 0 | 2 | 0 | 0 | 0 | 0 | 0 | 0 | 0 |

| transcript | gene | transcript length (nt) | max | mean | adipose | colon | heart | hypothalamus | kidney | liver | lung | ovary | skeletalmuscle | spleen | testes |
|---|---|---|---|---|---|---|---|---|---|---|---|---|---|---|---|
| NM_000831 | GRIK3 | 9219 | 4 | 0 | 0 | 0 | 0 | 4 | 0 | 0 | 0 | 0 | 0 | 0 | 0 |
| NM_000832 | GRIN1 | 3902 | 6 | 1 | 0 | 0 | 0 | 6 | 0 | 0 | 0 | 0 | 0 | 0 | 0 |
| NM_000833 | GRIN2A | 14444 | 9 | 1 | 0 | 0 | 0 | 9 | 0 | 0 | 0 | 0 | 0 | 0 | 0 |
| NM_000834 | GRIN2B | 5941 | 10 | 1 | 0 | 0 | 0 | 10 | 0 | 0 | 0 | 0 | 0 | 0 | 0 |
| NM_000835 | GRIN2C | 4264 | 0 | 0 | 0 | 0 | 0 | 0 | 0 | 0 | 0 | 0 | 0 | 0 | 0 |
| NM_000836 | GRIN2D | 5093 | 0 | 0 | 0 | 0 | 0 | 0 | 0 | 0 | 0 | 0 | 0 | 0 | 0 |
| NM_000837 | GRINA | 1968 | 9 | 5 | 4 | 2 | 4 | 8 | 6 | 9 | 4 | 3 | 2 | 9 | 3 |
| NM_000838 | GRM1 | 6854 | 2 | 0 | 0 | 0 | 0 | 2 | 0 | 0 | 0 | 0 | 0 | 0 | 0 |
| NM_000839 | GRM2 | 3355 | 0 | 0 | 0 | 0 | 0 | 0 | 0 | 0 | 0 | 0 | 0 | 0 | 0 |
| NM_000840 | GRM3 | 4260 | 4 | 0 | 0 | 0 | 0 | 4 | 0 | 0 | 0 | 0 | 0 | 0 | 0 |
| NM_000841 | GRM4 | 3879 | 0 | 0 | 0 | 0 | 0 | 0 | 0 | 0 | 0 | 0 | 0 | 0 | 0 |
| NM_000842 | GRM5 | 7926 | 5 | 1 | 0 | 0 | 0 | 5 | 0 | 0 | 0 | 0 | 0 | 0 | 0 |
| NM_000843 | GRM6 | 6141 | 0 | 0 | 0 | 0 | 0 | 0 | 0 | 0 | 0 | 0 | 0 | 0 | 0 |
| NM_000844 | GRM7 | 4147 | 2 | 0 | 0 | 0 | 0 | 2 | 0 | 0 | 0 | 0 | 0 | 0 | 0 |
| NM_000845 | GRM8 | 3559 | 3 | 0 | 0 | 0 | 0 | 3 | 0 | 1 | 0 | 0 | 0 | 0 | 0 |
| NM_000846 | GSTA2 | 1302 | 14 | 2 | 0 | 0 | 0 | 0 | 14 | 5 | 1 | 0 | 0 | 0 | 5 |
| NM_000847 | GSTA3 | 911 | 1 | 0 | 0 | 0 | 0 | 0 | 0 | 0 | 0 | 0 | 0 | 0 | 1 |
| NM_000848 | GSTM2 | 1200 | 2 | 1 | 1 | 1 | 0 | 2 | 0 | 0 | 0 | 1 | 1 | 0 | 0 |
| NM_000849 | GSTM3 | 4127 | 16 | 5 | 2 | 2 | 3 | 8 | 10 | 2 | 3 | 10 | 1 | 2 | 16 |
| NM_000850 | GSTM4 | 1425 | 1 | 1 | 1 | 1 | 1 | 1 | 1 | 1 | 0 | 1 | 1 | 1 | 1 |
| NM_000851 | GSTM5 | 1568 | 6 | 1 | 1 | 0 | 0 | 1 | 0 | 0 | 1 | 6 | 0 | 1 | 1 |
| NM_000852 | GSTP1 | 958 | 11 | 6 | 6 | 6 | 8 | 6 | 10 | 1 | 11 | 3 | 3 | 7 | 3 |
| NM_000853 | GSTT1 | 1060 | 1 | 1 | 0 | 1 | 0 | 0 | 1 | 1 | 1 | 0 | 1 | 0 | 1 |
| NM_000854 | GSTT2 | 1119 | 0 | 0 | 0 | 0 | 0 | 0 | 0 | 0 | 0 | 0 | 0 | 0 | 0 |
| NM_000855 | GUCY1A2 | 2954 | 4 | 2 | 1 | 0 | 2 | 4 | 1 | 0 | 2 | 1 | 1 | 2 | 2 |
| NM_000856 | GUCY1A3 | 4727 | 5 | 3 | 2 | 3 | 5 | 5 | 4 | 1 | 3 | 1 | 1 | 4 | 1 |
| NM_000857 | GUCY1B3 | 3248 | 26 | 5 | 2 | 2 | 4 | 26 | 4 | 1 | 4 | 2 | 2 | 6 | 2 |
| NM_000858 | GUK1 | 1152 | 3 | 2 | 1 | 1 | 1 | 3 | 2 | 3 | 2 | 1 | 0 | 2 | 1 |
| NM_000859 | HMGCR | 4580 | 17 | 6 | 2 | 4 | 3 | 17 | 6 | 5 | 5 | 6 | 1 | 6 | 8 |
| NM_000860 | HPGD | 2770 | 51 | 7 | 1 | 4 | 1 | 0 | 7 | 51 | 11 | 0 | 1 | 3 | 2 |
| NM_000861 | HRH1 | 4407 | 1 | 1 | 1 | 1 | 0 | 1 | 0 | 0 | 1 | 1 | 0 | 0 | 1 |
| NM_000862 | HSD3B1 | 1680 | 0 | 0 | 0 | 0 | 0 | 0 | 0 | 0 | 0 | 0 | 0 | 0 | 0 |
| NM_000863 | HTR1B | 1173 | 0 | 0 | 0 | 0 | 0 | 0 | 0 | 0 | 0 | 0 | 0 | 0 | 0 |
| NM_000864 | HTR1D | 2835 | 0 | 0 | 0 | 0 | 0 | 0 | 0 | 0 | 0 | 0 | 0 | 0 | 0 |
| NM_000865 | HTR1E | 2041 | 1 | 0 | 0 | 0 | 0 | 1 | 0 | 0 | 0 | 1 | 0 | 0 | 0 |
| NM_000866 | HTR1F | 3178 | 1 | 0 | 0 | 0 | 0 | 0 | 0 | 0 | 0 | 0 | 0 | 1 | 0 |
| NM_000867 | HTR2B | 2233 | 1 | 1 | 1 | 1 | 1 | 0 | 1 | 1 | 0 | 1 | 0 | 1 | 1 |
| NM_000868 | HTR2C | 4751 | 5 | 0 | 0 | 0 | 0 | 5 | 0 | 0 | 0 | 0 | 0 | 0 | 0 |
| NM_000869 | HTR3A | 2235 | 0 | 0 | 0 | 0 | 0 | 0 | 0 | 0 | 0 | 0 | 0 | 0 | 0 |
| NM_000870 | HTR4 | 2978 | 0 | 0 | 0 | 0 | 0 | 0 | 0 | 0 | 0 | 0 | 0 | 0 | 0 |
| NM_000871 | HTR6 | 1984 | 0 | 0 | 0 | 0 | 0 | 0 | 0 | 0 | 0 | 0 | 0 | 0 | 0 |
| NM_000872 | HTR7 | 3246 | 1 | 0 | 0 | 0 | 0 | 1 | 0 | 0 | 0 | 0 | 0 | 0 | 1 |
| NM_000873 | ICAM2 | 1212 | 3 | 1 | 2 | 1 | 1 | 1 | 0 | 1 | 1 | 0 | 0 | 3 | 0 |
| NM_000874 | IFNAR2 | 1411 | 7 | 2 | 2 | 1 | 2 | 1 | 1 | 2 | 4 | 2 | 1 | 7 | 1 |
| NM_000875 | IGF1R | 11242 | 6 | 3 | 1 | 3 | 3 | 3 | 5 | 0 | 2 | 6 | 2 | 2 | 4 |
| NM_000876 | IGF2R | 9091 | 19 | 10 | 10 | 6 | 9 | 4 | 11 | 10 | 9 | 7 | 16 | 19 | 10 |
| NM_000877 | IL1R1 | 4907 | 12 | 6 | 9 | 2 | 9 | 1 | 5 | 8 | 12 | 3 | 2 | 10 | 7 |
| NM_000878 | IL2RB | 4043 | 2 | 0 | 0 | 0 | 0 | 0 | 0 | 0 | 0 | 0 | 0 | 2 | 0 |
| NM_000879 | IL5 | 816 | 1 | 0 | 0 | 0 | 0 | 0 | 0 | 0 | 0 | 0 | 0 | 0 | 1 |
| NM_000880 | IL7 | 2078 | 2 | 1 | 1 | 1 | 1 | 1 | 1 | 2 | 2 | 1 | 0 | 2 | 1 |
| NM_000882 | IL12A | 1437 | 1 | 0 | 0 | 0 | 0 | 1 | 0 | 0 | 0 | 0 | 0 | 1 | 0 |
| NM_000883 | IMPDH1 | 2611 | 4 | 1 | 2 | 1 | 1 | 2 | 0 | 0 | 1 | 1 | 0 | 4 | 1 |
| NM_000884 | IMPDH2 | 1691 | 19 | 7 | 4 | 7 | 5 | 4 | 5 | 7 | 5 | 19 | 9 | 7 | 6 |
| NM_000885 | ITGA4 | 6076 | 17 | 2 | 1 | 2 | 1 | 0 | 1 | 0 | 3 | 1 | 0 | 17 | 0 |
| NM_000887 | ITGAX | 4649 | 3 | 1 | 3 | 0 | 0 | 0 | 0 | 0 | 3 | 0 | 0 | 3 | 0 |
| NM_000888 | ITGB6 | 2397 | 2 | 1 | 0 | 0 | 0 | 0 | 2 | 0 | 2 | 0 | 1 | 0 | 0 |
| NM_000889 | ITGB7 | 2771 | 1 | 0 | 0 | 0 | 0 | 0 | 0 | 0 | 0 | 0 | 0 | 1 | 0 |
| NM_000890 | KCNJ5 | 2899 | 1 | 1 | 1 | 0 | 1 | 0 | 0 | 0 | 0 | 0 | 0 | 1 | 0 |
| NM_000891 | KCNJ2 | 5386 | 5 | 2 | 1 | 1 | 3 | 2 | 0 | 1 | 2 | 0 | 4 | 5 | 1 |
| NM_000892 | KLKB1 | 2247 | 20 | 2 | 0 | 0 | 0 | 0 | 2 | 20 | 0 | 0 | 0 | 1 | 0 |
| NM_000893 | KNG1 | 2142 | 262 | 30 | 0 | 1 | 1 | 0 | 68 | 262 | 0 | 1 | 1 | 1 | 0 |
| NM_000894 | LHB | 523 | 0 | 0 | 0 | 0 | 0 | 0 | 0 | 0 | 0 | 0 | 0 | 0 | 0 |
| NM_000895 | LTA4H | 2060 | 43 | 20 | 15 | 10 | 14 | 14 | 15 | 13 | 43 | 35 | 12 | 31 | 15 |
| NM_000896 | CYP4F3 | 2988 | 22 | 3 | 0 | 0 | 0 | 0 | 7 | 22 | 1 | 0 | 0 | 1 | 0 |
| NM_000898 | MAOB | 2595 | 25 | 11 | 8 | 3 | 10 | 19 | 15 | 25 | 4 | 24 | 8 | 1 | 5 |
| NM_000899 | KITLG | 5434 | 8 | 3 | 2 | 1 | 2 | 3 | 6 | 0 | 8 | 2 | 1 | 1 | 5 |
| NM_000900 | MGP | 661 | 1,059 | 334 | 931 | 173 | 1,059 | 85 | 241 | 11 | 325 | 179 | 101 | 42 | 531 |
| NM_000901 | NR3C2 | 5897 | 6 | 3 | 1 | 6 | 2 | 4 | 4 | 2 | 3 | 5 | 2 | 2 | 2 |
| NM_000902 | MME | 5643 | 35 | 7 | 20 | 0 | 1 | 1 | 35 | 4 | 6 | 0 | 3 | 1 | 1 |
| NM_000903 | NQO1 | 2591 | 53 | 8 | 53 | 3 | 2 | 4 | 10 | 0 | 5 | 1 | 1 | 2 | 2 |
| NM_000904 | NQO2 | 1139 | 8 | 3 | 3 | 0 | 3 | 3 | 8 | 5 | 1 | 1 | 3 | 1 | 1 |
| NM_000905 | NPY | 560 | 5 | 1 | 0 | 0 | 0 | 5 | 1 | 0 | 0 | 0 | 0 | 0 | 0 |
| NM_000906 | NPR1 | 4185 | 6 | 2 | 6 | 1 | 2 | 0 | 3 | 0 | 3 | 2 | 0 | 0 | 1 |
| NM_000908 | NPR3 | 2644 | 14 | 4 | 12 | 1 | 4 | 1 | 14 | 1 | 6 | 2 | 0 | 1 | 1 |
| NM_000909 | NPY1R | 2759 | 11 | 2 | 5 | 0 | 1 | 1 | 5 | 1 | 1 | 0 | 0 | 11 | 1 |
| NM_000910 | NPY2R | 3626 | 3 | 0 | 0 | 0 | 0 | 3 | 0 | 0 | 0 | 0 | 0 | 0 | 1 |
| NM_000911 | OPRD1 | 1774 | 0 | 0 | 0 | 0 | 0 | 0 | 0 | 0 | 0 | 0 | 0 | 0 | 0 |

| transcript | gene | transcript length (nt) | max | mean | adipose | colon | heart | hypothalamus | kidney | liver | lung | ovary | skeletalmuscle | spleen | testes |
|---|---|---|---|---|---|---|---|---|---|---|---|---|---|---|---|
| NM_000912 | OPRK1 | 4959 | 1 | 0 | 0 | 0 | 0 | 1 | 0 | 0 | 0 | 0 | 0 | 0 | 0 |
| NM_000913 | OPRL1 | 3208 | 0 | 0 | 0 | 0 | 0 | 0 | 0 | 0 | 0 | 0 | 0 | 0 | 0 |
| NM_000914 | OPRM1 | 2178 | 1 | 0 | 0 | 0 | 0 | 1 | 0 | 0 | 0 | 0 | 0 | 0 | 0 |
| NM_000915 | OXT | 512 | 0 | 0 | 0 | 0 | 0 | 0 | 0 | 0 | 0 | 0 | 0 | 0 | 0 |
| NM_000916 | OXTR | 4360 | 1 | 0 | 0 | 0 | 0 | 0 | 0 | 0 | 1 | 1 | 0 | 0 | 0 |
| NM_000917 | P4HA1 | 2844 | 25 | 11 | 5 | 3 | 19 | 4 | 7 | 6 | 13 | 13 | 25 | 13 | 10 |
| NM_000918 | P4HB | 2578 | 287 | 53 | 33 | 25 | 45 | 13 | 33 | 287 | 28 | 18 | 21 | 61 | 14 |
| NM_000918 | P4HB | 2596 | 285 | 52 | 33 | 25 | 45 | 12 | 33 | 285 | 28 | 18 | 21 | 61 | 14 |
| NM_000919 | PAM | 3946 | 119 | 23 | 20 | 7 | 119 | 24 | 13 | 1 | 11 | 19 | 19 | 9 | 9 |
| NM_000920 | PC | 4004 | 7 | 1 | 2 | 0 | 0 | 1 | 2 | 7 | 0 | 0 | 0 | 0 | 0 |
| NM_000921 | PDE3A | 4124 | 15 | 2 | 1 | 3 | 15 | 1 | 2 | 0 | 1 | 1 | 1 | 1 | 1 |
| NM_000922 | PDE3B | 6088 | 22 | 5 | 22 | 2 | 2 | 1 | 2 | 8 | 4 | 0 | 0 | 7 | 5 |
| NM_000923 | PDE4C | 5346 | 0 | 0 | 0 | 0 | 0 | 0 | 0 | 0 | 0 | 0 | 0 | 0 | 0 |
| NM_000924 | PDE1B | 3401 | 4 | 1 | 2 | 0 | 1 | 4 | 0 | 0 | 0 | 2 | 0 | 2 | 0 |
| NM_000925 | PDHB | 1516 | 24 | 13 | 9 | 11 | 20 | 17 | 16 | 24 | 7 | 5 | 10 | 11 | 9 |
| NM_000926 | PGR | 13037 | 11 | 2 | 0 | 0 | 2 | 0 | 0 | 0 | 1 | 11 | 0 | 1 | 1 |
| NM_000927 | ABCB1 | 4872 | 11 | 3 | 0 | 2 | 1 | 6 | 11 | 10 | 1 | 1 | 0 | 2 | 1 |
| NM_000928 | PLA2G1B | 565 | 4 | 1 | 0 | 1 | 0 | 0 | 0 | 0 | 4 | 1 | 0 | 0 | 0 |
| NM_000929 | PLA2G5 | 1894 | 4 | 1 | 0 | 0 | 4 | 1 | 0 | 0 | 0 | 1 | 0 | 0 | 1 |
| NM_000930 | PLAT | 3173 | 8 | 3 | 1 | 3 | 3 | 1 | 8 | 0 | 3 | 4 | 1 | 0 | 5 |
| NM_000932 | PLCB3 | 3705 | 7 | 2 | 2 | 7 | 1 | 1 | 1 | 1 | 2 | 1 | 1 | 2 | 2 |
| NM_000933 | PLCB4 | 5527 | 3 | 1 | 1 | 1 | 3 | 3 | 1 | 0 | 2 | 1 | 1 | 0 | 3 |
| NM_000934 | SERPINF2 | 2284 | 95 | 10 | 0 | 0 | 1 | 0 | 10 | 95 | 1 | 1 | 0 | 1 | 2 |
| NM_000935 | PLOD2 | 3996 | 15 | 5 | 15 | 3 | 6 | 3 | 6 | 7 | 4 | 6 | 1 | 2 | 4 |
| NM_000936 | PNLIP | 1498 | 1 | 0 | 1 | 0 | 0 | 0 | 0 | 0 | 0 | 0 | 0 | 0 | 0 |
| NM_000937 | POLR2A | 6582 | 44 | 22 | 11 | 12 | 17 | 25 | 25 | 11 | 15 | 30 | 16 | 34 | 44 |
| NM_000938 | POLR2B | 3748 | 25 | 18 | 11 | 8 | 24 | 21 | 16 | 17 | 13 | 18 | 20 | 25 | 22 |
| NM_000939 | POMC | 1245 | 0 | 0 | 0 | 0 | 0 | 0 | 0 | 0 | 0 | 0 | 0 | 0 | 0 |
| NM_000940 | PON3 | 1191 | 58 | 6 | 0 | 0 | 0 | 0 | 0 | 58 | 2 | 2 | 0 | 0 | 2 |
| NM_000941 | POR | 2499 | 5 | 1 | 0 | 0 | 0 | 1 | 1 | 5 | 0 | 0 | 0 | 1 | 1 |
| NM_000942 | PPIB | 1028 | 105 | 37 | 25 | 19 | 41 | 19 | 41 | 105 | 31 | 39 | 7 | 54 | 29 |
| NM_000943 | PPIC | 1236 | 7 | 4 | 7 | 6 | 7 | 1 | 4 | 5 | 2 | 2 | 2 | 2 | 3 |
| NM_000944 | PPP3CA | 4676 | 97 | 21 | 16 | 5 | 12 | 97 | 7 | 10 | 14 | 17 | 22 | 17 | 17 |
| NM_000945 | PPP3R1 | 3011 | 56 | 15 | 11 | 5 | 8 | 56 | 8 | 9 | 13 | 11 | 12 | 17 | 12 |
| NM_000946 | PRIM1 | 1462 | 2 | 1 | 1 | 1 | 1 | 1 | 0 | 1 | 1 | 1 | 1 | 2 | 2 |
| NM_000947 | PRIM2 | 2282 | 1 | 0 | 0 | 0 | 0 | 0 | 0 | 0 | 0 | 1 | 0 | 1 | 1 |
| NM_000948 | PRL | 1356 | 0 | 0 | 0 | 0 | 0 | 0 | 0 | 0 | 0 | 0 | 0 | 0 | 0 |
| NM_000949 | PRLR | 11788 | 6 | 1 | 0 | 0 | 0 | 3 | 6 | 1 | 0 | 2 | 0 | 0 | 1 |
| NM_000950 | PRRG1 | 4583 | 7 | 2 | 2 | 1 | 3 | 7 | 3 | 1 | 2 | 1 | 3 | 2 | 1 |
| NM_000951 | PRRG2 | 1393 | 0 | 0 | 0 | 0 | 0 | 0 | 0 | 0 | 0 | 0 | 0 | 0 | 0 |
| NM_000952 | PTAFR | 4247 | 1 | 0 | 0 | 0 | 0 | 0 | 0 | 0 | 1 | 0 | 0 | 1 | 0 |
| NM_000953 | PTGDR | 2942 | 1 | 0 | 0 | 1 | 0 | 0 | 0 | 0 | 0 | 0 | 0 | 1 | 0 |
| NM_000954 | PTGDS | 812 | 68 | 18 | 1 | 4 | 46 | 61 | 3 | 0 | 9 | 1 | 1 | 9 | 68 |
| NM_000955 | PTGER1 | 1421 | 0 | 0 | 0 | 0 | 0 | 0 | 0 | 0 | 0 | 0 | 0 | 0 | 0 |
| NM_000956 | PTGER2 | 2478 | 2 | 0 | 0 | 0 | 0 | 0 | 0 | 0 | 0 | 1 | 0 | 2 | 0 |
| NM_000958 | PTGER4 | 3416 | 2 | 1 | 1 | 2 | 1 | 0 | 1 | 0 | 2 | 0 | 1 | 2 | 0 |
| NM_000959 | PTGFR | 5360 | 1 | 1 | 1 | 0 | 1 | 0 | 1 | 0 | 0 | 1 | 1 | 0 | 1 |
| NM_000960 | PTGIR | 2086 | 1 | 0 | 0 | 0 | 0 | 0 | 0 | 0 | 1 | 0 | 0 | 0 | 0 |
| NM_000961 | PTGIS | 5603 | 11 | 4 | 3 | 2 | 10 | 1 | 1 | 1 | 3 | 11 | 2 | 0 | 5 |
| NM_000962 | PTGS1 | 5093 | 5 | 1 | 1 | 5 | 2 | 1 | 1 | 0 | 1 | 0 | 0 | 1 | 1 |
| NM_000963 | PTGS2 | 4492 | 3 | 1 | 0 | 0 | 0 | 1 | 1 | 0 | 3 | 0 | 0 | 0 | 0 |
| NM_000964 | RARA | 3260 | 1 | 1 | 0 | 0 | 1 | 0 | 1 | 1 | 1 | 1 | 1 | 1 | 0 |
| NM_000965 | RARB | 3049 | 5 | 2 | 1 | 1 | 4 | 3 | 4 | 1 | 2 | 4 | 1 | 5 | 1 |
| NM_000966 | RARG | 2957 | 0 | 0 | 0 | 0 | 0 | 0 | 0 | 0 | 0 | 0 | 0 | 0 | 0 |
| NM_000967 | RPL3 | 1338 | 453 | 156 | 123 | 138 | 107 | 115 | 110 | 164 | 137 | 453 | 39 | 205 | 121 |
| NM_000968 | RPL4 | 1432 | 353 | 201 | 213 | 125 | 185 | 176 | 137 | 261 | 159 | 353 | 211 | 262 | 134 |
| NM_000969 | RPL5 | 1031 | 232 | 91 | 79 | 47 | 96 | 55 | 75 | 75 | 83 | 232 | 51 | 136 | 73 |
| NM_000970 | RPL6 | 954 | 134 | 65 | 50 | 23 | 77 | 43 | 53 | 81 | 56 | 134 | 59 | 104 | 39 |
| NM_000971 | RPL7 | 837 | 220 | 93 | 67 | 30 | 114 | 47 | 81 | 82 | 79 | 220 | 109 | 128 | 70 |
| NM_000972 | RPL7A | 890 | 111 | 47 | 25 | 35 | 48 | 23 | 37 | 35 | 42 | 111 | 48 | 78 | 35 |
| NM_000973 | RPL8 | 875 | 217 | 108 | 79 | 103 | 114 | 67 | 79 | 97 | 99 | 217 | 114 | 158 | 58 |
| NM_000975 | RPL11 | 609 | 138 | 67 | 63 | 48 | 60 | 35 | 48 | 83 | 78 | 138 | 49 | 94 | 42 |
| NM_000976 | RPL12 | 631 | 55 | 34 | 41 | 44 | 31 | 21 | 17 | 38 | 25 | 55 | 34 | 49 | 14 |
| NM_000977 | RPL13 | 1085 | 81 | 33 | 21 | 24 | 29 | 26 | 18 | 21 | 30 | 81 | 41 | 49 | 21 |
| NM_000978 | RPL23 | 577 | 104 | 46 | 52 | 31 | 44 | 32 | 33 | 49 | 41 | 104 | 44 | 56 | 21 |
| NM_000979 | RPL18 | 641 | 10 | 5 | 5 | 7 | 4 | 5 | 3 | 7 | 5 | 10 | 3 | 8 | 3 |
| NM_000980 | RPL18A | 618 | 0 | 0 | 0 | 0 | 0 | 0 | 0 | 0 | 0 | 0 | 0 | 0 | 0 |
| NM_000981 | RPL19 | 732 | 126 | 85 | 82 | 66 | 74 | 65 | 63 | 103 | 89 | 121 | 93 | 126 | 57 |
| NM_000982 | RPL21 | 566 | 0 | 0 | 0 | 0 | 0 | 0 | 0 | 0 | 0 | 0 | 0 | 0 | 0 |
| NM_000983 | RPL22 | 2085 | 162 | 63 | 46 | 25 | 63 | 30 | 45 | 50 | 59 | 162 | 74 | 94 | 50 |
| NM_000984 | RPL23A | 970 | 233 | 95 | 64 | 56 | 85 | 50 | 55 | 83 | 87 | 233 | 110 | 156 | 63 |
| NM_000985 | RPL17 | 929 | 87 | 51 | 80 | 20 | 59 | 61 | 45 | 87 | 36 | 73 | 19 | 50 | 31 |
| NM_000986 | RPL24 | 562 | 122 | 65 | 60 | 33 | 88 | 66 | 54 | 82 | 43 | 122 | 59 | 72 | 37 |
| NM_000987 | RPL26 | 582 | 343 | 188 | 178 | 95 | 216 | 139 | 148 | 185 | 193 | 343 | 190 | 273 | 108 |
| NM_000988 | RPL27 | 490 | 372 | 252 | 273 | 350 | 230 | 128 | 108 | 313 | 304 | 291 | 263 | 372 | 143 |
| NM_000989 | RPL30 | 505 | 542 | 242 | 227 | 146 | 222 | 128 | 122 | 278 | 205 | 542 | 277 | 358 | 160 |
| NM_000990 | RPL27A | 4879 | 13 | 7 | 6 | 5 | 6 | 5 | 5 | 9 | 7 | 12 | 6 | 13 | 4 |

| transcript | gene | transcript length (nt) | max | mean | adipose | colon | heart | hypothalamus | kidney | liver | lung | ovary | skeletalmuscle | spleen | testes |
|---|---|---|---|---|---|---|---|---|---|---|---|---|---|---|---|
| NM_000991 | RPL28 | 4205 | 14 | 7 | 3 | 7 | 6 | 4 | 4 | 8 | 7 | 14 | 5 | 14 | 3 |
| NM_000992 | RPL29 | 695 | 41 | 25 | 24 | 19 | 17 | 18 | 14 | 36 | 21 | 34 | 41 | 31 | 17 |
| NM_000993 | RPL31 | 507 | 381 | 202 | 218 | 126 | 181 | 231 | 123 | 252 | 143 | 381 | 169 | 283 | 116 |
| NM_000994 | RPL32 | 1651 | 41 | 23 | 20 | 14 | 23 | 18 | 16 | 24 | 18 | 41 | 32 | 37 | 14 |
| NM_000995 | RPL34 | 902 | 157 | 81 | 94 | 46 | 81 | 74 | 51 | 79 | 82 | 157 | 53 | 120 | 51 |
| NM_000996 | RPL35A | 483 | 243 | 123 | 183 | 114 | 97 | 165 | 48 | 156 | 62 | 243 | 72 | 124 | 87 |
| NM_000997 | RPL37 | 1570 | 40 | 21 | 25 | 18 | 18 | 14 | 14 | 23 | 18 | 40 | 21 | 31 | 11 |
| NM_000998 | RPL37A | 418 | 135 | 75 | 54 | 46 | 87 | 51 | 51 | 72 | 62 | 135 | 126 | 94 | 47 |
| NM_000999 | RPL38 | 392 | 137 | 75 | 56 | 40 | 73 | 66 | 40 | 82 | 53 | 137 | 126 | 89 | 56 |
| NM_001000 | RPL39 | 401 | 21 | 12 | 11 | 7 | 13 | 4 | 8 | 21 | 13 | 19 | 13 | 17 | 6 |
| NM_001001 | RPL36AL | 522 | 124 | 72 | 76 | 36 | 84 | 73 | 51 | 124 | 55 | 114 | 58 | 76 | 46 |
| NM_001001132 | ITSN1 | 5441 | 49 | 14 | 49 | 5 | 13 | 20 | 8 | 17 | 6 | 11 | 6 | 11 | 11 |
| NM_001001290 | SLC2A9 | 1868 | 3 | 1 | 0 | 0 | 0 | 0 | 3 | 1 | 1 | 0 | 0 | 0 | 0 |
| NM_001001317 | TRYX3 | 913 | 0 | 0 | 0 | 0 | 0 | 0 | 0 | 0 | 0 | 0 | 0 | 0 | 0 |
| NM_001001323 | ATP2B1 | 6933 | 37 | 11 | 4 | 4 | 6 | 37 | 9 | 4 | 10 | 6 | 8 | 17 | 19 |
| NM_001001325 | SPINK5L2 | 294 | 0 | 0 | 0 | 0 | 0 | 0 | 0 | 0 | 0 | 0 | 0 | 0 | 0 |
| NM_001001329 | PRKCSH | 2252 | 15 | 9 | 9 | 11 | 7 | 7 | 10 | 15 | 7 | 10 | 3 | 13 | 10 |
| NM_001001330 | REEP3 | 2294 | 6 | 4 | 4 | 2 | 4 | 4 | 4 | 6 | 4 | 2 | 2 | 4 | 4 |
| NM_001001331 | ATP2B2 | 8962 | 8 | 1 | 0 | 0 | 0 | 8 | 0 | 2 | 0 | 0 | 0 | 0 | 0 |
| NM_001001342 | BLOC1S2 | 2024 | 25 | 10 | 8 | 2 | 9 | 25 | 8 | 10 | 9 | 5 | 8 | 17 | 7 |
| NM_001001343 | C5orf40 | 2126 | 1 | 0 | 0 | 0 | 0 | 1 | 0 | 0 | 0 | 0 | 0 | 0 | 0 |
| NM_001001344 | ATP2B3 | 6420 | 3 | 0 | 0 | 0 | 0 | 3 | 0 | 0 | 0 | 0 | 0 | 0 | 0 |
| NM_001001346 | CLDN20 | 1206 | 3 | 2 | 1 | 2 | 2 | 1 | 3 | 1 | 1 | 2 | 1 | 3 | 2 |
| NM_001001349 | NKIRAS2 | 2384 | 3 | 2 | 1 | 1 | 3 | 1 | 1 | 1 | 2 | 1 | 2 | 2 | 3 |
| NM_001001389 | CD44 | 5603 | 30 | 12 | 22 | 10 | 10 | 15 | 3 | 4 | 24 | 4 | 5 | 30 | 6 |
| NM_001001390 | CD44 | 4985 | 33 | 13 | 25 | 11 | 12 | 17 | 3 | 4 | 25 | 4 | 5 | 33 | 7 |
| NM_001001391 | CD44 | 4589 | 35 | 14 | 27 | 10 | 12 | 18 | 3 | 4 | 25 | 5 | 6 | 35 | 7 |
| NM_001001392 | CD44 | 3969 | 25 | 9 | 17 | 6 | 9 | 11 | 3 | 3 | 19 | 3 | 3 | 25 | 5 |
| NM_001001394 | DNAJB3 | 1266 | 1 | 0 | 0 | 0 | 0 | 0 | 1 | 0 | 0 | 0 | 0 | 0 | 1 |
| NM_001001395 | LMO3 | 3450 | 15 | 2 | 2 | 1 | 1 | 15 | 0 | 0 | 4 | 0 | 0 | 0 | 1 |
| NM_001001396 | ATP2B4 | 8907 | 24 | 13 | 22 | 18 | 24 | 21 | 4 | 5 | 11 | 12 | 2 | 7 | 16 |
| NM_001001410 | C16orf42 | 1204 | 5 | 3 | 4 | 5 | 3 | 4 | 5 | 4 | 3 | 3 | 2 | 3 | 3 |
| NM_001001411 | ZNF676 | 2934 | 31 | 6 | 2 | 0 | 6 | 2 | 11 | 0 | 1 | 10 | 1 | 4 | 31 |
| NM_001001412 | CALHM1 | 3056 | 0 | 0 | 0 | 0 | 0 | 0 | 0 | 0 | 0 | 0 | 0 | 0 | 0 |
| NM_001001413 | GOLGA6L1 | 3822 | 7 | 1 | 0 | 0 | 0 | 0 | 0 | 0 | 0 | 0 | 0 | 0 | 7 |
| NM_001001414 | NCCRP1 | 1973 | 0 | 0 | 0 | 0 | 0 | 0 | 0 | 0 | 0 | 0 | 0 | 0 | 0 |
| NM_001001415 | ZNF429 | 2361 | 18 | 7 | 4 | 3 | 3 | 7 | 9 | 3 | 5 | 18 | 4 | 13 | 11 |
| NM_001001417 | TBC1D3B | 2114 | 0 | 0 | 0 | 0 | 0 | 0 | 0 | 0 | 0 | 0 | 0 | 0 | 0 |
| NM_001001418 | TBC1D3C | 2110 | 0 | 0 | 0 | 0 | 0 | 0 | 0 | 0 | 0 | 0 | 0 | 0 | 0 |
| NM_001001419 | SMAD5 | 7095 | 11 | 4 | 2 | 2 | 5 | 2 | 4 | 2 | 4 | 11 | 3 | 6 | 5 |
| NM_001001420 | SMAD5 | 6936 | 11 | 4 | 2 | 2 | 5 | 2 | 4 | 2 | 4 | 11 | 4 | 6 | 5 |
| NM_001001430 | TNNT2 | 1132 | 2,729 | 250 | 0 | 1 | 2,729 | 2 | 11 | 0 | 3 | 1 | 4 | 1 | 1 |
| NM_001001431 | TNNT2 | 1123 | 2,750 | 252 | 0 | 1 | 2,750 | 2 | 11 | 0 | 3 | 1 | 4 | 1 | 1 |
| NM_001001432 | TNNT2 | 1114 | 2,773 | 254 | 0 | 1 | 2,773 | 2 | 11 | 0 | 3 | 1 | 4 | 1 | 1 |
| NM_001001433 | STX16 | 4948 | 8 | 5 | 5 | 5 | 4 | 6 | 5 | 3 | 6 | 4 | 3 | 8 | 6 |
| NM_001001435 | CCL4L1 | 658 | 0 | 0 | 0 | 0 | 0 | 0 | 0 | 0 | 0 | 0 | 0 | 0 | 0 |
| NM_001001436 | C16orf87 | 1383 | 13 | 6 | 3 | 2 | 5 | 9 | 7 | 9 | 4 | 3 | 9 | 5 | 13 |
| NM_001001437 | CCL3L3 | 773 | 0 | 0 | 0 | 0 | 0 | 0 | 0 | 0 | 0 | 0 | 0 | 0 | 0 |
| NM_001001437 | CCL3L3 | 774 | 0 | 0 | 0 | 0 | 0 | 0 | 0 | 0 | 0 | 0 | 0 | 0 | 0 |
| NM_001001438 | LSS | 2691 | 1 | 1 | 1 | 1 | 1 | 1 | 1 | 1 | 1 | 1 | 1 | 0 | 1 |
| NM_001001479 | SLC35E4 | 2542 | 0 | 0 | 0 | 0 | 0 | 0 | 0 | 0 | 0 | 0 | 0 | 0 | 0 |
| NM_001001480 | KRTAP5-5 | 1127 | 0 | 0 | 0 | 0 | 0 | 0 | 0 | 0 | 0 | 0 | 0 | 0 | 0 |
| NM_001001481 | UBE2W | 4039 | 7 | 5 | 3 | 1 | 6 | 7 | 6 | 3 | 5 | 5 | 6 | 7 | 7 |
| NM_001001483 | ZDHHC13 | 2287 | 3 | 1 | 1 | 2 | 2 | 3 | 2 | 0 | 1 | 1 | 1 | 2 | 2 |
| NM_001001484 | PTER | 3793 | 3 | 1 | 1 | 0 | 1 | 3 | 3 | 1 | 1 | 0 | 0 | 2 | 1 |
| NM_001001485 | ATP2C1 | 3369 | 16 | 9 | 8 | 3 | 11 | 16 | 11 | 7 | 11 | 8 | 4 | 10 | 14 |
| NM_001001486 | ATP2C1 | 3494 | 16 | 9 | 8 | 3 | 11 | 16 | 12 | 7 | 11 | 8 | 4 | 10 | 14 |
| NM_001001487 | ATP2C1 | 3464 | 16 | 9 | 8 | 3 | 11 | 16 | 12 | 7 | 11 | 8 | 4 | 10 | 14 |
| NM_001001502 | SNCB | 1450 | 4 | 0 | 0 | 0 | 0 | 4 | 0 | 0 | 0 | 0 | 0 | 0 | 0 |
| NM_001001503 | NDUFV3 | 1035 | 7 | 3 | 2 | 3 | 7 | 4 | 3 | 4 | 1 | 1 | 5 | 2 | 1 |
| NM_001001520 | HDGF2 | 2309 | 14 | 8 | 5 | 7 | 6 | 12 | 7 | 5 | 5 | 11 | 14 | 7 | 13 |
| NM_001001521 | UGP2 | 2112 | 90 | 47 | 54 | 22 | 90 | 52 | 35 | 62 | 33 | 29 | 68 | 43 | 25 |
| NM_001001522 | TAGLN | 1557 | 13 | 4 | 4 | 13 | 6 | 3 | 2 | 1 | 2 | 4 | 1 | 3 | 2 |
| NM_001001523 | RORC | 3036 | 14 | 2 | 0 | 0 | 1 | 0 | 2 | 4 | 0 | 0 | 14 | 0 | 2 |
| NM_001001524 | TM6SF2 | 1483 | 0 | 0 | 0 | 0 | 0 | 0 | 0 | 0 | 0 | 0 | 0 | 0 | 0 |
| NM_001001547 | CD36 | 2065 | 715 | 107 | 715 | 18 | 194 | 2 | 4 | 14 | 38 | 5 | 40 | 137 | 7 |
| NM_001001548 | CD36 | 4727 | 343 | 51 | 343 | 9 | 88 | 1 | 2 | 6 | 18 | 3 | 19 | 65 | 3 |
| NM_001001549 | GRB10 | 4567 | 7 | 3 | 1 | 1 | 3 | 3 | 3 | 2 | 1 | 1 | 5 | 7 | 1 |
| NM_001001550 | GRB10 | 4785 | 7 | 3 | 1 | 1 | 3 | 3 | 4 | 2 | 2 | 1 | 5 | 7 | 1 |
| NM_001001551 | C9orf103 | 1614 | 8 | 3 | 4 | 1 | 1 | 6 | 7 | 8 | 1 | 2 | 1 | 3 | 3 |
| NM_001001552 | LEMD1 | 779 | 2 | 0 | 0 | 0 | 0 | 0 | 0 | 0 | 0 | 0 | 0 | 0 | 2 |
| NM_001001555 | GRB10 | 5032 | 7 | 3 | 1 | 1 | 3 | 3 | 3 | 2 | 2 | 1 | 5 | 7 | 1 |
| NM_001001556 | GALK2 | 3208 | 9 | 5 | 3 | 3 | 5 | 7 | 9 | 7 | 3 | 6 | 2 | 5 | 4 |
| NM_001001557 | GDF6 | 3701 | 0 | 0 | 0 | 0 | 0 | 0 | 0 | 0 | 0 | 0 | 0 | 0 | 0 |
| NM_001001560 | GGA1 | 2877 | 1 | 1 | 0 | 1 | 0 | 0 | 0 | 0 | 0 | 1 | 0 | 1 | 1 |
| NM_001001561 | GGA1 | 1696 | 2 | 1 | 1 | 2 | 0 | 0 | 0 | 0 | 0 | 1 | 0 | 1 | 1 |
| NM_001001563 | TIMM50 | 2572 | 2 | 1 | 1 | 1 | 2 | 2 | 2 | 2 | 1 | 1 | 2 | 1 | 2 |

| transcript | gene | transcript length (nt) | max | mean | adipose | colon | heart | hypothalamus | kidney | liver | lung | ovary | skeletalmuscle | spleen | testes |
|---|---|---|---|---|---|---|---|---|---|---|---|---|---|---|---|
| NM_001001567 | PDE9A | 1880 | 12 | 3 | 2 | 12 | 1 | 3 | 6 | 0 | 1 | 1 | 0 | 6 | 1 |
| NM_001001568 | PDE9A | 1731 | 13 | 3 | 2 | 13 | 1 | 3 | 6 | 0 | 1 | 1 | 0 | 7 | 2 |
| NM_001001569 | PDE9A | 1676 | 14 | 3 | 2 | 14 | 1 | 3 | 6 | 1 | 2 | 1 | 0 | 7 | 2 |
| NM_001001570 | PDE9A | 1901 | 12 | 3 | 2 | 12 | 1 | 3 | 6 | 0 | 1 | 1 | 0 | 6 | 1 |
| NM_001001571 | PDE9A | 1809 | 13 | 3 | 2 | 13 | 1 | 3 | 6 | 0 | 1 | 1 | 0 | 6 | 1 |
| NM_001001572 | PDE9A | 1687 | 14 | 3 | 2 | 14 | 1 | 3 | 6 | 1 | 1 | 1 | 0 | 7 | 2 |
| NM_001001573 | PDE9A | 1867 | 13 | 3 | 2 | 13 | 1 | 3 | 6 | 1 | 1 | 1 | 0 | 6 | 1 |
| NM_001001574 | PDE9A | 1802 | 13 | 3 | 2 | 13 | 1 | 3 | 6 | 0 | 1 | 1 | 0 | 6 | 1 |
| NM_001001575 | PDE9A | 1632 | 14 | 4 | 2 | 14 | 1 | 3 | 6 | 1 | 2 | 1 | 1 | 7 | 2 |
| NM_001001576 | PDE9A | 1591 | 11 | 3 | 2 | 11 | 1 | 3 | 6 | 1 | 1 | 1 | 1 | 7 | 2 |
| NM_001001577 | PDE9A | 1710 | 14 | 3 | 2 | 14 | 1 | 3 | 6 | 0 | 1 | 1 | 0 | 7 | 2 |
| NM_001001578 | PDE9A | 1754 | 13 | 3 | 2 | 13 | 1 | 3 | 6 | 0 | 1 | 1 | 0 | 7 | 2 |
| NM_001001579 | PDE9A | 1747 | 13 | 3 | 2 | 13 | 1 | 3 | 6 | 0 | 1 | 1 | 0 | 7 | 2 |
| NM_001001580 | PDE9A | 1547 | 12 | 3 | 2 | 12 | 1 | 3 | 6 | 1 | 1 | 1 | 1 | 7 | 2 |
| NM_001001581 | PDE9A | 1911 | 12 | 3 | 1 | 12 | 1 | 3 | 6 | 1 | 1 | 1 | 0 | 6 | 1 |
| NM_001001582 | PDE9A | 1989 | 12 | 3 | 1 | 12 | 1 | 3 | 6 | 1 | 1 | 1 | 0 | 6 | 1 |
| NM_001001583 | PDE9A | 1982 | 12 | 3 | 1 | 12 | 1 | 3 | 6 | 1 | 1 | 1 | 0 | 6 | 1 |
| NM_001001584 | PDE9A | 1808 | 13 | 3 | 2 | 13 | 1 | 3 | 6 | 0 | 1 | 1 | 0 | 6 | 1 |
| NM_001001585 | PDE9A | 1934 | 12 | 3 | 1 | 12 | 1 | 3 | 6 | 1 | 1 | 1 | 0 | 6 | 1 |
| NM_001001655 | ALKBH2 | 1047 | 3 | 1 | 1 | 1 | 1 | 2 | 1 | 3 | 1 | 1 | 1 | 2 | 1 |
| NM_001001656 | OR9A4 | 945 | 0 | 0 | 0 | 0 | 0 | 0 | 0 | 0 | 0 | 0 | 0 | 0 | 0 |
| NM_001001657 | OR2Y1 | 936 | 0 | 0 | 0 | 0 | 0 | 0 | 0 | 0 | 0 | 0 | 0 | 0 | 0 |
| NM_001001658 | OR9A2 | 933 | 0 | 0 | 0 | 0 | 0 | 0 | 0 | 0 | 0 | 0 | 0 | 0 | 0 |
| NM_001001659 | OR2A14 | 933 | 0 | 0 | 0 | 0 | 0 | 0 | 0 | 0 | 0 | 0 | 0 | 0 | 0 |
| NM_001001660 | LYRM5 | 1135 | 7 | 4 | 4 | 1 | 7 | 6 | 6 | 7 | 2 | 2 | 4 | 3 | 5 |
| NM_001001661 | ZNF425 | 3158 | 1 | 0 | 0 | 0 | 0 | 1 | 0 | 0 | 0 | 1 | 0 | 0 | 0 |
| NM_001001662 | ZNF782 | 3313 | 9 | 3 | 1 | 1 | 1 | 2 | 4 | 1 | 2 | 9 | 2 | 4 | 5 |
| NM_001001663 | TMEM211 | 507 | 0 | 0 | 0 | 0 | 0 | 0 | 0 | 0 | 0 | 0 | 0 | 0 | 0 |
| NM_001001664 | SPOPL | 5732 | 3 | 1 | 1 | 0 | 1 | 1 | 2 | 1 | 2 | 2 | 1 | 3 | 1 |
| NM_001001665 | CYP27C1 | 4685 | 0 | 0 | 0 | 0 | 0 | 0 | 0 | 0 | 0 | 0 | 0 | 0 | 0 |
| NM_001001666 | ANO7 | 891 | 0 | 0 | 0 | 0 | 0 | 0 | 0 | 0 | 0 | 0 | 0 | 0 | 0 |
| NM_001001667 | OR6V1 | 942 | 0 | 0 | 0 | 0 | 0 | 0 | 0 | 0 | 0 | 0 | 0 | 0 | 0 |
| NM_001001668 | ZNF470 | 7141 | 2 | 1 | 1 | 0 | 1 | 2 | 2 | 1 | 1 | 2 | 1 | 1 | 2 |
| NM_001001669 | FLJ41603 | 4865 | 2 | 1 | 0 | 1 | 2 | 2 | 1 | 0 | 0 | 0 | 1 | 0 | 1 |
| NM_001001670 | FLJ46321 | 4832 | 1 | 0 | 0 | 0 | 0 | 0 | 0 | 0 | 0 | 0 | 0 | 0 | 1 |
| NM_001001671 | MAP3K15 | 4697 | 1 | 0 | 0 | 0 | 0 | 0 | 1 | 0 | 0 | 0 | 0 | 0 | 0 |
| NM_001001673 | RNASE9 | 1049 | 0 | 0 | 0 | 0 | 0 | 0 | 0 | 0 | 0 | 0 | 0 | 0 | 0 |
| NM_001001674 | OR4F15 | 939 | 0 | 0 | 0 | 0 | 0 | 0 | 0 | 0 | 0 | 0 | 0 | 0 | 0 |
| NM_001001676 | LCN9 | 524 | 0 | 0 | 0 | 0 | 0 | 0 | 0 | 0 | 0 | 0 | 0 | 0 | 0 |
| NM_001001683 | MED11 | 830 | 7 | 4 | 3 | 2 | 5 | 4 | 7 | 6 | 3 | 2 | 4 | 6 | 3 |
| NM_001001694 | IL17REL | 3635 | 0 | 0 | 0 | 0 | 0 | 0 | 0 | 0 | 0 | 0 | 0 | 0 | 0 |
| NM_001001701 | C4orf3 | 2839 | 4 | 3 | 3 | 2 | 3 | 4 | 4 | 2 | 4 | 3 | 1 | 4 | 3 |
| NM_001001709 | C9orf170 | 3409 | 0 | 0 | 0 | 0 | 0 | 0 | 0 | 0 | 0 | 0 | 0 | 0 | 0 |
| NM_001001710 | FAM166A | 1124 | 1 | 0 | 0 | 0 | 0 | 0 | 0 | 0 | 0 | 0 | 0 | 0 | 1 |
| NM_001001711 | DDI1 | 2615 | 2 | 0 | 0 | 0 | 0 | 0 | 0 | 0 | 0 | 2 | 0 | 0 | 2 |
| NM_001001712 | LCN10 | 2017 | 0 | 0 | 0 | 0 | 0 | 0 | 0 | 0 | 0 | 0 | 0 | 0 | 0 |
| NM_001001713 | SH3BGR | 998 | 70 | 15 | 3 | 3 | 70 | 8 | 3 | 2 | 1 | 1 | 65 | 1 | 4 |
| NM_001001715 | FARP1 | 1321 | 2 | 1 | 0 | 0 | 0 | 1 | 2 | 1 | 0 | 1 | 0 | 1 | 1 |
| NM_001001716 | NFKBIB | 2206 | 4 | 3 | 2 | 2 | 4 | 3 | 4 | 4 | 2 | 1 | 2 | 3 | 2 |
| NM_001001722 | CDY2B | 1960 | 0 | 0 | 0 | 0 | 0 | 0 | 0 | 0 | 0 | 0 | 0 | 0 | 0 |
| NM_001001732 | CC2D2B | 1843 | 1 | 0 | 0 | 0 | 0 | 0 | 0 | 0 | 0 | 1 | 0 | 0 | 1 |
| NM_001001734 | ATP1A4 | 886 | 0 | 0 | 0 | 0 | 0 | 0 | 0 | 0 | 0 | 0 | 0 | 0 | 0 |
| NM_001001740 | RFWD2 | 2702 | 8 | 4 | 4 | 3 | 4 | 5 | 4 | 4 | 4 | 3 | 2 | 8 | 7 |
| NM_001001786 | BLID | 862 | 2 | 0 | 0 | 0 | 1 | 0 | 1 | 0 | 0 | 2 | 0 | 0 | 1 |
| NM_001001787 | ATP1B1 | 1556 | 725 | 166 | 20 | 49 | 173 | 399 | 725 | 86 | 60 | 48 | 173 | 32 | 63 |
| NM_001001788 | RAET1G | 1107 | 1 | 0 | 0 | 0 | 0 | 1 | 0 | 0 | 0 | 0 | 0 | 0 | 0 |
| NM_001001790 | TOMM5 | 746 | 9 | 5 | 6 | 3 | 6 | 8 | 7 | 9 | 3 | 2 | 2 | 7 | 3 |
| NM_001001791 | C10orf55 | 2506 | 0 | 0 | 0 | 0 | 0 | 0 | 0 | 0 | 0 | 0 | 0 | 0 | 0 |
| NM_001001794 | FAM116B | 1762 | 1 | 0 | 0 | 0 | 0 | 1 | 0 | 0 | 0 | 0 | 0 | 0 | 0 |
| NM_001001795 | MGC70857 | 1932 | 0 | 0 | 0 | 0 | 0 | 0 | 0 | 0 | 0 | 0 | 0 | 0 | 0 |
| NM_001001802 | OR2A42 | 915 | 0 | 0 | 0 | 0 | 0 | 0 | 0 | 0 | 0 | 0 | 0 | 0 | 0 |
| NM_001001821 | OR2T34 | 957 | 0 | 0 | 0 | 0 | 0 | 0 | 0 | 0 | 0 | 0 | 0 | 0 | 0 |
| NM_001001824 | OR2T27 | 954 | 0 | 0 | 0 | 0 | 0 | 0 | 0 | 0 | 0 | 0 | 0 | 0 | 0 |
| NM_001001827 | OR2T35 | 972 | 0 | 0 | 0 | 0 | 0 | 0 | 0 | 0 | 0 | 0 | 0 | 0 | 0 |
| NM_001001850 | STX19 | 1156 | 2 | 1 | 0 | 2 | 0 | 0 | 0 | 0 | 2 | 0 | 0 | 1 | 1 |
| NM_001001851 | ITIH5 | 3105 | 120 | 16 | 120 | 11 | 3 | 5 | 6 | 0 | 5 | 8 | 3 | 1 | 8 |
| NM_001001852 | PIM3 | 2378 | 6 | 4 | 4 | 2 | 3 | 3 | 6 | 6 | 6 | 2 | 5 | 5 | 2 |
| NM_001001872 | C14orf37 | 3082 | 13 | 5 | 2 | 1 | 3 | 13 | 11 | 0 | 1 | 10 | 1 | 1 | 10 |
| NM_001001874 | TPD52L3 | 1539 | 12 | 1 | 0 | 0 | 0 | 0 | 0 | 0 | 0 | 0 | 0 | 0 | 12 |
| NM_001001875 | TPD52L3 | 1349 | 12 | 1 | 0 | 0 | 0 | 0 | 0 | 0 | 0 | 0 | 0 | 0 | 12 |
| NM_001001877 | HSFY2 | 1038 | 0 | 0 | 0 | 0 | 0 | 0 | 0 | 0 | 0 | 0 | 0 | 0 | 0 |
| NM_001001888 | VCX3B | 1085 | 0 | 0 | 0 | 0 | 0 | 0 | 0 | 0 | 0 | 0 | 0 | 0 | 0 |
| NM_001001890 | RUNX1 | 7274 | 3 | 2 | 2 | 1 | 2 | 1 | 1 | 0 | 3 | 2 | 2 | 3 | 1 |
| NM_001001891 | ANO7 | 4128 | 1 | 0 | 0 | 1 | 0 | 0 | 0 | 0 | 0 | 0 | 0 | 0 | 0 |
| NM_001001894 | TTC3 | 7716 | 24 | 11 | 12 | 3 | 8 | 24 | 11 | 8 | 7 | 19 | 6 | 10 | 14 |
| NM_001001895 | UBASH3A | 2378 | 0 | 0 | 0 | 0 | 0 | 0 | 0 | 0 | 0 | 0 | 0 | 0 | 0 |
| NM_001001912 | OR4E2 | 942 | 0 | 0 | 0 | 0 | 0 | 0 | 0 | 0 | 0 | 0 | 0 | 0 | 0 |

| | | transcript | | | | | | hypothala | | | | | skeletalm | | |
|---|---|---|---|---|---|---|---|---|---|---|---|---|---|---|---|
| transcript | gene | length (nt) | max | mean | adipose | colon | heart | mus | kidney | liver | lung | ovary | uscle | spleen | testes |
| NM_001001913 | OR52N1 | 963 | 0 | 0 | 0 | 0 | 0 | 0 | 0 | 0 | 0 | 0 | 0 | 0 | 0 |
| NM_001001914 | OR2G3 | 930 | 0 | 0 | 0 | 0 | 0 | 0 | 0 | 0 | 0 | 0 | 0 | 0 | 0 |
| NM_001001915 | OR2G2 | 954 | 0 | 0 | 0 | 0 | 0 | 0 | 0 | 0 | 0 | 0 | 0 | 0 | 0 |
| NM_001001916 | OR52J3 | 936 | 0 | 0 | 0 | 0 | 0 | 0 | 0 | 0 | 0 | 0 | 0 | 0 | 0 |
| NM_001001917 | OR56A1 | 957 | 0 | 0 | 0 | 0 | 0 | 0 | 0 | 0 | 0 | 0 | 0 | 0 | 0 |
| NM_001001918 | OR14C36 | 939 | 0 | 0 | 0 | 0 | 0 | 0 | 0 | 0 | 0 | 0 | 0 | 0 | 0 |
| NM_001001919 | OR13C4 | 957 | 0 | 0 | 0 | 0 | 0 | 0 | 0 | 0 | 0 | 0 | 0 | 0 | 0 |
| NM_001001920 | OR4C15 | 1113 | 0 | 0 | 0 | 0 | 0 | 0 | 0 | 0 | 0 | 0 | 0 | 0 | 0 |
| NM_001001921 | OR5AS1 | 975 | 0 | 0 | 0 | 0 | 0 | 0 | 0 | 0 | 0 | 0 | 0 | 0 | 0 |
| NM_001001922 | OR52N5 | 975 | 0 | 0 | 0 | 0 | 0 | 0 | 0 | 0 | 0 | 0 | 0 | 0 | 0 |
| NM_001001923 | OR5C1 | 963 | 0 | 0 | 0 | 0 | 0 | 0 | 0 | 0 | 0 | 0 | 0 | 0 | 0 |
| NM_001001924 | MTUS1 | 6418 | 23 | 11 | 6 | 4 | 23 | 13 | 7 | 16 | 10 | 18 | 13 | 8 | 8 |
| NM_001001925 | MTUS1 | 6256 | 23 | 12 | 7 | 5 | 23 | 14 | 8 | 16 | 10 | 18 | 13 | 8 | 8 |
| NM_001001928 | PPARA | 9965 | 9 | 4 | 1 | 3 | 7 | 2 | 9 | 8 | 1 | 3 | 3 | 3 | 1 |
| NM_001001931 | MTUS1 | 4005 | 24 | 12 | 9 | 5 | 24 | 18 | 7 | 14 | 9 | 18 | 16 | 8 | 9 |
| NM_001001933 | LHX8 | 2373 | 0 | 0 | 0 | 0 | 0 | 0 | 0 | 0 | 0 | 0 | 0 | 0 | 0 |
| NM_001001936 | AFAP1L2 | 3976 | 10 | 2 | 1 | 1 | 0 | 2 | 2 | 0 | 0 | 2 | 0 | 10 | 1 |
| NM_001001937 | ATP5A1 | 1945 | 146 | 54 | 34 | 28 | 146 | 65 | 88 | 52 | 23 | 28 | 63 | 37 | 35 |
| NM_001001938 | C9orf47 | 4894 | 0 | 0 | 0 | 0 | 0 | 0 | 0 | 0 | 0 | 0 | 0 | 0 | 0 |
| NM_001001952 | OR5D18 | 942 | 0 | 0 | 0 | 0 | 0 | 0 | 0 | 0 | 0 | 0 | 0 | 0 | 0 |
| NM_001001953 | OR10G9 | 936 | 0 | 0 | 0 | 0 | 0 | 0 | 0 | 0 | 0 | 0 | 0 | 0 | 0 |
| NM_001001954 | OR5A2 | 975 | 0 | 0 | 0 | 0 | 0 | 0 | 0 | 0 | 0 | 0 | 0 | 0 | 0 |
| NM_001001955 | OR4C13 | 930 | 0 | 0 | 0 | 0 | 0 | 0 | 0 | 0 | 0 | 0 | 0 | 0 | 0 |
| NM_001001956 | OR13C9 | 957 | 0 | 0 | 0 | 0 | 0 | 0 | 0 | 0 | 0 | 0 | 0 | 0 | 0 |
| NM_001001957 | OR2W3 | 945 | 0 | 0 | 0 | 0 | 0 | 0 | 0 | 0 | 0 | 0 | 0 | 0 | 0 |
| NM_001001958 | OR7G3 | 939 | 0 | 0 | 0 | 0 | 0 | 0 | 0 | 0 | 0 | 0 | 0 | 0 | 0 |
| NM_001001959 | OR11L1 | 969 | 0 | 0 | 0 | 0 | 0 | 0 | 0 | 0 | 0 | 0 | 0 | 0 | 0 |
| NM_001001960 | OR5W2 | 933 | 0 | 0 | 0 | 0 | 0 | 0 | 0 | 0 | 0 | 0 | 0 | 0 | 0 |
| NM_001001961 | OR13C3 | 1044 | 0 | 0 | 0 | 0 | 0 | 0 | 0 | 0 | 0 | 0 | 0 | 0 | 0 |
| NM_001001963 | OR2L8 | 939 | 0 | 0 | 0 | 0 | 0 | 0 | 0 | 0 | 0 | 0 | 0 | 0 | 0 |
| NM_001001964 | OR2T11 | 951 | 0 | 0 | 0 | 0 | 0 | 0 | 0 | 0 | 0 | 0 | 0 | 0 | 0 |
| NM_001001965 | OR4D5 | 957 | 0 | 0 | 0 | 0 | 0 | 0 | 0 | 0 | 0 | 0 | 0 | 0 | 0 |
| NM_001001966 | OR14A16 | 930 | 0 | 0 | 0 | 0 | 0 | 0 | 0 | 0 | 0 | 0 | 0 | 0 | 0 |
| NM_001001967 | OR5D13 | 945 | 0 | 0 | 0 | 0 | 0 | 0 | 0 | 0 | 0 | 0 | 0 | 0 | 0 |
| NM_001001968 | OR6S1 | 996 | 0 | 0 | 0 | 0 | 0 | 0 | 0 | 0 | 0 | 0 | 0 | 0 | 0 |
| NM_001001971 | FAM13C | 3080 | 5 | 2 | 1 | 1 | 1 | 5 | 0 | 0 | 1 | 2 | 3 | 1 | 2 |
| NM_001001973 | ATP5C1 | 1146 | 32 | 18 | 16 | 15 | 31 | 27 | 24 | 32 | 8 | 8 | 13 | 16 | 11 |
| NM_001001974 | PLEKHA1 | 3750 | 13 | 7 | 4 | 2 | 10 | 7 | 13 | 6 | 8 | 6 | 7 | 5 | 6 |
| NM_001001975 | ATP5D | 700 | 2 | 1 | 0 | 1 | 0 | 1 | 0 | 1 | 0 | 0 | 2 | 0 | 0 |
| NM_001001976 | ATE1 | 2209 | 10 | 6 | 3 | 3 | 6 | 10 | 7 | 6 | 3 | 5 | 8 | 6 | 7 |
| NM_001001977 | ATP5E | 439 | 97 | 54 | 71 | 32 | 97 | 84 | 49 | 89 | 34 | 19 | 35 | 64 | 23 |
| NM_001001991 | PAMR1 | 2734 | 1 | 0 | 0 | 1 | 1 | 1 | 0 | 0 | 1 | 0 | 0 | 0 | 0 |
| NM_001001992 | USP16 | 2970 | 20 | 13 | 12 | 4 | 18 | 11 | 14 | 12 | 11 | 14 | 14 | 17 | 20 |
| NM_001001994 | GPM6B | 1509 | 67 | 7 | 0 | 1 | 2 | 67 | 1 | 0 | 2 | 0 | 0 | 5 | 1 |
| NM_001001995 | GPM6B | 3216 | 55 | 6 | 0 | 1 | 1 | 55 | 0 | 0 | 1 | 0 | 0 | 2 | 1 |
| NM_001001996 | GPM6B | 1775 | 107 | 11 | 0 | 2 | 2 | 107 | 1 | 0 | 2 | 0 | 0 | 5 | 1 |
| NM_001001998 | EXOSC10 | 2807 | 10 | 7 | 7 | 6 | 5 | 7 | 7 | 6 | 6 | 7 | 5 | 10 | 7 |
| NM_001002 | RPLP0 | 1204 | 145 | 79 | 86 | 75 | 76 | 38 | 38 | 145 | 65 | 125 | 68 | 100 | 49 |
| NM_001002000 | GMPR2 | 1951 | 6 | 4 | 3 | 2 | 3 | 5 | 6 | 4 | 4 | 4 | 2 | 6 | 3 |
| NM_001002001 | GMPR2 | 1892 | 7 | 4 | 4 | 2 | 3 | 5 | 6 | 4 | 4 | 4 | 2 | 7 | 3 |
| NM_001002002 | GMPR2 | 1626 | 8 | 5 | 4 | 3 | 4 | 5 | 7 | 4 | 5 | 5 | 3 | 8 | 4 |
| NM_001002006 | NT5C1B | 2862 | 2 | 0 | 0 | 0 | 0 | 0 | 0 | 0 | 0 | 0 | 0 | 0 | 2 |
| NM_001002010 | NT5C3 | 1688 | 4 | 3 | 2 | 1 | 2 | 3 | 3 | 2 | 2 | 3 | 4 | 4 | 2 |
| NM_001002017 | HCFC1R1 | 943 | 55 | 20 | 10 | 11 | 20 | 31 | 24 | 4 | 16 | 20 | 55 | 12 | 14 |
| NM_001002018 | HCFC1R1 | 788 | 66 | 23 | 12 | 13 | 24 | 37 | 28 | 5 | 19 | 23 | 66 | 15 | 16 |
| NM_001002019 | PUS1 | 1637 | 0 | 0 | 0 | 0 | 0 | 0 | 0 | 0 | 0 | 0 | 0 | 0 | 0 |
| NM_001002020 | PUS1 | 1666 | 0 | 0 | 0 | 0 | 0 | 0 | 0 | 0 | 0 | 0 | 0 | 0 | 0 |
| NM_001002026 | CLDN18 | 3350 | 15 | 1 | 0 | 0 | 0 | 0 | 0 | 0 | 15 | 0 | 0 | 0 | 0 |
| NM_001002027 | ATP5G1 | 576 | 2 | 1 | 1 | 1 | 2 | 1 | 1 | 1 | 0 | 0 | 0 | 1 | 0 |
| NM_001002029 | C4B | 5427 | 0 | 0 | 0 | 0 | 0 | 0 | 0 | 0 | 0 | 0 | 0 | 0 | 0 |
| NM_001002030 | ECHDC1 | 2339 | 24 | 10 | 24 | 4 | 8 | 12 | 15 | 15 | 12 | 5 | 5 | 9 | 6 |
| NM_001002031 | ATP5G2 | 1164 | 8 | 5 | 4 | 3 | 7 | 6 | 5 | 4 | 2 | 4 | 8 | 5 | 2 |
| NM_001002032 | HN1 | 1581 | 4 | 2 | 1 | 2 | 1 | 4 | 1 | 1 | 1 | 1 | 1 | 2 | 3 |
| NM_001002033 | HN1 | 1695 | 3 | 2 | 1 | 2 | 1 | 3 | 1 | 1 | 1 | 1 | 1 | 2 | 3 |
| NM_001002034 | FAM109B | 2332 | 0 | 0 | 0 | 0 | 0 | 0 | 0 | 0 | 0 | 0 | 0 | 0 | 0 |
| NM_001002035 | DEFB108B | 222 | 1 | 0 | 0 | 0 | 0 | 0 | 0 | 0 | 0 | 1 | 0 | 0 | 0 |
| NM_001002036 | ASTL | 1296 | 0 | 0 | 0 | 0 | 0 | 0 | 0 | 0 | 0 | 0 | 0 | 0 | 0 |
| NM_001002231 | KLK2 | 2872 | 0 | 0 | 0 | 0 | 0 | 0 | 0 | 0 | 0 | 0 | 0 | 0 | 0 |
| NM_001002233 | RAB11FIP1 | 4557 | 8 | 2 | 1 | 3 | 1 | 0 | 2 | 0 | 8 | 1 | 0 | 3 | 2 |
| NM_001002235 | SERPINA1 | 3199 | 4,250 | 400 | 0 | 7 | 9 | 4 | 54 | 4,250 | 19 | 10 | 6 | 27 | 9 |
| NM_001002236 | SERPINA1 | 3513 | 3,871 | 364 | 0 | 7 | 8 | 3 | 49 | 3,871 | 18 | 9 | 6 | 25 | 8 |
| NM_001002243 | AFTPH | 4010 | 9 | 5 | 3 | 3 | 3 | 5 | 9 | 4 | 6 | 4 | 3 | 9 | 6 |
| NM_001002244 | ANAPC11 | 1130 | 1 | 1 | 1 | 1 | 1 | 1 | 1 | 1 | 1 | 0 | 0 | 0 | 1 |
| NM_001002244 | ANAPC11 | 1140 | 1 | 1 | 1 | 1 | 1 | 1 | 1 | 1 | 1 | 0 | 0 | 0 | 1 |
| NM_001002245 | ANAPC11 | 799 | 1 | 1 | 0 | 0 | 1 | 1 | 1 | 1 | 1 | 0 | 0 | 1 | 1 |
| NM_001002246 | ANAPC11 | 862 | 2 | 1 | 0 | 0 | 1 | 2 | 1 | 1 | 1 | 0 | 1 | 1 | 1 |
| NM_001002247 | ANAPC11 | 807 | 2 | 1 | 1 | 1 | 1 | 2 | 1 | 1 | 1 | 0 | 1 | 1 | 1 |

| transcript | gene | transcript length (nt) | max | mean | adipose | colon | heart | hypothalamus | kidney | liver | lung | ovary | skeletalmuscle | spleen | testes |
|---|---|---|---|---|---|---|---|---|---|---|---|---|---|---|---|
| NM_001002248 | ANAPC11 | 870 | 2 | 1 | 1 | 1 | 1 | 2 | 1 | 1 | 1 | 0 | 1 | 1 | 1 |
| NM_001002249 | ANAPC11 | 916 | 1 | 0 | 0 | 0 | 0 | 1 | 0 | 1 | 0 | 0 | 0 | 0 | 0 |
| NM_001002251 | ARL6IP4 | 1351 | 34 | 24 | 22 | 17 | 34 | 29 | 21 | 21 | 19 | 26 | 27 | 32 | 20 |
| NM_001002252 | ARL6IP4 | 1399 | 33 | 24 | 21 | 16 | 33 | 28 | 20 | 21 | 18 | 26 | 26 | 31 | 20 |
| NM_001002254 | AWAT2 | 2124 | 0 | 0 | 0 | 0 | 0 | 0 | 0 | 0 | 0 | 0 | 0 | 0 | 0 |
| NM_001002255 | SUMO4 | 688 | 1 | 1 | 0 | 0 | 1 | 1 | 1 | 0 | 1 | 0 | 0 | 0 | 0 |
| NM_001002257 | LCLAT1 | 4854 | 9 | 4 | 1 | 2 | 9 | 4 | 5 | 5 | 2 | 3 | 2 | 3 | 6 |
| NM_001002258 | ATP5G3 | 2729 | 8 | 4 | 4 | 3 | 4 | 8 | 6 | 6 | 4 | 1 | 2 | 3 | 1 |
| NM_001002259 | CAPRIN2 | 4485 | 6 | 3 | 2 | 2 | 3 | 6 | 3 | 1 | 2 | 4 | 2 | 3 | 5 |
| NM_001002261 | ZFYVE27 | 3046 | 2 | 1 | 1 | 1 | 1 | 2 | 1 | 1 | 1 | 1 | 0 | 1 | 1 |
| NM_001002262 | ZFYVE27 | 3010 | 2 | 1 | 1 | 1 | 1 | 2 | 1 | 1 | 1 | 1 | 0 | 1 | 2 |
| NM_001002264 | EPSTI1 | 1573 | 16 | 7 | 9 | 3 | 7 | 3 | 6 | 2 | 8 | 7 | 5 | 16 | 6 |
| NM_001002265 | 08 Mar | 2589 | 11 | 6 | 4 | 4 | 7 | 5 | 5 | 6 | 4 | 9 | 3 | 9 | 11 |
| NM_001002266 | 08 Mar | 1985 | 14 | 8 | 5 | 5 | 9 | 6 | 6 | 8 | 5 | 12 | 3 | 11 | 14 |
| NM_001002269 | EXOSC3 | 1070 | 11 | 6 | 5 | 2 | 6 | 6 | 6 | 7 | 4 | 11 | 3 | 9 | 8 |
| NM_001002273 | FCGR2B | 1573 | 7 | 3 | 7 | 1 | 4 | 1 | 0 | 4 | 3 | 1 | 3 | 3 | 2 |
| NM_001002274 | FCGR2B | 1576 | 7 | 3 | 7 | 1 | 4 | 1 | 0 | 4 | 3 | 1 | 3 | 3 | 2 |
| NM_001002275 | FCGR2B | 1630 | 6 | 3 | 6 | 1 | 4 | 1 | 0 | 3 | 3 | 1 | 3 | 3 | 2 |
| NM_001002292 | GPR177 | 2067 | 11 | 6 | 4 | 6 | 5 | 10 | 11 | 2 | 5 | 6 | 1 | 5 | 8 |
| NM_001002294 | FMO3 | 2085 | 23 | 3 | 2 | 0 | 1 | 0 | 2 | 23 | 3 | 0 | 0 | 0 | 0 |
| NM_001002295 | GATA3 | 3070 | 3 | 1 | 0 | 0 | 0 | 0 | 3 | 0 | 1 | 0 | 0 | 1 | 0 |
| NM_001002296 | GOLGA7 | 2027 | 32 | 12 | 13 | 6 | 9 | 32 | 11 | 14 | 8 | 9 | 6 | 12 | 15 |
| NM_001002755 | NFU1 | 1089 | 15 | 7 | 11 | 2 | 15 | 8 | 9 | 5 | 4 | 4 | 13 | 6 | 5 |
| NM_001002756 | NFU1 | 1043 | 16 | 8 | 11 | 2 | 16 | 8 | 9 | 5 | 4 | 4 | 14 | 7 | 6 |
| NM_001002757 | NFU1 | 953 | 17 | 9 | 12 | 2 | 17 | 9 | 10 | 5 | 5 | 5 | 15 | 7 | 6 |
| NM_001002758 | PRY2 | 1238 | 0 | 0 | 0 | 0 | 0 | 0 | 0 | 0 | 0 | 0 | 0 | 0 | 0 |
| NM_001002759 | C10orf78 | 1432 | 2 | 1 | 1 | 1 | 1 | 2 | 0 | 0 | 1 | 1 | 1 | 1 | 1 |
| NM_001002760 | BPY2B | 1204 | 0 | 0 | 0 | 0 | 0 | 0 | 0 | 0 | 0 | 0 | 0 | 0 | 0 |
| NM_001002761 | BPY2C | 1204 | 0 | 0 | 0 | 0 | 0 | 0 | 0 | 0 | 0 | 0 | 0 | 0 | 0 |
| NM_001002762 | DNAJB12 | 4360 | 3 | 2 | 2 | 2 | 2 | 2 | 3 | 3 | 2 | 2 | 2 | 3 | 2 |
| NM_001002796 | MCTP1 | 3606 | 13 | 4 | 7 | 1 | 3 | 13 | 1 | 1 | 5 | 1 | 1 | 5 | 3 |
| NM_001002800 | SMC4 | 5124 | 20 | 7 | 4 | 2 | 7 | 2 | 3 | 3 | 9 | 5 | 4 | 15 | 20 |
| NM_001002810 | PDE4DIP | 1249 | 208 | 44 | 9 | 2 | 208 | 35 | 3 | 4 | 7 | 1 | 205 | 3 | 6 |
| NM_001002811 | PDE4DIP | 5346 | 81 | 20 | 15 | 6 | 51 | 22 | 9 | 11 | 7 | 3 | 81 | 7 | 4 |
| NM_001002812 | PDE4DIP | 4824 | 143 | 31 | 15 | 4 | 108 | 28 | 8 | 10 | 7 | 2 | 143 | 6 | 5 |
| NM_001002814 | RAB11FIP1 | 7839 | 15 | 4 | 2 | 6 | 2 | 1 | 3 | 1 | 15 | 1 | 1 | 5 | 4 |
| NM_001002836 | ZNF787 | 1928 | 1 | 0 | 0 | 1 | 1 | 0 | 0 | 1 | 0 | 0 | 1 | 1 | 0 |
| NM_001002837 | INPP5J | 2235 | 0 | 0 | 0 | 0 | 0 | 0 | 0 | 0 | 0 | 0 | 0 | 0 | 0 |
| NM_001002838 | WNK3 | 7573 | 14 | 2 | 0 | 0 | 1 | 2 | 1 | 4 | 0 | 3 | 0 | 0 | 14 |
| NM_001002840 | STAG3L1 | 1702 | 6 | 3 | 2 | 5 | 2 | 2 | 5 | 1 | 3 | 4 | 2 | 6 | 5 |
| NM_001002841 | MYL4 | 908 | 72 | 7 | 0 | 0 | 72 | 0 | 0 | 0 | 0 | 0 | 0 | 0 | 1 |
| NM_001002843 | ZNF280D | 4410 | 15 | 7 | 5 | 2 | 5 | 7 | 5 | 3 | 5 | 15 | 11 | 9 | 8 |
| NM_001002844 | ZNF280D | 2164 | 6 | 3 | 2 | 1 | 2 | 3 | 3 | 2 | 2 | 6 | 3 | 3 | 3 |
| NM_001002857 | ANXA2 | 1635 | 121 | 30 | 121 | 29 | 31 | 9 | 13 | 11 | 44 | 13 | 13 | 28 | 23 |
| NM_001002858 | ANXA2 | 1625 | 122 | 31 | 122 | 29 | 31 | 9 | 13 | 11 | 44 | 13 | 13 | 28 | 23 |
| NM_001002860 | BTBD7 | 8448 | 3 | 2 | 1 | 1 | 2 | 2 | 3 | 1 | 2 | 3 | 1 | 3 | 1 |
| NM_001002862 | DERL3 | 3097 | 0 | 0 | 0 | 0 | 0 | 0 | 0 | 0 | 0 | 0 | 0 | 0 | 0 |
| NM_001002876 | CENPM | 838 | 0 | 0 | 0 | 0 | 0 | 0 | 0 | 0 | 0 | 0 | 0 | 0 | 0 |
| NM_001002877 | THOC5 | 2546 | 5 | 4 | 4 | 4 | 4 | 5 | 3 | 3 | 3 | 4 | 4 | 4 | 4 |
| NM_001002878 | THOC5 | 2677 | 5 | 4 | 4 | 4 | 4 | 5 | 3 | 3 | 3 | 4 | 4 | 4 | 4 |
| NM_001002879 | THOC5 | 2561 | 5 | 4 | 4 | 4 | 4 | 5 | 3 | 3 | 3 | 4 | 4 | 4 | 4 |
| NM_001002880 | CBY1 | 1271 | 6 | 4 | 5 | 4 | 5 | 6 | 5 | 3 | 3 | 5 | 2 | 4 | 4 |
| NM_001002901 | FCRLB | 1768 | 0 | 0 | 0 | 0 | 0 | 0 | 0 | 0 | 0 | 0 | 0 | 0 | 0 |
| NM_001002905 | OR8G1 | 929 | 0 | 0 | 0 | 0 | 0 | 0 | 0 | 0 | 0 | 0 | 0 | 0 | 0 |
| NM_001002906 | XKRY2 | 1581 | 0 | 0 | 0 | 0 | 0 | 0 | 0 | 0 | 0 | 0 | 0 | 0 | 0 |
| NM_001002907 | OR8K1 | 960 | 0 | 0 | 0 | 0 | 0 | 0 | 0 | 0 | 0 | 0 | 0 | 0 | 0 |
| NM_001002909 | GPATCH8 | 6859 | 7 | 5 | 5 | 4 | 4 | 5 | 4 | 2 | 4 | 6 | 7 | 7 | 7 |
| NM_001002911 | GPR139 | 1238 | 0 | 0 | 0 | 0 | 0 | 0 | 0 | 0 | 0 | 0 | 0 | 0 | 0 |
| NM_001002912 | C1orf173 | 7159 | 13 | 2 | 0 | 0 | 0 | 13 | 0 | 0 | 3 | 0 | 0 | 0 | 4 |
| NM_001002913 | PTRH1 | 815 | 0 | 0 | 0 | 0 | 0 | 0 | 0 | 0 | 0 | 0 | 0 | 0 | 0 |
| NM_001002914 | KCTD11 | 3055 | 2 | 1 | 2 | 1 | 1 | 1 | 1 | 1 | 1 | 1 | 1 | 2 | 1 |
| NM_001002915 | IGFL2 | 898 | 1 | 0 | 0 | 0 | 0 | 0 | 0 | 0 | 0 | 0 | 0 | 0 | 1 |
| NM_001002916 | H2BFWT | 891 | 0 | 0 | 0 | 0 | 0 | 0 | 0 | 0 | 0 | 0 | 0 | 0 | 0 |
| NM_001002917 | OR8D1 | 927 | 0 | 0 | 0 | 0 | 0 | 0 | 0 | 0 | 0 | 0 | 0 | 0 | 0 |
| NM_001002918 | OR8D2 | 936 | 0 | 0 | 0 | 0 | 0 | 0 | 0 | 0 | 0 | 0 | 0 | 0 | 0 |
| NM_001002919 | FAM150B | 1398 | 14 | 3 | 1 | 2 | 1 | 3 | 4 | 1 | 3 | 14 | 0 | 3 | 7 |
| NM_001002920 | POTEA | 1838 | 2 | 0 | 0 | 0 | 0 | 0 | 0 | 0 | 0 | 0 | 0 | 0 | 2 |
| NM_001002923 | IGFL4 | 573 | 0 | 0 | 0 | 0 | 0 | 0 | 0 | 0 | 0 | 0 | 0 | 0 | 0 |
| NM_001002925 | OR5AP2 | 951 | 0 | 0 | 0 | 0 | 0 | 0 | 0 | 0 | 0 | 0 | 0 | 0 | 0 |
| NM_001002926 | TWISTNB | 3892 | 5 | 3 | 3 | 1 | 3 | 5 | 3 | 3 | 2 | 3 | 2 | 5 | 5 |
| NM_001003 | RPLP1 | 512 | 9 | 4 | 3 | 3 | 7 | 3 | 2 | 2 | 4 | 5 | 9 | 4 | 3 |
| NM_001003395 | TPD52L1 | 1391 | 4 | 2 | 1 | 0 | 2 | 3 | 2 | 4 | 0 | 1 | 3 | 0 | 1 |
| NM_001003396 | TPD52L1 | 1309 | 4 | 2 | 1 | 0 | 2 | 3 | 2 | 4 | 0 | 1 | 3 | 0 | 1 |
| NM_001003397 | TPD52L1 | 1270 | 4 | 2 | 1 | 1 | 2 | 3 | 2 | 4 | 0 | 1 | 3 | 0 | 1 |
| NM_001003398 | BICD1 | 3285 | 18 | 4 | 2 | 1 | 2 | 9 | 3 | 0 | 2 | 18 | 1 | 6 | 3 |
| NM_001003406 | CACNA1I | 9898 | 1 | 0 | 0 | 0 | 0 | 1 | 0 | 0 | 0 | 0 | 0 | 0 | 0 |
| NM_001003407 | ABLIM1 | 7609 | 48 | 15 | 13 | 6 | 48 | 11 | 13 | 7 | 9 | 37 | 8 | 5 | 15 |

| transcript | gene | transcript length (nt) | max | mean | adipose | colon | heart | hypothala mus | kidney | liver | lung | ovary | skeletalm uscle | spleen | testes |
|---|---|---|---|---|---|---|---|---|---|---|---|---|---|---|---|
| NM_001003408 | ABLIM1 | 7693 | 47 | 15 | 12 | 6 | 47 | 11 | 13 | 6 | 9 | 36 | 8 | 5 | 15 |
| NM_001003443 | OR56A3 | 948 | 0 | 0 | 0 | 0 | 0 | 0 | 0 | 0 | 0 | 0 | 0 | 0 | 0 |
| NM_001003652 | SMAD2 | 10531 | 6 | 4 | 4 | 2 | 3 | 4 | 4 | 2 | 4 | 6 | 2 | 5 | 6 |
| NM_001003656 | PPP2R2D | 1836 | 4 | 3 | 2 | 3 | 3 | 4 | 3 | 2 | 2 | 4 | 4 | 4 | 4 |
| NM_001003665 | C1orf95 | 4093 | 10 | 1 | 0 | 1 | 0 | 10 | 1 | 0 | 0 | 0 | 0 | 0 | 0 |
| NM_001003674 | C18orf1 | 8946 | 5 | 2 | 1 | 1 | 3 | 5 | 1 | 2 | 1 | 1 | 0 | 3 | 1 |
| NM_001003675 | C18orf1 | 8892 | 5 | 2 | 1 | 1 | 3 | 5 | 1 | 2 | 1 | 1 | 0 | 3 | 1 |
| NM_001003676 | C11orf49 | 1984 | 4 | 1 | 1 | 1 | 1 | 4 | 1 | 0 | 1 | 2 | 1 | 1 | 2 |
| NM_001003677 | C11orf49 | 1729 | 4 | 2 | 1 | 1 | 1 | 4 | 2 | 0 | 1 | 2 | 1 | 1 | 2 |
| NM_001003678 | C11orf49 | 1215 | 4 | 2 | 1 | 2 | 1 | 4 | 2 | 0 | 1 | 2 | 2 | 1 | 2 |
| NM_001003679 | LEPR | 5221 | 14 | 3 | 3 | 0 | 2 | 2 | 1 | 14 | 6 | 5 | 2 | 1 | 3 |
| NM_001003680 | LEPR | 3154 | 21 | 5 | 4 | 1 | 3 | 3 | 2 | 21 | 9 | 7 | 2 | 1 | 4 |
| NM_001003681 | HMGXB4 | 4135 | 6 | 2 | 2 | 1 | 2 | 2 | 2 | 1 | 1 | 3 | 2 | 2 | 6 |
| NM_001003682 | TMEM200B | 2461 | 4 | 1 | 1 | 1 | 0 | 0 | 1 | 0 | 1 | 3 | 0 | 1 | 4 |
| NM_001003683 | PDE1A | 2005 | 12 | 3 | 1 | 1 | 3 | 12 | 7 | 0 | 1 | 1 | 0 | 0 | 2 |
| NM_001003684 | UCRC | 975 | 19 | 9 | 8 | 10 | 17 | 19 | 9 | 15 | 3 | 2 | 11 | 5 | 6 |
| NM_001003688 | SMAD1 | 2877 | 5 | 3 | 2 | 2 | 2 | 4 | 3 | 3 | 3 | 4 | 4 | 5 | 3 |
| NM_001003690 | MAD2L1BP | 1547 | 1 | 1 | 0 | 1 | 1 | 1 | 1 | 0 | 0 | 0 | 0 | 1 | 0 |
| NM_001003692 | ZMAT5 | 918 | 0 | 0 | 0 | 0 | 0 | 0 | 0 | 0 | 0 | 0 | 0 | 0 | 0 |
| NM_001003693 | LY6G6F | 898 | 0 | 0 | 0 | 0 | 0 | 0 | 0 | 0 | 0 | 0 | 0 | 0 | 0 |
| NM_001003694 | BRPF1 | 4728 | 2 | 1 | 1 | 2 | 1 | 2 | 1 | 1 | 1 | 1 | 1 | 2 | 2 |
| NM_001003696 | ATP5J | 834 | 332 | 103 | 94 | 43 | 332 | 141 | 79 | 178 | 24 | 46 | 103 | 47 | 39 |
| NM_001003697 | ATP5J | 662 | 418 | 129 | 119 | 55 | 418 | 178 | 100 | 224 | 30 | 58 | 130 | 60 | 49 |
| NM_001003698 | RREB1 | 8567 | 6 | 4 | 4 | 4 | 2 | 1 | 4 | 5 | 3 | 4 | 3 | 6 | 4 |
| NM_001003699 | RREB1 | 8732 | 6 | 4 | 4 | 4 | 2 | 1 | 4 | 5 | 3 | 4 | 3 | 6 | 4 |
| NM_001003700 | RREB1 | 7934 | 6 | 4 | 4 | 4 | 2 | 1 | 4 | 5 | 3 | 4 | 3 | 6 | 4 |
| NM_001003701 | ATP5J | 1210 | 229 | 71 | 65 | 30 | 229 | 97 | 55 | 123 | 16 | 32 | 71 | 33 | 27 |
| NM_001003702 | ARHGEF5L | 2474 | 0 | 0 | 0 | 0 | 0 | 0 | 0 | 0 | 0 | 0 | 0 | 0 | 0 |
| NM_001003703 | ATP5J | 1296 | 214 | 66 | 61 | 28 | 214 | 91 | 51 | 115 | 15 | 30 | 66 | 30 | 25 |
| NM_001003704 | MTP18 | 936 | 1 | 0 | 0 | 1 | 0 | 0 | 1 | 1 | 0 | 0 | 0 | 1 | 0 |
| NM_001003712 | OSBPL8 | 7195 | 12 | 6 | 6 | 1 | 6 | 7 | 8 | 3 | 8 | 7 | 5 | 12 | 7 |
| NM_001003713 | ATP5J2 | 477 | 15 | 9 | 7 | 5 | 14 | 11 | 15 | 9 | 7 | 2 | 8 | 9 | 12 |
| NM_001003714 | ATP5J2 | 378 | 6 | 3 | 2 | 4 | 5 | 6 | 4 | 3 | 1 | 1 | 2 | 2 | 2 |
| NM_001003715 | RECQL5 | 1745 | 2 | 1 | 1 | 1 | 0 | 2 | 2 | 1 | 0 | 1 | 0 | 2 | 2 |
| NM_001003716 | RECQL5 | 3710 | 1 | 1 | 1 | 1 | 0 | 1 | 1 | 0 | 0 | 1 | 0 | 1 | 1 |
| NM_001003722 | GLE1 | 3342 | 7 | 4 | 4 | 3 | 4 | 3 | 5 | 5 | 3 | 5 | 4 | 7 | 5 |
| NM_001003745 | OR10A3 | 945 | 0 | 0 | 0 | 0 | 0 | 0 | 0 | 0 | 0 | 0 | 0 | 0 | 0 |
| NM_001003750 | OR8I2 | 933 | 0 | 0 | 0 | 0 | 0 | 0 | 0 | 0 | 0 | 0 | 0 | 0 | 0 |
| NM_001003760 | KLHL31 | 5743 | 31 | 5 | 9 | 0 | 31 | 0 | 0 | 0 | 0 | 0 | 13 | 0 | 0 |
| NM_001003785 | ATP5H | 550 | 776 | 319 | 274 | 182 | 776 | 460 | 402 | 452 | 149 | 120 | 359 | 204 | 132 |
| NM_001003786 | STRADA | 2224 | 5 | 2 | 1 | 2 | 1 | 5 | 2 | 1 | 2 | 2 | 1 | 4 | 3 |
| NM_001003787 | STRADA | 2335 | 7 | 3 | 1 | 3 | 2 | 7 | 2 | 1 | 2 | 2 | 1 | 4 | 4 |
| NM_001003788 | STRADA | 2306 | 7 | 3 | 2 | 3 | 2 | 7 | 2 | 1 | 2 | 2 | 1 | 4 | 4 |
| NM_001003789 | RABL2B | 2190 | 1 | 0 | 0 | 0 | 0 | 1 | 1 | 0 | 1 | 0 | 0 | 1 | 0 |
| NM_001003790 | ERLIN2 | 1751 | 3 | 1 | 2 | 1 | 1 | 1 | 3 | 1 | 1 | 2 | 1 | 2 | 2 |
| NM_001003791 | ERLIN2 | 1524 | 3 | 2 | 2 | 1 | 1 | 1 | 3 | 1 | 1 | 2 | 1 | 2 | 2 |
| NM_001003792 | RBMS3 | 2757 | 10 | 5 | 5 | 1 | 6 | 2 | 3 | 1 | 6 | 10 | 5 | 7 | 5 |
| NM_001003793 | RBMS3 | 2811 | 10 | 5 | 5 | 1 | 6 | 2 | 3 | 1 | 6 | 10 | 5 | 7 | 5 |
| NM_001003794 | MGLL | 4189 | 27 | 9 | 27 | 8 | 8 | 7 | 10 | 10 | 9 | 2 | 5 | 9 | 3 |
| NM_001003795 | GTF2IRD2B | 3585 | 0 | 0 | 0 | 0 | 0 | 0 | 0 | 0 | 0 | 0 | 0 | 0 | 0 |
| NM_001003796 | NHP2L1 | 1500 | 11 | 6 | 3 | 2 | 8 | 11 | 6 | 3 | 4 | 7 | 6 | 6 | 4 |
| NM_001003799 | TARP | 1027 | 3 | 0 | 0 | 0 | 0 | 0 | 0 | 0 | 1 | 0 | 0 | 3 | 0 |
| NM_001003800 | BICD2 | 6416 | 3 | 1 | 2 | 1 | 2 | 2 | 1 | 0 | 2 | 1 | 1 | 3 | 2 |
| NM_001003801 | SMARCD3 | 1683 | 32 | 10 | 2 | 2 | 17 | 12 | 5 | 0 | 5 | 15 | 32 | 7 | 12 |
| NM_001003802 | SMARCD3 | 1672 | 31 | 10 | 2 | 2 | 17 | 12 | 4 | 0 | 5 | 14 | 31 | 7 | 12 |
| NM_001003803 | ATP5S | 1567 | 3 | 2 | 1 | 0 | 3 | 3 | 3 | 2 | 1 | 1 | 3 | 3 | 3 |
| NM_001003805 | ATP5S | 1395 | 3 | 2 | 1 | 0 | 3 | 3 | 3 | 2 | 1 | 1 | 3 | 3 | 3 |
| NM_001003806 | TARP | 1027 | 3 | 0 | 0 | 0 | 0 | 0 | 0 | 0 | 1 | 0 | 0 | 3 | 0 |
| NM_001003809 | DLGAP1 | 2388 | 31 | 3 | 0 | 1 | 0 | 31 | 1 | 0 | 0 | 1 | 0 | 0 | 3 |
| NM_001003810 | HNRNPD | 2053 | 106 | 64 | 40 | 48 | 62 | 72 | 54 | 48 | 63 | 88 | 54 | 106 | 70 |
| NM_001003811 | TEX11 | 3140 | 4 | 0 | 0 | 0 | 0 | 0 | 0 | 0 | 0 | 0 | 0 | 0 | 4 |
| NM_001003818 | TRIM6 | 3409 | 2 | 1 | 1 | 0 | 0 | 0 | 2 | 0 | 0 | 1 | 0 | 1 | 1 |
| NM_001003819 | TRIM6-TRIM | 3457 | 0 | 0 | 0 | 0 | 0 | 0 | 0 | 0 | 0 | 0 | 0 | 0 | 0 |
| NM_001003827 | TRIM34 | 2310 | 0 | 0 | 0 | 0 | 0 | 0 | 0 | 0 | 0 | 0 | 0 | 0 | 0 |
| NM_001003828 | PARVB | 1787 | 4 | 1 | 1 | 0 | 1 | 1 | 1 | 1 | 1 | 1 | 4 | 2 | 0 |
| NM_001003841 | SLC6A19 | 5172 | 6 | 1 | 0 | 0 | 0 | 0 | 6 | 0 | 0 | 0 | 0 | 0 | 0 |
| NM_001003845 | SP5 | 1946 | 0 | 0 | 0 | 0 | 0 | 0 | 0 | 0 | 0 | 0 | 0 | 0 | 0 |
| NM_001003891 | MED15 | 3375 | 1 | 1 | 1 | 1 | 1 | 1 | 1 | 0 | 1 | 1 | 1 | 1 | 1 |
| NM_001003892 | DUPD1 | 663 | 1 | 0 | 0 | 0 | 0 | 0 | 0 | 0 | 0 | 0 | 1 | 0 | 0 |
| NM_001003894 | CDY1B | 2222 | 0 | 0 | 0 | 0 | 0 | 0 | 0 | 0 | 0 | 0 | 0 | 0 | 0 |
| NM_001003895 | CDY1B | 2365 | 0 | 0 | 0 | 0 | 0 | 0 | 0 | 0 | 0 | 0 | 0 | 0 | 0 |
| NM_001003895 | CDY1B | 2366 | 0 | 0 | 0 | 0 | 0 | 0 | 0 | 0 | 0 | 0 | 0 | 0 | 0 |
| NM_001003897 | MANBAL | 1198 | 5 | 2 | 2 | 2 | 2 | 5 | 3 | 2 | 1 | 1 | 0 | 2 | 3 |
| NM_001003927 | EVI2A | 3010 | 7 | 2 | 1 | 0 | 2 | 6 | 1 | 0 | 7 | 1 | 0 | 6 | 1 |
| NM_001003931 | PARP3 | 2355 | 5 | 3 | 5 | 3 | 3 | 3 | 5 | 3 | 2 | 2 | 1 | 4 | 1 |
| NM_001003936 | TXNDC8 | 490 | 1 | 0 | 0 | 0 | 0 | 0 | 0 | 0 | 0 | 0 | 0 | 0 | 1 |
| NM_001003937 | TSPYL6 | 3095 | 4 | 1 | 0 | 0 | 0 | 0 | 0 | 0 | 0 | 0 | 0 | 0 | 4 |

| transcript | gene | transcript length (nt) | max | mean | adipose | colon | heart | hypothalamus | kidney | liver | lung | ovary | skeletalmuscle | spleen | testes |
|---|---|---|---|---|---|---|---|---|---|---|---|---|---|---|---|
| NM_001003938 | HBM | 510 | 0 | 0 | 0 | 0 | 0 | 0 | 0 | 0 | 0 | 0 | 0 | 0 | 0 |
| NM_001003940 | BMF | 4687 | 2 | 1 | 1 | 1 | 0 | 0 | 0 | 0 | 1 | 1 | 0 | 2 | 0 |
| NM_001003941 | OGDH | 1811 | 35 | 15 | 14 | 12 | 35 | 18 | 25 | 11 | 8 | 4 | 27 | 10 | 7 |
| NM_001003942 | BMF | 4389 | 2 | 1 | 1 | 1 | 0 | 0 | 1 | 0 | 1 | 1 | 0 | 2 | 0 |
| NM_001003943 | BMF | 4291 | 2 | 1 | 1 | 1 | 0 | 0 | 1 | 0 | 1 | 1 | 0 | 2 | 0 |
| NM_001003954 | ANXA13 | 1593 | 2 | 0 | 0 | 2 | 0 | 0 | 0 | 1 | 0 | 0 | 0 | 0 | 0 |
| NM_001003962 | CAPNS1 | 1475 | 9 | 6 | 8 | 7 | 8 | 6 | 9 | 5 | 6 | 3 | 4 | 8 | 5 |
| NM_001004 | RPLP2 | 493 | 703 | 257 | 166 | 118 | 268 | 67 | 147 | 122 | 250 | 703 | 287 | 556 | 145 |
| NM_001004019 | FBLN2 | 4325 | 7 | 1 | 7 | 1 | 2 | 0 | 0 | 0 | 1 | 0 | 1 | 0 | 2 |
| NM_001004023 | DYRK3 | 2261 | 5 | 1 | 1 | 0 | 2 | 1 | 1 | 1 | 1 | 2 | 0 | 1 | 5 |
| NM_001004051 | GPRASP2 | 3572 | 9 | 2 | 0 | 1 | 1 | 9 | 1 | 1 | 0 | 3 | 0 | 1 | 1 |
| NM_001004052 | OR52B2 | 972 | 0 | 0 | 0 | 0 | 0 | 0 | 0 | 0 | 0 | 0 | 0 | 0 | 0 |
| NM_001004055 | LRRC29 | 1304 | 0 | 0 | 0 | 0 | 0 | 0 | 0 | 0 | 0 | 0 | 0 | 0 | 0 |
| NM_001004056 | GRK4 | 2218 | 4 | 1 | 0 | 0 | 1 | 2 | 0 | 0 | 0 | 0 | 0 | 0 | 4 |
| NM_001004057 | GRK4 | 2176 | 4 | 1 | 0 | 0 | 1 | 2 | 0 | 0 | 0 | 0 | 0 | 0 | 4 |
| NM_001004058 | OR8K5 | 924 | 0 | 0 | 0 | 0 | 0 | 0 | 0 | 0 | 0 | 0 | 0 | 0 | 0 |
| NM_001004059 | OR4S2 | 936 | 0 | 0 | 0 | 0 | 0 | 0 | 0 | 0 | 0 | 0 | 0 | 0 | 0 |
| NM_001004060 | NOMO2 | 3922 | 0 | 0 | 0 | 0 | 0 | 0 | 0 | 0 | 0 | 0 | 0 | 0 | 0 |
| NM_001004063 | OR4K1 | 936 | 0 | 0 | 0 | 0 | 0 | 0 | 0 | 0 | 0 | 0 | 0 | 0 | 0 |
| NM_001004064 | OR8J3 | 948 | 0 | 0 | 0 | 0 | 0 | 0 | 0 | 0 | 0 | 0 | 0 | 0 | 0 |
| NM_001004065 | AKAP2 | 6865 | 0 | 0 | 0 | 0 | 0 | 0 | 0 | 0 | 0 | 0 | 0 | 0 | 0 |
| NM_001004067 | NOMO3 | 4315 | 0 | 0 | 0 | 0 | 0 | 0 | 0 | 0 | 0 | 0 | 0 | 0 | 0 |
| NM_001004105 | GRK6 | 1946 | 2 | 1 | 0 | 1 | 0 | 1 | 0 | 0 | 1 | 0 | 0 | 2 | 0 |
| NM_001004106 | GRK6 | 2950 | 2 | 0 | 0 | 1 | 0 | 1 | 0 | 0 | 1 | 0 | 0 | 2 | 0 |
| NM_001004124 | OR4P4 | 939 | 0 | 0 | 0 | 0 | 0 | 0 | 0 | 0 | 0 | 0 | 0 | 0 | 0 |
| NM_001004125 | TUSC1 | 2470 | 1 | 1 | 0 | 1 | 1 | 1 | 1 | 1 | 0 | 1 | 0 | 1 | 1 |
| NM_001004127 | ALG11 | 2538 | 8 | 5 | 6 | 3 | 5 | 7 | 6 | 8 | 3 | 4 | 2 | 5 | 6 |
| NM_001004128 | QSOX1 | 2583 | 6 | 2 | 1 | 2 | 3 | 2 | 1 | 2 | 4 | 3 | 1 | 1 | 6 |
| NM_001004134 | OR10AD1 | 954 | 1 | 0 | 0 | 0 | 0 | 0 | 0 | 0 | 1 | 1 | 0 | 1 | 0 |
| NM_001004135 | OR2A12 | 933 | 0 | 0 | 0 | 0 | 0 | 0 | 0 | 0 | 0 | 0 | 0 | 0 | 0 |
| NM_001004136 | OR2T2 | 975 | 0 | 0 | 0 | 0 | 0 | 0 | 0 | 0 | 0 | 0 | 0 | 0 | 0 |
| NM_001004137 | OR52M1 | 954 | 0 | 0 | 0 | 0 | 0 | 0 | 0 | 0 | 0 | 0 | 0 | 0 | 0 |
| NM_001004195 | OR4F4 | 918 | 0 | 0 | 0 | 0 | 0 | 0 | 0 | 0 | 0 | 0 | 0 | 0 | 0 |
| NM_001004196 | CD200 | 2290 | 3 | 1 | 1 | 1 | 2 | 3 | 2 | 0 | 0 | 1 | 0 | 0 | 0 |
| NM_001004297 | OR13A1 | 2078 | 0 | 0 | 0 | 0 | 0 | 0 | 0 | 0 | 0 | 0 | 0 | 0 | 0 |
| NM_001004298 | C10orf90 | 3080 | 2 | 0 | 0 | 0 | 0 | 2 | 0 | 0 | 0 | 0 | 0 | 0 | 1 |
| NM_001004301 | ZNF813 | 4188 | 5 | 2 | 2 | 1 | 2 | 1 | 3 | 1 | 2 | 4 | 1 | 5 | 5 |
| NM_001004303 | C1orf168 | 3460 | 1 | 0 | 0 | 0 | 0 | 0 | 1 | 1 | 0 | 0 | 1 | 0 | 0 |
| NM_001004304 | ZNF740 | 4256 | 3 | 2 | 2 | 2 | 1 | 2 | 2 | 1 | 1 | 2 | 1 | 3 | 1 |
| NM_001004306 | CCDC144NL | 2806 | 2 | 0 | 0 | 0 | 0 | 0 | 0 | 0 | 0 | 0 | 0 | 0 | 2 |
| NM_001004308 | ZCCHC16 | 2958 | 0 | 0 | 0 | 0 | 0 | 0 | 0 | 0 | 0 | 0 | 0 | 0 | 0 |
| NM_001004309 | ZNF774 | 1838 | 1 | 1 | 0 | 1 | 1 | 1 | 0 | 0 | 0 | 1 | 1 | 0 | 1 |
| NM_001004310 | FCRL6 | 1943 | 0 | 0 | 0 | 0 | 0 | 0 | 0 | 0 | 0 | 0 | 0 | 0 | 0 |
| NM_001004311 | FIGLA | 722 | 0 | 0 | 0 | 0 | 0 | 0 | 0 | 0 | 0 | 0 | 0 | 0 | 0 |
| NM_001004312 | RTP2 | 1346 | 0 | 0 | 0 | 0 | 0 | 0 | 0 | 0 | 0 | 0 | 0 | 0 | 0 |
| NM_001004313 | TMEM220 | 3092 | 11 | 2 | 2 | 1 | 1 | 1 | 3 | 11 | 1 | 1 | 1 | 2 | 4 |
| NM_001004316 | LEKR1 | 2572 | 3 | 0 | 0 | 0 | 0 | 0 | 0 | 0 | 0 | 0 | 0 | 0 | 3 |
| NM_001004317 | LIN28B | 5504 | 1 | 0 | 0 | 0 | 0 | 0 | 0 | 0 | 0 | 0 | 0 | 0 | 1 |
| NM_001004318 | PAPL | 2959 | 0 | 0 | 0 | 0 | 0 | 0 | 0 | 0 | 0 | 0 | 0 | 0 | 0 |
| NM_001004319 | VHLL | 1013 | 0 | 0 | 0 | 0 | 0 | 0 | 0 | 0 | 0 | 0 | 0 | 0 | 0 |
| NM_001004320 | TMEM195 | 2475 | 6 | 1 | 0 | 0 | 0 | 0 | 1 | 6 | 0 | 0 | 0 | 0 | 1 |
| NM_001004323 | C7orf61 | 1004 | 1 | 0 | 0 | 0 | 0 | 0 | 0 | 0 | 0 | 0 | 0 | 0 | 1 |
| NM_001004325 | KRTAP5-2 | 1118 | 0 | 0 | 0 | 0 | 0 | 0 | 0 | 0 | 0 | 0 | 0 | 0 | 0 |
| NM_001004326 | SLC22A20 | 1686 | 0 | 0 | 0 | 0 | 0 | 0 | 0 | 0 | 0 | 0 | 0 | 0 | 0 |
| NM_001004328 | ZNF705A | 3438 | 0 | 0 | 0 | 0 | 0 | 0 | 0 | 0 | 0 | 0 | 0 | 0 | 0 |
| NM_001004329 | DBX2 | 2722 | 0 | 0 | 0 | 0 | 0 | 0 | 0 | 0 | 0 | 0 | 0 | 0 | 0 |
| NM_001004330 | PLEKHG7 | 3267 | 1 | 0 | 0 | 0 | 0 | 0 | 0 | 0 | 1 | 0 | 0 | 1 | 0 |
| NM_001004333 | RNASEK | 748 | 22 | 13 | 13 | 5 | 13 | 20 | 17 | 10 | 18 | 8 | 7 | 22 | 12 |
| NM_001004334 | GPR179 | 7980 | 1 | 0 | 0 | 0 | 0 | 1 | 0 | 0 | 0 | 0 | 0 | 0 | 0 |
| NM_001004339 | ZYG11A | 4271 | 1 | 0 | 0 | 0 | 0 | 0 | 1 | 0 | 0 | 0 | 0 | 0 | 1 |
| NM_001004340 | FCGR1B | 845 | 0 | 0 | 0 | 0 | 0 | 0 | 0 | 0 | 0 | 0 | 0 | 0 | 0 |
| NM_001004341 | ETV3L | 1976 | 0 | 0 | 0 | 0 | 0 | 0 | 0 | 0 | 0 | 0 | 0 | 0 | 0 |
| NM_001004342 | TRIM67 | 8522 | 1 | 0 | 0 | 1 | 0 | 0 | 0 | 0 | 0 | 0 | 0 | 0 | 0 |
| NM_001004343 | MAP1LC3C | 1192 | 2 | 1 | 2 | 0 | 0 | 0 | 0 | 0 | 2 | 1 | 1 | 0 | 0 |
| NM_001004351 | SPDYE3 | 1903 | 0 | 0 | 0 | 0 | 0 | 0 | 0 | 0 | 0 | 0 | 0 | 0 | 0 |
| NM_001004353 | C9orf173 | 1173 | 0 | 0 | 0 | 0 | 0 | 0 | 0 | 0 | 0 | 0 | 0 | 0 | 0 |
| NM_001004354 | NRARP | 2621 | 2 | 0 | 0 | 2 | 0 | 0 | 0 | 0 | 0 | 0 | 0 | 1 | 0 |
| NM_001004356 | FGFRL1 | 3215 | 5 | 2 | 2 | 1 | 3 | 2 | 3 | 5 | 1 | 3 | 1 | 1 | 1 |
| NM_001004358 | FGFRL1 | 3098 | 5 | 2 | 2 | 1 | 3 | 2 | 3 | 5 | 1 | 3 | 1 | 1 | 1 |
| NM_001004360 | DPP10 | 4464 | 5 | 0 | 0 | 0 | 0 | 5 | 0 | 0 | 0 | 0 | 0 | 0 | 0 |
| NM_001004416 | UMODL1 | 4878 | 0 | 0 | 0 | 0 | 0 | 0 | 0 | 0 | 0 | 0 | 0 | 0 | 0 |
| NM_001004419 | CLEC2D | 5249 | 8 | 3 | 2 | 1 | 1 | 1 | 1 | 1 | 4 | 5 | 1 | 8 | 2 |
| NM_001004426 | PLA2G6 | 3061 | 1 | 0 | 0 | 0 | 0 | 0 | 0 | 0 | 0 | 0 | 0 | 0 | 1 |
| NM_001004431 | METRNL | 1332 | 1 | 0 | 1 | 0 | 0 | 0 | 0 | 0 | 1 | 0 | 0 | 0 | 0 |
| NM_001004432 | LINGO4 | 2606 | 2 | 0 | 0 | 0 | 0 | 0 | 0 | 0 | 0 | 0 | 2 | 0 | 0 |
| NM_001004434 | SLC30A2 | 2473 | 2 | 0 | 0 | 0 | 0 | 0 | 2 | 0 | 0 | 0 | 0 | 0 | 0 |
| NM_001004439 | ITGA11 | 5009 | 2 | 1 | 1 | 1 | 2 | 0 | 1 | 0 | 0 | 1 | 0 | 1 | 1 |

| transcript | gene | transcript length (nt) | max | mean | adipose | colon | heart | hypothala mus | kidney | liver | lung | ovary | skeletalm uscle | spleen | testes |
|---|---|---|---|---|---|---|---|---|---|---|---|---|---|---|---|
| NM_001004440 | FAM19A3 | 1133 | 0 | 0 | 0 | 0 | 0 | 0 | 0 | 0 | 0 | 0 | 0 | 0 | 0 |
| NM_001004441 | ANKRD34B | 3934 | 1 | 0 | 0 | 0 | 0 | 1 | 0 | 0 | 0 | 0 | 0 | 0 | 0 |
| NM_001004450 | OR1B1 | 957 | 0 | 0 | 0 | 0 | 0 | 0 | 0 | 0 | 0 | 0 | 0 | 0 | 0 |
| NM_001004451 | OR1J1 | 969 | 0 | 0 | 0 | 0 | 0 | 0 | 0 | 0 | 0 | 0 | 0 | 0 | 0 |
| NM_001004452 | OR1J4 | 942 | 0 | 0 | 0 | 0 | 0 | 0 | 0 | 0 | 0 | 0 | 0 | 0 | 0 |
| NM_001004453 | OR1L6 | 934 | 0 | 0 | 0 | 0 | 0 | 0 | 0 | 0 | 0 | 0 | 0 | 0 | 0 |
| NM_001004454 | OR1L8 | 930 | 0 | 0 | 0 | 0 | 0 | 0 | 0 | 0 | 0 | 0 | 0 | 0 | 0 |
| NM_001004456 | OR1M1 | 942 | 0 | 0 | 0 | 0 | 0 | 0 | 0 | 0 | 0 | 0 | 0 | 0 | 0 |
| NM_001004457 | OR1N2 | 993 | 0 | 0 | 0 | 0 | 0 | 0 | 0 | 0 | 0 | 0 | 0 | 0 | 0 |
| NM_001004458 | OR1S1 | 978 | 0 | 0 | 0 | 0 | 0 | 0 | 0 | 0 | 0 | 0 | 0 | 0 | 0 |
| NM_001004459 | OR1S2 | 978 | 0 | 0 | 0 | 0 | 0 | 0 | 0 | 0 | 0 | 0 | 0 | 0 | 0 |
| NM_001004460 | OR10A2 | 912 | 0 | 0 | 0 | 0 | 0 | 0 | 0 | 0 | 0 | 0 | 0 | 0 | 0 |
| NM_001004461 | OR10A6 | 945 | 0 | 0 | 0 | 0 | 0 | 0 | 0 | 0 | 0 | 0 | 0 | 0 | 0 |
| NM_001004462 | OR10G4 | 936 | 0 | 0 | 0 | 0 | 0 | 0 | 0 | 0 | 0 | 0 | 0 | 0 | 0 |
| NM_001004463 | OR10G7 | 936 | 0 | 0 | 0 | 0 | 0 | 0 | 0 | 0 | 0 | 0 | 0 | 0 | 0 |
| NM_001004464 | OR10G8 | 936 | 0 | 0 | 0 | 0 | 0 | 0 | 0 | 0 | 0 | 0 | 0 | 0 | 0 |
| NM_001004465 | OR10H4 | 951 | 0 | 0 | 0 | 0 | 0 | 0 | 0 | 0 | 0 | 0 | 0 | 0 | 0 |
| NM_001004466 | OR10H5 | 948 | 0 | 0 | 0 | 0 | 0 | 0 | 0 | 0 | 0 | 0 | 0 | 0 | 0 |
| NM_001004467 | OR10J3 | 990 | 0 | 0 | 0 | 0 | 0 | 0 | 0 | 0 | 0 | 0 | 0 | 0 | 0 |
| NM_001004469 | OR10J5 | 930 | 1 | 0 | 0 | 0 | 0 | 0 | 0 | 1 | 0 | 0 | 0 | 0 | 0 |
| NM_001004470 | ST8SIA6 | 1398 | 2 | 1 | 0 | 0 | 2 | 1 | 0 | 1 | 1 | 0 | 0 | 0 | 1 |
| NM_001004471 | OR10Q1 | 960 | 0 | 0 | 0 | 0 | 0 | 0 | 0 | 0 | 0 | 0 | 0 | 0 | 0 |
| NM_001004472 | OR10R2 | 1008 | 0 | 0 | 0 | 0 | 0 | 0 | 0 | 0 | 0 | 0 | 0 | 0 | 0 |
| NM_001004473 | OR10K1 | 942 | 0 | 0 | 0 | 0 | 0 | 0 | 0 | 0 | 0 | 0 | 0 | 0 | 0 |
| NM_001004474 | OR10S1 | 996 | 0 | 0 | 0 | 0 | 0 | 0 | 0 | 0 | 0 | 0 | 0 | 0 | 0 |
| NM_001004475 | OR10T2 | 945 | 0 | 0 | 0 | 0 | 0 | 0 | 0 | 0 | 0 | 0 | 0 | 0 | 0 |
| NM_001004476 | OR10K2 | 939 | 0 | 0 | 0 | 0 | 0 | 0 | 0 | 0 | 0 | 0 | 0 | 0 | 0 |
| NM_001004477 | OR10X1 | 981 | 0 | 0 | 0 | 0 | 0 | 0 | 0 | 0 | 0 | 0 | 0 | 0 | 0 |
| NM_001004478 | OR10Z1 | 942 | 0 | 0 | 0 | 0 | 0 | 0 | 0 | 0 | 0 | 0 | 0 | 0 | 0 |
| NM_001004479 | OR11H4 | 975 | 0 | 0 | 0 | 0 | 0 | 0 | 0 | 0 | 0 | 0 | 0 | 0 | 0 |
| NM_001004480 | OR11H6 | 993 | 0 | 0 | 0 | 0 | 0 | 0 | 0 | 0 | 0 | 0 | 0 | 0 | 0 |
| NM_001004481 | OR13C2 | 957 | 0 | 0 | 0 | 0 | 0 | 0 | 0 | 0 | 0 | 0 | 0 | 0 | 0 |
| NM_001004482 | OR13C5 | 957 | 1 | 0 | 0 | 0 | 0 | 1 | 0 | 0 | 0 | 0 | 0 | 0 | 0 |
| NM_001004483 | OR13C8 | 963 | 0 | 0 | 0 | 0 | 0 | 0 | 0 | 0 | 0 | 0 | 0 | 0 | 0 |
| NM_001004484 | OR13D1 | 1041 | 1 | 0 | 0 | 0 | 0 | 0 | 0 | 0 | 0 | 0 | 0 | 1 | 1 |
| NM_001004485 | OR13F1 | 960 | 0 | 0 | 0 | 0 | 0 | 0 | 0 | 0 | 0 | 0 | 0 | 0 | 0 |
| NM_001004486 | OR13H1 | 927 | 0 | 0 | 0 | 0 | 0 | 0 | 0 | 0 | 0 | 0 | 0 | 0 | 0 |
| NM_001004487 | OR13J1 | 939 | 0 | 0 | 0 | 0 | 0 | 0 | 0 | 0 | 0 | 0 | 0 | 0 | 0 |
| NM_001004488 | OR2A25 | 933 | 0 | 0 | 0 | 0 | 0 | 0 | 0 | 0 | 0 | 0 | 0 | 0 | 0 |
| NM_001004489 | OR2AG1 | 951 | 0 | 0 | 0 | 0 | 0 | 0 | 0 | 0 | 0 | 0 | 0 | 0 | 0 |
| NM_001004490 | OR2AG2 | 951 | 0 | 0 | 0 | 0 | 0 | 0 | 0 | 0 | 0 | 0 | 0 | 0 | 0 |
| NM_001004491 | OR2AK2 | 1008 | 0 | 0 | 0 | 0 | 0 | 0 | 0 | 0 | 0 | 0 | 0 | 0 | 0 |
| NM_001004492 | OR2B11 | 954 | 0 | 0 | 0 | 0 | 0 | 0 | 0 | 0 | 0 | 0 | 0 | 0 | 0 |
| NM_001004684 | OR2D3 | 993 | 0 | 0 | 0 | 0 | 0 | 0 | 0 | 0 | 0 | 0 | 0 | 0 | 0 |
| NM_001004685 | OR2F2 | 954 | 0 | 0 | 0 | 0 | 0 | 0 | 0 | 0 | 0 | 0 | 0 | 0 | 0 |
| NM_001004686 | OR2L2 | 1134 | 0 | 0 | 0 | 0 | 0 | 0 | 0 | 0 | 0 | 0 | 0 | 0 | 0 |
| NM_001004687 | OR2L3 | 939 | 0 | 0 | 0 | 0 | 0 | 0 | 0 | 0 | 0 | 0 | 0 | 0 | 0 |
| NM_001004688 | OR2M2 | 1044 | 0 | 0 | 0 | 0 | 0 | 0 | 0 | 0 | 0 | 0 | 0 | 0 | 0 |
| NM_001004689 | OR2M3 | 939 | 0 | 0 | 0 | 0 | 0 | 0 | 0 | 0 | 0 | 0 | 0 | 0 | 0 |
| NM_001004690 | OR2M5 | 939 | 0 | 0 | 0 | 0 | 0 | 0 | 0 | 0 | 0 | 0 | 0 | 0 | 0 |
| NM_001004691 | OR2M7 | 939 | 0 | 0 | 0 | 0 | 0 | 0 | 0 | 0 | 0 | 0 | 0 | 0 | 0 |
| NM_001004692 | OR2T12 | 963 | 0 | 0 | 0 | 0 | 0 | 0 | 0 | 0 | 0 | 0 | 0 | 0 | 0 |
| NM_001004693 | OR2T10 | 939 | 0 | 0 | 0 | 0 | 0 | 0 | 0 | 0 | 0 | 0 | 0 | 0 | 0 |
| NM_001004694 | OR2T29 | 930 | 0 | 0 | 0 | 0 | 0 | 0 | 0 | 0 | 0 | 0 | 0 | 0 | 0 |
| NM_001004695 | OR2T33 | 963 | 0 | 0 | 0 | 0 | 0 | 0 | 0 | 0 | 0 | 0 | 0 | 0 | 0 |
| NM_001004696 | OR2T4 | 1047 | 0 | 0 | 0 | 0 | 0 | 0 | 0 | 0 | 0 | 0 | 0 | 0 | 0 |
| NM_001004697 | OR2T5 | 948 | 0 | 0 | 0 | 0 | 0 | 0 | 0 | 0 | 0 | 0 | 0 | 0 | 0 |
| NM_001004698 | OR2W5 | 963 | 0 | 0 | 0 | 0 | 0 | 0 | 0 | 0 | 0 | 0 | 0 | 0 | 0 |
| NM_001004699 | OR2Z1 | 945 | 0 | 0 | 0 | 0 | 0 | 0 | 0 | 0 | 0 | 0 | 0 | 0 | 0 |
| NM_001004700 | OR4C11 | 933 | 0 | 0 | 0 | 0 | 0 | 0 | 0 | 0 | 0 | 0 | 0 | 0 | 0 |
| NM_001004701 | OR4C16 | 933 | 0 | 0 | 0 | 0 | 0 | 0 | 0 | 0 | 0 | 0 | 0 | 0 | 0 |
| NM_001004702 | OR4C3 | 990 | 0 | 0 | 0 | 0 | 0 | 0 | 0 | 0 | 0 | 0 | 0 | 0 | 0 |
| NM_001004703 | OR4C46 | 930 | 0 | 0 | 0 | 0 | 0 | 0 | 0 | 0 | 0 | 0 | 0 | 0 | 0 |
| NM_001004704 | OR4C6 | 930 | 0 | 0 | 0 | 0 | 0 | 0 | 0 | 0 | 0 | 0 | 0 | 0 | 0 |
| NM_001004705 | OR4D10 | 936 | 0 | 0 | 0 | 0 | 0 | 0 | 0 | 0 | 0 | 0 | 0 | 0 | 0 |
| NM_001004706 | OR4D11 | 936 | 0 | 0 | 0 | 0 | 0 | 0 | 0 | 0 | 0 | 0 | 0 | 0 | 0 |
| NM_001004707 | OR4D2 | 924 | 0 | 0 | 0 | 0 | 0 | 0 | 0 | 0 | 0 | 0 | 0 | 0 | 0 |
| NM_001004708 | OR4D6 | 945 | 0 | 0 | 0 | 0 | 0 | 0 | 0 | 0 | 0 | 0 | 0 | 0 | 0 |
| NM_001004711 | OR4D9 | 945 | 0 | 0 | 0 | 0 | 0 | 0 | 0 | 0 | 0 | 0 | 0 | 0 | 0 |
| NM_001004712 | OR4K14 | 933 | 0 | 0 | 0 | 0 | 0 | 0 | 0 | 0 | 0 | 0 | 0 | 0 | 0 |
| NM_001004713 | OR1I1 | 1068 | 0 | 0 | 0 | 0 | 0 | 0 | 0 | 0 | 0 | 0 | 0 | 0 | 0 |
| NM_001004714 | OR4K13 | 915 | 0 | 0 | 0 | 0 | 0 | 0 | 0 | 0 | 0 | 0 | 0 | 0 | 0 |
| NM_001004715 | OR4K17 | 1032 | 0 | 0 | 0 | 0 | 0 | 0 | 0 | 0 | 0 | 0 | 0 | 0 | 0 |
| NM_001004717 | OR4L1 | 939 | 0 | 0 | 0 | 0 | 0 | 0 | 0 | 0 | 0 | 0 | 0 | 0 | 0 |
| NM_001004719 | OR4M2 | 942 | 0 | 0 | 0 | 0 | 0 | 0 | 0 | 0 | 0 | 0 | 0 | 0 | 0 |
| NM_001004720 | NCK2 | 2304 | 3 | 1 | 1 | 1 | 1 | 1 | 1 | 0 | 1 | 1 | 0 | 3 | 1 |
| NM_001004722 | NCK2 | 1959 | 3 | 1 | 1 | 1 | 1 | 2 | 1 | 0 | 1 | 1 | 1 | 3 | 1 |

| transcript | gene | transcript length (nt) | max | mean | adipose | colon | heart | hypothala mus | kidney | liver | lung | ovary | skeletalm uscle | spleen | testes |
|---|---|---|---|---|---|---|---|---|---|---|---|---|---|---|---|
| NM_001004723 | OR4N2 | 924 | 0 | 0 | 0 | 0 | 0 | 0 | 0 | 0 | 0 | 0 | 0 | 0 | 0 |
| NM_001004724 | OR4N5 | 927 | 0 | 0 | 0 | 0 | 0 | 0 | 0 | 0 | 0 | 0 | 0 | 0 | 0 |
| NM_001004725 | OR4S1 | 930 | 0 | 0 | 0 | 0 | 0 | 0 | 0 | 0 | 0 | 0 | 0 | 0 | 0 |
| NM_001004726 | OR4X1 | 918 | 0 | 0 | 0 | 0 | 0 | 0 | 0 | 0 | 0 | 0 | 0 | 0 | 0 |
| NM_001004727 | OR4X2 | 912 | 0 | 0 | 0 | 0 | 0 | 0 | 0 | 0 | 0 | 0 | 0 | 0 | 0 |
| NM_001004728 | OR5A1 | 948 | 0 | 0 | 0 | 0 | 0 | 0 | 0 | 0 | 0 | 0 | 0 | 0 | 0 |
| NM_001004729 | OR5AN1 | 936 | 0 | 0 | 0 | 0 | 0 | 0 | 0 | 0 | 0 | 0 | 0 | 0 | 0 |
| NM_001004730 | OR5AR1 | 933 | 0 | 0 | 0 | 0 | 0 | 0 | 0 | 0 | 0 | 0 | 0 | 0 | 0 |
| NM_001004731 | OR5AU1 | 1089 | 0 | 0 | 0 | 0 | 0 | 0 | 0 | 0 | 0 | 0 | 0 | 0 | 0 |
| NM_001004733 | OR5B12 | 945 | 0 | 0 | 0 | 0 | 0 | 0 | 0 | 0 | 0 | 0 | 0 | 0 | 0 |
| NM_001004734 | OR14I1 | 936 | 0 | 0 | 0 | 0 | 0 | 0 | 0 | 0 | 0 | 0 | 0 | 0 | 0 |
| NM_001004735 | OR5D14 | 945 | 0 | 0 | 0 | 0 | 0 | 0 | 0 | 0 | 0 | 0 | 0 | 0 | 0 |
| NM_001004736 | OR5K1 | 952 | 0 | 0 | 0 | 0 | 0 | 0 | 0 | 0 | 0 | 0 | 0 | 0 | 0 |
| NM_001004737 | OR5K2 | 951 | 0 | 0 | 0 | 0 | 0 | 0 | 0 | 0 | 0 | 0 | 0 | 0 | 0 |
| NM_001004738 | OR5L1 | 936 | 0 | 0 | 0 | 0 | 0 | 0 | 0 | 0 | 0 | 0 | 0 | 0 | 0 |
| NM_001004739 | OR5L2 | 936 | 0 | 0 | 0 | 0 | 0 | 0 | 0 | 0 | 0 | 0 | 0 | 0 | 0 |
| NM_001004740 | OR5M1 | 948 | 0 | 0 | 0 | 0 | 0 | 0 | 0 | 0 | 0 | 0 | 0 | 0 | 0 |
| NM_001004741 | OR5M10 | 948 | 0 | 0 | 0 | 0 | 0 | 0 | 0 | 0 | 0 | 0 | 0 | 0 | 0 |
| NM_001004742 | OR5M3 | 924 | 0 | 0 | 0 | 0 | 0 | 0 | 0 | 0 | 0 | 0 | 0 | 0 | 0 |
| NM_001004743 | OR5M9 | 933 | 0 | 0 | 0 | 0 | 0 | 0 | 0 | 0 | 0 | 0 | 0 | 0 | 0 |
| NM_001004744 | OR5R1 | 975 | 0 | 0 | 0 | 0 | 0 | 0 | 0 | 0 | 0 | 0 | 0 | 0 | 0 |
| NM_001004745 | OR5T1 | 981 | 0 | 0 | 0 | 0 | 0 | 0 | 0 | 0 | 0 | 0 | 0 | 0 | 0 |
| NM_001004746 | OR5T2 | 1080 | 0 | 0 | 0 | 0 | 0 | 0 | 0 | 0 | 0 | 0 | 0 | 0 | 0 |
| NM_001004747 | OR5T3 | 1023 | 0 | 0 | 0 | 0 | 0 | 0 | 0 | 0 | 0 | 0 | 0 | 0 | 0 |
| NM_001004748 | OR51A2 | 942 | 0 | 0 | 0 | 0 | 0 | 0 | 0 | 0 | 0 | 0 | 0 | 0 | 0 |
| NM_001004749 | OR51A7 | 939 | 0 | 0 | 0 | 0 | 0 | 0 | 0 | 0 | 0 | 0 | 0 | 0 | 0 |
| NM_001004750 | OR51B6 | 939 | 0 | 0 | 0 | 0 | 0 | 0 | 0 | 0 | 0 | 0 | 0 | 0 | 0 |
| NM_001004751 | OR51D1 | 975 | 0 | 0 | 0 | 0 | 0 | 0 | 0 | 0 | 0 | 0 | 0 | 0 | 0 |
| NM_001004752 | OR51F1 | 939 | 0 | 0 | 0 | 0 | 0 | 0 | 0 | 0 | 0 | 0 | 0 | 0 | 0 |
| NM_001004753 | OR51F2 | 1029 | 0 | 0 | 0 | 0 | 0 | 0 | 0 | 0 | 0 | 0 | 0 | 0 | 0 |
| NM_001004754 | OR51I2 | 939 | 1 | 0 | 0 | 0 | 0 | 0 | 0 | 0 | 0 | 1 | 0 | 0 | 0 |
| NM_001004755 | OR51L1 | 948 | 0 | 0 | 0 | 0 | 0 | 0 | 0 | 0 | 0 | 0 | 0 | 0 | 0 |
| NM_001004756 | OR51M1 | 981 | 0 | 0 | 0 | 0 | 0 | 0 | 0 | 0 | 0 | 0 | 0 | 0 | 0 |
| NM_001004757 | OR51Q1 | 954 | 1 | 0 | 0 | 0 | 0 | 0 | 0 | 0 | 0 | 1 | 0 | 0 | 0 |
| NM_001004758 | OR51S1 | 972 | 0 | 0 | 0 | 0 | 0 | 0 | 0 | 0 | 0 | 0 | 0 | 0 | 0 |
| NM_001004759 | OR51T1 | 1065 | 0 | 0 | 0 | 0 | 0 | 0 | 0 | 0 | 0 | 0 | 0 | 0 | 0 |
| NM_001004760 | OR51V1 | 966 | 0 | 0 | 0 | 0 | 0 | 0 | 0 | 0 | 0 | 0 | 0 | 0 | 0 |
| NM_001005 | RPS3 | 841 | 71 | 35 | 27 | 39 | 27 | 19 | 20 | 34 | 37 | 71 | 21 | 59 | 26 |
| NM_001005158 | SFMBT1 | 3614 | 4 | 1 | 0 | 1 | 1 | 1 | 2 | 1 | 1 | 1 | 2 | 1 | 4 |
| NM_001005159 | SFMBT1 | 3695 | 4 | 1 | 0 | 1 | 1 | 1 | 2 | 1 | 1 | 1 | 2 | 1 | 4 |
| NM_001005160 | OR52A5 | 951 | 0 | 0 | 0 | 0 | 0 | 0 | 0 | 0 | 0 | 0 | 0 | 0 | 0 |
| NM_001005161 | OR52B4 | 945 | 0 | 0 | 0 | 0 | 0 | 0 | 0 | 0 | 0 | 0 | 0 | 0 | 0 |
| NM_001005162 | OR52B6 | 1008 | 2 | 0 | 0 | 0 | 0 | 0 | 0 | 0 | 1 | 2 | 0 | 0 | 1 |
| NM_001005163 | OR52D1 | 957 | 0 | 0 | 0 | 0 | 0 | 0 | 0 | 0 | 0 | 0 | 0 | 0 | 0 |
| NM_001005164 | OR52E2 | 978 | 0 | 0 | 0 | 0 | 0 | 0 | 0 | 0 | 0 | 0 | 0 | 0 | 0 |
| NM_001005165 | OR52E4 | 939 | 0 | 0 | 0 | 0 | 0 | 0 | 0 | 0 | 0 | 0 | 0 | 0 | 0 |
| NM_001005167 | OR52E6 | 942 | 0 | 0 | 0 | 0 | 0 | 0 | 0 | 0 | 0 | 0 | 0 | 0 | 0 |
| NM_001005168 | OR52E8 | 954 | 0 | 0 | 0 | 0 | 0 | 0 | 0 | 0 | 0 | 0 | 0 | 0 | 0 |
| NM_001005169 | OR52I1 | 975 | 0 | 0 | 0 | 0 | 0 | 0 | 0 | 0 | 0 | 0 | 0 | 0 | 0 |
| NM_001005170 | OR52I2 | 1053 | 0 | 0 | 0 | 0 | 0 | 0 | 0 | 0 | 0 | 0 | 0 | 0 | 0 |
| NM_001005171 | OR52K1 | 945 | 0 | 0 | 0 | 0 | 0 | 0 | 0 | 0 | 0 | 0 | 0 | 0 | 0 |
| NM_001005172 | OR52K2 | 945 | 0 | 0 | 0 | 0 | 0 | 0 | 0 | 0 | 0 | 0 | 0 | 0 | 0 |
| NM_001005173 | OR52L1 | 1094 | 0 | 0 | 0 | 0 | 0 | 0 | 0 | 0 | 0 | 0 | 0 | 0 | 0 |
| NM_001005174 | OR52N2 | 966 | 0 | 0 | 0 | 0 | 0 | 0 | 0 | 0 | 0 | 0 | 0 | 0 | 0 |
| NM_001005175 | OR52N4 | 1036 | 0 | 0 | 0 | 0 | 0 | 0 | 0 | 0 | 0 | 0 | 0 | 0 | 0 |
| NM_001005176 | SP140 | 835 | 6 | 1 | 0 | 1 | 0 | 0 | 0 | 0 | 1 | 0 | 0 | 6 | 0 |
| NM_001005177 | OR52R1 | 947 | 0 | 0 | 0 | 0 | 0 | 0 | 0 | 0 | 0 | 0 | 0 | 0 | 0 |
| NM_001005178 | OR52W1 | 963 | 0 | 0 | 0 | 0 | 0 | 0 | 0 | 0 | 0 | 0 | 0 | 0 | 0 |
| NM_001005179 | OR56A4 | 1098 | 0 | 0 | 0 | 0 | 0 | 0 | 0 | 0 | 0 | 0 | 0 | 0 | 0 |
| NM_001005180 | OR56B1 | 975 | 0 | 0 | 0 | 0 | 0 | 0 | 0 | 0 | 0 | 0 | 0 | 0 | 0 |
| NM_001005181 | OR56B4 | 960 | 0 | 0 | 0 | 0 | 0 | 0 | 0 | 0 | 0 | 0 | 0 | 0 | 0 |
| NM_001005182 | OR6C1 | 939 | 0 | 0 | 0 | 0 | 0 | 0 | 0 | 0 | 0 | 0 | 0 | 0 | 0 |
| NM_001005183 | OR6C76 | 939 | 0 | 0 | 0 | 0 | 0 | 0 | 0 | 0 | 0 | 0 | 0 | 0 | 0 |
| NM_001005184 | OR6K6 | 1032 | 0 | 0 | 0 | 0 | 0 | 0 | 0 | 0 | 0 | 0 | 0 | 0 | 0 |
| NM_001005185 | OR6N1 | 939 | 0 | 0 | 0 | 0 | 0 | 0 | 0 | 0 | 0 | 0 | 0 | 0 | 0 |
| NM_001005186 | OR6Q1 | 954 | 0 | 0 | 0 | 0 | 0 | 0 | 0 | 0 | 0 | 0 | 0 | 0 | 0 |
| NM_001005187 | OR6T1 | 972 | 0 | 0 | 0 | 0 | 0 | 0 | 0 | 0 | 0 | 0 | 0 | 0 | 0 |
| NM_001005188 | OR6X1 | 939 | 0 | 0 | 0 | 0 | 0 | 0 | 0 | 0 | 0 | 0 | 0 | 0 | 0 |
| NM_001005189 | OR6Y1 | 978 | 0 | 0 | 0 | 0 | 0 | 0 | 0 | 0 | 0 | 0 | 0 | 0 | 0 |
| NM_001005190 | OR7A10 | 930 | 0 | 0 | 0 | 0 | 0 | 0 | 0 | 0 | 0 | 0 | 0 | 0 | 0 |
| NM_001005191 | OR7D4 | 939 | 0 | 0 | 0 | 0 | 0 | 0 | 0 | 0 | 0 | 0 | 0 | 0 | 0 |
| NM_001005192 | OR7G1 | 1002 | 0 | 0 | 0 | 0 | 0 | 0 | 0 | 0 | 0 | 0 | 0 | 0 | 0 |
| NM_001005193 | OR7G2 | 1038 | 0 | 0 | 0 | 0 | 0 | 0 | 0 | 0 | 0 | 0 | 0 | 0 | 0 |
| NM_001005194 | OR8A1 | 981 | 0 | 0 | 0 | 0 | 0 | 0 | 0 | 0 | 0 | 0 | 0 | 0 | 0 |
| NM_001005195 | OR8B12 | 933 | 0 | 0 | 0 | 0 | 0 | 0 | 0 | 0 | 0 | 0 | 0 | 0 | 0 |
| NM_001005196 | OR8B4 | 930 | 0 | 0 | 0 | 0 | 0 | 0 | 0 | 0 | 0 | 0 | 0 | 0 | 0 |
| NM_001005197 | OR8D4 | 945 | 0 | 0 | 0 | 0 | 0 | 0 | 0 | 0 | 0 | 0 | 0 | 0 | 0 |

| transcript | gene | transcript length (nt) | max | mean | adipose | colon | heart | hypothalamus | kidney | liver | lung | ovary | skeletalmuscle | spleen | testes |
|---|---|---|---|---|---|---|---|---|---|---|---|---|---|---|---|
| NM_001005198 | OR8G5 | 1041 | 0 | 0 | 0 | 0 | 0 | 0 | 0 | 0 | 0 | 0 | 0 | 0 | 0 |
| NM_001005199 | OR8H1 | 936 | 0 | 0 | 0 | 0 | 0 | 0 | 0 | 0 | 0 | 0 | 0 | 0 | 0 |
| NM_001005200 | OR8H2 | 939 | 0 | 0 | 0 | 0 | 0 | 0 | 0 | 0 | 0 | 0 | 0 | 0 | 0 |
| NM_001005201 | OR8H3 | 939 | 0 | 0 | 0 | 0 | 0 | 0 | 0 | 0 | 0 | 0 | 0 | 0 | 0 |
| NM_001005202 | OR8K3 | 939 | 0 | 0 | 0 | 0 | 0 | 0 | 0 | 0 | 0 | 0 | 0 | 0 | 0 |
| NM_001005203 | OR8S1 | 1080 | 0 | 0 | 0 | 0 | 0 | 0 | 0 | 0 | 0 | 0 | 0 | 0 | 0 |
| NM_001005204 | OR8U1 | 930 | 0 | 0 | 0 | 0 | 0 | 0 | 0 | 0 | 0 | 0 | 0 | 0 | 0 |
| NM_001005205 | OR8J1 | 951 | 0 | 0 | 0 | 0 | 0 | 0 | 0 | 0 | 0 | 0 | 0 | 0 | 0 |
| NM_001005207 | TRIM37 | 3618 | 12 | 3 | 2 | 1 | 2 | 12 | 2 | 2 | 1 | 2 | 1 | 2 | 8 |
| NM_001005209 | TMEM198 | 2218 | 0 | 0 | 0 | 0 | 0 | 0 | 0 | 0 | 0 | 0 | 0 | 0 | 0 |
| NM_001005210 | LRRC55 | 5407 | 1 | 0 | 0 | 0 | 0 | 1 | 0 | 0 | 0 | 0 | 0 | 0 | 0 |
| NM_001005211 | OR9I1 | 945 | 0 | 0 | 0 | 0 | 0 | 0 | 0 | 0 | 0 | 0 | 0 | 0 | 0 |
| NM_001005212 | OR9Q1 | 2438 | 0 | 0 | 0 | 0 | 0 | 0 | 0 | 0 | 0 | 0 | 0 | 0 | 0 |
| NM_001005213 | OR9G1 | 918 | 0 | 0 | 0 | 0 | 0 | 0 | 0 | 0 | 0 | 0 | 0 | 0 | 0 |
| NM_001005214 | LRRC52 | 1122 | 0 | 0 | 0 | 0 | 0 | 0 | 0 | 0 | 0 | 0 | 0 | 0 | 0 |
| NM_001005216 | OR2J3 | 936 | 0 | 0 | 0 | 0 | 0 | 0 | 0 | 0 | 0 | 0 | 0 | 0 | 0 |
| NM_001005217 | FRG2 | 2083 | 0 | 0 | 0 | 0 | 0 | 0 | 0 | 0 | 0 | 0 | 0 | 0 | 0 |
| NM_001005218 | OR5B21 | 930 | 0 | 0 | 0 | 0 | 0 | 0 | 0 | 0 | 0 | 0 | 0 | 0 | 0 |
| NM_001005221 | OR4F29 | 939 | 0 | 0 | 0 | 0 | 0 | 0 | 0 | 0 | 0 | 0 | 0 | 0 | 0 |
| NM_001005222 | OR52A4 | 915 | 0 | 0 | 0 | 0 | 0 | 0 | 0 | 0 | 0 | 0 | 0 | 0 | 0 |
| NM_001005224 | OR4F3 | 939 | 0 | 0 | 0 | 0 | 0 | 0 | 0 | 0 | 0 | 0 | 0 | 0 | 0 |
| NM_001005226 | OR2B3 | 1105 | 0 | 0 | 0 | 0 | 0 | 0 | 0 | 0 | 0 | 0 | 0 | 0 | 0 |
| NM_001005226 | OR2B3 | 1106 | 0 | 0 | 0 | 0 | 0 | 0 | 0 | 0 | 0 | 0 | 0 | 0 | 0 |
| NM_001005234 | OR1L3 | 975 | 0 | 0 | 0 | 0 | 0 | 0 | 0 | 0 | 0 | 0 | 0 | 0 | 0 |
| NM_001005235 | OR1L4 | 936 | 0 | 0 | 0 | 0 | 0 | 0 | 0 | 0 | 0 | 0 | 0 | 0 | 0 |
| NM_001005236 | OR1L1 | 931 | 0 | 0 | 0 | 0 | 0 | 0 | 0 | 0 | 0 | 0 | 0 | 0 | 0 |
| NM_001005237 | OR51G1 | 966 | 0 | 0 | 0 | 0 | 0 | 0 | 0 | 0 | 0 | 0 | 0 | 0 | 0 |
| NM_001005238 | OR51G2 | 945 | 0 | 0 | 0 | 0 | 0 | 0 | 0 | 0 | 0 | 0 | 0 | 0 | 0 |
| NM_001005239 | OR11H1 | 981 | 0 | 0 | 0 | 0 | 0 | 0 | 0 | 0 | 0 | 0 | 0 | 0 | 0 |
| NM_001005240 | OR4F17 | 918 | 0 | 0 | 0 | 0 | 0 | 0 | 0 | 0 | 0 | 0 | 0 | 0 | 0 |
| NM_001005241 | OR4N4 | 951 | 0 | 0 | 0 | 0 | 0 | 0 | 0 | 0 | 0 | 0 | 0 | 0 | 0 |
| NM_001005242 | PKP2 | 4307 | 36 | 6 | 0 | 6 | 36 | 2 | 2 | 8 | 1 | 4 | 1 | 2 | 3 |
| NM_001005243 | OR9K2 | 1008 | 0 | 0 | 0 | 0 | 0 | 0 | 0 | 0 | 0 | 0 | 0 | 0 | 0 |
| NM_001005245 | OR5M11 | 918 | 0 | 0 | 0 | 0 | 0 | 0 | 0 | 0 | 0 | 0 | 0 | 0 | 0 |
| NM_001005270 | OR4C12 | 930 | 0 | 0 | 0 | 0 | 0 | 0 | 0 | 0 | 0 | 0 | 0 | 0 | 0 |
| NM_001005271 | CHD3 | 7354 | 22 | 11 | 6 | 16 | 6 | 22 | 12 | 1 | 12 | 18 | 1 | 16 | 13 |
| NM_001005272 | OR4A5 | 948 | 0 | 0 | 0 | 0 | 0 | 0 | 0 | 0 | 0 | 0 | 0 | 0 | 0 |
| NM_001005273 | CHD3 | 7325 | 21 | 10 | 6 | 14 | 6 | 21 | 11 | 1 | 11 | 17 | 1 | 15 | 12 |
| NM_001005274 | OR4A16 | 987 | 0 | 0 | 0 | 0 | 0 | 0 | 0 | 0 | 0 | 0 | 0 | 0 | 0 |
| NM_001005275 | OR4A15 | 1035 | 0 | 0 | 0 | 0 | 0 | 0 | 0 | 0 | 0 | 0 | 0 | 0 | 0 |
| NM_001005276 | OR2AE1 | 972 | 0 | 0 | 0 | 0 | 0 | 0 | 0 | 0 | 0 | 0 | 0 | 0 | 0 |
| NM_001005277 | OR4F16 | 939 | 0 | 0 | 0 | 0 | 0 | 0 | 0 | 0 | 0 | 0 | 0 | 0 | 0 |
| NM_001005278 | OR6N2 | 954 | 0 | 0 | 0 | 0 | 0 | 0 | 0 | 0 | 0 | 0 | 0 | 0 | 0 |
| NM_001005279 | OR6K2 | 975 | 0 | 0 | 0 | 0 | 0 | 0 | 0 | 0 | 0 | 0 | 0 | 0 | 0 |
| NM_001005280 | OR10A7 | 951 | 0 | 0 | 0 | 0 | 0 | 0 | 0 | 0 | 0 | 0 | 0 | 0 | 0 |
| NM_001005281 | OR6B1 | 936 | 0 | 0 | 0 | 0 | 0 | 0 | 0 | 0 | 0 | 0 | 0 | 0 | 0 |
| NM_001005282 | OR5M8 | 936 | 0 | 0 | 0 | 0 | 0 | 0 | 0 | 0 | 0 | 0 | 0 | 0 | 0 |
| NM_001005283 | OR9Q2 | 945 | 0 | 0 | 0 | 0 | 0 | 0 | 0 | 0 | 0 | 0 | 0 | 0 | 0 |
| NM_001005284 | OR9G4 | 984 | 0 | 0 | 0 | 0 | 0 | 0 | 0 | 0 | 0 | 0 | 0 | 0 | 0 |
| NM_001005285 | OR2AT4 | 963 | 0 | 0 | 0 | 0 | 0 | 0 | 0 | 0 | 0 | 0 | 0 | 0 | 0 |
| NM_001005286 | OR6F1 | 927 | 0 | 0 | 0 | 0 | 0 | 0 | 0 | 0 | 0 | 0 | 0 | 0 | 0 |
| NM_001005287 | OR2A1 | 933 | 0 | 0 | 0 | 0 | 0 | 0 | 0 | 0 | 0 | 0 | 0 | 0 | 0 |
| NM_001005288 | OR51I1 | 945 | 0 | 0 | 0 | 0 | 0 | 0 | 0 | 0 | 0 | 0 | 0 | 0 | 0 |
| NM_001005289 | OR52H1 | 963 | 1 | 0 | 0 | 0 | 0 | 0 | 0 | 0 | 0 | 1 | 0 | 0 | 0 |
| NM_001005290 | PSRC1 | 1618 | 11 | 2 | 1 | 2 | 0 | 11 | 0 | 1 | 1 | 1 | 0 | 1 | 5 |
| NM_001005291 | SREBF1 | 4996 | 3 | 1 | 1 | 1 | 0 | 1 | 1 | 3 | 1 | 0 | 1 | 1 | 1 |
| NM_001005323 | OR5AK2 | 930 | 5 | 0 | 0 | 0 | 0 | 0 | 0 | 0 | 0 | 0 | 0 | 5 | 0 |
| NM_001005324 | OR10V1 | 930 | 0 | 0 | 0 | 0 | 0 | 0 | 0 | 0 | 0 | 0 | 0 | 0 | 0 |
| NM_001005325 | OR6M1 | 942 | 0 | 0 | 0 | 0 | 0 | 0 | 0 | 0 | 0 | 0 | 0 | 0 | 0 |
| NM_001005326 | OR4F6 | 939 | 0 | 0 | 0 | 0 | 0 | 0 | 0 | 0 | 0 | 0 | 0 | 0 | 0 |
| NM_001005327 | OR6K3 | 996 | 0 | 0 | 0 | 0 | 0 | 0 | 0 | 0 | 0 | 0 | 0 | 0 | 0 |
| NM_001005328 | OR2A7 | 933 | 1 | 0 | 0 | 0 | 0 | 0 | 1 | 0 | 0 | 0 | 0 | 1 | 0 |
| NM_001005329 | OR51A4 | 942 | 0 | 0 | 0 | 0 | 0 | 0 | 0 | 0 | 0 | 0 | 0 | 0 | 0 |
| NM_001005332 | MAGED1 | 2649 | 26 | 8 | 3 | 5 | 7 | 26 | 6 | 12 | 3 | 8 | 4 | 5 | 11 |
| NM_001005333 | MAGED1 | 2912 | 24 | 7 | 3 | 4 | 6 | 24 | 5 | 11 | 3 | 8 | 3 | 4 | 10 |
| NM_001005335 | HNRNPL | 1881 | 42 | 29 | 28 | 29 | 27 | 26 | 25 | 42 | 35 | 21 | 20 | 42 | 26 |
| NM_001005336 | DNM1 | 3252 | 39 | 4 | 2 | 1 | 1 | 39 | 1 | 0 | 0 | 0 | 1 | 0 | 1 |
| NM_001005337 | PKP1 | 5380 | 1 | 0 | 1 | 0 | 0 | 0 | 0 | 0 | 0 | 0 | 0 | 0 | 0 |
| NM_001005338 | OR5H1 | 942 | 0 | 0 | 0 | 0 | 0 | 0 | 0 | 0 | 0 | 0 | 0 | 0 | 0 |
| NM_001005339 | RGS10 | 904 | 2 | 1 | 2 | 2 | 1 | 2 | 1 | 0 | 2 | 1 | 1 | 2 | 0 |
| NM_001005340 | GPNMB | 2766 | 55 | 18 | 51 | 7 | 21 | 1 | 4 | 3 | 55 | 19 | 8 | 10 | 14 |
| NM_001005353 | AK3L1 | 2345 | 20 | 5 | 4 | 1 | 7 | 3 | 20 | 11 | 0 | 1 | 0 | 1 | 4 |
| NM_001005354 | PRR13 | 1045 | 48 | 17 | 13 | 19 | 10 | 17 | 48 | 17 | 16 | 7 | 6 | 32 | 7 |
| NM_001005356 | POTEG | 1926 | 0 | 0 | 0 | 0 | 0 | 0 | 0 | 0 | 0 | 0 | 0 | 0 | 0 |
| NM_001005360 | DNM2 | 3633 | 12 | 7 | 5 | 12 | 9 | 6 | 9 | 5 | 7 | 3 | 6 | 10 | 8 |
| NM_001005361 | DNM2 | 3633 | 12 | 7 | 5 | 12 | 9 | 6 | 9 | 5 | 7 | 3 | 6 | 10 | 8 |
| NM_001005362 | DNM2 | 3621 | 12 | 7 | 5 | 12 | 9 | 6 | 9 | 5 | 7 | 3 | 6 | 10 | 8 |

| transcript | gene | transcript length (nt) | max | mean | adipose | colon | heart | hypothalamus | kidney | liver | lung | ovary | skeletalmuscle | spleen | testes |
|---|---|---|---|---|---|---|---|---|---|---|---|---|---|---|---|
| NM_001005365 | POTEA | 1976 | 1 | 0 | 0 | 0 | 0 | 0 | 0 | 0 | 0 | 0 | 0 | 0 | 1 |
| NM_001005366 | KDM2B | 5225 | 1 | 1 | 1 | 1 | 0 | 1 | 0 | 1 | 1 | 1 | 1 | 1 | 1 |
| NM_001005367 | TTYH1 | 1983 | 2 | 0 | 0 | 0 | 0 | 2 | 0 | 0 | 0 | 0 | 0 | 0 | 0 |
| NM_001005368 | ZNF32 | 1202 | 11 | 6 | 7 | 2 | 8 | 11 | 7 | 3 | 3 | 8 | 6 | 7 | 5 |
| NM_001005369 | MTIF2 | 2949 | 16 | 7 | 7 | 3 | 11 | 8 | 9 | 16 | 4 | 4 | 5 | 8 | 6 |
| NM_001005373 | LRSAM1 | 3117 | 2 | 1 | 1 | 1 | 1 | 2 | 1 | 1 | 1 | 1 | 1 | 1 | 1 |
| NM_001005374 | LRSAM1 | 2849 | 3 | 1 | 1 | 1 | 1 | 3 | 1 | 1 | 1 | 1 | 1 | 1 | 1 |
| NM_001005375 | DAZ4 | 3820 | 0 | 0 | 0 | 0 | 0 | 0 | 0 | 0 | 0 | 0 | 0 | 0 | 0 |
| NM_001005376 | PLAUR | 1437 | 5 | 1 | 3 | 1 | 2 | 1 | 0 | 0 | 5 | 1 | 0 | 2 | 1 |
| NM_001005377 | PLAUR | 1413 | 4 | 1 | 1 | 1 | 2 | 1 | 0 | 0 | 4 | 1 | 0 | 2 | 1 |
| NM_001005386 | ACTR2 | 3932 | 76 | 28 | 25 | 11 | 27 | 38 | 22 | 21 | 30 | 22 | 14 | 76 | 23 |
| NM_001005388 | NFASC | 10333 | 9 | 2 | 0 | 1 | 1 | 9 | 2 | 0 | 0 | 2 | 0 | 1 | 0 |
| NM_001005389 | NFASC | 2508 | 3 | 1 | 0 | 1 | 0 | 3 | 1 | 0 | 0 | 1 | 0 | 0 | 0 |
| NM_001005404 | YPEL2 | 5232 | 8 | 6 | 7 | 3 | 6 | 7 | 6 | 5 | 7 | 3 | 8 | 7 | 8 |
| NM_001005405 | KRTAP5-11 | 1021 | 0 | 0 | 0 | 0 | 0 | 0 | 0 | 0 | 0 | 0 | 0 | 0 | 0 |
| NM_001005407 | CACNA1H | 8070 | 1 | 0 | 0 | 1 | 0 | 0 | 0 | 0 | 0 | 1 | 0 | 0 | 0 |
| NM_001005408 | NEURL4 | 5181 | 1 | 0 | 0 | 1 | 0 | 1 | 1 | 0 | 0 | 0 | 0 | 0 | 1 |
| NM_001005409 | SF3A1 | 4925 | 9 | 6 | 5 | 6 | 4 | 5 | 6 | 4 | 5 | 8 | 6 | 9 | 9 |
| NM_001005413 | ZWINT | 1519 | 4 | 1 | 1 | 4 | 1 | 0 | 1 | 0 | 1 | 1 | 0 | 2 | 3 |
| NM_001005415 | 02 Mar | 1380 | 2 | 1 | 1 | 1 | 2 | 1 | 1 | 1 | 1 | 0 | 1 | 1 | 0 |
| NM_001005416 | 02 Mar | 1170 | 2 | 1 | 1 | 1 | 2 | 1 | 1 | 1 | 1 | 0 | 2 | 2 | 0 |
| NM_001005417 | B4GALT2 | 1995 | 1 | 0 | 0 | 0 | 0 | 1 | 0 | 0 | 0 | 0 | 0 | 0 | 0 |
| NM_001005463 | EBF3 | 4361 | 4 | 1 | 4 | 0 | 1 | 0 | 0 | 0 | 0 | 0 | 1 | 0 | 1 |
| NM_001005464 | HIST2H3A | 507 | 0 | 0 | 0 | 0 | 0 | 0 | 0 | 0 | 0 | 0 | 0 | 0 | 0 |
| NM_001005465 | OR10G3 | 942 | 0 | 0 | 0 | 0 | 0 | 0 | 0 | 0 | 0 | 0 | 0 | 0 | 0 |
| NM_001005466 | OR10G2 | 933 | 0 | 0 | 0 | 0 | 0 | 0 | 0 | 0 | 0 | 0 | 0 | 0 | 0 |
| NM_001005467 | OR8B3 | 942 | 0 | 0 | 0 | 0 | 0 | 0 | 0 | 0 | 0 | 0 | 0 | 0 | 0 |
| NM_001005468 | OR8B2 | 942 | 0 | 0 | 0 | 0 | 0 | 0 | 0 | 0 | 0 | 0 | 0 | 0 | 0 |
| NM_001005469 | OR5B3 | 945 | 0 | 0 | 0 | 0 | 0 | 0 | 0 | 0 | 0 | 0 | 0 | 0 | 0 |
| NM_001005470 | OR4B1 | 930 | 0 | 0 | 0 | 0 | 0 | 0 | 0 | 0 | 0 | 0 | 0 | 0 | 0 |
| NM_001005471 | OR2T6 | 927 | 0 | 0 | 0 | 0 | 0 | 0 | 0 | 0 | 0 | 0 | 0 | 0 | 0 |
| NM_001005473 | PLCXD3 | 7739 | 13 | 3 | 1 | 0 | 13 | 5 | 3 | 1 | 1 | 9 | 0 | 0 | 1 |
| NM_001005474 | NFKBIZ | 3767 | 12 | 6 | 3 | 5 | 4 | 2 | 5 | 5 | 12 | 10 | 2 | 12 | 2 |
| NM_001005476 | PKP4 | 4461 | 20 | 6 | 4 | 3 | 6 | 17 | 20 | 4 | 4 | 4 | 2 | 3 | 4 |
| NM_001005479 | OR5H6 | 978 | 0 | 0 | 0 | 0 | 0 | 0 | 0 | 0 | 0 | 0 | 0 | 0 | 0 |
| NM_001005480 | OR2A2 | 957 | 0 | 0 | 0 | 0 | 0 | 0 | 0 | 0 | 0 | 0 | 0 | 0 | 0 |
| NM_001005482 | OR5H2 | 945 | 0 | 0 | 0 | 0 | 0 | 0 | 0 | 0 | 0 | 0 | 0 | 0 | 0 |
| NM_001005483 | OR4K5 | 972 | 0 | 0 | 0 | 0 | 0 | 0 | 0 | 0 | 0 | 0 | 0 | 0 | 0 |
| NM_001005484 | OR4F5 | 918 | 0 | 0 | 0 | 0 | 0 | 0 | 0 | 0 | 0 | 0 | 0 | 0 | 0 |
| NM_001005486 | OR4K15 | 1047 | 1 | 0 | 0 | 0 | 0 | 0 | 0 | 0 | 0 | 0 | 0 | 0 | 1 |
| NM_001005487 | OR13G1 | 924 | 1 | 0 | 0 | 0 | 0 | 0 | 0 | 0 | 0 | 0 | 0 | 0 | 1 |
| NM_001005489 | OR5B17 | 945 | 0 | 0 | 0 | 0 | 0 | 0 | 0 | 0 | 0 | 0 | 0 | 0 | 0 |
| NM_001005490 | OR6C74 | 939 | 0 | 0 | 0 | 0 | 0 | 0 | 0 | 0 | 0 | 0 | 0 | 0 | 0 |
| NM_001005491 | OR10AG1 | 906 | 0 | 0 | 0 | 0 | 0 | 0 | 0 | 0 | 0 | 0 | 0 | 0 | 0 |
| NM_001005492 | OR5J2 | 939 | 0 | 0 | 0 | 0 | 0 | 0 | 0 | 0 | 0 | 0 | 0 | 0 | 0 |
| NM_001005493 | OR6C6 | 945 | 0 | 0 | 0 | 0 | 0 | 0 | 0 | 0 | 0 | 0 | 0 | 0 | 0 |
| NM_001005494 | OR6C4 | 930 | 0 | 0 | 0 | 0 | 0 | 0 | 0 | 0 | 0 | 0 | 0 | 0 | 0 |
| NM_001005495 | OR2T3 | 957 | 0 | 0 | 0 | 0 | 0 | 0 | 0 | 0 | 0 | 0 | 0 | 0 | 0 |
| NM_001005496 | OR5D16 | 987 | 0 | 0 | 0 | 0 | 0 | 0 | 0 | 0 | 0 | 0 | 0 | 0 | 0 |
| NM_001005497 | OR6C75 | 939 | 0 | 0 | 0 | 0 | 0 | 0 | 0 | 0 | 0 | 0 | 0 | 0 | 0 |
| NM_001005498 | RHBDF2 | 3517 | 1 | 0 | 0 | 0 | 0 | 0 | 0 | 0 | 1 | 0 | 0 | 1 | 0 |
| NM_001005499 | OR6C70 | 939 | 0 | 0 | 0 | 0 | 0 | 0 | 0 | 0 | 0 | 0 | 0 | 0 | 0 |
| NM_001005500 | OR4M1 | 942 | 0 | 0 | 0 | 0 | 0 | 0 | 0 | 0 | 0 | 0 | 0 | 0 | 0 |
| NM_001005501 | OR4K2 | 945 | 0 | 0 | 0 | 0 | 0 | 0 | 0 | 0 | 0 | 0 | 0 | 0 | 0 |
| NM_001005502 | CPM | 6634 | 11 | 5 | 11 | 1 | 2 | 1 | 7 | 4 | 10 | 3 | 1 | 9 | 2 |
| NM_001005503 | OR11G2 | 1038 | 0 | 0 | 0 | 0 | 0 | 0 | 0 | 0 | 0 | 0 | 0 | 0 | 0 |
| NM_001005504 | OR4F21 | 939 | 0 | 0 | 0 | 0 | 0 | 0 | 0 | 0 | 0 | 0 | 0 | 0 | 0 |
| NM_001005505 | CACNA2D2 | 5342 | 3 | 1 | 0 | 0 | 0 | 1 | 0 | 0 | 3 | 0 | 0 | 0 | 1 |
| NM_001005512 | OR4A47 | 930 | 0 | 0 | 0 | 0 | 0 | 0 | 0 | 0 | 0 | 0 | 0 | 0 | 0 |
| NM_001005513 | OR4C45 | 919 | 0 | 0 | 0 | 0 | 0 | 0 | 0 | 0 | 0 | 0 | 0 | 0 | 0 |
| NM_001005514 | OR5H14 | 933 | 0 | 0 | 0 | 0 | 0 | 0 | 0 | 0 | 0 | 0 | 0 | 0 | 0 |
| NM_001005515 | OR5H15 | 942 | 0 | 0 | 0 | 0 | 0 | 0 | 0 | 0 | 0 | 0 | 0 | 0 | 0 |
| NM_001005516 | OR5K3 | 966 | 0 | 0 | 0 | 0 | 0 | 0 | 0 | 0 | 0 | 0 | 0 | 0 | 0 |
| NM_001005517 | OR5K4 | 966 | 0 | 0 | 0 | 0 | 0 | 0 | 0 | 0 | 0 | 0 | 0 | 0 | 0 |
| NM_001005518 | OR6C65 | 939 | 0 | 0 | 0 | 0 | 0 | 0 | 0 | 0 | 0 | 0 | 0 | 0 | 0 |
| NM_001005519 | OR6C68 | 954 | 0 | 0 | 0 | 0 | 0 | 0 | 0 | 0 | 0 | 0 | 0 | 0 | 0 |
| NM_001005522 | OR2T8 | 939 | 0 | 0 | 0 | 0 | 0 | 0 | 0 | 0 | 0 | 0 | 0 | 0 | 0 |
| NM_001005526 | SF3B1 | 618 | 39 | 23 | 21 | 13 | 21 | 19 | 23 | 21 | 25 | 24 | 16 | 39 | 29 |
| NM_001005527 | FAM19A4 | 2150 | 1 | 0 | 0 | 0 | 0 | 1 | 0 | 0 | 0 | 0 | 0 | 0 | 0 |
| NM_001005566 | OR5B2 | 930 | 0 | 0 | 0 | 0 | 0 | 0 | 0 | 0 | 0 | 0 | 0 | 0 | 0 |
| NM_001005567 | OR51B5 | 939 | 0 | 0 | 0 | 0 | 0 | 0 | 0 | 0 | 0 | 0 | 0 | 0 | 0 |
| NM_001005609 | EDA | 5274 | 1 | 0 | 1 | 0 | 1 | 0 | 1 | 0 | 0 | 1 | 0 | 0 | 0 |
| NM_001005610 | EDA | 1089 | 0 | 0 | 0 | 0 | 0 | 0 | 0 | 0 | 0 | 0 | 0 | 0 | 0 |
| NM_001005611 | EDA | 1233 | 0 | 0 | 0 | 0 | 0 | 0 | 0 | 0 | 0 | 0 | 0 | 0 | 0 |
| NM_001005612 | EDA | 1140 | 0 | 0 | 0 | 0 | 0 | 0 | 0 | 0 | 0 | 0 | 0 | 0 | 0 |
| NM_001005615 | EDA | 840 | 0 | 0 | 0 | 0 | 0 | 0 | 0 | 0 | 0 | 0 | 0 | 0 | 0 |
| NM_001005619 | ITGB4 | 5689 | 4 | 1 | 1 | 3 | 1 | 2 | 1 | 0 | 1 | 0 | 0 | 4 | 1 |

| transcript | gene | transcript length (nt) | max | mean | adipose | colon | heart | hypothalamus | kidney | liver | lung | ovary | skeletalmuscle | spleen | testes |
|---|---|---|---|---|---|---|---|---|---|---|---|---|---|---|---|
| NM_001005731 | ITGB4 | 5709 | 4 | 1 | 1 | 3 | 1 | 2 | 1 | 0 | 1 | 0 | 0 | 4 | 1 |
| NM_001005735 | CHEK2 | 1987 | 1 | 1 | 0 | 1 | 0 | 0 | 0 | 0 | 1 | 1 | 0 | 1 | 1 |
| NM_001005738 | FPR2 | 1913 | 38 | 6 | 0 | 0 | 6 | 2 | 0 | 3 | 20 | 0 | 0 | 38 | 1 |
| NM_001005739 | VPS54 | 3958 | 6 | 4 | 3 | 2 | 4 | 3 | 6 | 5 | 5 | 4 | 3 | 6 | 6 |
| NM_001005741 | GBA | 2414 | 1 | 1 | 1 | 1 | 1 | 1 | 1 | 1 | 0 | 0 | 0 | 1 | 1 |
| NM_001005742 | GBA | 2395 | 1 | 1 | 1 | 1 | 1 | 1 | 1 | 1 | 0 | 0 | 0 | 1 | 1 |
| NM_001005743 | NUMB | 3644 | 6 | 4 | 4 | 3 | 4 | 3 | 5 | 3 | 6 | 3 | 2 | 6 | 4 |
| NM_001005744 | NUMB | 3500 | 6 | 4 | 4 | 3 | 4 | 3 | 6 | 3 | 6 | 3 | 2 | 6 | 4 |
| NM_001005745 | NUMB | 3467 | 6 | 4 | 4 | 3 | 4 | 3 | 6 | 3 | 6 | 3 | 2 | 6 | 4 |
| NM_001005746 | CACNB4 | 7926 | 4 | 1 | 0 | 0 | 0 | 4 | 0 | 0 | 0 | 0 | 0 | 0 | 1 |
| NM_001005747 | CACNB4 | 8064 | 4 | 1 | 0 | 0 | 0 | 4 | 0 | 0 | 0 | 0 | 0 | 0 | 1 |
| NM_001005749 | GBA | 2553 | 1 | 1 | 1 | 1 | 0 | 1 | 1 | 1 | 0 | 0 | 0 | 1 | 1 |
| NM_001005750 | GBA | 2550 | 1 | 1 | 1 | 1 | 0 | 1 | 1 | 1 | 0 | 0 | 0 | 1 | 1 |
| NM_001005751 | FAM21A | 4672 | 0 | 0 | 0 | 0 | 0 | 0 | 0 | 0 | 0 | 0 | 0 | 0 | 0 |
| NM_001005752 | GJB3 | 1762 | 0 | 0 | 0 | 0 | 0 | 0 | 0 | 0 | 0 | 0 | 0 | 0 | 0 |
| NM_001005753 | VPS24 | 3111 | 12 | 8 | 10 | 6 | 12 | 10 | 10 | 2 | 8 | 7 | 9 | 8 | 7 |
| NM_001005781 | SUMO1 | 1078 | 3 | 2 | 1 | 1 | 2 | 3 | 2 | 3 | 1 | 1 | 1 | 1 | 1 |
| NM_001005782 | SUMO1 | 1452 | 2 | 1 | 1 | 1 | 1 | 2 | 2 | 2 | 1 | 1 | 1 | 1 | 1 |
| NM_001005783 | HAO2 | 1696 | 37 | 4 | 0 | 0 | 0 | 0 | 11 | 37 | 0 | 0 | 0 | 0 | 0 |
| NM_001005785 | DAZ2 | 3756 | 0 | 0 | 0 | 0 | 0 | 0 | 0 | 0 | 0 | 0 | 0 | 0 | 0 |
| NM_001005786 | DAZ2 | 3195 | 0 | 0 | 0 | 0 | 0 | 0 | 0 | 0 | 0 | 0 | 0 | 0 | 0 |
| NM_001005849 | SUMO2 | 994 | 2 | 2 | 2 | 1 | 2 | 2 | 2 | 1 | 1 | 2 | 1 | 1 | 2 |
| NM_001005850 | ZNF835 | 1680 | 1 | 1 | 0 | 1 | 0 | 1 | 0 | 0 | 0 | 1 | 0 | 0 | 1 |
| NM_001005851 | ZNF780B | 8663 | 2 | 1 | 1 | 1 | 1 | 2 | 2 | 0 | 1 | 2 | 1 | 2 | 1 |
| NM_001005852 | CYorf15A | 865 | 6 | 2 | 3 | 0 | 1 | 3 | 1 | 2 | 2 | 0 | 0 | 6 | 3 |
| NM_001005853 | OR6B2 | 939 | 0 | 0 | 0 | 0 | 0 | 0 | 0 | 0 | 0 | 0 | 0 | 0 | 0 |
| NM_001005855 | ATP8B2 | 1517 | 1 | 1 | 0 | 0 | 1 | 0 | 0 | 0 | 1 | 1 | 0 | 1 | 1 |
| NM_001005861 | RYK | 2940 | 21 | 8 | 5 | 3 | 10 | 3 | 7 | 3 | 8 | 21 | 6 | 10 | 16 |
| NM_001005862 | ERBB2 | 4816 | 5 | 2 | 0 | 5 | 3 | 1 | 4 | 1 | 2 | 1 | 1 | 1 | 1 |
| NM_001005909 | IP6K2 | 1795 | 11 | 6 | 6 | 8 | 4 | 8 | 5 | 3 | 4 | 8 | 3 | 7 | 11 |
| NM_001005910 | IP6K2 | 1240 | 8 | 4 | 2 | 7 | 3 | 3 | 8 | 1 | 2 | 6 | 2 | 5 | 5 |
| NM_001005911 | IP6K2 | 1237 | 8 | 4 | 2 | 7 | 3 | 3 | 8 | 1 | 2 | 6 | 2 | 5 | 5 |
| NM_001005914 | SEMA3B | 2923 | 2 | 1 | 0 | 1 | 0 | 2 | 1 | 0 | 1 | 0 | 0 | 0 | 0 |
| NM_001005915 | ERBB3 | 1050 | 1 | 0 | 0 | 1 | 0 | 0 | 1 | 1 | 0 | 0 | 0 | 0 | 0 |
| NM_001005918 | ATP7B | 6021 | 2 | 1 | 0 | 1 | 0 | 0 | 1 | 2 | 0 | 0 | 0 | 0 | 1 |
| NM_001005920 | JMJD8 | 2042 | 5 | 2 | 3 | 2 | 1 | 2 | 2 | 5 | 1 | 2 | 1 | 2 | 2 |
| NM_001005922 | KRTAP5-1 | 942 | 1 | 0 | 0 | 1 | 0 | 0 | 0 | 0 | 0 | 0 | 0 | 0 | 0 |
| NM_001006 | RPS3A | 905 | 75 | 36 | 31 | 18 | 36 | 24 | 34 | 31 | 37 | 75 | 26 | 63 | 26 |
| NM_001006109 | C3orf37 | 1793 | 3 | 2 | 2 | 2 | 3 | 3 | 2 | 2 | 2 | 2 | 2 | 2 | 2 |
| NM_001006114 | C21orf57 | 1181 | 3 | 1 | 1 | 2 | 1 | 2 | 1 | 1 | 1 | 1 | 1 | 3 | 2 |
| NM_001006115 | IP6K1 | 4117 | 3 | 1 | 1 | 1 | 1 | 3 | 1 | 1 | 1 | 1 | 0 | 1 | 2 |
| NM_001006117 | RBMY1J | 1880 | 0 | 0 | 0 | 0 | 0 | 0 | 0 | 0 | 0 | 0 | 0 | 0 | 0 |
| NM_001006117 | RBMY1J | 1881 | 0 | 0 | 0 | 0 | 0 | 0 | 0 | 0 | 0 | 0 | 0 | 0 | 0 |
| NM_001006118 | RBMY1E | 1881 | 0 | 0 | 0 | 0 | 0 | 0 | 0 | 0 | 0 | 0 | 0 | 0 | 0 |
| NM_001006120 | RBMY1D | 1881 | 0 | 0 | 0 | 0 | 0 | 0 | 0 | 0 | 0 | 0 | 0 | 0 | 0 |
| NM_001006121 | RBMY1B | 1887 | 0 | 0 | 0 | 0 | 0 | 0 | 0 | 0 | 0 | 0 | 0 | 0 | 0 |
| NM_001006600 | ERBB2IP | 6709 | 29 | 19 | 11 | 7 | 16 | 26 | 21 | 23 | 17 | 21 | 19 | 29 | 19 |
| NM_001006605 | FAM69A | 2599 | 12 | 4 | 5 | 1 | 2 | 12 | 1 | 7 | 1 | 1 | 2 | 1 | 3 | 5 |
| NM_001006607 | LRRC37A2 | 5175 | 0 | 0 | 0 | 0 | 0 | 0 | 0 | 0 | 0 | 0 | 0 | 0 | 0 |
| NM_001006610 | SIAH1 | 2383 | 2 | 1 | 1 | 1 | 1 | 2 | 1 | 1 | 1 | 2 | 2 | 1 | 2 |
| NM_001006612 | WBP5 | 1086 | 49 | 24 | 24 | 9 | 32 | 27 | 24 | 6 | 22 | 49 | 4 | 25 | 38 |
| NM_001006613 | WBP5 | 1010 | 51 | 24 | 23 | 9 | 33 | 27 | 25 | 6 | 22 | 51 | 4 | 26 | 40 |
| NM_001006614 | WBP5 | 1007 | 51 | 24 | 24 | 9 | 33 | 27 | 25 | 6 | 22 | 51 | 4 | 26 | 40 |
| NM_001006616 | TSPAN17 | 2556 | 1 | 1 | 0 | 1 | 1 | 1 | 1 | 1 | 0 | 0 | 1 | 1 | 1 |
| NM_001006617 | MAPKAP1 | 3395 | 5 | 4 | 4 | 3 | 5 | 5 | 4 | 5 | 3 | 3 | 2 | 4 | 3 |
| NM_001006618 | MAPKAP1 | 1587 | 5 | 4 | 4 | 3 | 5 | 3 | 4 | 5 | 3 | 3 | 3 | 4 | 4 |
| NM_001006619 | MAPKAP1 | 3254 | 5 | 4 | 4 | 3 | 5 | 5 | 4 | 5 | 3 | 3 | 2 | 4 | 3 |
| NM_001006620 | MAPKAP1 | 3067 | 4 | 3 | 4 | 2 | 4 | 4 | 4 | 4 | 2 | 3 | 2 | 4 | 3 |
| NM_001006621 | MAPKAP1 | 2977 | 4 | 3 | 3 | 2 | 4 | 4 | 3 | 3 | 2 | 2 | 2 | 3 | 2 |
| NM_001006622 | WDR33 | 1169 | 6 | 4 | 3 | 2 | 4 | 4 | 5 | 4 | 4 | 5 | 2 | 6 | 6 |
| NM_001006623 | WDR33 | 3574 | 3 | 2 | 1 | 1 | 2 | 1 | 2 | 1 | 2 | 3 | 1 | 3 | 2 |
| NM_001006624 | PDPN | 2636 | 3 | 1 | 0 | 1 | 3 | 2 | 0 | 0 | 2 | 3 | 2 | 0 | 1 |
| NM_001006625 | PDPN | 2630 | 3 | 1 | 0 | 1 | 3 | 2 | 0 | 0 | 2 | 3 | 2 | 0 | 1 |
| NM_001006626 | CHRM2 | 2571 | 6 | 1 | 0 | 1 | 6 | 0 | 0 | 0 | 0 | 0 | 0 | 0 | 0 |
| NM_001006627 | CHRM2 | 2704 | 6 | 1 | 0 | 1 | 6 | 0 | 0 | 0 | 0 | 0 | 0 | 0 | 0 |
| NM_001006628 | CHRM2 | 2456 | 6 | 1 | 0 | 1 | 6 | 0 | 0 | 0 | 0 | 0 | 0 | 0 | 0 |
| NM_001006629 | CHRM2 | 2387 | 6 | 1 | 0 | 1 | 6 | 0 | 0 | 0 | 0 | 0 | 0 | 0 | 0 |
| NM_001006630 | CHRM2 | 2782 | 6 | 1 | 0 | 1 | 6 | 0 | 0 | 0 | 0 | 0 | 0 | 0 | 0 |
| NM_001006631 | CHRM2 | 2474 | 6 | 1 | 0 | 1 | 6 | 0 | 0 | 0 | 0 | 0 | 0 | 0 | 0 |
| NM_001006632 | CHRM2 | 2452 | 6 | 1 | 0 | 1 | 6 | 0 | 0 | 0 | 0 | 0 | 0 | 0 | 0 |
| NM_001006634 | ARHGAP17 | 3478 | 9 | 4 | 4 | 4 | 3 | 2 | 3 | 2 | 3 | 3 | 3 | 9 | 5 |
| NM_001006636 | GTDC1 | 2693 | 5 | 2 | 3 | 0 | 2 | 5 | 1 | 1 | 2 | 2 | 1 | 4 | 3 |
| NM_001006638 | RAB37 | 2651 | 1 | 0 | 0 | 0 | 0 | 1 | 0 | 1 | 1 | 0 | 0 | 1 | 0 |
| NM_001006639 | TCEAL1 | 1194 | 19 | 9 | 5 | 4 | 8 | 18 | 5 | 6 | 6 | 19 | 7 | 7 | 11 |
| NM_001006640 | TCEAL1 | 1206 | 18 | 9 | 5 | 4 | 8 | 18 | 5 | 6 | 6 | 18 | 7 | 7 | 11 |
| NM_001006641 | SLC25A25 | 3433 | 14 | 3 | 1 | 1 | 1 | 4 | 3 | 14 | 2 | 3 | 4 | 2 | 1 |
| NM_001006642 | SLC25A25 | 3293 | 14 | 4 | 1 | 1 | 1 | 5 | 3 | 14 | 2 | 4 | 5 | 3 | 1 |

| transcript | gene | transcript length (nt) | max | mean | adipose | colon | heart | hypothalamus | kidney | liver | lung | ovary | skeletalmuscle | spleen | testes |
|---|---|---|---|---|---|---|---|---|---|---|---|---|---|---|---|
| NM_001006643 | SLC25A25 | 3264 | 14 | 4 | 1 | 1 | 1 | 5 | 3 | 14 | 2 | 4 | 5 | 3 | 1 |
| NM_001006655 | FAM149A | 2405 | 3 | 1 | 1 | 1 | 1 | 2 | 3 | 2 | 1 | 0 | 0 | 0 | 1 |
| NM_001006656 | ZNF473 | 4568 | 7 | 2 | 1 | 1 | 1 | 1 | 1 | 1 | 1 | 2 | 1 | 3 | 7 |
| NM_001006657 | WDR35 | 6944 | 4 | 2 | 1 | 1 | 1 | 3 | 3 | 1 | 1 | 3 | 1 | 1 | 4 |
| NM_001006658 | CR2 | 4168 | 5 | 0 | 0 | 0 | 0 | 0 | 0 | 0 | 0 | 0 | 0 | 5 | 0 |
| NM_001006665 | RPS6KA1 | 3112 | 3 | 1 | 1 | 2 | 0 | 1 | 1 | 1 | 2 | 0 | 0 | 3 | 0 |
| NM_001006666 | APOBEC3F | 805 | 1 | 0 | 0 | 0 | 0 | 0 | 0 | 0 | 0 | 1 | 0 | 0 | 0 |
| NM_001006681 | SPIN2B | 1259 | 2 | 1 | 1 | 1 | 1 | 2 | 1 | 0 | 0 | 1 | 1 | 1 | 1 |
| NM_001006682 | SPIN2B | 1207 | 2 | 1 | 1 | 1 | 1 | 2 | 1 | 0 | 0 | 1 | 1 | 1 | 1 |
| NM_001006683 | SPIN2B | 1204 | 2 | 1 | 1 | 1 | 1 | 2 | 1 | 0 | 0 | 1 | 1 | 1 | 1 |
| NM_001006684 | TCEAL8 | 1139 | 30 | 17 | 14 | 10 | 24 | 26 | 18 | 5 | 18 | 21 | 4 | 21 | 30 |
| NM_001006932 | RPS6KA2 | 5735 | 7 | 4 | 2 | 1 | 7 | 7 | 2 | 1 | 6 | 4 | 1 | 7 | 1 |
| NM_001006933 | TCEAL3 | 1125 | 111 | 45 | 15 | 10 | 63 | 111 | 35 | 8 | 32 | 84 | 9 | 58 | 71 |
| NM_001006935 | TCEAL4 | 1286 | 346 | 118 | 60 | 22 | 152 | 168 | 82 | 62 | 77 | 346 | 36 | 96 | 193 |
| NM_001006936 | TCEAL4 | 1285 | 347 | 118 | 60 | 22 | 152 | 168 | 82 | 62 | 77 | 347 | 36 | 96 | 193 |
| NM_001006937 | TCEAL4 | 1281 | 348 | 118 | 61 | 22 | 153 | 168 | 83 | 62 | 78 | 348 | 36 | 96 | 193 |
| NM_001006938 | TCEAL6 | 1532 | 10 | 1 | 0 | 0 | 0 | 10 | 0 | 0 | 0 | 0 | 0 | 0 | 6 |
| NM_001006939 | LRRC18 | 1619 | 1 | 0 | 0 | 0 | 0 | 0 | 0 | 0 | 0 | 0 | 0 | 1 | 1 |
| NM_001006941 | ALG3 | 1606 | 5 | 2 | 1 | 1 | 2 | 1 | 1 | 5 | 1 | 1 | 2 | 2 | 2 |
| NM_001006943 | EPHA8 | 1853 | 0 | 0 | 0 | 0 | 0 | 0 | 0 | 0 | 0 | 0 | 0 | 0 | 0 |
| NM_001006944 | RPS6KA4 | 3119 | 2 | 1 | 1 | 1 | 2 | 1 | 1 | 0 | 1 | 1 | 1 | 2 | 1 |
| NM_001006945 | C1orf103 | 1807 | 12 | 4 | 2 | 0 | 4 | 4 | 2 | 2 | 2 | 4 | 3 | 5 | 12 |
| NM_001006946 | SDC1 | 3293 | 27 | 4 | 0 | 3 | 0 | 0 | 12 | 27 | 4 | 0 | 0 | 1 | 1 |
| NM_001006947 | UHRF1BP1L | 2040 | 12 | 7 | 7 | 2 | 7 | 11 | 7 | 12 | 7 | 6 | 4 | 8 | 6 |
| NM_001007 | RPS4X | 956 | 935 | 300 | 169 | 182 | 300 | 133 | 236 | 179 | 284 | 935 | 321 | 381 | 179 |
| NM_001007022 | ODF2L | 2162 | 9 | 4 | 3 | 1 | 3 | 5 | 5 | 0 | 7 | 4 | 1 | 9 | 3 |
| NM_001007023 | DIO2 | 6121 | 8 | 1 | 0 | 0 | 3 | 8 | 1 | 0 | 0 | 1 | 1 | 0 | 1 |
| NM_001007024 | GOSR1 | 5231 | 7 | 5 | 4 | 3 | 4 | 7 | 5 | 6 | 3 | 6 | 3 | 5 | 5 |
| NM_001007025 | GOSR1 | 5209 | 7 | 5 | 4 | 3 | 4 | 7 | 5 | 6 | 3 | 6 | 3 | 5 | 5 |
| NM_001007026 | ATN1 | 4351 | 15 | 6 | 5 | 6 | 8 | 9 | 5 | 1 | 5 | 15 | 5 | 8 | 4 |
| NM_001007027 | ALG8 | 1743 | 5 | 2 | 2 | 1 | 2 | 2 | 3 | 5 | 2 | 2 | 1 | 3 | 3 |
| NM_001007033 | CLEC6A | 1613 | 0 | 0 | 0 | 0 | 0 | 0 | 0 | 0 | 0 | 0 | 0 | 0 | 0 |
| NM_001007067 | SDCBP | 2164 | 24 | 13 | 22 | 6 | 14 | 16 | 10 | 10 | 24 | 7 | 5 | 24 | 7 |
| NM_001007068 | SDCBP | 2149 | 24 | 13 | 22 | 6 | 14 | 16 | 10 | 10 | 24 | 7 | 5 | 24 | 7 |
| NM_001007069 | SDCBP | 2164 | 24 | 13 | 22 | 6 | 14 | 16 | 10 | 10 | 24 | 7 | 5 | 24 | 7 |
| NM_001007070 | SDCBP | 2161 | 24 | 13 | 22 | 6 | 14 | 16 | 10 | 10 | 24 | 7 | 5 | 24 | 7 |
| NM_001007072 | ZSCAN2 | 934 | 0 | 0 | 0 | 0 | 0 | 0 | 0 | 0 | 0 | 0 | 0 | 0 | 0 |
| NM_001007073 | RPL32 | 1750 | 39 | 22 | 19 | 14 | 21 | 17 | 15 | 23 | 17 | 39 | 30 | 35 | 13 |
| NM_001007074 | RPL32 | 1770 | 39 | 22 | 18 | 13 | 21 | 16 | 15 | 23 | 16 | 39 | 30 | 35 | 13 |
| NM_001007075 | KLHL5 | 3425 | 9 | 5 | 9 | 3 | 3 | 9 | 5 | 7 | 4 | 5 | 1 | 6 | 3 |
| NM_001007088 | ZNF182 | 3488 | 5 | 2 | 2 | 1 | 2 | 2 | 3 | 1 | 2 | 4 | 1 | 5 | 3 |
| NM_001007089 | RESP18 | 794 | 0 | 0 | 0 | 0 | 0 | 0 | 0 | 0 | 0 | 0 | 0 | 0 | 0 |
| NM_001007090 | C8orf48 | 1445 | 2 | 1 | 0 | 0 | 1 | 1 | 1 | 0 | 0 | 1 | 0 | 0 | 2 |
| NM_001007094 | ZNF37A | 7018 | 6 | 3 | 2 | 1 | 3 | 2 | 3 | 2 | 2 | 6 | 2 | 5 | 2 |
| NM_001007097 | NTRK2 | 7111 | 114 | 15 | 31 | 2 | 3 | 114 | 7 | 0 | 2 | 4 | 2 | 2 | 3 |
| NM_001007098 | SCP2 | 2045 | 16 | 4 | 3 | 1 | 3 | 2 | 4 | 16 | 2 | 2 | 2 | 2 | 3 |
| NM_001007099 | SCP2 | 1421 | 130 | 34 | 31 | 17 | 29 | 23 | 47 | 130 | 19 | 19 | 11 | 22 | 26 |
| NM_001007100 | SCP2 | 1412 | 131 | 34 | 31 | 17 | 29 | 23 | 48 | 131 | 20 | 19 | 11 | 22 | 26 |
| NM_001007101 | ZNF484 | 2798 | 12 | 6 | 4 | 1 | 5 | 7 | 6 | 2 | 5 | 12 | 4 | 11 | 7 |
| NM_001007102 | L3MBTL3 | 4125 | 7 | 3 | 2 | 1 | 2 | 3 | 2 | 1 | 3 | 5 | 2 | 7 | 7 |
| NM_001007122 | FSD2 | 2493 | 17 | 3 | 0 | 0 | 15 | 0 | 0 | 0 | 0 | 0 | 17 | 0 | 0 |
| NM_001007125 | C20orf201 | 868 | 0 | 0 | 0 | 0 | 0 | 0 | 0 | 0 | 0 | 0 | 0 | 0 | 0 |
| NM_001007139 | IGF2 | 5139 | 0 | 0 | 0 | 0 | 0 | 0 | 0 | 0 | 0 | 0 | 0 | 0 | 0 |
| NM_001007156 | NTRK3 | 3982 | 3 | 0 | 0 | 1 | 0 | 3 | 0 | 0 | 0 | 0 | 0 | 0 | 0 |
| NM_001007157 | PHF14 | 3409 | 14 | 7 | 7 | 4 | 5 | 13 | 6 | 5 | 5 | 14 | 3 | 8 | 11 |
| NM_001007169 | ZNF483 | 2425 | 5 | 1 | 0 | 0 | 0 | 5 | 1 | 0 | 1 | 1 | 0 | 0 | 1 |
| NM_001007176 | C8orf22 | 1265 | 12 | 1 | 0 | 0 | 0 | 0 | 0 | 0 | 0 | 0 | 12 | 0 | 0 |
| NM_001007188 | DEPDC5 | 2381 | 1 | 1 | 1 | 1 | 1 | 1 | 1 | 1 | 1 | 1 | 0 | 1 | 1 |
| NM_001007189 | C5orf53 | 2871 | 6 | 2 | 2 | 1 | 2 | 6 | 2 | 1 | 1 | 3 | 1 | 2 | 2 |
| NM_001007214 | CACYBP | 2875 | 21 | 7 | 6 | 3 | 8 | 21 | 7 | 4 | 4 | 7 | 4 | 8 | 7 |
| NM_001007225 | IGF2BP2 | 3542 | 3 | 2 | 1 | 3 | 3 | 0 | 2 | 0 | 3 | 1 | 1 | 2 | 2 |
| NM_001007226 | SPOP | 3090 | 8 | 5 | 5 | 4 | 4 | 8 | 3 | 5 | 4 | 5 | 4 | 5 | 5 |
| NM_001007227 | SPOP | 2968 | 8 | 5 | 5 | 4 | 5 | 8 | 3 | 5 | 4 | 6 | 4 | 5 | 5 |
| NM_001007228 | SPOP | 2916 | 8 | 5 | 5 | 4 | 5 | 8 | 3 | 5 | 4 | 6 | 4 | 5 | 5 |
| NM_001007229 | SPOP | 2967 | 8 | 5 | 5 | 4 | 5 | 8 | 3 | 5 | 4 | 6 | 4 | 5 | 5 |
| NM_001007230 | SPOP | 3019 | 8 | 5 | 5 | 4 | 5 | 8 | 3 | 5 | 4 | 6 | 4 | 5 | 5 |
| NM_001007231 | ARHGAP25 | 2982 | 11 | 2 | 2 | 1 | 1 | 1 | 1 | 1 | 3 | 2 | 0 | 11 | 1 |
| NM_001007232 | CARD17 | 466 | 0 | 0 | 0 | 0 | 0 | 0 | 0 | 0 | 0 | 0 | 0 | 0 | 0 |
| NM_001007237 | IGSF3 | 7193 | 4 | 1 | 0 | 1 | 1 | 1 | 4 | 0 | 1 | 0 | 0 | 0 | 2 |
| NM_001007239 | METTL13 | 2939 | 4 | 2 | 2 | 1 | 1 | 2 | 2 | 2 | 1 | 1 | 1 | 2 | 4 |
| NM_001007240 | GP2 | 2431 | 1 | 0 | 1 | 0 | 0 | 0 | 1 | 0 | 0 | 0 | 0 | 0 | 0 |
| NM_001007241 | GP2 | 1990 | 1 | 0 | 1 | 0 | 0 | 0 | 1 | 0 | 0 | 0 | 0 | 0 | 0 |
| NM_001007242 | GP2 | 1981 | 1 | 0 | 1 | 0 | 0 | 0 | 1 | 0 | 0 | 0 | 0 | 0 | 0 |
| NM_001007243 | STAR | 2641 | 41 | 6 | 0 | 0 | 0 | 1 | 0 | 0 | 0 | 41 | 0 | 0 | 22 |
| NM_001007245 | IFRD1 | 2471 | 4 | 3 | 3 | 2 | 4 | 2 | 3 | 1 | 4 | 3 | 3 | 3 | 4 |
| NM_001007246 | BRWD1 | 2481 | 15 | 4 | 1 | 1 | 3 | 15 | 3 | 2 | 4 | 4 | 3 | 4 | 5 |
| NM_001007248 | ZNF599 | 3115 | 1 | 0 | 0 | 0 | 0 | 0 | 0 | 0 | 0 | 1 | 0 | 0 | 1 |

| transcript | gene | transcript length (nt) | max | mean | adipose | colon | heart | hypothalamus | kidney | liver | lung | ovary | skeletalmuscle | spleen | testes |
|---|---|---|---|---|---|---|---|---|---|---|---|---|---|---|---|
| NM_001007249 | OR8G2 | 915 | 0 | 0 | 0 | 0 | 0 | 0 | 0 | 0 | 0 | 0 | 0 | 0 | 0 |
| NM_001007250 | SCP2 | 1291 | 113 | 29 | 27 | 12 | 25 | 19 | 40 | 113 | 17 | 17 | 11 | 19 | 21 |
| NM_001007255 | KLHDC9 | 1363 | 2 | 1 | 0 | 0 | 0 | 2 | 1 | 0 | 0 | 0 | 0 | 0 | 2 |
| NM_001007256 | KLHDC9 | 1083 | 3 | 1 | 0 | 0 | 0 | 2 | 2 | 0 | 1 | 0 | 0 | 0 | 3 |
| NM_001007257 | PRDM2 | 6196 | 11 | 7 | 5 | 4 | 7 | 10 | 4 | 3 | 5 | 11 | 8 | 9 | 8 |
| NM_001007259 | SETD4 | 1116 | 3 | 2 | 1 | 1 | 1 | 2 | 3 | 2 | 1 | 1 | 0 | 2 | 3 |
| NM_001007267 | PLA2R1 | 5160 | 4 | 1 | 1 | 1 | 1 | 1 | 4 | 0 | 1 | 4 | 0 | 2 | 1 |
| NM_001007269 | GEMIN7 | 1524 | 1 | 0 | 0 | 0 | 0 | 0 | 1 | 0 | 0 | 1 | 0 | 1 | 0 |
| NM_001007270 | GEMIN7 | 1561 | 1 | 0 | 0 | 0 | 0 | 0 | 1 | 0 | 0 | 0 | 0 | 1 | 0 |
| NM_001007271 | DUSP13 | 1771 | 0 | 0 | 0 | 0 | 0 | 0 | 0 | 0 | 0 | 0 | 0 | 0 | 0 |
| NM_001007272 | DUSP13 | 1046 | 1 | 0 | 0 | 0 | 0 | 0 | 0 | 0 | 0 | 0 | 1 | 0 | 0 |
| NM_001007273 | DUSP13 | 1345 | 1 | 0 | 0 | 0 | 0 | 0 | 0 | 0 | 0 | 0 | 1 | 0 | 0 |
| NM_001007277 | EI24 | 2149 | 92 | 22 | 19 | 17 | 16 | 22 | 18 | 92 | 12 | 11 | 14 | 14 | 10 |
| NM_001007278 | TRIM13 | 6954 | 5 | 3 | 2 | 1 | 3 | 2 | 3 | 1 | 3 | 4 | 3 | 5 | 4 |
| NM_001007279 | RASL10A | 1787 | 0 | 0 | 0 | 0 | 0 | 0 | 0 | 0 | 0 | 0 | 0 | 0 | 0 |
| NM_001007464 | RWDD1 | 1181 | 28 | 16 | 17 | 4 | 28 | 18 | 14 | 15 | 13 | 17 | 23 | 17 | 13 |
| NM_001007466 | TULP4 | 8618 | 14 | 6 | 5 | 4 | 5 | 14 | 5 | 3 | 4 | 6 | 5 | 6 | 6 |
| NM_001007467 | SFI1 | 4252 | 3 | 1 | 1 | 2 | 0 | 1 | 1 | 0 | 1 | 1 | 0 | 3 | 2 |
| NM_001007468 | SMARCB1 | 1676 | 13 | 8 | 4 | 6 | 8 | 12 | 8 | 6 | 3 | 10 | 3 | 13 | 12 |
| NM_001007469 | HCST | 504 | 1 | 0 | 1 | 0 | 0 | 1 | 0 | 0 | 1 | 0 | 0 | 1 | 0 |
| NM_001007470 | TRPM3 | 1362 | 15 | 3 | 0 | 0 | 0 | 15 | 13 | 0 | 0 | 1 | 0 | 0 | 2 |
| NM_001007471 | TRPM3 | 6271 | 6 | 1 | 0 | 0 | 0 | 6 | 6 | 0 | 0 | 0 | 0 | 0 | 1 |
| NM_001007523 | F8A2 | 1116 | 0 | 0 | 0 | 0 | 0 | 0 | 0 | 0 | 0 | 0 | 0 | 0 | 0 |
| NM_001007524 | F8A3 | 1116 | 0 | 0 | 0 | 0 | 0 | 0 | 0 | 0 | 0 | 0 | 0 | 0 | 0 |
| NM_001007525 | NWD1 | 8403 | 1 | 0 | 0 | 0 | 0 | 1 | 0 | 0 | 0 | 0 | 0 | 0 | 0 |
| NM_001007527 | LMBRD2 | 3237 | 17 | 5 | 3 | 2 | 4 | 17 | 4 | 6 | 2 | 3 | 4 | 5 | 4 |
| NM_001007530 | CDRT15 | 567 | 0 | 0 | 0 | 0 | 0 | 0 | 0 | 0 | 0 | 0 | 0 | 0 | 0 |
| NM_001007531 | NKAPL | 1639 | 8 | 2 | 1 | 0 | 3 | 1 | 0 | 0 | 0 | 1 | 2 | 1 | 8 |
| NM_001007532 | STH | 445 | 0 | 0 | 0 | 0 | 0 | 0 | 0 | 0 | 0 | 0 | 0 | 0 | 0 |
| NM_001007533 | DYSFIP1 | 799 | 5 | 0 | 0 | 0 | 0 | 0 | 0 | 0 | 0 | 0 | 5 | 0 | 0 |
| NM_001007537 | C1QTNF9B | 1002 | 0 | 0 | 0 | 0 | 0 | 0 | 0 | 0 | 0 | 0 | 0 | 0 | 0 |
| NM_001007538 | SHISA2 | 2889 | 1 | 0 | 0 | 0 | 0 | 0 | 0 | 0 | 1 | 0 | 0 | 0 | 0 |
| NM_001007540 | CDH29 | 2466 | 0 | 0 | 0 | 0 | 0 | 0 | 0 | 0 | 0 | 0 | 0 | 0 | 0 |
| NM_001007544 | C1orf186 | 1665 | 4 | 1 | 0 | 1 | 0 | 0 | 4 | 0 | 1 | 0 | 0 | 2 | 1 |
| NM_001007551 | CT45A5 | 1008 | 0 | 0 | 0 | 0 | 0 | 0 | 0 | 0 | 0 | 0 | 0 | 0 | 0 |
| NM_001007553 | CSDE1 | 4115 | 386 | 139 | 168 | 40 | 190 | 99 | 86 | 95 | 92 | 127 | 386 | 112 | 132 |
| NM_001007559 | SS18 | 3436 | 31 | 18 | 15 | 8 | 20 | 13 | 22 | 18 | 15 | 31 | 6 | 27 | 27 |
| NM_001007561 | IRGQ | 9687 | 5 | 2 | 1 | 2 | 2 | 5 | 2 | 2 | 1 | 2 | 2 | 2 | 1 |
| NM_001007563 | IGFBPL1 | 1093 | 2 | 1 | 0 | 0 | 0 | 2 | 0 | 0 | 0 | 0 | 0 | 0 | 2 |
| NM_001007565 | TFG | 1798 | 7 | 5 | 4 | 2 | 7 | 5 | 7 | 7 | 5 | 4 | 4 | 6 | 5 |
| NM_001007593 | SMPD1 | 2327 | 3 | 1 | 1 | 1 | 1 | 1 | 3 | 2 | 1 | 0 | 0 | 0 | 0 |
| NM_001007595 | C2CD4B | 1576 | 0 | 0 | 0 | 0 | 0 | 0 | 0 | 0 | 0 | 0 | 0 | 0 | 0 |
| NM_001007792 | NTRK1 | 2571 | 0 | 0 | 0 | 0 | 0 | 0 | 0 | 0 | 0 | 0 | 0 | 0 | 0 |
| NM_001007793 | BUB3 | 1537 | 12 | 6 | 4 | 4 | 5 | 6 | 5 | 3 | 6 | 7 | 3 | 12 | 7 |
| NM_001007794 | CEPT1 | 2251 | 27 | 9 | 10 | 4 | 5 | 10 | 8 | 11 | 9 | 9 | 2 | 27 | 9 |
| NM_001008 | RPS4Y1 | 897 | 69 | 29 | 47 | 0 | 30 | 43 | 14 | 54 | 23 | 0 | 6 | 69 | 34 |
| NM_001008211 | OPTN | 3597 | 190 | 41 | 31 | 8 | 52 | 29 | 23 | 15 | 17 | 34 | 190 | 21 | 34 |
| NM_001008212 | OPTN | 3528 | 194 | 42 | 32 | 9 | 53 | 30 | 23 | 16 | 18 | 35 | 194 | 21 | 35 |
| NM_001008213 | OPTN | 3582 | 191 | 42 | 31 | 8 | 52 | 29 | 23 | 16 | 17 | 34 | 191 | 21 | 34 |
| NM_001008215 | C2orf64 | 1662 | 4 | 2 | 1 | 1 | 2 | 4 | 3 | 1 | 2 | 2 | 1 | 4 | 2 |
| NM_001008216 | GALE | 1566 | 2 | 0 | 0 | 1 | 0 | 1 | 2 | 1 | 0 | 0 | 0 | 0 | 0 |
| NM_001008218 | AMY1B | 1838 | 0 | 0 | 0 | 0 | 0 | 0 | 0 | 0 | 0 | 0 | 0 | 0 | 0 |
| NM_001008219 | AMY1C | 1860 | 0 | 0 | 0 | 0 | 0 | 0 | 0 | 0 | 0 | 0 | 0 | 0 | 0 |
| NM_001008220 | CPLX2 | 4676 | 56 | 5 | 0 | 1 | 0 | 56 | 0 | 0 | 0 | 0 | 0 | 0 | 1 |
| NM_001008221 | AMY1A | 1781 | 0 | 0 | 0 | 0 | 0 | 0 | 0 | 0 | 0 | 0 | 0 | 0 | 0 |
| NM_001008222 | ZDHHC9 | 2897 | 4 | 2 | 1 | 1 | 1 | 4 | 3 | 4 | 2 | 0 | 0 | 1 | 1 |
| NM_001008223 | C1QL4 | 2065 | 0 | 0 | 0 | 0 | 0 | 0 | 0 | 0 | 0 | 0 | 0 | 0 | 0 |
| NM_001008224 | UACA | 7080 | 36 | 12 | 10 | 2 | 12 | 4 | 13 | 3 | 10 | 14 | 36 | 14 | 11 |
| NM_001008225 | CNOT4 | 2808 | 9 | 5 | 3 | 2 | 4 | 5 | 4 | 2 | 4 | 8 | 7 | 5 | 9 |
| NM_001008226 | FAM154B | 3130 | 1 | 0 | 0 | 0 | 0 | 0 | 0 | 0 | 1 | 0 | 0 | 0 | 0 |
| NM_001008228 | MOG | 2089 | 13 | 1 | 0 | 2 | 0 | 13 | 0 | 0 | 0 | 0 | 0 | 0 | 0 |
| NM_001008228 | MOG | 2093 | 13 | 1 | 0 | 2 | 0 | 13 | 0 | 0 | 0 | 0 | 0 | 0 | 0 |
| NM_001008228 | MOG | 2097 | 13 | 1 | 0 | 2 | 0 | 13 | 0 | 0 | 0 | 0 | 0 | 0 | 0 |
| NM_001008229 | MOG | 1770 | 15 | 2 | 0 | 2 | 0 | 15 | 0 | 0 | 0 | 0 | 0 | 0 | 0 |
| NM_001008229 | MOG | 1774 | 15 | 2 | 0 | 2 | 0 | 15 | 0 | 0 | 0 | 0 | 0 | 0 | 0 |
| NM_001008229 | MOG | 1778 | 15 | 2 | 0 | 2 | 0 | 15 | 0 | 0 | 0 | 0 | 0 | 0 | 0 |
| NM_001008237 | TTC32 | 819 | 1 | 0 | 0 | 0 | 0 | 1 | 1 | 1 | 0 | 0 | 0 | 0 | 1 |
| NM_001008239 | C18orf25 | 5282 | 4 | 3 | 2 | 2 | 3 | 3 | 2 | 1 | 2 | 3 | 4 | 3 | 4 |
| NM_001008269 | TMEM89 | 480 | 0 | 0 | 0 | 0 | 0 | 0 | 0 | 0 | 0 | 0 | 0 | 0 | 0 |
| NM_001008270 | PRSS37 | 1189 | 1 | 0 | 0 | 0 | 0 | 0 | 0 | 0 | 0 | 1 | 0 | 0 | 1 |
| NM_001008271 | SCXA | 606 | 0 | 0 | 0 | 0 | 0 | 0 | 0 | 0 | 0 | 0 | 0 | 0 | 0 |
| NM_001008272 | TAGLN3 | 1315 | 23 | 2 | 0 | 0 | 0 | 23 | 0 | 0 | 0 | 0 | 0 | 0 | 0 |
| NM_001008273 | TAGLN3 | 1200 | 26 | 2 | 0 | 0 | 0 | 26 | 0 | 0 | 0 | 0 | 0 | 0 | 0 |
| NM_001008274 | TRIM72 | 2152 | 1 | 0 | 0 | 0 | 0 | 0 | 0 | 0 | 0 | 0 | 1 | 0 | 0 |
| NM_001008387 | REG3G | 942 | 2 | 0 | 0 | 0 | 0 | 0 | 0 | 0 | 0 | 0 | 0 | 0 | 2 |
| NM_001008388 | CISD2 | 1840 | 5 | 2 | 2 | 1 | 1 | 4 | 4 | 5 | 1 | 2 | 0 | 3 | 2 |
| NM_001008389 | UMOD | 2252 | 187 | 17 | 0 | 0 | 0 | 0 | 187 | 0 | 0 | 0 | 0 | 0 | 0 |

| transcript | gene | transcript length (nt) | max | mean | adipose | colon | heart | hypothala mus | kidney | liver | lung | ovary | skeletalm uscle | spleen | testes |
|---|---|---|---|---|---|---|---|---|---|---|---|---|---|---|---|
| NM_001008390 | CGGBP1 | 4539 | 6 | 3 | 3 | 1 | 4 | 3 | 4 | 2 | 3 | 5 | 3 | 6 | 4 |
| NM_001008391 | CCDC73 | 4015 | 4 | 1 | 0 | 0 | 0 | 1 | 1 | 0 | 0 | 1 | 0 | 1 | 4 |
| NM_001008392 | CTDSPL | 4455 | 3 | 1 | 1 | 1 | 1 | 0 | 3 | 1 | 2 | 1 | 0 | 2 | 1 |
| NM_001008393 | C4orf46 | 3525 | 2 | 1 | 0 | 0 | 0 | 0 | 0 | 0 | 1 | 1 | 0 | 1 | 2 |
| NM_001008394 | EID3 | 1434 | 9 | 2 | 1 | 1 | 1 | 1 | 1 | 1 | 2 | 2 | 0 | 2 | 9 |
| NM_001008395 | C7orf59 | 612 | 3 | 1 | 3 | 2 | 1 | 2 | 2 | 1 | 0 | 1 | 0 | 2 | 1 |
| NM_001008396 | WDHD1 | 4641 | 2 | 1 | 1 | 0 | 0 | 1 | 1 | 0 | 1 | 1 | 0 | 1 | 2 |
| NM_001008397 | GPX8 | 3750 | 4 | 1 | 2 | 0 | 2 | 0 | 1 | 0 | 1 | 4 | 0 | 1 | 2 |
| NM_001008401 | ZNF761 | 4212 | 6 | 3 | 2 | 1 | 2 | 2 | 2 | 1 | 3 | 6 | 2 | 5 | 5 |
| NM_001008404 | C14orf180 | 1847 | 1 | 0 | 1 | 0 | 1 | 0 | 0 | 0 | 0 | 0 | 0 | 0 | 0 |
| NM_001008405 | BCAP29 | 5773 | 19 | 9 | 8 | 2 | 19 | 13 | 5 | 7 | 7 | 6 | 6 | 8 | 15 |
| NM_001008409 | TTLL9 | 1607 | 0 | 0 | 0 | 0 | 0 | 0 | 0 | 0 | 0 | 0 | 0 | 0 | 0 |
| NM_001008410 | STEAP3 | 3869 | 11 | 2 | 1 | 1 | 2 | 1 | 1 | 11 | 1 | 0 | 2 | 0 | 1 |
| NM_001008485 | SLC41A3 | 1800 | 3 | 1 | 0 | 1 | 1 | 3 | 1 | 0 | 0 | 2 | 1 | 1 | 2 |
| NM_001008486 | SLC41A3 | 1692 | 3 | 1 | 0 | 1 | 1 | 3 | 1 | 0 | 0 | 2 | 1 | 1 | 2 |
| NM_001008487 | SLC41A3 | 2268 | 3 | 1 | 1 | 1 | 1 | 3 | 1 | 0 | 0 | 1 | 1 | 1 | 2 |
| NM_001008489 | PHOSPHO2 | 1126 | 3 | 1 | 1 | 2 | 1 | 1 | 2 | 1 | 1 | 2 | 1 | 1 | 3 |
| NM_001008491 | 02 Sep | 3596 | 55 | 37 | 55 | 17 | 36 | 55 | 40 | 19 | 32 | 43 | 17 | 49 | 40 |
| NM_001008492 | 02 Sep | 3394 | 58 | 38 | 58 | 17 | 38 | 57 | 41 | 20 | 33 | 45 | 18 | 51 | 41 |
| NM_001008493 | ENAH | 13172 | 19 | 9 | 6 | 5 | 14 | 12 | 4 | 2 | 7 | 13 | 8 | 8 | 19 |
| NM_001008494 | ISX | 3383 | 2 | 0 | 0 | 2 | 0 | 0 | 0 | 0 | 0 | 0 | 0 | 0 | 0 |
| NM_001008495 | TMEM64 | 4819 | 6 | 3 | 2 | 1 | 2 | 2 | 3 | 3 | 3 | 3 | 2 | 3 | 6 |
| NM_001008496 | PIWIL3 | 3504 | 0 | 0 | 0 | 0 | 0 | 0 | 0 | 0 | 0 | 0 | 0 | 0 | 0 |
| NM_001008503 | OPRM1 | 1576 | 1 | 0 | 0 | 0 | 0 | 1 | 0 | 0 | 0 | 0 | 0 | 0 | 0 |
| NM_001008504 | OPRM1 | 1867 | 0 | 0 | 0 | 0 | 0 | 0 | 0 | 0 | 0 | 0 | 0 | 0 | 0 |
| NM_001008505 | OPRM1 | 1652 | 0 | 0 | 0 | 0 | 0 | 0 | 0 | 0 | 0 | 0 | 0 | 0 | 0 |
| NM_001008528 | MXRA7 | 2504 | 19 | 9 | 12 | 6 | 19 | 9 | 5 | 1 | 8 | 11 | 6 | 13 | 14 |
| NM_001008529 | MXRA7 | 1920 | 28 | 12 | 15 | 9 | 28 | 14 | 6 | 1 | 8 | 10 | 5 | 16 | 16 |
| NM_001008530 | LGMN | 2137 | 34 | 12 | 26 | 6 | 10 | 6 | 34 | 6 | 10 | 5 | 4 | 21 | 6 |
| NM_001008534 | AKAP14 | 661 | 3 | 0 | 0 | 0 | 0 | 0 | 0 | 0 | 1 | 0 | 0 | 0 | 3 |
| NM_001008535 | AKAP14 | 470 | 5 | 1 | 0 | 0 | 0 | 0 | 0 | 0 | 1 | 0 | 0 | 0 | 5 |
| NM_001008536 | TCHHL1 | 3603 | 0 | 0 | 0 | 0 | 0 | 0 | 0 | 0 | 0 | 0 | 0 | 0 | 0 |
| NM_001008537 | KIAA2022 | 11716 | 2 | 0 | 0 | 0 | 0 | 2 | 0 | 0 | 0 | 0 | 0 | 0 | 0 |
| NM_001008539 | SLC7A2 | 7568 | 24 | 6 | 1 | 0 | 6 | 1 | 2 | 24 | 2 | 9 | 16 | 2 | 6 |
| NM_001008540 | CXCR4 | 1895 | 15 | 2 | 2 | 1 | 0 | 0 | 0 | 0 | 6 | 1 | 0 | 15 | 1 |
| NM_001008541 | MXI1 | 3047 | 12 | 6 | 3 | 3 | 8 | 9 | 4 | 3 | 5 | 12 | 7 | 3 | 8 |
| NM_001008563 | USP20 | 4371 | 1 | 0 | 0 | 0 | 0 | 1 | 0 | 0 | 0 | 0 | 0 | 0 | 0 |
| NM_001008564 | NUPL1 | 4299 | 5 | 3 | 1 | 1 | 2 | 3 | 3 | 1 | 3 | 3 | 2 | 3 | 5 |
| NM_001008566 | TPST2 | 2002 | 1 | 0 | 0 | 0 | 0 | 0 | 0 | 1 | 1 | 0 | 0 | 0 | 0 |
| NM_001008656 | TCOF1 | 4981 | 4 | 2 | 2 | 2 | 2 | 3 | 1 | 1 | 1 | 2 | 2 | 4 | 3 |
| NM_001008657 | TCOF1 | 3860 | 3 | 2 | 1 | 1 | 1 | 2 | 1 | 0 | 1 | 2 | 1 | 3 | 2 |
| NM_001008660 | PICALM | 3723 | 32 | 16 | 19 | 4 | 16 | 15 | 22 | 12 | 17 | 12 | 7 | 32 | 15 |
| NM_001008661 | CCBL2 | 2152 | 11 | 7 | 5 | 2 | 4 | 10 | 6 | 10 | 6 | 8 | 2 | 11 | 9 |
| NM_001008662 | CCBL2 | 2052 | 11 | 7 | 5 | 2 | 5 | 11 | 7 | 10 | 6 | 8 | 2 | 11 | 10 |
| NM_001008693 | CST9 | 1687 | 0 | 0 | 0 | 0 | 0 | 0 | 0 | 0 | 0 | 0 | 0 | 0 | 0 |
| NM_001008695 | THAP7 | 1068 | 1 | 1 | 1 | 0 | 0 | 1 | 1 | 1 | 0 | 1 | 1 | 1 | 1 |
| NM_001008697 | TFIP11 | 2925 | 5 | 3 | 2 | 2 | 2 | 3 | 3 | 5 | 1 | 2 | 3 | 4 | 4 |
| NM_001008699 | IL4R | 1104 | 10 | 4 | 2 | 7 | 3 | 2 | 2 | 4 | 8 | 1 | 1 | 10 | 3 |
| NM_001008701 | LPHN1 | 7855 | 3 | 1 | 1 | 1 | 0 | 3 | 1 | 0 | 0 | 1 | 0 | 1 | 1 |
| NM_001008703 | C6orf1 | 954 | 1 | 0 | 1 | 0 | 1 | 1 | 1 | 0 | 0 | 0 | 0 | 0 | 0 |
| NM_001008704 | C6orf1 | 894 | 1 | 0 | 1 | 0 | 1 | 1 | 1 | 0 | 1 | 1 | 0 | 0 | 0 |
| NM_001008707 | EML1 | 4520 | 2 | 2 | 2 | 2 | 2 | 2 | 2 | 1 | 2 | 0 | 1 | 1 | 2 |
| NM_001008708 | CHAC2 | 1275 | 1 | 1 | 0 | 0 | 0 | 1 | 0 | 1 | 0 | 0 | 1 | 1 | 1 |
| NM_001008709 | PPP1CA | 1503 | 8 | 4 | 3 | 4 | 3 | 3 | 5 | 6 | 3 | 1 | 2 | 8 | 3 |
| NM_001008710 | RBPMS | 2948 | 7 | 4 | 4 | 3 | 7 | 1 | 7 | 2 | 4 | 7 | 1 | 4 | 4 |
| NM_001008711 | RBPMS | 3034 | 7 | 4 | 4 | 3 | 7 | 1 | 7 | 2 | 4 | 7 | 1 | 4 | 4 |
| NM_001008712 | RBPMS | 1414 | 19 | 11 | 13 | 9 | 19 | 2 | 18 | 8 | 10 | 19 | 3 | 9 | 9 |
| NM_001008723 | CCDC147 | 3313 | 2 | 1 | 0 | 0 | 1 | 1 | 0 | 0 | 1 | 0 | 0 | 0 | 2 |
| NM_001008726 | WDR89 | 3150 | 2 | 1 | 0 | 0 | 1 | 1 | 1 | 1 | 1 | 2 | 1 | 2 | 1 |
| NM_001008727 | ZNF121 | 1729 | 36 | 17 | 8 | 5 | 18 | 12 | 15 | 9 | 14 | 36 | 16 | 32 | 20 |
| NM_001008728 | HMGB4 | 677 | 26 | 2 | 0 | 0 | 0 | 0 | 0 | 0 | 0 | 0 | 0 | 0 | 26 |
| NM_001008735 | HMGB1L1 | 636 | 90 | 50 | 57 | 33 | 33 | 76 | 26 | 38 | 46 | 55 | 53 | 90 | 50 |
| NM_001008737 | LOC401052 | 1897 | 1 | 0 | 1 | 0 | 0 | 0 | 0 | 0 | 0 | 1 | 0 | 1 | 1 |
| NM_001008738 | FNIP1 | 6530 | 12 | 7 | 7 | 2 | 6 | 6 | 5 | 5 | 7 | 12 | 11 | 8 | 10 |
| NM_001008739 | C6orf226 | 552 | 0 | 0 | 0 | 0 | 0 | 0 | 0 | 0 | 0 | 0 | 0 | 0 | 0 |
| NM_001008740 | TMCO2 | 741 | 8 | 1 | 0 | 0 | 0 | 0 | 0 | 0 | 0 | 0 | 0 | 0 | 8 |
| NM_001008743 | SULT1C3 | 915 | 1 | 0 | 0 | 0 | 0 | 0 | 1 | 0 | 0 | 0 | 0 | 0 | 0 |
| NM_001008744 | TDP1 | 3524 | 2 | 1 | 1 | 1 | 0 | 1 | 1 | 0 | 1 | 1 | 0 | 2 | 2 |
| NM_001008747 | LOC441294 | 2355 | 0 | 0 | 0 | 0 | 0 | 0 | 0 | 0 | 0 | 0 | 0 | 0 | 0 |
| NM_001008749 | RAB19 | 952 | 1 | 0 | 0 | 1 | 0 | 0 | 1 | 0 | 0 | 0 | 0 | 0 | 0 |
| NM_001008756 | GCET2 | 3468 | 1 | 0 | 0 | 0 | 0 | 0 | 0 | 0 | 1 | 0 | 0 | 1 | 0 |
| NM_001008777 | FBXO47 | 2302 | 2 | 0 | 0 | 0 | 0 | 0 | 0 | 0 | 0 | 0 | 0 | 0 | 2 |
| NM_001008778 | SPDYC | 882 | 0 | 0 | 0 | 0 | 0 | 0 | 0 | 0 | 0 | 0 | 0 | 0 | 0 |
| NM_001008779 | SPDYA | 1208 | 3 | 0 | 0 | 0 | 0 | 0 | 0 | 0 | 0 | 0 | 0 | 0 | 3 |
| NM_001008781 | FAT3 | 19047 | 8 | 1 | 0 | 0 | 0 | 8 | 0 | 0 | 1 | 0 | 0 | 0 | 1 |
| NM_001008783 | SLC35D3 | 2358 | 0 | 0 | 0 | 0 | 0 | 0 | 0 | 0 | 0 | 0 | 0 | 0 | 0 |
| NM_001008784 | CD200R1L | 1296 | 0 | 0 | 0 | 0 | 0 | 0 | 0 | 0 | 0 | 0 | 0 | 0 | 0 |

| transcript | gene | transcript length (nt) | max | mean | adipose | colon | heart | hypothala mus | kidney | liver | lung | ovary | skeletalm uscle | spleen | testes |
|---|---|---|---|---|---|---|---|---|---|---|---|---|---|---|---|
| NM_001008800 | CCT3 | 1982 | 37 | 26 | 17 | 14 | 30 | 32 | 27 | 37 | 13 | 27 | 22 | 32 | 32 |
| NM_001008801 | ZNF468 | 3913 | 5 | 3 | 2 | 1 | 3 | 2 | 5 | 1 | 3 | 4 | 1 | 5 | 4 |
| NM_001008844 | DSP | 7933 | 96 | 20 | 0 | 16 | 96 | 7 | 26 | 50 | 8 | 5 | 1 | 6 | 4 |
| NM_001008860 | NIPA2 | 2448 | 2 | 1 | 1 | 1 | 1 | 1 | 1 | 1 | 1 | 1 | 0 | 2 | 0 |
| NM_001008883 | CCT3 | 2093 | 36 | 25 | 17 | 14 | 29 | 31 | 26 | 36 | 12 | 26 | 21 | 31 | 31 |
| NM_001008892 | NIPA2 | 2312 | 1 | 1 | 1 | 1 | 1 | 1 | 1 | 1 | 1 | 1 | 0 | 1 | 0 |
| NM_001008894 | NIPA2 | 2255 | 1 | 1 | 1 | 1 | 1 | 1 | 1 | 1 | 1 | 1 | 0 | 1 | 0 |
| NM_001008895 | CUL4A | 3787 | 23 | 9 | 6 | 4 | 15 | 5 | 8 | 7 | 5 | 6 | 23 | 8 | 10 |
| NM_001008896 | C1orf213 | 888 | 0 | 0 | 0 | 0 | 0 | 0 | 0 | 0 | 0 | 0 | 0 | 0 | 0 |
| NM_001008897 | TCP1 | 2367 | 41 | 28 | 23 | 13 | 33 | 41 | 28 | 35 | 17 | 31 | 26 | 27 | 36 |
| NM_001008901 | GTSF1L | 767 | 14 | 1 | 0 | 0 | 0 | 0 | 0 | 0 | 0 | 0 | 0 | 0 | 14 |
| NM_001008910 | STK16 | 2853 | 2 | 1 | 1 | 1 | 1 | 1 | 1 | 2 | 1 | 0 | 0 | 2 | 1 |
| NM_001008925 | RCHY1 | 4317 | 2 | 1 | 0 | 1 | 1 | 2 | 1 | 0 | 1 | 1 | 1 | 2 | 1 |
| NM_001008938 | CKAP5 | 6698 | 19 | 9 | 6 | 3 | 9 | 19 | 6 | 6 | 4 | 13 | 13 | 8 | 17 |
| NM_001008949 | ITPRIPL1 | 2056 | 0 | 0 | 0 | 0 | 0 | 0 | 0 | 0 | 0 | 0 | 0 | 0 | 0 |
| NM_001009 | RPS5 | 741 | 20 | 12 | 7 | 10 | 9 | 7 | 9 | 20 | 11 | 20 | 12 | 20 | 9 |
| NM_001009181 | STATH | 570 | 0 | 0 | 0 | 0 | 0 | 0 | 0 | 0 | 0 | 0 | 0 | 0 | 0 |
| NM_001009182 | SIP1 | 1314 | 1 | 1 | 0 | 1 | 1 | 1 | 1 | 0 | 1 | 1 | 1 | 1 | 1 |
| NM_001009183 | SIP1 | 1300 | 3 | 2 | 1 | 1 | 1 | 3 | 1 | 1 | 1 | 2 | 2 | 2 | 2 |
| NM_001009184 | GRINA | 1856 | 10 | 5 | 4 | 2 | 4 | 8 | 7 | 10 | 4 | 3 | 2 | 9 | 3 |
| NM_001009185 | ACSL6 | 3047 | 13 | 2 | 0 | 0 | 0 | 13 | 1 | 0 | 0 | 0 | 0 | 0 | 2 |
| NM_001009186 | CCT6A | 2529 | 28 | 19 | 13 | 8 | 23 | 25 | 24 | 19 | 14 | 13 | 21 | 25 | 28 |
| NM_001009552 | PPP2CB | 1970 | 53 | 19 | 13 | 9 | 16 | 53 | 18 | 20 | 15 | 26 | 7 | 13 | 17 |
| NM_001009554 | MFAP3L | 5827 | 7 | 2 | 0 | 0 | 0 | 4 | 6 | 7 | 0 | 0 | 0 | 0 | 1 |
| NM_001009555 | SH3D19 | 5273 | 29 | 14 | 29 | 12 | 21 | 6 | 9 | 13 | 17 | 22 | 7 | 3 | 11 |
| NM_001009562 | LST-3TM12 | 1923 | 0 | 0 | 0 | 0 | 0 | 0 | 0 | 0 | 0 | 0 | 0 | 0 | 0 |
| NM_001009565 | CDKL4 | 1567 | 2 | 1 | 0 | 0 | 0 | 1 | 0 | 0 | 1 | 1 | 1 | 0 | 2 |
| NM_001009566 | CLSTN1 | 5192 | 51 | 11 | 4 | 8 | 6 | 51 | 11 | 1 | 5 | 12 | 4 | 7 | 7 |
| NM_001009567 | MRC1L1 | 5171 | 0 | 0 | 0 | 0 | 0 | 0 | 0 | 0 | 0 | 0 | 0 | 0 | 0 |
| NM_001009568 | SMPDL3B | 1594 | 0 | 0 | 0 | 0 | 0 | 0 | 0 | 0 | 0 | 0 | 0 | 0 | 0 |
| NM_001009569 | MLLT10 | 4725 | 11 | 4 | 3 | 2 | 2 | 2 | 4 | 2 | 4 | 4 | 3 | 4 | 11 |
| NM_001009570 | CCT7 | 1323 | 19 | 14 | 12 | 9 | 17 | 19 | 12 | 19 | 7 | 9 | 17 | 14 | 16 |
| NM_001009571 | CADPS2 | 5931 | 5 | 2 | 1 | 1 | 2 | 3 | 5 | 2 | 3 | 2 | 0 | 1 | 1 |
| NM_001009584 | ARMCX6 | 1766 | 4 | 2 | 2 | 2 | 2 | 3 | 2 | 1 | 2 | 2 | 1 | 4 | 3 |
| NM_001009598 | RXRG | 623 | 3 | 1 | 0 | 1 | 0 | 3 | 0 | 0 | 0 | 0 | 2 | 0 | 0 |
| NM_001009606 | HS3ST6 | 1062 | 0 | 0 | 0 | 0 | 0 | 0 | 0 | 0 | 0 | 0 | 0 | 0 | 0 |
| NM_001009608 | C20orf94 | 1407 | 3 | 2 | 1 | 2 | 1 | 2 | 3 | 1 | 1 | 3 | 0 | 3 | 3 |
| NM_001009609 | SPANXN3 | 594 | 7 | 1 | 0 | 0 | 0 | 0 | 0 | 0 | 0 | 0 | 0 | 0 | 7 |
| NM_001009610 | FAM22D | 5543 | 1 | 0 | 0 | 1 | 0 | 0 | 0 | 0 | 0 | 1 | 0 | 0 | 0 |
| NM_001009611 | PRAMEF4 | 1840 | 0 | 0 | 0 | 0 | 0 | 0 | 0 | 0 | 0 | 0 | 0 | 0 | 0 |
| NM_001009612 | C20orf202 | 1604 | 0 | 0 | 0 | 0 | 0 | 0 | 0 | 0 | 0 | 0 | 0 | 0 | 0 |
| NM_001009613 | SPANXN4 | 431 | 17 | 2 | 0 | 0 | 0 | 0 | 0 | 0 | 0 | 0 | 0 | 0 | 17 |
| NM_001009614 | SPANXN1 | 613 | 0 | 0 | 0 | 0 | 0 | 0 | 0 | 0 | 0 | 0 | 0 | 0 | 0 |
| NM_001009615 | SPANXN2 | 1377 | 0 | 0 | 0 | 0 | 0 | 0 | 0 | 0 | 0 | 0 | 0 | 0 | 0 |
| NM_001009616 | SPANXN5 | 561 | 4 | 0 | 0 | 0 | 0 | 0 | 0 | 0 | 0 | 0 | 0 | 0 | 4 |
| NM_001009811 | AZI1 | 3511 | 1 | 0 | 0 | 1 | 1 | 0 | 0 | 0 | 0 | 1 | 0 | 1 | 1 |
| NM_001009812 | LBX2 | 1452 | 0 | 0 | 0 | 0 | 0 | 0 | 0 | 0 | 0 | 0 | 0 | 0 | 0 |
| NM_001009813 | MEIS3 | 1971 | 1 | 0 | 0 | 0 | 0 | 1 | 0 | 0 | 0 | 0 | 0 | 0 | 0 |
| NM_001009814 | KIAA0564 | 3440 | 8 | 5 | 4 | 3 | 6 | 4 | 8 | 6 | 2 | 4 | 8 | 4 | 4 |
| NM_001009824 | GHRHR | 1127 | 0 | 0 | 0 | 0 | 0 | 0 | 0 | 0 | 0 | 0 | 0 | 0 | 0 |
| NM_001009877 | BRD9 | 2520 | 5 | 3 | 3 | 4 | 3 | 5 | 3 | 1 | 2 | 3 | 3 | 4 | 4 |
| NM_001009880 | C22orf9 | 6215 | 5 | 2 | 2 | 5 | 1 | 3 | 1 | 1 | 2 | 1 | 1 | 2 | 1 |
| NM_001009881 | ZCCHC11 | 5831 | 15 | 8 | 3 | 2 | 7 | 5 | 8 | 4 | 8 | 15 | 10 | 12 | 13 |
| NM_001009882 | ZCCHC11 | 5816 | 15 | 8 | 3 | 2 | 7 | 5 | 8 | 4 | 8 | 15 | 10 | 12 | 13 |
| NM_001009894 | C12orf29 | 2874 | 1 | 0 | 0 | 0 | 1 | 1 | 0 | 0 | 0 | 1 | 0 | 1 | 0 |
| NM_001009899 | KIAA2018 | 13707 | 9 | 5 | 4 | 3 | 4 | 4 | 5 | 6 | 3 | 6 | 9 | 8 | 5 |
| NM_001009905 | B3GNTL1 | 1383 | 0 | 0 | 0 | 0 | 0 | 0 | 0 | 0 | 0 | 0 | 0 | 0 | 0 |
| NM_001009909 | LUZP2 | 5257 | 7 | 1 | 0 | 0 | 0 | 7 | 0 | 0 | 0 | 0 | 0 | 0 | 0 |
| NM_001009921 | VPS8 | 5047 | 5 | 3 | 2 | 1 | 5 | 3 | 2 | 1 | 3 | 3 | 4 | 4 | 3 |
| NM_001009922 | RCHY1 | 4292 | 2 | 1 | 0 | 1 | 1 | 2 | 1 | 0 | 1 | 1 | 1 | 2 | 1 |
| NM_001009923 | C20orf30 | 1564 | 13 | 10 | 11 | 4 | 12 | 8 | 13 | 13 | 10 | 9 | 8 | 13 | 9 |
| NM_001009924 | C20orf30 | 1782 | 12 | 9 | 10 | 4 | 10 | 7 | 12 | 11 | 9 | 8 | 7 | 11 | 8 |
| NM_001009925 | C20orf30 | 1458 | 14 | 11 | 12 | 5 | 13 | 9 | 14 | 14 | 11 | 10 | 8 | 14 | 9 |
| NM_001009931 | HRNR | 9623 | 1 | 0 | 0 | 1 | 0 | 0 | 1 | 0 | 0 | 1 | 0 | 0 | 1 |
| NM_001009932 | DNASE1L1 | 3008 | 3 | 1 | 0 | 0 | 1 | 0 | 1 | 0 | 1 | 0 | 3 | 1 | 0 |
| NM_001009933 | DNASE1L1 | 2750 | 3 | 1 | 0 | 0 | 1 | 0 | 1 | 0 | 1 | 0 | 3 | 1 | 0 |
| NM_001009934 | DNASE1L1 | 2665 | 3 | 1 | 0 | 0 | 1 | 0 | 1 | 0 | 1 | 0 | 3 | 1 | 0 |
| NM_001009936 | PHF19 | 988 | 1 | 0 | 0 | 0 | 0 | 0 | 0 | 0 | 0 | 0 | 0 | 1 | 1 |
| NM_001009941 | ANKRD16 | 1671 | 0 | 0 | 0 | 0 | 0 | 0 | 0 | 0 | 0 | 0 | 0 | 0 | 0 |
| NM_001009943 | ANKRD16 | 1592 | 0 | 0 | 0 | 0 | 0 | 0 | 0 | 0 | 0 | 0 | 0 | 0 | 0 |
| NM_001009944 | PKD1 | 14138 | 0 | 0 | 0 | 0 | 0 | 0 | 0 | 0 | 0 | 0 | 0 | 0 | 0 |
| NM_001009955 | SSBP3 | 2106 | 10 | 4 | 2 | 3 | 3 | 7 | 2 | 2 | 2 | 10 | 3 | 3 | 4 |
| NM_001009958 | ZNF655 | 1199 | 1 | 1 | 1 | 0 | 0 | 0 | 1 | 1 | 1 | 0 | 0 | 1 | 1 |
| NM_001009959 | ERMN | 3707 | 27 | 3 | 0 | 0 | 0 | 27 | 0 | 0 | 0 | 0 | 0 | 0 | 0 |
| NM_001009960 | ZNF655 | 4566 | 11 | 7 | 4 | 2 | 7 | 7 | 9 | 3 | 6 | 10 | 4 | 11 | 9 |
| NM_001009984 | C20orf194 | 6855 | 4 | 2 | 2 | 1 | 2 | 4 | 2 | 0 | 1 | 2 | 1 | 1 | 2 |
| NM_001009991 | SYTL3 | 2277 | 4 | 1 | 1 | 1 | 1 | 0 | 1 | 0 | 3 | 0 | 1 | 4 | 1 |

| transcript | gene | transcript length (nt) | max | mean | adipose | colon | heart | hypothalamus | kidney | liver | lung | ovary | skeletalmuscle | spleen | testes |
|---|---|---|---|---|---|---|---|---|---|---|---|---|---|---|---|
| NM_001009992 | ZNF648 | 3649 | 0 | 0 | 0 | 0 | 0 | 0 | 0 | 0 | 0 | 0 | 0 | 0 | 0 |
| NM_001009993 | FAM168B | 5406 | 12 | 7 | 6 | 6 | 7 | 12 | 9 | 6 | 5 | 6 | 5 | 7 | 8 |
| NM_001009994 | RIPPLY2 | 664 | 0 | 0 | 0 | 0 | 0 | 0 | 0 | 0 | 0 | 0 | 0 | 0 | 0 |
| NM_001009996 | DALRD3 | 2238 | 0 | 0 | 0 | 0 | 0 | 0 | 0 | 0 | 0 | 0 | 0 | 0 | 0 |
| NM_001009997 | C10orf62 | 1208 | 8 | 1 | 0 | 0 | 0 | 0 | 1 | 0 | 0 | 0 | 0 | 0 | 8 |
| NM_001009998 | SSBP4 | 1645 | 2 | 1 | 0 | 0 | 0 | 2 | 1 | 0 | 0 | 1 | 0 | 1 | 2 |
| NM_001009999 | KDM1A | 3107 | 17 | 8 | 4 | 5 | 6 | 12 | 6 | 4 | 6 | 17 | 7 | 7 | 14 |
| NM_001010 | RPS6 | 829 | 361 | 122 | 92 | 83 | 238 | 61 | 93 | 98 | 78 | 129 | 52 | 361 | 52 |
| NM_001010000 | ARHGAP28 | 5479 | 5 | 1 | 1 | 0 | 0 | 0 | 1 | 0 | 1 | 3 | 2 | 4 | 5 |
| NM_001010844 | IRAK1BP1 | 1157 | 2 | 1 | 0 | 0 | 1 | 1 | 2 | 0 | 1 | 2 | 0 | 1 | 2 |
| NM_001010845 | ACSM2A | 2964 | 21 | 3 | 0 | 0 | 0 | 0 | 21 | 9 | 0 | 0 | 0 | 0 | 0 |
| NM_001010846 | SHE | 6241 | 7 | 3 | 2 | 1 | 3 | 1 | 2 | 1 | 3 | 2 | 1 | 7 | 4 |
| NM_001010848 | NRG3 | 3692 | 4 | 0 | 0 | 0 | 0 | 4 | 0 | 0 | 0 | 0 | 0 | 0 | 0 |
| NM_001010851 | ZNF766 | 2975 | 14 | 7 | 5 | 2 | 7 | 5 | 7 | 2 | 6 | 14 | 6 | 12 | 11 |
| NM_001010852 | CLVS2 | 2010 | 10 | 1 | 0 | 0 | 0 | 10 | 0 | 0 | 0 | 0 | 0 | 0 | 0 |
| NM_001010853 | PM20D2 | 4708 | 6 | 2 | 0 | 1 | 3 | 1 | 2 | 1 | 1 | 3 | 6 | 1 | 1 |
| NM_001010854 | TTC7B | 3434 | 10 | 3 | 2 | 1 | 4 | 10 | 3 | 2 | 2 | 2 | 3 | 3 | 1 |
| NM_001010855 | PIK3R6 | 3040 | 1 | 0 | 0 | 0 | 0 | 0 | 0 | 0 | 0 | 0 | 0 | 1 | 0 |
| NM_001010857 | LELP1 | 523 | 10 | 1 | 0 | 0 | 0 | 0 | 0 | 0 | 0 | 0 | 0 | 0 | 10 |
| NM_001010858 | RNF187 | 3132 | 3 | 1 | 1 | 1 | 1 | 3 | 2 | 1 | 1 | 1 | 1 | 2 | 1 |
| NM_001010859 | C22orf42 | 805 | 0 | 0 | 0 | 0 | 0 | 0 | 0 | 0 | 0 | 0 | 0 | 0 | 0 |
| NM_001010860 | C14orf174 | 2025 | 17 | 3 | 0 | 0 | 1 | 5 | 5 | 0 | 2 | 2 | 0 | 1 | 17 |
| NM_001010861 | LPPR5 | 4000 | 2 | 0 | 0 | 0 | 0 | 2 | 0 | 0 | 0 | 0 | 0 | 0 | 0 |
| NM_001010862 | SPIN3 | 4459 | 2 | 1 | 1 | 1 | 0 | 2 | 1 | 0 | 0 | 1 | 0 | 1 | 1 |
| NM_001010863 | C10orf128 | 383 | 1 | 0 | 0 | 0 | 0 | 0 | 0 | 0 | 1 | 0 | 0 | 1 | 0 |
| NM_001010865 | EME2 | 1335 | 0 | 0 | 0 | 0 | 0 | 0 | 0 | 0 | 0 | 0 | 0 | 0 | 0 |
| NM_001010866 | TMEM201 | 3906 | 0 | 0 | 0 | 0 | 0 | 0 | 0 | 0 | 0 | 0 | 0 | 0 | 0 |
| NM_001010867 | C1orf69 | 7904 | 1 | 1 | 0 | 1 | 1 | 0 | 1 | 0 | 1 | 1 | 1 | 1 | 1 |
| NM_001010868 | C6orf163 | 1303 | 3 | 2 | 1 | 2 | 3 | 1 | 3 | 1 | 1 | 3 | 2 | 3 | 3 |
| NM_001010870 | TDRD6 | 8975 | 5 | 1 | 0 | 0 | 0 | 0 | 0 | 0 | 0 | 0 | 0 | 1 | 5 |
| NM_001010872 | FAM83B | 3166 | 3 | 1 | 0 | 3 | 1 | 0 | 3 | 0 | 0 | 0 | 1 | 0 | 0 |
| NM_001010873 | TSPO2 | 1012 | 0 | 0 | 0 | 0 | 0 | 0 | 0 | 0 | 0 | 0 | 0 | 0 | 0 |
| NM_001010874 | TECRL | 2806 | 127 | 12 | 0 | 0 | 127 | 0 | 0 | 0 | 0 | 0 | 4 | 0 | 0 |
| NM_001010875 | SLC25A30 | 3666 | 11 | 5 | 2 | 1 | 9 | 2 | 11 | 9 | 2 | 4 | 3 | 5 | 3 |
| NM_001010876 | C6orf191 | 545 | 0 | 0 | 0 | 0 | 0 | 0 | 0 | 0 | 0 | 0 | 0 | 0 | 0 |
| NM_001010877 | ZNF311 | 2698 | 1 | 0 | 0 | 0 | 0 | 0 | 0 | 0 | 0 | 1 | 0 | 1 | 1 |
| NM_001010878 | C16orf91 | 1071 | 1 | 0 | 0 | 0 | 0 | 1 | 0 | 1 | 1 | 0 | 0 | 0 | 1 |
| NM_001010879 | ZIK1 | 2676 | 1 | 1 | 0 | 0 | 1 | 1 | 0 | 0 | 0 | 1 | 0 | 1 | 1 |
| NM_001010880 | ZNF780A | 3674 | 5 | 2 | 2 | 1 | 2 | 3 | 2 | 1 | 1 | 5 | 1 | 5 | 2 |
| NM_001010883 | FAM102B | 5340 | 4 | 2 | 1 | 1 | 1 | 4 | 1 | 0 | 2 | 2 | 0 | 3 | 2 |
| NM_001010886 | C6orf127 | 477 | 0 | 0 | 0 | 0 | 0 | 0 | 0 | 0 | 0 | 0 | 0 | 0 | 0 |
| NM_001010887 | ACER2 | 2852 | 2 | 1 | 2 | 0 | 1 | 0 | 1 | 0 | 1 | 0 | 1 | 1 | 1 |
| NM_001010888 | ZC3H12B | 7165 | 1 | 0 | 0 | 0 | 0 | 1 | 0 | 0 | 0 | 1 | 0 | 0 | 0 |
| NM_001010889 | PRAMEF6 | 1720 | 0 | 0 | 0 | 0 | 0 | 0 | 0 | 0 | 0 | 0 | 0 | 0 | 0 |
| NM_001010890 | PRAMEF9 | 1863 | 0 | 0 | 0 | 0 | 0 | 0 | 0 | 0 | 0 | 0 | 0 | 0 | 0 |
| NM_001010891 | MTX3 | 7843 | 4 | 2 | 2 | 1 | 2 | 4 | 3 | 1 | 1 | 3 | 1 | 2 | 3 |
| NM_001010892 | RSPH4A | 2840 | 2 | 0 | 0 | 0 | 0 | 1 | 0 | 0 | 2 | 0 | 0 | 0 | 1 |
| NM_001010893 | SLC10A5 | 1317 | 1 | 0 | 0 | 0 | 0 | 0 | 0 | 1 | 0 | 0 | 0 | 0 | 0 |
| NM_001010895 | C9orf102 | 4647 | 5 | 3 | 2 | 1 | 2 | 4 | 2 | 2 | 2 | 5 | 1 | 4 | 4 |
| NM_001010896 | C10orf113 | 518 | 1 | 0 | 0 | 0 | 0 | 0 | 0 | 0 | 1 | 0 | 0 | 0 | 0 |
| NM_001010897 | SERP2 | 784 | 1 | 0 | 0 | 0 | 0 | 1 | 0 | 0 | 0 | 0 | 0 | 0 | 0 |
| NM_001010898 | SLC6A17 | 6402 | 7 | 1 | 0 | 0 | 0 | 7 | 0 | 0 | 0 | 0 | 0 | 0 | 0 |
| NM_001010903 | C6orf222 | 3735 | 0 | 0 | 0 | 0 | 0 | 0 | 0 | 0 | 0 | 0 | 0 | 0 | 0 |
| NM_001010904 | GLYATL3 | 2129 | 0 | 0 | 0 | 0 | 0 | 0 | 0 | 0 | 0 | 0 | 0 | 0 | 0 |
| NM_001010905 | C6orf58 | 1198 | 5 | 0 | 0 | 0 | 0 | 0 | 0 | 0 | 5 | 0 | 0 | 0 | 0 |
| NM_001010906 | C8orf80 | 3887 | 8 | 1 | 0 | 1 | 0 | 0 | 1 | 8 | 0 | 0 | 0 | 1 | 0 |
| NM_001010907 | C9orf153 | 409 | 1 | 0 | 0 | 0 | 0 | 0 | 0 | 0 | 0 | 0 | 0 | 0 | 1 |
| NM_001010908 | C1QL3 | 2493 | 7 | 1 | 0 | 0 | 0 | 7 | 1 | 0 | 0 | 0 | 0 | 1 | 0 |
| NM_001010909 | MUC21 | 3566 | 0 | 0 | 0 | 0 | 0 | 0 | 0 | 0 | 0 | 0 | 0 | 0 | 0 |
| NM_001010909 | MUC21 | 3656 | 0 | 0 | 0 | 0 | 0 | 0 | 0 | 0 | 0 | 0 | 0 | 0 | 0 |
| NM_001010911 | C10orf114 | 1989 | 2 | 1 | 0 | 0 | 0 | 1 | 2 | 0 | 0 | 1 | 0 | 0 | 1 |
| NM_001010912 | C10orf120 | 1064 | 2 | 0 | 0 | 0 | 0 | 0 | 0 | 0 | 0 | 0 | 0 | 0 | 2 |
| NM_001010915 | PTPLAD2 | 1416 | 1 | 0 | 1 | 0 | 0 | 0 | 0 | 0 | 1 | 1 | 0 | 1 | 0 |
| NM_001010917 | GOLGA7B | 2894 | 2 | 0 | 0 | 0 | 0 | 2 | 0 | 0 | 0 | 0 | 0 | 0 | 0 |
| NM_001010919 | FAM26F | 1109 | 8 | 1 | 0 | 0 | 0 | 1 | 0 | 0 | 0 | 0 | 0 | 8 | 0 |
| NM_001010922 | BCL2L15 | 4973 | 3 | 0 | 0 | 3 | 0 | 0 | 0 | 0 | 0 | 0 | 0 | 0 | 0 |
| NM_001010923 | THEMIS | 3865 | 1 | 0 | 0 | 0 | 0 | 0 | 0 | 0 | 1 | 0 | 0 | 1 | 0 |
| NM_001010924 | FAM171A1 | 3950 | 4 | 1 | 1 | 1 | 1 | 4 | 2 | 0 | 0 | 0 | 0 | 1 | 0 |
| NM_001010926 | HES5 | 1304 | 0 | 0 | 0 | 0 | 0 | 0 | 0 | 0 | 0 | 0 | 0 | 0 | 0 |
| NM_001010927 | TIAM2 | 2668 | 2 | 1 | 0 | 0 | 0 | 2 | 0 | 0 | 0 | 0 | 1 | 0 | 1 |
| NM_001010931 | HGF | 1289 | 7 | 3 | 1 | 7 | 2 | 1 | 2 | 5 | 4 | 1 | 0 | 3 | 2 |
| NM_001010932 | HGF | 2790 | 7 | 2 | 2 | 7 | 1 | 1 | 2 | 5 | 4 | 0 | 0 | 3 | 2 |
| NM_001010933 | HGF | 1274 | 7 | 3 | 1 | 7 | 2 | 1 | 2 | 5 | 4 | 1 | 0 | 3 | 2 |
| NM_001010934 | HGF | 2010 | 4 | 1 | 1 | 4 | 1 | 1 | 1 | 3 | 2 | 0 | 0 | 2 | 1 |
| NM_001010935 | RAP1A | 1906 | 23 | 11 | 8 | 6 | 19 | 8 | 11 | 7 | 16 | 8 | 9 | 23 | 9 |
| NM_001010938 | TNK2 | 4222 | 4 | 1 | 1 | 1 | 1 | 4 | 0 | 1 | 0 | 0 | 1 | 1 | 1 |
| NM_001010939 | LIPJ | 1483 | 0 | 0 | 0 | 0 | 0 | 0 | 0 | 0 | 0 | 0 | 0 | 0 | 0 |

| transcript | gene | transcript length (nt) | max | mean | adipose | colon | heart | hypothalamus | kidney | liver | lung | ovary | skeletalmuscle | spleen | testes |
|---|---|---|---|---|---|---|---|---|---|---|---|---|---|---|---|
| NM_001010940 | C9orf135 | 851 | 1 | 0 | 0 | 0 | 0 | 0 | 0 | 0 | 0 | 0 | 0 | 0 | 1 |
| NM_001010942 | RAP1B | 2098 | 208 | 95 | 113 | 33 | 85 | 69 | 80 | 72 | 135 | 76 | 40 | 208 | 140 |
| NM_001010969 | CYP4A22 | 1668 | 0 | 0 | 0 | 0 | 0 | 0 | 0 | 0 | 0 | 0 | 0 | 0 | 0 |
| NM_001010971 | SAMD13 | 1566 | 1 | 0 | 0 | 1 | 0 | 0 | 0 | 0 | 0 | 0 | 1 | 0 | 1 |
| NM_001010972 | ZYX | 2304 | 5 | 2 | 2 | 2 | 3 | 1 | 2 | 1 | 4 | 3 | 2 | 5 | 3 |
| NM_001010974 | BCAS4 | 1110 | 0 | 0 | 0 | 0 | 0 | 0 | 0 | 0 | 0 | 0 | 0 | 0 | 0 |
| NM_001010977 | C13orf39 | 1085 | 0 | 0 | 0 | 0 | 0 | 0 | 0 | 0 | 0 | 0 | 0 | 0 | 0 |
| NM_001010978 | LDLRAD1 | 785 | 0 | 0 | 0 | 0 | 0 | 0 | 0 | 0 | 0 | 0 | 0 | 0 | 0 |
| NM_001010979 | C1orf189 | 387 | 2 | 0 | 0 | 0 | 0 | 0 | 0 | 0 | 1 | 0 | 0 | 1 | 2 |
| NM_001010980 | C1orf130 | 3953 | 0 | 0 | 0 | 0 | 0 | 0 | 0 | 0 | 0 | 0 | 0 | 0 | 0 |
| NM_001010982 | AFMID | 1691 | 17 | 4 | 2 | 1 | 1 | 4 | 7 | 17 | 2 | 1 | 0 | 2 | 1 |
| NM_001010983 | GLT8D1 | 2324 | 3 | 1 | 1 | 1 | 1 | 2 | 2 | 3 | 1 | 2 | 1 | 1 | 1 |
| NM_001010984 | C1orf124 | 3838 | 2 | 1 | 1 | 0 | 1 | 1 | 1 | 1 | 1 | 1 | 2 | 1 | 1 |
| NM_001010985 | MYBPHL | 1372 | 0 | 0 | 0 | 0 | 0 | 0 | 0 | 0 | 0 | 0 | 0 | 0 | 0 |
| NM_001010986 | ATP11C | 6013 | 7 | 3 | 2 | 1 | 2 | 1 | 3 | 5 | 2 | 4 | 1 | 7 | 5 |
| NM_001010987 | IFIT1L | 1972 | 0 | 0 | 0 | 0 | 0 | 0 | 0 | 0 | 0 | 0 | 0 | 0 | 0 |
| NM_001010989 | HERPUD1 | 2173 | 12 | 7 | 4 | 4 | 6 | 5 | 10 | 4 | 9 | 10 | 7 | 12 | 3 |
| NM_001010990 | HERPUD1 | 2101 | 11 | 6 | 4 | 3 | 6 | 4 | 9 | 4 | 8 | 8 | 6 | 11 | 3 |
| NM_001011 | RPS7 | 728 | 222 | 107 | 123 | 63 | 93 | 90 | 77 | 155 | 73 | 222 | 60 | 151 | 73 |
| NM_001011513 | PDLIM5 | 5773 | 26 | 7 | 4 | 4 | 26 | 5 | 8 | 7 | 4 | 5 | 5 | 7 | 5 |
| NM_001011515 | PDLIM5 | 1993 | 30 | 6 | 1 | 1 | 27 | 1 | 1 | 1 | 1 | 1 | 30 | 2 | 2 |
| NM_001011516 | PDLIM5 | 1933 | 32 | 6 | 1 | 1 | 27 | 1 | 1 | 1 | 1 | 1 | 32 | 2 | 2 |
| NM_001011537 | FYTTD1 | 3654 | 11 | 6 | 5 | 2 | 9 | 5 | 5 | 5 | 6 | 5 | 10 | 7 | 11 |
| NM_001011543 | MAGEA10 | 1593 | 0 | 0 | 0 | 0 | 0 | 0 | 0 | 0 | 0 | 0 | 0 | 0 | 0 |
| NM_001011544 | MAGEA11 | 1885 | 1 | 0 | 0 | 0 | 0 | 0 | 0 | 0 | 0 | 0 | 0 | 0 | 1 |
| NM_001011546 | DSTN | 1746 | 348 | 136 | 85 | 110 | 348 | 133 | 127 | 66 | 175 | 147 | 52 | 75 | 180 |
| NM_001011547 | SLC5A9 | 3199 | 4 | 1 | 0 | 0 | 1 | 0 | 4 | 1 | 1 | 1 | 0 | 1 | 0 |
| NM_001011548 | MAGEA4 | 1724 | 2 | 0 | 0 | 0 | 0 | 0 | 0 | 0 | 0 | 0 | 0 | 0 | 2 |
| NM_001011549 | MAGEA4 | 1721 | 2 | 0 | 0 | 0 | 0 | 0 | 0 | 0 | 0 | 0 | 0 | 0 | 2 |
| NM_001011550 | MAGEA4 | 1712 | 2 | 0 | 0 | 0 | 0 | 0 | 0 | 0 | 0 | 0 | 0 | 0 | 2 |
| NM_001011551 | C1GALT1C1 | 1725 | 4 | 2 | 3 | 2 | 4 | 2 | 3 | 4 | 2 | 1 | 1 | 3 | 3 |
| NM_001011552 | SLC9A4 | 4138 | 2 | 0 | 0 | 0 | 0 | 0 | 2 | 0 | 0 | 0 | 0 | 0 | 0 |
| NM_001011553 | 07 Sep | 2518 | 190 | 70 | 75 | 26 | 77 | 190 | 34 | 32 | 51 | 86 | 38 | 87 | 77 |
| NM_001011554 | SLC13A3 | 4066 | 62 | 6 | 0 | 0 | 0 | 2 | 62 | 0 | 0 | 0 | 0 | 0 | 0 |
| NM_001011645 | AR | 1765 | 6 | 2 | 3 | 0 | 2 | 0 | 1 | 6 | 0 | 4 | 1 | 0 | 2 |
| NM_001011649 | CDK5RAP2 | 5993 | 16 | 8 | 7 | 4 | 10 | 6 | 4 | 10 | 6 | 12 | 16 | 8 | 11 |
| NM_001011655 | TMEM44 | 2348 | 4 | 1 | 1 | 4 | 1 | 1 | 1 | 0 | 2 | 2 | 0 | 3 | 1 |
| NM_001011657 | ZMAT1 | 3184 | 6 | 3 | 2 | 1 | 2 | 4 | 6 | 1 | 2 | 4 | 1 | 5 | 4 |
| NM_001011658 | TRAPPC2 | 2848 | 1 | 1 | 0 | 0 | 0 | 1 | 1 | 0 | 0 | 1 | 0 | 1 | 0 |
| NM_001011663 | PCGF6 | 2230 | 2 | 1 | 1 | 1 | 1 | 1 | 1 | 2 | 1 | 1 | 1 | 1 | 1 |
| NM_001011666 | CREB5 | 7772 | 4 | 1 | 2 | 1 | 2 | 3 | 1 | 0 | 2 | 0 | 1 | 4 | 1 |
| NM_001011667 | CHCHD7 | 1730 | 25 | 12 | 8 | 9 | 15 | 25 | 13 | 8 | 11 | 8 | 8 | 21 | 7 |
| NM_001011668 | CHCHD7 | 1686 | 26 | 13 | 8 | 9 | 16 | 26 | 13 | 8 | 11 | 9 | 8 | 22 | 7 |
| NM_001011669 | CHCHD7 | 1710 | 26 | 12 | 8 | 9 | 16 | 26 | 13 | 8 | 11 | 9 | 8 | 21 | 7 |
| NM_001011670 | CHCHD7 | 1582 | 28 | 13 | 9 | 9 | 17 | 28 | 14 | 8 | 12 | 9 | 9 | 23 | 8 |
| NM_001011671 | CHCHD7 | 1538 | 28 | 14 | 9 | 9 | 17 | 28 | 14 | 9 | 12 | 9 | 9 | 23 | 8 |
| NM_001011699 | HAUS8 | 1453 | 1 | 0 | 0 | 0 | 0 | 0 | 0 | 0 | 0 | 0 | 0 | 1 | 1 |
| NM_001011700 | MCCD1 | 709 | 2 | 0 | 0 | 0 | 0 | 0 | 2 | 0 | 0 | 0 | 0 | 0 | 0 |
| NM_001011703 | FAM125B | 2679 | 1 | 0 | 1 | 1 | 0 | 1 | 0 | 0 | 1 | 1 | 0 | 1 | 0 |
| NM_001011705 | LECT1 | 1444 | 1 | 0 | 0 | 0 | 0 | 1 | 0 | 0 | 0 | 0 | 0 | 0 | 1 |
| NM_001011708 | OLA1 | 4255 | 9 | 6 | 3 | 2 | 7 | 9 | 7 | 6 | 4 | 5 | 9 | 6 | 8 |
| NM_001011709 | PNLIPRP3 | 2308 | 0 | 0 | 0 | 0 | 0 | 0 | 0 | 0 | 0 | 0 | 0 | 0 | 0 |
| NM_001011713 | NAT12 | 4475 | 6 | 3 | 2 | 1 | 3 | 5 | 3 | 3 | 1 | 3 | 4 | 3 | 6 |
| NM_001011717 | NMS | 485 | 0 | 0 | 0 | 0 | 0 | 0 | 0 | 0 | 0 | 0 | 0 | 0 | 0 |
| NM_001011718 | XKR7 | 2910 | 0 | 0 | 0 | 0 | 0 | 0 | 0 | 0 | 0 | 0 | 0 | 0 | 0 |
| NM_001011719 | ARSH | 1689 | 0 | 0 | 0 | 0 | 0 | 0 | 0 | 0 | 0 | 0 | 0 | 0 | 0 |
| NM_001011720 | XKR9 | 3174 | 1 | 0 | 0 | 0 | 0 | 1 | 0 | 0 | 0 | 0 | 0 | 0 | 1 |
| NM_001011722 | ARHGEF10L | 4261 | 2 | 1 | 1 | 2 | 1 | 1 | 1 | 2 | 0 | 0 | 1 | 0 | 0 |
| NM_001011724 | HNRNPA1L2 | 2365 | 50 | 17 | 11 | 10 | 16 | 16 | 14 | 12 | 13 | 50 | 11 | 24 | 13 |
| NM_001011725 | HNRNPA1L2 | 2224 | 53 | 18 | 11 | 11 | 17 | 16 | 15 | 13 | 14 | 53 | 11 | 26 | 14 |
| NM_001011878 | DEFB121 | 282 | 0 | 0 | 0 | 0 | 0 | 0 | 0 | 0 | 0 | 0 | 0 | 0 | 0 |
| NM_001011880 | CLEC18B | 1943 | 0 | 0 | 0 | 0 | 0 | 0 | 0 | 0 | 0 | 0 | 0 | 0 | 0 |
| NM_001011885 | BTBD1 | 3202 | 59 | 22 | 15 | 6 | 41 | 31 | 16 | 13 | 11 | 10 | 59 | 19 | 24 |
| NM_001012 | RPS8 | 705 | 171 | 63 | 40 | 43 | 71 | 29 | 34 | 47 | 63 | 171 | 49 | 111 | 37 |
| NM_001012241 | MSL1 | 3817 | 14 | 10 | 8 | 8 | 8 | 14 | 6 | 5 | 12 | 9 | 9 | 14 | 12 |
| NM_001012264 | RNASE13 | 1607 | 0 | 0 | 0 | 0 | 0 | 0 | 0 | 0 | 0 | 0 | 0 | 0 | 0 |
| NM_001012267 | CENPP | 3413 | 0 | 0 | 0 | 0 | 0 | 0 | 0 | 0 | 0 | 0 | 0 | 0 | 0 |
| NM_001012270 | BIRC5 | 2513 | 0 | 0 | 0 | 0 | 0 | 0 | 0 | 0 | 0 | 0 | 0 | 0 | 0 |
| NM_001012271 | BIRC5 | 2700 | 0 | 0 | 0 | 0 | 0 | 0 | 0 | 0 | 0 | 0 | 0 | 0 | 0 |
| NM_001012276 | PRAMEF8 | 1843 | 0 | 0 | 0 | 0 | 0 | 0 | 0 | 0 | 0 | 0 | 0 | 0 | 0 |
| NM_001012277 | PRAMEF7 | 1425 | 0 | 0 | 0 | 0 | 0 | 0 | 0 | 0 | 0 | 0 | 0 | 0 | 0 |
| NM_001012279 | C6orf174 | 11817 | 2 | 0 | 0 | 0 | 0 | 2 | 0 | 0 | 0 | 0 | 0 | 0 | 0 |
| NM_001012288 | CRLF2 | 671 | 0 | 0 | 0 | 0 | 0 | 0 | 0 | 0 | 0 | 0 | 0 | 0 | 0 |
| NM_001012300 | MCRS1 | 1768 | 2 | 1 | 1 | 1 | 2 | 1 | 1 | 0 | 0 | 1 | 1 | 1 | 1 |
| NM_001012301 | ARSI | 3167 | 0 | 0 | 0 | 0 | 0 | 0 | 0 | 0 | 0 | 0 | 0 | 0 | 0 |
| NM_001012302 | ANO9 | 2875 | 0 | 0 | 0 | 0 | 0 | 0 | 0 | 0 | 0 | 0 | 0 | 0 | 0 |
| NM_001012320 | ZNF302 | 2600 | 7 | 4 | 3 | 1 | 6 | 5 | 4 | 2 | 3 | 6 | 5 | 7 | 5 |

| transcript | gene | transcript length (nt) | max | mean | adipose | colon | heart | hypothalamus | kidney | liver | lung | ovary | skeletalmuscle | spleen | testes |
|---|---|---|---|---|---|---|---|---|---|---|---|---|---|---|---|
| NM_001012321 | RPSA | 1095 | 3 | 2 | 2 | 3 | 1 | 2 | 1 | 3 | 1 | 2 | 1 | 2 | 1 |
| NM_001012329 | CTNNBIP1 | 2961 | 2 | 1 | 1 | 1 | 1 | 2 | 1 | 1 | 2 | 1 | 0 | 1 | 2 |
| NM_001012331 | NTRK1 | 2637 | 0 | 0 | 0 | 0 | 0 | 0 | 0 | 0 | 0 | 0 | 0 | 0 | 0 |
| NM_001012333 | MDK | 897 | 4 | 1 | 0 | 0 | 0 | 0 | 0 | 0 | 0 | 4 | 0 | 0 | 0 |
| NM_001012334 | MDK | 985 | 4 | 1 | 0 | 0 | 0 | 0 | 0 | 0 | 0 | 4 | 0 | 0 | 0 |
| NM_001012337 | ROPN1B | 1000 | 3 | 0 | 0 | 0 | 0 | 0 | 0 | 0 | 0 | 0 | 0 | 0 | 3 |
| NM_001012338 | NTRK3 | 2860 | 4 | 1 | 0 | 1 | 0 | 4 | 0 | 0 | 0 | 0 | 0 | 0 | 0 |
| NM_001012339 | DNAJC21 | 6073 | 13 | 8 | 7 | 3 | 12 | 6 | 8 | 8 | 7 | 6 | 12 | 8 | 13 |
| NM_001012361 | WDR31 | 2454 | 2 | 1 | 1 | 0 | 2 | 1 | 2 | 0 | 1 | 1 | 0 | 0 | 2 |
| NM_001012393 | OPCML | 6413 | 10 | 1 | 0 | 0 | 0 | 10 | 0 | 0 | 0 | 0 | 0 | 0 | 0 |
| NM_001012398 | AKTIP | 2222 | 11 | 6 | 5 | 1 | 10 | 11 | 6 | 5 | 3 | 4 | 7 | 4 | 7 |
| NM_001012409 | SGOL1 | 1943 | 2 | 0 | 1 | 0 | 0 | 0 | 0 | 0 | 0 | 0 | 0 | 1 | 2 |
| NM_001012410 | SGOL1 | 2338 | 2 | 0 | 1 | 0 | 0 | 0 | 0 | 0 | 0 | 0 | 0 | 1 | 2 |
| NM_001012411 | SGOL1 | 1187 | 1 | 0 | 0 | 0 | 0 | 0 | 0 | 0 | 0 | 0 | 0 | 0 | 1 |
| NM_001012412 | SGOL1 | 1582 | 1 | 0 | 0 | 0 | 0 | 0 | 0 | 0 | 0 | 0 | 0 | 0 | 1 |
| NM_001012413 | SGOL1 | 1136 | 0 | 0 | 0 | 0 | 0 | 0 | 0 | 0 | 0 | 0 | 0 | 0 | 0 |
| NM_001012414 | TRIM61 | 1578 | 1 | 0 | 0 | 0 | 0 | 0 | 0 | 0 | 0 | 1 | 0 | 1 | 1 |
| NM_001012415 | SOHLH1 | 1985 | 1 | 0 | 0 | 0 | 0 | 1 | 0 | 0 | 0 | 0 | 0 | 0 | 1 |
| NM_001012416 | KRTAP5-6 | 561 | 0 | 0 | 0 | 0 | 0 | 0 | 0 | 0 | 0 | 0 | 0 | 0 | 0 |
| NM_001012417 | MS4A13 | 925 | 0 | 0 | 0 | 0 | 0 | 0 | 0 | 0 | 0 | 0 | 0 | 0 | 0 |
| NM_001012418 | MYLK4 | 5760 | 1 | 0 | 0 | 0 | 0 | 1 | 1 | 0 | 0 | 0 | 0 | 0 | 0 |
| NM_001012419 | ANKRD20A3 | 3964 | 1 | 0 | 0 | 0 | 0 | 0 | 0 | 0 | 0 | 0 | 0 | 1 | 1 |
| NM_001012420 | GOLGA8G | 5256 | 0 | 0 | 0 | 0 | 0 | 0 | 0 | 0 | 0 | 0 | 0 | 0 | 0 |
| NM_001012421 | ANKRD20A2 | 3511 | 0 | 0 | 0 | 0 | 0 | 0 | 0 | 0 | 0 | 0 | 0 | 0 | 0 |
| NM_001012423 | GOLGA8E | 5243 | 0 | 0 | 0 | 0 | 0 | 0 | 0 | 0 | 0 | 0 | 0 | 0 | 0 |
| NM_001012425 | C1orf146 | 751 | 0 | 0 | 0 | 0 | 0 | 0 | 0 | 0 | 0 | 0 | 0 | 0 | 0 |
| NM_001012426 | FOXP4 | 5949 | 1 | 1 | 0 | 1 | 1 | 0 | 1 | 0 | 1 | 0 | 0 | 0 | 1 |
| NM_001012427 | FOXP4 | 5943 | 1 | 1 | 0 | 1 | 1 | 0 | 1 | 0 | 1 | 0 | 0 | 0 | 1 |
| NM_001012428 | ASB11 | 970 | 13 | 2 | 0 | 0 | 5 | 0 | 0 | 0 | 0 | 0 | 13 | 0 | 0 |
| NM_001012446 | C9orf128 | 2946 | 0 | 0 | 0 | 0 | 0 | 0 | 0 | 0 | 0 | 0 | 0 | 0 | 0 |
| NM_001012452 | GOLGA8F | 4070 | 0 | 0 | 0 | 0 | 0 | 0 | 0 | 0 | 0 | 0 | 0 | 0 | 0 |
| NM_001012454 | FAM71F2 | 1198 | 1 | 0 | 0 | 0 | 0 | 0 | 1 | 0 | 0 | 1 | 1 | 0 | 1 |
| NM_001012455 | ZSCAN23 | 3161 | 2 | 1 | 0 | 1 | 0 | 1 | 1 | 0 | 0 | 2 | 0 | 0 | 1 |
| NM_001012456 | SEC61G | 462 | 12 | 6 | 5 | 3 | 10 | 5 | 6 | 12 | 4 | 5 | 4 | 6 | 7 |
| NM_001012478 | U2AF2 | 3119 | 4 | 3 | 2 | 4 | 2 | 4 | 2 | 2 | 2 | 2 | 1 | 4 | 3 |
| NM_001012502 | C9orf117 | 1722 | 2 | 0 | 0 | 0 | 0 | 1 | 0 | 0 | 2 | 0 | 0 | 0 | 1 |
| NM_001012503 | KRTAP5-7 | 898 | 0 | 0 | 0 | 0 | 0 | 0 | 0 | 0 | 0 | 0 | 0 | 0 | 0 |
| NM_001012505 | FOXP1 | 892 | 8 | 4 | 2 | 5 | 2 | 5 | 2 | 1 | 4 | 8 | 2 | 8 | 4 |
| NM_001012506 | CCDC66 | 3115 | 9 | 5 | 5 | 2 | 6 | 4 | 6 | 1 | 5 | 7 | 6 | 9 | 8 |
| NM_001012507 | C6orf173 | 549 | 1 | 0 | 0 | 0 | 0 | 0 | 0 | 0 | 0 | 0 | 0 | 0 | 1 |
| NM_001012508 | SPRN | 3173 | 3 | 0 | 0 | 1 | 0 | 3 | 0 | 1 | 0 | 0 | 0 | 0 | 0 |
| NM_001012509 | SLC45A2 | 2754 | 0 | 0 | 0 | 0 | 0 | 0 | 0 | 0 | 0 | 0 | 0 | 0 | 0 |
| NM_001012511 | GOSR2 | 2220 | 3 | 2 | 1 | 1 | 1 | 2 | 3 | 2 | 1 | 2 | 1 | 3 | 1 |
| NM_001012512 | GRP | 826 | 1 | 0 | 0 | 0 | 0 | 1 | 0 | 0 | 0 | 0 | 0 | 0 | 0 |
| NM_001012513 | GRP | 828 | 1 | 0 | 0 | 0 | 0 | 1 | 0 | 0 | 0 | 0 | 0 | 0 | 0 |
| NM_001012514 | ITM2C | 1932 | 19 | 4 | 1 | 14 | 1 | 19 | 4 | 1 | 1 | 2 | 0 | 4 | 1 |
| NM_001012515 | FECH | 7278 | 5 | 2 | 1 | 1 | 2 | 2 | 5 | 3 | 1 | 1 | 2 | 1 | 1 |
| NM_001012516 | ITM2C | 1962 | 18 | 4 | 1 | 13 | 1 | 18 | 4 | 0 | 1 | 2 | 0 | 3 | 1 |
| NM_001012614 | CTBP1 | 2483 | 3 | 2 | 1 | 1 | 2 | 2 | 3 | 1 | 2 | 1 | 3 | 3 | 1 |
| NM_001012631 | IL32 | 1157 | 91 | 20 | 3 | 5 | 11 | 2 | 41 | 91 | 21 | 3 | 7 | 36 | 3 |
| NM_001012632 | IL32 | 956 | 110 | 24 | 3 | 6 | 13 | 3 | 49 | 110 | 25 | 4 | 9 | 44 | 3 |
| NM_001012633 | IL32 | 919 | 114 | 25 | 3 | 6 | 13 | 3 | 51 | 114 | 26 | 4 | 9 | 45 | 4 |
| NM_001012634 | IL32 | 897 | 111 | 25 | 3 | 6 | 13 | 3 | 51 | 111 | 26 | 4 | 9 | 45 | 4 |
| NM_001012635 | IL32 | 892 | 112 | 25 | 3 | 6 | 14 | 3 | 51 | 112 | 26 | 4 | 9 | 46 | 4 |
| NM_001012636 | IL32 | 880 | 120 | 27 | 4 | 6 | 14 | 3 | 53 | 120 | 27 | 4 | 9 | 47 | 4 |
| NM_001012642 | GRAMD2 | 3291 | 1 | 0 | 0 | 0 | 0 | 0 | 1 | 0 | 1 | 0 | 0 | 0 | 0 |
| NM_001012643 | MYPOP | 1887 | 3 | 1 | 0 | 0 | 1 | 3 | 1 | 0 | 1 | 1 | 1 | 2 | 1 |
| NM_001012644 | DUSP15 | 1212 | 1 | 0 | 0 | 0 | 0 | 0 | 1 | 0 | 0 | 0 | 0 | 0 | 1 |
| NM_001012659 | ARGFX | 5065 | 0 | 0 | 0 | 0 | 0 | 0 | 0 | 0 | 0 | 0 | 0 | 0 | 0 |
| NM_001012661 | SLC3A2 | 2398 | 17 | 5 | 3 | 1 | 3 | 4 | 17 | 6 | 4 | 3 | 4 | 5 | 5 |
| NM_001012662 | SLC3A2 | 2308 | 18 | 5 | 3 | 1 | 3 | 4 | 18 | 6 | 5 | 3 | 4 | 5 | 5 |
| NM_001012663 | SLC3A2 | 2212 | 19 | 5 | 3 | 1 | 3 | 5 | 19 | 6 | 5 | 3 | 5 | 6 | 5 |
| NM_001012664 | SLC3A2 | 2119 | 20 | 6 | 3 | 1 | 3 | 5 | 20 | 6 | 5 | 3 | 5 | 6 | 6 |
| NM_001012665 | DYNC2LI1 | 1398 | 14 | 6 | 5 | 2 | 4 | 13 | 11 | 2 | 6 | 8 | 2 | 3 | 14 |
| NM_001012706 | C14orf182 | 2686 | 0 | 0 | 0 | 0 | 0 | 0 | 0 | 0 | 0 | 0 | 0 | 0 | 0 |
| NM_001012708 | KRTAP5-3 | 899 | 0 | 0 | 0 | 0 | 0 | 0 | 0 | 0 | 0 | 0 | 0 | 0 | 0 |
| NM_001012709 | KRTAP5-4 | 1181 | 0 | 0 | 0 | 0 | 0 | 0 | 0 | 0 | 0 | 0 | 0 | 0 | 0 |
| NM_001012710 | KRTAP5-10 | 1058 | 0 | 0 | 0 | 0 | 0 | 0 | 0 | 0 | 0 | 0 | 0 | 0 | 0 |
| NM_001012715 | C9orf106 | 843 | 0 | 0 | 0 | 0 | 0 | 0 | 0 | 0 | 0 | 0 | 0 | 0 | 0 |
| NM_001012716 | C18orf56 | 843 | 0 | 0 | 0 | 0 | 0 | 0 | 0 | 0 | 0 | 0 | 0 | 0 | 0 |
| NM_001012718 | IL32 | 1093 | 96 | 21 | 3 | 5 | 11 | 3 | 43 | 96 | 22 | 3 | 8 | 38 | 3 |
| NM_001012720 | RGR | 1463 | 0 | 0 | 0 | 0 | 0 | 0 | 0 | 0 | 0 | 0 | 0 | 0 | 0 |
| NM_001012722 | RGR | 1349 | 0 | 0 | 0 | 0 | 0 | 0 | 0 | 0 | 0 | 0 | 0 | 0 | 0 |
| NM_001012727 | AGPAT2 | 1452 | 3 | 1 | 3 | 1 | 0 | 0 | 0 | 2 | 0 | 0 | 0 | 0 | 0 |
| NM_001012728 | DPRX | 648 | 0 | 0 | 0 | 0 | 0 | 0 | 0 | 0 | 0 | 0 | 0 | 0 | 0 |
| NM_001012729 | DUXA | 3346 | 0 | 0 | 0 | 0 | 0 | 0 | 0 | 0 | 0 | 0 | 0 | 0 | 0 |
| NM_001012732 | DCTD | 2042 | 10 | 6 | 6 | 4 | 5 | 5 | 7 | 10 | 5 | 6 | 3 | 8 | 5 |

| | | transcript | | | | | | hypothala | | | | | skeletalm | | |
|---|---|---|---|---|---|---|---|---|---|---|---|---|---|---|---|
| transcript | gene | length (nt) | max | mean | adipose | colon | heart | mus | kidney | liver | lung | ovary | uscle | spleen | testes |
| NM_001012750 | ABI1 | 3587 | 9 | 4 | 4 | 3 | 3 | 9 | 4 | 3 | 5 | 3 | 2 | 8 | 5 |
| NM_001012751 | ABI1 | 3500 | 10 | 4 | 4 | 4 | 3 | 10 | 4 | 3 | 5 | 3 | 2 | 8 | 5 |
| NM_001012752 | ABI1 | 3572 | 10 | 4 | 4 | 3 | 3 | 10 | 4 | 3 | 5 | 3 | 2 | 8 | 5 |
| NM_001012753 | ZNF763 | 2623 | 4 | 1 | 1 | 1 | 1 | 1 | 2 | 0 | 1 | 4 | 1 | 2 | 2 |
| NM_001012754 | NHLRC3 | 3549 | 11 | 7 | 11 | 4 | 5 | 6 | 9 | 5 | 6 | 7 | 3 | 11 | 7 |
| NM_001012755 | MCART6 | 6224 | 0 | 0 | 0 | 0 | 0 | 0 | 0 | 0 | 0 | 0 | 0 | 0 | 0 |
| NM_001012756 | ZNF260 | 5473 | 6 | 3 | 2 | 0 | 2 | 3 | 4 | 1 | 2 | 6 | 1 | 4 | 3 |
| NM_001012758 | NUDT17 | 1089 | 0 | 0 | 0 | 0 | 0 | 0 | 0 | 0 | 0 | 0 | 0 | 0 | 0 |
| NM_001012759 | CTU2 | 1723 | 0 | 0 | 0 | 0 | 0 | 0 | 0 | 0 | 0 | 0 | 0 | 0 | 0 |
| NM_001012761 | RGMB | 4578 | 6 | 2 | 1 | 2 | 2 | 6 | 1 | 2 | 1 | 6 | 4 | 2 | 1 |
| NM_001012762 | CTU2 | 1664 | 0 | 0 | 0 | 0 | 0 | 0 | 0 | 0 | 0 | 0 | 0 | 0 | 0 |
| NM_001012763 | GNRHR | 5709 | 1 | 0 | 0 | 0 | 0 | 0 | 1 | 0 | 0 | 1 | 0 | 1 | 1 |
| NM_001012957 | DISC1 | 6993 | 0 | 0 | 0 | 0 | 0 | 0 | 0 | 0 | 0 | 0 | 0 | 0 | 0 |
| NM_001012958 | DISC1 | 2636 | 0 | 0 | 0 | 0 | 0 | 0 | 0 | 0 | 0 | 0 | 0 | 0 | 0 |
| NM_001012959 | DISC1 | 2218 | 0 | 0 | 0 | 0 | 0 | 0 | 0 | 0 | 0 | 0 | 0 | 0 | 0 |
| NM_001012961 | C1orf84 | 1188 | 1 | 1 | 1 | 1 | 1 | 0 | 1 | 0 | 1 | 1 | 0 | 1 | 1 |
| NM_001012964 | KLK6 | 1526 | 3 | 0 | 0 | 0 | 0 | 3 | 0 | 0 | 0 | 0 | 0 | 0 | 0 |
| NM_001012965 | KLK6 | 1479 | 3 | 0 | 0 | 0 | 0 | 3 | 0 | 0 | 0 | 0 | 0 | 0 | 0 |
| NM_001012967 | DDX60L | 6754 | 4 | 2 | 3 | 1 | 2 | 1 | 1 | 1 | 2 | 2 | 1 | 4 | 2 |
| NM_001012968 | SPIN4 | 4112 | 1 | 1 | 0 | 1 | 0 | 1 | 1 | 0 | 1 | 1 | 0 | 1 | 1 |
| NM_001012969 | ADAL | 1616 | 5 | 2 | 2 | 1 | 5 | 4 | 4 | 2 | 1 | 1 | 3 | 1 | 5 |
| NM_001012970 | C1orf100 | 579 | 4 | 1 | 0 | 1 | 0 | 0 | 0 | 0 | 0 | 1 | 0 | 1 | 4 |
| NM_001012971 | C20orf106 | 671 | 1 | 0 | 0 | 0 | 0 | 0 | 0 | 0 | 0 | 0 | 0 | 0 | 1 |
| NM_001012973 | PLAC9 | 673 | 3 | 1 | 3 | 1 | 1 | 0 | 0 | 0 | 0 | 0 | 0 | 0 | 0 |
| NM_001012974 | C6orf154 | 2121 | 0 | 0 | 0 | 0 | 0 | 0 | 0 | 0 | 0 | 0 | 0 | 0 | 0 |
| NM_001012975 | RNASE10 | 651 | 0 | 0 | 0 | 0 | 0 | 0 | 0 | 0 | 0 | 0 | 0 | 0 | 0 |
| NM_001012977 | PABPC1L2A | 2175 | 1 | 0 | 0 | 1 | 0 | 0 | 0 | 0 | 0 | 0 | 0 | 0 | 0 |
| NM_001012978 | BEX5 | 818 | 47 | 6 | 0 | 1 | 1 | 47 | 4 | 0 | 4 | 2 | 0 | 2 | 4 |
| NM_001012979 | TCEAL5 | 1168 | 43 | 8 | 0 | 0 | 1 | 37 | 0 | 0 | 0 | 1 | 0 | 0 | 43 |
| NM_001012980 | SATL1 | 2151 | 0 | 0 | 0 | 0 | 0 | 0 | 0 | 0 | 0 | 0 | 0 | 0 | 0 |
| NM_001012981 | ZKSCAN2 | 7126 | 2 | 1 | 1 | 0 | 1 | 1 | 1 | 0 | 0 | 1 | 1 | 1 | 2 |
| NM_001012982 | C4orf44 | 1141 | 0 | 0 | 0 | 0 | 0 | 0 | 0 | 0 | 0 | 0 | 0 | 0 | 0 |
| NM_001012983 | FAM136B | 417 | 9 | 1 | 0 | 0 | 0 | 0 | 2 | 9 | 0 | 1 | 0 | 1 | 0 |
| NM_001012984 | C16orf86 | 1269 | 0 | 0 | 0 | 0 | 0 | 0 | 0 | 0 | 0 | 0 | 0 | 0 | 0 |
| NM_001012985 | C1orf31 | 641 | 2 | 1 | 1 | 0 | 1 | 2 | 1 | 2 | 0 | 0 | 1 | 1 | 0 |
| NM_001012989 | UBE2NL | 1185 | 1 | 0 | 0 | 0 | 0 | 0 | 1 | 0 | 0 | 0 | 1 | 0 | 1 |
| NM_001012990 | ARL13A | 1309 | 1 | 0 | 0 | 0 | 0 | 0 | 0 | 0 | 0 | 0 | 0 | 1 | 1 |
| NM_001012991 | C16orf88 | 1950 | 5 | 3 | 2 | 3 | 2 | 4 | 3 | 2 | 2 | 5 | 2 | 4 | 5 |
| NM_001012993 | C9orf152 | 2656 | 3 | 0 | 0 | 3 | 0 | 0 | 0 | 0 | 1 | 0 | 0 | 0 | 0 |
| NM_001012994 | SNX30 | 7606 | 5 | 2 | 1 | 2 | 1 | 4 | 5 | 1 | 4 | 2 | 0 | 2 | 3 |
| NM_001013 | RPS9 | 713 | 23 | 14 | 14 | 16 | 12 | 10 | 11 | 19 | 19 | 15 | 9 | 23 | 10 |
| NM_001013031 | SORCS1 | 7163 | 2 | 0 | 0 | 0 | 0 | 2 | 1 | 0 | 0 | 0 | 0 | 0 | 0 |
| NM_001013251 | SLC3A2 | 1987 | 21 | 6 | 4 | 1 | 3 | 5 | 21 | 7 | 5 | 3 | 5 | 6 | 6 |
| NM_001013253 | LSP1 | 1785 | 18 | 3 | 9 | 3 | 1 | 0 | 0 | 0 | 3 | 1 | 1 | 18 | 1 |
| NM_001013254 | LSP1 | 2015 | 16 | 3 | 8 | 3 | 1 | 0 | 0 | 0 | 3 | 1 | 0 | 16 | 1 |
| NM_001013255 | LSP1 | 1521 | 21 | 4 | 10 | 4 | 2 | 0 | 0 | 0 | 4 | 1 | 1 | 21 | 1 |
| NM_001013257 | BCAM | 2434 | 6 | 1 | 1 | 1 | 1 | 0 | 6 | 0 | 3 | 1 | 0 | 1 | 1 |
| NM_001013258 | ZNF789 | 533 | 2 | 1 | 0 | 2 | 0 | 1 | 1 | 0 | 0 | 1 | 0 | 1 | 1 |
| NM_001013354 | OR11H12 | 981 | 0 | 0 | 0 | 0 | 0 | 0 | 0 | 0 | 0 | 0 | 0 | 0 | 0 |
| NM_001013355 | OR2G6 | 951 | 0 | 0 | 0 | 0 | 0 | 0 | 0 | 0 | 0 | 0 | 0 | 0 | 0 |
| NM_001013356 | OR8U8 | 906 | 0 | 0 | 0 | 0 | 0 | 0 | 0 | 0 | 0 | 0 | 0 | 0 | 0 |
| NM_001013358 | OR9G9 | 918 | 0 | 0 | 0 | 0 | 0 | 0 | 0 | 0 | 0 | 0 | 0 | 0 | 0 |
| NM_001013398 | IGFBP3 | 2631 | 56 | 32 | 56 | 20 | 50 | 10 | 28 | 56 | 20 | 56 | 16 | 17 | 24 |
| NM_001013399 | CCNC | 2085 | 13 | 5 | 5 | 1 | 3 | 5 | 8 | 13 | 6 | 3 | 3 | 7 | 4 |
| NM_001013403 | CXorf66 | 1278 | 1 | 0 | 0 | 0 | 0 | 0 | 0 | 0 | 0 | 0 | 0 | 0 | 1 |
| NM_001013406 | KRIT1 | 4231 | 8 | 4 | 3 | 1 | 4 | 4 | 5 | 2 | 3 | 7 | 3 | 6 | 8 |
| NM_001013407 | PRAMEF5 | 1855 | 0 | 0 | 0 | 0 | 0 | 0 | 0 | 0 | 0 | 0 | 0 | 0 | 0 |
| NM_001013415 | FBXW7 | 3570 | 22 | 6 | 3 | 6 | 8 | 22 | 1 | 2 | 2 | 3 | 6 | 5 | 4 |
| NM_001013435 | PAGE5 | 540 | 2 | 0 | 0 | 0 | 0 | 0 | 0 | 0 | 0 | 0 | 0 | 0 | 2 |
| NM_001013436 | MPST | 1340 | 1 | 0 | 0 | 0 | 0 | 0 | 0 | 1 | 0 | 0 | 0 | 0 | 0 |
| NM_001013437 | SEH1L | 1884 | 5 | 3 | 2 | 2 | 2 | 5 | 3 | 3 | 2 | 2 | 2 | 3 | 3 |
| NM_001013438 | FXR1 | 2669 | 152 | 41 | 23 | 8 | 50 | 37 | 22 | 15 | 26 | 41 | 152 | 28 | 51 |
| NM_001013439 | FXR1 | 3105 | 141 | 37 | 21 | 7 | 45 | 33 | 19 | 13 | 23 | 36 | 141 | 25 | 45 |
| NM_001013442 | EPGN | 847 | 0 | 0 | 0 | 0 | 0 | 0 | 0 | 0 | 0 | 0 | 0 | 0 | 0 |
| NM_001013579 | AWAT1 | 1398 | 0 | 0 | 0 | 0 | 0 | 0 | 0 | 0 | 0 | 0 | 0 | 0 | 0 |
| NM_001013615 | C1orf190 | 1848 | 1 | 1 | 0 | 0 | 1 | 1 | 1 | 0 | 0 | 1 | 0 | 1 | 1 |
| NM_001013619 | AGPHD1 | 1235 | 5 | 2 | 2 | 1 | 1 | 3 | 5 | 2 | 1 | 3 | 2 | 1 | 1 |
| NM_001013620 | ALG10B | 10074 | 1 | 0 | 0 | 0 | 0 | 0 | 1 | 0 | 0 | 1 | 0 | 1 | 1 |
| NM_001013622 | FAM53A | 2626 | 0 | 0 | 0 | 0 | 0 | 0 | 0 | 0 | 0 | 0 | 0 | 0 | 0 |
| NM_001013623 | C6orf94 | 992 | 0 | 0 | 0 | 0 | 0 | 0 | 0 | 0 | 0 | 0 | 0 | 0 | 0 |
| NM_001013625 | C1orf192 | 774 | 20 | 9 | 8 | 5 | 8 | 20 | 13 | 9 | 4 | 6 | 12 | 5 | 7 |
| NM_001013626 | LRRC67 | 1083 | 1 | 0 | 0 | 0 | 0 | 0 | 0 | 0 | 0 | 0 | 0 | 0 | 1 |
| NM_001013627 | NHSL2 | 3676 | 3 | 1 | 1 | 1 | 1 | 3 | 1 | 0 | 1 | 1 | 1 | 2 | 3 |
| NM_001013628 | DCAF12L2 | 1421 | 0 | 0 | 0 | 0 | 0 | 0 | 0 | 0 | 0 | 0 | 0 | 0 | 0 |
| NM_001013630 | AADACL4 | 1575 | 0 | 0 | 0 | 0 | 0 | 0 | 0 | 0 | 0 | 0 | 0 | 0 | 0 |
| NM_001013631 | HNRNPCL1 | 1108 | 0 | 0 | 0 | 0 | 0 | 0 | 0 | 0 | 0 | 0 | 0 | 0 | 0 |
| NM_001013632 | TCTEX1D4 | 1264 | 0 | 0 | 0 | 0 | 0 | 0 | 0 | 0 | 0 | 0 | 0 | 0 | 0 |

| | | transcript | | | | | | hypothala | | | | | skeletalm | | |
|---|---|---|---|---|---|---|---|---|---|---|---|---|---|---|---|
| transcript | gene | length (nt) | max | mean | adipose | colon | heart | mus | kidney | liver | lung | ovary | uscle | spleen | testes |
| NM_001013635 | C12orf68 | 2344 | 2 | 0 | 0 | 0 | 0 | 2 | 0 | 0 | 0 | 0 | 0 | 0 | 0 |
| NM_001013638 | PRR25 | 1209 | 0 | 0 | 0 | 0 | 0 | 0 | 0 | 0 | 0 | 0 | 0 | 0 | 0 |
| NM_001013641 | TMEM82 | 1520 | 0 | 0 | 0 | 0 | 0 | 0 | 0 | 0 | 0 | 0 | 0 | 0 | 0 |
| NM_001013642 | TRNP1 | 1949 | 1 | 0 | 0 | 0 | 0 | 1 | 0 | 0 | 0 | 0 | 0 | 0 | 0 |
| NM_001013646 | C20orf107 | 694 | 2 | 0 | 0 | 0 | 0 | 0 | 0 | 0 | 0 | 0 | 0 | 0 | 2 |
| NM_001013647 | LOC646851 | 10322 | 1 | 0 | 0 | 0 | 0 | 0 | 0 | 0 | 0 | 0 | 0 | 0 | 1 |
| NM_001013649 | C2orf68 | 4265 | 3 | 2 | 1 | 2 | 1 | 1 | 2 | 1 | 1 | 3 | 2 | 2 | 2 |
| NM_001013650 | PRR23B | 1896 | 0 | 0 | 0 | 0 | 0 | 0 | 0 | 0 | 0 | 0 | 0 | 0 | 0 |
| NM_001013653 | LRRC26 | 1195 | 0 | 0 | 0 | 0 | 0 | 0 | 0 | 0 | 0 | 0 | 0 | 0 | 0 |
| NM_001013657 | C15orf58 | 1351 | 1 | 0 | 0 | 0 | 0 | 0 | 1 | 0 | 0 | 0 | 0 | 0 | 0 |
| NM_001013658 | C16orf38 | 1422 | 0 | 0 | 0 | 0 | 0 | 0 | 0 | 0 | 0 | 0 | 0 | 0 | 0 |
| NM_001013659 | ZNF793 | 7121 | 1 | 1 | 0 | 0 | 0 | 1 | 1 | 0 | 0 | 1 | 0 | 1 | 1 |
| NM_001013660 | FRRS1 | 2678 | 4 | 1 | 1 | 1 | 1 | 0 | 0 | 4 | 1 | 1 | 0 | 0 | 1 |
| NM_001013661 | VSIG8 | 1818 | 0 | 0 | 0 | 0 | 0 | 0 | 0 | 0 | 0 | 0 | 0 | 0 | 0 |
| NM_001013662 | C2orf90 | 3189 | 0 | 0 | 0 | 0 | 0 | 0 | 0 | 0 | 0 | 0 | 0 | 0 | 0 |
| NM_001013663 | C2orf79 | 572 | 14 | 7 | 6 | 7 | 4 | 10 | 7 | 8 | 7 | 3 | 3 | 5 | 14 |
| NM_001013672 | C17orf97 | 1837 | 4 | 1 | 0 | 1 | 0 | 3 | 1 | 1 | 1 | 1 | 1 | 1 | 4 |
| NM_001013674 | C1orf141 | 2143 | 10 | 1 | 0 | 0 | 0 | 1 | 0 | 0 | 1 | 1 | 0 | 0 | 10 |
| NM_001013690 | FIGNL2 | 4533 | 1 | 0 | 0 | 0 | 0 | 0 | 1 | 0 | 0 | 0 | 0 | 0 | 0 |
| NM_001013692 | PRAMEF3 | 2104 | 0 | 0 | 0 | 0 | 0 | 0 | 0 | 0 | 0 | 0 | 0 | 0 | 0 |
| NM_001013693 | LDLRAD2 | 4012 | 0 | 0 | 0 | 0 | 0 | 0 | 0 | 0 | 0 | 0 | 0 | 0 | 0 |
| NM_001013694 | SRRD | 1293 | 8 | 5 | 5 | 3 | 6 | 8 | 5 | 8 | 3 | 5 | 8 | 4 | 5 |
| NM_001013698 | C12orf69 | 2104 | 0 | 0 | 0 | 0 | 0 | 0 | 0 | 0 | 0 | 0 | 0 | 0 | 0 |
| NM_001013699 | H3F3C | 1057 | 4 | 1 | 1 | 0 | 1 | 1 | 1 | 0 | 1 | 2 | 1 | 3 | 4 |
| NM_001013703 | EIF2AK4 | 5512 | 5 | 3 | 5 | 2 | 4 | 4 | 4 | 3 | 3 | 3 | 2 | 4 | 4 |
| NM_001013706 | PLIN5 | 2441 | 1 | 0 | 0 | 0 | 1 | 0 | 1 | 0 | 0 | 0 | 1 | 0 | 0 |
| NM_001013717 | C5orf56 | 2025 | 2 | 1 | 1 | 1 | 1 | 0 | 1 | 0 | 2 | 2 | 1 | 2 | 0 |
| NM_001013732 | C6orf138 | 2850 | 0 | 0 | 0 | 0 | 0 | 0 | 0 | 0 | 0 | 0 | 0 | 0 | 0 |
| NM_001013734 | RFPL4B | 1900 | 1 | 0 | 0 | 0 | 0 | 0 | 0 | 0 | 0 | 0 | 0 | 0 | 1 |
| NM_001013735 | FOXB2 | 1299 | 0 | 0 | 0 | 0 | 0 | 0 | 0 | 0 | 0 | 0 | 0 | 0 | 0 |
| NM_001013736 | FAM47C | 3270 | 0 | 0 | 0 | 0 | 0 | 0 | 0 | 0 | 0 | 0 | 0 | 0 | 0 |
| NM_001013739 | STAG3L3 | 1258 | 0 | 0 | 0 | 0 | 0 | 0 | 0 | 0 | 0 | 0 | 0 | 0 | 0 |
| NM_001013742 | DGKK | 3917 | 0 | 0 | 0 | 0 | 0 | 0 | 0 | 0 | 0 | 0 | 0 | 0 | 0 |
| NM_001013743 | TMEM225 | 1098 | 2 | 0 | 0 | 0 | 0 | 0 | 0 | 0 | 0 | 0 | 0 | 0 | 2 |
| NM_001013746 | ZNF107 | 5236 | 19 | 5 | 1 | 1 | 2 | 2 | 4 | 0 | 4 | 10 | 1 | 19 | 6 |
| NM_001013836 | MAD1L1 | 2701 | 2 | 1 | 2 | 2 | 1 | 2 | 1 | 1 | 1 | 1 | 1 | 2 | 2 |
| NM_001013837 | MAD1L1 | 2698 | 2 | 1 | 2 | 2 | 1 | 2 | 1 | 1 | 1 | 1 | 1 | 2 | 2 |
| NM_001013838 | RLTPR | 4479 | 0 | 0 | 0 | 0 | 0 | 0 | 0 | 0 | 0 | 0 | 0 | 0 | 0 |
| NM_001013839 | EXOC7 | 4792 | 7 | 5 | 5 | 4 | 5 | 7 | 5 | 4 | 3 | 5 | 3 | 6 | 4 |
| NM_001013841 | STAP2 | 1399 | 4 | 1 | 0 | 4 | 0 | 0 | 2 | 1 | 0 | 1 | 0 | 0 | 0 |
| NM_001013842 | C8orf58 | 2053 | 1 | 0 | 0 | 0 | 0 | 0 | 0 | 0 | 0 | 0 | 0 | 0 | 1 |
| NM_001013843 | SLTM | 4093 | 36 | 21 | 22 | 10 | 25 | 19 | 23 | 16 | 15 | 19 | 21 | 36 | 22 |
| NM_001013845 | CXorf40B | 1340 | 0 | 0 | 0 | 0 | 0 | 0 | 0 | 0 | 0 | 0 | 0 | 0 | 0 |
| NM_001013848 | EXOC6 | 3564 | 9 | 5 | 2 | 2 | 3 | 7 | 3 | 5 | 5 | 3 | 7 | 7 | 9 |
| NM_001014 | RPS10 | 618 | 20 | 13 | 13 | 17 | 11 | 11 | 11 | 20 | 9 | 16 | 8 | 19 | 6 |
| NM_001014279 | C5orf39 | 1265 | 1 | 0 | 0 | 0 | 0 | 0 | 0 | 0 | 0 | 0 | 0 | 1 | 0 |
| NM_001014283 | DCUN1D2 | 3029 | 3 | 1 | 0 | 1 | 3 | 2 | 1 | 1 | 0 | 1 | 3 | 1 | 1 |
| NM_001014286 | FAM48A | 2946 | 7 | 3 | 2 | 2 | 2 | 1 | 3 | 1 | 2 | 3 | 2 | 5 | 7 |
| NM_001014291 | SPRR2G | 649 | 0 | 0 | 0 | 0 | 0 | 0 | 0 | 0 | 0 | 0 | 0 | 0 | 0 |
| NM_001014336 | IL31 | 904 | 0 | 0 | 0 | 0 | 0 | 0 | 0 | 0 | 0 | 0 | 0 | 0 | 0 |
| NM_001014342 | FLG2 | 9122 | 0 | 0 | 0 | 0 | 0 | 0 | 0 | 0 | 0 | 0 | 0 | 0 | 0 |
| NM_001014380 | KATNAL1 | 7484 | 7 | 3 | 3 | 2 | 5 | 7 | 2 | 1 | 2 | 2 | 2 | 2 | 3 |
| NM_001014431 | AKT1 | 2773 | 3 | 2 | 2 | 3 | 2 | 1 | 1 | 3 | 1 | 1 | 1 | 2 | 1 |
| NM_001014432 | AKT1 | 2857 | 3 | 2 | 2 | 3 | 2 | 1 | 1 | 3 | 1 | 1 | 1 | 1 | 1 |
| NM_001014433 | CUTA | 808 | 3 | 1 | 1 | 1 | 1 | 3 | 1 | 2 | 1 | 1 | 1 | 2 | 1 |
| NM_001014434 | LHX9 | 2078 | 1 | 0 | 0 | 0 | 0 | 0 | 0 | 0 | 0 | 1 | 0 | 0 | 1 |
| NM_001014435 | CA7 | 1700 | 1 | 0 | 0 | 1 | 0 | 0 | 0 | 0 | 0 | 0 | 0 | 0 | 0 |
| NM_001014436 | DBNL | 2191 | 13 | 5 | 5 | 4 | 5 | 6 | 4 | 4 | 5 | 2 | 2 | 13 | 3 |
| NM_001014437 | CARS | 2755 | 5 | 3 | 3 | 2 | 3 | 5 | 2 | 4 | 2 | 1 | 2 | 3 | 3 |
| NM_001014438 | CARS | 2802 | 5 | 2 | 3 | 2 | 3 | 5 | 2 | 4 | 2 | 1 | 2 | 3 | 3 |
| NM_001014440 | ODF3B | 970 | 0 | 0 | 0 | 0 | 0 | 0 | 0 | 0 | 0 | 0 | 0 | 0 | 0 |
| NM_001014442 | PCOTH | 804 | 0 | 0 | 0 | 0 | 0 | 0 | 0 | 0 | 0 | 0 | 0 | 0 | 0 |
| NM_001014443 | USP21 | 2354 | 1 | 0 | 0 | 1 | 0 | 0 | 0 | 0 | 0 | 0 | 0 | 1 | 1 |
| NM_001014444 | CRYM | 1242 | 10 | 2 | 0 | 1 | 4 | 10 | 3 | 0 | 0 | 0 | 0 | 0 | 0 |
| NM_001014445 | NLE1 | 2709 | 0 | 0 | 0 | 0 | 0 | 0 | 0 | 0 | 0 | 0 | 0 | 0 | 0 |
| NM_001014446 | OCIAD2 | 764 | 32 | 8 | 3 | 10 | 3 | 10 | 32 | 16 | 3 | 3 | 1 | 4 | 6 |
| NM_001014447 | CPZ | 2229 | 2 | 0 | 0 | 0 | 0 | 0 | 0 | 0 | 0 | 2 | 0 | 0 | 0 |
| NM_001014448 | CPZ | 2492 | 2 | 0 | 0 | 0 | 0 | 0 | 0 | 0 | 0 | 2 | 0 | 0 | 0 |
| NM_001014449 | DDX19B | 1712 | 3 | 2 | 2 | 1 | 2 | 1 | 2 | 3 | 1 | 2 | 2 | 3 | 3 |
| NM_001014450 | SPRR2F | 656 | 2 | 0 | 0 | 0 | 0 | 0 | 0 | 0 | 0 | 2 | 0 | 0 | 0 |
| NM_001014451 | DDX19B | 1722 | 3 | 2 | 2 | 1 | 3 | 1 | 2 | 3 | 2 | 2 | 2 | 3 | 3 |
| NM_001014763 | ETFB | 1301 | 10 | 4 | 7 | 3 | 7 | 4 | 5 | 10 | 2 | 2 | 4 | 3 | 3 |
| NM_001014764 | TMEM93 | 788 | 4 | 2 | 1 | 1 | 4 | 2 | 2 | 3 | 0 | 0 | 3 | 2 | 1 |
| NM_001014765 | FBXO44 | 3320 | 1 | 0 | 0 | 0 | 0 | 1 | 0 | 0 | 0 | 0 | 0 | 0 | 0 |
| NM_001014794 | ILK | 1751 | 17 | 6 | 7 | 17 | 7 | 4 | 5 | 3 | 5 | 4 | 4 | 7 | 4 |
| NM_001014795 | ILK | 1911 | 16 | 6 | 6 | 16 | 7 | 4 | 5 | 3 | 4 | 4 | 4 | 6 | 4 |
| NM_001014796 | DDR2 | 3244 | 16 | 3 | 16 | 3 | 3 | 2 | 1 | 0 | 2 | 5 | 1 | 1 | 2 |

| | | transcript | | | | | | hypothala | | | | | skeletalm | | |
|---|---|---|---|---|---|---|---|---|---|---|---|---|---|---|---|
| transcript | gene | length (nt) | max | mean | adipose | colon | heart | mus | kidney | liver | lung | ovary | uscle | spleen | testes |
| NM_001014797 | KCNMA1 | 11994 | 6 | 1 | 0 | 2 | 0 | 6 | 0 | 1 | 1 | 1 | 2 | 1 | 1 |
| NM_001014809 | CRMP1 | 3146 | 4 | 1 | 1 | 1 | 1 | 4 | 3 | 0 | 1 | 2 | 0 | 0 | 1 |
| NM_001014811 | ME3 | 2240 | 3 | 1 | 1 | 1 | 1 | 3 | 1 | 0 | 0 | 2 | 1 | 1 | 1 |
| NM_001014812 | FAM96A | 1042 | 32 | 8 | 5 | 5 | 9 | 5 | 10 | 32 | 7 | 2 | 3 | 11 | 4 |
| NM_001014830 | C14orf183 | 975 | 0 | 0 | 0 | 0 | 0 | 0 | 0 | 0 | 0 | 0 | 0 | 0 | 0 |
| NM_001014831 | PAK4 | 3064 | 1 | 1 | 0 | 0 | 0 | 0 | 1 | 0 | 1 | 1 | 0 | 0 | 1 |
| NM_001014832 | PAK4 | 2765 | 1 | 1 | 0 | 0 | 0 | 0 | 1 | 0 | 1 | 1 | 0 | 0 | 1 |
| NM_001014834 | PAK4 | 2306 | 2 | 1 | 0 | 0 | 1 | 0 | 2 | 1 | 1 | 1 | 0 | 0 | 1 |
| NM_001014835 | PAK4 | 2379 | 2 | 1 | 0 | 0 | 1 | 0 | 2 | 0 | 1 | 1 | 0 | 0 | 1 |
| NM_001014837 | CUTA | 1057 | 4 | 2 | 1 | 1 | 1 | 4 | 2 | 3 | 1 | 2 | 1 | 3 | 1 |
| NM_001014838 | CUTA | 1026 | 3 | 1 | 1 | 1 | 1 | 3 | 1 | 2 | 1 | 1 | 0 | 2 | 1 |
| NM_001014839 | NCDN | 3433 | 18 | 2 | 0 | 1 | 1 | 18 | 1 | 0 | 0 | 1 | 0 | 1 | 1 |
| NM_001014840 | CUTA | 936 | 3 | 1 | 1 | 1 | 1 | 3 | 2 | 2 | 1 | 1 | 0 | 2 | 1 |
| NM_001014841 | NCDN | 3365 | 18 | 2 | 0 | 1 | 1 | 18 | 1 | 0 | 0 | 1 | 0 | 1 | 1 |
| NM_001014842 | TM9SF1 | 2117 | 5 | 1 | 2 | 1 | 1 | 1 | 2 | 5 | 1 | 1 | 0 | 1 | 1 |
| NM_001014972 | ZNF638 | 6498 | 22 | 14 | 10 | 5 | 16 | 14 | 15 | 9 | 12 | 22 | 16 | 19 | 20 |
| NM_001014975 | CFH | 1824 | 302 | 44 | 17 | 6 | 55 | 7 | 7 | 302 | 19 | 44 | 8 | 7 | 14 |
| NM_001014977 | BANF2 | 445 | 0 | 0 | 0 | 0 | 0 | 0 | 0 | 0 | 0 | 0 | 0 | 0 | 0 |
| NM_001014979 | C16orf93 | 1829 | 1 | 0 | 0 | 0 | 0 | 0 | 0 | 0 | 0 | 0 | 0 | 0 | 1 |
| NM_001014980 | FAM132A | 1036 | 0 | 0 | 0 | 0 | 0 | 0 | 0 | 0 | 0 | 0 | 0 | 0 | 0 |
| NM_001014985 | GLTPD2 | 1023 | 0 | 0 | 0 | 0 | 0 | 0 | 0 | 0 | 0 | 0 | 0 | 0 | 0 |
| NM_001014986 | FOLH1 | 2542 | 3 | 1 | 0 | 0 | 0 | 3 | 1 | 1 | 0 | 0 | 0 | 0 | 0 |
| NM_001014987 | LAT | 1656 | 0 | 0 | 0 | 0 | 0 | 0 | 0 | 0 | 0 | 0 | 0 | 0 | 0 |
| NM_001014988 | LAT | 1653 | 0 | 0 | 0 | 0 | 0 | 0 | 0 | 0 | 0 | 0 | 0 | 0 | 0 |
| NM_001014989 | LAT | 1448 | 1 | 0 | 0 | 1 | 0 | 0 | 0 | 0 | 0 | 0 | 0 | 1 | 0 |
| NM_001014990 | MRPL22 | 3055 | 3 | 1 | 1 | 1 | 2 | 1 | 1 | 3 | 1 | 1 | 1 | 1 | 1 |
| NM_001014999 | GIYD1 | 1112 | 0 | 0 | 0 | 0 | 0 | 0 | 0 | 0 | 0 | 0 | 0 | 0 | 0 |
| NM_001015 | RPS11 | 609 | 61 | 29 | 20 | 21 | 23 | 13 | 20 | 29 | 26 | 56 | 30 | 61 | 19 |
| NM_001015000 | GIYD1 | 770 | 0 | 0 | 0 | 0 | 0 | 0 | 0 | 0 | 0 | 0 | 0 | 0 | 0 |
| NM_001015001 | CKMT1A | 1779 | 1 | 0 | 0 | 1 | 0 | 0 | 0 | 0 | 0 | 0 | 0 | 0 | 0 |
| NM_001015002 | LLGL2 | 1406 | 1 | 0 | 0 | 1 | 0 | 0 | 1 | 0 | 0 | 0 | 0 | 0 | 0 |
| NM_001015038 | PAGE2B | 493 | 0 | 0 | 0 | 0 | 0 | 0 | 0 | 0 | 0 | 0 | 0 | 0 | 0 |
| NM_001015045 | FAM13A | 4919 | 10 | 3 | 10 | 3 | 2 | 2 | 3 | 3 | 2 | 3 | 1 | 1 | 4 |
| NM_001015048 | BAG5 | 4840 | 13 | 6 | 6 | 3 | 6 | 7 | 7 | 3 | 6 | 5 | 4 | 9 | 13 |
| NM_001015049 | BAG5 | 5006 | 13 | 6 | 6 | 3 | 5 | 7 | 7 | 3 | 5 | 5 | 4 | 9 | 13 |
| NM_001015050 | ALG1L | 805 | 0 | 0 | 0 | 0 | 0 | 0 | 0 | 0 | 0 | 0 | 0 | 0 | 0 |
| NM_001015051 | RUNX2 | 5486 | 1 | 0 | 0 | 0 | 0 | 0 | 0 | 0 | 1 | 1 | 0 | 1 | 0 |
| NM_001015052 | MPG | 1089 | 0 | 0 | 0 | 0 | 0 | 0 | 0 | 0 | 0 | 0 | 0 | 0 | 0 |
| NM_001015053 | HDAC5 | 5327 | 1 | 1 | 1 | 1 | 1 | 1 | 1 | 0 | 1 | 1 | 1 | 1 | 1 |
| NM_001015054 | MPG | 1032 | 0 | 0 | 0 | 0 | 0 | 0 | 0 | 0 | 0 | 0 | 0 | 0 | 0 |
| NM_001015055 | RTKN | 2191 | 3 | 1 | 0 | 1 | 1 | 2 | 3 | 3 | 0 | 0 | 0 | 0 | 0 |
| NM_001015056 | RTKN | 2194 | 3 | 1 | 0 | 1 | 1 | 2 | 3 | 3 | 0 | 0 | 0 | 0 | 0 |
| NM_001015070 | KIR3DP1 | 1294 | 0 | 0 | 0 | 0 | 0 | 0 | 0 | 0 | 0 | 0 | 0 | 0 | 0 |
| NM_001015072 | UFSP1 | 996 | 0 | 0 | 0 | 0 | 0 | 0 | 0 | 0 | 0 | 0 | 0 | 0 | 0 |
| NM_001015508 | PURG | 2690 | 1 | 0 | 0 | 0 | 0 | 1 | 0 | 0 | 0 | 0 | 0 | 0 | 0 |
| NM_001015877 | PHF6 | 4432 | 13 | 4 | 2 | 1 | 2 | 4 | 3 | 1 | 2 | 13 | 1 | 6 | 6 |
| NM_001015878 | AURKC | 1250 | 14 | 3 | 1 | 0 | 1 | 1 | 1 | 0 | 1 | 0 | 1 | 7 | 14 |
| NM_001015879 | AURKC | 1109 | 16 | 3 | 1 | 0 | 1 | 1 | 1 | 0 | 1 | 0 | 1 | 8 | 16 |
| NM_001015880 | PAPSS2 | 3858 | 14 | 5 | 4 | 5 | 3 | 1 | 4 | 14 | 12 | 6 | 1 | 3 | 2 |
| NM_001015881 | TSC22D3 | 1747 | 20 | 8 | 7 | 5 | 5 | 5 | 4 | 4 | 17 | 3 | 9 | 20 | 4 |
| NM_001015882 | DNAJC25 | 2268 | 14 | 4 | 4 | 1 | 4 | 3 | 5 | 14 | 2 | 2 | 1 | 2 | 5 |
| NM_001015885 | RAE1 | 1836 | 4 | 1 | 1 | 1 | 1 | 1 | 1 | 1 | 1 | 1 | 1 | 2 | 4 |
| NM_001015887 | IGSF11 | 3431 | 2 | 1 | 0 | 0 | 0 | 2 | 1 | 0 | 0 | 2 | 1 | 0 | 2 |
| NM_001015891 | TAF9 | 1183 | 13 | 10 | 7 | 3 | 11 | 13 | 11 | 10 | 7 | 7 | 10 | 13 | 12 |
| NM_001015892 | TAF9 | 1462 | 10 | 6 | 6 | 3 | 8 | 9 | 4 | 8 | 3 | 3 | 4 | 6 | 10 |
| NM_001016 | RPS12 | 503 | 146 | 69 | 64 | 50 | 67 | 40 | 42 | 67 | 84 | 146 | 51 | 108 | 43 |
| NM_001017 | RPS13 | 529 | 191 | 106 | 83 | 46 | 125 | 61 | 64 | 93 | 98 | 191 | 149 | 154 | 103 |
| NM_001017361 | C6orf221 | 1022 | 0 | 0 | 0 | 0 | 0 | 0 | 0 | 0 | 0 | 0 | 0 | 0 | 0 |
| NM_001017363 | ARID3C | 1239 | 1 | 0 | 0 | 0 | 0 | 0 | 0 | 1 | 0 | 0 | 0 | 0 | 0 |
| NM_001017364 | C4BPB | 1112 | 51 | 5 | 0 | 0 | 0 | 0 | 0 | 51 | 0 | 3 | 0 | 0 | 0 |
| NM_001017365 | C4BPB | 952 | 27 | 3 | 0 | 0 | 0 | 0 | 0 | 27 | 1 | 2 | 0 | 0 | 0 |
| NM_001017366 | C4BPB | 949 | 27 | 3 | 0 | 0 | 0 | 0 | 0 | 27 | 1 | 2 | 0 | 0 | 0 |
| NM_001017367 | C4BPB | 951 | 27 | 3 | 0 | 0 | 0 | 0 | 0 | 27 | 1 | 2 | 0 | 0 | 0 |
| NM_001017368 | RFFL | 4121 | 4 | 2 | 1 | 2 | 1 | 2 | 3 | 4 | 2 | 1 | 2 | 3 | 2 |
| NM_001017369 | SC4MOL | 1853 | 6 | 2 | 0 | 1 | 0 | 3 | 2 | 6 | 2 | 1 | 0 | 1 | 2 |
| NM_001017370 | NHLRC3 | 3348 | 8 | 5 | 8 | 3 | 4 | 5 | 7 | 3 | 5 | 6 | 2 | 8 | 5 |
| NM_001017371 | SP3 | 3937 | 9 | 6 | 6 | 3 | 5 | 5 | 4 | 5 | 6 | 8 | 5 | 9 | 7 |
| NM_001017372 | SLC27A6 | 2838 | 6 | 1 | 0 | 0 | 4 | 0 | 0 | 0 | 0 | 2 | 0 | 0 | 6 |
| NM_001017373 | SAMD3 | 2095 | 2 | 1 | 0 | 0 | 0 | 1 | 0 | 0 | 1 | 0 | 0 | 2 | 1 |
| NM_001017388 | TLR10 | 3070 | 3 | 0 | 0 | 0 | 0 | 1 | 0 | 0 | 0 | 0 | 0 | 3 | 0 |
| NM_001017389 | SULT1A4 | 1567 | 0 | 0 | 0 | 0 | 0 | 0 | 0 | 0 | 0 | 0 | 0 | 0 | 0 |
| NM_001017389 | SULT1A4 | 1572 | 0 | 0 | 0 | 0 | 0 | 0 | 0 | 0 | 0 | 0 | 0 | 0 | 0 |
| NM_001017390 | SULT1A4 | 1384 | 0 | 0 | 0 | 0 | 0 | 0 | 0 | 0 | 0 | 0 | 0 | 0 | 0 |
| NM_001017390 | SULT1A4 | 1389 | 0 | 0 | 0 | 0 | 0 | 0 | 0 | 0 | 0 | 0 | 0 | 0 | 0 |
| NM_001017392 | SFRS14 | 4768 | 16 | 6 | 4 | 6 | 5 | 13 | 6 | 1 | 4 | 4 | 4 | 6 | 16 |
| NM_001017395 | TMCC1 | 6152 | 3 | 2 | 2 | 1 | 1 | 3 | 1 | 2 | 1 | 2 | 2 | 2 | 3 |
| NM_001017396 | ZNF2 | 3881 | 1 | 1 | 0 | 0 | 0 | 1 | 1 | 0 | 0 | 1 | 1 | 1 | 1 |

| transcript | gene | transcript length (nt) | max | mean | adipose | colon | heart | hypothalamus | kidney | liver | lung | ovary | skeletalmuscle | spleen | testes |
|---|---|---|---|---|---|---|---|---|---|---|---|---|---|---|---|
| NM_001017397 | TRIM36 | 692 | 1 | 0 | 0 | 0 | 0 | 1 | 0 | 0 | 0 | 0 | 0 | 0 | 1 |
| NM_001017398 | TRIM36 | 906 | 1 | 0 | 0 | 0 | 0 | 1 | 0 | 0 | 0 | 0 | 0 | 0 | 0 |
| NM_001017402 | LAMB3 | 4307 | 8 | 2 | 8 | 3 | 0 | 0 | 1 | 1 | 4 | 0 | 0 | 0 | 0 |
| NM_001017403 | LGR6 | 3458 | 0 | 0 | 0 | 0 | 0 | 0 | 0 | 0 | 0 | 0 | 0 | 0 | 0 |
| NM_001017404 | LGR6 | 3041 | 0 | 0 | 0 | 0 | 0 | 0 | 0 | 0 | 0 | 0 | 0 | 0 | 0 |
| NM_001017405 | MAEA | 2189 | 5 | 3 | 2 | 3 | 3 | 5 | 3 | 2 | 2 | 2 | 1 | 4 | 3 |
| NM_001017406 | S100PBP | 1483 | 34 | 10 | 7 | 8 | 5 | 9 | 6 | 2 | 7 | 14 | 4 | 9 | 34 |
| NM_001017408 | GOPC | 4579 | 6 | 3 | 3 | 2 | 4 | 6 | 3 | 3 | 2 | 4 | 3 | 4 | 4 |
| NM_001017415 | USP1 | 3509 | 23 | 9 | 6 | 2 | 7 | 9 | 6 | 4 | 7 | 13 | 6 | 13 | 23 |
| NM_001017416 | USP1 | 3414 | 24 | 9 | 7 | 2 | 7 | 9 | 6 | 4 | 7 | 14 | 6 | 13 | 24 |
| NM_001017417 | CT45A1 | 1008 | 0 | 0 | 0 | 0 | 0 | 0 | 0 | 0 | 0 | 0 | 0 | 0 | 0 |
| NM_001017418 | SPRR2B | 662 | 0 | 0 | 0 | 0 | 0 | 0 | 0 | 0 | 0 | 0 | 0 | 0 | 0 |
| NM_001017420 | ESCO2 | 3358 | 2 | 0 | 0 | 1 | 0 | 0 | 0 | 0 | 0 | 0 | 0 | 0 | 2 |
| NM_001017423 | ALDH18A1 | 3457 | 11 | 4 | 3 | 8 | 3 | 4 | 5 | 11 | 2 | 3 | 1 | 4 | 3 |
| NM_001017424 | KCNK2 | 3597 | 3 | 0 | 0 | 0 | 0 | 3 | 0 | 0 | 0 | 0 | 0 | 0 | 0 |
| NM_001017425 | KCNK2 | 3398 | 3 | 0 | 0 | 0 | 0 | 3 | 0 | 0 | 0 | 0 | 0 | 0 | 0 |
| NM_001017434 | PGBD2 | 2032 | 2 | 1 | 0 | 1 | 0 | 1 | 1 | 1 | 0 | 1 | 0 | 2 | 2 |
| NM_001017435 | CT45A3 | 1008 | 0 | 0 | 0 | 0 | 0 | 0 | 0 | 0 | 0 | 0 | 0 | 0 | 0 |
| NM_001017436 | CT45A4 | 1008 | 0 | 0 | 0 | 0 | 0 | 0 | 0 | 0 | 0 | 0 | 0 | 0 | 0 |
| NM_001017437 | CCDC157 | 3003 | 0 | 0 | 0 | 0 | 0 | 0 | 0 | 0 | 0 | 0 | 0 | 0 | 0 |
| NM_001017438 | CT45A6 | 1008 | 0 | 0 | 0 | 0 | 0 | 0 | 0 | 0 | 0 | 0 | 0 | 0 | 0 |
| NM_001017440 | CALN1 | 9471 | 7 | 1 | 0 | 0 | 0 | 7 | 0 | 0 | 0 | 0 | 0 | 0 | 0 |
| NM_001017520 | DNTT | 2037 | 1 | 0 | 0 | 0 | 0 | 0 | 0 | 0 | 1 | 0 | 0 | 0 | 0 |
| NM_001017523 | BTBD11 | 3900 | 2 | 0 | 0 | 0 | 0 | 2 | 2 | 0 | 0 | 0 | 0 | 0 | 0 |
| NM_001017524 | SIRT3 | 2756 | 0 | 0 | 0 | 0 | 0 | 0 | 0 | 0 | 0 | 0 | 0 | 0 | 0 |
| NM_001017526 | ARHGAP8 | 1725 | 0 | 0 | 0 | 0 | 0 | 0 | 0 | 0 | 0 | 0 | 0 | 0 | 0 |
| NM_001017528 | PRR5 | 1677 | 0 | 0 | 0 | 0 | 0 | 0 | 0 | 0 | 0 | 0 | 0 | 0 | 0 |
| NM_001017529 | PRR5 | 1560 | 0 | 0 | 0 | 0 | 0 | 0 | 0 | 0 | 0 | 0 | 0 | 0 | 0 |
| NM_001017530 | PRR5 | 1900 | 0 | 0 | 0 | 0 | 0 | 0 | 0 | 0 | 0 | 0 | 0 | 0 | 0 |
| NM_001017534 | CARD16 | 695 | 13 | 3 | 2 | 1 | 2 | 0 | 1 | 0 | 7 | 2 | 0 | 13 | 1 |
| NM_001017535 | VDR | 4775 | 4 | 1 | 0 | 4 | 0 | 0 | 4 | 0 | 1 | 0 | 0 | 1 | 0 |
| NM_001017915 | INPP5D | 4446 | 6 | 1 | 1 | 1 | 0 | 1 | 0 | 0 | 1 | 0 | 0 | 6 | 0 |
| NM_001017916 | CYB561 | 3154 | 2 | 1 | 1 | 1 | 1 | 2 | 2 | 1 | 1 | 0 | 0 | 0 | 0 |
| NM_001017917 | CYB561 | 2987 | 2 | 1 | 1 | 1 | 1 | 2 | 2 | 1 | 1 | 0 | 0 | 0 | 0 |
| NM_001017919 | RCCD1 | 2683 | 3 | 1 | 1 | 2 | 1 | 3 | 2 | 1 | 1 | 2 | 1 | 1 | 1 |
| NM_001017920 | DAPL1 | 541 | 0 | 0 | 0 | 0 | 0 | 0 | 0 | 0 | 0 | 0 | 0 | 0 | 0 |
| NM_001017921 | VMAC | 1670 | 1 | 0 | 0 | 1 | 0 | 0 | 0 | 0 | 0 | 0 | 0 | 0 | 0 |
| NM_001017922 | ERMAP | 3455 | 2 | 1 | 2 | 1 | 0 | 1 | 1 | 1 | 1 | 1 | 0 | 1 | 1 |
| NM_001017923 | C14orf28 | 2906 | 1 | 1 | 1 | 0 | 1 | 0 | 1 | 1 | 1 | 1 | 0 | 1 | 1 |
| NM_001017924 | LRIT2 | 3088 | 0 | 0 | 0 | 0 | 0 | 0 | 0 | 0 | 0 | 0 | 0 | 0 | 0 |
| NM_001017926 | ZHX1 | 5015 | 23 | 15 | 20 | 3 | 15 | 23 | 14 | 22 | 10 | 18 | 11 | 17 | 13 |
| NM_001017927 | C2orf76 | 950 | 3 | 1 | 1 | 0 | 1 | 3 | 1 | 1 | 0 | 1 | 0 | 1 | 1 |
| NM_001017928 | CCDC58 | 718 | 6 | 3 | 4 | 5 | 3 | 3 | 4 | 6 | 1 | 2 | 2 | 2 | 1 |
| NM_001017929 | FRMPD2 | 1883 | 0 | 0 | 0 | 0 | 0 | 0 | 0 | 0 | 0 | 0 | 0 | 0 | 0 |
| NM_001017930 | DCAF8L1 | 3457 | 0 | 0 | 0 | 0 | 0 | 0 | 0 | 0 | 0 | 0 | 0 | 0 | 0 |
| NM_001017931 | PAGE3 | 741 | 0 | 0 | 0 | 0 | 0 | 0 | 0 | 0 | 0 | 0 | 0 | 0 | 0 |
| NM_001017956 | OS9 | 2692 | 104 | 57 | 72 | 42 | 57 | 48 | 64 | 104 | 58 | 52 | 18 | 64 | 43 |
| NM_001017957 | OS9 | 2647 | 98 | 52 | 69 | 38 | 51 | 45 | 58 | 98 | 56 | 46 | 16 | 61 | 39 |
| NM_001017958 | OS9 | 2812 | 96 | 51 | 68 | 38 | 49 | 44 | 57 | 96 | 55 | 44 | 16 | 59 | 38 |
| NM_001017961 | FAM78B | 1598 | 0 | 0 | 0 | 0 | 0 | 0 | 0 | 0 | 0 | 0 | 0 | 0 | 0 |
| NM_001017962 | P4HA1 | 2844 | 25 | 11 | 5 | 3 | 19 | 4 | 7 | 7 | 13 | 13 | 25 | 13 | 10 |
| NM_001017963 | HSP90AA1 | 3884 | 398 | 180 | 166 | 45 | 267 | 398 | 174 | 109 | 179 | 105 | 107 | 149 | 286 |
| NM_001017964 | YDJC | 1337 | 1 | 1 | 0 | 1 | 0 | 1 | 1 | 1 | 0 | 0 | 0 | 1 | 0 |
| NM_001017967 | MARVELD3 | 2234 | 1 | 0 | 0 | 1 | 0 | 0 | 1 | 0 | 0 | 0 | 0 | 0 | 0 |
| NM_001017969 | KIAA2026 | 7204 | 9 | 5 | 3 | 2 | 5 | 5 | 5 | 1 | 4 | 8 | 5 | 7 | 9 |
| NM_001017970 | TMEM30B | 4442 | 3 | 1 | 0 | 3 | 1 | 0 | 2 | 1 | 2 | 0 | 0 | 0 | 1 |
| NM_001017971 | ATP6AP1L | 758 | 1 | 0 | 0 | 0 | 0 | 1 | 0 | 0 | 0 | 1 | 1 | 0 | 0 |
| NM_001017973 | P4HA2 | 2565 | 8 | 3 | 2 | 3 | 8 | 2 | 5 | 4 | 3 | 1 | 1 | 2 | 2 |
| NM_001017974 | P4HA2 | 2093 | 9 | 4 | 3 | 3 | 9 | 3 | 6 | 5 | 3 | 1 | 1 | 2 | 3 |
| NM_001017975 | HFM1 | 4931 | 2 | 0 | 0 | 0 | 0 | 0 | 0 | 0 | 0 | 1 | 1 | 0 | 2 |
| NM_001017977 | DCAF6 | 3233 | 127 | 34 | 18 | 10 | 59 | 25 | 16 | 30 | 14 | 20 | 127 | 17 | 41 |
| NM_001017978 | CXorf61 | 518 | 0 | 0 | 0 | 0 | 0 | 0 | 0 | 0 | 0 | 0 | 0 | 0 | 0 |
| NM_001017979 | RAB28 | 1716 | 3 | 2 | 1 | 1 | 3 | 3 | 2 | 1 | 2 | 2 | 1 | 2 | 3 |
| NM_001017980 | VMA21 | 4735 | 4 | 2 | 1 | 1 | 2 | 4 | 3 | 1 | 2 | 2 | 1 | 3 | 3 |
| NM_001017981 | RNF215 | 1920 | 1 | 0 | 0 | 0 | 0 | 1 | 0 | 0 | 0 | 0 | 0 | 0 | 0 |
| NM_001017986 | FCGR1B | 1121 | 0 | 0 | 0 | 0 | 0 | 0 | 0 | 0 | 0 | 0 | 0 | 0 | 0 |
| NM_001017987 | C5orf45 | 1036 | 1 | 0 | 0 | 0 | 0 | 0 | 0 | 0 | 0 | 1 | 0 | 1 | 0 |
| NM_001017989 | OPA3 | 1932 | 1 | 0 | 0 | 0 | 0 | 0 | 0 | 0 | 0 | 0 | 0 | 1 | 0 |
| NM_001017990 | H2AFB1 | 517 | 0 | 0 | 0 | 0 | 0 | 0 | 0 | 0 | 0 | 0 | 0 | 0 | 0 |
| NM_001017991 | H2AFB2 | 348 | 0 | 0 | 0 | 0 | 0 | 0 | 0 | 0 | 0 | 0 | 0 | 0 | 0 |
| NM_001017992 | ACTBL2 | 2794 | 0 | 0 | 0 | 0 | 0 | 0 | 0 | 0 | 0 | 0 | 0 | 0 | 0 |
| NM_001017995 | SH3PXD2B | 7775 | 6 | 3 | 2 | 2 | 3 | 3 | 1 | 1 | 4 | 6 | 1 | 5 | 3 |
| NM_001017998 | GNG10 | 1245 | 0 | 0 | 0 | 0 | 0 | 0 | 0 | 0 | 0 | 0 | 0 | 0 | 0 |
| NM_001017999 | KIAA1026 | 3653 | 8 | 2 | 1 | 0 | 1 | 3 | 2 | 0 | 1 | 1 | 0 | 8 | 1 |
| NM_001018 | RPS15 | 531 | 18 | 9 | 6 | 5 | 13 | 4 | 9 | 5 | 12 | 18 | 10 | 17 | 5 |
| NM_001018000 | KIAA1026 | 3842 | 8 | 1 | 1 | 0 | 0 | 3 | 2 | 0 | 0 | 1 | 0 | 8 | 1 |
| NM_001018001 | KIAA1026 | 3643 | 8 | 2 | 1 | 0 | 1 | 3 | 2 | 0 | 1 | 1 | 0 | 8 | 1 |

| transcript | gene | transcript length (nt) | max | mean | adipose | colon | heart | hypothalamus | kidney | liver | lung | ovary | skeletalmuscle | spleen | testes |
|---|---|---|---|---|---|---|---|---|---|---|---|---|---|---|---|
| NM_001018003 | SORBS3 | 2040 | 3 | 2 | 2 | 1 | 2 | 1 | 2 | 2 | 3 | 3 | 2 | 3 | 2 |
| NM_001018004 | TPM1 | 1788 | 583 | 85 | 13 | 83 | 583 | 17 | 32 | 6 | 17 | 41 | 85 | 30 | 27 |
| NM_001018005 | TPM1 | 1240 | 854 | 108 | 9 | 70 | 854 | 15 | 23 | 5 | 14 | 33 | 122 | 22 | 20 |
| NM_001018006 | TPM1 | 1788 | 466 | 71 | 13 | 76 | 466 | 16 | 31 | 6 | 16 | 39 | 67 | 29 | 25 |
| NM_001018007 | TPM1 | 1788 | 574 | 84 | 13 | 83 | 574 | 17 | 31 | 6 | 17 | 41 | 83 | 29 | 27 |
| NM_001018008 | TPM1 | 1566 | 635 | 81 | 7 | 54 | 635 | 13 | 19 | 4 | 11 | 25 | 89 | 17 | 16 |
| NM_001018009 | SH3BP5 | 2706 | 9 | 5 | 6 | 2 | 2 | 7 | 6 | 2 | 8 | 9 | 4 | 6 | 8 |
| NM_001018011 | ZBTB16 | 2251 | 15 | 8 | 11 | 5 | 7 | 5 | 7 | 8 | 12 | 4 | 15 | 8 | 5 |
| NM_001018016 | MUC1 | 1172 | 22 | 5 | 0 | 6 | 0 | 0 | 20 | 0 | 22 | 0 | 0 | 0 | 2 |
| NM_001018017 | MUC1 | 1145 | 22 | 5 | 0 | 6 | 0 | 0 | 21 | 0 | 22 | 0 | 0 | 0 | 2 |
| NM_001018020 | TPM1 | 1788 | 457 | 70 | 13 | 75 | 457 | 16 | 31 | 6 | 16 | 39 | 66 | 29 | 24 |
| NM_001018024 | MTCP1NB | 1012 | 1 | 1 | 1 | 0 | 1 | 1 | 0 | 1 | 0 | 0 | 0 | 0 | 0 |
| NM_001018025 | MTCP1 | 2320 | 0 | 0 | 0 | 0 | 0 | 0 | 0 | 0 | 0 | 0 | 0 | 0 | 0 |
| NM_001018036 | TSHR | 1181 | 2 | 0 | 0 | 0 | 0 | 0 | 0 | 2 | 0 | 1 | 0 | 0 | 0 |
| NM_001018037 | VPS13A | 11145 | 10 | 6 | 3 | 2 | 7 | 5 | 8 | 2 | 4 | 3 | 10 | 8 | 8 |
| NM_001018038 | VPS13A | 10176 | 11 | 6 | 3 | 3 | 8 | 5 | 9 | 3 | 4 | 3 | 11 | 8 | 9 |
| NM_001018041 | EPM2A | 1702 | 1 | 0 | 1 | 0 | 1 | 1 | 0 | 0 | 0 | 0 | 1 | 0 | 0 |
| NM_001018046 | YSK4 | 1711 | 0 | 0 | 0 | 0 | 0 | 0 | 0 | 0 | 0 | 0 | 0 | 0 | 0 |
| NM_001018049 | PAEP | 840 | 1 | 0 | 0 | 0 | 0 | 0 | 0 | 0 | 0 | 1 | 0 | 0 | 0 |
| NM_001018050 | POLR3H | 4469 | 0 | 0 | 0 | 0 | 0 | 0 | 0 | 0 | 0 | 0 | 0 | 0 | 0 |
| NM_001018052 | POLR3H | 4382 | 0 | 0 | 0 | 0 | 0 | 0 | 0 | 0 | 0 | 0 | 0 | 0 | 0 |
| NM_001018053 | PFKFB2 | 3529 | 19 | 9 | 2 | 10 | 10 | 15 | 19 | 3 | 7 | 2 | 16 | 8 | 3 |
| NM_001018054 | LRP8 | 4330 | 6 | 1 | 0 | 0 | 0 | 2 | 0 | 0 | 0 | 0 | 0 | 0 | 6 |
| NM_001018055 | BRCC3 | 2839 | 8 | 6 | 6 | 2 | 6 | 5 | 8 | 6 | 5 | 5 | 6 | 8 | 3 |
| NM_001018056 | VLDLR | 3552 | 25 | 8 | 14 | 1 | 23 | 5 | 5 | 0 | 2 | 25 | 4 | 3 | 4 |
| NM_001018057 | DKK3 | 2578 | 153 | 35 | 2 | 9 | 126 | 153 | 6 | 1 | 27 | 12 | 3 | 13 | 31 |
| NM_001018058 | TFEC | 6544 | 4 | 1 | 1 | 0 | 1 | 0 | 4 | 0 | 2 | 0 | 0 | 3 | 0 |
| NM_001018060 | AIFM3 | 2344 | 2 | 0 | 0 | 2 | 0 | 2 | 0 | 0 | 0 | 0 | 0 | 0 | 0 |
| NM_001018064 | NTRK2 | 5560 | 73 | 9 | 15 | 1 | 1 | 73 | 3 | 0 | 1 | 2 | 1 | 1 | 2 |
| NM_001018065 | NTRK2 | 8323 | 46 | 6 | 10 | 1 | 1 | 46 | 2 | 0 | 1 | 1 | 1 | 1 | 1 |
| NM_001018066 | NTRK2 | 8275 | 46 | 6 | 10 | 1 | 1 | 46 | 2 | 0 | 1 | 1 | 1 | 1 | 1 |
| NM_001018067 | SERBP1 | 6764 | 25 | 19 | 14 | 9 | 21 | 15 | 19 | 24 | 15 | 20 | 25 | 23 | 17 |
| NM_001018068 | SERBP1 | 6746 | 25 | 19 | 14 | 9 | 21 | 15 | 19 | 24 | 15 | 20 | 25 | 23 | 17 |
| NM_001018069 | SERBP1 | 6719 | 25 | 19 | 14 | 9 | 22 | 15 | 19 | 25 | 15 | 20 | 25 | 23 | 17 |
| NM_001018070 | CORO1B | 1903 | 2 | 1 | 1 | 1 | 1 | 0 | 1 | 0 | 1 | 0 | 0 | 2 | 0 |
| NM_001018071 | FRMPD2 | 5024 | 0 | 0 | 0 | 0 | 0 | 0 | 0 | 0 | 0 | 0 | 0 | 0 | 0 |
| NM_001018072 | BTBD11 | 5760 | 2 | 0 | 0 | 0 | 0 | 2 | 1 | 0 | 0 | 0 | 0 | 0 | 0 |
| NM_001018073 | PCK2 | 1714 | 15 | 3 | 2 | 2 | 1 | 1 | 9 | 15 | 1 | 0 | 0 | 2 | 1 |
| NM_001018074 | NR3C1 | 6614 | 11 | 6 | 8 | 2 | 5 | 5 | 5 | 5 | 7 | 6 | 11 | 8 | 4 |
| NM_001018075 | NR3C1 | 6517 | 11 | 6 | 9 | 2 | 5 | 5 | 5 | 5 | 7 | 6 | 11 | 8 | 5 |
| NM_001018076 | NR3C1 | 6410 | 11 | 6 | 9 | 2 | 6 | 5 | 5 | 5 | 7 | 6 | 11 | 8 | 5 |
| NM_001018077 | NR3C1 | 7286 | 10 | 5 | 8 | 2 | 5 | 4 | 4 | 5 | 6 | 5 | 10 | 7 | 4 |
| NM_001018078 | FPGS | 2475 | 1 | 1 | 0 | 0 | 1 | 0 | 0 | 1 | 1 | 1 | 0 | 1 | 1 |
| NM_001018080 | FSHB | 1906 | 0 | 0 | 0 | 0 | 0 | 0 | 0 | 0 | 0 | 0 | 0 | 0 | 0 |
| NM_001018081 | KIR2DL5B | 1632 | 0 | 0 | 0 | 0 | 0 | 0 | 0 | 0 | 0 | 0 | 0 | 0 | 0 |
| NM_001018082 | ADIG | 770 | 0 | 0 | 0 | 0 | 0 | 0 | 0 | 0 | 0 | 0 | 0 | 0 | 0 |
| NM_001018088 | VPS13C | 11141 | 21 | 11 | 10 | 4 | 10 | 11 | 11 | 5 | 16 | 14 | 10 | 21 | 12 |
| NM_001018089 | NARG2 | 7049 | 4 | 3 | 3 | 1 | 2 | 2 | 3 | 1 | 2 | 4 | 2 | 4 | 4 |
| NM_001018090 | GCOM1 | 4648 | 71 | 10 | 20 | 2 | 71 | 1 | 1 | 0 | 5 | 1 | 1 | 3 | 1 |
| NM_001018091 | GCOM1 | 4443 | 74 | 10 | 21 | 2 | 74 | 1 | 1 | 0 | 6 | 1 | 1 | 3 | 1 |
| NM_001018100 | GCOM1 | 2386 | 152 | 21 | 44 | 4 | 152 | 3 | 3 | 0 | 12 | 2 | 2 | 6 | 3 |
| NM_001018102 | GRINL1A | 3652 | 0 | 0 | 0 | 0 | 0 | 0 | 0 | 0 | 0 | 0 | 0 | 0 | 0 |
| NM_001018104 | FAHD1 | 1700 | 5 | 2 | 1 | 2 | 1 | 2 | 5 | 3 | 0 | 1 | 1 | 1 | 1 |
| NM_001018108 | SERF2 | 2594 | 5 | 4 | 5 | 4 | 5 | 4 | 4 | 4 | 3 | 2 | 2 | 5 | 3 |
| NM_001018109 | PIR | 1279 | 4 | 2 | 2 | 1 | 2 | 4 | 2 | 3 | 1 | 1 | 1 | 4 | 2 |
| NM_001018111 | PODXL | 5984 | 19 | 5 | 2 | 2 | 7 | 5 | 19 | 1 | 9 | 3 | 1 | 3 | 2 |
| NM_001018112 | FANCA | 1656 | 1 | 0 | 0 | 1 | 0 | 0 | 0 | 0 | 0 | 0 | 0 | 0 | 0 |
| NM_001018113 | FANCB | 3008 | 0 | 0 | 0 | 0 | 0 | 0 | 0 | 0 | 0 | 0 | 0 | 0 | 0 |
| NM_001018115 | FANCD2 | 5102 | 1 | 0 | 0 | 1 | 0 | 0 | 0 | 0 | 1 | 1 | 0 | 1 | 1 |
| NM_001018116 | MURC | 2625 | 125 | 16 | 0 | 0 | 49 | 1 | 0 | 0 | 0 | 1 | 125 | 0 | 3 |
| NM_001018136 | NME1-NME | 1052 | 0 | 0 | 0 | 0 | 0 | 0 | 0 | 0 | 0 | 0 | 0 | 0 | 0 |
| NM_001018137 | NME2 | 755 | 0 | 0 | 0 | 0 | 0 | 0 | 0 | 0 | 0 | 0 | 0 | 0 | 0 |
| NM_001018138 | NME2 | 708 | 0 | 0 | 0 | 0 | 0 | 0 | 0 | 0 | 0 | 0 | 0 | 0 | 0 |
| NM_001018139 | NME2 | 647 | 0 | 0 | 0 | 0 | 0 | 0 | 0 | 0 | 0 | 0 | 0 | 0 | 0 |
| NM_001018159 | NAE1 | 1911 | 17 | 9 | 8 | 3 | 10 | 14 | 8 | 8 | 6 | 10 | 7 | 9 | 17 |
| NM_001018160 | NAE1 | 1709 | 19 | 10 | 9 | 4 | 11 | 16 | 9 | 9 | 6 | 11 | 8 | 10 | 19 |
| NM_001018161 | PON2 | 1618 | 8 | 2 | 1 | 0 | 2 | 4 | 2 | 8 | 3 | 1 | 0 | 1 | 2 |
| NM_001018676 | FNTA | 1608 | 22 | 13 | 17 | 5 | 17 | 22 | 10 | 10 | 10 | 12 | 13 | 17 | 12 |
| NM_001018677 | FNTA | 1493 | 23 | 14 | 19 | 6 | 18 | 23 | 10 | 10 | 10 | 12 | 14 | 18 | 12 |
| NM_001018837 | HAX1 | 1031 | 25 | 13 | 8 | 6 | 25 | 16 | 9 | 23 | 5 | 8 | 17 | 10 | 11 |
| NM_001019 | RPS15A | 543 | 87 | 46 | 28 | 20 | 45 | 16 | 35 | 42 | 62 | 87 | 49 | 86 | 37 |
| NM_001020 | RPS16 | 590 | 13 | 9 | 7 | 10 | 8 | 3 | 8 | 13 | 8 | 12 | 11 | 13 | 5 |
| NM_001020658 | PUM1 | 5385 | 12 | 8 | 7 | 7 | 7 | 12 | 9 | 6 | 8 | 11 | 9 | 10 | 7 |
| NM_001020818 | MYADM | 3220 | 7 | 4 | 2 | 5 | 7 | 2 | 1 | 1 | 5 | 7 | 1 | 5 | 3 |
| NM_001020819 | MYADM | 3118 | 8 | 4 | 2 | 5 | 7 | 2 | 1 | 1 | 5 | 8 | 1 | 6 | 3 |
| NM_001020820 | MYADM | 3054 | 8 | 4 | 2 | 5 | 7 | 3 | 1 | 1 | 5 | 8 | 1 | 6 | 3 |
| NM_001020821 | MYADM | 3186 | 7 | 4 | 2 | 5 | 7 | 2 | 1 | 1 | 5 | 7 | 1 | 5 | 3 |

| transcript | gene | transcript length (nt) | max | mean | adipose | colon | heart | hypothalamus | kidney | liver | lung | ovary | skeletalmuscle | spleen | testes |
|---|---|---|---|---|---|---|---|---|---|---|---|---|---|---|---|
| NM_001020825 | NR3C1 | 4154 | 10 | 6 | 9 | 2 | 5 | 5 | 4 | 6 | 6 | 5 | 10 | 7 | 4 |
| NM_001021 | RPS17 | 485 | 386 | 237 | 229 | 238 | 220 | 142 | 157 | 386 | 219 | 336 | 179 | 365 | 133 |
| NM_001022 | RPS19 | 849 | 12 | 6 | 3 | 6 | 3 | 3 | 3 | 8 | 8 | 6 | 4 | 12 | 4 |
| NM_001023 | RPS20 | 839 | 122 | 69 | 81 | 27 | 68 | 46 | 50 | 57 | 79 | 122 | 56 | 109 | 67 |
| NM_001023560 | ZNF187 | 2816 | 9 | 4 | 3 | 2 | 3 | 5 | 3 | 1 | 2 | 9 | 3 | 5 | 5 |
| NM_001023561 | ZNF749 | 2929 | 1 | 1 | 1 | 1 | 1 | 1 | 1 | 1 | 0 | 1 | 0 | 1 | 1 |
| NM_001023563 | ZNF805 | 10125 | 3 | 1 | 1 | 1 | 1 | 1 | 1 | 1 | 1 | 2 | 1 | 3 | 2 |
| NM_001023567 | GOLGA8B | 4268 | 0 | 0 | 0 | 0 | 0 | 0 | 0 | 0 | 0 | 0 | 0 | 0 | 0 |
| NM_001023570 | IQCB1 | 2594 | 10 | 4 | 2 | 2 | 3 | 5 | 3 | 2 | 4 | 5 | 2 | 5 | 10 |
| NM_001023571 | IQCB1 | 2195 | 10 | 4 | 2 | 2 | 2 | 5 | 3 | 2 | 4 | 5 | 2 | 5 | 10 |
| NM_001023587 | ABCC5 | 1973 | 14 | 6 | 5 | 7 | 5 | 6 | 4 | 1 | 2 | 3 | 14 | 7 | 6 |
| NM_001024 | RPS21 | 396 | 5 | 3 | 5 | 4 | 2 | 4 | 3 | 4 | 3 | 5 | 1 | 3 | 2 |
| NM_001024024 | GCH1 | 1979 | 3 | 1 | 0 | 0 | 0 | 0 | 1 | 3 | 1 | 0 | 0 | 1 | 0 |
| NM_001024070 | GCH1 | 1924 | 2 | 0 | 0 | 0 | 0 | 0 | 1 | 2 | 1 | 0 | 0 | 1 | 0 |
| NM_001024071 | GCH1 | 1836 | 2 | 0 | 0 | 0 | 0 | 0 | 1 | 2 | 1 | 0 | 0 | 1 | 0 |
| NM_001024074 | HNMT | 739 | 4 | 2 | 4 | 1 | 1 | 1 | 3 | 2 | 3 | 4 | 1 | 2 | 1 |
| NM_001024075 | HNMT | 907 | 6 | 2 | 6 | 1 | 2 | 0 | 4 | 3 | 2 | 2 | 0 | 3 | 2 |
| NM_001024094 | NR3C1 | 6787 | 11 | 6 | 8 | 2 | 5 | 4 | 5 | 5 | 7 | 6 | 11 | 8 | 4 |
| NM_001024209 | SPRR2E | 678 | 0 | 0 | 0 | 0 | 0 | 0 | 0 | 0 | 0 | 0 | 0 | 0 | 0 |
| NM_001024210 | S100A13 | 896 | 21 | 10 | 17 | 8 | 21 | 11 | 10 | 3 | 9 | 10 | 10 | 7 | 6 |
| NM_001024211 | S100A13 | 612 | 31 | 15 | 24 | 11 | 31 | 15 | 14 | 5 | 13 | 14 | 14 | 10 | 9 |
| NM_001024212 | S100A13 | 583 | 33 | 15 | 26 | 12 | 33 | 16 | 15 | 5 | 14 | 15 | 15 | 10 | 9 |
| NM_001024213 | S100A13 | 551 | 35 | 16 | 27 | 13 | 35 | 17 | 16 | 5 | 15 | 16 | 16 | 11 | 10 |
| NM_001024215 | FBLIM1 | 1521 | 1 | 0 | 0 | 1 | 0 | 0 | 0 | 0 | 0 | 0 | 0 | 0 | 0 |
| NM_001024216 | FBLIM1 | 2779 | 1 | 0 | 0 | 0 | 1 | 0 | 0 | 0 | 0 | 0 | 0 | 0 | 0 |
| NM_001024218 | GPHN | 4203 | 7 | 3 | 1 | 2 | 2 | 5 | 6 | 7 | 1 | 2 | 2 | 2 | 2 |
| NM_001024226 | ARF1 | 1973 | 14 | 10 | 8 | 6 | 12 | 14 | 11 | 11 | 9 | 5 | 7 | 13 | 10 |
| NM_001024227 | ARF1 | 2007 | 13 | 10 | 8 | 6 | 12 | 13 | 11 | 11 | 9 | 5 | 7 | 13 | 9 |
| NM_001024228 | ARF1 | 1985 | 14 | 10 | 8 | 6 | 12 | 14 | 11 | 11 | 9 | 5 | 7 | 13 | 10 |
| NM_001024372 | BAALC | 2682 | 41 | 5 | 1 | 1 | 1 | 41 | 1 | 0 | 1 | 1 | 0 | 6 | 0 |
| NM_001024380 | TM2D2 | 3435 | 4 | 3 | 2 | 1 | 3 | 4 | 4 | 3 | 2 | 2 | 1 | 4 | 3 |
| NM_001024381 | TM2D2 | 3334 | 4 | 3 | 3 | 1 | 3 | 4 | 4 | 3 | 2 | 2 | 1 | 4 | 4 |
| NM_001024382 | HMBS | 1411 | 2 | 1 | 1 | 1 | 1 | 1 | 1 | 2 | 1 | 0 | 1 | 1 | 1 |
| NM_001024401 | SBK1 | 4992 | 1 | 0 | 0 | 0 | 0 | 1 | 0 | 0 | 0 | 0 | 0 | 0 | 0 |
| NM_001024452 | GLRA4 | 1674 | 0 | 0 | 0 | 0 | 0 | 0 | 0 | 0 | 0 | 0 | 0 | 0 | 0 |
| NM_001024455 | RGAG4 | 4789 | 3 | 1 | 1 | 1 | 1 | 3 | 1 | 1 | 1 | 1 | 0 | 1 | 3 |
| NM_001024457 | RGPD1 | 6692 | 0 | 0 | 0 | 0 | 0 | 0 | 0 | 0 | 0 | 0 | 0 | 0 | 0 |
| NM_001024457 | RGPD1 | 6695 | 0 | 0 | 0 | 0 | 0 | 0 | 0 | 0 | 0 | 0 | 0 | 0 | 0 |
| NM_001024463 | HISPPD2A | 4979 | 9 | 3 | 3 | 6 | 1 | 9 | 2 | 1 | 1 | 2 | 3 | 2 | 5 |
| NM_001024465 | SOD2 | 1024 | 792 | 181 | 66 | 17 | 221 | 52 | 213 | 313 | 137 | 27 | 792 | 97 | 53 |
| NM_001024466 | SOD2 | 907 | 883 | 201 | 73 | 19 | 247 | 57 | 237 | 346 | 153 | 30 | 883 | 108 | 60 |
| NM_001024593 | ZMYND17 | 2423 | 5 | 1 | 0 | 0 | 0 | 0 | 0 | 0 | 0 | 0 | 5 | 0 | 0 |
| NM_001024594 | C1orf53 | 681 | 0 | 0 | 0 | 0 | 0 | 0 | 0 | 0 | 0 | 0 | 0 | 0 | 0 |
| NM_001024596 | ZNF772 | 5418 | 2 | 1 | 1 | 1 | 1 | 2 | 2 | 0 | 1 | 2 | 1 | 2 | 2 |
| NM_001024598 | HES3 | 687 | 0 | 0 | 0 | 0 | 0 | 0 | 0 | 0 | 0 | 0 | 0 | 0 | 0 |
| NM_001024599 | HIST2H2BF | 1810 | 5 | 2 | 1 | 1 | 0 | 3 | 1 | 0 | 5 | 4 | 0 | 2 | 2 |
| NM_001024601 | C1orf227 | 504 | 0 | 0 | 0 | 0 | 0 | 0 | 0 | 0 | 0 | 0 | 0 | 0 | 0 |
| NM_001024607 | C7orf66 | 422 | 0 | 0 | 0 | 0 | 0 | 0 | 0 | 0 | 0 | 0 | 0 | 0 | 0 |
| NM_001024611 | LRRC66 | 3329 | 1 | 0 | 0 | 0 | 0 | 0 | 0 | 0 | 0 | 0 | 1 | 0 | 0 |
| NM_001024613 | FEZF1 | 2104 | 0 | 0 | 0 | 0 | 0 | 0 | 0 | 0 | 0 | 0 | 0 | 0 | 0 |
| NM_001024628 | NRP1 | 2478 | 18 | 8 | 18 | 3 | 10 | 3 | 15 | 3 | 8 | 9 | 2 | 11 | 5 |
| NM_001024629 | NRP1 | 2373 | 19 | 8 | 19 | 3 | 11 | 3 | 16 | 3 | 8 | 10 | 3 | 11 | 5 |
| NM_001024630 | RUNX2 | 5552 | 1 | 0 | 0 | 0 | 0 | 0 | 0 | 0 | 1 | 1 | 0 | 1 | 0 |
| NM_001024644 | XCR1 | 1251 | 1 | 0 | 0 | 0 | 0 | 0 | 0 | 0 | 0 | 0 | 0 | 1 | 0 |
| NM_001024647 | RAB3IP | 8839 | 7 | 2 | 0 | 1 | 2 | 4 | 7 | 0 | 1 | 0 | 1 | 1 | 2 |
| NM_001024649 | CANX | 4897 | 160 | 70 | 69 | 31 | 82 | 66 | 83 | 160 | 62 | 58 | 22 | 78 | 62 |
| NM_001024656 | ASPDH | 848 | 3 | 1 | 0 | 0 | 0 | 1 | 3 | 1 | 0 | 0 | 0 | 0 | 0 |
| NM_001024660 | KALRN | 10806 | 12 | 3 | 2 | 2 | 3 | 12 | 1 | 1 | 1 | 2 | 1 | 7 | 2 |
| NM_001024661 | PRAMEF13 | 2164 | 0 | 0 | 0 | 0 | 0 | 0 | 0 | 0 | 0 | 0 | 0 | 0 | 0 |
| NM_001024662 | RPL6 | 1130 | 113 | 55 | 42 | 20 | 65 | 37 | 46 | 69 | 47 | 113 | 50 | 88 | 33 |
| NM_001024666 | SH3KBP1 | 4454 | 7 | 3 | 7 | 3 | 3 | 5 | 2 | 2 | 4 | 2 | 1 | 6 | 2 |
| NM_001024668 | LETMD1 | 2025 | 3 | 2 | 2 | 2 | 1 | 3 | 2 | 1 | 1 | 2 | 1 | 2 | 1 |
| NM_001024674 | LIN52 | 1798 | 2 | 1 | 1 | 1 | 1 | 2 | 2 | 2 | 0 | 1 | 1 | 1 | 1 |
| NM_001024675 | C20orf134 | 2028 | 0 | 0 | 0 | 0 | 0 | 0 | 0 | 0 | 0 | 0 | 0 | 0 | 0 |
| NM_001024678 | LRRC24 | 1771 | 0 | 0 | 0 | 0 | 0 | 0 | 0 | 0 | 0 | 0 | 0 | 0 | 0 |
| NM_001024679 | C1orf68 | 908 | 0 | 0 | 0 | 0 | 0 | 0 | 0 | 0 | 0 | 0 | 0 | 0 | 0 |
| NM_001024680 | FBXO48 | 2712 | 0 | 0 | 0 | 0 | 0 | 0 | 0 | 0 | 0 | 0 | 0 | 0 | 0 |
| NM_001024683 | ZNF688 | 1135 | 1 | 1 | 1 | 1 | 1 | 1 | 1 | 1 | 0 | 1 | 1 | 1 | 1 |
| NM_001024732 | MECR | 2434 | 1 | 0 | 1 | 1 | 0 | 1 | 1 | 0 | 0 | 0 | 0 | 0 | 1 |
| NM_001024736 | CD276 | 3401 | 3 | 2 | 3 | 1 | 3 | 1 | 1 | 1 | 1 | 3 | 1 | 2 | 2 |
| NM_001024807 | APLP1 | 2450 | 25 | 2 | 0 | 0 | 0 | 25 | 0 | 0 | 0 | 0 | 0 | 0 | 0 |
| NM_001024808 | BCL7A | 3651 | 1 | 1 | 0 | 0 | 1 | 1 | 1 | 0 | 0 | 0 | 1 | 1 | 0 |
| NM_001024809 | RARA | 3405 | 1 | 1 | 0 | 0 | 1 | 0 | 1 | 1 | 1 | 1 | 0 | 1 | 0 |
| NM_001024822 | RNASE12 | 744 | 0 | 0 | 0 | 0 | 0 | 0 | 0 | 0 | 0 | 0 | 0 | 0 | 0 |
| NM_001024843 | TNRC6B | 15959 | 6 | 4 | 3 | 2 | 3 | 4 | 3 | 2 | 3 | 6 | 4 | 6 | 5 |
| NM_001024844 | CD82 | 1616 | 2 | 1 | 1 | 1 | 1 | 2 | 1 | 2 | 2 | 1 | 0 | 2 | 0 |
| NM_001024845 | SLC6A9 | 3206 | 1 | 0 | 0 | 0 | 0 | 1 | 0 | 0 | 0 | 0 | 0 | 0 | 0 |

| transcript | gene | transcript length (nt) | max | mean | adipose | colon | heart | hypothalamus | kidney | liver | lung | ovary | skeletalmuscle | spleen | testes |
|---|---|---|---|---|---|---|---|---|---|---|---|---|---|---|---|
| NM_001024847 | TGFBR2 | 4703 | 41 | 17 | 36 | 10 | 33 | 5 | 12 | 10 | 21 | 5 | 5 | 41 | 8 |
| NM_001024855 | ZNF197 | 2965 | 7 | 4 | 3 | 2 | 4 | 6 | 4 | 2 | 3 | 7 | 5 | 5 | 5 |
| NM_001024858 | SPTB | 10063 | 14 | 2 | 0 | 0 | 4 | 2 | 1 | 0 | 0 | 0 | 14 | 0 | 0 |
| NM_001024912 | CEACAM1 | 3444 | 6 | 2 | 1 | 6 | 0 | 0 | 3 | 4 | 1 | 0 | 0 | 1 | 0 |
| NM_001024916 | CBWD5 | 1572 | 0 | 0 | 0 | 0 | 0 | 0 | 0 | 0 | 0 | 0 | 0 | 0 | 0 |
| NM_001024921 | RPL9 | 838 | 87 | 40 | 42 | 21 | 41 | 33 | 32 | 43 | 35 | 87 | 25 | 55 | 23 |
| NM_001024924 | EXOC1 | 3582 | 11 | 6 | 7 | 3 | 6 | 11 | 6 | 3 | 5 | 6 | 3 | 7 | 8 |
| NM_001024934 | WASF1 | 3122 | 15 | 3 | 1 | 0 | 1 | 15 | 2 | 0 | 0 | 3 | 0 | 1 | 6 |
| NM_001024935 | WASF1 | 3075 | 16 | 3 | 1 | 0 | 1 | 16 | 2 | 0 | 0 | 3 | 0 | 1 | 6 |
| NM_001024936 | WASF1 | 2977 | 16 | 3 | 1 | 0 | 1 | 16 | 2 | 0 | 0 | 3 | 0 | 1 | 6 |
| NM_001024937 | MINK1 | 4712 | 12 | 4 | 2 | 6 | 2 | 12 | 3 | 1 | 2 | 5 | 1 | 3 | 3 |
| NM_001024938 | SLC2A11 | 2279 | 1 | 0 | 0 | 0 | 0 | 0 | 1 | 0 | 0 | 0 | 0 | 0 | 0 |
| NM_001024939 | SLC2A11 | 2335 | 1 | 0 | 0 | 0 | 0 | 0 | 1 | 0 | 0 | 0 | 0 | 0 | 0 |
| NM_001024940 | TRIM17 | 2047 | 1 | 0 | 0 | 0 | 0 | 1 | 0 | 0 | 0 | 0 | 0 | 0 | 1 |
| NM_001024941 | TRIM17 | 1969 | 1 | 0 | 0 | 0 | 0 | 1 | 0 | 0 | 0 | 0 | 0 | 0 | 1 |
| NM_001024943 | ASL | 2060 | 6 | 1 | 0 | 2 | 0 | 1 | 2 | 6 | 1 | 0 | 0 | 1 | 0 |
| NM_001024944 | ASL | 2000 | 5 | 1 | 0 | 1 | 0 | 0 | 2 | 5 | 1 | 0 | 0 | 1 | 0 |
| NM_001024946 | ASL | 1982 | 6 | 1 | 0 | 2 | 0 | 1 | 2 | 6 | 1 | 0 | 0 | 1 | 0 |
| NM_001024947 | YIPF5 | 3449 | 2 | 1 | 1 | 1 | 1 | 2 | 2 | 2 | 1 | 2 | 0 | 2 | 2 |
| NM_001024948 | FNBP1L | 5192 | 18 | 7 | 5 | 3 | 8 | 7 | 18 | 4 | 9 | 7 | 5 | 3 | 9 |
| NM_001024956 | SC5DL | 7019 | 11 | 3 | 2 | 1 | 2 | 8 | 4 | 11 | 2 | 2 | 1 | 2 | 4 |
| NM_001024957 | BRMS1 | 1355 | 8 | 5 | 4 | 5 | 4 | 3 | 8 | 5 | 4 | 2 | 3 | 7 | 6 |
| NM_001024959 | ARPC4 | 1557 | 56 | 28 | 30 | 24 | 24 | 43 | 27 | 23 | 26 | 19 | 14 | 56 | 24 |
| NM_001024960 | ARPC4 | 1412 | 61 | 31 | 33 | 27 | 27 | 47 | 29 | 25 | 28 | 21 | 15 | 61 | 27 |
| NM_001025 | RPS23 | 3314 | 29 | 17 | 12 | 15 | 17 | 23 | 13 | 15 | 13 | 29 | 18 | 23 | 14 |
| NM_001025068 | ARPP-21 | 2409 | 15 | 3 | 0 | 0 | 0 | 15 | 0 | 0 | 0 | 0 | 15 | 0 | 0 |
| NM_001025069 | ARPP-21 | 2418 | 15 | 3 | 0 | 0 | 0 | 15 | 0 | 0 | 0 | 0 | 15 | 0 | 0 |
| NM_001025070 | RPS14 | 780 | 38 | 27 | 27 | 26 | 22 | 17 | 22 | 24 | 33 | 38 | 26 | 34 | 22 |
| NM_001025071 | RPS14 | 786 | 38 | 26 | 27 | 26 | 21 | 17 | 22 | 24 | 33 | 38 | 26 | 34 | 22 |
| NM_001025076 | CUGBP2 | 8042 | 6 | 3 | 3 | 1 | 4 | 6 | 0 | 0 | 4 | 6 | 2 | 6 | 2 |
| NM_001025077 | CUGBP2 | 9239 | 6 | 3 | 3 | 1 | 3 | 6 | 0 | 0 | 4 | 5 | 2 | 6 | 2 |
| NM_001025079 | CD47 | 5313 | 11 | 5 | 3 | 2 | 4 | 11 | 3 | 3 | 11 | 4 | 2 | 8 | 4 |
| NM_001025081 | MBP | 2279 | 864 | 81 | 1 | 9 | 2 | 864 | 1 | 1 | 1 | 1 | 5 | 2 | 1 |
| NM_001025087 | BRUNOL4 | 4051 | 12 | 1 | 0 | 1 | 0 | 12 | 0 | 0 | 0 | 0 | 0 | 0 | 0 |
| NM_001025088 | BRUNOL4 | 4048 | 12 | 1 | 0 | 1 | 0 | 12 | 0 | 0 | 0 | 0 | 0 | 0 | 0 |
| NM_001025089 | BRUNOL4 | 3940 | 12 | 1 | 0 | 1 | 0 | 12 | 0 | 0 | 0 | 0 | 0 | 0 | 0 |
| NM_001025090 | MBP | 2201 | 894 | 83 | 1 | 9 | 2 | 894 | 1 | 1 | 1 | 1 | 5 | 2 | 1 |
| NM_001025091 | ABCF1 | 3464 | 24 | 16 | 13 | 10 | 17 | 15 | 15 | 24 | 9 | 10 | 23 | 16 | 19 |
| NM_001025091 | ABCF1 | 3465 | 24 | 16 | 13 | 10 | 17 | 15 | 15 | 24 | 9 | 10 | 23 | 16 | 19 |
| NM_001025092 | MBP | 2168 | 908 | 85 | 1 | 9 | 2 | 908 | 1 | 1 | 1 | 1 | 5 | 2 | 1 |
| NM_001025096 | PSMA8 | 1811 | 1 | 0 | 0 | 0 | 0 | 0 | 0 | 0 | 0 | 0 | 0 | 0 | 1 |
| NM_001025097 | PSMA8 | 1697 | 1 | 0 | 0 | 0 | 0 | 0 | 0 | 0 | 0 | 0 | 0 | 0 | 1 |
| NM_001025100 | MBP | 4889 | 132 | 15 | 3 | 3 | 3 | 132 | 6 | 3 | 4 | 3 | 5 | 7 | 2 |
| NM_001025101 | MBP | 2773 | 710 | 67 | 1 | 8 | 2 | 710 | 1 | 1 | 1 | 1 | 4 | 2 | 1 |
| NM_001025104 | CSN1S1 | 954 | 2 | 0 | 2 | 0 | 0 | 0 | 0 | 0 | 0 | 0 | 0 | 0 | 0 |
| NM_001025105 | CSNK1A1 | 3149 | 43 | 25 | 17 | 12 | 43 | 22 | 20 | 19 | 28 | 28 | 24 | 35 | 28 |
| NM_001025107 | ADAR | 6521 | 25 | 13 | 9 | 7 | 10 | 21 | 13 | 15 | 12 | 14 | 5 | 25 | 15 |
| NM_001025108 | AFF3 | 8120 | 5 | 2 | 2 | 1 | 1 | 5 | 1 | 0 | 2 | 4 | 0 | 5 | 2 |
| NM_001025109 | CD34 | 2616 | 5 | 3 | 5 | 2 | 5 | 2 | 3 | 0 | 2 | 2 | 3 | 1 | 3 |
| NM_001025158 | CD74 | 1138 | 175 | 40 | 33 | 19 | 19 | 23 | 33 | 13 | 90 | 15 | 11 | 175 | 10 |
| NM_001025159 | CD74 | 1503 | 623 | 157 | 209 | 94 | 60 | 113 | 120 | 71 | 304 | 52 | 39 | 623 | 46 |
| NM_001025160 | CD97 | 3086 | 5 | 1 | 1 | 1 | 1 | 0 | 0 | 0 | 1 | 0 | 1 | 5 | 0 |
| NM_001025161 | CYP2D6 | 1506 | 1 | 0 | 0 | 0 | 0 | 0 | 0 | 1 | 0 | 0 | 0 | 0 | 0 |
| NM_001025190 | MSLNL | 3162 | 0 | 0 | 0 | 0 | 0 | 0 | 0 | 0 | 0 | 0 | 0 | 0 | 0 |
| NM_001025194 | CES1 | 2009 | 109 | 18 | 59 | 3 | 5 | 0 | 0 | 109 | 10 | 1 | 1 | 8 | 1 |
| NM_001025195 | CES1 | 2012 | 109 | 18 | 59 | 3 | 5 | 0 | 0 | 109 | 10 | 1 | 1 | 8 | 1 |
| NM_001025197 | CHI3L2 | 1440 | 3 | 1 | 1 | 0 | 3 | 2 | 0 | 0 | 2 | 0 | 0 | 2 | 0 |
| NM_001025199 | CHI3L2 | 1500 | 3 | 1 | 0 | 0 | 3 | 2 | 0 | 0 | 1 | 0 | 0 | 2 | 0 |
| NM_001025200 | CTRB2 | 890 | 0 | 0 | 0 | 0 | 0 | 0 | 0 | 0 | 0 | 0 | 0 | 0 | 0 |
| NM_001025201 | CHN1 | 2509 | 63 | 7 | 1 | 1 | 3 | 63 | 1 | 0 | 2 | 1 | 0 | 1 | 5 |
| NM_001025202 | STAG3L2 | 1124 | 0 | 0 | 0 | 0 | 0 | 0 | 0 | 0 | 0 | 0 | 0 | 0 | 0 |
| NM_001025203 | U2AF1 | 953 | 45 | 29 | 21 | 23 | 31 | 31 | 29 | 23 | 31 | 33 | 21 | 45 | 24 |
| NM_001025204 | U2AF1 | 1020 | 43 | 27 | 20 | 22 | 29 | 29 | 27 | 22 | 29 | 31 | 20 | 43 | 22 |
| NM_001025205 | AP2M1 | 1934 | 97 | 34 | 34 | 24 | 39 | 97 | 30 | 31 | 21 | 20 | 11 | 35 | 30 |
| NM_001025231 | KPRP | 2523 | 0 | 0 | 0 | 0 | 0 | 0 | 0 | 0 | 0 | 0 | 0 | 0 | 0 |
| NM_001025232 | CLLU1OS | 686 | 0 | 0 | 0 | 0 | 0 | 0 | 0 | 0 | 0 | 0 | 0 | 0 | 0 |
| NM_001025233 | CLLU1 | 3897 | 0 | 0 | 0 | 0 | 0 | 0 | 0 | 0 | 0 | 0 | 0 | 0 | 0 |
| NM_001025234 | TSPAN4 | 1426 | 1 | 0 | 0 | 0 | 0 | 0 | 0 | 0 | 1 | 0 | 0 | 0 | 0 |
| NM_001025235 | TSPAN4 | 1425 | 1 | 0 | 0 | 0 | 0 | 0 | 0 | 0 | 1 | 0 | 0 | 0 | 0 |
| NM_001025236 | TSPAN4 | 1430 | 1 | 0 | 0 | 0 | 0 | 0 | 0 | 0 | 1 | 0 | 0 | 0 | 0 |
| NM_001025237 | TSPAN4 | 1462 | 1 | 0 | 0 | 0 | 0 | 0 | 0 | 0 | 1 | 0 | 0 | 0 | 0 |
| NM_001025238 | TSPAN4 | 1312 | 1 | 0 | 0 | 0 | 0 | 0 | 0 | 0 | 1 | 0 | 0 | 0 | 0 |
| NM_001025239 | TSPAN4 | 1332 | 1 | 0 | 0 | 0 | 0 | 0 | 0 | 0 | 1 | 0 | 0 | 0 | 0 |
| NM_001025242 | IRAK1 | 3481 | 3 | 2 | 2 | 2 | 1 | 1 | 2 | 3 | 2 | 0 | 1 | 2 | 1 |
| NM_001025243 | IRAK1 | 3334 | 3 | 2 | 2 | 2 | 1 | 1 | 2 | 3 | 1 | 0 | 1 | 2 | 1 |
| NM_001025247 | TAF5L | 4139 | 2 | 1 | 1 | 1 | 1 | 1 | 1 | 1 | 1 | 1 | 0 | 2 | 1 |
| NM_001025248 | DUT | 2146 | 2 | 1 | 1 | 1 | 1 | 1 | 2 | 0 | 2 | 1 | 1 | 2 | 2 |

| transcript | gene | transcript length (nt) | max | mean | adipose | colon | heart | hypothalamus | kidney | liver | lung | ovary | skeletalmuscle | spleen | testes |
|---|---|---|---|---|---|---|---|---|---|---|---|---|---|---|---|
| NM_001025249 | DUT | 1830 | 2 | 1 | 1 | 1 | 2 | 1 | 2 | 0 | 2 | 1 | 1 | 2 | 2 |
| NM_001025252 | TPD52 | 4243 | 11 | 4 | 1 | 10 | 1 | 11 | 7 | 4 | 3 | 0 | 1 | 4 | 1 |
| NM_001025253 | TPD52 | 4028 | 12 | 4 | 1 | 8 | 1 | 12 | 6 | 4 | 3 | 0 | 1 | 4 | 2 |
| NM_001025265 | CXorf65 | 566 | 3 | 1 | 0 | 0 | 0 | 0 | 0 | 0 | 1 | 0 | 0 | 2 | 3 |
| NM_001025266 | C3orf70 | 5901 | 4 | 1 | 0 | 2 | 0 | 4 | 0 | 0 | 0 | 1 | 0 | 0 | 3 |
| NM_001025290 | DPPA5 | 612 | 0 | 0 | 0 | 0 | 0 | 0 | 0 | 0 | 0 | 0 | 0 | 0 | 0 |
| NM_001025295 | IFITM5 | 733 | 0 | 0 | 0 | 0 | 0 | 0 | 0 | 0 | 0 | 0 | 0 | 0 | 0 |
| NM_001025300 | RAB12 | 2138 | 61 | 24 | 18 | 8 | 32 | 30 | 18 | 20 | 23 | 16 | 61 | 20 | 15 |
| NM_001025356 | ANO6 | 6104 | 22 | 13 | 22 | 5 | 13 | 5 | 11 | 13 | 14 | 21 | 13 | 9 | 13 |
| NM_001025357 | C8orf85 | 1784 | 8 | 1 | 0 | 0 | 0 | 0 | 0 | 0 | 0 | 0 | 0 | 0 | 8 |
| NM_001025366 | VEGFA | 3654 | 17 | 10 | 10 | 7 | 12 | 4 | 17 | 10 | 13 | 2 | 17 | 9 | 5 |
| NM_001025367 | VEGFA | 3585 | 18 | 10 | 10 | 7 | 12 | 4 | 17 | 10 | 13 | 2 | 18 | 9 | 5 |
| NM_001025368 | VEGFA | 3531 | 18 | 10 | 10 | 7 | 12 | 4 | 17 | 10 | 13 | 2 | 18 | 10 | 5 |
| NM_001025369 | VEGFA | 3496 | 18 | 10 | 10 | 7 | 13 | 4 | 18 | 10 | 14 | 2 | 18 | 10 | 5 |
| NM_001025370 | VEGFA | 3399 | 18 | 10 | 10 | 7 | 13 | 4 | 18 | 11 | 14 | 2 | 18 | 10 | 5 |
| NM_001025389 | AMPD3 | 4334 | 4 | 1 | 1 | 0 | 0 | 1 | 1 | 0 | 1 | 0 | 4 | 1 | 0 |
| NM_001025390 | AMPD3 | 4473 | 4 | 1 | 1 | 0 | 0 | 1 | 1 | 0 | 1 | 0 | 4 | 1 | 0 |
| NM_001025433 | NQO1 | 2489 | 55 | 8 | 55 | 3 | 2 | 4 | 10 | 0 | 5 | 1 | 1 | 2 | 2 |
| NM_001025434 | NQO1 | 2477 | 53 | 8 | 53 | 3 | 2 | 4 | 10 | 0 | 5 | 1 | 1 | 2 | 2 |
| NM_001025436 | SPAG16 | 1250 | 10 | 4 | 3 | 2 | 3 | 10 | 6 | 2 | 3 | 6 | 1 | 2 | 10 |
| NM_001025579 | NDEL1 | 2410 | 16 | 8 | 9 | 4 | 8 | 16 | 5 | 5 | 8 | 11 | 5 | 10 | 8 |
| NM_001025580 | AMMECR1 | 5306 | 5 | 1 | 1 | 1 | 1 | 1 | 2 | 1 | 1 | 5 | 1 | 3 | 2 |
| NM_001025591 | SCGBL | 427 | 0 | 0 | 0 | 0 | 0 | 0 | 0 | 0 | 0 | 0 | 0 | 0 | 0 |
| NM_001025593 | ARFIP1 | 2994 | 4 | 3 | 2 | 2 | 2 | 2 | 4 | 2 | 2 | 3 | 1 | 4 | 4 |
| NM_001025595 | ARFIP1 | 3090 | 4 | 3 | 2 | 2 | 2 | 2 | 4 | 2 | 2 | 3 | 1 | 4 | 4 |
| NM_001025596 | CUGBP1 | 2009 | 10 | 7 | 5 | 7 | 6 | 10 | 8 | 9 | 6 | 5 | 7 | 10 | 8 |
| NM_001025598 | ARHGAP30 | 4425 | 52 | 9 | 11 | 5 | 6 | 4 | 2 | 1 | 13 | 2 | 1 | 52 | 3 |
| NM_001025603 | RFX5 | 3611 | 3 | 1 | 1 | 1 | 1 | 1 | 1 | 1 | 1 | 1 | 0 | 3 | 1 |
| NM_001025604 | ARRDC2 | 2525 | 8 | 2 | 3 | 1 | 1 | 3 | 1 | 1 | 3 | 0 | 8 | 3 | 1 |
| NM_001025616 | ARHGAP24 | 4644 | 13 | 5 | 7 | 1 | 5 | 5 | 11 | 1 | 5 | 2 | 2 | 13 | 2 |
| NM_001025778 | VRK3 | 1950 | 2 | 1 | 0 | 1 | 0 | 1 | 2 | 1 | 1 | 1 | 0 | 1 | 2 |
| NM_001025780 | FAM108B1 | 3104 | 6 | 3 | 1 | 1 | 2 | 6 | 3 | 2 | 2 | 2 | 2 | 3 | 4 |
| NM_001025930 | TTLL3 | 3402 | 1 | 1 | 1 | 1 | 0 | 1 | 0 | 0 | 1 | 1 | 0 | 1 | 1 |
| NM_001026 | RPS24 | 631 | 634 | 282 | 358 | 141 | 263 | 291 | 166 | 329 | 212 | 634 | 247 | 269 | 186 |
| NM_001026213 | CYP11B1 | 3337 | 0 | 0 | 0 | 0 | 0 | 0 | 0 | 0 | 0 | 0 | 0 | 0 | 0 |
| NM_001026383 | CCDC7 | 1926 | 10 | 4 | 1 | 1 | 4 | 6 | 2 | 1 | 3 | 8 | 4 | 4 | 10 |
| NM_001028 | RPS25 | 491 | 145 | 70 | 65 | 43 | 66 | 50 | 44 | 72 | 49 | 145 | 93 | 98 | 42 |
| NM_001029 | RPS26 | 682 | 1 | 0 | 0 | 1 | 0 | 0 | 0 | 1 | 1 | 0 | 0 | 1 | 0 |
| NM_001029835 | CCM2 | 1881 | 4 | 2 | 2 | 1 | 1 | 3 | 1 | 4 | 1 | 0 | 1 | 4 | 2 |
| NM_001029839 | C3orf23 | 2178 | 2 | 1 | 1 | 1 | 1 | 1 | 2 | 2 | 1 | 1 | 1 | 1 | 1 |
| NM_001029840 | C3orf23 | 1996 | 2 | 1 | 1 | 1 | 1 | 1 | 2 | 2 | 1 | 1 | 1 | 2 | 1 |
| NM_001029851 | PDE8B | 3261 | 3 | 1 | 2 | 0 | 1 | 3 | 1 | 0 | 0 | 0 | 0 | 0 | 1 |
| NM_001029852 | PDE8B | 3387 | 3 | 1 | 2 | 0 | 1 | 3 | 1 | 0 | 0 | 0 | 0 | 0 | 1 |
| NM_001029853 | PDE8B | 3492 | 3 | 1 | 2 | 0 | 1 | 3 | 1 | 0 | 0 | 0 | 0 | 0 | 1 |
| NM_001029854 | PDE8B | 3411 | 3 | 1 | 2 | 0 | 1 | 3 | 1 | 0 | 0 | 0 | 0 | 0 | 1 |
| NM_001029858 | SLC35F1 | 4852 | 5 | 1 | 0 | 1 | 0 | 5 | 0 | 0 | 0 | 0 | 0 | 0 | 0 |
| NM_001029859 | KCTD21 | 3378 | 2 | 1 | 1 | 1 | 2 | 2 | 1 | 2 | 1 | 2 | 1 | 1 | 1 |
| NM_001029860 | FBXO43 | 3211 | 1 | 0 | 0 | 0 | 0 | 0 | 0 | 0 | 0 | 0 | 0 | 0 | 1 |
| NM_001029861 | NANOS2 | 1564 | 0 | 0 | 0 | 0 | 0 | 0 | 0 | 0 | 0 | 0 | 0 | 0 | 0 |
| NM_001029863 | C6orf120 | 4146 | 4 | 2 | 2 | 0 | 2 | 2 | 3 | 3 | 1 | 3 | 1 | 4 | 2 |
| NM_001029864 | KIAA1755 | 6412 | 0 | 0 | 0 | 0 | 0 | 0 | 0 | 0 | 0 | 0 | 0 | 0 | 0 |
| NM_001029865 | DBX1 | 1149 | 0 | 0 | 0 | 0 | 0 | 0 | 0 | 0 | 0 | 0 | 0 | 0 | 0 |
| NM_001029869 | PLAC8L1 | 725 | 0 | 0 | 0 | 0 | 0 | 0 | 0 | 0 | 0 | 0 | 0 | 0 | 0 |
| NM_001029870 | ANKRD56 | 2921 | 2 | 0 | 0 | 1 | 0 | 1 | 1 | 2 | 0 | 0 | 0 | 0 | 0 |
| NM_001029871 | RSPO4 | 2707 | 1 | 0 | 0 | 0 | 0 | 0 | 0 | 0 | 1 | 0 | 0 | 0 | 0 |
| NM_001029874 | REP15 | 1139 | 4 | 1 | 0 | 4 | 2 | 1 | 1 | 1 | 0 | 2 | 0 | 1 | 1 |
| NM_001029875 | RGS7BP | 3816 | 11 | 1 | 0 | 0 | 0 | 11 | 0 | 0 | 0 | 1 | 0 | 0 | 1 |
| NM_001029880 | SFMBT2 | 4363 | 3 | 1 | 1 | 1 | 1 | 2 | 1 | 0 | 1 | 1 | 1 | 3 | 1 |
| NM_001029881 | CIB4 | 745 | 0 | 0 | 0 | 0 | 0 | 0 | 0 | 0 | 0 | 0 | 0 | 0 | 0 |
| NM_001029882 | AHDC1 | 6496 | 2 | 1 | 0 | 1 | 1 | 1 | 1 | 0 | 1 | 2 | 2 | 1 | 1 |
| NM_001029883 | C2orf71 | 7044 | 0 | 0 | 0 | 0 | 0 | 0 | 0 | 0 | 0 | 0 | 0 | 0 | 0 |
| NM_001029884 | PLEKHG1 | 7237 | 18 | 3 | 2 | 1 | 2 | 2 | 2 | 1 | 3 | 2 | 1 | 18 | 4 |
| NM_001029885 | GLTPD1 | 2193 | 0 | 0 | 0 | 0 | 0 | 0 | 0 | 0 | 0 | 0 | 0 | 0 | 0 |
| NM_001029886 | PFN3 | 530 | 0 | 0 | 0 | 0 | 0 | 0 | 0 | 0 | 0 | 0 | 0 | 0 | 0 |
| NM_001029887 | HELT | 984 | 0 | 0 | 0 | 0 | 0 | 0 | 0 | 0 | 0 | 0 | 0 | 0 | 0 |
| NM_001029888 | FAM24A | 596 | 1 | 0 | 0 | 0 | 0 | 0 | 0 | 0 | 0 | 0 | 0 | 0 | 1 |
| NM_001029891 | PGAM4 | 1678 | 1 | 1 | 1 | 1 | 0 | 1 | 1 | 1 | 0 | 0 | 0 | 0 | 1 |
| NM_001029896 | WDR45 | 1625 | 8 | 5 | 6 | 3 | 8 | 7 | 6 | 5 | 3 | 4 | 5 | 5 | 3 |
| NM_001029939 | ZFAT | 5473 | 2 | 1 | 1 | 0 | 0 | 1 | 2 | 0 | 0 | 1 | 0 | 1 | 1 |
| NM_001029954 | CDNF | 1330 | 8 | 1 | 0 | 0 | 1 | 1 | 1 | 2 | 0 | 1 | 8 | 0 | 2 |
| NM_001029955 | DCAF4L1 | 4772 | 11 | 1 | 0 | 0 | 0 | 0 | 0 | 0 | 0 | 0 | 0 | 0 | 11 |
| NM_001029964 | TTLL13 | 1793 | 1 | 0 | 0 | 0 | 0 | 0 | 0 | 0 | 0 | 0 | 0 | 0 | 1 |
| NM_001029976 | ZNF16 | 2616 | 1 | 1 | 0 | 0 | 0 | 1 | 1 | 0 | 0 | 1 | 1 | 0 | 1 |
| NM_001029989 | KIAA0101 | 1323 | 0 | 0 | 0 | 0 | 0 | 0 | 0 | 0 | 0 | 0 | 0 | 0 | 0 |
| NM_001029991 | METT11D1 | 1786 | 4 | 3 | 2 | 4 | 3 | 2 | 3 | 3 | 1 | 2 | 2 | 3 | 2 |
| NM_001029996 | PCDP1 | 2946 | 6 | 1 | 0 | 0 | 0 | 2 | 3 | 0 | 2 | 0 | 0 | 0 | 6 |
| NM_001029997 | ZNF181 | 2951 | 4 | 2 | 1 | 0 | 4 | 2 | 3 | 1 | 1 | 4 | 2 | 3 | 3 |

|  |  | transcript |  |  |  |  |  | hypothala |  |  |  |  | skeletalm |  |  |
|---|---|---|---|---|---|---|---|---|---|---|---|---|---|---|---|
| transcript | gene | length (nt) | max | mean | adipose | colon | heart | mus | kidney | liver | lung | ovary | uscle | spleen | testes |
| NM_001029998 | SLC10A7 | 3783 | 2 | 1 | 1 | 1 | 1 | 1 | 1 | 1 | 1 | 1 | 0 | 2 | 1 |
| NM_001030 | RPS27 | 351 | 151 | 60 | 53 | 44 | 64 | 34 | 35 | 37 | 62 | 151 | 52 | 95 | 32 |
| NM_001030001 | RPS29 | 722 | 180 | 99 | 118 | 161 | 64 | 100 | 36 | 180 | 71 | 127 | 63 | 130 | 42 |
| NM_001030002 | GRB7 | 2093 | 0 | 0 | 0 | 0 | 0 | 0 | 0 | 0 | 0 | 0 | 0 | 0 | 0 |
| NM_001030003 | HNF4A | 1339 | 2 | 0 | 0 | 1 | 0 | 0 | 1 | 2 | 0 | 0 | 0 | 0 | 0 |
| NM_001030004 | HNF4A | 1192 | 2 | 0 | 0 | 1 | 0 | 0 | 1 | 2 | 0 | 0 | 0 | 0 | 0 |
| NM_001030005 | CPLX3 | 2012 | 2 | 0 | 0 | 0 | 0 | 2 | 0 | 0 | 0 | 0 | 0 | 1 | 0 |
| NM_001030006 | AP2B1 | 5763 | 28 | 10 | 7 | 7 | 9 | 28 | 13 | 5 | 8 | 9 | 5 | 10 | 12 |
| NM_001030007 | AP1G1 | 6848 | 10 | 6 | 4 | 3 | 4 | 6 | 10 | 7 | 5 | 4 | 5 | 7 | 7 |
| NM_001030009 | RPS15A | 477 | 99 | 52 | 31 | 22 | 51 | 18 | 40 | 48 | 70 | 99 | 56 | 98 | 42 |
| NM_001030010 | ALDH3B1 | 2705 | 2 | 1 | 1 | 0 | 1 | 0 | 1 | 0 | 2 | 0 | 0 | 2 | 0 |
| NM_001030013 | NPS | 322 | 0 | 0 | 0 | 0 | 0 | 0 | 0 | 0 | 0 | 0 | 0 | 0 | 0 |
| NM_001030015 | OPN4 | 2405 | 0 | 0 | 0 | 0 | 0 | 0 | 0 | 0 | 0 | 0 | 0 | 0 | 0 |
| NM_001030018 | APRT | 673 | 1 | 1 | 1 | 1 | 0 | 0 | 1 | 1 | 0 | 0 | 0 | 1 | 0 |
| NM_001030019 | SUNC1 | 1436 | 5 | 0 | 0 | 0 | 0 | 0 | 0 | 0 | 0 | 0 | 0 | 0 | 5 |
| NM_001030047 | KLK3 | 1906 | 0 | 0 | 0 | 0 | 0 | 0 | 0 | 0 | 0 | 0 | 0 | 0 | 0 |
| NM_001030048 | KLK3 | 1335 | 0 | 0 | 0 | 0 | 0 | 0 | 0 | 0 | 0 | 0 | 0 | 0 | 0 |
| NM_001030049 | KLK3 | 1341 | 0 | 0 | 0 | 0 | 0 | 0 | 0 | 0 | 0 | 0 | 0 | 0 | 0 |
| NM_001030050 | KLK3 | 555 | 0 | 0 | 0 | 0 | 0 | 0 | 0 | 0 | 0 | 0 | 0 | 0 | 0 |
| NM_001030051 | OPN5 | 3441 | 1 | 0 | 0 | 0 | 0 | 0 | 0 | 0 | 0 | 0 | 0 | 0 | 1 |
| NM_001030055 | ARHGAP5 | 9604 | 18 | 10 | 5 | 2 | 18 | 14 | 11 | 5 | 8 | 12 | 7 | 11 | 14 |
| NM_001030059 | PPAPDC1A | 1521 | 1 | 0 | 0 | 0 | 0 | 1 | 0 | 0 | 0 | 0 | 0 | 0 | 0 |
| NM_001030060 | SAMD5 | 6089 | 4 | 1 | 0 | 0 | 0 | 0 | 4 | 1 | 1 | 0 | 0 | 0 | 1 |
| NM_001030272 | ARNTL | 2740 | 6 | 3 | 2 | 1 | 2 | 3 | 2 | 6 | 2 | 2 | 4 | 4 | 3 |
| NM_001030273 | ARNTL | 2811 | 6 | 3 | 2 | 1 | 2 | 3 | 2 | 6 | 2 | 2 | 4 | 4 | 3 |
| NM_001030287 | ATF3 | 1898 | 25 | 9 | 19 | 2 | 17 | 6 | 5 | 6 | 25 | 6 | 5 | 3 | 5 |
| NM_001030288 | SPN | 6910 | 2 | 1 | 0 | 1 | 0 | 0 | 0 | 0 | 2 | 0 | 0 | 2 | 0 |
| NM_001030311 | CERKL | 3286 | 1 | 0 | 0 | 0 | 0 | 0 | 1 | 0 | 0 | 0 | 0 | 0 | 0 |
| NM_001030312 | CERKL | 2869 | 1 | 0 | 0 | 0 | 0 | 0 | 1 | 0 | 0 | 0 | 0 | 0 | 0 |
| NM_001030313 | CERKL | 3001 | 1 | 0 | 0 | 0 | 0 | 0 | 1 | 0 | 0 | 0 | 0 | 0 | 0 |
| NM_001031 | RPS28 | 382 | 17 | 7 | 2 | 3 | 6 | 3 | 8 | 17 | 7 | 8 | 7 | 16 | 2 |
| NM_001031615 | ALDH3B2 | 2494 | 0 | 0 | 0 | 0 | 0 | 0 | 0 | 0 | 0 | 0 | 0 | 0 | 0 |
| NM_001031617 | COX19 | 4891 | 2 | 1 | 1 | 1 | 1 | 2 | 1 | 1 | 1 | 1 | 1 | 2 | 1 |
| NM_001031618 | SPDYE2 | 1983 | 0 | 0 | 0 | 0 | 0 | 0 | 0 | 0 | 0 | 0 | 0 | 0 | 0 |
| NM_001031623 | ZNF451 | 5241 | 9 | 6 | 5 | 2 | 5 | 7 | 5 | 4 | 6 | 8 | 3 | 8 | 9 |
| NM_001031628 | SMAGP | 1087 | 3 | 1 | 1 | 3 | 1 | 0 | 1 | 0 | 2 | 1 | 0 | 1 | 0 |
| NM_001031665 | ZNF816A | 2697 | 6 | 3 | 2 | 1 | 3 | 2 | 4 | 0 | 2 | 6 | 1 | 6 | 5 |
| NM_001031666 | MS4A3 | 1346 | 1 | 0 | 0 | 0 | 0 | 0 | 0 | 0 | 1 | 0 | 0 | 0 | 0 |
| NM_001031672 | CYB5RL | 3533 | 1 | 0 | 0 | 1 | 0 | 0 | 0 | 0 | 0 | 0 | 0 | 0 | 0 |
| NM_001031677 | RAB24 | 1612 | 6 | 2 | 2 | 2 | 1 | 6 | 4 | 2 | 1 | 1 | 1 | 5 | 1 |
| NM_001031679 | MSRB3 | 4352 | 13 | 5 | 8 | 5 | 13 | 2 | 2 | 0 | 4 | 4 | 5 | 4 | 4 |
| NM_001031680 | RUNX3 | 4340 | 4 | 1 | 0 | 0 | 0 | 0 | 0 | 0 | 2 | 0 | 0 | 4 | 0 |
| NM_001031681 | CTNS | 4042 | 1 | 0 | 0 | 1 | 0 | 0 | 1 | 0 | 0 | 0 | 0 | 0 | 1 |
| NM_001031683 | IFIT3 | 2453 | 13 | 5 | 7 | 2 | 5 | 6 | 4 | 1 | 7 | 6 | 13 | 5 | 3 |
| NM_001031684 | SFRS7 | 2487 | 9 | 6 | 5 | 3 | 4 | 8 | 6 | 3 | 6 | 8 | 3 | 9 | 7 |
| NM_001031685 | TP53BP2 | 4657 | 8 | 5 | 2 | 2 | 6 | 8 | 4 | 3 | 7 | 6 | 4 | 6 | 7 |
| NM_001031689 | PLAA | 4953 | 7 | 5 | 4 | 2 | 6 | 5 | 5 | 7 | 3 | 3 | 7 | 5 | 6 |
| NM_001031690 | FAM131B | 4377 | 2 | 0 | 0 | 0 | 0 | 2 | 0 | 0 | 0 | 0 | 0 | 0 | 0 |
| NM_001031692 | LRRC17 | 2215 | 13 | 2 | 1 | 0 | 1 | 0 | 0 | 0 | 0 | 13 | 0 | 0 | 2 |
| NM_001031693 | HHLA3 | 802 | 4 | 1 | 1 | 1 | 0 | 4 | 2 | 2 | 1 | 2 | 1 | 2 | 1 |
| NM_001031694 | SCMH1 | 3286 | 3 | 2 | 1 | 1 | 3 | 1 | 2 | 1 | 2 | 2 | 2 | 2 | 2 |
| NM_001031695 | RBM9 | 6977 | 17 | 7 | 5 | 5 | 7 | 13 | 5 | 3 | 5 | 17 | 6 | 4 | 6 |
| NM_001031696 | PLD3 | 2181 | 17 | 6 | 9 | 4 | 4 | 17 | 2 | 2 | 6 | 5 | 2 | 10 | 4 |
| NM_001031698 | PRPF40B | 3154 | 2 | 1 | 1 | 1 | 1 | 1 | 1 | 0 | 0 | 1 | 1 | 1 | 2 |
| NM_001031700 | FAM198B | 4818 | 29 | 9 | 14 | 2 | 20 | 5 | 3 | 1 | 5 | 29 | 1 | 11 | 6 |
| NM_001031701 | NT5DC3 | 7214 | 7 | 2 | 1 | 6 | 2 | 7 | 1 | 1 | 1 | 1 | 2 | 1 | 2 |
| NM_001031702 | SEMA5B | 4707 | 0 | 0 | 0 | 0 | 0 | 0 | 0 | 0 | 0 | 0 | 0 | 0 | 0 |
| NM_001031703 | C3orf75 | 1349 | 2 | 1 | 1 | 1 | 1 | 2 | 2 | 1 | 1 | 1 | 0 | 1 | 2 |
| NM_001031705 | CXorf48 | 1872 | 1 | 0 | 0 | 0 | 0 | 0 | 0 | 0 | 0 | 0 | 0 | 0 | 1 |
| NM_001031709 | RNLS | 2415 | 1 | 0 | 0 | 0 | 0 | 0 | 0 | 0 | 0 | 0 | 1 | 0 | 0 |
| NM_001031710 | KLHL7 | 3175 | 6 | 2 | 1 | 0 | 6 | 4 | 2 | 1 | 1 | 2 | 1 | 1 | 3 |
| NM_001031711 | ERGIC1 | 2936 | 29 | 8 | 7 | 5 | 6 | 4 | 9 | 29 | 8 | 5 | 5 | 12 | 5 |
| NM_001031712 | TRMT11 | 1951 | 5 | 3 | 1 | 1 | 3 | 5 | 4 | 3 | 2 | 5 | 2 | 3 | 3 |
| NM_001031713 | CCDC90A | 1229 | 16 | 10 | 12 | 4 | 15 | 14 | 16 | 16 | 7 | 4 | 6 | 10 | 5 |
| NM_001031714 | INF2 | 4653 | 2 | 1 | 1 | 2 | 0 | 1 | 0 | 1 | 1 | 0 | 0 | 1 | 1 |
| NM_001031715 | IQCH | 4238 | 2 | 0 | 0 | 0 | 0 | 0 | 1 | 0 | 0 | 0 | 0 | 0 | 2 |
| NM_001031716 | OBFC2A | 3848 | 16 | 4 | 9 | 2 | 2 | 2 | 3 | 1 | 8 | 2 | 1 | 16 | 4 |
| NM_001031717 | CRELD1 | 2402 | 2 | 1 | 2 | 2 | 1 | 2 | 1 | 2 | 1 | 1 | 1 | 1 | 1 |
| NM_001031719 | DHRS12 | 1324 | 1 | 1 | 1 | 1 | 0 | 0 | 1 | 1 | 1 | 1 | 0 | 1 | 1 |
| NM_001031720 | GSTCD | 2150 | 1 | 1 | 0 | 0 | 0 | 1 | 1 | 0 | 1 | 1 | 0 | 1 | 1 |
| NM_001031721 | ZNF613 | 2292 | 3 | 2 | 1 | 1 | 2 | 2 | 2 | 1 | 1 | 2 | 1 | 3 | 2 |
| NM_001031723 | DNAJB14 | 2764 | 6 | 3 | 2 | 1 | 3 | 6 | 4 | 2 | 5 | 4 | 1 | 4 | 2 |
| NM_001031725 | DDX59 | 2320 | 5 | 3 | 2 | 1 | 3 | 2 | 2 | 2 | 2 | 2 | 5 | 4 | 2 |
| NM_001031726 | C19orf12 | 4412 | 2 | 1 | 2 | 0 | 1 | 2 | 1 | 2 | 0 | 1 | 1 | 1 | 1 |
| NM_001031727 | MRI1 | 3177 | 1 | 1 | 0 | 1 | 0 | 1 | 1 | 1 | 0 | 1 | 1 | 1 | 0 |
| NM_001031730 | TSPAN18 | 1182 | 1 | 1 | 0 | 1 | 1 | 0 | 1 | 0 | 0 | 0 | 0 | 0 | 0 |
| NM_001031732 | YTHDC1 | 6249 | 26 | 15 | 12 | 7 | 12 | 18 | 14 | 6 | 15 | 26 | 13 | 19 | 19 |

| transcript | gene | transcript length (nt) | max | mean | adipose | colon | heart | hypothalamus | kidney | liver | lung | ovary | skeletalmuscle | spleen | testes |
|---|---|---|---|---|---|---|---|---|---|---|---|---|---|---|---|
| NM_001031733 | CALML4 | 4494 | 3 | 2 | 1 | 3 | 2 | 1 | 3 | 1 | 2 | 2 | 1 | 3 | 2 |
| NM_001031734 | FDX1L | 842 | 1 | 0 | 0 | 0 | 0 | 1 | 1 | 1 | 0 | 0 | 0 | 0 | 0 |
| NM_001031735 | C19orf36 | 961 | 1 | 0 | 0 | 0 | 0 | 0 | 0 | 0 | 0 | 0 | 0 | 0 | 1 |
| NM_001031737 | CCDC78 | 1610 | 0 | 0 | 0 | 0 | 0 | 0 | 0 | 0 | 0 | 0 | 0 | 0 | 0 |
| NM_001031738 | TMEM150A | 1553 | 1 | 0 | 0 | 0 | 0 | 0 | 1 | 1 | 0 | 0 | 0 | 1 | 0 |
| NM_001031739 | ASB9 | 1699 | 2 | 0 | 0 | 0 | 0 | 0 | 1 | 1 | 0 | 0 | 0 | 1 | 2 |
| NM_001031740 | MANEAL | 2581 | 2 | 0 | 0 | 0 | 0 | 2 | 0 | 1 | 0 | 0 | 0 | 0 | 0 |
| NM_001031743 | C6orf165 | 2215 | 9 | 1 | 0 | 0 | 0 | 2 | 0 | 0 | 1 | 0 | 0 | 0 | 9 |
| NM_001031745 | RIBC1 | 1414 | 2 | 0 | 0 | 0 | 0 | 0 | 0 | 0 | 0 | 0 | 0 | 0 | 2 |
| NM_001031746 | C10orf72 | 6354 | 3 | 1 | 2 | 1 | 1 | 1 | 1 | 0 | 1 | 3 | 0 | 0 | 2 |
| NM_001031748 | C12orf40 | 2791 | 2 | 0 | 0 | 0 | 0 | 0 | 0 | 0 | 0 | 0 | 0 | 0 | 2 |
| NM_001031749 | LYPD5 | 2501 | 1 | 0 | 0 | 0 | 0 | 1 | 0 | 0 | 0 | 0 | 0 | 1 | 0 |
| NM_001031800 | TIPRL | 849 | 5 | 3 | 2 | 1 | 3 | 5 | 2 | 4 | 2 | 2 | 1 | 3 | 3 |
| NM_001031801 | LIMK2 | 2914 | 4 | 2 | 1 | 3 | 2 | 2 | 2 | 1 | 4 | 1 | 1 | 3 | 3 |
| NM_001031803 | LLGL2 | 3548 | 1 | 0 | 0 | 1 | 0 | 0 | 0 | 0 | 0 | 0 | 0 | 0 | 0 |
| NM_001031804 | MAF | 6877 | 11 | 7 | 8 | 3 | 4 | 4 | 11 | 4 | 7 | 11 | 6 | 11 | 6 |
| NM_001031806 | ALDH3A2 | 3823 | 8 | 4 | 8 | 3 | 3 | 4 | 6 | 8 | 4 | 2 | 4 | 2 | 3 |
| NM_001031807 | LBXCOR1 | 2766 | 0 | 0 | 0 | 0 | 0 | 0 | 0 | 0 | 0 | 0 | 0 | 0 | 0 |
| NM_001031809 | MS4A3 | 1517 | 0 | 0 | 0 | 0 | 0 | 0 | 0 | 0 | 0 | 0 | 0 | 0 | 0 |
| NM_001031812 | CSNK1G3 | 4365 | 5 | 3 | 2 | 2 | 2 | 4 | 2 | 3 | 3 | 4 | 1 | 5 | 5 |
| NM_001031827 | BOLA2 | 1012 | 0 | 0 | 0 | 0 | 0 | 0 | 0 | 0 | 0 | 0 | 0 | 0 | 0 |
| NM_001031834 | RAB40AL | 1029 | 0 | 0 | 0 | 0 | 0 | 0 | 0 | 0 | 0 | 0 | 0 | 0 | 0 |
| NM_001031835 | PHKB | 5603 | 6 | 4 | 4 | 2 | 5 | 4 | 5 | 3 | 3 | 3 | 6 | 5 | 5 |
| NM_001031836 | KCNU1 | 3742 | 2 | 0 | 0 | 0 | 0 | 0 | 0 | 0 | 0 | 0 | 0 | 0 | 2 |
| NM_001031847 | CPT1A | 2637 | 4 | 2 | 1 | 3 | 2 | 2 | 4 | 2 | 1 | 2 | 1 | 3 | 1 |
| NM_001031848 | SERPINB8 | 1268 | 1 | 1 | 1 | 1 | 1 | 0 | 0 | 1 | 1 | 0 | 0 | 1 | 0 |
| NM_001031849 | MASP1 | 1990 | 7 | 2 | 0 | 1 | 6 | 3 | 1 | 7 | 0 | 0 | 0 | 0 | 0 |
| NM_001031850 | PSG6 | 1698 | 0 | 0 | 0 | 0 | 0 | 0 | 0 | 0 | 0 | 0 | 0 | 0 | 0 |
| NM_001031853 | INSC | 2954 | 0 | 0 | 0 | 0 | 0 | 0 | 0 | 0 | 0 | 0 | 0 | 0 | 0 |
| NM_001031854 | ACCSL | 1820 | 0 | 0 | 0 | 0 | 0 | 0 | 0 | 0 | 0 | 0 | 0 | 0 | 0 |
| NM_001031855 | LONRF3 | 2607 | 7 | 2 | 1 | 1 | 2 | 1 | 1 | 4 | 4 | 1 | 7 | 1 | 2 |
| NM_001032 | RPS29 | 296 | 437 | 239 | 284 | 391 | 153 | 240 | 84 | 437 | 169 | 305 | 152 | 312 | 101 |
| NM_001032221 | STXBP1 | 3841 | 33 | 4 | 2 | 1 | 2 | 33 | 1 | 0 | 3 | 2 | 0 | 1 | 1 |
| NM_001032278 | MMP28 | 1021 | 1 | 0 | 1 | 0 | 0 | 1 | 0 | 0 | 1 | 0 | 0 | 0 | 1 |
| NM_001032279 | RCE1 | 1467 | 0 | 0 | 0 | 0 | 0 | 0 | 0 | 0 | 0 | 0 | 0 | 0 | 0 |
| NM_001032280 | TFAP2A | 3808 | 1 | 0 | 0 | 0 | 0 | 0 | 1 | 0 | 0 | 0 | 0 | 0 | 0 |
| NM_001032281 | TFPI | 1148 | 100 | 28 | 16 | 5 | 28 | 2 | 26 | 62 | 100 | 14 | 11 | 26 | 14 |
| NM_001032282 | KLF10 | 3231 | 37 | 11 | 7 | 5 | 12 | 3 | 11 | 6 | 10 | 11 | 37 | 13 | 9 |
| NM_001032283 | TMPO | 2701 | 16 | 7 | 3 | 6 | 4 | 5 | 5 | 9 | 7 | 11 | 4 | 16 | 7 |
| NM_001032284 | TMPO | 2374 | 11 | 5 | 2 | 4 | 2 | 4 | 4 | 4 | 5 | 8 | 3 | 11 | 4 |
| NM_001032287 | NR2C1 | 1870 | 10 | 6 | 6 | 6 | 5 | 5 | 5 | 5 | 4 | 6 | 5 | 6 | 10 |
| NM_001032288 | UBE2V1 | 2133 | 0 | 0 | 0 | 0 | 0 | 0 | 0 | 0 | 0 | 0 | 0 | 0 | 0 |
| NM_001032289 | SLC35A2 | 1726 | 1 | 0 | 0 | 0 | 0 | 1 | 1 | 1 | 0 | 0 | 0 | 0 | 0 |
| NM_001032290 | PSRC1 | 1811 | 10 | 2 | 0 | 2 | 0 | 10 | 0 | 1 | 1 | 1 | 0 | 1 | 4 |
| NM_001032291 | PSRC1 | 1721 | 11 | 2 | 0 | 2 | 0 | 11 | 0 | 1 | 1 | 1 | 0 | 1 | 4 |
| NM_001032292 | ZNF174 | 1574 | 2 | 1 | 1 | 1 | 2 | 2 | 1 | 1 | 1 | 1 | 1 | 2 | 2 |
| NM_001032293 | ZNF207 | 2230 | 66 | 29 | 16 | 17 | 30 | 21 | 32 | 21 | 34 | 33 | 24 | 66 | 29 |
| NM_001032295 | SERPING1 | 1814 | 571 | 92 | 60 | 20 | 54 | 12 | 23 | 571 | 64 | 90 | 27 | 45 | 40 |
| NM_001032296 | STK24 | 4624 | 6 | 4 | 3 | 4 | 3 | 4 | 5 | 2 | 4 | 3 | 2 | 6 | 5 |
| NM_001032363 | C1orf151 | 2589 | 4 | 2 | 2 | 2 | 4 | 4 | 3 | 2 | 2 | 1 | 1 | 2 | 1 |
| NM_001032364 | GGT1 | 2302 | 2 | 0 | 0 | 0 | 0 | 0 | 2 | 1 | 0 | 0 | 0 | 0 | 0 |
| NM_001032365 | GGT1 | 2346 | 2 | 0 | 0 | 0 | 0 | 0 | 2 | 1 | 0 | 0 | 0 | 0 | 0 |
| NM_001032367 | SPINT1 | 2332 | 11 | 3 | 1 | 11 | 0 | 1 | 10 | 0 | 5 | 0 | 0 | 1 | 1 |
| NM_001032372 | ZNF226 | 2603 | 8 | 4 | 2 | 2 | 3 | 5 | 4 | 1 | 2 | 8 | 3 | 7 | 5 |
| NM_001032373 | ZNF226 | 2567 | 8 | 4 | 2 | 2 | 4 | 5 | 4 | 1 | 2 | 8 | 3 | 7 | 5 |
| NM_001032374 | ZNF226 | 994 | 2 | 1 | 1 | 1 | 1 | 2 | 1 | 1 | 0 | 1 | 1 | 1 | 2 |
| NM_001032377 | SULT6B1 | 934 | 0 | 0 | 0 | 0 | 0 | 0 | 0 | 0 | 0 | 0 | 0 | 0 | 0 |
| NM_001032381 | PQBP1 | 978 | 11 | 8 | 8 | 9 | 6 | 11 | 8 | 11 | 7 | 9 | 6 | 8 | 6 |
| NM_001032382 | PQBP1 | 1046 | 11 | 8 | 7 | 9 | 6 | 10 | 7 | 11 | 7 | 8 | 5 | 8 | 6 |
| NM_001032383 | PQBP1 | 1057 | 11 | 8 | 7 | 9 | 6 | 10 | 7 | 11 | 7 | 8 | 5 | 8 | 6 |
| NM_001032384 | PQBP1 | 966 | 11 | 7 | 7 | 9 | 6 | 11 | 7 | 10 | 6 | 7 | 5 | 7 | 6 |
| NM_001032386 | SUOX | 2336 | 6 | 2 | 2 | 2 | 2 | 2 | 6 | 3 | 1 | 1 | 1 | 1 | 2 |
| NM_001032387 | SUOX | 2227 | 6 | 2 | 2 | 2 | 2 | 2 | 6 | 4 | 1 | 1 | 1 | 1 | 2 |
| NM_001032391 | LCMT1 | 1205 | 7 | 5 | 2 | 1 | 6 | 7 | 6 | 1 | 4 | 7 | 7 | 4 | 7 |
| NM_001032392 | PLGLB1 | 3010 | 0 | 0 | 0 | 0 | 0 | 0 | 0 | 0 | 0 | 0 | 0 | 0 | 0 |
| NM_001032392 | PLGLB1 | 3011 | 0 | 0 | 0 | 0 | 0 | 0 | 0 | 0 | 0 | 0 | 0 | 0 | 0 |
| NM_001032393 | HNRNPH2 | 2299 | 45 | 27 | 19 | 11 | 28 | 45 | 32 | 29 | 24 | 20 | 26 | 36 | 25 |
| NM_001032394 | GPR126 | 6877 | 7 | 2 | 1 | 1 | 1 | 0 | 2 | 7 | 3 | 0 | 0 | 3 | 1 |
| NM_001032395 | GPR126 | 6831 | 7 | 2 | 1 | 1 | 1 | 0 | 2 | 7 | 3 | 0 | 0 | 3 | 1 |
| NM_001032396 | PJA1 | 2338 | 18 | 6 | 8 | 3 | 4 | 18 | 4 | 1 | 3 | 7 | 1 | 5 | 14 |
| NM_001032409 | OAS1 | 1565 | 4 | 2 | 4 | 2 | 1 | 1 | 2 | 2 | 2 | 1 | 2 | 3 | 1 |
| NM_001032410 | USP16 | 3036 | 19 | 13 | 12 | 4 | 18 | 11 | 13 | 12 | 11 | 14 | 13 | 17 | 19 |
| NM_001032726 | RAB41 | 1028 | 0 | 0 | 0 | 0 | 0 | 0 | 0 | 0 | 0 | 0 | 0 | 0 | 0 |
| NM_001032730 | USP48 | 2412 | 7 | 4 | 4 | 3 | 4 | 5 | 4 | 2 | 4 | 7 | 2 | 7 | 5 |
| NM_001032731 | OAS2 | 2085 | 3 | 1 | 1 | 0 | 1 | 0 | 0 | 0 | 1 | 1 | 1 | 3 | 0 |
| NM_001032998 | KYNU | 1288 | 5 | 1 | 1 | 0 | 1 | 0 | 1 | 5 | 2 | 0 | 0 | 2 | 1 |
| NM_001032999 | CBFA2T2 | 7319 | 2 | 1 | 1 | 2 | 1 | 2 | 1 | 0 | 1 | 2 | 1 | 1 | 2 |

| transcript | gene | transcript length (nt) | max | mean | adipose | colon | heart | hypothalamus | kidney | liver | lung | ovary | skeletalmuscle | spleen | testes |
|---|---|---|---|---|---|---|---|---|---|---|---|---|---|---|---|
| NM_001033 | RRM1 | 3191 | 7 | 4 | 5 | 3 | 5 | 4 | 4 | 3 | 4 | 4 | 7 | 4 | 5 |
| NM_001033002 | RPAIN | 1679 | 6 | 3 | 2 | 2 | 5 | 6 | 3 | 2 | 3 | 2 | 4 | 3 | 4 |
| NM_001033017 | DEFB135 | 234 | 0 | 0 | 0 | 0 | 0 | 0 | 0 | 0 | 0 | 0 | 0 | 0 | 0 |
| NM_001033018 | DEFB136 | 237 | 0 | 0 | 0 | 0 | 0 | 0 | 0 | 0 | 0 | 0 | 0 | 0 | 0 |
| NM_001033019 | DEFB134 | 201 | 1 | 0 | 0 | 0 | 0 | 0 | 0 | 0 | 0 | 0 | 0 | 0 | 1 |
| NM_001033024 | FBXO7 | 1758 | 25 | 17 | 14 | 9 | 13 | 25 | 20 | 17 | 17 | 19 | 7 | 24 | 25 |
| NM_001033025 | EXTL2 | 2851 | 3 | 1 | 1 | 0 | 1 | 3 | 2 | 1 | 1 | 2 | 1 | 1 | 2 |
| NM_001033026 | C19orf6 | 2708 | 1 | 1 | 0 | 1 | 1 | 1 | 1 | 1 | 1 | 0 | 1 | 1 | 1 |
| NM_001033028 | CYFIP1 | 3334 | 8 | 5 | 6 | 4 | 5 | 5 | 5 | 5 | 5 | 4 | 2 | 8 | 4 |
| NM_001033030 | FAIM | 1526 | 6 | 3 | 6 | 1 | 2 | 5 | 4 | 3 | 2 | 2 | 1 | 2 | 2 |
| NM_001033031 | FAIM | 1152 | 8 | 4 | 8 | 2 | 2 | 7 | 5 | 4 | 3 | 3 | 1 | 2 | 3 |
| NM_001033032 | FAIM | 1092 | 9 | 4 | 9 | 2 | 2 | 7 | 5 | 4 | 3 | 3 | 1 | 2 | 3 |
| NM_001033044 | GLUL | 4363 | 194 | 78 | 63 | 19 | 83 | 139 | 15 | 46 | 107 | 32 | 107 | 194 | 49 |
| NM_001033045 | GPR155 | 7473 | 5 | 2 | 1 | 1 | 1 | 5 | 3 | 1 | 1 | 1 | 1 | 1 | 1 |
| NM_001033046 | C17orf62 | 2082 | 2 | 1 | 0 | 1 | 1 | 1 | 1 | 1 | 1 | 0 | 0 | 2 | 0 |
| NM_001033047 | NPNT | 4584 | 29 | 6 | 1 | 5 | 4 | 2 | 16 | 0 | 29 | 2 | 2 | 6 | 3 |
| NM_001033050 | MTERFD3 | 1775 | 5 | 4 | 5 | 2 | 5 | 5 | 5 | 5 | 2 | 3 | 2 | 3 | 5 |
| NM_001033053 | NLRP1 | 5098 | 4 | 2 | 3 | 2 | 1 | 0 | 0 | 0 | 3 | 1 | 1 | 4 | 1 |
| NM_001033054 | AIPL1 | 2782 | 0 | 0 | 0 | 0 | 0 | 0 | 0 | 0 | 0 | 0 | 0 | 0 | 0 |
| NM_001033055 | AIPL1 | 2791 | 0 | 0 | 0 | 0 | 0 | 0 | 0 | 0 | 0 | 0 | 0 | 0 | 0 |
| NM_001033056 | GLUL | 4065 | 208 | 83 | 68 | 20 | 90 | 149 | 16 | 49 | 115 | 34 | 115 | 208 | 53 |
| NM_001033057 | MAGI1 | 7062 | 5 | 3 | 3 | 4 | 3 | 4 | 4 | 4 | 4 | 1 | 1 | 5 | 3 |
| NM_001033059 | AMD1 | 3103 | 12 | 8 | 2 | 3 | 11 | 12 | 8 | 6 | 9 | 7 | 5 | 11 | 11 |
| NM_001033080 | TAAR2 | 1056 | 0 | 0 | 0 | 0 | 0 | 0 | 0 | 0 | 0 | 0 | 0 | 0 | 0 |
| NM_001033081 | MYCL1 | 3622 | 1 | 0 | 0 | 1 | 0 | 0 | 0 | 1 | 1 | 0 | 0 | 1 | 0 |
| NM_001033082 | MYCL1 | 3258 | 1 | 1 | 0 | 1 | 0 | 1 | 0 | 1 | 1 | 0 | 0 | 1 | 0 |
| NM_001033083 | PBLD | 1224 | 15 | 3 | 1 | 3 | 0 | 1 | 15 | 10 | 0 | 0 | 0 | 0 | 1 |
| NM_001033087 | MACROD2 | 4424 | 4 | 1 | 0 | 0 | 0 | 2 | 0 | 0 | 4 | 1 | 0 | 0 | 1 |
| NM_001033088 | NGRN | 1349 | 28 | 9 | 7 | 4 | 11 | 28 | 6 | 7 | 4 | 5 | 12 | 4 | 7 |
| NM_001033112 | PAIP2 | 1504 | 59 | 24 | 23 | 8 | 31 | 59 | 13 | 19 | 17 | 19 | 18 | 26 | 28 |
| NM_001033113 | ENTPD8 | 2222 | 0 | 0 | 0 | 0 | 0 | 0 | 0 | 0 | 0 | 0 | 0 | 0 | 0 |
| NM_001033117 | SRGAP3 | 8829 | 4 | 1 | 0 | 0 | 0 | 4 | 1 | 0 | 0 | 1 | 0 | 0 | 1 |
| NM_001033503 | SAR1B | 1365 | 178 | 49 | 38 | 16 | 42 | 53 | 35 | 178 | 19 | 16 | 92 | 27 | 28 |
| NM_001033504 | TMEM98 | 1719 | 2 | 1 | 0 | 1 | 1 | 1 | 1 | 0 | 1 | 2 | 0 | 0 | 1 |
| NM_001033505 | CSTF3 | 627 | 6 | 3 | 2 | 1 | 3 | 4 | 4 | 2 | 2 | 2 | 2 | 2 | 6 |
| NM_001033506 | CSTF3 | 749 | 5 | 2 | 2 | 1 | 2 | 4 | 3 | 1 | 2 | 2 | 1 | 1 | 5 |
| NM_001033507 | LMX1A | 2790 | 0 | 0 | 0 | 0 | 0 | 0 | 0 | 0 | 0 | 0 | 0 | 0 | 0 |
| NM_001033515 | LOC100132 | 1599 | 2 | 1 | 1 | 1 | 2 | 2 | 1 | 1 | 1 | 2 | 1 | 1 | 2 |
| NM_001033517 | SERINC4 | 1490 | 2 | 1 | 1 | 2 | 0 | 0 | 1 | 0 | 1 | 1 | 0 | 1 | 1 |
| NM_001033518 | WIPI2 | 4456 | 5 | 3 | 3 | 2 | 3 | 5 | 3 | 3 | 2 | 3 | 1 | 5 | 5 |
| NM_001033519 | WIPI2 | 4402 | 5 | 3 | 3 | 2 | 3 | 5 | 3 | 3 | 2 | 3 | 1 | 5 | 5 |
| NM_001033520 | WIPI2 | 4295 | 5 | 3 | 3 | 2 | 3 | 5 | 4 | 3 | 2 | 3 | 1 | 5 | 5 |
| NM_001033521 | CSTF1 | 2295 | 3 | 2 | 1 | 1 | 1 | 2 | 2 | 3 | 1 | 2 | 1 | 2 | 3 |
| NM_001033522 | CSTF1 | 2292 | 3 | 2 | 1 | 1 | 1 | 2 | 2 | 3 | 1 | 2 | 1 | 2 | 3 |
| NM_001033549 | C19orf62 | 1451 | 3 | 2 | 1 | 1 | 2 | 3 | 1 | 1 | 1 | 1 | 2 | 2 | 1 |
| NM_001033551 | TOM1L2 | 5658 | 6 | 2 | 2 | 2 | 4 | 6 | 3 | 1 | 1 | 1 | 2 | 1 | 3 |
| NM_001033553 | CYTSB | 3947 | 19 | 9 | 8 | 6 | 4 | 19 | 3 | 0 | 5 | 19 | 2 | 12 | 16 |
| NM_001033554 | CYTSB | 2535 | 29 | 13 | 13 | 9 | 6 | 28 | 5 | 0 | 8 | 29 | 4 | 18 | 25 |
| NM_001033555 | CYTSB | 3761 | 20 | 9 | 9 | 6 | 4 | 20 | 3 | 0 | 6 | 20 | 3 | 13 | 17 |
| NM_001033556 | PPM1B | 1497 | 67 | 41 | 35 | 18 | 38 | 53 | 29 | 56 | 34 | 28 | 67 | 52 | 44 |
| NM_001033557 | PPM1B | 3042 | 41 | 24 | 21 | 10 | 23 | 31 | 17 | 32 | 19 | 16 | 41 | 31 | 25 |
| NM_001033559 | DYX1C1 | 1887 | 5 | 1 | 1 | 0 | 0 | 2 | 2 | 0 | 2 | 1 | 0 | 1 | 5 |
| NM_001033560 | DYX1C1 | 1793 | 4 | 1 | 1 | 0 | 0 | 1 | 2 | 0 | 2 | 1 | 0 | 1 | 4 |
| NM_001033561 | PHF12 | 4475 | 3 | 2 | 1 | 1 | 2 | 1 | 1 | 1 | 3 | 2 | 2 | 3 | 2 |
| NM_001033564 | C6orf225 | 2671 | 6 | 2 | 0 | 0 | 2 | 6 | 2 | 0 | 1 | 2 | 0 | 0 | 4 |
| NM_001033566 | RHOT1 | 3213 | 23 | 13 | 9 | 5 | 18 | 23 | 13 | 7 | 15 | 13 | 12 | 16 | 15 |
| NM_001033568 | RHOT1 | 3309 | 22 | 13 | 9 | 5 | 18 | 22 | 12 | 6 | 15 | 13 | 12 | 16 | 15 |
| NM_001033569 | AMZ2 | 1427 | 10 | 4 | 7 | 1 | 3 | 10 | 3 | 3 | 3 | 3 | 4 | 3 | 8 |
| NM_001033570 | AMZ2 | 1423 | 10 | 4 | 7 | 1 | 3 | 10 | 3 | 3 | 3 | 3 | 4 | 3 | 8 |
| NM_001033571 | AMZ2 | 1399 | 10 | 4 | 7 | 1 | 4 | 10 | 4 | 3 | 3 | 3 | 4 | 3 | 8 |
| NM_001033572 | AMZ2 | 1395 | 10 | 4 | 7 | 1 | 4 | 10 | 4 | 3 | 3 | 3 | 4 | 3 | 8 |
| NM_001033574 | AMZ2 | 1806 | 8 | 4 | 6 | 1 | 3 | 8 | 4 | 2 | 3 | 2 | 3 | 3 | 7 |
| NM_001033575 | DUSP28 | 1555 | 0 | 0 | 0 | 0 | 0 | 0 | 0 | 0 | 0 | 0 | 0 | 0 | 0 |
| NM_001033576 | UNC45B | 5673 | 8 | 1 | 0 | 0 | 6 | 0 | 0 | 0 | 0 | 0 | 8 | 0 | 0 |
| NM_001033578 | SGK3 | 4231 | 2 | 1 | 1 | 1 | 1 | 2 | 2 | 2 | 2 | 1 | 1 | 2 | 2 |
| NM_001033580 | MYO19 | 2240 | 1 | 1 | 0 | 1 | 0 | 0 | 1 | 1 | 0 | 0 | 0 | 1 | 1 |
| NM_001033581 | PRKCZ | 2114 | 4 | 1 | 0 | 2 | 1 | 4 | 1 | 0 | 1 | 0 | 0 | 1 | 1 |
| NM_001033582 | PRKCZ | 2011 | 4 | 1 | 0 | 2 | 1 | 4 | 1 | 0 | 2 | 0 | 0 | 1 | 1 |
| NM_001033583 | ACOT9 | 1700 | 17 | 7 | 9 | 4 | 17 | 5 | 5 | 6 | 7 | 4 | 7 | 8 | 5 |
| NM_001033602 | MTUS2 | 6939 | 7 | 1 | 0 | 0 | 7 | 1 | 0 | 0 | 0 | 1 | 0 | 0 | 0 |
| NM_001033604 | BBS9 | 3914 | 6 | 3 | 2 | 1 | 3 | 3 | 3 | 1 | 3 | 6 | 1 | 3 | 5 |
| NM_001033605 | BBS9 | 4004 | 7 | 3 | 2 | 1 | 3 | 3 | 3 | 1 | 3 | 7 | 2 | 3 | 6 |
| NM_001033667 | LY9 | 1408 | 2 | 0 | 1 | 0 | 0 | 0 | 0 | 0 | 0 | 0 | 0 | 2 | 0 |
| NM_001033677 | CABP1 | 1604 | 7 | 1 | 0 | 0 | 0 | 7 | 1 | 0 | 0 | 0 | 0 | 0 | 0 |
| NM_001033678 | TRPT1 | 1052 | 3 | 1 | 1 | 0 | 2 | 1 | 1 | 1 | 0 | 0 | 3 | 1 | 1 |
| NM_001033714 | NOP2 | 2729 | 4 | 1 | 1 | 1 | 1 | 1 | 1 | 4 | 1 | 0 | 2 | 2 | 1 |
| NM_001033719 | ZNF404 | 1842 | 2 | 1 | 0 | 0 | 1 | 2 | 0 | 0 | 1 | 1 | 1 | 1 | 1 |

| transcript | gene | transcript length (nt) | max | mean | adipose | colon | heart | hypothalamus | kidney | liver | lung | ovary | skeletalmuscle | spleen | testes |
|---|---|---|---|---|---|---|---|---|---|---|---|---|---|---|---|
| NM_001033723 | ZNF704 | 4303 | 13 | 4 | 4 | 3 | 5 | 3 | 5 | 0 | 3 | 13 | 2 | 1 | 4 |
| NM_001033756 | VEGFA | 3465 | 18 | 10 | 10 | 7 | 13 | 4 | 18 | 11 | 14 | 2 | 18 | 10 | 5 |
| NM_001033853 | RPL3 | 1191 | 492 | 168 | 132 | 150 | 114 | 125 | 117 | 178 | 148 | 492 | 42 | 219 | 131 |
| NM_001033855 | DCLRE1C | 3700 | 4 | 1 | 1 | 1 | 1 | 1 | 1 | 0 | 1 | 1 | 1 | 4 | 1 |
| NM_001033857 | DCLRE1C | 3785 | 4 | 1 | 1 | 1 | 1 | 1 | 1 | 0 | 1 | 1 | 1 | 4 | 1 |
| NM_001033858 | DCLRE1C | 4107 | 3 | 1 | 1 | 1 | 1 | 1 | 1 | 0 | 1 | 1 | 1 | 3 | 1 |
| NM_001033859 | ACADVL | 2222 | 8 | 4 | 7 | 5 | 8 | 2 | 7 | 6 | 2 | 2 | 4 | 2 | 2 |
| NM_001033873 | SMAGP | 1076 | 3 | 1 | 1 | 3 | 1 | 0 | 1 | 0 | 2 | 1 | 0 | 1 | 0 |
| NM_001033886 | CXCL12 | 471 | 25 | 9 | 16 | 5 | 25 | 11 | 6 | 2 | 4 | 5 | 6 | 21 | 3 |
| NM_001033910 | TRAF5 | 3999 | 6 | 1 | 1 | 2 | 1 | 1 | 1 | 0 | 1 | 1 | 0 | 6 | 2 |
| NM_001033925 | TIAL1 | 3942 | 7 | 5 | 4 | 2 | 5 | 5 | 6 | 5 | 4 | 7 | 6 | 7 | 5 |
| NM_001033930 | UBA52 | 2811 | 17 | 10 | 5 | 9 | 7 | 9 | 6 | 7 | 8 | 15 | 14 | 17 | 8 |
| NM_001033952 | CALCA | 796 | 1 | 0 | 0 | 0 | 0 | 0 | 0 | 0 | 0 | 0 | 0 | 1 | 0 |
| NM_001033953 | CALCA | 915 | 0 | 0 | 0 | 0 | 0 | 0 | 0 | 0 | 0 | 0 | 0 | 0 | 0 |
| NM_001034 | RRM2 | 3266 | 7 | 2 | 3 | 6 | 1 | 0 | 1 | 0 | 3 | 1 | 0 | 7 | 3 |
| NM_001034023 | DMAP1 | 1585 | 2 | 1 | 1 | 1 | 1 | 1 | 1 | 1 | 1 | 1 | 1 | 2 | 2 |
| NM_001034024 | DMAP1 | 1545 | 2 | 1 | 1 | 1 | 1 | 1 | 1 | 1 | 1 | 1 | 1 | 2 | 2 |
| NM_001034025 | ERP29 | 1333 | 71 | 28 | 22 | 23 | 18 | 29 | 31 | 71 | 26 | 19 | 6 | 39 | 22 |
| NM_001034077 | HIST2H4B | 396 | 0 | 0 | 0 | 0 | 0 | 0 | 0 | 0 | 0 | 0 | 0 | 0 | 0 |
| NM_001034116 | EIF2B4 | 1763 | 4 | 2 | 2 | 3 | 4 | 3 | 3 | 3 | 1 | 1 | 3 | 2 | 2 |
| NM_001034172 | MCART2 | 1185 | 0 | 0 | 0 | 0 | 0 | 0 | 0 | 0 | 0 | 0 | 0 | 0 | 0 |
| NM_001034173 | ALDH1L2 | 7537 | 3 | 1 | 2 | 1 | 3 | 1 | 0 | 0 | 1 | 0 | 0 | 1 | 1 |
| NM_001034191 | PPA2 | 1619 | 6 | 3 | 3 | 4 | 5 | 4 | 6 | 5 | 3 | 2 | 1 | 4 | 2 |
| NM_001034194 | EXOSC9 | 1644 | 2 | 1 | 2 | 1 | 1 | 2 | 1 | 1 | 1 | 1 | 1 | 1 | 2 |
| NM_001034832 | SSX4B | 1213 | 0 | 0 | 0 | 0 | 0 | 0 | 0 | 0 | 0 | 0 | 0 | 0 | 0 |
| NM_001034833 | NHP2 | 747 | 4 | 2 | 2 | 2 | 2 | 3 | 3 | 4 | 2 | 2 | 2 | 3 | 2 |
| NM_001034836 | RDM1 | 815 | 1 | 0 | 0 | 0 | 0 | 0 | 0 | 0 | 0 | 1 | 0 | 1 | 1 |
| NM_001034837 | KCNIP1 | 2039 | 1 | 0 | 0 | 0 | 0 | 1 | 0 | 0 | 0 | 0 | 0 | 0 | 0 |
| NM_001034838 | KCNIP1 | 1504 | 2 | 0 | 0 | 0 | 0 | 2 | 0 | 0 | 0 | 0 | 0 | 0 | 0 |
| NM_001034841 | ITPRIPL2 | 7695 | 9 | 5 | 8 | 4 | 6 | 3 | 4 | 2 | 6 | 9 | 2 | 5 | 4 |
| NM_001034842 | PTCHD3 | 2528 | 0 | 0 | 0 | 0 | 0 | 0 | 0 | 0 | 0 | 0 | 0 | 0 | 0 |
| NM_001034845 | GALNTL6 | 2849 | 1 | 0 | 0 | 0 | 0 | 1 | 0 | 0 | 0 | 0 | 0 | 0 | 0 |
| NM_001034850 | FAM134B | 3235 | 51 | 12 | 3 | 1 | 26 | 14 | 19 | 4 | 3 | 1 | 51 | 1 | 6 |
| NM_001034852 | SMOC1 | 3669 | 6 | 1 | 0 | 0 | 0 | 3 | 0 | 6 | 0 | 0 | 0 | 2 | 2 |
| NM_001034853 | RPGR | 4718 | 5 | 2 | 1 | 2 | 2 | 1 | 2 | 1 | 5 | 2 | 1 | 3 | 3 |
| NM_001034914 | KCNIP3 | 2721 | 1 | 0 | 0 | 1 | 0 | 1 | 0 | 0 | 0 | 0 | 0 | 0 | 1 |
| NM_001034915 | ESRP1 | 3794 | 6 | 1 | 0 | 6 | 0 | 0 | 2 | 0 | 3 | 0 | 0 | 0 | 1 |
| NM_001034954 | SORBS1 | 7210 | 97 | 26 | 97 | 22 | 62 | 23 | 5 | 12 | 8 | 2 | 39 | 3 | 12 |
| NM_001034955 | SORBS1 | 6787 | 103 | 27 | 103 | 23 | 66 | 24 | 6 | 13 | 8 | 2 | 41 | 3 | 12 |
| NM_001034956 | SORBS1 | 6049 | 115 | 30 | 115 | 26 | 68 | 26 | 6 | 15 | 9 | 3 | 40 | 4 | 13 |
| NM_001034957 | SORBS1 | 5554 | 124 | 32 | 124 | 28 | 72 | 28 | 7 | 16 | 10 | 3 | 42 | 4 | 15 |
| NM_001034996 | RPL14 | 917 | 264 | 120 | 105 | 88 | 110 | 87 | 73 | 154 | 103 | 264 | 126 | 150 | 65 |
| NM_001035 | RYR2 | 16365 | 88 | 9 | 0 | 1 | 88 | 7 | 0 | 0 | 1 | 3 | 0 | 0 | 1 |
| NM_001035003 | KCNIP4 | 2402 | 7 | 1 | 0 | 0 | 0 | 7 | 1 | 0 | 0 | 1 | 0 | 0 | 0 |
| NM_001035004 | KCNIP4 | 2323 | 7 | 1 | 0 | 0 | 0 | 7 | 3 | 0 | 0 | 1 | 0 | 0 | 0 |
| NM_001035005 | C18orf32 | 1128 | 31 | 13 | 9 | 4 | 13 | 31 | 21 | 18 | 12 | 8 | 4 | 15 | 11 |
| NM_001035006 | RPL17 | 680 | 119 | 69 | 109 | 26 | 81 | 83 | 62 | 119 | 48 | 99 | 25 | 68 | 42 |
| NM_001035223 | RGL3 | 2543 | 3 | 1 | 1 | 1 | 0 | 1 | 3 | 0 | 1 | 0 | 0 | 2 | 0 |
| NM_001035230 | RABGAP1L | 775 | 11 | 4 | 2 | 2 | 10 | 11 | 5 | 2 | 4 | 5 | 1 | 4 | 3 |
| NM_001035235 | SRA1 | 1948 | 1 | 1 | 1 | 1 | 1 | 1 | 1 | 1 | 1 | 1 | 1 | 1 | 1 |
| NM_001035247 | UFD1L | 1730 | 5 | 3 | 4 | 2 | 3 | 3 | 2 | 5 | 1 | 2 | 2 | 3 | 2 |
| NM_001035254 | FAM102A | 3844 | 3 | 1 | 0 | 1 | 0 | 3 | 1 | 0 | 1 | 1 | 0 | 1 | 3 |
| NM_001035256 | POMC | 1295 | 0 | 0 | 0 | 0 | 0 | 0 | 0 | 0 | 0 | 0 | 0 | 0 | 0 |
| NM_001035258 | RPL38 | 357 | 149 | 81 | 62 | 43 | 80 | 71 | 44 | 89 | 57 | 149 | 138 | 97 | 61 |
| NM_001035260 | VPS26A | 2558 | 13 | 7 | 7 | 2 | 7 | 8 | 7 | 6 | 6 | 7 | 5 | 9 | 13 |
| NM_001035267 | RPL41 | 584 | 146 | 82 | 74 | 86 | 49 | 62 | 69 | 146 | 86 | 89 | 69 | 110 | 63 |
| NM_001035505 | BOLA3 | 465 | 3 | 1 | 0 | 1 | 3 | 2 | 2 | 1 | 0 | 1 | 0 | 0 | 0 |
| NM_001035507 | AGBL5 | 2437 | 10 | 2 | 1 | 1 | 1 | 1 | 2 | 1 | 1 | 1 | 0 | 1 | 10 |
| NM_001035511 | SDHC | 2688 | 10 | 6 | 6 | 4 | 7 | 6 | 10 | 8 | 3 | 3 | 5 | 4 | 4 |
| NM_001035512 | SDHC | 2750 | 10 | 5 | 6 | 4 | 7 | 6 | 10 | 8 | 3 | 3 | 5 | 4 | 4 |
| NM_001035513 | SDHC | 2693 | 10 | 6 | 6 | 4 | 7 | 6 | 10 | 8 | 3 | 3 | 5 | 4 | 4 |
| NM_001035516 | DMKN | 600 | 0 | 0 | 0 | 0 | 0 | 0 | 0 | 0 | 0 | 0 | 0 | 0 | 0 |
| NM_001035521 | GTF3C2 | 3844 | 1 | 1 | 1 | 1 | 1 | 1 | 1 | 1 | 1 | 1 | 1 | 1 | 1 |
| NM_001035534 | FAM129B | 3760 | 4 | 2 | 1 | 3 | 2 | 2 | 2 | 1 | 4 | 1 | 0 | 2 | 1 |
| NM_001036 | RYR3 | 15559 | 4 | 1 | 0 | 1 | 0 | 2 | 0 | 0 | 0 | 0 | 4 | 0 | 0 |
| NM_001036645 | HHLA3 | 899 | 4 | 2 | 1 | 1 | 0 | 4 | 2 | 2 | 1 | 2 | 1 | 2 | 2 |
| NM_001036646 | HHLA3 | 798 | 4 | 1 | 1 | 1 | 0 | 4 | 2 | 2 | 1 | 2 | 1 | 2 | 1 |
| NM_001037 | SCN1B | 1447 | 24 | 4 | 1 | 0 | 3 | 6 | 1 | 0 | 1 | 0 | 24 | 2 | 0 |
| NM_001037125 | UNKL | 1379 | 0 | 0 | 0 | 0 | 0 | 0 | 0 | 0 | 0 | 0 | 0 | 0 | 0 |
| NM_001037126 | EXOC4 | 1605 | 13 | 8 | 6 | 11 | 4 | 6 | 8 | 3 | 7 | 12 | 6 | 13 | 10 |
| NM_001037131 | AGAP1 | 4523 | 10 | 2 | 1 | 3 | 1 | 10 | 3 | 1 | 1 | 2 | 1 | 1 | 2 |
| NM_001037132 | NRCAM | 6659 | 17 | 2 | 1 | 0 | 0 | 17 | 2 | 0 | 0 | 1 | 0 | 4 | 1 |
| NM_001037133 | NRCAM | 2821 | 18 | 3 | 1 | 0 | 0 | 18 | 2 | 0 | 1 | 1 | 0 | 5 | 1 |
| NM_001037144 | CNTROB | 3771 | 4 | 2 | 2 | 2 | 1 | 2 | 2 | 1 | 3 | 1 | 1 | 2 | 4 |
| NM_001037160 | CYS1 | 2719 | 12 | 1 | 0 | 0 | 0 | 0 | 12 | 0 | 0 | 1 | 0 | 0 | 0 |
| NM_001037161 | ACOT1 | 1603 | 1 | 0 | 1 | 0 | 0 | 0 | 1 | 1 | 0 | 0 | 0 | 0 | 0 |
| NM_001037162 | ACOT6 | 1004 | 1 | 0 | 1 | 0 | 0 | 0 | 0 | 1 | 0 | 0 | 0 | 0 | 0 |

| transcript | gene | transcript length (nt) | max | mean | adipose | colon | heart | hypothalamus | kidney | liver | lung | ovary | skeletalmuscle | spleen | testes |
|---|---|---|---|---|---|---|---|---|---|---|---|---|---|---|---|
| NM_001037163 | C7orf70 | 2214 | 5 | 2 | 1 | 1 | 2 | 4 | 1 | 1 | 1 | 2 | 5 | 2 | 4 |
| NM_001037164 | ARL4A | 2956 | 4 | 2 | 2 | 1 | 2 | 2 | 1 | 1 | 1 | 1 | 1 | 3 | 4 |
| NM_001037165 | FOXK1 | 11191 | 3 | 2 | 1 | 2 | 1 | 3 | 2 | 0 | 1 | 2 | 2 | 3 | 2 |
| NM_001037171 | ACOT9 | 1727 | 16 | 7 | 9 | 4 | 16 | 5 | 5 | 6 | 7 | 4 | 7 | 8 | 5 |
| NM_001037172 | ACPL2 | 3007 | 2 | 1 | 1 | 1 | 2 | 2 | 1 | 0 | 1 | 2 | 1 | 1 | 2 |
| NM_001037174 | ARL5A | 2913 | 6 | 4 | 2 | 1 | 6 | 4 | 4 | 4 | 4 | 3 | 4 | 4 | 3 |
| NM_001037175 | SUSD4 | 1096 | 4 | 1 | 0 | 0 | 0 | 4 | 1 | 1 | 0 | 2 | 0 | 0 | 1 |
| NM_001037225 | C11orf85 | 835 | 7 | 1 | 0 | 0 | 0 | 0 | 0 | 0 | 0 | 0 | 0 | 0 | 7 |
| NM_001037228 | LOC285033 | 537 | 1 | 0 | 0 | 0 | 0 | 0 | 0 | 0 | 0 | 0 | 1 | 1 | 0 |
| NM_001037232 | ZNF829 | 1802 | 1 | 1 | 0 | 0 | 1 | 1 | 0 | 0 | 0 | 1 | 0 | 1 | 1 |
| NM_001037277 | GGPS1 | 2744 | 3 | 2 | 2 | 1 | 2 | 3 | 2 | 1 | 2 | 2 | 1 | 2 | 3 |
| NM_001037278 | GGPS1 | 2815 | 3 | 2 | 2 | 1 | 3 | 3 | 2 | 1 | 2 | 2 | 1 | 2 | 3 |
| NM_001037281 | PARD6A | 1260 | 0 | 0 | 0 | 0 | 0 | 0 | 0 | 0 | 0 | 0 | 0 | 0 | 0 |
| NM_001037283 | EIF3B | 3081 | 47 | 28 | 23 | 20 | 25 | 26 | 18 | 47 | 14 | 27 | 42 | 31 | 38 |
| NM_001037288 | CMTM5 | 1107 | 2 | 0 | 0 | 0 | 0 | 2 | 0 | 0 | 0 | 0 | 0 | 0 | 0 |
| NM_001037290 | BCO2 | 2797 | 6 | 1 | 0 | 0 | 6 | 1 | 0 | 3 | 0 | 1 | 1 | 0 | 1 |
| NM_001037293 | PALM2 | 9261 | 2 | 1 | 1 | 0 | 1 | 2 | 0 | 2 | 0 | 0 | 0 | 0 | 0 |
| NM_001037304 | THYN1 | 788 | 5 | 3 | 2 | 2 | 4 | 5 | 3 | 2 | 1 | 3 | 1 | 2 | 4 |
| NM_001037305 | THYN1 | 939 | 7 | 3 | 3 | 3 | 4 | 7 | 3 | 3 | 1 | 4 | 1 | 3 | 4 |
| NM_001037317 | LPPR5 | 4015 | 2 | 0 | 0 | 0 | 0 | 2 | 0 | 0 | 0 | 0 | 0 | 0 | 0 |
| NM_001037324 | ECE2 | 3147 | 1 | 0 | 0 | 0 | 0 | 1 | 0 | 0 | 0 | 0 | 0 | 0 | 0 |
| NM_001037325 | CCDC46 | 1311 | 13 | 3 | 3 | 0 | 4 | 4 | 1 | 0 | 3 | 3 | 2 | 3 | 13 |
| NM_001037328 | STAU1 | 3536 | 13 | 9 | 7 | 5 | 11 | 10 | 11 | 13 | 8 | 7 | 8 | 9 | 9 |
| NM_001037329 | CNGA4 | 1903 | 1 | 0 | 0 | 0 | 0 | 0 | 0 | 0 | 0 | 0 | 0 | 0 | 1 |
| NM_001037330 | TRIM16L | 1982 | 1 | 0 | 1 | 0 | 0 | 0 | 0 | 0 | 0 | 0 | 0 | 0 | 0 |
| NM_001037331 | C18orf62 | 1369 | 0 | 0 | 0 | 0 | 0 | 0 | 0 | 0 | 0 | 0 | 0 | 0 | 0 |
| NM_001037332 | CYFIP2 | 6791 | 32 | 7 | 0 | 2 | 2 | 32 | 21 | 8 | 2 | 1 | 1 | 8 | 1 |
| NM_001037333 | CYFIP2 | 6498 | 34 | 8 | 0 | 2 | 2 | 34 | 22 | 9 | 2 | 1 | 1 | 9 | 1 |
| NM_001037334 | USP14 | 4051 | 7 | 5 | 4 | 1 | 6 | 7 | 4 | 4 | 3 | 5 | 5 | 6 | 5 |
| NM_001037335 | PRIC285 | 10002 | 0 | 0 | 0 | 0 | 0 | 0 | 0 | 0 | 0 | 0 | 0 | 0 | 0 |
| NM_001037339 | PDE4B | 3876 | 22 | 8 | 3 | 1 | 8 | 22 | 1 | 2 | 8 | 2 | 12 | 20 | 4 |
| NM_001037340 | PDE4B | 4267 | 20 | 7 | 3 | 1 | 7 | 20 | 1 | 2 | 7 | 2 | 11 | 18 | 4 |
| NM_001037341 | PDE4B | 4303 | 19 | 7 | 3 | 1 | 7 | 19 | 1 | 2 | 7 | 2 | 11 | 18 | 4 |
| NM_001037343 | CDKL5 | 3427 | 10 | 2 | 1 | 1 | 1 | 10 | 2 | 1 | 2 | 2 | 1 | 1 | 3 |
| NM_001037442 | RUFY3 | 4417 | 22 | 6 | 3 | 3 | 3 | 22 | 7 | 3 | 4 | 6 | 2 | 5 | 11 |
| NM_001037494 | DYNLL1 | 809 | 193 | 57 | 57 | 59 | 35 | 193 | 38 | 52 | 32 | 34 | 13 | 40 | 78 |
| NM_001037495 | DYNLL1 | 736 | 212 | 63 | 63 | 64 | 38 | 212 | 42 | 57 | 36 | 37 | 14 | 43 | 85 |
| NM_001037497 | DEFB110 | 384 | 2 | 0 | 0 | 0 | 0 | 0 | 0 | 0 | 0 | 0 | 0 | 0 | 2 |
| NM_001037498 | DEFB112 | 342 | 1 | 0 | 0 | 0 | 0 | 0 | 0 | 0 | 0 | 0 | 0 | 0 | 1 |
| NM_001037499 | DEFB114 | 210 | 0 | 0 | 0 | 0 | 0 | 0 | 0 | 0 | 0 | 0 | 0 | 0 | 0 |
| NM_001037500 | DEFB124 | 216 | 1 | 0 | 0 | 0 | 0 | 0 | 0 | 0 | 1 | 0 | 0 | 0 | 0 |
| NM_001037525 | GALC | 1630 | 4 | 3 | 2 | 1 | 2 | 4 | 3 | 2 | 4 | 2 | 1 | 3 | 4 |
| NM_001037533 | GON4L | 7637 | 6 | 5 | 4 | 4 | 4 | 5 | 6 | 2 | 4 | 6 | 4 | 6 | 6 |
| NM_001037535 | SCML1 | 2678 | 4 | 1 | 0 | 0 | 1 | 1 | 1 | 1 | 1 | 1 | 1 | 1 | 4 |
| NM_001037536 | SCML1 | 2675 | 4 | 1 | 0 | 0 | 1 | 1 | 1 | 1 | 1 | 1 | 1 | 1 | 4 |
| NM_001037537 | PHYH | 1715 | 13 | 6 | 5 | 1 | 9 | 3 | 12 | 12 | 1 | 3 | 13 | 2 | 2 |
| NM_001037540 | SCML1 | 2908 | 4 | 1 | 0 | 0 | 1 | 1 | 1 | 1 | 1 | 1 | 1 | 1 | 4 |
| NM_001037553 | AGPAT3 | 6435 | 12 | 5 | 6 | 3 | 5 | 8 | 12 | 8 | 3 | 2 | 4 | 5 | 3 |
| NM_001037558 | HEPN1 | 1428 | 0 | 0 | 0 | 0 | 0 | 0 | 0 | 0 | 0 | 0 | 0 | 0 | 0 |
| NM_001037582 | SCD5 | 3103 | 40 | 7 | 0 | 1 | 1 | 40 | 6 | 0 | 2 | 14 | 0 | 1 | 11 |
| NM_001037631 | CTLA4 | 1867 | 0 | 0 | 0 | 0 | 0 | 0 | 0 | 0 | 0 | 0 | 0 | 0 | 0 |
| NM_001037633 | SIL1 | 1988 | 17 | 7 | 8 | 4 | 6 | 4 | 6 | 17 | 4 | 4 | 5 | 4 | 10 |
| NM_001037637 | BTF3 | 1249 | 8 | 5 | 6 | 4 | 4 | 5 | 3 | 7 | 4 | 8 | 4 | 5 | 2 |
| NM_001037639 | PARL | 1262 | 14 | 9 | 11 | 8 | 8 | 14 | 7 | 13 | 7 | 6 | 6 | 8 | 8 |
| NM_001037663 | EEF1B2 | 882 | 109 | 62 | 37 | 30 | 66 | 41 | 54 | 78 | 57 | 80 | 91 | 109 | 42 |
| NM_001037666 | GATSL3 | 1622 | 0 | 0 | 0 | 0 | 0 | 0 | 0 | 0 | 0 | 0 | 0 | 0 | 0 |
| NM_001037668 | DEFB107A | 389 | 0 | 0 | 0 | 0 | 0 | 0 | 0 | 0 | 0 | 0 | 0 | 0 | 0 |
| NM_001037671 | C12orf74 | 1377 | 0 | 0 | 0 | 0 | 0 | 0 | 0 | 0 | 0 | 0 | 0 | 0 | 0 |
| NM_001037675 | NBPF20 | 2928 | 1 | 1 | 1 | 1 | 0 | 1 | 0 | 1 | 1 | 1 | 0 | 1 | 1 |
| NM_001037728 | DEFB110 | 234 | 3 | 0 | 0 | 0 | 0 | 0 | 0 | 0 | 0 | 0 | 0 | 0 | 3 |
| NM_001037729 | DEFB113 | 249 | 0 | 0 | 0 | 0 | 0 | 0 | 0 | 0 | 0 | 0 | 0 | 0 | 0 |
| NM_001037730 | DEFB115 | 267 | 0 | 0 | 0 | 0 | 0 | 0 | 0 | 0 | 0 | 0 | 0 | 0 | 0 |
| NM_001037731 | DEFB116 | 309 | 0 | 0 | 0 | 0 | 0 | 0 | 0 | 0 | 0 | 0 | 0 | 0 | 0 |
| NM_001037732 | DEFB128 | 282 | 0 | 0 | 0 | 0 | 0 | 0 | 0 | 0 | 0 | 0 | 0 | 0 | 0 |
| NM_001037735 | ZNF630 | 2516 | 3 | 1 | 1 | 1 | 1 | 1 | 1 | 0 | 1 | 3 | 0 | 1 | 1 |
| NM_001037738 | NPM1 | 1345 | 63 | 29 | 22 | 18 | 25 | 24 | 24 | 30 | 31 | 63 | 19 | 32 | 27 |
| NM_001037763 | COL28A1 | 3515 | 1 | 0 | 0 | 1 | 0 | 0 | 0 | 0 | 0 | 0 | 1 | 0 | 0 |
| NM_001037804 | DEFB130 | 240 | 0 | 0 | 0 | 0 | 0 | 0 | 0 | 0 | 0 | 0 | 0 | 0 | 0 |
| NM_001037806 | NCKAP5L | 4900 | 0 | 0 | 0 | 0 | 0 | 0 | 0 | 0 | 0 | 0 | 0 | 0 | 0 |
| NM_001037808 | EIF3C | 3095 | 0 | 0 | 0 | 0 | 0 | 0 | 0 | 0 | 0 | 0 | 0 | 0 | 0 |
| NM_001037808 | EIF3C | 3098 | 0 | 0 | 0 | 0 | 0 | 0 | 0 | 0 | 0 | 0 | 0 | 0 | 0 |
| NM_001037811 | HSD17B10 | 936 | 7 | 2 | 1 | 1 | 2 | 1 | 4 | 7 | 1 | 1 | 1 | 2 | 1 |
| NM_001037813 | ZNF284 | 2208 | 5 | 3 | 2 | 1 | 3 | 3 | 2 | 1 | 2 | 4 | 1 | 5 | 2 |
| NM_001037814 | GAB4 | 2629 | 0 | 0 | 0 | 0 | 0 | 0 | 0 | 0 | 0 | 0 | 0 | 0 | 0 |
| NM_001037866 | RGPD6 | 3304 | 0 | 0 | 0 | 0 | 0 | 0 | 0 | 0 | 0 | 0 | 0 | 0 | 0 |
| NM_001037872 | REV1 | 4731 | 4 | 2 | 1 | 1 | 2 | 2 | 2 | 1 | 2 | 4 | 2 | 3 | 4 |
| NM_001037954 | DIXDC1 | 5895 | 5 | 3 | 4 | 2 | 3 | 5 | 3 | 1 | 2 | 3 | 2 | 1 | 4 |

| transcript | gene | transcript length (nt) | max | mean | adipose | colon | heart | hypothalamus | kidney | liver | lung | ovary | skeletalmuscle | spleen | testes |
|---|---|---|---|---|---|---|---|---|---|---|---|---|---|---|---|
| NM_001037967 | ALAS2 | 1915 | 1 | 0 | 0 | 0 | 0 | 0 | 0 | 0 | 1 | 0 | 0 | 0 | 0 |
| NM_001037968 | ALAS2 | 2022 | 0 | 0 | 0 | 0 | 0 | 0 | 0 | 0 | 0 | 0 | 0 | 0 | 0 |
| NM_001037984 | SLC38A10 | 4292 | 3 | 1 | 2 | 2 | 1 | 1 | 2 | 3 | 1 | 1 | 0 | 2 | 1 |
| NM_001038 | SCNN1A | 3327 | 18 | 4 | 0 | 9 | 0 | 0 | 18 | 1 | 10 | 0 | 0 | 0 | 1 |
| NM_001038493 | DLX1 | 2182 | 1 | 0 | 0 | 0 | 0 | 1 | 0 | 0 | 0 | 0 | 0 | 0 | 0 |
| NM_001038603 | MARVELD2 | 2145 | 4 | 1 | 0 | 2 | 0 | 0 | 3 | 4 | 2 | 0 | 0 | 0 | 0 |
| NM_001038603 | MARVELD2 | 2147 | 4 | 1 | 0 | 2 | 0 | 0 | 3 | 4 | 2 | 0 | 0 | 0 | 0 |
| NM_001038618 | NARF | 1779 | 4 | 2 | 2 | 2 | 3 | 3 | 2 | 2 | 2 | 2 | 2 | 4 | 3 |
| NM_001038628 | B3GALNT1 | 3641 | 6 | 2 | 0 | 0 | 5 | 6 | 3 | 0 | 1 | 2 | 0 | 1 | 2 |
| NM_001038633 | RSPO1 | 2985 | 0 | 0 | 0 | 0 | 0 | 0 | 0 | 0 | 0 | 0 | 0 | 0 | 0 |
| NM_001038640 | GOLGA6A | 3176 | 0 | 0 | 0 | 0 | 0 | 0 | 0 | 0 | 0 | 0 | 0 | 0 | 0 |
| NM_001038702 | CDC42SE2 | 3487 | 20 | 7 | 3 | 4 | 3 | 14 | 8 | 3 | 8 | 6 | 2 | 20 | 9 |
| NM_001038704 | C17orf47 | 2077 | 1 | 0 | 0 | 0 | 0 | 0 | 0 | 0 | 0 | 0 | 0 | 0 | 1 |
| NM_001038705 | GPR149 | 2323 | 0 | 0 | 0 | 0 | 0 | 0 | 0 | 0 | 0 | 0 | 0 | 0 | 0 |
| NM_001038707 | CDC42SE1 | 3183 | 16 | 5 | 3 | 3 | 4 | 3 | 4 | 2 | 8 | 2 | 1 | 16 | 3 |
| NM_001039 | SCNN1G | 3499 | 5 | 1 | 0 | 0 | 0 | 0 | 5 | 0 | 1 | 0 | 0 | 0 | 0 |
| NM_001039029 | LRTM2 | 3950 | 1 | 0 | 0 | 0 | 0 | 1 | 0 | 0 | 0 | 0 | 0 | 0 | 0 |
| NM_001039083 | ARL17B | 746 | 0 | 0 | 0 | 0 | 0 | 0 | 0 | 0 | 0 | 0 | 0 | 0 | 0 |
| NM_001039091 | PRPS2 | 2518 | 70 | 12 | 2 | 3 | 3 | 4 | 9 | 4 | 5 | 22 | 1 | 6 | 70 |
| NM_001039111 | TRIM71 | 3138 | 0 | 0 | 0 | 0 | 0 | 0 | 0 | 0 | 0 | 0 | 0 | 0 | 0 |
| NM_001039112 | FER1L6 | 6051 | 1 | 0 | 0 | 1 | 0 | 0 | 1 | 0 | 0 | 0 | 0 | 0 | 0 |
| NM_001039127 | ZNF718 | 2121 | 9 | 3 | 1 | 1 | 4 | 1 | 5 | 1 | 3 | 9 | 1 | 6 | 6 |
| NM_001039130 | ALOX15B | 2628 | 5 | 1 | 0 | 0 | 1 | 0 | 0 | 0 | 5 | 0 | 0 | 1 | 0 |
| NM_001039131 | ALOX15B | 2493 | 5 | 1 | 0 | 0 | 1 | 0 | 0 | 0 | 5 | 0 | 0 | 1 | 0 |
| NM_001039132 | ICAM4 | 1196 | 0 | 0 | 0 | 0 | 0 | 0 | 0 | 0 | 0 | 0 | 0 | 0 | 0 |
| NM_001039140 | C20orf27 | 1376 | 3 | 1 | 1 | 2 | 1 | 1 | 1 | 0 | 1 | 3 | 1 | 3 | 1 |
| NM_001039141 | TRIOBP | 10144 | 4 | 2 | 1 | 3 | 3 | 1 | 1 | 1 | 1 | 2 | 1 | 4 | 1 |
| NM_001039152 | RGS21 | 1793 | 0 | 0 | 0 | 0 | 0 | 0 | 0 | 0 | 0 | 0 | 0 | 0 | 0 |
| NM_001039165 | MRGPRE | 1296 | 0 | 0 | 0 | 0 | 0 | 0 | 0 | 0 | 0 | 0 | 0 | 0 | 0 |
| NM_001039178 | ATP5J2 | 360 | 6 | 3 | 3 | 4 | 5 | 6 | 5 | 3 | 2 | 1 | 2 | 2 | 2 |
| NM_001039182 | BOLA2B | 1012 | 0 | 0 | 0 | 0 | 0 | 0 | 0 | 0 | 0 | 0 | 0 | 0 | 0 |
| NM_001039199 | TTPAL | 6191 | 6 | 1 | 0 | 0 | 1 | 1 | 1 | 6 | 1 | 2 | 0 | 1 | 1 |
| NM_001039210 | ALG13 | 2934 | 3 | 2 | 1 | 1 | 1 | 1 | 2 | 1 | 2 | 3 | 0 | 3 | 2 |
| NM_001039211 | ATAD3C | 3859 | 1 | 0 | 0 | 0 | 0 | 0 | 0 | 1 | 0 | 0 | 0 | 0 | 0 |
| NM_001039213 | CEACAM16 | 1797 | 0 | 0 | 0 | 0 | 0 | 0 | 0 | 0 | 0 | 0 | 0 | 0 | 0 |
| NM_001039348 | EFEMP1 | 2723 | 102 | 33 | 70 | 8 | 102 | 17 | 17 | 2 | 45 | 47 | 10 | 19 | 22 |
| NM_001039349 | EFEMP1 | 2682 | 103 | 33 | 71 | 9 | 103 | 17 | 17 | 2 | 46 | 48 | 10 | 19 | 22 |
| NM_001039350 | DPP6 | 4561 | 18 | 2 | 0 | 1 | 0 | 18 | 1 | 0 | 0 | 0 | 0 | 0 | 3 |
| NM_001039355 | SLC25A29 | 2370 | 1 | 0 | 0 | 0 | 0 | 1 | 1 | 0 | 0 | 0 | 0 | 0 | 1 |
| NM_001039360 | ZBTB7C | 3762 | 4 | 1 | 0 | 4 | 0 | 1 | 0 | 0 | 1 | 1 | 0 | 2 | 1 |
| NM_001039361 | PRAMEF10 | 1518 | 0 | 0 | 0 | 0 | 0 | 0 | 0 | 0 | 0 | 0 | 0 | 0 | 0 |
| NM_001039362 | ATP6V1C2 | 3240 | 2 | 0 | 0 | 0 | 0 | 0 | 2 | 0 | 0 | 0 | 0 | 0 | 0 |
| NM_001039366 | ATP6V1E1 | 1339 | 182 | 56 | 50 | 20 | 71 | 182 | 64 | 33 | 36 | 25 | 37 | 49 | 44 |
| NM_001039367 | ATP6V1E1 | 1315 | 183 | 56 | 50 | 20 | 71 | 183 | 64 | 34 | 36 | 25 | 38 | 50 | 44 |
| NM_001039372 | HEPACAM2 | 2092 | 4 | 1 | 0 | 4 | 0 | 0 | 3 | 0 | 0 | 0 | 0 | 0 | 0 |
| NM_001039374 | KIAA1984 | 1704 | 2 | 1 | 0 | 2 | 0 | 0 | 0 | 0 | 0 | 0 | 0 | 0 | 2 |
| NM_001039375 | C20orf7 | 2206 | 5 | 3 | 2 | 2 | 4 | 5 | 4 | 3 | 1 | 1 | 2 | 2 | 2 |
| NM_001039380 | C10orf25 | 3078 | 2 | 1 | 1 | 0 | 0 | 0 | 0 | 2 | 0 | 0 | 0 | 0 | 1 |
| NM_001039395 | C9orf68 | 3741 | 1 | 0 | 0 | 0 | 0 | 1 | 0 | 0 | 1 | 0 | 0 | 0 | 1 |
| NM_001039396 | MPEG1 | 4512 | 62 | 11 | 17 | 7 | 6 | 2 | 1 | 11 | 12 | 3 | 2 | 62 | 2 |
| NM_001039397 | TBC1D28 | 1978 | 0 | 0 | 0 | 0 | 0 | 0 | 0 | 0 | 0 | 0 | 0 | 0 | 0 |
| NM_001039457 | ATP6V0B | 973 | 3 | 1 | 0 | 1 | 1 | 3 | 2 | 1 | 1 | 0 | 1 | 1 | 1 |
| NM_001039459 | ELMO1 | 2088 | 9 | 2 | 1 | 0 | 1 | 9 | 0 | 1 | 1 | 1 | 0 | 5 | 1 |
| NM_001039464 | C1orf175 | 4375 | 2 | 0 | 0 | 1 | 0 | 0 | 0 | 0 | 0 | 0 | 0 | 0 | 2 |
| NM_001039465 | SFRS5 | 1651 | 55 | 41 | 44 | 53 | 28 | 55 | 37 | 49 | 37 | 35 | 19 | 50 | 42 |
| NM_001039467 | RGS19 | 1492 | 1 | 0 | 0 | 0 | 0 | 0 | 0 | 0 | 0 | 0 | 0 | 1 | 0 |
| NM_001039469 | MARK2 | 4728 | 5 | 2 | 2 | 3 | 2 | 5 | 2 | 2 | 2 | 2 | 2 | 4 | 2 |
| NM_001039476 | C16orf35 | 2748 | 1 | 1 | 0 | 0 | 1 | 1 | 1 | 1 | 0 | 1 | 1 | 1 | 0 |
| NM_001039477 | C1orf38 | 1236 | 24 | 4 | 3 | 1 | 3 | 2 | 0 | 1 | 6 | 1 | 0 | 24 | 4 |
| NM_001039479 | KIAA0317 | 5445 | 4 | 2 | 1 | 1 | 2 | 3 | 3 | 2 | 2 | 1 | 1 | 3 | 4 |
| NM_001039481 | ETNK1 | 910 | 8 | 5 | 3 | 6 | 3 | 8 | 6 | 6 | 6 | 5 | 2 | 7 | 7 |
| NM_001039487 | NSUN5C | 1214 | 0 | 0 | 0 | 0 | 0 | 0 | 0 | 0 | 0 | 0 | 0 | 0 | 0 |
| NM_001039490 | CD151 | 1483 | 13 | 5 | 13 | 5 | 8 | 1 | 6 | 6 | 5 | 2 | 2 | 3 | 3 |
| NM_001039492 | FHL2 | 1521 | 123 | 18 | 2 | 3 | 123 | 2 | 3 | 0 | 1 | 50 | 3 | 4 | 12 |
| NM_001039496 | C11orf20 | 744 | 4 | 0 | 0 | 0 | 0 | 0 | 0 | 0 | 0 | 0 | 0 | 0 | 4 |
| NM_001039500 | VWA5B1 | 4475 | 0 | 0 | 0 | 0 | 0 | 0 | 0 | 0 | 0 | 0 | 0 | 0 | 0 |
| NM_001039503 | POL3S | 2028 | 0 | 0 | 0 | 0 | 0 | 0 | 0 | 0 | 0 | 0 | 0 | 0 | 0 |
| NM_001039508 | SIRPG | 1383 | 1 | 0 | 0 | 0 | 0 | 0 | 0 | 0 | 0 | 0 | 0 | 1 | 0 |
| NM_001039517 | C1orf104 | 1306 | 0 | 0 | 0 | 0 | 0 | 0 | 0 | 0 | 0 | 0 | 0 | 0 | 0 |
| NM_001039523 | CHRNA1 | 2056 | 20 | 2 | 0 | 0 | 0 | 0 | 0 | 0 | 0 | 0 | 20 | 0 | 0 |
| NM_001039535 | SKA1 | 2918 | 2 | 0 | 0 | 1 | 0 | 0 | 0 | 0 | 0 | 0 | 0 | 1 | 2 |
| NM_001039538 | MAP2 | 5844 | 146 | 15 | 1 | 2 | 2 | 146 | 1 | 1 | 7 | 3 | 1 | 1 | 4 |
| NM_001039547 | GK5 | 9816 | 2 | 1 | 1 | 1 | 1 | 1 | 1 | 1 | 1 | 2 | 0 | 1 | 2 |
| NM_001039548 | KLHL35 | 1936 | 0 | 0 | 0 | 0 | 0 | 0 | 0 | 0 | 0 | 0 | 0 | 0 | 0 |
| NM_001039550 | DNAJB2 | 1974 | 9 | 5 | 2 | 2 | 8 | 9 | 7 | 2 | 3 | 3 | 5 | 5 | 6 |
| NM_001039567 | RPS4Y2 | 888 | 0 | 0 | 0 | 0 | 0 | 0 | 0 | 0 | 0 | 0 | 0 | 0 | 0 |
| NM_001039569 | AP1S3 | 4002 | 0 | 0 | 0 | 0 | 0 | 0 | 0 | 0 | 0 | 0 | 0 | 0 | 0 |

| | | transcript | | | | | | hypothala | | | | | skeletalm | | |
|---|---|---|---|---|---|---|---|---|---|---|---|---|---|---|---|
| transcript | gene | length (nt) | max | mean | adipose | colon | heart | mus | kidney | liver | lung | ovary | uscle | spleen | testes |
| NM_001039570 | KREMEN1 | 6133 | 3 | 2 | 1 | 1 | 2 | 1 | 1 | 0 | 1 | 1 | 1 | 3 | 3 |
| NM_001039573 | SEC14L1 | 3013 | 49 | 21 | 17 | 6 | 22 | 26 | 21 | 8 | 33 | 14 | 14 | 49 | 23 |
| NM_001039574 | KCNC4 | 3010 | 4 | 1 | 1 | 1 | 1 | 4 | 1 | 0 | 0 | 1 | 0 | 1 | 1 |
| NM_001039575 | NSUN5B | 1837 | 0 | 0 | 0 | 0 | 0 | 0 | 0 | 0 | 0 | 0 | 0 | 0 | 0 |
| NM_001039576 | NSUN5B | 1313 | 0 | 0 | 0 | 0 | 0 | 0 | 0 | 0 | 0 | 0 | 0 | 0 | 0 |
| NM_001039577 | CCNL2 | 5268 | 3 | 2 | 2 | 3 | 1 | 2 | 2 | 1 | 2 | 1 | 1 | 3 | 2 |
| NM_001039580 | MAP9 | 7331 | 3 | 1 | 0 | 0 | 1 | 3 | 1 | 0 | 1 | 1 | 0 | 1 | 2 |
| NM_001039582 | PNCK | 1870 | 1 | 0 | 0 | 0 | 0 | 1 | 0 | 0 | 0 | 0 | 0 | 0 | 0 |
| NM_001039583 | PICK1 | 2109 | 2 | 1 | 1 | 1 | 1 | 2 | 2 | 1 | 1 | 1 | 1 | 1 | 1 |
| NM_001039584 | PICK1 | 2056 | 2 | 1 | 1 | 1 | 1 | 2 | 2 | 1 | 1 | 1 | 1 | 1 | 1 |
| NM_001039585 | PTGFR | 5431 | 1 | 1 | 1 | 0 | 1 | 0 | 1 | 0 | 0 | 1 | 1 | 0 | 1 |
| NM_001039589 | DPH2 | 1813 | 1 | 1 | 0 | 0 | 1 | 1 | 1 | 1 | 0 | 0 | 1 | 1 | 0 |
| NM_001039590 | USP9X | 12401 | 12 | 9 | 7 | 3 | 9 | 10 | 8 | 10 | 7 | 7 | 10 | 10 | 12 |
| NM_001039591 | USP9X | 12353 | 12 | 9 | 7 | 3 | 9 | 10 | 8 | 10 | 7 | 7 | 10 | 11 | 12 |
| NM_001039592 | SPAG8 | 1701 | 2 | 1 | 1 | 1 | 0 | 2 | 0 | 0 | 0 | 1 | 1 | 0 | 0 | 2 |
| NM_001039613 | IAH1 | 906 | 4 | 3 | 4 | 2 | 4 | 4 | 3 | 4 | 3 | 2 | 3 | 3 | 4 |
| NM_001039614 | C15orf59 | 1344 | 2 | 0 | 0 | 0 | 1 | 2 | 0 | 0 | 0 | 0 | 0 | 0 | 0 |
| NM_001039615 | ZNF705D | 3683 | 0 | 0 | 0 | 0 | 0 | 0 | 0 | 0 | 0 | 0 | 0 | 0 | 0 |
| NM_001039616 | SPRED3 | 543 | 2 | 1 | 0 | 1 | 1 | 2 | 0 | 0 | 1 | 0 | 0 | 0 | 0 |
| NM_001039617 | ZDHHC19 | 1337 | 0 | 0 | 0 | 0 | 0 | 0 | 0 | 0 | 0 | 0 | 0 | 0 | 0 |
| NM_001039618 | CREBZF | 7573 | 5 | 3 | 2 | 3 | 2 | 2 | 3 | 2 | 5 | 4 | 2 | 5 | 3 |
| NM_001039619 | PRMT5 | 2470 | 10 | 7 | 4 | 4 | 7 | 9 | 7 | 7 | 5 | 7 | 10 | 6 | 10 |
| NM_001039649 | ZMYM5 | 1219 | 4 | 2 | 2 | 3 | 1 | 2 | 2 | 1 | 1 | 3 | 1 | 2 | 4 |
| NM_001039650 | ZMYM5 | 1505 | 4 | 2 | 2 | 2 | 1 | 2 | 2 | 1 | 1 | 2 | 1 | 2 | 4 |
| NM_001039651 | C6orf26 | 912 | 3 | 1 | 0 | 1 | 1 | 1 | 3 | 0 | 0 | 0 | 2 | 0 | 1 |
| NM_001039651 | C6orf26 | 913 | 3 | 1 | 0 | 1 | 1 | 1 | 3 | 0 | 0 | 0 | 2 | 0 | 1 |
| NM_001039654 | ZNF550 | 1146 | 2 | 1 | 2 | 1 | 2 | 1 | 1 | 0 | 1 | 1 | 1 | 2 | 1 |
| NM_001039656 | MTL5 | 3625 | 0 | 0 | 0 | 0 | 0 | 0 | 0 | 0 | 0 | 0 | 0 | 0 | 0 |
| NM_001039659 | IL18BP | 1530 | 3 | 1 | 1 | 2 | 0 | 0 | 1 | 0 | 1 | 1 | 0 | 3 | 0 |
| NM_001039660 | IL18BP | 1418 | 3 | 1 | 1 | 1 | 0 | 0 | 1 | 0 | 1 | 1 | 0 | 3 | 0 |
| NM_001039661 | TIRAP | 2348 | 2 | 1 | 1 | 1 | 1 | 1 | 1 | 1 | 1 | 2 | 1 | 2 | 1 |
| NM_001039664 | TNFRSF25 | 894 | 2 | 1 | 1 | 2 | 0 | 1 | 1 | 0 | 1 | 0 | 0 | 1 | 0 |
| NM_001039667 | ANGPTL4 | 1789 | 5 | 1 | 5 | 0 | 1 | 2 | 0 | 5 | 0 | 0 | 0 | 0 | 0 |
| NM_001039670 | IFFO1 | 2681 | 1 | 1 | 1 | 1 | 1 | 1 | 0 | 0 | 1 | 0 | 1 | 1 | 1 |
| NM_001039671 | YIF1B | 2768 | 1 | 1 | 0 | 0 | 1 | 1 | 1 | 1 | 1 | 0 | 0 | 1 | 0 |
| NM_001039672 | YIF1B | 2786 | 1 | 1 | 0 | 0 | 1 | 1 | 1 | 1 | 1 | 0 | 0 | 1 | 0 |
| NM_001039673 | YIF1B | 2777 | 1 | 1 | 0 | 0 | 1 | 1 | 1 | 1 | 1 | 0 | 0 | 1 | 0 |
| NM_001039675 | UNC45A | 4002 | 3 | 2 | 3 | 3 | 2 | 2 | 3 | 2 | 2 | 2 | 1 | 3 | 2 |
| NM_001039690 | CHTF8 | 2914 | 13 | 8 | 8 | 5 | 8 | 8 | 10 | 9 | 6 | 9 | 5 | 13 | 8 |
| NM_001039693 | C2orf60 | 5370 | 2 | 1 | 1 | 0 | 1 | 1 | 1 | 1 | 1 | 1 | 0 | 2 | 1 |
| NM_001039697 | SNAPC3 | 3095 | 9 | 5 | 3 | 2 | 5 | 7 | 4 | 3 | 3 | 5 | 3 | 5 | 9 |
| NM_001039702 | OLAH | 1675 | 1 | 0 | 0 | 0 | 0 | 0 | 0 | 0 | 1 | 0 | 0 | 1 | 1 |
| NM_001039703 | NBPF10 | 10920 | 40 | 23 | 23 | 24 | 16 | 9 | 14 | 17 | 28 | 31 | 8 | 40 | 40 |
| NM_001039705 | TRO | 4643 | 5 | 1 | 0 | 0 | 0 | 5 | 0 | 0 | 0 | 2 | 0 | 0 | 0 |
| NM_001039706 | C7orf63 | 3900 | 1 | 0 | 0 | 0 | 0 | 0 | 1 | 0 | 1 | 0 | 0 | 0 | 0 |
| NM_001039707 | SDCCAG3 | 2379 | 5 | 1 | 1 | 1 | 1 | 1 | 1 | 5 | 1 | 1 | 1 | 2 | 1 |
| NM_001039708 | SDCCAG3 | 2160 | 5 | 2 | 1 | 1 | 1 | 1 | 1 | 5 | 1 | 1 | 1 | 2 | 1 |
| NM_001039709 | CBFA2T2 | 7449 | 2 | 1 | 1 | 2 | 1 | 2 | 1 | 0 | 1 | 2 | 1 | 1 | 2 |
| NM_001039711 | DEDD | 2328 | 2 | 1 | 1 | 1 | 1 | 1 | 2 | 1 | 1 | 2 | 2 | 2 | 1 |
| NM_001039712 | DEDD | 2299 | 2 | 2 | 1 | 1 | 2 | 1 | 2 | 1 | 1 | 2 | 2 | 2 | 2 |
| NM_001039715 | DIO1 | 1716 | 11 | 2 | 0 | 0 | 0 | 0 | 11 | 7 | 0 | 1 | 0 | 0 | 0 |
| NM_001039716 | DIO1 | 1660 | 14 | 2 | 0 | 0 | 0 | 0 | 14 | 11 | 0 | 1 | 0 | 0 | 0 |
| NM_001039717 | C4orf29 | 2187 | 3 | 1 | 1 | 1 | 3 | 2 | 1 | 2 | 1 | 1 | 1 | 1 | 1 |
| NM_001039724 | NOSTRIN | 1606 | 18 | 9 | 13 | 2 | 12 | 3 | 13 | 3 | 13 | 11 | 4 | 5 | 18 |
| NM_001039752 | SLC22A10 | 2574 | 1 | 0 | 0 | 0 | 0 | 0 | 0 | 1 | 0 | 0 | 0 | 0 | 0 |
| NM_001039753 | EML6 | 7850 | 2 | 0 | 0 | 0 | 0 | 2 | 0 | 0 | 0 | 0 | 0 | 1 | 1 |
| NM_001039762 | FAM196A | 4407 | 1 | 0 | 0 | 0 | 0 | 1 | 0 | 0 | 0 | 0 | 0 | 0 | 0 |
| NM_001039763 | TMEM232 | 2261 | 7 | 1 | 0 | 0 | 0 | 4 | 3 | 0 | 2 | 0 | 0 | 0 | 7 |
| NM_001039770 | TMPPE | 3970 | 2 | 1 | 1 | 1 | 0 | 1 | 2 | 1 | 1 | 0 | 0 | 1 | 0 |
| NM_001039771 | CBLN3 | 2344 | 0 | 0 | 0 | 0 | 0 | 0 | 0 | 0 | 0 | 0 | 0 | 0 | 0 |
| NM_001039775 | AIM1L | 2390 | 0 | 0 | 0 | 0 | 0 | 0 | 0 | 0 | 0 | 0 | 0 | 0 | 0 |
| NM_001039780 | CCNI2 | 2355 | 0 | 0 | 0 | 0 | 0 | 0 | 0 | 0 | 0 | 0 | 0 | 0 | 0 |
| NM_001039783 | TXNRD3IT1 | 1134 | 0 | 0 | 0 | 0 | 0 | 0 | 0 | 0 | 0 | 0 | 0 | 0 | 0 |
| NM_001039792 | HRCT1 | 950 | 3 | 1 | 3 | 1 | 1 | 0 | 0 | 0 | 0 | 0 | 0 | 0 | 1 |
| NM_001039802 | CDC42 | 2294 | 41 | 26 | 30 | 20 | 23 | 31 | 28 | 27 | 31 | 16 | 12 | 41 | 24 |
| NM_001039803 | CCRK | 2365 | 0 | 0 | 0 | 0 | 0 | 0 | 0 | 0 | 0 | 0 | 0 | 0 | 0 |
| NM_001039840 | CHIC1 | 6909 | 2 | 1 | 0 | 0 | 1 | 2 | 1 | 0 | 0 | 1 | 0 | 1 | 1 |
| NM_001039841 | ARHGAP11E | 1017 | 0 | 0 | 0 | 0 | 0 | 0 | 0 | 0 | 0 | 0 | 0 | 0 | 0 |
| NM_001039842 | C17orf90 | 620 | 0 | 0 | 0 | 0 | 0 | 0 | 0 | 0 | 0 | 0 | 0 | 0 | 0 |
| NM_001039844 | ACBD7 | 3370 | 6 | 1 | 0 | 1 | 0 | 6 | 0 | 0 | 0 | 0 | 0 | 0 | 0 |
| NM_001039845 | MDH1B | 2330 | 3 | 1 | 0 | 0 | 0 | 1 | 1 | 0 | 1 | 0 | 0 | 0 | 3 |
| NM_001039846 | C19orf36 | 1015 | 1 | 0 | 0 | 0 | 0 | 0 | 0 | 0 | 0 | 0 | 0 | 0 | 1 |
| NM_001039847 | GPX4 | 939 | 8 | 3 | 8 | 1 | 3 | 4 | 3 | 2 | 1 | 1 | 2 | 1 | 2 |
| NM_001039848 | GPX4 | 1006 | 7 | 2 | 7 | 1 | 2 | 4 | 3 | 2 | 1 | 1 | 2 | 1 | 2 |
| NM_001039876 | C19orf46 | 1541 | 0 | 0 | 0 | 0 | 0 | 0 | 0 | 0 | 0 | 0 | 0 | 0 | 0 |
| NM_001039877 | STRN4 | 3210 | 3 | 2 | 1 | 2 | 2 | 3 | 2 | 1 | 1 | 1 | 1 | 2 | 3 |
| NM_001039884 | ZNF826 | 4733 | 11 | 3 | 1 | 0 | 1 | 1 | 11 | 0 | 1 | 11 | 0 | 3 | 4 |

| transcript | gene | transcript length (nt) | max | mean | adipose | colon | heart | hypothalamus | kidney | liver | lung | ovary | skeletalmuscle | spleen | testes |
|---|---|---|---|---|---|---|---|---|---|---|---|---|---|---|---|
| NM_001039885 | FKRP | 3474 | 3 | 2 | 1 | 1 | 1 | 2 | 2 | 1 | 1 | 1 | 1 | 2 | 3 |
| NM_001039886 | ZNF808 | 3311 | 20 | 11 | 7 | 5 | 15 | 10 | 18 | 2 | 9 | 20 | 8 | 16 | 13 |
| NM_001039887 | C19orf55 | 2147 | 0 | 0 | 0 | 0 | 0 | 0 | 0 | 0 | 0 | 0 | 0 | 0 | 0 |
| NM_001039888 | ANKRD34A | 3612 | 1 | 0 | 0 | 0 | 0 | 1 | 0 | 0 | 0 | 0 | 0 | 0 | 0 |
| NM_001039891 | ZNF674 | 4090 | 1 | 1 | 0 | 0 | 0 | 1 | 1 | 0 | 0 | 1 | 1 | 1 | 1 |
| NM_001039905 | C15orf56 | 2125 | 0 | 0 | 0 | 0 | 0 | 0 | 0 | 0 | 0 | 0 | 0 | 0 | 0 |
| NM_001039916 | ZNF384 | 3286 | 4 | 2 | 1 | 2 | 2 | 1 | 2 | 1 | 2 | 3 | 2 | 4 | 3 |
| NM_001039917 | ZNF384 | 2989 | 4 | 2 | 1 | 2 | 2 | 1 | 2 | 1 | 2 | 4 | 2 | 4 | 3 |
| NM_001039918 | ZNF384 | 3069 | 4 | 2 | 1 | 2 | 2 | 1 | 2 | 1 | 2 | 4 | 2 | 4 | 3 |
| NM_001039919 | ZNF384 | 2941 | 4 | 2 | 1 | 2 | 2 | 1 | 2 | 1 | 2 | 4 | 2 | 4 | 3 |
| NM_001039920 | ZNF384 | 2824 | 4 | 2 | 2 | 2 | 3 | 1 | 2 | 1 | 2 | 4 | 3 | 4 | 3 |
| NM_001039933 | CD79B | 1264 | 5 | 1 | 0 | 0 | 0 | 0 | 0 | 0 | 0 | 0 | 0 | 5 | 0 |
| NM_001039935 | ANKRD55 | 1720 | 0 | 0 | 0 | 0 | 0 | 0 | 0 | 0 | 0 | 0 | 0 | 0 | 0 |
| NM_001039936 | CHN2 | 2833 | 13 | 3 | 0 | 2 | 2 | 7 | 2 | 13 | 1 | 5 | 0 | 4 | 1 |
| NM_001039937 | INTS6 | 6973 | 8 | 3 | 2 | 1 | 3 | 2 | 3 | 3 | 2 | 3 | 2 | 4 | 8 |
| NM_001039938 | INTS6 | 2493 | 3 | 2 | 1 | 1 | 1 | 0 | 2 | 2 | 2 | 3 | 1 | 2 | 3 |
| NM_001039948 | SGSM1 | 6176 | 2 | 0 | 0 | 0 | 0 | 2 | 0 | 0 | 0 | 0 | 0 | 0 | 0 |
| NM_001039958 | MESP2 | 1611 | 0 | 0 | 0 | 0 | 0 | 0 | 0 | 0 | 0 | 0 | 0 | 0 | 0 |
| NM_001039960 | SLC4A8 | 5218 | 10 | 2 | 0 | 0 | 0 | 10 | 1 | 0 | 0 | 2 | 0 | 0 | 3 |
| NM_001039966 | GPER | 2971 | 0 | 0 | 0 | 0 | 0 | 0 | 0 | 0 | 0 | 0 | 0 | 0 | 0 |
| NM_001039970 | PTPN5 | 3039 | 6 | 1 | 0 | 0 | 0 | 6 | 0 | 0 | 0 | 0 | 0 | 0 | 0 |
| NM_001039999 | FAM83G | 2930 | 0 | 0 | 0 | 0 | 0 | 0 | 0 | 0 | 0 | 0 | 0 | 0 | 0 |
| NM_001040 | SHBG | 1277 | 1 | 0 | 0 | 0 | 0 | 0 | 0 | 1 | 0 | 0 | 0 | 0 | 0 |
| NM_001040000 | MLLT4 | 7551 | 10 | 6 | 4 | 7 | 4 | 6 | 9 | 6 | 9 | 5 | 2 | 7 | 10 |
| NM_001040001 | MLLT4 | 7735 | 10 | 7 | 4 | 7 | 4 | 6 | 10 | 7 | 9 | 5 | 3 | 7 | 10 |
| NM_001040002 | MEOX1 | 2168 | 3 | 1 | 3 | 1 | 2 | 0 | 0 | 0 | 0 | 0 | 1 | 1 | 1 |
| NM_001040006 | TCERG1 | 4138 | 10 | 6 | 4 | 3 | 7 | 7 | 5 | 4 | 5 | 6 | 8 | 10 | 8 |
| NM_001040007 | RSPO4 | 2521 | 1 | 0 | 0 | 0 | 0 | 0 | 0 | 0 | 1 | 0 | 0 | 0 | 0 |
| NM_001040011 | C9orf119 | 981 | 3 | 1 | 1 | 1 | 1 | 1 | 1 | 1 | 1 | 1 | 0 | 0 | 3 |
| NM_001040020 | FAM3C | 2502 | 31 | 16 | 10 | 8 | 22 | 31 | 18 | 18 | 12 | 10 | 6 | 24 | 20 |
| NM_001040021 | CD14 | 1513 | 5 | 2 | 3 | 1 | 3 | 4 | 1 | 5 | 2 | 1 | 0 | 3 | 2 |
| NM_001040022 | SIRPA | 4201 | 8 | 2 | 2 | 1 | 2 | 8 | 1 | 3 | 1 | 1 | 1 | 2 | 0 |
| NM_001040023 | SIRPA | 4109 | 8 | 2 | 2 | 1 | 2 | 8 | 1 | 3 | 1 | 1 | 1 | 2 | 0 |
| NM_001040025 | ARL16 | 1207 | 3 | 2 | 2 | 2 | 1 | 3 | 3 | 1 | 1 | 1 | 0 | 2 | 1 |
| NM_001040031 | CD37 | 1209 | 31 | 5 | 2 | 3 | 2 | 2 | 1 | 1 | 7 | 2 | 1 | 31 | 1 |
| NM_001040032 | C8orf74 | 1036 | 1 | 0 | 0 | 0 | 0 | 0 | 0 | 0 | 0 | 0 | 0 | 0 | 1 |
| NM_001040033 | CD53 | 1572 | 35 | 6 | 5 | 1 | 5 | 3 | 1 | 2 | 13 | 1 | 0 | 35 | 1 |
| NM_001040034 | CD63 | 1011 | 301 | 128 | 144 | 74 | 301 | 68 | 114 | 72 | 175 | 85 | 88 | 188 | 96 |
| NM_001040056 | MAPK3 | 1979 | 4 | 2 | 2 | 4 | 2 | 4 | 1 | 0 | 1 | 1 | 1 | 1 | 1 |
| NM_001040057 | FAM133B | 2468 | 5 | 3 | 2 | 1 | 3 | 2 | 4 | 2 | 3 | 5 | 2 | 5 | 3 |
| NM_001040058 | SPP1 | 1625 | 318 | 38 | 48 | 0 | 6 | 45 | 318 | 1 | 1 | 2 | 0 | 0 | 1 |
| NM_001040059 | CD68 | 1774 | 189 | 50 | 108 | 14 | 36 | 16 | 18 | 42 | 87 | 15 | 12 | 189 | 10 |
| NM_001040060 | SPP1 | 1544 | 329 | 40 | 49 | 0 | 6 | 46 | 329 | 1 | 1 | 2 | 0 | 0 | 1 |
| NM_001040061 | C3orf72 | 4075 | 2 | 0 | 0 | 0 | 0 | 0 | 0 | 0 | 0 | 2 | 0 | 0 | 0 |
| NM_001040065 | FAM75A2 | 4226 | 0 | 0 | 0 | 0 | 0 | 0 | 0 | 0 | 0 | 0 | 0 | 0 | 0 |
| NM_001040071 | LOC650293 | 649 | 0 | 0 | 0 | 0 | 0 | 0 | 0 | 0 | 0 | 0 | 0 | 0 | 0 |
| NM_001040078 | LGALS9C | 1719 | 0 | 0 | 0 | 0 | 0 | 0 | 0 | 0 | 0 | 0 | 0 | 0 | 0 |
| NM_001040084 | ANXA8 | 2041 | 0 | 0 | 0 | 0 | 0 | 0 | 0 | 0 | 0 | 0 | 0 | 0 | 0 |
| NM_001040092 | ENPP2 | 3120 | 31 | 10 | 12 | 2 | 2 | 29 | 10 | 1 | 7 | 4 | 1 | 31 | 10 |
| NM_001040097 | MOSPD3 | 1104 | 1 | 1 | 1 | 1 | 1 | 1 | 1 | 1 | 1 | 1 | 0 | 1 | 1 |
| NM_001040098 | MOSPD3 | 1020 | 2 | 1 | 1 | 1 | 1 | 1 | 1 | 1 | 1 | 2 | 0 | 1 | 1 |
| NM_001040099 | MOSPD3 | 1173 | 1 | 1 | 1 | 1 | 1 | 1 | 1 | 1 | 1 | 1 | 0 | 1 | 1 |
| NM_001040100 | C3orf57 | 2306 | 1 | 0 | 0 | 0 | 0 | 1 | 0 | 0 | 0 | 0 | 0 | 0 | 0 |
| NM_001040101 | D4S234E | 2595 | 5 | 1 | 0 | 0 | 0 | 5 | 1 | 0 | 0 | 2 | 0 | 0 | 1 |
| NM_001040102 | OPALIN | 3519 | 7 | 1 | 0 | 0 | 0 | 7 | 0 | 0 | 0 | 0 | 0 | 0 | 0 |
| NM_001040103 | OPALIN | 3627 | 7 | 1 | 0 | 0 | 0 | 7 | 0 | 0 | 0 | 0 | 0 | 0 | 0 |
| NM_001040105 | MUC17 | 14350 | 1 | 0 | 0 | 1 | 0 | 0 | 0 | 0 | 0 | 0 | 0 | 0 | 0 |
| NM_001040107 | HVCN1 | 1736 | 3 | 1 | 1 | 0 | 1 | 1 | 0 | 0 | 1 | 1 | 0 | 3 | 2 |
| NM_001040108 | MLH3 | 7896 | 5 | 3 | 2 | 1 | 3 | 3 | 4 | 2 | 3 | 5 | 3 | 5 | 5 |
| NM_001040109 | MLN | 569 | 0 | 0 | 0 | 0 | 0 | 0 | 0 | 0 | 0 | 0 | 0 | 0 | 0 |
| NM_001040110 | NRF1 | 3513 | 1 | 1 | 0 | 1 | 0 | 1 | 0 | 0 | 1 | 1 | 1 | 1 | 1 |
| NM_001040113 | MYH11 | 6942 | 398 | 85 | 15 | 398 | 58 | 20 | 20 | 1 | 46 | 85 | 26 | 29 | 231 |
| NM_001040114 | MYH11 | 6903 | 400 | 85 | 15 | 400 | 59 | 20 | 20 | 1 | 46 | 86 | 26 | 29 | 232 |
| NM_001040118 | ARAP1 | 5136 | 3 | 1 | 2 | 2 | 1 | 1 | 1 | 1 | 2 | 0 | 0 | 3 | 1 |
| NM_001040125 | PQLC2 | 2255 | 0 | 0 | 0 | 0 | 0 | 0 | 0 | 0 | 0 | 0 | 0 | 0 | 0 |
| NM_001040126 | PQLC2 | 1643 | 0 | 0 | 0 | 0 | 0 | 0 | 0 | 0 | 0 | 0 | 0 | 0 | 0 |
| NM_001040129 | SPINK5L3 | 509 | 1 | 0 | 0 | 0 | 0 | 0 | 0 | 0 | 0 | 0 | 0 | 0 | 1 |
| NM_001040134 | PALM | 2742 | 5 | 2 | 3 | 1 | 2 | 5 | 2 | 0 | 2 | 3 | 1 | 2 | 2 |
| NM_001040135 | ACTR3B | 1989 | 4 | 1 | 0 | 0 | 0 | 4 | 1 | 1 | 0 | 0 | 1 | 1 | 1 |
| NM_001040138 | CKLF | 655 | 4 | 1 | 1 | 3 | 1 | 1 | 1 | 1 | 2 | 1 | 0 | 4 | 2 |
| NM_001040142 | SCN2A | 8794 | 38 | 4 | 0 | 0 | 0 | 38 | 2 | 0 | 0 | 0 | 0 | 0 | 0 |
| NM_001040143 | SCN2A | 8555 | 38 | 4 | 0 | 0 | 0 | 38 | 2 | 0 | 0 | 0 | 0 | 0 | 0 |
| NM_001040144 | CHTF8 | 2856 | 14 | 8 | 8 | 5 | 8 | 8 | 10 | 10 | 7 | 10 | 5 | 14 | 9 |
| NM_001040146 | CHTF8 | 2882 | 14 | 8 | 8 | 5 | 8 | 8 | 10 | 10 | 7 | 10 | 5 | 14 | 9 |
| NM_001040147 | SERPINB7 | 1973 | 0 | 0 | 0 | 0 | 0 | 0 | 0 | 0 | 0 | 0 | 0 | 0 | 0 |
| NM_001040151 | SCN3B | 5667 | 26 | 3 | 0 | 0 | 0 | 26 | 0 | 0 | 0 | 1 | 2 | 0 | 1 |
| NM_001040152 | PEG10 | 6617 | 35 | 8 | 3 | 1 | 0 | 35 | 2 | 0 | 4 | 19 | 0 | 1 | 25 |

| | | transcript | | | | | | hypothala | | | | | skeletalm | | |
|---|---|---|---|---|---|---|---|---|---|---|---|---|---|---|---|
| transcript | gene | length (nt) | max | mean | adipose | colon | heart | mus | kidney | liver | lung | ovary | uscle | spleen | testes |
| NM_001040153 | SLAIN1 | 2374 | 19 | 3 | 0 | 0 | 1 | 19 | 1 | 1 | 1 | 1 | 1 | 2 | 7 |
| NM_001040157 | KIAA1712 | 4534 | 2 | 1 | 1 | 0 | 1 | 1 | 1 | 1 | 1 | 2 | 1 | 1 | 2 |
| NM_001040158 | H2AFY | 1921 | 21 | 10 | 11 | 9 | 9 | 10 | 9 | 7 | 17 | 6 | 7 | 21 | 7 |
| NM_001040159 | SPOCK3 | 2979 | 34 | 3 | 0 | 1 | 0 | 34 | 0 | 0 | 0 | 1 | 0 | 0 | 1 |
| NM_001040160 | C16orf13 | 772 | 5 | 2 | 1 | 1 | 3 | 5 | 3 | 3 | 1 | 1 | 3 | 2 | 1 |
| NM_001040161 | C16orf13 | 549 | 7 | 3 | 1 | 1 | 4 | 7 | 4 | 4 | 2 | 2 | 4 | 3 | 1 |
| NM_001040162 | C16orf13 | 609 | 6 | 3 | 1 | 1 | 3 | 6 | 4 | 3 | 1 | 2 | 4 | 3 | 1 |
| NM_001040165 | C16orf13 | 712 | 5 | 2 | 1 | 1 | 3 | 5 | 3 | 3 | 1 | 1 | 3 | 2 | 1 |
| NM_001040167 | LFNG | 2374 | 1 | 0 | 0 | 1 | 0 | 0 | 0 | 0 | 0 | 0 | 0 | 1 | 0 |
| NM_001040168 | LFNG | 1393 | 0 | 0 | 0 | 0 | 0 | 0 | 0 | 0 | 0 | 0 | 0 | 0 | 0 |
| NM_001040169 | HTR4 | 1481 | 1 | 0 | 0 | 1 | 0 | 0 | 0 | 0 | 0 | 0 | 0 | 0 | 0 |
| NM_001040171 | HTR4 | 1350 | 1 | 0 | 0 | 1 | 0 | 0 | 0 | 0 | 0 | 0 | 0 | 0 | 0 |
| NM_001040172 | HTR4 | 3020 | 0 | 0 | 0 | 0 | 0 | 0 | 0 | 0 | 0 | 0 | 0 | 0 | 0 |
| NM_001040173 | HTR4 | 3098 | 0 | 0 | 0 | 0 | 0 | 0 | 0 | 0 | 0 | 0 | 0 | 0 | 0 |
| NM_001040174 | HTR4 | 1379 | 1 | 0 | 0 | 1 | 0 | 0 | 0 | 0 | 0 | 0 | 0 | 0 | 0 |
| NM_001040177 | AKR1E2 | 1607 | 2 | 1 | 0 | 1 | 0 | 1 | 0 | 0 | 1 | 0 | 0 | 1 | 2 |
| NM_001040179 | MCHR2 | 2368 | 0 | 0 | 0 | 0 | 0 | 0 | 0 | 0 | 0 | 0 | 0 | 0 | 0 |
| NM_001040181 | CLDND1 | 2184 | 41 | 8 | 4 | 3 | 6 | 41 | 3 | 4 | 6 | 5 | 2 | 7 | 9 |
| NM_001040182 | CLDND1 | 2228 | 40 | 8 | 4 | 3 | 6 | 40 | 3 | 4 | 6 | 4 | 2 | 7 | 9 |
| NM_001040183 | CLDND1 | 2179 | 41 | 8 | 4 | 3 | 6 | 41 | 3 | 4 | 6 | 5 | 2 | 7 | 9 |
| NM_001040185 | ZNF765 | 4577 | 23 | 6 | 3 | 2 | 4 | 2 | 4 | 2 | 4 | 23 | 2 | 10 | 9 |
| NM_001040192 | DNAJC28 | 1609 | 2 | 1 | 1 | 0 | 2 | 2 | 2 | 2 | 1 | 0 | 1 | 1 | 2 |
| NM_001040194 | AGTRAP | 1162 | 1 | 0 | 0 | 0 | 1 | 1 | 0 | 0 | 0 | 0 | 0 | 1 | 0 |
| NM_001040195 | AGTRAP | 1127 | 1 | 0 | 0 | 0 | 1 | 1 | 0 | 0 | 0 | 0 | 0 | 1 | 0 |
| NM_001040196 | AGTRAP | 1259 | 1 | 0 | 0 | 0 | 1 | 0 | 0 | 0 | 0 | 0 | 0 | 1 | 0 |
| NM_001040197 | AGTRAP | 1280 | 1 | 0 | 0 | 0 | 1 | 0 | 0 | 0 | 0 | 0 | 0 | 1 | 0 |
| NM_001040199 | CLDND1 | 2491 | 36 | 7 | 4 | 2 | 5 | 36 | 3 | 4 | 5 | 4 | 2 | 6 | 8 |
| NM_001040200 | CLDND1 | 1874 | 32 | 6 | 3 | 2 | 5 | 32 | 2 | 3 | 4 | 4 | 2 | 5 | 7 |
| NM_001040202 | PAQR3 | 3739 | 2 | 1 | 0 | 0 | 0 | 2 | 1 | 1 | 0 | 0 | 1 | 1 | 2 |
| NM_001040214 | NKAIN2 | 3315 | 3 | 0 | 0 | 0 | 0 | 3 | 0 | 0 | 0 | 0 | 0 | 0 | 0 |
| NM_001040217 | FAM63A | 2175 | 3 | 1 | 1 | 1 | 1 | 2 | 3 | 1 | 1 | 2 | 1 | 1 | 2 |
| NM_001040260 | DCLK2 | 3610 | 9 | 1 | 0 | 1 | 1 | 9 | 0 | 0 | 0 | 1 | 0 | 0 | 1 |
| NM_001040261 | DCLK2 | 3607 | 9 | 1 | 0 | 1 | 1 | 9 | 0 | 0 | 0 | 1 | 0 | 0 | 1 |
| NM_001040272 | ADAMTSL1 | 7817 | 1 | 0 | 0 | 1 | 0 | 0 | 0 | 0 | 0 | 0 | 0 | 0 | 0 |
| NM_001040273 | TYSND1 | 3485 | 0 | 0 | 0 | 0 | 0 | 0 | 0 | 0 | 0 | 0 | 0 | 0 | 0 |
| NM_001040274 | SYCP2L | 3127 | 1 | 0 | 0 | 0 | 0 | 1 | 0 | 0 | 1 | 0 | 0 | 0 | 1 |
| NM_001040275 | ESR2 | 2456 | 1 | 0 | 0 | 0 | 0 | 0 | 0 | 0 | 0 | 1 | 0 | 0 | 1 |
| NM_001040276 | ESR2 | 2731 | 1 | 0 | 0 | 0 | 0 | 0 | 0 | 0 | 0 | 1 | 0 | 0 | 0 |
| NM_001040280 | CD83 | 2474 | 4 | 2 | 3 | 1 | 0 | 3 | 2 | 0 | 4 | 1 | 0 | 3 | 1 |
| NM_001040282 | TBC1D3G | 2118 | 0 | 0 | 0 | 0 | 0 | 0 | 0 | 0 | 0 | 0 | 0 | 0 | 0 |
| NM_001040283 | DMRTC2 | 1820 | 1 | 0 | 0 | 0 | 0 | 0 | 0 | 0 | 0 | 0 | 0 | 0 | 1 |
| NM_001040284 | PAPD5 | 8110 | 4 | 3 | 2 | 1 | 2 | 3 | 3 | 3 | 3 | 4 | 2 | 4 | 4 |
| NM_001040285 | PAPD5 | 7969 | 4 | 3 | 2 | 1 | 2 | 3 | 3 | 3 | 3 | 4 | 2 | 4 | 4 |
| NM_001040402 | DCUN1D4 | 4265 | 18 | 10 | 8 | 4 | 10 | 15 | 11 | 10 | 7 | 15 | 6 | 11 | 18 |
| NM_001040410 | C9orf131 | 3286 | 2 | 0 | 0 | 0 | 0 | 0 | 0 | 0 | 0 | 0 | 1 | 0 | 2 |
| NM_001040411 | C9orf131 | 3183 | 2 | 0 | 0 | 0 | 0 | 0 | 0 | 0 | 0 | 0 | 1 | 0 | 2 |
| NM_001040412 | C9orf131 | 3258 | 2 | 0 | 0 | 0 | 0 | 0 | 0 | 0 | 0 | 0 | 1 | 0 | 2 |
| NM_001040424 | PRDM15 | 6654 | 1 | 1 | 0 | 1 | 1 | 1 | 1 | 0 | 1 | 1 | 1 | 1 | 1 |
| NM_001040425 | U2AF1L4 | 782 | 2 | 1 | 1 | 1 | 1 | 2 | 1 | 1 | 1 | 1 | 0 | 1 | 1 |
| NM_001040427 | HAGH | 1610 | 7 | 3 | 2 | 2 | 3 | 7 | 4 | 7 | 2 | 1 | 4 | 2 | 3 |
| NM_001040428 | SPATA7 | 1893 | 4 | 2 | 1 | 0 | 1 | 2 | 2 | 0 | 1 | 4 | 1 | 1 | 4 |
| NM_001040429 | PCDH17 | 8009 | 17 | 3 | 0 | 0 | 0 | 5 | 0 | 0 | 4 | 0 | 0 | 17 | 1 |
| NM_001040431 | CCDC56 | 753 | 7 | 3 | 2 | 3 | 3 | 3 | 7 | 6 | 1 | 2 | 2 | 2 | 1 |
| NM_001040432 | ZCWPW2 | 1659 | 1 | 0 | 0 | 0 | 1 | 1 | 0 | 0 | 0 | 1 | 1 | 0 | 1 |
| NM_001040433 | SPINK9 | 453 | 2 | 1 | 0 | 0 | 0 | 1 | 1 | 0 | 0 | 2 | 1 | 1 | 1 |
| NM_001040436 | YARS2 | 2161 | 3 | 2 | 2 | 2 | 2 | 3 | 2 | 3 | 1 | 3 | 1 | 3 | 2 |
| NM_001040437 | C6orf48 | 840 | 27 | 11 | 12 | 6 | 7 | 14 | 10 | 3 | 16 | 27 | 3 | 14 | 10 |
| NM_001040437 | C6orf48 | 842 | 26 | 11 | 12 | 6 | 7 | 14 | 10 | 3 | 16 | 26 | 3 | 14 | 10 |
| NM_001040438 | C6orf48 | 746 | 30 | 12 | 14 | 6 | 8 | 16 | 11 | 3 | 18 | 30 | 4 | 16 | 11 |
| NM_001040438 | C6orf48 | 748 | 29 | 12 | 14 | 6 | 8 | 16 | 11 | 3 | 18 | 29 | 4 | 15 | 11 |
| NM_001040439 | MAPK8IP3 | 5606 | 9 | 2 | 1 | 1 | 1 | 9 | 1 | 0 | 1 | 1 | 1 | 1 | 1 |
| NM_001040440 | CCDC112 | 2496 | 17 | 4 | 3 | 1 | 3 | 7 | 2 | 1 | 3 | 2 | 2 | 4 | 17 |
| NM_001040441 | ZBTB8A | 7333 | 1 | 0 | 1 | 0 | 0 | 1 | 0 | 0 | 1 | 1 | 0 | 0 | 1 |
| NM_001040442 | FABP6 | 663 | 11 | 2 | 0 | 5 | 0 | 11 | 0 | 0 | 2 | 0 | 0 | 0 | 1 |
| NM_001040443 | PHF11 | 1649 | 12 | 6 | 7 | 3 | 7 | 6 | 7 | 3 | 7 | 5 | 2 | 12 | 5 |
| NM_001040444 | PHF11 | 1471 | 13 | 6 | 8 | 3 | 8 | 7 | 7 | 3 | 8 | 6 | 3 | 13 | 6 |
| NM_001040445 | ASB1 | 6833 | 2 | 1 | 1 | 0 | 1 | 2 | 1 | 0 | 0 | 1 | 1 | 1 | 1 |
| NM_001040446 | MTMR12 | 5187 | 5 | 3 | 4 | 1 | 2 | 2 | 3 | 3 | 2 | 3 | 1 | 3 | 5 |
| NM_001040448 | DEFB131 | 213 | 2 | 0 | 0 | 0 | 0 | 0 | 1 | 0 | 0 | 2 | 0 | 1 | 0 |
| NM_001040450 | FAM63B | 4993 | 6 | 3 | 2 | 1 | 6 | 5 | 3 | 1 | 3 | 5 | 2 | 4 | 3 |
| NM_001040451 | RUFY1 | 2610 | 10 | 5 | 8 | 4 | 4 | 5 | 7 | 4 | 5 | 5 | 3 | 10 | 4 |
| NM_001040452 | RUFY1 | 2421 | 11 | 6 | 8 | 4 | 5 | 5 | 8 | 4 | 5 | 5 | 3 | 11 | 5 |
| NM_001040453 | FAM63B | 4990 | 6 | 3 | 2 | 1 | 6 | 5 | 3 | 1 | 3 | 5 | 2 | 4 | 3 |
| NM_001040454 | SLC26A6 | 2731 | 1 | 0 | 0 | 1 | 0 | 0 | 1 | 0 | 0 | 0 | 0 | 0 | 0 |
| NM_001040455 | SIDT2 | 3919 | 2 | 1 | 2 | 2 | 1 | 1 | 1 | 1 | 1 | 2 | 1 | 2 | 2 |
| NM_001040456 | RHBDD2 | 1756 | 3 | 1 | 0 | 0 | 0 | 3 | 1 | 1 | 1 | 1 | 0 | 1 | 0 |
| NM_001040457 | RHBDD2 | 1878 | 3 | 1 | 1 | 0 | 0 | 3 | 1 | 1 | 1 | 1 | 0 | 1 | 1 |

| transcript | gene | transcript length (nt) | max | mean | adipose | colon | heart | hypothala mus | kidney | liver | lung | ovary | skeletalm uscle | spleen | testes |
|---|---|---|---|---|---|---|---|---|---|---|---|---|---|---|---|
| NM_001040458 | ERAP1 | 5085 | 8 | 4 | 8 | 3 | 3 | 2 | 2 | 5 | 4 | 3 | 3 | 6 | 2 |
| NM_001040462 | BTNL8 | 2097 | 1 | 0 | 0 | 1 | 0 | 0 | 0 | 0 | 0 | 0 | 0 | 0 | 1 |
| NM_001040610 | EFTUD1 | 3532 | 4 | 2 | 2 | 1 | 3 | 2 | 2 | 2 | 1 | 1 | 2 | 2 | 4 |
| NM_001040612 | SSX4B | 1077 | 0 | 0 | 0 | 0 | 0 | 0 | 0 | 0 | 0 | 0 | 0 | 0 | 0 |
| NM_001040613 | TMEM70 | 2041 | 11 | 3 | 1 | 0 | 4 | 2 | 2 | 3 | 1 | 2 | 11 | 3 | 2 |
| NM_001040614 | LINS1 | 2573 | 3 | 1 | 1 | 2 | 1 | 1 | 1 | 1 | 1 | 2 | 1 | 3 | 2 |
| NM_001040615 | LINS1 | 2468 | 3 | 1 | 1 | 2 | 1 | 1 | 1 | 1 | 1 | 2 | 1 | 3 | 2 |
| NM_001040616 | LINS1 | 2464 | 3 | 1 | 1 | 2 | 1 | 1 | 1 | 1 | 1 | 2 | 1 | 3 | 2 |
| NM_001040619 | ATF3 | 2360 | 20 | 7 | 15 | 2 | 13 | 5 | 4 | 5 | 20 | 5 | 4 | 2 | 4 |
| NM_001040624 | NCALD | 3743 | 61 | 10 | 4 | 4 | 4 | 61 | 6 | 3 | 4 | 17 | 3 | 2 | 4 |
| NM_001040625 | NCALD | 3656 | 63 | 10 | 4 | 4 | 5 | 63 | 6 | 3 | 4 | 18 | 3 | 2 | 4 |
| NM_001040626 | NCALD | 3656 | 63 | 10 | 4 | 4 | 5 | 63 | 6 | 3 | 4 | 18 | 3 | 2 | 4 |
| NM_001040627 | NCALD | 3569 | 64 | 11 | 4 | 4 | 5 | 64 | 6 | 3 | 4 | 18 | 3 | 2 | 4 |
| NM_001040628 | NCALD | 3712 | 62 | 10 | 4 | 4 | 4 | 62 | 6 | 3 | 4 | 17 | 3 | 2 | 4 |
| NM_001040629 | NCALD | 3625 | 63 | 11 | 4 | 4 | 5 | 63 | 6 | 3 | 4 | 18 | 3 | 2 | 4 |
| NM_001040630 | NCALD | 3809 | 60 | 10 | 4 | 4 | 4 | 60 | 5 | 3 | 4 | 17 | 3 | 2 | 4 |
| NM_001040633 | PRKAG2 | 3138 | 16 | 4 | 2 | 2 | 16 | 6 | 5 | 4 | 3 | 2 | 1 | 4 | 3 |
| NM_001040647 | CRCP | 2702 | 6 | 4 | 3 | 3 | 3 | 5 | 4 | 6 | 3 | 3 | 2 | 5 | 3 |
| NM_001040648 | CRCP | 2570 | 5 | 3 | 3 | 3 | 3 | 4 | 3 | 5 | 2 | 3 | 1 | 4 | 2 |
| NM_001040649 | ACP1 | 658 | 15 | 7 | 7 | 5 | 6 | 13 | 6 | 15 | 4 | 4 | 6 | 4 | 8 |
| NM_001040651 | CD3D | 609 | 20 | 3 | 1 | 5 | 1 | 0 | 0 | 0 | 4 | 1 | 1 | 20 | 0 |
| NM_001040653 | ZXDC | 12600 | 2 | 1 | 1 | 1 | 1 | 1 | 2 | 1 | 1 | 2 | 2 | 1 | 1 |
| NM_001040655 | TTC23 | 3808 | 2 | 1 | 1 | 2 | 1 | 1 | 2 | 1 | 1 | 2 | 1 | 1 | 2 |
| NM_001040656 | TTC23 | 3491 | 3 | 2 | 1 | 2 | 1 | 1 | 2 | 1 | 2 | 2 | 1 | 1 | 3 |
| NM_001040657 | TTC23 | 3440 | 2 | 1 | 1 | 2 | 1 | 1 | 2 | 1 | 1 | 2 | 1 | 1 | 2 |
| NM_001040658 | TTC23 | 3367 | 2 | 1 | 1 | 2 | 1 | 1 | 2 | 1 | 2 | 2 | 1 | 1 | 2 |
| NM_001040659 | TTC23 | 3245 | 2 | 2 | 1 | 2 | 1 | 1 | 2 | 1 | 2 | 2 | 1 | 1 | 2 |
| NM_001040660 | TTC23 | 3686 | 3 | 2 | 1 | 2 | 1 | 1 | 2 | 1 | 1 | 2 | 1 | 1 | 3 |
| NM_001040661 | SLC29A4 | 2891 | 1 | 0 | 1 | 0 | 0 | 0 | 0 | 0 | 0 | 0 | 0 | 0 | 0 |
| NM_001040662 | C14orf138 | 1679 | 5 | 2 | 1 | 1 | 2 | 3 | 2 | 2 | 2 | 3 | 2 | 5 | 3 |
| NM_001040663 | GAGE1 | 810 | 0 | 0 | 0 | 0 | 0 | 0 | 0 | 0 | 0 | 0 | 0 | 0 | 0 |
| NM_001040664 | PPAN-P2RY1 | 2385 | 0 | 0 | 0 | 0 | 0 | 0 | 0 | 0 | 0 | 0 | 0 | 0 | 0 |
| NM_001040665 | STEAP2 | 6696 | 2 | 1 | 1 | 1 | 1 | 2 | 1 | 0 | 2 | 1 | 0 | 0 | 1 |
| NM_001040666 | STEAP2 | 2308 | 5 | 2 | 2 | 1 | 2 | 5 | 2 | 0 | 3 | 3 | 1 | 1 | 1 |
| NM_001040667 | HSF4 | 2604 | 0 | 0 | 0 | 0 | 0 | 0 | 0 | 0 | 0 | 0 | 0 | 0 | 0 |
| NM_001040668 | BCL2L12 | 1852 | 8 | 3 | 2 | 8 | 1 | 1 | 3 | 0 | 3 | 3 | 1 | 5 | 2 |
| NM_001040694 | INCENP | 4146 | 4 | 2 | 2 | 3 | 2 | 4 | 2 | 1 | 2 | 2 | 1 | 3 | 3 |
| NM_001040697 | UEVLD | 2119 | 5 | 3 | 4 | 2 | 3 | 4 | 4 | 4 | 4 | 3 | 3 | 5 | 4 |
| NM_001040701 | FUT6 | 3009 | 5 | 1 | 0 | 1 | 0 | 0 | 5 | 0 | 0 | 0 | 0 | 0 | 0 |
| NM_001040702 | DEFB104B | 281 | 0 | 0 | 0 | 0 | 0 | 0 | 0 | 0 | 0 | 0 | 0 | 0 | 0 |
| NM_001040703 | DEFB105B | 237 | 0 | 0 | 0 | 0 | 0 | 0 | 0 | 0 | 0 | 0 | 0 | 0 | 0 |
| NM_001040704 | DEFB106B | 303 | 0 | 0 | 0 | 0 | 0 | 0 | 0 | 0 | 0 | 0 | 0 | 0 | 0 |
| NM_001040705 | DEFB107B | 389 | 0 | 0 | 0 | 0 | 0 | 0 | 0 | 0 | 0 | 0 | 0 | 0 | 0 |
| NM_001040708 | HEY1 | 2305 | 7 | 1 | 2 | 1 | 1 | 7 | 0 | 0 | 2 | 0 | 2 | 0 | 1 |
| NM_001040709 | SYPL2 | 3779 | 4 | 1 | 0 | 0 | 0 | 0 | 1 | 0 | 0 | 0 | 4 | 0 | 1 |
| NM_001040710 | C2orf84 | 1774 | 2 | 0 | 0 | 0 | 0 | 0 | 0 | 0 | 0 | 0 | 0 | 0 | 2 |
| NM_001040712 | PTPRD | 8848 | 14 | 3 | 1 | 2 | 1 | 14 | 8 | 1 | 1 | 6 | 0 | 0 | 1 |
| NM_001040715 | KIAA0895L | 3400 | 1 | 1 | 0 | 1 | 0 | 1 | 1 | 0 | 1 | 1 | 0 | 0 | 1 |
| NM_001040716 | PC | 4192 | 6 | 1 | 2 | 0 | 0 | 1 | 2 | 6 | 0 | 0 | 0 | 0 | 0 |
| NM_001040874 | HIST2H2AA4 | 534 | 0 | 0 | 0 | 0 | 0 | 0 | 0 | 0 | 0 | 0 | 0 | 0 | 0 |
| NM_001040875 | GDAP1 | 3724 | 15 | 2 | 1 | 0 | 0 | 15 | 1 | 0 | 0 | 2 | 1 | 1 | 2 |
| NM_001040876 | ABCE1 | 4191 | 9 | 6 | 5 | 1 | 6 | 7 | 5 | 9 | 4 | 5 | 4 | 9 | 7 |
| NM_001041 | SI | 6010 | 2 | 0 | 0 | 2 | 0 | 0 | 0 | 0 | 0 | 0 | 0 | 0 | 0 |
| NM_001042 | SLC2A4 | 3149 | 12 | 2 | 2 | 2 | 6 | 0 | 0 | 1 | 1 | 0 | 12 | 0 | 1 |
| NM_001042351 | G6PD | 2270 | 2 | 1 | 1 | 1 | 0 | 1 | 0 | 0 | 1 | 0 | 0 | 1 | 2 |
| NM_001042353 | FAM110A | 1811 | 3 | 1 | 0 | 1 | 1 | 0 | 1 | 0 | 2 | 0 | 1 | 3 | 0 |
| NM_001042355 | CYLD | 8567 | 10 | 5 | 3 | 2 | 5 | 9 | 3 | 2 | 6 | 5 | 4 | 10 | 5 |
| NM_001042357 | PTPN20B | 2965 | 0 | 0 | 0 | 0 | 0 | 0 | 0 | 0 | 0 | 0 | 0 | 0 | 0 |
| NM_001042358 | PTPN20B | 2870 | 0 | 0 | 0 | 0 | 0 | 0 | 0 | 0 | 0 | 0 | 0 | 0 | 0 |
| NM_001042359 | PTPN20B | 2444 | 0 | 0 | 0 | 0 | 0 | 0 | 0 | 0 | 0 | 0 | 0 | 0 | 0 |
| NM_001042360 | PTPN20B | 2321 | 0 | 0 | 0 | 0 | 0 | 0 | 0 | 0 | 0 | 0 | 0 | 0 | 0 |
| NM_001042361 | PTPN20B | 2319 | 0 | 0 | 0 | 0 | 0 | 0 | 0 | 0 | 0 | 0 | 0 | 0 | 0 |
| NM_001042362 | PTPN20B | 2076 | 0 | 0 | 0 | 0 | 0 | 0 | 0 | 0 | 0 | 0 | 0 | 0 | 0 |
| NM_001042363 | PTPN20B | 2872 | 0 | 0 | 0 | 0 | 0 | 0 | 0 | 0 | 0 | 0 | 0 | 0 | 0 |
| NM_001042364 | PTPN20B | 2226 | 0 | 0 | 0 | 0 | 0 | 0 | 0 | 0 | 0 | 0 | 0 | 0 | 0 |
| NM_001042365 | PTPN20B | 1885 | 0 | 0 | 0 | 0 | 0 | 0 | 0 | 0 | 0 | 0 | 0 | 0 | 0 |
| NM_001042367 | C15orf60 | 925 | 3 | 0 | 0 | 0 | 0 | 0 | 0 | 0 | 0 | 0 | 0 | 0 | 3 |
| NM_001042368 | RALGDS | 3668 | 5 | 1 | 1 | 1 | 1 | 5 | 1 | 0 | 1 | 1 | 1 | 2 | 1 |
| NM_001042369 | TROVE2 | 2086 | 28 | 16 | 12 | 5 | 17 | 19 | 14 | 9 | 15 | 28 | 9 | 21 | 22 |
| NM_001042370 | TROVE2 | 1218 | 9 | 4 | 3 | 1 | 4 | 5 | 5 | 3 | 4 | 9 | 3 | 6 | 5 |
| NM_001042371 | PGP | 3136 | 1 | 0 | 0 | 0 | 1 | 0 | 0 | 0 | 0 | 0 | 0 | 0 | 0 |
| NM_001042376 | INS-IGF2 | 828 | 0 | 0 | 0 | 0 | 0 | 0 | 0 | 0 | 0 | 0 | 0 | 0 | 0 |
| NM_001042383 | CEP63 | 5326 | 12 | 7 | 6 | 2 | 9 | 7 | 6 | 4 | 4 | 5 | 11 | 7 | 12 |
| NM_001042384 | CEP63 | 1895 | 32 | 17 | 16 | 4 | 24 | 17 | 14 | 9 | 11 | 13 | 27 | 17 | 32 |
| NM_001042385 | PREPL | 4626 | 27 | 6 | 2 | 1 | 6 | 27 | 10 | 2 | 2 | 4 | 3 | 3 | 5 |
| NM_001042386 | PREPL | 4614 | 28 | 6 | 3 | 1 | 6 | 28 | 10 | 2 | 2 | 4 | 3 | 3 | 5 |
| NM_001042387 | PTPN20A | 2478 | 0 | 0 | 0 | 0 | 0 | 0 | 0 | 0 | 0 | 0 | 0 | 0 | 0 |

| transcript | gene | transcript length (nt) | max | mean | adipose | colon | heart | hypothalamus | kidney | liver | lung | ovary | skeletalmuscle | spleen | testes |
|---|---|---|---|---|---|---|---|---|---|---|---|---|---|---|---|
| NM_001042388 | PPP4R1 | 3966 | 10 | 7 | 5 | 4 | 8 | 6 | 6 | 5 | 9 | 7 | 4 | 10 | 10 |
| NM_001042389 | PTPN20A | 2965 | 0 | 0 | 0 | 0 | 0 | 0 | 0 | 0 | 0 | 0 | 0 | 0 | 0 |
| NM_001042390 | PTPN20A | 2870 | 0 | 0 | 0 | 0 | 0 | 0 | 0 | 0 | 0 | 0 | 0 | 0 | 0 |
| NM_001042391 | PTPN20A | 2444 | 0 | 0 | 0 | 0 | 0 | 0 | 0 | 0 | 0 | 0 | 0 | 0 | 0 |
| NM_001042392 | PTPN20A | 2321 | 0 | 0 | 0 | 0 | 0 | 0 | 0 | 0 | 0 | 0 | 0 | 0 | 0 |
| NM_001042393 | PTPN20A | 2319 | 0 | 0 | 0 | 0 | 0 | 0 | 0 | 0 | 0 | 0 | 0 | 0 | 0 |
| NM_001042394 | PTPN20A | 2076 | 0 | 0 | 0 | 0 | 0 | 0 | 0 | 0 | 0 | 0 | 0 | 0 | 0 |
| NM_001042395 | PTPN20A | 2872 | 0 | 0 | 0 | 0 | 0 | 0 | 0 | 0 | 0 | 0 | 0 | 0 | 0 |
| NM_001042396 | PTPN20A | 2226 | 0 | 0 | 0 | 0 | 0 | 0 | 0 | 0 | 0 | 0 | 0 | 0 | 0 |
| NM_001042397 | PTPN20A | 1885 | 0 | 0 | 0 | 0 | 0 | 0 | 0 | 0 | 0 | 0 | 0 | 0 | 0 |
| NM_001042399 | CCDC41 | 3172 | 11 | 5 | 3 | 2 | 6 | 4 | 5 | 2 | 3 | 8 | 4 | 5 | 11 |
| NM_001042400 | CEP63 | 5292 | 12 | 7 | 6 | 2 | 9 | 7 | 6 | 4 | 5 | 6 | 11 | 7 | 12 |
| NM_001042402 | NAAA | 1732 | 5 | 2 | 2 | 2 | 1 | 1 | 2 | 3 | 2 | 1 | 1 | 5 | 1 |
| NM_001042403 | USP44 | 3326 | 5 | 1 | 0 | 0 | 0 | 0 | 1 | 0 | 2 | 1 | 0 | 1 | 5 |
| NM_001042404 | CEP170 | 6890 | 19 | 8 | 7 | 1 | 8 | 19 | 3 | 2 | 7 | 10 | 7 | 11 | 9 |
| NM_001042405 | CEP170 | 6812 | 19 | 8 | 7 | 1 | 8 | 19 | 3 | 2 | 7 | 10 | 7 | 11 | 9 |
| NM_001042406 | HMGCLL1 | 2471 | 6 | 1 | 0 | 0 | 0 | 6 | 0 | 0 | 1 | 0 | 0 | 0 | 2 |
| NM_001042410 | ANKZF1 | 2506 | 3 | 2 | 2 | 3 | 2 | 2 | 3 | 1 | 2 | 2 | 2 | 3 | 3 |
| NM_001042412 | CYLD | 8591 | 10 | 5 | 3 | 2 | 5 | 9 | 3 | 2 | 6 | 5 | 4 | 10 | 5 |
| NM_001042413 | GLIS3 | 7650 | 2 | 1 | 0 | 0 | 1 | 1 | 2 | 0 | 1 | 2 | 0 | 1 | 1 |
| NM_001042414 | PSPC1 | 2064 | 8 | 5 | 3 | 4 | 3 | 8 | 5 | 2 | 3 | 5 | 4 | 6 | 7 |
| NM_001042415 | ZNF596 | 2805 | 11 | 3 | 2 | 1 | 2 | 2 | 3 | 1 | 1 | 3 | 2 | 3 | 11 |
| NM_001042416 | ZNF596 | 2875 | 11 | 3 | 2 | 1 | 2 | 2 | 3 | 1 | 1 | 3 | 2 | 3 | 11 |
| NM_001042417 | RUFY2 | 2686 | 11 | 5 | 4 | 3 | 3 | 11 | 4 | 1 | 5 | 8 | 3 | 4 | 6 |
| NM_001042422 | SLC16A3 | 2054 | 4 | 2 | 1 | 1 | 2 | 1 | 1 | 0 | 3 | 0 | 1 | 4 | 2 |
| NM_001042423 | SLC16A3 | 2033 | 4 | 2 | 1 | 1 | 2 | 1 | 1 | 0 | 3 | 0 | 1 | 4 | 3 |
| NM_001042424 | WHSC1 | 7572 | 6 | 3 | 2 | 4 | 2 | 4 | 1 | 1 | 2 | 1 | 1 | 3 | 6 |
| NM_001042425 | TFAP2A | 3150 | 0 | 0 | 0 | 0 | 0 | 0 | 0 | 0 | 0 | 0 | 0 | 0 | 0 |
| NM_001042426 | CENPA | 1350 | 2 | 0 | 0 | 2 | 0 | 0 | 0 | 0 | 0 | 0 | 0 | 1 | 0 |
| NM_001042427 | C10orf53 | 642 | 8 | 1 | 0 | 0 | 0 | 0 | 0 | 0 | 0 | 0 | 0 | 0 | 8 |
| NM_001042428 | ZNF205 | 1992 | 0 | 0 | 0 | 0 | 0 | 0 | 0 | 0 | 0 | 0 | 0 | 0 | 0 |
| NM_001042429 | FAM98B | 1101 | 4 | 2 | 3 | 1 | 2 | 4 | 3 | 1 | 2 | 2 | 2 | 3 | 2 |
| NM_001042430 | FAM164C | 1561 | 7 | 1 | 0 | 0 | 1 | 1 | 1 | 1 | 0 | 0 | 1 | 0 | 7 |
| NM_001042432 | CLN3 | 1913 | 1 | 1 | 1 | 1 | 0 | 0 | 1 | 1 | 0 | 0 | 0 | 1 | 0 |
| NM_001042437 | ST3GAL5 | 2236 | 9 | 3 | 2 | 1 | 1 | 9 | 1 | 1 | 4 | 2 | 2 | 5 | 4 |
| NM_001042439 | C20orf71 | 1001 | 2 | 0 | 0 | 0 | 0 | 0 | 0 | 0 | 0 | 0 | 0 | 0 | 2 |
| NM_001042440 | CAST | 4504 | 65 | 25 | 21 | 10 | 65 | 9 | 18 | 15 | 22 | 18 | 49 | 23 | 22 |
| NM_001042441 | CAST | 4570 | 64 | 24 | 20 | 10 | 64 | 9 | 18 | 15 | 22 | 17 | 49 | 23 | 21 |
| NM_001042442 | CAST | 4561 | 65 | 25 | 21 | 10 | 65 | 9 | 18 | 15 | 22 | 18 | 49 | 24 | 22 |
| NM_001042443 | CAST | 4355 | 68 | 26 | 22 | 10 | 68 | 9 | 19 | 16 | 23 | 18 | 52 | 25 | 23 |
| NM_001042444 | CAST | 4232 | 69 | 26 | 22 | 11 | 69 | 9 | 19 | 16 | 24 | 19 | 52 | 25 | 23 |
| NM_001042445 | CAST | 4250 | 69 | 26 | 22 | 11 | 69 | 9 | 19 | 16 | 24 | 19 | 53 | 25 | 23 |
| NM_001042446 | CAST | 4193 | 69 | 26 | 22 | 11 | 69 | 9 | 19 | 16 | 24 | 19 | 53 | 25 | 23 |
| NM_001042450 | SLC5A10 | 2091 | 2 | 0 | 0 | 0 | 0 | 0 | 2 | 0 | 0 | 0 | 0 | 0 | 0 |
| NM_001042452 | MST4 | 2859 | 2 | 1 | 0 | 1 | 0 | 0 | 1 | 0 | 1 | 0 | 0 | 2 | 1 |
| NM_001042453 | MST4 | 3105 | 1 | 0 | 0 | 0 | 0 | 0 | 0 | 0 | 1 | 0 | 0 | 1 | 1 |
| NM_001042454 | TGFB1I1 | 1804 | 1 | 0 | 0 | 1 | 1 | 0 | 0 | 0 | 0 | 1 | 0 | 0 | 0 |
| NM_001042459 | FILIP1L | 4211 | 71 | 29 | 23 | 5 | 71 | 6 | 8 | 4 | 16 | 66 | 52 | 41 | 26 |
| NM_001042461 | TRAPPC5 | 770 | 5 | 3 | 3 | 4 | 4 | 5 | 3 | 4 | 2 | 2 | 2 | 3 | 3 |
| NM_001042462 | TRAPPC5 | 679 | 5 | 3 | 3 | 4 | 4 | 5 | 3 | 5 | 2 | 2 | 2 | 3 | 3 |
| NM_001042463 | TMEM80 | 1623 | 1 | 0 | 0 | 1 | 0 | 0 | 1 | 0 | 0 | 0 | 0 | 1 | 0 |
| NM_001042465 | PSAP | 2831 | 140 | 77 | 126 | 28 | 77 | 111 | 66 | 58 | 93 | 53 | 41 | 140 | 52 |
| NM_001042466 | PSAP | 2828 | 140 | 77 | 126 | 28 | 77 | 111 | 66 | 58 | 94 | 53 | 41 | 140 | 52 |
| NM_001042467 | MLPH | 3672 | 2 | 1 | 0 | 1 | 1 | 0 | 0 | 1 | 2 | 0 | 0 | 0 | 0 |
| NM_001042469 | SUMF2 | 2009 | 26 | 7 | 4 | 4 | 10 | 6 | 5 | 4 | 3 | 3 | 26 | 4 | 3 |
| NM_001042470 | SUMF2 | 1802 | 29 | 7 | 4 | 4 | 11 | 7 | 6 | 4 | 3 | 3 | 29 | 4 | 3 |
| NM_001042471 | GSDMB | 1560 | 14 | 2 | 2 | 14 | 0 | 1 | 1 | 0 | 1 | 1 | 0 | 2 | 2 |
| NM_001042472 | ABHD12 | 1966 | 6 | 2 | 2 | 2 | 2 | 6 | 2 | 2 | 2 | 2 | 1 | 2 | 2 |
| NM_001042473 | ACBD5 | 3859 | 10 | 5 | 4 | 2 | 7 | 8 | 10 | 7 | 4 | 3 | 3 | 5 | 8 |
| NM_001042474 | ZNF565 | 1943 | 1 | 1 | 1 | 0 | 0 | 1 | 1 | 0 | 0 | 1 | 1 | 1 | 1 |
| NM_001042475 | C6orf204 | 3335 | 11 | 5 | 2 | 1 | 4 | 5 | 2 | 1 | 5 | 8 | 10 | 7 | 11 |
| NM_001042476 | CARHSP1 | 3013 | 3 | 2 | 1 | 1 | 1 | 2 | 3 | 2 | 2 | 3 | 0 | 2 | 2 |
| NM_001042478 | AJAP1 | 2070 | 1 | 0 | 0 | 0 | 0 | 1 | 0 | 0 | 0 | 0 | 0 | 0 | 0 |
| NM_001042479 | GEMIN8 | 3171 | 2 | 1 | 1 | 1 | 1 | 2 | 1 | 0 | 1 | 2 | 0 | 1 | 1 |
| NM_001042480 | GEMIN8 | 3089 | 2 | 1 | 1 | 1 | 1 | 2 | 1 | 0 | 1 | 2 | 0 | 1 | 1 |
| NM_001042481 | FRMD6 | 5035 | 4 | 1 | 2 | 1 | 1 | 1 | 1 | 0 | 1 | 3 | 1 | 2 | 4 |
| NM_001042482 | TPK1 | 2292 | 3 | 1 | 1 | 1 | 2 | 2 | 1 | 1 | 1 | 1 | 0 | 3 | 1 |
| NM_001042483 | NUPR1 | 912 | 15 | 9 | 15 | 8 | 8 | 4 | 8 | 12 | 12 | 10 | 5 | 4 | 9 |
| NM_001042486 | DLGAP4 | 3007 | 6 | 3 | 3 | 2 | 3 | 6 | 2 | 1 | 2 | 3 | 5 | 2 | 4 |
| NM_001042490 | GTF2H2D | 1803 | 0 | 0 | 0 | 0 | 0 | 0 | 0 | 0 | 0 | 0 | 0 | 0 | 0 |
| NM_001042492 | NF1 | 12424 | 7 | 3 | 2 | 2 | 4 | 7 | 4 | 3 | 3 | 4 | 2 | 3 | 4 |
| NM_001042493 | C6orf162 | 2434 | 2 | 1 | 1 | 1 | 1 | 2 | 2 | 1 | 1 | 2 | 1 | 1 | 2 |
| NM_001042494 | SLC12A6 | 7688 | 3 | 2 | 1 | 1 | 2 | 2 | 3 | 1 | 2 | 1 | 1 | 3 | 2 |
| NM_001042495 | SLC12A6 | 7363 | 4 | 2 | 1 | 1 | 2 | 3 | 3 | 1 | 2 | 1 | 1 | 4 | 3 |
| NM_001042496 | SLC12A6 | 7754 | 3 | 2 | 1 | 1 | 2 | 2 | 3 | 1 | 2 | 1 | 1 | 3 | 2 |
| NM_001042497 | SLC12A6 | 7457 | 4 | 2 | 1 | 1 | 2 | 3 | 3 | 1 | 2 | 1 | 1 | 4 | 3 |
| NM_001042498 | SLC35A2 | 2619 | 1 | 0 | 0 | 0 | 0 | 0 | 0 | 1 | 0 | 0 | 0 | 0 | 0 |

| | | transcript | | | | | | hypothala | | | | | skeletalm | | |
|---|---|---|---|---|---|---|---|---|---|---|---|---|---|---|---|
| transcript | gene | length (nt) | max | mean | adipose | colon | heart | mus | kidney | liver | lung | ovary | uscle | spleen | testes |
| NM_001042500 | DEFA1B | 498 | 0 | 0 | 0 | 0 | 0 | 0 | 0 | 0 | 0 | 0 | 0 | 0 | 0 |
| NM_001042506 | PABPC1L2B | 2200 | 1 | 0 | 0 | 0 | 0 | 1 | 0 | 0 | 0 | 0 | 0 | 0 | 0 |
| NM_001042507 | LGALS7B | 489 | 0 | 0 | 0 | 0 | 0 | 0 | 0 | 0 | 0 | 0 | 0 | 0 | 0 |
| NM_001042510 | ZNF706 | 2860 | 9 | 5 | 3 | 2 | 5 | 9 | 4 | 2 | 4 | 6 | 5 | 8 | 4 |
| NM_001042511 | ZNF706 | 2658 | 10 | 5 | 3 | 2 | 5 | 10 | 4 | 2 | 4 | 7 | 6 | 8 | 4 |
| NM_001042512 | FRMPD2 | 1869 | 0 | 0 | 0 | 0 | 0 | 0 | 0 | 0 | 0 | 0 | 0 | 0 | 0 |
| NM_001042515 | FRMPD2L2 | 1883 | 0 | 0 | 0 | 0 | 0 | 0 | 0 | 0 | 0 | 0 | 0 | 0 | 0 |
| NM_001042516 | FRMPD2L2 | 1869 | 0 | 0 | 0 | 0 | 0 | 0 | 0 | 0 | 0 | 0 | 0 | 0 | 0 |
| NM_001042517 | DIAPH3 | 4796 | 2 | 0 | 0 | 1 | 0 | 0 | 0 | 0 | 1 | 0 | 0 | 0 | 2 |
| NM_001042518 | SERPINA9 | 1541 | 0 | 0 | 0 | 0 | 0 | 0 | 0 | 0 | 0 | 0 | 0 | 0 | 0 |
| NM_001042519 | C2orf88 | 4035 | 4 | 2 | 1 | 2 | 3 | 4 | 2 | 1 | 1 | 1 | 2 | 1 | 3 |
| NM_001042520 | C2orf88 | 3991 | 4 | 2 | 1 | 2 | 3 | 4 | 2 | 2 | 1 | 1 | 2 | 1 | 3 |
| NM_001042521 | C2orf88 | 4003 | 4 | 2 | 1 | 2 | 3 | 4 | 2 | 1 | 1 | 1 | 2 | 1 | 3 |
| NM_001042522 | SPRED3 | 1323 | 1 | 0 | 0 | 0 | 0 | 1 | 0 | 0 | 0 | 0 | 0 | 0 | 0 |
| NM_001042524 | FRMPD2L1 | 1883 | 0 | 0 | 0 | 0 | 0 | 0 | 0 | 0 | 0 | 0 | 0 | 0 | 0 |
| NM_001042525 | FRMPD2L1 | 1869 | 0 | 0 | 0 | 0 | 0 | 0 | 0 | 0 | 0 | 0 | 0 | 0 | 0 |
| NM_001042529 | COASY | 2161 | 5 | 2 | 1 | 2 | 1 | 2 | 5 | 5 | 2 | 1 | 1 | 3 | 2 |
| NM_001042530 | COASY | 2417 | 5 | 2 | 1 | 2 | 1 | 2 | 5 | 5 | 2 | 1 | 1 | 3 | 1 |
| NM_001042531 | COASY | 1840 | 5 | 2 | 1 | 2 | 1 | 2 | 5 | 5 | 2 | 1 | 1 | 3 | 2 |
| NM_001042532 | COASY | 2292 | 5 | 2 | 1 | 2 | 1 | 2 | 5 | 5 | 2 | 1 | 1 | 3 | 1 |
| NM_001042533 | MINA | 5271 | 2 | 1 | 1 | 1 | 1 | 2 | 2 | 2 | 1 | 1 | 2 | 2 | 1 |
| NM_001042535 | AGAP3 | 2045 | 2 | 0 | 0 | 0 | 0 | 2 | 0 | 0 | 0 | 0 | 0 | 0 | 0 |
| NM_001042536 | INSC | 2878 | 0 | 0 | 0 | 0 | 0 | 0 | 0 | 0 | 0 | 0 | 0 | 0 | 0 |
| NM_001042537 | SLC9A6 | 4727 | 20 | 4 | 2 | 1 | 4 | 20 | 3 | 2 | 2 | 3 | 2 | 3 | 3 |
| NM_001042539 | MAZ | 2779 | 15 | 11 | 7 | 15 | 9 | 11 | 9 | 5 | 12 | 12 | 11 | 15 | 15 |
| NM_001042540 | NCBP2 | 2011 | 16 | 10 | 12 | 5 | 10 | 16 | 11 | 6 | 6 | 12 | 6 | 16 | 9 |
| NM_001042543 | GLRA3 | 3592 | 1 | 0 | 0 | 0 | 0 | 1 | 0 | 0 | 0 | 0 | 0 | 0 | 0 |
| NM_001042544 | LTBP4 | 5144 | 2 | 1 | 0 | 1 | 1 | 0 | 0 | 0 | 1 | 2 | 0 | 1 | 1 |
| NM_001042545 | LTBP4 | 4960 | 2 | 1 | 0 | 1 | 1 | 0 | 0 | 0 | 1 | 2 | 0 | 1 | 1 |
| NM_001042546 | ATPAF1 | 3925 | 17 | 6 | 4 | 2 | 17 | 12 | 7 | 6 | 2 | 4 | 6 | 3 | 4 |
| NM_001042548 | FEZ2 | 2095 | 35 | 14 | 18 | 7 | 12 | 20 | 9 | 5 | 8 | 9 | 35 | 14 | 14 |
| NM_001042549 | NSL1 | 13220 | 2 | 1 | 1 | 1 | 1 | 2 | 1 | 1 | 1 | 2 | 1 | 2 | 1 |
| NM_001042550 | SMC2 | 6048 | 3 | 2 | 2 | 1 | 2 | 2 | 2 | 1 | 1 | 2 | 1 | 3 | 3 |
| NM_001042551 | SMC2 | 5986 | 3 | 2 | 2 | 1 | 2 | 2 | 2 | 1 | 1 | 2 | 1 | 3 | 3 |
| NM_001042552 | TATDN3 | 2588 | 3 | 2 | 2 | 1 | 2 | 3 | 3 | 1 | 1 | 1 | 2 | 2 | 1 |
| NM_001042553 | TATDN3 | 2585 | 3 | 2 | 2 | 1 | 2 | 3 | 3 | 1 | 1 | 1 | 2 | 2 | 1 |
| NM_001042555 | FRS2 | 6857 | 4 | 2 | 2 | 1 | 2 | 3 | 2 | 1 | 1 | 4 | 1 | 2 | 3 |
| NM_001042559 | EIF4G2 | 3797 | 70 | 50 | 40 | 24 | 70 | 50 | 56 | 48 | 43 | 43 | 50 | 69 | 60 |
| NM_001042572 | CHD2 | 2234 | 32 | 19 | 12 | 11 | 16 | 15 | 17 | 9 | 26 | 32 | 16 | 28 | 22 |
| NM_001042573 | ENGASE | 4490 | 1 | 1 | 1 | 1 | 0 | 1 | 1 | 0 | 0 | 0 | 0 | 1 | 0 |
| NM_001042574 | CRTC3 | 5168 | 3 | 1 | 1 | 1 | 2 | 1 | 1 | 0 | 1 | 3 | 1 | 2 | 2 |
| NM_001042575 | TMPRSS7 | 2509 | 0 | 0 | 0 | 0 | 0 | 0 | 0 | 0 | 0 | 0 | 0 | 0 | 0 |
| NM_001042576 | RRBP1 | 3792 | 12 | 4 | 3 | 5 | 3 | 2 | 5 | 12 | 3 | 3 | 1 | 5 | 4 |
| NM_001042581 | SNUPN | 1698 | 7 | 3 | 5 | 3 | 2 | 7 | 3 | 3 | 1 | 2 | 1 | 4 | 5 |
| NM_001042583 | CD1E | 2027 | 0 | 0 | 0 | 0 | 0 | 0 | 0 | 0 | 0 | 0 | 0 | 0 | 0 |
| NM_001042584 | CD1E | 1959 | 0 | 0 | 0 | 0 | 0 | 0 | 0 | 0 | 0 | 0 | 0 | 0 | 0 |
| NM_001042585 | CD1E | 1862 | 0 | 0 | 0 | 0 | 0 | 0 | 0 | 0 | 0 | 0 | 0 | 0 | 0 |
| NM_001042586 | CD1E | 1460 | 0 | 0 | 0 | 0 | 0 | 0 | 0 | 0 | 0 | 0 | 0 | 0 | 0 |
| NM_001042587 | CD1E | 1392 | 0 | 0 | 0 | 0 | 0 | 0 | 0 | 0 | 0 | 0 | 0 | 0 | 0 |
| NM_001042588 | SNUPN | 1503 | 7 | 4 | 5 | 3 | 2 | 7 | 3 | 3 | 1 | 3 | 1 | 4 | 5 |
| NM_001042589 | TMEM8B | 3441 | 2 | 1 | 0 | 1 | 0 | 2 | 1 | 0 | 0 | 1 | 0 | 0 | 1 |
| NM_001042590 | TMEM8B | 3714 | 2 | 1 | 0 | 1 | 0 | 2 | 1 | 0 | 0 | 1 | 1 | 0 | 1 |
| NM_001042594 | POT1 | 3951 | 5 | 2 | 1 | 0 | 2 | 2 | 2 | 1 | 2 | 3 | 1 | 3 | 5 |
| NM_001042595 | TMEM91 | 971 | 2 | 1 | 1 | 2 | 1 | 1 | 1 | 0 | 1 | 2 | 0 | 2 | 1 |
| NM_001042599 | ERBB4 | 11875 | 7 | 2 | 0 | 0 | 2 | 7 | 5 | 0 | 1 | 0 | 1 | 0 | 6 |
| NM_001042600 | MAP4K1 | 2681 | 2 | 0 | 0 | 0 | 0 | 0 | 0 | 0 | 0 | 0 | 0 | 2 | 0 |
| NM_001042601 | TTC14 | 4786 | 9 | 4 | 4 | 2 | 3 | 4 | 5 | 2 | 5 | 7 | 1 | 9 | 6 |
| NM_001042603 | KDM5A | 10835 | 7 | 4 | 3 | 2 | 3 | 3 | 4 | 2 | 4 | 5 | 3 | 7 | 6 |
| NM_001042604 | XRN1 | 10053 | 9 | 5 | 3 | 2 | 4 | 3 | 4 | 5 | 7 | 7 | 7 | 9 | 6 |
| NM_001042610 | DBNDD1 | 2129 | 2 | 0 | 0 | 0 | 0 | 2 | 1 | 0 | 0 | 0 | 1 | 0 | 0 |
| NM_001042616 | PIGY | 1353 | 8 | 6 | 6 | 3 | 7 | 8 | 7 | 7 | 5 | 7 | 5 | 7 | 7 |
| NM_001042618 | PARP2 | 1848 | 9 | 4 | 3 | 2 | 3 | 9 | 3 | 2 | 2 | 4 | 5 | 3 | 6 |
| NM_001042625 | CAPSL | 971 | 3 | 1 | 0 | 0 | 0 | 2 | 0 | 0 | 3 | 0 | 0 | 0 | 1 |
| NM_001042631 | SDHAF1 | 1114 | 2 | 1 | 1 | 1 | 1 | 2 | 1 | 1 | 1 | 0 | 1 | 2 | 1 |
| NM_001042632 | SNX21 | 3166 | 1 | 1 | 1 | 1 | 1 | 1 | 1 | 0 | 1 | 1 | 1 | 1 | 1 |
| NM_001042633 | SNX21 | 3184 | 1 | 1 | 1 | 1 | 1 | 1 | 1 | 0 | 1 | 1 | 1 | 1 | 1 |
| NM_001042635 | NGDN | 1142 | 8 | 5 | 3 | 2 | 6 | 8 | 5 | 5 | 3 | 7 | 6 | 8 | 6 |
| NM_001042646 | TRAK1 | 5117 | 6 | 4 | 3 | 4 | 6 | 4 | 4 | 1 | 3 | 2 | 5 | 5 | 5 |
| NM_001042663 | PLEKHG5 | 4761 | 2 | 1 | 2 | 1 | 1 | 2 | 0 | 0 | 0 | 1 | 0 | 2 | 0 |
| NM_001042664 | PLEKHG5 | 4715 | 2 | 1 | 2 | 1 | 1 | 2 | 0 | 0 | 0 | 1 | 0 | 2 | 0 |
| NM_001042665 | PLEKHG5 | 4698 | 2 | 1 | 2 | 1 | 1 | 2 | 0 | 0 | 0 | 1 | 0 | 2 | 0 |
| NM_001042669 | ARHGAP24 | 4195 | 12 | 4 | 6 | 1 | 4 | 4 | 12 | 1 | 4 | 2 | 2 | 7 | 2 |
| NM_001042678 | RHOC | 1328 | 5 | 3 | 3 | 4 | 4 | 3 | 5 | 3 | 3 | 1 | 0 | 5 | 4 |
| NM_001042679 | RHOC | 1295 | 6 | 3 | 4 | 4 | 5 | 3 | 6 | 3 | 3 | 1 | 0 | 5 | 4 |
| NM_001042680 | C19orf28 | 2082 | 2 | 0 | 1 | 0 | 0 | 0 | 2 | 0 | 0 | 0 | 0 | 0 | 0 |
| NM_001042681 | RERE | 8009 | 22 | 12 | 7 | 11 | 12 | 22 | 12 | 3 | 8 | 13 | 13 | 13 | 18 |
| NM_001042682 | RERE | 6308 | 11 | 6 | 4 | 4 | 9 | 5 | 7 | 2 | 4 | 6 | 11 | 7 | 6 |

| transcript | gene | transcript length (nt) | max | mean | adipose | colon | heart | hypothalamus | kidney | liver | lung | ovary | skeletalmuscle | spleen | testes |
|---|---|---|---|---|---|---|---|---|---|---|---|---|---|---|---|
| NM_001042683 | SHPRH | 7346 | 8 | 3 | 2 | 1 | 2 | 2 | 4 | 1 | 3 | 8 | 3 | 5 | 5 |
| NM_001042685 | LGALS9B | 1243 | 0 | 0 | 0 | 0 | 0 | 0 | 0 | 0 | 0 | 0 | 0 | 0 | 0 |
| NM_001042690 | C4orf44 | 1670 | 0 | 0 | 0 | 0 | 0 | 0 | 0 | 0 | 0 | 0 | 0 | 0 | 0 |
| NM_001042692 | C2orf86 | 2439 | 4 | 3 | 3 | 1 | 4 | 2 | 3 | 1 | 2 | 4 | 2 | 2 | 4 |
| NM_001042693 | FAM159A | 698 | 0 | 0 | 0 | 0 | 0 | 0 | 0 | 0 | 0 | 0 | 0 | 0 | 0 |
| NM_001042697 | ZSWIM7 | 2007 | 5 | 3 | 3 | 3 | 3 | 4 | 3 | 2 | 3 | 4 | 2 | 5 | 3 |
| NM_001042698 | ZSWIM7 | 1877 | 5 | 3 | 3 | 3 | 3 | 4 | 3 | 2 | 3 | 4 | 2 | 5 | 3 |
| NM_001042702 | DFNB59 | 1521 | 2 | 1 | 1 | 0 | 0 | 0 | 1 | 0 | 0 | 1 | 0 | 0 | 2 |
| NM_001042704 | CLCNKA | 2533 | 1 | 0 | 0 | 0 | 0 | 0 | 1 | 0 | 0 | 0 | 0 | 0 | 0 |
| NM_001042705 | IQCJ | 885 | 0 | 0 | 0 | 0 | 0 | 0 | 0 | 0 | 0 | 0 | 0 | 0 | 0 |
| NM_001042706 | IQCJ | 1459 | 0 | 0 | 0 | 0 | 0 | 0 | 0 | 0 | 0 | 0 | 0 | 0 | 0 |
| NM_001042717 | GTPBP10 | 3175 | 3 | 2 | 2 | 1 | 2 | 2 | 2 | 2 | 1 | 3 | 2 | 3 | 3 |
| NM_001042723 | RYR1 | 15376 | 78 | 7 | 1 | 0 | 0 | 1 | 0 | 0 | 0 | 0 | 78 | 0 | 1 |
| NM_001042724 | PVRL2 | 2856 | 2 | 1 | 0 | 0 | 1 | 0 | 1 | 1 | 1 | 0 | 0 | 1 | 2 |
| NM_001042728 | RARG | 2705 | 0 | 0 | 0 | 0 | 0 | 0 | 0 | 0 | 0 | 0 | 0 | 0 | 0 |
| NM_001042729 | FGR | 2426 | 3 | 1 | 1 | 0 | 0 | 0 | 0 | 0 | 2 | 0 | 0 | 3 | 0 |
| NM_001042734 | SEC24B | 4541 | 15 | 10 | 7 | 3 | 9 | 9 | 7 | 15 | 9 | 13 | 12 | 12 | 11 |
| NM_001042747 | FGR | 2485 | 3 | 1 | 1 | 0 | 0 | 0 | 0 | 0 | 1 | 0 | 0 | 3 | 0 |
| NM_001042749 | STAG2 | 6269 | 15 | 8 | 5 | 2 | 6 | 6 | 8 | 5 | 11 | 14 | 8 | 15 | 6 |
| NM_001042750 | STAG2 | 6178 | 15 | 8 | 5 | 2 | 6 | 5 | 8 | 5 | 11 | 14 | 7 | 15 | 6 |
| NM_001042751 | STAG2 | 6067 | 15 | 8 | 5 | 2 | 6 | 5 | 8 | 5 | 11 | 13 | 7 | 15 | 6 |
| NM_001042758 | SRGAP2 | 3382 | 15 | 6 | 5 | 3 | 4 | 15 | 3 | 2 | 8 | 5 | 2 | 12 | 4 |
| NM_001042762 | FIGNL1 | 3531 | 2 | 1 | 1 | 1 | 1 | 1 | 1 | 1 | 1 | 2 | 1 | 1 | 1 |
| NM_001042771 | LCK | 2100 | 2 | 0 | 0 | 0 | 0 | 0 | 0 | 0 | 1 | 0 | 0 | 2 | 0 |
| NM_001042780 | TNNT3 | 1179 | 283 | 26 | 3 | 0 | 1 | 0 | 0 | 0 | 0 | 0 | 283 | 0 | 1 |
| NM_001042781 | TNNT3 | 1197 | 282 | 26 | 3 | 0 | 2 | 0 | 0 | 0 | 0 | 0 | 282 | 0 | 1 |
| NM_001042782 | TNNT3 | 1179 | 286 | 27 | 3 | 0 | 2 | 0 | 0 | 0 | 0 | 0 | 286 | 0 | 1 |
| NM_001042784 | CCDC158 | 3626 | 10 | 1 | 0 | 0 | 0 | 1 | 1 | 1 | 0 | 0 | 0 | 0 | 10 |
| NM_001043 | SLC6A2 | 3347 | 0 | 0 | 0 | 0 | 0 | 0 | 0 | 0 | 0 | 0 | 0 | 0 | 0 |
| NM_001043229 | METTL12 | 1382 | 1 | 0 | 0 | 0 | 0 | 0 | 0 | 0 | 0 | 0 | 0 | 1 | 0 |
| NM_001043318 | C14orf43 | 7656 | 4 | 2 | 2 | 2 | 1 | 2 | 1 | 0 | 2 | 2 | 1 | 4 | 2 |
| NM_001043351 | TPM3 | 3212 | 284 | 63 | 38 | 24 | 45 | 42 | 31 | 26 | 49 | 16 | 284 | 92 | 39 |
| NM_001043352 | TPM3 | 4539 | 161 | 42 | 28 | 19 | 31 | 32 | 23 | 20 | 37 | 12 | 161 | 70 | 29 |
| NM_001043353 | TPM3 | 4539 | 202 | 45 | 27 | 18 | 32 | 32 | 22 | 19 | 35 | 12 | 202 | 66 | 28 |
| NM_001044 | SLC6A3 | 3934 | 1 | 0 | 0 | 0 | 0 | 1 | 0 | 0 | 0 | 0 | 0 | 0 | 0 |
| NM_001044264 | MSMP | 672 | 2 | 1 | 1 | 1 | 1 | 1 | 2 | 1 | 1 | 1 | 1 | 2 | 1 |
| NM_001044305 | SMAP1 | 3331 | 6 | 2 | 2 | 1 | 2 | 6 | 1 | 1 | 1 | 1 | 2 | 1 | 2 |
| NM_001044369 | FAM69C | 2042 | 3 | 1 | 0 | 0 | 0 | 3 | 0 | 0 | 0 | 0 | 0 | 0 | 3 |
| NM_001044370 | MPPED1 | 2394 | 1 | 0 | 0 | 0 | 0 | 1 | 0 | 0 | 0 | 0 | 0 | 0 | 0 |
| NM_001044385 | ALS2CR4 | 5399 | 2 | 1 | 1 | 0 | 2 | 2 | 1 | 0 | 1 | 2 | 2 | 0 | 1 |
| NM_001044387 | ZNF557 | 5989 | 4 | 2 | 1 | 1 | 1 | 2 | 2 | 1 | 1 | 4 | 1 | 3 | 2 |
| NM_001044388 | ZNF557 | 5980 | 4 | 2 | 1 | 1 | 1 | 2 | 2 | 1 | 1 | 4 | 1 | 3 | 2 |
| NM_001044390 | MUC1 | 989 | 15 | 3 | 0 | 5 | 0 | 0 | 12 | 0 | 15 | 0 | 0 | 0 | 1 |
| NM_001044391 | MUC1 | 830 | 20 | 4 | 0 | 6 | 0 | 0 | 20 | 0 | 20 | 0 | 0 | 0 | 1 |
| NM_001044392 | MUC1 | 857 | 20 | 4 | 0 | 6 | 0 | 0 | 20 | 0 | 19 | 0 | 0 | 0 | 1 |
| NM_001044393 | MUC1 | 957 | 16 | 3 | 0 | 6 | 0 | 0 | 13 | 0 | 16 | 0 | 0 | 0 | 1 |
| NM_001044722 | CSNK1G3 | 4341 | 5 | 3 | 2 | 2 | 2 | 4 | 2 | 3 | 3 | 4 | 1 | 5 | 5 |
| NM_001044723 | CSNK1G3 | 4461 | 5 | 3 | 2 | 2 | 2 | 4 | 2 | 3 | 3 | 4 | 1 | 5 | 5 |
| NM_001045 | SLC6A4 | 4531 | 5 | 1 | 0 | 0 | 0 | 0 | 0 | 0 | 5 | 0 | 0 | 0 | 0 |
| NM_001045476 | WDR38 | 1252 | 0 | 0 | 0 | 0 | 0 | 0 | 0 | 0 | 0 | 0 | 0 | 0 | 0 |
| NM_001045477 | FAM22G | 4888 | 0 | 0 | 0 | 0 | 0 | 0 | 0 | 0 | 0 | 0 | 0 | 0 | 0 |
| NM_001045479 | S100A7L2 | 480 | 0 | 0 | 0 | 0 | 0 | 0 | 0 | 0 | 0 | 0 | 0 | 0 | 0 |
| NM_001045480 | PRAMEF16 | 1586 | 0 | 0 | 0 | 0 | 0 | 0 | 0 | 0 | 0 | 0 | 0 | 0 | 0 |
| NM_001045556 | SLA | 3459 | 29 | 5 | 3 | 1 | 4 | 2 | 1 | 2 | 7 | 0 | 1 | 29 | 1 |
| NM_001045557 | SLA | 3181 | 31 | 5 | 3 | 1 | 5 | 2 | 1 | 2 | 7 | 0 | 1 | 31 | 1 |
| NM_001046 | SLC12A2 | 6860 | 12 | 5 | 3 | 10 | 2 | 12 | 3 | 2 | 2 | 4 | 3 | 3 | 4 |
| NM_001047 | SRD5A1 | 2264 | 3 | 1 | 0 | 0 | 0 | 1 | 1 | 3 | 0 | 0 | 0 | 0 | 0 |
| NM_001047160 | NET1 | 3398 | 17 | 9 | 17 | 10 | 5 | 8 | 12 | 11 | 6 | 11 | 5 | 2 | 8 |
| NM_001047434 | DPH3 | 3989 | 3 | 2 | 2 | 1 | 3 | 2 | 2 | 3 | 2 | 1 | 3 | 3 | 2 |
| NM_001047980 | NBPF7 | 1887 | 5 | 2 | 3 | 2 | 1 | 0 | 2 | 1 | 2 | 5 | 1 | 3 | 3 |
| NM_001048 | SST | 632 | 6 | 1 | 0 | 1 | 0 | 6 | 0 | 0 | 0 | 0 | 0 | 0 | 0 |
| NM_001048164 | SLC7A3 | 2227 | 0 | 0 | 0 | 0 | 0 | 0 | 0 | 0 | 0 | 0 | 0 | 0 | 0 |
| NM_001048166 | STIL | 5019 | 2 | 0 | 0 | 1 | 0 | 0 | 0 | 0 | 0 | 0 | 0 | 0 | 2 |
| NM_001048171 | MUTYH | 1888 | 0 | 0 | 0 | 0 | 0 | 0 | 0 | 0 | 0 | 0 | 0 | 0 | 0 |
| NM_001048172 | MUTYH | 1779 | 1 | 0 | 0 | 0 | 0 | 0 | 0 | 0 | 0 | 0 | 0 | 1 | 0 |
| NM_001048173 | MUTYH | 1776 | 1 | 0 | 0 | 0 | 0 | 0 | 0 | 0 | 0 | 0 | 0 | 1 | 0 |
| NM_001048174 | MUTYH | 1695 | 1 | 0 | 0 | 0 | 0 | 0 | 0 | 0 | 0 | 0 | 0 | 1 | 0 |
| NM_001048181 | OPN1MW2 | 1281 | 0 | 0 | 0 | 0 | 0 | 0 | 0 | 0 | 0 | 0 | 0 | 0 | 0 |
| NM_001048183 | PHACTR4 | 6211 | 5 | 3 | 2 | 3 | 3 | 2 | 5 | 3 | 3 | 4 | 3 | 5 | 4 |
| NM_001048194 | RCC1 | 2532 | 0 | 0 | 0 | 0 | 0 | 0 | 0 | 0 | 0 | 0 | 0 | 0 | 0 |
| NM_001048195 | RCC1 | 2490 | 0 | 0 | 0 | 0 | 0 | 0 | 0 | 0 | 0 | 0 | 0 | 0 | 0 |
| NM_001048199 | SNHG3-RCC | 2715 | 0 | 0 | 0 | 0 | 0 | 0 | 0 | 0 | 0 | 0 | 0 | 0 | 0 |
| NM_001048200 | HIPK3 | 4680 | 36 | 18 | 14 | 5 | 36 | 12 | 16 | 10 | 17 | 21 | 31 | 21 | 20 |
| NM_001048201 | UHRF1 | 3900 | 1 | 0 | 0 | 0 | 0 | 0 | 0 | 0 | 0 | 0 | 0 | 1 | 0 |
| NM_001048205 | REC8 | 2331 | 1 | 0 | 0 | 1 | 0 | 1 | 1 | 0 | 0 | 0 | 0 | 1 | 1 |
| NM_001048209 | NTM | 3042 | 11 | 2 | 2 | 1 | 1 | 11 | 0 | 0 | 1 | 0 | 0 | 0 | 0 |
| NM_001048210 | CLCC1 | 4825 | 10 | 7 | 6 | 3 | 10 | 10 | 6 | 6 | 5 | 9 | 8 | 8 | 8 |

| transcript | gene | transcript length (nt) | max | mean | adipose | colon | heart | hypothalamus | kidney | liver | lung | ovary | skeletalmuscle | spleen | testes |
|---|---|---|---|---|---|---|---|---|---|---|---|---|---|---|---|
| NM_001048212 | CEMP1 | 1384 | 1 | 0 | 0 | 0 | 0 | 0 | 1 | 0 | 0 | 0 | 0 | 0 | 0 |
| NM_001048218 | SCYL1 | 2598 | 3 | 2 | 2 | 1 | 2 | 3 | 2 | 3 | 2 | 2 | 3 | 3 | 2 |
| NM_001048221 | DBNDD2 | 1141 | 1 | 0 | 0 | 0 | 0 | 1 | 0 | 0 | 0 | 0 | 0 | 0 | 0 |
| NM_001048222 | DBNDD2 | 1239 | 1 | 0 | 0 | 0 | 0 | 1 | 0 | 0 | 0 | 0 | 0 | 0 | 0 |
| NM_001048223 | DBNDD2 | 1097 | 0 | 0 | 0 | 0 | 0 | 0 | 0 | 0 | 0 | 0 | 0 | 0 | 0 |
| NM_001048224 | DBNDD2 | 1195 | 0 | 0 | 0 | 0 | 0 | 0 | 0 | 0 | 0 | 0 | 0 | 0 | 0 |
| NM_001048225 | DBNDD2 | 1146 | 0 | 0 | 0 | 0 | 0 | 0 | 0 | 0 | 0 | 0 | 0 | 0 | 0 |
| NM_001048226 | DBNDD2 | 1244 | 0 | 0 | 0 | 0 | 0 | 0 | 0 | 0 | 0 | 0 | 0 | 0 | 0 |
| NM_001048230 | ADORA1 | 2764 | 3 | 1 | 0 | 0 | 0 | 3 | 0 | 0 | 0 | 0 | 0 | 0 | 2 |
| NM_001048241 | UBL5 | 439 | 39 | 16 | 7 | 7 | 39 | 17 | 21 | 12 | 19 | 9 | 17 | 21 | 8 |
| NM_001048249 | C5orf43 | 2890 | 8 | 4 | 4 | 3 | 3 | 5 | 5 | 8 | 3 | 5 | 1 | 5 | 4 |
| NM_001048251 | CMTM3 | 2063 | 2 | 1 | 1 | 1 | 1 | 1 | 1 | 0 | 1 | 1 | 0 | 1 | 2 |
| NM_001048252 | CTXN3 | 1683 | 2 | 0 | 0 | 0 | 0 | 1 | 2 | 0 | 0 | 0 | 0 | 0 | 0 |
| NM_001048265 | C9orf116 | 722 | 1 | 0 | 0 | 0 | 0 | 1 | 1 | 0 | 1 | 0 | 0 | 0 | 1 |
| NM_001049 | SSTR1 | 4291 | 1 | 0 | 0 | 0 | 0 | 1 | 0 | 0 | 0 | 0 | 0 | 0 | 0 |
| NM_001050 | SSTR2 | 2964 | 3 | 1 | 0 | 0 | 0 | 3 | 2 | 0 | 0 | 0 | 0 | 2 | 0 |
| NM_001051 | SSTR3 | 2123 | 1 | 0 | 0 | 0 | 0 | 0 | 0 | 0 | 0 | 0 | 0 | 0 | 1 |
| NM_001052 | SSTR4 | 1258 | 0 | 0 | 0 | 0 | 0 | 0 | 0 | 0 | 0 | 0 | 0 | 0 | 0 |
| NM_001053 | SSTR5 | 1362 | 0 | 0 | 0 | 0 | 0 | 0 | 0 | 0 | 0 | 0 | 0 | 0 | 0 |
| NM_001054 | SULT1A2 | 1049 | 0 | 0 | 0 | 0 | 0 | 0 | 0 | 0 | 0 | 0 | 0 | 0 | 0 |
| NM_001055 | SULT1A1 | 1222 | 4 | 1 | 4 | 1 | 1 | 1 | 0 | 3 | 2 | 0 | 0 | 2 | 0 |
| NM_001056 | SULT1C2 | 2795 | 8 | 1 | 0 | 0 | 0 | 0 | 8 | 0 | 0 | 0 | 0 | 0 | 0 |
| NM_001057 | TACR2 | 2416 | 2 | 1 | 0 | 2 | 2 | 1 | 0 | 0 | 0 | 1 | 1 | 0 | 0 |
| NM_001058 | TACR1 | 4778 | 1 | 0 | 1 | 0 | 0 | 0 | 0 | 0 | 0 | 1 | 0 | 0 | 0 |
| NM_001059 | TACR3 | 1753 | 0 | 0 | 0 | 0 | 0 | 0 | 0 | 0 | 0 | 0 | 0 | 0 | 0 |
| NM_001060 | TBXA2R | 2601 | 2 | 1 | 0 | 0 | 1 | 0 | 1 | 1 | 1 | 0 | 0 | 2 | 0 |
| NM_001061 | TBXAS1 | 2064 | 37 | 7 | 5 | 2 | 5 | 4 | 1 | 4 | 13 | 2 | 1 | 37 | 1 |
| NM_001062 | TCN1 | 1567 | 2 | 0 | 0 | 0 | 0 | 0 | 0 | 0 | 2 | 0 | 0 | 0 | 0 |
| NM_001063 | TF | 2790 | 694 | 74 | 12 | 3 | 4 | 76 | 1 | 694 | 2 | 5 | 3 | 7 | 6 |
| NM_001064 | TKT | 2099 | 43 | 21 | 43 | 11 | 11 | 18 | 29 | 17 | 36 | 15 | 2 | 40 | 10 |
| NM_001065 | TNFRSF1A | 2204 | 9 | 5 | 5 | 3 | 7 | 3 | 6 | 8 | 7 | 5 | 4 | 9 | 3 |
| NM_001066 | TNFRSF1B | 3675 | 7 | 2 | 2 | 1 | 1 | 1 | 0 | 1 | 3 | 0 | 1 | 7 | 1 |
| NM_001067 | TOP2A | 5697 | 12 | 2 | 2 | 2 | 1 | 0 | 0 | 0 | 3 | 1 | 0 | 3 | 12 |
| NM_001068 | TOP2B | 5189 | 29 | 18 | 11 | 7 | 23 | 23 | 18 | 15 | 17 | 23 | 15 | 29 | 18 |
| NM_001069 | TUBB2A | 1619 | 51 | 7 | 8 | 4 | 1 | 51 | 2 | 5 | 1 | 0 | 0 | 1 | 1 |
| NM_001070 | TUBG1 | 1949 | 3 | 1 | 1 | 1 | 1 | 2 | 1 | 2 | 0 | 0 | 1 | 1 | 3 |
| NM_001071 | TYMS | 1583 | 15 | 6 | 7 | 12 | 4 | 5 | 5 | 3 | 4 | 4 | 0 | 9 | 15 |
| NM_001071775 | C13orf37 | 2412 | 4 | 2 | 1 | 0 | 2 | 4 | 2 | 1 | 2 | 1 | 2 | 2 | 2 |
| NM_001072 | UGT1A6 | 2484 | 11 | 2 | 0 | 0 | 0 | 0 | 11 | 7 | 0 | 0 | 0 | 0 | 0 |
| NM_001073 | UGT2B11 | 1710 | 0 | 0 | 0 | 0 | 0 | 0 | 0 | 0 | 0 | 0 | 0 | 0 | 0 |
| NM_001074 | UGT2B7 | 1887 | 84 | 12 | 0 | 0 | 0 | 0 | 84 | 50 | 0 | 0 | 0 | 0 | 1 |
| NM_001075 | UGT2B10 | 2754 | 3 | 0 | 0 | 0 | 0 | 0 | 0 | 3 | 0 | 0 | 0 | 0 | 0 |
| NM_001075098 | MOCS1 | 4111 | 2 | 1 | 1 | 1 | 1 | 1 | 2 | 2 | 1 | 0 | 1 | 0 | 1 |
| NM_001075099 | SKAP1 | 1573 | 5 | 2 | 1 | 1 | 0 | 0 | 5 | 1 | 3 | 0 | 0 | 5 | 3 |
| NM_001076 | UGT2B15 | 2138 | 3 | 0 | 0 | 0 | 0 | 0 | 0 | 3 | 0 | 0 | 0 | 0 | 0 |
| NM_001076 | UGT2B15 | 2140 | 3 | 0 | 0 | 0 | 0 | 0 | 0 | 3 | 0 | 0 | 0 | 0 | 0 |
| NM_001076552 | ACSS2 | 3018 | 6 | 3 | 4 | 4 | 3 | 1 | 6 | 2 | 2 | 1 | 5 | 1 | 1 |
| NM_001076674 | TMUB2 | 1990 | 1 | 1 | 1 | 1 | 1 | 1 | 1 | 0 | 1 | 1 | 1 | 1 | 1 |
| NM_001076675 | ZNF626 | 6088 | 12 | 3 | 1 | 1 | 1 | 4 | 3 | 0 | 1 | 12 | 1 | 4 | 4 |
| NM_001076677 | CLTA | 1158 | 16 | 8 | 10 | 7 | 6 | 16 | 10 | 8 | 7 | 7 | 3 | 10 | 7 |
| NM_001076678 | ZNF493 | 4952 | 20 | 5 | 3 | 1 | 3 | 4 | 7 | 1 | 5 | 20 | 3 | 8 | 5 |
| NM_001076680 | C17orf108 | 1481 | 6 | 1 | 2 | 0 | 1 | 6 | 2 | 0 | 0 | 2 | 1 | 1 | 1 |
| NM_001076682 | NCAM1 | 4898 | 28 | 5 | 0 | 2 | 17 | 28 | 0 | 0 | 0 | 4 | 1 | 0 | 2 |
| NM_001076683 | UBTF | 4603 | 13 | 8 | 7 | 9 | 7 | 10 | 10 | 5 | 5 | 9 | 8 | 13 | 7 |
| NM_001076684 | UBTF | 4572 | 13 | 8 | 8 | 9 | 7 | 10 | 10 | 5 | 5 | 9 | 8 | 13 | 7 |
| NM_001076778 | FAM107A | 3366 | 50 | 7 | 1 | 1 | 7 | 50 | 2 | 0 | 5 | 1 | 1 | 3 | 1 |
| NM_001076780 | PKD1L2 | 3426 | 0 | 0 | 0 | 0 | 0 | 0 | 0 | 0 | 0 | 0 | 0 | 0 | 0 |
| NM_001076781 | ZNF391 | 1596 | 5 | 2 | 3 | 1 | 1 | 5 | 2 | 0 | 1 | 5 | 1 | 2 | 5 |
| NM_001076785 | SLC7A6 | 6348 | 4 | 2 | 3 | 2 | 3 | 1 | 1 | 0 | 2 | 1 | 4 | 3 | 2 |
| NM_001076786 | QSER1 | 9269 | 8 | 3 | 2 | 1 | 3 | 3 | 3 | 1 | 3 | 8 | 3 | 4 | 5 |
| NM_001076787 | TP53I11 | 3648 | 7 | 3 | 3 | 4 | 5 | 3 | 1 | 0 | 3 | 1 | 1 | 7 | 1 |
| NM_001077 | UGT2B17 | 2076 | 8 | 1 | 0 | 8 | 0 | 0 | 0 | 0 | 0 | 0 | 0 | 0 | 0 |
| NM_001077180 | METTL9 | 3192 | 8 | 4 | 3 | 1 | 4 | 4 | 8 | 1 | 4 | 3 | 3 | 6 | 3 |
| NM_001077181 | CDC14B | 5131 | 4 | 3 | 4 | 1 | 4 | 3 | 4 | 4 | 1 | 2 | 1 | 1 | 3 |
| NM_001077182 | FSCN2 | 1585 | 0 | 0 | 0 | 0 | 0 | 0 | 0 | 0 | 0 | 0 | 0 | 0 | 0 |
| NM_001077183 | TSC2 | 5431 | 3 | 2 | 1 | 3 | 1 | 2 | 1 | 1 | 1 | 1 | 2 | 2 | 2 |
| NM_001077186 | MYH14 | 6813 | 6 | 2 | 2 | 6 | 3 | 2 | 1 | 2 | 2 | 0 | 6 | 0 | 0 |
| NM_001077188 | HS6ST2 | 4567 | 2 | 1 | 0 | 0 | 0 | 2 | 2 | 0 | 0 | 2 | 0 | 0 | 1 |
| NM_001077191 | GPBAR1 | 2002 | 0 | 0 | 0 | 0 | 0 | 0 | 0 | 0 | 0 | 0 | 0 | 0 | 0 |
| NM_001077194 | GPBAR1 | 1494 | 0 | 0 | 0 | 0 | 0 | 0 | 0 | 0 | 0 | 0 | 0 | 0 | 0 |
| NM_001077195 | ZNF436 | 4493 | 7 | 3 | 4 | 1 | 3 | 7 | 2 | 1 | 2 | 7 | 1 | 4 | 4 |
| NM_001077196 | PDE11A | 7779 | 1 | 1 | 1 | 0 | 0 | 0 | 0 | 1 | 1 | 0 | 1 | 0 | 0 |
| NM_001077197 | PDE11A | 8309 | 1 | 0 | 1 | 0 | 0 | 0 | 0 | 1 | 1 | 0 | 1 | 0 | 0 |
| NM_001077198 | ATG9A | 3773 | 5 | 2 | 1 | 2 | 3 | 4 | 2 | 3 | 2 | 1 | 5 | 2 | 3 |
| NM_001077199 | SFRS12 | 4013 | 14 | 9 | 10 | 3 | 9 | 13 | 11 | 5 | 9 | 11 | 8 | 12 | 14 |
| NM_001077203 | SENP7 | 4738 | 5 | 3 | 2 | 1 | 2 | 2 | 3 | 1 | 2 | 5 | 2 | 5 | 4 |
| NM_001077204 | CSPP1 | 4470 | 14 | 5 | 3 | 2 | 4 | 5 | 7 | 2 | 5 | 6 | 6 | 5 | 14 |

| transcript | gene | transcript length (nt) | max | mean | adipose | colon | heart | hypothalamus | kidney | liver | lung | ovary | skeletalmuscle | spleen | testes |
|---|---|---|---|---|---|---|---|---|---|---|---|---|---|---|---|
| NM_001077206 | SEC31A | 3898 | 23 | 13 | 11 | 5 | 15 | 9 | 15 | 13 | 9 | 23 | 9 | 13 | 14 |
| NM_001077207 | SEC31A | 4240 | 22 | 12 | 10 | 5 | 14 | 8 | 15 | 13 | 9 | 22 | 9 | 12 | 13 |
| NM_001077208 | SEC31A | 4195 | 22 | 12 | 11 | 5 | 14 | 8 | 14 | 13 | 9 | 22 | 9 | 12 | 13 |
| NM_001077238 | SPPL2B | 3814 | 0 | 0 | 0 | 0 | 0 | 0 | 0 | 0 | 0 | 0 | 0 | 0 | 0 |
| NM_001077239 | RNF214 | 2695 | 7 | 4 | 4 | 3 | 3 | 6 | 4 | 1 | 3 | 5 | 4 | 3 | 7 |
| NM_001077241 | SLC25A45 | 2231 | 1 | 0 | 0 | 0 | 0 | 0 | 0 | 0 | 0 | 0 | 0 | 1 | 0 |
| NM_001077242 | DEPDC7 | 1755 | 17 | 3 | 0 | 1 | 0 | 0 | 3 | 17 | 0 | 1 | 2 | 0 | 7 |
| NM_001077243 | GRIA4 | 5621 | 6 | 1 | 0 | 0 | 0 | 6 | 0 | 0 | 0 | 0 | 0 | 0 | 0 |
| NM_001077244 | GRIA4 | 3345 | 8 | 1 | 0 | 0 | 0 | 8 | 0 | 0 | 0 | 0 | 0 | 0 | 0 |
| NM_001077261 | NCOR2 | 8523 | 6 | 4 | 3 | 4 | 4 | 5 | 3 | 2 | 5 | 6 | 5 | 5 | 4 |
| NM_001077262 | UBXN11 | 1368 | 1 | 0 | 1 | 0 | 0 | 0 | 0 | 0 | 0 | 0 | 0 | 0 | 0 |
| NM_001077263 | TMPRSS13 | 3391 | 1 | 0 | 0 | 0 | 0 | 0 | 0 | 0 | 0 | 1 | 0 | 0 | 0 |
| NM_001077268 | ZFYVE19 | 2273 | 1 | 1 | 0 | 1 | 0 | 1 | 1 | 0 | 1 | 0 | 0 | 1 | 1 |
| NM_001077269 | WIPF1 | 4649 | 25 | 6 | 6 | 3 | 6 | 4 | 3 | 1 | 10 | 3 | 1 | 25 | 3 |
| NM_001077349 | ZNF682 | 3164 | 2 | 1 | 1 | 0 | 1 | 2 | 1 | 0 | 1 | 1 | 0 | 2 | 2 |
| NM_001077350 | C16orf35 | 2708 | 1 | 0 | 0 | 0 | 0 | 1 | 1 | 0 | 0 | 1 | 0 | 0 | 0 |
| NM_001077351 | RBM23 | 2604 | 14 | 8 | 5 | 6 | 6 | 9 | 9 | 7 | 7 | 10 | 6 | 14 | 11 |
| NM_001077352 | RBM23 | 2502 | 14 | 8 | 5 | 5 | 6 | 9 | 8 | 6 | 6 | 10 | 6 | 14 | 11 |
| NM_001077358 | PDE11A | 1869 | 2 | 0 | 1 | 0 | 0 | 0 | 1 | 1 | 0 | 0 | 2 | 0 | 0 |
| NM_001077365 | POMT1 | 3080 | 1 | 1 | 0 | 1 | 0 | 1 | 1 | 0 | 0 | 0 | 0 | 1 | 1 |
| NM_001077366 | POMT1 | 2928 | 1 | 1 | 0 | 1 | 0 | 1 | 1 | 0 | 0 | 1 | 0 | 1 | 1 |
| NM_001077394 | DPH5 | 1806 | 9 | 5 | 4 | 2 | 5 | 6 | 4 | 5 | 4 | 9 | 4 | 6 | 4 |
| NM_001077395 | DPH5 | 1785 | 9 | 5 | 4 | 2 | 5 | 6 | 4 | 6 | 4 | 9 | 4 | 6 | 4 |
| NM_001077397 | IRF2BP2 | 4615 | 8 | 5 | 3 | 4 | 4 | 5 | 4 | 3 | 4 | 8 | 5 | 4 | 4 |
| NM_001077399 | PNKD | 756 | 4 | 1 | 2 | 1 | 1 | 4 | 1 | 2 | 0 | 1 | 1 | 0 | 0 |
| NM_001077400 | FER1L5 | 2536 | 0 | 0 | 0 | 0 | 0 | 0 | 0 | 0 | 0 | 0 | 0 | 0 | 0 |
| NM_001077401 | ACVRL1 | 4126 | 5 | 2 | 2 | 3 | 2 | 1 | 1 | 1 | 5 | 0 | 0 | 4 | 0 |
| NM_001077415 | CRELD1 | 2208 | 2 | 1 | 1 | 1 | 1 | 2 | 1 | 1 | 0 | 0 | 1 | 1 | 1 |
| NM_001077416 | TMEM231 | 2954 | 2 | 1 | 0 | 0 | 1 | 2 | 1 | 0 | 1 | 1 | 0 | 0 | 1 |
| NM_001077418 | TMEM231 | 2891 | 2 | 1 | 0 | 0 | 1 | 2 | 1 | 0 | 1 | 1 | 0 | 0 | 1 |
| NM_001077419 | TMEM231 | 3014 | 2 | 1 | 0 | 0 | 1 | 2 | 1 | 0 | 1 | 1 | 0 | 0 | 1 |
| NM_001077427 | LYPD1 | 1974 | 8 | 1 | 0 | 0 | 0 | 8 | 1 | 0 | 0 | 0 | 0 | 0 | 0 |
| NM_001077440 | BCLAF1 | 7110 | 28 | 19 | 14 | 5 | 16 | 24 | 16 | 15 | 16 | 27 | 17 | 27 | 28 |
| NM_001077441 | BCLAF1 | 6744 | 19 | 13 | 10 | 4 | 11 | 17 | 11 | 11 | 12 | 18 | 11 | 19 | 19 |
| NM_001077442 | HNRNPC | 3225 | 37 | 22 | 14 | 13 | 18 | 32 | 22 | 20 | 22 | 22 | 17 | 37 | 23 |
| NM_001077443 | HNRNPC | 3186 | 38 | 22 | 14 | 13 | 18 | 32 | 22 | 20 | 23 | 22 | 18 | 38 | 24 |
| NM_001077445 | PHF16 | 4685 | 6 | 3 | 2 | 1 | 2 | 1 | 2 | 4 | 2 | 4 | 0 | 4 | 6 |
| NM_001077446 | TSEN34 | 2222 | 0 | 0 | 0 | 0 | 0 | 0 | 0 | 0 | 0 | 0 | 0 | 0 | 0 |
| NM_001077447 | PPCS | 1044 | 14 | 7 | 9 | 5 | 7 | 9 | 14 | 10 | 5 | 5 | 4 | 9 | 5 |
| NM_001077469 | NR1I3 | 1242 | 5 | 1 | 0 | 0 | 0 | 0 | 1 | 5 | 0 | 0 | 0 | 0 | 0 |
| NM_001077470 | NR1I3 | 1124 | 3 | 0 | 0 | 0 | 0 | 0 | 0 | 3 | 0 | 0 | 0 | 0 | 0 |
| NM_001077471 | NR1I3 | 1264 | 5 | 1 | 0 | 0 | 0 | 0 | 1 | 5 | 0 | 0 | 0 | 0 | 0 |
| NM_001077472 | NR1I3 | 1256 | 2 | 0 | 0 | 0 | 0 | 0 | 0 | 2 | 0 | 0 | 0 | 0 | 0 |
| NM_001077473 | NR1I3 | 1114 | 3 | 0 | 0 | 0 | 0 | 0 | 0 | 3 | 0 | 0 | 0 | 0 | 0 |
| NM_001077474 | NR1I3 | 1110 | 5 | 1 | 0 | 0 | 0 | 0 | 1 | 5 | 0 | 0 | 0 | 0 | 0 |
| NM_001077475 | NR1I3 | 970 | 3 | 0 | 0 | 0 | 0 | 0 | 0 | 3 | 0 | 0 | 0 | 0 | 0 |
| NM_001077476 | NR1I3 | 1102 | 3 | 0 | 0 | 0 | 0 | 0 | 0 | 3 | 0 | 0 | 0 | 0 | 0 |
| NM_001077477 | NR1I3 | 1087 | 3 | 0 | 0 | 0 | 0 | 0 | 0 | 3 | 0 | 0 | 0 | 0 | 0 |
| NM_001077478 | NR1I3 | 1239 | 5 | 1 | 0 | 0 | 0 | 0 | 1 | 5 | 0 | 0 | 0 | 0 | 0 |
| NM_001077479 | NR1I3 | 1241 | 2 | 0 | 0 | 0 | 0 | 0 | 0 | 2 | 0 | 0 | 0 | 0 | 0 |
| NM_001077480 | NR1I3 | 1393 | 4 | 1 | 0 | 0 | 0 | 0 | 1 | 4 | 0 | 0 | 0 | 0 | 0 |
| NM_001077481 | NR1I3 | 1279 | 5 | 1 | 0 | 0 | 0 | 0 | 1 | 5 | 0 | 0 | 0 | 0 | 0 |
| NM_001077482 | NR1I3 | 1408 | 4 | 1 | 0 | 0 | 0 | 0 | 1 | 4 | 0 | 0 | 0 | 0 | 0 |
| NM_001077484 | SLC38A1 | 8145 | 9 | 4 | 0 | 3 | 9 | 8 | 4 | 1 | 3 | 1 | 4 | 9 | 1 |
| NM_001077488 | GNAS | 1567 | 48 | 19 | 21 | 14 | 23 | 48 | 15 | 12 | 12 | 11 | 25 | 19 | 8 |
| NM_001077489 | GNAS | 1863 | 37 | 15 | 17 | 12 | 18 | 37 | 12 | 10 | 10 | 9 | 20 | 15 | 6 |
| NM_001077490 | GNAS | 3765 | 14 | 6 | 6 | 3 | 7 | 14 | 5 | 3 | 3 | 4 | 8 | 6 | 2 |
| NM_001077491 | KLK5 | 1428 | 0 | 0 | 0 | 0 | 0 | 0 | 0 | 0 | 0 | 0 | 0 | 0 | 0 |
| NM_001077492 | KLK5 | 1360 | 0 | 0 | 0 | 0 | 0 | 0 | 0 | 0 | 0 | 0 | 0 | 0 | 0 |
| NM_001077493 | NFKB2 | 3049 | 2 | 1 | 1 | 1 | 1 | 0 | 1 | 0 | 1 | 1 | 1 | 2 | 1 |
| NM_001077494 | NFKB2 | 3116 | 2 | 1 | 1 | 1 | 1 | 0 | 1 | 0 | 1 | 1 | 1 | 2 | 1 |
| NM_001077497 | PRR3 | 2778 | 1 | 1 | 0 | 0 | 0 | 0 | 1 | 0 | 1 | 1 | 0 | 1 | 1 |
| NM_001077498 | C17orf63 | 4162 | 2 | 1 | 1 | 1 | 1 | 1 | 2 | 1 | 1 | 2 | 2 | 1 | 2 |
| NM_001077500 | KLK10 | 3007 | 0 | 0 | 0 | 0 | 0 | 0 | 0 | 0 | 0 | 0 | 0 | 0 | 0 |
| NM_001077501 | LRRCC1 | 3615 | 7 | 2 | 1 | 1 | 2 | 2 | 2 | 0 | 3 | 3 | 1 | 3 | 7 |
| NM_001077503 | TAC4 | 624 | 2 | 1 | 0 | 1 | 0 | 0 | 1 | 0 | 1 | 2 | 1 | 2 | 2 |
| NM_001077504 | TAC4 | 597 | 2 | 1 | 0 | 1 | 0 | 0 | 1 | 0 | 1 | 2 | 1 | 2 | 2 |
| NM_001077505 | TAC4 | 564 | 2 | 1 | 0 | 1 | 0 | 0 | 1 | 0 | 1 | 2 | 1 | 2 | 2 |
| NM_001077506 | TAC4 | 657 | 2 | 1 | 0 | 1 | 0 | 0 | 1 | 0 | 1 | 2 | 1 | 2 | 1 |
| NM_001077511 | TCF19 | 3060 | 1 | 1 | 1 | 1 | 1 | 0 | 0 | 0 | 1 | 1 | 0 | 1 | 1 |
| NM_001077511 | TCF19 | 3061 | 1 | 1 | 1 | 1 | 1 | 0 | 0 | 0 | 1 | 1 | 0 | 1 | 1 |
| NM_001077516 | SLC39A7 | 2151 | 23 | 9 | 10 | 11 | 10 | 8 | 12 | 23 | 7 | 4 | 3 | 8 | 7 |
| NM_001077523 | AP3D1 | 4741 | 15 | 10 | 7 | 7 | 12 | 14 | 15 | 12 | 6 | 6 | 7 | 10 | 11 |
| NM_001077525 | MTMR14 | 2497 | 12 | 7 | 6 | 5 | 10 | 5 | 7 | 7 | 6 | 4 | 7 | 12 | 8 |
| NM_001077526 | MTMR14 | 2341 | 13 | 7 | 7 | 5 | 11 | 5 | 7 | 7 | 6 | 5 | 7 | 13 | 8 |
| NM_001077527 | JRK | 3580 | 2 | 1 | 1 | 1 | 1 | 1 | 1 | 0 | 1 | 1 | 1 | 2 | 1 |
| NM_001077528 | FBXO43 | 3255 | 1 | 0 | 0 | 0 | 0 | 0 | 0 | 0 | 0 | 0 | 0 | 0 | 1 |

| transcript | gene | transcript length (nt) | max | mean | adipose | colon | heart | hypothalamus | kidney | liver | lung | ovary | skeletalmuscle | spleen | testes |
|---|---|---|---|---|---|---|---|---|---|---|---|---|---|---|---|
| NM_001077594 | C14orf73 | 2579 | 0 | 0 | 0 | 0 | 0 | 0 | 0 | 0 | 0 | 0 | 0 | 0 | 0 |
| NM_001077619 | UBXN2B | 5068 | 5 | 2 | 1 | 1 | 3 | 4 | 3 | 2 | 2 | 2 | 1 | 5 | 3 |
| NM_001077620 | PRCD | 1994 | 0 | 0 | 0 | 0 | 0 | 0 | 0 | 0 | 0 | 0 | 0 | 0 | 0 |
| NM_001077621 | VPS37D | 1605 | 0 | 0 | 0 | 0 | 0 | 0 | 0 | 0 | 0 | 0 | 0 | 0 | 0 |
| NM_001077624 | ZNF846 | 2133 | 7 | 3 | 3 | 3 | 3 | 4 | 4 | 0 | 3 | 6 | 1 | 4 | 7 |
| NM_001077628 | APH1A | 1851 | 8 | 5 | 4 | 5 | 3 | 3 | 7 | 7 | 5 | 3 | 2 | 8 | 5 |
| NM_001077637 | LOC150786 | 3667 | 13 | 5 | 4 | 3 | 3 | 13 | 3 | 7 | 3 | 4 | 3 | 5 | 5 |
| NM_001077639 | FAM55D | 2163 | 6 | 1 | 0 | 6 | 0 | 0 | 0 | 0 | 0 | 0 | 0 | 0 | 0 |
| NM_001077653 | TBX20 | 1823 | 5 | 0 | 0 | 0 | 5 | 0 | 0 | 0 | 0 | 0 | 0 | 0 | 0 |
| NM_001077654 | TNFAIP8 | 1969 | 26 | 6 | 12 | 3 | 4 | 1 | 4 | 1 | 9 | 1 | 1 | 26 | 3 |
| NM_001077657 | C3orf43 | 1810 | 4 | 0 | 0 | 0 | 4 | 0 | 0 | 0 | 0 | 0 | 1 | 0 | 0 |
| NM_001077663 | URGCP | 3618 | 7 | 4 | 3 | 6 | 4 | 7 | 7 | 4 | 2 | 3 | 3 | 4 | 3 |
| NM_001077664 | URGCP | 3813 | 6 | 4 | 3 | 5 | 4 | 6 | 6 | 4 | 2 | 3 | 3 | 4 | 3 |
| NM_001077665 | AGAP6 | 2772 | 0 | 0 | 0 | 0 | 0 | 0 | 0 | 0 | 0 | 0 | 0 | 0 | 0 |
| NM_001077685 | AGAP7 | 2420 | 0 | 0 | 0 | 0 | 0 | 0 | 0 | 0 | 0 | 0 | 0 | 0 | 0 |
| NM_001077686 | AGAP8 | 2420 | 0 | 0 | 0 | 0 | 0 | 0 | 0 | 0 | 0 | 0 | 0 | 0 | 0 |
| NM_001077690 | ALG9 | 6138 | 5 | 3 | 5 | 1 | 3 | 3 | 2 | 4 | 2 | 4 | 2 | 3 | 4 |
| NM_001077691 | ALG9 | 6435 | 5 | 3 | 5 | 1 | 3 | 3 | 2 | 4 | 2 | 4 | 2 | 3 | 4 |
| NM_001077692 | ALG9 | 6414 | 5 | 3 | 5 | 1 | 3 | 3 | 2 | 4 | 2 | 4 | 2 | 3 | 4 |
| NM_001077693 | ECSCR | 397 | 2 | 1 | 1 | 0 | 2 | 0 | 0 | 0 | 1 | 1 | 1 | 2 | 0 |
| NM_001077697 | TSPY3 | 979 | 0 | 0 | 0 | 0 | 0 | 0 | 0 | 0 | 0 | 0 | 0 | 0 | 0 |
| NM_001077700 | MIER1 | 5551 | 13 | 7 | 5 | 4 | 6 | 5 | 6 | 6 | 10 | 10 | 4 | 11 | 13 |
| NM_001077701 | MIER1 | 5407 | 13 | 7 | 5 | 4 | 7 | 5 | 6 | 6 | 10 | 10 | 5 | 11 | 13 |
| NM_001077702 | MIER1 | 3815 | 18 | 10 | 7 | 6 | 9 | 8 | 9 | 8 | 14 | 14 | 6 | 15 | 18 |
| NM_001077703 | MIER1 | 3741 | 18 | 10 | 7 | 6 | 9 | 8 | 9 | 8 | 14 | 14 | 6 | 16 | 18 |
| NM_001077704 | MIER1 | 3597 | 19 | 11 | 7 | 6 | 10 | 8 | 9 | 8 | 14 | 14 | 6 | 16 | 19 |
| NM_001077706 | ECT2L | 4261 | 1 | 0 | 0 | 0 | 0 | 0 | 0 | 0 | 1 | 0 | 0 | 0 | 0 |
| NM_001077710 | FAM110C | 966 | 1 | 0 | 0 | 0 | 0 | 0 | 0 | 1 | 0 | 0 | 0 | 0 | 1 |
| NM_001077711 | KRTAP27-1 | 682 | 0 | 0 | 0 | 0 | 0 | 0 | 0 | 0 | 0 | 0 | 0 | 0 | 0 |
| NM_001078 | VCAM1 | 3102 | 249 | 35 | 8 | 3 | 5 | 9 | 54 | 17 | 13 | 16 | 3 | 249 | 6 |
| NM_001078166 | SFRS1 | 5643 | 17 | 9 | 7 | 6 | 8 | 8 | 11 | 10 | 9 | 9 | 7 | 17 | 10 |
| NM_001078170 | RGPD2 | 5498 | 0 | 0 | 0 | 0 | 0 | 0 | 0 | 0 | 0 | 0 | 0 | 0 | 0 |
| NM_001078171 | FAM127A | 1243 | 71 | 25 | 15 | 8 | 71 | 50 | 22 | 6 | 15 | 31 | 25 | 13 | 14 |
| NM_001078172 | FAM127B | 1259 | 5 | 3 | 2 | 2 | 5 | 4 | 4 | 1 | 2 | 5 | 3 | 4 | 2 |
| NM_001078173 | FAM127C | 2033 | 2 | 1 | 1 | 0 | 2 | 1 | 1 | 0 | 1 | 1 | 0 | 1 | 1 |
| NM_001078174 | SLC29A1 | 2326 | 5 | 2 | 4 | 2 | 5 | 1 | 2 | 1 | 2 | 1 | 3 | 2 | 1 |
| NM_001078175 | SLC29A1 | 2235 | 5 | 2 | 4 | 2 | 5 | 1 | 2 | 1 | 2 | 1 | 3 | 2 | 1 |
| NM_001078176 | SLC29A1 | 2128 | 6 | 2 | 4 | 2 | 6 | 1 | 2 | 1 | 3 | 1 | 3 | 2 | 1 |
| NM_001078177 | SLC29A1 | 2485 | 5 | 2 | 4 | 2 | 5 | 1 | 2 | 1 | 2 | 1 | 2 | 2 | 1 |
| NM_001078645 | CDC16 | 2293 | 7 | 5 | 6 | 2 | 4 | 4 | 5 | 6 | 3 | 6 | 3 | 6 | 7 |
| NM_001078650 | TMEM134 | 877 | 1 | 0 | 0 | 0 | 1 | 0 | 1 | 1 | 0 | 0 | 0 | 1 | 0 |
| NM_001078651 | TMEM134 | 895 | 1 | 0 | 0 | 0 | 1 | 0 | 1 | 1 | 0 | 0 | 0 | 1 | 0 |
| NM_001079 | ZAP70 | 2429 | 1 | 0 | 0 | 0 | 0 | 0 | 0 | 0 | 0 | 0 | 0 | 1 | 0 |
| NM_001079512 | FAM18A | 3339 | 0 | 0 | 0 | 0 | 0 | 0 | 0 | 0 | 0 | 0 | 0 | 0 | 0 |
| NM_001079514 | UBN1 | 5974 | 4 | 2 | 2 | 2 | 2 | 2 | 2 | 2 | 2 | 1 | 1 | 4 | 3 |
| NM_001079515 | TBCE | 1958 | 10 | 7 | 6 | 6 | 9 | 9 | 9 | 8 | 5 | 5 | 10 | 6 | 6 |
| NM_001079516 | ASAH2B | 632 | 0 | 0 | 0 | 0 | 0 | 0 | 0 | 0 | 0 | 0 | 0 | 0 | 0 |
| NM_001079518 | MED24 | 3698 | 2 | 1 | 1 | 2 | 1 | 2 | 1 | 1 | 1 | 1 | 1 | 1 | 1 |
| NM_001079519 | FAM177A1 | 3256 | 12 | 7 | 6 | 4 | 12 | 11 | 7 | 11 | 9 | 4 | 4 | 5 | 7 |
| NM_001079520 | DACT1 | 3750 | 3 | 1 | 1 | 1 | 1 | 2 | 1 | 0 | 1 | 3 | 0 | 1 | 1 |
| NM_001079521 | KTN1 | 4733 | 133 | 67 | 49 | 17 | 133 | 42 | 70 | 42 | 56 | 43 | 111 | 62 | 115 |
| NM_001079522 | KTN1 | 4704 | 134 | 68 | 50 | 17 | 134 | 42 | 70 | 42 | 56 | 43 | 112 | 62 | 116 |
| NM_001079524 | PAICS | 3318 | 15 | 7 | 3 | 4 | 5 | 9 | 8 | 15 | 3 | 7 | 4 | 8 | 9 |
| NM_001079525 | PAICS | 3339 | 15 | 7 | 3 | 4 | 5 | 9 | 8 | 15 | 3 | 7 | 4 | 8 | 9 |
| NM_001079526 | IKZF2 | 9492 | 2 | 1 | 0 | 0 | 1 | 1 | 1 | 0 | 1 | 1 | 1 | 2 | 1 |
| NM_001079527 | FAM153C | 1807 | 0 | 0 | 0 | 0 | 0 | 0 | 0 | 0 | 0 | 0 | 0 | 0 | 0 |
| NM_001079528 | ABCC6 | 718 | 1 | 0 | 0 | 0 | 0 | 0 | 1 | 0 | 0 | 0 | 0 | 0 | 0 |
| NM_001079529 | FAM153B | 2095 | 1 | 0 | 0 | 0 | 0 | 0 | 0 | 0 | 0 | 0 | 0 | 0 | 1 |
| NM_001079530 | CFC1B | 1160 | 0 | 0 | 0 | 0 | 0 | 0 | 0 | 0 | 0 | 0 | 0 | 0 | 0 |
| NM_001079533 | CPEB1 | 3092 | 3 | 1 | 0 | 0 | 0 | 3 | 0 | 0 | 0 | 0 | 0 | 0 | 3 |
| NM_001079534 | CPEB1 | 3077 | 3 | 1 | 0 | 0 | 0 | 3 | 0 | 0 | 0 | 0 | 0 | 0 | 3 |
| NM_001079535 | CPEB1 | 3178 | 3 | 1 | 0 | 0 | 0 | 3 | 0 | 0 | 0 | 0 | 0 | 0 | 3 |
| NM_001079536 | ART5 | 1249 | 4 | 1 | 0 | 0 | 1 | 0 | 0 | 0 | 0 | 2 | 3 | 0 | 4 |
| NM_001079537 | TRAPPC6B | 3351 | 12 | 6 | 4 | 2 | 5 | 12 | 5 | 5 | 5 | 5 | 4 | 7 | 8 |
| NM_001079538 | XAGE2B | 336 | 0 | 0 | 0 | 0 | 0 | 0 | 0 | 0 | 0 | 0 | 0 | 0 | 0 |
| NM_001079539 | XBP1 | 1794 | 106 | 29 | 12 | 34 | 21 | 14 | 18 | 106 | 39 | 14 | 8 | 49 | 9 |
| NM_001079559 | HNRNPUL2 | 2988 | 88 | 52 | 35 | 36 | 61 | 55 | 54 | 26 | 50 | 58 | 44 | 88 | 63 |
| NM_001079668 | NKX2-1 | 2183 | 2 | 0 | 0 | 0 | 0 | 0 | 0 | 0 | 2 | 0 | 0 | 0 | 0 |
| NM_001079669 | TMTC4 | 3544 | 3 | 1 | 0 | 1 | 1 | 3 | 2 | 1 | 1 | 1 | 1 | 1 | 1 |
| NM_001079670 | CAB39L | 3523 | 5 | 2 | 2 | 1 | 2 | 5 | 1 | 0 | 2 | 2 | 0 | 1 | 2 |
| NM_001079673 | FNDC3A | 6286 | 10 | 6 | 3 | 2 | 6 | 5 | 7 | 10 | 7 | 7 | 2 | 7 | 8 |
| NM_001079675 | ETV4 | 2392 | 0 | 0 | 0 | 0 | 0 | 0 | 0 | 0 | 0 | 0 | 0 | 0 | 0 |
| NM_001079684 | KIAA1191 | 2740 | 10 | 4 | 2 | 3 | 3 | 8 | 10 | 4 | 2 | 4 | 2 | 3 | 2 |
| NM_001079685 | KIAA1191 | 2719 | 10 | 4 | 2 | 3 | 3 | 8 | 10 | 5 | 2 | 4 | 2 | 3 | 2 |
| NM_001079691 | N4BP2L1 | 3039 | 3 | 1 | 1 | 0 | 0 | 2 | 1 | 3 | 2 | 2 | 1 | 3 | 1 |
| NM_001079692 | MS4A14 | 3301 | 12 | 2 | 1 | 0 | 1 | 0 | 0 | 0 | 1 | 0 | 0 | 1 | 12 |
| NM_001079802 | FKTN | 7456 | 1 | 1 | 1 | 0 | 1 | 1 | 1 | 0 | 0 | 1 | 0 | 1 | 1 |

| transcript | gene | transcript length (nt) | max | mean | adipose | colon | heart | hypothalamus | kidney | liver | lung | ovary | skeletalmuscle | spleen | testes |
|---|---|---|---|---|---|---|---|---|---|---|---|---|---|---|---|
| NM_001079803 | GAA | 3590 | 2 | 1 | 1 | 0 | 1 | 0 | 1 | 1 | 1 | 1 | 1 | 2 | 1 |
| NM_001079804 | GAA | 3510 | 2 | 1 | 1 | 0 | 1 | 0 | 1 | 1 | 1 | 1 | 1 | 2 | 1 |
| NM_001079807 | PGA3 | 1396 | 0 | 0 | 0 | 0 | 0 | 0 | 0 | 0 | 0 | 0 | 0 | 0 | 0 |
| NM_001079808 | PGA4 | 1395 | 0 | 0 | 0 | 0 | 0 | 0 | 0 | 0 | 0 | 0 | 0 | 0 | 0 |
| NM_001079809 | TMEM183B | 1689 | 0 | 0 | 0 | 0 | 0 | 0 | 0 | 0 | 0 | 0 | 0 | 0 | 0 |
| NM_001079810 | XIRP2 | 3223 | 495 | 68 | 0 | 0 | 244 | 0 | 0 | 0 | 0 | 0 | 495 | 1 | 3 |
| NM_001079811 | GLB1 | 2500 | 5 | 2 | 3 | 2 | 2 | 1 | 5 | 3 | 3 | 2 | 1 | 3 | 2 |
| NM_001079812 | DIAPH1 | 5763 | 29 | 13 | 6 | 8 | 13 | 6 | 16 | 29 | 15 | 5 | 18 | 14 | 7 |
| NM_001079817 | INSR | 9023 | 27 | 7 | 3 | 3 | 7 | 3 | 7 | 9 | 2 | 8 | 8 | 27 | 5 |
| NM_001079818 | ITGA6 | 5680 | 28 | 14 | 13 | 11 | 13 | 10 | 26 | 6 | 10 | 7 | 7 | 20 | 28 |
| NM_001079821 | NLRP3 | 3847 | 2 | 1 | 1 | 0 | 0 | 1 | 0 | 0 | 1 | 0 | 0 | 2 | 0 |
| NM_001079823 | LAMA2 | 9680 | 32 | 10 | 7 | 2 | 32 | 2 | 3 | 2 | 10 | 22 | 7 | 8 | 12 |
| NM_001079827 | CLRN2 | 823 | 0 | 0 | 0 | 0 | 0 | 0 | 0 | 0 | 0 | 0 | 0 | 0 | 0 |
| NM_001079839 | OCIAD1 | 2067 | 10 | 5 | 3 | 2 | 7 | 10 | 8 | 6 | 3 | 5 | 5 | 5 | 5 |
| NM_001079840 | OCIAD1 | 1851 | 11 | 5 | 3 | 2 | 5 | 11 | 6 | 6 | 2 | 4 | 4 | 5 | 4 |
| NM_001079841 | OCIAD1 | 1896 | 11 | 5 | 3 | 2 | 5 | 11 | 6 | 6 | 2 | 4 | 4 | 4 | 4 |
| NM_001079842 | OCIAD1 | 1914 | 11 | 5 | 3 | 2 | 5 | 11 | 6 | 5 | 2 | 4 | 4 | 4 | 4 |
| NM_001079843 | CASZ1 | 7933 | 1 | 0 | 0 | 1 | 1 | 0 | 0 | 0 | 1 | 0 | 1 | 0 | 0 |
| NM_001079846 | CREBBP | 10082 | 9 | 4 | 3 | 4 | 3 | 3 | 3 | 2 | 3 | 9 | 6 | 7 | 5 |
| NM_001079855 | GYG2 | 3302 | 8 | 1 | 8 | 1 | 0 | 1 | 0 | 2 | 0 | 0 | 0 | 0 | 0 |
| NM_001079858 | GPR64 | 4768 | 6 | 1 | 0 | 0 | 0 | 0 | 0 | 0 | 0 | 1 | 0 | 1 | 6 |
| NM_001079859 | GPR64 | 4733 | 6 | 1 | 0 | 0 | 0 | 0 | 0 | 0 | 0 | 1 | 0 | 1 | 6 |
| NM_001079860 | GPR64 | 4702 | 6 | 1 | 0 | 0 | 0 | 0 | 0 | 0 | 0 | 1 | 0 | 1 | 6 |
| NM_001079862 | DBI | 616 | 461 | 205 | 357 | 127 | 255 | 287 | 340 | 461 | 121 | 43 | 45 | 117 | 104 |
| NM_001079863 | DBI | 740 | 384 | 171 | 297 | 105 | 212 | 239 | 283 | 384 | 101 | 36 | 38 | 98 | 87 |
| NM_001079864 | TAX1BP1 | 3340 | 45 | 29 | 17 | 5 | 45 | 37 | 35 | 23 | 30 | 22 | 23 | 39 | 44 |
| NM_001079866 | BCS1L | 1454 | 1 | 1 | 0 | 1 | 0 | 1 | 1 | 1 | 0 | 0 | 1 | 0 | 0 |
| NM_001079867 | PXMP3 | 4347 | 2 | 1 | 1 | 1 | 2 | 2 | 2 | 1 | 1 | 1 | 1 | 2 | 1 |
| NM_001079870 | HAP1 | 3883 | 2 | 0 | 0 | 0 | 0 | 2 | 0 | 0 | 0 | 0 | 0 | 0 | 0 |
| NM_001079871 | HAP1 | 3859 | 2 | 0 | 0 | 0 | 0 | 2 | 0 | 0 | 0 | 0 | 0 | 0 | 0 |
| NM_001079872 | CUL4B | 5182 | 30 | 11 | 7 | 2 | 10 | 11 | 9 | 6 | 9 | 20 | 4 | 15 | 30 |
| NM_001079874 | VAV3 | 3108 | 20 | 3 | 1 | 5 | 1 | 1 | 20 | 0 | 1 | 0 | 0 | 5 | 1 |
| NM_001079877 | RASA4 | 5468 | 4 | 2 | 2 | 1 | 1 | 2 | 2 | 0 | 1 | 2 | 4 | 3 | 1 |
| NM_001079878 | CNGA3 | 3794 | 0 | 0 | 0 | 0 | 0 | 0 | 0 | 0 | 0 | 0 | 0 | 0 | 0 |
| NM_001079880 | PRKD2 | 3322 | 8 | 3 | 2 | 4 | 2 | 1 | 3 | 2 | 5 | 5 | 2 | 8 | 2 |
| NM_001079881 | PRKD2 | 3186 | 8 | 3 | 2 | 4 | 2 | 1 | 3 | 2 | 5 | 5 | 2 | 8 | 2 |
| NM_001079882 | PRKD2 | 2923 | 8 | 3 | 2 | 3 | 2 | 1 | 3 | 2 | 4 | 5 | 2 | 8 | 2 |
| NM_001079900 | SPACA5B | 595 | 0 | 0 | 0 | 0 | 0 | 0 | 0 | 0 | 0 | 0 | 0 | 0 | 0 |
| NM_001079906 | ZNF331 | 4131 | 10 | 3 | 2 | 1 | 3 | 3 | 2 | 1 | 4 | 10 | 1 | 3 | 2 |
| NM_001079907 | ZNF331 | 4151 | 10 | 3 | 3 | 1 | 3 | 3 | 2 | 1 | 4 | 10 | 1 | 3 | 2 |
| NM_001079910 | LRRIQ1 | 5444 | 2 | 0 | 0 | 0 | 0 | 1 | 1 | 0 | 1 | 0 | 0 | 0 | 2 |
| NM_001079911 | DMP1 | 2636 | 0 | 0 | 0 | 0 | 0 | 0 | 0 | 0 | 0 | 0 | 0 | 0 | 0 |
| NM_001079935 | OR7E24 | 1020 | 0 | 0 | 0 | 0 | 0 | 0 | 0 | 0 | 0 | 0 | 0 | 0 | 0 |
| NM_001080 | ALDH5A1 | 5131 | 7 | 2 | 2 | 1 | 2 | 7 | 4 | 4 | 1 | 1 | 2 | 1 | 1 |
| NM_001080113 | C14orf184 | 2596 | 0 | 0 | 0 | 0 | 0 | 0 | 0 | 0 | 0 | 0 | 0 | 0 | 0 |
| NM_001080114 | LDB3 | 4968 | 65 | 10 | 0 | 2 | 44 | 1 | 0 | 0 | 0 | 0 | 65 | 0 | 1 |
| NM_001080115 | LDB3 | 1695 | 12 | 2 | 0 | 0 | 9 | 0 | 0 | 0 | 0 | 0 | 12 | 0 | 0 |
| NM_001080116 | LDB3 | 1659 | 40 | 6 | 0 | 1 | 18 | 0 | 0 | 0 | 0 | 0 | 40 | 0 | 0 |
| NM_001080121 | PRNP | 2735 | 57 | 16 | 6 | 7 | 18 | 57 | 13 | 7 | 12 | 13 | 9 | 15 | 19 |
| NM_001080122 | PRNP | 2731 | 57 | 16 | 6 | 7 | 18 | 57 | 13 | 7 | 12 | 13 | 9 | 15 | 19 |
| NM_001080123 | PRNP | 2604 | 59 | 17 | 6 | 8 | 18 | 59 | 14 | 7 | 12 | 14 | 10 | 16 | 20 |
| NM_001080124 | CASP8 | 2747 | 9 | 3 | 4 | 3 | 1 | 1 | 3 | 2 | 5 | 2 | 0 | 9 | 2 |
| NM_001080125 | CASP8 | 2935 | 9 | 3 | 4 | 2 | 1 | 1 | 3 | 2 | 4 | 2 | 0 | 9 | 2 |
| NM_001080137 | CT47A10 | 1293 | 0 | 0 | 0 | 0 | 0 | 0 | 0 | 0 | 0 | 0 | 0 | 0 | 0 |
| NM_001080137 | CT47A10 | 1294 | 0 | 0 | 0 | 0 | 0 | 0 | 0 | 0 | 0 | 0 | 0 | 0 | 0 |
| NM_001080138 | CT47A9 | 1293 | 0 | 0 | 0 | 0 | 0 | 0 | 0 | 0 | 0 | 0 | 0 | 0 | 0 |
| NM_001080138 | CT47A9 | 1294 | 0 | 0 | 0 | 0 | 0 | 0 | 0 | 0 | 0 | 0 | 0 | 0 | 0 |
| NM_001080139 | CT47A8 | 1293 | 0 | 0 | 0 | 0 | 0 | 0 | 0 | 0 | 0 | 0 | 0 | 0 | 0 |
| NM_001080139 | CT47A8 | 1294 | 0 | 0 | 0 | 0 | 0 | 0 | 0 | 0 | 0 | 0 | 0 | 0 | 0 |
| NM_001080140 | CT47A7 | 1293 | 0 | 0 | 0 | 0 | 0 | 0 | 0 | 0 | 0 | 0 | 0 | 0 | 0 |
| NM_001080141 | CT47A6 | 1293 | 0 | 0 | 0 | 0 | 0 | 0 | 0 | 0 | 0 | 0 | 0 | 0 | 0 |
| NM_001080141 | CT47A6 | 1294 | 0 | 0 | 0 | 0 | 0 | 0 | 0 | 0 | 0 | 0 | 0 | 0 | 0 |
| NM_001080142 | CT47A5 | 1293 | 0 | 0 | 0 | 0 | 0 | 0 | 0 | 0 | 0 | 0 | 0 | 0 | 0 |
| NM_001080142 | CT47A5 | 1294 | 0 | 0 | 0 | 0 | 0 | 0 | 0 | 0 | 0 | 0 | 0 | 0 | 0 |
| NM_001080143 | CT47A4 | 1293 | 0 | 0 | 0 | 0 | 0 | 0 | 0 | 0 | 0 | 0 | 0 | 0 | 0 |
| NM_001080143 | CT47A4 | 1294 | 0 | 0 | 0 | 0 | 0 | 0 | 0 | 0 | 0 | 0 | 0 | 0 | 0 |
| NM_001080144 | CT47A3 | 1293 | 0 | 0 | 0 | 0 | 0 | 0 | 0 | 0 | 0 | 0 | 0 | 0 | 0 |
| NM_001080144 | CT47A3 | 1294 | 0 | 0 | 0 | 0 | 0 | 0 | 0 | 0 | 0 | 0 | 0 | 0 | 0 |
| NM_001080145 | CT47A2 | 1293 | 0 | 0 | 0 | 0 | 0 | 0 | 0 | 0 | 0 | 0 | 0 | 0 | 0 |
| NM_001080145 | CT47A2 | 1294 | 0 | 0 | 0 | 0 | 0 | 0 | 0 | 0 | 0 | 0 | 0 | 0 | 0 |
| NM_001080146 | CT47A1 | 1293 | 0 | 0 | 0 | 0 | 0 | 0 | 0 | 0 | 0 | 0 | 0 | 0 | 0 |
| NM_001080146 | CT47A1 | 1294 | 0 | 0 | 0 | 0 | 0 | 0 | 0 | 0 | 0 | 0 | 0 | 0 | 0 |
| NM_001080156 | ARHGAP9 | 2085 | 9 | 1 | 1 | 1 | 1 | 1 | 0 | 0 | 2 | 0 | 0 | 9 | 2 |
| NM_001080157 | ARHGAP9 | 2587 | 7 | 1 | 1 | 1 | 1 | 1 | 0 | 0 | 1 | 0 | 0 | 7 | 2 |
| NM_001080209 | TMEM200C | 1920 | 0 | 0 | 0 | 0 | 0 | 0 | 0 | 0 | 0 | 0 | 0 | 0 | 0 |
| NM_001080378 | PACRG | 1503 | 1 | 0 | 0 | 0 | 0 | 1 | 1 | 0 | 0 | 0 | 0 | 0 | 1 |
| NM_001080379 | PACRG | 1545 | 1 | 0 | 0 | 0 | 0 | 1 | 1 | 0 | 0 | 0 | 0 | 0 | 1 |

| transcript | gene | transcript length (nt) | max | mean | adipose | colon | heart | hypothalamus | kidney | liver | lung | ovary | skeletalmuscle | spleen | testes |
|---|---|---|---|---|---|---|---|---|---|---|---|---|---|---|---|
| NM_001080383 | GJC1 | 7640 | 3 | 1 | 1 | 3 | 2 | 0 | 0 | 0 | 1 | 1 | 0 | 0 | 1 |
| NM_001080391 | SP100 | 5455 | 27 | 8 | 8 | 3 | 10 | 3 | 5 | 6 | 11 | 9 | 5 | 27 | 5 |
| NM_001080392 | KIAA1147 | 7296 | 7 | 3 | 2 | 3 | 2 | 6 | 4 | 5 | 3 | 3 | 1 | 7 | 1 |
| NM_001080393 | GXYLT2 | 1544 | 11 | 3 | 3 | 2 | 5 | 2 | 0 | 0 | 2 | 11 | 0 | 0 | 3 |
| NM_001080394 | KIAA0146 | 3220 | 8 | 5 | 4 | 4 | 4 | 5 | 3 | 4 | 5 | 7 | 5 | 8 | 4 |
| NM_001080395 | AATK | 5311 | 6 | 1 | 0 | 0 | 0 | 6 | 0 | 0 | 0 | 0 | 0 | 0 | 0 |
| NM_001080396 | FAM155A | 3858 | 9 | 1 | 0 | 0 | 0 | 9 | 0 | 0 | 0 | 1 | 0 | 0 | 1 |
| NM_001080397 | SLC45A1 | 2401 | 0 | 0 | 0 | 0 | 0 | 0 | 0 | 0 | 0 | 0 | 0 | 0 | 0 |
| NM_001080398 | KIAA0368 | 7391 | 57 | 19 | 16 | 7 | 20 | 22 | 12 | 12 | 11 | 17 | 57 | 14 | 18 |
| NM_001080399 | OC90 | 1782 | 0 | 0 | 0 | 0 | 0 | 0 | 0 | 0 | 0 | 0 | 0 | 0 | 0 |
| NM_001080400 | PLIN4 | 6353 | 62 | 7 | 62 | 3 | 3 | 0 | 0 | 0 | 0 | 0 | 4 | 0 | 0 |
| NM_001080401 | FLJ40125 | 1714 | 0 | 0 | 0 | 0 | 0 | 0 | 0 | 0 | 0 | 0 | 0 | 0 | 0 |
| NM_001080402 | CCDC61 | 1825 | 0 | 0 | 0 | 0 | 0 | 0 | 0 | 0 | 0 | 0 | 0 | 0 | 0 |
| NM_001080404 | ZNF878 | 1737 | 1 | 0 | 0 | 0 | 0 | 0 | 1 | 0 | 0 | 0 | 0 | 0 | 1 |
| NM_001080405 | CEACAM18 | 1197 | 0 | 0 | 0 | 0 | 0 | 0 | 0 | 0 | 0 | 0 | 0 | 0 | 0 |
| NM_001080406 | C12orf71 | 966 | 1 | 0 | 0 | 0 | 0 | 0 | 0 | 0 | 0 | 1 | 0 | 0 | 0 |
| NM_001080407 | GLB1L3 | 2944 | 0 | 0 | 0 | 0 | 0 | 0 | 0 | 0 | 0 | 0 | 0 | 0 | 0 |
| NM_001080408 | PROX2 | 1779 | 0 | 0 | 0 | 0 | 0 | 0 | 0 | 0 | 0 | 0 | 0 | 0 | 0 |
| NM_001080409 | ZNF99 | 3111 | 4 | 1 | 0 | 0 | 0 | 0 | 1 | 0 | 0 | 1 | 0 | 0 | 4 |
| NM_001080410 | FBXO41 | 7121 | 2 | 0 | 0 | 0 | 0 | 2 | 0 | 0 | 0 | 0 | 0 | 0 | 0 |
| NM_001080411 | ZNF433 | 2321 | 7 | 2 | 1 | 0 | 2 | 2 | 5 | 1 | 1 | 3 | 1 | 2 | 7 |
| NM_001080412 | ZBTB38 | 8384 | 8 | 6 | 3 | 2 | 8 | 6 | 6 | 3 | 6 | 7 | 7 | 5 | 7 |
| NM_001080413 | NOBOX | 1725 | 0 | 0 | 0 | 0 | 0 | 0 | 0 | 0 | 0 | 0 | 0 | 0 | 0 |
| NM_001080414 | CCDC88C | 7488 | 4 | 1 | 1 | 2 | 0 | 1 | 1 | 0 | 2 | 0 | 0 | 4 | 1 |
| NM_001080415 | SR140 | 7464 | 9 | 5 | 5 | 2 | 5 | 5 | 5 | 4 | 4 | 7 | 5 | 9 | 9 |
| NM_001080416 | MYBL1 | 5188 | 5 | 2 | 1 | 1 | 1 | 2 | 0 | 0 | 1 | 0 | 1 | 5 | 5 |
| NM_001080417 | ZNF629 | 6087 | 3 | 2 | 1 | 2 | 2 | 2 | 2 | 1 | 1 | 3 | 1 | 1 | 2 |
| NM_001080418 | DLGAP3 | 3587 | 1 | 0 | 0 | 0 | 0 | 1 | 0 | 0 | 0 | 0 | 0 | 0 | 0 |
| NM_001080419 | UNK | 4049 | 5 | 2 | 2 | 2 | 2 | 2 | 2 | 1 | 2 | 4 | 2 | 5 | 2 |
| NM_001080420 | SHANK3 | 7145 | 6 | 2 | 2 | 1 | 2 | 6 | 2 | 1 | 2 | 1 | 1 | 6 | 0 |
| NM_001080421 | UNC13A | 10102 | 18 | 2 | 0 | 0 | 0 | 18 | 0 | 0 | 0 | 0 | 0 | 1 | 0 |
| NM_001080423 | GRIP2 | 3426 | 0 | 0 | 0 | 0 | 0 | 0 | 0 | 0 | 0 | 0 | 0 | 0 | 0 |
| NM_001080424 | KDM6B | 6704 | 3 | 2 | 0 | 2 | 1 | 1 | 1 | 1 | 2 | 3 | 1 | 3 | 1 |
| NM_001080425 | BEX4 | 1276 | 89 | 19 | 9 | 11 | 15 | 89 | 19 | 2 | 9 | 11 | 1 | 19 | 26 |
| NM_001080426 | DUSP27 | 4034 | 62 | 11 | 0 | 0 | 62 | 0 | 0 | 0 | 0 | 0 | 58 | 0 | 1 |
| NM_001080427 | THSD7B | 5845 | 1 | 0 | 1 | 0 | 0 | 1 | 0 | 0 | 0 | 0 | 0 | 0 | 0 |
| NM_001080429 | KIAA1543 | 4185 | 0 | 0 | 0 | 0 | 0 | 0 | 0 | 0 | 0 | 0 | 0 | 0 | 0 |
| NM_001080430 | TOX3 | 3121 | 1 | 0 | 0 | 0 | 0 | 1 | 1 | 0 | 0 | 0 | 0 | 0 | 0 |
| NM_001080431 | SLC45A4 | 3376 | 1 | 0 | 0 | 0 | 0 | 0 | 0 | 0 | 0 | 0 | 0 | 0 | 1 |
| NM_001080432 | FTO | 4291 | 12 | 4 | 5 | 2 | 4 | 12 | 4 | 3 | 3 | 4 | 4 | 3 | 3 |
| NM_001080433 | CCDC85A | 3983 | 4 | 1 | 1 | 0 | 1 | 4 | 0 | 0 | 1 | 0 | 0 | 0 | 0 |
| NM_001080434 | LMTK3 | 4972 | 2 | 0 | 0 | 0 | 0 | 2 | 0 | 0 | 0 | 0 | 0 | 0 | 0 |
| NM_001080435 | WHAMM | 4261 | 3 | 1 | 2 | 1 | 1 | 2 | 1 | 1 | 1 | 1 | 3 | 2 | 1 |
| NM_001080436 | WTIP | 2204 | 0 | 0 | 0 | 0 | 0 | 0 | 0 | 0 | 0 | 0 | 0 | 0 | 0 |
| NM_001080437 | SNED1 | 6834 | 2 | 1 | 1 | 1 | 2 | 1 | 1 | 0 | 1 | 2 | 1 | 2 | 2 |
| NM_001080439 | HSF5 | 4107 | 3 | 0 | 0 | 0 | 0 | 0 | 0 | 0 | 0 | 0 | 0 | 0 | 3 |
| NM_001080440 | OTOL1 | 1434 | 1 | 0 | 0 | 0 | 0 | 1 | 0 | 0 | 0 | 0 | 0 | 0 | 0 |
| NM_001080441 | TTC36 | 679 | 2 | 0 | 0 | 0 | 0 | 0 | 1 | 2 | 0 | 0 | 0 | 0 | 0 |
| NM_001080442 | SLC38A8 | 1308 | 0 | 0 | 0 | 0 | 0 | 0 | 0 | 0 | 0 | 0 | 0 | 0 | 0 |
| NM_001080443 | KIF18B | 2669 | 0 | 0 | 0 | 0 | 0 | 0 | 0 | 0 | 0 | 0 | 0 | 0 | 0 |
| NM_001080444 | IGSF5 | 2066 | 1 | 0 | 0 | 0 | 1 | 0 | 0 | 0 | 0 | 0 | 0 | 1 | 0 |
| NM_001080446 | C11orf94 | 368 | 1 | 0 | 0 | 0 | 0 | 0 | 0 | 0 | 0 | 0 | 0 | 0 | 1 |
| NM_001080447 | TTLL8 | 2505 | 0 | 0 | 0 | 0 | 0 | 0 | 0 | 0 | 0 | 0 | 0 | 0 | 0 |
| NM_001080448 | EPHA6 | 3677 | 9 | 1 | 0 | 1 | 0 | 9 | 0 | 0 | 0 | 2 | 0 | 0 | 2 |
| NM_001080449 | DNA2 | 4416 | 1 | 0 | 0 | 1 | 0 | 1 | 1 | 0 | 0 | 0 | 0 | 1 | 1 |
| NM_001080450 | BEND3 | 6660 | 0 | 0 | 0 | 0 | 0 | 0 | 0 | 0 | 0 | 0 | 0 | 0 | 0 |
| NM_001080451 | SERPINA11 | 1471 | 14 | 1 | 0 | 0 | 0 | 0 | 0 | 14 | 0 | 0 | 0 | 0 | 0 |
| NM_001080452 | GPR108 | 2076 | 1 | 1 | 1 | 0 | 1 | 0 | 1 | 1 | 1 | 0 | 0 | 1 | 1 |
| NM_001080453 | INTS1 | 6974 | 1 | 1 | 0 | 1 | 0 | 1 | 1 | 1 | 0 | 0 | 1 | 1 | 1 |
| NM_001080454 | ACSM4 | 1743 | 1 | 0 | 0 | 0 | 0 | 0 | 0 | 0 | 0 | 1 | 0 | 0 | 0 |
| NM_001080455 | SLC35F4 | 1779 | 0 | 0 | 0 | 0 | 0 | 0 | 0 | 0 | 0 | 0 | 0 | 0 | 0 |
| NM_001080456 | ZSCAN5B | 1625 | 0 | 0 | 0 | 0 | 0 | 0 | 0 | 0 | 0 | 0 | 0 | 0 | 0 |
| NM_001080457 | LRRC4B | 2957 | 2 | 0 | 0 | 0 | 2 | 0 | 0 | 0 | 0 | 0 | 0 | 0 | 0 |
| NM_001080458 | EVX2 | 1617 | 0 | 0 | 0 | 0 | 0 | 0 | 0 | 0 | 0 | 0 | 0 | 0 | 0 |
| NM_001080460 | LRRIQ4 | 1824 | 0 | 0 | 0 | 0 | 0 | 0 | 0 | 0 | 0 | 0 | 0 | 0 | 0 |
| NM_001080461 | UNCX | 1596 | 0 | 0 | 0 | 0 | 0 | 0 | 0 | 0 | 0 | 0 | 0 | 0 | 0 |
| NM_001080462 | TMEM202 | 1296 | 0 | 0 | 0 | 0 | 0 | 0 | 0 | 0 | 0 | 0 | 0 | 0 | 0 |
| NM_001080463 | DYNC2H1 | 13699 | 8 | 3 | 2 | 1 | 2 | 4 | 5 | 0 | 3 | 8 | 1 | 2 | 7 |
| NM_001080464 | ASPG | 1844 | 1 | 0 | 0 | 0 | 0 | 0 | 0 | 1 | 0 | 0 | 0 | 0 | 0 |
| NM_001080465 | C17orf98 | 566 | 1 | 0 | 0 | 0 | 0 | 0 | 0 | 0 | 0 | 0 | 0 | 0 | 1 |
| NM_001080466 | BTBD17 | 1677 | 0 | 0 | 0 | 0 | 0 | 0 | 0 | 0 | 0 | 0 | 0 | 0 | 0 |
| NM_001080467 | MYO5B | 9520 | 5 | 2 | 0 | 3 | 1 | 1 | 5 | 2 | 2 | 1 | 0 | 1 | 1 |
| NM_001080468 | SYCN | 482 | 1 | 0 | 0 | 0 | 0 | 0 | 0 | 0 | 0 | 0 | 0 | 0 | 1 |
| NM_001080469 | FBXO46 | 2990 | 0 | 0 | 0 | 0 | 0 | 0 | 0 | 0 | 0 | 0 | 0 | 0 | 0 |
| NM_001080470 | ZNF697 | 5080 | 4 | 1 | 2 | 1 | 1 | 1 | 4 | 1 | 2 | 2 | 0 | 1 | 1 |
| NM_001080471 | PEAR1 | 4866 | 2 | 1 | 1 | 0 | 1 | 0 | 1 | 0 | 1 | 1 | 0 | 2 | 1 |
| NM_001080472 | FITM2 | 958 | 3 | 1 | 2 | 1 | 3 | 1 | 1 | 1 | 0 | 0 | 1 | 0 | 1 |

| transcript | gene | transcript length (nt) | max | mean | adipose | colon | heart | hypothalamus | kidney | liver | lung | ovary | skeletalmuscle | spleen | testes |
|---|---|---|---|---|---|---|---|---|---|---|---|---|---|---|---|
| NM_001080473 | MFSD2B | 1508 | 0 | 0 | 0 | 0 | 0 | 0 | 0 | 0 | 0 | 0 | 0 | 0 | 0 |
| NM_001080474 | C2orf78 | 3045 | 2 | 0 | 0 | 0 | 0 | 0 | 0 | 0 | 0 | 0 | 0 | 0 | 2 |
| NM_001080475 | PLEKHM3 | 9806 | 2 | 1 | 1 | 1 | 1 | 2 | 1 | 1 | 1 | 1 | 1 | 1 | 1 |
| NM_001080476 | GRXCR1 | 991 | 0 | 0 | 0 | 0 | 0 | 0 | 0 | 0 | 0 | 0 | 0 | 0 | 0 |
| NM_001080477 | ODZ3 | 10810 | 5 | 1 | 1 | 0 | 0 | 5 | 0 | 0 | 0 | 0 | 1 | 2 | 1 |
| NM_001080478 | LRRC14B | 1573 | 1 | 0 | 0 | 0 | 0 | 0 | 0 | 0 | 0 | 0 | 1 | 0 | 0 |
| NM_001080479 | RGNEF | 5252 | 3 | 1 | 1 | 0 | 0 | 1 | 3 | 1 | 1 | 3 | 0 | 0 | 0 |
| NM_001080480 | MBOAT1 | 3275 | 4 | 1 | 2 | 4 | 1 | 1 | 1 | 1 | 1 | 3 | 0 | 1 | 1 |
| NM_001080481 | USP45 | 6385 | 2 | 1 | 0 | 0 | 1 | 1 | 1 | 0 | 1 | 1 | 1 | 2 | 1 |
| NM_001080482 | C9orf172 | 2931 | 0 | 0 | 0 | 0 | 0 | 0 | 0 | 0 | 0 | 0 | 0 | 0 | 0 |
| NM_001080483 | TMEM8C | 818 | 0 | 0 | 0 | 0 | 0 | 0 | 0 | 0 | 0 | 0 | 0 | 0 | 0 |
| NM_001080484 | KIAA1751 | 4710 | 0 | 0 | 0 | 0 | 0 | 0 | 0 | 0 | 0 | 0 | 0 | 0 | 0 |
| NM_001080485 | ZNF275 | 6417 | 9 | 2 | 2 | 1 | 1 | 3 | 1 | 3 | 1 | 9 | 1 | 3 | 4 |
| NM_001080486 | FOLR4 | 732 | 0 | 0 | 0 | 0 | 0 | 0 | 0 | 0 | 0 | 0 | 0 | 0 | 0 |
| NM_001080487 | PABPN1L | 870 | 0 | 0 | 0 | 0 | 0 | 0 | 0 | 0 | 0 | 0 | 0 | 0 | 0 |
| NM_001080488 | ONECUT3 | 1485 | 0 | 0 | 0 | 0 | 0 | 0 | 0 | 0 | 0 | 0 | 0 | 0 | 0 |
| NM_001080489 | GLOD5 | 735 | 1 | 0 | 0 | 0 | 0 | 0 | 1 | 0 | 0 | 0 | 0 | 0 | 0 |
| NM_001080490 | PLA2G4E | 2520 | 0 | 0 | 0 | 0 | 0 | 0 | 0 | 0 | 0 | 0 | 0 | 0 | 0 |
| NM_001080491 | USP6NL | 4512 | 7 | 3 | 3 | 2 | 2 | 3 | 2 | 3 | 3 | 3 | 1 | 7 | 6 |
| NM_001080492 | KLKBL4 | 1810 | 1 | 0 | 0 | 0 | 0 | 0 | 0 | 0 | 0 | 0 | 0 | 0 | 1 |
| NM_001080493 | ZNF823 | 2395 | 6 | 3 | 2 | 2 | 3 | 3 | 6 | 3 | 3 | 6 | 2 | 3 | 2 |
| NM_001080494 | TTC39A | 2740 | 3 | 1 | 0 | 1 | 0 | 0 | 3 | 0 | 0 | 0 | 0 | 0 | 2 |
| NM_001080495 | TNRC18 | 10570 | 2 | 1 | 1 | 2 | 1 | 1 | 1 | 1 | 1 | 2 | 0 | 1 | 1 |
| NM_001080496 | RGP1 | 1296 | 2 | 2 | 1 | 2 | 2 | 2 | 2 | 2 | 1 | 1 | 2 | 2 | 1 |
| NM_001080497 | MEGF9 | 6297 | 7 | 3 | 3 | 1 | 2 | 6 | 2 | 7 | 6 | 2 | 1 | 5 | 1 |
| NM_001080500 | VWC2L | 908 | 0 | 0 | 0 | 0 | 0 | 0 | 0 | 0 | 0 | 0 | 0 | 0 | 0 |
| NM_001080501 | TMEM223 | 937 | 2 | 1 | 1 | 1 | 1 | 1 | 1 | 2 | 0 | 0 | 1 | 2 | 1 |
| NM_001080503 | CCDC159 | 1062 | 3 | 1 | 1 | 1 | 2 | 1 | 2 | 0 | 1 | 2 | 0 | 3 | 2 |
| NM_001080504 | RBM44 | 4282 | 4 | 0 | 0 | 0 | 0 | 0 | 0 | 0 | 0 | 0 | 0 | 0 | 4 |
| NM_001080505 | SHISA3 | 1971 | 1 | 0 | 0 | 0 | 0 | 0 | 1 | 0 | 0 | 0 | 0 | 0 | 0 |
| NM_001080506 | TMEM150C | 1888 | 6 | 3 | 1 | 1 | 4 | 5 | 4 | 2 | 1 | 6 | 1 | 3 | 5 |
| NM_001080507 | OOEP | 677 | 0 | 0 | 0 | 0 | 0 | 0 | 0 | 0 | 0 | 0 | 0 | 0 | 0 |
| NM_001080508 | TBX18 | 4070 | 1 | 0 | 1 | 0 | 1 | 1 | 0 | 0 | 0 | 1 | 0 | 0 | 1 |
| NM_001080509 | TSPAN11 | 5561 | 1 | 0 | 0 | 1 | 0 | 1 | 0 | 0 | 0 | 1 | 0 | 0 | 0 |
| NM_001080510 | C17orf95 | 1099 | 3 | 2 | 1 | 1 | 2 | 1 | 2 | 2 | 2 | 1 | 1 | 3 | 2 |
| NM_001080511 | CLEC2L | 1288 | 1 | 0 | 0 | 0 | 0 | 1 | 0 | 0 | 0 | 0 | 0 | 0 | 0 |
| NM_001080512 | BICC1 | 3119 | 18 | 5 | 4 | 1 | 6 | 2 | 18 | 1 | 3 | 12 | 3 | 2 | 5 |
| NM_001080513 | CPN2 | 3027 | 22 | 2 | 0 | 0 | 0 | 0 | 3 | 22 | 0 | 0 | 0 | 0 | 0 |
| NM_001080514 | SCXB | 606 | 0 | 0 | 0 | 0 | 0 | 0 | 0 | 0 | 0 | 0 | 0 | 0 | 0 |
| NM_001080515 | FAM163B | 501 | 0 | 0 | 0 | 0 | 0 | 0 | 0 | 0 | 0 | 0 | 0 | 0 | 0 |
| NM_001080516 | GRXCR2 | 747 | 7 | 1 | 0 | 0 | 7 | 0 | 0 | 0 | 0 | 0 | 0 | 0 | 0 |
| NM_001080517 | SETD5 | 6827 | 11 | 7 | 6 | 7 | 6 | 6 | 6 | 5 | 9 | 11 | 5 | 10 | 11 |
| NM_001080518 | LIPK | 1200 | 0 | 0 | 0 | 0 | 0 | 0 | 0 | 0 | 0 | 0 | 0 | 0 | 0 |
| NM_001080519 | BAHCC1 | 10177 | 0 | 0 | 0 | 0 | 0 | 0 | 0 | 0 | 0 | 0 | 0 | 0 | 0 |
| NM_001080520 | DRGX | 807 | 0 | 0 | 0 | 0 | 0 | 0 | 0 | 0 | 0 | 0 | 0 | 0 | 0 |
| NM_001080521 | RASSF10 | 1848 | 0 | 0 | 0 | 0 | 0 | 0 | 0 | 0 | 0 | 0 | 0 | 0 | 0 |
| NM_001080522 | CC2D2A | 5240 | 5 | 2 | 2 | 1 | 2 | 2 | 4 | 1 | 2 | 5 | 1 | 2 | 3 |
| NM_001080523 | ARRDC5 | 1638 | 2 | 1 | 0 | 0 | 0 | 0 | 0 | 0 | 1 | 0 | 0 | 2 | 2 |
| NM_001080524 | C16orf90 | 904 | 0 | 0 | 0 | 0 | 0 | 0 | 0 | 0 | 0 | 0 | 0 | 0 | 0 |
| NM_001080525 | SPINK8 | 440 | 0 | 0 | 0 | 0 | 0 | 0 | 0 | 0 | 0 | 0 | 0 | 0 | 0 |
| NM_001080526 | FABP9 | 399 | 1 | 0 | 1 | 0 | 0 | 0 | 0 | 0 | 0 | 0 | 0 | 0 | 0 |
| NM_001080527 | MYO7B | 6715 | 3 | 0 | 0 | 3 | 0 | 0 | 2 | 0 | 0 | 0 | 0 | 1 | 0 |
| NM_001080528 | LOC646498 | 616 | 0 | 0 | 0 | 0 | 0 | 0 | 0 | 0 | 0 | 0 | 0 | 0 | 0 |
| NM_001080529 | WIPF3 | 3241 | 7 | 2 | 0 | 0 | 0 | 3 | 0 | 0 | 0 | 7 | 2 | 0 | 6 |
| NM_001080530 | SNX29 | 7003 | 6 | 2 | 2 | 1 | 1 | 1 | 6 | 0 | 1 | 2 | 1 | 2 | 1 |
| NM_001080531 | C4orf51 | 845 | 1 | 0 | 0 | 0 | 0 | 0 | 0 | 0 | 0 | 0 | 0 | 0 | 1 |
| NM_001080532 | TMC3 | 3303 | 0 | 0 | 0 | 0 | 0 | 0 | 0 | 0 | 0 | 0 | 0 | 0 | 0 |
| NM_001080533 | UNC119B | 4811 | 6 | 3 | 2 | 2 | 3 | 4 | 5 | 2 | 2 | 6 | 1 | 4 | 4 |
| NM_001080534 | UNC13C | 8140 | 13 | 3 | 0 | 0 | 0 | 12 | 1 | 0 | 0 | 1 | 13 | 0 | 0 |
| NM_001080537 | SNTN | 1584 | 6 | 1 | 0 | 0 | 0 | 0 | 0 | 0 | 6 | 0 | 0 | 0 | 1 |
| NM_001080538 | AKR1B15 | 951 | 0 | 0 | 0 | 0 | 0 | 0 | 0 | 0 | 0 | 0 | 0 | 0 | 0 |
| NM_001080539 | CCDC150 | 3685 | 1 | 0 | 0 | 0 | 0 | 0 | 1 | 0 | 0 | 0 | 0 | 0 | 1 |
| NM_001080540 | ISCA1L | 1881 | 0 | 0 | 0 | 0 | 0 | 0 | 0 | 0 | 0 | 0 | 0 | 0 | 0 |
| NM_001080541 | MGA | 11413 | 15 | 8 | 5 | 4 | 6 | 9 | 7 | 2 | 5 | 14 | 6 | 11 | 15 |
| NM_001080542 | FBF1 | 3862 | 2 | 0 | 0 | 0 | 0 | 1 | 0 | 0 | 0 | 0 | 0 | 0 | 2 |
| NM_001080543 | C19orf29 | 3624 | 1 | 0 | 0 | 1 | 0 | 0 | 0 | 0 | 0 | 0 | 0 | 0 | 0 |
| NM_001080545 | PPP1R1C | 1014 | 2 | 0 | 0 | 0 | 2 | 0 | 0 | 0 | 0 | 0 | 0 | 0 | 1 |
| NM_001080546 | TMEM218 | 2103 | 1 | 0 | 1 | 0 | 0 | 0 | 0 | 0 | 0 | 0 | 0 | 0 | 0 |
| NM_001080547 | SPI1 | 1408 | 6 | 1 | 2 | 1 | 1 | 1 | 0 | 1 | 2 | 0 | 0 | 6 | 0 |
| NM_001080551 | C9orf84 | 4612 | 2 | 1 | 0 | 0 | 0 | 0 | 1 | 0 | 1 | 1 | 0 | 1 | 2 |
| NM_001080554 | GSG1 | 2179 | 34 | 3 | 0 | 0 | 0 | 0 | 0 | 0 | 0 | 0 | 0 | 0 | 34 |
| NM_001080555 | GSG1 | 2055 | 36 | 3 | 0 | 0 | 0 | 0 | 0 | 0 | 0 | 0 | 0 | 0 | 36 |
| NM_001080556 | WDR16 | 1989 | 2 | 0 | 0 | 0 | 0 | 1 | 0 | 0 | 1 | 0 | 0 | 0 | 2 |
| NM_001080744 | DGKG | 5727 | 3 | 1 | 0 | 0 | 1 | 3 | 0 | 0 | 0 | 0 | 1 | 0 | 0 |
| NM_001080745 | DGKG | 5685 | 3 | 1 | 0 | 0 | 1 | 3 | 0 | 0 | 0 | 0 | 1 | 0 | 0 |
| NM_001080770 | KIR2DL4 | 1475 | 1 | 0 | 0 | 0 | 0 | 0 | 0 | 0 | 0 | 0 | 0 | 1 | 0 |
| NM_001080772 | KIR2DL4 | 1579 | 1 | 0 | 0 | 0 | 0 | 0 | 0 | 0 | 0 | 0 | 0 | 1 | 0 |

| transcript | gene | transcript length (nt) | max | mean | adipose | colon | heart | hypothalamus | kidney | liver | lung | ovary | skeletalmuscle | spleen | testes |
|---|---|---|---|---|---|---|---|---|---|---|---|---|---|---|---|
| NM_001080779 | MYO1C | 4952 | 14 | 5 | 14 | 6 | 7 | 1 | 4 | 3 | 4 | 2 | 3 | 3 | 3 |
| NM_001080790 | MAGEA9B | 1814 | 0 | 0 | 0 | 0 | 0 | 0 | 0 | 0 | 0 | 0 | 0 | 0 | 0 |
| NM_001080791 | C15orf57 | 1730 | 2 | 1 | 1 | 2 | 1 | 1 | 1 | 1 | 1 | 1 | 1 | 2 | 1 |
| NM_001080792 | C15orf57 | 1520 | 2 | 1 | 1 | 2 | 1 | 1 | 1 | 1 | 1 | 1 | 1 | 2 | 1 |
| NM_001080821 | ZNF799 | 2532 | 3 | 1 | 1 | 1 | 1 | 1 | 2 | 1 | 1 | 2 | 1 | 3 | 2 |
| NM_001080824 | C2orf89 | 1705 | 1 | 0 | 0 | 1 | 0 | 0 | 0 | 0 | 0 | 1 | 0 | 0 | 0 |
| NM_001080825 | TMEM120B | 7508 | 0 | 0 | 0 | 0 | 0 | 0 | 0 | 0 | 0 | 0 | 0 | 0 | 0 |
| NM_001080826 | PRAGMIN | 4627 | 0 | 0 | 0 | 0 | 0 | 0 | 0 | 0 | 0 | 0 | 0 | 0 | 0 |
| NM_001080830 | PRAMEF12 | 1785 | 0 | 0 | 0 | 0 | 0 | 0 | 0 | 0 | 0 | 0 | 0 | 0 | 0 |
| NM_001080835 | PRR21 | 1170 | 0 | 0 | 0 | 0 | 0 | 0 | 0 | 0 | 0 | 0 | 0 | 0 | 0 |
| NM_001080836 | MEIG1 | 625 | 2 | 0 | 0 | 0 | 0 | 0 | 0 | 0 | 0 | 0 | 0 | 0 | 2 |
| NM_001080837 | SEBOX | 665 | 0 | 0 | 0 | 0 | 0 | 0 | 0 | 0 | 0 | 0 | 0 | 0 | 0 |
| NM_001080843 | GSTT2B | 1094 | 0 | 0 | 0 | 0 | 0 | 0 | 0 | 0 | 0 | 0 | 0 | 0 | 0 |
| NM_001080848 | CSAG2 | 847 | 0 | 0 | 0 | 0 | 0 | 0 | 0 | 0 | 0 | 0 | 0 | 0 | 0 |
| NM_001080849 | DNLZ | 724 | 1 | 0 | 0 | 0 | 1 | 0 | 0 | 0 | 0 | 0 | 0 | 0 | 0 |
| NM_001080850 | CCDC30 | 3098 | 4 | 2 | 1 | 0 | 0 | 4 | 4 | 0 | 1 | 3 | 0 | 1 | 3 |
| NM_001080851 | DMRTC1B | 1100 | 0 | 0 | 0 | 0 | 0 | 0 | 0 | 0 | 0 | 0 | 0 | 0 | 0 |
| NM_001080855 | PXN | 3774 | 4 | 2 | 2 | 2 | 2 | 1 | 2 | 1 | 2 | 1 | 2 | 4 | 2 |
| NM_001080950 | MYO1C | 4721 | 15 | 5 | 15 | 6 | 7 | 1 | 4 | 3 | 5 | 2 | 3 | 4 | 3 |
| NM_001080951 | PLAGL1 | 3279 | 1 | 1 | 1 | 1 | 1 | 0 | 1 | 0 | 1 | 1 | 1 | 1 | 1 |
| NM_001080952 | PLAGL1 | 3420 | 1 | 1 | 1 | 1 | 1 | 0 | 1 | 0 | 1 | 1 | 1 | 1 | 1 |
| NM_001080953 | PLAGL1 | 3374 | 1 | 1 | 1 | 1 | 1 | 0 | 1 | 0 | 1 | 1 | 1 | 1 | 1 |
| NM_001080954 | PLAGL1 | 3373 | 1 | 1 | 1 | 1 | 1 | 0 | 1 | 0 | 1 | 1 | 0 | 1 | 1 |
| NM_001080955 | PLAGL1 | 2728 | 2 | 1 | 1 | 1 | 1 | 0 | 1 | 0 | 1 | 2 | 1 | 1 | 1 |
| NM_001080956 | PLAGL1 | 2912 | 2 | 1 | 1 | 1 | 1 | 0 | 1 | 0 | 1 | 2 | 1 | 1 | 1 |
| NM_001080975 | REPS2 | 7950 | 6 | 1 | 0 | 1 | 1 | 6 | 1 | 1 | 1 | 1 | 0 | 1 | 1 |
| NM_001080976 | DSE | 4005 | 10 | 4 | 7 | 1 | 5 | 3 | 3 | 2 | 6 | 4 | 2 | 10 | 3 |
| NM_001080978 | LILRB2 | 2921 | 6 | 1 | 1 | 0 | 1 | 0 | 0 | 0 | 1 | 0 | 0 | 6 | 0 |
| NM_001080997 | RTBDN | 1043 | 1 | 0 | 0 | 0 | 0 | 1 | 0 | 0 | 0 | 0 | 0 | 0 | 0 |
| NM_001080998 | FRG2B | 890 | 0 | 0 | 0 | 0 | 0 | 0 | 0 | 0 | 0 | 0 | 0 | 0 | 0 |
| NM_001081 | CUBN | 11933 | 49 | 5 | 0 | 0 | 0 | 0 | 49 | 0 | 0 | 0 | 0 | 0 | 0 |
| NM_001081003 | COMMD5 | 1406 | 0 | 0 | 0 | 0 | 0 | 0 | 0 | 0 | 0 | 0 | 0 | 0 | 0 |
| NM_001081004 | COMMD5 | 1304 | 0 | 0 | 0 | 0 | 0 | 0 | 0 | 0 | 0 | 0 | 0 | 0 | 0 |
| NM_001081438 | LILRB4 | 2082 | 3 | 1 | 3 | 0 | 1 | 1 | 0 | 1 | 2 | 0 | 0 | 2 | 0 |
| NM_001081442 | LILRB5 | 2267 | 3 | 1 | 3 | 1 | 1 | 0 | 0 | 1 | 1 | 0 | 1 | 2 | 0 |
| NM_001081443 | LILRB5 | 1967 | 3 | 1 | 3 | 1 | 1 | 0 | 0 | 1 | 1 | 0 | 1 | 3 | 0 |
| NM_001081450 | LILRB3 | 2825 | 2 | 0 | 0 | 0 | 1 | 0 | 0 | 0 | 1 | 0 | 0 | 2 | 0 |
| NM_001081455 | P2RY14 | 2678 | 2 | 1 | 2 | 1 | 1 | 0 | 0 | 0 | 2 | 0 | 1 | 1 | 0 |
| NM_001081461 | JMJD6 | 5423 | 3 | 1 | 1 | 1 | 3 | 1 | 1 | 1 | 1 | 1 | 2 | 1 | 1 |
| NM_001081472 | SPATA1 | 1981 | 2 | 1 | 0 | 0 | 1 | 0 | 1 | 0 | 1 | 1 | 0 | 1 | 2 |
| NM_001081491 | NXF1 | 4147 | 3 | 2 | 3 | 3 | 1 | 2 | 2 | 2 | 2 | 2 | 2 | 3 | 2 |
| NM_001081492 | KRT80 | 3894 | 0 | 0 | 0 | 0 | 0 | 0 | 0 | 0 | 0 | 0 | 0 | 0 | 0 |
| NM_001081550 | THOC2 | 5610 | 25 | 15 | 10 | 5 | 12 | 10 | 15 | 9 | 18 | 23 | 11 | 24 | 25 |
| NM_001081551 | DEFB103B | 437 | 0 | 0 | 0 | 0 | 0 | 0 | 0 | 0 | 0 | 0 | 0 | 0 | 0 |
| NM_001081552 | SPAG11A | 756 | 0 | 0 | 0 | 0 | 0 | 0 | 0 | 0 | 0 | 0 | 0 | 0 | 0 |
| NM_001081559 | CPSF4 | 1751 | 1 | 1 | 0 | 0 | 0 | 1 | 1 | 0 | 0 | 1 | 0 | 0 | 1 |
| NM_001081560 | DMPK | 2850 | 7 | 2 | 1 | 3 | 7 | 1 | 1 | 0 | 1 | 1 | 3 | 1 | 1 |
| NM_001081562 | DMPK | 2846 | 7 | 2 | 1 | 3 | 7 | 1 | 1 | 0 | 1 | 1 | 3 | 1 | 1 |
| NM_001081563 | DMPK | 3234 | 7 | 2 | 1 | 3 | 7 | 1 | 1 | 0 | 1 | 1 | 3 | 1 | 2 |
| NM_001081573 | GAB3 | 4740 | 3 | 1 | 1 | 0 | 1 | 0 | 0 | 0 | 1 | 0 | 0 | 3 | 1 |
| NM_001081637 | LILRB1 | 2799 | 5 | 1 | 0 | 0 | 0 | 0 | 0 | 0 | 1 | 0 | 0 | 5 | 0 |
| NM_001081638 | LILRB1 | 2856 | 5 | 1 | 0 | 0 | 0 | 0 | 0 | 0 | 1 | 0 | 0 | 5 | 0 |
| NM_001081639 | LILRB1 | 2856 | 5 | 1 | 0 | 0 | 0 | 0 | 0 | 0 | 1 | 0 | 0 | 5 | 0 |
| NM_001081640 | PRKDC | 13415 | 12 | 8 | 6 | 6 | 9 | 9 | 7 | 8 | 5 | 9 | 8 | 12 | 11 |
| NM_001081675 | KLHL38 | 1834 | 3 | 0 | 0 | 0 | 1 | 0 | 0 | 0 | 0 | 0 | 3 | 0 | 0 |
| NM_001081676 | SCN3A | 8978 | 5 | 1 | 0 | 0 | 0 | 5 | 1 | 0 | 0 | 0 | 0 | 0 | 0 |
| NM_001081677 | SCN3A | 8978 | 5 | 1 | 0 | 0 | 0 | 5 | 1 | 0 | 0 | 0 | 0 | 0 | 0 |
| NM_001081686 | G6PC2 | 2965 | 0 | 0 | 0 | 0 | 0 | 0 | 0 | 0 | 0 | 0 | 0 | 0 | 0 |
| NM_001081752 | ELN | 3331 | 4 | 1 | 2 | 1 | 4 | 1 | 1 | 0 | 2 | 1 | 1 | 1 | 1 |
| NM_001081753 | ELN | 3376 | 4 | 1 | 2 | 1 | 4 | 1 | 1 | 0 | 2 | 0 | 1 | 1 | 1 |
| NM_001081754 | ELN | 3433 | 4 | 1 | 2 | 1 | 4 | 0 | 1 | 0 | 2 | 0 | 1 | 1 | 1 |
| NM_001081755 | ELN | 3415 | 4 | 1 | 2 | 1 | 4 | 0 | 1 | 0 | 2 | 0 | 1 | 1 | 1 |
| NM_001081955 | RGS9 | 2513 | 1 | 0 | 0 | 0 | 0 | 1 | 0 | 0 | 0 | 0 | 0 | 0 | 0 |
| NM_001081976 | ADORA3 | 1163 | 2 | 1 | 0 | 0 | 1 | 1 | 0 | 0 | 1 | 0 | 0 | 2 | 1 |
| NM_001082 | CYP4F2 | 2360 | 23 | 3 | 0 | 0 | 0 | 0 | 6 | 23 | 0 | 0 | 0 | 0 | 0 |
| NM_001082480 | ZNF623 | 4046 | 10 | 6 | 4 | 5 | 4 | 9 | 4 | 2 | 3 | 10 | 3 | 8 | 8 |
| NM_001082486 | ACD | 2066 | 0 | 0 | 0 | 0 | 0 | 0 | 0 | 0 | 0 | 0 | 0 | 0 | 0 |
| NM_001082487 | ACD | 2018 | 0 | 0 | 0 | 0 | 0 | 0 | 0 | 0 | 0 | 0 | 0 | 0 | 0 |
| NM_001082488 | DHRS4L1 | 1249 | 0 | 0 | 0 | 0 | 0 | 0 | 0 | 0 | 0 | 0 | 0 | 0 | 0 |
| NM_001082533 | CA10 | 3370 | 9 | 1 | 0 | 0 | 0 | 9 | 1 | 0 | 0 | 0 | 0 | 0 | 0 |
| NM_001082534 | CA10 | 2935 | 10 | 1 | 0 | 0 | 0 | 10 | 1 | 0 | 0 | 0 | 0 | 0 | 0 |
| NM_001082537 | TCTN1 | 1961 | 1 | 1 | 0 | 0 | 1 | 0 | 1 | 0 | 0 | 1 | 0 | 1 | 1 |
| NM_001082538 | TCTN1 | 1976 | 1 | 1 | 0 | 0 | 1 | 0 | 1 | 0 | 0 | 1 | 0 | 1 | 1 |
| NM_001082575 | HRNBP3 | 2679 | 3 | 0 | 0 | 1 | 0 | 3 | 0 | 0 | 0 | 0 | 0 | 0 | 0 |
| NM_001082576 | RBM9 | 6965 | 17 | 7 | 5 | 5 | 7 | 13 | 5 | 3 | 5 | 17 | 6 | 4 | 6 |
| NM_001082577 | RBM9 | 6937 | 17 | 7 | 5 | 5 | 7 | 13 | 5 | 3 | 5 | 17 | 6 | 4 | 6 |
| NM_001082578 | RBM9 | 6937 | 16 | 6 | 4 | 5 | 7 | 11 | 5 | 3 | 4 | 16 | 5 | 4 | 5 |

| transcript | gene | transcript length (nt) | max | mean | adipose | colon | heart | hypothalamus | kidney | liver | lung | ovary | skeletalmuscle | spleen | testes |
|---|---|---|---|---|---|---|---|---|---|---|---|---|---|---|---|
| NM_001082579 | RBM9 | 6934 | 16 | 6 | 4 | 5 | 7 | 12 | 5 | 3 | 4 | 16 | 5 | 4 | 5 |
| NM_001082618 | CD33 | 1085 | 3 | 0 | 1 | 0 | 1 | 0 | 0 | 0 | 0 | 0 | 0 | 3 | 0 |
| NM_001082619 | PIH1D2 | 1248 | 3 | 1 | 0 | 0 | 0 | 1 | 1 | 0 | 0 | 0 | 0 | 0 | 3 |
| NM_001082959 | SCARB1 | 2613 | 4 | 1 | 1 | 0 | 0 | 1 | 0 | 4 | 0 | 1 | 0 | 0 | 0 |
| NM_001082967 | FAM19A5 | 2601 | 7 | 1 | 0 | 0 | 0 | 7 | 0 | 1 | 0 | 1 | 0 | 0 | 1 |
| NM_001082968 | TOM1L2 | 5808 | 6 | 2 | 2 | 2 | 4 | 6 | 3 | 1 | 1 | 1 | 2 | 1 | 3 |
| NM_001082969 | C11orf57 | 3183 | 4 | 3 | 2 | 1 | 3 | 3 | 3 | 2 | 2 | 4 | 2 | 4 | 4 |
| NM_001082970 | C11orf57 | 3738 | 3 | 2 | 2 | 1 | 3 | 3 | 3 | 1 | 2 | 3 | 2 | 3 | 3 |
| NM_001082971 | DDC | 2070 | 16 | 2 | 0 | 2 | 0 | 0 | 16 | 2 | 0 | 0 | 0 | 0 | 0 |
| NM_001082972 | CASC1 | 2638 | 8 | 2 | 0 | 0 | 0 | 3 | 2 | 0 | 2 | 1 | 0 | 0 | 8 |
| NM_001082973 | CASC1 | 2492 | 8 | 2 | 0 | 0 | 1 | 3 | 2 | 0 | 2 | 1 | 0 | 0 | 8 |
| NM_001083 | PDE5A | 6989 | 6 | 3 | 2 | 4 | 4 | 2 | 2 | 0 | 5 | 3 | 3 | 2 | 6 |
| NM_001083111 | GNRH1 | 1281 | 1 | 0 | 0 | 0 | 0 | 1 | 0 | 0 | 1 | 0 | 0 | 1 | 0 |
| NM_001083112 | GPD2 | 6029 | 8 | 3 | 2 | 2 | 2 | 7 | 3 | 0 | 4 | 2 | 8 | 4 | 2 |
| NM_001083113 | ZGPAT | 1905 | 3 | 2 | 1 | 2 | 1 | 2 | 3 | 3 | 1 | 1 | 1 | 2 | 1 |
| NM_001083116 | PRF1 | 2534 | 1 | 0 | 0 | 0 | 0 | 0 | 0 | 0 | 0 | 0 | 0 | 1 | 0 |
| NM_001083124 | FAM75A3 | 4223 | 0 | 0 | 0 | 0 | 0 | 0 | 0 | 0 | 0 | 0 | 0 | 0 | 0 |
| NM_001083308 | PYDC2 | 294 | 0 | 0 | 0 | 0 | 0 | 0 | 0 | 0 | 0 | 0 | 0 | 0 | 0 |
| NM_001083314 | CHMP1A | 2334 | 1 | 0 | 0 | 0 | 0 | 1 | 1 | 0 | 0 | 0 | 0 | 1 | 0 |
| NM_001083330 | ZNF133 | 2289 | 1 | 1 | 1 | 1 | 1 | 1 | 1 | 0 | 0 | 1 | 1 | 1 | 1 |
| NM_001083335 | ZFP112 | 3735 | 4 | 2 | 1 | 1 | 2 | 2 | 2 | 0 | 1 | 4 | 1 | 2 | 3 |
| NM_001083535 | C6orf182 | 2902 | 4 | 2 | 3 | 1 | 1 | 3 | 2 | 1 | 1 | 2 | 1 | 2 | 4 |
| NM_001083536 | FGD3 | 3453 | 2 | 0 | 0 | 0 | 0 | 0 | 0 | 0 | 1 | 0 | 0 | 2 | 0 |
| NM_001083537 | FAM86B1 | 2438 | 1 | 0 | 0 | 0 | 0 | 0 | 1 | 0 | 0 | 0 | 0 | 0 | 0 |
| NM_001083538 | POTEE | 3440 | 1 | 0 | 0 | 0 | 0 | 0 | 0 | 0 | 0 | 0 | 0 | 0 | 1 |
| NM_001083539 | KIR3DS1 | 1741 | 0 | 0 | 0 | 0 | 0 | 0 | 0 | 0 | 0 | 0 | 0 | 0 | 0 |
| NM_001083585 | RABEP1 | 5303 | 38 | 21 | 18 | 6 | 27 | 38 | 17 | 13 | 14 | 19 | 28 | 30 | 18 |
| NM_001083588 | E2F5 | 1732 | 2 | 1 | 0 | 0 | 0 | 0 | 1 | 0 | 0 | 0 | 0 | 2 | 1 |
| NM_001083589 | E2F5 | 1586 | 2 | 1 | 0 | 0 | 0 | 1 | 1 | 0 | 0 | 0 | 0 | 2 | 1 |
| NM_001083590 | TMEM214 | 2905 | 2 | 2 | 2 | 1 | 2 | 1 | 2 | 2 | 1 | 1 | 1 | 2 | 1 |
| NM_001083591 | CUGBP2 | 8036 | 6 | 3 | 3 | 1 | 4 | 6 | 0 | 0 | 4 | 6 | 2 | 6 | 2 |
| NM_001083592 | ROR1 | 2292 | 2 | 0 | 0 | 2 | 0 | 0 | 0 | 0 | 0 | 1 | 0 | 1 | 0 |
| NM_001083600 | NAT15 | 2628 | 5 | 3 | 2 | 3 | 3 | 5 | 5 | 5 | 2 | 3 | 1 | 4 | 3 |
| NM_001083601 | NAT15 | 2678 | 5 | 3 | 2 | 3 | 3 | 5 | 5 | 5 | 2 | 3 | 1 | 4 | 3 |
| NM_001083602 | PTCH1 | 8051 | 4 | 1 | 0 | 3 | 0 | 1 | 1 | 0 | 1 | 2 | 0 | 1 | 4 |
| NM_001083603 | PTCH1 | 7897 | 4 | 1 | 0 | 3 | 0 | 1 | 1 | 0 | 1 | 2 | 0 | 1 | 4 |
| NM_001083604 | PTCH1 | 7661 | 4 | 1 | 0 | 3 | 0 | 1 | 1 | 0 | 1 | 2 | 0 | 1 | 4 |
| NM_001083605 | PTCH1 | 7644 | 5 | 1 | 0 | 3 | 0 | 1 | 1 | 0 | 1 | 2 | 0 | 1 | 5 |
| NM_001083606 | PTCH1 | 7743 | 4 | 1 | 0 | 3 | 0 | 1 | 1 | 0 | 1 | 2 | 0 | 1 | 4 |
| NM_001083607 | PTCH1 | 7817 | 4 | 1 | 0 | 3 | 0 | 1 | 1 | 0 | 1 | 2 | 0 | 1 | 4 |
| NM_001083608 | NARF | 1477 | 4 | 3 | 2 | 2 | 3 | 3 | 2 | 2 | 2 | 2 | 2 | 4 | 4 |
| NM_001083612 | AGPHD1 | 848 | 7 | 3 | 3 | 2 | 2 | 4 | 7 | 2 | 1 | 4 | 4 | 1 | 2 |
| NM_001083613 | TMEM219 | 999 | 2 | 1 | 1 | 2 | 1 | 1 | 1 | 0 | 1 | 0 | 0 | 1 | 1 |
| NM_001083614 | EARS2 | 3962 | 1 | 1 | 1 | 1 | 1 | 1 | 1 | 1 | 0 | 1 | 0 | 1 | 1 |
| NM_001083615 | MYO3B | 5442 | 1 | 0 | 0 | 0 | 0 | 0 | 1 | 0 | 0 | 0 | 0 | 0 | 1 |
| NM_001083617 | RB1CC1 | 6658 | 15 | 10 | 6 | 2 | 14 | 10 | 8 | 7 | 10 | 10 | 15 | 11 | 15 |
| NM_001083619 | GRIA2 | 5754 | 43 | 4 | 0 | 0 | 0 | 43 | 0 | 0 | 0 | 0 | 0 | 0 | 0 |
| NM_001083620 | GRIA2 | 5265 | 46 | 4 | 0 | 0 | 0 | 46 | 0 | 0 | 0 | 0 | 0 | 0 | 0 |
| NM_001083621 | ZBTB40 | 8992 | 3 | 2 | 1 | 1 | 2 | 2 | 2 | 1 | 1 | 3 | 1 | 3 | 2 |
| NM_001083625 | ANKRD12 | 10459 | 14 | 8 | 6 | 2 | 7 | 11 | 8 | 4 | 10 | 10 | 8 | 14 | 11 |
| NM_001083885 | DFNB31 | 2889 | 1 | 0 | 0 | 0 | 0 | 1 | 0 | 0 | 0 | 0 | 0 | 0 | 1 |
| NM_001083893 | STRN3 | 4222 | 19 | 9 | 6 | 3 | 10 | 10 | 9 | 8 | 7 | 8 | 19 | 9 | 12 |
| NM_001083896 | SEBOX | 825 | 0 | 0 | 0 | 0 | 0 | 0 | 0 | 0 | 0 | 0 | 0 | 0 | 0 |
| NM_001083899 | GP6 | 2266 | 1 | 0 | 0 | 0 | 0 | 0 | 0 | 0 | 0 | 0 | 0 | 1 | 1 |
| NM_001083907 | BANK1 | 3212 | 31 | 4 | 4 | 1 | 0 | 0 | 1 | 0 | 2 | 0 | 0 | 31 | 2 |
| NM_001083908 | C14orf104 | 2819 | 2 | 1 | 1 | 1 | 1 | 1 | 2 | 2 | 1 | 1 | 1 | 2 | 1 |
| NM_001083909 | GPR123 | 4283 | 1 | 0 | 0 | 0 | 0 | 1 | 0 | 0 | 0 | 0 | 0 | 0 | 0 |
| NM_001083910 | SIRPB1 | 1761 | 2 | 1 | 0 | 0 | 1 | 2 | 0 | 1 | 2 | 0 | 1 | 2 | 0 |
| NM_001083913 | C10orf26 | 4109 | 7 | 5 | 6 | 3 | 4 | 3 | 4 | 7 | 4 | 4 | 5 | 6 | 4 |
| NM_001083914 | CTBP2 | 3441 | 9 | 6 | 4 | 8 | 6 | 7 | 7 | 1 | 7 | 9 | 3 | 7 | 7 |
| NM_001083924 | C1orf116 | 5316 | 6 | 1 | 0 | 1 | 0 | 0 | 1 | 0 | 6 | 0 | 0 | 0 | 0 |
| NM_001083926 | ASRGL1 | 2419 | 7 | 2 | 1 | 1 | 1 | 7 | 7 | 1 | 2 | 0 | 0 | 1 | 6 |
| NM_001083946 | C2orf56 | 1917 | 8 | 4 | 2 | 2 | 6 | 6 | 4 | 3 | 2 | 3 | 3 | 4 | 8 |
| NM_001083947 | TMPRSS4 | 3518 | 2 | 0 | 0 | 2 | 0 | 0 | 1 | 0 | 0 | 0 | 0 | 0 | 0 |
| NM_001083953 | THADA | 6326 | 3 | 2 | 1 | 1 | 2 | 1 | 2 | 3 | 2 | 3 | 1 | 3 | 3 |
| NM_001083956 | ZNF655 | 4844 | 10 | 6 | 4 | 2 | 7 | 7 | 9 | 2 | 6 | 9 | 4 | 10 | 9 |
| NM_001083961 | WDR62 | 4745 | 3 | 0 | 0 | 0 | 0 | 0 | 0 | 0 | 0 | 0 | 0 | 0 | 3 |
| NM_001083962 | TCF4 | 8332 | 16 | 8 | 8 | 2 | 8 | 11 | 2 | 1 | 13 | 16 | 5 | 14 | 7 |
| NM_001083963 | TDRKH | 2815 | 4 | 1 | 1 | 1 | 1 | 4 | 1 | 0 | 1 | 0 | 1 | 1 | 3 |
| NM_001083964 | TDRKH | 2705 | 3 | 1 | 1 | 1 | 1 | 3 | 1 | 0 | 1 | 0 | 1 | 1 | 3 |
| NM_001083965 | TDRKH | 2840 | 4 | 1 | 1 | 1 | 1 | 4 | 1 | 0 | 1 | 0 | 1 | 1 | 3 |
| NM_001084 | PLOD3 | 2977 | 3 | 2 | 2 | 2 | 2 | 1 | 2 | 3 | 2 | 1 | 1 | 3 | 2 |
| NM_001084392 | DDT | 620 | 0 | 0 | 0 | 0 | 0 | 0 | 0 | 0 | 0 | 0 | 0 | 0 | 0 |
| NM_001084393 | DDTL | 1635 | 1 | 0 | 0 | 0 | 0 | 0 | 1 | 0 | 1 | 0 | 0 | 1 | 0 |
| NM_001085 | SERPINA3 | 1590 | 5,181 | 498 | 2 | 9 | 67 | 78 | 32 | 5,181 | 21 | 19 | 10 | 41 | 20 |
| NM_001085049 | MRAS | 4013 | 21 | 7 | 21 | 2 | 17 | 17 | 3 | 0 | 2 | 4 | 3 | 2 | 3 |
| NM_001085347 | TOR2A | 1531 | 0 | 0 | 0 | 0 | 0 | 0 | 0 | 0 | 0 | 0 | 0 | 0 | 0 |

| transcript | gene | transcript length (nt) | max | mean | adipose | colon | heart | hypothalamus | kidney | liver | lung | ovary | skeletalmuscle | spleen | testes |
|---|---|---|---|---|---|---|---|---|---|---|---|---|---|---|---|
| NM_001085354 | MORC4 | 3764 | 5 | 2 | 2 | 1 | 1 | 1 | 1 | 4 | 2 | 2 | 1 | 1 | 5 |
| NM_001085357 | BTLA | 3071 | 2 | 0 | 0 | 0 | 0 | 0 | 0 | 0 | 0 | 0 | 0 | 2 | 0 |
| NM_001085365 | FAM128A | 693 | 2 | 1 | 0 | 0 | 2 | 1 | 0 | 0 | 0 | 0 | 1 | 1 | 1 |
| NM_001085366 | ZNF655 | 1372 | 1 | 0 | 0 | 0 | 0 | 0 | 1 | 1 | 1 | 0 | 0 | 1 | 1 |
| NM_001085367 | ZNF655 | 1412 | 2 | 1 | 1 | 1 | 1 | 1 | 2 | 1 | 1 | 0 | 0 | 2 | 1 |
| NM_001085368 | ZNF655 | 4671 | 11 | 7 | 4 | 2 | 7 | 7 | 9 | 3 | 6 | 10 | 4 | 11 | 9 |
| NM_001085372 | C11orf83 | 640 | 3 | 1 | 1 | 3 | 1 | 2 | 2 | 2 | 1 | 0 | 1 | 1 | 1 |
| NM_001085375 | C1orf226 | 4126 | 2 | 0 | 0 | 1 | 0 | 0 | 1 | 2 | 0 | 0 | 0 | 0 | 0 |
| NM_001085377 | MCC | 8679 | 14 | 3 | 2 | 1 | 3 | 2 | 2 | 4 | 2 | 14 | 1 | 2 | 2 |
| NM_001085382 | PSAPL1 | 4679 | 0 | 0 | 0 | 0 | 0 | 0 | 0 | 0 | 0 | 0 | 0 | 0 | 0 |
| NM_001085384 | ZNF154 | 2747 | 3 | 1 | 1 | 1 | 1 | 2 | 2 | 0 | 1 | 1 | 1 | 3 | 1 |
| NM_001085398 | TLX1NB | 1882 | 0 | 0 | 0 | 0 | 0 | 0 | 0 | 0 | 0 | 0 | 0 | 0 | 0 |
| NM_001085399 | RELL1 | 1327 | 19 | 9 | 12 | 7 | 13 | 7 | 6 | 2 | 16 | 6 | 9 | 19 | 7 |
| NM_001085400 | RELL1 | 3618 | 13 | 6 | 9 | 4 | 9 | 5 | 4 | 1 | 10 | 4 | 5 | 13 | 5 |
| NM_001085401 | C6orf201 | 1651 | 3 | 0 | 0 | 0 | 0 | 0 | 0 | 0 | 0 | 0 | 0 | 0 | 3 |
| NM_001085411 | C5orf33 | 3881 | 49 | 9 | 4 | 2 | 6 | 10 | 6 | 49 | 3 | 7 | 5 | 4 | 6 |
| NM_001085420 | PLSCR5 | 1969 | 0 | 0 | 0 | 0 | 0 | 0 | 0 | 0 | 0 | 0 | 0 | 0 | 0 |
| NM_001085423 | C17orf60 | 1448 | 2 | 0 | 1 | 0 | 1 | 0 | 0 | 0 | 0 | 0 | 0 | 2 | 0 |
| NM_001085425 | ARSA | 1949 | 1 | 0 | 0 | 0 | 0 | 0 | 1 | 0 | 0 | 0 | 0 | 0 | 0 |
| NM_001085426 | ARSA | 1901 | 1 | 0 | 0 | 0 | 0 | 0 | 1 | 0 | 0 | 0 | 0 | 0 | 0 |
| NM_001085427 | ARSA | 1856 | 1 | 0 | 0 | 0 | 0 | 0 | 1 | 0 | 0 | 0 | 0 | 0 | 0 |
| NM_001085428 | ARSA | 1664 | 1 | 0 | 0 | 0 | 0 | 0 | 1 | 0 | 0 | 0 | 0 | 0 | 0 |
| NM_001085429 | TMEM213 | 3390 | 7 | 1 | 0 | 0 | 0 | 0 | 7 | 0 | 0 | 0 | 0 | 0 | 0 |
| NM_001085430 | C17orf67 | 812 | 0 | 0 | 0 | 0 | 0 | 0 | 0 | 0 | 0 | 0 | 0 | 0 | 0 |
| NM_001085437 | C2orf54 | 2525 | 0 | 0 | 0 | 0 | 0 | 0 | 0 | 0 | 0 | 0 | 0 | 0 | 0 |
| NM_001085447 | C2orf77 | 2169 | 12 | 2 | 0 | 0 | 0 | 4 | 0 | 0 | 1 | 1 | 0 | 0 | 12 |
| NM_001085451 | LNP1 | 2217 | 1 | 0 | 0 | 0 | 0 | 1 | 0 | 0 | 0 | 0 | 0 | 0 | 1 |
| NM_001085452 | FAM75A1 | 4223 | 0 | 0 | 0 | 0 | 0 | 0 | 0 | 0 | 0 | 0 | 0 | 0 | 0 |
| NM_001085454 | GIT1 | 3760 | 2 | 1 | 0 | 0 | 1 | 2 | 1 | 0 | 1 | 0 | 0 | 1 | 2 |
| NM_001085455 | KRTAP24-1 | 1650 | 0 | 0 | 0 | 0 | 0 | 0 | 0 | 0 | 0 | 0 | 0 | 0 | 0 |
| NM_001085457 | CBWD6 | 1719 | 0 | 0 | 0 | 0 | 0 | 0 | 0 | 0 | 0 | 0 | 0 | 0 | 0 |
| NM_001085458 | CTNND1 | 6350 | 29 | 19 | 14 | 24 | 15 | 10 | 29 | 17 | 22 | 20 | 6 | 24 | 25 |
| NM_001085459 | CTNND1 | 6332 | 29 | 19 | 14 | 24 | 15 | 10 | 29 | 17 | 22 | 20 | 6 | 24 | 26 |
| NM_001085460 | CTNND1 | 6172 | 29 | 19 | 14 | 23 | 16 | 10 | 29 | 18 | 22 | 20 | 6 | 25 | 26 |
| NM_001085461 | CTNND1 | 6245 | 29 | 19 | 14 | 24 | 16 | 10 | 29 | 17 | 22 | 21 | 6 | 24 | 26 |
| NM_001085462 | CTNND1 | 6053 | 29 | 19 | 14 | 23 | 16 | 10 | 29 | 18 | 23 | 21 | 6 | 25 | 26 |
| NM_001085463 | CTNND1 | 5869 | 30 | 20 | 14 | 24 | 16 | 11 | 30 | 18 | 23 | 21 | 6 | 26 | 27 |
| NM_001085464 | CTNND1 | 5851 | 31 | 20 | 14 | 24 | 16 | 11 | 31 | 19 | 23 | 22 | 6 | 26 | 27 |
| NM_001085465 | CTNND1 | 5788 | 29 | 19 | 13 | 24 | 16 | 10 | 29 | 17 | 22 | 20 | 6 | 24 | 26 |
| NM_001085466 | CTNND1 | 5782 | 31 | 20 | 14 | 24 | 17 | 11 | 31 | 19 | 24 | 22 | 6 | 26 | 28 |
| NM_001085467 | CTNND1 | 5837 | 31 | 20 | 15 | 25 | 17 | 11 | 31 | 19 | 24 | 22 | 6 | 26 | 28 |
| NM_001085468 | CTNND1 | 5764 | 31 | 20 | 15 | 24 | 17 | 11 | 31 | 19 | 24 | 22 | 6 | 26 | 28 |
| NM_001085469 | CTNND1 | 5692 | 31 | 20 | 15 | 25 | 17 | 11 | 31 | 19 | 24 | 22 | 7 | 26 | 28 |
| NM_001085471 | FOXN3 | 7852 | 9 | 6 | 9 | 3 | 6 | 3 | 2 | 5 | 5 | 9 | 9 | 5 | 6 |
| NM_001085474 | LOC646627 | 435 | 1 | 0 | 0 | 1 | 0 | 0 | 0 | 0 | 0 | 0 | 0 | 0 | 0 |
| NM_001085476 | FOXD4L6 | 2725 | 0 | 0 | 0 | 0 | 0 | 0 | 0 | 0 | 0 | 0 | 0 | 0 | 0 |
| NM_001085479 | IQCF3 | 865 | 1 | 0 | 0 | 0 | 0 | 0 | 0 | 0 | 0 | 0 | 0 | 0 | 1 |
| NM_001085480 | FAM162B | 1035 | 1 | 0 | 0 | 0 | 0 | 0 | 0 | 0 | 1 | 0 | 0 | 0 | 1 |
| NM_001085481 | MAP1LC3B2 | 832 | 3 | 1 | 1 | 1 | 1 | 3 | 0 | 1 | 1 | 0 | 1 | 1 | 1 |
| NM_001085486 | SEPP1 | 2115 | 121 | 24 | 42 | 8 | 6 | 14 | 8 | 121 | 10 | 11 | 5 | 33 | 5 |
| NM_001085487 | MYSM1 | 2589 | 13 | 8 | 7 | 3 | 7 | 8 | 9 | 5 | 7 | 9 | 6 | 13 | 10 |
| NM_001085488 | TMEM150B | 875 | 0 | 0 | 0 | 0 | 0 | 0 | 0 | 0 | 0 | 0 | 0 | 0 | 0 |
| NM_001086 | AADAC | 1606 | 64 | 6 | 0 | 1 | 0 | 0 | 0 | 64 | 0 | 0 | 0 | 0 | 0 |
| NM_001086521 | C17orf89 | 560 | 5 | 2 | 3 | 2 | 3 | 4 | 2 | 5 | 0 | 1 | 1 | 2 | 0 |
| NM_001087 | AAMP | 1793 | 11 | 8 | 9 | 8 | 9 | 11 | 11 | 8 | 7 | 7 | 5 | 9 | 9 |
| NM_001088 | AANAT | 1025 | 0 | 0 | 0 | 0 | 0 | 0 | 0 | 0 | 0 | 0 | 0 | 0 | 0 |
| NM_001089 | ABCA3 | 6623 | 4 | 1 | 0 | 0 | 0 | 2 | 0 | 0 | 4 | 0 | 0 | 0 | 0 |
| NM_001089584 | C5orf49 | 1026 | 3 | 1 | 0 | 0 | 0 | 3 | 1 | 0 | 2 | 0 | 0 | 0 | 3 |
| NM_001089591 | UQCRHL | 538 | 141 | 57 | 50 | 68 | 141 | 66 | 68 | 76 | 32 | 23 | 44 | 38 | 26 |
| NM_001089593 | LOC642502 | 407 | 0 | 0 | 0 | 0 | 0 | 0 | 0 | 0 | 0 | 0 | 0 | 0 | 0 |
| NM_001090 | ABCF1 | 3350 | 23 | 15 | 12 | 10 | 17 | 15 | 15 | 23 | 9 | 10 | 23 | 15 | 18 |
| NM_001090 | ABCF1 | 3351 | 23 | 15 | 12 | 10 | 17 | 15 | 15 | 23 | 9 | 10 | 23 | 15 | 18 |
| NM_001091 | ABP1 | 2428 | 23 | 3 | 0 | 11 | 0 | 0 | 23 | 0 | 0 | 0 | 0 | 0 | 0 |
| NM_001092 | ABR | 5046 | 31 | 7 | 3 | 11 | 7 | 31 | 2 | 1 | 4 | 3 | 1 | 7 | 2 |
| NM_001093 | ACACB | 9249 | 37 | 10 | 37 | 9 | 7 | 2 | 7 | 21 | 1 | 3 | 18 | 3 | 3 |
| NM_001093725 | MEX3A | 6124 | 2 | 0 | 0 | 0 | 0 | 0 | 0 | 0 | 0 | 2 | 0 | 0 | 0 |
| NM_001093726 | SEPP1 | 2174 | 118 | 23 | 41 | 8 | 6 | 13 | 7 | 118 | 10 | 11 | 4 | 32 | 5 |
| NM_001093728 | TCP11 | 1929 | 8 | 1 | 0 | 0 | 0 | 0 | 0 | 0 | 0 | 0 | 0 | 0 | 8 |
| NM_001093729 | CCDC102B | 2817 | 11 | 3 | 3 | 1 | 2 | 3 | 2 | 0 | 11 | 1 | 0 | 2 | 3 |
| NM_001093730 | DYTN | 2051 | 0 | 0 | 0 | 0 | 0 | 0 | 0 | 0 | 0 | 0 | 0 | 0 | 0 |
| NM_001093755 | C5orf44 | 3103 | 6 | 3 | 2 | 1 | 3 | 6 | 3 | 3 | 2 | 2 | 2 | 3 | 4 |
| NM_001093756 | C5orf44 | 3082 | 6 | 3 | 2 | 1 | 3 | 6 | 3 | 3 | 2 | 2 | 2 | 3 | 4 |
| NM_001093767 | FAM195B | 877 | 2 | 1 | 1 | 1 | 1 | 2 | 1 | 0 | 1 | 0 | 1 | 1 | 1 |
| NM_001093770 | SFTPA1 | 2203 | 236 | 21 | 0 | 0 | 0 | 0 | 0 | 0 | 236 | 0 | 0 | 0 | 0 |
| NM_001093771 | TXNRD1 | 3835 | 27 | 14 | 12 | 4 | 27 | 11 | 10 | 21 | 18 | 8 | 10 | 15 | 13 |
| NM_001093772 | KIT | 5164 | 7 | 2 | 1 | 1 | 0 | 3 | 2 | 0 | 3 | 7 | 0 | 1 | 1 |
| NM_001094 | ACCN1 | 2690 | 3 | 0 | 0 | 0 | 0 | 3 | 0 | 0 | 0 | 0 | 0 | 0 | 0 |

| transcript | gene | transcript length (nt) | max | mean | adipose | colon | heart | hypothalamus | kidney | liver | lung | ovary | skeletalmuscle | spleen | testes |
|---|---|---|---|---|---|---|---|---|---|---|---|---|---|---|---|
| NM_001095 | ACCN2 | 3778 | 2 | 0 | 0 | 0 | 0 | 2 | 0 | 0 | 0 | 0 | 0 | 0 | 0 |
| NM_001096 | ACLY | 4353 | 11 | 5 | 6 | 4 | 4 | 11 | 9 | 5 | 4 | 3 | 2 | 8 | 5 |
| NM_001097 | ACR | 1370 | 4 | 0 | 0 | 0 | 0 | 0 | 0 | 0 | 0 | 0 | 0 | 0 | 4 |
| NM_001097577 | ANG | 740 | 83 | 9 | 1 | 1 | 1 | 0 | 2 | 83 | 1 | 3 | 1 | 1 | 3 |
| NM_001097579 | GPR34 | 1928 | 6 | 2 | 3 | 1 | 2 | 1 | 1 | 1 | 3 | 2 | 0 | 6 | 1 |
| NM_001097589 | SPRR3 | 902 | 0 | 0 | 0 | 0 | 0 | 0 | 0 | 0 | 0 | 0 | 0 | 0 | 0 |
| NM_001097590 | RPP38 | 1299 | 3 | 1 | 1 | 1 | 1 | 1 | 1 | 1 | 1 | 1 | 1 | 1 | 3 |
| NM_001097591 | XAGE1A | 777 | 0 | 0 | 0 | 0 | 0 | 0 | 0 | 0 | 0 | 0 | 0 | 0 | 0 |
| NM_001097592 | XAGE1A | 613 | 0 | 0 | 0 | 0 | 0 | 0 | 0 | 0 | 0 | 0 | 0 | 0 | 0 |
| NM_001097593 | XAGE1A | 395 | 0 | 0 | 0 | 0 | 0 | 0 | 0 | 0 | 0 | 0 | 0 | 0 | 0 |
| NM_001097594 | XAGE1B | 613 | 0 | 0 | 0 | 0 | 0 | 0 | 0 | 0 | 0 | 0 | 0 | 0 | 0 |
| NM_001097595 | XAGE1B | 777 | 0 | 0 | 0 | 0 | 0 | 0 | 0 | 0 | 0 | 0 | 0 | 0 | 0 |
| NM_001097596 | XAGE1B | 395 | 0 | 0 | 0 | 0 | 0 | 0 | 0 | 0 | 0 | 0 | 0 | 0 | 0 |
| NM_001097597 | XAGE1C | 613 | 0 | 0 | 0 | 0 | 0 | 0 | 0 | 0 | 0 | 0 | 0 | 0 | 0 |
| NM_001097598 | XAGE1C | 395 | 0 | 0 | 0 | 0 | 0 | 0 | 0 | 0 | 0 | 0 | 0 | 0 | 0 |
| NM_001097599 | TMEM22 | 1795 | 2 | 1 | 2 | 0 | 1 | 2 | 1 | 0 | 1 | 1 | 0 | 1 | 2 |
| NM_001097600 | TMEM22 | 1646 | 2 | 1 | 2 | 0 | 1 | 2 | 1 | 0 | 1 | 1 | 0 | 1 | 2 |
| NM_001097602 | XAGE1C | 777 | 0 | 0 | 0 | 0 | 0 | 0 | 0 | 0 | 0 | 0 | 0 | 0 | 0 |
| NM_001097603 | XAGE1E | 777 | 0 | 0 | 0 | 0 | 0 | 0 | 0 | 0 | 0 | 0 | 0 | 0 | 0 |
| NM_001097604 | XAGE1E | 613 | 0 | 0 | 0 | 0 | 0 | 0 | 0 | 0 | 0 | 0 | 0 | 0 | 0 |
| NM_001097605 | XAGE1E | 395 | 0 | 0 | 0 | 0 | 0 | 0 | 0 | 0 | 0 | 0 | 0 | 0 | 0 |
| NM_001097610 | LOC653486 | 358 | 0 | 0 | 0 | 0 | 0 | 0 | 0 | 0 | 0 | 0 | 0 | 0 | 0 |
| NM_001097611 | KNCN | 2392 | 0 | 0 | 0 | 0 | 0 | 0 | 0 | 0 | 0 | 0 | 0 | 0 | 0 |
| NM_001097612 | GPR89A | 1966 | 0 | 0 | 0 | 0 | 0 | 0 | 0 | 0 | 0 | 0 | 0 | 0 | 0 |
| NM_001097613 | GPR89A | 1986 | 0 | 0 | 0 | 0 | 0 | 0 | 0 | 0 | 0 | 0 | 0 | 0 | 0 |
| NM_001097615 | POLR2J3 | 1680 | 1 | 0 | 0 | 1 | 0 | 1 | 0 | 0 | 0 | 1 | 0 | 1 | 0 |
| NM_001097616 | GPR89C | 1569 | 0 | 0 | 0 | 0 | 0 | 0 | 0 | 0 | 0 | 0 | 0 | 0 | 0 |
| NM_001097620 | TMEM184A | 6276 | 0 | 0 | 0 | 0 | 0 | 0 | 0 | 0 | 0 | 0 | 0 | 0 | 0 |
| NM_001097622 | OCM | 696 | 0 | 0 | 0 | 0 | 0 | 0 | 0 | 0 | 0 | 0 | 0 | 0 | 0 |
| NM_001097633 | GCNT1 | 5478 | 2 | 1 | 1 | 1 | 1 | 0 | 0 | 0 | 1 | 0 | 0 | 2 | 0 |
| NM_001097634 | GCNT1 | 5974 | 2 | 1 | 1 | 1 | 1 | 0 | 0 | 0 | 1 | 0 | 0 | 2 | 0 |
| NM_001097635 | GCNT1 | 5514 | 2 | 1 | 1 | 1 | 1 | 0 | 0 | 0 | 1 | 0 | 0 | 2 | 0 |
| NM_001097636 | GCNT1 | 5607 | 2 | 1 | 1 | 1 | 1 | 0 | 0 | 0 | 1 | 0 | 0 | 2 | 0 |
| NM_001097638 | FUT2 | 3089 | 1 | 0 | 0 | 1 | 0 | 0 | 0 | 0 | 0 | 0 | 0 | 0 | 0 |
| NM_001097639 | FUT3 | 2242 | 3 | 0 | 0 | 3 | 0 | 0 | 0 | 0 | 0 | 0 | 0 | 0 | 0 |
| NM_001097640 | FUT3 | 2188 | 3 | 0 | 0 | 3 | 0 | 0 | 1 | 0 | 0 | 0 | 0 | 0 | 0 |
| NM_001097641 | FUT3 | 2049 | 2 | 0 | 0 | 2 | 0 | 0 | 0 | 0 | 0 | 0 | 0 | 0 | 0 |
| NM_001097642 | GJB1 | 1603 | 19 | 3 | 0 | 2 | 0 | 1 | 7 | 19 | 0 | 0 | 0 | 0 | 2 |
| NM_001097643 | TAS2R30 | 960 | 2 | 1 | 0 | 0 | 0 | 0 | 1 | 0 | 1 | 2 | 1 | 2 | 1 |
| NM_001098 | ACO2 | 2741 | 13 | 6 | 4 | 4 | 13 | 11 | 12 | 4 | 1 | 1 | 9 | 3 | 2 |
| NM_001098169 | BSX | 702 | 0 | 0 | 0 | 0 | 0 | 0 | 0 | 0 | 0 | 0 | 0 | 0 | 0 |
| NM_001098173 | PRDM7 | 3222 | 0 | 0 | 0 | 0 | 0 | 0 | 0 | 0 | 0 | 0 | 0 | 0 | 0 |
| NM_001098175 | ENTPD1 | 12615 | 4 | 2 | 1 | 1 | 2 | 2 | 2 | 1 | 2 | 2 | 1 | 4 | 1 |
| NM_001098199 | GPR1 | 2314 | 0 | 0 | 0 | 0 | 0 | 0 | 0 | 0 | 0 | 0 | 0 | 0 | 0 |
| NM_001098200 | GPR18 | 1469 | 11 | 2 | 1 | 1 | 1 | 0 | 2 | 1 | 2 | 2 | 1 | 11 | 4 |
| NM_001098201 | GPER | 2644 | 1 | 0 | 0 | 0 | 0 | 0 | 0 | 0 | 1 | 0 | 0 | 0 | 0 |
| NM_001098202 | HIC1 | 3054 | 0 | 0 | 0 | 0 | 0 | 0 | 0 | 0 | 0 | 0 | 0 | 0 | 0 |
| NM_001098204 | HNRNPF | 2632 | 21 | 12 | 7 | 9 | 10 | 10 | 14 | 17 | 13 | 9 | 7 | 21 | 12 |
| NM_001098205 | HNRNPF | 2672 | 21 | 12 | 7 | 9 | 9 | 10 | 14 | 17 | 13 | 9 | 6 | 21 | 12 |
| NM_001098206 | HNRNPF | 2649 | 21 | 12 | 7 | 9 | 9 | 10 | 14 | 17 | 13 | 9 | 6 | 21 | 12 |
| NM_001098207 | HNRNPF | 2676 | 21 | 11 | 7 | 9 | 9 | 10 | 14 | 17 | 13 | 9 | 6 | 21 | 12 |
| NM_001098208 | HNRNPF | 2755 | 20 | 11 | 7 | 9 | 9 | 10 | 14 | 16 | 13 | 8 | 6 | 20 | 11 |
| NM_001098209 | CTNNB1 | 3415 | 31 | 21 | 16 | 31 | 21 | 29 | 18 | 17 | 15 | 30 | 8 | 23 | 20 |
| NM_001098210 | CTNNB1 | 3256 | 30 | 18 | 13 | 30 | 18 | 25 | 15 | 14 | 12 | 24 | 8 | 20 | 17 |
| NM_001098211 | HRH1 | 4328 | 1 | 1 | 1 | 1 | 1 | 1 | 0 | 0 | 1 | 1 | 1 | 0 | 1 |
| NM_001098212 | HRH1 | 4278 | 1 | 1 | 1 | 1 | 1 | 1 | 0 | 0 | 1 | 1 | 1 | 0 | 1 |
| NM_001098213 | HRH1 | 4558 | 1 | 1 | 1 | 1 | 0 | 1 | 0 | 0 | 1 | 1 | 0 | 0 | 1 |
| NM_001098268 | LIG4 | 4060 | 9 | 4 | 2 | 1 | 6 | 9 | 4 | 5 | 3 | 2 | 2 | 6 | 6 |
| NM_001098270 | MGAT3 | 4971 | 3 | 0 | 1 | 0 | 0 | 3 | 0 | 0 | 0 | 0 | 0 | 0 | 0 |
| NM_001098272 | HMGCS1 | 3510 | 40 | 12 | 5 | 5 | 6 | 40 | 11 | 20 | 17 | 7 | 3 | 6 | 14 |
| NM_001098376 | PRAMEF15 | 1862 | 0 | 0 | 0 | 0 | 0 | 0 | 0 | 0 | 0 | 0 | 0 | 0 | 0 |
| NM_001098398 | COPA | 5666 | 17 | 11 | 11 | 8 | 12 | 12 | 13 | 17 | 9 | 8 | 6 | 15 | 9 |
| NM_001098402 | ZNF295 | 7479 | 2 | 1 | 1 | 0 | 1 | 1 | 1 | 1 | 1 | 2 | 1 | 2 | 2 |
| NM_001098403 | ZNF295 | 6876 | 2 | 1 | 1 | 0 | 1 | 1 | 1 | 1 | 1 | 2 | 1 | 2 | 2 |
| NM_001098405 | GAGE12F | 354 | 0 | 0 | 0 | 0 | 0 | 0 | 0 | 0 | 0 | 0 | 0 | 0 | 0 |
| NM_001098406 | GAGE12J | 564 | 0 | 0 | 0 | 0 | 0 | 0 | 0 | 0 | 0 | 0 | 0 | 0 | 0 |
| NM_001098407 | GAGE2D | 561 | 0 | 0 | 0 | 0 | 0 | 0 | 0 | 0 | 0 | 0 | 0 | 0 | 0 |
| NM_001098408 | GAGE12C | 564 | 0 | 0 | 0 | 0 | 0 | 0 | 0 | 0 | 0 | 0 | 0 | 0 | 0 |
| NM_001098409 | GAGE12G | 564 | 0 | 0 | 0 | 0 | 0 | 0 | 0 | 0 | 0 | 0 | 0 | 0 | 0 |
| NM_001098410 | GAGE12H | 564 | 0 | 0 | 0 | 0 | 0 | 0 | 0 | 0 | 0 | 0 | 0 | 0 | 0 |
| NM_001098411 | GAGE2B | 509 | 0 | 0 | 0 | 0 | 0 | 0 | 0 | 0 | 0 | 0 | 0 | 0 | 0 |
| NM_001098412 | GAGE13 | 524 | 0 | 0 | 0 | 0 | 0 | 0 | 0 | 0 | 0 | 0 | 0 | 0 | 0 |
| NM_001098413 | GAGE10 | 549 | 0 | 0 | 0 | 0 | 0 | 0 | 0 | 0 | 0 | 0 | 0 | 0 | 0 |
| NM_001098414 | ZNF621 | 7998 | 3 | 2 | 3 | 2 | 2 | 3 | 3 | 1 | 2 | 3 | 1 | 3 | 2 |
| NM_001098416 | HDAC7 | 4177 | 2 | 1 | 1 | 1 | 1 | 1 | 1 | 0 | 1 | 1 | 0 | 2 | 1 |
| NM_001098418 | GAGE12E | 564 | 0 | 0 | 0 | 0 | 0 | 0 | 0 | 0 | 0 | 0 | 0 | 0 | 0 |
| NM_001098424 | DLG1 | 4968 | 21 | 12 | 11 | 6 | 15 | 21 | 15 | 10 | 9 | 12 | 13 | 11 | 13 |

| transcript | gene | transcript length (nt) | max | mean | adipose | colon | heart | hypothala mus | kidney | liver | lung | ovary | skeletalm uscle | spleen | testes |
|---|---|---|---|---|---|---|---|---|---|---|---|---|---|---|---|
| NM_001098426 | SMARCD2 | 2770 | 12 | 6 | 6 | 6 | 5 | 2 | 4 | 8 | 5 | 6 | 3 | 12 | 9 |
| NM_001098475 | TDRD10 | 2529 | 1 | 0 | 0 | 0 | 0 | 0 | 0 | 0 | 0 | 0 | 0 | 0 | 1 |
| NM_001098477 | GRSF1 | 6467 | 15 | 9 | 6 | 5 | 11 | 15 | 9 | 7 | 6 | 7 | 13 | 9 | 9 |
| NM_001098478 | HLA-F | 1008 | 32 | 8 | 7 | 11 | 3 | 3 | 3 | 2 | 21 | 1 | 2 | 32 | 2 |
| NM_001098479 | HLA-F | 1574 | 21 | 5 | 4 | 7 | 2 | 2 | 2 | 1 | 14 | 1 | 1 | 21 | 1 |
| NM_001098482 | CRTC1 | 6992 | 2 | 0 | 0 | 0 | 0 | 2 | 0 | 0 | 0 | 0 | 0 | 0 | 1 |
| NM_001098483 | C10orf125 | 562 | 3 | 1 | 0 | 0 | 0 | 0 | 1 | 3 | 0 | 0 | 0 | 1 | 0 |
| NM_001098484 | SLC4A4 | 7692 | 51 | 7 | 1 | 5 | 2 | 12 | 51 | 3 | 2 | 0 | 0 | 0 | 0 |
| NM_001098486 | SLC17A3 | 1808 | 11 | 1 | 0 | 0 | 0 | 0 | 11 | 1 | 0 | 0 | 0 | 0 | 0 |
| NM_001098491 | ZNF419 | 2366 | 2 | 1 | 1 | 1 | 1 | 2 | 1 | 0 | 1 | 2 | 1 | 2 | 1 |
| NM_001098492 | ZNF419 | 2327 | 2 | 1 | 1 | 1 | 1 | 2 | 1 | 0 | 1 | 2 | 1 | 2 | 1 |
| NM_001098493 | ZNF419 | 2324 | 2 | 1 | 1 | 1 | 1 | 2 | 1 | 0 | 1 | 2 | 1 | 2 | 1 |
| NM_001098494 | ZNF419 | 2267 | 2 | 1 | 1 | 1 | 1 | 2 | 1 | 0 | 1 | 2 | 1 | 2 | 1 |
| NM_001098495 | ZNF419 | 2228 | 2 | 1 | 1 | 1 | 1 | 2 | 1 | 0 | 1 | 2 | 1 | 2 | 1 |
| NM_001098496 | ZNF419 | 2225 | 2 | 1 | 1 | 1 | 1 | 2 | 1 | 0 | 1 | 2 | 1 | 2 | 1 |
| NM_001098497 | SGSM1 | 6011 | 2 | 0 | 0 | 0 | 0 | 2 | 0 | 0 | 0 | 0 | 0 | 0 | 0 |
| NM_001098498 | SGSM1 | 5828 | 2 | 0 | 0 | 0 | 0 | 2 | 0 | 0 | 0 | 0 | 0 | 0 | 0 |
| NM_001098500 | KIAA1217 | 6123 | 25 | 10 | 7 | 5 | 6 | 9 | 11 | 4 | 6 | 8 | 25 | 5 | 22 |
| NM_001098501 | KIAA1217 | 5852 | 27 | 11 | 8 | 6 | 6 | 9 | 11 | 4 | 6 | 8 | 27 | 6 | 24 |
| NM_001098502 | CHCHD4 | 1434 | 9 | 6 | 6 | 5 | 9 | 9 | 7 | 7 | 2 | 5 | 7 | 7 | 4 |
| NM_001098503 | PTPRJ | 3174 | 7 | 2 | 1 | 1 | 1 | 2 | 3 | 2 | 2 | 0 | 0 | 7 | 0 |
| NM_001098504 | DDX17 | 4796 | 62 | 39 | 32 | 31 | 30 | 30 | 42 | 16 | 46 | 48 | 40 | 62 | 47 |
| NM_001098505 | DDX17 | 3655 | 54 | 31 | 25 | 21 | 25 | 24 | 37 | 12 | 40 | 38 | 32 | 54 | 37 |
| NM_001098506 | CEACAM21 | 1359 | 1 | 0 | 0 | 0 | 0 | 1 | 0 | 0 | 0 | 1 | 0 | 1 | 0 |
| NM_001098507 | ZNF207 | 2323 | 63 | 28 | 15 | 17 | 29 | 20 | 31 | 20 | 32 | 32 | 23 | 63 | 28 |
| NM_001098509 | SGSM2 | 4730 | 2 | 1 | 0 | 1 | 0 | 2 | 1 | 0 | 1 | 0 | 0 | 1 | 1 |
| NM_001098510 | PELI3 | 2672 | 2 | 0 | 0 | 0 | 0 | 2 | 0 | 0 | 0 | 0 | 1 | 0 | 0 |
| NM_001098511 | KIF2A | 3360 | 19 | 7 | 4 | 2 | 3 | 19 | 3 | 2 | 5 | 6 | 2 | 11 | 15 |
| NM_001098512 | PRKG1 | 3824 | 8 | 3 | 1 | 2 | 8 | 2 | 2 | 0 | 5 | 2 | 6 | 1 | 3 |
| NM_001098514 | C16orf89 | 1514 | 1 | 0 | 0 | 0 | 0 | 1 | 1 | 0 | 1 | 0 | 0 | 0 | 0 |
| NM_001098515 | MRGPRF | 2283 | 0 | 0 | 0 | 0 | 0 | 0 | 0 | 0 | 0 | 0 | 0 | 0 | 0 |
| NM_001098516 | MUC20 | 6445 | 2 | 0 | 0 | 1 | 0 | 0 | 2 | 0 | 0 | 0 | 0 | 0 | 0 |
| NM_001098517 | CADM1 | 4223 | 12 | 4 | 1 | 1 | 3 | 12 | 4 | 2 | 6 | 2 | 1 | 1 | 11 |
| NM_001098518 | GPR116 | 5814 | 16 | 5 | 3 | 0 | 6 | 2 | 9 | 3 | 16 | 1 | 2 | 12 | 1 |
| NM_001098519 | LRRC43 | 2025 | 1 | 0 | 0 | 0 | 0 | 1 | 0 | 0 | 0 | 0 | 0 | 0 | 1 |
| NM_001098520 | HTATIP2 | 1478 | 9 | 4 | 5 | 4 | 3 | 3 | 4 | 9 | 6 | 1 | 3 | 8 | 2 |
| NM_001098521 | HTATIP2 | 1406 | 10 | 5 | 5 | 4 | 3 | 3 | 5 | 10 | 7 | 1 | 3 | 8 | 2 |
| NM_001098522 | HTATIP2 | 1688 | 8 | 4 | 5 | 3 | 3 | 3 | 4 | 8 | 6 | 1 | 3 | 7 | 2 |
| NM_001098523 | HTATIP2 | 773 | 2 | 1 | 1 | 0 | 0 | 1 | 1 | 2 | 2 | 0 | 0 | 2 | 1 |
| NM_001098524 | FAM129C | 2222 | 2 | 0 | 0 | 0 | 0 | 0 | 0 | 0 | 0 | 0 | 0 | 2 | 0 |
| NM_001098525 | CKAP2 | 3736 | 7 | 2 | 2 | 1 | 2 | 3 | 2 | 1 | 2 | 2 | 1 | 5 | 7 |
| NM_001098526 | AMICA1 | 1957 | 10 | 2 | 2 | 2 | 1 | 0 | 0 | 0 | 3 | 0 | 0 | 10 | 0 |
| NM_001098527 | RFPL2 | 2410 | 0 | 0 | 0 | 0 | 0 | 0 | 0 | 0 | 0 | 0 | 0 | 0 | 0 |
| NM_001098529 | TXNDC2 | 2129 | 3 | 0 | 0 | 0 | 0 | 0 | 0 | 0 | 0 | 0 | 0 | 0 | 3 |
| NM_001098530 | TXNDC2 | 1660 | 4 | 0 | 0 | 0 | 0 | 0 | 0 | 0 | 0 | 0 | 0 | 0 | 4 |
| NM_001098531 | RAPGEF3 | 6217 | 1 | 0 | 0 | 0 | 0 | 1 | 1 | 0 | 0 | 1 | 0 | 0 | 0 |
| NM_001098532 | RAPGEF3 | 5981 | 1 | 0 | 0 | 0 | 0 | 1 | 1 | 0 | 0 | 1 | 0 | 0 | 0 |
| NM_001098533 | CDK10 | 1741 | 3 | 1 | 1 | 2 | 1 | 1 | 3 | 2 | 1 | 1 | 0 | 1 | 1 |
| NM_001098534 | BAT3 | 3644 | 4 | 2 | 2 | 2 | 2 | 2 | 2 | 2 | 2 | 2 | 3 | 3 | 4 |
| NM_001098534 | BAT3 | 3648 | 4 | 2 | 2 | 2 | 2 | 2 | 2 | 2 | 2 | 2 | 3 | 3 | 4 |
| NM_001098534 | BAT3 | 3656 | 4 | 2 | 2 | 2 | 2 | 2 | 2 | 2 | 2 | 2 | 3 | 3 | 4 |
| NM_001098535 | RFPL3 | 1488 | 0 | 0 | 0 | 0 | 0 | 0 | 0 | 0 | 0 | 0 | 0 | 0 | 0 |
| NM_001098536 | USP5 | 3190 | 11 | 4 | 3 | 3 | 4 | 11 | 5 | 4 | 2 | 2 | 4 | 3 | 6 |
| NM_001098537 | PNPLA7 | 4806 | 0 | 0 | 0 | 0 | 0 | 0 | 0 | 0 | 0 | 0 | 0 | 0 | 0 |
| NM_001098538 | PRR4 | 890 | 505 | 49 | 3 | 2 | 2 | 11 | 4 | 1 | 505 | 5 | 2 | 3 | 1 |
| NM_001098540 | HPSE | 1760 | 2 | 1 | 1 | 0 | 1 | 1 | 0 | 1 | 1 | 1 | 0 | 2 | 0 |
| NM_001098576 | TMBIM6 | 3254 | 53 | 21 | 15 | 12 | 12 | 19 | 53 | 44 | 20 | 9 | 7 | 23 | 13 |
| NM_001098577 | RPL31 | 1129 | 171 | 91 | 98 | 56 | 81 | 103 | 55 | 112 | 64 | 171 | 76 | 128 | 52 |
| NM_001098579 | MRVI1 | 6032 | 7 | 2 | 1 | 4 | 3 | 7 | 1 | 0 | 1 | 2 | 1 | 1 | 2 |
| NM_001098612 | SIGLEC14 | 2113 | 6 | 1 | 0 | 0 | 1 | 1 | 0 | 0 | 1 | 0 | 0 | 6 | 0 |
| NM_001098614 | PUS7L | 3948 | 3 | 2 | 1 | 0 | 2 | 2 | 2 | 1 | 1 | 3 | 1 | 3 | 2 |
| NM_001098615 | PUS7L | 4362 | 3 | 2 | 1 | 1 | 2 | 2 | 2 | 1 | 1 | 3 | 1 | 3 | 2 |
| NM_001098616 | C1orf43 | 1898 | 19 | 9 | 9 | 4 | 11 | 12 | 10 | 19 | 7 | 3 | 12 | 7 | 6 |
| NM_001098620 | PCBP2 | 3066 | 44 | 25 | 19 | 9 | 27 | 17 | 35 | 18 | 21 | 44 | 20 | 32 | 31 |
| NM_001098621 | C14orf109 | 1277 | 5 | 4 | 4 | 2 | 4 | 4 | 5 | 3 | 5 | 3 | 3 | 4 | 3 |
| NM_001098622 | NANOS3 | 946 | 0 | 0 | 0 | 0 | 0 | 0 | 0 | 0 | 0 | 0 | 0 | 0 | 0 |
| NM_001098623 | OBSCN | 24030 | 30 | 3 | 0 | 1 | 6 | 0 | 0 | 0 | 0 | 0 | 30 | 0 | 0 |
| NM_001098624 | MID1 | 6147 | 2 | 1 | 2 | 1 | 2 | 1 | 1 | 0 | 2 | 1 | 0 | 0 | 2 |
| NM_001098625 | JKAMP | 2356 | 5 | 3 | 3 | 1 | 3 | 5 | 4 | 3 | 3 | 2 | 2 | 4 | 5 |
| NM_001098626 | ZNF98 | 2260 | 1 | 0 | 0 | 0 | 0 | 0 | 0 | 0 | 0 | 0 | 0 | 0 | 1 |
| NM_001098627 | IRF5 | 2852 | 0 | 0 | 0 | 0 | 0 | 0 | 0 | 0 | 0 | 0 | 0 | 0 | 0 |
| NM_001098628 | IRF5 | 2804 | 0 | 0 | 0 | 0 | 0 | 0 | 0 | 0 | 0 | 0 | 0 | 0 | 0 |
| NM_001098629 | IRF5 | 2882 | 0 | 0 | 0 | 0 | 0 | 0 | 0 | 0 | 0 | 0 | 0 | 0 | 0 |
| NM_001098630 | IRF5 | 2834 | 0 | 0 | 0 | 0 | 0 | 0 | 0 | 0 | 0 | 0 | 0 | 0 | 0 |
| NM_001098631 | IRF5 | 2723 | 0 | 0 | 0 | 0 | 0 | 0 | 0 | 0 | 0 | 0 | 0 | 0 | 0 |
| NM_001098632 | AKT1S1 | 2018 | 5 | 3 | 2 | 3 | 3 | 2 | 3 | 4 | 1 | 2 | 5 | 2 | 3 |
| NM_001098633 | AKT1S1 | 1774 | 6 | 3 | 2 | 3 | 3 | 2 | 3 | 4 | 1 | 2 | 6 | 3 | 3 |

| transcript | gene | transcript length (nt) | max | mean | adipose | colon | heart | hypothalamus | kidney | liver | lung | ovary | skeletalmuscle | spleen | testes |
|---|---|---|---|---|---|---|---|---|---|---|---|---|---|---|---|
| NM_001098634 | RBM47 | 5141 | 10 | 3 | 1 | 4 | 1 | 0 | 10 | 4 | 3 | 0 | 0 | 4 | 2 |
| NM_001098635 | SEZ6 | 4196 | 1 | 0 | 0 | 0 | 0 | 1 | 0 | 0 | 0 | 0 | 0 | 0 | 0 |
| NM_001098637 | PWWP2B | 2309 | 0 | 0 | 0 | 0 | 0 | 0 | 0 | 0 | 0 | 0 | 0 | 0 | 0 |
| NM_001098638 | RNF169 | 7823 | 6 | 3 | 3 | 2 | 3 | 3 | 3 | 1 | 3 | 6 | 4 | 4 | 3 |
| NM_001098668 | SFTPA2 | 2200 | 365 | 33 | 0 | 0 | 0 | 0 | 0 | 0 | 365 | 0 | 0 | 0 | 0 |
| NM_001098668 | SFTPA2 | 2201 | 365 | 33 | 0 | 0 | 0 | 0 | 0 | 0 | 365 | 0 | 0 | 0 | 0 |
| NM_001098670 | RASGRP2 | 2244 | 0 | 0 | 0 | 0 | 0 | 0 | 0 | 0 | 0 | 0 | 0 | 0 | 0 |
| NM_001098671 | RASGRP2 | 2284 | 1 | 0 | 0 | 0 | 0 | 0 | 0 | 0 | 0 | 0 | 0 | 1 | 0 |
| NM_001098672 | HEPHL1 | 5951 | 0 | 0 | 0 | 0 | 0 | 0 | 0 | 0 | 0 | 0 | 0 | 0 | 0 |
| NM_001098673 | C12orf44 | 1266 | 1 | 1 | 1 | 1 | 1 | 1 | 1 | 1 | 1 | 0 | 1 | 1 | 1 |
| NM_001098721 | GNG4 | 4980 | 6 | 1 | 0 | 0 | 0 | 5 | 0 | 0 | 0 | 6 | 0 | 0 | 0 |
| NM_001098722 | GNG4 | 5106 | 6 | 1 | 0 | 0 | 0 | 5 | 0 | 0 | 0 | 6 | 0 | 0 | 0 |
| NM_001098725 | TCL1A | 1391 | 1 | 0 | 0 | 0 | 0 | 0 | 0 | 0 | 0 | 0 | 0 | 1 | 0 |
| NM_001098728 | GTF2H2C | 2105 | 0 | 0 | 0 | 0 | 0 | 0 | 0 | 0 | 0 | 0 | 0 | 0 | 0 |
| NM_001098729 | GTF2H2B | 2087 | 0 | 0 | 0 | 0 | 0 | 0 | 0 | 0 | 0 | 0 | 0 | 0 | 0 |
| NM_001098783 | RPP14 | 3500 | 4 | 2 | 1 | 2 | 3 | 2 | 2 | 3 | 1 | 2 | 4 | 2 | 2 |
| NM_001098784 | FAM89B | 1458 | 0 | 0 | 0 | 0 | 0 | 0 | 0 | 0 | 0 | 0 | 0 | 0 | 0 |
| NM_001098785 | FAM89B | 1448 | 0 | 0 | 0 | 0 | 0 | 0 | 0 | 0 | 0 | 0 | 0 | 0 | 0 |
| NM_001098786 | C7orf68 | 1298 | 3 | 1 | 3 | 1 | 1 | 2 | 1 | 0 | 1 | 1 | 0 | 0 | 1 |
| NM_001098787 | BET1L | 2815 | 2 | 1 | 1 | 2 | 1 | 1 | 2 | 1 | 1 | 2 | 1 | 2 | 1 |
| NM_001098790 | MID1IP1 | 2008 | 9 | 4 | 1 | 2 | 4 | 5 | 3 | 4 | 6 | 3 | 9 | 2 | 5 |
| NM_001098791 | MID1IP1 | 1904 | 9 | 4 | 1 | 1 | 5 | 4 | 3 | 4 | 6 | 3 | 9 | 2 | 5 |
| NM_001098794 | FAM160A2 | 3450 | 2 | 1 | 0 | 2 | 1 | 1 | 1 | 1 | 1 | 1 | 0 | 1 | 2 |
| NM_001098796 | TOX2 | 2417 | 1 | 0 | 0 | 0 | 0 | 1 | 0 | 0 | 1 | 0 | 0 | 0 | 1 |
| NM_001098797 | TOX2 | 2489 | 1 | 0 | 0 | 0 | 0 | 1 | 0 | 0 | 1 | 0 | 0 | 0 | 1 |
| NM_001098798 | TOX2 | 2412 | 1 | 0 | 0 | 0 | 0 | 1 | 0 | 0 | 1 | 0 | 0 | 0 | 1 |
| NM_001098800 | MAGED4 | 2495 | 0 | 0 | 0 | 0 | 0 | 0 | 0 | 0 | 0 | 0 | 0 | 0 | 0 |
| NM_001098801 | C18orf19 | 4384 | 3 | 1 | 1 | 1 | 3 | 2 | 2 | 2 | 1 | 1 | 2 | 1 | 1 |
| NM_001098802 | CEP78 | 2750 | 2 | 1 | 1 | 1 | 0 | 1 | 1 | 1 | 1 | 1 | 0 | 1 | 2 |
| NM_001098805 | ANKRD20A4 | 3517 | 0 | 0 | 0 | 0 | 0 | 0 | 0 | 0 | 0 | 0 | 0 | 0 | 0 |
| NM_001098806 | CCDC29 | 781 | 17 | 3 | 1 | 0 | 2 | 3 | 3 | 0 | 2 | 1 | 1 | 6 | 17 |
| NM_001098808 | C9orf129 | 1154 | 2 | 0 | 0 | 1 | 1 | 2 | 0 | 0 | 0 | 0 | 0 | 0 | 0 |
| NM_001098811 | 08 Sep | 2889 | 24 | 4 | 3 | 4 | 3 | 24 | 3 | 1 | 2 | 2 | 1 | 2 | 2 |
| NM_001098812 | 08 Sep | 4076 | 19 | 5 | 4 | 5 | 5 | 19 | 5 | 1 | 3 | 4 | 1 | 5 | 3 |
| NM_001098813 | 08 Sep | 4270 | 18 | 5 | 3 | 5 | 4 | 18 | 5 | 1 | 3 | 4 | 1 | 5 | 2 |
| NM_001098814 | SRL | 4214 | 22 | 4 | 0 | 0 | 22 | 0 | 0 | 0 | 0 | 0 | 20 | 0 | 0 |
| NM_001098815 | KIAA0748 | 1923 | 4 | 1 | 0 | 0 | 0 | 4 | 0 | 0 | 0 | 1 | 0 | 1 | 0 |
| NM_001098816 | ODZ4 | 13548 | 3 | 1 | 1 | 0 | 0 | 2 | 0 | 0 | 0 | 3 | 0 | 0 | 0 |
| NM_001098817 | INO80C | 1088 | 1 | 0 | 0 | 1 | 0 | 0 | 0 | 0 | 0 | 0 | 0 | 1 | 0 |
| NM_001098818 | PDE4C | 5034 | 0 | 0 | 0 | 0 | 0 | 0 | 0 | 0 | 0 | 0 | 0 | 0 | 0 |
| NM_001098819 | PDE4C | 5124 | 0 | 0 | 0 | 0 | 0 | 0 | 0 | 0 | 0 | 0 | 0 | 0 | 0 |
| NM_001098821 | TMEM91 | 1079 | 1 | 0 | 0 | 0 | 0 | 1 | 0 | 0 | 0 | 1 | 0 | 1 | 0 |
| NM_001098822 | TMEM91 | 931 | 2 | 1 | 0 | 0 | 1 | 1 | 0 | 0 | 0 | 1 | 0 | 2 | 0 |
| NM_001098823 | TMEM91 | 920 | 2 | 1 | 0 | 0 | 1 | 1 | 0 | 0 | 0 | 1 | 0 | 2 | 0 |
| NM_001098824 | TMEM91 | 908 | 2 | 1 | 0 | 0 | 1 | 1 | 0 | 0 | 0 | 1 | 0 | 2 | 0 |
| NM_001098825 | TMEM91 | 739 | 2 | 1 | 0 | 0 | 1 | 1 | 0 | 0 | 1 | 1 | 0 | 2 | 0 |
| NM_001098831 | MORN4 | 2333 | 8 | 2 | 1 | 0 | 1 | 8 | 5 | 0 | 1 | 1 | 0 | 0 | 1 |
| NM_001098832 | FAM104A | 2888 | 12 | 3 | 2 | 1 | 3 | 2 | 2 | 2 | 2 | 3 | 2 | 3 | 12 |
| NM_001098833 | ATXN7L3 | 3593 | 5 | 2 | 1 | 2 | 2 | 5 | 2 | 1 | 2 | 1 | 1 | 3 | 2 |
| NM_001098834 | GBX1 | 1324 | 0 | 0 | 0 | 0 | 0 | 0 | 0 | 0 | 0 | 0 | 0 | 0 | 0 |
| NM_001098835 | MS4A15 | 1959 | 0 | 0 | 0 | 0 | 0 | 0 | 0 | 0 | 0 | 0 | 0 | 0 | 0 |
| NM_001098843 | CXorf30 | 2862 | 0 | 0 | 0 | 0 | 0 | 0 | 0 | 0 | 0 | 0 | 0 | 0 | 0 |
| NM_001098844 | FAM23A | 5463 | 2 | 0 | 0 | 2 | 1 | 0 | 0 | 0 | 1 | 0 | 0 | 0 | 0 |
| NM_001098844 | FAM23A | 5464 | 2 | 0 | 0 | 2 | 1 | 0 | 0 | 0 | 1 | 0 | 0 | 0 | 0 |
| NM_001098845 | ANXA8L1 | 1954 | 0 | 0 | 0 | 0 | 0 | 0 | 0 | 0 | 0 | 0 | 0 | 0 | 0 |
| NM_001099 | ACPP | 3219 | 7 | 1 | 0 | 1 | 1 | 0 | 7 | 0 | 3 | 1 | 0 | 1 | 1 |
| NM_001099218 | RAD51AP2 | 3589 | 2 | 0 | 0 | 0 | 0 | 0 | 0 | 0 | 0 | 0 | 0 | 0 | 2 |
| NM_001099219 | KRTAP19-8 | 318 | 0 | 0 | 0 | 0 | 0 | 0 | 0 | 0 | 0 | 0 | 0 | 0 | 0 |
| NM_001099220 | ZNF862 | 6897 | 0 | 0 | 0 | 0 | 0 | 0 | 0 | 0 | 0 | 0 | 0 | 0 | 0 |
| NM_001099221 | TIFAB | 1273 | 0 | 0 | 0 | 0 | 0 | 0 | 0 | 0 | 0 | 0 | 0 | 0 | 0 |
| NM_001099222 | IFT74 | 2171 | 12 | 5 | 3 | 1 | 4 | 6 | 7 | 4 | 3 | 8 | 2 | 4 | 12 |
| NM_001099223 | IFT74 | 2127 | 13 | 5 | 3 | 1 | 4 | 6 | 7 | 4 | 3 | 8 | 2 | 5 | 13 |
| NM_001099224 | IFT74 | 1566 | 12 | 5 | 3 | 1 | 4 | 6 | 8 | 4 | 3 | 8 | 2 | 4 | 12 |
| NM_001099225 | CCDC43 | 2079 | 17 | 8 | 6 | 3 | 9 | 11 | 7 | 4 | 5 | 5 | 17 | 9 | 9 |
| NM_001099269 | ZNF506 | 3324 | 26 | 9 | 5 | 3 | 7 | 6 | 11 | 1 | 8 | 26 | 5 | 18 | 10 |
| NM_001099270 | ZBTB34 | 6563 | 3 | 2 | 2 | 1 | 2 | 2 | 1 | 0 | 2 | 3 | 1 | 3 | 2 |
| NM_001099271 | C5orf37 | 2161 | 1 | 1 | 0 | 0 | 0 | 1 | 1 | 1 | 1 | 1 | 1 | 1 | 1 |
| NM_001099272 | BTBD9 | 8749 | 3 | 1 | 1 | 1 | 1 | 3 | 1 | 0 | 1 | 2 | 0 | 1 | 1 |
| NM_001099274 | TINF2 | 1850 | 3 | 1 | 1 | 1 | 1 | 2 | 2 | 1 | 1 | 1 | 1 | 3 | 1 |
| NM_001099279 | FOXD4L2 | 3109 | 0 | 0 | 0 | 0 | 0 | 0 | 0 | 0 | 0 | 0 | 0 | 0 | 0 |
| NM_001099280 | HEATR7A | 1850 | 1 | 0 | 0 | 1 | 0 | 0 | 0 | 0 | 0 | 0 | 0 | 0 | 0 |
| NM_001099281 | HEATR7A | 1730 | 1 | 0 | 0 | 1 | 0 | 0 | 0 | 0 | 0 | 0 | 0 | 0 | 0 |
| NM_001099282 | ZNF239 | 2069 | 2 | 1 | 1 | 0 | 1 | 2 | 1 | 0 | 0 | 1 | 1 | 1 | 1 |
| NM_001099283 | ZNF239 | 1906 | 2 | 1 | 1 | 0 | 1 | 2 | 1 | 0 | 0 | 1 | 1 | 1 | 1 |
| NM_001099284 | ZNF239 | 2029 | 2 | 1 | 1 | 0 | 1 | 2 | 1 | 0 | 0 | 1 | 1 | 1 | 1 |
| NM_001099285 | PTMA | 1207 | 501 | 226 | 160 | 212 | 171 | 174 | 203 | 134 | 361 | 273 | 96 | 501 | 203 |
| NM_001099286 | FAM54A | 1793 | 2 | 0 | 0 | 0 | 0 | 0 | 0 | 0 | 0 | 0 | 0 | 1 | 2 |

| transcript | gene | transcript length (nt) | max | mean | adipose | colon | heart | hypothalamus | kidney | liver | lung | ovary | skeletalmuscle | spleen | testes |
|---|---|---|---|---|---|---|---|---|---|---|---|---|---|---|---|
| NM_001099287 | NIPAL4 | 3279 | 1 | 0 | 0 | 0 | 0 | 0 | 0 | 0 | 0 | 0 | 0 | 1 | 0 |
| NM_001099289 | SH3RF3 | 5278 | 2 | 1 | 1 | 0 | 1 | 2 | 0 | 0 | 1 | 0 | 0 | 1 | 1 |
| NM_001099293 | KIF4B | 4426 | 1 | 0 | 0 | 0 | 0 | 0 | 0 | 0 | 0 | 0 | 0 | 0 | 1 |
| NM_001099294 | KIAA1644 | 6741 | 5 | 1 | 0 | 1 | 0 | 5 | 0 | 0 | 0 | 0 | 0 | 0 | 0 |
| NM_001099334 | C2orf80 | 1214 | 11 | 1 | 0 | 0 | 0 | 11 | 0 | 0 | 0 | 0 | 0 | 0 | 0 |
| NM_001099335 | PHYHIP | 3337 | 15 | 2 | 0 | 0 | 0 | 15 | 0 | 0 | 0 | 0 | 0 | 1 | 1 |
| NM_001099336 | C12orf42 | 1462 | 1 | 0 | 0 | 0 | 0 | 0 | 0 | 0 | 0 | 0 | 0 | 0 | 1 |
| NM_001099337 | COQ10A | 1410 | 5 | 1 | 0 | 0 | 5 | 1 | 2 | 1 | 1 | 0 | 2 | 1 | 1 |
| NM_001099338 | FAM22A | 3288 | 1 | 0 | 0 | 1 | 0 | 0 | 0 | 0 | 0 | 0 | 0 | 0 | 0 |
| NM_001099339 | COMMD7 | 1908 | 1 | 1 | 1 | 0 | 1 | 1 | 1 | 0 | 1 | 0 | 0 | 1 | 0 |
| NM_001099400 | SGCE | 1708 | 5 | 2 | 2 | 1 | 2 | 2 | 1 | 0 | 1 | 5 | 1 | 2 | 3 |
| NM_001099401 | SGCE | 1775 | 5 | 2 | 2 | 1 | 2 | 2 | 1 | 0 | 1 | 5 | 1 | 2 | 3 |
| NM_001099402 | CCNK | 2606 | 8 | 4 | 3 | 3 | 5 | 4 | 4 | 3 | 4 | 5 | 4 | 7 | 8 |
| NM_001099403 | PRDM8 | 3129 | 3 | 1 | 0 | 0 | 0 | 3 | 0 | 0 | 0 | 1 | 0 | 0 | 0 |
| NM_001099404 | SCN5A | 8504 | 3 | 0 | 0 | 0 | 3 | 0 | 0 | 0 | 0 | 0 | 0 | 0 | 0 |
| NM_001099405 | SCN5A | 8450 | 3 | 0 | 0 | 0 | 3 | 0 | 0 | 0 | 0 | 0 | 0 | 0 | 0 |
| NM_001099406 | SLC39A6 | 1666 | 2 | 1 | 2 | 0 | 1 | 2 | 2 | 1 | 1 | 2 | 0 | 1 | 2 |
| NM_001099407 | C20orf12 | 3500 | 1 | 0 | 0 | 0 | 0 | 0 | 0 | 0 | 0 | 1 | 0 | 0 | 1 |
| NM_001099408 | EIF4E1B | 1974 | 1 | 0 | 0 | 0 | 0 | 1 | 0 | 0 | 0 | 0 | 0 | 0 | 0 |
| NM_001099409 | EHBP1L1 | 5180 | 62 | 18 | 10 | 22 | 30 | 6 | 5 | 3 | 13 | 6 | 62 | 34 | 7 |
| NM_001099410 | GPRASP1 | 5879 | 30 | 6 | 2 | 3 | 4 | 30 | 3 | 0 | 2 | 16 | 2 | 4 | 6 |
| NM_001099411 | GPRASP1 | 5811 | 30 | 6 | 2 | 3 | 4 | 30 | 3 | 0 | 2 | 16 | 2 | 4 | 6 |
| NM_001099412 | MYST3 | 9285 | 17 | 8 | 5 | 4 | 7 | 7 | 6 | 5 | 10 | 13 | 8 | 17 | 12 |
| NM_001099413 | MYST3 | 9241 | 17 | 9 | 5 | 4 | 7 | 7 | 6 | 5 | 11 | 13 | 8 | 17 | 12 |
| NM_001099414 | HAVCR1 | 1493 | 5 | 1 | 0 | 2 | 0 | 0 | 5 | 0 | 0 | 0 | 0 | 0 | 0 |
| NM_001099415 | POM121C | 5863 | 1 | 0 | 0 | 0 | 0 | 1 | 0 | 0 | 0 | 0 | 0 | 1 | 0 |
| NM_001099431 | CLEC1B | 871 | 3 | 0 | 0 | 0 | 0 | 0 | 0 | 3 | 0 | 0 | 0 | 1 | 0 |
| NM_001099432 | BCAS3 | 3609 | 6 | 3 | 2 | 2 | 2 | 6 | 3 | 1 | 3 | 3 | 3 | 2 | 4 |
| NM_001099433 | JAKMIP1 | 3011 | 31 | 3 | 0 | 1 | 0 | 31 | 0 | 0 | 1 | 0 | 0 | 1 | 4 |
| NM_001099434 | DCDC2B | 1349 | 1 | 0 | 0 | 0 | 0 | 0 | 0 | 0 | 0 | 1 | 0 | 0 | 0 |
| NM_001099435 | SPDYE5 | 2667 | 0 | 0 | 0 | 0 | 0 | 0 | 0 | 0 | 0 | 0 | 0 | 0 | 0 |
| NM_001099436 | ULK3 | 2635 | 1 | 0 | 0 | 1 | 0 | 1 | 1 | 0 | 0 | 0 | 0 | 0 | 0 |
| NM_001099437 | ZNF30 | 2587 | 2 | 1 | 1 | 1 | 1 | 2 | 1 | 1 | 0 | 1 | 0 | 1 | 1 |
| NM_001099438 | ZNF30 | 2653 | 2 | 1 | 1 | 1 | 1 | 2 | 1 | 1 | 0 | 1 | 0 | 1 | 1 |
| NM_001099439 | EPHA10 | 5511 | 1 | 0 | 0 | 1 | 0 | 0 | 0 | 0 | 0 | 0 | 0 | 0 | 0 |
| NM_001099455 | CPPED1 | 5717 | 3 | 2 | 1 | 0 | 1 | 1 | 3 | 2 | 1 | 1 | 0 | 3 | 2 |
| NM_001099456 | NPW | 1016 | 0 | 0 | 0 | 0 | 0 | 0 | 0 | 0 | 0 | 0 | 0 | 0 | 0 |
| NM_001099625 | FAM54B | 2005 | 13 | 5 | 4 | 3 | 13 | 6 | 5 | 4 | 2 | 5 | 6 | 4 | 7 |
| NM_001099626 | FAM54B | 2057 | 12 | 5 | 4 | 3 | 12 | 6 | 5 | 4 | 2 | 5 | 6 | 4 | 7 |
| NM_001099627 | FAM54B | 1896 | 12 | 5 | 4 | 3 | 12 | 6 | 5 | 4 | 2 | 5 | 6 | 4 | 7 |
| NM_001099638 | ZNF146 | 3782 | 42 | 23 | 14 | 5 | 29 | 20 | 23 | 16 | 20 | 42 | 25 | 33 | 22 |
| NM_001099639 | ZNF146 | 3741 | 43 | 23 | 14 | 5 | 29 | 20 | 23 | 16 | 20 | 43 | 26 | 34 | 23 |
| NM_001099640 | TMEM100 | 2253 | 9 | 2 | 3 | 0 | 2 | 1 | 0 | 2 | 9 | 1 | 0 | 0 | 1 |
| NM_001099642 | FTSJD1 | 4027 | 4 | 2 | 1 | 1 | 2 | 1 | 3 | 2 | 2 | 2 | 1 | 4 | 3 |
| NM_001099645 | RPL22L1 | 1962 | 27 | 11 | 4 | 3 | 20 | 6 | 5 | 16 | 12 | 13 | 4 | 27 | 9 |
| NM_001099646 | SLC47A2 | 2247 | 8 | 1 | 0 | 0 | 0 | 0 | 8 | 0 | 0 | 0 | 0 | 0 | 0 |
| NM_001099650 | GXYLT1 | 7395 | 5 | 3 | 2 | 1 | 2 | 2 | 3 | 3 | 3 | 3 | 1 | 4 | 5 |
| NM_001099652 | GPR137C | 3888 | 3 | 1 | 0 | 0 | 0 | 3 | 0 | 0 | 0 | 0 | 0 | 0 | 2 |
| NM_001099653 | FAM86C | 2090 | 1 | 0 | 0 | 0 | 0 | 0 | 1 | 0 | 0 | 0 | 1 | 0 | 0 |
| NM_001099658 | LRRN3 | 3620 | 5 | 1 | 1 | 0 | 1 | 2 | 1 | 0 | 1 | 2 | 1 | 1 | 5 |
| NM_001099660 | LRRN3 | 3727 | 5 | 1 | 1 | 0 | 1 | 2 | 1 | 0 | 1 | 2 | 1 | 1 | 5 |
| NM_001099661 | EIF3CL | 3050 | 0 | 0 | 0 | 0 | 0 | 0 | 0 | 0 | 0 | 0 | 0 | 0 | 0 |
| NM_001099661 | EIF3CL | 3053 | 0 | 0 | 0 | 0 | 0 | 0 | 0 | 0 | 0 | 0 | 0 | 0 | 0 |
| NM_001099666 | PTAR1 | 10051 | 4 | 2 | 2 | 1 | 2 | 3 | 4 | 1 | 2 | 4 | 2 | 3 | 3 |
| NM_001099667 | ARMS2 | 808 | 0 | 0 | 0 | 0 | 0 | 0 | 0 | 0 | 0 | 0 | 0 | 0 | 0 |
| NM_001099668 | HIGD1A | 2938 | 12 | 5 | 3 | 4 | 6 | 9 | 6 | 12 | 2 | 1 | 2 | 2 | 2 |
| NM_001099669 | HIGD1A | 2791 | 12 | 4 | 3 | 3 | 6 | 10 | 5 | 12 | 2 | 1 | 2 | 2 | 2 |
| NM_001099670 | C8orf59 | 1044 | 2 | 1 | 1 | 1 | 2 | 1 | 1 | 2 | 0 | 1 | 1 | 1 | 1 |
| NM_001099671 | C8orf59 | 1041 | 2 | 1 | 1 | 1 | 2 | 1 | 1 | 2 | 0 | 1 | 1 | 1 | 1 |
| NM_001099672 | C8orf59 | 957 | 2 | 1 | 1 | 1 | 2 | 1 | 2 | 2 | 1 | 1 | 1 | 1 | 1 |
| NM_001099673 | C8orf59 | 916 | 2 | 1 | 1 | 1 | 2 | 1 | 2 | 2 | 1 | 1 | 1 | 1 | 1 |
| NM_001099676 | C12orf56 | 1587 | 1 | 0 | 0 | 0 | 0 | 0 | 1 | 0 | 0 | 0 | 0 | 0 | 1 |
| NM_001099677 | C8orf79 | 9298 | 3 | 1 | 0 | 0 | 1 | 3 | 1 | 0 | 1 | 0 | 0 | 1 | 1 |
| NM_001099678 | LRRC58 | 7683 | 8 | 4 | 3 | 1 | 3 | 4 | 6 | 3 | 3 | 8 | 2 | 7 | 5 |
| NM_001099679 | TRIM32 | 3716 | 2 | 1 | 1 | 1 | 1 | 2 | 1 | 0 | 1 | 2 | 2 | 2 | 1 |
| NM_001099680 | MAGIX | 2622 | 0 | 0 | 0 | 0 | 0 | 0 | 0 | 0 | 0 | 0 | 0 | 0 | 0 |
| NM_001099681 | MAGIX | 1922 | 0 | 0 | 0 | 0 | 0 | 0 | 0 | 0 | 0 | 0 | 0 | 0 | 0 |
| NM_001099682 | MAGIX | 1907 | 0 | 0 | 0 | 0 | 0 | 0 | 0 | 0 | 0 | 0 | 0 | 0 | 0 |
| NM_001099684 | FAM156B | 1699 | 0 | 0 | 0 | 0 | 0 | 0 | 0 | 0 | 0 | 0 | 0 | 0 | 0 |
| NM_001099685 | RHOXF2B | 1309 | 0 | 0 | 0 | 0 | 0 | 0 | 0 | 0 | 0 | 0 | 0 | 0 | 0 |
| NM_001099686 | NXF2B | 3959 | 0 | 0 | 0 | 0 | 0 | 0 | 0 | 0 | 0 | 0 | 0 | 0 | 0 |
| NM_001099687 | TP53TG3B | 2861 | 0 | 0 | 0 | 0 | 0 | 0 | 0 | 0 | 0 | 0 | 0 | 0 | 0 |
| NM_001099687 | TP53TG3B | 2866 | 0 | 0 | 0 | 0 | 0 | 0 | 0 | 0 | 0 | 0 | 0 | 0 | 0 |
| NM_001099687 | TP53TG3B | 2867 | 0 | 0 | 0 | 0 | 0 | 0 | 0 | 0 | 0 | 0 | 0 | 0 | 0 |
| NM_001099691 | TGFA | 4260 | 5 | 1 | 0 | 2 | 0 | 3 | 3 | 1 | 1 | 0 | 0 | 5 | 0 |
| NM_001099692 | EIF5AL1 | 3836 | 1 | 0 | 0 | 0 | 0 | 0 | 0 | 1 | 0 | 0 | 0 | 0 | 1 |
| NM_001099693 | RPL31 | 785 | 246 | 131 | 141 | 81 | 117 | 150 | 80 | 164 | 92 | 246 | 109 | 185 | 75 |

| transcript | gene | transcript length (nt) | max | mean | adipose | colon | heart | hypothala mus | kidney | liver | lung | ovary | skeletalm uscle | spleen | testes |
|---|---|---|---|---|---|---|---|---|---|---|---|---|---|---|---|
| NM_001099694 | ZNF578 | 6741 | 3 | 1 | 0 | 0 | 1 | 1 | 1 | 0 | 1 | 2 | 0 | 1 | 3 |
| NM_001099695 | REPIN1 | 3167 | 3 | 2 | 2 | 3 | 1 | 2 | 3 | 2 | 1 | 1 | 2 | 2 | 1 |
| NM_001099696 | REPIN1 | 2969 | 3 | 2 | 2 | 3 | 1 | 2 | 3 | 2 | 1 | 1 | 2 | 2 | 1 |
| NM_001099697 | RSPH10B2 | 3106 | 0 | 0 | 0 | 0 | 0 | 0 | 0 | 0 | 0 | 0 | 0 | 0 | 0 |
| NM_001099733 | ADCYAP1 | 3187 | 2 | 0 | 0 | 0 | 0 | 2 | 0 | 0 | 0 | 0 | 0 | 0 | 1 |
| NM_001099735 | CKMT2 | 1478 | 41 | 6 | 1 | 0 | 41 | 0 | 1 | 0 | 1 | 1 | 13 | 1 | 1 |
| NM_001099736 | CKMT2 | 1482 | 41 | 5 | 1 | 0 | 41 | 0 | 1 | 0 | 1 | 1 | 13 | 1 | 1 |
| NM_001099737 | MORG1 | 1452 | 0 | 0 | 0 | 0 | 0 | 0 | 0 | 0 | 0 | 0 | 0 | 0 | 0 |
| NM_001099743 | GOLSYN | 3235 | 11 | 2 | 1 | 1 | 1 | 11 | 1 | 5 | 1 | 1 | 0 | 0 | 1 |
| NM_001099744 | GOLSYN | 3238 | 11 | 2 | 1 | 1 | 1 | 11 | 1 | 5 | 1 | 1 | 0 | 0 | 1 |
| NM_001099745 | GOLSYN | 3074 | 11 | 2 | 2 | 2 | 1 | 11 | 1 | 6 | 1 | 1 | 0 | 0 | 1 |
| NM_001099746 | GOLSYN | 2788 | 12 | 2 | 2 | 2 | 1 | 12 | 1 | 6 | 2 | 1 | 0 | 0 | 1 |
| NM_001099747 | GOLSYN | 2992 | 12 | 2 | 2 | 2 | 1 | 12 | 1 | 6 | 1 | 1 | 0 | 0 | 1 |
| NM_001099748 | GOLSYN | 2995 | 12 | 2 | 2 | 2 | 1 | 12 | 1 | 6 | 1 | 1 | 0 | 0 | 1 |
| NM_001099749 | GOLSYN | 2709 | 13 | 3 | 2 | 2 | 1 | 13 | 1 | 6 | 2 | 1 | 0 | 0 | 1 |
| NM_001099750 | GOLSYN | 3116 | 11 | 2 | 1 | 2 | 1 | 11 | 1 | 6 | 1 | 1 | 0 | 0 | 1 |
| NM_001099751 | GOLSYN | 3081 | 11 | 2 | 2 | 2 | 1 | 11 | 1 | 6 | 1 | 1 | 0 | 0 | 1 |
| NM_001099752 | GOLSYN | 3084 | 11 | 2 | 2 | 2 | 1 | 11 | 1 | 6 | 1 | 1 | 0 | 0 | 1 |
| NM_001099753 | GOLSYN | 2882 | 12 | 2 | 2 | 2 | 1 | 12 | 1 | 6 | 2 | 1 | 0 | 0 | 1 |
| NM_001099754 | GOLSYN | 2885 | 12 | 2 | 2 | 2 | 1 | 12 | 1 | 6 | 2 | 1 | 0 | 0 | 1 |
| NM_001099755 | GOLSYN | 2680 | 13 | 3 | 2 | 2 | 1 | 13 | 1 | 6 | 2 | 1 | 0 | 0 | 1 |
| NM_001099756 | GOLSYN | 2956 | 12 | 2 | 2 | 2 | 1 | 12 | 1 | 6 | 2 | 1 | 0 | 0 | 1 |
| NM_001099771 | POTEF | 4337 | 1 | 0 | 0 | 0 | 0 | 0 | 0 | 0 | 0 | 0 | 0 | 0 | 1 |
| NM_001099772 | CYP4B1 | 2158 | 4 | 1 | 1 | 0 | 0 | 0 | 0 | 0 | 4 | 0 | 0 | 0 | 0 |
| NM_001099773 | CYP11A1 | 2004 | 11 | 2 | 0 | 0 | 0 | 0 | 0 | 0 | 0 | 11 | 0 | 3 | 4 |
| NM_001099777 | C3orf55 | 1077 | 3 | 0 | 3 | 0 | 0 | 0 | 0 | 0 | 1 | 0 | 0 | 0 | 0 |
| NM_001099780 | PSMB11 | 1894 | 0 | 0 | 0 | 0 | 0 | 0 | 0 | 0 | 0 | 0 | 0 | 0 | 0 |
| NM_001099781 | GGT5 | 2497 | 1 | 0 | 0 | 0 | 1 | 0 | 1 | 0 | 0 | 0 | 0 | 1 | 0 |
| NM_001099782 | GGT5 | 2398 | 1 | 0 | 0 | 0 | 1 | 0 | 1 | 0 | 0 | 0 | 0 | 1 | 0 |
| NM_001099783 | C4orf33 | 1589 | 2 | 1 | 1 | 0 | 0 | 1 | 2 | 0 | 1 | 1 | 0 | 2 | 1 |
| NM_001099784 | FBXL19 | 3796 | 2 | 1 | 0 | 1 | 0 | 2 | 0 | 0 | 0 | 0 | 0 | 0 | 2 |
| NM_001099786 | ICAM2 | 1335 | 3 | 1 | 2 | 1 | 1 | 1 | 0 | 1 | 1 | 0 | 0 | 3 | 0 |
| NM_001099787 | ICAM2 | 1253 | 3 | 1 | 2 | 1 | 1 | 1 | 0 | 1 | 1 | 0 | 0 | 3 | 0 |
| NM_001099788 | ICAM2 | 1218 | 3 | 1 | 2 | 1 | 1 | 1 | 0 | 1 | 1 | 0 | 0 | 3 | 0 |
| NM_001099789 | ICAM2 | 1136 | 3 | 1 | 2 | 1 | 1 | 1 | 0 | 1 | 1 | 0 | 0 | 3 | 0 |
| NM_001099790 | PRAMEF19 | 2123 | 0 | 0 | 0 | 0 | 0 | 0 | 0 | 0 | 0 | 0 | 0 | 0 | 0 |
| NM_001099850 | PRAMEF18 | 2123 | 0 | 0 | 0 | 0 | 0 | 0 | 0 | 0 | 0 | 0 | 0 | 0 | 0 |
| NM_001099851 | PRAMEF17 | 1557 | 0 | 0 | 0 | 0 | 0 | 0 | 0 | 0 | 0 | 0 | 0 | 0 | 0 |
| NM_001099852 | PRAMEF20 | 1597 | 0 | 0 | 0 | 0 | 0 | 0 | 0 | 0 | 0 | 0 | 0 | 0 | 0 |
| NM_001099854 | PRAMEF14 | 2100 | 0 | 0 | 0 | 0 | 0 | 0 | 0 | 0 | 0 | 0 | 0 | 0 | 0 |
| NM_001099855 | MCF2 | 3819 | 6 | 1 | 0 | 0 | 0 | 1 | 0 | 0 | 0 | 0 | 0 | 0 | 6 |
| NM_001099856 | IKBKG | 2068 | 1 | 0 | 0 | 0 | 0 | 0 | 0 | 1 | 0 | 0 | 0 | 1 | 0 |
| NM_001099857 | IKBKG | 2259 | 1 | 0 | 0 | 0 | 0 | 0 | 0 | 1 | 0 | 0 | 0 | 1 | 0 |
| NM_001099858 | C7orf25 | 1825 | 4 | 2 | 2 | 1 | 3 | 3 | 3 | 1 | 2 | 2 | 1 | 3 | 4 |
| NM_001099921 | MAGEB16 | 1818 | 0 | 0 | 0 | 0 | 0 | 0 | 0 | 0 | 0 | 0 | 0 | 0 | 0 |
| NM_001099922 | ALG13 | 4161 | 4 | 2 | 2 | 1 | 1 | 1 | 1 | 1 | 2 | 2 | 1 | 3 | 4 |
| NM_001099951 | FAM166B | 889 | 6 | 1 | 0 | 0 | 0 | 0 | 0 | 0 | 1 | 0 | 6 | 0 | 0 |
| NM_001099952 | ITPR1 | 10096 | 19 | 8 | 3 | 4 | 6 | 13 | 9 | 1 | 7 | 19 | 1 | 12 | 10 |
| NM_001100 | ACTA1 | 1492 | 6,246 | 683 | 1 | 0 | 1,261 | 1 | 0 | 0 | 0 | 0 | 6,246 | 0 | 7 |
| NM_001100108 | ABL2 | 11927 | 2 | 2 | 2 | 1 | 2 | 2 | 1 | 1 | 2 | 2 | 1 | 2 | 2 |
| NM_001100111 | LOC286238 | 682 | 1 | 0 | 0 | 0 | 0 | 0 | 0 | 0 | 0 | 0 | 0 | 0 | 1 |
| NM_001100112 | MYH2 | 6086 | 405 | 37 | 1 | 0 | 0 | 0 | 0 | 0 | 0 | 0 | 405 | 0 | 0 |
| NM_001100114 | PRAMEF21 | 1508 | 0 | 0 | 0 | 0 | 0 | 0 | 0 | 0 | 0 | 0 | 0 | 0 | 0 |
| NM_001100117 | RIMS2 | 5592 | 10 | 1 | 0 | 0 | 0 | 10 | 0 | 0 | 0 | 0 | 0 | 0 | 2 |
| NM_001100118 | XRCC3 | 2545 | 1 | 0 | 0 | 1 | 0 | 0 | 0 | 0 | 0 | 0 | 0 | 0 | 0 |
| NM_001100119 | XRCC3 | 2639 | 0 | 0 | 0 | 0 | 0 | 0 | 0 | 0 | 0 | 0 | 0 | 0 | 0 |
| NM_001100120 | ECE2 | 3372 | 1 | 0 | 0 | 0 | 0 | 1 | 0 | 0 | 0 | 0 | 0 | 0 | 0 |
| NM_001100121 | ECE2 | 3234 | 1 | 0 | 0 | 0 | 0 | 1 | 0 | 0 | 0 | 0 | 0 | 0 | 0 |
| NM_001100122 | SBNO2 | 4593 | 1 | 0 | 0 | 0 | 0 | 0 | 0 | 0 | 0 | 0 | 0 | 1 | 0 |
| NM_001100159 | C7orf57 | 2125 | 1 | 0 | 0 | 0 | 0 | 0 | 0 | 0 | 0 | 0 | 0 | 0 | 1 |
| NM_001100161 | XPO7 | 4890 | 9 | 6 | 5 | 4 | 6 | 9 | 8 | 4 | 4 | 6 | 4 | 7 | 7 |
| NM_001100162 | XPO7 | 4819 | 9 | 6 | 5 | 4 | 6 | 9 | 8 | 4 | 4 | 6 | 4 | 7 | 7 |
| NM_001100163 | MRVI1 | 6313 | 7 | 2 | 1 | 4 | 3 | 7 | 1 | 0 | 1 | 2 | 0 | 1 | 2 |
| NM_001100164 | PHACTR2 | 9633 | 9 | 5 | 5 | 1 | 7 | 4 | 3 | 2 | 7 | 6 | 3 | 9 | 8 |
| NM_001100165 | PHACTR2 | 9393 | 9 | 5 | 5 | 1 | 7 | 4 | 3 | 3 | 7 | 6 | 3 | 9 | 8 |
| NM_001100166 | PHACTR2 | 9291 | 9 | 5 | 5 | 1 | 7 | 4 | 3 | 3 | 7 | 6 | 3 | 9 | 8 |
| NM_001100167 | MRVI1 | 6011 | 7 | 2 | 1 | 4 | 3 | 7 | 1 | 0 | 1 | 2 | 0 | 1 | 2 |
| NM_001100168 | CCR5 | 3426 | 4 | 1 | 2 | 1 | 1 | 1 | 0 | 0 | 2 | 1 | 0 | 4 | 0 |
| NM_001100169 | IMMT | 2998 | 23 | 14 | 13 | 8 | 23 | 16 | 18 | 18 | 6 | 8 | 17 | 14 | 14 |
| NM_001100170 | IMMT | 2968 | 23 | 14 | 13 | 8 | 23 | 16 | 18 | 18 | 6 | 8 | 17 | 14 | 14 |
| NM_001100176 | HOOK2 | 2604 | 8 | 3 | 8 | 4 | 3 | 1 | 5 | 1 | 1 | 1 | 1 | 1 | 2 |
| NM_001100388 | C11orf88 | 714 | 3 | 1 | 0 | 0 | 0 | 2 | 0 | 0 | 3 | 0 | 0 | 0 | 3 |
| NM_001100389 | TMEM192 | 4495 | 12 | 6 | 4 | 2 | 5 | 5 | 11 | 12 | 3 | 9 | 3 | 5 | 5 |
| NM_001100390 | IQCE | 6796 | 1 | 1 | 1 | 1 | 1 | 1 | 1 | 0 | 1 | 0 | 0 | 0 | 1 |
| NM_001100391 | RALYL | 1981 | 16 | 2 | 0 | 0 | 0 | 16 | 6 | 0 | 0 | 0 | 0 | 1 | 0 |
| NM_001100392 | RALYL | 2224 | 14 | 2 | 0 | 0 | 0 | 14 | 5 | 0 | 0 | 0 | 0 | 1 | 0 |
| NM_001100393 | RALYL | 2192 | 15 | 2 | 0 | 0 | 0 | 15 | 5 | 0 | 0 | 0 | 0 | 1 | 0 |

| transcript | gene | transcript length (nt) | max | mean | adipose | colon | heart | hypothalamus | kidney | liver | lung | ovary | skeletalmuscle | spleen | testes |
|---|---|---|---|---|---|---|---|---|---|---|---|---|---|---|---|
| NM_001100396 | C2orf73 | 1921 | 3 | 0 | 0 | 0 | 0 | 0 | 0 | 0 | 0 | 0 | 0 | 0 | 3 |
| NM_001100397 | RAPGEF4 | 4107 | 31 | 4 | 1 | 0 | 2 | 31 | 1 | 1 | 3 | 2 | 0 | 0 | 2 |
| NM_001100398 | RAP1GAP2 | 6618 | 2 | 0 | 0 | 1 | 1 | 2 | 0 | 0 | 0 | 0 | 0 | 0 | 0 |
| NM_001100399 | PDS5A | 7176 | 15 | 9 | 7 | 4 | 8 | 7 | 10 | 10 | 10 | 12 | 7 | 15 | 9 |
| NM_001100400 | PDS5A | 2728 | 12 | 8 | 5 | 4 | 7 | 6 | 8 | 8 | 9 | 10 | 6 | 12 | 9 |
| NM_001100407 | C17orf62 | 2132 | 2 | 1 | 0 | 1 | 1 | 1 | 1 | 1 | 1 | 0 | 0 | 2 | 0 |
| NM_001100408 | C17orf62 | 2040 | 2 | 1 | 0 | 1 | 1 | 1 | 1 | 1 | 1 | 0 | 0 | 2 | 0 |
| NM_001100409 | SENP6 | 6621 | 10 | 6 | 4 | 2 | 6 | 6 | 6 | 4 | 6 | 9 | 5 | 9 | 10 |
| NM_001100411 | FAM184A | 3505 | 11 | 3 | 1 | 0 | 1 | 7 | 4 | 1 | 4 | 3 | 1 | 1 | 11 |
| NM_001100412 | RNASEN | 5386 | 8 | 5 | 3 | 4 | 5 | 8 | 5 | 3 | 3 | 8 | 4 | 6 | 4 |
| NM_001100417 | UBR7 | 3560 | 3 | 2 | 1 | 1 | 2 | 3 | 2 | 2 | 1 | 1 | 1 | 2 | 3 |
| NM_001100418 | C19orf60 | 880 | 1 | 1 | 1 | 1 | 0 | 1 | 1 | 0 | 0 | 1 | 1 | 1 | 0 |
| NM_001100419 | C19orf60 | 814 | 1 | 1 | 1 | 1 | 0 | 1 | 1 | 0 | 0 | 1 | 1 | 1 | 0 |
| NM_001100420 | C21orf91 | 5433 | 5 | 2 | 1 | 0 | 1 | 3 | 1 | 1 | 2 | 2 | 1 | 2 | 5 |
| NM_001100421 | C21orf91 | 5370 | 5 | 2 | 1 | 0 | 1 | 3 | 1 | 1 | 2 | 2 | 1 | 2 | 5 |
| NM_001100422 | SPATS2L | 6321 | 5 | 3 | 2 | 2 | 3 | 3 | 5 | 3 | 2 | 3 | 2 | 3 | 3 |
| NM_001100423 | SPATS2L | 6271 | 4 | 3 | 2 | 1 | 3 | 2 | 4 | 2 | 2 | 3 | 2 | 3 | 3 |
| NM_001100424 | SPATS2L | 6114 | 4 | 2 | 2 | 1 | 3 | 2 | 4 | 2 | 1 | 2 | 2 | 2 | 1 |
| NM_001100425 | KIAA0895 | 4224 | 2 | 1 | 0 | 0 | 0 | 1 | 1 | 0 | 0 | 2 | 0 | 0 | 2 |
| NM_001100426 | RAP1GDS1 | 3761 | 12 | 3 | 2 | 1 | 2 | 12 | 3 | 3 | 2 | 2 | 0 | 5 | 3 |
| NM_001100427 | RAP1GDS1 | 3758 | 12 | 3 | 2 | 1 | 2 | 12 | 3 | 3 | 2 | 2 | 0 | 5 | 3 |
| NM_001100428 | RAP1GDS1 | 3614 | 12 | 3 | 2 | 1 | 2 | 12 | 3 | 3 | 2 | 2 | 0 | 5 | 4 |
| NM_001100429 | RAP1GDS1 | 3611 | 12 | 3 | 2 | 1 | 2 | 12 | 3 | 3 | 2 | 2 | 0 | 5 | 4 |
| NM_001100430 | RAP1GDS1 | 3485 | 12 | 3 | 2 | 1 | 2 | 12 | 3 | 3 | 2 | 2 | 0 | 6 | 4 |
| NM_001100431 | VSIG4 | 1570 | 20 | 6 | 9 | 1 | 18 | 4 | 2 | 6 | 20 | 1 | 2 | 6 | 2 |
| NM_001100588 | RC3H2 | 3991 | 14 | 8 | 5 | 4 | 9 | 14 | 7 | 5 | 8 | 7 | 6 | 12 | 9 |
| NM_001100590 | KIAA0232 | 7773 | 13 | 7 | 4 | 2 | 12 | 13 | 7 | 4 | 4 | 5 | 11 | 7 | 7 |
| NM_001100592 | ATP6V0E2 | 2565 | 4 | 1 | 0 | 0 | 0 | 4 | 1 | 1 | 0 | 0 | 0 | 0 | 0 |
| NM_001100594 | SNRK | 5153 | 23 | 10 | 12 | 3 | 7 | 8 | 9 | 5 | 20 | 7 | 6 | 23 | 7 |
| NM_001100595 | SKA2 | 2970 | 12 | 7 | 7 | 3 | 12 | 11 | 7 | 5 | 8 | 7 | 2 | 8 | 11 |
| NM_001100598 | ZNF707 | 2212 | 2 | 1 | 0 | 0 | 0 | 0 | 0 | 0 | 0 | 0 | 0 | 2 | 1 |
| NM_001100599 | ZNF707 | 2114 | 1 | 0 | 0 | 0 | 0 | 0 | 0 | 0 | 0 | 0 | 0 | 0 | 1 |
| NM_001100600 | MMD2 | 2416 | 2 | 0 | 0 | 0 | 0 | 2 | 0 | 0 | 0 | 0 | 0 | 0 | 1 |
| NM_001100603 | KDELR2 | 2607 | 31 | 8 | 6 | 5 | 7 | 3 | 8 | 31 | 5 | 6 | 2 | 6 | 6 |
| NM_001100607 | SERPINA10 | 2108 | 103 | 10 | 0 | 0 | 0 | 0 | 0 | 103 | 0 | 0 | 0 | 0 | 0 |
| NM_001100619 | CABLES1 | 5005 | 2 | 1 | 2 | 0 | 0 | 2 | 1 | 1 | 1 | 0 | 0 | 1 | 0 |
| NM_001100620 | TROAP | 738 | 0 | 0 | 0 | 0 | 0 | 0 | 0 | 0 | 0 | 0 | 0 | 0 | 0 |
| NM_001100621 | C17orf80 | 3531 | 6 | 3 | 2 | 1 | 3 | 3 | 3 | 1 | 1 | 3 | 3 | 3 | 6 |
| NM_001100622 | C17orf80 | 2100 | 9 | 4 | 3 | 1 | 4 | 3 | 4 | 2 | 2 | 4 | 5 | 4 | 9 |
| NM_001100623 | PLEKHB2 | 4308 | 31 | 13 | 7 | 7 | 10 | 31 | 27 | 5 | 14 | 4 | 3 | 19 | 12 |
| NM_001100624 | CENPN | 4644 | 1 | 0 | 0 | 0 | 1 | 0 | 0 | 1 | 0 | 0 | 0 | 1 | 0 |
| NM_001100625 | CENPN | 2262 | 1 | 0 | 0 | 0 | 1 | 0 | 0 | 1 | 0 | 0 | 0 | 1 | 0 |
| NM_001100626 | ERLIN1 | 3195 | 20 | 5 | 4 | 3 | 3 | 2 | 5 | 20 | 3 | 3 | 1 | 5 | 4 |
| NM_001100631 | PRAMEF22 | 1446 | 0 | 0 | 0 | 0 | 0 | 0 | 0 | 0 | 0 | 0 | 0 | 0 | 0 |
| NM_001100812 | CXCL16 | 1451 | 11 | 4 | 4 | 1 | 2 | 2 | 2 | 3 | 10 | 2 | 0 | 11 | 4 |
| NM_001100814 | TMEM55B | 1714 | 2 | 1 | 1 | 1 | 1 | 2 | 1 | 1 | 1 | 1 | 1 | 1 | 1 |
| NM_001100816 | MED7 | 1384 | 17 | 9 | 9 | 5 | 8 | 12 | 10 | 6 | 7 | 10 | 6 | 10 | 17 |
| NM_001100817 | TCEB3CL | 1641 | 0 | 0 | 0 | 0 | 0 | 0 | 0 | 0 | 0 | 0 | 0 | 0 | 0 |
| NM_001100818 | PID1 | 2611 | 8 | 2 | 2 | 1 | 2 | 2 | 1 | 8 | 1 | 2 | 1 | 1 | 1 |
| NM_001100819 | MOBKL3 | 2800 | 13 | 8 | 5 | 4 | 12 | 13 | 6 | 6 | 7 | 7 | 8 | 11 | 8 |
| NM_001100829 | TMEM170B | 8362 | 3 | 1 | 1 | 0 | 1 | 3 | 1 | 1 | 1 | 1 | 1 | 1 | 1 |
| NM_001100873 | C16orf46 | 1911 | 2 | 1 | 0 | 0 | 1 | 2 | 1 | 1 | 0 | 1 | 0 | 1 | 2 |
| NM_001100874 | NHEDC1 | 1731 | 2 | 0 | 0 | 0 | 0 | 0 | 0 | 0 | 0 | 0 | 0 | 0 | 2 |
| NM_001100875 | 10 Mar | 3054 | 4 | 0 | 0 | 0 | 0 | 0 | 0 | 0 | 0 | 0 | 0 | 0 | 4 |
| NM_001100876 | PHYHD1 | 2137 | 2 | 1 | 0 | 0 | 0 | 1 | 1 | 2 | 0 | 0 | 0 | 0 | 0 |
| NM_001100877 | PHYHD1 | 1182 | 3 | 1 | 1 | 1 | 1 | 1 | 1 | 3 | 1 | 1 | 1 | 0 | 0 |
| NM_001100878 | C8orf73 | 3250 | 0 | 0 | 0 | 0 | 0 | 0 | 0 | 0 | 0 | 0 | 0 | 0 | 0 |
| NM_001100879 | ST20 | 1011 | 0 | 0 | 0 | 0 | 0 | 0 | 0 | 0 | 0 | 0 | 0 | 0 | 0 |
| NM_001100880 | ST20 | 626 | 0 | 0 | 0 | 0 | 0 | 0 | 0 | 0 | 0 | 0 | 0 | 0 | 0 |
| NM_001100909 | MS4A13 | 805 | 0 | 0 | 0 | 0 | 0 | 0 | 0 | 0 | 0 | 0 | 0 | 0 | 0 |
| NM_001100910 | FAM72B | 2374 | 0 | 0 | 0 | 0 | 0 | 0 | 0 | 0 | 0 | 0 | 0 | 0 | 0 |
| NM_001100912 | BEND7 | 1907 | 6 | 2 | 2 | 2 | 2 | 1 | 6 | 2 | 2 | 2 | 3 | 1 | 5 |
| NM_001100913 | PACS2 | 6332 | 4 | 2 | 2 | 2 | 1 | 4 | 1 | 1 | 1 | 1 | 3 | 2 | 2 |
| NM_001100915 | KCTD19 | 2911 | 5 | 0 | 0 | 0 | 0 | 0 | 0 | 0 | 0 | 0 | 0 | 0 | 5 |
| NM_001100916 | MBOAT4 | 1663 | 1 | 0 | 0 | 0 | 0 | 0 | 0 | 0 | 0 | 0 | 0 | 1 | 0 |
| NM_001100917 | TSPAN19 | 1020 | 0 | 0 | 0 | 0 | 0 | 0 | 0 | 0 | 0 | 0 | 0 | 0 | 0 |
| NM_001101 | ACTB | 1812 | 172 | 83 | 107 | 120 | 67 | 141 | 69 | 55 | 70 | 52 | 20 | 172 | 44 |
| NM_001101311 | TMEM176B | 1305 | 38 | 9 | 3 | 7 | 5 | 1 | 25 | 38 | 3 | 3 | 1 | 8 | 2 |
| NM_001101312 | TMEM176B | 1297 | 38 | 9 | 3 | 7 | 5 | 1 | 25 | 38 | 3 | 3 | 1 | 9 | 2 |
| NM_001101313 | TMEM176B | 1222 | 40 | 10 | 4 | 7 | 6 | 2 | 27 | 40 | 4 | 3 | 1 | 9 | 2 |
| NM_001101314 | TMEM176B | 1186 | 42 | 10 | 4 | 7 | 6 | 2 | 28 | 42 | 4 | 3 | 1 | 9 | 3 |
| NM_001101320 | SERPINE3 | 1441 | 0 | 0 | 0 | 0 | 0 | 0 | 0 | 0 | 0 | 0 | 0 | 0 | 0 |
| NM_001101330 | LOC728819 | 1170 | 1 | 0 | 1 | 0 | 0 | 0 | 0 | 0 | 0 | 0 | 0 | 0 | 1 |
| NM_001101337 | C3orf79 | 580 | 0 | 0 | 0 | 0 | 0 | 0 | 0 | 0 | 0 | 0 | 0 | 0 | 0 |
| NM_001101338 | LOC169834 | 2379 | 5 | 2 | 1 | 0 | 3 | 5 | 2 | 0 | 0 | 2 | 0 | 1 | 4 |
| NM_001101339 | C12orf77 | 1509 | 0 | 0 | 0 | 0 | 0 | 0 | 0 | 0 | 0 | 0 | 0 | 0 | 0 |
| NM_001101340 | C19orf76 | 2112 | 1 | 0 | 0 | 0 | 0 | 0 | 0 | 0 | 0 | 0 | 0 | 1 | 0 |

| transcript | gene | transcript length (nt) | max | mean | adipose | colon | heart | hypothala mus | kidney | liver | lung | ovary | skeletalm uscle | spleen | testes |
|---|---|---|---|---|---|---|---|---|---|---|---|---|---|---|---|
| NM_001101341 | SFTA3 | 1487 | 40 | 4 | 0 | 0 | 0 | 1 | 0 | 0 | 40 | 0 | 0 | 0 | 0 |
| NM_001101357 | CCDC160 | 1299 | 2 | 0 | 0 | 0 | 0 | 1 | 2 | 0 | 0 | 0 | 0 | 0 | 1 |
| NM_001101362 | LOC390594 | 1377 | 0 | 0 | 0 | 0 | 0 | 0 | 0 | 0 | 0 | 0 | 0 | 0 | 0 |
| NM_001101372 | IGLON5 | 3100 | 1 | 0 | 0 | 0 | 0 | 1 | 0 | 0 | 0 | 0 | 0 | 0 | 0 |
| NM_001101376 | FAM183A | 517 | 2 | 0 | 0 | 0 | 0 | 2 | 0 | 0 | 2 | 0 | 0 | 0 | 1 |
| NM_001101387 | PIRT | 3466 | 1 | 0 | 0 | 0 | 0 | 1 | 0 | 0 | 0 | 0 | 0 | 0 | 0 |
| NM_001101389 | CLDN25 | 690 | 0 | 0 | 0 | 0 | 0 | 0 | 0 | 0 | 0 | 0 | 0 | 0 | 0 |
| NM_001101391 | LINGO3 | 2130 | 0 | 0 | 0 | 0 | 0 | 0 | 0 | 0 | 0 | 0 | 0 | 0 | 0 |
| NM_001101401 | SBK2 | 1047 | 0 | 0 | 0 | 0 | 0 | 0 | 0 | 0 | 0 | 0 | 0 | 0 | 0 |
| NM_001101404 | SH2D7 | 1892 | 1 | 0 | 0 | 1 | 0 | 0 | 0 | 0 | 0 | 0 | 0 | 0 | 0 |
| NM_001101417 | ISPD | 5374 | 2 | 1 | 0 | 0 | 1 | 2 | 1 | 0 | 0 | 1 | 0 | 0 | 1 |
| NM_001101419 | ZNF541 | 4631 | 1 | 0 | 0 | 0 | 0 | 0 | 0 | 0 | 0 | 0 | 0 | 0 | 1 |
| NM_001101421 | MYO1H | 4258 | 0 | 0 | 0 | 0 | 0 | 0 | 0 | 0 | 0 | 0 | 0 | 0 | 0 |
| NM_001101426 | ISPD | 5524 | 2 | 1 | 0 | 0 | 1 | 2 | 1 | 0 | 0 | 1 | 0 | 0 | 1 |
| NM_001101648 | NPC1L1 | 4966 | 2 | 0 | 0 | 0 | 0 | 0 | 0 | 2 | 0 | 0 | 0 | 0 | 0 |
| NM_001101653 | COMMD9 | 2848 | 4 | 2 | 2 | 1 | 2 | 4 | 3 | 2 | 2 | 2 | 2 | 3 | 2 |
| NM_001101654 | CXXC1 | 2966 | 2 | 1 | 1 | 2 | 1 | 1 | 2 | 1 | 1 | 1 | 1 | 2 | 2 |
| NM_001101662 | NRD1 | 3820 | 33 | 13 | 9 | 4 | 16 | 12 | 8 | 14 | 8 | 7 | 33 | 13 | 13 |
| NM_001101663 | NBPF11 | 3409 | 0 | 0 | 0 | 0 | 0 | 0 | 0 | 0 | 0 | 0 | 0 | 0 | 0 |
| NM_001101667 | ACOX3 | 2806 | 3 | 1 | 0 | 1 | 2 | 2 | 1 | 2 | 1 | 3 | 0 | 1 | 1 |
| NM_001101669 | INPP4B | 4024 | 3 | 2 | 1 | 1 | 3 | 2 | 1 | 0 | 2 | 2 | 3 | 3 | 3 |
| NM_001101672 | ZNF219 | 2946 | 5 | 1 | 5 | 2 | 2 | 1 | 1 | 1 | 1 | 1 | 1 | 1 | 1 |
| NM_001101676 | SAMD12 | 8869 | 5 | 1 | 0 | 1 | 1 | 5 | 2 | 0 | 1 | 0 | 0 | 1 | 1 |
| NM_001101677 | SOHLH1 | 1413 | 2 | 0 | 0 | 0 | 0 | 2 | 0 | 0 | 0 | 0 | 0 | 0 | 1 |
| NM_001101800 | FAM13B | 5470 | 21 | 9 | 6 | 5 | 9 | 10 | 6 | 2 | 9 | 13 | 4 | 18 | 21 |
| NM_001101801 | FAM13B | 5344 | 22 | 10 | 6 | 5 | 9 | 10 | 7 | 2 | 10 | 14 | 4 | 18 | 22 |
| NM_001101802 | PHF21A | 7316 | 5 | 3 | 2 | 2 | 3 | 3 | 2 | 1 | 3 | 3 | 1 | 5 | 4 |
| NM_001102 | ACTN1 | 3725 | 22 | 10 | 13 | 22 | 17 | 8 | 5 | 9 | 11 | 6 | 2 | 14 | 8 |
| NM_001102366 | C14orf159 | 3145 | 5 | 3 | 2 | 3 | 5 | 3 | 5 | 1 | 1 | 2 | 5 | 3 | 3 |
| NM_001102367 | C14orf159 | 2911 | 5 | 3 | 2 | 3 | 5 | 3 | 5 | 1 | 2 | 2 | 5 | 3 | 3 |
| NM_001102368 | C14orf159 | 2744 | 6 | 3 | 2 | 3 | 6 | 3 | 5 | 1 | 2 | 2 | 5 | 3 | 3 |
| NM_001102369 | C14orf159 | 2744 | 6 | 3 | 2 | 3 | 6 | 3 | 5 | 1 | 1 | 2 | 5 | 3 | 3 |
| NM_001102371 | FOXRED2 | 4971 | 2 | 1 | 0 | 0 | 0 | 2 | 1 | 0 | 0 | 1 | 0 | 1 | 1 |
| NM_001102386 | GNAT3 | 1065 | 0 | 0 | 0 | 0 | 0 | 0 | 0 | 0 | 0 | 0 | 0 | 0 | 0 |
| NM_001102396 | SIKE1 | 5503 | 3 | 2 | 2 | 1 | 2 | 3 | 3 | 2 | 2 | 2 | 1 | 3 | 2 |
| NM_001102397 | HNRNPR | 2566 | 70 | 46 | 33 | 22 | 47 | 56 | 42 | 42 | 45 | 57 | 31 | 70 | 58 |
| NM_001102398 | HNRNPR | 2741 | 67 | 44 | 32 | 21 | 45 | 54 | 40 | 40 | 43 | 55 | 30 | 67 | 56 |
| NM_001102399 | HNRNPR | 2575 | 69 | 45 | 33 | 21 | 47 | 56 | 41 | 41 | 45 | 56 | 31 | 69 | 58 |
| NM_001102401 | C8orf41 | 2185 | 2 | 1 | 1 | 1 | 2 | 1 | 1 | 1 | 1 | 1 | 1 | 1 | 2 |
| NM_001102402 | PCTP | 2274 | 20 | 5 | 4 | 4 | 3 | 2 | 5 | 20 | 3 | 2 | 2 | 4 | 2 |
| NM_001102406 | ALPK1 | 5360 | 2 | 1 | 1 | 1 | 1 | 1 | 2 | 0 | 2 | 1 | 1 | 2 | 1 |
| NM_001102416 | KNG1 | 2725 | 156 | 18 | 0 | 0 | 0 | 0 | 39 | 156 | 0 | 1 | 0 | 1 | 0 |
| NM_001102420 | ZFAND5 | 5868 | 21 | 9 | 5 | 2 | 7 | 10 | 7 | 8 | 8 | 14 | 21 | 12 | 10 |
| NM_001102421 | ZFAND5 | 5731 | 16 | 7 | 4 | 1 | 6 | 6 | 5 | 6 | 7 | 11 | 16 | 9 | 7 |
| NM_001102426 | TBC1D8 | 4147 | 11 | 5 | 5 | 2 | 6 | 4 | 4 | 5 | 4 | 2 | 6 | 11 | 7 |
| NM_001102445 | RGS4 | 3480 | 59 | 6 | 0 | 1 | 1 | 59 | 0 | 0 | 0 | 2 | 0 | 0 | 1 |
| NM_001102450 | RGS8 | 912 | 1 | 0 | 0 | 0 | 0 | 1 | 0 | 0 | 0 | 0 | 0 | 0 | 0 |
| NM_001102454 | ZNF219 | 2936 | 5 | 1 | 5 | 2 | 2 | 1 | 1 | 1 | 1 | 1 | 1 | 1 | 1 |
| NM_001102467 | AQP12B | 1114 | 0 | 0 | 0 | 0 | 0 | 0 | 0 | 0 | 0 | 0 | 0 | 0 | 0 |
| NM_001102469 | LIPN | 1197 | 1 | 0 | 0 | 0 | 0 | 0 | 0 | 0 | 1 | 0 | 0 | 0 | 0 |
| NM_001102470 | ADH6 | 2803 | 17 | 2 | 0 | 1 | 0 | 0 | 4 | 17 | 0 | 0 | 0 | 0 | 0 |
| NM_001102559 | PPAPDC1B | 2128 | 4 | 1 | 1 | 1 | 1 | 1 | 2 | 4 | 1 | 1 | 0 | 2 | 1 |
| NM_001102560 | PPAPDC1B | 771 | 2 | 1 | 1 | 1 | 1 | 1 | 1 | 1 | 0 | 2 | 0 | 1 | 1 |
| NM_001102562 | 11 Mar | 1528 | 2 | 0 | 0 | 0 | 0 | 0 | 0 | 0 | 0 | 0 | 0 | 0 | 2 |
| NM_001102564 | C14orf179 | 833 | 2 | 1 | 1 | 1 | 1 | 2 | 2 | 1 | 1 | 1 | 0 | 1 | 2 |
| NM_001102566 | PCP4L1 | 1424 | 6 | 1 | 0 | 1 | 0 | 6 | 0 | 1 | 1 | 1 | 0 | 0 | 1 |
| NM_001102575 | SNX18 | 5219 | 19 | 7 | 3 | 1 | 9 | 3 | 6 | 3 | 7 | 16 | 4 | 19 | 10 |
| NM_001102576 | CSAG1 | 659 | 0 | 0 | 0 | 0 | 0 | 0 | 0 | 0 | 0 | 0 | 0 | 0 | 0 |
| NM_001102592 | C1orf59 | 1698 | 15 | 3 | 2 | 2 | 1 | 7 | 1 | 0 | 1 | 1 | 1 | 3 | 15 |
| NM_001102594 | DTX2 | 2699 | 0 | 0 | 0 | 0 | 0 | 0 | 0 | 0 | 0 | 0 | 0 | 0 | 0 |
| NM_001102595 | DTX2 | 2534 | 0 | 0 | 0 | 0 | 0 | 0 | 0 | 0 | 0 | 0 | 0 | 0 | 0 |
| NM_001102596 | DTX2 | 2122 | 0 | 0 | 0 | 0 | 0 | 0 | 0 | 0 | 0 | 0 | 0 | 0 | 0 |
| NM_001102597 | CEACAM20 | 1810 | 0 | 0 | 0 | 0 | 0 | 0 | 0 | 0 | 0 | 0 | 0 | 0 | 0 |
| NM_001102598 | CEACAM20 | 1495 | 0 | 0 | 0 | 0 | 0 | 0 | 0 | 0 | 0 | 0 | 0 | 0 | 0 |
| NM_001102599 | CEACAM20 | 1531 | 0 | 0 | 0 | 0 | 0 | 0 | 0 | 0 | 0 | 0 | 0 | 0 | 0 |
| NM_001102600 | CEACAM20 | 1774 | 0 | 0 | 0 | 0 | 0 | 0 | 0 | 0 | 0 | 0 | 0 | 0 | 0 |
| NM_001102601 | CCDC163P | 953 | 1 | 0 | 0 | 0 | 0 | 1 | 0 | 0 | 0 | 0 | 0 | 0 | 0 |
| NM_001102603 | ZNF160 | 4336 | 28 | 10 | 7 | 3 | 7 | 8 | 7 | 2 | 6 | 16 | 4 | 28 | 18 |
| NM_001102605 | IMPDH1 | 2581 | 4 | 1 | 2 | 1 | 1 | 2 | 0 | 0 | 1 | 1 | 0 | 4 | 1 |
| NM_001102608 | COL6A6 | 8470 | 5 | 1 | 0 | 0 | 1 | 0 | 0 | 0 | 5 | 0 | 0 | 1 | 0 |
| NM_001102609 | C5orf58 | 510 | 10 | 1 | 0 | 0 | 0 | 0 | 0 | 0 | 0 | 0 | 0 | 0 | 10 |
| NM_001102610 | TUBGCP5 | 3442 | 3 | 2 | 1 | 1 | 1 | 2 | 2 | 2 | 1 | 1 | 1 | 1 | 3 |
| NM_001102612 | LOC145814 | 1139 | 3 | 0 | 0 | 0 | 0 | 0 | 0 | 0 | 0 | 0 | 3 | 0 | 0 |
| NM_001102614 | AMAC1L3 | 1195 | 0 | 0 | 0 | 0 | 0 | 0 | 0 | 0 | 0 | 0 | 0 | 0 | 0 |
| NM_001102651 | ZNF554 | 3698 | 2 | 1 | 1 | 1 | 1 | 2 | 1 | 0 | 0 | 1 | 1 | 1 | 1 |
| NM_001102653 | OTUD4 | 7068 | 21 | 10 | 4 | 4 | 10 | 6 | 8 | 5 | 10 | 9 | 18 | 14 | 21 |
| NM_001102654 | NTF3 | 1335 | 1 | 0 | 0 | 0 | 0 | 0 | 0 | 0 | 0 | 1 | 0 | 1 | 0 |

| transcript | gene | transcript length (nt) | max | mean | adipose | colon | heart | hypothalamus | kidney | liver | lung | ovary | skeletalmuscle | spleen | testes |
|---|---|---|---|---|---|---|---|---|---|---|---|---|---|---|---|
| NM_001102657 | ZNF836 | 3333 | 13 | 4 | 2 | 1 | 2 | 2 | 3 | 1 | 3 | 13 | 1 | 7 | 4 |
| NM_001102658 | CT62 | 1831 | 2 | 0 | 0 | 0 | 0 | 0 | 0 | 0 | 0 | 0 | 0 | 0 | 2 |
| NM_001102659 | LOC200726 | 1376 | 0 | 0 | 0 | 0 | 0 | 0 | 0 | 0 | 0 | 0 | 0 | 0 | 0 |
| NM_001102663 | NBPF16 | 3831 | 1 | 0 | 0 | 0 | 0 | 0 | 0 | 0 | 0 | 0 | 0 | 1 | 1 |
| NM_001102664 | EPN2 | 3776 | 2 | 1 | 0 | 0 | 1 | 2 | 0 | 0 | 1 | 1 | 0 | 1 | 1 |
| NM_001102667 | PSMA4 | 1145 | 42 | 20 | 21 | 9 | 23 | 22 | 21 | 42 | 16 | 10 | 14 | 26 | 21 |
| NM_001102668 | PSMA4 | 1058 | 24 | 15 | 14 | 4 | 19 | 18 | 16 | 24 | 12 | 9 | 11 | 20 | 16 |
| NM_001102669 | RRAS2 | 2153 | 7 | 5 | 6 | 2 | 7 | 2 | 6 | 3 | 4 | 5 | 5 | 6 | 6 |
| NM_001103 | ACTN2 | 4528 | 508 | 80 | 1 | 0 | 364 | 7 | 0 | 0 | 1 | 0 | 508 | 0 | 3 |
| NM_001103146 | GIGYF2 | 7814 | 19 | 12 | 9 | 7 | 12 | 12 | 13 | 7 | 9 | 13 | 13 | 15 | 19 |
| NM_001103147 | GIGYF2 | 7947 | 19 | 12 | 9 | 7 | 12 | 12 | 13 | 7 | 9 | 13 | 13 | 15 | 19 |
| NM_001103148 | GIGYF2 | 7730 | 19 | 12 | 10 | 7 | 12 | 12 | 13 | 7 | 9 | 13 | 13 | 15 | 19 |
| NM_001103149 | PNMAL1 | 3491 | 28 | 4 | 0 | 3 | 1 | 28 | 2 | 0 | 0 | 2 | 0 | 0 | 7 |
| NM_001103150 | PNMA5 | 3214 | 4 | 0 | 0 | 0 | 0 | 4 | 0 | 0 | 0 | 0 | 0 | 0 | 1 |
| NM_001103151 | PNMA5 | 3114 | 4 | 0 | 0 | 0 | 0 | 4 | 0 | 0 | 0 | 0 | 0 | 0 | 1 |
| NM_001103154 | ARL17B | 488 | 0 | 0 | 0 | 0 | 0 | 0 | 0 | 0 | 0 | 0 | 0 | 0 | 0 |
| NM_001103160 | SH2D5 | 3705 | 2 | 0 | 0 | 0 | 0 | 2 | 0 | 0 | 0 | 0 | 0 | 0 | 0 |
| NM_001103161 | SH2D5 | 3834 | 2 | 0 | 0 | 0 | 0 | 2 | 0 | 0 | 0 | 0 | 0 | 0 | 0 |
| NM_001103167 | ZGLP1 | 1980 | 0 | 0 | 0 | 0 | 0 | 0 | 0 | 0 | 0 | 0 | 0 | 0 | 0 |
| NM_001103169 | AADACL3 | 3832 | 0 | 0 | 0 | 0 | 0 | 0 | 0 | 0 | 0 | 0 | 0 | 0 | 0 |
| NM_001103170 | AADACL3 | 4049 | 0 | 0 | 0 | 0 | 0 | 0 | 0 | 0 | 0 | 0 | 0 | 0 | 0 |
| NM_001103175 | CCDC64B | 1811 | 1 | 0 | 0 | 1 | 0 | 0 | 0 | 0 | 0 | 0 | 0 | 0 | 0 |
| NM_001103176 | CNPY1 | 2234 | 0 | 0 | 0 | 0 | 0 | 0 | 0 | 0 | 0 | 0 | 0 | 0 | 0 |
| NM_001103184 | FMN1 | 3757 | 5 | 2 | 2 | 4 | 2 | 3 | 3 | 1 | 4 | 0 | 1 | 2 | 5 |
| NM_001104 | ACTN3 | 2857 | 6 | 1 | 0 | 0 | 0 | 0 | 0 | 0 | 0 | 0 | 6 | 0 | 0 |
| NM_001104544 | FAM70A | 3425 | 4 | 1 | 0 | 0 | 0 | 3 | 0 | 0 | 0 | 4 | 0 | 0 | 0 |
| NM_001104545 | FAM70A | 3173 | 4 | 1 | 0 | 0 | 0 | 3 | 0 | 0 | 0 | 4 | 0 | 0 | 0 |
| NM_001104546 | RPP30 | 4272 | 2 | 1 | 1 | 0 | 1 | 2 | 1 | 1 | 0 | 2 | 1 | 2 | 2 |
| NM_001104548 | LOC642587 | 890 | 0 | 0 | 0 | 0 | 0 | 0 | 0 | 0 | 0 | 0 | 0 | 0 | 0 |
| NM_001104554 | PAQR5 | 5476 | 5 | 1 | 0 | 1 | 0 | 0 | 5 | 0 | 0 | 0 | 0 | 0 | 0 |
| NM_001104558 | ELOVL7 | 3830 | 4 | 1 | 1 | 0 | 1 | 3 | 4 | 0 | 1 | 0 | 0 | 1 | 0 |
| NM_001104577 | GPR172B | 2415 | 0 | 0 | 0 | 0 | 0 | 0 | 0 | 0 | 0 | 0 | 0 | 0 | 0 |
| NM_001104586 | ALS2CR8 | 5171 | 2 | 1 | 1 | 0 | 0 | 1 | 1 | 0 | 1 | 1 | 1 | 2 | 2 |
| NM_001104587 | SLFN11 | 5277 | 2 | 1 | 2 | 1 | 2 | 1 | 0 | 0 | 2 | 2 | 0 | 2 | 1 |
| NM_001104588 | SLFN11 | 5195 | 2 | 1 | 2 | 1 | 2 | 1 | 0 | 0 | 2 | 2 | 0 | 2 | 1 |
| NM_001104589 | SLFN11 | 5117 | 2 | 1 | 2 | 1 | 2 | 1 | 0 | 0 | 2 | 2 | 0 | 2 | 1 |
| NM_001104590 | SLFN11 | 5107 | 3 | 1 | 2 | 1 | 2 | 1 | 0 | 0 | 3 | 2 | 0 | 2 | 1 |
| NM_001104595 | FAM118A | 3240 | 8 | 3 | 2 | 4 | 3 | 3 | 2 | 1 | 3 | 3 | 4 | 6 | 8 |
| NM_001104629 | C4orf19 | 3638 | 1 | 0 | 0 | 0 | 0 | 0 | 1 | 1 | 0 | 0 | 0 | 0 | 0 |
| NM_001104631 | PDE4D | 8227 | 20 | 4 | 1 | 2 | 7 | 4 | 5 | 0 | 4 | 0 | 20 | 2 | 1 |
| NM_001104647 | SLC25A36 | 4661 | 5 | 3 | 2 | 1 | 4 | 2 | 4 | 0 | 3 | 4 | 3 | 3 | 5 |
| NM_001104925 | PCMTD2 | 3831 | 12 | 6 | 3 | 3 | 7 | 6 | 7 | 3 | 6 | 12 | 6 | 9 | 8 |
| NM_001105 | ACVR1 | 3044 | 8 | 4 | 3 | 2 | 5 | 4 | 8 | 5 | 4 | 6 | 3 | 4 | 4 |
| NM_001105069 | ACSM2B | 2406 | 15 | 2 | 0 | 0 | 0 | 0 | 15 | 6 | 0 | 0 | 0 | 0 | 0 |
| NM_001105077 | MECOM | 4900 | 14 | 3 | 2 | 3 | 4 | 0 | 14 | 0 | 6 | 1 | 1 | 3 | 2 |
| NM_001105078 | MECOM | 4891 | 14 | 3 | 2 | 3 | 4 | 0 | 14 | 0 | 6 | 1 | 1 | 3 | 2 |
| NM_001105079 | FBRS | 2811 | 2 | 1 | 0 | 1 | 1 | 1 | 1 | 0 | 1 | 1 | 1 | 2 | 1 |
| NM_001105192 | TLE3 | 5322 | 5 | 2 | 3 | 3 | 1 | 2 | 2 | 1 | 3 | 2 | 1 | 5 | 2 |
| NM_001105193 | FAM123C | 6236 | 1 | 0 | 0 | 0 | 0 | 1 | 0 | 0 | 0 | 0 | 0 | 0 | 0 |
| NM_001105194 | FAM123C | 6172 | 1 | 0 | 0 | 0 | 0 | 1 | 0 | 0 | 0 | 0 | 0 | 0 | 0 |
| NM_001105195 | FAM123C | 6169 | 1 | 0 | 0 | 0 | 0 | 1 | 0 | 0 | 0 | 0 | 0 | 0 | 0 |
| NM_001105198 | TMEM177 | 1738 | 1 | 0 | 0 | 0 | 0 | 0 | 1 | 1 | 0 | 0 | 0 | 0 | 1 |
| NM_001105199 | TMEM177 | 1346 | 1 | 0 | 0 | 0 | 0 | 1 | 1 | 1 | 0 | 0 | 0 | 0 | 1 |
| NM_001105203 | RUSC1 | 3485 | 5 | 1 | 1 | 0 | 0 | 5 | 1 | 0 | 0 | 1 | 0 | 0 | 0 |
| NM_001105204 | RUSC1 | 3167 | 5 | 1 | 1 | 0 | 0 | 5 | 1 | 0 | 0 | 1 | 0 | 0 | 0 |
| NM_001105205 | RUSC1 | 2507 | 1 | 0 | 0 | 0 | 0 | 1 | 0 | 0 | 0 | 0 | 0 | 0 | 0 |
| NM_001105206 | LAMA4 | 7287 | 22 | 4 | 22 | 4 | 4 | 2 | 1 | 0 | 4 | 5 | 1 | 0 | 3 |
| NM_001105207 | LAMA4 | 7283 | 22 | 4 | 22 | 4 | 4 | 2 | 1 | 0 | 4 | 5 | 1 | 0 | 3 |
| NM_001105208 | LAMA4 | 676 | 15 | 3 | 15 | 5 | 2 | 2 | 0 | 0 | 3 | 3 | 0 | 0 | 2 |
| NM_001105209 | LAMA4 | 693 | 14 | 3 | 14 | 5 | 2 | 2 | 0 | 0 | 3 | 3 | 0 | 0 | 2 |
| NM_001105214 | ASH2L | 2647 | 9 | 4 | 3 | 2 | 4 | 7 | 4 | 3 | 3 | 5 | 4 | 5 | 9 |
| NM_001105243 | PCDH19 | 9615 | 6 | 1 | 1 | 1 | 0 | 6 | 0 | 0 | 0 | 1 | 0 | 0 | 0 |
| NM_001105244 | PTPRM | 5600 | 14 | 7 | 14 | 3 | 13 | 8 | 8 | 2 | 9 | 6 | 7 | 6 | 6 |
| NM_001105247 | ARMC5 | 3615 | 1 | 0 | 0 | 0 | 0 | 1 | 0 | 0 | 0 | 0 | 0 | 0 | 0 |
| NM_001105248 | TMC5 | 4916 | 9 | 1 | 0 | 4 | 0 | 0 | 0 | 0 | 9 | 0 | 0 | 0 | 2 |
| NM_001105249 | TMC5 | 4742 | 10 | 1 | 0 | 4 | 0 | 0 | 0 | 0 | 10 | 0 | 0 | 0 | 2 |
| NM_001105250 | NRXN3 | 4814 | 10 | 1 | 0 | 1 | 1 | 10 | 1 | 0 | 1 | 0 | 0 | 0 | 1 |
| NM_001105251 | ZFYVE16 | 7224 | 11 | 7 | 5 | 2 | 8 | 7 | 7 | 5 | 7 | 11 | 4 | 9 | 11 |
| NM_001105281 | FABP12 | 423 | 0 | 0 | 0 | 0 | 0 | 0 | 0 | 0 | 0 | 0 | 0 | 0 | 0 |
| NM_001105513 | TTC21A | 4258 | 5 | 1 | 0 | 0 | 0 | 0 | 0 | 0 | 1 | 0 | 0 | 0 | 5 |
| NM_001105515 | ABCC4 | 3063 | 6 | 2 | 0 | 2 | 1 | 2 | 6 | 0 | 2 | 2 | 1 | 2 | 2 |
| NM_001105516 | ACOXL | 2714 | 1 | 0 | 0 | 0 | 0 | 0 | 0 | 0 | 1 | 0 | 0 | 0 | 0 |
| NM_001105517 | FAM58B | 988 | 0 | 0 | 0 | 0 | 0 | 0 | 0 | 0 | 0 | 0 | 0 | 0 | 0 |
| NM_001105518 | C1orf26 | 3795 | 15 | 4 | 2 | 1 | 3 | 3 | 3 | 1 | 4 | 9 | 3 | 4 | 15 |
| NM_001105519 | C2orf70 | 695 | 1 | 0 | 0 | 0 | 0 | 0 | 0 | 0 | 0 | 0 | 0 | 0 | 1 |
| NM_001105520 | C17orf100 | 1559 | 0 | 0 | 0 | 0 | 0 | 0 | 0 | 0 | 0 | 0 | 0 | 0 | 0 |
| NM_001105521 | JAKMIP3 | 6487 | 2 | 0 | 0 | 0 | 0 | 2 | 0 | 0 | 0 | 1 | 0 | 0 | 0 |

| transcript | gene | transcript length (nt) | max | mean | adipose | colon | heart | hypothalamus | kidney | liver | lung | ovary | skeletalmuscle | spleen | testes |
|---|---|---|---|---|---|---|---|---|---|---|---|---|---|---|---|
| NM_001105522 | LOC729384 | 1649 | 0 | 0 | 0 | 0 | 0 | 0 | 0 | 0 | 0 | 0 | 0 | 0 | 0 |
| NM_001105528 | C18orf34 | 3355 | 4 | 1 | 0 | 0 | 0 | 0 | 1 | 0 | 0 | 2 | 0 | 1 | 4 |
| NM_001105529 | ATP8A1 | 8223 | 8 | 2 | 0 | 1 | 1 | 8 | 1 | 0 | 3 | 3 | 3 | 3 | 1 |
| NM_001105530 | CAP1 | 2755 | 49 | 23 | 31 | 20 | 18 | 27 | 21 | 23 | 30 | 9 | 5 | 49 | 20 |
| NM_001105531 | FAM135A | 5729 | 11 | 6 | 3 | 3 | 5 | 9 | 7 | 2 | 5 | 8 | 3 | 6 | 11 |
| NM_001105533 | C7orf58 | 2828 | 8 | 3 | 8 | 2 | 2 | 0 | 1 | 2 | 3 | 8 | 3 | 4 | 2 |
| NM_001105537 | ZNF142 | 5922 | 1 | 1 | 0 | 1 | 1 | 1 | 1 | 0 | 0 | 1 | 1 | 1 | 1 |
| NM_001105538 | MYBBP1A | 4105 | 1 | 1 | 1 | 1 | 1 | 1 | 1 | 1 | 1 | 0 | 0 | 1 | 1 |
| NM_001105539 | ZBTB10 | 6061 | 10 | 5 | 3 | 2 | 5 | 4 | 5 | 3 | 6 | 10 | 5 | 6 | 7 |
| NM_001105540 | DGKZ | 4086 | 2 | 0 | 0 | 0 | 0 | 2 | 0 | 0 | 0 | 0 | 0 | 0 | 0 |
| NM_001105541 | NEFM | 2514 | 391 | 37 | 0 | 5 | 1 | 391 | 2 | 0 | 0 | 2 | 1 | 0 | 10 |
| NM_001105543 | PLXNA4 | 3408 | 2 | 0 | 2 | 0 | 1 | 1 | 0 | 0 | 0 | 1 | 0 | 1 | 0 |
| NM_001105544 | ECHDC1 | 2117 | 26 | 11 | 26 | 4 | 9 | 13 | 17 | 16 | 13 | 5 | 5 | 10 | 7 |
| NM_001105545 | ECHDC1 | 2064 | 27 | 12 | 27 | 4 | 9 | 14 | 17 | 17 | 13 | 5 | 5 | 10 | 7 |
| NM_001105549 | ZNF83 | 2942 | 17 | 9 | 9 | 2 | 8 | 6 | 15 | 1 | 11 | 17 | 2 | 17 | 12 |
| NM_001105550 | ZNF83 | 2826 | 18 | 9 | 9 | 2 | 8 | 6 | 15 | 1 | 12 | 18 | 2 | 17 | 13 |
| NM_001105551 | ZNF83 | 2852 | 18 | 9 | 9 | 2 | 8 | 6 | 15 | 1 | 12 | 18 | 2 | 17 | 13 |
| NM_001105552 | ZNF83 | 2728 | 18 | 10 | 10 | 2 | 9 | 6 | 16 | 1 | 12 | 18 | 2 | 18 | 13 |
| NM_001105553 | ZNF83 | 2880 | 16 | 9 | 8 | 2 | 8 | 6 | 14 | 1 | 11 | 16 | 2 | 16 | 11 |
| NM_001105554 | ZNF83 | 2517 | 19 | 10 | 10 | 2 | 9 | 7 | 16 | 1 | 12 | 19 | 2 | 18 | 13 |
| NM_001105556 | C1orf38 | 2720 | 21 | 4 | 4 | 1 | 2 | 2 | 0 | 1 | 6 | 1 | 0 | 21 | 3 |
| NM_001105558 | WEE2 | 3061 | 0 | 0 | 0 | 0 | 0 | 0 | 0 | 0 | 0 | 0 | 0 | 0 | 0 |
| NM_001105562 | UBE4B | 5904 | 8 | 5 | 4 | 4 | 8 | 7 | 4 | 4 | 3 | 7 | 7 | 5 | 4 |
| NM_001105563 | CCHCR1 | 2850 | 2 | 1 | 1 | 1 | 0 | 0 | 1 | 0 | 0 | 1 | 0 | 0 | 2 |
| NM_001105564 | CCHCR1 | 2958 | 2 | 1 | 1 | 1 | 0 | 0 | 1 | 0 | 0 | 0 | 0 | 0 | 2 |
| NM_001105565 | SMTNL1 | 2062 | 107 | 10 | 0 | 0 | 0 | 0 | 0 | 0 | 0 | 0 | 107 | 1 | 0 |
| NM_001105566 | KIF13A | 5835 | 16 | 8 | 10 | 6 | 13 | 6 | 5 | 4 | 7 | 9 | 16 | 4 | 5 |
| NM_001105567 | KIF13A | 5796 | 15 | 8 | 10 | 6 | 13 | 6 | 5 | 4 | 7 | 9 | 15 | 4 | 5 |
| NM_001105568 | KIF13A | 5858 | 15 | 8 | 10 | 6 | 13 | 6 | 5 | 4 | 7 | 9 | 15 | 4 | 5 |
| NM_001105569 | MSGN1 | 582 | 0 | 0 | 0 | 0 | 0 | 0 | 0 | 0 | 0 | 0 | 0 | 0 | 0 |
| NM_001105570 | NUDT19 | 2967 | 0 | 0 | 0 | 0 | 0 | 0 | 0 | 0 | 0 | 0 | 0 | 0 | 0 |
| NM_001105571 | DHRS7C | 986 | 2 | 0 | 0 | 0 | 2 | 0 | 0 | 0 | 0 | 0 | 2 | 0 | 0 |
| NM_001105572 | PLA2G2C | 453 | 0 | 0 | 0 | 0 | 0 | 0 | 0 | 0 | 0 | 0 | 0 | 0 | 0 |
| NM_001105573 | FBXO45 | 5733 | 3 | 1 | 1 | 1 | 1 | 3 | 1 | 1 | 1 | 1 | 1 | 1 | 1 |
| NM_001105574 | HMX3 | 1074 | 0 | 0 | 0 | 0 | 0 | 0 | 0 | 0 | 0 | 0 | 0 | 0 | 0 |
| NM_001105575 | TRIM75 | 1407 | 0 | 0 | 0 | 0 | 0 | 0 | 0 | 0 | 0 | 0 | 0 | 0 | 0 |
| NM_001105576 | ANKRD58 | 1589 | 0 | 0 | 0 | 0 | 0 | 0 | 0 | 0 | 0 | 0 | 0 | 0 | 0 |
| NM_001105577 | PRHOXNB | 522 | 2 | 0 | 2 | 0 | 0 | 0 | 0 | 0 | 0 | 0 | 0 | 0 | 0 |
| NM_001105578 | SYCE2 | 933 | 7 | 1 | 0 | 0 | 0 | 0 | 0 | 0 | 0 | 0 | 0 | 0 | 7 |
| NM_001105579 | TMEM90A | 2607 | 0 | 0 | 0 | 0 | 0 | 0 | 0 | 0 | 0 | 0 | 0 | 0 | 0 |
| NM_001105580 | GABRR3 | 1404 | 0 | 0 | 0 | 0 | 0 | 0 | 0 | 0 | 0 | 0 | 0 | 0 | 0 |
| NM_001105581 | LRRC30 | 906 | 1 | 0 | 0 | 0 | 0 | 0 | 0 | 0 | 0 | 0 | 1 | 0 | 0 |
| NM_001105659 | LRRIQ3 | 2862 | 1 | 0 | 0 | 0 | 0 | 0 | 0 | 0 | 0 | 0 | 0 | 0 | 1 |
| NM_001105662 | USP17 | 1593 | 0 | 0 | 0 | 0 | 0 | 0 | 0 | 0 | 0 | 0 | 0 | 0 | 0 |
| NM_001105663 | NUDT7 | 1093 | 3 | 1 | 1 | 1 | 3 | 1 | 1 | 2 | 1 | 1 | 0 | 1 | 1 |
| NM_001105669 | TTC24 | 2005 | 0 | 0 | 0 | 0 | 0 | 0 | 0 | 0 | 0 | 0 | 0 | 0 | 0 |
| NM_001105677 | UGT2A2 | 1587 | 0 | 0 | 0 | 0 | 0 | 0 | 0 | 0 | 0 | 0 | 0 | 0 | 0 |
| NM_001106 | ACVR2B | 11373 | 3 | 1 | 1 | 1 | 1 | 2 | 1 | 1 | 1 | 3 | 2 | 1 | 2 |
| NM_001107 | ACYP1 | 607 | 2 | 1 | 1 | 1 | 0 | 2 | 1 | 0 | 1 | 0 | 1 | 0 | 0 |
| NM_001109 | ADAM8 | 3299 | 0 | 0 | 0 | 0 | 0 | 0 | 0 | 0 | 0 | 0 | 0 | 0 | 0 |
| NM_001109619 | HIGD1C | 294 | 1 | 0 | 0 | 0 | 0 | 0 | 0 | 0 | 0 | 1 | 1 | 0 | 1 |
| NM_001109659 | C16orf92 | 815 | 0 | 0 | 0 | 0 | 0 | 0 | 0 | 0 | 0 | 0 | 0 | 0 | 0 |
| NM_001109660 | C16orf92 | 895 | 0 | 0 | 0 | 0 | 0 | 0 | 0 | 0 | 0 | 0 | 0 | 0 | 0 |
| NM_001109662 | C12orf51 | 14089 | 7 | 2 | 1 | 2 | 1 | 7 | 2 | 1 | 2 | 2 | 1 | 2 | 2 |
| NM_001109689 | ZNF250 | 6349 | 2 | 1 | 0 | 1 | 1 | 1 | 1 | 0 | 1 | 2 | 1 | 1 | 1 |
| NM_001109754 | PTPRB | 11329 | 17 | 5 | 4 | 2 | 7 | 2 | 6 | 6 | 10 | 3 | 2 | 17 | 2 |
| NM_001109760 | C17orf70 | 3629 | 0 | 0 | 0 | 0 | 0 | 0 | 0 | 0 | 0 | 0 | 0 | 0 | 0 |
| NM_001109763 | GSG1L | 4922 | 2 | 0 | 0 | 0 | 0 | 2 | 0 | 0 | 0 | 0 | 0 | 0 | 0 |
| NM_001109809 | ZFP57 | 1868 | 0 | 0 | 0 | 0 | 0 | 0 | 0 | 0 | 0 | 0 | 0 | 0 | 0 |
| NM_001109809 | ZFP57 | 1870 | 0 | 0 | 0 | 0 | 0 | 0 | 0 | 0 | 0 | 0 | 0 | 0 | 0 |
| NM_001109878 | TBX22 | 2355 | 3 | 0 | 0 | 0 | 0 | 0 | 0 | 0 | 0 | 0 | 0 | 0 | 3 |
| NM_001109879 | TBX22 | 2351 | 3 | 0 | 0 | 0 | 0 | 0 | 0 | 0 | 0 | 0 | 0 | 0 | 3 |
| NM_001109891 | MAPK3 | 1744 | 5 | 2 | 2 | 5 | 2 | 5 | 1 | 0 | 1 | 1 | 1 | 1 | 1 |
| NM_001109903 | RNFT2 | 2898 | 2 | 0 | 0 | 0 | 0 | 2 | 0 | 0 | 0 | 0 | 0 | 0 | 0 |
| NM_001109938 | C6orf136 | 1435 | 3 | 2 | 1 | 2 | 3 | 2 | 3 | 1 | 1 | 1 | 2 | 1 | 1 |
| NM_001109974 | SYNPO | 4608 | 61 | 12 | 9 | 3 | 28 | 18 | 2 | 2 | 3 | 2 | 61 | 4 | 3 |
| NM_001109977 | FAM160A1 | 4576 | 7 | 1 | 0 | 1 | 2 | 0 | 2 | 0 | 1 | 1 | 7 | 0 | 1 |
| NM_001109997 | KLHL33 | 1863 | 2 | 0 | 0 | 0 | 0 | 0 | 0 | 0 | 0 | 0 | 2 | 0 | 0 |
| NM_001110 | ADAM10 | 3927 | 27 | 11 | 8 | 4 | 12 | 11 | 12 | 9 | 13 | 8 | 5 | 27 | 12 |
| NM_001110199 | SRRM3 | 3615 | 0 | 0 | 0 | 0 | 0 | 0 | 0 | 0 | 0 | 0 | 0 | 0 | 0 |
| NM_001110213 | PRH2 | 3177 | 1 | 0 | 0 | 0 | 0 | 0 | 1 | 0 | 1 | 1 | 0 | 1 | 0 |
| NM_001110215 | CENPM | 541 | 0 | 0 | 0 | 0 | 0 | 0 | 0 | 0 | 0 | 0 | 0 | 0 | 0 |
| NM_001110219 | GJB6 | 2140 | 13 | 1 | 0 | 0 | 0 | 13 | 0 | 0 | 0 | 0 | 0 | 0 | 0 |
| NM_001110220 | GJB6 | 2030 | 14 | 1 | 0 | 0 | 0 | 14 | 0 | 0 | 0 | 0 | 0 | 0 | 0 |
| NM_001110221 | GJB6 | 1906 | 15 | 1 | 0 | 0 | 0 | 15 | 0 | 0 | 0 | 0 | 0 | 0 | 0 |
| NM_001110303 | USP20 | 4334 | 1 | 0 | 0 | 0 | 0 | 1 | 0 | 0 | 0 | 0 | 0 | 0 | 0 |
| NM_001110354 | ZP3 | 1300 | 0 | 0 | 0 | 0 | 0 | 0 | 0 | 0 | 0 | 0 | 0 | 0 | 0 |

| | | transcript | | | | | | hypothala | | | | | skeletalm | | |
|---|---|---|---|---|---|---|---|---|---|---|---|---|---|---|---|
| transcript | gene | length (nt) | max | mean | adipose | colon | heart | mus | kidney | liver | lung | ovary | uscle | spleen | testes |
| NM_001110356 | RNASE9 | 1101 | 0 | 0 | 0 | 0 | 0 | 0 | 0 | 0 | 0 | 0 | 0 | 0 | 0 |
| NM_001110357 | RNASE9 | 1084 | 0 | 0 | 0 | 0 | 0 | 0 | 0 | 0 | 0 | 0 | 0 | 0 | 0 |
| NM_001110358 | RNASE9 | 1137 | 0 | 0 | 0 | 0 | 0 | 0 | 0 | 0 | 0 | 0 | 0 | 0 | 0 |
| NM_001110359 | RNASE9 | 1247 | 0 | 0 | 0 | 0 | 0 | 0 | 0 | 0 | 0 | 0 | 0 | 0 | 0 |
| NM_001110360 | RNASE9 | 1212 | 0 | 0 | 0 | 0 | 0 | 0 | 0 | 0 | 0 | 0 | 0 | 0 | 0 |
| NM_001110361 | RNASE9 | 1172 | 0 | 0 | 0 | 0 | 0 | 0 | 0 | 0 | 0 | 0 | 0 | 0 | 0 |
| NM_001110503 | TMEM87A | 1716 | 3 | 2 | 2 | 1 | 1 | 3 | 1 | 2 | 2 | 2 | 1 | 1 | 3 |
| NM_001110514 | EBF4 | 2911 | 0 | 0 | 0 | 0 | 0 | 0 | 0 | 0 | 0 | 0 | 0 | 0 | 0 |
| NM_001110533 | C1orf177 | 1702 | 0 | 0 | 0 | 0 | 0 | 0 | 0 | 0 | 0 | 0 | 0 | 0 | 0 |
| NM_001110556 | FLNA | 8510 | 28 | 8 | 11 | 28 | 7 | 3 | 3 | 1 | 7 | 8 | 2 | 10 | 7 |
| NM_001110781 | LOC728661 | 6003 | 1 | 1 | 0 | 1 | 0 | 1 | 1 | 0 | 0 | 1 | 0 | 1 | 0 |
| NM_001110792 | MECP2 | 1695 | 3 | 2 | 1 | 1 | 2 | 3 | 1 | 1 | 2 | 2 | 1 | 3 | 2 |
| NM_001110798 | NARG1L | 1826 | 3 | 2 | 1 | 0 | 1 | 2 | 1 | 1 | 1 | 2 | 2 | 2 | 3 |
| NM_001110822 | TDRD12 | 1617 | 1 | 0 | 0 | 0 | 0 | 0 | 0 | 0 | 0 | 0 | 0 | 0 | 1 |
| NM_001111 | ADAR | 6629 | 24 | 13 | 9 | 7 | 10 | 21 | 13 | 15 | 12 | 14 | 5 | 24 | 15 |
| NM_001111018 | NAV2 | 10670 | 2 | 1 | 1 | 2 | 2 | 2 | 2 | 2 | 2 | 1 | 0 | 0 | 1 |
| NM_001111019 | NAV2 | 7842 | 2 | 1 | 1 | 2 | 2 | 2 | 2 | 2 | 1 | 1 | 0 | 0 | 1 |
| NM_001111020 | SUPT5H | 3710 | 35 | 18 | 14 | 15 | 14 | 24 | 13 | 15 | 12 | 17 | 16 | 20 | 35 |
| NM_001111031 | ACVR1C | 8761 | 6 | 1 | 6 | 0 | 0 | 1 | 0 | 0 | 0 | 0 | 0 | 0 | 0 |
| NM_001111032 | ACVR1C | 8636 | 6 | 1 | 6 | 0 | 0 | 1 | 0 | 0 | 0 | 0 | 0 | 0 | 0 |
| NM_001111033 | ACVR1C | 8405 | 6 | 1 | 6 | 0 | 0 | 1 | 0 | 0 | 0 | 0 | 0 | 0 | 0 |
| NM_001111034 | ACP5 | 1608 | 10 | 2 | 5 | 1 | 0 | 0 | 5 | 1 | 10 | 2 | 0 | 3 | 0 |
| NM_001111035 | ACP5 | 1631 | 10 | 2 | 5 | 1 | 0 | 0 | 5 | 1 | 10 | 2 | 0 | 3 | 0 |
| NM_001111036 | ACP5 | 1558 | 10 | 3 | 5 | 1 | 0 | 0 | 5 | 1 | 10 | 2 | 0 | 3 | 0 |
| NM_001111038 | FBXL13 | 2804 | 4 | 1 | 0 | 0 | 0 | 1 | 1 | 0 | 1 | 2 | 0 | 1 | 4 |
| NM_001111039 | ZNF187 | 2813 | 9 | 4 | 3 | 2 | 3 | 5 | 3 | 1 | 2 | 9 | 3 | 5 | 5 |
| NM_001111045 | CCNA1 | 1962 | 5 | 1 | 0 | 0 | 0 | 2 | 0 | 0 | 0 | 0 | 0 | 0 | 5 |
| NM_001111046 | CCNA1 | 1844 | 6 | 1 | 0 | 0 | 0 | 2 | 0 | 0 | 0 | 0 | 0 | 0 | 6 |
| NM_001111047 | CCNA1 | 1841 | 6 | 1 | 0 | 0 | 0 | 2 | 0 | 0 | 0 | 0 | 0 | 0 | 6 |
| NM_001111061 | NHLH2 | 2500 | 0 | 0 | 0 | 0 | 0 | 0 | 0 | 0 | 0 | 0 | 0 | 0 | 0 |
| NM_001111067 | ACVR1 | 2863 | 8 | 5 | 3 | 3 | 6 | 4 | 8 | 5 | 4 | 6 | 3 | 4 | 4 |
| NM_001111077 | EZR | 3115 | 127 | 65 | 17 | 65 | 85 | 53 | 127 | 25 | 121 | 29 | 15 | 90 | 87 |
| NM_001111097 | LYN | 3029 | 59 | 11 | 7 | 3 | 7 | 6 | 3 | 7 | 18 | 3 | 1 | 59 | 5 |
| NM_001111101 | CNRIP1 | 1295 | 1 | 0 | 1 | 0 | 0 | 1 | 0 | 0 | 0 | 1 | 0 | 0 | 0 |
| NM_001111112 | UBE2K | 5114 | 17 | 10 | 8 | 6 | 10 | 17 | 11 | 17 | 9 | 7 | 8 | 11 | 9 |
| NM_001111113 | UBE2K | 5090 | 15 | 9 | 7 | 6 | 9 | 15 | 10 | 15 | 8 | 7 | 7 | 10 | 9 |
| NM_001111125 | IQSEC2 | 6004 | 3 | 0 | 0 | 0 | 0 | 3 | 0 | 0 | 0 | 0 | 0 | 0 | 0 |
| NM_001111270 | GEFT | 2246 | 6 | 2 | 1 | 4 | 1 | 6 | 0 | 0 | 1 | 2 | 0 | 2 | 1 |
| NM_001111283 | IGF1 | 7370 | 8 | 3 | 8 | 1 | 6 | 0 | 1 | 3 | 1 | 4 | 1 | 2 | 6 |
| NM_001111284 | IGF1 | 7204 | 8 | 3 | 8 | 1 | 6 | 0 | 1 | 3 | 1 | 4 | 1 | 2 | 6 |
| NM_001111285 | IGF1 | 949 | 15 | 5 | 15 | 2 | 10 | 0 | 1 | 9 | 2 | 5 | 1 | 3 | 10 |
| NM_001111298 | PPIL6 | 3505 | 2 | 1 | 0 | 0 | 0 | 1 | 1 | 0 | 1 | 1 | 0 | 0 | 2 |
| NM_001111307 | PDE4A | 4779 | 3 | 1 | 1 | 1 | 1 | 3 | 1 | 0 | 1 | 0 | 1 | 1 | 1 |
| NM_001111308 | PDE4A | 4593 | 3 | 1 | 1 | 1 | 1 | 3 | 1 | 0 | 1 | 0 | 1 | 1 | 1 |
| NM_001111309 | PDE4A | 4488 | 3 | 1 | 1 | 1 | 1 | 3 | 1 | 0 | 1 | 0 | 1 | 1 | 1 |
| NM_001111319 | CLDN22 | 2708 | 0 | 0 | 0 | 0 | 0 | 0 | 0 | 0 | 0 | 0 | 0 | 0 | 0 |
| NM_001111322 | DDX54 | 4381 | 3 | 2 | 1 | 1 | 2 | 1 | 2 | 3 | 1 | 1 | 3 | 2 | 1 |
| NM_001112 | ADARB1 | 6907 | 3 | 1 | 1 | 1 | 1 | 2 | 1 | 0 | 3 | 1 | 1 | 2 | 1 |
| NM_001112704 | VAX1 | 1968 | 0 | 0 | 0 | 0 | 0 | 0 | 0 | 0 | 0 | 0 | 0 | 0 | 0 |
| NM_001112706 | SCIN | 3246 | 15 | 3 | 2 | 3 | 0 | 7 | 15 | 0 | 1 | 0 | 0 | 0 | 0 |
| NM_001112707 | TLK2 | 5343 | 16 | 10 | 8 | 8 | 8 | 8 | 7 | 4 | 9 | 14 | 11 | 13 | 16 |
| NM_001112717 | LIMCH1 | 6087 | 77 | 29 | 15 | 3 | 59 | 30 | 13 | 4 | 51 | 14 | 49 | 77 | 6 |
| NM_001112718 | LIMCH1 | 6084 | 77 | 29 | 15 | 3 | 59 | 30 | 13 | 4 | 51 | 14 | 49 | 77 | 6 |
| NM_001112719 | LIMCH1 | 5741 | 78 | 29 | 15 | 3 | 59 | 29 | 13 | 4 | 52 | 14 | 49 | 78 | 6 |
| NM_001112720 | LIMCH1 | 5705 | 78 | 29 | 15 | 3 | 59 | 29 | 13 | 4 | 52 | 14 | 50 | 78 | 6 |
| NM_001112724 | STK32A | 1693 | 1 | 0 | 0 | 0 | 0 | 1 | 1 | 0 | 0 | 0 | 0 | 0 | 0 |
| NM_001112726 | KIAA0284 | 6681 | 2 | 1 | 0 | 1 | 0 | 2 | 0 | 0 | 0 | 0 | 0 | 0 | 1 |
| NM_001112732 | MCF2L | 5260 | 2 | 1 | 1 | 1 | 1 | 2 | 1 | 0 | 1 | 0 | 0 | 0 | 0 |
| NM_001112734 | ZNF397OS | 4128 | 3 | 2 | 1 | 1 | 2 | 2 | 3 | 0 | 1 | 2 | 2 | 3 | 2 |
| NM_001112736 | C3orf63 | 8296 | 14 | 7 | 4 | 2 | 6 | 6 | 7 | 3 | 7 | 10 | 3 | 14 | 12 |
| NM_001112741 | KCNC1 | 3134 | 5 | 1 | 0 | 0 | 0 | 5 | 0 | 0 | 0 | 0 | 0 | 0 | 0 |
| NM_001112800 | SLC8A1 | 6038 | 79 | 13 | 3 | 5 | 79 | 30 | 11 | 1 | 4 | 2 | 1 | 10 | 2 |
| NM_001112801 | SLC8A1 | 6014 | 77 | 13 | 3 | 5 | 77 | 30 | 11 | 1 | 4 | 2 | 1 | 11 | 2 |
| NM_001112802 | SLC8A1 | 6032 | 77 | 13 | 3 | 5 | 77 | 30 | 11 | 1 | 4 | 2 | 1 | 11 | 2 |
| NM_001112808 | TNNI3K | 3269 | 7 | 1 | 0 | 0 | 7 | 0 | 0 | 0 | 0 | 1 | 0 | 0 | 1 |
| NM_001112812 | GRIA4 | 3213 | 8 | 1 | 0 | 0 | 0 | 8 | 0 | 0 | 0 | 0 | 0 | 0 | 0 |
| NM_001113178 | NFAT5 | 14154 | 9 | 6 | 5 | 3 | 5 | 8 | 9 | 3 | 9 | 7 | 4 | 9 | 8 |
| NM_001113182 | BRD2 | 4794 | 37 | 22 | 16 | 17 | 19 | 23 | 20 | 14 | 20 | 30 | 17 | 37 | 31 |
| NM_001113201 | NACA | 1108 | 239 | 103 | 99 | 37 | 103 | 112 | 56 | 108 | 75 | 239 | 106 | 110 | 85 |
| NM_001113202 | NACA | 1041 | 254 | 109 | 105 | 38 | 110 | 119 | 59 | 113 | 79 | 254 | 112 | 116 | 89 |
| NM_001113203 | NACA | 6630 | 96 | 27 | 17 | 7 | 46 | 19 | 10 | 18 | 13 | 40 | 96 | 19 | 14 |
| NM_001113205 | TSTD1 | 673 | 8 | 5 | 3 | 6 | 4 | 8 | 8 | 5 | 6 | 3 | 2 | 2 | 5 |
| NM_001113206 | TSTD1 | 464 | 12 | 7 | 4 | 8 | 5 | 12 | 12 | 7 | 9 | 4 | 3 | 4 | 8 |
| NM_001113207 | TSTD1 | 587 | 10 | 5 | 3 | 7 | 4 | 10 | 9 | 5 | 7 | 3 | 3 | 3 | 6 |
| NM_001113226 | NTNG1 | 3351 | 4 | 1 | 0 | 0 | 0 | 4 | 1 | 0 | 1 | 0 | 0 | 0 | 0 |
| NM_001113228 | NTNG1 | 3174 | 4 | 1 | 0 | 0 | 0 | 4 | 1 | 0 | 1 | 0 | 0 | 0 | 0 |
| NM_001113239 | HIPK2 | 15143 | 22 | 11 | 11 | 4 | 18 | 22 | 19 | 14 | 6 | 3 | 6 | 13 | 2 |

| transcript | gene | transcript length (nt) | max | mean | adipose | colon | heart | hypothala mus | kidney | liver | lung | ovary | skeletalm uscle | spleen | testes |
|---|---|---|---|---|---|---|---|---|---|---|---|---|---|---|---|
| NM_001113324 | C17orf106 | 994 | 1 | 0 | 0 | 1 | 0 | 0 | 1 | 1 | 0 | 0 | 0 | 0 | 0 |
| NM_001113347 | ECE1 | 5241 | 5 | 3 | 3 | 3 | 4 | 2 | 2 | 3 | 3 | 3 | 1 | 5 | 3 |
| NM_001113348 | ECE1 | 5136 | 5 | 3 | 3 | 3 | 4 | 2 | 2 | 3 | 3 | 4 | 1 | 5 | 3 |
| NM_001113349 | ECE1 | 5087 | 5 | 3 | 3 | 3 | 4 | 2 | 2 | 3 | 3 | 4 | 1 | 5 | 3 |
| NM_001113361 | TBC1D14 | 4887 | 9 | 5 | 3 | 4 | 5 | 7 | 9 | 3 | 4 | 4 | 4 | 8 | 5 |
| NM_001113363 | TBC1D14 | 4153 | 10 | 6 | 4 | 4 | 5 | 8 | 10 | 3 | 4 | 4 | 4 | 9 | 6 |
| NM_001113378 | FANCI | 4749 | 4 | 1 | 1 | 1 | 0 | 0 | 0 | 0 | 1 | 1 | 0 | 1 | 4 |
| NM_001113380 | RGS4 | 3055 | 65 | 7 | 0 | 1 | 2 | 65 | 0 | 0 | 0 | 2 | 0 | 0 | 1 |
| NM_001113381 | RGS4 | 2924 | 21 | 2 | 0 | 0 | 0 | 21 | 0 | 0 | 0 | 1 | 0 | 0 | 0 |
| NM_001113382 | FER1L5 | 6477 | 0 | 0 | 0 | 0 | 0 | 0 | 0 | 0 | 0 | 0 | 0 | 0 | 0 |
| NM_001113397 | ZNF385B | 2759 | 3 | 1 | 0 | 0 | 1 | 2 | 2 | 2 | 3 | 1 | 1 | 0 | 2 |
| NM_001113398 | ZNF385B | 2619 | 3 | 1 | 0 | 0 | 1 | 2 | 2 | 2 | 3 | 1 | 1 | 0 | 3 |
| NM_001113402 | AMN1 | 2110 | 4 | 2 | 1 | 0 | 2 | 4 | 1 | 1 | 1 | 2 | 1 | 3 | 4 |
| NM_001113407 | LDB1 | 2104 | 12 | 6 | 4 | 6 | 5 | 8 | 7 | 2 | 5 | 12 | 4 | 7 | 8 |
| NM_001113410 | PSG11 | 1175 | 0 | 0 | 0 | 0 | 0 | 0 | 0 | 0 | 0 | 0 | 0 | 0 | 0 |
| NM_001113411 | FGGY | 2086 | 4 | 1 | 1 | 1 | 1 | 1 | 2 | 4 | 1 | 1 | 3 | 1 | 1 |
| NM_001113434 | C17orf51 | 7911 | 2 | 1 | 0 | 0 | 0 | 2 | 0 | 0 | 0 | 0 | 0 | 1 | 1 |
| NM_001113475 | C14orf148 | 1777 | 0 | 0 | 0 | 0 | 0 | 0 | 0 | 0 | 0 | 0 | 0 | 0 | 0 |
| NM_001113482 | MANEAL | 2858 | 2 | 0 | 0 | 0 | 0 | 2 | 0 | 1 | 0 | 0 | 0 | 0 | 0 |
| NM_001113490 | AMOT | 6945 | 7 | 3 | 3 | 1 | 1 | 6 | 4 | 1 | 1 | 2 | 7 | 1 | 3 |
| NM_001113491 | 09 Sep | 3825 | 14 | 6 | 5 | 8 | 4 | 6 | 5 | 6 | 5 | 5 | 3 | 14 | 7 |
| NM_001113492 | 09 Sep | 4000 | 13 | 6 | 5 | 7 | 4 | 6 | 5 | 6 | 5 | 5 | 3 | 13 | 7 |
| NM_001113493 | 09 Sep | 3986 | 13 | 6 | 5 | 7 | 4 | 6 | 5 | 6 | 5 | 5 | 3 | 13 | 7 |
| NM_001113494 | 09 Sep | 3939 | 14 | 6 | 5 | 8 | 4 | 6 | 5 | 6 | 5 | 5 | 3 | 14 | 7 |
| NM_001113495 | 09 Sep | 3639 | 14 | 6 | 5 | 8 | 4 | 6 | 5 | 6 | 5 | 5 | 3 | 14 | 7 |
| NM_001113496 | 09 Sep | 3234 | 16 | 7 | 5 | 9 | 4 | 7 | 5 | 7 | 5 | 6 | 3 | 16 | 8 |
| NM_001113498 | MDGA2 | 5373 | 3 | 0 | 0 | 0 | 0 | 3 | 0 | 0 | 0 | 0 | 0 | 0 | 1 |
| NM_001113511 | ARHGEF7 | 5525 | 9 | 3 | 3 | 2 | 2 | 9 | 2 | 1 | 2 | 2 | 2 | 5 | 3 |
| NM_001113512 | ARHGEF7 | 5375 | 9 | 3 | 3 | 2 | 2 | 9 | 2 | 1 | 2 | 2 | 3 | 5 | 3 |
| NM_001113513 | ARHGEF7 | 4946 | 7 | 4 | 4 | 3 | 4 | 6 | 3 | 2 | 3 | 4 | 4 | 7 | 4 |
| NM_001113523 | PARP15 | 2692 | 8 | 1 | 1 | 1 | 0 | 0 | 0 | 0 | 1 | 0 | 1 | 8 | 0 |
| NM_001113525 | ZNF276 | 4620 | 1 | 0 | 0 | 1 | 0 | 0 | 0 | 0 | 0 | 0 | 0 | 0 | 0 |
| NM_001113528 | METT5D1 | 4345 | 4 | 2 | 1 | 1 | 2 | 2 | 2 | 1 | 2 | 4 | 2 | 2 | 1 |
| NM_001113534 | FOLR2 | 1125 | 3 | 1 | 3 | 0 | 2 | 0 | 0 | 0 | 1 | 0 | 0 | 1 | 1 |
| NM_001113535 | FOLR2 | 1119 | 3 | 1 | 3 | 0 | 2 | 0 | 0 | 0 | 1 | 0 | 0 | 1 | 1 |
| NM_001113536 | FOLR2 | 1113 | 3 | 1 | 3 | 0 | 2 | 0 | 0 | 0 | 1 | 0 | 0 | 1 | 1 |
| NM_001113541 | FAM75A5 | 4226 | 0 | 0 | 0 | 0 | 0 | 0 | 0 | 0 | 0 | 0 | 0 | 0 | 0 |
| NM_001113546 | LIMA1 | 3741 | 35 | 11 | 35 | 13 | 7 | 4 | 12 | 4 | 11 | 7 | 4 | 13 | 8 |
| NM_001113547 | LIMA1 | 3622 | 36 | 11 | 36 | 13 | 7 | 4 | 12 | 4 | 12 | 7 | 4 | 13 | 8 |
| NM_001113561 | RNF180 | 4944 | 4 | 2 | 1 | 1 | 3 | 4 | 4 | 1 | 2 | 3 | 1 | 2 | 3 |
| NM_001113567 | C17orf76 | 2785 | 1 | 0 | 0 | 1 | 0 | 0 | 0 | 0 | 0 | 0 | 0 | 1 | 1 |
| NM_001113575 | CDKL3 | 2125 | 3 | 1 | 0 | 0 | 0 | 1 | 1 | 0 | 0 | 1 | 1 | 1 | 3 |
| NM_001113738 | ARL17A | 5273 | 4 | 1 | 1 | 2 | 1 | 1 | 2 | 0 | 1 | 3 | 1 | 4 | 1 |
| NM_001113755 | TYMP | 1670 | 3 | 1 | 2 | 0 | 1 | 1 | 0 | 2 | 1 | 0 | 0 | 3 | 0 |
| NM_001113756 | TYMP | 1586 | 3 | 1 | 3 | 0 | 1 | 2 | 1 | 3 | 2 | 0 | 0 | 3 | 0 |
| NM_001114 | ADCY7 | 6138 | 6 | 2 | 2 | 2 | 1 | 1 | 1 | 0 | 2 | 3 | 1 | 6 | 1 |
| NM_001114086 | CLIC5 | 5976 | 19 | 5 | 1 | 2 | 19 | 1 | 7 | 0 | 6 | 0 | 17 | 6 | 0 |
| NM_001114089 | ENTPD6 | 2693 | 2 | 1 | 0 | 1 | 1 | 2 | 1 | 1 | 0 | 0 | 0 | 0 | 1 |
| NM_001114090 | NGEF | 2812 | 5 | 1 | 0 | 1 | 0 | 5 | 1 | 1 | 0 | 0 | 0 | 0 | 0 |
| NM_001114091 | CDC27 | 5610 | 4 | 3 | 3 | 1 | 4 | 4 | 3 | 3 | 3 | 3 | 3 | 4 | 4 |
| NM_001114092 | THUMPD3 | 3775 | 8 | 4 | 4 | 1 | 4 | 4 | 5 | 4 | 3 | 4 | 3 | 4 | 8 |
| NM_001114093 | LSM14A | 3772 | 26 | 18 | 12 | 7 | 19 | 15 | 19 | 15 | 16 | 25 | 19 | 26 | 22 |
| NM_001114094 | BLNK | 1754 | 10 | 2 | 1 | 3 | 0 | 1 | 3 | 5 | 1 | 0 | 0 | 10 | 1 |
| NM_001114099 | SEZ6L2 | 3303 | 8 | 1 | 0 | 1 | 0 | 8 | 0 | 0 | 0 | 1 | 0 | 0 | 1 |
| NM_001114100 | SEZ6L2 | 3210 | 8 | 1 | 0 | 1 | 0 | 8 | 0 | 0 | 0 | 1 | 0 | 0 | 1 |
| NM_001114101 | C1QC | 1191 | 73 | 14 | 14 | 3 | 15 | 13 | 2 | 10 | 17 | 2 | 2 | 73 | 4 |
| NM_001114106 | SLC44A3 | 2393 | 6 | 2 | 0 | 5 | 1 | 1 | 6 | 0 | 1 | 1 | 0 | 0 | 1 |
| NM_001114107 | PDLIM3 | 1672 | 77 | 9 | 0 | 3 | 14 | 1 | 0 | 0 | 2 | 2 | 77 | 3 | 2 |
| NM_001114108 | TTC22 | 1979 | 0 | 0 | 0 | 0 | 0 | 0 | 0 | 0 | 0 | 0 | 0 | 0 | 0 |
| NM_001114118 | C17orf85 | 3764 | 4 | 3 | 2 | 3 | 3 | 3 | 3 | 2 | 3 | 4 | 2 | 4 | 4 |
| NM_001114120 | DEPDC1 | 5333 | 2 | 0 | 0 | 0 | 0 | 0 | 0 | 0 | 0 | 0 | 0 | 0 | 2 |
| NM_001114121 | CHEK1 | 2678 | 2 | 1 | 1 | 1 | 1 | 1 | 1 | 0 | 1 | 1 | 0 | 1 | 2 |
| NM_001114122 | CHEK1 | 2748 | 2 | 1 | 1 | 1 | 0 | 1 | 1 | 0 | 1 | 1 | 0 | 1 | 2 |
| NM_001114123 | ELK1 | 2917 | 2 | 1 | 0 | 0 | 0 | 2 | 0 | 1 | 1 | 2 | 1 | 1 | 1 |
| NM_001114132 | NBEAL1 | 9058 | 5 | 3 | 4 | 2 | 3 | 2 | 5 | 2 | 5 | 4 | 2 | 3 | 3 |
| NM_001114133 | SYNPO2L | 4918 | 32 | 5 | 0 | 0 | 24 | 0 | 0 | 0 | 0 | 0 | 32 | 0 | 0 |
| NM_001114134 | EPB42 | 2450 | 2 | 0 | 2 | 0 | 0 | 0 | 0 | 0 | 1 | 0 | 0 | 0 | 0 |
| NM_001114135 | EPB49 | 2552 | 11 | 3 | 2 | 1 | 4 | 11 | 5 | 1 | 2 | 0 | 1 | 1 | 1 |
| NM_001114136 | EPB49 | 2509 | 12 | 3 | 2 | 1 | 4 | 12 | 5 | 1 | 2 | 0 | 1 | 1 | 1 |
| NM_001114137 | EPB49 | 2602 | 11 | 3 | 2 | 1 | 4 | 11 | 5 | 1 | 2 | 0 | 1 | 1 | 1 |
| NM_001114138 | EPB49 | 2574 | 11 | 3 | 2 | 1 | 4 | 11 | 5 | 1 | 2 | 0 | 1 | 1 | 1 |
| NM_001114139 | EPB49 | 2501 | 12 | 3 | 2 | 1 | 4 | 12 | 5 | 1 | 2 | 0 | 1 | 1 | 1 |
| NM_001114171 | FOSB | 3668 | 60 | 7 | 2 | 2 | 2 | 1 | 2 | 3 | 3 | 60 | 0 | 1 | 0 |
| NM_001114172 | PIK3R3 | 5176 | 10 | 4 | 6 | 1 | 10 | 4 | 3 | 1 | 4 | 2 | 2 | 8 | 9 |
| NM_001114173 | CTSC | 6091 | 8 | 2 | 2 | 1 | 3 | 1 | 2 | 1 | 4 | 1 | 1 | 8 | 2 |
| NM_001114175 | GABRA2 | 2772 | 7 | 1 | 0 | 0 | 0 | 7 | 0 | 0 | 0 | 0 | 0 | 0 | 0 |
| NM_001114176 | AEBP2 | 5099 | 5 | 3 | 2 | 2 | 3 | 4 | 3 | 1 | 3 | 5 | 1 | 4 | 5 |

| transcript | gene | transcript length (nt) | max | mean | adipose | colon | heart | hypothala mus | kidney | liver | lung | ovary | skeletalm uscle | spleen | testes |
|---|---|---|---|---|---|---|---|---|---|---|---|---|---|---|---|
| NM_001114182 | IRAK4 | 4341 | 2 | 1 | 1 | 0 | 1 | 1 | 1 | 1 | 1 | 1 | 0 | 2 | 1 |
| NM_001114183 | GRIA1 | 5730 | 13 | 1 | 0 | 0 | 0 | 13 | 0 | 0 | 1 | 0 | 0 | 0 | 0 |
| NM_001114184 | MTRF1L | 3741 | 9 | 4 | 3 | 1 | 7 | 4 | 5 | 5 | 3 | 3 | 4 | 9 | 3 |
| NM_001114185 | MVK | 2064 | 5 | 1 | 1 | 1 | 0 | 1 | 2 | 5 | 0 | 1 | 0 | 1 | 2 |
| NM_001114309 | ELF3 | 3247 | 13 | 3 | 0 | 13 | 2 | 0 | 8 | 2 | 6 | 0 | 0 | 0 | 0 |
| NM_001114329 | GRM1 | 6939 | 2 | 0 | 0 | 0 | 0 | 2 | 0 | 0 | 0 | 0 | 0 | 0 | 0 |
| NM_001114331 | CLCN7 | 4153 | 1 | 1 | 1 | 0 | 0 | 1 | 1 | 1 | 0 | 0 | 0 | 1 | 0 |
| NM_001114357 | C4orf47 | 974 | 1 | 0 | 0 | 0 | 0 | 0 | 0 | 0 | 0 | 0 | 0 | 0 | 1 |
| NM_001114377 | FOXP3 | 2277 | 0 | 0 | 0 | 0 | 0 | 0 | 0 | 0 | 0 | 0 | 0 | 0 | 0 |
| NM_001114380 | ITGAL | 4957 | 5 | 1 | 0 | 1 | 0 | 0 | 0 | 0 | 1 | 0 | 0 | 5 | 0 |
| NM_001114382 | TSC2 | 5563 | 3 | 1 | 1 | 3 | 1 | 2 | 1 | 1 | 1 | 1 | 2 | 2 | 2 |
| NM_001114387 | TMPRSS11A | 2419 | 0 | 0 | 0 | 0 | 0 | 0 | 0 | 0 | 0 | 0 | 0 | 0 | 0 |
| NM_001114393 | PAPD4 | 3248 | 7 | 4 | 4 | 2 | 4 | 3 | 5 | 2 | 4 | 5 | 2 | 7 | 5 |
| NM_001114394 | PAPD4 | 3523 | 6 | 4 | 3 | 2 | 3 | 2 | 4 | 2 | 3 | 5 | 2 | 6 | 5 |
| NM_001114395 | CNTLN | 4871 | 6 | 2 | 2 | 1 | 2 | 1 | 2 | 4 | 2 | 6 | 1 | 3 | 4 |
| NM_001114396 | KLRD1 | 3195 | 7 | 1 | 0 | 0 | 0 | 0 | 0 | 0 | 2 | 0 | 0 | 7 | 0 |
| NM_001114397 | DHX36 | 3569 | 13 | 7 | 4 | 2 | 6 | 8 | 6 | 7 | 6 | 8 | 8 | 8 | 13 |
| NM_001114403 | UPLP | 1340 | 0 | 0 | 0 | 0 | 0 | 0 | 0 | 0 | 0 | 0 | 0 | 0 | 0 |
| NM_001114598 | ASPDH | 1044 | 3 | 0 | 0 | 0 | 0 | 1 | 3 | 1 | 0 | 0 | 0 | 0 | 0 |
| NM_001114600 | C1orf144 | 3634 | 5 | 4 | 3 | 3 | 5 | 3 | 4 | 4 | 3 | 4 | 3 | 5 | 3 |
| NM_001114614 | MFGE8 | 1833 | 6 | 2 | 2 | 1 | 6 | 0 | 1 | 0 | 2 | 2 | 3 | 1 | 2 |
| NM_001114617 | MGAT1 | 3084 | 5 | 3 | 4 | 2 | 3 | 2 | 2 | 5 | 3 | 2 | 1 | 5 | 2 |
| NM_001114618 | MGAT1 | 3170 | 5 | 3 | 4 | 2 | 3 | 2 | 2 | 5 | 3 | 2 | 1 | 5 | 2 |
| NM_001114619 | MGAT1 | 2647 | 4 | 2 | 3 | 1 | 2 | 1 | 1 | 3 | 3 | 1 | 1 | 4 | 1 |
| NM_001114620 | MGAT1 | 2858 | 4 | 2 | 3 | 1 | 2 | 1 | 1 | 3 | 2 | 1 | 1 | 4 | 1 |
| NM_001114632 | JMJD7 | 1422 | 0 | 0 | 0 | 0 | 0 | 0 | 0 | 0 | 0 | 0 | 0 | 0 | 0 |
| NM_001114633 | PLA2G4B | 2735 | 0 | 0 | 0 | 0 | 0 | 0 | 0 | 0 | 0 | 0 | 0 | 0 | 0 |
| NM_001114634 | PLAG1 | 7233 | 1 | 0 | 0 | 0 | 0 | 0 | 0 | 0 | 1 | 1 | 0 | 1 | 1 |
| NM_001114635 | PLAG1 | 6874 | 1 | 0 | 0 | 0 | 0 | 0 | 0 | 0 | 0 | 0 | 0 | 1 | 1 |
| NM_001114636 | FANCL | 1732 | 19 | 7 | 4 | 3 | 5 | 7 | 4 | 3 | 7 | 19 | 5 | 10 | 15 |
| NM_001114726 | PRRT4 | 3428 | 0 | 0 | 0 | 0 | 0 | 0 | 0 | 0 | 0 | 0 | 0 | 0 | 0 |
| NM_001114734 | PABPC4L | 4943 | 0 | 0 | 0 | 0 | 0 | 0 | 0 | 0 | 0 | 0 | 0 | 0 | 0 |
| NM_001114735 | BCL2A1 | 943 | 16 | 4 | 3 | 0 | 2 | 3 | 0 | 0 | 15 | 0 | 0 | 16 | 1 |
| NM_001114748 | C1orf70 | 1117 | 1 | 0 | 0 | 0 | 0 | 1 | 0 | 0 | 0 | 0 | 0 | 0 | 0 |
| NM_001114752 | CD55 | 2914 | 32 | 10 | 3 | 3 | 13 | 3 | 2 | 4 | 32 | 16 | 12 | 17 | 4 |
| NM_001114753 | ENG | 3060 | 5 | 2 | 2 | 1 | 5 | 1 | 2 | 1 | 2 | 3 | 1 | 4 | 3 |
| NM_001114759 | ZNF683 | 1653 | 1 | 0 | 0 | 0 | 0 | 0 | 0 | 0 | 0 | 0 | 0 | 0 | 1 |
| NM_001114937 | SH2D1A | 2492 | 2 | 0 | 0 | 0 | 0 | 0 | 0 | 0 | 1 | 0 | 0 | 2 | 0 |
| NM_001114938 | CCDC17 | 2152 | 0 | 0 | 0 | 0 | 0 | 0 | 0 | 0 | 0 | 0 | 0 | 0 | 0 |
| NM_001114974 | SMTNL2 | 2285 | 13 | 2 | 0 | 0 | 1 | 0 | 4 | 0 | 0 | 0 | 13 | 0 | 0 |
| NM_001114978 | TP63 | 4815 | 8 | 1 | 0 | 0 | 0 | 0 | 0 | 0 | 0 | 0 | 8 | 0 | 1 |
| NM_001114979 | TP63 | 2870 | 11 | 1 | 0 | 0 | 0 | 0 | 0 | 0 | 0 | 0 | 11 | 0 | 1 |
| NM_001114980 | TP63 | 4679 | 8 | 1 | 0 | 0 | 0 | 0 | 0 | 0 | 1 | 0 | 8 | 0 | 1 |
| NM_001114981 | TP63 | 4585 | 8 | 1 | 0 | 0 | 0 | 0 | 0 | 0 | 1 | 0 | 8 | 0 | 1 |
| NM_001114982 | TP63 | 2640 | 11 | 1 | 0 | 0 | 0 | 0 | 0 | 0 | 0 | 0 | 11 | 0 | 1 |
| NM_001115 | ADCY8 | 4101 | 3 | 0 | 0 | 0 | 0 | 3 | 0 | 0 | 0 | 0 | 0 | 0 | 0 |
| NM_001115007 | LIN54 | 5213 | 1 | 1 | 0 | 0 | 1 | 1 | 1 | 1 | 1 | 1 | 1 | 1 | 1 |
| NM_001115008 | LIN54 | 5463 | 1 | 1 | 1 | 1 | 1 | 1 | 1 | 1 | 1 | 1 | 1 | 1 | 1 |
| NM_001115016 | KIAA1310 | 5224 | 5 | 3 | 2 | 3 | 3 | 3 | 4 | 2 | 3 | 4 | 5 | 4 | 4 |
| NM_001115113 | WDR26 | 6824 | 14 | 10 | 10 | 5 | 10 | 8 | 8 | 7 | 14 | 8 | 10 | 14 | 11 |
| NM_001115116 | ANKRD53 | 1993 | 0 | 0 | 0 | 0 | 0 | 0 | 0 | 0 | 0 | 0 | 0 | 0 | 0 |
| NM_001115131 | C6 | 3661 | 101 | 12 | 1 | 0 | 21 | 0 | 3 | 101 | 1 | 1 | 1 | 1 | 4 |
| NM_001115152 | CD300LD | 644 | 0 | 0 | 0 | 0 | 0 | 0 | 0 | 0 | 0 | 0 | 0 | 0 | 0 |
| NM_001115156 | GDI2 | 2277 | 89 | 54 | 43 | 26 | 53 | 42 | 51 | 77 | 47 | 55 | 41 | 89 | 67 |
| NM_001116 | ADCY9 | 7725 | 4 | 2 | 1 | 1 | 3 | 2 | 3 | 3 | 2 | 2 | 4 | 1 | 2 |
| NM_001117 | ADCYAP1 | 3159 | 2 | 0 | 0 | 0 | 0 | 2 | 0 | 0 | 0 | 0 | 0 | 0 | 1 |
| NM_001118 | ADCYAP1R1 | 1643 | 9 | 1 | 0 | 0 | 0 | 9 | 0 | 0 | 0 | 0 | 0 | 0 | 0 |
| NM_001118885 | GLRA2 | 3017 | 2 | 0 | 0 | 0 | 0 | 2 | 0 | 0 | 0 | 0 | 0 | 0 | 0 |
| NM_001118886 | GLRA2 | 3445 | 2 | 0 | 0 | 0 | 0 | 2 | 0 | 0 | 0 | 0 | 0 | 0 | 0 |
| NM_001118887 | ANGPT2 | 5266 | 4 | 2 | 2 | 0 | 4 | 1 | 1 | 1 | 1 | 3 | 4 | 3 | 2 |
| NM_001118888 | ANGPT2 | 5113 | 4 | 2 | 2 | 0 | 4 | 1 | 1 | 0 | 1 | 3 | 4 | 3 | 1 |
| NM_001118890 | GLRX | 1079 | 47 | 21 | 17 | 5 | 47 | 10 | 47 | 27 | 20 | 2 | 37 | 19 | 2 |
| NM_001119 | ADD1 | 3941 | 39 | 21 | 19 | 15 | 24 | 39 | 17 | 12 | 14 | 27 | 21 | 26 | 21 |
| NM_001120 | MFSD10 | 1958 | 2 | 1 | 1 | 1 | 1 | 1 | 1 | 0 | 1 | 1 | 0 | 2 | 0 |
| NM_001121 | ADD3 | 4348 | 28 | 12 | 9 | 7 | 16 | 28 | 20 | 2 | 10 | 11 | 3 | 10 | 17 |
| NM_001122 | PLIN2 | 1980 | 27 | 11 | 27 | 2 | 21 | 1 | 13 | 21 | 8 | 2 | 11 | 7 | 7 |
| NM_001122606 | LAMP2 | 3752 | 11 | 6 | 4 | 2 | 5 | 10 | 11 | 9 | 6 | 5 | 6 | 7 | 6 |
| NM_001122607 | RUNX1 | 2722 | 2 | 1 | 1 | 1 | 1 | 1 | 0 | 1 | 2 | 1 | 0 | 1 | 0 |
| NM_001122608 | HCCS | 2286 | 14 | 7 | 4 | 5 | 14 | 7 | 7 | 7 | 4 | 4 | 8 | 7 | 7 |
| NM_001122630 | CDKN1C | 1763 | 1 | 0 | 0 | 0 | 0 | 0 | 1 | 0 | 0 | 1 | 0 | 0 | 0 |
| NM_001122631 | CDKN1C | 1758 | 1 | 0 | 0 | 0 | 0 | 0 | 1 | 0 | 0 | 1 | 0 | 0 | 0 |
| NM_001122633 | CPS1 | 5722 | 210 | 20 | 1 | 1 | 1 | 1 | 1 | 210 | 0 | 2 | 1 | 1 | 1 |
| NM_001122634 | CPS1 | 4770 | 216 | 20 | 1 | 1 | 1 | 1 | 1 | 216 | 0 | 1 | 1 | 1 | 2 |
| NM_001122636 | GALNT9 | 2663 | 1 | 0 | 0 | 0 | 0 | 1 | 0 | 0 | 0 | 0 | 0 | 0 | 0 |
| NM_001122646 | FAM178B | 2685 | 0 | 0 | 0 | 0 | 0 | 0 | 0 | 0 | 0 | 0 | 0 | 0 | 0 |
| NM_001122659 | EDNRB | 4306 | 13 | 6 | 4 | 1 | 8 | 9 | 7 | 6 | 10 | 1 | 1 | 13 | 2 |
| NM_001122665 | DDX3Y | 4648 | 28 | 10 | 10 | 0 | 9 | 11 | 4 | 11 | 12 | 0 | 1 | 22 | 28 |

| transcript | gene | transcript length (nt) | max | mean | adipose | colon | heart | hypothala mus | kidney | liver | lung | ovary | skeletalm uscle | spleen | testes |
|---|---|---|---|---|---|---|---|---|---|---|---|---|---|---|---|
| NM_001122670 | PHKA1 | 6122 | 5 | 1 | 0 | 0 | 1 | 1 | 1 | 0 | 0 | 1 | 5 | 1 | 1 |
| NM_001122671 | CCBL1 | 1919 | 1 | 0 | 0 | 0 | 0 | 1 | 0 | 0 | 0 | 0 | 0 | 0 | 0 |
| NM_001122672 | CCBL1 | 1775 | 1 | 0 | 0 | 0 | 0 | 1 | 0 | 0 | 0 | 0 | 0 | 0 | 0 |
| NM_001122674 | ABCD3 | 967 | 2 | 1 | 0 | 0 | 1 | 1 | 2 | 2 | 1 | 1 | 0 | 0 | 1 |
| NM_001122679 | ODZ2 | 9645 | 12 | 2 | 0 | 0 | 12 | 5 | 0 | 0 | 0 | 0 | 0 | 0 | 1 |
| NM_001122681 | SH3BP2 | 9068 | 2 | 1 | 0 | 0 | 0 | 0 | 2 | 0 | 0 | 0 | 0 | 2 | 0 |
| NM_001122716 | CXorf64 | 1227 | 0 | 0 | 0 | 0 | 0 | 0 | 0 | 0 | 0 | 0 | 0 | 0 | 0 |
| NM_001122731 | MICAL3 | 3369 | 6 | 3 | 2 | 6 | 3 | 4 | 3 | 2 | 1 | 3 | 2 | 4 | 4 |
| NM_001122740 | ESR1 | 6356 | 3 | 1 | 1 | 1 | 0 | 0 | 1 | 1 | 0 | 3 | 0 | 0 | 1 |
| NM_001122741 | ESR1 | 6313 | 3 | 1 | 1 | 1 | 0 | 0 | 1 | 1 | 0 | 3 | 0 | 0 | 1 |
| NM_001122742 | ESR1 | 6465 | 3 | 1 | 1 | 1 | 0 | 0 | 1 | 1 | 0 | 3 | 0 | 0 | 1 |
| NM_001122752 | SERPINI1 | 1677 | 38 | 4 | 0 | 0 | 1 | 38 | 2 | 0 | 0 | 1 | 0 | 1 | 3 |
| NM_001122757 | POU1F1 | 1340 | 0 | 0 | 0 | 0 | 0 | 0 | 0 | 0 | 0 | 0 | 0 | 0 | 0 |
| NM_001122764 | PPOX | 1768 | 0 | 0 | 0 | 0 | 0 | 0 | 0 | 0 | 0 | 0 | 0 | 0 | 0 |
| NM_001122769 | LCA5 | 4563 | 8 | 3 | 2 | 0 | 2 | 4 | 2 | 0 | 3 | 6 | 2 | 2 | 8 |
| NM_001122770 | ZBTB37 | 2300 | 4 | 2 | 3 | 1 | 1 | 3 | 3 | 1 | 2 | 3 | 1 | 4 | 3 |
| NM_001122772 | AGAP2 | 4920 | 4 | 0 | 0 | 1 | 0 | 4 | 0 | 0 | 0 | 0 | 0 | 0 | 0 |
| NM_001122779 | FAM124B | 2469 | 1 | 0 | 1 | 0 | 0 | 0 | 0 | 0 | 0 | 0 | 0 | 0 | 0 |
| NM_001122819 | KIF17 | 3775 | 1 | 0 | 0 | 0 | 0 | 1 | 0 | 0 | 0 | 0 | 0 | 0 | 1 |
| NM_001122821 | SET | 2846 | 96 | 55 | 50 | 46 | 52 | 96 | 49 | 51 | 50 | 52 | 37 | 74 | 51 |
| NM_001122823 | GTF3C5 | 2441 | 6 | 3 | 2 | 3 | 3 | 2 | 4 | 2 | 3 | 4 | 4 | 5 | 6 |
| NM_001122824 | TACC1 | 6572 | 17 | 9 | 12 | 5 | 9 | 12 | 6 | 4 | 10 | 7 | 3 | 17 | 10 |
| NM_001122825 | ESRP1 | 3545 | 6 | 1 | 0 | 6 | 0 | 0 | 2 | 0 | 4 | 0 | 0 | 0 | 1 |
| NM_001122826 | ESRP1 | 3684 | 6 | 1 | 0 | 6 | 0 | 0 | 2 | 0 | 4 | 0 | 0 | 0 | 1 |
| NM_001122827 | ESRP1 | 3533 | 6 | 1 | 0 | 6 | 0 | 0 | 2 | 0 | 4 | 0 | 0 | 0 | 1 |
| NM_001122833 | STK31 | 3261 | 10 | 1 | 0 | 0 | 0 | 0 | 0 | 0 | 0 | 0 | 0 | 0 | 10 |
| NM_001122834 | HHAT | 3559 | 1 | 0 | 0 | 0 | 0 | 0 | 1 | 0 | 0 | 1 | 0 | 1 | 1 |
| NM_001122835 | TTC13 | 3117 | 3 | 1 | 2 | 1 | 1 | 3 | 1 | 1 | 1 | 1 | 1 | 2 | 1 |
| NM_001122837 | RAG1AP1 | 1219 | 11 | 6 | 6 | 8 | 5 | 4 | 7 | 11 | 8 | 2 | 1 | 11 | 5 |
| NM_001122838 | NAPEPLD | 5363 | 4 | 2 | 1 | 1 | 1 | 4 | 4 | 1 | 1 | 3 | 1 | 4 | 4 |
| NM_001122839 | RAG1AP1 | 1181 | 12 | 6 | 6 | 9 | 5 | 4 | 7 | 12 | 8 | 2 | 1 | 12 | 5 |
| NM_001122841 | FRMD1 | 2864 | 1 | 0 | 0 | 1 | 0 | 0 | 0 | 0 | 0 | 0 | 0 | 0 | 0 |
| NM_001122842 | NCOA7 | 5422 | 16 | 5 | 4 | 1 | 3 | 16 | 7 | 2 | 6 | 4 | 2 | 7 | 4 |
| NM_001122847 | SLC6A12 | 3129 | 7 | 1 | 0 | 0 | 0 | 0 | 6 | 7 | 0 | 0 | 0 | 0 | 0 |
| NM_001122848 | SLC6A12 | 3255 | 5 | 1 | 0 | 0 | 0 | 0 | 5 | 4 | 0 | 0 | 0 | 0 | 0 |
| NM_001122853 | MYOZ3 | 3831 | 3 | 0 | 0 | 0 | 0 | 0 | 0 | 0 | 0 | 0 | 3 | 0 | 1 |
| NM_001122870 | PPM1M | 1792 | 1 | 1 | 1 | 1 | 1 | 1 | 0 | 0 | 1 | 1 | 0 | 1 | 0 |
| NM_001122890 | GGT6 | 2630 | 2 | 0 | 0 | 2 | 0 | 0 | 2 | 0 | 0 | 0 | 0 | 0 | 0 |
| NM_001122898 | CD99 | 1189 | 83 | 37 | 69 | 20 | 50 | 32 | 12 | 24 | 24 | 31 | 22 | 40 | 83 |
| NM_001122951 | DARC | 1090 | 10 | 4 | 5 | 1 | 6 | 1 | 1 | 0 | 7 | 1 | 4 | 10 | 5 |
| NM_001122955 | BSCL2 | 2014 | 12 | 2 | 1 | 1 | 1 | 12 | 2 | 1 | 1 | 1 | 1 | 1 | 4 |
| NM_001122956 | DBNL | 2218 | 12 | 5 | 5 | 3 | 5 | 6 | 4 | 4 | 5 | 2 | 2 | 12 | 3 |
| NM_001122957 | BCKDK | 2174 | 1 | 0 | 0 | 0 | 1 | 1 | 0 | 1 | 0 | 0 | 1 | 0 | 0 |
| NM_001122961 | C1orf194 | 731 | 11 | 1 | 0 | 0 | 0 | 1 | 0 | 0 | 1 | 0 | 0 | 0 | 11 |
| NM_001122962 | SIRPB2 | 2833 | 2 | 1 | 0 | 0 | 1 | 0 | 0 | 0 | 1 | 0 | 0 | 2 | 0 |
| NM_001122964 | SMEK2 | 4458 | 20 | 14 | 17 | 7 | 10 | 13 | 17 | 17 | 13 | 15 | 8 | 20 | 15 |
| NM_001122965 | RPTN | 3569 | 0 | 0 | 0 | 0 | 0 | 0 | 0 | 0 | 0 | 0 | 0 | 0 | 0 |
| NM_001122979 | FCAMR | 1897 | 14 | 1 | 0 | 0 | 0 | 0 | 14 | 0 | 0 | 0 | 0 | 0 | 0 |
| NM_001122980 | FCAMR | 2616 | 9 | 1 | 0 | 0 | 0 | 0 | 9 | 0 | 0 | 0 | 0 | 0 | 0 |
| NM_001123 | ADK | 2082 | 15 | 4 | 3 | 1 | 4 | 5 | 4 | 15 | 4 | 2 | 2 | 4 | 5 |
| NM_001123040 | C3orf71 | 1598 | 1 | 0 | 0 | 0 | 0 | 1 | 0 | 0 | 0 | 0 | 0 | 0 | 0 |
| NM_001123041 | CCR2 | 2672 | 4 | 1 | 0 | 0 | 1 | 0 | 0 | 0 | 1 | 0 | 0 | 4 | 0 |
| NM_001123065 | C7orf65 | 2911 | 0 | 0 | 0 | 0 | 0 | 0 | 0 | 0 | 0 | 0 | 0 | 0 | 0 |
| NM_001123066 | MAPT | 6815 | 22 | 3 | 1 | 1 | 1 | 22 | 3 | 0 | 0 | 0 | 4 | 0 | 0 |
| NM_001123067 | MAPT | 5723 | 27 | 4 | 2 | 1 | 1 | 27 | 3 | 0 | 0 | 0 | 5 | 0 | 0 |
| NM_001123068 | PPIAL4G | 738 | 0 | 0 | 0 | 0 | 0 | 0 | 0 | 0 | 0 | 0 | 0 | 0 | 0 |
| NM_001123168 | FAM72A | 1867 | 0 | 0 | 0 | 0 | 0 | 0 | 0 | 0 | 0 | 0 | 0 | 0 | 0 |
| NM_001123225 | C22orf41 | 442 | 13 | 1 | 0 | 0 | 0 | 1 | 1 | 0 | 0 | 0 | 0 | 0 | 13 |
| NM_001123226 | MTO1 | 3044 | 9 | 4 | 3 | 2 | 4 | 4 | 5 | 9 | 3 | 5 | 3 | 6 | 5 |
| NM_001123228 | TMEM14E | 1293 | 8 | 3 | 2 | 3 | 3 | 1 | 2 | 1 | 6 | 5 | 4 | 8 | 3 |
| NM_001123329 | ZBTB1 | 3838 | 19 | 6 | 5 | 2 | 6 | 4 | 6 | 3 | 6 | 6 | 4 | 19 | 7 |
| NM_001123355 | PPP6C | 4350 | 6 | 4 | 3 | 2 | 4 | 5 | 4 | 3 | 3 | 2 | 6 | 4 | 4 |
| NM_001123363 | RGPD6 | 7639 | 0 | 0 | 0 | 0 | 0 | 0 | 0 | 0 | 0 | 0 | 0 | 0 | 0 |
| NM_001123364 | C6orf186 | 1101 | 1 | 0 | 0 | 1 | 0 | 0 | 1 | 0 | 0 | 1 | 0 | 0 | 0 |
| NM_001123366 | HMSD | 625 | 0 | 0 | 0 | 0 | 0 | 0 | 0 | 0 | 0 | 0 | 0 | 0 | 0 |
| NM_001123369 | PPP6C | 4173 | 6 | 4 | 3 | 2 | 4 | 5 | 4 | 3 | 3 | 3 | 6 | 5 | 4 |
| NM_001123375 | HIST2H3D | 411 | 1 | 0 | 0 | 0 | 0 | 0 | 0 | 0 | 0 | 1 | 0 | 0 | 0 |
| NM_001123376 | TMEM72 | 1085 | 1 | 0 | 0 | 0 | 0 | 0 | 1 | 0 | 0 | 0 | 0 | 0 | 0 |
| NM_001123377 | PARK7 | 903 | 52 | 30 | 32 | 16 | 42 | 52 | 29 | 51 | 16 | 22 | 27 | 25 | 22 |
| NM_001123378 | ADSL | 1386 | 37 | 12 | 7 | 8 | 12 | 7 | 10 | 14 | 5 | 10 | 37 | 11 | 10 |
| NM_001123383 | BCOR | 6392 | 3 | 2 | 1 | 2 | 2 | 2 | 2 | 1 | 2 | 3 | 3 | 3 | 3 |
| NM_001123384 | BCOR | 6338 | 3 | 2 | 1 | 2 | 2 | 2 | 2 | 1 | 2 | 3 | 3 | 3 | 3 |
| NM_001123385 | BCOR | 6423 | 3 | 2 | 1 | 2 | 2 | 2 | 2 | 1 | 2 | 3 | 3 | 3 | 3 |
| NM_001123387 | KRTAP2-1 | 773 | 0 | 0 | 0 | 0 | 0 | 0 | 0 | 0 | 0 | 0 | 0 | 0 | 0 |
| NM_001123391 | TBC1D3 | 2305 | 0 | 0 | 0 | 0 | 0 | 0 | 0 | 0 | 0 | 0 | 0 | 0 | 0 |
| NM_001123392 | TBC1D3H | 2305 | 0 | 0 | 0 | 0 | 0 | 0 | 0 | 0 | 0 | 0 | 0 | 0 | 0 |
| NM_001123395 | CLDN19 | 3602 | 1 | 0 | 0 | 0 | 0 | 0 | 1 | 0 | 0 | 0 | 0 | 0 | 0 |

| transcript | gene | transcript length (nt) | max | mean | adipose | colon | heart | hypothalamus | kidney | liver | lung | ovary | skeletalmuscle | spleen | testes |
|---|---|---|---|---|---|---|---|---|---|---|---|---|---|---|---|
| NM_001123396 | CCR2 | 2335 | 2 | 0 | 0 | 0 | 0 | 0 | 0 | 0 | 1 | 0 | 0 | 2 | 0 |
| NM_001124 | ADM | 1449 | 15 | 7 | 11 | 1 | 5 | 2 | 10 | 2 | 13 | 3 | 15 | 9 | 2 |
| NM_001124756 | PABPC1L | 2129 | 1 | 0 | 0 | 0 | 0 | 0 | 1 | 0 | 0 | 0 | 0 | 0 | 0 |
| NM_001124758 | SPNS2 | 4349 | 0 | 0 | 0 | 0 | 0 | 0 | 0 | 0 | 0 | 0 | 0 | 0 | 0 |
| NM_001124759 | FRG2C | 2069 | 0 | 0 | 0 | 0 | 0 | 0 | 0 | 0 | 0 | 0 | 0 | 0 | 0 |
| NM_001124767 | LOC440957 | 449 | 19 | 7 | 8 | 6 | 19 | 6 | 11 | 4 | 6 | 8 | 6 | 4 | 5 |
| NM_001125 | ADPRH | 3439 | 6 | 2 | 4 | 1 | 2 | 1 | 1 | 1 | 3 | 1 | 2 | 6 | 2 |
| NM_001126 | ADSS | 2757 | 11 | 6 | 3 | 2 | 4 | 8 | 8 | 4 | 7 | 9 | 2 | 9 | 11 |
| NM_001126044 | PTGER3 | 2055 | 3 | 1 | 3 | 0 | 0 | 0 | 3 | 0 | 0 | 0 | 0 | 0 | 0 |
| NM_001126049 | KILLIN | 4277 | 1 | 0 | 0 | 0 | 0 | 0 | 0 | 0 | 0 | 0 | 0 | 1 | 0 |
| NM_001126050 | HDGF | 2198 | 25 | 11 | 9 | 13 | 10 | 10 | 14 | 25 | 11 | 6 | 10 | 11 | 7 |
| NM_001126051 | HDGF | 2173 | 26 | 12 | 9 | 13 | 10 | 10 | 14 | 26 | 11 | 6 | 10 | 11 | 7 |
| NM_001126054 | CASK | 8227 | 3 | 1 | 1 | 1 | 1 | 3 | 2 | 1 | 1 | 2 | 0 | 1 | 1 |
| NM_001126055 | CASK | 8224 | 3 | 1 | 1 | 1 | 1 | 3 | 2 | 1 | 1 | 2 | 0 | 1 | 1 |
| NM_001126056 | DMKN | 1551 | 1 | 0 | 0 | 0 | 0 | 1 | 1 | 0 | 1 | 0 | 0 | 0 | 0 |
| NM_001126057 | DMKN | 1497 | 1 | 0 | 0 | 0 | 0 | 1 | 1 | 0 | 1 | 0 | 0 | 0 | 0 |
| NM_001126058 | DMKN | 1461 | 1 | 0 | 0 | 0 | 0 | 1 | 1 | 0 | 1 | 0 | 0 | 0 | 0 |
| NM_001126059 | DMKN | 972 | 1 | 0 | 0 | 0 | 0 | 0 | 1 | 0 | 1 | 1 | 0 | 0 | 0 |
| NM_001126060 | NOS1AP | 5559 | 2 | 0 | 0 | 0 | 0 | 2 | 0 | 0 | 0 | 0 | 0 | 0 | 0 |
| NM_001126061 | DMKN | 1041 | 1 | 0 | 0 | 0 | 0 | 0 | 1 | 0 | 1 | 1 | 0 | 0 | 0 |
| NM_001126063 | KHDC1L | 633 | 0 | 0 | 0 | 0 | 0 | 0 | 0 | 0 | 0 | 0 | 0 | 0 | 0 |
| NM_001126102 | HP | 1254 | 76,051 | 7,105 | 10 | 72 | 318 | 0 | 118 | 76,051 | 222 | 321 | 262 | 423 | 360 |
| NM_001126103 | RACGAP1 | 3326 | 5 | 2 | 2 | 2 | 1 | 2 | 1 | 1 | 2 | 1 | 1 | 2 | 5 |
| NM_001126104 | RACGAP1 | 3206 | 5 | 2 | 2 | 3 | 1 | 2 | 1 | 1 | 2 | 1 | 1 | 2 | 5 |
| NM_001126105 | SLC7A7 | 2130 | 36 | 9 | 4 | 2 | 5 | 2 | 36 | 3 | 10 | 2 | 0 | 27 | 3 |
| NM_001126106 | SLC7A7 | 2265 | 34 | 8 | 4 | 2 | 5 | 2 | 34 | 3 | 9 | 2 | 0 | 26 | 3 |
| NM_001126107 | SLC12A3 | 5564 | 20 | 2 | 0 | 0 | 0 | 0 | 20 | 0 | 0 | 0 | 0 | 0 | 0 |
| NM_001126108 | SLC12A3 | 5540 | 20 | 2 | 0 | 0 | 0 | 0 | 20 | 0 | 0 | 0 | 0 | 0 | 0 |
| NM_001126111 | OSGIN2 | 4245 | 5 | 3 | 3 | 1 | 2 | 5 | 2 | 3 | 3 | 5 | 2 | 3 | 2 |
| NM_001126112 | TP53 | 2583 | 4 | 2 | 1 | 4 | 1 | 1 | 1 | 1 | 1 | 2 | 1 | 4 | 2 |
| NM_001126113 | TP53 | 2646 | 4 | 2 | 1 | 3 | 1 | 1 | 1 | 1 | 1 | 2 | 1 | 4 | 2 |
| NM_001126114 | TP53 | 2719 | 4 | 2 | 1 | 3 | 1 | 1 | 1 | 1 | 1 | 2 | 1 | 4 | 2 |
| NM_001126115 | TP53 | 2271 | 4 | 2 | 1 | 3 | 1 | 1 | 1 | 1 | 1 | 2 | 1 | 4 | 3 |
| NM_001126116 | TP53 | 2404 | 4 | 2 | 1 | 3 | 1 | 1 | 1 | 1 | 1 | 2 | 1 | 4 | 2 |
| NM_001126117 | TP53 | 2331 | 4 | 2 | 1 | 3 | 1 | 1 | 1 | 1 | 1 | 2 | 1 | 4 | 3 |
| NM_001126121 | SLC25A19 | 1658 | 2 | 1 | 0 | 1 | 1 | 1 | 0 | 0 | 0 | 0 | 0 | 1 | 2 |
| NM_001126122 | SLC25A19 | 1568 | 2 | 1 | 0 | 1 | 1 | 1 | 0 | 0 | 0 | 0 | 0 | 1 | 2 |
| NM_001126123 | ENOSF1 | 1968 | 3 | 1 | 0 | 1 | 1 | 0 | 3 | 1 | 1 | 1 | 0 | 1 | 1 |
| NM_001126128 | PROK2 | 1613 | 1 | 0 | 0 | 0 | 0 | 0 | 0 | 0 | 0 | 0 | 0 | 1 | 0 |
| NM_001126129 | GINS3 | 2391 | 1 | 0 | 1 | 0 | 1 | 0 | 0 | 0 | 0 | 0 | 0 | 1 | 1 |
| NM_001126130 | GINS3 | 2040 | 1 | 1 | 1 | 0 | 1 | 0 | 0 | 0 | 0 | 0 | 0 | 1 | 1 |
| NM_001126131 | POLG | 4436 | 4 | 2 | 2 | 2 | 2 | 1 | 2 | 2 | 2 | 1 | 2 | 4 | 2 |
| NM_001126132 | TNNT1 | 1000 | 440 | 41 | 1 | 0 | 1 | 2 | 0 | 0 | 0 | 0 | 440 | 0 | 2 |
| NM_001126133 | TNNT1 | 967 | 447 | 41 | 1 | 0 | 1 | 2 | 0 | 0 | 0 | 0 | 447 | 0 | 2 |
| NM_001126181 | NRGN | 1218 | 85 | 10 | 1 | 2 | 1 | 85 | 1 | 0 | 6 | 1 | 0 | 6 | 1 |
| NM_001126240 | TP73 | 2822 | 0 | 0 | 0 | 0 | 0 | 0 | 0 | 0 | 0 | 0 | 0 | 0 | 0 |
| NM_001126241 | TP73 | 2728 | 0 | 0 | 0 | 0 | 0 | 0 | 0 | 0 | 0 | 0 | 0 | 0 | 0 |
| NM_001126242 | TP73 | 2673 | 0 | 0 | 0 | 0 | 0 | 0 | 0 | 0 | 0 | 0 | 0 | 0 | 0 |
| NM_001126328 | LNX1 | 3080 | 3 | 1 | 1 | 1 | 1 | 3 | 3 | 1 | 0 | 0 | 0 | 0 | 1 |
| NM_001126334 | FOXD4L5 | 3109 | 0 | 0 | 0 | 0 | 0 | 0 | 0 | 0 | 0 | 0 | 0 | 0 | 0 |
| NM_001126335 | SLC7A9 | 1688 | 5 | 1 | 0 | 0 | 0 | 0 | 5 | 1 | 0 | 0 | 0 | 0 | 0 |
| NM_001126336 | VCAN | 4193 | 20 | 5 | 5 | 1 | 20 | 3 | 2 | 1 | 8 | 2 | 1 | 8 | 3 |
| NM_001126337 | TUFT1 | 3036 | 7 | 2 | 2 | 3 | 1 | 2 | 7 | 1 | 2 | 2 | 1 | 1 | 2 |
| NM_001126339 | THTPA | 1797 | 1 | 1 | 1 | 1 | 1 | 1 | 1 | 0 | 0 | 0 | 0 | 1 | 1 |
| NM_001126340 | ORAI2 | 10769 | 1 | 0 | 0 | 1 | 0 | 0 | 0 | 0 | 0 | 0 | 0 | 1 | 0 |
| NM_001126493 | LOC402644 | 477 | 46 | 17 | 20 | 12 | 8 | 46 | 6 | 34 | 10 | 15 | 5 | 22 | 10 |
| NM_001127 | AP1B1 | 4176 | 9 | 5 | 4 | 4 | 4 | 7 | 5 | 7 | 4 | 2 | 2 | 9 | 3 |
| NM_001127173 | CADM3 | 3559 | 16 | 2 | 2 | 2 | 1 | 16 | 0 | 0 | 0 | 0 | 0 | 1 | 0 |
| NM_001127174 | MRO | 4888 | 19 | 3 | 1 | 1 | 0 | 7 | 3 | 0 | 0 | 2 | 0 | 1 | 19 |
| NM_001127175 | MRO | 4999 | 19 | 3 | 1 | 1 | 0 | 7 | 3 | 0 | 0 | 2 | 0 | 1 | 19 |
| NM_001127176 | MRO | 5155 | 21 | 3 | 1 | 1 | 0 | 7 | 3 | 0 | 0 | 2 | 0 | 1 | 21 |
| NM_001127178 | PIGG | 3250 | 3 | 2 | 2 | 2 | 1 | 2 | 3 | 2 | 2 | 2 | 1 | 3 | 2 |
| NM_001127179 | MYO7A | 4051 | 9 | 2 | 0 | 1 | 0 | 1 | 3 | 4 | 0 | 0 | 0 | 1 | 9 |
| NM_001127180 | MYO7A | 7339 | 6 | 1 | 1 | 1 | 0 | 1 | 2 | 2 | 0 | 0 | 0 | 1 | 6 |
| NM_001127181 | CENPL | 2923 | 1 | 0 | 0 | 0 | 0 | 0 | 0 | 0 | 0 | 0 | 0 | 1 | 0 |
| NM_001127182 | CEP55 | 2516 | 7 | 1 | 2 | 1 | 1 | 0 | 0 | 0 | 1 | 1 | 0 | 2 | 7 |
| NM_001127183 | CFLAR | 1963 | 44 | 24 | 26 | 15 | 42 | 9 | 22 | 14 | 44 | 10 | 34 | 40 | 12 |
| NM_001127184 | CFLAR | 1359 | 57 | 31 | 34 | 19 | 55 | 12 | 27 | 18 | 57 | 13 | 43 | 51 | 15 |
| NM_001127190 | CSK | 2221 | 4 | 1 | 1 | 1 | 1 | 1 | 0 | 2 | 1 | 0 | 0 | 4 | 1 |
| NM_001127192 | CNBP | 3384 | 123 | 51 | 29 | 13 | 76 | 35 | 34 | 45 | 50 | 59 | 123 | 53 | 46 |
| NM_001127193 | CNBP | 3381 | 123 | 51 | 29 | 13 | 76 | 35 | 34 | 45 | 50 | 59 | 123 | 53 | 46 |
| NM_001127194 | CNBP | 3363 | 123 | 52 | 29 | 13 | 76 | 35 | 34 | 45 | 50 | 60 | 123 | 53 | 46 |
| NM_001127195 | CNBP | 3360 | 124 | 52 | 29 | 13 | 76 | 35 | 34 | 45 | 51 | 60 | 124 | 53 | 46 |
| NM_001127196 | CNBP | 3357 | 124 | 52 | 29 | 13 | 76 | 35 | 35 | 45 | 51 | 60 | 124 | 53 | 47 |
| NM_001127197 | ELF4 | 4096 | 2 | 1 | 1 | 2 | 0 | 0 | 0 | 0 | 1 | 1 | 0 | 2 | 0 |
| NM_001127198 | TMC6 | 2891 | 1 | 0 | 0 | 0 | 0 | 0 | 0 | 0 | 0 | 0 | 0 | 1 | 0 |
| NM_001127199 | GAGE12D | 585 | 0 | 0 | 0 | 0 | 0 | 0 | 0 | 0 | 0 | 0 | 0 | 0 | 0 |

| transcript | gene | transcript length (nt) | max | mean | adipose | colon | heart | hypothala mus | kidney | liver | lung | ovary | skeletalm uscle | spleen | testes |
|---|---|---|---|---|---|---|---|---|---|---|---|---|---|---|---|
| NM_001127200 | GAGE2E | 517 | 0 | 0 | 0 | 0 | 0 | 0 | 0 | 0 | 0 | 0 | 0 | 0 | 0 |
| NM_001127200 | GAGE2E | 520 | 0 | 0 | 0 | 0 | 0 | 0 | 0 | 0 | 0 | 0 | 0 | 0 | 0 |
| NM_001127202 | PCID2 | 1868 | 6 | 3 | 3 | 2 | 4 | 4 | 2 | 3 | 2 | 3 | 3 | 4 | 6 |
| NM_001127203 | PCID2 | 1703 | 7 | 3 | 4 | 2 | 4 | 4 | 2 | 3 | 2 | 3 | 3 | 4 | 7 |
| NM_001127204 | HMOX2 | 1849 | 21 | 10 | 5 | 4 | 20 | 16 | 14 | 21 | 4 | 3 | 2 | 16 | 10 |
| NM_001127205 | HMOX2 | 1753 | 22 | 11 | 5 | 4 | 21 | 17 | 14 | 22 | 5 | 3 | 3 | 17 | 11 |
| NM_001127206 | HMOX2 | 1713 | 23 | 11 | 6 | 4 | 21 | 17 | 15 | 23 | 5 | 3 | 3 | 17 | 11 |
| NM_001127207 | SMARCAL1 | 3083 | 2 | 1 | 1 | 1 | 1 | 1 | 1 | 0 | 1 | 2 | 1 | 1 | 1 |
| NM_001127208 | TET2 | 9677 | 5 | 2 | 2 | 1 | 2 | 2 | 3 | 1 | 3 | 4 | 3 | 5 | 2 |
| NM_001127211 | KIAA1598 | 3943 | 41 | 10 | 8 | 4 | 4 | 41 | 10 | 11 | 9 | 4 | 1 | 7 | 15 |
| NM_001127212 | GAGE2A | 542 | 0 | 0 | 0 | 0 | 0 | 0 | 0 | 0 | 0 | 0 | 0 | 0 | 0 |
| NM_001127213 | LY6E | 1171 | 7 | 3 | 2 | 1 | 3 | 3 | 3 | 7 | 4 | 4 | 2 | 6 | 2 |
| NM_001127214 | ACSF3 | 2284 | 0 | 0 | 0 | 0 | 0 | 0 | 0 | 0 | 0 | 0 | 0 | 0 | 0 |
| NM_001127215 | GFI1 | 2701 | 1 | 0 | 0 | 0 | 0 | 0 | 0 | 0 | 0 | 0 | 0 | 1 | 0 |
| NM_001127216 | GFI1 | 2694 | 1 | 0 | 0 | 0 | 0 | 0 | 0 | 0 | 0 | 0 | 0 | 1 | 0 |
| NM_001127217 | SMAD9 | 2353 | 7 | 3 | 1 | 2 | 3 | 6 | 2 | 0 | 2 | 7 | 3 | 1 | 3 |
| NM_001127218 | POLD2 | 1775 | 2 | 1 | 1 | 1 | 2 | 1 | 1 | 2 | 1 | 1 | 2 | 1 | 0 |
| NM_001127219 | ACCS | 1877 | 1 | 1 | 1 | 1 | 0 | 0 | 1 | 0 | 1 | 0 | 0 | 1 | 1 |
| NM_001127221 | CACNA1A | 8625 | 1 | 0 | 0 | 0 | 0 | 1 | 0 | 0 | 0 | 0 | 0 | 0 | 0 |
| NM_001127222 | CACNA1A | 8627 | 1 | 0 | 0 | 0 | 0 | 1 | 0 | 0 | 0 | 0 | 0 | 0 | 0 |
| NM_001127223 | CD59 | 7785 | 24 | 12 | 10 | 4 | 24 | 12 | 18 | 8 | 21 | 6 | 10 | 10 | 9 |
| NM_001127225 | CD59 | 7678 | 24 | 12 | 11 | 4 | 24 | 13 | 18 | 9 | 22 | 6 | 10 | 10 | 9 |
| NM_001127226 | CD59 | 7613 | 24 | 12 | 11 | 4 | 24 | 13 | 19 | 9 | 22 | 7 | 10 | 10 | 9 |
| NM_001127227 | CD59 | 7557 | 24 | 13 | 11 | 4 | 24 | 13 | 19 | 9 | 22 | 7 | 10 | 10 | 9 |
| NM_001127228 | CBX1 | 2232 | 27 | 11 | 7 | 6 | 10 | 27 | 8 | 5 | 6 | 16 | 9 | 9 | 14 |
| NM_001127229 | AURKAIP1 | 909 | 10 | 6 | 6 | 7 | 7 | 7 | 7 | 10 | 2 | 1 | 5 | 4 | 5 |
| NM_001127230 | AURKAIP1 | 812 | 12 | 6 | 7 | 7 | 8 | 8 | 8 | 12 | 3 | 2 | 6 | 5 | 5 |
| NM_001127231 | AUTS2 | 5936 | 5 | 3 | 1 | 3 | 2 | 5 | 4 | 2 | 4 | 5 | 4 | 2 | 2 |
| NM_001127232 | AUTS2 | 1648 | 2 | 1 | 1 | 1 | 1 | 2 | 0 | 0 | 1 | 2 | 0 | 1 | 1 |
| NM_001127235 | GPBP1 | 3589 | 30 | 15 | 9 | 5 | 13 | 17 | 13 | 7 | 16 | 21 | 13 | 20 | 30 |
| NM_001127236 | GPBP1 | 2286 | 38 | 18 | 12 | 6 | 14 | 20 | 15 | 8 | 20 | 23 | 15 | 23 | 38 |
| NM_001127240 | BBC3 | 1827 | 3 | 1 | 0 | 1 | 0 | 1 | 0 | 0 | 1 | 1 | 3 | 1 | 0 |
| NM_001127241 | BBC3 | 1538 | 3 | 1 | 0 | 1 | 0 | 1 | 0 | 0 | 1 | 1 | 3 | 2 | 1 |
| NM_001127242 | BBC3 | 1347 | 3 | 1 | 1 | 1 | 0 | 2 | 0 | 0 | 2 | 1 | 3 | 2 | 1 |
| NM_001127244 | LRRC8A | 4623 | 4 | 2 | 1 | 1 | 2 | 4 | 2 | 2 | 3 | 2 | 1 | 3 | 2 |
| NM_001127245 | LRRC8A | 4247 | 5 | 2 | 1 | 2 | 2 | 5 | 2 | 2 | 3 | 2 | 1 | 4 | 2 |
| NM_001127255 | NLRP7 | 3421 | 0 | 0 | 0 | 0 | 0 | 0 | 0 | 0 | 0 | 0 | 0 | 0 | 0 |
| NM_001127257 | SLC39A10 | 5371 | 21 | 6 | 3 | 1 | 4 | 21 | 5 | 1 | 4 | 6 | 1 | 11 | 8 |
| NM_001127258 | HHIPL1 | 3462 | 0 | 0 | 0 | 0 | 0 | 0 | 0 | 0 | 0 | 0 | 0 | 0 | 0 |
| NM_001127266 | TMEM129 | 2837 | 0 | 0 | 0 | 0 | 0 | 0 | 0 | 0 | 0 | 0 | 0 | 0 | 0 |
| NM_001127320 | UBAP2L | 3410 | 17 | 12 | 10 | 8 | 12 | 14 | 11 | 8 | 12 | 12 | 10 | 16 | 17 |
| NM_001127321 | CBX5 | 11505 | 19 | 9 | 5 | 5 | 8 | 19 | 12 | 4 | 6 | 17 | 3 | 9 | 10 |
| NM_001127322 | CBX5 | 11703 | 19 | 9 | 5 | 5 | 8 | 19 | 11 | 4 | 6 | 17 | 3 | 9 | 10 |
| NM_001127323 | GRM8 | 3860 | 3 | 0 | 0 | 0 | 0 | 3 | 0 | 1 | 0 | 0 | 0 | 0 | 0 |
| NM_001127325 | MAD2L2 | 1152 | 1 | 1 | 0 | 0 | 1 | 1 | 1 | 1 | 1 | 0 | 1 | 1 | 1 |
| NM_001127328 | ACADM | 2615 | 32 | 14 | 12 | 4 | 32 | 10 | 29 | 17 | 4 | 7 | 15 | 11 | 9 |
| NM_001127345 | GAGE12B | 465 | 0 | 0 | 0 | 0 | 0 | 0 | 0 | 0 | 0 | 0 | 0 | 0 | 0 |
| NM_001127350 | MED18 | 1813 | 1 | 0 | 0 | 0 | 0 | 0 | 0 | 0 | 0 | 0 | 0 | 1 | 0 |
| NM_001127357 | PHTF2 | 5008 | 5 | 2 | 2 | 1 | 2 | 2 | 1 | 1 | 2 | 1 | 5 | 3 | 2 |
| NM_001127358 | PHTF2 | 5085 | 5 | 2 | 2 | 1 | 2 | 2 | 1 | 1 | 2 | 1 | 5 | 3 | 2 |
| NM_001127359 | PHTF2 | 2548 | 5 | 2 | 3 | 1 | 2 | 2 | 1 | 1 | 2 | 2 | 5 | 4 | 2 |
| NM_001127360 | PHTF2 | 2625 | 5 | 2 | 2 | 1 | 2 | 2 | 1 | 1 | 2 | 2 | 5 | 4 | 2 |
| NM_001127361 | RNF19B | 2603 | 15 | 6 | 3 | 5 | 4 | 8 | 6 | 1 | 7 | 4 | 3 | 6 | 15 |
| NM_001127362 | NR2C1 | 2114 | 8 | 5 | 6 | 5 | 4 | 5 | 4 | 5 | 4 | 5 | 5 | 5 | 8 |
| NM_001127364 | C7orf46 | 1364 | 3 | 1 | 0 | 1 | 0 | 3 | 1 | 1 | 1 | 0 | 0 | 0 | 3 |
| NM_001127365 | C7orf46 | 1190 | 2 | 1 | 0 | 0 | 0 | 2 | 1 | 1 | 1 | 0 | 0 | 0 | 2 |
| NM_001127366 | PAX3 | 3338 | 0 | 0 | 0 | 0 | 0 | 0 | 0 | 0 | 0 | 0 | 0 | 0 | 0 |
| NM_001127370 | CDCA7L | 2961 | 2 | 1 | 0 | 1 | 2 | 1 | 0 | 0 | 2 | 2 | 0 | 2 | 2 |
| NM_001127371 | CDCA7L | 2769 | 2 | 1 | 0 | 1 | 1 | 1 | 0 | 0 | 1 | 2 | 0 | 1 | 2 |
| NM_001127372 | ZNF84 | 6935 | 8 | 3 | 2 | 1 | 2 | 5 | 4 | 1 | 2 | 8 | 2 | 4 | 4 |
| NM_001127380 | SAA2 | 1196 | 490 | 51 | 32 | 1 | 5 | 0 | 1 | 490 | 11 | 2 | 11 | 2 | 2 |
| NM_001127381 | PPP2R2B | 2092 | 13 | 1 | 0 | 0 | 0 | 13 | 0 | 0 | 0 | 0 | 0 | 0 | 1 |
| NM_001127383 | CYBRD1 | 4148 | 39 | 16 | 39 | 7 | 19 | 7 | 5 | 2 | 36 | 29 | 9 | 7 | 20 |
| NM_001127384 | CTNNA3 | 3174 | 8 | 2 | 0 | 0 | 8 | 3 | 0 | 0 | 0 | 1 | 4 | 0 | 1 |
| NM_001127385 | CTXN3 | 1492 | 3 | 0 | 0 | 0 | 0 | 1 | 3 | 0 | 0 | 0 | 0 | 0 | 0 |
| NM_001127386 | LOC728410 | 1604 | 0 | 0 | 0 | 0 | 0 | 0 | 0 | 0 | 0 | 0 | 0 | 0 | 0 |
| NM_001127386 | LOC728410 | 1611 | 0 | 0 | 0 | 0 | 0 | 0 | 0 | 0 | 0 | 0 | 0 | 0 | 0 |
| NM_001127387 | LOC653543 | 1601 | 0 | 0 | 0 | 0 | 0 | 0 | 0 | 0 | 0 | 0 | 0 | 0 | 0 |
| NM_001127387 | LOC653543 | 1611 | 0 | 0 | 0 | 0 | 0 | 0 | 0 | 0 | 0 | 0 | 0 | 0 | 0 |
| NM_001127388 | LOC653544 | 1601 | 0 | 0 | 0 | 0 | 0 | 0 | 0 | 0 | 0 | 0 | 0 | 0 | 0 |
| NM_001127388 | LOC653544 | 1611 | 0 | 0 | 0 | 0 | 0 | 0 | 0 | 0 | 0 | 0 | 0 | 0 | 0 |
| NM_001127389 | LOC653545 | 1601 | 0 | 0 | 0 | 0 | 0 | 0 | 0 | 0 | 0 | 0 | 0 | 0 | 0 |
| NM_001127389 | LOC653545 | 1611 | 0 | 0 | 0 | 0 | 0 | 0 | 0 | 0 | 0 | 0 | 0 | 0 | 0 |
| NM_001127391 | ALS2CR12 | 1944 | 2 | 1 | 0 | 0 | 1 | 1 | 1 | 0 | 1 | 0 | 0 | 1 | 2 |
| NM_001127392 | C11orf9 | 5926 | 2 | 0 | 0 | 0 | 0 | 2 | 0 | 0 | 1 | 0 | 0 | 0 | 0 |
| NM_001127393 | C14orf2 | 921 | 11 | 4 | 4 | 4 | 11 | 10 | 5 | 4 | 2 | 2 | 3 | 2 | 2 |
| NM_001127394 | TSEN15 | 1816 | 7 | 4 | 4 | 1 | 6 | 6 | 4 | 2 | 4 | 3 | 4 | 7 | 5 |

| transcript | gene | transcript length (nt) | max | mean | adipose | colon | heart | hypothalamus | kidney | liver | lung | ovary | skeletalmuscle | spleen | testes |
|---|---|---|---|---|---|---|---|---|---|---|---|---|---|---|---|
| NM_001127395 | FAM119A | 4805 | 3 | 1 | 1 | 1 | 1 | 1 | 1 | 1 | 1 | 1 | 0 | 3 | 1 |
| NM_001127396 | STXBP2 | 1881 | 3 | 1 | 2 | 3 | 1 | 0 | 3 | 2 | 3 | 0 | 0 | 3 | 2 |
| NM_001127397 | ERLEC1 | 3043 | 9 | 6 | 5 | 2 | 8 | 9 | 5 | 5 | 6 | 5 | 3 | 6 | 8 |
| NM_001127398 | ERLEC1 | 2959 | 10 | 6 | 5 | 2 | 9 | 10 | 5 | 5 | 7 | 5 | 3 | 7 | 9 |
| NM_001127399 | YPEL5 | 2320 | 63 | 40 | 29 | 13 | 41 | 57 | 38 | 24 | 46 | 45 | 25 | 57 | 63 |
| NM_001127400 | YPEL5 | 2556 | 58 | 37 | 27 | 12 | 37 | 52 | 35 | 22 | 42 | 42 | 23 | 52 | 58 |
| NM_001127401 | YPEL5 | 2617 | 57 | 36 | 26 | 12 | 37 | 51 | 34 | 21 | 42 | 41 | 23 | 51 | 57 |
| NM_001127441 | CPA5 | 2062 | 0 | 0 | 0 | 0 | 0 | 0 | 0 | 0 | 0 | 0 | 0 | 0 | 0 |
| NM_001127442 | CPA5 | 1868 | 0 | 0 | 0 | 0 | 0 | 0 | 0 | 0 | 0 | 0 | 0 | 0 | 0 |
| NM_001127443 | CD36 | 1810 | 827 | 123 | 827 | 21 | 222 | 2 | 4 | 16 | 43 | 6 | 46 | 157 | 8 |
| NM_001127444 | CD36 | 1985 | 747 | 111 | 747 | 19 | 202 | 2 | 4 | 14 | 39 | 6 | 42 | 143 | 7 |
| NM_001127445 | CPA6 | 1491 | 1 | 0 | 0 | 1 | 0 | 0 | 0 | 0 | 0 | 0 | 0 | 0 | 1 |
| NM_001127448 | ABAT | 4908 | 45 | 9 | 0 | 1 | 3 | 22 | 19 | 45 | 1 | 2 | 0 | 1 | 5 |
| NM_001127453 | DFNA5 | 2250 | 5 | 2 | 2 | 1 | 2 | 3 | 2 | 1 | 2 | 3 | 1 | 0 | 5 |
| NM_001127454 | DFNA5 | 2265 | 5 | 2 | 1 | 1 | 1 | 3 | 2 | 1 | 1 | 3 | 1 | 0 | 5 |
| NM_001127457 | CRY2 | 4065 | 3 | 2 | 1 | 1 | 2 | 3 | 1 | 2 | 1 | 1 | 3 | 1 | 2 |
| NM_001127458 | CRLS1 | 3652 | 7 | 2 | 1 | 1 | 2 | 1 | 3 | 4 | 7 | 1 | 1 | 1 | 1 |
| NM_001127460 | PAN2 | 5372 | 2 | 1 | 1 | 2 | 1 | 1 | 2 | 2 | 1 | 1 | 1 | 2 | 2 |
| NM_001127461 | NLRP3 | 4318 | 2 | 1 | 1 | 0 | 0 | 0 | 0 | 0 | 1 | 0 | 0 | 2 | 0 |
| NM_001127462 | NLRP3 | 4284 | 2 | 1 | 1 | 0 | 0 | 0 | 0 | 0 | 1 | 0 | 0 | 2 | 0 |
| NM_001127464 | ZNF469 | 13203 | 0 | 0 | 0 | 0 | 0 | 0 | 0 | 0 | 0 | 0 | 0 | 0 | 0 |
| NM_001127482 | C13orf1 | 3085 | 4 | 2 | 1 | 1 | 2 | 4 | 2 | 2 | 1 | 1 | 3 | 1 | 1 |
| NM_001127487 | FLNC | 9039 | 72 | 12 | 1 | 7 | 72 | 1 | 0 | 0 | 1 | 1 | 51 | 0 | 1 |
| NM_001127489 | FXYD2 | 2734 | 10 | 1 | 0 | 0 | 0 | 0 | 10 | 0 | 0 | 0 | 0 | 0 | 0 |
| NM_001127491 | ITGB2 | 2912 | 9 | 3 | 6 | 1 | 3 | 1 | 0 | 1 | 6 | 1 | 1 | 9 | 0 |
| NM_001127493 | ANK2 | 8082 | 52 | 10 | 4 | 2 | 12 | 52 | 25 | 0 | 1 | 8 | 4 | 4 | 3 |
| NM_001127496 | SPRY4 | 4941 | 2 | 1 | 1 | 1 | 1 | 1 | 1 | 1 | 2 | 0 | 0 | 1 | 0 |
| NM_001127500 | MET | 6676 | 5 | 2 | 1 | 3 | 1 | 1 | 5 | 4 | 3 | 0 | 2 | 1 | 1 |
| NM_001127501 | ALPL | 2558 | 8 | 3 | 1 | 0 | 4 | 3 | 6 | 8 | 6 | 0 | 0 | 8 | 0 |
| NM_001127505 | ASAH1 | 2485 | 141 | 35 | 141 | 8 | 52 | 22 | 42 | 17 | 43 | 7 | 10 | 37 | 9 |
| NM_001127507 | BANK1 | 3044 | 33 | 4 | 4 | 1 | 0 | 0 | 1 | 0 | 2 | 0 | 0 | 33 | 2 |
| NM_001127510 | APC | 10838 | 28 | 6 | 3 | 2 | 5 | 28 | 4 | 2 | 3 | 5 | 5 | 5 | 4 |
| NM_001127511 | APC | 11025 | 28 | 6 | 3 | 2 | 5 | 28 | 4 | 2 | 3 | 5 | 4 | 5 | 4 |
| NM_001127582 | ING4 | 1448 | 5 | 2 | 3 | 2 | 1 | 5 | 2 | 2 | 1 | 2 | 1 | 3 | 3 |
| NM_001127583 | ING4 | 1439 | 5 | 2 | 3 | 2 | 1 | 5 | 2 | 2 | 1 | 2 | 1 | 3 | 3 |
| NM_001127584 | ING4 | 1436 | 5 | 2 | 3 | 2 | 1 | 5 | 2 | 2 | 1 | 2 | 1 | 3 | 3 |
| NM_001127585 | ING4 | 1376 | 4 | 2 | 2 | 2 | 1 | 4 | 2 | 1 | 1 | 2 | 1 | 3 | 2 |
| NM_001127586 | ING4 | 1300 | 5 | 2 | 3 | 2 | 1 | 5 | 2 | 2 | 1 | 3 | 1 | 4 | 3 |
| NM_001127592 | FCGR3A | 2305 | 38 | 7 | 3 | 0 | 9 | 8 | 1 | 6 | 9 | 0 | 0 | 38 | 1 |
| NM_001127593 | FCGR3A | 2171 | 41 | 7 | 3 | 0 | 10 | 8 | 1 | 7 | 9 | 0 | 0 | 41 | 1 |
| NM_001127595 | FCGR3A | 2153 | 41 | 7 | 3 | 0 | 10 | 8 | 1 | 7 | 10 | 0 | 0 | 41 | 1 |
| NM_001127596 | FCGR3A | 2150 | 41 | 7 | 3 | 0 | 10 | 8 | 1 | 7 | 10 | 0 | 0 | 41 | 1 |
| NM_001127598 | IGF2 | 4839 | 0 | 0 | 0 | 0 | 0 | 0 | 0 | 0 | 0 | 0 | 0 | 0 | 0 |
| NM_001127603 | C9orf95 | 1121 | 2 | 1 | 1 | 0 | 1 | 1 | 2 | 1 | 2 | 2 | 1 | 2 | 1 |
| NM_001127605 | LIPA | 2657 | 84 | 16 | 34 | 2 | 6 | 8 | 6 | 10 | 19 | 7 | 2 | 84 | 4 |
| NM_001127608 | FAM189A2 | 2107 | 7 | 2 | 0 | 0 | 1 | 5 | 0 | 0 | 2 | 5 | 7 | 0 | 0 |
| NM_001127610 | BAAT | 3377 | 48 | 5 | 0 | 0 | 0 | 0 | 0 | 48 | 0 | 0 | 0 | 0 | 0 |
| NM_001127612 | PAX6 | 6880 | 1 | 0 | 0 | 0 | 0 | 1 | 0 | 0 | 0 | 0 | 0 | 0 | 0 |
| NM_001127615 | ENOX1 | 2658 | 2 | 1 | 0 | 0 | 1 | 2 | 1 | 0 | 0 | 1 | 1 | 0 | 1 |
| NM_001127617 | ALDOA | 1451 | 318 | 73 | 54 | 39 | 115 | 92 | 54 | 14 | 31 | 24 | 318 | 43 | 22 |
| NM_001127621 | GALE | 1607 | 2 | 0 | 0 | 1 | 0 | 1 | 2 | 1 | 0 | 0 | 0 | 0 | 0 |
| NM_001127628 | FBP1 | 1493 | 57 | 8 | 6 | 2 | 0 | 1 | 16 | 57 | 5 | 1 | 0 | 2 | 0 |
| NM_001127641 | LAMB3 | 4035 | 9 | 2 | 9 | 3 | 0 | 0 | 1 | 1 | 4 | 0 | 0 | 0 | 0 |
| NM_001127643 | GABRA1 | 4263 | 10 | 1 | 0 | 0 | 0 | 10 | 0 | 0 | 0 | 0 | 0 | 0 | 0 |
| NM_001127644 | GABRA1 | 4412 | 10 | 1 | 0 | 0 | 0 | 10 | 0 | 0 | 0 | 0 | 0 | 0 | 0 |
| NM_001127645 | GABRA1 | 4000 | 10 | 1 | 0 | 0 | 0 | 10 | 0 | 0 | 0 | 0 | 0 | 0 | 0 |
| NM_001127646 | GABRA1 | 4067 | 10 | 1 | 0 | 0 | 0 | 10 | 0 | 0 | 0 | 0 | 0 | 0 | 0 |
| NM_001127647 | GABRA1 | 4223 | 10 | 1 | 0 | 0 | 0 | 10 | 0 | 0 | 0 | 0 | 0 | 0 | 0 |
| NM_001127648 | GABRA1 | 4022 | 10 | 1 | 0 | 0 | 0 | 10 | 0 | 0 | 0 | 0 | 0 | 0 | 0 |
| NM_001127649 | PEX26 | 2741 | 1 | 0 | 0 | 1 | 0 | 0 | 1 | 0 | 0 | 0 | 0 | 0 | 0 |
| NM_001127651 | NCF2 | 2239 | 16 | 5 | 9 | 1 | 4 | 2 | 1 | 1 | 16 | 1 | 1 | 16 | 1 |
| NM_001127656 | CSRP3 | 1282 | 266 | 28 | 0 | 0 | 266 | 0 | 0 | 0 | 0 | 0 | 41 | 0 | 0 |
| NM_001127660 | MFN2 | 4532 | 11 | 4 | 3 | 3 | 11 | 6 | 5 | 4 | 2 | 2 | 6 | 3 | 3 |
| NM_001127662 | GSN | 2562 | 32 | 7 | 32 | 8 | 17 | 4 | 2 | 2 | 3 | 3 | 5 | 2 | 4 |
| NM_001127663 | GSN | 2767 | 30 | 7 | 30 | 7 | 16 | 3 | 2 | 2 | 3 | 3 | 4 | 2 | 3 |
| NM_001127664 | GSN | 2734 | 30 | 7 | 30 | 7 | 16 | 4 | 2 | 2 | 3 | 3 | 4 | 2 | 3 |
| NM_001127665 | GSN | 2729 | 30 | 7 | 30 | 7 | 16 | 3 | 2 | 2 | 3 | 3 | 4 | 2 | 3 |
| NM_001127666 | GSN | 2726 | 31 | 7 | 31 | 7 | 16 | 4 | 2 | 2 | 3 | 3 | 4 | 2 | 3 |
| NM_001127667 | GSN | 2717 | 31 | 7 | 31 | 7 | 16 | 4 | 2 | 2 | 3 | 3 | 5 | 2 | 3 |
| NM_001127668 | KCNE1 | 3182 | 1 | 0 | 0 | 0 | 1 | 0 | 1 | 0 | 1 | 0 | 0 | 0 | 1 |
| NM_001127669 | KCNE1 | 3154 | 1 | 0 | 0 | 0 | 1 | 0 | 1 | 0 | 1 | 0 | 0 | 0 | 1 |
| NM_001127670 | KCNE1 | 3375 | 1 | 0 | 0 | 0 | 1 | 0 | 1 | 0 | 1 | 0 | 0 | 0 | 1 |
| NM_001127671 | LIFR | 10258 | 6 | 4 | 3 | 0 | 4 | 5 | 5 | 3 | 6 | 5 | 3 | 4 | 4 |
| NM_001127687 | ADIPOR1 | 2127 | 12 | 7 | 4 | 3 | 9 | 7 | 7 | 12 | 8 | 4 | 6 | 11 | 8 |
| NM_001127688 | BEX4 | 1166 | 97 | 21 | 10 | 11 | 16 | 97 | 20 | 2 | 10 | 12 | 1 | 21 | 29 |
| NM_001127691 | RHD | 2545 | 1 | 0 | 0 | 0 | 0 | 0 | 0 | 0 | 1 | 0 | 0 | 1 | 0 |
| NM_001127692 | PCCA | 2415 | 23 | 8 | 5 | 3 | 7 | 8 | 23 | 17 | 3 | 8 | 3 | 6 | 7 |

| transcript | gene | transcript length (nt) | max | mean | adipose | colon | heart | hypothalamus | kidney | liver | lung | ovary | skeletalmuscle | spleen | testes |
|---|---|---|---|---|---|---|---|---|---|---|---|---|---|---|---|
| NM_001127695 | CTSA | 2286 | 15 | 5 | 6 | 4 | 4 | 3 | 15 | 5 | 4 | 3 | 2 | 7 | 3 |
| NM_001127696 | ATXN3 | 6878 | 4 | 2 | 2 | 1 | 2 | 3 | 2 | 1 | 2 | 4 | 1 | 4 | 4 |
| NM_001127697 | ATXN3 | 6769 | 4 | 2 | 2 | 1 | 2 | 2 | 2 | 1 | 2 | 4 | 1 | 3 | 3 |
| NM_001127698 | SPINK5 | 3729 | 1 | 0 | 0 | 0 | 0 | 0 | 0 | 0 | 1 | 0 | 0 | 0 | 0 |
| NM_001127699 | SPINK5 | 2915 | 1 | 0 | 0 | 0 | 0 | 0 | 0 | 0 | 1 | 0 | 0 | 0 | 0 |
| NM_001127700 | SERPINA1 | 3236 | 4,201 | 395 | 0 | 7 | 9 | 4 | 53 | 4,201 | 19 | 10 | 6 | 26 | 9 |
| NM_001127701 | SERPINA1 | 3532 | 3,850 | 362 | 0 | 7 | 8 | 3 | 49 | 3,850 | 18 | 9 | 6 | 25 | 8 |
| NM_001127702 | SERPINA1 | 3340 | 4,072 | 383 | 0 | 7 | 9 | 3 | 52 | 4,072 | 18 | 9 | 6 | 26 | 9 |
| NM_001127703 | SERPINA1 | 3495 | 3,891 | 366 | 0 | 7 | 8 | 3 | 49 | 3,891 | 18 | 9 | 6 | 25 | 8 |
| NM_001127704 | SERPINA1 | 3492 | 3,895 | 366 | 0 | 7 | 8 | 3 | 49 | 3,895 | 18 | 9 | 6 | 25 | 8 |
| NM_001127705 | SERPINA1 | 3510 | 3,875 | 364 | 0 | 7 | 8 | 3 | 49 | 3,875 | 18 | 9 | 6 | 25 | 8 |
| NM_001127706 | SERPINA1 | 3303 | 4,117 | 387 | 0 | 7 | 9 | 4 | 52 | 4,117 | 19 | 9 | 6 | 26 | 9 |
| NM_001127707 | SERPINA1 | 3300 | 4,121 | 387 | 0 | 7 | 9 | 4 | 52 | 4,121 | 19 | 9 | 6 | 26 | 9 |
| NM_001127708 | PRG4 | 4921 | 214 | 26 | 31 | 2 | 4 | 2 | 1 | 214 | 6 | 5 | 12 | 2 | 4 |
| NM_001127709 | PRG4 | 4765 | 227 | 27 | 33 | 2 | 4 | 2 | 1 | 227 | 6 | 5 | 13 | 2 | 4 |
| NM_001127710 | PRG4 | 4642 | 227 | 27 | 33 | 2 | 4 | 2 | 1 | 227 | 6 | 5 | 13 | 2 | 4 |
| NM_001127711 | TMEM14B | 870 | 131 | 69 | 67 | 20 | 131 | 53 | 86 | 54 | 53 | 63 | 84 | 79 | 66 |
| NM_001127713 | ATL1 | 2814 | 14 | 2 | 0 | 0 | 0 | 14 | 1 | 0 | 0 | 1 | 0 | 1 | 1 |
| NM_001127714 | HIVEP3 | 8789 | 1 | 0 | 1 | 0 | 0 | 1 | 0 | 0 | 1 | 0 | 0 | 1 | 0 |
| NM_001127715 | STXBP5 | 6433 | 11 | 4 | 2 | 3 | 3 | 11 | 2 | 1 | 3 | 2 | 3 | 5 | 5 |
| NM_001127716 | ETFA | 1207 | 80 | 27 | 20 | 10 | 80 | 16 | 35 | 56 | 13 | 10 | 26 | 21 | 13 |
| NM_001127717 | LAMA3 | 10337 | 4 | 1 | 1 | 2 | 1 | 0 | 0 | 0 | 4 | 1 | 0 | 0 | 1 |
| NM_001127718 | LAMA3 | 5449 | 6 | 1 | 1 | 2 | 1 | 0 | 1 | 0 | 6 | 1 | 0 | 1 | 2 |
| NM_001127889 | SPG21 | 1618 | 3 | 2 | 2 | 1 | 2 | 1 | 3 | 3 | 2 | 2 | 1 | 3 | 2 |
| NM_001127890 | SPG21 | 1717 | 3 | 2 | 1 | 1 | 2 | 1 | 3 | 2 | 2 | 2 | 1 | 3 | 2 |
| NM_001127891 | MMP2 | 3379 | 37 | 15 | 24 | 10 | 20 | 1 | 3 | 1 | 18 | 37 | 11 | 11 | 26 |
| NM_001127892 | SALL1 | 5253 | 6 | 2 | 0 | 1 | 0 | 4 | 6 | 5 | 0 | 1 | 0 | 0 | 1 |
| NM_001127893 | CEACAM19 | 2245 | 1 | 0 | 0 | 1 | 0 | 0 | 0 | 0 | 0 | 0 | 0 | 0 | 0 |
| NM_001127895 | CHST8 | 2226 | 0 | 0 | 0 | 0 | 0 | 0 | 0 | 0 | 0 | 0 | 0 | 0 | 0 |
| NM_001127896 | CHST8 | 2137 | 0 | 0 | 0 | 0 | 0 | 0 | 0 | 0 | 0 | 0 | 0 | 0 | 0 |
| NM_001127897 | RPGRIP1L | 5942 | 3 | 1 | 1 | 0 | 1 | 2 | 1 | 0 | 1 | 2 | 0 | 1 | 3 |
| NM_001127898 | CLCN5 | 9849 | 9 | 1 | 0 | 0 | 0 | 0 | 9 | 1 | 1 | 1 | 0 | 1 | 1 |
| NM_001127899 | CLCN5 | 10108 | 9 | 1 | 0 | 0 | 0 | 0 | 9 | 1 | 1 | 1 | 0 | 1 | 1 |
| NM_001128 | AP1G1 | 6839 | 10 | 6 | 4 | 3 | 4 | 6 | 10 | 7 | 5 | 4 | 5 | 7 | 7 |
| NM_001128076 | SPDYE4 | 923 | 0 | 0 | 0 | 0 | 0 | 0 | 0 | 0 | 0 | 0 | 0 | 0 | 0 |
| NM_001128077 | KRTAP20-3 | 273 | 0 | 0 | 0 | 0 | 0 | 0 | 0 | 0 | 0 | 0 | 0 | 0 | 0 |
| NM_001128085 | ASPA | 1351 | 5 | 1 | 1 | 0 | 0 | 2 | 5 | 0 | 1 | 1 | 0 | 1 | 1 |
| NM_001128126 | AP4S1 | 1214 | 6 | 2 | 2 | 0 | 2 | 6 | 2 | 1 | 2 | 3 | 1 | 3 | 4 |
| NM_001128127 | GK | 3707 | 3 | 1 | 0 | 0 | 0 | 0 | 3 | 3 | 1 | 0 | 0 | 1 | 0 |
| NM_001128128 | ZEB1 | 5650 | 24 | 14 | 18 | 4 | 24 | 24 | 5 | 4 | 17 | 10 | 23 | 9 | 13 |
| NM_001128141 | DPEP1 | 1648 | 10 | 1 | 0 | 0 | 0 | 0 | 10 | 0 | 0 | 0 | 0 | 0 | 1 |
| NM_001128142 | RWDD3 | 1136 | 5 | 3 | 3 | 2 | 3 | 5 | 4 | 3 | 2 | 3 | 2 | 4 | 5 |
| NM_001128143 | PMS1 | 3404 | 4 | 2 | 2 | 0 | 2 | 3 | 2 | 2 | 1 | 3 | 2 | 3 | 4 |
| NM_001128144 | PMS1 | 3035 | 3 | 2 | 2 | 0 | 2 | 2 | 2 | 1 | 1 | 3 | 2 | 3 | 3 |
| NM_001128147 | NF1 | 2939 | 4 | 2 | 1 | 1 | 3 | 4 | 3 | 2 | 2 | 4 | 2 | 2 | 4 |
| NM_001128148 | TFRC | 5083 | 39 | 20 | 25 | 11 | 21 | 24 | 21 | 10 | 32 | 39 | 5 | 12 | 17 |
| NM_001128149 | ATXN7 | 7292 | 3 | 2 | 1 | 1 | 2 | 1 | 2 | 1 | 2 | 2 | 2 | 3 | 2 |
| NM_001128150 | ORMDL1 | 2072 | 12 | 6 | 5 | 2 | 6 | 6 | 6 | 4 | 6 | 5 | 3 | 12 | 8 |
| NM_001128159 | VPS53 | 10284 | 9 | 3 | 2 | 2 | 2 | 9 | 3 | 2 | 2 | 2 | 2 | 3 | 3 |
| NM_001128160 | UBP1 | 4040 | 11 | 6 | 5 | 7 | 8 | 7 | 6 | 6 | 6 | 6 | 3 | 11 | 6 |
| NM_001128161 | UBP1 | 3767 | 11 | 7 | 5 | 8 | 8 | 7 | 6 | 6 | 6 | 7 | 3 | 11 | 7 |
| NM_001128164 | ATXN1 | 10587 | 6 | 3 | 2 | 2 | 2 | 6 | 2 | 1 | 3 | 3 | 4 | 3 | 2 |
| NM_001128165 | FBLN7 | 2161 | 0 | 0 | 0 | 0 | 0 | 0 | 0 | 0 | 0 | 0 | 0 | 0 | 0 |
| NM_001128166 | PAK3 | 2577 | 33 | 4 | 0 | 0 | 0 | 33 | 0 | 0 | 0 | 1 | 0 | 1 | 5 |
| NM_001128167 | PAK3 | 2504 | 34 | 4 | 0 | 0 | 0 | 34 | 0 | 0 | 0 | 1 | 0 | 1 | 5 |
| NM_001128168 | PAK3 | 2268 | 38 | 4 | 1 | 0 | 0 | 38 | 0 | 0 | 0 | 1 | 0 | 1 | 5 |
| NM_001128172 | PAK3 | 2223 | 39 | 4 | 1 | 0 | 0 | 39 | 0 | 0 | 0 | 1 | 0 | 1 | 6 |
| NM_001128173 | PAK3 | 2205 | 39 | 4 | 1 | 0 | 0 | 39 | 0 | 0 | 0 | 2 | 0 | 1 | 6 |
| NM_001128174 | UGT8 | 2906 | 28 | 5 | 0 | 6 | 0 | 28 | 12 | 0 | 1 | 0 | 0 | 2 | 2 |
| NM_001128175 | DTNA | 1720 | 25 | 5 | 2 | 1 | 14 | 25 | 1 | 0 | 1 | 2 | 7 | 2 | 4 |
| NM_001128176 | THRB | 7465 | 4 | 1 | 2 | 1 | 1 | 4 | 1 | 2 | 1 | 0 | 2 | 1 | 0 |
| NM_001128177 | THRB | 7538 | 4 | 1 | 2 | 1 | 1 | 4 | 1 | 2 | 1 | 0 | 2 | 1 | 0 |
| NM_001128178 | NPHP1 | 2582 | 6 | 2 | 1 | 1 | 2 | 3 | 3 | 0 | 2 | 2 | 3 | 1 | 6 |
| NM_001128179 | NPHP1 | 2393 | 6 | 2 | 1 | 1 | 2 | 3 | 3 | 0 | 2 | 2 | 4 | 1 | 6 |
| NM_001128202 | C10orf122 | 922 | 1 | 0 | 0 | 0 | 0 | 0 | 0 | 0 | 0 | 0 | 0 | 0 | 1 |
| NM_001128203 | PLA2G16 | 1101 | 31 | 7 | 31 | 1 | 7 | 10 | 7 | 8 | 3 | 3 | 4 | 1 | 4 |
| NM_001128204 | SULF1 | 5548 | 9 | 3 | 2 | 3 | 9 | 4 | 4 | 0 | 2 | 4 | 1 | 1 | 5 |
| NM_001128205 | SULF1 | 5716 | 9 | 3 | 2 | 3 | 9 | 4 | 4 | 0 | 2 | 4 | 1 | 1 | 5 |
| NM_001128206 | SULF1 | 5554 | 9 | 3 | 2 | 3 | 9 | 4 | 4 | 0 | 2 | 4 | 1 | 1 | 5 |
| NM_001128208 | LEPROTL1 | 821 | 1 | 0 | 0 | 0 | 0 | 1 | 0 | 0 | 0 | 0 | 0 | 0 | 0 |
| NM_001128209 | SGCD | 9743 | 9 | 2 | 1 | 0 | 9 | 1 | 0 | 0 | 0 | 4 | 5 | 0 | 1 |
| NM_001128210 | SPRED2 | 4089 | 2 | 1 | 1 | 1 | 1 | 2 | 1 | 1 | 1 | 2 | 1 | 1 | 1 |
| NM_001128211 | NUDCD1 | 3853 | 3 | 2 | 1 | 0 | 2 | 2 | 2 | 1 | 1 | 2 | 1 | 3 | 3 |
| NM_001128212 | WDSUB1 | 1838 | 2 | 1 | 1 | 1 | 1 | 1 | 2 | 0 | 1 | 1 | 1 | 2 | 1 |
| NM_001128213 | WDSUB1 | 1928 | 2 | 1 | 1 | 1 | 1 | 1 | 2 | 0 | 1 | 1 | 1 | 2 | 1 |
| NM_001128214 | KCTD6 | 1559 | 3 | 1 | 1 | 0 | 1 | 3 | 2 | 1 | 1 | 1 | 1 | 1 | 1 |
| NM_001128215 | LIPM | 1484 | 0 | 0 | 0 | 0 | 0 | 0 | 0 | 0 | 0 | 0 | 0 | 0 | 0 |

| transcript | gene | transcript length (nt) | max | mean | adipose | colon | heart | hypothalamus | kidney | liver | lung | ovary | skeletalmuscle | spleen | testes |
|---|---|---|---|---|---|---|---|---|---|---|---|---|---|---|---|
| NM_001128217 | PSIP1 | 3373 | 59 | 21 | 11 | 4 | 13 | 59 | 12 | 5 | 13 | 42 | 13 | 27 | 33 |
| NM_001128219 | VGLL4 | 3783 | 4 | 2 | 1 | 1 | 2 | 1 | 2 | 0 | 2 | 4 | 0 | 2 | 4 |
| NM_001128220 | VGLL4 | 3182 | 4 | 2 | 1 | 1 | 2 | 2 | 2 | 0 | 2 | 4 | 0 | 2 | 4 |
| NM_001128221 | VGLL4 | 3425 | 4 | 2 | 1 | 1 | 2 | 1 | 2 | 0 | 2 | 4 | 0 | 2 | 4 |
| NM_001128223 | ZNF717 | 3068 | 6 | 3 | 1 | 1 | 2 | 3 | 3 | 1 | 2 | 6 | 2 | 3 | 5 |
| NM_001128224 | TMCC1 | 5470 | 3 | 2 | 2 | 1 | 1 | 3 | 1 | 2 | 1 | 2 | 2 | 2 | 3 |
| NM_001128225 | SLC39A13 | 2420 | 1 | 1 | 0 | 1 | 1 | 0 | 1 | 0 | 1 | 0 | 0 | 1 | 1 |
| NM_001128226 | DIS3 | 7554 | 4 | 3 | 2 | 1 | 3 | 3 | 2 | 2 | 3 | 3 | 2 | 3 | 4 |
| NM_001128227 | GNE | 5285 | 23 | 5 | 3 | 4 | 2 | 4 | 3 | 23 | 2 | 4 | 2 | 3 | 4 |
| NM_001128228 | C9orf75 | 2448 | 1 | 0 | 0 | 1 | 0 | 1 | 0 | 0 | 0 | 0 | 0 | 0 | 0 |
| NM_001128301 | LYRM1 | 1633 | 8 | 5 | 5 | 2 | 6 | 4 | 6 | 8 | 3 | 7 | 3 | 4 | 4 |
| NM_001128302 | LYRM1 | 1401 | 9 | 4 | 4 | 1 | 6 | 4 | 6 | 9 | 3 | 5 | 3 | 3 | 3 |
| NM_001128303 | C13orf31 | 4263 | 8 | 4 | 4 | 2 | 4 | 5 | 3 | 2 | 4 | 2 | 1 | 5 | 8 |
| NM_001128304 | PLSCR4 | 3431 | 10 | 5 | 6 | 2 | 5 | 5 | 4 | 3 | 7 | 8 | 2 | 6 | 10 |
| NM_001128305 | PLSCR4 | 3393 | 10 | 5 | 6 | 2 | 5 | 5 | 4 | 3 | 7 | 8 | 2 | 6 | 10 |
| NM_001128306 | PLSCR4 | 3035 | 11 | 6 | 7 | 2 | 5 | 5 | 4 | 3 | 8 | 9 | 2 | 7 | 11 |
| NM_001128309 | TSPAN14 | 5210 | 7 | 3 | 4 | 2 | 2 | 2 | 3 | 2 | 5 | 3 | 1 | 7 | 3 |
| NM_001128310 | SPARCL1 | 3006 | 303 | 108 | 105 | 14 | 250 | 303 | 19 | 3 | 140 | 112 | 66 | 57 | 114 |
| NM_001128324 | ANUBL1 | 3271 | 12 | 2 | 1 | 1 | 2 | 1 | 2 | 2 | 1 | 2 | 1 | 2 | 12 |
| NM_001128325 | SPON2 | 1787 | 2 | 1 | 1 | 1 | 0 | 0 | 1 | 1 | 1 | 2 | 0 | 0 | 0 |
| NM_001128326 | BSPH1 | 654 | 1 | 0 | 0 | 0 | 0 | 0 | 0 | 0 | 0 | 0 | 0 | 0 | 1 |
| NM_001128423 | MPV17L | 2309 | 5 | 3 | 1 | 1 | 1 | 4 | 4 | 5 | 1 | 3 | 4 | 2 | 2 |
| NM_001128424 | FAM198B | 4794 | 29 | 9 | 15 | 2 | 20 | 6 | 3 | 1 | 5 | 29 | 1 | 12 | 6 |
| NM_001128425 | MUTYH | 1930 | 0 | 0 | 0 | 0 | 0 | 0 | 0 | 0 | 0 | 0 | 0 | 0 | 0 |
| NM_001128426 | AP1AR | 2809 | 10 | 6 | 3 | 3 | 4 | 10 | 8 | 4 | 5 | 6 | 3 | 9 | 8 |
| NM_001128427 | FSTL5 | 4827 | 3 | 0 | 0 | 0 | 0 | 3 | 0 | 0 | 0 | 0 | 0 | 0 | 0 |
| NM_001128428 | FSTL5 | 4800 | 3 | 0 | 0 | 0 | 0 | 3 | 0 | 0 | 0 | 0 | 0 | 0 | 0 |
| NM_001128429 | SMARCAD1 | 5100 | 4 | 2 | 1 | 0 | 1 | 2 | 2 | 1 | 2 | 3 | 1 | 3 | 4 |
| NM_001128430 | SMARCAD1 | 5022 | 4 | 2 | 1 | 1 | 1 | 2 | 3 | 1 | 2 | 3 | 1 | 3 | 4 |
| NM_001128431 | SLC39A14 | 4690 | 45 | 8 | 1 | 2 | 17 | 3 | 6 | 45 | 2 | 2 | 2 | 3 | 6 |
| NM_001128432 | GBA3 | 1226 | 16 | 2 | 0 | 1 | 0 | 0 | 16 | 5 | 0 | 0 | 0 | 2 | 0 |
| NM_001128588 | SLC14A1 | 4128 | 2 | 0 | 0 | 0 | 1 | 2 | 1 | 0 | 1 | 0 | 0 | 0 | 0 |
| NM_001128590 | CYP21A2 | 2024 | 0 | 0 | 0 | 0 | 0 | 0 | 0 | 0 | 0 | 0 | 0 | 0 | 0 |
| NM_001128591 | PSMG4 | 821 | 14 | 9 | 8 | 7 | 9 | 12 | 8 | 10 | 5 | 7 | 14 | 10 | 10 |
| NM_001128592 | PSMG4 | 938 | 12 | 8 | 7 | 6 | 7 | 11 | 7 | 9 | 4 | 6 | 12 | 9 | 8 |
| NM_001128595 | TC2N | 4841 | 9 | 3 | 1 | 2 | 4 | 1 | 3 | 1 | 9 | 1 | 1 | 6 | 4 |
| NM_001128596 | TC2N | 4981 | 8 | 3 | 1 | 2 | 4 | 0 | 3 | 1 | 8 | 1 | 1 | 5 | 4 |
| NM_001128598 | KRTAP25-1 | 370 | 0 | 0 | 0 | 0 | 0 | 0 | 0 | 0 | 0 | 0 | 0 | 0 | 0 |
| NM_001128600 | LCE6A | 642 | 0 | 0 | 0 | 0 | 0 | 0 | 0 | 0 | 0 | 0 | 0 | 0 | 0 |
| NM_001128602 | RASGRP1 | 4920 | 13 | 2 | 1 | 1 | 0 | 13 | 3 | 0 | 2 | 1 | 0 | 4 | 2 |
| NM_001128608 | MAPKBP1 | 7250 | 2 | 1 | 1 | 1 | 0 | 1 | 1 | 0 | 1 | 1 | 0 | 1 | 2 |
| NM_001128610 | USP8 | 5687 | 14 | 7 | 5 | 3 | 7 | 8 | 8 | 6 | 6 | 7 | 5 | 10 | 14 |
| NM_001128611 | USP8 | 5849 | 13 | 7 | 5 | 3 | 7 | 8 | 8 | 6 | 6 | 7 | 4 | 10 | 13 |
| NM_001128612 | NUDT22 | 988 | 1 | 1 | 0 | 1 | 0 | 1 | 1 | 1 | 0 | 0 | 0 | 0 | 1 |
| NM_001128613 | NUDT22 | 1019 | 1 | 0 | 0 | 1 | 0 | 1 | 1 | 1 | 0 | 0 | 0 | 0 | 0 |
| NM_001128615 | ARHGEF3 | 3639 | 7 | 4 | 1 | 1 | 3 | 7 | 6 | 0 | 6 | 4 | 2 | 5 | 7 |
| NM_001128616 | ARHGEF3 | 4055 | 6 | 3 | 1 | 1 | 2 | 6 | 6 | 0 | 6 | 4 | 2 | 4 | 6 |
| NM_001128617 | REPS1 | 3470 | 5 | 3 | 1 | 1 | 2 | 3 | 2 | 5 | 1 | 3 | 1 | 4 | 5 |
| NM_001128618 | C9orf57 | 1497 | 0 | 0 | 0 | 0 | 0 | 0 | 0 | 0 | 0 | 0 | 0 | 0 | 0 |
| NM_001128619 | LUZP6 | 3884 | 0 | 0 | 0 | 0 | 0 | 0 | 0 | 0 | 0 | 0 | 0 | 0 | 0 |
| NM_001128620 | PAK1 | 3479 | 42 | 10 | 4 | 12 | 3 | 42 | 7 | 2 | 6 | 8 | 6 | 9 | 8 |
| NM_001128626 | SPIRE1 | 5444 | 14 | 5 | 4 | 1 | 9 | 14 | 5 | 1 | 5 | 4 | 6 | 3 | 6 |
| NM_001128627 | SPIRE1 | 5100 | 15 | 6 | 4 | 1 | 10 | 15 | 5 | 1 | 6 | 4 | 7 | 3 | 7 |
| NM_001128628 | PAK6 | 3879 | 1 | 0 | 0 | 0 | 0 | 1 | 0 | 0 | 0 | 0 | 0 | 0 | 1 |
| NM_001128629 | PAK6 | 3796 | 1 | 0 | 0 | 0 | 0 | 1 | 0 | 0 | 0 | 0 | 0 | 0 | 1 |
| NM_001128631 | DCAKD | 1963 | 4 | 1 | 2 | 2 | 1 | 4 | 1 | 1 | 1 | 2 | 1 | 1 | 1 |
| NM_001128633 | RIMBP3C | 5793 | 0 | 0 | 0 | 0 | 0 | 0 | 0 | 0 | 0 | 0 | 0 | 0 | 0 |
| NM_001128635 | RIMBP3B | 5416 | 0 | 0 | 0 | 0 | 0 | 0 | 0 | 0 | 0 | 0 | 0 | 0 | 0 |
| NM_001128636 | ELFN1 | 3742 | 1 | 0 | 0 | 0 | 1 | 1 | 0 | 1 | 0 | 0 | 0 | 0 | 0 |
| NM_001128826 | FREQ | 4860 | 5 | 1 | 0 | 1 | 0 | 5 | 1 | 0 | 0 | 0 | 0 | 0 | 0 |
| NM_001128827 | DLG4 | 3445 | 8 | 1 | 0 | 0 | 0 | 8 | 0 | 0 | 0 | 0 | 0 | 0 | 0 |
| NM_001128829 | CA1 | 1284 | 11 | 1 | 0 | 11 | 0 | 0 | 0 | 0 | 1 | 0 | 0 | 0 | 0 |
| NM_001128830 | CA1 | 1286 | 11 | 1 | 0 | 11 | 0 | 0 | 0 | 0 | 1 | 0 | 0 | 0 | 0 |
| NM_001128831 | CA1 | 1209 | 11 | 1 | 0 | 11 | 0 | 0 | 0 | 0 | 1 | 0 | 0 | 0 | 0 |
| NM_001128833 | ZBTB4 | 5866 | 7 | 4 | 5 | 3 | 5 | 7 | 4 | 1 | 5 | 6 | 3 | 5 | 4 |
| NM_001128834 | PLP1 | 3135 | 251 | 24 | 0 | 3 | 2 | 251 | 0 | 0 | 0 | 1 | 0 | 0 | 9 |
| NM_001128835 | TRAPPC2 | 1600 | 1 | 1 | 0 | 0 | 0 | 1 | 1 | 0 | 1 | 1 | 0 | 1 | 1 |
| NM_001128839 | CACNA1D | 7636 | 1 | 1 | 0 | 0 | 0 | 1 | 1 | 0 | 1 | 0 | 0 | 1 | 1 |
| NM_001128840 | CACNA1D | 7708 | 1 | 1 | 0 | 0 | 0 | 1 | 1 | 0 | 1 | 0 | 0 | 1 | 1 |
| NM_001128843 | TNIP3 | 2428 | 3 | 0 | 0 | 0 | 0 | 0 | 0 | 0 | 3 | 0 | 0 | 1 | 0 |
| NM_001128844 | SMARCA4 | 5770 | 9 | 4 | 3 | 4 | 3 | 9 | 5 | 3 | 2 | 3 | 3 | 4 | 4 |
| NM_001128845 | SMARCA4 | 5320 | 9 | 4 | 3 | 5 | 4 | 9 | 6 | 3 | 3 | 3 | 3 | 5 | 4 |
| NM_001128846 | SMARCA4 | 5317 | 9 | 4 | 3 | 5 | 4 | 9 | 6 | 3 | 3 | 3 | 3 | 5 | 4 |
| NM_001128847 | SMARCA4 | 5311 | 9 | 4 | 3 | 5 | 4 | 9 | 6 | 3 | 3 | 3 | 3 | 5 | 5 |
| NM_001128848 | SMARCA4 | 5308 | 9 | 4 | 3 | 5 | 4 | 9 | 6 | 3 | 3 | 3 | 3 | 5 | 5 |
| NM_001128849 | SMARCA4 | 5580 | 9 | 4 | 3 | 5 | 3 | 9 | 5 | 3 | 3 | 3 | 3 | 4 | 4 |
| NM_001128850 | RRAD | 1476 | 2 | 0 | 0 | 0 | 1 | 0 | 0 | 0 | 0 | 0 | 2 | 0 | 0 |

| transcript | gene | transcript length (nt) | max | mean | adipose | colon | heart | hypothalamus | kidney | liver | lung | ovary | skeletalmuscle | spleen | testes |
|---|---|---|---|---|---|---|---|---|---|---|---|---|---|---|---|
| NM_001128852 | SRRT | 3010 | 38 | 15 | 9 | 16 | 15 | 10 | 14 | 11 | 9 | 13 | 13 | 20 | 38 |
| NM_001128853 | SRRT | 3001 | 38 | 15 | 9 | 16 | 15 | 10 | 14 | 12 | 10 | 13 | 13 | 20 | 38 |
| NM_001128854 | SRRT | 2998 | 38 | 15 | 9 | 16 | 15 | 10 | 14 | 12 | 10 | 13 | 13 | 20 | 38 |
| NM_001128855 | GTPBP3 | 2509 | 0 | 0 | 0 | 0 | 0 | 0 | 0 | 0 | 0 | 0 | 0 | 0 | 0 |
| NM_001128911 | PCBP2 | 3168 | 43 | 24 | 19 | 8 | 26 | 17 | 34 | 18 | 20 | 43 | 20 | 31 | 30 |
| NM_001128912 | PCBP2 | 3156 | 43 | 24 | 19 | 9 | 26 | 17 | 34 | 18 | 20 | 43 | 20 | 31 | 30 |
| NM_001128913 | PCBP2 | 3078 | 44 | 25 | 19 | 9 | 27 | 17 | 34 | 18 | 21 | 44 | 20 | 32 | 31 |
| NM_001128914 | PCBP2 | 3027 | 45 | 25 | 20 | 9 | 28 | 17 | 35 | 18 | 21 | 45 | 21 | 32 | 32 |
| NM_001128915 | CYP24A1 | 3068 | 8 | 1 | 0 | 0 | 0 | 0 | 8 | 0 | 0 | 0 | 0 | 0 | 0 |
| NM_001128916 | TOMM40 | 1710 | 2 | 2 | 1 | 1 | 2 | 2 | 2 | 2 | 2 | 1 | 2 | 2 | 1 |
| NM_001128917 | TOMM40 | 1802 | 2 | 2 | 1 | 1 | 2 | 2 | 2 | 2 | 2 | 1 | 2 | 2 | 1 |
| NM_001128918 | MARK3 | 3530 | 20 | 12 | 7 | 7 | 20 | 13 | 10 | 7 | 14 | 12 | 11 | 14 | 15 |
| NM_001128919 | MARK3 | 3503 | 20 | 12 | 7 | 7 | 20 | 13 | 10 | 7 | 14 | 12 | 11 | 14 | 15 |
| NM_001128920 | MARK3 | 3410 | 21 | 12 | 7 | 7 | 21 | 13 | 10 | 7 | 14 | 12 | 11 | 14 | 16 |
| NM_001128921 | MARK3 | 3248 | 21 | 12 | 7 | 7 | 21 | 13 | 10 | 7 | 14 | 12 | 11 | 14 | 16 |
| NM_001128922 | LRRC32 | 4207 | 5 | 2 | 2 | 1 | 2 | 1 | 2 | 0 | 5 | 1 | 1 | 3 | 1 |
| NM_001128923 | SH3D19 | 5192 | 29 | 14 | 29 | 12 | 22 | 6 | 9 | 14 | 18 | 23 | 7 | 3 | 11 |
| NM_001128924 | SH3D19 | 4195 | 34 | 16 | 34 | 14 | 26 | 7 | 11 | 16 | 21 | 27 | 8 | 4 | 14 |
| NM_001128925 | CYP2C18 | 2368 | 23 | 2 | 0 | 1 | 0 | 0 | 0 | 23 | 0 | 0 | 0 | 0 | 0 |
| NM_001128926 | FAM71F2 | 1105 | 1 | 0 | 0 | 0 | 0 | 0 | 1 | 0 | 0 | 1 | 1 | 0 | 1 |
| NM_001128928 | INPP1 | 2163 | 8 | 3 | 2 | 2 | 4 | 8 | 2 | 3 | 3 | 2 | 2 | 4 | 7 |
| NM_001128929 | ROBO2 | 5535 | 7 | 1 | 0 | 0 | 0 | 7 | 0 | 0 | 2 | 4 | 0 | 1 | 2 |
| NM_001128930 | ENTPD4 | 2966 | 6 | 3 | 2 | 2 | 4 | 6 | 3 | 2 | 3 | 3 | 1 | 6 | 5 |
| NM_001128931 | NAF1 | 1635 | 7 | 3 | 2 | 1 | 2 | 3 | 2 | 2 | 3 | 7 | 7 | 3 | 3 |
| NM_001128932 | CYP4F11 | 2973 | 17 | 2 | 0 | 1 | 0 | 1 | 2 | 17 | 0 | 0 | 0 | 0 | 0 |
| NM_001128933 | SYNPO2 | 9935 | 141 | 24 | 11 | 19 | 35 | 6 | 3 | 1 | 5 | 16 | 141 | 6 | 15 |
| NM_001128934 | SYNPO2 | 4851 | 158 | 27 | 13 | 25 | 39 | 7 | 4 | 1 | 5 | 18 | 158 | 8 | 17 |
| NM_001129 | AEBP1 | 4081 | 24 | 11 | 12 | 8 | 20 | 5 | 7 | 1 | 12 | 24 | 4 | 11 | 18 |
| NM_001129727 | PLEKHG4 | 4591 | 0 | 0 | 0 | 0 | 0 | 0 | 0 | 0 | 0 | 0 | 0 | 0 | 0 |
| NM_001129728 | PLEKHG4 | 4567 | 0 | 0 | 0 | 0 | 0 | 0 | 0 | 0 | 0 | 0 | 0 | 0 | 0 |
| NM_001129729 | PLEKHG4 | 4478 | 0 | 0 | 0 | 0 | 0 | 0 | 0 | 0 | 0 | 0 | 0 | 0 | 0 |
| NM_001129730 | ZNF837 | 2033 | 0 | 0 | 0 | 0 | 0 | 0 | 0 | 0 | 0 | 0 | 0 | 0 | 0 |
| NM_001129731 | PLEKHG4 | 4481 | 0 | 0 | 0 | 0 | 0 | 0 | 0 | 0 | 0 | 0 | 0 | 0 | 0 |
| NM_001129742 | CALHM3 | 1652 | 0 | 0 | 0 | 0 | 0 | 0 | 0 | 0 | 0 | 0 | 0 | 0 | 0 |
| NM_001129758 | DPEP3 | 1735 | 1 | 0 | 0 | 0 | 0 | 0 | 0 | 0 | 0 | 0 | 0 | 0 | 1 |
| NM_001129765 | NSDHL | 1630 | 16 | 6 | 6 | 3 | 7 | 8 | 4 | 16 | 3 | 6 | 2 | 4 | 6 |
| NM_001129778 | GRAPL | 967 | 0 | 0 | 0 | 0 | 0 | 0 | 0 | 0 | 0 | 0 | 0 | 0 | 0 |
| NM_001129819 | CYB5R3 | 2199 | 18 | 9 | 15 | 5 | 13 | 9 | 7 | 18 | 5 | 6 | 5 | 6 | 7 |
| NM_001129820 | SLFN14 | 2882 | 0 | 0 | 0 | 0 | 0 | 0 | 0 | 0 | 0 | 0 | 0 | 0 | 0 |
| NM_001129826 | CSAG3 | 770 | 0 | 0 | 0 | 0 | 0 | 0 | 0 | 0 | 0 | 0 | 0 | 0 | 0 |
| NM_001129827 | CACNA1C | 13624 | 3 | 1 | 0 | 2 | 3 | 2 | 0 | 0 | 1 | 0 | 0 | 1 | 0 |
| NM_001129828 | CSAG3 | 796 | 0 | 0 | 0 | 0 | 0 | 0 | 0 | 0 | 0 | 0 | 0 | 0 | 0 |
| NM_001129829 | CACNA1C | 13603 | 3 | 1 | 0 | 2 | 3 | 2 | 0 | 0 | 1 | 0 | 0 | 1 | 0 |
| NM_001129830 | CACNA1C | 13585 | 3 | 1 | 0 | 2 | 3 | 2 | 0 | 0 | 1 | 0 | 0 | 1 | 0 |
| NM_001129831 | CACNA1C | 13564 | 3 | 1 | 0 | 2 | 3 | 2 | 0 | 0 | 1 | 0 | 0 | 1 | 0 |
| NM_001129832 | CACNA1C | 13540 | 3 | 1 | 0 | 2 | 3 | 2 | 0 | 0 | 1 | 0 | 0 | 1 | 0 |
| NM_001129833 | CACNA1C | 13537 | 3 | 1 | 0 | 2 | 3 | 2 | 0 | 0 | 1 | 0 | 0 | 1 | 0 |
| NM_001129834 | CACNA1C | 13537 | 3 | 1 | 0 | 2 | 3 | 2 | 0 | 0 | 1 | 0 | 0 | 1 | 0 |
| NM_001129835 | CACNA1C | 13537 | 3 | 1 | 0 | 2 | 3 | 2 | 0 | 0 | 1 | 0 | 0 | 1 | 0 |
| NM_001129836 | CACNA1C | 13531 | 3 | 1 | 0 | 2 | 3 | 2 | 0 | 0 | 1 | 0 | 0 | 1 | 0 |
| NM_001129837 | CACNA1C | 13504 | 3 | 1 | 0 | 2 | 3 | 2 | 0 | 0 | 1 | 0 | 0 | 1 | 0 |
| NM_001129838 | CACNA1C | 13504 | 3 | 1 | 0 | 2 | 3 | 2 | 0 | 0 | 1 | 0 | 0 | 1 | 0 |
| NM_001129839 | CACNA1C | 13498 | 3 | 1 | 0 | 2 | 3 | 2 | 0 | 0 | 1 | 0 | 0 | 1 | 0 |
| NM_001129840 | CACNA1C | 13480 | 3 | 1 | 0 | 2 | 3 | 2 | 0 | 0 | 1 | 0 | 0 | 1 | 0 |
| NM_001129841 | CACNA1C | 13480 | 3 | 1 | 0 | 2 | 3 | 2 | 0 | 0 | 1 | 0 | 0 | 1 | 0 |
| NM_001129842 | CACNA1C | 13480 | 3 | 1 | 0 | 2 | 3 | 2 | 0 | 0 | 1 | 0 | 0 | 1 | 0 |
| NM_001129843 | CACNA1C | 13480 | 3 | 1 | 0 | 2 | 3 | 2 | 0 | 0 | 1 | 0 | 0 | 1 | 0 |
| NM_001129844 | CACNA1C | 13471 | 3 | 1 | 0 | 2 | 3 | 2 | 0 | 0 | 1 | 0 | 0 | 1 | 0 |
| NM_001129846 | CACNA1C | 13447 | 3 | 1 | 0 | 2 | 3 | 2 | 0 | 0 | 1 | 0 | 0 | 1 | 0 |
| NM_001129883 | PATE3 | 1334 | 1 | 0 | 0 | 0 | 0 | 0 | 0 | 0 | 0 | 0 | 0 | 0 | 1 |
| NM_001129884 | TEX264 | 1418 | 2 | 1 | 1 | 1 | 1 | 1 | 2 | 1 | 1 | 1 | 0 | 1 | 1 |
| NM_001129885 | CPSF4L | 647 | 0 | 0 | 0 | 0 | 0 | 0 | 0 | 0 | 0 | 0 | 0 | 0 | 0 |
| NM_001129887 | DNAJC2 | 2053 | 8 | 5 | 3 | 1 | 5 | 5 | 4 | 4 | 3 | 5 | 5 | 6 | 8 |
| NM_001129888 | CYHR1 | 1162 | 1 | 0 | 1 | 1 | 0 | 1 | 1 | 0 | 0 | 0 | 0 | 0 | 1 |
| NM_001129889 | DCT | 2397 | 0 | 0 | 0 | 0 | 0 | 0 | 0 | 0 | 0 | 0 | 0 | 0 | 0 |
| NM_001129890 | LRRC69 | 1323 | 2 | 1 | 1 | 1 | 1 | 1 | 1 | 0 | 1 | 1 | 1 | 2 | 2 |
| NM_001129891 | FAM196B | 3548 | 1 | 0 | 0 | 0 | 0 | 0 | 0 | 0 | 0 | 0 | 0 | 1 | 0 |
| NM_001129895 | HGC6.3 | 1016 | 0 | 0 | 0 | 0 | 0 | 0 | 0 | 0 | 0 | 0 | 0 | 0 | 0 |
| NM_001129896 | SYTL4 | 3856 | 23 | 5 | 3 | 3 | 2 | 4 | 2 | 3 | 3 | 23 | 1 | 2 | 9 |
| NM_001129897 | NUSAP1 | 2303 | 3 | 1 | 1 | 3 | 1 | 0 | 0 | 1 | 2 | 2 | 1 | 2 | 2 |
| NM_001129898 | ZNF673 | 2442 | 4 | 2 | 1 | 1 | 1 | 2 | 2 | 1 | 2 | 3 | 1 | 4 | 2 |
| NM_001129899 | ZNF673 | 2486 | 4 | 2 | 1 | 1 | 1 | 2 | 2 | 1 | 2 | 3 | 1 | 4 | 2 |
| NM_001129900 | ZNF673 | 2471 | 4 | 2 | 1 | 1 | 1 | 2 | 2 | 1 | 2 | 3 | 1 | 4 | 2 |
| NM_001129907 | FLJ41562 | 3202 | 0 | 0 | 0 | 0 | 0 | 0 | 0 | 0 | 0 | 0 | 0 | 0 | 0 |
| NM_001129908 | FAM198A | 3431 | 1 | 0 | 0 | 0 | 0 | 0 | 0 | 0 | 0 | 1 | 0 | 0 | 0 |
| NM_001129979 | SYCE1L | 877 | 0 | 0 | 0 | 0 | 0 | 0 | 0 | 0 | 0 | 0 | 0 | 0 | 0 |
| NM_001129981 | ANKRD2 | 1352 | 13 | 1 | 0 | 0 | 4 | 0 | 0 | 0 | 0 | 0 | 13 | 0 | 0 |

| transcript | gene | transcript length (nt) | max | mean | adipose | colon | heart | hypothalamus | kidney | liver | lung | ovary | skeletalmuscle | spleen | testes |
|---|---|---|---|---|---|---|---|---|---|---|---|---|---|---|---|
| NM_001129993 | KIAA1841 | 4576 | 8 | 3 | 4 | 2 | 3 | 8 | 1 | 1 | 3 | 2 | 2 | 2 | 3 |
| NM_001129994 | KCTD15 | 3933 | 1 | 0 | 0 | 1 | 1 | 0 | 0 | 0 | 0 | 1 | 0 | 1 | 0 |
| NM_001129995 | KCTD15 | 3930 | 1 | 0 | 0 | 1 | 1 | 0 | 0 | 0 | 0 | 1 | 0 | 1 | 0 |
| NM_001129996 | ZNF222 | 1650 | 3 | 1 | 1 | 1 | 1 | 3 | 1 | 1 | 1 | 2 | 0 | 3 | 2 |
| NM_001129998 | CLEC12B | 1278 | 6 | 1 | 0 | 0 | 0 | 0 | 0 | 0 | 0 | 0 | 0 | 0 | 6 |
| NM_001130 | AES | 1676 | 58 | 42 | 36 | 47 | 57 | 57 | 30 | 25 | 33 | 31 | 45 | 58 | 41 |
| NM_001130001 | C3orf55 | 1421 | 2 | 0 | 2 | 0 | 0 | 0 | 0 | 0 | 0 | 0 | 0 | 0 | 0 |
| NM_001130002 | C3orf55 | 1510 | 2 | 0 | 2 | 0 | 0 | 0 | 0 | 0 | 0 | 0 | 0 | 0 | 0 |
| NM_001130003 | SYNPR | 2594 | 15 | 1 | 0 | 0 | 0 | 15 | 0 | 0 | 0 | 0 | 0 | 0 | 0 |
| NM_001130004 | ACTN1 | 3791 | 22 | 10 | 13 | 22 | 17 | 8 | 5 | 8 | 11 | 6 | 2 | 14 | 8 |
| NM_001130005 | ACTN1 | 3710 | 22 | 10 | 13 | 22 | 17 | 8 | 5 | 9 | 11 | 6 | 2 | 14 | 8 |
| NM_001130006 | GSPT1 | 7150 | 13 | 10 | 8 | 4 | 13 | 10 | 10 | 12 | 8 | 9 | 13 | 12 | 10 |
| NM_001130007 | GSPT1 | 7174 | 13 | 10 | 8 | 4 | 13 | 10 | 10 | 12 | 8 | 9 | 13 | 12 | 10 |
| NM_001130009 | GEN1 | 6367 | 3 | 1 | 0 | 1 | 0 | 0 | 1 | 1 | 1 | 0 | 0 | 2 | 3 |
| NM_001130010 | C15orf41 | 2477 | 5 | 1 | 0 | 0 | 5 | 0 | 0 | 0 | 0 | 1 | 1 | 0 | 0 |
| NM_001130011 | TEX101 | 1163 | 15 | 1 | 0 | 0 | 0 | 0 | 0 | 0 | 0 | 0 | 0 | 0 | 15 |
| NM_001130012 | SLC9A3R2 | 2175 | 147 | 49 | 54 | 31 | 78 | 38 | 32 | 147 | 61 | 20 | 14 | 42 | 18 |
| NM_001130014 | PSG5 | 1572 | 0 | 0 | 0 | 0 | 0 | 0 | 0 | 0 | 0 | 0 | 0 | 0 | 0 |
| NM_001130015 | ANKRD33 | 1779 | 0 | 0 | 0 | 0 | 0 | 0 | 0 | 0 | 0 | 0 | 0 | 0 | 0 |
| NM_001130016 | ART3 | 1572 | 39 | 7 | 0 | 0 | 16 | 2 | 0 | 0 | 0 | 0 | 39 | 1 | 13 |
| NM_001130017 | ART3 | 1533 | 38 | 6 | 0 | 0 | 16 | 2 | 0 | 0 | 0 | 0 | 38 | 1 | 13 |
| NM_001130020 | ATP6V0A1 | 4143 | 25 | 8 | 4 | 6 | 4 | 25 | 15 | 4 | 7 | 5 | 9 | 7 | 7 |
| NM_001130021 | ATP6V0A1 | 4140 | 25 | 8 | 4 | 6 | 4 | 25 | 15 | 4 | 7 | 5 | 9 | 7 | 7 |
| NM_001130022 | ZNF680 | 2288 | 1 | 0 | 0 | 0 | 1 | 1 | 0 | 0 | 1 | 1 | 0 | 1 | 1 |
| NM_001130025 | FAM115C | 2974 | 1 | 0 | 0 | 0 | 0 | 0 | 0 | 0 | 1 | 1 | 0 | 1 | 0 |
| NM_001130026 | FAM115C | 2337 | 1 | 0 | 0 | 0 | 0 | 0 | 0 | 0 | 1 | 1 | 0 | 1 | 0 |
| NM_001130028 | CLK3 | 2696 | 2 | 1 | 1 | 2 | 1 | 1 | 1 | 1 | 1 | 1 | 1 | 2 | 2 |
| NM_001130029 | RELL2 | 2155 | 0 | 0 | 0 | 0 | 0 | 0 | 0 | 0 | 0 | 0 | 0 | 0 | 0 |
| NM_001130031 | ZNF562 | 5784 | 5 | 3 | 3 | 2 | 4 | 5 | 3 | 2 | 2 | 5 | 2 | 5 | 3 |
| NM_001130032 | ZNF562 | 5731 | 5 | 3 | 3 | 2 | 4 | 5 | 3 | 2 | 2 | 5 | 2 | 5 | 3 |
| NM_001130033 | PLEKHB1 | 2275 | 52 | 6 | 0 | 1 | 1 | 52 | 2 | 0 | 1 | 1 | 0 | 1 | 2 |
| NM_001130034 | PLEKHB1 | 2121 | 56 | 6 | 0 | 1 | 1 | 56 | 2 | 0 | 1 | 1 | 0 | 1 | 3 |
| NM_001130035 | PLEKHB1 | 2016 | 59 | 6 | 0 | 1 | 1 | 59 | 2 | 0 | 1 | 1 | 0 | 1 | 3 |
| NM_001130036 | PLEKHB1 | 2148 | 55 | 6 | 0 | 1 | 1 | 55 | 2 | 0 | 1 | 1 | 0 | 1 | 3 |
| NM_001130037 | ELMOD1 | 2967 | 15 | 2 | 0 | 0 | 1 | 15 | 1 | 0 | 0 | 0 | 0 | 0 | 1 |
| NM_001130040 | SHC1 | 3481 | 9 | 5 | 5 | 4 | 7 | 2 | 5 | 8 | 5 | 4 | 2 | 9 | 3 |
| NM_001130041 | SHC1 | 3166 | 10 | 5 | 5 | 4 | 8 | 3 | 5 | 9 | 6 | 5 | 2 | 10 | 4 |
| NM_001130042 | CRYZ | 2303 | 27 | 5 | 1 | 1 | 4 | 2 | 27 | 11 | 2 | 3 | 1 | 3 | 4 |
| NM_001130043 | CRYZ | 2162 | 28 | 6 | 1 | 1 | 4 | 2 | 28 | 12 | 3 | 3 | 1 | 3 | 4 |
| NM_001130045 | TTLL10 | 2259 | 0 | 0 | 0 | 0 | 0 | 0 | 0 | 0 | 0 | 0 | 0 | 0 | 0 |
| NM_001130046 | CCL20 | 839 | 1 | 0 | 0 | 0 | 0 | 0 | 0 | 0 | 1 | 0 | 0 | 0 | 0 |
| NM_001130047 | TBCEL | 3902 | 8 | 4 | 3 | 2 | 4 | 6 | 4 | 3 | 5 | 8 | 1 | 5 | 6 |
| NM_001130048 | DOCK9 | 7639 | 9 | 4 | 3 | 3 | 5 | 9 | 3 | 2 | 9 | 4 | 2 | 3 | 4 |
| NM_001130049 | DOCK9 | 3979 | 10 | 5 | 3 | 3 | 5 | 8 | 4 | 2 | 10 | 5 | 2 | 3 | 5 |
| NM_001130050 | DOCK9 | 4096 | 10 | 5 | 3 | 3 | 5 | 8 | 3 | 2 | 10 | 5 | 2 | 3 | 4 |
| NM_001130053 | EEF1D | 2350 | 23 | 14 | 10 | 15 | 18 | 6 | 11 | 16 | 14 | 20 | 16 | 23 | 9 |
| NM_001130054 | EEF1D | 2464 | 22 | 14 | 10 | 15 | 17 | 5 | 10 | 16 | 14 | 19 | 15 | 22 | 9 |
| NM_001130055 | EEF1D | 1421 | 38 | 24 | 17 | 25 | 30 | 9 | 17 | 27 | 24 | 33 | 27 | 38 | 15 |
| NM_001130056 | EEF1D | 1173 | 44 | 27 | 19 | 29 | 34 | 10 | 19 | 32 | 27 | 38 | 30 | 44 | 17 |
| NM_001130057 | EEF1D | 1179 | 46 | 29 | 20 | 31 | 36 | 11 | 21 | 33 | 29 | 40 | 32 | 46 | 18 |
| NM_001130058 | SLC44A5 | 3894 | 0 | 0 | 0 | 0 | 0 | 0 | 0 | 0 | 0 | 0 | 0 | 0 | 0 |
| NM_001130059 | ATF7 | 6584 | 4 | 3 | 2 | 2 | 3 | 2 | 2 | 1 | 3 | 4 | 3 | 3 | 3 |
| NM_001130060 | ATF7 | 6625 | 4 | 3 | 2 | 2 | 3 | 2 | 2 | 1 | 3 | 4 | 3 | 3 | 3 |
| NM_001130063 | GRM2 | 1931 | 0 | 0 | 0 | 0 | 0 | 0 | 0 | 0 | 0 | 0 | 0 | 0 | 0 |
| NM_001130064 | GAP43 | 2104 | 24 | 2 | 0 | 1 | 0 | 24 | 0 | 0 | 0 | 0 | 0 | 0 | 0 |
| NM_001130065 | MYO9B | 7623 | 8 | 3 | 3 | 4 | 2 | 2 | 1 | 2 | 3 | 1 | 0 | 8 | 7 |
| NM_001130067 | TRIM2 | 6792 | 14 | 3 | 1 | 2 | 1 | 14 | 5 | 1 | 1 | 2 | 0 | 3 | 1 |
| NM_001130069 | SUMF2 | 1909 | 28 | 7 | 4 | 4 | 10 | 6 | 6 | 4 | 3 | 3 | 28 | 4 | 3 |
| NM_001130071 | EPN1 | 2947 | 2 | 1 | 1 | 1 | 1 | 1 | 2 | 1 | 1 | 1 | 2 | 2 | 2 |
| NM_001130072 | EPN1 | 2484 | 3 | 2 | 1 | 2 | 2 | 2 | 2 | 2 | 1 | 1 | 2 | 3 | 2 |
| NM_001130079 | MOV10 | 3521 | 3 | 1 | 1 | 1 | 0 | 0 | 2 | 1 | 1 | 1 | 0 | 2 | 3 |
| NM_001130080 | IFI27 | 648 | 7 | 3 | 4 | 4 | 2 | 3 | 1 | 1 | 3 | 1 | 1 | 7 | 1 |
| NM_001130081 | PLD1 | 5477 | 16 | 5 | 3 | 3 | 8 | 4 | 5 | 3 | 3 | 16 | 1 | 10 | 3 |
| NM_001130082 | PLXNB1 | 7143 | 2 | 1 | 0 | 1 | 0 | 2 | 2 | 1 | 0 | 0 | 0 | 0 | 0 |
| NM_001130083 | ABLIM2 | 3761 | 21 | 3 | 0 | 0 | 1 | 3 | 0 | 0 | 0 | 0 | 21 | 2 | 0 |
| NM_001130084 | ABLIM2 | 3659 | 22 | 3 | 0 | 0 | 1 | 3 | 0 | 0 | 0 | 0 | 22 | 3 | 0 |
| NM_001130085 | ABLIM2 | 3542 | 22 | 3 | 0 | 0 | 1 | 3 | 0 | 0 | 0 | 0 | 22 | 3 | 0 |
| NM_001130086 | ABLIM2 | 3503 | 21 | 3 | 0 | 0 | 1 | 3 | 0 | 0 | 0 | 0 | 21 | 3 | 0 |
| NM_001130087 | ABLIM2 | 3419 | 21 | 3 | 0 | 0 | 1 | 3 | 0 | 0 | 0 | 0 | 21 | 3 | 0 |
| NM_001130088 | ABLIM2 | 2577 | 7 | 1 | 0 | 0 | 0 | 1 | 0 | 0 | 0 | 0 | 7 | 1 | 0 |
| NM_001130089 | KARS | 2193 | 23 | 12 | 10 | 7 | 13 | 12 | 10 | 13 | 8 | 9 | 23 | 13 | 14 |
| NM_001130090 | LRRC48 | 2155 | 1 | 0 | 0 | 0 | 0 | 1 | 1 | 0 | 0 | 0 | 0 | 0 | 1 |
| NM_001130091 | LRRC48 | 2136 | 1 | 0 | 0 | 0 | 0 | 1 | 1 | 0 | 0 | 0 | 0 | 0 | 0 |
| NM_001130092 | LRRC48 | 2047 | 1 | 0 | 0 | 0 | 0 | 1 | 1 | 0 | 0 | 0 | 0 | 0 | 0 |
| NM_001130099 | KIFC3 | 3339 | 1 | 0 | 0 | 0 | 1 | 1 | 1 | 0 | 0 | 0 | 0 | 0 | 0 |
| NM_001130100 | KIFC3 | 3395 | 1 | 0 | 0 | 0 | 1 | 1 | 1 | 0 | 0 | 0 | 0 | 0 | 0 |
| NM_001130101 | NR1H3 | 1539 | 8 | 3 | 8 | 3 | 1 | 2 | 2 | 6 | 2 | 1 | 1 | 6 | 2 |

| transcript | gene | transcript length (nt) | max | mean | adipose | colon | heart | hypothalamus | kidney | liver | lung | ovary | skeletalmuscle | spleen | testes |
|---|---|---|---|---|---|---|---|---|---|---|---|---|---|---|---|
| NM_001130102 | NR1H3 | 1557 | 8 | 3 | 8 | 3 | 1 | 1 | 2 | 6 | 2 | 1 | 1 | 6 | 2 |
| NM_001130103 | COL13A1 | 3137 | 1 | 0 | 0 | 0 | 0 | 0 | 0 | 0 | 1 | 0 | 0 | 1 | 0 |
| NM_001130105 | COL4A3BP | 5494 | 11 | 7 | 11 | 3 | 5 | 8 | 5 | 4 | 7 | 7 | 7 | 9 | 8 |
| NM_001130106 | HSPBP1 | 1799 | 1 | 0 | 0 | 0 | 0 | 1 | 0 | 0 | 0 | 1 | 0 | 0 | 0 |
| NM_001130107 | KLC1 | 2513 | 97 | 22 | 17 | 16 | 24 | 97 | 9 | 7 | 12 | 15 | 17 | 17 | 15 |
| NM_001130110 | SETBP1 | 1785 | 4 | 2 | 2 | 3 | 1 | 1 | 4 | 2 | 2 | 3 | 2 | 3 | 3 |
| NM_001130111 | FAM108A1 | 1563 | 5 | 2 | 2 | 2 | 2 | 5 | 1 | 1 | 2 | 1 | 1 | 2 | 1 |
| NM_001130112 | RFC5 | 2230 | 2 | 2 | 1 | 1 | 1 | 2 | 2 | 2 | 1 | 2 | 1 | 2 | 2 |
| NM_001130113 | RFC5 | 2163 | 3 | 2 | 1 | 1 | 1 | 2 | 2 | 2 | 1 | 2 | 1 | 3 | 2 |
| NM_001130136 | ISLR2 | 4817 | 0 | 0 | 0 | 0 | 0 | 0 | 0 | 0 | 0 | 0 | 0 | 0 | 0 |
| NM_001130137 | ISLR2 | 4553 | 0 | 0 | 0 | 0 | 0 | 0 | 0 | 0 | 0 | 0 | 0 | 0 | 0 |
| NM_001130138 | ISLR2 | 4261 | 0 | 0 | 0 | 0 | 0 | 0 | 0 | 0 | 0 | 0 | 0 | 0 | 0 |
| NM_001130140 | ERAP2 | 5253 | 12 | 3 | 4 | 2 | 2 | 1 | 3 | 3 | 5 | 3 | 1 | 12 | 1 |
| NM_001130141 | PCBP3 | 1911 | 2 | 1 | 1 | 0 | 1 | 2 | 0 | 0 | 0 | 1 | 0 | 2 | 1 |
| NM_001130142 | VWA5A | 3536 | 4 | 2 | 2 | 2 | 1 | 4 | 2 | 0 | 2 | 1 | 1 | 1 | 2 |
| NM_001130143 | PPP1R14D | 873 | 2 | 0 | 0 | 2 | 0 | 0 | 0 | 0 | 0 | 0 | 0 | 0 | 0 |
| NM_001130144 | LTBP3 | 4701 | 4 | 2 | 2 | 2 | 2 | 2 | 2 | 1 | 2 | 4 | 1 | 1 | 1 |
| NM_001130145 | YAP1 | 5368 | 25 | 11 | 14 | 5 | 12 | 4 | 10 | 7 | 14 | 25 | 9 | 6 | 11 |
| NM_001130146 | CCDC77 | 2313 | 3 | 2 | 1 | 1 | 1 | 2 | 1 | 1 | 1 | 2 | 1 | 2 | 3 |
| NM_001130147 | CCDC77 | 2340 | 3 | 2 | 1 | 1 | 1 | 2 | 1 | 1 | 1 | 2 | 1 | 2 | 3 |
| NM_001130148 | CCDC77 | 2286 | 3 | 2 | 1 | 1 | 1 | 2 | 1 | 1 | 1 | 2 | 1 | 2 | 3 |
| NM_001130156 | MLF1 | 2249 | 10 | 2 | 0 | 0 | 5 | 2 | 1 | 0 | 1 | 1 | 1 | 0 | 10 |
| NM_001130157 | MLF1 | 2322 | 10 | 2 | 0 | 0 | 4 | 2 | 1 | 0 | 1 | 1 | 1 | 0 | 10 |
| NM_001130158 | MYO1B | 5095 | 80 | 13 | 7 | 4 | 6 | 3 | 8 | 80 | 17 | 7 | 2 | 7 | 5 |
| NM_001130159 | STOX1 | 1221 | 4 | 1 | 1 | 0 | 0 | 4 | 1 | 0 | 1 | 1 | 0 | 0 | 1 |
| NM_001130160 | STOX1 | 1021 | 2 | 0 | 1 | 0 | 0 | 2 | 0 | 0 | 0 | 0 | 0 | 0 | 0 |
| NM_001130161 | STOX1 | 3306 | 6 | 1 | 2 | 1 | 0 | 6 | 1 | 0 | 1 | 0 | 0 | 0 | 1 |
| NM_001130162 | STOX1 | 3111 | 6 | 1 | 2 | 1 | 0 | 6 | 1 | 0 | 1 | 0 | 0 | 0 | 1 |
| NM_001130167 | PSG8 | 2031 | 0 | 0 | 0 | 0 | 0 | 0 | 0 | 0 | 0 | 0 | 0 | 0 | 0 |
| NM_001130168 | PSG8 | 1665 | 0 | 0 | 0 | 0 | 0 | 0 | 0 | 0 | 0 | 0 | 0 | 0 | 0 |
| NM_001130172 | MYB | 3306 | 7 | 1 | 0 | 7 | 0 | 0 | 0 | 0 | 1 | 0 | 0 | 1 | 0 |
| NM_001130173 | MYB | 3678 | 6 | 1 | 0 | 6 | 0 | 0 | 0 | 0 | 1 | 0 | 0 | 1 | 0 |
| NM_001130182 | DNAJA4 | 3047 | 118 | 21 | 4 | 5 | 118 | 34 | 6 | 0 | 12 | 3 | 15 | 7 | 23 |
| NM_001130183 | DNAJA4 | 2874 | 121 | 21 | 4 | 5 | 121 | 34 | 6 | 0 | 12 | 3 | 15 | 7 | 24 |
| NM_001130404 | PRR20B | 1847 | 0 | 0 | 0 | 0 | 0 | 0 | 0 | 0 | 0 | 0 | 0 | 0 | 0 |
| NM_001130405 | PRR20C | 1847 | 0 | 0 | 0 | 0 | 0 | 0 | 0 | 0 | 0 | 0 | 0 | 0 | 0 |
| NM_001130406 | PRR20D | 1847 | 0 | 0 | 0 | 0 | 0 | 0 | 0 | 0 | 0 | 0 | 0 | 0 | 0 |
| NM_001130407 | PRR20E | 1847 | 0 | 0 | 0 | 0 | 0 | 0 | 0 | 0 | 0 | 0 | 0 | 0 | 0 |
| NM_001130410 | ACAA1 | 1540 | 33 | 11 | 9 | 6 | 9 | 6 | 33 | 32 | 5 | 3 | 2 | 9 | 3 |
| NM_001130413 | SCNN1D | 2684 | 0 | 0 | 0 | 0 | 0 | 0 | 0 | 0 | 0 | 0 | 0 | 0 | 0 |
| NM_001130414 | APBA2 | 3601 | 12 | 1 | 0 | 0 | 0 | 12 | 0 | 0 | 0 | 0 | 0 | 2 | 1 |
| NM_001130415 | APOLD1 | 4737 | 28 | 7 | 28 | 1 | 9 | 9 | 1 | 4 | 4 | 2 | 7 | 6 | 2 |
| NM_001130417 | SLC8A3 | 2954 | 5 | 1 | 0 | 0 | 0 | 4 | 0 | 0 | 0 | 0 | 5 | 0 | 0 |
| NM_001130420 | SMARCC2 | 5402 | 18 | 9 | 11 | 8 | 7 | 18 | 8 | 4 | 8 | 11 | 7 | 10 | 10 |
| NM_001130438 | SPTAN1 | 7874 | 90 | 26 | 28 | 21 | 26 | 90 | 22 | 11 | 15 | 21 | 5 | 23 | 30 |
| NM_001130440 | SRP9 | 1620 | 83 | 51 | 36 | 19 | 68 | 83 | 61 | 56 | 41 | 44 | 44 | 64 | 44 |
| NM_001130442 | HRAS | 1152 | 3 | 1 | 1 | 3 | 1 | 2 | 1 | 1 | 0 | 0 | 1 | 1 | 1 |
| NM_001130445 | SSFA2 | 5292 | 21 | 12 | 7 | 7 | 9 | 11 | 12 | 16 | 16 | 5 | 6 | 15 | 21 |
| NM_001130446 | C10orf131 | 1175 | 1 | 0 | 0 | 0 | 0 | 0 | 0 | 0 | 0 | 0 | 0 | 0 | 1 |
| NM_001130447 | HAUS2 | 3851 | 3 | 2 | 2 | 1 | 2 | 3 | 1 | 1 | 2 | 1 | 1 | 2 | 2 |
| NM_001130448 | C15orf62 | 2487 | 0 | 0 | 0 | 0 | 0 | 0 | 0 | 0 | 0 | 0 | 0 | 0 | 0 |
| NM_001130455 | DYSF | 6657 | 8 | 3 | 1 | 1 | 4 | 1 | 1 | 2 | 3 | 1 | 5 | 8 | 1 |
| NM_001130475 | THAP5 | 3306 | 6 | 4 | 2 | 0 | 4 | 5 | 5 | 3 | 4 | 6 | 4 | 6 | 6 |
| NM_001130478 | LY6H | 981 | 16 | 2 | 0 | 1 | 0 | 16 | 0 | 0 | 0 | 0 | 0 | 0 | 0 |
| NM_001130480 | VRK2 | 1987 | 4 | 2 | 3 | 1 | 3 | 2 | 2 | 3 | 2 | 2 | 2 | 4 | 2 |
| NM_001130481 | VRK2 | 1899 | 4 | 2 | 3 | 1 | 3 | 2 | 2 | 3 | 2 | 2 | 2 | 4 | 2 |
| NM_001130482 | VRK2 | 1933 | 4 | 2 | 3 | 1 | 3 | 2 | 2 | 3 | 2 | 2 | 2 | 4 | 2 |
| NM_001130483 | VRK2 | 2036 | 4 | 3 | 3 | 1 | 4 | 2 | 2 | 3 | 2 | 2 | 3 | 4 | 2 |
| NM_001130487 | NDRG4 | 3560 | 38 | 6 | 0 | 1 | 21 | 38 | 0 | 0 | 1 | 0 | 0 | 2 | 6 |
| NM_001130514 | C19orf69 | 946 | 3 | 0 | 0 | 0 | 0 | 0 | 3 | 0 | 0 | 0 | 0 | 0 | 0 |
| NM_001130517 | MPST | 1516 | 1 | 0 | 0 | 0 | 0 | 0 | 0 | 1 | 0 | 0 | 0 | 0 | 0 |
| NM_001130518 | CSGALNACT | 4282 | 6 | 3 | 2 | 1 | 6 | 4 | 1 | 1 | 3 | 5 | 1 | 3 | 1 |
| NM_001130519 | ZNF195 | 2336 | 11 | 6 | 3 | 3 | 7 | 9 | 9 | 3 | 3 | 8 | 7 | 11 | 7 |
| NM_001130520 | ZNF195 | 2405 | 11 | 6 | 3 | 3 | 6 | 9 | 9 | 3 | 3 | 8 | 7 | 11 | 7 |
| NM_001130523 | CSDE1 | 4216 | 392 | 137 | 164 | 39 | 190 | 97 | 84 | 93 | 90 | 124 | 392 | 109 | 129 |
| NM_001130524 | AP1M1 | 2391 | 2 | 1 | 1 | 1 | 1 | 2 | 2 | 2 | 1 | 1 | 1 | 2 | 1 |
| NM_001130528 | SPAG9 | 8272 | 20 | 13 | 11 | 5 | 11 | 20 | 15 | 8 | 15 | 12 | 19 | 10 | 19 |
| NM_001130674 | CALU | 3410 | 121 | 33 | 37 | 12 | 121 | 20 | 18 | 23 | 30 | 24 | 19 | 33 | 29 |
| NM_001130675 | CLGN | 2822 | 9 | 2 | 0 | 0 | 9 | 1 | 0 | 1 | 0 | 0 | 0 | 0 | 8 |
| NM_001130677 | C17orf96 | 3229 | 2 | 0 | 0 | 0 | 0 | 2 | 0 | 1 | 0 | 0 | 0 | 0 | 1 |
| NM_001130678 | EIF4E | 3406 | 7 | 4 | 3 | 2 | 4 | 7 | 5 | 3 | 4 | 3 | 3 | 6 | 5 |
| NM_001130679 | EIF4E | 4842 | 5 | 3 | 2 | 2 | 3 | 5 | 4 | 2 | 3 | 2 | 2 | 4 | 4 |
| NM_001130682 | GUCY1A3 | 4633 | 5 | 3 | 2 | 3 | 5 | 5 | 4 | 1 | 3 | 2 | 1 | 4 | 1 |
| NM_001130683 | GUCY1A3 | 9312 | 7 | 3 | 2 | 2 | 7 | 5 | 5 | 1 | 4 | 2 | 1 | 5 | 2 |
| NM_001130684 | GUCY1A3 | 4573 | 5 | 3 | 2 | 3 | 5 | 5 | 4 | 1 | 3 | 2 | 1 | 4 | 1 |
| NM_001130685 | GUCY1A3 | 4422 | 5 | 2 | 1 | 2 | 5 | 4 | 3 | 1 | 3 | 1 | 1 | 3 | 1 |
| NM_001130686 | GUCY1A3 | 1658 | 11 | 6 | 4 | 7 | 10 | 11 | 7 | 3 | 7 | 3 | 2 | 8 | 2 |

| transcript | gene | transcript length (nt) | max | mean | adipose | colon | heart | hypothalamus | kidney | liver | lung | ovary | skeletalmuscle | spleen | testes |
|---|---|---|---|---|---|---|---|---|---|---|---|---|---|---|---|
| NM_001130687 | GUCY1A3 | 2354 | 8 | 4 | 3 | 5 | 8 | 8 | 6 | 2 | 5 | 2 | 1 | 6 | 2 |
| NM_001130688 | HMGB2 | 1467 | 48 | 24 | 26 | 29 | 21 | 15 | 13 | 4 | 48 | 20 | 15 | 39 | 33 |
| NM_001130689 | HMGB2 | 1454 | 49 | 24 | 27 | 29 | 21 | 15 | 13 | 4 | 49 | 20 | 15 | 39 | 34 |
| NM_001130690 | PDE10A | 8189 | 4 | 2 | 0 | 0 | 4 | 2 | 3 | 0 | 0 | 2 | 0 | 2 | 2 |
| NM_001130691 | PPP3CA | 4646 | 98 | 21 | 16 | 5 | 12 | 98 | 7 | 10 | 14 | 17 | 22 | 17 | 17 |
| NM_001130692 | PPP3CA | 4520 | 100 | 22 | 17 | 5 | 12 | 100 | 7 | 10 | 14 | 18 | 22 | 17 | 17 |
| NM_001130698 | TRPC3 | 3548 | 0 | 0 | 0 | 0 | 0 | 0 | 0 | 0 | 0 | 0 | 0 | 0 | 0 |
| NM_001130699 | IPCEF1 | 6858 | 4 | 1 | 0 | 0 | 0 | 4 | 1 | 0 | 1 | 0 | 0 | 2 | 0 |
| NM_001130700 | IPCEF1 | 6818 | 4 | 1 | 0 | 0 | 0 | 4 | 1 | 0 | 1 | 0 | 0 | 2 | 0 |
| NM_001130701 | STYX | 4615 | 5 | 3 | 3 | 1 | 2 | 2 | 2 | 5 | 2 | 3 | 3 | 4 | 4 |
| NM_001130702 | BSCL2 | 2038 | 11 | 2 | 1 | 1 | 1 | 11 | 2 | 1 | 1 | 1 | 1 | 1 | 4 |
| NM_001130703 | ADAM29 | 3243 | 4 | 0 | 0 | 0 | 0 | 0 | 0 | 0 | 0 | 0 | 0 | 0 | 4 |
| NM_001130704 | ADAM29 | 3218 | 4 | 0 | 0 | 0 | 0 | 0 | 0 | 0 | 0 | 0 | 0 | 0 | 4 |
| NM_001130705 | ADAM29 | 3137 | 4 | 0 | 0 | 0 | 0 | 0 | 0 | 0 | 0 | 0 | 0 | 0 | 4 |
| NM_001130706 | C14orf93 | 2193 | 2 | 1 | 1 | 1 | 0 | 1 | 1 | 0 | 1 | 2 | 1 | 1 | 1 |
| NM_001130708 | C14orf93 | 2114 | 2 | 1 | 1 | 1 | 0 | 1 | 1 | 0 | 1 | 2 | 1 | 1 | 1 |
| NM_001130709 | RUFY3 | 1821 | 44 | 11 | 5 | 5 | 4 | 44 | 11 | 5 | 6 | 10 | 4 | 6 | 17 |
| NM_001130710 | LSM5 | 2187 | 2 | 1 | 1 | 1 | 1 | 1 | 1 | 1 | 1 | 1 | 1 | 2 | 1 |
| NM_001130711 | CLEC2A | 916 | 0 | 0 | 0 | 0 | 0 | 0 | 0 | 0 | 0 | 0 | 0 | 0 | 0 |
| NM_001130713 | LEF1 | 3518 | 4 | 1 | 0 | 1 | 0 | 1 | 1 | 0 | 1 | 1 | 0 | 4 | 2 |
| NM_001130714 | LEF1 | 3477 | 4 | 1 | 0 | 1 | 0 | 1 | 1 | 0 | 1 | 1 | 0 | 4 | 2 |
| NM_001130715 | PLAC8 | 1003 | 40 | 8 | 1 | 23 | 2 | 1 | 1 | 6 | 10 | 0 | 0 | 40 | 0 |
| NM_001130716 | PLAC8 | 1432 | 29 | 5 | 1 | 16 | 1 | 1 | 0 | 5 | 7 | 0 | 0 | 29 | 0 |
| NM_001130720 | AHCYL2 | 5052 | 11 | 4 | 1 | 9 | 1 | 11 | 5 | 1 | 3 | 1 | 3 | 1 | 3 |
| NM_001130721 | ELOVL6 | 3188 | 5 | 1 | 0 | 1 | 1 | 2 | 1 | 5 | 0 | 0 | 0 | 0 | 0 |
| NM_001130722 | AHCYL2 | 5049 | 11 | 4 | 1 | 9 | 1 | 11 | 5 | 1 | 3 | 1 | 3 | 1 | 3 |
| NM_001130723 | AHCYL2 | 5046 | 11 | 4 | 1 | 9 | 1 | 11 | 5 | 1 | 3 | 1 | 3 | 1 | 3 |
| NM_001130725 | PSMB5 | 940 | 21 | 11 | 14 | 7 | 12 | 21 | 9 | 21 | 5 | 4 | 12 | 7 | 5 |
| NM_001130726 | CCDC149 | 2288 | 13 | 6 | 4 | 3 | 7 | 13 | 4 | 4 | 5 | 8 | 3 | 4 | 6 |
| NM_001130727 | PACRGL | 1778 | 1 | 1 | 1 | 1 | 0 | 1 | 1 | 0 | 0 | 0 | 0 | 1 | 1 |
| NM_001130820 | RABL5 | 2337 | 6 | 2 | 1 | 1 | 2 | 4 | 4 | 2 | 1 | 2 | 1 | 1 | 6 |
| NM_001130821 | RABL5 | 2458 | 5 | 2 | 1 | 1 | 2 | 4 | 4 | 1 | 1 | 2 | 1 | 1 | 5 |
| NM_001130822 | RABL5 | 2350 | 5 | 2 | 1 | 1 | 2 | 5 | 4 | 1 | 1 | 2 | 1 | 1 | 5 |
| NM_001130823 | DNMT1 | 5399 | 5 | 3 | 3 | 3 | 2 | 3 | 2 | 2 | 3 | 2 | 1 | 5 | 5 |
| NM_001130824 | SUPT5H | 3635 | 36 | 18 | 14 | 15 | 14 | 25 | 13 | 15 | 13 | 17 | 16 | 20 | 36 |
| NM_001130825 | SUPT5H | 3698 | 35 | 18 | 14 | 15 | 14 | 24 | 13 | 15 | 12 | 17 | 16 | 20 | 35 |
| NM_001130829 | CDC2 | 1926 | 0 | 0 | 0 | 0 | 0 | 0 | 0 | 0 | 0 | 0 | 0 | 0 | 0 |
| NM_001130831 | GAS7 | 8024 | 18 | 4 | 4 | 2 | 7 | 18 | 0 | 0 | 2 | 2 | 1 | 4 | 4 |
| NM_001130834 | LDB2 | 2683 | 23 | 8 | 9 | 1 | 8 | 11 | 6 | 4 | 16 | 7 | 3 | 23 | 5 |
| NM_001130841 | RTCD1 | 2621 | 9 | 6 | 7 | 4 | 8 | 8 | 9 | 5 | 5 | 3 | 6 | 8 | 5 |
| NM_001130842 | ZNF286A | 5456 | 2 | 1 | 0 | 0 | 1 | 1 | 1 | 0 | 1 | 2 | 0 | 1 | 1 |
| NM_001130845 | BCL6 | 3659 | 42 | 11 | 13 | 2 | 13 | 9 | 4 | 6 | 10 | 7 | 42 | 11 | 7 |
| NM_001130846 | AIFM1 | 1923 | 26 | 7 | 2 | 3 | 13 | 2 | 26 | 12 | 2 | 1 | 4 | 4 | 7 |
| NM_001130847 | AIFM1 | 2416 | 29 | 9 | 3 | 4 | 15 | 3 | 29 | 19 | 2 | 2 | 6 | 4 | 8 |
| NM_001130848 | PITPNM1 | 4222 | 0 | 0 | 0 | 0 | 0 | 0 | 0 | 0 | 0 | 0 | 0 | 0 | 0 |
| NM_001130849 | CAB39 | 3950 | 17 | 11 | 10 | 7 | 12 | 17 | 11 | 8 | 13 | 6 | 13 | 15 | 9 |
| NM_001130850 | CAB39 | 3694 | 18 | 11 | 10 | 6 | 12 | 18 | 11 | 8 | 12 | 6 | 14 | 15 | 9 |
| NM_001130851 | CDKN3 | 769 | 24 | 3 | 1 | 1 | 1 | 1 | 0 | 0 | 1 | 1 | 0 | 1 | 24 |
| NM_001130852 | CBLC | 1452 | 0 | 0 | 0 | 0 | 0 | 0 | 0 | 0 | 0 | 0 | 0 | 0 | 0 |
| NM_001130858 | HISPPD2A | 5696 | 8 | 3 | 2 | 5 | 1 | 8 | 2 | 1 | 1 | 2 | 3 | 2 | 5 |
| NM_001130859 | HISPPD2A | 5616 | 8 | 3 | 2 | 5 | 1 | 8 | 2 | 1 | 1 | 2 | 3 | 2 | 5 |
| NM_001130860 | HEPH | 4435 | 8 | 2 | 0 | 8 | 2 | 2 | 1 | 0 | 1 | 2 | 0 | 2 | 1 |
| NM_001130861 | CLDN5 | 2314 | 2 | 0 | 0 | 0 | 0 | 0 | 0 | 0 | 2 | 0 | 1 | 0 | 0 |
| NM_001130862 | RAD51AP1 | 2268 | 1 | 0 | 1 | 1 | 0 | 0 | 0 | 0 | 1 | 0 | 0 | 1 | 1 |
| NM_001130863 | ENPP2 | 3194 | 30 | 10 | 12 | 2 | 2 | 29 | 10 | 1 | 7 | 4 | 1 | 30 | 10 |
| NM_001130864 | PWWP2A | 3367 | 1 | 1 | 0 | 0 | 1 | 1 | 1 | 1 | 1 | 1 | 1 | 1 | 1 |
| NM_001130865 | DMRT2 | 2244 | 4 | 1 | 2 | 0 | 0 | 0 | 4 | 0 | 0 | 0 | 0 | 0 | 0 |
| NM_001130867 | ERCC2 | 1733 | 1 | 0 | 0 | 1 | 0 | 1 | 1 | 1 | 0 | 0 | 0 | 1 | 1 |
| NM_001130910 | CCRL2 | 1592 | 1 | 0 | 1 | 0 | 0 | 0 | 0 | 0 | 1 | 0 | 0 | 1 | 0 |
| NM_001130912 | SUGT1 | 1786 | 4 | 3 | 2 | 1 | 3 | 4 | 4 | 2 | 2 | 3 | 2 | 2 | 3 |
| NM_001130913 | ZNF720 | 2773 | 2 | 1 | 1 | 1 | 1 | 1 | 1 | 1 | 1 | 1 | 0 | 2 | 1 |
| NM_001130914 | BTG3 | 1592 | 5 | 2 | 1 | 1 | 2 | 3 | 1 | 2 | 4 | 2 | 0 | 2 | 5 |
| NM_001130915 | MAMSTR | 1579 | 14 | 2 | 0 | 1 | 1 | 2 | 0 | 0 | 1 | 2 | 14 | 2 | 3 |
| NM_001130916 | TGFBR1 | 6241 | 12 | 6 | 5 | 2 | 5 | 12 | 7 | 2 | 6 | 6 | 3 | 12 | 5 |
| NM_001130917 | LILRA2 | 1753 | 1 | 0 | 0 | 0 | 0 | 0 | 0 | 0 | 0 | 0 | 0 | 1 | 0 |
| NM_001130918 | TTLL6 | 3453 | 1 | 0 | 0 | 1 | 0 | 0 | 1 | 0 | 0 | 0 | 0 | 0 | 1 |
| NM_001130919 | RABL2B | 2186 | 1 | 0 | 0 | 0 | 0 | 1 | 1 | 0 | 1 | 0 | 0 | 1 | 0 |
| NM_001130920 | RABL2B | 2345 | 1 | 0 | 0 | 0 | 0 | 1 | 1 | 0 | 1 | 0 | 0 | 1 | 0 |
| NM_001130921 | RABL2B | 2438 | 1 | 1 | 0 | 1 | 0 | 1 | 1 | 0 | 1 | 1 | 0 | 1 | 1 |
| NM_001130922 | RABL2B | 2187 | 1 | 0 | 0 | 0 | 0 | 1 | 1 | 0 | 1 | 0 | 0 | 1 | 0 |
| NM_001130923 | RABL2B | 2372 | 1 | 0 | 0 | 0 | 0 | 1 | 1 | 0 | 1 | 0 | 0 | 1 | 0 |
| NM_001130924 | TMEM201 | 3852 | 0 | 0 | 0 | 0 | 0 | 0 | 0 | 0 | 0 | 0 | 0 | 0 | 0 |
| NM_001130926 | MEF2A | 5464 | 14 | 6 | 4 | 1 | 14 | 7 | 4 | 3 | 6 | 7 | 10 | 9 | 5 |
| NM_001130927 | MEF2A | 5284 | 14 | 6 | 4 | 1 | 14 | 7 | 4 | 3 | 6 | 7 | 10 | 9 | 6 |
| NM_001130928 | MEF2A | 5083 | 15 | 7 | 4 | 1 | 15 | 7 | 4 | 3 | 6 | 8 | 11 | 10 | 6 |
| NM_001130929 | PL-5283 | 2497 | 6 | 4 | 4 | 2 | 5 | 4 | 4 | 3 | 4 | 4 | 3 | 4 | 6 |
| NM_001130955 | ARHGEF18 | 5740 | 4 | 1 | 0 | 1 | 0 | 1 | 1 | 1 | 1 | 1 | 0 | 4 | 1 |

| transcript | gene | transcript length (nt) | max | mean | adipose | colon | heart | hypothalamus | kidney | liver | lung | ovary | skeletalmuscle | spleen | testes |
|---|---|---|---|---|---|---|---|---|---|---|---|---|---|---|---|
| NM_001130957 | C1orf101 | 3192 | 6 | 1 | 0 | 0 | 0 | 1 | 1 | 0 | 1 | 1 | 0 | 0 | 6 |
| NM_001130958 | FABP6 | 743 | 10 | 2 | 0 | 4 | 0 | 10 | 0 | 0 | 3 | 0 | 0 | 0 | 1 |
| NM_001130960 | PLCH1 | 6445 | 2 | 0 | 0 | 0 | 0 | 1 | 1 | 0 | 1 | 0 | 0 | 0 | 2 |
| NM_001130961 | PLCH1 | 6473 | 1 | 0 | 0 | 0 | 0 | 1 | 1 | 0 | 1 | 0 | 0 | 0 | 1 |
| NM_001130963 | TMEM194A | 5616 | 2 | 1 | 1 | 1 | 0 | 1 | 1 | 0 | 1 | 1 | 0 | 2 | 1 |
| NM_001130964 | PLCD1 | 2963 | 4 | 1 | 1 | 1 | 0 | 1 | 1 | 0 | 1 | 0 | 0 | 1 | 4 |
| NM_001130965 | UNC84A | 4008 | 2 | 1 | 1 | 1 | 2 | 1 | 1 | 1 | 2 | 2 | 2 | 2 | 2 |
| NM_001130966 | TBXAS1 | 2367 | 33 | 6 | 5 | 2 | 4 | 3 | 1 | 4 | 11 | 2 | 1 | 33 | 1 |
| NM_001130967 | ZNF385A | 2336 | 1 | 1 | 1 | 0 | 1 | 1 | 0 | 0 | 1 | 0 | 1 | 1 | 0 |
| NM_001130968 | ZNF385A | 2093 | 1 | 1 | 1 | 0 | 1 | 1 | 0 | 0 | 1 | 0 | 1 | 1 | 0 |
| NM_001130969 | NELF | 3647 | 5 | 1 | 0 | 1 | 0 | 5 | 1 | 1 | 0 | 0 | 0 | 0 | 1 |
| NM_001130970 | NELF | 3578 | 5 | 1 | 0 | 1 | 0 | 5 | 1 | 1 | 0 | 0 | 0 | 0 | 1 |
| NM_001130971 | NELF | 3572 | 5 | 1 | 0 | 1 | 0 | 5 | 1 | 1 | 0 | 0 | 0 | 0 | 1 |
| NM_001130976 | DYSF | 6847 | 8 | 3 | 1 | 1 | 4 | 1 | 1 | 2 | 3 | 1 | 5 | 8 | 1 |
| NM_001130977 | DYSF | 6910 | 8 | 3 | 1 | 1 | 4 | 1 | 1 | 2 | 3 | 1 | 5 | 8 | 1 |
| NM_001130978 | DYSF | 6952 | 8 | 3 | 1 | 1 | 4 | 1 | 1 | 2 | 3 | 1 | 5 | 8 | 1 |
| NM_001130979 | DYSF | 6982 | 8 | 2 | 1 | 1 | 4 | 1 | 1 | 2 | 3 | 1 | 5 | 8 | 1 |
| NM_001130980 | DYSF | 6940 | 8 | 3 | 1 | 1 | 4 | 1 | 1 | 2 | 3 | 1 | 5 | 8 | 1 |
| NM_001130981 | DYSF | 7003 | 8 | 2 | 1 | 1 | 4 | 1 | 1 | 2 | 3 | 1 | 5 | 8 | 1 |
| NM_001130982 | DYSF | 6750 | 8 | 3 | 1 | 1 | 4 | 1 | 1 | 2 | 3 | 1 | 5 | 8 | 1 |
| NM_001130983 | DYSF | 6720 | 8 | 3 | 1 | 1 | 4 | 1 | 1 | 2 | 3 | 1 | 5 | 8 | 1 |
| NM_001130984 | DYSF | 6678 | 8 | 3 | 1 | 1 | 4 | 1 | 1 | 2 | 3 | 1 | 5 | 8 | 1 |
| NM_001130985 | DYSF | 6708 | 8 | 3 | 1 | 1 | 4 | 1 | 1 | 2 | 3 | 1 | 5 | 8 | 1 |
| NM_001130986 | DYSF | 6615 | 8 | 3 | 1 | 1 | 5 | 1 | 2 | 2 | 3 | 1 | 5 | 8 | 1 |
| NM_001130987 | DYSF | 6771 | 8 | 3 | 1 | 1 | 4 | 1 | 1 | 2 | 3 | 1 | 5 | 8 | 1 |
| NM_001130991 | HYOU1 | 4478 | 45 | 9 | 3 | 5 | 7 | 9 | 8 | 45 | 5 | 2 | 1 | 7 | 5 |
| NM_001130992 | RBP1 | 1486 | 38 | 6 | 1 | 2 | 1 | 8 | 1 | 4 | 2 | 38 | 0 | 2 | 8 |
| NM_001130993 | RBP1 | 666 | 84 | 14 | 1 | 4 | 2 | 18 | 3 | 9 | 4 | 84 | 1 | 6 | 18 |
| NM_001130996 | HMGCR | 4421 | 18 | 6 | 2 | 4 | 3 | 18 | 6 | 5 | 6 | 6 | 1 | 6 | 8 |
| NM_001130997 | FAM58A | 1228 | 1 | 0 | 0 | 0 | 0 | 1 | 0 | 0 | 0 | 0 | 0 | 0 | 0 |
| NM_001131 | CRISP1 | 1876 | 68 | 6 | 0 | 0 | 0 | 0 | 0 | 0 | 0 | 0 | 0 | 0 | 68 |
| NM_001131005 | MEF2C | 6130 | 38 | 12 | 8 | 3 | 8 | 33 | 3 | 1 | 8 | 10 | 38 | 17 | 6 |
| NM_001131007 | KIAA0922 | 5017 | 7 | 2 | 2 | 1 | 1 | 0 | 1 | 3 | 2 | 1 | 4 | 7 | 1 |
| NM_001131008 | PTPN12 | 3107 | 26 | 11 | 12 | 3 | 14 | 7 | 9 | 6 | 21 | 10 | 6 | 26 | 10 |
| NM_001131009 | PTPN12 | 3011 | 27 | 12 | 13 | 3 | 14 | 7 | 9 | 7 | 22 | 10 | 6 | 27 | 10 |
| NM_001131010 | SATB1 | 3908 | 8 | 4 | 2 | 3 | 5 | 8 | 3 | 1 | 4 | 3 | 8 | 7 | 3 |
| NM_001131015 | CIZ1 | 2863 | 23 | 14 | 16 | 13 | 12 | 17 | 10 | 5 | 11 | 23 | 14 | 15 | 22 |
| NM_001131016 | CIZ1 | 2991 | 22 | 14 | 15 | 13 | 12 | 17 | 9 | 4 | 10 | 22 | 14 | 15 | 21 |
| NM_001131017 | CIZ1 | 2808 | 23 | 15 | 17 | 13 | 13 | 18 | 10 | 5 | 11 | 23 | 15 | 16 | 23 |
| NM_001131018 | CIZ1 | 2751 | 24 | 15 | 17 | 13 | 13 | 18 | 10 | 5 | 11 | 24 | 15 | 16 | 23 |
| NM_001131019 | GFAP | 1819 | 97 | 9 | 0 | 1 | 0 | 97 | 0 | 0 | 0 | 0 | 0 | 0 | 0 |
| NM_001131023 | PEX5 | 3305 | 5 | 3 | 2 | 1 | 2 | 5 | 4 | 4 | 2 | 2 | 3 | 2 | 5 |
| NM_001131024 | PEX5 | 3149 | 5 | 3 | 2 | 1 | 2 | 5 | 4 | 4 | 2 | 2 | 3 | 2 | 5 |
| NM_001131025 | PEX5 | 3726 | 5 | 3 | 2 | 1 | 2 | 5 | 4 | 4 | 2 | 2 | 3 | 1 | 4 |
| NM_001131026 | PEX5 | 2689 | 3 | 1 | 1 | 0 | 1 | 3 | 2 | 2 | 1 | 1 | 1 | 1 | 3 |
| NM_001131027 | PDLIM4 | 2175 | 0 | 0 | 0 | 0 | 0 | 0 | 0 | 0 | 0 | 0 | 0 | 0 | 0 |
| NM_001131028 | ATG10 | 2421 | 2 | 2 | 1 | 1 | 2 | 2 | 2 | 1 | 1 | 2 | 1 | 2 | 2 |
| NM_001131034 | RNF212 | 2617 | 1 | 0 | 0 | 0 | 0 | 0 | 1 | 0 | 0 | 1 | 0 | 0 | 1 |
| NM_001131035 | ZCCHC9 | 1431 | 5 | 2 | 3 | 1 | 2 | 2 | 2 | 5 | 2 | 3 | 1 | 3 | 3 |
| NM_001131036 | ZCCHC9 | 1403 | 5 | 2 | 3 | 1 | 2 | 2 | 2 | 5 | 2 | 3 | 1 | 3 | 3 |
| NM_001131039 | PEX19 | 3559 | 9 | 4 | 9 | 2 | 4 | 6 | 4 | 6 | 2 | 2 | 3 | 3 | 3 |
| NM_001131055 | HRH2 | 2552 | 2 | 0 | 0 | 0 | 2 | 1 | 0 | 0 | 1 | 0 | 0 | 1 | 0 |
| NM_001131062 | SAP30L | 6101 | 3 | 2 | 2 | 1 | 2 | 2 | 2 | 2 | 1 | 3 | 2 | 3 | 2 |
| NM_001131063 | SAP30L | 6086 | 3 | 2 | 2 | 1 | 2 | 2 | 2 | 2 | 1 | 3 | 2 | 3 | 2 |
| NM_001131064 | ACP2 | 730 | 6 | 1 | 2 | 1 | 1 | 2 | 1 | 6 | 1 | 0 | 0 | 1 | 0 |
| NM_001131065 | RFESD | 2799 | 1 | 1 | 0 | 0 | 1 | 0 | 0 | 1 | 0 | 1 | 1 | 0 | 1 |
| NM_001131066 | RFESD | 1644 | 2 | 1 | 1 | 0 | 1 | 0 | 0 | 1 | 0 | 1 | 1 | 1 | 2 |
| NM_001133 | AFM | 1997 | 42 | 5 | 0 | 0 | 0 | 0 | 12 | 42 | 0 | 0 | 0 | 0 | 0 |
| NM_001134 | AFP | 2032 | 0 | 0 | 0 | 0 | 0 | 0 | 0 | 0 | 0 | 0 | 0 | 0 | 0 |
| NM_001134194 | ACPP | 1825 | 10 | 2 | 1 | 1 | 1 | 0 | 10 | 0 | 4 | 2 | 0 | 3 | 2 |
| NM_001134222 | IRX2 | 2301 | 1 | 0 | 0 | 0 | 0 | 0 | 1 | 0 | 1 | 0 | 0 | 0 | 0 |
| NM_001134223 | USP46 | 8039 | 3 | 1 | 0 | 0 | 2 | 3 | 1 | 0 | 0 | 1 | 1 | 1 | 1 |
| NM_001134224 | INPP4A | 6751 | 5 | 1 | 1 | 1 | 1 | 5 | 1 | 0 | 1 | 1 | 1 | 3 | 1 |
| NM_001134225 | INPP4A | 6736 | 5 | 1 | 1 | 1 | 1 | 5 | 1 | 0 | 1 | 1 | 1 | 3 | 1 |
| NM_001134231 | NT5DC2 | 1815 | 5 | 2 | 2 | 2 | 5 | 2 | 2 | 1 | 1 | 4 | 1 | 2 | 3 |
| NM_001134232 | TMEM106B | 6319 | 6 | 5 | 3 | 1 | 5 | 6 | 6 | 5 | 3 | 5 | 5 | 5 | 6 |
| NM_001134233 | C1orf204 | 2501 | 0 | 0 | 0 | 0 | 0 | 0 | 0 | 0 | 0 | 0 | 0 | 0 | 0 |
| NM_001134285 | ESRRG | 5311 | 6 | 1 | 0 | 0 | 3 | 2 | 6 | 0 | 0 | 0 | 2 | 0 | 0 |
| NM_001134296 | AP3M2 | 3670 | 10 | 3 | 2 | 1 | 1 | 10 | 2 | 0 | 2 | 2 | 1 | 2 | 4 |
| NM_001134316 | PRR22 | 1392 | 1 | 0 | 0 | 0 | 0 | 0 | 0 | 0 | 0 | 0 | 0 | 0 | 1 |
| NM_001134321 | FAM127B | 707 | 8 | 5 | 3 | 3 | 7 | 7 | 5 | 2 | 2 | 8 | 5 | 6 | 3 |
| NM_001134335 | EIF2AK1 | 4460 | 13 | 7 | 5 | 5 | 5 | 13 | 7 | 12 | 4 | 5 | 5 | 7 | 6 |
| NM_001134336 | FAM40B | 2877 | 1 | 0 | 0 | 0 | 1 | 1 | 1 | 0 | 0 | 0 | 1 | 0 | 1 |
| NM_001134337 | RNF24 | 3228 | 2 | 1 | 1 | 0 | 1 | 2 | 1 | 0 | 1 | 1 | 0 | 1 | 1 |
| NM_001134338 | RNF24 | 3297 | 2 | 1 | 1 | 0 | 1 | 2 | 1 | 0 | 2 | 1 | 0 | 2 | 1 |
| NM_001134340 | PSMG3 | 779 | 3 | 1 | 1 | 3 | 1 | 2 | 3 | 1 | 2 | 0 | 1 | 2 | 1 |
| NM_001134363 | RBM20 | 7233 | 8 | 1 | 0 | 0 | 8 | 0 | 0 | 0 | 0 | 0 | 3 | 0 | 1 |

| transcript | gene | transcript length (nt) | max | mean | adipose | colon | heart | hypothalamus | kidney | liver | lung | ovary | skeletalmuscle | spleen | testes |
|---|---|---|---|---|---|---|---|---|---|---|---|---|---|---|---|
| NM_001134364 | MAP4 | 6036 | 40 | 17 | 23 | 8 | 35 | 21 | 11 | 8 | 9 | 10 | 40 | 11 | 16 |
| NM_001134365 | MAP4 | 4845 | 103 | 17 | 5 | 3 | 50 | 5 | 3 | 2 | 3 | 3 | 103 | 3 | 6 |
| NM_001134366 | GAD2 | 2419 | 6 | 1 | 0 | 0 | 0 | 6 | 0 | 0 | 0 | 0 | 0 | 0 | 0 |
| NM_001134367 | SLC6A6 | 6502 | 6 | 3 | 2 | 1 | 3 | 2 | 3 | 0 | 4 | 6 | 1 | 5 | 1 |
| NM_001134368 | SLC6A6 | 1269 | 2 | 1 | 1 | 0 | 1 | 1 | 1 | 0 | 1 | 2 | 0 | 1 | 0 |
| NM_001134369 | LRRFIP2 | 2923 | 21 | 12 | 8 | 8 | 21 | 10 | 9 | 16 | 10 | 12 | 11 | 17 | 15 |
| NM_001134373 | NT5C2 | 5263 | 12 | 8 | 5 | 5 | 6 | 9 | 9 | 5 | 10 | 8 | 8 | 10 | 12 |
| NM_001134374 | SPATC1 | 1895 | 1 | 0 | 0 | 0 | 0 | 0 | 0 | 0 | 0 | 0 | 0 | 0 | 1 |
| NM_001134375 | CCNJ | 4136 | 3 | 1 | 1 | 1 | 1 | 1 | 1 | 1 | 1 | 2 | 1 | 3 | 2 |
| NM_001134376 | CCNJ | 4100 | 3 | 1 | 1 | 1 | 1 | 1 | 1 | 1 | 1 | 2 | 1 | 3 | 2 |
| NM_001134380 | TBC1D5 | 6615 | 10 | 5 | 4 | 2 | 4 | 6 | 5 | 2 | 5 | 7 | 2 | 10 | 5 |
| NM_001134381 | TBC1D5 | 6518 | 10 | 5 | 4 | 2 | 4 | 6 | 5 | 2 | 5 | 8 | 2 | 10 | 5 |
| NM_001134382 | IQSEC1 | 7212 | 8 | 3 | 2 | 1 | 3 | 8 | 2 | 3 | 2 | 2 | 1 | 5 | 2 |
| NM_001134387 | ZDHHC4 | 1552 | 4 | 2 | 1 | 1 | 1 | 2 | 2 | 2 | 1 | 2 | 1 | 2 | 4 |
| NM_001134388 | ZDHHC4 | 1461 | 4 | 2 | 2 | 1 | 2 | 3 | 2 | 2 | 1 | 2 | 1 | 2 | 4 |
| NM_001134389 | ZDHHC4 | 1330 | 5 | 2 | 2 | 1 | 2 | 3 | 2 | 3 | 1 | 2 | 1 | 2 | 5 |
| NM_001134395 | C7orf50 | 1283 | 8 | 4 | 3 | 4 | 6 | 8 | 7 | 3 | 4 | 5 | 4 | 3 | 2 |
| NM_001134396 | C7orf50 | 1264 | 8 | 5 | 3 | 4 | 6 | 8 | 7 | 3 | 4 | 5 | 4 | 4 | 2 |
| NM_001134398 | VAV2 | 4850 | 2 | 1 | 0 | 1 | 0 | 1 | 2 | 1 | 1 | 0 | 0 | 1 | 0 |
| NM_001134400 | CYB561D1 | 4998 | 1 | 0 | 0 | 0 | 0 | 0 | 1 | 0 | 0 | 1 | 0 | 1 | 0 |
| NM_001134402 | CYB561D1 | 4804 | 1 | 0 | 0 | 0 | 0 | 0 | 1 | 0 | 0 | 1 | 0 | 1 | 0 |
| NM_001134403 | CYB561D1 | 5036 | 1 | 0 | 0 | 0 | 0 | 0 | 1 | 0 | 0 | 0 | 0 | 1 | 0 |
| NM_001134404 | CYB561D1 | 5113 | 1 | 0 | 0 | 0 | 0 | 0 | 1 | 0 | 0 | 0 | 0 | 1 | 0 |
| NM_001134405 | RUNDC3B | 4049 | 4 | 1 | 0 | 1 | 1 | 4 | 1 | 2 | 0 | 1 | 0 | 1 | 2 |
| NM_001134406 | RUNDC3B | 3902 | 4 | 1 | 0 | 1 | 1 | 4 | 1 | 2 | 0 | 1 | 0 | 1 | 2 |
| NM_001134407 | GRIN2A | 14682 | 8 | 1 | 0 | 0 | 0 | 8 | 0 | 0 | 0 | 0 | 0 | 0 | 0 |
| NM_001134408 | GRIN2A | 4734 | 14 | 1 | 0 | 0 | 0 | 14 | 0 | 0 | 0 | 0 | 0 | 0 | 1 |
| NM_001134415 | TBCCD1 | 3062 | 1 | 1 | 0 | 0 | 0 | 0 | 1 | 1 | 0 | 1 | 0 | 1 | 1 |
| NM_001134418 | LEPREL1 | 3065 | 21 | 5 | 1 | 2 | 3 | 1 | 17 | 1 | 6 | 8 | 0 | 21 | 2 |
| NM_001134419 | CDC7 | 3184 | 4 | 1 | 0 | 0 | 0 | 2 | 1 | 0 | 1 | 1 | 0 | 2 | 4 |
| NM_001134420 | CDC7 | 3312 | 4 | 1 | 0 | 0 | 1 | 2 | 1 | 0 | 1 | 1 | 0 | 2 | 4 |
| NM_001134422 | CDV3 | 4195 | 20 | 11 | 13 | 3 | 14 | 7 | 8 | 8 | 10 | 7 | 20 | 18 | 10 |
| NM_001134423 | CDV3 | 3633 | 22 | 12 | 15 | 4 | 16 | 8 | 9 | 9 | 11 | 8 | 22 | 21 | 11 |
| NM_001134430 | TOR2A | 1402 | 0 | 0 | 0 | 0 | 0 | 0 | 0 | 0 | 0 | 0 | 0 | 0 | 0 |
| NM_001134431 | TOR2A | 2242 | 0 | 0 | 0 | 0 | 0 | 0 | 0 | 0 | 0 | 0 | 0 | 0 | 0 |
| NM_001134432 | AZI2 | 1445 | 19 | 13 | 18 | 5 | 12 | 15 | 12 | 13 | 19 | 13 | 7 | 15 | 14 |
| NM_001134433 | AZI2 | 1441 | 19 | 13 | 18 | 5 | 12 | 15 | 12 | 13 | 19 | 13 | 7 | 15 | 14 |
| NM_001134434 | SPOCK2 | 1283 | 5 | 1 | 0 | 0 | 0 | 5 | 1 | 0 | 1 | 0 | 0 | 1 | 0 |
| NM_001134435 | IQCG | 1981 | 10 | 3 | 3 | 1 | 2 | 5 | 3 | 1 | 4 | 4 | 1 | 2 | 10 |
| NM_001134437 | PHLDB2 | 5938 | 19 | 5 | 7 | 1 | 19 | 2 | 7 | 4 | 4 | 2 | 1 | 4 | 2 |
| NM_001134438 | PHLDB2 | 6125 | 20 | 5 | 7 | 1 | 20 | 2 | 7 | 3 | 4 | 2 | 1 | 4 | 2 |
| NM_001134439 | PHLDB2 | 5886 | 21 | 5 | 7 | 1 | 21 | 2 | 8 | 4 | 4 | 2 | 1 | 4 | 2 |
| NM_001134440 | ZNF502 | 3267 | 4 | 2 | 2 | 0 | 2 | 2 | 2 | 0 | 1 | 4 | 2 | 2 | 4 |
| NM_001134441 | ZNF502 | 3173 | 4 | 2 | 2 | 1 | 2 | 2 | 2 | 0 | 1 | 4 | 2 | 2 | 4 |
| NM_001134442 | ZNF502 | 3169 | 4 | 2 | 2 | 1 | 2 | 2 | 2 | 0 | 1 | 4 | 2 | 2 | 4 |
| NM_001134445 | DDAH1 | 4017 | 44 | 14 | 2 | 10 | 7 | 34 | 44 | 16 | 9 | 5 | 9 | 7 | 14 |
| NM_001134450 | TMEM130 | 3064 | 19 | 2 | 0 | 1 | 0 | 19 | 0 | 0 | 0 | 0 | 0 | 0 | 3 |
| NM_001134451 | TMEM130 | 2722 | 21 | 2 | 0 | 1 | 0 | 21 | 0 | 0 | 0 | 0 | 0 | 0 | 3 |
| NM_001134453 | DSG4 | 3637 | 0 | 0 | 0 | 0 | 0 | 0 | 0 | 0 | 0 | 0 | 0 | 0 | 0 |
| NM_001134456 | FAM55C | 8911 | 5 | 2 | 1 | 1 | 4 | 4 | 1 | 0 | 2 | 2 | 5 | 4 | 2 |
| NM_001134462 | NOTO | 1447 | 0 | 0 | 0 | 0 | 0 | 0 | 0 | 0 | 0 | 0 | 0 | 0 | 0 |
| NM_001134468 | C7orf53 | 1462 | 2 | 1 | 1 | 0 | 1 | 0 | 1 | 1 | 1 | 0 | 2 | 1 | 1 |
| NM_001134470 | C3orf58 | 3400 | 4 | 2 | 1 | 1 | 1 | 2 | 1 | 3 | 2 | 4 | 1 | 2 | 1 |
| NM_001134473 | KIAA0182 | 7202 | 4 | 3 | 4 | 2 | 2 | 2 | 3 | 2 | 2 | 3 | 4 | 2 | 4 |
| NM_001134476 | LRRC8B | 7735 | 7 | 2 | 1 | 0 | 1 | 7 | 1 | 0 | 2 | 1 | 1 | 2 | 2 |
| NM_001134477 | PARN | 2990 | 8 | 5 | 6 | 4 | 4 | 7 | 5 | 6 | 5 | 5 | 3 | 7 | 8 |
| NM_001134478 | PLCXD2 | 7725 | 2 | 0 | 0 | 0 | 0 | 2 | 0 | 0 | 0 | 0 | 0 | 0 | 0 |
| NM_001134479 | LRRC8D | 3785 | 6 | 3 | 2 | 1 | 2 | 6 | 5 | 5 | 1 | 2 | 1 | 6 | 5 |
| NM_001134484 | TOMM5 | 903 | 7 | 4 | 5 | 3 | 5 | 7 | 5 | 7 | 2 | 2 | 2 | 5 | 2 |
| NM_001134485 | TOMM5 | 846 | 8 | 5 | 5 | 3 | 5 | 7 | 6 | 8 | 3 | 2 | 2 | 6 | 3 |
| NM_001134486 | GBP5 | 3943 | 6 | 1 | 1 | 1 | 0 | 0 | 0 | 0 | 3 | 1 | 0 | 6 | 1 |
| NM_001134492 | HS2ST1 | 1577 | 14 | 6 | 5 | 2 | 4 | 14 | 5 | 8 | 4 | 6 | 2 | 9 | 7 |
| NM_001134493 | TOMM6 | 854 | 20 | 13 | 9 | 4 | 20 | 15 | 19 | 15 | 8 | 12 | 12 | 20 | 10 |
| NM_001134647 | AFAP1 | 7770 | 1 | 1 | 1 | 1 | 1 | 1 | 1 | 0 | 1 | 1 | 1 | 1 | 1 |
| NM_001134649 | EIF4E3 | 6205 | 7 | 5 | 6 | 2 | 5 | 6 | 3 | 2 | 5 | 5 | 7 | 7 | 4 |
| NM_001134650 | EIF4E3 | 6083 | 7 | 5 | 6 | 2 | 5 | 6 | 3 | 2 | 5 | 6 | 7 | 7 | 4 |
| NM_001134651 | EIF4E3 | 6074 | 7 | 5 | 6 | 2 | 5 | 6 | 3 | 2 | 5 | 6 | 7 | 7 | 4 |
| NM_001134655 | ZNF213 | 3264 | 0 | 0 | 0 | 0 | 0 | 0 | 0 | 0 | 0 | 0 | 0 | 0 | 0 |
| NM_001134656 | ZNF662 | 5524 | 2 | 1 | 0 | 0 | 1 | 1 | 1 | 0 | 0 | 2 | 0 | 1 | 2 |
| NM_001134657 | PRR23C | 2791 | 0 | 0 | 0 | 0 | 0 | 0 | 0 | 0 | 0 | 0 | 0 | 0 | 0 |
| NM_001134658 | TMEM20 | 2402 | 1 | 0 | 0 | 0 | 0 | 0 | 0 | 0 | 0 | 0 | 0 | 0 | 1 |
| NM_001134659 | PRR23A | 2306 | 0 | 0 | 0 | 0 | 0 | 0 | 0 | 0 | 0 | 0 | 0 | 0 | 0 |
| NM_001134663 | SAMD13 | 1560 | 1 | 0 | 0 | 0 | 0 | 0 | 0 | 0 | 0 | 0 | 1 | 0 | 1 |
| NM_001134664 | SAMD13 | 1404 | 1 | 0 | 0 | 1 | 0 | 0 | 0 | 0 | 0 | 0 | 1 | 0 | 1 |
| NM_001134665 | RG9MTD2 | 3536 | 2 | 1 | 1 | 0 | 1 | 1 | 1 | 1 | 1 | 1 | 1 | 1 | 2 |
| NM_001134666 | RG9MTD2 | 3525 | 2 | 1 | 1 | 0 | 1 | 1 | 1 | 1 | 1 | 1 | 1 | 1 | 2 |
| NM_001134670 | DHDPSL | 1991 | 3 | 1 | 0 | 0 | 1 | 0 | 3 | 1 | 0 | 0 | 0 | 0 | 0 |

| | | transcript | | | | | | hypothala | | | | | skeletalm | | |
|---|---|---|---|---|---|---|---|---|---|---|---|---|---|---|---|
| transcript | gene | length (nt) | max | mean | adipose | colon | heart | mus | kidney | liver | lung | ovary | uscle | spleen | testes |
| NM_001134671 | DERL1 | 3101 | 7 | 4 | 3 | 2 | 5 | 3 | 3 | 7 | 3 | 3 | 4 | 6 | 4 |
| NM_001134672 | C10orf84 | 2565 | 12 | 7 | 11 | 3 | 6 | 12 | 7 | 7 | 5 | 6 | 6 | 8 | 8 |
| NM_001134673 | NFIA | 9481 | 15 | 10 | 10 | 4 | 15 | 9 | 8 | 9 | 7 | 15 | 7 | 9 | 13 |
| NM_001134693 | LOC100128[illegible] | 485 | 13 | 7 | 11 | 10 | 13 | 8 | 6 | 11 | 5 | 4 | 4 | 5 | 3 |
| NM_001134707 | SARDH | 3216 | 1 | 0 | 0 | 0 | 0 | 0 | 0 | 1 | 0 | 0 | 0 | 0 | 0 |
| NM_001134709 | DEK | 2767 | 32 | 18 | 12 | 6 | 23 | 10 | 15 | 9 | 26 | 26 | 24 | 32 | 18 |
| NM_001134734 | C1orf94 | 3002 | 0 | 0 | 0 | 0 | 0 | 0 | 0 | 0 | 0 | 0 | 0 | 0 | 0 |
| NM_001134738 | BCL6 | 3083 | 47 | 13 | 15 | 2 | 15 | 11 | 4 | 7 | 12 | 8 | 47 | 13 | 8 |
| NM_001134742 | SLC4A4 | 7595 | 47 | 6 | 1 | 4 | 2 | 11 | 47 | 2 | 2 | 0 | 0 | 0 | 0 |
| NM_001134745 | LRRTM4 | 3151 | 6 | 1 | 0 | 0 | 0 | 6 | 0 | 0 | 0 | 0 | 0 | 0 | 0 |
| NM_001134758 | ARFRP1 | 2614 | 3 | 2 | 2 | 3 | 1 | 2 | 2 | 2 | 1 | 1 | 1 | 3 | 2 |
| NM_001134759 | CRYZ | 1973 | 30 | 6 | 1 | 1 | 4 | 2 | 30 | 13 | 3 | 3 | 1 | 3 | 4 |
| NM_001134771 | SLC12A5 | 6041 | 17 | 2 | 0 | 0 | 0 | 17 | 0 | 0 | 0 | 0 | 0 | 0 | 0 |
| NM_001134772 | STX16 | 4934 | 8 | 5 | 5 | 5 | 4 | 6 | 5 | 3 | 6 | 4 | 3 | 8 | 6 |
| NM_001134773 | STX16 | 4895 | 8 | 5 | 5 | 5 | 4 | 7 | 5 | 3 | 6 | 4 | 3 | 8 | 6 |
| NM_001134774 | KLC2 | 2723 | 3 | 1 | 0 | 0 | 1 | 3 | 0 | 0 | 0 | 0 | 1 | 0 | 1 |
| NM_001134775 | KLC2 | 3015 | 3 | 1 | 0 | 0 | 1 | 3 | 0 | 0 | 0 | 0 | 1 | 0 | 1 |
| NM_001134776 | KLC2 | 2894 | 3 | 1 | 0 | 0 | 1 | 3 | 0 | 0 | 0 | 0 | 1 | 0 | 1 |
| NM_001134778 | PBX3 | 2786 | 8 | 3 | 2 | 2 | 4 | 2 | 1 | 2 | 2 | 8 | 1 | 2 | 3 |
| NM_001134779 | IPO11 | 4448 | 10 | 3 | 2 | 1 | 3 | 3 | 3 | 2 | 2 | 3 | 2 | 3 | 10 |
| NM_001134793 | HYLS1 | 2058 | 2 | 1 | 0 | 1 | 1 | 1 | 1 | 1 | 0 | 1 | 0 | 1 | 2 |
| NM_001134830 | AHI1 | 5499 | 16 | 6 | 4 | 2 | 4 | 8 | 6 | 1 | 6 | 10 | 3 | 5 | 16 |
| NM_001134831 | AHI1 | 5646 | 16 | 6 | 4 | 2 | 4 | 7 | 6 | 1 | 5 | 9 | 3 | 4 | 16 |
| NM_001134832 | AHI1 | 3657 | 23 | 8 | 5 | 2 | 6 | 9 | 7 | 1 | 8 | 14 | 4 | 6 | 23 |
| NM_001134836 | SIRPB2 | 2539 | 2 | 1 | 0 | 0 | 1 | 0 | 0 | 0 | 1 | 0 | 0 | 2 | 0 |
| NM_001134848 | CCDC152 | 3491 | 3 | 1 | 2 | 1 | 1 | 1 | 1 | 1 | 2 | 2 | 0 | 3 | 1 |
| NM_001134851 | TCF7 | 2837 | 3 | 1 | 0 | 1 | 0 | 0 | 0 | 0 | 2 | 1 | 0 | 3 | 0 |
| NM_001134852 | TCF7 | 2787 | 3 | 1 | 0 | 1 | 0 | 0 | 0 | 0 | 2 | 1 | 0 | 3 | 1 |
| NM_001134855 | TRIM17 | 1782 | 1 | 0 | 0 | 0 | 0 | 1 | 0 | 0 | 0 | 0 | 0 | 0 | 1 |
| NM_001134870 | KIAA1949 | 2951 | 13 | 5 | 7 | 4 | 6 | 5 | 1 | 2 | 8 | 4 | 2 | 13 | 3 |
| NM_001134870 | KIAA1949 | 2952 | 13 | 5 | 7 | 4 | 6 | 5 | 1 | 2 | 8 | 4 | 2 | 13 | 3 |
| NM_001134875 | C14orf80 | 1709 | 0 | 0 | 0 | 0 | 0 | 0 | 0 | 0 | 0 | 0 | 0 | 0 | 0 |
| NM_001134876 | C14orf80 | 1610 | 0 | 0 | 0 | 0 | 0 | 0 | 0 | 0 | 0 | 0 | 0 | 0 | 0 |
| NM_001134877 | C14orf80 | 1537 | 0 | 0 | 0 | 0 | 0 | 0 | 0 | 0 | 0 | 0 | 0 | 0 | 0 |
| NM_001134878 | KIF9 | 3602 | 2 | 1 | 0 | 0 | 0 | 1 | 1 | 1 | 0 | 0 | 0 | 0 | 2 |
| NM_001134888 | RTL1 | 4193 | 0 | 0 | 0 | 0 | 0 | 0 | 0 | 0 | 0 | 0 | 0 | 0 | 0 |
| NM_001134937 | FIP1L1 | 2439 | 42 | 23 | 19 | 11 | 18 | 38 | 15 | 15 | 17 | 31 | 14 | 30 | 42 |
| NM_001134938 | FIP1L1 | 2235 | 45 | 24 | 21 | 12 | 19 | 41 | 16 | 16 | 18 | 34 | 15 | 32 | 45 |
| NM_001134939 | OAZ3 | 851 | 17 | 2 | 1 | 0 | 0 | 0 | 0 | 0 | 0 | 0 | 0 | 1 | 17 |
| NM_001134941 | GHRL | 525 | 0 | 0 | 0 | 0 | 0 | 0 | 0 | 0 | 0 | 0 | 0 | 0 | 0 |
| NM_001134944 | GHRL | 477 | 0 | 0 | 0 | 0 | 0 | 0 | 0 | 0 | 0 | 0 | 0 | 0 | 0 |
| NM_001134945 | GHRL | 474 | 0 | 0 | 0 | 0 | 0 | 0 | 0 | 0 | 0 | 0 | 0 | 0 | 0 |
| NM_001134946 | GHRL | 360 | 0 | 0 | 0 | 0 | 0 | 0 | 0 | 0 | 0 | 0 | 0 | 0 | 0 |
| NM_001134999 | FERMT2 | 3355 | 25 | 11 | 25 | 5 | 16 | 9 | 9 | 11 | 9 | 9 | 5 | 11 | 8 |
| NM_001135 | ACAN | 8543 | 0 | 0 | 0 | 0 | 0 | 0 | 0 | 0 | 0 | 0 | 0 | 0 | 0 |
| NM_001135000 | FERMT2 | 2160 | 33 | 14 | 33 | 7 | 20 | 12 | 11 | 15 | 11 | 11 | 7 | 15 | 11 |
| NM_001135004 | DNAJB5 | 2502 | 11 | 4 | 1 | 4 | 11 | 3 | 2 | 0 | 1 | 1 | 10 | 2 | 4 |
| NM_001135005 | DNAJB5 | 2796 | 10 | 3 | 1 | 3 | 10 | 2 | 2 | 0 | 1 | 0 | 9 | 2 | 4 |
| NM_001135021 | ELMOD3 | 2599 | 4 | 1 | 4 | 1 | 1 | 1 | 1 | 1 | 1 | 0 | 0 | 1 | 1 |
| NM_001135022 | ELMOD3 | 2415 | 5 | 1 | 5 | 1 | 1 | 1 | 1 | 1 | 1 | 1 | 0 | 1 | 1 |
| NM_001135023 | ELMOD3 | 2371 | 5 | 1 | 5 | 1 | 1 | 1 | 1 | 1 | 1 | 1 | 0 | 1 | 1 |
| NM_001135024 | PDHX | 2658 | 18 | 9 | 7 | 3 | 18 | 12 | 8 | 7 | 5 | 6 | 11 | 7 | 11 |
| NM_001135031 | GFI1B | 1652 | 0 | 0 | 0 | 0 | 0 | 0 | 0 | 0 | 0 | 0 | 0 | 0 | 0 |
| NM_001135032 | FAM176A | 1855 | 10 | 1 | 0 | 0 | 0 | 0 | 2 | 10 | 1 | 0 | 0 | 2 | 1 |
| NM_001135036 | FAM18B2 | 4226 | 2 | 1 | 0 | 1 | 0 | 0 | 1 | 0 | 1 | 2 | 0 | 1 | 1 |
| NM_001135037 | MFAP3 | 4588 | 4 | 2 | 2 | 1 | 3 | 2 | 2 | 3 | 2 | 2 | 1 | 4 | 2 |
| NM_001135040 | DCTN1 | 4417 | 37 | 14 | 11 | 9 | 19 | 37 | 10 | 6 | 8 | 11 | 10 | 12 | 23 |
| NM_001135041 | DCTN1 | 4147 | 37 | 14 | 11 | 9 | 19 | 37 | 10 | 6 | 8 | 11 | 10 | 12 | 23 |
| NM_001135044 | MAPK9 | 2369 | 5 | 2 | 1 | 1 | 1 | 5 | 1 | 1 | 1 | 1 | 1 | 2 | 2 |
| NM_001135047 | JDP2 | 3783 | 12 | 5 | 7 | 2 | 9 | 4 | 3 | 2 | 5 | 3 | 2 | 12 | 2 |
| NM_001135048 | JDP2 | 3775 | 12 | 5 | 7 | 2 | 9 | 4 | 3 | 2 | 5 | 3 | 2 | 12 | 2 |
| NM_001135049 | JDP2 | 3942 | 11 | 4 | 6 | 2 | 8 | 4 | 3 | 2 | 5 | 3 | 2 | 11 | 2 |
| NM_001135050 | IGSF9 | 4044 | 0 | 0 | 0 | 0 | 0 | 0 | 0 | 0 | 0 | 0 | 0 | 0 | 0 |
| NM_001135051 | FAM160B1 | 2864 | 3 | 2 | 2 | 1 | 2 | 1 | 1 | 3 | 2 | 2 | 1 | 2 | 2 |
| NM_001135052 | SYK | 4994 | 8 | 2 | 3 | 2 | 1 | 1 | 2 | 0 | 2 | 0 | 0 | 8 | 2 |
| NM_001135053 | SIGIRR | 1670 | 1 | 0 | 0 | 0 | 0 | 0 | 1 | 0 | 0 | 0 | 0 | 0 | 0 |
| NM_001135054 | SIGIRR | 1743 | 1 | 0 | 0 | 1 | 0 | 0 | 1 | 0 | 0 | 0 | 0 | 0 | 0 |
| NM_001135055 | TKT | 2889 | 11 | 5 | 11 | 3 | 3 | 4 | 7 | 4 | 8 | 3 | 0 | 8 | 2 |
| NM_001135056 | TKT | 1850 | 48 | 24 | 48 | 12 | 13 | 20 | 33 | 20 | 40 | 17 | 3 | 46 | 11 |
| NM_001135057 | LRRC15 | 5939 | 0 | 0 | 0 | 0 | 0 | 0 | 0 | 0 | 0 | 0 | 0 | 0 | 0 |
| NM_001135058 | COCH | 2882 | 3 | 1 | 0 | 0 | 0 | 3 | 1 | 0 | 0 | 0 | 1 | 1 | 1 |
| NM_001135086 | TESSP1 | 956 | 0 | 0 | 0 | 0 | 0 | 0 | 0 | 0 | 0 | 0 | 0 | 0 | 0 |
| NM_001135091 | MUC15 | 3391 | 4 | 1 | 0 | 0 | 0 | 0 | 3 | 0 | 2 | 0 | 0 | 0 | 4 |
| NM_001135092 | MUC15 | 3241 | 4 | 1 | 0 | 0 | 0 | 0 | 3 | 0 | 2 | 0 | 0 | 0 | 4 |
| NM_001135095 | FNDC3B | 6939 | 10 | 7 | 9 | 4 | 9 | 2 | 6 | 7 | 8 | 10 | 5 | 8 | 5 |
| NM_001135098 | UPP2 | 2408 | 2 | 0 | 0 | 0 | 0 | 0 | 2 | 1 | 0 | 0 | 0 | 0 | 0 |
| NM_001135099 | TMPRSS2 | 3240 | 9 | 2 | 0 | 9 | 0 | 0 | 6 | 3 | 4 | 0 | 0 | 0 | 0 |

| transcript | gene | transcript length (nt) | max | mean | adipose | colon | heart | hypothalamus | kidney | liver | lung | ovary | skeletalmuscle | spleen | testes |
|---|---|---|---|---|---|---|---|---|---|---|---|---|---|---|---|
| NM_001135101 | CRELD2 | 1575 | 12 | 2 | 1 | 2 | 2 | 1 | 1 | 12 | 1 | 0 | 0 | 2 | 1 |
| NM_001135105 | KCNK16 | 1408 | 0 | 0 | 0 | 0 | 0 | 0 | 0 | 0 | 0 | 0 | 0 | 0 | 0 |
| NM_001135106 | KCNK16 | 947 | 0 | 0 | 0 | 0 | 0 | 0 | 0 | 0 | 0 | 0 | 0 | 0 | 0 |
| NM_001135107 | KCNK16 | 1106 | 0 | 0 | 0 | 0 | 0 | 0 | 0 | 0 | 0 | 0 | 0 | 0 | 0 |
| NM_001135109 | RIC3 | 5266 | 3 | 1 | 0 | 0 | 1 | 3 | 1 | 0 | 0 | 1 | 0 | 1 | 1 |
| NM_001135110 | DNAJA3 | 2591 | 13 | 4 | 2 | 3 | 4 | 4 | 5 | 13 | 1 | 2 | 5 | 3 | 4 |
| NM_001135111 | KCNK17 | 1716 | 0 | 0 | 0 | 0 | 0 | 0 | 0 | 0 | 0 | 0 | 0 | 0 | 0 |
| NM_001135113 | IGFL2 | 610 | 1 | 0 | 0 | 0 | 0 | 0 | 0 | 0 | 0 | 0 | 0 | 0 | 1 |
| NM_001135146 | SLC39A8 | 3170 | 49 | 9 | 6 | 6 | 8 | 3 | 4 | 10 | 49 | 3 | 1 | 3 | 1 |
| NM_001135147 | SLC39A8 | 4080 | 5 | 1 | 0 | 0 | 1 | 0 | 0 | 1 | 5 | 0 | 0 | 0 | 0 |
| NM_001135148 | SLC39A8 | 2607 | 16 | 3 | 2 | 1 | 4 | 1 | 2 | 4 | 16 | 1 | 0 | 2 | 1 |
| NM_001135153 | SLC39A14 | 4731 | 44 | 8 | 1 | 2 | 17 | 3 | 6 | 44 | 2 | 2 | 2 | 3 | 6 |
| NM_001135154 | SLC39A14 | 1742 | 8 | 2 | 0 | 2 | 2 | 1 | 1 | 8 | 1 | 0 | 1 | 0 | 1 |
| NM_001135155 | DPF1 | 2325 | 1 | 0 | 0 | 0 | 0 | 1 | 0 | 0 | 0 | 0 | 0 | 0 | 0 |
| NM_001135156 | DPF1 | 2302 | 1 | 0 | 0 | 0 | 0 | 1 | 0 | 0 | 0 | 0 | 0 | 0 | 0 |
| NM_001135161 | COMT | 2260 | 13 | 6 | 4 | 3 | 7 | 8 | 6 | 13 | 5 | 5 | 5 | 6 | 4 |
| NM_001135162 | COMT | 2277 | 13 | 6 | 4 | 3 | 7 | 8 | 6 | 13 | 5 | 5 | 5 | 6 | 4 |
| NM_001135163 | SIGLEC11 | 2875 | 2 | 0 | 0 | 0 | 0 | 0 | 0 | 0 | 0 | 2 | 0 | 1 | 0 |
| NM_001135167 | ALDH3A1 | 1771 | 2 | 0 | 0 | 1 | 0 | 0 | 0 | 0 | 2 | 0 | 0 | 0 | 0 |
| NM_001135168 | ALDH3A1 | 1926 | 1 | 0 | 0 | 1 | 0 | 0 | 0 | 0 | 1 | 0 | 0 | 0 | 0 |
| NM_001135170 | C1QTNF7 | 4517 | 6 | 2 | 3 | 1 | 1 | 0 | 2 | 0 | 2 | 6 | 1 | 3 | 2 |
| NM_001135171 | C1QTNF7 | 4464 | 6 | 2 | 3 | 1 | 1 | 0 | 2 | 0 | 2 | 6 | 1 | 3 | 2 |
| NM_001135175 | NEDD1 | 3780 | 3 | 1 | 1 | 0 | 1 | 1 | 1 | 0 | 1 | 1 | 2 | 2 | 3 |
| NM_001135176 | NEDD1 | 3699 | 3 | 1 | 1 | 0 | 1 | 1 | 1 | 0 | 1 | 1 | 2 | 2 | 3 |
| NM_001135177 | NEDD1 | 3713 | 3 | 1 | 1 | 0 | 1 | 1 | 1 | 0 | 1 | 1 | 2 | 2 | 3 |
| NM_001135178 | ZNF397 | 2643 | 8 | 4 | 4 | 2 | 3 | 5 | 4 | 1 | 3 | 8 | 3 | 8 | 4 |
| NM_001135179 | ZDHHC3 | 12601 | 2 | 1 | 2 | 2 | 1 | 1 | 2 | 1 | 1 | 1 | 1 | 2 | 2 |
| NM_001135180 | ZDHHC3 | 3897 | 4 | 2 | 3 | 3 | 2 | 2 | 3 | 2 | 2 | 2 | 1 | 3 | 4 |
| NM_001135181 | SLC5A9 | 3274 | 4 | 1 | 0 | 0 | 1 | 0 | 4 | 1 | 1 | 1 | 0 | 1 | 0 |
| NM_001135186 | ABI3 | 2091 | 3 | 1 | 0 | 0 | 0 | 1 | 0 | 1 | 1 | 0 | 0 | 3 | 0 |
| NM_001135187 | AGFG1 | 8732 | 4 | 2 | 3 | 1 | 3 | 3 | 2 | 2 | 3 | 2 | 1 | 4 | 3 |
| NM_001135188 | AGFG1 | 8660 | 4 | 2 | 3 | 1 | 3 | 3 | 2 | 2 | 3 | 2 | 1 | 4 | 3 |
| NM_001135189 | AGFG1 | 8546 | 4 | 2 | 3 | 1 | 3 | 3 | 2 | 2 | 3 | 2 | 1 | 4 | 3 |
| NM_001135190 | ARAP1 | 4731 | 6 | 4 | 6 | 6 | 3 | 2 | 3 | 3 | 4 | 5 | 1 | 6 | 3 |
| NM_001135191 | ASAP2 | 5586 | 8 | 3 | 4 | 4 | 3 | 4 | 8 | 1 | 3 | 3 | 1 | 1 | 7 |
| NM_001135195 | SLC39A5 | 1963 | 7 | 1 | 0 | 3 | 0 | 0 | 7 | 3 | 0 | 0 | 0 | 0 | 0 |
| NM_001135196 | C10orf71 | 5286 | 30 | 3 | 0 | 0 | 6 | 0 | 0 | 0 | 0 | 0 | 30 | 0 | 0 |
| NM_001135197 | CCDC36 | 2696 | 1 | 0 | 0 | 0 | 0 | 0 | 0 | 0 | 0 | 0 | 0 | 0 | 1 |
| NM_001135208 | FKBP2 | 645 | 57 | 17 | 9 | 10 | 17 | 26 | 18 | 57 | 8 | 9 | 3 | 16 | 11 |
| NM_001135211 | FKBP6 | 1544 | 2 | 0 | 0 | 0 | 0 | 0 | 0 | 0 | 0 | 0 | 0 | 0 | 2 |
| NM_001135212 | FKBP7 | 2899 | 9 | 3 | 2 | 1 | 4 | 1 | 1 | 2 | 2 | 9 | 1 | 1 | 4 |
| NM_001135213 | GIT2 | 5387 | 10 | 4 | 4 | 3 | 6 | 4 | 3 | 2 | 3 | 6 | 3 | 10 | 4 |
| NM_001135214 | GIT2 | 5531 | 10 | 4 | 4 | 3 | 5 | 4 | 3 | 2 | 4 | 6 | 3 | 10 | 4 |
| NM_001135215 | ZNF323 | 3551 | 3 | 1 | 1 | 1 | 1 | 1 | 1 | 1 | 1 | 3 | 1 | 1 | 3 |
| NM_001135216 | ZNF323 | 3047 | 3 | 1 | 1 | 1 | 1 | 2 | 1 | 1 | 1 | 3 | 1 | 1 | 3 |
| NM_001135217 | LRRC23 | 1799 | 2 | 1 | 1 | 0 | 1 | 2 | 1 | 0 | 2 | 1 | 1 | 0 | 2 |
| NM_001135218 | TAF12 | 1448 | 5 | 4 | 4 | 2 | 5 | 3 | 3 | 4 | 3 | 4 | 4 | 5 | 5 |
| NM_001135219 | PIP5KL1 | 1546 | 0 | 0 | 0 | 0 | 0 | 0 | 0 | 0 | 0 | 0 | 0 | 0 | 0 |
| NM_001135239 | LDHA | 2034 | 317 | 98 | 126 | 43 | 146 | 84 | 93 | 317 | 62 | 20 | 52 | 80 | 58 |
| NM_001135240 | C1orf226 | 984 | 2 | 0 | 0 | 1 | 0 | 0 | 1 | 2 | 0 | 0 | 0 | 0 | 0 |
| NM_001135241 | AKR1C2 | 943 | 10 | 2 | 8 | 0 | 0 | 0 | 0 | 10 | 0 | 0 | 0 | 0 | 1 |
| NM_001135242 | NDRG1 | 3478 | 68 | 26 | 20 | 24 | 22 | 30 | 68 | 6 | 36 | 27 | 7 | 24 | 25 |
| NM_001135243 | TCOF1 | 5095 | 4 | 2 | 2 | 2 | 2 | 3 | 1 | 1 | 1 | 2 | 2 | 4 | 3 |
| NM_001135244 | TCOF1 | 4984 | 4 | 2 | 2 | 2 | 2 | 3 | 1 | 1 | 1 | 2 | 2 | 4 | 3 |
| NM_001135245 | TCOF1 | 4867 | 4 | 2 | 2 | 2 | 2 | 3 | 1 | 1 | 1 | 2 | 2 | 4 | 3 |
| NM_001135247 | MFAP2 | 1079 | 3 | 1 | 1 | 1 | 1 | 0 | 0 | 0 | 1 | 3 | 0 | 0 | 1 |
| NM_001135248 | MFAP2 | 1098 | 3 | 1 | 1 | 1 | 1 | 0 | 0 | 0 | 1 | 3 | 0 | 0 | 1 |
| NM_001135254 | PAX7 | 6052 | 0 | 0 | 0 | 0 | 0 | 0 | 0 | 0 | 0 | 0 | 0 | 0 | 0 |
| NM_001135255 | RBBP4 | 7938 | 24 | 10 | 4 | 5 | 9 | 9 | 8 | 5 | 7 | 15 | 8 | 15 | 24 |
| NM_001135256 | RBBP4 | 7841 | 24 | 10 | 4 | 5 | 10 | 9 | 8 | 5 | 7 | 15 | 8 | 16 | 24 |
| NM_001135553 | MKNK1 | 2675 | 6 | 3 | 4 | 2 | 4 | 3 | 2 | 2 | 2 | 1 | 2 | 6 | 2 |
| NM_001135554 | EPB41L2 | 3458 | 47 | 26 | 47 | 12 | 22 | 43 | 11 | 6 | 23 | 39 | 6 | 34 | 38 |
| NM_001135555 | EPB41L2 | 3426 | 47 | 26 | 47 | 12 | 22 | 43 | 11 | 6 | 23 | 40 | 6 | 34 | 38 |
| NM_001135556 | DYNC1I1 | 2994 | 12 | 1 | 0 | 1 | 1 | 12 | 0 | 0 | 0 | 0 | 0 | 0 | 0 |
| NM_001135557 | DYNC1I1 | 2934 | 12 | 1 | 0 | 1 | 1 | 12 | 0 | 0 | 0 | 0 | 0 | 0 | 0 |
| NM_001135564 | HSF2 | 2643 | 18 | 7 | 2 | 2 | 11 | 8 | 3 | 1 | 4 | 6 | 14 | 5 | 18 |
| NM_001135565 | HDHD1A | 2209 | 2 | 1 | 1 | 1 | 2 | 1 | 2 | 1 | 1 | 1 | 2 | 1 | 1 |
| NM_001135570 | C12orf73 | 1693 | 1 | 1 | 0 | 0 | 0 | 1 | 0 | 1 | 0 | 1 | 0 | 0 | 1 |
| NM_001135575 | LOC221710 | 4686 | 13 | 3 | 1 | 1 | 2 | 13 | 1 | 3 | 1 | 2 | 2 | 2 | 2 |
| NM_001135580 | C19orf71 | 670 | 1 | 0 | 0 | 0 | 0 | 0 | 1 | 0 | 0 | 0 | 0 | 0 | 0 |
| NM_001135581 | SLC1A4 | 3965 | 6 | 2 | 1 | 1 | 1 | 6 | 0 | 1 | 1 | 1 | 2 | 2 | 1 |
| NM_001135585 | SLC2A5 | 1279 | 1 | 1 | 0 | 0 | 0 | 1 | 1 | 0 | 0 | 0 | 0 | 0 | 1 |
| NM_001135586 | C5orf24 | 5082 | 7 | 4 | 2 | 2 | 4 | 4 | 7 | 4 | 4 | 4 | 2 | 5 | 5 |
| NM_001135589 | GDAP2 | 2531 | 4 | 2 | 2 | 1 | 2 | 4 | 3 | 2 | 2 | 3 | 1 | 3 | 4 |
| NM_001135590 | ZNF577 | 2931 | 15 | 7 | 4 | 3 | 4 | 9 | 6 | 1 | 4 | 15 | 6 | 7 | 14 |
| NM_001135592 | RPS27A | 703 | 54 | 30 | 29 | 20 | 31 | 26 | 21 | 33 | 27 | 54 | 18 | 53 | 14 |
| NM_001135597 | CCDC88A | 9811 | 18 | 7 | 10 | 1 | 7 | 18 | 3 | 3 | 7 | 5 | 3 | 10 | 14 |

| | | transcript | | | | | | hypothala | | | | | skeletalm | | |
|---|---|---|---|---|---|---|---|---|---|---|---|---|---|---|---|
| transcript | gene | length (nt) | max | mean | adipose | colon | heart | mus | kidney | liver | lung | ovary | uscle | spleen | testes |
| NM_001135598 | C2orf63 | 4898 | 1 | 1 | 0 | 0 | 0 | 1 | 1 | 0 | 1 | 1 | 0 | 1 | 1 |
| NM_001135599 | TGFB2 | 5237 | 5 | 2 | 0 | 0 | 5 | 4 | 1 | 0 | 2 | 3 | 2 | 1 | 1 |
| NM_001135602 | GLB1 | 2194 | 4 | 2 | 3 | 1 | 2 | 1 | 4 | 3 | 2 | 2 | 0 | 3 | 1 |
| NM_001135604 | ESM1 | 1938 | 2 | 0 | 0 | 0 | 0 | 0 | 2 | 0 | 1 | 0 | 0 | 1 | 0 |
| NM_001135608 | ARHGAP26 | 8875 | 6 | 2 | 1 | 2 | 4 | 6 | 2 | 0 | 2 | 1 | 1 | 4 | 3 |
| NM_001135610 | PRDM2 | 2700 | 4 | 1 | 1 | 1 | 1 | 4 | 0 | 0 | 1 | 2 | 0 | 1 | 1 |
| NM_001135629 | KLRAQ1 | 3169 | 17 | 8 | 7 | 4 | 6 | 11 | 17 | 2 | 7 | 9 | 3 | 11 | 10 |
| NM_001135630 | KLRAQ1 | 3208 | 17 | 8 | 7 | 4 | 6 | 11 | 17 | 2 | 7 | 9 | 3 | 11 | 10 |
| NM_001135635 | C11orf68 | 1557 | 1 | 1 | 0 | 0 | 1 | 1 | 0 | 0 | 1 | 1 | 1 | 1 | 1 |
| NM_001135636 | PIP5K1A | 3686 | 3 | 1 | 1 | 1 | 2 | 1 | 1 | 1 | 1 | 1 | 1 | 1 | 3 |
| NM_001135637 | PIP5K1A | 3620 | 3 | 1 | 1 | 1 | 2 | 1 | 1 | 1 | 1 | 1 | 1 | 1 | 3 |
| NM_001135638 | PIP5K1A | 3806 | 3 | 1 | 1 | 1 | 1 | 1 | 1 | 1 | 1 | 1 | 1 | 1 | 3 |
| NM_001135639 | CNGB1 | 1632 | 0 | 0 | 0 | 0 | 0 | 0 | 0 | 0 | 0 | 0 | 0 | 0 | 0 |
| NM_001135642 | INPP5K | 3371 | 1 | 1 | 1 | 1 | 1 | 1 | 1 | 1 | 1 | 1 | 1 | 1 | 1 |
| NM_001135643 | DCTN4 | 3907 | 5 | 3 | 2 | 1 | 3 | 5 | 3 | 2 | 3 | 4 | 4 | 3 | 4 |
| NM_001135644 | DCTN4 | 4054 | 4 | 3 | 2 | 1 | 3 | 4 | 3 | 2 | 3 | 3 | 4 | 3 | 4 |
| NM_001135647 | FAM53C | 4417 | 4 | 2 | 1 | 2 | 2 | 2 | 1 | 1 | 2 | 1 | 2 | 2 | 4 |
| NM_001135648 | PTPRK | 6155 | 10 | 5 | 2 | 5 | 5 | 9 | 6 | 10 | 5 | 6 | 2 | 3 | 2 |
| NM_001135649 | FOXI3 | 1262 | 0 | 0 | 0 | 0 | 0 | 0 | 0 | 0 | 0 | 0 | 0 | 0 | 0 |
| NM_001135650 | EEF1E1 | 607 | 6 | 3 | 2 | 2 | 3 | 6 | 6 | 4 | 2 | 2 | 2 | 3 | 5 |
| NM_001135651 | EIF2AK2 | 2696 | 10 | 6 | 7 | 2 | 7 | 8 | 6 | 4 | 5 | 10 | 5 | 8 | 6 |
| NM_001135652 | EIF2AK2 | 2266 | 11 | 7 | 8 | 2 | 8 | 9 | 7 | 5 | 6 | 11 | 6 | 9 | 7 |
| NM_001135653 | PABPC4 | 3204 | 82 | 26 | 16 | 9 | 38 | 10 | 10 | 19 | 15 | 34 | 82 | 24 | 31 |
| NM_001135654 | PABPC4 | 3117 | 85 | 27 | 17 | 10 | 39 | 10 | 10 | 19 | 15 | 35 | 85 | 25 | 31 |
| NM_001135655 | LY6H | 960 | 16 | 2 | 0 | 1 | 0 | 16 | 0 | 0 | 0 | 0 | 0 | 0 | 0 |
| NM_001135659 | NRXN1 | 9559 | 27 | 3 | 0 | 1 | 1 | 27 | 0 | 0 | 0 | 0 | 0 | 0 | 1 |
| NM_001135662 | RAB7L1 | 3206 | 12 | 3 | 2 | 1 | 3 | 2 | 12 | 3 | 2 | 1 | 1 | 5 | 1 |
| NM_001135663 | RAB7L1 | 3421 | 11 | 3 | 2 | 1 | 3 | 2 | 11 | 3 | 2 | 1 | 1 | 5 | 1 |
| NM_001135664 | RAB7L1 | 3053 | 13 | 3 | 2 | 1 | 3 | 3 | 13 | 4 | 2 | 1 | 1 | 5 | 1 |
| NM_001135669 | XPR1 | 8299 | 8 | 3 | 2 | 1 | 8 | 4 | 4 | 1 | 2 | 2 | 1 | 3 | 7 |
| NM_001135670 | MSRA | 1362 | 11 | 2 | 1 | 1 | 2 | 2 | 11 | 3 | 1 | 1 | 1 | 3 | 1 |
| NM_001135671 | MSRA | 1631 | 12 | 3 | 1 | 1 | 2 | 3 | 12 | 4 | 1 | 1 | 1 | 3 | 1 |
| NM_001135673 | ATL2 | 2887 | 11 | 6 | 2 | 3 | 5 | 5 | 7 | 7 | 5 | 10 | 11 | 8 | 6 |
| NM_001135674 | C8orf40 | 1468 | 4 | 3 | 3 | 1 | 3 | 4 | 3 | 4 | 2 | 3 | 3 | 3 | 2 |
| NM_001135675 | C8orf40 | 1076 | 5 | 4 | 5 | 2 | 5 | 5 | 4 | 5 | 2 | 4 | 4 | 4 | 3 |
| NM_001135676 | C8orf40 | 761 | 7 | 6 | 7 | 2 | 7 | 7 | 6 | 7 | 3 | 6 | 6 | 6 | 4 |
| NM_001135685 | LTK | 2737 | 0 | 0 | 0 | 0 | 0 | 0 | 0 | 0 | 0 | 0 | 0 | 0 | 0 |
| NM_001135686 | TARM1 | 897 | 0 | 0 | 0 | 0 | 0 | 0 | 0 | 0 | 0 | 0 | 0 | 0 | 0 |
| NM_001135690 | PENK | 1334 | 8 | 1 | 1 | 1 | 0 | 3 | 0 | 0 | 1 | 1 | 0 | 0 | 8 |
| NM_001135691 | SLC18A1 | 2956 | 0 | 0 | 0 | 0 | 0 | 0 | 0 | 0 | 0 | 0 | 0 | 0 | 0 |
| NM_001135694 | VDAC3 | 1431 | 44 | 18 | 10 | 9 | 34 | 26 | 16 | 9 | 8 | 8 | 44 | 13 | 19 |
| NM_001135697 | SGCA | 1060 | 12 | 1 | 0 | 0 | 2 | 0 | 0 | 0 | 0 | 0 | 12 | 0 | 0 |
| NM_001135699 | YWHAZ | 3009 | 120 | 45 | 43 | 23 | 42 | 120 | 33 | 18 | 44 | 40 | 16 | 71 | 41 |
| NM_001135700 | YWHAZ | 2963 | 122 | 45 | 43 | 24 | 42 | 122 | 34 | 18 | 45 | 41 | 16 | 72 | 42 |
| NM_001135701 | YWHAZ | 3012 | 120 | 45 | 43 | 23 | 42 | 120 | 33 | 18 | 44 | 40 | 16 | 70 | 41 |
| NM_001135702 | YWHAZ | 3031 | 119 | 44 | 42 | 23 | 41 | 119 | 33 | 18 | 44 | 40 | 15 | 70 | 41 |
| NM_001135703 | LRP12 | 4052 | 5 | 2 | 2 | 1 | 5 | 5 | 2 | 0 | 2 | 2 | 2 | 3 | 2 |
| NM_001135704 | ACBD4 | 2028 | 3 | 1 | 1 | 1 | 0 | 1 | 1 | 3 | 0 | 1 | 0 | 0 | 0 |
| NM_001135705 | ACBD4 | 1865 | 3 | 1 | 1 | 1 | 0 | 1 | 1 | 3 | 0 | 1 | 0 | 0 | 0 |
| NM_001135706 | ACBD4 | 1903 | 3 | 1 | 1 | 1 | 0 | 1 | 1 | 3 | 0 | 1 | 0 | 0 | 0 |
| NM_001135707 | ACBD4 | 1500 | 3 | 1 | 1 | 1 | 0 | 1 | 1 | 3 | 0 | 1 | 0 | 1 | 0 |
| NM_001135721 | PIWIL2 | 3997 | 4 | 1 | 0 | 0 | 0 | 0 | 0 | 0 | 0 | 0 | 0 | 0 | 4 |
| NM_001135726 | HMBOX1 | 3173 | 6 | 4 | 3 | 3 | 4 | 6 | 3 | 2 | 4 | 4 | 2 | 5 | 5 |
| NM_001135729 | TOM1 | 2317 | 6 | 3 | 2 | 1 | 2 | 4 | 3 | 3 | 2 | 1 | 6 | 2 | 2 |
| NM_001135730 | TOM1 | 2252 | 6 | 2 | 2 | 1 | 2 | 4 | 2 | 3 | 2 | 1 | 6 | 2 | 2 |
| NM_001135731 | ZMAT4 | 2234 | 3 | 0 | 0 | 0 | 0 | 3 | 0 | 0 | 0 | 0 | 0 | 0 | 0 |
| NM_001135732 | TOM1 | 2390 | 6 | 2 | 2 | 1 | 2 | 4 | 2 | 3 | 2 | 1 | 6 | 2 | 2 |
| NM_001135733 | TP53INP1 | 5651 | 7 | 3 | 1 | 1 | 1 | 0 | 2 | 5 | 4 | 3 | 1 | 7 | 5 |
| NM_001135734 | ZNF384 | 3177 | 4 | 2 | 2 | 2 | 2 | 1 | 3 | 1 | 2 | 4 | 2 | 4 | 3 |
| NM_001135740 | PNCK | 1504 | 1 | 0 | 0 | 0 | 0 | 1 | 0 | 0 | 0 | 0 | 0 | 0 | 0 |
| NM_001135745 | ALS2 | 2890 | 7 | 4 | 3 | 3 | 4 | 6 | 3 | 7 | 2 | 3 | 3 | 3 | 3 |
| NM_001135746 | NEIL2 | 2202 | 5 | 2 | 3 | 1 | 2 | 5 | 2 | 2 | 1 | 1 | 2 | 2 | 3 |
| NM_001135747 | NEIL2 | 2062 | 5 | 2 | 3 | 1 | 2 | 5 | 2 | 2 | 1 | 2 | 2 | 2 | 3 |
| NM_001135748 | NEIL2 | 2016 | 5 | 2 | 3 | 1 | 1 | 5 | 2 | 2 | 1 | 1 | 2 | 2 | 2 |
| NM_001135750 | PSMG4 | 448 | 0 | 0 | 0 | 0 | 0 | 0 | 0 | 0 | 0 | 0 | 0 | 0 | 0 |
| NM_001135751 | DERL3 | 1067 | 0 | 0 | 0 | 0 | 0 | 0 | 0 | 0 | 0 | 0 | 0 | 0 | 0 |
| NM_001135752 | ECD | 2335 | 4 | 3 | 3 | 2 | 2 | 4 | 3 | 3 | 2 | 3 | 2 | 2 | 3 |
| NM_001135753 | ECD | 2107 | 4 | 3 | 3 | 2 | 2 | 4 | 4 | 3 | 2 | 3 | 2 | 3 | 4 |
| NM_001135765 | ATP2A2 | 8231 | 97 | 25 | 7 | 8 | 97 | 32 | 11 | 11 | 10 | 10 | 58 | 14 | 15 |
| NM_001135768 | PVR | 5766 | 18 | 8 | 4 | 6 | 18 | 8 | 7 | 16 | 6 | 4 | 5 | 5 | 5 |
| NM_001135769 | PVR | 5742 | 18 | 8 | 4 | 6 | 18 | 8 | 7 | 16 | 6 | 4 | 5 | 5 | 5 |
| NM_001135770 | PVR | 1642 | 2 | 1 | 0 | 0 | 2 | 1 | 0 | 1 | 0 | 0 | 0 | 0 | 0 |
| NM_001135771 | RPN2 | 2457 | 20 | 8 | 7 | 5 | 9 | 6 | 9 | 20 | 5 | 4 | 4 | 10 | 7 |
| NM_001135772 | LRP5L | 1663 | 0 | 0 | 0 | 0 | 0 | 0 | 0 | 0 | 0 | 0 | 0 | 0 | 0 |
| NM_001135773 | SPATA2 | 4138 | 2 | 1 | 0 | 1 | 1 | 2 | 1 | 0 | 1 | 1 | 1 | 2 | 2 |
| NM_001135774 | SYN3 | 3141 | 2 | 0 | 0 | 0 | 0 | 2 | 0 | 0 | 0 | 0 | 0 | 0 | 0 |
| NM_001135775 | C9orf7 | 2629 | 1 | 0 | 0 | 1 | 0 | 0 | 1 | 0 | 0 | 0 | 0 | 1 | 0 |

| transcript | gene | transcript length (nt) | max | mean | adipose | colon | heart | hypothalamus | kidney | liver | lung | ovary | skeletalmuscle | spleen | testes |
|---|---|---|---|---|---|---|---|---|---|---|---|---|---|---|---|
| NM_001135776 | ZBTB43 | 5847 | 9 | 4 | 2 | 2 | 5 | 4 | 3 | 2 | 3 | 4 | 9 | 3 | 6 |
| NM_001135789 | PPIAL4C | 783 | 0 | 0 | 0 | 0 | 0 | 0 | 0 | 0 | 0 | 0 | 0 | 0 | 0 |
| NM_001135805 | SYT1 | 4823 | 134 | 13 | 0 | 4 | 0 | 134 | 1 | 0 | 0 | 2 | 0 | 1 | 1 |
| NM_001135806 | SYT1 | 4409 | 145 | 14 | 0 | 4 | 0 | 145 | 1 | 0 | 0 | 2 | 0 | 1 | 1 |
| NM_001135811 | FAM60A | 3111 | 14 | 6 | 2 | 3 | 4 | 2 | 9 | 1 | 7 | 14 | 1 | 13 | 10 |
| NM_001135812 | FAM60A | 3007 | 14 | 6 | 2 | 3 | 4 | 2 | 9 | 1 | 8 | 14 | 1 | 14 | 10 |
| NM_001135816 | PCOTH | 700 | 0 | 0 | 0 | 0 | 0 | 0 | 0 | 0 | 0 | 0 | 0 | 0 | 0 |
| NM_001135820 | TMEM2 | 6334 | 16 | 6 | 4 | 4 | 4 | 3 | 4 | 13 | 15 | 1 | 2 | 16 | 4 |
| NM_001135821 | FDPS | 1481 | 4 | 2 | 1 | 2 | 2 | 4 | 3 | 3 | 2 | 1 | 1 | 2 | 1 |
| NM_001135822 | FDPS | 1338 | 5 | 2 | 2 | 2 | 2 | 5 | 3 | 3 | 2 | 1 | 1 | 2 | 1 |
| NM_001135823 | SSPN | 4346 | 5 | 2 | 3 | 0 | 3 | 2 | 1 | 0 | 1 | 4 | 5 | 1 | 3 |
| NM_001135824 | RNF185 | 2628 | 7 | 3 | 2 | 2 | 3 | 3 | 6 | 7 | 2 | 4 | 3 | 3 | 3 |
| NM_001135825 | RNF185 | 3043 | 7 | 4 | 2 | 2 | 3 | 3 | 6 | 7 | 3 | 3 | 3 | 4 | 3 |
| NM_001135844 | SIRPB1 | 2240 | 1 | 0 | 0 | 0 | 0 | 0 | 0 | 0 | 0 | 0 | 0 | 1 | 0 |
| NM_001135861 | PHPT1 | 1249 | 3 | 2 | 1 | 3 | 3 | 3 | 2 | 1 | 1 | 1 | 3 | 1 | 2 |
| NM_001135862 | PHF21B | 3497 | 0 | 0 | 0 | 0 | 0 | 0 | 0 | 0 | 0 | 0 | 0 | 0 | 0 |
| NM_001135863 | C12orf72 | 2107 | 0 | 0 | 0 | 0 | 0 | 0 | 0 | 0 | 0 | 0 | 0 | 0 | 0 |
| NM_001135864 | C12orf72 | 2088 | 0 | 0 | 0 | 0 | 0 | 0 | 0 | 0 | 0 | 0 | 0 | 0 | 0 |
| NM_001135865 | LOC100132[illegible] | 3803 | 0 | 0 | 0 | 0 | 0 | 0 | 0 | 0 | 0 | 0 | 0 | 0 | 0 |
| NM_001135911 | PIK3IP1 | 2393 | 14 | 6 | 11 | 5 | 2 | 7 | 3 | 1 | 10 | 4 | 3 | 14 | 6 |
| NM_001135914 | KCP | 4732 | 0 | 0 | 0 | 0 | 0 | 0 | 0 | 0 | 0 | 0 | 0 | 0 | 0 |
| NM_001135917 | DOLPP1 | 2046 | 1 | 1 | 1 | 1 | 0 | 1 | 1 | 1 | 0 | 0 | 0 | 1 | 1 |
| NM_001135919 | SLC46A3 | 2798 | 7 | 2 | 1 | 1 | 2 | 1 | 2 | 7 | 1 | 1 | 0 | 4 | 2 |
| NM_001135920 | KIAA1432 | 3603 | 5 | 3 | 3 | 2 | 2 | 2 | 2 | 2 | 3 | 3 | 3 | 5 | 3 |
| NM_001135924 | VWDE | 5583 | 0 | 0 | 0 | 0 | 0 | 0 | 0 | 0 | 0 | 0 | 0 | 0 | 0 |
| NM_001135934 | POSTN | 3208 | 28 | 8 | 28 | 8 | 25 | 0 | 4 | 1 | 7 | 2 | 4 | 0 | 8 |
| NM_001135935 | POSTN | 3214 | 28 | 8 | 28 | 8 | 25 | 0 | 4 | 1 | 7 | 2 | 4 | 0 | 8 |
| NM_001135936 | POSTN | 3124 | 29 | 8 | 29 | 8 | 25 | 0 | 4 | 1 | 7 | 2 | 4 | 0 | 8 |
| NM_001135937 | SMAD2 | 10443 | 6 | 4 | 4 | 2 | 3 | 4 | 4 | 2 | 4 | 6 | 2 | 5 | 6 |
| NM_001135940 | MYOT | 1789 | 91 | 9 | 0 | 0 | 8 | 1 | 0 | 0 | 0 | 0 | 91 | 0 | 0 |
| NM_001135943 | MADD | 5731 | 7 | 2 | 1 | 2 | 2 | 7 | 2 | 2 | 2 | 1 | 1 | 3 | 2 |
| NM_001135944 | MADD | 5698 | 7 | 2 | 1 | 2 | 2 | 7 | 2 | 2 | 2 | 1 | 1 | 3 | 2 |
| NM_001135947 | URM1 | 1726 | 1 | 0 | 0 | 0 | 1 | 0 | 1 | 1 | 0 | 0 | 0 | 1 | 0 |
| NM_001135953 | GKAP1 | 1719 | 24 | 4 | 2 | 1 | 3 | 4 | 2 | 1 | 1 | 3 | 6 | 1 | 24 |
| NM_001135954 | UCK1 | 2083 | 8 | 5 | 5 | 3 | 6 | 8 | 6 | 6 | 3 | 5 | 6 | 5 | 5 |
| NM_001135955 | TRPC4 | 3182 | 3 | 1 | 0 | 0 | 1 | 3 | 0 | 0 | 1 | 1 | 0 | 0 | 0 |
| NM_001135956 | TRPC4 | 2987 | 3 | 1 | 0 | 0 | 1 | 3 | 0 | 0 | 1 | 1 | 0 | 0 | 0 |
| NM_001135957 | TRPC4 | 3011 | 3 | 1 | 0 | 0 | 1 | 3 | 0 | 0 | 1 | 1 | 0 | 0 | 0 |
| NM_001135958 | TRPC4 | 2915 | 3 | 1 | 0 | 0 | 1 | 3 | 0 | 0 | 1 | 1 | 0 | 0 | 0 |
| NM_001135993 | TTC39C | 5239 | 14 | 3 | 2 | 1 | 1 | 2 | 1 | 14 | 4 | 1 | 0 | 2 | 3 |
| NM_001135995 | ATXN3L | 1749 | 2 | 0 | 0 | 0 | 0 | 0 | 0 | 0 | 0 | 0 | 0 | 0 | 2 |
| NM_001135998 | NDUFB11 | 579 | 13 | 7 | 3 | 6 | 12 | 8 | 6 | 13 | 3 | 5 | 13 | 6 | 2 |
| NM_001135999 | RUSC2 | 5635 | 3 | 1 | 1 | 1 | 1 | 3 | 1 | 0 | 0 | 1 | 1 | 1 | 1 |
| NM_001136 | AGER | 1413 | 49 | 5 | 0 | 0 | 0 | 0 | 0 | 0 | 49 | 0 | 0 | 0 | 0 |
| NM_001136000 | ABL2 | 11630 | 2 | 2 | 2 | 1 | 2 | 2 | 1 | 1 | 2 | 2 | 1 | 2 | 2 |
| NM_001136001 | ABL2 | 2139 | 4 | 3 | 4 | 2 | 2 | 4 | 2 | 1 | 4 | 3 | 1 | 3 | 2 |
| NM_001136002 | TMEM229A | 2553 | 0 | 0 | 0 | 0 | 0 | 0 | 0 | 0 | 0 | 0 | 0 | 0 | 0 |
| NM_001136003 | C2CD4D | 1713 | 0 | 0 | 0 | 0 | 0 | 0 | 0 | 0 | 0 | 0 | 0 | 0 | 0 |
| NM_001136004 | MICAL3 | 8073 | 3 | 2 | 1 | 3 | 2 | 2 | 1 | 1 | 0 | 1 | 1 | 2 | 2 |
| NM_001136005 | GART | 3536 | 7 | 3 | 3 | 2 | 4 | 4 | 3 | 7 | 2 | 2 | 3 | 4 | 4 |
| NM_001136006 | GART | 3499 | 7 | 4 | 3 | 2 | 4 | 4 | 3 | 7 | 2 | 2 | 3 | 5 | 4 |
| NM_001136007 | FXYD3 | 1578 | 12 | 1 | 0 | 12 | 0 | 0 | 0 | 0 | 1 | 0 | 0 | 0 | 0 |
| NM_001136008 | FXYD3 | 778 | 8 | 1 | 0 | 8 | 0 | 0 | 0 | 0 | 0 | 0 | 0 | 0 | 0 |
| NM_001136009 | FXYD3 | 843 | 10 | 1 | 0 | 10 | 0 | 0 | 0 | 0 | 0 | 0 | 0 | 0 | 0 |
| NM_001136010 | FXYD3 | 759 | 12 | 1 | 0 | 12 | 0 | 0 | 0 | 0 | 0 | 0 | 0 | 0 | 0 |
| NM_001136011 | FXYD3 | 1456 | 11 | 1 | 0 | 11 | 0 | 0 | 0 | 0 | 1 | 0 | 0 | 0 | 0 |
| NM_001136012 | FXYD3 | 1450 | 11 | 1 | 0 | 11 | 0 | 0 | 0 | 0 | 1 | 0 | 0 | 0 | 0 |
| NM_001136015 | ANXA2 | 1658 | 120 | 30 | 120 | 28 | 30 | 9 | 13 | 11 | 43 | 13 | 12 | 28 | 22 |
| NM_001136017 | CCND3 | 2059 | 5 | 1 | 1 | 1 | 1 | 2 | 1 | 1 | 2 | 1 | 0 | 5 | 1 |
| NM_001136018 | EPHX1 | 1660 | 25 | 6 | 9 | 1 | 2 | 4 | 6 | 25 | 4 | 7 | 2 | 2 | 5 |
| NM_001136019 | FCGRT | 1893 | 5 | 3 | 5 | 5 | 2 | 1 | 3 | 2 | 3 | 2 | 1 | 3 | 1 |
| NM_001136020 | ICA1 | 1737 | 7 | 2 | 2 | 2 | 7 | 4 | 2 | 0 | 1 | 0 | 0 | 2 | 2 |
| NM_001136021 | NFATC2 | 2973 | 7 | 2 | 2 | 2 | 1 | 2 | 1 | 1 | 1 | 1 | 1 | 7 | 2 |
| NM_001136022 | NFATC4 | 5670 | 1 | 1 | 0 | 1 | 1 | 0 | 0 | 0 | 1 | 1 | 0 | 0 | 1 |
| NM_001136023 | NFE2 | 1645 | 2 | 0 | 0 | 0 | 0 | 0 | 0 | 0 | 1 | 0 | 0 | 2 | 0 |
| NM_001136024 | NHS | 8226 | 5 | 1 | 2 | 1 | 1 | 2 | 5 | 0 | 1 | 1 | 0 | 1 | 3 |
| NM_001136025 | PLS3 | 3135 | 28 | 13 | 13 | 2 | 15 | 9 | 15 | 19 | 28 | 16 | 5 | 18 | 8 |
| NM_001136027 | VRK2 | 1929 | 4 | 2 | 3 | 1 | 3 | 2 | 2 | 3 | 2 | 2 | 3 | 4 | 2 |
| NM_001136029 | DEPDC5 | 4956 | 1 | 1 | 0 | 1 | 0 | 1 | 1 | 1 | 0 | 1 | 0 | 1 | 1 |
| NM_001136030 | KIAA0748 | 2010 | 4 | 1 | 0 | 0 | 0 | 4 | 0 | 0 | 0 | 1 | 0 | 1 | 0 |
| NM_001136031 | ATG7 | 4963 | 1 | 0 | 1 | 0 | 0 | 1 | 0 | 0 | 1 | 0 | 0 | 1 | 1 |
| NM_001136032 | KLK11 | 1179 | 0 | 0 | 0 | 0 | 0 | 0 | 0 | 0 | 0 | 0 | 0 | 0 | 0 |
| NM_001136033 | PUF60 | 1836 | 13 | 7 | 7 | 8 | 4 | 13 | 7 | 13 | 3 | 3 | 5 | 6 | 7 |
| NM_001136035 | TRMT1 | 2144 | 1 | 1 | 1 | 1 | 0 | 1 | 1 | 1 | 0 | 1 | 0 | 1 | 0 |
| NM_001136036 | ZNF692 | 2065 | 2 | 1 | 0 | 1 | 1 | 1 | 1 | 0 | 1 | 1 | 1 | 1 | 2 |
| NM_001136037 | LIMS2 | 2117 | 1 | 0 | 0 | 1 | 1 | 1 | 0 | 0 | 0 | 0 | 0 | 0 | 0 |
| NM_001136038 | ZNF415 | 2529 | 24 | 8 | 6 | 3 | 12 | 18 | 5 | 1 | 2 | 11 | 3 | 9 | 24 |

| transcript | gene | transcript length (nt) | max | mean | adipose | colon | heart | hypothalamus | kidney | liver | lung | ovary | skeletalmuscle | spleen | testes |
|---|---|---|---|---|---|---|---|---|---|---|---|---|---|---|---|
| NM_001136039 | NIF3L1 | 1479 | 2 | 1 | 1 | 1 | 1 | 1 | 1 | 1 | 1 | 1 | 1 | 2 | 1 |
| NM_001136040 | CPSF7 | 3611 | 5 | 4 | 3 | 3 | 3 | 5 | 4 | 3 | 3 | 5 | 5 | 5 | 5 |
| NM_001136041 | HDAC11 | 2816 | 2 | 0 | 0 | 1 | 0 | 2 | 1 | 0 | 0 | 0 | 0 | 0 | 0 |
| NM_001136042 | AARSD1 | 2172 | 9 | 5 | 4 | 3 | 7 | 8 | 4 | 5 | 2 | 4 | 9 | 3 | 6 |
| NM_001136043 | C15orf44 | 1853 | 2 | 2 | 1 | 1 | 1 | 2 | 2 | 2 | 1 | 2 | 1 | 2 | 2 |
| NM_001136044 | TMUB1 | 1334 | 4 | 2 | 1 | 2 | 2 | 2 | 1 | 4 | 1 | 1 | 2 | 2 | 1 |
| NM_001136046 | ZMYND15 | 2411 | 2 | 0 | 1 | 0 | 0 | 0 | 0 | 0 | 0 | 0 | 0 | 1 | 2 |
| NM_001136049 | LMLN | 7152 | 2 | 1 | 1 | 0 | 1 | 1 | 1 | 0 | 1 | 1 | 0 | 1 | 2 |
| NM_001136050 | DHRS1 | 1478 | 1 | 1 | 1 | 1 | 0 | 0 | 1 | 1 | 0 | 1 | 0 | 1 | 0 |
| NM_001136051 | LRIG3 | 3660 | 3 | 1 | 1 | 1 | 0 | 0 | 1 | 1 | 1 | 3 | 1 | 1 | 1 |
| NM_001136052 | CPT1C | 2785 | 1 | 0 | 0 | 0 | 0 | 1 | 0 | 0 | 0 | 0 | 0 | 0 | 0 |
| NM_001136053 | TPRA1 | 1831 | 1 | 0 | 0 | 0 | 0 | 0 | 0 | 0 | 0 | 0 | 0 | 1 | 0 |
| NM_001136103 | TMEM132C | 4946 | 2 | 1 | 2 | 0 | 1 | 1 | 0 | 0 | 0 | 1 | 0 | 0 | 1 |
| NM_001136105 | C11orf93 | 1414 | 1 | 0 | 0 | 1 | 0 | 0 | 1 | 0 | 0 | 0 | 0 | 0 | 0 |
| NM_001136106 | BEAN | 2766 | 0 | 0 | 0 | 0 | 0 | 0 | 0 | 0 | 0 | 0 | 0 | 0 | 0 |
| NM_001136107 | METTL11B | 938 | 0 | 0 | 0 | 0 | 0 | 0 | 0 | 0 | 0 | 0 | 0 | 0 | 0 |
| NM_001136108 | R3HCC1 | 1581 | 16 | 7 | 8 | 4 | 7 | 16 | 7 | 7 | 4 | 6 | 7 | 7 | 7 |
| NM_001136109 | CASP5 | 1275 | 2 | 0 | 0 | 0 | 0 | 0 | 0 | 0 | 0 | 0 | 0 | 2 | 0 |
| NM_001136110 | CASP5 | 1023 | 1 | 0 | 0 | 0 | 0 | 0 | 0 | 0 | 0 | 0 | 0 | 1 | 0 |
| NM_001136111 | CASP5 | 913 | 1 | 0 | 0 | 0 | 0 | 0 | 0 | 0 | 0 | 0 | 0 | 1 | 0 |
| NM_001136112 | CASP5 | 1488 | 2 | 0 | 0 | 0 | 0 | 0 | 0 | 0 | 0 | 0 | 0 | 2 | 0 |
| NM_001136113 | POMT1 | 3319 | 1 | 1 | 0 | 1 | 0 | 1 | 1 | 0 | 0 | 0 | 0 | 1 | 1 |
| NM_001136114 | POMT1 | 2820 | 1 | 1 | 0 | 1 | 0 | 1 | 1 | 0 | 0 | 0 | 0 | 1 | 1 |
| NM_001136115 | FBXO28 | 5253 | 3 | 2 | 2 | 1 | 2 | 3 | 3 | 2 | 1 | 3 | 3 | 3 | 3 |
| NM_001136116 | ZNF879 | 2603 | 3 | 1 | 1 | 0 | 1 | 1 | 2 | 0 | 1 | 3 | 1 | 2 | 3 |
| NM_001136123 | FAM178A | 4121 | 35 | 13 | 6 | 3 | 8 | 11 | 7 | 4 | 7 | 30 | 14 | 18 | 35 |
| NM_001136125 | CCND3 | 1834 | 5 | 2 | 1 | 1 | 1 | 2 | 1 | 1 | 3 | 1 | 1 | 5 | 1 |
| NM_001136126 | CCND3 | 1843 | 5 | 2 | 1 | 1 | 1 | 2 | 1 | 1 | 3 | 1 | 1 | 5 | 1 |
| NM_001136127 | DNM3 | 7631 | 23 | 3 | 0 | 0 | 0 | 23 | 1 | 0 | 1 | 1 | 0 | 1 | 1 |
| NM_001136129 | APP | 3239 | 218 | 80 | 33 | 50 | 71 | 218 | 132 | 47 | 64 | 75 | 16 | 79 | 92 |
| NM_001136130 | APP | 3464 | 204 | 75 | 31 | 47 | 67 | 204 | 125 | 45 | 60 | 71 | 15 | 75 | 86 |
| NM_001136134 | RPL28 | 4399 | 14 | 7 | 3 | 7 | 6 | 3 | 4 | 8 | 7 | 14 | 5 | 14 | 3 |
| NM_001136135 | RPL28 | 894 | 17 | 8 | 4 | 8 | 7 | 3 | 6 | 9 | 9 | 16 | 7 | 17 | 4 |
| NM_001136136 | RPL28 | 594 | 6 | 3 | 4 | 3 | 3 | 2 | 2 | 5 | 4 | 6 | 2 | 5 | 1 |
| NM_001136137 | RPL28 | 682 | 5 | 3 | 3 | 3 | 2 | 2 | 2 | 4 | 3 | 5 | 1 | 4 | 1 |
| NM_001136138 | RPS6KC1 | 4154 | 10 | 4 | 3 | 1 | 4 | 10 | 3 | 2 | 3 | 6 | 3 | 5 | 10 |
| NM_001136139 | TCF3 | 4074 | 2 | 1 | 0 | 1 | 1 | 1 | 1 | 0 | 1 | 1 | 0 | 2 | 2 |
| NM_001136140 | CMPK1 | 2782 | 66 | 36 | 34 | 17 | 44 | 25 | 39 | 66 | 47 | 23 | 26 | 47 | 27 |
| NM_001136152 | ALG1L2 | 968 | 0 | 0 | 0 | 0 | 0 | 0 | 0 | 0 | 0 | 0 | 0 | 0 | 0 |
| NM_001136153 | ATF6B | 2605 | 4 | 2 | 2 | 2 | 2 | 2 | 2 | 2 | 3 | 2 | 1 | 4 | 2 |
| NM_001136153 | ATF6B | 2607 | 4 | 2 | 2 | 2 | 2 | 2 | 2 | 2 | 3 | 2 | 1 | 4 | 2 |
| NM_001136154 | ERG | 5106 | 15 | 4 | 5 | 0 | 5 | 1 | 3 | 2 | 6 | 3 | 2 | 15 | 2 |
| NM_001136155 | ERG | 4719 | 17 | 5 | 5 | 1 | 6 | 1 | 3 | 2 | 7 | 3 | 2 | 17 | 2 |
| NM_001136156 | ZNF507 | 7716 | 4 | 2 | 1 | 1 | 2 | 3 | 2 | 2 | 1 | 3 | 3 | 3 | 4 |
| NM_001136157 | OTUD5 | 2908 | 18 | 10 | 5 | 7 | 10 | 16 | 11 | 5 | 10 | 14 | 9 | 18 | 9 |
| NM_001136158 | OTUD5 | 2682 | 11 | 5 | 4 | 5 | 3 | 11 | 5 | 3 | 3 | 5 | 3 | 6 | 3 |
| NM_001136159 | OTUD5 | 2408 | 22 | 12 | 6 | 8 | 12 | 19 | 14 | 6 | 12 | 17 | 11 | 22 | 11 |
| NM_001136160 | C8orf45 | 2043 | 2 | 0 | 0 | 0 | 0 | 0 | 0 | 0 | 0 | 0 | 0 | 0 | 2 |
| NM_001136161 | C8orf45 | 2369 | 2 | 0 | 0 | 0 | 0 | 0 | 0 | 0 | 0 | 0 | 0 | 0 | 2 |
| NM_001136177 | EGR2 | 2792 | 3 | 1 | 0 | 0 | 0 | 0 | 0 | 0 | 0 | 3 | 0 | 0 | 1 |
| NM_001136178 | EGR2 | 2847 | 2 | 0 | 0 | 0 | 0 | 0 | 0 | 0 | 0 | 2 | 0 | 0 | 0 |
| NM_001136179 | EGR2 | 2875 | 3 | 1 | 0 | 0 | 0 | 0 | 0 | 0 | 0 | 3 | 0 | 0 | 1 |
| NM_001136180 | HSBP1L1 | 748 | 1 | 1 | 0 | 0 | 0 | 1 | 1 | 1 | 0 | 0 | 0 | 1 | 0 |
| NM_001136190 | LAX1 | 3005 | 3 | 1 | 0 | 1 | 0 | 0 | 0 | 0 | 1 | 0 | 0 | 3 | 0 |
| NM_001136191 | KANK2 | 5207 | 34 | 19 | 27 | 30 | 34 | 6 | 8 | 9 | 19 | 32 | 6 | 16 | 19 |
| NM_001136193 | FASTKD2 | 6926 | 4 | 3 | 2 | 1 | 3 | 4 | 3 | 4 | 1 | 3 | 3 | 2 | 2 |
| NM_001136194 | FASTKD2 | 6941 | 4 | 3 | 2 | 1 | 3 | 4 | 3 | 4 | 1 | 3 | 3 | 2 | 2 |
| NM_001136195 | TNPO2 | 5091 | 8 | 2 | 2 | 2 | 2 | 8 | 2 | 1 | 1 | 2 | 1 | 2 | 3 |
| NM_001136196 | TNPO2 | 4948 | 9 | 2 | 2 | 2 | 2 | 9 | 2 | 1 | 1 | 2 | 1 | 2 | 3 |
| NM_001136197 | FZR1 | 3170 | 0 | 0 | 0 | 0 | 0 | 0 | 0 | 0 | 0 | 0 | 0 | 0 | 0 |
| NM_001136198 | FZR1 | 3446 | 1 | 1 | 0 | 1 | 0 | 1 | 1 | 1 | 1 | 1 | 0 | 1 | 1 |
| NM_001136199 | GRAMD1A | 2607 | 1 | 0 | 0 | 0 | 0 | 0 | 0 | 0 | 1 | 1 | 0 | 1 | 1 |
| NM_001136200 | C10orf32 | 2435 | 4 | 2 | 1 | 1 | 2 | 3 | 3 | 4 | 2 | 2 | 1 | 4 | 3 |
| NM_001136201 | ISOC2 | 1122 | 5 | 1 | 1 | 1 | 1 | 1 | 2 | 5 | 0 | 0 | 0 | 1 | 1 |
| NM_001136202 | ISOC2 | 912 | 6 | 1 | 1 | 1 | 1 | 1 | 2 | 6 | 0 | 0 | 0 | 1 | 1 |
| NM_001136203 | CCDC124 | 986 | 3 | 2 | 3 | 3 | 3 | 3 | 3 | 3 | 1 | 1 | 2 | 2 | 1 |
| NM_001136204 | RCC2 | 3950 | 5 | 2 | 2 | 3 | 2 | 2 | 2 | 1 | 3 | 2 | 1 | 5 | 3 |
| NM_001136205 | KCTD1 | 2167 | 24 | 7 | 2 | 3 | 6 | 24 | 16 | 2 | 2 | 10 | 2 | 3 | 5 |
| NM_001136213 | POTEH | 2037 | 0 | 0 | 0 | 0 | 0 | 0 | 0 | 0 | 0 | 0 | 0 | 0 | 0 |
| NM_001136214 | CLEC18A | 1834 | 0 | 0 | 0 | 0 | 0 | 0 | 0 | 0 | 0 | 0 | 0 | 0 | 0 |
| NM_001136215 | ARTN | 1794 | 0 | 0 | 0 | 0 | 0 | 0 | 0 | 0 | 0 | 0 | 0 | 0 | 0 |
| NM_001136216 | TMEM51 | 2015 | 2 | 1 | 0 | 1 | 0 | 0 | 2 | 0 | 0 | 1 | 0 | 1 | 0 |
| NM_001136217 | TMEM51 | 1942 | 2 | 1 | 0 | 1 | 0 | 0 | 2 | 0 | 0 | 1 | 0 | 1 | 0 |
| NM_001136218 | TMEM51 | 1984 | 2 | 1 | 0 | 1 | 0 | 0 | 2 | 0 | 0 | 1 | 0 | 1 | 0 |
| NM_001136219 | FCGR2A | 2428 | 16 | 5 | 6 | 1 | 11 | 5 | 2 | 1 | 13 | 3 | 1 | 16 | 1 |
| NM_001136223 | RCOR3 | 4470 | 3 | 2 | 1 | 1 | 1 | 1 | 2 | 1 | 2 | 3 | 3 | 2 | 3 |
| NM_001136224 | RCOR3 | 4228 | 3 | 2 | 1 | 1 | 1 | 1 | 2 | 0 | 2 | 3 | 3 | 2 | 3 |

| transcript | gene | transcript length (nt) | max | mean | adipose | colon | heart | hypothalamus | kidney | liver | lung | ovary | skeletalmuscle | spleen | testes |
|---|---|---|---|---|---|---|---|---|---|---|---|---|---|---|---|
| NM_001136225 | RCOR3 | 1864 | 4 | 2 | 2 | 2 | 2 | 2 | 2 | 1 | 2 | 3 | 4 | 2 | 3 |
| NM_001136230 | HSD17B13 | 2287 | 38 | 4 | 3 | 0 | 0 | 0 | 0 | 38 | 0 | 0 | 0 | 0 | 0 |
| NM_001136232 | SEC13 | 1465 | 8 | 5 | 4 | 4 | 4 | 5 | 5 | 8 | 3 | 3 | 4 | 6 | 3 |
| NM_001136233 | FAM48B2 | 2453 | 0 | 0 | 0 | 0 | 0 | 0 | 0 | 0 | 0 | 0 | 0 | 0 | 0 |
| NM_001136234 | FAM48B1 | 2663 | 0 | 0 | 0 | 0 | 0 | 0 | 0 | 0 | 0 | 0 | 0 | 0 | 0 |
| NM_001136238 | TMPPE | 3455 | 2 | 1 | 1 | 1 | 0 | 1 | 2 | 1 | 1 | 1 | 0 | 2 | 0 |
| NM_001136239 | PRDM6 | 3026 | 2 | 0 | 0 | 1 | 1 | 0 | 0 | 0 | 1 | 2 | 0 | 0 | 0 |
| NM_001136257 | SGMS2 | 5952 | 4 | 1 | 1 | 1 | 3 | 1 | 2 | 2 | 4 | 0 | 0 | 1 | 1 |
| NM_001136258 | SGMS2 | 6144 | 3 | 1 | 1 | 1 | 3 | 1 | 2 | 2 | 3 | 0 | 0 | 1 | 1 |
| NM_001136262 | LOC552889 | 3673 | 19 | 8 | 4 | 4 | 6 | 19 | 9 | 5 | 5 | 11 | 4 | 9 | 7 |
| NM_001136263 | C2CD4C | 3123 | 1 | 0 | 0 | 0 | 0 | 1 | 0 | 0 | 0 | 0 | 0 | 0 | 0 |
| NM_001136264 | DAZAP2 | 2098 | 16 | 8 | 6 | 5 | 7 | 8 | 13 | 8 | 10 | 4 | 3 | 16 | 6 |
| NM_001136265 | IFFO2 | 5834 | 3 | 1 | 1 | 1 | 1 | 3 | 3 | 1 | 2 | 1 | 1 | 2 | 1 |
| NM_001136266 | DAZAP2 | 2087 | 16 | 8 | 6 | 5 | 7 | 8 | 13 | 8 | 10 | 4 | 3 | 16 | 6 |
| NM_001136267 | DAZAP2 | 2068 | 16 | 8 | 6 | 5 | 8 | 8 | 13 | 8 | 10 | 4 | 3 | 16 | 6 |
| NM_001136268 | DAZAP2 | 1918 | 17 | 9 | 6 | 6 | 8 | 9 | 14 | 9 | 11 | 5 | 3 | 17 | 6 |
| NM_001136269 | DAZAP2 | 1219 | 15 | 7 | 4 | 7 | 6 | 9 | 10 | 8 | 7 | 4 | 3 | 15 | 5 |
| NM_001136271 | NKX2-6 | 716 | 0 | 0 | 0 | 0 | 0 | 0 | 0 | 0 | 0 | 0 | 0 | 0 | 0 |
| NM_001136273 | ZFP92 | 1250 | 0 | 0 | 0 | 0 | 0 | 0 | 0 | 0 | 0 | 0 | 0 | 0 | 0 |
| NM_001136275 | AKIRIN1 | 2589 | 15 | 7 | 4 | 4 | 8 | 11 | 8 | 8 | 5 | 4 | 2 | 10 | 15 |
| NM_001136469 | TMEM108 | 3786 | 1 | 0 | 0 | 0 | 0 | 1 | 0 | 0 | 0 | 0 | 0 | 0 | 0 |
| NM_001136472 | LITAF | 2467 | 38 | 18 | 23 | 11 | 15 | 12 | 19 | 29 | 26 | 11 | 6 | 38 | 6 |
| NM_001136473 | LITAF | 2565 | 37 | 17 | 22 | 11 | 14 | 12 | 18 | 28 | 25 | 10 | 5 | 37 | 6 |
| NM_001136474 | VASH2 | 4271 | 0 | 0 | 0 | 0 | 0 | 0 | 0 | 0 | 0 | 0 | 0 | 0 | 0 |
| NM_001136475 | VASH2 | 3987 | 0 | 0 | 0 | 0 | 0 | 0 | 0 | 0 | 0 | 0 | 0 | 0 | 0 |
| NM_001136478 | ANP32E | 3329 | 29 | 16 | 11 | 6 | 17 | 29 | 17 | 12 | 18 | 12 | 14 | 23 | 13 |
| NM_001136479 | ANP32E | 3128 | 28 | 16 | 11 | 5 | 17 | 28 | 17 | 12 | 18 | 12 | 14 | 23 | 13 |
| NM_001136482 | C19orf38 | 1089 | 0 | 0 | 0 | 0 | 0 | 0 | 0 | 0 | 0 | 0 | 0 | 0 | 0 |
| NM_001136483 | C17orf105 | 827 | 2 | 0 | 0 | 0 | 0 | 0 | 0 | 0 | 0 | 0 | 0 | 0 | 2 |
| NM_001136485 | C11orf86 | 1184 | 0 | 0 | 0 | 0 | 0 | 0 | 0 | 0 | 0 | 0 | 0 | 0 | 0 |
| NM_001136486 | TRIM64 | 1531 | 0 | 0 | 0 | 0 | 0 | 0 | 0 | 0 | 0 | 0 | 0 | 0 | 0 |
| NM_001136493 | MFSD2A | 2200 | 6 | 1 | 0 | 0 | 0 | 0 | 0 | 6 | 1 | 0 | 0 | 0 | 1 |
| NM_001136494 | C1orf198 | 3819 | 4 | 2 | 2 | 1 | 3 | 4 | 1 | 0 | 3 | 2 | 3 | 1 | 1 |
| NM_001136495 | C1orf198 | 3543 | 4 | 2 | 2 | 1 | 3 | 4 | 1 | 0 | 3 | 2 | 3 | 1 | 1 |
| NM_001136497 | BTF3L4 | 2278 | 10 | 6 | 4 | 2 | 6 | 10 | 4 | 3 | 4 | 9 | 8 | 5 | 6 |
| NM_001136498 | CISD3 | 2636 | 1 | 0 | 0 | 1 | 0 | 0 | 1 | 1 | 0 | 0 | 0 | 0 | 0 |
| NM_001136499 | ZNF841 | 3876 | 14 | 5 | 4 | 2 | 4 | 4 | 5 | 1 | 5 | 14 | 2 | 10 | 8 |
| NM_001136501 | ZNF844 | 2834 | 22 | 8 | 6 | 3 | 7 | 5 | 15 | 1 | 5 | 22 | 9 | 9 | 13 |
| NM_001136503 | C19orf77 | 908 | 110 | 10 | 0 | 2 | 0 | 0 | 110 | 0 | 0 | 0 | 0 | 0 | 2 |
| NM_001136504 | SYT2 | 7614 | 2 | 0 | 0 | 1 | 0 | 2 | 0 | 0 | 0 | 1 | 0 | 0 | 0 |
| NM_001136505 | CCDC79 | 2445 | 4 | 0 | 0 | 0 | 0 | 0 | 0 | 0 | 0 | 0 | 0 | 0 | 4 |
| NM_001136506 | SLC22A24 | 2096 | 2 | 0 | 0 | 0 | 0 | 0 | 2 | 0 | 0 | 0 | 0 | 0 | 0 |
| NM_001136507 | MBD3L5 | 772 | 0 | 0 | 0 | 0 | 0 | 0 | 0 | 0 | 0 | 0 | 0 | 0 | 0 |
| NM_001136508 | C1orf185 | 909 | 1 | 0 | 0 | 0 | 0 | 0 | 0 | 0 | 0 | 0 | 0 | 0 | 1 |
| NM_001136509 | ZNF843 | 2013 | 0 | 0 | 0 | 0 | 0 | 0 | 0 | 0 | 0 | 0 | 0 | 0 | 0 |
| NM_001136528 | SERPINE2 | 2256 | 23 | 5 | 1 | 1 | 6 | 11 | 6 | 0 | 1 | 23 | 0 | 3 | 7 |
| NM_001136529 | SERPINE2 | 2674 | 19 | 4 | 1 | 1 | 5 | 9 | 5 | 0 | 0 | 19 | 0 | 2 | 6 |
| NM_001136530 | SERPINE2 | 2186 | 24 | 6 | 1 | 1 | 6 | 11 | 6 | 0 | 1 | 24 | 0 | 3 | 7 |
| NM_001136533 | DCAF8L2 | 2012 | 10 | 1 | 1 | 0 | 0 | 0 | 1 | 0 | 1 | 0 | 0 | 0 | 10 |
| NM_001136534 | TMEM233 | 1573 | 3 | 0 | 0 | 0 | 0 | 0 | 0 | 0 | 0 | 0 | 3 | 0 | 0 |
| NM_001136536 | DTHD1 | 2754 | 5 | 1 | 0 | 0 | 0 | 0 | 0 | 0 | 5 | 0 | 0 | 3 | 1 |
| NM_001136537 | BTBD19 | 1572 | 0 | 0 | 0 | 0 | 0 | 0 | 0 | 0 | 0 | 0 | 0 | 0 | 0 |
| NM_001136538 | ACAD10 | 4106 | 4 | 1 | 2 | 2 | 1 | 1 | 4 | 2 | 1 | 1 | 1 | 1 | 1 |
| NM_001136540 | APOL1 | 2901 | 20 | 6 | 6 | 3 | 5 | 3 | 4 | 20 | 8 | 4 | 2 | 10 | 3 |
| NM_001136541 | APOL1 | 2808 | 21 | 6 | 6 | 2 | 5 | 3 | 5 | 21 | 8 | 4 | 2 | 10 | 3 |
| NM_001136543 | LYSMD1 | 1839 | 2 | 1 | 1 | 1 | 1 | 2 | 1 | 1 | 1 | 1 | 1 | 1 | 1 |
| NM_001136554 | TLK1 | 5289 | 22 | 12 | 10 | 5 | 7 | 16 | 13 | 10 | 9 | 14 | 5 | 19 | 22 |
| NM_001136555 | TLK1 | 5060 | 17 | 9 | 7 | 3 | 5 | 11 | 10 | 8 | 7 | 11 | 4 | 15 | 17 |
| NM_001136557 | GPR107 | 7078 | 6 | 4 | 3 | 3 | 4 | 6 | 4 | 3 | 3 | 3 | 2 | 4 | 4 |
| NM_001136558 | GPR107 | 6991 | 6 | 4 | 3 | 3 | 4 | 6 | 5 | 3 | 3 | 3 | 2 | 4 | 4 |
| NM_001136561 | LOC440563 | 1007 | 0 | 0 | 0 | 0 | 0 | 0 | 0 | 0 | 0 | 0 | 0 | 0 | 0 |
| NM_001136562 | AKAP2 | 6797 | 0 | 0 | 0 | 0 | 0 | 0 | 0 | 0 | 0 | 0 | 0 | 0 | 0 |
| NM_001136566 | RAD21L1 | 1808 | 7 | 1 | 0 | 0 | 0 | 0 | 0 | 0 | 0 | 0 | 0 | 0 | 7 |
| NM_001136570 | FAM47E | 1541 | 4 | 1 | 1 | 0 | 0 | 4 | 2 | 0 | 1 | 0 | 1 | 0 | 1 |
| NM_001136571 | ZAR1L | 1103 | 0 | 0 | 0 | 0 | 0 | 0 | 0 | 0 | 0 | 0 | 0 | 0 | 0 |
| NM_001136572 | FAM90A7 | 1956 | 0 | 0 | 0 | 0 | 0 | 0 | 0 | 0 | 0 | 0 | 0 | 0 | 0 |
| NM_001136574 | LANCL1 | 4513 | 37 | 9 | 7 | 2 | 9 | 37 | 10 | 3 | 5 | 6 | 5 | 8 | 10 |
| NM_001136575 | LANCL1 | 4508 | 37 | 9 | 7 | 2 | 9 | 37 | 10 | 3 | 5 | 6 | 5 | 8 | 10 |
| NM_001137548 | FAM25C | 332 | 0 | 0 | 0 | 0 | 0 | 0 | 0 | 0 | 0 | 0 | 0 | 0 | 0 |
| NM_001137548 | FAM25C | 333 | 0 | 0 | 0 | 0 | 0 | 0 | 0 | 0 | 0 | 0 | 0 | 0 | 0 |
| NM_001137548 | FAM25C | 335 | 0 | 0 | 0 | 0 | 0 | 0 | 0 | 0 | 0 | 0 | 0 | 0 | 0 |
| NM_001137549 | FAM25G | 332 | 0 | 0 | 0 | 0 | 0 | 0 | 0 | 0 | 0 | 0 | 0 | 0 | 0 |
| NM_001137549 | FAM25G | 333 | 0 | 0 | 0 | 0 | 0 | 0 | 0 | 0 | 0 | 0 | 0 | 0 | 0 |
| NM_001137549 | FAM25G | 335 | 0 | 0 | 0 | 0 | 0 | 0 | 0 | 0 | 0 | 0 | 0 | 0 | 0 |
| NM_001137550 | LRRFIP1 | 4100 | 20 | 6 | 5 | 4 | 20 | 5 | 3 | 2 | 7 | 3 | 13 | 6 | 3 |
| NM_001137551 | LRRFIP1 | 3590 | 15 | 6 | 5 | 4 | 15 | 4 | 3 | 2 | 7 | 3 | 10 | 6 | 3 |
| NM_001137552 | LRRFIP1 | 4492 | 20 | 10 | 9 | 9 | 14 | 3 | 16 | 11 | 16 | 4 | 5 | 20 | 5 |

| transcript | gene | transcript length (nt) | max | mean | adipose | colon | heart | hypothalamus | kidney | liver | lung | ovary | skeletalmuscle | spleen | testes |
|---|---|---|---|---|---|---|---|---|---|---|---|---|---|---|---|
| NM_001137553 | LRRFIP1 | 4324 | 21 | 10 | 9 | 9 | 12 | 3 | 16 | 12 | 17 | 4 | 3 | 21 | 5 |
| NM_001137554 | MCTS1 | 9598 | 2 | 1 | 1 | 1 | 1 | 2 | 1 | 1 | 1 | 1 | 1 | 2 | 1 |
| NM_001137556 | FAM25B | 332 | 0 | 0 | 0 | 0 | 0 | 0 | 0 | 0 | 0 | 0 | 0 | 0 | 0 |
| NM_001137556 | FAM25B | 333 | 0 | 0 | 0 | 0 | 0 | 0 | 0 | 0 | 0 | 0 | 0 | 0 | 0 |
| NM_001137556 | FAM25B | 335 | 0 | 0 | 0 | 0 | 0 | 0 | 0 | 0 | 0 | 0 | 0 | 0 | 0 |
| NM_001137559 | ANAPC5 | 2362 | 5 | 4 | 4 | 3 | 3 | 5 | 4 | 5 | 2 | 2 | 4 | 5 | 5 |
| NM_001137560 | TMEM151B | 4619 | 5 | 1 | 0 | 0 | 0 | 5 | 0 | 0 | 0 | 0 | 0 | 0 | 0 |
| NM_001137601 | ZBTB42 | 3634 | 1 | 0 | 0 | 0 | 0 | 0 | 0 | 0 | 1 | 0 | 0 | 0 | 0 |
| NM_001137602 | MEMO1 | 1742 | 5 | 2 | 1 | 1 | 2 | 2 | 2 | 2 | 2 | 2 | 3 | 3 | 5 |
| NM_001137604 | POLR1B | 5241 | 2 | 1 | 1 | 1 | 1 | 1 | 1 | 2 | 0 | 1 | 1 | 2 | 2 |
| NM_001137605 | PARVG | 2148 | 2 | 0 | 0 | 0 | 0 | 0 | 0 | 0 | 1 | 0 | 0 | 2 | 0 |
| NM_001137606 | PARVG | 1967 | 2 | 0 | 0 | 0 | 0 | 0 | 0 | 0 | 1 | 0 | 0 | 2 | 0 |
| NM_001137608 | ZNF732 | 2179 | 6 | 1 | 0 | 0 | 0 | 0 | 0 | 0 | 0 | 2 | 0 | 0 | 6 |
| NM_001137610 | FAM86B2 | 1265 | 0 | 0 | 0 | 0 | 0 | 0 | 0 | 0 | 0 | 0 | 0 | 0 | 0 |
| NM_001137664 | ANAPC7 | 2208 | 3 | 2 | 2 | 1 | 1 | 3 | 2 | 1 | 1 | 1 | 2 | 2 | 2 |
| NM_001137667 | CASP8AP2 | 6723 | 5 | 3 | 3 | 1 | 3 | 4 | 3 | 2 | 3 | 4 | 4 | 5 | 5 |
| NM_001137668 | CASP8AP2 | 6590 | 5 | 3 | 3 | 1 | 3 | 4 | 3 | 2 | 3 | 4 | 4 | 5 | 5 |
| NM_001137669 | RGSL1 | 3758 | 1 | 0 | 0 | 0 | 0 | 0 | 0 | 0 | 0 | 0 | 0 | 0 | 1 |
| NM_001137671 | POTEC | 2242 | 0 | 0 | 0 | 0 | 0 | 0 | 0 | 0 | 0 | 0 | 0 | 0 | 0 |
| NM_001137673 | ILF3 | 3740 | 12 | 8 | 6 | 7 | 9 | 8 | 8 | 5 | 7 | 10 | 8 | 12 | 11 |
| NM_001137674 | ZNF860 | 3098 | 2 | 1 | 0 | 0 | 0 | 0 | 1 | 0 | 0 | 1 | 0 | 2 | 2 |
| NM_001137675 | ATXN1L | 7876 | 3 | 2 | 3 | 2 | 2 | 2 | 3 | 1 | 2 | 2 | 1 | 3 | 2 |
| NM_001138 | AGRP | 764 | 2 | 0 | 0 | 0 | 0 | 2 | 0 | 0 | 0 | 0 | 0 | 0 | 0 |
| NM_001139 | ALOX12B | 2502 | 0 | 0 | 0 | 0 | 0 | 0 | 0 | 0 | 0 | 0 | 0 | 0 | 0 |
| NM_001139441 | BCAP31 | 1342 | 49 | 20 | 49 | 13 | 12 | 21 | 29 | 20 | 18 | 8 | 5 | 21 | 20 |
| NM_001139442 | TTLL11 | 3252 | 1 | 0 | 0 | 0 | 0 | 1 | 0 | 0 | 0 | 0 | 1 | 0 | 1 |
| NM_001139443 | BEST1 | 2832 | 1 | 1 | 1 | 0 | 1 | 1 | 0 | 0 | 1 | 0 | 0 | 1 | 1 |
| NM_001139444 | BET3L | 1106 | 0 | 0 | 0 | 0 | 0 | 0 | 0 | 0 | 0 | 0 | 0 | 0 | 0 |
| NM_001139456 | SVOPL | 1803 | 0 | 0 | 0 | 0 | 0 | 0 | 0 | 0 | 0 | 0 | 0 | 0 | 0 |
| NM_001139457 | BCAP31 | 1817 | 36 | 15 | 36 | 10 | 9 | 16 | 22 | 14 | 13 | 6 | 3 | 16 | 15 |
| NM_001139459 | CNST | 2544 | 4 | 2 | 1 | 1 | 2 | 4 | 1 | 3 | 2 | 1 | 3 | 3 | 3 |
| NM_001139466 | TBL1X | 5595 | 3 | 2 | 1 | 1 | 1 | 2 | 2 | 2 | 1 | 2 | 0 | 2 | 3 |
| NM_001139467 | TBL1X | 5450 | 3 | 2 | 1 | 1 | 1 | 2 | 2 | 2 | 1 | 2 | 0 | 2 | 3 |
| NM_001139468 | TBL1X | 5585 | 3 | 2 | 1 | 1 | 1 | 2 | 2 | 2 | 1 | 2 | 0 | 2 | 3 |
| NM_001139488 | RASGRP3 | 4780 | 5 | 2 | 2 | 1 | 5 | 3 | 1 | 1 | 1 | 0 | 2 | 3 | 0 |
| NM_001139490 | TRAF3IP1 | 3976 | 19 | 9 | 9 | 5 | 6 | 11 | 8 | 5 | 7 | 9 | 11 | 7 | 19 |
| NM_001139498 | FGF13 | 2142 | 6 | 1 | 1 | 0 | 0 | 6 | 1 | 0 | 0 | 0 | 1 | 1 | 1 |
| NM_001139499 | LSM5 | 2426 | 1 | 1 | 1 | 1 | 1 | 1 | 1 | 1 | 1 | 1 | 1 | 1 | 1 |
| NM_001139500 | FGF13 | 2310 | 6 | 1 | 1 | 0 | 0 | 6 | 1 | 0 | 0 | 0 | 2 | 1 | 1 |
| NM_001139501 | FGF13 | 2420 | 5 | 1 | 1 | 0 | 0 | 5 | 1 | 0 | 0 | 0 | 2 | 0 | 1 |
| NM_001139502 | FGF13 | 2063 | 6 | 1 | 1 | 0 | 0 | 6 | 1 | 0 | 0 | 0 | 2 | 1 | 1 |
| NM_001139510 | ECHDC1 | 2115 | 20 | 9 | 20 | 3 | 8 | 10 | 14 | 13 | 10 | 5 | 5 | 9 | 6 |
| NM_001139514 | DACH2 | 2425 | 1 | 0 | 0 | 0 | 0 | 1 | 0 | 0 | 0 | 0 | 0 | 0 | 0 |
| NM_001139515 | DACH2 | 1803 | 1 | 0 | 0 | 0 | 0 | 1 | 0 | 0 | 0 | 0 | 0 | 0 | 0 |
| NM_001139517 | PRKRA | 1905 | 20 | 10 | 5 | 2 | 14 | 14 | 10 | 4 | 7 | 10 | 20 | 8 | 13 |
| NM_001139518 | PRKRA | 1626 | 23 | 11 | 6 | 3 | 17 | 17 | 11 | 5 | 8 | 12 | 23 | 9 | 16 |
| NM_001140 | ALOX15 | 2684 | 1 | 0 | 0 | 0 | 0 | 0 | 0 | 0 | 1 | 0 | 0 | 0 | 0 |
| NM_001141 | ALOX15B | 2715 | 5 | 1 | 0 | 0 | 0 | 0 | 0 | 0 | 5 | 0 | 0 | 1 | 0 |
| NM_001141917 | C9orf144B | 4296 | 2 | 0 | 0 | 0 | 0 | 0 | 0 | 0 | 0 | 0 | 0 | 0 | 2 |
| NM_001141919 | XG | 2929 | 1 | 0 | 1 | 0 | 0 | 0 | 0 | 0 | 0 | 0 | 1 | 0 | 0 |
| NM_001141920 | XG | 2887 | 1 | 0 | 1 | 0 | 0 | 0 | 0 | 0 | 0 | 0 | 1 | 0 | 0 |
| NM_001141936 | C4orf48 | 483 | 0 | 0 | 0 | 0 | 0 | 0 | 0 | 0 | 0 | 0 | 0 | 0 | 0 |
| NM_001141945 | ACTA2 | 1756 | 422 | 157 | 60 | 229 | 422 | 43 | 58 | 14 | 100 | 271 | 67 | 207 | 253 |
| NM_001141947 | CCDC66 | 3134 | 9 | 5 | 5 | 2 | 6 | 4 | 6 | 1 | 5 | 7 | 6 | 9 | 8 |
| NM_001141968 | TPTE2 | 1820 | 1 | 0 | 0 | 0 | 0 | 0 | 0 | 0 | 0 | 0 | 0 | 0 | 1 |
| NM_001141969 | DAXX | 2615 | 6 | 3 | 3 | 3 | 2 | 2 | 2 | 1 | 3 | 1 | 2 | 6 | 6 |
| NM_001141969 | DAXX | 2617 | 6 | 3 | 3 | 3 | 2 | 2 | 2 | 1 | 3 | 1 | 2 | 6 | 6 |
| NM_001141970 | DAXX | 2546 | 6 | 3 | 3 | 3 | 2 | 3 | 2 | 1 | 3 | 1 | 2 | 6 | 6 |
| NM_001141970 | DAXX | 2548 | 6 | 3 | 3 | 3 | 2 | 3 | 2 | 1 | 3 | 1 | 2 | 6 | 6 |
| NM_001141972 | ATPBD4 | 2380 | 1 | 0 | 0 | 0 | 1 | 0 | 1 | 0 | 0 | 1 | 0 | 0 | 1 |
| NM_001141973 | ATP13A2 | 3981 | 2 | 0 | 0 | 0 | 0 | 2 | 0 | 0 | 0 | 0 | 0 | 1 | 0 |
| NM_001141974 | ATP13A2 | 3694 | 2 | 0 | 0 | 0 | 0 | 2 | 0 | 0 | 0 | 0 | 0 | 1 | 0 |
| NM_001141979 | TP53BP1 | 6208 | 18 | 7 | 5 | 5 | 7 | 18 | 8 | 1 | 5 | 10 | 3 | 7 | 15 |
| NM_001141980 | TP53BP1 | 6214 | 18 | 7 | 5 | 5 | 7 | 18 | 7 | 1 | 5 | 10 | 3 | 7 | 15 |
| NM_001142 | AMELX | 793 | 0 | 0 | 0 | 0 | 0 | 0 | 0 | 0 | 0 | 0 | 0 | 0 | 0 |
| NM_001142269 | GGCX | 3076 | 8 | 2 | 1 | 1 | 1 | 1 | 2 | 8 | 1 | 1 | 1 | 2 | 1 |
| NM_001142270 | DHRS9 | 1742 | 4 | 1 | 2 | 1 | 4 | 2 | 0 | 0 | 1 | 1 | 0 | 2 | 1 |
| NM_001142271 | DHRS9 | 1465 | 5 | 1 | 3 | 1 | 5 | 2 | 0 | 0 | 2 | 1 | 0 | 2 | 1 |
| NM_001142272 | RAB11FIP3 | 3205 | 23 | 5 | 2 | 3 | 3 | 8 | 23 | 3 | 2 | 3 | 3 | 3 | 6 |
| NM_001142273 | CLASP1 | 7913 | 7 | 4 | 3 | 2 | 5 | 7 | 4 | 2 | 3 | 4 | 5 | 5 | 4 |
| NM_001142274 | CLASP1 | 7889 | 7 | 4 | 3 | 2 | 5 | 7 | 4 | 2 | 3 | 4 | 5 | 5 | 4 |
| NM_001142275 | NIPA1 | 6384 | 12 | 3 | 1 | 1 | 2 | 12 | 4 | 2 | 3 | 1 | 1 | 3 | 3 |
| NM_001142276 | APLP2 | 3775 | 144 | 81 | 91 | 41 | 107 | 100 | 144 | 90 | 127 | 67 | 20 | 60 | 48 |
| NM_001142277 | APLP2 | 3607 | 149 | 84 | 94 | 43 | 110 | 103 | 149 | 93 | 131 | 69 | 21 | 62 | 50 |
| NM_001142278 | APLP2 | 3088 | 124 | 70 | 73 | 35 | 96 | 89 | 124 | 74 | 104 | 64 | 19 | 54 | 41 |
| NM_001142279 | RNASEH2B | 1589 | 3 | 1 | 1 | 1 | 2 | 2 | 1 | 1 | 2 | 2 | 1 | 3 | 2 |
| NM_001142281 | ARCN1 | 3749 | 58 | 42 | 36 | 21 | 58 | 43 | 37 | 51 | 36 | 43 | 22 | 51 | 58 |

| transcript | gene | transcript length (nt) | max | mean | adipose | colon | heart | hypothalamus | kidney | liver | lung | ovary | skeletalmuscle | spleen | testes |
|---|---|---|---|---|---|---|---|---|---|---|---|---|---|---|---|
| NM_001142282 | RPS24 | 656 | 610 | 271 | 344 | 136 | 253 | 280 | 159 | 317 | 204 | 610 | 238 | 259 | 179 |
| NM_001142283 | RPS24 | 674 | 594 | 265 | 335 | 133 | 248 | 273 | 156 | 309 | 200 | 594 | 235 | 253 | 175 |
| NM_001142284 | RPS24 | 671 | 597 | 266 | 337 | 134 | 249 | 274 | 156 | 310 | 201 | 597 | 236 | 255 | 176 |
| NM_001142285 | RPS24 | 2814 | 141 | 63 | 79 | 32 | 59 | 65 | 37 | 73 | 47 | 141 | 55 | 60 | 42 |
| NM_001142286 | SMC6 | 5259 | 14 | 4 | 3 | 1 | 4 | 3 | 3 | 3 | 3 | 3 | 3 | 8 | 14 |
| NM_001142287 | SEMA4D | 4400 | 10 | 4 | 1 | 1 | 1 | 10 | 4 | 0 | 2 | 2 | 8 | 9 | 3 |
| NM_001142288 | COX4NB | 1866 | 5 | 3 | 2 | 1 | 5 | 4 | 3 | 2 | 1 | 2 | 3 | 3 | 2 |
| NM_001142289 | MGRN1 | 3869 | 3 | 2 | 1 | 1 | 2 | 3 | 3 | 2 | 2 | 3 | 2 | 3 | 2 |
| NM_001142290 | MGRN1 | 6469 | 2 | 1 | 1 | 1 | 1 | 2 | 2 | 1 | 1 | 2 | 2 | 2 | 1 |
| NM_001142291 | MGRN1 | 6406 | 2 | 1 | 1 | 1 | 1 | 2 | 2 | 1 | 1 | 2 | 1 | 2 | 1 |
| NM_001142292 | LMAN2L | 2440 | 3 | 2 | 2 | 2 | 3 | 2 | 3 | 3 | 1 | 3 | 2 | 2 | 2 |
| NM_001142293 | ARFGAP3 | 2701 | 13 | 8 | 5 | 3 | 9 | 8 | 11 | 11 | 8 | 6 | 5 | 10 | 13 |
| NM_001142294 | SPG20 | 4820 | 11 | 7 | 9 | 2 | 6 | 11 | 8 | 2 | 6 | 10 | 5 | 7 | 9 |
| NM_001142295 | SPG20 | 4944 | 11 | 7 | 9 | 2 | 6 | 11 | 8 | 2 | 5 | 10 | 4 | 7 | 9 |
| NM_001142296 | SPG20 | 4996 | 11 | 7 | 9 | 2 | 6 | 11 | 8 | 2 | 5 | 10 | 4 | 7 | 9 |
| NM_001142298 | SQSTM1 | 2913 | 27 | 15 | 24 | 9 | 11 | 10 | 16 | 15 | 17 | 9 | 27 | 12 | 11 |
| NM_001142299 | SQSTM1 | 2830 | 28 | 15 | 25 | 10 | 11 | 10 | 16 | 16 | 18 | 9 | 28 | 13 | 11 |
| NM_001142300 | CCNYL1 | 3756 | 2 | 1 | 2 | 1 | 1 | 1 | 2 | 2 | 1 | 1 | 0 | 1 | 1 |
| NM_001142301 | TMEM67 | 4656 | 4 | 1 | 1 | 0 | 4 | 2 | 2 | 0 | 1 | 2 | 0 | 1 | 3 |
| NM_001142302 | CCDC113 | 5102 | 2 | 1 | 0 | 0 | 0 | 2 | 0 | 0 | 1 | 0 | 0 | 0 | 1 |
| NM_001142303 | MS4A2 | 1106 | 2 | 1 | 1 | 2 | 0 | 0 | 0 | 0 | 2 | 0 | 0 | 1 | 1 |
| NM_001142305 | ZNF771 | 1441 | 2 | 1 | 1 | 0 | 1 | 2 | 2 | 2 | 1 | 1 | 1 | 0 | 1 |
| NM_001142306 | C5orf60 | 1193 | 0 | 0 | 0 | 0 | 0 | 0 | 0 | 0 | 0 | 0 | 0 | 0 | 0 |
| NM_001142307 | GTF2H1 | 3515 | 13 | 6 | 5 | 2 | 7 | 6 | 6 | 6 | 4 | 6 | 4 | 8 | 13 |
| NM_001142310 | TMEM169 | 3460 | 1 | 0 | 0 | 0 | 0 | 1 | 1 | 0 | 0 | 0 | 0 | 0 | 0 |
| NM_001142311 | TMEM169 | 3374 | 1 | 0 | 0 | 0 | 0 | 1 | 1 | 0 | 0 | 0 | 0 | 0 | 0 |
| NM_001142312 | TMEM169 | 3368 | 1 | 0 | 0 | 0 | 0 | 1 | 1 | 0 | 0 | 0 | 0 | 0 | 0 |
| NM_001142314 | DUSP19 | 5210 | 1 | 1 | 0 | 0 | 1 | 1 | 1 | 0 | 0 | 1 | 0 | 1 | 1 |
| NM_001142315 | LMO2 | 1595 | 15 | 5 | 6 | 1 | 3 | 15 | 5 | 2 | 5 | 2 | 4 | 9 | 2 |
| NM_001142316 | LMO2 | 1695 | 13 | 4 | 5 | 1 | 3 | 13 | 4 | 2 | 4 | 2 | 4 | 8 | 2 |
| NM_001142317 | TXNL4B | 2490 | 2 | 1 | 1 | 1 | 1 | 1 | 1 | 1 | 1 | 1 | 0 | 1 | 2 |
| NM_001142318 | TXNL4B | 2388 | 2 | 1 | 1 | 1 | 1 | 1 | 1 | 1 | 1 | 1 | 0 | 1 | 2 |
| NM_001142319 | C2orf44 | 3776 | 1 | 0 | 0 | 0 | 0 | 1 | 0 | 0 | 0 | 0 | 0 | 0 | 1 |
| NM_001142324 | SLC18A1 | 2860 | 0 | 0 | 0 | 0 | 0 | 0 | 0 | 0 | 0 | 0 | 0 | 0 | 0 |
| NM_001142325 | SLC18A1 | 2822 | 0 | 0 | 0 | 0 | 0 | 0 | 0 | 0 | 0 | 0 | 0 | 0 | 0 |
| NM_001142326 | DMTF1 | 3682 | 9 | 5 | 4 | 3 | 4 | 5 | 5 | 1 | 5 | 6 | 2 | 9 | 9 |
| NM_001142327 | DMTF1 | 3799 | 9 | 5 | 3 | 3 | 4 | 5 | 5 | 1 | 5 | 6 | 2 | 9 | 9 |
| NM_001142333 | A2BP1 | 4694 | 8 | 2 | 0 | 0 | 3 | 8 | 0 | 0 | 0 | 0 | 8 | 0 | 0 |
| NM_001142334 | A2BP1 | 4007 | 10 | 2 | 0 | 0 | 4 | 10 | 0 | 0 | 0 | 0 | 10 | 0 | 0 |
| NM_001142339 | GNAL | 3402 | 14 | 3 | 1 | 1 | 2 | 14 | 1 | 0 | 1 | 2 | 3 | 1 | 2 |
| NM_001142343 | CMKLR1 | 5495 | 18 | 4 | 6 | 2 | 4 | 1 | 1 | 2 | 5 | 1 | 1 | 18 | 2 |
| NM_001142344 | CMKLR1 | 5282 | 18 | 4 | 5 | 2 | 4 | 1 | 1 | 2 | 5 | 1 | 1 | 18 | 2 |
| NM_001142345 | CMKLR1 | 5109 | 19 | 4 | 5 | 2 | 4 | 1 | 1 | 2 | 5 | 1 | 1 | 19 | 2 |
| NM_001142348 | SCN4B | 4175 | 1 | 0 | 1 | 0 | 0 | 1 | 0 | 0 | 0 | 0 | 0 | 0 | 0 |
| NM_001142349 | SCN4B | 4514 | 2 | 0 | 1 | 0 | 0 | 2 | 0 | 0 | 0 | 0 | 0 | 0 | 0 |
| NM_001142350 | THOC6 | 1285 | 1 | 0 | 0 | 1 | 0 | 0 | 0 | 0 | 0 | 0 | 0 | 0 | 0 |
| NM_001142351 | ST6GAL2 | 6785 | 2 | 0 | 0 | 0 | 1 | 2 | 0 | 0 | 0 | 1 | 0 | 0 | 2 |
| NM_001142352 | ST6GAL2 | 1656 | 6 | 1 | 0 | 0 | 1 | 6 | 0 | 0 | 0 | 1 | 0 | 0 | 3 |
| NM_001142353 | PPP3CB | 3149 | 90 | 29 | 26 | 11 | 25 | 90 | 13 | 8 | 14 | 25 | 63 | 19 | 24 |
| NM_001142354 | PPP3CB | 3119 | 91 | 29 | 26 | 11 | 25 | 91 | 13 | 8 | 14 | 25 | 63 | 19 | 24 |
| NM_001142355 | NIF3L1 | 1428 | 2 | 1 | 1 | 1 | 1 | 1 | 1 | 1 | 1 | 1 | 1 | 2 | 1 |
| NM_001142356 | NIF3L1 | 1275 | 1 | 1 | 1 | 0 | 0 | 1 | 1 | 1 | 0 | 1 | 1 | 1 | 1 |
| NM_001142364 | ALG5 | 1121 | 11 | 4 | 3 | 2 | 3 | 4 | 5 | 11 | 3 | 3 | 1 | 4 | 3 |
| NM_001142368 | GSTM2 | 1477 | 1 | 1 | 1 | 1 | 0 | 1 | 0 | 0 | 0 | 1 | 1 | 0 | 0 |
| NM_001142370 | PTPN18 | 3347 | 31 | 9 | 12 | 10 | 7 | 5 | 9 | 6 | 8 | 5 | 2 | 31 | 4 |
| NM_001142386 | PDK3 | 2148 | 8 | 4 | 1 | 3 | 4 | 6 | 4 | 2 | 3 | 4 | 1 | 5 | 8 |
| NM_001142389 | PNPLA4 | 2752 | 3 | 1 | 1 | 1 | 1 | 1 | 3 | 1 | 1 | 1 | 2 | 0 | 1 |
| NM_001142391 | SLC10A3 | 1806 | 2 | 1 | 0 | 1 | 1 | 0 | 2 | 0 | 1 | 2 | 1 | 1 | 1 |
| NM_001142392 | SLC10A3 | 1893 | 2 | 1 | 0 | 1 | 1 | 0 | 2 | 0 | 1 | 2 | 1 | 1 | 1 |
| NM_001142393 | NEDD9 | 4730 | 17 | 5 | 5 | 3 | 5 | 1 | 6 | 3 | 17 | 2 | 5 | 7 | 4 |
| NM_001142395 | PRRG1 | 4492 | 7 | 2 | 2 | 1 | 3 | 7 | 3 | 1 | 2 | 1 | 3 | 2 | 1 |
| NM_001142397 | KIAA0427 | 5810 | 4 | 2 | 4 | 1 | 1 | 3 | 1 | 1 | 1 | 1 | 1 | 1 | 1 |
| NM_001142398 | FAHD1 | 1581 | 5 | 2 | 1 | 2 | 1 | 2 | 5 | 3 | 0 | 1 | 1 | 1 | 1 |
| NM_001142401 | CD164 | 3071 | 35 | 22 | 34 | 8 | 23 | 9 | 35 | 19 | 23 | 17 | 11 | 31 | 30 |
| NM_001142402 | CD164 | 3053 | 34 | 21 | 34 | 8 | 22 | 9 | 34 | 19 | 22 | 16 | 10 | 30 | 29 |
| NM_001142403 | CD164 | 2514 | 41 | 26 | 41 | 10 | 27 | 10 | 41 | 22 | 27 | 20 | 13 | 37 | 35 |
| NM_001142404 | CD164 | 2418 | 39 | 25 | 39 | 9 | 26 | 10 | 39 | 22 | 26 | 19 | 12 | 35 | 34 |
| NM_001142405 | SLMO1 | 1718 | 0 | 0 | 0 | 0 | 0 | 0 | 0 | 0 | 0 | 0 | 0 | 0 | 0 |
| NM_001142407 | CRISP2 | 1345 | 9 | 1 | 0 | 0 | 0 | 0 | 0 | 0 | 0 | 0 | 0 | 0 | 9 |
| NM_001142408 | CRISP2 | 1402 | 8 | 1 | 0 | 0 | 0 | 0 | 0 | 0 | 0 | 0 | 0 | 0 | 8 |
| NM_001142413 | C9orf47 | 4837 | 0 | 0 | 0 | 0 | 0 | 0 | 0 | 0 | 0 | 0 | 0 | 0 | 0 |
| NM_001142414 | CRCP | 2644 | 6 | 4 | 3 | 3 | 3 | 5 | 4 | 6 | 3 | 3 | 2 | 5 | 3 |
| NM_001142415 | AIMP1 | 2521 | 10 | 8 | 7 | 2 | 9 | 6 | 10 | 7 | 6 | 9 | 10 | 9 | 9 |
| NM_001142416 | AIMP1 | 2582 | 10 | 7 | 7 | 2 | 9 | 6 | 9 | 7 | 5 | 9 | 10 | 8 | 8 |
| NM_001142417 | CRISP2 | 1382 | 8 | 1 | 0 | 0 | 0 | 0 | 0 | 0 | 0 | 0 | 0 | 0 | 8 |
| NM_001142418 | MORF4L2 | 2032 | 48 | 31 | 15 | 9 | 42 | 48 | 26 | 27 | 23 | 47 | 23 | 27 | 48 |
| NM_001142419 | MORF4L2 | 1965 | 49 | 31 | 15 | 10 | 43 | 49 | 26 | 28 | 23 | 48 | 24 | 28 | 48 |

| transcript | gene | transcript length (nt) | max | mean | adipose | colon | heart | hypothalamus | kidney | liver | lung | ovary | skeletalmuscle | spleen | testes |
|---|---|---|---|---|---|---|---|---|---|---|---|---|---|---|---|
| NM_001142420 | MORF4L2 | 1812 | 51 | 31 | 14 | 10 | 44 | 50 | 25 | 26 | 21 | 51 | 25 | 28 | 45 |
| NM_001142421 | MORF4L2 | 1914 | 50 | 32 | 16 | 10 | 44 | 50 | 27 | 29 | 24 | 49 | 25 | 29 | 49 |
| NM_001142422 | MORF4L2 | 2014 | 48 | 30 | 15 | 9 | 42 | 48 | 26 | 28 | 22 | 46 | 23 | 28 | 47 |
| NM_001142423 | MORF4L2 | 1932 | 51 | 32 | 15 | 10 | 44 | 51 | 27 | 29 | 24 | 49 | 24 | 29 | 50 |
| NM_001142424 | MORF4L2 | 1981 | 50 | 31 | 15 | 10 | 43 | 50 | 26 | 28 | 24 | 48 | 24 | 28 | 49 |
| NM_001142425 | MORF4L2 | 1952 | 50 | 32 | 15 | 10 | 43 | 50 | 27 | 28 | 24 | 49 | 24 | 28 | 50 |
| NM_001142426 | MORF4L2 | 1830 | 51 | 31 | 14 | 10 | 44 | 51 | 26 | 26 | 22 | 51 | 25 | 28 | 46 |
| NM_001142427 | MORF4L2 | 1934 | 49 | 32 | 15 | 10 | 43 | 49 | 27 | 29 | 23 | 48 | 24 | 29 | 49 |
| NM_001142428 | MORF4L2 | 1963 | 49 | 31 | 15 | 10 | 43 | 49 | 26 | 28 | 23 | 48 | 24 | 28 | 48 |
| NM_001142429 | MORF4L2 | 2001 | 49 | 31 | 15 | 9 | 42 | 49 | 26 | 28 | 23 | 48 | 23 | 28 | 49 |
| NM_001142430 | MORF4L2 | 1879 | 50 | 30 | 13 | 9 | 43 | 50 | 25 | 25 | 21 | 50 | 24 | 27 | 45 |
| NM_001142431 | MORF4L2 | 1861 | 49 | 30 | 13 | 9 | 43 | 49 | 25 | 25 | 21 | 49 | 24 | 27 | 44 |
| NM_001142432 | MORF4L2 | 1983 | 48 | 31 | 15 | 9 | 42 | 48 | 26 | 28 | 23 | 47 | 24 | 28 | 48 |
| NM_001142434 | MGEA5 | 5043 | 53 | 31 | 40 | 22 | 32 | 53 | 29 | 9 | 31 | 30 | 20 | 34 | 40 |
| NM_001142435 | CRISP2 | 1365 | 8 | 1 | 0 | 0 | 0 | 0 | 0 | 0 | 0 | 0 | 0 | 0 | 8 |
| NM_001142443 | EDC3 | 4068 | 2 | 1 | 1 | 1 | 1 | 1 | 1 | 1 | 1 | 1 | 0 | 1 | 2 |
| NM_001142444 | EDC3 | 3853 | 2 | 1 | 1 | 1 | 1 | 1 | 1 | 1 | 1 | 1 | 0 | 1 | 2 |
| NM_001142445 | ANK1 | 3235 | 7 | 1 | 0 | 0 | 3 | 1 | 0 | 0 | 0 | 0 | 7 | 0 | 0 |
| NM_001142446 | ANK1 | 8477 | 2 | 0 | 0 | 0 | 1 | 1 | 0 | 0 | 0 | 0 | 2 | 0 | 0 |
| NM_001142447 | ATP1B4 | 3891 | 11 | 1 | 0 | 0 | 1 | 0 | 0 | 0 | 0 | 0 | 11 | 0 | 0 |
| NM_001142448 | SPNS1 | 1975 | 0 | 0 | 0 | 0 | 0 | 0 | 0 | 0 | 0 | 0 | 0 | 0 | 0 |
| NM_001142449 | SPNS1 | 1990 | 0 | 0 | 0 | 0 | 0 | 0 | 0 | 0 | 0 | 0 | 0 | 0 | 0 |
| NM_001142450 | SPNS1 | 2125 | 0 | 0 | 0 | 0 | 0 | 0 | 0 | 0 | 0 | 0 | 0 | 0 | 0 |
| NM_001142451 | SPNS1 | 2056 | 0 | 0 | 0 | 0 | 0 | 0 | 0 | 0 | 0 | 0 | 0 | 0 | 0 |
| NM_001142459 | ASB10 | 1934 | 0 | 0 | 0 | 0 | 0 | 0 | 0 | 0 | 0 | 0 | 0 | 0 | 0 |
| NM_001142460 | ASB10 | 1820 | 0 | 0 | 0 | 0 | 0 | 0 | 0 | 0 | 0 | 0 | 0 | 0 | 0 |
| NM_001142462 | OSR2 | 1869 | 4 | 1 | 0 | 0 | 0 | 0 | 0 | 0 | 0 | 4 | 0 | 0 | 2 |
| NM_001142463 | DKC1 | 2561 | 35 | 19 | 12 | 10 | 21 | 17 | 16 | 18 | 12 | 22 | 14 | 33 | 35 |
| NM_001142464 | ECSIT | 1535 | 1 | 1 | 0 | 1 | 1 | 1 | 1 | 1 | 0 | 1 | 1 | 1 | 0 |
| NM_001142465 | ECSIT | 1042 | 1 | 1 | 1 | 1 | 1 | 1 | 1 | 1 | 0 | 1 | 1 | 1 | 0 |
| NM_001142466 | GPT2 | 3873 | 15 | 4 | 5 | 1 | 1 | 7 | 4 | 15 | 0 | 0 | 4 | 0 | 3 |
| NM_001142467 | HES4 | 1037 | 0 | 0 | 0 | 0 | 0 | 0 | 0 | 0 | 0 | 0 | 0 | 0 | 0 |
| NM_001142468 | C6orf203 | 1434 | 12 | 8 | 6 | 4 | 12 | 11 | 12 | 5 | 3 | 8 | 7 | 7 | 7 |
| NM_001142469 | C16orf45 | 2049 | 13 | 3 | 2 | 1 | 4 | 13 | 2 | 1 | 1 | 2 | 2 | 1 | 3 |
| NM_001142470 | C6orf203 | 1517 | 12 | 7 | 6 | 4 | 11 | 10 | 12 | 5 | 2 | 7 | 7 | 7 | 7 |
| NM_001142471 | INTS12 | 1590 | 2 | 1 | 1 | 0 | 2 | 2 | 1 | 2 | 1 | 1 | 1 | 1 | 1 |
| NM_001142472 | FAIM3 | 3044 | 6 | 1 | 0 | 1 | 0 | 0 | 0 | 0 | 0 | 0 | 0 | 6 | 0 |
| NM_001142473 | FAIM3 | 2750 | 5 | 1 | 0 | 0 | 0 | 0 | 0 | 0 | 0 | 0 | 0 | 5 | 0 |
| NM_001142474 | C5orf13 | 2117 | 4 | 2 | 1 | 2 | 1 | 4 | 3 | 0 | 1 | 4 | 1 | 3 | 1 |
| NM_001142475 | C5orf13 | 2147 | 4 | 2 | 1 | 2 | 1 | 4 | 3 | 0 | 1 | 4 | 1 | 3 | 1 |
| NM_001142476 | C5orf13 | 2199 | 4 | 2 | 1 | 1 | 1 | 4 | 3 | 0 | 1 | 4 | 1 | 3 | 1 |
| NM_001142477 | C5orf13 | 2090 | 5 | 2 | 1 | 2 | 1 | 5 | 3 | 0 | 1 | 4 | 1 | 3 | 1 |
| NM_001142478 | C5orf13 | 2066 | 5 | 2 | 1 | 2 | 1 | 5 | 3 | 0 | 1 | 4 | 1 | 3 | 1 |
| NM_001142479 | C5orf13 | 2103 | 5 | 2 | 1 | 2 | 1 | 5 | 3 | 0 | 1 | 4 | 1 | 3 | 1 |
| NM_001142480 | C5orf13 | 2097 | 5 | 2 | 1 | 2 | 1 | 5 | 3 | 0 | 1 | 4 | 1 | 3 | 1 |
| NM_001142481 | C5orf13 | 2006 | 5 | 2 | 1 | 2 | 1 | 5 | 3 | 0 | 1 | 4 | 1 | 3 | 1 |
| NM_001142482 | C5orf13 | 2013 | 5 | 2 | 1 | 2 | 1 | 5 | 3 | 0 | 1 | 4 | 1 | 3 | 1 |
| NM_001142483 | C5orf13 | 2302 | 4 | 2 | 1 | 1 | 1 | 4 | 3 | 0 | 1 | 4 | 0 | 3 | 1 |
| NM_001142495 | MYO5A | 12141 | 22 | 4 | 6 | 1 | 2 | 22 | 2 | 0 | 3 | 2 | 1 | 4 | 2 |
| NM_001142497 | MLKL | 1882 | 5 | 1 | 1 | 1 | 1 | 1 | 1 | 1 | 1 | 0 | 0 | 5 | 1 |
| NM_001142498 | SIRT1 | 3587 | 7 | 3 | 2 | 1 | 2 | 2 | 3 | 2 | 3 | 5 | 4 | 4 | 7 |
| NM_001142499 | FLYWCH2 | 932 | 1 | 0 | 0 | 0 | 0 | 1 | 1 | 0 | 0 | 0 | 0 | 1 | 0 |
| NM_001142500 | FLYWCH2 | 1022 | 1 | 0 | 0 | 0 | 0 | 0 | 1 | 0 | 0 | 0 | 0 | 1 | 0 |
| NM_001142501 | MON1A | 1743 | 1 | 0 | 1 | 0 | 0 | 1 | 0 | 0 | 0 | 0 | 0 | 0 | 0 |
| NM_001142502 | PPP1R13L | 3121 | 1 | 0 | 0 | 0 | 1 | 0 | 0 | 0 | 0 | 0 | 0 | 0 | 0 |
| NM_001142503 | STARD8 | 4939 | 2 | 1 | 0 | 0 | 1 | 0 | 1 | 1 | 1 | 0 | 0 | 2 | 0 |
| NM_001142504 | STARD8 | 4822 | 2 | 1 | 1 | 0 | 1 | 0 | 1 | 1 | 2 | 0 | 0 | 2 | 0 |
| NM_001142505 | ABCG4 | 3609 | 0 | 0 | 0 | 0 | 0 | 0 | 0 | 0 | 0 | 0 | 0 | 0 | 0 |
| NM_001142519 | FAM111A | 3759 | 5 | 2 | 3 | 1 | 2 | 1 | 3 | 1 | 4 | 2 | 1 | 5 | 3 |
| NM_001142520 | FAM111A | 3782 | 5 | 2 | 3 | 1 | 2 | 1 | 3 | 1 | 4 | 2 | 1 | 5 | 3 |
| NM_001142521 | FAM111A | 3595 | 5 | 2 | 4 | 1 | 2 | 1 | 3 | 1 | 4 | 2 | 1 | 5 | 3 |
| NM_001142522 | FBXO5 | 2251 | 3 | 1 | 1 | 1 | 1 | 1 | 1 | 1 | 1 | 2 | 0 | 3 | 1 |
| NM_001142523 | IRAK3 | 8151 | 15 | 3 | 3 | 1 | 4 | 1 | 1 | 1 | 7 | 1 | 1 | 15 | 1 |
| NM_001142524 | BHLHB9 | 4084 | 5 | 1 | 1 | 0 | 1 | 5 | 1 | 0 | 0 | 1 | 0 | 1 | 1 |
| NM_001142525 | BHLHB9 | 4080 | 5 | 1 | 1 | 0 | 1 | 5 | 1 | 0 | 0 | 1 | 0 | 1 | 1 |
| NM_001142526 | BHLHB9 | 4016 | 5 | 1 | 1 | 0 | 1 | 5 | 1 | 0 | 0 | 1 | 0 | 1 | 2 |
| NM_001142527 | BHLHB9 | 4068 | 5 | 1 | 0 | 0 | 1 | 5 | 1 | 0 | 0 | 1 | 0 | 1 | 1 |
| NM_001142528 | BHLHB9 | 4000 | 5 | 1 | 0 | 0 | 1 | 5 | 1 | 0 | 0 | 1 | 0 | 1 | 1 |
| NM_001142529 | BHLHB9 | 4033 | 5 | 1 | 0 | 0 | 1 | 5 | 1 | 0 | 0 | 1 | 0 | 1 | 1 |
| NM_001142530 | BHLHB9 | 3938 | 5 | 1 | 0 | 0 | 1 | 5 | 1 | 0 | 0 | 1 | 0 | 1 | 1 |
| NM_001142531 | SH2D3C | 2546 | 1 | 0 | 0 | 0 | 0 | 0 | 0 | 0 | 1 | 0 | 0 | 1 | 0 |
| NM_001142532 | SH2D3C | 2795 | 1 | 0 | 0 | 0 | 0 | 0 | 0 | 0 | 0 | 0 | 0 | 1 | 0 |
| NM_001142533 | SH2D3C | 2683 | 1 | 0 | 0 | 0 | 0 | 0 | 0 | 0 | 0 | 0 | 0 | 1 | 0 |
| NM_001142534 | SH2D3C | 2655 | 1 | 0 | 0 | 0 | 0 | 0 | 0 | 0 | 0 | 0 | 0 | 1 | 0 |
| NM_001142535 | LGALS12 | 1736 | 2 | 0 | 2 | 0 | 0 | 0 | 0 | 0 | 0 | 0 | 0 | 0 | 0 |
| NM_001142536 | LGALS12 | 1706 | 2 | 0 | 2 | 0 | 0 | 0 | 0 | 0 | 0 | 0 | 0 | 0 | 0 |
| NM_001142537 | LGALS12 | 1823 | 2 | 0 | 2 | 0 | 0 | 0 | 0 | 0 | 0 | 0 | 0 | 0 | 0 |

| | | transcript | | | | | | hypothala | | | | | skeletalm | | |
|---|---|---|---|---|---|---|---|---|---|---|---|---|---|---|---|
| transcript | gene | length (nt) | max | mean | adipose | colon | heart | mus | kidney | liver | lung | ovary | uscle | spleen | testes |
| NM_001142538 | LGALS12 | 1796 | 2 | 0 | 2 | 0 | 0 | 0 | 0 | 0 | 0 | 0 | 0 | 0 | 0 |
| NM_001142540 | SLC35B3 | 2062 | 6 | 4 | 5 | 4 | 3 | 2 | 5 | 5 | 4 | 2 | 1 | 6 | 3 |
| NM_001142541 | SLC35B3 | 1851 | 6 | 4 | 5 | 4 | 4 | 3 | 5 | 6 | 4 | 2 | 1 | 6 | 4 |
| NM_001142544 | CTF1 | 1660 | 1 | 0 | 0 | 0 | 0 | 0 | 0 | 0 | 0 | 1 | 0 | 0 | 0 |
| NM_001142545 | ADCK1 | 2029 | 1 | 0 | 0 | 0 | 0 | 0 | 1 | 0 | 0 | 0 | 0 | 0 | 0 |
| NM_001142546 | LUZP1 | 8421 | 5 | 3 | 3 | 2 | 5 | 5 | 3 | 1 | 3 | 2 | 1 | 4 | 2 |
| NM_001142547 | RPUSD3 | 1181 | 1 | 0 | 0 | 0 | 0 | 0 | 1 | 0 | 0 | 0 | 0 | 0 | 0 |
| NM_001142548 | RAD54L | 2549 | 1 | 0 | 0 | 0 | 0 | 0 | 0 | 0 | 0 | 0 | 0 | 0 | 1 |
| NM_001142549 | DDX4 | 2777 | 42 | 4 | 0 | 0 | 0 | 0 | 0 | 0 | 0 | 0 | 0 | 0 | 42 |
| NM_001142550 | WDR47 | 4375 | 19 | 4 | 2 | 1 | 3 | 19 | 2 | 1 | 2 | 2 | 3 | 2 | 5 |
| NM_001142551 | WDR47 | 4351 | 19 | 4 | 2 | 1 | 3 | 19 | 2 | 1 | 2 | 2 | 3 | 2 | 5 |
| NM_001142552 | DCHS2 | 5064 | 1 | 0 | 0 | 1 | 0 | 1 | 0 | 0 | 0 | 0 | 0 | 0 | 0 |
| NM_001142553 | DCHS2 | 2553 | 1 | 0 | 0 | 0 | 0 | 1 | 0 | 0 | 0 | 0 | 0 | 0 | 0 |
| NM_001142554 | TRMT1 | 2178 | 1 | 1 | 1 | 1 | 0 | 1 | 1 | 1 | 0 | 1 | 0 | 1 | 0 |
| NM_001142555 | ADCK4 | 2042 | 0 | 0 | 0 | 0 | 0 | 0 | 0 | 0 | 0 | 0 | 0 | 0 | 0 |
| NM_001142556 | HMMR | 3139 | 5 | 1 | 1 | 0 | 1 | 0 | 0 | 0 | 1 | 0 | 0 | 2 | 5 |
| NM_001142557 | HMMR | 2912 | 5 | 1 | 1 | 1 | 1 | 0 | 0 | 0 | 1 | 0 | 0 | 2 | 5 |
| NM_001142560 | C1orf66 | 1783 | 1 | 0 | 0 | 1 | 0 | 1 | 0 | 0 | 0 | 0 | 0 | 0 | 1 |
| NM_001142564 | CNGA1 | 2865 | 2 | 1 | 0 | 1 | 0 | 0 | 1 | 1 | 0 | 0 | 0 | 2 | 0 |
| NM_001142565 | CPSF7 | 3584 | 5 | 4 | 3 | 3 | 3 | 5 | 4 | 3 | 3 | 5 | 5 | 5 | 5 |
| NM_001142568 | BBX | 8896 | 41 | 12 | 7 | 2 | 10 | 10 | 12 | 3 | 9 | 15 | 4 | 14 | 41 |
| NM_001142569 | C1orf106 | 4154 | 1 | 0 | 0 | 1 | 0 | 0 | 1 | 0 | 0 | 0 | 0 | 0 | 0 |
| NM_001142571 | RAD51L3 | 2461 | 1 | 1 | 1 | 1 | 0 | 1 | 1 | 0 | 0 | 1 | 0 | 1 | 1 |
| NM_001142572 | ZNF669 | 1722 | 3 | 1 | 1 | 1 | 1 | 1 | 1 | 1 | 1 | 2 | 1 | 3 | 2 |
| NM_001142573 | IMPDH1 | 2380 | 4 | 1 | 2 | 1 | 2 | 2 | 0 | 0 | 2 | 1 | 0 | 4 | 1 |
| NM_001142574 | IMPDH1 | 2365 | 4 | 1 | 2 | 1 | 2 | 2 | 0 | 0 | 2 | 1 | 0 | 4 | 1 |
| NM_001142575 | IMPDH1 | 2305 | 4 | 1 | 1 | 1 | 1 | 2 | 0 | 0 | 1 | 1 | 0 | 4 | 1 |
| NM_001142576 | IMPDH1 | 2511 | 4 | 1 | 2 | 1 | 1 | 2 | 0 | 0 | 2 | 1 | 0 | 4 | 1 |
| NM_001142577 | ZNF780A | 3600 | 5 | 2 | 2 | 1 | 2 | 3 | 2 | 1 | 1 | 5 | 1 | 5 | 2 |
| NM_001142578 | ZNF780A | 3597 | 5 | 2 | 2 | 1 | 2 | 3 | 2 | 1 | 1 | 5 | 1 | 5 | 2 |
| NM_001142579 | ZNF780A | 4249 | 1 | 1 | 0 | 0 | 0 | 1 | 0 | 1 | 0 | 1 | 0 | 1 | 0 |
| NM_001142587 | NFYC | 2084 | 4 | 3 | 2 | 3 | 3 | 3 | 3 | 2 | 2 | 3 | 3 | 4 | 2 |
| NM_001142588 | NFYC | 2144 | 4 | 3 | 2 | 3 | 3 | 3 | 3 | 2 | 2 | 3 | 3 | 4 | 2 |
| NM_001142589 | NFYC | 1973 | 4 | 3 | 2 | 3 | 3 | 3 | 3 | 2 | 2 | 3 | 3 | 4 | 2 |
| NM_001142590 | NFYC | 2362 | 3 | 2 | 2 | 2 | 2 | 2 | 3 | 2 | 2 | 3 | 3 | 3 | 2 |
| NM_001142593 | ITPK1 | 3250 | 5 | 2 | 4 | 2 | 2 | 5 | 2 | 1 | 1 | 1 | 1 | 3 | 2 |
| NM_001142594 | ITPK1 | 2803 | 1 | 1 | 1 | 1 | 1 | 1 | 1 | 0 | 0 | 0 | 0 | 1 | 1 |
| NM_001142595 | P4HA1 | 2937 | 24 | 10 | 5 | 3 | 18 | 4 | 7 | 6 | 13 | 12 | 24 | 12 | 9 |
| NM_001142596 | P4HA1 | 2790 | 25 | 11 | 5 | 3 | 19 | 4 | 7 | 6 | 13 | 13 | 25 | 13 | 10 |
| NM_001142597 | CLP1 | 1653 | 2 | 1 | 1 | 1 | 1 | 1 | 1 | 2 | 1 | 1 | 1 | 2 | 1 |
| NM_001142598 | P4HA2 | 2227 | 9 | 3 | 2 | 3 | 9 | 3 | 6 | 4 | 3 | 1 | 1 | 2 | 2 |
| NM_001142599 | P4HA2 | 2233 | 9 | 3 | 2 | 3 | 9 | 3 | 6 | 4 | 3 | 1 | 1 | 2 | 2 |
| NM_001142600 | SLC26A9 | 4088 | 1 | 0 | 0 | 0 | 1 | 0 | 1 | 0 | 1 | 0 | 0 | 0 | 0 |
| NM_001142601 | SPHK1 | 1821 | 0 | 0 | 0 | 0 | 0 | 0 | 0 | 0 | 0 | 0 | 0 | 0 | 0 |
| NM_001142602 | SPHK1 | 1866 | 0 | 0 | 0 | 0 | 0 | 0 | 0 | 0 | 0 | 0 | 0 | 0 | 0 |
| NM_001142603 | KIAA0141 | 4164 | 2 | 1 | 1 | 1 | 2 | 1 | 2 | 1 | 1 | 2 | 1 | 2 | 1 |
| NM_001142604 | PPT1 | 2195 | 9 | 3 | 2 | 0 | 2 | 3 | 2 | 1 | 3 | 2 | 1 | 9 | 2 |
| NM_001142605 | EFTUD2 | 4058 | 5 | 3 | 3 | 3 | 2 | 5 | 2 | 3 | 2 | 2 | 3 | 4 | 3 |
| NM_001142610 | ULK2 | 3897 | 6 | 2 | 3 | 1 | 1 | 6 | 1 | 0 | 1 | 4 | 1 | 2 | 5 |
| NM_001142614 | EHBP1 | 4870 | 18 | 8 | 18 | 2 | 4 | 12 | 6 | 10 | 3 | 15 | 3 | 3 | 10 |
| NM_001142615 | EHBP1 | 4968 | 18 | 8 | 18 | 2 | 4 | 12 | 6 | 10 | 3 | 15 | 3 | 3 | 10 |
| NM_001142616 | EHBP1 | 4949 | 18 | 8 | 18 | 2 | 4 | 12 | 6 | 10 | 3 | 15 | 3 | 3 | 10 |
| NM_001142617 | STRA6 | 2861 | 0 | 0 | 0 | 0 | 0 | 0 | 0 | 0 | 0 | 0 | 0 | 0 | 0 |
| NM_001142618 | STRA6 | 2843 | 0 | 0 | 0 | 0 | 0 | 0 | 0 | 0 | 0 | 0 | 0 | 0 | 0 |
| NM_001142619 | STRA6 | 2834 | 0 | 0 | 0 | 0 | 0 | 0 | 0 | 0 | 0 | 0 | 0 | 0 | 0 |
| NM_001142620 | STRA6 | 1938 | 0 | 0 | 0 | 0 | 0 | 0 | 0 | 0 | 0 | 0 | 0 | 0 | 0 |
| NM_001142621 | TGFBRAP1 | 2962 | 3 | 2 | 2 | 1 | 2 | 2 | 2 | 1 | 1 | 2 | 1 | 3 | 1 |
| NM_001142622 | GHDC | 2328 | 4 | 2 | 1 | 1 | 1 | 1 | 4 | 1 | 2 | 2 | 1 | 3 | 1 |
| NM_001142623 | GHDC | 2534 | 4 | 1 | 1 | 1 | 1 | 1 | 4 | 1 | 2 | 2 | 1 | 3 | 1 |
| NM_001142624 | RAB34 | 1290 | 3 | 1 | 1 | 1 | 2 | 1 | 1 | 0 | 1 | 3 | 0 | 1 | 1 |
| NM_001142625 | RAB34 | 1099 | 3 | 1 | 2 | 1 | 2 | 1 | 1 | 0 | 2 | 3 | 1 | 1 | 1 |
| NM_001142626 | TSHR | 1242 | 2 | 0 | 0 | 0 | 0 | 0 | 0 | 2 | 0 | 0 | 0 | 0 | 0 |
| NM_001142627 | SEC61A2 | 2091 | 4 | 1 | 0 | 0 | 1 | 4 | 1 | 0 | 0 | 1 | 1 | 1 | 1 |
| NM_001142628 | SEC61A2 | 2462 | 7 | 2 | 0 | 1 | 1 | 7 | 1 | 2 | 1 | 1 | 1 | 2 | 2 |
| NM_001142633 | PIK3R5 | 4490 | 3 | 1 | 1 | 0 | 1 | 0 | 0 | 0 | 1 | 0 | 0 | 3 | 0 |
| NM_001142634 | SPDYA | 1740 | 2 | 0 | 0 | 0 | 0 | 0 | 0 | 0 | 0 | 0 | 0 | 0 | 2 |
| NM_001142640 | TNRC6C | 9771 | 4 | 2 | 2 | 1 | 2 | 4 | 1 | 1 | 1 | 3 | 2 | 2 | 2 |
| NM_001142641 | FBRSL1 | 4545 | 1 | 1 | 0 | 1 | 1 | 1 | 1 | 0 | 1 | 1 | 0 | 1 | 1 |
| NM_001142643 | CASKIN2 | 4486 | 1 | 0 | 0 | 0 | 1 | 0 | 1 | 0 | 1 | 0 | 0 | 1 | 0 |
| NM_001142644 | SPHKAP | 6913 | 7 | 1 | 0 | 0 | 7 | 6 | 0 | 0 | 0 | 0 | 0 | 0 | 0 |
| NM_001142645 | TMEM194B | 3636 | 1 | 0 | 0 | 0 | 0 | 0 | 0 | 1 | 0 | 1 | 0 | 1 | 1 |
| NM_001142646 | TPRA1 | 1741 | 1 | 0 | 0 | 0 | 0 | 0 | 0 | 0 | 0 | 0 | 0 | 1 | 0 |
| NM_001142648 | SAR1A | 3064 | 17 | 9 | 17 | 4 | 11 | 10 | 9 | 8 | 9 | 8 | 5 | 9 | 7 |
| NM_001142649 | ANO5 | 6658 | 17 | 4 | 1 | 1 | 15 | 5 | 3 | 2 | 1 | 0 | 17 | 1 | 2 |
| NM_001142650 | HNRPLL | 2779 | 9 | 5 | 4 | 3 | 4 | 7 | 4 | 3 | 6 | 7 | 3 | 4 | 9 |
| NM_001142651 | NEURL1B | 6405 | 8 | 4 | 3 | 6 | 8 | 5 | 3 | 2 | 4 | 7 | 1 | 0 | 3 |
| NM_001142653 | AARSD1 | 1905 | 5 | 1 | 0 | 1 | 3 | 1 | 0 | 0 | 0 | 0 | 5 | 0 | 0 |

| transcript | gene | transcript length (nt) | max | mean | adipose | colon | heart | hypothalamus | kidney | liver | lung | ovary | skeletal muscle | spleen | testes |
|---|---|---|---|---|---|---|---|---|---|---|---|---|---|---|---|
| NM_001142654 | AARSD1 | 1890 | 5 | 1 | 0 | 1 | 2 | 1 | 0 | 0 | 0 | 0 | 5 | 0 | 0 |
| NM_001142673 | KIAA0652 | 4723 | 4 | 2 | 2 | 1 | 3 | 4 | 2 | 2 | 1 | 2 | 1 | 3 | 4 |
| NM_001142674 | CHID1 | 3427 | 2 | 1 | 1 | 1 | 1 | 1 | 1 | 2 | 1 | 1 | 1 | 2 | 1 |
| NM_001142675 | CHID1 | 3537 | 2 | 1 | 1 | 1 | 1 | 1 | 1 | 2 | 1 | 1 | 1 | 2 | 1 |
| NM_001142676 | CHID1 | 3398 | 2 | 1 | 1 | 1 | 1 | 1 | 1 | 2 | 1 | 1 | 1 | 2 | 1 |
| NM_001142677 | CHID1 | 3230 | 2 | 1 | 1 | 1 | 1 | 1 | 2 | 2 | 1 | 1 | 1 | 2 | 1 |
| NM_001142678 | ANO6 | 5951 | 21 | 12 | 21 | 4 | 12 | 5 | 11 | 12 | 13 | 21 | 13 | 9 | 12 |
| NM_001142679 | ANO6 | 3591 | 21 | 12 | 21 | 5 | 12 | 5 | 11 | 12 | 14 | 20 | 12 | 9 | 12 |
| NM_001142680 | ANO6 | 3448 | 22 | 13 | 22 | 5 | 13 | 6 | 12 | 13 | 14 | 21 | 13 | 10 | 12 |
| NM_001142682 | CCDC121 | 2763 | 2 | 1 | 1 | 0 | 1 | 1 | 1 | 0 | 1 | 1 | 1 | 1 | 2 |
| NM_001142683 | CCDC121 | 2757 | 2 | 1 | 1 | 0 | 1 | 1 | 1 | 0 | 1 | 1 | 1 | 1 | 2 |
| NM_001142684 | ZMYM5 | 3268 | 3 | 2 | 2 | 1 | 1 | 2 | 1 | 1 | 1 | 2 | 1 | 2 | 3 |
| NM_001142685 | RICS | 10110 | 8 | 2 | 1 | 2 | 0 | 8 | 2 | 1 | 1 | 2 | 1 | 1 | 2 |
| NM_001142699 | DLG2 | 8043 | 21 | 2 | 0 | 0 | 1 | 21 | 1 | 0 | 0 | 2 | 0 | 0 | 1 |
| NM_001142700 | DLG2 | 7078 | 21 | 2 | 0 | 0 | 1 | 21 | 0 | 0 | 0 | 2 | 0 | 0 | 1 |
| NM_001142702 | DLG2 | 6120 | 20 | 2 | 0 | 0 | 0 | 20 | 0 | 0 | 0 | 1 | 0 | 0 | 1 |
| NM_001142703 | FAM111B | 3392 | 2 | 0 | 1 | 0 | 1 | 0 | 0 | 0 | 1 | 0 | 0 | 2 | 0 |
| NM_001142704 | FAM111B | 3347 | 2 | 0 | 1 | 0 | 1 | 0 | 0 | 0 | 1 | 0 | 0 | 2 | 0 |
| NM_001142705 | C11orf58 | 4064 | 30 | 21 | 19 | 9 | 23 | 30 | 17 | 29 | 18 | 21 | 19 | 25 | 21 |
| NM_001142725 | ERI2 | 3499 | 3 | 1 | 1 | 1 | 1 | 1 | 2 | 2 | 1 | 2 | 0 | 1 | 3 |
| NM_001142726 | C1orf122 | 1185 | 0 | 0 | 0 | 0 | 0 | 0 | 0 | 0 | 0 | 0 | 0 | 0 | 0 |
| NM_001142730 | KCTD1 | 3431 | 16 | 4 | 1 | 2 | 4 | 16 | 10 | 1 | 1 | 6 | 2 | 2 | 3 |
| NM_001142733 | ASB14 | 3175 | 2 | 1 | 1 | 1 | 1 | 0 | 1 | 0 | 1 | 1 | 2 | 1 | 1 |
| NM_001142749 | KIAA1324L | 6837 | 4 | 1 | 2 | 1 | 1 | 4 | 1 | 0 | 3 | 0 | 0 | 2 | 1 |
| NM_001142761 | C15orf23 | 2032 | 9 | 3 | 2 | 3 | 1 | 3 | 1 | 5 | 2 | 2 | 2 | 2 | 9 |
| NM_001142762 | C15orf23 | 1694 | 9 | 3 | 2 | 3 | 1 | 4 | 1 | 4 | 2 | 2 | 2 | 2 | 9 |
| NM_001142763 | PCDH15 | 7042 | 1 | 0 | 0 | 0 | 0 | 1 | 0 | 0 | 0 | 0 | 0 | 0 | 0 |
| NM_001142764 | PCDH15 | 7027 | 1 | 0 | 0 | 0 | 0 | 1 | 0 | 0 | 0 | 0 | 0 | 0 | 0 |
| NM_001142765 | PCDH15 | 6814 | 1 | 0 | 0 | 0 | 0 | 1 | 0 | 0 | 0 | 0 | 0 | 0 | 0 |
| NM_001142766 | PCDH15 | 7012 | 1 | 0 | 0 | 0 | 0 | 1 | 0 | 0 | 0 | 0 | 0 | 0 | 0 |
| NM_001142767 | PCDH15 | 6901 | 1 | 0 | 0 | 0 | 0 | 1 | 0 | 0 | 0 | 0 | 0 | 0 | 0 |
| NM_001142768 | PCDH15 | 6961 | 1 | 0 | 0 | 0 | 0 | 1 | 0 | 0 | 0 | 0 | 0 | 0 | 0 |
| NM_001142769 | PCDH15 | 5767 | 1 | 0 | 0 | 0 | 0 | 1 | 0 | 0 | 0 | 0 | 0 | 0 | 0 |
| NM_001142770 | PCDH15 | 5802 | 1 | 0 | 0 | 0 | 0 | 1 | 0 | 0 | 0 | 0 | 0 | 0 | 0 |
| NM_001142771 | PCDH15 | 9248 | 1 | 0 | 0 | 0 | 0 | 1 | 0 | 0 | 0 | 0 | 0 | 0 | 0 |
| NM_001142772 | PCDH15 | 9233 | 1 | 0 | 0 | 0 | 0 | 1 | 0 | 0 | 0 | 0 | 0 | 0 | 0 |
| NM_001142773 | PCDH15 | 6952 | 1 | 0 | 0 | 0 | 0 | 1 | 0 | 0 | 0 | 0 | 0 | 0 | 0 |
| NM_001142776 | CHAC1 | 1425 | 1 | 0 | 0 | 0 | 0 | 0 | 0 | 0 | 0 | 0 | 1 | 0 | 0 |
| NM_001142777 | HSD3B7 | 2215 | 2 | 1 | 1 | 0 | 0 | 0 | 1 | 2 | 0 | 0 | 0 | 0 | 0 |
| NM_001142778 | HSD3B7 | 2022 | 2 | 1 | 1 | 0 | 0 | 0 | 1 | 2 | 0 | 0 | 0 | 1 | 0 |
| NM_001142782 | MAGI3 | 6526 | 11 | 4 | 2 | 4 | 3 | 6 | 6 | 4 | 11 | 5 | 1 | 3 | 3 |
| NM_001142784 | IL11RA | 1706 | 2 | 1 | 2 | 0 | 2 | 1 | 0 | 0 | 0 | 0 | 1 | 0 | 1 |
| NM_001142795 | DENND1B | 2687 | 3 | 2 | 2 | 1 | 2 | 2 | 3 | 2 | 2 | 2 | 1 | 2 | 1 |
| NM_001142797 | CXCR3 | 1867 | 0 | 0 | 0 | 0 | 0 | 0 | 0 | 0 | 0 | 0 | 0 | 0 | 0 |
| NM_001142798 | C17orf49 | 881 | 2 | 1 | 1 | 1 | 1 | 1 | 0 | 1 | 1 | 1 | 1 | 2 | 1 |
| NM_001142799 | C17orf49 | 775 | 2 | 1 | 1 | 1 | 1 | 1 | 0 | 1 | 1 | 1 | 1 | 2 | 1 |
| NM_001142800 | EYS | 10556 | 1 | 0 | 0 | 0 | 0 | 0 | 0 | 0 | 0 | 1 | 0 | 1 | 0 |
| NM_001142801 | EYS | 5444 | 0 | 0 | 0 | 0 | 0 | 0 | 0 | 0 | 0 | 0 | 0 | 0 | 0 |
| NM_001142805 | SLC6A8 | 3535 | 3 | 1 | 1 | 1 | 2 | 2 | 3 | 0 | 0 | 0 | 2 | 1 | 1 |
| NM_001142806 | SLC6A8 | 3114 | 4 | 1 | 1 | 1 | 2 | 2 | 4 | 0 | 0 | 1 | 2 | 1 | 1 |
| NM_001142807 | ACOXL | 2373 | 1 | 0 | 0 | 0 | 0 | 0 | 0 | 0 | 1 | 0 | 0 | 0 | 1 |
| NM_001142808 | ACOXL | 2501 | 1 | 0 | 0 | 0 | 0 | 0 | 0 | 0 | 1 | 0 | 0 | 0 | 0 |
| NM_001142853 | HES6 | 1362 | 0 | 0 | 0 | 0 | 0 | 0 | 0 | 0 | 0 | 0 | 0 | 0 | 0 |
| NM_001142854 | C21orf56 | 2315 | 0 | 0 | 0 | 0 | 0 | 0 | 0 | 0 | 0 | 0 | 0 | 0 | 0 |
| NM_001142857 | EFCAB1 | 2286 | 0 | 0 | 0 | 0 | 0 | 0 | 0 | 0 | 0 | 0 | 0 | 0 | 0 |
| NM_001142861 | SMAD6 | 1283 | 0 | 0 | 0 | 0 | 0 | 0 | 0 | 0 | 0 | 0 | 0 | 0 | 0 |
| NM_001142864 | FAM38A | 7832 | 2 | 1 | 2 | 1 | 1 | 0 | 1 | 1 | 1 | 0 | 0 | 2 | 0 |
| NM_001142883 | IP6K3 | 2822 | 7 | 1 | 0 | 0 | 1 | 0 | 0 | 1 | 0 | 0 | 7 | 0 | 0 |
| NM_001142885 | TMOD2 | 9074 | 40 | 5 | 1 | 1 | 2 | 40 | 1 | 0 | 2 | 3 | 1 | 5 | 2 |
| NM_001142886 | EPHX3 | 1771 | 0 | 0 | 0 | 0 | 0 | 0 | 0 | 0 | 0 | 0 | 0 | 0 | 0 |
| NM_001142928 | LRRC61 | 1962 | 1 | 0 | 0 | 0 | 0 | 0 | 1 | 1 | 0 | 0 | 0 | 1 | 0 |
| NM_001142929 | CHAT | 2458 | 0 | 0 | 0 | 0 | 0 | 0 | 0 | 0 | 0 | 0 | 0 | 0 | 0 |
| NM_001142930 | API5 | 3770 | 14 | 9 | 6 | 4 | 7 | 12 | 10 | 13 | 7 | 9 | 7 | 14 | 11 |
| NM_001142931 | API5 | 3603 | 14 | 9 | 6 | 4 | 7 | 12 | 11 | 13 | 7 | 9 | 7 | 14 | 11 |
| NM_001142933 | CHAT | 2528 | 0 | 0 | 0 | 0 | 0 | 0 | 0 | 0 | 0 | 0 | 0 | 0 | 0 |
| NM_001142934 | CHAT | 2528 | 0 | 0 | 0 | 0 | 0 | 0 | 0 | 0 | 0 | 0 | 0 | 0 | 0 |
| NM_001142935 | MXD3 | 2662 | 0 | 0 | 0 | 0 | 0 | 0 | 0 | 0 | 0 | 0 | 0 | 0 | 0 |
| NM_001142936 | DAGLB | 2522 | 1 | 1 | 0 | 1 | 1 | 1 | 1 | 1 | 1 | 1 | 1 | 1 | 1 |
| NM_001142946 | C11orf21 | 2262 | 0 | 0 | 0 | 0 | 0 | 0 | 0 | 0 | 0 | 0 | 0 | 0 | 0 |
| NM_001142958 | FBXO15 | 1721 | 1 | 0 | 0 | 0 | 0 | 1 | 0 | 0 | 0 | 0 | 0 | 0 | 1 |
| NM_001142961 | LPAR5 | 2695 | 1 | 0 | 0 | 1 | 0 | 1 | 0 | 0 | 0 | 0 | 0 | 1 | 0 |
| NM_001142964 | C22orf46 | 4975 | 12 | 8 | 12 | 3 | 10 | 12 | 7 | 3 | 4 | 12 | 3 | 9 | 10 |
| NM_001142966 | GREB1L | 6050 | 2 | 1 | 0 | 0 | 0 | 1 | 0 | 0 | 0 | 1 | 1 | 0 | 2 |
| NM_001143 | AMELY | 802 | 0 | 0 | 0 | 0 | 0 | 0 | 0 | 0 | 0 | 0 | 0 | 0 | 0 |
| NM_001143667 | ZBED5 | 2706 | 11 | 6 | 4 | 2 | 9 | 7 | 8 | 2 | 5 | 11 | 7 | 11 | 7 |
| NM_001143668 | AMIGO2 | 3058 | 8 | 3 | 2 | 2 | 2 | 3 | 2 | 1 | 3 | 7 | 0 | 5 | 8 |
| NM_001143674 | BRP44 | 793 | 130 | 36 | 31 | 24 | 45 | 46 | 36 | 130 | 10 | 10 | 22 | 9 | 34 |

| transcript | gene | transcript length (nt) | max | mean | adipose | colon | heart | hypothalamus | kidney | liver | lung | ovary | skeletalmuscle | spleen | testes |
|---|---|---|---|---|---|---|---|---|---|---|---|---|---|---|---|
| NM_001143676 | SGK1 | 3200 | 6 | 3 | 2 | 2 | 1 | 4 | 4 | 6 | 3 | 5 | 1 | 2 | 2 |
| NM_001143677 | SGK1 | 2451 | 7 | 4 | 3 | 2 | 2 | 5 | 6 | 7 | 4 | 7 | 1 | 2 | 2 |
| NM_001143678 | SGK1 | 2630 | 7 | 3 | 3 | 2 | 2 | 4 | 5 | 7 | 4 | 6 | 1 | 2 | 2 |
| NM_001143679 | GSTK1 | 1225 | 12 | 7 | 10 | 9 | 8 | 5 | 12 | 10 | 4 | 2 | 4 | 9 | 3 |
| NM_001143680 | GSTK1 | 1021 | 14 | 8 | 12 | 10 | 10 | 6 | 14 | 12 | 5 | 2 | 4 | 10 | 4 |
| NM_001143681 | GSTK1 | 928 | 15 | 9 | 13 | 11 | 10 | 6 | 15 | 13 | 5 | 2 | 4 | 10 | 4 |
| NM_001143682 | CALCOCO1 | 2790 | 13 | 8 | 13 | 9 | 12 | 8 | 6 | 2 | 9 | 6 | 12 | 8 | 7 |
| NM_001143685 | CES7 | 2174 | 0 | 0 | 0 | 0 | 0 | 0 | 0 | 0 | 0 | 0 | 0 | 0 | 0 |
| NM_001143687 | DCST1 | 2204 | 0 | 0 | 0 | 0 | 0 | 0 | 0 | 0 | 0 | 0 | 0 | 0 | 0 |
| NM_001143688 | DIS3L | 3765 | 5 | 4 | 5 | 2 | 4 | 4 | 3 | 3 | 2 | 5 | 4 | 4 | 5 |
| NM_001143757 | LRRC27 | 8205 | 1 | 0 | 0 | 0 | 0 | 0 | 0 | 0 | 0 | 0 | 0 | 0 | 1 |
| NM_001143758 | LRRC27 | 1784 | 1 | 0 | 0 | 0 | 0 | 1 | 0 | 0 | 0 | 0 | 0 | 0 | 1 |
| NM_001143759 | LRRC27 | 1855 | 1 | 0 | 0 | 0 | 0 | 1 | 0 | 0 | 0 | 0 | 0 | 0 | 1 |
| NM_001143760 | EIF5A | 1254 | 7 | 3 | 2 | 2 | 2 | 3 | 3 | 7 | 2 | 1 | 2 | 3 | 2 |
| NM_001143761 | EIF5A | 1282 | 7 | 3 | 2 | 2 | 2 | 3 | 3 | 7 | 2 | 1 | 2 | 3 | 2 |
| NM_001143762 | EIF5A | 1338 | 7 | 2 | 2 | 2 | 2 | 3 | 3 | 7 | 2 | 1 | 2 | 3 | 2 |
| NM_001143763 | SYCE1 | 1452 | 8 | 1 | 0 | 0 | 0 | 2 | 0 | 0 | 0 | 0 | 0 | 0 | 8 |
| NM_001143764 | SYCE1 | 1309 | 7 | 1 | 0 | 0 | 0 | 2 | 0 | 0 | 0 | 0 | 0 | 0 | 7 |
| NM_001143766 | ZNF438 | 3293 | 4 | 3 | 4 | 1 | 3 | 2 | 1 | 1 | 3 | 2 | 4 | 4 | 4 |
| NM_001143767 | ZNF438 | 3106 | 4 | 3 | 4 | 1 | 3 | 3 | 1 | 1 | 3 | 2 | 4 | 4 | 4 |
| NM_001143768 | ZNF438 | 3164 | 4 | 3 | 4 | 1 | 3 | 2 | 1 | 1 | 3 | 2 | 4 | 4 | 4 |
| NM_001143769 | ZNF438 | 3418 | 4 | 3 | 4 | 1 | 3 | 2 | 1 | 1 | 3 | 2 | 4 | 4 | 4 |
| NM_001143770 | ZNF438 | 3344 | 4 | 3 | 4 | 1 | 3 | 3 | 1 | 1 | 3 | 2 | 4 | 4 | 4 |
| NM_001143771 | ZNF438 | 3315 | 4 | 3 | 4 | 1 | 3 | 2 | 1 | 1 | 3 | 2 | 4 | 4 | 4 |
| NM_001143773 | FAM13C | 3547 | 5 | 1 | 1 | 1 | 1 | 5 | 0 | 0 | 1 | 2 | 2 | 1 | 2 |
| NM_001143774 | PHYHIPL | 3358 | 16 | 2 | 0 | 0 | 0 | 16 | 2 | 0 | 0 | 0 | 0 | 0 | 3 |
| NM_001143775 | DULLARD | 1713 | 11 | 6 | 6 | 4 | 6 | 5 | 6 | 3 | 6 | 4 | 11 | 10 | 4 |
| NM_001143778 | ASAP3 | 4102 | 2 | 1 | 0 | 1 | 1 | 1 | 1 | 1 | 1 | 2 | 1 | 0 | 1 |
| NM_001143779 | IFT81 | 2729 | 9 | 3 | 2 | 1 | 2 | 4 | 4 | 1 | 3 | 6 | 2 | 2 | 9 |
| NM_001143780 | SLC25A39 | 1610 | 5 | 2 | 1 | 2 | 1 | 2 | 5 | 5 | 2 | 1 | 1 | 2 | 1 |
| NM_001143781 | FKBP11 | 543 | 4 | 1 | 1 | 1 | 2 | 2 | 1 | 4 | 1 | 1 | 0 | 3 | 0 |
| NM_001143782 | FKBP11 | 986 | 4 | 1 | 0 | 1 | 1 | 4 | 1 | 4 | 1 | 1 | 0 | 3 | 0 |
| NM_001143783 | FES | 2511 | 3 | 1 | 0 | 1 | 0 | 0 | 0 | 0 | 1 | 0 | 0 | 3 | 0 |
| NM_001143784 | FES | 2475 | 4 | 1 | 0 | 1 | 1 | 0 | 0 | 1 | 1 | 0 | 0 | 4 | 0 |
| NM_001143785 | FES | 2420 | 4 | 1 | 0 | 1 | 1 | 0 | 0 | 0 | 1 | 0 | 0 | 4 | 0 |
| NM_001143787 | PPHLN1 | 3412 | 24 | 14 | 10 | 7 | 15 | 24 | 12 | 11 | 9 | 16 | 12 | 19 | 20 |
| NM_001143788 | PPHLN1 | 1605 | 49 | 27 | 19 | 13 | 29 | 49 | 22 | 21 | 18 | 32 | 23 | 35 | 39 |
| NM_001143789 | PPHLN1 | 1440 | 54 | 30 | 21 | 14 | 32 | 54 | 24 | 23 | 19 | 34 | 25 | 39 | 42 |
| NM_001143804 | PHOSPHO1 | 2173 | 2 | 0 | 0 | 0 | 0 | 0 | 0 | 0 | 0 | 0 | 0 | 2 | 1 |
| NM_001143805 | BDNF | 3827 | 4 | 1 | 0 | 0 | 4 | 3 | 0 | 0 | 1 | 1 | 0 | 0 | 0 |
| NM_001143806 | BDNF | 4042 | 4 | 1 | 0 | 0 | 4 | 2 | 0 | 0 | 1 | 1 | 0 | 0 | 0 |
| NM_001143807 | BDNF | 3928 | 4 | 1 | 0 | 0 | 4 | 3 | 0 | 0 | 1 | 1 | 0 | 0 | 0 |
| NM_001143808 | BDNF | 3764 | 4 | 1 | 0 | 0 | 4 | 3 | 0 | 0 | 1 | 1 | 0 | 0 | 0 |
| NM_001143809 | BDNF | 3774 | 4 | 1 | 0 | 0 | 4 | 3 | 0 | 0 | 1 | 1 | 0 | 0 | 0 |
| NM_001143810 | BDNF | 4057 | 4 | 1 | 0 | 0 | 4 | 2 | 0 | 0 | 1 | 1 | 0 | 0 | 0 |
| NM_001143811 | BDNF | 4174 | 4 | 1 | 0 | 0 | 4 | 2 | 0 | 0 | 1 | 1 | 0 | 0 | 0 |
| NM_001143812 | BDNF | 3916 | 4 | 1 | 0 | 0 | 4 | 3 | 0 | 0 | 1 | 1 | 0 | 0 | 0 |
| NM_001143813 | BDNF | 3998 | 4 | 1 | 0 | 0 | 4 | 3 | 0 | 0 | 1 | 1 | 0 | 0 | 0 |
| NM_001143814 | BDNF | 4123 | 4 | 1 | 0 | 0 | 4 | 2 | 0 | 0 | 1 | 1 | 0 | 0 | 0 |
| NM_001143815 | BDNF | 3876 | 4 | 1 | 0 | 0 | 4 | 3 | 0 | 0 | 1 | 1 | 0 | 0 | 0 |
| NM_001143816 | BDNF | 4521 | 4 | 1 | 0 | 0 | 4 | 2 | 0 | 0 | 1 | 1 | 0 | 0 | 0 |
| NM_001143818 | SERPINB2 | 2162 | 0 | 0 | 0 | 0 | 0 | 0 | 0 | 0 | 0 | 0 | 0 | 0 | 0 |
| NM_001143819 | TPCN1 | 5347 | 4 | 2 | 1 | 2 | 2 | 1 | 4 | 1 | 1 | 2 | 1 | 1 | 1 |
| NM_001143820 | ETS1 | 5143 | 56 | 12 | 10 | 4 | 10 | 2 | 7 | 4 | 26 | 8 | 4 | 56 | 4 |
| NM_001143821 | PLEKHA5 | 4412 | 24 | 10 | 5 | 4 | 18 | 11 | 22 | 6 | 7 | 24 | 2 | 2 | 13 |
| NM_001143823 | ZFP161 | 3527 | 6 | 3 | 3 | 2 | 2 | 3 | 3 | 2 | 2 | 3 | 2 | 6 | 3 |
| NM_001143824 | SLC38A4 | 3791 | 54 | 5 | 0 | 0 | 0 | 0 | 1 | 54 | 0 | 0 | 2 | 0 | 0 |
| NM_001143826 | MAPRE2 | 3120 | 20 | 4 | 2 | 2 | 7 | 20 | 2 | 2 | 2 | 3 | 3 | 4 | 2 |
| NM_001143827 | MAPRE2 | 3266 | 21 | 5 | 2 | 2 | 7 | 21 | 2 | 2 | 2 | 3 | 4 | 4 | 2 |
| NM_001143828 | HRH4 | 3403 | 0 | 0 | 0 | 0 | 0 | 0 | 0 | 0 | 0 | 0 | 0 | 0 | 0 |
| NM_001143829 | CCDC68 | 4027 | 4 | 2 | 0 | 1 | 2 | 1 | 1 | 2 | 3 | 4 | 0 | 1 | 4 |
| NM_001143830 | GAS2 | 2232 | 4 | 1 | 0 | 0 | 1 | 1 | 1 | 4 | 0 | 1 | 0 | 0 | 1 |
| NM_001143831 | GRM5 | 7854 | 6 | 1 | 0 | 0 | 0 | 6 | 0 | 0 | 0 | 0 | 0 | 0 | 0 |
| NM_001143832 | LEUTX | 670 | 0 | 0 | 0 | 0 | 0 | 0 | 0 | 0 | 0 | 0 | 0 | 0 | 0 |
| NM_001143833 | IQCF6 | 432 | 1 | 0 | 0 | 0 | 0 | 0 | 0 | 0 | 0 | 0 | 0 | 0 | 1 |
| NM_001143835 | NFRKB | 5051 | 3 | 2 | 1 | 2 | 1 | 2 | 2 | 1 | 1 | 2 | 2 | 2 | 3 |
| NM_001143836 | NOX4 | 4258 | 21 | 3 | 2 | 0 | 3 | 0 | 21 | 0 | 1 | 1 | 1 | 0 | 1 |
| NM_001143837 | NOX4 | 4603 | 20 | 3 | 2 | 0 | 3 | 0 | 20 | 0 | 1 | 1 | 1 | 0 | 1 |
| NM_001143838 | SLC13A5 | 3142 | 41 | 4 | 0 | 0 | 0 | 0 | 0 | 41 | 0 | 0 | 0 | 1 | 0 |
| NM_001143839 | PDE2A | 4131 | 4 | 1 | 1 | 0 | 0 | 3 | 0 | 0 | 0 | 0 | 0 | 4 | 0 |
| NM_001143841 | TMEM106C | 1459 | 13 | 7 | 4 | 13 | 8 | 7 | 11 | 3 | 6 | 5 | 2 | 13 | 7 |
| NM_001143842 | TMEM106C | 1587 | 12 | 7 | 4 | 12 | 7 | 6 | 11 | 3 | 5 | 4 | 2 | 12 | 7 |
| NM_001143843 | TMEM106C | 1530 | 13 | 7 | 4 | 12 | 8 | 6 | 11 | 3 | 5 | 5 | 2 | 13 | 7 |
| NM_001143850 | TCTN2 | 2906 | 2 | 1 | 0 | 0 | 0 | 1 | 1 | 0 | 1 | 2 | 0 | 0 | 1 |
| NM_001143852 | TCHP | 3095 | 5 | 3 | 2 | 3 | 3 | 1 | 3 | 1 | 2 | 2 | 2 | 5 | 4 |
| NM_001143853 | WIBG | 1087 | 1 | 1 | 1 | 1 | 1 | 1 | 1 | 1 | 1 | 1 | 0 | 1 | 1 |
| NM_001143854 | RPH3A | 4590 | 10 | 1 | 0 | 3 | 0 | 10 | 0 | 1 | 0 | 0 | 0 | 0 | 0 |

| transcript | gene | transcript length (nt) | max | mean | adipose | colon | heart | hypothalamus | kidney | liver | lung | ovary | skeletalmuscle | spleen | testes |
|---|---|---|---|---|---|---|---|---|---|---|---|---|---|---|---|
| NM_001143883 | PPIAL4B | 783 | 0 | 0 | 0 | 0 | 0 | 0 | 0 | 0 | 0 | 0 | 0 | 0 | 0 |
| NM_001143885 | PPP1R12A | 5581 | 20 | 12 | 9 | 5 | 12 | 16 | 10 | 4 | 14 | 15 | 9 | 18 | 20 |
| NM_001143886 | PPP1R12A | 5336 | 21 | 13 | 10 | 5 | 13 | 17 | 10 | 4 | 15 | 16 | 9 | 20 | 21 |
| NM_001143887 | COPS2 | 4108 | 20 | 13 | 11 | 2 | 17 | 15 | 9 | 12 | 10 | 16 | 20 | 12 | 18 |
| NM_001143888 | BSDC1 | 2907 | 3 | 2 | 2 | 2 | 1 | 3 | 1 | 2 | 1 | 2 | 1 | 2 | 2 |
| NM_001143889 | BSDC1 | 2688 | 4 | 2 | 2 | 2 | 1 | 4 | 1 | 2 | 1 | 2 | 1 | 2 | 2 |
| NM_001143890 | BSDC1 | 2571 | 4 | 2 | 2 | 2 | 1 | 4 | 1 | 2 | 1 | 2 | 1 | 2 | 2 |
| NM_001143905 | C12orf65 | 1572 | 1 | 1 | 1 | 1 | 1 | 1 | 1 | 1 | 1 | 1 | 1 | 1 | 1 |
| NM_001143906 | TRAFD1 | 3178 | 7 | 4 | 3 | 4 | 5 | 4 | 3 | 2 | 7 | 4 | 2 | 7 | 6 |
| NM_001143909 | LOC729020 | 2130 | 0 | 0 | 0 | 0 | 0 | 0 | 0 | 0 | 0 | 0 | 0 | 0 | 0 |
| NM_001143912 | FAM76A | 3457 | 3 | 2 | 1 | 1 | 2 | 2 | 2 | 1 | 1 | 2 | 2 | 2 | 3 |
| NM_001143913 | FAM76A | 3370 | 3 | 2 | 1 | 1 | 2 | 2 | 2 | 1 | 2 | 2 | 2 | 2 | 3 |
| NM_001143914 | FAM76A | 3268 | 3 | 2 | 1 | 1 | 2 | 2 | 2 | 1 | 2 | 2 | 2 | 2 | 3 |
| NM_001143915 | FAM76A | 3115 | 3 | 2 | 1 | 1 | 2 | 2 | 2 | 1 | 2 | 2 | 2 | 2 | 3 |
| NM_001143917 | LYSMD2 | 1100 | 11 | 3 | 2 | 1 | 1 | 11 | 1 | 3 | 2 | 1 | 2 | 5 | 1 |
| NM_001143919 | LTB4R | 2705 | 2 | 0 | 0 | 0 | 0 | 0 | 0 | 0 | 1 | 0 | 0 | 2 | 0 |
| NM_001143935 | CROT | 3320 | 2 | 1 | 1 | 1 | 1 | 1 | 2 | 1 | 0 | 1 | 1 | 1 | 1 |
| NM_001143936 | C15orf61 | 1294 | 1 | 0 | 0 | 0 | 0 | 1 | 0 | 0 | 0 | 0 | 1 | 1 | 0 |
| NM_001143937 | PSMA1 | 926 | 5 | 3 | 2 | 1 | 5 | 3 | 4 | 3 | 4 | 2 | 3 | 5 | 2 |
| NM_001143938 | ZNF534 | 2045 | 1 | 0 | 0 | 0 | 0 | 0 | 0 | 0 | 0 | 0 | 0 | 0 | 1 |
| NM_001143939 | ZNF534 | 2084 | 1 | 0 | 0 | 0 | 0 | 0 | 0 | 0 | 0 | 0 | 0 | 0 | 1 |
| NM_001143940 | LGSN | 5212 | 1 | 0 | 0 | 0 | 0 | 0 | 1 | 0 | 0 | 0 | 0 | 0 | 0 |
| NM_001143941 | RBM24 | 2446 | 42 | 6 | 0 | 0 | 17 | 1 | 0 | 0 | 0 | 0 | 42 | 0 | 1 |
| NM_001143942 | RBM24 | 2462 | 43 | 6 | 0 | 0 | 17 | 1 | 0 | 0 | 0 | 0 | 43 | 0 | 1 |
| NM_001143943 | EFCAB2 | 3772 | 4 | 2 | 1 | 1 | 2 | 4 | 2 | 1 | 1 | 1 | 1 | 1 | 2 |
| NM_001143944 | LEMD2 | 2358 | 2 | 1 | 1 | 1 | 1 | 1 | 2 | 1 | 2 | 2 | 1 | 2 | 2 |
| NM_001143945 | GAS6 | 2170 | 6 | 3 | 1 | 2 | 6 | 3 | 2 | 2 | 3 | 2 | 3 | 1 | 6 |
| NM_001143946 | GAS6 | 2505 | 5 | 2 | 1 | 2 | 5 | 2 | 2 | 2 | 3 | 2 | 3 | 1 | 5 |
| NM_001143947 | UNC93A | 1984 | 1 | 0 | 0 | 0 | 0 | 0 | 0 | 1 | 0 | 0 | 0 | 0 | 0 |
| NM_001143948 | C6orf105 | 1856 | 7 | 1 | 0 | 7 | 0 | 1 | 1 | 0 | 1 | 0 | 0 | 1 | 1 |
| NM_001143957 | GPR63 | 2197 | 0 | 0 | 0 | 0 | 0 | 0 | 0 | 0 | 0 | 0 | 0 | 0 | 0 |
| NM_001143958 | TMEM30A | 4436 | 45 | 23 | 16 | 7 | 22 | 45 | 25 | 24 | 36 | 18 | 12 | 33 | 21 |
| NM_001143959 | C2orf74 | 887 | 35 | 14 | 25 | 2 | 15 | 35 | 16 | 10 | 8 | 13 | 5 | 8 | 21 |
| NM_001143960 | C2orf74 | 860 | 36 | 15 | 26 | 2 | 16 | 36 | 17 | 10 | 8 | 13 | 5 | 8 | 22 |
| NM_001143962 | CAPN8 | 2101 | 5 | 1 | 0 | 5 | 0 | 0 | 0 | 0 | 2 | 0 | 0 | 0 | 0 |
| NM_001143963 | L1CAM | 4999 | 4 | 1 | 0 | 1 | 0 | 4 | 1 | 0 | 0 | 0 | 0 | 0 | 0 |
| NM_001143964 | TBC1D7 | 1260 | 1 | 0 | 0 | 0 | 0 | 0 | 1 | 0 | 0 | 0 | 0 | 0 | 0 |
| NM_001143965 | TBC1D7 | 1120 | 1 | 0 | 0 | 0 | 0 | 0 | 1 | 0 | 0 | 0 | 0 | 0 | 0 |
| NM_001143966 | TBC1D7 | 1039 | 1 | 0 | 0 | 0 | 0 | 0 | 1 | 0 | 0 | 0 | 0 | 0 | 0 |
| NM_001143967 | EFCAB8 | 3960 | 0 | 0 | 0 | 0 | 0 | 0 | 0 | 0 | 0 | 0 | 0 | 0 | 0 |
| NM_001143968 | ARL5C | 940 | 0 | 0 | 0 | 0 | 0 | 0 | 0 | 0 | 0 | 0 | 0 | 0 | 0 |
| NM_001143970 | CDYL | 3309 | 12 | 8 | 6 | 7 | 8 | 9 | 7 | 4 | 7 | 8 | 9 | 12 | 12 |
| NM_001143971 | CDYL | 3233 | 13 | 8 | 7 | 7 | 8 | 9 | 7 | 4 | 7 | 9 | 10 | 12 | 13 |
| NM_001143973 | TCTN3 | 2294 | 2 | 1 | 1 | 1 | 0 | 1 | 2 | 1 | 0 | 1 | 0 | 1 | 1 |
| NM_001143974 | ASAH2 | 2311 | 1 | 0 | 0 | 0 | 0 | 0 | 0 | 0 | 0 | 0 | 0 | 0 | 1 |
| NM_001143975 | UBTFL1 | 1181 | 0 | 0 | 0 | 0 | 0 | 0 | 0 | 0 | 0 | 0 | 0 | 0 | 0 |
| NM_001143976 | WEE1 | 3216 | 15 | 6 | 2 | 6 | 15 | 2 | 5 | 4 | 6 | 8 | 4 | 5 | 9 |
| NM_001143978 | ZCCHC18 | 3045 | 0 | 0 | 0 | 0 | 0 | 0 | 0 | 0 | 0 | 0 | 0 | 0 | 0 |
| NM_001143979 | NDE1 | 3932 | 7 | 2 | 1 | 7 | 2 | 2 | 1 | 0 | 2 | 2 | 1 | 3 | 3 |
| NM_001143980 | CCDC154 | 2190 | 0 | 0 | 0 | 0 | 0 | 0 | 0 | 0 | 0 | 0 | 0 | 0 | 0 |
| NM_001143981 | CHRDL1 | 4100 | 133 | 21 | 133 | 19 | 6 | 12 | 5 | 0 | 24 | 1 | 3 | 17 | 12 |
| NM_001143982 | CHRDL1 | 4097 | 133 | 21 | 133 | 19 | 6 | 12 | 5 | 0 | 24 | 1 | 3 | 17 | 12 |
| NM_001143983 | CHRDL1 | 3857 | 138 | 22 | 138 | 20 | 7 | 13 | 5 | 0 | 24 | 1 | 4 | 17 | 12 |
| NM_001143984 | PTPMT1 | 2547 | 3 | 2 | 2 | 1 | 2 | 2 | 2 | 3 | 1 | 1 | 1 | 2 | 2 |
| NM_001143985 | BANF1 | 1094 | 4 | 3 | 4 | 4 | 4 | 4 | 3 | 4 | 2 | 2 | 3 | 4 | 2 |
| NM_001143986 | TLE6 | 2008 | 1 | 0 | 0 | 0 | 0 | 0 | 0 | 1 | 0 | 0 | 0 | 0 | 0 |
| NM_001143987 | NBPF6 | 2221 | 0 | 0 | 0 | 0 | 0 | 0 | 0 | 0 | 0 | 0 | 0 | 0 | 0 |
| NM_001143988 | NBPF6 | 2134 | 0 | 0 | 0 | 0 | 0 | 0 | 0 | 0 | 0 | 0 | 0 | 0 | 0 |
| NM_001143989 | NBPF4 | 2499 | 0 | 0 | 0 | 0 | 0 | 0 | 0 | 0 | 0 | 0 | 0 | 0 | 0 |
| NM_001143990 | WRAP53 | 1817 | 0 | 0 | 0 | 0 | 0 | 0 | 0 | 0 | 0 | 0 | 0 | 0 | 0 |
| NM_001143991 | WRAP53 | 1827 | 0 | 0 | 0 | 0 | 0 | 0 | 0 | 0 | 0 | 0 | 0 | 0 | 0 |
| NM_001143992 | WRAP53 | 1876 | 0 | 0 | 0 | 0 | 0 | 0 | 0 | 0 | 0 | 0 | 0 | 0 | 0 |
| NM_001143993 | RASSF7 | 1728 | 0 | 0 | 0 | 0 | 0 | 0 | 0 | 0 | 0 | 0 | 0 | 0 | 0 |
| NM_001143994 | RASSF7 | 1453 | 0 | 0 | 0 | 0 | 0 | 0 | 0 | 0 | 0 | 0 | 0 | 0 | 0 |
| NM_001143995 | LPXN | 1877 | 4 | 1 | 1 | 0 | 0 | 0 | 1 | 0 | 2 | 1 | 0 | 4 | 0 |
| NM_001143996 | OGDHL | 3590 | 9 | 1 | 0 | 0 | 0 | 3 | 9 | 1 | 0 | 0 | 0 | 0 | 0 |
| NM_001143997 | OGDHL | 3385 | 9 | 1 | 0 | 0 | 0 | 4 | 9 | 1 | 0 | 0 | 0 | 0 | 0 |
| NM_001143998 | SEC14L1 | 5542 | 48 | 22 | 16 | 7 | 21 | 28 | 27 | 9 | 36 | 13 | 11 | 48 | 23 |
| NM_001143999 | SEC14L1 | 5538 | 48 | 22 | 16 | 7 | 21 | 28 | 27 | 9 | 36 | 13 | 11 | 48 | 23 |
| NM_001144 | AMFR | 3594 | 15 | 6 | 3 | 2 | 4 | 6 | 9 | 15 | 2 | 5 | 8 | 5 | 7 |
| NM_001144000 | AGAP5 | 2425 | 0 | 0 | 0 | 0 | 0 | 0 | 0 | 0 | 0 | 0 | 0 | 0 | 0 |
| NM_001144001 | SEC14L1 | 5330 | 50 | 23 | 17 | 7 | 22 | 29 | 28 | 9 | 37 | 14 | 12 | 50 | 24 |
| NM_001144002 | CTPS2 | 4321 | 3 | 1 | 1 | 1 | 1 | 1 | 1 | 1 | 1 | 3 | 1 | 1 | 1 |
| NM_001144003 | SLITRK2 | 3922 | 4 | 1 | 0 | 0 | 0 | 4 | 0 | 0 | 0 | 0 | 0 | 1 | 0 |
| NM_001144004 | SLITRK2 | 3910 | 4 | 1 | 0 | 0 | 0 | 4 | 0 | 0 | 0 | 0 | 0 | 1 | 0 |
| NM_001144005 | SLITRK2 | 3932 | 4 | 1 | 0 | 0 | 0 | 4 | 0 | 0 | 0 | 0 | 0 | 1 | 0 |
| NM_001144006 | SLITRK2 | 3996 | 4 | 1 | 0 | 0 | 0 | 4 | 0 | 0 | 0 | 0 | 0 | 1 | 0 |

| transcript | gene | transcript length (nt) | max | mean | adipose | colon | heart | hypothalamus | kidney | liver | lung | ovary | skeletalmuscle | spleen | testes |
|---|---|---|---|---|---|---|---|---|---|---|---|---|---|---|---|
| NM_001144007 | SLITRK2 | 3998 | 4 | 1 | 0 | 0 | 0 | 4 | 0 | 0 | 0 | 0 | 0 | 1 | 0 |
| NM_001144008 | SLITRK2 | 4008 | 4 | 1 | 0 | 0 | 0 | 4 | 0 | 0 | 0 | 0 | 0 | 1 | 0 |
| NM_001144009 | SLITRK2 | 3849 | 4 | 1 | 0 | 0 | 0 | 4 | 0 | 0 | 0 | 0 | 0 | 1 | 0 |
| NM_001144010 | SLITRK2 | 3986 | 4 | 1 | 0 | 0 | 0 | 4 | 0 | 0 | 0 | 0 | 0 | 1 | 0 |
| NM_001144012 | TMX2 | 1553 | 35 | 17 | 13 | 19 | 17 | 23 | 22 | 35 | 13 | 8 | 7 | 14 | 16 |
| NM_001144013 | RGPD3 | 5873 | 0 | 0 | 0 | 0 | 0 | 0 | 0 | 0 | 0 | 0 | 0 | 0 | 0 |
| NM_001144026 | NDOR1 | 4876 | 0 | 0 | 0 | 0 | 0 | 0 | 0 | 0 | 0 | 0 | 0 | 0 | 0 |
| NM_001144027 | NDOR1 | 4725 | 0 | 0 | 0 | 0 | 0 | 0 | 0 | 0 | 0 | 0 | 0 | 0 | 0 |
| NM_001144028 | NDOR1 | 4828 | 0 | 0 | 0 | 0 | 0 | 0 | 0 | 0 | 0 | 0 | 0 | 0 | 0 |
| NM_001144030 | NAT10 | 3825 | 5 | 3 | 2 | 2 | 3 | 5 | 3 | 3 | 2 | 4 | 3 | 5 | 4 |
| NM_001144032 | PPIAL4E | 783 | 0 | 0 | 0 | 0 | 0 | 0 | 0 | 0 | 0 | 0 | 0 | 0 | 0 |
| NM_001144033 | STOML3 | 1997 | 1 | 0 | 0 | 0 | 0 | 1 | 0 | 0 | 1 | 0 | 0 | 0 | 0 |
| NM_001144034 | TMEM25 | 2397 | 2 | 1 | 0 | 0 | 0 | 2 | 1 | 0 | 0 | 1 | 0 | 0 | 1 |
| NM_001144035 | TMEM25 | 2394 | 2 | 1 | 0 | 0 | 0 | 2 | 1 | 0 | 0 | 1 | 0 | 0 | 1 |
| NM_001144036 | TMEM25 | 2085 | 1 | 0 | 0 | 0 | 0 | 1 | 1 | 0 | 0 | 1 | 0 | 0 | 1 |
| NM_001144037 | TMEM25 | 1681 | 2 | 1 | 0 | 0 | 0 | 2 | 1 | 0 | 0 | 1 | 0 | 0 | 1 |
| NM_001144038 | TMEM25 | 1546 | 2 | 1 | 0 | 0 | 0 | 2 | 1 | 0 | 0 | 1 | 0 | 0 | 1 |
| NM_001144058 | NTM | 3407 | 10 | 1 | 2 | 1 | 1 | 10 | 0 | 0 | 1 | 0 | 0 | 0 | 0 |
| NM_001144059 | NTM | 1960 | 4 | 1 | 1 | 1 | 0 | 4 | 0 | 0 | 0 | 0 | 0 | 0 | 0 |
| NM_001144060 | NHSL1 | 7010 | 8 | 3 | 1 | 5 | 3 | 3 | 5 | 8 | 3 | 1 | 0 | 2 | 1 |
| NM_001144061 | COPB1 | 3429 | 10 | 6 | 6 | 3 | 6 | 5 | 8 | 10 | 5 | 5 | 3 | 7 | 7 |
| NM_001144062 | COPB1 | 3368 | 11 | 6 | 6 | 3 | 7 | 5 | 8 | 11 | 6 | 5 | 3 | 8 | 7 |
| NM_001144063 | OSBPL5 | 3683 | 1 | 0 | 0 | 1 | 0 | 0 | 0 | 0 | 0 | 0 | 0 | 1 | 0 |
| NM_001144064 | CXorf51 | 433 | 6 | 1 | 0 | 0 | 0 | 0 | 0 | 0 | 0 | 0 | 0 | 0 | 6 |
| NM_001144068 | ZNF772 | 5295 | 3 | 1 | 1 | 1 | 1 | 2 | 2 | 0 | 1 | 2 | 1 | 2 | 3 |
| NM_001144070 | ABCC3 | 1883 | 3 | 1 | 1 | 2 | 0 | 0 | 1 | 3 | 0 | 0 | 0 | 1 | 0 |
| NM_001144071 | LDHAL6A | 1990 | 1 | 0 | 0 | 0 | 0 | 0 | 0 | 0 | 0 | 0 | 0 | 0 | 1 |
| NM_001144072 | UBAC2 | 2681 | 5 | 3 | 3 | 2 | 2 | 3 | 3 | 3 | 3 | 2 | 2 | 4 | 5 |
| NM_001144073 | CHORDC1 | 3325 | 5 | 2 | 2 | 1 | 3 | 4 | 1 | 1 | 2 | 3 | 2 | 5 | 3 |
| NM_001144074 | DET1 | 2273 | 1 | 1 | 0 | 1 | 0 | 1 | 1 | 0 | 0 | 1 | 1 | 1 | 1 |
| NM_001144382 | PLCL2 | 4141 | 12 | 7 | 4 | 3 | 12 | 9 | 6 | 2 | 3 | 11 | 12 | 11 | 5 |
| NM_001144663 | CDH17 | 3699 | 22 | 2 | 0 | 22 | 0 | 0 | 0 | 0 | 0 | 0 | 0 | 0 | 0 |
| NM_001144755 | MYBL1 | 5008 | 5 | 2 | 1 | 1 | 1 | 2 | 0 | 0 | 1 | 0 | 1 | 5 | 5 |
| NM_001144756 | NPFFR2 | 2052 | 0 | 0 | 0 | 0 | 0 | 0 | 0 | 0 | 0 | 0 | 0 | 0 | 0 |
| NM_001144757 | SCG5 | 1244 | 36 | 4 | 0 | 1 | 0 | 36 | 0 | 0 | 0 | 1 | 0 | 0 | 1 |
| NM_001144758 | PHLDB1 | 5472 | 17 | 5 | 17 | 5 | 5 | 7 | 3 | 0 | 4 | 3 | 2 | 3 | 3 |
| NM_001144759 | PHLDB1 | 5298 | 17 | 5 | 17 | 6 | 5 | 7 | 3 | 1 | 4 | 3 | 2 | 3 | 3 |
| NM_001144760 | PPBPL1 | 379 | 0 | 0 | 0 | 0 | 0 | 0 | 0 | 0 | 0 | 0 | 0 | 0 | 0 |
| NM_001144761 | TLE2 | 2490 | 1 | 1 | 1 | 1 | 1 | 0 | 1 | 0 | 1 | 0 | 0 | 1 | 1 |
| NM_001144762 | TLE2 | 2281 | 1 | 1 | 1 | 1 | 1 | 0 | 1 | 0 | 1 | 0 | 0 | 1 | 1 |
| NM_001144763 | EXPH5 | 10143 | 4 | 1 | 0 | 1 | 0 | 1 | 4 | 4 | 3 | 0 | 0 | 0 | 0 |
| NM_001144765 | EXPH5 | 9829 | 4 | 1 | 0 | 1 | 0 | 1 | 4 | 4 | 3 | 0 | 0 | 0 | 0 |
| NM_001144766 | DNAJC7 | 1846 | 38 | 20 | 15 | 7 | 24 | 38 | 20 | 18 | 13 | 18 | 20 | 25 | 23 |
| NM_001144767 | UGT2B10 | 2502 | 2 | 0 | 0 | 0 | 0 | 0 | 0 | 2 | 0 | 0 | 0 | 0 | 0 |
| NM_001144769 | DST | 18074 | 51 | 23 | 16 | 9 | 50 | 51 | 12 | 11 | 23 | 22 | 27 | 19 | 13 |
| NM_001144770 | DST | 17758 | 52 | 24 | 16 | 10 | 50 | 52 | 12 | 11 | 24 | 23 | 28 | 19 | 13 |
| NM_001144771 | DST | 17640 | 53 | 24 | 16 | 10 | 51 | 53 | 12 | 11 | 24 | 23 | 28 | 19 | 14 |
| NM_001144772 | NSMAF | 3507 | 8 | 4 | 5 | 2 | 4 | 3 | 3 | 1 | 4 | 4 | 2 | 8 | 3 |
| NM_001144773 | CSRP1 | 1960 | 23 | 7 | 3 | 23 | 3 | 11 | 4 | 1 | 7 | 12 | 1 | 7 | 5 |
| NM_001144774 | ELAVL4 | 2059 | 8 | 1 | 0 | 0 | 0 | 8 | 0 | 0 | 0 | 0 | 0 | 0 | 1 |
| NM_001144775 | ELAVL4 | 1884 | 9 | 1 | 0 | 0 | 0 | 9 | 0 | 0 | 0 | 0 | 0 | 0 | 1 |
| NM_001144776 | ELAVL4 | 1873 | 9 | 1 | 0 | 0 | 0 | 9 | 0 | 0 | 0 | 0 | 0 | 0 | 1 |
| NM_001144777 | ELAVL4 | 1951 | 9 | 1 | 0 | 0 | 0 | 9 | 0 | 0 | 0 | 0 | 0 | 0 | 1 |
| NM_001144822 | CD58 | 1394 | 1 | 1 | 1 | 0 | 1 | 0 | 1 | 0 | 1 | 0 | 0 | 1 | 1 |
| NM_001144823 | DENND4A | 7336 | 8 | 5 | 3 | 1 | 4 | 6 | 4 | 5 | 5 | 8 | 7 | 8 | 7 |
| NM_001144824 | ZNF234 | 4480 | 6 | 3 | 2 | 1 | 3 | 3 | 2 | 1 | 2 | 6 | 2 | 4 | 2 |
| NM_001144825 | RUNDC3A | 2046 | 8 | 1 | 0 | 0 | 0 | 8 | 0 | 0 | 0 | 0 | 0 | 0 | 0 |
| NM_001144826 | RUNDC3A | 1842 | 32 | 3 | 0 | 1 | 0 | 32 | 0 | 0 | 0 | 0 | 0 | 0 | 0 |
| NM_001144827 | RPUSD4 | 2418 | 10 | 3 | 2 | 2 | 6 | 2 | 2 | 3 | 1 | 2 | 10 | 3 | 1 |
| NM_001144829 | FMO5 | 1980 | 26 | 4 | 0 | 3 | 0 | 0 | 6 | 26 | 8 | 1 | 0 | 1 | 2 |
| NM_001144830 | FMO5 | 2499 | 38 | 6 | 1 | 4 | 1 | 0 | 9 | 38 | 13 | 2 | 1 | 2 | 2 |
| NM_001144831 | PHB2 | 1441 | 8 | 4 | 3 | 3 | 4 | 3 | 4 | 6 | 3 | 8 | 5 | 5 | 2 |
| NM_001144832 | TTC39A | 2714 | 3 | 1 | 0 | 1 | 0 | 0 | 3 | 0 | 1 | 0 | 0 | 0 | 2 |
| NM_001144856 | PLEKHG6 | 2956 | 2 | 0 | 1 | 2 | 0 | 0 | 0 | 0 | 0 | 0 | 0 | 0 | 0 |
| NM_001144857 | PLEKHG6 | 3124 | 2 | 0 | 1 | 2 | 0 | 0 | 0 | 0 | 0 | 0 | 0 | 0 | 0 |
| NM_001144867 | CCNL2 | 3838 | 3 | 2 | 2 | 3 | 2 | 2 | 2 | 1 | 2 | 1 | 1 | 2 | 2 |
| NM_001144868 | CCNL2 | 3128 | 3 | 2 | 2 | 3 | 1 | 2 | 2 | 1 | 1 | 1 | 1 | 2 | 1 |
| NM_001144869 | LIPT2 | 959 | 2 | 1 | 0 | 0 | 0 | 0 | 2 | 1 | 1 | 1 | 1 | 1 | 1 |
| NM_001144871 | C11orf90 | 603 | 1 | 0 | 0 | 0 | 0 | 1 | 0 | 0 | 0 | 0 | 0 | 0 | 0 |
| NM_001144872 | CCDC42B | 1513 | 0 | 0 | 0 | 0 | 0 | 0 | 0 | 0 | 0 | 0 | 0 | 0 | 0 |
| NM_001144874 | PATE4 | 1985 | 2 | 0 | 0 | 0 | 0 | 0 | 0 | 0 | 0 | 0 | 0 | 0 | 2 |
| NM_001144875 | DOK3 | 2326 | 11 | 1 | 1 | 1 | 1 | 1 | 0 | 0 | 1 | 0 | 0 | 11 | 0 |
| NM_001144876 | DOK3 | 2102 | 12 | 2 | 1 | 1 | 1 | 1 | 0 | 0 | 2 | 0 | 0 | 12 | 1 |
| NM_001144877 | SCAI | 12098 | 3 | 1 | 1 | 1 | 0 | 3 | 1 | 0 | 1 | 2 | 1 | 1 | 1 |
| NM_001144878 | IMPA1 | 3597 | 10 | 5 | 2 | 2 | 7 | 8 | 7 | 5 | 5 | 3 | 2 | 6 | 10 |
| NM_001144879 | IMPA1 | 3287 | 11 | 6 | 2 | 2 | 8 | 9 | 7 | 5 | 5 | 3 | 2 | 6 | 11 |
| NM_001144881 | PRICKLE1 | 4253 | 5 | 2 | 1 | 1 | 3 | 5 | 1 | 0 | 1 | 3 | 1 | 4 | 2 |

| transcript | gene | transcript length (nt) | max | mean | adipose | colon | heart | hypothala mus | kidney | liver | lung | ovary | skeletalm uscle | spleen | testes |
|---|---|---|---|---|---|---|---|---|---|---|---|---|---|---|---|
| NM_001144882 | PRICKLE1 | 4252 | 5 | 2 | 1 | 1 | 3 | 5 | 1 | 0 | 1 | 3 | 1 | 4 | 2 |
| NM_001144883 | PRICKLE1 | 4249 | 5 | 2 | 1 | 1 | 3 | 5 | 1 | 0 | 1 | 3 | 1 | 4 | 2 |
| NM_001144884 | SLC30A7 | 7900 | 6 | 3 | 2 | 1 | 2 | 2 | 4 | 5 | 5 | 3 | 1 | 6 | 3 |
| NM_001144885 | CITED1 | 941 | 15 | 2 | 0 | 0 | 0 | 3 | 0 | 0 | 0 | 0 | 0 | 0 | 15 |
| NM_001144886 | CITED1 | 893 | 16 | 2 | 0 | 0 | 0 | 3 | 0 | 0 | 0 | 0 | 0 | 0 | 16 |
| NM_001144887 | CITED1 | 820 | 17 | 2 | 0 | 0 | 0 | 3 | 0 | 0 | 0 | 0 | 0 | 0 | 17 |
| NM_001144888 | BAIAP2 | 3306 | 3 | 1 | 0 | 1 | 0 | 2 | 2 | 3 | 2 | 0 | 0 | 1 | 1 |
| NM_001144889 | SLC23A3 | 2392 | 2 | 0 | 0 | 0 | 0 | 0 | 2 | 0 | 0 | 0 | 0 | 0 | 0 |
| NM_001144890 | SLC23A3 | 2416 | 2 | 0 | 0 | 0 | 0 | 0 | 2 | 0 | 0 | 0 | 0 | 0 | 0 |
| NM_001144891 | MBIP | 1645 | 15 | 8 | 5 | 2 | 5 | 11 | 8 | 8 | 15 | 11 | 3 | 8 | 9 |
| NM_001144892 | FGF1 | 3660 | 45 | 7 | 6 | 1 | 7 | 45 | 16 | 0 | 1 | 0 | 1 | 1 | 1 |
| NM_001144893 | CD209 | 3919 | 5 | 2 | 5 | 1 | 5 | 1 | 1 | 1 | 1 | 1 | 0 | 2 | 1 |
| NM_001144894 | CD209 | 4195 | 5 | 2 | 5 | 1 | 4 | 1 | 1 | 1 | 1 | 1 | 0 | 1 | 1 |
| NM_001144895 | CD209 | 4051 | 5 | 2 | 5 | 1 | 5 | 1 | 1 | 1 | 1 | 1 | 0 | 2 | 1 |
| NM_001144896 | CD209 | 4255 | 5 | 2 | 5 | 1 | 4 | 1 | 1 | 1 | 1 | 1 | 0 | 1 | 1 |
| NM_001144897 | CD209 | 4309 | 5 | 2 | 5 | 1 | 4 | 1 | 1 | 1 | 1 | 1 | 0 | 1 | 1 |
| NM_001144899 | CD209 | 3844 | 4 | 1 | 3 | 1 | 4 | 0 | 1 | 1 | 1 | 1 | 0 | 2 | 1 |
| NM_001144900 | SMCR7 | 2517 | 1 | 0 | 0 | 0 | 0 | 0 | 1 | 0 | 0 | 0 | 1 | 0 | 0 |
| NM_001144903 | TM6SF1 | 1815 | 4 | 1 | 1 | 0 | 1 | 4 | 1 | 0 | 1 | 1 | 2 | 2 | 1 |
| NM_001144904 | CLEC4M | 1781 | 1 | 0 | 0 | 0 | 0 | 0 | 0 | 1 | 0 | 0 | 0 | 0 | 0 |
| NM_001144905 | CLEC4M | 1862 | 1 | 0 | 0 | 0 | 0 | 0 | 0 | 1 | 0 | 0 | 0 | 0 | 0 |
| NM_001144906 | CLEC4M | 1526 | 1 | 0 | 0 | 0 | 0 | 0 | 0 | 1 | 0 | 0 | 0 | 0 | 0 |
| NM_001144907 | CLEC4M | 1733 | 1 | 0 | 0 | 0 | 0 | 0 | 0 | 1 | 0 | 0 | 0 | 0 | 0 |
| NM_001144908 | CLEC4M | 1545 | 1 | 0 | 0 | 0 | 0 | 0 | 0 | 1 | 0 | 0 | 0 | 0 | 0 |
| NM_001144909 | CLEC4M | 1796 | 1 | 0 | 0 | 0 | 0 | 0 | 0 | 1 | 0 | 0 | 0 | 0 | 0 |
| NM_001144910 | CLEC4M | 1865 | 1 | 0 | 0 | 0 | 0 | 0 | 0 | 1 | 0 | 0 | 0 | 0 | 0 |
| NM_001144911 | CLEC4M | 1737 | 1 | 0 | 0 | 0 | 0 | 0 | 0 | 1 | 0 | 0 | 0 | 0 | 0 |
| NM_001144912 | ATG7 | 4804 | 1 | 0 | 1 | 0 | 0 | 1 | 0 | 0 | 1 | 0 | 0 | 1 | 1 |
| NM_001144913 | FGFR2 | 2777 | 12 | 4 | 1 | 7 | 0 | 12 | 9 | 3 | 2 | 4 | 0 | 0 | 2 |
| NM_001144914 | FGFR2 | 3806 | 9 | 3 | 1 | 6 | 0 | 9 | 8 | 2 | 2 | 3 | 0 | 0 | 2 |
| NM_001144915 | FGFR2 | 3693 | 10 | 3 | 1 | 5 | 0 | 10 | 8 | 2 | 2 | 3 | 0 | 0 | 1 |
| NM_001144916 | FGFR2 | 4088 | 9 | 3 | 1 | 5 | 0 | 9 | 7 | 2 | 2 | 3 | 0 | 0 | 2 |
| NM_001144917 | FGFR2 | 4291 | 8 | 3 | 1 | 5 | 0 | 8 | 7 | 2 | 2 | 3 | 0 | 0 | 1 |
| NM_001144918 | FGFR2 | 4288 | 9 | 3 | 1 | 5 | 0 | 9 | 7 | 2 | 2 | 3 | 0 | 0 | 1 |
| NM_001144919 | FGFR2 | 3007 | 11 | 3 | 1 | 6 | 0 | 11 | 8 | 3 | 2 | 3 | 0 | 0 | 2 |
| NM_001144920 | TTC26 | 2017 | 2 | 1 | 1 | 0 | 1 | 1 | 1 | 0 | 1 | 1 | 0 | 0 | 2 |
| NM_001144923 | TTC26 | 2058 | 2 | 1 | 1 | 0 | 1 | 1 | 1 | 0 | 1 | 1 | 0 | 0 | 2 |
| NM_001144924 | LOC81691 | 2682 | 44 | 4 | 0 | 1 | 1 | 1 | 0 | 0 | 0 | 1 | 0 | 0 | 44 |
| NM_001144925 | MX1 | 3391 | 12 | 5 | 4 | 2 | 7 | 5 | 2 | 2 | 4 | 9 | 7 | 12 | 2 |
| NM_001144927 | NKIRAS2 | 2488 | 3 | 2 | 1 | 1 | 3 | 1 | 1 | 1 | 2 | 1 | 1 | 2 | 2 |
| NM_001144928 | NKIRAS2 | 2301 | 3 | 1 | 1 | 1 | 3 | 1 | 1 | 1 | 2 | 1 | 1 | 2 | 2 |
| NM_001144929 | NKIRAS2 | 2372 | 3 | 1 | 1 | 1 | 3 | 1 | 1 | 1 | 2 | 1 | 1 | 2 | 2 |
| NM_001144931 | LOC391322 | 563 | 0 | 0 | 0 | 0 | 0 | 0 | 0 | 0 | 0 | 0 | 0 | 0 | 0 |
| NM_001144932 | PSMB5 | 1378 | 18 | 9 | 11 | 6 | 10 | 16 | 8 | 18 | 5 | 4 | 10 | 6 | 4 |
| NM_001144933 | EFCAB3 | 1691 | 1 | 0 | 0 | 0 | 0 | 0 | 0 | 0 | 0 | 0 | 0 | 0 | 1 |
| NM_001144934 | FGF1 | 3866 | 43 | 7 | 5 | 1 | 7 | 43 | 16 | 0 | 1 | 0 | 1 | 1 | 1 |
| NM_001144935 | FGF1 | 3772 | 44 | 7 | 6 | 1 | 7 | 44 | 16 | 0 | 1 | 0 | 1 | 1 | 1 |
| NM_001144936 | C11orf95 | 5581 | 1 | 1 | 0 | 0 | 1 | 1 | 1 | 0 | 0 | 1 | 0 | 1 | 1 |
| NM_001144937 | FNDC7 | 3058 | 0 | 0 | 0 | 0 | 0 | 0 | 0 | 0 | 0 | 0 | 0 | 0 | 0 |
| NM_001144939 | VMO1 | 803 | 1 | 0 | 0 | 0 | 0 | 0 | 0 | 0 | 0 | 0 | 0 | 1 | 0 |
| NM_001144940 | VMO1 | 747 | 1 | 0 | 0 | 0 | 0 | 0 | 0 | 0 | 0 | 0 | 0 | 1 | 0 |
| NM_001144941 | VMO1 | 651 | 1 | 0 | 0 | 0 | 0 | 0 | 0 | 0 | 0 | 0 | 0 | 1 | 0 |
| NM_001144942 | RAB34 | 1742 | 2 | 1 | 1 | 1 | 1 | 1 | 1 | 0 | 1 | 2 | 0 | 1 | 1 |
| NM_001144943 | RAB34 | 1314 | 3 | 1 | 1 | 1 | 2 | 1 | 1 | 0 | 1 | 3 | 0 | 1 | 1 |
| NM_001144944 | MYL12B | 1023 | 35 | 24 | 31 | 15 | 25 | 35 | 32 | 34 | 26 | 13 | 13 | 28 | 16 |
| NM_001144945 | MYL12B | 945 | 38 | 26 | 33 | 17 | 27 | 38 | 35 | 37 | 28 | 14 | 14 | 30 | 18 |
| NM_001144946 | MYL12B | 950 | 37 | 26 | 33 | 17 | 27 | 37 | 35 | 37 | 28 | 14 | 14 | 30 | 18 |
| NM_001144950 | SSC5D | 4909 | 5 | 2 | 3 | 5 | 1 | 1 | 0 | 0 | 1 | 5 | 1 | 3 | 1 |
| NM_001144951 | GLYCTK | 1371 | 0 | 0 | 0 | 0 | 0 | 0 | 0 | 0 | 0 | 0 | 0 | 0 | 0 |
| NM_001144952 | SDK2 | 10721 | 1 | 0 | 0 | 0 | 0 | 0 | 0 | 0 | 0 | 0 | 0 | 1 | 1 |
| NM_001144954 | C5orf47 | 2521 | 0 | 0 | 0 | 0 | 0 | 0 | 0 | 0 | 0 | 0 | 0 | 0 | 0 |
| NM_001144955 | DTWD1 | 2621 | 3 | 1 | 1 | 0 | 1 | 1 | 1 | 1 | 1 | 3 | 1 | 2 | 1 |
| NM_001144956 | C1orf230 | 455 | 1 | 0 | 0 | 0 | 0 | 1 | 0 | 0 | 0 | 0 | 0 | 0 | 0 |
| NM_001144958 | EFCAB4B | 2795 | 3 | 1 | 0 | 3 | 0 | 0 | 0 | 0 | 0 | 0 | 0 | 1 | 0 |
| NM_001144959 | EFCAB4B | 2231 | 3 | 1 | 0 | 3 | 0 | 0 | 0 | 0 | 1 | 0 | 0 | 1 | 0 |
| NM_001144960 | C3orf16 | 2471 | 0 | 0 | 0 | 0 | 0 | 0 | 0 | 0 | 0 | 0 | 0 | 0 | 0 |
| NM_001144961 | NFKBIL1 | 1437 | 1 | 1 | 0 | 1 | 1 | 1 | 1 | 0 | 1 | 1 | 1 | 1 | 1 |
| NM_001144962 | NFKBIL1 | 1429 | 1 | 1 | 0 | 1 | 1 | 1 | 1 | 0 | 1 | 1 | 1 | 1 | 1 |
| NM_001144963 | NFKBIL1 | 1384 | 1 | 1 | 0 | 1 | 0 | 1 | 1 | 0 | 1 | 1 | 1 | 1 | 1 |
| NM_001144964 | NEDD4L | 8436 | 7 | 3 | 1 | 2 | 2 | 5 | 7 | 1 | 5 | 1 | 2 | 1 | 1 |
| NM_001144965 | NEDD4L | 8388 | 7 | 3 | 1 | 3 | 2 | 5 | 7 | 1 | 5 | 1 | 2 | 1 | 1 |
| NM_001144966 | NEDD4L | 8300 | 8 | 3 | 1 | 3 | 2 | 5 | 8 | 1 | 5 | 1 | 2 | 1 | 1 |
| NM_001144967 | NEDD4L | 8473 | 7 | 3 | 1 | 2 | 2 | 5 | 7 | 1 | 5 | 1 | 2 | 1 | 1 |
| NM_001144968 | NEDD4L | 8378 | 7 | 3 | 1 | 3 | 2 | 5 | 7 | 1 | 5 | 1 | 2 | 1 | 1 |
| NM_001144969 | NEDD4L | 8318 | 8 | 3 | 1 | 3 | 2 | 5 | 8 | 1 | 5 | 1 | 2 | 1 | 1 |
| NM_001144970 | NEDD4L | 8346 | 7 | 3 | 1 | 3 | 2 | 5 | 7 | 1 | 5 | 1 | 2 | 1 | 1 |
| NM_001144971 | NEDD4L | 8406 | 7 | 3 | 1 | 3 | 2 | 5 | 7 | 1 | 5 | 1 | 2 | 1 | 1 |

| transcript | gene | transcript length (nt) | max | mean | adipose | colon | heart | hypothala mus | kidney | liver | lung | ovary | skeletalm uscle | spleen | testes |
|---|---|---|---|---|---|---|---|---|---|---|---|---|---|---|---|
| NM_001144972 | SNX20 | 2197 | 0 | 0 | 0 | 0 | 0 | 0 | 0 | 0 | 0 | 0 | 0 | 0 | 0 |
| NM_001144978 | MTHFD2L | 2357 | 3 | 1 | 0 | 0 | 1 | 1 | 0 | 1 | 0 | 1 | 1 | 1 | 3 |
| NM_001144981 | C13orf38 | 895 | 13 | 2 | 0 | 0 | 0 | 1 | 1 | 0 | 0 | 0 | 0 | 0 | 13 |
| NM_001144982 | C13orf38 | 938 | 10 | 1 | 0 | 0 | 0 | 1 | 1 | 0 | 0 | 0 | 0 | 0 | 10 |
| NM_001144983 | C13orf38 | 934 | 10 | 1 | 0 | 0 | 0 | 1 | 1 | 0 | 0 | 0 | 0 | 0 | 10 |
| NM_001144984 | C13orf38 | 897 | 10 | 1 | 0 | 0 | 0 | 1 | 1 | 0 | 0 | 0 | 0 | 0 | 10 |
| NM_001144985 | C13orf38 | 815 | 12 | 1 | 1 | 0 | 0 | 1 | 1 | 0 | 0 | 0 | 0 | 0 | 12 |
| NM_001144986 | C13orf38 | 811 | 12 | 1 | 1 | 0 | 0 | 1 | 1 | 0 | 0 | 0 | 0 | 0 | 12 |
| NM_001144989 | ZNF814 | 6283 | 4 | 2 | 1 | 2 | 1 | 1 | 2 | 1 | 1 | 2 | 1 | 4 | 3 |
| NM_001144990 | KIAA1239 | 7476 | 2 | 0 | 0 | 0 | 0 | 2 | 0 | 0 | 0 | 0 | 0 | 0 | 0 |
| NM_001144994 | C2orf72 | 3577 | 3 | 0 | 0 | 1 | 0 | 1 | 0 | 3 | 0 | 0 | 0 | 0 | 0 |
| NM_001144995 | CCDC85C | 5670 | 1 | 0 | 0 | 1 | 0 | 0 | 0 | 1 | 0 | 0 | 0 | 0 | 0 |
| NM_001144996 | ITGA7 | 4132 | 9 | 2 | 9 | 1 | 5 | 1 | 1 | 0 | 0 | 0 | 2 | 0 | 0 |
| NM_001144997 | ITGA7 | 3657 | 6 | 1 | 6 | 1 | 4 | 1 | 1 | 0 | 0 | 0 | 2 | 0 | 0 |
| NM_001144999 | ITGAV | 6747 | 19 | 9 | 9 | 2 | 19 | 11 | 15 | 3 | 9 | 10 | 4 | 10 | 9 |
| NM_001145 | ANG | 1220 | 51 | 5 | 1 | 1 | 1 | 0 | 1 | 51 | 1 | 2 | 0 | 0 | 2 |
| NM_001145000 | ITGAV | 6923 | 19 | 9 | 9 | 2 | 19 | 10 | 14 | 3 | 9 | 9 | 4 | 10 | 9 |
| NM_001145001 | NEK6 | 2796 | 11 | 5 | 6 | 5 | 3 | 7 | 7 | 11 | 4 | 3 | 2 | 6 | 3 |
| NM_001145004 | GOLGA6L6 | 4091 | 4 | 0 | 0 | 0 | 0 | 0 | 0 | 0 | 0 | 0 | 0 | 0 | 4 |
| NM_001145006 | MUC7 | 2540 | 0 | 0 | 0 | 0 | 0 | 0 | 0 | 0 | 0 | 0 | 0 | 0 | 0 |
| NM_001145007 | MUC7 | 2479 | 0 | 0 | 0 | 0 | 0 | 0 | 0 | 0 | 0 | 0 | 0 | 0 | 0 |
| NM_001145008 | BTN3A1 | 3276 | 32 | 9 | 6 | 6 | 6 | 3 | 5 | 2 | 19 | 16 | 4 | 32 | 6 |
| NM_001145009 | BTN3A1 | 4189 | 25 | 8 | 5 | 5 | 4 | 2 | 4 | 1 | 15 | 13 | 3 | 25 | 5 |
| NM_001145010 | C12orf70 | 1271 | 0 | 0 | 0 | 0 | 0 | 0 | 0 | 0 | 0 | 0 | 0 | 0 | 0 |
| NM_001145011 | LOC342346 | 3425 | 0 | 0 | 0 | 0 | 0 | 0 | 0 | 0 | 0 | 0 | 0 | 0 | 0 |
| NM_001145014 | RFPL4A | 906 | 0 | 0 | 0 | 0 | 0 | 0 | 0 | 0 | 0 | 0 | 0 | 0 | 0 |
| NM_001145017 | SLC36A3 | 2983 | 0 | 0 | 0 | 0 | 0 | 0 | 0 | 0 | 0 | 0 | 0 | 0 | 0 |
| NM_001145018 | CCDC153 | 766 | 1 | 0 | 1 | 0 | 0 | 0 | 0 | 0 | 1 | 0 | 0 | 0 | 0 |
| NM_001145020 | C6orf52 | 619 | 1 | 0 | 0 | 0 | 0 | 0 | 1 | 0 | 0 | 0 | 0 | 0 | 1 |
| NM_001145023 | SCRN2 | 1832 | 2 | 1 | 1 | 1 | 0 | 1 | 2 | 0 | 0 | 0 | 0 | 0 | 0 |
| NM_001145024 | C11orf34 | 1541 | 0 | 0 | 0 | 0 | 0 | 0 | 0 | 0 | 0 | 0 | 0 | 0 | 0 |
| NM_001145025 | EDEM2 | 1802 | 10 | 3 | 3 | 2 | 3 | 3 | 3 | 10 | 2 | 1 | 1 | 5 | 2 |
| NM_001145026 | PTPRQ | 7550 | 1 | 0 | 0 | 0 | 0 | 0 | 1 | 0 | 1 | 0 | 1 | 0 | 0 |
| NM_001145027 | SLFN12L | 2749 | 4 | 1 | 0 | 0 | 0 | 0 | 0 | 0 | 1 | 0 | 0 | 4 | 0 |
| NM_001145028 | PALM3 | 2258 | 41 | 9 | 0 | 1 | 0 | 5 | 41 | 25 | 7 | 0 | 0 | 0 | 14 |
| NM_001145029 | ANKRD30B | 4617 | 4 | 0 | 0 | 1 | 0 | 0 | 0 | 0 | 0 | 0 | 0 | 0 | 4 |
| NM_001145030 | C3orf77 | 5333 | 2 | 0 | 0 | 0 | 0 | 0 | 0 | 0 | 0 | 0 | 0 | 0 | 2 |
| NM_001145031 | PLAU | 2660 | 4 | 1 | 2 | 0 | 1 | 0 | 4 | 0 | 1 | 0 | 1 | 3 | 0 |
| NM_001145033 | LOC387763 | 1327 | 2 | 1 | 1 | 0 | 1 | 1 | 0 | 0 | 2 | 2 | 0 | 0 | 1 |
| NM_001145036 | COG2 | 2956 | 6 | 4 | 4 | 4 | 5 | 4 | 5 | 5 | 2 | 3 | 4 | 6 | 6 |
| NM_001145044 | SLCO3A1 | 2853 | 1 | 1 | 0 | 0 | 1 | 1 | 0 | 0 | 1 | 0 | 0 | 1 | 1 |
| NM_001145045 | ZNF286B | 5325 | 0 | 0 | 0 | 0 | 0 | 0 | 0 | 0 | 0 | 0 | 0 | 0 | 0 |
| NM_001145047 | GPN1 | 1793 | 7 | 4 | 5 | 2 | 4 | 7 | 5 | 4 | 3 | 3 | 4 | 5 | 4 |
| NM_001145048 | GPN1 | 1725 | 7 | 4 | 5 | 2 | 4 | 7 | 5 | 4 | 3 | 3 | 4 | 6 | 4 |
| NM_001145049 | GPN1 | 1618 | 7 | 5 | 6 | 2 | 4 | 7 | 6 | 5 | 3 | 3 | 4 | 6 | 5 |
| NM_001145051 | LOC388946 | 679 | 0 | 0 | 0 | 0 | 0 | 0 | 0 | 0 | 0 | 0 | 0 | 0 | 0 |
| NM_001145053 | EFCAB5 | 3743 | 4 | 1 | 0 | 0 | 0 | 0 | 0 | 0 | 0 | 0 | 0 | 0 | 4 |
| NM_001145054 | C2orf81 | 2229 | 0 | 0 | 0 | 0 | 0 | 0 | 0 | 0 | 0 | 0 | 0 | 0 | 0 |
| NM_001145055 | IL18BP | 1038 | 4 | 1 | 1 | 2 | 1 | 1 | 1 | 0 | 2 | 1 | 0 | 4 | 1 |
| NM_001145056 | SLC44A2 | 3374 | 25 | 9 | 6 | 6 | 6 | 8 | 9 | 3 | 12 | 3 | 5 | 25 | 21 |
| NM_001145057 | IL18BP | 1356 | 3 | 1 | 1 | 2 | 0 | 0 | 1 | 0 | 1 | 1 | 0 | 3 | 0 |
| NM_001145059 | IQCF5 | 511 | 1 | 0 | 0 | 0 | 0 | 0 | 0 | 0 | 0 | 0 | 0 | 0 | 1 |
| NM_001145063 | GATSL1 | 990 | 0 | 0 | 0 | 0 | 0 | 0 | 0 | 0 | 0 | 0 | 0 | 0 | 0 |
| NM_001145064 | GATSL2 | 990 | 0 | 0 | 0 | 0 | 0 | 0 | 0 | 0 | 0 | 0 | 0 | 0 | 0 |
| NM_001145065 | FAM190A | 6204 | 1 | 1 | 0 | 0 | 0 | 1 | 1 | 0 | 0 | 0 | 0 | 1 | 1 |
| NM_001145073 | USP27X | 2697 | 5 | 2 | 1 | 1 | 1 | 5 | 1 | 1 | 1 | 4 | 0 | 2 | 1 |
| NM_001145076 | EML4 | 5373 | 9 | 4 | 3 | 3 | 2 | 2 | 5 | 6 | 6 | 4 | 1 | 9 | 5 |
| NM_001145077 | LRRC10B | 2213 | 0 | 0 | 0 | 0 | 0 | 0 | 0 | 0 | 0 | 0 | 0 | 0 | 0 |
| NM_001145078 | ZNF805 | 9998 | 3 | 1 | 1 | 1 | 1 | 1 | 1 | 1 | 1 | 3 | 1 | 3 | 2 |
| NM_001145079 | COG6 | 5257 | 1 | 1 | 1 | 0 | 1 | 1 | 1 | 0 | 1 | 1 | 0 | 1 | 1 |
| NM_001145080 | C17orf104 | 4678 | 11 | 1 | 0 | 0 | 0 | 0 | 0 | 0 | 0 | 0 | 0 | 0 | 11 |
| NM_001145082 | ZNF619 | 2511 | 2 | 1 | 1 | 1 | 1 | 1 | 1 | 1 | 1 | 2 | 1 | 2 | 1 |
| NM_001145083 | ZNF619 | 2192 | 2 | 1 | 1 | 2 | 1 | 1 | 1 | 1 | 1 | 2 | 1 | 2 | 1 |
| NM_001145088 | WDR67 | 3214 | 2 | 1 | 1 | 0 | 1 | 1 | 1 | 0 | 1 | 1 | 1 | 2 | 1 |
| NM_001145093 | ZNF619 | 2346 | 2 | 1 | 1 | 2 | 1 | 1 | 1 | 1 | 1 | 2 | 1 | 2 | 1 |
| NM_001145094 | ZNF619 | 2417 | 2 | 1 | 1 | 2 | 1 | 1 | 1 | 1 | 1 | 2 | 1 | 2 | 1 |
| NM_001145095 | HHLA1 | 4105 | 0 | 0 | 0 | 0 | 0 | 0 | 0 | 0 | 0 | 0 | 0 | 0 | 0 |
| NM_001145097 | SKIL | 3369 | 12 | 8 | 6 | 3 | 8 | 10 | 8 | 6 | 12 | 11 | 6 | 9 | 10 |
| NM_001145098 | SKIL | 3019 | 13 | 9 | 7 | 3 | 9 | 11 | 9 | 7 | 13 | 12 | 7 | 10 | 11 |
| NM_001145099 | SLC2A6 | 2359 | 0 | 0 | 0 | 0 | 0 | 0 | 0 | 0 | 0 | 0 | 0 | 0 | 0 |
| NM_001145101 | BTBD18 | 3047 | 1 | 0 | 0 | 0 | 0 | 0 | 0 | 0 | 0 | 1 | 0 | 0 | 0 |
| NM_001145102 | SMAD3 | 5999 | 4 | 2 | 2 | 2 | 1 | 2 | 2 | 1 | 2 | 4 | 3 | 2 | 1 |
| NM_001145103 | SMAD3 | 5810 | 4 | 2 | 2 | 2 | 2 | 2 | 2 | 1 | 2 | 4 | 3 | 2 | 1 |
| NM_001145104 | SMAD3 | 5443 | 4 | 2 | 2 | 2 | 2 | 3 | 2 | 1 | 2 | 4 | 3 | 2 | 1 |
| NM_001145106 | FIBCD1 | 3118 | 0 | 0 | 0 | 0 | 0 | 0 | 0 | 0 | 0 | 0 | 0 | 0 | 0 |
| NM_001145107 | NELL2 | 3385 | 26 | 3 | 0 | 0 | 0 | 26 | 0 | 0 | 1 | 0 | 0 | 1 | 0 |
| NM_001145108 | NELL2 | 3705 | 24 | 2 | 0 | 0 | 0 | 24 | 0 | 0 | 1 | 0 | 0 | 1 | 0 |

| | | transcript | | | | | | hypothala | | | | | skeletalm | | |
|---|---|---|---|---|---|---|---|---|---|---|---|---|---|---|---|
| transcript | gene | length (nt) | max | mean | adipose | colon | heart | mus | kidney | liver | lung | ovary | uscle | spleen | testes |
| NM_001145109 | NELL2 | 3210 | 27 | 3 | 0 | 0 | 0 | 27 | 0 | 0 | 1 | 0 | 0 | 1 | 0 |
| NM_001145110 | NELL2 | 3271 | 27 | 3 | 0 | 0 | 0 | 27 | 0 | 0 | 1 | 0 | 0 | 1 | 0 |
| NM_001145112 | PATL2 | 1950 | 1 | 0 | 0 | 1 | 0 | 0 | 0 | 0 | 0 | 0 | 0 | 1 | 0 |
| NM_001145113 | MYADML2 | 2451 | 1 | 0 | 0 | 0 | 0 | 0 | 0 | 0 | 0 | 0 | 1 | 0 | 0 |
| NM_001145114 | RRP12 | 4242 | 3 | 2 | 1 | 1 | 2 | 2 | 1 | 1 | 1 | 1 | 3 | 2 | 2 |
| NM_001145115 | PPP1R3G | 1736 | 0 | 0 | 0 | 0 | 0 | 0 | 0 | 0 | 0 | 0 | 0 | 0 | 0 |
| NM_001145118 | GRID2IP | 4631 | 0 | 0 | 0 | 0 | 0 | 0 | 0 | 0 | 0 | 0 | 0 | 0 | 0 |
| NM_001145121 | TCP10L2 | 2184 | 0 | 0 | 0 | 0 | 0 | 0 | 0 | 0 | 0 | 0 | 0 | 0 | 0 |
| NM_001145122 | CAPN14 | 3820 | 0 | 0 | 0 | 0 | 0 | 0 | 0 | 0 | 0 | 0 | 0 | 0 | 0 |
| NM_001145123 | C7orf16 | 1644 | 0 | 0 | 0 | 0 | 0 | 0 | 0 | 0 | 0 | 0 | 0 | 0 | 0 |
| NM_001145124 | FAM75C1 | 3778 | 0 | 0 | 0 | 0 | 0 | 0 | 0 | 0 | 0 | 0 | 0 | 0 | 0 |
| NM_001145125 | MKRN1 | 1679 | 31 | 18 | 8 | 14 | 21 | 18 | 14 | 7 | 16 | 20 | 23 | 29 | 31 |
| NM_001145127 | EVPLL | 1989 | 0 | 0 | 0 | 0 | 0 | 0 | 0 | 0 | 0 | 0 | 0 | 0 | 0 |
| NM_001145128 | AKD1 | 6326 | 4 | 1 | 1 | 0 | 1 | 3 | 1 | 0 | 1 | 1 | 1 | 2 | 4 |
| NM_001145129 | ZNF389 | 1639 | 1 | 0 | 0 | 0 | 0 | 1 | 0 | 0 | 0 | 0 | 1 | 1 | 1 |
| NM_001145132 | C5orf52 | 695 | 3 | 0 | 0 | 0 | 0 | 0 | 0 | 0 | 0 | 0 | 0 | 0 | 3 |
| NM_001145134 | CPT1B | 2577 | 0 | 0 | 0 | 0 | 0 | 0 | 0 | 0 | 0 | 0 | 0 | 0 | 0 |
| NM_001145135 | CPT1B | 2679 | 0 | 0 | 0 | 0 | 0 | 0 | 0 | 0 | 0 | 0 | 0 | 0 | 0 |
| NM_001145136 | CPT1B | 2436 | 0 | 0 | 0 | 0 | 0 | 0 | 0 | 0 | 0 | 0 | 0 | 0 | 0 |
| NM_001145137 | CPT1B | 2703 | 0 | 0 | 0 | 0 | 0 | 0 | 0 | 0 | 0 | 0 | 0 | 0 | 0 |
| NM_001145138 | RELA | 2568 | 2 | 1 | 1 | 1 | 1 | 1 | 1 | 2 | 1 | 2 | 2 | 2 | 1 |
| NM_001145139 | CXorf49B | 1850 | 0 | 0 | 0 | 0 | 0 | 0 | 0 | 0 | 0 | 0 | 0 | 0 | 0 |
| NM_001145140 | CXorf49 | 1850 | 0 | 0 | 0 | 0 | 0 | 0 | 0 | 0 | 0 | 0 | 0 | 0 | 0 |
| NM_001145143 | HTR3D | 1608 | 0 | 0 | 0 | 0 | 0 | 0 | 0 | 0 | 0 | 0 | 0 | 0 | 0 |
| NM_001145144 | SLC1A5 | 1735 | 7 | 3 | 7 | 3 | 2 | 3 | 3 | 1 | 3 | 2 | 0 | 4 | 2 |
| NM_001145145 | SLC1A5 | 1925 | 6 | 3 | 6 | 3 | 2 | 3 | 3 | 1 | 2 | 2 | 0 | 4 | 2 |
| NM_001145146 | CRHR1 | 2663 | 0 | 0 | 0 | 0 | 0 | 0 | 0 | 0 | 0 | 0 | 0 | 0 | 0 |
| NM_001145147 | CRHR1 | 2456 | 0 | 0 | 0 | 0 | 0 | 0 | 0 | 0 | 0 | 0 | 0 | 0 | 0 |
| NM_001145148 | CRHR1 | 2534 | 0 | 0 | 0 | 0 | 0 | 0 | 0 | 0 | 0 | 0 | 0 | 0 | 0 |
| NM_001145149 | VAMP7 | 2531 | 4 | 3 | 3 | 1 | 3 | 3 | 4 | 4 | 3 | 2 | 1 | 4 | 3 |
| NM_001145152 | VPS37A | 4509 | 2 | 1 | 1 | 0 | 2 | 1 | 2 | 1 | 1 | 1 | 2 | 2 | 2 |
| NM_001145153 | TMEM71 | 1947 | 2 | 0 | 0 | 0 | 1 | 0 | 0 | 0 | 1 | 0 | 0 | 2 | 0 |
| NM_001145154 | DNAH14 | 1995 | 7 | 2 | 1 | 0 | 1 | 1 | 4 | 2 | 1 | 7 | 0 | 1 | 7 |
| NM_001145155 | NR2F2 | 3867 | 20 | 6 | 2 | 2 | 3 | 2 | 7 | 2 | 4 | 20 | 2 | 14 | 7 |
| NM_001145156 | NR2F2 | 3499 | 21 | 6 | 3 | 2 | 3 | 2 | 7 | 2 | 5 | 21 | 2 | 15 | 8 |
| NM_001145157 | NR2F2 | 3960 | 19 | 6 | 2 | 2 | 3 | 2 | 7 | 2 | 4 | 19 | 2 | 13 | 7 |
| NM_001145158 | HBS1L | 7030 | 4 | 2 | 2 | 1 | 3 | 3 | 2 | 4 | 1 | 2 | 4 | 2 | 2 |
| NM_001145159 | INTS9 | 2696 | 1 | 0 | 0 | 0 | 0 | 0 | 0 | 0 | 0 | 0 | 0 | 1 | 1 |
| NM_001145160 | TPM4 | 2630 | 153 | 58 | 53 | 35 | 73 | 32 | 36 | 10 | 100 | 73 | 12 | 153 | 57 |
| NM_001145161 | UBE2QL1 | 2000 | 3 | 1 | 0 | 0 | 1 | 3 | 0 | 0 | 0 | 0 | 2 | 0 | 0 |
| NM_001145165 | DOHH | 2031 | 4 | 2 | 1 | 1 | 1 | 4 | 2 | 2 | 1 | 1 | 1 | 1 | 1 |
| NM_001145166 | PRMT3 | 2545 | 2 | 1 | 1 | 1 | 2 | 2 | 2 | 1 | 1 | 2 | 1 | 2 | 1 |
| NM_001145167 | PRMT3 | 2595 | 2 | 1 | 1 | 1 | 2 | 2 | 2 | 1 | 1 | 2 | 1 | 2 | 1 |
| NM_001145168 | GPR113 | 3239 | 0 | 0 | 0 | 0 | 0 | 0 | 0 | 0 | 0 | 0 | 0 | 0 | 0 |
| NM_001145169 | GPR113 | 4396 | 0 | 0 | 0 | 0 | 0 | 0 | 0 | 0 | 0 | 0 | 0 | 0 | 0 |
| NM_001145176 | SHISA7 | 6035 | 1 | 0 | 0 | 0 | 0 | 1 | 0 | 0 | 0 | 0 | 0 | 0 | 0 |
| NM_001145191 | LOC285550 | 4377 | 4 | 2 | 3 | 1 | 2 | 4 | 3 | 1 | 2 | 3 | 1 | 3 | 3 |
| NM_001145194 | LOC642597 | 2302 | 1 | 0 | 0 | 0 | 0 | 1 | 0 | 0 | 0 | 0 | 0 | 0 | 0 |
| NM_001145195 | SLC39A12 | 2808 | 7 | 1 | 0 | 0 | 0 | 7 | 0 | 0 | 0 | 0 | 0 | 0 | 0 |
| NM_001145196 | FAM75A6 | 4212 | 0 | 0 | 0 | 0 | 0 | 0 | 0 | 0 | 0 | 0 | 0 | 0 | 0 |
| NM_001145197 | FLJ43859 | 4923 | 0 | 0 | 0 | 0 | 0 | 0 | 0 | 0 | 0 | 0 | 0 | 0 | 0 |
| NM_001145199 | C12orf75 | 1365 | 3 | 1 | 0 | 0 | 0 | 0 | 3 | 0 | 1 | 0 | 1 | 0 | 2 |
| NM_001145201 | PRAP1 | 750 | 8 | 1 | 0 | 1 | 0 | 0 | 2 | 8 | 0 | 0 | 0 | 0 | 0 |
| NM_001145204 | SHISA9 | 6726 | 4 | 0 | 0 | 0 | 0 | 4 | 0 | 0 | 0 | 0 | 0 | 0 | 0 |
| NM_001145205 | SHISA9 | 4180 | 1 | 0 | 0 | 0 | 0 | 1 | 0 | 0 | 0 | 0 | 0 | 0 | 0 |
| NM_001145206 | KIAA1671 | 10492 | 3 | 2 | 1 | 3 | 1 | 2 | 3 | 2 | 1 | 2 | 2 | 1 | 1 |
| NM_001145207 | HBS1L | 2807 | 3 | 3 | 2 | 1 | 3 | 3 | 3 | 3 | 2 | 3 | 3 | 2 | 3 |
| NM_001145208 | DEPDC1B | 2317 | 1 | 0 | 0 | 0 | 0 | 0 | 0 | 0 | 0 | 0 | 0 | 0 | 1 |
| NM_001145210 | LOC441869 | 2016 | 1 | 0 | 1 | 0 | 0 | 1 | 0 | 0 | 0 | 0 | 0 | 0 | 0 |
| NM_001145211 | SLCO2B1 | 4038 | 10 | 3 | 2 | 1 | 2 | 4 | 2 | 10 | 5 | 1 | 1 | 7 | 1 |
| NM_001145212 | SLCO2B1 | 3904 | 8 | 3 | 2 | 1 | 2 | 4 | 2 | 8 | 4 | 1 | 1 | 6 | 1 |
| NM_001145224 | GOLGA6D | 3447 | 0 | 0 | 0 | 0 | 0 | 0 | 0 | 0 | 0 | 0 | 0 | 0 | 0 |
| NM_001145248 | FAM157A | 1275 | 0 | 0 | 0 | 0 | 0 | 0 | 0 | 0 | 0 | 0 | 0 | 0 | 0 |
| NM_001145249 | FAM157B | 1278 | 0 | 0 | 0 | 0 | 0 | 0 | 0 | 0 | 0 | 0 | 0 | 0 | 0 |
| NM_001145250 | SP9 | 1455 | 0 | 0 | 0 | 0 | 0 | 0 | 0 | 0 | 0 | 0 | 0 | 0 | 0 |
| NM_001145251 | SDR16C6 | 972 | 1 | 0 | 0 | 0 | 0 | 0 | 0 | 0 | 0 | 0 | 0 | 0 | 1 |
| NM_001145252 | CFP | 1597 | 1 | 0 | 0 | 0 | 0 | 0 | 0 | 0 | 0 | 0 | 0 | 1 | 0 |
| NM_001145255 | IKBKG | 1961 | 1 | 0 | 0 | 0 | 0 | 0 | 0 | 1 | 0 | 0 | 0 | 1 | 0 |
| NM_001145256 | IRAK4 | 4195 | 2 | 1 | 1 | 0 | 1 | 1 | 1 | 1 | 1 | 1 | 0 | 2 | 1 |
| NM_001145257 | IRAK4 | 4147 | 2 | 1 | 1 | 0 | 1 | 1 | 1 | 1 | 1 | 1 | 0 | 2 | 1 |
| NM_001145258 | IRAK4 | 3977 | 2 | 1 | 1 | 0 | 1 | 1 | 1 | 1 | 1 | 1 | 0 | 2 | 1 |
| NM_001145260 | NCOA4 | 3737 | 36 | 17 | 21 | 11 | 18 | 14 | 17 | 36 | 12 | 8 | 11 | 32 | 10 |
| NM_001145261 | NCOA4 | 3648 | 37 | 18 | 22 | 11 | 19 | 14 | 17 | 37 | 12 | 9 | 11 | 33 | 10 |
| NM_001145262 | NCOA4 | 3577 | 38 | 18 | 22 | 11 | 19 | 14 | 18 | 38 | 13 | 9 | 12 | 34 | 11 |
| NM_001145263 | NCOA4 | 3551 | 38 | 18 | 22 | 11 | 19 | 14 | 18 | 38 | 13 | 9 | 12 | 34 | 11 |
| NM_001145264 | ROBLD3 | 591 | 30 | 10 | 12 | 5 | 9 | 13 | 7 | 30 | 4 | 3 | 6 | 12 | 3 |
| NM_001145265 | SLC35C1 | 3213 | 2 | 0 | 0 | 1 | 0 | 0 | 0 | 2 | 0 | 0 | 0 | 0 | 0 |

| | | transcript | | | | | | hypothala | | | | | skeletalm | | |
|---|---|---|---|---|---|---|---|---|---|---|---|---|---|---|---|
| transcript | gene | length (nt) | max | mean | adipose | colon | heart | mus | kidney | liver | lung | ovary | uscle | spleen | testes |
| NM_001145266 | SLC35C1 | 3116 | 1 | 0 | 0 | 1 | 0 | 0 | 0 | 1 | 0 | 0 | 0 | 0 | 0 |
| NM_001145268 | FAM185A | 2257 | 7 | 4 | 3 | 2 | 3 | 6 | 4 | 3 | 2 | 7 | 4 | 5 | 3 |
| NM_001145269 | FAM185A | 1906 | 8 | 4 | 3 | 2 | 3 | 7 | 4 | 3 | 2 | 8 | 5 | 5 | 3 |
| NM_001145271 | ADAMDEC1 | 2438 | 42 | 5 | 0 | 42 | 0 | 0 | 0 | 0 | 3 | 0 | 0 | 5 | 0 |
| NM_001145272 | ADAMDEC1 | 2229 | 18 | 2 | 0 | 18 | 0 | 0 | 0 | 0 | 1 | 0 | 0 | 2 | 0 |
| NM_001145275 | ZFY | 4997 | 2 | 1 | 1 | 0 | 1 | 1 | 0 | 1 | 1 | 0 | 0 | 2 | 1 |
| NM_001145276 | ZFY | 4749 | 3 | 1 | 2 | 0 | 2 | 2 | 1 | 2 | 1 | 0 | 1 | 3 | 3 |
| NM_001145277 | NECAP2 | 2003 | 6 | 2 | 2 | 1 | 2 | 1 | 2 | 1 | 2 | 2 | 0 | 6 | 3 |
| NM_001145278 | NECAP2 | 2001 | 6 | 2 | 2 | 1 | 2 | 1 | 2 | 1 | 2 | 2 | 0 | 6 | 3 |
| NM_001145279 | OPRM1 | 2738 | 0 | 0 | 0 | 0 | 0 | 0 | 0 | 0 | 0 | 0 | 0 | 0 | 0 |
| NM_001145280 | OPRM1 | 2169 | 1 | 0 | 0 | 0 | 0 | 1 | 0 | 0 | 0 | 0 | 0 | 0 | 0 |
| NM_001145281 | OPRM1 | 1710 | 1 | 0 | 0 | 0 | 0 | 1 | 0 | 0 | 0 | 0 | 0 | 0 | 0 |
| NM_001145282 | OPRM1 | 1500 | 1 | 0 | 0 | 0 | 0 | 1 | 0 | 0 | 0 | 0 | 0 | 0 | 0 |
| NM_001145283 | OPRM1 | 2404 | 0 | 0 | 0 | 0 | 0 | 0 | 0 | 0 | 0 | 0 | 0 | 0 | 0 |
| NM_001145284 | OPRM1 | 2569 | 0 | 0 | 0 | 0 | 0 | 0 | 0 | 0 | 0 | 0 | 0 | 0 | 0 |
| NM_001145285 | OPRM1 | 1438 | 1 | 0 | 0 | 0 | 0 | 1 | 0 | 0 | 0 | 0 | 0 | 0 | 0 |
| NM_001145286 | OPRM1 | 1589 | 1 | 0 | 0 | 0 | 0 | 1 | 0 | 0 | 0 | 0 | 0 | 0 | 0 |
| NM_001145287 | OPRM1 | 2949 | 0 | 0 | 0 | 0 | 0 | 0 | 0 | 0 | 0 | 0 | 0 | 0 | 0 |
| NM_001145288 | SLC17A8 | 3832 | 0 | 0 | 0 | 0 | 0 | 0 | 0 | 0 | 0 | 0 | 0 | 0 | 0 |
| NM_001145289 | AKR7L | 2115 | 2 | 1 | 0 | 1 | 0 | 0 | 2 | 1 | 0 | 0 | 0 | 0 | 0 |
| NM_001145290 | SLC37A2 | 4138 | 2 | 1 | 1 | 1 | 0 | 0 | 0 | 0 | 1 | 0 | 0 | 2 | 0 |
| NM_001145291 | PDE6B | 3399 | 2 | 1 | 0 | 0 | 0 | 2 | 1 | 0 | 0 | 0 | 0 | 1 | 0 |
| NM_001145292 | PDE6B | 2781 | 3 | 1 | 0 | 0 | 0 | 3 | 1 | 0 | 1 | 0 | 0 | 1 | 0 |
| NM_001145293 | NGLY1 | 2534 | 6 | 4 | 3 | 2 | 4 | 3 | 3 | 4 | 2 | 2 | 4 | 5 | 6 |
| NM_001145294 | NGLY1 | 2528 | 6 | 3 | 2 | 2 | 3 | 3 | 3 | 4 | 2 | 2 | 4 | 4 | 6 |
| NM_001145295 | NGLY1 | 2410 | 6 | 3 | 3 | 2 | 3 | 3 | 3 | 4 | 2 | 2 | 5 | 4 | 6 |
| NM_001145297 | EXOC7 | 4945 | 7 | 5 | 5 | 4 | 5 | 7 | 5 | 4 | 3 | 5 | 2 | 5 | 4 |
| NM_001145298 | EXOC7 | 4768 | 7 | 5 | 5 | 4 | 5 | 7 | 5 | 4 | 3 | 5 | 3 | 6 | 4 |
| NM_001145299 | EXOC7 | 4861 | 7 | 5 | 5 | 4 | 5 | 7 | 5 | 4 | 3 | 5 | 3 | 5 | 4 |
| NM_001145301 | RARA | 3371 | 1 | 1 | 0 | 0 | 1 | 0 | 1 | 1 | 1 | 1 | 1 | 1 | 0 |
| NM_001145302 | RARA | 3080 | 1 | 1 | 0 | 0 | 1 | 0 | 1 | 1 | 1 | 1 | 0 | 1 | 0 |
| NM_001145303 | TMC4 | 2478 | 1 | 0 | 0 | 1 | 0 | 0 | 1 | 0 | 1 | 0 | 0 | 0 | 0 |
| NM_001145304 | KIAA1683 | 4403 | 3 | 0 | 0 | 0 | 0 | 0 | 0 | 0 | 0 | 3 | 0 | 0 | 1 |
| NM_001145305 | KIAA1683 | 3881 | 3 | 1 | 0 | 0 | 0 | 0 | 0 | 0 | 0 | 3 | 0 | 0 | 2 |
| NM_001145306 | CDK6 | 11715 | 2 | 1 | 1 | 1 | 2 | 1 | 2 | 1 | 2 | 1 | 0 | 2 | 1 |
| NM_001145307 | LRTOMT | 983 | 3 | 1 | 1 | 0 | 0 | 2 | 1 | 1 | 1 | 1 | 0 | 0 | 3 |
| NM_001145308 | LRTOMT | 3599 | 1 | 0 | 0 | 0 | 0 | 0 | 0 | 0 | 0 | 0 | 0 | 0 | 1 |
| NM_001145309 | LRTOMT | 3820 | 1 | 0 | 0 | 0 | 0 | 0 | 0 | 0 | 0 | 0 | 0 | 0 | 1 |
| NM_001145310 | LRTOMT | 3704 | 1 | 0 | 0 | 0 | 0 | 0 | 0 | 0 | 0 | 0 | 0 | 0 | 1 |
| NM_001145311 | PLIN1 | 2894 | 200 | 20 | 200 | 8 | 3 | 0 | 0 | 1 | 0 | 0 | 2 | 0 | 1 |
| NM_001145312 | ETV3 | 2002 | 1 | 1 | 1 | 1 | 1 | 1 | 0 | 0 | 1 | 1 | 0 | 1 | 1 |
| NM_001145313 | FSD1L | 2099 | 12 | 3 | 2 | 1 | 2 | 12 | 2 | 0 | 2 | 1 | 8 | 2 | 4 |
| NM_001145314 | KIAA1712 | 3290 | 1 | 1 | 0 | 0 | 1 | 1 | 1 | 0 | 0 | 1 | 1 | 1 | 1 |
| NM_001145315 | DSN1 | 2248 | 2 | 1 | 1 | 1 | 0 | 1 | 0 | 0 | 1 | 0 | 0 | 1 | 2 |
| NM_001145316 | DSN1 | 2440 | 2 | 1 | 1 | 0 | 0 | 1 | 0 | 0 | 1 | 0 | 0 | 1 | 2 |
| NM_001145317 | DSN1 | 1927 | 3 | 1 | 1 | 1 | 0 | 1 | 0 | 0 | 1 | 0 | 0 | 1 | 3 |
| NM_001145318 | DSN1 | 2199 | 1 | 0 | 1 | 0 | 0 | 1 | 0 | 0 | 0 | 0 | 0 | 1 | 1 |
| NM_001145319 | PLS1 | 3703 | 3 | 1 | 0 | 3 | 0 | 1 | 3 | 2 | 1 | 0 | 0 | 0 | 0 |
| NM_001145320 | ADAMTSL2 | 4067 | 1 | 0 | 0 | 0 | 0 | 0 | 0 | 1 | 0 | 0 | 0 | 0 | 0 |
| NM_001145335 | UBE2Q2 | 2979 | 7 | 4 | 5 | 2 | 6 | 5 | 4 | 1 | 5 | 5 | 3 | 6 | 7 |
| NM_001145337 | MDM2 | 6991 | 11 | 6 | 5 | 2 | 5 | 4 | 5 | 8 | 8 | 7 | 11 | 10 | 6 |
| NM_001145338 | ZBTB22 | 2645 | 1 | 1 | 0 | 1 | 1 | 1 | 1 | 0 | 1 | 1 | 0 | 1 | 1 |
| NM_001145339 | MDM2 | 7198 | 11 | 6 | 5 | 2 | 5 | 4 | 5 | 7 | 8 | 7 | 11 | 10 | 5 |
| NM_001145340 | MDM2 | 6544 | 8 | 5 | 4 | 2 | 4 | 3 | 4 | 6 | 6 | 6 | 8 | 8 | 4 |
| NM_001145343 | ZNF566 | 5252 | 3 | 1 | 1 | 1 | 1 | 1 | 1 | 1 | 1 | 3 | 1 | 2 | 1 |
| NM_001145344 | ZNF566 | 5249 | 3 | 1 | 1 | 1 | 1 | 1 | 1 | 1 | 1 | 3 | 1 | 2 | 1 |
| NM_001145345 | ZNF566 | 5261 | 3 | 1 | 1 | 1 | 1 | 1 | 1 | 1 | 1 | 3 | 1 | 2 | 1 |
| NM_001145346 | RBMXL3 | 3469 | 0 | 0 | 0 | 0 | 0 | 0 | 0 | 0 | 0 | 0 | 0 | 0 | 0 |
| NM_001145347 | ZNF576 | 1863 | 2 | 1 | 1 | 1 | 1 | 2 | 1 | 2 | 1 | 1 | 1 | 1 | 1 |
| NM_001145348 | JMJD5 | 2521 | 4 | 1 | 0 | 0 | 0 | 0 | 0 | 4 | 0 | 0 | 0 | 1 | 0 |
| NM_001145349 | IPP | 2106 | 1 | 0 | 0 | 0 | 0 | 1 | 1 | 0 | 0 | 1 | 1 | 1 | 0 |
| NM_001145350 | C20orf173 | 1059 | 0 | 0 | 0 | 0 | 0 | 0 | 0 | 0 | 0 | 0 | 0 | 0 | 0 |
| NM_001145353 | ELF1 | 3521 | 46 | 15 | 9 | 5 | 15 | 4 | 11 | 7 | 23 | 15 | 10 | 46 | 15 |
| NM_001145354 | MKLN1 | 11254 | 8 | 5 | 4 | 2 | 4 | 5 | 6 | 4 | 5 | 7 | 4 | 6 | 8 |
| NM_001145355 | CBWD1 | 1820 | 2 | 1 | 1 | 0 | 1 | 1 | 2 | 1 | 1 | 1 | 0 | 1 | 1 |
| NM_001145356 | CBWD1 | 1665 | 2 | 1 | 1 | 0 | 1 | 1 | 2 | 1 | 1 | 1 | 0 | 1 | 1 |
| NM_001145357 | SIN3A | 6602 | 9 | 5 | 3 | 3 | 4 | 5 | 4 | 3 | 5 | 6 | 4 | 7 | 9 |
| NM_001145358 | SIN3A | 6777 | 9 | 5 | 3 | 3 | 4 | 5 | 4 | 3 | 5 | 6 | 4 | 7 | 9 |
| NM_001145363 | ABHD11 | 779 | 1 | 0 | 0 | 1 | 0 | 0 | 0 | 0 | 0 | 0 | 0 | 0 | 0 |
| NM_001145364 | ABHD11 | 1296 | 1 | 0 | 0 | 1 | 0 | 0 | 1 | 0 | 0 | 0 | 0 | 0 | 0 |
| NM_001145365 | ZNF652 | 11491 | 7 | 5 | 4 | 4 | 3 | 5 | 4 | 5 | 5 | 7 | 3 | 7 | 6 |
| NM_001145368 | PTPN3 | 6581 | 6 | 2 | 1 | 1 | 3 | 1 | 6 | 4 | 1 | 0 | 5 | 0 | 1 |
| NM_001145369 | PTPN3 | 8765 | 5 | 2 | 1 | 1 | 2 | 1 | 5 | 3 | 1 | 0 | 4 | 0 | 1 |
| NM_001145370 | PTPN3 | 8630 | 5 | 2 | 1 | 1 | 2 | 1 | 5 | 3 | 1 | 0 | 4 | 0 | 1 |
| NM_001145371 | PTPN3 | 6199 | 6 | 2 | 1 | 1 | 3 | 1 | 6 | 4 | 1 | 0 | 5 | 0 | 1 |
| NM_001145372 | PTPN3 | 6064 | 6 | 2 | 1 | 1 | 3 | 1 | 6 | 4 | 1 | 0 | 6 | 0 | 1 |
| NM_001145373 | OTUD1 | 3111 | 8 | 2 | 2 | 1 | 8 | 1 | 2 | 0 | 3 | 2 | 3 | 3 | 2 |

| transcript | gene | transcript length (nt) | max | mean | adipose | colon | heart | hypothalamus | kidney | liver | lung | ovary | skeletalmuscle | spleen | testes |
|---|---|---|---|---|---|---|---|---|---|---|---|---|---|---|---|
| NM_001145374 | ALKBH2 | 1197 | 3 | 1 | 1 | 1 | 1 | 2 | 1 | 3 | 1 | 1 | 1 | 2 | 1 |
| NM_001145375 | ALKBH2 | 944 | 3 | 1 | 1 | 1 | 1 | 2 | 1 | 3 | 1 | 1 | 1 | 2 | 1 |
| NM_001145391 | OXSM | 1277 | 2 | 1 | 0 | 1 | 0 | 0 | 1 | 2 | 0 | 0 | 0 | 1 | 1 |
| NM_001145392 | TSEN2 | 2132 | 3 | 2 | 1 | 3 | 2 | 3 | 2 | 1 | 1 | 3 | 2 | 2 | 2 |
| NM_001145393 | TSEN2 | 2022 | 3 | 2 | 1 | 3 | 2 | 3 | 2 | 1 | 1 | 3 | 2 | 2 | 2 |
| NM_001145394 | TSEN2 | 1923 | 3 | 2 | 1 | 3 | 2 | 3 | 2 | 1 | 1 | 3 | 2 | 1 | 2 |
| NM_001145395 | TSEN2 | 2696 | 3 | 1 | 1 | 3 | 1 | 2 | 2 | 1 | 1 | 2 | 1 | 1 | 1 |
| NM_001145396 | ALDH16A1 | 2952 | 0 | 0 | 0 | 0 | 0 | 0 | 0 | 0 | 0 | 0 | 0 | 0 | 0 |
| NM_001145398 | TEF | 4331 | 3 | 1 | 1 | 1 | 1 | 3 | 1 | 1 | 1 | 2 | 1 | 1 | 1 |
| NM_001145399 | MPPED2 | 5693 | 1 | 0 | 0 | 0 | 0 | 1 | 1 | 0 | 0 | 0 | 0 | 0 | 0 |
| NM_001145400 | ADAD2 | 2058 | 0 | 0 | 0 | 0 | 0 | 0 | 0 | 0 | 0 | 0 | 0 | 0 | 0 |
| NM_001145402 | FAM71E2 | 3182 | 0 | 0 | 0 | 0 | 0 | 0 | 0 | 0 | 0 | 0 | 0 | 0 | 0 |
| NM_001145404 | ZNF506 | 3228 | 27 | 10 | 5 | 3 | 8 | 7 | 12 | 1 | 8 | 27 | 5 | 19 | 11 |
| NM_001145408 | NONO | 3216 | 28 | 14 | 11 | 11 | 12 | 11 | 15 | 13 | 13 | 28 | 9 | 20 | 15 |
| NM_001145409 | NONO | 3045 | 30 | 15 | 11 | 11 | 13 | 12 | 16 | 13 | 13 | 30 | 9 | 21 | 16 |
| NM_001145410 | NONO | 2882 | 31 | 16 | 12 | 12 | 14 | 12 | 17 | 14 | 14 | 31 | 10 | 22 | 17 |
| NM_001145411 | CCDC110 | 2787 | 10 | 1 | 0 | 0 | 0 | 1 | 0 | 0 | 0 | 1 | 1 | 0 | 10 |
| NM_001145412 | NFE2L2 | 2739 | 18 | 10 | 6 | 4 | 10 | 7 | 11 | 8 | 10 | 18 | 8 | 11 | 11 |
| NM_001145413 | NFE2L2 | 2718 | 19 | 10 | 6 | 4 | 10 | 8 | 11 | 8 | 10 | 19 | 8 | 11 | 12 |
| NM_001145414 | CHM | 2834 | 2 | 1 | 1 | 1 | 1 | 2 | 1 | 1 | 1 | 1 | 0 | 1 | 1 |
| NM_001145415 | SETDB1 | 4446 | 6 | 3 | 2 | 3 | 2 | 2 | 3 | 2 | 2 | 4 | 2 | 4 | 6 |
| NM_001145416 | TMEM205 | 797 | 6 | 2 | 1 | 1 | 3 | 5 | 4 | 6 | 1 | 1 | 2 | 1 | 1 |
| NM_001145417 | MSL2 | 3895 | 13 | 6 | 3 | 3 | 5 | 4 | 4 | 2 | 4 | 13 | 6 | 12 | 6 |
| NM_001145418 | TTC28 | 11793 | 4 | 2 | 2 | 1 | 1 | 1 | 2 | 1 | 2 | 4 | 1 | 1 | 2 |
| NM_001145425 | NR1D2 | 5057 | 18 | 8 | 8 | 3 | 4 | 10 | 5 | 6 | 5 | 18 | 13 | 6 | 7 |
| NM_001145426 | CSDA | 1765 | 1,757 | 255 | 62 | 31 | 530 | 31 | 36 | 38 | 80 | 42 | 1,757 | 73 | 129 |
| NM_001145427 | SNX18 | 5375 | 18 | 7 | 3 | 1 | 9 | 3 | 6 | 3 | 7 | 16 | 3 | 18 | 10 |
| NM_001145432 | C4orf52 | 1013 | 9 | 6 | 5 | 2 | 8 | 5 | 8 | 7 | 4 | 6 | 8 | 4 | 9 |
| NM_001145434 | ZNF880 | 2260 | 26 | 11 | 6 | 2 | 12 | 12 | 14 | 2 | 10 | 26 | 10 | 12 | 14 |
| NM_001145436 | LSS | 4225 | 1 | 1 | 1 | 1 | 1 | 1 | 1 | 1 | 1 | 1 | 0 | 0 | 1 |
| NM_001145437 | LSS | 4390 | 1 | 1 | 1 | 1 | 1 | 1 | 1 | 1 | 1 | 1 | 0 | 0 | 1 |
| NM_001145438 | PGAP2 | 1841 | 1 | 1 | 0 | 1 | 1 | 0 | 1 | 1 | 1 | 0 | 0 | 1 | 1 |
| NM_001145439 | PGAP2 | 1595 | 1 | 1 | 0 | 1 | 1 | 0 | 1 | 1 | 1 | 0 | 0 | 1 | 1 |
| NM_001145440 | TYW1B | 3157 | 0 | 0 | 0 | 0 | 0 | 0 | 0 | 0 | 0 | 0 | 0 | 0 | 0 |
| NM_001145441 | TYW1B | 1604 | 0 | 0 | 0 | 0 | 0 | 0 | 0 | 0 | 0 | 0 | 0 | 0 | 0 |
| NM_001145442 | P704P | 6666 | 3 | 0 | 0 | 0 | 0 | 0 | 0 | 0 | 1 | 0 | 0 | 0 | 3 |
| NM_001145443 | PFKFB3 | 4221 | 30 | 9 | 30 | 2 | 2 | 11 | 6 | 4 | 7 | 1 | 25 | 9 | 3 |
| NM_001145444 | SFRS15 | 4224 | 12 | 7 | 5 | 4 | 6 | 7 | 6 | 6 | 6 | 8 | 6 | 12 | 11 |
| NM_001145445 | SFRS15 | 4203 | 12 | 7 | 5 | 4 | 6 | 7 | 6 | 6 | 6 | 9 | 6 | 12 | 11 |
| NM_001145446 | ZNF200 | 3179 | 3 | 1 | 1 | 1 | 1 | 1 | 1 | 1 | 1 | 1 | 0 | 2 | 3 |
| NM_001145447 | ZNF200 | 3384 | 2 | 1 | 1 | 0 | 1 | 1 | 1 | 1 | 1 | 1 | 0 | 2 | 2 |
| NM_001145448 | ZNF200 | 3303 | 3 | 1 | 1 | 0 | 1 | 1 | 1 | 1 | 1 | 1 | 0 | 2 | 3 |
| NM_001145450 | MORN2 | 739 | 19 | 6 | 4 | 3 | 8 | 10 | 11 | 2 | 5 | 4 | 1 | 3 | 19 |
| NM_001145451 | LOC100271 | 4996 | 0 | 0 | 0 | 0 | 0 | 0 | 0 | 0 | 0 | 0 | 0 | 0 | 0 |
| NM_001145453 | GFRA1 | 9216 | 5 | 2 | 1 | 1 | 1 | 2 | 1 | 5 | 1 | 1 | 1 | 1 | 1 |
| NM_001145454 | STMN1 | 2237 | 515 | 91 | 50 | 34 | 29 | 515 | 23 | 4 | 34 | 96 | 8 | 40 | 168 |
| NM_001145457 | NCR1 | 1150 | 1 | 0 | 0 | 0 | 0 | 0 | 0 | 0 | 0 | 0 | 0 | 1 | 0 |
| NM_001145458 | NCR1 | 1102 | 0 | 0 | 0 | 0 | 0 | 0 | 0 | 0 | 0 | 0 | 0 | 0 | 0 |
| NM_001145459 | HOPX | 1189 | 15 | 3 | 0 | 0 | 3 | 6 | 0 | 0 | 15 | 0 | 0 | 1 | 3 |
| NM_001145460 | HOPX | 1673 | 11 | 2 | 0 | 0 | 2 | 5 | 0 | 0 | 11 | 0 | 0 | 0 | 2 |
| NM_001145461 | YIF1B | 2762 | 1 | 1 | 0 | 0 | 1 | 1 | 1 | 1 | 1 | 0 | 0 | 1 | 0 |
| NM_001145462 | YIF1B | 2738 | 1 | 1 | 0 | 0 | 1 | 1 | 1 | 1 | 1 | 0 | 0 | 1 | 0 |
| NM_001145463 | YIF1B | 2854 | 1 | 1 | 0 | 0 | 1 | 1 | 0 | 1 | 1 | 0 | 0 | 1 | 0 |
| NM_001145464 | EXOG | 2996 | 2 | 1 | 1 | 1 | 2 | 2 | 1 | 0 | 1 | 1 | 0 | 2 | 1 |
| NM_001145465 | LOC360030 | 907 | 0 | 0 | 0 | 0 | 0 | 0 | 0 | 0 | 0 | 0 | 0 | 0 | 0 |
| NM_001145466 | NCR3 | 1109 | 0 | 0 | 0 | 0 | 0 | 0 | 0 | 0 | 0 | 0 | 0 | 0 | 0 |
| NM_001145467 | NCR3 | 875 | 0 | 0 | 0 | 0 | 0 | 0 | 0 | 0 | 0 | 0 | 0 | 0 | 0 |
| NM_001145468 | CYTSA | 6673 | 11 | 7 | 5 | 4 | 7 | 8 | 6 | 4 | 6 | 8 | 5 | 8 | 11 |
| NM_001145472 | LOXHD1 | 3945 | 1 | 0 | 0 | 0 | 0 | 0 | 0 | 0 | 0 | 0 | 0 | 0 | 1 |
| NM_001145473 | LOXHD1 | 1956 | 1 | 0 | 0 | 0 | 0 | 0 | 0 | 0 | 0 | 0 | 0 | 0 | 1 |
| NM_001145474 | C1orf223 | 849 | 5 | 0 | 0 | 0 | 0 | 0 | 0 | 0 | 0 | 0 | 0 | 0 | 5 |
| NM_001145475 | FAM186A | 7265 | 1 | 0 | 0 | 0 | 0 | 0 | 0 | 0 | 0 | 0 | 0 | 0 | 1 |
| NM_001145511 | NFIA | 9325 | 14 | 9 | 9 | 3 | 13 | 8 | 7 | 8 | 6 | 14 | 7 | 8 | 12 |
| NM_001145512 | NFIA | 9214 | 14 | 9 | 10 | 3 | 14 | 8 | 7 | 8 | 6 | 14 | 7 | 9 | 12 |
| NM_001145513 | SCRN1 | 5275 | 29 | 6 | 4 | 2 | 4 | 29 | 5 | 0 | 3 | 5 | 1 | 4 | 8 |
| NM_001145514 | SCRN1 | 5207 | 30 | 6 | 4 | 2 | 4 | 30 | 5 | 0 | 3 | 5 | 1 | 4 | 8 |
| NM_001145515 | SCRN1 | 5107 | 29 | 6 | 4 | 2 | 4 | 29 | 5 | 0 | 3 | 5 | 1 | 4 | 8 |
| NM_001145520 | RAI14 | 5043 | 36 | 9 | 3 | 2 | 7 | 4 | 9 | 6 | 10 | 6 | 1 | 36 | 11 |
| NM_001145521 | RAI14 | 5156 | 35 | 8 | 3 | 2 | 7 | 4 | 9 | 6 | 10 | 6 | 1 | 35 | 11 |
| NM_001145522 | RAI14 | 4898 | 36 | 9 | 3 | 2 | 7 | 4 | 9 | 6 | 10 | 6 | 1 | 36 | 11 |
| NM_001145523 | RAI14 | 4917 | 31 | 7 | 3 | 2 | 6 | 4 | 8 | 6 | 8 | 5 | 1 | 31 | 9 |
| NM_001145524 | YPEL3 | 939 | 21 | 10 | 6 | 4 | 8 | 20 | 4 | 2 | 10 | 12 | 21 | 15 | 4 |
| NM_001145525 | RAI14 | 5337 | 34 | 8 | 3 | 2 | 7 | 4 | 8 | 6 | 9 | 6 | 1 | 34 | 10 |
| NM_001145526 | AFMID | 1700 | 17 | 4 | 2 | 1 | 1 | 4 | 7 | 17 | 2 | 1 | 0 | 2 | 1 |
| NM_001145527 | EHMT1 | 2706 | 6 | 4 | 3 | 5 | 2 | 3 | 3 | 2 | 3 | 4 | 3 | 6 | 6 |
| NM_001145529 | LOC283999 | 2271 | 1 | 0 | 0 | 0 | 0 | 1 | 0 | 0 | 0 | 0 | 0 | 0 | 0 |
| NM_001145531 | C7orf71 | 1990 | 0 | 0 | 0 | 0 | 0 | 0 | 0 | 0 | 0 | 0 | 0 | 0 | 0 |

| transcript | gene | transcript length (nt) | max | mean | adipose | colon | heart | hypothalamus | kidney | liver | lung | ovary | skeletalmuscle | spleen | testes |
|---|---|---|---|---|---|---|---|---|---|---|---|---|---|---|---|
| NM_001145536 | C17orf107 | 3082 | 0 | 0 | 0 | 0 | 0 | 0 | 0 | 0 | 0 | 0 | 0 | 0 | 0 |
| NM_001145541 | TCP11L1 | 2632 | 4 | 1 | 1 | 2 | 1 | 4 | 1 | 1 | 1 | 1 | 1 | 2 | 1 |
| NM_001145542 | ZSCAN18 | 2573 | 8 | 2 | 2 | 1 | 2 | 8 | 2 | 0 | 1 | 3 | 1 | 2 | 2 |
| NM_001145543 | ZSCAN18 | 2684 | 8 | 2 | 2 | 1 | 2 | 8 | 2 | 0 | 1 | 3 | 1 | 2 | 2 |
| NM_001145544 | ZSCAN18 | 2218 | 10 | 2 | 2 | 1 | 2 | 10 | 2 | 0 | 1 | 3 | 1 | 2 | 3 |
| NM_001145545 | C16orf82 | 2268 | 10 | 1 | 0 | 0 | 0 | 0 | 0 | 0 | 0 | 0 | 0 | 0 | 10 |
| NM_001145546 | GALP | 889 | 0 | 0 | 0 | 0 | 0 | 0 | 0 | 0 | 0 | 0 | 0 | 0 | 0 |
| NM_001145547 | RBM17 | 3751 | 39 | 25 | 26 | 16 | 31 | 27 | 19 | 18 | 26 | 20 | 26 | 39 | 28 |
| NM_001145548 | ZDHHC7 | 3273 | 12 | 8 | 9 | 10 | 10 | 7 | 8 | 5 | 8 | 10 | 8 | 12 | 5 |
| NM_001145549 | TXNDC5 | 2741 | 20 | 9 | 7 | 8 | 9 | 3 | 8 | 20 | 8 | 8 | 1 | 16 | 5 |
| NM_001145553 | FLJ25363 | 1493 | 0 | 0 | 0 | 0 | 0 | 0 | 0 | 0 | 0 | 0 | 0 | 0 | 0 |
| NM_001145635 | TRIM45 | 3527 | 1 | 0 | 0 | 0 | 0 | 0 | 0 | 0 | 0 | 0 | 1 | 0 | 1 |
| NM_001145636 | C1orf228 | 1685 | 1 | 0 | 0 | 0 | 0 | 0 | 0 | 0 | 0 | 0 | 0 | 0 | 1 |
| NM_001145638 | GPSM1 | 3633 | 0 | 0 | 0 | 0 | 0 | 0 | 0 | 0 | 0 | 0 | 0 | 0 | 0 |
| NM_001145639 | GPSM1 | 2057 | 0 | 0 | 0 | 0 | 0 | 0 | 0 | 0 | 0 | 0 | 0 | 0 | 0 |
| NM_001145640 | ZFR2 | 867 | 1 | 0 | 0 | 0 | 0 | 1 | 0 | 0 | 0 | 0 | 0 | 0 | 0 |
| NM_001145641 | SRRM5 | 2397 | 9 | 1 | 0 | 1 | 0 | 0 | 1 | 0 | 0 | 1 | 1 | 1 | 9 |
| NM_001145642 | KIAA0226 | 6903 | 4 | 2 | 1 | 1 | 2 | 4 | 1 | 1 | 2 | 2 | 1 | 4 | 4 |
| NM_001145643 | PHGR1 | 433 | 13 | 1 | 0 | 13 | 0 | 0 | 0 | 0 | 0 | 0 | 0 | 0 | 0 |
| NM_001145645 | TNFSF13B | 1146 | 2 | 1 | 1 | 0 | 1 | 0 | 0 | 0 | 2 | 0 | 0 | 2 | 0 |
| NM_001145646 | APH1B | 4069 | 5 | 2 | 1 | 0 | 1 | 3 | 1 | 1 | 2 | 2 | 1 | 5 | 5 |
| NM_001145647 | ZNF709 | 4845 | 5 | 1 | 0 | 0 | 1 | 2 | 2 | 0 | 1 | 5 | 0 | 2 | 2 |
| NM_001145648 | RASGRF1 | 6344 | 9 | 1 | 0 | 0 | 0 | 9 | 0 | 0 | 0 | 0 | 0 | 0 | 0 |
| NM_001145649 | ZNF529 | 5226 | 1 | 1 | 0 | 0 | 1 | 1 | 1 | 0 | 1 | 1 | 1 | 1 | 1 |
| NM_001145650 | ZNF529 | 5166 | 1 | 1 | 0 | 0 | 1 | 1 | 1 | 0 | 1 | 1 | 1 | 1 | 1 |
| NM_001145652 | C6orf141 | 1694 | 1 | 0 | 0 | 0 | 0 | 0 | 0 | 0 | 0 | 0 | 0 | 0 | 1 |
| NM_001145657 | RAP1GAP | 3494 | 10 | 2 | 0 | 2 | 1 | 9 | 10 | 2 | 1 | 0 | 0 | 0 | 0 |
| NM_001145658 | RAP1GAP | 3300 | 10 | 2 | 0 | 2 | 1 | 9 | 10 | 2 | 1 | 0 | 0 | 0 | 0 |
| NM_001145659 | CTAGE9 | 2575 | 0 | 0 | 0 | 0 | 0 | 0 | 0 | 0 | 0 | 0 | 0 | 0 | 0 |
| NM_001145661 | GATA2 | 3475 | 1 | 0 | 1 | 0 | 0 | 0 | 1 | 0 | 1 | 0 | 0 | 0 | 0 |
| NM_001145662 | GATA2 | 3254 | 1 | 0 | 1 | 0 | 0 | 0 | 1 | 0 | 1 | 0 | 0 | 0 | 0 |
| NM_001145664 | RFX8 | 1729 | 0 | 0 | 0 | 0 | 0 | 0 | 0 | 0 | 0 | 0 | 0 | 0 | 0 |
| NM_001145665 | ZNF181 | 2948 | 4 | 2 | 1 | 0 | 4 | 2 | 3 | 1 | 1 | 4 | 2 | 3 | 3 |
| NM_001145666 | GLG1 | 8255 | 23 | 13 | 12 | 8 | 14 | 17 | 14 | 9 | 8 | 23 | 10 | 14 | 15 |
| NM_001145667 | GLG1 | 9327 | 21 | 12 | 11 | 7 | 13 | 16 | 13 | 9 | 8 | 21 | 10 | 13 | 14 |
| NM_001145668 | CTXN2 | 2695 | 1 | 0 | 0 | 0 | 0 | 1 | 0 | 0 | 0 | 0 | 0 | 0 | 0 |
| NM_001145670 | SORBS2 | 4928 | 49 | 8 | 1 | 4 | 49 | 6 | 4 | 8 | 2 | 3 | 2 | 3 | 2 |
| NM_001145671 | SORBS2 | 4684 | 49 | 8 | 1 | 4 | 49 | 6 | 4 | 7 | 2 | 3 | 2 | 4 | 2 |
| NM_001145672 | SORBS2 | 4355 | 52 | 8 | 1 | 4 | 52 | 6 | 5 | 8 | 2 | 3 | 2 | 4 | 2 |
| NM_001145673 | SORBS2 | 5241 | 46 | 7 | 1 | 4 | 46 | 5 | 4 | 6 | 2 | 2 | 2 | 3 | 2 |
| NM_001145674 | SORBS2 | 5528 | 32 | 5 | 1 | 3 | 32 | 4 | 3 | 4 | 1 | 2 | 2 | 3 | 1 |
| NM_001145675 | SORBS2 | 3992 | 44 | 7 | 1 | 4 | 44 | 5 | 4 | 6 | 2 | 3 | 2 | 4 | 2 |
| NM_001145678 | C5orf36 | 7240 | 1 | 1 | 0 | 0 | 0 | 1 | 1 | 0 | 1 | 1 | 0 | 1 | 1 |
| NM_001145710 | LOC375190 | 1356 | 6 | 3 | 2 | 1 | 2 | 3 | 2 | 0 | 3 | 6 | 4 | 2 | 4 |
| NM_001145712 | LOC389493 | 599 | 5 | 1 | 0 | 0 | 0 | 1 | 1 | 0 | 0 | 0 | 0 | 0 | 5 |
| NM_001145713 | SAE1 | 2318 | 8 | 3 | 4 | 4 | 2 | 8 | 1 | 3 | 2 | 3 | 2 | 4 | 3 |
| NM_001145714 | SAE1 | 2388 | 8 | 3 | 4 | 4 | 2 | 8 | 1 | 3 | 2 | 3 | 2 | 4 | 3 |
| NM_001145715 | KPNA7 | 1685 | 0 | 0 | 0 | 0 | 0 | 0 | 0 | 0 | 0 | 0 | 0 | 0 | 0 |
| NM_001145716 | PNPLA1 | 2359 | 0 | 0 | 0 | 0 | 0 | 0 | 0 | 0 | 0 | 0 | 0 | 0 | 0 |
| NM_001145717 | PNPLA1 | 2478 | 0 | 0 | 0 | 0 | 0 | 0 | 0 | 0 | 0 | 0 | 0 | 0 | 0 |
| NM_001145718 | CT47B1 | 1327 | 0 | 0 | 0 | 0 | 0 | 0 | 0 | 0 | 0 | 0 | 0 | 0 | 0 |
| NM_001145719 | SH2D4B | 3487 | 0 | 0 | 0 | 0 | 0 | 0 | 0 | 0 | 0 | 0 | 0 | 0 | 0 |
| NM_001145720 | ZBTB8B | 4016 | 1 | 0 | 0 | 0 | 0 | 1 | 0 | 0 | 0 | 0 | 0 | 0 | 0 |
| NM_001145721 | HOMER3 | 1418 | 1 | 0 | 0 | 0 | 0 | 1 | 1 | 0 | 0 | 0 | 0 | 1 | 0 |
| NM_001145722 | HOMER3 | 1849 | 1 | 0 | 0 | 0 | 0 | 0 | 1 | 0 | 0 | 0 | 0 | 1 | 0 |
| NM_001145724 | HOMER3 | 1287 | 1 | 0 | 0 | 0 | 0 | 0 | 1 | 0 | 0 | 0 | 0 | 1 | 0 |
| NM_001145725 | LYAR | 1468 | 14 | 4 | 2 | 1 | 5 | 2 | 1 | 2 | 2 | 3 | 4 | 5 | 14 |
| NM_001145727 | IFLTD1 | 1283 | 0 | 0 | 0 | 0 | 0 | 0 | 0 | 0 | 0 | 0 | 0 | 0 | 0 |
| NM_001145728 | IFLTD1 | 1847 | 1 | 0 | 0 | 0 | 0 | 0 | 0 | 0 | 0 | 0 | 0 | 0 | 1 |
| NM_001145729 | IFLTD1 | 1522 | 1 | 0 | 0 | 0 | 0 | 0 | 0 | 0 | 0 | 0 | 0 | 0 | 1 |
| NM_001145734 | CDC20B | 2858 | 3 | 0 | 0 | 0 | 0 | 0 | 0 | 0 | 0 | 0 | 0 | 0 | 3 |
| NM_001145769 | XPC | 3610 | 6 | 5 | 5 | 3 | 2 | 6 | 6 | 4 | 4 | 6 | 3 | 6 | 5 |
| NM_001145770 | GPR56 | 3806 | 28 | 5 | 2 | 2 | 2 | 14 | 28 | 0 | 2 | 0 | 0 | 1 | 9 |
| NM_001145771 | GPR56 | 3981 | 26 | 5 | 2 | 2 | 2 | 14 | 26 | 0 | 2 | 0 | 0 | 1 | 8 |
| NM_001145772 | GPR56 | 3989 | 26 | 5 | 2 | 2 | 2 | 14 | 26 | 0 | 2 | 0 | 0 | 1 | 8 |
| NM_001145773 | GPR56 | 3860 | 27 | 5 | 2 | 2 | 2 | 14 | 27 | 0 | 2 | 0 | 0 | 1 | 9 |
| NM_001145774 | GPR56 | 4043 | 26 | 5 | 2 | 2 | 2 | 14 | 26 | 0 | 2 | 0 | 0 | 1 | 8 |
| NM_001145775 | FKBP5 | 3940 | 103 | 43 | 21 | 9 | 42 | 8 | 41 | 83 | 43 | 28 | 62 | 103 | 33 |
| NM_001145776 | FKBP5 | 3827 | 107 | 44 | 22 | 9 | 44 | 8 | 42 | 85 | 44 | 28 | 64 | 107 | 34 |
| NM_001145777 | FKBP5 | 7384 | 14 | 6 | 4 | 2 | 6 | 1 | 7 | 11 | 7 | 4 | 8 | 14 | 4 |
| NM_001145783 | LOC729991 | 1497 | 2 | 0 | 0 | 0 | 0 | 2 | 0 | 0 | 0 | 0 | 0 | 0 | 0 |
| NM_001145784 | LOC729991 | 1432 | 0 | 0 | 0 | 0 | 0 | 0 | 0 | 0 | 0 | 0 | 0 | 0 | 0 |
| NM_001145785 | MEF2B | 1450 | 0 | 0 | 0 | 0 | 0 | 0 | 0 | 0 | 0 | 0 | 0 | 0 | 0 |
| NM_001145794 | ANTXR2 | 2304 | 17 | 7 | 13 | 6 | 5 | 1 | 3 | 8 | 7 | 17 | 2 | 5 | 6 |
| NM_001145795 | SH2B1 | 2935 | 3 | 1 | 1 | 3 | 1 | 2 | 1 | 0 | 1 | 2 | 1 | 2 | 2 |
| NM_001145796 | SH2B1 | 3035 | 2 | 1 | 1 | 2 | 1 | 2 | 1 | 0 | 1 | 2 | 1 | 2 | 2 |
| NM_001145797 | SH2B1 | 3088 | 2 | 1 | 1 | 2 | 1 | 2 | 1 | 0 | 1 | 2 | 1 | 2 | 2 |

| transcript | gene | transcript length (nt) | max | mean | adipose | colon | heart | hypothalamus | kidney | liver | lung | ovary | skeletalmuscle | spleen | testes |
|---|---|---|---|---|---|---|---|---|---|---|---|---|---|---|---|
| NM_001145798 | CACNB4 | 7790 | 4 | 1 | 0 | 0 | 0 | 4 | 0 | 0 | 0 | 0 | 0 | 0 | 1 |
| NM_001145805 | IRGM | 1657 | 0 | 0 | 0 | 0 | 0 | 0 | 0 | 0 | 0 | 0 | 0 | 0 | 0 |
| NM_001145808 | ITGAM | 4743 | 3 | 1 | 3 | 0 | 2 | 1 | 0 | 0 | 2 | 0 | 0 | 3 | 0 |
| NM_001145809 | MYH14 | 6912 | 6 | 2 | 2 | 6 | 3 | 2 | 1 | 2 | 2 | 0 | 6 | 0 | 0 |
| NM_001145811 | SOX6 | 8956 | 2 | 1 | 0 | 0 | 1 | 1 | 2 | 0 | 0 | 0 | 1 | 0 | 1 |
| NM_001145812 | SH2B1 | 3134 | 2 | 1 | 1 | 2 | 1 | 2 | 1 | 0 | 1 | 2 | 1 | 2 | 2 |
| NM_001145815 | AMDHD2 | 2076 | 0 | 0 | 0 | 0 | 0 | 0 | 0 | 0 | 0 | 0 | 0 | 0 | 0 |
| NM_001145816 | HPGD | 2606 | 36 | 5 | 1 | 4 | 0 | 0 | 4 | 36 | 7 | 0 | 0 | 2 | 1 |
| NM_001145819 | SOX6 | 8918 | 2 | 1 | 0 | 0 | 1 | 1 | 2 | 0 | 0 | 0 | 1 | 0 | 1 |
| NM_001145822 | TPM2 | 1024 | 1,036 | 148 | 35 | 221 | 128 | 20 | 16 | 2 | 39 | 50 | 1,036 | 24 | 58 |
| NM_001145829 | TNNI2 | 739 | 896 | 82 | 2 | 0 | 0 | 0 | 0 | 0 | 1 | 0 | 896 | 1 | 1 |
| NM_001145838 | MTFR1 | 2502 | 12 | 5 | 3 | 3 | 4 | 3 | 12 | 12 | 2 | 2 | 2 | 4 | 6 |
| NM_001145839 | MTFR1 | 1471 | 17 | 7 | 4 | 5 | 5 | 4 | 17 | 17 | 4 | 3 | 3 | 5 | 9 |
| NM_001145841 | TNNI2 | 700 | 943 | 86 | 2 | 0 | 0 | 0 | 0 | 0 | 2 | 0 | 943 | 1 | 1 |
| NM_001145844 | ROBO1 | 7494 | 7 | 3 | 4 | 1 | 3 | 7 | 1 | 1 | 2 | 5 | 1 | 5 | 5 |
| NM_001145845 | ROBO1 | 7329 | 7 | 3 | 4 | 1 | 3 | 7 | 1 | 1 | 2 | 5 | 1 | 5 | 5 |
| NM_001145847 | PROM1 | 4257 | 3 | 1 | 0 | 2 | 0 | 0 | 3 | 0 | 1 | 0 | 0 | 0 | 1 |
| NM_001145848 | PROM1 | 3973 | 3 | 1 | 0 | 2 | 0 | 0 | 3 | 0 | 1 | 0 | 0 | 0 | 1 |
| NM_001145849 | PROM1 | 3908 | 3 | 1 | 0 | 3 | 0 | 0 | 3 | 0 | 1 | 0 | 0 | 0 | 1 |
| NM_001145850 | PROM1 | 3884 | 3 | 1 | 0 | 3 | 0 | 0 | 3 | 0 | 1 | 0 | 0 | 0 | 1 |
| NM_001145851 | PROM1 | 3881 | 3 | 1 | 0 | 3 | 0 | 0 | 3 | 0 | 1 | 0 | 0 | 0 | 1 |
| NM_001145852 | PROM1 | 3857 | 3 | 1 | 0 | 3 | 0 | 0 | 3 | 0 | 1 | 0 | 0 | 0 | 1 |
| NM_001145853 | WFS1 | 3635 | 1 | 0 | 1 | 0 | 0 | 1 | 0 | 0 | 0 | 1 | 0 | 1 | 1 |
| NM_001145855 | SH3BP2 | 9210 | 2 | 1 | 0 | 0 | 0 | 0 | 2 | 0 | 0 | 0 | 0 | 2 | 0 |
| NM_001145856 | SH3BP2 | 9157 | 2 | 1 | 0 | 0 | 0 | 0 | 2 | 0 | 0 | 0 | 0 | 2 | 0 |
| NM_001145860 | POP1 | 4744 | 1 | 0 | 0 | 0 | 1 | 1 | 1 | 1 | 0 | 1 | 0 | 1 | 1 |
| NM_001145861 | POP1 | 4684 | 1 | 0 | 0 | 0 | 1 | 1 | 1 | 1 | 0 | 1 | 0 | 1 | 1 |
| NM_001145862 | MTMR11 | 2858 | 5 | 1 | 0 | 5 | 1 | 0 | 2 | 0 | 1 | 2 | 0 | 1 | 1 |
| NM_001145873 | CD8A | 3053 | 6 | 1 | 0 | 0 | 0 | 0 | 0 | 0 | 1 | 0 | 0 | 6 | 0 |
| NM_001145901 | SARS2 | 2065 | 0 | 0 | 0 | 0 | 0 | 0 | 0 | 0 | 0 | 0 | 0 | 0 | 0 |
| NM_001145903 | C2 | 2441 | 55 | 8 | 5 | 2 | 3 | 3 | 2 | 55 | 6 | 1 | 1 | 10 | 1 |
| NM_001145909 | TANC1 | 7486 | 4 | 2 | 1 | 2 | 4 | 2 | 2 | 1 | 3 | 2 | 0 | 1 | 1 |
| NM_001145923 | SCAPER | 4850 | 11 | 6 | 3 | 2 | 7 | 11 | 5 | 2 | 4 | 7 | 5 | 6 | 9 |
| NM_001145928 | SAP130 | 4173 | 6 | 2 | 1 | 1 | 1 | 2 | 1 | 1 | 1 | 2 | 1 | 2 | 6 |
| NM_001145933 | TKTL1 | 2633 | 16 | 1 | 0 | 0 | 0 | 0 | 0 | 0 | 0 | 0 | 0 | 0 | 16 |
| NM_001145934 | TKTL1 | 2578 | 16 | 2 | 0 | 0 | 0 | 0 | 0 | 0 | 0 | 0 | 0 | 0 | 16 |
| NM_001145938 | MMP1 | 1903 | 3 | 0 | 0 | 0 | 0 | 0 | 0 | 0 | 0 | 0 | 0 | 3 | 0 |
| NM_001145939 | PAFAH1B3 | 1098 | 0 | 0 | 0 | 0 | 0 | 0 | 0 | 0 | 0 | 0 | 0 | 0 | 0 |
| NM_001145940 | PAFAH1B3 | 859 | 0 | 0 | 0 | 0 | 0 | 0 | 0 | 0 | 0 | 0 | 0 | 0 | 0 |
| NM_001145944 | SLCO1C1 | 3170 | 1 | 0 | 0 | 0 | 0 | 1 | 0 | 0 | 0 | 0 | 0 | 0 | 0 |
| NM_001145945 | SLCO1C1 | 3203 | 1 | 0 | 0 | 0 | 0 | 1 | 0 | 0 | 0 | 0 | 0 | 0 | 0 |
| NM_001145946 | SLCO1C1 | 3453 | 1 | 0 | 0 | 0 | 0 | 1 | 0 | 0 | 0 | 0 | 0 | 0 | 0 |
| NM_001145951 | TIMM8A | 551 | 1 | 0 | 0 | 1 | 0 | 0 | 0 | 1 | 0 | 0 | 0 | 0 | 0 |
| NM_001145961 | SLC12A4 | 4747 | 5 | 3 | 3 | 2 | 5 | 2 | 2 | 3 | 3 | 2 | 2 | 2 | 3 |
| NM_001145962 | SLC12A4 | 4670 | 5 | 3 | 3 | 2 | 5 | 2 | 2 | 3 | 3 | 2 | 2 | 2 | 3 |
| NM_001145963 | SLC12A4 | 4644 | 5 | 3 | 3 | 2 | 5 | 2 | 2 | 3 | 3 | 2 | 2 | 2 | 3 |
| NM_001145964 | SLC12A4 | 4635 | 5 | 3 | 3 | 2 | 5 | 2 | 2 | 3 | 3 | 2 | 2 | 2 | 3 |
| NM_001145966 | MKI67 | 11416 | 7 | 2 | 4 | 5 | 2 | 0 | 0 | 0 | 3 | 2 | 0 | 7 | 2 |
| NM_001145971 | RDH13 | 1931 | 1 | 0 | 0 | 1 | 1 | 1 | 1 | 0 | 0 | 0 | 0 | 0 | 1 |
| NM_001145975 | SLC13A2 | 2502 | 3 | 0 | 0 | 0 | 0 | 0 | 3 | 0 | 0 | 0 | 0 | 0 | 0 |
| NM_001145976 | SLC13A2 | 2226 | 4 | 0 | 0 | 0 | 0 | 0 | 4 | 0 | 0 | 0 | 0 | 0 | 0 |
| NM_001145998 | SLC15A2 | 5430 | 3 | 1 | 0 | 0 | 0 | 2 | 3 | 0 | 2 | 0 | 0 | 1 | 2 |
| NM_001146 | ANGPT1 | 4338 | 10 | 3 | 10 | 0 | 2 | 1 | 2 | 0 | 7 | 2 | 3 | 2 | 1 |
| NM_001146003 | NEK11 | 2782 | 6 | 2 | 2 | 1 | 1 | 5 | 3 | 0 | 2 | 3 | 1 | 1 | 6 |
| NM_001146006 | IGFALS | 2198 | 0 | 0 | 0 | 0 | 0 | 0 | 0 | 0 | 0 | 0 | 0 | 0 | 0 |
| NM_001146008 | SLCO5A1 | 3733 | 2 | 0 | 0 | 0 | 2 | 0 | 0 | 0 | 0 | 0 | 2 | 0 | 0 |
| NM_001146009 | SLCO5A1 | 3412 | 2 | 0 | 0 | 0 | 2 | 0 | 0 | 0 | 0 | 0 | 2 | 0 | 0 |
| NM_001146015 | DLGAP5 | 3082 | 4 | 1 | 0 | 1 | 0 | 0 | 0 | 0 | 1 | 0 | 0 | 1 | 4 |
| NM_001146016 | TJAP1 | 2751 | 1 | 1 | 0 | 1 | 0 | 1 | 1 | 0 | 0 | 1 | 1 | 1 | 1 |
| NM_001146017 | TJAP1 | 2716 | 1 | 1 | 0 | 1 | 0 | 1 | 1 | 0 | 0 | 1 | 1 | 1 | 1 |
| NM_001146018 | TJAP1 | 2778 | 1 | 1 | 0 | 1 | 0 | 1 | 1 | 0 | 0 | 1 | 1 | 1 | 1 |
| NM_001146019 | TJAP1 | 2721 | 1 | 1 | 0 | 1 | 0 | 1 | 1 | 0 | 0 | 1 | 1 | 1 | 1 |
| NM_001146020 | TJAP1 | 2572 | 1 | 1 | 1 | 1 | 0 | 1 | 1 | 0 | 0 | 1 | 1 | 1 | 1 |
| NM_001146028 | JPH4 | 4278 | 6 | 1 | 0 | 0 | 0 | 6 | 0 | 0 | 0 | 1 | 0 | 0 | 0 |
| NM_001146029 | SEMA7A | 3333 | 3 | 1 | 0 | 0 | 0 | 1 | 0 | 0 | 0 | 0 | 0 | 3 | 2 |
| NM_001146030 | SEMA7A | 3228 | 3 | 1 | 0 | 0 | 0 | 1 | 0 | 0 | 0 | 0 | 0 | 3 | 2 |
| NM_001146032 | FCHO2 | 4881 | 7 | 3 | 3 | 1 | 2 | 1 | 3 | 1 | 6 | 3 | 2 | 7 | 3 |
| NM_001146033 | OR56A5 | 940 | 0 | 0 | 0 | 0 | 0 | 0 | 0 | 0 | 0 | 0 | 0 | 0 | 0 |
| NM_001146036 | SLC14A1 | 4057 | 2 | 1 | 0 | 0 | 1 | 2 | 1 | 0 | 1 | 0 | 0 | 0 | 0 |
| NM_001146037 | SLC14A1 | 4225 | 2 | 0 | 0 | 0 | 1 | 2 | 1 | 0 | 1 | 0 | 0 | 0 | 0 |
| NM_001146039 | GORAB | 1705 | 1 | 0 | 0 | 0 | 0 | 0 | 0 | 0 | 0 | 0 | 0 | 1 | 0 |
| NM_001146040 | GLRA1 | 1820 | 0 | 0 | 0 | 0 | 0 | 0 | 0 | 0 | 0 | 0 | 0 | 0 | 0 |
| NM_001146041 | KRTAP4-9 | 1098 | 0 | 0 | 0 | 0 | 0 | 0 | 0 | 0 | 0 | 0 | 0 | 0 | 0 |
| NM_001146051 | HSDL1 | 3503 | 5 | 2 | 1 | 1 | 2 | 5 | 2 | 1 | 1 | 3 | 1 | 2 | 3 |
| NM_001146054 | SNCA | 3210 | 26 | 3 | 1 | 1 | 1 | 26 | 0 | 0 | 1 | 1 | 0 | 1 | 0 |
| NM_001146055 | SNCA | 3021 | 28 | 3 | 1 | 1 | 1 | 28 | 0 | 0 | 1 | 1 | 0 | 1 | 1 |
| NM_001146056 | MBOAT7 | 2392 | 2 | 1 | 0 | 1 | 0 | 2 | 1 | 0 | 1 | 1 | 0 | 1 | 2 |

| transcript | gene | transcript length (nt) | max | mean | adipose | colon | heart | hypothalamus | kidney | liver | lung | ovary | skeletalmuscle | spleen | testes |
|---|---|---|---|---|---|---|---|---|---|---|---|---|---|---|---|
| NM_001146068 | CAPN2 | 3264 | 19 | 9 | 12 | 8 | 9 | 4 | 15 | 3 | 19 | 8 | 7 | 6 | 10 |
| NM_001146069 | MFSD10 | 1756 | 2 | 1 | 1 | 1 | 1 | 1 | 1 | 0 | 1 | 1 | 0 | 2 | 0 |
| NM_001146070 | TDRD3 | 2579 | 7 | 4 | 4 | 2 | 6 | 4 | 5 | 2 | 3 | 7 | 5 | 4 | 5 |
| NM_001146071 | TDRD3 | 3019 | 6 | 4 | 3 | 2 | 5 | 4 | 4 | 2 | 2 | 6 | 4 | 3 | 4 |
| NM_001146075 | RPAP3 | 3584 | 6 | 4 | 3 | 1 | 4 | 5 | 3 | 3 | 2 | 5 | 5 | 6 | 6 |
| NM_001146076 | RPAP3 | 3527 | 6 | 4 | 2 | 1 | 4 | 5 | 3 | 3 | 2 | 5 | 4 | 6 | 6 |
| NM_001146077 | CLDN14 | 1451 | 0 | 0 | 0 | 0 | 0 | 0 | 0 | 0 | 0 | 0 | 0 | 0 | 0 |
| NM_001146078 | CLDN14 | 1328 | 0 | 0 | 0 | 0 | 0 | 0 | 0 | 0 | 0 | 0 | 0 | 0 | 0 |
| NM_001146079 | CLDN14 | 1750 | 1 | 0 | 0 | 0 | 0 | 0 | 0 | 1 | 0 | 0 | 0 | 0 | 0 |
| NM_001146082 | MBOAT7 | 2020 | 1 | 0 | 0 | 0 | 0 | 1 | 0 | 0 | 0 | 0 | 0 | 0 | 0 |
| NM_001146083 | MBOAT7 | 2471 | 2 | 1 | 0 | 1 | 0 | 2 | 1 | 0 | 1 | 1 | 0 | 1 | 2 |
| NM_001146094 | MTMR15 | 2841 | 2 | 1 | 1 | 1 | 1 | 2 | 2 | 1 | 0 | 2 | 1 | 1 | 1 |
| NM_001146095 | MTMR15 | 2738 | 2 | 1 | 1 | 1 | 1 | 2 | 2 | 1 | 0 | 2 | 1 | 1 | 1 |
| NM_001146096 | MTMR15 | 2854 | 2 | 1 | 1 | 1 | 1 | 2 | 2 | 1 | 0 | 2 | 1 | 1 | 1 |
| NM_001146099 | NEK3 | 2344 | 6 | 2 | 3 | 2 | 2 | 1 | 3 | 1 | 2 | 1 | 1 | 2 | 6 |
| NM_001146102 | PARP9 | 3159 | 13 | 6 | 4 | 2 | 5 | 3 | 5 | 13 | 7 | 4 | 6 | 12 | 4 |
| NM_001146103 | PARP9 | 3236 | 13 | 6 | 4 | 2 | 5 | 3 | 5 | 13 | 7 | 4 | 6 | 11 | 4 |
| NM_001146104 | PARP9 | 3235 | 13 | 6 | 4 | 2 | 5 | 3 | 5 | 13 | 7 | 4 | 6 | 11 | 4 |
| NM_001146105 | PARP9 | 3054 | 14 | 6 | 4 | 2 | 5 | 3 | 5 | 14 | 7 | 4 | 6 | 12 | 4 |
| NM_001146106 | PARP9 | 6801 | 6 | 3 | 2 | 1 | 2 | 1 | 2 | 6 | 3 | 2 | 3 | 5 | 2 |
| NM_001146108 | PTGR1 | 1405 | 67 | 14 | 6 | 5 | 8 | 3 | 40 | 67 | 6 | 8 | 2 | 4 | 6 |
| NM_001146109 | PTGR1 | 1219 | 52 | 11 | 5 | 4 | 6 | 2 | 28 | 52 | 4 | 6 | 2 | 3 | 5 |
| NM_001146110 | MIER1 | 5628 | 12 | 7 | 5 | 4 | 6 | 5 | 6 | 6 | 10 | 10 | 4 | 11 | 12 |
| NM_001146111 | MIER1 | 3875 | 18 | 10 | 7 | 6 | 9 | 7 | 9 | 8 | 13 | 13 | 6 | 15 | 18 |
| NM_001146112 | MIER1 | 5586 | 13 | 7 | 5 | 4 | 6 | 5 | 6 | 6 | 10 | 10 | 4 | 11 | 13 |
| NM_001146113 | MIER1 | 3595 | 15 | 8 | 5 | 5 | 8 | 6 | 7 | 6 | 12 | 11 | 5 | 13 | 15 |
| NM_001146114 | DIP2A | 3606 | 1 | 1 | 0 | 1 | 1 | 1 | 1 | 0 | 1 | 0 | 0 | 1 | 1 |
| NM_001146115 | DIP2A | 2740 | 1 | 1 | 1 | 1 | 1 | 1 | 1 | 0 | 1 | 0 | 1 | 1 | 1 |
| NM_001146116 | DIP2A | 7278 | 2 | 1 | 1 | 1 | 1 | 1 | 1 | 0 | 1 | 1 | 1 | 2 | 1 |
| NM_001146151 | AVPR2 | 1730 | 0 | 0 | 0 | 0 | 0 | 0 | 0 | 0 | 0 | 0 | 0 | 0 | 0 |
| NM_001146152 | CYP51A1 | 2933 | 42 | 18 | 6 | 7 | 12 | 28 | 19 | 31 | 17 | 19 | 5 | 10 | 42 |
| NM_001146154 | PTGR2 | 2502 | 4 | 2 | 2 | 1 | 4 | 4 | 4 | 2 | 1 | 1 | 2 | 1 | 2 |
| NM_001146155 | PTGR2 | 2550 | 4 | 2 | 2 | 1 | 4 | 4 | 3 | 2 | 1 | 1 | 2 | 1 | 2 |
| NM_001146156 | GSK3B | 7077 | 17 | 8 | 5 | 6 | 8 | 17 | 8 | 3 | 6 | 8 | 7 | 11 | 8 |
| NM_001146157 | FAM25A | 361 | 0 | 0 | 0 | 0 | 0 | 0 | 0 | 0 | 0 | 0 | 0 | 0 | 0 |
| NM_001146158 | MOGS | 2568 | 2 | 1 | 1 | 2 | 2 | 1 | 1 | 2 | 1 | 1 | 1 | 2 | 1 |
| NM_001146160 | TATDN1 | 920 | 10 | 7 | 7 | 3 | 6 | 10 | 6 | 8 | 5 | 9 | 8 | 7 | 8 |
| NM_001146162 | TRIM77 | 1351 | 0 | 0 | 0 | 0 | 0 | 0 | 0 | 0 | 0 | 0 | 0 | 0 | 0 |
| NM_001146169 | TATDN3 | 2575 | 3 | 2 | 2 | 1 | 2 | 3 | 3 | 1 | 1 | 1 | 2 | 2 | 1 |
| NM_001146170 | TATDN3 | 2525 | 3 | 2 | 2 | 1 | 2 | 3 | 3 | 1 | 1 | 1 | 2 | 2 | 1 |
| NM_001146171 | TATDN3 | 2609 | 3 | 2 | 2 | 1 | 2 | 3 | 3 | 1 | 1 | 1 | 2 | 2 | 1 |
| NM_001146172 | SLAMF9 | 881 | 1 | 0 | 1 | 0 | 0 | 0 | 0 | 0 | 0 | 0 | 0 | 0 | 0 |
| NM_001146173 | SLAMF9 | 745 | 1 | 0 | 1 | 0 | 0 | 0 | 0 | 0 | 0 | 0 | 0 | 0 | 0 |
| NM_001146175 | ZNF414 | 1403 | 1 | 0 | 0 | 0 | 0 | 0 | 0 | 0 | 0 | 1 | 0 | 1 | 0 |
| NM_001146178 | IP6K2 | 1307 | 7 | 4 | 2 | 7 | 2 | 3 | 7 | 1 | 2 | 6 | 2 | 4 | 5 |
| NM_001146179 | IP6K2 | 1304 | 7 | 4 | 2 | 7 | 2 | 3 | 7 | 1 | 2 | 6 | 2 | 4 | 5 |
| NM_001146181 | LOC649330 | 1002 | 0 | 0 | 0 | 0 | 0 | 0 | 0 | 0 | 0 | 0 | 0 | 0 | 0 |
| NM_001146184 | PEG3 | 8683 | 151 | 22 | 1 | 1 | 2 | 52 | 1 | 1 | 1 | 151 | 0 | 1 | 28 |
| NM_001146185 | PEG3 | 8369 | 156 | 23 | 1 | 1 | 2 | 54 | 2 | 2 | 1 | 156 | 0 | 1 | 29 |
| NM_001146186 | PEG3 | 8716 | 150 | 22 | 1 | 1 | 2 | 52 | 1 | 1 | 1 | 150 | 0 | 1 | 28 |
| NM_001146187 | PEG3 | 8375 | 156 | 23 | 1 | 1 | 2 | 54 | 2 | 2 | 1 | 156 | 0 | 1 | 29 |
| NM_001146188 | TOX3 | 3123 | 1 | 0 | 0 | 1 | 0 | 1 | 1 | 0 | 0 | 0 | 0 | 0 | 0 |
| NM_001146189 | ZNF407 | 5848 | 9 | 5 | 3 | 1 | 4 | 4 | 5 | 2 | 4 | 9 | 4 | 8 | 6 |
| NM_001146190 | ZNF407 | 5597 | 10 | 5 | 4 | 1 | 4 | 4 | 5 | 2 | 4 | 10 | 4 | 9 | 6 |
| NM_001146191 | MPZL1 | 4559 | 9 | 4 | 4 | 3 | 5 | 3 | 3 | 2 | 4 | 5 | 1 | 4 | 9 |
| NM_001146192 | ZMYND12 | 1600 | 3 | 1 | 0 | 1 | 0 | 2 | 1 | 1 | 1 | 0 | 0 | 0 | 3 |
| NM_001146197 | LOC643677 | 21468 | 2 | 0 | 0 | 0 | 0 | 0 | 0 | 0 | 0 | 0 | 0 | 0 | 2 |
| NM_001146202 | RASGRP4 | 3176 | 1 | 0 | 0 | 0 | 0 | 0 | 0 | 0 | 0 | 0 | 0 | 1 | 0 |
| NM_001146203 | RASGRP4 | 2942 | 1 | 0 | 0 | 0 | 0 | 0 | 0 | 0 | 0 | 0 | 0 | 1 | 0 |
| NM_001146204 | RASGRP4 | 3011 | 1 | 0 | 0 | 0 | 0 | 0 | 0 | 0 | 0 | 0 | 0 | 1 | 0 |
| NM_001146205 | RASGRP4 | 3116 | 1 | 0 | 0 | 0 | 0 | 0 | 0 | 0 | 0 | 0 | 0 | 1 | 0 |
| NM_001146206 | RASGRP4 | 2651 | 1 | 0 | 0 | 0 | 0 | 0 | 0 | 0 | 0 | 0 | 0 | 1 | 0 |
| NM_001146207 | RASGRP4 | 2927 | 1 | 0 | 0 | 0 | 0 | 0 | 0 | 0 | 0 | 0 | 0 | 1 | 0 |
| NM_001146209 | PDE2A | 4181 | 4 | 1 | 1 | 0 | 0 | 3 | 0 | 0 | 0 | 0 | 0 | 4 | 0 |
| NM_001146210 | SPDYE6 | 3144 | 1 | 0 | 0 | 0 | 0 | 0 | 1 | 0 | 0 | 1 | 0 | 1 | 0 |
| NM_001146213 | TBC1D15 | 5743 | 6 | 4 | 4 | 1 | 3 | 4 | 3 | 4 | 5 | 4 | 4 | 6 | 4 |
| NM_001146214 | TBC1D15 | 5835 | 6 | 4 | 4 | 1 | 3 | 4 | 3 | 4 | 4 | 4 | 4 | 6 | 4 |
| NM_001146216 | TACC1 | 7747 | 16 | 8 | 12 | 5 | 9 | 12 | 6 | 3 | 10 | 7 | 3 | 16 | 9 |
| NM_001146218 | WRB | 1462 | 8 | 2 | 1 | 1 | 2 | 8 | 2 | 2 | 2 | 2 | 1 | 2 | 4 |
| NM_001146220 | ZNF226 | 885 | 2 | 1 | 1 | 1 | 1 | 2 | 2 | 1 | 0 | 1 | 1 | 1 | 2 |
| NM_001146221 | LOC1001338 | 1095 | 1 | 0 | 0 | 0 | 0 | 0 | 0 | 0 | 0 | 0 | 0 | 0 | 1 |
| NM_001146225 | KRT72 | 1888 | 1 | 0 | 0 | 0 | 0 | 0 | 0 | 0 | 0 | 0 | 0 | 0 | 1 |
| NM_001146226 | KRT72 | 1889 | 1 | 0 | 0 | 0 | 0 | 0 | 0 | 0 | 0 | 0 | 0 | 0 | 1 |
| NM_001146227 | RPS20 | 1013 | 92 | 52 | 61 | 21 | 51 | 27 | 37 | 42 | 62 | 92 | 41 | 85 | 52 |
| NM_001146254 | PODNL1 | 3307 | 0 | 0 | 0 | 0 | 0 | 0 | 0 | 0 | 0 | 0 | 0 | 0 | 0 |
| NM_001146255 | PODNL1 | 3040 | 0 | 0 | 0 | 0 | 0 | 0 | 0 | 0 | 0 | 0 | 0 | 0 | 0 |
| NM_001146256 | ZDHHC15 | 6014 | 0 | 0 | 0 | 0 | 0 | 0 | 0 | 0 | 0 | 0 | 0 | 0 | 0 |

| transcript | gene | transcript length (nt) | max | mean | adipose | colon | heart | hypothalamus | kidney | liver | lung | ovary | skeletalmuscle | spleen | testes |
|---|---|---|---|---|---|---|---|---|---|---|---|---|---|---|---|
| NM_001146257 | ZDHHC15 | 1312 | 0 | 0 | 0 | 0 | 0 | 0 | 0 | 0 | 0 | 0 | 0 | 0 | 0 |
| NM_001146258 | PIP4K2C | 2929 | 2 | 1 | 0 | 1 | 0 | 2 | 2 | 1 | 1 | 0 | 0 | 1 | 1 |
| NM_001146259 | PIP4K2C | 3164 | 2 | 1 | 1 | 1 | 0 | 2 | 2 | 1 | 1 | 1 | 0 | 1 | 1 |
| NM_001146260 | PIP4K2C | 3074 | 2 | 1 | 1 | 1 | 0 | 2 | 2 | 1 | 1 | 1 | 0 | 1 | 2 |
| NM_001146261 | SYT14 | 5162 | 2 | 0 | 0 | 0 | 0 | 2 | 0 | 0 | 0 | 0 | 0 | 0 | 1 |
| NM_001146262 | SYT14 | 5041 | 2 | 0 | 0 | 0 | 0 | 2 | 0 | 0 | 0 | 0 | 0 | 0 | 1 |
| NM_001146264 | SYT14 | 5105 | 2 | 0 | 0 | 0 | 0 | 2 | 0 | 0 | 0 | 0 | 0 | 0 | 1 |
| NM_001146265 | GPR85 | 4936 | 1 | 0 | 0 | 0 | 0 | 1 | 0 | 0 | 0 | 0 | 0 | 0 | 0 |
| NM_001146266 | GPR85 | 4826 | 1 | 0 | 0 | 0 | 0 | 1 | 0 | 0 | 0 | 0 | 0 | 0 | 0 |
| NM_001146267 | GPR85 | 4763 | 1 | 0 | 0 | 0 | 0 | 1 | 0 | 0 | 1 | 0 | 0 | 0 | 0 |
| NM_001146273 | TMEM64 | 4663 | 6 | 3 | 2 | 1 | 2 | 2 | 3 | 3 | 3 | 3 | 2 | 3 | 6 |
| NM_001146274 | TCF7L2 | 4019 | 6 | 3 | 3 | 3 | 2 | 1 | 2 | 1 | 3 | 6 | 1 | 5 | 3 |
| NM_001146276 | NCEH1 | 4314 | 4 | 2 | 3 | 0 | 2 | 4 | 2 | 1 | 1 | 0 | 0 | 2 | 0 |
| NM_001146277 | NCEH1 | 4280 | 4 | 2 | 3 | 0 | 2 | 4 | 2 | 1 | 1 | 0 | 0 | 2 | 0 |
| NM_001146278 | NCEH1 | 4061 | 4 | 2 | 3 | 0 | 2 | 4 | 2 | 1 | 1 | 0 | 0 | 2 | 0 |
| NM_001146279 | SHBG | 1223 | 1 | 0 | 0 | 0 | 0 | 0 | 0 | 1 | 0 | 0 | 0 | 0 | 0 |
| NM_001146280 | SHBG | 1069 | 1 | 0 | 0 | 0 | 0 | 0 | 0 | 1 | 0 | 0 | 0 | 0 | 0 |
| NM_001146281 | SHBG | 932 | 1 | 0 | 0 | 0 | 0 | 0 | 0 | 1 | 0 | 0 | 0 | 0 | 0 |
| NM_001146282 | SRP54 | 2187 | 22 | 10 | 8 | 3 | 9 | 12 | 11 | 22 | 8 | 6 | 6 | 11 | 13 |
| NM_001146283 | TCF7L2 | 4018 | 6 | 3 | 3 | 3 | 2 | 1 | 2 | 1 | 3 | 6 | 1 | 5 | 3 |
| NM_001146284 | TCF7L2 | 3916 | 6 | 3 | 3 | 3 | 2 | 2 | 2 | 1 | 3 | 6 | 1 | 5 | 3 |
| NM_001146285 | TCF7L2 | 3950 | 6 | 3 | 3 | 3 | 2 | 2 | 2 | 1 | 3 | 6 | 1 | 5 | 3 |
| NM_001146286 | TCF7L2 | 3877 | 6 | 3 | 3 | 3 | 2 | 2 | 2 | 1 | 3 | 6 | 1 | 5 | 3 |
| NM_001146288 | AIFM3 | 2429 | 2 | 0 | 0 | 2 | 0 | 2 | 0 | 0 | 0 | 0 | 0 | 0 | 0 |
| NM_001146289 | LEPRE1 | 2705 | 1 | 1 | 1 | 0 | 1 | 0 | 0 | 1 | 0 | 1 | 0 | 1 | 1 |
| NM_001146291 | ZNF674 | 4072 | 1 | 1 | 0 | 0 | 0 | 1 | 1 | 0 | 0 | 1 | 1 | 1 | 1 |
| NM_001146312 | MYOCD | 6950 | 4 | 1 | 0 | 1 | 4 | 0 | 0 | 0 | 0 | 2 | 0 | 0 | 1 |
| NM_001146313 | MYOCD | 2344 | 2 | 0 | 0 | 0 | 2 | 0 | 0 | 0 | 0 | 1 | 0 | 0 | 0 |
| NM_001146314 | ABHD14B | 2242 | 9 | 4 | 5 | 3 | 1 | 1 | 9 | 5 | 4 | 4 | 1 | 4 | 2 |
| NM_001146316 | SPSB2 | 1202 | 0 | 0 | 0 | 0 | 0 | 0 | 0 | 0 | 0 | 0 | 0 | 0 | 0 |
| NM_001146319 | GRAMD3 | 2783 | 2 | 1 | 0 | 0 | 1 | 2 | 1 | 1 | 0 | 2 | 0 | 1 | 1 |
| NM_001146320 | GRAMD3 | 2820 | 2 | 1 | 0 | 0 | 1 | 2 | 1 | 1 | 1 | 2 | 0 | 1 | 1 |
| NM_001146321 | GRAMD3 | 2975 | 2 | 1 | 0 | 0 | 1 | 2 | 1 | 1 | 1 | 2 | 0 | 1 | 1 |
| NM_001146322 | GRAMD3 | 2594 | 2 | 1 | 0 | 0 | 2 | 2 | 2 | 1 | 1 | 2 | 0 | 1 | 1 |
| NM_001146326 | ZIM2 | 2167 | 1 | 0 | 0 | 0 | 0 | 0 | 0 | 0 | 0 | 1 | 0 | 0 | 1 |
| NM_001146327 | ZIM2 | 2254 | 1 | 0 | 0 | 0 | 0 | 0 | 0 | 0 | 0 | 1 | 0 | 0 | 1 |
| NM_001146333 | SUMF2 | 1896 | 28 | 6 | 4 | 3 | 10 | 5 | 5 | 3 | 3 | 3 | 28 | 4 | 2 |
| NM_001146334 | NACAD | 4760 | 1 | 0 | 0 | 0 | 0 | 1 | 0 | 0 | 0 | 1 | 0 | 0 | 0 |
| NM_001146335 | SLC6A15 | 4351 | 3 | 0 | 0 | 0 | 0 | 3 | 0 | 0 | 0 | 0 | 0 | 0 | 0 |
| NM_001146336 | TMEM114 | 643 | 0 | 0 | 0 | 0 | 0 | 0 | 0 | 0 | 0 | 0 | 0 | 0 | 0 |
| NM_001146337 | AFAP1L1 | 4081 | 8 | 3 | 8 | 1 | 6 | 1 | 1 | 1 | 4 | 2 | 2 | 3 | 2 |
| NM_001146339 | VSTM2B | 1243 | 3 | 0 | 0 | 0 | 0 | 3 | 0 | 0 | 0 | 0 | 0 | 0 | 0 |
| NM_001146340 | NKX1-2 | 970 | 0 | 0 | 0 | 0 | 0 | 0 | 0 | 0 | 0 | 0 | 0 | 0 | 0 |
| NM_001146341 | ANKRD34C | 5423 | 0 | 0 | 0 | 0 | 0 | 0 | 0 | 0 | 0 | 0 | 0 | 0 | 0 |
| NM_001146343 | PQLC1 | 2272 | 7 | 2 | 1 | 1 | 1 | 3 | 3 | 7 | 1 | 1 | 1 | 2 | 1 |
| NM_001146344 | PRAMEF11 | 1839 | 0 | 0 | 0 | 0 | 0 | 0 | 0 | 0 | 0 | 0 | 0 | 0 | 0 |
| NM_001146345 | PQLC1 | 2489 | 6 | 2 | 1 | 1 | 1 | 3 | 3 | 6 | 1 | 1 | 1 | 1 | 1 |
| NM_001146590 | AGXT2L1 | 2099 | 45 | 9 | 0 | 1 | 9 | 45 | 1 | 37 | 0 | 0 | 0 | 1 | 0 |
| NM_001146627 | AGXT2L1 | 2020 | 47 | 9 | 0 | 1 | 10 | 47 | 1 | 39 | 0 | 0 | 0 | 1 | 0 |
| NM_001146683 | C14orf176 | 1525 | 1 | 0 | 0 | 1 | 0 | 0 | 0 | 0 | 0 | 0 | 0 | 0 | 0 |
| NM_001146684 | RNF222 | 3064 | 0 | 0 | 0 | 0 | 0 | 0 | 0 | 0 | 0 | 0 | 0 | 0 | 0 |
| NM_001146685 | TMEM88B | 491 | 3 | 0 | 0 | 0 | 0 | 3 | 0 | 0 | 0 | 0 | 0 | 0 | 0 |
| NM_001146686 | LOC647309 | 3624 | 1 | 0 | 0 | 0 | 0 | 0 | 1 | 1 | 0 | 0 | 0 | 0 | 0 |
| NM_001146688 | KDM3A | 4620 | 11 | 5 | 4 | 3 | 5 | 5 | 4 | 2 | 6 | 7 | 5 | 7 | 11 |
| NM_001146693 | LOC100130 | 931 | 1 | 0 | 0 | 1 | 0 | 0 | 1 | 0 | 0 | 0 | 0 | 1 | 0 |
| NM_001146694 | KDM4C | 4602 | 7 | 3 | 3 | 4 | 3 | 4 | 3 | 2 | 3 | 3 | 2 | 7 | 4 |
| NM_001146695 | KDM4C | 3835 | 7 | 4 | 3 | 4 | 3 | 4 | 3 | 2 | 3 | 3 | 2 | 7 | 4 |
| NM_001146696 | KDM4C | 3407 | 8 | 4 | 3 | 5 | 3 | 4 | 4 | 3 | 4 | 4 | 2 | 8 | 5 |
| NM_001146698 | RBM19 | 3634 | 3 | 2 | 2 | 2 | 2 | 2 | 2 | 2 | 2 | 3 | 2 | 3 | 3 |
| NM_001146699 | RBM19 | 4491 | 3 | 2 | 2 | 2 | 2 | 2 | 2 | 2 | 1 | 3 | 2 | 3 | 2 |
| NM_001146702 | KDM5C | 6095 | 3 | 2 | 1 | 3 | 2 | 2 | 2 | 1 | 2 | 2 | 2 | 2 | 2 |
| NM_001146705 | KDM5D | 5579 | 3 | 1 | 1 | 0 | 1 | 1 | 1 | 0 | 1 | 0 | 0 | 3 | 2 |
| NM_001146706 | KDM5D | 5315 | 3 | 1 | 1 | 0 | 1 | 1 | 1 | 0 | 1 | 0 | 0 | 3 | 2 |
| NM_001146726 | TIMD4 | 1285 | 3 | 1 | 0 | 0 | 0 | 0 | 1 | 2 | 0 | 0 | 0 | 3 | 3 |
| NM_001146728 | HRASLS5 | 3063 | 17 | 3 | 17 | 1 | 1 | 1 | 1 | 0 | 1 | 1 | 0 | 0 | 11 |
| NM_001146729 | HRASLS5 | 3083 | 17 | 3 | 17 | 1 | 1 | 1 | 1 | 0 | 1 | 1 | 0 | 0 | 11 |
| NM_001147 | ANGPT2 | 5269 | 4 | 2 | 2 | 0 | 4 | 1 | 1 | 1 | 1 | 3 | 4 | 3 | 2 |
| NM_001148 | ANK2 | 14243 | 53 | 9 | 3 | 2 | 7 | 53 | 20 | 0 | 1 | 6 | 3 | 3 | 2 |
| NM_001149 | ANK3 | 5072 | 37 | 13 | 1 | 3 | 37 | 28 | 32 | 2 | 5 | 6 | 13 | 8 | 7 |
| NM_001150 | ANPEP | 3650 | 29 | 6 | 2 | 10 | 1 | 0 | 29 | 21 | 1 | 0 | 0 | 1 | 0 |
| NM_001151 | SLC25A4 | 4419 | 141 | 23 | 3 | 4 | 141 | 23 | 11 | 7 | 3 | 2 | 51 | 2 | 6 |
| NM_001152 | SLC25A5 | 1349 | 42 | 17 | 10 | 18 | 15 | 16 | 42 | 28 | 18 | 8 | 3 | 20 | 6 |
| NM_001153 | ANXA4 | 2142 | 18 | 8 | 9 | 10 | 4 | 4 | 17 | 18 | 9 | 5 | 3 | 4 | 3 |
| NM_001153446 | SCOC | 1866 | 11 | 4 | 2 | 1 | 5 | 11 | 7 | 3 | 2 | 2 | 2 | 3 | 2 |
| NM_001153484 | SCOC | 1959 | 10 | 3 | 2 | 1 | 5 | 10 | 6 | 3 | 2 | 2 | 2 | 3 | 2 |
| NM_001153552 | SCOC | 1875 | 10 | 3 | 2 | 1 | 5 | 10 | 6 | 3 | 2 | 2 | 2 | 3 | 2 |
| NM_001153585 | SCOC | 1999 | 10 | 3 | 2 | 1 | 5 | 10 | 6 | 3 | 2 | 2 | 2 | 3 | 2 |

| transcript | gene | transcript length (nt) | max | mean | adipose | colon | heart | hypothalamus | kidney | liver | lung | ovary | skeletalmuscle | spleen | testes |
|---|---|---|---|---|---|---|---|---|---|---|---|---|---|---|---|
| NM_001153635 | SCOC | 1863 | 11 | 4 | 2 | 1 | 5 | 11 | 7 | 3 | 2 | 2 | 2 | 3 | 2 |
| NM_001153663 | SCOC | 1956 | 10 | 3 | 2 | 1 | 5 | 10 | 6 | 3 | 2 | 2 | 2 | 3 | 2 |
| NM_001153690 | SCOC | 1915 | 10 | 3 | 2 | 1 | 5 | 10 | 6 | 3 | 2 | 2 | 2 | 3 | 2 |
| NM_001154 | ANXA5 | 1599 | 110 | 36 | 110 | 14 | 44 | 13 | 30 | 22 | 49 | 24 | 10 | 48 | 34 |
| NM_001154458 | 12 Sep | 1203 | 4 | 0 | 0 | 0 | 0 | 0 | 0 | 0 | 0 | 0 | 0 | 0 | 4 |
| NM_001155 | ANXA6 | 2915 | 25 | 13 | 21 | 6 | 13 | 18 | 6 | 25 | 6 | 13 | 12 | 15 | 6 |
| NM_001156 | ANXA7 | 2138 | 24 | 17 | 19 | 8 | 20 | 19 | 24 | 24 | 13 | 10 | 23 | 17 | 10 |
| NM_001156474 | CCDC81 | 2780 | 2 | 1 | 0 | 0 | 0 | 0 | 0 | 0 | 1 | 0 | 0 | 0 | 2 |
| NM_001157 | ANXA11 | 2485 | 7 | 4 | 7 | 5 | 7 | 3 | 5 | 2 | 3 | 3 | 3 | 3 | 2 |
| NM_001158 | AOC2 | 2585 | 1 | 0 | 1 | 0 | 0 | 0 | 0 | 0 | 0 | 0 | 0 | 0 | 0 |
| NM_001158261 | CCDC42 | 1157 | 15 | 1 | 0 | 0 | 0 | 0 | 0 | 0 | 0 | 0 | 0 | 0 | 15 |
| NM_001159 | AOX1 | 4933 | 154 | 19 | 8 | 1 | 4 | 1 | 22 | 154 | 3 | 7 | 2 | 3 | 9 |
| NM_001159279 | ZNF716 | 5210 | 1 | 0 | 0 | 0 | 0 | 0 | 0 | 0 | 0 | 0 | 0 | 0 | 1 |
| NM_001159280 | ADAL | 3512 | 4 | 2 | 2 | 1 | 4 | 3 | 3 | 2 | 1 | 1 | 2 | 1 | 3 |
| NM_001159285 | ADAD1 | 1904 | 12 | 1 | 0 | 0 | 0 | 0 | 0 | 0 | 0 | 0 | 0 | 0 | 12 |
| NM_001159286 | HDAC10 | 2644 | 0 | 0 | 0 | 0 | 0 | 0 | 0 | 0 | 0 | 0 | 0 | 0 | 0 |
| NM_001159287 | TPI1 | 1458 | 66 | 38 | 23 | 27 | 61 | 66 | 51 | 48 | 21 | 19 | 44 | 31 | 23 |
| NM_001159291 | MICAL1 | 3384 | 2 | 1 | 1 | 2 | 1 | 1 | 1 | 0 | 1 | 0 | 0 | 2 | 1 |
| NM_001159293 | ZNF737 | 8390 | 25 | 5 | 2 | 1 | 2 | 7 | 6 | 0 | 3 | 25 | 1 | 6 | 5 |
| NM_001159295 | ADAD1 | 2001 | 11 | 1 | 0 | 0 | 0 | 0 | 0 | 0 | 0 | 0 | 0 | 0 | 11 |
| NM_001159296 | KLF8 | 4819 | 2 | 1 | 2 | 1 | 1 | 1 | 2 | 0 | 1 | 1 | 0 | 1 | 1 |
| NM_001159322 | PLA2G4C | 2492 | 3 | 1 | 0 | 1 | 1 | 2 | 1 | 0 | 0 | 0 | 3 | 2 | 0 |
| NM_001159323 | PLA2G4C | 2566 | 3 | 1 | 0 | 1 | 1 | 2 | 1 | 0 | 0 | 0 | 3 | 1 | 0 |
| NM_001159330 | ARHGAP27 | 3486 | 1 | 0 | 0 | 1 | 0 | 0 | 1 | 0 | 0 | 0 | 0 | 1 | 0 |
| NM_001159352 | REG4 | 1516 | 1 | 0 | 0 | 1 | 0 | 0 | 0 | 0 | 0 | 0 | 0 | 0 | 0 |
| NM_001159353 | REG4 | 1176 | 0 | 0 | 0 | 0 | 0 | 0 | 0 | 0 | 0 | 0 | 0 | 0 | 0 |
| NM_001159377 | MTHFSD | 3037 | 3 | 2 | 1 | 1 | 1 | 1 | 2 | 1 | 1 | 2 | 2 | 2 | 3 |
| NM_001159378 | MTHFSD | 3037 | 3 | 2 | 1 | 1 | 1 | 1 | 2 | 1 | 1 | 2 | 2 | 2 | 3 |
| NM_001159379 | MTHFSD | 3034 | 3 | 2 | 1 | 1 | 1 | 1 | 2 | 1 | 1 | 2 | 2 | 2 | 3 |
| NM_001159380 | MTHFSD | 3001 | 3 | 2 | 1 | 1 | 1 | 1 | 2 | 1 | 1 | 2 | 2 | 2 | 3 |
| NM_001159387 | B4GALNT2 | 1806 | 6 | 1 | 0 | 6 | 0 | 0 | 1 | 0 | 0 | 0 | 0 | 0 | 0 |
| NM_001159388 | B4GALNT2 | 1819 | 6 | 1 | 0 | 6 | 0 | 0 | 1 | 0 | 0 | 0 | 0 | 0 | 0 |
| NM_001159390 | GUK1 | 987 | 3 | 1 | 1 | 1 | 1 | 3 | 1 | 3 | 1 | 1 | 0 | 2 | 1 |
| NM_001159391 | GUK1 | 901 | 4 | 2 | 1 | 1 | 1 | 3 | 1 | 4 | 1 | 1 | 0 | 2 | 1 |
| NM_001159397 | FCRL1 | 2969 | 2 | 0 | 0 | 0 | 0 | 0 | 0 | 0 | 0 | 0 | 0 | 2 | 0 |
| NM_001159398 | FCRL1 | 3093 | 2 | 0 | 0 | 0 | 0 | 0 | 0 | 0 | 0 | 0 | 0 | 2 | 0 |
| NM_001159484 | NUP210L | 5428 | 3 | 1 | 0 | 0 | 0 | 0 | 0 | 0 | 0 | 1 | 1 | 1 | 3 |
| NM_001159491 | BRI3 | 699 | 1 | 0 | 0 | 0 | 0 | 0 | 0 | 0 | 0 | 0 | 1 | 0 | 0 |
| NM_001159495 | BANF2 | 369 | 0 | 0 | 0 | 0 | 0 | 0 | 0 | 0 | 0 | 0 | 0 | 0 | 0 |
| NM_001159508 | IVD | 4575 | 4 | 1 | 1 | 1 | 1 | 2 | 2 | 4 | 0 | 1 | 0 | 1 | 1 |
| NM_001159522 | ZNF727 | 1677 | 22 | 6 | 3 | 2 | 3 | 7 | 5 | 0 | 2 | 22 | 3 | 8 | 11 |
| NM_001159524 | ZNF735 | 1237 | 0 | 0 | 0 | 0 | 0 | 0 | 0 | 0 | 0 | 0 | 0 | 0 | 0 |
| NM_001159531 | BEGAIN | 2748 | 1 | 0 | 0 | 0 | 0 | 1 | 0 | 0 | 0 | 0 | 0 | 0 | 0 |
| NM_001159542 | POU5F1B | 1583 | 0 | 0 | 0 | 0 | 0 | 0 | 0 | 0 | 0 | 0 | 0 | 0 | 0 |
| NM_001159545 | RFPL2 | 1452 | 0 | 0 | 0 | 0 | 0 | 0 | 0 | 0 | 0 | 0 | 0 | 0 | 0 |
| NM_001159546 | RFPL2 | 1481 | 0 | 0 | 0 | 0 | 0 | 0 | 0 | 0 | 0 | 0 | 0 | 0 | 0 |
| NM_001159547 | BEND4 | 8777 | 4 | 1 | 0 | 0 | 0 | 1 | 0 | 0 | 0 | 0 | 0 | 1 | 4 |
| NM_001159554 | P2RX6 | 2666 | 1 | 0 | 1 | 0 | 0 | 0 | 0 | 0 | 0 | 0 | 1 | 0 | 0 |
| NM_001159560 | BEX5 | 860 | 44 | 6 | 0 | 1 | 1 | 44 | 4 | 0 | 4 | 2 | 0 | 2 | 4 |
| NM_001159575 | SCNN1A | 3198 | 19 | 4 | 0 | 9 | 0 | 0 | 19 | 1 | 11 | 0 | 0 | 0 | 1 |
| NM_001159576 | SCNN1A | 3479 | 21 | 4 | 0 | 11 | 0 | 1 | 21 | 1 | 12 | 0 | 0 | 0 | 2 |
| NM_001159587 | CD109 | 9401 | 4 | 2 | 4 | 1 | 2 | 1 | 3 | 0 | 4 | 1 | 1 | 1 | 1 |
| NM_001159588 | CD109 | 9221 | 4 | 1 | 4 | 1 | 2 | 1 | 2 | 0 | 3 | 1 | 1 | 1 | 1 |
| NM_001159596 | BIVM | 3200 | 3 | 2 | 1 | 1 | 2 | 1 | 3 | 2 | 1 | 2 | 1 | 2 | 1 |
| NM_001159597 | SH3YL1 | 1729 | 9 | 4 | 3 | 6 | 1 | 6 | 9 | 1 | 6 | 3 | 1 | 3 | 2 |
| NM_001159601 | RAB28 | 1789 | 3 | 2 | 1 | 1 | 3 | 3 | 2 | 1 | 2 | 2 | 1 | 2 | 3 |
| NM_001159629 | SLC27A2 | 2231 | 163 | 20 | 0 | 4 | 0 | 1 | 41 | 163 | 2 | 0 | 0 | 1 | 2 |
| NM_001159642 | BNIPL | 2412 | 1 | 0 | 0 | 0 | 0 | 0 | 0 | 0 | 1 | 0 | 0 | 0 | 0 |
| NM_001159643 | MCTP2 | 7454 | 6 | 1 | 0 | 2 | 1 | 0 | 1 | 0 | 3 | 0 | 0 | 6 | 1 |
| NM_001159644 | MCTP2 | 1996 | 13 | 3 | 0 | 3 | 1 | 1 | 1 | 1 | 5 | 1 | 0 | 13 | 1 |
| NM_001159651 | BOD1 | 1737 | 23 | 12 | 10 | 6 | 16 | 10 | 10 | 7 | 7 | 20 | 23 | 12 | 15 |
| NM_001159673 | SYNCRIP | 6764 | 20 | 14 | 11 | 5 | 15 | 11 | 14 | 13 | 11 | 20 | 16 | 20 | 14 |
| NM_001159674 | SYNCRIP | 6787 | 20 | 14 | 11 | 5 | 15 | 11 | 14 | 13 | 11 | 20 | 16 | 20 | 14 |
| NM_001159675 | SYNCRIP | 2560 | 35 | 23 | 22 | 12 | 28 | 20 | 24 | 26 | 16 | 27 | 24 | 35 | 19 |
| NM_001159676 | SYNCRIP | 6795 | 20 | 14 | 11 | 5 | 15 | 11 | 14 | 13 | 11 | 20 | 16 | 20 | 14 |
| NM_001159677 | SYNCRIP | 7327 | 21 | 15 | 12 | 7 | 17 | 12 | 15 | 13 | 12 | 21 | 17 | 21 | 14 |
| NM_001159694 | EMCN | 3996 | 5 | 1 | 2 | 0 | 3 | 0 | 5 | 0 | 3 | 1 | 1 | 0 | 1 |
| NM_001159699 | FHL1 | 2442 | 176 | 28 | 39 | 7 | 62 | 8 | 2 | 1 | 7 | 3 | 176 | 2 | 5 |
| NM_001159700 | FHL1 | 2437 | 176 | 28 | 39 | 7 | 62 | 8 | 2 | 1 | 7 | 3 | 176 | 2 | 5 |
| NM_001159701 | FHL1 | 2313 | 186 | 30 | 41 | 7 | 65 | 8 | 2 | 1 | 8 | 3 | 186 | 2 | 6 |
| NM_001159702 | FHL1 | 2706 | 159 | 26 | 35 | 6 | 56 | 7 | 2 | 1 | 7 | 2 | 159 | 2 | 5 |
| NM_001159703 | FHL1 | 2319 | 179 | 29 | 40 | 7 | 62 | 8 | 2 | 1 | 7 | 3 | 179 | 2 | 6 |
| NM_001159704 | FHL1 | 2888 | 149 | 24 | 33 | 6 | 52 | 6 | 1 | 1 | 6 | 2 | 149 | 2 | 5 |
| NM_001159707 | BTNL8 | 1749 | 1 | 0 | 0 | 1 | 0 | 0 | 0 | 0 | 0 | 0 | 0 | 0 | 1 |
| NM_001159708 | BTNL8 | 2206 | 1 | 0 | 0 | 1 | 0 | 0 | 0 | 0 | 0 | 0 | 0 | 0 | 1 |
| NM_001159709 | BTNL8 | 1800 | 1 | 0 | 0 | 1 | 0 | 0 | 0 | 0 | 0 | 0 | 0 | 0 | 1 |
| NM_001159710 | BTNL8 | 1600 | 1 | 0 | 0 | 1 | 0 | 0 | 0 | 0 | 0 | 0 | 0 | 0 | 1 |

| transcript | gene | transcript length (nt) | max | mean | adipose | colon | heart | hypothala mus | kidney | liver | lung | ovary | skeletalm uscle | spleen | testes |
|---|---|---|---|---|---|---|---|---|---|---|---|---|---|---|---|
| NM_001159726 | TSPO2 | 822 | 0 | 0 | 0 | 0 | 0 | 0 | 0 | 0 | 0 | 0 | 0 | 0 | 0 |
| NM_001159727 | PLBD2 | 2736 | 4 | 2 | 4 | 2 | 2 | 4 | 2 | 2 | 2 | 2 | 1 | 2 | 2 |
| NM_001159728 | SEPSECS | 5781 | 4 | 1 | 1 | 0 | 1 | 1 | 2 | 4 | 1 | 2 | 1 | 1 | 2 |
| NM_001159736 | BUD13 | 1802 | 10 | 6 | 5 | 5 | 5 | 5 | 4 | 4 | 5 | 7 | 6 | 9 | 10 |
| NM_001159740 | LTA | 1459 | 1 | 0 | 0 | 0 | 0 | 0 | 0 | 0 | 0 | 0 | 0 | 1 | 0 |
| NM_001159746 | ABR | 5602 | 28 | 6 | 3 | 10 | 6 | 28 | 2 | 1 | 4 | 3 | 1 | 6 | 2 |
| NM_001159747 | CC2D2B | 2113 | 1 | 0 | 0 | 0 | 0 | 0 | 0 | 0 | 0 | 0 | 0 | 0 | 1 |
| NM_001159749 | RIOK2 | 2226 | 3 | 2 | 2 | 1 | 3 | 3 | 2 | 3 | 1 | 3 | 2 | 3 | 3 |
| NM_001159767 | BZW2 | 1873 | 14 | 3 | 1 | 1 | 14 | 2 | 2 | 2 | 2 | 1 | 9 | 2 | 1 |
| NM_001159770 | SLC39A11 | 2782 | 5 | 2 | 3 | 2 | 2 | 2 | 5 | 3 | 3 | 1 | 1 | 3 | 2 |
| NM_001159772 | CANT1 | 3479 | 5 | 2 | 2 | 5 | 2 | 2 | 2 | 1 | 2 | 2 | 1 | 4 | 3 |
| NM_001159773 | CANT1 | 3340 | 5 | 2 | 2 | 5 | 2 | 2 | 2 | 1 | 2 | 2 | 1 | 4 | 3 |
| NM_001159846 | MPND | 1506 | 2 | 1 | 0 | 1 | 0 | 0 | 2 | 0 | 1 | 0 | 0 | 0 | 0 |
| NM_001159860 | ZNF583 | 2538 | 6 | 3 | 2 | 0 | 3 | 4 | 2 | 1 | 1 | 6 | 2 | 4 | 4 |
| NM_001159861 | ZNF583 | 2543 | 6 | 3 | 2 | 0 | 3 | 4 | 2 | 1 | 1 | 6 | 2 | 4 | 4 |
| NM_001159878 | ZPBP | 1221 | 6 | 1 | 0 | 0 | 0 | 0 | 0 | 0 | 0 | 0 | 0 | 0 | 6 |
| NM_001159920 | FLT1 | 6499 | 9 | 4 | 4 | 1 | 9 | 3 | 4 | 4 | 9 | 1 | 4 | 7 | 2 |
| NM_001159927 | FBXW12 | 1462 | 0 | 0 | 0 | 0 | 0 | 0 | 0 | 0 | 0 | 0 | 0 | 0 | 0 |
| NM_001159929 | FBXW12 | 1471 | 0 | 0 | 0 | 0 | 0 | 0 | 0 | 0 | 0 | 0 | 0 | 0 | 0 |
| NM_001159936 | EBNA1BP2 | 1503 | 11 | 7 | 4 | 3 | 7 | 11 | 11 | 10 | 4 | 4 | 4 | 7 | 9 |
| NM_001159944 | EVI5L | 3734 | 2 | 0 | 0 | 0 | 0 | 2 | 1 | 0 | 0 | 0 | 0 | 0 | 1 |
| NM_001159969 | EPS15 | 4350 | 23 | 11 | 12 | 4 | 10 | 23 | 7 | 6 | 11 | 9 | 10 | 14 | 12 |
| NM_001159995 | NRG1 | 2427 | 3 | 1 | 0 | 1 | 0 | 3 | 2 | 2 | 1 | 0 | 0 | 0 | 1 |
| NM_001159996 | NRG1 | 2411 | 2 | 1 | 0 | 1 | 0 | 2 | 1 | 1 | 0 | 0 | 0 | 0 | 0 |
| NM_001159999 | NRG1 | 2478 | 3 | 1 | 0 | 1 | 0 | 3 | 2 | 2 | 1 | 0 | 0 | 0 | 1 |
| NM_001160 | APAF1 | 7029 | 5 | 2 | 1 | 2 | 1 | 2 | 1 | 1 | 3 | 3 | 1 | 5 | 1 |
| NM_001160001 | NRG1 | 2376 | 3 | 1 | 0 | 1 | 0 | 3 | 2 | 2 | 1 | 0 | 0 | 0 | 1 |
| NM_001160002 | NRG1 | 1636 | 3 | 1 | 0 | 1 | 0 | 3 | 2 | 2 | 1 | 1 | 0 | 0 | 1 |
| NM_001160004 | NRG1 | 3211 | 2 | 1 | 0 | 1 | 0 | 2 | 1 | 2 | 0 | 0 | 0 | 0 | 1 |
| NM_001160005 | NRG1 | 1623 | 3 | 1 | 0 | 1 | 0 | 3 | 2 | 2 | 1 | 1 | 0 | 0 | 1 |
| NM_001160007 | NRG1 | 1585 | 3 | 1 | 0 | 1 | 0 | 3 | 2 | 2 | 1 | 1 | 0 | 0 | 1 |
| NM_001160008 | NRG1 | 2359 | 3 | 1 | 0 | 1 | 0 | 3 | 2 | 2 | 1 | 0 | 0 | 0 | 1 |
| NM_001160011 | ST6GALNAC | 1146 | 2 | 1 | 1 | 0 | 1 | 1 | 1 | 1 | 2 | 1 | 0 | 1 | 0 |
| NM_001160030 | FLT1 | 2968 | 16 | 7 | 6 | 1 | 16 | 4 | 7 | 7 | 12 | 2 | 5 | 12 | 4 |
| NM_001160031 | FLT1 | 1910 | 12 | 5 | 4 | 1 | 11 | 3 | 5 | 6 | 12 | 1 | 3 | 9 | 2 |
| NM_001160033 | SGOL2 | 4535 | 9 | 2 | 1 | 0 | 1 | 1 | 1 | 0 | 1 | 2 | 0 | 2 | 9 |
| NM_001160036 | RHOBTB2 | 5261 | 5 | 2 | 1 | 1 | 2 | 5 | 1 | 0 | 4 | 1 | 1 | 2 | 1 |
| NM_001160037 | RHOBTB2 | 4957 | 5 | 2 | 1 | 1 | 2 | 5 | 1 | 0 | 5 | 1 | 1 | 2 | 2 |
| NM_001160042 | IQCC | 2293 | 1 | 0 | 0 | 1 | 0 | 1 | 0 | 0 | 0 | 1 | 0 | 0 | 1 |
| NM_001160045 | GLI1 | 3396 | 0 | 0 | 0 | 0 | 0 | 0 | 0 | 0 | 0 | 0 | 0 | 0 | 0 |
| NM_001160046 | SGOL2 | 4029 | 11 | 2 | 1 | 0 | 1 | 1 | 1 | 0 | 1 | 2 | 0 | 2 | 11 |
| NM_001160047 | TXNDC16 | 4625 | 17 | 6 | 3 | 2 | 4 | 8 | 3 | 1 | 7 | 11 | 6 | 9 | 17 |
| NM_001160094 | ACOT13 | 4391 | 5 | 3 | 3 | 2 | 4 | 2 | 5 | 5 | 1 | 2 | 2 | 2 | 2 |
| NM_001160100 | CLDN10 | 2601 | 4 | 0 | 0 | 0 | 0 | 1 | 4 | 0 | 0 | 0 | 0 | 0 | 0 |
| NM_001160102 | LPO | 2729 | 0 | 0 | 0 | 0 | 0 | 0 | 0 | 0 | 0 | 0 | 0 | 0 | 0 |
| NM_001160103 | ZC3H14 | 4190 | 17 | 8 | 6 | 4 | 6 | 9 | 7 | 12 | 4 | 9 | 4 | 6 | 17 |
| NM_001160104 | ZC3H14 | 4175 | 17 | 8 | 6 | 4 | 6 | 9 | 7 | 12 | 4 | 9 | 4 | 6 | 17 |
| NM_001160109 | NOS3 | 2030 | 6 | 1 | 0 | 0 | 1 | 0 | 1 | 0 | 0 | 0 | 0 | 6 | 0 |
| NM_001160110 | NOS3 | 2376 | 5 | 1 | 0 | 0 | 0 | 0 | 1 | 0 | 0 | 0 | 0 | 5 | 0 |
| NM_001160111 | NOS3 | 2536 | 5 | 1 | 0 | 0 | 0 | 0 | 1 | 0 | 0 | 0 | 0 | 5 | 0 |
| NM_001160113 | C15orf40 | 998 | 0 | 0 | 0 | 0 | 0 | 0 | 0 | 0 | 0 | 0 | 0 | 0 | 0 |
| NM_001160114 | C15orf40 | 979 | 0 | 0 | 0 | 0 | 0 | 0 | 0 | 0 | 0 | 0 | 0 | 0 | 0 |
| NM_001160115 | C15orf40 | 552 | 1 | 0 | 0 | 0 | 0 | 0 | 0 | 0 | 0 | 0 | 0 | 1 | 1 |
| NM_001160116 | C15orf40 | 586 | 1 | 0 | 0 | 0 | 0 | 0 | 0 | 0 | 0 | 0 | 0 | 1 | 0 |
| NM_001160124 | KLF6 | 4535 | 23 | 13 | 17 | 6 | 11 | 7 | 10 | 14 | 23 | 11 | 19 | 16 | 7 |
| NM_001160125 | KLF6 | 4537 | 24 | 14 | 19 | 6 | 11 | 8 | 10 | 16 | 24 | 12 | 20 | 17 | 8 |
| NM_001160130 | KCNQ5 | 6553 | 10 | 2 | 0 | 0 | 0 | 7 | 0 | 0 | 0 | 0 | 10 | 1 | 0 |
| NM_001160132 | KCNQ5 | 6610 | 10 | 2 | 0 | 0 | 0 | 7 | 0 | 0 | 0 | 0 | 10 | 1 | 0 |
| NM_001160133 | KCNQ5 | 6637 | 10 | 2 | 0 | 0 | 0 | 7 | 0 | 0 | 0 | 0 | 10 | 1 | 0 |
| NM_001160134 | KCNQ5 | 6250 | 10 | 2 | 0 | 0 | 0 | 7 | 0 | 0 | 0 | 0 | 10 | 1 | 0 |
| NM_001160138 | C7orf63 | 3846 | 1 | 0 | 0 | 0 | 0 | 0 | 1 | 0 | 1 | 0 | 0 | 0 | 0 |
| NM_001160147 | DDHD1 | 12896 | 2 | 1 | 1 | 0 | 1 | 2 | 1 | 0 | 1 | 2 | 0 | 2 | 2 |
| NM_001160148 | DDHD1 | 12959 | 2 | 1 | 1 | 0 | 1 | 2 | 1 | 0 | 1 | 2 | 0 | 2 | 2 |
| NM_001160154 | MGAT4A | 3709 | 7 | 3 | 2 | 3 | 2 | 6 | 7 | 1 | 6 | 1 | 1 | 3 | 1 |
| NM_001160160 | SCN5A | 8405 | 3 | 0 | 0 | 0 | 3 | 0 | 0 | 0 | 0 | 0 | 0 | 0 | 0 |
| NM_001160161 | SCN5A | 8342 | 3 | 0 | 0 | 0 | 3 | 0 | 0 | 0 | 0 | 0 | 0 | 0 | 0 |
| NM_001160166 | HRH4 | 3503 | 0 | 0 | 0 | 0 | 0 | 0 | 0 | 0 | 0 | 0 | 0 | 0 | 0 |
| NM_001160167 | PRR5L | 3963 | 7 | 1 | 1 | 2 | 0 | 1 | 0 | 0 | 1 | 2 | 0 | 7 | 0 |
| NM_001160168 | PRR5L | 3362 | 7 | 1 | 1 | 2 | 0 | 1 | 1 | 0 | 1 | 2 | 0 | 7 | 0 |
| NM_001160169 | PRR5L | 3572 | 7 | 1 | 1 | 2 | 0 | 1 | 1 | 0 | 1 | 2 | 0 | 7 | 0 |
| NM_001160170 | NAT1 | 2332 | 1 | 0 | 0 | 1 | 0 | 0 | 0 | 0 | 1 | 0 | 0 | 0 | 0 |
| NM_001160171 | NAT1 | 2182 | 1 | 0 | 0 | 1 | 0 | 0 | 0 | 1 | 1 | 0 | 0 | 0 | 0 |
| NM_001160172 | NAT1 | 2103 | 1 | 0 | 0 | 1 | 0 | 0 | 1 | 1 | 1 | 0 | 0 | 0 | 0 |
| NM_001160173 | NAT1 | 1948 | 1 | 0 | 0 | 1 | 0 | 0 | 1 | 1 | 1 | 0 | 0 | 0 | 0 |
| NM_001160174 | NAT1 | 2021 | 1 | 0 | 0 | 0 | 0 | 0 | 0 | 1 | 1 | 0 | 0 | 0 | 0 |
| NM_001160175 | NAT1 | 2066 | 1 | 0 | 0 | 1 | 0 | 0 | 1 | 1 | 1 | 0 | 0 | 0 | 0 |
| NM_001160176 | NAT1 | 1916 | 1 | 0 | 0 | 1 | 0 | 0 | 1 | 1 | 1 | 0 | 0 | 0 | 0 |

| transcript | gene | transcript length (nt) | max | mean | adipose | colon | heart | hypothalamus | kidney | liver | lung | ovary | skeletalmuscle | spleen | testes |
|---|---|---|---|---|---|---|---|---|---|---|---|---|---|---|---|
| NM_001160179 | NAT1 | 2008 | 1 | 0 | 0 | 0 | 0 | 0 | 1 | 1 | 1 | 0 | 0 | 0 | 0 |
| NM_001160183 | ZNF138 | 2716 | 9 | 5 | 3 | 1 | 4 | 9 | 6 | 2 | 3 | 8 | 5 | 9 | 6 |
| NM_001160184 | PLEKHN1 | 2293 | 0 | 0 | 0 | 0 | 0 | 0 | 0 | 0 | 0 | 0 | 0 | 0 | 0 |
| NM_001160210 | SLC25A13 | 3192 | 14 | 3 | 1 | 1 | 1 | 1 | 4 | 14 | 1 | 1 | 1 | 2 | 2 |
| NM_001160213 | PMFBP1 | 3674 | 2 | 0 | 0 | 0 | 0 | 0 | 0 | 0 | 0 | 0 | 0 | 0 | 2 |
| NM_001160223 | RNF170 | 4116 | 2 | 1 | 1 | 0 | 1 | 2 | 2 | 1 | 1 | 1 | 1 | 1 | 1 |
| NM_001160224 | RNF170 | 2003 | 0 | 0 | 0 | 0 | 0 | 0 | 0 | 0 | 0 | 0 | 0 | 0 | 0 |
| NM_001160225 | RNF170 | 3765 | 2 | 1 | 1 | 0 | 1 | 2 | 2 | 1 | 1 | 2 | 1 | 1 | 1 |
| NM_001160226 | CNR1 | 5859 | 7 | 1 | 1 | 0 | 0 | 7 | 0 | 0 | 1 | 0 | 0 | 1 | 1 |
| NM_001160227 | SPG11 | 7448 | 9 | 5 | 4 | 3 | 5 | 4 | 6 | 4 | 5 | 5 | 4 | 9 | 6 |
| NM_001160230 | ADARB1 | 3468 | 4 | 2 | 2 | 1 | 2 | 3 | 1 | 0 | 4 | 1 | 1 | 2 | 1 |
| NM_001160233 | ATP1A1 | 3763 | 108 | 30 | 8 | 36 | 31 | 43 | 108 | 24 | 25 | 11 | 3 | 16 | 22 |
| NM_001160234 | ATP1A1 | 3572 | 113 | 31 | 9 | 38 | 33 | 45 | 113 | 26 | 26 | 12 | 3 | 17 | 23 |
| NM_001160243 | RPAIN | 2654 | 4 | 3 | 3 | 4 | 3 | 4 | 3 | 1 | 3 | 3 | 2 | 3 | 4 |
| NM_001160244 | RPAIN | 1538 | 6 | 3 | 2 | 2 | 5 | 6 | 2 | 2 | 3 | 2 | 4 | 3 | 4 |
| NM_001160246 | RPAIN | 1362 | 7 | 4 | 3 | 3 | 6 | 7 | 3 | 2 | 4 | 2 | 5 | 3 | 4 |
| NM_001160258 | CNR1 | 5897 | 7 | 1 | 1 | 0 | 0 | 7 | 0 | 0 | 1 | 0 | 0 | 1 | 1 |
| NM_001160259 | CNR1 | 5772 | 7 | 1 | 1 | 0 | 0 | 7 | 0 | 0 | 1 | 0 | 0 | 1 | 1 |
| NM_001160260 | CNR1 | 8970 | 5 | 1 | 1 | 1 | 0 | 5 | 0 | 0 | 1 | 0 | 0 | 1 | 1 |
| NM_001160264 | FEZF1 | 1954 | 0 | 0 | 0 | 0 | 0 | 0 | 0 | 0 | 0 | 0 | 0 | 0 | 0 |
| NM_001160266 | RPAIN | 1474 | 6 | 3 | 3 | 3 | 5 | 6 | 3 | 2 | 3 | 2 | 4 | 3 | 4 |
| NM_001160267 | RPAIN | 2478 | 5 | 3 | 3 | 5 | 4 | 4 | 3 | 2 | 3 | 4 | 3 | 3 | 4 |
| NM_001160277 | CERKL | 3154 | 1 | 0 | 0 | 0 | 0 | 0 | 1 | 0 | 0 | 0 | 0 | 0 | 0 |
| NM_001160300 | PANX2 | 2966 | 1 | 0 | 0 | 0 | 0 | 1 | 0 | 0 | 0 | 0 | 0 | 0 | 0 |
| NM_001160301 | DPYD | 1788 | 4 | 1 | 1 | 1 | 1 | 1 | 1 | 2 | 2 | 2 | 0 | 4 | 1 |
| NM_001160302 | SYNJ1 | 7059 | 10 | 2 | 1 | 0 | 1 | 10 | 1 | 0 | 1 | 1 | 1 | 2 | 1 |
| NM_001160305 | SETD6 | 2978 | 3 | 2 | 2 | 2 | 2 | 2 | 3 | 3 | 2 | 2 | 3 | 3 | 3 |
| NM_001160306 | SYNJ1 | 6942 | 10 | 2 | 1 | 0 | 1 | 10 | 1 | 0 | 1 | 1 | 1 | 2 | 1 |
| NM_001160308 | SETDB2 | 5619 | 6 | 3 | 2 | 1 | 3 | 2 | 3 | 1 | 3 | 4 | 1 | 4 | 6 |
| NM_001160325 | OR6P1 | 953 | 0 | 0 | 0 | 0 | 0 | 0 | 0 | 0 | 0 | 0 | 0 | 0 | 0 |
| NM_001160328 | SYT3 | 2842 | 2 | 0 | 0 | 0 | 0 | 2 | 0 | 0 | 0 | 0 | 0 | 0 | 0 |
| NM_001160329 | SYT3 | 2478 | 2 | 0 | 0 | 0 | 0 | 2 | 0 | 0 | 0 | 0 | 0 | 0 | 0 |
| NM_001160331 | NFASC | 9942 | 9 | 2 | 0 | 1 | 1 | 9 | 2 | 0 | 0 | 2 | 0 | 1 | 0 |
| NM_001160332 | NFASC | 10135 | 9 | 2 | 0 | 1 | 1 | 9 | 2 | 0 | 1 | 2 | 0 | 1 | 0 |
| NM_001160333 | NFASC | 2490 | 3 | 1 | 0 | 1 | 0 | 3 | 1 | 0 | 0 | 1 | 0 | 0 | 0 |
| NM_001160354 | LY6K | 1955 | 0 | 0 | 0 | 0 | 0 | 0 | 0 | 0 | 0 | 0 | 0 | 0 | 0 |
| NM_001160355 | LY6K | 1676 | 0 | 0 | 0 | 0 | 0 | 0 | 0 | 0 | 0 | 0 | 0 | 0 | 0 |
| NM_001160364 | TMC7 | 4495 | 1 | 0 | 0 | 1 | 0 | 1 | 0 | 0 | 0 | 0 | 0 | 0 | 1 |
| NM_001160367 | CDK10 | 1759 | 2 | 1 | 1 | 2 | 1 | 1 | 2 | 2 | 1 | 1 | 0 | 1 | 1 |
| NM_001160372 | TRAPPC9 | 4299 | 5 | 3 | 2 | 3 | 2 | 5 | 2 | 1 | 2 | 3 | 4 | 4 | 3 |
| NM_001160389 | TRPT1 | 1058 | 3 | 1 | 1 | 0 | 2 | 1 | 1 | 1 | 0 | 0 | 3 | 1 | 1 |
| NM_001160390 | TRPT1 | 1040 | 3 | 1 | 1 | 0 | 2 | 1 | 1 | 1 | 0 | 0 | 3 | 1 | 1 |
| NM_001160392 | TRPT1 | 941 | 4 | 1 | 1 | 1 | 2 | 1 | 1 | 1 | 0 | 0 | 4 | 1 | 1 |
| NM_001160393 | TRPT1 | 850 | 4 | 1 | 1 | 1 | 2 | 1 | 2 | 1 | 0 | 0 | 4 | 1 | 1 |
| NM_001160407 | TLCD1 | 886 | 2 | 1 | 0 | 1 | 1 | 1 | 1 | 2 | 1 | 0 | 0 | 1 | 1 |
| NM_001160408 | TULP3 | 2001 | 6 | 2 | 1 | 1 | 1 | 1 | 4 | 0 | 2 | 6 | 1 | 3 | 2 |
| NM_001160417 | ZBP1 | 2295 | 1 | 0 | 0 | 1 | 0 | 0 | 0 | 0 | 0 | 0 | 0 | 0 | 0 |
| NM_001160418 | ZBP1 | 2073 | 1 | 0 | 0 | 1 | 0 | 0 | 0 | 0 | 0 | 0 | 0 | 0 | 0 |
| NM_001160419 | ZBP1 | 1220 | 1 | 0 | 0 | 1 | 0 | 0 | 0 | 0 | 0 | 0 | 0 | 0 | 0 |
| NM_001160423 | IGF2BP1 | 8350 | 1 | 0 | 0 | 0 | 0 | 0 | 0 | 0 | 0 | 0 | 0 | 0 | 1 |
| NM_001160706 | SCEL | 3098 | 5 | 0 | 0 | 0 | 0 | 0 | 0 | 0 | 5 | 0 | 0 | 0 | 0 |
| NM_001160708 | POLR3B | 4118 | 4 | 2 | 2 | 1 | 2 | 2 | 3 | 4 | 1 | 3 | 3 | 2 | 3 |
| NM_001161 | NUDT2 | 1060 | 4 | 2 | 2 | 2 | 2 | 4 | 1 | 2 | 1 | 2 | 2 | 2 | 3 |
| NM_001161009 | ARMC10 | 2524 | 8 | 5 | 3 | 2 | 5 | 8 | 5 | 5 | 4 | 8 | 2 | 6 | 7 |
| NM_001161010 | ARMC10 | 2452 | 8 | 5 | 3 | 2 | 5 | 8 | 5 | 5 | 4 | 8 | 2 | 7 | 7 |
| NM_001161011 | ARMC10 | 2452 | 8 | 5 | 3 | 2 | 5 | 8 | 5 | 5 | 4 | 8 | 2 | 7 | 7 |
| NM_001161012 | ARMC10 | 2347 | 9 | 5 | 4 | 2 | 5 | 9 | 6 | 6 | 5 | 9 | 2 | 7 | 7 |
| NM_001161013 | ARMC10 | 2275 | 9 | 6 | 4 | 2 | 5 | 9 | 6 | 6 | 5 | 9 | 2 | 7 | 7 |
| NM_001161330 | SSH1 | 2346 | 3 | 2 | 2 | 1 | 2 | 2 | 2 | 1 | 2 | 2 | 2 | 3 | 2 |
| NM_001161331 | SSH1 | 2438 | 2 | 2 | 2 | 1 | 2 | 2 | 1 | 1 | 2 | 2 | 2 | 2 | 2 |
| NM_001161334 | HIST2H2BF | 999 | 2 | 1 | 0 | 1 | 0 | 2 | 0 | 0 | 2 | 1 | 0 | 1 | 1 |
| NM_001161342 | TMEM171 | 1263 | 1 | 0 | 0 | 1 | 0 | 0 | 1 | 0 | 0 | 0 | 0 | 1 | 0 |
| NM_001161344 | CHFR | 3281 | 2 | 1 | 1 | 1 | 1 | 1 | 1 | 1 | 1 | 1 | 1 | 2 | 1 |
| NM_001161345 | CHFR | 3278 | 2 | 1 | 1 | 1 | 1 | 1 | 1 | 1 | 1 | 1 | 1 | 2 | 1 |
| NM_001161346 | CHFR | 3245 | 2 | 1 | 1 | 1 | 1 | 1 | 1 | 1 | 1 | 0 | 1 | 2 | 1 |
| NM_001161347 | CHFR | 3005 | 2 | 1 | 1 | 1 | 1 | 1 | 1 | 1 | 1 | 1 | 1 | 2 | 1 |
| NM_001161352 | KCNMA1 | 6276 | 6 | 3 | 1 | 6 | 1 | 6 | 0 | 3 | 2 | 3 | 3 | 5 | 2 |
| NM_001161353 | KCNMA1 | 6225 | 6 | 3 | 1 | 6 | 1 | 6 | 0 | 3 | 2 | 3 | 3 | 5 | 2 |
| NM_001161354 | PLEKHA4 | 2787 | 36 | 15 | 14 | 9 | 36 | 8 | 9 | 14 | 8 | 28 | 11 | 13 | 16 |
| NM_001161357 | FCHO1 | 3225 | 2 | 0 | 0 | 0 | 0 | 2 | 0 | 0 | 0 | 0 | 0 | 0 | 0 |
| NM_001161358 | FCHO1 | 3121 | 2 | 0 | 0 | 0 | 0 | 2 | 0 | 0 | 0 | 0 | 0 | 0 | 0 |
| NM_001161359 | FCHO1 | 3051 | 2 | 0 | 0 | 0 | 0 | 2 | 0 | 0 | 0 | 0 | 0 | 0 | 0 |
| NM_001161363 | NPTN | 2441 | 19 | 8 | 4 | 6 | 13 | 19 | 9 | 3 | 6 | 6 | 2 | 10 | 11 |
| NM_001161364 | NPTN | 2093 | 22 | 9 | 5 | 7 | 15 | 22 | 10 | 4 | 8 | 6 | 3 | 12 | 13 |
| NM_001161368 | SEC14L4 | 2147 | 6 | 1 | 0 | 0 | 0 | 0 | 0 | 6 | 2 | 0 | 0 | 0 | 1 |
| NM_001161376 | C6orf136 | 1978 | 2 | 1 | 1 | 2 | 2 | 2 | 2 | 0 | 1 | 0 | 2 | 1 | 1 |
| NM_001161403 | LIMS2 | 2051 | 1 | 0 | 0 | 1 | 1 | 1 | 0 | 0 | 0 | 0 | 0 | 0 | 0 |

| | | transcript | | | | | | hypothala | | | | | skeletalm | | |
|---|---|---|---|---|---|---|---|---|---|---|---|---|---|---|---|
| transcript | gene | length (nt) | max | mean | adipose | colon | heart | mus | kidney | liver | lung | ovary | uscle | spleen | testes |
| NM_001161404 | LIMS2 | 2341 | 1 | 0 | 0 | 1 | 0 | 1 | 0 | 0 | 0 | 0 | 0 | 0 | 0 |
| NM_001161407 | NDFIP2 | 4588 | 16 | 4 | 1 | 2 | 2 | 16 | 5 | 4 | 2 | 1 | 2 | 2 | 2 |
| NM_001161415 | GPR17 | 2598 | 1 | 0 | 0 | 1 | 0 | 1 | 0 | 0 | 0 | 0 | 0 | 0 | 0 |
| NM_001161416 | GPR17 | 2533 | 1 | 0 | 0 | 1 | 0 | 1 | 0 | 0 | 0 | 0 | 0 | 0 | 0 |
| NM_001161417 | GPR17 | 2304 | 1 | 0 | 0 | 1 | 0 | 1 | 0 | 0 | 0 | 0 | 0 | 0 | 0 |
| NM_001161425 | ZNF610 | 2263 | 6 | 2 | 1 | 0 | 1 | 4 | 2 | 0 | 1 | 6 | 1 | 2 | 4 |
| NM_001161426 | ZNF610 | 2150 | 6 | 2 | 1 | 0 | 1 | 4 | 2 | 0 | 1 | 6 | 1 | 2 | 5 |
| NM_001161427 | ZNF610 | 2134 | 6 | 2 | 1 | 0 | 1 | 4 | 2 | 0 | 1 | 6 | 1 | 1 | 4 |
| NM_001161429 | RANBP3L | 2617 | 5 | 1 | 0 | 0 | 0 | 5 | 4 | 0 | 0 | 1 | 0 | 0 | 2 |
| NM_001161440 | PTPRH | 3393 | 2 | 0 | 0 | 2 | 0 | 0 | 0 | 1 | 0 | 0 | 0 | 2 | 0 |
| NM_001161441 | SH2D2A | 1673 | 1 | 0 | 0 | 0 | 0 | 0 | 0 | 0 | 0 | 0 | 0 | 1 | 0 |
| NM_001161442 | SH2D2A | 1612 | 1 | 0 | 0 | 0 | 0 | 0 | 0 | 0 | 0 | 0 | 0 | 1 | 0 |
| NM_001161443 | SH2D2A | 1522 | 1 | 0 | 0 | 0 | 0 | 0 | 0 | 0 | 0 | 0 | 0 | 1 | 0 |
| NM_001161444 | SH2D2A | 1428 | 1 | 0 | 0 | 0 | 0 | 0 | 0 | 0 | 0 | 0 | 0 | 1 | 0 |
| NM_001161452 | CYBASC3 | 2554 | 1 | 1 | 1 | 0 | 0 | 1 | 1 | 1 | 1 | 1 | 0 | 1 | 1 |
| NM_001161454 | CYBASC3 | 3209 | 1 | 1 | 1 | 0 | 0 | 1 | 1 | 1 | 0 | 1 | 0 | 1 | 1 |
| NM_001161465 | JMJD4 | 2546 | 0 | 0 | 0 | 0 | 0 | 0 | 0 | 0 | 0 | 0 | 0 | 0 | 0 |
| NM_001161473 | ALDH3B1 | 2855 | 2 | 1 | 1 | 0 | 1 | 0 | 1 | 0 | 2 | 0 | 0 | 2 | 0 |
| NM_001161476 | WDR25 | 1988 | 1 | 1 | 0 | 1 | 0 | 1 | 1 | 0 | 1 | 1 | 1 | 1 | 0 |
| NM_001161482 | ZMYND11 | 4297 | 23 | 14 | 11 | 5 | 19 | 23 | 16 | 8 | 11 | 17 | 13 | 15 | 15 |
| NM_001161498 | FLJ44817 | 1812 | 0 | 0 | 0 | 0 | 0 | 0 | 0 | 0 | 0 | 0 | 0 | 0 | 0 |
| NM_001161499 | ZNF611 | 4515 | 6 | 3 | 2 | 1 | 2 | 4 | 6 | 1 | 3 | 6 | 1 | 5 | 4 |
| NM_001161500 | ZNF611 | 4415 | 6 | 3 | 2 | 1 | 2 | 4 | 6 | 1 | 3 | 6 | 1 | 5 | 4 |
| NM_001161501 | ZNF611 | 4378 | 6 | 3 | 2 | 1 | 2 | 4 | 6 | 1 | 3 | 6 | 1 | 5 | 4 |
| NM_001161504 | ALDH4A1 | 3241 | 10 | 2 | 1 | 0 | 1 | 1 | 10 | 6 | 0 | 0 | 1 | 0 | 0 |
| NM_001161520 | COG5 | 5666 | 7 | 5 | 5 | 4 | 7 | 5 | 4 | 3 | 4 | 6 | 4 | 5 | 6 |
| NM_001161521 | KLHL2 | 3013 | 28 | 9 | 5 | 3 | 5 | 28 | 7 | 15 | 9 | 2 | 2 | 16 | 6 |
| NM_001161522 | KLHL2 | 2906 | 29 | 9 | 5 | 3 | 5 | 29 | 7 | 15 | 8 | 2 | 2 | 16 | 6 |
| NM_001161527 | TNIP2 | 1688 | 9 | 6 | 8 | 9 | 5 | 4 | 5 | 6 | 4 | 3 | 4 | 8 | 7 |
| NM_001161528 | LOC401387 | 2661 | 1 | 0 | 0 | 0 | 0 | 0 | 0 | 0 | 0 | 0 | 0 | 0 | 1 |
| NM_001161529 | CSF2RA | 1981 | 5 | 1 | 2 | 1 | 1 | 1 | 0 | 0 | 4 | 1 | 0 | 5 | 1 |
| NM_001161530 | CSF2RA | 1787 | 6 | 2 | 2 | 1 | 1 | 1 | 1 | 0 | 4 | 1 | 0 | 6 | 1 |
| NM_001161531 | CSF2RA | 1510 | 6 | 2 | 2 | 1 | 1 | 1 | 1 | 0 | 4 | 1 | 0 | 6 | 1 |
| NM_001161532 | CSF2RA | 1622 | 6 | 2 | 2 | 1 | 1 | 1 | 1 | 0 | 4 | 1 | 0 | 6 | 1 |
| NM_001161533 | RBM34 | 905 | 5 | 3 | 4 | 2 | 3 | 5 | 4 | 4 | 2 | 3 | 3 | 4 | 4 |
| NM_001161546 | LOC389333 | 3251 | 1 | 0 | 0 | 0 | 0 | 0 | 0 | 0 | 0 | 0 | 1 | 0 | 0 |
| NM_001161560 | TNIK | 5782 | 18 | 5 | 1 | 4 | 4 | 18 | 3 | 3 | 4 | 1 | 5 | 2 | 11 |
| NM_001161561 | TNIK | 5719 | 18 | 5 | 1 | 4 | 4 | 18 | 3 | 3 | 4 | 1 | 5 | 2 | 11 |
| NM_001161562 | TNIK | 5695 | 18 | 5 | 1 | 4 | 4 | 18 | 3 | 3 | 4 | 1 | 5 | 2 | 11 |
| NM_001161563 | TNIK | 5641 | 19 | 5 | 1 | 4 | 4 | 19 | 3 | 3 | 4 | 1 | 5 | 2 | 11 |
| NM_001161564 | TNIK | 5617 | 19 | 5 | 1 | 4 | 4 | 19 | 3 | 3 | 4 | 1 | 5 | 2 | 11 |
| NM_001161565 | TNIK | 5554 | 19 | 5 | 1 | 4 | 4 | 19 | 4 | 3 | 4 | 1 | 5 | 2 | 11 |
| NM_001161566 | TNIK | 5530 | 19 | 5 | 1 | 4 | 4 | 19 | 4 | 3 | 4 | 1 | 5 | 2 | 11 |
| NM_001161572 | MAFF | 2463 | 14 | 5 | 10 | 2 | 8 | 3 | 1 | 3 | 14 | 5 | 8 | 3 | 3 |
| NM_001161573 | MAFF | 2221 | 15 | 6 | 10 | 2 | 8 | 4 | 2 | 4 | 15 | 6 | 9 | 3 | 3 |
| NM_001161574 | MAFF | 2370 | 14 | 6 | 10 | 2 | 8 | 3 | 1 | 3 | 14 | 5 | 8 | 3 | 3 |
| NM_001161575 | LRRC36 | 2130 | 3 | 0 | 0 | 0 | 0 | 0 | 0 | 0 | 0 | 0 | 0 | 0 | 3 |
| NM_001161576 | SAMD4A | 6262 | 11 | 4 | 5 | 2 | 11 | 5 | 2 | 1 | 3 | 1 | 5 | 2 | 2 |
| NM_001161577 | SAMD4A | 5570 | 11 | 4 | 5 | 2 | 11 | 5 | 2 | 1 | 3 | 1 | 5 | 2 | 1 |
| NM_001161580 | WDR51A | 2051 | 0 | 0 | 0 | 0 | 0 | 0 | 0 | 0 | 0 | 0 | 0 | 0 | 0 |
| NM_001161581 | WDR51A | 2266 | 0 | 0 | 0 | 0 | 0 | 0 | 0 | 0 | 0 | 0 | 0 | 0 | 0 |
| NM_001161584 | TEX10 | 3006 | 6 | 3 | 2 | 1 | 2 | 3 | 2 | 1 | 2 | 4 | 3 | 4 | 6 |
| NM_001161585 | C9orf11 | 948 | 1 | 0 | 0 | 0 | 0 | 0 | 0 | 0 | 0 | 0 | 0 | 0 | 1 |
| NM_001161586 | ME3 | 2311 | 3 | 1 | 1 | 1 | 1 | 3 | 1 | 0 | 0 | 2 | 1 | 1 | 1 |
| NM_001161587 | GYS1 | 3425 | 10 | 3 | 3 | 2 | 7 | 2 | 3 | 1 | 2 | 1 | 10 | 2 | 1 |
| NM_001161616 | RGL3 | 2561 | 3 | 1 | 1 | 1 | 0 | 1 | 3 | 0 | 1 | 0 | 0 | 2 | 0 |
| NM_001161619 | DUS3L | 1383 | 1 | 0 | 0 | 0 | 0 | 0 | 0 | 0 | 0 | 0 | 0 | 1 | 0 |
| NM_001161625 | NXNL2 | 804 | 0 | 0 | 0 | 0 | 0 | 0 | 0 | 0 | 0 | 0 | 0 | 0 | 0 |
| NM_001161630 | KDM4DL | 2339 | 0 | 0 | 0 | 0 | 0 | 0 | 0 | 0 | 0 | 0 | 0 | 0 | 0 |
| NM_001161656 | MYB | 3669 | 6 | 1 | 0 | 6 | 0 | 0 | 0 | 0 | 1 | 0 | 0 | 1 | 0 |
| NM_001161657 | MYB | 3060 | 7 | 1 | 0 | 7 | 0 | 0 | 0 | 0 | 1 | 0 | 0 | 1 | 0 |
| NM_001161658 | MYB | 3630 | 6 | 1 | 0 | 6 | 0 | 0 | 0 | 0 | 1 | 0 | 0 | 1 | 0 |
| NM_001161659 | MYB | 3204 | 7 | 1 | 0 | 7 | 0 | 0 | 0 | 0 | 1 | 0 | 0 | 1 | 0 |
| NM_001161660 | MYB | 3210 | 7 | 1 | 0 | 7 | 0 | 0 | 0 | 0 | 1 | 0 | 0 | 1 | 0 |
| NM_001161661 | WWC1 | 6737 | 12 | 2 | 0 | 1 | 0 | 3 | 12 | 2 | 3 | 0 | 0 | 0 | 0 |
| NM_001161662 | WWC1 | 6734 | 12 | 2 | 0 | 1 | 0 | 3 | 12 | 2 | 3 | 0 | 0 | 0 | 0 |
| NM_001161664 | RSPH4A | 2704 | 2 | 0 | 0 | 0 | 0 | 1 | 0 | 0 | 2 | 0 | 0 | 0 | 1 |
| NM_001161683 | OTOA | 3465 | 0 | 0 | 0 | 0 | 0 | 0 | 0 | 0 | 0 | 0 | 0 | 0 | 0 |
| NM_001161705 | BFSP1 | 2062 | 2 | 0 | 0 | 0 | 0 | 1 | 0 | 0 | 0 | 1 | 0 | 0 | 2 |
| NM_001161706 | FXN | 980 | 2 | 1 | 2 | 1 | 2 | 1 | 1 | 2 | 0 | 2 | 2 | 2 | 2 |
| NM_001161707 | KIRREL3 | 2534 | 0 | 0 | 0 | 0 | 0 | 0 | 0 | 0 | 0 | 0 | 0 | 0 | 0 |
| NM_001161708 | SYNC | 3413 | 13 | 3 | 3 | 2 | 4 | 1 | 0 | 0 | 1 | 1 | 13 | 1 | 2 |
| NM_001161725 | PPP2R5C | 4481 | 22 | 12 | 7 | 5 | 22 | 18 | 8 | 6 | 11 | 14 | 8 | 22 | 14 |
| NM_001161726 | PPP2R5C | 4436 | 22 | 12 | 7 | 5 | 22 | 19 | 9 | 7 | 11 | 14 | 8 | 22 | 14 |
| NM_001161727 | PLA2G2A | 968 | 641 | 122 | 19 | 26 | 572 | 1 | 6 | 641 | 9 | 12 | 25 | 3 | 21 |
| NM_001161728 | PLA2G2A | 888 | 699 | 132 | 21 | 28 | 624 | 1 | 7 | 699 | 10 | 13 | 27 | 4 | 23 |
| NM_001161729 | PLA2G2A | 922 | 673 | 128 | 20 | 28 | 601 | 1 | 6 | 673 | 10 | 13 | 26 | 3 | 22 |

| transcript | gene | transcript length (nt) | max | mean | adipose | colon | heart | hypothala mus | kidney | liver | lung | ovary | skeletalm uscle | spleen | testes |
|---|---|---|---|---|---|---|---|---|---|---|---|---|---|---|---|
| NM_001161748 | LIM2 | 857 | 0 | 0 | 0 | 0 | 0 | 0 | 0 | 0 | 0 | 0 | 0 | 0 | 0 |
| NM_001161766 | AHCY | 2371 | 45 | 15 | 10 | 9 | 11 | 12 | 24 | 45 | 7 | 16 | 10 | 11 | 15 |
| NM_001161772 | HTR3A | 1994 | 0 | 0 | 0 | 0 | 0 | 0 | 0 | 0 | 0 | 0 | 0 | 0 | 0 |
| NM_001161778 | PDP1 | 4233 | 12 | 4 | 2 | 1 | 10 | 12 | 4 | 1 | 2 | 3 | 5 | 4 | 6 |
| NM_001161779 | PDP1 | 4358 | 11 | 4 | 2 | 1 | 10 | 11 | 4 | 1 | 2 | 3 | 4 | 4 | 6 |
| NM_001161780 | PDP1 | 4232 | 12 | 4 | 2 | 1 | 10 | 12 | 4 | 1 | 2 | 3 | 5 | 4 | 6 |
| NM_001161781 | PDP1 | 4219 | 12 | 4 | 2 | 1 | 10 | 12 | 4 | 1 | 2 | 3 | 5 | 4 | 6 |
| NM_001161808 | GPR144 | 2891 | 0 | 0 | 0 | 0 | 0 | 0 | 0 | 0 | 0 | 0 | 0 | 0 | 0 |
| NM_001161812 | DAOA | 923 | 0 | 0 | 0 | 0 | 0 | 0 | 0 | 0 | 0 | 0 | 0 | 0 | 0 |
| NM_001161814 | DAOA | 1008 | 0 | 0 | 0 | 0 | 0 | 0 | 0 | 0 | 0 | 0 | 0 | 0 | 0 |
| NM_001161819 | MYO1B | 4935 | 83 | 14 | 7 | 4 | 6 | 3 | 8 | 83 | 18 | 7 | 2 | 7 | 5 |
| NM_001161834 | C7orf72 | 1910 | 0 | 0 | 0 | 0 | 0 | 0 | 0 | 0 | 0 | 0 | 0 | 0 | 0 |
| NM_001161841 | SULF2 | 4238 | 35 | 8 | 4 | 5 | 8 | 8 | 10 | 7 | 3 | 35 | 2 | 3 | 8 |
| NM_001162367 | LOC10012 | 484 | 0 | 0 | 0 | 0 | 0 | 0 | 0 | 0 | 0 | 0 | 0 | 0 | 0 |
| NM_001162371 | LOC728392 | 1716 | 18 | 2 | 1 | 0 | 0 | 18 | 0 | 0 | 0 | 1 | 0 | 1 | 1 |
| NM_001162383 | ARHGEF2 | 4182 | 6 | 3 | 4 | 3 | 3 | 6 | 1 | 0 | 4 | 2 | 1 | 6 | 1 |
| NM_001162384 | ARHGEF2 | 4179 | 6 | 3 | 4 | 3 | 3 | 6 | 1 | 0 | 4 | 2 | 1 | 6 | 1 |
| NM_001162407 | CLK1 | 2212 | 80 | 42 | 48 | 22 | 27 | 25 | 25 | 13 | 80 | 60 | 56 | 60 | 49 |
| NM_001162422 | ETS1 | 4594 | 59 | 13 | 11 | 4 | 10 | 2 | 7 | 4 | 27 | 8 | 4 | 59 | 4 |
| NM_001162426 | TSC1 | 8613 | 4 | 3 | 2 | 3 | 3 | 4 | 2 | 1 | 1 | 4 | 2 | 2 | 3 |
| NM_001162427 | TSC1 | 8463 | 4 | 3 | 2 | 3 | 3 | 4 | 2 | 1 | 1 | 4 | 2 | 2 | 3 |
| NM_001162429 | PDCD6IP | 5954 | 8 | 6 | 7 | 4 | 7 | 6 | 8 | 7 | 6 | 6 | 5 | 8 | 7 |
| NM_001162435 | LOC100287 | 999 | 0 | 0 | 0 | 0 | 0 | 0 | 0 | 0 | 0 | 0 | 0 | 0 | 0 |
| NM_001162483 | C2orf83 | 2087 | 0 | 0 | 0 | 0 | 0 | 0 | 0 | 0 | 0 | 0 | 0 | 0 | 0 |
| NM_001162490 | ARL13A | 1535 | 1 | 0 | 0 | 0 | 0 | 0 | 0 | 0 | 0 | 0 | 0 | 1 | 1 |
| NM_001162491 | ARL13A | 1128 | 1 | 0 | 0 | 0 | 0 | 0 | 0 | 0 | 0 | 0 | 0 | 1 | 1 |
| NM_001162495 | C21orf62 | 4119 | 1 | 0 | 0 | 0 | 0 | 0 | 1 | 0 | 0 | 1 | 0 | 0 | 0 |
| NM_001162496 | C21orf62 | 3963 | 1 | 0 | 0 | 0 | 0 | 0 | 1 | 0 | 0 | 1 | 0 | 0 | 0 |
| NM_001162497 | LPAR6 | 2519 | 7 | 4 | 6 | 1 | 4 | 2 | 4 | 1 | 6 | 4 | 2 | 7 | 3 |
| NM_001162498 | LPAR6 | 2470 | 7 | 4 | 6 | 1 | 4 | 3 | 4 | 1 | 7 | 4 | 2 | 7 | 2 |
| NM_001162499 | CAND2 | 4592 | 9 | 2 | 0 | 0 | 2 | 1 | 0 | 0 | 0 | 0 | 9 | 0 | 3 |
| NM_001162501 | TNRC6B | 18279 | 7 | 5 | 3 | 3 | 4 | 5 | 4 | 3 | 4 | 7 | 5 | 7 | 7 |
| NM_001162505 | TMEM189 | 2286 | 1 | 1 | 1 | 1 | 1 | 1 | 0 | 1 | 1 | 0 | 1 | 1 | 0 |
| NM_001162529 | FAM135A | 5932 | 10 | 5 | 2 | 3 | 5 | 8 | 6 | 2 | 5 | 8 | 3 | 5 | 10 |
| NM_001162530 | C1orf113 | 2519 | 0 | 0 | 0 | 0 | 0 | 0 | 0 | 0 | 0 | 0 | 0 | 0 | 0 |
| NM_001162536 | RBMXL1 | 5086 | 5 | 2 | 1 | 1 | 2 | 2 | 1 | 2 | 2 | 3 | 1 | 3 | 5 |
| NM_001162861 | COX11 | 825 | 6 | 3 | 2 | 1 | 2 | 6 | 4 | 4 | 2 | 1 | 2 | 2 | 2 |
| NM_001162862 | COX11 | 811 | 6 | 3 | 2 | 1 | 2 | 6 | 4 | 4 | 2 | 1 | 2 | 2 | 2 |
| NM_001162893 | KIAA0040 | 4721 | 8 | 3 | 5 | 1 | 3 | 2 | 3 | 2 | 5 | 1 | 1 | 8 | 1 |
| NM_001162894 | KIAA0040 | 4644 | 9 | 3 | 5 | 1 | 3 | 2 | 3 | 2 | 5 | 1 | 1 | 9 | 1 |
| NM_001162895 | KIAA0040 | 4510 | 9 | 3 | 5 | 1 | 3 | 2 | 3 | 2 | 5 | 1 | 1 | 9 | 1 |
| NM_001162900 | TMEM217 | 1992 | 1 | 0 | 0 | 0 | 0 | 0 | 0 | 0 | 0 | 0 | 0 | 0 | 1 |
| NM_001162914 | LOC100130 | 1319 | 0 | 0 | 0 | 0 | 0 | 0 | 0 | 0 | 0 | 0 | 0 | 0 | 0 |
| NM_001162916 | TYW3 | 3335 | 3 | 2 | 1 | 1 | 2 | 3 | 3 | 2 | 1 | 2 | 1 | 2 | 2 |
| NM_001162951 | SYTL2 | 4052 | 8 | 4 | 2 | 7 | 2 | 7 | 8 | 1 | 4 | 2 | 2 | 3 | 3 |
| NM_001162952 | SYTL2 | 2640 | 9 | 4 | 2 | 5 | 2 | 9 | 7 | 1 | 4 | 2 | 2 | 2 | 3 |
| NM_001162953 | SYTL2 | 4055 | 8 | 4 | 2 | 7 | 2 | 7 | 8 | 1 | 4 | 2 | 2 | 3 | 3 |
| NM_001162995 | LOC643008 | 1548 | 0 | 0 | 0 | 0 | 0 | 0 | 0 | 0 | 0 | 0 | 0 | 0 | 0 |
| NM_001162997 | LOC100130 | 826 | 1 | 0 | 0 | 0 | 0 | 0 | 1 | 0 | 0 | 0 | 0 | 0 | 1 |
| NM_001163 | APBA1 | 6584 | 11 | 2 | 0 | 1 | 0 | 11 | 0 | 1 | 0 | 1 | 0 | 1 | 1 |
| NM_001163022 | PLK1S1 | 2033 | 19 | 8 | 5 | 4 | 7 | 9 | 6 | 2 | 5 | 19 | 8 | 6 | 17 |
| NM_001163023 | PLK1S1 | 1943 | 16 | 7 | 4 | 3 | 6 | 8 | 5 | 2 | 4 | 16 | 7 | 5 | 13 |
| NM_001163034 | RPTOR | 6382 | 2 | 1 | 1 | 2 | 1 | 2 | 1 | 1 | 1 | 1 | 2 | 2 | 1 |
| NM_001163075 | C17orf99 | 1089 | 0 | 0 | 0 | 0 | 0 | 0 | 0 | 0 | 0 | 0 | 0 | 0 | 0 |
| NM_001163079 | CECR6 | 3961 | 1 | 0 | 0 | 0 | 0 | 1 | 0 | 0 | 0 | 0 | 0 | 0 | 0 |
| NM_001163120 | RDM1 | 809 | 1 | 0 | 0 | 0 | 0 | 0 | 0 | 0 | 0 | 1 | 0 | 1 | 1 |
| NM_001163121 | RDM1 | 1064 | 2 | 1 | 1 | 0 | 1 | 1 | 1 | 0 | 0 | 2 | 0 | 2 | 1 |
| NM_001163122 | RDM1 | 963 | 2 | 1 | 1 | 1 | 1 | 1 | 1 | 0 | 1 | 2 | 0 | 2 | 2 |
| NM_001163124 | RDM1 | 708 | 1 | 0 | 0 | 0 | 0 | 0 | 0 | 0 | 0 | 1 | 0 | 1 | 1 |
| NM_001163125 | RDM1 | 545 | 1 | 0 | 0 | 0 | 0 | 0 | 1 | 0 | 1 | 1 | 0 | 1 | 1 |
| NM_001163130 | RDM1 | 1062 | 2 | 1 | 1 | 0 | 1 | 1 | 1 | 0 | 0 | 2 | 0 | 2 | 1 |
| NM_001163147 | ETV1 | 6540 | 4 | 1 | 0 | 0 | 2 | 4 | 0 | 0 | 4 | 0 | 0 | 1 | 1 |
| NM_001163148 | ETV1 | 6348 | 5 | 1 | 0 | 0 | 3 | 5 | 0 | 0 | 4 | 0 | 0 | 1 | 2 |
| NM_001163149 | ETV1 | 6219 | 5 | 1 | 0 | 0 | 3 | 5 | 0 | 0 | 4 | 0 | 0 | 1 | 2 |
| NM_001163150 | ETV1 | 6285 | 4 | 1 | 0 | 0 | 2 | 4 | 0 | 0 | 3 | 0 | 0 | 1 | 1 |
| NM_001163151 | ETV1 | 6231 | 4 | 1 | 0 | 0 | 2 | 4 | 0 | 0 | 3 | 0 | 0 | 1 | 1 |
| NM_001163152 | ETV1 | 6096 | 4 | 1 | 0 | 0 | 2 | 4 | 0 | 0 | 3 | 0 | 0 | 1 | 1 |
| NM_001163213 | FGFR3 | 4293 | 4 | 1 | 0 | 1 | 0 | 4 | 2 | 1 | 0 | 0 | 0 | 0 | 1 |
| NM_001163257 | PLXNB3 | 6377 | 0 | 0 | 0 | 0 | 0 | 0 | 0 | 0 | 0 | 0 | 0 | 0 | 0 |
| NM_001163258 | FAM63A | 2238 | 3 | 2 | 1 | 2 | 1 | 2 | 3 | 1 | 1 | 2 | 1 | 1 | 2 |
| NM_001163259 | FAM63A | 2140 | 3 | 1 | 1 | 1 | 1 | 2 | 3 | 1 | 1 | 2 | 1 | 1 | 2 |
| NM_001163260 | FAM63A | 2051 | 3 | 1 | 1 | 1 | 1 | 2 | 3 | 1 | 1 | 2 | 1 | 1 | 2 |
| NM_001163264 | PCYT1B | 5491 | 2 | 0 | 0 | 0 | 0 | 2 | 0 | 0 | 0 | 0 | 0 | 0 | 1 |
| NM_001163265 | PCYT1B | 5223 | 2 | 0 | 0 | 0 | 0 | 2 | 0 | 0 | 0 | 0 | 0 | 0 | 1 |
| NM_001163278 | ODZ1 | 12893 | 3 | 1 | 1 | 0 | 0 | 3 | 1 | 1 | 0 | 0 | 0 | 0 | 0 |
| NM_001163279 | ODZ1 | 12890 | 3 | 1 | 1 | 0 | 0 | 3 | 1 | 1 | 0 | 0 | 0 | 0 | 0 |
| NM_001163280 | HTATSF1 | 3019 | 32 | 18 | 17 | 6 | 25 | 32 | 17 | 8 | 9 | 18 | 29 | 12 | 29 |

| transcript | gene | transcript length (nt) | max | mean | adipose | colon | heart | hypothalamus | kidney | liver | lung | ovary | skeletalmuscle | spleen | testes |
|---|---|---|---|---|---|---|---|---|---|---|---|---|---|---|---|
| NM_001163285 | EWSR1 | 2659 | 40 | 24 | 19 | 20 | 25 | 24 | 25 | 28 | 19 | 16 | 18 | 40 | 30 |
| NM_001163286 | EWSR1 | 2494 | 39 | 23 | 18 | 18 | 24 | 21 | 25 | 25 | 19 | 15 | 18 | 39 | 29 |
| NM_001163287 | EWSR1 | 1571 | 33 | 20 | 13 | 18 | 20 | 19 | 20 | 24 | 14 | 15 | 16 | 33 | 25 |
| NM_001163296 | MARK2 | 4521 | 5 | 3 | 2 | 3 | 2 | 5 | 2 | 2 | 2 | 2 | 2 | 5 | 2 |
| NM_001163297 | MARK2 | 4491 | 5 | 3 | 2 | 3 | 2 | 5 | 2 | 2 | 2 | 2 | 2 | 5 | 2 |
| NM_001163315 | FBXL17 | 5194 | 6 | 2 | 2 | 1 | 3 | 6 | 2 | 2 | 2 | 2 | 3 | 2 | 2 |
| NM_001163321 | CCDC120 | 3593 | 0 | 0 | 0 | 0 | 0 | 0 | 0 | 0 | 0 | 0 | 0 | 0 | 0 |
| NM_001163322 | CCDC120 | 3541 | 0 | 0 | 0 | 0 | 0 | 0 | 0 | 0 | 0 | 0 | 0 | 0 | 0 |
| NM_001163323 | CCDC120 | 4023 | 0 | 0 | 0 | 0 | 0 | 0 | 0 | 0 | 0 | 0 | 0 | 0 | 0 |
| NM_001163334 | SYTL5 | 4704 | 2 | 0 | 0 | 1 | 0 | 2 | 0 | 1 | 0 | 0 | 0 | 0 | 0 |
| NM_001163335 | SYTL5 | 4816 | 2 | 0 | 0 | 1 | 0 | 2 | 0 | 1 | 0 | 0 | 0 | 0 | 0 |
| NM_001163377 | QPCTL | 2021 | 0 | 0 | 0 | 0 | 0 | 0 | 0 | 0 | 0 | 0 | 0 | 0 | 0 |
| NM_001163380 | RAB2B | 2854 | 2 | 1 | 1 | 0 | 1 | 2 | 1 | 1 | 1 | 1 | 0 | 1 | 1 |
| NM_001163391 | ZSCAN12 | 3518 | 5 | 2 | 2 | 1 | 2 | 2 | 2 | 1 | 2 | 4 | 1 | 5 | 3 |
| NM_001163417 | FAM172A | 4595 | 3 | 2 | 2 | 1 | 1 | 2 | 2 | 1 | 1 | 3 | 0 | 2 | 2 |
| NM_001163418 | FAM172A | 4403 | 3 | 1 | 2 | 1 | 1 | 2 | 2 | 1 | 1 | 3 | 0 | 2 | 2 |
| NM_001163423 | ZNF580 | 1025 | 5 | 3 | 2 | 3 | 3 | 5 | 4 | 1 | 2 | 4 | 1 | 4 | 2 |
| NM_001163424 | MYEOV2 | 446 | 2 | 1 | 1 | 1 | 1 | 2 | 1 | 1 | 1 | 0 | 0 | 1 | 0 |
| NM_001163435 | TBCK | 3541 | 4 | 2 | 1 | 0 | 1 | 2 | 3 | 0 | 2 | 4 | 1 | 3 | 2 |
| NM_001163436 | TBCK | 3362 | 4 | 2 | 1 | 1 | 1 | 2 | 3 | 0 | 2 | 4 | 1 | 3 | 3 |
| NM_001163437 | TBCK | 3424 | 4 | 2 | 1 | 0 | 1 | 2 | 3 | 0 | 2 | 4 | 1 | 3 | 2 |
| NM_001163438 | LOC644538 | 1533 | 11 | 3 | 2 | 0 | 4 | 2 | 1 | 0 | 1 | 2 | 1 | 5 | 11 |
| NM_001163446 | CPA4 | 2717 | 0 | 0 | 0 | 0 | 0 | 0 | 0 | 0 | 0 | 0 | 0 | 0 | 0 |
| NM_001163474 | ZNF746 | 3800 | 1 | 1 | 0 | 0 | 0 | 1 | 1 | 0 | 0 | 1 | 0 | 1 | 1 |
| NM_001163484 | DCAF11 | 3853 | 18 | 5 | 3 | 3 | 4 | 5 | 5 | 18 | 2 | 2 | 3 | 4 | 2 |
| NM_001163499 | FBXO24 | 1993 | 11 | 1 | 0 | 0 | 0 | 0 | 0 | 0 | 0 | 0 | 0 | 0 | 11 |
| NM_001163508 | DCAF4 | 2533 | 5 | 3 | 2 | 2 | 1 | 5 | 4 | 1 | 1 | 3 | 2 | 3 | 3 |
| NM_001163509 | DCAF4 | 2522 | 5 | 3 | 2 | 2 | 2 | 5 | 4 | 1 | 1 | 3 | 2 | 3 | 3 |
| NM_001163523 | ITPRIPL1 | 1946 | 0 | 0 | 0 | 0 | 0 | 0 | 0 | 0 | 0 | 0 | 0 | 0 | 0 |
| NM_001163524 | ITPRIPL1 | 1813 | 0 | 0 | 0 | 0 | 0 | 0 | 0 | 0 | 0 | 0 | 0 | 0 | 0 |
| NM_001163541 | SATL1 | 2192 | 0 | 0 | 0 | 0 | 0 | 0 | 0 | 0 | 0 | 0 | 0 | 0 | 0 |
| NM_001163544 | SYNRG | 7936 | 6 | 3 | 2 | 2 | 3 | 4 | 4 | 2 | 2 | 3 | 2 | 6 | 4 |
| NM_001163545 | SYNRG | 7933 | 6 | 3 | 2 | 2 | 3 | 4 | 4 | 2 | 2 | 3 | 2 | 6 | 4 |
| NM_001163546 | SYNRG | 7765 | 6 | 3 | 2 | 2 | 3 | 4 | 4 | 2 | 3 | 3 | 2 | 6 | 4 |
| NM_001163547 | SYNRG | 7552 | 6 | 3 | 2 | 2 | 2 | 4 | 4 | 2 | 2 | 3 | 2 | 6 | 4 |
| NM_001163558 | PRL | 1027 | 0 | 0 | 0 | 0 | 0 | 0 | 0 | 0 | 0 | 0 | 0 | 0 | 0 |
| NM_001163560 | C16orf73 | 1885 | 4 | 0 | 0 | 0 | 0 | 0 | 0 | 0 | 0 | 0 | 0 | 0 | 4 |
| NM_001163561 | C2orf61 | 889 | 0 | 0 | 0 | 0 | 0 | 0 | 0 | 0 | 0 | 0 | 0 | 0 | 0 |
| NM_001163629 | C1orf129 | 3164 | 0 | 0 | 0 | 0 | 0 | 0 | 0 | 0 | 0 | 0 | 0 | 0 | 0 |
| NM_001163636 | GTF2I | 4461 | 9 | 6 | 5 | 3 | 5 | 5 | 9 | 5 | 4 | 7 | 6 | 7 | 5 |
| NM_001163646 | HTR3D | 1758 | 0 | 0 | 0 | 0 | 0 | 0 | 0 | 0 | 0 | 0 | 0 | 0 | 0 |
| NM_001163673 | WDR81 | 3184 | 0 | 0 | 0 | 0 | 0 | 0 | 0 | 0 | 0 | 0 | 0 | 0 | 0 |
| NM_001163677 | KCNMB3 | 1109 | 1 | 0 | 0 | 0 | 0 | 0 | 0 | 0 | 0 | 0 | 0 | 1 | 0 |
| NM_001163678 | SHOX2 | 3114 | 0 | 0 | 0 | 0 | 0 | 0 | 0 | 0 | 0 | 0 | 0 | 0 | 0 |
| NM_001163692 | LOC390595 | 1382 | 0 | 0 | 0 | 0 | 0 | 0 | 0 | 0 | 0 | 0 | 0 | 0 | 0 |
| NM_001163724 | LOC388588 | 545 | 1 | 0 | 0 | 0 | 0 | 0 | 0 | 0 | 0 | 0 | 0 | 0 | 1 |
| NM_001163735 | MYO19 | 4400 | 1 | 0 | 0 | 1 | 0 | 0 | 1 | 1 | 0 | 0 | 0 | 1 | 0 |
| NM_001163771 | COL11A2 | 1206 | 0 | 0 | 0 | 0 | 0 | 0 | 0 | 0 | 0 | 0 | 0 | 0 | 0 |
| NM_001163771 | COL11A2 | 1207 | 0 | 0 | 0 | 0 | 0 | 0 | 0 | 0 | 0 | 0 | 0 | 0 | 0 |
| NM_001163788 | ROD1 | 7234 | 21 | 10 | 6 | 6 | 6 | 4 | 13 | 5 | 15 | 9 | 3 | 21 | 17 |
| NM_001163790 | ROD1 | 7268 | 21 | 9 | 6 | 6 | 6 | 4 | 13 | 5 | 15 | 9 | 3 | 21 | 17 |
| NM_001163809 | WDR81 | 6869 | 1 | 0 | 0 | 0 | 0 | 0 | 0 | 0 | 0 | 0 | 0 | 1 | 0 |
| NM_001163811 | WDR81 | 3320 | 0 | 0 | 0 | 0 | 0 | 0 | 0 | 0 | 0 | 0 | 0 | 0 | 0 |
| NM_001163812 | C10orf2 | 3665 | 5 | 3 | 1 | 2 | 3 | 3 | 3 | 3 | 1 | 3 | 4 | 4 | 5 |
| NM_001163813 | C10orf2 | 1781 | 3 | 2 | 1 | 1 | 2 | 2 | 2 | 2 | 0 | 1 | 3 | 2 | 3 |
| NM_001163814 | C10orf2 | 1825 | 3 | 2 | 1 | 1 | 2 | 2 | 2 | 2 | 0 | 1 | 3 | 2 | 3 |
| NM_001163817 | DHCR7 | 2642 | 23 | 5 | 1 | 1 | 2 | 7 | 2 | 23 | 2 | 3 | 1 | 3 | 6 |
| NM_001163857 | C22orf33 | 1048 | 17 | 2 | 0 | 0 | 0 | 0 | 0 | 0 | 0 | 0 | 0 | 0 | 17 |
| NM_001163922 | VSIG10L | 3672 | 0 | 0 | 0 | 0 | 0 | 0 | 0 | 0 | 0 | 0 | 0 | 0 | 0 |
| NM_001163925 | LRTM2 | 3986 | 1 | 0 | 0 | 0 | 0 | 1 | 0 | 0 | 0 | 0 | 0 | 0 | 0 |
| NM_001163926 | LRTM2 | 3953 | 1 | 0 | 0 | 0 | 0 | 1 | 0 | 0 | 0 | 0 | 0 | 0 | 0 |
| NM_001163940 | PYGL | 2743 | 28 | 8 | 28 | 3 | 5 | 3 | 2 | 13 | 6 | 3 | 1 | 19 | 3 |
| NM_001163941 | ABCB5 | 5400 | 1 | 0 | 0 | 0 | 0 | 0 | 0 | 0 | 0 | 0 | 0 | 0 | 1 |
| NM_001163942 | ABCB5 | 2246 | 1 | 0 | 1 | 0 | 0 | 0 | 0 | 0 | 0 | 0 | 0 | 0 | 1 |
| NM_001163989 | RAB37 | 2800 | 1 | 0 | 0 | 0 | 0 | 0 | 0 | 1 | 1 | 0 | 0 | 1 | 0 |
| NM_001163990 | RAB37 | 2540 | 1 | 0 | 0 | 0 | 0 | 1 | 0 | 1 | 1 | 0 | 0 | 1 | 0 |
| NM_001163991 | FAM170A | 1233 | 2 | 0 | 0 | 0 | 0 | 0 | 0 | 0 | 0 | 0 | 0 | 0 | 2 |
| NM_001163993 | ABCB5 | 1724 | 2 | 0 | 1 | 0 | 0 | 0 | 0 | 0 | 0 | 0 | 0 | 0 | 2 |
| NM_001163999 | MECOM | 4811 | 15 | 3 | 2 | 3 | 4 | 0 | 15 | 0 | 6 | 1 | 1 | 3 | 2 |
| NM_001164 | APBB1 | 2642 | 12 | 2 | 0 | 1 | 2 | 12 | 1 | 0 | 1 | 1 | 1 | 1 | 1 |
| NM_001164000 | MECOM | 5740 | 13 | 3 | 2 | 3 | 4 | 0 | 13 | 0 | 6 | 1 | 1 | 2 | 1 |
| NM_001164093 | COPS7A | 2036 | 3 | 1 | 1 | 0 | 1 | 3 | 2 | 1 | 0 | 0 | 1 | 1 | 1 |
| NM_001164094 | COPS7A | 1901 | 3 | 1 | 1 | 0 | 1 | 3 | 2 | 1 | 0 | 0 | 1 | 1 | 1 |
| NM_001164095 | COPS7A | 1877 | 3 | 1 | 1 | 0 | 1 | 3 | 2 | 1 | 0 | 0 | 1 | 1 | 1 |
| NM_001164097 | VCAN | 9455 | 54 | 15 | 24 | 3 | 54 | 13 | 4 | 1 | 20 | 6 | 6 | 24 | 8 |
| NM_001164098 | VCAN | 7154 | 27 | 7 | 7 | 1 | 27 | 8 | 2 | 1 | 9 | 4 | 2 | 11 | 3 |
| NM_001164104 | MOV10L1 | 3818 | 4 | 0 | 0 | 0 | 0 | 0 | 0 | 0 | 0 | 1 | 0 | 0 | 4 |

| transcript | gene | transcript length (nt) | max | mean | adipose | colon | heart | hypothalamus | kidney | liver | lung | ovary | skeletalmuscle | spleen | testes |
|---|---|---|---|---|---|---|---|---|---|---|---|---|---|---|---|
| NM_001164105 | MOV10L1 | 3941 | 4 | 0 | 0 | 0 | 0 | 0 | 0 | 0 | 0 | 1 | 0 | 0 | 4 |
| NM_001164106 | MOV10L1 | 1341 | 1 | 0 | 0 | 0 | 0 | 0 | 0 | 0 | 0 | 0 | 0 | 0 | 1 |
| NM_001164114 | CASS4 | 2993 | 1 | 0 | 1 | 0 | 0 | 0 | 0 | 0 | 1 | 0 | 0 | 1 | 0 |
| NM_001164115 | CASS4 | 1844 | 1 | 0 | 0 | 0 | 0 | 0 | 0 | 0 | 1 | 0 | 0 | 1 | 0 |
| NM_001164116 | CASS4 | 3179 | 1 | 0 | 0 | 0 | 0 | 0 | 0 | 0 | 1 | 0 | 0 | 1 | 0 |
| NM_001164144 | ZNF828 | 3814 | 4 | 3 | 2 | 2 | 2 | 4 | 3 | 2 | 1 | 4 | 2 | 4 | 3 |
| NM_001164145 | ZNF828 | 3778 | 5 | 3 | 2 | 2 | 2 | 4 | 3 | 2 | 1 | 5 | 2 | 4 | 3 |
| NM_001164154 | TUBB8 | 1604 | 0 | 0 | 0 | 0 | 0 | 0 | 0 | 0 | 0 | 0 | 0 | 0 | 0 |
| NM_001164160 | SAPS3 | 5121 | 22 | 15 | 9 | 6 | 17 | 9 | 16 | 9 | 13 | 20 | 17 | 22 | 22 |
| NM_001164161 | SAPS3 | 5103 | 22 | 15 | 9 | 6 | 18 | 9 | 16 | 9 | 13 | 20 | 17 | 22 | 22 |
| NM_001164162 | SAPS3 | 5085 | 22 | 15 | 9 | 6 | 18 | 9 | 16 | 9 | 13 | 20 | 17 | 22 | 22 |
| NM_001164163 | SAPS3 | 5016 | 23 | 15 | 9 | 6 | 18 | 9 | 16 | 9 | 13 | 20 | 17 | 23 | 22 |
| NM_001164164 | SAPS3 | 3402 | 24 | 16 | 9 | 7 | 20 | 10 | 18 | 10 | 15 | 21 | 18 | 24 | 24 |
| NM_001164165 | LOC100302( | 1960 | 0 | 0 | 0 | 0 | 0 | 0 | 0 | 0 | 0 | 0 | 0 | 0 | 0 |
| NM_001164178 | ENTPD1 | 12514 | 4 | 2 | 1 | 1 | 2 | 2 | 2 | 1 | 2 | 2 | 1 | 4 | 1 |
| NM_001164179 | ENTPD1 | 12616 | 3 | 1 | 1 | 1 | 1 | 2 | 2 | 1 | 2 | 1 | 1 | 3 | 1 |
| NM_001164181 | ENTPD1 | 12386 | 4 | 2 | 1 | 1 | 2 | 2 | 2 | 1 | 2 | 2 | 1 | 4 | 1 |
| NM_001164182 | ENTPD1 | 12588 | 4 | 2 | 1 | 1 | 2 | 2 | 2 | 1 | 2 | 1 | 1 | 4 | 1 |
| NM_001164183 | ENTPD1 | 12470 | 4 | 2 | 1 | 1 | 1 | 2 | 2 | 1 | 2 | 1 | 1 | 4 | 1 |
| NM_001164189 | PLIN3 | 2337 | 3 | 2 | 1 | 2 | 2 | 3 | 1 | 1 | 2 | 2 | 2 | 3 | 2 |
| NM_001164194 | PLIN3 | 2304 | 3 | 2 | 1 | 2 | 2 | 3 | 1 | 1 | 2 | 2 | 2 | 3 | 2 |
| NM_001164211 | LRCH1 | 5579 | 4 | 2 | 2 | 1 | 2 | 1 | 3 | 1 | 2 | 2 | 1 | 4 | 2 |
| NM_001164213 | LRCH1 | 4715 | 5 | 3 | 3 | 2 | 2 | 1 | 4 | 2 | 3 | 3 | 1 | 5 | 2 |
| NM_001164232 | DDHD2 | 4531 | 11 | 5 | 7 | 3 | 5 | 11 | 5 | 2 | 4 | 6 | 3 | 4 | 6 |
| NM_001164234 | DDHD2 | 1882 | 9 | 4 | 7 | 3 | 4 | 9 | 5 | 1 | 4 | 5 | 2 | 4 | 5 |
| NM_001164238 | NLGN4Y | 880 | 2 | 0 | 0 | 0 | 0 | 2 | 0 | 0 | 0 | 0 | 0 | 0 | 0 |
| NM_001164239 | DHX16 | 3139 | 7 | 4 | 4 | 5 | 3 | 4 | 4 | 6 | 3 | 2 | 4 | 5 | 7 |
| NM_001164245 | C1orf27 | 3826 | 5 | 3 | 3 | 1 | 3 | 3 | 5 | 5 | 3 | 3 | 2 | 4 | 4 |
| NM_001164246 | C1orf27 | 3799 | 5 | 3 | 3 | 1 | 3 | 3 | 5 | 5 | 3 | 3 | 2 | 4 | 4 |
| NM_001164257 | C17orf72 | 3105 | 0 | 0 | 0 | 0 | 0 | 0 | 0 | 0 | 0 | 0 | 0 | 0 | 0 |
| NM_001164261 | PPIAL4D | 783 | 0 | 0 | 0 | 0 | 0 | 0 | 0 | 0 | 0 | 0 | 0 | 0 | 0 |
| NM_001164262 | PPIAL4F | 783 | 0 | 0 | 0 | 0 | 0 | 0 | 0 | 0 | 0 | 0 | 0 | 0 | 0 |
| NM_001164266 | LTBP3 | 4556 | 4 | 2 | 2 | 2 | 2 | 2 | 2 | 1 | 2 | 4 | 1 | 2 | 2 |
| NM_001164267 | WDR46 | 2201 | 4 | 2 | 2 | 2 | 2 | 2 | 2 | 2 | 2 | 2 | 3 | 4 | 2 |
| NM_001164269 | RAD51 | 2145 | 1 | 0 | 0 | 1 | 0 | 0 | 0 | 0 | 0 | 0 | 0 | 0 | 1 |
| NM_001164270 | RAD51 | 2175 | 2 | 0 | 0 | 1 | 0 | 0 | 0 | 0 | 0 | 0 | 0 | 1 | 2 |
| NM_001164271 | DLC1 | 5786 | 11 | 4 | 7 | 1 | 6 | 4 | 3 | 4 | 11 | 2 | 1 | 7 | 2 |
| NM_001164273 | MGA | 12040 | 14 | 7 | 5 | 4 | 6 | 9 | 7 | 2 | 5 | 13 | 5 | 10 | 14 |
| NM_001164276 | ZNF44 | 2792 | 8 | 4 | 3 | 3 | 3 | 2 | 7 | 3 | 4 | 8 | 3 | 6 | 7 |
| NM_001164277 | SLC37A4 | 2607 | 5 | 1 | 0 | 1 | 0 | 0 | 3 | 5 | 0 | 0 | 1 | 1 | 1 |
| NM_001164278 | SLC37A4 | 2357 | 5 | 1 | 0 | 1 | 0 | 0 | 4 | 5 | 0 | 0 | 1 | 1 | 1 |
| NM_001164279 | SLC37A4 | 2033 | 4 | 1 | 0 | 1 | 0 | 0 | 4 | 4 | 0 | 0 | 1 | 1 | 1 |
| NM_001164280 | SLC37A4 | 2049 | 6 | 1 | 0 | 1 | 0 | 0 | 4 | 6 | 0 | 0 | 1 | 1 | 1 |
| NM_001164281 | TRAF3IP2 | 2662 | 2 | 1 | 2 | 2 | 1 | 1 | 1 | 0 | 1 | 1 | 1 | 1 | 1 |
| NM_001164282 | TRAF3IP2 | 1135 | 1 | 1 | 1 | 1 | 1 | 1 | 1 | 0 | 1 | 1 | 1 | 1 | 1 |
| NM_001164283 | TRAF3IP2 | 905 | 2 | 1 | 2 | 1 | 1 | 1 | 1 | 0 | 1 | 1 | 1 | 1 | 1 |
| NM_001164309 | ZNF415 | 2402 | 26 | 9 | 6 | 3 | 13 | 19 | 5 | 1 | 3 | 12 | 3 | 10 | 26 |
| NM_001164310 | FAM166B | 958 | 6 | 1 | 0 | 0 | 0 | 0 | 0 | 0 | 1 | 0 | 6 | 0 | 0 |
| NM_001164313 | ZBTB24 | 1245 | 8 | 4 | 4 | 2 | 2 | 4 | 4 | 3 | 3 | 4 | 3 | 8 | 4 |
| NM_001164315 | ANKRD36 | 6081 | 10 | 4 | 2 | 2 | 2 | 2 | 2 | 0 | 4 | 10 | 1 | 6 | 9 |
| NM_001164317 | FLNB | 9560 | 10 | 4 | 3 | 10 | 3 | 2 | 6 | 5 | 4 | 4 | 1 | 5 | 6 |
| NM_001164318 | FLNB | 9434 | 10 | 4 | 3 | 10 | 3 | 2 | 6 | 5 | 4 | 4 | 1 | 5 | 7 |
| NM_001164319 | FLNB | 9395 | 10 | 4 | 3 | 10 | 3 | 2 | 6 | 5 | 4 | 4 | 1 | 5 | 7 |
| NM_001164326 | BRD8 | 2967 | 32 | 13 | 11 | 13 | 8 | 23 | 9 | 8 | 7 | 12 | 8 | 12 | 32 |
| NM_001164328 | GOLGA8F | 4511 | 0 | 0 | 0 | 0 | 0 | 0 | 0 | 0 | 0 | 0 | 0 | 0 | 0 |
| NM_001164342 | ZBTB20 | 3311 | 8 | 3 | 3 | 2 | 4 | 3 | 5 | 3 | 2 | 8 | 3 | 3 | 4 |
| NM_001164343 | ZBTB20 | 3773 | 8 | 3 | 3 | 2 | 4 | 3 | 5 | 3 | 2 | 8 | 4 | 3 | 4 |
| NM_001164344 | ZBTB20 | 3737 | 8 | 4 | 3 | 2 | 4 | 3 | 5 | 3 | 2 | 8 | 3 | 2 | 4 |
| NM_001164345 | ZBTB20 | 3691 | 8 | 4 | 3 | 2 | 4 | 3 | 5 | 3 | 2 | 8 | 3 | 3 | 4 |
| NM_001164346 | ZBTB20 | 3330 | 8 | 3 | 3 | 2 | 4 | 3 | 5 | 3 | 2 | 8 | 3 | 3 | 4 |
| NM_001164347 | ZBTB20 | 3352 | 8 | 3 | 3 | 2 | 4 | 3 | 5 | 3 | 2 | 8 | 3 | 3 | 4 |
| NM_001164356 | THAP4 | 784 | 2 | 1 | 0 | 1 | 1 | 1 | 1 | 0 | 0 | 1 | 2 | 1 | 1 |
| NM_001164372 | GPN3 | 1604 | 3 | 2 | 2 | 1 | 3 | 2 | 2 | 3 | 2 | 2 | 2 | 2 | 2 |
| NM_001164375 | C10orf105 | 4836 | 1 | 0 | 0 | 0 | 0 | 1 | 0 | 0 | 0 | 0 | 0 | 0 | 0 |
| NM_001164377 | MRGPRG | 868 | 0 | 0 | 0 | 0 | 0 | 0 | 0 | 0 | 0 | 0 | 0 | 0 | 0 |
| NM_001164379 | FAM180B | 1403 | 2 | 1 | 2 | 2 | 1 | 1 | 0 | 0 | 1 | 1 | 0 | 0 | 1 |
| NM_001164380 | STAU2 | 3055 | 9 | 4 | 3 | 2 | 7 | 9 | 4 | 3 | 3 | 2 | 5 | 4 | 3 |
| NM_001164381 | STAU2 | 2924 | 9 | 4 | 3 | 2 | 7 | 9 | 4 | 3 | 3 | 3 | 5 | 4 | 3 |
| NM_001164382 | STAU2 | 2878 | 9 | 4 | 3 | 2 | 7 | 9 | 4 | 3 | 3 | 3 | 5 | 4 | 3 |
| NM_001164383 | STAU2 | 2449 | 8 | 4 | 3 | 2 | 7 | 8 | 4 | 3 | 3 | 3 | 5 | 4 | 3 |
| NM_001164384 | STAU2 | 4210 | 8 | 3 | 2 | 1 | 5 | 8 | 4 | 2 | 3 | 3 | 3 | 4 | 3 |
| NM_001164385 | STAU2 | 3967 | 8 | 4 | 2 | 1 | 5 | 8 | 4 | 3 | 3 | 3 | 3 | 4 | 3 |
| NM_001164386 | RAPGEF6 | 8379 | 4 | 2 | 1 | 1 | 1 | 2 | 2 | 1 | 2 | 2 | 2 | 4 | 3 |
| NM_001164387 | RAPGEF6 | 8079 | 4 | 2 | 1 | 1 | 2 | 3 | 2 | 1 | 2 | 2 | 2 | 4 | 3 |
| NM_001164388 | RAPGEF6 | 8064 | 4 | 2 | 1 | 1 | 2 | 3 | 2 | 1 | 2 | 2 | 2 | 4 | 3 |
| NM_001164389 | RAPGEF6 | 4507 | 6 | 3 | 2 | 2 | 2 | 4 | 2 | 1 | 4 | 3 | 3 | 6 | 6 |
| NM_001164390 | RAPGEF6 | 3111 | 7 | 4 | 2 | 2 | 3 | 5 | 3 | 1 | 4 | 4 | 3 | 7 | 7 |

| transcript | gene | transcript length (nt) | max | mean | adipose | colon | heart | hypothala mus | kidney | liver | lung | ovary | skeletalm uscle | spleen | testes |
|---|---|---|---|---|---|---|---|---|---|---|---|---|---|---|---|
| NM_001164391 | MTF2 | 4046 | 4 | 2 | 1 | 1 | 1 | 1 | 2 | 1 | 2 | 3 | 2 | 4 | 4 |
| NM_001164392 | MTF2 | 3955 | 4 | 2 | 1 | 1 | 1 | 1 | 2 | 1 | 2 | 3 | 1 | 3 | 4 |
| NM_001164393 | MTF2 | 3845 | 5 | 2 | 1 | 1 | 1 | 1 | 2 | 1 | 2 | 3 | 2 | 4 | 5 |
| NM_001164397 | TRIM64B | 1533 | 0 | 0 | 0 | 0 | 0 | 0 | 0 | 0 | 0 | 0 | 0 | 0 | 0 |
| NM_001164399 | C14orf38 | 2609 | 1 | 0 | 0 | 0 | 0 | 0 | 0 | 0 | 0 | 0 | 0 | 0 | 1 |
| NM_001164404 | GOLGA6C | 5379 | 1 | 0 | 0 | 0 | 0 | 0 | 0 | 0 | 0 | 0 | 0 | 0 | 1 |
| NM_001164405 | BHLHA9 | 707 | 0 | 0 | 0 | 0 | 0 | 0 | 0 | 0 | 0 | 0 | 0 | 0 | 0 |
| NM_001164407 | TLCD2 | 5901 | 81 | 20 | 81 | 22 | 20 | 4 | 8 | 31 | 21 | 7 | 5 | 12 | 9 |
| NM_001164410 | CDK5 | 1093 | 30 | 5 | 3 | 2 | 2 | 30 | 3 | 4 | 2 | 1 | 1 | 5 | 2 |
| NM_001164415 | HSFX2 | 1966 | 0 | 0 | 0 | 0 | 0 | 0 | 0 | 0 | 0 | 0 | 0 | 0 | 0 |
| NM_001164416 | H2BFM | 1881 | 0 | 0 | 0 | 0 | 0 | 0 | 0 | 0 | 0 | 0 | 0 | 0 | 0 |
| NM_001164417 | SSX2B | 1302 | 0 | 0 | 0 | 0 | 0 | 0 | 0 | 0 | 0 | 0 | 0 | 0 | 0 |
| NM_001164417 | SSX2B | 1306 | 0 | 0 | 0 | 0 | 0 | 0 | 0 | 0 | 0 | 0 | 0 | 0 | 0 |
| NM_001164419 | MBD3L4 | 772 | 0 | 0 | 0 | 0 | 0 | 0 | 0 | 0 | 0 | 0 | 0 | 0 | 0 |
| NM_001164425 | MBD3L3 | 772 | 0 | 0 | 0 | 0 | 0 | 0 | 0 | 0 | 0 | 0 | 0 | 0 | 0 |
| NM_001164431 | C20orf95 | 2838 | 0 | 0 | 0 | 0 | 0 | 0 | 0 | 0 | 0 | 0 | 0 | 0 | 0 |
| NM_001164434 | KRTAP22-2 | 292 | 0 | 0 | 0 | 0 | 0 | 0 | 0 | 0 | 0 | 0 | 0 | 0 | 0 |
| NM_001164435 | KRTAP21-3 | 253 | 0 | 0 | 0 | 0 | 0 | 0 | 0 | 0 | 0 | 0 | 0 | 0 | 0 |
| NM_001164436 | TMEM212 | 1881 | 1 | 0 | 0 | 0 | 0 | 0 | 0 | 0 | 1 | 1 | 0 | 0 | 0 |
| NM_001164440 | ANKRD33B | 9332 | 3 | 1 | 1 | 0 | 0 | 1 | 3 | 0 | 0 | 0 | 1 | 2 | 0 |
| NM_001164442 | FAM159B | 967 | 2 | 0 | 0 | 0 | 0 | 2 | 0 | 0 | 0 | 0 | 0 | 0 | 0 |
| NM_001164443 | ANKRD31 | 6035 | 4 | 0 | 0 | 0 | 0 | 0 | 0 | 0 | 0 | 0 | 0 | 0 | 4 |
| NM_001164444 | CBY3 | 810 | 0 | 0 | 0 | 0 | 0 | 0 | 0 | 0 | 0 | 0 | 0 | 0 | 0 |
| NM_001164446 | C6orf132 | 6741 | 1 | 0 | 0 | 1 | 0 | 0 | 1 | 0 | 1 | 0 | 0 | 0 | 0 |
| NM_001164447 | FAM90A10 | 1394 | 0 | 0 | 0 | 0 | 0 | 0 | 0 | 0 | 0 | 0 | 0 | 0 | 0 |
| NM_001164448 | FAM90A9 | 1394 | 0 | 0 | 0 | 0 | 0 | 0 | 0 | 0 | 0 | 0 | 0 | 0 | 0 |
| NM_001164449 | FAM90A19 | 1394 | 0 | 0 | 0 | 0 | 0 | 0 | 0 | 0 | 0 | 0 | 0 | 0 | 0 |
| NM_001164450 | FAM90A8 | 1394 | 0 | 0 | 0 | 0 | 0 | 0 | 0 | 0 | 0 | 0 | 0 | 0 | 0 |
| NM_001164451 | FAM90A18 | 1394 | 0 | 0 | 0 | 0 | 0 | 0 | 0 | 0 | 0 | 0 | 0 | 0 | 0 |
| NM_001164452 | FAM90A14 | 1394 | 0 | 0 | 0 | 0 | 0 | 0 | 0 | 0 | 0 | 0 | 0 | 0 | 0 |
| NM_001164453 | FAM90A20 | 1394 | 0 | 0 | 0 | 0 | 0 | 0 | 0 | 0 | 0 | 0 | 0 | 0 | 0 |
| NM_001164455 | FAM90A5 | 1394 | 0 | 0 | 0 | 0 | 0 | 0 | 0 | 0 | 0 | 0 | 0 | 0 | 0 |
| NM_001164456 | FAM90A13 | 1394 | 0 | 0 | 0 | 0 | 0 | 0 | 0 | 0 | 0 | 0 | 0 | 0 | 0 |
| NM_001164457 | ZNF705G | 924 | 0 | 0 | 0 | 0 | 0 | 0 | 0 | 0 | 0 | 0 | 0 | 0 | 0 |
| NM_001164458 | ACTR3C | 1280 | 1 | 0 | 0 | 0 | 0 | 0 | 1 | 1 | 0 | 0 | 0 | 0 | 0 |
| NM_001164459 | ACTR3C | 1218 | 1 | 0 | 0 | 1 | 0 | 0 | 1 | 1 | 0 | 0 | 0 | 0 | 0 |
| NM_001164460 | MGC87042 | 1299 | 0 | 0 | 0 | 0 | 0 | 0 | 0 | 0 | 0 | 0 | 0 | 0 | 0 |
| NM_001164462 | MUC12 | 16320 | 90 | 8 | 0 | 90 | 0 | 0 | 0 | 0 | 0 | 0 | 0 | 0 | 0 |
| NM_001164463 | RGPD8 | 7298 | 0 | 0 | 0 | 0 | 0 | 0 | 0 | 0 | 0 | 0 | 0 | 0 | 0 |
| NM_001164464 | TRIM43B | 1668 | 0 | 0 | 0 | 0 | 0 | 0 | 0 | 0 | 0 | 0 | 0 | 0 | 0 |
| NM_001164465 | GOLGA6L10 | 3529 | 1 | 0 | 0 | 0 | 0 | 0 | 0 | 0 | 0 | 0 | 0 | 0 | 1 |
| NM_001164465 | GOLGA6L10 | 3545 | 1 | 0 | 0 | 0 | 0 | 0 | 0 | 0 | 0 | 0 | 0 | 0 | 1 |
| NM_001164467 | LOC653548 | 1601 | 0 | 0 | 0 | 0 | 0 | 0 | 0 | 0 | 0 | 0 | 0 | 0 | 0 |
| NM_001164467 | LOC653548 | 1611 | 0 | 0 | 0 | 0 | 0 | 0 | 0 | 0 | 0 | 0 | 0 | 0 | 0 |
| NM_001164468 | TMED7-TIC/ | 3508 | 0 | 0 | 0 | 0 | 0 | 0 | 0 | 0 | 0 | 0 | 0 | 0 | 0 |
| NM_001164469 | TMED7-TIC/ | 3626 | 0 | 0 | 0 | 0 | 0 | 0 | 0 | 0 | 0 | 0 | 0 | 0 | 0 |
| NM_001164470 | MS4A12 | 1021 | 6 | 1 | 0 | 6 | 0 | 0 | 0 | 0 | 0 | 0 | 0 | 0 | 0 |
| NM_001164471 | TSPY4 | 1131 | 1 | 0 | 0 | 0 | 0 | 0 | 0 | 0 | 0 | 0 | 0 | 0 | 1 |
| NM_001164471 | TSPY4 | 1132 | 1 | 0 | 0 | 0 | 0 | 0 | 0 | 0 | 0 | 0 | 0 | 0 | 1 |
| NM_001164473 | FNBP1L | 4061 | 20 | 8 | 6 | 4 | 10 | 9 | 20 | 4 | 10 | 9 | 6 | 4 | 11 |
| NM_001164475 | SLC41A3 | 1969 | 3 | 1 | 1 | 1 | 1 | 3 | 1 | 0 | 0 | 2 | 1 | 1 | 2 |
| NM_001164478 | FLJ44606 | 965 | 1 | 0 | 0 | 0 | 0 | 0 | 0 | 0 | 0 | 0 | 0 | 1 | 1 |
| NM_001164479 | FLJ44606 | 5679 | 1 | 0 | 0 | 0 | 0 | 0 | 0 | 0 | 0 | 0 | 0 | 1 | 1 |
| NM_001164484 | FAM170B | 1411 | 0 | 0 | 0 | 0 | 0 | 0 | 0 | 0 | 0 | 0 | 0 | 0 | 0 |
| NM_001164489 | ADAM8 | 3132 | 0 | 0 | 0 | 0 | 0 | 0 | 0 | 0 | 0 | 0 | 0 | 0 | 0 |
| NM_001164490 | ADAM8 | 3026 | 0 | 0 | 0 | 0 | 0 | 0 | 0 | 0 | 0 | 0 | 0 | 0 | 0 |
| NM_001164496 | WDR52 | 10259 | 2 | 1 | 1 | 1 | 0 | 1 | 1 | 0 | 1 | 1 | 0 | 1 | 2 |
| NM_001164501 | EIF4ENIF1 | 3676 | 11 | 6 | 4 | 5 | 4 | 9 | 6 | 2 | 4 | 6 | 5 | 6 | 11 |
| NM_001164502 | EIF4ENIF1 | 3178 | 4 | 2 | 2 | 2 | 1 | 3 | 2 | 1 | 1 | 3 | 2 | 3 | 4 |
| NM_001164507 | NEB | 26200 | 1,461 | 134 | 3 | 0 | 1 | 0 | 0 | 0 | 0 | 0 | 1,461 | 0 | 4 |
| NM_001164508 | NEB | 26200 | 1,461 | 134 | 3 | 0 | 1 | 0 | 0 | 0 | 0 | 0 | 1,461 | 0 | 4 |
| NM_001164527 | ZNF135 | 690 | 2 | 1 | 0 | 0 | 0 | 1 | 1 | 0 | 0 | 2 | 0 | 1 | 1 |
| NM_001164529 | ZNF135 | 2936 | 5 | 1 | 1 | 1 | 0 | 2 | 1 | 0 | 0 | 5 | 1 | 1 | 2 |
| NM_001164530 | ZNF135 | 2455 | 4 | 1 | 1 | 1 | 0 | 2 | 1 | 0 | 0 | 4 | 1 | 1 | 2 |
| NM_001164537 | DISC1 | 7153 | 0 | 0 | 0 | 0 | 0 | 0 | 0 | 0 | 0 | 0 | 0 | 0 | 0 |
| NM_001164538 | DISC1 | 2963 | 0 | 0 | 0 | 0 | 0 | 0 | 0 | 0 | 0 | 0 | 0 | 0 | 0 |
| NM_001164539 | DISC1 | 2673 | 0 | 0 | 0 | 0 | 0 | 0 | 0 | 0 | 0 | 0 | 0 | 0 | 0 |
| NM_001164540 | DISC1 | 6691 | 0 | 0 | 0 | 0 | 0 | 0 | 0 | 0 | 0 | 0 | 0 | 0 | 0 |
| NM_001164541 | DISC1 | 2902 | 0 | 0 | 0 | 0 | 0 | 0 | 0 | 0 | 0 | 0 | 0 | 0 | 0 |
| NM_001164542 | DISC1 | 2710 | 0 | 0 | 0 | 0 | 0 | 0 | 0 | 0 | 0 | 0 | 0 | 0 | 0 |
| NM_001164544 | DISC1 | 2761 | 0 | 0 | 0 | 0 | 0 | 0 | 0 | 0 | 0 | 0 | 0 | 0 | 0 |
| NM_001164545 | DISC1 | 2658 | 0 | 0 | 0 | 0 | 0 | 0 | 0 | 0 | 0 | 0 | 0 | 0 | 0 |
| NM_001164546 | DISC1 | 2363 | 0 | 0 | 0 | 0 | 0 | 0 | 0 | 0 | 0 | 0 | 0 | 0 | 0 |
| NM_001164547 | DISC1 | 2616 | 0 | 0 | 0 | 0 | 0 | 0 | 0 | 0 | 0 | 0 | 0 | 0 | 0 |
| NM_001164548 | DISC1 | 2603 | 0 | 0 | 0 | 0 | 0 | 0 | 0 | 0 | 0 | 0 | 0 | 0 | 0 |
| NM_001164549 | DISC1 | 2277 | 0 | 0 | 0 | 0 | 0 | 0 | 0 | 0 | 0 | 0 | 0 | 0 | 0 |
| NM_001164550 | DISC1 | 2832 | 0 | 0 | 0 | 0 | 0 | 0 | 0 | 0 | 0 | 0 | 0 | 0 | 0 |

| | | transcript | | | | | | hypothala | | | | | skeletalm | | |
|---|---|---|---|---|---|---|---|---|---|---|---|---|---|---|---|
| transcript | gene | length (nt) | max | mean | adipose | colon | heart | mus | kidney | liver | lung | ovary | uscle | spleen | testes |
| NM_001164551 | DISC1 | 1863 | 0 | 0 | 0 | 0 | 0 | 0 | 0 | 0 | 0 | 0 | 0 | 0 | 0 |
| NM_001164552 | DISC1 | 2783 | 0 | 0 | 0 | 0 | 0 | 0 | 0 | 0 | 0 | 0 | 0 | 0 | 0 |
| NM_001164553 | DISC1 | 2706 | 0 | 0 | 0 | 0 | 0 | 0 | 0 | 0 | 0 | 0 | 0 | 0 | 0 |
| NM_001164554 | DISC1 | 1707 | 0 | 0 | 0 | 0 | 0 | 0 | 0 | 0 | 0 | 0 | 0 | 0 | 0 |
| NM_001164555 | DISC1 | 1793 | 0 | 0 | 0 | 0 | 0 | 0 | 0 | 0 | 0 | 0 | 0 | 0 | 0 |
| NM_001164556 | DISC1 | 1553 | 0 | 0 | 0 | 0 | 0 | 0 | 0 | 0 | 0 | 0 | 0 | 0 | 0 |
| NM_001164579 | C16orf52 | 4440 | 5 | 2 | 1 | 1 | 2 | 5 | 2 | 2 | 2 | 3 | 1 | 3 | 3 |
| NM_001164586 | IGFN1 | 11809 | 26 | 3 | 1 | 0 | 0 | 1 | 0 | 0 | 0 | 0 | 26 | 0 | 2 |
| NM_001164595 | PDZRN4 | 3811 | 4 | 1 | 1 | 2 | 0 | 2 | 0 | 0 | 0 | 2 | 0 | 0 | 4 |
| NM_001164603 | ASXL1 | 1068 | 4 | 3 | 3 | 2 | 2 | 3 | 3 | 3 | 3 | 4 | 2 | 3 | 2 |
| NM_001164605 | FXYD5 | 897 | 46 | 13 | 20 | 8 | 13 | 4 | 4 | 4 | 27 | 5 | 5 | 46 | 6 |
| NM_001164615 | FZD6 | 3766 | 5 | 3 | 3 | 1 | 3 | 1 | 5 | 1 | 5 | 3 | 1 | 3 | 4 |
| NM_001164616 | FZD6 | 3678 | 5 | 3 | 3 | 1 | 3 | 1 | 5 | 1 | 5 | 3 | 1 | 3 | 4 |
| NM_001164617 | GPC3 | 2383 | 7 | 2 | 7 | 1 | 1 | 1 | 5 | 0 | 7 | 2 | 1 | 0 | 1 |
| NM_001164618 | GPC3 | 2266 | 7 | 2 | 7 | 1 | 1 | 1 | 5 | 0 | 7 | 2 | 1 | 0 | 1 |
| NM_001164619 | GPC3 | 2152 | 8 | 2 | 7 | 1 | 1 | 1 | 5 | 0 | 8 | 2 | 1 | 0 | 1 |
| NM_001164623 | EIF2C2 | 3398 | 3 | 2 | 2 | 3 | 2 | 2 | 2 | 1 | 3 | 3 | 3 | 3 | 2 |
| NM_001164629 | GTDC1 | 2660 | 5 | 2 | 3 | 0 | 2 | 5 | 1 | 1 | 2 | 2 | 1 | 4 | 3 |
| NM_001164637 | FLJ35220 | 2681 | 1 | 0 | 0 | 0 | 0 | 0 | 1 | 0 | 0 | 0 | 0 | 0 | 0 |
| NM_001164638 | FLJ35220 | 1270 | 0 | 0 | 0 | 0 | 0 | 0 | 0 | 0 | 0 | 0 | 0 | 0 | 0 |
| NM_001164644 | SFTPA1 | 2173 | 241 | 22 | 0 | 0 | 0 | 0 | 0 | 0 | 241 | 0 | 0 | 0 | 0 |
| NM_001164645 | SFTPA1 | 2059 | 252 | 23 | 0 | 0 | 0 | 0 | 0 | 0 | 252 | 0 | 0 | 0 | 0 |
| NM_001164646 | SFTPA1 | 2067 | 253 | 23 | 0 | 0 | 0 | 0 | 0 | 0 | 253 | 0 | 0 | 0 | 0 |
| NM_001164647 | SFTPA1 | 2143 | 243 | 22 | 0 | 0 | 0 | 0 | 0 | 0 | 243 | 0 | 0 | 0 | 0 |
| NM_001164664 | MAST4 | 10723 | 6 | 3 | 3 | 1 | 6 | 2 | 4 | 1 | 3 | 4 | 3 | 2 | 2 |
| NM_001164665 | KIAA1549 | 12425 | 1 | 0 | 0 | 0 | 0 | 1 | 1 | 0 | 0 | 1 | 0 | 0 | 0 |
| NM_001164673 | DOK7 | 2554 | 0 | 0 | 0 | 0 | 0 | 0 | 0 | 0 | 0 | 0 | 0 | 0 | 0 |
| NM_001164674 | SUMF1 | 2086 | 2 | 1 | 2 | 1 | 0 | 1 | 2 | 1 | 0 | 1 | 0 | 1 | 1 |
| NM_001164675 | SUMF1 | 2101 | 2 | 1 | 1 | 1 | 0 | 1 | 2 | 1 | 0 | 1 | 0 | 1 | 1 |
| NM_001164680 | CCR3 | 1776 | 0 | 0 | 0 | 0 | 0 | 0 | 0 | 0 | 0 | 0 | 0 | 0 | 0 |
| NM_001164685 | THEMIS | 3982 | 1 | 0 | 0 | 0 | 0 | 0 | 0 | 0 | 1 | 0 | 0 | 1 | 0 |
| NM_001164687 | THEMIS | 3829 | 1 | 0 | 0 | 0 | 0 | 0 | 0 | 0 | 1 | 0 | 0 | 1 | 0 |
| NM_001164688 | RD3 | 4263 | 0 | 0 | 0 | 0 | 0 | 0 | 0 | 0 | 0 | 0 | 0 | 0 | 0 |
| NM_001164690 | AMHR2 | 1859 | 0 | 0 | 0 | 0 | 0 | 0 | 0 | 0 | 0 | 0 | 0 | 0 | 0 |
| NM_001164691 | AMHR2 | 1578 | 0 | 0 | 0 | 0 | 0 | 0 | 0 | 0 | 0 | 0 | 0 | 0 | 0 |
| NM_001164692 | LTB4R2 | 1540 | 0 | 0 | 0 | 0 | 0 | 0 | 0 | 0 | 0 | 0 | 0 | 0 | 0 |
| NM_001164693 | LTB4R2 | 1073 | 1 | 0 | 0 | 1 | 0 | 0 | 0 | 0 | 0 | 0 | 0 | 1 | 0 |
| NM_001164694 | IYD | 7513 | 4 | 1 | 0 | 0 | 0 | 0 | 4 | 2 | 0 | 0 | 0 | 0 | 0 |
| NM_001164695 | IYD | 7462 | 4 | 1 | 0 | 0 | 0 | 0 | 4 | 2 | 0 | 0 | 0 | 0 | 0 |
| NM_001164710 | AMT | 2069 | 1 | 0 | 0 | 1 | 0 | 0 | 1 | 1 | 0 | 0 | 0 | 0 | 0 |
| NM_001164711 | AMT | 2033 | 2 | 1 | 0 | 2 | 1 | 1 | 2 | 2 | 1 | 1 | 0 | 0 | 1 |
| NM_001164712 | AMT | 1954 | 3 | 1 | 0 | 2 | 1 | 1 | 2 | 3 | 1 | 1 | 0 | 0 | 1 |
| NM_001164715 | ZNF605 | 6185 | 4 | 2 | 1 | 1 | 3 | 2 | 4 | 1 | 1 | 4 | 2 | 3 | 2 |
| NM_001164716 | PYGM | 3351 | 127 | 12 | 1 | 1 | 3 | 1 | 0 | 0 | 0 | 0 | 127 | 0 | 1 |
| NM_001164719 | TGFB1I1 | 1773 | 1 | 0 | 0 | 1 | 1 | 0 | 0 | 0 | 0 | 1 | 0 | 0 | 0 |
| NM_001164720 | CC2D2A | 1524 | 2 | 1 | 0 | 0 | 0 | 1 | 2 | 0 | 1 | 1 | 0 | 0 | 1 |
| NM_001164721 | PTAFR | 4191 | 1 | 0 | 0 | 0 | 0 | 0 | 0 | 0 | 1 | 0 | 0 | 1 | 0 |
| NM_001164722 | PTAFR | 4099 | 1 | 0 | 0 | 0 | 0 | 0 | 0 | 0 | 1 | 0 | 0 | 1 | 0 |
| NM_001164723 | PTAFR | 4047 | 1 | 0 | 0 | 0 | 0 | 0 | 0 | 0 | 1 | 0 | 0 | 1 | 0 |
| NM_001164730 | REEP1 | 3735 | 25 | 4 | 0 | 1 | 2 | 11 | 1 | 0 | 1 | 0 | 1 | 1 | 25 |
| NM_001164731 | REEP1 | 3778 | 24 | 4 | 0 | 1 | 2 | 10 | 1 | 0 | 1 | 0 | 1 | 1 | 24 |
| NM_001164732 | REEP1 | 3616 | 25 | 4 | 0 | 1 | 3 | 11 | 1 | 0 | 1 | 0 | 1 | 1 | 25 |
| NM_001164737 | CALCR | 3696 | 0 | 0 | 0 | 0 | 0 | 0 | 0 | 0 | 0 | 0 | 0 | 0 | 0 |
| NM_001164738 | CALCR | 3610 | 0 | 0 | 0 | 0 | 0 | 0 | 0 | 0 | 0 | 0 | 0 | 0 | 0 |
| NM_001164741 | ARHGAP4 | 3371 | 2 | 1 | 1 | 1 | 1 | 0 | 0 | 0 | 1 | 0 | 0 | 2 | 0 |
| NM_001164746 | RASSF8 | 5862 | 6 | 4 | 3 | 1 | 5 | 6 | 6 | 1 | 4 | 6 | 3 | 2 | 5 |
| NM_001164747 | RASSF8 | 5607 | 7 | 4 | 3 | 1 | 5 | 7 | 6 | 1 | 4 | 7 | 3 | 2 | 5 |
| NM_001164748 | RASSF8 | 5541 | 7 | 4 | 3 | 1 | 5 | 7 | 6 | 1 | 4 | 7 | 3 | 2 | 5 |
| NM_001164749 | NPAS3 | 5932 | 3 | 0 | 0 | 0 | 0 | 3 | 0 | 0 | 0 | 1 | 0 | 0 | 0 |
| NM_001164750 | ASPH | 5153 | 75 | 15 | 75 | 4 | 12 | 9 | 9 | 10 | 11 | 10 | 4 | 10 | 6 |
| NM_001164751 | ASPH | 3621 | 78 | 16 | 78 | 5 | 15 | 14 | 11 | 9 | 14 | 10 | 5 | 11 | 6 |
| NM_001164752 | ASPH | 3564 | 78 | 16 | 78 | 5 | 15 | 14 | 11 | 9 | 14 | 10 | 5 | 11 | 6 |
| NM_001164753 | ASPH | 3495 | 56 | 12 | 56 | 4 | 13 | 11 | 9 | 6 | 11 | 7 | 4 | 9 | 4 |
| NM_001164754 | ASPH | 3717 | 75 | 15 | 75 | 5 | 15 | 14 | 10 | 9 | 13 | 9 | 5 | 10 | 6 |
| NM_001164755 | ASPH | 3645 | 54 | 12 | 54 | 4 | 12 | 10 | 9 | 6 | 11 | 7 | 4 | 9 | 4 |
| NM_001164756 | ASPH | 1694 | 36 | 9 | 36 | 3 | 8 | 12 | 6 | 4 | 8 | 4 | 4 | 6 | 3 |
| NM_001164757 | NOS1AP | 4449 | 3 | 1 | 0 | 1 | 0 | 3 | 0 | 1 | 0 | 0 | 0 | 0 | 0 |
| NM_001164758 | PRKAR1B | 2488 | 14 | 2 | 0 | 1 | 1 | 14 | 1 | 1 | 1 | 1 | 0 | 1 | 1 |
| NM_001164759 | PRKAR1B | 2423 | 15 | 2 | 0 | 1 | 1 | 15 | 1 | 1 | 1 | 1 | 0 | 1 | 1 |
| NM_001164760 | PRKAR1B | 2533 | 14 | 2 | 0 | 1 | 1 | 14 | 1 | 1 | 1 | 1 | 0 | 1 | 1 |
| NM_001164761 | PRKAR1B | 2554 | 14 | 2 | 0 | 1 | 1 | 14 | 1 | 1 | 1 | 1 | 0 | 1 | 1 |
| NM_001164762 | PRKAR1B | 2494 | 14 | 2 | 0 | 1 | 1 | 14 | 1 | 1 | 1 | 1 | 0 | 1 | 1 |
| NM_001164766 | ZFHX3 | 12774 | 4 | 2 | 2 | 2 | 2 | 2 | 2 | 2 | 2 | 2 | 4 | 2 | 2 |
| NM_001164771 | SLC7A2 | 7564 | 24 | 6 | 1 | 0 | 6 | 1 | 2 | 24 | 2 | 9 | 16 | 2 | 6 |
| NM_001164773 | BCAT2 | 1322 | 0 | 0 | 0 | 0 | 0 | 0 | 0 | 0 | 0 | 0 | 0 | 0 | 0 |
| NM_001164774 | ATXN3 | 6239 | 2 | 1 | 1 | 1 | 1 | 1 | 1 | 0 | 1 | 2 | 0 | 2 | 2 |
| NM_001164775 | ATXN3 | 6338 | 3 | 2 | 1 | 1 | 1 | 1 | 1 | 0 | 2 | 3 | 1 | 3 | 2 |

| transcript | gene | transcript length (nt) | max | mean | adipose | colon | heart | hypothalamus | kidney | liver | lung | ovary | skeletalmuscle | spleen | testes |
|---|---|---|---|---|---|---|---|---|---|---|---|---|---|---|---|
| NM_001164776 | ATXN3 | 6284 | 2 | 1 | 1 | 1 | 1 | 1 | 1 | 0 | 1 | 2 | 0 | 2 | 2 |
| NM_001164777 | ATXN3 | 6119 | 2 | 1 | 1 | 0 | 1 | 1 | 1 | 0 | 1 | 2 | 0 | 1 | 1 |
| NM_001164778 | ATXN3 | 6437 | 3 | 2 | 2 | 1 | 2 | 2 | 2 | 1 | 2 | 3 | 1 | 3 | 3 |
| NM_001164779 | ATXN3 | 6559 | 3 | 2 | 1 | 1 | 1 | 2 | 2 | 1 | 2 | 3 | 1 | 3 | 2 |
| NM_001164780 | ATXN3 | 6626 | 3 | 2 | 1 | 1 | 1 | 2 | 1 | 0 | 2 | 3 | 1 | 3 | 2 |
| NM_001164781 | ATXN3 | 6712 | 4 | 2 | 2 | 1 | 2 | 2 | 2 | 1 | 2 | 4 | 1 | 3 | 3 |
| NM_001164782 | ATXN3 | 6074 | 2 | 1 | 1 | 0 | 1 | 1 | 1 | 0 | 1 | 2 | 0 | 2 | 1 |
| NM_001164783 | BCKDHA | 1770 | 1 | 1 | 1 | 1 | 1 | 1 | 1 | 1 | 0 | 0 | 1 | 1 | 1 |
| NM_001164796 | SLC25A26 | 1753 | 4 | 2 | 2 | 1 | 2 | 4 | 2 | 2 | 1 | 2 | 2 | 2 | 2 |
| NM_001164803 | RBMX | 2052 | 40 | 19 | 11 | 11 | 16 | 8 | 16 | 6 | 18 | 29 | 22 | 40 | 34 |
| NM_001164811 | PET117 | 1156 | 1 | 1 | 0 | 1 | 1 | 1 | 1 | 1 | 0 | 0 | 0 | 1 | 0 |
| NM_001164814 | ACIN1 | 4896 | 22 | 14 | 14 | 16 | 13 | 10 | 15 | 8 | 10 | 17 | 14 | 18 | 22 |
| NM_001164815 | ACIN1 | 4815 | 23 | 15 | 14 | 17 | 14 | 10 | 15 | 9 | 11 | 18 | 14 | 19 | 23 |
| NM_001164816 | ACIN1 | 2454 | 15 | 9 | 9 | 10 | 10 | 7 | 11 | 4 | 8 | 9 | 8 | 12 | 15 |
| NM_001164817 | ACIN1 | 2516 | 15 | 9 | 9 | 10 | 9 | 7 | 10 | 4 | 8 | 8 | 8 | 11 | 15 |
| NM_001164821 | DCAF17 | 5625 | 3 | 2 | 1 | 1 | 2 | 2 | 2 | 1 | 1 | 2 | 1 | 3 | 3 |
| NM_001164824 | C1orf212 | 6009 | 2 | 1 | 1 | 1 | 1 | 1 | 1 | 2 | 1 | 1 | 0 | 1 | 1 |
| NM_001164825 | C1orf212 | 5902 | 2 | 1 | 1 | 1 | 1 | 1 | 1 | 2 | 1 | 1 | 0 | 1 | 1 |
| NM_001164829 | C1orf210 | 1402 | 1 | 0 | 0 | 1 | 0 | 0 | 1 | 0 | 0 | 0 | 0 | 0 | 0 |
| NM_001164830 | CA1 | 1196 | 12 | 1 | 0 | 12 | 0 | 0 | 0 | 0 | 1 | 0 | 0 | 0 | 0 |
| NM_001164831 | FXYD6 | 1746 | 16 | 4 | 2 | 2 | 1 | 16 | 5 | 1 | 1 | 6 | 0 | 4 | 5 |
| NM_001164832 | FXYD6 | 2151 | 13 | 3 | 1 | 2 | 1 | 13 | 4 | 1 | 1 | 5 | 0 | 3 | 4 |
| NM_001164835 | L1TD1 | 3987 | 8 | 1 | 0 | 2 | 0 | 0 | 0 | 0 | 1 | 0 | 0 | 2 | 8 |
| NM_001164836 | FXYD6 | 2227 | 13 | 3 | 1 | 2 | 1 | 13 | 4 | 0 | 1 | 5 | 0 | 3 | 4 |
| NM_001164837 | FXYD6 | 2130 | 13 | 3 | 1 | 2 | 1 | 13 | 4 | 1 | 1 | 5 | 0 | 3 | 4 |
| NM_001164839 | C3orf24 | 864 | 1 | 0 | 0 | 0 | 0 | 0 | 0 | 0 | 0 | 0 | 0 | 0 | 1 |
| NM_001164840 | LYRM4 | 794 | 1 | 0 | 0 | 0 | 0 | 1 | 0 | 0 | 0 | 0 | 0 | 1 | 1 |
| NM_001164841 | LYRM4 | 1610 | 2 | 1 | 1 | 0 | 1 | 1 | 1 | 0 | 0 | 1 | 0 | 2 | 1 |
| NM_001164883 | CTNNA2 | 3854 | 26 | 3 | 0 | 0 | 0 | 26 | 0 | 0 | 0 | 0 | 0 | 0 | 5 |
| NM_001165 | BIRC3 | 5229 | 28 | 5 | 2 | 2 | 5 | 1 | 3 | 1 | 13 | 1 | 1 | 28 | 4 |
| NM_001165030 | TMEM41B | 837 | 1 | 0 | 0 | 0 | 0 | 1 | 1 | 0 | 1 | 0 | 0 | 0 | 0 |
| NM_001165031 | DTYMK | 1137 | 1 | 1 | 1 | 0 | 0 | 1 | 0 | 1 | 0 | 1 | 0 | 1 | 0 |
| NM_001165032 | RNF182 | 3495 | 2 | 0 | 0 | 0 | 0 | 2 | 0 | 0 | 1 | 0 | 0 | 0 | 1 |
| NM_001165033 | RNF182 | 3274 | 2 | 0 | 0 | 0 | 0 | 2 | 0 | 0 | 1 | 0 | 0 | 0 | 1 |
| NM_001165034 | RNF182 | 3382 | 2 | 0 | 0 | 0 | 0 | 2 | 0 | 0 | 1 | 0 | 0 | 0 | 1 |
| NM_001165035 | FBLN2 | 4581 | 7 | 1 | 7 | 1 | 2 | 0 | 0 | 0 | 0 | 0 | 1 | 0 | 2 |
| NM_001165036 | OGDH | 4289 | 22 | 9 | 8 | 6 | 22 | 10 | 17 | 6 | 5 | 2 | 16 | 6 | 4 |
| NM_001165037 | GABRA5 | 2763 | 15 | 1 | 0 | 0 | 0 | 15 | 0 | 0 | 0 | 0 | 0 | 0 | 0 |
| NM_001165038 | GFRA2 | 3013 | 1 | 0 | 0 | 0 | 0 | 0 | 0 | 0 | 0 | 0 | 0 | 1 | 0 |
| NM_001165039 | GFRA2 | 2929 | 1 | 0 | 0 | 0 | 0 | 0 | 0 | 0 | 0 | 0 | 0 | 1 | 0 |
| NM_001165136 | HERC6 | 3779 | 5 | 3 | 2 | 1 | 3 | 4 | 2 | 1 | 2 | 5 | 4 | 2 | 3 |
| NM_001165255 | MNX1 | 1620 | 0 | 0 | 0 | 0 | 0 | 0 | 0 | 0 | 0 | 0 | 0 | 0 | 0 |
| NM_001165257 | SCG3 | 3256 | 51 | 5 | 0 | 1 | 0 | 51 | 0 | 0 | 0 | 0 | 0 | 0 | 0 |
| NM_001165258 | TMEM14C | 1183 | 48 | 29 | 28 | 11 | 48 | 17 | 43 | 25 | 30 | 30 | 21 | 40 | 29 |
| NM_001165412 | NFKB1 | 4083 | 22 | 10 | 8 | 9 | 9 | 5 | 8 | 8 | 11 | 11 | 8 | 22 | 8 |
| NM_001165413 | SERPINE1 | 3135 | 11 | 4 | 1 | 0 | 8 | 1 | 1 | 11 | 10 | 5 | 1 | 4 | 1 |
| NM_001165414 | LDHA | 2305 | 281 | 87 | 111 | 38 | 129 | 74 | 82 | 281 | 54 | 18 | 46 | 71 | 52 |
| NM_001165415 | LDHA | 1939 | 329 | 102 | 130 | 44 | 152 | 87 | 96 | 329 | 64 | 21 | 53 | 83 | 60 |
| NM_001165416 | LDHA | 2084 | 308 | 95 | 121 | 42 | 142 | 81 | 90 | 308 | 60 | 19 | 50 | 77 | 56 |
| NM_001165417 | SLC25A11 | 1778 | 3 | 2 | 1 | 1 | 3 | 3 | 3 | 2 | 1 | 1 | 3 | 1 | 1 |
| NM_001165418 | SLC25A11 | 1658 | 3 | 2 | 1 | 1 | 2 | 3 | 2 | 2 | 1 | 1 | 3 | 1 | 1 |
| NM_001165877 | ATP5L2 | 799 | 0 | 0 | 0 | 0 | 0 | 0 | 0 | 0 | 0 | 0 | 0 | 0 | 0 |
| NM_001165881 | ZNF268 | 1460 | 6 | 3 | 2 | 1 | 2 | 3 | 4 | 2 | 2 | 6 | 1 | 5 | 5 |
| NM_001165882 | ZNF268 | 1467 | 6 | 3 | 2 | 1 | 2 | 3 | 4 | 2 | 2 | 6 | 1 | 5 | 5 |
| NM_001165884 | ZNF268 | 1580 | 5 | 3 | 2 | 1 | 2 | 3 | 3 | 2 | 2 | 5 | 1 | 4 | 4 |
| NM_001165887 | ZNF268 | 1424 | 5 | 3 | 2 | 1 | 2 | 3 | 3 | 2 | 2 | 5 | 1 | 4 | 4 |
| NM_001165893 | NPAS3 | 5842 | 3 | 0 | 0 | 0 | 0 | 3 | 0 | 0 | 0 | 1 | 0 | 0 | 0 |
| NM_001165895 | CD320 | 1125 | 0 | 0 | 0 | 0 | 0 | 0 | 0 | 0 | 0 | 0 | 0 | 0 | 0 |
| NM_001165899 | PDE4D | 8118 | 21 | 4 | 1 | 2 | 7 | 4 | 5 | 0 | 4 | 0 | 21 | 2 | 1 |
| NM_001165903 | STX1A | 2079 | 7 | 1 | 0 | 0 | 0 | 7 | 0 | 0 | 0 | 0 | 0 | 0 | 0 |
| NM_001165920 | SERPINF2 | 2262 | 96 | 10 | 0 | 0 | 1 | 0 | 10 | 96 | 1 | 1 | 0 | 1 | 2 |
| NM_001165921 | SERPINF2 | 2092 | 103 | 11 | 0 | 0 | 1 | 0 | 10 | 103 | 1 | 1 | 0 | 1 | 2 |
| NM_001165923 | HNF1B | 2757 | 18 | 2 | 0 | 4 | 0 | 0 | 18 | 2 | 1 | 0 | 0 | 0 | 1 |
| NM_001165924 | EMX2 | 2710 | 15 | 3 | 2 | 0 | 0 | 7 | 15 | 0 | 0 | 4 | 1 | 0 | 5 |
| NM_001165927 | MKS1 | 2387 | 1 | 1 | 0 | 0 | 0 | 1 | 1 | 0 | 1 | 1 | 0 | 0 | 1 |
| NM_001165928 | DAG1 | 5881 | 7 | 4 | 2 | 3 | 7 | 3 | 4 | 7 | 2 | 3 | 7 | 2 | 7 |
| NM_001165931 | RRM2 | 3434 | 6 | 2 | 3 | 6 | 1 | 0 | 1 | 0 | 3 | 1 | 0 | 6 | 3 |
| NM_001165933 | RGS9 | 1656 | 2 | 0 | 0 | 0 | 0 | 2 | 0 | 0 | 0 | 0 | 0 | 0 | 0 |
| NM_001165937 | STARD3 | 2834 | 1 | 1 | 1 | 1 | 1 | 1 | 1 | 1 | 1 | 1 | 0 | 1 | 1 |
| NM_001165938 | STARD3 | 2780 | 1 | 1 | 1 | 1 | 1 | 1 | 1 | 1 | 1 | 1 | 0 | 1 | 1 |
| NM_001165945 | CLCNKB | 2131 | 2 | 0 | 0 | 0 | 0 | 0 | 2 | 0 | 0 | 0 | 0 | 0 | 0 |
| NM_001165946 | IDE | 4480 | 4 | 2 | 1 | 0 | 2 | 1 | 2 | 3 | 1 | 1 | 4 | 2 | 3 |
| NM_001165947 | HTR2A | 2824 | 2 | 0 | 0 | 0 | 0 | 2 | 0 | 0 | 0 | 0 | 0 | 0 | 0 |
| NM_001165958 | GSDMB | 1597 | 14 | 2 | 2 | 14 | 0 | 0 | 1 | 0 | 1 | 1 | 0 | 2 | 2 |
| NM_001165959 | GSDMB | 1561 | 14 | 2 | 2 | 14 | 0 | 1 | 1 | 0 | 1 | 1 | 0 | 2 | 2 |
| NM_001165960 | ALOXE3 | 3396 | 0 | 0 | 0 | 0 | 0 | 0 | 0 | 0 | 0 | 0 | 0 | 0 | 0 |
| NM_001165962 | ELAC2 | 3683 | 3 | 2 | 1 | 1 | 3 | 2 | 3 | 2 | 1 | 2 | 2 | 3 | 2 |

| transcript | gene | transcript length (nt) | max | mean | adipose | colon | heart | hypothalamus | kidney | liver | lung | ovary | skeletalmuscle | spleen | testes |
|---|---|---|---|---|---|---|---|---|---|---|---|---|---|---|---|
| NM_001165963 | SCN1A | 8132 | 6 | 1 | 0 | 0 | 0 | 6 | 0 | 0 | 1 | 0 | 0 | 0 | 0 |
| NM_001165964 | SCN1A | 8048 | 6 | 1 | 0 | 0 | 0 | 6 | 0 | 0 | 1 | 0 | 0 | 0 | 0 |
| NM_001165966 | PITPNM3 | 7042 | 6 | 1 | 0 | 1 | 0 | 4 | 0 | 0 | 0 | 2 | 0 | 6 | 0 |
| NM_001165967 | HES7 | 1682 | 2 | 0 | 0 | 0 | 1 | 0 | 1 | 0 | 0 | 0 | 0 | 2 | 0 |
| NM_001165969 | STRADA | 2157 | 5 | 2 | 1 | 2 | 1 | 5 | 2 | 1 | 2 | 2 | 1 | 4 | 3 |
| NM_001165970 | STRADA | 2112 | 5 | 2 | 1 | 2 | 1 | 5 | 2 | 1 | 2 | 2 | 1 | 3 | 2 |
| NM_001165972 | NRG3 | 3689 | 4 | 0 | 0 | 0 | 0 | 4 | 0 | 0 | 0 | 0 | 0 | 0 | 0 |
| NM_001165973 | NRG3 | 3348 | 4 | 0 | 0 | 0 | 0 | 4 | 0 | 0 | 0 | 0 | 0 | 0 | 0 |
| NM_001165974 | UROC1 | 3445 | 1 | 0 | 0 | 0 | 0 | 0 | 0 | 1 | 0 | 0 | 0 | 0 | 0 |
| NM_001165975 | PDE1B | 3218 | 4 | 1 | 2 | 0 | 1 | 4 | 0 | 0 | 0 | 2 | 0 | 2 | 0 |
| NM_001165977 | PROM2 | 2921 | 0 | 0 | 0 | 0 | 0 | 0 | 0 | 0 | 0 | 0 | 0 | 0 | 0 |
| NM_001165978 | PROM2 | 4730 | 3 | 0 | 0 | 1 | 0 | 0 | 3 | 0 | 0 | 0 | 0 | 0 | 0 |
| NM_001165979 | PLCE1 | 6453 | 4 | 2 | 1 | 4 | 3 | 2 | 2 | 0 | 2 | 1 | 2 | 4 | 1 |
| NM_001165993 | GDPD1 | 1779 | 3 | 0 | 0 | 0 | 0 | 3 | 0 | 0 | 0 | 0 | 0 | 0 | 1 |
| NM_001165994 | GDPD1 | 1149 | 4 | 1 | 0 | 0 | 0 | 4 | 0 | 0 | 0 | 0 | 0 | 0 | 1 |
| NM_001166 | BIRC2 | 3743 | 19 | 12 | 10 | 5 | 12 | 11 | 12 | 10 | 14 | 13 | 8 | 16 | 19 |
| NM_001166002 | APOBEC3H | 1044 | 0 | 0 | 0 | 0 | 0 | 0 | 0 | 0 | 0 | 0 | 0 | 0 | 0 |
| NM_001166003 | APOBEC3H | 1141 | 0 | 0 | 0 | 0 | 0 | 0 | 0 | 0 | 0 | 0 | 0 | 0 | 0 |
| NM_001166004 | APOBEC3H | 639 | 0 | 0 | 0 | 0 | 0 | 0 | 0 | 0 | 0 | 0 | 0 | 0 | 0 |
| NM_001166005 | EPB41 | 5956 | 11 | 6 | 7 | 6 | 3 | 3 | 5 | 6 | 8 | 6 | 4 | 11 | 5 |
| NM_001166006 | EPB41 | 2403 | 14 | 8 | 9 | 8 | 4 | 4 | 7 | 9 | 12 | 7 | 5 | 14 | 7 |
| NM_001166007 | EPB41 | 5777 | 11 | 6 | 7 | 6 | 3 | 3 | 6 | 7 | 8 | 6 | 4 | 11 | 5 |
| NM_001166010 | PECI | 1404 | 53 | 21 | 20 | 11 | 30 | 10 | 39 | 18 | 7 | 22 | 53 | 11 | 12 |
| NM_001166012 | ZNF397OS | 4332 | 3 | 2 | 1 | 1 | 2 | 2 | 3 | 0 | 1 | 2 | 2 | 2 | 2 |
| NM_001166017 | SKA3 | 2799 | 0 | 0 | 0 | 0 | 0 | 0 | 0 | 0 | 0 | 0 | 0 | 0 | 0 |
| NM_001166018 | POU6F2 | 2216 | 1 | 0 | 0 | 0 | 0 | 1 | 0 | 0 | 0 | 0 | 0 | 0 | 1 |
| NM_001166019 | AP1B1 | 4086 | 9 | 5 | 4 | 4 | 4 | 7 | 5 | 7 | 4 | 2 | 2 | 9 | 3 |
| NM_001166034 | SBSN | 1985 | 0 | 0 | 0 | 0 | 0 | 0 | 0 | 0 | 0 | 0 | 0 | 0 | 0 |
| NM_001166035 | SBSN | 722 | 0 | 0 | 0 | 0 | 0 | 0 | 0 | 0 | 0 | 0 | 0 | 0 | 0 |
| NM_001166036 | ZNF260 | 5467 | 6 | 3 | 2 | 0 | 2 | 3 | 4 | 1 | 2 | 6 | 1 | 4 | 3 |
| NM_001166037 | ZNF260 | 5359 | 6 | 3 | 2 | 0 | 3 | 3 | 4 | 1 | 2 | 6 | 1 | 4 | 3 |
| NM_001166038 | ZNF260 | 5340 | 6 | 3 | 2 | 0 | 3 | 3 | 4 | 1 | 2 | 6 | 1 | 4 | 3 |
| NM_001166049 | ERCC1 | 3310 | 1 | 1 | 1 | 0 | 1 | 1 | 0 | 0 | 0 | 1 | 1 | 1 | 1 |
| NM_001166050 | APBB2 | 8971 | 5 | 2 | 3 | 1 | 2 | 4 | 2 | 1 | 2 | 5 | 1 | 2 | 2 |
| NM_001166051 | APBB2 | 6992 | 4 | 2 | 3 | 1 | 2 | 4 | 2 | 1 | 2 | 4 | 1 | 2 | 2 |
| NM_001166052 | APBB2 | 6986 | 4 | 2 | 3 | 1 | 2 | 4 | 2 | 1 | 2 | 4 | 1 | 2 | 2 |
| NM_001166053 | APBB2 | 6987 | 4 | 2 | 3 | 1 | 2 | 4 | 2 | 1 | 2 | 4 | 1 | 2 | 2 |
| NM_001166054 | APBB2 | 6982 | 4 | 2 | 3 | 1 | 2 | 4 | 2 | 1 | 2 | 4 | 1 | 2 | 2 |
| NM_001166055 | EDNRA | 3823 | 7 | 2 | 1 | 0 | 3 | 0 | 1 | 0 | 2 | 7 | 1 | 3 | 3 |
| NM_001166056 | PEPD | 1887 | 18 | 5 | 3 | 1 | 2 | 2 | 18 | 14 | 2 | 2 | 1 | 4 | 1 |
| NM_001166057 | PEPD | 1818 | 20 | 6 | 4 | 1 | 2 | 4 | 20 | 18 | 2 | 2 | 1 | 5 | 1 |
| NM_001166058 | RXFP2 | 2730 | 0 | 0 | 0 | 0 | 0 | 0 | 0 | 0 | 0 | 0 | 0 | 0 | 0 |
| NM_001166059 | HMGCL | 1394 | 6 | 2 | 1 | 1 | 1 | 1 | 4 | 6 | 1 | 1 | 1 | 1 | 1 |
| NM_001166060 | GLRB | 3041 | 10 | 1 | 1 | 0 | 1 | 10 | 0 | 0 | 0 | 0 | 0 | 0 | 1 |
| NM_001166061 | GLRB | 2765 | 11 | 1 | 1 | 0 | 1 | 11 | 0 | 0 | 0 | 0 | 1 | 0 | 1 |
| NM_001166102 | NDUFV1 | 1604 | 30 | 13 | 9 | 16 | 25 | 16 | 30 | 13 | 6 | 5 | 11 | 9 | 7 |
| NM_001166103 | SPINT2 | 1630 | 11 | 5 | 0 | 11 | 2 | 8 | 11 | 0 | 7 | 2 | 0 | 3 | 7 |
| NM_001166104 | NR3C2 | 5546 | 6 | 3 | 1 | 6 | 2 | 4 | 5 | 3 | 3 | 5 | 2 | 2 | 2 |
| NM_001166105 | TADA2A | 1630 | 3 | 2 | 1 | 1 | 2 | 3 | 2 | 1 | 1 | 2 | 3 | 3 | 3 |
| NM_001166106 | CALM1 | 4230 | 272 | 60 | 52 | 27 | 52 | 272 | 42 | 26 | 33 | 21 | 42 | 46 | 48 |
| NM_001166107 | HMGCS2 | 2339 | 12 | 3 | 0 | 9 | 1 | 0 | 4 | 12 | 0 | 1 | 0 | 0 | 4 |
| NM_001166108 | PALLD | 5859 | 112 | 29 | 15 | 17 | 112 | 8 | 14 | 3 | 25 | 33 | 26 | 41 | 29 |
| NM_001166109 | PALLD | 4927 | 131 | 35 | 18 | 20 | 131 | 9 | 17 | 3 | 30 | 39 | 30 | 49 | 35 |
| NM_001166110 | PALLD | 4426 | 142 | 38 | 19 | 21 | 142 | 10 | 18 | 4 | 33 | 43 | 31 | 54 | 39 |
| NM_001166111 | PNPLA6 | 4573 | 1 | 0 | 0 | 0 | 0 | 1 | 0 | 0 | 1 | 0 | 0 | 1 | 0 |
| NM_001166112 | PNPLA6 | 4508 | 1 | 0 | 0 | 0 | 0 | 1 | 0 | 0 | 1 | 0 | 0 | 1 | 0 |
| NM_001166113 | PNPLA6 | 4460 | 1 | 0 | 0 | 0 | 0 | 1 | 0 | 0 | 1 | 0 | 0 | 1 | 0 |
| NM_001166114 | PNPLA6 | 4389 | 1 | 0 | 0 | 0 | 0 | 1 | 0 | 0 | 1 | 0 | 0 | 1 | 0 |
| NM_001166116 | TMOD1 | 3259 | 16 | 3 | 0 | 1 | 16 | 2 | 0 | 1 | 1 | 2 | 12 | 0 | 1 |
| NM_001166118 | NT5C3 | 1635 | 5 | 3 | 2 | 1 | 2 | 3 | 3 | 2 | 2 | 3 | 5 | 4 | 2 |
| NM_001166119 | LEF1 | 2642 | 5 | 1 | 0 | 2 | 0 | 1 | 1 | 0 | 2 | 1 | 0 | 5 | 2 |
| NM_001166120 | HSD3B2 | 1785 | 6 | 1 | 0 | 0 | 0 | 0 | 0 | 0 | 0 | 6 | 0 | 0 | 1 |
| NM_001166131 | EME1 | 2354 | 0 | 0 | 0 | 0 | 0 | 0 | 0 | 0 | 0 | 0 | 0 | 0 | 0 |
| NM_001166133 | FRAS1 | 7215 | 3 | 1 | 0 | 1 | 0 | 1 | 3 | 0 | 1 | 0 | 0 | 0 | 0 |
| NM_001166135 | RBM28 | 2385 | 10 | 8 | 10 | 6 | 9 | 8 | 10 | 6 | 5 | 10 | 7 | 9 | 10 |
| NM_001166136 | EVC2 | 4847 | 3 | 1 | 1 | 1 | 1 | 0 | 1 | 0 | 1 | 3 | 0 | 1 | 2 |
| NM_001166137 | LOC645961 | 3760 | 0 | 0 | 0 | 0 | 0 | 0 | 0 | 0 | 0 | 0 | 0 | 0 | 0 |
| NM_001166139 | LCORL | 5042 | 2 | 1 | 1 | 1 | 1 | 1 | 1 | 1 | 1 | 2 | 1 | 1 | 1 |
| NM_001166158 | PDHX | 2306 | 7 | 3 | 3 | 1 | 7 | 6 | 4 | 3 | 2 | 2 | 4 | 2 | 3 |
| NM_001166159 | NDUFS2 | 2064 | 16 | 10 | 8 | 7 | 16 | 12 | 14 | 16 | 4 | 6 | 10 | 8 | 5 |
| NM_001166160 | PPP1R9A | 10517 | 8 | 2 | 1 | 1 | 2 | 8 | 2 | 0 | 1 | 2 | 1 | 1 | 3 |
| NM_001166161 | PPP1R9A | 10217 | 8 | 2 | 1 | 1 | 2 | 8 | 2 | 0 | 1 | 2 | 1 | 1 | 3 |
| NM_001166162 | PPP1R9A | 10088 | 7 | 2 | 1 | 1 | 2 | 7 | 2 | 0 | 1 | 2 | 1 | 1 | 3 |
| NM_001166163 | PPP1R9A | 9599 | 8 | 2 | 1 | 1 | 3 | 8 | 2 | 0 | 1 | 2 | 1 | 1 | 4 |
| NM_001166167 | NEK6 | 2578 | 12 | 6 | 6 | 5 | 4 | 7 | 7 | 12 | 4 | 4 | 2 | 6 | 3 |
| NM_001166168 | NEK6 | 2573 | 12 | 6 | 7 | 5 | 4 | 7 | 7 | 12 | 4 | 4 | 2 | 6 | 3 |
| NM_001166169 | NEK6 | 2582 | 12 | 6 | 6 | 5 | 4 | 7 | 7 | 12 | 4 | 4 | 2 | 6 | 3 |

| transcript | gene | transcript length (nt) | max | mean | adipose | colon | heart | hypothalamus | kidney | liver | lung | ovary | skeletalmuscle | spleen | testes |
|---|---|---|---|---|---|---|---|---|---|---|---|---|---|---|---|
| NM_001166170 | NEK6 | 2580 | 12 | 6 | 6 | 5 | 4 | 7 | 7 | 12 | 4 | 4 | 2 | 6 | 3 |
| NM_001166171 | NEK6 | 2745 | 12 | 5 | 6 | 5 | 4 | 7 | 7 | 12 | 4 | 3 | 2 | 6 | 3 |
| NM_001166175 | NKX2-5 | 1961 | 9 | 1 | 0 | 0 | 9 | 0 | 0 | 0 | 0 | 0 | 0 | 1 | 0 |
| NM_001166176 | NKX2-5 | 1924 | 10 | 1 | 0 | 0 | 10 | 0 | 0 | 0 | 0 | 0 | 0 | 1 | 0 |
| NM_001166208 | SYNPO | 5172 | 54 | 11 | 8 | 3 | 25 | 16 | 2 | 2 | 3 | 2 | 54 | 4 | 3 |
| NM_001166209 | SYNPO | 5105 | 55 | 11 | 8 | 3 | 25 | 16 | 2 | 2 | 3 | 2 | 55 | 4 | 3 |
| NM_001166212 | CLCF1 | 1759 | 0 | 0 | 0 | 0 | 0 | 0 | 0 | 0 | 0 | 0 | 0 | 0 | 0 |
| NM_001166215 | S1PR5 | 2456 | 4 | 1 | 0 | 0 | 0 | 4 | 0 | 0 | 1 | 0 | 0 | 2 | 0 |
| NM_001166217 | MSL3L2 | 1992 | 6 | 1 | 1 | 1 | 1 | 0 | 0 | 1 | 0 | 0 | 0 | 1 | 6 |
| NM_001166220 | TBX20 | 1372 | 3 | 0 | 0 | 0 | 3 | 0 | 0 | 0 | 0 | 0 | 0 | 0 | 0 |
| NM_001166221 | UTP14A | 2403 | 10 | 6 | 5 | 3 | 7 | 5 | 5 | 6 | 3 | 8 | 6 | 10 | 3 |
| NM_001166222 | ATPGD1 | 3967 | 4 | 0 | 0 | 0 | 0 | 4 | 0 | 0 | 0 | 0 | 0 | 0 | 0 |
| NM_001166226 | CEP120 | 4629 | 8 | 4 | 3 | 1 | 4 | 4 | 3 | 1 | 3 | 7 | 3 | 8 | 7 |
| NM_001166237 | GSDMD | 2313 | 1 | 0 | 0 | 0 | 0 | 0 | 0 | 1 | 0 | 0 | 0 | 1 | 0 |
| NM_001166239 | CGREF1 | 1778 | 3 | 1 | 1 | 0 | 0 | 3 | 0 | 0 | 0 | 0 | 0 | 0 | 0 |
| NM_001166240 | CGREF1 | 1122 | 0 | 0 | 0 | 0 | 0 | 0 | 0 | 0 | 0 | 0 | 0 | 0 | 0 |
| NM_001166241 | CGREF1 | 1407 | 0 | 0 | 0 | 0 | 0 | 0 | 0 | 0 | 0 | 0 | 0 | 0 | 0 |
| NM_001166242 | C22orf39 | 1537 | 1 | 1 | 1 | 0 | 1 | 1 | 1 | 0 | 1 | 1 | 1 | 1 | 1 |
| NM_001166243 | FHIT | 1077 | 5 | 1 | 0 | 1 | 1 | 5 | 2 | 0 | 0 | 0 | 0 | 0 | 1 |
| NM_001166244 | HPSE2 | 4174 | 4 | 1 | 1 | 1 | 0 | 4 | 0 | 0 | 1 | 0 | 0 | 0 | 1 |
| NM_001166245 | HPSE2 | 4012 | 5 | 1 | 1 | 1 | 0 | 5 | 0 | 0 | 1 | 0 | 0 | 0 | 0 |
| NM_001166246 | HPSE2 | 4418 | 4 | 1 | 1 | 1 | 0 | 4 | 0 | 0 | 0 | 0 | 0 | 0 | 1 |
| NM_001166247 | GRIK2 | 4723 | 4 | 1 | 0 | 0 | 1 | 4 | 0 | 0 | 0 | 0 | 0 | 0 | 1 |
| NM_001166252 | LPPR4 | 4846 | 19 | 2 | 0 | 0 | 0 | 19 | 0 | 0 | 0 | 0 | 0 | 0 | 1 |
| NM_001166253 | TBXAS1 | 2202 | 35 | 6 | 5 | 2 | 5 | 4 | 1 | 4 | 12 | 2 | 1 | 35 | 1 |
| NM_001166254 | TBXAS1 | 2114 | 36 | 7 | 5 | 2 | 5 | 4 | 1 | 4 | 13 | 2 | 1 | 36 | 1 |
| NM_001166260 | TRIP13 | 1482 | 1 | 0 | 0 | 0 | 0 | 0 | 0 | 0 | 0 | 0 | 0 | 0 | 1 |
| NM_001166261 | MTIF3 | 999 | 67 | 31 | 35 | 8 | 67 | 22 | 37 | 28 | 18 | 21 | 30 | 32 | 39 |
| NM_001166262 | MTIF3 | 1069 | 63 | 29 | 33 | 7 | 63 | 20 | 34 | 26 | 17 | 20 | 28 | 30 | 36 |
| NM_001166263 | MTIF3 | 1098 | 61 | 28 | 32 | 7 | 61 | 20 | 34 | 25 | 16 | 20 | 27 | 29 | 35 |
| NM_001166264 | LTBP1 | 5164 | 23 | 6 | 2 | 2 | 23 | 2 | 4 | 2 | 6 | 7 | 3 | 2 | 11 |
| NM_001166265 | LTBP1 | 5131 | 24 | 6 | 2 | 3 | 24 | 2 | 4 | 2 | 6 | 7 | 3 | 2 | 11 |
| NM_001166266 | LTBP1 | 5005 | 24 | 6 | 2 | 3 | 24 | 2 | 4 | 2 | 6 | 8 | 3 | 2 | 11 |
| NM_001166269 | HAUS4 | 1637 | 8 | 4 | 7 | 4 | 4 | 3 | 3 | 2 | 5 | 7 | 3 | 8 | 3 |
| NM_001166270 | HAUS4 | 1494 | 9 | 5 | 7 | 4 | 4 | 3 | 3 | 2 | 5 | 8 | 3 | 9 | 3 |
| NM_001166271 | SPATA13 | 8457 | 11 | 2 | 1 | 1 | 1 | 3 | 2 | 2 | 2 | 2 | 0 | 11 | 1 |
| NM_001166276 | ARHGAP25 | 2922 | 9 | 2 | 2 | 1 | 1 | 1 | 0 | 1 | 3 | 2 | 0 | 9 | 1 |
| NM_001166277 | ARHGAP25 | 2862 | 11 | 2 | 2 | 1 | 1 | 1 | 1 | 1 | 3 | 2 | 0 | 11 | 1 |
| NM_001166278 | DLG3 | 4531 | 5 | 2 | 0 | 2 | 1 | 5 | 2 | 0 | 1 | 2 | 0 | 3 | 1 |
| NM_001166279 | PAN2 | 5298 | 2 | 1 | 1 | 2 | 1 | 1 | 2 | 2 | 1 | 1 | 1 | 2 | 2 |
| NM_001166280 | MUSK | 2496 | 3 | 1 | 0 | 1 | 1 | 0 | 0 | 0 | 1 | 0 | 3 | 0 | 1 |
| NM_001166281 | MUSK | 2466 | 3 | 1 | 0 | 1 | 1 | 0 | 0 | 0 | 1 | 0 | 3 | 0 | 1 |
| NM_001166283 | RGMA | 3199 | 3 | 1 | 1 | 1 | 1 | 3 | 0 | 0 | 0 | 1 | 2 | 0 | 1 |
| NM_001166284 | CCT7 | 1674 | 18 | 12 | 10 | 9 | 15 | 18 | 10 | 17 | 6 | 8 | 15 | 13 | 14 |
| NM_001166285 | CCT7 | 2031 | 27 | 20 | 15 | 13 | 25 | 27 | 16 | 26 | 11 | 14 | 25 | 21 | 23 |
| NM_001166286 | RGMA | 3059 | 3 | 1 | 1 | 1 | 1 | 3 | 0 | 0 | 0 | 1 | 2 | 0 | 1 |
| NM_001166287 | RGMA | 3173 | 3 | 1 | 1 | 1 | 1 | 3 | 0 | 0 | 0 | 1 | 2 | 0 | 1 |
| NM_001166288 | RGMA | 3057 | 3 | 1 | 1 | 1 | 1 | 3 | 0 | 0 | 0 | 1 | 2 | 0 | 1 |
| NM_001166289 | RGMA | 3077 | 3 | 1 | 1 | 1 | 1 | 3 | 0 | 0 | 0 | 1 | 2 | 0 | 2 |
| NM_001166290 | KCNJ11 | 2754 | 1 | 0 | 0 | 0 | 0 | 0 | 0 | 0 | 0 | 0 | 1 | 0 | 0 |
| NM_001166292 | PTCH2 | 4298 | 0 | 0 | 0 | 0 | 0 | 0 | 0 | 0 | 0 | 0 | 0 | 0 | 0 |
| NM_001166293 | SSX2IP | 5847 | 15 | 4 | 0 | 1 | 3 | 12 | 5 | 3 | 1 | 1 | 1 | 2 | 15 |
| NM_001166294 | SSX2IP | 5715 | 14 | 4 | 0 | 1 | 2 | 12 | 5 | 3 | 1 | 1 | 1 | 2 | 14 |
| NM_001166295 | SSX2IP | 5843 | 14 | 4 | 0 | 1 | 2 | 12 | 5 | 3 | 1 | 1 | 1 | 2 | 14 |
| NM_001166301 | DHX40 | 3435 | 9 | 5 | 4 | 2 | 4 | 5 | 6 | 2 | 5 | 7 | 3 | 5 | 9 |
| NM_001166304 | PIGX | 3091 | 5 | 2 | 2 | 2 | 2 | 5 | 3 | 1 | 2 | 2 | 1 | 2 | 2 |
| NM_001166305 | TMEM44 | 2489 | 4 | 1 | 1 | 4 | 1 | 1 | 1 | 0 | 2 | 1 | 0 | 3 | 1 |
| NM_001166306 | TMEM44 | 2344 | 4 | 1 | 1 | 4 | 1 | 1 | 1 | 0 | 2 | 2 | 0 | 3 | 1 |
| NM_001166340 | SENP8 | 1679 | 3 | 1 | 0 | 0 | 1 | 2 | 2 | 0 | 1 | 2 | 2 | 1 | 3 |
| NM_001166343 | MFNG | 2034 | 0 | 0 | 0 | 0 | 0 | 0 | 0 | 0 | 0 | 0 | 0 | 0 | 0 |
| NM_001166345 | MDFIC | 5312 | 21 | 7 | 21 | 3 | 8 | 2 | 5 | 2 | 8 | 6 | 3 | 13 | 2 |
| NM_001166346 | MDFIC | 890 | 63 | 17 | 63 | 9 | 24 | 7 | 15 | 7 | 20 | 13 | 7 | 21 | 6 |
| NM_001166347 | SLC26A11 | 2921 | 0 | 0 | 0 | 0 | 0 | 0 | 0 | 0 | 0 | 0 | 0 | 0 | 0 |
| NM_001166348 | SLC26A11 | 2851 | 0 | 0 | 0 | 0 | 0 | 0 | 0 | 0 | 0 | 0 | 0 | 0 | 0 |
| NM_001166349 | SLC26A11 | 2721 | 1 | 0 | 0 | 0 | 0 | 1 | 0 | 0 | 0 | 0 | 0 | 0 | 0 |
| NM_001166355 | LFNG | 2268 | 1 | 0 | 0 | 1 | 0 | 0 | 0 | 0 | 1 | 0 | 0 | 1 | 0 |
| NM_001166356 | SHMT2 | 2264 | 12 | 2 | 1 | 2 | 1 | 1 | 2 | 12 | 1 | 1 | 1 | 3 | 1 |
| NM_001166357 | SHMT2 | 2356 | 10 | 2 | 1 | 2 | 1 | 1 | 2 | 10 | 1 | 1 | 1 | 3 | 1 |
| NM_001166358 | SHMT2 | 2255 | 11 | 2 | 1 | 2 | 1 | 1 | 2 | 11 | 1 | 1 | 1 | 3 | 1 |
| NM_001166359 | SHMT2 | 2148 | 11 | 2 | 1 | 2 | 1 | 1 | 2 | 11 | 1 | 1 | 1 | 3 | 1 |
| NM_001166373 | 01 Mar | 5502 | 7 | 1 | 1 | 0 | 1 | 2 | 0 | 0 | 2 | 1 | 0 | 7 | 1 |
| NM_001166386 | MAGEA12 | 1859 | 0 | 0 | 0 | 0 | 0 | 0 | 0 | 0 | 0 | 0 | 0 | 0 | 0 |
| NM_001166387 | MAGEA12 | 1767 | 0 | 0 | 0 | 0 | 0 | 0 | 0 | 0 | 0 | 0 | 0 | 0 | 0 |
| NM_001166392 | TPBG | 2878 | 1 | 0 | 0 | 0 | 0 | 0 | 1 | 0 | 0 | 0 | 0 | 1 | 0 |
| NM_001166393 | MTUS1 | 3650 | 26 | 14 | 9 | 5 | 26 | 20 | 7 | 15 | 10 | 20 | 18 | 9 | 10 |
| NM_001166395 | CHST4 | 2174 | 1 | 0 | 0 | 0 | 0 | 0 | 0 | 1 | 0 | 0 | 0 | 0 | 0 |
| NM_001166400 | MAGEA8 | 2112 | 0 | 0 | 0 | 0 | 0 | 0 | 0 | 0 | 0 | 0 | 0 | 0 | 0 |

| | | transcript | | | | | | hypothala | | | | | skeletalm | | |
|---|---|---|---|---|---|---|---|---|---|---|---|---|---|---|---|
| transcript | gene | length (nt) | max | mean | adipose | colon | heart | mus | kidney | liver | lung | ovary | uscle | spleen | testes |
| NM_001166401 | MAGEA8 | 2084 | 0 | 0 | 0 | 0 | 0 | 0 | 0 | 0 | 0 | 0 | 0 | 0 | 0 |
| NM_001166412 | SMOC2 | 3116 | 35 | 8 | 3 | 3 | 9 | 2 | 2 | 1 | 3 | 35 | 5 | 1 | 22 |
| NM_001166415 | EHHADH | 3971 | 26 | 5 | 4 | 1 | 2 | 1 | 23 | 26 | 1 | 1 | 0 | 1 | 1 |
| NM_001166417 | SSX2IP | 6025 | 14 | 4 | 0 | 1 | 2 | 12 | 5 | 3 | 1 | 1 | 1 | 2 | 14 |
| NM_001166418 | HDAC8 | 1740 | 2 | 1 | 1 | 1 | 1 | 2 | 2 | 1 | 1 | 2 | 1 | 2 | 1 |
| NM_001166419 | HDAC8 | 2392 | 3 | 2 | 2 | 1 | 1 | 2 | 3 | 2 | 2 | 3 | 1 | 2 | 2 |
| NM_001166420 | HDAC8 | 1085 | 1 | 0 | 1 | 0 | 0 | 1 | 1 | 0 | 1 | 0 | 0 | 1 | 0 |
| NM_001166422 | HDAC8 | 993 | 1 | 0 | 1 | 0 | 0 | 1 | 1 | 0 | 1 | 0 | 0 | 1 | 0 |
| NM_001166424 | PGC | 1151 | 21 | 2 | 0 | 0 | 0 | 0 | 0 | 0 | 21 | 0 | 0 | 0 | 0 |
| NM_001166425 | GNAI2 | 2143 | 156 | 73 | 93 | 32 | 85 | 83 | 30 | 56 | 94 | 80 | 27 | 156 | 63 |
| NM_001166426 | WDR13 | 2460 | 3 | 2 | 1 | 1 | 2 | 2 | 2 | 3 | 1 | 2 | 1 | 1 | 1 |
| NM_001166434 | ITIH1 | 2741 | 51 | 5 | 0 | 0 | 0 | 0 | 0 | 51 | 0 | 0 | 0 | 0 | 0 |
| NM_001166435 | ITIH1 | 2486 | 24 | 2 | 0 | 0 | 0 | 0 | 0 | 24 | 0 | 0 | 0 | 0 | 0 |
| NM_001166436 | ITIH1 | 2377 | 25 | 2 | 0 | 0 | 0 | 0 | 0 | 25 | 0 | 0 | 0 | 0 | 0 |
| NM_001166448 | HDAC8 | 2041 | 3 | 2 | 2 | 1 | 2 | 2 | 3 | 2 | 2 | 3 | 1 | 2 | 2 |
| NM_001166449 | ITIH4 | 3192 | 47 | 5 | 0 | 0 | 1 | 0 | 0 | 47 | 0 | 0 | 1 | 1 | 1 |
| NM_001166451 | KNG1 | 2034 | 267 | 31 | 0 | 1 | 1 | 0 | 68 | 267 | 0 | 1 | 1 | 1 | 0 |
| NM_001166460 | MPP1 | 1995 | 2 | 1 | 1 | 0 | 1 | 1 | 2 | 1 | 2 | 0 | 1 | 2 | 1 |
| NM_001166461 | MPP1 | 1986 | 2 | 1 | 1 | 0 | 1 | 1 | 2 | 1 | 2 | 0 | 1 | 2 | 1 |
| NM_001166462 | MPP1 | 2195 | 2 | 1 | 1 | 0 | 1 | 1 | 2 | 1 | 2 | 0 | 1 | 2 | 1 |
| NM_001166478 | DEFB133 | 186 | 0 | 0 | 0 | 0 | 0 | 0 | 0 | 0 | 0 | 0 | 0 | 0 | 0 |
| NM_001166496 | SLC16A1 | 4376 | 10 | 4 | 2 | 5 | 10 | 3 | 2 | 5 | 1 | 3 | 3 | 2 | 10 |
| NM_001166498 | HPSE | 1574 | 2 | 1 | 1 | 0 | 1 | 1 | 0 | 1 | 1 | 1 | 0 | 2 | 1 |
| NM_001166504 | ADH7 | 2101 | 0 | 0 | 0 | 0 | 0 | 0 | 0 | 0 | 0 | 0 | 0 | 0 | 0 |
| NM_001166533 | DDX4 | 2452 | 45 | 4 | 0 | 0 | 0 | 0 | 0 | 0 | 0 | 0 | 0 | 0 | 45 |
| NM_001166534 | DDX4 | 2378 | 42 | 4 | 0 | 0 | 0 | 0 | 0 | 0 | 0 | 0 | 0 | 0 | 42 |
| NM_001166538 | LST1 | 554 | 6 | 1 | 0 | 0 | 0 | 1 | 0 | 0 | 1 | 0 | 0 | 6 | 0 |
| NM_001166550 | IDS | 5808 | 33 | 6 | 2 | 1 | 4 | 33 | 3 | 1 | 4 | 4 | 2 | 5 | 2 |
| NM_001166579 | AANAT | 1912 | 0 | 0 | 0 | 0 | 0 | 0 | 0 | 0 | 0 | 0 | 0 | 0 | 0 |
| NM_001166588 | EIF2B3 | 1564 | 3 | 1 | 3 | 1 | 1 | 2 | 1 | 2 | 1 | 1 | 2 | 1 | 2 |
| NM_001166598 | APOA5 | 1907 | 20 | 2 | 0 | 0 | 0 | 0 | 0 | 20 | 0 | 0 | 0 | 0 | 0 |
| NM_001166599 | FAM122B | 4335 | 16 | 5 | 2 | 3 | 4 | 7 | 3 | 1 | 3 | 7 | 1 | 6 | 16 |
| NM_001166600 | FAM122B | 2698 | 25 | 7 | 4 | 4 | 6 | 10 | 5 | 2 | 4 | 10 | 2 | 10 | 25 |
| NM_001166621 | TRAPPC1 | 759 | 19 | 10 | 10 | 9 | 11 | 19 | 10 | 10 | 8 | 7 | 5 | 15 | 7 |
| NM_001166624 | CFHR3 | 1457 | 5 | 0 | 0 | 0 | 0 | 0 | 0 | 5 | 0 | 0 | 0 | 0 | 0 |
| NM_001166660 | NLGN3 | 3863 | 10 | 1 | 0 | 1 | 0 | 10 | 0 | 0 | 0 | 2 | 0 | 0 | 2 |
| NM_001166663 | CD244 | 2525 | 2 | 0 | 0 | 0 | 0 | 0 | 0 | 0 | 0 | 0 | 0 | 2 | 0 |
| NM_001166664 | CD244 | 2234 | 2 | 0 | 0 | 0 | 0 | 0 | 0 | 0 | 0 | 0 | 0 | 2 | 0 |
| NM_001166686 | PFKM | 3526 | 24 | 4 | 0 | 1 | 7 | 6 | 2 | 0 | 1 | 2 | 24 | 1 | 2 |
| NM_001166687 | PFKM | 3078 | 27 | 5 | 1 | 1 | 8 | 6 | 2 | 0 | 1 | 2 | 27 | 1 | 2 |
| NM_001166688 | PFKM | 3167 | 26 | 5 | 0 | 1 | 8 | 6 | 2 | 0 | 1 | 2 | 26 | 1 | 2 |
| NM_001166691 | TTK | 3019 | 8 | 1 | 0 | 0 | 0 | 0 | 0 | 0 | 0 | 0 | 0 | 1 | 8 |
| NM_001166692 | C11orf91 | 903 | 0 | 0 | 0 | 0 | 0 | 0 | 0 | 0 | 0 | 0 | 0 | 0 | 0 |
| NM_001166693 | AFF1 | 9284 | 22 | 10 | 9 | 7 | 10 | 4 | 17 | 6 | 9 | 12 | 22 | 11 | 7 |
| NM_001166694 | CHRNA3 | 2027 | 1 | 0 | 0 | 1 | 0 | 1 | 0 | 0 | 0 | 0 | 0 | 0 | 1 |
| NM_001166695 | SLC1A3 | 3652 | 47 | 6 | 6 | 1 | 6 | 47 | 1 | 0 | 3 | 1 | 1 | 4 | 1 |
| NM_001166696 | SLC1A3 | 1348 | 35 | 5 | 6 | 0 | 4 | 35 | 0 | 0 | 3 | 1 | 1 | 3 | 1 |
| NM_001166698 | FAM13C | 3456 | 5 | 1 | 1 | 1 | 1 | 5 | 0 | 0 | 1 | 2 | 3 | 1 | 2 |
| NM_001166699 | FAM104B | 1067 | 8 | 4 | 2 | 3 | 4 | 8 | 4 | 4 | 3 | 5 | 4 | 3 | 5 |
| NM_001166700 | FAM104B | 1229 | 5 | 3 | 2 | 2 | 4 | 5 | 3 | 3 | 3 | 4 | 3 | 3 | 5 |
| NM_001166701 | FAM104B | 1226 | 5 | 3 | 2 | 2 | 4 | 5 | 3 | 3 | 3 | 4 | 3 | 3 | 5 |
| NM_001166702 | FAM104B | 1327 | 5 | 3 | 2 | 2 | 4 | 5 | 3 | 3 | 3 | 4 | 3 | 2 | 5 |
| NM_001166703 | FAM104B | 1405 | 5 | 3 | 2 | 2 | 3 | 5 | 3 | 2 | 3 | 4 | 3 | 2 | 4 |
| NM_001166704 | FAM104B | 1233 | 5 | 3 | 2 | 2 | 4 | 5 | 3 | 3 | 3 | 4 | 3 | 3 | 5 |
| NM_001167 | XIAP | 8413 | 7 | 4 | 3 | 2 | 4 | 5 | 6 | 3 | 5 | 4 | 4 | 7 | 4 |
| NM_001167574 | UPK3A | 682 | 0 | 0 | 0 | 0 | 0 | 0 | 0 | 0 | 0 | 0 | 0 | 0 | 0 |
| NM_001167575 | C9orf82 | 2968 | 10 | 6 | 4 | 4 | 4 | 5 | 7 | 10 | 3 | 4 | 3 | 7 | 9 |
| NM_001167576 | TRPC7 | 2587 | 0 | 0 | 0 | 0 | 0 | 0 | 0 | 0 | 0 | 0 | 0 | 0 | 0 |
| NM_001167577 | TRPC7 | 2752 | 0 | 0 | 0 | 0 | 0 | 0 | 0 | 0 | 0 | 0 | 0 | 0 | 0 |
| NM_001167579 | SLC34A1 | 1914 | 5 | 0 | 0 | 0 | 0 | 0 | 5 | 0 | 0 | 0 | 0 | 0 | 0 |
| NM_001167580 | SV2B | 10769 | 15 | 2 | 0 | 0 | 0 | 15 | 0 | 0 | 0 | 0 | 0 | 0 | 0 |
| NM_001167582 | RBM6 | 2225 | 33 | 21 | 18 | 19 | 19 | 18 | 23 | 9 | 18 | 22 | 15 | 33 | 33 |
| NM_001167583 | ZFAT | 4532 | 2 | 1 | 1 | 0 | 0 | 1 | 2 | 0 | 0 | 1 | 0 | 1 | 1 |
| NM_001167594 | CTSA | 2187 | 15 | 5 | 6 | 4 | 4 | 3 | 15 | 5 | 4 | 3 | 2 | 8 | 3 |
| NM_001167595 | AMACR | 2601 | 41 | 7 | 2 | 4 | 2 | 8 | 41 | 8 | 1 | 2 | 1 | 1 | 2 |
| NM_001167596 | AMACR | 1347 | 7 | 2 | 1 | 1 | 2 | 1 | 7 | 3 | 0 | 0 | 1 | 0 | 0 |
| NM_001167597 | AMACR | 1186 | 7 | 2 | 1 | 1 | 2 | 1 | 7 | 3 | 1 | 0 | 1 | 0 | 0 |
| NM_001167598 | AMACR | 945 | 9 | 1 | 0 | 1 | 0 | 1 | 9 | 2 | 0 | 0 | 0 | 0 | 0 |
| NM_001167599 | NEU4 | 2353 | 0 | 0 | 0 | 0 | 0 | 0 | 0 | 0 | 0 | 0 | 0 | 0 | 0 |
| NM_001167600 | NEU4 | 2033 | 0 | 0 | 0 | 0 | 0 | 0 | 0 | 0 | 0 | 0 | 0 | 0 | 0 |
| NM_001167601 | NEU4 | 2036 | 0 | 0 | 0 | 0 | 0 | 0 | 0 | 0 | 0 | 0 | 0 | 0 | 0 |
| NM_001167602 | NEU4 | 2531 | 0 | 0 | 0 | 0 | 0 | 0 | 0 | 0 | 0 | 0 | 0 | 0 | 0 |
| NM_001167604 | XPNPEP1 | 2467 | 2 | 1 | 1 | 2 | 1 | 2 | 2 | 1 | 1 | 1 | 1 | 2 | 1 |
| NM_001167605 | KLK11 | 1266 | 0 | 0 | 0 | 0 | 0 | 0 | 0 | 0 | 0 | 0 | 0 | 0 | 0 |
| NM_001167606 | RAB35 | 2820 | 1 | 1 | 1 | 1 | 1 | 1 | 1 | 1 | 1 | 1 | 0 | 1 | 1 |
| NM_001167607 | GM2A | 3476 | 7 | 3 | 7 | 1 | 3 | 4 | 2 | 1 | 4 | 3 | 1 | 6 | 2 |
| NM_001167608 | RHBDD1 | 5155 | 4 | 3 | 2 | 2 | 3 | 3 | 3 | 3 | 2 | 4 | 2 | 4 | 3 |

| transcript | gene | transcript length (nt) | max | mean | adipose | colon | heart | hypothala mus | kidney | liver | lung | ovary | skeletalm uscle | spleen | testes |
|---|---|---|---|---|---|---|---|---|---|---|---|---|---|---|---|
| NM_001167609 | GLI1 | 3477 | 0 | 0 | 0 | 0 | 0 | 0 | 0 | 0 | 0 | 0 | 0 | 0 | 0 |
| NM_001167614 | LEMD3 | 4793 | 3 | 2 | 1 | 2 | 2 | 3 | 2 | 1 | 3 | 2 | 1 | 3 | 3 |
| NM_001167617 | MLH1 | 2455 | 7 | 5 | 5 | 3 | 6 | 6 | 4 | 5 | 3 | 4 | 5 | 7 | 7 |
| NM_001167618 | MLH1 | 2455 | 7 | 5 | 5 | 3 | 6 | 6 | 4 | 5 | 3 | 4 | 5 | 7 | 7 |
| NM_001167619 | MLH1 | 2368 | 8 | 5 | 5 | 3 | 6 | 6 | 4 | 6 | 3 | 4 | 6 | 7 | 8 |
| NM_001167621 | ATXN10 | 3137 | 8 | 4 | 2 | 1 | 5 | 8 | 4 | 4 | 3 | 5 | 3 | 5 | 4 |
| NM_001167623 | CACNA1C | 13480 | 3 | 1 | 0 | 2 | 3 | 2 | 0 | 0 | 1 | 0 | 0 | 1 | 0 |
| NM_001167624 | CACNA1C | 13585 | 3 | 1 | 0 | 2 | 3 | 2 | 0 | 0 | 1 | 0 | 0 | 1 | 0 |
| NM_001167625 | CACNA1C | 13660 | 3 | 1 | 0 | 2 | 3 | 2 | 0 | 0 | 1 | 0 | 0 | 1 | 0 |
| NM_001167667 | CDH18 | 2976 | 10 | 1 | 0 | 0 | 0 | 10 | 0 | 0 | 0 | 0 | 0 | 0 | 1 |
| NM_001167670 | LOC100288 | 700 | 1 | 0 | 0 | 0 | 0 | 0 | 0 | 0 | 0 | 0 | 0 | 0 | 1 |
| NM_001167671 | LPP | 18220 | 13 | 7 | 6 | 10 | 13 | 2 | 9 | 2 | 9 | 7 | 5 | 9 | 9 |
| NM_001167672 | LPP | 17746 | 13 | 7 | 6 | 10 | 13 | 2 | 9 | 2 | 9 | 7 | 5 | 10 | 9 |
| NM_001167674 | CADM2 | 9578 | 22 | 2 | 1 | 1 | 1 | 22 | 0 | 0 | 0 | 0 | 0 | 0 | 0 |
| NM_001167675 | CADM2 | 9485 | 22 | 2 | 1 | 0 | 1 | 22 | 0 | 0 | 0 | 0 | 0 | 0 | 0 |
| NM_001167676 | LOC100128 | 699 | 0 | 0 | 0 | 0 | 0 | 0 | 0 | 0 | 0 | 0 | 0 | 0 | 0 |
| NM_001167681 | FLI1 | 3926 | 12 | 2 | 3 | 1 | 2 | 1 | 1 | 1 | 3 | 1 | 1 | 12 | 1 |
| NM_001167733 | VARS2 | 3377 | 1 | 1 | 1 | 1 | 0 | 1 | 1 | 1 | 0 | 0 | 1 | 1 | 1 |
| NM_001167734 | VARS2 | 3609 | 1 | 1 | 1 | 1 | 0 | 1 | 1 | 1 | 0 | 0 | 1 | 1 | 1 |
| NM_001167738 | NAV1 | 11683 | 5 | 2 | 4 | 1 | 3 | 5 | 1 | 0 | 1 | 1 | 1 | 1 | 2 |
| NM_001167740 | SMYD3 | 1635 | 2 | 1 | 0 | 1 | 1 | 2 | 1 | 1 | 1 | 1 | 1 | 1 | 1 |
| NM_001167741 | MTX3 | 7862 | 4 | 2 | 1 | 1 | 2 | 4 | 3 | 1 | 1 | 3 | 1 | 2 | 3 |
| NM_001167749 | ADCY10 | 4966 | 1 | 0 | 0 | 0 | 0 | 0 | 0 | 0 | 0 | 0 | 0 | 0 | 1 |
| NM_001167816 | C20orf26 | 1946 | 1 | 0 | 0 | 0 | 0 | 0 | 0 | 0 | 0 | 0 | 1 | 0 | 0 |
| NM_001167819 | FHL1 | 2374 | 181 | 29 | 40 | 7 | 64 | 8 | 2 | 1 | 8 | 3 | 181 | 2 | 6 |
| NM_001167820 | BLCAP | 2205 | 47 | 15 | 12 | 7 | 13 | 30 | 13 | 10 | 6 | 11 | 47 | 10 | 8 |
| NM_001167821 | BLCAP | 2035 | 51 | 16 | 13 | 8 | 14 | 33 | 14 | 11 | 6 | 11 | 51 | 10 | 8 |
| NM_001167822 | BLCAP | 2041 | 51 | 16 | 13 | 8 | 14 | 33 | 14 | 11 | 6 | 11 | 51 | 10 | 8 |
| NM_001167823 | BLCAP | 2108 | 49 | 16 | 13 | 8 | 14 | 32 | 13 | 10 | 6 | 11 | 49 | 10 | 8 |
| NM_001167827 | TFEB | 2187 | 1 | 0 | 0 | 0 | 0 | 0 | 0 | 0 | 0 | 0 | 1 | 0 | 0 |
| NM_001167830 | TMED5 | 6114 | 22 | 10 | 9 | 2 | 12 | 7 | 14 | 22 | 9 | 5 | 10 | 12 | 6 |
| NM_001167856 | SBNO1 | 10981 | 22 | 7 | 4 | 3 | 6 | 9 | 6 | 5 | 4 | 7 | 5 | 7 | 22 |
| NM_001167857 | PPP1R12B | 1819 | 14 | 4 | 3 | 2 | 14 | 6 | 2 | 1 | 2 | 2 | 12 | 1 | 2 |
| NM_001167858 | PPP1R12B | 1706 | 9 | 3 | 2 | 1 | 9 | 5 | 1 | 1 | 1 | 1 | 8 | 1 | 1 |
| NM_001167865 | NSMCE4A | 1396 | 7 | 5 | 7 | 5 | 5 | 4 | 5 | 5 | 3 | 4 | 4 | 7 | 2 |
| NM_001167867 | ATP5SL | 1771 | 4 | 2 | 2 | 2 | 3 | 3 | 3 | 4 | 2 | 1 | 1 | 2 | 1 |
| NM_001167868 | ATP5SL | 1608 | 4 | 2 | 2 | 2 | 4 | 3 | 3 | 4 | 2 | 1 | 1 | 2 | 1 |
| NM_001167869 | ATP5SL | 1562 | 4 | 2 | 2 | 2 | 4 | 3 | 3 | 4 | 2 | 1 | 1 | 2 | 1 |
| NM_001167870 | ATP5SL | 1481 | 5 | 3 | 2 | 2 | 4 | 3 | 3 | 5 | 2 | 1 | 1 | 2 | 1 |
| NM_001167871 | ATP5SL | 1644 | 4 | 2 | 2 | 2 | 3 | 3 | 3 | 4 | 2 | 1 | 1 | 2 | 1 |
| NM_001167880 | LHPP | 1592 | 4 | 1 | 1 | 2 | 1 | 4 | 2 | 2 | 1 | 0 | 0 | 1 | 1 |
| NM_001167882 | ANKRD50 | 7519 | 6 | 3 | 2 | 1 | 3 | 5 | 2 | 2 | 3 | 6 | 1 | 2 | 6 |
| NM_001167890 | EGFL6 | 2431 | 15 | 2 | 15 | 0 | 0 | 0 | 0 | 0 | 2 | 0 | 0 | 0 | 0 |
| NM_001167902 | LOC145814 | 1483 | 2 | 0 | 0 | 0 | 0 | 0 | 0 | 0 | 0 | 0 | 2 | 0 | 0 |
| NM_001167903 | MFSD1 | 2244 | 11 | 5 | 7 | 1 | 5 | 2 | 5 | 4 | 8 | 3 | 1 | 11 | 2 |
| NM_001167911 | VEPH1 | 3984 | 5 | 1 | 0 | 0 | 0 | 0 | 5 | 0 | 5 | 0 | 0 | 1 | 1 |
| NM_001167912 | VEPH1 | 4119 | 5 | 1 | 0 | 0 | 0 | 0 | 5 | 0 | 5 | 0 | 0 | 0 | 1 |
| NM_001167915 | VEPH1 | 1664 | 3 | 1 | 0 | 0 | 0 | 0 | 3 | 0 | 3 | 0 | 0 | 0 | 0 |
| NM_001167916 | VEPH1 | 1402 | 4 | 1 | 0 | 0 | 0 | 0 | 3 | 0 | 4 | 0 | 0 | 0 | 0 |
| NM_001167917 | VEPH1 | 992 | 4 | 1 | 0 | 0 | 0 | 0 | 4 | 0 | 4 | 0 | 0 | 0 | 1 |
| NM_001167924 | C3orf26 | 1089 | 23 | 9 | 7 | 2 | 11 | 23 | 6 | 9 | 4 | 7 | 16 | 7 | 13 |
| NM_001167928 | IL1RAP | 4947 | 15 | 3 | 1 | 0 | 3 | 2 | 1 | 15 | 4 | 1 | 0 | 6 | 1 |
| NM_001167929 | IL1RAP | 4696 | 15 | 3 | 1 | 0 | 3 | 2 | 1 | 15 | 4 | 1 | 0 | 6 | 1 |
| NM_001167930 | IL1RAP | 2005 | 6 | 1 | 0 | 0 | 1 | 1 | 0 | 6 | 1 | 0 | 0 | 1 | 0 |
| NM_001167931 | IL1RAP | 2910 | 9 | 2 | 1 | 0 | 1 | 2 | 0 | 9 | 2 | 0 | 0 | 3 | 0 |
| NM_001167934 | CCM2 | 1719 | 2 | 1 | 1 | 1 | 1 | 2 | 0 | 2 | 0 | 0 | 0 | 2 | 1 |
| NM_001167935 | CCM2 | 1620 | 4 | 2 | 2 | 1 | 1 | 3 | 1 | 4 | 1 | 0 | 1 | 4 | 2 |
| NM_001167940 | CADPS2 | 6066 | 5 | 2 | 1 | 1 | 2 | 3 | 5 | 2 | 3 | 2 | 0 | 1 | 1 |
| NM_001167941 | WDR76 | 3917 | 1 | 0 | 0 | 0 | 0 | 0 | 0 | 0 | 0 | 0 | 0 | 1 | 1 |
| NM_001167942 | TNFAIP8L1 | 3845 | 4 | 1 | 1 | 2 | 1 | 1 | 2 | 4 | 1 | 1 | 1 | 3 | 1 |
| NM_001167945 | CACNB2 | 4187 | 5 | 2 | 0 | 2 | 4 | 5 | 1 | 0 | 1 | 3 | 0 | 1 | 1 |
| NM_001167947 | TIMM17B | 1099 | 1 | 1 | 1 | 0 | 1 | 1 | 1 | 1 | 0 | 0 | 1 | 1 | 0 |
| NM_001167959 | TMEM178 | 1347 | 13 | 2 | 1 | 1 | 1 | 13 | 5 | 0 | 1 | 1 | 1 | 0 | 1 |
| NM_001167961 | KCTD7 | 3708 | 2 | 1 | 0 | 0 | 0 | 2 | 1 | 0 | 0 | 1 | 0 | 1 | 1 |
| NM_001167962 | SLC26A5 | 2588 | 0 | 0 | 0 | 0 | 0 | 0 | 0 | 0 | 0 | 0 | 0 | 0 | 0 |
| NM_001167965 | WDR65 | 3034 | 1 | 0 | 0 | 0 | 0 | 0 | 1 | 0 | 0 | 0 | 0 | 0 | 1 |
| NM_001167966 | WDR65 | 2969 | 1 | 0 | 0 | 0 | 0 | 0 | 1 | 0 | 0 | 0 | 0 | 0 | 1 |
| NM_001167970 | TRMT2B | 3163 | 1 | 1 | 1 | 1 | 1 | 1 | 1 | 1 | 1 | 1 | 1 | 1 | 1 |
| NM_001167971 | TRMT2B | 3144 | 1 | 1 | 1 | 1 | 1 | 1 | 1 | 1 | 1 | 1 | 1 | 1 | 1 |
| NM_001167972 | TRMT2B | 3106 | 1 | 1 | 1 | 1 | 1 | 1 | 1 | 1 | 1 | 1 | 1 | 1 | 1 |
| NM_001167985 | INCA1 | 1220 | 1 | 0 | 0 | 1 | 0 | 1 | 0 | 0 | 0 | 1 | 0 | 1 | 1 |
| NM_001167986 | INCA1 | 1552 | 1 | 0 | 0 | 1 | 0 | 1 | 0 | 0 | 0 | 0 | 0 | 1 | 1 |
| NM_001167987 | INCA1 | 1265 | 1 | 0 | 0 | 1 | 0 | 1 | 0 | 0 | 0 | 1 | 0 | 1 | 1 |
| NM_001167989 | PQBP1 | 976 | 11 | 8 | 8 | 9 | 6 | 11 | 8 | 11 | 7 | 9 | 6 | 8 | 7 |
| NM_001167990 | PQBP1 | 935 | 12 | 9 | 8 | 10 | 7 | 11 | 8 | 12 | 7 | 9 | 6 | 9 | 7 |
| NM_001167992 | PQBP1 | 574 | 1 | 0 | 0 | 0 | 0 | 0 | 0 | 0 | 0 | 0 | 0 | 1 | 0 |
| NM_001168 | BIRC5 | 2631 | 0 | 0 | 0 | 0 | 0 | 0 | 0 | 0 | 0 | 0 | 0 | 0 | 0 |

| transcript | gene | transcript length (nt) | max | mean | adipose | colon | heart | hypothalamus | kidney | liver | lung | ovary | skeletalmuscle | spleen | testes |
|---|---|---|---|---|---|---|---|---|---|---|---|---|---|---|---|
| NM_001168214 | LOC401097 | 2577 | 2 | 0 | 0 | 0 | 0 | 2 | 0 | 0 | 0 | 0 | 0 | 0 | 0 |
| NM_001168215 | TMEM92 | 2765 | 0 | 0 | 0 | 0 | 0 | 0 | 0 | 0 | 0 | 0 | 0 | 0 | 0 |
| NM_001168216 | ALS2CR11 | 1768 | 4 | 0 | 0 | 0 | 0 | 0 | 0 | 0 | 0 | 0 | 0 | 0 | 4 |
| NM_001168217 | ALS2CR11 | 3257 | 3 | 0 | 0 | 0 | 0 | 0 | 0 | 0 | 0 | 0 | 0 | 0 | 3 |
| NM_001168221 | ALS2CR11 | 5703 | 4 | 0 | 0 | 0 | 0 | 0 | 0 | 0 | 0 | 0 | 0 | 0 | 4 |
| NM_001168222 | TBC1D17 | 2272 | 2 | 1 | 0 | 1 | 1 | 1 | 1 | 1 | 1 | 1 | 2 | 0 | 1 |
| NM_001168235 | FREM3 | 6621 | 0 | 0 | 0 | 0 | 0 | 0 | 0 | 0 | 0 | 0 | 0 | 0 | 0 |
| NM_001168236 | ABL2 | 12163 | 2 | 2 | 2 | 1 | 1 | 2 | 1 | 1 | 2 | 2 | 1 | 2 | 2 |
| NM_001168237 | ABL2 | 11917 | 2 | 2 | 2 | 1 | 1 | 2 | 1 | 1 | 2 | 2 | 1 | 2 | 2 |
| NM_001168238 | ABL2 | 11854 | 2 | 2 | 2 | 1 | 1 | 2 | 1 | 1 | 2 | 2 | 1 | 2 | 2 |
| NM_001168239 | ABL2 | 11567 | 2 | 2 | 2 | 1 | 2 | 2 | 1 | 1 | 2 | 2 | 1 | 2 | 2 |
| NM_001168241 | FAM59B | 4160 | 1 | 0 | 0 | 0 | 0 | 1 | 0 | 0 | 0 | 0 | 0 | 0 | 0 |
| NM_001168243 | C4orf48 | 433 | 0 | 0 | 0 | 0 | 0 | 0 | 0 | 0 | 0 | 0 | 0 | 0 | 0 |
| NM_001168247 | SLFNL1 | 2117 | 2 | 0 | 0 | 0 | 0 | 0 | 0 | 0 | 0 | 0 | 0 | 0 | 2 |
| NM_001168249 | COL11A1 | 6942 | 1 | 0 | 0 | 0 | 0 | 1 | 0 | 0 | 0 | 1 | 0 | 1 | 0 |
| NM_001168251 | PAN3 | 5632 | 8 | 4 | 3 | 2 | 2 | 2 | 4 | 1 | 5 | 5 | 2 | 8 | 5 |
| NM_001168254 | OCIAD1 | 2023 | 11 | 6 | 3 | 2 | 7 | 11 | 9 | 6 | 4 | 5 | 5 | 5 | 5 |
| NM_001168271 | GPR156 | 4181 | 1 | 0 | 0 | 0 | 0 | 0 | 0 | 0 | 0 | 0 | 0 | 0 | 1 |
| NM_001168272 | ITPR1 | 10195 | 19 | 8 | 3 | 4 | 6 | 13 | 9 | 1 | 7 | 19 | 1 | 12 | 10 |
| NM_001168278 | WWTR1 | 5030 | 30 | 16 | 12 | 7 | 19 | 11 | 23 | 5 | 24 | 30 | 6 | 17 | 17 |
| NM_001168280 | WWTR1 | 4977 | 30 | 16 | 12 | 7 | 19 | 11 | 23 | 5 | 24 | 30 | 6 | 17 | 18 |
| NM_001168298 | CXCR2 | 2661 | 7 | 1 | 0 | 0 | 0 | 0 | 0 | 1 | 2 | 0 | 0 | 7 | 0 |
| NM_001168299 | KLHL13 | 3204 | 3 | 1 | 0 | 0 | 0 | 2 | 3 | 0 | 0 | 1 | 0 | 0 | 1 |
| NM_001168300 | KLHL13 | 3452 | 3 | 1 | 0 | 0 | 0 | 2 | 3 | 0 | 0 | 1 | 0 | 0 | 1 |
| NM_001168301 | KLHL13 | 3102 | 3 | 1 | 0 | 0 | 0 | 2 | 3 | 0 | 0 | 1 | 0 | 0 | 1 |
| NM_001168302 | KLHL13 | 3396 | 3 | 1 | 0 | 0 | 0 | 1 | 3 | 0 | 0 | 1 | 0 | 0 | 1 |
| NM_001168303 | KLHL13 | 3322 | 3 | 1 | 0 | 0 | 0 | 1 | 3 | 0 | 0 | 1 | 0 | 0 | 1 |
| NM_001168316 | UGT3A2 | 2281 | 1 | 0 | 0 | 0 | 0 | 0 | 0 | 0 | 0 | 0 | 0 | 0 | 1 |
| NM_001168319 | EDN1 | 2096 | 3 | 1 | 0 | 0 | 1 | 2 | 1 | 0 | 3 | 0 | 0 | 1 | 1 |
| NM_001168320 | TSPAN9 | 4283 | 5 | 2 | 1 | 1 | 4 | 1 | 5 | 3 | 2 | 2 | 0 | 1 | 1 |
| NM_001168322 | CD300LG | 1398 | 2 | 0 | 2 | 0 | 0 | 0 | 0 | 0 | 0 | 0 | 0 | 0 | 1 |
| NM_001168323 | CD300LG | 1296 | 2 | 0 | 2 | 0 | 0 | 0 | 0 | 0 | 0 | 0 | 0 | 0 | 1 |
| NM_001168324 | CD300LG | 995 | 3 | 0 | 3 | 0 | 0 | 0 | 0 | 0 | 0 | 0 | 0 | 0 | 1 |
| NM_001168325 | TESC | 936 | 1 | 0 | 0 | 0 | 1 | 1 | 0 | 0 | 0 | 0 | 0 | 0 | 0 |
| NM_001168331 | NDUFB4 | 1560 | 2 | 1 | 1 | 1 | 2 | 1 | 2 | 1 | 1 | 1 | 1 | 1 | 1 |
| NM_001168335 | ME2 | 4450 | 17 | 7 | 4 | 3 | 12 | 9 | 5 | 3 | 5 | 4 | 7 | 17 | 9 |
| NM_001168338 | PLG | 1198 | 35 | 4 | 0 | 0 | 0 | 0 | 12 | 35 | 0 | 1 | 0 | 0 | 0 |
| NM_001168339 | TTC39B | 3254 | 4 | 1 | 1 | 1 | 1 | 1 | 4 | 1 | 2 | 2 | 1 | 1 | 1 |
| NM_001168340 | TTC39B | 3221 | 4 | 1 | 1 | 1 | 1 | 1 | 4 | 1 | 2 | 1 | 1 | 1 | 1 |
| NM_001168341 | TTC39B | 3053 | 4 | 1 | 1 | 1 | 1 | 2 | 4 | 1 | 2 | 1 | 1 | 1 | 1 |
| NM_001168342 | TTC39B | 3135 | 4 | 1 | 1 | 1 | 1 | 2 | 4 | 1 | 2 | 2 | 1 | 1 | 1 |
| NM_001168344 | RREB1 | 8531 | 6 | 4 | 4 | 4 | 2 | 1 | 4 | 5 | 3 | 4 | 3 | 6 | 4 |
| NM_001168347 | NSUN5 | 2371 | 0 | 0 | 0 | 0 | 0 | 0 | 0 | 0 | 0 | 0 | 0 | 0 | 0 |
| NM_001168348 | NSUN5 | 2332 | 0 | 0 | 0 | 0 | 0 | 0 | 0 | 0 | 0 | 0 | 0 | 0 | 0 |
| NM_001168355 | OSMR | 1739 | 25 | 16 | 14 | 5 | 22 | 15 | 14 | 22 | 21 | 11 | 8 | 25 | 14 |
| NM_001168357 | PLA2G7 | 1747 | 34 | 7 | 25 | 1 | 2 | 1 | 0 | 1 | 14 | 4 | 0 | 34 | 1 |
| NM_001168359 | TDRD6 | 8885 | 5 | 1 | 0 | 0 | 0 | 0 | 0 | 0 | 0 | 0 | 0 | 1 | 5 |
| NM_001168360 | PCDH11X | 8347 | 0 | 0 | 0 | 0 | 0 | 0 | 0 | 0 | 0 | 0 | 0 | 0 | 0 |
| NM_001168361 | PCDH11X | 8208 | 0 | 0 | 0 | 0 | 0 | 0 | 0 | 0 | 0 | 0 | 0 | 0 | 0 |
| NM_001168362 | PCDH11X | 8260 | 0 | 0 | 0 | 0 | 0 | 0 | 0 | 0 | 0 | 0 | 0 | 0 | 0 |
| NM_001168363 | PCDH11X | 8317 | 0 | 0 | 0 | 0 | 0 | 0 | 0 | 0 | 0 | 0 | 0 | 0 | 0 |
| NM_001168364 | KRTCAP3 | 954 | 2 | 0 | 0 | 2 | 0 | 0 | 0 | 0 | 1 | 0 | 0 | 0 | 0 |
| NM_001168368 | GALNTL1 | 4309 | 1 | 0 | 0 | 0 | 1 | 1 | 0 | 0 | 0 | 0 | 0 | 0 | 0 |
| NM_001168370 | CUL7 | 5506 | 2 | 1 | 1 | 1 | 1 | 1 | 2 | 0 | 1 | 2 | 1 | 1 | 1 |
| NM_001168374 | KIAA0319 | 6896 | 2 | 0 | 0 | 0 | 0 | 2 | 0 | 0 | 0 | 0 | 0 | 0 | 0 |
| NM_001168375 | KIAA0319 | 6780 | 3 | 0 | 0 | 0 | 0 | 3 | 0 | 0 | 0 | 0 | 0 | 0 | 0 |
| NM_001168376 | KIAA0319 | 6620 | 3 | 0 | 0 | 0 | 0 | 3 | 0 | 0 | 0 | 0 | 0 | 0 | 0 |
| NM_001168377 | KIAA0319 | 6622 | 2 | 0 | 0 | 0 | 0 | 2 | 0 | 0 | 0 | 0 | 0 | 0 | 0 |
| NM_001168378 | ZIC4 | 4152 | 0 | 0 | 0 | 0 | 0 | 0 | 0 | 0 | 0 | 0 | 0 | 0 | 0 |
| NM_001168379 | ZIC4 | 3979 | 0 | 0 | 0 | 0 | 0 | 0 | 0 | 0 | 0 | 0 | 0 | 0 | 0 |
| NM_001168385 | ALG13 | 3031 | 3 | 2 | 1 | 1 | 1 | 1 | 2 | 1 | 2 | 3 | 0 | 3 | 2 |
| NM_001168388 | CITED2 | 1790 | 3 | 2 | 1 | 0 | 1 | 1 | 1 | 0 | 3 | 3 | 1 | 3 | 1 |
| NM_001168389 | CITED2 | 1773 | 3 | 1 | 1 | 0 | 1 | 1 | 1 | 0 | 2 | 3 | 1 | 3 | 1 |
| NM_001168390 | C10orf105 | 4738 | 1 | 0 | 0 | 0 | 0 | 1 | 0 | 0 | 0 | 0 | 0 | 0 | 0 |
| NM_001168393 | C5orf41 | 2464 | 10 | 6 | 5 | 2 | 4 | 5 | 4 | 3 | 8 | 8 | 5 | 9 | 10 |
| NM_001168394 | C5orf41 | 2468 | 10 | 6 | 5 | 2 | 4 | 5 | 4 | 3 | 8 | 8 | 5 | 9 | 10 |
| NM_001168398 | SLC35A1 | 1723 | 2 | 1 | 1 | 1 | 1 | 1 | 2 | 1 | 1 | 2 | 1 | 2 | 1 |
| NM_001168399 | BEX2 | 1077 | 90 | 13 | 0 | 2 | 5 | 90 | 9 | 0 | 3 | 13 | 4 | 5 | 17 |
| NM_001168400 | BEX2 | 1074 | 90 | 13 | 0 | 2 | 5 | 90 | 9 | 0 | 3 | 13 | 4 | 5 | 17 |
| NM_001168401 | BEX2 | 819 | 118 | 18 | 0 | 3 | 6 | 118 | 12 | 0 | 5 | 17 | 5 | 7 | 22 |
| NM_001168407 | RIMS1 | 5780 | 11 | 2 | 0 | 0 | 0 | 11 | 0 | 0 | 0 | 0 | 0 | 0 | 5 |
| NM_001168408 | RIMS1 | 5195 | 6 | 1 | 0 | 0 | 0 | 6 | 0 | 0 | 0 | 0 | 0 | 0 | 1 |
| NM_001168409 | RIMS1 | 5052 | 6 | 1 | 0 | 0 | 0 | 6 | 0 | 0 | 0 | 0 | 0 | 0 | 1 |
| NM_001168410 | RIMS1 | 5339 | 6 | 1 | 0 | 0 | 0 | 6 | 0 | 0 | 0 | 0 | 0 | 0 | 1 |
| NM_001168411 | RIMS1 | 3279 | 2 | 0 | 0 | 0 | 0 | 2 | 0 | 0 | 0 | 0 | 0 | 0 | 0 |
| NM_001168412 | TSPAN3 | 3659 | 29 | 13 | 28 | 13 | 8 | 29 | 12 | 4 | 12 | 8 | 13 | 9 | 8 |
| NM_001168465 | MAP7D2 | 4149 | 24 | 3 | 0 | 1 | 0 | 24 | 2 | 0 | 0 | 0 | 0 | 0 | 4 |

| transcript | gene | transcript length (nt) | max | mean | adipose | colon | heart | hypothalamus | kidney | liver | lung | ovary | skeletalmuscle | spleen | testes |
|---|---|---|---|---|---|---|---|---|---|---|---|---|---|---|---|
| NM_001168466 | MAP7D2 | 3891 | 26 | 3 | 0 | 1 | 0 | 26 | 2 | 0 | 0 | 0 | 0 | 0 | 4 |
| NM_001168467 | MAP7D2 | 3863 | 26 | 3 | 0 | 1 | 0 | 26 | 2 | 0 | 0 | 0 | 0 | 0 | 4 |
| NM_001168468 | PDZD3 | 2287 | 2 | 0 | 0 | 1 | 0 | 0 | 2 | 0 | 0 | 0 | 0 | 0 | 0 |
| NM_001168474 | TAF7L | 2186 | 20 | 2 | 0 | 0 | 0 | 1 | 0 | 0 | 0 | 0 | 0 | 0 | 20 |
| NM_001168478 | ARMCX5 | 2785 | 3 | 2 | 1 | 2 | 1 | 2 | 2 | 3 | 2 | 3 | 1 | 2 | 2 |
| NM_001168479 | ARMCX5 | 2901 | 3 | 2 | 1 | 1 | 1 | 2 | 2 | 2 | 1 | 3 | 1 | 2 | 2 |
| NM_001168480 | ARMCX5 | 2895 | 3 | 2 | 1 | 1 | 1 | 2 | 2 | 2 | 1 | 3 | 1 | 2 | 2 |
| NM_001168482 | ARMCX5 | 2684 | 2 | 1 | 1 | 1 | 1 | 2 | 1 | 1 | 1 | 1 | 0 | 1 | 2 |
| NM_001168485 | ARMCX5 | 2782 | 2 | 1 | 1 | 1 | 1 | 2 | 1 | 1 | 1 | 1 | 0 | 1 | 1 |
| NM_001168499 | CNDP2 | 4946 | 14 | 3 | 2 | 1 | 2 | 4 | 14 | 5 | 3 | 1 | 0 | 5 | 1 |
| NM_001168530 | ASB9 | 1730 | 2 | 0 | 0 | 0 | 0 | 0 | 1 | 1 | 0 | 0 | 0 | 1 | 2 |
| NM_001168531 | ASB9 | 1947 | 2 | 0 | 0 | 0 | 0 | 0 | 1 | 1 | 0 | 0 | 0 | 1 | 2 |
| NM_001168551 | TMEM48 | 4703 | 3 | 2 | 1 | 2 | 1 | 2 | 2 | 2 | 1 | 2 | 1 | 3 | 3 |
| NM_001168618 | C11orf60 | 1722 | 5 | 2 | 2 | 2 | 2 | 5 | 3 | 1 | 2 | 4 | 1 | 2 | 3 |
| NM_001168647 | CNKSR2 | 5668 | 14 | 2 | 0 | 0 | 0 | 14 | 0 | 0 | 1 | 0 | 0 | 1 | 1 |
| NM_001168648 | CNKSR2 | 4502 | 25 | 3 | 0 | 1 | 0 | 25 | 0 | 0 | 1 | 0 | 0 | 1 | 1 |
| NM_001168649 | CNKSR2 | 4355 | 26 | 3 | 0 | 1 | 0 | 26 | 0 | 0 | 1 | 0 | 0 | 1 | 1 |
| NM_001168683 | CXorf15 | 3998 | 12 | 5 | 5 | 2 | 4 | 4 | 4 | 4 | 4 | 12 | 5 | 6 | 7 |
| NM_001168724 | TMEM135 | 9013 | 2 | 1 | 2 | 1 | 1 | 2 | 1 | 1 | 1 | 2 | 1 | 1 | 2 |
| NM_001169 | AQP8 | 1308 | 6 | 1 | 0 | 6 | 0 | 0 | 0 | 0 | 0 | 0 | 0 | 0 | 0 |
| NM_001169106 | FAM21C | 4521 | 1 | 0 | 1 | 0 | 0 | 0 | 1 | 0 | 1 | 0 | 0 | 1 | 1 |
| NM_001169107 | FAM21C | 4419 | 1 | 0 | 1 | 0 | 0 | 0 | 1 | 0 | 1 | 1 | 0 | 1 | 1 |
| NM_001169109 | SCO2 | 1049 | 0 | 0 | 0 | 0 | 0 | 0 | 0 | 0 | 0 | 0 | 0 | 0 | 0 |
| NM_001169110 | SCO2 | 1000 | 0 | 0 | 0 | 0 | 0 | 0 | 0 | 0 | 0 | 0 | 0 | 0 | 0 |
| NM_001169111 | SCO2 | 1018 | 0 | 0 | 0 | 0 | 0 | 0 | 0 | 0 | 0 | 0 | 0 | 0 | 0 |
| NM_001169117 | STIM2 | 5222 | 6 | 3 | 2 | 1 | 2 | 4 | 2 | 2 | 3 | 6 | 1 | 6 | 2 |
| NM_001169118 | STIM2 | 5178 | 6 | 3 | 2 | 1 | 2 | 4 | 2 | 2 | 3 | 6 | 1 | 6 | 2 |
| NM_001169122 | AFF2 | 13640 | 2 | 1 | 0 | 0 | 0 | 2 | 0 | 0 | 1 | 1 | 0 | 0 | 2 |
| NM_001169123 | AFF2 | 13715 | 2 | 1 | 0 | 0 | 0 | 2 | 0 | 0 | 1 | 1 | 0 | 0 | 2 |
| NM_001169124 | AFF2 | 13640 | 2 | 1 | 0 | 0 | 0 | 2 | 0 | 0 | 1 | 1 | 0 | 0 | 2 |
| NM_001169125 | AFF2 | 13628 | 2 | 1 | 0 | 0 | 0 | 2 | 0 | 0 | 1 | 1 | 0 | 0 | 2 |
| NM_001169126 | TMIGD2 | 1250 | 0 | 0 | 0 | 0 | 0 | 0 | 0 | 0 | 0 | 0 | 0 | 0 | 0 |
| NM_001169154 | CXorf41 | 1926 | 0 | 0 | 0 | 0 | 0 | 0 | 0 | 0 | 0 | 0 | 0 | 0 | 0 |
| NM_001169574 | CXorf58 | 1541 | 0 | 0 | 0 | 0 | 0 | 0 | 0 | 0 | 0 | 0 | 0 | 0 | 0 |
| NM_001170 | AQP7 | 1256 | 14 | 2 | 14 | 1 | 1 | 0 | 2 | 1 | 0 | 0 | 1 | 0 | 0 |
| NM_001170330 | C4orf3 | 2959 | 4 | 3 | 3 | 2 | 2 | 4 | 4 | 2 | 3 | 3 | 1 | 3 | 3 |
| NM_001170331 | LANCL3 | 3248 | 1 | 0 | 0 | 0 | 0 | 1 | 0 | 0 | 0 | 0 | 0 | 0 | 0 |
| NM_001170402 | CDC20B | 2984 | 3 | 0 | 0 | 0 | 0 | 0 | 0 | 0 | 0 | 0 | 0 | 0 | 3 |
| NM_001170406 | CDK1 | 1754 | 0 | 0 | 0 | 0 | 0 | 0 | 0 | 0 | 0 | 0 | 0 | 0 | 0 |
| NM_001170407 | CDK1 | 1742 | 0 | 0 | 0 | 0 | 0 | 0 | 0 | 0 | 0 | 0 | 0 | 0 | 0 |
| NM_001170414 | TJP2 | 4085 | 7 | 4 | 4 | 2 | 3 | 7 | 4 | 4 | 4 | 3 | 1 | 1 | 5 |
| NM_001170415 | TJP2 | 4459 | 7 | 4 | 5 | 2 | 4 | 7 | 5 | 5 | 4 | 3 | 1 | 1 | 5 |
| NM_001170416 | TJP2 | 4500 | 7 | 4 | 5 | 2 | 4 | 7 | 5 | 5 | 4 | 3 | 1 | 1 | 5 |
| NM_001170417 | MOG | 1810 | 14 | 2 | 0 | 2 | 0 | 14 | 0 | 0 | 0 | 0 | 0 | 0 | 0 |
| NM_001170417 | MOG | 1814 | 14 | 2 | 0 | 2 | 0 | 14 | 0 | 0 | 0 | 0 | 0 | 0 | 0 |
| NM_001170417 | MOG | 1818 | 14 | 2 | 0 | 2 | 0 | 14 | 0 | 0 | 0 | 0 | 0 | 0 | 0 |
| NM_001170418 | MOG | 1741 | 14 | 2 | 0 | 2 | 0 | 14 | 0 | 0 | 0 | 0 | 0 | 0 | 0 |
| NM_001170418 | MOG | 1745 | 14 | 2 | 0 | 2 | 0 | 14 | 0 | 0 | 0 | 0 | 0 | 0 | 0 |
| NM_001170418 | MOG | 1749 | 14 | 2 | 0 | 2 | 0 | 14 | 0 | 0 | 0 | 0 | 0 | 0 | 0 |
| NM_001170423 | PRSS35 | 2604 | 2 | 0 | 0 | 0 | 1 | 1 | 1 | 0 | 0 | 2 | 0 | 0 | 1 |
| NM_001170460 | CDK16 | 3016 | 7 | 5 | 3 | 5 | 6 | 7 | 6 | 2 | 3 | 2 | 6 | 3 | 6 |
| NM_001170464 | CDK17 | 2487 | 10 | 4 | 2 | 1 | 3 | 10 | 2 | 2 | 5 | 3 | 2 | 7 | 5 |
| NM_001170543 | PGAM5 | 2848 | 3 | 1 | 1 | 1 | 1 | 2 | 2 | 3 | 1 | 0 | 1 | 1 | 1 |
| NM_001170544 | PGAM5 | 2845 | 3 | 1 | 1 | 1 | 1 | 2 | 2 | 3 | 1 | 0 | 1 | 1 | 1 |
| NM_001170553 | VSIG1 | 3235 | 27 | 3 | 0 | 0 | 0 | 0 | 0 | 0 | 0 | 0 | 0 | 0 | 27 |
| NM_001170628 | AFF2 | 12279 | 2 | 1 | 0 | 0 | 0 | 2 | 0 | 0 | 1 | 1 | 0 | 0 | 2 |
| NM_001170629 | CHD8 | 8229 | 7 | 4 | 3 | 3 | 3 | 6 | 4 | 3 | 3 | 6 | 4 | 7 | 6 |
| NM_001170630 | TJP2 | 4718 | 5 | 3 | 3 | 2 | 2 | 5 | 3 | 3 | 4 | 3 | 1 | 1 | 4 |
| NM_001170631 | FCAMR | 2782 | 11 | 1 | 0 | 0 | 0 | 0 | 11 | 0 | 0 | 0 | 0 | 0 | 0 |
| NM_001170633 | C12orf56 | 2067 | 1 | 0 | 0 | 0 | 0 | 0 | 1 | 0 | 0 | 0 | 0 | 0 | 1 |
| NM_001170637 | SRGAP2 | 5970 | 10 | 4 | 3 | 2 | 3 | 10 | 2 | 1 | 5 | 3 | 1 | 8 | 3 |
| NM_001170670 | ZNHIT6 | 6079 | 6 | 3 | 4 | 2 | 4 | 6 | 4 | 2 | 2 | 4 | 2 | 4 | 3 |
| NM_001171 | ABCC6 | 5117 | 1 | 0 | 0 | 0 | 0 | 0 | 1 | 1 | 0 | 0 | 0 | 0 | 0 |
| NM_001172 | ARG2 | 1980 | 10 | 2 | 1 | 0 | 2 | 3 | 10 | 1 | 1 | 3 | 2 | 1 | 1 |
| NM_001173 | ARHGAP5 | 9601 | 18 | 10 | 5 | 2 | 18 | 14 | 11 | 5 | 8 | 12 | 7 | 11 | 14 |
| NM_001175 | ARHGDIB | 1216 | 345 | 66 | 82 | 20 | 67 | 29 | 23 | 22 | 89 | 14 | 12 | 345 | 20 |
| NM_001176 | ARHGDIG | 942 | 1 | 0 | 0 | 0 | 0 | 1 | 0 | 0 | 0 | 0 | 0 | 0 | 0 |
| NM_001177 | ARL1 | 3198 | 8 | 5 | 5 | 2 | 6 | 8 | 7 | 5 | 4 | 5 | 4 | 4 | 6 |
| NM_001178 | ARNTL | 2795 | 6 | 3 | 2 | 1 | 2 | 3 | 2 | 6 | 2 | 2 | 4 | 4 | 3 |
| NM_001179 | ART3 | 1539 | 40 | 7 | 0 | 0 | 16 | 2 | 0 | 0 | 0 | 0 | 40 | 1 | 13 |
| NM_001181 | ASGR2 | 1454 | 28 | 3 | 0 | 0 | 0 | 0 | 0 | 28 | 0 | 0 | 0 | 0 | 0 |
| NM_001182 | ALDH7A1 | 3551 | 30 | 12 | 7 | 6 | 6 | 30 | 23 | 21 | 4 | 22 | 2 | 2 | 8 |
| NM_001183 | ATP6AP1 | 2099 | 6 | 2 | 2 | 1 | 2 | 6 | 4 | 1 | 3 | 1 | 1 | 4 | 2 |
| NM_001184 | ATR | 8251 | 5 | 2 | 3 | 1 | 2 | 3 | 3 | 2 | 2 | 2 | 1 | 5 | 4 |
| NM_001185 | AZGP1 | 1263 | 219 | 27 | 2 | 1 | 18 | 1 | 37 | 219 | 10 | 1 | 1 | 2 | 5 |
| NM_001186 | BACH1 | 5641 | 17 | 9 | 5 | 3 | 7 | 10 | 7 | 7 | 15 | 10 | 12 | 17 | 10 |
| NM_001187 | BAGE | 951 | 0 | 0 | 0 | 0 | 0 | 0 | 0 | 0 | 0 | 0 | 0 | 0 | 0 |

| transcript | gene | transcript length (nt) | max | mean | adipose | colon | heart | hypothalamus | kidney | liver | lung | ovary | skeletalmuscle | spleen | testes |
|---|---|---|---|---|---|---|---|---|---|---|---|---|---|---|---|
| NM_001188 | BAK1 | 2193 | 2 | 1 | 1 | 2 | 1 | 1 | 1 | 0 | 1 | 1 | 0 | 2 | 1 |
| NM_001189 | NKX3-2 | 2241 | 0 | 0 | 0 | 0 | 0 | 0 | 0 | 0 | 0 | 0 | 0 | 0 | 0 |
| NM_001190 | BCAT2 | 1598 | 1 | 0 | 0 | 0 | 0 | 0 | 1 | 0 | 1 | 0 | 0 | 0 | 0 |
| NM_001191 | BCL2L1 | 2370 | 8 | 5 | 4 | 3 | 4 | 7 | 8 | 5 | 7 | 2 | 3 | 6 | 4 |
| NM_001192 | TNFRSF17 | 994 | 2 | 1 | 0 | 2 | 0 | 0 | 0 | 0 | 0 | 1 | 0 | 2 | 0 |
| NM_001194 | HCN2 | 3408 | 3 | 0 | 0 | 0 | 0 | 3 | 0 | 0 | 0 | 0 | 0 | 0 | 0 |
| NM_001195 | BFSP1 | 2205 | 2 | 0 | 0 | 0 | 0 | 0 | 0 | 0 | 0 | 1 | 0 | 0 | 2 |
| NM_001196 | BID | 2198 | 2 | 1 | 1 | 0 | 1 | 1 | 1 | 2 | 0 | 0 | 1 | 2 | 0 |
| NM_001197 | BIK | 950 | 0 | 0 | 0 | 0 | 0 | 0 | 0 | 0 | 0 | 0 | 0 | 0 | 0 |
| NM_001198 | PRDM1 | 5165 | 8 | 2 | 2 | 2 | 1 | 1 | 0 | 1 | 6 | 2 | 0 | 8 | 1 |
| NM_001199 | BMP1 | 2715 | 1 | 0 | 0 | 0 | 0 | 0 | 0 | 0 | 0 | 0 | 0 | 1 | 0 |
| NM_001200 | BMP2 | 3150 | 3 | 1 | 1 | 3 | 0 | 0 | 0 | 0 | 1 | 0 | 0 | 2 | 0 |
| NM_001201 | BMP3 | 5734 | 1 | 0 | 0 | 1 | 0 | 0 | 0 | 0 | 1 | 1 | 0 | 0 | 0 |
| NM_001202 | BMP4 | 1941 | 1 | 1 | 0 | 1 | 1 | 0 | 0 | 0 | 0 | 1 | 1 | 0 | 0 |
| NM_001203 | BMPR1B | 5560 | 4 | 1 | 0 | 0 | 1 | 4 | 2 | 0 | 1 | 2 | 0 | 0 | 1 |
| NM_001204 | BMPR2 | 12070 | 14 | 8 | 5 | 3 | 11 | 13 | 7 | 4 | 14 | 8 | 4 | 10 | 7 |
| NM_001205 | BNIP1 | 1257 | 2 | 1 | 1 | 1 | 1 | 2 | 1 | 1 | 0 | 1 | 0 | 1 | 1 |
| NM_001206 | KLF9 | 5208 | 22 | 13 | 12 | 4 | 13 | 10 | 10 | 19 | 21 | 9 | 22 | 13 | 9 |
| NM_001207 | BTF3 | 1008 | 9 | 6 | 7 | 4 | 5 | 6 | 3 | 9 | 5 | 9 | 4 | 5 | 2 |
| NM_001211 | BUB1B | 3742 | 6 | 1 | 1 | 1 | 1 | 0 | 0 | 0 | 1 | 0 | 0 | 1 | 6 |
| NM_001212 | C1QBP | 1163 | 58 | 36 | 17 | 21 | 51 | 47 | 43 | 58 | 19 | 18 | 35 | 47 | 37 |
| NM_001214 | C16orf3 | 994 | 1 | 0 | 0 | 0 | 0 | 0 | 1 | 0 | 0 | 0 | 0 | 0 | 0 |
| NM_001215 | CA6 | 1336 | 0 | 0 | 0 | 0 | 0 | 0 | 0 | 0 | 0 | 0 | 0 | 0 | 0 |
| NM_001216 | CA9 | 1554 | 0 | 0 | 0 | 0 | 0 | 0 | 0 | 0 | 0 | 0 | 0 | 0 | 0 |
| NM_001217 | CA11 | 1653 | 136 | 17 | 2 | 8 | 2 | 136 | 4 | 0 | 4 | 16 | 1 | 4 | 8 |
| NM_001218 | CA12 | 3975 | 27 | 3 | 0 | 7 | 0 | 1 | 27 | 0 | 0 | 0 | 0 | 0 | 0 |
| NM_001219 | CALU | 3410 | 137 | 36 | 39 | 13 | 137 | 22 | 20 | 24 | 32 | 26 | 19 | 37 | 32 |
| NM_001220 | CAMK2B | 4586 | 11 | 2 | 1 | 0 | 2 | 11 | 0 | 1 | 6 | 0 | 5 | 0 | 1 |
| NM_001221 | CAMK2D | 5870 | 8 | 3 | 2 | 2 | 6 | 8 | 2 | 2 | 3 | 2 | 5 | 2 | 2 |
| NM_001222 | CAMK2G | 3624 | 11 | 3 | 2 | 5 | 1 | 11 | 4 | 1 | 2 | 1 | 4 | 2 | 2 |
| NM_001223 | CASP1 | 1292 | 10 | 3 | 3 | 3 | 2 | 2 | 1 | 1 | 6 | 1 | 1 | 10 | 1 |
| NM_001225 | CASP4 | 1319 | 23 | 8 | 8 | 4 | 7 | 4 | 6 | 11 | 14 | 6 | 2 | 23 | 3 |
| NM_001226 | CASP6 | 1661 | 2 | 1 | 1 | 2 | 1 | 1 | 2 | 2 | 1 | 1 | 0 | 1 | 1 |
| NM_001227 | CASP7 | 2589 | 10 | 5 | 4 | 8 | 5 | 2 | 5 | 4 | 8 | 5 | 3 | 10 | 4 |
| NM_001228 | CASP8 | 2911 | 9 | 3 | 4 | 3 | 1 | 1 | 3 | 2 | 4 | 2 | 0 | 9 | 2 |
| NM_001229 | CASP9 | 1989 | 1 | 1 | 0 | 1 | 0 | 1 | 1 | 0 | 0 | 1 | 0 | 0 | 1 |
| NM_001230 | CASP10 | 5777 | 4 | 1 | 2 | 1 | 1 | 0 | 1 | 1 | 3 | 1 | 0 | 4 | 1 |
| NM_001231 | CASQ1 | 1956 | 63 | 6 | 0 | 0 | 3 | 1 | 0 | 0 | 0 | 0 | 63 | 0 | 0 |
| NM_001232 | CASQ2 | 2700 | 64 | 7 | 0 | 1 | 64 | 0 | 0 | 0 | 0 | 0 | 13 | 0 | 0 |
| NM_001233 | CAV2 | 3301 | 10 | 4 | 10 | 0 | 8 | 1 | 1 | 1 | 9 | 3 | 1 | 5 | 2 |
| NM_001234 | CAV3 | 1325 | 3 | 0 | 0 | 0 | 1 | 0 | 0 | 0 | 0 | 0 | 3 | 0 | 0 |
| NM_001235 | SERPINH1 | 2204 | 9 | 4 | 5 | 3 | 9 | 1 | 3 | 1 | 6 | 7 | 1 | 8 | 4 |
| NM_001236 | CBR3 | 1112 | 2 | 1 | 2 | 1 | 1 | 1 | 0 | 0 | 0 | 2 | 0 | 1 | 0 |
| NM_001237 | CCNA2 | 2798 | 1 | 0 | 1 | 1 | 0 | 0 | 0 | 0 | 0 | 0 | 0 | 1 | 1 |
| NM_001238 | CCNE1 | 1951 | 1 | 0 | 0 | 0 | 0 | 1 | 0 | 0 | 0 | 0 | 0 | 0 | 1 |
| NM_001239 | CCNH | 1378 | 10 | 6 | 8 | 2 | 4 | 6 | 5 | 5 | 7 | 10 | 3 | 6 | 8 |
| NM_001240 | CCNT1 | 2568 | 4 | 2 | 2 | 1 | 2 | 4 | 2 | 2 | 1 | 3 | 2 | 4 | 3 |
| NM_001241 | CCNT2 | 4490 | 5 | 3 | 2 | 1 | 2 | 3 | 3 | 2 | 3 | 4 | 2 | 5 | 5 |
| NM_001242 | CD27 | 1320 | 2 | 0 | 0 | 0 | 0 | 0 | 0 | 0 | 0 | 0 | 0 | 2 | 0 |
| NM_001243 | TNFRSF8 | 3686 | 0 | 0 | 0 | 0 | 0 | 0 | 0 | 0 | 0 | 0 | 0 | 0 | 0 |
| NM_001244 | TNFSF8 | 1979 | 3 | 1 | 1 | 0 | 0 | 0 | 0 | 0 | 1 | 0 | 0 | 3 | 0 |
| NM_001245 | SIGLEC6 | 2076 | 0 | 0 | 0 | 0 | 0 | 0 | 0 | 0 | 0 | 0 | 0 | 0 | 0 |
| NM_001246 | ENTPD2 | 2031 | 1 | 0 | 0 | 0 | 0 | 1 | 0 | 0 | 0 | 0 | 0 | 0 | 0 |
| NM_001247 | ENTPD6 | 2762 | 2 | 1 | 0 | 1 | 1 | 2 | 1 | 0 | 0 | 0 | 0 | 0 | 1 |
| NM_001248 | ENTPD3 | 2793 | 2 | 1 | 0 | 0 | 0 | 2 | 0 | 0 | 0 | 1 | 0 | 0 | 1 |
| NM_001249 | ENTPD5 | 2046 | 25 | 7 | 3 | 12 | 2 | 2 | 25 | 25 | 1 | 1 | 1 | 2 | 2 |
| NM_001250 | CD40 | 1616 | 3 | 1 | 2 | 1 | 1 | 1 | 1 | 1 | 2 | 1 | 1 | 3 | 1 |
| NM_001251 | CD68 | 1856 | 190 | 51 | 115 | 15 | 37 | 16 | 19 | 45 | 89 | 16 | 12 | 190 | 11 |
| NM_001252 | CD70 | 913 | 0 | 0 | 0 | 0 | 0 | 0 | 0 | 0 | 0 | 0 | 0 | 0 | 0 |
| NM_001253 | CDC5L | 3000 | 25 | 16 | 12 | 4 | 18 | 20 | 11 | 14 | 11 | 17 | 25 | 18 | 23 |
| NM_001254 | CDC6 | 3053 | 4 | 1 | 1 | 2 | 1 | 0 | 0 | 0 | 1 | 1 | 0 | 3 | 4 |
| NM_001255 | CDC20 | 1674 | 1 | 0 | 0 | 1 | 0 | 0 | 0 | 0 | 0 | 0 | 0 | 1 | 1 |
| NM_001256 | CDC27 | 5592 | 4 | 3 | 3 | 1 | 4 | 4 | 3 | 3 | 3 | 3 | 3 | 4 | 4 |
| NM_001257 | CDH13 | 3826 | 7 | 2 | 1 | 0 | 7 | 4 | 0 | 0 | 1 | 0 | 2 | 0 | 1 |
| NM_001258 | CDK3 | 1570 | 1 | 0 | 0 | 1 | 0 | 0 | 0 | 0 | 0 | 0 | 0 | 0 | 1 |
| NM_001259 | CDK6 | 11610 | 2 | 1 | 1 | 1 | 2 | 1 | 2 | 1 | 2 | 2 | 0 | 2 | 1 |
| NM_001260 | CDK8 | 1769 | 6 | 4 | 4 | 4 | 4 | 6 | 4 | 5 | 3 | 3 | 3 | 4 | 6 |
| NM_001261 | CDK9 | 2472 | 7 | 5 | 4 | 4 | 4 | 6 | 3 | 7 | 4 | 5 | 2 | 7 | 5 |
| NM_001262 | CDKN2C | 2087 | 7 | 1 | 7 | 1 | 1 | 0 | 0 | 0 | 1 | 1 | 0 | 1 | 0 |
| NM_001263 | CDS1 | 4538 | 2 | 1 | 0 | 1 | 0 | 2 | 2 | 0 | 1 | 1 | 0 | 0 | 2 |
| NM_001264 | CDSN | 2546 | 0 | 0 | 0 | 0 | 0 | 0 | 0 | 0 | 0 | 0 | 0 | 0 | 0 |
| NM_001264 | CDSN | 2549 | 0 | 0 | 0 | 0 | 0 | 0 | 0 | 0 | 0 | 0 | 0 | 0 | 0 |
| NM_001265 | CDX2 | 2090 | 4 | 0 | 0 | 4 | 0 | 0 | 0 | 0 | 0 | 0 | 0 | 0 | 0 |
| NM_001266 | CES1 | 2006 | 109 | 18 | 59 | 3 | 5 | 0 | 0 | 109 | 10 | 1 | 1 | 8 | 1 |
| NM_001267 | CHAD | 1695 | 3 | 1 | 0 | 2 | 0 | 1 | 3 | 1 | 1 | 1 | 1 | 0 | 0 |
| NM_001268 | RCBTB2 | 3175 | 3 | 2 | 2 | 1 | 1 | 2 | 2 | 1 | 1 | 3 | 1 | 2 | 2 |
| NM_001269 | RCC1 | 2439 | 0 | 0 | 0 | 0 | 0 | 0 | 0 | 0 | 0 | 0 | 0 | 0 | 0 |

| transcript | gene | transcript length (nt) | max | mean | adipose | colon | heart | hypothalamus | kidney | liver | lung | ovary | skeletalmuscle | spleen | testes |
|---|---|---|---|---|---|---|---|---|---|---|---|---|---|---|---|
| NM_001270 | CHD1 | 6457 | 15 | 8 | 5 | 3 | 6 | 4 | 7 | 6 | 10 | 12 | 5 | 15 | 10 |
| NM_001271 | CHD2 | 9364 | 23 | 15 | 11 | 11 | 11 | 14 | 13 | 8 | 21 | 23 | 14 | 23 | 17 |
| NM_001273 | CHD4 | 6497 | 17 | 12 | 10 | 10 | 16 | 16 | 12 | 11 | 10 | 13 | 8 | 17 | 14 |
| NM_001274 | CHEK1 | 2035 | 3 | 1 | 1 | 2 | 1 | 1 | 1 | 0 | 1 | 1 | 0 | 1 | 3 |
| NM_001275 | CHGA | 2045 | 9 | 3 | 0 | 9 | 0 | 9 | 0 | 0 | 0 | 0 | 0 | 0 | 8 |
| NM_001276 | CHI3L1 | 1837 | 397 | 42 | 11 | 1 | 13 | 6 | 6 | 397 | 8 | 3 | 4 | 5 | 7 |
| NM_001277 | CHKA | 2717 | 11 | 4 | 2 | 3 | 2 | 5 | 3 | 3 | 3 | 3 | 1 | 5 | 11 |
| NM_001278 | CHUK | 3524 | 5 | 3 | 3 | 5 | 3 | 3 | 4 | 5 | 2 | 2 | 2 | 5 | 3 |
| NM_001279 | CIDEA | 1050 | 4 | 0 | 4 | 0 | 0 | 0 | 0 | 0 | 0 | 0 | 0 | 0 | 0 |
| NM_001280 | CIRBP | 1397 | 138 | 41 | 21 | 8 | 42 | 54 | 38 | 16 | 23 | 138 | 39 | 33 | 36 |
| NM_001281 | TBCB | 1475 | 3 | 1 | 1 | 1 | 1 | 3 | 1 | 1 | 0 | 0 | 0 | 1 | 0 |
| NM_001282 | AP2B1 | 5721 | 28 | 10 | 7 | 7 | 9 | 28 | 13 | 5 | 8 | 9 | 5 | 10 | 12 |
| NM_001283 | AP1S1 | 1297 | 7 | 1 | 1 | 2 | 0 | 7 | 2 | 1 | 1 | 1 | 0 | 1 | 1 |
| NM_001284 | AP3S1 | 1289 | 16 | 8 | 10 | 5 | 8 | 16 | 4 | 7 | 6 | 7 | 3 | 9 | 8 |
| NM_001285 | CLCA1 | 3120 | 78 | 7 | 0 | 78 | 0 | 0 | 0 | 0 | 0 | 0 | 0 | 0 | 0 |
| NM_001286 | CLCN6 | 5644 | 4 | 1 | 1 | 1 | 1 | 4 | 1 | 0 | 1 | 1 | 1 | 1 | 1 |
| NM_001287 | CLCN7 | 4225 | 1 | 1 | 1 | 1 | 0 | 1 | 1 | 1 | 0 | 0 | 0 | 1 | 0 |
| NM_001288 | CLIC1 | 1251 | 119 | 52 | 66 | 63 | 75 | 28 | 62 | 26 | 62 | 41 | 11 | 119 | 22 |
| NM_001289 | CLIC2 | 2686 | 9 | 2 | 2 | 0 | 2 | 1 | 1 | 1 | 6 | 2 | 1 | 9 | 1 |
| NM_001290 | LDB2 | 2540 | 24 | 9 | 10 | 2 | 8 | 12 | 7 | 4 | 17 | 7 | 3 | 24 | 5 |
| NM_001293 | CLNS1A | 1372 | 48 | 28 | 21 | 15 | 25 | 48 | 17 | 37 | 19 | 41 | 19 | 35 | 27 |
| NM_001294 | CLPTM1 | 2469 | 9 | 5 | 3 | 4 | 4 | 8 | 6 | 9 | 3 | 3 | 4 | 5 | 6 |
| NM_001295 | CCR1 | 2676 | 9 | 2 | 3 | 0 | 3 | 3 | 1 | 1 | 3 | 1 | 1 | 9 | 0 |
| NM_001296 | CCBP2 | 2960 | 6 | 1 | 0 | 0 | 0 | 1 | 1 | 6 | 1 | 0 | 0 | 1 | 1 |
| NM_001297 | CNGB1 | 5645 | 1 | 0 | 0 | 0 | 0 | 1 | 0 | 0 | 0 | 0 | 0 | 0 | 0 |
| NM_001298 | CNGA3 | 3848 | 1 | 0 | 0 | 1 | 0 | 0 | 0 | 0 | 0 | 0 | 0 | 0 | 0 |
| NM_001299 | CNN1 | 1586 | 98 | 15 | 2 | 98 | 28 | 4 | 2 | 0 | 6 | 5 | 3 | 1 | 15 |
| NM_001300 | KLF6 | 4661 | 24 | 13 | 18 | 6 | 11 | 7 | 10 | 15 | 24 | 12 | 20 | 17 | 8 |
| NM_001302 | CORT | 935 | 0 | 0 | 0 | 0 | 0 | 0 | 0 | 0 | 0 | 0 | 0 | 0 | 0 |
| NM_001303 | COX10 | 3002 | 2 | 2 | 1 | 2 | 2 | 1 | 2 | 1 | 1 | 1 | 2 | 1 | 2 |
| NM_001304 | CPD | 8025 | 17 | 8 | 6 | 3 | 7 | 9 | 9 | 17 | 11 | 4 | 3 | 9 | 13 |
| NM_001305 | CLDN4 | 1823 | 2 | 1 | 0 | 2 | 0 | 0 | 2 | 0 | 2 | 0 | 0 | 0 | 0 |
| NM_001306 | CLDN3 | 1274 | 1 | 0 | 0 | 1 | 0 | 0 | 0 | 0 | 0 | 0 | 0 | 0 | 0 |
| NM_001307 | CLDN7 | 1540 | 11 | 2 | 0 | 11 | 0 | 0 | 2 | 1 | 2 | 0 | 0 | 0 | 0 |
| NM_001308 | CPN1 | 1756 | 11 | 1 | 0 | 0 | 0 | 0 | 0 | 11 | 0 | 0 | 0 | 0 | 0 |
| NM_001310 | CREBL2 | 3748 | 10 | 6 | 7 | 2 | 4 | 6 | 7 | 6 | 6 | 7 | 1 | 10 | 6 |
| NM_001311 | CRIP1 | 478 | 4 | 1 | 2 | 4 | 1 | 1 | 1 | 0 | 3 | 0 | 0 | 1 | 1 |
| NM_001312 | CRIP2 | 1206 | 8 | 2 | 1 | 0 | 8 | 1 | 2 | 1 | 1 | 0 | 0 | 2 | 1 |
| NM_001313 | CRMP1 | 3037 | 4 | 1 | 1 | 1 | 1 | 4 | 3 | 0 | 1 | 3 | 0 | 0 | 1 |
| NM_001315 | MAPK14 | 4353 | 9 | 4 | 3 | 4 | 4 | 3 | 4 | 4 | 5 | 3 | 3 | 9 | 3 |
| NM_001316 | CSE1L | 3561 | 20 | 8 | 5 | 3 | 7 | 10 | 8 | 10 | 6 | 6 | 8 | 8 | 20 |
| NM_001317 | CSH1 | 879 | 0 | 0 | 0 | 0 | 0 | 0 | 0 | 0 | 0 | 0 | 0 | 0 | 0 |
| NM_001318 | CSHL1 | 661 | 1 | 0 | 0 | 0 | 0 | 1 | 0 | 0 | 0 | 0 | 0 | 0 | 0 |
| NM_001319 | CSNK1G2 | 2904 | 6 | 3 | 2 | 3 | 2 | 4 | 3 | 1 | 3 | 2 | 1 | 6 | 5 |
| NM_001320 | CSNK2B | 1128 | 7 | 4 | 3 | 2 | 3 | 7 | 3 | 4 | 2 | 4 | 4 | 4 | 4 |
| NM_001321 | CSRP2 | 901 | 34 | 17 | 12 | 7 | 22 | 21 | 34 | 10 | 22 | 12 | 7 | 19 | 25 |
| NM_001322 | CST2 | 694 | 0 | 0 | 0 | 0 | 0 | 0 | 0 | 0 | 0 | 0 | 0 | 0 | 0 |
| NM_001323 | CST6 | 609 | 0 | 0 | 0 | 0 | 0 | 0 | 0 | 0 | 0 | 0 | 0 | 0 | 0 |
| NM_001324 | CSTF1 | 2323 | 3 | 2 | 1 | 1 | 1 | 2 | 2 | 3 | 1 | 2 | 1 | 2 | 3 |
| NM_001325 | CSTF2 | 2016 | 3 | 1 | 1 | 1 | 1 | 2 | 1 | 1 | 1 | 1 | 0 | 1 | 3 |
| NM_001326 | CSTF3 | 2823 | 8 | 4 | 3 | 1 | 3 | 5 | 4 | 3 | 2 | 3 | 2 | 4 | 8 |
| NM_001327 | CTAG1B | 747 | 0 | 0 | 0 | 0 | 0 | 0 | 0 | 0 | 0 | 0 | 0 | 0 | 0 |
| NM_001328 | CTBP1 | 2288 | 3 | 2 | 1 | 1 | 2 | 2 | 3 | 1 | 2 | 1 | 2 | 3 | 1 |
| NM_001329 | CTBP2 | 3428 | 9 | 6 | 4 | 8 | 6 | 7 | 7 | 1 | 7 | 9 | 3 | 7 | 7 |
| NM_001330 | CTF1 | 1663 | 1 | 0 | 0 | 0 | 0 | 0 | 0 | 0 | 0 | 1 | 0 | 0 | 0 |
| NM_001331 | CTNND1 | 6269 | 27 | 18 | 13 | 23 | 15 | 9 | 27 | 16 | 21 | 19 | 6 | 23 | 24 |
| NM_001332 | CTNND2 | 5436 | 18 | 2 | 0 | 0 | 1 | 18 | 0 | 0 | 0 | 0 | 0 | 2 | 1 |
| NM_001333 | CTSL2 | 1495 | 15 | 2 | 0 | 0 | 0 | 0 | 3 | 0 | 0 | 0 | 0 | 0 | 15 |
| NM_001334 | CTSO | 2933 | 7 | 4 | 4 | 1 | 3 | 2 | 7 | 6 | 5 | 5 | 2 | 7 | 4 |
| NM_001335 | CTSW | 1296 | 1 | 0 | 0 | 0 | 0 | 0 | 0 | 0 | 0 | 0 | 0 | 1 | 0 |
| NM_001336 | CTSZ | 1499 | 54 | 20 | 54 | 10 | 26 | 10 | 12 | 34 | 28 | 11 | 4 | 31 | 5 |
| NM_001337 | CX3CR1 | 3108 | 5 | 1 | 0 | 0 | 0 | 5 | 0 | 0 | 0 | 0 | 0 | 0 | 0 |
| NM_001338 | CXADR | 2472 | 3 | 1 | 0 | 1 | 1 | 2 | 1 | 2 | 1 | 0 | 0 | 0 | 3 |
| NM_001340 | CYLC2 | 2171 | 5 | 1 | 0 | 0 | 0 | 0 | 0 | 0 | 0 | 0 | 0 | 0 | 5 |
| NM_001343 | DAB2 | 4597 | 30 | 10 | 16 | 3 | 9 | 3 | 30 | 2 | 10 | 9 | 3 | 14 | 6 |
| NM_001344 | DAD1 | 697 | 9 | 5 | 3 | 2 | 5 | 5 | 9 | 7 | 5 | 1 | 2 | 7 | 4 |
| NM_001345 | DGKA | 2727 | 8 | 2 | 0 | 3 | 2 | 2 | 1 | 0 | 3 | 2 | 0 | 8 | 1 |
| NM_001346 | DGKG | 5802 | 3 | 1 | 0 | 0 | 1 | 3 | 0 | 0 | 0 | 0 | 1 | 0 | 0 |
| NM_001347 | DGKQ | 4636 | 0 | 0 | 0 | 0 | 0 | 0 | 0 | 0 | 0 | 0 | 0 | 0 | 0 |
| NM_001348 | DAPK3 | 2105 | 1 | 1 | 1 | 1 | 1 | 1 | 0 | 0 | 1 | 0 | 1 | 1 | 1 |
| NM_001349 | DARS | 2322 | 23 | 17 | 13 | 6 | 22 | 16 | 19 | 18 | 14 | 20 | 19 | 23 | 13 |
| NM_001350 | DAXX | 2612 | 6 | 3 | 3 | 3 | 2 | 2 | 2 | 1 | 3 | 1 | 2 | 6 | 6 |
| NM_001350 | DAXX | 2614 | 6 | 3 | 3 | 3 | 2 | 2 | 2 | 1 | 3 | 1 | 2 | 6 | 6 |
| NM_001351 | DAZL | 3048 | 5 | 0 | 0 | 0 | 0 | 0 | 0 | 0 | 0 | 0 | 0 | 0 | 5 |
| NM_001352 | DBP | 1643 | 1 | 0 | 0 | 0 | 0 | 1 | 0 | 0 | 0 | 1 | 0 | 0 | 0 |
| NM_001353 | AKR1C1 | 1379 | 6 | 1 | 4 | 0 | 1 | 0 | 0 | 6 | 0 | 0 | 0 | 0 | 0 |
| NM_001354 | AKR1C2 | 1590 | 34 | 7 | 32 | 0 | 1 | 0 | 0 | 34 | 1 | 0 | 2 | 0 | 1 |

| transcript | gene | transcript length (nt) | max | mean | adipose | colon | heart | hypothalamus | kidney | liver | lung | ovary | skeletalmuscle | spleen | testes |
|---|---|---|---|---|---|---|---|---|---|---|---|---|---|---|---|
| NM_001355 | DDT | 671 | 0 | 0 | 0 | 0 | 0 | 0 | 0 | 0 | 0 | 0 | 0 | 0 | 0 |
| NM_001356 | DDX3X | 5396 | 23 | 15 | 9 | 6 | 18 | 10 | 18 | 17 | 19 | 19 | 15 | 23 | 12 |
| NM_001357 | DHX9 | 4291 | 12 | 8 | 6 | 4 | 7 | 10 | 10 | 8 | 6 | 9 | 4 | 12 | 11 |
| NM_001358 | DHX15 | 3015 | 32 | 23 | 16 | 15 | 25 | 25 | 24 | 26 | 21 | 26 | 16 | 32 | 27 |
| NM_001359 | DECR1 | 1251 | 13 | 5 | 6 | 2 | 11 | 4 | 6 | 13 | 2 | 2 | 2 | 4 | 4 |
| NM_001360 | DHCR7 | 2665 | 23 | 5 | 1 | 1 | 2 | 7 | 2 | 23 | 2 | 3 | 1 | 3 | 6 |
| NM_001361 | DHODH | 2066 | 9 | 2 | 1 | 1 | 1 | 1 | 1 | 9 | 1 | 2 | 1 | 1 | 1 |
| NM_001362 | DIO3 | 2102 | 2 | 0 | 0 | 0 | 0 | 0 | 0 | 0 | 1 | 2 | 0 | 0 | 1 |
| NM_001363 | DKC1 | 2576 | 35 | 19 | 12 | 10 | 21 | 17 | 16 | 18 | 12 | 22 | 14 | 33 | 35 |
| NM_001364 | DLG2 | 7719 | 22 | 3 | 0 | 0 | 1 | 22 | 1 | 0 | 0 | 2 | 0 | 0 | 1 |
| NM_001365 | DLG4 | 4320 | 6 | 1 | 0 | 0 | 0 | 6 | 0 | 0 | 0 | 0 | 0 | 0 | 1 |
| NM_001369 | DNAH5 | 15573 | 3 | 0 | 0 | 0 | 0 | 1 | 0 | 1 | 3 | 0 | 0 | 0 | 0 |
| NM_001370 | DNAH6 | 12795 | 2 | 0 | 0 | 0 | 0 | 1 | 1 | 0 | 1 | 1 | 0 | 0 | 2 |
| NM_001371 | DNAH8 | 14472 | 3 | 0 | 0 | 0 | 0 | 0 | 0 | 0 | 0 | 0 | 0 | 1 | 3 |
| NM_001372 | DNAH9 | 13750 | 2 | 0 | 0 | 0 | 0 | 1 | 0 | 0 | 2 | 0 | 0 | 0 | 1 |
| NM_001373 | DNAH14 | 13788 | 3 | 1 | 0 | 0 | 0 | 0 | 1 | 0 | 0 | 2 | 0 | 0 | 3 |
| NM_001374 | DNASE1L2 | 1326 | 0 | 0 | 0 | 0 | 0 | 0 | 0 | 0 | 0 | 0 | 0 | 0 | 0 |
| NM_001375 | DNASE2 | 2008 | 1 | 1 | 1 | 1 | 1 | 1 | 0 | 1 | 1 | 0 | 0 | 1 | 0 |
| NM_001376 | DYNC1H1 | 14340 | 49 | 21 | 21 | 14 | 24 | 49 | 16 | 11 | 14 | 15 | 26 | 20 | 21 |
| NM_001378 | DYNC1I2 | 2602 | 47 | 22 | 23 | 10 | 27 | 47 | 23 | 12 | 15 | 27 | 12 | 22 | 23 |
| NM_001379 | DNMT1 | 5351 | 5 | 3 | 3 | 3 | 2 | 3 | 2 | 2 | 3 | 2 | 1 | 5 | 5 |
| NM_001380 | DOCK1 | 6751 | 6 | 3 | 3 | 3 | 3 | 4 | 6 | 2 | 3 | 6 | 1 | 4 | 3 |
| NM_001381 | DOK1 | 1890 | 2 | 1 | 2 | 1 | 0 | 0 | 0 | 0 | 1 | 0 | 0 | 2 | 1 |
| NM_001382 | DPAGT1 | 2142 | 3 | 1 | 1 | 0 | 1 | 1 | 1 | 3 | 1 | 1 | 0 | 1 | 1 |
| NM_001383 | DPH1 | 2195 | 1 | 0 | 0 | 0 | 0 | 1 | 1 | 1 | 0 | 0 | 0 | 0 | 0 |
| NM_001384 | DPH2 | 2497 | 1 | 0 | 0 | 0 | 1 | 1 | 1 | 1 | 0 | 0 | 1 | 1 | 0 |
| NM_001385 | DPYS | 2123 | 26 | 5 | 0 | 0 | 0 | 0 | 24 | 26 | 0 | 0 | 0 | 0 | 0 |
| NM_001386 | DPYSL2 | 4540 | 139 | 24 | 17 | 7 | 22 | 139 | 8 | 3 | 22 | 21 | 6 | 13 | 10 |
| NM_001387 | DPYSL3 | 5050 | 24 | 8 | 5 | 8 | 8 | 15 | 1 | 0 | 4 | 24 | 11 | 5 | 8 |
| NM_001388 | DRG2 | 1888 | 1 | 1 | 0 | 1 | 0 | 1 | 1 | 1 | 1 | 0 | 0 | 1 | 0 |
| NM_001389 | DSCAM | 7109 | 1 | 0 | 0 | 0 | 0 | 1 | 0 | 0 | 0 | 0 | 0 | 0 | 0 |
| NM_001390 | DTNA | 6343 | 17 | 3 | 1 | 1 | 4 | 17 | 0 | 0 | 1 | 1 | 4 | 1 | 2 |
| NM_001391 | DTNA | 4365 | 18 | 3 | 1 | 1 | 7 | 18 | 0 | 0 | 1 | 1 | 4 | 1 | 2 |
| NM_001392 | DTNA | 1733 | 24 | 5 | 2 | 1 | 13 | 24 | 1 | 0 | 1 | 2 | 7 | 1 | 4 |
| NM_001393 | ECM2 | 3163 | 26 | 7 | 26 | 1 | 3 | 5 | 4 | 4 | 3 | 16 | 2 | 0 | 9 |
| NM_001394 | DUSP4 | 2498 | 0 | 0 | 0 | 0 | 0 | 0 | 0 | 0 | 0 | 0 | 0 | 0 | 0 |
| NM_001395 | DUSP9 | 2380 | 1 | 0 | 0 | 0 | 0 | 0 | 1 | 0 | 0 | 0 | 0 | 0 | 0 |
| NM_001396 | DYRK1A | 5212 | 11 | 7 | 4 | 4 | 8 | 8 | 6 | 4 | 6 | 9 | 11 | 9 | 10 |
| NM_001397 | ECE1 | 5096 | 5 | 3 | 3 | 3 | 4 | 2 | 2 | 3 | 3 | 4 | 1 | 5 | 3 |
| NM_001398 | ECH1 | 1263 | 47 | 17 | 15 | 11 | 47 | 8 | 26 | 17 | 10 | 7 | 30 | 16 | 7 |
| NM_001399 | EDA | 5280 | 1 | 0 | 1 | 0 | 1 | 0 | 1 | 0 | 0 | 1 | 0 | 0 | 0 |
| NM_001400 | S1PR1 | 3050 | 6 | 3 | 3 | 1 | 3 | 3 | 1 | 2 | 6 | 0 | 1 | 6 | 1 |
| NM_001401 | LPAR1 | 3090 | 2 | 1 | 1 | 1 | 1 | 2 | 1 | 0 | 1 | 1 | 1 | 2 | 1 |
| NM_001402 | EEF1A1 | 3512 | 420 | 176 | 143 | 89 | 135 | 82 | 192 | 114 | 235 | 420 | 59 | 286 | 178 |
| NM_001404 | EEF1G | 1538 | 104 | 73 | 72 | 89 | 62 | 53 | 61 | 69 | 58 | 100 | 90 | 104 | 50 |
| NM_001405 | EFNA2 | 642 | 0 | 0 | 0 | 0 | 0 | 0 | 0 | 0 | 0 | 0 | 0 | 0 | 0 |
| NM_001406 | EFNB3 | 3219 | 2 | 0 | 0 | 0 | 0 | 2 | 0 | 0 | 0 | 0 | 0 | 0 | 0 |
| NM_001407 | CELSR3 | 11962 | 0 | 0 | 0 | 0 | 0 | 0 | 0 | 0 | 0 | 0 | 0 | 0 | 0 |
| NM_001408 | CELSR2 | 10540 | 4 | 1 | 0 | 0 | 0 | 4 | 1 | 0 | 0 | 0 | 0 | 0 | 2 |
| NM_001409 | MEGF6 | 7439 | 1 | 0 | 1 | 0 | 1 | 0 | 0 | 0 | 1 | 0 | 0 | 1 | 1 |
| NM_001410 | MEGF8 | 10966 | 5 | 2 | 2 | 1 | 2 | 5 | 2 | 1 | 0 | 1 | 1 | 2 | 1 |
| NM_001412 | EIF1AX | 4431 | 24 | 17 | 13 | 8 | 20 | 19 | 18 | 24 | 13 | 22 | 24 | 14 | 14 |
| NM_001414 | EIF2B1 | 1870 | 9 | 6 | 8 | 5 | 5 | 9 | 5 | 8 | 3 | 5 | 5 | 7 | 7 |
| NM_001415 | EIF2S3 | 3468 | 70 | 30 | 25 | 19 | 27 | 18 | 27 | 24 | 29 | 70 | 27 | 42 | 23 |
| NM_001416 | EIF4A1 | 1844 | 47 | 27 | 22 | 13 | 35 | 26 | 23 | 47 | 25 | 25 | 15 | 42 | 23 |
| NM_001417 | EIF4B | 4028 | 138 | 72 | 57 | 36 | 67 | 48 | 58 | 45 | 64 | 138 | 126 | 85 | 70 |
| NM_001418 | EIF4G2 | 3911 | 69 | 49 | 39 | 23 | 69 | 49 | 54 | 47 | 42 | 42 | 49 | 67 | 59 |
| NM_001419 | ELAVL1 | 6058 | 6 | 3 | 3 | 2 | 4 | 4 | 3 | 2 | 3 | 3 | 3 | 5 | 6 |
| NM_001420 | ELAVL3 | 4682 | 15 | 1 | 0 | 0 | 0 | 15 | 0 | 0 | 0 | 0 | 0 | 0 | 0 |
| NM_001421 | ELF4 | 4178 | 2 | 1 | 1 | 2 | 0 | 0 | 0 | 0 | 1 | 1 | 0 | 2 | 0 |
| NM_001422 | ELF5 | 2302 | 2 | 0 | 0 | 0 | 0 | 0 | 2 | 0 | 1 | 0 | 0 | 0 | 0 |
| NM_001423 | EMP1 | 2803 | 53 | 17 | 53 | 12 | 36 | 9 | 9 | 1 | 25 | 7 | 10 | 20 | 8 |
| NM_001424 | EMP2 | 5180 | 38 | 9 | 5 | 5 | 10 | 2 | 2 | 13 | 38 | 14 | 2 | 4 | 4 |
| NM_001425 | EMP3 | 829 | 29 | 13 | 29 | 18 | 18 | 10 | 5 | 6 | 19 | 10 | 5 | 17 | 9 |
| NM_001426 | EN1 | 2962 | 1 | 0 | 0 | 0 | 0 | 0 | 0 | 0 | 1 | 0 | 0 | 0 | 0 |
| NM_001427 | EN2 | 3395 | 0 | 0 | 0 | 0 | 0 | 0 | 0 | 0 | 0 | 0 | 0 | 0 | 0 |
| NM_001428 | ENO1 | 1812 | 42 | 23 | 25 | 18 | 14 | 39 | 42 | 37 | 20 | 8 | 4 | 27 | 13 |
| NM_001429 | EP300 | 8761 | 13 | 8 | 6 | 9 | 6 | 7 | 7 | 5 | 7 | 12 | 7 | 12 | 13 |
| NM_001430 | EPAS1 | 5166 | 61 | 14 | 16 | 4 | 17 | 7 | 12 | 10 | 61 | 4 | 3 | 10 | 7 |
| NM_001431 | EPB41L2 | 4454 | 50 | 26 | 50 | 14 | 23 | 39 | 12 | 6 | 25 | 40 | 7 | 35 | 37 |
| NM_001432 | EREG | 4628 | 1 | 0 | 0 | 0 | 0 | 0 | 0 | 0 | 1 | 0 | 0 | 1 | 0 |
| NM_001433 | ERN1 | 4005 | 2 | 1 | 1 | 1 | 1 | 0 | 1 | 2 | 2 | 1 | 0 | 2 | 1 |
| NM_001436 | FBL | 1164 | 64 | 31 | 18 | 33 | 24 | 21 | 20 | 20 | 23 | 64 | 26 | 47 | 47 |
| NM_001437 | ESR2 | 2169 | 1 | 0 | 0 | 0 | 0 | 0 | 0 | 0 | 0 | 1 | 0 | 0 | 1 |
| NM_001438 | ESRRG | 5236 | 6 | 1 | 0 | 0 | 3 | 2 | 6 | 0 | 0 | 0 | 2 | 0 | 0 |
| NM_001439 | EXTL2 | 2887 | 3 | 1 | 1 | 0 | 1 | 3 | 2 | 1 | 1 | 2 | 1 | 1 | 1 |
| NM_001440 | EXTL3 | 6172 | 4 | 2 | 1 | 1 | 3 | 4 | 1 | 1 | 1 | 1 | 1 | 3 | 2 |

| transcript | gene | transcript length (nt) | max | mean | adipose | colon | heart | hypothalamus | kidney | liver | lung | ovary | skeletalmuscle | spleen | testes |
|---|---|---|---|---|---|---|---|---|---|---|---|---|---|---|---|
| NM_001441 | FAAH | 2095 | 2 | 1 | 0 | 1 | 0 | 2 | 2 | 1 | 0 | 0 | 0 | 0 | 1 |
| NM_001442 | FABP4 | 838 | 1,103 | 112 | 1,103 | 30 | 39 | 1 | 4 | 0 | 22 | 3 | 20 | 7 | 4 |
| NM_001443 | FABP1 | 489 | 1,499 | 289 | 0 | 1,427 | 1 | 0 | 233 | 1,499 | 1 | 4 | 3 | 4 | 4 |
| NM_001444 | FABP5 | 660 | 200 | 45 | 200 | 32 | 42 | 100 | 4 | 2 | 34 | 7 | 13 | 54 | 10 |
| NM_001445 | FABP6 | 578 | 12 | 2 | 0 | 5 | 1 | 12 | 0 | 0 | 2 | 0 | 0 | 0 | 2 |
| NM_001446 | FABP7 | 999 | 18 | 2 | 0 | 0 | 0 | 18 | 0 | 0 | 0 | 0 | 1 | 0 | 0 |
| NM_001447 | FAT2 | 14536 | 0 | 0 | 0 | 0 | 0 | 0 | 0 | 0 | 0 | 0 | 0 | 0 | 0 |
| NM_001448 | GPC4 | 3714 | 5 | 3 | 5 | 2 | 3 | 2 | 5 | 0 | 5 | 2 | 1 | 2 | 2 |
| NM_001449 | FHL1 | 2506 | 171 | 28 | 38 | 7 | 60 | 7 | 2 | 1 | 7 | 3 | 171 | 2 | 5 |
| NM_001450 | FHL2 | 1704 | 110 | 16 | 2 | 3 | 110 | 2 | 2 | 0 | 1 | 45 | 2 | 4 | 11 |
| NM_001451 | FOXF1 | 2562 | 1 | 0 | 0 | 0 | 0 | 0 | 0 | 0 | 1 | 0 | 0 | 0 | 0 |
| NM_001452 | FOXF2 | 2187 | 1 | 0 | 0 | 1 | 0 | 0 | 0 | 0 | 0 | 0 | 0 | 0 | 0 |
| NM_001453 | FOXC1 | 3449 | 4 | 1 | 1 | 0 | 3 | 2 | 4 | 0 | 1 | 1 | 1 | 0 | 1 |
| NM_001454 | FOXJ1 | 2638 | 1 | 0 | 0 | 0 | 0 | 0 | 0 | 0 | 1 | 0 | 0 | 0 | 1 |
| NM_001455 | FOXO3 | 7341 | 3 | 1 | 1 | 1 | 1 | 1 | 1 | 1 | 2 | 2 | 3 | 2 | 1 |
| NM_001456 | FLNA | 8486 | 28 | 8 | 11 | 28 | 7 | 3 | 3 | 1 | 7 | 8 | 2 | 10 | 7 |
| NM_001457 | FLNB | 9467 | 10 | 4 | 3 | 10 | 3 | 2 | 6 | 5 | 4 | 4 | 1 | 5 | 7 |
| NM_001458 | FLNC | 9138 | 71 | 12 | 1 | 7 | 71 | 1 | 0 | 0 | 1 | 1 | 50 | 0 | 1 |
| NM_001459 | FLT3LG | 1036 | 1 | 0 | 0 | 1 | 0 | 0 | 0 | 0 | 1 | 1 | 1 | 0 | 0 |
| NM_001460 | FMO2 | 5181 | 18 | 3 | 4 | 0 | 9 | 1 | 2 | 1 | 18 | 1 | 3 | 0 | 1 |
| NM_001461 | FMO5 | 2632 | 37 | 6 | 1 | 4 | 1 | 0 | 9 | 37 | 12 | 2 | 1 | 2 | 2 |
| NM_001462 | FPR2 | 2011 | 36 | 6 | 0 | 0 | 6 | 2 | 0 | 3 | 19 | 0 | 0 | 36 | 1 |
| NM_001463 | FRZB | 2035 | 16 | 4 | 0 | 1 | 4 | 4 | 3 | 0 | 3 | 4 | 6 | 16 | 3 |
| NM_001464 | ADAM2 | 2642 | 2 | 0 | 0 | 0 | 0 | 0 | 0 | 0 | 0 | 0 | 0 | 0 | 2 |
| NM_001465 | FYB | 4828 | 33 | 6 | 4 | 2 | 4 | 5 | 2 | 2 | 10 | 1 | 1 | 33 | 2 |
| NM_001466 | FZD2 | 1983 | 0 | 0 | 0 | 0 | 0 | 0 | 0 | 0 | 0 | 0 | 0 | 0 | 0 |
| NM_001467 | SLC37A4 | 2103 | 6 | 1 | 0 | 1 | 0 | 0 | 4 | 6 | 0 | 0 | 1 | 1 | 1 |
| NM_001468 | GAGE1 | 953 | 0 | 0 | 0 | 0 | 0 | 0 | 0 | 0 | 0 | 0 | 0 | 0 | 0 |
| NM_001469 | XRCC6 | 2133 | 17 | 13 | 12 | 8 | 14 | 17 | 11 | 14 | 10 | 12 | 13 | 14 | 17 |
| NM_001470 | GABBR1 | 4537 | 13 | 2 | 0 | 1 | 1 | 13 | 0 | 0 | 1 | 1 | 0 | 1 | 1 |
| NM_001472 | GAGE2C | 538 | 0 | 0 | 0 | 0 | 0 | 0 | 0 | 0 | 0 | 0 | 0 | 0 | 0 |
| NM_001474 | GAGE4 | 526 | 0 | 0 | 0 | 0 | 0 | 0 | 0 | 0 | 0 | 0 | 0 | 0 | 0 |
| NM_001475 | GAGE5 | 522 | 0 | 0 | 0 | 0 | 0 | 0 | 0 | 0 | 0 | 0 | 0 | 0 | 0 |
| NM_001476 | GAGE6 | 525 | 0 | 0 | 0 | 0 | 0 | 0 | 0 | 0 | 0 | 0 | 0 | 0 | 0 |
| NM_001477 | GAGE12I | 502 | 0 | 0 | 0 | 0 | 0 | 0 | 0 | 0 | 0 | 0 | 0 | 0 | 0 |
| NM_001478 | B4GALNT1 | 3010 | 1 | 0 | 0 | 0 | 0 | 1 | 0 | 0 | 0 | 0 | 0 | 0 | 0 |
| NM_001480 | GALR1 | 2785 | 0 | 0 | 0 | 0 | 0 | 0 | 0 | 0 | 0 | 0 | 0 | 0 | 0 |
| NM_001481 | GAS8 | 3185 | 4 | 2 | 1 | 1 | 2 | 3 | 4 | 1 | 1 | 2 | 1 | 1 | 3 |
| NM_001482 | GATM | 2586 | 124 | 21 | 1 | 1 | 2 | 9 | 124 | 62 | 1 | 13 | 3 | 5 | 13 |
| NM_001483 | GBAS | 1974 | 9 | 3 | 3 | 1 | 9 | 3 | 3 | 0 | 2 | 2 | 9 | 2 | 2 |
| NM_001485 | GBX2 | 1335 | 0 | 0 | 0 | 0 | 0 | 0 | 0 | 0 | 0 | 0 | 0 | 0 | 0 |
| NM_001486 | GCKR | 2186 | 10 | 1 | 0 | 0 | 0 | 0 | 0 | 10 | 0 | 0 | 0 | 0 | 0 |
| NM_001487 | BLOC1S1 | 572 | 102 | 59 | 58 | 59 | 56 | 102 | 48 | 88 | 30 | 42 | 69 | 72 | 21 |
| NM_001488 | TADA2A | 1840 | 2 | 2 | 1 | 1 | 2 | 2 | 2 | 1 | 1 | 2 | 2 | 2 | 2 |
| NM_001489 | NR6A1 | 1887 | 3 | 0 | 0 | 0 | 0 | 0 | 0 | 0 | 0 | 0 | 0 | 0 | 3 |
| NM_001490 | GCNT1 | 5596 | 2 | 1 | 1 | 1 | 1 | 0 | 0 | 0 | 1 | 0 | 0 | 2 | 0 |
| NM_001491 | GCNT2 | 4672 | 2 | 1 | 0 | 1 | 2 | 0 | 2 | 1 | 1 | 0 | 0 | 1 | 0 |
| NM_001492 | GDF1 | 2558 | 0 | 0 | 0 | 0 | 0 | 0 | 0 | 0 | 0 | 0 | 0 | 0 | 0 |
| NM_001493 | GDI1 | 2482 | 29 | 7 | 2 | 2 | 6 | 29 | 7 | 2 | 6 | 5 | 4 | 7 | 7 |
| NM_001494 | GDI2 | 2412 | 84 | 51 | 41 | 24 | 50 | 40 | 48 | 73 | 44 | 52 | 39 | 84 | 63 |
| NM_001495 | GFRA2 | 3330 | 1 | 0 | 0 | 0 | 0 | 0 | 0 | 0 | 0 | 0 | 0 | 1 | 0 |
| NM_001496 | GFRA3 | 1931 | 0 | 0 | 0 | 0 | 0 | 0 | 0 | 0 | 0 | 0 | 0 | 0 | 0 |
| NM_001497 | B4GALT1 | 4199 | 37 | 16 | 11 | 9 | 16 | 4 | 16 | 37 | 16 | 12 | 5 | 24 | 30 |
| NM_001498 | GCLC | 3716 | 8 | 3 | 4 | 1 | 1 | 3 | 3 | 8 | 2 | 1 | 1 | 6 | 2 |
| NM_001499 | GLE1 | 2257 | 6 | 4 | 4 | 3 | 3 | 2 | 4 | 4 | 2 | 4 | 3 | 5 | 6 |
| NM_001500 | GMDS | 1672 | 20 | 5 | 2 | 20 | 2 | 4 | 4 | 9 | 3 | 2 | 1 | 3 | 3 |
| NM_001501 | GNRH2 | 423 | 0 | 0 | 0 | 0 | 0 | 0 | 0 | 0 | 0 | 0 | 0 | 0 | 0 |
| NM_001502 | GP2 | 2422 | 1 | 0 | 1 | 0 | 0 | 0 | 1 | 0 | 0 | 0 | 0 | 0 | 0 |
| NM_001503 | GPLD1 | 3489 | 13 | 3 | 6 | 1 | 0 | 1 | 1 | 13 | 1 | 1 | 0 | 1 | 2 |
| NM_001504 | CXCR3 | 1624 | 0 | 0 | 0 | 0 | 0 | 0 | 0 | 0 | 0 | 0 | 0 | 0 | 0 |
| NM_001505 | GPER | 2847 | 0 | 0 | 0 | 0 | 0 | 0 | 0 | 0 | 0 | 0 | 0 | 0 | 0 |
| NM_001506 | GPR32 | 1071 | 0 | 0 | 0 | 0 | 0 | 0 | 0 | 0 | 0 | 0 | 0 | 0 | 0 |
| NM_001507 | MLNR | 1239 | 0 | 0 | 0 | 0 | 0 | 0 | 0 | 0 | 0 | 0 | 0 | 0 | 0 |
| NM_001508 | GPR39 | 2821 | 0 | 0 | 0 | 0 | 0 | 0 | 0 | 0 | 0 | 0 | 0 | 0 | 0 |
| NM_001509 | GPX5 | 1452 | 0 | 0 | 0 | 0 | 0 | 0 | 0 | 0 | 0 | 0 | 0 | 0 | 0 |
| NM_001510 | GRID2 | 3282 | 2 | 0 | 0 | 0 | 0 | 2 | 0 | 0 | 0 | 0 | 0 | 0 | 2 |
| NM_001511 | CXCL1 | 1103 | 26 | 3 | 0 | 1 | 2 | 1 | 1 | 0 | 6 | 0 | 0 | 26 | 0 |
| NM_001512 | GSTA4 | 1340 | 10 | 4 | 2 | 1 | 2 | 10 | 3 | 1 | 6 | 6 | 2 | 2 | 6 |
| NM_001513 | GSTZ1 | 1425 | 2 | 1 | 1 | 0 | 0 | 0 | 1 | 2 | 0 | 0 | 1 | 1 | 1 |
| NM_001514 | GTF2B | 1645 | 6 | 4 | 3 | 3 | 3 | 5 | 3 | 5 | 4 | 4 | 2 | 6 | 4 |
| NM_001515 | GTF2H2 | 1951 | 0 | 0 | 0 | 0 | 0 | 0 | 0 | 0 | 0 | 0 | 0 | 0 | 0 |
| NM_001516 | GTF2H3 | 1505 | 3 | 2 | 1 | 1 | 1 | 3 | 1 | 3 | 1 | 2 | 1 | 1 | 2 |
| NM_001517 | GTF2H4 | 1716 | 1 | 0 | 1 | 0 | 0 | 0 | 0 | 1 | 0 | 0 | 0 | 0 | 0 |
| NM_001518 | GTF2I | 4406 | 9 | 6 | 5 | 3 | 5 | 5 | 9 | 5 | 4 | 7 | 6 | 7 | 5 |
| NM_001519 | BRF1 | 3660 | 1 | 0 | 0 | 0 | 0 | 1 | 1 | 0 | 0 | 0 | 0 | 0 | 0 |
| NM_001520 | GTF3C1 | 7107 | 4 | 3 | 2 | 2 | 3 | 4 | 3 | 2 | 2 | 3 | 3 | 3 | 4 |
| NM_001521 | GTF3C2 | 3920 | 2 | 1 | 1 | 2 | 1 | 1 | 2 | 1 | 1 | 1 | 1 | 2 | 2 |

| transcript | gene | transcript length (nt) | max | mean | adipose | colon | heart | hypothalamus | kidney | liver | lung | ovary | skeletalmuscle | spleen | testes |
|---|---|---|---|---|---|---|---|---|---|---|---|---|---|---|---|
| NM_001522 | GUCY2F | 3698 | 1 | 0 | 0 | 1 | 0 | 0 | 0 | 0 | 0 | 0 | 0 | 0 | 0 |
| NM_001523 | HAS1 | 2087 | 0 | 0 | 0 | 0 | 0 | 0 | 0 | 0 | 0 | 0 | 0 | 0 | 0 |
| NM_001524 | HCRT | 577 | 0 | 0 | 0 | 0 | 0 | 0 | 0 | 0 | 0 | 0 | 0 | 0 | 0 |
| NM_001525 | HCRTR1 | 2001 | 0 | 0 | 0 | 0 | 0 | 0 | 0 | 0 | 0 | 0 | 0 | 0 | 0 |
| NM_001526 | HCRTR2 | 1814 | 1 | 0 | 0 | 0 | 0 | 1 | 0 | 0 | 0 | 0 | 0 | 0 | 0 |
| NM_001527 | HDAC2 | 6644 | 17 | 8 | 6 | 3 | 8 | 9 | 7 | 5 | 6 | 10 | 5 | 9 | 17 |
| NM_001528 | HGFAC | 2038 | 0 | 0 | 0 | 0 | 0 | 0 | 0 | 0 | 0 | 0 | 0 | 0 | 0 |
| NM_001530 | HIF1A | 4059 | 52 | 19 | 10 | 3 | 21 | 14 | 39 | 6 | 27 | 18 | 4 | 52 | 20 |
| NM_001531 | MR1 | 1263 | 4 | 2 | 4 | 1 | 2 | 2 | 2 | 2 | 2 | 2 | 1 | 4 | 1 |
| NM_001532 | SLC29A2 | 2515 | 1 | 0 | 0 | 1 | 0 | 0 | 0 | 0 | 0 | 0 | 1 | 0 | 0 |
| NM_001533 | HNRNPL | 2115 | 38 | 26 | 25 | 26 | 24 | 23 | 22 | 38 | 31 | 19 | 18 | 37 | 23 |
| NM_001535 | PRMT2 | 2131 | 4 | 2 | 2 | 2 | 1 | 4 | 2 | 0 | 2 | 2 | 0 | 3 | 2 |
| NM_001536 | PRMT1 | 1360 | 10 | 6 | 3 | 5 | 7 | 7 | 4 | 4 | 4 | 6 | 10 | 10 | 8 |
| NM_001537 | HSBP1 | 1891 | 24 | 12 | 8 | 10 | 12 | 24 | 14 | 11 | 10 | 8 | 5 | 13 | 19 |
| NM_001538 | HSF4 | 2514 | 0 | 0 | 0 | 0 | 0 | 0 | 0 | 0 | 0 | 0 | 0 | 0 | 0 |
| NM_001539 | DNAJA1 | 1538 | 156 | 62 | 49 | 20 | 58 | 156 | 52 | 61 | 59 | 50 | 23 | 59 | 92 |
| NM_001540 | HSPB1 | 896 | 61 | 18 | 11 | 16 | 61 | 7 | 17 | 2 | 11 | 8 | 55 | 8 | 5 |
| NM_001541 | HSPB2 | 841 | 3 | 1 | 1 | 0 | 3 | 1 | 0 | 0 | 0 | 0 | 2 | 0 | 0 |
| NM_001542 | IGSF3 | 7253 | 4 | 1 | 0 | 1 | 1 | 1 | 4 | 0 | 1 | 0 | 0 | 0 | 2 |
| NM_001543 | NDST1 | 8029 | 4 | 1 | 1 | 1 | 1 | 2 | 1 | 2 | 1 | 1 | 0 | 4 | 1 |
| NM_001544 | ICAM4 | 1273 | 0 | 0 | 0 | 0 | 0 | 0 | 0 | 0 | 0 | 0 | 0 | 0 | 0 |
| NM_001545 | ICT1 | 879 | 11 | 5 | 4 | 2 | 8 | 6 | 6 | 11 | 2 | 2 | 7 | 4 | 2 |
| NM_001546 | ID4 | 2344 | 21 | 11 | 16 | 2 | 15 | 14 | 11 | 1 | 9 | 20 | 2 | 8 | 21 |
| NM_001547 | IFIT2 | 3495 | 2 | 1 | 2 | 0 | 1 | 2 | 2 | 0 | 2 | 2 | 2 | 2 | 1 |
| NM_001548 | IFIT1 | 1860 | 13 | 5 | 5 | 1 | 5 | 11 | 5 | 1 | 2 | 6 | 13 | 6 | 3 |
| NM_001549 | IFIT3 | 2541 | 13 | 5 | 7 | 2 | 4 | 5 | 4 | 1 | 7 | 6 | 13 | 5 | 3 |
| NM_001550 | IFRD1 | 2257 | 4 | 3 | 3 | 2 | 4 | 3 | 3 | 2 | 4 | 4 | 4 | 3 | 4 |
| NM_001551 | IGBP1 | 1660 | 7 | 4 | 2 | 3 | 4 | 3 | 4 | 1 | 3 | 7 | 6 | 5 | 4 |
| NM_001552 | IGFBP4 | 2236 | 15 | 8 | 5 | 4 | 10 | 1 | 14 | 15 | 5 | 10 | 1 | 12 | 6 |
| NM_001553 | IGFBP7 | 1110 | 22 | 11 | 14 | 4 | 21 | 12 | 22 | 5 | 5 | 6 | 2 | 17 | 10 |
| NM_001554 | CYR61 | 2291 | 58 | 30 | 16 | 3 | 51 | 12 | 26 | 19 | 53 | 58 | 24 | 56 | 11 |
| NM_001555 | IGSF1 | 4537 | 1 | 0 | 0 | 0 | 0 | 1 | 0 | 0 | 0 | 0 | 0 | 0 | 1 |
| NM_001556 | IKBKB | 3916 | 4 | 2 | 2 | 4 | 1 | 1 | 2 | 1 | 2 | 1 | 1 | 4 | 2 |
| NM_001557 | CXCR2 | 2869 | 7 | 1 | 0 | 0 | 0 | 0 | 0 | 1 | 2 | 0 | 0 | 7 | 0 |
| NM_001558 | IL10RA | 3654 | 10 | 2 | 3 | 1 | 1 | 1 | 0 | 1 | 3 | 0 | 0 | 10 | 0 |
| NM_001559 | IL12RB2 | 4040 | 1 | 0 | 0 | 0 | 0 | 1 | 0 | 0 | 0 | 0 | 1 | 0 | 0 |
| NM_001560 | IL13RA1 | 4006 | 36 | 15 | 12 | 8 | 15 | 8 | 12 | 36 | 17 | 15 | 8 | 18 | 11 |
| NM_001561 | TNFRSF9 | 1884 | 1 | 0 | 0 | 0 | 0 | 0 | 0 | 0 | 0 | 0 | 0 | 1 | 0 |
| NM_001562 | IL18 | 1145 | 9 | 2 | 2 | 1 | 1 | 1 | 2 | 1 | 4 | 1 | 1 | 9 | 1 |
| NM_001563 | IMPG1 | 3268 | 0 | 0 | 0 | 0 | 0 | 0 | 0 | 0 | 0 | 0 | 0 | 0 | 0 |
| NM_001564 | ING2 | 1116 | 80 | 19 | 9 | 4 | 10 | 19 | 11 | 8 | 9 | 80 | 11 | 15 | 30 |
| NM_001565 | CXCL10 | 1175 | 2 | 0 | 1 | 0 | 0 | 0 | 0 | 0 | 2 | 0 | 0 | 1 | 1 |
| NM_001566 | INPP4A | 3323 | 7 | 2 | 2 | 2 | 2 | 7 | 1 | 0 | 2 | 2 | 1 | 5 | 1 |
| NM_001567 | INPPL1 | 4713 | 2 | 1 | 1 | 1 | 2 | 1 | 1 | 0 | 1 | 1 | 1 | 2 | 1 |
| NM_001568 | EIF3E | 1508 | 54 | 25 | 20 | 9 | 25 | 12 | 19 | 23 | 27 | 54 | 31 | 33 | 25 |
| NM_001569 | IRAK1 | 3571 | 3 | 2 | 2 | 2 | 1 | 1 | 2 | 3 | 2 | 0 | 1 | 2 | 1 |
| NM_001570 | IRAK2 | 3469 | 1 | 0 | 1 | 0 | 0 | 0 | 0 | 0 | 1 | 0 | 1 | 0 | 0 |
| NM_001571 | IRF3 | 1613 | 1 | 0 | 0 | 1 | 0 | 1 | 0 | 0 | 1 | 0 | 0 | 1 | 0 |
| NM_001572 | IRF7 | 1972 | 0 | 0 | 0 | 0 | 0 | 0 | 0 | 0 | 0 | 0 | 0 | 0 | 0 |
| NM_001584 | MPPED2 | 2401 | 2 | 0 | 0 | 0 | 1 | 1 | 2 | 0 | 0 | 0 | 0 | 0 | 0 |
| NM_001586 | TEX28 | 1525 | 0 | 0 | 0 | 0 | 0 | 0 | 0 | 0 | 0 | 0 | 0 | 0 | 0 |
| NM_001587 | OCRL | 5130 | 14 | 5 | 3 | 3 | 3 | 9 | 3 | 2 | 2 | 7 | 3 | 3 | 14 |
| NM_001604 | PAX6 | 6888 | 1 | 0 | 0 | 0 | 0 | 1 | 0 | 0 | 0 | 0 | 0 | 0 | 0 |
| NM_001605 | AARS | 3344 | 36 | 13 | 12 | 8 | 12 | 36 | 12 | 23 | 6 | 6 | 11 | 11 | 8 |
| NM_001606 | ABCA2 | 8159 | 5 | 1 | 1 | 0 | 0 | 5 | 0 | 0 | 0 | 0 | 0 | 0 | 0 |
| NM_001607 | ACAA1 | 1819 | 29 | 10 | 8 | 5 | 8 | 6 | 29 | 28 | 5 | 3 | 2 | 8 | 3 |
| NM_001608 | ACADL | 2490 | 3 | 1 | 1 | 0 | 1 | 1 | 3 | 1 | 1 | 1 | 1 | 0 | 1 |
| NM_001609 | ACADSB | 5941 | 58 | 11 | 4 | 2 | 9 | 6 | 23 | 58 | 3 | 3 | 5 | 3 | 3 |
| NM_001610 | ACP2 | 2203 | 4 | 2 | 2 | 1 | 1 | 2 | 2 | 4 | 2 | 0 | 1 | 2 | 1 |
| NM_001611 | ACP5 | 1474 | 11 | 3 | 5 | 1 | 0 | 0 | 6 | 1 | 11 | 2 | 0 | 3 | 0 |
| NM_001612 | ACRV1 | 1324 | 2 | 0 | 0 | 0 | 0 | 0 | 0 | 0 | 0 | 0 | 0 | 0 | 2 |
| NM_001613 | ACTA2 | 1397 | 530 | 197 | 75 | 288 | 530 | 54 | 73 | 17 | 126 | 341 | 84 | 261 | 318 |
| NM_001614 | ACTG1 | 1919 | 50 | 26 | 22 | 16 | 20 | 40 | 27 | 19 | 25 | 50 | 10 | 41 | 17 |
| NM_001615 | ACTG2 | 1331 | 294 | 34 | 3 | 294 | 14 | 6 | 4 | 0 | 9 | 15 | 5 | 2 | 22 |
| NM_001616 | ACVR2A | 5244 | 5 | 3 | 2 | 2 | 2 | 5 | 3 | 2 | 3 | 4 | 4 | 3 | 5 |
| NM_001617 | ADD2 | 3940 | 23 | 2 | 0 | 1 | 0 | 23 | 0 | 0 | 0 | 0 | 0 | 0 | 0 |
| NM_001618 | PARP1 | 3985 | 26 | 12 | 9 | 8 | 14 | 26 | 7 | 11 | 6 | 7 | 7 | 20 | 13 |
| NM_001619 | ADRBK1 | 3444 | 3 | 1 | 1 | 1 | 1 | 2 | 1 | 1 | 2 | 0 | 0 | 3 | 0 |
| NM_001620 | AHNAK | 18815 | 155 | 57 | 155 | 62 | 61 | 24 | 29 | 14 | 65 | 63 | 70 | 38 | 43 |
| NM_001621 | AHR | 6247 | 6 | 3 | 3 | 1 | 3 | 1 | 1 | 4 | 6 | 2 | 1 | 5 | 2 |
| NM_001622 | AHSG | 1574 | 38 | 4 | 0 | 0 | 0 | 0 | 0 | 38 | 0 | 0 | 0 | 1 | 0 |
| NM_001623 | AIF1 | 639 | 112 | 23 | 24 | 4 | 23 | 37 | 9 | 7 | 27 | 4 | 2 | 112 | 5 |
| NM_001624 | AIM1 | 7552 | 7 | 3 | 5 | 4 | 1 | 1 | 5 | 2 | 3 | 1 | 0 | 7 | 1 |
| NM_001625 | AK2 | 961 | 18 | 9 | 7 | 6 | 15 | 5 | 18 | 11 | 5 | 5 | 9 | 9 | 5 |
| NM_001626 | AKT2 | 5263 | 2 | 1 | 1 | 1 | 1 | 0 | 1 | 2 | 1 | 1 | 1 | 1 | 1 |
| NM_001627 | ALCAM | 4741 | 20 | 6 | 7 | 1 | 3 | 17 | 5 | 7 | 20 | 6 | 0 | 2 | 2 |
| NM_001628 | AKR1B1 | 1398 | 2 | 1 | 1 | 0 | 2 | 2 | 2 | 0 | 1 | 1 | 2 | 1 | 1 |

| transcript | gene | transcript length (nt) | max | mean | adipose | colon | heart | hypothalamus | kidney | liver | lung | ovary | skeletalmuscle | spleen | testes |
|---|---|---|---|---|---|---|---|---|---|---|---|---|---|---|---|
| NM_001629 | ALOX5AP | 873 | 14 | 5 | 5 | 2 | 6 | 7 | 1 | 1 | 14 | 1 | 1 | 13 | 2 |
| NM_001630 | ANXA8L2 | 2029 | 0 | 0 | 0 | 0 | 0 | 0 | 0 | 0 | 0 | 0 | 0 | 0 | 0 |
| NM_001631 | ALPI | 2550 | 0 | 0 | 0 | 0 | 0 | 0 | 0 | 0 | 0 | 0 | 0 | 0 | 0 |
| NM_001632 | ALPP | 2867 | 1 | 0 | 0 | 0 | 0 | 0 | 0 | 0 | 1 | 0 | 0 | 0 | 0 |
| NM_001633 | AMBP | 1433 | 664 | 63 | 0 | 3 | 4 | 0 | 2 | 664 | 1 | 3 | 2 | 4 | 4 |
| NM_001634 | AMD1 | 3421 | 12 | 7 | 2 | 3 | 11 | 12 | 8 | 6 | 8 | 6 | 4 | 10 | 10 |
| NM_001635 | AMPH | 3271 | 13 | 2 | 0 | 0 | 1 | 13 | 0 | 0 | 0 | 0 | 0 | 1 | 0 |
| NM_001636 | SLC25A6 | 1484 | 25 | 13 | 10 | 14 | 11 | 9 | 20 | 4 | 12 | 25 | 5 | 23 | 7 |
| NM_001637 | AOAH | 2378 | 16 | 2 | 1 | 1 | 1 | 1 | 1 | 1 | 2 | 0 | 0 | 16 | 0 |
| NM_001638 | APOF | 1689 | 11 | 1 | 0 | 0 | 0 | 0 | 0 | 11 | 0 | 0 | 0 | 0 | 0 |
| NM_001639 | APCS | 930 | 354 | 33 | 0 | 0 | 1 | 0 | 1 | 354 | 1 | 3 | 0 | 2 | 1 |
| NM_001640 | APEH | 2758 | 3 | 2 | 1 | 2 | 2 | 2 | 3 | 3 | 1 | 1 | 2 | 2 | 1 |
| NM_001641 | APEX1 | 1548 | 6 | 4 | 4 | 3 | 3 | 6 | 5 | 5 | 3 | 6 | 3 | 6 | 3 |
| NM_001642 | APLP2 | 3811 | 143 | 81 | 90 | 41 | 106 | 99 | 143 | 89 | 126 | 66 | 20 | 60 | 47 |
| NM_001643 | APOA2 | 473 | 1,308 | 122 | 0 | 1 | 5 | 0 | 1 | 1,308 | 3 | 10 | 3 | 9 | 4 |
| NM_001644 | APOBEC1 | 892 | 0 | 0 | 0 | 0 | 0 | 0 | 0 | 0 | 0 | 0 | 0 | 0 | 0 |
| NM_001645 | APOC1 | 461 | 42 | 4 | 1 | 0 | 0 | 1 | 0 | 42 | 2 | 0 | 0 | 2 | 1 |
| NM_001646 | APOC4 | 613 | 5 | 1 | 0 | 0 | 0 | 0 | 0 | 5 | 0 | 0 | 0 | 0 | 0 |
| NM_001647 | APOD | 1130 | 103 | 28 | 44 | 3 | 54 | 49 | 3 | 0 | 16 | 7 | 103 | 0 | 30 |
| NM_001648 | KLK3 | 1464 | 0 | 0 | 0 | 0 | 0 | 0 | 0 | 0 | 0 | 0 | 0 | 0 | 0 |
| NM_001649 | SHROOM2 | 7445 | 1 | 0 | 0 | 0 | 0 | 1 | 0 | 1 | 1 | 1 | 0 | 0 | 1 |
| NM_001650 | AQP4 | 5203 | 135 | 15 | 0 | 1 | 2 | 135 | 0 | 0 | 22 | 0 | 3 | 0 | 0 |
| NM_001651 | AQP5 | 1821 | 1 | 0 | 0 | 0 | 0 | 0 | 0 | 0 | 0 | 0 | 0 | 0 | 1 |
| NM_001652 | AQP6 | 2654 | 8 | 1 | 0 | 0 | 0 | 0 | 8 | 0 | 0 | 0 | 0 | 0 | 0 |
| NM_001654 | ARAF | 2417 | 3 | 2 | 2 | 2 | 2 | 1 | 2 | 3 | 2 | 2 | 3 | 3 | 1 |
| NM_001655 | ARCN1 | 4013 | 58 | 41 | 36 | 20 | 58 | 43 | 37 | 51 | 35 | 43 | 22 | 50 | 57 |
| NM_001656 | TRIM23 | 3900 | 6 | 2 | 1 | 1 | 2 | 6 | 2 | 1 | 1 | 2 | 2 | 2 | 3 |
| NM_001657 | AREG | 1238 | 6 | 2 | 4 | 1 | 6 | 0 | 0 | 0 | 3 | 2 | 4 | 5 | 2 |
| NM_001658 | ARF1 | 1888 | 14 | 10 | 8 | 7 | 12 | 14 | 11 | 12 | 9 | 5 | 8 | 14 | 10 |
| NM_001659 | ARF3 | 3537 | 38 | 10 | 6 | 12 | 5 | 38 | 12 | 4 | 8 | 5 | 1 | 10 | 7 |
| NM_001660 | ARF4 | 1740 | 36 | 25 | 28 | 14 | 29 | 22 | 23 | 35 | 26 | 24 | 9 | 27 | 36 |
| NM_001661 | ARL4D | 1585 | 1 | 0 | 0 | 0 | 0 | 0 | 1 | 0 | 0 | 0 | 0 | 1 | 0 |
| NM_001662 | ARF5 | 978 | 3 | 2 | 2 | 2 | 2 | 3 | 3 | 2 | 2 | 1 | 1 | 2 | 1 |
| NM_001663 | ARF6 | 3939 | 18 | 6 | 4 | 4 | 6 | 4 | 8 | 6 | 7 | 5 | 3 | 18 | 6 |
| NM_001664 | RHOA | 1921 | 66 | 37 | 66 | 28 | 32 | 27 | 31 | 25 | 57 | 18 | 24 | 55 | 42 |
| NM_001665 | RHOG | 1319 | 3 | 1 | 0 | 0 | 1 | 1 | 0 | 0 | 1 | 0 | 0 | 3 | 0 |
| NM_001666 | ARHGAP4 | 3251 | 2 | 1 | 1 | 1 | 1 | 0 | 0 | 0 | 1 | 0 | 0 | 2 | 0 |
| NM_001667 | ARL2 | 978 | 8 | 4 | 6 | 3 | 4 | 8 | 5 | 1 | 3 | 1 | 4 | 3 | 3 |
| NM_001668 | ARNT | 4824 | 10 | 7 | 6 | 4 | 6 | 4 | 6 | 8 | 8 | 10 | 7 | 9 | 10 |
| NM_001669 | ARSD | 5159 | 9 | 3 | 2 | 3 | 1 | 1 | 9 | 4 | 2 | 3 | 0 | 2 | 2 |
| NM_001670 | ARVCF | 4019 | 0 | 0 | 0 | 0 | 0 | 0 | 0 | 0 | 0 | 0 | 0 | 0 | 0 |
| NM_001671 | ASGR1 | 1503 | 51 | 5 | 1 | 0 | 0 | 0 | 0 | 51 | 1 | 0 | 0 | 1 | 1 |
| NM_001672 | ASIP | 584 | 3 | 0 | 0 | 0 | 3 | 0 | 0 | 0 | 0 | 2 | 0 | 0 | 0 |
| NM_001673 | ASNS | 2067 | 15 | 3 | 1 | 1 | 4 | 15 | 2 | 2 | 2 | 1 | 1 | 4 | 4 |
| NM_001674 | ATF3 | 2048 | 23 | 8 | 17 | 2 | 15 | 5 | 5 | 6 | 23 | 6 | 5 | 3 | 5 |
| NM_001675 | ATF4 | 2022 | 9 | 4 | 9 | 5 | 6 | 5 | 3 | 7 | 4 | 2 | 4 | 4 | 2 |
| NM_001676 | ATP12A | 3565 | 1 | 0 | 0 | 0 | 0 | 0 | 1 | 0 | 0 | 0 | 0 | 0 | 0 |
| NM_001677 | ATP1B1 | 2200 | 573 | 129 | 15 | 36 | 134 | 301 | 573 | 66 | 49 | 37 | 135 | 24 | 50 |
| NM_001678 | ATP1B2 | 3332 | 39 | 4 | 0 | 1 | 1 | 39 | 1 | 0 | 0 | 3 | 0 | 1 | 1 |
| NM_001679 | ATP1B3 | 1853 | 13 | 7 | 6 | 5 | 9 | 10 | 10 | 2 | 13 | 5 | 2 | 10 | 8 |
| NM_001680 | FXYD2 | 571 | 43 | 4 | 0 | 0 | 0 | 0 | 43 | 1 | 0 | 0 | 0 | 0 | 0 |
| NM_001681 | ATP2A2 | 4344 | 184 | 38 | 9 | 11 | 184 | 37 | 10 | 14 | 8 | 8 | 114 | 12 | 13 |
| NM_001682 | ATP2B1 | 6779 | 38 | 12 | 4 | 5 | 6 | 38 | 9 | 4 | 10 | 6 | 9 | 18 | 19 |
| NM_001683 | ATP2B2 | 8827 | 8 | 1 | 0 | 0 | 0 | 8 | 0 | 2 | 0 | 0 | 0 | 0 | 0 |
| NM_001684 | ATP2B4 | 8729 | 25 | 13 | 22 | 19 | 25 | 21 | 4 | 5 | 11 | 12 | 2 | 7 | 17 |
| NM_001685 | ATP5J | 1160 | 239 | 74 | 68 | 31 | 239 | 102 | 57 | 128 | 17 | 33 | 74 | 34 | 28 |
| NM_001686 | ATP5B | 1843 | 65 | 23 | 17 | 15 | 65 | 27 | 37 | 28 | 9 | 8 | 24 | 15 | 11 |
| NM_001687 | ATP5D | 996 | 1 | 0 | 0 | 0 | 0 | 1 | 0 | 0 | 0 | 0 | 1 | 0 | 0 |
| NM_001688 | ATP5F1 | 2101 | 55 | 22 | 11 | 12 | 55 | 22 | 32 | 21 | 11 | 12 | 26 | 19 | 19 |
| NM_001689 | ATP5G3 | 2639 | 8 | 4 | 4 | 3 | 4 | 8 | 6 | 6 | 4 | 1 | 2 | 3 | 1 |
| NM_001690 | ATP6V1A | 4549 | 37 | 12 | 8 | 4 | 4 | 37 | 27 | 11 | 9 | 4 | 2 | 14 | 8 |
| NM_001692 | ATP6V1B1 | 1939 | 3 | 0 | 0 | 0 | 0 | 0 | 3 | 0 | 0 | 0 | 0 | 0 | 0 |
| NM_001693 | ATP6V1B2 | 3044 | 54 | 16 | 14 | 5 | 10 | 54 | 26 | 8 | 17 | 5 | 7 | 20 | 14 |
| NM_001694 | ATP6V0C | 1093 | 48 | 23 | 11 | 7 | 24 | 48 | 45 | 16 | 23 | 15 | 19 | 30 | 17 |
| NM_001695 | ATP6V1C1 | 5704 | 21 | 7 | 5 | 2 | 4 | 21 | 10 | 6 | 5 | 6 | 2 | 9 | 5 |
| NM_001696 | ATP6V1E1 | 1405 | 178 | 55 | 49 | 20 | 69 | 178 | 63 | 32 | 35 | 24 | 37 | 48 | 43 |
| NM_001697 | ATP5O | 806 | 130 | 44 | 31 | 26 | 130 | 54 | 47 | 38 | 19 | 31 | 57 | 30 | 24 |
| NM_001698 | AUH | 1588 | 6 | 2 | 1 | 2 | 2 | 3 | 6 | 1 | 2 | 1 | 1 | 1 | 1 |
| NM_001699 | AXL | 4710 | 5 | 3 | 3 | 2 | 3 | 3 | 1 | 1 | 3 | 2 | 1 | 5 | 4 |
| NM_001700 | AZU1 | 912 | 0 | 0 | 0 | 0 | 0 | 0 | 0 | 0 | 0 | 0 | 0 | 0 | 0 |
| NM_001701 | BAAT | 3478 | 47 | 5 | 0 | 0 | 0 | 0 | 0 | 47 | 0 | 1 | 0 | 1 | 1 |
| NM_001702 | BAI1 | 5528 | 2 | 0 | 0 | 0 | 0 | 2 | 0 | 0 | 0 | 0 | 0 | 0 | 0 |
| NM_001703 | BAI2 | 5415 | 2 | 0 | 0 | 0 | 0 | 2 | 0 | 0 | 0 | 0 | 0 | 0 | 0 |
| NM_001704 | BAI3 | 5637 | 15 | 2 | 0 | 0 | 1 | 15 | 0 | 0 | 1 | 0 | 0 | 1 | 1 |
| NM_001706 | BCL6 | 3529 | 42 | 11 | 13 | 2 | 13 | 10 | 4 | 6 | 10 | 7 | 42 | 11 | 7 |
| NM_001707 | BCL7B | 1667 | 7 | 4 | 5 | 4 | 7 | 6 | 4 | 5 | 2 | 2 | 4 | 5 | 4 |
| NM_001708 | OPN1SW | 1098 | 0 | 0 | 0 | 0 | 0 | 0 | 0 | 0 | 0 | 0 | 0 | 0 | 0 |

| transcript | gene | transcript length (nt) | max | mean | adipose | colon | heart | hypothalamus | kidney | liver | lung | ovary | skeletalmuscle | spleen | testes |
|---|---|---|---|---|---|---|---|---|---|---|---|---|---|---|---|
| NM_001709 | BDNF | 4016 | 4 | 1 | 0 | 0 | 4 | 3 | 0 | 0 | 1 | 1 | 0 | 0 | 0 |
| NM_001710 | CFB | 2627 | 213 | 22 | 1 | 1 | 4 | 1 | 5 | 213 | 3 | 2 | 2 | 2 | 2 |
| NM_001711 | BGN | 2444 | 17 | 6 | 4 | 1 | 17 | 2 | 7 | 4 | 7 | 6 | 1 | 13 | 4 |
| NM_001712 | CEACAM1 | 3497 | 6 | 2 | 1 | 6 | 0 | 0 | 3 | 4 | 1 | 0 | 0 | 1 | 0 |
| NM_001713 | BHMT | 2500 | 117 | 16 | 0 | 0 | 0 | 0 | 117 | 62 | 0 | 0 | 0 | 1 | 0 |
| NM_001714 | BICD1 | 3589 | 17 | 4 | 2 | 1 | 2 | 9 | 3 | 0 | 1 | 17 | 1 | 6 | 3 |
| NM_001715 | BLK | 2590 | 3 | 0 | 0 | 0 | 0 | 0 | 0 | 0 | 0 | 0 | 0 | 3 | 0 |
| NM_001716 | CXCR5 | 2828 | 3 | 0 | 0 | 0 | 0 | 0 | 0 | 0 | 0 | 0 | 0 | 3 | 0 |
| NM_001717 | BNC1 | 4612 | 2 | 0 | 0 | 0 | 0 | 0 | 0 | 0 | 0 | 0 | 0 | 0 | 2 |
| NM_001718 | BMP6 | 3105 | 5 | 1 | 1 | 0 | 1 | 2 | 2 | 1 | 2 | 5 | 1 | 1 | 1 |
| NM_001719 | BMP7 | 4031 | 3 | 1 | 0 | 0 | 1 | 3 | 1 | 0 | 0 | 0 | 0 | 0 | 0 |
| NM_001720 | BMP8B | 3773 | 0 | 0 | 0 | 0 | 0 | 0 | 0 | 0 | 0 | 0 | 0 | 0 | 0 |
| NM_001721 | BMX | 2521 | 1 | 0 | 0 | 0 | 1 | 0 | 1 | 0 | 1 | 0 | 0 | 0 | 1 |
| NM_001722 | POLR3D | 1929 | 3 | 2 | 1 | 2 | 3 | 2 | 1 | 1 | 1 | 2 | 3 | 2 | 2 |
| NM_001723 | DST | 8993 | 18 | 9 | 5 | 4 | 15 | 18 | 5 | 4 | 11 | 10 | 11 | 8 | 4 |
| NM_001724 | BPGM | 1782 | 6 | 2 | 2 | 1 | 2 | 6 | 4 | 2 | 2 | 1 | 1 | 2 | 2 |
| NM_001725 | BPI | 1845 | 3 | 1 | 0 | 0 | 0 | 0 | 2 | 0 | 3 | 0 | 0 | 1 | 1 |
| NM_001726 | BRDT | 3142 | 28 | 3 | 0 | 0 | 0 | 0 | 0 | 0 | 0 | 0 | 0 | 0 | 28 |
| NM_001727 | BRS3 | 1413 | 0 | 0 | 0 | 0 | 0 | 0 | 0 | 0 | 0 | 0 | 0 | 0 | 0 |
| NM_001728 | BSG | 2005 | 7 | 4 | 4 | 6 | 7 | 7 | 5 | 3 | 2 | 2 | 2 | 2 | 4 |
| NM_001729 | BTC | 1323 | 0 | 0 | 0 | 0 | 0 | 0 | 0 | 0 | 0 | 0 | 0 | 0 | 0 |
| NM_001730 | KLF5 | 3350 | 19 | 3 | 0 | 19 | 1 | 2 | 2 | 1 | 6 | 1 | 3 | 0 | 2 |
| NM_001731 | BTG1 | 4678 | 31 | 9 | 9 | 3 | 7 | 2 | 4 | 5 | 15 | 11 | 3 | 31 | 5 |
| NM_001732 | BTN1A1 | 2851 | 0 | 0 | 0 | 0 | 0 | 0 | 0 | 0 | 0 | 0 | 0 | 0 | 0 |
| NM_001733 | C1R | 2192 | 133 | 28 | 17 | 6 | 35 | 4 | 9 | 133 | 18 | 29 | 7 | 28 | 24 |
| NM_001734 | C1S | 2721 | 601 | 131 | 124 | 34 | 105 | 17 | 32 | 601 | 80 | 166 | 38 | 136 | 110 |
| NM_001735 | C5 | 5464 | 66 | 7 | 0 | 0 | 0 | 0 | 1 | 66 | 1 | 1 | 0 | 1 | 1 |
| NM_001736 | C5AR1 | 2341 | 3 | 1 | 2 | 0 | 1 | 1 | 0 | 0 | 3 | 0 | 0 | 3 | 0 |
| NM_001737 | C9 | 2692 | 518 | 48 | 0 | 1 | 1 | 0 | 1 | 518 | 0 | 2 | 0 | 2 | 1 |
| NM_001738 | CA1 | 1263 | 11 | 1 | 0 | 11 | 0 | 0 | 0 | 0 | 1 | 0 | 0 | 0 | 0 |
| NM_001739 | CA5A | 1084 | 12 | 1 | 0 | 0 | 0 | 0 | 0 | 12 | 0 | 0 | 0 | 0 | 0 |
| NM_001740 | CALB2 | 1460 | 9 | 2 | 9 | 0 | 0 | 6 | 0 | 0 | 0 | 1 | 0 | 0 | 1 |
| NM_001741 | CALCA | 774 | 1 | 0 | 0 | 0 | 0 | 0 | 0 | 0 | 0 | 0 | 0 | 1 | 0 |
| NM_001742 | CALCR | 3577 | 0 | 0 | 0 | 0 | 0 | 0 | 0 | 0 | 0 | 0 | 0 | 0 | 0 |
| NM_001743 | CALM2 | 1301 | 440 | 128 | 95 | 56 | 124 | 440 | 90 | 78 | 94 | 86 | 36 | 178 | 137 |
| NM_001744 | CAMK4 | 2106 | 18 | 2 | 0 | 1 | 0 | 18 | 0 | 0 | 2 | 1 | 0 | 2 | 2 |
| NM_001745 | CAMLG | 2199 | 5 | 3 | 2 | 1 | 2 | 4 | 2 | 1 | 3 | 5 | 3 | 2 | 4 |
| NM_001746 | CANX | 4950 | 160 | 70 | 69 | 32 | 81 | 65 | 82 | 160 | 62 | 58 | 22 | 77 | 61 |
| NM_001747 | CAPG | 1442 | 11 | 4 | 11 | 3 | 2 | 1 | 7 | 0 | 9 | 1 | 0 | 4 | 1 |
| NM_001748 | CAPN2 | 3488 | 18 | 9 | 11 | 8 | 8 | 4 | 14 | 3 | 18 | 8 | 6 | 6 | 10 |
| NM_001749 | CAPNS1 | 1478 | 9 | 6 | 8 | 7 | 8 | 6 | 9 | 4 | 6 | 3 | 4 | 8 | 5 |
| NM_001750 | CAST | 4627 | 64 | 24 | 21 | 10 | 64 | 9 | 18 | 15 | 22 | 17 | 49 | 23 | 21 |
| NM_001751 | CARS | 2506 | 5 | 3 | 3 | 2 | 3 | 5 | 2 | 4 | 2 | 1 | 2 | 3 | 3 |
| NM_001752 | CAT | 2299 | 52 | 15 | 52 | 9 | 8 | 6 | 28 | 31 | 10 | 5 | 8 | 11 | 3 |
| NM_001753 | CAV1 | 2704 | 97 | 27 | 97 | 14 | 40 | 3 | 3 | 2 | 59 | 31 | 13 | 24 | 13 |
| NM_001754 | RUNX1 | 5967 | 4 | 2 | 2 | 1 | 2 | 1 | 1 | 0 | 4 | 2 | 2 | 4 | 1 |
| NM_001755 | CBFB | 3133 | 6 | 3 | 3 | 2 | 4 | 4 | 3 | 2 | 4 | 3 | 5 | 6 | 3 |
| NM_001756 | SERPINA6 | 1492 | 40 | 4 | 0 | 0 | 0 | 0 | 4 | 40 | 0 | 0 | 0 | 0 | 0 |
| NM_001757 | CBR1 | 1245 | 41 | 15 | 9 | 11 | 8 | 41 | 25 | 31 | 7 | 6 | 3 | 11 | 8 |
| NM_001759 | CCND2 | 6521 | 30 | 9 | 14 | 11 | 30 | 15 | 3 | 0 | 7 | 6 | 1 | 10 | 7 |
| NM_001760 | CCND3 | 2050 | 5 | 1 | 1 | 1 | 1 | 2 | 1 | 1 | 2 | 1 | 0 | 5 | 1 |
| NM_001761 | CCNF | 4287 | 1 | 0 | 0 | 1 | 0 | 1 | 0 | 0 | 0 | 0 | 0 | 0 | 0 |
| NM_001762 | CCT6A | 2664 | 27 | 18 | 13 | 7 | 21 | 23 | 23 | 18 | 13 | 12 | 19 | 24 | 27 |
| NM_001763 | CD1A | 2095 | 0 | 0 | 0 | 0 | 0 | 0 | 0 | 0 | 0 | 0 | 0 | 0 | 0 |
| NM_001764 | CD1B | 1396 | 0 | 0 | 0 | 0 | 0 | 0 | 0 | 0 | 0 | 0 | 0 | 0 | 0 |
| NM_001765 | CD1C | 2592 | 7 | 1 | 0 | 1 | 0 | 0 | 0 | 0 | 0 | 0 | 0 | 7 | 0 |
| NM_001766 | CD1D | 3783 | 1 | 0 | 0 | 0 | 0 | 0 | 0 | 1 | 0 | 0 | 0 | 1 | 0 |
| NM_001767 | CD2 | 1531 | 5 | 1 | 0 | 1 | 0 | 0 | 0 | 0 | 3 | 0 | 0 | 5 | 0 |
| NM_001768 | CD8A | 2911 | 6 | 1 | 0 | 0 | 0 | 0 | 0 | 0 | 1 | 0 | 0 | 6 | 0 |
| NM_001769 | CD9 | 1231 | 44 | 23 | 23 | 41 | 21 | 29 | 29 | 15 | 44 | 8 | 8 | 21 | 13 |
| NM_001770 | CD19 | 1950 | 1 | 0 | 0 | 0 | 0 | 0 | 0 | 0 | 0 | 0 | 0 | 1 | 0 |
| NM_001771 | CD22 | 3285 | 12 | 2 | 0 | 1 | 0 | 1 | 0 | 0 | 0 | 6 | 0 | 12 | 0 |
| NM_001772 | CD33 | 1466 | 3 | 1 | 1 | 0 | 1 | 1 | 0 | 0 | 0 | 0 | 0 | 3 | 0 |
| NM_001773 | CD34 | 2811 | 5 | 3 | 5 | 2 | 5 | 1 | 3 | 0 | 2 | 2 | 3 | 1 | 3 |
| NM_001774 | CD37 | 1243 | 31 | 5 | 2 | 3 | 2 | 2 | 1 | 1 | 7 | 2 | 1 | 31 | 1 |
| NM_001775 | CD38 | 1491 | 17 | 4 | 2 | 1 | 5 | 6 | 0 | 4 | 5 | 1 | 2 | 17 | 0 |
| NM_001776 | ENTPD1 | 12739 | 4 | 2 | 1 | 1 | 2 | 2 | 2 | 1 | 2 | 1 | 1 | 4 | 1 |
| NM_001777 | CD47 | 5346 | 11 | 5 | 3 | 2 | 4 | 11 | 3 | 3 | 11 | 4 | 2 | 8 | 4 |
| NM_001778 | CD48 | 1070 | 31 | 4 | 2 | 2 | 1 | 0 | 0 | 1 | 8 | 1 | 0 | 31 | 1 |
| NM_001779 | CD58 | 1152 | 1 | 1 | 1 | 0 | 1 | 0 | 1 | 0 | 1 | 0 | 0 | 1 | 1 |
| NM_001780 | CD63 | 1017 | 299 | 127 | 143 | 73 | 299 | 68 | 113 | 71 | 174 | 85 | 88 | 187 | 95 |
| NM_001781 | CD69 | 1674 | 13 | 3 | 2 | 1 | 1 | 2 | 1 | 0 | 13 | 1 | 0 | 12 | 2 |
| NM_001782 | CD72 | 1548 | 26 | 3 | 1 | 1 | 0 | 0 | 0 | 0 | 1 | 0 | 0 | 26 | 0 |
| NM_001783 | CD79A | 1258 | 40 | 4 | 0 | 3 | 0 | 0 | 0 | 0 | 2 | 0 | 0 | 40 | 0 |
| NM_001784 | CD97 | 2954 | 5 | 1 | 1 | 1 | 1 | 0 | 0 | 0 | 2 | 0 | 1 | 5 | 0 |
| NM_001785 | CDA | 959 | 1 | 0 | 0 | 1 | 0 | 0 | 1 | 1 | 1 | 0 | 0 | 1 | 0 |
| NM_001786 | CDK1 | 1907 | 0 | 0 | 0 | 0 | 0 | 0 | 0 | 0 | 0 | 0 | 0 | 0 | 0 |

| | | transcript | | | | | | hypothala | | | | | skeletalm | | |
|---|---|---|---|---|---|---|---|---|---|---|---|---|---|---|---|
| transcript | gene | length (nt) | max | mean | adipose | colon | heart | mus | kidney | liver | lung | ovary | uscle | spleen | testes |
| NM_001788 | 07 Sep | 2519 | 190 | 70 | 75 | 26 | 77 | 190 | 34 | 32 | 51 | 86 | 38 | 87 | 77 |
| NM_001789 | CDC25A | 3706 | 1 | 0 | 0 | 1 | 0 | 0 | 0 | 0 | 0 | 0 | 0 | 0 | 1 |
| NM_001790 | CDC25C | 2115 | 4 | 0 | 0 | 1 | 0 | 0 | 0 | 0 | 0 | 0 | 0 | 0 | 4 |
| NM_001791 | CDC42 | 2168 | 43 | 27 | 32 | 22 | 25 | 33 | 29 | 28 | 33 | 17 | 13 | 43 | 25 |
| NM_001792 | CDH2 | 4367 | 37 | 8 | 0 | 0 | 37 | 9 | 4 | 8 | 0 | 3 | 0 | 3 | 18 |
| NM_001793 | CDH3 | 4276 | 6 | 1 | 0 | 0 | 0 | 1 | 1 | 0 | 1 | 6 | 0 | 3 | 0 |
| NM_001794 | CDH4 | 3060 | 1 | 0 | 0 | 0 | 0 | 1 | 0 | 0 | 0 | 0 | 0 | 1 | 0 |
| NM_001795 | CDH5 | 4128 | 9 | 3 | 5 | 1 | 4 | 2 | 1 | 1 | 9 | 1 | 2 | 4 | 1 |
| NM_001796 | CDH8 | 2929 | 9 | 1 | 0 | 0 | 0 | 9 | 0 | 0 | 0 | 0 | 0 | 0 | 0 |
| NM_001797 | CDH11 | 3648 | 61 | 12 | 5 | 2 | 5 | 7 | 3 | 0 | 8 | 61 | 1 | 32 | 5 |
| NM_001798 | CDK2 | 2293 | 4 | 2 | 2 | 3 | 2 | 1 | 1 | 3 | 4 | 2 | 1 | 4 | 3 |
| NM_001799 | CDK7 | 1517 | 2 | 1 | 1 | 0 | 1 | 1 | 2 | 1 | 1 | 1 | 1 | 2 | 2 |
| NM_001800 | CDKN2D | 1399 | 4 | 1 | 0 | 0 | 1 | 4 | 0 | 0 | 1 | 0 | 0 | 2 | 1 |
| NM_001801 | CDO1 | 1614 | 22 | 4 | 10 | 1 | 2 | 5 | 1 | 22 | 1 | 2 | 1 | 0 | 3 |
| NM_001802 | CDR2 | 2702 | 11 | 5 | 4 | 4 | 3 | 6 | 6 | 3 | 8 | 3 | 1 | 7 | 11 |
| NM_001803 | CD52 | 505 | 110 | 22 | 47 | 5 | 2 | 1 | 1 | 2 | 24 | 1 | 1 | 110 | 44 |
| NM_001804 | CDX1 | 1772 | 2 | 0 | 0 | 2 | 0 | 0 | 0 | 0 | 0 | 0 | 0 | 0 | 0 |
| NM_001805 | CEBPE | 1201 | 0 | 0 | 0 | 0 | 0 | 0 | 0 | 0 | 0 | 0 | 0 | 0 | 0 |
| NM_001806 | CEBPG | 3736 | 11 | 6 | 6 | 5 | 7 | 8 | 4 | 10 | 5 | 2 | 11 | 5 | 5 |
| NM_001807 | CEL | 2384 | 0 | 0 | 0 | 0 | 0 | 0 | 0 | 0 | 0 | 0 | 0 | 0 | 0 |
| NM_001809 | CENPA | 1429 | 2 | 0 | 0 | 2 | 0 | 0 | 0 | 0 | 0 | 0 | 0 | 1 | 0 |
| NM_001810 | CENPB | 2840 | 6 | 4 | 6 | 4 | 4 | 5 | 5 | 4 | 5 | 2 | 3 | 5 | 3 |
| NM_001812 | CENPC1 | 3349 | 32 | 11 | 5 | 2 | 8 | 8 | 10 | 4 | 10 | 32 | 7 | 21 | 20 |
| NM_001813 | CENPE | 8612 | 3 | 1 | 1 | 1 | 1 | 1 | 0 | 0 | 1 | 1 | 0 | 2 | 3 |
| NM_001814 | CTSC | 1907 | 48 | 16 | 19 | 9 | 16 | 9 | 26 | 8 | 24 | 3 | 3 | 48 | 12 |
| NM_001815 | CEACAM3 | 1151 | 0 | 0 | 0 | 0 | 0 | 0 | 0 | 0 | 0 | 0 | 0 | 0 | 0 |
| NM_001816 | CEACAM8 | 2303 | 2 | 0 | 0 | 0 | 0 | 0 | 0 | 0 | 2 | 0 | 0 | 0 | 0 |
| NM_001817 | CEACAM4 | 1205 | 1 | 0 | 0 | 0 | 0 | 0 | 0 | 0 | 0 | 0 | 0 | 1 | 0 |
| NM_001818 | AKR1C4 | 1194 | 36 | 3 | 0 | 0 | 0 | 0 | 0 | 36 | 0 | 0 | 0 | 0 | 0 |
| NM_001819 | CHGB | 2648 | 177 | 23 | 0 | 7 | 22 | 177 | 27 | 0 | 0 | 12 | 1 | 0 | 9 |
| NM_001821 | CHML | 7066 | 4 | 2 | 1 | 1 | 1 | 2 | 1 | 1 | 1 | 3 | 1 | 2 | 4 |
| NM_001822 | CHN1 | 2712 | 59 | 7 | 1 | 1 | 3 | 59 | 1 | 0 | 2 | 1 | 0 | 1 | 4 |
| NM_001823 | CKB | 1431 | 18 | 5 | 1 | 15 | 7 | 18 | 6 | 0 | 1 | 1 | 1 | 0 | 3 |
| NM_001824 | CKM | 1557 | 343 | 36 | 0 | 0 | 58 | 0 | 0 | 0 | 0 | 0 | 343 | 0 | 0 |
| NM_001825 | CKMT2 | 1642 | 38 | 5 | 1 | 0 | 38 | 0 | 1 | 0 | 1 | 1 | 11 | 1 | 1 |
| NM_001826 | CKS1B | 815 | 7 | 3 | 7 | 6 | 1 | 3 | 2 | 5 | 3 | 2 | 1 | 3 | 3 |
| NM_001827 | CKS2 | 613 | 26 | 6 | 4 | 9 | 2 | 2 | 5 | 5 | 7 | 2 | 1 | 7 | 26 |
| NM_001828 | CLC | 630 | 1 | 0 | 0 | 0 | 0 | 0 | 0 | 0 | 1 | 0 | 0 | 0 | 0 |
| NM_001829 | CLCN3 | 3975 | 34 | 13 | 10 | 10 | 12 | 34 | 13 | 10 | 7 | 10 | 9 | 11 | 22 |
| NM_001830 | CLCN4 | 6788 | 8 | 2 | 0 | 0 | 2 | 8 | 1 | 1 | 1 | 0 | 3 | 1 | 1 |
| NM_001831 | CLU | 2842 | 201 | 60 | 83 | 12 | 15 | 201 | 24 | 172 | 15 | 13 | 7 | 10 | 106 |
| NM_001832 | CLPS | 539 | 0 | 0 | 0 | 0 | 0 | 0 | 0 | 0 | 0 | 0 | 0 | 0 | 0 |
| NM_001833 | CLTA | 1104 | 15 | 8 | 10 | 7 | 6 | 15 | 10 | 9 | 7 | 7 | 3 | 10 | 7 |
| NM_001834 | CLTB | 1131 | 21 | 11 | 11 | 16 | 21 | 19 | 9 | 9 | 7 | 5 | 12 | 7 | 6 |
| NM_001835 | CLTCL1 | 5341 | 16 | 2 | 1 | 0 | 1 | 1 | 0 | 0 | 0 | 0 | 16 | 0 | 2 |
| NM_001836 | CMA1 | 784 | 1 | 0 | 1 | 0 | 0 | 0 | 0 | 0 | 0 | 0 | 0 | 0 | 0 |
| NM_001837 | CCR3 | 1795 | 0 | 0 | 0 | 0 | 0 | 0 | 0 | 0 | 0 | 0 | 0 | 0 | 0 |
| NM_001838 | CCR7 | 2172 | 2 | 0 | 0 | 0 | 0 | 0 | 0 | 0 | 0 | 0 | 0 | 2 | 0 |
| NM_001839 | CNN3 | 2066 | 43 | 27 | 22 | 10 | 30 | 43 | 42 | 35 | 36 | 27 | 5 | 20 | 22 |
| NM_001841 | CNR2 | 1774 | 1 | 0 | 0 | 0 | 0 | 0 | 0 | 0 | 0 | 0 | 0 | 1 | 0 |
| NM_001842 | CNTFR | 1927 | 1 | 1 | 1 | 0 | 0 | 1 | 0 | 0 | 0 | 0 | 1 | 1 | 0 |
| NM_001843 | CNTN1 | 3427 | 64 | 8 | 0 | 1 | 1 | 64 | 3 | 0 | 1 | 10 | 0 | 1 | 4 |
| NM_001844 | COL2A1 | 5087 | 0 | 0 | 0 | 0 | 0 | 0 | 0 | 0 | 0 | 0 | 0 | 0 | 0 |
| NM_001845 | COL4A1 | 6532 | 26 | 8 | 18 | 5 | 15 | 2 | 6 | 1 | 9 | 5 | 2 | 26 | 2 |
| NM_001846 | COL4A2 | 6263 | 68 | 22 | 33 | 18 | 36 | 4 | 16 | 2 | 24 | 22 | 6 | 68 | 9 |
| NM_001847 | COL4A6 | 6572 | 1 | 0 | 0 | 0 | 0 | 0 | 0 | 0 | 0 | 1 | 0 | 0 | 0 |
| NM_001848 | COL6A1 | 4225 | 23 | 9 | 23 | 8 | 13 | 1 | 3 | 1 | 8 | 22 | 3 | 9 | 6 |
| NM_001849 | COL6A2 | 3439 | 12 | 4 | 12 | 7 | 5 | 1 | 1 | 0 | 3 | 5 | 1 | 3 | 3 |
| NM_001850 | COL8A1 | 2658 | 4 | 1 | 2 | 0 | 4 | 0 | 1 | 0 | 3 | 1 | 0 | 0 | 1 |
| NM_001851 | COL9A1 | 3782 | 1 | 0 | 0 | 0 | 0 | 1 | 0 | 0 | 0 | 0 | 0 | 0 | 0 |
| NM_001852 | COL9A2 | 2831 | 0 | 0 | 0 | 0 | 0 | 0 | 0 | 0 | 0 | 0 | 0 | 0 | 0 |
| NM_001853 | COL9A3 | 2485 | 2 | 0 | 0 | 0 | 0 | 2 | 0 | 0 | 0 | 0 | 0 | 0 | 0 |
| NM_001854 | COL11A1 | 7291 | 1 | 0 | 0 | 0 | 0 | 1 | 0 | 0 | 0 | 1 | 0 | 1 | 0 |
| NM_001855 | COL15A1 | 5273 | 17 | 4 | 17 | 4 | 7 | 0 | 2 | 0 | 1 | 4 | 1 | 1 | 6 |
| NM_001856 | COL16A1 | 5584 | 4 | 1 | 1 | 1 | 1 | 1 | 0 | 0 | 1 | 4 | 0 | 0 | 1 |
| NM_001858 | COL19A1 | 8725 | 0 | 0 | 0 | 0 | 0 | 0 | 0 | 0 | 0 | 0 | 0 | 0 | 0 |
| NM_001859 | SLC31A1 | 4777 | 9 | 3 | 2 | 1 | 2 | 2 | 4 | 9 | 2 | 1 | 0 | 3 | 2 |
| NM_001860 | SLC31A2 | 1774 | 6 | 2 | 2 | 1 | 1 | 1 | 2 | 3 | 2 | 0 | 0 | 6 | 1 |
| NM_001861 | COX4I1 | 801 | 101 | 46 | 43 | 51 | 101 | 64 | 44 | 44 | 22 | 27 | 48 | 41 | 17 |
| NM_001862 | COX5B | 505 | 67 | 35 | 38 | 45 | 54 | 67 | 40 | 58 | 14 | 6 | 29 | 20 | 15 |
| NM_001863 | COX6B1 | 558 | 40 | 21 | 22 | 23 | 40 | 33 | 21 | 26 | 11 | 8 | 24 | 14 | 7 |
| NM_001864 | COX7A1 | 762 | 4 | 1 | 1 | 0 | 4 | 0 | 0 | 0 | 0 | 0 | 4 | 0 | 0 |
| NM_001865 | COX7A2 | 700 | 16 | 8 | 7 | 6 | 11 | 16 | 10 | 14 | 4 | 2 | 2 | 5 | 9 |
| NM_001866 | COX7B | 456 | 64 | 19 | 15 | 11 | 64 | 21 | 31 | 27 | 7 | 5 | 20 | 9 | 3 |
| NM_001867 | COX7C | 407 | 413 | 140 | 64 | 107 | 413 | 169 | 187 | 127 | 69 | 93 | 140 | 111 | 60 |
| NM_001868 | CPA1 | 1428 | 3 | 0 | 3 | 1 | 0 | 0 | 0 | 0 | 0 | 0 | 0 | 0 | 0 |
| NM_001869 | CPA2 | 1327 | 1 | 0 | 1 | 0 | 0 | 0 | 0 | 0 | 0 | 0 | 0 | 0 | 0 |

| transcript | gene | transcript length (nt) | max | mean | adipose | colon | heart | hypothalamus | kidney | liver | lung | ovary | skeletalmuscle | spleen | testes |
|---|---|---|---|---|---|---|---|---|---|---|---|---|---|---|---|
| NM_001870 | CPA3 | 1682 | 6 | 2 | 4 | 2 | 1 | 0 | 0 | 0 | 6 | 1 | 0 | 0 | 3 |
| NM_001871 | CPB1 | 1460 | 2 | 0 | 2 | 0 | 0 | 0 | 0 | 0 | 0 | 0 | 0 | 2 | 0 |
| NM_001872 | CPB2 | 1766 | 149 | 14 | 0 | 0 | 1 | 0 | 0 | 149 | 1 | 1 | 1 | 2 | 1 |
| NM_001873 | CPE | 2428 | 244 | 37 | 23 | 8 | 18 | 244 | 9 | 2 | 9 | 11 | 6 | 1 | 78 |
| NM_001874 | CPM | 6666 | 11 | 5 | 11 | 1 | 2 | 1 | 7 | 4 | 10 | 3 | 1 | 9 | 2 |
| NM_001875 | CPS1 | 5757 | 209 | 20 | 1 | 1 | 1 | 1 | 1 | 209 | 0 | 2 | 1 | 1 | 1 |
| NM_001876 | CPT1A | 5253 | 6 | 3 | 2 | 4 | 3 | 2 | 6 | 3 | 2 | 3 | 1 | 4 | 2 |
| NM_001877 | CR2 | 3991 | 5 | 1 | 0 | 0 | 0 | 0 | 0 | 0 | 0 | 0 | 0 | 5 | 0 |
| NM_001878 | CRABP2 | 966 | 12 | 2 | 12 | 3 | 1 | 6 | 1 | 0 | 0 | 2 | 1 | 1 | 1 |
| NM_001879 | MASP1 | 4353 | 6 | 1 | 0 | 1 | 2 | 1 | 1 | 6 | 0 | 0 | 0 | 0 | 0 |
| NM_001880 | ATF2 | 2111 | 7 | 5 | 4 | 2 | 4 | 7 | 5 | 4 | 4 | 7 | 3 | 6 | 6 |
| NM_001881 | CREM | 1257 | 4 | 1 | 1 | 0 | 1 | 1 | 1 | 1 | 1 | 1 | 1 | 1 | 4 |
| NM_001882 | CRHBP | 1838 | 6 | 1 | 1 | 0 | 1 | 2 | 3 | 6 | 0 | 0 | 0 | 2 | 0 |
| NM_001883 | CRHR2 | 1693 | 0 | 0 | 0 | 0 | 0 | 0 | 0 | 0 | 0 | 0 | 0 | 0 | 0 |
| NM_001884 | HAPLN1 | 4678 | 0 | 0 | 0 | 0 | 0 | 0 | 0 | 0 | 0 | 0 | 0 | 0 | 0 |
| NM_001885 | CRYAB | 691 | 453 | 84 | 86 | 7 | 453 | 62 | 141 | 0 | 4 | 2 | 158 | 7 | 5 |
| NM_001886 | CRYBA4 | 811 | 0 | 0 | 0 | 0 | 0 | 0 | 0 | 0 | 0 | 0 | 0 | 0 | 0 |
| NM_001887 | CRYBB1 | 921 | 0 | 0 | 0 | 0 | 0 | 0 | 0 | 0 | 0 | 0 | 0 | 0 | 0 |
| NM_001888 | CRYM | 1290 | 10 | 2 | 0 | 0 | 4 | 10 | 3 | 0 | 0 | 0 | 0 | 0 | 0 |
| NM_001889 | CRYZ | 2264 | 27 | 5 | 1 | 1 | 4 | 2 | 27 | 12 | 3 | 3 | 1 | 3 | 4 |
| NM_001890 | CSN1S1 | 981 | 2 | 0 | 2 | 0 | 0 | 0 | 0 | 0 | 0 | 0 | 0 | 0 | 0 |
| NM_001891 | CSN2 | 1067 | 0 | 0 | 0 | 0 | 0 | 0 | 0 | 0 | 0 | 0 | 0 | 0 | 0 |
| NM_001892 | CSNK1A1 | 3065 | 44 | 26 | 18 | 12 | 44 | 23 | 20 | 20 | 29 | 28 | 24 | 36 | 29 |
| NM_001893 | CSNK1D | 1981 | 8 | 5 | 4 | 5 | 4 | 8 | 5 | 2 | 4 | 4 | 3 | 7 | 5 |
| NM_001894 | CSNK1E | 2642 | 2 | 1 | 1 | 1 | 1 | 2 | 1 | 0 | 1 | 1 | 1 | 1 | 1 |
| NM_001895 | CSNK2A1 | 2719 | 3 | 2 | 2 | 2 | 3 | 3 | 3 | 1 | 2 | 2 | 2 | 3 | 3 |
| NM_001896 | CSNK2A2 | 1658 | 9 | 4 | 3 | 2 | 4 | 3 | 3 | 3 | 3 | 7 | 4 | 5 | 9 |
| NM_001897 | CSPG4 | 8290 | 1 | 0 | 1 | 1 | 0 | 0 | 0 | 0 | 0 | 0 | 1 | 0 | 0 |
| NM_001898 | CST1 | 760 | 0 | 0 | 0 | 0 | 0 | 0 | 0 | 0 | 0 | 0 | 0 | 0 | 0 |
| NM_001899 | CST4 | 736 | 0 | 0 | 0 | 0 | 0 | 0 | 0 | 0 | 0 | 0 | 0 | 0 | 0 |
| NM_001900 | CST5 | 749 | 0 | 0 | 0 | 0 | 0 | 0 | 0 | 0 | 0 | 0 | 0 | 0 | 0 |
| NM_001901 | CTGF | 2343 | 110 | 24 | 6 | 3 | 45 | 13 | 23 | 6 | 15 | 24 | 5 | 110 | 9 |
| NM_001902 | CTH | 1805 | 12 | 2 | 0 | 0 | 0 | 0 | 1 | 12 | 0 | 4 | 0 | 0 | 1 |
| NM_001903 | CTNNA1 | 3761 | 44 | 24 | 24 | 16 | 44 | 20 | 38 | 18 | 32 | 21 | 12 | 27 | 19 |
| NM_001904 | CTNNB1 | 3720 | 28 | 20 | 15 | 28 | 20 | 28 | 18 | 16 | 14 | 28 | 8 | 22 | 19 |
| NM_001905 | CTPS | 3199 | 10 | 3 | 1 | 2 | 3 | 4 | 2 | 10 | 1 | 1 | 2 | 3 | 4 |
| NM_001906 | CTRB1 | 872 | 0 | 0 | 0 | 0 | 0 | 0 | 0 | 0 | 0 | 0 | 0 | 0 | 0 |
| NM_001907 | CTRL | 1169 | 0 | 0 | 0 | 0 | 0 | 0 | 0 | 0 | 0 | 0 | 0 | 0 | 0 |
| NM_001908 | CTSB | 3782 | 160 | 75 | 160 | 19 | 116 | 38 | 120 | 65 | 94 | 20 | 21 | 151 | 20 |
| NM_001909 | CTSD | 2120 | 28 | 14 | 28 | 7 | 12 | 6 | 23 | 17 | 22 | 4 | 8 | 20 | 7 |
| NM_001910 | CTSE | 2228 | 12 | 1 | 0 | 0 | 0 | 0 | 0 | 0 | 12 | 0 | 0 | 0 | 0 |
| NM_001911 | CTSG | 924 | 3 | 1 | 3 | 1 | 0 | 0 | 0 | 0 | 2 | 0 | 0 | 0 | 0 |
| NM_001912 | CTSL1 | 1712 | 86 | 38 | 63 | 12 | 40 | 21 | 46 | 69 | 43 | 15 | 7 | 86 | 16 |
| NM_001913 | CUX1 | 2924 | 11 | 7 | 6 | 8 | 11 | 7 | 10 | 5 | 6 | 8 | 6 | 5 | 4 |
| NM_001914 | CYB5A | 832 | 76 | 14 | 13 | 7 | 4 | 3 | 26 | 76 | 12 | 4 | 1 | 4 | 4 |
| NM_001915 | CYB561 | 2984 | 2 | 1 | 1 | 1 | 1 | 2 | 2 | 1 | 1 | 0 | 0 | 0 | 0 |
| NM_001916 | CYC1 | 1210 | 6 | 3 | 1 | 4 | 6 | 3 | 3 | 4 | 1 | 1 | 4 | 1 | 1 |
| NM_001917 | DAO | 1596 | 5 | 1 | 0 | 0 | 0 | 1 | 5 | 5 | 0 | 0 | 0 | 0 | 0 |
| NM_001918 | DBT | 10815 | 3 | 2 | 1 | 1 | 3 | 2 | 3 | 3 | 1 | 2 | 2 | 1 | 2 |
| NM_001919 | DCI | 1027 | 6 | 2 | 1 | 1 | 3 | 2 | 3 | 6 | 1 | 1 | 3 | 1 | 0 |
| NM_001920 | DCN | 2288 | 60 | 23 | 39 | 8 | 42 | 5 | 11 | 6 | 36 | 60 | 14 | 1 | 32 |
| NM_001921 | DCTD | 2019 | 11 | 6 | 6 | 4 | 5 | 5 | 7 | 11 | 5 | 6 | 3 | 8 | 5 |
| NM_001922 | DCT | 2298 | 0 | 0 | 0 | 0 | 0 | 0 | 0 | 0 | 0 | 0 | 0 | 0 | 0 |
| NM_001923 | DDB1 | 4338 | 11 | 8 | 6 | 5 | 6 | 11 | 9 | 8 | 5 | 7 | 8 | 7 | 10 |
| NM_001924 | GADD45A | 1355 | 81 | 29 | 20 | 6 | 25 | 35 | 81 | 24 | 26 | 14 | 51 | 21 | 23 |
| NM_001925 | DEFA4 | 542 | 4 | 0 | 0 | 0 | 0 | 0 | 0 | 0 | 4 | 0 | 0 | 0 | 0 |
| NM_001926 | DEFA6 | 465 | 22 | 2 | 0 | 22 | 0 | 0 | 0 | 0 | 0 | 0 | 0 | 0 | 0 |
| NM_001927 | DES | 2248 | 1,676 | 306 | 9 | 293 | 1,676 | 5 | 6 | 0 | 34 | 11 | 1,260 | 7 | 68 |
| NM_001928 | CFD | 1159 | 4 | 1 | 4 | 1 | 2 | 0 | 0 | 0 | 1 | 0 | 0 | 0 | 1 |
| NM_001930 | DHPS | 1325 | 2 | 1 | 1 | 1 | 1 | 2 | 1 | 1 | 1 | 1 | 2 | 2 | 1 |
| NM_001931 | DLAT | 4344 | 20 | 8 | 8 | 4 | 20 | 10 | 9 | 6 | 4 | 4 | 10 | 6 | 5 |
| NM_001932 | MPP3 | 2826 | 2 | 0 | 0 | 0 | 2 | 1 | 0 | 0 | 1 | 0 | 0 | 0 | 1 |
| NM_001933 | DLST | 2842 | 9 | 5 | 5 | 4 | 8 | 4 | 9 | 7 | 4 | 4 | 5 | 5 | 4 |
| NM_001934 | DLX4 | 1391 | 0 | 0 | 0 | 0 | 0 | 0 | 0 | 0 | 0 | 0 | 0 | 0 | 0 |
| NM_001935 | DPP4 | 3900 | 17 | 3 | 2 | 1 | 1 | 0 | 17 | 2 | 4 | 2 | 1 | 3 | 1 |
| NM_001936 | DPP6 | 4450 | 18 | 2 | 0 | 1 | 0 | 18 | 1 | 0 | 0 | 0 | 0 | 0 | 3 |
| NM_001937 | DPT | 1703 | 131 | 17 | 131 | 23 | 14 | 0 | 2 | 1 | 4 | 2 | 4 | 0 | 11 |
| NM_001938 | DR1 | 3208 | 9 | 6 | 3 | 3 | 6 | 6 | 6 | 4 | 5 | 6 | 5 | 9 | 9 |
| NM_001939 | DRP2 | 7118 | 1 | 0 | 0 | 0 | 0 | 1 | 0 | 0 | 0 | 0 | 0 | 0 | 0 |
| NM_001940 | ATN1 | 4344 | 15 | 6 | 5 | 6 | 8 | 9 | 5 | 1 | 5 | 15 | 5 | 8 | 4 |
| NM_001941 | DSC3 | 6935 | 1 | 0 | 0 | 0 | 0 | 0 | 0 | 0 | 0 | 0 | 0 | 0 | 1 |
| NM_001942 | DSG1 | 5446 | 0 | 0 | 0 | 0 | 0 | 0 | 0 | 0 | 0 | 0 | 0 | 0 | 0 |
| NM_001943 | DSG2 | 5653 | 16 | 6 | 6 | 12 | 11 | 1 | 16 | 5 | 5 | 9 | 1 | 0 | 2 |
| NM_001944 | DSG3 | 5551 | 0 | 0 | 0 | 0 | 0 | 0 | 0 | 0 | 0 | 0 | 0 | 0 | 0 |
| NM_001945 | HBEGF | 2358 | 3 | 1 | 1 | 0 | 2 | 1 | 1 | 0 | 3 | 0 | 1 | 0 | 1 |
| NM_001946 | DUSP6 | 2820 | 14 | 6 | 9 | 3 | 5 | 4 | 5 | 7 | 10 | 4 | 1 | 14 | 2 |
| NM_001947 | DUSP7 | 3231 | 4 | 2 | 1 | 1 | 2 | 2 | 1 | 0 | 2 | 1 | 2 | 4 | 2 |

| transcript | gene | transcript length (nt) | max | mean | adipose | colon | heart | hypothalamus | kidney | liver | lung | ovary | skeletalmuscle | spleen | testes |
|---|---|---|---|---|---|---|---|---|---|---|---|---|---|---|---|
| NM_001948 | DUT | 1874 | 2 | 1 | 1 | 1 | 2 | 1 | 2 | 0 | 2 | 1 | 1 | 2 | 2 |
| NM_001949 | E2F3 | 5009 | 2 | 1 | 1 | 1 | 1 | 1 | 1 | 1 | 1 | 0 | 0 | 2 | 1 |
| NM_001950 | E2F4 | 2100 | 3 | 1 | 1 | 1 | 1 | 1 | 2 | 1 | 1 | 1 | 1 | 3 | 2 |
| NM_001951 | E2F5 | 1735 | 2 | 1 | 0 | 0 | 0 | 0 | 1 | 0 | 0 | 0 | 0 | 2 | 1 |
| NM_001953 | TYMP | 1658 | 3 | 1 | 2 | 0 | 1 | 1 | 0 | 2 | 1 | 0 | 0 | 3 | 0 |
| NM_001954 | DDR1 | 3824 | 14 | 3 | 1 | 5 | 2 | 14 | 6 | 0 | 3 | 1 | 2 | 1 | 3 |
| NM_001955 | EDN1 | 2099 | 3 | 1 | 0 | 0 | 1 | 2 | 1 | 0 | 3 | 0 | 0 | 1 | 1 |
| NM_001956 | EDN2 | 1240 | 0 | 0 | 0 | 0 | 0 | 0 | 0 | 0 | 0 | 0 | 0 | 0 | 0 |
| NM_001957 | EDNRA | 4150 | 7 | 2 | 1 | 0 | 3 | 0 | 1 | 0 | 2 | 7 | 1 | 3 | 3 |
| NM_001958 | EEF1A2 | 1843 | 48 | 9 | 0 | 0 | 26 | 19 | 0 | 1 | 0 | 0 | 48 | 0 | 0 |
| NM_001959 | EEF1B2 | 1110 | 87 | 50 | 30 | 24 | 53 | 33 | 43 | 62 | 46 | 64 | 72 | 87 | 33 |
| NM_001960 | EEF1D | 1245 | 43 | 27 | 19 | 29 | 34 | 10 | 20 | 31 | 27 | 38 | 30 | 43 | 17 |
| NM_001961 | EEF2 | 3158 | 80 | 49 | 38 | 35 | 59 | 33 | 38 | 53 | 37 | 78 | 80 | 53 | 34 |
| NM_001962 | EFNA5 | 5335 | 1 | 1 | 1 | 0 | 1 | 1 | 1 | 0 | 0 | 1 | 0 | 1 | 0 |
| NM_001963 | EGF | 4886 | 24 | 3 | 0 | 0 | 1 | 0 | 24 | 0 | 0 | 0 | 6 | 0 | 0 |
| NM_001964 | EGR1 | 3136 | 115 | 17 | 2 | 3 | 6 | 3 | 15 | 15 | 12 | 115 | 5 | 3 | 3 |
| NM_001965 | EGR4 | 2372 | 0 | 0 | 0 | 0 | 0 | 0 | 0 | 0 | 0 | 0 | 0 | 0 | 0 |
| NM_001966 | EHHADH | 3848 | 27 | 6 | 4 | 1 | 2 | 1 | 23 | 27 | 1 | 1 | 0 | 1 | 1 |
| NM_001967 | EIF4A2 | 1890 | 384 | 181 | 105 | 72 | 192 | 384 | 110 | 56 | 145 | 268 | 292 | 183 | 182 |
| NM_001968 | EIF4E | 4749 | 5 | 3 | 2 | 2 | 3 | 5 | 4 | 2 | 3 | 2 | 2 | 4 | 4 |
| NM_001969 | EIF5 | 5790 | 47 | 17 | 16 | 7 | 17 | 22 | 16 | 47 | 8 | 7 | 8 | 18 | 16 |
| NM_001970 | EIF5A | 1353 | 7 | 3 | 2 | 2 | 2 | 3 | 3 | 7 | 2 | 1 | 2 | 3 | 2 |
| NM_001971 | CELA1 | 952 | 0 | 0 | 0 | 0 | 0 | 0 | 0 | 0 | 0 | 0 | 0 | 0 | 0 |
| NM_001972 | ELANE | 924 | 1 | 0 | 0 | 0 | 0 | 0 | 0 | 0 | 1 | 0 | 0 | 0 | 0 |
| NM_001973 | ELK4 | 2985 | 6 | 3 | 2 | 2 | 2 | 2 | 3 | 2 | 3 | 3 | 2 | 6 | 2 |
| NM_001974 | EMR1 | 3123 | 4 | 0 | 0 | 0 | 0 | 0 | 0 | 0 | 0 | 0 | 0 | 4 | 0 |
| NM_001975 | ENO2 | 2423 | 42 | 5 | 0 | 1 | 1 | 42 | 0 | 0 | 1 | 1 | 0 | 1 | 1 |
| NM_001976 | ENO3 | 1523 | 309 | 33 | 1 | 1 | 43 | 0 | 1 | 8 | 0 | 0 | 309 | 1 | 2 |
| NM_001977 | ENPEP | 4995 | 59 | 8 | 2 | 1 | 3 | 1 | 59 | 5 | 4 | 1 | 2 | 14 | 1 |
| NM_001978 | EPB49 | 2773 | 10 | 2 | 2 | 1 | 3 | 10 | 4 | 1 | 2 | 0 | 1 | 1 | 1 |
| NM_001979 | EPHX2 | 2196 | 10 | 2 | 1 | 2 | 1 | 1 | 10 | 6 | 1 | 1 | 0 | 1 | 1 |
| NM_001980 | STX2 | 3329 | 5 | 3 | 4 | 2 | 4 | 3 | 2 | 2 | 3 | 3 | 1 | 3 | 5 |
| NM_001981 | EPS15 | 5220 | 20 | 11 | 12 | 4 | 10 | 20 | 8 | 6 | 11 | 10 | 10 | 15 | 14 |
| NM_001982 | ERBB3 | 5511 | 7 | 2 | 0 | 7 | 0 | 2 | 5 | 5 | 3 | 0 | 0 | 0 | 0 |
| NM_001983 | ERCC1 | 3382 | 1 | 1 | 1 | 0 | 1 | 1 | 0 | 0 | 0 | 1 | 1 | 1 | 1 |
| NM_001984 | ESD | 1192 | 18 | 11 | 10 | 7 | 11 | 12 | 17 | 18 | 8 | 10 | 7 | 10 | 11 |
| NM_001985 | ETFB | 916 | 15 | 6 | 10 | 4 | 11 | 5 | 7 | 15 | 2 | 3 | 5 | 4 | 4 |
| NM_001986 | ETV4 | 2259 | 0 | 0 | 0 | 0 | 0 | 0 | 0 | 0 | 0 | 0 | 0 | 0 | 0 |
| NM_001987 | ETV6 | 5978 | 4 | 2 | 1 | 1 | 1 | 1 | 1 | 1 | 2 | 1 | 1 | 4 | 1 |
| NM_001988 | EVPL | 6587 | 2 | 1 | 0 | 2 | 0 | 0 | 2 | 0 | 2 | 0 | 0 | 0 | 0 |
| NM_001989 | EVX1 | 1858 | 0 | 0 | 0 | 0 | 0 | 0 | 0 | 0 | 0 | 0 | 0 | 0 | 0 |
| NM_001990 | EYA3 | 2045 | 9 | 5 | 3 | 3 | 4 | 4 | 6 | 2 | 3 | 6 | 4 | 6 | 9 |
| NM_001991 | EZH1 | 4646 | 6 | 4 | 5 | 2 | 3 | 5 | 4 | 1 | 3 | 6 | 4 | 5 | 4 |
| NM_001992 | F2R | 3810 | 9 | 2 | 1 | 0 | 2 | 0 | 2 | 1 | 2 | 1 | 0 | 9 | 1 |
| NM_001993 | F3 | 2351 | 30 | 8 | 10 | 3 | 5 | 30 | 2 | 0 | 13 | 9 | 1 | 3 | 10 |
| NM_001994 | F13B | 2217 | 13 | 1 | 0 | 0 | 0 | 0 | 0 | 13 | 0 | 0 | 0 | 0 | 0 |
| NM_001995 | ACSL1 | 3805 | 212 | 51 | 124 | 6 | 36 | 14 | 49 | 212 | 26 | 4 | 44 | 36 | 10 |
| NM_001996 | FBLN1 | 2295 | 4 | 2 | 2 | 4 | 3 | 2 | 1 | 0 | 4 | 2 | 1 | 1 | 2 |
| NM_001997 | FAU | 560 | 8 | 5 | 5 | 5 | 5 | 4 | 5 | 8 | 7 | 3 | 4 | 7 | 4 |
| NM_001998 | FBLN2 | 4184 | 7 | 1 | 7 | 1 | 2 | 1 | 0 | 0 | 1 | 0 | 1 | 0 | 2 |
| NM_001999 | FBN2 | 10724 | 1 | 0 | 0 | 0 | 1 | 0 | 0 | 0 | 0 | 0 | 0 | 0 | 1 |
| NM_002000 | FCAR | 1671 | 7 | 2 | 1 | 0 | 1 | 1 | 0 | 0 | 7 | 0 | 0 | 6 | 0 |
| NM_002001 | FCER1A | 1165 | 0 | 0 | 0 | 0 | 0 | 0 | 0 | 0 | 0 | 0 | 0 | 0 | 0 |
| NM_002002 | FCER2 | 1614 | 5 | 1 | 1 | 0 | 0 | 0 | 0 | 0 | 0 | 0 | 0 | 5 | 0 |
| NM_002003 | FCN1 | 1283 | 8 | 2 | 1 | 0 | 2 | 0 | 0 | 0 | 5 | 1 | 0 | 8 | 0 |
| NM_002004 | FDPS | 1536 | 4 | 2 | 1 | 2 | 1 | 4 | 3 | 3 | 2 | 1 | 1 | 2 | 1 |
| NM_002005 | FES | 2781 | 3 | 1 | 0 | 0 | 0 | 0 | 0 | 0 | 1 | 0 | 0 | 3 | 0 |
| NM_002006 | FGF2 | 6774 | 4 | 1 | 2 | 0 | 1 | 4 | 1 | 0 | 1 | 2 | 0 | 0 | 1 |
| NM_002007 | FGF4 | 1220 | 0 | 0 | 0 | 0 | 0 | 0 | 0 | 0 | 0 | 0 | 0 | 0 | 0 |
| NM_002009 | FGF7 | 3935 | 17 | 6 | 10 | 1 | 17 | 0 | 2 | 0 | 15 | 10 | 3 | 0 | 5 |
| NM_002010 | FGF9 | 4530 | 5 | 1 | 0 | 0 | 1 | 1 | 5 | 0 | 0 | 1 | 0 | 0 | 0 |
| NM_002011 | FGFR4 | 3025 | 2 | 1 | 0 | 1 | 0 | 0 | 2 | 1 | 1 | 0 | 0 | 1 | 0 |
| NM_002012 | FHIT | 1090 | 5 | 1 | 0 | 1 | 1 | 5 | 2 | 0 | 0 | 0 | 0 | 0 | 1 |
| NM_002013 | FKBP3 | 1346 | 29 | 13 | 7 | 3 | 19 | 25 | 11 | 11 | 6 | 9 | 29 | 9 | 11 |
| NM_002014 | FKBP4 | 3739 | 6 | 3 | 2 | 3 | 3 | 6 | 5 | 2 | 3 | 3 | 4 | 3 | 4 |
| NM_002015 | FOXO1 | 5738 | 19 | 6 | 6 | 2 | 3 | 2 | 3 | 3 | 5 | 19 | 18 | 6 | 4 |
| NM_002016 | FLG | 12747 | 0 | 0 | 0 | 0 | 0 | 0 | 0 | 0 | 0 | 0 | 0 | 0 | 0 |
| NM_002017 | FLI1 | 3979 | 12 | 3 | 3 | 1 | 3 | 1 | 1 | 1 | 3 | 1 | 1 | 12 | 1 |
| NM_002018 | FLII | 4160 | 9 | 4 | 4 | 4 | 5 | 3 | 3 | 3 | 3 | 1 | 9 | 5 | 3 |
| NM_002019 | FLT1 | 7123 | 15 | 7 | 6 | 1 | 15 | 4 | 8 | 7 | 10 | 2 | 4 | 13 | 4 |
| NM_002020 | FLT4 | 4504 | 1 | 0 | 0 | 0 | 0 | 0 | 0 | 0 | 0 | 0 | 0 | 1 | 0 |
| NM_002021 | FMO1 | 2126 | 17 | 2 | 1 | 0 | 0 | 0 | 17 | 0 | 0 | 1 | 0 | 0 | 1 |
| NM_002022 | FMO4 | 2139 | 11 | 2 | 1 | 1 | 1 | 0 | 11 | 5 | 1 | 1 | 0 | 2 | 1 |
| NM_002023 | FMOD | 2986 | 10 | 3 | 10 | 2 | 4 | 1 | 1 | 0 | 2 | 6 | 2 | 2 | 2 |
| NM_002024 | FMR1 | 4365 | 20 | 13 | 8 | 5 | 10 | 20 | 19 | 10 | 12 | 19 | 5 | 18 | 16 |
| NM_002025 | AFF2 | 13745 | 2 | 1 | 0 | 0 | 0 | 2 | 0 | 0 | 1 | 1 | 0 | 0 | 2 |
| NM_002026 | FN1 | 8433 | 193 | 59 | 34 | 56 | 130 | 23 | 10 | 193 | 101 | 64 | 10 | 6 | 16 |

| transcript | gene | transcript length (nt) | max | mean | adipose | colon | heart | hypothalamus | kidney | liver | lung | ovary | skeletalmuscle | spleen | testes |
|---|---|---|---|---|---|---|---|---|---|---|---|---|---|---|---|
| NM_002027 | FNTA | 1694 | 21 | 13 | 17 | 5 | 16 | 21 | 10 | 10 | 10 | 11 | 12 | 17 | 11 |
| NM_002028 | FNTB | 2819 | 2 | 1 | 1 | 1 | 1 | 2 | 1 | 1 | 1 | 1 | 1 | 1 | 1 |
| NM_002029 | FPR1 | 1317 | 13 | 3 | 0 | 0 | 3 | 6 | 0 | 1 | 5 | 0 | 0 | 13 | 1 |
| NM_002030 | FPR3 | 2517 | 15 | 4 | 15 | 2 | 3 | 0 | 1 | 1 | 8 | 1 | 1 | 4 | 2 |
| NM_002031 | FRK | 2849 | 7 | 2 | 0 | 2 | 2 | 1 | 7 | 5 | 1 | 1 | 1 | 0 | 2 |
| NM_002032 | FTH1 | 1229 | 107 | 48 | 107 | 31 | 44 | 53 | 66 | 35 | 75 | 18 | 17 | 63 | 21 |
| NM_002033 | FUT4 | 6046 | 1 | 0 | 0 | 1 | 0 | 0 | 1 | 0 | 1 | 1 | 0 | 1 | 0 |
| NM_002034 | FUT5 | 1988 | 0 | 0 | 0 | 0 | 0 | 0 | 0 | 0 | 0 | 0 | 0 | 0 | 0 |
| NM_002035 | KDSR | 5198 | 10 | 6 | 4 | 2 | 6 | 5 | 8 | 10 | 4 | 8 | 3 | 5 | 6 |
| NM_002036 | DARC | 1301 | 8 | 3 | 5 | 1 | 5 | 2 | 1 | 0 | 6 | 1 | 4 | 8 | 4 |
| NM_002037 | FYN | 2650 | 14 | 5 | 7 | 2 | 4 | 14 | 2 | 1 | 7 | 4 | 1 | 10 | 5 |
| NM_002038 | IFI6 | 836 | 8 | 4 | 5 | 0 | 4 | 6 | 1 | 2 | 5 | 4 | 8 | 3 | 2 |
| NM_002039 | GAB1 | 7819 | 4 | 3 | 1 | 4 | 3 | 3 | 2 | 1 | 2 | 4 | 2 | 2 | 3 |
| NM_002040 | GABPA | 5180 | 10 | 6 | 6 | 2 | 5 | 4 | 5 | 6 | 5 | 8 | 4 | 10 | 9 |
| NM_002041 | GABPB1 | 1647 | 6 | 3 | 2 | 1 | 2 | 3 | 2 | 2 | 3 | 4 | 2 | 6 | 5 |
| NM_002042 | GABRR1 | 3161 | 0 | 0 | 0 | 0 | 0 | 0 | 0 | 0 | 0 | 0 | 0 | 0 | 0 |
| NM_002043 | GABRR2 | 1631 | 0 | 0 | 0 | 0 | 0 | 0 | 0 | 0 | 0 | 0 | 0 | 0 | 0 |
| NM_002044 | GALK2 | 3121 | 9 | 5 | 3 | 3 | 5 | 7 | 9 | 7 | 4 | 6 | 3 | 6 | 4 |
| NM_002045 | GAP43 | 1708 | 30 | 3 | 0 | 1 | 0 | 30 | 0 | 0 | 0 | 0 | 0 | 0 | 0 |
| NM_002046 | GAPDH | 1310 | 195 | 61 | 25 | 21 | 108 | 66 | 55 | 65 | 26 | 26 | 195 | 49 | 30 |
| NM_002047 | GARS | 2749 | 30 | 16 | 12 | 7 | 17 | 30 | 17 | 29 | 10 | 10 | 11 | 15 | 18 |
| NM_002048 | GAS1 | 2828 | 6 | 2 | 4 | 1 | 3 | 1 | 1 | 0 | 1 | 6 | 1 | 1 | 3 |
| NM_002049 | GATA1 | 1479 | 0 | 0 | 0 | 0 | 0 | 0 | 0 | 0 | 0 | 0 | 0 | 0 | 0 |
| NM_002051 | GATA3 | 3067 | 3 | 1 | 0 | 0 | 0 | 0 | 3 | 0 | 1 | 0 | 0 | 1 | 0 |
| NM_002052 | GATA4 | 3408 | 10 | 2 | 0 | 0 | 7 | 0 | 0 | 3 | 0 | 10 | 0 | 0 | 5 |
| NM_002053 | GBP1 | 3050 | 6 | 3 | 3 | 1 | 2 | 3 | 3 | 4 | 6 | 2 | 3 | 6 | 1 |
| NM_002054 | GCG | 1128 | 10 | 1 | 0 | 10 | 0 | 0 | 0 | 0 | 0 | 0 | 0 | 0 | 0 |
| NM_002055 | GFAP | 3081 | 595 | 55 | 0 | 7 | 1 | 595 | 0 | 0 | 0 | 1 | 1 | 1 | 1 |
| NM_002056 | GFPT1 | 8641 | 20 | 14 | 18 | 15 | 19 | 10 | 15 | 13 | 14 | 12 | 7 | 11 | 20 |
| NM_002059 | GH2 | 821 | 0 | 0 | 0 | 0 | 0 | 0 | 0 | 0 | 0 | 0 | 0 | 0 | 0 |
| NM_002060 | GJA4 | 1622 | 1 | 0 | 0 | 0 | 1 | 0 | 0 | 0 | 0 | 0 | 0 | 0 | 0 |
| NM_002061 | GCLM | 3074 | 5 | 2 | 2 | 1 | 1 | 2 | 2 | 5 | 1 | 0 | 1 | 1 | 1 |
| NM_002062 | GLP1R | 3123 | 0 | 0 | 0 | 0 | 0 | 0 | 0 | 0 | 0 | 0 | 0 | 0 | 0 |
| NM_002063 | GLRA2 | 3445 | 2 | 0 | 0 | 0 | 0 | 2 | 0 | 0 | 0 | 0 | 0 | 0 | 0 |
| NM_002064 | GLRX | 1647 | 31 | 14 | 11 | 3 | 31 | 7 | 31 | 17 | 13 | 2 | 25 | 13 | 2 |
| NM_002065 | GLUL | 4719 | 179 | 72 | 58 | 17 | 77 | 129 | 14 | 42 | 99 | 29 | 99 | 179 | 46 |
| NM_002066 | GML | 723 | 0 | 0 | 0 | 0 | 0 | 0 | 0 | 0 | 0 | 0 | 0 | 0 | 0 |
| NM_002067 | GNA11 | 1599 | 2 | 1 | 1 | 2 | 1 | 2 | 1 | 1 | 1 | 1 | 1 | 0 | 1 |
| NM_002068 | GNA15 | 2132 | 0 | 0 | 0 | 0 | 0 | 0 | 0 | 0 | 0 | 0 | 0 | 0 | 0 |
| NM_002069 | GNAI1 | 3318 | 10 | 3 | 6 | 1 | 1 | 10 | 3 | 3 | 2 | 2 | 1 | 2 | 4 |
| NM_002070 | GNAI2 | 2200 | 157 | 73 | 93 | 33 | 84 | 83 | 31 | 57 | 95 | 78 | 26 | 157 | 63 |
| NM_002071 | GNAL | 4027 | 12 | 2 | 1 | 1 | 1 | 12 | 1 | 0 | 1 | 1 | 3 | 1 | 2 |
| NM_002072 | GNAQ | 2169 | 26 | 10 | 9 | 6 | 7 | 26 | 10 | 5 | 15 | 12 | 4 | 8 | 10 |
| NM_002073 | GNAZ | 3208 | 15 | 2 | 2 | 2 | 1 | 15 | 1 | 0 | 0 | 2 | 0 | 2 | 2 |
| NM_002074 | GNB1 | 3128 | 58 | 25 | 24 | 16 | 24 | 58 | 24 | 19 | 23 | 19 | 7 | 35 | 25 |
| NM_002075 | GNB3 | 1923 | 0 | 0 | 0 | 0 | 0 | 0 | 0 | 0 | 0 | 0 | 0 | 0 | 0 |
| NM_002076 | GNS | 5128 | 17 | 9 | 16 | 2 | 10 | 4 | 17 | 7 | 9 | 10 | 3 | 13 | 5 |
| NM_002077 | GOLGA1 | 4881 | 3 | 2 | 2 | 1 | 2 | 1 | 2 | 2 | 1 | 2 | 1 | 2 | 3 |
| NM_002078 | GOLGA4 | 7702 | 56 | 26 | 26 | 5 | 56 | 15 | 34 | 22 | 19 | 17 | 38 | 25 | 33 |
| NM_002079 | GOT1 | 2126 | 30 | 10 | 1 | 3 | 30 | 20 | 14 | 20 | 1 | 1 | 12 | 2 | 2 |
| NM_002080 | GOT2 | 2447 | 20 | 9 | 1 | 3 | 20 | 12 | 12 | 18 | 2 | 2 | 12 | 4 | 5 |
| NM_002081 | GPC1 | 3686 | 3 | 1 | 2 | 0 | 3 | 2 | 0 | 1 | 0 | 1 | 1 | 1 | 3 |
| NM_002082 | GRK6 | 2952 | 2 | 0 | 0 | 1 | 0 | 1 | 0 | 0 | 1 | 0 | 0 | 2 | 0 |
| NM_002083 | GPX2 | 994 | 24 | 3 | 0 | 5 | 0 | 0 | 2 | 24 | 0 | 0 | 0 | 1 | 0 |
| NM_002084 | GPX3 | 1761 | 1,414 | 206 | 68 | 11 | 262 | 8 | 1,414 | 86 | 194 | 20 | 93 | 82 | 23 |
| NM_002085 | GPX4 | 917 | 8 | 3 | 8 | 1 | 3 | 4 | 3 | 2 | 1 | 1 | 2 | 1 | 2 |
| NM_002086 | GRB2 | 3303 | 19 | 9 | 9 | 6 | 10 | 19 | 8 | 8 | 11 | 4 | 4 | 19 | 5 |
| NM_002087 | GRN | 2305 | 7 | 3 | 6 | 4 | 2 | 1 | 3 | 3 | 4 | 1 | 1 | 7 | 1 |
| NM_002088 | GRIK5 | 3528 | 1 | 0 | 0 | 0 | 0 | 1 | 0 | 0 | 0 | 0 | 0 | 0 | 0 |
| NM_002089 | CXCL2 | 1203 | 53 | 8 | 2 | 1 | 5 | 2 | 2 | 53 | 10 | 2 | 7 | 1 | 2 |
| NM_002090 | CXCL3 | 1153 | 3 | 0 | 0 | 0 | 0 | 0 | 0 | 0 | 3 | 0 | 0 | 0 | 0 |
| NM_002091 | GRP | 847 | 1 | 0 | 0 | 0 | 0 | 1 | 0 | 0 | 0 | 0 | 0 | 0 | 0 |
| NM_002092 | GRSF1 | 6631 | 15 | 9 | 6 | 5 | 11 | 15 | 8 | 7 | 6 | 6 | 12 | 9 | 9 |
| NM_002093 | GSK3B | 7116 | 17 | 8 | 5 | 6 | 8 | 17 | 7 | 3 | 6 | 8 | 7 | 11 | 8 |
| NM_002094 | GSPT1 | 7153 | 13 | 10 | 8 | 4 | 13 | 10 | 10 | 12 | 8 | 9 | 13 | 12 | 10 |
| NM_002095 | GTF2E2 | 1536 | 17 | 10 | 13 | 5 | 9 | 10 | 6 | 14 | 6 | 13 | 3 | 12 | 17 |
| NM_002096 | GTF2F1 | 2551 | 9 | 6 | 4 | 5 | 6 | 8 | 9 | 4 | 5 | 7 | 3 | 8 | 7 |
| NM_002097 | GTF3A | 1404 | 31 | 13 | 11 | 3 | 31 | 9 | 12 | 24 | 9 | 4 | 14 | 19 | 8 |
| NM_002098 | GUCA1B | 2269 | 0 | 0 | 0 | 0 | 0 | 0 | 0 | 0 | 0 | 0 | 0 | 0 | 0 |
| NM_002099 | GYPA | 2660 | 0 | 0 | 0 | 0 | 0 | 0 | 0 | 0 | 0 | 0 | 0 | 0 | 0 |
| NM_002100 | GYPB | 527 | 0 | 0 | 0 | 0 | 0 | 0 | 0 | 0 | 0 | 0 | 0 | 0 | 0 |
| NM_002101 | GYPC | 1071 | 50 | 19 | 50 | 9 | 28 | 4 | 7 | 4 | 18 | 23 | 17 | 43 | 9 |
| NM_002102 | GYPE | 497 | 1 | 0 | 0 | 0 | 1 | 0 | 0 | 0 | 0 | 1 | 0 | 0 | 0 |
| NM_002103 | GYS1 | 3617 | 10 | 3 | 3 | 2 | 7 | 2 | 3 | 1 | 2 | 1 | 10 | 2 | 1 |
| NM_002104 | GZMK | 1045 | 3 | 0 | 0 | 0 | 0 | 0 | 0 | 0 | 1 | 0 | 0 | 3 | 0 |
| NM_002105 | H2AFX | 1593 | 6 | 2 | 1 | 3 | 2 | 6 | 1 | 1 | 2 | 3 | 1 | 4 | 3 |
| NM_002106 | H2AFZ | 947 | 9 | 6 | 4 | 5 | 5 | 9 | 5 | 5 | 6 | 4 | 4 | 9 | 5 |

| | | transcript | | | | | | hypothala | | | | | skeletalm | | |
|---|---|---|---|---|---|---|---|---|---|---|---|---|---|---|---|
| transcript | gene | length (nt) | max | mean | adipose | colon | heart | mus | kidney | liver | lung | ovary | uscle | spleen | testes |
| NM_002107 | H3F3A | 1049 | 0 | 0 | 0 | 0 | 0 | 0 | 0 | 0 | 0 | 0 | 0 | 0 | 0 |
| NM_002108 | HAL | 3139 | 20 | 2 | 0 | 0 | 0 | 0 | 0 | 20 | 0 | 0 | 0 | 1 | 0 |
| NM_002109 | HARS | 1964 | 15 | 5 | 4 | 3 | 5 | 9 | 4 | 15 | 2 | 3 | 3 | 4 | 4 |
| NM_002110 | HCK | 2087 | 7 | 1 | 1 | 0 | 1 | 1 | 0 | 0 | 1 | 0 | 0 | 7 | 0 |
| NM_002111 | HTT | 13475 | 4 | 2 | 2 | 2 | 2 | 4 | 2 | 2 | 2 | 2 | 2 | 3 | 2 |
| NM_002112 | HDC | 2406 | 3 | 0 | 0 | 1 | 0 | 3 | 0 | 0 | 1 | 0 | 0 | 0 | 0 |
| NM_002113 | CFHR1 | 1297 | 34 | 3 | 0 | 0 | 0 | 0 | 0 | 34 | 0 | 0 | 0 | 0 | 0 |
| NM_002114 | HIVEP1 | 8874 | 3 | 2 | 2 | 1 | 2 | 3 | 2 | 2 | 2 | 3 | 2 | 3 | 3 |
| NM_002115 | HK3 | 3068 | 2 | 0 | 1 | 0 | 0 | 0 | 0 | 0 | 1 | 0 | 0 | 2 | 0 |
| NM_002116 | HLA-A | 1548 | 169 | 50 | 61 | 26 | 30 | 17 | 50 | 37 | 98 | 25 | 25 | 169 | 12 |
| NM_002116 | HLA-A | 1550 | 169 | 50 | 61 | 26 | 30 | 17 | 50 | 37 | 98 | 25 | 25 | 169 | 12 |
| NM_002117 | HLA-C | 1512 | 35 | 10 | 5 | 11 | 6 | 2 | 11 | 10 | 22 | 3 | 3 | 35 | 2 |
| NM_002117 | HLA-C | 1525 | 34 | 10 | 5 | 11 | 6 | 2 | 11 | 10 | 22 | 3 | 3 | 34 | 2 |
| NM_002118 | HLA-DMB | 1358 | 5 | 2 | 4 | 1 | 1 | 1 | 1 | 0 | 5 | 0 | 0 | 5 | 0 |
| NM_002119 | HLA-DOA | 3484 | 7 | 1 | 1 | 1 | 1 | 1 | 1 | 0 | 3 | 1 | 0 | 7 | 0 |
| NM_002120 | HLA-DOB | 1372 | 2 | 0 | 0 | 0 | 0 | 0 | 0 | 0 | 0 | 0 | 0 | 2 | 0 |
| NM_002121 | HLA-DPB1 | 1501 | 30 | 8 | 12 | 7 | 4 | 5 | 4 | 4 | 20 | 3 | 2 | 30 | 2 |
| NM_002122 | HLA-DQA1 | 1542 | 7 | 2 | 2 | 2 | 0 | 1 | 1 | 0 | 4 | 1 | 0 | 7 | 0 |
| NM_002123 | HLA-DQB1 | 1224 | 21 | 4 | 2 | 1 | 1 | 2 | 4 | 1 | 11 | 1 | 1 | 21 | 2 |
| NM_002124 | HLA-DRB1 | 1138 | 21 | 6 | 7 | 5 | 1 | 4 | 4 | 6 | 13 | 2 | 1 | 21 | 1 |
| NM_002124 | HLA-DRB1 | 1165 | 20 | 6 | 7 | 5 | 1 | 4 | 4 | 6 | 12 | 1 | 1 | 20 | 1 |
| NM_002125 | HLA-DRB5 | 1168 | 24 | 6 | 3 | 4 | 1 | 2 | 4 | 1 | 24 | 1 | 1 | 21 | 2 |
| NM_002126 | HLF | 5599 | 10 | 4 | 1 | 1 | 3 | 10 | 4 | 10 | 2 | 9 | 1 | 2 | 2 |
| NM_002127 | HLA-G | 1578 | 0 | 0 | 0 | 0 | 0 | 0 | 0 | 0 | 0 | 0 | 0 | 0 | 0 |
| NM_002128 | HMGB1 | 3426 | 32 | 19 | 19 | 10 | 21 | 21 | 18 | 12 | 21 | 17 | 17 | 32 | 19 |
| NM_002129 | HMGB2 | 1527 | 47 | 23 | 25 | 27 | 20 | 15 | 13 | 4 | 47 | 19 | 15 | 37 | 32 |
| NM_002130 | HMGCS1 | 3451 | 40 | 12 | 5 | 5 | 6 | 40 | 11 | 20 | 17 | 8 | 3 | 6 | 14 |
| NM_002131 | HMGA1 | 1959 | 5 | 2 | 2 | 3 | 1 | 2 | 1 | 1 | 2 | 0 | 3 | 5 | 4 |
| NM_002133 | HMOX1 | 1550 | 65 | 11 | 4 | 2 | 2 | 2 | 5 | 15 | 16 | 6 | 3 | 65 | 2 |
| NM_002134 | HMOX2 | 1731 | 22 | 11 | 6 | 4 | 21 | 17 | 15 | 22 | 5 | 3 | 2 | 17 | 11 |
| NM_002135 | NR4A1 | 2670 | 22 | 5 | 1 | 1 | 5 | 2 | 4 | 1 | 8 | 22 | 4 | 1 | 2 |
| NM_002136 | HNRNPA1 | 1769 | 13 | 4 | 2 | 1 | 5 | 4 | 4 | 2 | 3 | 13 | 4 | 6 | 3 |
| NM_002137 | HNRNPA2B1 | 3666 | 397 | 211 | 103 | 84 | 293 | 138 | 249 | 190 | 229 | 196 | 210 | 397 | 230 |
| NM_002138 | HNRNPD | 2110 | 109 | 65 | 40 | 51 | 63 | 72 | 55 | 48 | 64 | 89 | 55 | 109 | 72 |
| NM_002139 | RBMX | 2079 | 5 | 2 | 1 | 1 | 2 | 2 | 2 | 1 | 1 | 5 | 3 | 3 | 1 |
| NM_002140 | HNRNPK | 2978 | 31 | 21 | 18 | 11 | 23 | 24 | 23 | 21 | 21 | 20 | 15 | 31 | 21 |
| NM_002141 | HOXA4 | 1728 | 3 | 1 | 1 | 2 | 0 | 1 | 2 | 0 | 1 | 1 | 3 | 2 | 1 |
| NM_002143 | HPCA | 1406 | 18 | 2 | 0 | 0 | 0 | 18 | 0 | 0 | 0 | 0 | 0 | 0 | 0 |
| NM_002144 | HOXB1 | 1014 | 0 | 0 | 0 | 0 | 0 | 0 | 0 | 0 | 0 | 0 | 0 | 0 | 0 |
| NM_002145 | HOXB2 | 1602 | 2 | 0 | 0 | 0 | 0 | 0 | 2 | 0 | 0 | 0 | 0 | 1 | 0 |
| NM_002146 | HOXB3 | 3618 | 1 | 0 | 1 | 1 | 0 | 0 | 1 | 0 | 0 | 0 | 0 | 0 | 0 |
| NM_002147 | HOXB5 | 1821 | 1 | 0 | 0 | 1 | 0 | 0 | 1 | 0 | 0 | 0 | 0 | 0 | 0 |
| NM_002148 | HOXD10 | 1804 | 6 | 1 | 0 | 3 | 0 | 0 | 6 | 0 | 0 | 1 | 0 | 0 | 1 |
| NM_002149 | HPCAL1 | 1752 | 2 | 1 | 1 | 1 | 1 | 2 | 1 | 1 | 2 | 0 | 0 | 1 | 1 |
| NM_002150 | HPD | 1423 | 60 | 8 | 0 | 0 | 1 | 0 | 24 | 60 | 0 | 1 | 1 | 1 | 1 |
| NM_002151 | HPN | 1783 | 1 | 0 | 0 | 0 | 0 | 0 | 1 | 1 | 0 | 0 | 0 | 0 | 0 |
| NM_002152 | HRC | 2384 | 258 | 34 | 2 | 1 | 258 | 1 | 1 | 1 | 1 | 1 | 110 | 0 | 2 |
| NM_002153 | HSD17B2 | 1433 | 13 | 1 | 0 | 3 | 0 | 0 | 0 | 13 | 0 | 0 | 0 | 0 | 0 |
| NM_002154 | HSPA4 | 3385 | 51 | 29 | 17 | 7 | 35 | 43 | 33 | 34 | 17 | 21 | 28 | 33 | 51 |
| NM_002155 | HSPA6 | 2652 | 0 | 0 | 0 | 0 | 0 | 0 | 0 | 0 | 0 | 0 | 0 | 0 | 0 |
| NM_002156 | HSPD1 | 2321 | 168 | 46 | 26 | 19 | 39 | 35 | 79 | 168 | 30 | 32 | 20 | 35 | 26 |
| NM_002157 | HSPE1 | 952 | 758 | 159 | 89 | 71 | 160 | 221 | 152 | 758 | 48 | 51 | 57 | 84 | 54 |
| NM_002158 | FOXN2 | 5463 | 6 | 3 | 3 | 1 | 2 | 3 | 2 | 3 | 3 | 2 | 2 | 6 | 3 |
| NM_002159 | HTN1 | 537 | 0 | 0 | 0 | 0 | 0 | 0 | 0 | 0 | 0 | 0 | 0 | 0 | 0 |
| NM_002160 | TNC | 7599 | 14 | 3 | 2 | 8 | 14 | 2 | 4 | 0 | 3 | 0 | 0 | 4 | 1 |
| NM_002161 | IARS | 4406 | 9 | 6 | 5 | 3 | 8 | 9 | 7 | 9 | 4 | 4 | 8 | 7 | 6 |
| NM_002162 | ICAM3 | 1780 | 1 | 0 | 0 | 0 | 0 | 0 | 0 | 0 | 1 | 0 | 0 | 0 | 0 |
| NM_002163 | IRF8 | 2661 | 28 | 5 | 7 | 5 | 2 | 4 | 1 | 1 | 5 | 1 | 0 | 28 | 1 |
| NM_002164 | IDO1 | 1569 | 2 | 0 | 0 | 0 | 0 | 0 | 0 | 0 | 2 | 0 | 1 | 1 | 0 |
| NM_002165 | ID1 | 983 | 17 | 9 | 11 | 12 | 15 | 7 | 7 | 17 | 9 | 6 | 2 | 8 | 4 |
| NM_002166 | ID2 | 1364 | 4 | 2 | 1 | 2 | 1 | 3 | 3 | 4 | 2 | 1 | 0 | 1 | 1 |
| NM_002167 | ID3 | 1288 | 1 | 1 | 1 | 1 | 1 | 1 | 1 | 0 | 1 | 1 | 0 | 0 | 0 |
| NM_002168 | IDH2 | 1731 | 32 | 14 | 3 | 9 | 28 | 11 | 25 | 32 | 4 | 2 | 27 | 5 | 2 |
| NM_002169 | IFNA5 | 570 | 0 | 0 | 0 | 0 | 0 | 0 | 0 | 0 | 0 | 0 | 0 | 0 | 0 |
| NM_002170 | IFNA8 | 1039 | 0 | 0 | 0 | 0 | 0 | 0 | 0 | 0 | 0 | 0 | 0 | 0 | 0 |
| NM_002171 | IFNA10 | 963 | 0 | 0 | 0 | 0 | 0 | 0 | 0 | 0 | 0 | 0 | 0 | 0 | 0 |
| NM_002172 | IFNA14 | 778 | 0 | 0 | 0 | 0 | 0 | 0 | 0 | 0 | 0 | 0 | 0 | 0 | 0 |
| NM_002173 | IFNA16 | 939 | 0 | 0 | 0 | 0 | 0 | 0 | 0 | 0 | 0 | 0 | 0 | 0 | 0 |
| NM_002175 | IFNA21 | 1024 | 0 | 0 | 0 | 0 | 0 | 0 | 0 | 0 | 0 | 0 | 0 | 0 | 0 |
| NM_002176 | IFNB1 | 840 | 0 | 0 | 0 | 0 | 0 | 0 | 0 | 0 | 0 | 0 | 0 | 0 | 0 |
| NM_002177 | IFNW1 | 1514 | 0 | 0 | 0 | 0 | 0 | 0 | 0 | 0 | 0 | 0 | 0 | 0 | 0 |
| NM_002178 | IGFBP6 | 966 | 12 | 5 | 12 | 4 | 8 | 5 | 3 | 1 | 4 | 5 | 4 | 5 | 7 |
| NM_002180 | IGHMBP2 | 3954 | 0 | 0 | 0 | 0 | 0 | 0 | 0 | 0 | 0 | 0 | 0 | 0 | 0 |
| NM_002181 | IHH | 2019 | 1 | 0 | 0 | 1 | 0 | 0 | 0 | 0 | 0 | 0 | 0 | 0 | 0 |
| NM_002182 | IL1RAP | 4783 | 15 | 3 | 1 | 0 | 3 | 2 | 1 | 15 | 4 | 1 | 0 | 6 | 1 |
| NM_002183 | IL3RA | 1710 | 2 | 1 | 2 | 1 | 2 | 1 | 1 | 0 | 2 | 2 | 1 | 2 | 1 |
| NM_002184 | IL6ST | 3228 | 48 | 28 | 30 | 5 | 32 | 20 | 27 | 48 | 35 | 25 | 17 | 45 | 22 |

| | | transcript | | | | | | hypothala | | | | | skeletalm | | |
|---|---|---|---|---|---|---|---|---|---|---|---|---|---|---|---|
| transcript | gene | length (nt) | max | mean | adipose | colon | heart | mus | kidney | liver | lung | ovary | uscle | spleen | testes |
| NM_002185 | IL7R | 1804 | 39 | 8 | 3 | 4 | 2 | 0 | 1 | 1 | 39 | 1 | 0 | 34 | 3 |
| NM_002186 | IL9R | 2153 | 0 | 0 | 0 | 0 | 0 | 0 | 0 | 0 | 0 | 0 | 0 | 0 | 0 |
| NM_002187 | IL12B | 2347 | 0 | 0 | 0 | 0 | 0 | 0 | 0 | 0 | 0 | 0 | 0 | 0 | 0 |
| NM_002188 | IL13 | 1282 | 2 | 0 | 0 | 0 | 0 | 0 | 0 | 0 | 0 | 0 | 0 | 0 | 2 |
| NM_002189 | IL15RA | 1610 | 2 | 1 | 1 | 0 | 1 | 0 | 0 | 2 | 2 | 0 | 0 | 2 | 0 |
| NM_002190 | IL17A | 1859 | 0 | 0 | 0 | 0 | 0 | 0 | 0 | 0 | 0 | 0 | 0 | 0 | 0 |
| NM_002191 | INHA | 1423 | 3 | 0 | 0 | 0 | 0 | 0 | 0 | 0 | 0 | 1 | 0 | 0 | 3 |
| NM_002192 | INHBA | 2175 | 6 | 2 | 0 | 0 | 3 | 1 | 1 | 1 | 6 | 2 | 0 | 0 | 2 |
| NM_002193 | INHBB | 3203 | 7 | 2 | 7 | 0 | 1 | 1 | 0 | 0 | 1 | 1 | 1 | 0 | 5 |
| NM_002194 | INPP1 | 2019 | 8 | 4 | 3 | 2 | 4 | 8 | 2 | 4 | 3 | 2 | 2 | 4 | 7 |
| NM_002195 | INSL4 | 615 | 0 | 0 | 0 | 0 | 0 | 0 | 0 | 0 | 0 | 0 | 0 | 0 | 0 |
| NM_002196 | INSM1 | 2828 | 0 | 0 | 0 | 0 | 0 | 0 | 0 | 0 | 0 | 0 | 0 | 0 | 0 |
| NM_002197 | ACO1 | 3548 | 41 | 15 | 41 | 5 | 11 | 5 | 35 | 35 | 5 | 6 | 5 | 6 | 12 |
| NM_002198 | IRF1 | 3553 | 3 | 1 | 1 | 1 | 0 | 0 | 1 | 0 | 3 | 0 | 0 | 3 | 0 |
| NM_002199 | IRF2 | 2286 | 12 | 5 | 4 | 4 | 3 | 4 | 3 | 5 | 6 | 6 | 3 | 12 | 3 |
| NM_002200 | IRF5 | 2852 | 0 | 0 | 0 | 0 | 0 | 0 | 0 | 0 | 0 | 0 | 0 | 0 | 0 |
| NM_002201 | ISG20 | 956 | 9 | 2 | 1 | 3 | 1 | 1 | 0 | 3 | 7 | 1 | 1 | 9 | 1 |
| NM_002202 | ISL1 | 2717 | 0 | 0 | 0 | 0 | 0 | 0 | 0 | 0 | 0 | 0 | 0 | 0 | 0 |
| NM_002203 | ITGA2 | 7869 | 2 | 1 | 0 | 1 | 0 | 1 | 2 | 0 | 1 | 0 | 0 | 2 | 0 |
| NM_002204 | ITGA3 | 5027 | 2 | 1 | 1 | 1 | 1 | 1 | 2 | 0 | 2 | 1 | 0 | 1 | 0 |
| NM_002205 | ITGA5 | 4250 | 6 | 3 | 2 | 6 | 6 | 1 | 1 | 2 | 4 | 2 | 2 | 2 | 1 |
| NM_002206 | ITGA7 | 4120 | 9 | 2 | 9 | 1 | 5 | 1 | 1 | 0 | 0 | 0 | 2 | 0 | 0 |
| NM_002207 | ITGA9 | 3962 | 13 | 4 | 2 | 4 | 6 | 2 | 1 | 4 | 2 | 3 | 1 | 13 | 4 |
| NM_002208 | ITGAE | 3861 | 1 | 1 | 1 | 1 | 1 | 1 | 0 | 0 | 1 | 0 | 0 | 1 | 1 |
| NM_002209 | ITGAL | 5209 | 5 | 1 | 0 | 1 | 0 | 0 | 0 | 0 | 1 | 0 | 0 | 5 | 0 |
| NM_002210 | ITGAV | 7031 | 18 | 9 | 9 | 2 | 18 | 10 | 14 | 3 | 9 | 9 | 3 | 10 | 9 |
| NM_002211 | ITGB1 | 3870 | 120 | 57 | 120 | 23 | 112 | 15 | 43 | 61 | 59 | 46 | 27 | 80 | 40 |
| NM_002212 | EIF6 | 1084 | 4 | 2 | 1 | 2 | 1 | 1 | 4 | 2 | 1 | 1 | 1 | 2 | 1 |
| NM_002213 | ITGB5 | 3372 | 12 | 3 | 12 | 3 | 4 | 2 | 3 | 4 | 2 | 1 | 1 | 4 | 1 |
| NM_002214 | ITGB8 | 8777 | 12 | 3 | 0 | 0 | 1 | 6 | 12 | 0 | 1 | 2 | 0 | 0 | 8 |
| NM_002215 | ITIH1 | 2910 | 50 | 5 | 0 | 0 | 0 | 0 | 0 | 50 | 0 | 0 | 0 | 0 | 0 |
| NM_002216 | ITIH2 | 3189 | 171 | 16 | 0 | 0 | 0 | 1 | 0 | 171 | 0 | 1 | 1 | 1 | 0 |
| NM_002217 | ITIH3 | 3037 | 105 | 10 | 0 | 0 | 0 | 0 | 0 | 105 | 1 | 0 | 1 | 2 | 1 |
| NM_002218 | ITIH4 | 3282 | 46 | 5 | 0 | 0 | 1 | 0 | 1 | 46 | 0 | 0 | 1 | 1 | 1 |
| NM_002220 | ITPKA | 1773 | 1 | 0 | 0 | 0 | 0 | 1 | 0 | 0 | 0 | 0 | 0 | 0 | 0 |
| NM_002221 | ITPKB | 6162 | 7 | 2 | 1 | 2 | 1 | 7 | 2 | 0 | 2 | 1 | 0 | 3 | 1 |
| NM_002222 | ITPR1 | 10051 | 19 | 8 | 3 | 4 | 6 | 13 | 9 | 1 | 7 | 19 | 1 | 12 | 10 |
| NM_002223 | ITPR2 | 12568 | 12 | 7 | 9 | 3 | 5 | 8 | 11 | 12 | 5 | 7 | 2 | 11 | 6 |
| NM_002224 | ITPR3 | 9026 | 4 | 2 | 1 | 4 | 1 | 1 | 1 | 0 | 3 | 1 | 0 | 4 | 3 |
| NM_002225 | IVD | 4665 | 4 | 1 | 1 | 1 | 1 | 2 | 2 | 4 | 0 | 1 | 1 | 1 | 1 |
| NM_002226 | JAG2 | 5077 | 0 | 0 | 0 | 0 | 0 | 0 | 0 | 0 | 0 | 0 | 0 | 0 | 0 |
| NM_002227 | JAK1 | 5053 | 62 | 30 | 31 | 10 | 31 | 20 | 26 | 21 | 33 | 36 | 23 | 62 | 34 |
| NM_002228 | JUN | 3323 | 77 | 31 | 58 | 17 | 21 | 25 | 15 | 18 | 55 | 77 | 32 | 9 | 16 |
| NM_002229 | JUNB | 1816 | 7 | 2 | 1 | 1 | 1 | 1 | 1 | 1 | 4 | 7 | 1 | 2 | 0 |
| NM_002230 | JUP | 3508 | 2 | 1 | 0 | 1 | 1 | 0 | 2 | 1 | 1 | 0 | 0 | 0 | 0 |
| NM_002231 | CD82 | 1691 | 2 | 1 | 1 | 1 | 1 | 1 | 1 | 2 | 2 | 1 | 0 | 2 | 0 |
| NM_002232 | KCNA3 | 3346 | 3 | 1 | 0 | 0 | 0 | 1 | 0 | 0 | 1 | 0 | 0 | 3 | 0 |
| NM_002233 | KCNA4 | 3613 | 3 | 0 | 0 | 0 | 0 | 3 | 0 | 0 | 0 | 0 | 0 | 0 | 0 |
| NM_002234 | KCNA5 | 2865 | 4 | 1 | 0 | 0 | 4 | 1 | 0 | 0 | 1 | 0 | 0 | 0 | 0 |
| NM_002235 | KCNA6 | 5977 | 1 | 0 | 0 | 0 | 0 | 1 | 0 | 0 | 0 | 0 | 0 | 0 | 0 |
| NM_002236 | KCNF1 | 2289 | 1 | 0 | 0 | 0 | 0 | 1 | 0 | 0 | 0 | 0 | 0 | 0 | 0 |
| NM_002237 | KCNG1 | 2220 | 1 | 0 | 0 | 0 | 0 | 1 | 0 | 0 | 0 | 0 | 0 | 0 | 0 |
| NM_002238 | KCNH1 | 8025 | 2 | 0 | 0 | 0 | 0 | 2 | 0 | 0 | 0 | 0 | 0 | 0 | 0 |
| NM_002239 | KCNJ3 | 2890 | 3 | 1 | 0 | 0 | 1 | 3 | 1 | 0 | 0 | 0 | 0 | 0 | 0 |
| NM_002240 | KCNJ6 | 2483 | 4 | 0 | 0 | 0 | 0 | 4 | 0 | 0 | 0 | 0 | 0 | 0 | 0 |
| NM_002241 | KCNJ10 | 4440 | 14 | 2 | 0 | 0 | 0 | 14 | 8 | 0 | 0 | 0 | 0 | 4 | 0 |
| NM_002242 | KCNJ13 | 3261 | 2 | 1 | 0 | 0 | 0 | 2 | 1 | 0 | 1 | 1 | 0 | 1 | 1 |
| NM_002243 | KCNJ15 | 2865 | 31 | 3 | 0 | 0 | 0 | 0 | 31 | 0 | 2 | 0 | 0 | 2 | 0 |
| NM_002245 | KCNK1 | 2161 | 2 | 1 | 0 | 0 | 1 | 2 | 1 | 0 | 0 | 0 | 0 | 0 | 0 |
| NM_002246 | KCNK3 | 3978 | 1 | 0 | 0 | 0 | 0 | 0 | 0 | 0 | 1 | 0 | 0 | 0 | 0 |
| NM_002247 | KCNMA1 | 6102 | 6 | 3 | 1 | 6 | 1 | 6 | 0 | 3 | 2 | 3 | 3 | 5 | 2 |
| NM_002248 | KCNN1 | 2657 | 0 | 0 | 0 | 0 | 0 | 0 | 0 | 0 | 0 | 0 | 0 | 0 | 0 |
| NM_002249 | KCNN3 | 3049 | 3 | 1 | 0 | 1 | 0 | 3 | 1 | 0 | 0 | 0 | 1 | 1 | 0 |
| NM_002250 | KCNN4 | 2240 | 1 | 0 | 0 | 1 | 0 | 0 | 0 | 0 | 0 | 0 | 0 | 1 | 0 |
| NM_002251 | KCNS1 | 4539 | 1 | 0 | 0 | 0 | 0 | 1 | 0 | 0 | 0 | 0 | 0 | 0 | 0 |
| NM_002252 | KCNS3 | 2330 | 6 | 2 | 0 | 2 | 1 | 2 | 2 | 1 | 5 | 1 | 6 | 0 | 2 |
| NM_002253 | KDR | 6054 | 9 | 5 | 5 | 2 | 6 | 5 | 7 | 6 | 9 | 3 | 2 | 4 | 3 |
| NM_002254 | KIF3C | 5431 | 11 | 1 | 0 | 0 | 0 | 11 | 0 | 0 | 0 | 1 | 0 | 0 | 1 |
| NM_002255 | KIR2DL4 | 1579 | 1 | 0 | 0 | 0 | 0 | 0 | 0 | 0 | 0 | 0 | 0 | 1 | 0 |
| NM_002255 | KIR2DL4 | 1580 | 1 | 0 | 0 | 0 | 0 | 0 | 0 | 0 | 0 | 0 | 0 | 1 | 0 |
| NM_002256 | KISS1 | 714 | 0 | 0 | 0 | 0 | 0 | 0 | 0 | 0 | 0 | 0 | 0 | 0 | 0 |
| NM_002257 | KLK1 | 874 | 2 | 0 | 0 | 2 | 0 | 0 | 1 | 0 | 0 | 0 | 0 | 0 | 0 |
| NM_002258 | KLRB1 | 740 | 16 | 3 | 2 | 2 | 0 | 0 | 0 | 1 | 5 | 1 | 0 | 16 | 1 |
| NM_002259 | KLRC1 | 1379 | 0 | 0 | 0 | 0 | 0 | 0 | 0 | 0 | 0 | 0 | 0 | 0 | 0 |
| NM_002260 | KLRC2 | 1215 | 0 | 0 | 0 | 0 | 0 | 0 | 0 | 0 | 0 | 0 | 0 | 0 | 0 |
| NM_002261 | KLRC3 | 1025 | 1 | 0 | 0 | 0 | 0 | 0 | 0 | 0 | 1 | 0 | 0 | 1 | 0 |
| NM_002262 | KLRD1 | 3258 | 7 | 1 | 0 | 0 | 0 | 0 | 0 | 0 | 2 | 0 | 0 | 7 | 0 |

| transcript | gene | transcript length (nt) | max | mean | adipose | colon | heart | hypothalamus | kidney | liver | lung | ovary | skeletalmuscle | spleen | testes |
|---|---|---|---|---|---|---|---|---|---|---|---|---|---|---|---|
| NM_002263 | KIFC1 | 2692 | 2 | 1 | 1 | 2 | 0 | 0 | 0 | 0 | 1 | 1 | 0 | 1 | 1 |
| NM_002263 | KIFC1 | 2704 | 2 | 1 | 1 | 2 | 0 | 0 | 0 | 0 | 1 | 1 | 0 | 1 | 1 |
| NM_002264 | KPNA1 | 6889 | 12 | 6 | 5 | 2 | 8 | 7 | 4 | 8 | 5 | 5 | 12 | 6 | 5 |
| NM_002265 | KPNB1 | 4201 | 42 | 29 | 28 | 13 | 33 | 36 | 23 | 30 | 21 | 32 | 27 | 39 | 42 |
| NM_002266 | KPNA2 | 1980 | 19 | 4 | 2 | 2 | 5 | 5 | 2 | 4 | 2 | 1 | 1 | 5 | 19 |
| NM_002267 | KPNA3 | 4473 | 12 | 4 | 3 | 1 | 6 | 4 | 2 | 4 | 3 | 2 | 12 | 3 | 6 |
| NM_002268 | KPNA4 | 3802 | 14 | 7 | 9 | 2 | 9 | 8 | 4 | 5 | 5 | 3 | 14 | 7 | 7 |
| NM_002269 | KPNA5 | 2158 | 9 | 4 | 3 | 1 | 3 | 7 | 2 | 1 | 3 | 9 | 3 | 5 | 9 |
| NM_002270 | TNPO1 | 8559 | 8 | 6 | 5 | 2 | 7 | 5 | 5 | 4 | 5 | 7 | 4 | 8 | 7 |
| NM_002271 | IPO5 | 6002 | 26 | 14 | 10 | 3 | 19 | 17 | 10 | 9 | 7 | 16 | 23 | 11 | 26 |
| NM_002272 | KRT4 | 2576 | 1 | 0 | 0 | 0 | 0 | 0 | 0 | 0 | 1 | 0 | 0 | 0 | 0 |
| NM_002273 | KRT8 | 1796 | 133 | 25 | 1 | 133 | 15 | 1 | 33 | 59 | 20 | 3 | 0 | 3 | 4 |
| NM_002274 | KRT13 | 1689 | 1 | 0 | 0 | 0 | 0 | 0 | 0 | 0 | 0 | 0 | 0 | 0 | 1 |
| NM_002275 | KRT15 | 1839 | 2 | 0 | 0 | 1 | 0 | 0 | 0 | 0 | 2 | 0 | 0 | 0 | 1 |
| NM_002276 | KRT19 | 1471 | 11 | 2 | 0 | 11 | 0 | 0 | 3 | 0 | 4 | 1 | 0 | 0 | 0 |
| NM_002277 | KRT31 | 1595 | 0 | 0 | 0 | 0 | 0 | 0 | 0 | 0 | 0 | 0 | 0 | 0 | 0 |
| NM_002278 | KRT32 | 2005 | 0 | 0 | 0 | 0 | 0 | 0 | 0 | 0 | 0 | 0 | 0 | 0 | 0 |
| NM_002279 | KRT33B | 1602 | 0 | 0 | 0 | 0 | 0 | 0 | 0 | 0 | 0 | 0 | 0 | 0 | 0 |
| NM_002280 | KRT35 | 1671 | 0 | 0 | 0 | 0 | 0 | 0 | 0 | 0 | 0 | 0 | 0 | 0 | 0 |
| NM_002281 | KRT81 | 1910 | 0 | 0 | 0 | 0 | 0 | 0 | 0 | 0 | 0 | 0 | 0 | 0 | 0 |
| NM_002282 | KRT83 | 1875 | 0 | 0 | 0 | 0 | 0 | 0 | 0 | 0 | 0 | 0 | 0 | 0 | 0 |
| NM_002283 | KRT85 | 2491 | 0 | 0 | 0 | 0 | 0 | 0 | 0 | 0 | 0 | 0 | 0 | 0 | 0 |
| NM_002284 | KRT86 | 2091 | 0 | 0 | 0 | 0 | 0 | 0 | 0 | 0 | 0 | 0 | 0 | 0 | 0 |
| NM_002285 | AFF3 | 8137 | 5 | 2 | 2 | 1 | 1 | 5 | 1 | 0 | 2 | 4 | 0 | 5 | 2 |
| NM_002286 | LAG3 | 1992 | 5 | 1 | 0 | 1 | 0 | 0 | 0 | 0 | 0 | 4 | 0 | 5 | 2 |
| NM_002287 | LAIR1 | 2816 | 3 | 1 | 3 | 0 | 1 | 1 | 0 | 0 | 1 | 0 | 0 | 3 | 1 |
| NM_002288 | LAIR2 | 699 | 1 | 0 | 0 | 0 | 0 | 0 | 0 | 0 | 1 | 0 | 0 | 1 | 0 |
| NM_002289 | LALBA | 728 | 0 | 0 | 0 | 0 | 0 | 0 | 0 | 0 | 0 | 0 | 0 | 0 | 0 |
| NM_002290 | LAMA4 | 7266 | 22 | 4 | 22 | 4 | 4 | 2 | 1 | 0 | 4 | 5 | 1 | 0 | 3 |
| NM_002291 | LAMB1 | 5846 | 52 | 22 | 17 | 10 | 37 | 6 | 37 | 11 | 27 | 21 | 5 | 52 | 16 |
| NM_002292 | LAMB2 | 5808 | 8 | 4 | 7 | 3 | 8 | 2 | 4 | 3 | 4 | 7 | 2 | 3 | 4 |
| NM_002293 | LAMC1 | 7889 | 50 | 21 | 50 | 12 | 44 | 4 | 13 | 7 | 23 | 29 | 23 | 8 | 23 |
| NM_002294 | LAMP2 | 6588 | 10 | 5 | 3 | 1 | 4 | 7 | 10 | 7 | 5 | 4 | 4 | 5 | 5 |
| NM_002295 | RPSA | 1146 | 3 | 2 | 2 | 2 | 1 | 1 | 1 | 3 | 1 | 2 | 1 | 2 | 1 |
| NM_002296 | LBR | 3792 | 10 | 4 | 3 | 6 | 3 | 2 | 3 | 5 | 4 | 3 | 2 | 10 | 3 |
| NM_002297 | LCN1 | 779 | 0 | 0 | 0 | 0 | 0 | 0 | 0 | 0 | 0 | 0 | 0 | 0 | 0 |
| NM_002298 | LCP1 | 3789 | 77 | 15 | 31 | 2 | 10 | 4 | 2 | 10 | 24 | 2 | 2 | 77 | 5 |
| NM_002299 | LCT | 6274 | 0 | 0 | 0 | 0 | 0 | 0 | 0 | 0 | 0 | 0 | 0 | 0 | 0 |
| NM_002300 | LDHB | 1303 | 348 | 144 | 230 | 40 | 332 | 303 | 348 | 8 | 58 | 82 | 42 | 66 | 79 |
| NM_002301 | LDHC | 1283 | 3 | 0 | 0 | 0 | 0 | 0 | 0 | 0 | 0 | 0 | 0 | 0 | 3 |
| NM_002302 | LECT2 | 1077 | 5 | 1 | 0 | 0 | 0 | 0 | 0 | 5 | 0 | 0 | 0 | 0 | 1 |
| NM_002303 | LEPR | 4248 | 16 | 4 | 3 | 0 | 2 | 2 | 1 | 16 | 7 | 5 | 2 | 1 | 3 |
| NM_002304 | LFNG | 2056 | 1 | 0 | 0 | 1 | 0 | 0 | 0 | 0 | 1 | 0 | 0 | 1 | 0 |
| NM_002305 | LGALS1 | 558 | 7 | 2 | 7 | 2 | 2 | 1 | 0 | 1 | 2 | 1 | 1 | 1 | 1 |
| NM_002306 | LGALS3 | 1063 | 278 | 98 | 257 | 278 | 117 | 36 | 35 | 8 | 98 | 116 | 36 | 35 | 61 |
| NM_002307 | LGALS7 | 494 | 0 | 0 | 0 | 0 | 0 | 0 | 0 | 0 | 0 | 0 | 0 | 0 | 0 |
| NM_002308 | LGALS9 | 1668 | 3 | 1 | 1 | 2 | 1 | 0 | 0 | 0 | 1 | 0 | 0 | 3 | 0 |
| NM_002309 | LIF | 3919 | 1 | 0 | 0 | 1 | 0 | 0 | 0 | 0 | 0 | 0 | 0 | 0 | 0 |
| NM_002310 | LIFR | 10090 | 6 | 4 | 3 | 0 | 4 | 5 | 5 | 3 | 6 | 5 | 3 | 5 | 4 |
| NM_002311 | LIG3 | 3059 | 6 | 3 | 2 | 2 | 3 | 5 | 3 | 2 | 2 | 5 | 4 | 4 | 6 |
| NM_002312 | LIG4 | 4098 | 9 | 4 | 2 | 1 | 6 | 9 | 4 | 5 | 3 | 2 | 2 | 6 | 6 |
| NM_002313 | ABLIM1 | 7586 | 48 | 15 | 12 | 6 | 48 | 11 | 13 | 7 | 9 | 37 | 8 | 5 | 15 |
| NM_002314 | LIMK1 | 3332 | 4 | 1 | 1 | 1 | 1 | 4 | 1 | 0 | 1 | 0 | 1 | 2 | 1 |
| NM_002315 | LMO1 | 1274 | 0 | 0 | 0 | 0 | 0 | 0 | 0 | 0 | 0 | 0 | 0 | 0 | 0 |
| NM_002316 | LMX1B | 5750 | 0 | 0 | 0 | 0 | 0 | 0 | 0 | 0 | 0 | 0 | 0 | 0 | 0 |
| NM_002317 | LOX | 3905 | 3 | 1 | 3 | 0 | 3 | 0 | 1 | 0 | 2 | 1 | 1 | 1 | 1 |
| NM_002318 | LOXL2 | 3810 | 2 | 1 | 2 | 1 | 0 | 0 | 0 | 0 | 0 | 1 | 0 | 1 | 0 |
| NM_002319 | LRCH4 | 3201 | 1 | 0 | 0 | 0 | 0 | 0 | 0 | 0 | 0 | 0 | 0 | 1 | 1 |
| NM_002332 | LRP1 | 14888 | 27 | 9 | 27 | 6 | 12 | 8 | 2 | 9 | 7 | 13 | 3 | 7 | 6 |
| NM_002333 | LRP3 | 3692 | 1 | 0 | 0 | 0 | 0 | 1 | 0 | 0 | 0 | 0 | 0 | 0 | 0 |
| NM_002334 | LRP4 | 7980 | 6 | 1 | 0 | 2 | 1 | 6 | 0 | 0 | 0 | 1 | 2 | 1 | 0 |
| NM_002335 | LRP5 | 5128 | 2 | 1 | 1 | 1 | 1 | 0 | 1 | 2 | 1 | 1 | 0 | 0 | 0 |
| NM_002336 | LRP6 | 10083 | 10 | 5 | 4 | 3 | 4 | 4 | 7 | 6 | 6 | 10 | 3 | 3 | 5 |
| NM_002337 | LRPAP1 | 1570 | 30 | 15 | 11 | 5 | 30 | 20 | 22 | 13 | 10 | 15 | 10 | 15 | 10 |
| NM_002338 | LSAMP | 9477 | 17 | 2 | 0 | 1 | 1 | 17 | 0 | 0 | 2 | 1 | 0 | 0 | 1 |
| NM_002339 | LSP1 | 1699 | 19 | 4 | 9 | 3 | 1 | 0 | 0 | 0 | 3 | 1 | 1 | 19 | 1 |
| NM_002340 | LSS | 4258 | 1 | 1 | 1 | 1 | 1 | 1 | 1 | 1 | 1 | 1 | 0 | 0 | 1 |
| NM_002341 | LTB | 893 | 68 | 7 | 0 | 4 | 1 | 0 | 1 | 0 | 5 | 0 | 0 | 68 | 0 |
| NM_002342 | LTBR | 2105 | 22 | 9 | 9 | 8 | 8 | 3 | 12 | 22 | 9 | 6 | 4 | 12 | 6 |
| NM_002343 | LTF | 2361 | 16 | 3 | 1 | 0 | 1 | 0 | 3 | 0 | 16 | 1 | 0 | 6 | 2 |
| NM_002344 | LTK | 3127 | 0 | 0 | 0 | 0 | 0 | 0 | 0 | 0 | 0 | 0 | 0 | 0 | 0 |
| NM_002345 | LUM | 2116 | 14 | 5 | 14 | 4 | 9 | 1 | 4 | 1 | 13 | 8 | 1 | 0 | 4 |
| NM_002346 | LY6E | 1176 | 7 | 3 | 2 | 1 | 3 | 3 | 3 | 7 | 4 | 4 | 2 | 6 | 2 |
| NM_002347 | LY6H | 924 | 17 | 2 | 0 | 1 | 0 | 17 | 0 | 0 | 0 | 0 | 0 | 0 | 0 |
| NM_002348 | LY9 | 2457 | 2 | 0 | 0 | 0 | 0 | 0 | 0 | 0 | 0 | 0 | 0 | 2 | 0 |
| NM_002349 | LY75 | 6925 | 12 | 2 | 1 | 2 | 1 | 1 | 2 | 0 | 3 | 1 | 0 | 12 | 1 |
| NM_002350 | LYN | 3092 | 70 | 13 | 9 | 4 | 8 | 8 | 4 | 9 | 21 | 3 | 1 | 70 | 5 |

| transcript | gene | transcript length (nt) | max | mean | adipose | colon | heart | hypothalamus | kidney | liver | lung | ovary | skeletalmuscle | spleen | testes |
|---|---|---|---|---|---|---|---|---|---|---|---|---|---|---|---|
| NM_002351 | SH2D1A | 2501 | 2 | 0 | 0 | 0 | 0 | 0 | 0 | 0 | 1 | 0 | 0 | 2 | 0 |
| NM_002353 | TACSTD2 | 2072 | 15 | 3 | 0 | 0 | 0 | 0 | 15 | 1 | 10 | 0 | 0 | 0 | 1 |
| NM_002354 | EPCAM | 1716 | 63 | 14 | 0 | 63 | 0 | 2 | 62 | 1 | 17 | 1 | 0 | 1 | 7 |
| NM_002355 | M6PR | 2454 | 40 | 19 | 23 | 13 | 16 | 19 | 18 | 18 | 17 | 9 | 7 | 40 | 24 |
| NM_002356 | MARCKS | 4291 | 14 | 6 | 6 | 8 | 4 | 14 | 4 | 1 | 9 | 8 | 2 | 12 | 3 |
| NM_002357 | MXD1 | 5586 | 6 | 2 | 1 | 2 | 2 | 1 | 1 | 1 | 6 | 2 | 1 | 6 | 1 |
| NM_002358 | MAD2L1 | 1438 | 15 | 5 | 4 | 5 | 2 | 7 | 2 | 1 | 4 | 4 | 2 | 8 | 15 |
| NM_002359 | MAFG | 5038 | 2 | 1 | 0 | 1 | 1 | 2 | 1 | 1 | 1 | 0 | 1 | 1 | 1 |
| NM_002360 | MAFK | 3350 | 3 | 1 | 1 | 2 | 3 | 1 | 1 | 0 | 3 | 1 | 2 | 1 | 1 |
| NM_002361 | MAG | 2380 | 2 | 0 | 0 | 0 | 0 | 2 | 0 | 0 | 0 | 0 | 0 | 0 | 0 |
| NM_002362 | MAGEA4 | 1727 | 2 | 0 | 0 | 0 | 0 | 0 | 0 | 0 | 0 | 0 | 0 | 0 | 2 |
| NM_002363 | MAGEB1 | 1864 | 8 | 1 | 0 | 0 | 0 | 0 | 0 | 0 | 0 | 0 | 0 | 0 | 8 |
| NM_002364 | MAGEB2 | 1610 | 2 | 0 | 0 | 0 | 0 | 0 | 0 | 0 | 0 | 0 | 0 | 0 | 2 |
| NM_002365 | MAGEB3 | 2305 | 2 | 0 | 0 | 0 | 0 | 0 | 0 | 0 | 0 | 0 | 0 | 0 | 2 |
| NM_002367 | MAGEB4 | 2239 | 7 | 1 | 0 | 0 | 0 | 0 | 0 | 0 | 0 | 0 | 0 | 0 | 7 |
| NM_002370 | MAGOH | 681 | 3 | 2 | 2 | 2 | 2 | 2 | 2 | 1 | 2 | 2 | 1 | 3 | 1 |
| NM_002371 | MAL | 1056 | 10 | 2 | 0 | 0 | 0 | 7 | 10 | 0 | 1 | 0 | 0 | 0 | 0 |
| NM_002372 | MAN2A1 | 5128 | 8 | 5 | 3 | 2 | 3 | 6 | 6 | 8 | 6 | 4 | 2 | 7 | 6 |
| NM_002373 | MAP1A | 10275 | 90 | 11 | 4 | 3 | 9 | 90 | 1 | 0 | 1 | 3 | 2 | 1 | 5 |
| NM_002374 | MAP2 | 9445 | 165 | 17 | 1 | 2 | 2 | 165 | 1 | 1 | 5 | 2 | 1 | 0 | 3 |
| NM_002375 | MAP4 | 6327 | 38 | 17 | 22 | 8 | 33 | 20 | 11 | 8 | 9 | 10 | 38 | 10 | 15 |
| NM_002376 | MARK3 | 3458 | 21 | 12 | 7 | 7 | 21 | 13 | 10 | 7 | 14 | 12 | 11 | 14 | 15 |
| NM_002377 | MAS1 | 1134 | 1 | 0 | 0 | 0 | 0 | 1 | 0 | 0 | 0 | 0 | 0 | 0 | 0 |
| NM_002378 | MATK | 2079 | 1 | 0 | 1 | 0 | 0 | 1 | 0 | 0 | 0 | 0 | 0 | 0 | 0 |
| NM_002379 | MATN1 | 3828 | 0 | 0 | 0 | 0 | 0 | 0 | 0 | 0 | 0 | 0 | 0 | 0 | 0 |
| NM_002380 | MATN2 | 4108 | 13 | 4 | 5 | 3 | 3 | 3 | 3 | 3 | 1 | 13 | 0 | 1 | 6 |
| NM_002381 | MATN3 | 2583 | 0 | 0 | 0 | 0 | 0 | 0 | 0 | 0 | 0 | 0 | 0 | 0 | 0 |
| NM_002382 | MAX | 2002 | 19 | 10 | 10 | 10 | 9 | 11 | 9 | 5 | 11 | 14 | 5 | 19 | 10 |
| NM_002383 | MAZ | 2554 | 17 | 12 | 7 | 16 | 10 | 12 | 10 | 6 | 13 | 13 | 12 | 16 | 17 |
| NM_002384 | MBD1 | 2954 | 1 | 1 | 1 | 1 | 1 | 1 | 1 | 1 | 0 | 1 | 1 | 1 | 1 |
| NM_002385 | MBP | 2246 | 876 | 82 | 1 | 9 | 2 | 876 | 1 | 1 | 1 | 1 | 5 | 2 | 1 |
| NM_002386 | MC1R | 3099 | 0 | 0 | 0 | 0 | 0 | 0 | 0 | 0 | 0 | 0 | 0 | 0 | 0 |
| NM_002387 | MCC | 8223 | 15 | 4 | 2 | 1 | 3 | 2 | 2 | 4 | 3 | 15 | 1 | 3 | 2 |
| NM_002388 | MCM3 | 3111 | 13 | 7 | 7 | 8 | 7 | 6 | 7 | 5 | 6 | 9 | 4 | 13 | 10 |
| NM_002389 | CD46 | 3371 | 30 | 18 | 29 | 7 | 18 | 10 | 30 | 25 | 25 | 13 | 8 | 23 | 14 |
| NM_002390 | ADAM11 | 4402 | 2 | 0 | 0 | 0 | 0 | 2 | 0 | 0 | 0 | 0 | 0 | 0 | 0 |
| NM_002391 | MDK | 854 | 5 | 1 | 1 | 0 | 0 | 0 | 0 | 0 | 0 | 5 | 0 | 0 | 0 |
| NM_002392 | MDM2 | 7363 | 11 | 6 | 5 | 2 | 5 | 4 | 5 | 8 | 8 | 7 | 11 | 10 | 6 |
| NM_002393 | MDM4 | 10072 | 10 | 6 | 5 | 4 | 4 | 5 | 5 | 2 | 5 | 8 | 6 | 10 | 6 |
| NM_002394 | SLC3A2 | 2305 | 18 | 5 | 3 | 1 | 3 | 4 | 18 | 6 | 5 | 3 | 4 | 5 | 5 |
| NM_002395 | ME1 | 3358 | 14 | 5 | 14 | 1 | 5 | 8 | 1 | 4 | 4 | 3 | 5 | 4 | 7 |
| NM_002396 | ME2 | 4620 | 17 | 7 | 4 | 3 | 12 | 9 | 5 | 3 | 5 | 4 | 7 | 17 | 9 |
| NM_002397 | MEF2C | 6457 | 35 | 13 | 9 | 4 | 10 | 35 | 4 | 1 | 11 | 11 | 34 | 21 | 7 |
| NM_002398 | MEIS1 | 3181 | 12 | 4 | 1 | 4 | 5 | 2 | 3 | 1 | 4 | 12 | 1 | 6 | 4 |
| NM_002399 | MEIS2 | 3047 | 7 | 4 | 2 | 2 | 7 | 6 | 4 | 1 | 2 | 7 | 0 | 4 | 3 |
| NM_002401 | MAP3K3 | 4733 | 4 | 2 | 2 | 1 | 3 | 1 | 1 | 1 | 2 | 2 | 1 | 4 | 1 |
| NM_002402 | MEST | 2459 | 33 | 6 | 33 | 2 | 2 | 8 | 2 | 4 | 3 | 2 | 0 | 8 | 4 |
| NM_002403 | MFAP2 | 1101 | 3 | 1 | 1 | 1 | 1 | 0 | 0 | 0 | 1 | 3 | 0 | 0 | 1 |
| NM_002404 | MFAP4 | 1813 | 21 | 7 | 13 | 10 | 6 | 2 | 3 | 1 | 21 | 10 | 3 | 3 | 6 |
| NM_002405 | MFNG | 2129 | 0 | 0 | 0 | 0 | 0 | 0 | 0 | 0 | 0 | 0 | 0 | 0 | 0 |
| NM_002406 | MGAT1 | 2926 | 4 | 2 | 3 | 1 | 2 | 1 | 1 | 3 | 2 | 1 | 1 | 4 | 1 |
| NM_002407 | SCGB2A1 | 521 | 4 | 0 | 0 | 0 | 0 | 0 | 0 | 0 | 0 | 0 | 0 | 0 | 4 |
| NM_002408 | MGAT2 | 2711 | 6 | 3 | 2 | 2 | 3 | 2 | 3 | 4 | 2 | 2 | 1 | 6 | 6 |
| NM_002409 | MGAT3 | 5086 | 3 | 0 | 1 | 0 | 0 | 3 | 0 | 0 | 0 | 0 | 0 | 0 | 0 |
| NM_002410 | MGAT5 | 2421 | 7 | 3 | 1 | 2 | 4 | 5 | 2 | 0 | 2 | 2 | 0 | 7 | 2 |
| NM_002411 | SCGB2A2 | 502 | 1 | 0 | 0 | 0 | 0 | 0 | 0 | 0 | 0 | 1 | 0 | 0 | 0 |
| NM_002412 | MGMT | 1265 | 2 | 1 | 1 | 1 | 0 | 1 | 1 | 2 | 1 | 0 | 1 | 1 | 0 |
| NM_002413 | MGST2 | 800 | 4 | 2 | 2 | 1 | 4 | 2 | 2 | 4 | 1 | 1 | 1 | 2 | 2 |
| NM_002414 | CD99 | 1237 | 80 | 36 | 67 | 20 | 48 | 31 | 11 | 23 | 23 | 30 | 22 | 39 | 80 |
| NM_002415 | MIF | 561 | 100 | 57 | 18 | 22 | 86 | 70 | 97 | 55 | 69 | 53 | 14 | 100 | 46 |
| NM_002416 | CXCL9 | 2545 | 2 | 0 | 0 | 0 | 0 | 0 | 0 | 0 | 1 | 0 | 0 | 2 | 0 |
| NM_002417 | MKI67 | 12496 | 7 | 2 | 4 | 5 | 2 | 0 | 0 | 0 | 3 | 2 | 0 | 7 | 2 |
| NM_002418 | MLN | 572 | 0 | 0 | 0 | 0 | 0 | 0 | 0 | 0 | 0 | 0 | 0 | 0 | 0 |
| NM_002419 | MAP3K11 | 3573 | 2 | 1 | 1 | 1 | 1 | 0 | 1 | 2 | 1 | 0 | 0 | 1 | 0 |
| NM_002420 | TRPM1 | 5414 | 0 | 0 | 0 | 0 | 0 | 0 | 0 | 0 | 0 | 0 | 0 | 0 | 0 |
| NM_002421 | MMP1 | 2055 | 2 | 0 | 0 | 0 | 0 | 0 | 0 | 0 | 0 | 0 | 0 | 2 | 0 |
| NM_002422 | MMP3 | 1828 | 1 | 0 | 0 | 0 | 0 | 0 | 0 | 0 | 0 | 0 | 1 | 0 | 1 |
| NM_002423 | MMP7 | 1119 | 47 | 6 | 2 | 0 | 0 | 0 | 47 | 1 | 3 | 3 | 0 | 0 | 7 |
| NM_002424 | MMP8 | 3056 | 6 | 1 | 0 | 0 | 3 | 0 | 0 | 0 | 4 | 0 | 0 | 6 | 0 |
| NM_002425 | MMP10 | 1743 | 1 | 0 | 0 | 0 | 0 | 0 | 0 | 0 | 1 | 0 | 0 | 0 | 0 |
| NM_002426 | MMP12 | 1875 | 7 | 1 | 0 | 1 | 0 | 0 | 0 | 0 | 0 | 0 | 0 | 7 | 0 |
| NM_002427 | MMP13 | 2715 | 0 | 0 | 0 | 0 | 0 | 0 | 0 | 0 | 0 | 0 | 0 | 0 | 0 |
| NM_002428 | MMP15 | 4437 | 1 | 0 | 0 | 1 | 0 | 0 | 0 | 0 | 0 | 0 | 0 | 0 | 1 |
| NM_002429 | MMP19 | 3254 | 3 | 1 | 2 | 0 | 1 | 0 | 0 | 0 | 1 | 2 | 0 | 3 | 0 |
| NM_002430 | MN1 | 7556 | 2 | 0 | 0 | 0 | 0 | 1 | 0 | 0 | 0 | 0 | 2 | 0 | 0 |
| NM_002431 | MNAT1 | 1372 | 17 | 8 | 5 | 2 | 8 | 10 | 9 | 4 | 5 | 8 | 17 | 7 | 9 |
| NM_002432 | MNDA | 1667 | 20 | 4 | 3 | 0 | 4 | 2 | 1 | 1 | 10 | 1 | 1 | 20 | 2 |

| transcript | gene | transcript length (nt) | max | mean | adipose | colon | heart | hypothalamus | kidney | liver | lung | ovary | skeletalmuscle | spleen | testes |
|---|---|---|---|---|---|---|---|---|---|---|---|---|---|---|---|
| NM_002433 | MOG | 1908 | 14 | 2 | 0 | 2 | 0 | 14 | 0 | 0 | 0 | 0 | 0 | 0 | 0 |
| NM_002433 | MOG | 1912 | 14 | 2 | 0 | 2 | 0 | 14 | 0 | 0 | 0 | 0 | 0 | 0 | 0 |
| NM_002433 | MOG | 1916 | 14 | 2 | 0 | 2 | 0 | 14 | 0 | 0 | 0 | 0 | 0 | 0 | 0 |
| NM_002434 | MPG | 1282 | 0 | 0 | 0 | 0 | 0 | 0 | 0 | 0 | 0 | 0 | 0 | 0 | 0 |
| NM_002435 | MPI | 1771 | 2 | 1 | 1 | 1 | 1 | 2 | 1 | 1 | 0 | 1 | 1 | 1 | 1 |
| NM_002436 | MPP1 | 2046 | 2 | 1 | 1 | 0 | 1 | 1 | 2 | 1 | 2 | 0 | 1 | 2 | 1 |
| NM_002437 | MPV17 | 1007 | 7 | 4 | 3 | 2 | 6 | 5 | 7 | 1 | 4 | 4 | 3 | 4 | 2 |
| NM_002438 | MRC1 | 5171 | 0 | 0 | 0 | 0 | 0 | 0 | 0 | 0 | 0 | 0 | 0 | 0 | 0 |
| NM_002439 | MSH3 | 4620 | 3 | 2 | 2 | 1 | 1 | 2 | 3 | 2 | 1 | 2 | 1 | 2 | 1 |
| NM_002440 | MSH4 | 3203 | 1 | 0 | 0 | 0 | 0 | 0 | 0 | 0 | 0 | 0 | 1 | 0 | 1 |
| NM_002441 | MSH5 | 2467 | 1 | 1 | 1 | 1 | 0 | 0 | 1 | 0 | 0 | 1 | 0 | 1 | 1 |
| NM_002441 | MSH5 | 2933 | 1 | 1 | 0 | 1 | 0 | 0 | 1 | 0 | 0 | 1 | 0 | 1 | 1 |
| NM_002442 | MSI1 | 2950 | 1 | 0 | 0 | 0 | 0 | 1 | 0 | 0 | 0 | 0 | 0 | 0 | 0 |
| NM_002443 | MSMB | 569 | 11 | 1 | 0 | 0 | 0 | 0 | 0 | 1 | 11 | 0 | 0 | 0 | 0 |
| NM_002444 | MSN | 3970 | 106 | 47 | 57 | 16 | 67 | 22 | 62 | 28 | 106 | 22 | 12 | 102 | 22 |
| NM_002445 | MSR1 | 2960 | 22 | 6 | 22 | 1 | 9 | 1 | 1 | 4 | 15 | 3 | 1 | 5 | 2 |
| NM_002446 | MAP3K10 | 3435 | 1 | 0 | 0 | 0 | 0 | 1 | 0 | 0 | 0 | 0 | 0 | 0 | 0 |
| NM_002447 | MST1R | 4771 | 0 | 0 | 0 | 0 | 0 | 0 | 0 | 0 | 0 | 0 | 0 | 0 | 0 |
| NM_002448 | MSX1 | 1937 | 1 | 0 | 1 | 0 | 0 | 1 | 0 | 0 | 0 | 0 | 0 | 0 | 0 |
| NM_002449 | MSX2 | 2208 | 1 | 0 | 0 | 0 | 0 | 0 | 0 | 0 | 0 | 0 | 0 | 0 | 1 |
| NM_002451 | MTAP | 4921 | 3 | 2 | 2 | 1 | 1 | 1 | 2 | 1 | 1 | 3 | 1 | 3 | 2 |
| NM_002452 | NUDT1 | 662 | 1 | 0 | 0 | 0 | 0 | 0 | 0 | 0 | 0 | 0 | 0 | 1 | 0 |
| NM_002453 | MTIF2 | 2528 | 19 | 9 | 8 | 3 | 13 | 9 | 11 | 19 | 5 | 5 | 6 | 9 | 6 |
| NM_002454 | MTRR | 3298 | 7 | 4 | 3 | 1 | 3 | 2 | 5 | 7 | 4 | 3 | 5 | 4 | 3 |
| NM_002455 | MTX1 | 1632 | 1 | 1 | 1 | 1 | 0 | 1 | 1 | 1 | 0 | 0 | 0 | 1 | 1 |
| NM_002456 | MUC1 | 1199 | 22 | 5 | 0 | 6 | 0 | 0 | 20 | 0 | 22 | 0 | 0 | 0 | 2 |
| NM_002457 | MUC2 | 8623 | 1,234 | 112 | 0 | 1,234 | 0 | 0 | 0 | 0 | 0 | 0 | 0 | 0 | 0 |
| NM_002458 | MUC5B | 17924 | 13 | 2 | 0 | 13 | 0 | 0 | 0 | 0 | 5 | 3 | 0 | 0 | 0 |
| NM_002460 | IRF4 | 5314 | 2 | 0 | 0 | 1 | 0 | 0 | 0 | 0 | 0 | 0 | 0 | 2 | 0 |
| NM_002461 | MVD | 1795 | 0 | 0 | 0 | 0 | 0 | 0 | 0 | 0 | 0 | 0 | 0 | 0 | 0 |
| NM_002462 | MX1 | 2965 | 14 | 6 | 5 | 2 | 8 | 6 | 3 | 2 | 5 | 10 | 8 | 14 | 3 |
| NM_002463 | MX2 | 2961 | 5 | 1 | 2 | 0 | 2 | 1 | 0 | 0 | 1 | 1 | 2 | 5 | 1 |
| NM_002465 | MYBPC1 | 3936 | 2,117 | 194 | 4 | 0 | 0 | 3 | 0 | 0 | 0 | 0 | 2,117 | 0 | 5 |
| NM_002466 | MYBL2 | 2713 | 2 | 0 | 0 | 1 | 0 | 0 | 0 | 0 | 0 | 0 | 0 | 2 | 1 |
| NM_002467 | MYC | 2364 | 8 | 4 | 3 | 3 | 3 | 1 | 1 | 8 | 2 | 6 | 4 | 6 | 2 |
| NM_002468 | MYD88 | 2847 | 9 | 3 | 2 | 2 | 2 | 1 | 3 | 5 | 3 | 2 | 1 | 9 | 2 |
| NM_002469 | MYF6 | 1294 | 59 | 5 | 0 | 0 | 0 | 0 | 0 | 0 | 0 | 0 | 59 | 0 | 0 |
| NM_002470 | MYH3 | 6017 | 5 | 1 | 0 | 1 | 0 | 0 | 0 | 0 | 0 | 0 | 5 | 0 | 1 |
| NM_002471 | MYH6 | 5937 | 720 | 66 | 0 | 0 | 720 | 0 | 0 | 0 | 0 | 0 | 9 | 0 | 0 |
| NM_002472 | MYH8 | 6038 | 4 | 0 | 0 | 0 | 0 | 0 | 1 | 0 | 0 | 0 | 4 | 0 | 0 |
| NM_002473 | MYH9 | 7504 | 58 | 23 | 25 | 24 | 25 | 11 | 21 | 13 | 39 | 14 | 7 | 58 | 13 |
| NM_002474 | MYH11 | 6882 | 401 | 85 | 15 | 401 | 59 | 20 | 20 | 1 | 46 | 86 | 26 | 29 | 232 |
| NM_002475 | MYL6B | 869 | 8 | 2 | 1 | 0 | 2 | 3 | 2 | 1 | 1 | 2 | 8 | 1 | 7 |
| NM_002476 | MYL4 | 855 | 81 | 7 | 0 | 0 | 81 | 0 | 0 | 0 | 0 | 0 | 0 | 0 | 1 |
| NM_002477 | MYL5 | 661 | 0 | 0 | 0 | 0 | 0 | 0 | 0 | 0 | 0 | 0 | 0 | 0 | 0 |
| NM_002478 | MYOD1 | 1806 | 0 | 0 | 0 | 0 | 0 | 0 | 0 | 0 | 0 | 0 | 0 | 0 | 0 |
| NM_002479 | MYOG | 1744 | 1 | 0 | 0 | 0 | 0 | 0 | 0 | 0 | 1 | 0 | 1 | 0 | 0 |
| NM_002480 | PPP1R12A | 5725 | 19 | 12 | 9 | 5 | 12 | 16 | 9 | 4 | 14 | 14 | 9 | 18 | 19 |
| NM_002481 | PPP1R12B | 11108 | 41 | 9 | 3 | 12 | 41 | 6 | 3 | 1 | 5 | 9 | 11 | 5 | 5 |
| NM_002482 | NASP | 3223 | 28 | 13 | 9 | 9 | 17 | 11 | 8 | 7 | 13 | 12 | 7 | 18 | 28 |
| NM_002483 | CEACAM6 | 2631 | 67 | 7 | 0 | 6 | 0 | 0 | 0 | 0 | 67 | 0 | 0 | 0 | 0 |
| NM_002484 | NUBP1 | 1232 | 1 | 1 | 1 | 1 | 1 | 0 | 1 | 1 | 1 | 1 | 1 | 1 | 1 |
| NM_002485 | NBN | 4621 | 4 | 2 | 2 | 0 | 2 | 2 | 3 | 2 | 2 | 3 | 1 | 4 | 2 |
| NM_002486 | NCBP1 | 5380 | 11 | 6 | 4 | 4 | 6 | 7 | 5 | 6 | 4 | 7 | 5 | 9 | 11 |
| NM_002487 | NDN | 1897 | 33 | 12 | 15 | 5 | 14 | 33 | 4 | 0 | 4 | 27 | 3 | 12 | 12 |
| NM_002488 | NDUFA2 | 586 | 2 | 1 | 1 | 2 | 1 | 1 | 2 | 1 | 1 | 1 | 0 | 1 | 1 |
| NM_002489 | NDUFA4 | 823 | 100 | 31 | 15 | 8 | 100 | 78 | 25 | 25 | 11 | 11 | 38 | 19 | 11 |
| NM_002490 | NDUFA6 | 1184 | 26 | 10 | 8 | 6 | 26 | 15 | 13 | 13 | 4 | 4 | 13 | 7 | 5 |
| NM_002491 | NDUFB3 | 755 | 6 | 2 | 2 | 1 | 6 | 3 | 4 | 3 | 1 | 0 | 1 | 2 | 2 |
| NM_002492 | NDUFB5 | 1059 | 17 | 8 | 8 | 5 | 13 | 17 | 12 | 12 | 5 | 4 | 6 | 5 | 4 |
| NM_002493 | NDUFB6 | 862 | 2 | 1 | 0 | 0 | 1 | 2 | 1 | 1 | 0 | 0 | 1 | 1 | 0 |
| NM_002494 | NDUFC1 | 412 | 314 | 115 | 50 | 46 | 314 | 134 | 174 | 93 | 53 | 82 | 134 | 106 | 78 |
| NM_002495 | NDUFS4 | 676 | 28 | 14 | 13 | 8 | 17 | 28 | 14 | 18 | 6 | 9 | 21 | 7 | 10 |
| NM_002496 | NDUFS8 | 794 | 2 | 1 | 1 | 1 | 2 | 0 | 2 | 0 | 0 | 0 | 2 | 1 | 0 |
| NM_002497 | NEK2 | 2130 | 9 | 1 | 0 | 1 | 0 | 0 | 0 | 0 | 0 | 0 | 0 | 1 | 9 |
| NM_002498 | NEK3 | 2396 | 7 | 2 | 3 | 2 | 2 | 1 | 3 | 1 | 2 | 1 | 1 | 2 | 7 |
| NM_002499 | NEO1 | 7036 | 6 | 3 | 1 | 5 | 1 | 6 | 4 | 1 | 2 | 4 | 3 | 1 | 4 |
| NM_002500 | NEUROD1 | 2630 | 2 | 0 | 0 | 0 | 0 | 1 | 0 | 1 | 2 | 0 | 0 | 0 | 1 |
| NM_002501 | NFIX | 5576 | 17 | 6 | 9 | 5 | 6 | 11 | 1 | 2 | 5 | 8 | 17 | 2 | 4 |
| NM_002502 | NFKB2 | 3042 | 2 | 1 | 1 | 1 | 1 | 0 | 1 | 0 | 1 | 1 | 1 | 2 | 1 |
| NM_002503 | NFKBIB | 1191 | 1 | 1 | 1 | 1 | 1 | 0 | 1 | 1 | 0 | 0 | 1 | 1 | 1 |
| NM_002504 | NFX1 | 4603 | 4 | 3 | 4 | 2 | 3 | 4 | 2 | 3 | 2 | 4 | 3 | 4 | 3 |
| NM_002505 | NFYA | 6218 | 4 | 2 | 1 | 1 | 1 | 2 | 2 | 0 | 2 | 4 | 2 | 3 | 3 |
| NM_002506 | NGF | 1049 | 1 | 0 | 0 | 0 | 1 | 0 | 0 | 0 | 0 | 1 | 0 | 0 | 0 |
| NM_002507 | NGFR | 3410 | 5 | 1 | 1 | 1 | 5 | 1 | 1 | 1 | 0 | 0 | 0 | 2 | 2 |
| NM_002508 | NID1 | 5881 | 19 | 9 | 7 | 6 | 12 | 2 | 8 | 15 | 7 | 11 | 2 | 19 | 6 |
| NM_002509 | NKX2-2 | 2088 | 1 | 0 | 0 | 0 | 0 | 1 | 0 | 0 | 0 | 0 | 0 | 0 | 0 |

| | | transcript | | | | | | hypothala | | | | | skeletalm | | |
|---|---|---|---|---|---|---|---|---|---|---|---|---|---|---|---|
| transcript | gene | length (nt) | max | mean | adipose | colon | heart | mus | kidney | liver | lung | ovary | uscle | spleen | testes |
| NM_002510 | GPNMB | 2730 | 56 | 18 | 51 | 7 | 21 | 1 | 4 | 3 | 56 | 20 | 8 | 10 | 14 |
| NM_002511 | NMBR | 1354 | 0 | 0 | 0 | 0 | 0 | 0 | 0 | 0 | 0 | 0 | 0 | 0 | 0 |
| NM_002512 | NME2 | 822 | 0 | 0 | 0 | 0 | 0 | 0 | 0 | 0 | 0 | 0 | 0 | 0 | 0 |
| NM_002513 | NME3 | 1014 | 0 | 0 | 0 | 0 | 0 | 0 | 0 | 0 | 0 | 0 | 0 | 0 | 0 |
| NM_002514 | NOV | 2601 | 5 | 1 | 1 | 0 | 5 | 3 | 1 | 0 | 0 | 0 | 1 | 0 | 1 |
| NM_002515 | NOVA1 | 3918 | 4 | 1 | 2 | 0 | 1 | 4 | 1 | 0 | 0 | 1 | 1 | 0 | 1 |
| NM_002516 | NOVA2 | 1877 | 2 | 0 | 0 | 0 | 0 | 2 | 0 | 0 | 0 | 0 | 0 | 0 | 0 |
| NM_002517 | NPAS1 | 2043 | 0 | 0 | 0 | 0 | 0 | 0 | 0 | 0 | 0 | 0 | 0 | 0 | 0 |
| NM_002518 | NPAS2 | 4004 | 3 | 1 | 0 | 1 | 3 | 3 | 2 | 2 | 1 | 0 | 1 | 1 | 1 |
| NM_002519 | NPAT | 5937 | 10 | 4 | 2 | 1 | 3 | 3 | 3 | 2 | 5 | 7 | 3 | 10 | 6 |
| NM_002520 | NPM1 | 1448 | 71 | 31 | 23 | 18 | 28 | 24 | 27 | 30 | 34 | 71 | 20 | 35 | 29 |
| NM_002521 | NPPB | 698 | 54 | 5 | 0 | 0 | 54 | 0 | 0 | 0 | 0 | 0 | 0 | 0 | 0 |
| NM_002522 | NPTX1 | 5437 | 5 | 1 | 0 | 1 | 0 | 5 | 0 | 0 | 0 | 0 | 0 | 0 | 0 |
| NM_002523 | NPTX2 | 2713 | 8 | 2 | 0 | 0 | 2 | 7 | 0 | 0 | 0 | 1 | 0 | 4 | 8 |
| NM_002524 | NRAS | 4461 | 8 | 4 | 3 | 3 | 3 | 5 | 6 | 5 | 4 | 3 | 1 | 8 | 4 |
| NM_002525 | NRD1 | 4024 | 32 | 12 | 8 | 4 | 15 | 11 | 8 | 14 | 7 | 7 | 32 | 13 | 12 |
| NM_002526 | NT5E | 4057 | 7 | 3 | 4 | 3 | 2 | 3 | 3 | 4 | 1 | 7 | 1 | 4 | 5 |
| NM_002527 | NTF3 | 1168 | 1 | 0 | 0 | 0 | 0 | 0 | 0 | 0 | 0 | 1 | 0 | 1 | 0 |
| NM_002528 | NTHL1 | 1067 | 0 | 0 | 0 | 0 | 0 | 0 | 0 | 0 | 0 | 0 | 0 | 0 | 0 |
| NM_002529 | NTRK1 | 2655 | 0 | 0 | 0 | 0 | 0 | 0 | 0 | 0 | 0 | 0 | 0 | 0 | 0 |
| NM_002530 | NTRK3 | 2818 | 4 | 1 | 0 | 1 | 0 | 4 | 0 | 0 | 0 | 0 | 0 | 0 | 0 |
| NM_002531 | NTSR1 | 4132 | 0 | 0 | 0 | 0 | 0 | 0 | 0 | 0 | 0 | 0 | 0 | 0 | 0 |
| NM_002532 | NUP88 | 2358 | 9 | 5 | 3 | 2 | 4 | 4 | 4 | 8 | 3 | 2 | 7 | 6 | 9 |
| NM_002533 | NVL | 2906 | 4 | 2 | 2 | 2 | 1 | 2 | 3 | 1 | 2 | 4 | 1 | 4 | 3 |
| NM_002534 | OAS1 | 1470 | 4 | 2 | 4 | 2 | 1 | 1 | 1 | 2 | 2 | 1 | 1 | 3 | 1 |
| NM_002535 | OAS2 | 3647 | 3 | 1 | 1 | 0 | 1 | 0 | 0 | 0 | 1 | 1 | 1 | 3 | 0 |
| NM_002536 | TBC1D25 | 3812 | 1 | 1 | 0 | 0 | 1 | 1 | 0 | 0 | 0 | 0 | 0 | 1 | 1 |
| NM_002537 | OAZ2 | 1916 | 34 | 20 | 13 | 9 | 19 | 34 | 13 | 18 | 13 | 17 | 23 | 34 | 22 |
| NM_002538 | OCLN | 1312 | 9 | 3 | 0 | 4 | 0 | 1 | 8 | 6 | 9 | 0 | 0 | 0 | 2 |
| NM_002538 | OCLN | 1314 | 9 | 3 | 0 | 4 | 0 | 1 | 8 | 6 | 9 | 0 | 0 | 0 | 2 |
| NM_002538 | OCLN | 2638 | 5 | 1 | 0 | 2 | 0 | 0 | 4 | 3 | 5 | 0 | 0 | 0 | 1 |
| NM_002538 | OCLN | 2640 | 5 | 1 | 0 | 2 | 0 | 0 | 4 | 3 | 5 | 0 | 0 | 0 | 1 |
| NM_002539 | ODC1 | 2062 | 15 | 9 | 4 | 6 | 9 | 5 | 14 | 15 | 4 | 6 | 13 | 10 | 14 |
| NM_002540 | ODF2 | 3708 | 17 | 3 | 2 | 2 | 2 | 2 | 2 | 1 | 2 | 2 | 1 | 3 | 17 |
| NM_002541 | OGDH | 4301 | 22 | 9 | 8 | 6 | 22 | 10 | 17 | 6 | 5 | 2 | 16 | 6 | 4 |
| NM_002542 | OGG1 | 1634 | 1 | 0 | 0 | 0 | 0 | 0 | 1 | 0 | 0 | 0 | 0 | 0 | 0 |
| NM_002543 | OLR1 | 2516 | 12 | 2 | 1 | 0 | 0 | 6 | 2 | 0 | 12 | 0 | 0 | 0 | 0 |
| NM_002544 | OMG | 1894 | 38 | 4 | 1 | 1 | 1 | 38 | 1 | 0 | 2 | 2 | 0 | 1 | 1 |
| NM_002545 | OPCML | 6300 | 10 | 1 | 0 | 0 | 0 | 10 | 0 | 0 | 0 | 0 | 0 | 0 | 0 |
| NM_002546 | TNFRSF11B | 2346 | 13 | 2 | 1 | 0 | 13 | 0 | 3 | 0 | 1 | 0 | 0 | 0 | 0 |
| NM_002547 | OPHN1 | 7531 | 4 | 2 | 1 | 1 | 2 | 4 | 2 | 0 | 2 | 2 | 1 | 1 | 1 |
| NM_002548 | OR1D2 | 939 | 0 | 0 | 0 | 0 | 0 | 0 | 0 | 0 | 0 | 0 | 0 | 0 | 0 |
| NM_002550 | OR3A1 | 948 | 0 | 0 | 0 | 0 | 0 | 0 | 0 | 0 | 0 | 0 | 0 | 0 | 0 |
| NM_002551 | OR3A2 | 1085 | 0 | 0 | 0 | 0 | 0 | 0 | 0 | 0 | 0 | 0 | 0 | 0 | 0 |
| NM_002552 | ORC4L | 2818 | 14 | 7 | 4 | 2 | 6 | 14 | 9 | 7 | 5 | 10 | 6 | 8 | 11 |
| NM_002553 | ORC5L | 1926 | 5 | 2 | 1 | 1 | 1 | 2 | 2 | 2 | 1 | 1 | 1 | 3 | 5 |
| NM_002555 | SLC22A18 | 1538 | 0 | 0 | 0 | 0 | 0 | 0 | 0 | 0 | 0 | 0 | 0 | 0 | 0 |
| NM_002556 | OSBP | 5083 | 41 | 18 | 14 | 12 | 13 | 17 | 25 | 41 | 12 | 14 | 16 | 14 | 16 |
| NM_002557 | OVGP1 | 2242 | 0 | 0 | 0 | 0 | 0 | 0 | 0 | 0 | 0 | 0 | 0 | 0 | 0 |
| NM_002558 | P2RX1 | 2864 | 0 | 0 | 0 | 0 | 0 | 0 | 0 | 0 | 0 | 0 | 0 | 0 | 0 |
| NM_002559 | P2RX3 | 1349 | 0 | 0 | 0 | 0 | 0 | 0 | 0 | 0 | 0 | 0 | 0 | 0 | 0 |
| NM_002560 | P2RX4 | 2028 | 2 | 1 | 2 | 2 | 1 | 1 | 2 | 1 | 1 | 2 | 0 | 2 | 1 |
| NM_002561 | P2RX5 | 2193 | 8 | 1 | 0 | 0 | 1 | 1 | 0 | 0 | 0 | 0 | 1 | 8 | 0 |
| NM_002562 | P2RX7 | 3137 | 2 | 0 | 1 | 1 | 0 | 2 | 0 | 0 | 0 | 1 | 0 | 0 | 0 |
| NM_002563 | P2RY1 | 3108 | 1 | 1 | 0 | 1 | 0 | 1 | 0 | 0 | 1 | 1 | 0 | 1 | 0 |
| NM_002564 | P2RY2 | 2524 | 1 | 0 | 0 | 0 | 1 | 0 | 0 | 0 | 0 | 0 | 1 | 1 | 0 |
| NM_002565 | P2RY4 | 1639 | 0 | 0 | 0 | 0 | 0 | 0 | 0 | 0 | 0 | 0 | 0 | 0 | 0 |
| NM_002566 | P2RY11 | 1976 | 0 | 0 | 0 | 0 | 0 | 0 | 0 | 0 | 0 | 0 | 0 | 0 | 0 |
| NM_002567 | PEBP1 | 1491 | 72 | 30 | 17 | 7 | 39 | 63 | 72 | 49 | 13 | 22 | 23 | 14 | 16 |
| NM_002568 | PABPC1 | 2860 | 119 | 52 | 47 | 31 | 38 | 23 | 30 | 43 | 67 | 90 | 16 | 119 | 62 |
| NM_002569 | FURIN | 4180 | 19 | 4 | 2 | 1 | 4 | 2 | 2 | 19 | 3 | 1 | 1 | 5 | 1 |
| NM_002570 | PCSK6 | 4552 | 26 | 5 | 0 | 1 | 2 | 3 | 0 | 26 | 0 | 1 | 0 | 16 | 3 |
| NM_002571 | PAEP | 811 | 1 | 0 | 0 | 0 | 0 | 0 | 0 | 0 | 0 | 1 | 0 | 0 | 0 |
| NM_002572 | PAFAH1B2 | 1191 | 51 | 28 | 25 | 18 | 25 | 51 | 26 | 22 | 25 | 24 | 11 | 31 | 50 |
| NM_002573 | PAFAH1B3 | 1076 | 0 | 0 | 0 | 0 | 0 | 0 | 0 | 0 | 0 | 0 | 0 | 0 | 0 |
| NM_002574 | PRDX1 | 1234 | 114 | 59 | 43 | 26 | 64 | 69 | 114 | 79 | 58 | 29 | 51 | 73 | 39 |
| NM_002575 | SERPINB2 | 1904 | 0 | 0 | 0 | 0 | 0 | 0 | 0 | 0 | 0 | 0 | 0 | 0 | 0 |
| NM_002576 | PAK1 | 3430 | 42 | 10 | 4 | 12 | 3 | 42 | 7 | 2 | 6 | 8 | 6 | 9 | 9 |
| NM_002577 | PAK2 | 6139 | 9 | 4 | 3 | 2 | 4 | 2 | 3 | 4 | 3 | 3 | 3 | 9 | 4 |
| NM_002578 | PAK3 | 2754 | 33 | 4 | 0 | 0 | 0 | 33 | 0 | 0 | 0 | 1 | 0 | 1 | 5 |
| NM_002579 | PALM | 2874 | 5 | 2 | 3 | 1 | 2 | 5 | 2 | 0 | 2 | 3 | 1 | 2 | 2 |
| NM_002580 | REG3A | 807 | 6 | 1 | 0 | 6 | 0 | 0 | 0 | 0 | 0 | 0 | 0 | 0 | 0 |
| NM_002581 | PAPPA | 10970 | 5 | 1 | 0 | 0 | 1 | 0 | 5 | 0 | 0 | 0 | 0 | 0 | 1 |
| NM_002582 | PARN | 3077 | 7 | 5 | 5 | 4 | 4 | 7 | 5 | 5 | 5 | 5 | 3 | 7 | 7 |
| NM_002583 | PAWR | 1951 | 25 | 12 | 4 | 14 | 9 | 5 | 16 | 11 | 16 | 25 | 2 | 8 | 21 |
| NM_002584 | PAX7 | 2259 | 0 | 0 | 0 | 0 | 0 | 0 | 0 | 0 | 0 | 0 | 0 | 0 | 0 |
| NM_002585 | PBX1 | 6683 | 9 | 4 | 4 | 3 | 3 | 6 | 5 | 0 | 3 | 9 | 8 | 2 | 4 |

| transcript | gene | transcript length (nt) | max | mean | adipose | colon | heart | hypothalamus | kidney | liver | lung | ovary | skeletalmuscle | spleen | testes |
|---|---|---|---|---|---|---|---|---|---|---|---|---|---|---|---|
| NM_002586 | PBX2 | 3210 | 5 | 2 | 1 | 2 | 2 | 2 | 2 | 2 | 4 | 2 | 1 | 5 | 3 |
| NM_002586 | PBX2 | 3213 | 5 | 2 | 1 | 2 | 2 | 2 | 2 | 2 | 4 | 2 | 1 | 5 | 3 |
| NM_002587 | PCDH1 | 3796 | 4 | 2 | 1 | 3 | 1 | 4 | 3 | 2 | 2 | 0 | 0 | 0 | 0 |
| NM_002588 | PCDHGC3 | 4726 | 5 | 1 | 1 | 1 | 1 | 5 | 0 | 0 | 2 | 1 | 0 | 1 | 1 |
| NM_002589 | PCDH7 | 4646 | 7 | 2 | 0 | 1 | 3 | 7 | 0 | 0 | 1 | 1 | 0 | 4 | 1 |
| NM_002590 | PCDH8 | 4002 | 2 | 0 | 0 | 0 | 0 | 2 | 0 | 0 | 0 | 0 | 0 | 0 | 0 |
| NM_002591 | PCK1 | 2685 | 178 | 30 | 9 | 11 | 1 | 0 | 128 | 178 | 1 | 1 | 0 | 1 | 0 |
| NM_002592 | PCNA | 1355 | 12 | 6 | 7 | 12 | 5 | 8 | 4 | 7 | 4 | 3 | 3 | 9 | 6 |
| NM_002593 | PCOLCE | 1620 | 1 | 0 | 1 | 0 | 1 | 0 | 0 | 0 | 0 | 0 | 0 | 0 | 0 |
| NM_002594 | PCSK2 | 4744 | 10 | 1 | 0 | 0 | 0 | 10 | 0 | 0 | 1 | 2 | 0 | 0 | 3 |
| NM_002595 | CDK17 | 4121 | 7 | 3 | 2 | 1 | 3 | 7 | 2 | 2 | 5 | 2 | 2 | 6 | 4 |
| NM_002596 | CDK18 | 3183 | 8 | 2 | 1 | 2 | 8 | 7 | 3 | 1 | 1 | 1 | 0 | 1 | 0 |
| NM_002597 | PDC | 1210 | 0 | 0 | 0 | 0 | 0 | 0 | 0 | 0 | 0 | 0 | 0 | 0 | 0 |
| NM_002598 | PDCD2 | 1311 | 8 | 5 | 5 | 3 | 5 | 5 | 6 | 8 | 4 | 3 | 3 | 8 | 4 |
| NM_002599 | PDE2A | 4311 | 3 | 1 | 1 | 0 | 0 | 3 | 0 | 0 | 0 | 0 | 0 | 3 | 0 |
| NM_002600 | PDE4B | 4258 | 20 | 7 | 3 | 1 | 7 | 20 | 1 | 2 | 7 | 2 | 11 | 18 | 4 |
| NM_002601 | PDE6D | 1118 | 2 | 1 | 1 | 0 | 1 | 2 | 1 | 1 | 1 | 0 | 0 | 1 | 0 |
| NM_002602 | PDE6G | 1023 | 0 | 0 | 0 | 0 | 0 | 0 | 0 | 0 | 0 | 0 | 0 | 0 | 0 |
| NM_002603 | PDE7A | 3135 | 7 | 3 | 1 | 2 | 6 | 1 | 4 | 0 | 4 | 1 | 5 | 7 | 2 |
| NM_002604 | PDE7A | 2977 | 7 | 3 | 1 | 2 | 4 | 1 | 3 | 1 | 3 | 1 | 4 | 7 | 2 |
| NM_002605 | PDE8A | 3917 | 4 | 3 | 4 | 3 | 2 | 4 | 4 | 4 | 2 | 3 | 1 | 2 | 2 |
| NM_002606 | PDE9A | 2060 | 12 | 3 | 1 | 12 | 1 | 2 | 5 | 0 | 1 | 1 | 0 | 6 | 1 |
| NM_002607 | PDGFA | 2805 | 4 | 2 | 1 | 1 | 2 | 4 | 1 | 1 | 2 | 0 | 1 | 3 | 1 |
| NM_002608 | PDGFB | 3375 | 3 | 1 | 2 | 1 | 1 | 1 | 1 | 0 | 3 | 0 | 1 | 1 | 0 |
| NM_002609 | PDGFRB | 5714 | 4 | 2 | 4 | 1 | 2 | 1 | 1 | 0 | 2 | 4 | 1 | 3 | 2 |
| NM_002610 | PDK1 | 4576 | 6 | 3 | 2 | 2 | 6 | 2 | 1 | 2 | 3 | 3 | 2 | 4 | 3 |
| NM_002611 | PDK2 | 2290 | 6 | 4 | 2 | 3 | 5 | 5 | 6 | 2 | 1 | 4 | 6 | 2 | 3 |
| NM_002612 | PDK4 | 3700 | 336 | 80 | 67 | 6 | 268 | 7 | 42 | 10 | 26 | 39 | 336 | 55 | 21 |
| NM_002613 | PDPK1 | 7236 | 6 | 3 | 2 | 4 | 3 | 6 | 4 | 2 | 2 | 3 | 3 | 4 | 3 |
| NM_002614 | PDZK1 | 2107 | 33 | 3 | 0 | 0 | 0 | 0 | 33 | 3 | 0 | 0 | 0 | 0 | 0 |
| NM_002615 | SERPINF1 | 1542 | 37 | 14 | 36 | 4 | 13 | 8 | 2 | 37 | 4 | 10 | 8 | 9 | 26 |
| NM_002616 | PER1 | 4709 | 5 | 3 | 2 | 2 | 2 | 1 | 1 | 2 | 3 | 4 | 5 | 3 | 2 |
| NM_002617 | PEX10 | 2014 | 4 | 2 | 2 | 1 | 1 | 4 | 2 | 2 | 1 | 1 | 1 | 1 | 2 |
| NM_002618 | PEX13 | 4514 | 8 | 4 | 3 | 2 | 4 | 3 | 5 | 8 | 3 | 4 | 5 | 4 | 4 |
| NM_002619 | PF4 | 476 | 4 | 1 | 0 | 0 | 0 | 0 | 0 | 0 | 1 | 0 | 0 | 4 | 0 |
| NM_002620 | PF4V1 | 741 | 0 | 0 | 0 | 0 | 0 | 0 | 0 | 0 | 0 | 0 | 0 | 0 | 0 |
| NM_002621 | CFP | 1713 | 1 | 0 | 0 | 0 | 0 | 0 | 0 | 0 | 0 | 0 | 0 | 1 | 0 |
| NM_002622 | PFDN1 | 1324 | 37 | 16 | 18 | 10 | 20 | 37 | 13 | 9 | 8 | 12 | 17 | 14 | 16 |
| NM_002623 | PFDN4 | 1346 | 10 | 4 | 4 | 2 | 4 | 10 | 3 | 2 | 2 | 2 | 2 | 4 | 4 |
| NM_002624 | PFDN5 | 687 | 49 | 30 | 35 | 15 | 32 | 36 | 23 | 22 | 28 | 45 | 21 | 49 | 26 |
| NM_002625 | PFKFB1 | 1733 | 6 | 1 | 6 | 0 | 0 | 0 | 0 | 2 | 0 | 0 | 2 | 0 | 0 |
| NM_002626 | PFKL | 2913 | 3 | 1 | 1 | 2 | 1 | 2 | 3 | 1 | 1 | 1 | 0 | 1 | 1 |
| NM_002627 | PFKP | 2616 | 9 | 2 | 1 | 2 | 5 | 9 | 2 | 0 | 1 | 1 | 2 | 1 | 3 |
| NM_002628 | PFN2 | 1784 | 25 | 7 | 3 | 2 | 5 | 25 | 10 | 1 | 2 | 5 | 12 | 3 | 5 |
| NM_002629 | PGAM1 | 1720 | 30 | 13 | 13 | 8 | 17 | 30 | 15 | 18 | 10 | 6 | 2 | 17 | 13 |
| NM_002630 | PGC | 1374 | 25 | 2 | 0 | 0 | 0 | 0 | 0 | 0 | 25 | 0 | 0 | 0 | 0 |
| NM_002631 | PGD | 1937 | 17 | 6 | 17 | 3 | 4 | 6 | 4 | 7 | 7 | 3 | 1 | 10 | 7 |
| NM_002632 | PGF | 1730 | 2 | 1 | 0 | 0 | 2 | 1 | 0 | 1 | 0 | 1 | 1 | 0 | 0 |
| NM_002633 | PGM1 | 2473 | 52 | 14 | 12 | 7 | 28 | 4 | 7 | 27 | 5 | 5 | 52 | 3 | 3 |
| NM_002634 | PHB | 1826 | 11 | 5 | 4 | 4 | 5 | 3 | 11 | 9 | 3 | 2 | 2 | 3 | 3 |
| NM_002635 | SLC25A3 | 1677 | 67 | 27 | 20 | 15 | 67 | 22 | 34 | 29 | 14 | 24 | 38 | 21 | 11 |
| NM_002636 | PHF1 | 2188 | 2 | 1 | 1 | 1 | 1 | 1 | 1 | 0 | 1 | 2 | 1 | 1 | 1 |
| NM_002637 | PHKA1 | 6161 | 5 | 1 | 0 | 0 | 1 | 1 | 1 | 0 | 0 | 1 | 5 | 1 | 1 |
| NM_002638 | PI3 | 553 | 3 | 0 | 0 | 1 | 0 | 0 | 0 | 0 | 3 | 0 | 0 | 0 | 0 |
| NM_002639 | SERPINB5 | 2633 | 0 | 0 | 0 | 0 | 0 | 0 | 0 | 0 | 0 | 0 | 0 | 0 | 0 |
| NM_002640 | SERPINB8 | 3373 | 1 | 1 | 1 | 0 | 1 | 0 | 0 | 1 | 1 | 0 | 0 | 1 | 0 |
| NM_002641 | PIGA | 3605 | 2 | 1 | 0 | 0 | 1 | 1 | 1 | 1 | 2 | 1 | 0 | 1 | 1 |
| NM_002642 | PIGC | 1500 | 7 | 4 | 3 | 2 | 4 | 4 | 5 | 2 | 4 | 7 | 3 | 6 | 6 |
| NM_002643 | PIGF | 1028 | 0 | 0 | 0 | 0 | 0 | 0 | 0 | 0 | 0 | 0 | 0 | 0 | 0 |
| NM_002644 | PIGR | 4273 | 325 | 39 | 0 | 325 | 0 | 0 | 53 | 3 | 43 | 1 | 0 | 0 | 0 |
| NM_002645 | PIK3C2A | 8288 | 15 | 8 | 7 | 2 | 9 | 10 | 11 | 8 | 10 | 6 | 3 | 11 | 15 |
| NM_002646 | PIK3C2B | 7606 | 2 | 1 | 2 | 2 | 1 | 1 | 1 | 1 | 1 | 0 | 0 | 1 | 0 |
| NM_002647 | PIK3C3 | 3067 | 21 | 12 | 10 | 5 | 10 | 20 | 11 | 9 | 8 | 16 | 8 | 18 | 21 |
| NM_002648 | PIM1 | 2707 | 31 | 15 | 13 | 6 | 21 | 18 | 7 | 8 | 16 | 26 | 16 | 31 | 8 |
| NM_002649 | PIK3CG | 5379 | 5 | 1 | 1 | 1 | 1 | 0 | 0 | 0 | 1 | 0 | 0 | 5 | 1 |
| NM_002650 | PI4KA | 3038 | 13 | 3 | 1 | 2 | 1 | 13 | 1 | 3 | 1 | 1 | 1 | 3 | 2 |
| NM_002651 | PI4KB | 3471 | 3 | 2 | 2 | 2 | 2 | 3 | 2 | 1 | 1 | 2 | 1 | 2 | 2 |
| NM_002652 | PIP | 576 | 3 | 0 | 0 | 0 | 0 | 0 | 0 | 0 | 3 | 0 | 0 | 0 | 0 |
| NM_002653 | PITX1 | 2383 | 0 | 0 | 0 | 0 | 0 | 0 | 0 | 0 | 0 | 0 | 0 | 0 | 0 |
| NM_002654 | PKM2 | 2458 | 84 | 38 | 35 | 25 | 57 | 84 | 48 | 4 | 28 | 15 | 42 | 45 | 36 |
| NM_002655 | PLAG1 | 7338 | 1 | 0 | 0 | 0 | 0 | 0 | 0 | 0 | 1 | 1 | 0 | 1 | 1 |
| NM_002656 | PLAGL1 | 2944 | 2 | 1 | 1 | 1 | 1 | 0 | 1 | 0 | 1 | 2 | 1 | 1 | 1 |
| NM_002657 | PLAGL2 | 5656 | 1 | 1 | 0 | 1 | 1 | 0 | 1 | 0 | 1 | 1 | 1 | 1 | 1 |
| NM_002658 | PLAU | 2375 | 4 | 1 | 2 | 0 | 1 | 0 | 4 | 0 | 1 | 0 | 1 | 4 | 0 |
| NM_002659 | PLAUR | 1548 | 4 | 1 | 1 | 1 | 2 | 1 | 0 | 0 | 4 | 1 | 0 | 2 | 1 |
| NM_002660 | PLCG1 | 5205 | 7 | 5 | 4 | 7 | 5 | 6 | 4 | 2 | 4 | 5 | 2 | 6 | 6 |
| NM_002661 | PLCG2 | 4241 | 9 | 2 | 2 | 2 | 1 | 1 | 6 | 1 | 2 | 0 | 0 | 9 | 1 |

| transcript | gene | transcript length (nt) | max | mean | adipose | colon | heart | hypothalamus | kidney | liver | lung | ovary | skeletalmuscle | spleen | testes |
|---|---|---|---|---|---|---|---|---|---|---|---|---|---|---|---|
| NM_002662 | PLD1 | 5591 | 16 | 5 | 3 | 3 | 8 | 4 | 5 | 3 | 3 | 16 | 1 | 10 | 3 |
| NM_002663 | PLD2 | 3471 | 1 | 0 | 1 | 0 | 0 | 0 | 0 | 0 | 0 | 1 | 0 | 0 | 0 |
| NM_002664 | PLEK | 2852 | 52 | 12 | 22 | 2 | 10 | 8 | 2 | 3 | 20 | 2 | 2 | 52 | 3 |
| NM_002665 | PLGLB2 | 3014 | 0 | 0 | 0 | 0 | 0 | 0 | 0 | 0 | 0 | 0 | 0 | 0 | 0 |
| NM_002665 | PLGLB2 | 3015 | 0 | 0 | 0 | 0 | 0 | 0 | 0 | 0 | 0 | 0 | 0 | 0 | 0 |
| NM_002666 | PLIN1 | 2965 | 208 | 20 | 208 | 9 | 4 | 0 | 1 | 1 | 0 | 0 | 2 | 0 | 1 |
| NM_002667 | PLN | 1714 | 169 | 19 | 1 | 1 | 169 | 1 | 2 | 0 | 3 | 3 | 23 | 1 | 5 |
| NM_002668 | PLP2 | 929 | 54 | 26 | 17 | 35 | 54 | 5 | 9 | 17 | 39 | 30 | 34 | 33 | 18 |
| NM_002669 | PLRG1 | 1783 | 6 | 4 | 4 | 2 | 4 | 5 | 5 | 6 | 3 | 4 | 2 | 6 | 4 |
| NM_002670 | PLS1 | 3719 | 3 | 1 | 0 | 3 | 0 | 1 | 3 | 2 | 1 | 0 | 0 | 0 | 0 |
| NM_002673 | PLXNB1 | 7310 | 2 | 1 | 0 | 1 | 0 | 1 | 2 | 1 | 0 | 0 | 0 | 0 | 0 |
| NM_002674 | PMCH | 757 | 0 | 0 | 0 | 0 | 0 | 0 | 0 | 0 | 0 | 0 | 0 | 0 | 0 |
| NM_002675 | PML | 2238 | 4 | 1 | 1 | 1 | 2 | 0 | 1 | 0 | 3 | 2 | 1 | 4 | 1 |
| NM_002676 | PMM1 | 1283 | 7 | 4 | 2 | 3 | 4 | 5 | 7 | 3 | 5 | 7 | 2 | 2 | 5 |
| NM_002677 | PMP2 | 3566 | 47 | 4 | 0 | 0 | 0 | 47 | 0 | 0 | 0 | 0 | 0 | 0 | 0 |
| NM_002685 | EXOSC10 | 2732 | 10 | 7 | 8 | 6 | 5 | 7 | 8 | 6 | 6 | 8 | 5 | 10 | 7 |
| NM_002686 | PNMT | 1157 | 0 | 0 | 0 | 0 | 0 | 0 | 0 | 0 | 0 | 0 | 0 | 0 | 0 |
| NM_002687 | PNN | 3606 | 47 | 30 | 20 | 16 | 26 | 29 | 30 | 19 | 30 | 45 | 24 | 47 | 41 |
| NM_002688 | 05 Sep | 3525 | 7 | 1 | 0 | 0 | 2 | 7 | 0 | 0 | 0 | 0 | 0 | 0 | 0 |
| NM_002689 | POLA2 | 2477 | 1 | 1 | 1 | 1 | 0 | 1 | 0 | 0 | 1 | 1 | 0 | 1 | 1 |
| NM_002690 | POLB | 1259 | 11 | 5 | 4 | 1 | 5 | 8 | 3 | 11 | 3 | 4 | 5 | 3 | 9 |
| NM_002691 | POLD1 | 3447 | 0 | 0 | 0 | 0 | 0 | 0 | 0 | 0 | 0 | 0 | 0 | 0 | 0 |
| NM_002692 | POLE2 | 1689 | 1 | 0 | 0 | 0 | 0 | 0 | 0 | 1 | 0 | 0 | 0 | 0 | 1 |
| NM_002693 | POLG | 4448 | 4 | 2 | 2 | 2 | 2 | 1 | 2 | 2 | 2 | 1 | 2 | 4 | 2 |
| NM_002695 | POLR2E | 2866 | 3 | 2 | 3 | 1 | 1 | 2 | 2 | 2 | 1 | 1 | 1 | 3 | 1 |
| NM_002696 | POLR2G | 835 | 3 | 1 | 1 | 1 | 1 | 3 | 1 | 2 | 0 | 1 | 1 | 1 | 1 |
| NM_002697 | POU2F1 | 2673 | 9 | 4 | 2 | 3 | 3 | 2 | 3 | 2 | 4 | 6 | 3 | 9 | 6 |
| NM_002698 | POU2F2 | 4497 | 3 | 1 | 0 | 0 | 0 | 1 | 0 | 0 | 1 | 0 | 0 | 3 | 0 |
| NM_002699 | POU3F1 | 2928 | 0 | 0 | 0 | 0 | 0 | 0 | 0 | 0 | 0 | 0 | 0 | 0 | 0 |
| NM_002700 | POU4F3 | 1182 | 0 | 0 | 0 | 0 | 0 | 0 | 0 | 0 | 0 | 0 | 0 | 0 | 0 |
| NM_002701 | POU5F1 | 1401 | 0 | 0 | 0 | 0 | 0 | 0 | 0 | 0 | 0 | 0 | 0 | 0 | 0 |
| NM_002702 | POU6F1 | 4698 | 1 | 0 | 0 | 0 | 0 | 1 | 0 | 0 | 0 | 1 | 0 | 0 | 0 |
| NM_002703 | PPAT | 3745 | 7 | 3 | 1 | 0 | 2 | 4 | 3 | 2 | 1 | 5 | 2 | 3 | 7 |
| NM_002704 | PPBP | 700 | 7 | 1 | 0 | 0 | 0 | 0 | 0 | 0 | 2 | 0 | 0 | 7 | 0 |
| NM_002705 | PPL | 6238 | 8 | 4 | 8 | 2 | 3 | 3 | 4 | 5 | 7 | 2 | 4 | 0 | 4 |
| NM_002706 | PPM1B | 2622 | 47 | 28 | 24 | 12 | 26 | 36 | 20 | 37 | 23 | 19 | 47 | 36 | 29 |
| NM_002707 | PPM1G | 2153 | 45 | 18 | 16 | 13 | 12 | 16 | 16 | 23 | 13 | 9 | 15 | 18 | 45 |
| NM_002708 | PPP1CA | 1470 | 8 | 4 | 3 | 4 | 4 | 3 | 6 | 6 | 3 | 1 | 2 | 8 | 3 |
| NM_002709 | PPP1CB | 4986 | 102 | 52 | 31 | 19 | 84 | 50 | 36 | 23 | 53 | 55 | 102 | 61 | 56 |
| NM_002710 | PPP1CC | 2483 | 22 | 13 | 11 | 10 | 22 | 11 | 7 | 7 | 8 | 16 | 17 | 16 | 14 |
| NM_002711 | PPP1R3A | 4296 | 33 | 5 | 0 | 0 | 18 | 0 | 0 | 0 | 0 | 0 | 33 | 0 | 0 |
| NM_002712 | PPP1R7 | 1293 | 33 | 16 | 16 | 8 | 17 | 33 | 14 | 16 | 9 | 9 | 15 | 14 | 24 |
| NM_002713 | PPP1R8 | 2590 | 4 | 3 | 2 | 2 | 2 | 3 | 2 | 2 | 2 | 3 | 2 | 3 | 4 |
| NM_002714 | PPP1R10 | 4519 | 21 | 10 | 5 | 11 | 5 | 8 | 10 | 7 | 7 | 21 | 14 | 16 | 10 |
| NM_002715 | PPP2CA | 2643 | 25 | 11 | 6 | 6 | 16 | 25 | 10 | 9 | 7 | 8 | 9 | 13 | 11 |
| NM_002716 | PPP2R1B | 5605 | 12 | 3 | 12 | 2 | 1 | 1 | 2 | 5 | 1 | 2 | 1 | 1 | 2 |
| NM_002717 | PPP2R2A | 2363 | 14 | 9 | 8 | 3 | 11 | 13 | 8 | 5 | 9 | 9 | 10 | 9 | 14 |
| NM_002718 | PPP2R3A | 6743 | 14 | 5 | 2 | 1 | 8 | 3 | 14 | 0 | 2 | 5 | 10 | 1 | 6 |
| NM_002719 | PPP2R5C | 4432 | 22 | 12 | 7 | 5 | 22 | 18 | 8 | 6 | 11 | 14 | 8 | 22 | 14 |
| NM_002720 | PPP4C | 1331 | 4 | 2 | 2 | 3 | 1 | 2 | 2 | 4 | 2 | 0 | 1 | 2 | 2 |
| NM_002721 | PPP6C | 4239 | 6 | 4 | 3 | 2 | 4 | 5 | 4 | 3 | 3 | 3 | 6 | 4 | 4 |
| NM_002722 | PPY | 457 | 0 | 0 | 0 | 0 | 0 | 0 | 0 | 0 | 0 | 0 | 0 | 0 | 0 |
| NM_002723 | PRB4 | 915 | 8 | 1 | 0 | 0 | 0 | 0 | 0 | 0 | 8 | 0 | 0 | 0 | 0 |
| NM_002725 | PRELP | 5819 | 19 | 10 | 11 | 5 | 16 | 3 | 3 | 3 | 19 | 19 | 9 | 12 | 7 |
| NM_002726 | PREP | 2849 | 15 | 8 | 5 | 9 | 8 | 8 | 6 | 10 | 5 | 6 | 15 | 8 | 13 |
| NM_002727 | SRGN | 1254 | 29 | 7 | 5 | 1 | 7 | 3 | 3 | 2 | 22 | 1 | 1 | 29 | 1 |
| NM_002728 | PRG2 | 857 | 7 | 1 | 0 | 0 | 0 | 3 | 0 | 0 | 7 | 0 | 0 | 1 | 0 |
| NM_002729 | HHEX | 1759 | 7 | 2 | 1 | 1 | 1 | 1 | 1 | 5 | 2 | 1 | 1 | 7 | 1 |
| NM_002730 | PRKACA | 2673 | 8 | 5 | 3 | 2 | 8 | 7 | 3 | 6 | 2 | 5 | 6 | 7 | 3 |
| NM_002731 | PRKACB | 4513 | 21 | 4 | 2 | 2 | 2 | 21 | 3 | 1 | 2 | 2 | 1 | 6 | 2 |
| NM_002732 | PRKACG | 1584 | 0 | 0 | 0 | 0 | 0 | 0 | 0 | 0 | 0 | 0 | 0 | 0 | 0 |
| NM_002733 | PRKAG1 | 1692 | 5 | 4 | 5 | 2 | 5 | 5 | 5 | 5 | 2 | 2 | 5 | 4 | 4 |
| NM_002734 | PRKAR1A | 3619 | 110 | 70 | 55 | 21 | 105 | 110 | 66 | 39 | 70 | 61 | 88 | 77 | 77 |
| NM_002735 | PRKAR1B | 2512 | 14 | 2 | 0 | 1 | 1 | 14 | 1 | 1 | 1 | 1 | 0 | 1 | 1 |
| NM_002736 | PRKAR2B | 3661 | 48 | 10 | 48 | 3 | 6 | 12 | 2 | 1 | 3 | 14 | 0 | 10 | 12 |
| NM_002737 | PRKCA | 8770 | 13 | 3 | 1 | 3 | 1 | 13 | 4 | 2 | 1 | 3 | 1 | 3 | 2 |
| NM_002738 | PRKCB | 8012 | 31 | 6 | 1 | 2 | 1 | 31 | 0 | 1 | 2 | 0 | 0 | 19 | 3 |
| NM_002739 | PRKCG | 3128 | 6 | 1 | 0 | 0 | 0 | 6 | 0 | 0 | 0 | 0 | 0 | 0 | 0 |
| NM_002740 | PRKCI | 4884 | 3 | 1 | 1 | 1 | 1 | 2 | 2 | 1 | 1 | 1 | 1 | 1 | 3 |
| NM_002741 | PKN1 | 3080 | 3 | 1 | 2 | 1 | 2 | 2 | 1 | 1 | 2 | 1 | 1 | 3 | 2 |
| NM_002742 | PRKD1 | 3677 | 8 | 3 | 2 | 1 | 2 | 2 | 6 | 1 | 3 | 3 | 1 | 1 | 8 |
| NM_002743 | PRKCSH | 2261 | 15 | 9 | 9 | 11 | 6 | 7 | 10 | 15 | 6 | 10 | 3 | 13 | 10 |
| NM_002744 | PRKCZ | 2326 | 3 | 1 | 0 | 2 | 0 | 3 | 1 | 0 | 1 | 0 | 0 | 1 | 1 |
| NM_002745 | MAPK1 | 5915 | 27 | 9 | 5 | 4 | 9 | 27 | 9 | 7 | 7 | 6 | 6 | 12 | 9 |
| NM_002746 | MAPK3 | 1876 | 5 | 2 | 2 | 5 | 2 | 4 | 1 | 0 | 1 | 1 | 1 | 1 | 1 |
| NM_002747 | MAPK4 | 4736 | 8 | 2 | 0 | 1 | 8 | 6 | 1 | 0 | 0 | 0 | 0 | 0 | 1 |
| NM_002748 | MAPK6 | 4224 | 13 | 8 | 4 | 4 | 6 | 12 | 7 | 13 | 8 | 5 | 9 | 8 | 13 |

| transcript | gene | transcript length (nt) | max | mean | adipose | colon | heart | hypothalamus | kidney | liver | lung | ovary | skeletalmuscle | spleen | testes |
|---|---|---|---|---|---|---|---|---|---|---|---|---|---|---|---|
| NM_002749 | MAPK7 | 2938 | 1 | 0 | 0 | 0 | 0 | 0 | 0 | 0 | 0 | 0 | 0 | 1 | 0 |
| NM_002750 | MAPK8 | 1417 | 8 | 5 | 2 | 8 | 4 | 7 | 5 | 5 | 4 | 8 | 3 | 4 | 6 |
| NM_002751 | MAPK11 | 2388 | 0 | 0 | 0 | 0 | 0 | 0 | 0 | 0 | 0 | 0 | 0 | 0 | 0 |
| NM_002752 | MAPK9 | 4333 | 12 | 4 | 3 | 2 | 4 | 12 | 3 | 2 | 2 | 2 | 3 | 4 | 3 |
| NM_002753 | MAPK10 | 3727 | 30 | 4 | 3 | 1 | 1 | 30 | 4 | 0 | 1 | 3 | 0 | 1 | 4 |
| NM_002754 | MAPK13 | 1888 | 6 | 2 | 1 | 6 | 0 | 1 | 2 | 0 | 3 | 0 | 0 | 1 | 4 |
| NM_002755 | MAP2K1 | 2586 | 15 | 5 | 5 | 2 | 6 | 15 | 4 | 8 | 5 | 2 | 5 | 6 | 2 |
| NM_002756 | MAP2K3 | 2303 | 3 | 2 | 1 | 1 | 2 | 1 | 2 | 2 | 2 | 0 | 2 | 3 | 0 |
| NM_002757 | MAP2K5 | 2334 | 2 | 1 | 1 | 1 | 1 | 2 | 1 | 1 | 1 | 1 | 1 | 1 | 2 |
| NM_002758 | MAP2K6 | 1869 | 6 | 1 | 1 | 2 | 1 | 1 | 1 | 0 | 1 | 1 | 6 | 1 | 0 |
| NM_002759 | EIF2AK2 | 2930 | 9 | 6 | 6 | 2 | 6 | 7 | 6 | 4 | 5 | 9 | 5 | 7 | 5 |
| NM_002761 | PRM1 | 409 | 928 | 85 | 0 | 0 | 0 | 1 | 0 | 1 | 0 | 0 | 0 | 0 | 928 |
| NM_002762 | PRM2 | 683 | 151 | 14 | 0 | 0 | 0 | 0 | 0 | 0 | 0 | 0 | 0 | 0 | 151 |
| NM_002763 | PROX1 | 3071 | 19 | 4 | 0 | 1 | 4 | 2 | 2 | 19 | 0 | 3 | 2 | 6 | 1 |
| NM_002764 | PRPS1 | 2156 | 5 | 2 | 2 | 1 | 1 | 5 | 2 | 3 | 1 | 2 | 2 | 2 | 2 |
| NM_002765 | PRPS2 | 2509 | 70 | 12 | 3 | 3 | 3 | 4 | 9 | 4 | 5 | 22 | 1 | 6 | 70 |
| NM_002766 | PRPSAP1 | 2359 | 14 | 7 | 7 | 6 | 5 | 13 | 6 | 14 | 6 | 5 | 2 | 5 | 10 |
| NM_002767 | PRPSAP2 | 1890 | 5 | 3 | 1 | 2 | 2 | 5 | 4 | 3 | 2 | 3 | 1 | 3 | 3 |
| NM_002768 | CHMP1A | 2354 | 1 | 0 | 0 | 0 | 0 | 1 | 1 | 0 | 0 | 0 | 0 | 1 | 0 |
| NM_002769 | PRSS1 | 817 | 1 | 0 | 1 | 1 | 0 | 0 | 0 | 0 | 0 | 0 | 0 | 0 | 0 |
| NM_002770 | PRSS2 | 802 | 1 | 0 | 1 | 1 | 0 | 0 | 0 | 0 | 0 | 0 | 0 | 0 | 0 |
| NM_002771 | PRSS3 | 808 | 1 | 0 | 0 | 1 | 0 | 1 | 0 | 0 | 0 | 0 | 0 | 0 | 0 |
| NM_002772 | PRSS7 | 3943 | 0 | 0 | 0 | 0 | 0 | 0 | 0 | 0 | 0 | 0 | 0 | 0 | 0 |
| NM_002773 | PRSS8 | 1940 | 2 | 1 | 0 | 2 | 0 | 0 | 2 | 1 | 1 | 0 | 0 | 0 | 0 |
| NM_002774 | KLK6 | 1511 | 3 | 0 | 0 | 0 | 0 | 3 | 0 | 0 | 0 | 0 | 0 | 0 | 0 |
| NM_002775 | HTRA1 | 2120 | 39 | 12 | 28 | 3 | 6 | 22 | 8 | 12 | 4 | 39 | 2 | 4 | 5 |
| NM_002776 | KLK10 | 3106 | 0 | 0 | 0 | 0 | 0 | 0 | 0 | 0 | 0 | 0 | 0 | 0 | 0 |
| NM_002777 | PRTN3 | 1001 | 0 | 0 | 0 | 0 | 0 | 0 | 0 | 0 | 0 | 0 | 0 | 0 | 0 |
| NM_002778 | PSAP | 2822 | 141 | 77 | 126 | 28 | 77 | 112 | 66 | 59 | 94 | 53 | 41 | 141 | 52 |
| NM_002779 | PSD | 4183 | 1 | 0 | 0 | 0 | 0 | 1 | 0 | 0 | 0 | 0 | 0 | 0 | 0 |
| NM_002780 | PSG4 | 2032 | 0 | 0 | 0 | 0 | 0 | 0 | 0 | 0 | 0 | 0 | 0 | 0 | 0 |
| NM_002781 | PSG5 | 1640 | 0 | 0 | 0 | 0 | 0 | 0 | 0 | 0 | 0 | 0 | 0 | 0 | 0 |
| NM_002782 | PSG6 | 1391 | 0 | 0 | 0 | 0 | 0 | 0 | 0 | 0 | 0 | 0 | 0 | 0 | 0 |
| NM_002783 | PSG7 | 2038 | 0 | 0 | 0 | 0 | 0 | 0 | 0 | 0 | 0 | 0 | 0 | 0 | 0 |
| NM_002784 | PSG9 | 1706 | 0 | 0 | 0 | 0 | 0 | 0 | 0 | 0 | 0 | 0 | 0 | 0 | 0 |
| NM_002785 | PSG11 | 1541 | 0 | 0 | 0 | 0 | 0 | 0 | 0 | 0 | 0 | 0 | 0 | 0 | 0 |
| NM_002786 | PSMA1 | 1253 | 11 | 6 | 5 | 2 | 11 | 7 | 7 | 7 | 6 | 4 | 8 | 10 | 5 |
| NM_002787 | PSMA2 | 1464 | 18 | 7 | 9 | 4 | 6 | 8 | 6 | 18 | 4 | 3 | 5 | 7 | 8 |
| NM_002788 | PSMA3 | 927 | 12 | 6 | 6 | 2 | 8 | 7 | 7 | 12 | 5 | 4 | 5 | 9 | 4 |
| NM_002789 | PSMA4 | 1221 | 39 | 19 | 20 | 8 | 22 | 21 | 20 | 39 | 15 | 9 | 13 | 25 | 19 |
| NM_002790 | PSMA5 | 974 | 12 | 6 | 6 | 4 | 6 | 12 | 7 | 12 | 3 | 3 | 5 | 6 | 5 |
| NM_002791 | PSMA6 | 1018 | 23 | 15 | 13 | 7 | 22 | 16 | 12 | 23 | 14 | 8 | 22 | 16 | 17 |
| NM_002792 | PSMA7 | 984 | 19 | 12 | 13 | 8 | 17 | 11 | 12 | 14 | 11 | 4 | 19 | 13 | 5 |
| NM_002793 | PSMB1 | 916 | 15 | 9 | 11 | 5 | 10 | 11 | 8 | 15 | 5 | 5 | 10 | 9 | 5 |
| NM_002794 | PSMB2 | 845 | 81 | 33 | 29 | 20 | 27 | 51 | 27 | 81 | 29 | 18 | 27 | 33 | 25 |
| NM_002795 | PSMB3 | 754 | 13 | 5 | 3 | 4 | 4 | 5 | 4 | 13 | 3 | 1 | 3 | 6 | 3 |
| NM_002796 | PSMB4 | 925 | 9 | 5 | 5 | 5 | 4 | 6 | 5 | 9 | 3 | 3 | 4 | 5 | 3 |
| NM_002797 | PSMB5 | 1293 | 17 | 9 | 11 | 6 | 10 | 17 | 8 | 17 | 5 | 3 | 10 | 6 | 4 |
| NM_002798 | PSMB6 | 826 | 64 | 36 | 29 | 18 | 64 | 62 | 34 | 58 | 16 | 16 | 49 | 36 | 18 |
| NM_002799 | PSMB7 | 984 | 4 | 3 | 2 | 1 | 4 | 4 | 3 | 4 | 2 | 2 | 3 | 2 | 3 |
| NM_002800 | PSMB9 | 1048 | 6 | 2 | 1 | 2 | 2 | 1 | 1 | 1 | 3 | 1 | 1 | 6 | 0 |
| NM_002800 | PSMB9 | 1050 | 6 | 2 | 1 | 2 | 2 | 1 | 1 | 1 | 3 | 1 | 1 | 6 | 0 |
| NM_002801 | PSMB10 | 979 | 3 | 2 | 2 | 2 | 2 | 3 | 1 | 1 | 2 | 0 | 1 | 3 | 1 |
| NM_002802 | PSMC1 | 1586 | 70 | 41 | 43 | 14 | 60 | 56 | 31 | 50 | 21 | 28 | 70 | 40 | 39 |
| NM_002803 | PSMC2 | 1527 | 43 | 20 | 25 | 8 | 22 | 30 | 18 | 43 | 11 | 9 | 23 | 19 | 17 |
| NM_002804 | PSMC3 | 1580 | 26 | 14 | 13 | 11 | 18 | 20 | 12 | 26 | 7 | 7 | 21 | 8 | 7 |
| NM_002805 | PSMC5 | 1320 | 13 | 6 | 6 | 2 | 7 | 9 | 8 | 13 | 3 | 2 | 7 | 6 | 5 |
| NM_002806 | PSMC6 | 1592 | 36 | 19 | 10 | 3 | 36 | 17 | 21 | 12 | 15 | 23 | 27 | 26 | 15 |
| NM_002807 | PSMD1 | 3227 | 41 | 20 | 19 | 5 | 24 | 41 | 15 | 30 | 9 | 11 | 27 | 18 | 19 |
| NM_002808 | PSMD2 | 2922 | 20 | 13 | 13 | 7 | 14 | 20 | 11 | 20 | 6 | 6 | 18 | 11 | 15 |
| NM_002809 | PSMD3 | 2147 | 8 | 5 | 3 | 3 | 4 | 7 | 3 | 7 | 3 | 2 | 8 | 5 | 5 |
| NM_002810 | PSMD4 | 1331 | 7 | 4 | 5 | 2 | 4 | 5 | 2 | 6 | 2 | 2 | 7 | 3 | 3 |
| NM_002811 | PSMD7 | 1662 | 50 | 24 | 22 | 10 | 50 | 31 | 19 | 33 | 16 | 8 | 41 | 21 | 19 |
| NM_002812 | PSMD8 | 1539 | 23 | 13 | 15 | 6 | 23 | 14 | 10 | 16 | 6 | 5 | 18 | 12 | 12 |
| NM_002813 | PSMD9 | 2343 | 2 | 1 | 1 | 1 | 1 | 1 | 2 | 2 | 1 | 1 | 0 | 1 | 1 |
| NM_002814 | PSMD10 | 1546 | 9 | 3 | 2 | 2 | 2 | 9 | 4 | 2 | 3 | 2 | 1 | 3 | 5 |
| NM_002815 | PSMD11 | 1581 | 25 | 16 | 13 | 8 | 18 | 14 | 18 | 24 | 13 | 7 | 25 | 18 | 13 |
| NM_002816 | PSMD12 | 1797 | 24 | 16 | 11 | 4 | 21 | 18 | 14 | 24 | 12 | 13 | 22 | 20 | 21 |
| NM_002817 | PSMD13 | 1757 | 5 | 3 | 3 | 3 | 3 | 4 | 3 | 5 | 2 | 1 | 4 | 5 | 3 |
| NM_002818 | PSME2 | 829 | 37 | 12 | 7 | 8 | 11 | 12 | 11 | 37 | 8 | 5 | 4 | 19 | 4 |
| NM_002819 | PTBP1 | 3281 | 21 | 11 | 7 | 12 | 8 | 5 | 11 | 16 | 13 | 12 | 4 | 21 | 12 |
| NM_002820 | PTHLH | 1873 | 1 | 0 | 0 | 0 | 0 | 1 | 0 | 0 | 0 | 0 | 0 | 0 | 0 |
| NM_002821 | PTK7 | 4249 | 3 | 1 | 0 | 0 | 0 | 0 | 1 | 0 | 1 | 3 | 0 | 1 | 1 |
| NM_002822 | TWF1 | 3045 | 12 | 6 | 5 | 2 | 6 | 4 | 12 | 9 | 6 | 6 | 2 | 9 | 8 |
| NM_002823 | PTMA | 1204 | 503 | 227 | 160 | 212 | 171 | 174 | 204 | 134 | 362 | 274 | 96 | 503 | 203 |
| NM_002824 | PTMS | 1150 | 105 | 51 | 45 | 56 | 34 | 52 | 105 | 63 | 70 | 41 | 10 | 43 | 37 |
| NM_002825 | PTN | 1542 | 61 | 9 | 0 | 3 | 7 | 61 | 4 | 0 | 2 | 2 | 0 | 3 | 23 |

| transcript | gene | transcript length (nt) | max | mean | adipose | colon | heart | hypothalamus | kidney | liver | lung | ovary | skeletalmuscle | spleen | testes |
|---|---|---|---|---|---|---|---|---|---|---|---|---|---|---|---|
| NM_002826 | QSOX1 | 3316 | 5 | 2 | 1 | 2 | 2 | 2 | 1 | 2 | 3 | 2 | 1 | 1 | 5 |
| NM_002827 | PTPN1 | 3316 | 10 | 4 | 3 | 2 | 4 | 3 | 6 | 2 | 6 | 2 | 1 | 10 | 3 |
| NM_002828 | PTPN2 | 2472 | 16 | 7 | 5 | 3 | 7 | 8 | 5 | 5 | 9 | 10 | 4 | 16 | 7 |
| NM_002829 | PTPN3 | 6716 | 6 | 2 | 1 | 1 | 3 | 1 | 6 | 4 | 1 | 0 | 5 | 0 | 1 |
| NM_002830 | PTPN4 | 3943 | 8 | 4 | 3 | 1 | 4 | 7 | 6 | 1 | 3 | 6 | 3 | 5 | 8 |
| NM_002831 | PTPN6 | 2246 | 3 | 1 | 0 | 1 | 0 | 0 | 0 | 0 | 1 | 0 | 0 | 3 | 0 |
| NM_002832 | PTPN7 | 2805 | 1 | 0 | 0 | 0 | 0 | 0 | 0 | 0 | 0 | 0 | 0 | 1 | 1 |
| NM_002833 | PTPN9 | 3938 | 3 | 2 | 1 | 2 | 2 | 3 | 2 | 2 | 2 | 3 | 1 | 3 | 2 |
| NM_002834 | PTPN11 | 6283 | 31 | 21 | 31 | 7 | 29 | 30 | 16 | 31 | 15 | 13 | 25 | 18 | 16 |
| NM_002835 | PTPN12 | 3225 | 25 | 11 | 12 | 3 | 14 | 7 | 9 | 6 | 21 | 10 | 5 | 25 | 10 |
| NM_002836 | PTPRA | 3620 | 17 | 5 | 5 | 4 | 3 | 17 | 5 | 2 | 4 | 6 | 2 | 6 | 5 |
| NM_002837 | PTPRB | 10661 | 17 | 6 | 4 | 2 | 7 | 2 | 6 | 6 | 10 | 3 | 2 | 17 | 2 |
| NM_002838 | PTPRC | 5330 | 72 | 12 | 7 | 3 | 6 | 4 | 2 | 2 | 30 | 2 | 1 | 72 | 3 |
| NM_002839 | PTPRD | 10078 | 13 | 3 | 1 | 2 | 1 | 13 | 8 | 1 | 1 | 6 | 0 | 0 | 2 |
| NM_002840 | PTPRF | 7733 | 4 | 2 | 4 | 4 | 0 | 3 | 2 | 4 | 2 | 1 | 0 | 0 | 1 |
| NM_002841 | PTPRG | 6648 | 7 | 4 | 4 | 2 | 5 | 4 | 7 | 3 | 6 | 4 | 2 | 5 | 6 |
| NM_002842 | PTPRH | 3927 | 2 | 0 | 0 | 2 | 0 | 0 | 0 | 1 | 0 | 0 | 0 | 2 | 0 |
| NM_002843 | PTPRJ | 7849 | 20 | 5 | 3 | 2 | 3 | 6 | 7 | 4 | 5 | 1 | 0 | 20 | 1 |
| NM_002844 | PTPRK | 6086 | 10 | 5 | 2 | 5 | 5 | 9 | 6 | 10 | 5 | 6 | 2 | 3 | 2 |
| NM_002845 | PTPRM | 5561 | 14 | 8 | 14 | 3 | 13 | 8 | 8 | 2 | 9 | 6 | 7 | 6 | 6 |
| NM_002846 | PTPRN | 3632 | 7 | 1 | 0 | 0 | 0 | 7 | 0 | 0 | 0 | 0 | 0 | 0 | 0 |
| NM_002847 | PTPRN2 | 4827 | 8 | 1 | 0 | 1 | 0 | 8 | 0 | 0 | 0 | 0 | 0 | 1 | 0 |
| NM_002848 | PTPRO | 5069 | 4 | 1 | 0 | 0 | 0 | 4 | 3 | 0 | 0 | 0 | 0 | 0 | 0 |
| NM_002849 | PTPRR | 3490 | 4 | 0 | 0 | 0 | 0 | 4 | 0 | 0 | 0 | 0 | 0 | 0 | 0 |
| NM_002850 | PTPRS | 7347 | 6 | 2 | 6 | 1 | 2 | 5 | 2 | 1 | 3 | 2 | 1 | 1 | 2 |
| NM_002851 | PTPRZ1 | 8159 | 21 | 2 | 0 | 0 | 0 | 21 | 0 | 0 | 0 | 0 | 0 | 0 | 0 |
| NM_002852 | PTX3 | 1938 | 11 | 2 | 0 | 0 | 11 | 1 | 1 | 0 | 4 | 0 | 0 | 3 | 0 |
| NM_002853 | RAD1 | 4682 | 2 | 1 | 1 | 1 | 2 | 2 | 1 | 1 | 1 | 1 | 1 | 1 | 2 |
| NM_002854 | PVALB | 572 | 14 | 3 | 0 | 1 | 0 | 14 | 13 | 0 | 0 | 0 | 0 | 1 | 0 |
| NM_002855 | PVRL1 | 5480 | 5 | 1 | 0 | 1 | 1 | 5 | 1 | 0 | 1 | 1 | 0 | 1 | 0 |
| NM_002856 | PVRL2 | 2151 | 3 | 1 | 1 | 1 | 2 | 1 | 1 | 2 | 2 | 1 | 1 | 1 | 3 |
| NM_002857 | PEX19 | 3669 | 9 | 4 | 9 | 2 | 4 | 7 | 4 | 6 | 2 | 2 | 3 | 3 | 3 |
| NM_002858 | ABCD3 | 3616 | 19 | 7 | 4 | 4 | 9 | 7 | 16 | 19 | 5 | 2 | 4 | 5 | 8 |
| NM_002859 | PXN | 3672 | 4 | 2 | 2 | 2 | 2 | 1 | 3 | 1 | 2 | 1 | 2 | 4 | 2 |
| NM_002860 | ALDH18A1 | 3463 | 11 | 4 | 3 | 8 | 3 | 4 | 5 | 11 | 2 | 3 | 1 | 4 | 3 |
| NM_002861 | PCYT2 | 1850 | 8 | 3 | 3 | 1 | 2 | 3 | 4 | 8 | 1 | 1 | 1 | 1 | 5 |
| NM_002861 | PCYT2 | 1860 | 8 | 3 | 3 | 1 | 2 | 3 | 4 | 8 | 1 | 1 | 1 | 1 | 5 |
| NM_002862 | PYGB | 4131 | 7 | 3 | 3 | 6 | 6 | 7 | 2 | 1 | 2 | 2 | 0 | 2 | 3 |
| NM_002863 | PYGL | 2845 | 27 | 8 | 27 | 3 | 5 | 3 | 2 | 13 | 6 | 3 | 1 | 19 | 3 |
| NM_002864 | PZP | 4610 | 2 | 0 | 0 | 0 | 0 | 0 | 0 | 2 | 1 | 0 | 0 | 1 | 0 |
| NM_002865 | RAB2A | 1148 | 120 | 50 | 76 | 25 | 56 | 120 | 34 | 65 | 30 | 31 | 46 | 34 | 34 |
| NM_002866 | RAB3A | 1501 | 27 | 3 | 0 | 1 | 2 | 27 | 1 | 0 | 0 | 3 | 0 | 0 | 0 |
| NM_002867 | RAB3B | 1549 | 6 | 1 | 0 | 0 | 0 | 6 | 0 | 0 | 0 | 1 | 0 | 0 | 0 |
| NM_002868 | RAB5B | 3294 | 16 | 10 | 7 | 7 | 12 | 16 | 10 | 6 | 10 | 11 | 9 | 11 | 16 |
| NM_002869 | RAB6A | 3408 | 7 | 3 | 2 | 1 | 3 | 7 | 3 | 3 | 3 | 3 | 3 | 5 | 2 |
| NM_002870 | RAB13 | 1153 | 81 | 53 | 81 | 33 | 65 | 34 | 66 | 70 | 56 | 43 | 15 | 69 | 54 |
| NM_002871 | RABIF | 2429 | 1 | 0 | 0 | 0 | 0 | 0 | 1 | 0 | 0 | 0 | 0 | 0 | 1 |
| NM_002872 | RAC2 | 1468 | 20 | 3 | 2 | 1 | 1 | 1 | 0 | 0 | 3 | 0 | 0 | 20 | 0 |
| NM_002873 | RAD17 | 3164 | 8 | 3 | 1 | 1 | 3 | 2 | 3 | 1 | 2 | 3 | 2 | 3 | 8 |
| NM_002874 | RAD23B | 4130 | 45 | 25 | 30 | 10 | 27 | 25 | 15 | 45 | 18 | 19 | 36 | 22 | 26 |
| NM_002875 | RAD51 | 2297 | 2 | 0 | 0 | 1 | 0 | 0 | 0 | 0 | 0 | 0 | 0 | 1 | 2 |
| NM_002876 | RAD51C | 591 | 3 | 1 | 1 | 0 | 2 | 3 | 1 | 0 | 0 | 1 | 1 | 2 | 2 |
| NM_002877 | RAD51L1 | 1858 | 1 | 0 | 1 | 0 | 0 | 0 | 0 | 0 | 0 | 1 | 0 | 1 | 0 |
| NM_002878 | RAD51L3 | 2401 | 1 | 1 | 1 | 1 | 0 | 1 | 1 | 0 | 0 | 1 | 0 | 1 | 1 |
| NM_002880 | RAF1 | 3275 | 13 | 8 | 5 | 6 | 13 | 5 | 7 | 12 | 6 | 7 | 13 | 9 | 8 |
| NM_002881 | RALB | 2261 | 14 | 8 | 8 | 4 | 10 | 9 | 11 | 5 | 8 | 6 | 4 | 14 | 8 |
| NM_002882 | RANBP1 | 884 | 95 | 50 | 34 | 41 | 38 | 76 | 30 | 90 | 30 | 35 | 24 | 60 | 95 |
| NM_002883 | RANGAP1 | 3021 | 9 | 3 | 2 | 3 | 2 | 9 | 3 | 3 | 2 | 1 | 1 | 2 | 7 |
| NM_002884 | RAP1A | 1812 | 23 | 11 | 8 | 6 | 19 | 8 | 11 | 7 | 16 | 8 | 9 | 23 | 9 |
| NM_002885 | RAP1GAP | 3333 | 10 | 2 | 0 | 2 | 1 | 9 | 10 | 2 | 1 | 0 | 0 | 0 | 0 |
| NM_002886 | RAP2B | 6235 | 4 | 2 | 2 | 1 | 2 | 4 | 1 | 1 | 3 | 3 | 0 | 4 | 2 |
| NM_002887 | RARS | 2138 | 7 | 5 | 4 | 3 | 7 | 4 | 6 | 6 | 5 | 5 | 3 | 7 | 5 |
| NM_002888 | RARRES1 | 883 | 6 | 2 | 1 | 3 | 6 | 1 | 2 | 1 | 2 | 2 | 2 | 1 | 3 |
| NM_002889 | RARRES2 | 740 | 94 | 20 | 49 | 16 | 3 | 5 | 7 | 94 | 15 | 12 | 2 | 9 | 5 |
| NM_002890 | RASA1 | 4295 | 12 | 5 | 4 | 1 | 3 | 4 | 4 | 1 | 5 | 12 | 2 | 9 | 5 |
| NM_002891 | RASGRF1 | 6392 | 9 | 1 | 0 | 0 | 0 | 9 | 0 | 0 | 0 | 0 | 0 | 0 | 0 |
| NM_002892 | ARID4A | 5772 | 11 | 6 | 6 | 2 | 6 | 8 | 5 | 4 | 7 | 8 | 3 | 11 | 7 |
| NM_002893 | RBBP7 | 2017 | 89 | 40 | 27 | 19 | 49 | 46 | 27 | 25 | 23 | 64 | 30 | 40 | 89 |
| NM_002894 | RBBP8 | 3271 | 10 | 4 | 2 | 3 | 2 | 3 | 10 | 3 | 4 | 2 | 1 | 4 | 8 |
| NM_002895 | RBL1 | 4270 | 4 | 2 | 1 | 1 | 1 | 1 | 1 | 1 | 2 | 3 | 3 | 4 | 3 |
| NM_002896 | RBM4 | 1644 | 10 | 6 | 4 | 7 | 5 | 6 | 5 | 2 | 6 | 9 | 7 | 10 | 9 |
| NM_002897 | RBMS1 | 4276 | 10 | 4 | 6 | 2 | 6 | 1 | 4 | 1 | 7 | 5 | 2 | 10 | 3 |
| NM_002898 | RBMS2 | 8486 | 14 | 5 | 5 | 3 | 5 | 1 | 4 | 1 | 14 | 6 | 1 | 7 | 3 |
| NM_002899 | RBP1 | 898 | 108 | 17 | 2 | 5 | 3 | 23 | 3 | 13 | 4 | 108 | 1 | 7 | 19 |
| NM_002900 | RBP3 | 4276 | 0 | 0 | 0 | 0 | 0 | 0 | 0 | 0 | 0 | 0 | 0 | 0 | 0 |
| NM_002901 | RCN1 | 2515 | 77 | 28 | 27 | 3 | 33 | 30 | 12 | 77 | 17 | 41 | 10 | 24 | 33 |
| NM_002902 | RCN2 | 2212 | 23 | 10 | 5 | 4 | 12 | 23 | 10 | 5 | 8 | 9 | 6 | 10 | 17 |

| transcript | gene | transcript length (nt) | max | mean | adipose | colon | heart | hypothalamus | kidney | liver | lung | ovary | skeletalmuscle | spleen | testes |
|---|---|---|---|---|---|---|---|---|---|---|---|---|---|---|---|
| NM_002903 | RCVRN | 1175 | 0 | 0 | 0 | 0 | 0 | 0 | 0 | 0 | 0 | 0 | 0 | 0 | 0 |
| NM_002904 | RDBP | 1561 | 5 | 3 | 3 | 2 | 3 | 4 | 3 | 1 | 2 | 3 | 2 | 3 | 5 |
| NM_002905 | RDH5 | 1336 | 11 | 1 | 11 | 1 | 1 | 0 | 1 | 2 | 0 | 0 | 0 | 0 | 0 |
| NM_002906 | RDX | 4490 | 59 | 42 | 33 | 12 | 46 | 41 | 42 | 45 | 46 | 52 | 50 | 40 | 59 |
| NM_002907 | RECQL | 3778 | 8 | 4 | 5 | 2 | 4 | 2 | 3 | 3 | 5 | 2 | 2 | 8 | 3 |
| NM_002908 | REL | 2592 | 31 | 11 | 10 | 5 | 6 | 8 | 4 | 4 | 17 | 16 | 5 | 31 | 12 |
| NM_002909 | REG1A | 808 | 3 | 0 | 0 | 3 | 0 | 0 | 1 | 0 | 0 | 0 | 0 | 0 | 0 |
| NM_002910 | RENBP | 1470 | 1 | 0 | 0 | 0 | 0 | 0 | 1 | 0 | 0 | 0 | 0 | 1 | 0 |
| NM_002911 | UPF1 | 5345 | 3 | 2 | 1 | 2 | 1 | 2 | 2 | 2 | 2 | 1 | 1 | 3 | 3 |
| NM_002912 | REV3L | 10701 | 6 | 4 | 5 | 2 | 4 | 3 | 4 | 2 | 4 | 4 | 5 | 4 | 6 |
| NM_002913 | RFC1 | 4883 | 4 | 3 | 3 | 1 | 2 | 4 | 4 | 3 | 3 | 4 | 1 | 4 | 4 |
| NM_002914 | RFC2 | 1592 | 2 | 1 | 1 | 1 | 0 | 1 | 1 | 2 | 1 | 0 | 0 | 1 | 1 |
| NM_002915 | RFC3 | 2393 | 3 | 1 | 1 | 1 | 1 | 1 | 1 | 1 | 1 | 2 | 1 | 2 | 3 |
| NM_002916 | RFC4 | 1391 | 4 | 2 | 2 | 2 | 2 | 3 | 2 | 2 | 2 | 3 | 2 | 4 | 4 |
| NM_002917 | RFNG | 1828 | 1 | 0 | 0 | 0 | 0 | 0 | 0 | 0 | 0 | 1 | 1 | 0 | 0 |
| NM_002918 | RFX1 | 4376 | 0 | 0 | 0 | 0 | 0 | 0 | 0 | 0 | 0 | 0 | 0 | 0 | 0 |
| NM_002919 | RFX3 | 3275 | 5 | 2 | 1 | 1 | 1 | 5 | 1 | 1 | 2 | 3 | 2 | 3 | 3 |
| NM_002920 | RFX4 | 2188 | 5 | 1 | 0 | 0 | 0 | 4 | 0 | 0 | 0 | 0 | 0 | 0 | 5 |
| NM_002921 | RGR | 1475 | 0 | 0 | 0 | 0 | 0 | 0 | 0 | 0 | 0 | 0 | 0 | 0 | 0 |
| NM_002922 | RGS1 | 1403 | 4 | 1 | 3 | 1 | 0 | 4 | 0 | 1 | 3 | 1 | 0 | 2 | 1 |
| NM_002923 | RGS2 | 1349 | 38 | 8 | 3 | 2 | 6 | 8 | 3 | 2 | 14 | 8 | 1 | 38 | 6 |
| NM_002924 | RGS7 | 2440 | 6 | 1 | 0 | 0 | 0 | 6 | 0 | 0 | 0 | 0 | 0 | 0 | 2 |
| NM_002925 | RGS10 | 853 | 3 | 1 | 2 | 2 | 1 | 2 | 1 | 0 | 2 | 2 | 1 | 3 | 0 |
| NM_002926 | RGS12 | 6240 | 1 | 1 | 1 | 1 | 1 | 1 | 1 | 1 | 1 | 1 | 1 | 1 | 1 |
| NM_002927 | RGS13 | 1497 | 1 | 0 | 0 | 0 | 0 | 0 | 0 | 0 | 1 | 0 | 0 | 0 | 1 |
| NM_002928 | RGS16 | 2432 | 1 | 0 | 0 | 0 | 0 | 1 | 0 | 0 | 1 | 0 | 0 | 0 | 0 |
| NM_002929 | GRK1 | 1091 | 0 | 0 | 0 | 0 | 0 | 0 | 0 | 0 | 0 | 0 | 0 | 0 | 0 |
| NM_002930 | RIT2 | 1093 | 1 | 0 | 0 | 0 | 0 | 1 | 0 | 0 | 0 | 0 | 0 | 0 | 0 |
| NM_002931 | RING1 | 1738 | 3 | 2 | 1 | 1 | 1 | 3 | 2 | 1 | 1 | 2 | 1 | 2 | 2 |
| NM_002933 | RNASE1 | 819 | 28 | 9 | 13 | 3 | 11 | 3 | 3 | 1 | 16 | 3 | 6 | 6 | 28 |
| NM_002934 | RNASE2 | 735 | 9 | 3 | 0 | 3 | 9 | 2 | 0 | 1 | 7 | 0 | 0 | 7 | 1 |
| NM_002935 | RNASE3 | 716 | 1 | 0 | 0 | 1 | 0 | 0 | 0 | 0 | 1 | 0 | 0 | 0 | 0 |
| NM_002936 | RNASEH1 | 1635 | 8 | 6 | 6 | 4 | 7 | 8 | 6 | 8 | 4 | 4 | 5 | 7 | 6 |
| NM_002937 | RNASE4 | 1791 | 63 | 13 | 13 | 4 | 7 | 2 | 8 | 63 | 10 | 20 | 3 | 4 | 13 |
| NM_002938 | RNF4 | 2946 | 5 | 3 | 3 | 2 | 3 | 5 | 3 | 2 | 2 | 3 | 1 | 5 | 3 |
| NM_002939 | RNH1 | 2038 | 2 | 1 | 1 | 1 | 2 | 1 | 2 | 1 | 1 | 1 | 1 | 2 | 1 |
| NM_002940 | ABCE1 | 4194 | 9 | 6 | 5 | 1 | 6 | 7 | 5 | 9 | 4 | 5 | 4 | 8 | 7 |
| NM_002941 | ROBO1 | 6839 | 7 | 3 | 4 | 1 | 3 | 7 | 1 | 1 | 2 | 6 | 1 | 5 | 6 |
| NM_002942 | ROBO2 | 6237 | 6 | 1 | 0 | 0 | 0 | 6 | 0 | 0 | 2 | 3 | 0 | 1 | 2 |
| NM_002943 | RORA | 10972 | 17 | 5 | 1 | 1 | 2 | 3 | 4 | 10 | 4 | 4 | 17 | 4 | 8 |
| NM_002944 | ROS1 | 7368 | 6 | 1 | 0 | 1 | 0 | 0 | 0 | 0 | 6 | 0 | 0 | 0 | 0 |
| NM_002945 | RPA1 | 4345 | 6 | 4 | 4 | 3 | 5 | 6 | 3 | 3 | 3 | 4 | 6 | 5 | 5 |
| NM_002946 | RPA2 | 1720 | 4 | 2 | 2 | 1 | 2 | 3 | 2 | 2 | 2 | 2 | 3 | 3 | 4 |
| NM_002947 | RPA3 | 1594 | 3 | 2 | 1 | 1 | 2 | 2 | 3 | 2 | 2 | 2 | 1 | 3 | 2 |
| NM_002948 | RPL15 | 1991 | 77 | 44 | 40 | 30 | 40 | 39 | 29 | 49 | 40 | 77 | 36 | 62 | 44 |
| NM_002949 | MRPL12 | 1000 | 1 | 0 | 0 | 1 | 1 | 1 | 1 | 1 | 0 | 0 | 0 | 0 | 0 |
| NM_002950 | RPN1 | 2341 | 56 | 17 | 10 | 7 | 18 | 11 | 20 | 56 | 12 | 10 | 7 | 23 | 14 |
| NM_002951 | RPN2 | 2505 | 26 | 10 | 9 | 7 | 11 | 8 | 11 | 26 | 7 | 5 | 4 | 14 | 9 |
| NM_002952 | RPS2 | 942 | 141 | 63 | 35 | 49 | 64 | 23 | 30 | 82 | 52 | 141 | 80 | 108 | 33 |
| NM_002953 | RPS6KA1 | 3199 | 2 | 1 | 1 | 2 | 0 | 1 | 1 | 1 | 2 | 0 | 0 | 2 | 0 |
| NM_002954 | RPS27A | 733 | 52 | 28 | 28 | 18 | 30 | 25 | 20 | 32 | 26 | 52 | 17 | 51 | 13 |
| NM_002956 | CLIP1 | 5880 | 67 | 15 | 12 | 4 | 19 | 12 | 11 | 9 | 11 | 6 | 67 | 11 | 8 |
| NM_002957 | RXRA | 5523 | 16 | 5 | 4 | 3 | 4 | 4 | 3 | 16 | 2 | 4 | 9 | 6 | 2 |
| NM_002958 | RYK | 2931 | 21 | 8 | 5 | 3 | 10 | 3 | 7 | 3 | 8 | 21 | 6 | 10 | 16 |
| NM_002959 | SORT1 | 7018 | 24 | 12 | 16 | 4 | 22 | 24 | 11 | 5 | 11 | 1 | 12 | 6 | 19 |
| NM_002960 | S100A3 | 738 | 3 | 1 | 1 | 0 | 1 | 0 | 1 | 0 | 3 | 0 | 0 | 1 | 0 |
| NM_002961 | S100A4 | 512 | 31 | 5 | 31 | 3 | 4 | 0 | 1 | 1 | 7 | 0 | 2 | 6 | 2 |
| NM_002962 | S100A5 | 710 | 0 | 0 | 0 | 0 | 0 | 0 | 0 | 0 | 0 | 0 | 0 | 0 | 0 |
| NM_002963 | S100A7 | 439 | 0 | 0 | 0 | 0 | 0 | 0 | 0 | 0 | 0 | 0 | 0 | 0 | 0 |
| NM_002964 | S100A8 | 408 | 253 | 37 | 1 | 0 | 45 | 5 | 1 | 4 | 97 | 0 | 1 | 253 | 3 |
| NM_002965 | S100A9 | 577 | 389 | 72 | 12 | 1 | 119 | 41 | 5 | 32 | 172 | 2 | 5 | 389 | 15 |
| NM_002966 | S100A10 | 1055 | 104 | 50 | 92 | 47 | 77 | 15 | 49 | 8 | 104 | 58 | 18 | 33 | 45 |
| NM_002967 | SAFB | 2988 | 12 | 9 | 7 | 9 | 9 | 11 | 8 | 6 | 7 | 9 | 10 | 12 | 10 |
| NM_002968 | SALL1 | 5143 | 6 | 2 | 0 | 1 | 0 | 4 | 6 | 5 | 0 | 1 | 0 | 0 | 1 |
| NM_002969 | MAPK12 | 1754 | 11 | 2 | 0 | 0 | 1 | 2 | 1 | 0 | 0 | 1 | 11 | 0 | 0 |
| NM_002970 | SAT1 | 1062 | 208 | 64 | 50 | 24 | 92 | 27 | 60 | 52 | 208 | 14 | 6 | 133 | 32 |
| NM_002971 | SATB1 | 5423 | 8 | 4 | 2 | 3 | 5 | 7 | 3 | 1 | 5 | 3 | 8 | 7 | 3 |
| NM_002972 | SBF1 | 8015 | 1 | 1 | 1 | 1 | 0 | 1 | 1 | 0 | 0 | 0 | 1 | 0 | 1 |
| NM_002973 | ATXN2 | 4702 | 8 | 4 | 3 | 3 | 5 | 8 | 4 | 3 | 3 | 5 | 3 | 4 | 6 |
| NM_002974 | SERPINB4 | 1719 | 0 | 0 | 0 | 0 | 0 | 0 | 0 | 0 | 0 | 0 | 0 | 0 | 0 |
| NM_002975 | CLEC11A | 1407 | 1 | 1 | 1 | 1 | 1 | 0 | 0 | 0 | 0 | 1 | 0 | 1 | 1 |
| NM_002976 | SCN7A | 5727 | 19 | 5 | 0 | 1 | 8 | 1 | 1 | 0 | 19 | 17 | 1 | 3 | 3 |
| NM_002977 | SCN9A | 9753 | 4 | 1 | 1 | 0 | 0 | 1 | 1 | 0 | 0 | 0 | 0 | 2 | 4 |
| NM_002978 | SCNN1D | 2503 | 0 | 0 | 0 | 0 | 0 | 0 | 0 | 0 | 0 | 0 | 0 | 0 | 0 |
| NM_002979 | SCP2 | 2671 | 81 | 20 | 17 | 10 | 17 | 14 | 28 | 81 | 11 | 11 | 7 | 13 | 16 |
| NM_002980 | SCTR | 1823 | 0 | 0 | 0 | 0 | 0 | 0 | 0 | 0 | 0 | 0 | 0 | 0 | 0 |
| NM_002981 | CCL1 | 542 | 0 | 0 | 0 | 0 | 0 | 0 | 0 | 0 | 0 | 0 | 0 | 0 | 0 |

| transcript | gene | transcript length (nt) | max | mean | adipose | colon | heart | hypothalamus | kidney | liver | lung | ovary | skeletalmuscle | spleen | testes |
|---|---|---|---|---|---|---|---|---|---|---|---|---|---|---|---|
| NM_002982 | CCL2 | 747 | 19 | 6 | 4 | 2 | 14 | 16 | 11 | 1 | 19 | 1 | 3 | 0 | 1 |
| NM_002983 | CCL3 | 796 | 0 | 0 | 0 | 0 | 0 | 0 | 0 | 0 | 0 | 0 | 0 | 0 | 0 |
| NM_002984 | CCL4 | 667 | 0 | 0 | 0 | 0 | 0 | 0 | 0 | 0 | 0 | 0 | 0 | 0 | 0 |
| NM_002985 | CCL5 | 1229 | 11 | 2 | 1 | 3 | 0 | 1 | 0 | 1 | 6 | 1 | 1 | 11 | 0 |
| NM_002986 | CCL11 | 925 | 1 | 0 | 0 | 1 | 1 | 0 | 0 | 0 | 0 | 0 | 0 | 0 | 0 |
| NM_002987 | CCL17 | 615 | 0 | 0 | 0 | 0 | 0 | 0 | 0 | 0 | 0 | 0 | 0 | 0 | 0 |
| NM_002988 | CCL18 | 770 | 10 | 1 | 0 | 1 | 0 | 0 | 0 | 0 | 10 | 0 | 0 | 0 | 0 |
| NM_002989 | CCL21 | 878 | 5 | 1 | 0 | 2 | 1 | 0 | 1 | 0 | 3 | 1 | 0 | 5 | 0 |
| NM_002990 | CCL22 | 2894 | 1 | 0 | 0 | 0 | 0 | 0 | 0 | 0 | 0 | 0 | 0 | 1 | 0 |
| NM_002991 | CCL24 | 360 | 2 | 0 | 0 | 0 | 0 | 0 | 0 | 0 | 0 | 0 | 0 | 2 | 0 |
| NM_002993 | CXCL6 | 1659 | 15 | 2 | 0 | 0 | 2 | 0 | 0 | 1 | 2 | 0 | 0 | 15 | 0 |
| NM_002994 | CXCL5 | 2458 | 2 | 0 | 0 | 0 | 1 | 1 | 0 | 0 | 2 | 0 | 0 | 1 | 0 |
| NM_002995 | XCL1 | 1222 | 0 | 0 | 0 | 0 | 0 | 0 | 0 | 0 | 0 | 0 | 0 | 0 | 0 |
| NM_002996 | CX3CL1 | 3273 | 4 | 1 | 1 | 0 | 1 | 4 | 2 | 0 | 4 | 0 | 1 | 0 | 1 |
| NM_002997 | SDC1 | 3201 | 27 | 4 | 0 | 3 | 0 | 0 | 12 | 27 | 4 | 0 | 0 | 1 | 1 |
| NM_002998 | SDC2 | 3485 | 21 | 9 | 13 | 3 | 10 | 6 | 8 | 21 | 6 | 17 | 2 | 6 | 7 |
| NM_002999 | SDC4 | 2612 | 47 | 20 | 3 | 9 | 14 | 11 | 40 | 47 | 39 | 4 | 11 | 11 | 28 |
| NM_003000 | SDHB | 1145 | 19 | 7 | 4 | 6 | 13 | 7 | 10 | 19 | 4 | 2 | 6 | 5 | 4 |
| NM_003001 | SDHC | 2852 | 9 | 5 | 6 | 4 | 7 | 6 | 9 | 8 | 3 | 3 | 5 | 4 | 4 |
| NM_003002 | SDHD | 1364 | 7 | 4 | 2 | 2 | 6 | 2 | 7 | 5 | 2 | 2 | 4 | 4 | 1 |
| NM_003003 | SEC14L1 | 5489 | 49 | 22 | 16 | 7 | 21 | 29 | 27 | 9 | 36 | 13 | 11 | 49 | 23 |
| NM_003004 | SECTM1 | 2282 | 1 | 0 | 0 | 0 | 0 | 0 | 0 | 0 | 0 | 0 | 0 | 1 | 0 |
| NM_003005 | SELP | 3171 | 4 | 1 | 4 | 1 | 3 | 0 | 1 | 1 | 2 | 1 | 0 | 1 | 1 |
| NM_003006 | SELPLG | 2534 | 4 | 1 | 1 | 0 | 0 | 1 | 0 | 1 | 2 | 0 | 0 | 4 | 0 |
| NM_003007 | SEMG1 | 1663 | 0 | 0 | 0 | 0 | 0 | 0 | 0 | 0 | 0 | 0 | 0 | 0 | 0 |
| NM_003008 | SEMG2 | 1982 | 0 | 0 | 0 | 0 | 0 | 0 | 0 | 0 | 0 | 0 | 0 | 0 | 0 |
| NM_003009 | SEPW1 | 880 | 11 | 4 | 3 | 4 | 8 | 11 | 2 | 3 | 3 | 2 | 6 | 4 | 2 |
| NM_003010 | MAP2K4 | 3743 | 9 | 2 | 1 | 1 | 2 | 9 | 2 | 2 | 1 | 1 | 1 | 2 | 2 |
| NM_003011 | SET | 2919 | 93 | 54 | 49 | 44 | 51 | 93 | 47 | 50 | 48 | 50 | 36 | 72 | 49 |
| NM_003012 | SFRP1 | 4466 | 11 | 4 | 5 | 2 | 3 | 3 | 11 | 0 | 0 | 5 | 0 | 1 | 8 |
| NM_003013 | SFRP2 | 1990 | 18 | 5 | 10 | 18 | 5 | 7 | 1 | 0 | 7 | 0 | 2 | 0 | 6 |
| NM_003014 | SFRP4 | 2973 | 20 | 4 | 10 | 1 | 1 | 1 | 1 | 0 | 2 | 20 | 0 | 3 | 5 |
| NM_003015 | SFRP5 | 1883 | 1 | 0 | 0 | 0 | 0 | 0 | 0 | 1 | 0 | 0 | 0 | 0 | 0 |
| NM_003016 | SFRS2 | 2882 | 4 | 2 | 2 | 2 | 2 | 2 | 2 | 2 | 2 | 2 | 2 | 4 | 2 |
| NM_003017 | SFRS3 | 3144 | 14 | 10 | 13 | 8 | 9 | 12 | 10 | 6 | 11 | 11 | 8 | 14 | 12 |
| NM_003018 | SFTPC | 983 | 85 | 8 | 0 | 0 | 0 | 0 | 0 | 0 | 85 | 0 | 0 | 0 | 0 |
| NM_003019 | SFTPD | 1283 | 15 | 1 | 0 | 0 | 0 | 0 | 0 | 0 | 15 | 0 | 0 | 0 | 0 |
| NM_003020 | SCG5 | 1241 | 36 | 4 | 0 | 1 | 0 | 36 | 0 | 0 | 0 | 1 | 0 | 0 | 1 |
| NM_003021 | SGTA | 2315 | 1 | 1 | 0 | 1 | 1 | 1 | 1 | 1 | 0 | 1 | 1 | 1 | 1 |
| NM_003022 | SH3BGRL | 2058 | 25 | 12 | 18 | 8 | 8 | 22 | 9 | 4 | 13 | 12 | 3 | 25 | 14 |
| NM_003023 | SH3BP2 | 9209 | 2 | 1 | 0 | 0 | 0 | 0 | 2 | 0 | 0 | 0 | 0 | 2 | 0 |
| NM_003024 | ITSN1 | 6439 | 36 | 11 | 36 | 4 | 9 | 17 | 6 | 13 | 4 | 8 | 4 | 8 | 8 |
| NM_003025 | SH3GL1 | 2443 | 2 | 1 | 1 | 1 | 1 | 1 | 1 | 1 | 1 | 1 | 0 | 2 | 2 |
| NM_003026 | SH3GL2 | 2726 | 67 | 7 | 0 | 1 | 2 | 67 | 6 | 0 | 1 | 0 | 0 | 0 | 4 |
| NM_003027 | SH3GL3 | 1691 | 32 | 4 | 0 | 0 | 0 | 32 | 0 | 0 | 1 | 0 | 0 | 0 | 8 |
| NM_003028 | SHB | 6021 | 1 | 1 | 0 | 1 | 1 | 1 | 1 | 1 | 1 | 0 | 0 | 0 | 0 |
| NM_003029 | SHC1 | 3169 | 10 | 5 | 5 | 4 | 8 | 3 | 5 | 9 | 6 | 5 | 2 | 10 | 4 |
| NM_003030 | SHOX2 | 3222 | 0 | 0 | 0 | 0 | 0 | 0 | 0 | 0 | 0 | 0 | 0 | 0 | 0 |
| NM_003031 | SIAH1 | 2003 | 3 | 2 | 2 | 1 | 1 | 2 | 2 | 1 | 1 | 3 | 2 | 2 | 2 |
| NM_003032 | ST6GAL1 | 4281 | 75 | 12 | 2 | 4 | 5 | 8 | 11 | 75 | 5 | 2 | 1 | 14 | 2 |
| NM_003033 | ST3GAL1 | 6971 | 9 | 4 | 2 | 1 | 9 | 1 | 8 | 8 | 3 | 2 | 3 | 7 | 2 |
| NM_003034 | ST8SIA1 | 9714 | 2 | 0 | 0 | 0 | 0 | 2 | 0 | 0 | 0 | 0 | 0 | 0 | 0 |
| NM_003035 | STIL | 5016 | 2 | 0 | 0 | 1 | 0 | 0 | 0 | 0 | 0 | 0 | 0 | 0 | 2 |
| NM_003036 | SKI | 5706 | 3 | 2 | 1 | 1 | 2 | 2 | 1 | 1 | 2 | 3 | 1 | 2 | 2 |
| NM_003037 | SLAMF1 | 2001 | 2 | 0 | 0 | 0 | 0 | 0 | 0 | 0 | 1 | 0 | 0 | 2 | 0 |
| NM_003038 | SLC1A4 | 4638 | 6 | 1 | 1 | 1 | 1 | 6 | 0 | 1 | 1 | 1 | 2 | 2 | 1 |
| NM_003039 | SLC2A5 | 2436 | 4 | 1 | 0 | 1 | 1 | 4 | 2 | 0 | 0 | 0 | 1 | 1 | 2 |
| NM_003040 | SLC4A2 | 4142 | 3 | 1 | 1 | 1 | 1 | 1 | 2 | 3 | 1 | 1 | 0 | 2 | 1 |
| NM_003041 | SLC5A2 | 2273 | 0 | 0 | 0 | 0 | 0 | 0 | 0 | 0 | 0 | 0 | 0 | 0 | 0 |
| NM_003042 | SLC6A1 | 4494 | 7 | 1 | 0 | 0 | 0 | 7 | 0 | 2 | 0 | 0 | 0 | 0 | 0 |
| NM_003043 | SLC6A6 | 6508 | 6 | 3 | 2 | 1 | 3 | 2 | 3 | 0 | 4 | 6 | 1 | 5 | 1 |
| NM_003044 | SLC6A12 | 3370 | 5 | 1 | 0 | 0 | 0 | 0 | 5 | 4 | 0 | 0 | 0 | 0 | 0 |
| NM_003045 | SLC7A1 | 7342 | 6 | 2 | 1 | 2 | 3 | 6 | 2 | 0 | 1 | 1 | 0 | 4 | 3 |
| NM_003046 | SLC7A2 | 7561 | 24 | 6 | 1 | 0 | 6 | 1 | 2 | 24 | 1 | 9 | 16 | 2 | 6 |
| NM_003047 | SLC9A1 | 4571 | 4 | 2 | 1 | 4 | 2 | 2 | 2 | 0 | 1 | 1 | 0 | 3 | 1 |
| NM_003048 | SLC9A2 | 5442 | 2 | 0 | 0 | 2 | 0 | 0 | 1 | 0 | 0 | 0 | 0 | 0 | 1 |
| NM_003049 | SLC10A1 | 1631 | 29 | 4 | 0 | 1 | 1 | 0 | 1 | 29 | 1 | 3 | 1 | 3 | 1 |
| NM_003051 | SLC16A1 | 3909 | 11 | 5 | 2 | 5 | 11 | 4 | 2 | 6 | 1 | 3 | 4 | 2 | 11 |
| NM_003052 | SLC34A1 | 2585 | 13 | 1 | 0 | 0 | 0 | 0 | 13 | 0 | 0 | 0 | 0 | 0 | 0 |
| NM_003053 | SLC18A1 | 2752 | 0 | 0 | 0 | 0 | 0 | 0 | 0 | 0 | 0 | 0 | 0 | 0 | 0 |
| NM_003054 | SLC18A2 | 1894 | 1 | 0 | 1 | 0 | 0 | 1 | 0 | 0 | 0 | 0 | 0 | 0 | 0 |
| NM_003055 | SLC18A3 | 2420 | 0 | 0 | 0 | 0 | 0 | 0 | 0 | 0 | 0 | 0 | 0 | 0 | 0 |
| NM_003057 | SLC22A1 | 1906 | 47 | 4 | 0 | 0 | 0 | 0 | 0 | 47 | 0 | 0 | 0 | 0 | 0 |
| NM_003058 | SLC22A2 | 2510 | 21 | 2 | 0 | 0 | 0 | 0 | 21 | 0 | 0 | 0 | 0 | 0 | 0 |
| NM_003059 | SLC22A4 | 2192 | 3 | 1 | 1 | 2 | 1 | 1 | 3 | 0 | 1 | 1 | 2 | 1 | 1 |
| NM_003060 | SLC22A5 | 3280 | 5 | 1 | 0 | 1 | 1 | 0 | 5 | 0 | 0 | 0 | 2 | 0 | 1 |
| NM_003061 | SLIT1 | 7931 | 2 | 0 | 0 | 0 | 0 | 2 | 0 | 0 | 0 | 0 | 0 | 0 | 0 |

| transcript | gene | transcript length (nt) | max | mean | adipose | colon | heart | hypothalamus | kidney | liver | lung | ovary | skeletalmuscle | spleen | testes |
|---|---|---|---|---|---|---|---|---|---|---|---|---|---|---|---|
| NM_003062 | SLIT3 | 5380 | 6 | 2 | 6 | 2 | 2 | 2 | 2 | 0 | 2 | 2 | 1 | 1 | 1 |
| NM_003063 | SLN | 730 | 178 | 18 | 0 | 0 | 19 | 1 | 0 | 0 | 0 | 0 | 178 | 0 | 1 |
| NM_003064 | SLPI | 598 | 9 | 1 | 0 | 0 | 0 | 1 | 0 | 2 | 9 | 0 | 3 | 0 | 0 |
| NM_003068 | SNAI2 | 2096 | 87 | 19 | 11 | 9 | 23 | 2 | 10 | 7 | 8 | 87 | 4 | 26 | 18 |
| NM_003069 | SMARCA1 | 4086 | 91 | 24 | 8 | 3 | 22 | 29 | 16 | 21 | 12 | 44 | 10 | 9 | 91 |
| NM_003070 | SMARCA2 | 5756 | 28 | 15 | 13 | 5 | 13 | 23 | 12 | 6 | 11 | 28 | 14 | 20 | 25 |
| NM_003071 | HLTF | 5351 | 17 | 4 | 2 | 1 | 3 | 4 | 3 | 2 | 2 | 4 | 1 | 2 | 17 |
| NM_003072 | SMARCA4 | 5694 | 9 | 4 | 3 | 4 | 3 | 9 | 5 | 3 | 2 | 3 | 3 | 4 | 4 |
| NM_003073 | SMARCB1 | 1703 | 13 | 8 | 4 | 6 | 8 | 12 | 8 | 6 | 3 | 10 | 3 | 13 | 12 |
| NM_003074 | SMARCC1 | 5757 | 17 | 9 | 6 | 7 | 7 | 6 | 11 | 7 | 7 | 14 | 7 | 14 | 17 |
| NM_003075 | SMARCC2 | 5588 | 17 | 9 | 10 | 8 | 7 | 17 | 8 | 4 | 8 | 11 | 7 | 10 | 9 |
| NM_003076 | SMARCD1 | 3429 | 8 | 5 | 5 | 4 | 4 | 6 | 7 | 3 | 5 | 4 | 2 | 8 | 7 |
| NM_003077 | SMARCD2 | 2707 | 13 | 6 | 6 | 6 | 5 | 2 | 4 | 8 | 5 | 6 | 3 | 13 | 9 |
| NM_003078 | SMARCD3 | 1711 | 30 | 10 | 2 | 2 | 16 | 12 | 4 | 0 | 5 | 14 | 30 | 7 | 12 |
| NM_003079 | SMARCE1 | 2417 | 84 | 45 | 30 | 16 | 57 | 26 | 49 | 25 | 36 | 68 | 37 | 84 | 68 |
| NM_003080 | SMPD2 | 1680 | 1 | 0 | 0 | 0 | 0 | 0 | 0 | 0 | 0 | 0 | 0 | 0 | 1 |
| NM_003081 | SNAP25 | 2053 | 145 | 14 | 0 | 2 | 0 | 145 | 0 | 0 | 0 | 1 | 0 | 0 | 1 |
| NM_003082 | SNAPC1 | 2656 | 7 | 3 | 2 | 1 | 4 | 2 | 3 | 1 | 4 | 3 | 2 | 4 | 7 |
| NM_003083 | SNAPC2 | 1550 | 1 | 0 | 0 | 1 | 0 | 1 | 0 | 0 | 0 | 1 | 0 | 0 | 1 |
| NM_003085 | SNCB | 1293 | 5 | 0 | 0 | 0 | 0 | 5 | 0 | 0 | 0 | 0 | 0 | 0 | 0 |
| NM_003086 | SNAPC4 | 4650 | 1 | 0 | 0 | 0 | 0 | 0 | 0 | 0 | 0 | 0 | 0 | 1 | 1 |
| NM_003087 | SNCG | 826 | 14 | 3 | 14 | 1 | 1 | 10 | 0 | 0 | 0 | 0 | 0 | 2 | 0 |
| NM_003088 | FSCN1 | 2777 | 1 | 0 | 0 | 0 | 0 | 1 | 0 | 0 | 0 | 1 | 0 | 1 | 0 |
| NM_003089 | SNRNP70 | 1923 | 16 | 11 | 9 | 16 | 7 | 10 | 13 | 10 | 13 | 7 | 7 | 15 | 13 |
| NM_003090 | SNRPA1 | 1059 | 3 | 2 | 2 | 1 | 2 | 2 | 3 | 3 | 2 | 1 | 1 | 3 | 3 |
| NM_003091 | SNRPB | 1153 | 1 | 1 | 1 | 1 | 1 | 1 | 1 | 1 | 1 | 0 | 0 | 1 | 1 |
| NM_003092 | SNRPB2 | 1281 | 9 | 6 | 5 | 2 | 8 | 7 | 7 | 5 | 4 | 6 | 4 | 9 | 6 |
| NM_003093 | SNRPC | 1216 | 10 | 5 | 3 | 4 | 10 | 7 | 5 | 7 | 4 | 3 | 5 | 5 | 6 |
| NM_003094 | SNRPE | 1547 | 11 | 7 | 6 | 6 | 7 | 11 | 7 | 10 | 4 | 11 | 5 | 8 | 6 |
| NM_003095 | SNRPF | 793 | 38 | 14 | 9 | 12 | 13 | 13 | 17 | 38 | 11 | 10 | 4 | 22 | 9 |
| NM_003096 | SNRPG | 579 | 3 | 2 | 1 | 1 | 3 | 0 | 3 | 2 | 2 | 1 | 1 | 3 | 1 |
| NM_003097 | SNRPN | 1326 | 0 | 0 | 0 | 0 | 0 | 0 | 0 | 0 | 0 | 0 | 0 | 0 | 0 |
| NM_003098 | SNTA1 | 2340 | 4 | 1 | 1 | 0 | 3 | 3 | 1 | 0 | 0 | 1 | 4 | 0 | 1 |
| NM_003099 | SNX1 | 8221 | 6 | 4 | 6 | 2 | 4 | 3 | 5 | 2 | 5 | 5 | 1 | 5 | 4 |
| NM_003100 | SNX2 | 2068 | 33 | 15 | 12 | 4 | 16 | 10 | 18 | 7 | 15 | 22 | 11 | 33 | 19 |
| NM_003101 | SOAT1 | 3393 | 19 | 9 | 11 | 5 | 6 | 6 | 10 | 7 | 19 | 8 | 3 | 16 | 14 |
| NM_003102 | SOD3 | 1529 | 4 | 2 | 2 | 2 | 4 | 1 | 3 | 0 | 2 | 1 | 1 | 1 | 3 |
| NM_003104 | SORD | 2526 | 9 | 1 | 0 | 0 | 0 | 1 | 5 | 9 | 0 | 0 | 0 | 0 | 1 |
| NM_003105 | SORL1 | 10924 | 20 | 7 | 1 | 6 | 2 | 20 | 7 | 9 | 6 | 3 | 0 | 14 | 4 |
| NM_003106 | SOX2 | 2502 | 26 | 3 | 0 | 1 | 0 | 26 | 0 | 0 | 2 | 0 | 0 | 0 | 3 |
| NM_003107 | SOX4 | 4878 | 9 | 2 | 1 | 1 | 1 | 0 | 2 | 0 | 2 | 9 | 1 | 1 | 2 |
| NM_003108 | SOX11 | 8719 | 1 | 0 | 0 | 0 | 0 | 1 | 0 | 0 | 1 | 0 | 0 | 0 | 0 |
| NM_003109 | SP1 | 7592 | 8 | 5 | 4 | 4 | 4 | 2 | 6 | 2 | 5 | 6 | 5 | 8 | 6 |
| NM_003110 | SP2 | 3112 | 2 | 1 | 1 | 1 | 1 | 1 | 1 | 1 | 1 | 2 | 1 | 2 | 1 |
| NM_003111 | SP3 | 3920 | 9 | 6 | 6 | 3 | 5 | 5 | 4 | 5 | 6 | 8 | 5 | 9 | 7 |
| NM_003112 | SP4 | 5800 | 4 | 1 | 0 | 0 | 1 | 1 | 1 | 0 | 1 | 4 | 2 | 3 | 2 |
| NM_003113 | SP100 | 3668 | 28 | 10 | 11 | 4 | 13 | 4 | 7 | 6 | 14 | 11 | 5 | 28 | 6 |
| NM_003114 | SPAG1 | 3852 | 1 | 1 | 0 | 1 | 0 | 1 | 1 | 1 | 1 | 0 | 0 | 1 | 1 |
| NM_003115 | UAP1 | 2327 | 12 | 4 | 4 | 4 | 4 | 3 | 2 | 12 | 4 | 6 | 2 | 1 | 6 |
| NM_003116 | SPAG4 | 1452 | 0 | 0 | 0 | 0 | 0 | 0 | 0 | 0 | 0 | 0 | 0 | 0 | 0 |
| NM_003117 | SPAM1 | 2381 | 1 | 0 | 0 | 0 | 0 | 0 | 0 | 0 | 0 | 0 | 0 | 0 | 1 |
| NM_003118 | SPARC | 3140 | 314 | 81 | 314 | 28 | 61 | 100 | 33 | 16 | 64 | 77 | 12 | 57 | 133 |
| NM_003119 | SPG7 | 3082 | 4 | 2 | 2 | 2 | 3 | 4 | 3 | 2 | 1 | 2 | 2 | 2 | 2 |
| NM_003120 | SPI1 | 1405 | 6 | 1 | 2 | 1 | 1 | 1 | 0 | 1 | 2 | 0 | 0 | 6 | 0 |
| NM_003121 | SPIB | 1470 | 0 | 0 | 0 | 0 | 0 | 0 | 0 | 0 | 0 | 0 | 0 | 0 | 0 |
| NM_003122 | SPINK1 | 441 | 40 | 9 | 0 | 40 | 0 | 0 | 18 | 35 | 0 | 1 | 0 | 1 | 0 |
| NM_003123 | SPN | 6866 | 2 | 1 | 0 | 1 | 0 | 0 | 0 | 0 | 2 | 0 | 0 | 2 | 0 |
| NM_003124 | SPR | 1459 | 10 | 4 | 4 | 2 | 3 | 1 | 7 | 10 | 2 | 1 | 3 | 3 | 3 |
| NM_003125 | SPRR1B | 619 | 0 | 0 | 0 | 0 | 0 | 0 | 0 | 0 | 0 | 0 | 0 | 0 | 0 |
| NM_003126 | SPTA1 | 8017 | 1 | 0 | 1 | 0 | 0 | 0 | 0 | 0 | 0 | 0 | 0 | 0 | 0 |
| NM_003127 | SPTAN1 | 7859 | 90 | 26 | 28 | 21 | 26 | 90 | 22 | 11 | 15 | 21 | 5 | 23 | 30 |
| NM_003128 | SPTBN1 | 10221 | 176 | 74 | 176 | 40 | 74 | 125 | 60 | 54 | 84 | 51 | 18 | 74 | 60 |
| NM_003129 | SQLE | 2990 | 4 | 1 | 0 | 0 | 1 | 3 | 1 | 1 | 2 | 1 | 0 | 2 | 4 |
| NM_003130 | SRI | 2010 | 17 | 5 | 4 | 7 | 5 | 17 | 6 | 1 | 5 | 4 | 1 | 5 | 5 |
| NM_003131 | SRF | 4318 | 7 | 3 | 1 | 3 | 7 | 2 | 2 | 1 | 3 | 3 | 4 | 5 | 3 |
| NM_003132 | SRM | 1273 | 2 | 1 | 1 | 0 | 2 | 2 | 1 | 2 | 1 | 1 | 2 | 1 | 1 |
| NM_003133 | SRP9 | 1496 | 90 | 55 | 39 | 21 | 73 | 90 | 66 | 61 | 44 | 48 | 48 | 69 | 48 |
| NM_003134 | SRP14 | 1126 | 21 | 13 | 16 | 8 | 18 | 21 | 16 | 12 | 9 | 9 | 9 | 14 | 10 |
| NM_003135 | SRP19 | 886 | 13 | 8 | 8 | 2 | 12 | 13 | 7 | 11 | 5 | 4 | 5 | 10 | 10 |
| NM_003136 | SRP54 | 2279 | 27 | 12 | 9 | 4 | 10 | 15 | 13 | 27 | 10 | 8 | 7 | 13 | 16 |
| NM_003137 | SRPK1 | 4361 | 20 | 10 | 4 | 9 | 7 | 8 | 9 | 12 | 10 | 6 | 9 | 13 | 20 |
| NM_003139 | SRPR | 2945 | 62 | 23 | 17 | 12 | 26 | 20 | 22 | 62 | 21 | 13 | 18 | 27 | 18 |
| NM_003140 | SRY | 897 | 0 | 0 | 0 | 0 | 0 | 0 | 0 | 0 | 0 | 0 | 0 | 0 | 0 |
| NM_003141 | TRIM21 | 1929 | 5 | 2 | 3 | 2 | 2 | 2 | 2 | 3 | 2 | 2 | 2 | 5 | 2 |
| NM_003142 | SSB | 1643 | 33 | 23 | 29 | 4 | 33 | 31 | 26 | 15 | 18 | 23 | 15 | 33 | 24 |
| NM_003143 | SSBP1 | 628 | 15 | 11 | 7 | 4 | 13 | 12 | 14 | 12 | 6 | 13 | 9 | 15 | 12 |
| NM_003144 | SSR1 | 9858 | 16 | 7 | 6 | 3 | 7 | 8 | 8 | 16 | 6 | 7 | 3 | 9 | 7 |

| transcript | gene | transcript length (nt) | max | mean | adipose | colon | heart | hypothala mus | kidney | liver | lung | ovary | skeletalm uscle | spleen | testes |
|---|---|---|---|---|---|---|---|---|---|---|---|---|---|---|---|
| NM_003145 | SSR2 | 1124 | 22 | 12 | 11 | 5 | 11 | 8 | 16 | 18 | 11 | 22 | 5 | 18 | 10 |
| NM_003146 | SSRP1 | 2819 | 11 | 8 | 6 | 8 | 7 | 10 | 7 | 8 | 7 | 10 | 11 | 11 | 10 |
| NM_003147 | SSX2 | 1448 | 0 | 0 | 0 | 0 | 0 | 0 | 0 | 0 | 0 | 0 | 0 | 0 | 0 |
| NM_003147 | SSX2 | 1452 | 0 | 0 | 0 | 0 | 0 | 0 | 0 | 0 | 0 | 0 | 0 | 0 | 0 |
| NM_003149 | STAC | 2963 | 0 | 0 | 0 | 0 | 0 | 0 | 0 | 0 | 0 | 0 | 0 | 0 | 0 |
| NM_003150 | STAT3 | 4948 | 29 | 13 | 8 | 8 | 21 | 8 | 13 | 29 | 16 | 6 | 11 | 19 | 9 |
| NM_003151 | STAT4 | 2761 | 3 | 1 | 1 | 0 | 1 | 2 | 0 | 0 | 2 | 0 | 0 | 3 | 2 |
| NM_003152 | STAT5A | 4298 | 6 | 2 | 6 | 1 | 2 | 1 | 1 | 1 | 2 | 1 | 2 | 3 | 1 |
| NM_003153 | STAT6 | 3977 | 15 | 7 | 9 | 8 | 6 | 2 | 10 | 6 | 8 | 7 | 2 | 15 | 5 |
| NM_003154 | STATH | 600 | 0 | 0 | 0 | 0 | 0 | 0 | 0 | 0 | 0 | 0 | 0 | 0 | 0 |
| NM_003155 | STC1 | 3877 | 2 | 1 | 1 | 0 | 2 | 1 | 2 | 0 | 1 | 0 | 0 | 0 | 1 |
| NM_003156 | STIM1 | 4038 | 9 | 6 | 6 | 3 | 3 | 9 | 4 | 9 | 4 | 5 | 9 | 9 | 8 |
| NM_003157 | NEK4 | 3707 | 6 | 3 | 2 | 2 | 2 | 4 | 3 | 2 | 2 | 3 | 2 | 3 | 6 |
| NM_003159 | CDKL5 | 3431 | 10 | 2 | 1 | 1 | 1 | 10 | 2 | 1 | 2 | 2 | 1 | 1 | 3 |
| NM_003160 | AURKC | 1114 | 16 | 3 | 1 | 0 | 1 | 1 | 1 | 0 | 1 | 0 | 1 | 8 | 16 |
| NM_003161 | RPS6KB1 | 5318 | 4 | 3 | 3 | 3 | 3 | 3 | 2 | 3 | 2 | 4 | 4 | 4 | 2 |
| NM_003162 | STRN | 3479 | 13 | 7 | 6 | 4 | 13 | 9 | 5 | 4 | 6 | 6 | 4 | 10 | 9 |
| NM_003164 | STX5 | 1794 | 2 | 1 | 1 | 1 | 1 | 1 | 2 | 2 | 2 | 1 | 1 | 2 | 1 |
| NM_003165 | STXBP1 | 3967 | 32 | 4 | 2 | 1 | 2 | 32 | 1 | 0 | 3 | 2 | 0 | 1 | 1 |
| NM_003166 | SULT1A3 | 1593 | 0 | 0 | 0 | 0 | 0 | 0 | 0 | 0 | 0 | 0 | 0 | 0 | 0 |
| NM_003166 | SULT1A3 | 1597 | 0 | 0 | 0 | 0 | 0 | 0 | 0 | 0 | 0 | 0 | 0 | 0 | 0 |
| NM_003167 | SULT2A1 | 1987 | 73 | 7 | 0 | 2 | 0 | 0 | 0 | 73 | 0 | 0 | 0 | 0 | 0 |
| NM_003168 | SUPT4H1 | 1489 | 18 | 10 | 8 | 5 | 11 | 9 | 12 | 8 | 10 | 10 | 6 | 18 | 11 |
| NM_003169 | SUPT5H | 3776 | 34 | 17 | 13 | 15 | 13 | 24 | 12 | 15 | 12 | 16 | 15 | 19 | 34 |
| NM_003170 | SUPT6H | 5877 | 26 | 20 | 15 | 13 | 19 | 21 | 18 | 22 | 16 | 26 | 24 | 25 | 25 |
| NM_003171 | SUPV3L1 | 2474 | 3 | 2 | 1 | 1 | 2 | 2 | 2 | 3 | 0 | 1 | 1 | 2 | 2 |
| NM_003172 | SURF1 | 1037 | 4 | 2 | 1 | 1 | 2 | 2 | 4 | 4 | 1 | 1 | 1 | 2 | 2 |
| NM_003173 | SUV39H1 | 2729 | 1 | 1 | 1 | 1 | 0 | 1 | 0 | 1 | 0 | 0 | 1 | 1 | 1 |
| NM_003174 | SVIL | 6716 | 237 | 40 | 9 | 39 | 56 | 6 | 11 | 3 | 11 | 42 | 237 | 6 | 23 |
| NM_003175 | XCL2 | 565 | 0 | 0 | 0 | 0 | 0 | 0 | 0 | 0 | 0 | 0 | 0 | 0 | 0 |
| NM_003176 | SYCP1 | 3490 | 12 | 1 | 0 | 0 | 0 | 0 | 0 | 0 | 0 | 0 | 0 | 0 | 12 |
| NM_003177 | SYK | 5063 | 8 | 2 | 3 | 2 | 1 | 1 | 2 | 0 | 2 | 0 | 0 | 8 | 2 |
| NM_003178 | SYN2 | 3884 | 34 | 3 | 1 | 0 | 0 | 34 | 0 | 0 | 0 | 0 | 0 | 0 | 1 |
| NM_003179 | SYP | 2433 | 119 | 11 | 0 | 1 | 0 | 119 | 0 | 0 | 0 | 0 | 0 | 0 | 0 |
| NM_003180 | SYT5 | 1825 | 4 | 0 | 0 | 0 | 0 | 4 | 0 | 0 | 0 | 0 | 0 | 0 | 0 |
| NM_003181 | T | 2518 | 0 | 0 | 0 | 0 | 0 | 0 | 0 | 0 | 0 | 0 | 0 | 0 | 0 |
| NM_003182 | TAC1 | 1188 | 1 | 0 | 0 | 0 | 0 | 1 | 0 | 0 | 0 | 0 | 0 | 1 | 0 |
| NM_003183 | ADAM17 | 3553 | 9 | 4 | 3 | 1 | 4 | 3 | 3 | 2 | 5 | 5 | 2 | 6 | 9 |
| NM_003184 | TAF2 | 5020 | 4 | 2 | 2 | 1 | 2 | 2 | 2 | 2 | 1 | 2 | 1 | 3 | 4 |
| NM_003185 | TAF4 | 4628 | 5 | 2 | 1 | 2 | 1 | 1 | 2 | 2 | 2 | 3 | 2 | 3 | 5 |
| NM_003186 | TAGLN | 1160 | 17 | 5 | 6 | 17 | 8 | 4 | 3 | 1 | 3 | 6 | 2 | 4 | 2 |
| NM_003187 | TAF9 | 1225 | 11 | 7 | 7 | 4 | 9 | 11 | 5 | 9 | 3 | 3 | 5 | 7 | 11 |
| NM_003188 | MAP3K7 | 2912 | 5 | 3 | 2 | 1 | 3 | 3 | 3 | 2 | 3 | 3 | 2 | 5 | 3 |
| NM_003189 | TAL1 | 5002 | 2 | 1 | 0 | 0 | 1 | 1 | 0 | 0 | 2 | 0 | 0 | 2 | 0 |
| NM_003190 | TAPBP | 3756 | 7 | 3 | 2 | 3 | 4 | 2 | 3 | 2 | 5 | 3 | 2 | 7 | 1 |
| NM_003190 | TAPBP | 3763 | 7 | 3 | 2 | 3 | 4 | 2 | 3 | 2 | 5 | 3 | 2 | 7 | 1 |
| NM_003190 | TAPBP | 3765 | 7 | 3 | 2 | 3 | 4 | 2 | 3 | 2 | 5 | 3 | 2 | 7 | 1 |
| NM_003192 | TBCC | 1651 | 2 | 1 | 1 | 1 | 1 | 2 | 1 | 1 | 1 | 0 | 1 | 2 | 1 |
| NM_003193 | TBCE | 1910 | 10 | 7 | 6 | 6 | 9 | 10 | 9 | 8 | 5 | 5 | 10 | 6 | 6 |
| NM_003194 | TBP | 1846 | 4 | 1 | 1 | 1 | 1 | 1 | 1 | 1 | 1 | 1 | 1 | 2 | 4 |
| NM_003195 | TCEA2 | 1723 | 1 | 0 | 0 | 0 | 0 | 1 | 0 | 0 | 0 | 0 | 0 | 0 | 0 |
| NM_003196 | TCEA3 | 1586 | 40 | 9 | 1 | 12 | 6 | 2 | 7 | 11 | 3 | 8 | 40 | 1 | 2 |
| NM_003198 | TCEB3 | 4942 | 10 | 4 | 4 | 2 | 4 | 3 | 4 | 10 | 4 | 3 | 3 | 5 | 4 |
| NM_003199 | TCF4 | 8320 | 16 | 8 | 8 | 2 | 8 | 11 | 2 | 1 | 13 | 16 | 5 | 14 | 7 |
| NM_003200 | TCF3 | 4445 | 2 | 1 | 0 | 1 | 0 | 1 | 0 | 0 | 0 | 1 | 0 | 2 | 2 |
| NM_003201 | TFAM | 1936 | 9 | 6 | 3 | 2 | 6 | 5 | 8 | 7 | 5 | 6 | 4 | 9 | 7 |
| NM_003202 | TCF7 | 3276 | 3 | 1 | 0 | 1 | 0 | 0 | 0 | 0 | 1 | 1 | 0 | 3 | 0 |
| NM_003203 | C2orf3 | 4434 | 4 | 2 | 2 | 1 | 2 | 2 | 3 | 3 | 1 | 3 | 4 | 2 | 3 |
| NM_003204 | NFE2L1 | 4874 | 67 | 28 | 30 | 16 | 38 | 31 | 34 | 16 | 13 | 17 | 67 | 22 | 23 |
| NM_003205 | TCF12 | 4714 | 9 | 5 | 5 | 3 | 5 | 6 | 5 | 4 | 5 | 9 | 4 | 7 | 7 |
| NM_003206 | TCF21 | 1267 | 20 | 5 | 0 | 4 | 3 | 0 | 3 | 0 | 13 | 20 | 0 | 12 | 1 |
| NM_003211 | TDG | 3243 | 4 | 2 | 1 | 1 | 4 | 1 | 2 | 2 | 3 | 3 | 1 | 4 | 2 |
| NM_003212 | TDGF1 | 2059 | 0 | 0 | 0 | 0 | 0 | 0 | 0 | 0 | 0 | 0 | 0 | 0 | 0 |
| NM_003213 | TEAD4 | 1777 | 5 | 1 | 0 | 0 | 2 | 0 | 1 | 0 | 1 | 0 | 5 | 1 | 0 |
| NM_003214 | TEAD3 | 2991 | 3 | 1 | 0 | 3 | 2 | 0 | 1 | 0 | 1 | 2 | 1 | 1 | 1 |
| NM_003215 | TEC | 3620 | 4 | 2 | 1 | 1 | 2 | 0 | 2 | 1 | 3 | 1 | 1 | 4 | 4 |
| NM_003216 | TEF | 4364 | 3 | 1 | 1 | 1 | 1 | 3 | 1 | 0 | 1 | 2 | 1 | 1 | 1 |
| NM_003217 | TMBIM6 | 2884 | 61 | 24 | 18 | 14 | 14 | 22 | 61 | 51 | 23 | 10 | 8 | 27 | 15 |
| NM_003218 | TERF1 | 2900 | 6 | 3 | 3 | 1 | 3 | 6 | 5 | 3 | 3 | 5 | 2 | 4 | 4 |
| NM_003220 | TFAP2A | 3300 | 1 | 0 | 0 | 0 | 0 | 0 | 1 | 0 | 0 | 0 | 0 | 0 | 0 |
| NM_003221 | TFAP2B | 5770 | 1 | 0 | 0 | 0 | 0 | 0 | 1 | 0 | 0 | 0 | 0 | 0 | 0 |
| NM_003222 | TFAP2C | 2865 | 1 | 0 | 0 | 0 | 0 | 0 | 0 | 0 | 1 | 0 | 0 | 0 | 0 |
| NM_003223 | TFAP4 | 2139 | 1 | 1 | 0 | 1 | 0 | 0 | 0 | 0 | 1 | 1 | 0 | 1 | 1 |
| NM_003224 | ARFRP1 | 2543 | 3 | 2 | 2 | 3 | 2 | 2 | 2 | 2 | 1 | 1 | 1 | 3 | 2 |
| NM_003225 | TFF1 | 492 | 1 | 0 | 0 | 1 | 0 | 0 | 0 | 0 | 0 | 0 | 0 | 0 | 0 |
| NM_003226 | TFF3 | 1053 | 4 | 0 | 0 | 4 | 0 | 0 | 0 | 0 | 0 | 0 | 0 | 0 | 0 |
| NM_003227 | TFR2 | 2888 | 14 | 1 | 0 | 0 | 0 | 0 | 0 | 14 | 0 | 0 | 0 | 0 | 0 |

| transcript | gene | transcript length (nt) | max | mean | adipose | colon | heart | hypothalamus | kidney | liver | lung | ovary | skeletalmuscle | spleen | testes |
|---|---|---|---|---|---|---|---|---|---|---|---|---|---|---|---|
| NM_003234 | TFRC | 5224 | 38 | 19 | 24 | 11 | 20 | 23 | 20 | 10 | 32 | 38 | 5 | 12 | 16 |
| NM_003235 | TG | 8453 | 0 | 0 | 0 | 0 | 0 | 0 | 0 | 0 | 0 | 0 | 0 | 0 | 0 |
| NM_003236 | TGFA | 4263 | 5 | 1 | 0 | 2 | 0 | 3 | 3 | 1 | 1 | 0 | 0 | 5 | 0 |
| NM_003238 | TGFB2 | 5153 | 5 | 2 | 0 | 0 | 5 | 4 | 1 | 0 | 2 | 3 | 2 | 1 | 1 |
| NM_003239 | TGFB3 | 3183 | 8 | 3 | 3 | 1 | 6 | 4 | 2 | 2 | 4 | 2 | 8 | 1 | 5 |
| NM_003240 | LEFTY2 | 2019 | 3 | 0 | 0 | 0 | 0 | 0 | 0 | 0 | 0 | 3 | 0 | 0 | 1 |
| NM_003241 | TGM4 | 2999 | 0 | 0 | 0 | 0 | 0 | 0 | 0 | 0 | 0 | 0 | 0 | 0 | 0 |
| NM_003242 | TGFBR2 | 4628 | 42 | 17 | 37 | 10 | 33 | 5 | 12 | 11 | 21 | 5 | 6 | 42 | 8 |
| NM_003243 | TGFBR3 | 6416 | 21 | 9 | 11 | 2 | 7 | 2 | 6 | 3 | 9 | 21 | 5 | 20 | 9 |
| NM_003244 | TGIF1 | 1603 | 7 | 4 | 3 | 5 | 2 | 2 | 4 | 5 | 4 | 7 | 2 | 6 | 5 |
| NM_003245 | TGM3 | 2677 | 0 | 0 | 0 | 0 | 0 | 0 | 0 | 0 | 0 | 0 | 0 | 0 | 0 |
| NM_003246 | THBS1 | 5790 | 129 | 35 | 16 | 12 | 129 | 9 | 26 | 8 | 73 | 32 | 5 | 69 | 12 |
| NM_003247 | THBS2 | 5809 | 7 | 2 | 2 | 1 | 7 | 3 | 1 | 0 | 2 | 5 | 1 | 1 | 2 |
| NM_003248 | THBS4 | 3352 | 15 | 5 | 9 | 5 | 14 | 6 | 0 | 0 | 0 | 3 | 15 | 0 | 5 |
| NM_003249 | THOP1 | 2550 | 2 | 1 | 0 | 1 | 1 | 2 | 1 | 1 | 0 | 0 | 1 | 1 | 1 |
| NM_003250 | THRA | 2441 | 7 | 3 | 2 | 2 | 3 | 7 | 2 | 0 | 2 | 4 | 3 | 1 | 2 |
| NM_003251 | THRSP | 1174 | 24 | 3 | 24 | 1 | 1 | 0 | 1 | 6 | 0 | 0 | 1 | 0 | 0 |
| NM_003252 | TIAL1 | 3891 | 7 | 5 | 4 | 2 | 5 | 5 | 6 | 6 | 5 | 7 | 6 | 7 | 5 |
| NM_003253 | TIAM1 | 7198 | 6 | 1 | 1 | 1 | 1 | 6 | 0 | 0 | 1 | 1 | 0 | 1 | 3 |
| NM_003254 | TIMP1 | 916 | 235 | 60 | 25 | 11 | 235 | 14 | 23 | 42 | 60 | 105 | 5 | 78 | 65 |
| NM_003255 | TIMP2 | 3655 | 54 | 18 | 30 | 11 | 12 | 25 | 6 | 3 | 13 | 54 | 4 | 17 | 22 |
| NM_003256 | TIMP4 | 1429 | 17 | 2 | 17 | 0 | 2 | 0 | 0 | 0 | 0 | 0 | 1 | 1 | 0 |
| NM_003257 | TJP1 | 7164 | 34 | 17 | 12 | 6 | 24 | 24 | 14 | 9 | 19 | 17 | 10 | 14 | 34 |
| NM_003258 | TK1 | 1595 | 1 | 0 | 0 | 1 | 0 | 0 | 1 | 0 | 1 | 0 | 0 | 1 | 0 |
| NM_003259 | ICAM5 | 3002 | 1 | 0 | 0 | 0 | 0 | 1 | 0 | 0 | 0 | 0 | 0 | 0 | 0 |
| NM_003260 | TLE2 | 2704 | 1 | 1 | 1 | 1 | 1 | 0 | 1 | 0 | 1 | 0 | 0 | 1 | 1 |
| NM_003262 | SEC62 | 6541 | 22 | 12 | 16 | 3 | 9 | 22 | 13 | 18 | 10 | 11 | 4 | 15 | 11 |
| NM_003263 | TLR1 | 2851 | 6 | 1 | 1 | 0 | 1 | 1 | 1 | 1 | 2 | 2 | 0 | 6 | 0 |
| NM_003264 | TLR2 | 3402 | 6 | 2 | 1 | 0 | 3 | 1 | 1 | 1 | 5 | 0 | 0 | 6 | 1 |
| NM_003265 | TLR3 | 3041 | 2 | 1 | 1 | 0 | 0 | 0 | 2 | 1 | 1 | 1 | 0 | 1 | 0 |
| NM_003268 | TLR5 | 4267 | 1 | 0 | 0 | 0 | 0 | 0 | 0 | 0 | 1 | 1 | 0 | 1 | 0 |
| NM_003269 | NR2E1 | 3257 | 1 | 0 | 0 | 0 | 0 | 1 | 0 | 0 | 0 | 0 | 0 | 0 | 0 |
| NM_003270 | TSPAN6 | 2069 | 21 | 9 | 8 | 7 | 3 | 5 | 15 | 21 | 4 | 19 | 1 | 5 | 11 |
| NM_003271 | TSPAN4 | 1412 | 1 | 0 | 0 | 0 | 0 | 0 | 0 | 0 | 1 | 0 | 0 | 0 | 0 |
| NM_003272 | GPR137B | 2041 | 3 | 1 | 1 | 1 | 2 | 2 | 3 | 0 | 1 | 1 | 0 | 3 | 1 |
| NM_003273 | TM7SF2 | 1586 | 2 | 1 | 1 | 1 | 1 | 2 | 1 | 1 | 0 | 1 | 0 | 0 | 1 |
| NM_003274 | TRAPPC10 | 6969 | 8 | 4 | 3 | 4 | 3 | 6 | 5 | 4 | 3 | 2 | 2 | 8 | 4 |
| NM_003275 | TMOD1 | 3336 | 16 | 3 | 0 | 1 | 16 | 2 | 0 | 1 | 1 | 2 | 12 | 0 | 1 |
| NM_003276 | TMPO | 2490 | 5 | 2 | 1 | 2 | 1 | 2 | 2 | 3 | 2 | 4 | 1 | 5 | 2 |
| NM_003277 | CLDN5 | 1702 | 2 | 0 | 0 | 0 | 0 | 0 | 0 | 0 | 2 | 0 | 1 | 0 | 0 |
| NM_003278 | CLEC3B | 852 | 16 | 3 | 16 | 5 | 2 | 1 | 2 | 1 | 3 | 0 | 1 | 5 | 3 |
| NM_003279 | TNNC2 | 681 | 1,084 | 99 | 2 | 0 | 0 | 1 | 0 | 0 | 0 | 0 | 1,084 | 0 | 0 |
| NM_003280 | TNNC1 | 695 | 447 | 70 | 2 | 0 | 316 | 1 | 1 | 0 | 3 | 1 | 447 | 0 | 2 |
| NM_003281 | TNNI1 | 6143 | 45 | 4 | 0 | 0 | 0 | 0 | 0 | 0 | 0 | 0 | 45 | 0 | 0 |
| NM_003282 | TNNI2 | 731 | 906 | 83 | 2 | 0 | 0 | 0 | 0 | 0 | 1 | 0 | 906 | 1 | 1 |
| NM_003283 | TNNT1 | 1048 | 420 | 39 | 0 | 0 | 1 | 2 | 0 | 0 | 0 | 0 | 420 | 0 | 2 |
| NM_003284 | TNP1 | 401 | 1,457 | 133 | 0 | 0 | 0 | 1 | 0 | 0 | 0 | 0 | 0 | 0 | 1,457 |
| NM_003285 | TNR | 5190 | 10 | 1 | 0 | 0 | 0 | 10 | 0 | 0 | 0 | 0 | 0 | 0 | 0 |
| NM_003286 | TOP1 | 3733 | 28 | 15 | 11 | 5 | 14 | 15 | 18 | 15 | 22 | 16 | 8 | 28 | 16 |
| NM_003287 | TPD52L1 | 1370 | 4 | 2 | 1 | 0 | 2 | 3 | 2 | 4 | 0 | 1 | 3 | 0 | 1 |
| NM_003288 | TPD52L2 | 2342 | 4 | 3 | 4 | 1 | 4 | 2 | 3 | 1 | 2 | 2 | 2 | 4 | 3 |
| NM_003289 | TPM2 | 1327 | 1,669 | 201 | 29 | 189 | 152 | 18 | 13 | 1 | 32 | 41 | 1,669 | 20 | 49 |
| NM_003290 | TPM4 | 2645 | 148 | 56 | 53 | 32 | 70 | 32 | 36 | 10 | 98 | 72 | 12 | 148 | 55 |
| NM_003291 | TPP2 | 4710 | 9 | 6 | 5 | 3 | 5 | 4 | 5 | 5 | 5 | 5 | 7 | 8 | 9 |
| NM_003292 | TPR | 9706 | 28 | 14 | 12 | 4 | 15 | 12 | 12 | 8 | 11 | 17 | 14 | 19 | 28 |
| NM_003294 | TPSAB1 | 1194 | 0 | 0 | 0 | 0 | 0 | 0 | 0 | 0 | 0 | 0 | 0 | 0 | 0 |
| NM_003295 | TPT1 | 829 | 812 | 387 | 325 | 164 | 381 | 174 | 309 | 520 | 389 | 493 | 812 | 481 | 211 |
| NM_003296 | CRISP2 | 1408 | 8 | 1 | 0 | 0 | 0 | 0 | 0 | 0 | 0 | 0 | 0 | 0 | 8 |
| NM_003297 | NR2C1 | 4015 | 5 | 3 | 4 | 3 | 3 | 3 | 3 | 3 | 2 | 4 | 3 | 4 | 5 |
| NM_003298 | NR2C2 | 8330 | 5 | 3 | 2 | 3 | 3 | 5 | 3 | 2 | 3 | 4 | 3 | 5 | 4 |
| NM_003299 | HSP90B1 | 2780 | 2,545 | 648 | 305 | 209 | 1,072 | 335 | 444 | 2,545 | 444 | 356 | 103 | 885 | 429 |
| NM_003300 | TRAF3 | 2428 | 6 | 3 | 2 | 3 | 2 | 4 | 2 | 2 | 2 | 1 | 1 | 6 | 3 |
| NM_003301 | TRHR | 1339 | 1 | 0 | 0 | 0 | 0 | 1 | 0 | 0 | 0 | 0 | 1 | 0 | 0 |
| NM_003302 | TRIP6 | 1752 | 1 | 1 | 1 | 1 | 0 | 0 | 1 | 0 | 1 | 1 | 0 | 0 | 1 |
| NM_003304 | TRPC1 | 4068 | 3 | 1 | 1 | 0 | 1 | 3 | 0 | 0 | 1 | 3 | 0 | 1 | 2 |
| NM_003305 | TRPC3 | 3330 | 0 | 0 | 0 | 0 | 0 | 0 | 0 | 0 | 0 | 0 | 0 | 0 | 0 |
| NM_003306 | TRPC4 | 3449 | 3 | 1 | 0 | 0 | 0 | 3 | 0 | 0 | 1 | 1 | 0 | 0 | 0 |
| NM_003307 | TRPM2 | 5876 | 1 | 0 | 0 | 0 | 0 | 1 | 0 | 0 | 0 | 0 | 0 | 0 | 0 |
| NM_003308 | TSPY1 | 1161 | 0 | 0 | 0 | 0 | 0 | 0 | 0 | 0 | 0 | 0 | 0 | 0 | 0 |
| NM_003309 | TSPYL1 | 5259 | 29 | 9 | 4 | 5 | 7 | 29 | 12 | 6 | 6 | 9 | 10 | 7 | 9 |
| NM_003310 | TSSC1 | 1720 | 1 | 1 | 0 | 0 | 0 | 1 | 1 | 0 | 0 | 0 | 0 | 1 | 1 |
| NM_003311 | PHLDA2 | 920 | 2 | 0 | 1 | 2 | 0 | 1 | 0 | 1 | 1 | 0 | 0 | 0 | 0 |
| NM_003312 | TST | 1128 | 9 | 2 | 1 | 4 | 3 | 1 | 5 | 9 | 1 | 2 | 1 | 1 | 0 |
| NM_003313 | TSTA3 | 1358 | 2 | 1 | 0 | 1 | 0 | 0 | 1 | 2 | 0 | 0 | 0 | 1 | 1 |
| NM_003314 | TTC1 | 1407 | 52 | 25 | 26 | 10 | 32 | 52 | 24 | 21 | 17 | 22 | 19 | 30 | 26 |
| NM_003315 | DNAJC7 | 2078 | 34 | 18 | 14 | 7 | 22 | 34 | 18 | 17 | 12 | 16 | 18 | 22 | 20 |
| NM_003316 | TTC3 | 9007 | 21 | 10 | 11 | 4 | 8 | 21 | 10 | 7 | 7 | 18 | 6 | 9 | 13 |

| transcript | gene | transcript length (nt) | max | mean | adipose | colon | heart | hypothala mus | kidney | liver | lung | ovary | skeletalm uscle | spleen | testes |
|---|---|---|---|---|---|---|---|---|---|---|---|---|---|---|---|
| NM_003317 | NKX2-1 | 2338 | 3 | 0 | 0 | 0 | 0 | 0 | 0 | 0 | 3 | 0 | 0 | 0 | 0 |
| NM_003318 | TTK | 3010 | 8 | 1 | 0 | 0 | 0 | 0 | 0 | 0 | 0 | 0 | 0 | 1 | 8 |
| NM_003319 | TTN | 82024 | 1,452 | 167 | 5 | 0 | 374 | 0 | 0 | 0 | 2 | 1 | 1,452 | 1 | 5 |
| NM_003320 | TUB | 6414 | 6 | 1 | 0 | 0 | 1 | 6 | 2 | 0 | 0 | 2 | 0 | 0 | 1 |
| NM_003321 | TUFM | 2071 | 12 | 6 | 5 | 6 | 7 | 8 | 9 | 12 | 3 | 3 | 6 | 6 | 5 |
| NM_003322 | TULP1 | 2094 | 0 | 0 | 0 | 0 | 0 | 0 | 0 | 0 | 0 | 0 | 0 | 0 | 0 |
| NM_003323 | TULP2 | 1760 | 1 | 0 | 0 | 0 | 0 | 0 | 0 | 0 | 0 | 0 | 0 | 0 | 1 |
| NM_003324 | TULP3 | 3106 | 8 | 3 | 2 | 2 | 3 | 2 | 6 | 1 | 3 | 8 | 2 | 3 | 3 |
| NM_003325 | HIRA | 4013 | 2 | 1 | 1 | 2 | 1 | 1 | 2 | 2 | 1 | 1 | 1 | 2 | 1 |
| NM_003326 | TNFSF4 | 3492 | 1 | 0 | 0 | 0 | 0 | 0 | 0 | 0 | 1 | 0 | 0 | 1 | 1 |
| NM_003327 | TNFRSF4 | 1068 | 0 | 0 | 0 | 0 | 0 | 0 | 0 | 0 | 0 | 0 | 0 | 0 | 0 |
| NM_003328 | TXK | 2914 | 2 | 0 | 0 | 0 | 0 | 0 | 0 | 0 | 1 | 0 | 0 | 2 | 0 |
| NM_003329 | TXN | 508 | 7 | 4 | 3 | 6 | 2 | 6 | 7 | 7 | 3 | 1 | 1 | 2 | 1 |
| NM_003330 | TXNRD1 | 3907 | 27 | 13 | 12 | 4 | 27 | 11 | 10 | 20 | 18 | 8 | 10 | 15 | 13 |
| NM_003331 | TYK2 | 4248 | 2 | 1 | 1 | 1 | 1 | 1 | 1 | 1 | 1 | 1 | 1 | 2 | 0 |
| NM_003332 | TYROBP | 566 | 102 | 32 | 68 | 7 | 28 | 11 | 8 | 16 | 86 | 8 | 5 | 102 | 7 |
| NM_003333 | UBA52 | 2789 | 17 | 10 | 5 | 9 | 7 | 9 | 6 | 7 | 8 | 16 | 14 | 17 | 8 |
| NM_003334 | UBA1 | 3599 | 7 | 5 | 3 | 4 | 5 | 6 | 7 | 4 | 4 | 4 | 5 | 6 | 6 |
| NM_003335 | UBA7 | 3313 | 1 | 0 | 0 | 0 | 0 | 0 | 0 | 0 | 0 | 0 | 0 | 1 | 0 |
| NM_003336 | UBE2A | 1796 | 15 | 10 | 11 | 5 | 15 | 13 | 10 | 7 | 10 | 6 | 8 | 13 | 10 |
| NM_003337 | UBE2B | 2631 | 8 | 3 | 2 | 2 | 5 | 3 | 2 | 2 | 3 | 3 | 8 | 4 | 3 |
| NM_003338 | UBE2D1 | 2651 | 3 | 1 | 0 | 0 | 2 | 1 | 0 | 0 | 1 | 1 | 3 | 2 | 0 |
| NM_003339 | UBE2D2 | 2702 | 13 | 8 | 4 | 5 | 10 | 13 | 6 | 4 | 5 | 7 | 9 | 9 | 12 |
| NM_003340 | UBE2D3 | 2370 | 75 | 51 | 36 | 22 | 73 | 46 | 43 | 57 | 52 | 48 | 55 | 75 | 54 |
| NM_003341 | UBE2E1 | 1466 | 20 | 12 | 14 | 10 | 14 | 20 | 10 | 8 | 12 | 10 | 9 | 17 | 11 |
| NM_003342 | UBE2G1 | 4208 | 21 | 6 | 3 | 2 | 6 | 3 | 4 | 5 | 3 | 3 | 21 | 6 | 5 |
| NM_003343 | UBE2G2 | 2910 | 31 | 20 | 15 | 11 | 31 | 12 | 24 | 18 | 18 | 16 | 26 | 30 | 18 |
| NM_003344 | UBE2H | 2728 | 22 | 14 | 12 | 7 | 22 | 16 | 13 | 10 | 15 | 13 | 13 | 15 | 19 |
| NM_003345 | UBE2I | 1221 | 11 | 6 | 3 | 4 | 7 | 6 | 5 | 4 | 5 | 8 | 4 | 11 | 9 |
| NM_003347 | UBE2L3 | 2907 | 46 | 29 | 35 | 19 | 36 | 46 | 22 | 38 | 18 | 19 | 22 | 36 | 29 |
| NM_003348 | UBE2N | 2552 | 8 | 4 | 2 | 3 | 3 | 8 | 3 | 3 | 2 | 3 | 3 | 4 | 5 |
| NM_003350 | UBE2V2 | 1524 | 9 | 3 | 3 | 1 | 3 | 9 | 3 | 4 | 2 | 2 | 2 | 2 | 2 |
| NM_003352 | SUMO1 | 1527 | 2 | 1 | 1 | 1 | 1 | 2 | 1 | 2 | 1 | 1 | 1 | 1 | 1 |
| NM_003353 | UCN | 615 | 0 | 0 | 0 | 0 | 0 | 0 | 0 | 0 | 0 | 0 | 0 | 0 | 0 |
| NM_003355 | UCP2 | 1646 | 67 | 11 | 14 | 4 | 4 | 4 | 3 | 1 | 9 | 2 | 12 | 67 | 2 |
| NM_003356 | UCP3 | 2423 | 14 | 1 | 0 | 0 | 0 | 0 | 0 | 0 | 0 | 0 | 14 | 0 | 0 |
| NM_003357 | SCGB1A1 | 425 | 49 | 4 | 0 | 0 | 0 | 0 | 0 | 0 | 49 | 0 | 0 | 0 | 0 |
| NM_003358 | UGCG | 1631 | 6 | 2 | 2 | 1 | 2 | 2 | 4 | 1 | 3 | 1 | 1 | 6 | 2 |
| NM_003359 | UGDH | 3189 | 92 | 15 | 6 | 13 | 7 | 3 | 14 | 92 | 7 | 9 | 2 | 5 | 4 |
| NM_003360 | UGT8 | 2898 | 26 | 5 | 0 | 6 | 0 | 26 | 12 | 0 | 1 | 0 | 0 | 2 | 2 |
| NM_003361 | UMOD | 2315 | 184 | 17 | 0 | 0 | 0 | 0 | 184 | 0 | 0 | 0 | 0 | 0 | 0 |
| NM_003362 | UNG | 2064 | 3 | 2 | 1 | 1 | 2 | 2 | 3 | 1 | 1 | 1 | 1 | 2 | 2 |
| NM_003363 | USP4 | 4120 | 6 | 4 | 3 | 3 | 4 | 4 | 4 | 3 | 4 | 4 | 2 | 6 | 4 |
| NM_003364 | UPP1 | 1777 | 1 | 0 | 0 | 0 | 0 | 0 | 0 | 0 | 0 | 0 | 0 | 1 | 0 |
| NM_003365 | UQCRC1 | 1617 | 15 | 7 | 4 | 8 | 15 | 7 | 10 | 8 | 2 | 2 | 10 | 3 | 3 |
| NM_003366 | UQCRC2 | 1674 | 39 | 17 | 13 | 11 | 39 | 23 | 22 | 15 | 8 | 7 | 25 | 12 | 7 |
| NM_003367 | USF2 | 1709 | 15 | 8 | 7 | 6 | 9 | 7 | 5 | 6 | 7 | 8 | 8 | 15 | 4 |
| NM_003368 | USP1 | 3939 | 21 | 8 | 6 | 1 | 6 | 8 | 5 | 4 | 6 | 12 | 6 | 11 | 21 |
| NM_003369 | UVRAG | 5166 | 4 | 2 | 4 | 1 | 1 | 2 | 2 | 1 | 2 | 1 | 2 | 3 | 2 |
| NM_003370 | VASP | 2290 | 6 | 2 | 1 | 2 | 1 | 1 | 1 | 1 | 2 | 0 | 0 | 6 | 1 |
| NM_003371 | VAV2 | 4733 | 2 | 1 | 0 | 1 | 0 | 1 | 2 | 1 | 1 | 0 | 0 | 1 | 0 |
| NM_003372 | VBP1 | 1626 | 15 | 8 | 5 | 2 | 15 | 14 | 9 | 6 | 5 | 6 | 11 | 10 | 7 |
| NM_003373 | VCL | 5282 | 48 | 24 | 25 | 30 | 43 | 8 | 16 | 5 | 21 | 18 | 19 | 30 | 48 |
| NM_003374 | VDAC1 | 1806 | 178 | 68 | 32 | 42 | 119 | 87 | 72 | 77 | 28 | 29 | 178 | 49 | 31 |
| NM_003375 | VDAC2 | 1503 | 26 | 13 | 11 | 10 | 26 | 19 | 13 | 12 | 8 | 9 | 21 | 9 | 11 |
| NM_003376 | VEGFA | 3603 | 17 | 10 | 10 | 7 | 12 | 4 | 17 | 10 | 13 | 2 | 17 | 9 | 5 |
| NM_003377 | VEGFB | 1118 | 59 | 25 | 59 | 11 | 47 | 31 | 21 | 6 | 16 | 29 | 18 | 21 | 13 |
| NM_003378 | VGF | 2553 | 8 | 1 | 0 | 0 | 0 | 8 | 0 | 0 | 0 | 0 | 0 | 0 | 0 |
| NM_003379 | EZR | 3149 | 125 | 64 | 16 | 64 | 84 | 53 | 125 | 24 | 120 | 28 | 15 | 89 | 86 |
| NM_003380 | VIM | 2136 | 1,539 | 380 | 857 | 71 | 401 | 114 | 82 | 27 | 259 | 1,539 | 153 | 363 | 314 |
| NM_003381 | VIP | 1584 | 1 | 0 | 0 | 1 | 0 | 1 | 0 | 0 | 0 | 0 | 0 | 0 | 0 |
| NM_003382 | VIPR2 | 3944 | 0 | 0 | 0 | 0 | 0 | 0 | 0 | 0 | 0 | 0 | 0 | 0 | 0 |
| NM_003383 | VLDLR | 3636 | 25 | 8 | 15 | 1 | 24 | 5 | 5 | 0 | 2 | 25 | 4 | 3 | 4 |
| NM_003384 | VRK1 | 1703 | 10 | 4 | 3 | 2 | 2 | 5 | 2 | 1 | 3 | 4 | 1 | 7 | 10 |
| NM_003385 | VSNL1 | 2005 | 245 | 24 | 0 | 4 | 6 | 245 | 0 | 1 | 0 | 1 | 0 | 0 | 1 |
| NM_003386 | ZAN | 8668 | 0 | 0 | 0 | 0 | 0 | 0 | 0 | 0 | 0 | 0 | 0 | 0 | 0 |
| NM_003387 | WIPF1 | 4585 | 26 | 6 | 6 | 3 | 6 | 4 | 3 | 1 | 11 | 3 | 1 | 26 | 3 |
| NM_003388 | CLIP2 | 5563 | 4 | 1 | 1 | 2 | 0 | 4 | 0 | 0 | 1 | 1 | 0 | 1 | 0 |
| NM_003389 | CORO2A | 5641 | 2 | 1 | 0 | 1 | 0 | 0 | 0 | 0 | 1 | 0 | 0 | 1 | 2 |
| NM_003390 | WEE1 | 3356 | 14 | 6 | 2 | 6 | 14 | 2 | 4 | 4 | 6 | 8 | 3 | 5 | 9 |
| NM_003391 | WNT2 | 2904 | 0 | 0 | 0 | 0 | 0 | 0 | 0 | 0 | 0 | 0 | 0 | 0 | 0 |
| NM_003392 | WNT5A | 5838 | 3 | 1 | 0 | 0 | 0 | 1 | 0 | 0 | 1 | 3 | 0 | 1 | 0 |
| NM_003393 | WNT8B | 1996 | 0 | 0 | 0 | 0 | 0 | 0 | 0 | 0 | 0 | 0 | 0 | 0 | 0 |
| NM_003394 | WNT10B | 2271 | 1 | 0 | 0 | 0 | 0 | 1 | 0 | 0 | 0 | 0 | 0 | 0 | 0 |
| NM_003395 | WNT9A | 1240 | 0 | 0 | 0 | 0 | 0 | 0 | 0 | 0 | 0 | 0 | 0 | 0 | 0 |
| NM_003396 | WNT9B | 1464 | 0 | 0 | 0 | 0 | 0 | 0 | 0 | 0 | 0 | 0 | 0 | 0 | 0 |
| NM_003399 | XPNPEP2 | 3333 | 12 | 1 | 0 | 1 | 0 | 0 | 12 | 0 | 0 | 0 | 0 | 0 | 0 |

| transcript | gene | transcript length (nt) | max | mean | adipose | colon | heart | hypothalamus | kidney | liver | lung | ovary | skeletalmuscle | spleen | testes |
|---|---|---|---|---|---|---|---|---|---|---|---|---|---|---|---|
| NM_003400 | XPO1 | 4830 | 31 | 17 | 10 | 6 | 19 | 15 | 15 | 8 | 15 | 22 | 18 | 24 | 31 |
| NM_003401 | XRCC4 | 1649 | 4 | 2 | 1 | 1 | 3 | 2 | 2 | 1 | 1 | 1 | 0 | 3 | 4 |
| NM_003403 | YY1 | 2592 | 8 | 5 | 3 | 4 | 4 | 4 | 5 | 5 | 7 | 3 | 4 | 8 | 6 |
| NM_003404 | YWHAB | 3105 | 95 | 33 | 31 | 15 | 30 | 95 | 34 | 23 | 25 | 16 | 15 | 52 | 25 |
| NM_003405 | YWHAH | 1793 | 443 | 68 | 43 | 34 | 38 | 443 | 27 | 28 | 41 | 14 | 19 | 47 | 19 |
| NM_003406 | YWHAZ | 2992 | 121 | 45 | 43 | 24 | 42 | 121 | 34 | 18 | 45 | 40 | 16 | 71 | 41 |
| NM_003407 | ZFP36 | 1745 | 19 | 6 | 2 | 3 | 4 | 3 | 4 | 3 | 19 | 14 | 8 | 7 | 2 |
| NM_003408 | ZFP37 | 2752 | 2 | 1 | 0 | 0 | 0 | 1 | 1 | 0 | 0 | 2 | 0 | 1 | 0 |
| NM_003409 | ZFP161 | 3497 | 6 | 3 | 3 | 2 | 2 | 3 | 3 | 2 | 2 | 3 | 2 | 6 | 3 |
| NM_003410 | ZFX | 5802 | 15 | 8 | 6 | 5 | 6 | 6 | 6 | 5 | 6 | 15 | 13 | 8 | 9 |
| NM_003411 | ZFY | 5239 | 4 | 2 | 2 | 0 | 2 | 2 | 1 | 2 | 2 | 0 | 1 | 4 | 3 |
| NM_003412 | ZIC1 | 5231 | 8 | 1 | 0 | 0 | 0 | 8 | 0 | 0 | 0 | 0 | 1 | 0 | 0 |
| NM_003413 | ZIC3 | 3939 | 0 | 0 | 0 | 0 | 0 | 0 | 0 | 0 | 0 | 0 | 0 | 0 | 0 |
| NM_003414 | ZNF267 | 3266 | 20 | 8 | 6 | 1 | 7 | 6 | 6 | 3 | 11 | 7 | 4 | 20 | 11 |
| NM_003415 | ZNF268 | 1519 | 5 | 3 | 2 | 1 | 2 | 3 | 4 | 2 | 2 | 5 | 1 | 4 | 5 |
| NM_003416 | ZNF7 | 2251 | 9 | 5 | 5 | 4 | 4 | 5 | 4 | 3 | 3 | 7 | 6 | 6 | 9 |
| NM_003417 | ZNF264 | 12162 | 3 | 2 | 1 | 1 | 2 | 2 | 2 | 1 | 2 | 3 | 1 | 3 | 2 |
| NM_003418 | CNBP | 3378 | 123 | 51 | 29 | 13 | 76 | 35 | 34 | 45 | 50 | 59 | 123 | 53 | 46 |
| NM_003419 | ZNF345 | 3112 | 1 | 1 | 1 | 0 | 0 | 0 | 1 | 0 | 1 | 1 | 0 | 1 | 1 |
| NM_003420 | ZNF35 | 2649 | 5 | 2 | 1 | 1 | 3 | 2 | 2 | 1 | 1 | 2 | 2 | 5 | 5 |
| NM_003421 | ZNF37A | 6544 | 6 | 3 | 2 | 1 | 3 | 3 | 3 | 2 | 2 | 6 | 2 | 5 | 2 |
| NM_003422 | MZF1 | 2603 | 0 | 0 | 0 | 0 | 0 | 0 | 0 | 0 | 0 | 0 | 0 | 0 | 0 |
| NM_003423 | ZNF43 | 5217 | 23 | 7 | 4 | 1 | 4 | 6 | 6 | 1 | 6 | 23 | 2 | 12 | 11 |
| NM_003425 | ZNF45 | 3154 | 5 | 3 | 2 | 2 | 3 | 2 | 3 | 1 | 2 | 5 | 1 | 4 | 3 |
| NM_003426 | ZNF74 | 3868 | 0 | 0 | 0 | 0 | 0 | 0 | 0 | 0 | 0 | 0 | 0 | 0 | 0 |
| NM_003427 | ZNF76 | 2653 | 1 | 1 | 0 | 1 | 1 | 1 | 1 | 0 | 1 | 0 | 1 | 1 | 0 |
| NM_003428 | ZNF84 | 7021 | 8 | 3 | 2 | 1 | 2 | 5 | 4 | 1 | 2 | 8 | 2 | 4 | 4 |
| NM_003429 | ZNF85 | 2310 | 12 | 4 | 2 | 1 | 2 | 3 | 4 | 0 | 2 | 12 | 1 | 5 | 8 |
| NM_003430 | ZNF91 | 5396 | 218 | 78 | 40 | 16 | 140 | 78 | 84 | 15 | 43 | 99 | 218 | 63 | 66 |
| NM_003431 | ZNF124 | 1674 | 28 | 11 | 5 | 3 | 7 | 10 | 17 | 3 | 11 | 28 | 4 | 20 | 7 |
| NM_003432 | ZNF131 | 2402 | 13 | 6 | 4 | 2 | 3 | 6 | 4 | 4 | 4 | 8 | 6 | 7 | 13 |
| NM_003433 | ZNF132 | 3031 | 1 | 1 | 0 | 1 | 1 | 1 | 1 | 0 | 0 | 1 | 0 | 0 | 1 |
| NM_003434 | ZNF133 | 2701 | 2 | 1 | 1 | 1 | 1 | 1 | 1 | 1 | 1 | 2 | 1 | 1 | 2 |
| NM_003435 | ZNF134 | 2248 | 7 | 4 | 3 | 2 | 4 | 6 | 4 | 2 | 3 | 6 | 3 | 7 | 7 |
| NM_003436 | ZNF135 | 3393 | 4 | 1 | 1 | 1 | 0 | 2 | 1 | 0 | 0 | 4 | 1 | 1 | 2 |
| NM_003437 | ZNF136 | 2971 | 7 | 3 | 2 | 1 | 2 | 4 | 4 | 1 | 3 | 7 | 2 | 5 | 5 |
| NM_003439 | ZKSCAN1 | 5494 | 13 | 8 | 5 | 8 | 5 | 9 | 8 | 13 | 5 | 9 | 3 | 8 | 12 |
| NM_003440 | ZNF140 | 3065 | 8 | 4 | 2 | 1 | 3 | 3 | 5 | 3 | 3 | 8 | 2 | 6 | 6 |
| NM_003441 | ZNF141 | 1642 | 36 | 12 | 6 | 3 | 8 | 6 | 11 | 3 | 13 | 36 | 6 | 22 | 20 |
| NM_003442 | ZNF143 | 2946 | 3 | 2 | 2 | 1 | 2 | 2 | 2 | 2 | 2 | 2 | 3 | 3 | 3 |
| NM_003443 | ZBTB17 | 2751 | 2 | 1 | 1 | 1 | 1 | 1 | 1 | 1 | 1 | 1 | 1 | 2 | 1 |
| NM_003445 | ZNF155 | 2673 | 4 | 2 | 1 | 1 | 2 | 2 | 2 | 1 | 1 | 2 | 0 | 4 | 2 |
| NM_003446 | ZNF157 | 1695 | 2 | 0 | 0 | 0 | 0 | 2 | 1 | 0 | 0 | 1 | 0 | 1 | 1 |
| NM_003447 | ZNF165 | 2634 | 4 | 1 | 0 | 0 | 0 | 1 | 1 | 1 | 1 | 0 | 0 | 1 | 4 |
| NM_003449 | TRIM26 | 3488 | 5 | 3 | 2 | 3 | 2 | 2 | 5 | 3 | 3 | 1 | 1 | 3 | 3 |
| NM_003450 | ZNF174 | 2482 | 3 | 2 | 1 | 1 | 1 | 3 | 1 | 1 | 1 | 2 | 1 | 2 | 2 |
| NM_003451 | ZNF177 | 1835 | 4 | 1 | 1 | 0 | 1 | 2 | 1 | 0 | 1 | 4 | 0 | 1 | 4 |
| NM_003452 | ZNF189 | 3168 | 19 | 11 | 7 | 3 | 13 | 15 | 11 | 5 | 5 | 16 | 6 | 19 | 18 |
| NM_003453 | ZMYM2 | 5217 | 19 | 9 | 6 | 5 | 8 | 12 | 9 | 5 | 9 | 11 | 4 | 12 | 19 |
| NM_003454 | ZNF200 | 3387 | 2 | 1 | 1 | 0 | 1 | 1 | 1 | 1 | 1 | 1 | 0 | 2 | 2 |
| NM_003455 | ZNF202 | 4055 | 1 | 1 | 1 | 1 | 1 | 1 | 1 | 0 | 1 | 1 | 1 | 1 | 1 |
| NM_003456 | ZNF205 | 2060 | 0 | 0 | 0 | 0 | 0 | 0 | 0 | 0 | 0 | 0 | 0 | 0 | 0 |
| NM_003457 | ZNF207 | 2275 | 65 | 29 | 16 | 17 | 29 | 20 | 31 | 20 | 33 | 32 | 24 | 65 | 28 |
| NM_003458 | BSN | 15959 | 5 | 1 | 0 | 0 | 0 | 5 | 0 | 0 | 0 | 0 | 0 | 0 | 0 |
| NM_003459 | SLC30A3 | 2093 | 1 | 0 | 0 | 0 | 0 | 1 | 0 | 0 | 0 | 0 | 0 | 0 | 1 |
| NM_003460 | ZP2 | 2266 | 0 | 0 | 0 | 0 | 0 | 0 | 0 | 0 | 0 | 0 | 0 | 0 | 0 |
| NM_003461 | ZYX | 2307 | 5 | 2 | 2 | 2 | 3 | 1 | 2 | 1 | 4 | 3 | 2 | 5 | 3 |
| NM_003462 | DNALI1 | 2649 | 6 | 2 | 0 | 1 | 1 | 3 | 2 | 3 | 2 | 2 | 0 | 1 | 6 |
| NM_003463 | PTP4A1 | 5082 | 37 | 12 | 4 | 5 | 7 | 8 | 7 | 35 | 9 | 6 | 37 | 5 | 5 |
| NM_003465 | CHIT1 | 2307 | 4 | 1 | 4 | 0 | 0 | 0 | 0 | 0 | 2 | 0 | 0 | 0 | 0 |
| NM_003466 | PAX8 | 4055 | 7 | 1 | 0 | 0 | 0 | 0 | 7 | 0 | 0 | 0 | 0 | 0 | 0 |
| NM_003467 | CXCR4 | 1674 | 17 | 3 | 2 | 1 | 0 | 0 | 0 | 0 | 7 | 1 | 0 | 17 | 1 |
| NM_003468 | FZD5 | 6564 | 5 | 2 | 1 | 5 | 1 | 1 | 2 | 5 | 2 | 2 | 0 | 1 | 0 |
| NM_003469 | SCG2 | 2476 | 117 | 11 | 0 | 3 | 1 | 117 | 0 | 0 | 0 | 2 | 0 | 0 | 0 |
| NM_003470 | USP7 | 5412 | 19 | 14 | 9 | 10 | 13 | 19 | 10 | 13 | 10 | 15 | 16 | 19 | 18 |
| NM_003471 | KCNAB1 | 3835 | 6 | 2 | 2 | 1 | 2 | 6 | 1 | 1 | 1 | 1 | 2 | 1 | 2 |
| NM_003472 | DEK | 2871 | 46 | 29 | 23 | 12 | 36 | 19 | 21 | 18 | 36 | 39 | 38 | 46 | 30 |
| NM_003473 | STAM | 2966 | 10 | 5 | 4 | 3 | 7 | 10 | 5 | 2 | 5 | 6 | 7 | 5 | 7 |
| NM_003474 | ADAM12 | 6086 | 6 | 1 | 6 | 0 | 0 | 1 | 0 | 0 | 0 | 2 | 0 | 0 | 0 |
| NM_003475 | RASSF7 | 1536 | 0 | 0 | 0 | 0 | 0 | 0 | 0 | 0 | 0 | 0 | 0 | 0 | 0 |
| NM_003476 | CSRP3 | 1341 | 255 | 27 | 0 | 0 | 255 | 0 | 0 | 0 | 0 | 0 | 40 | 0 | 0 |
| NM_003477 | PDHX | 2987 | 16 | 8 | 6 | 3 | 16 | 11 | 7 | 6 | 5 | 5 | 10 | 6 | 10 |
| NM_003478 | CUL5 | 6387 | 15 | 7 | 4 | 2 | 7 | 8 | 9 | 6 | 5 | 7 | 15 | 7 | 7 |
| NM_003480 | MFAP5 | 2882 | 16 | 3 | 16 | 2 | 6 | 0 | 1 | 0 | 1 | 0 | 4 | 0 | 1 |
| NM_003481 | USP5 | 3115 | 11 | 4 | 4 | 3 | 4 | 11 | 5 | 4 | 2 | 2 | 4 | 3 | 6 |
| NM_003482 | MLL2 | 19419 | 4 | 2 | 1 | 3 | 2 | 3 | 2 | 1 | 2 | 2 | 3 | 4 | 2 |
| NM_003483 | HMGA2 | 4140 | 0 | 0 | 0 | 0 | 0 | 0 | 0 | 0 | 0 | 0 | 0 | 0 | 0 |

| | | transcript | | | | | | hypothala | | | | | skeletalm | | |
|---|---|---|---|---|---|---|---|---|---|---|---|---|---|---|---|
| transcript | gene | length (nt) | max | mean | adipose | colon | heart | mus | kidney | liver | lung | ovary | uscle | spleen | testes |
| NM_003484 | HMGA2 | 1529 | 0 | 0 | 0 | 0 | 0 | 0 | 0 | 0 | 0 | 0 | 0 | 0 | 0 |
| NM_003485 | GPR68 | 2814 | 0 | 0 | 0 | 0 | 0 | 0 | 0 | 0 | 0 | 0 | 0 | 0 | 0 |
| NM_003486 | SLC7A5 | 4543 | 1 | 0 | 0 | 0 | 0 | 1 | 0 | 0 | 0 | 0 | 0 | 0 | 1 |
| NM_003487 | TAF15 | 2145 | 156 | 104 | 97 | 66 | 116 | 122 | 87 | 107 | 81 | 86 | 101 | 156 | 130 |
| NM_003488 | AKAP1 | 3968 | 13 | 7 | 6 | 13 | 10 | 5 | 6 | 9 | 3 | 2 | 11 | 2 | 9 |
| NM_003489 | NRIP1 | 7556 | 10 | 6 | 5 | 2 | 4 | 5 | 7 | 7 | 3 | 8 | 10 | 8 | 5 |
| NM_003490 | SYN3 | 3041 | 2 | 0 | 0 | 0 | 0 | 2 | 0 | 0 | 0 | 0 | 0 | 0 | 0 |
| NM_003491 | ARD1A | 882 | 2 | 1 | 2 | 1 | 2 | 2 | 2 | 1 | 1 | 1 | 1 | 1 | 1 |
| NM_003492 | TMEM187 | 1667 | 1 | 1 | 0 | 0 | 1 | 1 | 1 | 1 | 0 | 1 | 1 | 1 | 1 |
| NM_003493 | HIST3H3 | 481 | 0 | 0 | 0 | 0 | 0 | 0 | 0 | 0 | 0 | 0 | 0 | 0 | 0 |
| NM_003494 | DYSF | 6890 | 8 | 3 | 1 | 1 | 4 | 1 | 1 | 2 | 3 | 1 | 5 | 8 | 1 |
| NM_003495 | HIST1H4I | 370 | 1 | 0 | 0 | 1 | 0 | 0 | 0 | 1 | 0 | 0 | 0 | 0 | 0 |
| NM_003496 | TRRAP | 12590 | 4 | 2 | 1 | 2 | 2 | 2 | 2 | 2 | 1 | 3 | 1 | 3 | 4 |
| NM_003498 | SNN | 3274 | 23 | 6 | 9 | 1 | 6 | 23 | 3 | 1 | 3 | 3 | 8 | 10 | 2 |
| NM_003500 | ACOX2 | 2308 | 14 | 3 | 3 | 2 | 3 | 1 | 5 | 14 | 0 | 2 | 1 | 0 | 1 |
| NM_003501 | ACOX3 | 2874 | 3 | 1 | 0 | 1 | 2 | 2 | 1 | 2 | 1 | 3 | 0 | 1 | 1 |
| NM_003502 | AXIN1 | 3448 | 3 | 1 | 1 | 3 | 1 | 1 | 1 | 1 | 1 | 1 | 1 | 2 | 1 |
| NM_003503 | CDC7 | 3218 | 4 | 1 | 0 | 0 | 1 | 2 | 1 | 0 | 1 | 1 | 0 | 2 | 4 |
| NM_003504 | CDC45L | 1910 | 1 | 0 | 0 | 0 | 0 | 0 | 0 | 0 | 0 | 0 | 0 | 0 | 1 |
| NM_003505 | FZD1 | 4350 | 3 | 1 | 0 | 1 | 1 | 1 | 3 | 0 | 2 | 3 | 0 | 1 | 1 |
| NM_003506 | FZD6 | 3793 | 5 | 3 | 3 | 1 | 3 | 1 | 5 | 1 | 5 | 3 | 1 | 3 | 4 |
| NM_003507 | FZD7 | 3851 | 14 | 4 | 2 | 5 | 4 | 2 | 4 | 0 | 1 | 14 | 3 | 1 | 3 |
| NM_003508 | FZD9 | 2342 | 0 | 0 | 0 | 0 | 0 | 0 | 0 | 0 | 0 | 0 | 0 | 0 | 0 |
| NM_003509 | HIST1H2AI | 469 | 1 | 0 | 0 | 1 | 0 | 0 | 0 | 0 | 0 | 0 | 0 | 0 | 0 |
| NM_003510 | HIST1H2AK | 460 | 0 | 0 | 0 | 0 | 0 | 0 | 0 | 0 | 0 | 0 | 0 | 0 | 0 |
| NM_003511 | HIST1H2AL | 470 | 11 | 4 | 2 | 9 | 1 | 1 | 0 | 0 | 6 | 2 | 0 | 11 | 7 |
| NM_003512 | HIST1H2AC | 546 | 14 | 7 | 7 | 14 | 3 | 8 | 7 | 4 | 14 | 5 | 3 | 8 | 6 |
| NM_003513 | HIST1H2AB | 477 | 1 | 0 | 0 | 1 | 0 | 0 | 0 | 0 | 0 | 0 | 0 | 0 | 1 |
| NM_003514 | HIST1H2AM | 487 | 12 | 2 | 4 | 12 | 0 | 1 | 0 | 1 | 1 | 1 | 0 | 3 | 1 |
| NM_003516 | HIST2H2AA3 | 534 | 0 | 0 | 0 | 0 | 0 | 0 | 0 | 0 | 0 | 0 | 0 | 0 | 0 |
| NM_003517 | HIST2H2AC | 437 | 5 | 2 | 5 | 3 | 2 | 1 | 4 | 1 | 2 | 2 | 1 | 2 | 1 |
| NM_003518 | HIST1H2BG | 444 | 9 | 4 | 2 | 6 | 1 | 5 | 3 | 1 | 9 | 1 | 1 | 7 | 6 |
| NM_003519 | HIST1H2BL | 453 | 1 | 0 | 0 | 1 | 0 | 0 | 0 | 0 | 0 | 1 | 0 | 0 | 0 |
| NM_003520 | HIST1H2BN | 449 | 1 | 0 | 0 | 1 | 0 | 0 | 0 | 0 | 0 | 0 | 0 | 0 | 0 |
| NM_003521 | HIST1H2BM | 446 | 1 | 1 | 0 | 1 | 0 | 0 | 0 | 0 | 1 | 0 | 0 | 1 | 0 |
| NM_003522 | HIST1H2BF | 430 | 15 | 4 | 2 | 15 | 2 | 2 | 0 | 0 | 4 | 3 | 1 | 11 | 4 |
| NM_003523 | HIST1H2BE | 435 | 2 | 0 | 0 | 2 | 0 | 0 | 0 | 0 | 1 | 0 | 0 | 0 | 1 |
| NM_003524 | HIST1H2BH | 425 | 4 | 1 | 1 | 4 | 0 | 0 | 0 | 0 | 1 | 1 | 0 | 4 | 1 |
| NM_003525 | HIST1H2BI | 437 | 58 | 15 | 15 | 58 | 5 | 3 | 3 | 0 | 13 | 11 | 1 | 39 | 13 |
| NM_003526 | HIST1H2BC | 438 | 10 | 3 | 4 | 10 | 1 | 3 | 3 | 2 | 5 | 1 | 1 | 5 | 4 |
| NM_003527 | HIST1H2BO | 467 | 4 | 1 | 1 | 4 | 0 | 0 | 0 | 0 | 3 | 0 | 0 | 2 | 1 |
| NM_003528 | HIST2H2BE | 2223 | 4 | 1 | 1 | 0 | 4 | 1 | 1 | 1 | 3 | 1 | 2 | 1 | 1 |
| NM_003529 | HIST1H3A | 469 | 6 | 1 | 1 | 2 | 0 | 1 | 1 | 1 | 0 | 1 | 0 | 1 | 6 |
| NM_003530 | HIST1H3D | 864 | 0 | 0 | 0 | 0 | 0 | 0 | 0 | 0 | 0 | 0 | 0 | 0 | 0 |
| NM_003531 | HIST1H3C | 459 | 4 | 1 | 1 | 4 | 0 | 0 | 0 | 0 | 0 | 0 | 0 | 1 | 2 |
| NM_003532 | HIST1H3E | 462 | 0 | 0 | 0 | 0 | 0 | 0 | 0 | 0 | 0 | 0 | 0 | 0 | 0 |
| NM_003533 | HIST1H3I | 477 | 9 | 2 | 2 | 9 | 1 | 0 | 0 | 1 | 2 | 1 | 0 | 5 | 1 |
| NM_003534 | HIST1H3G | 467 | 1 | 0 | 0 | 1 | 0 | 0 | 0 | 0 | 0 | 0 | 0 | 0 | 0 |
| NM_003535 | HIST1H3J | 478 | 2 | 1 | 1 | 1 | 0 | 0 | 0 | 0 | 1 | 0 | 0 | 2 | 0 |
| NM_003536 | HIST1H3H | 473 | 0 | 0 | 0 | 0 | 0 | 0 | 0 | 0 | 0 | 0 | 0 | 0 | 0 |
| NM_003537 | HIST1H3B | 472 | 8 | 3 | 3 | 8 | 1 | 0 | 0 | 0 | 4 | 0 | 0 | 6 | 7 |
| NM_003538 | HIST1H4A | 372 | 6 | 3 | 2 | 4 | 2 | 1 | 1 | 1 | 4 | 6 | 1 | 4 | 3 |
| NM_003539 | HIST1H4D | 367 | 9 | 5 | 4 | 8 | 1 | 8 | 3 | 2 | 4 | 4 | 0 | 9 | 9 |
| NM_003540 | HIST1H4F | 368 | 18 | 5 | 3 | 7 | 1 | 2 | 2 | 0 | 4 | 1 | 0 | 14 | 18 |
| NM_003541 | HIST1H4K | 354 | 0 | 0 | 0 | 0 | 0 | 0 | 0 | 0 | 0 | 0 | 0 | 0 | 0 |
| NM_003542 | HIST1H4C | 390 | 11 | 4 | 2 | 8 | 1 | 11 | 5 | 5 | 3 | 1 | 1 | 3 | 7 |
| NM_003543 | HIST1H4H | 374 | 36 | 15 | 13 | 36 | 7 | 17 | 11 | 2 | 14 | 21 | 8 | 19 | 21 |
| NM_003544 | HIST1H4B | 357 | 4 | 2 | 2 | 4 | 1 | 1 | 3 | 1 | 1 | 2 | 0 | 2 | 3 |
| NM_003545 | HIST1H4E | 377 | 60 | 22 | 16 | 38 | 12 | 60 | 14 | 6 | 19 | 40 | 2 | 20 | 21 |
| NM_003546 | HIST1H4L | 364 | 44 | 9 | 6 | 44 | 3 | 1 | 1 | 0 | 7 | 4 | 1 | 24 | 6 |
| NM_003547 | HIST1H4G | 367 | 0 | 0 | 0 | 0 | 0 | 0 | 0 | 0 | 0 | 0 | 0 | 0 | 0 |
| NM_003548 | HIST2H4A | 396 | 0 | 0 | 0 | 0 | 0 | 0 | 0 | 0 | 0 | 0 | 0 | 0 | 0 |
| NM_003549 | HYAL3 | 1942 | 0 | 0 | 0 | 0 | 0 | 0 | 0 | 0 | 0 | 0 | 0 | 0 | 0 |
| NM_003550 | MAD1L1 | 2738 | 2 | 1 | 2 | 2 | 1 | 2 | 1 | 1 | 1 | 1 | 1 | 2 | 2 |
| NM_003551 | NME5 | 1226 | 4 | 1 | 0 | 0 | 0 | 4 | 3 | 0 | 1 | 1 | 0 | 0 | 4 |
| NM_003552 | OR1D4 | 993 | 0 | 0 | 0 | 0 | 0 | 0 | 0 | 0 | 0 | 0 | 0 | 0 | 0 |
| NM_003553 | OR1E1 | 945 | 0 | 0 | 0 | 0 | 0 | 0 | 0 | 0 | 0 | 0 | 0 | 0 | 0 |
| NM_003554 | OR1E2 | 972 | 0 | 0 | 0 | 0 | 0 | 0 | 0 | 0 | 0 | 0 | 0 | 0 | 0 |
| NM_003555 | OR1G1 | 942 | 0 | 0 | 0 | 0 | 0 | 0 | 0 | 0 | 0 | 0 | 0 | 0 | 0 |
| NM_003557 | PIP5K1A | 3767 | 3 | 1 | 1 | 1 | 1 | 1 | 1 | 1 | 1 | 1 | 1 | 1 | 3 |
| NM_003558 | PIP5K1B | 2654 | 11 | 4 | 1 | 5 | 11 | 10 | 2 | 0 | 5 | 0 | 1 | 1 | 10 |
| NM_003559 | PIP4K2B | 5732 | 16 | 5 | 4 | 2 | 4 | 16 | 3 | 2 | 2 | 6 | 6 | 5 | 3 |
| NM_003560 | PLA2G6 | 3223 | 0 | 0 | 0 | 0 | 0 | 0 | 0 | 0 | 0 | 0 | 0 | 0 | 0 |
| NM_003561 | PLA2G10 | 997 | 2 | 0 | 0 | 2 | 0 | 0 | 0 | 0 | 1 | 0 | 0 | 0 | 0 |
| NM_003562 | SLC25A11 | 1811 | 3 | 2 | 1 | 1 | 3 | 3 | 3 | 2 | 1 | 1 | 3 | 1 | 1 |
| NM_003563 | SPOP | 3038 | 8 | 5 | 5 | 4 | 5 | 8 | 3 | 5 | 4 | 6 | 4 | 5 | 5 |
| NM_003564 | TAGLN2 | 1360 | 26 | 10 | 7 | 8 | 10 | 3 | 9 | 10 | 25 | 3 | 3 | 26 | 10 |

| | | transcript | | | | | | hypothala | | | | | skeletalm | | |
|---|---|---|---|---|---|---|---|---|---|---|---|---|---|---|---|
| transcript | gene | length (nt) | max | mean | adipose | colon | heart | mus | kidney | liver | lung | ovary | uscle | spleen | testes |
| NM_003565 | ULK1 | 5221 | 1 | 0 | 0 | 0 | 0 | 1 | 0 | 0 | 0 | 0 | 1 | 0 | 0 |
| NM_003566 | EEA1 | 8003 | 16 | 11 | 16 | 2 | 14 | 12 | 9 | 6 | 10 | 11 | 15 | 13 | 10 |
| NM_003567 | BCAR3 | 3170 | 6 | 3 | 4 | 4 | 3 | 2 | 5 | 2 | 2 | 2 | 6 | 2 | 4 |
| NM_003568 | ANXA9 | 1824 | 4 | 1 | 1 | 1 | 1 | 2 | 4 | 1 | 0 | 1 | 0 | 0 | 1 |
| NM_003569 | STX7 | 4270 | 13 | 7 | 8 | 3 | 7 | 13 | 9 | 1 | 7 | 8 | 4 | 13 | 4 |
| NM_003571 | BFSP2 | 1626 | 0 | 0 | 0 | 0 | 0 | 0 | 0 | 0 | 0 | 0 | 0 | 0 | 0 |
| NM_003573 | LTBP4 | 5033 | 2 | 1 | 0 | 1 | 1 | 0 | 0 | 0 | 1 | 2 | 0 | 1 | 1 |
| NM_003574 | VAPA | 6994 | 15 | 9 | 6 | 5 | 11 | 15 | 8 | 8 | 9 | 12 | 9 | 10 | 10 |
| NM_003575 | ZNF282 | 3721 | 2 | 2 | 1 | 2 | 1 | 1 | 1 | 1 | 1 | 2 | 1 | 2 | 1 |
| NM_003576 | STK24 | 4539 | 6 | 4 | 3 | 5 | 3 | 4 | 5 | 2 | 4 | 3 | 2 | 6 | 5 |
| NM_003577 | UTF1 | 1157 | 0 | 0 | 0 | 0 | 0 | 0 | 0 | 0 | 0 | 0 | 0 | 0 | 0 |
| NM_003578 | SOAT2 | 2073 | 1 | 0 | 0 | 0 | 0 | 0 | 0 | 0 | 0 | 0 | 0 | 1 | 0 |
| NM_003579 | RAD54L | 3146 | 0 | 0 | 0 | 0 | 0 | 0 | 0 | 0 | 0 | 0 | 0 | 0 | 0 |
| NM_003580 | NSMAF | 3567 | 8 | 3 | 5 | 2 | 4 | 3 | 3 | 1 | 4 | 4 | 2 | 8 | 3 |
| NM_003581 | NCK2 | 2681 | 3 | 1 | 1 | 1 | 1 | 1 | 1 | 0 | 1 | 1 | 1 | 3 | 1 |
| NM_003582 | DYRK3 | 2167 | 5 | 1 | 1 | 0 | 2 | 1 | 1 | 1 | 1 | 2 | 0 | 1 | 5 |
| NM_003583 | DYRK2 | 6010 | 8 | 4 | 3 | 5 | 7 | 3 | 3 | 1 | 3 | 4 | 8 | 6 | 3 |
| NM_003584 | DUSP11 | 1638 | 1 | 1 | 1 | 1 | 1 | 1 | 1 | 0 | 1 | 1 | 0 | 1 | 1 |
| NM_003585 | DOC2B | 2023 | 3 | 1 | 0 | 0 | 0 | 3 | 0 | 0 | 0 | 0 | 0 | 1 | 0 |
| NM_003586 | DOC2A | 2066 | 6 | 1 | 0 | 1 | 0 | 6 | 0 | 0 | 0 | 0 | 0 | 0 | 1 |
| NM_003587 | DHX16 | 3460 | 6 | 4 | 4 | 5 | 3 | 4 | 4 | 5 | 2 | 2 | 4 | 5 | 6 |
| NM_003588 | CUL4B | 5349 | 29 | 11 | 7 | 2 | 9 | 10 | 9 | 6 | 8 | 19 | 4 | 14 | 29 |
| NM_003589 | CUL4A | 3725 | 23 | 9 | 6 | 4 | 15 | 5 | 8 | 8 | 5 | 6 | 23 | 9 | 10 |
| NM_003590 | CUL3 | 6784 | 27 | 12 | 6 | 3 | 12 | 12 | 10 | 8 | 8 | 10 | 18 | 13 | 27 |
| NM_003591 | CUL2 | 2881 | 11 | 7 | 5 | 2 | 6 | 9 | 5 | 7 | 4 | 5 | 9 | 9 | 11 |
| NM_003592 | CUL1 | 3209 | 28 | 14 | 8 | 5 | 16 | 14 | 9 | 12 | 10 | 15 | 16 | 17 | 28 |
| NM_003593 | FOXN1 | 2697 | 0 | 0 | 0 | 0 | 0 | 0 | 0 | 0 | 0 | 0 | 0 | 0 | 0 |
| NM_003594 | TTF2 | 4854 | 4 | 2 | 3 | 4 | 2 | 2 | 2 | 1 | 2 | 2 | 1 | 3 | 3 |
| NM_003595 | TPST2 | 1894 | 1 | 0 | 1 | 0 | 0 | 0 | 0 | 1 | 1 | 0 | 0 | 0 | 0 |
| NM_003596 | TPST1 | 2050 | 10 | 4 | 3 | 1 | 2 | 10 | 4 | 9 | 4 | 6 | 0 | 5 | 5 |
| NM_003597 | KLF11 | 4030 | 4 | 2 | 3 | 1 | 2 | 0 | 4 | 3 | 1 | 4 | 1 | 3 | 4 |
| NM_003598 | TEAD2 | 2161 | 2 | 1 | 1 | 1 | 2 | 0 | 1 | 0 | 2 | 1 | 0 | 1 | 1 |
| NM_003599 | SUPT3H | 1128 | 4 | 1 | 1 | 1 | 1 | 4 | 2 | 0 | 1 | 2 | 0 | 1 | 4 |
| NM_003600 | AURKA | 2339 | 3 | 1 | 0 | 2 | 1 | 0 | 0 | 1 | 1 | 1 | 0 | 1 | 3 |
| NM_003601 | SMARCA5 | 3851 | 115 | 54 | 26 | 18 | 48 | 81 | 36 | 23 | 57 | 67 | 35 | 84 | 115 |
| NM_003602 | FKBP6 | 1570 | 2 | 0 | 0 | 0 | 0 | 0 | 0 | 0 | 0 | 0 | 0 | 0 | 2 |
| NM_003603 | SORBS2 | 4939 | 49 | 8 | 1 | 4 | 49 | 6 | 4 | 7 | 2 | 3 | 2 | 3 | 2 |
| NM_003604 | IRS4 | 3881 | 1 | 0 | 0 | 1 | 0 | 1 | 0 | 0 | 0 | 1 | 0 | 0 | 0 |
| NM_003607 | CDC42BPA | 10506 | 12 | 7 | 7 | 3 | 8 | 12 | 10 | 4 | 6 | 8 | 3 | 6 | 11 |
| NM_003608 | GPR65 | 1754 | 11 | 2 | 2 | 0 | 1 | 1 | 1 | 0 | 4 | 1 | 0 | 11 | 0 |
| NM_003609 | HIRIP3 | 2344 | 23 | 13 | 10 | 7 | 13 | 17 | 11 | 8 | 9 | 15 | 15 | 13 | 23 |
| NM_003610 | RAE1 | 1812 | 4 | 1 | 1 | 1 | 1 | 1 | 1 | 1 | 1 | 1 | 1 | 2 | 4 |
| NM_003611 | OFD1 | 3651 | 8 | 4 | 3 | 2 | 3 | 3 | 5 | 2 | 5 | 6 | 2 | 8 | 6 |
| NM_003612 | SEMA7A | 3375 | 3 | 1 | 0 | 0 | 0 | 1 | 0 | 0 | 0 | 0 | 0 | 3 | 2 |
| NM_003613 | CILP | 4468 | 4 | 1 | 4 | 0 | 1 | 0 | 0 | 0 | 0 | 0 | 1 | 0 | 1 |
| NM_003614 | GALR3 | 1157 | 0 | 0 | 0 | 0 | 0 | 0 | 0 | 0 | 0 | 0 | 0 | 0 | 0 |
| NM_003615 | SLC4A7 | 7757 | 6 | 3 | 2 | 2 | 3 | 6 | 3 | 1 | 4 | 2 | 2 | 4 | 4 |
| NM_003616 | SIP1 | 1359 | 3 | 1 | 1 | 1 | 1 | 3 | 1 | 1 | 1 | 2 | 2 | 2 | 2 |
| NM_003617 | RGS5 | 5828 | 23 | 8 | 8 | 2 | 23 | 7 | 10 | 1 | 11 | 4 | 10 | 2 | 8 |
| NM_003618 | MAP4K3 | 4113 | 21 | 12 | 13 | 3 | 12 | 14 | 13 | 6 | 9 | 19 | 21 | 9 | 17 |
| NM_003619 | PRSS12 | 3349 | 7 | 2 | 0 | 3 | 0 | 1 | 0 | 0 | 3 | 2 | 0 | 0 | 7 |
| NM_003620 | PPM1D | 3163 | 2 | 1 | 1 | 1 | 1 | 1 | 1 | 1 | 1 | 2 | 1 | 1 | 1 |
| NM_003621 | PPFIBP2 | 3561 | 5 | 3 | 3 | 3 | 1 | 2 | 4 | 5 | 1 | 3 | 3 | 4 | 4 |
| NM_003622 | PPFIBP1 | 6076 | 44 | 17 | 7 | 5 | 44 | 12 | 20 | 9 | 18 | 36 | 5 | 15 | 16 |
| NM_003624 | RANBP3 | 3385 | 5 | 3 | 2 | 3 | 3 | 5 | 3 | 2 | 2 | 3 | 2 | 4 | 5 |
| NM_003625 | PPFIA2 | 4052 | 29 | 3 | 0 | 0 | 1 | 29 | 1 | 0 | 0 | 2 | 0 | 0 | 3 |
| NM_003626 | PPFIA1 | 5213 | 10 | 7 | 9 | 6 | 7 | 10 | 9 | 5 | 7 | 6 | 5 | 8 | 10 |
| NM_003627 | SLC43A1 | 2498 | 27 | 4 | 1 | 2 | 1 | 0 | 1 | 27 | 1 | 2 | 2 | 1 | 1 |
| NM_003628 | PKP4 | 4590 | 19 | 6 | 4 | 3 | 6 | 16 | 19 | 4 | 4 | 4 | 2 | 3 | 4 |
| NM_003629 | PIK3R3 | 5675 | 9 | 4 | 5 | 1 | 9 | 4 | 3 | 1 | 4 | 2 | 2 | 7 | 8 |
| NM_003630 | PEX3 | 2770 | 8 | 3 | 2 | 1 | 3 | 3 | 4 | 8 | 1 | 3 | 1 | 2 | 4 |
| NM_003631 | PARG | 4253 | 10 | 7 | 5 | 3 | 10 | 10 | 9 | 5 | 5 | 9 | 8 | 8 | 9 |
| NM_003632 | CNTNAP1 | 5453 | 19 | 3 | 1 | 2 | 4 | 19 | 0 | 0 | 1 | 1 | 0 | 1 | 1 |
| NM_003633 | ENC1 | 5517 | 53 | 6 | 1 | 1 | 1 | 53 | 1 | 1 | 1 | 7 | 1 | 1 | 3 |
| NM_003634 | NIPSNAP1 | 2054 | 31 | 10 | 3 | 5 | 4 | 19 | 24 | 31 | 3 | 7 | 1 | 6 | 5 |
| NM_003635 | NDST2 | 3996 | 2 | 1 | 0 | 1 | 1 | 1 | 1 | 0 | 1 | 1 | 0 | 2 | 1 |
| NM_003636 | KCNAB2 | 3171 | 3 | 1 | 1 | 0 | 0 | 3 | 1 | 0 | 1 | 0 | 0 | 2 | 0 |
| NM_003637 | ITGA10 | 5170 | 2 | 0 | 0 | 0 | 2 | 0 | 0 | 0 | 2 | 0 | 1 | 0 | 0 |
| NM_003638 | ITGA8 | 3261 | 17 | 5 | 1 | 1 | 17 | 2 | 4 | 0 | 10 | 3 | 1 | 11 | 5 |
| NM_003639 | IKBKG | 2103 | 1 | 0 | 0 | 0 | 0 | 0 | 0 | 1 | 0 | 0 | 0 | 1 | 0 |
| NM_003640 | IKBKAP | 6114 | 4 | 2 | 2 | 1 | 2 | 4 | 2 | 3 | 1 | 3 | 1 | 2 | 3 |
| NM_003641 | IFITM1 | 717 | 79 | 31 | 32 | 10 | 36 | 24 | 16 | 16 | 23 | 37 | 31 | 79 | 33 |
| NM_003642 | HAT1 | 1669 | 15 | 9 | 6 | 3 | 13 | 9 | 9 | 7 | 10 | 7 | 15 | 12 | 13 |
| NM_003643 | GCM1 | 2762 | 1 | 0 | 0 | 0 | 0 | 0 | 1 | 0 | 0 | 0 | 0 | 0 | 0 |
| NM_003644 | GAS7 | 7773 | 17 | 4 | 4 | 2 | 7 | 17 | 0 | 0 | 3 | 1 | 1 | 4 | 5 |
| NM_003645 | SLC27A2 | 2390 | 153 | 18 | 0 | 3 | 0 | 1 | 39 | 153 | 1 | 0 | 0 | 1 | 2 |
| NM_003646 | DGKZ | 3651 | 2 | 0 | 0 | 0 | 0 | 2 | 0 | 0 | 0 | 0 | 0 | 0 | 0 |

| transcript | gene | transcript length (nt) | max | mean | adipose | colon | heart | hypothalamus | kidney | liver | lung | ovary | skeletalmuscle | spleen | testes |
|---|---|---|---|---|---|---|---|---|---|---|---|---|---|---|---|
| NM_003647 | DGKE | 7766 | 2 | 1 | 0 | 1 | 1 | 2 | 1 | 0 | 1 | 1 | 0 | 2 | 2 |
| NM_003648 | DGKD | 6229 | 3 | 1 | 0 | 2 | 3 | 1 | 0 | 0 | 1 | 1 | 3 | 2 | 2 |
| NM_003649 | DDO | 1709 | 4 | 2 | 1 | 0 | 4 | 2 | 3 | 4 | 0 | 0 | 1 | 0 | 1 |
| NM_003650 | CST7 | 927 | 7 | 1 | 0 | 1 | 1 | 0 | 0 | 1 | 2 | 0 | 0 | 7 | 0 |
| NM_003651 | CSDA | 1972 | 1,580 | 230 | 57 | 28 | 476 | 29 | 32 | 35 | 72 | 38 | 1,580 | 66 | 117 |
| NM_003652 | CPZ | 2196 | 2 | 0 | 0 | 0 | 0 | 0 | 0 | 0 | 0 | 2 | 0 | 0 | 0 |
| NM_003653 | COPS3 | 1636 | 12 | 6 | 4 | 2 | 7 | 6 | 4 | 6 | 3 | 2 | 10 | 6 | 12 |
| NM_003654 | CHST1 | 2718 | 1 | 0 | 0 | 0 | 0 | 1 | 0 | 0 | 0 | 0 | 0 | 0 | 0 |
| NM_003655 | CBX4 | 2649 | 2 | 1 | 0 | 1 | 1 | 2 | 1 | 1 | 1 | 1 | 0 | 2 | 1 |
| NM_003656 | CAMK1 | 1483 | 2 | 1 | 1 | 0 | 1 | 2 | 1 | 2 | 1 | 1 | 0 | 1 | 1 |
| NM_003657 | BCAS1 | 3475 | 52 | 6 | 0 | 13 | 0 | 52 | 1 | 0 | 1 | 0 | 0 | 0 | 2 |
| NM_003658 | BARX2 | 1767 | 0 | 0 | 0 | 0 | 0 | 0 | 0 | 0 | 0 | 0 | 0 | 0 | 0 |
| NM_003659 | AGPS | 7665 | 5 | 3 | 4 | 2 | 3 | 3 | 4 | 3 | 3 | 3 | 2 | 4 | 5 |
| NM_003660 | PPFIA3 | 4718 | 2 | 0 | 0 | 0 | 0 | 2 | 0 | 0 | 0 | 0 | 0 | 0 | 0 |
| NM_003661 | APOL1 | 2862 | 20 | 6 | 6 | 3 | 5 | 3 | 4 | 20 | 8 | 4 | 2 | 10 | 3 |
| NM_003662 | PIR | 1313 | 4 | 2 | 2 | 1 | 2 | 4 | 2 | 3 | 1 | 1 | 1 | 4 | 2 |
| NM_003663 | CGGBP1 | 4437 | 6 | 3 | 3 | 1 | 4 | 4 | 4 | 2 | 3 | 5 | 3 | 6 | 4 |
| NM_003664 | AP3B1 | 3986 | 8 | 5 | 6 | 2 | 5 | 5 | 8 | 3 | 5 | 4 | 2 | 8 | 7 |
| NM_003665 | FCN3 | 1032 | 7 | 1 | 0 | 0 | 1 | 0 | 0 | 2 | 7 | 0 | 0 | 0 | 0 |
| NM_003666 | BLZF1 | 2316 | 8 | 4 | 3 | 1 | 5 | 4 | 5 | 3 | 2 | 4 | 3 | 5 | 8 |
| NM_003667 | LGR5 | 2879 | 3 | 0 | 0 | 0 | 0 | 0 | 0 | 0 | 0 | 0 | 3 | 0 | 0 |
| NM_003668 | MAPKAPK5 | 2038 | 6 | 5 | 4 | 4 | 5 | 6 | 5 | 5 | 4 | 4 | 5 | 6 | 6 |
| NM_003670 | BHLHE40 | 3032 | 23 | 8 | 7 | 2 | 4 | 8 | 11 | 12 | 9 | 5 | 23 | 5 | 6 |
| NM_003671 | CDC14B | 5472 | 4 | 2 | 4 | 1 | 4 | 3 | 3 | 4 | 1 | 2 | 0 | 1 | 3 |
| NM_003672 | CDC14A | 4270 | 3 | 1 | 1 | 1 | 1 | 1 | 2 | 0 | 3 | 2 | 1 | 3 | 2 |
| NM_003673 | TCAP | 962 | 644 | 117 | 1 | 0 | 644 | 0 | 0 | 0 | 0 | 0 | 642 | 0 | 2 |
| NM_003675 | PRPF18 | 1705 | 14 | 12 | 10 | 5 | 10 | 14 | 14 | 13 | 10 | 14 | 10 | 14 | 13 |
| NM_003676 | DEGS1 | 2045 | 6 | 4 | 4 | 1 | 6 | 5 | 4 | 2 | 5 | 3 | 1 | 6 | 5 |
| NM_003677 | DENR | 3061 | 20 | 13 | 11 | 6 | 13 | 19 | 14 | 12 | 9 | 15 | 12 | 20 | 14 |
| NM_003678 | THOC5 | 2430 | 6 | 4 | 4 | 4 | 5 | 6 | 3 | 3 | 3 | 4 | 4 | 4 | 4 |
| NM_003679 | KMO | 5015 | 4 | 1 | 0 | 0 | 0 | 0 | 1 | 4 | 0 | 0 | 0 | 1 | 0 |
| NM_003680 | YARS | 3117 | 20 | 9 | 6 | 4 | 8 | 20 | 5 | 18 | 8 | 4 | 6 | 8 | 9 |
| NM_003681 | PDXK | 7381 | 4 | 2 | 3 | 1 | 1 | 4 | 3 | 1 | 1 | 1 | 0 | 1 | 2 |
| NM_003682 | MADD | 6016 | 8 | 2 | 1 | 2 | 2 | 8 | 2 | 2 | 2 | 1 | 1 | 3 | 2 |
| NM_003683 | RRP1 | 1807 | 2 | 1 | 1 | 1 | 1 | 2 | 1 | 1 | 1 | 1 | 1 | 1 | 1 |
| NM_003684 | MKNK1 | 2798 | 6 | 3 | 3 | 2 | 4 | 2 | 2 | 2 | 2 | 1 | 1 | 6 | 2 |
| NM_003685 | KHSRP | 3250 | 9 | 6 | 5 | 6 | 5 | 9 | 5 | 7 | 3 | 6 | 5 | 7 | 9 |
| NM_003686 | EXO1 | 3120 | 2 | 0 | 0 | 0 | 0 | 0 | 0 | 0 | 0 | 0 | 0 | 1 | 2 |
| NM_003687 | PDLIM4 | 2293 | 0 | 0 | 0 | 0 | 0 | 0 | 0 | 0 | 0 | 0 | 0 | 0 | 0 |
| NM_003688 | CASK | 8296 | 3 | 1 | 1 | 1 | 1 | 3 | 2 | 1 | 1 | 2 | 0 | 1 | 1 |
| NM_003689 | AKR7A2 | 1359 | 1 | 0 | 0 | 0 | 0 | 1 | 1 | 1 | 0 | 0 | 0 | 0 | 0 |
| NM_003690 | PRKRA | 1825 | 21 | 10 | 5 | 3 | 15 | 15 | 10 | 4 | 7 | 11 | 21 | 8 | 14 |
| NM_003692 | TMEFF1 | 2285 | 11 | 1 | 0 | 0 | 0 | 11 | 0 | 0 | 0 | 1 | 0 | 0 | 2 |
| NM_003693 | SCARF1 | 3443 | 2 | 0 | 1 | 0 | 0 | 0 | 0 | 0 | 1 | 0 | 0 | 2 | 0 |
| NM_003695 | LY6D | 802 | 0 | 0 | 0 | 0 | 0 | 0 | 0 | 0 | 0 | 0 | 0 | 0 | 0 |
| NM_003696 | OR6A2 | 1384 | 0 | 0 | 0 | 0 | 0 | 0 | 0 | 0 | 0 | 0 | 0 | 0 | 0 |
| NM_003697 | OR5F1 | 945 | 0 | 0 | 0 | 0 | 0 | 0 | 0 | 0 | 0 | 0 | 0 | 0 | 0 |
| NM_003700 | OR2D2 | 927 | 0 | 0 | 0 | 0 | 0 | 0 | 0 | 0 | 0 | 0 | 0 | 0 | 0 |
| NM_003701 | TNFSF11 | 2198 | 0 | 0 | 0 | 0 | 0 | 0 | 0 | 0 | 0 | 0 | 0 | 0 | 0 |
| NM_003702 | RGS20 | 1703 | 2 | 0 | 0 | 0 | 0 | 2 | 0 | 0 | 1 | 0 | 0 | 0 | 0 |
| NM_003703 | NOP14 | 2917 | 18 | 9 | 8 | 6 | 9 | 7 | 8 | 18 | 6 | 9 | 13 | 12 | 9 |
| NM_003704 | FAM193A | 4733 | 13 | 7 | 4 | 5 | 6 | 5 | 6 | 2 | 7 | 11 | 10 | 9 | 13 |
| NM_003705 | SLC25A12 | 3995 | 5 | 2 | 1 | 1 | 5 | 4 | 1 | 0 | 1 | 1 | 4 | 1 | 1 |
| NM_003706 | PLA2G4C | 2523 | 3 | 1 | 0 | 1 | 1 | 2 | 1 | 0 | 0 | 0 | 3 | 2 | 0 |
| NM_003707 | RUVBL1 | 1767 | 5 | 2 | 2 | 1 | 2 | 4 | 2 | 2 | 2 | 1 | 1 | 5 | 3 |
| NM_003708 | RDH16 | 1723 | 29 | 3 | 0 | 0 | 0 | 0 | 0 | 29 | 0 | 0 | 0 | 1 | 0 |
| NM_003709 | KLF7 | 1639 | 4 | 2 | 2 | 1 | 2 | 2 | 2 | 0 | 3 | 3 | 1 | 4 | 1 |
| NM_003710 | SPINT1 | 2426 | 11 | 3 | 1 | 11 | 0 | 1 | 9 | 0 | 5 | 0 | 0 | 1 | 1 |
| NM_003711 | PPAP2A | 1622 | 20 | 8 | 20 | 6 | 12 | 6 | 7 | 19 | 4 | 3 | 3 | 4 | 5 |
| NM_003712 | PPAP2C | 1310 | 0 | 0 | 0 | 0 | 0 | 0 | 0 | 0 | 0 | 0 | 0 | 0 | 0 |
| NM_003713 | PPAP2B | 3294 | 13 | 8 | 7 | 2 | 10 | 6 | 12 | 7 | 8 | 9 | 4 | 6 | 13 |
| NM_003714 | STC2 | 5343 | 2 | 1 | 0 | 0 | 1 | 0 | 0 | 0 | 2 | 0 | 0 | 2 | 0 |
| NM_003715 | USO1 | 3831 | 74 | 23 | 13 | 4 | 32 | 12 | 16 | 24 | 12 | 23 | 74 | 16 | 24 |
| NM_003716 | CADPS | 5481 | 18 | 2 | 0 | 1 | 1 | 18 | 0 | 0 | 0 | 0 | 0 | 0 | 0 |
| NM_003717 | NPFF | 592 | 1 | 0 | 0 | 0 | 0 | 0 | 0 | 0 | 0 | 1 | 0 | 0 | 0 |
| NM_003718 | CDK13 | 6973 | 5 | 3 | 3 | 3 | 3 | 3 | 3 | 2 | 3 | 5 | 3 | 5 | 5 |
| NM_003719 | PDE8B | 3552 | 3 | 1 | 2 | 0 | 1 | 3 | 1 | 0 | 0 | 0 | 0 | 0 | 1 |
| NM_003720 | PSMG1 | 1128 | 6 | 3 | 2 | 2 | 2 | 3 | 4 | 5 | 1 | 2 | 1 | 3 | 6 |
| NM_003721 | RFXANK | 1438 | 3 | 1 | 1 | 1 | 1 | 1 | 1 | 1 | 1 | 1 | 1 | 3 | 1 |
| NM_003722 | TP63 | 4909 | 8 | 1 | 0 | 0 | 0 | 0 | 0 | 0 | 0 | 0 | 8 | 0 | 1 |
| NM_003724 | JRK | 9118 | 1 | 1 | 0 | 1 | 0 | 0 | 1 | 0 | 0 | 1 | 0 | 1 | 1 |
| NM_003725 | HSD17B6 | 1512 | 173 | 19 | 0 | 1 | 1 | 3 | 0 | 173 | 7 | 3 | 0 | 13 | 4 |
| NM_003726 | SKAP1 | 1576 | 5 | 2 | 1 | 1 | 0 | 0 | 5 | 1 | 3 | 0 | 0 | 5 | 3 |
| NM_003728 | UNC5C | 3646 | 4 | 1 | 1 | 1 | 1 | 4 | 0 | 0 | 1 | 1 | 0 | 1 | 2 |
| NM_003729 | RTCD1 | 2582 | 9 | 6 | 7 | 4 | 8 | 8 | 9 | 5 | 5 | 4 | 6 | 8 | 5 |
| NM_003730 | RNASET2 | 1250 | 26 | 5 | 3 | 2 | 3 | 4 | 2 | 2 | 6 | 4 | 0 | 26 | 1 |
| NM_003731 | SSNA1 | 941 | 2 | 1 | 2 | 1 | 1 | 1 | 1 | 1 | 1 | 0 | 1 | 2 | 2 |

| transcript | gene | transcript length (nt) | max | mean | adipose | colon | heart | hypothalamus | kidney | liver | lung | ovary | skeletalmuscle | spleen | testes |
|---|---|---|---|---|---|---|---|---|---|---|---|---|---|---|---|
| NM_003732 | EIF4EBP3 | 691 | 0 | 0 | 0 | 0 | 0 | 0 | 0 | 0 | 0 | 0 | 0 | 0 | 0 |
| NM_003733 | OASL | 1820 | 0 | 0 | 0 | 0 | 0 | 0 | 0 | 0 | 0 | 0 | 0 | 0 | 0 |
| NM_003734 | AOC3 | 4025 | 8 | 1 | 8 | 1 | 0 | 0 | 1 | 0 | 3 | 0 | 0 | 0 | 0 |
| NM_003735 | PCDHGA12 | 4774 | 1 | 0 | 0 | 0 | 0 | 1 | 0 | 0 | 0 | 0 | 0 | 0 | 0 |
| NM_003736 | PCDHGB4 | 4578 | 1 | 0 | 0 | 0 | 1 | 1 | 0 | 0 | 0 | 1 | 0 | 0 | 1 |
| NM_003737 | DCHS1 | 10750 | 1 | 0 | 0 | 1 | 0 | 0 | 0 | 0 | 0 | 1 | 0 | 1 | 0 |
| NM_003738 | PTCH2 | 3773 | 0 | 0 | 0 | 0 | 0 | 0 | 0 | 0 | 0 | 0 | 0 | 0 | 0 |
| NM_003739 | AKR1C3 | 1224 | 23 | 6 | 18 | 4 | 2 | 1 | 10 | 23 | 2 | 1 | 2 | 0 | 1 |
| NM_003740 | KCNK5 | 3783 | 5 | 1 | 0 | 1 | 0 | 0 | 5 | 0 | 0 | 0 | 0 | 0 | 0 |
| NM_003741 | CHRD | 3521 | 0 | 0 | 0 | 0 | 0 | 0 | 0 | 0 | 0 | 0 | 0 | 0 | 0 |
| NM_003742 | ABCB11 | 4775 | 9 | 1 | 0 | 0 | 0 | 0 | 0 | 9 | 0 | 0 | 0 | 0 | 0 |
| NM_003743 | NCOA1 | 6894 | 12 | 7 | 4 | 2 | 6 | 12 | 6 | 4 | 5 | 8 | 10 | 8 | 8 |
| NM_003744 | NUMB | 3611 | 6 | 4 | 4 | 3 | 4 | 3 | 5 | 3 | 6 | 3 | 2 | 6 | 4 |
| NM_003745 | SOCS1 | 1216 | 0 | 0 | 0 | 0 | 0 | 0 | 0 | 0 | 0 | 0 | 0 | 0 | 0 |
| NM_003746 | DYNLL1 | 722 | 216 | 64 | 64 | 66 | 39 | 216 | 43 | 58 | 36 | 38 | 15 | 44 | 87 |
| NM_003747 | TNKS | 9599 | 5 | 2 | 2 | 1 | 2 | 4 | 2 | 1 | 2 | 4 | 3 | 2 | 5 |
| NM_003748 | ALDH4A1 | 3381 | 9 | 2 | 1 | 0 | 1 | 1 | 9 | 6 | 0 | 0 | 1 | 0 | 0 |
| NM_003749 | IRS2 | 6998 | 5 | 3 | 4 | 1 | 3 | 5 | 1 | 4 | 3 | 4 | 3 | 2 | 1 |
| NM_003750 | EIF3A | 5305 | 128 | 75 | 77 | 25 | 102 | 61 | 64 | 74 | 59 | 74 | 128 | 90 | 77 |
| NM_003751 | EIF3B | 3006 | 48 | 29 | 23 | 21 | 25 | 26 | 18 | 48 | 15 | 28 | 43 | 32 | 39 |
| NM_003752 | EIF3C | 3046 | 0 | 0 | 0 | 0 | 0 | 0 | 0 | 0 | 0 | 0 | 0 | 0 | 0 |
| NM_003752 | EIF3C | 3049 | 0 | 0 | 0 | 0 | 0 | 0 | 0 | 0 | 0 | 0 | 0 | 0 | 0 |
| NM_003753 | EIF3D | 1949 | 71 | 34 | 20 | 24 | 38 | 16 | 24 | 14 | 25 | 61 | 45 | 71 | 36 |
| NM_003754 | EIF3F | 1256 | 8 | 4 | 4 | 4 | 4 | 3 | 3 | 4 | 4 | 8 | 5 | 7 | 3 |
| NM_003755 | EIF3G | 1127 | 44 | 22 | 17 | 16 | 22 | 18 | 13 | 19 | 17 | 28 | 44 | 25 | 21 |
| NM_003756 | EIF3H | 1275 | 74 | 38 | 26 | 11 | 51 | 33 | 29 | 27 | 30 | 74 | 54 | 47 | 40 |
| NM_003757 | EIF3I | 1458 | 7 | 5 | 4 | 3 | 6 | 6 | 5 | 7 | 3 | 5 | 6 | 4 | 3 |
| NM_003758 | EIF3J | 2558 | 21 | 11 | 10 | 3 | 13 | 14 | 11 | 21 | 7 | 6 | 15 | 10 | 10 |
| NM_003759 | SLC4A4 | 7639 | 53 | 7 | 1 | 4 | 2 | 12 | 53 | 2 | 2 | 0 | 0 | 0 | 0 |
| NM_003760 | EIF4G3 | 6177 | 23 | 11 | 7 | 6 | 9 | 23 | 8 | 6 | 6 | 7 | 10 | 12 | 22 |
| NM_003761 | VAMP8 | 742 | 25 | 13 | 17 | 14 | 7 | 7 | 22 | 19 | 21 | 1 | 2 | 25 | 2 |
| NM_003762 | VAMP4 | 2126 | 13 | 6 | 5 | 2 | 5 | 10 | 5 | 3 | 8 | 3 | 2 | 7 | 13 |
| NM_003763 | STX16 | 4883 | 8 | 5 | 5 | 5 | 5 | 7 | 5 | 3 | 6 | 4 | 3 | 8 | 6 |
| NM_003764 | STX11 | 5504 | 3 | 1 | 1 | 0 | 1 | 0 | 0 | 0 | 2 | 0 | 0 | 3 | 0 |
| NM_003765 | STX10 | 750 | 5 | 3 | 3 | 3 | 3 | 2 | 2 | 3 | 3 | 2 | 1 | 5 | 2 |
| NM_003766 | BECN1 | 2143 | 7 | 6 | 6 | 5 | 7 | 7 | 7 | 6 | 4 | 3 | 6 | 7 | 5 |
| NM_003768 | PEA15 | 2467 | 129 | 35 | 45 | 12 | 60 | 129 | 15 | 4 | 24 | 14 | 10 | 38 | 33 |
| NM_003769 | SFRS9 | 1163 | 15 | 9 | 8 | 6 | 6 | 11 | 10 | 12 | 10 | 8 | 2 | 15 | 13 |
| NM_003770 | KRT37 | 1718 | 0 | 0 | 0 | 0 | 0 | 0 | 0 | 0 | 0 | 0 | 0 | 0 | 0 |
| NM_003771 | KRT36 | 1644 | 0 | 0 | 0 | 0 | 0 | 0 | 0 | 0 | 0 | 0 | 0 | 0 | 0 |
| NM_003772 | JRKL | 3570 | 3 | 2 | 2 | 1 | 2 | 2 | 2 | 1 | 2 | 3 | 1 | 3 | 3 |
| NM_003773 | HYAL2 | 1998 | 4 | 1 | 1 | 0 | 1 | 0 | 1 | 1 | 1 | 0 | 0 | 4 | 1 |
| NM_003774 | GALNT4 | 5346 | 4 | 2 | 1 | 4 | 1 | 1 | 2 | 1 | 1 | 2 | 0 | 4 | 1 |
| NM_003775 | S1PR4 | 1565 | 0 | 0 | 0 | 0 | 0 | 0 | 0 | 0 | 0 | 0 | 0 | 0 | 0 |
| NM_003776 | MRPL40 | 774 | 43 | 22 | 15 | 8 | 36 | 18 | 21 | 43 | 11 | 15 | 41 | 19 | 15 |
| NM_003777 | DNAH11 | 14189 | 1 | 0 | 0 | 0 | 0 | 0 | 0 | 0 | 1 | 0 | 1 | 0 | 1 |
| NM_003778 | B4GALT4 | 2261 | 5 | 2 | 1 | 3 | 2 | 1 | 5 | 2 | 1 | 2 | 1 | 2 | 2 |
| NM_003779 | B4GALT3 | 1949 | 2 | 1 | 1 | 1 | 1 | 1 | 1 | 1 | 1 | 1 | 1 | 2 | 1 |
| NM_003780 | B4GALT2 | 2209 | 1 | 0 | 0 | 0 | 0 | 1 | 0 | 0 | 0 | 0 | 0 | 0 | 0 |
| NM_003781 | B3GALNT1 | 3318 | 7 | 2 | 0 | 0 | 6 | 7 | 3 | 0 | 1 | 2 | 0 | 1 | 2 |
| NM_003782 | B3GALT4 | 1686 | 1 | 0 | 1 | 1 | 0 | 0 | 0 | 0 | 1 | 0 | 0 | 1 | 1 |
| NM_003783 | B3GALT2 | 3548 | 16 | 3 | 0 | 1 | 14 | 16 | 0 | 0 | 1 | 3 | 0 | 0 | 1 |
| NM_003784 | SERPINB7 | 2235 | 0 | 0 | 0 | 0 | 0 | 0 | 0 | 0 | 0 | 0 | 0 | 0 | 0 |
| NM_003785 | PAGE1 | 664 | 7 | 1 | 0 | 0 | 0 | 0 | 0 | 0 | 0 | 0 | 0 | 0 | 7 |
| NM_003786 | ABCC3 | 5165 | 8 | 2 | 1 | 5 | 0 | 0 | 2 | 8 | 1 | 0 | 0 | 2 | 0 |
| NM_003787 | NOL4 | 3888 | 5 | 1 | 0 | 0 | 0 | 5 | 0 | 0 | 0 | 0 | 0 | 0 | 1 |
| NM_003789 | TRADD | 1482 | 1 | 0 | 0 | 0 | 0 | 0 | 0 | 0 | 1 | 0 | 0 | 1 | 0 |
| NM_003790 | TNFRSF25 | 1622 | 0 | 0 | 0 | 0 | 0 | 0 | 0 | 0 | 0 | 0 | 0 | 0 | 0 |
| NM_003791 | MBTPS1 | 4346 | 9 | 6 | 5 | 4 | 9 | 5 | 6 | 7 | 4 | 7 | 2 | 7 | 5 |
| NM_003792 | EDF1 | 640 | 55 | 35 | 27 | 29 | 37 | 37 | 50 | 55 | 24 | 26 | 30 | 38 | 31 |
| NM_003793 | CTSF | 2014 | 3 | 2 | 2 | 1 | 1 | 3 | 2 | 1 | 1 | 2 | 2 | 1 | 2 |
| NM_003794 | SNX4 | 2524 | 13 | 6 | 5 | 2 | 5 | 10 | 13 | 12 | 4 | 4 | 4 | 5 | 6 |
| NM_003795 | SNX3 | 1463 | 14 | 10 | 12 | 3 | 14 | 11 | 9 | 14 | 9 | 7 | 7 | 14 | 11 |
| NM_003796 | C19orf2 | 2275 | 61 | 39 | 30 | 21 | 40 | 47 | 32 | 31 | 35 | 48 | 46 | 43 | 61 |
| NM_003797 | EED | 1996 | 5 | 3 | 3 | 3 | 3 | 3 | 3 | 2 | 3 | 3 | 1 | 5 | 4 |
| NM_003798 | CTNNAL1 | 2445 | 40 | 15 | 7 | 2 | 24 | 3 | 10 | 10 | 16 | 40 | 10 | 10 | 33 |
| NM_003799 | RNMT | 6203 | 11 | 5 | 4 | 2 | 6 | 11 | 4 | 3 | 3 | 6 | 4 | 6 | 5 |
| NM_003800 | RNGTT | 4463 | 3 | 2 | 1 | 1 | 1 | 3 | 2 | 1 | 1 | 2 | 1 | 3 | 3 |
| NM_003801 | GPAA1 | 2067 | 1 | 0 | 1 | 0 | 0 | 1 | 1 | 1 | 0 | 0 | 0 | 0 | 0 |
| NM_003802 | MYH13 | 5992 | 0 | 0 | 0 | 0 | 0 | 0 | 0 | 0 | 0 | 0 | 0 | 0 | 0 |
| NM_003803 | MYOM1 | 5847 | 141 | 22 | 3 | 1 | 87 | 2 | 0 | 4 | 1 | 1 | 141 | 0 | 3 |
| NM_003804 | RIPK1 | 3864 | 9 | 6 | 7 | 4 | 5 | 3 | 7 | 9 | 5 | 4 | 2 | 9 | 6 |
| NM_003805 | CRADD | 1188 | 6 | 2 | 1 | 1 | 4 | 1 | 3 | 6 | 1 | 1 | 1 | 1 | 1 |
| NM_003806 | HRK | 697 | 0 | 0 | 0 | 0 | 0 | 0 | 0 | 0 | 0 | 0 | 0 | 0 | 0 |
| NM_003807 | TNFSF14 | 1476 | 1 | 0 | 0 | 0 | 0 | 0 | 0 | 0 | 0 | 0 | 0 | 1 | 0 |
| NM_003808 | TNFSF13 | 2276 | 1 | 0 | 0 | 0 | 0 | 0 | 1 | 0 | 0 | 0 | 0 | 0 | 0 |
| NM_003809 | TNFSF12 | 1386 | 1 | 0 | 0 | 0 | 0 | 0 | 0 | 0 | 0 | 0 | 0 | 1 | 0 |

| transcript | gene | transcript length (nt) | max | mean | adipose | colon | heart | hypothalamus | kidney | liver | lung | ovary | skeletalmuscle | spleen | testes |
|---|---|---|---|---|---|---|---|---|---|---|---|---|---|---|---|
| NM_003810 | TNFSF10 | 1759 | 36 | 10 | 11 | 2 | 6 | 3 | 36 | 5 | 22 | 5 | 6 | 15 | 3 |
| NM_003811 | TNFSF9 | 1665 | 0 | 0 | 0 | 0 | 0 | 0 | 0 | 0 | 0 | 0 | 0 | 0 | 0 |
| NM_003812 | ADAM23 | 3050 | 16 | 3 | 1 | 0 | 10 | 16 | 0 | 0 | 0 | 1 | 1 | 0 | 2 |
| NM_003813 | ADAM21 | 2406 | 0 | 0 | 0 | 0 | 0 | 0 | 0 | 0 | 0 | 0 | 0 | 0 | 0 |
| NM_003814 | ADAM20 | 2792 | 1 | 0 | 0 | 0 | 0 | 1 | 0 | 0 | 0 | 1 | 1 | 0 | 1 |
| NM_003815 | ADAM15 | 2820 | 1 | 0 | 0 | 0 | 1 | 1 | 0 | 0 | 1 | 0 | 0 | 0 | 0 |
| NM_003816 | ADAM9 | 4098 | 38 | 19 | 20 | 5 | 38 | 11 | 23 | 6 | 36 | 11 | 10 | 28 | 21 |
| NM_003817 | ADAM7 | 2594 | 29 | 3 | 0 | 0 | 0 | 0 | 0 | 0 | 0 | 0 | 0 | 0 | 29 |
| NM_003818 | CDS2 | 2704 | 19 | 7 | 5 | 5 | 9 | 19 | 7 | 7 | 6 | 5 | 2 | 8 | 10 |
| NM_003819 | PABPC4 | 3156 | 83 | 27 | 17 | 9 | 39 | 10 | 10 | 19 | 15 | 35 | 83 | 24 | 31 |
| NM_003820 | TNFRSF14 | 1706 | 3 | 1 | 1 | 2 | 0 | 0 | 2 | 0 | 2 | 1 | 0 | 3 | 0 |
| NM_003821 | RIPK2 | 2585 | 2 | 1 | 1 | 0 | 1 | 1 | 1 | 1 | 2 | 1 | 1 | 2 | 1 |
| NM_003822 | NR5A2 | 4906 | 5 | 1 | 0 | 1 | 1 | 0 | 0 | 5 | 1 | 2 | 0 | 0 | 1 |
| NM_003823 | TNFRSF6B | 1122 | 4 | 1 | 0 | 0 | 0 | 1 | 0 | 0 | 2 | 0 | 0 | 4 | 0 |
| NM_003824 | FADD | 1853 | 15 | 10 | 10 | 11 | 9 | 11 | 12 | 10 | 8 | 10 | 4 | 15 | 10 |
| NM_003825 | SNAP23 | 2307 | 41 | 15 | 8 | 9 | 15 | 5 | 15 | 8 | 20 | 15 | 10 | 41 | 21 |
| NM_003826 | NAPG | 3711 | 15 | 4 | 2 | 2 | 5 | 15 | 5 | 3 | 2 | 2 | 2 | 4 | 3 |
| NM_003827 | NAPA | 1842 | 5 | 2 | 2 | 1 | 2 | 5 | 2 | 5 | 2 | 1 | 3 | 2 | 2 |
| NM_003828 | MTMR1 | 2732 | 6 | 4 | 2 | 2 | 3 | 6 | 3 | 4 | 3 | 3 | 3 | 6 | 6 |
| NM_003829 | MPDZ | 7438 | 11 | 7 | 5 | 2 | 11 | 11 | 7 | 8 | 5 | 8 | 5 | 5 | 9 |
| NM_003830 | SIGLEC5 | 2497 | 1 | 0 | 0 | 0 | 0 | 0 | 0 | 0 | 0 | 0 | 0 | 1 | 0 |
| NM_003831 | RIOK3 | 4033 | 6 | 4 | 3 | 2 | 5 | 3 | 5 | 4 | 5 | 3 | 6 | 5 | 5 |
| NM_003833 | MATN4 | 2253 | 0 | 0 | 0 | 0 | 0 | 0 | 0 | 0 | 0 | 0 | 0 | 0 | 0 |
| NM_003834 | RGS11 | 2376 | 0 | 0 | 0 | 0 | 0 | 0 | 0 | 0 | 0 | 0 | 0 | 0 | 0 |
| NM_003835 | RGS9 | 2522 | 1 | 0 | 0 | 0 | 0 | 1 | 0 | 0 | 0 | 0 | 0 | 0 | 0 |
| NM_003836 | DLK1 | 1531 | 2 | 0 | 0 | 0 | 0 | 0 | 0 | 0 | 0 | 2 | 0 | 0 | 1 |
| NM_003837 | FBP2 | 1304 | 7 | 1 | 0 | 0 | 0 | 1 | 0 | 0 | 0 | 0 | 7 | 0 | 0 |
| NM_003838 | FPGT | 3426 | 6 | 4 | 3 | 2 | 4 | 3 | 5 | 2 | 3 | 4 | 1 | 6 | 6 |
| NM_003839 | TNFRSF11A | 3126 | 2 | 0 | 1 | 2 | 0 | 0 | 0 | 0 | 0 | 0 | 0 | 0 | 0 |
| NM_003840 | TNFRSF10D | 3532 | 2 | 1 | 0 | 0 | 0 | 0 | 1 | 2 | 1 | 1 | 0 | 1 | 1 |
| NM_003841 | TNFRSF10C | 1387 | 28 | 4 | 2 | 1 | 2 | 2 | 1 | 2 | 8 | 1 | 1 | 28 | 2 |
| NM_003842 | TNFRSF10B | 4152 | 3 | 1 | 1 | 1 | 1 | 0 | 1 | 3 | 2 | 1 | 1 | 2 | 1 |
| NM_003843 | SCEL | 3164 | 4 | 0 | 0 | 0 | 0 | 0 | 0 | 0 | 4 | 0 | 0 | 0 | 0 |
| NM_003844 | TNFRSF10A | 1749 | 3 | 1 | 1 | 1 | 1 | 0 | 2 | 2 | 3 | 0 | 1 | 3 | 0 |
| NM_003845 | DYRK4 | 1845 | 2 | 1 | 1 | 0 | 1 | 1 | 0 | 0 | 0 | 1 | 0 | 1 | 2 |
| NM_003846 | PEX11B | 1610 | 1 | 0 | 0 | 0 | 0 | 1 | 1 | 0 | 0 | 0 | 0 | 0 | 0 |
| NM_003847 | PEX11A | 1160 | 10 | 4 | 10 | 6 | 3 | 3 | 7 | 6 | 2 | 4 | 2 | 2 | 4 |
| NM_003848 | SUCLG2 | 2352 | 29 | 11 | 7 | 14 | 10 | 3 | 17 | 29 | 6 | 6 | 10 | 10 | 5 |
| NM_003849 | SUCLG1 | 1452 | 44 | 12 | 11 | 7 | 23 | 8 | 44 | 10 | 3 | 4 | 9 | 6 | 5 |
| NM_003850 | SUCLA2 | 2164 | 28 | 14 | 16 | 4 | 28 | 25 | 21 | 7 | 5 | 7 | 20 | 8 | 9 |
| NM_003851 | CREG1 | 2025 | 58 | 27 | 48 | 8 | 23 | 23 | 14 | 44 | 29 | 17 | 12 | 58 | 18 |
| NM_003852 | TRIM24 | 3902 | 12 | 4 | 1 | 1 | 3 | 3 | 3 | 3 | 3 | 12 | 3 | 4 | 6 |
| NM_003853 | IL18RAP | 2667 | 7 | 1 | 0 | 0 | 0 | 0 | 0 | 1 | 3 | 0 | 0 | 7 | 0 |
| NM_003854 | IL1RL2 | 1964 | 2 | 1 | 0 | 1 | 0 | 1 | 1 | 2 | 0 | 0 | 0 | 0 | 0 |
| NM_003855 | IL18R1 | 3521 | 12 | 3 | 1 | 1 | 2 | 0 | 2 | 2 | 12 | 0 | 0 | 8 | 1 |
| NM_003856 | IL1RL1 | 2507 | 25 | 4 | 0 | 1 | 5 | 2 | 7 | 2 | 25 | 0 | 0 | 1 | 1 |
| NM_003857 | GALR2 | 1298 | 0 | 0 | 0 | 0 | 0 | 0 | 0 | 0 | 0 | 0 | 0 | 0 | 0 |
| NM_003858 | CCNK | 1933 | 10 | 6 | 3 | 4 | 7 | 5 | 5 | 3 | 6 | 6 | 6 | 9 | 10 |
| NM_003859 | DPM1 | 1047 | 18 | 12 | 9 | 5 | 17 | 11 | 11 | 12 | 9 | 10 | 10 | 17 | 18 |
| NM_003860 | BANF1 | 1151 | 4 | 3 | 4 | 4 | 4 | 4 | 3 | 4 | 2 | 2 | 3 | 4 | 2 |
| NM_003861 | DCAF5 | 5984 | 7 | 5 | 5 | 3 | 7 | 6 | 5 | 5 | 4 | 4 | 7 | 5 | 7 |
| NM_003862 | FGF18 | 1516 | 0 | 0 | 0 | 0 | 0 | 0 | 0 | 0 | 0 | 0 | 0 | 0 | 0 |
| NM_003863 | DPM2 | 1546 | 1 | 0 | 0 | 0 | 0 | 0 | 1 | 0 | 1 | 0 | 0 | 0 | 0 |
| NM_003864 | SAP30 | 1109 | 13 | 8 | 5 | 3 | 12 | 11 | 4 | 3 | 10 | 7 | 3 | 13 | 13 |
| NM_003865 | HESX1 | 1173 | 1 | 0 | 0 | 0 | 0 | 0 | 0 | 0 | 0 | 0 | 0 | 0 | 1 |
| NM_003866 | INPP4B | 4123 | 3 | 2 | 1 | 1 | 3 | 2 | 1 | 0 | 2 | 2 | 3 | 3 | 3 |
| NM_003867 | FGF17 | 1222 | 0 | 0 | 0 | 0 | 0 | 0 | 0 | 0 | 0 | 0 | 0 | 0 | 0 |
| NM_003868 | FGF16 | 351 | 1 | 0 | 0 | 1 | 0 | 0 | 0 | 0 | 0 | 0 | 0 | 0 | 0 |
| NM_003869 | CES2 | 3938 | 14 | 4 | 1 | 7 | 3 | 2 | 9 | 14 | 1 | 1 | 2 | 1 | 1 |
| NM_003870 | IQGAP1 | 7210 | 67 | 26 | 31 | 14 | 29 | 8 | 24 | 7 | 45 | 29 | 14 | 67 | 23 |
| NM_003872 | NRP2 | 6647 | 8 | 3 | 5 | 3 | 1 | 3 | 1 | 1 | 3 | 8 | 1 | 2 | 1 |
| NM_003873 | NRP1 | 5880 | 23 | 10 | 23 | 3 | 13 | 4 | 18 | 5 | 9 | 13 | 4 | 15 | 6 |
| NM_003874 | CD84 | 3270 | 6 | 2 | 4 | 1 | 1 | 1 | 0 | 1 | 3 | 0 | 0 | 6 | 1 |
| NM_003875 | GMPS | 2456 | 40 | 12 | 7 | 5 | 10 | 14 | 9 | 10 | 9 | 11 | 7 | 13 | 40 |
| NM_003876 | TMEM11 | 1396 | 11 | 6 | 7 | 4 | 9 | 4 | 5 | 6 | 3 | 4 | 11 | 6 | 4 |
| NM_003877 | SOCS2 | 2210 | 3 | 1 | 1 | 1 | 2 | 1 | 2 | 1 | 3 | 1 | 0 | 1 | 2 |
| NM_003878 | GGH | 1488 | 8 | 2 | 1 | 1 | 1 | 2 | 5 | 8 | 1 | 0 | 0 | 1 | 2 |
| NM_003879 | CFLAR | 2202 | 39 | 22 | 24 | 13 | 37 | 8 | 19 | 13 | 39 | 9 | 31 | 36 | 11 |
| NM_003880 | WISP3 | 1239 | 0 | 0 | 0 | 0 | 0 | 0 | 0 | 0 | 0 | 0 | 0 | 0 | 0 |
| NM_003881 | WISP2 | 1404 | 12 | 4 | 12 | 2 | 7 | 0 | 0 | 0 | 7 | 5 | 2 | 0 | 6 |
| NM_003882 | WISP1 | 2798 | 1 | 0 | 0 | 0 | 0 | 0 | 0 | 0 | 0 | 1 | 0 | 1 | 0 |
| NM_003883 | HDAC3 | 1945 | 4 | 3 | 3 | 2 | 2 | 4 | 3 | 3 | 2 | 3 | 2 | 4 | 2 |
| NM_003884 | KAT2B | 4824 | 7 | 4 | 3 | 1 | 7 | 7 | 3 | 4 | 3 | 4 | 6 | 5 | 1 |
| NM_003885 | CDK5R1 | 3854 | 4 | 0 | 0 | 0 | 0 | 4 | 0 | 0 | 0 | 0 | 0 | 1 | 0 |
| NM_003886 | AKAP4 | 2881 | 22 | 2 | 0 | 0 | 0 | 0 | 0 | 0 | 0 | 0 | 0 | 0 | 22 |
| NM_003887 | ASAP2 | 5721 | 8 | 3 | 4 | 4 | 3 | 4 | 8 | 1 | 3 | 3 | 1 | 1 | 7 |
| NM_003888 | ALDH1A2 | 3383 | 7 | 2 | 1 | 0 | 2 | 1 | 2 | 0 | 2 | 3 | 1 | 1 | 7 |

| transcript | gene | transcript length (nt) | max | mean | adipose | colon | heart | hypothalamus | kidney | liver | lung | ovary | skeletalmuscle | spleen | testes |
|---|---|---|---|---|---|---|---|---|---|---|---|---|---|---|---|
| NM_003889 | NR1I2 | 4417 | 4 | 1 | 0 | 1 | 0 | 0 | 0 | 4 | 0 | 0 | 0 | 0 | 0 |
| NM_003890 | FCGBP | 16390 | 11 | 1 | 0 | 11 | 1 | 0 | 0 | 0 | 0 | 0 | 0 | 0 | 0 |
| NM_003891 | PROZ | 1485 | 9 | 1 | 0 | 0 | 0 | 0 | 3 | 9 | 0 | 0 | 0 | 0 | 0 |
| NM_003893 | LDB1 | 2297 | 11 | 6 | 4 | 5 | 5 | 7 | 7 | 2 | 4 | 11 | 3 | 6 | 8 |
| NM_003895 | SYNJ1 | 7075 | 10 | 2 | 1 | 0 | 1 | 10 | 1 | 0 | 1 | 1 | 1 | 2 | 1 |
| NM_003896 | ST3GAL5 | 2382 | 8 | 3 | 2 | 1 | 1 | 8 | 1 | 1 | 4 | 2 | 2 | 5 | 4 |
| NM_003897 | IER3 | 1240 | 23 | 9 | 2 | 7 | 19 | 5 | 14 | 10 | 23 | 7 | 3 | 9 | 4 |
| NM_003898 | SYNJ2 | 6738 | 5 | 1 | 0 | 2 | 1 | 5 | 1 | 1 | 0 | 1 | 0 | 1 | 1 |
| NM_003899 | ARHGEF7 | 5059 | 7 | 4 | 4 | 3 | 4 | 6 | 3 | 2 | 3 | 3 | 4 | 7 | 4 |
| NM_003900 | SQSTM1 | 2902 | 27 | 15 | 24 | 9 | 11 | 10 | 16 | 15 | 17 | 9 | 27 | 12 | 11 |
| NM_003901 | SGPL1 | 5767 | 8 | 3 | 2 | 3 | 2 | 2 | 5 | 5 | 3 | 2 | 1 | 8 | 6 |
| NM_003902 | FUBP1 | 2884 | 31 | 20 | 13 | 14 | 21 | 23 | 20 | 12 | 18 | 24 | 21 | 31 | 21 |
| NM_003903 | CDC16 | 2211 | 7 | 5 | 6 | 2 | 4 | 4 | 5 | 6 | 4 | 6 | 3 | 6 | 7 |
| NM_003904 | ZNF259 | 1778 | 7 | 4 | 3 | 2 | 4 | 7 | 3 | 3 | 2 | 3 | 3 | 5 | 4 |
| NM_003905 | NAE1 | 1813 | 18 | 10 | 9 | 4 | 10 | 15 | 8 | 9 | 6 | 10 | 7 | 10 | 18 |
| NM_003906 | MCM3AP | 6113 | 4 | 3 | 3 | 3 | 2 | 3 | 3 | 3 | 2 | 2 | 3 | 4 | 3 |
| NM_003907 | EIF2B5 | 2898 | 2 | 2 | 1 | 1 | 2 | 2 | 2 | 2 | 1 | 1 | 2 | 2 | 1 |
| NM_003908 | EIF2S2 | 2592 | 36 | 25 | 20 | 9 | 36 | 24 | 23 | 32 | 27 | 15 | 26 | 33 | 29 |
| NM_003909 | CPNE3 | 4864 | 12 | 7 | 6 | 3 | 11 | 5 | 12 | 9 | 8 | 10 | 4 | 9 | 6 |
| NM_003910 | BUD31 | 1043 | 19 | 11 | 11 | 7 | 8 | 19 | 12 | 11 | 9 | 10 | 4 | 12 | 15 |
| NM_003913 | PRPF4B | 7482 | 18 | 9 | 7 | 4 | 9 | 8 | 9 | 7 | 10 | 13 | 8 | 18 | 11 |
| NM_003914 | CCNA1 | 1965 | 5 | 1 | 0 | 0 | 0 | 2 | 0 | 0 | 0 | 0 | 0 | 0 | 5 |
| NM_003915 | CPNE1 | 2191 | 3 | 2 | 2 | 1 | 1 | 1 | 2 | 2 | 2 | 2 | 1 | 3 | 1 |
| NM_003916 | AP1S2 | 2283 | 9 | 3 | 1 | 1 | 9 | 3 | 1 | 0 | 2 | 1 | 4 | 5 | 3 |
| NM_003917 | AP1G2 | 2876 | 2 | 1 | 1 | 2 | 0 | 0 | 1 | 0 | 1 | 1 | 0 | 2 | 1 |
| NM_003918 | GYG2 | 3395 | 8 | 1 | 8 | 1 | 0 | 1 | 0 | 2 | 0 | 0 | 0 | 0 | 0 |
| NM_003919 | SGCE | 1700 | 5 | 2 | 2 | 1 | 2 | 2 | 1 | 0 | 1 | 5 | 1 | 2 | 3 |
| NM_003920 | TIMELESS | 5137 | 4 | 2 | 2 | 2 | 1 | 1 | 1 | 4 | 1 | 1 | 1 | 3 | 2 |
| NM_003921 | BCL10 | 3257 | 5 | 3 | 2 | 3 | 2 | 2 | 2 | 2 | 3 | 4 | 1 | 5 | 3 |
| NM_003922 | HERC1 | 15197 | 13 | 8 | 6 | 4 | 8 | 13 | 5 | 3 | 6 | 9 | 9 | 12 | 8 |
| NM_003923 | FOXH1 | 2181 | 0 | 0 | 0 | 0 | 0 | 0 | 0 | 0 | 0 | 0 | 0 | 0 | 0 |
| NM_003924 | PHOX2B | 3030 | 0 | 0 | 0 | 0 | 0 | 0 | 0 | 0 | 0 | 0 | 0 | 0 | 0 |
| NM_003925 | MBD4 | 2470 | 9 | 5 | 4 | 1 | 6 | 5 | 5 | 5 | 5 | 4 | 3 | 9 | 5 |
| NM_003926 | MBD3 | 2559 | 1 | 1 | 1 | 0 | 0 | 1 | 0 | 1 | 0 | 0 | 0 | 0 | 0 |
| NM_003927 | MBD2 | 2584 | 31 | 14 | 28 | 8 | 16 | 8 | 13 | 11 | 15 | 9 | 7 | 31 | 11 |
| NM_003929 | RAB7L1 | 3307 | 12 | 3 | 2 | 1 | 3 | 2 | 12 | 3 | 2 | 1 | 1 | 5 | 1 |
| NM_003930 | SKAP2 | 3970 | 16 | 5 | 6 | 3 | 3 | 5 | 7 | 2 | 7 | 3 | 3 | 16 | 4 |
| NM_003931 | WASF1 | 3220 | 15 | 2 | 1 | 0 | 1 | 15 | 1 | 0 | 0 | 3 | 0 | 1 | 5 |
| NM_003932 | ST13 | 3194 | 114 | 39 | 31 | 12 | 34 | 34 | 42 | 25 | 20 | 114 | 48 | 26 | 41 |
| NM_003933 | BAIAP3 | 4523 | 1 | 0 | 0 | 0 | 0 | 1 | 0 | 0 | 0 | 0 | 0 | 0 | 0 |
| NM_003934 | FUBP3 | 3157 | 17 | 10 | 10 | 6 | 10 | 14 | 11 | 17 | 7 | 10 | 4 | 12 | 14 |
| NM_003935 | TOP3B | 3107 | 1 | 0 | 0 | 0 | 0 | 0 | 0 | 1 | 0 | 0 | 0 | 0 | 0 |
| NM_003936 | CDK5R2 | 2480 | 3 | 0 | 0 | 0 | 0 | 3 | 0 | 0 | 0 | 0 | 0 | 0 | 0 |
| NM_003937 | KYNU | 1672 | 8 | 2 | 1 | 0 | 2 | 0 | 2 | 8 | 2 | 0 | 0 | 3 | 1 |
| NM_003938 | AP3D1 | 4863 | 14 | 10 | 7 | 7 | 12 | 14 | 14 | 12 | 6 | 6 | 7 | 9 | 11 |
| NM_003939 | BTRC | 6013 | 14 | 4 | 2 | 2 | 5 | 14 | 3 | 2 | 2 | 5 | 3 | 4 | 7 |
| NM_003940 | USP13 | 7931 | 31 | 5 | 1 | 0 | 9 | 2 | 1 | 1 | 1 | 3 | 31 | 1 | 3 |
| NM_003941 | WASL | 4422 | 19 | 10 | 4 | 5 | 8 | 19 | 12 | 10 | 7 | 15 | 8 | 9 | 16 |
| NM_003942 | RPS6KA4 | 3137 | 2 | 1 | 1 | 1 | 2 | 1 | 1 | 0 | 1 | 1 | 1 | 2 | 1 |
| NM_003943 | STBD1 | 2453 | 24 | 8 | 16 | 4 | 17 | 3 | 2 | 12 | 5 | 2 | 24 | 2 | 4 |
| NM_003944 | SELENBP1 | 1705 | 31 | 7 | 9 | 31 | 2 | 2 | 6 | 12 | 5 | 1 | 2 | 2 | 1 |
| NM_003945 | ATP6V0E1 | 882 | 41 | 20 | 21 | 8 | 23 | 10 | 41 | 18 | 23 | 11 | 7 | 32 | 21 |
| NM_003946 | NOL3 | 1384 | 1 | 0 | 1 | 0 | 1 | 0 | 0 | 0 | 0 | 0 | 1 | 0 | 0 |
| NM_003947 | KALRN | 6508 | 18 | 4 | 2 | 2 | 4 | 18 | 1 | 0 | 1 | 2 | 1 | 7 | 2 |
| NM_003948 | CDKL2 | 4715 | 3 | 1 | 0 | 1 | 0 | 3 | 3 | 0 | 1 | 0 | 0 | 0 | 2 |
| NM_003950 | F2RL3 | 2731 | 2 | 1 | 1 | 1 | 0 | 0 | 0 | 0 | 2 | 0 | 0 | 1 | 0 |
| NM_003951 | SLC25A14 | 1595 | 3 | 1 | 0 | 0 | 0 | 3 | 1 | 0 | 0 | 1 | 0 | 0 | 1 |
| NM_003952 | RPS6KB2 | 1770 | 1 | 1 | 1 | 1 | 0 | 0 | 0 | 1 | 0 | 0 | 0 | 1 | 0 |
| NM_003953 | MPZL1 | 5009 | 9 | 4 | 4 | 3 | 5 | 3 | 3 | 2 | 4 | 4 | 1 | 4 | 9 |
| NM_003954 | MAP3K14 | 4467 | 1 | 0 | 0 | 0 | 0 | 0 | 0 | 0 | 0 | 0 | 1 | 1 | 0 |
| NM_003955 | SOCS3 | 2731 | 4 | 2 | 3 | 0 | 3 | 3 | 1 | 2 | 3 | 2 | 1 | 4 | 0 |
| NM_003956 | CH25H | 1378 | 1 | 0 | 0 | 0 | 0 | 1 | 0 | 0 | 1 | 0 | 0 | 0 | 0 |
| NM_003957 | BRSK2 | 3502 | 2 | 0 | 0 | 0 | 0 | 2 | 0 | 0 | 0 | 0 | 0 | 0 | 0 |
| NM_003958 | RNF8 | 5631 | 4 | 2 | 1 | 1 | 2 | 4 | 2 | 1 | 1 | 2 | 1 | 1 | 4 |
| NM_003959 | HIP1R | 4473 | 1 | 1 | 0 | 1 | 0 | 1 | 1 | 1 | 0 | 0 | 0 | 0 | 0 |
| NM_003960 | NAT8 | 1073 | 21 | 3 | 0 | 0 | 0 | 0 | 21 | 1 | 0 | 2 | 0 | 4 | 1 |
| NM_003961 | RHBDL1 | 1559 | 0 | 0 | 0 | 0 | 0 | 0 | 0 | 0 | 0 | 0 | 0 | 0 | 0 |
| NM_003963 | TM4SF5 | 708 | 1 | 0 | 0 | 0 | 0 | 0 | 0 | 1 | 0 | 0 | 0 | 0 | 0 |
| NM_003965 | CCRL2 | 1729 | 1 | 0 | 1 | 0 | 0 | 0 | 0 | 0 | 1 | 0 | 0 | 1 | 0 |
| NM_003966 | SEMA5A | 11808 | 4 | 2 | 1 | 2 | 3 | 4 | 4 | 0 | 3 | 1 | 0 | 2 | 1 |
| NM_003967 | TAAR5 | 1147 | 0 | 0 | 0 | 0 | 0 | 0 | 0 | 0 | 0 | 0 | 0 | 0 | 0 |
| NM_003968 | UBA3 | 2119 | 12 | 7 | 5 | 3 | 12 | 9 | 6 | 5 | 8 | 5 | 5 | 12 | 8 |
| NM_003969 | UBE2M | 1524 | 1 | 1 | 0 | 0 | 1 | 1 | 1 | 0 | 0 | 0 | 1 | 1 | 1 |
| NM_003970 | MYOM2 | 5011 | 78 | 11 | 0 | 0 | 78 | 1 | 1 | 0 | 0 | 0 | 43 | 0 | 1 |
| NM_003971 | SPAG9 | 8230 | 20 | 13 | 11 | 5 | 11 | 20 | 15 | 8 | 15 | 12 | 19 | 10 | 19 |
| NM_003972 | BTAF1 | 7038 | 5 | 3 | 2 | 2 | 2 | 2 | 3 | 2 | 3 | 4 | 2 | 5 | 4 |
| NM_003973 | RPL14 | 835 | 290 | 132 | 115 | 96 | 121 | 96 | 80 | 169 | 113 | 290 | 138 | 165 | 71 |

| transcript | gene | transcript length (nt) | max | mean | adipose | colon | heart | hypothalamus | kidney | liver | lung | ovary | skeletalmuscle | spleen | testes |
|---|---|---|---|---|---|---|---|---|---|---|---|---|---|---|---|
| NM_003974 | DOK2 | 1770 | 4 | 1 | 3 | 1 | 1 | 0 | 0 | 0 | 3 | 0 | 0 | 4 | 0 |
| NM_003975 | SH2D2A | 1643 | 1 | 0 | 0 | 0 | 0 | 0 | 0 | 0 | 0 | 0 | 0 | 1 | 0 |
| NM_003976 | ARTN | 1435 | 0 | 0 | 0 | 0 | 0 | 0 | 0 | 0 | 0 | 0 | 0 | 0 | 0 |
| NM_003977 | AIP | 1232 | 3 | 1 | 1 | 2 | 1 | 3 | 2 | 1 | 1 | 1 | 0 | 3 | 1 |
| NM_003978 | PSTPIP1 | 1855 | 1 | 0 | 1 | 0 | 0 | 0 | 0 | 0 | 1 | 0 | 0 | 1 | 0 |
| NM_003979 | GPRC5A | 2851 | 10 | 1 | 0 | 1 | 0 | 0 | 0 | 0 | 10 | 1 | 0 | 0 | 0 |
| NM_003980 | MAP7 | 3990 | 23 | 8 | 3 | 11 | 1 | 23 | 22 | 7 | 4 | 1 | 0 | 2 | 14 |
| NM_003981 | PRC1 | 3113 | 3 | 1 | 1 | 2 | 1 | 1 | 0 | 0 | 1 | 1 | 0 | 1 | 3 |
| NM_003982 | SLC7A7 | 2509 | 31 | 7 | 4 | 2 | 4 | 2 | 31 | 3 | 8 | 2 | 0 | 23 | 3 |
| NM_003983 | SLC7A6 | 6261 | 4 | 2 | 4 | 2 | 3 | 1 | 1 | 0 | 2 | 1 | 4 | 3 | 2 |
| NM_003984 | SLC13A2 | 2355 | 4 | 0 | 0 | 0 | 0 | 0 | 4 | 0 | 0 | 0 | 0 | 0 | 0 |
| NM_003985 | TNK1 | 2835 | 1 | 0 | 0 | 1 | 0 | 0 | 0 | 0 | 0 | 0 | 0 | 0 | 0 |
| NM_003986 | BBOX1 | 1886 | 31 | 3 | 0 | 0 | 0 | 2 | 31 | 1 | 0 | 0 | 0 | 0 | 0 |
| NM_003987 | PAX2 | 4126 | 3 | 0 | 0 | 0 | 0 | 0 | 3 | 0 | 0 | 0 | 0 | 0 | 0 |
| NM_003988 | PAX2 | 4140 | 2 | 0 | 0 | 0 | 0 | 0 | 2 | 0 | 0 | 0 | 0 | 0 | 0 |
| NM_003989 | PAX2 | 4038 | 2 | 0 | 0 | 0 | 0 | 0 | 2 | 0 | 0 | 0 | 0 | 0 | 0 |
| NM_003990 | PAX2 | 4107 | 3 | 0 | 0 | 0 | 0 | 0 | 3 | 0 | 0 | 0 | 0 | 0 | 0 |
| NM_003991 | EDNRB | 2530 | 6 | 3 | 3 | 1 | 3 | 6 | 4 | 3 | 5 | 0 | 0 | 6 | 1 |
| NM_003992 | CLK3 | 1846 | 3 | 2 | 1 | 3 | 2 | 2 | 1 | 1 | 2 | 2 | 2 | 2 | 2 |
| NM_003993 | CLK2 | 2137 | 3 | 2 | 1 | 2 | 1 | 2 | 2 | 1 | 1 | 2 | 1 | 3 | 1 |
| NM_003994 | KITLG | 5350 | 8 | 3 | 2 | 1 | 2 | 3 | 6 | 0 | 8 | 2 | 1 | 1 | 5 |
| NM_003995 | NPR2 | 3430 | 1 | 1 | 1 | 1 | 1 | 1 | 1 | 0 | 1 | 1 | 1 | 1 | 1 |
| NM_003996 | GPX5 | 1334 | 0 | 0 | 0 | 0 | 0 | 0 | 0 | 0 | 0 | 0 | 0 | 0 | 0 |
| NM_003998 | NFKB1 | 4086 | 22 | 10 | 8 | 9 | 9 | 5 | 8 | 8 | 11 | 11 | 8 | 22 | 8 |
| NM_003999 | OSMR | 5538 | 14 | 8 | 6 | 2 | 13 | 7 | 8 | 10 | 10 | 5 | 4 | 14 | 7 |
| NM_004000 | CHI3L2 | 1470 | 3 | 1 | 1 | 0 | 3 | 2 | 0 | 0 | 2 | 0 | 0 | 2 | 0 |
| NM_004001 | FCGR2B | 1633 | 6 | 3 | 6 | 1 | 4 | 1 | 0 | 3 | 3 | 1 | 3 | 3 | 2 |
| NM_004004 | GJB2 | 2330 | 5 | 1 | 0 | 3 | 0 | 3 | 3 | 5 | 0 | 0 | 0 | 0 | 0 |
| NM_004006 | DMD | 13993 | 68 | 12 | 8 | 3 | 30 | 6 | 1 | 3 | 3 | 9 | 68 | 0 | 5 |
| NM_004007 | DMD | 13718 | 69 | 13 | 8 | 3 | 30 | 6 | 1 | 3 | 3 | 9 | 69 | 0 | 5 |
| NM_004009 | DMD | 14000 | 68 | 12 | 8 | 3 | 30 | 6 | 1 | 3 | 3 | 9 | 68 | 0 | 5 |
| NM_004010 | DMD | 14083 | 67 | 12 | 8 | 3 | 29 | 6 | 1 | 3 | 3 | 9 | 67 | 0 | 5 |
| NM_004011 | DMD | 9771 | 38 | 8 | 5 | 2 | 18 | 4 | 1 | 4 | 2 | 5 | 38 | 0 | 4 |
| NM_004012 | DMD | 9914 | 38 | 7 | 5 | 2 | 18 | 4 | 1 | 4 | 2 | 5 | 38 | 0 | 3 |
| NM_004013 | DMD | 7410 | 30 | 6 | 4 | 1 | 15 | 4 | 1 | 5 | 2 | 5 | 30 | 0 | 3 |
| NM_004014 | DMD | 5623 | 18 | 5 | 3 | 1 | 10 | 3 | 1 | 6 | 1 | 4 | 18 | 0 | 3 |
| NM_004015 | DMD | 4623 | 20 | 5 | 4 | 1 | 11 | 4 | 1 | 7 | 2 | 4 | 20 | 0 | 4 |
| NM_004016 | DMD | 4591 | 20 | 5 | 4 | 1 | 11 | 4 | 1 | 7 | 2 | 4 | 20 | 0 | 4 |
| NM_004017 | DMD | 4584 | 20 | 5 | 4 | 1 | 11 | 4 | 1 | 7 | 2 | 4 | 20 | 0 | 4 |
| NM_004018 | DMD | 4552 | 20 | 5 | 4 | 1 | 11 | 4 | 1 | 7 | 2 | 4 | 20 | 0 | 4 |
| NM_004019 | DMD | 1571 | 11 | 3 | 3 | 1 | 4 | 3 | 1 | 5 | 1 | 2 | 11 | 0 | 2 |
| NM_004020 | DMD | 7050 | 31 | 6 | 4 | 1 | 15 | 4 | 1 | 5 | 2 | 5 | 31 | 0 | 4 |
| NM_004021 | DMD | 7378 | 30 | 6 | 4 | 1 | 15 | 4 | 1 | 5 | 2 | 5 | 30 | 0 | 3 |
| NM_004022 | DMD | 7339 | 30 | 6 | 4 | 1 | 15 | 4 | 1 | 5 | 2 | 5 | 30 | 0 | 3 |
| NM_004023 | DMD | 7048 | 31 | 6 | 4 | 1 | 15 | 4 | 1 | 5 | 2 | 5 | 31 | 0 | 4 |
| NM_004027 | INPP4A | 6634 | 5 | 2 | 1 | 1 | 1 | 5 | 1 | 0 | 1 | 1 | 1 | 3 | 1 |
| NM_004028 | AQP4 | 5123 | 137 | 15 | 0 | 1 | 2 | 137 | 0 | 0 | 22 | 0 | 3 | 0 | 0 |
| NM_004029 | IRF7 | 1885 | 0 | 0 | 0 | 0 | 0 | 0 | 0 | 0 | 0 | 0 | 0 | 0 | 0 |
| NM_004031 | IRF7 | 2051 | 0 | 0 | 0 | 0 | 0 | 0 | 0 | 0 | 0 | 0 | 0 | 0 | 0 |
| NM_004032 | DDO | 1532 | 4 | 2 | 1 | 1 | 4 | 2 | 4 | 4 | 0 | 0 | 1 | 1 | 1 |
| NM_004033 | ANXA6 | 2897 | 25 | 13 | 21 | 6 | 13 | 18 | 6 | 25 | 6 | 13 | 12 | 15 | 6 |
| NM_004034 | ANXA7 | 2204 | 24 | 16 | 19 | 8 | 20 | 18 | 24 | 23 | 12 | 10 | 22 | 16 | 9 |
| NM_004035 | ACOX1 | 7510 | 14 | 6 | 9 | 5 | 5 | 6 | 7 | 14 | 3 | 2 | 5 | 4 | 4 |
| NM_004036 | ADCY3 | 4397 | 2 | 1 | 1 | 1 | 1 | 2 | 0 | 0 | 1 | 1 | 0 | 1 | 1 |
| NM_004037 | AMPD2 | 3903 | 3 | 1 | 0 | 1 | 1 | 3 | 1 | 1 | 1 | 1 | 0 | 2 | 0 |
| NM_004038 | AMY1A | 1862 | 0 | 0 | 0 | 0 | 0 | 0 | 0 | 0 | 0 | 0 | 0 | 0 | 0 |
| NM_004039 | ANXA2 | 1554 | 128 | 32 | 128 | 30 | 32 | 9 | 14 | 12 | 46 | 14 | 13 | 30 | 24 |
| NM_004040 | RHOB | 2367 | 9 | 5 | 5 | 3 | 5 | 7 | 5 | 9 | 9 | 6 | 2 | 4 | 3 |
| NM_004041 | ARRB1 | 2204 | 6 | 3 | 4 | 3 | 2 | 5 | 1 | 1 | 5 | 1 | 1 | 6 | 1 |
| NM_004042 | ARSF | 2012 | 1 | 0 | 0 | 0 | 0 | 0 | 1 | 0 | 0 | 0 | 0 | 0 | 0 |
| NM_004043 | ASMT | 1334 | 0 | 0 | 0 | 0 | 0 | 0 | 0 | 0 | 0 | 0 | 0 | 0 | 0 |
| NM_004044 | ATIC | 2084 | 5 | 3 | 2 | 2 | 2 | 5 | 3 | 5 | 1 | 2 | 2 | 4 | 3 |
| NM_004045 | ATOX1 | 489 | 27 | 14 | 12 | 7 | 13 | 25 | 27 | 23 | 11 | 5 | 4 | 19 | 7 |
| NM_004046 | ATP5A1 | 1890 | 150 | 56 | 35 | 29 | 150 | 67 | 90 | 54 | 24 | 28 | 65 | 38 | 35 |
| NM_004047 | ATP6V0B | 1022 | 3 | 1 | 0 | 1 | 1 | 3 | 2 | 1 | 1 | 0 | 1 | 1 | 1 |
| NM_004048 | B2M | 987 | 1,291 | 432 | 626 | 338 | 247 | 233 | 222 | 569 | 814 | 132 | 135 | 1,291 | 146 |
| NM_004049 | BCL2A1 | 887 | 17 | 4 | 3 | 0 | 2 | 3 | 0 | 0 | 16 | 0 | 0 | 17 | 1 |
| NM_004050 | BCL2L2 | 3564 | 22 | 9 | 16 | 9 | 9 | 22 | 12 | 3 | 8 | 3 | 3 | 6 | 12 |
| NM_004051 | BDH1 | 3394 | 2 | 1 | 0 | 2 | 1 | 1 | 1 | 2 | 1 | 0 | 0 | 0 | 0 |
| NM_004052 | BNIP3 | 1534 | 33 | 17 | 20 | 5 | 20 | 30 | 18 | 33 | 10 | 8 | 28 | 5 | 10 |
| NM_004053 | BYSL | 1990 | 2 | 1 | 1 | 1 | 2 | 2 | 1 | 2 | 0 | 1 | 2 | 1 | 1 |
| NM_004054 | C3AR1 | 1955 | 3 | 1 | 2 | 0 | 1 | 1 | 0 | 0 | 3 | 0 | 0 | 2 | 1 |
| NM_004055 | CAPN5 | 4390 | 4 | 1 | 0 | 4 | 0 | 1 | 2 | 3 | 0 | 1 | 0 | 0 | 2 |
| NM_004056 | CA8 | 2278 | 6 | 1 | 1 | 1 | 1 | 3 | 1 | 0 | 1 | 0 | 0 | 1 | 6 |
| NM_004057 | S100G | 453 | 0 | 0 | 0 | 0 | 0 | 0 | 0 | 0 | 0 | 0 | 0 | 0 | 0 |
| NM_004058 | CAPS | 1609 | 1 | 0 | 0 | 0 | 0 | 0 | 0 | 0 | 1 | 0 | 0 | 0 | 0 |
| NM_004059 | CCBL1 | 1925 | 1 | 0 | 0 | 0 | 0 | 1 | 0 | 0 | 0 | 0 | 0 | 0 | 0 |

| transcript | gene | transcript length (nt) | max | mean | adipose | colon | heart | hypothalamus | kidney | liver | lung | ovary | skeletalmuscle | spleen | testes |
|---|---|---|---|---|---|---|---|---|---|---|---|---|---|---|---|
| NM_004060 | CCNG1 | 2468 | 9 | 5 | 3 | 2 | 7 | 3 | 8 | 5 | 4 | 9 | 6 | 4 | 5 |
| NM_004061 | CDH12 | 4344 | 2 | 1 | 0 | 0 | 0 | 2 | 0 | 1 | 0 | 0 | 0 | 0 | 0 |
| NM_004062 | CDH16 | 2833 | 10 | 1 | 0 | 0 | 0 | 0 | 10 | 0 | 0 | 0 | 0 | 0 | 0 |
| NM_004063 | CDH17 | 3698 | 22 | 2 | 0 | 22 | 0 | 0 | 0 | 0 | 0 | 0 | 0 | 0 | 0 |
| NM_004064 | CDKN1B | 2413 | 17 | 9 | 7 | 6 | 6 | 11 | 5 | 5 | 15 | 9 | 9 | 17 | 14 |
| NM_004065 | CDR1 | 1299 | 214 | 32 | 65 | 7 | 19 | 214 | 3 | 0 | 10 | 5 | 8 | 10 | 9 |
| NM_004066 | CETN1 | 1156 | 5 | 0 | 0 | 0 | 0 | 0 | 0 | 0 | 0 | 0 | 0 | 0 | 5 |
| NM_004067 | CHN2 | 3437 | 13 | 3 | 1 | 2 | 2 | 6 | 2 | 13 | 1 | 4 | 0 | 4 | 1 |
| NM_004068 | AP2M1 | 1941 | 97 | 34 | 33 | 24 | 39 | 97 | 30 | 31 | 21 | 20 | 11 | 34 | 30 |
| NM_004069 | AP2S1 | 890 | 2 | 1 | 1 | 1 | 1 | 2 | 1 | 1 | 1 | 0 | 1 | 1 | 0 |
| NM_004070 | CLCNKA | 2536 | 1 | 0 | 0 | 0 | 0 | 0 | 1 | 0 | 0 | 0 | 0 | 0 | 0 |
| NM_004071 | CLK1 | 1932 | 84 | 46 | 52 | 24 | 30 | 29 | 28 | 15 | 84 | 65 | 63 | 66 | 54 |
| NM_004072 | CMKLR1 | 5419 | 18 | 4 | 5 | 2 | 4 | 1 | 1 | 2 | 5 | 1 | 1 | 18 | 2 |
| NM_004073 | PLK3 | 2358 | 1 | 0 | 0 | 0 | 0 | 0 | 0 | 0 | 1 | 0 | 0 | 0 | 0 |
| NM_004074 | COX8A | 507 | 4 | 2 | 1 | 3 | 4 | 4 | 3 | 2 | 1 | 1 | 2 | 1 | 1 |
| NM_004075 | CRY1 | 3258 | 6 | 2 | 1 | 1 | 3 | 3 | 3 | 2 | 3 | 1 | 1 | 2 | 6 |
| NM_004076 | CRYBB3 | 853 | 0 | 0 | 0 | 0 | 0 | 0 | 0 | 0 | 0 | 0 | 0 | 0 | 0 |
| NM_004077 | CS | 2976 | 7 | 3 | 4 | 2 | 7 | 3 | 4 | 2 | 2 | 1 | 5 | 2 | 1 |
| NM_004078 | CSRP1 | 1954 | 42 | 16 | 6 | 42 | 11 | 28 | 11 | 3 | 22 | 15 | 4 | 21 | 13 |
| NM_004079 | CTSS | 4099 | 67 | 14 | 18 | 8 | 7 | 2 | 2 | 7 | 34 | 3 | 2 | 67 | 3 |
| NM_004080 | DGKB | 6684 | 8 | 1 | 0 | 0 | 0 | 8 | 0 | 0 | 0 | 0 | 0 | 0 | 0 |
| NM_004081 | DAZ1 | 4419 | 0 | 0 | 0 | 0 | 0 | 0 | 0 | 0 | 0 | 0 | 0 | 0 | 0 |
| NM_004082 | DCTN1 | 4492 | 37 | 14 | 11 | 9 | 18 | 37 | 10 | 6 | 8 | 11 | 10 | 12 | 22 |
| NM_004083 | DDIT3 | 911 | 3 | 2 | 2 | 1 | 2 | 3 | 3 | 1 | 2 | 2 | 2 | 2 | 2 |
| NM_004084 | DEFA1 | 486 | 0 | 0 | 0 | 0 | 0 | 0 | 0 | 0 | 0 | 0 | 0 | 0 | 0 |
| NM_004085 | TIMM8A | 1442 | 13 | 5 | 2 | 3 | 8 | 7 | 6 | 13 | 2 | 5 | 3 | 5 | 5 |
| NM_004086 | COCH | 2558 | 3 | 1 | 0 | 0 | 0 | 3 | 1 | 0 | 0 | 0 | 1 | 1 | 1 |
| NM_004087 | DLG1 | 5034 | 21 | 13 | 12 | 6 | 16 | 21 | 15 | 11 | 9 | 13 | 13 | 11 | 14 |
| NM_004088 | DNTT | 2040 | 1 | 0 | 0 | 0 | 0 | 0 | 0 | 0 | 1 | 0 | 0 | 0 | 0 |
| NM_004089 | TSC22D3 | 1952 | 18 | 7 | 6 | 4 | 4 | 4 | 4 | 4 | 16 | 3 | 8 | 18 | 4 |
| NM_004090 | DUSP3 | 4122 | 11 | 4 | 2 | 1 | 11 | 5 | 5 | 5 | 2 | 1 | 7 | 4 | 2 |
| NM_004091 | E2F2 | 5192 | 1 | 0 | 0 | 1 | 0 | 0 | 0 | 0 | 0 | 0 | 0 | 0 | 0 |
| NM_004092 | ECHS1 | 1329 | 83 | 19 | 22 | 10 | 11 | 12 | 43 | 83 | 4 | 4 | 5 | 4 | 7 |
| NM_004093 | EFNB2 | 4319 | 3 | 2 | 1 | 3 | 2 | 3 | 1 | 1 | 2 | 1 | 0 | 2 | 1 |
| NM_004094 | EIF2S1 | 4165 | 6 | 4 | 4 | 1 | 4 | 5 | 3 | 6 | 3 | 3 | 4 | 4 | 5 |
| NM_004095 | EIF4EBP1 | 859 | 5 | 2 | 2 | 1 | 2 | 1 | 2 | 5 | 1 | 2 | 5 | 3 | 0 |
| NM_004096 | EIF4EBP2 | 7529 | 6 | 4 | 6 | 3 | 4 | 3 | 6 | 3 | 3 | 4 | 3 | 5 | 3 |
| NM_004097 | EMX1 | 2188 | 1 | 0 | 0 | 0 | 0 | 0 | 1 | 0 | 0 | 0 | 0 | 0 | 0 |
| NM_004098 | EMX2 | 2896 | 14 | 3 | 2 | 0 | 0 | 6 | 14 | 0 | 0 | 4 | 1 | 0 | 4 |
| NM_004099 | STOM | 3074 | 150 | 60 | 58 | 13 | 76 | 41 | 41 | 150 | 82 | 28 | 32 | 107 | 29 |
| NM_004100 | EYA4 | 5693 | 6 | 1 | 0 | 0 | 3 | 0 | 1 | 0 | 0 | 1 | 6 | 1 | 1 |
| NM_004101 | F2RL2 | 3410 | 1 | 0 | 0 | 1 | 0 | 0 | 1 | 0 | 1 | 0 | 0 | 1 | 0 |
| NM_004102 | FABP3 | 1097 | 169 | 22 | 3 | 1 | 169 | 22 | 14 | 0 | 1 | 2 | 25 | 3 | 2 |
| NM_004103 | PTK2B | 4555 | 9 | 3 | 2 | 3 | 1 | 9 | 2 | 1 | 3 | 1 | 0 | 8 | 2 |
| NM_004104 | FASN | 8458 | 4 | 1 | 4 | 1 | 1 | 1 | 0 | 2 | 2 | 0 | 0 | 0 | 0 |
| NM_004105 | EFEMP1 | 2918 | 95 | 31 | 66 | 8 | 95 | 16 | 16 | 2 | 42 | 44 | 9 | 18 | 21 |
| NM_004106 | FCER1G | 591 | 91 | 21 | 22 | 1 | 27 | 11 | 4 | 9 | 53 | 3 | 3 | 91 | 3 |
| NM_004107 | FCGRT | 1629 | 5 | 3 | 5 | 4 | 2 | 1 | 3 | 3 | 3 | 1 | 1 | 4 | 1 |
| NM_004108 | FCN2 | 1057 | 18 | 2 | 0 | 0 | 0 | 0 | 0 | 18 | 0 | 0 | 0 | 0 | 0 |
| NM_004109 | FDX1 | 3219 | 2 | 1 | 1 | 0 | 1 | 1 | 2 | 2 | 1 | 1 | 1 | 1 | 1 |
| NM_004110 | FDXR | 1901 | 5 | 1 | 1 | 1 | 0 | 1 | 1 | 2 | 0 | 2 | 1 | 2 | 5 |
| NM_004111 | FEN1 | 2249 | 6 | 3 | 3 | 3 | 2 | 6 | 2 | 2 | 2 | 2 | 4 | 5 | 4 |
| NM_004112 | FGF11 | 2715 | 1 | 0 | 0 | 0 | 0 | 1 | 0 | 0 | 0 | 0 | 0 | 0 | 0 |
| NM_004113 | FGF12 | 2906 | 13 | 3 | 0 | 0 | 13 | 13 | 0 | 0 | 0 | 0 | 0 | 3 | 0 |
| NM_004114 | FGF13 | 2675 | 5 | 1 | 1 | 0 | 0 | 5 | 1 | 0 | 0 | 0 | 1 | 0 | 1 |
| NM_004115 | FGF14 | 2720 | 2 | 0 | 0 | 0 | 1 | 2 | 0 | 0 | 1 | 0 | 0 | 0 | 0 |
| NM_004116 | FKBP1B | 949 | 9 | 2 | 0 | 1 | 1 | 9 | 1 | 3 | 1 | 1 | 0 | 0 | 1 |
| NM_004117 | FKBP5 | 3782 | 108 | 45 | 22 | 10 | 44 | 8 | 42 | 86 | 44 | 29 | 65 | 108 | 34 |
| NM_004118 | FOXS1 | 1318 | 0 | 0 | 0 | 0 | 0 | 0 | 0 | 0 | 0 | 0 | 0 | 0 | 0 |
| NM_004119 | FLT3 | 3842 | 2 | 0 | 0 | 0 | 0 | 0 | 1 | 0 | 1 | 0 | 0 | 2 | 0 |
| NM_004120 | GBP2 | 2595 | 24 | 10 | 12 | 5 | 7 | 3 | 6 | 4 | 24 | 12 | 11 | 15 | 10 |
| NM_004121 | GGT5 | 2494 | 1 | 0 | 0 | 0 | 1 | 0 | 1 | 0 | 0 | 0 | 0 | 1 | 0 |
| NM_004122 | GHSR | 870 | 0 | 0 | 0 | 0 | 0 | 0 | 0 | 0 | 0 | 0 | 0 | 0 | 0 |
| NM_004123 | GIP | 711 | 1 | 0 | 0 | 1 | 0 | 0 | 0 | 0 | 0 | 0 | 0 | 0 | 0 |
| NM_004124 | GMFB | 4125 | 16 | 5 | 5 | 2 | 5 | 16 | 6 | 4 | 4 | 4 | 1 | 6 | 4 |
| NM_004125 | DNAJC25-GI | 1448 | 0 | 0 | 0 | 0 | 0 | 0 | 0 | 0 | 0 | 0 | 0 | 0 | 0 |
| NM_004126 | GNG11 | 954 | 7 | 2 | 7 | 1 | 2 | 2 | 2 | 1 | 3 | 4 | 1 | 3 | 1 |
| NM_004127 | GPS1 | 1864 | 2 | 1 | 1 | 1 | 1 | 2 | 2 | 1 | 1 | 1 | 2 | 1 | 2 |
| NM_004128 | GTF2F2 | 1453 | 8 | 5 | 4 | 2 | 7 | 8 | 4 | 4 | 3 | 5 | 4 | 6 | 8 |
| NM_004130 | GYG1 | 1805 | 21 | 6 | 3 | 2 | 11 | 4 | 2 | 2 | 3 | 3 | 21 | 7 | 10 |
| NM_004131 | GZMB | 923 | 5 | 1 | 0 | 0 | 0 | 0 | 0 | 0 | 1 | 0 | 0 | 5 | 0 |
| NM_004132 | HABP2 | 3018 | 35 | 4 | 0 | 0 | 0 | 0 | 8 | 35 | 0 | 0 | 0 | 0 | 0 |
| NM_004133 | HNF4G | 4093 | 3 | 1 | 0 | 1 | 0 | 0 | 3 | 3 | 0 | 0 | 0 | 0 | 0 |
| NM_004134 | HSPA9 | 2850 | 102 | 44 | 29 | 12 | 66 | 48 | 65 | 102 | 21 | 25 | 49 | 32 | 39 |
| NM_004135 | IDH3G | 1474 | 2 | 2 | 2 | 1 | 2 | 2 | 2 | 2 | 1 | 1 | 1 | 2 | 1 |
| NM_004136 | IREB2 | 6367 | 14 | 7 | 5 | 2 | 6 | 6 | 8 | 7 | 6 | 9 | 6 | 14 | 11 |
| NM_004137 | KCNMB1 | 1517 | 1 | 0 | 0 | 1 | 0 | 0 | 0 | 0 | 0 | 0 | 0 | 0 | 1 |

| transcript | gene | transcript length (nt) | max | mean | adipose | colon | heart | hypothalamus | kidney | liver | lung | ovary | skeletalmuscle | spleen | testes |
|---|---|---|---|---|---|---|---|---|---|---|---|---|---|---|---|
| NM_004138 | KRT33A | 1252 | 0 | 0 | 0 | 0 | 0 | 0 | 0 | 0 | 0 | 0 | 0 | 0 | 0 |
| NM_004139 | LBP | 1823 | 278 | 26 | 1 | 1 | 1 | 0 | 3 | 278 | 0 | 1 | 1 | 2 | 1 |
| NM_004140 | LLGL1 | 4224 | 1 | 0 | 0 | 0 | 0 | 1 | 0 | 0 | 0 | 0 | 0 | 0 | 0 |
| NM_004143 | CITED1 | 1241 | 11 | 1 | 0 | 0 | 0 | 2 | 0 | 0 | 0 | 0 | 0 | 0 | 11 |
| NM_004145 | MYO9B | 7611 | 8 | 3 | 3 | 4 | 2 | 2 | 1 | 2 | 3 | 1 | 0 | 8 | 7 |
| NM_004146 | NDUFB7 | 543 | 11 | 6 | 9 | 6 | 7 | 9 | 11 | 6 | 3 | 2 | 7 | 4 | 4 |
| NM_004147 | DRG1 | 1450 | 15 | 6 | 4 | 3 | 7 | 6 | 5 | 6 | 3 | 5 | 12 | 3 | 15 |
| NM_004148 | NINJ1 | 1281 | 2 | 1 | 1 | 0 | 2 | 1 | 1 | 2 | 1 | 1 | 1 | 1 | 1 |
| NM_004152 | OAZ1 | 1146 | 18 | 10 | 11 | 6 | 13 | 15 | 9 | 11 | 13 | 3 | 8 | 18 | 6 |
| NM_004153 | ORC1L | 3157 | 1 | 0 | 0 | 1 | 0 | 0 | 0 | 0 | 0 | 0 | 0 | 0 | 0 |
| NM_004154 | P2RY6 | 2400 | 2 | 0 | 0 | 0 | 0 | 0 | 0 | 0 | 0 | 0 | 0 | 2 | 0 |
| NM_004155 | SERPINB9 | 4143 | 10 | 3 | 1 | 1 | 2 | 2 | 1 | 2 | 5 | 1 | 0 | 10 | 5 |
| NM_004156 | PPP2CB | 1790 | 58 | 21 | 14 | 10 | 18 | 58 | 20 | 22 | 17 | 28 | 8 | 14 | 18 |
| NM_004157 | PRKAR2A | 2381 | 10 | 5 | 4 | 4 | 5 | 4 | 4 | 8 | 3 | 3 | 10 | 4 | 6 |
| NM_004158 | PSPN | 471 | 0 | 0 | 0 | 0 | 0 | 0 | 0 | 0 | 0 | 0 | 0 | 0 | 0 |
| NM_004159 | PSMB8 | 1583 | 4 | 1 | 1 | 1 | 1 | 1 | 1 | 2 | 2 | 1 | 0 | 4 | 0 |
| NM_004160 | PYY | 1052 | 2 | 0 | 0 | 2 | 0 | 0 | 0 | 0 | 0 | 0 | 0 | 0 | 0 |
| NM_004161 | RAB1A | 2642 | 55 | 35 | 39 | 18 | 41 | 50 | 41 | 55 | 30 | 25 | 14 | 35 | 31 |
| NM_004162 | RAB5A | 2526 | 18 | 9 | 7 | 3 | 12 | 18 | 10 | 9 | 11 | 8 | 5 | 11 | 9 |
| NM_004163 | RAB27B | 1335 | 9 | 1 | 0 | 1 | 0 | 9 | 0 | 0 | 2 | 0 | 0 | 1 | 1 |
| NM_004164 | RBP2 | 688 | 14 | 1 | 0 | 14 | 0 | 0 | 1 | 0 | 1 | 0 | 0 | 0 | 0 |
| NM_004165 | RRAD | 1467 | 2 | 0 | 0 | 0 | 1 | 0 | 0 | 0 | 0 | 0 | 2 | 0 | 0 |
| NM_004168 | SDHA | 2389 | 10 | 5 | 4 | 5 | 10 | 6 | 6 | 8 | 2 | 3 | 6 | 3 | 3 |
| NM_004169 | SHMT1 | 2519 | 23 | 5 | 3 | 1 | 1 | 1 | 19 | 23 | 1 | 1 | 1 | 3 | 2 |
| NM_004170 | SLC1A1 | 3722 | 9 | 2 | 0 | 1 | 0 | 2 | 6 | 9 | 3 | 0 | 0 | 0 | 1 |
| NM_004171 | SLC1A2 | 12006 | 149 | 15 | 0 | 1 | 1 | 149 | 0 | 10 | 0 | 0 | 0 | 0 | 0 |
| NM_004172 | SLC1A3 | 4170 | 41 | 6 | 6 | 1 | 5 | 41 | 1 | 0 | 3 | 1 | 1 | 3 | 1 |
| NM_004173 | SLC7A4 | 2313 | 0 | 0 | 0 | 0 | 0 | 0 | 0 | 0 | 0 | 0 | 0 | 0 | 0 |
| NM_004174 | SLC9A3 | 2777 | 2 | 0 | 0 | 1 | 0 | 0 | 2 | 0 | 0 | 0 | 0 | 0 | 0 |
| NM_004175 | SNRPD3 | 1385 | 22 | 9 | 7 | 5 | 7 | 10 | 9 | 22 | 5 | 6 | 7 | 10 | 6 |
| NM_004176 | SREBF1 | 4906 | 3 | 1 | 1 | 1 | 0 | 1 | 1 | 3 | 1 | 0 | 1 | 1 | 1 |
| NM_004177 | STX3 | 2941 | 12 | 4 | 2 | 6 | 2 | 2 | 12 | 1 | 6 | 3 | 1 | 5 | 8 |
| NM_004178 | TARBP2 | 1407 | 1 | 0 | 0 | 0 | 0 | 0 | 0 | 1 | 0 | 0 | 0 | 0 | 1 |
| NM_004179 | TPH1 | 1815 | 1 | 0 | 0 | 1 | 0 | 0 | 0 | 0 | 0 | 0 | 0 | 0 | 0 |
| NM_004180 | TANK | 2089 | 12 | 7 | 7 | 2 | 8 | 7 | 5 | 8 | 10 | 6 | 8 | 12 | 6 |
| NM_004181 | UCHL1 | 1123 | 79 | 9 | 9 | 2 | 1 | 79 | 4 | 0 | 1 | 3 | 1 | 0 | 2 |
| NM_004182 | UXT | 575 | 9 | 5 | 5 | 4 | 4 | 2 | 8 | 9 | 5 | 6 | 3 | 8 | 6 |
| NM_004183 | BEST1 | 2683 | 1 | 1 | 1 | 0 | 1 | 1 | 0 | 0 | 1 | 0 | 0 | 1 | 1 |
| NM_004184 | WARS | 2884 | 19 | 10 | 8 | 4 | 15 | 16 | 8 | 5 | 19 | 3 | 8 | 19 | 6 |
| NM_004185 | WNT2B | 2016 | 5 | 1 | 0 | 1 | 0 | 1 | 0 | 0 | 1 | 5 | 0 | 0 | 3 |
| NM_004186 | SEMA3F | 3516 | 3 | 0 | 0 | 0 | 0 | 0 | 0 | 0 | 0 | 0 | 0 | 3 | 0 |
| NM_004187 | KDM5C | 6861 | 3 | 2 | 1 | 3 | 1 | 2 | 2 | 1 | 2 | 2 | 2 | 2 | 2 |
| NM_004188 | GFI1B | 1790 | 0 | 0 | 0 | 0 | 0 | 0 | 0 | 0 | 0 | 0 | 0 | 0 | 0 |
| NM_004189 | SOX14 | 818 | 0 | 0 | 0 | 0 | 0 | 0 | 0 | 0 | 0 | 0 | 0 | 0 | 0 |
| NM_004190 | LIPF | 1497 | 0 | 0 | 0 | 0 | 0 | 0 | 0 | 0 | 0 | 0 | 0 | 0 | 0 |
| NM_004192 | ASMTL | 2107 | 2 | 1 | 1 | 1 | 1 | 2 | 1 | 1 | 0 | 1 | 1 | 1 | 1 |
| NM_004193 | GBF1 | 6376 | 9 | 6 | 4 | 5 | 6 | 9 | 6 | 5 | 4 | 5 | 7 | 6 | 7 |
| NM_004194 | ADAM22 | 2744 | 9 | 1 | 1 | 0 | 0 | 9 | 1 | 0 | 0 | 1 | 0 | 0 | 1 |
| NM_004195 | TNFRSF18 | 1200 | 0 | 0 | 0 | 0 | 0 | 0 | 0 | 0 | 0 | 0 | 0 | 0 | 0 |
| NM_004196 | CDKL1 | 1177 | 3 | 1 | 1 | 0 | 0 | 0 | 3 | 0 | 1 | 1 | 0 | 1 | 0 |
| NM_004197 | STK19 | 444 | 5 | 2 | 1 | 1 | 1 | 4 | 5 | 2 | 1 | 2 | 0 | 3 | 1 |
| NM_004197 | STK19 | 1620 | 1 | 1 | 0 | 0 | 0 | 1 | 1 | 1 | 0 | 1 | 0 | 1 | 0 |
| NM_004198 | CHRNA6 | 2073 | 0 | 0 | 0 | 0 | 0 | 0 | 0 | 0 | 0 | 0 | 0 | 0 | 0 |
| NM_004199 | P4HA2 | 2571 | 8 | 3 | 2 | 3 | 8 | 2 | 5 | 4 | 3 | 1 | 1 | 2 | 2 |
| NM_004200 | SYT7 | 4532 | 8 | 1 | 0 | 1 | 0 | 3 | 0 | 8 | 0 | 0 | 0 | 0 | 0 |
| NM_004202 | TMSB4Y | 1667 | 1 | 0 | 0 | 0 | 0 | 1 | 0 | 0 | 0 | 0 | 0 | 0 | 1 |
| NM_004203 | PKMYT1 | 2105 | 0 | 0 | 0 | 0 | 0 | 0 | 0 | 0 | 0 | 0 | 0 | 0 | 0 |
| NM_004204 | PIGQ | 2921 | 0 | 0 | 0 | 0 | 0 | 0 | 0 | 0 | 0 | 0 | 0 | 0 | 0 |
| NM_004205 | USP2 | 3733 | 2 | 1 | 0 | 0 | 2 | 0 | 2 | 0 | 0 | 0 | 2 | 0 | 1 |
| NM_004206 | SEC22C | 1506 | 3 | 1 | 1 | 1 | 1 | 3 | 1 | 1 | 1 | 1 | 0 | 1 | 1 |
| NM_004207 | SLC16A3 | 2035 | 4 | 2 | 1 | 1 | 2 | 1 | 1 | 0 | 3 | 0 | 1 | 4 | 3 |
| NM_004208 | AIFM1 | 2214 | 32 | 10 | 4 | 4 | 16 | 3 | 32 | 21 | 3 | 2 | 6 | 5 | 9 |
| NM_004209 | SYNGR3 | 2029 | 15 | 1 | 0 | 0 | 0 | 15 | 0 | 0 | 0 | 0 | 0 | 0 | 0 |
| NM_004210 | NEURL | 4314 | 1 | 0 | 0 | 0 | 0 | 1 | 0 | 0 | 0 | 0 | 1 | 0 | 0 |
| NM_004211 | SLC6A5 | 2863 | 0 | 0 | 0 | 0 | 0 | 0 | 0 | 0 | 0 | 0 | 0 | 0 | 0 |
| NM_004212 | SLC28A2 | 2463 | 1 | 0 | 0 | 1 | 0 | 0 | 0 | 0 | 0 | 0 | 0 | 0 | 0 |
| NM_004213 | SLC28A1 | 2748 | 6 | 1 | 0 | 0 | 0 | 0 | 6 | 2 | 0 | 0 | 0 | 0 | 0 |
| NM_004214 | FIBP | 1429 | 2 | 1 | 1 | 0 | 1 | 2 | 1 | 2 | 0 | 0 | 1 | 1 | 1 |
| NM_004215 | EBAG9 | 1641 | 5 | 3 | 2 | 1 | 4 | 5 | 4 | 2 | 2 | 3 | 4 | 4 | 4 |
| NM_004217 | AURKB | 1236 | 4 | 1 | 1 | 4 | 0 | 0 | 0 | 0 | 1 | 0 | 0 | 2 | 0 |
| NM_004218 | RAB11B | 1628 | 20 | 12 | 7 | 7 | 18 | 20 | 12 | 9 | 8 | 13 | 11 | 13 | 12 |
| NM_004219 | PTTG1 | 712 | 4 | 1 | 1 | 1 | 0 | 1 | 0 | 0 | 1 | 0 | 0 | 1 | 4 |
| NM_004220 | ZNF213 | 3283 | 0 | 0 | 0 | 0 | 0 | 0 | 0 | 0 | 0 | 0 | 0 | 0 | 0 |
| NM_004221 | IL32 | 1090 | 97 | 21 | 3 | 5 | 11 | 3 | 43 | 97 | 22 | 3 | 8 | 38 | 3 |
| NM_004223 | UBE2L6 | 1260 | 25 | 10 | 6 | 3 | 6 | 13 | 6 | 25 | 13 | 7 | 5 | 22 | 5 |
| NM_004224 | GPR50 | 2010 | 0 | 0 | 0 | 0 | 0 | 0 | 0 | 0 | 0 | 0 | 0 | 0 | 0 |
| NM_004225 | MFHAS1 | 5255 | 2 | 1 | 0 | 1 | 1 | 1 | 1 | 1 | 1 | 0 | 0 | 2 | 1 |

| transcript | gene | transcript length (nt) | max | mean | adipose | colon | heart | hypothalamus | kidney | liver | lung | ovary | skeletalmuscle | spleen | testes |
|---|---|---|---|---|---|---|---|---|---|---|---|---|---|---|---|
| NM_004226 | STK17B | 5306 | 23 | 4 | 3 | 1 | 2 | 1 | 1 | 1 | 7 | 1 | 1 | 23 | 2 |
| NM_004227 | CYTH3 | 4467 | 4 | 2 | 3 | 2 | 3 | 3 | 1 | 1 | 2 | 2 | 1 | 2 | 4 |
| NM_004228 | CYTH2 | 4603 | 4 | 2 | 2 | 3 | 1 | 4 | 2 | 1 | 2 | 2 | 1 | 2 | 3 |
| NM_004229 | MED14 | 6768 | 3 | 2 | 1 | 2 | 2 | 2 | 2 | 2 | 1 | 2 | 3 | 2 | 1 |
| NM_004230 | S1PR2 | 3584 | 1 | 0 | 0 | 0 | 1 | 0 | 0 | 0 | 1 | 0 | 0 | 0 | 0 |
| NM_004231 | ATP6V1F | 691 | 136 | 60 | 71 | 28 | 56 | 136 | 94 | 31 | 55 | 30 | 36 | 65 | 57 |
| NM_004232 | SOCS6 | 5846 | 3 | 2 | 1 | 1 | 2 | 1 | 2 | 2 | 1 | 3 | 3 | 1 | 2 |
| NM_004233 | CD83 | 2477 | 4 | 2 | 3 | 1 | 0 | 3 | 2 | 0 | 4 | 1 | 0 | 3 | 1 |
| NM_004234 | ZNF235 | 3190 | 4 | 2 | 1 | 1 | 1 | 2 | 1 | 0 | 2 | 3 | 1 | 4 | 3 |
| NM_004235 | KLF4 | 2933 | 10 | 5 | 9 | 7 | 5 | 2 | 1 | 2 | 7 | 10 | 3 | 8 | 6 |
| NM_004236 | COPS2 | 4087 | 20 | 13 | 12 | 2 | 17 | 15 | 9 | 12 | 10 | 16 | 20 | 12 | 18 |
| NM_004237 | TRIP13 | 2390 | 2 | 0 | 0 | 1 | 0 | 0 | 0 | 0 | 0 | 0 | 0 | 0 | 2 |
| NM_004238 | TRIP12 | 6428 | 46 | 26 | 21 | 10 | 28 | 29 | 22 | 23 | 17 | 26 | 25 | 34 | 46 |
| NM_004239 | TRIP11 | 7283 | 10 | 7 | 5 | 1 | 9 | 5 | 10 | 5 | 5 | 9 | 8 | 10 | 9 |
| NM_004240 | TRIP10 | 2011 | 11 | 4 | 4 | 5 | 5 | 1 | 3 | 1 | 3 | 3 | 11 | 1 | 3 |
| NM_004241 | JMJD1C | 8393 | 14 | 8 | 7 | 3 | 7 | 6 | 8 | 5 | 11 | 14 | 6 | 14 | 12 |
| NM_004242 | HMGN3 | 921 | 15 | 8 | 7 | 3 | 14 | 10 | 15 | 6 | 9 | 4 | 7 | 6 | 13 |
| NM_004244 | CD163 | 4192 | 284 | 89 | 62 | 8 | 184 | 8 | 27 | 85 | 265 | 10 | 19 | 284 | 30 |
| NM_004245 | TGM5 | 2521 | 0 | 0 | 0 | 0 | 0 | 0 | 0 | 0 | 0 | 0 | 0 | 0 | 0 |
| NM_004246 | GLP2R | 1662 | 0 | 0 | 0 | 0 | 0 | 0 | 0 | 0 | 0 | 0 | 0 | 0 | 0 |
| NM_004247 | EFTUD2 | 4167 | 13 | 9 | 8 | 9 | 9 | 13 | 8 | 10 | 7 | 7 | 10 | 12 | 11 |
| NM_004248 | PRLHR | 1980 | 0 | 0 | 0 | 0 | 0 | 0 | 0 | 0 | 0 | 0 | 0 | 0 | 0 |
| NM_004249 | RAB28 | 1811 | 3 | 2 | 1 | 1 | 3 | 3 | 2 | 1 | 2 | 2 | 1 | 2 | 3 |
| NM_004251 | RAB9A | 1081 | 6 | 4 | 5 | 2 | 6 | 4 | 5 | 3 | 5 | 4 | 2 | 6 | 3 |
| NM_004252 | SLC9A3R1 | 2003 | 13 | 3 | 0 | 2 | 1 | 3 | 13 | 7 | 1 | 1 | 0 | 2 | 1 |
| NM_004254 | SLC22A8 | 2178 | 19 | 2 | 0 | 0 | 0 | 1 | 19 | 0 | 0 | 0 | 0 | 0 | 0 |
| NM_004255 | COX5A | 769 | 69 | 21 | 10 | 19 | 69 | 15 | 20 | 20 | 9 | 4 | 42 | 12 | 12 |
| NM_004256 | SLC22A13 | 2560 | 1 | 0 | 0 | 0 | 0 | 0 | 1 | 0 | 0 | 0 | 0 | 0 | 0 |
| NM_004257 | TGFBRAP1 | 2965 | 3 | 2 | 2 | 1 | 2 | 2 | 2 | 1 | 1 | 2 | 1 | 3 | 1 |
| NM_004258 | CD101 | 3547 | 0 | 0 | 0 | 0 | 0 | 0 | 0 | 0 | 0 | 0 | 0 | 0 | 0 |
| NM_004259 | RECQL5 | 3684 | 1 | 1 | 0 | 1 | 0 | 1 | 1 | 0 | 0 | 1 | 0 | 1 | 1 |
| NM_004260 | RECQL4 | 3803 | 0 | 0 | 0 | 0 | 0 | 0 | 0 | 0 | 0 | 0 | 0 | 0 | 0 |
| NM_004261 | 15 Sep | 1840 | 33 | 19 | 18 | 5 | 18 | 22 | 23 | 27 | 17 | 18 | 8 | 33 | 24 |
| NM_004262 | TMPRSS11D | 2783 | 1 | 0 | 0 | 0 | 0 | 0 | 1 | 0 | 0 | 1 | 0 | 1 | 0 |
| NM_004263 | SEMA4F | 4273 | 1 | 0 | 0 | 0 | 0 | 1 | 0 | 0 | 0 | 0 | 0 | 0 | 0 |
| NM_004264 | MED21 | 1753 | 8 | 5 | 2 | 2 | 6 | 5 | 5 | 2 | 5 | 6 | 5 | 4 | 8 |
| NM_004265 | FADS2 | 3149 | 6 | 2 | 1 | 1 | 2 | 6 | 1 | 1 | 2 | 1 | 0 | 1 | 3 |
| NM_004267 | CHST2 | 3043 | 2 | 0 | 0 | 0 | 0 | 2 | 0 | 0 | 0 | 0 | 0 | 2 | 0 |
| NM_004268 | MED17 | 3497 | 2 | 1 | 1 | 1 | 1 | 1 | 1 | 1 | 1 | 2 | 1 | 2 | 2 |
| NM_004269 | MED27 | 1384 | 1 | 0 | 0 | 0 | 0 | 0 | 0 | 0 | 0 | 0 | 0 | 1 | 0 |
| NM_004270 | MED7 | 1100 | 21 | 11 | 12 | 6 | 11 | 15 | 13 | 7 | 9 | 12 | 8 | 13 | 21 |
| NM_004271 | LY86 | 879 | 3 | 1 | 1 | 0 | 0 | 1 | 0 | 0 | 1 | 0 | 0 | 3 | 0 |
| NM_004272 | HOMER1 | 4214 | 18 | 5 | 0 | 1 | 10 | 18 | 4 | 0 | 2 | 5 | 6 | 1 | 6 |
| NM_004273 | CHST3 | 6972 | 3 | 1 | 1 | 0 | 2 | 1 | 1 | 0 | 0 | 2 | 1 | 0 | 3 |
| NM_004274 | AKAP6 | 10387 | 22 | 6 | 0 | 1 | 22 | 22 | 1 | 0 | 1 | 1 | 20 | 0 | 2 |
| NM_004275 | MED20 | 2478 | 6 | 4 | 3 | 4 | 6 | 3 | 6 | 2 | 2 | 5 | 3 | 6 | 5 |
| NM_004276 | CABP1 | 998 | 11 | 1 | 0 | 0 | 0 | 11 | 2 | 0 | 0 | 0 | 0 | 0 | 0 |
| NM_004277 | SLC25A27 | 2959 | 5 | 1 | 1 | 1 | 1 | 5 | 2 | 1 | 1 | 2 | 1 | 0 | 2 |
| NM_004278 | PIGL | 1152 | 1 | 0 | 0 | 0 | 0 | 0 | 0 | 0 | 0 | 1 | 0 | 0 | 1 |
| NM_004279 | PMPCB | 3919 | 16 | 12 | 8 | 5 | 16 | 13 | 16 | 15 | 9 | 12 | 8 | 13 | 16 |
| NM_004280 | EEF1E1 | 1086 | 11 | 5 | 3 | 2 | 6 | 11 | 10 | 5 | 3 | 4 | 2 | 6 | 6 |
| NM_004281 | BAG3 | 2569 | 28 | 8 | 6 | 3 | 20 | 11 | 7 | 3 | 6 | 3 | 28 | 3 | 4 |
| NM_004282 | BAG2 | 2050 | 11 | 5 | 3 | 5 | 11 | 3 | 2 | 3 | 4 | 10 | 6 | 2 | 7 |
| NM_004283 | RAB3D | 1879 | 1 | 0 | 0 | 0 | 0 | 0 | 0 | 0 | 1 | 0 | 0 | 1 | 0 |
| NM_004284 | CHD1L | 3010 | 9 | 4 | 3 | 4 | 2 | 5 | 3 | 9 | 3 | 2 | 2 | 8 | 5 |
| NM_004285 | H6PD | 9115 | 9 | 3 | 2 | 2 | 2 | 1 | 2 | 9 | 2 | 5 | 3 | 3 | 3 |
| NM_004286 | GTPBP1 | 5047 | 4 | 2 | 1 | 3 | 2 | 3 | 2 | 1 | 4 | 2 | 2 | 3 | 2 |
| NM_004287 | GOSR2 | 3319 | 3 | 2 | 1 | 1 | 2 | 2 | 3 | 2 | 1 | 1 | 1 | 3 | 2 |
| NM_004288 | CYTIP | 2209 | 13 | 2 | 1 | 1 | 1 | 0 | 0 | 1 | 7 | 0 | 0 | 13 | 1 |
| NM_004289 | NFE2L3 | 3708 | 4 | 1 | 1 | 1 | 1 | 0 | 1 | 1 | 2 | 2 | 1 | 4 | 1 |
| NM_004290 | RNF14 | 3115 | 17 | 10 | 14 | 5 | 10 | 15 | 14 | 8 | 5 | 6 | 4 | 9 | 17 |
| NM_004291 | CARTPT | 915 | 8 | 1 | 0 | 1 | 0 | 8 | 0 | 0 | 0 | 0 | 0 | 0 | 1 |
| NM_004292 | RIN1 | 2684 | 0 | 0 | 0 | 0 | 0 | 0 | 0 | 0 | 0 | 0 | 0 | 0 | 0 |
| NM_004293 | GDA | 5430 | 9 | 2 | 0 | 1 | 0 | 9 | 8 | 2 | 0 | 0 | 0 | 0 | 0 |
| NM_004294 | MTRF1 | 2174 | 2 | 1 | 1 | 1 | 1 | 1 | 2 | 1 | 1 | 1 | 1 | 2 | 2 |
| NM_004295 | TRAF4 | 2902 | 2 | 0 | 0 | 0 | 0 | 0 | 2 | 1 | 0 | 1 | 0 | 1 | 0 |
| NM_004296 | RGS6 | 5736 | 2 | 1 | 1 | 0 | 1 | 2 | 0 | 0 | 0 | 0 | 0 | 0 | 0 |
| NM_004297 | GNA14 | 2490 | 2 | 1 | 0 | 0 | 1 | 1 | 1 | 0 | 1 | 1 | 0 | 2 | 0 |
| NM_004298 | NUP155 | 4200 | 8 | 2 | 2 | 1 | 2 | 2 | 2 | 1 | 1 | 3 | 2 | 3 | 8 |
| NM_004299 | ABCB7 | 2387 | 5 | 3 | 3 | 2 | 4 | 3 | 4 | 5 | 2 | 4 | 4 | 3 | 3 |
| NM_004300 | ACP1 | 1549 | 43 | 29 | 16 | 10 | 36 | 31 | 39 | 37 | 29 | 24 | 20 | 32 | 43 |
| NM_004301 | ACTL6A | 1858 | 34 | 16 | 12 | 14 | 16 | 10 | 13 | 14 | 13 | 21 | 11 | 23 | 34 |
| NM_004302 | ACVR1B | 4526 | 8 | 3 | 1 | 3 | 2 | 5 | 8 | 4 | 2 | 1 | 2 | 3 | 3 |
| NM_004304 | ALK | 6220 | 1 | 0 | 0 | 1 | 0 | 0 | 0 | 0 | 0 | 0 | 0 | 0 | 0 |
| NM_004305 | BIN1 | 2204 | 43 | 8 | 3 | 2 | 1 | 20 | 6 | 1 | 1 | 2 | 43 | 4 | 1 |
| NM_004306 | ANXA13 | 1470 | 2 | 0 | 0 | 2 | 0 | 0 | 0 | 1 | 0 | 0 | 0 | 0 | 1 |
| NM_004307 | APBB2 | 8974 | 5 | 2 | 3 | 1 | 2 | 4 | 2 | 1 | 2 | 5 | 1 | 2 | 2 |

| transcript | gene | transcript length (nt) | max | mean | adipose | colon | heart | hypothalamus | kidney | liver | lung | ovary | skeletalmuscle | spleen | testes |
|---|---|---|---|---|---|---|---|---|---|---|---|---|---|---|---|
| NM_004308 | ARHGAP1 | 3344 | 2 | 1 | 1 | 1 | 2 | 1 | 1 | 0 | 1 | 2 | 1 | 2 | 1 |
| NM_004309 | ARHGDIA | 1862 | 18 | 11 | 11 | 9 | 11 | 18 | 12 | 8 | 11 | 6 | 6 | 18 | 7 |
| NM_004310 | RHOH | 1999 | 4 | 0 | 0 | 0 | 0 | 0 | 0 | 0 | 0 | 0 | 0 | 4 | 0 |
| NM_004311 | ARL3 | 3870 | 6 | 2 | 1 | 1 | 3 | 6 | 3 | 1 | 2 | 3 | 1 | 1 | 2 |
| NM_004312 | ARR3 | 1309 | 0 | 0 | 0 | 0 | 0 | 0 | 0 | 0 | 0 | 0 | 0 | 0 | 0 |
| NM_004313 | ARRB2 | 1919 | 44 | 11 | 8 | 4 | 11 | 9 | 6 | 2 | 21 | 5 | 4 | 44 | 4 |
| NM_004314 | ART1 | 1312 | 1 | 0 | 0 | 0 | 0 | 0 | 0 | 0 | 0 | 0 | 1 | 0 | 0 |
| NM_004315 | ASAH1 | 2551 | 139 | 35 | 139 | 8 | 51 | 21 | 41 | 17 | 42 | 7 | 10 | 37 | 9 |
| NM_004316 | ASCL1 | 2484 | 1 | 0 | 0 | 0 | 0 | 1 | 0 | 0 | 0 | 0 | 0 | 0 | 0 |
| NM_004317 | ASNA1 | 1280 | 3 | 2 | 1 | 1 | 1 | 3 | 3 | 1 | 1 | 1 | 1 | 2 | 2 |
| NM_004318 | ASPH | 5348 | 73 | 14 | 73 | 4 | 12 | 9 | 9 | 9 | 10 | 9 | 4 | 10 | 6 |
| NM_004319 | ASTN1 | 7314 | 5 | 1 | 0 | 0 | 0 | 5 | 0 | 0 | 0 | 0 | 0 | 0 | 0 |
| NM_004320 | ATP2A1 | 3536 | 129 | 12 | 0 | 0 | 0 | 0 | 0 | 0 | 0 | 0 | 129 | 0 | 0 |
| NM_004321 | KIF1A | 8816 | 37 | 4 | 0 | 1 | 0 | 37 | 0 | 0 | 0 | 0 | 0 | 0 | 1 |
| NM_004322 | BAD | 1223 | 1 | 0 | 1 | 1 | 0 | 1 | 0 | 0 | 0 | 0 | 0 | 0 | 0 |
| NM_004323 | BAG1 | 3874 | 18 | 7 | 3 | 4 | 12 | 4 | 10 | 4 | 6 | 4 | 18 | 9 | 8 |
| NM_004324 | BAX | 891 | 1 | 0 | 0 | 0 | 0 | 0 | 0 | 0 | 0 | 0 | 0 | 1 | 0 |
| NM_004326 | BCL9 | 6276 | 3 | 1 | 1 | 2 | 1 | 2 | 2 | 0 | 1 | 3 | 1 | 1 | 2 |
| NM_004327 | BCR | 6927 | 2 | 1 | 1 | 1 | 1 | 2 | 1 | 1 | 1 | 0 | 0 | 1 | 2 |
| NM_004328 | BCS1L | 1663 | 1 | 1 | 1 | 1 | 0 | 1 | 1 | 1 | 0 | 1 | 1 | 0 | 0 |
| NM_004329 | BMPR1A | 3616 | 12 | 6 | 6 | 6 | 9 | 7 | 5 | 3 | 5 | 10 | 5 | 3 | 12 |
| NM_004330 | BNIP2 | 2517 | 17 | 9 | 9 | 3 | 12 | 5 | 5 | 5 | 13 | 9 | 6 | 17 | 10 |
| NM_004331 | BNIP3L | 3500 | 32 | 15 | 19 | 3 | 16 | 17 | 14 | 5 | 18 | 32 | 6 | 14 | 25 |
| NM_004332 | BPHL | 1546 | 7 | 1 | 1 | 0 | 1 | 1 | 7 | 1 | 0 | 1 | 1 | 1 | 1 |
| NM_004333 | BRAF | 2946 | 13 | 8 | 5 | 11 | 6 | 12 | 8 | 4 | 8 | 8 | 4 | 8 | 13 |
| NM_004334 | BST1 | 1480 | 1 | 0 | 1 | 0 | 1 | 0 | 1 | 0 | 1 | 1 | 0 | 1 | 0 |
| NM_004335 | BST2 | 939 | 207 | 70 | 86 | 18 | 30 | 29 | 24 | 140 | 50 | 207 | 23 | 117 | 46 |
| NM_004336 | BUB1 | 3500 | 4 | 1 | 0 | 1 | 0 | 0 | 0 | 0 | 1 | 0 | 0 | 1 | 4 |
| NM_004337 | OSGIN2 | 4199 | 5 | 3 | 3 | 1 | 2 | 5 | 2 | 3 | 3 | 5 | 2 | 3 | 2 |
| NM_004338 | C18orf1 | 8086 | 5 | 2 | 1 | 1 | 3 | 5 | 1 | 3 | 1 | 1 | 0 | 3 | 1 |
| NM_004339 | PTTG1IP | 2736 | 32 | 19 | 15 | 16 | 26 | 20 | 24 | 32 | 22 | 11 | 5 | 21 | 19 |
| NM_004341 | CAD | 7108 | 1 | 0 | 0 | 1 | 0 | 0 | 0 | 0 | 0 | 1 | 0 | 1 | 1 |
| NM_004342 | CALD1 | 4466 | 132 | 63 | 48 | 29 | 89 | 22 | 55 | 47 | 51 | 132 | 20 | 101 | 97 |
| NM_004343 | CALR | 1911 | 622 | 165 | 151 | 103 | 167 | 120 | 151 | 622 | 112 | 75 | 30 | 199 | 89 |
| NM_004344 | CETN2 | 1080 | 7 | 3 | 3 | 1 | 2 | 7 | 6 | 2 | 2 | 3 | 0 | 2 | 3 |
| NM_004345 | CAMP | 714 | 99 | 12 | 1 | 1 | 1 | 2 | 0 | 1 | 25 | 0 | 1 | 5 | 99 |
| NM_004346 | CASP3 | 2673 | 3 | 1 | 1 | 1 | 1 | 1 | 1 | 1 | 2 | 1 | 1 | 3 | 1 |
| NM_004347 | CASP5 | 1449 | 2 | 0 | 0 | 0 | 0 | 0 | 0 | 0 | 0 | 0 | 0 | 2 | 0 |
| NM_004348 | RUNX2 | 5701 | 1 | 0 | 0 | 0 | 0 | 0 | 0 | 0 | 1 | 1 | 0 | 1 | 0 |
| NM_004349 | RUNX1T1 | 3463 | 6 | 3 | 4 | 2 | 5 | 5 | 1 | 0 | 3 | 4 | 3 | 1 | 6 |
| NM_004350 | RUNX3 | 4270 | 4 | 1 | 0 | 0 | 0 | 0 | 0 | 0 | 2 | 0 | 0 | 4 | 0 |
| NM_004352 | CBLN1 | 2025 | 2 | 0 | 0 | 1 | 1 | 1 | 0 | 0 | 0 | 0 | 0 | 0 | 2 |
| NM_004354 | CCNG2 | 5474 | 2 | 1 | 1 | 1 | 1 | 1 | 1 | 0 | 1 | 2 | 1 | 2 | 1 |
| NM_004355 | CD74 | 1311 | 710 | 178 | 237 | 105 | 68 | 127 | 136 | 80 | 344 | 59 | 44 | 710 | 52 |
| NM_004356 | CD81 | 1497 | 16 | 10 | 13 | 4 | 12 | 10 | 7 | 12 | 13 | 6 | 2 | 13 | 16 |
| NM_004357 | CD151 | 1549 | 13 | 5 | 13 | 4 | 8 | 1 | 5 | 5 | 5 | 2 | 2 | 3 | 3 |
| NM_004358 | CDC25B | 3632 | 3 | 1 | 1 | 1 | 1 | 1 | 1 | 0 | 3 | 1 | 1 | 2 | 1 |
| NM_004359 | CDC34 | 1445 | 2 | 1 | 1 | 2 | 2 | 2 | 1 | 2 | 1 | 1 | 2 | 1 | 2 |
| NM_004360 | CDH1 | 4815 | 12 | 3 | 0 | 12 | 0 | 0 | 7 | 8 | 5 | 0 | 0 | 1 | 1 |
| NM_004361 | CDH7 | 2828 | 2 | 0 | 0 | 0 | 0 | 2 | 0 | 0 | 0 | 0 | 0 | 0 | 0 |
| NM_004362 | CLGN | 2738 | 9 | 2 | 0 | 0 | 9 | 1 | 0 | 1 | 0 | 0 | 0 | 0 | 8 |
| NM_004363 | CEACAM5 | 3600 | 17 | 2 | 0 | 17 | 0 | 0 | 0 | 0 | 0 | 0 | 0 | 0 | 0 |
| NM_004364 | CEBPA | 2589 | 5 | 1 | 5 | 1 | 0 | 0 | 0 | 3 | 1 | 0 | 0 | 0 | 0 |
| NM_004365 | CETN3 | 981 | 38 | 12 | 8 | 7 | 10 | 18 | 10 | 6 | 11 | 10 | 6 | 11 | 38 |
| NM_004366 | CLCN2 | 2875 | 1 | 0 | 0 | 0 | 0 | 0 | 0 | 0 | 0 | 0 | 0 | 0 | 1 |
| NM_004367 | CCR6 | 3216 | 2 | 0 | 0 | 0 | 0 | 0 | 0 | 0 | 0 | 0 | 0 | 2 | 0 |
| NM_004368 | CNN2 | 2458 | 7 | 2 | 2 | 1 | 2 | 0 | 1 | 1 | 5 | 4 | 0 | 7 | 1 |
| NM_004369 | COL6A3 | 10581 | 56 | 20 | 56 | 17 | 36 | 2 | 3 | 2 | 19 | 44 | 10 | 17 | 16 |
| NM_004370 | COL12A1 | 11579 | 56 | 12 | 25 | 6 | 9 | 1 | 7 | 0 | 8 | 56 | 5 | 4 | 7 |
| NM_004371 | COPA | 5639 | 17 | 11 | 11 | 8 | 12 | 12 | 13 | 17 | 9 | 9 | 6 | 15 | 9 |
| NM_004373 | COX6A1 | 545 | 271 | 101 | 77 | 109 | 79 | 271 | 151 | 143 | 62 | 50 | 14 | 96 | 57 |
| NM_004374 | COX6C | 425 | 225 | 94 | 88 | 62 | 225 | 211 | 75 | 116 | 39 | 44 | 69 | 49 | 55 |
| NM_004375 | COX11 | 2414 | 5 | 2 | 2 | 1 | 3 | 3 | 5 | 4 | 2 | 2 | 2 | 2 | 2 |
| NM_004376 | COX15 | 6450 | 4 | 2 | 3 | 1 | 2 | 2 | 4 | 2 | 2 | 3 | 2 | 3 | 3 |
| NM_004377 | CPT1B | 2879 | 0 | 0 | 0 | 0 | 0 | 0 | 0 | 0 | 0 | 0 | 0 | 0 | 0 |
| NM_004378 | CRABP1 | 772 | 1 | 0 | 0 | 1 | 0 | 1 | 0 | 0 | 0 | 0 | 0 | 1 | 0 |
| NM_004379 | CREB1 | 9735 | 5 | 2 | 2 | 1 | 2 | 2 | 2 | 1 | 3 | 4 | 1 | 5 | 2 |
| NM_004380 | CREBBP | 10196 | 9 | 4 | 3 | 4 | 3 | 3 | 3 | 2 | 3 | 9 | 6 | 7 | 5 |
| NM_004381 | ATF6B | 2614 | 4 | 2 | 2 | 2 | 2 | 2 | 2 | 2 | 3 | 2 | 1 | 4 | 2 |
| NM_004381 | ATF6B | 2616 | 4 | 2 | 2 | 2 | 2 | 2 | 2 | 2 | 3 | 2 | 1 | 4 | 2 |
| NM_004382 | CRHR1 | 2576 | 0 | 0 | 0 | 0 | 0 | 0 | 0 | 0 | 0 | 0 | 0 | 0 | 0 |
| NM_004383 | CSK | 2740 | 5 | 1 | 1 | 1 | 1 | 1 | 1 | 2 | 1 | 1 | 0 | 5 | 1 |
| NM_004384 | CSNK1G3 | 4437 | 5 | 3 | 2 | 2 | 2 | 4 | 2 | 3 | 3 | 4 | 1 | 5 | 5 |
| NM_004385 | VCAN | 12416 | 50 | 14 | 21 | 3 | 50 | 13 | 4 | 1 | 18 | 6 | 5 | 22 | 7 |
| NM_004386 | NCAN | 6406 | 12 | 1 | 0 | 0 | 0 | 12 | 0 | 0 | 0 | 0 | 0 | 0 | 0 |
| NM_004387 | NKX2-5 | 1669 | 11 | 1 | 0 | 0 | 11 | 0 | 0 | 0 | 0 | 0 | 0 | 2 | 0 |
| NM_004388 | CTBS | 3099 | 3 | 2 | 1 | 1 | 1 | 1 | 3 | 3 | 2 | 1 | 0 | 3 | 1 |

| transcript | gene | transcript length (nt) | max | mean | adipose | colon | heart | hypothalamus | kidney | liver | lung | ovary | skeletalmuscle | spleen | testes |
|---|---|---|---|---|---|---|---|---|---|---|---|---|---|---|---|
| NM_004389 | CTNNA2 | 3989 | 26 | 3 | 0 | 0 | 0 | 26 | 0 | 0 | 0 | 0 | 0 | 0 | 5 |
| NM_004390 | CTSH | 1485 | 45 | 10 | 6 | 3 | 3 | 5 | 45 | 4 | 28 | 2 | 1 | 9 | 1 |
| NM_004391 | CYP8B1 | 3950 | 58 | 6 | 0 | 1 | 1 | 0 | 4 | 58 | 0 | 1 | 1 | 1 | 1 |
| NM_004392 | DACH1 | 4633 | 2 | 1 | 0 | 1 | 0 | 1 | 2 | 0 | 1 | 0 | 1 | 2 | 2 |
| NM_004393 | DAG1 | 5520 | 8 | 5 | 3 | 3 | 8 | 4 | 4 | 7 | 2 | 4 | 7 | 2 | 8 |
| NM_004394 | DAP | 2342 | 20 | 6 | 4 | 4 | 5 | 4 | 10 | 20 | 6 | 6 | 1 | 7 | 4 |
| NM_004395 | DBN1 | 2942 | 16 | 7 | 9 | 5 | 8 | 16 | 8 | 0 | 3 | 8 | 3 | 4 | 9 |
| NM_004396 | DDX5 | 3759 | 98 | 60 | 73 | 34 | 59 | 66 | 62 | 57 | 70 | 45 | 25 | 98 | 68 |
| NM_004397 | DDX6 | 6226 | 22 | 13 | 12 | 5 | 13 | 20 | 10 | 6 | 11 | 16 | 10 | 22 | 16 |
| NM_004398 | DDX10 | 3191 | 20 | 9 | 5 | 2 | 8 | 11 | 10 | 7 | 5 | 14 | 7 | 12 | 20 |
| NM_004399 | DDX11 | 3777 | 0 | 0 | 0 | 0 | 0 | 0 | 0 | 0 | 0 | 0 | 0 | 0 | 0 |
| NM_004400 | DDX12 | 3894 | 0 | 0 | 0 | 0 | 0 | 0 | 0 | 0 | 0 | 0 | 0 | 0 | 0 |
| NM_004401 | DFFA | 2038 | 3 | 2 | 2 | 1 | 2 | 2 | 2 | 1 | 1 | 2 | 2 | 3 | 2 |
| NM_004402 | DFFB | 3028 | 1 | 0 | 0 | 1 | 0 | 0 | 1 | 0 | 0 | 1 | 0 | 1 | 0 |
| NM_004403 | DFNA5 | 2495 | 4 | 2 | 1 | 1 | 1 | 3 | 2 | 1 | 1 | 3 | 1 | 0 | 4 |
| NM_004404 | 02 Sep | 3309 | 59 | 39 | 59 | 18 | 39 | 59 | 42 | 20 | 34 | 46 | 19 | 52 | 42 |
| NM_004405 | DLX2 | 2304 | 0 | 0 | 0 | 0 | 0 | 0 | 0 | 0 | 0 | 0 | 0 | 0 | 0 |
| NM_004406 | DMBT1 | 5802 | 4 | 0 | 0 | 1 | 0 | 0 | 0 | 0 | 4 | 0 | 0 | 0 | 0 |
| NM_004407 | DMP1 | 2684 | 0 | 0 | 0 | 0 | 0 | 0 | 0 | 0 | 0 | 0 | 0 | 0 | 0 |
| NM_004408 | DNM1 | 3215 | 39 | 4 | 2 | 1 | 1 | 39 | 1 | 0 | 0 | 0 | 1 | 0 | 1 |
| NM_004409 | DMPK | 2865 | 7 | 2 | 1 | 3 | 7 | 1 | 1 | 0 | 1 | 1 | 3 | 1 | 1 |
| NM_004411 | DYNC1I1 | 3045 | 11 | 1 | 0 | 1 | 1 | 11 | 0 | 0 | 0 | 0 | 0 | 0 | 0 |
| NM_004412 | TRDMT1 | 7281 | 2 | 0 | 0 | 0 | 0 | 0 | 1 | 0 | 0 | 2 | 0 | 1 | 0 |
| NM_004413 | DPEP1 | 1736 | 9 | 1 | 0 | 0 | 0 | 0 | 9 | 0 | 0 | 0 | 0 | 0 | 1 |
| NM_004414 | RCAN1 | 2429 | 42 | 19 | 11 | 8 | 42 | 26 | 35 | 39 | 16 | 5 | 8 | 10 | 7 |
| NM_004415 | DSP | 9730 | 149 | 29 | 0 | 19 | 149 | 10 | 38 | 70 | 12 | 8 | 1 | 10 | 6 |
| NM_004416 | DTX1 | 3288 | 2 | 1 | 1 | 0 | 0 | 1 | 0 | 2 | 0 | 0 | 0 | 2 | 0 |
| NM_004417 | DUSP1 | 2024 | 24 | 9 | 7 | 2 | 5 | 3 | 4 | 17 | 24 | 14 | 5 | 9 | 5 |
| NM_004418 | DUSP2 | 1685 | 1 | 0 | 0 | 0 | 0 | 0 | 0 | 0 | 0 | 0 | 0 | 1 | 0 |
| NM_004419 | DUSP5 | 2528 | 4 | 2 | 2 | 1 | 1 | 2 | 1 | 1 | 2 | 1 | 0 | 4 | 2 |
| NM_004420 | DUSP8 | 4473 | 1 | 0 | 0 | 0 | 0 | 1 | 0 | 0 | 0 | 0 | 0 | 0 | 0 |
| NM_004421 | DVL1 | 2924 | 2 | 1 | 1 | 1 | 1 | 2 | 0 | 1 | 1 | 1 | 2 | 1 | 1 |
| NM_004422 | DVL2 | 3016 | 4 | 2 | 1 | 1 | 2 | 1 | 2 | 1 | 1 | 4 | 2 | 2 | 3 |
| NM_004423 | DVL3 | 5062 | 6 | 4 | 3 | 4 | 6 | 4 | 4 | 2 | 3 | 6 | 4 | 5 | 4 |
| NM_004424 | E4F1 | 2573 | 1 | 0 | 0 | 1 | 0 | 1 | 0 | 1 | 0 | 0 | 0 | 1 | 0 |
| NM_004425 | ECM1 | 2143 | 4 | 2 | 4 | 1 | 3 | 1 | 1 | 3 | 1 | 1 | 1 | 1 | 2 |
| NM_004426 | PHC1 | 5176 | 4 | 1 | 1 | 1 | 2 | 1 | 1 | 0 | 1 | 2 | 1 | 2 | 4 |
| NM_004427 | PHC2 | 2566 | 8 | 4 | 2 | 2 | 4 | 4 | 4 | 6 | 4 | 2 | 4 | 8 | 2 |
| NM_004428 | EFNA1 | 1566 | 12 | 3 | 2 | 3 | 2 | 2 | 3 | 12 | 6 | 1 | 1 | 1 | 0 |
| NM_004429 | EFNB1 | 3330 | 2 | 1 | 2 | 2 | 2 | 1 | 2 | 1 | 2 | 1 | 0 | 2 | 1 |
| NM_004430 | EGR3 | 4334 | 7 | 1 | 0 | 0 | 1 | 2 | 0 | 0 | 1 | 7 | 0 | 1 | 0 |
| NM_004431 | EPHA2 | 3946 | 1 | 0 | 0 | 0 | 1 | 0 | 1 | 1 | 1 | 0 | 0 | 1 | 0 |
| NM_004432 | ELAVL2 | 3807 | 19 | 3 | 0 | 0 | 0 | 11 | 0 | 0 | 0 | 0 | 0 | 0 | 19 |
| NM_004433 | ELF3 | 3133 | 13 | 3 | 0 | 13 | 2 | 0 | 8 | 3 | 6 | 0 | 0 | 0 | 0 |
| NM_004434 | EML1 | 4463 | 2 | 2 | 2 | 2 | 2 | 2 | 2 | 1 | 2 | 0 | 1 | 1 | 2 |
| NM_004435 | ENDOG | 1145 | 0 | 0 | 0 | 0 | 0 | 0 | 0 | 0 | 0 | 0 | 0 | 0 | 0 |
| NM_004436 | ENSA | 1239 | 19 | 10 | 8 | 9 | 10 | 19 | 11 | 10 | 11 | 8 | 8 | 12 | 5 |
| NM_004437 | EPB41 | 5818 | 11 | 6 | 7 | 6 | 3 | 3 | 5 | 6 | 8 | 5 | 4 | 11 | 5 |
| NM_004438 | EPHA4 | 6348 | 13 | 3 | 1 | 2 | 3 | 13 | 1 | 0 | 1 | 3 | 1 | 1 | 7 |
| NM_004439 | EPHA5 | 7857 | 10 | 1 | 0 | 0 | 0 | 10 | 0 | 0 | 0 | 1 | 0 | 0 | 2 |
| NM_004440 | EPHA7 | 6644 | 5 | 2 | 0 | 2 | 1 | 5 | 5 | 0 | 0 | 1 | 0 | 5 | 2 |
| NM_004441 | EPHB1 | 4514 | 2 | 1 | 0 | 1 | 0 | 1 | 0 | 2 | 0 | 0 | 1 | 0 | 2 |
| NM_004442 | EPHB2 | 4869 | 3 | 1 | 1 | 3 | 0 | 1 | 0 | 0 | 0 | 1 | 0 | 0 | 1 |
| NM_004443 | EPHB3 | 4220 | 1 | 0 | 0 | 1 | 0 | 0 | 0 | 0 | 0 | 0 | 0 | 0 | 0 |
| NM_004444 | EPHB4 | 4351 | 9 | 3 | 2 | 3 | 3 | 0 | 3 | 5 | 2 | 3 | 0 | 9 | 2 |
| NM_004445 | EPHB6 | 4043 | 3 | 1 | 0 | 0 | 1 | 3 | 0 | 0 | 1 | 1 | 0 | 0 | 1 |
| NM_004446 | EPRS | 5015 | 14 | 9 | 9 | 3 | 11 | 13 | 8 | 14 | 6 | 9 | 13 | 11 | 8 |
| NM_004447 | EPS8 | 4081 | 25 | 16 | 24 | 7 | 19 | 8 | 25 | 10 | 13 | 24 | 8 | 16 | 17 |
| NM_004448 | ERBB2 | 4624 | 7 | 2 | 1 | 7 | 3 | 1 | 6 | 2 | 3 | 2 | 2 | 1 | 1 |
| NM_004449 | ERG | 5034 | 15 | 4 | 5 | 0 | 5 | 1 | 3 | 2 | 6 | 3 | 2 | 15 | 2 |
| NM_004450 | ERH | 801 | 26 | 14 | 14 | 8 | 16 | 26 | 12 | 21 | 8 | 13 | 8 | 18 | 10 |
| NM_004451 | ESRRA | 2221 | 4 | 1 | 1 | 1 | 4 | 1 | 2 | 2 | 1 | 0 | 2 | 1 | 0 |
| NM_004452 | ESRRB | 3012 | 0 | 0 | 0 | 0 | 0 | 0 | 0 | 0 | 0 | 0 | 0 | 0 | 0 |
| NM_004453 | ETFDH | 2348 | 18 | 7 | 8 | 4 | 14 | 3 | 9 | 18 | 2 | 2 | 6 | 4 | 3 |
| NM_004454 | ETV5 | 4102 | 5 | 2 | 2 | 1 | 2 | 5 | 1 | 1 | 2 | 1 | 1 | 5 | 2 |
| NM_004455 | EXTL1 | 4014 | 1 | 0 | 0 | 0 | 0 | 1 | 0 | 0 | 0 | 0 | 0 | 0 | 0 |
| NM_004456 | EZH2 | 2685 | 4 | 1 | 1 | 4 | 0 | 0 | 0 | 0 | 1 | 1 | 1 | 1 | 4 |
| NM_004457 | ACSL3 | 4361 | 9 | 5 | 3 | 1 | 5 | 9 | 6 | 7 | 5 | 3 | 5 | 5 | 7 |
| NM_004458 | ACSL4 | 5023 | 28 | 14 | 16 | 2 | 13 | 17 | 17 | 2 | 20 | 8 | 15 | 16 | 28 |
| NM_004459 | BPTF | 10863 | 22 | 12 | 8 | 6 | 11 | 11 | 12 | 6 | 11 | 19 | 10 | 20 | 22 |
| NM_004460 | FAP | 2780 | 1 | 0 | 1 | 0 | 0 | 0 | 0 | 0 | 0 | 0 | 0 | 0 | 0 |
| NM_004461 | FARSA | 1853 | 3 | 1 | 1 | 1 | 1 | 3 | 1 | 1 | 1 | 1 | 1 | 2 | 1 |
| NM_004462 | FDFT1 | 2106 | 21 | 10 | 9 | 7 | 6 | 21 | 7 | 10 | 10 | 7 | 10 | 7 | 16 |
| NM_004463 | FGD1 | 4275 | 1 | 0 | 0 | 1 | 0 | 1 | 0 | 0 | 0 | 1 | 0 | 1 | 0 |
| NM_004464 | FGF5 | 5389 | 0 | 0 | 0 | 0 | 0 | 0 | 0 | 0 | 0 | 0 | 0 | 0 | 0 |
| NM_004465 | FGF10 | 627 | 3 | 1 | 2 | 0 | 1 | 0 | 1 | 0 | 1 | 3 | 0 | 0 | 3 |
| NM_004466 | GPC5 | 2878 | 6 | 1 | 0 | 0 | 0 | 6 | 1 | 0 | 1 | 0 | 0 | 0 | 3 |

| | | transcript | | | | | | hypothala | | | | | skeletalm | | |
|---|---|---|---|---|---|---|---|---|---|---|---|---|---|---|---|
| transcript | gene | length (nt) | max | mean | adipose | colon | heart | mus | kidney | liver | lung | ovary | uscle | spleen | testes |
| NM_004467 | FGL1 | 1249 | 1,393 | 128 | 0 | 2 | 1 | 0 | 1 | 1,393 | 1 | 3 | 2 | 3 | 3 |
| NM_004468 | FHL3 | 1704 | 22 | 2 | 0 | 0 | 1 | 1 | 0 | 0 | 0 | 1 | 22 | 1 | 0 |
| NM_004469 | FIGF | 2111 | 6 | 1 | 1 | 0 | 2 | 0 | 0 | 0 | 6 | 0 | 4 | 0 | 1 |
| NM_004470 | FKBP2 | 730 | 51 | 15 | 8 | 8 | 15 | 22 | 16 | 51 | 7 | 8 | 2 | 14 | 9 |
| NM_004472 | FOXD1 | 2268 | 0 | 0 | 0 | 0 | 0 | 0 | 0 | 0 | 0 | 0 | 0 | 0 | 0 |
| NM_004473 | FOXE1 | 3461 | 0 | 0 | 0 | 0 | 0 | 0 | 0 | 0 | 0 | 0 | 0 | 0 | 0 |
| NM_004474 | FOXD2 | 4675 | 0 | 0 | 0 | 0 | 0 | 0 | 0 | 0 | 0 | 0 | 0 | 0 | 0 |
| NM_004475 | FLOT2 | 2632 | 31 | 12 | 5 | 7 | 16 | 10 | 20 | 14 | 11 | 4 | 8 | 31 | 6 |
| NM_004476 | FOLH1 | 2635 | 3 | 1 | 0 | 0 | 0 | 3 | 1 | 1 | 0 | 0 | 0 | 0 | 0 |
| NM_004477 | FRG1 | 1043 | 4 | 3 | 3 | 1 | 3 | 4 | 3 | 3 | 2 | 3 | 2 | 4 | 2 |
| NM_004479 | FUT7 | 2414 | 0 | 0 | 0 | 0 | 0 | 0 | 0 | 0 | 0 | 0 | 0 | 0 | 0 |
| NM_004480 | FUT8 | 2957 | 10 | 4 | 2 | 1 | 2 | 10 | 3 | 0 | 2 | 4 | 1 | 6 | 7 |
| NM_004481 | GALNT2 | 4459 | 27 | 6 | 5 | 3 | 4 | 3 | 4 | 27 | 3 | 4 | 4 | 3 | 5 |
| NM_004482 | GALNT3 | 3267 | 6 | 2 | 0 | 2 | 0 | 0 | 2 | 0 | 3 | 1 | 0 | 6 | 3 |
| NM_004483 | GCSH | 1161 | 43 | 12 | 11 | 3 | 13 | 10 | 27 | 43 | 4 | 7 | 7 | 4 | 6 |
| NM_004484 | GPC3 | 2314 | 7 | 2 | 7 | 1 | 1 | 1 | 5 | 0 | 7 | 2 | 1 | 0 | 1 |
| NM_004485 | GNG4 | 4868 | 6 | 1 | 0 | 0 | 0 | 5 | 0 | 0 | 0 | 6 | 0 | 0 | 0 |
| NM_004486 | GOLGA2 | 4260 | 10 | 7 | 9 | 6 | 10 | 9 | 6 | 5 | 5 | 6 | 5 | 5 | 7 |
| NM_004487 | GOLGB1 | 11185 | 31 | 21 | 22 | 8 | 30 | 22 | 31 | 14 | 17 | 28 | 10 | 24 | 30 |
| NM_004488 | GP5 | 3493 | 0 | 0 | 0 | 0 | 0 | 0 | 0 | 0 | 0 | 0 | 0 | 0 | 0 |
| NM_004489 | GPS2 | 1157 | 16 | 12 | 11 | 12 | 8 | 11 | 15 | 14 | 9 | 6 | 15 | 13 | 16 |
| NM_004490 | GRB14 | 2387 | 2 | 1 | 0 | 0 | 1 | 0 | 2 | 2 | 0 | 1 | 0 | 0 | 2 |
| NM_004491 | GRLF1 | 8888 | 11 | 5 | 4 | 3 | 5 | 11 | 4 | 8 | 2 | 4 | 2 | 3 | 4 |
| NM_004492 | GTF2A2 | 1580 | 10 | 4 | 3 | 2 | 7 | 4 | 4 | 4 | 3 | 3 | 3 | 5 | 10 |
| NM_004493 | HSD17B10 | 963 | 6 | 2 | 1 | 1 | 2 | 1 | 4 | 6 | 1 | 1 | 1 | 2 | 1 |
| NM_004494 | HDGF | 2387 | 23 | 11 | 8 | 12 | 9 | 9 | 13 | 23 | 10 | 5 | 9 | 10 | 7 |
| NM_004495 | NRG1 | 1687 | 3 | 1 | 0 | 1 | 0 | 3 | 2 | 2 | 1 | 1 | 0 | 0 | 1 |
| NM_004496 | FOXA1 | 3110 | 3 | 1 | 0 | 2 | 0 | 0 | 0 | 3 | 1 | 0 | 0 | 0 | 0 |
| NM_004497 | FOXA3 | 1989 | 1 | 0 | 0 | 0 | 0 | 0 | 0 | 1 | 0 | 0 | 0 | 0 | 0 |
| NM_004498 | ONECUT1 | 1925 | 5 | 1 | 0 | 1 | 0 | 0 | 0 | 5 | 0 | 0 | 0 | 0 | 0 |
| NM_004499 | HNRNPAB | 1675 | 23 | 13 | 10 | 13 | 15 | 11 | 14 | 23 | 9 | 6 | 11 | 18 | 8 |
| NM_004500 | HNRNPC | 3212 | 38 | 22 | 14 | 13 | 18 | 32 | 22 | 20 | 22 | 22 | 17 | 38 | 24 |
| NM_004501 | HNRNPU | 6789 | 68 | 41 | 23 | 16 | 44 | 32 | 44 | 24 | 45 | 66 | 45 | 68 | 49 |
| NM_004502 | HOXB7 | 1366 | 4 | 1 | 4 | 4 | 0 | 0 | 4 | 0 | 0 | 0 | 0 | 1 | 1 |
| NM_004503 | HOXC6 | 1681 | 1 | 0 | 1 | 0 | 0 | 0 | 1 | 0 | 0 | 1 | 1 | 0 | 1 |
| NM_004504 | AGFG1 | 8666 | 4 | 2 | 3 | 1 | 3 | 3 | 2 | 2 | 3 | 2 | 1 | 4 | 3 |
| NM_004505 | USP6 | 7971 | 1 | 0 | 0 | 0 | 0 | 0 | 0 | 0 | 0 | 0 | 1 | 0 | 1 |
| NM_004506 | HSF2 | 2697 | 17 | 7 | 2 | 1 | 11 | 8 | 3 | 1 | 4 | 6 | 14 | 5 | 17 |
| NM_004507 | HUS1 | 2143 | 3 | 2 | 1 | 1 | 2 | 2 | 2 | 3 | 2 | 2 | 1 | 2 | 1 |
| NM_004508 | IDI1 | 2136 | 12 | 4 | 2 | 2 | 3 | 12 | 3 | 8 | 4 | 2 | 4 | 5 | 3 |
| NM_004509 | SP110 | 2504 | 32 | 8 | 6 | 3 | 6 | 5 | 4 | 3 | 8 | 10 | 5 | 32 | 3 |
| NM_004510 | SP110 | 2082 | 38 | 9 | 7 | 4 | 7 | 6 | 5 | 4 | 10 | 12 | 6 | 38 | 4 |
| NM_004512 | IL11RA | 1723 | 2 | 1 | 2 | 0 | 2 | 1 | 0 | 0 | 0 | 0 | 1 | 0 | 1 |
| NM_004513 | IL16 | 6162 | 6 | 1 | 1 | 1 | 0 | 0 | 0 | 0 | 2 | 0 | 0 | 6 | 1 |
| NM_004514 | FOXK2 | 5262 | 2 | 2 | 1 | 2 | 2 | 2 | 1 | 1 | 1 | 1 | 2 | 2 | 2 |
| NM_004515 | ILF2 | 1612 | 9 | 5 | 4 | 2 | 4 | 5 | 9 | 5 | 4 | 4 | 3 | 7 | 7 |
| NM_004516 | ILF3 | 3728 | 12 | 8 | 6 | 7 | 9 | 8 | 8 | 5 | 7 | 10 | 8 | 12 | 11 |
| NM_004517 | ILK | 1797 | 16 | 6 | 7 | 16 | 7 | 4 | 5 | 3 | 4 | 4 | 4 | 6 | 4 |
| NM_004518 | KCNQ2 | 3167 | 4 | 0 | 0 | 0 | 0 | 4 | 0 | 0 | 0 | 0 | 0 | 0 | 0 |
| NM_004519 | KCNQ3 | 3097 | 2 | 0 | 0 | 0 | 0 | 2 | 0 | 0 | 0 | 0 | 0 | 0 | 0 |
| NM_004520 | KIF2A | 3246 | 20 | 7 | 4 | 2 | 4 | 20 | 4 | 2 | 6 | 7 | 3 | 11 | 16 |
| NM_004521 | KIF5B | 5889 | 54 | 30 | 36 | 8 | 54 | 34 | 25 | 15 | 31 | 20 | 31 | 38 | 38 |
| NM_004522 | KIF5C | 6859 | 46 | 6 | 0 | 1 | 0 | 46 | 1 | 0 | 0 | 1 | 0 | 1 | 11 |
| NM_004523 | KIF11 | 5076 | 4 | 1 | 1 | 1 | 0 | 0 | 0 | 0 | 1 | 0 | 0 | 1 | 4 |
| NM_004524 | LLGL2 | 3505 | 1 | 0 | 0 | 1 | 0 | 0 | 0 | 0 | 0 | 0 | 0 | 0 | 0 |
| NM_004525 | LRP2 | 15735 | 75 | 7 | 0 | 0 | 0 | 1 | 75 | 0 | 1 | 0 | 0 | 0 | 1 |
| NM_004526 | MCM2 | 3434 | 1 | 1 | 1 | 1 | 0 | 0 | 0 | 0 | 0 | 0 | 0 | 1 | 1 |
| NM_004527 | MEOX1 | 2314 | 2 | 1 | 2 | 1 | 2 | 0 | 0 | 0 | 0 | 0 | 1 | 1 | 1 |
| NM_004528 | MGST3 | 661 | 11 | 6 | 8 | 4 | 11 | 9 | 10 | 3 | 3 | 3 | 4 | 2 | 3 |
| NM_004529 | MLLT3 | 3439 | 7 | 3 | 2 | 2 | 2 | 7 | 3 | 0 | 3 | 4 | 1 | 2 | 2 |
| NM_004530 | MMP2 | 3526 | 35 | 14 | 23 | 10 | 20 | 1 | 3 | 1 | 18 | 35 | 10 | 10 | 25 |
| NM_004531 | MOCS2 | 1518 | 47 | 27 | 16 | 8 | 45 | 43 | 47 | 45 | 17 | 18 | 15 | 17 | 25 |
| NM_004532 | MUC4 | 4400 | 4 | 0 | 0 | 4 | 0 | 0 | 0 | 0 | 1 | 0 | 0 | 0 | 0 |
| NM_004533 | MYBPC2 | 3598 | 43 | 4 | 0 | 0 | 0 | 0 | 0 | 0 | 0 | 0 | 43 | 0 | 0 |
| NM_004535 | MYT1 | 5535 | 1 | 0 | 0 | 0 | 0 | 1 | 0 | 0 | 0 | 0 | 0 | 0 | 0 |
| NM_004536 | NAIP | 4473 | 26 | 6 | 3 | 2 | 6 | 5 | 3 | 2 | 6 | 5 | 1 | 26 | 4 |
| NM_004536 | NAIP | 4474 | 26 | 6 | 3 | 2 | 6 | 5 | 3 | 2 | 6 | 5 | 1 | 26 | 4 |
| NM_004536 | NAIP | 6551 | 18 | 4 | 2 | 2 | 4 | 4 | 2 | 1 | 4 | 3 | 1 | 18 | 3 |
| NM_004537 | NAP1L1 | 5125 | 131 | 55 | 30 | 12 | 71 | 53 | 27 | 15 | 66 | 131 | 56 | 84 | 59 |
| NM_004538 | NAP1L3 | 2677 | 48 | 6 | 0 | 1 | 2 | 48 | 1 | 0 | 1 | 4 | 0 | 1 | 13 |
| NM_004539 | NARS | 2865 | 42 | 22 | 20 | 12 | 26 | 39 | 42 | 24 | 13 | 12 | 14 | 22 | 14 |
| NM_004540 | NCAM2 | 3699 | 47 | 6 | 0 | 1 | 3 | 47 | 0 | 0 | 3 | 1 | 0 | 1 | 13 |
| NM_004541 | NDUFA1 | 486 | 123 | 44 | 35 | 41 | 123 | 53 | 62 | 48 | 22 | 12 | 39 | 35 | 16 |
| NM_004542 | NDUFA3 | 353 | 19 | 10 | 6 | 8 | 19 | 18 | 10 | 13 | 5 | 5 | 18 | 8 | 5 |
| NM_004543 | NEB | 20596 | 1,655 | 151 | 3 | 0 | 2 | 0 | 0 | 0 | 0 | 0 | 1,655 | 0 | 4 |
| NM_004544 | NDUFA10 | 4914 | 15 | 8 | 8 | 9 | 14 | 15 | 9 | 9 | 4 | 6 | 7 | 5 | 7 |
| NM_004545 | NDUFB1 | 419 | 51 | 17 | 17 | 9 | 51 | 21 | 24 | 14 | 10 | 5 | 14 | 13 | 5 |

| | | transcript | | | | | | hypothala | | | | | skeletalm | | |
|---|---|---|---|---|---|---|---|---|---|---|---|---|---|---|---|
| transcript | gene | length (nt) | max | mean | adipose | colon | heart | mus | kidney | liver | lung | ovary | uscle | spleen | testes |
| NM_004546 | NDUFB2 | 495 | 32 | 13 | 12 | 12 | 32 | 22 | 21 | 16 | 5 | 3 | 12 | 8 | 5 |
| NM_004547 | NDUFB4 | 610 | 5 | 2 | 2 | 1 | 5 | 2 | 3 | 3 | 1 | 1 | 2 | 1 | 1 |
| NM_004548 | NDUFB10 | 697 | 145 | 57 | 43 | 43 | 145 | 61 | 49 | 64 | 21 | 24 | 119 | 34 | 24 |
| NM_004549 | NDUFC2 | 2472 | 42 | 25 | 21 | 15 | 39 | 41 | 29 | 42 | 16 | 17 | 23 | 20 | 17 |
| NM_004550 | NDUFS2 | 2041 | 16 | 10 | 8 | 7 | 16 | 12 | 14 | 16 | 4 | 6 | 10 | 8 | 6 |
| NM_004551 | NDUFS3 | 959 | 4 | 2 | 2 | 2 | 2 | 4 | 2 | 2 | 0 | 1 | 2 | 2 | 1 |
| NM_004552 | NDUFS5 | 511 | 575 | 243 | 246 | 209 | 575 | 532 | 158 | 232 | 87 | 152 | 258 | 108 | 115 |
| NM_004553 | NDUFS6 | 544 | 11 | 6 | 4 | 7 | 11 | 9 | 7 | 11 | 3 | 3 | 6 | 4 | 1 |
| NM_004554 | NFATC4 | 4929 | 1 | 0 | 0 | 0 | 1 | 0 | 0 | 0 | 0 | 1 | 0 | 0 | 1 |
| NM_004555 | NFATC3 | 4005 | 14 | 9 | 7 | 5 | 6 | 6 | 8 | 8 | 10 | 11 | 12 | 13 | 14 |
| NM_004556 | NFKBIE | 2581 | 5 | 1 | 1 | 1 | 1 | 2 | 1 | 0 | 1 | 1 | 0 | 5 | 1 |
| NM_004557 | NOTCH4 | 6745 | 0 | 0 | 0 | 0 | 0 | 0 | 0 | 0 | 0 | 0 | 0 | 0 | 0 |
| NM_004557 | NOTCH4 | 6751 | 0 | 0 | 0 | 0 | 0 | 0 | 0 | 0 | 0 | 0 | 0 | 0 | 0 |
| NM_004558 | NRTN | 1104 | 2 | 0 | 0 | 1 | 0 | 0 | 2 | 1 | 0 | 0 | 0 | 0 | 0 |
| NM_004559 | YBX1 | 1546 | 246 | 156 | 206 | 107 | 203 | 47 | 103 | 154 | 146 | 83 | 246 | 207 | 212 |
| NM_004560 | ROR2 | 4091 | 2 | 1 | 0 | 1 | 1 | 0 | 0 | 0 | 0 | 2 | 0 | 1 | 1 |
| NM_004561 | OVOL1 | 2993 | 1 | 0 | 0 | 1 | 0 | 0 | 1 | 0 | 0 | 0 | 0 | 0 | 1 |
| NM_004562 | PARK2 | 4073 | 3 | 2 | 2 | 1 | 3 | 3 | 3 | 0 | 1 | 2 | 3 | 0 | 2 |
| NM_004563 | PCK2 | 2221 | 16 | 3 | 2 | 2 | 0 | 1 | 10 | 16 | 1 | 0 | 0 | 2 | 1 |
| NM_004564 | PET112L | 2011 | 2 | 1 | 1 | 1 | 1 | 2 | 1 | 1 | 0 | 1 | 1 | 1 | 1 |
| NM_004565 | PEX14 | 1929 | 9 | 5 | 5 | 3 | 5 | 9 | 4 | 7 | 4 | 3 | 3 | 3 | 5 |
| NM_004566 | PFKFB3 | 4547 | 28 | 8 | 28 | 2 | 1 | 10 | 5 | 4 | 7 | 1 | 23 | 8 | 2 |
| NM_004567 | PFKFB4 | 3490 | 1 | 0 | 0 | 0 | 0 | 0 | 0 | 0 | 0 | 0 | 0 | 0 | 1 |
| NM_004568 | SERPINB6 | 1646 | 12 | 9 | 10 | 10 | 12 | 9 | 8 | 5 | 8 | 7 | 6 | 12 | 12 |
| NM_004569 | PIGH | 1438 | 1 | 1 | 1 | 1 | 1 | 0 | 1 | 1 | 1 | 1 | 1 | 1 | 1 |
| NM_004570 | PIK3C2G | 4844 | 3 | 0 | 0 | 0 | 0 | 0 | 1 | 3 | 0 | 0 | 0 | 0 | 0 |
| NM_004571 | PKNOX1 | 4976 | 1 | 1 | 0 | 1 | 0 | 1 | 1 | 0 | 0 | 1 | 1 | 1 | 1 |
| NM_004572 | PKP2 | 4439 | 35 | 6 | 0 | 6 | 35 | 2 | 2 | 8 | 1 | 4 | 1 | 2 | 3 |
| NM_004573 | PLCB2 | 4677 | 12 | 3 | 3 | 4 | 2 | 2 | 1 | 0 | 3 | 0 | 0 | 12 | 1 |
| NM_004574 | 04 Sep | 1736 | 53 | 7 | 2 | 1 | 5 | 53 | 2 | 4 | 3 | 2 | 1 | 5 | 2 |
| NM_004575 | POU4F2 | 3141 | 0 | 0 | 0 | 0 | 0 | 0 | 0 | 0 | 0 | 0 | 0 | 0 | 0 |
| NM_004576 | PPP2R2B | 2299 | 12 | 1 | 0 | 0 | 0 | 12 | 0 | 0 | 0 | 0 | 0 | 0 | 1 |
| NM_004577 | PSPH | 2118 | 16 | 8 | 2 | 2 | 2 | 4 | 15 | 4 | 4 | 15 | 9 | 16 | 13 |
| NM_004578 | RAB4A | 1843 | 6 | 3 | 2 | 2 | 5 | 6 | 2 | 5 | 1 | 2 | 2 | 2 | 2 |
| NM_004579 | MAP4K2 | 2955 | 1 | 0 | 0 | 0 | 0 | 1 | 0 | 0 | 1 | 0 | 0 | 1 | 0 |
| NM_004580 | RAB27A | 2816 | 17 | 5 | 2 | 4 | 3 | 2 | 3 | 17 | 9 | 2 | 1 | 9 | 3 |
| NM_004581 | RABGGTA | 2203 | 1 | 1 | 1 | 1 | 1 | 1 | 1 | 1 | 0 | 0 | 0 | 1 | 0 |
| NM_004582 | RABGGTB | 1496 | 12 | 7 | 6 | 4 | 6 | 8 | 9 | 12 | 6 | 3 | 6 | 8 | 5 |
| NM_004583 | RAB5C | 1655 | 9 | 4 | 5 | 2 | 3 | 6 | 9 | 6 | 4 | 2 | 1 | 8 | 3 |
| NM_004584 | RAD9A | 2122 | 0 | 0 | 0 | 0 | 0 | 0 | 0 | 0 | 0 | 0 | 0 | 0 | 0 |
| NM_004585 | RARRES3 | 765 | 13 | 4 | 3 | 2 | 1 | 4 | 13 | 6 | 8 | 3 | 1 | 4 | 2 |
| NM_004586 | RPS6KA3 | 7710 | 16 | 9 | 6 | 7 | 7 | 5 | 8 | 11 | 13 | 5 | 13 | 16 | 5 |
| NM_004587 | RRBP1 | 3715 | 13 | 4 | 3 | 5 | 3 | 2 | 5 | 13 | 3 | 3 | 1 | 5 | 4 |
| NM_004588 | SCN2B | 4922 | 8 | 1 | 0 | 0 | 0 | 8 | 0 | 0 | 0 | 0 | 1 | 0 | 0 |
| NM_004589 | SCO1 | 1754 | 11 | 7 | 6 | 6 | 8 | 8 | 9 | 11 | 3 | 4 | 4 | 6 | 7 |
| NM_004590 | CCL16 | 1497 | 4 | 0 | 0 | 0 | 0 | 0 | 0 | 4 | 0 | 0 | 0 | 0 | 0 |
| NM_004591 | CCL20 | 842 | 1 | 0 | 0 | 0 | 0 | 0 | 0 | 0 | 1 | 0 | 0 | 0 | 0 |
| NM_004592 | SFRS8 | 3245 | 8 | 5 | 4 | 5 | 4 | 4 | 4 | 2 | 5 | 6 | 7 | 8 | 6 |
| NM_004593 | TRA2B | 4287 | 16 | 10 | 9 | 5 | 9 | 10 | 8 | 10 | 9 | 13 | 7 | 16 | 11 |
| NM_004594 | SLC9A5 | 3735 | 1 | 0 | 0 | 0 | 0 | 0 | 0 | 0 | 0 | 0 | 0 | 1 | 0 |
| NM_004595 | SMS | 1688 | 61 | 24 | 13 | 9 | 30 | 24 | 42 | 9 | 23 | 61 | 13 | 26 | 16 |
| NM_004596 | SNRPA | 1606 | 14 | 8 | 4 | 6 | 8 | 4 | 7 | 3 | 9 | 12 | 9 | 14 | 8 |
| NM_004597 | SNRPD2 | 752 | 5 | 3 | 3 | 4 | 3 | 5 | 3 | 2 | 2 | 2 | 2 | 4 | 3 |
| NM_004598 | SPOCK1 | 4827 | 17 | 4 | 3 | 1 | 5 | 17 | 4 | 0 | 0 | 1 | 4 | 1 | 11 |
| NM_004599 | SREBF2 | 4306 | 8 | 3 | 2 | 3 | 3 | 8 | 4 | 3 | 4 | 2 | 1 | 3 | 4 |
| NM_004600 | TROVE2 | 3616 | 25 | 13 | 9 | 3 | 14 | 15 | 13 | 7 | 13 | 25 | 8 | 19 | 19 |
| NM_004602 | STAU1 | 3518 | 13 | 9 | 7 | 5 | 11 | 10 | 11 | 13 | 8 | 7 | 9 | 10 | 9 |
| NM_004603 | STX1A | 2125 | 8 | 1 | 0 | 0 | 0 | 8 | 0 | 0 | 0 | 0 | 0 | 0 | 1 |
| NM_004604 | STX4 | 1386 | 5 | 3 | 5 | 3 | 3 | 4 | 2 | 3 | 2 | 2 | 2 | 3 | 2 |
| NM_004605 | SULT2B1 | 1253 | 0 | 0 | 0 | 0 | 0 | 0 | 0 | 0 | 0 | 0 | 0 | 0 | 0 |
| NM_004606 | TAF1 | 7692 | 7 | 4 | 4 | 4 | 3 | 4 | 3 | 1 | 4 | 6 | 4 | 7 | 6 |
| NM_004607 | TBCA | 679 | 126 | 76 | 43 | 35 | 94 | 85 | 100 | 126 | 71 | 84 | 43 | 86 | 72 |
| NM_004608 | TBX6 | 1800 | 0 | 0 | 0 | 0 | 0 | 0 | 0 | 0 | 0 | 0 | 0 | 0 | 0 |
| NM_004609 | TCF15 | 1227 | 16 | 4 | 15 | 0 | 16 | 1 | 1 | 0 | 1 | 1 | 7 | 0 | 1 |
| NM_004610 | TCP10 | 1273 | 0 | 0 | 0 | 0 | 0 | 0 | 0 | 0 | 0 | 0 | 0 | 0 | 0 |
| NM_004612 | TGFBR1 | 6474 | 12 | 6 | 5 | 2 | 5 | 12 | 6 | 2 | 6 | 6 | 3 | 12 | 4 |
| NM_004613 | TGM2 | 3921 | 47 | 11 | 1 | 2 | 47 | 3 | 6 | 32 | 13 | 1 | 1 | 9 | 2 |
| NM_004614 | TK2 | 3675 | 30 | 11 | 30 | 5 | 8 | 15 | 9 | 13 | 6 | 10 | 4 | 8 | 11 |
| NM_004615 | TSPAN7 | 1806 | 29 | 6 | 2 | 2 | 3 | 29 | 3 | 1 | 4 | 2 | 9 | 6 | 1 |
| NM_004616 | TSPAN8 | 1130 | 22 | 3 | 0 | 22 | 0 | 0 | 3 | 1 | 2 | 2 | 3 | 0 | 0 |
| NM_004617 | TM4SF4 | 1428 | 20 | 2 | 0 | 0 | 0 | 0 | 0 | 20 | 0 | 0 | 0 | 0 | 0 |
| NM_004618 | TOP3A | 4116 | 3 | 2 | 1 | 2 | 1 | 2 | 1 | 2 | 2 | 2 | 1 | 3 | 3 |
| NM_004619 | TRAF5 | 3976 | 6 | 1 | 1 | 2 | 1 | 1 | 1 | 0 | 1 | 1 | 0 | 6 | 2 |
| NM_004620 | TRAF6 | 2481 | 3 | 2 | 1 | 1 | 2 | 2 | 2 | 1 | 2 | 2 | 1 | 3 | 2 |
| NM_004621 | TRPC6 | 4612 | 2 | 0 | 0 | 0 | 0 | 0 | 0 | 0 | 2 | 1 | 0 | 0 | 0 |
| NM_004622 | TSN | 3407 | 7 | 4 | 3 | 2 | 4 | 7 | 4 | 4 | 2 | 4 | 3 | 4 | 5 |
| NM_004623 | TTC4 | 2012 | 7 | 4 | 3 | 2 | 5 | 6 | 4 | 4 | 2 | 5 | 7 | 4 | 7 |

| | | transcript | | | | | | hypothala | | | | | skeletalm | | |
|---|---|---|---|---|---|---|---|---|---|---|---|---|---|---|---|
| transcript | gene | length (nt) | max | mean | adipose | colon | heart | mus | kidney | liver | lung | ovary | uscle | spleen | testes |
| NM_004624 | VIPR1 | 2791 | 11 | 2 | 2 | 5 | 1 | 2 | 0 | 5 | 11 | 0 | 0 | 1 | 0 |
| NM_004625 | WNT7A | 1714 | 0 | 0 | 0 | 0 | 0 | 0 | 0 | 0 | 0 | 0 | 0 | 0 | 0 |
| NM_004626 | WNT11 | 1927 | 1 | 0 | 1 | 0 | 0 | 0 | 0 | 0 | 0 | 0 | 0 | 0 | 0 |
| NM_004627 | WRB | 1572 | 7 | 2 | 1 | 1 | 2 | 7 | 2 | 2 | 2 | 2 | 1 | 2 | 4 |
| NM_004628 | XPC | 3721 | 6 | 5 | 5 | 3 | 2 | 6 | 6 | 4 | 4 | 6 | 3 | 6 | 5 |
| NM_004629 | FANCG | 2631 | 0 | 0 | 0 | 0 | 0 | 0 | 0 | 0 | 0 | 0 | 0 | 0 | 0 |
| NM_004630 | SF1 | 3509 | 14 | 10 | 7 | 7 | 10 | 11 | 9 | 5 | 6 | 14 | 11 | 13 | 13 |
| NM_004631 | LRP8 | 4507 | 6 | 1 | 0 | 0 | 0 | 2 | 0 | 0 | 0 | 0 | 0 | 0 | 6 |
| NM_004632 | DAP3 | 1582 | 7 | 5 | 5 | 3 | 7 | 5 | 5 | 7 | 5 | 2 | 5 | 6 | 7 |
| NM_004633 | IL1R2 | 1456 | 16 | 3 | 0 | 2 | 3 | 0 | 1 | 2 | 8 | 0 | 0 | 16 | 0 |
| NM_004634 | BRPF1 | 4710 | 2 | 1 | 1 | 2 | 1 | 2 | 1 | 1 | 1 | 1 | 1 | 2 | 2 |
| NM_004635 | MAPKAPK3 | 2500 | 5 | 1 | 1 | 0 | 5 | 0 | 1 | 1 | 1 | 0 | 2 | 1 | 0 |
| NM_004636 | SEMA3B | 2926 | 2 | 1 | 0 | 1 | 0 | 2 | 1 | 0 | 1 | 0 | 0 | 0 | 0 |
| NM_004637 | RAB7A | 2232 | 69 | 43 | 49 | 30 | 50 | 58 | 41 | 38 | 44 | 27 | 33 | 69 | 36 |
| NM_004639 | BAT3 | 3816 | 3 | 2 | 2 | 2 | 2 | 2 | 2 | 2 | 1 | 2 | 3 | 3 | 3 |
| NM_004639 | BAT3 | 3820 | 3 | 2 | 2 | 2 | 2 | 2 | 2 | 2 | 1 | 2 | 3 | 3 | 3 |
| NM_004640 | BAT1 | 2133 | 24 | 15 | 9 | 13 | 14 | 13 | 13 | 9 | 15 | 24 | 11 | 22 | 20 |
| NM_004641 | MLLT10 | 5121 | 10 | 3 | 2 | 2 | 2 | 2 | 3 | 2 | 4 | 3 | 3 | 4 | 10 |
| NM_004642 | CDK2AP1 | 1613 | 36 | 15 | 16 | 6 | 20 | 36 | 12 | 8 | 11 | 9 | 6 | 17 | 18 |
| NM_004643 | PABPN1 | 3077 | 17 | 11 | 10 | 9 | 11 | 11 | 11 | 11 | 9 | 11 | 7 | 17 | 17 |
| NM_004644 | AP3B2 | 3704 | 26 | 3 | 0 | 0 | 0 | 26 | 0 | 0 | 1 | 0 | 0 | 0 | 2 |
| NM_004645 | COIL | 2633 | 23 | 6 | 3 | 3 | 4 | 5 | 4 | 4 | 3 | 6 | 4 | 7 | 23 |
| NM_004646 | NPHS1 | 5024 | 3 | 0 | 0 | 0 | 0 | 0 | 3 | 0 | 0 | 0 | 0 | 0 | 0 |
| NM_004647 | DPF1 | 2223 | 1 | 0 | 0 | 0 | 0 | 1 | 0 | 0 | 0 | 0 | 0 | 0 | 0 |
| NM_004649 | C21orf33 | 1667 | 5 | 2 | 2 | 1 | 3 | 3 | 5 | 5 | 1 | 1 | 1 | 1 | 2 |
| NM_004650 | PNPLA4 | 2847 | 3 | 1 | 1 | 1 | 1 | 1 | 3 | 1 | 1 | 1 | 2 | 0 | 1 |
| NM_004651 | USP11 | 3290 | 15 | 3 | 1 | 1 | 2 | 15 | 2 | 1 | 1 | 5 | 2 | 3 | 4 |
| NM_004653 | KDM5D | 5486 | 3 | 1 | 1 | 0 | 1 | 1 | 1 | 0 | 1 | 0 | 0 | 3 | 2 |
| NM_004654 | USP9Y | 10040 | 4 | 2 | 2 | 0 | 3 | 4 | 2 | 1 | 2 | 0 | 0 | 4 | 4 |
| NM_004655 | AXIN2 | 4230 | 4 | 2 | 1 | 4 | 2 | 2 | 0 | 0 | 2 | 3 | 2 | 2 | 2 |
| NM_004656 | BAP1 | 3584 | 5 | 3 | 2 | 3 | 3 | 5 | 4 | 3 | 2 | 1 | 2 | 3 | 5 |
| NM_004657 | SDPR | 3250 | 14 | 4 | 9 | 2 | 4 | 1 | 2 | 1 | 8 | 3 | 1 | 14 | 1 |
| NM_004658 | RASAL1 | 3124 | 0 | 0 | 0 | 0 | 0 | 0 | 0 | 0 | 0 | 0 | 0 | 0 | 0 |
| NM_004660 | DDX3Y | 4488 | 29 | 10 | 11 | 0 | 9 | 11 | 5 | 11 | 13 | 0 | 1 | 22 | 29 |
| NM_004661 | CDC23 | 3155 | 6 | 4 | 4 | 3 | 4 | 5 | 5 | 5 | 3 | 5 | 3 | 6 | 6 |
| NM_004662 | DNAH9 | 3147 | 1 | 0 | 0 | 0 | 0 | 0 | 0 | 0 | 1 | 0 | 0 | 0 | 1 |
| NM_004663 | RAB11A | 2393 | 43 | 25 | 20 | 13 | 26 | 43 | 26 | 14 | 20 | 19 | 15 | 42 | 34 |
| NM_004664 | LIN7A | 1230 | 3 | 2 | 0 | 0 | 2 | 3 | 3 | 3 | 2 | 1 | 0 | 2 | 2 |
| NM_004665 | VNN2 | 2004 | 8 | 1 | 0 | 0 | 1 | 0 | 0 | 0 | 5 | 0 | 0 | 8 | 0 |
| NM_004666 | VNN1 | 3842 | 17 | 2 | 0 | 0 | 1 | 0 | 2 | 17 | 3 | 0 | 0 | 3 | 0 |
| NM_004667 | HERC2 | 15332 | 4 | 2 | 2 | 3 | 2 | 4 | 2 | 2 | 1 | 2 | 2 | 3 | 2 |
| NM_004668 | MGAM | 6483 | 5 | 1 | 0 | 0 | 0 | 0 | 5 | 0 | 1 | 0 | 0 | 5 | 1 |
| NM_004669 | CLIC3 | 813 | 0 | 0 | 0 | 0 | 0 | 0 | 0 | 0 | 0 | 0 | 0 | 0 | 0 |
| NM_004670 | PAPSS2 | 3843 | 14 | 5 | 4 | 5 | 3 | 1 | 4 | 14 | 12 | 6 | 1 | 3 | 2 |
| NM_004671 | PIAS2 | 2360 | 5 | 2 | 2 | 1 | 2 | 3 | 3 | 1 | 2 | 3 | 2 | 3 | 5 |
| NM_004672 | MAP3K6 | 4328 | 1 | 0 | 0 | 1 | 0 | 0 | 0 | 0 | 0 | 0 | 0 | 0 | 0 |
| NM_004673 | ANGPTL1 | 3546 | 13 | 6 | 7 | 2 | 10 | 1 | 5 | 3 | 5 | 12 | 9 | 4 | 13 |
| NM_004674 | ASH2L | 2584 | 9 | 5 | 4 | 2 | 4 | 7 | 4 | 3 | 3 | 6 | 4 | 5 | 9 |
| NM_004675 | DIRAS3 | 1631 | 14 | 3 | 0 | 0 | 1 | 13 | 2 | 1 | 0 | 14 | 1 | 1 | 2 |
| NM_004676 | PRY | 1238 | 0 | 0 | 0 | 0 | 0 | 0 | 0 | 0 | 0 | 0 | 0 | 0 | 0 |
| NM_004677 | XKRY | 1581 | 0 | 0 | 0 | 0 | 0 | 0 | 0 | 0 | 0 | 0 | 0 | 0 | 0 |
| NM_004678 | BPY2 | 1204 | 0 | 0 | 0 | 0 | 0 | 0 | 0 | 0 | 0 | 0 | 0 | 0 | 0 |
| NM_004679 | VCY | 549 | 0 | 0 | 0 | 0 | 0 | 0 | 0 | 0 | 0 | 0 | 0 | 0 | 0 |
| NM_004680 | CDY1 | 2365 | 0 | 0 | 0 | 0 | 0 | 0 | 0 | 0 | 0 | 0 | 0 | 0 | 0 |
| NM_004680 | CDY1 | 2366 | 0 | 0 | 0 | 0 | 0 | 0 | 0 | 0 | 0 | 0 | 0 | 0 | 0 |
| NM_004681 | EIF1AY | 1390 | 18 | 6 | 7 | 0 | 18 | 8 | 2 | 8 | 4 | 0 | 1 | 14 | 6 |
| NM_004683 | RGN | 1615 | 20 | 3 | 1 | 0 | 1 | 2 | 6 | 20 | 1 | 1 | 0 | 0 | 1 |
| NM_004684 | SPARCL1 | 2904 | 314 | 111 | 109 | 14 | 259 | 314 | 20 | 3 | 145 | 116 | 69 | 59 | 118 |
| NM_004685 | MTMR6 | 5214 | 9 | 5 | 4 | 1 | 5 | 9 | 4 | 4 | 4 | 4 | 3 | 7 | 6 |
| NM_004686 | MTMR7 | 5287 | 3 | 1 | 0 | 0 | 0 | 3 | 0 | 0 | 0 | 1 | 0 | 1 | 1 |
| NM_004687 | MTMR4 | 5829 | 4 | 2 | 1 | 1 | 1 | 4 | 2 | 3 | 1 | 1 | 0 | 3 | 4 |
| NM_004688 | NMI | 1479 | 8 | 3 | 4 | 2 | 3 | 2 | 3 | 4 | 3 | 3 | 2 | 8 | 3 |
| NM_004689 | MTA1 | 2840 | 2 | 2 | 1 | 2 | 2 | 2 | 1 | 1 | 1 | 2 | 1 | 2 | 2 |
| NM_004690 | LATS1 | 4755 | 8 | 4 | 3 | 3 | 3 | 4 | 4 | 2 | 4 | 8 | 3 | 6 | 6 |
| NM_004691 | ATP6V0D1 | 1670 | 8 | 4 | 3 | 4 | 2 | 8 | 5 | 3 | 3 | 1 | 3 | 5 | 2 |
| NM_004693 | KRT75 | 2125 | 0 | 0 | 0 | 0 | 0 | 0 | 0 | 0 | 0 | 0 | 0 | 0 | 0 |
| NM_004694 | SLC16A6 | 2657 | 1 | 1 | 0 | 0 | 0 | 1 | 0 | 0 | 1 | 0 | 1 | 1 | 1 |
| NM_004695 | SLC16A5 | 1925 | 1 | 0 | 0 | 0 | 0 | 0 | 1 | 0 | 0 | 0 | 0 | 0 | 0 |
| NM_004696 | SLC16A4 | 2529 | 13 | 2 | 0 | 1 | 1 | 1 | 13 | 0 | 2 | 3 | 0 | 1 | 2 |
| NM_004697 | PRPF4 | 2735 | 3 | 2 | 2 | 1 | 3 | 2 | 3 | 3 | 1 | 3 | 2 | 3 | 2 |
| NM_004698 | PRPF3 | 2463 | 17 | 11 | 10 | 9 | 9 | 8 | 9 | 4 | 10 | 14 | 9 | 17 | 17 |
| NM_004699 | FAM50A | 1339 | 24 | 17 | 20 | 16 | 24 | 24 | 15 | 15 | 15 | 9 | 17 | 21 | 13 |
| NM_004700 | KCNQ4 | 2335 | 0 | 0 | 0 | 0 | 0 | 0 | 0 | 0 | 0 | 0 | 0 | 0 | 0 |
| NM_004701 | CCNB2 | 1513 | 11 | 2 | 1 | 2 | 1 | 0 | 0 | 0 | 1 | 0 | 0 | 1 | 11 |
| NM_004703 | RABEP1 | 5402 | 38 | 20 | 18 | 6 | 26 | 38 | 16 | 13 | 14 | 19 | 28 | 30 | 18 |
| NM_004704 | RRP9 | 1543 | 1 | 1 | 1 | 1 | 1 | 1 | 1 | 1 | 1 | 0 | 1 | 1 | 1 |
| NM_004705 | PRKRIR | 3190 | 18 | 10 | 9 | 4 | 10 | 9 | 9 | 7 | 8 | 11 | 9 | 18 | 17 |

| transcript | gene | transcript length (nt) | max | mean | adipose | colon | heart | hypothalamus | kidney | liver | lung | ovary | skeletalmuscle | spleen | testes |
|---|---|---|---|---|---|---|---|---|---|---|---|---|---|---|---|
| NM_004706 | ARHGEF1 | 3213 | 1 | 0 | 0 | 1 | 0 | 0 | 0 | 0 | 1 | 0 | 0 | 1 | 0 |
| NM_004707 | ATG12 | 2358 | 7 | 3 | 5 | 2 | 3 | 7 | 2 | 5 | 3 | 2 | 2 | 4 | 3 |
| NM_004708 | PDCD5 | 565 | 17 | 8 | 9 | 4 | 17 | 13 | 6 | 12 | 5 | 3 | 7 | 6 | 6 |
| NM_004709 | CXorf1 | 2443 | 0 | 0 | 0 | 0 | 0 | 0 | 0 | 0 | 0 | 0 | 0 | 0 | 0 |
| NM_004710 | SYNGR2 | 1694 | 33 | 18 | 16 | 17 | 19 | 15 | 15 | 20 | 28 | 4 | 28 | 33 | 7 |
| NM_004711 | SYNGR1 | 4438 | 21 | 2 | 0 | 1 | 0 | 21 | 1 | 0 | 0 | 2 | 0 | 0 | 1 |
| NM_004712 | HGS | 2910 | 5 | 2 | 2 | 2 | 1 | 5 | 2 | 2 | 1 | 1 | 2 | 2 | 2 |
| NM_004713 | SDCCAG1 | 4121 | 20 | 13 | 11 | 5 | 16 | 19 | 15 | 5 | 14 | 13 | 14 | 20 | 16 |
| NM_004714 | DYRK1B | 2535 | 4 | 1 | 0 | 0 | 1 | 1 | 0 | 0 | 1 | 1 | 4 | 1 | 1 |
| NM_004715 | CTDP1 | 3750 | 1 | 1 | 1 | 1 | 1 | 1 | 1 | 1 | 1 | 0 | 1 | 1 | 1 |
| NM_004716 | PCSK7 | 3484 | 18 | 7 | 3 | 18 | 15 | 3 | 4 | 2 | 5 | 16 | 4 | 6 | 6 |
| NM_004717 | DGKI | 4780 | 2 | 0 | 0 | 0 | 0 | 2 | 0 | 0 | 0 | 0 | 0 | 0 | 0 |
| NM_004718 | COX7A2L | 1115 | 21 | 10 | 9 | 6 | 10 | 21 | 15 | 10 | 7 | 11 | 5 | 10 | 8 |
| NM_004719 | SFRS2IP | 7596 | 36 | 20 | 20 | 5 | 24 | 11 | 19 | 17 | 21 | 26 | 22 | 36 | 19 |
| NM_004720 | LPAR2 | 1794 | 3 | 0 | 0 | 1 | 0 | 0 | 0 | 0 | 1 | 0 | 0 | 0 | 3 |
| NM_004721 | MAP3K13 | 3557 | 9 | 4 | 1 | 3 | 1 | 7 | 9 | 5 | 3 | 4 | 1 | 1 | 5 |
| NM_004722 | AP4M1 | 1868 | 0 | 0 | 0 | 0 | 0 | 0 | 0 | 0 | 0 | 0 | 0 | 0 | 0 |
| NM_004723 | ARHGEF2 | 4107 | 6 | 3 | 4 | 3 | 3 | 6 | 1 | 0 | 4 | 2 | 1 | 6 | 1 |
| NM_004724 | ZW10 | 2884 | 9 | 5 | 4 | 2 | 4 | 4 | 4 | 6 | 3 | 6 | 9 | 7 | 8 |
| NM_004725 | BUB3 | 2764 | 9 | 4 | 3 | 2 | 4 | 4 | 4 | 2 | 5 | 6 | 3 | 9 | 6 |
| NM_004726 | REPS2 | 7953 | 6 | 1 | 0 | 1 | 1 | 6 | 1 | 1 | 1 | 1 | 0 | 1 | 1 |
| NM_004727 | SLC24A1 | 5769 | 2 | 1 | 1 | 0 | 0 | 1 | 1 | 0 | 1 | 2 | 1 | 2 | 1 |
| NM_004728 | DDX21 | 4153 | 24 | 14 | 12 | 4 | 15 | 16 | 10 | 24 | 13 | 7 | 13 | 23 | 17 |
| NM_004729 | ZBED1 | 4507 | 3 | 2 | 2 | 2 | 3 | 2 | 3 | 3 | 1 | 2 | 3 | 3 | 3 |
| NM_004730 | ETF1 | 3689 | 12 | 8 | 10 | 3 | 12 | 8 | 8 | 8 | 8 | 5 | 7 | 10 | 10 |
| NM_004731 | SLC16A7 | 3541 | 7 | 3 | 6 | 0 | 7 | 1 | 5 | 1 | 2 | 1 | 1 | 4 | 1 |
| NM_004732 | KCNAB3 | 1608 | 0 | 0 | 0 | 0 | 0 | 0 | 0 | 0 | 0 | 0 | 0 | 0 | 0 |
| NM_004733 | SLC33A1 | 2659 | 4 | 2 | 1 | 1 | 2 | 2 | 3 | 4 | 2 | 2 | 1 | 2 | 2 |
| NM_004734 | DCLK1 | 8079 | 29 | 4 | 4 | 1 | 2 | 29 | 0 | 0 | 0 | 2 | 1 | 1 | 1 |
| NM_004735 | LRRFIP1 | 4420 | 20 | 10 | 9 | 9 | 12 | 3 | 16 | 11 | 17 | 4 | 4 | 20 | 5 |
| NM_004736 | XPR1 | 8494 | 8 | 3 | 2 | 1 | 8 | 4 | 4 | 1 | 2 | 2 | 1 | 3 | 7 |
| NM_004737 | LARGE | 4201 | 7 | 3 | 1 | 3 | 6 | 7 | 1 | 1 | 1 | 3 | 3 | 2 | 1 |
| NM_004738 | VAPB | 3675 | 26 | 17 | 12 | 10 | 21 | 26 | 21 | 22 | 12 | 17 | 22 | 16 | 15 |
| NM_004739 | MTA2 | 3060 | 7 | 3 | 3 | 2 | 3 | 3 | 3 | 2 | 3 | 3 | 1 | 7 | 4 |
| NM_004740 | TIAF1 | 2089 | 0 | 0 | 0 | 0 | 0 | 0 | 0 | 0 | 0 | 0 | 0 | 0 | 0 |
| NM_004741 | NOLC1 | 3936 | 13 | 6 | 3 | 4 | 6 | 13 | 5 | 8 | 4 | 6 | 7 | 9 | 7 |
| NM_004742 | MAGI1 | 6177 | 4 | 2 | 2 | 3 | 2 | 3 | 3 | 3 | 3 | 1 | 1 | 4 | 2 |
| NM_004744 | LRAT | 4888 | 1 | 0 | 0 | 0 | 0 | 1 | 0 | 0 | 0 | 1 | 0 | 0 | 1 |
| NM_004745 | DLGAP2 | 10095 | 1 | 0 | 0 | 0 | 0 | 1 | 0 | 0 | 0 | 0 | 0 | 0 | 0 |
| NM_004746 | DLGAP1 | 3347 | 23 | 2 | 0 | 1 | 0 | 23 | 0 | 0 | 0 | 0 | 0 | 0 | 2 |
| NM_004747 | DLG5 | 7479 | 4 | 2 | 2 | 2 | 1 | 2 | 1 | 1 | 1 | 3 | 1 | 1 | 4 |
| NM_004748 | CCPG1 | 6666 | 37 | 18 | 24 | 3 | 25 | 14 | 18 | 37 | 20 | 9 | 12 | 21 | 15 |
| NM_004749 | TBRG4 | 2247 | 2 | 1 | 0 | 1 | 1 | 1 | 1 | 2 | 1 | 1 | 1 | 1 | 1 |
| NM_004750 | CRLF1 | 1802 | 0 | 0 | 0 | 0 | 0 | 0 | 0 | 0 | 0 | 0 | 0 | 0 | 0 |
| NM_004751 | GCNT3 | 2221 | 10 | 1 | 0 | 10 | 0 | 0 | 2 | 0 | 0 | 0 | 0 | 0 | 0 |
| NM_004752 | GCM2 | 2365 | 0 | 0 | 0 | 0 | 0 | 0 | 0 | 0 | 0 | 0 | 0 | 0 | 0 |
| NM_004753 | DHRS3 | 1806 | 22 | 6 | 12 | 4 | 4 | 3 | 6 | 22 | 6 | 4 | 4 | 3 | 4 |
| NM_004755 | RPS6KA5 | 3872 | 6 | 3 | 1 | 2 | 2 | 6 | 1 | 1 | 2 | 4 | 6 | 3 | 3 |
| NM_004756 | NUMBL | 3559 | 1 | 0 | 1 | 1 | 0 | 1 | 0 | 0 | 0 | 1 | 0 | 0 | 1 |
| NM_004757 | AIMP1 | 3143 | 8 | 6 | 5 | 1 | 8 | 5 | 8 | 6 | 4 | 8 | 8 | 7 | 7 |
| NM_004758 | BZRAP1 | 7692 | 6 | 1 | 0 | 1 | 0 | 6 | 0 | 0 | 1 | 1 | 0 | 1 | 0 |
| NM_004759 | MAPKAPK2 | 3589 | 20 | 7 | 3 | 4 | 20 | 3 | 8 | 5 | 7 | 5 | 9 | 11 | 5 |
| NM_004760 | STK17A | 3933 | 10 | 3 | 2 | 1 | 5 | 2 | 2 | 1 | 4 | 4 | 2 | 10 | 2 |
| NM_004761 | RGL2 | 3352 | 1 | 0 | 0 | 0 | 0 | 0 | 0 | 0 | 0 | 0 | 0 | 1 | 0 |
| NM_004762 | CYTH1 | 3311 | 16 | 6 | 5 | 7 | 5 | 6 | 3 | 4 | 10 | 3 | 6 | 16 | 4 |
| NM_004763 | ITGB1BP1 | 1928 | 22 | 5 | 22 | 2 | 3 | 8 | 4 | 2 | 2 | 4 | 2 | 4 | 3 |
| NM_004764 | PIWIL1 | 3406 | 15 | 1 | 0 | 0 | 0 | 0 | 0 | 0 | 0 | 0 | 0 | 0 | 15 |
| NM_004765 | BCL7C | 858 | 1 | 0 | 0 | 0 | 0 | 0 | 1 | 0 | 0 | 0 | 0 | 1 | 0 |
| NM_004766 | COPB2 | 3123 | 26 | 15 | 17 | 9 | 16 | 14 | 17 | 26 | 13 | 11 | 5 | 19 | 15 |
| NM_004767 | GPR37L1 | 2459 | 4 | 0 | 0 | 0 | 0 | 4 | 0 | 0 | 0 | 0 | 0 | 0 | 0 |
| NM_004768 | SFRS11 | 2775 | 43 | 25 | 25 | 8 | 25 | 24 | 28 | 10 | 28 | 26 | 19 | 35 | 43 |
| NM_004769 | ACCN3 | 2068 | 0 | 0 | 0 | 0 | 0 | 0 | 0 | 0 | 0 | 0 | 0 | 0 | 0 |
| NM_004770 | KCNB2 | 3582 | 3 | 0 | 0 | 0 | 0 | 3 | 0 | 0 | 0 | 0 | 0 | 1 | 0 |
| NM_004771 | MMP20 | 1956 | 1 | 0 | 0 | 0 | 0 | 0 | 0 | 0 | 0 | 0 | 0 | 0 | 1 |
| NM_004772 | C5orf13 | 1994 | 6 | 2 | 1 | 2 | 2 | 6 | 4 | 0 | 1 | 5 | 1 | 3 | 1 |
| NM_004773 | ZNHIT3 | 953 | 5 | 3 | 2 | 2 | 2 | 5 | 3 | 1 | 2 | 2 | 3 | 2 | 5 |
| NM_004774 | MED1 | 8148 | 6 | 3 | 2 | 2 | 3 | 3 | 3 | 2 | 2 | 4 | 2 | 6 | 3 |
| NM_004775 | B4GALT6 | 4814 | 4 | 1 | 1 | 0 | 0 | 4 | 1 | 0 | 0 | 1 | 0 | 0 | 1 |
| NM_004776 | B4GALT5 | 4727 | 9 | 5 | 3 | 3 | 6 | 5 | 6 | 3 | 5 | 3 | 8 | 9 | 6 |
| NM_004778 | GPR44 | 2910 | 0 | 0 | 0 | 0 | 0 | 0 | 0 | 0 | 0 | 0 | 0 | 0 | 0 |
| NM_004779 | CNOT8 | 2483 | 12 | 7 | 6 | 4 | 6 | 8 | 5 | 3 | 11 | 5 | 3 | 12 | 12 |
| NM_004780 | TCEAL1 | 1206 | 18 | 9 | 5 | 4 | 8 | 18 | 5 | 6 | 6 | 18 | 7 | 7 | 11 |
| NM_004781 | VAMP3 | 2205 | 8 | 5 | 8 | 3 | 6 | 6 | 5 | 3 | 6 | 5 | 4 | 6 | 6 |
| NM_004782 | SNAP29 | 4259 | 3 | 2 | 2 | 2 | 2 | 2 | 2 | 3 | 2 | 1 | 2 | 3 | 3 |
| NM_004783 | TAOK2 | 4625 | 3 | 2 | 2 | 2 | 1 | 3 | 1 | 1 | 1 | 1 | 1 | 2 | 2 |
| NM_004784 | NDST3 | 5944 | 2 | 0 | 0 | 0 | 0 | 2 | 0 | 0 | 0 | 0 | 0 | 1 | 0 |
| NM_004785 | SLC9A3R2 | 2142 | 149 | 49 | 55 | 32 | 79 | 39 | 33 | 149 | 62 | 20 | 14 | 42 | 18 |

| transcript | gene | transcript length (nt) | max | mean | adipose | colon | heart | hypothalamus | kidney | liver | lung | ovary | skeletalmuscle | spleen | testes |
|---|---|---|---|---|---|---|---|---|---|---|---|---|---|---|---|
| NM_004786 | TXNL1 | 1324 | 10 | 7 | 6 | 4 | 8 | 10 | 7 | 8 | 6 | 7 | 9 | 7 | 7 |
| NM_004787 | SLIT2 | 4950 | 9 | 3 | 3 | 1 | 2 | 5 | 5 | 0 | 7 | 1 | 1 | 9 | 2 |
| NM_004788 | UBE4A | 6122 | 15 | 8 | 7 | 4 | 9 | 12 | 7 | 15 | 5 | 7 | 9 | 9 | 8 |
| NM_004789 | LHX2 | 2416 | 1 | 0 | 0 | 0 | 0 | 1 | 0 | 0 | 0 | 0 | 0 | 0 | 0 |
| NM_004790 | SLC22A6 | 2196 | 9 | 1 | 0 | 0 | 0 | 0 | 9 | 0 | 0 | 0 | 0 | 0 | 0 |
| NM_004791 | ITGBL1 | 2494 | 11 | 3 | 5 | 1 | 11 | 2 | 2 | 1 | 9 | 1 | 2 | 1 | 2 |
| NM_004792 | PPIG | 2706 | 70 | 49 | 43 | 11 | 49 | 70 | 45 | 49 | 40 | 61 | 46 | 67 | 57 |
| NM_004793 | LONP1 | 3111 | 5 | 2 | 1 | 1 | 2 | 2 | 2 | 5 | 1 | 1 | 2 | 2 | 2 |
| NM_004794 | RAB33A | 1108 | 1 | 0 | 0 | 0 | 0 | 1 | 0 | 0 | 0 | 0 | 0 | 0 | 0 |
| NM_004795 | KL | 5005 | 27 | 3 | 1 | 0 | 0 | 2 | 27 | 0 | 1 | 0 | 0 | 2 | 0 |
| NM_004796 | NRXN3 | 6130 | 8 | 1 | 0 | 1 | 1 | 8 | 0 | 0 | 1 | 0 | 0 | 0 | 0 |
| NM_004797 | ADIPOQ | 4578 | 129 | 13 | 129 | 6 | 2 | 0 | 0 | 0 | 0 | 0 | 1 | 0 | 0 |
| NM_004798 | KIF3B | 6100 | 11 | 5 | 5 | 4 | 3 | 11 | 11 | 6 | 4 | 4 | 1 | 4 | 6 |
| NM_004799 | ZFYVE9 | 4915 | 12 | 6 | 4 | 3 | 4 | 12 | 6 | 3 | 4 | 10 | 7 | 3 | 9 |
| NM_004800 | TM9SF2 | 2389 | 47 | 22 | 18 | 15 | 21 | 22 | 28 | 47 | 19 | 17 | 8 | 27 | 24 |
| NM_004801 | NRXN1 | 9349 | 28 | 3 | 0 | 1 | 1 | 28 | 0 | 0 | 0 | 0 | 0 | 0 | 1 |
| NM_004802 | OTOF | 4953 | 1 | 0 | 0 | 0 | 0 | 1 | 0 | 0 | 0 | 0 | 0 | 0 | 0 |
| NM_004803 | SLC22A14 | 1977 | 0 | 0 | 0 | 0 | 0 | 0 | 0 | 0 | 0 | 0 | 0 | 0 | 0 |
| NM_004804 | CIAO1 | 4036 | 4 | 3 | 2 | 3 | 3 | 4 | 4 | 3 | 2 | 2 | 2 | 3 | 2 |
| NM_004805 | POLR2D | 2324 | 7 | 3 | 1 | 1 | 3 | 3 | 2 | 2 | 1 | 3 | 3 | 3 | 7 |
| NM_004807 | HS6ST1 | 3952 | 4 | 2 | 1 | 1 | 1 | 4 | 3 | 2 | 1 | 2 | 1 | 3 | 3 |
| NM_004808 | NMT2 | 5004 | 9 | 5 | 9 | 3 | 2 | 4 | 3 | 6 | 2 | 7 | 3 | 3 | 9 |
| NM_004809 | STOML1 | 1986 | 0 | 0 | 0 | 0 | 0 | 0 | 0 | 0 | 0 | 0 | 0 | 0 | 0 |
| NM_004810 | GRAP2 | 1552 | 2 | 0 | 0 | 0 | 0 | 0 | 0 | 0 | 1 | 0 | 0 | 2 | 0 |
| NM_004811 | LPXN | 1887 | 4 | 1 | 1 | 0 | 0 | 1 | 1 | 0 | 2 | 1 | 0 | 4 | 0 |
| NM_004813 | PEX16 | 1907 | 1 | 0 | 0 | 0 | 0 | 1 | 1 | 1 | 0 | 0 | 0 | 0 | 0 |
| NM_004814 | SNRNP40 | 1624 | 6 | 5 | 4 | 3 | 4 | 6 | 4 | 5 | 3 | 5 | 5 | 6 | 5 |
| NM_004815 | ARHGAP29 | 9117 | 7 | 3 | 4 | 0 | 3 | 2 | 6 | 3 | 5 | 2 | 2 | 7 | 3 |
| NM_004816 | FAM189A2 | 2423 | 6 | 2 | 0 | 0 | 1 | 4 | 0 | 0 | 2 | 4 | 6 | 0 | 0 |
| NM_004817 | TJP2 | 4725 | 7 | 4 | 5 | 2 | 3 | 7 | 4 | 5 | 4 | 3 | 1 | 1 | 5 |
| NM_004818 | DDX23 | 3267 | 13 | 10 | 9 | 9 | 8 | 11 | 8 | 12 | 9 | 7 | 8 | 13 | 10 |
| NM_004819 | SYMPK | 4188 | 3 | 2 | 1 | 2 | 1 | 2 | 1 | 3 | 1 | 1 | 2 | 2 | 3 |
| NM_004820 | CYP7B1 | 2395 | 7 | 2 | 2 | 0 | 1 | 4 | 3 | 4 | 2 | 1 | 0 | 7 | 1 |
| NM_004821 | HAND1 | 1737 | 0 | 0 | 0 | 0 | 0 | 0 | 0 | 0 | 0 | 0 | 0 | 0 | 0 |
| NM_004822 | NTN1 | 5954 | 2 | 0 | 0 | 1 | 2 | 0 | 0 | 1 | 0 | 0 | 0 | 0 | 0 |
| NM_004823 | KCNK6 | 2655 | 1 | 0 | 1 | 1 | 1 | 0 | 0 | 0 | 0 | 0 | 0 | 1 | 0 |
| NM_004824 | CDYL | 3460 | 12 | 8 | 6 | 7 | 8 | 9 | 7 | 4 | 7 | 8 | 9 | 11 | 12 |
| NM_004825 | CDY2A | 1960 | 0 | 0 | 0 | 0 | 0 | 0 | 0 | 0 | 0 | 0 | 0 | 0 | 0 |
| NM_004826 | ECEL1 | 2859 | 0 | 0 | 0 | 0 | 0 | 0 | 0 | 0 | 0 | 0 | 0 | 0 | 0 |
| NM_004827 | ABCG2 | 4431 | 1 | 0 | 0 | 0 | 0 | 1 | 0 | 0 | 0 | 1 | 0 | 0 | 0 |
| NM_004828 | NCR2 | 941 | 0 | 0 | 0 | 0 | 0 | 0 | 0 | 0 | 0 | 0 | 0 | 0 | 0 |
| NM_004829 | NCR1 | 1153 | 1 | 0 | 0 | 0 | 0 | 0 | 0 | 0 | 0 | 0 | 0 | 1 | 0 |
| NM_004830 | MED23 | 5227 | 4 | 2 | 2 | 2 | 1 | 1 | 3 | 1 | 2 | 3 | 1 | 4 | 3 |
| NM_004831 | MED26 | 3184 | 2 | 1 | 1 | 0 | 0 | 0 | 0 | 0 | 0 | 1 | 0 | 1 | 2 |
| NM_004832 | GSTO1 | 785 | 6 | 3 | 4 | 1 | 6 | 4 | 3 | 6 | 3 | 1 | 2 | 3 | 1 |
| NM_004833 | AIM2 | 1484 | 6 | 1 | 0 | 0 | 0 | 0 | 0 | 0 | 1 | 0 | 0 | 6 | 0 |
| NM_004834 | MAP4K4 | 6997 | 32 | 15 | 17 | 6 | 21 | 32 | 7 | 11 | 11 | 13 | 7 | 14 | 23 |
| NM_004835 | AGTR1 | 2330 | 5 | 1 | 2 | 0 | 1 | 0 | 1 | 5 | 1 | 0 | 1 | 1 | 0 |
| NM_004836 | EIF2AK3 | 4548 | 3 | 2 | 1 | 2 | 3 | 2 | 2 | 2 | 2 | 2 | 1 | 3 | 2 |
| NM_004837 | GGPS1 | 2908 | 3 | 2 | 2 | 1 | 3 | 3 | 2 | 1 | 2 | 2 | 1 | 2 | 3 |
| NM_004838 | HOMER3 | 1577 | 1 | 0 | 0 | 0 | 0 | 1 | 0 | 0 | 0 | 0 | 0 | 1 | 0 |
| NM_004839 | HOMER2 | 1949 | 25 | 9 | 1 | 1 | 10 | 13 | 4 | 24 | 3 | 3 | 25 | 5 | 5 |
| NM_004840 | ARHGEF6 | 5270 | 17 | 6 | 17 | 2 | 7 | 6 | 1 | 1 | 8 | 6 | 7 | 10 | 3 |
| NM_004841 | RASAL2 | 9815 | 3 | 2 | 2 | 1 | 3 | 3 | 2 | 1 | 2 | 1 | 1 | 1 | 2 |
| NM_004842 | AKAP7 | 2257 | 2 | 1 | 0 | 1 | 1 | 2 | 0 | 2 | 1 | 1 | 0 | 1 | 1 |
| NM_004843 | IL27RA | 3258 | 2 | 1 | 1 | 0 | 1 | 0 | 0 | 0 | 1 | 0 | 0 | 2 | 0 |
| NM_004844 | SH3BP5 | 2793 | 11 | 7 | 8 | 3 | 3 | 9 | 7 | 2 | 11 | 11 | 4 | 7 | 10 |
| NM_004845 | PCYT1B | 5548 | 2 | 0 | 0 | 0 | 0 | 2 | 0 | 0 | 0 | 0 | 0 | 0 | 1 |
| NM_004846 | EIF4E2 | 1009 | 52 | 23 | 23 | 24 | 17 | 28 | 28 | 52 | 14 | 12 | 15 | 22 | 17 |
| NM_004847 | AIF1 | 1489 | 49 | 10 | 11 | 2 | 10 | 16 | 4 | 3 | 12 | 2 | 1 | 49 | 2 |
| NM_004848 | C1orf38 | 1647 | 19 | 3 | 3 | 1 | 2 | 2 | 0 | 1 | 5 | 1 | 0 | 19 | 3 |
| NM_004849 | ATG5 | 3244 | 5 | 3 | 2 | 1 | 3 | 3 | 4 | 3 | 4 | 4 | 3 | 5 | 5 |
| NM_004850 | ROCK2 | 6401 | 33 | 18 | 22 | 6 | 20 | 20 | 15 | 10 | 19 | 19 | 23 | 33 | 16 |
| NM_004851 | NAPSA | 1392 | 3 | 0 | 0 | 0 | 0 | 0 | 2 | 0 | 3 | 0 | 0 | 0 | 0 |
| NM_004852 | ONECUT2 | 16122 | 2 | 0 | 0 | 0 | 0 | 0 | 0 | 2 | 0 | 0 | 0 | 0 | 0 |
| NM_004853 | STX8 | 968 | 6 | 4 | 5 | 4 | 6 | 6 | 4 | 3 | 4 | 4 | 3 | 5 | 5 |
| NM_004854 | CHST10 | 2815 | 2 | 1 | 0 | 0 | 0 | 2 | 0 | 0 | 0 | 1 | 0 | 1 | 1 |
| NM_004855 | PIGB | 2197 | 2 | 1 | 1 | 1 | 1 | 1 | 2 | 1 | 1 | 1 | 1 | 2 | 1 |
| NM_004856 | KIF23 | 3306 | 4 | 1 | 1 | 1 | 0 | 0 | 1 | 0 | 1 | 1 | 0 | 1 | 4 |
| NM_004857 | AKAP5 | 6487 | 9 | 1 | 0 | 1 | 0 | 9 | 1 | 0 | 1 | 0 | 0 | 1 | 0 |
| NM_004858 | SLC4A8 | 4001 | 10 | 2 | 0 | 0 | 0 | 10 | 1 | 0 | 0 | 2 | 0 | 0 | 3 |
| NM_004859 | CLTC | 8575 | 38 | 20 | 18 | 8 | 17 | 38 | 30 | 25 | 14 | 12 | 7 | 25 | 21 |
| NM_004860 | FXR2 | 2988 | 7 | 4 | 2 | 2 | 7 | 7 | 3 | 2 | 2 | 2 | 7 | 3 | 4 |
| NM_004861 | GAL3ST1 | 1791 | 0 | 0 | 0 | 0 | 0 | 0 | 0 | 0 | 0 | 0 | 0 | 0 | 0 |
| NM_004862 | LITAF | 2630 | 36 | 17 | 22 | 11 | 14 | 11 | 17 | 27 | 24 | 10 | 5 | 36 | 5 |
| NM_004863 | SPTLC2 | 7234 | 12 | 4 | 4 | 3 | 3 | 7 | 5 | 2 | 3 | 4 | 1 | 12 | 3 |
| NM_004864 | GDF15 | 1200 | 0 | 0 | 0 | 0 | 0 | 0 | 0 | 0 | 0 | 0 | 0 | 0 | 0 |

| transcript | gene | transcript length (nt) | max | mean | adipose | colon | heart | hypothala mus | kidney | liver | lung | ovary | skeletalm uscle | spleen | testes |
|---|---|---|---|---|---|---|---|---|---|---|---|---|---|---|---|
| NM_004865 | TBPL1 | 1319 | 3 | 1 | 0 | 0 | 1 | 2 | 0 | 0 | 1 | 1 | 0 | 1 | 3 |
| NM_004866 | SCAMP1 | 6244 | 3 | 1 | 1 | 0 | 1 | 3 | 2 | 0 | 1 | 2 | 1 | 1 | 2 |
| NM_004867 | ITM2A | 1832 | 20 | 8 | 12 | 4 | 10 | 9 | 3 | 1 | 10 | 20 | 3 | 4 | 9 |
| NM_004869 | VPS4B | 3378 | 6 | 3 | 4 | 2 | 2 | 4 | 4 | 4 | 3 | 3 | 1 | 6 | 4 |
| NM_004870 | MPDU1 | 1616 | 7 | 2 | 2 | 3 | 2 | 2 | 5 | 7 | 2 | 1 | 0 | 3 | 1 |
| NM_004871 | GOSR1 | 5215 | 7 | 5 | 4 | 3 | 4 | 7 | 5 | 6 | 3 | 6 | 3 | 5 | 5 |
| NM_004872 | TMEM59 | 1696 | 16 | 10 | 8 | 6 | 10 | 13 | 15 | 16 | 11 | 6 | 5 | 12 | 12 |
| NM_004873 | BAG5 | 4847 | 13 | 6 | 6 | 3 | 6 | 7 | 7 | 3 | 6 | 5 | 4 | 9 | 13 |
| NM_004874 | BAG4 | 2182 | 5 | 2 | 3 | 1 | 1 | 5 | 2 | 2 | 1 | 3 | 1 | 2 | 2 |
| NM_004875 | POLR1C | 1107 | 2 | 1 | 0 | 1 | 1 | 1 | 2 | 1 | 0 | 1 | 1 | 1 | 1 |
| NM_004877 | GMFG | 656 | 46 | 10 | 16 | 5 | 6 | 4 | 2 | 4 | 18 | 1 | 1 | 46 | 2 |
| NM_004878 | PTGES | 1786 | 7 | 2 | 5 | 5 | 2 | 2 | 1 | 0 | 1 | 1 | 1 | 1 | 7 |
| NM_004879 | EI24 | 2261 | 87 | 21 | 18 | 16 | 15 | 21 | 17 | 87 | 12 | 10 | 13 | 14 | 9 |
| NM_004881 | TP53I3 | 1675 | 2 | 1 | 1 | 2 | 1 | 0 | 2 | 1 | 2 | 1 | 0 | 1 | 2 |
| NM_004882 | CIR1 | 1793 | 29 | 15 | 8 | 2 | 18 | 7 | 15 | 3 | 20 | 23 | 17 | 29 | 26 |
| NM_004883 | NRG2 | 3020 | 0 | 0 | 0 | 0 | 0 | 0 | 0 | 0 | 0 | 0 | 0 | 0 | 0 |
| NM_004884 | IGDCC3 | 4479 | 0 | 0 | 0 | 0 | 0 | 0 | 0 | 0 | 0 | 0 | 0 | 0 | 0 |
| NM_004885 | NPFFR2 | 2056 | 0 | 0 | 0 | 0 | 0 | 0 | 0 | 0 | 0 | 0 | 0 | 0 | 0 |
| NM_004886 | APBA3 | 2158 | 0 | 0 | 0 | 0 | 0 | 0 | 0 | 0 | 0 | 0 | 0 | 0 | 0 |
| NM_004887 | CXCL14 | 1975 | 47 | 10 | 20 | 7 | 3 | 4 | 47 | 4 | 8 | 1 | 6 | 5 | 4 |
| NM_004888 | ATP6V1G1 | 1611 | 21 | 14 | 9 | 6 | 14 | 19 | 16 | 19 | 12 | 10 | 9 | 21 | 16 |
| NM_004889 | ATP5J2 | 495 | 14 | 9 | 7 | 5 | 13 | 10 | 14 | 8 | 6 | 2 | 7 | 8 | 12 |
| NM_004890 | SPAG7 | 1025 | 35 | 21 | 19 | 11 | 35 | 35 | 23 | 26 | 13 | 11 | 30 | 17 | 14 |
| NM_004891 | MRPL33 | 518 | 42 | 15 | 13 | 7 | 42 | 18 | 17 | 19 | 9 | 3 | 14 | 12 | 12 |
| NM_004892 | SEC22B | 1821 | 25 | 19 | 24 | 10 | 20 | 19 | 20 | 25 | 22 | 19 | 14 | 23 | 18 |
| NM_004893 | H2AFY | 1883 | 21 | 10 | 11 | 9 | 9 | 10 | 9 | 8 | 17 | 6 | 7 | 21 | 7 |
| NM_004894 | C14orf2 | 652 | 16 | 6 | 6 | 5 | 16 | 13 | 7 | 6 | 2 | 2 | 4 | 3 | 2 |
| NM_004895 | NLRP3 | 4455 | 2 | 1 | 1 | 0 | 0 | 0 | 0 | 0 | 1 | 0 | 0 | 2 | 0 |
| NM_004896 | VPS26A | 2701 | 18 | 10 | 9 | 3 | 11 | 10 | 10 | 9 | 9 | 10 | 7 | 13 | 18 |
| NM_004897 | MINPP1 | 2847 | 1 | 0 | 0 | 0 | 0 | 1 | 1 | 1 | 0 | 0 | 0 | 0 | 0 |
| NM_004898 | CLOCK | 5801 | 14 | 8 | 6 | 2 | 9 | 10 | 7 | 6 | 7 | 7 | 9 | 10 | 14 |
| NM_004899 | BRE | 1890 | 9 | 7 | 6 | 7 | 7 | 9 | 9 | 6 | 4 | 5 | 5 | 8 | 7 |
| NM_004900 | APOBEC3B | 1536 | 1 | 0 | 0 | 1 | 0 | 0 | 0 | 0 | 0 | 0 | 0 | 0 | 0 |
| NM_004901 | ENTPD4 | 2882 | 6 | 3 | 2 | 3 | 5 | 6 | 3 | 2 | 3 | 3 | 1 | 6 | 5 |
| NM_004902 | RBM39 | 2791 | 104 | 61 | 45 | 36 | 51 | 50 | 66 | 26 | 74 | 87 | 56 | 104 | 76 |
| NM_004904 | CREB5 | 8213 | 4 | 1 | 2 | 1 | 2 | 3 | 1 | 0 | 2 | 0 | 1 | 4 | 1 |
| NM_004905 | PRDX6 | 1670 | 55 | 27 | 24 | 9 | 42 | 13 | 21 | 55 | 14 | 13 | 44 | 20 | 37 |
| NM_004906 | WTAP | 2121 | 53 | 41 | 39 | 29 | 35 | 53 | 25 | 38 | 47 | 41 | 39 | 49 | 49 |
| NM_004907 | IER2 | 2046 | 7 | 2 | 2 | 1 | 1 | 2 | 3 | 4 | 3 | 7 | 1 | 2 | 1 |
| NM_004909 | CSAG2 | 796 | 0 | 0 | 0 | 0 | 0 | 0 | 0 | 0 | 0 | 0 | 0 | 0 | 0 |
| NM_004910 | PITPNM1 | 4225 | 0 | 0 | 0 | 0 | 0 | 0 | 0 | 0 | 0 | 0 | 0 | 0 | 0 |
| NM_004911 | PDIA4 | 2952 | 155 | 23 | 8 | 13 | 15 | 6 | 11 | 155 | 10 | 7 | 2 | 23 | 9 |
| NM_004912 | KRIT1 | 4870 | 7 | 4 | 3 | 1 | 4 | 3 | 5 | 2 | 3 | 6 | 3 | 6 | 7 |
| NM_004913 | C16orf7 | 2749 | 0 | 0 | 0 | 0 | 0 | 0 | 0 | 0 | 0 | 0 | 0 | 0 | 0 |
| NM_004914 | RAB36 | 3822 | 0 | 0 | 0 | 0 | 0 | 0 | 0 | 0 | 0 | 0 | 0 | 0 | 0 |
| NM_004915 | ABCG1 | 2995 | 16 | 4 | 1 | 3 | 3 | 4 | 1 | 1 | 6 | 2 | 7 | 16 | 2 |
| NM_004917 | KLK4 | 1347 | 0 | 0 | 0 | 0 | 0 | 0 | 0 | 0 | 0 | 0 | 0 | 0 | 0 |
| NM_004918 | TCL1B | 1144 | 0 | 0 | 0 | 0 | 0 | 0 | 0 | 0 | 0 | 0 | 0 | 0 | 0 |
| NM_004922 | SEC24C | 4530 | 8 | 6 | 5 | 4 | 6 | 6 | 6 | 7 | 4 | 4 | 5 | 8 | 6 |
| NM_004923 | MTL5 | 2535 | 1 | 0 | 0 | 0 | 0 | 0 | 0 | 0 | 0 | 0 | 0 | 0 | 1 |
| NM_004924 | ACTN4 | 3893 | 16 | 9 | 9 | 12 | 6 | 9 | 15 | 9 | 11 | 6 | 3 | 16 | 4 |
| NM_004925 | AQP3 | 1822 | 32 | 7 | 1 | 1 | 2 | 0 | 32 | 18 | 12 | 1 | 0 | 9 | 1 |
| NM_004926 | ZFP36L1 | 3022 | 45 | 21 | 19 | 10 | 15 | 13 | 20 | 17 | 38 | 45 | 13 | 23 | 22 |
| NM_004927 | MRPL49 | 2089 | 3 | 2 | 1 | 1 | 1 | 2 | 3 | 3 | 1 | 1 | 1 | 2 | 2 |
| NM_004928 | C21orf2 | 2233 | 1 | 1 | 0 | 1 | 0 | 1 | 1 | 0 | 0 | 1 | 0 | 1 | 1 |
| NM_004929 | CALB1 | 2531 | 93 | 10 | 0 | 0 | 0 | 17 | 93 | 0 | 0 | 0 | 0 | 0 | 0 |
| NM_004930 | CAPZB | 1710 | 61 | 34 | 25 | 23 | 39 | 33 | 31 | 19 | 33 | 38 | 29 | 61 | 41 |
| NM_004931 | CD8B | 1424 | 1 | 0 | 0 | 0 | 0 | 0 | 0 | 0 | 0 | 0 | 0 | 1 | 0 |
| NM_004932 | CDH6 | 4521 | 29 | 4 | 2 | 0 | 1 | 3 | 29 | 0 | 2 | 1 | 1 | 0 | 1 |
| NM_004933 | CDH15 | 2859 | 0 | 0 | 0 | 0 | 0 | 0 | 0 | 0 | 0 | 0 | 0 | 0 | 0 |
| NM_004934 | CDH18 | 3085 | 10 | 1 | 0 | 0 | 0 | 10 | 0 | 0 | 0 | 0 | 0 | 0 | 1 |
| NM_004935 | CDK5 | 1193 | 28 | 5 | 3 | 2 | 2 | 28 | 3 | 4 | 2 | 1 | 1 | 4 | 2 |
| NM_004936 | CDKN2B | 3861 | 31 | 5 | 15 | 31 | 1 | 0 | 0 | 1 | 3 | 0 | 2 | 3 | 1 |
| NM_004937 | CTNS | 4431 | 1 | 0 | 0 | 1 | 0 | 0 | 1 | 0 | 0 | 0 | 0 | 0 | 1 |
| NM_004938 | DAPK1 | 5938 | 6 | 3 | 2 | 3 | 1 | 5 | 3 | 3 | 5 | 6 | 0 | 6 | 2 |
| NM_004939 | DDX1 | 2706 | 18 | 11 | 11 | 4 | 18 | 18 | 13 | 12 | 5 | 8 | 14 | 9 | 10 |
| NM_004941 | DHX8 | 4200 | 5 | 3 | 3 | 3 | 2 | 3 | 3 | 3 | 2 | 2 | 2 | 4 | 5 |
| NM_004942 | DEFB4 | 336 | 0 | 0 | 0 | 0 | 0 | 0 | 0 | 0 | 0 | 0 | 0 | 0 | 0 |
| NM_004943 | DMWD | 3308 | 3 | 2 | 1 | 2 | 2 | 3 | 1 | 0 | 1 | 3 | 1 | 2 | 2 |
| NM_004944 | DNASE1L3 | 1463 | 5 | 1 | 0 | 1 | 0 | 0 | 2 | 4 | 0 | 0 | 0 | 5 | 0 |
| NM_004945 | DNM2 | 3621 | 12 | 7 | 5 | 12 | 9 | 6 | 9 | 5 | 7 | 3 | 6 | 10 | 8 |
| NM_004946 | DOCK2 | 6095 | 18 | 3 | 4 | 2 | 2 | 2 | 0 | 1 | 5 | 1 | 0 | 18 | 1 |
| NM_004947 | DOCK3 | 8714 | 10 | 1 | 0 | 0 | 0 | 10 | 0 | 0 | 0 | 0 | 0 | 0 | 1 |
| NM_004948 | DSC1 | 4254 | 3 | 0 | 0 | 0 | 3 | 0 | 0 | 0 | 0 | 0 | 0 | 0 | 0 |
| NM_004949 | DSC2 | 5245 | 10 | 4 | 3 | 10 | 8 | 2 | 5 | 4 | 4 | 1 | 1 | 7 | 1 |
| NM_004950 | EPYC | 1537 | 0 | 0 | 0 | 0 | 0 | 0 | 0 | 0 | 0 | 0 | 0 | 0 | 0 |
| NM_004951 | GPR183 | 1785 | 12 | 4 | 5 | 1 | 3 | 3 | 1 | 1 | 12 | 2 | 1 | 12 | 2 |

| | | transcript | | | | | | hypothala | | | | | skeletalm | | |
|---|---|---|---|---|---|---|---|---|---|---|---|---|---|---|---|
| transcript | gene | length (nt) | max | mean | adipose | colon | heart | mus | kidney | liver | lung | ovary | uscle | spleen | testes |
| NM_004952 | EFNA3 | 1782 | 1 | 0 | 0 | 0 | 0 | 1 | 0 | 0 | 0 | 0 | 0 | 0 | 0 |
| NM_004953 | EIF4G1 | 4868 | 70 | 33 | 26 | 17 | 39 | 26 | 31 | 70 | 25 | 17 | 47 | 33 | 28 |
| NM_004954 | MARK2 | 4536 | 5 | 3 | 2 | 3 | 2 | 5 | 2 | 2 | 2 | 2 | 2 | 5 | 2 |
| NM_004955 | SLC29A1 | 2323 | 5 | 2 | 4 | 2 | 5 | 1 | 2 | 1 | 2 | 1 | 3 | 2 | 1 |
| NM_004956 | ETV1 | 6806 | 5 | 1 | 0 | 0 | 2 | 5 | 0 | 0 | 4 | 0 | 0 | 1 | 2 |
| NM_004957 | FPGS | 2487 | 1 | 0 | 0 | 0 | 0 | 0 | 0 | 1 | 1 | 0 | 0 | 0 | 0 |
| NM_004958 | MTOR | 8724 | 7 | 3 | 2 | 7 | 3 | 5 | 3 | 5 | 1 | 1 | 3 | 3 | 4 |
| NM_004959 | NR5A1 | 3095 | 5 | 1 | 0 | 0 | 0 | 0 | 0 | 0 | 0 | 1 | 0 | 5 | 1 |
| NM_004960 | FUS | 5117 | 28 | 19 | 10 | 15 | 19 | 28 | 24 | 12 | 13 | 27 | 15 | 26 | 25 |
| NM_004961 | GABRE | 3152 | 3 | 1 | 3 | 1 | 1 | 1 | 0 | 0 | 0 | 0 | 0 | 0 | 0 |
| NM_004962 | GDF10 | 2674 | 1 | 0 | 1 | 0 | 0 | 0 | 0 | 0 | 0 | 0 | 0 | 0 | 1 |
| NM_004963 | GUCY2C | 3841 | 4 | 0 | 0 | 4 | 0 | 0 | 0 | 0 | 0 | 0 | 0 | 0 | 0 |
| NM_004964 | HDAC1 | 2091 | 19 | 9 | 5 | 19 | 6 | 5 | 8 | 9 | 7 | 9 | 1 | 16 | 10 |
| NM_004965 | HMGN1 | 1304 | 15 | 9 | 6 | 8 | 9 | 6 | 13 | 8 | 11 | 11 | 2 | 15 | 11 |
| NM_004966 | HNRNPF | 2633 | 21 | 12 | 7 | 9 | 10 | 10 | 14 | 17 | 13 | 9 | 7 | 21 | 12 |
| NM_004967 | IBSP | 1591 | 1 | 0 | 0 | 0 | 0 | 1 | 0 | 0 | 0 | 0 | 0 | 0 | 0 |
| NM_004968 | ICA1 | 2385 | 5 | 2 | 2 | 2 | 5 | 3 | 2 | 0 | 1 | 0 | 0 | 1 | 2 |
| NM_004969 | IDE | 5896 | 6 | 3 | 2 | 1 | 4 | 2 | 3 | 5 | 2 | 2 | 6 | 3 | 3 |
| NM_004970 | IGFALS | 2084 | 0 | 0 | 0 | 0 | 0 | 0 | 0 | 0 | 0 | 0 | 0 | 0 | 0 |
| NM_004972 | JAK2 | 5284 | 24 | 13 | 7 | 4 | 19 | 6 | 8 | 2 | 16 | 13 | 20 | 24 | 19 |
| NM_004973 | JARID2 | 5756 | 4 | 2 | 1 | 1 | 1 | 2 | 1 | 0 | 2 | 2 | 1 | 4 | 4 |
| NM_004974 | KCNA2 | 2125 | 7 | 1 | 1 | 0 | 0 | 7 | 0 | 0 | 0 | 0 | 0 | 0 | 0 |
| NM_004975 | KCNB1 | 3756 | 6 | 1 | 2 | 1 | 0 | 6 | 0 | 1 | 0 | 0 | 1 | 0 | 1 |
| NM_004976 | KCNC1 | 6791 | 1 | 0 | 0 | 0 | 0 | 1 | 0 | 0 | 0 | 0 | 0 | 0 | 0 |
| NM_004977 | KCNC3 | 3176 | 0 | 0 | 0 | 0 | 0 | 0 | 0 | 0 | 0 | 0 | 0 | 0 | 0 |
| NM_004978 | KCNC4 | 3700 | 4 | 1 | 0 | 1 | 1 | 4 | 1 | 0 | 0 | 1 | 0 | 1 | 1 |
| NM_004979 | KCND1 | 4720 | 0 | 0 | 0 | 0 | 0 | 0 | 0 | 0 | 0 | 0 | 0 | 0 | 0 |
| NM_004980 | KCND3 | 2693 | 1 | 0 | 0 | 0 | 1 | 1 | 0 | 0 | 0 | 0 | 0 | 0 | 0 |
| NM_004981 | KCNJ4 | 1902 | 4 | 1 | 0 | 0 | 2 | 4 | 0 | 0 | 0 | 0 | 0 | 0 | 0 |
| NM_004982 | KCNJ8 | 2381 | 5 | 1 | 1 | 0 | 5 | 1 | 1 | 4 | 1 | 1 | 1 | 1 | 0 |
| NM_004983 | KCNJ9 | 3029 | 28 | 3 | 0 | 1 | 0 | 28 | 0 | 0 | 0 | 0 | 0 | 0 | 0 |
| NM_004984 | KIF5A | 3897 | 58 | 6 | 0 | 2 | 0 | 58 | 0 | 0 | 0 | 0 | 0 | 0 | 1 |
| NM_004985 | KRAS | 5297 | 13 | 7 | 3 | 3 | 6 | 13 | 7 | 6 | 7 | 6 | 4 | 11 | 8 |
| NM_004986 | KTN1 | 4556 | 137 | 69 | 51 | 17 | 137 | 43 | 71 | 43 | 58 | 44 | 113 | 64 | 119 |
| NM_004987 | LIMS1 | 1531 | 4 | 3 | 3 | 1 | 4 | 1 | 3 | 2 | 4 | 3 | 4 | 4 | 3 |
| NM_004988 | MAGEA1 | 1709 | 1 | 0 | 0 | 0 | 0 | 0 | 0 | 0 | 0 | 0 | 0 | 0 | 1 |
| NM_004990 | MARS | 2795 | 11 | 5 | 4 | 3 | 4 | 11 | 4 | 8 | 3 | 1 | 3 | 6 | 5 |
| NM_004991 | MECOM | 5533 | 17 | 4 | 3 | 5 | 4 | 0 | 17 | 0 | 7 | 1 | 2 | 3 | 2 |
| NM_004992 | MECP2 | 10241 | 2 | 1 | 1 | 1 | 1 | 2 | 1 | 0 | 1 | 2 | 1 | 2 | 1 |
| NM_004993 | ATXN3 | 6923 | 4 | 2 | 2 | 1 | 2 | 3 | 2 | 1 | 2 | 4 | 1 | 4 | 4 |
| NM_004994 | MMP9 | 2336 | 2 | 1 | 2 | 0 | 0 | 0 | 0 | 0 | 1 | 0 | 0 | 2 | 0 |
| NM_004995 | MMP14 | 3538 | 6 | 3 | 6 | 3 | 3 | 1 | 2 | 1 | 4 | 4 | 1 | 3 | 2 |
| NM_004996 | ABCC1 | 6563 | 3 | 1 | 1 | 1 | 1 | 1 | 1 | 0 | 1 | 1 | 1 | 2 | 3 |
| NM_004997 | MYBPH | 1806 | 2 | 0 | 0 | 0 | 0 | 0 | 0 | 0 | 0 | 0 | 2 | 0 | 0 |
| NM_004998 | MYO1E | 4715 | 11 | 6 | 7 | 7 | 4 | 4 | 8 | 11 | 5 | 4 | 3 | 7 | 5 |
| NM_004999 | MYO6 | 8662 | 15 | 4 | 2 | 2 | 1 | 4 | 15 | 2 | 5 | 2 | 1 | 2 | 7 |
| NM_005000 | NDUFA5 | 1550 | 38 | 15 | 6 | 2 | 36 | 38 | 24 | 11 | 7 | 8 | 18 | 9 | 10 |
| NM_005001 | NDUFA7 | 535 | 84 | 31 | 10 | 10 | 76 | 16 | 41 | 19 | 22 | 15 | 84 | 35 | 17 |
| NM_005002 | NDUFA9 | 1269 | 28 | 13 | 6 | 10 | 28 | 18 | 18 | 17 | 4 | 5 | 15 | 9 | 5 |
| NM_005003 | NDUFAB1 | 667 | 24 | 9 | 7 | 6 | 24 | 13 | 11 | 13 | 2 | 2 | 8 | 6 | 4 |
| NM_005004 | NDUFB8 | 686 | 70 | 29 | 24 | 38 | 42 | 70 | 31 | 40 | 17 | 10 | 15 | 20 | 13 |
| NM_005005 | NDUFB9 | 719 | 45 | 25 | 23 | 17 | 45 | 33 | 37 | 33 | 12 | 6 | 39 | 15 | 21 |
| NM_005006 | NDUFS1 | 3417 | 46 | 21 | 13 | 9 | 46 | 30 | 29 | 26 | 7 | 9 | 31 | 14 | 15 |
| NM_005007 | NFKBIL1 | 1482 | 1 | 1 | 0 | 1 | 1 | 1 | 1 | 0 | 1 | 1 | 1 | 1 | 1 |
| NM_005008 | NHP2L1 | 1581 | 11 | 5 | 2 | 2 | 8 | 11 | 6 | 3 | 4 | 6 | 6 | 5 | 4 |
| NM_005009 | NME4 | 1007 | 1 | 1 | 1 | 1 | 1 | 0 | 0 | 0 | 0 | 0 | 0 | 0 | 0 |
| NM_005010 | NRCAM | 6296 | 18 | 2 | 1 | 0 | 0 | 18 | 2 | 0 | 0 | 1 | 0 | 4 | 1 |
| NM_005011 | NRF1 | 3530 | 1 | 1 | 0 | 1 | 0 | 1 | 0 | 0 | 1 | 1 | 1 | 1 | 1 |
| NM_005012 | ROR1 | 3382 | 3 | 1 | 1 | 3 | 1 | 0 | 0 | 0 | 1 | 2 | 0 | 1 | 1 |
| NM_005013 | NUCB2 | 1612 | 75 | 28 | 18 | 5 | 48 | 18 | 17 | 75 | 29 | 23 | 7 | 25 | 47 |
| NM_005014 | OMD | 2542 | 1 | 1 | 1 | 0 | 1 | 0 | 0 | 0 | 1 | 1 | 1 | 1 | 1 |
| NM_005015 | OXA1L | 1719 | 20 | 13 | 10 | 8 | 16 | 9 | 14 | 15 | 12 | 12 | 20 | 18 | 10 |
| NM_005016 | PCBP2 | 3171 | 43 | 24 | 19 | 8 | 26 | 17 | 33 | 18 | 20 | 43 | 20 | 31 | 30 |
| NM_005017 | PCYT1A | 1583 | 5 | 3 | 5 | 2 | 4 | 2 | 3 | 2 | 2 | 2 | 2 | 4 | 2 |
| NM_005018 | PDCD1 | 2112 | 0 | 0 | 0 | 0 | 0 | 0 | 0 | 0 | 0 | 0 | 0 | 0 | 0 |
| NM_005019 | PDE1A | 2414 | 10 | 2 | 1 | 0 | 3 | 10 | 6 | 0 | 1 | 1 | 0 | 0 | 2 |
| NM_005020 | PDE1C | 2694 | 20 | 3 | 1 | 1 | 20 | 9 | 2 | 0 | 2 | 1 | 0 | 0 | 2 |
| NM_005021 | ENPP3 | 3165 | 3 | 1 | 0 | 2 | 0 | 0 | 3 | 1 | 0 | 0 | 0 | 0 | 0 |
| NM_005022 | PFN1 | 807 | 80 | 32 | 35 | 45 | 30 | 13 | 38 | 34 | 42 | 10 | 10 | 80 | 12 |
| NM_005023 | PGGT1B | 2727 | 3 | 2 | 3 | 2 | 2 | 2 | 2 | 2 | 2 | 2 | 1 | 3 | 2 |
| NM_005024 | SERPINB10 | 1194 | 1 | 0 | 0 | 0 | 0 | 0 | 0 | 0 | 0 | 0 | 0 | 1 | 0 |
| NM_005025 | SERPINI1 | 1910 | 32 | 4 | 0 | 0 | 1 | 32 | 2 | 0 | 0 | 0 | 0 | 1 | 2 |
| NM_005026 | PIK3CD | 5411 | 2 | 0 | 0 | 0 | 0 | 0 | 0 | 0 | 0 | 0 | 0 | 2 | 0 |
| NM_005027 | PIK3R2 | 3938 | 6 | 2 | 1 | 3 | 2 | 6 | 2 | 2 | 1 | 3 | 2 | 2 | 2 |
| NM_005028 | PIP4K2A | 3833 | 18 | 5 | 3 | 2 | 3 | 18 | 3 | 2 | 5 | 2 | 1 | 15 | 2 |
| NM_005029 | PITX3 | 1388 | 0 | 0 | 0 | 0 | 0 | 0 | 0 | 0 | 0 | 0 | 0 | 0 | 0 |
| NM_005030 | PLK1 | 2169 | 1 | 0 | 1 | 1 | 0 | 0 | 0 | 0 | 0 | 0 | 0 | 0 | 1 |

| transcript | gene | transcript length (nt) | max | mean | adipose | colon | heart | hypothalamus | kidney | liver | lung | ovary | skeletalmuscle | spleen | testes |
|---|---|---|---|---|---|---|---|---|---|---|---|---|---|---|---|
| NM_005031 | FXYD1 | 547 | 18 | 5 | 1 | 2 | 11 | 4 | 1 | 6 | 3 | 3 | 18 | 1 | 1 |
| NM_005032 | PLS3 | 3392 | 25 | 12 | 12 | 2 | 13 | 8 | 13 | 18 | 25 | 14 | 5 | 16 | 8 |
| NM_005033 | EXOSC9 | 1593 | 2 | 1 | 2 | 1 | 1 | 2 | 1 | 1 | 1 | 1 | 1 | 1 | 2 |
| NM_005034 | POLR2K | 971 | 28 | 14 | 14 | 6 | 14 | 28 | 14 | 15 | 11 | 10 | 7 | 18 | 13 |
| NM_005035 | POLRMT | 3800 | 1 | 0 | 0 | 0 | 0 | 0 | 1 | 0 | 0 | 0 | 1 | 0 | 1 |
| NM_005036 | PPARA | 10049 | 9 | 4 | 1 | 3 | 7 | 2 | 9 | 8 | 1 | 3 | 3 | 3 | 1 |
| NM_005037 | PPARG | 1818 | 23 | 6 | 23 | 19 | 3 | 1 | 4 | 2 | 4 | 4 | 1 | 5 | 2 |
| NM_005038 | PPID | 1834 | 14 | 10 | 8 | 5 | 14 | 14 | 10 | 8 | 6 | 7 | 14 | 13 | 11 |
| NM_005039 | PRB1 | 1173 | 0 | 0 | 0 | 0 | 0 | 0 | 0 | 0 | 0 | 0 | 0 | 0 | 0 |
| NM_005040 | PRCP | 2143 | 10 | 4 | 4 | 1 | 5 | 5 | 10 | 5 | 5 | 4 | 1 | 6 | 3 |
| NM_005041 | PRF1 | 2508 | 1 | 0 | 0 | 0 | 0 | 0 | 0 | 0 | 0 | 0 | 0 | 1 | 0 |
| NM_005042 | PRH2 | 4323 | 1 | 0 | 0 | 0 | 0 | 0 | 1 | 0 | 1 | 1 | 0 | 1 | 0 |
| NM_005044 | PRKX | 6043 | 2 | 1 | 0 | 1 | 0 | 1 | 2 | 0 | 1 | 1 | 0 | 2 | 2 |
| NM_005045 | RELN | 11569 | 6 | 1 | 0 | 1 | 0 | 6 | 0 | 6 | 0 | 1 | 0 | 0 | 1 |
| NM_005046 | KLK7 | 1927 | 1 | 0 | 0 | 0 | 0 | 1 | 0 | 0 | 0 | 0 | 0 | 0 | 0 |
| NM_005047 | PSMD5 | 3386 | 5 | 4 | 4 | 2 | 3 | 4 | 3 | 5 | 3 | 4 | 2 | 5 | 5 |
| NM_005048 | PTH2R | 2713 | 0 | 0 | 0 | 0 | 0 | 0 | 0 | 0 | 0 | 0 | 0 | 0 | 0 |
| NM_005049 | PWP2 | 3260 | 2 | 1 | 1 | 1 | 1 | 2 | 1 | 1 | 1 | 1 | 1 | 2 | 1 |
| NM_005050 | ABCD4 | 3157 | 4 | 2 | 2 | 2 | 2 | 2 | 4 | 2 | 1 | 1 | 1 | 3 | 1 |
| NM_005051 | QARS | 2430 | 14 | 9 | 11 | 14 | 8 | 9 | 9 | 12 | 6 | 9 | 8 | 10 | 5 |
| NM_005052 | RAC3 | 1060 | 0 | 0 | 0 | 0 | 0 | 0 | 0 | 0 | 0 | 0 | 0 | 0 | 0 |
| NM_005053 | RAD23A | 1793 | 40 | 9 | 7 | 5 | 9 | 9 | 6 | 8 | 5 | 3 | 40 | 6 | 6 |
| NM_005054 | RGPD5 | 7165 | 0 | 0 | 0 | 0 | 0 | 0 | 0 | 0 | 0 | 0 | 0 | 0 | 0 |
| NM_005055 | RAPSN | 1664 | 2 | 0 | 0 | 0 | 0 | 0 | 0 | 0 | 0 | 0 | 2 | 0 | 0 |
| NM_005056 | KDM5A | 10932 | 7 | 4 | 3 | 2 | 3 | 3 | 4 | 2 | 4 | 5 | 2 | 7 | 6 |
| NM_005057 | RBBP5 | 3683 | 6 | 4 | 3 | 3 | 3 | 6 | 5 | 4 | 3 | 4 | 4 | 5 | 4 |
| NM_005058 | RBMY1A1 | 1887 | 0 | 0 | 0 | 0 | 0 | 0 | 0 | 0 | 0 | 0 | 0 | 0 | 0 |
| NM_005059 | RLN2 | 889 | 0 | 0 | 0 | 0 | 0 | 0 | 0 | 0 | 0 | 0 | 0 | 0 | 0 |
| NM_005060 | RORC | 3066 | 14 | 2 | 0 | 0 | 1 | 0 | 2 | 4 | 0 | 0 | 14 | 0 | 2 |
| NM_005061 | RPL3L | 1529 | 26 | 3 | 0 | 0 | 11 | 0 | 0 | 0 | 0 | 0 | 26 | 0 | 0 |
| NM_005063 | SCD | 5468 | 173 | 32 | 173 | 11 | 11 | 71 | 5 | 32 | 21 | 7 | 6 | 7 | 13 |
| NM_005064 | CCL23 | 625 | 2 | 0 | 0 | 0 | 1 | 0 | 0 | 1 | 1 | 0 | 0 | 2 | 0 |
| NM_005065 | SEL1L | 6577 | 30 | 11 | 10 | 4 | 11 | 11 | 10 | 30 | 8 | 7 | 6 | 14 | 7 |
| NM_005066 | SFPQ | 3071 | 86 | 62 | 57 | 61 | 53 | 86 | 59 | 45 | 35 | 74 | 46 | 84 | 79 |
| NM_005067 | SIAH2 | 2620 | 2 | 1 | 1 | 1 | 1 | 1 | 0 | 2 | 1 | 1 | 0 | 1 | 1 |
| NM_005068 | SIM1 | 3995 | 5 | 1 | 1 | 0 | 0 | 0 | 5 | 0 | 0 | 0 | 2 | 0 | 0 |
| NM_005069 | SIM2 | 4177 | 1 | 0 | 0 | 0 | 0 | 0 | 1 | 0 | 0 | 0 | 0 | 0 | 0 |
| NM_005070 | SLC4A3 | 4220 | 8 | 1 | 0 | 0 | 8 | 4 | 0 | 0 | 0 | 2 | 0 | 0 | 0 |
| NM_005071 | SLC1A6 | 1719 | 1 | 0 | 0 | 0 | 0 | 1 | 0 | 0 | 0 | 0 | 0 | 0 | 1 |
| NM_005072 | SLC12A4 | 4765 | 5 | 3 | 3 | 2 | 5 | 2 | 2 | 3 | 3 | 2 | 2 | 2 | 3 |
| NM_005073 | SLC15A1 | 3125 | 3 | 1 | 0 | 0 | 0 | 0 | 3 | 3 | 0 | 0 | 0 | 0 | 0 |
| NM_005074 | SLC17A1 | 1843 | 13 | 1 | 0 | 0 | 0 | 0 | 13 | 0 | 0 | 0 | 0 | 0 | 0 |
| NM_005076 | CNTN2 | 7637 | 8 | 1 | 0 | 0 | 0 | 8 | 0 | 0 | 0 | 0 | 0 | 0 | 0 |
| NM_005077 | TLE1 | 3269 | 2 | 1 | 1 | 1 | 0 | 1 | 0 | 1 | 0 | 1 | 1 | 2 | 1 |
| NM_005078 | TLE3 | 5331 | 5 | 2 | 3 | 3 | 1 | 2 | 2 | 1 | 3 | 2 | 1 | 5 | 2 |
| NM_005079 | TPD52 | 3959 | 12 | 4 | 1 | 8 | 1 | 12 | 7 | 4 | 3 | 0 | 1 | 4 | 2 |
| NM_005080 | XBP1 | 1820 | 105 | 29 | 12 | 33 | 21 | 14 | 18 | 105 | 39 | 14 | 8 | 48 | 9 |
| NM_005082 | TRIM25 | 5744 | 4 | 2 | 2 | 2 | 1 | 1 | 1 | 2 | 2 | 2 | 1 | 4 | 2 |
| NM_005084 | PLA2G7 | 1839 | 32 | 7 | 23 | 1 | 2 | 1 | 0 | 1 | 13 | 3 | 0 | 32 | 1 |
| NM_005085 | NUP214 | 6601 | 9 | 4 | 3 | 3 | 3 | 3 | 5 | 2 | 2 | 6 | 4 | 6 | 9 |
| NM_005086 | SSPN | 4529 | 5 | 2 | 3 | 1 | 3 | 3 | 1 | 0 | 1 | 4 | 5 | 1 | 3 |
| NM_005087 | FXR1 | 2761 | 158 | 42 | 23 | 8 | 50 | 37 | 22 | 15 | 26 | 41 | 158 | 28 | 51 |
| NM_005088 | SFRS17A | 3208 | 3 | 2 | 2 | 2 | 1 | 1 | 2 | 0 | 2 | 2 | 1 | 3 | 2 |
| NM_005089 | ZRSR2 | 1511 | 14 | 10 | 11 | 8 | 10 | 12 | 8 | 4 | 12 | 11 | 11 | 14 | 7 |
| NM_005090 | JMJD7-PLA2 | 3367 | 0 | 0 | 0 | 0 | 0 | 0 | 0 | 0 | 0 | 0 | 0 | 0 | 0 |
| NM_005091 | PGLYRP1 | 690 | 0 | 0 | 0 | 0 | 0 | 0 | 0 | 0 | 0 | 0 | 0 | 0 | 0 |
| NM_005092 | TNFSF18 | 748 | 0 | 0 | 0 | 0 | 0 | 0 | 0 | 0 | 0 | 0 | 0 | 0 | 0 |
| NM_005093 | CBFA2T2 | 7737 | 2 | 1 | 1 | 2 | 1 | 2 | 1 | 0 | 1 | 2 | 1 | 1 | 2 |
| NM_005094 | SLC27A4 | 2948 | 1 | 0 | 0 | 0 | 0 | 1 | 1 | 1 | 0 | 0 | 0 | 0 | 0 |
| NM_005095 | ZMYM4 | 6994 | 9 | 5 | 4 | 2 | 5 | 7 | 5 | 2 | 4 | 9 | 4 | 7 | 9 |
| NM_005096 | ZMYM3 | 5517 | 2 | 1 | 0 | 1 | 0 | 1 | 1 | 0 | 1 | 2 | 0 | 1 | 2 |
| NM_005097 | LGI1 | 2366 | 6 | 1 | 0 | 0 | 0 | 6 | 0 | 0 | 0 | 0 | 0 | 0 | 0 |
| NM_005098 | MSC | 2058 | 1 | 0 | 1 | 0 | 0 | 0 | 0 | 0 | 0 | 0 | 0 | 0 | 0 |
| NM_005099 | ADAMTS4 | 4332 | 5 | 2 | 3 | 1 | 5 | 4 | 1 | 1 | 5 | 3 | 1 | 1 | 1 |
| NM_005100 | AKAP12 | 8432 | 75 | 30 | 39 | 11 | 18 | 41 | 18 | 5 | 30 | 62 | 7 | 24 | 75 |
| NM_005101 | ISG15 | 666 | 2 | 1 | 1 | 0 | 2 | 1 | 0 | 1 | 1 | 0 | 2 | 0 | 0 |
| NM_005102 | FEZ2 | 2014 | 36 | 14 | 18 | 7 | 12 | 20 | 10 | 6 | 8 | 9 | 36 | 14 | 15 |
| NM_005103 | FEZ1 | 1674 | 55 | 7 | 2 | 2 | 4 | 55 | 1 | 1 | 3 | 3 | 1 | 4 | 5 |
| NM_005104 | BRD2 | 4894 | 36 | 22 | 15 | 16 | 18 | 23 | 19 | 14 | 20 | 29 | 17 | 36 | 30 |
| NM_005105 | RBM8A | 2787 | 8 | 5 | 4 | 2 | 5 | 7 | 6 | 5 | 4 | 4 | 8 | 8 | 6 |
| NM_005107 | EXOG | 3146 | 2 | 1 | 1 | 1 | 2 | 2 | 1 | 0 | 1 | 1 | 0 | 2 | 1 |
| NM_005108 | XYLB | 3683 | 7 | 2 | 1 | 1 | 0 | 0 | 7 | 5 | 0 | 2 | 0 | 1 | 1 |
| NM_005109 | OXSR1 | 4523 | 8 | 6 | 6 | 3 | 8 | 4 | 8 | 4 | 6 | 5 | 7 | 8 | 8 |
| NM_005110 | GFPT2 | 3011 | 7 | 3 | 4 | 3 | 7 | 4 | 1 | 0 | 4 | 5 | 2 | 1 | 5 |
| NM_005112 | WDR1 | 2723 | 7 | 4 | 4 | 4 | 6 | 6 | 7 | 3 | 4 | 4 | 2 | 6 | 3 |
| NM_005113 | GOLGA5 | 2891 | 19 | 10 | 10 | 3 | 9 | 10 | 8 | 17 | 6 | 8 | 5 | 11 | 19 |
| NM_005114 | HS3ST1 | 1965 | 22 | 3 | 1 | 0 | 1 | 1 | 4 | 0 | 1 | 22 | 0 | 2 | 3 |

| transcript | gene | transcript length (nt) | max | mean | adipose | colon | heart | hypothalamus | kidney | liver | lung | ovary | skeletalmuscle | spleen | testes |
|---|---|---|---|---|---|---|---|---|---|---|---|---|---|---|---|
| NM_005115 | MVP | 2793 | 17 | 8 | 6 | 17 | 5 | 3 | 15 | 6 | 9 | 4 | 2 | 9 | 7 |
| NM_005116 | SLC23A2 | 6944 | 17 | 6 | 3 | 2 | 2 | 17 | 3 | 10 | 3 | 13 | 1 | 9 | 5 |
| NM_005117 | FGF19 | 2137 | 0 | 0 | 0 | 0 | 0 | 0 | 0 | 0 | 0 | 0 | 0 | 0 | 0 |
| NM_005118 | TNFSF15 | 1993 | 1 | 0 | 0 | 0 | 0 | 0 | 1 | 0 | 0 | 0 | 0 | 0 | 0 |
| NM_005119 | THRAP3 | 4431 | 35 | 25 | 21 | 12 | 25 | 22 | 20 | 32 | 20 | 26 | 35 | 32 | 35 |
| NM_005120 | MED12 | 6969 | 4 | 2 | 2 | 2 | 1 | 2 | 2 | 1 | 2 | 4 | 1 | 4 | 3 |
| NM_005121 | MED13 | 10465 | 9 | 6 | 4 | 3 | 5 | 6 | 6 | 5 | 6 | 7 | 7 | 9 | 7 |
| NM_005122 | NR1I3 | 1381 | 4 | 1 | 0 | 0 | 0 | 0 | 1 | 4 | 0 | 0 | 0 | 0 | 0 |
| NM_005123 | NR1H4 | 2143 | 61 | 9 | 0 | 5 | 0 | 0 | 21 | 61 | 0 | 9 | 0 | 0 | 0 |
| NM_005124 | NUP153 | 5687 | 4 | 2 | 2 | 1 | 2 | 2 | 2 | 2 | 3 | 3 | 3 | 4 | 3 |
| NM_005125 | CCS | 1032 | 13 | 5 | 3 | 5 | 5 | 6 | 5 | 13 | 3 | 5 | 3 | 5 | 5 |
| NM_005126 | NR1D2 | 5259 | 17 | 7 | 7 | 3 | 4 | 10 | 5 | 5 | 5 | 17 | 12 | 5 | 6 |
| NM_005127 | CLEC2B | 2038 | 17 | 6 | 7 | 0 | 5 | 1 | 1 | 1 | 16 | 11 | 7 | 17 | 3 |
| NM_005128 | DOPEY2 | 7679 | 2 | 1 | 1 | 2 | 1 | 1 | 1 | 2 | 1 | 0 | 1 | 1 | 0 |
| NM_005130 | FGFBP1 | 1182 | 1 | 0 | 0 | 1 | 0 | 0 | 0 | 0 | 0 | 0 | 0 | 0 | 0 |
| NM_005131 | THOC1 | 2120 | 8 | 3 | 3 | 1 | 3 | 3 | 4 | 2 | 2 | 4 | 2 | 5 | 8 |
| NM_005132 | REC8 | 2230 | 1 | 0 | 0 | 1 | 0 | 1 | 1 | 0 | 0 | 1 | 0 | 1 | 1 |
| NM_005133 | RCE1 | 1471 | 0 | 0 | 0 | 0 | 0 | 0 | 0 | 0 | 0 | 0 | 0 | 0 | 0 |
| NM_005134 | PPP4R1 | 3915 | 10 | 7 | 5 | 4 | 8 | 6 | 6 | 5 | 9 | 7 | 4 | 10 | 10 |
| NM_005135 | SLC12A6 | 7327 | 4 | 2 | 1 | 1 | 2 | 3 | 3 | 1 | 2 | 1 | 1 | 4 | 3 |
| NM_005137 | DGCR2 | 4484 | 18 | 7 | 4 | 5 | 7 | 18 | 7 | 5 | 5 | 12 | 3 | 7 | 6 |
| NM_005138 | SCO2 | 991 | 0 | 0 | 0 | 0 | 0 | 0 | 0 | 0 | 0 | 0 | 0 | 0 | 0 |
| NM_005139 | ANXA3 | 1618 | 9 | 2 | 1 | 1 | 3 | 2 | 3 | 0 | 9 | 0 | 1 | 3 | 1 |
| NM_005140 | CNGA2 | 3285 | 0 | 0 | 0 | 0 | 0 | 0 | 0 | 0 | 0 | 0 | 0 | 0 | 0 |
| NM_005141 | FGB | 1951 | 6,807 | 639 | 0 | 10 | 29 | 0 | 17 | 6,807 | 13 | 40 | 23 | 49 | 34 |
| NM_005142 | GIF | 1513 | 0 | 0 | 0 | 0 | 0 | 0 | 0 | 0 | 0 | 0 | 0 | 0 | 0 |
| NM_005143 | HP | 1431 | 66,644 | 6,226 | 9 | 63 | 279 | 0 | 104 | 66,644 | 194 | 282 | 230 | 371 | 316 |
| NM_005144 | HR | 5514 | 1 | 0 | 0 | 1 | 0 | 0 | 0 | 0 | 0 | 0 | 0 | 0 | 1 |
| NM_005146 | SART1 | 3601 | 7 | 4 | 4 | 4 | 4 | 5 | 3 | 3 | 3 | 4 | 4 | 7 | 5 |
| NM_005147 | DNAJA3 | 2708 | 12 | 4 | 2 | 3 | 4 | 4 | 5 | 12 | 1 | 2 | 5 | 3 | 4 |
| NM_005148 | UNC119 | 1384 | 2 | 1 | 1 | 1 | 0 | 2 | 1 | 1 | 0 | 0 | 0 | 1 | 2 |
| NM_005149 | TBX19 | 2822 | 0 | 0 | 0 | 0 | 0 | 0 | 0 | 0 | 0 | 0 | 0 | 0 | 0 |
| NM_005151 | USP14 | 4156 | 7 | 4 | 4 | 1 | 6 | 7 | 4 | 4 | 3 | 5 | 5 | 5 | 5 |
| NM_005153 | USP10 | 3378 | 4 | 3 | 2 | 2 | 3 | 4 | 3 | 3 | 2 | 2 | 3 | 4 | 3 |
| NM_005154 | USP8 | 5547 | 14 | 7 | 6 | 3 | 7 | 8 | 8 | 6 | 6 | 7 | 5 | 11 | 14 |
| NM_005155 | PPT2 | 2202 | 2 | 1 | 1 | 1 | 1 | 1 | 1 | 0 | 0 | 2 | 0 | 0 | 1 |
| NM_005156 | ROD1 | 7259 | 21 | 9 | 6 | 6 | 6 | 4 | 13 | 5 | 15 | 9 | 3 | 21 | 17 |
| NM_005157 | ABL1 | 5384 | 8 | 5 | 6 | 8 | 6 | 5 | 4 | 2 | 5 | 8 | 3 | 6 | 5 |
| NM_005158 | ABL2 | 11939 | 2 | 2 | 2 | 1 | 2 | 2 | 1 | 1 | 2 | 2 | 1 | 2 | 2 |
| NM_005159 | ACTC1 | 3693 | 310 | 30 | 0 | 0 | 310 | 0 | 0 | 0 | 0 | 0 | 15 | 0 | 1 |
| NM_005160 | ADRBK2 | 9055 | 7 | 2 | 7 | 1 | 1 | 4 | 1 | 1 | 1 | 1 | 0 | 6 | 1 |
| NM_005161 | APLNR | 3874 | 6 | 1 | 1 | 1 | 1 | 6 | 0 | 0 | 0 | 0 | 0 | 3 | 0 |
| NM_005163 | AKT1 | 2988 | 3 | 1 | 2 | 3 | 2 | 1 | 1 | 3 | 1 | 1 | 1 | 1 | 1 |
| NM_005164 | ABCD2 | 5341 | 7 | 1 | 7 | 0 | 1 | 2 | 0 | 0 | 0 | 0 | 0 | 0 | 0 |
| NM_005165 | ALDOC | 1651 | 85 | 13 | 25 | 2 | 17 | 85 | 0 | 10 | 1 | 3 | 0 | 2 | 2 |
| NM_005166 | APLP1 | 2447 | 25 | 2 | 0 | 0 | 0 | 25 | 0 | 0 | 0 | 0 | 0 | 0 | 0 |
| NM_005167 | PPM1J | 1714 | 1 | 0 | 0 | 0 | 0 | 0 | 0 | 0 | 0 | 0 | 0 | 0 | 1 |
| NM_005168 | RND3 | 2683 | 61 | 13 | 10 | 3 | 15 | 7 | 5 | 61 | 7 | 4 | 3 | 24 | 4 |
| NM_005169 | PHOX2A | 1699 | 0 | 0 | 0 | 0 | 0 | 0 | 0 | 0 | 0 | 0 | 0 | 0 | 0 |
| NM_005170 | ASCL2 | 1849 | 3 | 1 | 0 | 3 | 0 | 0 | 0 | 0 | 0 | 0 | 0 | 1 | 0 |
| NM_005171 | ATF1 | 2439 | 6 | 4 | 3 | 1 | 4 | 3 | 4 | 4 | 3 | 5 | 5 | 6 | 4 |
| NM_005172 | ATOH1 | 1065 | 0 | 0 | 0 | 0 | 0 | 0 | 0 | 0 | 0 | 0 | 0 | 0 | 0 |
| NM_005173 | ATP2A3 | 4698 | 2 | 0 | 0 | 2 | 0 | 0 | 0 | 0 | 0 | 0 | 0 | 2 | 0 |
| NM_005174 | ATP5C1 | 1109 | 33 | 19 | 17 | 15 | 32 | 28 | 25 | 33 | 8 | 8 | 13 | 17 | 11 |
| NM_005175 | ATP5G1 | 646 | 2 | 1 | 1 | 2 | 1 | 1 | 1 | 1 | 0 | 0 | 0 | 1 | 0 |
| NM_005176 | ATP5G2 | 884 | 10 | 6 | 5 | 4 | 10 | 8 | 7 | 6 | 3 | 5 | 10 | 7 | 3 |
| NM_005177 | ATP6V0A1 | 4122 | 25 | 8 | 4 | 6 | 4 | 25 | 15 | 4 | 7 | 5 | 9 | 7 | 7 |
| NM_005178 | BCL3 | 1864 | 2 | 1 | 0 | 1 | 1 | 0 | 1 | 1 | 1 | 0 | 0 | 2 | 0 |
| NM_005180 | BMI1 | 3566 | 18 | 11 | 8 | 3 | 10 | 11 | 8 | 10 | 9 | 11 | 11 | 18 | 17 |
| NM_005181 | CA3 | 1749 | 326 | 31 | 0 | 0 | 1 | 0 | 0 | 0 | 7 | 0 | 326 | 0 | 2 |
| NM_005182 | CA7 | 1552 | 1 | 0 | 0 | 1 | 0 | 0 | 0 | 0 | 0 | 0 | 0 | 0 | 0 |
| NM_005183 | CACNA1F | 6070 | 0 | 0 | 0 | 0 | 0 | 0 | 0 | 0 | 0 | 0 | 0 | 0 | 0 |
| NM_005184 | CALM3 | 2259 | 214 | 44 | 29 | 21 | 43 | 214 | 31 | 12 | 22 | 32 | 23 | 29 | 29 |
| NM_005185 | CALML3 | 1302 | 2 | 0 | 0 | 0 | 0 | 0 | 2 | 0 | 0 | 0 | 0 | 0 | 0 |
| NM_005186 | CAPN1 | 3007 | 1 | 1 | 1 | 1 | 1 | 1 | 1 | 1 | 1 | 1 | 1 | 1 | 0 |
| NM_005187 | CBFA2T3 | 4265 | 3 | 1 | 0 | 0 | 1 | 1 | 0 | 1 | 1 | 0 | 1 | 3 | 0 |
| NM_005188 | CBL | 11227 | 5 | 2 | 1 | 1 | 1 | 2 | 1 | 1 | 2 | 1 | 1 | 5 | 3 |
| NM_005189 | CBX2 | 4179 | 0 | 0 | 0 | 0 | 0 | 0 | 0 | 0 | 0 | 0 | 0 | 0 | 0 |
| NM_005190 | CCNC | 2307 | 12 | 5 | 5 | 1 | 3 | 5 | 8 | 12 | 5 | 3 | 3 | 7 | 4 |
| NM_005191 | CD80 | 2757 | 3 | 0 | 0 | 0 | 0 | 0 | 0 | 0 | 0 | 0 | 0 | 3 | 0 |
| NM_005192 | CDKN3 | 889 | 21 | 2 | 1 | 1 | 1 | 1 | 0 | 0 | 1 | 1 | 0 | 1 | 21 |
| NM_005193 | CDX4 | 855 | 0 | 0 | 0 | 0 | 0 | 0 | 0 | 0 | 0 | 0 | 0 | 0 | 0 |
| NM_005194 | CEBPB | 1837 | 6 | 2 | 3 | 0 | 2 | 1 | 1 | 6 | 3 | 1 | 3 | 3 | 1 |
| NM_005195 | CEBPD | 1251 | 19 | 10 | 9 | 2 | 14 | 5 | 7 | 12 | 14 | 11 | 16 | 19 | 4 |
| NM_005197 | FOXN3 | 7903 | 9 | 5 | 9 | 3 | 6 | 3 | 2 | 5 | 5 | 9 | 9 | 5 | 5 |
| NM_005198 | CHKB | 1629 | 0 | 0 | 0 | 0 | 0 | 0 | 0 | 0 | 0 | 0 | 0 | 0 | 0 |
| NM_005199 | CHRNG | 2187 | 0 | 0 | 0 | 0 | 0 | 0 | 0 | 0 | 0 | 0 | 0 | 0 | 0 |

| transcript | gene | transcript length (nt) | max | mean | adipose | colon | heart | hypothalamus | kidney | liver | lung | ovary | skeletalmuscle | spleen | testes |
|---|---|---|---|---|---|---|---|---|---|---|---|---|---|---|---|
| NM_005201 | CCR8 | 1487 | 0 | 0 | 0 | 0 | 0 | 0 | 0 | 0 | 0 | 0 | 0 | 0 | 0 |
| NM_005202 | COL8A2 | 4446 | 0 | 0 | 0 | 0 | 0 | 0 | 0 | 0 | 0 | 0 | 0 | 0 | 0 |
| NM_005203 | COL13A1 | 3136 | 1 | 0 | 0 | 0 | 0 | 0 | 0 | 0 | 1 | 0 | 0 | 1 | 0 |
| NM_005204 | MAP3K8 | 3096 | 14 | 4 | 2 | 1 | 5 | 2 | 2 | 1 | 10 | 2 | 3 | 14 | 2 |
| NM_005205 | COX6A2 | 409 | 22 | 3 | 0 | 0 | 16 | 0 | 0 | 0 | 0 | 0 | 22 | 0 | 0 |
| NM_005206 | CRK | 2227 | 11 | 6 | 8 | 4 | 6 | 7 | 5 | 11 | 6 | 4 | 4 | 6 | 6 |
| NM_005207 | CRKL | 5326 | 7 | 5 | 2 | 3 | 5 | 7 | 4 | 4 | 4 | 5 | 7 | 6 | 4 |
| NM_005208 | CRYBA1 | 790 | 0 | 0 | 0 | 0 | 0 | 0 | 0 | 0 | 0 | 0 | 0 | 0 | 0 |
| NM_005209 | CRYBA2 | 685 | 0 | 0 | 0 | 0 | 0 | 0 | 0 | 0 | 0 | 0 | 0 | 0 | 0 |
| NM_005210 | CRYGB | 626 | 0 | 0 | 0 | 0 | 0 | 0 | 0 | 0 | 0 | 0 | 0 | 0 | 0 |
| NM_005211 | CSF1R | 3988 | 28 | 5 | 6 | 2 | 3 | 8 | 1 | 3 | 3 | 1 | 1 | 28 | 2 |
| NM_005212 | CSN3 | 808 | 0 | 0 | 0 | 0 | 0 | 0 | 0 | 0 | 0 | 0 | 0 | 0 | 0 |
| NM_005213 | CSTA | 827 | 8 | 3 | 8 | 0 | 2 | 1 | 0 | 7 | 5 | 3 | 0 | 4 | 0 |
| NM_005214 | CTLA4 | 1977 | 0 | 0 | 0 | 0 | 0 | 0 | 0 | 0 | 0 | 0 | 0 | 0 | 0 |
| NM_005215 | DCC | 10210 | 1 | 0 | 0 | 0 | 0 | 1 | 0 | 0 | 0 | 0 | 0 | 0 | 0 |
| NM_005216 | DDOST | 2126 | 12 | 7 | 5 | 5 | 10 | 5 | 7 | 12 | 5 | 6 | 3 | 10 | 6 |
| NM_005217 | DEFA3 | 487 | 0 | 0 | 0 | 0 | 0 | 0 | 0 | 0 | 0 | 0 | 0 | 0 | 0 |
| NM_005218 | DEFB1 | 461 | 180 | 20 | 3 | 1 | 0 | 0 | 180 | 36 | 0 | 1 | 1 | 0 | 0 |
| NM_005219 | DIAPH1 | 5790 | 29 | 12 | 6 | 8 | 13 | 6 | 16 | 29 | 15 | 5 | 18 | 14 | 7 |
| NM_005220 | DLX3 | 2602 | 0 | 0 | 0 | 0 | 0 | 0 | 0 | 0 | 0 | 0 | 0 | 0 | 0 |
| NM_005221 | DLX5 | 1424 | 3 | 1 | 0 | 0 | 1 | 3 | 0 | 0 | 0 | 3 | 0 | 0 | 3 |
| NM_005222 | DLX6 | 1791 | 1 | 0 | 0 | 0 | 0 | 1 | 0 | 0 | 0 | 0 | 0 | 0 | 1 |
| NM_005223 | DNASE1 | 3098 | 2 | 0 | 0 | 1 | 0 | 0 | 2 | 0 | 0 | 0 | 0 | 0 | 0 |
| NM_005224 | ARID3A | 2810 | 1 | 0 | 1 | 1 | 0 | 0 | 1 | 0 | 1 | 0 | 0 | 1 | 0 |
| NM_005225 | E2F1 | 2700 | 0 | 0 | 0 | 0 | 0 | 0 | 0 | 0 | 0 | 0 | 0 | 0 | 0 |
| NM_005226 | S1PR3 | 4206 | 5 | 2 | 1 | 0 | 5 | 1 | 3 | 1 | 1 | 1 | 1 | 5 | 1 |
| NM_005227 | EFNA4 | 1257 | 1 | 0 | 0 | 1 | 0 | 0 | 0 | 0 | 0 | 0 | 0 | 0 | 0 |
| NM_005228 | EGFR | 5600 | 14 | 5 | 5 | 5 | 2 | 2 | 4 | 14 | 3 | 6 | 2 | 4 | 5 |
| NM_005229 | ELK1 | 2811 | 2 | 1 | 0 | 0 | 0 | 2 | 0 | 1 | 1 | 2 | 1 | 1 | 1 |
| NM_005230 | ELK3 | 2151 | 6 | 2 | 3 | 1 | 2 | 1 | 1 | 1 | 3 | 1 | 1 | 6 | 2 |
| NM_005231 | CTTN | 3281 | 31 | 19 | 23 | 15 | 22 | 26 | 31 | 17 | 14 | 19 | 9 | 14 | 21 |
| NM_005232 | EPHA1 | 3362 | 2 | 0 | 0 | 1 | 0 | 0 | 0 | 2 | 0 | 0 | 0 | 0 | 0 |
| NM_005233 | EPHA3 | 5809 | 2 | 1 | 1 | 1 | 2 | 1 | 0 | 0 | 0 | 0 | 0 | 0 | 0 |
| NM_005234 | NR2F6 | 1804 | 6 | 2 | 1 | 3 | 2 | 1 | 2 | 6 | 1 | 1 | 1 | 0 | 1 |
| NM_005235 | ERBB4 | 11923 | 7 | 2 | 0 | 0 | 2 | 7 | 5 | 0 | 1 | 0 | 1 | 0 | 6 |
| NM_005236 | ERCC4 | 6761 | 4 | 2 | 1 | 0 | 1 | 2 | 2 | 1 | 1 | 2 | 1 | 2 | 4 |
| NM_005238 | ETS1 | 5242 | 56 | 12 | 10 | 4 | 10 | 2 | 7 | 4 | 25 | 8 | 4 | 56 | 4 |
| NM_005239 | ETS2 | 3671 | 40 | 12 | 4 | 5 | 10 | 7 | 6 | 15 | 16 | 4 | 20 | 40 | 4 |
| NM_005240 | ETV3 | 1411 | 2 | 0 | 0 | 0 | 0 | 0 | 0 | 0 | 0 | 2 | 0 | 1 | 0 |
| NM_005241 | MECOM | 4835 | 15 | 3 | 2 | 3 | 4 | 0 | 15 | 0 | 6 | 1 | 1 | 3 | 2 |
| NM_005242 | F2RL1 | 2882 | 2 | 0 | 0 | 1 | 0 | 0 | 2 | 1 | 0 | 0 | 0 | 0 | 0 |
| NM_005243 | EWSR1 | 2662 | 40 | 24 | 19 | 20 | 25 | 24 | 25 | 28 | 19 | 16 | 18 | 40 | 30 |
| NM_005244 | EYA2 | 2700 | 1 | 1 | 1 | 1 | 0 | 1 | 1 | 0 | 1 | 0 | 0 | 0 | 1 |
| NM_005245 | FAT1 | 14764 | 15 | 7 | 12 | 8 | 7 | 7 | 15 | 10 | 3 | 5 | 1 | 1 | 8 |
| NM_005246 | FER | 2950 | 15 | 8 | 7 | 3 | 7 | 13 | 7 | 2 | 8 | 14 | 3 | 10 | 15 |
| NM_005247 | FGF3 | 1548 | 0 | 0 | 0 | 0 | 0 | 0 | 0 | 0 | 0 | 0 | 0 | 0 | 0 |
| NM_005248 | FGR | 2442 | 3 | 1 | 1 | 0 | 0 | 0 | 0 | 0 | 2 | 0 | 0 | 3 | 0 |
| NM_005249 | FOXG1 | 2585 | 7 | 1 | 0 | 0 | 0 | 7 | 0 | 0 | 0 | 0 | 0 | 0 | 0 |
| NM_005250 | FOXL1 | 3190 | 1 | 0 | 0 | 0 | 1 | 0 | 0 | 0 | 0 | 0 | 0 | 0 | 1 |
| NM_005251 | FOXC2 | 1679 | 0 | 0 | 0 | 0 | 0 | 0 | 0 | 0 | 0 | 0 | 0 | 0 | 0 |
| NM_005252 | FOS | 2156 | 285 | 54 | 28 | 31 | 15 | 42 | 35 | 72 | 54 | 285 | 8 | 13 | 13 |
| NM_005253 | FOSL2 | 3991 | 8 | 4 | 2 | 4 | 5 | 3 | 3 | 1 | 8 | 6 | 5 | 7 | 2 |
| NM_005254 | GABPB1 | 3052 | 5 | 2 | 2 | 1 | 2 | 2 | 2 | 2 | 2 | 3 | 2 | 5 | 4 |
| NM_005255 | GAK | 4531 | 3 | 2 | 1 | 3 | 1 | 1 | 3 | 1 | 2 | 1 | 1 | 2 | 2 |
| NM_005256 | GAS2 | 2023 | 4 | 1 | 0 | 0 | 1 | 1 | 1 | 4 | 0 | 1 | 0 | 0 | 1 |
| NM_005257 | GATA6 | 3494 | 8 | 2 | 0 | 1 | 6 | 0 | 1 | 1 | 1 | 8 | 0 | 3 | 2 |
| NM_005258 | GCHFR | 713 | 3 | 1 | 1 | 1 | 0 | 1 | 1 | 3 | 1 | 1 | 0 | 1 | 0 |
| NM_005259 | MSTN | 2819 | 0 | 0 | 0 | 0 | 0 | 0 | 0 | 0 | 0 | 0 | 0 | 0 | 0 |
| NM_005260 | GDF9 | 2020 | 0 | 0 | 0 | 0 | 0 | 0 | 0 | 0 | 0 | 0 | 0 | 0 | 0 |
| NM_005261 | GEM | 2181 | 10 | 4 | 6 | 3 | 10 | 4 | 3 | 1 | 4 | 2 | 2 | 4 | 4 |
| NM_005262 | GFER | 2409 | 1 | 0 | 1 | 1 | 0 | 1 | 1 | 0 | 0 | 0 | 1 | 1 | 0 |
| NM_005263 | GFI1 | 2787 | 1 | 0 | 0 | 0 | 0 | 0 | 0 | 0 | 0 | 0 | 0 | 1 | 0 |
| NM_005264 | GFRA1 | 9561 | 5 | 2 | 1 | 1 | 1 | 2 | 1 | 5 | 1 | 1 | 1 | 1 | 1 |
| NM_005265 | GGT1 | 2413 | 2 | 0 | 0 | 0 | 0 | 0 | 2 | 1 | 0 | 0 | 0 | 0 | 0 |
| NM_005266 | GJA5 | 3177 | 2 | 1 | 1 | 0 | 2 | 0 | 2 | 0 | 2 | 1 | 0 | 1 | 0 |
| NM_005267 | GJA8 | 1364 | 1 | 0 | 0 | 0 | 0 | 0 | 0 | 0 | 0 | 0 | 0 | 0 | 1 |
| NM_005268 | GJB5 | 1281 | 0 | 0 | 0 | 0 | 0 | 0 | 0 | 0 | 0 | 0 | 0 | 0 | 0 |
| NM_005269 | GLI1 | 3600 | 0 | 0 | 0 | 0 | 0 | 0 | 0 | 0 | 0 | 0 | 0 | 0 | 0 |
| NM_005270 | GLI2 | 6769 | 1 | 0 | 0 | 0 | 0 | 0 | 0 | 0 | 0 | 1 | 0 | 0 | 1 |
| NM_005271 | GLUD1 | 3476 | 30 | 7 | 4 | 1 | 4 | 18 | 9 | 30 | 3 | 4 | 2 | 4 | 2 |
| NM_005272 | GNAT2 | 1365 | 1 | 0 | 0 | 0 | 0 | 0 | 0 | 0 | 0 | 0 | 0 | 1 | 0 |
| NM_005273 | GNB2 | 1663 | 2 | 1 | 1 | 1 | 1 | 2 | 1 | 1 | 1 | 1 | 0 | 2 | 1 |
| NM_005274 | GNG5 | 806 | 70 | 31 | 18 | 10 | 70 | 8 | 37 | 27 | 44 | 19 | 32 | 55 | 21 |
| NM_005275 | GNL1 | 3007 | 5 | 2 | 2 | 1 | 1 | 5 | 1 | 1 | 2 | 3 | 1 | 2 | 2 |
| NM_005276 | GPD1 | 2876 | 81 | 11 | 81 | 3 | 2 | 2 | 16 | 13 | 1 | 0 | 7 | 0 | 0 |
| NM_005277 | GPM6A | 3016 | 42 | 4 | 0 | 1 | 0 | 42 | 1 | 0 | 1 | 0 | 0 | 1 | 0 |
| NM_005278 | GPM6B | 1655 | 115 | 12 | 0 | 2 | 2 | 115 | 1 | 0 | 2 | 0 | 0 | 5 | 1 |

| transcript | gene | transcript length (nt) | max | mean | adipose | colon | heart | hypothalamus | kidney | liver | lung | ovary | skeletalmuscle | spleen | testes |
|---|---|---|---|---|---|---|---|---|---|---|---|---|---|---|---|
| NM_005279 | GPR1 | 2318 | 0 | 0 | 0 | 0 | 0 | 0 | 0 | 0 | 0 | 0 | 0 | 0 | 0 |
| NM_005281 | GPR3 | 2110 | 1 | 0 | 0 | 0 | 0 | 1 | 0 | 0 | 0 | 0 | 0 | 0 | 0 |
| NM_005282 | GPR4 | 3045 | 5 | 2 | 2 | 0 | 4 | 2 | 1 | 0 | 5 | 1 | 1 | 2 | 1 |
| NM_005283 | XCR1 | 1373 | 1 | 0 | 0 | 0 | 0 | 0 | 0 | 0 | 0 | 0 | 0 | 1 | 0 |
| NM_005284 | GPR6 | 1626 | 0 | 0 | 0 | 0 | 0 | 0 | 0 | 0 | 0 | 0 | 0 | 0 | 0 |
| NM_005285 | NPBWR1 | 987 | 0 | 0 | 0 | 0 | 0 | 0 | 0 | 0 | 0 | 0 | 0 | 0 | 0 |
| NM_005286 | NPBWR2 | 1002 | 0 | 0 | 0 | 0 | 0 | 0 | 0 | 0 | 0 | 0 | 0 | 0 | 0 |
| NM_005288 | GPR12 | 4846 | 1 | 0 | 0 | 0 | 0 | 1 | 0 | 0 | 0 | 0 | 0 | 0 | 0 |
| NM_005290 | GPR15 | 1083 | 1 | 0 | 0 | 1 | 0 | 0 | 0 | 0 | 0 | 0 | 0 | 1 | 0 |
| NM_005291 | GPR17 | 2369 | 1 | 0 | 0 | 1 | 0 | 1 | 0 | 0 | 0 | 0 | 0 | 0 | 0 |
| NM_005292 | GPR18 | 1661 | 10 | 2 | 1 | 1 | 1 | 0 | 2 | 0 | 2 | 2 | 1 | 10 | 3 |
| NM_005293 | GPR20 | 1526 | 0 | 0 | 0 | 0 | 0 | 0 | 0 | 0 | 0 | 0 | 0 | 0 | 0 |
| NM_005294 | GPR21 | 1050 | 0 | 0 | 0 | 0 | 0 | 0 | 0 | 0 | 0 | 0 | 0 | 0 | 0 |
| NM_005295 | GPR22 | 2966 | 2 | 1 | 0 | 0 | 2 | 2 | 0 | 0 | 0 | 1 | 0 | 1 | 1 |
| NM_005296 | LPAR4 | 2615 | 0 | 0 | 0 | 0 | 0 | 0 | 0 | 0 | 0 | 0 | 0 | 0 | 0 |
| NM_005297 | MCHR1 | 2423 | 2 | 0 | 0 | 0 | 0 | 1 | 0 | 0 | 0 | 2 | 0 | 0 | 0 |
| NM_005298 | GPR25 | 1086 | 0 | 0 | 0 | 0 | 0 | 0 | 0 | 0 | 0 | 0 | 0 | 0 | 0 |
| NM_005299 | GPR31 | 960 | 0 | 0 | 0 | 0 | 0 | 0 | 0 | 0 | 0 | 0 | 0 | 0 | 0 |
| NM_005300 | GPR34 | 1852 | 6 | 2 | 3 | 1 | 2 | 1 | 1 | 1 | 3 | 2 | 0 | 6 | 1 |
| NM_005301 | GPR35 | 1875 | 1 | 0 | 0 | 1 | 0 | 0 | 0 | 0 | 0 | 0 | 0 | 0 | 0 |
| NM_005302 | GPR37 | 2958 | 6 | 1 | 0 | 0 | 0 | 6 | 0 | 1 | 0 | 0 | 0 | 1 | 3 |
| NM_005303 | FFAR1 | 923 | 0 | 0 | 0 | 0 | 0 | 0 | 0 | 0 | 0 | 0 | 0 | 0 | 0 |
| NM_005304 | FFAR3 | 1670 | 0 | 0 | 0 | 0 | 0 | 0 | 0 | 0 | 0 | 0 | 0 | 0 | 0 |
| NM_005306 | FFAR2 | 2051 | 3 | 1 | 1 | 0 | 0 | 0 | 0 | 0 | 2 | 0 | 0 | 3 | 0 |
| NM_005308 | GRK5 | 2558 | 10 | 4 | 3 | 4 | 6 | 2 | 1 | 2 | 9 | 3 | 1 | 10 | 1 |
| NM_005309 | GPT | 1886 | 0 | 0 | 0 | 0 | 0 | 0 | 0 | 0 | 0 | 0 | 0 | 0 | 0 |
| NM_005310 | GRB7 | 2237 | 0 | 0 | 0 | 0 | 0 | 0 | 0 | 0 | 0 | 0 | 0 | 0 | 0 |
| NM_005311 | GRB10 | 4705 | 8 | 3 | 1 | 1 | 3 | 4 | 4 | 2 | 2 | 1 | 5 | 8 | 1 |
| NM_005312 | RAPGEF1 | 6085 | 10 | 5 | 5 | 4 | 5 | 5 | 4 | 3 | 5 | 3 | 10 | 9 | 2 |
| NM_005313 | PDIA3 | 3054 | 97 | 31 | 19 | 14 | 32 | 20 | 28 | 97 | 21 | 26 | 7 | 43 | 29 |
| NM_005314 | GRPR | 2681 | 0 | 0 | 0 | 0 | 0 | 0 | 0 | 0 | 0 | 0 | 0 | 0 | 0 |
| NM_005315 | GSC2 | 618 | 0 | 0 | 0 | 0 | 0 | 0 | 0 | 0 | 0 | 0 | 0 | 0 | 0 |
| NM_005316 | GTF2H1 | 3308 | 13 | 6 | 5 | 2 | 7 | 6 | 6 | 7 | 4 | 6 | 4 | 9 | 13 |
| NM_005317 | GZMM | 947 | 0 | 0 | 0 | 0 | 0 | 0 | 0 | 0 | 0 | 0 | 0 | 0 | 0 |
| NM_005318 | H1F0 | 2330 | 12 | 9 | 5 | 7 | 11 | 8 | 12 | 11 | 9 | 10 | 12 | 8 | 9 |
| NM_005319 | HIST1H1C | 732 | 8 | 5 | 4 | 8 | 3 | 2 | 4 | 2 | 7 | 6 | 3 | 7 | 7 |
| NM_005320 | HIST1H1D | 777 | 83 | 23 | 14 | 30 | 9 | 9 | 6 | 1 | 41 | 16 | 3 | 83 | 38 |
| NM_005321 | HIST1H1E | 785 | 3 | 1 | 1 | 3 | 1 | 1 | 1 | 1 | 1 | 1 | 0 | 2 | 1 |
| NM_005322 | HIST1H1B | 790 | 34 | 8 | 6 | 34 | 4 | 1 | 0 | 0 | 12 | 4 | 0 | 20 | 4 |
| NM_005323 | HIST1H1T | 725 | 28 | 3 | 0 | 0 | 0 | 0 | 0 | 0 | 0 | 0 | 0 | 0 | 28 |
| NM_005324 | H3F3B | 2705 | 77 | 39 | 29 | 21 | 32 | 48 | 28 | 28 | 34 | 77 | 30 | 58 | 42 |
| NM_005325 | HIST1H1A | 781 | 8 | 1 | 0 | 0 | 1 | 1 | 1 | 1 | 0 | 2 | 0 | 0 | 8 |
| NM_005326 | HAGH | 1514 | 8 | 3 | 2 | 2 | 3 | 7 | 5 | 8 | 2 | 1 | 4 | 2 | 3 |
| NM_005327 | HADH | 1912 | 19 | 10 | 19 | 10 | 18 | 5 | 15 | 17 | 3 | 5 | 9 | 4 | 7 |
| NM_005328 | HAS2 | 3275 | 1 | 0 | 0 | 0 | 1 | 0 | 0 | 0 | 0 | 0 | 0 | 0 | 0 |
| NM_005329 | HAS3 | 4219 | 0 | 0 | 0 | 0 | 0 | 0 | 0 | 0 | 0 | 0 | 0 | 0 | 0 |
| NM_005330 | HBE1 | 816 | 0 | 0 | 0 | 0 | 0 | 0 | 0 | 0 | 0 | 0 | 0 | 0 | 0 |
| NM_005331 | HBQ1 | 651 | 0 | 0 | 0 | 0 | 0 | 0 | 0 | 0 | 0 | 0 | 0 | 0 | 0 |
| NM_005332 | HBZ | 589 | 0 | 0 | 0 | 0 | 0 | 0 | 0 | 0 | 0 | 0 | 0 | 0 | 0 |
| NM_005333 | HCCS | 2344 | 14 | 7 | 4 | 4 | 14 | 7 | 7 | 7 | 4 | 4 | 8 | 7 | 6 |
| NM_005334 | HCFC1 | 8426 | 2 | 1 | 1 | 1 | 1 | 2 | 1 | 1 | 1 | 1 | 1 | 2 | 1 |
| NM_005335 | HCLS1 | 2033 | 221 | 38 | 28 | 10 | 36 | 16 | 9 | 22 | 50 | 6 | 5 | 221 | 9 |
| NM_005336 | HDLBP | 6516 | 67 | 33 | 37 | 18 | 43 | 26 | 23 | 67 | 21 | 30 | 48 | 22 | 33 |
| NM_005337 | NCKAP1L | 3893 | 13 | 3 | 6 | 2 | 3 | 1 | 1 | 1 | 5 | 1 | 0 | 13 | 1 |
| NM_005338 | HIP1 | 7239 | 5 | 2 | 2 | 1 | 2 | 3 | 3 | 2 | 3 | 2 | 1 | 5 | 2 |
| NM_005339 | UBE2K | 5243 | 17 | 10 | 8 | 6 | 10 | 17 | 11 | 17 | 9 | 7 | 7 | 11 | 9 |
| NM_005340 | HINT1 | 782 | 37 | 14 | 12 | 14 | 11 | 37 | 12 | 37 | 7 | 5 | 5 | 10 | 6 |
| NM_005341 | ZBTB48 | 2338 | 1 | 0 | 0 | 0 | 0 | 0 | 1 | 0 | 0 | 0 | 0 | 1 | 1 |
| NM_005342 | HMGB3 | 3556 | 13 | 4 | 1 | 3 | 2 | 4 | 3 | 13 | 4 | 2 | 2 | 5 | 3 |
| NM_005343 | HRAS | 1044 | 3 | 1 | 1 | 3 | 1 | 3 | 1 | 1 | 1 | 0 | 1 | 1 | 1 |
| NM_005345 | HSPA1A | 2429 | 33 | 14 | 15 | 6 | 30 | 12 | 10 | 2 | 33 | 7 | 14 | 12 | 7 |
| NM_005346 | HSPA1B | 2520 | 9 | 4 | 3 | 3 | 6 | 6 | 5 | 1 | 9 | 4 | 4 | 2 | 3 |
| NM_005346 | HSPA1B | 2523 | 9 | 4 | 3 | 3 | 6 | 6 | 5 | 1 | 9 | 4 | 4 | 2 | 3 |
| NM_005346 | HSPA1B | 2524 | 9 | 4 | 3 | 3 | 6 | 6 | 5 | 1 | 9 | 4 | 4 | 2 | 3 |
| NM_005347 | HSPA5 | 3965 | 159 | 61 | 53 | 22 | 159 | 59 | 53 | 94 | 56 | 31 | 17 | 74 | 60 |
| NM_005348 | HSP90AA1 | 3363 | 459 | 208 | 192 | 53 | 309 | 459 | 201 | 126 | 206 | 121 | 123 | 172 | 330 |
| NM_005349 | RBPJ | 2388 | 10 | 7 | 8 | 2 | 7 | 9 | 5 | 4 | 9 | 9 | 7 | 10 | 8 |
| NM_005353 | ITGAD | 3912 | 2 | 0 | 0 | 0 | 0 | 0 | 0 | 0 | 0 | 0 | 2 | 1 | 0 |
| NM_005354 | JUND | 1870 | 5 | 2 | 2 | 1 | 2 | 2 | 1 | 1 | 4 | 5 | 1 | 2 | 2 |
| NM_005355 | KIF25 | 1354 | 0 | 0 | 0 | 0 | 0 | 0 | 0 | 0 | 0 | 0 | 0 | 0 | 0 |
| NM_005356 | LCK | 2117 | 2 | 0 | 0 | 0 | 0 | 0 | 0 | 0 | 1 | 0 | 0 | 2 | 0 |
| NM_005357 | LIPE | 3806 | 6 | 1 | 6 | 0 | 0 | 0 | 0 | 0 | 0 | 0 | 0 | 0 | 2 |
| NM_005358 | LMO7 | 7235 | 46 | 15 | 0 | 7 | 46 | 10 | 18 | 11 | 40 | 3 | 19 | 2 | 9 |
| NM_005359 | SMAD4 | 8769 | 7 | 4 | 2 | 3 | 4 | 3 | 4 | 3 | 4 | 7 | 6 | 6 | 5 |
| NM_005360 | MAF | 2646 | 3 | 2 | 2 | 1 | 1 | 1 | 3 | 1 | 1 | 2 | 2 | 2 | 1 |
| NM_005361 | MAGEA2 | 1979 | 0 | 0 | 0 | 0 | 0 | 0 | 0 | 0 | 0 | 0 | 0 | 0 | 0 |
| NM_005362 | MAGEA3 | 1724 | 0 | 0 | 0 | 0 | 0 | 0 | 0 | 0 | 0 | 0 | 0 | 0 | 0 |

| | | transcript | | | | | | hypothala | | | | | skeletalm | | |
|---|---|---|---|---|---|---|---|---|---|---|---|---|---|---|---|
| transcript | gene | length (nt) | max | mean | adipose | colon | heart | mus | kidney | liver | lung | ovary | uscle | spleen | testes |
| NM_005363 | MAGEA6 | 1698 | 0 | 0 | 0 | 0 | 0 | 0 | 0 | 0 | 0 | 0 | 0 | 0 | 0 |
| NM_005364 | MAGEA8 | 1833 | 0 | 0 | 0 | 0 | 0 | 0 | 0 | 0 | 0 | 0 | 0 | 0 | 0 |
| NM_005365 | MAGEA9 | 1807 | 0 | 0 | 0 | 0 | 0 | 0 | 0 | 0 | 0 | 0 | 0 | 0 | 0 |
| NM_005366 | MAGEA11 | 1884 | 1 | 0 | 0 | 0 | 0 | 0 | 0 | 0 | 0 | 0 | 0 | 0 | 1 |
| NM_005367 | MAGEA12 | 1661 | 0 | 0 | 0 | 0 | 0 | 0 | 0 | 0 | 0 | 0 | 0 | 0 | 0 |
| NM_005368 | MB | 1078 | 498 | 76 | 1 | 1 | 498 | 0 | 0 | 0 | 0 | 0 | 337 | 0 | 0 |
| NM_005369 | MCF2 | 3772 | 6 | 1 | 0 | 0 | 0 | 2 | 0 | 0 | 0 | 1 | 0 | 0 | 6 |
| NM_005370 | RAB8A | 2177 | 18 | 8 | 6 | 6 | 4 | 5 | 8 | 18 | 7 | 6 | 2 | 18 | 9 |
| NM_005371 | METTL1 | 1307 | 1 | 0 | 0 | 0 | 0 | 0 | 1 | 0 | 0 | 0 | 0 | 0 | 0 |
| NM_005372 | MOS | 1041 | 0 | 0 | 0 | 0 | 0 | 0 | 0 | 0 | 0 | 0 | 0 | 0 | 0 |
| NM_005373 | MPL | 3645 | 1 | 0 | 0 | 0 | 0 | 0 | 0 | 0 | 1 | 1 | 0 | 0 | 0 |
| NM_005374 | MPP2 | 4412 | 1 | 0 | 0 | 0 | 0 | 1 | 0 | 0 | 0 | 0 | 0 | 0 | 0 |
| NM_005375 | MYB | 3314 | 7 | 1 | 0 | 7 | 0 | 0 | 0 | 0 | 1 | 0 | 0 | 1 | 0 |
| NM_005376 | MYCL1 | 2038 | 0 | 0 | 0 | 0 | 0 | 0 | 0 | 0 | 0 | 0 | 0 | 0 | 0 |
| NM_005378 | MYCN | 2605 | 0 | 0 | 0 | 0 | 0 | 0 | 0 | 0 | 0 | 0 | 0 | 0 | 0 |
| NM_005379 | MYO1A | 3613 | 7 | 1 | 0 | 7 | 0 | 0 | 0 | 0 | 0 | 0 | 0 | 0 | 0 |
| NM_005380 | NBL1 | 2021 | 21 | 6 | 4 | 10 | 3 | 9 | 3 | 0 | 10 | 21 | 1 | 2 | 6 |
| NM_005381 | NCL | 2717 | 259 | 204 | 194 | 88 | 226 | 229 | 179 | 240 | 144 | 259 | 210 | 255 | 221 |
| NM_005382 | NEFM | 3271 | 303 | 29 | 0 | 4 | 1 | 303 | 2 | 0 | 0 | 2 | 1 | 0 | 7 |
| NM_005383 | NEU2 | 1142 | 0 | 0 | 0 | 0 | 0 | 0 | 0 | 0 | 0 | 0 | 0 | 0 | 0 |
| NM_005384 | NFIL3 | 2085 | 56 | 24 | 19 | 5 | 33 | 15 | 11 | 56 | 31 | 18 | 35 | 15 | 20 |
| NM_005385 | NKTR | 7302 | 11 | 6 | 5 | 3 | 5 | 4 | 7 | 2 | 8 | 9 | 4 | 11 | 10 |
| NM_005386 | NNAT | 1302 | 18 | 3 | 3 | 3 | 0 | 18 | 1 | 0 | 0 | 1 | 3 | 1 | 1 |
| NM_005387 | NUP98 | 3819 | 14 | 7 | 4 | 4 | 7 | 5 | 5 | 5 | 7 | 9 | 8 | 10 | 14 |
| NM_005388 | PDCL | 2743 | 5 | 3 | 3 | 3 | 2 | 4 | 3 | 2 | 4 | 3 | 1 | 5 | 4 |
| NM_005389 | PCMT1 | 1733 | 72 | 27 | 19 | 11 | 37 | 72 | 14 | 32 | 14 | 11 | 37 | 18 | 32 |
| NM_005390 | PDHA2 | 1387 | 14 | 2 | 0 | 0 | 1 | 0 | 1 | 1 | 0 | 0 | 0 | 0 | 14 |
| NM_005391 | PDK3 | 1521 | 9 | 4 | 1 | 4 | 5 | 7 | 5 | 3 | 4 | 5 | 1 | 5 | 9 |
| NM_005392 | PHF2 | 5349 | 2 | 1 | 1 | 1 | 1 | 1 | 1 | 1 | 1 | 1 | 1 | 2 | 1 |
| NM_005393 | PLXNB3 | 6211 | 0 | 0 | 0 | 0 | 0 | 0 | 0 | 0 | 0 | 0 | 0 | 0 | 0 |
| NM_005396 | PNLIPRP2 | 1481 | 1 | 0 | 0 | 1 | 0 | 0 | 0 | 0 | 0 | 0 | 0 | 0 | 0 |
| NM_005397 | PODXL | 5888 | 19 | 5 | 2 | 2 | 7 | 5 | 19 | 1 | 9 | 3 | 1 | 3 | 2 |
| NM_005398 | PPP1R3C | 2571 | 43 | 7 | 1 | 2 | 11 | 6 | 2 | 6 | 2 | 1 | 43 | 0 | 1 |
| NM_005399 | PRKAB2 | 5423 | 9 | 3 | 1 | 1 | 4 | 2 | 2 | 3 | 2 | 2 | 9 | 2 | 2 |
| NM_005400 | PRKCE | 5520 | 10 | 3 | 1 | 1 | 1 | 10 | 3 | 3 | 5 | 1 | 1 | 1 | 2 |
| NM_005401 | PTPN14 | 4089 | 10 | 5 | 5 | 3 | 7 | 2 | 7 | 0 | 4 | 5 | 10 | 4 | 7 |
| NM_005402 | RALA | 2794 | 9 | 6 | 7 | 4 | 6 | 9 | 4 | 4 | 8 | 5 | 3 | 6 | 6 |
| NM_005406 | ROCK1 | 6648 | 40 | 19 | 19 | 6 | 27 | 9 | 16 | 14 | 25 | 20 | 10 | 40 | 23 |
| NM_005407 | SALL2 | 4912 | 4 | 1 | 0 | 1 | 0 | 4 | 1 | 0 | 1 | 2 | 0 | 1 | 1 |
| NM_005408 | CCL13 | 851 | 2 | 0 | 1 | 2 | 1 | 0 | 0 | 0 | 2 | 0 | 0 | 0 | 0 |
| NM_005409 | CXCL11 | 1484 | 2 | 1 | 0 | 0 | 0 | 2 | 0 | 0 | 2 | 0 | 2 | 2 | 0 |
| NM_005410 | SEPP1 | 2086 | 122 | 24 | 43 | 8 | 6 | 14 | 8 | 122 | 10 | 11 | 5 | 34 | 5 |
| NM_005411 | SFTPA1 | 2214 | 237 | 22 | 0 | 0 | 0 | 0 | 0 | 0 | 237 | 0 | 0 | 0 | 0 |
| NM_005412 | SHMT2 | 2294 | 11 | 2 | 1 | 2 | 1 | 1 | 2 | 11 | 1 | 1 | 1 | 3 | 1 |
| NM_005413 | SIX3 | 2522 | 0 | 0 | 0 | 0 | 0 | 0 | 0 | 0 | 0 | 0 | 0 | 0 | 0 |
| NM_005414 | SKIL | 3510 | 11 | 8 | 6 | 3 | 8 | 10 | 8 | 6 | 11 | 10 | 6 | 9 | 9 |
| NM_005415 | SLC20A1 | 3284 | 7 | 4 | 3 | 4 | 3 | 7 | 4 | 3 | 5 | 2 | 2 | 7 | 4 |
| NM_005416 | SPRR3 | 986 | 0 | 0 | 0 | 0 | 0 | 0 | 0 | 0 | 0 | 0 | 0 | 0 | 0 |
| NM_005417 | SRC | 4099 | 5 | 1 | 1 | 3 | 1 | 1 | 1 | 0 | 1 | 1 | 0 | 1 | 5 |
| NM_005418 | ST5 | 4544 | 5 | 2 | 1 | 2 | 2 | 2 | 1 | 1 | 2 | 4 | 1 | 4 | 5 |
| NM_005419 | STAT2 | 4425 | 4 | 2 | 3 | 2 | 2 | 3 | 2 | 2 | 2 | 2 | 1 | 4 | 2 |
| NM_005420 | SULT1E1 | 1780 | 1 | 0 | 0 | 0 | 0 | 0 | 0 | 1 | 0 | 0 | 0 | 0 | 0 |
| NM_005421 | TAL2 | 646 | 0 | 0 | 0 | 0 | 0 | 0 | 0 | 0 | 0 | 0 | 0 | 0 | 0 |
| NM_005422 | TECTA | 6468 | 0 | 0 | 0 | 0 | 0 | 0 | 0 | 0 | 0 | 0 | 0 | 0 | 0 |
| NM_005423 | TFF2 | 705 | 0 | 0 | 0 | 0 | 0 | 0 | 0 | 0 | 0 | 0 | 0 | 0 | 0 |
| NM_005424 | TIE1 | 3882 | 1 | 1 | 1 | 0 | 1 | 0 | 0 | 1 | 1 | 0 | 0 | 1 | 0 |
| NM_005425 | TNP2 | 597 | 2 | 0 | 0 | 0 | 0 | 0 | 0 | 0 | 0 | 0 | 0 | 0 | 2 |
| NM_005426 | TP53BP2 | 4789 | 8 | 5 | 2 | 2 | 5 | 8 | 4 | 3 | 6 | 6 | 4 | 6 | 7 |
| NM_005427 | TP73 | 2845 | 0 | 0 | 0 | 0 | 0 | 0 | 0 | 0 | 0 | 0 | 0 | 0 | 0 |
| NM_005428 | VAV1 | 2888 | 11 | 2 | 2 | 2 | 1 | 2 | 0 | 1 | 2 | 0 | 0 | 11 | 1 |
| NM_005429 | VEGFC | 2079 | 2 | 1 | 2 | 0 | 1 | 0 | 1 | 0 | 1 | 1 | 1 | 1 | 1 |
| NM_005430 | WNT1 | 2283 | 0 | 0 | 0 | 0 | 0 | 0 | 0 | 0 | 0 | 0 | 0 | 0 | 0 |
| NM_005431 | XRCC2 | 3069 | 1 | 0 | 0 | 0 | 0 | 0 | 0 | 0 | 0 | 1 | 0 | 1 | 1 |
| NM_005432 | XRCC3 | 2602 | 1 | 0 | 0 | 1 | 0 | 0 | 0 | 0 | 0 | 0 | 0 | 0 | 0 |
| NM_005433 | YES1 | 4685 | 35 | 16 | 10 | 4 | 12 | 7 | 19 | 24 | 17 | 13 | 6 | 35 | 24 |
| NM_005434 | MALL | 3053 | 3 | 1 | 0 | 3 | 1 | 0 | 1 | 0 | 2 | 0 | 0 | 0 | 0 |
| NM_005435 | ARHGEF5 | 5488 | 2 | 1 | 1 | 2 | 0 | 0 | 1 | 1 | 1 | 1 | 0 | 1 | 0 |
| NM_005436 | CCDC6 | 6215 | 4 | 3 | 2 | 2 | 3 | 4 | 3 | 2 | 3 | 3 | 2 | 4 | 4 |
| NM_005437 | NCOA4 | 3491 | 39 | 19 | 23 | 11 | 20 | 14 | 18 | 39 | 13 | 9 | 12 | 35 | 11 |
| NM_005438 | FOSL1 | 1668 | 1 | 0 | 0 | 0 | 1 | 0 | 0 | 0 | 1 | 0 | 0 | 0 | 0 |
| NM_005439 | MLF2 | 1390 | 8 | 3 | 2 | 2 | 3 | 8 | 3 | 5 | 1 | 1 | 1 | 2 | 1 |
| NM_005440 | RND2 | 4152 | 8 | 1 | 0 | 1 | 0 | 8 | 0 | 1 | 0 | 1 | 0 | 0 | 3 |
| NM_005441 | CHAF1B | 2279 | 2 | 1 | 0 | 1 | 0 | 1 | 0 | 0 | 0 | 1 | 2 | 1 | 1 |
| NM_005442 | EOMES | 2736 | 4 | 1 | 0 | 0 | 0 | 0 | 0 | 0 | 1 | 0 | 0 | 4 | 0 |
| NM_005443 | PAPSS1 | 2542 | 28 | 9 | 9 | 5 | 6 | 28 | 9 | 1 | 11 | 10 | 2 | 8 | 12 |
| NM_005444 | RQCD1 | 900 | 4 | 2 | 2 | 1 | 2 | 4 | 1 | 2 | 1 | 3 | 2 | 3 | 4 |
| NM_005445 | SMC3 | 4112 | 23 | 10 | 8 | 2 | 9 | 9 | 8 | 6 | 8 | 15 | 9 | 15 | 23 |

| transcript | gene | transcript length (nt) | max | mean | adipose | colon | heart | hypothalamus | kidney | liver | lung | ovary | skeletalmuscle | spleen | testes |
|---|---|---|---|---|---|---|---|---|---|---|---|---|---|---|---|
| NM_005446 | P2RX6 | 2722 | 1 | 0 | 1 | 0 | 0 | 0 | 0 | 0 | 0 | 0 | 1 | 1 | 0 |
| NM_005447 | RASSF9 | 1796 | 2 | 1 | 1 | 0 | 2 | 1 | 2 | 1 | 2 | 2 | 1 | 2 | 1 |
| NM_005448 | BMP15 | 1261 | 0 | 0 | 0 | 0 | 0 | 0 | 0 | 0 | 0 | 0 | 0 | 0 | 0 |
| NM_005449 | FAIM3 | 3086 | 6 | 1 | 0 | 1 | 0 | 0 | 0 | 0 | 0 | 0 | 0 | 6 | 0 |
| NM_005450 | NOG | 1892 | 1 | 0 | 0 | 0 | 0 | 1 | 0 | 0 | 0 | 0 | 0 | 0 | 0 |
| NM_005451 | PDLIM7 | 1709 | 9 | 4 | 1 | 9 | 6 | 1 | 2 | 0 | 3 | 6 | 2 | 5 | 4 |
| NM_005452 | WDR46 | 2363 | 4 | 2 | 2 | 2 | 2 | 2 | 2 | 2 | 2 | 2 | 3 | 4 | 2 |
| NM_005453 | ZBTB22 | 2663 | 1 | 1 | 0 | 1 | 1 | 1 | 1 | 0 | 1 | 1 | 0 | 1 | 1 |
| NM_005454 | CER1 | 1205 | 0 | 0 | 0 | 0 | 0 | 0 | 0 | 0 | 0 | 0 | 0 | 0 | 0 |
| NM_005455 | ZRANB2 | 2918 | 10 | 6 | 5 | 3 | 5 | 6 | 6 | 2 | 7 | 5 | 5 | 7 | 10 |
| NM_005456 | MAPK8IP1 | 3050 | 3 | 0 | 0 | 0 | 0 | 3 | 0 | 0 | 0 | 0 | 0 | 0 | 0 |
| NM_005458 | GABBR2 | 5482 | 14 | 2 | 0 | 0 | 0 | 14 | 0 | 0 | 2 | 0 | 0 | 0 | 1 |
| NM_005459 | GUCA1C | 925 | 1 | 0 | 0 | 0 | 0 | 0 | 0 | 0 | 0 | 0 | 0 | 0 | 1 |
| NM_005460 | SNCAIP | 3569 | 8 | 1 | 1 | 0 | 0 | 1 | 0 | 0 | 0 | 8 | 0 | 3 | 2 |
| NM_005461 | MAFB | 3360 | 9 | 2 | 2 | 0 | 2 | 1 | 1 | 1 | 3 | 1 | 0 | 9 | 0 |
| NM_005462 | MAGEC1 | 4319 | 1 | 0 | 0 | 0 | 0 | 0 | 0 | 0 | 0 | 0 | 0 | 0 | 1 |
| NM_005465 | AKT3 | 3588 | 23 | 8 | 4 | 3 | 5 | 23 | 6 | 1 | 8 | 15 | 2 | 8 | 12 |
| NM_005466 | MED6 | 1344 | 11 | 6 | 4 | 3 | 5 | 11 | 5 | 4 | 6 | 8 | 6 | 6 | 7 |
| NM_005467 | NAALAD2 | 3196 | 8 | 1 | 1 | 0 | 0 | 1 | 0 | 0 | 0 | 3 | 0 | 1 | 8 |
| NM_005468 | NAALADL1 | 2687 | 0 | 0 | 0 | 0 | 0 | 0 | 0 | 0 | 0 | 0 | 0 | 0 | 0 |
| NM_005469 | ACOT8 | 1157 | 1 | 0 | 0 | 0 | 0 | 1 | 0 | 0 | 0 | 0 | 1 | 0 | 1 |
| NM_005470 | ABI1 | 3668 | 9 | 4 | 4 | 3 | 3 | 9 | 4 | 3 | 5 | 3 | 2 | 8 | 4 |
| NM_005471 | GNPDA1 | 2297 | 4 | 2 | 2 | 0 | 1 | 2 | 4 | 1 | 1 | 2 | 1 | 3 | 2 |
| NM_005472 | KCNE3 | 3070 | 4 | 2 | 4 | 3 | 1 | 0 | 3 | 0 | 3 | 1 | 0 | 3 | 1 |
| NM_005474 | HDAC5 | 5324 | 1 | 1 | 1 | 1 | 1 | 1 | 1 | 0 | 1 | 1 | 1 | 1 | 1 |
| NM_005475 | SH2B3 | 5405 | 15 | 4 | 5 | 2 | 7 | 3 | 2 | 2 | 5 | 3 | 2 | 15 | 3 |
| NM_005476 | GNE | 5307 | 22 | 5 | 3 | 4 | 2 | 4 | 3 | 22 | 2 | 4 | 2 | 3 | 3 |
| NM_005477 | HCN4 | 7227 | 1 | 0 | 0 | 0 | 1 | 0 | 0 | 0 | 0 | 0 | 0 | 0 | 0 |
| NM_005478 | INSL5 | 715 | 0 | 0 | 0 | 0 | 0 | 0 | 0 | 0 | 0 | 0 | 0 | 0 | 0 |
| NM_005479 | FRAT1 | 2651 | 4 | 1 | 0 | 1 | 0 | 0 | 0 | 1 | 2 | 1 | 1 | 4 | 1 |
| NM_005480 | TROAP | 2618 | 0 | 0 | 0 | 0 | 0 | 0 | 0 | 0 | 0 | 0 | 0 | 0 | 0 |
| NM_005481 | MED16 | 2924 | 0 | 0 | 0 | 0 | 0 | 0 | 0 | 0 | 0 | 0 | 0 | 0 | 0 |
| NM_005482 | PIGK | 4625 | 7 | 4 | 3 | 1 | 7 | 5 | 6 | 4 | 4 | 4 | 3 | 7 | 5 |
| NM_005483 | CHAF1A | 3343 | 5 | 2 | 2 | 5 | 1 | 2 | 1 | 1 | 1 | 2 | 2 | 3 | 4 |
| NM_005484 | PARP2 | 1887 | 9 | 4 | 3 | 2 | 3 | 9 | 3 | 2 | 2 | 4 | 5 | 3 | 6 |
| NM_005485 | PARP3 | 2360 | 5 | 3 | 5 | 3 | 3 | 3 | 5 | 3 | 2 | 2 | 1 | 4 | 1 |
| NM_005486 | TOM1L1 | 2340 | 6 | 2 | 1 | 1 | 0 | 1 | 6 | 5 | 1 | 1 | 0 | 0 | 1 |
| NM_005488 | TOM1 | 2387 | 6 | 2 | 2 | 1 | 2 | 4 | 2 | 3 | 2 | 1 | 6 | 2 | 2 |
| NM_005489 | SH2D3C | 2654 | 1 | 0 | 0 | 0 | 0 | 0 | 0 | 0 | 0 | 0 | 0 | 1 | 0 |
| NM_005490 | SH2D3A | 2354 | 2 | 0 | 0 | 2 | 0 | 0 | 1 | 0 | 1 | 0 | 0 | 0 | 0 |
| NM_005491 | MAMLD1 | 4605 | 2 | 0 | 0 | 0 | 0 | 1 | 0 | 0 | 0 | 2 | 0 | 0 | 2 |
| NM_005492 | CST8 | 890 | 2 | 0 | 0 | 0 | 0 | 0 | 0 | 0 | 0 | 0 | 0 | 0 | 2 |
| NM_005493 | RANBP9 | 3113 | 20 | 9 | 6 | 4 | 7 | 15 | 5 | 7 | 7 | 8 | 9 | 7 | 20 |
| NM_005494 | DNAJB6 | 1559 | 37 | 20 | 13 | 11 | 23 | 28 | 14 | 6 | 16 | 15 | 37 | 22 | 34 |
| NM_005495 | SLC17A4 | 3599 | 8 | 1 | 0 | 4 | 0 | 0 | 8 | 3 | 0 | 0 | 0 | 0 | 0 |
| NM_005496 | SMC4 | 5279 | 19 | 6 | 4 | 2 | 7 | 2 | 3 | 3 | 8 | 5 | 4 | 14 | 19 |
| NM_005497 | GJC1 | 7644 | 3 | 1 | 1 | 3 | 2 | 0 | 0 | 0 | 1 | 1 | 0 | 0 | 1 |
| NM_005498 | AP1M2 | 1750 | 4 | 1 | 0 | 2 | 0 | 0 | 4 | 0 | 1 | 0 | 0 | 0 | 0 |
| NM_005499 | UBA2 | 2665 | 16 | 9 | 13 | 3 | 8 | 10 | 6 | 7 | 8 | 9 | 11 | 10 | 16 |
| NM_005500 | SAE1 | 2533 | 7 | 3 | 4 | 3 | 2 | 7 | 1 | 3 | 2 | 3 | 2 | 3 | 3 |
| NM_005501 | ITGA3 | 4885 | 2 | 1 | 1 | 1 | 1 | 1 | 2 | 0 | 2 | 0 | 0 | 1 | 0 |
| NM_005502 | ABCA1 | 10411 | 14 | 5 | 9 | 3 | 3 | 3 | 2 | 14 | 5 | 4 | 4 | 8 | 5 |
| NM_005503 | APBA2 | 3637 | 12 | 1 | 0 | 0 | 0 | 12 | 0 | 0 | 0 | 0 | 0 | 2 | 1 |
| NM_005504 | BCAT1 | 8266 | 11 | 4 | 5 | 1 | 3 | 6 | 1 | 0 | 11 | 6 | 1 | 3 | 8 |
| NM_005505 | SCARB1 | 2742 | 4 | 1 | 1 | 0 | 0 | 1 | 0 | 4 | 0 | 1 | 0 | 0 | 0 |
| NM_005506 | SCARB2 | 4748 | 36 | 23 | 26 | 9 | 21 | 26 | 18 | 36 | 22 | 23 | 21 | 31 | 19 |
| NM_005507 | CFL1 | 1257 | 110 | 50 | 57 | 72 | 39 | 110 | 37 | 56 | 43 | 18 | 9 | 81 | 30 |
| NM_005508 | CCR4 | 1657 | 1 | 0 | 0 | 0 | 0 | 0 | 0 | 0 | 1 | 0 | 0 | 1 | 0 |
| NM_005509 | DMXL1 | 11173 | 7 | 4 | 3 | 1 | 3 | 5 | 7 | 2 | 3 | 4 | 2 | 5 | 4 |
| NM_005510 | DOM3Z | 1590 | 1 | 1 | 0 | 1 | 0 | 1 | 1 | 1 | 0 | 1 | 1 | 1 | 1 |
| NM_005511 | MLANA | 1524 | 1 | 0 | 0 | 0 | 0 | 0 | 0 | 0 | 0 | 0 | 0 | 1 | 0 |
| NM_005512 | LRRC32 | 4312 | 5 | 2 | 2 | 1 | 2 | 1 | 2 | 0 | 5 | 1 | 1 | 3 | 1 |
| NM_005513 | GTF2E1 | 3033 | 7 | 4 | 2 | 2 | 3 | 3 | 3 | 4 | 3 | 4 | 2 | 7 | 7 |
| NM_005514 | HLA-B | 1572 | 575 | 139 | 109 | 36 | 123 | 40 | 76 | 50 | 318 | 80 | 64 | 575 | 57 |
| NM_005515 | MNX1 | 2176 | 0 | 0 | 0 | 0 | 0 | 0 | 0 | 0 | 0 | 0 | 0 | 0 | 0 |
| NM_005516 | HLA-E | 1685 | 69 | 24 | 28 | 18 | 14 | 16 | 16 | 16 | 69 | 8 | 9 | 58 | 9 |
| NM_005517 | HMGN2 | 1969 | 5 | 4 | 3 | 5 | 3 | 4 | 4 | 2 | 4 | 5 | 3 | 4 | 4 |
| NM_005518 | HMGCS2 | 2465 | 12 | 3 | 0 | 8 | 1 | 0 | 4 | 12 | 0 | 1 | 0 | 0 | 4 |
| NM_005519 | HMX2 | 1628 | 0 | 0 | 0 | 0 | 0 | 0 | 0 | 0 | 0 | 0 | 0 | 0 | 0 |
| NM_005520 | HNRNPH1 | 2249 | 59 | 37 | 32 | 31 | 36 | 30 | 43 | 18 | 40 | 45 | 27 | 59 | 41 |
| NM_005521 | TLX1 | 2106 | 3 | 0 | 0 | 0 | 0 | 0 | 0 | 0 | 0 | 0 | 0 | 3 | 0 |
| NM_005522 | HOXA1 | 2547 | 0 | 0 | 0 | 0 | 0 | 0 | 0 | 0 | 0 | 0 | 0 | 0 | 0 |
| NM_005523 | HOXA11 | 2653 | 1 | 0 | 0 | 1 | 0 | 0 | 0 | 0 | 0 | 0 | 0 | 0 | 0 |
| NM_005524 | HES1 | 1453 | 6 | 3 | 2 | 2 | 2 | 1 | 6 | 5 | 3 | 4 | 5 | 2 | 2 |
| NM_005525 | HSD11B1 | 1405 | 230 | 25 | 14 | 1 | 4 | 2 | 1 | 230 | 6 | 3 | 1 | 6 | 8 |
| NM_005526 | HSF1 | 2152 | 3 | 2 | 1 | 2 | 2 | 1 | 1 | 1 | 1 | 2 | 3 | 3 | 3 |
| NM_005527 | HSPA1L | 2537 | 6 | 1 | 0 | 0 | 1 | 2 | 1 | 0 | 1 | 1 | 1 | 1 | 6 |

| | | transcript | | | | | | hypothala | | | | | skeletalm | | |
|---|---|---|---|---|---|---|---|---|---|---|---|---|---|---|---|
| transcript | gene | length (nt) | max | mean | adipose | colon | heart | mus | kidney | liver | lung | ovary | uscle | spleen | testes |
| NM_005528 | DNAJC4 | 1285 | 10 | 4 | 2 | 3 | 6 | 3 | 5 | 3 | 3 | 6 | 3 | 6 | 10 |
| NM_005529 | HSPG2 | 14288 | 2 | 1 | 2 | 2 | 2 | 0 | 1 | 0 | 1 | 1 | 0 | 1 | 1 |
| NM_005530 | IDH3A | 2665 | 17 | 6 | 4 | 3 | 17 | 13 | 7 | 4 | 3 | 2 | 7 | 5 | 7 |
| NM_005531 | IFI16 | 2734 | 46 | 15 | 18 | 3 | 16 | 14 | 5 | 3 | 22 | 20 | 7 | 46 | 14 |
| NM_005532 | IFI27 | 652 | 7 | 3 | 4 | 4 | 2 | 3 | 1 | 0 | 3 | 1 | 1 | 7 | 1 |
| NM_005533 | IFI35 | 1222 | 3 | 1 | 2 | 1 | 2 | 1 | 2 | 2 | 1 | 1 | 1 | 3 | 1 |
| NM_005534 | IFNGR2 | 2221 | 8 | 3 | 3 | 2 | 3 | 3 | 3 | 2 | 5 | 3 | 2 | 8 | 3 |
| NM_005535 | IL12RB1 | 2100 | 0 | 0 | 0 | 0 | 0 | 0 | 0 | 0 | 0 | 0 | 0 | 0 | 0 |
| NM_005536 | IMPA1 | 3396 | 11 | 5 | 2 | 2 | 8 | 9 | 7 | 5 | 5 | 3 | 2 | 6 | 11 |
| NM_005537 | ING1 | 2843 | 3 | 1 | 1 | 1 | 1 | 1 | 1 | 1 | 1 | 3 | 1 | 2 | 1 |
| NM_005538 | INHBC | 1990 | 7 | 1 | 0 | 0 | 0 | 0 | 0 | 7 | 0 | 0 | 0 | 0 | 0 |
| NM_005539 | INPP5A | 2938 | 9 | 3 | 1 | 2 | 9 | 3 | 2 | 1 | 2 | 3 | 5 | 3 | 4 |
| NM_005540 | INPP5B | 4454 | 4 | 2 | 2 | 2 | 2 | 2 | 2 | 1 | 3 | 2 | 1 | 4 | 2 |
| NM_005541 | INPP5D | 4443 | 6 | 1 | 1 | 1 | 0 | 1 | 0 | 0 | 1 | 0 | 0 | 6 | 0 |
| NM_005542 | INSIG1 | 3008 | 10 | 2 | 2 | 0 | 1 | 2 | 1 | 10 | 3 | 1 | 0 | 1 | 1 |
| NM_005543 | INSL3 | 742 | 4 | 1 | 0 | 0 | 0 | 0 | 0 | 0 | 0 | 2 | 0 | 0 | 4 |
| NM_005544 | IRS1 | 8736 | 6 | 2 | 1 | 2 | 1 | 1 | 1 | 2 | 1 | 6 | 2 | 1 | 1 |
| NM_005545 | ISLR | 2295 | 3 | 1 | 1 | 0 | 0 | 0 | 0 | 0 | 0 | 3 | 0 | 1 | 1 |
| NM_005546 | ITK | 4366 | 6 | 1 | 0 | 1 | 0 | 0 | 0 | 0 | 2 | 0 | 0 | 6 | 0 |
| NM_005547 | IVL | 2135 | 0 | 0 | 0 | 0 | 0 | 0 | 0 | 0 | 0 | 0 | 0 | 0 | 0 |
| NM_005548 | KARS | 2013 | 26 | 13 | 11 | 8 | 14 | 13 | 11 | 14 | 9 | 10 | 26 | 14 | 15 |
| NM_005549 | KCNA10 | 1959 | 0 | 0 | 0 | 0 | 0 | 0 | 0 | 0 | 0 | 0 | 0 | 0 | 0 |
| NM_005550 | KIFC3 | 3366 | 1 | 0 | 0 | 0 | 1 | 1 | 1 | 0 | 0 | 0 | 0 | 0 | 0 |
| NM_005551 | KLK2 | 2835 | 0 | 0 | 0 | 0 | 0 | 0 | 0 | 0 | 0 | 0 | 0 | 0 | 0 |
| NM_005552 | KLC1 | 2603 | 94 | 22 | 16 | 16 | 23 | 94 | 9 | 7 | 12 | 14 | 16 | 16 | 14 |
| NM_005553 | KRTAP5-9 | 1188 | 0 | 0 | 0 | 0 | 0 | 0 | 0 | 0 | 0 | 0 | 0 | 0 | 0 |
| NM_005554 | KRT6A | 2450 | 0 | 0 | 0 | 0 | 0 | 0 | 0 | 0 | 0 | 0 | 0 | 0 | 0 |
| NM_005555 | KRT6B | 2282 | 0 | 0 | 0 | 0 | 0 | 0 | 0 | 0 | 0 | 0 | 0 | 0 | 0 |
| NM_005556 | KRT7 | 1702 | 19 | 3 | 0 | 0 | 0 | 0 | 6 | 1 | 19 | 0 | 0 | 0 | 1 |
| NM_005557 | KRT16 | 1717 | 0 | 0 | 0 | 0 | 0 | 0 | 0 | 0 | 0 | 0 | 0 | 0 | 0 |
| NM_005558 | LAD1 | 2839 | 6 | 1 | 0 | 6 | 0 | 0 | 4 | 1 | 1 | 0 | 0 | 0 | 0 |
| NM_005559 | LAMA1 | 9514 | 5 | 1 | 0 | 0 | 0 | 1 | 1 | 0 | 0 | 1 | 0 | 0 | 5 |
| NM_005560 | LAMA5 | 11426 | 0 | 0 | 0 | 0 | 0 | 0 | 0 | 0 | 0 | 0 | 0 | 0 | 0 |
| NM_005561 | LAMP1 | 2540 | 59 | 41 | 44 | 21 | 35 | 36 | 58 | 59 | 45 | 33 | 17 | 54 | 43 |
| NM_005562 | LAMC2 | 5623 | 3 | 1 | 0 | 2 | 0 | 0 | 1 | 0 | 3 | 0 | 0 | 0 | 0 |
| NM_005563 | STMN1 | 1526 | 983 | 164 | 92 | 60 | 48 | 983 | 41 | 8 | 57 | 156 | 14 | 67 | 275 |
| NM_005564 | LCN2 | 822 | 19 | 3 | 0 | 2 | 1 | 1 | 2 | 3 | 19 | 2 | 0 | 5 | 2 |
| NM_005565 | LCP2 | 2422 | 22 | 4 | 4 | 2 | 3 | 3 | 1 | 2 | 10 | 1 | 1 | 22 | 1 |
| NM_005566 | LDHA | 2208 | 293 | 91 | 116 | 40 | 135 | 77 | 86 | 293 | 57 | 19 | 48 | 74 | 54 |
| NM_005567 | LGALS3BP | 2259 | 3 | 2 | 3 | 3 | 3 | 2 | 2 | 1 | 2 | 2 | 1 | 1 | 2 |
| NM_005568 | LHX1 | 2283 | 0 | 0 | 0 | 0 | 0 | 0 | 0 | 0 | 0 | 0 | 0 | 0 | 0 |
| NM_005569 | LIMK2 | 3701 | 4 | 2 | 1 | 3 | 1 | 2 | 2 | 1 | 4 | 1 | 1 | 4 | 3 |
| NM_005570 | LMAN1 | 4833 | 37 | 11 | 5 | 2 | 13 | 5 | 12 | 37 | 9 | 9 | 6 | 10 | 10 |
| NM_005572 | LMNA | 2070 | 6 | 3 | 6 | 5 | 3 | 1 | 2 | 1 | 3 | 2 | 1 | 2 | 2 |
| NM_005573 | LMNB1 | 2882 | 10 | 3 | 3 | 9 | 2 | 3 | 1 | 1 | 5 | 2 | 0 | 10 | 2 |
| NM_005574 | LMO2 | 2293 | 10 | 3 | 4 | 1 | 2 | 10 | 3 | 1 | 3 | 2 | 3 | 6 | 1 |
| NM_005575 | LNPEP | 4470 | 22 | 11 | 22 | 8 | 11 | 9 | 9 | 3 | 12 | 8 | 12 | 17 | 8 |
| NM_005576 | LOXL1 | 2361 | 2 | 1 | 1 | 1 | 2 | 0 | 1 | 0 | 0 | 1 | 0 | 1 | 0 |
| NM_005577 | LPA | 6489 | 4 | 0 | 0 | 0 | 0 | 0 | 0 | 4 | 0 | 0 | 0 | 0 | 0 |
| NM_005578 | LPP | 18277 | 13 | 7 | 6 | 10 | 13 | 2 | 9 | 2 | 9 | 7 | 5 | 10 | 9 |
| NM_005581 | BCAM | 2431 | 6 | 2 | 1 | 1 | 1 | 0 | 6 | 1 | 3 | 1 | 1 | 1 | 1 |
| NM_005582 | CD180 | 2715 | 17 | 2 | 2 | 1 | 1 | 1 | 0 | 0 | 2 | 0 | 0 | 17 | 0 |
| NM_005583 | LYL1 | 1490 | 0 | 0 | 0 | 0 | 0 | 0 | 0 | 0 | 0 | 0 | 0 | 0 | 0 |
| NM_005584 | MAB21L1 | 2513 | 1 | 0 | 1 | 0 | 0 | 0 | 0 | 0 | 0 | 1 | 1 | 0 | 0 |
| NM_005585 | SMAD6 | 2876 | 1 | 0 | 0 | 0 | 0 | 0 | 0 | 0 | 1 | 0 | 0 | 0 | 0 |
| NM_005586 | MDFI | 1617 | 1 | 0 | 1 | 0 | 0 | 0 | 0 | 0 | 1 | 1 | 0 | 0 | 0 |
| NM_005587 | MEF2A | 5552 | 14 | 6 | 3 | 1 | 14 | 6 | 4 | 3 | 6 | 7 | 10 | 9 | 5 |
| NM_005588 | MEP1A | 2929 | 5 | 0 | 0 | 5 | 0 | 0 | 0 | 0 | 0 | 0 | 0 | 0 | 0 |
| NM_005589 | ALDH6A1 | 2180 | 38 | 10 | 4 | 2 | 4 | 11 | 38 | 30 | 3 | 4 | 2 | 2 | 8 |
| NM_005590 | MRE11A | 5163 | 5 | 3 | 2 | 1 | 2 | 2 | 2 | 1 | 2 | 5 | 2 | 5 | 4 |
| NM_005591 | MRE11A | 5140 | 5 | 3 | 2 | 1 | 2 | 2 | 2 | 1 | 2 | 5 | 2 | 5 | 4 |
| NM_005592 | MUSK | 2754 | 2 | 1 | 0 | 1 | 1 | 0 | 0 | 0 | 1 | 0 | 2 | 0 | 1 |
| NM_005593 | MYF5 | 1520 | 0 | 0 | 0 | 0 | 0 | 0 | 0 | 0 | 0 | 0 | 0 | 0 | 0 |
| NM_005594 | NACA | 1082 | 245 | 105 | 102 | 38 | 106 | 115 | 58 | 110 | 77 | 245 | 108 | 113 | 87 |
| NM_005595 | NFIA | 9389 | 15 | 10 | 10 | 4 | 15 | 9 | 8 | 9 | 7 | 15 | 7 | 10 | 13 |
| NM_005596 | NFIB | 8159 | 16 | 9 | 14 | 3 | 16 | 8 | 9 | 5 | 8 | 11 | 7 | 6 | 7 |
| NM_005597 | NFIC | 2185 | 40 | 16 | 17 | 13 | 19 | 9 | 9 | 21 | 10 | 21 | 40 | 11 | 12 |
| NM_005598 | NHLH1 | 2559 | 0 | 0 | 0 | 0 | 0 | 0 | 0 | 0 | 0 | 0 | 0 | 0 | 0 |
| NM_005599 | NHLH2 | 2511 | 1 | 0 | 0 | 0 | 0 | 1 | 0 | 0 | 0 | 0 | 0 | 0 | 0 |
| NM_005600 | NIT1 | 1362 | 3 | 1 | 1 | 1 | 1 | 2 | 3 | 2 | 1 | 1 | 1 | 2 | 1 |
| NM_005601 | NKG7 | 826 | 0 | 0 | 0 | 0 | 0 | 0 | 0 | 0 | 0 | 0 | 0 | 0 | 0 |
| NM_005602 | CLDN11 | 2760 | 47 | 9 | 1 | 2 | 1 | 31 | 0 | 1 | 2 | 12 | 0 | 1 | 47 |
| NM_005603 | ATP8B1 | 5817 | 20 | 5 | 1 | 20 | 6 | 1 | 5 | 7 | 6 | 2 | 2 | 5 | 3 |
| NM_005604 | POU3F2 | 4087 | 3 | 0 | 0 | 0 | 0 | 3 | 0 | 0 | 0 | 0 | 0 | 0 | 0 |
| NM_005605 | PPP3CC | 2188 | 12 | 6 | 5 | 3 | 8 | 3 | 3 | 3 | 6 | 3 | 12 | 7 | 9 |
| NM_005606 | LGMN | 2019 | 36 | 13 | 27 | 6 | 10 | 6 | 36 | 6 | 11 | 5 | 4 | 22 | 6 |
| NM_005607 | PTK2 | 4442 | 18 | 8 | 4 | 7 | 7 | 18 | 9 | 4 | 6 | 9 | 5 | 5 | 11 |

| | | transcript | | | | | | hypothala | | | | | skeletalm | | |
|---|---|---|---|---|---|---|---|---|---|---|---|---|---|---|---|
| transcript | gene | length (nt) | max | mean | adipose | colon | heart | mus | kidney | liver | lung | ovary | uscle | spleen | testes |
| NM_005608 | PTPRCAP | 907 | 17 | 2 | 0 | 2 | 0 | 0 | 0 | 0 | 2 | 0 | 0 | 17 | 0 |
| NM_005609 | PYGM | 3616 | 124 | 12 | 1 | 1 | 3 | 1 | 0 | 0 | 0 | 0 | 124 | 0 | 1 |
| NM_005610 | RBBP4 | 7941 | 24 | 10 | 4 | 5 | 9 | 9 | 8 | 5 | 7 | 15 | 8 | 15 | 24 |
| NM_005611 | RBL2 | 4886 | 21 | 12 | 9 | 6 | 11 | 8 | 12 | 8 | 14 | 12 | 9 | 21 | 17 |
| NM_005612 | REST | 3663 | 17 | 10 | 10 | 3 | 9 | 4 | 11 | 6 | 8 | 16 | 5 | 15 | 17 |
| NM_005613 | RGS4 | 3371 | 60 | 6 | 0 | 1 | 1 | 60 | 0 | 0 | 0 | 2 | 0 | 0 | 1 |
| NM_005614 | RHEB | 2075 | 5 | 3 | 2 | 1 | 4 | 5 | 3 | 2 | 2 | 2 | 4 | 3 | 3 |
| NM_005615 | RNASE6 | 1061 | 8 | 2 | 3 | 1 | 2 | 0 | 1 | 0 | 2 | 1 | 0 | 8 | 1 |
| NM_005617 | RPS14 | 569 | 52 | 36 | 37 | 36 | 29 | 24 | 30 | 32 | 45 | 52 | 35 | 47 | 29 |
| NM_005618 | DLL1 | 3310 | 3 | 1 | 1 | 1 | 0 | 0 | 0 | 0 | 0 | 0 | 0 | 3 | 0 |
| NM_005619 | RTN2 | 2282 | 2 | 0 | 0 | 0 | 0 | 0 | 0 | 0 | 0 | 0 | 2 | 0 | 0 |
| NM_005620 | S100A11 | 594 | 21 | 9 | 18 | 5 | 12 | 3 | 11 | 2 | 14 | 4 | 1 | 21 | 2 |
| NM_005621 | S100A12 | 466 | 159 | 25 | 2 | 0 | 29 | 5 | 0 | 10 | 58 | 0 | 1 | 159 | 5 |
| NM_005622 | ACSM3 | 2133 | 6 | 1 | 0 | 1 | 0 | 0 | 6 | 3 | 1 | 4 | 0 | 0 | 1 |
| NM_005623 | CCL8 | 1250 | 2 | 1 | 2 | 2 | 1 | 1 | 1 | 0 | 1 | 1 | 0 | 0 | 0 |
| NM_005624 | CCL25 | 891 | 0 | 0 | 0 | 0 | 0 | 0 | 0 | 0 | 0 | 0 | 0 | 0 | 0 |
| NM_005625 | SDCBP | 2167 | 24 | 13 | 22 | 6 | 14 | 16 | 10 | 10 | 24 | 7 | 4 | 24 | 7 |
| NM_005626 | SFRS4 | 2529 | 42 | 28 | 21 | 21 | 31 | 25 | 23 | 20 | 37 | 31 | 30 | 42 | 26 |
| NM_005627 | SGK1 | 2407 | 8 | 4 | 3 | 2 | 2 | 5 | 6 | 8 | 4 | 7 | 1 | 2 | 2 |
| NM_005628 | SLC1A5 | 2871 | 4 | 2 | 4 | 2 | 1 | 2 | 2 | 1 | 2 | 2 | 0 | 3 | 1 |
| NM_005629 | SLC6A8 | 3565 | 3 | 1 | 1 | 1 | 2 | 2 | 3 | 0 | 0 | 0 | 2 | 1 | 1 |
| NM_005630 | SLCO2A1 | 4223 | 5 | 2 | 0 | 1 | 3 | 0 | 3 | 1 | 5 | 0 | 0 | 4 | 0 |
| NM_005631 | SMO | 3737 | 1 | 0 | 0 | 0 | 0 | 0 | 1 | 0 | 0 | 0 | 0 | 0 | 0 |
| NM_005632 | SOLH | 4744 | 0 | 0 | 0 | 0 | 0 | 0 | 0 | 0 | 0 | 0 | 0 | 0 | 0 |
| NM_005633 | SOS1 | 8318 | 10 | 5 | 9 | 2 | 5 | 5 | 5 | 3 | 4 | 10 | 5 | 6 | 7 |
| NM_005634 | SOX3 | 2074 | 0 | 0 | 0 | 0 | 0 | 0 | 0 | 0 | 0 | 0 | 0 | 0 | 0 |
| NM_005635 | SSX1 | 1253 | 1 | 0 | 0 | 0 | 0 | 0 | 0 | 0 | 0 | 0 | 0 | 0 | 1 |
| NM_005636 | SSX4 | 1217 | 0 | 0 | 0 | 0 | 0 | 0 | 0 | 0 | 0 | 0 | 0 | 0 | 0 |
| NM_005637 | SS18 | 3343 | 32 | 19 | 16 | 8 | 21 | 13 | 23 | 18 | 16 | 32 | 6 | 28 | 28 |
| NM_005638 | VAMP7 | 2654 | 4 | 3 | 3 | 1 | 3 | 3 | 4 | 3 | 3 | 2 | 1 | 4 | 3 |
| NM_005639 | SYT1 | 4809 | 134 | 13 | 0 | 4 | 0 | 134 | 1 | 0 | 0 | 2 | 0 | 1 | 1 |
| NM_005640 | TAF4B | 4751 | 4 | 1 | 1 | 1 | 1 | 2 | 1 | 0 | 1 | 2 | 1 | 3 | 4 |
| NM_005641 | TAF6 | 2737 | 4 | 2 | 1 | 2 | 2 | 4 | 2 | 1 | 1 | 2 | 1 | 2 | 2 |
| NM_005642 | TAF7 | 2295 | 55 | 36 | 23 | 10 | 54 | 46 | 24 | 24 | 23 | 43 | 53 | 38 | 55 |
| NM_005643 | TAF11 | 1533 | 7 | 4 | 3 | 3 | 4 | 4 | 3 | 3 | 3 | 3 | 3 | 5 | 7 |
| NM_005644 | TAF12 | 1111 | 6 | 5 | 5 | 2 | 6 | 4 | 4 | 5 | 4 | 5 | 5 | 6 | 6 |
| NM_005645 | TAF13 | 577 | 40 | 18 | 25 | 12 | 21 | 40 | 17 | 30 | 12 | 5 | 10 | 10 | 12 |
| NM_005646 | TARBP1 | 5130 | 1 | 1 | 1 | 1 | 1 | 1 | 1 | 0 | 1 | 0 | 1 | 1 | 1 |
| NM_005647 | TBL1X | 5714 | 3 | 2 | 1 | 1 | 1 | 2 | 2 | 2 | 1 | 2 | 0 | 2 | 3 |
| NM_005648 | TCEB1 | 655 | 59 | 30 | 30 | 13 | 56 | 59 | 22 | 37 | 20 | 12 | 20 | 23 | 38 |
| NM_005649 | ZNF354A | 2555 | 3 | 2 | 1 | 0 | 2 | 2 | 3 | 1 | 1 | 2 | 1 | 3 | 3 |
| NM_005650 | TCF20 | 5789 | 10 | 6 | 3 | 7 | 4 | 9 | 6 | 4 | 5 | 8 | 3 | 10 | 7 |
| NM_005650 | TCF20 | 7407 | 8 | 5 | 3 | 5 | 3 | 7 | 5 | 3 | 4 | 6 | 2 | 8 | 6 |
| NM_005651 | TDO2 | 1693 | 63 | 6 | 0 | 0 | 0 | 0 | 0 | 63 | 0 | 1 | 0 | 1 | 0 |
| NM_005652 | TERF2 | 2896 | 15 | 8 | 6 | 6 | 7 | 15 | 6 | 7 | 5 | 8 | 9 | 9 | 9 |
| NM_005653 | TFCP2 | 2372 | 3 | 2 | 2 | 2 | 2 | 3 | 3 | 1 | 1 | 2 | 2 | 3 | 2 |
| NM_005654 | NR2F1 | 3197 | 4 | 1 | 0 | 0 | 0 | 1 | 2 | 0 | 2 | 4 | 0 | 0 | 1 |
| NM_005655 | KLF10 | 2950 | 41 | 12 | 7 | 5 | 13 | 3 | 12 | 7 | 11 | 12 | 41 | 14 | 10 |
| NM_005656 | TMPRSS2 | 3202 | 9 | 2 | 0 | 9 | 0 | 0 | 6 | 3 | 5 | 0 | 0 | 0 | 0 |
| NM_005657 | TP53BP1 | 6234 | 18 | 7 | 5 | 5 | 7 | 18 | 7 | 1 | 5 | 10 | 3 | 7 | 15 |
| NM_005658 | TRAF1 | 4449 | 1 | 0 | 0 | 1 | 0 | 0 | 0 | 0 | 1 | 0 | 0 | 1 | 0 |
| NM_005659 | UFD1L | 1783 | 6 | 3 | 4 | 2 | 3 | 3 | 2 | 6 | 1 | 2 | 2 | 3 | 2 |
| NM_005660 | SLC35A2 | 1451 | 0 | 0 | 0 | 0 | 0 | 0 | 0 | 0 | 0 | 0 | 0 | 0 | 0 |
| NM_005662 | VDAC3 | 1428 | 44 | 18 | 10 | 9 | 34 | 26 | 16 | 9 | 8 | 8 | 44 | 13 | 19 |
| NM_005663 | WHSC2 | 2463 | 0 | 0 | 0 | 0 | 0 | 0 | 0 | 0 | 0 | 0 | 0 | 0 | 0 |
| NM_005664 | MKRN3 | 2713 | 0 | 0 | 0 | 0 | 0 | 0 | 0 | 0 | 0 | 0 | 0 | 0 | 0 |
| NM_005665 | EVI5 | 7403 | 6 | 3 | 2 | 1 | 4 | 4 | 3 | 5 | 3 | 3 | 1 | 3 | 6 |
| NM_005666 | CFHR2 | 1036 | 15 | 1 | 0 | 0 | 0 | 0 | 0 | 15 | 0 | 0 | 0 | 0 | 0 |
| NM_005667 | RNF103 | 3477 | 8 | 3 | 2 | 2 | 5 | 5 | 3 | 8 | 2 | 3 | 2 | 3 | 3 |
| NM_005668 | ST8SIA4 | 6319 | 12 | 3 | 2 | 1 | 3 | 3 | 0 | 1 | 7 | 1 | 1 | 12 | 1 |
| NM_005669 | REEP5 | 3116 | 10 | 4 | 4 | 1 | 6 | 10 | 7 | 4 | 3 | 3 | 3 | 4 | 2 |
| NM_005670 | EPM2A | 3465 | 7 | 2 | 2 | 1 | 3 | 2 | 2 | 1 | 0 | 1 | 7 | 0 | 1 |
| NM_005671 | UBXN8 | 1439 | 8 | 2 | 2 | 0 | 0 | 2 | 2 | 4 | 1 | 8 | 0 | 2 | 3 |
| NM_005672 | PSCA | 1020 | 0 | 0 | 0 | 0 | 0 | 0 | 0 | 0 | 0 | 0 | 0 | 0 | 0 |
| NM_005674 | ZNF239 | 2388 | 2 | 1 | 1 | 0 | 1 | 2 | 1 | 0 | 0 | 1 | 1 | 1 | 1 |
| NM_005675 | DGCR6 | 1214 | 0 | 0 | 0 | 0 | 0 | 0 | 0 | 0 | 0 | 0 | 0 | 0 | 0 |
| NM_005676 | RBM10 | 3384 | 3 | 2 | 1 | 2 | 1 | 3 | 1 | 2 | 1 | 2 | 1 | 2 | 2 |
| NM_005677 | COLQ | 3005 | 0 | 0 | 0 | 0 | 0 | 0 | 0 | 0 | 0 | 0 | 0 | 0 | 0 |
| NM_005678 | SNURF | 1326 | 0 | 0 | 0 | 0 | 0 | 0 | 0 | 0 | 0 | 0 | 0 | 0 | 0 |
| NM_005679 | TAF1C | 3924 | 1 | 1 | 1 | 1 | 1 | 1 | 1 | 0 | 1 | 1 | 0 | 1 | 1 |
| NM_005680 | TAF1B | 2297 | 4 | 2 | 2 | 1 | 2 | 3 | 3 | 1 | 2 | 3 | 1 | 3 | 4 |
| NM_005681 | TAF1A | 1879 | 4 | 2 | 1 | 1 | 1 | 2 | 1 | 1 | 1 | 3 | 1 | 3 | 4 |
| NM_005682 | GPR56 | 3824 | 28 | 5 | 2 | 2 | 2 | 14 | 28 | 0 | 2 | 0 | 0 | 1 | 9 |
| NM_005683 | GPR55 | 3828 | 4 | 1 | 0 | 1 | 0 | 0 | 0 | 0 | 0 | 0 | 0 | 2 | 4 |
| NM_005684 | GPR52 | 1472 | 4 | 2 | 0 | 1 | 2 | 2 | 2 | 0 | 1 | 3 | 1 | 4 | 1 |
| NM_005685 | GTF2IRD1 | 3063 | 2 | 1 | 0 | 1 | 1 | 0 | 1 | 0 | 0 | 1 | 2 | 0 | 0 |
| NM_005686 | SOX13 | 4073 | 1 | 1 | 1 | 1 | 0 | 0 | 1 | 0 | 1 | 1 | 0 | 1 | 1 |

| transcript | gene | transcript length (nt) | max | mean | adipose | colon | heart | hypothalamus | kidney | liver | lung | ovary | skeletalmuscle | spleen | testes |
|---|---|---|---|---|---|---|---|---|---|---|---|---|---|---|---|
| NM_005687 | FARSB | 2233 | 15 | 8 | 4 | 4 | 7 | 15 | 5 | 8 | 4 | 10 | 10 | 6 | 10 |
| NM_005688 | ABCC5 | 5843 | 9 | 4 | 4 | 4 | 5 | 6 | 3 | 1 | 2 | 2 | 9 | 5 | 5 |
| NM_005689 | ABCB6 | 2972 | 3 | 1 | 1 | 1 | 1 | 2 | 1 | 3 | 1 | 1 | 1 | 1 | 2 |
| NM_005690 | DNM1L | 4501 | 11 | 5 | 3 | 1 | 8 | 11 | 6 | 3 | 3 | 5 | 4 | 6 | 7 |
| NM_005691 | ABCC9 | 4670 | 16 | 4 | 4 | 1 | 7 | 2 | 2 | 16 | 2 | 2 | 8 | 1 | 2 |
| NM_005692 | ABCF2 | 2250 | 9 | 5 | 5 | 3 | 7 | 9 | 5 | 4 | 3 | 3 | 9 | 5 | 5 |
| NM_005693 | NR1H3 | 1719 | 7 | 3 | 7 | 3 | 1 | 1 | 2 | 6 | 2 | 1 | 1 | 6 | 2 |
| NM_005694 | COX17 | 423 | 30 | 12 | 15 | 5 | 30 | 17 | 11 | 17 | 6 | 3 | 13 | 6 | 5 |
| NM_005697 | SCAMP2 | 1291 | 4 | 2 | 2 | 2 | 2 | 1 | 4 | 2 | 2 | 1 | 1 | 3 | 2 |
| NM_005698 | SCAMP3 | 1591 | 4 | 2 | 3 | 2 | 2 | 3 | 3 | 4 | 2 | 2 | 2 | 3 | 2 |
| NM_005700 | DPP3 | 2684 | 1 | 1 | 1 | 1 | 0 | 1 | 1 | 1 | 0 | 0 | 0 | 1 | 0 |
| NM_005701 | SNUPN | 1803 | 6 | 3 | 5 | 3 | 2 | 6 | 3 | 3 | 1 | 2 | 1 | 3 | 4 |
| NM_005702 | ERAL1 | 1825 | 2 | 1 | 1 | 2 | 1 | 1 | 2 | 2 | 1 | 1 | 1 | 2 | 2 |
| NM_005704 | PTPRU | 5594 | 1 | 1 | 0 | 0 | 1 | 1 | 0 | 1 | 1 | 0 | 1 | 0 | 0 |
| NM_005706 | TSSC4 | 1424 | 0 | 0 | 0 | 0 | 0 | 0 | 0 | 0 | 0 | 0 | 0 | 0 | 0 |
| NM_005707 | PDCD7 | 2851 | 6 | 3 | 2 | 3 | 3 | 4 | 3 | 1 | 3 | 4 | 2 | 4 | 6 |
| NM_005708 | GPC6 | 7096 | 3 | 1 | 2 | 1 | 1 | 1 | 2 | 3 | 1 | 3 | 0 | 2 | 1 |
| NM_005709 | USH1C | 2236 | 8 | 1 | 0 | 6 | 0 | 2 | 8 | 0 | 0 | 0 | 0 | 0 | 0 |
| NM_005710 | PQBP1 | 1111 | 9 | 7 | 7 | 8 | 5 | 9 | 6 | 9 | 5 | 7 | 4 | 6 | 5 |
| NM_005711 | EDIL3 | 2974 | 21 | 3 | 1 | 4 | 3 | 21 | 1 | 0 | 3 | 2 | 0 | 1 | 1 |
| NM_005713 | COL4A3BP | 5395 | 11 | 7 | 11 | 3 | 5 | 8 | 6 | 4 | 8 | 7 | 7 | 10 | 8 |
| NM_005714 | KCNK7 | 1563 | 0 | 0 | 0 | 0 | 0 | 0 | 0 | 0 | 0 | 0 | 0 | 0 | 0 |
| NM_005715 | UST | 4391 | 2 | 1 | 1 | 0 | 1 | 2 | 0 | 0 | 1 | 2 | 0 | 2 | 1 |
| NM_005716 | GIPC1 | 1929 | 6 | 2 | 4 | 6 | 1 | 3 | 3 | 2 | 3 | 1 | 1 | 2 | 1 |
| NM_005717 | ARPC5 | 1982 | 35 | 13 | 14 | 8 | 13 | 14 | 9 | 10 | 22 | 8 | 3 | 35 | 13 |
| NM_005718 | ARPC4 | 1976 | 44 | 22 | 23 | 19 | 19 | 34 | 21 | 18 | 20 | 15 | 11 | 44 | 19 |
| NM_005719 | ARPC3 | 884 | 136 | 55 | 57 | 29 | 59 | 60 | 53 | 52 | 67 | 37 | 22 | 136 | 36 |
| NM_005720 | ARPC1B | 1500 | 28 | 7 | 6 | 3 | 9 | 2 | 5 | 4 | 12 | 1 | 1 | 28 | 3 |
| NM_005721 | ACTR3 | 2712 | 53 | 22 | 21 | 9 | 28 | 22 | 21 | 17 | 25 | 15 | 8 | 53 | 19 |
| NM_005722 | ACTR2 | 3917 | 76 | 28 | 25 | 11 | 27 | 38 | 22 | 21 | 30 | 22 | 14 | 76 | 23 |
| NM_005723 | TSPAN5 | 3396 | 7 | 2 | 0 | 0 | 0 | 7 | 1 | 0 | 0 | 6 | 0 | 2 | 3 |
| NM_005724 | TSPAN3 | 3851 | 30 | 14 | 29 | 13 | 8 | 30 | 13 | 5 | 13 | 9 | 13 | 10 | 9 |
| NM_005725 | TSPAN2 | 3211 | 3 | 1 | 1 | 2 | 3 | 1 | 2 | 0 | 1 | 1 | 0 | 1 | 1 |
| NM_005726 | TSFM | 2028 | 3 | 2 | 1 | 2 | 2 | 3 | 3 | 3 | 1 | 1 | 1 | 1 | 2 |
| NM_005727 | TSPAN1 | 1628 | 32 | 5 | 0 | 14 | 0 | 0 | 32 | 0 | 5 | 0 | 0 | 0 | 2 |
| NM_005729 | PPIF | 2200 | 7 | 2 | 1 | 1 | 3 | 3 | 3 | 7 | 1 | 1 | 1 | 2 | 2 |
| NM_005730 | CTDSP2 | 5020 | 18 | 12 | 12 | 15 | 11 | 9 | 10 | 10 | 14 | 16 | 11 | 18 | 8 |
| NM_005731 | ARPC2 | 1410 | 53 | 23 | 27 | 13 | 27 | 27 | 18 | 15 | 27 | 14 | 14 | 53 | 18 |
| NM_005732 | RAD50 | 5865 | 17 | 12 | 11 | 3 | 13 | 11 | 12 | 10 | 8 | 17 | 12 | 14 | 16 |
| NM_005733 | KIF20A | 3470 | 1 | 0 | 0 | 0 | 0 | 0 | 0 | 0 | 0 | 0 | 0 | 0 | 1 |
| NM_005734 | HIPK3 | 4743 | 36 | 19 | 14 | 5 | 36 | 12 | 16 | 10 | 17 | 21 | 31 | 22 | 20 |
| NM_005735 | ACTR1B | 2258 | 3 | 2 | 1 | 2 | 3 | 3 | 2 | 2 | 1 | 2 | 2 | 2 | 1 |
| NM_005736 | ACTR1A | 2872 | 12 | 5 | 6 | 5 | 6 | 12 | 4 | 3 | 4 | 2 | 6 | 6 | 4 |
| NM_005737 | ARL4C | 4008 | 7 | 2 | 1 | 0 | 1 | 3 | 1 | 1 | 2 | 0 | 0 | 7 | 0 |
| NM_005738 | ARL4A | 3161 | 4 | 2 | 1 | 1 | 1 | 2 | 1 | 1 | 1 | 1 | 1 | 2 | 4 |
| NM_005739 | RASGRP1 | 5025 | 13 | 2 | 1 | 1 | 0 | 13 | 3 | 0 | 2 | 1 | 0 | 4 | 2 |
| NM_005740 | DNAL4 | 1487 | 4 | 1 | 1 | 1 | 1 | 4 | 2 | 0 | 1 | 1 | 0 | 1 | 4 |
| NM_005741 | ZNF263 | 3281 | 4 | 3 | 2 | 2 | 2 | 4 | 3 | 2 | 3 | 4 | 2 | 4 | 4 |
| NM_005742 | PDIA6 | 2328 | 29 | 8 | 5 | 4 | 9 | 5 | 8 | 29 | 5 | 3 | 2 | 9 | 5 |
| NM_005744 | ARIH1 | 5251 | 11 | 8 | 6 | 4 | 9 | 9 | 6 | 5 | 7 | 11 | 11 | 10 | 11 |
| NM_005745 | BCAP31 | 1413 | 47 | 19 | 47 | 13 | 12 | 20 | 28 | 19 | 17 | 7 | 4 | 20 | 19 |
| NM_005746 | NAMPT | 4581 | 101 | 44 | 17 | 4 | 97 | 18 | 16 | 101 | 84 | 15 | 22 | 97 | 16 |
| NM_005747 | CELA3A | 905 | 0 | 0 | 0 | 0 | 0 | 0 | 0 | 0 | 0 | 0 | 0 | 0 | 0 |
| NM_005748 | YAF2 | 4095 | 2 | 1 | 1 | 0 | 2 | 2 | 1 | 1 | 0 | 0 | 2 | 1 | 1 |
| NM_005749 | TOB1 | 1827 | 26 | 10 | 4 | 5 | 7 | 6 | 13 | 26 | 7 | 9 | 21 | 5 | 6 |
| NM_005750 | C4orf6 | 872 | 1 | 0 | 0 | 0 | 0 | 0 | 0 | 0 | 0 | 0 | 0 | 0 | 1 |
| NM_005751 | AKAP9 | 12462 | 26 | 17 | 10 | 5 | 16 | 15 | 19 | 15 | 16 | 26 | 26 | 20 | 18 |
| NM_005752 | CLEC3A | 1935 | 0 | 0 | 0 | 0 | 0 | 0 | 0 | 0 | 0 | 0 | 0 | 0 | 0 |
| NM_005754 | G3BP1 | 2835 | 9 | 6 | 5 | 3 | 6 | 4 | 7 | 9 | 5 | 6 | 8 | 9 | 7 |
| NM_005755 | EBI3 | 1137 | 0 | 0 | 0 | 0 | 0 | 0 | 0 | 0 | 0 | 0 | 0 | 0 | 0 |
| NM_005756 | GPR64 | 4766 | 6 | 1 | 0 | 0 | 0 | 0 | 0 | 0 | 0 | 1 | 0 | 1 | 6 |
| NM_005759 | ABI2 | 6480 | 17 | 5 | 1 | 2 | 5 | 17 | 4 | 1 | 3 | 6 | 1 | 4 | 7 |
| NM_005760 | CEBPZ | 3327 | 15 | 9 | 7 | 2 | 10 | 8 | 10 | 8 | 6 | 10 | 13 | 13 | 15 |
| NM_005761 | PLXNC1 | 5120 | 9 | 3 | 1 | 1 | 1 | 4 | 0 | 0 | 4 | 4 | 0 | 9 | 5 |
| NM_005762 | TRIM28 | 2959 | 3 | 2 | 1 | 2 | 1 | 1 | 1 | 1 | 1 | 3 | 2 | 2 | 2 |
| NM_005763 | AASS | 5869 | 18 | 7 | 2 | 1 | 13 | 3 | 13 | 18 | 6 | 10 | 2 | 5 | 4 |
| NM_005764 | PDZK1IP1 | 894 | 15 | 1 | 0 | 0 | 0 | 0 | 15 | 0 | 0 | 0 | 0 | 0 | 0 |
| NM_005765 | ATP6AP2 | 2036 | 22 | 13 | 15 | 5 | 12 | 22 | 21 | 12 | 17 | 10 | 3 | 17 | 11 |
| NM_005766 | FARP1 | 4926 | 8 | 3 | 1 | 2 | 1 | 4 | 8 | 6 | 2 | 2 | 0 | 2 | 2 |
| NM_005767 | LPAR6 | 3001 | 6 | 3 | 5 | 1 | 3 | 2 | 3 | 1 | 5 | 3 | 2 | 6 | 2 |
| NM_005768 | LPCAT3 | 2297 | 10 | 4 | 2 | 3 | 3 | 2 | 6 | 10 | 4 | 3 | 4 | 4 | 2 |
| NM_005769 | CHST4 | 2122 | 1 | 0 | 0 | 0 | 0 | 0 | 0 | 1 | 0 | 0 | 0 | 0 | 0 |
| NM_005771 | DHRS9 | 2864 | 2 | 1 | 1 | 1 | 2 | 1 | 0 | 0 | 1 | 0 | 0 | 1 | 0 |
| NM_005772 | RCL1 | 2169 | 10 | 3 | 2 | 1 | 2 | 1 | 3 | 10 | 2 | 4 | 4 | 3 | 2 |
| NM_005773 | ZNF256 | 2210 | 5 | 3 | 2 | 1 | 4 | 4 | 2 | 0 | 1 | 5 | 2 | 4 | 4 |
| NM_005775 | SORBS3 | 3123 | 2 | 2 | 2 | 1 | 2 | 1 | 1 | 1 | 2 | 2 | 1 | 2 | 2 |
| NM_005776 | CNIH | 1423 | 4 | 2 | 2 | 2 | 3 | 2 | 2 | 4 | 2 | 2 | 1 | 2 | 2 |

| transcript | gene | transcript length (nt) | max | mean | adipose | colon | heart | hypothalamus | kidney | liver | lung | ovary | skeletalmuscle | spleen | testes |
|---|---|---|---|---|---|---|---|---|---|---|---|---|---|---|---|
| NM_005777 | RBM6 | 3621 | 43 | 27 | 23 | 27 | 23 | 22 | 30 | 12 | 24 | 33 | 21 | 43 | 41 |
| NM_005778 | RBM5 | 3099 | 35 | 26 | 29 | 33 | 19 | 29 | 21 | 15 | 19 | 33 | 23 | 35 | 32 |
| NM_005779 | LHFPL2 | 5045 | 6 | 3 | 4 | 2 | 4 | 3 | 1 | 1 | 6 | 6 | 1 | 2 | 5 |
| NM_005780 | LHFP | 2158 | 17 | 9 | 17 | 4 | 15 | 10 | 5 | 1 | 11 | 9 | 4 | 12 | 15 |
| NM_005781 | TNK2 | 4476 | 4 | 1 | 1 | 1 | 0 | 4 | 0 | 1 | 0 | 0 | 1 | 1 | 1 |
| NM_005782 | THOC4 | 1095 | 4 | 2 | 2 | 3 | 2 | 3 | 2 | 1 | 2 | 1 | 1 | 4 | 3 |
| NM_005783 | TXNDC9 | 1530 | 3 | 2 | 1 | 1 | 3 | 2 | 3 | 2 | 1 | 1 | 1 | 2 | 1 |
| NM_005785 | RNF41 | 3268 | 6 | 3 | 2 | 1 | 4 | 6 | 2 | 2 | 2 | 2 | 2 | 3 | 2 |
| NM_005786 | TSHZ1 | 4967 | 4 | 2 | 1 | 2 | 1 | 2 | 2 | 1 | 1 | 4 | 1 | 2 | 2 |
| NM_005787 | ALG3 | 1533 | 6 | 2 | 1 | 2 | 2 | 1 | 2 | 6 | 1 | 1 | 2 | 2 | 2 |
| NM_005788 | PRMT3 | 2731 | 2 | 1 | 1 | 1 | 2 | 2 | 2 | 1 | 1 | 2 | 1 | 2 | 1 |
| NM_005789 | PSME3 | 3173 | 7 | 4 | 3 | 2 | 5 | 6 | 7 | 5 | 3 | 3 | 3 | 7 | 4 |
| NM_005791 | MPHOSPH1 | 2483 | 18 | 13 | 9 | 3 | 17 | 13 | 13 | 13 | 8 | 18 | 16 | 17 | 14 |
| NM_005792 | MPHOSPH6 | 1101 | 26 | 10 | 6 | 5 | 10 | 26 | 9 | 9 | 7 | 6 | 4 | 15 | 13 |
| NM_005793 | NME6 | 1169 | 1 | 0 | 0 | 0 | 0 | 0 | 0 | 0 | 0 | 1 | 0 | 1 | 1 |
| NM_005794 | DHRS2 | 1676 | 1 | 0 | 0 | 0 | 0 | 0 | 0 | 1 | 0 | 1 | 0 | 1 | 0 |
| NM_005795 | CALCRL | 4996 | 14 | 3 | 1 | 0 | 4 | 1 | 2 | 1 | 14 | 4 | 1 | 2 | 1 |
| NM_005796 | NUTF2 | 886 | 2 | 1 | 1 | 1 | 2 | 2 | 2 | 1 | 1 | 1 | 2 | 1 | 1 |
| NM_005797 | MPZL2 | 2616 | 10 | 3 | 1 | 2 | 3 | 3 | 3 | 3 | 10 | 1 | 1 | 1 | 2 |
| NM_005798 | TRIM13 | 6755 | 5 | 3 | 2 | 1 | 3 | 2 | 3 | 1 | 3 | 4 | 3 | 5 | 4 |
| NM_005800 | USPL1 | 3904 | 7 | 4 | 3 | 1 | 3 | 3 | 3 | 2 | 4 | 5 | 3 | 6 | 7 |
| NM_005801 | EIF1 | 1326 | 138 | 71 | 54 | 20 | 101 | 60 | 69 | 43 | 96 | 54 | 138 | 88 | 59 |
| NM_005802 | TOPORS | 4148 | 14 | 7 | 5 | 1 | 7 | 7 | 8 | 5 | 6 | 14 | 4 | 10 | 12 |
| NM_005803 | FLOT1 | 1830 | 24 | 12 | 12 | 8 | 19 | 24 | 12 | 13 | 9 | 4 | 6 | 16 | 6 |
| NM_005804 | DDX39 | 1495 | 2 | 1 | 2 | 2 | 1 | 1 | 1 | 2 | 1 | 0 | 0 | 2 | 2 |
| NM_005805 | PSMD14 | 1715 | 23 | 11 | 12 | 3 | 13 | 20 | 11 | 23 | 6 | 5 | 11 | 11 | 11 |
| NM_005806 | OLIG2 | 2488 | 1 | 0 | 0 | 0 | 0 | 1 | 0 | 0 | 0 | 0 | 0 | 0 | 0 |
| NM_005807 | PRG4 | 5044 | 215 | 26 | 31 | 2 | 4 | 2 | 1 | 215 | 6 | 5 | 12 | 2 | 4 |
| NM_005808 | CTDSPL | 4422 | 3 | 1 | 1 | 1 | 1 | 0 | 3 | 1 | 2 | 1 | 0 | 2 | 1 |
| NM_005809 | PRDX2 | 1008 | 16 | 6 | 7 | 6 | 10 | 16 | 8 | 8 | 2 | 5 | 5 | 2 | 2 |
| NM_005810 | KLRG1 | 1788 | 0 | 0 | 0 | 0 | 0 | 0 | 0 | 0 | 0 | 0 | 0 | 0 | 0 |
| NM_005811 | GDF11 | 4259 | 11 | 4 | 1 | 2 | 11 | 9 | 3 | 1 | 2 | 4 | 1 | 8 | 3 |
| NM_005813 | PRKD3 | 5902 | 14 | 6 | 5 | 2 | 5 | 4 | 5 | 3 | 7 | 8 | 5 | 14 | 10 |
| NM_005814 | GPA33 | 2793 | 19 | 2 | 0 | 19 | 0 | 0 | 0 | 0 | 0 | 0 | 0 | 0 | 0 |
| NM_005815 | ZNF443 | 2664 | 2 | 1 | 0 | 1 | 1 | 1 | 2 | 1 | 1 | 2 | 1 | 1 | 1 |
| NM_005816 | CD96 | 4433 | 3 | 1 | 0 | 0 | 0 | 0 | 0 | 0 | 1 | 0 | 0 | 3 | 0 |
| NM_005817 | PLIN3 | 2340 | 3 | 2 | 1 | 2 | 2 | 3 | 1 | 1 | 2 | 2 | 2 | 3 | 2 |
| NM_005819 | STX6 | 4535 | 3 | 2 | 2 | 1 | 1 | 3 | 2 | 0 | 2 | 2 | 1 | 3 | 2 |
| NM_005822 | RCAN2 | 3318 | 37 | 8 | 2 | 2 | 37 | 25 | 4 | 0 | 3 | 4 | 10 | 1 | 4 |
| NM_005823 | MSLN | 2052 | 2 | 0 | 0 | 0 | 0 | 0 | 0 | 0 | 2 | 0 | 0 | 0 | 0 |
| NM_005824 | LRRC17 | 2268 | 13 | 2 | 1 | 0 | 1 | 0 | 0 | 0 | 0 | 13 | 0 | 0 | 2 |
| NM_005826 | HNRNPR | 2732 | 67 | 44 | 32 | 21 | 45 | 54 | 40 | 40 | 43 | 55 | 30 | 67 | 57 |
| NM_005827 | SLC35B1 | 1186 | 11 | 4 | 2 | 1 | 6 | 5 | 4 | 11 | 1 | 1 | 2 | 3 | 2 |
| NM_005828 | DCAF7 | 6354 | 9 | 5 | 3 | 5 | 3 | 7 | 7 | 3 | 5 | 4 | 2 | 9 | 6 |
| NM_005829 | AP3S2 | 5934 | 7 | 4 | 4 | 3 | 5 | 7 | 5 | 5 | 2 | 3 | 4 | 3 | 4 |
| NM_005830 | MRPS31 | 1339 | 13 | 9 | 6 | 4 | 13 | 13 | 10 | 13 | 5 | 11 | 7 | 9 | 9 |
| NM_005831 | CALCOCO2 | 3282 | 165 | 37 | 25 | 12 | 165 | 16 | 19 | 21 | 27 | 27 | 35 | 28 | 31 |
| NM_005832 | KCNMB2 | 2499 | 2 | 0 | 0 | 0 | 0 | 1 | 1 | 0 | 0 | 2 | 0 | 0 | 0 |
| NM_005833 | RABEPK | 1433 | 9 | 3 | 3 | 1 | 2 | 4 | 2 | 9 | 1 | 2 | 2 | 2 | 2 |
| NM_005834 | TIMM17B | 949 | 1 | 1 | 1 | 0 | 1 | 1 | 1 | 1 | 1 | 0 | 1 | 1 | 0 |
| NM_005835 | SLC17A2 | 2268 | 7 | 1 | 0 | 0 | 0 | 0 | 0 | 7 | 0 | 0 | 0 | 0 | 0 |
| NM_005836 | HRSP12 | 1011 | 52 | 10 | 3 | 1 | 5 | 7 | 33 | 52 | 2 | 1 | 2 | 2 | 4 |
| NM_005837 | POP7 | 932 | 5 | 2 | 1 | 1 | 1 | 5 | 2 | 4 | 1 | 1 | 2 | 2 | 1 |
| NM_005838 | GLYAT | 1145 | 21 | 5 | 12 | 0 | 0 | 0 | 19 | 21 | 0 | 0 | 0 | 0 | 0 |
| NM_005839 | SRRM1 | 3934 | 17 | 12 | 11 | 9 | 10 | 12 | 13 | 11 | 10 | 15 | 9 | 17 | 15 |
| NM_005840 | SPRY3 | 9038 | 1 | 0 | 0 | 0 | 0 | 0 | 0 | 0 | 0 | 1 | 0 | 0 | 0 |
| NM_005841 | SPRY1 | 2485 | 20 | 8 | 20 | 6 | 14 | 3 | 4 | 2 | 10 | 6 | 12 | 6 | 8 |
| NM_005842 | SPRY2 | 2110 | 8 | 4 | 4 | 3 | 4 | 8 | 2 | 3 | 2 | 2 | 3 | 6 | 1 |
| NM_005843 | STAM2 | 5701 | 2 | 1 | 1 | 0 | 2 | 2 | 2 | 1 | 1 | 2 | 1 | 2 | 2 |
| NM_005845 | ABCC4 | 5843 | 6 | 2 | 0 | 2 | 1 | 2 | 6 | 0 | 2 | 2 | 1 | 1 | 2 |
| NM_005847 | SLC23A1 | 2343 | 6 | 1 | 0 | 0 | 0 | 0 | 6 | 2 | 0 | 0 | 0 | 0 | 0 |
| NM_005848 | DENND4A | 7200 | 9 | 6 | 3 | 1 | 4 | 6 | 4 | 5 | 6 | 9 | 7 | 8 | 7 |
| NM_005849 | IGSF6 | 1028 | 33 | 5 | 5 | 1 | 1 | 2 | 1 | 2 | 5 | 2 | 1 | 33 | 1 |
| NM_005850 | SF3B4 | 1549 | 6 | 3 | 1 | 2 | 3 | 2 | 3 | 2 | 2 | 4 | 2 | 6 | 5 |
| NM_005851 | CDK2AP2 | 1378 | 9 | 3 | 2 | 3 | 9 | 2 | 5 | 4 | 2 | 1 | 1 | 5 | 1 |
| NM_005852 | CHD3 | 7223 | 18 | 9 | 5 | 12 | 4 | 18 | 9 | 1 | 9 | 13 | 1 | 12 | 9 |
| NM_005853 | IRX5 | 2062 | 6 | 1 | 2 | 0 | 6 | 0 | 2 | 0 | 3 | 0 | 1 | 0 | 0 |
| NM_005854 | RAMP2 | 785 | 3 | 1 | 2 | 1 | 1 | 1 | 1 | 0 | 3 | 0 | 0 | 3 | 1 |
| NM_005855 | RAMP1 | 909 | 1 | 0 | 0 | 0 | 1 | 0 | 0 | 0 | 0 | 0 | 0 | 0 | 0 |
| NM_005856 | RAMP3 | 1347 | 1 | 0 | 0 | 0 | 0 | 0 | 0 | 0 | 1 | 0 | 0 | 0 | 0 |
| NM_005857 | ZMPSTE24 | 3154 | 36 | 22 | 17 | 8 | 29 | 17 | 34 | 36 | 20 | 21 | 12 | 24 | 24 |
| NM_005858 | AKAP8 | 3531 | 9 | 7 | 6 | 6 | 7 | 6 | 7 | 5 | 6 | 6 | 9 | 9 | 9 |
| NM_005859 | PURA | 2614 | 6 | 3 | 2 | 1 | 3 | 6 | 3 | 1 | 2 | 3 | 2 | 3 | 2 |
| NM_005860 | FSTL3 | 2511 | 8 | 2 | 3 | 0 | 8 | 1 | 1 | 1 | 3 | 1 | 0 | 1 | 6 |
| NM_005861 | STUB1 | 1602 | 1 | 0 | 0 | 0 | 0 | 1 | 1 | 0 | 0 | 0 | 0 | 0 | 0 |
| NM_005862 | STAG1 | 5166 | 8 | 4 | 4 | 2 | 4 | 2 | 4 | 3 | 4 | 8 | 6 | 6 | 7 |
| NM_005863 | NET1 | 3281 | 18 | 9 | 18 | 12 | 5 | 8 | 13 | 12 | 6 | 12 | 5 | 2 | 8 |

| transcript | gene | transcript length (nt) | max | mean | adipose | colon | heart | hypothalamus | kidney | liver | lung | ovary | skeletalmuscle | spleen | testes |
|---|---|---|---|---|---|---|---|---|---|---|---|---|---|---|---|
| NM_005864 | EFS | 3118 | 1 | 0 | 0 | 0 | 0 | 0 | 0 | 0 | 0 | 1 | 0 | 0 | 0 |
| NM_005865 | PRSS16 | 2864 | 0 | 0 | 0 | 0 | 0 | 0 | 0 | 0 | 0 | 0 | 0 | 0 | 0 |
| NM_005866 | SIGMAR1 | 1655 | 33 | 8 | 6 | 5 | 7 | 7 | 6 | 33 | 4 | 5 | 2 | 6 | 6 |
| NM_005867 | DSCR4 | 1098 | 0 | 0 | 0 | 0 | 0 | 0 | 0 | 0 | 0 | 0 | 0 | 0 | 0 |
| NM_005868 | BET1 | 3061 | 1 | 1 | 1 | 0 | 1 | 1 | 1 | 1 | 1 | 1 | 0 | 1 | 1 |
| NM_005869 | SDCCAG10 | 2080 | 11 | 6 | 6 | 2 | 9 | 11 | 6 | 4 | 4 | 9 | 5 | 7 | 8 |
| NM_005870 | SAP18 | 2300 | 56 | 31 | 23 | 17 | 36 | 56 | 42 | 27 | 19 | 24 | 35 | 28 | 33 |
| NM_005871 | SMNDC1 | 2029 | 22 | 15 | 10 | 6 | 18 | 10 | 12 | 8 | 20 | 20 | 18 | 22 | 22 |
| NM_005872 | BCAS2 | 1301 | 30 | 12 | 11 | 2 | 14 | 30 | 12 | 14 | 6 | 11 | 10 | 12 | 13 |
| NM_005873 | RGS19 | 1592 | 1 | 0 | 0 | 0 | 0 | 0 | 0 | 0 | 0 | 0 | 0 | 1 | 0 |
| NM_005874 | LILRB2 | 2924 | 6 | 1 | 1 | 0 | 1 | 0 | 0 | 0 | 1 | 0 | 0 | 6 | 0 |
| NM_005875 | EIF1B | 1012 | 56 | 18 | 8 | 6 | 56 | 29 | 12 | 9 | 12 | 15 | 15 | 17 | 14 |
| NM_005876 | SPEG | 10650 | 6 | 1 | 0 | 1 | 2 | 1 | 0 | 0 | 0 | 0 | 6 | 0 | 0 |
| NM_005877 | SF3A1 | 5120 | 9 | 6 | 5 | 5 | 4 | 5 | 5 | 3 | 5 | 8 | 6 | 9 | 9 |
| NM_005879 | TRAIP | 2024 | 0 | 0 | 0 | 0 | 0 | 0 | 0 | 0 | 0 | 0 | 0 | 0 | 0 |
| NM_005880 | DNAJA2 | 3048 | 49 | 32 | 23 | 11 | 44 | 45 | 27 | 33 | 19 | 30 | 38 | 35 | 49 |
| NM_005881 | BCKDK | 2081 | 1 | 1 | 0 | 0 | 1 | 1 | 0 | 1 | 0 | 0 | 1 | 0 | 0 |
| NM_005882 | MAEA | 2066 | 4 | 3 | 1 | 2 | 3 | 4 | 3 | 2 | 2 | 2 | 1 | 4 | 3 |
| NM_005883 | APC2 | 10151 | 1 | 0 | 0 | 0 | 0 | 1 | 0 | 0 | 0 | 0 | 0 | 0 | 0 |
| NM_005884 | PAK4 | 2838 | 1 | 1 | 0 | 0 | 0 | 0 | 1 | 0 | 1 | 1 | 0 | 0 | 1 |
| NM_005885 | 06 Mar | 4611 | 26 | 17 | 11 | 10 | 13 | 26 | 16 | 12 | 17 | 22 | 23 | 17 | 16 |
| NM_005886 | KATNB1 | 2646 | 1 | 0 | 0 | 0 | 0 | 1 | 0 | 0 | 0 | 0 | 0 | 0 | 1 |
| NM_005888 | SLC25A3 | 1680 | 70 | 27 | 19 | 15 | 70 | 21 | 33 | 29 | 14 | 24 | 40 | 20 | 11 |
| NM_005891 | ACAT2 | 1551 | 46 | 13 | 6 | 7 | 5 | 28 | 11 | 46 | 9 | 11 | 2 | 7 | 14 |
| NM_005892 | FMNL1 | 3961 | 6 | 2 | 2 | 1 | 1 | 2 | 0 | 0 | 3 | 0 | 0 | 6 | 0 |
| NM_005893 | CCIN | 1943 | 4 | 0 | 0 | 0 | 0 | 0 | 0 | 0 | 0 | 0 | 0 | 0 | 4 |
| NM_005894 | CD5L | 2237 | 228 | 21 | 0 | 0 | 0 | 0 | 0 | 7 | 1 | 0 | 0 | 228 | 0 |
| NM_005895 | GOLGA3 | 9252 | 3 | 2 | 3 | 2 | 3 | 3 | 2 | 2 | 1 | 2 | 1 | 2 | 1 |
| NM_005896 | IDH1 | 2329 | 110 | 31 | 44 | 20 | 16 | 11 | 25 | 110 | 29 | 22 | 4 | 34 | 26 |
| NM_005897 | IPP | 3251 | 1 | 1 | 1 | 0 | 1 | 1 | 1 | 0 | 0 | 1 | 1 | 1 | 1 |
| NM_005898 | CAPRIN1 | 5562 | 30 | 16 | 14 | 9 | 14 | 20 | 17 | 11 | 13 | 18 | 9 | 21 | 30 |
| NM_005899 | NBR1 | 4598 | 17 | 12 | 10 | 8 | 14 | 13 | 14 | 10 | 9 | 13 | 17 | 15 | 13 |
| NM_005900 | SMAD1 | 3053 | 5 | 3 | 2 | 2 | 2 | 4 | 3 | 3 | 2 | 4 | 3 | 5 | 3 |
| NM_005901 | SMAD2 | 10384 | 6 | 4 | 4 | 2 | 3 | 4 | 4 | 2 | 4 | 6 | 2 | 6 | 6 |
| NM_005902 | SMAD3 | 6235 | 4 | 2 | 2 | 2 | 2 | 2 | 2 | 1 | 2 | 4 | 3 | 2 | 1 |
| NM_005903 | SMAD5 | 7011 | 11 | 4 | 2 | 2 | 5 | 2 | 4 | 2 | 4 | 11 | 4 | 6 | 5 |
| NM_005904 | SMAD7 | 3087 | 3 | 2 | 2 | 2 | 3 | 2 | 3 | 1 | 3 | 1 | 2 | 2 | 1 |
| NM_005905 | SMAD9 | 2242 | 6 | 3 | 1 | 2 | 2 | 6 | 2 | 0 | 2 | 6 | 2 | 1 | 3 |
| NM_005906 | MAK | 3830 | 3 | 1 | 0 | 0 | 0 | 0 | 0 | 0 | 1 | 1 | 0 | 1 | 3 |
| NM_005907 | MAN1A1 | 4121 | 44 | 15 | 22 | 5 | 10 | 4 | 21 | 44 | 16 | 7 | 5 | 22 | 6 |
| NM_005908 | MANBA | 3311 | 12 | 5 | 6 | 3 | 5 | 4 | 5 | 3 | 7 | 5 | 2 | 12 | 3 |
| NM_005909 | MAP1B | 11979 | 250 | 39 | 32 | 7 | 49 | 250 | 7 | 2 | 8 | 11 | 6 | 4 | 51 |
| NM_005910 | MAPT | 5810 | 26 | 4 | 2 | 1 | 1 | 26 | 3 | 0 | 0 | 0 | 5 | 0 | 0 |
| NM_005911 | MAT2A | 2819 | 14 | 8 | 5 | 5 | 9 | 9 | 14 | 6 | 5 | 8 | 6 | 10 | 8 |
| NM_005912 | MC4R | 1438 | 1 | 0 | 0 | 0 | 0 | 1 | 0 | 0 | 0 | 0 | 0 | 0 | 0 |
| NM_005913 | MC5R | 978 | 0 | 0 | 0 | 0 | 0 | 0 | 0 | 0 | 0 | 0 | 0 | 0 | 0 |
| NM_005914 | MCM4 | 3533 | 5 | 2 | 2 | 2 | 1 | 3 | 2 | 1 | 1 | 1 | 1 | 2 | 5 |
| NM_005915 | MCM6 | 3751 | 5 | 2 | 3 | 3 | 2 | 3 | 1 | 1 | 2 | 2 | 1 | 4 | 5 |
| NM_005916 | MCM7 | 2821 | 5 | 3 | 4 | 5 | 1 | 1 | 1 | 1 | 2 | 3 | 3 | 4 | 5 |
| NM_005917 | MDH1 | 1268 | 75 | 22 | 37 | 7 | 75 | 53 | 23 | 16 | 5 | 3 | 14 | 7 | 6 |
| NM_005918 | MDH2 | 1305 | 28 | 13 | 5 | 6 | 23 | 15 | 14 | 26 | 5 | 5 | 28 | 11 | 7 |
| NM_005919 | LOC729991- | 1951 | 0 | 0 | 0 | 0 | 0 | 0 | 0 | 0 | 0 | 0 | 0 | 0 | 0 |
| NM_005920 | MEF2D | 5874 | 13 | 5 | 3 | 3 | 8 | 8 | 2 | 2 | 4 | 6 | 13 | 5 | 5 |
| NM_005921 | MAP3K1 | 7511 | 11 | 3 | 3 | 1 | 2 | 2 | 5 | 2 | 3 | 4 | 1 | 11 | 5 |
| NM_005922 | MAP3K4 | 5484 | 7 | 4 | 3 | 2 | 4 | 5 | 4 | 2 | 3 | 6 | 4 | 5 | 7 |
| NM_005923 | MAP3K5 | 5195 | 18 | 6 | 3 | 2 | 6 | 11 | 4 | 3 | 5 | 18 | 1 | 8 | 9 |
| NM_005924 | MEOX2 | 2371 | 13 | 3 | 13 | 1 | 4 | 1 | 0 | 0 | 4 | 1 | 4 | 0 | 3 |
| NM_005925 | MEP1B | 2311 | 1 | 0 | 0 | 1 | 0 | 0 | 0 | 0 | 0 | 0 | 0 | 0 | 0 |
| NM_005926 | MFAP1 | 2063 | 35 | 26 | 17 | 11 | 34 | 33 | 28 | 18 | 20 | 32 | 22 | 32 | 35 |
| NM_005927 | MFAP3 | 5049 | 4 | 2 | 2 | 1 | 3 | 2 | 2 | 2 | 2 | 2 | 1 | 4 | 2 |
| NM_005928 | MFGE8 | 1989 | 6 | 2 | 2 | 1 | 6 | 0 | 1 | 0 | 2 | 2 | 3 | 1 | 2 |
| NM_005929 | MFI2 | 2417 | 0 | 0 | 0 | 0 | 0 | 0 | 0 | 0 | 0 | 0 | 0 | 0 | 0 |
| NM_005930 | CTAGE5 | 3680 | 7 | 3 | 1 | 1 | 1 | 1 | 7 | 6 | 2 | 1 | 2 | 2 | 2 |
| NM_005931 | MICB | 2475 | 1 | 0 | 0 | 0 | 0 | 0 | 0 | 0 | 0 | 0 | 0 | 1 | 0 |
| NM_005931 | MICB | 2481 | 1 | 0 | 0 | 0 | 0 | 0 | 0 | 0 | 0 | 0 | 0 | 1 | 0 |
| NM_005931 | MICB | 2483 | 1 | 0 | 0 | 0 | 0 | 0 | 0 | 0 | 0 | 0 | 0 | 1 | 0 |
| NM_005932 | MIPEP | 2401 | 4 | 1 | 1 | 1 | 2 | 1 | 4 | 1 | 1 | 0 | 1 | 2 | 2 |
| NM_005933 | MLL | 14982 | 12 | 6 | 4 | 4 | 4 | 7 | 4 | 1 | 7 | 12 | 5 | 9 | 6 |
| NM_005934 | MLLT1 | 4505 | 1 | 1 | 0 | 1 | 1 | 1 | 1 | 1 | 1 | 1 | 1 | 1 | 0 |
| NM_005935 | AFF1 | 9378 | 22 | 10 | 9 | 7 | 10 | 4 | 17 | 6 | 9 | 12 | 22 | 11 | 7 |
| NM_005936 | MLLT4 | 4981 | 12 | 8 | 5 | 8 | 5 | 8 | 12 | 8 | 10 | 7 | 3 | 8 | 12 |
| NM_005937 | MLLT6 | 7500 | 3 | 2 | 1 | 3 | 2 | 2 | 2 | 0 | 2 | 2 | 2 | 2 | 1 |
| NM_005938 | FOXO4 | 3321 | 10 | 5 | 5 | 4 | 4 | 6 | 3 | 2 | 4 | 6 | 7 | 4 | 10 |
| NM_005940 | MMP11 | 2261 | 2 | 0 | 0 | 0 | 0 | 0 | 0 | 0 | 0 | 2 | 0 | 0 | 0 |
| NM_005941 | MMP16 | 6335 | 3 | 1 | 0 | 0 | 0 | 3 | 0 | 0 | 0 | 1 | 0 | 0 | 1 |
| NM_005942 | MOCS1 | 4136 | 2 | 1 | 1 | 1 | 1 | 1 | 2 | 2 | 1 | 1 | 1 | 0 | 1 |
| NM_005943 | MOCS1 | 4151 | 2 | 1 | 1 | 1 | 1 | 1 | 2 | 2 | 1 | 1 | 1 | 0 | 1 |

| transcript | gene | transcript length (nt) | max | mean | adipose | colon | heart | hypothalamus | kidney | liver | lung | ovary | skeletalmuscle | spleen | testes |
|---|---|---|---|---|---|---|---|---|---|---|---|---|---|---|---|
| NM_005944 | CD200 | 2215 | 4 | 1 | 1 | 1 | 2 | 4 | 2 | 0 | 0 | 1 | 0 | 0 | 0 |
| NM_005946 | MT1A | 396 | 2 | 0 | 0 | 0 | 0 | 0 | 0 | 2 | 0 | 0 | 0 | 0 | 0 |
| NM_005947 | MT1B | 380 | 0 | 0 | 0 | 0 | 0 | 0 | 0 | 0 | 0 | 0 | 0 | 0 | 0 |
| NM_005949 | MT1F | 441 | 73 | 18 | 5 | 23 | 3 | 28 | 73 | 41 | 6 | 1 | 0 | 12 | 4 |
| NM_005950 | MT1G | 396 | 29 | 7 | 0 | 12 | 0 | 2 | 29 | 28 | 1 | 0 | 1 | 0 | 0 |
| NM_005951 | MT1H | 397 | 1 | 0 | 0 | 0 | 0 | 0 | 1 | 0 | 0 | 0 | 0 | 0 | 0 |
| NM_005952 | MT1X | 404 | 33 | 7 | 2 | 2 | 4 | 6 | 6 | 33 | 1 | 2 | 15 | 2 | 1 |
| NM_005953 | MT2A | 418 | 391 | 54 | 8 | 7 | 27 | 31 | 21 | 391 | 26 | 8 | 38 | 37 | 5 |
| NM_005954 | MT3 | 582 | 58 | 5 | 0 | 1 | 0 | 58 | 0 | 0 | 0 | 0 | 0 | 0 | 0 |
| NM_005955 | MTF1 | 7972 | 2 | 1 | 1 | 1 | 1 | 1 | 1 | 1 | 1 | 2 | 1 | 2 | 2 |
| NM_005956 | MTHFD1 | 3448 | 114 | 28 | 37 | 11 | 19 | 18 | 30 | 114 | 9 | 14 | 32 | 17 | 7 |
| NM_005957 | MTHFR | 7150 | 2 | 1 | 0 | 1 | 1 | 1 | 1 | 1 | 1 | 1 | 1 | 2 | 1 |
| NM_005958 | MTNR1A | 1105 | 0 | 0 | 0 | 0 | 0 | 0 | 0 | 0 | 0 | 0 | 0 | 0 | 0 |
| NM_005959 | MTNR1B | 1662 | 0 | 0 | 0 | 0 | 0 | 0 | 0 | 0 | 0 | 0 | 0 | 0 | 0 |
| NM_005961 | MUC6 | 8003 | 15 | 4 | 3 | 4 | 2 | 1 | 15 | 1 | 1 | 4 | 2 | 8 | 2 |
| NM_005962 | MXI1 | 3272 | 11 | 5 | 3 | 3 | 7 | 8 | 3 | 3 | 5 | 11 | 6 | 2 | 7 |
| NM_005963 | MYH1 | 6023 | 225 | 21 | 1 | 0 | 0 | 0 | 0 | 0 | 0 | 0 | 225 | 0 | 0 |
| NM_005964 | MYH10 | 7619 | 23 | 11 | 9 | 2 | 23 | 20 | 16 | 4 | 23 | 18 | 2 | 3 | 7 |
| NM_005966 | NAB1 | 4469 | 9 | 7 | 9 | 5 | 9 | 9 | 4 | 8 | 6 | 7 | 2 | 6 | 9 |
| NM_005967 | NAB2 | 2711 | 2 | 1 | 1 | 1 | 0 | 1 | 1 | 1 | 1 | 1 | 0 | 1 | 2 |
| NM_005968 | HNRNPM | 2531 | 23 | 16 | 13 | 14 | 16 | 15 | 15 | 21 | 12 | 17 | 12 | 23 | 17 |
| NM_005969 | NAP1L4 | 2518 | 21 | 11 | 8 | 6 | 15 | 21 | 11 | 8 | 8 | 9 | 13 | 11 | 13 |
| NM_005971 | FXYD3 | 1372 | 11 | 1 | 0 | 11 | 0 | 0 | 0 | 0 | 1 | 0 | 0 | 0 | 0 |
| NM_005972 | PPYR1 | 1956 | 0 | 0 | 0 | 0 | 0 | 0 | 0 | 0 | 0 | 0 | 0 | 0 | 0 |
| NM_005973 | PRCC | 2123 | 3 | 2 | 1 | 1 | 2 | 3 | 1 | 1 | 1 | 1 | 2 | 3 | 1 |
| NM_005975 | PTK6 | 2509 | 1 | 0 | 0 | 1 | 0 | 0 | 0 | 0 | 0 | 0 | 0 | 0 | 0 |
| NM_005977 | RNF6 | 3584 | 21 | 12 | 9 | 3 | 11 | 21 | 11 | 11 | 9 | 8 | 9 | 17 | 19 |
| NM_005978 | S100A2 | 962 | 4 | 0 | 0 | 0 | 0 | 0 | 4 | 0 | 1 | 0 | 0 | 0 | 0 |
| NM_005979 | S100A13 | 688 | 28 | 13 | 22 | 10 | 28 | 14 | 12 | 4 | 12 | 13 | 13 | 9 | 8 |
| NM_005980 | S100P | 510 | 1 | 0 | 0 | 0 | 0 | 0 | 0 | 0 | 1 | 0 | 0 | 1 | 0 |
| NM_005981 | TSPAN31 | 1740 | 2 | 1 | 1 | 1 | 2 | 2 | 2 | 1 | 1 | 2 | 1 | 2 | 1 |
| NM_005982 | SIX1 | 2687 | 27 | 3 | 2 | 0 | 0 | 0 | 0 | 0 | 2 | 0 | 27 | 0 | 1 |
| NM_005983 | SKP2 | 1594 | 1 | 0 | 1 | 0 | 0 | 0 | 0 | 0 | 0 | 1 | 0 | 1 | 0 |
| NM_005984 | SLC25A1 | 1599 | 1 | 0 | 1 | 0 | 0 | 0 | 1 | 1 | 0 | 0 | 0 | 0 | 0 |
| NM_005985 | SNAI1 | 1692 | 1 | 0 | 1 | 0 | 0 | 0 | 0 | 0 | 0 | 0 | 0 | 0 | 0 |
| NM_005986 | SOX1 | 4108 | 1 | 0 | 0 | 0 | 0 | 1 | 0 | 0 | 0 | 0 | 0 | 0 | 0 |
| NM_005987 | SPRR1A | 648 | 0 | 0 | 0 | 0 | 0 | 0 | 0 | 0 | 0 | 0 | 0 | 0 | 0 |
| NM_005988 | SPRR2A | 677 | 0 | 0 | 0 | 0 | 0 | 0 | 0 | 0 | 0 | 0 | 0 | 0 | 0 |
| NM_005989 | AKR1D1 | 2692 | 23 | 2 | 0 | 0 | 0 | 0 | 0 | 23 | 0 | 0 | 0 | 0 | 0 |
| NM_005990 | STK10 | 6019 | 16 | 5 | 6 | 3 | 3 | 2 | 2 | 2 | 5 | 1 | 1 | 16 | 9 |
| NM_005992 | TBX1 | 1534 | 10 | 1 | 0 | 0 | 0 | 0 | 0 | 0 | 0 | 0 | 10 | 0 | 3 |
| NM_005993 | TBCD | 4432 | 2 | 1 | 1 | 1 | 1 | 2 | 1 | 1 | 1 | 1 | 0 | 1 | 1 |
| NM_005994 | TBX2 | 3380 | 1 | 0 | 0 | 0 | 0 | 0 | 1 | 0 | 1 | 0 | 0 | 0 | 0 |
| NM_005995 | TBX10 | 1548 | 0 | 0 | 0 | 0 | 0 | 0 | 0 | 0 | 0 | 0 | 0 | 0 | 0 |
| NM_005996 | TBX3 | 4723 | 5 | 2 | 2 | 1 | 1 | 1 | 1 | 4 | 3 | 5 | 1 | 1 | 1 |
| NM_005997 | VPS72 | 1324 | 5 | 3 | 3 | 4 | 3 | 5 | 3 | 2 | 2 | 3 | 4 | 4 | 3 |
| NM_005998 | CCT3 | 2096 | 36 | 25 | 17 | 14 | 29 | 31 | 26 | 36 | 12 | 26 | 21 | 31 | 31 |
| NM_005999 | TSNAX | 2649 | 2 | 1 | 1 | 0 | 1 | 2 | 2 | 1 | 1 | 1 | 2 | 2 | 1 |
| NM_006000 | TUBA4A | 1478 | 62 | 20 | 1 | 10 | 28 | 57 | 16 | 16 | 7 | 2 | 62 | 18 | 4 |
| NM_006001 | TUBA3C | 1487 | 15 | 1 | 0 | 0 | 0 | 0 | 0 | 0 | 0 | 0 | 0 | 0 | 15 |
| NM_006002 | UCHL3 | 843 | 3 | 2 | 1 | 1 | 3 | 2 | 2 | 3 | 1 | 1 | 1 | 2 | 2 |
| NM_006003 | UQCRFS1 | 1224 | 34 | 16 | 11 | 13 | 34 | 20 | 24 | 27 | 6 | 5 | 21 | 8 | 12 |
| NM_006004 | UQCRH | 526 | 15 | 4 | 2 | 2 | 15 | 5 | 4 | 2 | 2 | 2 | 3 | 3 | 1 |
| NM_006005 | WFS1 | 3639 | 1 | 0 | 1 | 0 | 0 | 1 | 0 | 0 | 0 | 1 | 0 | 1 | 1 |
| NM_006006 | ZBTB16 | 2406 | 16 | 8 | 10 | 6 | 7 | 5 | 7 | 8 | 13 | 4 | 16 | 8 | 4 |
| NM_006007 | ZFAND5 | 5728 | 16 | 7 | 4 | 1 | 6 | 6 | 5 | 6 | 7 | 11 | 16 | 9 | 7 |
| NM_006009 | TUBA1A | 1665 | 30 | 5 | 2 | 3 | 4 | 30 | 3 | 0 | 2 | 3 | 0 | 4 | 4 |
| NM_006010 | MANF | 927 | 108 | 43 | 23 | 23 | 72 | 42 | 30 | 108 | 35 | 23 | 11 | 63 | 47 |
| NM_006011 | ST8SIA2 | 5626 | 0 | 0 | 0 | 0 | 0 | 0 | 0 | 0 | 0 | 0 | 0 | 0 | 0 |
| NM_006012 | CLPP | 1151 | 2 | 1 | 1 | 1 | 1 | 1 | 1 | 1 | 1 | 1 | 1 | 2 | 1 |
| NM_006013 | RPL10 | 2318 | 3 | 2 | 3 | 2 | 2 | 2 | 2 | 3 | 2 | 3 | 2 | 3 | 1 |
| NM_006014 | LAGE3 | 1816 | 0 | 0 | 0 | 0 | 0 | 0 | 0 | 0 | 0 | 0 | 0 | 0 | 0 |
| NM_006015 | ARID1A | 8585 | 19 | 9 | 5 | 7 | 9 | 10 | 9 | 5 | 8 | 14 | 8 | 19 | 10 |
| NM_006016 | CD164 | 3110 | 35 | 22 | 34 | 8 | 23 | 9 | 35 | 18 | 23 | 17 | 11 | 31 | 29 |
| NM_006017 | PROM1 | 3977 | 3 | 1 | 0 | 2 | 0 | 0 | 3 | 0 | 1 | 0 | 0 | 0 | 1 |
| NM_006018 | GPR109B | 2137 | 1 | 0 | 0 | 0 | 0 | 0 | 0 | 0 | 0 | 0 | 0 | 1 | 0 |
| NM_006019 | TCIRG1 | 2681 | 1 | 0 | 0 | 0 | 0 | 0 | 0 | 0 | 0 | 0 | 0 | 1 | 0 |
| NM_006020 | ALKBH1 | 2585 | 2 | 1 | 1 | 1 | 2 | 1 | 1 | 1 | 1 | 1 | 2 | 2 | 2 |
| NM_006022 | TSC22D1 | 1753 | 88 | 30 | 23 | 21 | 29 | 88 | 25 | 11 | 36 | 27 | 28 | 9 | 39 |
| NM_006023 | CDC123 | 1546 | 3 | 2 | 2 | 1 | 3 | 2 | 2 | 3 | 1 | 1 | 1 | 3 | 2 |
| NM_006024 | TAX1BP1 | 3466 | 43 | 28 | 17 | 5 | 43 | 36 | 34 | 23 | 29 | 21 | 22 | 37 | 42 |
| NM_006025 | P11 | 2321 | 0 | 0 | 0 | 0 | 0 | 0 | 0 | 0 | 0 | 0 | 0 | 0 | 0 |
| NM_006026 | H1FX | 1506 | 1 | 0 | 1 | 1 | 0 | 1 | 0 | 0 | 1 | 0 | 0 | 1 | 1 |
| NM_006027 | EXO1 | 3122 | 2 | 0 | 0 | 0 | 0 | 0 | 0 | 0 | 0 | 0 | 0 | 1 | 2 |
| NM_006028 | HTR3B | 1817 | 0 | 0 | 0 | 0 | 0 | 0 | 0 | 0 | 0 | 0 | 0 | 0 | 0 |
| NM_006029 | PNMA1 | 2643 | 36 | 8 | 6 | 4 | 4 | 36 | 7 | 1 | 3 | 8 | 1 | 7 | 12 |
| NM_006030 | CACNA2D2 | 5336 | 3 | 1 | 0 | 0 | 0 | 1 | 0 | 0 | 3 | 0 | 0 | 0 | 1 |

| transcript | gene | transcript length (nt) | max | mean | adipose | colon | heart | hypothalamus | kidney | liver | lung | ovary | skeletalmuscle | spleen | testes |
|---|---|---|---|---|---|---|---|---|---|---|---|---|---|---|---|
| NM_006031 | PCNT | 10560 | 43 | 7 | 3 | 3 | 7 | 3 | 2 | 2 | 2 | 3 | 43 | 4 | 6 |
| NM_006032 | CPNE6 | 2010 | 12 | 1 | 0 | 0 | 0 | 12 | 1 | 0 | 0 | 0 | 0 | 0 | 0 |
| NM_006033 | LIPG | 4141 | 7 | 1 | 0 | 1 | 0 | 0 | 1 | 7 | 1 | 1 | 0 | 0 | 2 |
| NM_006034 | TP53I11 | 3518 | 7 | 3 | 3 | 5 | 5 | 3 | 1 | 0 | 3 | 1 | 1 | 7 | 1 |
| NM_006035 | CDC42BPB | 6701 | 14 | 8 | 8 | 6 | 8 | 14 | 9 | 7 | 5 | 7 | 3 | 8 | 9 |
| NM_006036 | PREPL | 4812 | 27 | 6 | 3 | 1 | 6 | 27 | 10 | 2 | 2 | 4 | 3 | 3 | 5 |
| NM_006037 | HDAC4 | 8976 | 2 | 1 | 0 | 1 | 0 | 1 | 0 | 0 | 0 | 0 | 2 | 1 | 0 |
| NM_006038 | SPATA2 | 4054 | 2 | 1 | 0 | 1 | 1 | 2 | 1 | 0 | 1 | 1 | 1 | 2 | 2 |
| NM_006039 | MRC2 | 5982 | 1 | 1 | 1 | 1 | 1 | 0 | 0 | 0 | 1 | 1 | 0 | 0 | 0 |
| NM_006040 | HS3ST4 | 3203 | 6 | 1 | 0 | 0 | 0 | 6 | 0 | 0 | 0 | 0 | 0 | 0 | 0 |
| NM_006041 | HS3ST3B1 | 2032 | 3 | 0 | 0 | 0 | 0 | 0 | 0 | 3 | 0 | 0 | 0 | 1 | 0 |
| NM_006042 | HS3ST3A1 | 2527 | 0 | 0 | 0 | 0 | 0 | 0 | 0 | 0 | 0 | 0 | 0 | 0 | 0 |
| NM_006043 | HS3ST2 | 1952 | 1 | 0 | 0 | 0 | 0 | 1 | 0 | 0 | 1 | 0 | 0 | 0 | 0 |
| NM_006044 | HDAC6 | 4099 | 5 | 2 | 1 | 2 | 1 | 2 | 5 | 3 | 1 | 1 | 1 | 2 | 3 |
| NM_006045 | ATP9A | 7580 | 32 | 7 | 11 | 6 | 4 | 32 | 3 | 2 | 2 | 5 | 2 | 2 | 9 |
| NM_006047 | RBM12 | 6630 | 18 | 10 | 6 | 6 | 10 | 9 | 9 | 6 | 8 | 17 | 7 | 18 | 14 |
| NM_006048 | UBE4B | 5517 | 8 | 5 | 4 | 4 | 8 | 7 | 4 | 4 | 3 | 7 | 7 | 5 | 4 |
| NM_006049 | SNAPC5 | 785 | 7 | 3 | 4 | 2 | 3 | 6 | 3 | 7 | 1 | 1 | 1 | 3 | 2 |
| NM_006051 | APBB3 | 2158 | 0 | 0 | 0 | 0 | 0 | 0 | 0 | 0 | 0 | 0 | 0 | 0 | 0 |
| NM_006052 | DSCR3 | 3251 | 6 | 4 | 3 | 3 | 6 | 4 | 6 | 5 | 4 | 3 | 4 | 6 | 2 |
| NM_006053 | TCIRG1 | 2461 | 1 | 0 | 0 | 0 | 0 | 0 | 0 | 0 | 1 | 0 | 0 | 1 | 0 |
| NM_006054 | RTN3 | 2567 | 72 | 14 | 20 | 5 | 6 | 72 | 8 | 10 | 9 | 5 | 4 | 8 | 5 |
| NM_006055 | LANCL1 | 4602 | 36 | 9 | 7 | 2 | 9 | 36 | 10 | 3 | 5 | 6 | 5 | 8 | 10 |
| NM_006056 | NMUR1 | 3274 | 0 | 0 | 0 | 0 | 0 | 0 | 0 | 0 | 0 | 0 | 0 | 0 | 0 |
| NM_006057 | B3GALT5 | 2762 | 1 | 0 | 0 | 1 | 0 | 0 | 0 | 0 | 0 | 0 | 0 | 0 | 0 |
| NM_006058 | TNIP1 | 3268 | 25 | 8 | 25 | 6 | 7 | 3 | 8 | 6 | 7 | 3 | 9 | 13 | 4 |
| NM_006059 | LAMC3 | 6098 | 1 | 0 | 0 | 0 | 0 | 0 | 0 | 0 | 0 | 0 | 0 | 1 | 1 |
| NM_006060 | IKZF1 | 6188 | 20 | 3 | 1 | 1 | 1 | 2 | 1 | 1 | 4 | 1 | 0 | 20 | 1 |
| NM_006061 | CRISP3 | 2088 | 2 | 0 | 0 | 0 | 0 | 0 | 0 | 0 | 2 | 0 | 0 | 1 | 0 |
| NM_006062 | SMYD5 | 2567 | 1 | 0 | 0 | 0 | 0 | 1 | 1 | 0 | 0 | 0 | 0 | 0 | 1 |
| NM_006063 | KBTBD10 | 2464 | 688 | 65 | 1 | 0 | 24 | 0 | 0 | 0 | 0 | 0 | 688 | 0 | 3 |
| NM_006064 | RRAGB | 2123 | 8 | 3 | 2 | 2 | 2 | 8 | 3 | 0 | 2 | 3 | 2 | 3 | 4 |
| NM_006065 | SIRPB1 | 2412 | 2 | 1 | 0 | 0 | 0 | 1 | 0 | 1 | 1 | 0 | 0 | 2 | 0 |
| NM_006066 | AKR1A1 | 1535 | 20 | 6 | 4 | 3 | 4 | 3 | 20 | 11 | 4 | 2 | 2 | 6 | 3 |
| NM_006067 | COX4NB | 1961 | 4 | 3 | 2 | 1 | 4 | 4 | 3 | 3 | 1 | 2 | 2 | 3 | 2 |
| NM_006068 | TLR6 | 5882 | 2 | 0 | 0 | 0 | 0 | 0 | 0 | 0 | 1 | 0 | 0 | 2 | 0 |
| NM_006070 | TFG | 2088 | 6 | 4 | 3 | 2 | 6 | 4 | 6 | 6 | 4 | 3 | 3 | 6 | 4 |
| NM_006071 | PKDREJ | 7660 | 1 | 0 | 0 | 0 | 0 | 0 | 0 | 0 | 0 | 0 | 0 | 0 | 1 |
| NM_006072 | CCL26 | 562 | 0 | 0 | 0 | 0 | 0 | 0 | 0 | 0 | 0 | 0 | 0 | 0 | 0 |
| NM_006073 | TRDN | 4475 | 59 | 7 | 0 | 0 | 18 | 1 | 0 | 0 | 0 | 0 | 59 | 0 | 1 |
| NM_006074 | TRIM22 | 2989 | 25 | 7 | 7 | 3 | 6 | 4 | 2 | 2 | 11 | 6 | 4 | 25 | 5 |
| NM_006076 | AGFG2 | 4811 | 3 | 1 | 0 | 1 | 1 | 1 | 1 | 3 | 1 | 1 | 0 | 1 | 2 |
| NM_006077 | CBARA1 | 2425 | 17 | 10 | 10 | 5 | 8 | 17 | 14 | 13 | 9 | 11 | 10 | 10 | 8 |
| NM_006078 | CACNG2 | 1548 | 2 | 0 | 0 | 0 | 0 | 2 | 0 | 0 | 0 | 0 | 0 | 0 | 0 |
| NM_006079 | CITED2 | 1929 | 3 | 1 | 1 | 1 | 1 | 1 | 1 | 0 | 2 | 3 | 1 | 3 | 1 |
| NM_006080 | SEMA3A | 5659 | 9 | 1 | 1 | 1 | 1 | 2 | 0 | 0 | 1 | 1 | 0 | 9 | 1 |
| NM_006082 | TUBA1B | 1752 | 76 | 26 | 23 | 15 | 36 | 76 | 23 | 15 | 19 | 13 | 7 | 36 | 22 |
| NM_006083 | IK | 1935 | 82 | 60 | 54 | 23 | 67 | 66 | 49 | 43 | 52 | 65 | 81 | 79 | 82 |
| NM_006084 | IRF9 | 1678 | 4 | 2 | 2 | 2 | 1 | 3 | 2 | 2 | 1 | 2 | 2 | 4 | 2 |
| NM_006085 | BPNT1 | 2461 | 4 | 2 | 1 | 2 | 2 | 3 | 4 | 2 | 1 | 2 | 0 | 2 | 2 |
| NM_006086 | TUBB3 | 1711 | 18 | 2 | 0 | 0 | 0 | 18 | 0 | 0 | 0 | 0 | 0 | 0 | 1 |
| NM_006087 | TUBB4 | 2287 | 83 | 9 | 0 | 2 | 0 | 83 | 0 | 0 | 0 | 1 | 0 | 2 | 8 |
| NM_006088 | TUBB2C | 1591 | 24 | 10 | 6 | 12 | 15 | 14 | 16 | 10 | 7 | 2 | 3 | 5 | 24 |
| NM_006089 | SCML2 | 4198 | 1 | 0 | 0 | 0 | 0 | 0 | 0 | 0 | 0 | 0 | 0 | 0 | 1 |
| NM_006090 | CEPT1 | 2213 | 27 | 10 | 10 | 4 | 5 | 10 | 8 | 12 | 9 | 9 | 2 | 27 | 9 |
| NM_006091 | CORO2B | 3303 | 17 | 3 | 2 | 1 | 3 | 17 | 2 | 0 | 1 | 5 | 1 | 2 | 1 |
| NM_006092 | NOD1 | 4496 | 2 | 1 | 1 | 1 | 1 | 0 | 0 | 0 | 1 | 1 | 0 | 2 | 1 |
| NM_006093 | PRG3 | 868 | 1 | 0 | 0 | 0 | 0 | 0 | 0 | 0 | 1 | 0 | 0 | 0 | 0 |
| NM_006094 | DLC1 | 6044 | 10 | 4 | 7 | 1 | 6 | 4 | 3 | 4 | 10 | 2 | 1 | 6 | 2 |
| NM_006095 | ATP8A1 | 8268 | 7 | 2 | 0 | 1 | 1 | 7 | 1 | 0 | 3 | 2 | 2 | 2 | 1 |
| NM_006096 | NDRG1 | 3081 | 76 | 29 | 23 | 27 | 25 | 33 | 76 | 6 | 41 | 30 | 8 | 27 | 28 |
| NM_006097 | MYL9 | 1187 | 134 | 40 | 57 | 134 | 87 | 13 | 14 | 3 | 23 | 35 | 14 | 19 | 41 |
| NM_006098 | GNB2L1 | 1109 | 80 | 37 | 30 | 31 | 34 | 18 | 28 | 43 | 32 | 80 | 34 | 55 | 22 |
| NM_006099 | PIAS3 | 2902 | 2 | 1 | 1 | 1 | 1 | 2 | 1 | 0 | 1 | 2 | 1 | 1 | 2 |
| NM_006100 | ST3GAL6 | 2094 | 7 | 2 | 1 | 0 | 7 | 1 | 2 | 7 | 1 | 1 | 1 | 1 | 1 |
| NM_006101 | NDC80 | 2171 | 3 | 1 | 1 | 0 | 0 | 0 | 0 | 0 | 1 | 1 | 0 | 2 | 3 |
| NM_006103 | WFDC2 | 566 | 2 | 1 | 0 | 1 | 0 | 0 | 1 | 0 | 2 | 0 | 0 | 0 | 1 |
| NM_006105 | RAPGEF3 | 5751 | 1 | 0 | 1 | 0 | 0 | 1 | 1 | 0 | 0 | 1 | 0 | 0 | 0 |
| NM_006106 | YAP1 | 5218 | 25 | 11 | 14 | 4 | 13 | 4 | 10 | 6 | 14 | 25 | 9 | 6 | 12 |
| NM_006107 | LUC7L3 | 2010 | 55 | 35 | 30 | 15 | 31 | 39 | 39 | 16 | 40 | 45 | 27 | 48 | 55 |
| NM_006108 | SPON1 | 5383 | 7 | 3 | 4 | 2 | 2 | 4 | 2 | 0 | 4 | 7 | 0 | 3 | 1 |
| NM_006109 | PRMT5 | 2385 | 10 | 7 | 4 | 5 | 7 | 9 | 8 | 8 | 5 | 7 | 10 | 7 | 10 |
| NM_006110 | CD2BP2 | 3420 | 4 | 2 | 2 | 2 | 2 | 4 | 2 | 2 | 1 | 2 | 1 | 3 | 2 |
| NM_006111 | ACAA2 | 1934 | 92 | 28 | 36 | 20 | 33 | 8 | 65 | 92 | 8 | 9 | 13 | 11 | 11 |
| NM_006112 | PPIE | 1300 | 5 | 3 | 3 | 3 | 2 | 3 | 3 | 3 | 2 | 3 | 2 | 3 | 5 |
| NM_006113 | VAV3 | 4769 | 13 | 2 | 1 | 3 | 1 | 1 | 13 | 0 | 1 | 0 | 0 | 4 | 1 |
| NM_006114 | TOMM40 | 1707 | 2 | 2 | 1 | 1 | 2 | 2 | 2 | 2 | 2 | 1 | 2 | 2 | 1 |

| | | transcript | | | | | | hypothala | | | | | skeletalm | | |
|---|---|---|---|---|---|---|---|---|---|---|---|---|---|---|---|
| transcript | gene | length (nt) | max | mean | adipose | colon | heart | mus | kidney | liver | lung | ovary | uscle | spleen | testes |
| NM_006115 | PRAME | 2145 | 17 | 2 | 0 | 0 | 0 | 0 | 0 | 0 | 0 | 1 | 0 | 0 | 17 |
| NM_006116 | MAP3K7IP1 | 3224 | 2 | 1 | 1 | 1 | 1 | 2 | 1 | 1 | 1 | 1 | 1 | 1 | 1 |
| NM_006117 | PECI | 1393 | 54 | 21 | 20 | 11 | 30 | 10 | 39 | 18 | 7 | 22 | 54 | 12 | 12 |
| NM_006118 | HAX1 | 1175 | 24 | 13 | 8 | 6 | 24 | 16 | 9 | 24 | 6 | 8 | 17 | 10 | 11 |
| NM_006119 | FGF8 | 1017 | 0 | 0 | 0 | 0 | 0 | 0 | 0 | 0 | 0 | 0 | 0 | 0 | 0 |
| NM_006120 | HLA-DMA | 1122 | 11 | 3 | 6 | 3 | 2 | 2 | 3 | 1 | 7 | 1 | 1 | 11 | 1 |
| NM_006120 | HLA-DMA | 1125 | 11 | 3 | 6 | 3 | 2 | 2 | 3 | 1 | 7 | 1 | 1 | 11 | 1 |
| NM_006121 | KRT1 | 2451 | 0 | 0 | 0 | 0 | 0 | 0 | 0 | 0 | 0 | 0 | 0 | 0 | 0 |
| NM_006122 | MAN2A2 | 6269 | 14 | 6 | 7 | 4 | 7 | 14 | 4 | 6 | 4 | 2 | 8 | 8 | 4 |
| NM_006123 | IDS | 1406 | 31 | 5 | 2 | 2 | 3 | 31 | 3 | 1 | 4 | 4 | 2 | 5 | 2 |
| NM_006125 | ARHGAP6 | 3632 | 2 | 1 | 1 | 1 | 1 | 1 | 1 | 0 | 1 | 2 | 1 | 1 | 1 |
| NM_006128 | BMP1 | 3081 | 1 | 0 | 0 | 1 | 0 | 0 | 0 | 0 | 0 | 0 | 0 | 0 | 0 |
| NM_006129 | BMP1 | 3801 | 1 | 0 | 0 | 0 | 0 | 0 | 0 | 0 | 0 | 0 | 0 | 1 | 0 |
| NM_006133 | DAGLA | 5752 | 1 | 0 | 0 | 0 | 0 | 1 | 0 | 0 | 0 | 0 | 0 | 0 | 0 |
| NM_006134 | TMEM50B | 2332 | 8 | 6 | 4 | 4 | 6 | 8 | 7 | 2 | 7 | 7 | 4 | 7 | 7 |
| NM_006135 | CAPZA1 | 2740 | 18 | 6 | 7 | 3 | 5 | 6 | 5 | 8 | 7 | 3 | 2 | 18 | 6 |
| NM_006136 | CAPZA2 | 2356 | 54 | 32 | 28 | 9 | 54 | 38 | 38 | 32 | 29 | 15 | 38 | 42 | 24 |
| NM_006137 | CD7 | 1283 | 1 | 0 | 0 | 0 | 0 | 0 | 0 | 0 | 0 | 0 | 0 | 1 | 0 |
| NM_006138 | MS4A3 | 1655 | 0 | 0 | 0 | 0 | 0 | 0 | 0 | 0 | 0 | 0 | 0 | 0 | 0 |
| NM_006139 | CD28 | 3807 | 7 | 2 | 7 | 1 | 2 | 0 | 1 | 1 | 2 | 1 | 0 | 6 | 1 |
| NM_006140 | CSF2RA | 1853 | 6 | 2 | 2 | 1 | 1 | 1 | 1 | 0 | 4 | 1 | 0 | 6 | 1 |
| NM_006141 | DYNC1LI2 | 4336 | 25 | 11 | 14 | 8 | 16 | 25 | 11 | 4 | 10 | 10 | 7 | 10 | 9 |
| NM_006142 | SFN | 1315 | 4 | 1 | 0 | 4 | 0 | 0 | 0 | 0 | 2 | 0 | 0 | 0 | 0 |
| NM_006143 | GPR19 | 1742 | 3 | 0 | 0 | 0 | 0 | 3 | 0 | 0 | 0 | 0 | 0 | 0 | 0 |
| NM_006144 | GZMA | 896 | 1 | 0 | 0 | 0 | 0 | 0 | 0 | 0 | 1 | 0 | 0 | 1 | 0 |
| NM_006145 | DNAJB1 | 2233 | 9 | 5 | 6 | 4 | 4 | 7 | 6 | 4 | 9 | 5 | 2 | 3 | 8 |
| NM_006147 | IRF6 | 2170 | 6 | 3 | 0 | 6 | 3 | 0 | 5 | 5 | 3 | 1 | 0 | 1 | 3 |
| NM_006148 | LASP1 | 4108 | 22 | 10 | 12 | 12 | 8 | 7 | 12 | 9 | 12 | 11 | 2 | 22 | 7 |
| NM_006149 | LGALS4 | 1279 | 25 | 2 | 0 | 25 | 0 | 0 | 0 | 1 | 0 | 0 | 0 | 0 | 0 |
| NM_006150 | PRICKLE3 | 1990 | 1 | 0 | 0 | 1 | 1 | 0 | 0 | 0 | 0 | 0 | 1 | 0 | 0 |
| NM_006151 | LPO | 2978 | 0 | 0 | 0 | 0 | 0 | 0 | 0 | 0 | 0 | 0 | 0 | 0 | 0 |
| NM_006152 | LRMP | 2809 | 11 | 2 | 1 | 2 | 1 | 3 | 2 | 1 | 3 | 1 | 0 | 11 | 0 |
| NM_006153 | NCK1 | 1914 | 12 | 7 | 12 | 3 | 6 | 6 | 5 | 7 | 9 | 7 | 7 | 11 | 8 |
| NM_006154 | NEDD4 | 5758 | 10 | 3 | 1 | 1 | 2 | 1 | 3 | 6 | 2 | 2 | 10 | 3 | 1 |
| NM_006155 | 02 Sep | 3495 | 56 | 37 | 56 | 17 | 37 | 56 | 41 | 19 | 32 | 44 | 18 | 50 | 41 |
| NM_006156 | NEDD8 | 625 | 107 | 65 | 47 | 38 | 107 | 89 | 79 | 53 | 58 | 41 | 57 | 93 | 58 |
| NM_006157 | NELL1 | 3245 | 2 | 0 | 0 | 0 | 0 | 2 | 1 | 0 | 0 | 0 | 0 | 0 | 0 |
| NM_006158 | NEFL | 3587 | 58 | 5 | 0 | 1 | 0 | 58 | 1 | 0 | 0 | 0 | 0 | 0 | 0 |
| NM_006159 | NELL2 | 3312 | 27 | 3 | 0 | 0 | 0 | 27 | 0 | 0 | 1 | 0 | 0 | 1 | 0 |
| NM_006160 | NEUROD2 | 3032 | 3 | 1 | 1 | 0 | 0 | 1 | 0 | 1 | 3 | 0 | 0 | 0 | 1 |
| NM_006161 | NEUROG1 | 1668 | 0 | 0 | 0 | 0 | 0 | 0 | 0 | 0 | 0 | 0 | 0 | 0 | 0 |
| NM_006162 | NFATC1 | 4724 | 2 | 1 | 0 | 0 | 1 | 0 | 0 | 0 | 1 | 0 | 2 | 2 | 1 |
| NM_006163 | NFE2 | 1657 | 2 | 0 | 0 | 0 | 0 | 0 | 0 | 0 | 1 | 0 | 0 | 2 | 0 |
| NM_006164 | NFE2L2 | 2853 | 15 | 8 | 6 | 3 | 9 | 7 | 10 | 7 | 8 | 15 | 8 | 10 | 11 |
| NM_006165 | NFRKB | 5046 | 3 | 2 | 1 | 2 | 1 | 2 | 2 | 1 | 1 | 2 | 2 | 2 | 3 |
| NM_006166 | NFYB | 3482 | 7 | 4 | 3 | 1 | 7 | 5 | 4 | 3 | 6 | 3 | 3 | 5 | 7 |
| NM_006167 | NKX3-1 | 3280 | 19 | 3 | 0 | 0 | 3 | 1 | 0 | 1 | 1 | 1 | 2 | 0 | 19 |
| NM_006168 | NKX6-1 | 1116 | 0 | 0 | 0 | 0 | 0 | 0 | 0 | 0 | 0 | 0 | 0 | 0 | 0 |
| NM_006169 | NNMT | 1578 | 40 | 6 | 3 | 1 | 4 | 0 | 2 | 40 | 3 | 1 | 6 | 4 | 1 |
| NM_006170 | NOP2 | 2661 | 3 | 1 | 1 | 1 | 1 | 1 | 1 | 3 | 1 | 0 | 2 | 2 | 1 |
| NM_006172 | NPPA | 852 | 311 | 29 | 0 | 1 | 311 | 0 | 0 | 0 | 0 | 0 | 0 | 0 | 0 |
| NM_006174 | NPY5R | 1843 | 2 | 1 | 2 | 0 | 0 | 1 | 1 | 0 | 0 | 0 | 0 | 2 | 1 |
| NM_006175 | NRAP | 5403 | 290 | 44 | 0 | 0 | 189 | 0 | 0 | 0 | 0 | 0 | 290 | 0 | 1 |
| NM_006176 | NRGN | 1215 | 85 | 10 | 1 | 2 | 1 | 85 | 1 | 0 | 6 | 1 | 0 | 6 | 1 |
| NM_006177 | NRL | 1974 | 0 | 0 | 0 | 0 | 0 | 0 | 0 | 0 | 0 | 0 | 0 | 0 | 0 |
| NM_006178 | NSF | 3983 | 73 | 11 | 4 | 4 | 4 | 73 | 5 | 5 | 5 | 2 | 4 | 7 | 6 |
| NM_006179 | NTF4 | 999 | 0 | 0 | 0 | 0 | 0 | 0 | 0 | 0 | 0 | 0 | 0 | 0 | 0 |
| NM_006180 | NTRK2 | 5608 | 73 | 9 | 15 | 1 | 1 | 73 | 3 | 0 | 1 | 2 | 1 | 1 | 2 |
| NM_006181 | NTN3 | 1986 | 0 | 0 | 0 | 0 | 0 | 0 | 0 | 0 | 0 | 0 | 0 | 0 | 0 |
| NM_006182 | DDR2 | 3164 | 16 | 3 | 16 | 3 | 3 | 2 | 1 | 0 | 2 | 5 | 1 | 1 | 2 |
| NM_006183 | NTS | 1232 | 5 | 1 | 0 | 0 | 1 | 5 | 0 | 0 | 2 | 0 | 0 | 0 | 0 |
| NM_006184 | NUCB1 | 2243 | 55 | 19 | 24 | 12 | 20 | 12 | 24 | 55 | 16 | 8 | 5 | 21 | 9 |
| NM_006185 | NUMA1 | 7182 | 13 | 9 | 9 | 12 | 6 | 7 | 9 | 4 | 7 | 13 | 10 | 9 | 10 |
| NM_006186 | NR4A2 | 3531 | 15 | 2 | 1 | 0 | 1 | 2 | 1 | 1 | 2 | 15 | 1 | 0 | 1 |
| NM_006187 | OAS3 | 6628 | 3 | 1 | 3 | 1 | 2 | 1 | 1 | 1 | 1 | 2 | 2 | 2 | 1 |
| NM_006188 | OCM2 | 587 | 0 | 0 | 0 | 0 | 0 | 0 | 0 | 0 | 0 | 0 | 0 | 0 | 0 |
| NM_006189 | OMP | 492 | 0 | 0 | 0 | 0 | 0 | 0 | 0 | 0 | 0 | 0 | 0 | 0 | 0 |
| NM_006190 | ORC2L | 3121 | 9 | 4 | 3 | 3 | 4 | 4 | 4 | 2 | 3 | 6 | 3 | 5 | 9 |
| NM_006191 | PA2G4 | 2626 | 2 | 2 | 2 | 2 | 2 | 2 | 2 | 2 | 2 | 1 | 2 | 2 | 1 |
| NM_006192 | PAX1 | 2838 | 0 | 0 | 0 | 0 | 0 | 0 | 0 | 0 | 0 | 0 | 0 | 0 | 0 |
| NM_006193 | PAX4 | 2010 | 0 | 0 | 0 | 0 | 0 | 0 | 0 | 0 | 0 | 0 | 0 | 0 | 0 |
| NM_006194 | PAX9 | 3104 | 0 | 0 | 0 | 0 | 0 | 0 | 0 | 0 | 0 | 0 | 0 | 0 | 0 |
| NM_006195 | PBX3 | 2872 | 8 | 3 | 2 | 2 | 4 | 2 | 1 | 2 | 2 | 8 | 1 | 2 | 3 |
| NM_006196 | PCBP1 | 1748 | 42 | 20 | 14 | 7 | 23 | 14 | 25 | 16 | 19 | 18 | 42 | 32 | 15 |
| NM_006197 | PCM1 | 8783 | 54 | 29 | 17 | 6 | 33 | 29 | 20 | 17 | 22 | 39 | 51 | 30 | 54 |
| NM_006198 | PCP4 | 556 | 159 | 23 | 0 | 13 | 3 | 159 | 54 | 0 | 0 | 1 | 0 | 1 | 16 |
| NM_006200 | PCSK5 | 3350 | 5 | 2 | 3 | 5 | 2 | 2 | 3 | 0 | 1 | 5 | 1 | 1 | 3 |

| transcript | gene | transcript length (nt) | max | mean | adipose | colon | heart | hypothala mus | kidney | liver | lung | ovary | skeletalm uscle | spleen | testes |
|---|---|---|---|---|---|---|---|---|---|---|---|---|---|---|---|
| NM_006201 | CDK16 | 3151 | 7 | 4 | 3 | 5 | 5 | 7 | 5 | 2 | 3 | 2 | 6 | 3 | 5 |
| NM_006202 | PDE4A | 4261 | 4 | 1 | 1 | 1 | 1 | 4 | 1 | 0 | 1 | 0 | 1 | 1 | 1 |
| NM_006203 | PDE4D | 7770 | 22 | 5 | 1 | 2 | 8 | 4 | 6 | 0 | 4 | 0 | 22 | 2 | 1 |
| NM_006204 | PDE6C | 2969 | 0 | 0 | 0 | 0 | 0 | 0 | 0 | 0 | 0 | 0 | 0 | 0 | 0 |
| NM_006205 | PDE6H | 725 | 0 | 0 | 0 | 0 | 0 | 0 | 0 | 0 | 0 | 0 | 0 | 0 | 0 |
| NM_006206 | PDGFRA | 6574 | 44 | 15 | 10 | 7 | 14 | 6 | 7 | 6 | 13 | 44 | 3 | 42 | 12 |
| NM_006207 | PDGFRL | 1502 | 3 | 1 | 3 | 1 | 1 | 0 | 0 | 0 | 1 | 2 | 1 | 0 | 1 |
| NM_006208 | ENPP1 | 7442 | 4 | 1 | 3 | 0 | 1 | 0 | 2 | 4 | 1 | 1 | 0 | 0 | 2 |
| NM_006209 | ENPP2 | 3276 | 30 | 10 | 12 | 2 | 2 | 28 | 10 | 1 | 7 | 4 | 1 | 30 | 9 |
| NM_006210 | PEG3 | 8759 | 149 | 22 | 1 | 1 | 2 | 52 | 1 | 1 | 1 | 149 | 0 | 1 | 28 |
| NM_006211 | PENK | 1201 | 9 | 2 | 1 | 1 | 0 | 3 | 0 | 0 | 1 | 2 | 0 | 0 | 9 |
| NM_006212 | PFKFB2 | 7073 | 13 | 5 | 1 | 6 | 6 | 9 | 13 | 2 | 5 | 1 | 8 | 5 | 2 |
| NM_006213 | PHKG1 | 1431 | 10 | 1 | 0 | 1 | 2 | 1 | 0 | 0 | 0 | 0 | 10 | 0 | 0 |
| NM_006214 | PHYH | 1610 | 14 | 6 | 5 | 1 | 9 | 3 | 13 | 13 | 1 | 4 | 14 | 2 | 2 |
| NM_006215 | SERPINA4 | 1661 | 18 | 2 | 0 | 0 | 0 | 0 | 0 | 18 | 0 | 0 | 0 | 0 | 0 |
| NM_006216 | SERPINE2 | 2259 | 23 | 5 | 1 | 1 | 6 | 11 | 6 | 0 | 1 | 23 | 0 | 3 | 7 |
| NM_006217 | SERPINI2 | 1430 | 1 | 0 | 0 | 0 | 0 | 1 | 0 | 0 | 1 | 0 | 0 | 0 | 0 |
| NM_006218 | PIK3CA | 3709 | 5 | 3 | 3 | 4 | 3 | 3 | 2 | 2 | 3 | 3 | 2 | 5 | 3 |
| NM_006219 | PIK3CB | 3213 | 11 | 7 | 6 | 4 | 7 | 11 | 11 | 5 | 7 | 5 | 6 | 9 | 6 |
| NM_006221 | PIN1 | 997 | 4 | 1 | 0 | 0 | 1 | 4 | 1 | 1 | 0 | 0 | 0 | 0 | 0 |
| NM_006223 | PIN4 | 1071 | 4 | 3 | 2 | 2 | 4 | 4 | 4 | 3 | 1 | 3 | 3 | 3 | 2 |
| NM_006224 | PITPNA | 3641 | 14 | 6 | 4 | 5 | 6 | 14 | 6 | 5 | 6 | 4 | 2 | 9 | 6 |
| NM_006225 | PLCD1 | 2670 | 4 | 1 | 1 | 1 | 0 | 1 | 1 | 0 | 1 | 0 | 0 | 1 | 4 |
| NM_006226 | PLCL1 | 6606 | 15 | 4 | 2 | 1 | 15 | 11 | 9 | 0 | 2 | 4 | 4 | 1 | 1 |
| NM_006227 | PLTP | 1745 | 71 | 24 | 17 | 5 | 71 | 22 | 12 | 5 | 44 | 27 | 10 | 31 | 15 |
| NM_006228 | PNOC | 1196 | 0 | 0 | 0 | 0 | 0 | 0 | 0 | 0 | 0 | 0 | 0 | 0 | 0 |
| NM_006229 | PNLIPRP1 | 1480 | 0 | 0 | 0 | 0 | 0 | 0 | 0 | 0 | 0 | 0 | 0 | 0 | 0 |
| NM_006230 | POLD2 | 1602 | 3 | 1 | 1 | 1 | 2 | 1 | 1 | 3 | 1 | 1 | 2 | 1 | 0 |
| NM_006231 | POLE | 7840 | 2 | 1 | 1 | 1 | 0 | 0 | 0 | 1 | 0 | 0 | 1 | 2 | 1 |
| NM_006232 | POLR2H | 821 | 10 | 6 | 4 | 6 | 4 | 5 | 8 | 10 | 4 | 4 | 4 | 8 | 5 |
| NM_006233 | POLR2I | 872 | 2 | 1 | 1 | 1 | 1 | 2 | 1 | 1 | 1 | 1 | 1 | 0 | 1 |
| NM_006234 | POLR2J | 976 | 14 | 8 | 4 | 5 | 12 | 7 | 6 | 9 | 6 | 8 | 14 | 9 | 7 |
| NM_006235 | POU2AF1 | 3032 | 10 | 1 | 0 | 1 | 0 | 0 | 0 | 0 | 1 | 0 | 0 | 10 | 0 |
| NM_006236 | POU3F3 | 1503 | 0 | 0 | 0 | 0 | 0 | 0 | 0 | 0 | 0 | 0 | 0 | 0 | 0 |
| NM_006237 | POU4F1 | 3814 | 0 | 0 | 0 | 0 | 0 | 0 | 0 | 0 | 0 | 0 | 0 | 0 | 0 |
| NM_006238 | PPARD | 3734 | 2 | 1 | 0 | 1 | 1 | 1 | 1 | 0 | 1 | 1 | 0 | 2 | 1 |
| NM_006239 | PPEF2 | 3413 | 0 | 0 | 0 | 0 | 0 | 0 | 0 | 0 | 0 | 0 | 0 | 0 | 0 |
| NM_006240 | PPEF1 | 2872 | 2 | 0 | 0 | 0 | 0 | 1 | 0 | 0 | 0 | 0 | 0 | 0 | 2 |
| NM_006241 | PPP1R2 | 3465 | 6 | 4 | 2 | 1 | 4 | 6 | 3 | 2 | 4 | 3 | 6 | 4 | 4 |
| NM_006242 | PPP1R3D | 3466 | 2 | 1 | 0 | 0 | 1 | 1 | 1 | 0 | 0 | 1 | 1 | 2 | 1 |
| NM_006243 | PPP2R5A | 3128 | 26 | 12 | 26 | 5 | 17 | 8 | 10 | 13 | 17 | 6 | 9 | 19 | 7 |
| NM_006244 | PPP2R5B | 2710 | 2 | 1 | 1 | 1 | 0 | 2 | 1 | 1 | 0 | 0 | 0 | 0 | 0 |
| NM_006245 | PPP2R5D | 2951 | 7 | 4 | 2 | 3 | 5 | 7 | 3 | 3 | 2 | 5 | 5 | 4 | 6 |
| NM_006246 | PPP2R5E | 3366 | 12 | 8 | 5 | 3 | 10 | 11 | 7 | 6 | 7 | 8 | 8 | 12 | 8 |
| NM_006247 | PPP5C | 2062 | 33 | 18 | 5 | 14 | 31 | 33 | 16 | 11 | 8 | 9 | 23 | 28 | 17 |
| NM_006248 | PRB2 | 1426 | 0 | 0 | 0 | 0 | 0 | 0 | 0 | 0 | 0 | 0 | 0 | 0 | 0 |
| NM_006249 | PRB3 | 1091 | 2 | 0 | 0 | 0 | 0 | 0 | 0 | 0 | 2 | 0 | 0 | 0 | 0 |
| NM_006250 | PRH1 | 1203 | 0 | 0 | 0 | 0 | 0 | 0 | 0 | 0 | 0 | 0 | 0 | 0 | 0 |
| NM_006251 | PRKAA1 | 5085 | 17 | 9 | 7 | 5 | 8 | 6 | 10 | 17 | 10 | 7 | 5 | 12 | 11 |
| NM_006252 | PRKAA2 | 9352 | 18 | 4 | 0 | 1 | 12 | 2 | 5 | 0 | 1 | 1 | 18 | 1 | 1 |
| NM_006253 | PRKAB1 | 2401 | 3 | 1 | 0 | 1 | 1 | 1 | 3 | 1 | 1 | 0 | 0 | 2 | 1 |
| NM_006254 | PRKCD | 2835 | 4 | 2 | 2 | 2 | 2 | 2 | 1 | 0 | 2 | 1 | 0 | 4 | 1 |
| NM_006255 | PRKCH | 3506 | 6 | 2 | 2 | 1 | 1 | 1 | 1 | 1 | 4 | 1 | 1 | 6 | 1 |
| NM_006256 | PKN2 | 6104 | 9 | 5 | 5 | 2 | 4 | 3 | 5 | 3 | 5 | 9 | 3 | 6 | 6 |
| NM_006257 | PRKCQ | 3260 | 12 | 2 | 0 | 1 | 0 | 1 | 1 | 0 | 1 | 0 | 12 | 2 | 0 |
| NM_006258 | PRKG1 | 3792 | 8 | 3 | 1 | 2 | 8 | 2 | 2 | 0 | 5 | 2 | 6 | 1 | 3 |
| NM_006259 | PRKG2 | 3328 | 1 | 0 | 1 | 1 | 0 | 1 | 0 | 0 | 0 | 0 | 0 | 0 | 0 |
| NM_006260 | DNAJC3 | 5580 | 30 | 10 | 8 | 3 | 12 | 7 | 11 | 30 | 8 | 6 | 4 | 13 | 8 |
| NM_006261 | PROP1 | 1464 | 0 | 0 | 0 | 0 | 0 | 0 | 0 | 0 | 0 | 0 | 0 | 0 | 0 |
| NM_006262 | PRPH | 1833 | 1 | 0 | 0 | 1 | 0 | 1 | 0 | 0 | 0 | 0 | 0 | 0 | 0 |
| NM_006263 | PSME1 | 995 | 39 | 19 | 18 | 15 | 18 | 11 | 27 | 24 | 18 | 14 | 12 | 39 | 9 |
| NM_006264 | PTPN13 | 8505 | 10 | 4 | 3 | 1 | 2 | 5 | 9 | 0 | 6 | 10 | 1 | 2 | 9 |
| NM_006265 | RAD21 | 3749 | 51 | 27 | 20 | 10 | 28 | 44 | 19 | 23 | 27 | 23 | 23 | 33 | 51 |
| NM_006266 | RALGDS | 3632 | 5 | 1 | 1 | 1 | 1 | 5 | 1 | 0 | 1 | 1 | 1 | 2 | 1 |
| NM_006267 | RANBP2 | 11711 | 14 | 9 | 6 | 2 | 10 | 9 | 8 | 10 | 8 | 11 | 11 | 11 | 14 |
| NM_006268 | DPF2 | 2409 | 2 | 1 | 1 | 1 | 1 | 1 | 1 | 1 | 1 | 1 | 1 | 2 | 2 |
| NM_006269 | RP1 | 7100 | 1 | 0 | 0 | 0 | 0 | 0 | 0 | 0 | 1 | 0 | 0 | 0 | 0 |
| NM_006270 | RRAS | 983 | 7 | 3 | 7 | 2 | 5 | 1 | 2 | 1 | 5 | 1 | 1 | 4 | 2 |
| NM_006271 | S100A1 | 594 | 123 | 24 | 10 | 3 | 123 | 42 | 25 | 1 | 1 | 0 | 52 | 1 | 3 |
| NM_006272 | S100B | 1135 | 123 | 16 | 33 | 4 | 1 | 123 | 0 | 0 | 1 | 0 | 1 | 7 | 1 |
| NM_006273 | CCL7 | 805 | 0 | 0 | 0 | 0 | 0 | 0 | 0 | 0 | 0 | 0 | 0 | 0 | 0 |
| NM_006274 | CCL19 | 684 | 5 | 1 | 2 | 2 | 5 | 1 | 1 | 0 | 1 | 0 | 0 | 1 | 0 |
| NM_006275 | SFRS6 | 3744 | 18 | 11 | 7 | 6 | 8 | 8 | 15 | 13 | 10 | 11 | 5 | 18 | 16 |
| NM_006277 | ITSN2 | 6112 | 10 | 6 | 6 | 2 | 5 | 6 | 6 | 4 | 7 | 4 | 5 | 10 | 6 |
| NM_006278 | ST3GAL4 | 1743 | 10 | 5 | 3 | 3 | 7 | 6 | 5 | 3 | 4 | 7 | 0 | 8 | 10 |
| NM_006279 | ST3GAL3 | 2243 | 7 | 3 | 2 | 1 | 3 | 5 | 2 | 2 | 1 | 3 | 7 | 2 | 2 |
| NM_006280 | SSR4 | 637 | 6 | 2 | 2 | 2 | 2 | 2 | 2 | 6 | 3 | 1 | 1 | 4 | 1 |

| transcript | gene | transcript length (nt) | max | mean | adipose | colon | heart | hypothalamus | kidney | liver | lung | ovary | skeletalmuscle | spleen | testes |
|---|---|---|---|---|---|---|---|---|---|---|---|---|---|---|---|
| NM_006281 | STK3 | 2826 | 15 | 8 | 8 | 2 | 8 | 5 | 10 | 7 | 8 | 13 | 6 | 8 | 15 |
| NM_006282 | STK4 | 6330 | 31 | 9 | 6 | 5 | 5 | 9 | 4 | 4 | 14 | 7 | 3 | 31 | 10 |
| NM_006283 | TACC1 | 7802 | 16 | 8 | 12 | 5 | 8 | 12 | 6 | 3 | 10 | 7 | 3 | 16 | 9 |
| NM_006284 | TAF10 | 762 | 13 | 6 | 3 | 2 | 9 | 6 | 6 | 9 | 6 | 4 | 7 | 13 | 5 |
| NM_006285 | TESK1 | 2516 | 3 | 1 | 2 | 1 | 1 | 2 | 1 | 1 | 1 | 1 | 3 | 2 | 2 |
| NM_006286 | TFDP2 | 1280 | 25 | 12 | 9 | 6 | 13 | 7 | 17 | 6 | 7 | 23 | 7 | 8 | 25 |
| NM_006287 | TFPI | 3915 | 42 | 12 | 7 | 2 | 12 | 1 | 13 | 28 | 42 | 6 | 5 | 11 | 7 |
| NM_006288 | THY1 | 2396 | 47 | 7 | 6 | 3 | 1 | 47 | 17 | 0 | 1 | 1 | 0 | 3 | 2 |
| NM_006289 | TLN1 | 8434 | 31 | 15 | 31 | 16 | 19 | 9 | 11 | 10 | 13 | 11 | 7 | 30 | 10 |
| NM_006290 | TNFAIP3 | 4432 | 24 | 8 | 6 | 4 | 3 | 2 | 2 | 2 | 22 | 24 | 3 | 15 | 6 |
| NM_006291 | TNFAIP2 | 4175 | 2 | 1 | 2 | 1 | 1 | 0 | 1 | 0 | 1 | 0 | 0 | 1 | 0 |
| NM_006292 | TSG101 | 1534 | 8 | 6 | 5 | 3 | 7 | 6 | 7 | 6 | 4 | 4 | 6 | 7 | 8 |
| NM_006293 | TYRO3 | 3949 | 7 | 2 | 2 | 0 | 0 | 7 | 1 | 0 | 0 | 5 | 0 | 2 | 5 |
| NM_006294 | UQCRB | 4419 | 57 | 18 | 12 | 7 | 57 | 19 | 17 | 10 | 11 | 17 | 22 | 14 | 7 |
| NM_006295 | VARS | 1524 | 3 | 2 | 1 | 2 | 2 | 2 | 2 | 3 | 1 | 1 | 3 | 2 | 2 |
| NM_006295 | VARS | 4292 | 1 | 1 | 0 | 1 | 1 | 1 | 1 | 1 | 0 | 0 | 1 | 1 | 1 |
| NM_006296 | VRK2 | 2034 | 4 | 2 | 2 | 1 | 3 | 2 | 2 | 3 | 2 | 2 | 2 | 4 | 2 |
| NM_006297 | XRCC1 | 2102 | 2 | 1 | 2 | 1 | 1 | 2 | 1 | 0 | 1 | 2 | 1 | 2 | 1 |
| NM_006298 | ZNF192 | 5362 | 6 | 3 | 2 | 2 | 2 | 3 | 3 | 1 | 2 | 6 | 2 | 4 | 5 |
| NM_006299 | ZNF193 | 1634 | 3 | 2 | 1 | 2 | 1 | 1 | 2 | 0 | 1 | 2 | 1 | 2 | 3 |
| NM_006300 | ZNF230 | 4132 | 2 | 1 | 1 | 0 | 1 | 1 | 1 | 0 | 1 | 1 | 0 | 2 | 1 |
| NM_006301 | MAP3K12 | 3364 | 3 | 1 | 1 | 1 | 1 | 3 | 1 | 0 | 1 | 1 | 0 | 0 | 1 |
| NM_006302 | MOGS | 2895 | 2 | 1 | 1 | 2 | 1 | 1 | 1 | 2 | 1 | 1 | 1 | 2 | 1 |
| NM_006303 | AIMP2 | 1219 | 1 | 1 | 0 | 0 | 1 | 0 | 1 | 0 | 0 | 0 | 1 | 1 | 1 |
| NM_006304 | SHFM1 | 498 | 39 | 26 | 31 | 19 | 39 | 31 | 25 | 36 | 21 | 25 | 18 | 22 | 24 |
| NM_006305 | ANP32A | 2464 | 39 | 24 | 23 | 18 | 26 | 39 | 32 | 23 | 30 | 12 | 16 | 36 | 14 |
| NM_006306 | SMC1A | 9725 | 15 | 10 | 7 | 7 | 11 | 8 | 11 | 11 | 9 | 11 | 9 | 15 | 8 |
| NM_006307 | SRPX | 1852 | 22 | 5 | 11 | 2 | 22 | 2 | 1 | 2 | 4 | 2 | 2 | 1 | 8 |
| NM_006308 | HSPB3 | 763 | 24 | 3 | 0 | 0 | 24 | 1 | 0 | 0 | 0 | 0 | 5 | 0 | 0 |
| NM_006309 | LRRFIP2 | 3814 | 17 | 10 | 6 | 6 | 17 | 8 | 7 | 12 | 8 | 10 | 9 | 13 | 12 |
| NM_006310 | NPEPPS | 4339 | 10 | 6 | 4 | 4 | 5 | 10 | 7 | 3 | 7 | 7 | 10 | 7 | 7 |
| NM_006311 | NCOR1 | 7915 | 16 | 11 | 9 | 9 | 9 | 13 | 13 | 10 | 9 | 13 | 11 | 16 | 13 |
| NM_006312 | NCOR2 | 8709 | 6 | 4 | 3 | 4 | 4 | 5 | 3 | 2 | 5 | 6 | 5 | 5 | 4 |
| NM_006313 | USP15 | 4611 | 13 | 7 | 5 | 2 | 7 | 5 | 4 | 8 | 8 | 6 | 6 | 13 | 8 |
| NM_006314 | CNKSR1 | 2536 | 1 | 0 | 0 | 1 | 0 | 0 | 0 | 0 | 0 | 0 | 1 | 0 | 0 |
| NM_006315 | PCGF3 | 5665 | 3 | 2 | 1 | 2 | 2 | 2 | 2 | 1 | 2 | 3 | 1 | 3 | 2 |
| NM_006317 | BASP1 | 1797 | 20 | 4 | 1 | 1 | 2 | 20 | 1 | 1 | 5 | 3 | 0 | 13 | 1 |
| NM_006319 | CDIPT | 1874 | 1 | 1 | 1 | 1 | 1 | 1 | 1 | 1 | 1 | 1 | 0 | 1 | 0 |
| NM_006320 | PGRMC2 | 1849 | 33 | 15 | 33 | 8 | 13 | 16 | 16 | 29 | 10 | 11 | 6 | 9 | 11 |
| NM_006321 | ARIH2 | 4062 | 9 | 5 | 4 | 4 | 8 | 6 | 5 | 5 | 3 | 4 | 9 | 5 | 5 |
| NM_006322 | TUBGCP3 | 3890 | 3 | 2 | 2 | 1 | 2 | 3 | 3 | 3 | 1 | 3 | 1 | 3 | 3 |
| NM_006323 | SEC24B | 4646 | 14 | 10 | 7 | 3 | 9 | 9 | 7 | 14 | 8 | 13 | 11 | 12 | 11 |
| NM_006324 | CFDP1 | 1279 | 49 | 29 | 20 | 16 | 19 | 37 | 40 | 27 | 22 | 35 | 12 | 49 | 48 |
| NM_006325 | RAN | 1069 | 71 | 36 | 27 | 24 | 43 | 71 | 28 | 44 | 21 | 24 | 23 | 47 | 48 |
| NM_006327 | TIMM23 | 1168 | 10 | 6 | 6 | 6 | 8 | 10 | 6 | 7 | 4 | 4 | 6 | 5 | 7 |
| NM_006328 | RBM14 | 5400 | 2 | 1 | 0 | 2 | 0 | 0 | 1 | 0 | 1 | 1 | 0 | 1 | 0 |
| NM_006329 | FBLN5 | 2633 | 34 | 17 | 16 | 5 | 31 | 4 | 19 | 5 | 24 | 21 | 7 | 34 | 26 |
| NM_006330 | LYPLA1 | 2574 | 6 | 3 | 2 | 1 | 4 | 2 | 5 | 6 | 2 | 2 | 3 | 4 | 6 |
| NM_006331 | EMG1 | 1049 | 6 | 4 | 4 | 3 | 4 | 5 | 4 | 6 | 3 | 2 | 2 | 4 | 3 |
| NM_006332 | IFI30 | 1032 | 23 | 7 | 14 | 2 | 6 | 2 | 4 | 4 | 21 | 1 | 0 | 23 | 1 |
| NM_006333 | C1D | 1207 | 6 | 3 | 3 | 1 | 3 | 6 | 4 | 4 | 3 | 2 | 2 | 4 | 3 |
| NM_006334 | OLFM1 | 1178 | 56 | 6 | 1 | 2 | 1 | 56 | 0 | 0 | 1 | 1 | 1 | 5 | 2 |
| NM_006335 | TIMM17A | 1657 | 5 | 2 | 1 | 1 | 4 | 4 | 3 | 5 | 1 | 1 | 2 | 2 | 2 |
| NM_006336 | ZER1 | 4283 | 3 | 1 | 0 | 1 | 1 | 3 | 1 | 0 | 0 | 1 | 1 | 0 | 1 |
| NM_006337 | MCRS1 | 1920 | 2 | 1 | 1 | 1 | 2 | 1 | 1 | 1 | 0 | 1 | 0 | 1 | 1 |
| NM_006338 | LRRN2 | 3206 | 1 | 0 | 0 | 0 | 0 | 1 | 0 | 0 | 0 | 0 | 0 | 0 | 0 |
| NM_006339 | HMG20B | 1584 | 10 | 6 | 7 | 10 | 5 | 4 | 8 | 6 | 5 | 6 | 2 | 7 | 7 |
| NM_006340 | BAIAP2 | 3352 | 3 | 1 | 0 | 1 | 0 | 2 | 2 | 3 | 2 | 0 | 0 | 1 | 1 |
| NM_006341 | MAD2L2 | 1119 | 1 | 1 | 0 | 0 | 1 | 1 | 1 | 1 | 1 | 0 | 1 | 1 | 1 |
| NM_006342 | TACC3 | 2780 | 3 | 1 | 1 | 1 | 0 | 0 | 0 | 0 | 1 | 0 | 0 | 2 | 3 |
| NM_006343 | MERTK | 3626 | 14 | 4 | 2 | 1 | 3 | 2 | 3 | 3 | 5 | 2 | 1 | 14 | 5 |
| NM_006344 | CLEC10A | 1699 | 3 | 1 | 2 | 2 | 3 | 0 | 0 | 0 | 2 | 0 | 1 | 3 | 1 |
| NM_006345 | SLC30A9 | 3261 | 10 | 5 | 2 | 2 | 7 | 10 | 10 | 4 | 4 | 4 | 4 | 5 | 5 |
| NM_006346 | PIBF1 | 3113 | 11 | 5 | 4 | 1 | 5 | 5 | 5 | 3 | 5 | 8 | 4 | 7 | 11 |
| NM_006347 | PPIH | 800 | 1 | 1 | 1 | 1 | 0 | 1 | 1 | 1 | 0 | 1 | 0 | 1 | 1 |
| NM_006348 | COG5 | 4880 | 10 | 7 | 6 | 5 | 10 | 7 | 5 | 3 | 5 | 9 | 6 | 7 | 8 |
| NM_006349 | ZNHIT1 | 1189 | 3 | 1 | 1 | 1 | 1 | 2 | 2 | 3 | 0 | 1 | 0 | 1 | 1 |
| NM_006350 | FST | 1367 | 3 | 1 | 1 | 0 | 1 | 0 | 0 | 3 | 0 | 0 | 1 | 0 | 1 |
| NM_006351 | TIMM44 | 1830 | 3 | 2 | 1 | 2 | 3 | 2 | 2 | 2 | 1 | 1 | 3 | 2 | 1 |
| NM_006352 | ZNF238 | 4199 | 14 | 4 | 1 | 1 | 2 | 14 | 2 | 3 | 2 | 1 | 6 | 3 | 5 |
| NM_006353 | HMGN4 | 1968 | 10 | 5 | 2 | 3 | 7 | 3 | 6 | 1 | 4 | 6 | 3 | 9 | 10 |
| NM_006354 | TADA3 | 2234 | 2 | 1 | 0 | 1 | 1 | 2 | 1 | 1 | 1 | 1 | 1 | 2 | 1 |
| NM_006355 | TRIM38 | 3270 | 13 | 5 | 4 | 3 | 4 | 1 | 11 | 3 | 6 | 4 | 2 | 13 | 4 |
| NM_006356 | ATP5H | 622 | 749 | 308 | 262 | 173 | 749 | 435 | 398 | 437 | 145 | 115 | 348 | 198 | 128 |
| NM_006357 | UBE2E3 | 1543 | 16 | 10 | 8 | 5 | 9 | 16 | 11 | 8 | 7 | 8 | 15 | 9 | 14 |
| NM_006358 | SLC25A17 | 2263 | 6 | 2 | 1 | 1 | 1 | 3 | 2 | 2 | 1 | 6 | 1 | 2 | 3 |
| NM_006359 | SLC9A6 | 4631 | 14 | 3 | 1 | 1 | 3 | 14 | 2 | 1 | 2 | 2 | 2 | 2 | 2 |

| transcript | gene | transcript length (nt) | max | mean | adipose | colon | heart | hypothalamus | kidney | liver | lung | ovary | skeletalmuscle | spleen | testes |
|---|---|---|---|---|---|---|---|---|---|---|---|---|---|---|---|
| NM_006360 | EIF3M | 1237 | 21 | 15 | 9 | 10 | 16 | 14 | 15 | 14 | 17 | 20 | 11 | 19 | 21 |
| NM_006361 | HOXB13 | 3037 | 1 | 0 | 0 | 1 | 0 | 0 | 0 | 0 | 0 | 0 | 0 | 0 | 0 |
| NM_006362 | NXF1 | 2346 | 5 | 3 | 4 | 5 | 2 | 3 | 3 | 2 | 3 | 3 | 2 | 4 | 3 |
| NM_006363 | SEC23B | 3413 | 7 | 4 | 2 | 2 | 2 | 5 | 7 | 5 | 4 | 2 | 0 | 6 | 5 |
| NM_006364 | SEC23A | 3841 | 19 | 9 | 8 | 6 | 15 | 7 | 6 | 19 | 6 | 8 | 7 | 9 | 8 |
| NM_006365 | C1orf61 | 879 | 3 | 0 | 0 | 0 | 0 | 3 | 0 | 0 | 0 | 0 | 0 | 0 | 0 |
| NM_006366 | CAP2 | 3027 | 48 | 8 | 4 | 1 | 9 | 23 | 1 | 0 | 0 | 1 | 48 | 0 | 1 |
| NM_006367 | CAP1 | 2773 | 48 | 23 | 30 | 20 | 18 | 27 | 21 | 22 | 30 | 9 | 5 | 48 | 20 |
| NM_006368 | CREB3 | 1851 | 3 | 2 | 2 | 1 | 2 | 3 | 2 | 2 | 2 | 2 | 1 | 2 | 2 |
| NM_006369 | LRRC41 | 2948 | 4 | 2 | 2 | 1 | 2 | 3 | 3 | 2 | 2 | 2 | 2 | 2 | 4 |
| NM_006370 | VTI1B | 1331 | 40 | 20 | 40 | 16 | 22 | 33 | 22 | 25 | 11 | 9 | 10 | 16 | 11 |
| NM_006371 | CRTAP | 6651 | 37 | 12 | 37 | 6 | 9 | 10 | 11 | 4 | 11 | 13 | 9 | 8 | 11 |
| NM_006372 | SYNCRIP | 3194 | 35 | 23 | 22 | 15 | 30 | 20 | 25 | 24 | 18 | 26 | 23 | 35 | 20 |
| NM_006373 | VAT1 | 2758 | 14 | 7 | 14 | 5 | 5 | 6 | 7 | 4 | 7 | 11 | 2 | 5 | 8 |
| NM_006374 | STK25 | 2141 | 6 | 2 | 1 | 1 | 2 | 3 | 3 | 1 | 1 | 2 | 6 | 2 | 2 |
| NM_006375 | ENOX2 | 4093 | 5 | 3 | 2 | 1 | 4 | 3 | 2 | 3 | 2 | 2 | 2 | 5 | 4 |
| NM_006377 | UNC13B | 6370 | 5 | 3 | 3 | 3 | 2 | 3 | 5 | 4 | 4 | 1 | 2 | 2 | 2 |
| NM_006378 | SEMA4D | 4628 | 11 | 4 | 1 | 1 | 1 | 10 | 5 | 0 | 3 | 2 | 7 | 11 | 3 |
| NM_006379 | SEMA3C | 5174 | 16 | 5 | 16 | 2 | 5 | 2 | 2 | 0 | 4 | 9 | 2 | 2 | 6 |
| NM_006380 | APPBP2 | 6468 | 7 | 3 | 2 | 1 | 2 | 4 | 3 | 2 | 2 | 4 | 2 | 3 | 7 |
| NM_006382 | CDRT1 | 2763 | 0 | 0 | 0 | 0 | 0 | 0 | 0 | 0 | 0 | 0 | 0 | 0 | 0 |
| NM_006383 | CIB2 | 1547 | 0 | 0 | 0 | 0 | 0 | 0 | 0 | 0 | 0 | 0 | 0 | 0 | 0 |
| NM_006384 | CIB1 | 977 | 3 | 1 | 1 | 1 | 2 | 1 | 3 | 2 | 2 | 1 | 0 | 2 | 1 |
| NM_006385 | ZNF211 | 2512 | 6 | 3 | 2 | 2 | 3 | 4 | 3 | 1 | 2 | 4 | 2 | 6 | 3 |
| NM_006386 | DDX17 | 4790 | 63 | 39 | 32 | 31 | 30 | 30 | 42 | 16 | 46 | 48 | 40 | 63 | 47 |
| NM_006387 | CHERP | 4095 | 1 | 1 | 0 | 1 | 1 | 1 | 1 | 1 | 1 | 1 | 1 | 1 | 1 |
| NM_006388 | KAT5 | 2215 | 4 | 2 | 2 | 2 | 2 | 3 | 3 | 2 | 1 | 3 | 3 | 3 | 4 |
| NM_006389 | HYOU1 | 4602 | 44 | 9 | 3 | 4 | 7 | 9 | 8 | 44 | 4 | 2 | 1 | 7 | 5 |
| NM_006390 | IPO8 | 5156 | 13 | 9 | 7 | 6 | 7 | 9 | 12 | 9 | 7 | 11 | 6 | 13 | 11 |
| NM_006391 | IPO7 | 6191 | 35 | 20 | 13 | 9 | 34 | 23 | 18 | 19 | 19 | 16 | 35 | 18 | 15 |
| NM_006392 | NOP56 | 1937 | 16 | 9 | 8 | 9 | 8 | 16 | 8 | 10 | 6 | 8 | 9 | 14 | 9 |
| NM_006393 | NEBL | 9213 | 116 | 13 | 0 | 0 | 116 | 16 | 5 | 0 | 8 | 1 | 0 | 0 | 1 |
| NM_006395 | ATG7 | 5044 | 1 | 0 | 1 | 0 | 0 | 1 | 0 | 0 | 0 | 0 | 0 | 1 | 1 |
| NM_006396 | SSSCA1 | 654 | 2 | 1 | 1 | 1 | 1 | 2 | 1 | 1 | 1 | 1 | 1 | 1 | 1 |
| NM_006397 | RNASEH2A | 1142 | 1 | 0 | 1 | 0 | 0 | 1 | 0 | 0 | 0 | 1 | 1 | 1 | 1 |
| NM_006398 | UBD | 989 | 18 | 3 | 1 | 2 | 0 | 0 | 5 | 1 | 5 | 0 | 2 | 18 | 0 |
| NM_006399 | BATF | 933 | 4 | 1 | 1 | 1 | 1 | 1 | 0 | 0 | 2 | 0 | 0 | 4 | 0 |
| NM_006400 | DCTN2 | 1717 | 15 | 7 | 9 | 6 | 9 | 15 | 6 | 6 | 4 | 6 | 6 | 7 | 7 |
| NM_006401 | ANP32B | 1616 | 99 | 69 | 83 | 66 | 62 | 73 | 46 | 48 | 87 | 90 | 40 | 99 | 65 |
| NM_006402 | HBXIP | 848 | 11 | 4 | 4 | 2 | 7 | 6 | 11 | 3 | 3 | 1 | 3 | 5 | 1 |
| NM_006403 | NEDD9 | 4535 | 17 | 5 | 5 | 2 | 5 | 1 | 6 | 3 | 17 | 2 | 5 | 7 | 5 |
| NM_006404 | PROCR | 1450 | 16 | 8 | 15 | 6 | 12 | 4 | 6 | 2 | 7 | 6 | 2 | 16 | 7 |
| NM_006405 | TM9SF1 | 2437 | 6 | 2 | 2 | 1 | 1 | 1 | 2 | 6 | 1 | 1 | 0 | 1 | 1 |
| NM_006406 | PRDX4 | 921 | 34 | 8 | 5 | 2 | 10 | 3 | 3 | 34 | 4 | 5 | 1 | 8 | 9 |
| NM_006407 | ARL6IP5 | 2128 | 25 | 13 | 15 | 3 | 25 | 17 | 11 | 5 | 14 | 8 | 16 | 18 | 10 |
| NM_006408 | AGR2 | 981 | 28 | 3 | 0 | 28 | 0 | 0 | 0 | 0 | 3 | 0 | 0 | 0 | 0 |
| NM_006409 | ARPC1A | 1593 | 49 | 28 | 23 | 24 | 34 | 49 | 24 | 32 | 20 | 35 | 12 | 31 | 23 |
| NM_006410 | HTATIP2 | 1373 | 10 | 5 | 6 | 4 | 3 | 3 | 5 | 10 | 7 | 1 | 3 | 9 | 3 |
| NM_006411 | AGPAT1 | 2234 | 3 | 2 | 1 | 2 | 2 | 3 | 3 | 2 | 2 | 3 | 2 | 3 | 3 |
| NM_006412 | AGPAT2 | 1548 | 3 | 1 | 3 | 1 | 0 | 0 | 0 | 2 | 1 | 0 | 0 | 0 | 0 |
| NM_006413 | RPP30 | 2353 | 2 | 1 | 1 | 1 | 2 | 2 | 1 | 1 | 1 | 2 | 1 | 2 | 1 |
| NM_006414 | RPP38 | 1180 | 3 | 1 | 1 | 1 | 1 | 1 | 1 | 1 | 1 | 1 | 1 | 2 | 3 |
| NM_006415 | SPTLC1 | 2780 | 5 | 3 | 2 | 2 | 3 | 4 | 4 | 2 | 3 | 5 | 1 | 5 | 4 |
| NM_006416 | SLC35A1 | 1906 | 2 | 1 | 1 | 1 | 1 | 1 | 2 | 0 | 1 | 2 | 0 | 2 | 1 |
| NM_006417 | IFI44 | 1726 | 8 | 4 | 8 | 1 | 5 | 2 | 2 | 2 | 6 | 5 | 3 | 8 | 3 |
| NM_006418 | OLFM4 | 2815 | 54 | 7 | 0 | 54 | 1 | 0 | 3 | 0 | 6 | 1 | 1 | 6 | 4 |
| NM_006419 | CXCL13 | 1205 | 80 | 8 | 0 | 0 | 0 | 0 | 0 | 2 | 1 | 0 | 0 | 80 | 0 |
| NM_006420 | ARFGEF2 | 9004 | 9 | 5 | 4 | 4 | 6 | 6 | 7 | 9 | 3 | 3 | 3 | 7 | 7 |
| NM_006421 | ARFGEF1 | 7212 | 15 | 7 | 5 | 3 | 6 | 6 | 10 | 5 | 8 | 6 | 4 | 10 | 15 |
| NM_006422 | AKAP3 | 3020 | 18 | 2 | 0 | 0 | 0 | 0 | 1 | 0 | 0 | 0 | 0 | 0 | 18 |
| NM_006423 | RABAC1 | 806 | 2 | 1 | 1 | 1 | 2 | 2 | 1 | 2 | 1 | 1 | 0 | 2 | 1 |
| NM_006424 | SLC34A2 | 4151 | 107 | 11 | 0 | 0 | 0 | 0 | 11 | 0 | 107 | 1 | 0 | 0 | 0 |
| NM_006425 | SLU7 | 3560 | 23 | 13 | 11 | 3 | 15 | 23 | 10 | 8 | 10 | 12 | 13 | 20 | 18 |
| NM_006426 | DPYSL4 | 2719 | 1 | 0 | 0 | 0 | 1 | 1 | 0 | 0 | 0 | 0 | 0 | 0 | 0 |
| NM_006427 | SIVA1 | 771 | 1 | 0 | 1 | 0 | 1 | 1 | 0 | 0 | 0 | 0 | 0 | 0 | 0 |
| NM_006428 | MRPL28 | 1099 | 7 | 3 | 3 | 3 | 3 | 7 | 3 | 5 | 1 | 1 | 2 | 4 | 2 |
| NM_006429 | CCT7 | 1935 | 28 | 21 | 15 | 14 | 26 | 28 | 16 | 27 | 11 | 15 | 26 | 22 | 24 |
| NM_006430 | CCT4 | 2334 | 15 | 8 | 7 | 3 | 8 | 15 | 7 | 8 | 4 | 10 | 6 | 8 | 8 |
| NM_006431 | CCT2 | 1954 | 10 | 6 | 3 | 3 | 9 | 7 | 6 | 5 | 4 | 4 | 4 | 7 | 10 |
| NM_006432 | NPC2 | 897 | 8 | 3 | 4 | 1 | 4 | 2 | 4 | 1 | 8 | 4 | 1 | 5 | 4 |
| NM_006433 | GNLY | 744 | 2 | 0 | 0 | 0 | 0 | 0 | 0 | 0 | 2 | 0 | 0 | 2 | 0 |
| NM_006434 | SORBS1 | 5386 | 126 | 32 | 126 | 28 | 73 | 28 | 7 | 16 | 10 | 3 | 42 | 4 | 15 |
| NM_006435 | IFITM2 | 730 | 71 | 19 | 5 | 2 | 28 | 5 | 11 | 28 | 33 | 11 | 9 | 71 | 6 |
| NM_006437 | PARP4 | 5469 | 16 | 7 | 7 | 6 | 6 | 3 | 11 | 6 | 8 | 8 | 2 | 16 | 6 |
| NM_006438 | COLEC10 | 1696 | 8 | 1 | 0 | 0 | 0 | 1 | 0 | 8 | 0 | 0 | 0 | 0 | 0 |
| NM_006439 | MAB21L2 | 2767 | 50 | 5 | 0 | 50 | 1 | 1 | 1 | 1 | 1 | 1 | 0 | 1 | 0 |
| NM_006440 | TXNRD2 | 1964 | 1 | 0 | 0 | 0 | 0 | 0 | 1 | 1 | 0 | 0 | 0 | 0 | 0 |

| transcript | gene | transcript length (nt) | max | mean | adipose | colon | heart | hypothalamus | kidney | liver | lung | ovary | skeletalmuscle | spleen | testes |
|---|---|---|---|---|---|---|---|---|---|---|---|---|---|---|---|
| NM_006441 | MTHFS | 886 | 277 | 42 | 23 | 15 | 10 | 16 | 37 | 277 | 14 | 9 | 8 | 21 | 27 |
| NM_006442 | DRAP1 | 1004 | 26 | 16 | 13 | 9 | 26 | 22 | 12 | 11 | 17 | 10 | 20 | 19 | 19 |
| NM_006443 | C6orf108 | 646 | 2 | 1 | 2 | 1 | 0 | 1 | 2 | 1 | 0 | 0 | 1 | 1 | 0 |
| NM_006444 | SMC2 | 5977 | 3 | 2 | 2 | 1 | 2 | 2 | 2 | 1 | 1 | 2 | 1 | 3 | 3 |
| NM_006445 | PRPF8 | 7295 | 21 | 15 | 9 | 11 | 14 | 18 | 11 | 12 | 12 | 20 | 21 | 20 | 17 |
| NM_006446 | SLCO1B1 | 2785 | 15 | 1 | 0 | 0 | 0 | 0 | 0 | 15 | 0 | 0 | 0 | 0 | 0 |
| NM_006447 | USP16 | 2973 | 20 | 13 | 12 | 4 | 18 | 11 | 14 | 12 | 11 | 14 | 14 | 17 | 20 |
| NM_006449 | CDC42EP3 | 3981 | 6 | 3 | 2 | 1 | 6 | 1 | 2 | 1 | 4 | 2 | 6 | 3 | 2 |
| NM_006451 | PAIP1 | 2795 | 15 | 8 | 8 | 3 | 13 | 7 | 10 | 10 | 6 | 6 | 9 | 9 | 15 |
| NM_006452 | PAICS | 3339 | 15 | 7 | 3 | 4 | 5 | 9 | 8 | 15 | 3 | 7 | 4 | 8 | 9 |
| NM_006453 | TBL3 | 2594 | 0 | 0 | 0 | 0 | 0 | 0 | 0 | 0 | 0 | 0 | 0 | 0 | 0 |
| NM_006454 | MXD4 | 3773 | 1 | 0 | 1 | 0 | 0 | 0 | 1 | 0 | 0 | 0 | 0 | 1 | 0 |
| NM_006455 | SC65 | 2602 | 0 | 0 | 0 | 0 | 0 | 0 | 0 | 0 | 0 | 0 | 0 | 0 | 0 |
| NM_006456 | ST6GALNAC | 2105 | 2 | 1 | 0 | 0 | 0 | 0 | 2 | 0 | 2 | 0 | 1 | 0 | 1 |
| NM_006457 | PDLIM5 | 6100 | 25 | 7 | 4 | 4 | 25 | 4 | 7 | 7 | 4 | 4 | 4 | 7 | 5 |
| NM_006458 | TRIM3 | 3037 | 1 | 0 | 0 | 0 | 0 | 1 | 0 | 0 | 0 | 0 | 0 | 0 | 0 |
| NM_006459 | ERLIN1 | 3439 | 18 | 4 | 3 | 2 | 3 | 2 | 4 | 18 | 3 | 3 | 1 | 4 | 4 |
| NM_006460 | HEXIM1 | 4785 | 6 | 4 | 3 | 3 | 3 | 4 | 5 | 3 | 5 | 3 | 2 | 4 | 6 |
| NM_006461 | SPAG5 | 3808 | 9 | 2 | 1 | 2 | 0 | 1 | 9 | 2 | 1 | 1 | 0 | 1 | 7 |
| NM_006462 | RBCK1 | 2617 | 1 | 0 | 0 | 1 | 0 | 0 | 1 | 1 | 0 | 0 | 0 | 1 | 0 |
| NM_006463 | STAMBP | 2089 | 5 | 3 | 2 | 2 | 2 | 5 | 4 | 3 | 3 | 3 | 3 | 4 | 4 |
| NM_006464 | TGOLN2 | 6316 | 32 | 16 | 16 | 10 | 17 | 15 | 32 | 19 | 16 | 10 | 6 | 26 | 12 |
| NM_006465 | ARID3B | 4241 | 1 | 0 | 0 | 1 | 0 | 0 | 0 | 0 | 1 | 0 | 0 | 1 | 1 |
| NM_006466 | POLR3F | 2153 | 5 | 3 | 2 | 1 | 4 | 5 | 4 | 2 | 2 | 2 | 2 | 4 | 5 |
| NM_006467 | POLR3G | 3285 | 1 | 0 | 0 | 0 | 0 | 1 | 0 | 0 | 0 | 0 | 0 | 0 | 1 |
| NM_006468 | POLR3C | 1851 | 6 | 4 | 3 | 3 | 3 | 5 | 4 | 3 | 2 | 5 | 3 | 6 | 6 |
| NM_006469 | IVNS1ABP | 4197 | 37 | 15 | 11 | 8 | 14 | 10 | 37 | 8 | 21 | 15 | 6 | 21 | 12 |
| NM_006470 | TRIM16 | 2901 | 0 | 0 | 0 | 0 | 0 | 0 | 0 | 0 | 0 | 0 | 0 | 0 | 0 |
| NM_006471 | MYL12A | 1229 | 300 | 66 | 44 | 15 | 300 | 15 | 25 | 34 | 38 | 15 | 156 | 67 | 16 |
| NM_006472 | TXNIP | 2927 | 163 | 83 | 118 | 49 | 123 | 15 | 53 | 22 | 132 | 38 | 163 | 155 | 52 |
| NM_006473 | TAF6L | 2015 | 1 | 0 | 0 | 1 | 0 | 1 | 1 | 1 | 0 | 0 | 0 | 0 | 0 |
| NM_006474 | PDPN | 2812 | 3 | 1 | 0 | 1 | 3 | 2 | 0 | 0 | 2 | 3 | 2 | 0 | 1 |
| NM_006475 | POSTN | 3379 | 27 | 7 | 27 | 7 | 23 | 0 | 4 | 0 | 7 | 2 | 4 | 0 | 8 |
| NM_006476 | ATP5L | 1338 | 207 | 77 | 54 | 65 | 207 | 74 | 98 | 65 | 65 | 53 | 58 | 74 | 36 |
| NM_006477 | RASL10A | 1493 | 0 | 0 | 0 | 0 | 0 | 0 | 0 | 0 | 0 | 0 | 0 | 0 | 0 |
| NM_006478 | GAS2L1 | 2527 | 1 | 0 | 0 | 0 | 0 | 1 | 0 | 0 | 0 | 0 | 0 | 0 | 1 |
| NM_006479 | RAD51AP1 | 2217 | 1 | 0 | 1 | 1 | 0 | 0 | 0 | 0 | 1 | 0 | 0 | 1 | 1 |
| NM_006480 | RGS14 | 2406 | 1 | 0 | 0 | 0 | 0 | 0 | 1 | 0 | 0 | 0 | 0 | 0 | 0 |
| NM_006482 | DYRK2 | 6159 | 8 | 4 | 3 | 4 | 7 | 3 | 3 | 1 | 3 | 4 | 8 | 6 | 3 |
| NM_006483 | DYRK1B | 2415 | 5 | 1 | 0 | 0 | 1 | 1 | 1 | 0 | 1 | 1 | 5 | 1 | 1 |
| NM_006484 | DYRK1B | 2451 | 5 | 1 | 0 | 0 | 1 | 1 | 0 | 0 | 1 | 1 | 5 | 1 | 1 |
| NM_006485 | FBLN1 | 2662 | 4 | 2 | 2 | 3 | 3 | 1 | 1 | 0 | 4 | 1 | 1 | 0 | 2 |
| NM_006486 | FBLN1 | 2947 | 9 | 4 | 4 | 5 | 7 | 2 | 2 | 0 | 9 | 3 | 2 | 1 | 4 |
| NM_006487 | FBLN1 | 2485 | 4 | 2 | 2 | 3 | 3 | 2 | 1 | 0 | 4 | 1 | 1 | 0 | 2 |
| NM_006488 | KHK | 2415 | 8 | 1 | 0 | 0 | 0 | 0 | 8 | 4 | 0 | 0 | 0 | 0 | 1 |
| NM_006489 | NOVA1 | 3846 | 4 | 1 | 2 | 0 | 1 | 4 | 1 | 0 | 0 | 1 | 1 | 0 | 1 |
| NM_006491 | NOVA1 | 1210 | 4 | 1 | 2 | 1 | 1 | 4 | 1 | 0 | 0 | 2 | 2 | 0 | 1 |
| NM_006492 | ALX3 | 1478 | 0 | 0 | 0 | 0 | 0 | 0 | 0 | 0 | 0 | 0 | 0 | 0 | 0 |
| NM_006493 | CLN5 | 2799 | 3 | 1 | 2 | 0 | 1 | 1 | 3 | 2 | 1 | 1 | 0 | 1 | 1 |
| NM_006494 | ERF | 2705 | 2 | 1 | 1 | 1 | 1 | 1 | 1 | 1 | 1 | 1 | 0 | 1 | 2 |
| NM_006495 | EVI2B | 1995 | 139 | 23 | 16 | 4 | 13 | 5 | 3 | 8 | 55 | 8 | 3 | 139 | 5 |
| NM_006496 | GNAI3 | 4730 | 7 | 4 | 5 | 2 | 4 | 4 | 4 | 5 | 4 | 4 | 1 | 7 | 5 |
| NM_006497 | HIC1 | 3157 | 0 | 0 | 0 | 0 | 0 | 0 | 0 | 0 | 0 | 0 | 0 | 0 | 0 |
| NM_006498 | LGALS2 | 528 | 10 | 1 | 0 | 2 | 0 | 0 | 10 | 0 | 0 | 0 | 0 | 0 | 0 |
| NM_006499 | LGALS8 | 2996 | 31 | 12 | 12 | 4 | 20 | 11 | 9 | 13 | 9 | 11 | 2 | 31 | 13 |
| NM_006500 | MCAM | 3332 | 21 | 3 | 21 | 3 | 4 | 2 | 1 | 0 | 2 | 1 | 1 | 2 | 1 |
| NM_006502 | POLH | 8412 | 2 | 1 | 1 | 1 | 1 | 1 | 1 | 1 | 1 | 1 | 1 | 2 | 1 |
| NM_006503 | PSMC4 | 1435 | 6 | 3 | 2 | 3 | 2 | 6 | 3 | 4 | 1 | 1 | 2 | 2 | 1 |
| NM_006504 | PTPRE | 5374 | 6 | 3 | 4 | 2 | 4 | 5 | 1 | 1 | 3 | 0 | 0 | 6 | 2 |
| NM_006505 | PVR | 5901 | 18 | 8 | 4 | 6 | 18 | 8 | 7 | 16 | 6 | 4 | 5 | 5 | 5 |
| NM_006506 | RASA2 | 2592 | 10 | 3 | 2 | 2 | 2 | 3 | 2 | 1 | 4 | 5 | 1 | 10 | 4 |
| NM_006507 | REG1B | 806 | 2 | 0 | 0 | 2 | 0 | 0 | 0 | 0 | 0 | 0 | 0 | 0 | 0 |
| NM_006509 | RELB | 2266 | 0 | 0 | 0 | 0 | 0 | 0 | 0 | 0 | 0 | 0 | 0 | 0 | 0 |
| NM_006510 | TRIM27 | 2969 | 4 | 2 | 1 | 2 | 1 | 3 | 2 | 4 | 2 | 2 | 1 | 4 | 2 |
| NM_006511 | RSC1A1 | 1854 | 9 | 3 | 3 | 1 | 2 | 2 | 3 | 4 | 3 | 1 | 9 | 4 | 2 |
| NM_006512 | SAA4 | 634 | 64 | 6 | 0 | 1 | 0 | 0 | 0 | 64 | 0 | 0 | 0 | 1 | 0 |
| NM_006513 | SARS | 1895 | 78 | 36 | 24 | 22 | 39 | 78 | 25 | 62 | 29 | 27 | 29 | 29 | 33 |
| NM_006514 | SCN10A | 5874 | 0 | 0 | 0 | 0 | 0 | 0 | 0 | 0 | 0 | 0 | 0 | 0 | 0 |
| NM_006515 | SETMAR | 2141 | 3 | 2 | 2 | 1 | 1 | 2 | 2 | 1 | 1 | 3 | 2 | 2 | 2 |
| NM_006516 | SLC2A1 | 3670 | 4 | 1 | 0 | 1 | 2 | 4 | 2 | 0 | 1 | 1 | 0 | 1 | 1 |
| NM_006517 | SLC16A2 | 4372 | 3 | 1 | 1 | 1 | 1 | 3 | 2 | 3 | 1 | 2 | 0 | 1 | 1 |
| NM_006519 | DYNLT1 | 695 | 5 | 3 | 2 | 3 | 3 | 3 | 5 | 3 | 3 | 2 | 1 | 4 | 4 |
| NM_006520 | DYNLT3 | 2215 | 6 | 3 | 3 | 0 | 4 | 6 | 4 | 5 | 4 | 2 | 1 | 5 | 3 |
| NM_006521 | TFE3 | 3393 | 7 | 4 | 7 | 2 | 4 | 5 | 4 | 2 | 5 | 3 | 3 | 6 | 3 |
| NM_006522 | WNT6 | 1700 | 0 | 0 | 0 | 0 | 0 | 0 | 0 | 0 | 0 | 0 | 0 | 0 | 0 |
| NM_006524 | ZNF138 | 2501 | 10 | 5 | 3 | 1 | 4 | 10 | 6 | 2 | 3 | 9 | 5 | 9 | 6 |
| NM_006526 | ZNF217 | 5635 | 12 | 4 | 3 | 3 | 2 | 1 | 4 | 3 | 5 | 4 | 1 | 12 | 5 |

| transcript | gene | transcript length (nt) | max | mean | adipose | colon | heart | hypothalamus | kidney | liver | lung | ovary | skeletalmuscle | spleen | testes |
|---|---|---|---|---|---|---|---|---|---|---|---|---|---|---|---|
| NM_006527 | SLBP | 1725 | 36 | 13 | 9 | 7 | 8 | 15 | 12 | 9 | 14 | 11 | 6 | 19 | 36 |
| NM_006528 | TFPI2 | 1170 | 35 | 7 | 4 | 2 | 35 | 4 | 5 | 1 | 4 | 15 | 1 | 1 | 1 |
| NM_006529 | GLRA3 | 3637 | 1 | 0 | 0 | 0 | 0 | 1 | 0 | 0 | 0 | 0 | 0 | 0 | 0 |
| NM_006530 | YEATS4 | 1385 | 4 | 1 | 1 | 1 | 1 | 1 | 2 | 1 | 1 | 2 | 1 | 2 | 4 |
| NM_006531 | IFT88 | 2931 | 10 | 3 | 1 | 1 | 2 | 3 | 6 | 4 | 2 | 4 | 1 | 3 | 10 |
| NM_006532 | ELL | 4026 | 1 | 1 | 0 | 1 | 1 | 1 | 1 | 1 | 1 | 1 | 1 | 1 | 1 |
| NM_006533 | MIA | 613 | 0 | 0 | 0 | 0 | 0 | 0 | 0 | 0 | 0 | 0 | 0 | 0 | 0 |
| NM_006534 | NCOA3 | 7921 | 23 | 8 | 5 | 4 | 9 | 4 | 6 | 6 | 9 | 10 | 8 | 23 | 7 |
| NM_006536 | CLCA2 | 4024 | 1 | 0 | 0 | 0 | 0 | 0 | 0 | 0 | 1 | 0 | 0 | 0 | 0 |
| NM_006537 | USP3 | 2330 | 8 | 4 | 3 | 3 | 2 | 3 | 4 | 4 | 6 | 6 | 1 | 8 | 3 |
| NM_006538 | BCL2L11 | 4919 | 6 | 2 | 1 | 1 | 1 | 1 | 2 | 2 | 2 | 2 | 2 | 6 | 2 |
| NM_006539 | CACNG3 | 2701 | 12 | 1 | 0 | 2 | 0 | 12 | 0 | 0 | 0 | 0 | 0 | 0 | 0 |
| NM_006540 | NCOA2 | 6157 | 6 | 3 | 2 | 2 | 3 | 4 | 4 | 4 | 2 | 4 | 2 | 4 | 6 |
| NM_006541 | GLRX3 | 1244 | 13 | 8 | 9 | 4 | 13 | 6 | 8 | 9 | 6 | 6 | 5 | 9 | 12 |
| NM_006542 | SPHAR | 1122 | 1 | 0 | 0 | 0 | 1 | 1 | 1 | 1 | 0 | 1 | 1 | 0 | 0 |
| NM_006544 | EXOC5 | 8511 | 9 | 5 | 5 | 1 | 5 | 7 | 4 | 4 | 5 | 4 | 3 | 7 | 9 |
| NM_006545 | TUSC4 | 1664 | 2 | 1 | 1 | 1 | 0 | 2 | 1 | 1 | 1 | 0 | 1 | 1 | 0 |
| NM_006546 | IGF2BP1 | 8769 | 1 | 0 | 0 | 0 | 0 | 0 | 0 | 0 | 0 | 0 | 0 | 0 | 1 |
| NM_006547 | IGF2BP3 | 4159 | 2 | 0 | 0 | 0 | 0 | 0 | 0 | 0 | 0 | 1 | 0 | 1 | 2 |
| NM_006548 | IGF2BP2 | 3671 | 3 | 2 | 1 | 3 | 3 | 0 | 2 | 0 | 3 | 1 | 1 | 2 | 2 |
| NM_006549 | CAMKK2 | 5603 | 6 | 2 | 2 | 1 | 1 | 6 | 2 | 2 | 2 | 2 | 0 | 4 | 2 |
| NM_006551 | SCGB1D2 | 438 | 1 | 0 | 0 | 0 | 0 | 0 | 0 | 0 | 0 | 0 | 0 | 0 | 1 |
| NM_006552 | SCGB1D1 | 429 | 0 | 0 | 0 | 0 | 0 | 0 | 0 | 0 | 0 | 0 | 0 | 0 | 0 |
| NM_006553 | SLMO1 | 1715 | 0 | 0 | 0 | 0 | 0 | 0 | 0 | 0 | 0 | 0 | 0 | 0 | 0 |
| NM_006554 | MTX2 | 1386 | 10 | 6 | 4 | 4 | 9 | 10 | 9 | 5 | 3 | 5 | 5 | 5 | 10 |
| NM_006555 | YKT6 | 2766 | 6 | 3 | 4 | 3 | 3 | 6 | 4 | 2 | 2 | 1 | 2 | 3 | 3 |
| NM_006556 | PMVK | 1299 | 5 | 3 | 2 | 2 | 3 | 4 | 3 | 5 | 2 | 1 | 1 | 3 | 2 |
| NM_006557 | DMRT2 | 2660 | 4 | 1 | 1 | 0 | 0 | 0 | 4 | 0 | 0 | 0 | 0 | 0 | 0 |
| NM_006558 | KHDRBS3 | 1956 | 14 | 4 | 3 | 1 | 3 | 13 | 4 | 4 | 2 | 1 | 4 | 1 | 14 |
| NM_006559 | KHDRBS1 | 2685 | 37 | 18 | 13 | 10 | 17 | 20 | 15 | 12 | 13 | 37 | 15 | 24 | 22 |
| NM_006560 | CUGBP1 | 2142 | 9 | 7 | 5 | 7 | 5 | 9 | 8 | 8 | 5 | 5 | 6 | 9 | 8 |
| NM_006561 | CUGBP2 | 7988 | 7 | 3 | 3 | 1 | 4 | 6 | 0 | 0 | 4 | 6 | 2 | 7 | 2 |
| NM_006562 | LBX1 | 1285 | 0 | 0 | 0 | 0 | 0 | 0 | 0 | 0 | 0 | 0 | 0 | 0 | 0 |
| NM_006563 | KLF1 | 1614 | 0 | 0 | 0 | 0 | 0 | 0 | 0 | 0 | 0 | 0 | 0 | 0 | 0 |
| NM_006564 | CXCR6 | 1953 | 2 | 1 | 0 | 1 | 0 | 0 | 0 | 0 | 2 | 1 | 1 | 2 | 0 |
| NM_006565 | CTCF | 3787 | 16 | 10 | 7 | 7 | 8 | 11 | 10 | 8 | 8 | 12 | 10 | 16 | 10 |
| NM_006566 | CD226 | 2637 | 9 | 1 | 0 | 1 | 0 | 1 | 1 | 1 | 2 | 1 | 1 | 9 | 1 |
| NM_006567 | FARS2 | 1841 | 3 | 3 | 2 | 2 | 3 | 3 | 3 | 3 | 2 | 3 | 2 | 3 | 2 |
| NM_006568 | CGRRF1 | 1323 | 9 | 4 | 3 | 2 | 3 | 6 | 4 | 2 | 3 | 3 | 3 | 3 | 9 |
| NM_006569 | CGREF1 | 1929 | 3 | 0 | 1 | 0 | 0 | 3 | 0 | 0 | 0 | 0 | 0 | 0 | 0 |
| NM_006570 | RRAGA | 1650 | 28 | 15 | 11 | 11 | 17 | 28 | 13 | 15 | 11 | 16 | 15 | 15 | 15 |
| NM_006571 | DCTN6 | 1031 | 15 | 8 | 8 | 3 | 10 | 15 | 8 | 8 | 7 | 5 | 5 | 7 | 9 |
| NM_006572 | GNA13 | 6309 | 15 | 7 | 7 | 5 | 5 | 7 | 5 | 4 | 13 | 7 | 6 | 15 | 8 |
| NM_006573 | TNFSF13B | 1204 | 2 | 1 | 1 | 0 | 1 | 0 | 0 | 0 | 2 | 0 | 0 | 2 | 0 |
| NM_006574 | CSPG5 | 2158 | 17 | 2 | 0 | 0 | 0 | 17 | 0 | 0 | 0 | 0 | 0 | 0 | 0 |
| NM_006575 | MAP4K5 | 4355 | 12 | 5 | 3 | 2 | 4 | 4 | 4 | 2 | 7 | 12 | 3 | 9 | 5 |
| NM_006576 | AVIL | 2711 | 1 | 0 | 0 | 1 | 0 | 0 | 1 | 0 | 0 | 0 | 1 | 0 | 0 |
| NM_006577 | B3GNT2 | 2749 | 5 | 2 | 3 | 1 | 2 | 2 | 5 | 2 | 4 | 1 | 1 | 4 | 3 |
| NM_006578 | GNB5 | 2979 | 2 | 1 | 0 | 0 | 0 | 2 | 1 | 0 | 1 | 1 | 0 | 1 | 1 |
| NM_006579 | EBP | 1157 | 23 | 5 | 1 | 6 | 3 | 3 | 5 | 23 | 3 | 2 | 0 | 3 | 2 |
| NM_006580 | CLDN16 | 1494 | 15 | 1 | 0 | 0 | 0 | 0 | 15 | 0 | 0 | 0 | 0 | 0 | 0 |
| NM_006581 | FUT9 | 12798 | 8 | 1 | 0 | 0 | 0 | 8 | 0 | 0 | 0 | 0 | 0 | 0 | 0 |
| NM_006582 | GMEB1 | 1960 | 9 | 4 | 4 | 4 | 3 | 4 | 4 | 2 | 3 | 5 | 4 | 9 | 6 |
| NM_006583 | RRH | 1556 | 0 | 0 | 0 | 0 | 0 | 0 | 0 | 0 | 0 | 0 | 0 | 0 | 0 |
| NM_006584 | CCT6B | 1816 | 13 | 2 | 0 | 0 | 0 | 1 | 1 | 1 | 0 | 1 | 1 | 0 | 13 |
| NM_006585 | CCT8 | 1894 | 18 | 11 | 8 | 5 | 12 | 18 | 12 | 11 | 9 | 8 | 11 | 15 | 13 |
| NM_006586 | CNPY3 | 1765 | 6 | 3 | 4 | 2 | 2 | 4 | 2 | 4 | 3 | 2 | 1 | 6 | 2 |
| NM_006587 | CORIN | 4942 | 4 | 1 | 0 | 0 | 4 | 0 | 0 | 0 | 0 | 0 | 0 | 1 | 0 |
| NM_006588 | SULT1C4 | 1664 | 4 | 1 | 0 | 0 | 0 | 3 | 3 | 0 | 1 | 4 | 0 | 0 | 0 |
| NM_006589 | FAM189B | 3190 | 0 | 0 | 0 | 0 | 0 | 0 | 0 | 0 | 0 | 0 | 0 | 0 | 0 |
| NM_006590 | USP39 | 2209 | 4 | 3 | 2 | 2 | 4 | 3 | 3 | 3 | 3 | 3 | 4 | 4 | 3 |
| NM_006591 | POLD3 | 3430 | 3 | 1 | 1 | 1 | 1 | 2 | 2 | 1 | 1 | 2 | 1 | 2 | 3 |
| NM_006593 | TBR1 | 3186 | 3 | 0 | 0 | 0 | 0 | 3 | 0 | 0 | 0 | 0 | 0 | 0 | 0 |
| NM_006594 | AP4B1 | 2484 | 4 | 2 | 2 | 2 | 2 | 3 | 2 | 2 | 2 | 2 | 1 | 4 | 2 |
| NM_006595 | API5 | 3765 | 14 | 9 | 6 | 4 | 7 | 12 | 11 | 13 | 7 | 9 | 7 | 14 | 11 |
| NM_006597 | HSPA8 | 2261 | 695 | 398 | 296 | 158 | 668 | 695 | 439 | 103 | 295 | 293 | 381 | 584 | 467 |
| NM_006598 | SLC12A7 | 5304 | 3 | 1 | 1 | 2 | 2 | 0 | 3 | 1 | 1 | 0 | 0 | 2 | 3 |
| NM_006599 | NFAT5 | 14103 | 9 | 6 | 5 | 3 | 5 | 8 | 9 | 3 | 9 | 7 | 4 | 9 | 8 |
| NM_006600 | NUDC | 1327 | 36 | 23 | 14 | 14 | 30 | 36 | 18 | 29 | 23 | 20 | 29 | 24 | 22 |
| NM_006601 | PTGES3 | 1916 | 28 | 18 | 10 | 9 | 18 | 23 | 19 | 14 | 18 | 28 | 19 | 23 | 20 |
| NM_006602 | TCFL5 | 2456 | 10 | 2 | 1 | 1 | 1 | 4 | 2 | 1 | 1 | 3 | 1 | 2 | 10 |
| NM_006603 | STAG2 | 6002 | 15 | 8 | 5 | 2 | 6 | 5 | 8 | 5 | 11 | 13 | 7 | 15 | 6 |
| NM_006604 | RFPL3 | 1214 | 0 | 0 | 0 | 0 | 0 | 0 | 0 | 0 | 0 | 0 | 0 | 0 | 0 |
| NM_006605 | RFPL2 | 1496 | 0 | 0 | 0 | 0 | 0 | 0 | 0 | 0 | 0 | 0 | 0 | 0 | 0 |
| NM_006606 | RBBP9 | 3857 | 2 | 1 | 1 | 0 | 1 | 1 | 2 | 2 | 1 | 1 | 0 | 1 | 1 |
| NM_006607 | PTTG2 | 576 | 1 | 0 | 0 | 0 | 0 | 0 | 0 | 0 | 0 | 0 | 0 | 0 | 1 |
| NM_006608 | PHTF1 | 3244 | 6 | 2 | 1 | 1 | 2 | 3 | 3 | 2 | 3 | 1 | 1 | 4 | 6 |

| transcript | gene | transcript length (nt) | max | mean | adipose | colon | heart | hypothalamus | kidney | liver | lung | ovary | skeletalmuscle | spleen | testes |
|---|---|---|---|---|---|---|---|---|---|---|---|---|---|---|---|
| NM_006609 | MAP3K2 | 4941 | 10 | 7 | 7 | 3 | 4 | 6 | 7 | 7 | 9 | 9 | 9 | 10 | 10 |
| NM_006610 | MASP2 | 2444 | 8 | 1 | 0 | 0 | 0 | 0 | 0 | 8 | 0 | 1 | 0 | 1 | 0 |
| NM_006612 | KIF1C | 4186 | 137 | 49 | 62 | 35 | 112 | 28 | 41 | 47 | 26 | 13 | 137 | 26 | 16 |
| NM_006613 | GRAP | 2033 | 2 | 0 | 0 | 0 | 0 | 0 | 0 | 0 | 0 | 0 | 0 | 2 | 0 |
| NM_006614 | CHL1 | 7649 | 32 | 5 | 2 | 1 | 2 | 32 | 3 | 1 | 2 | 7 | 0 | 1 | 1 |
| NM_006615 | CAPN9 | 2362 | 1 | 0 | 0 | 1 | 0 | 1 | 0 | 0 | 0 | 0 | 0 | 0 | 0 |
| NM_006617 | NES | 5557 | 41 | 11 | 6 | 3 | 41 | 9 | 11 | 1 | 9 | 3 | 8 | 21 | 5 |
| NM_006618 | KDM5B | 6383 | 23 | 6 | 2 | 5 | 3 | 4 | 3 | 2 | 5 | 8 | 1 | 5 | 23 |
| NM_006620 | HBS1L | 7158 | 4 | 2 | 2 | 1 | 3 | 3 | 2 | 4 | 1 | 2 | 4 | 2 | 2 |
| NM_006621 | AHCYL1 | 4022 | 53 | 13 | 13 | 6 | 8 | 53 | 19 | 12 | 7 | 6 | 6 | 8 | 8 |
| NM_006622 | PLK2 | 2785 | 59 | 15 | 8 | 2 | 14 | 32 | 4 | 13 | 10 | 10 | 5 | 59 | 13 |
| NM_006623 | PHGDH | 2014 | 9 | 3 | 5 | 1 | 1 | 9 | 3 | 5 | 2 | 3 | 0 | 1 | 2 |
| NM_006624 | ZMYND11 | 4403 | 22 | 14 | 11 | 5 | 19 | 22 | 15 | 8 | 11 | 17 | 13 | 15 | 15 |
| NM_006625 | SFRS13A | 1842 | 12 | 8 | 7 | 4 | 9 | 10 | 7 | 12 | 6 | 8 | 5 | 11 | 8 |
| NM_006626 | ZBTB6 | 4106 | 2 | 1 | 1 | 0 | 1 | 1 | 1 | 1 | 1 | 2 | 1 | 2 | 2 |
| NM_006627 | POP4 | 2594 | 6 | 4 | 4 | 1 | 6 | 5 | 5 | 5 | 3 | 3 | 4 | 6 | 5 |
| NM_006628 | ARPP19 | 5162 | 43 | 17 | 16 | 8 | 16 | 43 | 16 | 11 | 15 | 15 | 12 | 18 | 20 |
| NM_006630 | ZNF234 | 4598 | 5 | 2 | 2 | 1 | 3 | 3 | 2 | 1 | 2 | 5 | 2 | 4 | 2 |
| NM_006631 | ZNF266 | 3601 | 7 | 3 | 4 | 3 | 3 | 2 | 3 | 1 | 4 | 4 | 2 | 7 | 4 |
| NM_006632 | SLC17A3 | 1574 | 12 | 1 | 0 | 0 | 0 | 0 | 12 | 1 | 0 | 0 | 0 | 0 | 0 |
| NM_006633 | IQGAP2 | 5769 | 49 | 12 | 10 | 5 | 4 | 1 | 17 | 49 | 9 | 5 | 1 | 18 | 10 |
| NM_006634 | VAMP5 | 665 | 3 | 2 | 3 | 1 | 2 | 2 | 1 | 3 | 2 | 1 | 2 | 3 | 0 |
| NM_006635 | ZNF460 | 3849 | 19 | 11 | 7 | 5 | 11 | 11 | 11 | 3 | 12 | 19 | 12 | 16 | 12 |
| NM_006636 | MTHFD2 | 2187 | 4 | 2 | 1 | 1 | 2 | 2 | 1 | 0 | 2 | 0 | 3 | 4 | 1 |
| NM_006637 | OR5I1 | 945 | 0 | 0 | 0 | 0 | 0 | 0 | 0 | 0 | 0 | 0 | 0 | 0 | 0 |
| NM_006638 | RPP40 | 1159 | 1 | 0 | 0 | 0 | 0 | 1 | 0 | 1 | 0 | 0 | 0 | 1 | 0 |
| NM_006639 | CYSLTR1 | 1514 | 2 | 0 | 1 | 0 | 0 | 0 | 0 | 0 | 0 | 0 | 0 | 2 | 0 |
| NM_006640 | 09 Sep | 4457 | 13 | 5 | 4 | 7 | 3 | 5 | 4 | 5 | 4 | 5 | 2 | 13 | 6 |
| NM_006641 | CCR9 | 2462 | 1 | 0 | 0 | 0 | 0 | 0 | 0 | 0 | 0 | 0 | 0 | 1 | 0 |
| NM_006642 | SDCCAG8 | 2604 | 17 | 9 | 7 | 3 | 10 | 17 | 8 | 4 | 10 | 10 | 6 | 11 | 10 |
| NM_006643 | SDCCAG3 | 2310 | 5 | 1 | 1 | 1 | 1 | 1 | 1 | 5 | 1 | 1 | 1 | 2 | 1 |
| NM_006644 | HSPH1 | 3667 | 24 | 9 | 4 | 2 | 11 | 24 | 8 | 2 | 14 | 6 | 4 | 10 | 19 |
| NM_006645 | STARD10 | 1988 | 11 | 3 | 0 | 6 | 1 | 1 | 2 | 11 | 1 | 1 | 2 | 1 | 2 |
| NM_006646 | WASF3 | 4832 | 12 | 3 | 5 | 0 | 1 | 12 | 1 | 1 | 1 | 2 | 0 | 1 | 2 |
| NM_006647 | NOXA1 | 1633 | 0 | 0 | 0 | 0 | 0 | 0 | 0 | 0 | 0 | 0 | 0 | 0 | 0 |
| NM_006648 | WNK2 | 6834 | 3 | 1 | 0 | 3 | 3 | 3 | 0 | 0 | 0 | 0 | 1 | 0 | 1 |
| NM_006649 | UTP14A | 2559 | 9 | 5 | 5 | 3 | 6 | 5 | 5 | 6 | 3 | 8 | 5 | 9 | 3 |
| NM_006650 | CPLX2 | 4726 | 56 | 5 | 0 | 1 | 0 | 56 | 0 | 0 | 0 | 0 | 0 | 0 | 1 |
| NM_006651 | CPLX1 | 2140 | 6 | 1 | 0 | 0 | 0 | 6 | 0 | 0 | 0 | 0 | 0 | 1 | 0 |
| NM_006652 | SPINT3 | 476 | 11 | 1 | 0 | 0 | 0 | 0 | 0 | 0 | 0 | 0 | 0 | 1 | 11 |
| NM_006653 | FRS3 | 2166 | 0 | 0 | 0 | 0 | 0 | 0 | 0 | 0 | 0 | 0 | 0 | 0 | 0 |
| NM_006654 | FRS2 | 6864 | 4 | 2 | 2 | 1 | 2 | 3 | 2 | 1 | 1 | 4 | 1 | 2 | 2 |
| NM_006656 | NEU3 | 2748 | 2 | 1 | 0 | 1 | 1 | 1 | 1 | 1 | 1 | 1 | 2 | 1 | 1 |
| NM_006657 | FTCD | 1905 | 1 | 0 | 0 | 0 | 0 | 0 | 1 | 0 | 0 | 0 | 0 | 0 | 0 |
| NM_006658 | C7orf16 | 1797 | 0 | 0 | 0 | 0 | 0 | 0 | 0 | 0 | 0 | 0 | 0 | 0 | 0 |
| NM_006659 | TUBGCP2 | 3198 | 9 | 6 | 5 | 5 | 5 | 9 | 7 | 4 | 5 | 7 | 6 | 8 | 7 |
| NM_006660 | CLPX | 2351 | 31 | 14 | 8 | 5 | 14 | 13 | 15 | 31 | 10 | 11 | 14 | 12 | 15 |
| NM_006661 | PDE10A | 8317 | 4 | 2 | 1 | 0 | 4 | 2 | 4 | 0 | 0 | 2 | 0 | 2 | 2 |
| NM_006662 | SRCAP | 10474 | 5 | 3 | 2 | 4 | 3 | 3 | 4 | 2 | 2 | 3 | 2 | 5 | 4 |
| NM_006663 | PPP1R13L | 3119 | 1 | 0 | 0 | 0 | 1 | 0 | 0 | 0 | 0 | 0 | 0 | 0 | 0 |
| NM_006664 | CCL27 | 446 | 0 | 0 | 0 | 0 | 0 | 0 | 0 | 0 | 0 | 0 | 0 | 0 | 0 |
| NM_006665 | HPSE | 1796 | 2 | 1 | 1 | 0 | 1 | 1 | 0 | 1 | 1 | 1 | 0 | 2 | 0 |
| NM_006666 | RUVBL2 | 1488 | 15 | 7 | 5 | 5 | 7 | 11 | 4 | 11 | 3 | 5 | 8 | 6 | 15 |
| NM_006667 | PGRMC1 | 1915 | 72 | 27 | 17 | 10 | 12 | 48 | 49 | 72 | 18 | 27 | 5 | 15 | 24 |
| NM_006668 | CYP46A1 | 2077 | 2 | 0 | 0 | 0 | 0 | 2 | 0 | 0 | 0 | 0 | 0 | 0 | 0 |
| NM_006669 | LILRB1 | 2968 | 5 | 1 | 0 | 0 | 0 | 0 | 0 | 0 | 1 | 0 | 0 | 5 | 0 |
| NM_006670 | TPBG | 3389 | 1 | 0 | 0 | 0 | 0 | 0 | 0 | 0 | 0 | 0 | 0 | 1 | 0 |
| NM_006671 | SLC1A7 | 2677 | 0 | 0 | 0 | 0 | 0 | 0 | 0 | 0 | 0 | 0 | 0 | 0 | 0 |
| NM_006672 | SLC22A7 | 2553 | 5 | 1 | 0 | 0 | 0 | 0 | 5 | 3 | 0 | 0 | 0 | 0 | 0 |
| NM_006674 | HCP5 | 2537 | 9 | 2 | 1 | 2 | 1 | 0 | 2 | 1 | 4 | 1 | 1 | 9 | 0 |
| NM_006675 | TSPAN9 | 4350 | 5 | 2 | 1 | 1 | 4 | 1 | 5 | 3 | 2 | 2 | 0 | 1 | 1 |
| NM_006676 | USP20 | 4511 | 1 | 0 | 0 | 0 | 0 | 1 | 0 | 0 | 0 | 0 | 0 | 0 | 0 |
| NM_006677 | USP19 | 4401 | 2 | 1 | 1 | 1 | 1 | 1 | 2 | 1 | 1 | 1 | 2 | 1 | 1 |
| NM_006678 | CD300C | 1517 | 0 | 0 | 0 | 0 | 0 | 0 | 0 | 0 | 0 | 0 | 0 | 0 | 0 |
| NM_006680 | ME3 | 2088 | 3 | 1 | 1 | 1 | 1 | 3 | 1 | 0 | 0 | 2 | 1 | 1 | 1 |
| NM_006681 | NMU | 809 | 1 | 0 | 0 | 0 | 0 | 1 | 0 | 0 | 1 | 0 | 0 | 0 | 0 |
| NM_006682 | FGL2 | 4268 | 60 | 13 | 11 | 8 | 13 | 6 | 13 | 8 | 16 | 6 | 3 | 60 | 4 |
| NM_006683 | FAM12A | 879 | 9 | 1 | 0 | 0 | 0 | 0 | 0 | 0 | 0 | 0 | 0 | 0 | 9 |
| NM_006684 | CFHR4 | 1292 | 5 | 0 | 0 | 0 | 0 | 0 | 0 | 5 | 0 | 0 | 0 | 0 | 0 |
| NM_006685 | SMR3B | 785 | 0 | 0 | 0 | 0 | 0 | 0 | 0 | 0 | 0 | 0 | 0 | 0 | 0 |
| NM_006686 | ACTL7B | 1405 | 1 | 0 | 0 | 0 | 0 | 0 | 0 | 0 | 0 | 0 | 0 | 0 | 1 |
| NM_006687 | ACTL7A | 1433 | 16 | 1 | 0 | 0 | 0 | 0 | 0 | 0 | 0 | 0 | 0 | 0 | 16 |
| NM_006688 | C1QL1 | 1500 | 0 | 0 | 0 | 0 | 0 | 0 | 0 | 0 | 0 | 0 | 0 | 0 | 0 |
| NM_006690 | MMP24 | 4171 | 1 | 0 | 0 | 1 | 0 | 1 | 1 | 0 | 1 | 0 | 0 | 0 | 0 |
| NM_006691 | LYVE1 | 2500 | 56 | 16 | 19 | 4 | 40 | 3 | 5 | 18 | 12 | 4 | 8 | 56 | 9 |
| NM_006693 | CPSF4 | 1826 | 1 | 1 | 0 | 1 | 0 | 1 | 1 | 0 | 1 | 1 | 0 | 0 | 1 |
| NM_006694 | JTB | 1574 | 5 | 2 | 2 | 2 | 3 | 3 | 2 | 5 | 1 | 1 | 1 | 3 | 1 |

| | | transcript | | | | | | hypothala | | | | | skeletalm | | |
|---|---|---|---|---|---|---|---|---|---|---|---|---|---|---|---|
| transcript | gene | length (nt) | max | mean | adipose | colon | heart | mus | kidney | liver | lung | ovary | uscle | spleen | testes |
| NM_006695 | RUNDC3A | 1857 | 32 | 3 | 0 | 1 | 0 | 32 | 0 | 0 | 0 | 0 | 0 | 0 | 0 |
| NM_006696 | BRD8 | 3235 | 31 | 13 | 11 | 12 | 8 | 22 | 9 | 7 | 7 | 12 | 7 | 12 | 31 |
| NM_006698 | BLCAP | 2072 | 50 | 16 | 13 | 8 | 14 | 32 | 14 | 11 | 6 | 11 | 50 | 10 | 8 |
| NM_006699 | MAN1A2 | 5388 | 38 | 19 | 11 | 6 | 15 | 37 | 16 | 17 | 15 | 28 | 9 | 22 | 38 |
| NM_006700 | TRAFD1 | 2647 | 9 | 5 | 4 | 4 | 6 | 4 | 3 | 3 | 8 | 4 | 2 | 9 | 8 |
| NM_006701 | TXNL4A | 1387 | 13 | 6 | 6 | 3 | 8 | 13 | 6 | 9 | 3 | 3 | 4 | 5 | 4 |
| NM_006702 | PNPLA6 | 4472 | 1 | 0 | 0 | 0 | 0 | 1 | 0 | 0 | 1 | 0 | 0 | 1 | 0 |
| NM_006703 | NUDT3 | 1354 | 63 | 20 | 15 | 8 | 21 | 63 | 19 | 10 | 12 | 19 | 22 | 19 | 14 |
| NM_006704 | SUGT1 | 1690 | 5 | 3 | 2 | 1 | 3 | 5 | 4 | 2 | 2 | 3 | 2 | 3 | 3 |
| NM_006705 | GADD45G | 1063 | 2 | 0 | 0 | 0 | 0 | 0 | 0 | 2 | 1 | 1 | 0 | 0 | 0 |
| NM_006706 | TCERG1 | 4201 | 10 | 6 | 4 | 3 | 7 | 7 | 5 | 4 | 5 | 6 | 8 | 10 | 8 |
| NM_006708 | GLO1 | 2046 | 7 | 4 | 2 | 2 | 4 | 7 | 5 | 4 | 4 | 3 | 4 | 5 | 4 |
| NM_006709 | EHMT2 | 3967 | 4 | 2 | 2 | 2 | 1 | 4 | 2 | 1 | 1 | 3 | 1 | 3 | 3 |
| NM_006709 | EHMT2 | 3968 | 4 | 2 | 2 | 2 | 1 | 4 | 2 | 1 | 1 | 3 | 1 | 3 | 3 |
| NM_006710 | COPS8 | 2258 | 26 | 9 | 8 | 4 | 12 | 26 | 7 | 5 | 5 | 7 | 12 | 6 | 11 |
| NM_006711 | RNPS1 | 2012 | 44 | 21 | 11 | 15 | 24 | 44 | 14 | 8 | 13 | 22 | 30 | 23 | 31 |
| NM_006712 | FASTK | 1852 | 0 | 0 | 0 | 0 | 0 | 0 | 0 | 0 | 0 | 0 | 0 | 0 | 0 |
| NM_006713 | SUB1 | 3507 | 42 | 13 | 19 | 4 | 14 | 42 | 9 | 16 | 7 | 8 | 6 | 12 | 10 |
| NM_006714 | SMPDL3A | 2194 | 13 | 4 | 1 | 3 | 2 | 1 | 5 | 7 | 4 | 2 | 4 | 13 | 1 |
| NM_006715 | MAN2C1 | 3254 | 1 | 0 | 0 | 1 | 0 | 0 | 1 | 0 | 1 | 0 | 1 | 1 | 0 |
| NM_006716 | DBF4 | 3907 | 4 | 1 | 0 | 0 | 0 | 0 | 0 | 0 | 0 | 1 | 0 | 1 | 4 |
| NM_006717 | SPIN1 | 4534 | 14 | 6 | 4 | 3 | 5 | 14 | 8 | 2 | 5 | 10 | 1 | 5 | 10 |
| NM_006718 | PLAGL1 | 3406 | 1 | 1 | 1 | 1 | 1 | 0 | 1 | 0 | 1 | 1 | 1 | 1 | 1 |
| NM_006720 | ABLIM1 | 6839 | 51 | 17 | 13 | 7 | 51 | 11 | 14 | 7 | 10 | 40 | 8 | 5 | 16 |
| NM_006721 | ADK | 2018 | 15 | 5 | 3 | 1 | 4 | 5 | 4 | 15 | 4 | 2 | 3 | 4 | 5 |
| NM_006722 | MITF | 4679 | 15 | 5 | 6 | 1 | 9 | 2 | 6 | 1 | 2 | 8 | 15 | 1 | 4 |
| NM_006724 | MAP3K4 | 5334 | 7 | 4 | 3 | 2 | 5 | 5 | 4 | 2 | 3 | 6 | 4 | 5 | 7 |
| NM_006725 | CD6 | 3288 | 1 | 0 | 0 | 0 | 0 | 0 | 0 | 0 | 1 | 0 | 0 | 1 | 0 |
| NM_006726 | LRBA | 10344 | 11 | 5 | 3 | 4 | 3 | 3 | 11 | 5 | 5 | 5 | 2 | 6 | 5 |
| NM_006727 | CDH10 | 3261 | 5 | 0 | 0 | 0 | 0 | 5 | 0 | 0 | 0 | 0 | 0 | 0 | 0 |
| NM_006729 | DIAPH2 | 4983 | 8 | 3 | 4 | 1 | 3 | 2 | 1 | 3 | 4 | 4 | 4 | 3 | 8 |
| NM_006730 | DNASE1L1 | 3147 | 3 | 1 | 1 | 1 | 2 | 0 | 1 | 0 | 1 | 1 | 3 | 2 | 0 |
| NM_006731 | FKTN | 7364 | 1 | 1 | 1 | 0 | 1 | 1 | 1 | 0 | 0 | 1 | 0 | 1 | 1 |
| NM_006732 | FOSB | 3776 | 58 | 7 | 2 | 2 | 2 | 1 | 2 | 3 | 3 | 58 | 0 | 1 | 0 |
| NM_006733 | CENPI | 2563 | 1 | 0 | 1 | 1 | 0 | 0 | 0 | 0 | 0 | 0 | 0 | 0 | 1 |
| NM_006734 | HIVEP2 | 9724 | 11 | 4 | 2 | 1 | 4 | 11 | 2 | 1 | 4 | 5 | 7 | 5 | 3 |
| NM_006735 | HOXA2 | 1778 | 1 | 0 | 0 | 1 | 0 | 0 | 0 | 0 | 0 | 0 | 0 | 0 | 0 |
| NM_006736 | DNAJB2 | 3125 | 6 | 3 | 2 | 2 | 5 | 6 | 4 | 1 | 2 | 2 | 3 | 3 | 4 |
| NM_006737 | KIR3DL2 | 1877 | 1 | 0 | 0 | 0 | 0 | 0 | 0 | 0 | 0 | 0 | 0 | 1 | 0 |
| NM_006738 | AKAP13 | 13302 | 22 | 10 | 6 | 7 | 22 | 4 | 5 | 6 | 18 | 9 | 7 | 18 | 10 |
| NM_006739 | MCM5 | 2546 | 4 | 1 | 1 | 2 | 1 | 1 | 1 | 0 | 1 | 2 | 1 | 4 | 1 |
| NM_006741 | PPP1R1A | 1834 | 48 | 11 | 32 | 2 | 8 | 6 | 15 | 7 | 0 | 1 | 48 | 0 | 1 |
| NM_006742 | PSKH1 | 3481 | 1 | 0 | 0 | 0 | 0 | 0 | 0 | 0 | 0 | 0 | 0 | 0 | 1 |
| NM_006743 | RBM3 | 1588 | 26 | 13 | 26 | 5 | 15 | 9 | 15 | 8 | 14 | 10 | 9 | 15 | 14 |
| NM_006744 | RBP4 | 929 | 179 | 19 | 14 | 2 | 1 | 1 | 4 | 179 | 1 | 2 | 1 | 2 | 0 |
| NM_006745 | SC4MOL | 2139 | 15 | 4 | 1 | 1 | 1 | 9 | 3 | 15 | 3 | 1 | 0 | 1 | 3 |
| NM_006746 | SCML1 | 2827 | 4 | 1 | 0 | 0 | 1 | 1 | 1 | 1 | 1 | 1 | 1 | 1 | 4 |
| NM_006747 | SIPA1 | 3501 | 4 | 1 | 0 | 0 | 1 | 0 | 0 | 1 | 1 | 0 | 0 | 4 | 0 |
| NM_006748 | SLA | 2825 | 35 | 5 | 4 | 1 | 5 | 2 | 1 | 2 | 8 | 1 | 1 | 35 | 1 |
| NM_006749 | SLC20A2 | 3656 | 6 | 2 | 1 | 1 | 5 | 5 | 2 | 3 | 1 | 1 | 6 | 1 | 3 |
| NM_006750 | SNTB2 | 9771 | 5 | 3 | 5 | 1 | 3 | 2 | 4 | 1 | 3 | 4 | 1 | 4 | 4 |
| NM_006751 | SSFA2 | 5174 | 21 | 11 | 7 | 6 | 9 | 11 | 12 | 16 | 16 | 5 | 6 | 15 | 21 |
| NM_006753 | SURF6 | 2317 | 5 | 3 | 2 | 3 | 2 | 5 | 3 | 2 | 1 | 3 | 2 | 3 | 2 |
| NM_006754 | SYPL1 | 2161 | 17 | 11 | 8 | 6 | 10 | 7 | 16 | 7 | 13 | 11 | 16 | 17 | 14 |
| NM_006755 | TALDO1 | 1243 | 25 | 15 | 20 | 9 | 16 | 15 | 19 | 22 | 19 | 8 | 10 | 25 | 7 |
| NM_006756 | TCEA1 | 2777 | 22 | 15 | 10 | 6 | 15 | 11 | 17 | 17 | 17 | 21 | 11 | 22 | 20 |
| NM_006757 | TNNT3 | 1203 | 281 | 26 | 3 | 0 | 2 | 0 | 0 | 0 | 0 | 0 | 281 | 0 | 1 |
| NM_006758 | U2AF1 | 953 | 45 | 28 | 21 | 23 | 31 | 31 | 29 | 23 | 31 | 33 | 21 | 45 | 24 |
| NM_006759 | UGP2 | 2168 | 45 | 23 | 37 | 7 | 45 | 18 | 16 | 44 | 12 | 11 | 38 | 17 | 11 |
| NM_006760 | UPK2 | 933 | 0 | 0 | 0 | 0 | 0 | 0 | 0 | 0 | 0 | 0 | 0 | 0 | 0 |
| NM_006761 | YWHAE | 1825 | 313 | 140 | 128 | 57 | 169 | 313 | 140 | 128 | 100 | 111 | 157 | 113 | 127 |
| NM_006762 | LAPTM5 | 2240 | 41 | 8 | 13 | 2 | 6 | 6 | 1 | 4 | 15 | 2 | 1 | 41 | 1 |
| NM_006763 | BTG2 | 2711 | 24 | 7 | 5 | 5 | 8 | 6 | 6 | 1 | 8 | 24 | 6 | 10 | 3 |
| NM_006764 | IFRD2 | 2107 | 1 | 0 | 0 | 0 | 0 | 0 | 0 | 1 | 0 | 0 | 0 | 0 | 0 |
| NM_006765 | TUSC3 | 1587 | 8 | 3 | 1 | 1 | 4 | 6 | 3 | 1 | 3 | 6 | 0 | 2 | 8 |
| NM_006766 | MYST3 | 9153 | 17 | 9 | 5 | 4 | 7 | 7 | 6 | 5 | 10 | 13 | 8 | 17 | 12 |
| NM_006767 | LZTR1 | 4315 | 2 | 1 | 2 | 1 | 1 | 2 | 1 | 1 | 1 | 1 | 0 | 1 | 1 |
| NM_006768 | BRAP | 4026 | 12 | 5 | 3 | 3 | 4 | 6 | 3 | 3 | 3 | 6 | 5 | 5 | 12 |
| NM_006769 | LMO4 | 5402 | 13 | 3 | 2 | 1 | 2 | 13 | 2 | 1 | 2 | 2 | 2 | 3 | 3 |
| NM_006770 | MARCO | 1835 | 9 | 3 | 3 | 0 | 9 | 1 | 0 | 8 | 9 | 0 | 1 | 4 | 1 |
| NM_006771 | KRT38 | 2837 | 0 | 0 | 0 | 0 | 0 | 0 | 0 | 0 | 0 | 0 | 0 | 0 | 0 |
| NM_006772 | SYNGAP1 | 6010 | 5 | 1 | 0 | 1 | 0 | 5 | 0 | 0 | 0 | 1 | 0 | 1 | 1 |
| NM_006773 | DDX18 | 3765 | 8 | 5 | 4 | 3 | 4 | 7 | 4 | 8 | 4 | 5 | 4 | 8 | 5 |
| NM_006774 | INMT | 2559 | 14 | 4 | 2 | 1 | 8 | 0 | 3 | 3 | 14 | 1 | 0 | 2 | 6 |
| NM_006775 | QKI | 4729 | 46 | 21 | 19 | 4 | 35 | 46 | 10 | 11 | 16 | 14 | 30 | 21 | 23 |
| NM_006777 | ZBTB33 | 5209 | 6 | 3 | 2 | 1 | 2 | 6 | 2 | 3 | 3 | 4 | 2 | 4 | 4 |
| NM_006778 | TRIM10 | 3544 | 1 | 0 | 0 | 0 | 0 | 0 | 1 | 0 | 0 | 0 | 0 | 0 | 0 |

| transcript | gene | transcript length (nt) | max | mean | adipose | colon | heart | hypothalamus | kidney | liver | lung | ovary | skeletalmuscle | spleen | testes |
|---|---|---|---|---|---|---|---|---|---|---|---|---|---|---|---|
| NM_006779 | CDC42EP2 | 1981 | 1 | 0 | 0 | 0 | 1 | 0 | 0 | 0 | 0 | 1 | 0 | 1 | 0 |
| NM_006781 | C6orf10 | 2128 | 4 | 1 | 0 | 0 | 0 | 0 | 0 | 0 | 0 | 1 | 0 | 0 | 4 |
| NM_006781 | C6orf10 | 2148 | 4 | 1 | 0 | 0 | 0 | 0 | 0 | 0 | 0 | 1 | 0 | 0 | 4 |
| NM_006782 | ZFPL1 | 1386 | 2 | 1 | 1 | 1 | 1 | 2 | 1 | 1 | 1 | 1 | 1 | 1 | 1 |
| NM_006783 | GJB6 | 2072 | 14 | 1 | 0 | 0 | 0 | 14 | 0 | 0 | 0 | 0 | 0 | 0 | 0 |
| NM_006784 | WDR3 | 3877 | 8 | 5 | 4 | 2 | 4 | 6 | 3 | 7 | 2 | 5 | 5 | 6 | 8 |
| NM_006785 | MALT1 | 5029 | 5 | 2 | 1 | 1 | 2 | 1 | 4 | 2 | 3 | 3 | 2 | 5 | 3 |
| NM_006786 | UTS2 | 569 | 1 | 0 | 0 | 0 | 0 | 0 | 0 | 0 | 1 | 0 | 0 | 0 | 0 |
| NM_006788 | RALBP1 | 4354 | 13 | 8 | 9 | 4 | 9 | 10 | 13 | 6 | 9 | 8 | 6 | 12 | 8 |
| NM_006789 | APOBEC2 | 1633 | 16 | 3 | 0 | 0 | 9 | 0 | 0 | 0 | 1 | 0 | 16 | 1 | 0 |
| NM_006790 | MYOT | 2271 | 96 | 9 | 0 | 0 | 6 | 1 | 0 | 0 | 0 | 0 | 96 | 0 | 1 |
| NM_006791 | MORF4L1 | 1825 | 73 | 40 | 36 | 15 | 48 | 73 | 38 | 28 | 36 | 40 | 33 | 48 | 51 |
| NM_006792 | MORF4 | 708 | 11 | 4 | 5 | 2 | 4 | 11 | 3 | 4 | 2 | 4 | 4 | 5 | 3 |
| NM_006793 | PRDX3 | 1591 | 25 | 12 | 10 | 4 | 22 | 10 | 25 | 23 | 8 | 5 | 10 | 13 | 7 |
| NM_006794 | GPR75 | 2115 | 2 | 1 | 0 | 0 | 0 | 2 | 0 | 0 | 0 | 1 | 0 | 1 | 0 |
| NM_006795 | EHD1 | 3457 | 5 | 2 | 1 | 1 | 1 | 1 | 2 | 1 | 2 | 1 | 1 | 5 | 2 |
| NM_006796 | AFG3L2 | 2963 | 16 | 10 | 7 | 9 | 16 | 12 | 15 | 14 | 5 | 7 | 13 | 7 | 8 |
| NM_006798 | UGT2A1 | 2594 | 0 | 0 | 0 | 0 | 0 | 0 | 0 | 0 | 0 | 0 | 0 | 0 | 0 |
| NM_006799 | PRSS21 | 1163 | 0 | 0 | 0 | 0 | 0 | 0 | 0 | 0 | 0 | 0 | 0 | 0 | 0 |
| NM_006800 | MSL3 | 2289 | 9 | 4 | 2 | 2 | 3 | 5 | 2 | 3 | 4 | 3 | 3 | 9 | 5 |
| NM_006801 | KDELR1 | 1557 | 6 | 3 | 2 | 2 | 4 | 2 | 4 | 3 | 3 | 6 | 2 | 3 | 4 |
| NM_006802 | SF3A3 | 2836 | 16 | 10 | 9 | 6 | 9 | 12 | 11 | 9 | 7 | 13 | 8 | 16 | 14 |
| NM_006803 | AP3M2 | 3611 | 11 | 3 | 2 | 1 | 1 | 11 | 2 | 0 | 2 | 2 | 1 | 2 | 4 |
| NM_006804 | STARD3 | 2834 | 1 | 1 | 1 | 1 | 1 | 1 | 1 | 1 | 1 | 1 | 0 | 1 | 1 |
| NM_006805 | HNRNPA0 | 2967 | 7 | 4 | 3 | 3 | 4 | 5 | 3 | 2 | 5 | 7 | 3 | 4 | 4 |
| NM_006806 | BTG3 | 1460 | 6 | 2 | 1 | 1 | 2 | 3 | 1 | 2 | 5 | 2 | 0 | 2 | 6 |
| NM_006807 | CBX1 | 2422 | 25 | 10 | 7 | 6 | 10 | 25 | 8 | 4 | 5 | 15 | 8 | 8 | 13 |
| NM_006808 | SEC61B | 562 | 4 | 2 | 3 | 1 | 1 | 1 | 2 | 4 | 1 | 2 | 0 | 2 | 1 |
| NM_006809 | TOMM34 | 2049 | 12 | 4 | 2 | 2 | 2 | 10 | 3 | 2 | 1 | 4 | 2 | 2 | 12 |
| NM_006810 | PDIA5 | 1862 | 9 | 3 | 2 | 3 | 2 | 1 | 3 | 9 | 2 | 2 | 0 | 2 | 4 |
| NM_006811 | SERINC3 | 2592 | 32 | 22 | 21 | 9 | 22 | 32 | 14 | 17 | 26 | 23 | 18 | 30 | 29 |
| NM_006812 | OS9 | 2857 | 103 | 55 | 71 | 41 | 55 | 47 | 63 | 103 | 57 | 51 | 17 | 63 | 42 |
| NM_006813 | PNRC1 | 2133 | 32 | 18 | 13 | 9 | 16 | 14 | 9 | 23 | 23 | 32 | 25 | 20 | 16 |
| NM_006814 | PSMF1 | 3695 | 4 | 2 | 1 | 1 | 2 | 3 | 2 | 4 | 2 | 2 | 3 | 3 | 3 |
| NM_006815 | TMED2 | 2126 | 115 | 28 | 30 | 20 | 19 | 27 | 17 | 115 | 17 | 13 | 9 | 19 | 16 |
| NM_006816 | LMAN2 | 1831 | 10 | 3 | 2 | 2 | 2 | 2 | 3 | 10 | 2 | 1 | 0 | 2 | 3 |
| NM_006817 | ERP29 | 1472 | 66 | 26 | 20 | 21 | 17 | 26 | 28 | 66 | 24 | 18 | 6 | 36 | 21 |
| NM_006818 | MLLT11 | 2158 | 50 | 8 | 0 | 1 | 27 | 50 | 1 | 0 | 1 | 3 | 3 | 1 | 3 |
| NM_006819 | STIP1 | 2201 | 16 | 9 | 6 | 5 | 12 | 16 | 7 | 4 | 7 | 7 | 10 | 11 | 15 |
| NM_006820 | IFI44L | 5874 | 3 | 2 | 3 | 0 | 2 | 2 | 1 | 0 | 1 | 3 | 3 | 2 | 1 |
| NM_006821 | ACOT2 | 1767 | 5 | 2 | 4 | 1 | 2 | 1 | 3 | 5 | 1 | 1 | 1 | 1 | 1 |
| NM_006822 | RAB40B | 1758 | 17 | 3 | 1 | 2 | 2 | 17 | 4 | 1 | 1 | 2 | 2 | 1 | 1 |
| NM_006823 | PKIA | 2184 | 12 | 2 | 0 | 0 | 7 | 6 | 0 | 0 | 0 | 0 | 12 | 0 | 0 |
| NM_006824 | EBNA1BP2 | 1386 | 12 | 7 | 4 | 3 | 8 | 12 | 12 | 11 | 4 | 5 | 4 | 8 | 10 |
| NM_006825 | CKAP4 | 3035 | 7 | 4 | 3 | 5 | 7 | 3 | 3 | 3 | 4 | 4 | 1 | 4 | 3 |
| NM_006826 | YWHAQ | 2166 | 45 | 20 | 16 | 11 | 23 | 45 | 19 | 20 | 18 | 19 | 7 | 20 | 27 |
| NM_006827 | TMED10 | 4144 | 50 | 27 | 25 | 15 | 33 | 28 | 22 | 50 | 23 | 29 | 17 | 24 | 33 |
| NM_006828 | ASCC3 | 7592 | 7 | 4 | 3 | 1 | 4 | 4 | 3 | 6 | 3 | 4 | 3 | 7 | 6 |
| NM_006829 | C10orf116 | 672 | 28 | 5 | 28 | 3 | 3 | 3 | 4 | 2 | 6 | 3 | 3 | 1 | 2 |
| NM_006830 | UQCR | 1335 | 5 | 2 | 3 | 2 | 5 | 3 | 3 | 4 | 1 | 1 | 2 | 2 | 1 |
| NM_006831 | CLP1 | 1845 | 2 | 1 | 1 | 1 | 1 | 1 | 1 | 2 | 1 | 1 | 0 | 2 | 1 |
| NM_006832 | FERMT2 | 3334 | 26 | 11 | 26 | 5 | 16 | 9 | 9 | 11 | 9 | 9 | 5 | 12 | 8 |
| NM_006833 | COPS6 | 1425 | 21 | 11 | 13 | 8 | 13 | 21 | 10 | 14 | 6 | 8 | 11 | 9 | 10 |
| NM_006834 | RAB32 | 1203 | 2 | 1 | 2 | 0 | 1 | 1 | 1 | 2 | 1 | 1 | 0 | 2 | 1 |
| NM_006835 | CCNI | 1875 | 9 | 7 | 6 | 4 | 8 | 7 | 8 | 3 | 9 | 8 | 4 | 9 | 8 |
| NM_006836 | GCN1L1 | 8668 | 3 | 2 | 2 | 3 | 2 | 2 | 3 | 3 | 2 | 2 | 3 | 3 | 2 |
| NM_006837 | COPS5 | 1489 | 15 | 7 | 7 | 5 | 10 | 15 | 6 | 10 | 4 | 3 | 7 | 7 | 7 |
| NM_006838 | METAP2 | 3504 | 17 | 12 | 12 | 6 | 13 | 16 | 11 | 17 | 9 | 12 | 10 | 9 | 17 |
| NM_006839 | IMMT | 3001 | 23 | 14 | 13 | 8 | 23 | 16 | 18 | 18 | 6 | 8 | 17 | 14 | 13 |
| NM_006840 | LILRB5 | 2264 | 3 | 1 | 3 | 1 | 1 | 0 | 0 | 1 | 1 | 0 | 1 | 2 | 0 |
| NM_006841 | SLC38A3 | 2453 | 3 | 0 | 0 | 0 | 0 | 0 | 0 | 3 | 0 | 0 | 0 | 0 | 0 |
| NM_006842 | SF3B2 | 2894 | 39 | 26 | 22 | 17 | 24 | 35 | 28 | 19 | 19 | 33 | 17 | 35 | 39 |
| NM_006843 | SDS | 1604 | 10 | 1 | 0 | 0 | 0 | 0 | 0 | 10 | 0 | 0 | 0 | 0 | 0 |
| NM_006844 | ILVBL | 2298 | 2 | 1 | 1 | 2 | 2 | 2 | 2 | 2 | 0 | 1 | 0 | 1 | 1 |
| NM_006845 | KIF2C | 2880 | 4 | 1 | 0 | 1 | 0 | 0 | 0 | 0 | 0 | 0 | 0 | 0 | 4 |
| NM_006846 | SPINK5 | 3639 | 1 | 0 | 0 | 0 | 1 | 0 | 0 | 0 | 1 | 0 | 0 | 0 | 0 |
| NM_006847 | LILRB4 | 2085 | 3 | 1 | 3 | 0 | 1 | 1 | 0 | 1 | 2 | 0 | 0 | 2 | 0 |
| NM_006848 | CCDC85B | 1232 | 3 | 1 | 1 | 1 | 1 | 3 | 0 | 0 | 0 | 0 | 0 | 1 | 0 |
| NM_006849 | PDIA2 | 1726 | 0 | 0 | 0 | 0 | 0 | 0 | 0 | 0 | 0 | 0 | 0 | 0 | 0 |
| NM_006850 | IL24 | 1975 | 1 | 0 | 0 | 0 | 1 | 0 | 0 | 0 | 0 | 0 | 0 | 1 | 0 |
| NM_006851 | GLIPR1 | 3907 | 6 | 2 | 5 | 1 | 3 | 1 | 1 | 1 | 4 | 2 | 1 | 6 | 1 |
| NM_006852 | TLK2 | 5439 | 16 | 10 | 8 | 8 | 8 | 8 | 7 | 4 | 9 | 14 | 11 | 13 | 16 |
| NM_006853 | KLK11 | 1193 | 0 | 0 | 0 | 0 | 0 | 0 | 0 | 0 | 0 | 0 | 0 | 0 | 0 |
| NM_006854 | KDELR2 | 2860 | 29 | 7 | 5 | 5 | 6 | 3 | 7 | 29 | 5 | 6 | 2 | 6 | 5 |
| NM_006855 | KDELR3 | 1705 | 7 | 3 | 3 | 2 | 7 | 1 | 2 | 4 | 2 | 4 | 1 | 2 | 3 |
| NM_006856 | ATF7 | 6688 | 4 | 3 | 2 | 2 | 3 | 2 | 2 | 1 | 3 | 4 | 3 | 3 | 3 |
| NM_006857 | SNRNP27 | 1404 | 9 | 4 | 3 | 1 | 5 | 9 | 3 | 2 | 3 | 3 | 5 | 4 | 3 |

| transcript | gene | transcript length (nt) | max | mean | adipose | colon | heart | hypothalamus | kidney | liver | lung | ovary | skeletalmuscle | spleen | testes |
|---|---|---|---|---|---|---|---|---|---|---|---|---|---|---|---|
| NM_006858 | TMED1 | 1357 | 0 | 0 | 0 | 0 | 0 | 0 | 0 | 0 | 0 | 0 | 0 | 0 | 0 |
| NM_006859 | LIAS | 1728 | 3 | 2 | 1 | 1 | 2 | 1 | 2 | 3 | 1 | 2 | 2 | 1 | 2 |
| NM_006860 | RABL4 | 1021 | 1 | 1 | 0 | 1 | 1 | 1 | 1 | 1 | 1 | 1 | 0 | 0 | 1 |
| NM_006861 | RAB35 | 2945 | 3 | 2 | 2 | 2 | 1 | 2 | 2 | 1 | 1 | 1 | 1 | 3 | 2 |
| NM_006862 | TDRKH | 2329 | 4 | 1 | 1 | 1 | 1 | 4 | 1 | 0 | 1 | 0 | 1 | 1 | 2 |
| NM_006863 | LILRA1 | 1910 | 1 | 0 | 0 | 0 | 0 | 0 | 0 | 0 | 0 | 0 | 0 | 1 | 0 |
| NM_006864 | LILRB3 | 2822 | 2 | 0 | 0 | 0 | 1 | 0 | 0 | 0 | 1 | 0 | 0 | 2 | 0 |
| NM_006865 | LILRA3 | 1576 | 1 | 0 | 0 | 0 | 0 | 0 | 0 | 0 | 0 | 0 | 0 | 1 | 0 |
| NM_006866 | LILRA2 | 1702 | 1 | 0 | 0 | 0 | 0 | 0 | 0 | 0 | 0 | 0 | 0 | 1 | 0 |
| NM_006867 | RBPMS | 3154 | 7 | 4 | 4 | 3 | 7 | 1 | 7 | 2 | 3 | 7 | 1 | 4 | 3 |
| NM_006868 | RAB31 | 3994 | 22 | 11 | 7 | 2 | 12 | 22 | 4 | 2 | 17 | 16 | 2 | 18 | 21 |
| NM_006869 | ADAP1 | 2322 | 1 | 0 | 0 | 1 | 0 | 1 | 0 | 0 | 0 | 0 | 0 | 0 | 0 |
| NM_006870 | DSTN | 1614 | 375 | 147 | 91 | 119 | 375 | 143 | 137 | 71 | 188 | 158 | 56 | 81 | 194 |
| NM_006871 | RIPK3 | 1921 | 1 | 0 | 1 | 1 | 0 | 0 | 0 | 0 | 0 | 0 | 0 | 1 | 0 |
| NM_006872 | GTF2A1L | 1668 | 0 | 0 | 0 | 0 | 0 | 0 | 0 | 0 | 0 | 0 | 0 | 0 | 0 |
| NM_006873 | STON1 | 5812 | 4 | 1 | 1 | 1 | 1 | 1 | 1 | 0 | 1 | 4 | 0 | 3 | 1 |
| NM_006874 | ELF2 | 3171 | 7 | 5 | 4 | 2 | 5 | 4 | 4 | 3 | 5 | 5 | 5 | 7 | 7 |
| NM_006875 | PIM2 | 2185 | 6 | 1 | 0 | 1 | 0 | 1 | 0 | 0 | 2 | 1 | 0 | 6 | 1 |
| NM_006876 | B3GNT1 | 2070 | 19 | 3 | 1 | 2 | 4 | 19 | 3 | 1 | 2 | 3 | 2 | 1 | 2 |
| NM_006877 | GMPR | 1515 | 11 | 3 | 1 | 0 | 4 | 2 | 1 | 0 | 1 | 3 | 11 | 1 | 6 |
| NM_006878 | MDM2 | 6619 | 9 | 5 | 4 | 2 | 4 | 3 | 4 | 6 | 6 | 5 | 9 | 9 | 5 |
| NM_006879 | MDM2 | 6149 | 8 | 4 | 4 | 1 | 3 | 3 | 3 | 5 | 6 | 4 | 7 | 8 | 4 |
| NM_006881 | MDM2 | 6546 | 9 | 5 | 4 | 2 | 4 | 3 | 4 | 6 | 7 | 5 | 9 | 9 | 5 |
| NM_006882 | MDM2 | 6409 | 8 | 5 | 4 | 1 | 4 | 3 | 4 | 6 | 6 | 5 | 8 | 8 | 4 |
| NM_006883 | SHOX | 1951 | 0 | 0 | 0 | 0 | 0 | 0 | 0 | 0 | 0 | 0 | 0 | 0 | 0 |
| NM_006884 | SHOX2 | 3150 | 0 | 0 | 0 | 0 | 0 | 0 | 0 | 0 | 0 | 0 | 0 | 0 | 0 |
| NM_006885 | ZFHX3 | 16062 | 3 | 2 | 2 | 2 | 2 | 1 | 1 | 1 | 2 | 1 | 3 | 2 | 2 |
| NM_006886 | ATP5E | 417 | 102 | 57 | 75 | 34 | 102 | 88 | 51 | 93 | 36 | 20 | 37 | 68 | 24 |
| NM_006887 | ZFP36L2 | 3693 | 37 | 15 | 11 | 9 | 12 | 7 | 10 | 20 | 18 | 37 | 10 | 30 | 7 |
| NM_006888 | CALM1 | 4254 | 271 | 60 | 51 | 27 | 52 | 271 | 42 | 26 | 33 | 21 | 42 | 46 | 48 |
| NM_006889 | CD86 | 2721 | 7 | 2 | 3 | 1 | 2 | 2 | 0 | 1 | 3 | 1 | 0 | 7 | 1 |
| NM_006890 | CEACAM7 | 2289 | 31 | 3 | 0 | 31 | 0 | 0 | 0 | 0 | 0 | 0 | 0 | 0 | 0 |
| NM_006891 | CRYGD | 707 | 0 | 0 | 0 | 0 | 0 | 0 | 0 | 0 | 0 | 0 | 0 | 0 | 0 |
| NM_006892 | DNMT3B | 4336 | 1 | 0 | 0 | 0 | 0 | 0 | 0 | 0 | 0 | 0 | 0 | 0 | 1 |
| NM_006893 | LGTN | 2096 | 1 | 1 | 1 | 1 | 1 | 1 | 1 | 1 | 1 | 1 | 1 | 1 | 1 |
| NM_006894 | FMO3 | 2068 | 24 | 3 | 2 | 0 | 1 | 0 | 2 | 24 | 3 | 0 | 0 | 0 | 0 |
| NM_006895 | HNMT | 3367 | 11 | 5 | 11 | 3 | 4 | 2 | 7 | 5 | 4 | 6 | 1 | 6 | 3 |
| NM_006896 | HOXA7 | 2015 | 1 | 0 | 0 | 0 | 0 | 0 | 1 | 0 | 0 | 0 | 1 | 0 | 0 |
| NM_006897 | HOXC9 | 1541 | 4 | 1 | 3 | 0 | 0 | 0 | 2 | 0 | 0 | 2 | 4 | 0 | 1 |
| NM_006898 | HOXD3 | 2299 | 1 | 0 | 0 | 0 | 0 | 0 | 1 | 0 | 0 | 0 | 0 | 0 | 0 |
| NM_006899 | IDH3B | 1523 | 13 | 6 | 5 | 4 | 13 | 11 | 9 | 6 | 3 | 4 | 8 | 5 | 4 |
| NM_006900 | IFNA13 | 705 | 0 | 0 | 0 | 0 | 0 | 0 | 0 | 0 | 0 | 0 | 0 | 0 | 0 |
| NM_006901 | MYO9A | 8680 | 18 | 8 | 4 | 3 | 6 | 12 | 11 | 2 | 7 | 11 | 6 | 9 | 18 |
| NM_006902 | PRRX1 | 4071 | 19 | 6 | 12 | 0 | 10 | 3 | 0 | 0 | 4 | 5 | 19 | 1 | 11 |
| NM_006903 | PPA2 | 1586 | 6 | 4 | 3 | 4 | 5 | 4 | 6 | 5 | 3 | 2 | 1 | 4 | 2 |
| NM_006904 | PRKDC | 13508 | 12 | 8 | 6 | 6 | 9 | 9 | 7 | 7 | 5 | 9 | 8 | 12 | 11 |
| NM_006905 | PSG1 | 2276 | 0 | 0 | 0 | 0 | 0 | 0 | 0 | 0 | 0 | 0 | 0 | 0 | 0 |
| NM_006906 | PTPN5 | 3135 | 6 | 1 | 0 | 0 | 0 | 6 | 0 | 0 | 0 | 0 | 0 | 0 | 0 |
| NM_006907 | PYCR1 | 2059 | 0 | 0 | 0 | 0 | 0 | 0 | 0 | 0 | 0 | 0 | 0 | 0 | 0 |
| NM_006907 | PYCR1 | 2072 | 0 | 0 | 0 | 0 | 0 | 0 | 0 | 0 | 0 | 0 | 0 | 0 | 0 |
| NM_006908 | RAC1 | 2341 | 50 | 29 | 25 | 21 | 32 | 50 | 28 | 18 | 31 | 26 | 26 | 36 | 23 |
| NM_006909 | RASGRF2 | 4078 | 13 | 3 | 3 | 1 | 1 | 13 | 2 | 0 | 2 | 6 | 1 | 1 | 1 |
| NM_006910 | RBBP6 | 6836 | 9 | 5 | 3 | 1 | 5 | 5 | 5 | 3 | 4 | 8 | 6 | 9 | 8 |
| NM_006911 | RLN1 | 967 | 0 | 0 | 0 | 0 | 0 | 0 | 0 | 0 | 0 | 0 | 0 | 0 | 0 |
| NM_006912 | RIT1 | 3425 | 17 | 7 | 5 | 2 | 7 | 4 | 11 | 3 | 11 | 7 | 3 | 17 | 7 |
| NM_006913 | RNF5 | 1137 | 0 | 0 | 0 | 0 | 0 | 0 | 0 | 0 | 0 | 0 | 0 | 0 | 0 |
| NM_006914 | RORB | 3604 | 4 | 1 | 0 | 0 | 1 | 4 | 0 | 0 | 0 | 0 | 0 | 0 | 0 |
| NM_006915 | RP2 | 3829 | 5 | 2 | 2 | 1 | 1 | 1 | 1 | 2 | 3 | 1 | 0 | 5 | 2 |
| NM_006916 | RPE | 2130 | 3 | 2 | 2 | 1 | 2 | 3 | 3 | 2 | 2 | 2 | 1 | 3 | 3 |
| NM_006917 | RXRG | 1842 | 2 | 1 | 1 | 1 | 0 | 2 | 0 | 0 | 1 | 0 | 2 | 0 | 0 |
| NM_006918 | SC5DL | 6946 | 11 | 4 | 2 | 1 | 2 | 8 | 4 | 11 | 2 | 2 | 1 | 2 | 4 |
| NM_006919 | SERPINB3 | 1777 | 0 | 0 | 0 | 0 | 0 | 0 | 0 | 0 | 0 | 0 | 0 | 0 | 0 |
| NM_006920 | SCN1A | 8100 | 6 | 1 | 0 | 0 | 0 | 6 | 0 | 0 | 1 | 0 | 0 | 0 | 0 |
| NM_006922 | SCN3A | 9125 | 5 | 1 | 0 | 0 | 0 | 5 | 1 | 0 | 0 | 0 | 0 | 0 | 0 |
| NM_006923 | SDF2 | 1075 | 6 | 4 | 3 | 4 | 4 | 5 | 6 | 5 | 4 | 3 | 2 | 6 | 4 |
| NM_006924 | SFRS1 | 5443 | 17 | 9 | 7 | 6 | 8 | 8 | 11 | 10 | 8 | 9 | 7 | 17 | 9 |
| NM_006925 | SFRS5 | 1517 | 60 | 44 | 48 | 58 | 30 | 60 | 41 | 54 | 40 | 38 | 20 | 54 | 45 |
| NM_006927 | ST3GAL2 | 4450 | 1 | 0 | 0 | 0 | 0 | 0 | 0 | 0 | 0 | 0 | 0 | 1 | 0 |
| NM_006928 | SILV | 2126 | 0 | 0 | 0 | 0 | 0 | 0 | 0 | 0 | 0 | 0 | 0 | 0 | 0 |
| NM_006929 | SKIV2L | 4170 | 1 | 1 | 0 | 1 | 0 | 1 | 1 | 0 | 0 | 0 | 0 | 1 | 1 |
| NM_006930 | SKP1 | 2699 | 72 | 36 | 28 | 12 | 46 | 72 | 37 | 30 | 23 | 34 | 39 | 29 | 47 |
| NM_006931 | SLC2A3 | 3937 | 25 | 7 | 10 | 2 | 4 | 8 | 2 | 1 | 25 | 3 | 4 | 10 | 4 |
| NM_006932 | SMTN | 3129 | 11 | 3 | 1 | 11 | 3 | 1 | 2 | 1 | 2 | 1 | 1 | 7 | 2 |
| NM_006933 | SLC5A3 | 11576 | 16 | 6 | 8 | 3 | 2 | 5 | 16 | 2 | 3 | 6 | 1 | 3 | 12 |
| NM_006934 | SLC6A9 | 3213 | 1 | 0 | 0 | 0 | 0 | 1 | 0 | 0 | 0 | 0 | 0 | 0 | 0 |
| NM_006936 | SUMO3 | 1807 | 11 | 6 | 5 | 3 | 6 | 11 | 6 | 5 | 6 | 4 | 5 | 8 | 5 |
| NM_006937 | SUMO2 | 1066 | 3 | 2 | 2 | 1 | 2 | 3 | 2 | 1 | 2 | 2 | 1 | 2 | 3 |

| transcript | gene | transcript length (nt) | max | mean | adipose | colon | heart | hypothala mus | kidney | liver | lung | ovary | skeletalm uscle | spleen | testes |
|---|---|---|---|---|---|---|---|---|---|---|---|---|---|---|---|
| NM_006938 | SNRPD1 | 1600 | 10 | 6 | 4 | 4 | 7 | 10 | 7 | 5 | 4 | 6 | 5 | 9 | 6 |
| NM_006939 | SOS2 | 5313 | 8 | 5 | 4 | 2 | 6 | 4 | 6 | 3 | 4 | 5 | 6 | 8 | 7 |
| NM_006940 | SOX5 | 4316 | 4 | 2 | 2 | 0 | 0 | 4 | 1 | 2 | 1 | 3 | 0 | 1 | 3 |
| NM_006941 | SOX10 | 2862 | 2 | 0 | 0 | 0 | 0 | 2 | 0 | 0 | 0 | 0 | 0 | 0 | 0 |
| NM_006942 | SOX15 | 1394 | 0 | 0 | 0 | 0 | 0 | 0 | 0 | 0 | 0 | 0 | 0 | 0 | 0 |
| NM_006943 | SOX12 | 4629 | 1 | 0 | 0 | 1 | 0 | 0 | 1 | 0 | 0 | 1 | 0 | 0 | 0 |
| NM_006944 | SPP2 | 1019 | 10 | 1 | 0 | 0 | 0 | 0 | 0 | 10 | 0 | 0 | 0 | 0 | 0 |
| NM_006945 | SPRR2D | 682 | 0 | 0 | 0 | 0 | 0 | 0 | 0 | 0 | 0 | 0 | 0 | 0 | 0 |
| NM_006946 | SPTBN2 | 7866 | 9 | 2 | 0 | 0 | 0 | 9 | 5 | 2 | 0 | 0 | 0 | 0 | 3 |
| NM_006947 | SRP72 | 3876 | 15 | 10 | 8 | 3 | 9 | 14 | 8 | 15 | 8 | 11 | 6 | 13 | 10 |
| NM_006948 | HSPA13 | 3986 | 6 | 2 | 1 | 1 | 2 | 6 | 2 | 2 | 2 | 2 | 1 | 2 | 3 |
| NM_006949 | STXBP2 | 1890 | 3 | 1 | 2 | 3 | 1 | 0 | 3 | 2 | 3 | 0 | 0 | 3 | 2 |
| NM_006950 | SYN1 | 3210 | 11 | 1 | 0 | 0 | 0 | 11 | 0 | 0 | 0 | 0 | 0 | 0 | 0 |
| NM_006951 | TAF5 | 3268 | 4 | 1 | 1 | 1 | 1 | 1 | 1 | 1 | 1 | 1 | 1 | 2 | 4 |
| NM_006952 | UPK1B | 2058 | 1 | 0 | 0 | 0 | 0 | 0 | 1 | 0 | 0 | 0 | 0 | 0 | 0 |
| NM_006953 | UPK3A | 1045 | 0 | 0 | 0 | 0 | 0 | 0 | 0 | 0 | 0 | 0 | 0 | 0 | 0 |
| NM_006954 | ZNF33A | 6121 | 11 | 6 | 4 | 1 | 5 | 6 | 8 | 3 | 4 | 11 | 7 | 10 | 5 |
| NM_006955 | ZNF33B | 5958 | 7 | 3 | 1 | 1 | 2 | 3 | 6 | 2 | 3 | 7 | 2 | 4 | 3 |
| NM_006956 | ZNF12 | 4949 | 8 | 4 | 3 | 2 | 4 | 4 | 4 | 1 | 3 | 8 | 2 | 7 | 5 |
| NM_006958 | ZNF16 | 2530 | 1 | 1 | 0 | 0 | 1 | 1 | 1 | 0 | 0 | 1 | 1 | 0 | 1 |
| NM_006959 | ZNF17 | 2678 | 3 | 2 | 1 | 1 | 2 | 2 | 2 | 0 | 1 | 3 | 1 | 3 | 2 |
| NM_006961 | ZNF19 | 2743 | 3 | 1 | 1 | 0 | 0 | 1 | 1 | 0 | 1 | 1 | 0 | 1 | 3 |
| NM_006962 | ZNF182 | 3582 | 5 | 2 | 2 | 1 | 2 | 2 | 3 | 1 | 2 | 4 | 1 | 5 | 3 |
| NM_006963 | ZNF22 | 2203 | 12 | 6 | 4 | 2 | 8 | 5 | 6 | 5 | 5 | 9 | 4 | 12 | 6 |
| NM_006965 | ZNF24 | 6311 | 13 | 8 | 4 | 2 | 9 | 9 | 8 | 6 | 8 | 10 | 8 | 13 | 10 |
| NM_006969 | ZNF28 | 4675 | 8 | 4 | 3 | 1 | 4 | 2 | 5 | 1 | 3 | 7 | 2 | 8 | 7 |
| NM_006973 | ZNF32 | 1159 | 11 | 6 | 8 | 2 | 9 | 11 | 7 | 4 | 3 | 8 | 7 | 7 | 5 |
| NM_006974 | ZNF33A | 6118 | 11 | 6 | 4 | 1 | 5 | 6 | 8 | 3 | 4 | 11 | 7 | 10 | 5 |
| NM_006977 | ZBTB25 | 1577 | 1 | 1 | 1 | 1 | 1 | 1 | 1 | 1 | 1 | 1 | 0 | 1 | 1 |
| NM_006978 | RNF113A | 1295 | 22 | 9 | 4 | 8 | 9 | 8 | 7 | 9 | 10 | 9 | 7 | 22 | 7 |
| NM_006979 | SLC39A7 | 2454 | 21 | 9 | 10 | 10 | 9 | 7 | 11 | 21 | 7 | 4 | 2 | 8 | 7 |
| NM_006980 | MTERF | 2163 | 2 | 1 | 1 | 0 | 1 | 1 | 2 | 1 | 1 | 1 | 0 | 2 | 2 |
| NM_006981 | NR4A3 | 5634 | 18 | 4 | 1 | 0 | 9 | 2 | 1 | 1 | 7 | 18 | 8 | 1 | 1 |
| NM_006982 | ALX1 | 1293 | 0 | 0 | 0 | 0 | 0 | 0 | 0 | 0 | 0 | 0 | 0 | 0 | 0 |
| NM_006983 | MMP23B | 1240 | 0 | 0 | 0 | 0 | 0 | 0 | 0 | 0 | 0 | 0 | 0 | 0 | 0 |
| NM_006984 | CLDN10 | 2490 | 3 | 0 | 0 | 0 | 0 | 1 | 3 | 0 | 0 | 0 | 0 | 0 | 0 |
| NM_006985 | NPIP | 1102 | 1 | 0 | 0 | 0 | 0 | 0 | 0 | 0 | 0 | 1 | 0 | 0 | 0 |
| NM_006986 | MAGED1 | 2744 | 26 | 8 | 3 | 4 | 6 | 26 | 6 | 12 | 3 | 8 | 3 | 5 | 11 |
| NM_006987 | RPH3AL | 2583 | 1 | 0 | 0 | 1 | 0 | 0 | 0 | 1 | 0 | 0 | 0 | 1 | 0 |
| NM_006988 | ADAMTS1 | 4651 | 56 | 16 | 15 | 2 | 33 | 7 | 13 | 5 | 20 | 56 | 12 | 7 | 10 |
| NM_006989 | RASA4 | 5606 | 4 | 2 | 2 | 1 | 1 | 2 | 2 | 0 | 1 | 2 | 4 | 3 | 1 |
| NM_006990 | WASF2 | 4270 | 24 | 11 | 11 | 9 | 10 | 7 | 8 | 4 | 13 | 16 | 4 | 24 | 11 |
| NM_006991 | ZNF197 | 4336 | 6 | 3 | 3 | 2 | 3 | 5 | 3 | 1 | 3 | 6 | 3 | 4 | 4 |
| NM_006992 | LRRC23 | 1329 | 3 | 1 | 1 | 0 | 1 | 2 | 1 | 0 | 2 | 1 | 1 | 0 | 3 |
| NM_006993 | NPM3 | 876 | 1 | 1 | 1 | 1 | 0 | 0 | 1 | 1 | 0 | 0 | 0 | 1 | 1 |
| NM_006994 | BTN3A3 | 3001 | 11 | 3 | 5 | 2 | 2 | 2 | 2 | 1 | 4 | 4 | 1 | 11 | 2 |
| NM_006995 | BTN2A2 | 3563 | 3 | 1 | 1 | 1 | 1 | 1 | 1 | 0 | 3 | 1 | 0 | 3 | 2 |
| NM_006996 | SLC19A2 | 3636 | 11 | 2 | 1 | 0 | 3 | 0 | 2 | 2 | 1 | 1 | 11 | 1 | 2 |
| NM_006997 | TACC2 | 3902 | 34 | 8 | 4 | 7 | 15 | 5 | 7 | 1 | 11 | 1 | 34 | 1 | 2 |
| NM_006998 | SCGN | 1479 | 1 | 0 | 0 | 0 | 1 | 0 | 0 | 0 | 0 | 0 | 0 | 0 | 0 |
| NM_006999 | POLS | 3861 | 4 | 3 | 3 | 2 | 2 | 3 | 2 | 3 | 3 | 4 | 2 | 4 | 3 |
| NM_007000 | UPK1A | 1223 | 0 | 0 | 0 | 0 | 0 | 0 | 0 | 0 | 0 | 0 | 0 | 0 | 0 |
| NM_007001 | SLC35D2 | 1625 | 6 | 2 | 1 | 2 | 2 | 1 | 6 | 6 | 3 | 2 | 1 | 2 | 2 |
| NM_007002 | ADRM1 | 1405 | 4 | 2 | 1 | 1 | 2 | 1 | 2 | 3 | 1 | 1 | 4 | 2 | 3 |
| NM_007003 | PAGE4 | 493 | 7 | 1 | 0 | 0 | 0 | 1 | 0 | 0 | 0 | 1 | 0 | 0 | 7 |
| NM_007005 | TLE4 | 4747 | 7 | 2 | 1 | 1 | 1 | 3 | 1 | 0 | 2 | 3 | 3 | 3 | 7 |
| NM_007006 | NUDT21 | 4407 | 18 | 9 | 5 | 3 | 9 | 18 | 10 | 4 | 6 | 13 | 6 | 12 | 11 |
| NM_007007 | CPSF6 | 6616 | 20 | 10 | 4 | 5 | 7 | 11 | 9 | 4 | 11 | 20 | 9 | 15 | 18 |
| NM_007008 | RTN4 | 1779 | 306 | 99 | 191 | 32 | 49 | 306 | 142 | 64 | 69 | 35 | 112 | 58 | 37 |
| NM_007009 | ZPBP | 1224 | 6 | 1 | 0 | 0 | 0 | 0 | 0 | 0 | 0 | 0 | 0 | 0 | 6 |
| NM_007010 | DDX52 | 3766 | 5 | 4 | 3 | 1 | 4 | 3 | 5 | 5 | 3 | 5 | 3 | 5 | 5 |
| NM_007011 | ABHD2 | 9133 | 31 | 9 | 4 | 5 | 10 | 13 | 6 | 31 | 8 | 6 | 2 | 10 | 5 |
| NM_007013 | WWP1 | 4105 | 24 | 14 | 12 | 4 | 23 | 8 | 15 | 24 | 10 | 9 | 19 | 17 | 16 |
| NM_007014 | WWP2 | 4502 | 4 | 2 | 2 | 2 | 2 | 2 | 3 | 2 | 2 | 2 | 2 | 4 | 3 |
| NM_007015 | LECT1 | 1447 | 1 | 0 | 0 | 0 | 0 | 1 | 0 | 0 | 0 | 0 | 0 | 0 | 1 |
| NM_007017 | SOX30 | 2772 | 4 | 0 | 0 | 0 | 0 | 0 | 0 | 0 | 0 | 0 | 0 | 0 | 4 |
| NM_007018 | CEP110 | 7431 | 11 | 4 | 3 | 2 | 2 | 2 | 2 | 1 | 4 | 4 | 1 | 10 | 11 |
| NM_007019 | UBE2C | 810 | 2 | 0 | 0 | 2 | 0 | 0 | 0 | 0 | 0 | 0 | 0 | 2 | 1 |
| NM_007021 | C10orf10 | 2060 | 27 | 8 | 20 | 3 | 4 | 4 | 2 | 2 | 27 | 2 | 6 | 5 | 9 |
| NM_007022 | CYB561D2 | 1200 | 1 | 0 | 0 | 0 | 0 | 0 | 1 | 0 | 0 | 0 | 0 | 1 | 0 |
| NM_007023 | RAPGEF4 | 4348 | 31 | 4 | 1 | 0 | 2 | 31 | 1 | 1 | 3 | 2 | 0 | 0 | 2 |
| NM_007024 | TMEM115 | 2095 | 1 | 1 | 0 | 1 | 1 | 0 | 1 | 1 | 0 | 1 | 0 | 1 | 1 |
| NM_007026 | DUSP14 | 1508 | 2 | 1 | 2 | 0 | 1 | 2 | 1 | 1 | 1 | 1 | 0 | 0 | 1 |
| NM_007027 | TOPBP1 | 5346 | 7 | 3 | 3 | 2 | 3 | 4 | 2 | 2 | 3 | 4 | 2 | 6 | 7 |
| NM_007028 | TRIM31 | 2027 | 5 | 0 | 0 | 5 | 0 | 0 | 0 | 0 | 0 | 0 | 0 | 0 | 0 |
| NM_007029 | STMN2 | 708 | 139 | 13 | 0 | 3 | 0 | 139 | 0 | 0 | 0 | 0 | 0 | 0 | 0 |
| NM_007030 | TPPP | 6022 | 28 | 3 | 0 | 1 | 1 | 28 | 1 | 0 | 1 | 0 | 1 | 1 | 1 |

| transcript | gene | transcript length (nt) | max | mean | adipose | colon | heart | hypothalamus | kidney | liver | lung | ovary | skeletalmuscle | spleen | testes |
|---|---|---|---|---|---|---|---|---|---|---|---|---|---|---|---|
| NM_007031 | HSF2BP | 1899 | 5 | 1 | 0 | 0 | 0 | 0 | 0 | 0 | 0 | 0 | 0 | 0 | 5 |
| NM_007032 | TRIOBP | 4739 | 6 | 3 | 3 | 4 | 6 | 1 | 3 | 1 | 2 | 3 | 1 | 6 | 2 |
| NM_007033 | RER1 | 3100 | 13 | 5 | 3 | 4 | 4 | 4 | 6 | 13 | 3 | 4 | 4 | 5 | 5 |
| NM_007034 | DNAJB4 | 2237 | 51 | 19 | 15 | 2 | 51 | 21 | 11 | 6 | 20 | 17 | 24 | 27 | 16 |
| NM_007035 | KERA | 2530 | 0 | 0 | 0 | 0 | 0 | 0 | 0 | 0 | 0 | 0 | 0 | 0 | 0 |
| NM_007036 | ESM1 | 2088 | 2 | 0 | 0 | 0 | 0 | 0 | 2 | 0 | 1 | 0 | 0 | 1 | 0 |
| NM_007037 | ADAMTS8 | 3663 | 1 | 0 | 0 | 0 | 0 | 0 | 0 | 0 | 1 | 0 | 0 | 0 | 0 |
| NM_007038 | ADAMTS5 | 9662 | 6 | 2 | 3 | 0 | 1 | 0 | 1 | 0 | 0 | 6 | 1 | 6 | 1 |
| NM_007039 | PTPN21 | 6215 | 4 | 2 | 2 | 2 | 3 | 1 | 2 | 1 | 3 | 4 | 1 | 1 | 4 |
| NM_007040 | HNRNPUL1 | 4201 | 16 | 10 | 8 | 9 | 11 | 10 | 8 | 7 | 9 | 15 | 9 | 16 | 11 |
| NM_007041 | ATE1 | 2209 | 11 | 6 | 3 | 3 | 7 | 11 | 7 | 7 | 4 | 5 | 8 | 6 | 7 |
| NM_007042 | RPP14 | 3264 | 4 | 2 | 1 | 2 | 3 | 2 | 3 | 3 | 1 | 2 | 4 | 2 | 2 |
| NM_007043 | KRR1 | 3357 | 12 | 8 | 7 | 3 | 7 | 12 | 7 | 9 | 6 | 8 | 10 | 10 | 7 |
| NM_007044 | KATNA1 | 1521 | 5 | 2 | 1 | 1 | 2 | 2 | 2 | 1 | 2 | 3 | 2 | 2 | 5 |
| NM_007045 | FGFR1OP | 1885 | 31 | 8 | 2 | 3 | 5 | 6 | 10 | 7 | 3 | 7 | 2 | 7 | 31 |
| NM_007046 | EMILIN1 | 3930 | 9 | 4 | 2 | 8 | 5 | 1 | 2 | 1 | 3 | 9 | 0 | 8 | 3 |
| NM_007047 | BTN3A2 | 3812 | 13 | 4 | 4 | 4 | 2 | 2 | 2 | 2 | 6 | 3 | 1 | 13 | 1 |
| NM_007048 | BTN3A1 | 3432 | 30 | 9 | 6 | 5 | 5 | 3 | 5 | 2 | 18 | 15 | 4 | 30 | 6 |
| NM_007049 | BTN2A1 | 2884 | 10 | 5 | 4 | 7 | 5 | 6 | 3 | 1 | 6 | 3 | 2 | 10 | 6 |
| NM_007050 | PTPRT | 12637 | 6 | 1 | 0 | 0 | 0 | 6 | 0 | 0 | 0 | 0 | 0 | 0 | 0 |
| NM_007051 | FAF1 | 2583 | 14 | 8 | 7 | 5 | 10 | 11 | 7 | 8 | 5 | 7 | 14 | 6 | 10 |
| NM_007052 | NOX1 | 2529 | 3 | 0 | 0 | 3 | 0 | 0 | 0 | 0 | 0 | 0 | 0 | 0 | 0 |
| NM_007053 | CD160 | 1559 | 4 | 1 | 0 | 1 | 0 | 0 | 0 | 0 | 1 | 0 | 0 | 4 | 0 |
| NM_007054 | KIF3A | 6257 | 40 | 6 | 1 | 1 | 3 | 40 | 4 | 0 | 3 | 4 | 1 | 2 | 10 |
| NM_007055 | POLR3A | 5599 | 3 | 1 | 1 | 1 | 1 | 3 | 1 | 1 | 1 | 1 | 1 | 1 | 2 |
| NM_007056 | SFRS16 | 2234 | 2 | 1 | 1 | 2 | 0 | 1 | 1 | 0 | 1 | 1 | 1 | 2 | 1 |
| NM_007057 | ZWINT | 1660 | 3 | 1 | 1 | 3 | 1 | 0 | 1 | 0 | 1 | 1 | 0 | 2 | 3 |
| NM_007058 | CAPN11 | 2728 | 2 | 0 | 0 | 0 | 0 | 0 | 0 | 0 | 0 | 0 | 0 | 0 | 2 |
| NM_007059 | KPTN | 1688 | 0 | 0 | 0 | 0 | 0 | 0 | 0 | 0 | 0 | 0 | 0 | 0 | 0 |
| NM_007062 | PWP1 | 1853 | 7 | 4 | 7 | 3 | 2 | 7 | 4 | 5 | 3 | 4 | 2 | 5 | 5 |
| NM_007064 | KALRN | 5751 | 5 | 2 | 1 | 1 | 2 | 5 | 1 | 1 | 1 | 1 | 1 | 5 | 2 |
| NM_007065 | CDC37 | 1671 | 40 | 22 | 15 | 13 | 35 | 17 | 23 | 13 | 22 | 17 | 27 | 40 | 26 |
| NM_007066 | PKIG | 1181 | 14 | 7 | 5 | 6 | 14 | 9 | 7 | 5 | 14 | 4 | 5 | 4 | 8 |
| NM_007067 | MYST2 | 3556 | 19 | 7 | 4 | 4 | 6 | 9 | 6 | 3 | 5 | 9 | 7 | 7 | 19 |
| NM_007068 | DMC1 | 2269 | 2 | 0 | 0 | 0 | 0 | 0 | 0 | 0 | 0 | 0 | 0 | 0 | 2 |
| NM_007069 | PLA2G16 | 1385 | 25 | 6 | 25 | 1 | 6 | 8 | 6 | 7 | 2 | 3 | 3 | 1 | 4 |
| NM_007072 | HHLA2 | 2660 | 3 | 1 | 0 | 3 | 0 | 0 | 2 | 0 | 1 | 0 | 0 | 0 | 0 |
| NM_007073 | BVES | 5435 | 12 | 3 | 1 | 1 | 11 | 1 | 1 | 0 | 1 | 0 | 12 | 0 | 3 |
| NM_007074 | CORO1A | 1620 | 2 | 0 | 0 | 0 | 0 | 1 | 0 | 0 | 0 | 0 | 0 | 2 | 0 |
| NM_007075 | WDR45 | 1895 | 7 | 4 | 5 | 3 | 7 | 6 | 5 | 4 | 3 | 4 | 4 | 4 | 3 |
| NM_007076 | FICD | 1651 | 1 | 0 | 0 | 0 | 0 | 1 | 0 | 1 | 0 | 0 | 0 | 0 | 1 |
| NM_007077 | AP4S1 | 1782 | 2 | 1 | 0 | 0 | 1 | 2 | 1 | 0 | 1 | 1 | 0 | 1 | 1 |
| NM_007078 | LDB3 | 5298 | 52 | 9 | 0 | 1 | 38 | 1 | 0 | 0 | 0 | 0 | 52 | 0 | 1 |
| NM_007079 | PTP4A3 | 1257 | 14 | 3 | 1 | 0 | 14 | 1 | 1 | 0 | 1 | 1 | 11 | 1 | 1 |
| NM_007080 | LSM6 | 787 | 21 | 12 | 15 | 6 | 12 | 20 | 9 | 12 | 9 | 12 | 6 | 21 | 14 |
| NM_007081 | RABL2B | 2183 | 1 | 0 | 0 | 0 | 0 | 1 | 1 | 0 | 1 | 0 | 0 | 1 | 0 |
| NM_007082 | RABL2A | 1126 | 0 | 0 | 0 | 0 | 0 | 0 | 0 | 0 | 0 | 0 | 0 | 0 | 0 |
| NM_007083 | NUDT6 | 1167 | 0 | 0 | 0 | 0 | 0 | 0 | 0 | 0 | 0 | 0 | 0 | 0 | 0 |
| NM_007084 | SOX21 | 2511 | 0 | 0 | 0 | 0 | 0 | 0 | 0 | 0 | 0 | 0 | 0 | 0 | 0 |
| NM_007085 | FSTL1 | 3839 | 84 | 29 | 84 | 18 | 67 | 12 | 13 | 5 | 22 | 34 | 10 | 29 | 28 |
| NM_007086 | WDHD1 | 4734 | 2 | 1 | 1 | 0 | 0 | 1 | 1 | 0 | 1 | 1 | 0 | 1 | 2 |
| NM_007088 | CALB2 | 1366 | 9 | 2 | 9 | 0 | 0 | 6 | 0 | 0 | 0 | 1 | 0 | 0 | 1 |
| NM_007096 | CLTA | 1194 | 16 | 8 | 10 | 6 | 6 | 16 | 9 | 8 | 7 | 7 | 3 | 9 | 7 |
| NM_007097 | CLTB | 1185 | 20 | 11 | 10 | 15 | 20 | 18 | 8 | 9 | 6 | 5 | 11 | 6 | 6 |
| NM_007098 | CLTCL1 | 5512 | 16 | 2 | 1 | 0 | 1 | 1 | 0 | 0 | 0 | 0 | 16 | 0 | 2 |
| NM_007099 | ACP1 | 1549 | 43 | 29 | 16 | 10 | 35 | 31 | 39 | 37 | 29 | 24 | 20 | 32 | 43 |
| NM_007100 | ATP5I | 360 | 75 | 29 | 30 | 28 | 75 | 39 | 40 | 28 | 15 | 12 | 25 | 17 | 14 |
| NM_007101 | SARDH | 3375 | 1 | 0 | 0 | 0 | 0 | 0 | 0 | 1 | 0 | 0 | 0 | 0 | 0 |
| NM_007102 | GUCA2B | 597 | 0 | 0 | 0 | 0 | 0 | 0 | 0 | 0 | 0 | 0 | 0 | 0 | 0 |
| NM_007103 | NDUFV1 | 1631 | 29 | 13 | 9 | 16 | 24 | 16 | 29 | 12 | 6 | 5 | 11 | 9 | 7 |
| NM_007104 | RPL10A | 719 | 46 | 19 | 17 | 14 | 14 | 11 | 14 | 22 | 16 | 46 | 22 | 31 | 10 |
| NM_007105 | SLC22A18AS | 1546 | 1 | 0 | 0 | 0 | 0 | 0 | 1 | 0 | 0 | 0 | 0 | 0 | 0 |
| NM_007106 | UBL3 | 4345 | 9 | 5 | 4 | 2 | 3 | 9 | 5 | 6 | 6 | 5 | 3 | 6 | 5 |
| NM_007107 | SSR3 | 3064 | 37 | 17 | 15 | 6 | 29 | 18 | 13 | 37 | 12 | 17 | 9 | 16 | 18 |
| NM_007108 | TCEB2 | 990 | 2 | 2 | 2 | 2 | 2 | 2 | 2 | 2 | 2 | 1 | 2 | 2 | 2 |
| NM_007109 | TCF19 | 3306 | 1 | 1 | 1 | 1 | 1 | 0 | 0 | 0 | 1 | 1 | 0 | 1 | 1 |
| NM_007109 | TCF19 | 3307 | 1 | 1 | 1 | 1 | 1 | 0 | 0 | 0 | 1 | 1 | 0 | 1 | 1 |
| NM_007110 | TEP1 | 10693 | 3 | 1 | 1 | 3 | 1 | 1 | 1 | 1 | 1 | 1 | 1 | 3 | 1 |
| NM_007111 | TFDP1 | 2649 | 3 | 2 | 3 | 2 | 2 | 2 | 2 | 2 | 1 | 2 | 2 | 3 | 3 |
| NM_007112 | THBS3 | 3147 | 2 | 1 | 1 | 2 | 1 | 1 | 1 | 0 | 1 | 1 | 0 | 1 | 1 |
| NM_007113 | TCHH | 6900 | 6 | 1 | 0 | 0 | 0 | 0 | 0 | 0 | 0 | 0 | 0 | 6 | 1 |
| NM_007114 | TMF1 | 6879 | 16 | 10 | 6 | 2 | 11 | 7 | 10 | 6 | 14 | 13 | 11 | 15 | 16 |
| NM_007115 | TNFAIP6 | 1423 | 3 | 1 | 1 | 0 | 2 | 0 | 0 | 0 | 3 | 0 | 0 | 1 | 0 |
| NM_007117 | TRH | 2007 | 1 | 0 | 0 | 0 | 0 | 1 | 0 | 0 | 0 | 0 | 0 | 0 | 0 |
| NM_007118 | TRIO | 10227 | 6 | 4 | 4 | 6 | 3 | 5 | 2 | 2 | 3 | 5 | 2 | 5 | 4 |
| NM_007120 | UGT1A4 | 2374 | 7 | 1 | 0 | 0 | 0 | 0 | 1 | 7 | 0 | 0 | 0 | 0 | 0 |
| NM_007121 | NR1H2 | 2067 | 4 | 2 | 2 | 2 | 2 | 2 | 3 | 2 | 4 | 1 | 2 | 3 | 2 |

| transcript | gene | transcript length (nt) | max | mean | adipose | colon | heart | hypothalamus | kidney | liver | lung | ovary | skeletalmuscle | spleen | testes |
|---|---|---|---|---|---|---|---|---|---|---|---|---|---|---|---|
| NM_007122 | USF1 | 1797 | 6 | 3 | 4 | 2 | 2 | 5 | 2 | 2 | 3 | 2 | 2 | 6 | 2 |
| NM_007123 | USH2A | 6314 | 0 | 0 | 0 | 0 | 0 | 0 | 0 | 0 | 0 | 0 | 0 | 0 | 0 |
| NM_007124 | UTRN | 12431 | 53 | 26 | 39 | 9 | 26 | 14 | 19 | 8 | 40 | 40 | 13 | 53 | 20 |
| NM_007125 | UTY | 6522 | 4 | 1 | 2 | 0 | 1 | 2 | 1 | 1 | 1 | 0 | 0 | 4 | 3 |
| NM_007126 | VCP | 3859 | 41 | 27 | 28 | 15 | 37 | 36 | 23 | 41 | 19 | 17 | 32 | 32 | 19 |
| NM_007127 | VIL1 | 2738 | 9 | 1 | 0 | 9 | 0 | 0 | 3 | 0 | 0 | 0 | 0 | 0 | 0 |
| NM_007128 | VPREB1 | 641 | 0 | 0 | 0 | 0 | 0 | 0 | 0 | 0 | 0 | 0 | 0 | 0 | 0 |
| NM_007129 | ZIC2 | 2682 | 1 | 0 | 0 | 0 | 0 | 1 | 0 | 0 | 0 | 0 | 0 | 0 | 0 |
| NM_007130 | ZNF41 | 4375 | 4 | 2 | 2 | 1 | 4 | 3 | 3 | 1 | 2 | 2 | 4 | 4 | 2 |
| NM_007131 | ZNF75D | 5538 | 4 | 2 | 1 | 1 | 2 | 2 | 3 | 1 | 2 | 2 | 1 | 4 | 1 |
| NM_007134 | ZNF135 | 3331 | 4 | 1 | 1 | 1 | 0 | 2 | 1 | 0 | 0 | 4 | 1 | 1 | 2 |
| NM_007135 | ZNF79 | 2086 | 2 | 1 | 1 | 1 | 1 | 1 | 1 | 1 | 1 | 2 | 2 | 1 | 2 |
| NM_007136 | ZNF80 | 2945 | 0 | 0 | 0 | 0 | 0 | 0 | 0 | 0 | 0 | 0 | 0 | 0 | 0 |
| NM_007137 | ZNF81 | 7933 | 2 | 1 | 1 | 0 | 1 | 1 | 2 | 0 | 1 | 2 | 0 | 2 | 1 |
| NM_007138 | ZNF90 | 3753 | 7 | 2 | 0 | 1 | 1 | 1 | 2 | 0 | 1 | 7 | 0 | 2 | 2 |
| NM_007139 | ZNF92 | 2987 | 11 | 5 | 2 | 1 | 3 | 6 | 5 | 2 | 4 | 10 | 3 | 11 | 9 |
| NM_007144 | PCGF2 | 2604 | 1 | 0 | 0 | 0 | 0 | 0 | 1 | 0 | 1 | 1 | 0 | 0 | 0 |
| NM_007145 | ZNF146 | 3856 | 41 | 22 | 13 | 5 | 29 | 19 | 23 | 16 | 20 | 41 | 25 | 33 | 22 |
| NM_007146 | VEZF1 | 4633 | 8 | 4 | 2 | 2 | 5 | 3 | 4 | 2 | 4 | 7 | 8 | 5 | 4 |
| NM_007147 | ZNF175 | 3785 | 3 | 2 | 1 | 1 | 1 | 3 | 2 | 1 | 1 | 3 | 1 | 3 | 2 |
| NM_007148 | RNF112 | 3225 | 2 | 0 | 0 | 1 | 0 | 2 | 0 | 0 | 0 | 0 | 0 | 0 | 0 |
| NM_007149 | ZNF184 | 3101 | 7 | 3 | 2 | 1 | 2 | 7 | 4 | 1 | 2 | 7 | 2 | 4 | 5 |
| NM_007150 | ZNF185 | 4312 | 1 | 0 | 0 | 0 | 0 | 0 | 1 | 0 | 1 | 0 | 0 | 0 | 1 |
| NM_007152 | ZNF195 | 2189 | 12 | 7 | 4 | 3 | 7 | 10 | 10 | 3 | 3 | 8 | 8 | 12 | 7 |
| NM_007153 | ZNF208 | 9086 | 8 | 3 | 1 | 1 | 4 | 1 | 4 | 0 | 1 | 4 | 0 | 4 | 8 |
| NM_007155 | ZP3 | 1477 | 0 | 0 | 0 | 0 | 0 | 0 | 0 | 0 | 0 | 0 | 0 | 0 | 0 |
| NM_007156 | ZXDA | 5202 | 3 | 1 | 1 | 1 | 1 | 1 | 1 | 1 | 1 | 3 | 1 | 2 | 1 |
| NM_007157 | ZXDB | 5642 | 4 | 2 | 1 | 1 | 2 | 2 | 1 | 3 | 2 | 4 | 2 | 2 | 1 |
| NM_007158 | CSDE1 | 4023 | 395 | 142 | 172 | 41 | 194 | 101 | 88 | 97 | 94 | 129 | 395 | 114 | 135 |
| NM_007159 | SLMAP | 4420 | 48 | 14 | 6 | 5 | 48 | 9 | 11 | 6 | 9 | 14 | 18 | 10 | 20 |
| NM_007160 | OR2H2 | 1063 | 0 | 0 | 0 | 0 | 0 | 0 | 0 | 0 | 0 | 0 | 0 | 0 | 0 |
| NM_007161 | LST1 | 647 | 6 | 1 | 0 | 0 | 0 | 1 | 0 | 0 | 1 | 0 | 0 | 6 | 0 |
| NM_007162 | TFEB | 2353 | 1 | 0 | 0 | 0 | 0 | 0 | 0 | 0 | 0 | 0 | 1 | 0 | 0 |
| NM_007163 | SLC14A2 | 4155 | 0 | 0 | 0 | 0 | 0 | 0 | 0 | 0 | 0 | 0 | 0 | 0 | 0 |
| NM_007165 | SF3A2 | 1650 | 4 | 2 | 2 | 3 | 1 | 2 | 1 | 0 | 2 | 4 | 2 | 3 | 3 |
| NM_007166 | PICALM | 3849 | 31 | 15 | 19 | 4 | 15 | 14 | 21 | 12 | 17 | 12 | 7 | 31 | 14 |
| NM_007167 | ZMYM6 | 5144 | 2 | 1 | 1 | 1 | 1 | 1 | 2 | 1 | 1 | 2 | 1 | 2 | 2 |
| NM_007168 | ABCA8 | 5720 | 8 | 4 | 5 | 1 | 8 | 2 | 2 | 2 | 3 | 8 | 2 | 4 | 8 |
| NM_007169 | PEMT | 1008 | 2 | 1 | 2 | 0 | 0 | 0 | 0 | 2 | 0 | 0 | 0 | 0 | 0 |
| NM_007170 | TESK2 | 3076 | 2 | 1 | 1 | 1 | 1 | 2 | 0 | 1 | 1 | 2 | 2 | 1 | 2 |
| NM_007171 | POMT1 | 3146 | 1 | 1 | 0 | 1 | 0 | 1 | 1 | 0 | 0 | 0 | 0 | 1 | 1 |
| NM_007172 | NUP50 | 5223 | 17 | 8 | 5 | 5 | 5 | 10 | 6 | 14 | 8 | 7 | 4 | 17 | 11 |
| NM_007173 | PRSS23 | 3804 | 7 | 4 | 4 | 3 | 5 | 2 | 5 | 2 | 7 | 6 | 2 | 2 | 7 |
| NM_007174 | CIT | 8576 | 6 | 1 | 0 | 1 | 0 | 6 | 2 | 0 | 1 | 0 | 0 | 1 | 1 |
| NM_007175 | ERLIN2 | 4821 | 11 | 6 | 5 | 3 | 4 | 6 | 11 | 10 | 3 | 7 | 3 | 5 | 6 |
| NM_007176 | C14orf1 | 1530 | 5 | 2 | 1 | 2 | 1 | 2 | 2 | 5 | 2 | 1 | 1 | 1 | 5 |
| NM_007177 | FAM107A | 3464 | 49 | 7 | 1 | 1 | 7 | 49 | 2 | 0 | 5 | 1 | 1 | 3 | 1 |
| NM_007178 | STRAP | 1905 | 35 | 21 | 16 | 6 | 35 | 35 | 22 | 20 | 14 | 16 | 20 | 20 | 22 |
| NM_007179 | INSL6 | 708 | 0 | 0 | 0 | 0 | 0 | 0 | 0 | 0 | 0 | 0 | 0 | 0 | 0 |
| NM_007180 | TREH | 1853 | 1 | 0 | 0 | 0 | 0 | 0 | 1 | 0 | 0 | 0 | 0 | 0 | 0 |
| NM_007181 | MAP4K1 | 2779 | 2 | 0 | 0 | 0 | 0 | 0 | 0 | 0 | 0 | 0 | 0 | 2 | 0 |
| NM_007182 | RASSF1 | 1949 | 3 | 1 | 3 | 1 | 1 | 1 | 1 | 1 | 2 | 1 | 1 | 3 | 1 |
| NM_007183 | PKP3 | 2809 | 1 | 0 | 0 | 1 | 0 | 0 | 0 | 0 | 0 | 0 | 0 | 0 | 0 |
| NM_007184 | NISCH | 5239 | 6 | 2 | 1 | 2 | 1 | 6 | 2 | 1 | 2 | 2 | 1 | 2 | 6 |
| NM_007185 | TNRC4 | 3297 | 5 | 0 | 0 | 0 | 0 | 5 | 0 | 0 | 0 | 0 | 0 | 0 | 0 |
| NM_007186 | CEP250 | 8348 | 4 | 2 | 2 | 2 | 2 | 4 | 1 | 1 | 1 | 3 | 1 | 4 | 3 |
| NM_007187 | WBP4 | 2333 | 6 | 4 | 4 | 1 | 6 | 6 | 3 | 2 | 3 | 3 | 6 | 3 | 5 |
| NM_007188 | ABCB8 | 4683 | 0 | 0 | 0 | 0 | 0 | 0 | 0 | 0 | 0 | 0 | 0 | 0 | 0 |
| NM_007189 | ABCF2 | 3547 | 6 | 4 | 3 | 2 | 5 | 6 | 3 | 3 | 2 | 2 | 6 | 4 | 4 |
| NM_007190 | SEC23IP | 4243 | 4 | 3 | 2 | 1 | 2 | 2 | 3 | 4 | 2 | 2 | 2 | 4 | 4 |
| NM_007191 | WIF1 | 2008 | 9 | 1 | 0 | 0 | 0 | 5 | 0 | 0 | 9 | 0 | 0 | 0 | 0 |
| NM_007192 | SUPT16H | 4683 | 18 | 10 | 10 | 5 | 9 | 15 | 10 | 8 | 7 | 14 | 6 | 14 | 18 |
| NM_007193 | ANXA10 | 1428 | 7 | 1 | 0 | 0 | 0 | 0 | 0 | 7 | 0 | 0 | 0 | 1 | 0 |
| NM_007194 | CHEK2 | 1858 | 2 | 1 | 0 | 1 | 0 | 1 | 0 | 0 | 1 | 1 | 0 | 2 | 1 |
| NM_007195 | POLI | 6058 | 3 | 2 | 2 | 1 | 1 | 3 | 3 | 1 | 1 | 2 | 1 | 2 | 3 |
| NM_007196 | KLK8 | 1013 | 0 | 0 | 0 | 0 | 0 | 0 | 0 | 0 | 0 | 0 | 0 | 0 | 0 |
| NM_007197 | FZD10 | 3254 | 0 | 0 | 0 | 0 | 0 | 0 | 0 | 0 | 0 | 0 | 0 | 0 | 0 |
| NM_007198 | PROSC | 2568 | 9 | 5 | 5 | 3 | 6 | 9 | 7 | 9 | 3 | 3 | 4 | 5 | 3 |
| NM_007199 | IRAK3 | 8334 | 15 | 3 | 2 | 1 | 4 | 1 | 1 | 1 | 7 | 1 | 1 | 15 | 1 |
| NM_007200 | AKAP13 | 13290 | 22 | 10 | 6 | 7 | 22 | 4 | 5 | 6 | 18 | 9 | 7 | 18 | 10 |
| NM_007202 | AKAP10 | 2916 | 9 | 5 | 5 | 3 | 3 | 9 | 4 | 2 | 5 | 5 | 2 | 8 | 7 |
| NM_007203 | PALM2-AKA | 7506 | 0 | 0 | 0 | 0 | 0 | 0 | 0 | 0 | 0 | 0 | 0 | 0 | 0 |
| NM_007204 | DDX20 | 3509 | 20 | 5 | 2 | 1 | 3 | 3 | 3 | 3 | 3 | 4 | 3 | 5 | 20 |
| NM_007207 | DUSP10 | 2619 | 4 | 1 | 2 | 1 | 1 | 1 | 0 | 4 | 2 | 1 | 3 | 1 | 1 |
| NM_007208 | MRPL3 | 1733 | 6 | 3 | 3 | 2 | 3 | 5 | 4 | 6 | 2 | 2 | 2 | 3 | 3 |
| NM_007209 | RPL35 | 460 | 112 | 73 | 60 | 48 | 79 | 53 | 47 | 89 | 63 | 112 | 107 | 101 | 48 |
| NM_007210 | GALNT6 | 4520 | 1 | 0 | 0 | 0 | 0 | 1 | 0 | 0 | 0 | 0 | 0 | 1 | 0 |

| transcript | gene | transcript length (nt) | max | mean | adipose | colon | heart | hypothalamus | kidney | liver | lung | ovary | skeletalmuscle | spleen | testes |
|---|---|---|---|---|---|---|---|---|---|---|---|---|---|---|---|
| NM_007211 | RASSF8 | 2317 | 13 | 7 | 7 | 1 | 7 | 13 | 9 | 2 | 6 | 11 | 5 | 3 | 8 |
| NM_007212 | RNF2 | 3551 | 3 | 1 | 1 | 1 | 1 | 2 | 1 | 1 | 1 | 2 | 1 | 2 | 3 |
| NM_007213 | PRAF2 | 1263 | 12 | 4 | 2 | 2 | 5 | 12 | 3 | 1 | 2 | 5 | 1 | 4 | 4 |
| NM_007214 | SEC63 | 6500 | 27 | 15 | 13 | 3 | 14 | 16 | 12 | 22 | 12 | 27 | 7 | 13 | 21 |
| NM_007215 | POLG2 | 1592 | 2 | 1 | 1 | 2 | 1 | 1 | 1 | 1 | 1 | 1 | 1 | 1 | 2 |
| NM_007216 | HPS5 | 4723 | 10 | 3 | 1 | 1 | 2 | 1 | 1 | 10 | 2 | 2 | 1 | 3 | 5 |
| NM_007217 | PDCD10 | 1438 | 29 | 18 | 17 | 8 | 22 | 25 | 19 | 15 | 17 | 11 | 8 | 29 | 25 |
| NM_007218 | RNF139 | 3312 | 5 | 2 | 2 | 1 | 2 | 2 | 3 | 2 | 2 | 3 | 1 | 3 | 5 |
| NM_007219 | RNF24 | 3358 | 1 | 1 | 1 | 0 | 1 | 1 | 1 | 0 | 1 | 1 | 0 | 1 | 1 |
| NM_007220 | CA5B | 6032 | 4 | 1 | 0 | 0 | 1 | 1 | 1 | 0 | 1 | 2 | 0 | 1 | 4 |
| NM_007221 | PMF1 | 1082 | 2 | 1 | 1 | 1 | 1 | 2 | 1 | 1 | 1 | 1 | 1 | 1 | 0 |
| NM_007222 | ZHX1 | 4920 | 24 | 16 | 21 | 3 | 16 | 24 | 15 | 23 | 10 | 18 | 12 | 18 | 13 |
| NM_007223 | GPR176 | 2816 | 1 | 0 | 0 | 0 | 0 | 1 | 0 | 0 | 0 | 0 | 0 | 0 | 0 |
| NM_007224 | NXPH4 | 1803 | 0 | 0 | 0 | 0 | 0 | 0 | 0 | 0 | 0 | 0 | 0 | 0 | 0 |
| NM_007225 | NXPH3 | 5550 | 1 | 0 | 0 | 1 | 1 | 1 | 0 | 0 | 0 | 1 | 0 | 0 | 1 |
| NM_007226 | NXPH2 | 2559 | 2 | 0 | 0 | 0 | 0 | 2 | 1 | 0 | 0 | 0 | 0 | 0 | 0 |
| NM_007227 | GPR45 | 1725 | 0 | 0 | 0 | 0 | 0 | 0 | 0 | 0 | 0 | 0 | 0 | 0 | 0 |
| NM_007229 | PACSIN2 | 2109 | 18 | 11 | 6 | 14 | 10 | 11 | 16 | 9 | 15 | 8 | 4 | 18 | 8 |
| NM_007231 | SLC6A14 | 4535 | 4 | 0 | 0 | 0 | 0 | 0 | 0 | 0 | 4 | 0 | 0 | 0 | 0 |
| NM_007232 | HRH3 | 2680 | 0 | 0 | 0 | 0 | 0 | 0 | 0 | 0 | 0 | 0 | 0 | 0 | 0 |
| NM_007234 | DCTN3 | 827 | 10 | 4 | 5 | 3 | 4 | 10 | 3 | 5 | 2 | 2 | 3 | 2 | 2 |
| NM_007235 | XPOT | 3867 | 17 | 10 | 11 | 3 | 10 | 15 | 8 | 17 | 9 | 6 | 6 | 13 | 12 |
| NM_007236 | CHP | 3228 | 13 | 5 | 6 | 3 | 4 | 5 | 6 | 13 | 4 | 2 | 3 | 3 | 2 |
| NM_007237 | SP140 | 3250 | 11 | 1 | 0 | 1 | 0 | 0 | 0 | 0 | 1 | 0 | 0 | 11 | 0 |
| NM_007238 | PXMP4 | 5724 | 1 | 1 | 1 | 0 | 1 | 1 | 1 | 0 | 1 | 1 | 0 | 1 | 0 |
| NM_007240 | DUSP12 | 1249 | 6 | 3 | 1 | 2 | 2 | 3 | 4 | 2 | 3 | 2 | 1 | 6 | 4 |
| NM_007241 | SNF8 | 1213 | 11 | 6 | 5 | 4 | 8 | 7 | 6 | 11 | 4 | 3 | 3 | 7 | 5 |
| NM_007242 | DDX19B | 1815 | 3 | 2 | 2 | 1 | 3 | 1 | 3 | 3 | 2 | 2 | 2 | 3 | 3 |
| NM_007243 | NRM | 1419 | 1 | 0 | 0 | 0 | 0 | 0 | 0 | 0 | 0 | 0 | 0 | 1 | 0 |
| NM_007244 | PRR4 | 564 | 797 | 78 | 4 | 3 | 4 | 17 | 7 | 1 | 797 | 7 | 3 | 5 | 2 |
| NM_007245 | ATXN2L | 4367 | 17 | 8 | 3 | 5 | 7 | 7 | 7 | 4 | 11 | 9 | 6 | 14 | 17 |
| NM_007246 | KLHL2 | 3136 | 27 | 9 | 5 | 3 | 5 | 27 | 6 | 14 | 8 | 2 | 2 | 16 | 6 |
| NM_007247 | SYNRG | 8170 | 6 | 3 | 2 | 2 | 2 | 4 | 4 | 2 | 2 | 3 | 2 | 6 | 4 |
| NM_007249 | KLF12 | 10909 | 4 | 2 | 1 | 1 | 2 | 3 | 3 | 3 | 1 | 3 | 1 | 4 | 2 |
| NM_007250 | KLF8 | 5419 | 2 | 1 | 2 | 1 | 1 | 1 | 2 | 0 | 1 | 1 | 0 | 1 | 1 |
| NM_007252 | POU6F2 | 2324 | 1 | 0 | 0 | 0 | 0 | 1 | 0 | 0 | 0 | 0 | 0 | 0 | 1 |
| NM_007253 | CYP4F8 | 1905 | 0 | 0 | 0 | 0 | 0 | 0 | 0 | 0 | 0 | 0 | 0 | 0 | 0 |
| NM_007254 | PNKP | 1716 | 2 | 1 | 1 | 2 | 1 | 1 | 1 | 1 | 1 | 1 | 1 | 2 | 1 |
| NM_007255 | B4GALT7 | 1725 | 0 | 0 | 0 | 0 | 0 | 0 | 0 | 0 | 0 | 0 | 0 | 0 | 0 |
| NM_007256 | SLCO2B1 | 4336 | 9 | 3 | 3 | 1 | 3 | 4 | 2 | 9 | 5 | 1 | 1 | 8 | 1 |
| NM_007257 | PNMA2 | 4846 | 53 | 6 | 0 | 1 | 1 | 53 | 2 | 0 | 2 | 0 | 0 | 0 | 1 |
| NM_007259 | VPS45 | 2817 | 6 | 3 | 5 | 2 | 4 | 6 | 4 | 2 | 2 | 2 | 2 | 3 | 3 |
| NM_007260 | LYPLA2 | 1624 | 2 | 1 | 0 | 1 | 0 | 1 | 2 | 2 | 0 | 0 | 0 | 1 | 0 |
| NM_007261 | CD300A | 1889 | 3 | 1 | 1 | 0 | 0 | 1 | 0 | 0 | 0 | 0 | 0 | 3 | 0 |
| NM_007262 | PARK7 | 961 | 48 | 28 | 30 | 15 | 39 | 48 | 27 | 48 | 15 | 21 | 25 | 23 | 20 |
| NM_007263 | COPE | 1134 | 6 | 2 | 1 | 1 | 1 | 3 | 3 | 4 | 2 | 1 | 1 | 3 | 6 |
| NM_007264 | GPR182 | 1570 | 4 | 1 | 0 | 0 | 0 | 0 | 0 | 0 | 0 | 0 | 0 | 4 | 0 |
| NM_007265 | ECD | 2236 | 4 | 3 | 3 | 2 | 2 | 4 | 3 | 3 | 2 | 3 | 2 | 3 | 4 |
| NM_007266 | GPN1 | 1832 | 7 | 4 | 5 | 2 | 4 | 7 | 5 | 4 | 3 | 3 | 4 | 5 | 4 |
| NM_007267 | TMC6 | 2789 | 1 | 0 | 0 | 0 | 0 | 0 | 0 | 0 | 0 | 0 | 0 | 1 | 0 |
| NM_007268 | VSIG4 | 1852 | 24 | 8 | 10 | 1 | 24 | 4 | 2 | 7 | 24 | 1 | 2 | 7 | 3 |
| NM_007269 | STXBP3 | 2503 | 12 | 7 | 5 | 5 | 7 | 12 | 9 | 5 | 8 | 6 | 4 | 12 | 7 |
| NM_007270 | FKBP9 | 3473 | 7 | 3 | 7 | 3 | 6 | 2 | 4 | 1 | 4 | 4 | 2 | 3 | 2 |
| NM_007271 | STK38 | 3576 | 14 | 8 | 12 | 11 | 9 | 3 | 9 | 8 | 10 | 5 | 4 | 14 | 7 |
| NM_007272 | CTRC | 899 | 0 | 0 | 0 | 0 | 0 | 0 | 0 | 0 | 0 | 0 | 0 | 0 | 0 |
| NM_007273 | PHB2 | 1416 | 8 | 4 | 3 | 4 | 4 | 4 | 5 | 7 | 3 | 8 | 5 | 5 | 2 |
| NM_007274 | ACOT7 | 1805 | 3 | 0 | 0 | 0 | 0 | 3 | 1 | 0 | 0 | 0 | 0 | 0 | 0 |
| NM_007275 | TUSC2 | 1682 | 2 | 1 | 0 | 0 | 1 | 2 | 1 | 0 | 0 | 0 | 0 | 1 | 0 |
| NM_007276 | CBX3 | 1811 | 10 | 7 | 7 | 3 | 7 | 8 | 9 | 9 | 7 | 6 | 4 | 10 | 8 |
| NM_007277 | EXOC3 | 2762 | 10 | 7 | 6 | 7 | 6 | 10 | 6 | 3 | 4 | 5 | 9 | 6 | 8 |
| NM_007278 | GABARAP | 893 | 30 | 21 | 13 | 7 | 23 | 25 | 28 | 15 | 22 | 22 | 24 | 30 | 19 |
| NM_007279 | U2AF2 | 3131 | 4 | 3 | 2 | 4 | 2 | 4 | 2 | 2 | 2 | 2 | 1 | 4 | 3 |
| NM_007280 | OIP5 | 1236 | 5 | 1 | 1 | 2 | 0 | 0 | 0 | 0 | 0 | 1 | 2 | 1 | 5 |
| NM_007281 | SCRG1 | 902 | 30 | 4 | 0 | 1 | 2 | 30 | 0 | 0 | 1 | 1 | 0 | 1 | 12 |
| NM_007282 | RNF13 | 2964 | 10 | 5 | 4 | 1 | 5 | 5 | 7 | 5 | 7 | 3 | 3 | 10 | 5 |
| NM_007283 | MGLL | 4614 | 24 | 8 | 24 | 7 | 8 | 7 | 9 | 9 | 8 | 2 | 5 | 8 | 2 |
| NM_007284 | TWF2 | 1625 | 3 | 2 | 3 | 1 | 2 | 3 | 1 | 1 | 1 | 1 | 2 | 2 | 1 |
| NM_007285 | GABARAPL2 | 1002 | 67 | 24 | 17 | 7 | 40 | 67 | 23 | 13 | 18 | 14 | 22 | 25 | 23 |
| NM_007286 | SYNPO | 5397 | 24 | 9 | 24 | 4 | 16 | 5 | 5 | 4 | 7 | 4 | 20 | 8 | 6 |
| NM_007287 | MME | 5619 | 35 | 7 | 20 | 0 | 1 | 1 | 35 | 4 | 6 | 0 | 3 | 1 | 1 |
| NM_007288 | MME | 5665 | 35 | 7 | 20 | 0 | 1 | 1 | 35 | 4 | 6 | 0 | 3 | 1 | 1 |
| NM_007289 | MME | 5710 | 36 | 7 | 20 | 0 | 1 | 1 | 36 | 4 | 6 | 0 | 3 | 1 | 1 |
| NM_007292 | ACOX1 | 7510 | 14 | 6 | 9 | 5 | 5 | 6 | 7 | 14 | 3 | 2 | 5 | 4 | 4 |
| NM_007293 | C4A | 5426 | 0 | 0 | 0 | 0 | 0 | 0 | 0 | 0 | 0 | 0 | 0 | 0 | 0 |
| NM_007294 | BRCA1 | 7206 | 7 | 2 | 2 | 7 | 1 | 1 | 0 | 3 | 1 | 1 | 1 | 2 | 4 |
| NM_007297 | BRCA1 | 7114 | 8 | 2 | 2 | 8 | 1 | 1 | 1 | 3 | 1 | 1 | 1 | 2 | 4 |
| NM_007298 | BRCA1 | 3681 | 6 | 2 | 2 | 4 | 1 | 1 | 1 | 6 | 1 | 1 | 1 | 2 | 5 |

| transcript | gene | transcript length (nt) | max | mean | adipose | colon | heart | hypothalamus | kidney | liver | lung | ovary | skeletalmuscle | spleen | testes |
|---|---|---|---|---|---|---|---|---|---|---|---|---|---|---|---|
| NM_007299 | BRCA1 | 3782 | 5 | 2 | 2 | 4 | 1 | 1 | 1 | 5 | 1 | 1 | 1 | 2 | 5 |
| NM_007300 | BRCA1 | 7269 | 7 | 2 | 2 | 7 | 1 | 1 | 1 | 3 | 1 | 1 | 1 | 2 | 4 |
| NM_007308 | SNCA | 3126 | 26 | 3 | 1 | 1 | 1 | 26 | 0 | 0 | 1 | 1 | 0 | 1 | 1 |
| NM_007309 | DIAPH2 | 3782 | 9 | 4 | 5 | 2 | 4 | 3 | 2 | 3 | 4 | 5 | 5 | 3 | 9 |
| NM_007310 | COMT | 2033 | 14 | 6 | 4 | 3 | 8 | 9 | 7 | 14 | 5 | 5 | 5 | 7 | 4 |
| NM_007311 | TSPO | 655 | 1 | 0 | 1 | 0 | 0 | 0 | 0 | 0 | 0 | 0 | 0 | 0 | 0 |
| NM_007312 | HYAL1 | 2518 | 8 | 2 | 0 | 0 | 2 | 0 | 4 | 8 | 1 | 0 | 0 | 3 | 0 |
| NM_007313 | ABL1 | 5881 | 8 | 5 | 5 | 8 | 5 | 4 | 4 | 2 | 4 | 8 | 3 | 6 | 4 |
| NM_007314 | ABL2 | 12226 | 2 | 2 | 2 | 1 | 1 | 2 | 1 | 1 | 2 | 2 | 1 | 2 | 2 |
| NM_007315 | STAT1 | 4308 | 26 | 15 | 15 | 6 | 14 | 12 | 18 | 15 | 18 | 12 | 22 | 26 | 11 |
| NM_007316 | AGRP | 467 | 3 | 1 | 0 | 0 | 0 | 3 | 1 | 0 | 0 | 0 | 0 | 0 | 1 |
| NM_007317 | KIF22 | 2095 | 3 | 1 | 1 | 3 | 0 | 1 | 1 | 0 | 1 | 1 | 0 | 1 | 1 |
| NM_007318 | PSEN1 | 6077 | 6 | 4 | 3 | 3 | 3 | 6 | 5 | 3 | 5 | 5 | 2 | 6 | 6 |
| NM_007320 | RANBP3 | 3196 | 5 | 3 | 3 | 3 | 4 | 5 | 3 | 3 | 2 | 3 | 3 | 4 | 5 |
| NM_007322 | RANBP3 | 3400 | 5 | 3 | 2 | 3 | 3 | 5 | 3 | 2 | 2 | 3 | 2 | 4 | 5 |
| NM_007323 | ZFYVE9 | 2625 | 14 | 7 | 5 | 3 | 5 | 14 | 8 | 4 | 5 | 12 | 9 | 3 | 11 |
| NM_007324 | ZFYVE9 | 4738 | 12 | 6 | 4 | 3 | 4 | 12 | 6 | 3 | 4 | 10 | 7 | 3 | 9 |
| NM_007325 | GRIA3 | 5182 | 17 | 2 | 0 | 0 | 0 | 17 | 1 | 0 | 0 | 1 | 0 | 0 | 1 |
| NM_007326 | CYB5R3 | 1954 | 21 | 10 | 17 | 6 | 15 | 10 | 8 | 21 | 5 | 6 | 5 | 7 | 8 |
| NM_007327 | GRIN1 | 4376 | 6 | 1 | 0 | 0 | 0 | 6 | 0 | 0 | 0 | 0 | 0 | 0 | 0 |
| NM_007328 | KLRC1 | 1325 | 0 | 0 | 0 | 0 | 0 | 0 | 0 | 0 | 0 | 0 | 0 | 0 | 0 |
| NM_007329 | DMBT1 | 7686 | 3 | 0 | 0 | 1 | 0 | 0 | 0 | 0 | 3 | 0 | 0 | 0 | 0 |
| NM_007331 | WHSC1 | 5172 | 7 | 3 | 2 | 4 | 3 | 5 | 2 | 1 | 3 | 1 | 1 | 3 | 7 |
| NM_007332 | TRPA1 | 5190 | 0 | 0 | 0 | 0 | 0 | 0 | 0 | 0 | 0 | 0 | 0 | 0 | 0 |
| NM_007333 | KLRC3 | 843 | 2 | 0 | 0 | 0 | 0 | 0 | 0 | 0 | 1 | 0 | 0 | 2 | 0 |
| NM_007334 | KLRD1 | 3165 | 6 | 1 | 0 | 0 | 0 | 0 | 0 | 0 | 1 | 0 | 0 | 6 | 0 |
| NM_007335 | DLEC1 | 5614 | 4 | 1 | 0 | 0 | 0 | 0 | 0 | 0 | 1 | 0 | 0 | 0 | 4 |
| NM_007337 | DLEC1 | 5490 | 4 | 1 | 0 | 0 | 0 | 0 | 0 | 0 | 1 | 0 | 0 | 0 | 4 |
| NM_007341 | SH3BGR | 1227 | 57 | 12 | 2 | 2 | 57 | 6 | 2 | 1 | 1 | 1 | 53 | 1 | 4 |
| NM_007342 | NUPL2 | 1796 | 4 | 3 | 2 | 2 | 2 | 4 | 3 | 3 | 1 | 2 | 1 | 4 | 4 |
| NM_007343 | PRSS3 | 966 | 1 | 0 | 0 | 1 | 0 | 0 | 0 | 0 | 0 | 0 | 0 | 0 | 0 |
| NM_007344 | TTF1 | 3135 | 2 | 1 | 1 | 1 | 2 | 1 | 1 | 0 | 1 | 1 | 1 | 1 | 1 |
| NM_007345 | ZNF236 | 8123 | 1 | 1 | 0 | 1 | 1 | 1 | 1 | 0 | 1 | 1 | 1 | 1 | 1 |
| NM_007346 | OGFR | 2420 | 2 | 1 | 1 | 1 | 1 | 1 | 1 | 1 | 1 | 1 | 1 | 2 | 1 |
| NM_007347 | AP4E1 | 6682 | 2 | 1 | 1 | 0 | 2 | 1 | 1 | 1 | 1 | 1 | 1 | 2 | 2 |
| NM_007348 | ATF6 | 2488 | 15 | 8 | 7 | 4 | 7 | 9 | 8 | 15 | 8 | 6 | 4 | 13 | 10 |
| NM_007349 | PAXIP1 | 3712 | 3 | 1 | 1 | 1 | 1 | 1 | 2 | 1 | 1 | 2 | 1 | 2 | 3 |
| NM_007350 | PHLDA1 | 5913 | 9 | 4 | 1 | 1 | 2 | 4 | 4 | 7 | 4 | 8 | 1 | 9 | 2 |
| NM_007351 | MMRN1 | 4969 | 5 | 2 | 1 | 1 | 2 | 0 | 0 | 3 | 5 | 3 | 2 | 1 | 5 |
| NM_007352 | CELA3B | 1003 | 0 | 0 | 0 | 0 | 0 | 0 | 0 | 0 | 0 | 0 | 0 | 0 | 0 |
| NM_007353 | GNA12 | 4384 | 5 | 2 | 3 | 1 | 2 | 3 | 2 | 2 | 2 | 3 | 1 | 5 | 4 |
| NM_007354 | C3orf27 | 2697 | 0 | 0 | 0 | 0 | 0 | 0 | 0 | 0 | 0 | 0 | 0 | 0 | 0 |
| NM_007355 | HSP90AB1 | 2538 | 417 | 222 | 169 | 79 | 308 | 417 | 141 | 132 | 148 | 387 | 272 | 202 | 185 |
| NM_007356 | LAMB4 | 5858 | 0 | 0 | 0 | 0 | 0 | 0 | 0 | 0 | 0 | 0 | 0 | 0 | 0 |
| NM_007357 | COG2 | 2959 | 6 | 4 | 4 | 4 | 5 | 4 | 5 | 5 | 2 | 3 | 4 | 6 | 6 |
| NM_007358 | MTF2 | 4128 | 4 | 2 | 1 | 1 | 1 | 1 | 2 | 1 | 2 | 3 | 2 | 4 | 4 |
| NM_007359 | CASC3 | 4180 | 10 | 7 | 7 | 6 | 5 | 10 | 6 | 4 | 6 | 7 | 6 | 8 | 9 |
| NM_007360 | KLRK1 | 1576 | 22 | 4 | 1 | 2 | 1 | 0 | 0 | 1 | 8 | 2 | 1 | 22 | 2 |
| NM_007361 | NID2 | 5046 | 6 | 2 | 2 | 1 | 3 | 1 | 1 | 0 | 1 | 6 | 1 | 0 | 1 |
| NM_007362 | NCBP2 | 2170 | 15 | 9 | 11 | 5 | 9 | 15 | 11 | 6 | 6 | 12 | 6 | 15 | 9 |
| NM_007363 | NONO | 3102 | 29 | 15 | 11 | 11 | 13 | 11 | 16 | 13 | 13 | 29 | 9 | 20 | 16 |
| NM_007364 | TMED3 | 1388 | 3 | 2 | 2 | 2 | 2 | 3 | 2 | 1 | 2 | 2 | 0 | 3 | 3 |
| NM_007365 | PADI2 | 4363 | 29 | 7 | 0 | 13 | 0 | 24 | 1 | 0 | 1 | 0 | 29 | 2 | 0 |
| NM_007366 | PLA2R1 | 4876 | 5 | 2 | 1 | 1 | 1 | 1 | 5 | 0 | 1 | 5 | 0 | 2 | 2 |
| NM_007367 | RALY | 1527 | 5 | 4 | 3 | 4 | 5 | 5 | 4 | 3 | 4 | 4 | 4 | 5 | 4 |
| NM_007368 | RASA3 | 4200 | 4 | 2 | 4 | 2 | 2 | 4 | 1 | 0 | 2 | 1 | 2 | 4 | 1 |
| NM_007370 | RFC5 | 2104 | 3 | 2 | 1 | 1 | 1 | 2 | 2 | 2 | 1 | 2 | 1 | 3 | 2 |
| NM_007371 | BRD3 | 5630 | 2 | 1 | 1 | 1 | 1 | 2 | 1 | 1 | 1 | 1 | 1 | 2 | 2 |
| NM_007372 | DDX42 | 3993 | 42 | 28 | 25 | 17 | 29 | 32 | 22 | 17 | 20 | 39 | 28 | 38 | 42 |
| NM_007373 | SHOC2 | 3946 | 39 | 19 | 19 | 5 | 18 | 28 | 15 | 13 | 17 | 21 | 13 | 25 | 39 |
| NM_007374 | SIX6 | 1475 | 0 | 0 | 0 | 0 | 0 | 0 | 0 | 0 | 0 | 0 | 0 | 0 | 0 |
| NM_007375 | TARDBP | 4217 | 9 | 6 | 5 | 4 | 6 | 6 | 7 | 6 | 7 | 6 | 5 | 9 | 7 |
| NM_009585 | AGTR1 | 2272 | 5 | 1 | 2 | 0 | 1 | 0 | 1 | 5 | 1 | 0 | 1 | 1 | 0 |
| NM_009586 | SIM2 | 2414 | 1 | 0 | 0 | 0 | 0 | 0 | 1 | 0 | 0 | 0 | 0 | 0 | 0 |
| NM_009587 | LGALS9 | 1764 | 3 | 1 | 1 | 2 | 1 | 0 | 0 | 0 | 1 | 0 | 0 | 3 | 0 |
| NM_009588 | LTB | 847 | 72 | 8 | 0 | 4 | 1 | 0 | 1 | 0 | 5 | 0 | 0 | 72 | 0 |
| NM_009589 | ARSD | 2236 | 2 | 1 | 0 | 2 | 0 | 0 | 2 | 1 | 1 | 1 | 0 | 0 | 1 |
| NM_009590 | AOC2 | 2666 | 1 | 0 | 1 | 0 | 0 | 0 | 0 | 0 | 0 | 0 | 0 | 0 | 0 |
| NM_012062 | DNM1L | 4612 | 11 | 5 | 3 | 1 | 8 | 11 | 6 | 3 | 3 | 5 | 4 | 6 | 7 |
| NM_012063 | DNM1L | 4534 | 11 | 5 | 3 | 1 | 8 | 11 | 6 | 3 | 3 | 5 | 4 | 6 | 7 |
| NM_012064 | MIP | 2004 | 0 | 0 | 0 | 0 | 0 | 0 | 0 | 0 | 0 | 0 | 0 | 0 | 0 |
| NM_012067 | AKR7A3 | 1241 | 5 | 1 | 0 | 1 | 0 | 0 | 5 | 2 | 0 | 0 | 0 | 0 | 0 |
| NM_012068 | ATF5 | 2259 | 79 | 9 | 2 | 2 | 2 | 1 | 2 | 79 | 2 | 2 | 1 | 2 | 2 |
| NM_012069 | ATP1B4 | 3879 | 11 | 1 | 0 | 0 | 1 | 0 | 0 | 0 | 0 | 0 | 11 | 0 | 0 |
| NM_012071 | COMMD3 | 946 | 8 | 5 | 4 | 4 | 8 | 4 | 4 | 6 | 5 | 4 | 5 | 6 | 6 |
| NM_012072 | CD93 | 6701 | 8 | 3 | 4 | 1 | 5 | 1 | 2 | 1 | 8 | 1 | 1 | 6 | 1 |
| NM_012073 | CCT5 | 3403 | 15 | 10 | 10 | 6 | 11 | 15 | 10 | 13 | 5 | 5 | 9 | 12 | 10 |

| transcript | gene | transcript length (nt) | max | mean | adipose | colon | heart | hypothalamus | kidney | liver | lung | ovary | skeletalmuscle | spleen | testes |
|---|---|---|---|---|---|---|---|---|---|---|---|---|---|---|---|
| NM_012074 | DPF3 | 2271 | 3 | 1 | 0 | 1 | 2 | 2 | 1 | 0 | 0 | 2 | 3 | 1 | 1 |
| NM_012079 | DGAT1 | 3703 | 1 | 0 | 0 | 1 | 0 | 0 | 0 | 0 | 0 | 0 | 0 | 0 | 0 |
| NM_012079 | DGAT1 | 3705 | 1 | 0 | 0 | 1 | 0 | 0 | 0 | 0 | 0 | 0 | 0 | 0 | 0 |
| NM_012080 | HDHD1A | 2140 | 3 | 1 | 1 | 1 | 2 | 1 | 2 | 1 | 1 | 1 | 3 | 1 | 1 |
| NM_012081 | ELL2 | 6046 | 14 | 7 | 5 | 1 | 14 | 7 | 3 | 12 | 8 | 2 | 5 | 10 | 6 |
| NM_012082 | ZFPM2 | 4478 | 11 | 2 | 2 | 0 | 3 | 2 | 0 | 0 | 1 | 11 | 2 | 1 | 2 |
| NM_012083 | FRAT2 | 2205 | 9 | 3 | 1 | 4 | 2 | 2 | 1 | 2 | 5 | 1 | 1 | 9 | 2 |
| NM_012084 | GLUD2 | 2335 | 21 | 5 | 2 | 1 | 2 | 11 | 6 | 21 | 2 | 3 | 1 | 2 | 4 |
| NM_012086 | GTF3C3 | 2938 | 4 | 3 | 3 | 1 | 2 | 3 | 3 | 2 | 2 | 3 | 3 | 3 | 4 |
| NM_012087 | GTF3C5 | 2420 | 6 | 3 | 2 | 3 | 3 | 2 | 4 | 2 | 3 | 4 | 4 | 5 | 6 |
| NM_012088 | PGLS | 1034 | 1 | 1 | 1 | 1 | 1 | 1 | 1 | 1 | 1 | 1 | 0 | 1 | 1 |
| NM_012089 | ABCB10 | 3857 | 2 | 1 | 1 | 1 | 1 | 1 | 1 | 2 | 1 | 0 | 1 | 1 | 0 |
| NM_012090 | MACF1 | 17821 | 42 | 23 | 20 | 10 | 27 | 32 | 21 | 12 | 42 | 27 | 9 | 29 | 24 |
| NM_012091 | ADAT1 | 2727 | 1 | 1 | 1 | 1 | 1 | 1 | 1 | 1 | 0 | 0 | 0 | 1 | 1 |
| NM_012092 | ICOS | 2641 | 0 | 0 | 0 | 0 | 0 | 0 | 0 | 0 | 0 | 0 | 0 | 0 | 0 |
| NM_012093 | AK5 | 3936 | 119 | 11 | 0 | 1 | 0 | 119 | 0 | 0 | 0 | 1 | 0 | 1 | 1 |
| NM_012094 | PRDX5 | 884 | 7 | 3 | 2 | 7 | 4 | 4 | 5 | 3 | 4 | 2 | 2 | 3 | 1 |
| NM_012095 | AP3M1 | 5127 | 4 | 3 | 3 | 1 | 3 | 3 | 2 | 3 | 2 | 3 | 2 | 4 | 3 |
| NM_012096 | APPL1 | 6060 | 10 | 6 | 4 | 2 | 8 | 7 | 6 | 4 | 5 | 10 | 7 | 8 | 7 |
| NM_012097 | ARL5A | 3169 | 5 | 3 | 2 | 1 | 5 | 3 | 4 | 3 | 4 | 2 | 3 | 4 | 3 |
| NM_012098 | ANGPTL2 | 3572 | 11 | 4 | 11 | 3 | 5 | 2 | 1 | 0 | 1 | 5 | 6 | 7 | 2 |
| NM_012099 | CD3EAP | 3286 | 1 | 0 | 0 | 0 | 0 | 0 | 0 | 0 | 0 | 1 | 0 | 0 | 0 |
| NM_012100 | DNPEP | 2438 | 1 | 1 | 1 | 1 | 1 | 1 | 1 | 1 | 1 | 1 | 1 | 1 | 1 |
| NM_012101 | TRIM29 | 3019 | 1 | 0 | 0 | 0 | 0 | 0 | 0 | 0 | 1 | 0 | 0 | 0 | 1 |
| NM_012102 | RERE | 8194 | 22 | 12 | 7 | 11 | 11 | 22 | 12 | 3 | 8 | 13 | 13 | 13 | 17 |
| NM_012104 | BACE1 | 5834 | 12 | 4 | 3 | 1 | 2 | 12 | 3 | 3 | 2 | 4 | 2 | 3 | 3 |
| NM_012105 | BACE2 | 2993 | 4 | 2 | 1 | 2 | 2 | 1 | 4 | 1 | 2 | 1 | 1 | 3 | 1 |
| NM_012106 | ARL2BP | 2100 | 15 | 10 | 10 | 4 | 13 | 12 | 10 | 7 | 8 | 11 | 5 | 15 | 15 |
| NM_012108 | STAP1 | 1511 | 15 | 2 | 0 | 0 | 0 | 0 | 5 | 0 | 1 | 0 | 0 | 15 | 0 |
| NM_012109 | TMEM59L | 1619 | 3 | 0 | 0 | 0 | 0 | 3 | 0 | 0 | 0 | 0 | 0 | 0 | 0 |
| NM_012110 | CHIC2 | 1123 | 3 | 1 | 1 | 1 | 1 | 1 | 1 | 1 | 3 | 1 | 0 | 2 | 3 |
| NM_012111 | AHSA1 | 1392 | 35 | 21 | 16 | 20 | 30 | 33 | 16 | 18 | 15 | 15 | 16 | 21 | 35 |
| NM_012112 | TPX2 | 3662 | 10 | 2 | 2 | 4 | 1 | 1 | 0 | 1 | 2 | 1 | 0 | 2 | 10 |
| NM_012113 | CA14 | 1739 | 3 | 0 | 0 | 0 | 1 | 3 | 0 | 0 | 0 | 0 | 0 | 0 | 0 |
| NM_012114 | CASP14 | 777 | 0 | 0 | 0 | 0 | 0 | 0 | 0 | 0 | 0 | 0 | 0 | 0 | 0 |
| NM_012115 | CASP8AP2 | 6762 | 5 | 3 | 3 | 1 | 3 | 4 | 3 | 2 | 3 | 4 | 4 | 5 | 5 |
| NM_012116 | CBLC | 1590 | 0 | 0 | 0 | 0 | 0 | 0 | 0 | 0 | 0 | 0 | 0 | 0 | 0 |
| NM_012117 | CBX5 | 11551 | 20 | 10 | 5 | 6 | 9 | 20 | 12 | 4 | 7 | 18 | 4 | 11 | 12 |
| NM_012118 | CCRN4L | 1954 | 3 | 1 | 1 | 1 | 1 | 1 | 1 | 3 | 0 | 0 | 1 | 1 | 1 |
| NM_012119 | CCRK | 2302 | 0 | 0 | 0 | 0 | 0 | 0 | 0 | 0 | 0 | 0 | 0 | 0 | 0 |
| NM_012120 | CD2AP | 5409 | 11 | 5 | 3 | 4 | 3 | 2 | 11 | 3 | 6 | 3 | 2 | 7 | 6 |
| NM_012121 | CDC42EP4 | 3101 | 4 | 2 | 2 | 2 | 2 | 3 | 2 | 2 | 2 | 4 | 1 | 2 | 2 |
| NM_012123 | MTO1 | 2924 | 9 | 5 | 3 | 2 | 4 | 5 | 5 | 9 | 3 | 5 | 3 | 6 | 6 |
| NM_012124 | CHORDC1 | 3382 | 5 | 2 | 2 | 1 | 3 | 4 | 1 | 1 | 2 | 3 | 2 | 5 | 2 |
| NM_012125 | CHRM5 | 3039 | 0 | 0 | 0 | 0 | 0 | 0 | 0 | 0 | 0 | 0 | 0 | 0 | 0 |
| NM_012127 | CIZ1 | 3032 | 22 | 14 | 15 | 12 | 12 | 16 | 9 | 4 | 10 | 22 | 14 | 14 | 21 |
| NM_012128 | CLCA4 | 3208 | 12 | 1 | 0 | 12 | 0 | 1 | 0 | 0 | 0 | 0 | 0 | 0 | 0 |
| NM_012129 | CLDN12 | 3469 | 10 | 5 | 2 | 2 | 8 | 5 | 8 | 10 | 3 | 6 | 3 | 2 | 6 |
| NM_012130 | CLDN14 | 1216 | 0 | 0 | 0 | 0 | 0 | 0 | 0 | 0 | 0 | 0 | 0 | 0 | 0 |
| NM_012131 | CLDN17 | 675 | 0 | 0 | 0 | 0 | 0 | 0 | 0 | 0 | 0 | 0 | 0 | 0 | 0 |
| NM_012133 | COPG2 | 1732 | 9 | 4 | 9 | 2 | 2 | 7 | 2 | 2 | 2 | 5 | 3 | 3 | 6 |
| NM_012134 | LMOD1 | 3967 | 41 | 15 | 14 | 41 | 21 | 2 | 4 | 0 | 14 | 17 | 13 | 10 | 30 |
| NM_012135 | FAM50B | 1618 | 2 | 1 | 0 | 0 | 1 | 2 | 1 | 1 | 0 | 1 | 2 | 1 | 1 |
| NM_012137 | DDAH1 | 3985 | 45 | 14 | 2 | 10 | 7 | 34 | 45 | 16 | 9 | 4 | 9 | 7 | 14 |
| NM_012138 | AATF | 2141 | 21 | 14 | 13 | 11 | 13 | 17 | 14 | 15 | 11 | 14 | 12 | 21 | 17 |
| NM_012139 | SERGEF | 1493 | 8 | 2 | 2 | 2 | 1 | 8 | 2 | 1 | 2 | 3 | 1 | 2 | 2 |
| NM_012140 | SLC25A10 | 1885 | 2 | 0 | 1 | 1 | 0 | 0 | 1 | 2 | 0 | 0 | 0 | 0 | 0 |
| NM_012141 | INTS6 | 7465 | 8 | 3 | 2 | 1 | 3 | 2 | 3 | 3 | 2 | 3 | 2 | 4 | 8 |
| NM_012142 | CCNDBP1 | 2503 | 13 | 8 | 10 | 4 | 9 | 12 | 7 | 7 | 7 | 3 | 7 | 13 | 6 |
| NM_012143 | TFIP11 | 2848 | 5 | 3 | 2 | 2 | 2 | 3 | 3 | 5 | 1 | 2 | 3 | 4 | 4 |
| NM_012144 | DNAI1 | 2521 | 5 | 1 | 0 | 0 | 0 | 1 | 0 | 0 | 2 | 0 | 0 | 0 | 5 |
| NM_012145 | DTYMK | 1053 | 1 | 1 | 1 | 0 | 0 | 1 | 0 | 1 | 0 | 1 | 0 | 1 | 1 |
| NM_012151 | F8A1 | 1702 | 7 | 4 | 5 | 2 | 4 | 7 | 5 | 7 | 2 | 2 | 3 | 5 | 2 |
| NM_012152 | LPAR3 | 1721 | 2 | 0 | 0 | 0 | 1 | 0 | 0 | 0 | 1 | 0 | 0 | 0 | 2 |
| NM_012153 | EHF | 3617 | 14 | 3 | 0 | 14 | 0 | 0 | 7 | 0 | 11 | 1 | 0 | 0 | 2 |
| NM_012154 | EIF2C2 | 3500 | 3 | 2 | 2 | 3 | 2 | 2 | 1 | 1 | 3 | 3 | 3 | 3 | 2 |
| NM_012155 | EML2 | 2248 | 3 | 1 | 1 | 1 | 1 | 3 | 1 | 1 | 1 | 0 | 0 | 1 | 0 |
| NM_012156 | EPB41L1 | 6276 | 11 | 2 | 4 | 1 | 1 | 11 | 5 | 0 | 1 | 2 | 0 | 1 | 2 |
| NM_012157 | FBXL2 | 2362 | 6 | 1 | 1 | 1 | 0 | 6 | 1 | 0 | 1 | 2 | 0 | 0 | 2 |
| NM_012158 | FBXL3 | 3548 | 8 | 5 | 4 | 2 | 5 | 4 | 5 | 2 | 4 | 7 | 4 | 7 | 8 |
| NM_012159 | FBXL21 | 1814 | 1 | 0 | 0 | 0 | 0 | 0 | 1 | 0 | 0 | 0 | 0 | 0 | 0 |
| NM_012160 | FBXL4 | 2777 | 5 | 2 | 2 | 2 | 2 | 2 | 4 | 1 | 2 | 5 | 2 | 3 | 3 |
| NM_012161 | FBXL5 | 3371 | 25 | 16 | 17 | 5 | 14 | 18 | 25 | 13 | 15 | 14 | 14 | 20 | 22 |
| NM_012162 | FBXL6 | 1745 | 0 | 0 | 0 | 0 | 0 | 0 | 0 | 0 | 0 | 0 | 0 | 0 | 0 |
| NM_012163 | LRRC29 | 1469 | 0 | 0 | 0 | 0 | 0 | 0 | 0 | 0 | 0 | 0 | 0 | 0 | 0 |
| NM_012164 | FBXW2 | 9186 | 7 | 4 | 4 | 2 | 3 | 4 | 4 | 4 | 4 | 3 | 2 | 7 | 3 |
| NM_012166 | FBXO10 | 4575 | 1 | 0 | 0 | 0 | 0 | 1 | 0 | 0 | 0 | 0 | 1 | 1 | 0 |

| transcript | gene | transcript length (nt) | max | mean | adipose | colon | heart | hypothalamus | kidney | liver | lung | ovary | skeletalmuscle | spleen | testes |
|---|---|---|---|---|---|---|---|---|---|---|---|---|---|---|---|
| NM_012167 | FBXO11 | 2154 | 21 | 13 | 10 | 4 | 11 | 17 | 10 | 7 | 12 | 21 | 12 | 18 | 21 |
| NM_012168 | FBXO2 | 1536 | 6 | 1 | 0 | 0 | 0 | 6 | 0 | 0 | 0 | 0 | 0 | 0 | 1 |
| NM_012170 | FBXO22 | 1822 | 1 | 1 | 1 | 0 | 1 | 0 | 1 | 1 | 1 | 1 | 1 | 1 | 0 |
| NM_012171 | TSPAN17 | 2588 | 1 | 1 | 0 | 1 | 1 | 1 | 1 | 1 | 0 | 0 | 1 | 1 | 1 |
| NM_012172 | FBXO24 | 2143 | 12 | 1 | 0 | 0 | 0 | 0 | 0 | 0 | 0 | 0 | 0 | 0 | 12 |
| NM_012173 | FBXO25 | 2364 | 3 | 1 | 1 | 1 | 1 | 2 | 1 | 2 | 1 | 1 | 1 | 1 | 3 |
| NM_012174 | FBXW8 | 4657 | 2 | 1 | 1 | 1 | 1 | 1 | 1 | 1 | 1 | 2 | 0 | 1 | 1 |
| NM_012175 | FBXO3 | 2408 | 14 | 9 | 5 | 4 | 12 | 13 | 14 | 7 | 7 | 7 | 13 | 9 | 9 |
| NM_012176 | FBXO4 | 1509 | 2 | 1 | 1 | 0 | 1 | 2 | 1 | 1 | 1 | 1 | 0 | 2 | 1 |
| NM_012177 | FBXO5 | 2091 | 2 | 1 | 1 | 0 | 1 | 1 | 1 | 1 | 1 | 2 | 0 | 2 | 1 |
| NM_012179 | FBXO7 | 2153 | 21 | 14 | 12 | 7 | 10 | 21 | 16 | 14 | 14 | 16 | 6 | 20 | 20 |
| NM_012180 | FBXO8 | 2563 | 6 | 3 | 3 | 2 | 4 | 3 | 4 | 6 | 3 | 3 | 1 | 4 | 3 |
| NM_012181 | FKBP8 | 1766 | 13 | 6 | 6 | 4 | 5 | 13 | 6 | 5 | 7 | 4 | 2 | 7 | 7 |
| NM_012182 | FOXB1 | 1459 | 0 | 0 | 0 | 0 | 0 | 0 | 0 | 0 | 0 | 0 | 0 | 0 | 0 |
| NM_012183 | FOXD3 | 2068 | 0 | 0 | 0 | 0 | 0 | 0 | 0 | 0 | 0 | 0 | 0 | 0 | 0 |
| NM_012184 | FOXD4L1 | 2067 | 0 | 0 | 0 | 0 | 0 | 0 | 0 | 0 | 0 | 0 | 0 | 0 | 0 |
| NM_012186 | FOXE3 | 1981 | 0 | 0 | 0 | 0 | 0 | 0 | 0 | 0 | 0 | 0 | 0 | 0 | 0 |
| NM_012188 | FOXI1 | 2296 | 4 | 0 | 0 | 0 | 0 | 0 | 4 | 0 | 0 | 0 | 0 | 0 | 0 |
| NM_012189 | CABYR | 2333 | 17 | 2 | 0 | 0 | 0 | 2 | 0 | 0 | 0 | 0 | 0 | 0 | 17 |
| NM_012190 | ALDH1L1 | 3125 | 13 | 4 | 4 | 1 | 2 | 3 | 13 | 13 | 0 | 1 | 3 | 0 | 1 |
| NM_012191 | NAT6 | 1195 | 10 | 4 | 2 | 1 | 3 | 5 | 3 | 1 | 3 | 4 | 6 | 4 | 10 |
| NM_012192 | FXC1 | 2842 | 7 | 5 | 4 | 3 | 4 | 6 | 7 | 3 | 5 | 6 | 3 | 7 | 5 |
| NM_012193 | FZD4 | 7383 | 15 | 4 | 15 | 2 | 4 | 1 | 5 | 1 | 3 | 3 | 2 | 2 | 2 |
| NM_012194 | C11orf41 | 11638 | 10 | 1 | 0 | 0 | 0 | 10 | 0 | 0 | 0 | 0 | 0 | 0 | 0 |
| NM_012196 | GAGE8 | 517 | 0 | 0 | 0 | 0 | 0 | 0 | 0 | 0 | 0 | 0 | 0 | 0 | 0 |
| NM_012196 | GAGE8 | 520 | 0 | 0 | 0 | 0 | 0 | 0 | 0 | 0 | 0 | 0 | 0 | 0 | 0 |
| NM_012197 | RABGAP1 | 4999 | 23 | 12 | 6 | 5 | 15 | 17 | 10 | 3 | 10 | 13 | 12 | 13 | 23 |
| NM_012198 | GCA | 3223 | 14 | 3 | 1 | 1 | 2 | 4 | 1 | 1 | 3 | 1 | 0 | 14 | 1 |
| NM_012199 | EIF2C1 | 7478 | 4 | 3 | 3 | 3 | 3 | 4 | 3 | 2 | 2 | 3 | 1 | 4 | 3 |
| NM_012200 | B3GAT3 | 1442 | 0 | 0 | 0 | 0 | 0 | 0 | 0 | 0 | 0 | 0 | 0 | 0 | 0 |
| NM_012201 | GLG1 | 8288 | 23 | 13 | 12 | 8 | 14 | 17 | 14 | 9 | 8 | 23 | 10 | 14 | 15 |
| NM_012202 | GNG3 | 902 | 82 | 8 | 0 | 1 | 0 | 82 | 0 | 0 | 0 | 0 | 0 | 1 | 0 |
| NM_012203 | GRHPR | 1235 | 21 | 5 | 3 | 2 | 3 | 4 | 6 | 21 | 1 | 3 | 3 | 3 | 3 |
| NM_012204 | GTF3C4 | 3865 | 4 | 2 | 2 | 2 | 2 | 4 | 2 | 2 | 1 | 4 | 1 | 2 | 4 |
| NM_012205 | HAAO | 1284 | 6 | 1 | 1 | 1 | 0 | 0 | 3 | 6 | 0 | 1 | 0 | 0 | 1 |
| NM_012206 | HAVCR1 | 1841 | 5 | 1 | 0 | 2 | 0 | 0 | 5 | 0 | 0 | 0 | 0 | 0 | 0 |
| NM_012207 | HNRNPH3 | 2412 | 111 | 74 | 52 | 39 | 88 | 88 | 68 | 26 | 79 | 111 | 68 | 105 | 94 |
| NM_012208 | HARS2 | 2490 | 9 | 7 | 7 | 6 | 8 | 7 | 8 | 6 | 4 | 5 | 6 | 9 | 8 |
| NM_012210 | TRIM32 | 3719 | 2 | 1 | 1 | 1 | 1 | 2 | 1 | 0 | 1 | 2 | 2 | 2 | 1 |
| NM_012212 | PTGR1 | 1245 | 75 | 16 | 7 | 5 | 9 | 3 | 45 | 75 | 6 | 8 | 3 | 5 | 7 |
| NM_012213 | MLYCD | 2195 | 4 | 1 | 1 | 1 | 4 | 1 | 2 | 1 | 0 | 1 | 2 | 1 | 1 |
| NM_012214 | MGAT4A | 8382 | 5 | 2 | 1 | 2 | 2 | 4 | 5 | 1 | 4 | 1 | 1 | 2 | 1 |
| NM_012215 | MGEA5 | 5202 | 52 | 30 | 39 | 22 | 31 | 52 | 28 | 8 | 31 | 29 | 20 | 33 | 39 |
| NM_012216 | MID2 | 7343 | 1 | 1 | 1 | 0 | 1 | 1 | 1 | 1 | 1 | 1 | 1 | 1 | 1 |
| NM_012217 | TPSD1 | 855 | 0 | 0 | 0 | 0 | 0 | 0 | 0 | 0 | 0 | 0 | 0 | 0 | 0 |
| NM_012218 | ILF3 | 6107 | 14 | 10 | 6 | 9 | 10 | 12 | 9 | 6 | 7 | 13 | 11 | 14 | 12 |
| NM_012219 | MRAS | 4454 | 19 | 6 | 19 | 1 | 16 | 15 | 3 | 0 | 2 | 4 | 3 | 2 | 3 |
| NM_012222 | MUTYH | 1921 | 0 | 0 | 0 | 0 | 0 | 0 | 0 | 0 | 0 | 0 | 0 | 0 | 0 |
| NM_012223 | MYO1B | 4921 | 82 | 14 | 7 | 4 | 6 | 3 | 8 | 82 | 18 | 7 | 2 | 7 | 5 |
| NM_012224 | NEK1 | 5501 | 9 | 4 | 3 | 1 | 5 | 7 | 4 | 2 | 3 | 7 | 3 | 5 | 9 |
| NM_012225 | NUBP2 | 1382 | 0 | 0 | 0 | 0 | 0 | 0 | 0 | 0 | 0 | 0 | 0 | 0 | 0 |
| NM_012226 | P2RX2 | 1614 | 0 | 0 | 0 | 0 | 0 | 0 | 0 | 0 | 0 | 0 | 0 | 0 | 0 |
| NM_012227 | GTPBP6 | 1180 | 0 | 0 | 0 | 0 | 0 | 0 | 0 | 0 | 0 | 0 | 0 | 0 | 0 |
| NM_012228 | MSRB2 | 1811 | 7 | 3 | 4 | 2 | 7 | 6 | 3 | 2 | 2 | 3 | 4 | 3 | 2 |
| NM_012229 | NT5C2 | 5361 | 12 | 8 | 5 | 5 | 6 | 9 | 9 | 5 | 9 | 8 | 8 | 10 | 12 |
| NM_012230 | POMZP3 | 1552 | 0 | 0 | 0 | 0 | 0 | 0 | 0 | 0 | 0 | 0 | 0 | 0 | 0 |
| NM_012231 | PRDM2 | 7955 | 9 | 5 | 4 | 3 | 5 | 9 | 3 | 2 | 4 | 8 | 6 | 6 | 6 |
| NM_012232 | PTRF | 3632 | 68 | 15 | 68 | 14 | 15 | 6 | 5 | 2 | 16 | 12 | 9 | 15 | 8 |
| NM_012233 | RAB3GAP1 | 4166 | 19 | 11 | 8 | 5 | 14 | 19 | 10 | 5 | 9 | 12 | 11 | 13 | 13 |
| NM_012234 | RYBP | 4444 | 6 | 3 | 2 | 1 | 2 | 3 | 3 | 2 | 6 | 5 | 1 | 5 | 6 |
| NM_012235 | SCAP | 4255 | 6 | 4 | 2 | 3 | 3 | 4 | 4 | 6 | 2 | 4 | 2 | 4 | 6 |
| NM_012236 | SCMH1 | 3249 | 3 | 2 | 1 | 1 | 3 | 1 | 2 | 1 | 2 | 3 | 2 | 2 | 3 |
| NM_012237 | SIRT2 | 1963 | 3 | 1 | 1 | 0 | 1 | 3 | 1 | 0 | 1 | 1 | 2 | 1 | 0 |
| NM_012238 | SIRT1 | 4093 | 8 | 4 | 3 | 2 | 3 | 3 | 3 | 2 | 4 | 7 | 4 | 5 | 8 |
| NM_012239 | SIRT3 | 2902 | 0 | 0 | 0 | 0 | 0 | 0 | 0 | 0 | 0 | 0 | 0 | 0 | 0 |
| NM_012240 | SIRT4 | 1171 | 0 | 0 | 0 | 0 | 0 | 0 | 0 | 0 | 0 | 0 | 0 | 0 | 0 |
| NM_012241 | SIRT5 | 3917 | 3 | 2 | 1 | 1 | 3 | 2 | 2 | 2 | 1 | 1 | 1 | 2 | 1 |
| NM_012242 | DKK1 | 1805 | 0 | 0 | 0 | 0 | 0 | 0 | 0 | 0 | 0 | 0 | 0 | 0 | 0 |
| NM_012243 | SLC35A3 | 2149 | 4 | 2 | 2 | 3 | 2 | 2 | 4 | 3 | 3 | 3 | 1 | 2 | 2 |
| NM_012244 | SLC7A8 | 4216 | 83 | 14 | 3 | 4 | 2 | 4 | 83 | 2 | 6 | 13 | 3 | 20 | 10 |
| NM_012245 | SNW1 | 2125 | 17 | 14 | 16 | 6 | 15 | 15 | 12 | 10 | 12 | 15 | 13 | 17 | 17 |
| NM_012247 | SEPHS1 | 2599 | 4 | 2 | 2 | 1 | 2 | 2 | 2 | 4 | 1 | 2 | 1 | 2 | 2 |
| NM_012248 | SEPHS2 | 2273 | 89 | 21 | 8 | 10 | 13 | 14 | 49 | 89 | 8 | 6 | 7 | 17 | 7 |
| NM_012249 | RHOQ | 4542 | 62 | 18 | 39 | 3 | 62 | 10 | 11 | 5 | 15 | 7 | 20 | 18 | 10 |
| NM_012250 | RRAS2 | 2345 | 6 | 4 | 6 | 2 | 6 | 2 | 5 | 3 | 3 | 4 | 4 | 6 | 6 |
| NM_012252 | TFEC | 6631 | 4 | 1 | 1 | 0 | 1 | 0 | 4 | 0 | 2 | 0 | 0 | 3 | 0 |
| NM_012253 | TKTL1 | 2651 | 16 | 1 | 0 | 0 | 0 | 0 | 0 | 0 | 0 | 0 | 0 | 0 | 16 |

| transcript | gene | transcript length (nt) | max | mean | adipose | colon | heart | hypothalamus | kidney | liver | lung | ovary | skeletalmuscle | spleen | testes |
|---|---|---|---|---|---|---|---|---|---|---|---|---|---|---|---|
| NM_012254 | SLC27A5 | 2363 | 17 | 2 | 0 | 0 | 0 | 1 | 0 | 17 | 0 | 0 | 0 | 0 | 0 |
| NM_012255 | XRN2 | 3435 | 31 | 16 | 9 | 4 | 20 | 8 | 18 | 15 | 17 | 20 | 14 | 31 | 21 |
| NM_012256 | ZNF212 | 2766 | 5 | 3 | 2 | 3 | 2 | 2 | 3 | 1 | 2 | 4 | 2 | 5 | 5 |
| NM_012257 | HBP1 | 2829 | 8 | 5 | 8 | 2 | 4 | 3 | 4 | 3 | 7 | 7 | 4 | 6 | 5 |
| NM_012258 | HEY1 | 2293 | 7 | 1 | 2 | 1 | 1 | 7 | 0 | 0 | 2 | 0 | 2 | 0 | 1 |
| NM_012259 | HEY2 | 2672 | 9 | 3 | 1 | 1 | 9 | 3 | 1 | 2 | 1 | 4 | 5 | 1 | 3 |
| NM_012260 | HACL1 | 2029 | 4 | 2 | 3 | 1 | 1 | 1 | 2 | 4 | 1 | 1 | 1 | 1 | 1 |
| NM_012261 | C20orf103 | 1776 | 4 | 1 | 0 | 0 | 0 | 4 | 0 | 0 | 0 | 3 | 0 | 0 | 0 |
| NM_012262 | HS2ST1 | 6757 | 5 | 3 | 2 | 1 | 2 | 5 | 3 | 3 | 2 | 3 | 1 | 4 | 4 |
| NM_012263 | TTLL1 | 1770 | 2 | 1 | 1 | 0 | 0 | 2 | 0 | 0 | 0 | 0 | 0 | 0 | 1 |
| NM_012264 | TMEM184B | 3587 | 8 | 4 | 4 | 3 | 5 | 8 | 3 | 1 | 7 | 2 | 3 | 5 | 2 |
| NM_012265 | RHBDD3 | 1738 | 1 | 0 | 0 | 0 | 0 | 0 | 0 | 1 | 0 | 0 | 0 | 0 | 0 |
| NM_012266 | DNAJB5 | 2357 | 12 | 4 | 1 | 4 | 12 | 3 | 2 | 0 | 1 | 1 | 11 | 2 | 5 |
| NM_012267 | HSPBP1 | 1908 | 1 | 0 | 0 | 0 | 0 | 1 | 0 | 0 | 0 | 0 | 0 | 0 | 0 |
| NM_012268 | PLD3 | 2405 | 16 | 5 | 8 | 3 | 3 | 16 | 2 | 2 | 6 | 4 | 2 | 9 | 3 |
| NM_012269 | HYAL4 | 2406 | 0 | 0 | 0 | 0 | 0 | 0 | 0 | 0 | 0 | 0 | 0 | 0 | 0 |
| NM_012272 | PRPF40B | 3602 | 2 | 1 | 0 | 1 | 1 | 1 | 1 | 0 | 0 | 1 | 1 | 1 | 2 |
| NM_012274 | CXorf27 | 501 | 5 | 1 | 0 | 0 | 0 | 0 | 0 | 0 | 0 | 0 | 0 | 0 | 5 |
| NM_012275 | IL1F5 | 2695 | 0 | 0 | 0 | 0 | 0 | 0 | 0 | 0 | 0 | 0 | 0 | 0 | 0 |
| NM_012276 | LILRA4 | 1708 | 1 | 0 | 0 | 0 | 0 | 1 | 0 | 0 | 0 | 0 | 0 | 1 | 0 |
| NM_012278 | ITGB1BP2 | 1235 | 6 | 1 | 0 | 0 | 6 | 0 | 0 | 0 | 0 | 0 | 1 | 0 | 0 |
| NM_012279 | ZNF346 | 3073 | 2 | 1 | 0 | 1 | 1 | 1 | 1 | 0 | 1 | 2 | 1 | 1 | 1 |
| NM_012280 | FTSJ1 | 1951 | 1 | 1 | 0 | 0 | 1 | 1 | 1 | 1 | 1 | 1 | 0 | 1 | 1 |
| NM_012281 | KCND2 | 5333 | 5 | 1 | 1 | 0 | 0 | 5 | 0 | 0 | 0 | 2 | 0 | 0 | 1 |
| NM_012282 | KCNE1L | 1465 | 0 | 0 | 0 | 0 | 0 | 0 | 0 | 0 | 0 | 0 | 0 | 0 | 0 |
| NM_012283 | KCNG2 | 1401 | 0 | 0 | 0 | 0 | 0 | 0 | 0 | 0 | 0 | 0 | 0 | 0 | 0 |
| NM_012284 | KCNH3 | 3853 | 1 | 0 | 0 | 0 | 0 | 1 | 0 | 0 | 0 | 0 | 0 | 0 | 0 |
| NM_012285 | KCNH4 | 3923 | 0 | 0 | 0 | 0 | 0 | 0 | 0 | 0 | 0 | 0 | 0 | 0 | 0 |
| NM_012286 | MORF4L2 | 1983 | 49 | 31 | 15 | 10 | 43 | 49 | 26 | 28 | 24 | 48 | 24 | 28 | 49 |
| NM_012287 | ACAP2 | 7161 | 11 | 5 | 5 | 2 | 4 | 5 | 4 | 3 | 5 | 8 | 2 | 11 | 7 |
| NM_012288 | TRAM2 | 7057 | 2 | 1 | 1 | 1 | 1 | 0 | 1 | 2 | 1 | 1 | 0 | 2 | 1 |
| NM_012289 | KEAP1 | 2563 | 4 | 2 | 1 | 1 | 2 | 1 | 1 | 2 | 1 | 1 | 4 | 2 | 1 |
| NM_012290 | TLK1 | 5730 | 21 | 11 | 10 | 5 | 7 | 15 | 12 | 10 | 8 | 13 | 5 | 18 | 21 |
| NM_012291 | ESPL1 | 6623 | 0 | 0 | 0 | 0 | 0 | 0 | 0 | 0 | 0 | 0 | 0 | 0 | 0 |
| NM_012292 | HMHA1 | 4264 | 1 | 0 | 0 | 0 | 0 | 0 | 0 | 0 | 1 | 0 | 0 | 1 | 0 |
| NM_012293 | PXDN | 6821 | 7 | 2 | 7 | 2 | 3 | 0 | 1 | 1 | 2 | 3 | 0 | 3 | 1 |
| NM_012294 | RAPGEF5 | 6622 | 18 | 5 | 2 | 1 | 2 | 12 | 1 | 2 | 6 | 1 | 1 | 18 | 2 |
| NM_012295 | CABIN1 | 7222 | 2 | 1 | 0 | 1 | 1 | 1 | 1 | 0 | 1 | 1 | 0 | 1 | 2 |
| NM_012296 | GAB2 | 6058 | 13 | 7 | 9 | 4 | 9 | 11 | 3 | 1 | 9 | 13 | 2 | 10 | 4 |
| NM_012297 | G3BP2 | 4275 | 54 | 23 | 13 | 9 | 22 | 54 | 23 | 19 | 19 | 16 | 26 | 25 | 25 |
| NM_012298 | CAND2 | 4241 | 10 | 2 | 0 | 0 | 2 | 1 | 0 | 0 | 0 | 0 | 10 | 0 | 4 |
| NM_012300 | FBXW11 | 4575 | 9 | 4 | 2 | 1 | 5 | 9 | 3 | 3 | 2 | 4 | 3 | 4 | 3 |
| NM_012301 | MAGI2 | 6880 | 8 | 2 | 1 | 1 | 1 | 8 | 2 | 0 | 1 | 2 | 0 | 1 | 1 |
| NM_012302 | LPHN2 | 5706 | 16 | 7 | 5 | 1 | 7 | 4 | 7 | 10 | 16 | 10 | 2 | 12 | 3 |
| NM_012304 | FBXL7 | 4562 | 2 | 1 | 1 | 1 | 1 | 1 | 0 | 0 | 0 | 2 | 0 | 0 | 1 |
| NM_012305 | AP2A2 | 4616 | 9 | 4 | 4 | 3 | 3 | 9 | 4 | 4 | 3 | 2 | 2 | 7 | 3 |
| NM_012306 | FAIM2 | 4703 | 15 | 1 | 0 | 0 | 0 | 15 | 0 | 0 | 0 | 0 | 0 | 0 | 0 |
| NM_012307 | EPB41L3 | 4446 | 60 | 19 | 21 | 6 | 14 | 60 | 22 | 2 | 12 | 7 | 5 | 29 | 31 |
| NM_012308 | KDM2A | 7369 | 9 | 5 | 4 | 4 | 5 | 4 | 5 | 4 | 5 | 5 | 5 | 9 | 7 |
| NM_012309 | SHANK2 | 10449 | 2 | 1 | 0 | 1 | 0 | 2 | 1 | 1 | 1 | 0 | 0 | 0 | 0 |
| NM_012310 | KIF4A | 4447 | 1 | 0 | 0 | 0 | 0 | 0 | 0 | 0 | 0 | 0 | 0 | 1 | 0 |
| NM_012311 | KIN | 1906 | 5 | 3 | 3 | 1 | 3 | 4 | 3 | 2 | 2 | 3 | 2 | 5 | 5 |
| NM_012312 | KIR2DS2 | 1559 | 0 | 0 | 0 | 0 | 0 | 0 | 0 | 0 | 0 | 0 | 0 | 0 | 0 |
| NM_012313 | KIR2DS3 | 1113 | 0 | 0 | 0 | 0 | 0 | 0 | 0 | 0 | 0 | 0 | 0 | 0 | 0 |
| NM_012314 | KIR2DS4 | 1564 | 0 | 0 | 0 | 0 | 0 | 0 | 0 | 0 | 0 | 0 | 0 | 0 | 0 |
| NM_012315 | KLK9 | 1438 | 0 | 0 | 0 | 0 | 0 | 0 | 0 | 0 | 0 | 0 | 0 | 0 | 0 |
| NM_012316 | KPNA6 | 7372 | 6 | 5 | 4 | 3 | 6 | 6 | 5 | 4 | 4 | 3 | 6 | 5 | 6 |
| NM_012317 | LDOC1 | 1380 | 21 | 8 | 4 | 2 | 8 | 21 | 12 | 0 | 5 | 20 | 3 | 6 | 8 |
| NM_012318 | LETM1 | 5462 | 8 | 5 | 6 | 6 | 6 | 7 | 8 | 7 | 2 | 2 | 3 | 5 | 6 |
| NM_012319 | SLC39A6 | 3620 | 8 | 5 | 6 | 1 | 4 | 7 | 6 | 2 | 3 | 8 | 2 | 5 | 8 |
| NM_012320 | PLA2G15 | 2723 | 1 | 1 | 1 | 0 | 1 | 1 | 1 | 0 | 0 | 0 | 1 | 1 | 0 |
| NM_012321 | LSM4 | 1147 | 3 | 1 | 1 | 2 | 1 | 2 | 3 | 1 | 1 | 1 | 1 | 2 | 1 |
| NM_012322 | LSM5 | 2250 | 2 | 1 | 1 | 1 | 1 | 1 | 1 | 1 | 1 | 1 | 1 | 2 | 1 |
| NM_012323 | MAFF | 2437 | 14 | 5 | 10 | 2 | 8 | 3 | 1 | 3 | 14 | 5 | 8 | 3 | 3 |
| NM_012324 | MAPK8IP2 | 3363 | 3 | 0 | 0 | 0 | 0 | 3 | 0 | 0 | 0 | 0 | 0 | 0 | 0 |
| NM_012325 | MAPRE1 | 2622 | 39 | 25 | 24 | 14 | 34 | 28 | 28 | 8 | 28 | 22 | 12 | 39 | 39 |
| NM_012326 | MAPRE3 | 1870 | 24 | 5 | 3 | 2 | 4 | 24 | 2 | 1 | 2 | 3 | 7 | 1 | 3 |
| NM_012327 | PIGN | 4798 | 2 | 1 | 1 | 1 | 1 | 1 | 2 | 1 | 1 | 2 | 1 | 2 | 1 |
| NM_012328 | DNAJB9 | 2371 | 25 | 13 | 5 | 2 | 23 | 14 | 16 | 25 | 11 | 9 | 11 | 11 | 13 |
| NM_012329 | MMD | 2723 | 18 | 4 | 18 | 1 | 2 | 7 | 1 | 1 | 3 | 1 | 1 | 5 | 5 |
| NM_012330 | MYST4 | 8550 | 12 | 6 | 6 | 3 | 5 | 6 | 5 | 2 | 4 | 12 | 5 | 6 | 12 |
| NM_012331 | MSRA | 1482 | 13 | 3 | 1 | 1 | 2 | 3 | 13 | 4 | 1 | 1 | 1 | 3 | 1 |
| NM_012333 | MYCBP | 2091 | 8 | 4 | 3 | 3 | 4 | 2 | 5 | 4 | 5 | 3 | 3 | 8 | 6 |
| NM_012334 | MYO10 | 11430 | 8 | 4 | 2 | 4 | 1 | 8 | 5 | 2 | 6 | 5 | 2 | 5 | 8 |
| NM_012335 | MYO1F | 3826 | 7 | 1 | 2 | 1 | 1 | 1 | 0 | 1 | 3 | 1 | 0 | 7 | 0 |
| NM_012336 | NARF | 1621 | 4 | 3 | 2 | 2 | 3 | 3 | 2 | 2 | 2 | 2 | 2 | 4 | 4 |
| NM_012337 | CCDC19 | 1795 | 6 | 1 | 0 | 0 | 0 | 1 | 0 | 0 | 3 | 0 | 0 | 0 | 6 |

| transcript | gene | transcript length (nt) | max | mean | adipose | colon | heart | hypothalamus | kidney | liver | lung | ovary | skeletalmuscle | spleen | testes |
|---|---|---|---|---|---|---|---|---|---|---|---|---|---|---|---|
| NM_012338 | TSPAN12 | 2564 | 12 | 4 | 2 | 2 | 6 | 1 | 12 | 3 | 6 | 1 | 0 | 9 | 1 |
| NM_012339 | TSPAN15 | 1699 | 2 | 1 | 2 | 1 | 0 | 2 | 1 | 0 | 1 | 0 | 0 | 2 | 1 |
| NM_012340 | NFATC2 | 3254 | 6 | 2 | 2 | 2 | 1 | 2 | 1 | 1 | 1 | 1 | 1 | 6 | 2 |
| NM_012341 | GTPBP4 | 2517 | 11 | 8 | 7 | 3 | 10 | 10 | 7 | 9 | 5 | 4 | 10 | 9 | 11 |
| NM_012342 | BAMBI | 1724 | 20 | 5 | 1 | 0 | 6 | 3 | 3 | 3 | 2 | 20 | 0 | 5 | 6 |
| NM_012343 | NNT | 4678 | 20 | 7 | 7 | 2 | 20 | 6 | 11 | 12 | 3 | 2 | 9 | 4 | 2 |
| NM_012344 | NTSR2 | 1608 | 1 | 0 | 0 | 0 | 0 | 1 | 0 | 0 | 0 | 0 | 0 | 0 | 0 |
| NM_012345 | NUFIP1 | 3487 | 3 | 2 | 1 | 0 | 1 | 2 | 2 | 1 | 0 | 2 | 2 | 2 | 3 |
| NM_012346 | NUP62 | 3240 | 5 | 3 | 2 | 3 | 2 | 3 | 2 | 3 | 2 | 3 | 3 | 5 | 3 |
| NM_012347 | FBXO9 | 4437 | 6 | 4 | 4 | 2 | 3 | 6 | 5 | 6 | 3 | 2 | 1 | 5 | 4 |
| NM_012351 | OR10J1 | 1088 | 0 | 0 | 0 | 0 | 0 | 0 | 0 | 0 | 0 | 0 | 0 | 0 | 0 |
| NM_012352 | OR1A2 | 930 | 0 | 0 | 0 | 0 | 0 | 0 | 0 | 0 | 0 | 0 | 0 | 0 | 0 |
| NM_012353 | OR1C1 | 945 | 2 | 0 | 0 | 0 | 0 | 0 | 0 | 0 | 0 | 0 | 0 | 0 | 2 |
| NM_012360 | OR1F1 | 939 | 0 | 0 | 0 | 0 | 0 | 0 | 0 | 0 | 0 | 0 | 0 | 0 | 0 |
| NM_012363 | OR1N1 | 936 | 0 | 0 | 0 | 0 | 0 | 0 | 0 | 0 | 0 | 0 | 0 | 0 | 0 |
| NM_012364 | OR1Q1 | 945 | 0 | 0 | 0 | 0 | 0 | 0 | 0 | 0 | 0 | 0 | 0 | 0 | 0 |
| NM_012365 | OR2A5 | 936 | 0 | 0 | 0 | 0 | 0 | 0 | 0 | 0 | 0 | 0 | 0 | 0 | 0 |
| NM_012367 | OR2B6 | 942 | 0 | 0 | 0 | 0 | 0 | 0 | 0 | 0 | 0 | 0 | 0 | 0 | 0 |
| NM_012368 | OR2C1 | 1036 | 0 | 0 | 0 | 0 | 0 | 0 | 0 | 0 | 0 | 0 | 0 | 0 | 0 |
| NM_012369 | OR2F1 | 1089 | 0 | 0 | 0 | 0 | 0 | 0 | 0 | 0 | 0 | 0 | 0 | 0 | 0 |
| NM_012373 | OR3A3 | 966 | 0 | 0 | 0 | 0 | 0 | 0 | 0 | 0 | 0 | 0 | 0 | 0 | 0 |
| NM_012374 | OR4D1 | 933 | 0 | 0 | 0 | 0 | 0 | 0 | 0 | 0 | 0 | 0 | 0 | 0 | 0 |
| NM_012375 | OR52A1 | 939 | 0 | 0 | 0 | 0 | 0 | 0 | 0 | 0 | 0 | 0 | 0 | 0 | 0 |
| NM_012377 | OR7C2 | 960 | 0 | 0 | 0 | 0 | 0 | 0 | 0 | 0 | 0 | 0 | 0 | 0 | 0 |
| NM_012378 | OR8B8 | 936 | 0 | 0 | 0 | 0 | 0 | 0 | 0 | 0 | 0 | 0 | 0 | 0 | 0 |
| NM_012381 | ORC3L | 2507 | 6 | 3 | 3 | 1 | 3 | 6 | 3 | 2 | 2 | 4 | 4 | 3 | 6 |
| NM_012382 | TTC33 | 5506 | 5 | 2 | 1 | 1 | 2 | 5 | 2 | 1 | 1 | 2 | 2 | 2 | 2 |
| NM_012383 | OSTF1 | 1313 | 19 | 6 | 6 | 3 | 6 | 6 | 5 | 4 | 12 | 3 | 1 | 19 | 5 |
| NM_012384 | GMEB2 | 4210 | 1 | 1 | 0 | 1 | 1 | 1 | 1 | 1 | 1 | 1 | 0 | 1 | 1 |
| NM_012385 | NUPR1 | 858 | 16 | 9 | 16 | 8 | 9 | 4 | 9 | 12 | 13 | 10 | 5 | 4 | 10 |
| NM_012387 | PADI4 | 2265 | 2 | 0 | 0 | 0 | 0 | 0 | 0 | 0 | 1 | 0 | 0 | 2 | 0 |
| NM_012388 | PLDN | 3941 | 37 | 20 | 10 | 6 | 23 | 20 | 20 | 11 | 20 | 23 | 14 | 37 | 32 |
| NM_012390 | SMR3A | 598 | 0 | 0 | 0 | 0 | 0 | 0 | 0 | 0 | 0 | 0 | 0 | 0 | 0 |
| NM_012391 | SPDEF | 1894 | 1 | 0 | 0 | 1 | 0 | 0 | 0 | 0 | 0 | 0 | 0 | 0 | 0 |
| NM_012392 | PEF1 | 1610 | 13 | 7 | 6 | 5 | 8 | 13 | 7 | 7 | 7 | 6 | 4 | 10 | 7 |
| NM_012393 | PFAS | 5384 | 2 | 1 | 1 | 1 | 1 | 1 | 1 | 1 | 0 | 1 | 1 | 2 | 1 |
| NM_012394 | PFDN2 | 642 | 79 | 31 | 33 | 13 | 36 | 79 | 24 | 46 | 16 | 15 | 28 | 26 | 25 |
| NM_012395 | CDK14 | 4953 | 9 | 4 | 2 | 1 | 3 | 9 | 6 | 4 | 3 | 4 | 1 | 5 | 3 |
| NM_012396 | PHLDA3 | 1516 | 1 | 0 | 1 | 0 | 0 | 0 | 0 | 0 | 0 | 0 | 0 | 0 | 0 |
| NM_012397 | SERPINB13 | 3179 | 0 | 0 | 0 | 0 | 0 | 0 | 0 | 0 | 0 | 0 | 0 | 0 | 0 |
| NM_012398 | PIP5K1C | 5047 | 1 | 1 | 0 | 1 | 1 | 1 | 0 | 0 | 0 | 0 | 0 | 1 | 1 |
| NM_012399 | PITPNB | 2925 | 36 | 19 | 18 | 10 | 26 | 36 | 12 | 26 | 16 | 17 | 9 | 19 | 18 |
| NM_012400 | PLA2G2D | 2628 | 1 | 0 | 0 | 0 | 0 | 0 | 0 | 0 | 0 | 0 | 0 | 1 | 0 |
| NM_012401 | PLXNB2 | 6317 | 6 | 3 | 2 | 2 | 2 | 1 | 5 | 4 | 3 | 3 | 0 | 6 | 3 |
| NM_012402 | ARFIP2 | 2580 | 2 | 1 | 1 | 1 | 1 | 2 | 1 | 1 | 1 | 1 | 0 | 1 | 1 |
| NM_012403 | ANP32C | 705 | 69 | 37 | 43 | 35 | 33 | 69 | 35 | 42 | 43 | 15 | 19 | 51 | 23 |
| NM_012404 | ANP32D | 396 | 0 | 0 | 0 | 0 | 0 | 0 | 0 | 0 | 0 | 0 | 0 | 0 | 0 |
| NM_012405 | ICMT | 4815 | 4 | 2 | 2 | 1 | 3 | 2 | 4 | 2 | 2 | 1 | 3 | 2 | 1 |
| NM_012406 | PRDM4 | 4187 | 4 | 2 | 2 | 2 | 2 | 3 | 4 | 1 | 2 | 3 | 1 | 4 | 3 |
| NM_012407 | PICK1 | 2207 | 2 | 1 | 1 | 1 | 1 | 2 | 1 | 1 | 1 | 1 | 1 | 1 | 1 |
| NM_012408 | ZMYND8 | 4568 | 22 | 12 | 7 | 8 | 10 | 13 | 12 | 4 | 9 | 13 | 10 | 22 | 19 |
| NM_012409 | PRND | 3980 | 32 | 3 | 0 | 0 | 0 | 0 | 0 | 0 | 0 | 0 | 0 | 0 | 32 |
| NM_012410 | SEZ6L2 | 3342 | 8 | 1 | 0 | 1 | 0 | 8 | 0 | 0 | 0 | 1 | 0 | 0 | 1 |
| NM_012411 | PTPN22 | 2347 | 5 | 1 | 1 | 0 | 1 | 0 | 0 | 0 | 2 | 0 | 0 | 5 | 1 |
| NM_012412 | H2AFV | 1429 | 4 | 2 | 2 | 2 | 4 | 4 | 3 | 3 | 2 | 3 | 2 | 2 | 1 |
| NM_012413 | QPCT | 1703 | 1 | 0 | 0 | 0 | 0 | 1 | 0 | 0 | 0 | 0 | 0 | 1 | 0 |
| NM_012414 | RAB3GAP2 | 7329 | 6 | 4 | 4 | 1 | 4 | 6 | 4 | 3 | 3 | 4 | 3 | 5 | 4 |
| NM_012415 | RAD54B | 3041 | 1 | 0 | 0 | 0 | 0 | 0 | 0 | 0 | 0 | 0 | 0 | 0 | 1 |
| NM_012416 | RANBP6 | 4600 | 6 | 3 | 2 | 1 | 4 | 6 | 3 | 2 | 1 | 2 | 2 | 3 | 3 |
| NM_012417 | PITPNC1 | 1989 | 11 | 3 | 4 | 3 | 3 | 11 | 2 | 1 | 3 | 2 | 1 | 6 | 3 |
| NM_012418 | FSCN2 | 1513 | 0 | 0 | 0 | 0 | 0 | 0 | 0 | 0 | 0 | 0 | 0 | 0 | 0 |
| NM_012419 | RGS17 | 1466 | 2 | 0 | 0 | 0 | 0 | 2 | 0 | 0 | 0 | 0 | 0 | 0 | 0 |
| NM_012420 | IFIT5 | 4033 | 3 | 2 | 1 | 1 | 1 | 3 | 2 | 1 | 2 | 2 | 1 | 3 | 1 |
| NM_012421 | RLF | 6250 | 9 | 5 | 4 | 1 | 5 | 5 | 4 | 2 | 5 | 8 | 6 | 7 | 9 |
| NM_012423 | RPL13A | 1126 | 7 | 3 | 2 | 2 | 3 | 1 | 3 | 3 | 4 | 7 | 2 | 7 | 2 |
| NM_012424 | RPS6KC1 | 4190 | 10 | 4 | 3 | 1 | 4 | 9 | 3 | 2 | 3 | 6 | 3 | 5 | 10 |
| NM_012425 | RSU1 | 3753 | 9 | 5 | 5 | 3 | 6 | 3 | 6 | 3 | 5 | 5 | 2 | 9 | 3 |
| NM_012426 | SF3B3 | 9719 | 10 | 6 | 4 | 4 | 7 | 8 | 6 | 5 | 4 | 10 | 6 | 9 | 7 |
| NM_012427 | KLK5 | 1563 | 0 | 0 | 0 | 0 | 0 | 0 | 0 | 0 | 0 | 0 | 0 | 0 | 0 |
| NM_012428 | NPTN | 2453 | 19 | 8 | 4 | 6 | 13 | 19 | 9 | 3 | 6 | 5 | 2 | 10 | 11 |
| NM_012429 | SEC14L2 | 4266 | 8 | 1 | 0 | 0 | 0 | 2 | 1 | 8 | 0 | 1 | 0 | 0 | 1 |
| NM_012430 | SEC22A | 3414 | 2 | 1 | 1 | 1 | 1 | 1 | 1 | 1 | 1 | 2 | 1 | 1 | 2 |
| NM_012431 | SEMA3E | 6474 | 3 | 0 | 0 | 0 | 0 | 3 | 0 | 0 | 1 | 0 | 0 | 0 | 0 |
| NM_012432 | SETDB1 | 4443 | 6 | 3 | 2 | 3 | 2 | 2 | 3 | 2 | 2 | 4 | 2 | 4 | 6 |
| NM_012433 | SF3B1 | 4292 | 69 | 41 | 35 | 18 | 47 | 33 | 41 | 35 | 42 | 46 | 39 | 69 | 48 |
| NM_012434 | SLC17A5 | 3314 | 7 | 2 | 2 | 1 | 2 | 2 | 7 | 4 | 3 | 1 | 1 | 2 | 2 |
| NM_012435 | SHC2 | 2494 | 0 | 0 | 0 | 0 | 0 | 0 | 0 | 0 | 0 | 0 | 0 | 0 | 0 |

| transcript | gene | transcript length (nt) | max | mean | adipose | colon | heart | hypothalamus | kidney | liver | lung | ovary | skeletalmuscle | spleen | testes |
|---|---|---|---|---|---|---|---|---|---|---|---|---|---|---|---|
| NM_012437 | SNAPIN | 1034 | 3 | 2 | 2 | 1 | 3 | 3 | 2 | 2 | 2 | 1 | 3 | 3 | 3 |
| NM_012443 | SPAG6 | 2594 | 8 | 1 | 0 | 0 | 0 | 2 | 0 | 0 | 2 | 0 | 0 | 0 | 8 |
| NM_012444 | SPO11 | 1817 | 0 | 0 | 0 | 0 | 0 | 0 | 0 | 0 | 0 | 0 | 0 | 0 | 0 |
| NM_012445 | SPON2 | 1848 | 2 | 1 | 1 | 1 | 0 | 0 | 1 | 1 | 1 | 2 | 0 | 0 | 0 |
| NM_012446 | SSBP2 | 1948 | 8 | 4 | 3 | 1 | 6 | 8 | 3 | 0 | 4 | 8 | 4 | 4 | 4 |
| NM_012447 | STAG3 | 4205 | 3 | 1 | 0 | 0 | 0 | 0 | 0 | 0 | 0 | 1 | 1 | 1 | 3 |
| NM_012448 | STAT5B | 5094 | 3 | 2 | 3 | 2 | 2 | 2 | 2 | 1 | 2 | 3 | 3 | 3 | 2 |
| NM_012449 | STEAP1 | 1313 | 1 | 0 | 1 | 0 | 0 | 0 | 0 | 1 | 0 | 0 | 0 | 0 | 0 |
| NM_012450 | SLC13A4 | 2894 | 9 | 1 | 0 | 0 | 0 | 9 | 0 | 0 | 0 | 0 | 0 | 0 | 1 |
| NM_012451 | SYNGR4 | 1009 | 0 | 0 | 0 | 0 | 0 | 0 | 0 | 0 | 0 | 0 | 0 | 0 | 0 |
| NM_012452 | TNFRSF13B | 1358 | 44 | 5 | 0 | 7 | 0 | 0 | 0 | 0 | 2 | 0 | 0 | 44 | 0 |
| NM_012453 | TBL2 | 3041 | 10 | 3 | 2 | 2 | 2 | 2 | 2 | 2 | 2 | 4 | 1 | 2 | 10 |
| NM_012454 | TIAM2 | 5916 | 1 | 0 | 0 | 0 | 0 | 1 | 0 | 0 | 0 | 0 | 1 | 0 | 1 |
| NM_012455 | PSD4 | 5039 | 2 | 1 | 0 | 1 | 0 | 0 | 1 | 2 | 1 | 0 | 0 | 2 | 1 |
| NM_012456 | TIMM10 | 674 | 14 | 5 | 5 | 2 | 6 | 14 | 6 | 10 | 2 | 2 | 3 | 4 | 3 |
| NM_012458 | TIMM13 | 1956 | 10 | 4 | 3 | 2 | 4 | 5 | 4 | 10 | 2 | 3 | 2 | 4 | 3 |
| NM_012459 | TIMM8B | 810 | 6 | 3 | 3 | 2 | 6 | 4 | 3 | 4 | 2 | 1 | 2 | 3 | 2 |
| NM_012460 | TIMM9 | 1078 | 15 | 9 | 7 | 3 | 10 | 12 | 8 | 9 | 5 | 15 | 11 | 9 | 10 |
| NM_012461 | TINF2 | 2177 | 3 | 2 | 2 | 2 | 2 | 2 | 2 | 1 | 1 | 1 | 1 | 3 | 2 |
| NM_012463 | ATP6V0A2 | 6539 | 3 | 2 | 1 | 1 | 2 | 1 | 2 | 2 | 2 | 1 | 2 | 3 | 3 |
| NM_012464 | TLL1 | 6654 | 1 | 0 | 1 | 0 | 0 | 1 | 1 | 0 | 1 | 0 | 0 | 0 | 0 |
| NM_012465 | TLL2 | 6753 | 0 | 0 | 0 | 0 | 0 | 0 | 0 | 0 | 0 | 0 | 0 | 0 | 0 |
| NM_012466 | TSPAN16 | 1040 | 2 | 0 | 0 | 0 | 0 | 0 | 0 | 0 | 0 | 0 | 0 | 0 | 2 |
| NM_012467 | TPSG1 | 1104 | 0 | 0 | 0 | 0 | 0 | 0 | 0 | 0 | 0 | 0 | 0 | 0 | 0 |
| NM_012469 | PRPF6 | 3101 | 21 | 15 | 12 | 11 | 13 | 20 | 14 | 10 | 10 | 19 | 17 | 17 | 21 |
| NM_012470 | TNPO3 | 3669 | 4 | 3 | 3 | 2 | 2 | 3 | 4 | 3 | 2 | 2 | 3 | 4 | 4 |
| NM_012471 | TRPC5 | 5840 | 1 | 0 | 0 | 0 | 0 | 1 | 0 | 0 | 0 | 0 | 0 | 0 | 0 |
| NM_012472 | LRRC6 | 1582 | 15 | 5 | 3 | 1 | 2 | 10 | 11 | 1 | 4 | 5 | 0 | 2 | 15 |
| NM_012473 | TXN2 | 1336 | 4 | 2 | 2 | 3 | 3 | 4 | 3 | 3 | 1 | 2 | 2 | 3 | 1 |
| NM_012474 | UCK2 | 1246 | 4 | 2 | 2 | 2 | 4 | 4 | 1 | 3 | 1 | 2 | 2 | 3 | 2 |
| NM_012475 | USP21 | 2213 | 1 | 0 | 0 | 1 | 0 | 0 | 0 | 0 | 0 | 0 | 0 | 1 | 0 |
| NM_012476 | VAX2 | 1144 | 0 | 0 | 0 | 0 | 0 | 0 | 0 | 0 | 0 | 0 | 0 | 0 | 0 |
| NM_012477 | WBP1 | 1165 | 2 | 1 | 2 | 1 | 1 | 2 | 2 | 1 | 1 | 1 | 1 | 2 | 1 |
| NM_012478 | WBP2 | 1944 | 23 | 8 | 5 | 4 | 10 | 23 | 7 | 6 | 8 | 4 | 8 | 9 | 5 |
| NM_012479 | YWHAG | 3747 | 143 | 42 | 64 | 11 | 50 | 143 | 20 | 22 | 19 | 18 | 63 | 31 | 20 |
| NM_012481 | IKZF3 | 2436 | 46 | 5 | 0 | 3 | 1 | 0 | 1 | 0 | 6 | 1 | 0 | 46 | 2 |
| NM_012482 | ZNF281 | 3521 | 6 | 3 | 2 | 1 | 2 | 4 | 3 | 6 | 4 | 2 | 2 | 6 | 2 |
| NM_012483 | GNLY | 988 | 2 | 0 | 0 | 0 | 0 | 0 | 0 | 0 | 1 | 0 | 0 | 2 | 0 |
| NM_012484 | HMMR | 3136 | 5 | 1 | 1 | 0 | 1 | 0 | 0 | 0 | 1 | 0 | 0 | 2 | 5 |
| NM_012485 | HMMR | 3091 | 5 | 1 | 1 | 0 | 1 | 0 | 0 | 0 | 1 | 0 | 0 | 2 | 5 |
| NM_012486 | PSEN2 | 2295 | 2 | 1 | 2 | 1 | 1 | 2 | 2 | 1 | 1 | 1 | 1 | 2 | 2 |
| NM_013227 | ACAN | 8840 | 0 | 0 | 0 | 0 | 0 | 0 | 0 | 0 | 0 | 0 | 0 | 0 | 0 |
| NM_013229 | APAF1 | 7158 | 5 | 2 | 1 | 2 | 1 | 2 | 1 | 1 | 3 | 3 | 1 | 5 | 1 |
| NM_013230 | CD24 | 2180 | 63 | 9 | 0 | 20 | 0 | 2 | 63 | 1 | 5 | 0 | 0 | 4 | 2 |
| NM_013231 | FLRT2 | 7179 | 9 | 2 | 1 | 1 | 1 | 3 | 1 | 0 | 0 | 9 | 1 | 3 | 3 |
| NM_013232 | PDCD6 | 1135 | 6 | 2 | 2 | 1 | 2 | 6 | 2 | 2 | 1 | 2 | 1 | 4 | 2 |
| NM_013233 | STK39 | 3274 | 12 | 3 | 3 | 3 | 4 | 12 | 2 | 0 | 2 | 2 | 0 | 3 | 5 |
| NM_013234 | EIF3K | 879 | 22 | 14 | 17 | 16 | 22 | 21 | 16 | 15 | 8 | 7 | 18 | 12 | 6 |
| NM_013235 | RNASEN | 5421 | 8 | 5 | 3 | 4 | 5 | 8 | 6 | 3 | 3 | 8 | 4 | 6 | 4 |
| NM_013236 | ATXN10 | 3329 | 10 | 5 | 2 | 2 | 5 | 10 | 4 | 5 | 3 | 6 | 3 | 5 | 5 |
| NM_013237 | PRELID1 | 1191 | 3 | 1 | 0 | 2 | 0 | 1 | 1 | 3 | 0 | 0 | 0 | 2 | 0 |
| NM_013238 | DNAJC15 | 2776 | 11 | 5 | 3 | 3 | 11 | 8 | 10 | 3 | 3 | 1 | 4 | 6 | 5 |
| NM_013239 | PPP2R3B | 2061 | 0 | 0 | 0 | 0 | 0 | 0 | 0 | 0 | 0 | 0 | 0 | 0 | 0 |
| NM_013240 | N6AMT1 | 4865 | 2 | 1 | 1 | 1 | 1 | 1 | 1 | 0 | 1 | 2 | 1 | 1 | 2 |
| NM_013241 | FHOD1 | 3854 | 1 | 1 | 0 | 1 | 0 | 0 | 0 | 0 | 1 | 0 | 1 | 1 | 0 |
| NM_013242 | C16orf80 | 1291 | 3 | 1 | 1 | 1 | 1 | 3 | 1 | 2 | 1 | 1 | 0 | 1 | 2 |
| NM_013243 | SCG3 | 3309 | 50 | 5 | 0 | 1 | 0 | 50 | 0 | 0 | 0 | 0 | 0 | 0 | 0 |
| NM_013244 | MGAT4C | 1934 | 4 | 1 | 0 | 0 | 1 | 4 | 0 | 0 | 0 | 0 | 0 | 0 | 1 |
| NM_013245 | VPS4A | 2176 | 26 | 13 | 10 | 8 | 14 | 20 | 15 | 8 | 7 | 7 | 26 | 12 | 10 |
| NM_013246 | CLCF1 | 1842 | 0 | 0 | 0 | 0 | 0 | 0 | 0 | 0 | 0 | 0 | 0 | 0 | 0 |
| NM_013247 | HTRA2 | 2550 | 0 | 0 | 0 | 0 | 0 | 0 | 0 | 0 | 0 | 0 | 0 | 0 | 0 |
| NM_013248 | NXT1 | 1117 | 2 | 1 | 0 | 1 | 1 | 0 | 1 | 0 | 1 | 1 | 1 | 2 | 1 |
| NM_013249 | ZNF214 | 2668 | 5 | 1 | 1 | 0 | 1 | 2 | 2 | 1 | 1 | 5 | 1 | 0 | 3 |
| NM_013250 | ZNF215 | 3658 | 0 | 0 | 0 | 0 | 0 | 0 | 0 | 0 | 0 | 0 | 0 | 0 | 0 |
| NM_013251 | TAC3 | 816 | 0 | 0 | 0 | 0 | 0 | 0 | 0 | 0 | 0 | 0 | 0 | 0 | 0 |
| NM_013252 | CLEC5A | 3511 | 1 | 0 | 0 | 0 | 0 | 0 | 0 | 0 | 1 | 0 | 0 | 1 | 0 |
| NM_013253 | DKK3 | 2746 | 143 | 33 | 2 | 9 | 118 | 143 | 5 | 1 | 25 | 12 | 2 | 12 | 29 |
| NM_013254 | TBK1 | 2982 | 8 | 5 | 3 | 1 | 4 | 5 | 4 | 6 | 6 | 4 | 3 | 8 | 7 |
| NM_013255 | MKLN1 | 11182 | 8 | 5 | 4 | 2 | 4 | 5 | 6 | 4 | 5 | 7 | 4 | 6 | 8 |
| NM_013256 | ZNF180 | 3121 | 3 | 1 | 1 | 0 | 1 | 1 | 2 | 1 | 1 | 3 | 1 | 2 | 1 |
| NM_013257 | SGK3 | 4146 | 2 | 1 | 1 | 1 | 1 | 2 | 2 | 2 | 2 | 1 | 1 | 2 | 2 |
| NM_013258 | PYCARD | 927 | 1 | 0 | 0 | 0 | 0 | 0 | 0 | 0 | 1 | 0 | 0 | 0 | 0 |
| NM_013259 | TAGLN3 | 1489 | 21 | 2 | 0 | 0 | 0 | 21 | 0 | 0 | 0 | 0 | 0 | 0 | 0 |
| NM_013260 | SAP30BP | 2519 | 7 | 3 | 4 | 5 | 3 | 7 | 2 | 4 | 2 | 2 | 2 | 3 | 2 |
| NM_013261 | PPARGC1A | 6318 | 17 | 5 | 1 | 4 | 11 | 4 | 17 | 13 | 1 | 0 | 5 | 0 | 2 |
| NM_013262 | MYLIP | 3071 | 9 | 4 | 4 | 2 | 2 | 2 | 4 | 1 | 4 | 5 | 4 | 9 | 4 |
| NM_013263 | BRD7 | 2204 | 58 | 29 | 23 | 17 | 27 | 36 | 26 | 18 | 22 | 31 | 26 | 38 | 58 |

| transcript | gene | transcript length (nt) | max | mean | adipose | colon | heart | hypothalamus | kidney | liver | lung | ovary | skeletalmuscle | spleen | testes |
|---|---|---|---|---|---|---|---|---|---|---|---|---|---|---|---|
| NM_013264 | DDX25 | 1822 | 3 | 1 | 0 | 0 | 0 | 3 | 0 | 0 | 0 | 0 | 0 | 0 | 3 |
| NM_013265 | C11orf2 | 2510 | 8 | 4 | 3 | 3 | 5 | 5 | 4 | 4 | 3 | 8 | 4 | 4 | 2 |
| NM_013266 | CTNNA3 | 3248 | 8 | 2 | 0 | 0 | 8 | 3 | 0 | 0 | 0 | 1 | 4 | 0 | 1 |
| NM_013267 | GLS2 | 2623 | 4 | 1 | 0 | 0 | 0 | 2 | 0 | 4 | 0 | 0 | 0 | 0 | 0 |
| NM_013268 | LGALS13 | 595 | 0 | 0 | 0 | 0 | 0 | 0 | 0 | 0 | 0 | 0 | 0 | 0 | 0 |
| NM_013269 | CLEC2D | 5167 | 8 | 3 | 2 | 1 | 1 | 1 | 1 | 1 | 4 | 5 | 1 | 8 | 2 |
| NM_013270 | PRSS50 | 1347 | 1 | 0 | 0 | 0 | 0 | 0 | 0 | 0 | 0 | 0 | 0 | 0 | 1 |
| NM_013271 | PCSK1N | 973 | 4 | 1 | 0 | 0 | 0 | 4 | 2 | 0 | 0 | 0 | 0 | 0 | 0 |
| NM_013272 | SLCO3A1 | 5106 | 2 | 1 | 1 | 0 | 2 | 1 | 1 | 0 | 2 | 1 | 0 | 2 | 1 |
| NM_013274 | POLL | 2677 | 1 | 0 | 0 | 1 | 0 | 0 | 0 | 0 | 0 | 0 | 0 | 0 | 1 |
| NM_013275 | ANKRD11 | 9304 | 12 | 8 | 5 | 5 | 9 | 10 | 6 | 3 | 6 | 11 | 9 | 12 | 10 |
| NM_013276 | SHPK | 3838 | 3 | 1 | 1 | 2 | 1 | 1 | 3 | 3 | 1 | 1 | 0 | 1 | 1 |
| NM_013277 | RACGAP1 | 3315 | 5 | 2 | 2 | 2 | 1 | 2 | 1 | 1 | 2 | 1 | 1 | 2 | 5 |
| NM_013278 | IL17C | 1045 | 0 | 0 | 0 | 0 | 0 | 0 | 0 | 0 | 0 | 0 | 0 | 0 | 0 |
| NM_013279 | C11orf9 | 5745 | 2 | 0 | 0 | 0 | 0 | 2 | 0 | 0 | 1 | 0 | 0 | 0 | 0 |
| NM_013280 | FLRT1 | 3247 | 1 | 0 | 0 | 0 | 0 | 1 | 0 | 0 | 0 | 0 | 0 | 0 | 0 |
| NM_013281 | FLRT3 | 3773 | 5 | 1 | 0 | 0 | 0 | 1 | 5 | 1 | 2 | 2 | 1 | 0 | 1 |
| NM_013282 | UHRF1 | 4064 | 1 | 0 | 0 | 0 | 0 | 0 | 0 | 0 | 0 | 0 | 0 | 1 | 0 |
| NM_013283 | MAT2B | 2072 | 24 | 11 | 6 | 6 | 11 | 11 | 15 | 8 | 12 | 14 | 8 | 24 | 11 |
| NM_013284 | POLM | 2620 | 1 | 0 | 0 | 0 | 0 | 0 | 0 | 0 | 0 | 0 | 0 | 1 | 0 |
| NM_013285 | GNL2 | 2401 | 21 | 12 | 10 | 3 | 17 | 10 | 11 | 13 | 10 | 10 | 21 | 14 | 18 |
| NM_013286 | RBM15B | 6606 | 2 | 1 | 1 | 1 | 1 | 1 | 1 | 1 | 1 | 2 | 1 | 2 | 1 |
| NM_013289 | KIR3DL1 | 1901 | 0 | 0 | 0 | 0 | 0 | 0 | 0 | 0 | 0 | 0 | 0 | 0 | 0 |
| NM_013290 | PSMC3IP | 1318 | 12 | 3 | 3 | 2 | 3 | 3 | 2 | 0 | 3 | 4 | 3 | 3 | 12 |
| NM_013291 | CPSF1 | 4494 | 2 | 1 | 1 | 2 | 0 | 1 | 1 | 1 | 1 | 0 | 0 | 1 | 1 |
| NM_013292 | MYLPF | 655 | 183 | 17 | 0 | 0 | 0 | 0 | 0 | 0 | 0 | 0 | 183 | 0 | 0 |
| NM_013293 | TRA2A | 1839 | 17 | 9 | 7 | 9 | 8 | 9 | 9 | 7 | 8 | 13 | 5 | 17 | 10 |
| NM_013296 | GPSM2 | 3033 | 5 | 2 | 1 | 2 | 1 | 5 | 1 | 0 | 2 | 1 | 2 | 1 | 3 |
| NM_013299 | SAC3D1 | 1567 | 2 | 1 | 1 | 1 | 0 | 1 | 1 | 1 | 1 | 1 | 1 | 1 | 2 |
| NM_013300 | C12orf24 | 1521 | 6 | 2 | 1 | 1 | 2 | 6 | 1 | 1 | 1 | 3 | 1 | 3 | 5 |
| NM_013301 | CCDC106 | 1992 | 0 | 0 | 0 | 0 | 0 | 0 | 0 | 0 | 0 | 0 | 0 | 0 | 0 |
| NM_013302 | EEF2K | 7412 | 3 | 2 | 3 | 2 | 3 | 1 | 1 | 2 | 2 | 2 | 2 | 3 | 1 |
| NM_013304 | ZDHHC1 | 2008 | 0 | 0 | 0 | 0 | 0 | 0 | 0 | 0 | 0 | 0 | 0 | 0 | 0 |
| NM_013305 | ST8SIA5 | 2623 | 5 | 1 | 0 | 0 | 1 | 5 | 0 | 0 | 0 | 0 | 0 | 0 | 0 |
| NM_013306 | SNX15 | 1909 | 1 | 1 | 1 | 1 | 0 | 1 | 1 | 0 | 0 | 1 | 0 | 1 | 0 |
| NM_013308 | GPR171 | 1795 | 1 | 0 | 0 | 0 | 0 | 0 | 0 | 0 | 0 | 0 | 0 | 1 | 0 |
| NM_013309 | SLC30A4 | 4285 | 11 | 4 | 3 | 3 | 3 | 6 | 5 | 1 | 2 | 5 | 1 | 5 | 11 |
| NM_013310 | C2orf27A | 1219 | 0 | 0 | 0 | 0 | 0 | 0 | 0 | 0 | 0 | 0 | 0 | 0 | 0 |
| NM_013312 | HOOK2 | 2610 | 8 | 3 | 8 | 4 | 3 | 1 | 5 | 1 | 1 | 1 | 1 | 1 | 2 |
| NM_013313 | YPEL1 | 4284 | 1 | 0 | 0 | 0 | 0 | 1 | 0 | 0 | 0 | 0 | 0 | 1 | 1 |
| NM_013314 | BLNK | 1823 | 10 | 2 | 1 | 3 | 0 | 1 | 3 | 5 | 1 | 0 | 0 | 10 | 1 |
| NM_013316 | CNOT4 | 3315 | 6 | 3 | 2 | 2 | 3 | 4 | 3 | 1 | 3 | 5 | 4 | 4 | 6 |
| NM_013318 | BAT2L1 | 11059 | 13 | 5 | 4 | 5 | 4 | 13 | 5 | 2 | 4 | 8 | 3 | 7 | 4 |
| NM_013319 | UBIAD1 | 3653 | 1 | 1 | 0 | 0 | 1 | 1 | 1 | 1 | 0 | 1 | 1 | 1 | 1 |
| NM_013320 | HCFC2 | 5734 | 3 | 1 | 1 | 1 | 1 | 2 | 1 | 1 | 1 | 2 | 2 | 2 | 3 |
| NM_013321 | SNX8 | 1493 | 1 | 0 | 0 | 0 | 0 | 0 | 0 | 0 | 0 | 0 | 0 | 1 | 1 |
| NM_013322 | SNX10 | 2578 | 20 | 8 | 8 | 1 | 2 | 20 | 19 | 16 | 11 | 1 | 0 | 10 | 1 |
| NM_013323 | SNX11 | 2315 | 1 | 1 | 1 | 1 | 0 | 1 | 1 | 0 | 1 | 0 | 0 | 1 | 1 |
| NM_013324 | CISH | 2267 | 3 | 1 | 1 | 0 | 2 | 1 | 3 | 1 | 1 | 1 | 1 | 0 | 0 |
| NM_013325 | ATG4B | 2877 | 1 | 0 | 0 | 1 | 0 | 1 | 1 | 0 | 0 | 0 | 0 | 0 | 1 |
| NM_013326 | C18orf8 | 2171 | 5 | 3 | 2 | 2 | 3 | 4 | 4 | 5 | 3 | 2 | 1 | 5 | 4 |
| NM_013327 | PARVB | 1692 | 4 | 1 | 1 | 0 | 1 | 1 | 1 | 1 | 1 | 1 | 4 | 2 | 0 |
| NM_013328 | PYCR2 | 1693 | 10 | 6 | 3 | 3 | 10 | 9 | 7 | 4 | 6 | 5 | 6 | 9 | 5 |
| NM_013329 | C21orf66 | 2808 | 6 | 4 | 4 | 2 | 3 | 2 | 4 | 2 | 4 | 4 | 2 | 5 | 6 |
| NM_013330 | NME7 | 1623 | 13 | 8 | 4 | 4 | 8 | 13 | 9 | 4 | 7 | 13 | 3 | 7 | 12 |
| NM_013332 | C7orf68 | 1412 | 2 | 1 | 2 | 0 | 1 | 2 | 1 | 0 | 1 | 1 | 0 | 0 | 1 |
| NM_013333 | EPN1 | 2406 | 3 | 2 | 1 | 2 | 2 | 2 | 2 | 2 | 1 | 1 | 2 | 3 | 2 |
| NM_013334 | GMPPB | 1642 | 1 | 0 | 0 | 1 | 0 | 0 | 0 | 1 | 1 | 0 | 0 | 0 | 0 |
| NM_013335 | GMPPA | 1522 | 1 | 0 | 0 | 0 | 0 | 0 | 0 | 1 | 0 | 0 | 0 | 0 | 0 |
| NM_013336 | SEC61A1 | 3636 | 14 | 5 | 5 | 3 | 6 | 3 | 7 | 14 | 5 | 3 | 2 | 7 | 5 |
| NM_013337 | TIMM22 | 1673 | 2 | 1 | 1 | 1 | 1 | 2 | 2 | 1 | 0 | 1 | 1 | 2 | 1 |
| NM_013338 | ALG5 | 1211 | 26 | 8 | 7 | 7 | 6 | 8 | 10 | 26 | 6 | 6 | 3 | 8 | 7 |
| NM_013339 | ALG6 | 3370 | 1 | 0 | 0 | 0 | 0 | 0 | 1 | 0 | 1 | 0 | 0 | 1 | 0 |
| NM_013340 | PCDHB1 | 2534 | 0 | 0 | 0 | 0 | 0 | 0 | 0 | 0 | 0 | 0 | 0 | 0 | 0 |
| NM_013341 | OLA1 | 4356 | 9 | 6 | 3 | 2 | 7 | 8 | 7 | 6 | 4 | 5 | 9 | 6 | 8 |
| NM_013342 | TFPT | 1205 | 9 | 3 | 3 | 3 | 3 | 9 | 2 | 3 | 2 | 1 | 2 | 3 | 2 |
| NM_013345 | GPR132 | 3636 | 3 | 1 | 0 | 1 | 0 | 0 | 0 | 0 | 1 | 0 | 0 | 3 | 0 |
| NM_013346 | SNX12 | 642 | 12 | 7 | 8 | 10 | 6 | 12 | 9 | 6 | 7 | 6 | 3 | 10 | 7 |
| NM_013347 | RPA4 | 1439 | 1 | 0 | 0 | 0 | 0 | 1 | 0 | 0 | 1 | 1 | 1 | 0 | 1 |
| NM_013348 | KCNJ14 | 3049 | 0 | 0 | 0 | 0 | 0 | 0 | 0 | 0 | 0 | 0 | 0 | 0 | 0 |
| NM_013349 | NENF | 947 | 7 | 2 | 3 | 1 | 2 | 7 | 2 | 2 | 1 | 1 | 1 | 1 | 1 |
| NM_013351 | TBX21 | 2572 | 0 | 0 | 0 | 0 | 0 | 0 | 0 | 0 | 0 | 0 | 0 | 0 | 0 |
| NM_013352 | DSE | 4039 | 9 | 4 | 7 | 1 | 5 | 3 | 3 | 2 | 6 | 4 | 2 | 9 | 3 |
| NM_013353 | TMOD4 | 1322 | 73 | 7 | 0 | 0 | 0 | 0 | 0 | 0 | 0 | 0 | 73 | 0 | 0 |
| NM_013354 | CNOT7 | 2646 | 7 | 5 | 3 | 2 | 5 | 7 | 6 | 7 | 4 | 6 | 6 | 7 | 7 |
| NM_013355 | PKN3 | 3377 | 0 | 0 | 0 | 0 | 0 | 0 | 0 | 0 | 0 | 0 | 0 | 0 | 0 |
| NM_013356 | SLC16A8 | 1896 | 0 | 0 | 0 | 0 | 0 | 0 | 0 | 0 | 0 | 0 | 0 | 0 | 0 |

| transcript | gene | transcript length (nt) | max | mean | adipose | colon | heart | hypothalamus | kidney | liver | lung | ovary | skeletalmuscle | spleen | testes |
|---|---|---|---|---|---|---|---|---|---|---|---|---|---|---|---|
| NM_013357 | PURG | 1961 | 2 | 0 | 0 | 0 | 0 | 2 | 0 | 0 | 0 | 0 | 0 | 0 | 0 |
| NM_013358 | PADI1 | 3847 | 0 | 0 | 0 | 0 | 0 | 0 | 0 | 0 | 0 | 0 | 0 | 0 | 0 |
| NM_013359 | ZNF221 | 2426 | 10 | 4 | 4 | 1 | 3 | 6 | 3 | 1 | 3 | 10 | 2 | 4 | 5 |
| NM_013360 | ZNF222 | 1618 | 3 | 1 | 1 | 1 | 1 | 3 | 1 | 1 | 1 | 2 | 0 | 3 | 2 |
| NM_013361 | ZNF223 | 2414 | 3 | 2 | 1 | 1 | 1 | 3 | 2 | 0 | 1 | 3 | 0 | 2 | 2 |
| NM_013362 | ZNF225 | 2731 | 7 | 3 | 2 | 1 | 3 | 3 | 3 | 1 | 2 | 7 | 1 | 5 | 3 |
| NM_013363 | PCOLCE2 | 1910 | 30 | 5 | 30 | 2 | 15 | 1 | 3 | 1 | 3 | 1 | 2 | 1 | 1 |
| NM_013364 | PNMA3 | 3751 | 3 | 0 | 0 | 0 | 0 | 3 | 0 | 0 | 0 | 0 | 0 | 0 | 1 |
| NM_013365 | GGA1 | 3138 | 1 | 1 | 0 | 1 | 0 | 0 | 0 | 0 | 0 | 1 | 0 | 1 | 1 |
| NM_013366 | ANAPC2 | 2700 | 1 | 1 | 1 | 1 | 0 | 1 | 1 | 1 | 0 | 1 | 1 | 1 | 1 |
| NM_013367 | ANAPC4 | 2672 | 16 | 9 | 7 | 4 | 8 | 6 | 10 | 8 | 9 | 12 | 7 | 15 | 16 |
| NM_013368 | SERTAD3 | 1518 | 1 | 1 | 0 | 1 | 0 | 0 | 1 | 0 | 1 | 1 | 1 | 1 | 0 |
| NM_013369 | DNMT3L | 1705 | 0 | 0 | 0 | 0 | 0 | 0 | 0 | 0 | 0 | 0 | 0 | 0 | 0 |
| NM_013370 | OSGIN1 | 2391 | 2 | 1 | 0 | 0 | 0 | 0 | 1 | 2 | 0 | 0 | 0 | 0 | 1 |
| NM_013371 | IL19 | 1831 | 0 | 0 | 0 | 0 | 0 | 0 | 0 | 0 | 0 | 0 | 0 | 0 | 0 |
| NM_013372 | GREM1 | 4134 | 3 | 1 | 1 | 3 | 1 | 1 | 1 | 0 | 1 | 1 | 0 | 1 | 1 |
| NM_013373 | ZDHHC8 | 3455 | 0 | 0 | 0 | 0 | 0 | 0 | 0 | 0 | 0 | 0 | 0 | 0 | 0 |
| NM_013374 | PDCD6IP | 5939 | 9 | 6 | 7 | 4 | 7 | 6 | 8 | 7 | 6 | 6 | 5 | 9 | 7 |
| NM_013375 | ABT1 | 2254 | 2 | 1 | 1 | 0 | 1 | 1 | 1 | 1 | 1 | 1 | 0 | 2 | 1 |
| NM_013376 | SERTAD1 | 1204 | 0 | 0 | 0 | 0 | 0 | 0 | 0 | 0 | 0 | 0 | 0 | 0 | 0 |
| NM_013377 | PDZRN4 | 3243 | 5 | 1 | 1 | 2 | 0 | 2 | 1 | 0 | 0 | 2 | 0 | 0 | 5 |
| NM_013378 | VPREB3 | 582 | 1 | 0 | 0 | 0 | 0 | 0 | 0 | 0 | 0 | 0 | 0 | 1 | 0 |
| NM_013379 | DPP7 | 1617 | 1 | 1 | 1 | 0 | 1 | 1 | 1 | 0 | 1 | 0 | 1 | 1 | 1 |
| NM_013380 | ZFP112 | 3686 | 4 | 2 | 1 | 1 | 2 | 2 | 2 | 0 | 1 | 4 | 1 | 2 | 3 |
| NM_013381 | TRHDE | 5551 | 2 | 1 | 2 | 0 | 0 | 2 | 2 | 0 | 1 | 2 | 0 | 2 | 1 |
| NM_013382 | POMT2 | 4872 | 2 | 1 | 0 | 0 | 0 | 1 | 1 | 0 | 0 | 0 | 1 | 0 | 2 |
| NM_013385 | CYTH4 | 3137 | 3 | 1 | 2 | 1 | 1 | 1 | 0 | 0 | 2 | 0 | 0 | 3 | 0 |
| NM_013386 | SLC25A24 | 3476 | 6 | 3 | 3 | 2 | 3 | 1 | 3 | 1 | 5 | 3 | 1 | 6 | 2 |
| NM_013387 | UCRC | 916 | 20 | 10 | 9 | 10 | 18 | 20 | 9 | 16 | 3 | 2 | 12 | 5 | 6 |
| NM_013388 | PREB | 2164 | 5 | 3 | 5 | 2 | 3 | 3 | 3 | 5 | 2 | 2 | 3 | 4 | 3 |
| NM_013389 | NPC1L1 | 5047 | 2 | 0 | 0 | 0 | 0 | 0 | 0 | 2 | 0 | 0 | 0 | 0 | 0 |
| NM_013390 | TMEM2 | 6523 | 16 | 6 | 4 | 4 | 4 | 3 | 4 | 12 | 14 | 1 | 2 | 16 | 4 |
| NM_013391 | DMGDH | 3083 | 30 | 6 | 3 | 0 | 1 | 1 | 30 | 25 | 0 | 1 | 0 | 0 | 1 |
| NM_013392 | NRBP1 | 2199 | 38 | 27 | 26 | 19 | 29 | 31 | 20 | 32 | 18 | 33 | 27 | 29 | 38 |
| NM_013393 | FTSJ2 | 1601 | 5 | 3 | 3 | 2 | 2 | 4 | 4 | 4 | 2 | 2 | 2 | 5 | 3 |
| NM_013396 | USP25 | 4968 | 29 | 11 | 9 | 3 | 12 | 11 | 10 | 6 | 10 | 9 | 29 | 12 | 15 |
| NM_013397 | PRICKLE4 | 1646 | 1 | 0 | 0 | 0 | 0 | 0 | 0 | 0 | 0 | 0 | 0 | 1 | 0 |
| NM_013398 | ZNF224 | 2466 | 12 | 5 | 5 | 3 | 4 | 5 | 6 | 1 | 6 | 8 | 3 | 12 | 6 |
| NM_013399 | C16orf5 | 2741 | 2 | 1 | 0 | 1 | 1 | 2 | 1 | 0 | 0 | 1 | 0 | 1 | 1 |
| NM_013400 | REPIN1 | 3292 | 3 | 2 | 2 | 3 | 1 | 2 | 2 | 2 | 1 | 1 | 2 | 2 | 1 |
| NM_013401 | RAB3IL1 | 2284 | 3 | 1 | 1 | 1 | 2 | 1 | 2 | 0 | 1 | 1 | 1 | 3 | 3 |
| NM_013402 | FADS1 | 4414 | 21 | 9 | 10 | 2 | 12 | 21 | 4 | 11 | 11 | 8 | 2 | 6 | 10 |
| NM_013403 | STRN4 | 3189 | 3 | 2 | 1 | 2 | 2 | 3 | 2 | 1 | 1 | 1 | 1 | 2 | 3 |
| NM_013404 | MSLN | 2076 | 1 | 0 | 0 | 0 | 0 | 0 | 0 | 0 | 1 | 0 | 0 | 0 | 0 |
| NM_013406 | DHPS | 1184 | 2 | 1 | 1 | 1 | 1 | 2 | 1 | 1 | 1 | 1 | 1 | 2 | 1 |
| NM_013407 | DHPS | 1157 | 2 | 1 | 1 | 1 | 1 | 2 | 1 | 1 | 1 | 1 | 2 | 2 | 1 |
| NM_013409 | FST | 1103 | 4 | 1 | 2 | 1 | 1 | 0 | 0 | 4 | 0 | 1 | 1 | 0 | 1 |
| NM_013410 | AK3L1 | 2192 | 21 | 5 | 5 | 1 | 8 | 3 | 21 | 11 | 0 | 1 | 1 | 1 | 4 |
| NM_013411 | AK2 | 3606 | 12 | 5 | 5 | 5 | 4 | 3 | 12 | 7 | 4 | 3 | 3 | 6 | 4 |
| NM_013412 | RABL2A | 2170 | 1 | 0 | 0 | 0 | 0 | 1 | 0 | 0 | 0 | 1 | 0 | 0 | 0 |
| NM_013416 | NCF4 | 1643 | 4 | 1 | 1 | 1 | 1 | 1 | 0 | 0 | 1 | 0 | 0 | 4 | 0 |
| NM_013417 | IARS | 4510 | 9 | 6 | 5 | 3 | 8 | 9 | 7 | 9 | 4 | 4 | 8 | 7 | 6 |
| NM_013423 | ARHGAP6 | 3947 | 2 | 1 | 1 | 1 | 1 | 1 | 1 | 0 | 1 | 2 | 1 | 1 | 1 |
| NM_013427 | ARHGAP6 | 5118 | 1 | 1 | 0 | 0 | 0 | 0 | 1 | 0 | 1 | 1 | 1 | 1 | 1 |
| NM_013430 | GGT1 | 2363 | 2 | 0 | 0 | 0 | 0 | 0 | 2 | 1 | 0 | 0 | 0 | 0 | 0 |
| NM_013431 | KLRC4 | 928 | 2 | 0 | 0 | 0 | 0 | 0 | 0 | 0 | 1 | 0 | 0 | 2 | 0 |
| NM_013432 | NFKBIL2 | 4515 | 0 | 0 | 0 | 0 | 0 | 0 | 0 | 0 | 0 | 0 | 0 | 0 | 0 |
| NM_013433 | TNPO2 | 4904 | 9 | 3 | 2 | 2 | 2 | 9 | 3 | 1 | 1 | 2 | 1 | 2 | 3 |
| NM_013434 | KCNIP3 | 2914 | 1 | 0 | 0 | 1 | 0 | 1 | 0 | 0 | 0 | 0 | 0 | 0 | 1 |
| NM_013435 | RAX | 3197 | 0 | 0 | 0 | 0 | 0 | 0 | 0 | 0 | 0 | 0 | 0 | 0 | 0 |
| NM_013436 | NCKAP1 | 4614 | 19 | 10 | 7 | 4 | 17 | 19 | 13 | 6 | 8 | 10 | 9 | 6 | 10 |
| NM_013437 | LRP12 | 4109 | 5 | 2 | 2 | 1 | 5 | 5 | 2 | 0 | 2 | 2 | 2 | 3 | 2 |
| NM_013438 | UBQLN1 | 4168 | 45 | 21 | 17 | 7 | 21 | 45 | 18 | 24 | 17 | 14 | 21 | 22 | 22 |
| NM_013439 | PILRA | 1316 | 1 | 0 | 1 | 0 | 0 | 0 | 0 | 0 | 1 | 0 | 0 | 1 | 0 |
| NM_013440 | PILRB | 3632 | 7 | 4 | 3 | 7 | 2 | 2 | 7 | 1 | 3 | 4 | 3 | 7 | 5 |
| NM_013441 | RCAN3 | 1699 | 10 | 3 | 2 | 6 | 2 | 3 | 3 | 0 | 6 | 1 | 0 | 10 | 3 |
| NM_013442 | STOML2 | 1277 | 18 | 9 | 4 | 3 | 18 | 9 | 11 | 13 | 4 | 8 | 10 | 9 | 7 |
| NM_013443 | ST6GALNAC | 2398 | 9 | 6 | 6 | 8 | 6 | 9 | 4 | 5 | 7 | 7 | 4 | 9 | 5 |
| NM_013444 | UBQLN2 | 3417 | 12 | 5 | 3 | 2 | 7 | 12 | 4 | 3 | 5 | 8 | 6 | 6 | 4 |
| NM_013445 | GAD1 | 1255 | 3 | 0 | 0 | 0 | 0 | 3 | 0 | 0 | 0 | 0 | 0 | 0 | 0 |
| NM_013446 | MKRN1 | 3151 | 23 | 14 | 8 | 13 | 15 | 13 | 12 | 7 | 13 | 14 | 16 | 21 | 23 |
| NM_013447 | EMR2 | 6771 | 6 | 1 | 0 | 0 | 0 | 0 | 0 | 0 | 1 | 0 | 0 | 6 | 0 |
| NM_013448 | BAZ1A | 6016 | 26 | 10 | 7 | 3 | 7 | 3 | 4 | 6 | 19 | 5 | 6 | 26 | 20 |
| NM_013449 | BAZ2A | 8934 | 7 | 4 | 3 | 5 | 3 | 3 | 4 | 2 | 6 | 3 | 2 | 7 | 7 |
| NM_013450 | BAZ2B | 8145 | 24 | 11 | 8 | 4 | 8 | 11 | 10 | 4 | 13 | 24 | 8 | 12 | 16 |
| NM_013451 | MYOF | 6843 | 8 | 4 | 8 | 4 | 4 | 2 | 3 | 0 | 6 | 4 | 1 | 5 | 3 |
| NM_013452 | VCX | 967 | 4 | 0 | 0 | 0 | 0 | 0 | 0 | 0 | 0 | 0 | 0 | 0 | 4 |

| transcript | gene | transcript length (nt) | max | mean | adipose | colon | heart | hypothala mus | kidney | liver | lung | ovary | skeletalm uscle | spleen | testes |
|---|---|---|---|---|---|---|---|---|---|---|---|---|---|---|---|
| NM_013453 | SPANXA1 | 384 | 0 | 0 | 0 | 0 | 0 | 0 | 0 | 0 | 0 | 0 | 0 | 0 | 0 |
| NM_013936 | OR12D2 | 1033 | 0 | 0 | 0 | 0 | 0 | 0 | 0 | 0 | 0 | 0 | 0 | 0 | 0 |
| NM_013937 | OR11A1 | 2229 | 0 | 0 | 0 | 0 | 0 | 0 | 0 | 0 | 0 | 0 | 0 | 0 | 0 |
| NM_013938 | OR10H3 | 951 | 0 | 0 | 0 | 0 | 0 | 0 | 0 | 0 | 0 | 0 | 0 | 0 | 0 |
| NM_013939 | OR10H2 | 1029 | 0 | 0 | 0 | 0 | 0 | 0 | 0 | 0 | 0 | 0 | 0 | 0 | 0 |
| NM_013940 | OR10H1 | 1120 | 0 | 0 | 0 | 0 | 0 | 0 | 0 | 0 | 0 | 0 | 0 | 0 | 0 |
| NM_013941 | OR10C1 | 939 | 0 | 0 | 0 | 0 | 0 | 0 | 0 | 0 | 0 | 0 | 0 | 0 | 0 |
| NM_013942 | PAX3 | 1080 | 0 | 0 | 0 | 0 | 0 | 0 | 0 | 0 | 0 | 0 | 0 | 0 | 0 |
| NM_013943 | CLIC4 | 4435 | 128 | 40 | 30 | 13 | 128 | 38 | 58 | 14 | 59 | 25 | 13 | 30 | 31 |
| NM_013945 | PAX7 | 2253 | 0 | 0 | 0 | 0 | 0 | 0 | 0 | 0 | 0 | 0 | 0 | 0 | 0 |
| NM_013951 | PAX8 | 3866 | 7 | 1 | 0 | 0 | 0 | 0 | 7 | 0 | 0 | 0 | 0 | 0 | 0 |
| NM_013952 | PAX8 | 3976 | 7 | 1 | 0 | 0 | 0 | 0 | 7 | 0 | 0 | 0 | 0 | 0 | 0 |
| NM_013953 | PAX8 | 3745 | 7 | 1 | 0 | 0 | 0 | 0 | 7 | 0 | 0 | 0 | 0 | 0 | 0 |
| NM_013955 | NOX1 | 2382 | 3 | 0 | 0 | 3 | 0 | 0 | 0 | 0 | 0 | 0 | 0 | 0 | 0 |
| NM_013956 | NRG1 | 3093 | 2 | 1 | 0 | 1 | 0 | 2 | 1 | 2 | 0 | 0 | 0 | 0 | 1 |
| NM_013957 | NRG1 | 3069 | 2 | 1 | 0 | 1 | 0 | 2 | 1 | 2 | 0 | 0 | 0 | 0 | 1 |
| NM_013958 | NRG1 | 1725 | 3 | 1 | 0 | 1 | 0 | 3 | 2 | 2 | 1 | 1 | 0 | 0 | 1 |
| NM_013959 | NRG1 | 2359 | 2 | 1 | 0 | 1 | 0 | 2 | 1 | 0 | 0 | 0 | 0 | 0 | 0 |
| NM_013960 | NRG1 | 3220 | 2 | 1 | 0 | 1 | 0 | 2 | 1 | 2 | 0 | 0 | 0 | 0 | 1 |
| NM_013962 | NRG1 | 1986 | 2 | 1 | 0 | 1 | 0 | 2 | 2 | 2 | 0 | 0 | 0 | 0 | 1 |
| NM_013964 | NRG1 | 3078 | 2 | 1 | 0 | 1 | 0 | 2 | 1 | 2 | 0 | 0 | 0 | 0 | 1 |
| NM_013974 | DDAH2 | 1331 | 20 | 8 | 20 | 9 | 6 | 8 | 7 | 5 | 8 | 7 | 2 | 9 | 4 |
| NM_013975 | LIG3 | 3701 | 5 | 3 | 1 | 2 | 3 | 4 | 3 | 2 | 2 | 4 | 3 | 4 | 5 |
| NM_013976 | GCDH | 1590 | 4 | 1 | 1 | 1 | 2 | 1 | 2 | 4 | 0 | 1 | 1 | 1 | 0 |
| NM_013978 | BNIP1 | 1155 | 1 | 1 | 1 | 0 | 1 | 1 | 1 | 1 | 0 | 0 | 0 | 1 | 1 |
| NM_013979 | BNIP1 | 1386 | 1 | 1 | 1 | 0 | 1 | 1 | 1 | 1 | 0 | 1 | 0 | 1 | 1 |
| NM_013980 | BNIP1 | 1284 | 1 | 1 | 1 | 0 | 1 | 1 | 1 | 1 | 0 | 0 | 0 | 1 | 1 |
| NM_013981 | NRG2 | 3002 | 0 | 0 | 0 | 0 | 0 | 0 | 0 | 0 | 0 | 0 | 0 | 0 | 0 |
| NM_013982 | NRG2 | 3044 | 0 | 0 | 0 | 0 | 0 | 0 | 0 | 0 | 0 | 0 | 0 | 0 | 0 |
| NM_013983 | NRG2 | 3026 | 0 | 0 | 0 | 0 | 0 | 0 | 0 | 0 | 0 | 0 | 0 | 0 | 0 |
| NM_013986 | EWSR1 | 2677 | 39 | 24 | 19 | 20 | 25 | 24 | 25 | 28 | 19 | 16 | 18 | 39 | 30 |
| NM_013987 | PARK2 | 3989 | 3 | 2 | 2 | 1 | 3 | 3 | 3 | 0 | 1 | 2 | 3 | 1 | 2 |
| NM_013988 | PARK2 | 3626 | 3 | 1 | 1 | 1 | 3 | 2 | 3 | 0 | 1 | 1 | 3 | 0 | 2 |
| NM_013989 | DIO2 | 6695 | 7 | 1 | 0 | 0 | 2 | 7 | 1 | 0 | 0 | 1 | 1 | 0 | 1 |
| NM_013992 | PAX8 | 3643 | 8 | 1 | 0 | 0 | 0 | 0 | 8 | 0 | 0 | 0 | 0 | 0 | 0 |
| NM_013993 | DDR1 | 3861 | 14 | 3 | 1 | 5 | 2 | 14 | 6 | 0 | 3 | 1 | 2 | 1 | 3 |
| NM_013994 | DDR1 | 3662 | 14 | 3 | 1 | 5 | 2 | 14 | 6 | 0 | 3 | 1 | 2 | 1 | 3 |
| NM_013995 | LAMP2 | 4073 | 25 | 14 | 10 | 4 | 12 | 25 | 16 | 13 | 15 | 13 | 20 | 16 | 15 |
| NM_013996 | TAC1 | 1134 | 1 | 0 | 0 | 0 | 0 | 1 | 0 | 0 | 0 | 0 | 0 | 0 | 0 |
| NM_013997 | TAC1 | 1143 | 1 | 0 | 0 | 0 | 0 | 1 | 0 | 0 | 0 | 0 | 0 | 1 | 0 |
| NM_013998 | TAC1 | 1089 | 1 | 0 | 0 | 0 | 0 | 1 | 0 | 0 | 0 | 0 | 0 | 0 | 0 |
| NM_013999 | MEOX1 | 2141 | 2 | 1 | 2 | 1 | 2 | 0 | 0 | 0 | 0 | 0 | 1 | 1 | 1 |
| NM_014000 | VCL | 5486 | 46 | 23 | 24 | 29 | 41 | 8 | 16 | 4 | 20 | 17 | 19 | 29 | 46 |
| NM_014001 | GGA3 | 3747 | 4 | 2 | 2 | 2 | 2 | 4 | 2 | 1 | 2 | 2 | 2 | 4 | 2 |
| NM_014002 | IKBKE | 3263 | 1 | 0 | 0 | 1 | 0 | 0 | 0 | 0 | 0 | 0 | 0 | 1 | 0 |
| NM_014003 | DHX38 | 4454 | 9 | 7 | 7 | 7 | 8 | 8 | 6 | 7 | 6 | 8 | 7 | 9 | 9 |
| NM_014004 | PCDHGA8 | 3488 | 1 | 0 | 0 | 0 | 1 | 0 | 0 | 0 | 0 | 0 | 0 | 0 | 0 |
| NM_014005 | PCDHA9 | 6388 | 0 | 0 | 0 | 0 | 0 | 0 | 0 | 0 | 0 | 0 | 0 | 0 | 0 |
| NM_014007 | ZBTB43 | 5970 | 8 | 4 | 2 | 2 | 5 | 4 | 2 | 2 | 3 | 4 | 8 | 3 | 6 |
| NM_014008 | CCDC22 | 2715 | 0 | 0 | 0 | 0 | 0 | 0 | 0 | 0 | 0 | 0 | 0 | 0 | 0 |
| NM_014009 | FOXP3 | 2382 | 0 | 0 | 0 | 0 | 0 | 0 | 0 | 0 | 0 | 0 | 0 | 0 | 0 |
| NM_014010 | ASTN2 | 4591 | 5 | 1 | 0 | 0 | 1 | 3 | 1 | 0 | 0 | 0 | 0 | 0 | 5 |
| NM_014011 | SOCS5 | 4422 | 7 | 3 | 3 | 1 | 3 | 4 | 3 | 2 | 3 | 7 | 2 | 5 | 5 |
| NM_014012 | REM1 | 1656 | 0 | 0 | 0 | 0 | 0 | 0 | 0 | 0 | 0 | 0 | 0 | 0 | 0 |
| NM_014014 | SNRNP200 | 7173 | 26 | 14 | 8 | 10 | 12 | 11 | 15 | 10 | 12 | 18 | 16 | 19 | 26 |
| NM_014015 | DEXI | 1501 | 7 | 3 | 2 | 2 | 4 | 4 | 2 | 7 | 2 | 2 | 2 | 4 | 2 |
| NM_014016 | SACM1L | 3707 | 11 | 7 | 6 | 3 | 6 | 9 | 9 | 6 | 7 | 5 | 3 | 11 | 7 |
| NM_014017 | ROBLD3 | 681 | 29 | 10 | 12 | 5 | 10 | 12 | 8 | 29 | 5 | 3 | 6 | 11 | 4 |
| NM_014018 | MRPS28 | 707 | 39 | 19 | 22 | 12 | 24 | 31 | 30 | 39 | 9 | 6 | 15 | 13 | 13 |
| NM_014020 | TMEM176B | 1429 | 35 | 8 | 3 | 6 | 5 | 1 | 23 | 35 | 3 | 3 | 1 | 8 | 2 |
| NM_014021 | SSX2IP | 5897 | 15 | 4 | 0 | 1 | 2 | 12 | 5 | 3 | 1 | 1 | 1 | 2 | 15 |
| NM_014023 | WDR37 | 4611 | 4 | 1 | 1 | 1 | 2 | 4 | 1 | 1 | 1 | 1 | 1 | 3 | 1 |
| NM_014026 | DCPS | 1479 | 2 | 1 | 1 | 0 | 1 | 1 | 1 | 2 | 1 | 0 | 1 | 1 | 0 |
| NM_014028 | OSTM1 | 4467 | 10 | 6 | 9 | 2 | 9 | 7 | 5 | 3 | 5 | 3 | 4 | 10 | 6 |
| NM_014030 | GIT1 | 3733 | 2 | 1 | 0 | 0 | 1 | 2 | 1 | 0 | 1 | 0 | 0 | 1 | 2 |
| NM_014031 | SLC27A6 | 2616 | 7 | 1 | 0 | 0 | 4 | 0 | 0 | 0 | 0 | 2 | 0 | 0 | 7 |
| NM_014033 | METTL7A | 3390 | 38 | 15 | 23 | 9 | 8 | 13 | 21 | 38 | 17 | 10 | 5 | 14 | 10 |
| NM_014034 | ASF1A | 2064 | 4 | 2 | 1 | 0 | 2 | 4 | 2 | 1 | 2 | 3 | 2 | 4 | 3 |
| NM_014035 | SNX24 | 2136 | 3 | 1 | 1 | 0 | 2 | 2 | 3 | 1 | 2 | 1 | 0 | 1 | 1 |
| NM_014037 | SLC6A16 | 2933 | 3 | 0 | 0 | 0 | 0 | 0 | 0 | 0 | 0 | 1 | 0 | 0 | 3 |
| NM_014038 | BZW2 | 1857 | 14 | 4 | 1 | 1 | 14 | 2 | 2 | 2 | 2 | 1 | 9 | 2 | 1 |
| NM_014039 | C11orf54 | 2390 | 57 | 11 | 3 | 4 | 5 | 4 | 57 | 22 | 5 | 6 | 5 | 5 | 5 |
| NM_014041 | SPCS1 | 1084 | 20 | 12 | 4 | 5 | 14 | 15 | 20 | 17 | 7 | 11 | 11 | 13 | 13 |
| NM_014042 | C11orf51 | 882 | 1 | 0 | 0 | 0 | 0 | 0 | 0 | 0 | 1 | 0 | 0 | 1 | 0 |
| NM_014043 | CHMP2B | 2628 | 8 | 6 | 5 | 5 | 7 | 8 | 6 | 6 | 6 | 5 | 4 | 8 | 6 |
| NM_014044 | UNC50 | 1142 | 27 | 18 | 18 | 9 | 17 | 27 | 20 | 19 | 15 | 13 | 9 | 20 | 27 |
| NM_014045 | LRP10 | 3250 | 2 | 1 | 1 | 2 | 1 | 1 | 2 | 1 | 1 | 1 | 0 | 1 | 1 |

| transcript | gene | transcript length (nt) | max | mean | adipose | colon | heart | hypothalamus | kidney | liver | lung | ovary | skeletalmuscle | spleen | testes |
|---|---|---|---|---|---|---|---|---|---|---|---|---|---|---|---|
| NM_014046 | MRPS18B | 1534 | 23 | 12 | 8 | 8 | 17 | 11 | 14 | 23 | 7 | 8 | 17 | 12 | 6 |
| NM_014047 | C19orf53 | 909 | 7 | 4 | 4 | 2 | 3 | 7 | 2 | 6 | 2 | 3 | 3 | 3 | 3 |
| NM_014048 | MKL2 | 8634 | 11 | 3 | 3 | 1 | 3 | 11 | 2 | 1 | 3 | 2 | 2 | 2 | 4 |
| NM_014049 | ACAD9 | 2575 | 9 | 5 | 9 | 5 | 4 | 7 | 5 | 7 | 2 | 4 | 5 | 4 | 4 |
| NM_014050 | MRPL42 | 2019 | 4 | 3 | 1 | 1 | 3 | 3 | 4 | 3 | 3 | 2 | 2 | 3 | 3 |
| NM_014051 | TMEM14A | 1014 | 3 | 1 | 1 | 0 | 0 | 3 | 1 | 1 | 1 | 0 | 0 | 0 | 0 |
| NM_014053 | FLVCR1 | 3431 | 1 | 1 | 0 | 1 | 0 | 1 | 1 | 0 | 1 | 1 | 0 | 1 | 1 |
| NM_014055 | IFT81 | 3147 | 7 | 3 | 2 | 1 | 2 | 3 | 3 | 1 | 3 | 5 | 2 | 2 | 7 |
| NM_014056 | HIGD1A | 2767 | 12 | 4 | 3 | 3 | 6 | 10 | 5 | 12 | 2 | 1 | 2 | 2 | 2 |
| NM_014057 | OGN | 2785 | 28 | 6 | 10 | 2 | 8 | 6 | 1 | 0 | 3 | 28 | 1 | 1 | 4 |
| NM_014058 | TMPRSS11E | 2136 | 0 | 0 | 0 | 0 | 0 | 0 | 0 | 0 | 0 | 0 | 0 | 0 | 0 |
| NM_014059 | C13orf15 | 1108 | 4 | 1 | 4 | 0 | 0 | 1 | 0 | 0 | 3 | 0 | 0 | 1 | 0 |
| NM_014060 | MCTS1 | 9786 | 2 | 1 | 1 | 1 | 1 | 1 | 1 | 1 | 1 | 1 | 1 | 2 | 1 |
| NM_014061 | MAGEH1 | 1462 | 7 | 2 | 1 | 1 | 1 | 7 | 2 | 1 | 1 | 1 | 1 | 1 | 6 |
| NM_014062 | NOB1 | 1716 | 4 | 3 | 2 | 2 | 2 | 3 | 2 | 3 | 2 | 4 | 2 | 4 | 2 |
| NM_014063 | DBNL | 2194 | 13 | 5 | 5 | 4 | 5 | 6 | 4 | 4 | 5 | 2 | 2 | 13 | 3 |
| NM_014064 | METTL11A | 1002 | 6 | 3 | 1 | 1 | 5 | 3 | 3 | 2 | 2 | 1 | 4 | 3 | 6 |
| NM_014065 | ASTE1 | 2662 | 1 | 0 | 0 | 0 | 1 | 0 | 1 | 0 | 0 | 1 | 0 | 1 | 1 |
| NM_014066 | COMMD5 | 1459 | 0 | 0 | 0 | 0 | 0 | 0 | 0 | 0 | 0 | 0 | 0 | 0 | 0 |
| NM_014067 | MACROD1 | 1256 | 3 | 1 | 0 | 1 | 1 | 0 | 2 | 0 | 0 | 0 | 3 | 0 | 0 |
| NM_014068 | PSORS1C1 | 859 | 1 | 0 | 0 | 0 | 0 | 0 | 0 | 0 | 0 | 0 | 0 | 0 | 1 |
| NM_014068 | PSORS1C1 | 861 | 1 | 0 | 0 | 0 | 0 | 0 | 0 | 0 | 0 | 0 | 0 | 0 | 1 |
| NM_014069 | PSORS1C2 | 1151 | 0 | 0 | 0 | 0 | 0 | 0 | 0 | 0 | 0 | 0 | 0 | 0 | 0 |
| NM_014069 | PSORS1C2 | 1152 | 0 | 0 | 0 | 0 | 0 | 0 | 0 | 0 | 0 | 0 | 0 | 0 | 0 |
| NM_014070 | C6orf15 | 1136 | 0 | 0 | 0 | 0 | 0 | 0 | 0 | 0 | 0 | 0 | 0 | 0 | 0 |
| NM_014071 | NCOA6 | 7062 | 5 | 4 | 3 | 5 | 4 | 5 | 5 | 2 | 4 | 5 | 3 | 5 | 5 |
| NM_014077 | FAM32A | 1449 | 28 | 15 | 17 | 13 | 14 | 23 | 14 | 28 | 11 | 9 | 6 | 21 | 12 |
| NM_014078 | MRPL13 | 1110 | 4 | 2 | 2 | 2 | 3 | 2 | 3 | 4 | 1 | 1 | 1 | 2 | 1 |
| NM_014079 | KLF15 | 2525 | 8 | 3 | 2 | 1 | 4 | 2 | 5 | 8 | 3 | 3 | 3 | 1 | 2 |
| NM_014080 | DUOX2 | 6346 | 0 | 0 | 0 | 0 | 0 | 0 | 0 | 0 | 0 | 0 | 0 | 0 | 0 |
| NM_014089 | NUPL1 | 4335 | 5 | 3 | 1 | 1 | 2 | 3 | 3 | 1 | 3 | 3 | 2 | 3 | 5 |
| NM_014096 | SLC43A3 | 2724 | 6 | 1 | 2 | 0 | 1 | 0 | 0 | 6 | 1 | 1 | 1 | 2 | 1 |
| NM_014098 | PRDX3 | 1537 | 26 | 13 | 10 | 4 | 22 | 10 | 26 | 23 | 8 | 5 | 11 | 13 | 7 |
| NM_014106 | ZNF770 | 5403 | 7 | 5 | 3 | 1 | 5 | 6 | 7 | 6 | 3 | 7 | 5 | 6 | 5 |
| NM_014109 | ATAD2 | 5564 | 10 | 3 | 2 | 1 | 4 | 2 | 1 | 3 | 4 | 4 | 2 | 4 | 10 |
| NM_014110 | PPP1R8 | 2336 | 5 | 3 | 2 | 2 | 3 | 4 | 3 | 2 | 2 | 4 | 2 | 4 | 5 |
| NM_014112 | TRPS1 | 9990 | 4 | 2 | 2 | 1 | 2 | 4 | 3 | 1 | 2 | 3 | 1 | 2 | 2 |
| NM_014117 | C16orf72 | 3979 | 9 | 5 | 4 | 2 | 6 | 6 | 4 | 5 | 5 | 5 | 5 | 9 | 6 |
| NM_014138 | FAM156A | 2249 | 0 | 0 | 0 | 0 | 0 | 0 | 0 | 0 | 0 | 0 | 0 | 0 | 0 |
| NM_014139 | SCN11A | 6500 | 0 | 0 | 0 | 0 | 0 | 0 | 0 | 0 | 0 | 0 | 0 | 0 | 0 |
| NM_014140 | SMARCAL1 | 3269 | 1 | 1 | 1 | 1 | 1 | 1 | 1 | 0 | 1 | 1 | 1 | 1 | 1 |
| NM_014141 | CNTNAP2 | 9890 | 10 | 1 | 0 | 0 | 0 | 10 | 0 | 0 | 0 | 1 | 0 | 0 | 0 |
| NM_014142 | NUDT5 | 1204 | 11 | 4 | 5 | 3 | 2 | 3 | 5 | 11 | 3 | 2 | 0 | 5 | 3 |
| NM_014143 | CD274 | 1540 | 9 | 2 | 1 | 0 | 7 | 1 | 0 | 1 | 9 | 1 | 1 | 7 | 1 |
| NM_014145 | C20orf30 | 1689 | 12 | 9 | 10 | 4 | 11 | 8 | 12 | 12 | 10 | 8 | 7 | 12 | 8 |
| NM_014146 | LAT2 | 2002 | 8 | 2 | 0 | 1 | 1 | 3 | 0 | 1 | 2 | 0 | 1 | 8 | 0 |
| NM_014149 | WDR91 | 4619 | 2 | 1 | 0 | 0 | 0 | 1 | 2 | 0 | 1 | 1 | 0 | 1 | 0 |
| NM_014153 | ZC3H7A | 3845 | 6 | 4 | 4 | 2 | 3 | 3 | 4 | 3 | 4 | 3 | 4 | 6 | 2 |
| NM_014154 | ARMC8 | 3163 | 6 | 4 | 3 | 2 | 3 | 6 | 3 | 2 | 3 | 4 | 4 | 5 | 4 |
| NM_014155 | ZBTB44 | 9340 | 6 | 4 | 2 | 1 | 3 | 3 | 5 | 2 | 3 | 6 | 6 | 5 | 5 |
| NM_014157 | CCDC113 | 5264 | 3 | 1 | 0 | 0 | 0 | 3 | 0 | 0 | 1 | 0 | 0 | 0 | 1 |
| NM_014159 | SETD2 | 8431 | 29 | 17 | 13 | 7 | 15 | 14 | 17 | 12 | 15 | 29 | 18 | 26 | 26 |
| NM_014160 | MKRN2 | 2765 | 4 | 2 | 1 | 1 | 2 | 3 | 2 | 1 | 2 | 2 | 2 | 2 | 4 |
| NM_014161 | MRPL18 | 977 | 4 | 2 | 2 | 2 | 3 | 4 | 2 | 3 | 1 | 2 | 1 | 3 | 2 |
| NM_014164 | FXYD5 | 894 | 46 | 13 | 20 | 8 | 13 | 4 | 4 | 4 | 27 | 5 | 5 | 46 | 6 |
| NM_014165 | NDUFAF4 | 2410 | 4 | 2 | 1 | 0 | 4 | 4 | 3 | 2 | 0 | 2 | 2 | 2 | 2 |
| NM_014166 | MED4 | 2022 | 11 | 8 | 8 | 3 | 10 | 11 | 7 | 8 | 6 | 7 | 7 | 9 | 11 |
| NM_014167 | CCDC59 | 1611 | 3 | 2 | 1 | 0 | 2 | 2 | 2 | 2 | 1 | 2 | 2 | 2 | 3 |
| NM_014168 | METTL5 | 938 | 5 | 3 | 4 | 2 | 5 | 5 | 3 | 3 | 2 | 1 | 2 | 4 | 3 |
| NM_014169 | CHMP4A | 1505 | 22 | 14 | 21 | 14 | 13 | 18 | 12 | 12 | 13 | 12 | 6 | 22 | 12 |
| NM_014170 | GTPBP8 | 1357 | 1 | 1 | 1 | 0 | 1 | 1 | 1 | 1 | 1 | 1 | 1 | 1 | 1 |
| NM_014171 | CRIPT | 1917 | 2 | 1 | 1 | 1 | 1 | 2 | 1 | 1 | 1 | 1 | 1 | 1 | 1 |
| NM_014172 | PHPT1 | 1194 | 3 | 2 | 1 | 3 | 3 | 3 | 2 | 1 | 1 | 1 | 3 | 1 | 2 |
| NM_014173 | C19orf62 | 1413 | 3 | 2 | 1 | 1 | 2 | 3 | 1 | 1 | 1 | 1 | 2 | 2 | 1 |
| NM_014174 | THYN1 | 1321 | 6 | 3 | 2 | 2 | 3 | 6 | 2 | 2 | 1 | 3 | 1 | 2 | 3 |
| NM_014175 | MRPL15 | 1749 | 11 | 5 | 4 | 2 | 11 | 8 | 7 | 9 | 1 | 2 | 7 | 4 | 2 |
| NM_014176 | UBE2T | 907 | 3 | 1 | 0 | 1 | 1 | 3 | 0 | 0 | 1 | 0 | 1 | 2 | 2 |
| NM_014177 | C18orf55 | 1534 | 24 | 10 | 8 | 5 | 24 | 13 | 11 | 9 | 3 | 6 | 10 | 10 | 7 |
| NM_014178 | STXBP6 | 1959 | 6 | 2 | 1 | 1 | 5 | 6 | 1 | 0 | 5 | 0 | 1 | 0 | 2 |
| NM_014180 | MRPL22 | 3173 | 3 | 2 | 2 | 1 | 2 | 1 | 2 | 3 | 1 | 1 | 1 | 2 | 2 |
| NM_014181 | HSPC159 | 3827 | 6 | 2 | 1 | 1 | 2 | 3 | 2 | 1 | 2 | 1 | 6 | 2 | 2 |
| NM_014182 | ORMDL2 | 1334 | 2 | 1 | 1 | 1 | 0 | 1 | 2 | 2 | 1 | 0 | 0 | 2 | 1 |
| NM_014183 | DYNLRB1 | 685 | 42 | 18 | 17 | 11 | 21 | 42 | 23 | 12 | 12 | 13 | 16 | 16 | 13 |
| NM_014184 | CNIH4 | 623 | 3 | 1 | 2 | 1 | 1 | 1 | 2 | 3 | 2 | 1 | 1 | 2 | 1 |
| NM_014186 | COMMD9 | 2974 | 3 | 2 | 2 | 1 | 2 | 3 | 3 | 2 | 2 | 2 | 2 | 3 | 2 |
| NM_014187 | TMEM208 | 794 | 6 | 2 | 2 | 2 | 2 | 4 | 3 | 6 | 2 | 1 | 1 | 2 | 1 |
| NM_014188 | SSU72 | 1303 | 22 | 15 | 10 | 7 | 20 | 18 | 16 | 16 | 9 | 12 | 22 | 20 | 11 |

| transcript | gene | transcript length (nt) | max | mean | adipose | colon | heart | hypothalamus | kidney | liver | lung | ovary | skeletalmuscle | spleen | testes |
|---|---|---|---|---|---|---|---|---|---|---|---|---|---|---|---|
| NM_014189 | ADD1 | 4034 | 38 | 21 | 18 | 15 | 24 | 38 | 16 | 12 | 13 | 27 | 20 | 26 | 21 |
| NM_014190 | ADD1 | 3975 | 39 | 21 | 19 | 15 | 24 | 39 | 16 | 12 | 13 | 27 | 20 | 26 | 21 |
| NM_014191 | SCN8A | 7207 | 20 | 2 | 0 | 1 | 0 | 20 | 1 | 0 | 0 | 0 | 0 | 0 | 1 |
| NM_014203 | AP2A1 | 3499 | 5 | 3 | 2 | 1 | 2 | 4 | 2 | 2 | 2 | 2 | 3 | 3 | 5 |
| NM_014205 | ZNHIT2 | 1296 | 0 | 0 | 0 | 0 | 0 | 0 | 0 | 0 | 0 | 0 | 0 | 0 | 0 |
| NM_014206 | C11orf10 | 411 | 39 | 22 | 17 | 12 | 39 | 31 | 20 | 32 | 15 | 24 | 14 | 26 | 14 |
| NM_014207 | CD5 | 3157 | 1 | 0 | 0 | 0 | 0 | 0 | 0 | 0 | 0 | 0 | 0 | 1 | 0 |
| NM_014208 | DSPP | 4331 | 0 | 0 | 0 | 0 | 0 | 0 | 0 | 0 | 0 | 0 | 0 | 0 | 0 |
| NM_014209 | ETV2 | 1487 | 0 | 0 | 0 | 0 | 0 | 0 | 0 | 0 | 0 | 0 | 0 | 0 | 0 |
| NM_014210 | EVI2A | 2869 | 7 | 2 | 2 | 0 | 2 | 6 | 1 | 0 | 7 | 1 | 0 | 6 | 1 |
| NM_014211 | GABRP | 3307 | 0 | 0 | 0 | 0 | 0 | 0 | 0 | 0 | 0 | 0 | 0 | 0 | 0 |
| NM_014212 | HOXC11 | 2037 | 0 | 0 | 0 | 0 | 0 | 0 | 0 | 0 | 0 | 0 | 0 | 0 | 0 |
| NM_014213 | HOXD9 | 1885 | 1 | 0 | 0 | 0 | 0 | 0 | 1 | 0 | 0 | 1 | 0 | 1 | 1 |
| NM_014214 | IMPA2 | 1500 | 26 | 7 | 5 | 10 | 4 | 1 | 26 | 9 | 4 | 3 | 14 | 4 | 1 |
| NM_014215 | INSRR | 4147 | 0 | 0 | 0 | 0 | 0 | 0 | 0 | 0 | 0 | 0 | 0 | 0 | 0 |
| NM_014216 | ITPK1 | 3371 | 4 | 2 | 4 | 2 | 2 | 4 | 2 | 1 | 1 | 1 | 1 | 2 | 2 |
| NM_014217 | KCNK2 | 3274 | 3 | 0 | 0 | 0 | 0 | 3 | 0 | 0 | 0 | 0 | 0 | 0 | 0 |
| NM_014218 | KIR2DL1 | 1598 | 0 | 0 | 0 | 0 | 0 | 0 | 0 | 0 | 0 | 0 | 0 | 0 | 0 |
| NM_014219 | KIR2DL2 | 1587 | 0 | 0 | 0 | 0 | 0 | 0 | 0 | 0 | 0 | 0 | 0 | 0 | 0 |
| NM_014220 | TM4SF1 | 1691 | 76 | 25 | 21 | 7 | 43 | 19 | 18 | 14 | 76 | 15 | 14 | 38 | 15 |
| NM_014222 | NDUFA8 | 843 | 14 | 5 | 3 | 4 | 14 | 11 | 6 | 6 | 2 | 2 | 5 | 3 | 4 |
| NM_014223 | NFYC | 2087 | 4 | 3 | 2 | 3 | 3 | 3 | 3 | 2 | 2 | 3 | 3 | 4 | 2 |
| NM_014224 | PGA5 | 1359 | 0 | 0 | 0 | 0 | 0 | 0 | 0 | 0 | 0 | 0 | 0 | 0 | 0 |
| NM_014225 | PPP2R1A | 2365 | 15 | 7 | 3 | 2 | 15 | 13 | 11 | 3 | 4 | 7 | 7 | 6 | 4 |
| NM_014226 | RAGE | 1934 | 7 | 2 | 1 | 1 | 1 | 3 | 2 | 1 | 1 | 2 | 0 | 0 | 7 |
| NM_014227 | SLC5A4 | 2018 | 0 | 0 | 0 | 0 | 0 | 0 | 0 | 0 | 0 | 0 | 0 | 0 | 0 |
| NM_014228 | SLC6A7 | 3739 | 2 | 0 | 0 | 1 | 0 | 2 | 0 | 0 | 0 | 0 | 0 | 0 | 0 |
| NM_014229 | SLC6A11 | 1991 | 3 | 0 | 0 | 0 | 0 | 3 | 0 | 0 | 0 | 0 | 0 | 0 | 0 |
| NM_014230 | SRP68 | 2515 | 24 | 17 | 12 | 8 | 19 | 24 | 15 | 20 | 12 | 17 | 24 | 17 | 15 |
| NM_014231 | VAMP1 | 2735 | 5 | 1 | 0 | 1 | 0 | 5 | 1 | 0 | 1 | 1 | 1 | 2 | 1 |
| NM_014232 | VAMP2 | 2155 | 116 | 19 | 9 | 7 | 6 | 116 | 12 | 3 | 10 | 8 | 7 | 14 | 15 |
| NM_014233 | UBTF | 4571 | 14 | 8 | 8 | 9 | 7 | 10 | 10 | 5 | 5 | 9 | 8 | 14 | 7 |
| NM_014234 | HSD17B8 | 989 | 1 | 0 | 0 | 0 | 0 | 0 | 1 | 1 | 0 | 0 | 0 | 0 | 0 |
| NM_014235 | UBL4A | 2305 | 1 | 0 | 0 | 0 | 1 | 1 | 0 | 1 | 0 | 0 | 1 | 1 | 0 |
| NM_014236 | GNPAT | 2680 | 10 | 5 | 3 | 2 | 7 | 4 | 5 | 3 | 3 | 4 | 10 | 6 | 5 |
| NM_014237 | ADAM18 | 2248 | 1 | 0 | 0 | 0 | 0 | 0 | 0 | 0 | 0 | 0 | 0 | 0 | 1 |
| NM_014238 | KSR1 | 4491 | 1 | 1 | 0 | 0 | 0 | 1 | 1 | 0 | 1 | 0 | 1 | 1 | 1 |
| NM_014239 | EIF2B2 | 1540 | 2 | 1 | 1 | 1 | 1 | 1 | 2 | 1 | 1 | 1 | 2 | 2 | 1 |
| NM_014240 | LIMD1 | 6284 | 3 | 1 | 1 | 2 | 2 | 0 | 1 | 1 | 3 | 1 | 1 | 2 | 1 |
| NM_014241 | PTPLA | 1307 | 5 | 1 | 0 | 1 | 5 | 0 | 0 | 0 | 1 | 0 | 1 | 0 | 0 |
| NM_014243 | ADAMTS3 | 5821 | 1 | 0 | 0 | 0 | 0 | 0 | 0 | 0 | 0 | 1 | 0 | 0 | 1 |
| NM_014244 | ADAMTS2 | 3654 | 2 | 1 | 2 | 1 | 1 | 0 | 0 | 1 | 1 | 1 | 0 | 2 | 0 |
| NM_014245 | RNF7 | 1599 | 7 | 4 | 3 | 2 | 7 | 4 | 4 | 3 | 3 | 6 | 4 | 6 | 5 |
| NM_014246 | CELSR1 | 11389 | 3 | 1 | 0 | 0 | 1 | 0 | 3 | 0 | 1 | 0 | 0 | 0 | 0 |
| NM_014247 | RAPGEF2 | 6820 | 15 | 6 | 5 | 3 | 6 | 15 | 6 | 7 | 6 | 4 | 4 | 9 | 4 |
| NM_014248 | RBX1 | 521 | 14 | 7 | 5 | 4 | 14 | 11 | 6 | 7 | 5 | 6 | 7 | 8 | 5 |
| NM_014249 | NR2E3 | 1996 | 0 | 0 | 0 | 0 | 0 | 0 | 0 | 0 | 0 | 0 | 0 | 0 | 0 |
| NM_014251 | SLC25A13 | 3190 | 14 | 3 | 1 | 1 | 1 | 1 | 4 | 14 | 1 | 1 | 1 | 2 | 2 |
| NM_014252 | SLC25A15 | 4020 | 15 | 2 | 0 | 1 | 0 | 0 | 1 | 15 | 0 | 0 | 0 | 1 | 1 |
| NM_014253 | ODZ1 | 12872 | 3 | 1 | 1 | 0 | 0 | 3 | 1 | 1 | 0 | 0 | 0 | 0 | 0 |
| NM_014254 | TMEM5 | 1451 | 2 | 1 | 1 | 1 | 2 | 2 | 2 | 1 | 1 | 1 | 1 | 1 | 2 |
| NM_014255 | CNPY2 | 1209 | 5 | 2 | 2 | 2 | 2 | 3 | 2 | 5 | 1 | 2 | 0 | 2 | 1 |
| NM_014256 | B3GNT3 | 2720 | 2 | 0 | 0 | 2 | 0 | 0 | 1 | 0 | 0 | 0 | 0 | 0 | 0 |
| NM_014257 | CLEC4M | 1934 | 1 | 0 | 0 | 0 | 0 | 0 | 0 | 1 | 0 | 0 | 0 | 0 | 0 |
| NM_014258 | SYCP2 | 5480 | 10 | 1 | 0 | 0 | 0 | 0 | 1 | 0 | 0 | 0 | 0 | 1 | 10 |
| NM_014260 | PFDN6 | 594 | 20 | 9 | 7 | 5 | 11 | 20 | 11 | 12 | 5 | 5 | 10 | 9 | 6 |
| NM_014262 | LEPREL2 | 2637 | 1 | 0 | 0 | 0 | 0 | 0 | 0 | 0 | 0 | 1 | 0 | 0 | 0 |
| NM_014263 | YME1L1 | 3856 | 22 | 16 | 13 | 6 | 20 | 14 | 15 | 20 | 13 | 16 | 18 | 19 | 22 |
| NM_014264 | PLK4 | 3814 | 3 | 1 | 1 | 1 | 0 | 0 | 0 | 0 | 0 | 0 | 0 | 1 | 3 |
| NM_014265 | ADAM28 | 3220 | 6 | 1 | 0 | 2 | 0 | 2 | 1 | 0 | 2 | 0 | 0 | 6 | 1 |
| NM_014266 | HCST | 507 | 1 | 0 | 1 | 0 | 0 | 1 | 0 | 0 | 1 | 0 | 0 | 1 | 0 |
| NM_014267 | C11orf58 | 3980 | 31 | 22 | 19 | 10 | 23 | 31 | 17 | 29 | 18 | 22 | 19 | 26 | 22 |
| NM_014268 | MAPRE2 | 3208 | 20 | 4 | 2 | 2 | 7 | 20 | 2 | 2 | 2 | 3 | 3 | 4 | 2 |
| NM_014269 | ADAM29 | 3324 | 4 | 0 | 0 | 0 | 0 | 0 | 0 | 0 | 0 | 0 | 0 | 0 | 4 |
| NM_014270 | SLC7A9 | 1774 | 5 | 0 | 0 | 0 | 0 | 0 | 5 | 1 | 0 | 0 | 0 | 0 | 0 |
| NM_014271 | IL1RAPL1 | 2679 | 7 | 1 | 0 | 0 | 1 | 7 | 0 | 0 | 0 | 1 | 0 | 0 | 0 |
| NM_014272 | ADAMTS7 | 5490 | 0 | 0 | 0 | 0 | 0 | 0 | 0 | 0 | 0 | 0 | 0 | 0 | 0 |
| NM_014275 | MGAT4B | 2462 | 3 | 1 | 1 | 2 | 1 | 1 | 1 | 3 | 1 | 1 | 0 | 1 | 1 |
| NM_014276 | RBPJL | 2491 | 0 | 0 | 0 | 0 | 0 | 0 | 0 | 0 | 0 | 0 | 0 | 0 | 0 |
| NM_014278 | HSPA4L | 3270 | 22 | 5 | 1 | 0 | 3 | 13 | 7 | 4 | 2 | 1 | 1 | 1 | 22 |
| NM_014279 | OLFM1 | 2874 | 33 | 4 | 1 | 1 | 1 | 33 | 0 | 0 | 1 | 0 | 1 | 3 | 2 |
| NM_014280 | DNAJC8 | 1764 | 24 | 14 | 14 | 6 | 15 | 24 | 15 | 13 | 10 | 13 | 9 | 20 | 12 |
| NM_014281 | PUF60 | 1836 | 13 | 7 | 7 | 8 | 4 | 13 | 7 | 13 | 3 | 3 | 5 | 6 | 7 |
| NM_014282 | HABP4 | 2688 | 32 | 7 | 8 | 2 | 8 | 32 | 8 | 2 | 3 | 3 | 3 | 2 | 5 |
| NM_014283 | C1orf9 | 5655 | 20 | 7 | 3 | 2 | 7 | 8 | 7 | 3 | 5 | 9 | 2 | 7 | 20 |
| NM_014284 | NCDN | 3705 | 16 | 2 | 0 | 1 | 1 | 16 | 1 | 0 | 0 | 1 | 0 | 1 | 1 |
| NM_014285 | EXOSC2 | 2194 | 5 | 3 | 3 | 3 | 2 | 5 | 3 | 4 | 2 | 4 | 2 | 4 | 3 |

| | | transcript | | | | | | hypothala | | | | | skeletalm | | |
|---|---|---|---|---|---|---|---|---|---|---|---|---|---|---|---|
| transcript | gene | length (nt) | max | mean | adipose | colon | heart | mus | kidney | liver | lung | ovary | uscle | spleen | testes |
| NM_014286 | FREQ | 4991 | 5 | 1 | 0 | 1 | 0 | 5 | 1 | 0 | 0 | 0 | 0 | 0 | 0 |
| NM_014287 | NOMO1 | 4247 | 1 | 0 | 0 | 0 | 1 | 1 | 0 | 0 | 0 | 0 | 0 | 0 | 0 |
| NM_014288 | ITGB3BP | 879 | 2 | 1 | 1 | 0 | 1 | 1 | 1 | 0 | 1 | 1 | 1 | 2 | 1 |
| NM_014289 | CAPN6 | 3594 | 2 | 0 | 0 | 1 | 0 | 0 | 1 | 0 | 0 | 0 | 0 | 0 | 2 |
| NM_014290 | TDRD7 | 3762 | 14 | 5 | 3 | 3 | 5 | 7 | 3 | 3 | 4 | 3 | 3 | 6 | 14 |
| NM_014291 | GCAT | 1459 | 1 | 0 | 0 | 1 | 1 | 1 | 0 | 1 | 0 | 0 | 0 | 0 | 1 |
| NM_014292 | CBX6 | 3268 | 16 | 3 | 1 | 2 | 3 | 16 | 4 | 1 | 1 | 4 | 1 | 4 | 1 |
| NM_014293 | NPTXR | 5814 | 8 | 1 | 0 | 0 | 1 | 8 | 0 | 0 | 0 | 0 | 0 | 0 | 0 |
| NM_014294 | TRAM1 | 3097 | 26 | 12 | 7 | 3 | 9 | 4 | 15 | 26 | 22 | 7 | 3 | 20 | 12 |
| NM_014296 | CAPN7 | 4368 | 5 | 3 | 2 | 1 | 3 | 2 | 3 | 2 | 3 | 5 | 3 | 4 | 5 |
| NM_014297 | ETHE1 | 963 | 1 | 0 | 0 | 1 | 0 | 0 | 0 | 1 | 0 | 0 | 0 | 0 | 0 |
| NM_014298 | QPRT | 1554 | 9 | 2 | 1 | 1 | 1 | 1 | 9 | 5 | 0 | 2 | 0 | 1 | 0 |
| NM_014299 | BRD4 | 4635 | 5 | 4 | 2 | 3 | 4 | 5 | 3 | 2 | 4 | 4 | 3 | 5 | 4 |
| NM_014300 | SEC11A | 1383 | 33 | 22 | 29 | 13 | 23 | 12 | 21 | 28 | 22 | 33 | 20 | 25 | 20 |
| NM_014301 | ISCU | 1172 | 5 | 2 | 3 | 0 | 4 | 5 | 4 | 1 | 1 | 2 | 2 | 2 | 2 |
| NM_014302 | SEC61G | 466 | 12 | 6 | 5 | 3 | 10 | 5 | 6 | 12 | 4 | 5 | 4 | 6 | 7 |
| NM_014303 | PES1 | 2262 | 6 | 4 | 4 | 3 | 3 | 5 | 6 | 6 | 2 | 3 | 3 | 5 | 6 |
| NM_014305 | TGDS | 1902 | 1 | 1 | 0 | 0 | 1 | 0 | 1 | 1 | 1 | 1 | 0 | 1 | 1 |
| NM_014306 | C22orf28 | 2066 | 14 | 7 | 6 | 7 | 6 | 9 | 9 | 14 | 4 | 5 | 5 | 7 | 5 |
| NM_014308 | PIK3R5 | 4432 | 3 | 1 | 1 | 0 | 1 | 0 | 0 | 0 | 1 | 0 | 0 | 3 | 0 |
| NM_014309 | RBM9 | 6928 | 17 | 7 | 5 | 5 | 8 | 13 | 5 | 3 | 5 | 17 | 6 | 4 | 6 |
| NM_014310 | RASD2 | 2972 | 1 | 0 | 0 | 0 | 0 | 1 | 0 | 0 | 0 | 0 | 0 | 0 | 0 |
| NM_014311 | SMUG1 | 1570 | 1 | 1 | 1 | 1 | 0 | 1 | 1 | 0 | 0 | 0 | 1 | 1 | 1 |
| NM_014312 | VSIG2 | 1121 | 1 | 0 | 0 | 1 | 0 | 0 | 0 | 0 | 0 | 0 | 0 | 0 | 0 |
| NM_014313 | TMEM50A | 2280 | 7 | 4 | 4 | 3 | 4 | 4 | 4 | 4 | 4 | 3 | 2 | 7 | 2 |
| NM_014314 | DDX58 | 4756 | 4 | 3 | 2 | 1 | 2 | 2 | 3 | 2 | 2 | 4 | 4 | 4 | 2 |
| NM_014315 | KLHDC2 | 1806 | 18 | 11 | 8 | 6 | 12 | 12 | 14 | 18 | 7 | 10 | 10 | 9 | 13 |
| NM_014316 | CARHSP1 | 2711 | 3 | 2 | 1 | 1 | 1 | 2 | 3 | 2 | 3 | 3 | 0 | 2 | 2 |
| NM_014317 | PDSS1 | 1618 | 2 | 1 | 0 | 1 | 0 | 1 | 1 | 2 | 0 | 0 | 0 | 1 | 0 |
| NM_014319 | LEMD3 | 4796 | 3 | 2 | 1 | 2 | 2 | 3 | 2 | 1 | 3 | 2 | 1 | 3 | 3 |
| NM_014320 | HEBP2 | 1282 | 41 | 14 | 41 | 10 | 20 | 6 | 10 | 26 | 6 | 6 | 17 | 8 | 4 |
| NM_014321 | ORC6L | 1628 | 1 | 0 | 0 | 0 | 0 | 0 | 0 | 0 | 0 | 0 | 0 | 0 | 1 |
| NM_014322 | OPN3 | 2632 | 0 | 0 | 0 | 0 | 0 | 0 | 0 | 0 | 0 | 0 | 0 | 0 | 0 |
| NM_014323 | PATZ1 | 3812 | 1 | 1 | 0 | 1 | 0 | 1 | 1 | 0 | 1 | 1 | 1 | 1 | 1 |
| NM_014324 | AMACR | 3350 | 34 | 5 | 2 | 3 | 2 | 6 | 34 | 7 | 1 | 1 | 1 | 1 | 1 |
| NM_014325 | CORO1C | 3813 | 20 | 10 | 20 | 9 | 10 | 10 | 7 | 9 | 14 | 7 | 5 | 9 | 7 |
| NM_014326 | DAPK2 | 2628 | 3 | 1 | 1 | 2 | 2 | 1 | 0 | 0 | 2 | 0 | 3 | 1 | 0 |
| NM_014328 | RUSC1 | 2060 | 1 | 0 | 0 | 0 | 0 | 1 | 0 | 0 | 0 | 0 | 0 | 0 | 0 |
| NM_014329 | EDC4 | 4727 | 1 | 1 | 0 | 1 | 0 | 1 | 1 | 1 | 0 | 1 | 1 | 1 | 1 |
| NM_014330 | PPP1R15A | 2383 | 22 | 8 | 9 | 4 | 8 | 4 | 4 | 2 | 22 | 8 | 11 | 7 | 6 |
| NM_014331 | SLC7A11 | 9648 | 12 | 1 | 0 | 0 | 0 | 12 | 0 | 0 | 1 | 0 | 0 | 0 | 1 |
| NM_014332 | SMPX | 886 | 46 | 7 | 0 | 0 | 46 | 0 | 0 | 0 | 0 | 0 | 34 | 0 | 0 |
| NM_014333 | CADM1 | 4307 | 11 | 4 | 1 | 1 | 3 | 11 | 4 | 2 | 6 | 2 | 1 | 1 | 11 |
| NM_014334 | C9orf4 | 1189 | 15 | 2 | 0 | 0 | 0 | 15 | 0 | 0 | 0 | 2 | 0 | 0 | 0 |
| NM_014335 | EID1 | 2091 | 58 | 27 | 20 | 6 | 58 | 45 | 24 | 14 | 22 | 37 | 20 | 25 | 30 |
| NM_014336 | AIPL1 | 2971 | 0 | 0 | 0 | 0 | 0 | 0 | 0 | 0 | 0 | 0 | 0 | 0 | 0 |
| NM_014337 | PPIL2 | 4102 | 1 | 1 | 1 | 1 | 0 | 1 | 1 | 1 | 0 | 1 | 1 | 1 | 1 |
| NM_014338 | PISD | 2677 | 5 | 2 | 5 | 1 | 2 | 4 | 2 | 1 | 2 | 1 | 1 | 4 | 3 |
| NM_014339 | IL17RA | 3413 | 1 | 0 | 0 | 0 | 0 | 0 | 0 | 0 | 0 | 0 | 0 | 1 | 0 |
| NM_014341 | MTCH1 | 1881 | 21 | 6 | 4 | 4 | 9 | 21 | 7 | 4 | 4 | 4 | 2 | 5 | 4 |
| NM_014342 | MTCH2 | 2627 | 45 | 11 | 11 | 6 | 9 | 9 | 17 | 45 | 3 | 3 | 7 | 6 | 10 |
| NM_014343 | CLDN15 | 1258 | 1 | 0 | 0 | 1 | 0 | 0 | 0 | 0 | 0 | 0 | 0 | 0 | 0 |
| NM_014344 | FJX1 | 2685 | 0 | 0 | 0 | 0 | 0 | 0 | 0 | 0 | 0 | 0 | 0 | 0 | 0 |
| NM_014345 | ZNF318 | 8006 | 12 | 4 | 2 | 2 | 3 | 3 | 4 | 3 | 2 | 4 | 2 | 7 | 12 |
| NM_014346 | TBC1D22A | 2144 | 3 | 1 | 2 | 2 | 1 | 1 | 2 | 1 | 2 | 1 | 1 | 3 | 1 |
| NM_014347 | ZNF324 | 3000 | 0 | 0 | 0 | 0 | 0 | 0 | 0 | 0 | 0 | 0 | 0 | 0 | 0 |
| NM_014350 | TNFAIP8 | 2005 | 25 | 6 | 11 | 3 | 4 | 1 | 4 | 1 | 9 | 1 | 1 | 25 | 3 |
| NM_014351 | SULT4A1 | 2465 | 35 | 3 | 0 | 1 | 0 | 35 | 0 | 0 | 0 | 0 | 0 | 0 | 1 |
| NM_014352 | POU2F3 | 2965 | 0 | 0 | 0 | 0 | 0 | 0 | 0 | 0 | 0 | 0 | 0 | 0 | 0 |
| NM_014353 | RAB26 | 1624 | 0 | 0 | 0 | 0 | 0 | 0 | 0 | 0 | 0 | 0 | 0 | 0 | 0 |
| NM_014357 | LCE2B | 609 | 0 | 0 | 0 | 0 | 0 | 0 | 0 | 0 | 0 | 0 | 0 | 0 | 0 |
| NM_014358 | CLEC4E | 2158 | 6 | 1 | 0 | 0 | 1 | 0 | 0 | 1 | 5 | 0 | 0 | 6 | 0 |
| NM_014359 | OPTC | 1444 | 0 | 0 | 0 | 0 | 0 | 0 | 0 | 0 | 0 | 0 | 0 | 0 | 0 |
| NM_014360 | NKX2-8 | 1803 | 0 | 0 | 0 | 0 | 0 | 0 | 0 | 0 | 0 | 0 | 0 | 0 | 0 |
| NM_014361 | CNTN5 | 4164 | 3 | 0 | 0 | 0 | 0 | 3 | 0 | 0 | 0 | 0 | 0 | 0 | 0 |
| NM_014362 | HIBCH | 1727 | 27 | 9 | 10 | 6 | 6 | 7 | 27 | 18 | 3 | 3 | 2 | 5 | 9 |
| NM_014363 | SACS | 15639 | 10 | 4 | 1 | 1 | 5 | 10 | 1 | 0 | 2 | 4 | 7 | 4 | 4 |
| NM_014364 | GAPDHS | 1492 | 1 | 0 | 0 | 0 | 0 | 0 | 0 | 0 | 0 | 0 | 0 | 0 | 1 |
| NM_014365 | HSPB8 | 2002 | 34 | 7 | 2 | 3 | 34 | 8 | 3 | 0 | 2 | 1 | 20 | 2 | 1 |
| NM_014366 | GNL3 | 2042 | 13 | 8 | 7 | 4 | 9 | 9 | 5 | 13 | 7 | 5 | 9 | 10 | 9 |
| NM_014367 | FAM162A | 838 | 25 | 15 | 9 | 16 | 25 | 22 | 21 | 25 | 5 | 13 | 8 | 6 | 10 |
| NM_014368 | LHX6 | 3360 | 0 | 0 | 0 | 0 | 0 | 0 | 0 | 0 | 0 | 0 | 0 | 0 | 0 |
| NM_014369 | PTPN18 | 3668 | 28 | 8 | 11 | 10 | 6 | 5 | 9 | 6 | 8 | 4 | 2 | 28 | 4 |
| NM_014370 | SRPK3 | 1948 | 2 | 0 | 0 | 0 | 0 | 0 | 0 | 0 | 0 | 0 | 2 | 0 | 0 |
| NM_014371 | AKAP8L | 2114 | 20 | 10 | 6 | 12 | 11 | 14 | 8 | 2 | 10 | 7 | 10 | 7 | 20 |
| NM_014372 | RNF11 | 3074 | 9 | 5 | 5 | 2 | 5 | 9 | 5 | 3 | 4 | 6 | 4 | 4 | 3 |
| NM_014373 | GPR160 | 2011 | 3 | 1 | 0 | 1 | 0 | 0 | 3 | 0 | 1 | 0 | 0 | 2 | 0 |

| transcript | gene | transcript length (nt) | max | mean | adipose | colon | heart | hypothalamus | kidney | liver | lung | ovary | skeletalmuscle | spleen | testes |
|---|---|---|---|---|---|---|---|---|---|---|---|---|---|---|---|
| NM_014374 | REPIN1 | 2876 | 3 | 2 | 2 | 3 | 1 | 2 | 3 | 2 | 1 | 1 | 2 | 3 | 1 |
| NM_014375 | FETUB | 1627 | 8 | 1 | 0 | 0 | 0 | 0 | 1 | 8 | 0 | 0 | 0 | 0 | 0 |
| NM_014376 | CYFIP2 | 6615 | 33 | 7 | 0 | 2 | 2 | 33 | 21 | 9 | 2 | 1 | 1 | 9 | 1 |
| NM_014377 | DNAJC2 | 2212 | 8 | 5 | 3 | 1 | 6 | 5 | 5 | 5 | 3 | 5 | 5 | 6 | 8 |
| NM_014379 | KCNV1 | 2929 | 3 | 0 | 0 | 0 | 0 | 3 | 0 | 0 | 0 | 0 | 0 | 0 | 0 |
| NM_014380 | NGFRAP1 | 891 | 188 | 48 | 23 | 20 | 45 | 188 | 47 | 9 | 30 | 63 | 4 | 28 | 76 |
| NM_014381 | MLH3 | 7824 | 6 | 3 | 2 | 1 | 3 | 3 | 4 | 2 | 3 | 5 | 3 | 5 | 6 |
| NM_014382 | ATP2C1 | 4740 | 13 | 8 | 7 | 3 | 10 | 13 | 10 | 6 | 10 | 8 | 4 | 9 | 12 |
| NM_014383 | ZBTB32 | 1960 | 1 | 0 | 0 | 0 | 0 | 0 | 0 | 0 | 0 | 0 | 0 | 0 | 1 |
| NM_014384 | ACAD8 | 2201 | 3 | 2 | 2 | 2 | 2 | 3 | 3 | 2 | 1 | 1 | 2 | 1 | 2 |
| NM_014385 | SIGLEC7 | 1754 | 6 | 1 | 1 | 0 | 0 | 0 | 0 | 1 | 1 | 0 | 0 | 6 | 0 |
| NM_014386 | PKD2L2 | 2218 | 1 | 0 | 0 | 0 | 0 | 0 | 0 | 0 | 0 | 0 | 0 | 0 | 1 |
| NM_014387 | LAT | 1743 | 0 | 0 | 0 | 0 | 0 | 0 | 0 | 0 | 0 | 0 | 0 | 0 | 0 |
| NM_014388 | C1orf107 | 8463 | 2 | 1 | 1 | 1 | 1 | 2 | 1 | 2 | 0 | 2 | 1 | 1 | 1 |
| NM_014389 | PELP1 | 3674 | 12 | 5 | 5 | 5 | 4 | 12 | 4 | 2 | 3 | 5 | 5 | 6 | 9 |
| NM_014390 | SND1 | 3508 | 26 | 16 | 17 | 12 | 14 | 13 | 12 | 26 | 14 | 19 | 7 | 20 | 19 |
| NM_014391 | ANKRD1 | 1974 | 3,864 | 364 | 1 | 0 | 3,864 | 0 | 0 | 0 | 5 | 0 | 126 | 1 | 2 |
| NM_014392 | D4S234E | 3124 | 4 | 1 | 0 | 0 | 0 | 4 | 0 | 0 | 0 | 2 | 0 | 0 | 1 |
| NM_014393 | STAU2 | 4146 | 8 | 4 | 2 | 1 | 5 | 8 | 4 | 3 | 3 | 3 | 3 | 4 | 3 |
| NM_014394 | GHITM | 2449 | 57 | 31 | 19 | 15 | 57 | 49 | 47 | 48 | 19 | 13 | 26 | 23 | 24 |
| NM_014395 | DAPP1 | 2937 | 2 | 0 | 0 | 0 | 0 | 0 | 0 | 0 | 0 | 0 | 0 | 2 | 0 |
| NM_014396 | VPS41 | 4894 | 14 | 7 | 9 | 4 | 5 | 14 | 8 | 4 | 7 | 8 | 2 | 10 | 9 |
| NM_014397 | NEK6 | 2631 | 12 | 5 | 6 | 5 | 4 | 7 | 7 | 12 | 4 | 3 | 2 | 6 | 3 |
| NM_014398 | LAMP3 | 3341 | 27 | 3 | 0 | 1 | 0 | 0 | 0 | 0 | 27 | 0 | 0 | 2 | 2 |
| NM_014399 | TSPAN13 | 1902 | 26 | 8 | 5 | 6 | 4 | 11 | 1 | 26 | 15 | 1 | 1 | 12 | 2 |
| NM_014400 | LYPD3 | 1706 | 0 | 0 | 0 | 0 | 0 | 0 | 0 | 0 | 0 | 0 | 0 | 0 | 0 |
| NM_014402 | UQCRQ | 1585 | 47 | 13 | 11 | 10 | 19 | 15 | 14 | 47 | 5 | 3 | 8 | 5 | 5 |
| NM_014404 | CACNG5 | 891 | 0 | 0 | 0 | 0 | 0 | 0 | 0 | 0 | 0 | 0 | 0 | 0 | 0 |
| NM_014405 | CACNG4 | 3397 | 1 | 0 | 0 | 0 | 0 | 1 | 0 | 0 | 0 | 0 | 0 | 0 | 0 |
| NM_014406 | CCT8L2 | 2053 | 0 | 0 | 0 | 0 | 0 | 0 | 0 | 0 | 0 | 0 | 0 | 0 | 0 |
| NM_014407 | KCNMB3 | 1490 | 2 | 1 | 1 | 0 | 1 | 0 | 1 | 0 | 0 | 0 | 0 | 2 | 1 |
| NM_014408 | TRAPPC3 | 1262 | 4 | 3 | 3 | 2 | 3 | 4 | 4 | 3 | 3 | 1 | 1 | 3 | 2 |
| NM_014409 | TAF5L | 3116 | 1 | 1 | 1 | 1 | 1 | 1 | 1 | 1 | 1 | 1 | 1 | 1 | 1 |
| NM_014410 | CLUL1 | 1938 | 2 | 0 | 0 | 0 | 0 | 2 | 1 | 0 | 0 | 0 | 0 | 0 | 1 |
| NM_014412 | CACYBP | 3060 | 20 | 7 | 6 | 3 | 7 | 20 | 6 | 3 | 4 | 7 | 4 | 8 | 7 |
| NM_014413 | EIF2AK1 | 4463 | 13 | 7 | 5 | 5 | 5 | 13 | 7 | 12 | 4 | 5 | 5 | 7 | 6 |
| NM_014415 | ZBTB11 | 5119 | 6 | 4 | 2 | 1 | 3 | 6 | 3 | 2 | 4 | 4 | 4 | 6 | 5 |
| NM_014417 | BBC3 | 1843 | 3 | 1 | 0 | 1 | 0 | 1 | 0 | 0 | 1 | 1 | 3 | 1 | 0 |
| NM_014419 | DKKL1 | 1031 | 1 | 0 | 0 | 0 | 0 | 0 | 0 | 0 | 0 | 0 | 0 | 0 | 1 |
| NM_014420 | DKK4 | 818 | 0 | 0 | 0 | 0 | 0 | 0 | 0 | 0 | 0 | 0 | 0 | 0 | 0 |
| NM_014421 | DKK2 | 3637 | 1 | 0 | 1 | 0 | 1 | 1 | 0 | 0 | 1 | 1 | 0 | 0 | 0 |
| NM_014423 | AFF4 | 9580 | 23 | 14 | 9 | 6 | 13 | 16 | 15 | 8 | 13 | 23 | 14 | 17 | 20 |
| NM_014424 | HSPB7 | 2892 | 103 | 13 | 6 | 1 | 103 | 0 | 1 | 0 | 1 | 0 | 27 | 0 | 1 |
| NM_014425 | INVS | 3880 | 3 | 2 | 2 | 2 | 1 | 1 | 3 | 2 | 2 | 3 | 1 | 2 | 2 |
| NM_014426 | SNX5 | 2058 | 36 | 15 | 12 | 4 | 12 | 10 | 24 | 10 | 13 | 20 | 11 | 36 | 11 |
| NM_014427 | CPNE7 | 2656 | 0 | 0 | 0 | 0 | 0 | 0 | 0 | 0 | 0 | 0 | 0 | 0 | 0 |
| NM_014428 | TJP3 | 2859 | 3 | 0 | 0 | 3 | 0 | 0 | 1 | 1 | 1 | 0 | 0 | 0 | 0 |
| NM_014429 | MORC1 | 3765 | 6 | 1 | 0 | 0 | 0 | 0 | 0 | 0 | 0 | 0 | 0 | 0 | 6 |
| NM_014430 | CIDEB | 2242 | 12 | 5 | 4 | 3 | 4 | 5 | 4 | 12 | 2 | 3 | 5 | 5 | 3 |
| NM_014431 | KIAA1274 | 4569 | 1 | 0 | 0 | 0 | 0 | 1 | 0 | 0 | 0 | 0 | 0 | 1 | 0 |
| NM_014432 | IL20RA | 3682 | 1 | 0 | 0 | 0 | 0 | 0 | 0 | 0 | 0 | 1 | 0 | 0 | 1 |
| NM_014433 | RTDR1 | 1252 | 0 | 0 | 0 | 0 | 0 | 0 | 0 | 0 | 0 | 0 | 0 | 0 | 0 |
| NM_014434 | NDOR1 | 4849 | 0 | 0 | 0 | 0 | 0 | 0 | 0 | 0 | 0 | 0 | 0 | 0 | 0 |
| NM_014435 | NAAA | 1863 | 9 | 2 | 2 | 1 | 1 | 2 | 2 | 3 | 1 | 1 | 1 | 9 | 0 |
| NM_014437 | SLC39A1 | 2427 | 9 | 5 | 5 | 3 | 6 | 3 | 8 | 9 | 6 | 4 | 2 | 8 | 3 |
| NM_014438 | IL1F8 | 1182 | 0 | 0 | 0 | 0 | 0 | 0 | 0 | 0 | 0 | 0 | 0 | 0 | 0 |
| NM_014439 | IL1F7 | 771 | 0 | 0 | 0 | 0 | 0 | 0 | 0 | 0 | 0 | 0 | 0 | 0 | 0 |
| NM_014440 | IL1F6 | 477 | 0 | 0 | 0 | 0 | 0 | 0 | 0 | 0 | 0 | 0 | 0 | 0 | 0 |
| NM_014441 | SIGLEC9 | 1690 | 3 | 1 | 1 | 0 | 0 | 1 | 0 | 1 | 1 | 0 | 0 | 3 | 0 |
| NM_014442 | SIGLEC8 | 2949 | 6 | 1 | 0 | 0 | 0 | 1 | 0 | 1 | 1 | 0 | 0 | 6 | 0 |
| NM_014443 | IL17B | 695 | 0 | 0 | 0 | 0 | 0 | 0 | 0 | 0 | 0 | 0 | 0 | 0 | 0 |
| NM_014444 | TUBGCP4 | 3069 | 4 | 1 | 1 | 1 | 1 | 4 | 1 | 0 | 1 | 2 | 1 | 1 | 1 |
| NM_014445 | SERP1 | 3172 | 17 | 9 | 6 | 4 | 6 | 8 | 10 | 13 | 13 | 7 | 2 | 17 | 10 |
| NM_014447 | ARFIP1 | 2859 | 4 | 3 | 2 | 2 | 2 | 2 | 4 | 3 | 2 | 3 | 1 | 4 | 4 |
| NM_014448 | ARHGEF16 | 2904 | 1 | 0 | 0 | 1 | 0 | 0 | 0 | 0 | 0 | 0 | 0 | 0 | 0 |
| NM_014449 | GPR162 | 1514 | 2 | 0 | 0 | 0 | 0 | 2 | 0 | 0 | 0 | 0 | 0 | 0 | 0 |
| NM_014450 | SIT1 | 1236 | 0 | 0 | 0 | 0 | 0 | 0 | 0 | 0 | 0 | 0 | 0 | 0 | 0 |
| NM_014451 | BBS9 | 3899 | 6 | 3 | 2 | 1 | 3 | 3 | 3 | 1 | 3 | 6 | 1 | 3 | 5 |
| NM_014452 | TNFRSF21 | 3634 | 9 | 4 | 5 | 4 | 2 | 9 | 8 | 1 | 4 | 2 | 1 | 7 | 1 |
| NM_014453 | CHMP2A | 908 | 70 | 46 | 58 | 46 | 49 | 70 | 49 | 68 | 30 | 25 | 44 | 42 | 24 |
| NM_014454 | SESN1 | 2759 | 22 | 8 | 8 | 3 | 7 | 6 | 4 | 3 | 7 | 6 | 22 | 12 | 15 |
| NM_014455 | RNF115 | 1673 | 9 | 3 | 1 | 1 | 9 | 1 | 2 | 2 | 1 | 2 | 7 | 2 | 3 |
| NM_014456 | PDCD4 | 3571 | 177 | 53 | 39 | 50 | 33 | 28 | 24 | 22 | 37 | 177 | 14 | 101 | 58 |
| NM_014458 | KLHL20 | 3464 | 5 | 3 | 4 | 2 | 4 | 4 | 3 | 2 | 2 | 4 | 3 | 4 | 5 |
| NM_014460 | CSDC2 | 2530 | 18 | 6 | 0 | 3 | 18 | 7 | 8 | 0 | 1 | 11 | 0 | 0 | 12 |
| NM_014461 | CNTN6 | 3530 | 1 | 0 | 0 | 0 | 0 | 1 | 0 | 0 | 1 | 0 | 0 | 0 | 0 |
| NM_014462 | LSM1 | 920 | 9 | 5 | 6 | 2 | 9 | 6 | 6 | 7 | 3 | 3 | 2 | 7 | 5 |

| transcript | gene | transcript length (nt) | max | mean | adipose | colon | heart | hypothalamus | kidney | liver | lung | ovary | skeletalmuscle | spleen | testes |
|---|---|---|---|---|---|---|---|---|---|---|---|---|---|---|---|
| NM_014463 | LSM3 | 695 | 31 | 20 | 26 | 13 | 23 | 27 | 25 | 31 | 10 | 10 | 11 | 18 | 21 |
| NM_014464 | TINAG | 1804 | 13 | 1 | 0 | 1 | 0 | 0 | 13 | 0 | 0 | 0 | 0 | 0 | 0 |
| NM_014465 | SULT1B1 | 1307 | 16 | 3 | 0 | 6 | 1 | 0 | 0 | 16 | 1 | 0 | 0 | 7 | 0 |
| NM_014466 | TEKT2 | 1509 | 7 | 1 | 0 | 0 | 0 | 2 | 0 | 0 | 2 | 0 | 0 | 0 | 7 |
| NM_014467 | SRPX2 | 2190 | 12 | 3 | 12 | 1 | 4 | 1 | 1 | 1 | 4 | 5 | 1 | 4 | 2 |
| NM_014468 | VENTX | 2411 | 0 | 0 | 0 | 0 | 0 | 0 | 0 | 0 | 0 | 0 | 0 | 0 | 0 |
| NM_014469 | RBMXL2 | 2215 | 2 | 0 | 0 | 0 | 0 | 0 | 0 | 0 | 0 | 0 | 0 | 0 | 2 |
| NM_014470 | RND1 | 1653 | 3 | 1 | 0 | 0 | 0 | 3 | 0 | 3 | 3 | 0 | 0 | 0 | 0 |
| NM_014471 | SPINK4 | 368 | 4 | 0 | 0 | 4 | 0 | 0 | 0 | 0 | 0 | 0 | 0 | 0 | 0 |
| NM_014472 | C10orf28 | 3371 | 7 | 3 | 3 | 2 | 2 | 2 | 3 | 1 | 2 | 2 | 2 | 4 | 7 |
| NM_014473 | DIMT1L | 1557 | 9 | 4 | 3 | 2 | 7 | 4 | 4 | 3 | 4 | 5 | 3 | 9 | 5 |
| NM_014474 | SMPDL3B | 1873 | 0 | 0 | 0 | 0 | 0 | 0 | 0 | 0 | 0 | 0 | 0 | 0 | 0 |
| NM_014475 | DHDH | 1081 | 0 | 0 | 0 | 0 | 0 | 0 | 0 | 0 | 0 | 0 | 0 | 0 | 0 |
| NM_014476 | PDLIM3 | 1816 | 75 | 8 | 0 | 2 | 10 | 1 | 0 | 0 | 1 | 1 | 75 | 2 | 1 |
| NM_014477 | TP53TG5 | 981 | 1 | 0 | 0 | 0 | 0 | 1 | 0 | 0 | 0 | 0 | 0 | 0 | 1 |
| NM_014478 | CRCP | 2801 | 6 | 4 | 3 | 3 | 3 | 5 | 4 | 6 | 3 | 3 | 2 | 4 | 3 |
| NM_014479 | ADAMDEC1 | 2348 | 44 | 5 | 0 | 44 | 0 | 0 | 0 | 0 | 3 | 0 | 0 | 5 | 0 |
| NM_014480 | ZNF544 | 3510 | 4 | 2 | 2 | 2 | 2 | 3 | 3 | 1 | 1 | 3 | 1 | 3 | 4 |
| NM_014481 | APEX2 | 1977 | 1 | 0 | 0 | 0 | 0 | 0 | 1 | 1 | 0 | 1 | 0 | 1 | 0 |
| NM_014482 | BMP10 | 1584 | 140 | 13 | 0 | 0 | 140 | 0 | 0 | 1 | 0 | 0 | 0 | 0 | 0 |
| NM_014483 | RBMS3 | 1598 | 15 | 7 | 8 | 2 | 9 | 3 | 4 | 2 | 9 | 15 | 7 | 10 | 7 |
| NM_014484 | MOCS3 | 2458 | 1 | 0 | 0 | 0 | 0 | 1 | 0 | 1 | 0 | 0 | 0 | 0 | 1 |
| NM_014485 | HPGDS | 1615 | 18 | 4 | 18 | 5 | 3 | 4 | 2 | 0 | 4 | 2 | 2 | 1 | 3 |
| NM_014487 | ZNF330 | 1890 | 27 | 10 | 10 | 6 | 27 | 7 | 9 | 13 | 10 | 7 | 10 | 9 | 7 |
| NM_014488 | RAB30 | 1602 | 11 | 4 | 8 | 3 | 3 | 11 | 1 | 1 | 2 | 5 | 4 | 6 | 4 |
| NM_014489 | PGAP2 | 1966 | 1 | 1 | 0 | 1 | 1 | 0 | 1 | 1 | 1 | 0 | 0 | 1 | 1 |
| NM_014491 | FOXP2 | 3633 | 3 | 1 | 1 | 2 | 1 | 1 | 1 | 1 | 1 | 3 | 3 | 0 | 1 |
| NM_014494 | TNRC6A | 8422 | 9 | 5 | 6 | 3 | 4 | 4 | 5 | 3 | 3 | 9 | 4 | 6 | 6 |
| NM_014495 | ANGPTL3 | 2126 | 95 | 10 | 0 | 0 | 0 | 0 | 15 | 95 | 0 | 1 | 0 | 1 | 1 |
| NM_014496 | RPS6KA6 | 2575 | 3 | 1 | 0 | 1 | 0 | 3 | 2 | 0 | 0 | 2 | 0 | 0 | 2 |
| NM_014497 | ZNF638 | 6508 | 22 | 14 | 10 | 5 | 16 | 15 | 15 | 9 | 12 | 22 | 16 | 19 | 20 |
| NM_014498 | GOLIM4 | 2838 | 57 | 28 | 20 | 14 | 57 | 35 | 32 | 50 | 24 | 21 | 15 | 29 | 15 |
| NM_014499 | P2RY10 | 1790 | 2 | 0 | 0 | 0 | 0 | 0 | 0 | 0 | 1 | 0 | 0 | 2 | 0 |
| NM_014500 | HTATSF1 | 2770 | 35 | 20 | 19 | 6 | 27 | 35 | 19 | 9 | 9 | 20 | 31 | 13 | 32 |
| NM_014501 | UBE2S | 1191 | 6 | 1 | 0 | 1 | 2 | 2 | 0 | 1 | 1 | 1 | 0 | 1 | 6 |
| NM_014502 | PRPF19 | 2339 | 19 | 7 | 5 | 5 | 5 | 19 | 6 | 10 | 4 | 6 | 5 | 10 | 5 |
| NM_014503 | UTP20 | 9010 | 4 | 2 | 2 | 1 | 2 | 1 | 2 | 2 | 1 | 3 | 2 | 3 | 4 |
| NM_014504 | RABGEF1 | 3807 | 8 | 3 | 3 | 1 | 3 | 5 | 3 | 2 | 3 | 3 | 2 | 4 | 8 |
| NM_014505 | KCNMB4 | 1631 | 8 | 1 | 0 | 0 | 0 | 8 | 0 | 0 | 1 | 0 | 0 | 0 | 1 |
| NM_014506 | TOR1B | 2769 | 4 | 2 | 2 | 3 | 2 | 2 | 2 | 4 | 2 | 2 | 1 | 3 | 2 |
| NM_014507 | MCAT | 1841 | 1 | 0 | 0 | 1 | 0 | 1 | 1 | 0 | 0 | 0 | 0 | 0 | 1 |
| NM_014508 | APOBEC3C | 1109 | 21 | 10 | 16 | 10 | 9 | 3 | 2 | 1 | 9 | 16 | 2 | 20 | 21 |
| NM_014509 | SERHL2 | 1374 | 0 | 0 | 0 | 0 | 0 | 0 | 0 | 0 | 0 | 0 | 0 | 0 | 0 |
| NM_014510 | PCLO | 17095 | 16 | 2 | 1 | 1 | 1 | 16 | 3 | 0 | 0 | 0 | 0 | 0 | 2 |
| NM_014511 | KIR2DL3 | 1302 | 0 | 0 | 0 | 0 | 0 | 0 | 0 | 0 | 0 | 0 | 0 | 0 | 0 |
| NM_014512 | KIR2DS1 | 1101 | 0 | 0 | 0 | 0 | 0 | 0 | 0 | 0 | 0 | 0 | 0 | 0 | 0 |
| NM_014513 | KIR2DS5 | 1601 | 0 | 0 | 0 | 0 | 0 | 0 | 0 | 0 | 0 | 0 | 0 | 0 | 0 |
| NM_014515 | CNOT2 | 3280 | 7 | 5 | 4 | 2 | 4 | 3 | 4 | 3 | 5 | 7 | 5 | 7 | 5 |
| NM_014516 | CNOT3 | 2855 | 3 | 2 | 1 | 2 | 2 | 2 | 2 | 1 | 2 | 2 | 1 | 3 | 3 |
| NM_014517 | UBP1 | 4148 | 10 | 6 | 5 | 7 | 7 | 7 | 6 | 6 | 6 | 6 | 3 | 10 | 6 |
| NM_014518 | ZNF229 | 4964 | 2 | 1 | 1 | 0 | 1 | 2 | 1 | 0 | 0 | 2 | 0 | 1 | 1 |
| NM_014519 | ZNF232 | 2078 | 3 | 2 | 1 | 2 | 1 | 2 | 1 | 2 | 1 | 3 | 3 | 2 | 2 |
| NM_014520 | MYBBP1A | 4568 | 1 | 1 | 1 | 1 | 0 | 1 | 1 | 1 | 1 | 0 | 0 | 1 | 1 |
| NM_014521 | SH3BP4 | 5183 | 3 | 1 | 0 | 0 | 1 | 1 | 2 | 3 | 1 | 1 | 0 | 1 | 1 |
| NM_014522 | PCDH11X | 4603 | 0 | 0 | 0 | 0 | 0 | 0 | 0 | 0 | 0 | 0 | 0 | 0 | 0 |
| NM_014547 | TMOD3 | 2117 | 22 | 11 | 10 | 6 | 9 | 6 | 12 | 8 | 22 | 11 | 4 | 20 | 7 |
| NM_014548 | TMOD2 | 9182 | 43 | 6 | 1 | 1 | 3 | 43 | 1 | 0 | 2 | 3 | 1 | 5 | 2 |
| NM_014550 | CARD10 | 3899 | 1 | 0 | 0 | 1 | 0 | 0 | 1 | 0 | 0 | 0 | 0 | 0 | 1 |
| NM_014551 | NCAPH2 | 1425 | 0 | 0 | 0 | 0 | 0 | 0 | 0 | 0 | 0 | 0 | 0 | 0 | 0 |
| NM_014553 | TFCP2L1 | 9284 | 11 | 2 | 0 | 4 | 0 | 0 | 11 | 0 | 1 | 0 | 0 | 0 | 0 |
| NM_014554 | SENP1 | 4726 | 4 | 1 | 1 | 0 | 0 | 1 | 1 | 0 | 1 | 1 | 1 | 1 | 4 |
| NM_014555 | TRPM5 | 3928 | 0 | 0 | 0 | 0 | 0 | 0 | 0 | 0 | 0 | 0 | 0 | 0 | 0 |
| NM_014562 | OTX1 | 2174 | 0 | 0 | 0 | 0 | 0 | 0 | 0 | 0 | 0 | 0 | 0 | 0 | 0 |
| NM_014563 | TRAPPC2 | 2706 | 1 | 0 | 0 | 0 | 0 | 1 | 1 | 0 | 0 | 1 | 0 | 1 | 0 |
| NM_014564 | LHX3 | 2403 | 0 | 0 | 0 | 0 | 0 | 0 | 0 | 0 | 0 | 0 | 0 | 0 | 0 |
| NM_014565 | OR1A1 | 930 | 0 | 0 | 0 | 0 | 0 | 0 | 0 | 0 | 0 | 0 | 0 | 0 | 0 |
| NM_014566 | OR1D5 | 939 | 0 | 0 | 0 | 0 | 0 | 0 | 0 | 0 | 0 | 0 | 0 | 0 | 0 |
| NM_014567 | BCAR1 | 3217 | 1 | 1 | 0 | 1 | 1 | 1 | 1 | 0 | 1 | 0 | 0 | 1 | 1 |
| NM_014568 | GALNT5 | 3131 | 6 | 1 | 0 | 4 | 1 | 0 | 0 | 0 | 6 | 0 | 0 | 0 | 0 |
| NM_014569 | ZKSCAN5 | 4344 | 3 | 1 | 1 | 1 | 1 | 2 | 1 | 0 | 1 | 1 | 1 | 1 | 3 |
| NM_014570 | ARFGAP3 | 2833 | 12 | 8 | 5 | 3 | 9 | 8 | 11 | 10 | 7 | 6 | 5 | 9 | 12 |
| NM_014571 | HEYL | 4114 | 3 | 1 | 0 | 0 | 3 | 1 | 1 | 0 | 1 | 1 | 0 | 1 | 3 |
| NM_014572 | LATS2 | 5542 | 4 | 2 | 2 | 1 | 3 | 1 | 2 | 1 | 4 | 1 | 2 | 3 | 2 |
| NM_014573 | TMEM97 | 2568 | 6 | 1 | 0 | 0 | 0 | 1 | 1 | 4 | 1 | 1 | 1 | 1 | 6 |
| NM_014574 | STRN3 | 3970 | 20 | 9 | 6 | 3 | 10 | 9 | 8 | 7 | 6 | 7 | 20 | 9 | 10 |
| NM_014575 | SCHIP1 | 2106 | 24 | 7 | 1 | 1 | 19 | 24 | 2 | 1 | 3 | 0 | 20 | 1 | 1 |
| NM_014576 | A1CF | 2113 | 43 | 5 | 0 | 2 | 0 | 0 | 7 | 43 | 0 | 0 | 0 | 0 | 0 |

| transcript | gene | transcript length (nt) | max | mean | adipose | colon | heart | hypothalamus | kidney | liver | lung | ovary | skeletalmuscle | spleen | testes |
|---|---|---|---|---|---|---|---|---|---|---|---|---|---|---|---|
| NM_014577 | BRD1 | 4608 | 5 | 3 | 2 | 4 | 2 | 2 | 2 | 1 | 2 | 4 | 2 | 4 | 5 |
| NM_014578 | RHOD | 1132 | 1 | 0 | 0 | 0 | 0 | 0 | 0 | 1 | 0 | 0 | 0 | 0 | 0 |
| NM_014579 | SLC39A2 | 1409 | 0 | 0 | 0 | 0 | 0 | 0 | 0 | 0 | 0 | 0 | 0 | 0 | 0 |
| NM_014580 | SLC2A8 | 2116 | 1 | 0 | 0 | 0 | 0 | 1 | 0 | 1 | 0 | 0 | 0 | 0 | 1 |
| NM_014581 | OBP2B | 689 | 0 | 0 | 0 | 0 | 0 | 0 | 0 | 0 | 0 | 0 | 0 | 0 | 0 |
| NM_014582 | OBP2A | 689 | 0 | 0 | 0 | 0 | 0 | 0 | 0 | 0 | 0 | 0 | 0 | 0 | 0 |
| NM_014583 | LMCD1 | 1734 | 36 | 10 | 9 | 1 | 36 | 3 | 3 | 0 | 12 | 8 | 21 | 12 | 5 |
| NM_014584 | ERO1L | 3310 | 3 | 2 | 1 | 1 | 3 | 2 | 2 | 3 | 3 | 1 | 1 | 3 | 3 |
| NM_014585 | SLC40A1 | 3355 | 101 | 26 | 14 | 6 | 9 | 2 | 12 | 42 | 22 | 55 | 8 | 101 | 13 |
| NM_014586 | HUNK | 7385 | 1 | 0 | 0 | 0 | 0 | 0 | 1 | 0 | 0 | 0 | 0 | 0 | 0 |
| NM_014587 | SOX8 | 3048 | 1 | 0 | 0 | 0 | 0 | 1 | 0 | 0 | 0 | 0 | 0 | 0 | 0 |
| NM_014588 | VSX1 | 1931 | 0 | 0 | 0 | 0 | 0 | 0 | 0 | 0 | 0 | 0 | 0 | 0 | 0 |
| NM_014589 | PLA2G2E | 487 | 0 | 0 | 0 | 0 | 0 | 0 | 0 | 0 | 0 | 0 | 0 | 0 | 0 |
| NM_014591 | KCNIP2 | 2589 | 4 | 1 | 4 | 0 | 4 | 1 | 0 | 0 | 0 | 0 | 0 | 0 | 0 |
| NM_014592 | KCNIP1 | 2006 | 1 | 0 | 0 | 0 | 0 | 1 | 0 | 0 | 0 | 0 | 0 | 0 | 0 |
| NM_014593 | CXXC1 | 2954 | 2 | 1 | 1 | 2 | 1 | 1 | 2 | 1 | 1 | 1 | 1 | 2 | 2 |
| NM_014594 | ZNF354C | 2373 | 10 | 3 | 2 | 1 | 3 | 4 | 3 | 0 | 2 | 10 | 2 | 4 | 3 |
| NM_014595 | NT5C | 941 | 6 | 3 | 4 | 6 | 3 | 3 | 4 | 3 | 2 | 2 | 2 | 5 | 2 |
| NM_014596 | ZNRD1 | 743 | 2 | 1 | 1 | 0 | 1 | 1 | 1 | 1 | 0 | 1 | 1 | 2 | 0 |
| NM_014596 | ZNRD1 | 744 | 2 | 1 | 1 | 0 | 1 | 1 | 1 | 1 | 0 | 1 | 1 | 2 | 0 |
| NM_014597 | DNTTIP2 | 2380 | 10 | 6 | 5 | 1 | 10 | 7 | 6 | 6 | 6 | 6 | 8 | 9 | 8 |
| NM_014598 | SOCS7 | 2153 | 2 | 1 | 0 | 1 | 1 | 1 | 0 | 0 | 0 | 1 | 0 | 0 | 2 |
| NM_014599 | MAGED2 | 2060 | 15 | 8 | 5 | 5 | 9 | 14 | 12 | 3 | 5 | 15 | 4 | 6 | 7 |
| NM_014600 | EHD3 | 3905 | 5 | 1 | 0 | 0 | 1 | 5 | 1 | 1 | 0 | 0 | 0 | 1 | 0 |
| NM_014601 | EHD2 | 3584 | 12 | 3 | 12 | 3 | 4 | 1 | 1 | 1 | 4 | 5 | 2 | 5 | 2 |
| NM_014602 | PIK3R4 | 5039 | 4 | 2 | 2 | 2 | 2 | 4 | 3 | 3 | 2 | 3 | 2 | 3 | 3 |
| NM_014603 | CDR2L | 3533 | 5 | 2 | 2 | 2 | 4 | 5 | 2 | 1 | 2 | 1 | 1 | 1 | 3 |
| NM_014604 | TAX1BP3 | 1381 | 1 | 1 | 1 | 1 | 1 | 0 | 1 | 0 | 1 | 1 | 0 | 1 | 1 |
| NM_014606 | HERC3 | 4894 | 4 | 2 | 2 | 2 | 3 | 4 | 2 | 1 | 2 | 2 | 2 | 3 | 1 |
| NM_014607 | UBXN4 | 4014 | 15 | 9 | 7 | 3 | 10 | 9 | 12 | 11 | 9 | 8 | 8 | 11 | 15 |
| NM_014608 | CYFIP1 | 4429 | 8 | 5 | 6 | 4 | 5 | 5 | 6 | 5 | 6 | 4 | 2 | 8 | 4 |
| NM_014611 | MDN1 | 17400 | 6 | 4 | 2 | 2 | 3 | 4 | 3 | 5 | 2 | 4 | 4 | 5 | 6 |
| NM_014612 | FAM120A | 4957 | 7 | 5 | 4 | 4 | 5 | 4 | 6 | 7 | 4 | 3 | 4 | 7 | 3 |
| NM_014613 | FAF2 | 4515 | 10 | 6 | 4 | 4 | 5 | 6 | 5 | 9 | 4 | 7 | 4 | 7 | 10 |
| NM_014614 | PSME4 | 7099 | 12 | 7 | 4 | 2 | 12 | 3 | 4 | 8 | 5 | 4 | 12 | 8 | 11 |
| NM_014615 | KIAA0182 | 7393 | 4 | 3 | 4 | 2 | 2 | 2 | 3 | 2 | 2 | 3 | 3 | 2 | 4 |
| NM_014616 | ATP11B | 7320 | 7 | 4 | 3 | 2 | 4 | 2 | 4 | 4 | 4 | 5 | 2 | 7 | 4 |
| NM_014617 | CRYGA | 606 | 0 | 0 | 0 | 0 | 0 | 0 | 0 | 0 | 0 | 0 | 0 | 0 | 0 |
| NM_014618 | DBC1 | 3196 | 15 | 2 | 0 | 0 | 0 | 15 | 0 | 0 | 0 | 0 | 0 | 0 | 0 |
| NM_014619 | GRIK4 | 2871 | 1 | 0 | 0 | 0 | 0 | 1 | 0 | 0 | 0 | 0 | 0 | 0 | 0 |
| NM_014620 | HOXC4 | 2300 | 1 | 0 | 0 | 0 | 0 | 0 | 1 | 0 | 0 | 0 | 0 | 0 | 0 |
| NM_014621 | HOXD4 | 1296 | 1 | 0 | 0 | 0 | 0 | 0 | 1 | 0 | 0 | 0 | 0 | 0 | 0 |
| NM_014622 | VWA5A | 3421 | 4 | 2 | 2 | 2 | 1 | 4 | 2 | 0 | 2 | 1 | 1 | 1 | 2 |
| NM_014623 | MEA1 | 866 | 25 | 11 | 12 | 9 | 8 | 25 | 10 | 13 | 5 | 7 | 3 | 11 | 20 |
| NM_014624 | S100A6 | 683 | 72 | 31 | 72 | 45 | 39 | 12 | 36 | 3 | 59 | 21 | 14 | 26 | 12 |
| NM_014625 | NPHS2 | 1871 | 9 | 1 | 0 | 0 | 0 | 0 | 9 | 0 | 0 | 0 | 0 | 0 | 0 |
| NM_014626 | TAAR2 | 944 | 0 | 0 | 0 | 0 | 0 | 0 | 0 | 0 | 0 | 0 | 0 | 0 | 0 |
| NM_014628 | MAD2L1BP | 1279 | 1 | 1 | 1 | 1 | 1 | 1 | 1 | 1 | 0 | 0 | 1 | 1 | 1 |
| NM_014629 | ARHGEF10 | 5591 | 17 | 5 | 5 | 2 | 7 | 7 | 3 | 0 | 7 | 5 | 1 | 4 | 17 |
| NM_014630 | ZNF592 | 8179 | 2 | 1 | 1 | 1 | 1 | 1 | 1 | 1 | 1 | 1 | 1 | 2 | 1 |
| NM_014631 | SH3PXD2A | 11247 | 7 | 4 | 3 | 5 | 5 | 5 | 2 | 1 | 3 | 7 | 3 | 6 | 2 |
| NM_014632 | MICAL2 | 3905 | 14 | 4 | 1 | 8 | 5 | 6 | 1 | 3 | 5 | 2 | 1 | 2 | 14 |
| NM_014633 | CTR9 | 4309 | 20 | 12 | 9 | 3 | 12 | 11 | 13 | 15 | 6 | 14 | 6 | 20 | 20 |
| NM_014634 | PPM1F | 5151 | 1 | 0 | 0 | 0 | 0 | 1 | 1 | 0 | 1 | 0 | 0 | 1 | 0 |
| NM_014636 | RALGPS1 | 6336 | 2 | 0 | 0 | 0 | 0 | 2 | 1 | 0 | 0 | 0 | 0 | 0 | 0 |
| NM_014637 | MTFR1 | 2733 | 11 | 4 | 3 | 3 | 3 | 2 | 11 | 11 | 2 | 2 | 2 | 3 | 5 |
| NM_014638 | PLCH2 | 4837 | 1 | 0 | 0 | 0 | 0 | 1 | 0 | 0 | 0 | 0 | 0 | 0 | 0 |
| NM_014639 | TTC37 | 5154 | 11 | 8 | 8 | 2 | 10 | 8 | 10 | 7 | 7 | 9 | 10 | 11 | 7 |
| NM_014640 | TTLL4 | 4994 | 1 | 1 | 0 | 1 | 0 | 1 | 0 | 0 | 1 | 0 | 0 | 1 | 1 |
| NM_014641 | MDC1 | 7368 | 8 | 4 | 2 | 4 | 3 | 3 | 4 | 2 | 3 | 5 | 2 | 5 | 8 |
| NM_014643 | ZNF516 | 6555 | 3 | 1 | 0 | 1 | 0 | 1 | 1 | 0 | 1 | 3 | 1 | 1 | 3 |
| NM_014644 | PDE4DIP | 8363 | 119 | 24 | 11 | 4 | 84 | 19 | 6 | 6 | 6 | 2 | 119 | 5 | 3 |
| NM_014646 | LPIN2 | 6229 | 6 | 2 | 1 | 1 | 1 | 3 | 3 | 6 | 2 | 2 | 0 | 3 | 4 |
| NM_014647 | KIAA0430 | 7728 | 6 | 3 | 3 | 3 | 2 | 4 | 3 | 2 | 3 | 5 | 2 | 6 | 3 |
| NM_014648 | DZIP3 | 5541 | 8 | 3 | 1 | 0 | 2 | 8 | 2 | 0 | 1 | 2 | 2 | 2 | 7 |
| NM_014649 | SAFB2 | 3318 | 19 | 13 | 7 | 9 | 12 | 13 | 18 | 8 | 9 | 16 | 10 | 17 | 19 |
| NM_014650 | ZNF432 | 2573 | 5 | 2 | 1 | 1 | 2 | 2 | 3 | 0 | 2 | 5 | 1 | 3 | 4 |
| NM_014652 | IPO13 | 4026 | 3 | 1 | 1 | 1 | 1 | 3 | 2 | 1 | 0 | 0 | 2 | 1 | 1 |
| NM_014653 | WSCD2 | 4635 | 3 | 0 | 0 | 0 | 0 | 3 | 0 | 0 | 0 | 0 | 0 | 0 | 0 |
| NM_014654 | SDC3 | 5121 | 10 | 2 | 1 | 2 | 2 | 7 | 1 | 1 | 1 | 1 | 0 | 10 | 0 |
| NM_014655 | SLC25A44 | 3631 | 5 | 2 | 1 | 2 | 2 | 5 | 5 | 5 | 1 | 1 | 1 | 2 | 2 |
| NM_014656 | KIAA0040 | 4584 | 9 | 3 | 5 | 1 | 3 | 2 | 3 | 2 | 5 | 1 | 1 | 9 | 1 |
| NM_014657 | KIAA0406 | 3914 | 3 | 2 | 1 | 1 | 2 | 2 | 2 | 3 | 1 | 3 | 2 | 3 | 3 |
| NM_014659 | HISPPD2A | 5621 | 8 | 3 | 2 | 5 | 1 | 8 | 2 | 1 | 1 | 2 | 3 | 2 | 5 |
| NM_014660 | PHF14 | 4259 | 11 | 6 | 6 | 3 | 4 | 11 | 5 | 4 | 4 | 11 | 2 | 6 | 8 |
| NM_014661 | FAM53B | 5740 | 3 | 1 | 1 | 1 | 1 | 1 | 1 | 0 | 1 | 3 | 1 | 3 | 2 |
| NM_014662 | DEPDC5 | 5379 | 1 | 1 | 0 | 1 | 0 | 1 | 1 | 1 | 0 | 1 | 0 | 1 | 1 |

| transcript | gene | transcript length (nt) | max | mean | adipose | colon | heart | hypothalamus | kidney | liver | lung | ovary | skeletalmuscle | spleen | testes |
|---|---|---|---|---|---|---|---|---|---|---|---|---|---|---|---|
| NM_014663 | KDM4A | 4509 | 6 | 4 | 4 | 5 | 3 | 4 | 5 | 5 | 3 | 6 | 2 | 6 | 3 |
| NM_014665 | LRRC14 | 5323 | 0 | 0 | 0 | 0 | 0 | 0 | 0 | 0 | 0 | 0 | 0 | 0 | 0 |
| NM_014666 | CLINT1 | 3425 | 26 | 18 | 14 | 10 | 19 | 13 | 26 | 24 | 16 | 16 | 18 | 21 | 17 |
| NM_014667 | VGLL4 | 3725 | 3 | 2 | 1 | 1 | 1 | 1 | 2 | 0 | 2 | 3 | 0 | 2 | 3 |
| NM_014668 | GREB1 | 8482 | 37 | 4 | 0 | 0 | 0 | 1 | 0 | 0 | 0 | 37 | 0 | 0 | 4 |
| NM_014669 | NUP93 | 2736 | 4 | 2 | 1 | 2 | 2 | 4 | 2 | 1 | 2 | 1 | 1 | 3 | 4 |
| NM_014670 | BZW1 | 2798 | 25 | 9 | 8 | 3 | 8 | 9 | 8 | 25 | 7 | 6 | 3 | 8 | 10 |
| NM_014671 | UBE3C | 5181 | 8 | 6 | 5 | 4 | 7 | 6 | 6 | 8 | 4 | 5 | 6 | 7 | 7 |
| NM_014672 | KIAA0391 | 2610 | 5 | 3 | 2 | 2 | 2 | 4 | 5 | 5 | 3 | 1 | 1 | 3 | 4 |
| NM_014673 | TTC35 | 1238 | 17 | 8 | 9 | 4 | 7 | 17 | 8 | 6 | 8 | 5 | 6 | 8 | 8 |
| NM_014674 | EDEM1 | 6152 | 16 | 4 | 2 | 2 | 2 | 2 | 3 | 16 | 6 | 2 | 1 | 8 | 3 |
| NM_014675 | CROCC | 6656 | 0 | 0 | 0 | 0 | 0 | 0 | 0 | 0 | 0 | 0 | 0 | 0 | 0 |
| NM_014676 | PUM1 | 5379 | 12 | 8 | 7 | 7 | 7 | 12 | 9 | 6 | 8 | 11 | 9 | 10 | 7 |
| NM_014677 | RIMS2 | 5215 | 10 | 1 | 0 | 0 | 0 | 10 | 0 | 0 | 0 | 0 | 0 | 0 | 2 |
| NM_014678 | SAPS2 | 4004 | 4 | 2 | 1 | 2 | 2 | 4 | 2 | 3 | 1 | 1 | 2 | 2 | 2 |
| NM_014679 | CEP57 | 3158 | 3 | 2 | 1 | 1 | 1 | 1 | 2 | 3 | 2 | 3 | 1 | 2 | 2 |
| NM_014680 | KIAA0100 | 7431 | 11 | 6 | 7 | 5 | 6 | 8 | 11 | 8 | 4 | 4 | 5 | 9 | 5 |
| NM_014681 | DHX34 | 4375 | 0 | 0 | 0 | 0 | 0 | 0 | 0 | 0 | 0 | 0 | 0 | 0 | 0 |
| NM_014682 | ST18 | 6330 | 3 | 0 | 0 | 0 | 0 | 3 | 0 | 0 | 0 | 0 | 0 | 0 | 0 |
| NM_014683 | ULK2 | 9138 | 3 | 1 | 1 | 1 | 1 | 3 | 1 | 0 | 1 | 2 | 1 | 1 | 2 |
| NM_014685 | HERPUD1 | 2176 | 12 | 7 | 4 | 3 | 6 | 5 | 10 | 4 | 9 | 10 | 7 | 12 | 3 |
| NM_014686 | KIAA0355 | 6683 | 2 | 1 | 1 | 1 | 1 | 1 | 1 | 1 | 2 | 1 | 1 | 2 | 2 |
| NM_014687 | KIAA0226 | 6732 | 4 | 2 | 1 | 1 | 2 | 4 | 1 | 1 | 2 | 2 | 1 | 4 | 4 |
| NM_014688 | USP6NL | 4739 | 6 | 3 | 2 | 2 | 2 | 3 | 2 | 3 | 3 | 2 | 1 | 6 | 6 |
| NM_014689 | DOCK10 | 7431 | 12 | 3 | 2 | 1 | 1 | 5 | 1 | 1 | 2 | 7 | 0 | 12 | 1 |
| NM_014690 | FAM131B | 4270 | 2 | 0 | 0 | 0 | 0 | 2 | 0 | 0 | 0 | 0 | 0 | 0 | 0 |
| NM_014691 | AQR | 5080 | 6 | 4 | 4 | 3 | 3 | 4 | 5 | 5 | 3 | 5 | 2 | 6 | 6 |
| NM_014692 | SEC14L5 | 6456 | 1 | 0 | 0 | 0 | 0 | 1 | 0 | 0 | 0 | 0 | 0 | 0 | 0 |
| NM_014693 | ECE2 | 3483 | 1 | 0 | 0 | 0 | 0 | 1 | 0 | 0 | 0 | 0 | 0 | 0 | 0 |
| NM_014694 | ADAMTSL2 | 3725 | 1 | 0 | 0 | 0 | 0 | 0 | 1 | 1 | 0 | 0 | 0 | 0 | 0 |
| NM_014695 | CCDC144A | 5828 | 2 | 1 | 0 | 0 | 0 | 1 | 0 | 0 | 0 | 1 | 0 | 1 | 2 |
| NM_014696 | GPRIN2 | 1823 | 1 | 0 | 0 | 1 | 0 | 0 | 0 | 0 | 1 | 0 | 0 | 0 | 0 |
| NM_014697 | NOS1AP | 4464 | 3 | 1 | 0 | 1 | 0 | 3 | 0 | 1 | 0 | 0 | 0 | 0 | 0 |
| NM_014698 | TMEM63A | 4452 | 5 | 2 | 1 | 3 | 1 | 3 | 5 | 3 | 2 | 1 | 1 | 3 | 1 |
| NM_014699 | ZNF646 | 6201 | 2 | 1 | 1 | 1 | 0 | 1 | 0 | 0 | 0 | 1 | 0 | 2 | 2 |
| NM_014700 | RAB11FIP3 | 4251 | 15 | 4 | 1 | 2 | 2 | 6 | 15 | 2 | 1 | 2 | 2 | 3 | 4 |
| NM_014701 | SECISBP2L | 6779 | 37 | 12 | 10 | 3 | 10 | 37 | 12 | 6 | 16 | 11 | 6 | 11 | 14 |
| NM_014702 | KIAA0408 | 8123 | 0 | 0 | 0 | 0 | 0 | 0 | 0 | 0 | 0 | 0 | 0 | 0 | 0 |
| NM_014703 | VPRBP | 5825 | 10 | 4 | 4 | 3 | 3 | 4 | 4 | 3 | 3 | 3 | 4 | 5 | 10 |
| NM_014704 | KIAA0562 | 6458 | 5 | 4 | 5 | 3 | 4 | 4 | 5 | 4 | 2 | 4 | 3 | 4 | 4 |
| NM_014705 | DOCK4 | 8336 | 8 | 2 | 1 | 1 | 2 | 8 | 2 | 2 | 6 | 3 | 1 | 1 | 1 |
| NM_014706 | SART3 | 4370 | 8 | 6 | 4 | 5 | 5 | 8 | 5 | 4 | 4 | 6 | 5 | 8 | 8 |
| NM_014707 | HDAC9 | 4238 | 4 | 2 | 2 | 1 | 2 | 4 | 0 | 0 | 1 | 1 | 2 | 4 | 2 |
| NM_014708 | KNTC1 | 6965 | 5 | 1 | 1 | 1 | 1 | 1 | 1 | 0 | 1 | 1 | 0 | 2 | 5 |
| NM_014709 | USP34 | 11310 | 19 | 12 | 9 | 5 | 11 | 12 | 12 | 8 | 11 | 16 | 10 | 18 | 19 |
| NM_014710 | GPRASP1 | 5970 | 30 | 6 | 2 | 3 | 4 | 30 | 3 | 0 | 2 | 16 | 2 | 4 | 6 |
| NM_014711 | CP110 | 5501 | 6 | 2 | 1 | 0 | 2 | 6 | 1 | 1 | 2 | 3 | 1 | 2 | 6 |
| NM_014712 | SETD1A | 6447 | 2 | 1 | 1 | 1 | 1 | 1 | 1 | 1 | 1 | 2 | 2 | 2 | 2 |
| NM_014713 | LAPTM4A | 1764 | 41 | 24 | 26 | 14 | 27 | 11 | 38 | 32 | 22 | 25 | 10 | 18 | 41 |
| NM_014714 | IFT140 | 5267 | 4 | 1 | 1 | 1 | 1 | 2 | 2 | 0 | 2 | 2 | 0 | 1 | 4 |
| NM_014715 | RICS | 9442 | 8 | 2 | 1 | 2 | 0 | 8 | 2 | 1 | 1 | 2 | 1 | 1 | 2 |
| NM_014716 | ACAP1 | 2509 | 2 | 0 | 0 | 1 | 0 | 0 | 0 | 0 | 1 | 0 | 0 | 2 | 0 |
| NM_014717 | ZNF536 | 4935 | 4 | 0 | 0 | 0 | 0 | 4 | 0 | 0 | 0 | 0 | 0 | 0 | 1 |
| NM_014718 | CLSTN3 | 4002 | 10 | 2 | 1 | 1 | 0 | 10 | 3 | 1 | 1 | 1 | 0 | 2 | 0 |
| NM_014719 | FAM115A | 4076 | 11 | 5 | 5 | 2 | 5 | 11 | 4 | 8 | 3 | 8 | 3 | 3 | 4 |
| NM_014720 | SLK | 5606 | 23 | 17 | 14 | 6 | 23 | 16 | 18 | 8 | 21 | 22 | 15 | 18 | 23 |
| NM_014721 | PHACTR2 | 9531 | 9 | 5 | 5 | 1 | 7 | 4 | 3 | 2 | 7 | 6 | 3 | 9 | 8 |
| NM_014722 | FAM65B | 5471 | 16 | 3 | 0 | 1 | 1 | 4 | 1 | 1 | 6 | 1 | 4 | 16 | 1 |
| NM_014723 | SNPH | 4994 | 2 | 0 | 0 | 0 | 0 | 2 | 0 | 0 | 0 | 0 | 0 | 0 | 0 |
| NM_014725 | STARD8 | 4857 | 2 | 1 | 1 | 0 | 1 | 0 | 1 | 1 | 1 | 0 | 0 | 2 | 0 |
| NM_014726 | TBKBP1 | 4132 | 1 | 0 | 0 | 0 | 0 | 1 | 0 | 0 | 0 | 0 | 0 | 0 | 0 |
| NM_014727 | MLL4 | 8469 | 2 | 1 | 1 | 2 | 1 | 1 | 1 | 0 | 1 | 1 | 1 | 1 | 2 |
| NM_014728 | FRMPD4 | 6352 | 6 | 1 | 0 | 0 | 0 | 6 | 0 | 0 | 0 | 0 | 0 | 0 | 0 |
| NM_014729 | TOX | 4131 | 2 | 1 | 0 | 1 | 0 | 2 | 1 | 0 | 1 | 1 | 0 | 1 | 2 |
| NM_014730 | MLEC | 6349 | 35 | 16 | 15 | 21 | 21 | 12 | 26 | 35 | 6 | 11 | 4 | 15 | 13 |
| NM_014731 | ProSAPiP1 | 5247 | 3 | 0 | 0 | 0 | 0 | 3 | 1 | 0 | 0 | 0 | 0 | 0 | 0 |
| NM_014732 | KIAA0513 | 7353 | 14 | 2 | 0 | 2 | 1 | 14 | 1 | 1 | 1 | 0 | 0 | 2 | 1 |
| NM_014733 | ZFYVE16 | 7096 | 11 | 7 | 5 | 2 | 8 | 7 | 7 | 5 | 7 | 11 | 4 | 9 | 11 |
| NM_014734 | KIAA0247 | 5391 | 4 | 2 | 1 | 1 | 1 | 2 | 2 | 1 | 3 | 1 | 1 | 4 | 1 |
| NM_014735 | PHF16 | 4932 | 6 | 2 | 2 | 1 | 2 | 1 | 2 | 4 | 1 | 3 | 0 | 4 | 6 |
| NM_014736 | KIAA0101 | 1486 | 0 | 0 | 0 | 0 | 0 | 0 | 0 | 0 | 0 | 0 | 0 | 0 | 0 |
| NM_014737 | RASSF2 | 5426 | 16 | 6 | 3 | 2 | 3 | 16 | 1 | 1 | 7 | 4 | 0 | 16 | 10 |
| NM_014738 | KIAA0195 | 5464 | 2 | 1 | 1 | 2 | 1 | 2 | 1 | 1 | 1 | 1 | 2 | 1 | 1 |
| NM_014739 | BCLAF1 | 7263 | 28 | 19 | 14 | 5 | 16 | 24 | 16 | 15 | 16 | 27 | 17 | 27 | 28 |
| NM_014740 | EIF4A3 | 1686 | 5 | 3 | 2 | 2 | 2 | 2 | 4 | 3 | 4 | 5 | 2 | 5 | 5 |
| NM_014741 | KIAA0652 | 4612 | 4 | 2 | 2 | 1 | 3 | 4 | 2 | 2 | 1 | 2 | 1 | 3 | 4 |
| NM_014742 | TM9SF4 | 3978 | 3 | 2 | 1 | 2 | 2 | 3 | 3 | 3 | 2 | 2 | 1 | 3 | 2 |

| transcript | gene | transcript length (nt) | max | mean | adipose | colon | heart | hypothala mus | kidney | liver | lung | ovary | skeletalm uscle | spleen | testes |
|---|---|---|---|---|---|---|---|---|---|---|---|---|---|---|---|
| NM_014743 | KIAA0232 | 7857 | 13 | 7 | 4 | 2 | 12 | 13 | 7 | 4 | 4 | 5 | 11 | 7 | 7 |
| NM_014744 | TBC1D5 | 7853 | 8 | 4 | 3 | 2 | 4 | 5 | 4 | 2 | 4 | 6 | 2 | 8 | 4 |
| NM_014746 | RNF144A | 5739 | 4 | 1 | 1 | 1 | 1 | 2 | 1 | 0 | 1 | 4 | 1 | 4 | 1 |
| NM_014747 | RIMS3 | 7229 | 4 | 0 | 0 | 0 | 0 | 4 | 0 | 0 | 0 | 0 | 0 | 0 | 0 |
| NM_014748 | SNX17 | 2043 | 7 | 4 | 5 | 1 | 4 | 6 | 4 | 7 | 3 | 3 | 2 | 7 | 4 |
| NM_014749 | KIAA0586 | 5591 | 6 | 3 | 1 | 1 | 2 | 2 | 2 | 2 | 2 | 4 | 2 | 4 | 6 |
| NM_014750 | DLGAP5 | 2991 | 4 | 1 | 0 | 1 | 0 | 0 | 0 | 0 | 1 | 0 | 0 | 1 | 4 |
| NM_014751 | MTSS1 | 5007 | 15 | 5 | 5 | 2 | 4 | 6 | 6 | 7 | 4 | 2 | 4 | 15 | 6 |
| NM_014752 | SPCS2 | 2708 | 1 | 1 | 0 | 1 | 0 | 1 | 1 | 1 | 1 | 1 | 1 | 1 | 0 |
| NM_014753 | BMS1 | 8046 | 7 | 4 | 3 | 2 | 4 | 4 | 4 | 4 | 3 | 3 | 4 | 5 | 7 |
| NM_014754 | PTDSS1 | 2504 | 6 | 3 | 3 | 2 | 6 | 5 | 4 | 6 | 3 | 2 | 1 | 5 | 2 |
| NM_014755 | SERTAD2 | 5547 | 6 | 2 | 1 | 1 | 2 | 1 | 1 | 1 | 2 | 2 | 1 | 6 | 2 |
| NM_014756 | CKAP5 | 6518 | 19 | 9 | 6 | 3 | 9 | 19 | 6 | 6 | 5 | 13 | 13 | 8 | 17 |
| NM_014757 | MAML1 | 5721 | 4 | 2 | 1 | 2 | 2 | 1 | 2 | 1 | 2 | 3 | 2 | 4 | 3 |
| NM_014758 | SNX19 | 6078 | 9 | 6 | 8 | 3 | 7 | 9 | 7 | 7 | 3 | 4 | 3 | 7 | 6 |
| NM_014759 | PHYHIP | 3215 | 15 | 2 | 0 | 0 | 0 | 15 | 0 | 0 | 0 | 0 | 0 | 1 | 1 |
| NM_014760 | TATDN2 | 4906 | 5 | 3 | 3 | 3 | 3 | 5 | 3 | 2 | 2 | 3 | 1 | 4 | 2 |
| NM_014761 | KIAA0174 | 2349 | 23 | 14 | 14 | 13 | 13 | 15 | 15 | 8 | 13 | 16 | 9 | 23 | 16 |
| NM_014762 | DHCR24 | 4286 | 66 | 13 | 7 | 4 | 2 | 16 | 4 | 66 | 16 | 8 | 7 | 12 | 7 |
| NM_014763 | MRPL19 | 7827 | 6 | 3 | 3 | 1 | 4 | 3 | 6 | 4 | 2 | 3 | 4 | 4 | 3 |
| NM_014764 | DAZAP2 | 2164 | 16 | 8 | 5 | 5 | 7 | 8 | 12 | 8 | 10 | 4 | 3 | 16 | 6 |
| NM_014765 | TOMM20 | 3383 | 56 | 21 | 11 | 8 | 23 | 56 | 20 | 12 | 18 | 32 | 18 | 16 | 20 |
| NM_014766 | SCRN1 | 5267 | 29 | 6 | 4 | 2 | 4 | 29 | 5 | 0 | 3 | 5 | 1 | 4 | 8 |
| NM_014767 | SPOCK2 | 5328 | 16 | 3 | 0 | 1 | 0 | 16 | 3 | 0 | 7 | 0 | 0 | 3 | 3 |
| NM_014770 | AGAP2 | 3938 | 5 | 1 | 0 | 1 | 0 | 5 | 0 | 0 | 0 | 0 | 0 | 1 | 0 |
| NM_014771 | RNF40 | 4232 | 9 | 6 | 4 | 5 | 5 | 6 | 6 | 4 | 4 | 7 | 3 | 7 | 9 |
| NM_014772 | KIAA0427 | 5730 | 5 | 2 | 5 | 1 | 1 | 3 | 1 | 1 | 1 | 1 | 1 | 1 | 1 |
| NM_014773 | KIAA0141 | 4957 | 2 | 1 | 1 | 1 | 2 | 1 | 2 | 1 | 1 | 1 | 1 | 2 | 1 |
| NM_014774 | KIAA0494 | 5767 | 19 | 14 | 13 | 8 | 14 | 16 | 16 | 13 | 13 | 14 | 10 | 19 | 14 |
| NM_014775 | SFI1 | 4159 | 3 | 1 | 1 | 2 | 0 | 1 | 1 | 0 | 1 | 1 | 0 | 3 | 2 |
| NM_014776 | GIT2 | 5381 | 10 | 4 | 4 | 3 | 6 | 4 | 3 | 2 | 3 | 6 | 3 | 10 | 4 |
| NM_014777 | URB2 | 5613 | 1 | 0 | 0 | 0 | 0 | 1 | 0 | 1 | 0 | 0 | 1 | 1 | 1 |
| NM_014779 | TSC22D2 | 3885 | 2 | 1 | 1 | 1 | 1 | 1 | 1 | 1 | 1 | 1 | 1 | 1 | 2 |
| NM_014780 | CUL7 | 5499 | 2 | 1 | 1 | 1 | 1 | 1 | 2 | 0 | 1 | 2 | 1 | 1 | 1 |
| NM_014781 | RB1CC1 | 6667 | 15 | 10 | 6 | 2 | 14 | 10 | 8 | 7 | 10 | 10 | 15 | 11 | 15 |
| NM_014782 | ARMCX2 | 2793 | 3 | 1 | 1 | 0 | 1 | 3 | 1 | 0 | 1 | 1 | 0 | 1 | 1 |
| NM_014783 | ARHGAP11A | 5616 | 2 | 1 | 1 | 1 | 0 | 0 | 0 | 0 | 1 | 1 | 0 | 2 | 2 |
| NM_014784 | ARHGEF11 | 6769 | 5 | 2 | 2 | 3 | 2 | 5 | 2 | 1 | 1 | 2 | 1 | 2 | 3 |
| NM_014786 | ARHGEF17 | 7812 | 1 | 0 | 0 | 0 | 1 | 1 | 0 | 0 | 0 | 0 | 0 | 0 | 0 |
| NM_014787 | DNAJC6 | 5750 | 25 | 3 | 0 | 0 | 1 | 25 | 1 | 0 | 0 | 0 | 0 | 0 | 0 |
| NM_014788 | TRIM14 | 4454 | 7 | 2 | 1 | 2 | 1 | 1 | 3 | 3 | 2 | 1 | 0 | 7 | 1 |
| NM_014789 | ZNF623 | 3947 | 11 | 6 | 4 | 5 | 4 | 9 | 4 | 2 | 3 | 11 | 3 | 8 | 8 |
| NM_014790 | JAKMIP2 | 3338 | 8 | 1 | 0 | 0 | 0 | 8 | 0 | 0 | 0 | 0 | 0 | 0 | 0 |
| NM_014791 | MELK | 2439 | 6 | 1 | 1 | 2 | 1 | 0 | 0 | 0 | 1 | 0 | 0 | 1 | 6 |
| NM_014793 | LCMT2 | 2918 | 1 | 1 | 0 | 0 | 0 | 1 | 1 | 1 | 0 | 1 | 0 | 1 | 1 |
| NM_014795 | ZEB2 | 5560 | 35 | 20 | 31 | 5 | 23 | 33 | 10 | 4 | 21 | 30 | 7 | 35 | 19 |
| NM_014797 | ZBTB24 | 5597 | 2 | 1 | 1 | 1 | 1 | 1 | 1 | 1 | 1 | 1 | 1 | 2 | 1 |
| NM_014798 | PLEKHM1 | 5299 | 2 | 1 | 1 | 1 | 1 | 1 | 1 | 1 | 1 | 1 | 1 | 2 | 0 |
| NM_014799 | HEPH | 4230 | 8 | 2 | 0 | 8 | 2 | 2 | 1 | 0 | 1 | 2 | 0 | 2 | 1 |
| NM_014800 | ELMO1 | 3686 | 7 | 2 | 2 | 1 | 1 | 6 | 1 | 1 | 2 | 1 | 0 | 7 | 1 |
| NM_014801 | PCNXL2 | 6817 | 7 | 1 | 1 | 1 | 0 | 7 | 1 | 0 | 1 | 2 | 0 | 1 | 1 |
| NM_014802 | KIAA0528 | 4407 | 4 | 3 | 2 | 2 | 2 | 4 | 3 | 1 | 2 | 4 | 1 | 4 | 3 |
| NM_014803 | ZNF518A | 8295 | 6 | 2 | 1 | 1 | 2 | 2 | 3 | 1 | 2 | 3 | 1 | 3 | 6 |
| NM_014804 | KIAA0753 | 4685 | 2 | 1 | 1 | 1 | 0 | 1 | 1 | 0 | 0 | 1 | 0 | 1 | 2 |
| NM_014805 | EPM2AIP1 | 7438 | 3 | 2 | 1 | 1 | 2 | 3 | 2 | 1 | 2 | 3 | 2 | 3 | 3 |
| NM_014806 | RUSC2 | 5321 | 3 | 1 | 1 | 1 | 1 | 3 | 1 | 0 | 0 | 1 | 1 | 1 | 1 |
| NM_014807 | C2CD2L | 3344 | 2 | 0 | 0 | 1 | 0 | 2 | 1 | 0 | 0 | 0 | 0 | 1 | 0 |
| NM_014808 | FARP2 | 3997 | 4 | 2 | 2 | 4 | 1 | 3 | 2 | 1 | 2 | 3 | 1 | 3 | 4 |
| NM_014809 | KIAA0319 | 6805 | 2 | 0 | 0 | 0 | 0 | 2 | 0 | 0 | 0 | 0 | 0 | 0 | 0 |
| NM_014810 | CEP350 | 13456 | 10 | 6 | 4 | 2 | 5 | 5 | 5 | 4 | 5 | 7 | 10 | 9 | 10 |
| NM_014811 | KIAA0649 | 4932 | 2 | 1 | 0 | 1 | 1 | 2 | 1 | 0 | 1 | 0 | 0 | 1 | 1 |
| NM_014812 | CEP170 | 7184 | 19 | 8 | 7 | 1 | 8 | 19 | 3 | 2 | 7 | 10 | 7 | 11 | 10 |
| NM_014813 | LRIG2 | 4015 | 5 | 2 | 2 | 2 | 2 | 3 | 3 | 1 | 2 | 5 | 1 | 4 | 3 |
| NM_014814 | PSMD6 | 1308 | 19 | 11 | 11 | 6 | 14 | 13 | 15 | 14 | 8 | 5 | 8 | 11 | 19 |
| NM_014815 | MED24 | 3715 | 2 | 1 | 1 | 2 | 1 | 2 | 1 | 1 | 1 | 1 | 1 | 1 | 1 |
| NM_014817 | TRIL | 5056 | 3 | 1 | 0 | 0 | 0 | 3 | 1 | 0 | 0 | 1 | 0 | 0 | 0 |
| NM_014818 | TRIM66 | 9751 | 1 | 1 | 0 | 1 | 0 | 1 | 1 | 0 | 0 | 0 | 0 | 1 | 1 |
| NM_014819 | PJA2 | 4869 | 80 | 28 | 24 | 7 | 27 | 80 | 19 | 17 | 24 | 30 | 16 | 30 | 31 |
| NM_014820 | TOMM70A | 4360 | 16 | 10 | 11 | 4 | 10 | 16 | 10 | 16 | 5 | 8 | 12 | 8 | 8 |
| NM_014822 | SEC24D | 4063 | 10 | 4 | 3 | 2 | 5 | 2 | 2 | 10 | 4 | 4 | 1 | 7 | 4 |
| NM_014824 | FCHSD2 | 4490 | 16 | 6 | 4 | 2 | 6 | 7 | 3 | 3 | 6 | 8 | 2 | 16 | 9 |
| NM_014825 | URB1 | 10808 | 2 | 1 | 1 | 1 | 1 | 2 | 1 | 2 | 1 | 2 | 1 | 1 | 2 |
| NM_014826 | CDC42BPA | 10263 | 11 | 7 | 6 | 3 | 7 | 11 | 9 | 4 | 6 | 7 | 3 | 5 | 10 |
| NM_014827 | ZC3H11A | 4778 | 17 | 10 | 10 | 5 | 10 | 8 | 11 | 8 | 11 | 12 | 8 | 17 | 14 |
| NM_014828 | TOX4 | 4524 | 7 | 5 | 4 | 3 | 6 | 6 | 4 | 3 | 4 | 4 | 7 | 5 | 5 |
| NM_014829 | DDX46 | 5679 | 12 | 8 | 8 | 4 | 9 | 9 | 9 | 7 | 7 | 9 | 9 | 12 | 10 |
| NM_014830 | ZBTB39 | 6170 | 2 | 1 | 0 | 1 | 1 | 1 | 1 | 1 | 1 | 1 | 0 | 2 | 2 |

| transcript | gene | transcript length (nt) | max | mean | adipose | colon | heart | hypothalamus | kidney | liver | lung | ovary | skeletalmuscle | spleen | testes |
|---|---|---|---|---|---|---|---|---|---|---|---|---|---|---|---|
| NM_014831 | TRANK1 | 10483 | 3 | 1 | 1 | 3 | 1 | 1 | 1 | 1 | 2 | 2 | 0 | 3 | 1 |
| NM_014832 | TBC1D4 | 6363 | 23 | 9 | 8 | 2 | 14 | 3 | 11 | 2 | 4 | 20 | 23 | 8 | 5 |
| NM_014834 | LRRC37A | 5177 | 0 | 0 | 0 | 0 | 0 | 0 | 0 | 0 | 0 | 0 | 0 | 0 | 0 |
| NM_014835 | OSBPL2 | 3935 | 10 | 5 | 5 | 6 | 4 | 10 | 5 | 4 | 5 | 4 | 2 | 7 | 5 |
| NM_014836 | RHOBTB1 | 4497 | 9 | 3 | 3 | 1 | 3 | 2 | 9 | 1 | 2 | 5 | 5 | 4 | 4 |
| NM_014838 | ZBED4 | 6886 | 2 | 1 | 1 | 1 | 1 | 1 | 1 | 1 | 1 | 1 | 1 | 2 | 1 |
| NM_014839 | LPPR4 | 5020 | 19 | 2 | 0 | 0 | 0 | 19 | 0 | 0 | 0 | 0 | 0 | 0 | 1 |
| NM_014840 | NUAK1 | 6821 | 6 | 2 | 2 | 1 | 4 | 6 | 1 | 1 | 1 | 1 | 2 | 0 | 1 |
| NM_014841 | SNAP91 | 4449 | 21 | 2 | 0 | 0 | 0 | 21 | 0 | 0 | 0 | 0 | 0 | 0 | 1 |
| NM_014844 | TECPR2 | 8607 | 5 | 2 | 1 | 1 | 3 | 5 | 2 | 1 | 1 | 2 | 2 | 2 | 4 |
| NM_014845 | FIG4 | 3105 | 5 | 3 | 2 | 1 | 2 | 5 | 3 | 1 | 3 | 2 | 2 | 5 | 3 |
| NM_014846 | KIAA0196 | 4165 | 8 | 4 | 5 | 2 | 4 | 5 | 6 | 4 | 4 | 5 | 3 | 8 | 4 |
| NM_014847 | UBAP2L | 3989 | 16 | 11 | 9 | 7 | 11 | 13 | 10 | 7 | 11 | 11 | 9 | 14 | 16 |
| NM_014848 | SV2B | 11342 | 31 | 3 | 1 | 1 | 1 | 31 | 0 | 0 | 0 | 0 | 0 | 0 | 0 |
| NM_014849 | SV2A | 4410 | 30 | 3 | 0 | 1 | 0 | 30 | 0 | 0 | 0 | 1 | 0 | 0 | 1 |
| NM_014850 | SRGAP3 | 8901 | 4 | 1 | 0 | 0 | 0 | 4 | 1 | 0 | 0 | 1 | 0 | 0 | 1 |
| NM_014851 | KLHL21 | 4487 | 8 | 2 | 1 | 1 | 4 | 2 | 2 | 1 | 1 | 3 | 8 | 2 | 2 |
| NM_014853 | SGSM2 | 4867 | 3 | 1 | 1 | 1 | 0 | 3 | 1 | 0 | 1 | 0 | 0 | 1 | 1 |
| NM_014855 | KIAA0415 | 2890 | 0 | 0 | 0 | 0 | 0 | 0 | 0 | 0 | 0 | 0 | 0 | 0 | 0 |
| NM_014856 | DENND4B | 5688 | 1 | 0 | 0 | 0 | 0 | 1 | 0 | 0 | 1 | 0 | 1 | 1 | 0 |
| NM_014857 | RABGAP1L | 2928 | 23 | 8 | 4 | 4 | 23 | 11 | 9 | 4 | 5 | 8 | 2 | 9 | 6 |
| NM_014858 | TMCC2 | 3792 | 2 | 0 | 0 | 0 | 0 | 2 | 0 | 0 | 0 | 0 | 0 | 0 | 0 |
| NM_014859 | RICH2 | 4256 | 11 | 2 | 0 | 5 | 1 | 11 | 1 | 0 | 3 | 2 | 0 | 0 | 1 |
| NM_014860 | SUPT7L | 4261 | 6 | 3 | 3 | 1 | 3 | 6 | 3 | 2 | 2 | 3 | 2 | 5 | 3 |
| NM_014861 | ATP2C2 | 3385 | 8 | 1 | 0 | 8 | 0 | 1 | 0 | 0 | 2 | 0 | 0 | 0 | 0 |
| NM_014862 | ARNT2 | 6558 | 15 | 2 | 0 | 1 | 0 | 15 | 2 | 0 | 0 | 1 | 0 | 0 | 2 |
| NM_014864 | FAM20B | 5945 | 7 | 4 | 5 | 2 | 6 | 7 | 3 | 3 | 2 | 3 | 3 | 3 | 2 |
| NM_014865 | NCAPD2 | 4806 | 6 | 3 | 3 | 4 | 2 | 2 | 2 | 1 | 3 | 2 | 1 | 6 | 4 |
| NM_014866 | SEC16A | 9042 | 5 | 2 | 2 | 2 | 2 | 2 | 2 | 5 | 2 | 2 | 1 | 3 | 2 |
| NM_014867 | KBTBD11 | 6717 | 9 | 2 | 0 | 1 | 1 | 9 | 3 | 1 | 1 | 0 | 0 | 4 | 1 |
| NM_014868 | RNF10 | 3847 | 5 | 3 | 2 | 2 | 5 | 3 | 4 | 3 | 3 | 2 | 3 | 5 | 3 |
| NM_014869 | IQSEC1 | 5071 | 9 | 3 | 2 | 1 | 3 | 9 | 1 | 3 | 2 | 2 | 1 | 5 | 1 |
| NM_014870 | ZBTB40 | 8704 | 3 | 2 | 1 | 1 | 2 | 2 | 2 | 1 | 1 | 3 | 1 | 3 | 2 |
| NM_014871 | PAN2 | 5289 | 2 | 1 | 1 | 2 | 1 | 1 | 2 | 2 | 1 | 1 | 1 | 2 | 2 |
| NM_014872 | ZBTB5 | 4647 | 3 | 1 | 1 | 1 | 1 | 1 | 2 | 1 | 1 | 2 | 1 | 2 | 3 |
| NM_014873 | LPGAT1 | 7781 | 17 | 10 | 13 | 3 | 9 | 16 | 8 | 17 | 6 | 5 | 4 | 15 | 9 |
| NM_014874 | MFN2 | 4677 | 12 | 5 | 3 | 3 | 12 | 6 | 5 | 5 | 2 | 2 | 6 | 3 | 3 |
| NM_014875 | KIF14 | 7277 | 1 | 0 | 0 | 0 | 0 | 0 | 0 | 0 | 0 | 0 | 0 | 1 | 1 |
| NM_014876 | JOSD1 | 3434 | 8 | 4 | 3 | 4 | 5 | 6 | 3 | 3 | 4 | 8 | 2 | 6 | 4 |
| NM_014877 | HELZ | 13830 | 12 | 7 | 6 | 3 | 6 | 5 | 6 | 4 | 7 | 11 | 6 | 10 | 12 |
| NM_014878 | KIAA0020 | 2219 | 19 | 10 | 9 | 4 | 10 | 9 | 19 | 8 | 8 | 13 | 6 | 14 | 14 |
| NM_014879 | P2RY14 | 2488 | 3 | 1 | 3 | 1 | 1 | 0 | 0 | 0 | 2 | 0 | 1 | 1 | 0 |
| NM_014880 | CD302 | 3977 | 24 | 7 | 6 | 1 | 4 | 2 | 3 | 24 | 10 | 7 | 2 | 9 | 5 |
| NM_014881 | DCLRE1A | 4468 | 2 | 1 | 1 | 1 | 1 | 2 | 2 | 2 | 1 | 1 | 1 | 2 | 2 |
| NM_014882 | ARHGAP25 | 2928 | 9 | 2 | 2 | 1 | 1 | 1 | 0 | 1 | 3 | 2 | 0 | 9 | 1 |
| NM_014883 | FAM13A | 5858 | 7 | 3 | 7 | 2 | 1 | 2 | 3 | 2 | 2 | 3 | 1 | 1 | 4 |
| NM_014884 | SFRS14 | 3657 | 21 | 8 | 5 | 8 | 6 | 17 | 7 | 2 | 5 | 6 | 5 | 7 | 21 |
| NM_014885 | ANAPC10 | 871 | 5 | 3 | 3 | 1 | 2 | 5 | 3 | 1 | 2 | 4 | 3 | 3 | 3 |
| NM_014886 | NSA2 | 1104 | 141 | 61 | 43 | 24 | 57 | 39 | 45 | 48 | 47 | 141 | 63 | 83 | 81 |
| NM_014887 | N4BP2L2 | 2839 | 94 | 55 | 37 | 23 | 53 | 41 | 57 | 22 | 67 | 94 | 58 | 81 | 67 |
| NM_014888 | FAM3C | 2542 | 31 | 16 | 10 | 8 | 22 | 31 | 18 | 18 | 12 | 10 | 6 | 24 | 20 |
| NM_014889 | PITRM1 | 3456 | 5 | 4 | 2 | 2 | 5 | 4 | 4 | 5 | 2 | 3 | 4 | 3 | 5 |
| NM_014890 | FILIP1L | 3274 | 90 | 37 | 30 | 6 | 90 | 8 | 10 | 5 | 21 | 84 | 63 | 53 | 33 |
| NM_014891 | PDAP1 | 2383 | 28 | 16 | 12 | 10 | 24 | 17 | 15 | 16 | 9 | 12 | 28 | 17 | 13 |
| NM_014892 | RBM16 | 5004 | 21 | 14 | 8 | 7 | 17 | 13 | 13 | 6 | 11 | 21 | 21 | 16 | 21 |
| NM_014893 | NLGN4Y | 5409 | 4 | 1 | 0 | 0 | 0 | 4 | 0 | 0 | 0 | 0 | 0 | 0 | 1 |
| NM_014895 | KIAA1009 | 5138 | 10 | 4 | 3 | 1 | 3 | 3 | 4 | 1 | 3 | 6 | 2 | 5 | 10 |
| NM_014897 | ZNF652 | 11346 | 7 | 5 | 4 | 4 | 3 | 5 | 4 | 5 | 5 | 7 | 3 | 7 | 6 |
| NM_014898 | ZFP30 | 4611 | 3 | 1 | 1 | 1 | 1 | 2 | 2 | 0 | 1 | 3 | 1 | 2 | 2 |
| NM_014899 | RHOBTB3 | 5566 | 22 | 10 | 17 | 3 | 9 | 13 | 8 | 7 | 5 | 22 | 7 | 6 | 14 |
| NM_014900 | COBLL1 | 4921 | 30 | 10 | 21 | 1 | 4 | 4 | 30 | 21 | 6 | 6 | 2 | 8 | 4 |
| NM_014901 | RNF44 | 4153 | 1 | 0 | 0 | 1 | 0 | 1 | 0 | 0 | 0 | 0 | 0 | 1 | 0 |
| NM_014902 | DLGAP4 | 4881 | 6 | 2 | 2 | 2 | 2 | 6 | 1 | 1 | 1 | 2 | 3 | 1 | 2 |
| NM_014903 | NAV3 | 9758 | 6 | 2 | 2 | 0 | 1 | 5 | 0 | 0 | 1 | 6 | 0 | 3 | 1 |
| NM_014904 | RAB11FIP2 | 6059 | 7 | 4 | 3 | 1 | 3 | 7 | 5 | 1 | 2 | 4 | 2 | 4 | 6 |
| NM_014905 | GLS | 4338 | 27 | 7 | 3 | 2 | 5 | 16 | 27 | 0 | 7 | 3 | 2 | 9 | 4 |
| NM_014906 | PPM1E | 6537 | 4 | 0 | 0 | 0 | 0 | 4 | 0 | 0 | 0 | 0 | 0 | 0 | 1 |
| NM_014907 | FRMPD1 | 4916 | 2 | 0 | 0 | 0 | 0 | 0 | 0 | 0 | 0 | 0 | 0 | 0 | 2 |
| NM_014908 | DOLK | 2204 | 1 | 1 | 1 | 0 | 1 | 1 | 1 | 1 | 0 | 1 | 0 | 1 | 1 |
| NM_014909 | VASH1 | 6030 | 3 | 1 | 1 | 1 | 2 | 2 | 1 | 0 | 1 | 2 | 0 | 3 | 1 |
| NM_014910 | ZNF507 | 7622 | 4 | 2 | 1 | 1 | 2 | 3 | 2 | 2 | 1 | 3 | 3 | 3 | 4 |
| NM_014911 | AAK1 | 21265 | 3 | 1 | 1 | 1 | 2 | 3 | 2 | 0 | 1 | 1 | 1 | 2 | 1 |
| NM_014912 | CPEB3 | 5840 | 3 | 1 | 0 | 0 | 1 | 3 | 1 | 2 | 1 | 1 | 3 | 1 | 1 |
| NM_014913 | ADNP2 | 5387 | 6 | 3 | 2 | 1 | 2 | 3 | 3 | 2 | 2 | 4 | 1 | 3 | 6 |
| NM_014914 | AGAP1 | 4364 | 10 | 2 | 1 | 3 | 1 | 10 | 3 | 1 | 1 | 2 | 1 | 1 | 2 |
| NM_014915 | ANKRD26 | 6779 | 3 | 2 | 1 | 0 | 2 | 2 | 3 | 1 | 1 | 2 | 2 | 2 | 2 |
| NM_014916 | LMTK2 | 8943 | 5 | 2 | 0 | 2 | 2 | 5 | 3 | 2 | 2 | 1 | 2 | 2 | 2 |

| | | transcript | | | | | | hypothala | | | | | skeletalm | | |
|---|---|---|---|---|---|---|---|---|---|---|---|---|---|---|---|
| transcript | gene | length (nt) | max | mean | adipose | colon | heart | mus | kidney | liver | lung | ovary | uscle | spleen | testes |
| NM_014917 | NTNG1 | 2932 | 4 | 1 | 0 | 0 | 0 | 4 | 1 | 0 | 1 | 0 | 0 | 0 | 0 |
| NM_014918 | CHSY1 | 4549 | 9 | 4 | 3 | 1 | 5 | 4 | 3 | 1 | 6 | 7 | 2 | 9 | 8 |
| NM_014920 | ICK | 6127 | 2 | 1 | 1 | 1 | 1 | 1 | 1 | 2 | 2 | 2 | 1 | 2 | 2 |
| NM_014921 | LPHN1 | 7840 | 3 | 1 | 1 | 1 | 0 | 3 | 0 | 0 | 0 | 0 | 0 | 1 | 0 |
| NM_014922 | NLRP1 | 5481 | 3 | 1 | 2 | 1 | 1 | 0 | 0 | 0 | 3 | 1 | 1 | 3 | 1 |
| NM_014923 | FNDC3A | 5900 | 10 | 6 | 3 | 2 | 6 | 5 | 7 | 10 | 7 | 7 | 2 | 7 | 8 |
| NM_014924 | KIAA0831 | 4742 | 4 | 3 | 3 | 2 | 3 | 2 | 2 | 1 | 2 | 4 | 3 | 3 | 4 |
| NM_014925 | R3HDM2 | 3974 | 10 | 7 | 4 | 4 | 8 | 10 | 7 | 6 | 5 | 8 | 8 | 8 | 10 |
| NM_014926 | SLITRK3 | 4222 | 3 | 1 | 0 | 1 | 0 | 3 | 0 | 1 | 0 | 0 | 0 | 0 | 0 |
| NM_014927 | CNKSR2 | 5758 | 15 | 2 | 0 | 0 | 0 | 15 | 0 | 0 | 1 | 0 | 0 | 1 | 1 |
| NM_014929 | FASTKD2 | 6833 | 5 | 3 | 2 | 1 | 3 | 4 | 3 | 5 | 1 | 3 | 3 | 2 | 2 |
| NM_014930 | ZNF510 | 5154 | 5 | 3 | 2 | 1 | 2 | 4 | 2 | 1 | 2 | 5 | 2 | 5 | 3 |
| NM_014931 | SAPS1 | 3637 | 20 | 9 | 3 | 7 | 9 | 13 | 7 | 6 | 7 | 5 | 6 | 20 | 15 |
| NM_014932 | NLGN1 | 4918 | 11 | 2 | 1 | 0 | 2 | 11 | 3 | 0 | 0 | 1 | 0 | 0 | 2 |
| NM_014933 | SEC31A | 4237 | 22 | 12 | 11 | 5 | 14 | 8 | 15 | 13 | 9 | 22 | 9 | 12 | 13 |
| NM_014934 | DZIP1 | 7431 | 4 | 2 | 1 | 0 | 1 | 3 | 3 | 0 | 1 | 4 | 0 | 0 | 4 |
| NM_014935 | PLEKHA6 | 7399 | 7 | 2 | 1 | 5 | 1 | 5 | 7 | 2 | 0 | 0 | 1 | 0 | 1 |
| NM_014936 | ENPP4 | 4680 | 7 | 4 | 2 | 1 | 7 | 6 | 6 | 1 | 5 | 1 | 5 | 5 | 4 |
| NM_014937 | INPP5F | 4932 | 15 | 3 | 2 | 1 | 2 | 15 | 3 | 0 | 2 | 2 | 2 | 2 | 3 |
| NM_014938 | MLXIP | 5368 | 6 | 3 | 1 | 6 | 2 | 1 | 1 | 1 | 2 | 4 | 6 | 4 | 2 |
| NM_014939 | KIAA1012 | 6254 | 7 | 4 | 3 | 1 | 5 | 5 | 5 | 5 | 3 | 4 | 4 | 6 | 7 |
| NM_014940 | MON1B | 3312 | 2 | 1 | 1 | 1 | 1 | 2 | 2 | 1 | 1 | 1 | 0 | 2 | 1 |
| NM_014941 | MORC2 | 4467 | 3 | 2 | 1 | 1 | 2 | 2 | 1 | 2 | 1 | 2 | 2 | 2 | 3 |
| NM_014942 | ANKRD6 | 5190 | 3 | 1 | 1 | 0 | 1 | 3 | 1 | 0 | 1 | 2 | 0 | 1 | 1 |
| NM_014943 | ZHX2 | 4359 | 7 | 3 | 1 | 1 | 4 | 3 | 2 | 2 | 4 | 7 | 6 | 6 | 2 |
| NM_014944 | CLSTN1 | 5162 | 51 | 11 | 4 | 8 | 6 | 51 | 11 | 1 | 5 | 13 | 4 | 7 | 7 |
| NM_014945 | ABLIM3 | 4335 | 32 | 10 | 26 | 3 | 14 | 11 | 8 | 32 | 3 | 1 | 5 | 4 | 1 |
| NM_014946 | SPAST | 5213 | 5 | 2 | 2 | 1 | 2 | 5 | 1 | 2 | 2 | 3 | 2 | 4 | 3 |
| NM_014947 | FOXJ3 | 5231 | 11 | 7 | 5 | 4 | 7 | 8 | 6 | 3 | 6 | 8 | 11 | 8 | 8 |
| NM_014948 | UBOX5 | 4329 | 1 | 0 | 0 | 0 | 0 | 1 | 0 | 0 | 0 | 1 | 1 | 1 | 0 |
| NM_014949 | KIAA0907 | 2945 | 7 | 5 | 4 | 6 | 4 | 5 | 5 | 1 | 5 | 6 | 4 | 7 | 7 |
| NM_014950 | ZBTB1 | 4119 | 21 | 7 | 6 | 3 | 6 | 5 | 7 | 3 | 7 | 6 | 4 | 21 | 7 |
| NM_014951 | ZNF365 | 4208 | 6 | 1 | 0 | 0 | 0 | 6 | 0 | 0 | 0 | 0 | 0 | 0 | 0 |
| NM_014952 | BAHD1 | 4526 | 1 | 1 | 0 | 0 | 1 | 1 | 1 | 0 | 1 | 1 | 0 | 1 | 1 |
| NM_014953 | DIS3 | 7644 | 4 | 3 | 2 | 1 | 3 | 3 | 2 | 2 | 3 | 4 | 2 | 3 | 4 |
| NM_014954 | RPH3A | 4578 | 10 | 1 | 0 | 3 | 0 | 10 | 0 | 1 | 0 | 0 | 0 | 0 | 0 |
| NM_014955 | METTL13 | 3082 | 3 | 2 | 1 | 1 | 1 | 2 | 2 | 2 | 1 | 1 | 1 | 2 | 3 |
| NM_014956 | CEP164 | 5628 | 3 | 1 | 1 | 1 | 1 | 1 | 1 | 0 | 1 | 1 | 0 | 1 | 3 |
| NM_014957 | DENND3 | 5443 | 4 | 2 | 1 | 2 | 1 | 2 | 1 | 0 | 4 | 1 | 1 | 4 | 1 |
| NM_014959 | CARD8 | 5233 | 6 | 2 | 2 | 1 | 2 | 1 | 1 | 0 | 3 | 1 | 1 | 6 | 1 |
| NM_014960 | ARSG | 2770 | 1 | 1 | 0 | 0 | 0 | 1 | 1 | 0 | 1 | 1 | 1 | 1 | 1 |
| NM_014961 | RUFY3 | 4263 | 25 | 5 | 2 | 2 | 2 | 25 | 5 | 2 | 3 | 5 | 2 | 3 | 8 |
| NM_014962 | BTBD3 | 4857 | 19 | 5 | 1 | 3 | 5 | 19 | 4 | 2 | 3 | 4 | 2 | 4 | 8 |
| NM_014963 | SBNO2 | 4925 | 1 | 0 | 0 | 0 | 0 | 0 | 0 | 0 | 0 | 0 | 0 | 1 | 0 |
| NM_014964 | EPN2 | 4835 | 4 | 1 | 1 | 1 | 2 | 4 | 1 | 0 | 1 | 1 | 1 | 1 | 1 |
| NM_014965 | TRAK1 | 4614 | 5 | 4 | 3 | 5 | 5 | 3 | 4 | 1 | 4 | 1 | 5 | 5 | 5 |
| NM_014966 | DHX30 | 4115 | 5 | 3 | 2 | 2 | 2 | 5 | 2 | 2 | 1 | 2 | 2 | 2 | 5 |
| NM_014967 | MTMR15 | 4887 | 3 | 2 | 1 | 1 | 2 | 3 | 2 | 1 | 1 | 2 | 2 | 2 | 2 |
| NM_014969 | WDR47 | 4354 | 19 | 4 | 2 | 1 | 3 | 19 | 2 | 1 | 2 | 2 | 3 | 2 | 5 |
| NM_014970 | KIFAP3 | 2997 | 17 | 5 | 2 | 2 | 7 | 17 | 4 | 1 | 2 | 6 | 3 | 3 | 9 |
| NM_014971 | EFR3B | 7458 | 5 | 1 | 0 | 0 | 0 | 5 | 0 | 0 | 0 | 0 | 0 | 1 | 0 |
| NM_014972 | TCF25 | 2259 | 39 | 15 | 14 | 16 | 10 | 39 | 10 | 16 | 9 | 12 | 7 | 13 | 20 |
| NM_014974 | DIP2C | 7894 | 2 | 1 | 1 | 1 | 2 | 2 | 2 | 1 | 0 | 1 | 1 | 1 | 1 |
| NM_014975 | MAST1 | 4922 | 2 | 0 | 0 | 0 | 0 | 2 | 0 | 0 | 0 | 0 | 0 | 0 | 0 |
| NM_014976 | PDCD11 | 6416 | 5 | 3 | 2 | 3 | 3 | 3 | 3 | 3 | 2 | 3 | 3 | 4 | 5 |
| NM_014977 | ACIN1 | 4937 | 22 | 14 | 14 | 16 | 13 | 10 | 15 | 8 | 10 | 17 | 13 | 18 | 22 |
| NM_014978 | SORCS3 | 5757 | 2 | 0 | 0 | 0 | 0 | 2 | 0 | 0 | 0 | 0 | 0 | 0 | 0 |
| NM_014979 | SV2C | 2362 | 6 | 1 | 0 | 1 | 0 | 6 | 0 | 0 | 0 | 0 | 0 | 0 | 0 |
| NM_014980 | STXBP5L | 9365 | 8 | 1 | 0 | 0 | 0 | 8 | 0 | 0 | 0 | 3 | 0 | 0 | 1 |
| NM_014981 | MYH15 | 7074 | 1 | 0 | 0 | 0 | 0 | 1 | 0 | 0 | 0 | 0 | 0 | 0 | 0 |
| NM_014982 | PCNX | 12919 | 8 | 5 | 4 | 2 | 6 | 5 | 4 | 3 | 5 | 7 | 4 | 8 | 7 |
| NM_014983 | HMGXB3 | 5405 | 2 | 1 | 1 | 1 | 1 | 1 | 1 | 1 | 1 | 1 | 1 | 2 | 2 |
| NM_014984 | AZI1 | 3619 | 1 | 0 | 0 | 1 | 1 | 0 | 0 | 0 | 0 | 1 | 0 | 1 | 1 |
| NM_014985 | CEP152 | 5097 | 5 | 2 | 2 | 1 | 1 | 1 | 3 | 0 | 2 | 2 | 1 | 3 | 5 |
| NM_014987 | IGSF9B | 7309 | 0 | 0 | 0 | 0 | 0 | 0 | 0 | 0 | 0 | 0 | 0 | 0 | 0 |
| NM_014988 | LIMCH1 | 6165 | 76 | 29 | 15 | 3 | 59 | 30 | 13 | 4 | 51 | 14 | 49 | 76 | 6 |
| NM_014989 | RIMS1 | 7585 | 10 | 1 | 0 | 0 | 0 | 10 | 0 | 0 | 0 | 0 | 0 | 0 | 4 |
| NM_014990 | RALGAPA1 | 7864 | 9 | 5 | 3 | 3 | 4 | 9 | 5 | 3 | 4 | 8 | 5 | 5 | 7 |
| NM_014991 | WDFY3 | 14311 | 11 | 6 | 4 | 2 | 5 | 11 | 9 | 2 | 5 | 7 | 3 | 9 | 7 |
| NM_014992 | DAAM1 | 4256 | 9 | 5 | 8 | 2 | 9 | 8 | 3 | 5 | 4 | 5 | 2 | 9 | 5 |
| NM_014994 | MAPKBP1 | 7232 | 2 | 1 | 1 | 1 | 0 | 1 | 1 | 0 | 1 | 1 | 0 | 1 | 2 |
| NM_014996 | PLCH1 | 6128 | 2 | 0 | 0 | 0 | 0 | 1 | 1 | 0 | 1 | 0 | 0 | 0 | 2 |
| NM_014997 | KLHDC10 | 4471 | 7 | 5 | 4 | 2 | 5 | 7 | 6 | 5 | 4 | 6 | 4 | 4 | 6 |
| NM_014999 | RAB21 | 2627 | 41 | 18 | 9 | 3 | 41 | 17 | 17 | 10 | 17 | 15 | 31 | 19 | 14 |
| NM_015000 | STK38L | 5094 | 31 | 9 | 9 | 4 | 31 | 9 | 6 | 2 | 9 | 7 | 5 | 10 | 11 |
| NM_015001 | SPEN | 12227 | 17 | 10 | 7 | 5 | 10 | 10 | 9 | 5 | 10 | 17 | 13 | 16 | 12 |
| NM_015002 | FBXO21 | 4212 | 35 | 13 | 3 | 4 | 11 | 17 | 14 | 8 | 7 | 25 | 8 | 12 | 35 |

| transcript | gene | transcript length (nt) | max | mean | adipose | colon | heart | hypothalamus | kidney | liver | lung | ovary | skeletalmuscle | spleen | testes |
|---|---|---|---|---|---|---|---|---|---|---|---|---|---|---|---|
| NM_015004 | EXOSC7 | 1036 | 2 | 1 | 1 | 1 | 2 | 2 | 2 | 1 | 1 | 1 | 2 | 1 | 2 |
| NM_015005 | KIAA0284 | 6549 | 2 | 1 | 0 | 1 | 0 | 2 | 0 | 0 | 0 | 0 | 0 | 0 | 1 |
| NM_015008 | TMCC1 | 5556 | 3 | 2 | 2 | 1 | 1 | 3 | 1 | 2 | 1 | 1 | 2 | 1 | 3 |
| NM_015009 | PDZRN3 | 4161 | 12 | 4 | 5 | 4 | 8 | 2 | 2 | 0 | 1 | 12 | 3 | 0 | 3 |
| NM_015011 | MYO16 | 6874 | 2 | 0 | 0 | 0 | 0 | 2 | 0 | 0 | 0 | 0 | 0 | 0 | 0 |
| NM_015012 | TMEM41B | 3444 | 5 | 3 | 1 | 2 | 3 | 3 | 3 | 3 | 5 | 2 | 2 | 3 | 3 |
| NM_015013 | KDM1A | 3035 | 16 | 7 | 4 | 5 | 5 | 11 | 5 | 4 | 6 | 16 | 6 | 7 | 13 |
| NM_015014 | RBM34 | 1852 | 10 | 6 | 6 | 3 | 8 | 10 | 6 | 6 | 4 | 7 | 5 | 8 | 7 |
| NM_015015 | KDM4B | 5603 | 1 | 1 | 0 | 1 | 1 | 1 | 1 | 1 | 0 | 1 | 1 | 1 | 1 |
| NM_015016 | MAST3 | 5893 | 4 | 1 | 0 | 1 | 0 | 4 | 0 | 0 | 0 | 0 | 0 | 1 | 0 |
| NM_015017 | USP33 | 4504 | 12 | 7 | 9 | 2 | 7 | 12 | 8 | 3 | 8 | 9 | 5 | 9 | 9 |
| NM_015018 | DOPEY1 | 7899 | 6 | 3 | 1 | 1 | 2 | 5 | 3 | 2 | 2 | 3 | 2 | 3 | 6 |
| NM_015020 | PHLPP2 | 7919 | 4 | 2 | 1 | 4 | 1 | 4 | 2 | 1 | 2 | 2 | 1 | 1 | 4 |
| NM_015021 | ZNF292 | 10100 | 14 | 6 | 3 | 2 | 5 | 5 | 6 | 2 | 6 | 14 | 5 | 8 | 8 |
| NM_015023 | WDTC1 | 4800 | 2 | 1 | 1 | 1 | 1 | 2 | 1 | 1 | 1 | 1 | 2 | 1 | 1 |
| NM_015024 | XPO7 | 4863 | 9 | 6 | 5 | 4 | 6 | 9 | 8 | 4 | 4 | 6 | 4 | 7 | 7 |
| NM_015025 | MYT1L | 7136 | 17 | 2 | 0 | 0 | 0 | 17 | 0 | 0 | 0 | 0 | 0 | 0 | 1 |
| NM_015026 | MON2 | 10379 | 5 | 3 | 3 | 2 | 3 | 4 | 4 | 4 | 3 | 5 | 2 | 5 | 4 |
| NM_015027 | PDXDC1 | 4011 | 7 | 3 | 3 | 4 | 3 | 2 | 6 | 7 | 3 | 2 | 2 | 3 | 4 |
| NM_015028 | TNIK | 5806 | 18 | 5 | 1 | 4 | 4 | 18 | 3 | 3 | 4 | 1 | 5 | 2 | 11 |
| NM_015029 | POP1 | 4672 | 1 | 1 | 0 | 0 | 1 | 1 | 1 | 1 | 0 | 1 | 0 | 1 | 1 |
| NM_015030 | FRYL | 11706 | 6 | 4 | 3 | 6 | 4 | 4 | 4 | 2 | 4 | 3 | 2 | 6 | 3 |
| NM_015032 | PDS5B | 7469 | 10 | 5 | 3 | 2 | 3 | 10 | 4 | 2 | 6 | 10 | 4 | 7 | 7 |
| NM_015033 | FNBP1 | 5425 | 15 | 5 | 4 | 5 | 4 | 7 | 3 | 2 | 6 | 5 | 2 | 15 | 7 |
| NM_015035 | ZHX3 | 9991 | 10 | 5 | 5 | 4 | 5 | 9 | 10 | 7 | 3 | 6 | 3 | 3 | 6 |
| NM_015036 | ENDOD1 | 4650 | 21 | 8 | 4 | 3 | 5 | 21 | 10 | 1 | 4 | 5 | 7 | 15 | 9 |
| NM_015037 | KIAA0913 | 5835 | 2 | 1 | 1 | 1 | 1 | 1 | 2 | 1 | 1 | 1 | 1 | 2 | 2 |
| NM_015038 | KIAA0754 | 6979 | 5 | 3 | 5 | 2 | 2 | 2 | 4 | 1 | 3 | 4 | 1 | 4 | 4 |
| NM_015039 | NMNAT2 | 5656 | 14 | 3 | 2 | 6 | 2 | 14 | 0 | 0 | 0 | 0 | 0 | 0 | 3 |
| NM_015040 | PIKFYVE | 9901 | 8 | 3 | 3 | 1 | 3 | 4 | 4 | 2 | 3 | 3 | 2 | 8 | 5 |
| NM_015041 | CLUAP1 | 1661 | 16 | 8 | 6 | 4 | 6 | 15 | 9 | 3 | 6 | 10 | 5 | 4 | 16 |
| NM_015042 | ZNF609 | 8617 | 5 | 3 | 2 | 3 | 2 | 4 | 2 | 2 | 3 | 5 | 1 | 4 | 4 |
| NM_015043 | TBC1D9B | 5114 | 9 | 4 | 6 | 3 | 3 | 9 | 4 | 4 | 2 | 4 | 2 | 4 | 3 |
| NM_015044 | GGA2 | 5186 | 7 | 3 | 2 | 2 | 2 | 2 | 5 | 2 | 3 | 3 | 2 | 7 | 2 |
| NM_015045 | WAPAL | 6302 | 22 | 11 | 7 | 4 | 9 | 9 | 10 | 10 | 9 | 12 | 11 | 14 | 22 |
| NM_015046 | SETX | 11016 | 23 | 10 | 6 | 3 | 8 | 8 | 8 | 7 | 9 | 11 | 6 | 23 | 20 |
| NM_015047 | KIAA0090 | 4238 | 7 | 4 | 4 | 3 | 4 | 7 | 5 | 5 | 2 | 4 | 3 | 4 | 6 |
| NM_015048 | SETD1B | 8194 | 1 | 1 | 1 | 1 | 1 | 1 | 1 | 0 | 1 | 1 | 1 | 1 | 1 |
| NM_015049 | TRAK2 | 6527 | 25 | 11 | 15 | 8 | 25 | 16 | 12 | 6 | 11 | 5 | 4 | 12 | 6 |
| NM_015050 | FTSJD2 | 4124 | 5 | 2 | 1 | 2 | 2 | 2 | 2 | 1 | 1 | 1 | 1 | 2 | 5 |
| NM_015051 | ERP44 | 4860 | 27 | 11 | 9 | 5 | 14 | 8 | 10 | 27 | 11 | 7 | 5 | 15 | 11 |
| NM_015052 | HECW1 | 6839 | 3 | 1 | 0 | 0 | 0 | 3 | 2 | 0 | 0 | 0 | 0 | 0 | 0 |
| NM_015053 | PPFIA4 | 5045 | 1 | 0 | 0 | 0 | 0 | 1 | 0 | 0 | 0 | 0 | 0 | 0 | 0 |
| NM_015054 | UHRF1BP1L | 5175 | 7 | 4 | 4 | 1 | 4 | 7 | 4 | 7 | 4 | 3 | 2 | 5 | 4 |
| NM_015055 | SWAP70 | 4847 | 39 | 10 | 12 | 3 | 12 | 7 | 8 | 3 | 13 | 6 | 4 | 39 | 6 |
| NM_015056 | RRP1B | 5087 | 3 | 2 | 1 | 1 | 1 | 1 | 1 | 2 | 1 | 3 | 2 | 2 | 2 |
| NM_015057 | MYCBP2 | 14735 | 19 | 9 | 15 | 3 | 7 | 19 | 5 | 5 | 7 | 10 | 4 | 17 | 8 |
| NM_015058 | KIAA0564 | 7160 | 6 | 4 | 3 | 2 | 5 | 3 | 6 | 4 | 2 | 3 | 6 | 3 | 3 |
| NM_015059 | TLN2 | 11649 | 6 | 2 | 5 | 2 | 2 | 6 | 3 | 0 | 0 | 1 | 2 | 1 | 1 |
| NM_015060 | AVL9 | 2353 | 6 | 3 | 2 | 3 | 2 | 6 | 2 | 3 | 2 | 2 | 2 | 4 | 2 |
| NM_015061 | KDM4C | 4669 | 9 | 5 | 4 | 5 | 4 | 5 | 4 | 3 | 4 | 4 | 3 | 9 | 5 |
| NM_015062 | PPRC1 | 5338 | 2 | 2 | 1 | 1 | 2 | 2 | 1 | 1 | 1 | 2 | 2 | 2 | 2 |
| NM_015063 | SLC8A2 | 5110 | 4 | 0 | 0 | 0 | 0 | 4 | 0 | 0 | 0 | 0 | 0 | 0 | 0 |
| NM_015065 | EXPH5 | 10187 | 4 | 1 | 0 | 2 | 0 | 1 | 4 | 4 | 4 | 1 | 0 | 0 | 0 |
| NM_015068 | PEG10 | 6617 | 35 | 8 | 3 | 1 | 0 | 35 | 2 | 0 | 4 | 19 | 0 | 1 | 25 |
| NM_015069 | ZNF423 | 4818 | 2 | 1 | 1 | 1 | 1 | 2 | 0 | 0 | 1 | 1 | 2 | 1 | 1 |
| NM_015070 | ZC3H13 | 6409 | 66 | 41 | 37 | 11 | 40 | 66 | 32 | 34 | 29 | 52 | 35 | 54 | 60 |
| NM_015071 | ARHGAP26 | 9040 | 6 | 2 | 1 | 3 | 4 | 6 | 2 | 0 | 2 | 1 | 1 | 4 | 3 |
| NM_015072 | TTLL5 | 4684 | 9 | 3 | 1 | 2 | 2 | 3 | 2 | 1 | 2 | 2 | 1 | 2 | 9 |
| NM_015073 | SIPA1L3 | 7983 | 2 | 1 | 0 | 2 | 0 | 0 | 1 | 0 | 1 | 0 | 0 | 1 | 1 |
| NM_015074 | KIF1B | 10581 | 32 | 9 | 4 | 4 | 8 | 32 | 6 | 5 | 4 | 4 | 15 | 4 | 7 |
| NM_015075 | IQSEC2 | 5238 | 3 | 1 | 0 | 1 | 0 | 3 | 0 | 0 | 0 | 0 | 0 | 0 | 0 |
| NM_015076 | CDK19 | 6139 | 3 | 2 | 1 | 1 | 1 | 3 | 1 | 1 | 2 | 3 | 1 | 3 | 3 |
| NM_015077 | SARM1 | 7000 | 0 | 0 | 0 | 0 | 0 | 0 | 0 | 0 | 0 | 0 | 0 | 0 | 0 |
| NM_015078 | MCF2L2 | 4772 | 4 | 1 | 0 | 0 | 0 | 4 | 0 | 0 | 0 | 0 | 0 | 1 | 0 |
| NM_015079 | TBC1D2B | 6015 | 4 | 2 | 4 | 2 | 1 | 1 | 1 | 2 | 2 | 4 | 1 | 4 | 1 |
| NM_015080 | NRXN2 | 6600 | 4 | 1 | 0 | 0 | 0 | 4 | 0 | 0 | 0 | 0 | 0 | 0 | 0 |
| NM_015082 | FSTL4 | 5382 | 1 | 0 | 0 | 0 | 0 | 1 | 0 | 0 | 0 | 0 | 0 | 0 | 0 |
| NM_015083 | CDK12 | 8263 | 7 | 4 | 3 | 3 | 3 | 2 | 4 | 4 | 4 | 4 | 2 | 6 | 7 |
| NM_015084 | MRPS27 | 2781 | 14 | 9 | 8 | 5 | 12 | 14 | 8 | 12 | 4 | 11 | 9 | 7 | 7 |
| NM_015085 | RAP1GAP2 | 6663 | 2 | 0 | 0 | 1 | 1 | 2 | 0 | 0 | 0 | 0 | 0 | 0 | 0 |
| NM_015086 | DDN | 3744 | 14 | 2 | 0 | 0 | 0 | 14 | 1 | 0 | 0 | 0 | 4 | 0 | 0 |
| NM_015087 | SPG20 | 5013 | 11 | 7 | 9 | 2 | 6 | 11 | 8 | 2 | 5 | 10 | 4 | 7 | 9 |
| NM_015088 | TNRC6B | 17950 | 7 | 5 | 3 | 3 | 4 | 5 | 4 | 3 | 4 | 7 | 5 | 7 | 7 |
| NM_015089 | CUL9 | 7771 | 2 | 1 | 1 | 1 | 0 | 2 | 1 | 0 | 1 | 1 | 0 | 1 | 1 |
| NM_015090 | NFASC | 10120 | 9 | 2 | 0 | 1 | 1 | 9 | 2 | 0 | 1 | 2 | 0 | 1 | 0 |
| NM_015091 | FAM179B | 6242 | 6 | 2 | 1 | 1 | 2 | 6 | 3 | 1 | 1 | 4 | 2 | 2 | 3 |

| transcript | gene | transcript length (nt) | max | mean | adipose | colon | heart | hypothalamus | kidney | liver | lung | ovary | skeletalmuscle | spleen | testes |
|---|---|---|---|---|---|---|---|---|---|---|---|---|---|---|---|
| NM_015092 | SMG1 | 16064 | 11 | 4 | 3 | 3 | 4 | 3 | 4 | 2 | 3 | 3 | 11 | 5 | 4 |
| NM_015093 | MAP3K7IP2 | 4154 | 13 | 9 | 7 | 4 | 12 | 11 | 8 | 6 | 9 | 13 | 11 | 12 | 9 |
| NM_015094 | HIC2 | 6796 | 1 | 0 | 0 | 0 | 0 | 0 | 0 | 0 | 0 | 1 | 0 | 1 | 1 |
| NM_015097 | CLASP2 | 7260 | 20 | 6 | 3 | 2 | 5 | 20 | 4 | 2 | 4 | 6 | 5 | 6 | 5 |
| NM_015099 | CAMTA2 | 4475 | 2 | 1 | 1 | 1 | 1 | 2 | 1 | 1 | 1 | 0 | 1 | 1 | 0 |
| NM_015100 | POGZ | 6617 | 10 | 5 | 2 | 3 | 4 | 4 | 5 | 1 | 4 | 10 | 4 | 7 | 7 |
| NM_015101 | GLT25D2 | 5182 | 2 | 0 | 0 | 0 | 0 | 2 | 0 | 0 | 0 | 0 | 0 | 0 | 0 |
| NM_015102 | NPHP4 | 5002 | 0 | 0 | 0 | 0 | 0 | 0 | 0 | 0 | 0 | 0 | 0 | 0 | 0 |
| NM_015103 | PLXND1 | 6977 | 6 | 3 | 6 | 2 | 3 | 2 | 2 | 1 | 3 | 1 | 1 | 5 | 1 |
| NM_015104 | ATG2A | 6372 | 0 | 0 | 0 | 0 | 0 | 0 | 0 | 0 | 0 | 0 | 0 | 0 | 0 |
| NM_015106 | RAD54L2 | 4705 | 2 | 2 | 2 | 2 | 1 | 2 | 2 | 2 | 2 | 1 | 1 | 2 | 2 |
| NM_015107 | PHF8 | 6057 | 7 | 2 | 1 | 2 | 2 | 1 | 2 | 4 | 2 | 3 | 1 | 3 | 7 |
| NM_015110 | SMC5 | 5965 | 21 | 10 | 5 | 3 | 12 | 5 | 8 | 8 | 10 | 21 | 12 | 14 | 16 |
| NM_015111 | N4BP3 | 5987 | 0 | 0 | 0 | 0 | 0 | 0 | 0 | 0 | 0 | 0 | 0 | 0 | 0 |
| NM_015112 | MAST2 | 5738 | 5 | 2 | 1 | 2 | 3 | 2 | 1 | 0 | 1 | 1 | 5 | 0 | 2 |
| NM_015113 | ZZEF1 | 11395 | 3 | 1 | 1 | 3 | 1 | 1 | 1 | 1 | 1 | 1 | 1 | 3 | 1 |
| NM_015114 | ANKLE2 | 4458 | 6 | 4 | 3 | 3 | 4 | 3 | 3 | 1 | 4 | 3 | 3 | 5 | 6 |
| NM_015115 | DCUN1D4 | 4160 | 18 | 11 | 8 | 4 | 10 | 16 | 11 | 10 | 7 | 16 | 6 | 11 | 18 |
| NM_015116 | LRCH1 | 5474 | 4 | 2 | 2 | 1 | 2 | 1 | 3 | 1 | 2 | 2 | 1 | 4 | 2 |
| NM_015117 | ZC3H3 | 3277 | 1 | 0 | 0 | 1 | 0 | 0 | 1 | 0 | 0 | 0 | 0 | 0 | 0 |
| NM_015120 | ALMS1 | 12922 | 28 | 9 | 4 | 3 | 6 | 6 | 6 | 2 | 5 | 25 | 6 | 7 | 28 |
| NM_015122 | FCHO1 | 3215 | 2 | 0 | 0 | 0 | 0 | 2 | 0 | 0 | 0 | 0 | 0 | 0 | 0 |
| NM_015123 | FRMD4B | 5261 | 9 | 3 | 4 | 2 | 3 | 3 | 4 | 4 | 3 | 2 | 1 | 9 | 1 |
| NM_015124 | GRAMD4 | 4316 | 2 | 1 | 1 | 2 | 2 | 1 | 1 | 1 | 1 | 1 | 0 | 1 | 0 |
| NM_015125 | CIC | 5473 | 1 | 1 | 0 | 0 | 0 | 1 | 1 | 0 | 0 | 1 | 0 | 1 | 1 |
| NM_015127 | CLCC1 | 4685 | 10 | 7 | 6 | 3 | 10 | 10 | 6 | 6 | 5 | 10 | 8 | 8 | 8 |
| NM_015129 | 06 Sep | 3386 | 16 | 5 | 3 | 5 | 2 | 6 | 4 | 4 | 3 | 2 | 1 | 14 | 16 |
| NM_015130 | TBC1D9 | 5486 | 17 | 6 | 10 | 2 | 3 | 13 | 6 | 2 | 5 | 5 | 1 | 17 | 3 |
| NM_015131 | WDR43 | 3501 | 9 | 6 | 7 | 3 | 6 | 7 | 5 | 9 | 4 | 6 | 8 | 7 | 6 |
| NM_015132 | SNX13 | 6370 | 16 | 8 | 7 | 3 | 9 | 8 | 9 | 5 | 8 | 9 | 9 | 10 | 16 |
| NM_015133 | MAPK8IP3 | 5624 | 9 | 2 | 1 | 1 | 1 | 9 | 1 | 0 | 1 | 1 | 1 | 1 | 1 |
| NM_015134 | MPRIP | 3837 | 11 | 6 | 6 | 5 | 10 | 11 | 4 | 2 | 4 | 6 | 5 | 7 | 6 |
| NM_015135 | NUP205 | 6255 | 12 | 6 | 5 | 4 | 4 | 5 | 5 | 5 | 5 | 7 | 5 | 11 | 12 |
| NM_015136 | STAB1 | 7926 | 4 | 1 | 2 | 1 | 2 | 0 | 1 | 1 | 1 | 0 | 0 | 4 | 0 |
| NM_015137 | EFR3A | 5296 | 19 | 8 | 7 | 3 | 8 | 19 | 7 | 11 | 7 | 6 | 7 | 9 | 7 |
| NM_015138 | RTF1 | 5020 | 9 | 5 | 3 | 2 | 5 | 9 | 5 | 4 | 4 | 6 | 6 | 6 | 8 |
| NM_015139 | SLC35D1 | 6459 | 17 | 4 | 1 | 5 | 1 | 1 | 4 | 17 | 1 | 3 | 2 | 2 | 2 |
| NM_015140 | TTLL12 | 3353 | 3 | 1 | 1 | 2 | 1 | 1 | 1 | 2 | 1 | 2 | 1 | 2 | 3 |
| NM_015141 | GPD1L | 3914 | 13 | 4 | 2 | 2 | 13 | 5 | 3 | 0 | 3 | 1 | 13 | 2 | 2 |
| NM_015143 | METAP1 | 2770 | 6 | 4 | 2 | 3 | 6 | 3 | 5 | 3 | 3 | 3 | 4 | 5 | 6 |
| NM_015144 | ZCCHC14 | 6911 | 5 | 2 | 1 | 2 | 2 | 2 | 3 | 2 | 2 | 5 | 2 | 2 | 5 |
| NM_015146 | 08 Sep | 4344 | 18 | 5 | 3 | 5 | 4 | 18 | 5 | 1 | 3 | 4 | 1 | 4 | 2 |
| NM_015147 | CEP68 | 5862 | 3 | 2 | 2 | 1 | 2 | 3 | 2 | 0 | 2 | 3 | 3 | 3 | 2 |
| NM_015148 | PASK | 4530 | 1 | 0 | 0 | 0 | 0 | 0 | 0 | 0 | 0 | 0 | 0 | 0 | 1 |
| NM_015149 | RGL1 | 5113 | 19 | 7 | 6 | 2 | 6 | 13 | 6 | 3 | 7 | 7 | 2 | 19 | 7 |
| NM_015150 | RFTN1 | 3002 | 14 | 5 | 8 | 2 | 6 | 4 | 2 | 2 | 4 | 7 | 2 | 14 | 2 |
| NM_015151 | DIP2A | 7290 | 2 | 1 | 1 | 1 | 1 | 1 | 1 | 0 | 1 | 1 | 1 | 2 | 1 |
| NM_015153 | PHF3 | 6947 | 41 | 22 | 16 | 5 | 17 | 19 | 18 | 14 | 20 | 41 | 24 | 31 | 32 |
| NM_015154 | MESDC2 | 4243 | 5 | 3 | 4 | 2 | 3 | 4 | 4 | 5 | 3 | 3 | 1 | 4 | 4 |
| NM_015155 | LARP4B | 5640 | 6 | 4 | 3 | 3 | 4 | 4 | 4 | 2 | 4 | 4 | 4 | 6 | 5 |
| NM_015156 | RCOR1 | 5519 | 3 | 2 | 1 | 2 | 1 | 1 | 1 | 1 | 3 | 1 | 1 | 3 | 3 |
| NM_015157 | PHLDB1 | 5695 | 16 | 5 | 16 | 5 | 5 | 7 | 3 | 0 | 4 | 3 | 2 | 3 | 3 |
| NM_015158 | KANK1 | 5062 | 19 | 9 | 17 | 5 | 19 | 7 | 11 | 17 | 4 | 6 | 6 | 3 | 8 |
| NM_015159 | FAM168A | 1767 | 13 | 4 | 2 | 3 | 3 | 13 | 3 | 2 | 2 | 4 | 2 | 4 | 3 |
| NM_015160 | PMPCA | 2080 | 4 | 2 | 1 | 1 | 3 | 2 | 2 | 4 | 1 | 1 | 2 | 2 | 2 |
| NM_015161 | ARL6IP1 | 2264 | 59 | 22 | 17 | 11 | 8 | 59 | 20 | 22 | 17 | 19 | 5 | 25 | 38 |
| NM_015162 | ACSBG1 | 2830 | 2 | 0 | 0 | 0 | 0 | 2 | 0 | 0 | 0 | 0 | 0 | 0 | 0 |
| NM_015163 | TRIM9 | 4926 | 3 | 0 | 0 | 0 | 0 | 3 | 0 | 0 | 0 | 0 | 0 | 0 | 0 |
| NM_015164 | PLEKHM2 | 4120 | 2 | 1 | 1 | 1 | 1 | 1 | 1 | 0 | 1 | 1 | 2 | 1 | 1 |
| NM_015166 | MLC1 | 3960 | 7 | 1 | 0 | 0 | 0 | 7 | 0 | 0 | 0 | 0 | 0 | 0 | 0 |
| NM_015167 | JMJD6 | 1822 | 5 | 3 | 3 | 3 | 5 | 3 | 2 | 2 | 2 | 2 | 3 | 4 | 3 |
| NM_015168 | ZC3H4 | 6116 | 6 | 4 | 4 | 5 | 3 | 4 | 3 | 2 | 3 | 5 | 3 | 6 | 4 |
| NM_015169 | RRS1 | 1706 | 8 | 4 | 2 | 1 | 4 | 4 | 3 | 6 | 2 | 3 | 8 | 4 | 2 |
| NM_015170 | SULF1 | 5710 | 9 | 3 | 2 | 3 | 9 | 4 | 4 | 0 | 2 | 4 | 1 | 1 | 5 |
| NM_015171 | XPO6 | 4422 | 10 | 3 | 2 | 3 | 2 | 4 | 3 | 2 | 4 | 2 | 1 | 10 | 4 |
| NM_015172 | BAT2L2 | 10383 | 42 | 23 | 19 | 10 | 20 | 24 | 21 | 13 | 26 | 29 | 15 | 42 | 35 |
| NM_015173 | TBC1D1 | 5688 | 17 | 8 | 4 | 5 | 15 | 5 | 17 | 5 | 5 | 7 | 5 | 11 | 8 |
| NM_015174 | ZFR2 | 4757 | 0 | 0 | 0 | 0 | 0 | 0 | 0 | 0 | 0 | 0 | 0 | 0 | 0 |
| NM_015175 | NBEAL2 | 8827 | 1 | 0 | 0 | 0 | 0 | 0 | 0 | 0 | 1 | 0 | 0 | 1 | 0 |
| NM_015176 | FBXO28 | 5449 | 3 | 2 | 2 | 1 | 2 | 2 | 2 | 2 | 1 | 3 | 3 | 3 | 3 |
| NM_015177 | DTX4 | 5795 | 2 | 1 | 1 | 2 | 0 | 2 | 1 | 1 | 2 | 1 | 0 | 1 | 0 |
| NM_015178 | RHOBTB2 | 5449 | 4 | 2 | 0 | 1 | 2 | 4 | 1 | 0 | 4 | 1 | 1 | 2 | 1 |
| NM_015179 | RRP12 | 4425 | 3 | 2 | 1 | 1 | 2 | 2 | 1 | 1 | 1 | 1 | 3 | 2 | 2 |
| NM_015180 | SYNE2 | 21813 | 32 | 12 | 5 | 4 | 10 | 2 | 32 | 3 | 10 | 20 | 11 | 14 | 22 |
| NM_015183 | MAST4 | 9916 | 7 | 3 | 4 | 1 | 7 | 3 | 4 | 1 | 3 | 4 | 3 | 2 | 2 |
| NM_015184 | PLCL2 | 3952 | 13 | 7 | 4 | 3 | 13 | 9 | 7 | 2 | 3 | 11 | 12 | 11 | 6 |
| NM_015185 | ARHGEF9 | 5450 | 26 | 5 | 2 | 3 | 6 | 26 | 4 | 1 | 1 | 5 | 1 | 3 | 2 |

| transcript | gene | transcript length (nt) | max | mean | adipose | colon | heart | hypothalamus | kidney | liver | lung | ovary | skeletalmuscle | spleen | testes |
|---|---|---|---|---|---|---|---|---|---|---|---|---|---|---|---|
| NM_015186 | VPS13A | 10055 | 10 | 6 | 3 | 3 | 7 | 5 | 9 | 2 | 4 | 3 | 10 | 8 | 9 |
| NM_015187 | SEL1L3 | 4549 | 19 | 4 | 2 | 6 | 2 | 2 | 3 | 1 | 7 | 1 | 0 | 19 | 1 |
| NM_015188 | TBC1D12 | 5749 | 3 | 1 | 1 | 1 | 1 | 3 | 1 | 0 | 1 | 1 | 1 | 1 | 1 |
| NM_015189 | EXOC6B | 2594 | 51 | 20 | 18 | 11 | 41 | 51 | 11 | 5 | 13 | 29 | 11 | 12 | 15 |
| NM_015190 | DNAJC9 | 1437 | 5 | 3 | 3 | 3 | 2 | 4 | 3 | 2 | 2 | 3 | 2 | 5 | 4 |
| NM_015191 | SIK2 | 5678 | 8 | 4 | 8 | 2 | 4 | 4 | 4 | 4 | 4 | 4 | 5 | 3 | 2 |
| NM_015192 | PLCB1 | 6705 | 14 | 3 | 1 | 1 | 2 | 14 | 4 | 2 | 1 | 1 | 2 | 1 | 2 |
| NM_015193 | ARC | 2943 | 0 | 0 | 0 | 0 | 0 | 0 | 0 | 0 | 0 | 0 | 0 | 0 | 0 |
| NM_015194 | MYO1D | 5182 | 18 | 5 | 4 | 18 | 5 | 4 | 4 | 2 | 6 | 6 | 1 | 3 | 4 |
| NM_015196 | KIAA0922 | 5016 | 7 | 2 | 2 | 1 | 1 | 0 | 1 | 3 | 2 | 1 | 4 | 7 | 1 |
| NM_015197 | PACS2 | 6320 | 4 | 2 | 2 | 2 | 1 | 4 | 1 | 1 | 1 | 1 | 3 | 2 | 2 |
| NM_015198 | COBL | 5310 | 8 | 2 | 0 | 2 | 2 | 8 | 3 | 3 | 2 | 0 | 3 | 0 | 0 |
| NM_015199 | ANKRD28 | 6338 | 7 | 4 | 3 | 1 | 3 | 4 | 4 | 4 | 3 | 5 | 7 | 3 | 4 |
| NM_015200 | PDS5A | 6712 | 16 | 10 | 8 | 4 | 9 | 8 | 11 | 11 | 11 | 13 | 7 | 16 | 10 |
| NM_015201 | BOP1 | 2373 | 1 | 0 | 0 | 0 | 0 | 1 | 0 | 1 | 0 | 0 | 1 | 1 | 0 |
| NM_015202 | KIAA0556 | 6623 | 1 | 1 | 0 | 1 | 1 | 1 | 1 | 0 | 1 | 1 | 0 | 1 | 1 |
| NM_015203 | RPRD2 | 7818 | 10 | 5 | 3 | 2 | 4 | 5 | 6 | 3 | 3 | 10 | 6 | 6 | 5 |
| NM_015204 | THSD7A | 10583 | 4 | 1 | 1 | 0 | 1 | 2 | 4 | 0 | 1 | 1 | 0 | 1 | 1 |
| NM_015205 | ATP11A | 8795 | 6 | 3 | 1 | 1 | 5 | 2 | 4 | 2 | 6 | 1 | 1 | 3 | 4 |
| NM_015206 | KIAA1024 | 6742 | 1 | 0 | 0 | 0 | 0 | 1 | 0 | 0 | 0 | 0 | 0 | 0 | 0 |
| NM_015207 | OTUD3 | 6514 | 5 | 2 | 1 | 1 | 1 | 2 | 2 | 0 | 1 | 2 | 1 | 1 | 5 |
| NM_015208 | ANKRD12 | 10528 | 19 | 10 | 8 | 2 | 10 | 19 | 9 | 4 | 11 | 13 | 11 | 15 | 14 |
| NM_015209 | KIAA1026 | 3905 | 7 | 1 | 1 | 0 | 0 | 2 | 2 | 0 | 0 | 1 | 0 | 7 | 1 |
| NM_015210 | KIAA0802 | 6092 | 3 | 1 | 0 | 3 | 1 | 2 | 1 | 0 | 0 | 0 | 0 | 0 | 3 |
| NM_015213 | DENND5A | 4964 | 14 | 7 | 9 | 3 | 12 | 14 | 3 | 2 | 7 | 5 | 2 | 9 | 6 |
| NM_015214 | DDHD2 | 4708 | 11 | 5 | 6 | 3 | 4 | 11 | 5 | 1 | 4 | 6 | 3 | 4 | 6 |
| NM_015215 | CAMTA1 | 8442 | 7 | 1 | 0 | 1 | 1 | 7 | 1 | 1 | 1 | 1 | 1 | 1 | 1 |
| NM_015216 | HISPPD1 | 5280 | 13 | 7 | 5 | 3 | 9 | 6 | 8 | 5 | 7 | 13 | 2 | 13 | 8 |
| NM_015219 | EXOC7 | 4699 | 8 | 5 | 5 | 4 | 5 | 8 | 5 | 4 | 3 | 5 | 3 | 6 | 4 |
| NM_015221 | DNMBP | 6400 | 4 | 3 | 3 | 4 | 2 | 1 | 3 | 2 | 2 | 3 | 2 | 2 | 4 |
| NM_015224 | C3orf63 | 7247 | 15 | 7 | 5 | 2 | 6 | 6 | 7 | 3 | 7 | 11 | 3 | 15 | 13 |
| NM_015225 | PRUNE2 | 12585 | 11 | 4 | 1 | 8 | 6 | 11 | 10 | 0 | 2 | 1 | 3 | 1 | 6 |
| NM_015226 | CLEC16A | 6889 | 5 | 2 | 1 | 2 | 2 | 2 | 2 | 2 | 2 | 1 | 2 | 3 | 5 |
| NM_015227 | POFUT2 | 4823 | 3 | 2 | 2 | 2 | 2 | 2 | 1 | 1 | 1 | 3 | 1 | 1 | 3 |
| NM_015229 | KIAA0664 | 5224 | 3 | 1 | 1 | 1 | 1 | 1 | 2 | 3 | 0 | 0 | 2 | 1 | 1 |
| NM_015230 | ARAP2 | 7360 | 10 | 4 | 1 | 1 | 1 | 10 | 2 | 2 | 6 | 1 | 0 | 9 | 7 |
| NM_015231 | NUP160 | 5383 | 8 | 5 | 4 | 3 | 4 | 3 | 5 | 4 | 4 | 7 | 4 | 8 | 6 |
| NM_015232 | IQSEC3 | 2699 | 1 | 0 | 0 | 0 | 0 | 1 | 0 | 0 | 0 | 0 | 0 | 0 | 0 |
| NM_015233 | MTUS2 | 3991 | 11 | 1 | 0 | 0 | 11 | 1 | 0 | 0 | 0 | 1 | 0 | 0 | 0 |
| NM_015234 | GPR116 | 5754 | 17 | 5 | 3 | 0 | 6 | 2 | 9 | 3 | 17 | 1 | 2 | 12 | 1 |
| NM_015235 | CSTF2T | 4110 | 13 | 8 | 6 | 4 | 8 | 13 | 7 | 6 | 4 | 8 | 7 | 12 | 7 |
| NM_015236 | LPHN3 | 6125 | 19 | 3 | 1 | 2 | 1 | 19 | 0 | 0 | 1 | 1 | 1 | 0 | 3 |
| NM_015237 | KIAA1107 | 4555 | 9 | 1 | 1 | 0 | 0 | 9 | 0 | 0 | 0 | 1 | 1 | 0 | 1 |
| NM_015238 | WWC1 | 6719 | 12 | 2 | 0 | 1 | 0 | 3 | 12 | 2 | 3 | 0 | 0 | 0 | 0 |
| NM_015239 | AGTPBP1 | 4419 | 12 | 4 | 2 | 1 | 6 | 12 | 3 | 1 | 5 | 2 | 2 | 6 | 4 |
| NM_015241 | MICAL3 | 9442 | 4 | 2 | 1 | 4 | 2 | 2 | 2 | 2 | 1 | 2 | 1 | 2 | 3 |
| NM_015242 | ARAP1 | 4947 | 6 | 4 | 6 | 6 | 3 | 2 | 3 | 2 | 4 | 5 | 1 | 6 | 2 |
| NM_015243 | VPS13B | 2822 | 8 | 4 | 4 | 3 | 3 | 4 | 4 | 2 | 4 | 8 | 2 | 8 | 6 |
| NM_015245 | ANKS1A | 6351 | 4 | 2 | 1 | 2 | 1 | 1 | 2 | 1 | 2 | 2 | 0 | 3 | 4 |
| NM_015246 | MGRN1 | 3932 | 3 | 2 | 1 | 1 | 2 | 3 | 3 | 2 | 2 | 3 | 3 | 3 | 2 |
| NM_015247 | CYLD | 8713 | 10 | 5 | 3 | 2 | 5 | 9 | 3 | 2 | 6 | 5 | 4 | 10 | 5 |
| NM_015250 | BICD2 | 4625 | 3 | 2 | 2 | 1 | 3 | 2 | 2 | 1 | 2 | 2 | 2 | 3 | 2 |
| NM_015251 | ATMIN | 4866 | 17 | 9 | 6 | 3 | 9 | 17 | 8 | 8 | 4 | 9 | 8 | 9 | 14 |
| NM_015252 | EHBP1 | 5162 | 17 | 8 | 17 | 2 | 4 | 12 | 6 | 10 | 3 | 14 | 2 | 3 | 10 |
| NM_015253 | WSCD1 | 5821 | 4 | 1 | 0 | 1 | 0 | 2 | 1 | 0 | 0 | 0 | 1 | 4 | 0 |
| NM_015254 | KIF13B | 8766 | 9 | 4 | 3 | 6 | 2 | 4 | 9 | 6 | 4 | 3 | 1 | 5 | 5 |
| NM_015255 | UBR2 | 5268 | 17 | 12 | 9 | 6 | 13 | 9 | 11 | 12 | 14 | 13 | 15 | 17 | 10 |
| NM_015256 | ACSL6 | 3047 | 13 | 2 | 0 | 0 | 0 | 13 | 1 | 0 | 0 | 0 | 0 | 0 | 2 |
| NM_015257 | TMEM194A | 5397 | 2 | 1 | 1 | 1 | 0 | 1 | 1 | 0 | 1 | 1 | 1 | 2 | 1 |
| NM_015258 | FKBP15 | 4336 | 15 | 6 | 8 | 4 | 6 | 5 | 5 | 3 | 6 | 5 | 4 | 15 | 5 |
| NM_015259 | ICOSLG | 3238 | 1 | 0 | 0 | 0 | 0 | 1 | 1 | 0 | 0 | 0 | 0 | 0 | 0 |
| NM_015260 | SIN3B | 5129 | 1 | 1 | 1 | 1 | 0 | 1 | 0 | 0 | 0 | 0 | 0 | 1 | 1 |
| NM_015261 | NCAPD3 | 5605 | 3 | 1 | 1 | 2 | 1 | 1 | 1 | 2 | 1 | 1 | 0 | 2 | 3 |
| NM_015262 | FAM21C | 4644 | 1 | 0 | 1 | 0 | 0 | 0 | 1 | 0 | 1 | 0 | 0 | 1 | 1 |
| NM_015263 | DMXL2 | 10578 | 15 | 5 | 6 | 3 | 3 | 15 | 5 | 1 | 7 | 3 | 1 | 8 | 6 |
| NM_015264 | C22orf9 | 6287 | 5 | 2 | 2 | 5 | 1 | 3 | 1 | 1 | 2 | 1 | 1 | 2 | 1 |
| NM_015265 | SATB2 | 5052 | 8 | 2 | 1 | 8 | 0 | 8 | 2 | 1 | 0 | 1 | 1 | 0 | 2 |
| NM_015266 | SLC9A8 | 6255 | 1 | 0 | 0 | 0 | 0 | 0 | 1 | 0 | 0 | 0 | 0 | 1 | 1 |
| NM_015267 | CUX2 | 6841 | 4 | 0 | 0 | 0 | 0 | 1 | 0 | 4 | 0 | 0 | 0 | 0 | 0 |
| NM_015268 | DNAJC13 | 7547 | 18 | 11 | 10 | 4 | 13 | 9 | 14 | 7 | 9 | 14 | 8 | 18 | 14 |
| NM_015269 | ZCCHC11 | 5828 | 15 | 8 | 3 | 2 | 7 | 5 | 8 | 4 | 8 | 15 | 10 | 12 | 13 |
| NM_015270 | ADCY6 | 6566 | 3 | 1 | 3 | 2 | 2 | 1 | 2 | 0 | 1 | 1 | 0 | 0 | 1 |
| NM_015271 | TRIM2 | 6757 | 15 | 3 | 1 | 2 | 1 | 15 | 5 | 1 | 1 | 2 | 0 | 3 | 1 |
| NM_015272 | RPGRIP1L | 6182 | 3 | 1 | 1 | 0 | 1 | 2 | 1 | 0 | 1 | 2 | 0 | 1 | 3 |
| NM_015274 | MAN2B2 | 4242 | 4 | 2 | 3 | 1 | 3 | 3 | 3 | 2 | 2 | 3 | 1 | 4 | 1 |
| NM_015275 | KIAA1033 | 5806 | 8 | 4 | 3 | 1 | 3 | 2 | 5 | 3 | 4 | 3 | 2 | 8 | 3 |
| NM_015276 | USP22 | 5220 | 19 | 10 | 5 | 6 | 10 | 19 | 10 | 7 | 6 | 13 | 11 | 11 | 10 |

| transcript | gene | transcript length (nt) | max | mean | adipose | colon | heart | hypothalamus | kidney | liver | lung | ovary | skeletalmuscle | spleen | testes |
|---|---|---|---|---|---|---|---|---|---|---|---|---|---|---|---|
| NM_015277 | NEDD4L | 8413 | 7 | 3 | 1 | 2 | 2 | 5 | 7 | 1 | 5 | 1 | 2 | 1 | 1 |
| NM_015278 | SASH1 | 7709 | 9 | 4 | 9 | 2 | 4 | 7 | 5 | 2 | 7 | 2 | 3 | 6 | 3 |
| NM_015282 | CLASP1 | 8090 | 7 | 4 | 3 | 2 | 5 | 7 | 4 | 2 | 3 | 4 | 5 | 5 | 4 |
| NM_015283 | DPY19L1 | 4865 | 3 | 2 | 1 | 1 | 2 | 3 | 1 | 2 | 2 | 1 | 1 | 3 | 3 |
| NM_015284 | KIAA0467 | 10217 | 1 | 1 | 1 | 1 | 0 | 0 | 1 | 0 | 0 | 1 | 0 | 1 | 0 |
| NM_015285 | WDR7 | 7282 | 8 | 3 | 2 | 1 | 2 | 8 | 2 | 2 | 2 | 2 | 2 | 3 | 3 |
| NM_015286 | SYNM | 6418 | 137 | 35 | 27 | 78 | 88 | 26 | 2 | 1 | 7 | 3 | 137 | 1 | 13 |
| NM_015288 | PHF15 | 6463 | 6 | 2 | 1 | 2 | 2 | 3 | 3 | 1 | 2 | 3 | 1 | 6 | 2 |
| NM_015289 | VPS39 | 4832 | 4 | 2 | 2 | 2 | 2 | 4 | 3 | 1 | 2 | 2 | 1 | 3 | 4 |
| NM_015291 | DNAJC16 | 6025 | 8 | 5 | 3 | 2 | 4 | 6 | 8 | 5 | 3 | 4 | 4 | 5 | 5 |
| NM_015292 | ESYT1 | 4173 | 11 | 4 | 11 | 3 | 3 | 3 | 3 | 6 | 3 | 3 | 1 | 5 | 4 |
| NM_015293 | SYNE1 | 10835 | 19 | 9 | 6 | 2 | 8 | 14 | 9 | 3 | 9 | 14 | 4 | 19 | 9 |
| NM_015294 | TRIM37 | 4516 | 17 | 4 | 2 | 1 | 3 | 17 | 3 | 2 | 2 | 2 | 2 | 3 | 10 |
| NM_015295 | SMCHD1 | 8672 | 21 | 7 | 4 | 3 | 6 | 4 | 4 | 2 | 12 | 8 | 5 | 21 | 10 |
| NM_015296 | DOCK9 | 7522 | 9 | 4 | 3 | 3 | 5 | 9 | 3 | 2 | 9 | 4 | 2 | 3 | 4 |
| NM_015297 | KIAA1045 | 5847 | 7 | 1 | 0 | 0 | 0 | 7 | 0 | 0 | 0 | 0 | 0 | 0 | 4 |
| NM_015299 | KHNYN | 6295 | 5 | 3 | 2 | 4 | 2 | 1 | 2 | 2 | 3 | 4 | 2 | 5 | 3 |
| NM_015302 | HAUS5 | 4309 | 0 | 0 | 0 | 0 | 0 | 0 | 0 | 0 | 0 | 0 | 0 | 0 | 0 |
| NM_015303 | VPS8 | 5041 | 5 | 3 | 2 | 1 | 5 | 3 | 2 | 1 | 3 | 3 | 4 | 4 | 3 |
| NM_015305 | ANGEL1 | 4111 | 1 | 1 | 0 | 0 | 0 | 1 | 1 | 0 | 1 | 1 | 0 | 1 | 1 |
| NM_015306 | USP24 | 10801 | 11 | 6 | 6 | 3 | 6 | 4 | 6 | 4 | 4 | 5 | 11 | 9 | 4 |
| NM_015307 | FAM189A1 | 4707 | 3 | 0 | 0 | 1 | 0 | 3 | 0 | 0 | 0 | 0 | 0 | 0 | 0 |
| NM_015308 | FNBP4 | 4112 | 20 | 12 | 11 | 9 | 11 | 8 | 11 | 6 | 14 | 14 | 12 | 20 | 14 |
| NM_015310 | PSD3 | 11687 | 21 | 5 | 1 | 2 | 6 | 21 | 1 | 6 | 1 | 9 | 0 | 5 | 3 |
| NM_015311 | OBSL1 | 5840 | 2 | 1 | 1 | 0 | 2 | 2 | 1 | 1 | 0 | 2 | 1 | 0 | 2 |
| NM_015312 | KIAA1109 | 15574 | 15 | 7 | 5 | 3 | 5 | 15 | 8 | 3 | 7 | 10 | 5 | 10 | 9 |
| NM_015313 | ARHGEF12 | 9501 | 26 | 14 | 7 | 6 | 19 | 18 | 26 | 19 | 15 | 10 | 11 | 12 | 9 |
| NM_015314 | KIAA0895 | 4321 | 1 | 1 | 0 | 0 | 0 | 1 | 1 | 0 | 0 | 1 | 0 | 0 | 1 |
| NM_015315 | LARP1 | 6591 | 18 | 10 | 10 | 7 | 10 | 18 | 10 | 11 | 6 | 8 | 8 | 11 | 8 |
| NM_015316 | PPP1R13B | 4959 | 3 | 2 | 0 | 1 | 3 | 2 | 2 | 1 | 1 | 2 | 1 | 2 | 3 |
| NM_015317 | PUM2 | 6111 | 24 | 14 | 9 | 8 | 13 | 17 | 12 | 12 | 13 | 21 | 9 | 24 | 17 |
| NM_015318 | ARHGEF18 | 5219 | 4 | 1 | 0 | 1 | 1 | 1 | 1 | 1 | 1 | 1 | 0 | 4 | 1 |
| NM_015319 | TENC1 | 4951 | 8 | 5 | 6 | 2 | 7 | 2 | 4 | 3 | 6 | 6 | 3 | 8 | 4 |
| NM_015320 | ARHGEF4 | 3666 | 4 | 1 | 0 | 0 | 0 | 4 | 0 | 0 | 0 | 1 | 0 | 0 | 0 |
| NM_015321 | CRTC1 | 6944 | 2 | 0 | 0 | 0 | 0 | 2 | 0 | 0 | 0 | 0 | 0 | 0 | 1 |
| NM_015322 | FEM1B | 2559 | 10 | 5 | 3 | 2 | 5 | 10 | 4 | 4 | 3 | 4 | 8 | 5 | 5 |
| NM_015323 | KIAA0776 | 4205 | 10 | 7 | 5 | 1 | 8 | 8 | 10 | 8 | 7 | 9 | 8 | 9 | 9 |
| NM_015324 | RRP8 | 1674 | 1 | 1 | 1 | 1 | 1 | 1 | 1 | 1 | 1 | 0 | 1 | 1 | 1 |
| NM_015325 | KIAA0947 | 7918 | 6 | 4 | 3 | 1 | 4 | 6 | 4 | 3 | 3 | 5 | 5 | 6 | 5 |
| NM_015326 | SRGAP2 | 5974 | 10 | 4 | 3 | 2 | 3 | 10 | 2 | 1 | 5 | 3 | 1 | 8 | 3 |
| NM_015327 | SMG5 | 4563 | 6 | 3 | 2 | 3 | 3 | 3 | 4 | 6 | 2 | 1 | 1 | 2 | 2 |
| NM_015328 | AHCYL2 | 5055 | 11 | 4 | 1 | 9 | 1 | 11 | 5 | 1 | 3 | 1 | 3 | 1 | 3 |
| NM_015329 | KIAA0892 | 4853 | 1 | 1 | 0 | 1 | 0 | 0 | 1 | 0 | 0 | 0 | 1 | 1 | 1 |
| NM_015330 | CYTSA | 6777 | 11 | 7 | 5 | 4 | 7 | 8 | 6 | 4 | 6 | 7 | 5 | 8 | 11 |
| NM_015331 | NCSTN | 2935 | 10 | 6 | 5 | 3 | 6 | 4 | 9 | 5 | 7 | 5 | 3 | 10 | 7 |
| NM_015332 | NUDCD3 | 4917 | 8 | 2 | 2 | 2 | 2 | 8 | 2 | 1 | 1 | 1 | 2 | 2 | 2 |
| NM_015335 | MED13L | 9376 | 13 | 8 | 6 | 5 | 6 | 5 | 6 | 4 | 8 | 13 | 9 | 12 | 9 |
| NM_015336 | ZDHHC17 | 4771 | 14 | 5 | 6 | 2 | 4 | 14 | 4 | 2 | 4 | 6 | 4 | 6 | 7 |
| NM_015338 | ASXL1 | 7038 | 3 | 2 | 2 | 2 | 1 | 2 | 2 | 1 | 2 | 3 | 2 | 3 | 2 |
| NM_015339 | ADNP | 4713 | 17 | 9 | 6 | 5 | 6 | 11 | 9 | 5 | 7 | 17 | 8 | 14 | 15 |
| NM_015340 | LARS2 | 4203 | 5 | 2 | 1 | 3 | 2 | 4 | 5 | 4 | 1 | 2 | 1 | 3 | 3 |
| NM_015341 | NCAPH | 4495 | 4 | 1 | 1 | 1 | 0 | 0 | 0 | 0 | 0 | 0 | 0 | 1 | 4 |
| NM_015342 | PPWD1 | 2095 | 19 | 10 | 9 | 6 | 9 | 10 | 10 | 6 | 9 | 15 | 8 | 19 | 12 |
| NM_015343 | DULLARD | 1780 | 10 | 5 | 6 | 4 | 4 | 5 | 5 | 3 | 5 | 4 | 10 | 9 | 4 |
| NM_015344 | LEPROTL1 | 2712 | 11 | 5 | 3 | 2 | 6 | 5 | 6 | 3 | 8 | 5 | 1 | 11 | 7 |
| NM_015345 | DAAM2 | 6215 | 7 | 2 | 2 | 1 | 1 | 7 | 1 | 1 | 1 | 1 | 1 | 4 | 2 |
| NM_015346 | ZFYVE26 | 9675 | 1 | 1 | 1 | 1 | 1 | 1 | 1 | 1 | 1 | 1 | 1 | 1 | 1 |
| NM_015347 | RIMBP2 | 6322 | 6 | 1 | 0 | 0 | 0 | 6 | 0 | 0 | 0 | 0 | 0 | 0 | 0 |
| NM_015348 | TMEM131 | 6642 | 10 | 6 | 3 | 3 | 6 | 5 | 6 | 4 | 4 | 7 | 7 | 10 | 6 |
| NM_015349 | KIAA0240 | 6515 | 5 | 3 | 2 | 2 | 2 | 3 | 2 | 1 | 2 | 5 | 2 | 4 | 3 |
| NM_015350 | LRRC8B | 7792 | 7 | 2 | 1 | 0 | 1 | 7 | 1 | 0 | 2 | 1 | 1 | 2 | 2 |
| NM_015351 | TTC9 | 5217 | 3 | 1 | 0 | 0 | 0 | 3 | 0 | 0 | 1 | 0 | 1 | 1 | 0 |
| NM_015352 | POFUT1 | 5219 | 7 | 3 | 4 | 2 | 4 | 1 | 4 | 7 | 1 | 3 | 1 | 3 | 4 |
| NM_015353 | KCTD2 | 3698 | 3 | 1 | 1 | 1 | 1 | 3 | 1 | 0 | 0 | 1 | 1 | 1 | 2 |
| NM_015354 | NUP188 | 5689 | 6 | 2 | 1 | 1 | 2 | 2 | 2 | 2 | 2 | 2 | 1 | 3 | 6 |
| NM_015355 | SUZ12 | 4477 | 6 | 3 | 2 | 1 | 2 | 2 | 3 | 2 | 3 | 3 | 2 | 5 | 6 |
| NM_015356 | SCRIB | 5143 | 1 | 0 | 0 | 1 | 0 | 0 | 0 | 0 | 0 | 0 | 0 | 0 | 1 |
| NM_015358 | MORC3 | 4245 | 19 | 11 | 8 | 3 | 9 | 10 | 7 | 4 | 13 | 19 | 11 | 19 | 14 |
| NM_015359 | SLC39A14 | 4690 | 45 | 8 | 1 | 3 | 17 | 3 | 6 | 45 | 2 | 2 | 2 | 3 | 6 |
| NM_015360 | SKIV2L2 | 4204 | 13 | 8 | 6 | 4 | 8 | 9 | 8 | 6 | 7 | 8 | 8 | 9 | 13 |
| NM_015361 | R3HDM1 | 4647 | 26 | 9 | 6 | 4 | 6 | 26 | 6 | 6 | 5 | 7 | 8 | 12 | 16 |
| NM_015362 | C17orf81 | 1389 | 4 | 2 | 1 | 2 | 2 | 2 | 1 | 1 | 1 | 1 | 1 | 1 | 4 |
| NM_015363 | ZIM2 | 2091 | 1 | 0 | 0 | 0 | 0 | 0 | 0 | 0 | 0 | 1 | 0 | 0 | 1 |
| NM_015364 | LY96 | 590 | 17 | 4 | 4 | 1 | 6 | 4 | 1 | 2 | 6 | 4 | 1 | 17 | 2 |
| NM_015365 | AMMECR1 | 5417 | 5 | 1 | 1 | 1 | 1 | 1 | 2 | 1 | 1 | 5 | 1 | 3 | 2 |
| NM_015366 | PRR5 | 2017 | 0 | 0 | 0 | 0 | 0 | 0 | 0 | 0 | 0 | 0 | 0 | 0 | 0 |
| NM_015367 | BCL2L13 | 3365 | 19 | 10 | 10 | 5 | 14 | 12 | 15 | 8 | 6 | 5 | 19 | 9 | 9 |

| transcript | gene | transcript length (nt) | max | mean | adipose | colon | heart | hypothalamus | kidney | liver | lung | ovary | skeletalmuscle | spleen | testes |
|---|---|---|---|---|---|---|---|---|---|---|---|---|---|---|---|
| NM_015368 | PANX1 | 2768 | 12 | 7 | 5 | 2 | 10 | 10 | 4 | 8 | 6 | 6 | 10 | 7 | 12 |
| NM_015370 | C22orf31 | 1070 | 0 | 0 | 0 | 0 | 0 | 0 | 0 | 0 | 0 | 0 | 0 | 0 | 0 |
| NM_015372 | C22orf24 | 1247 | 0 | 0 | 0 | 0 | 0 | 0 | 0 | 0 | 0 | 0 | 0 | 0 | 0 |
| NM_015373 | CBY1 | 1131 | 7 | 5 | 6 | 4 | 6 | 7 | 6 | 4 | 4 | 5 | 3 | 4 | 5 |
| NM_015374 | UNC84B | 3908 | 7 | 5 | 6 | 3 | 6 | 5 | 5 | 7 | 6 | 4 | 5 | 7 | 5 |
| NM_015375 | DSTYK | 7908 | 4 | 2 | 1 | 1 | 1 | 4 | 2 | 1 | 1 | 2 | 1 | 2 | 2 |
| NM_015376 | RASGRP3 | 4335 | 6 | 2 | 2 | 1 | 6 | 3 | 2 | 1 | 1 | 0 | 2 | 4 | 0 |
| NM_015378 | VPS13D | 16328 | 9 | 6 | 4 | 4 | 9 | 7 | 6 | 4 | 5 | 5 | 6 | 6 | 5 |
| NM_015379 | BRI3 | 791 | 10 | 6 | 6 | 2 | 9 | 3 | 7 | 6 | 7 | 3 | 9 | 10 | 4 |
| NM_015380 | SAMM50 | 1755 | 4 | 2 | 2 | 1 | 3 | 2 | 2 | 3 | 1 | 1 | 4 | 2 | 2 |
| NM_015381 | FAM19A5 | 2638 | 7 | 1 | 0 | 0 | 0 | 7 | 0 | 1 | 0 | 1 | 0 | 0 | 1 |
| NM_015382 | HECTD1 | 9134 | 38 | 17 | 15 | 7 | 17 | 18 | 14 | 21 | 11 | 13 | 38 | 14 | 17 |
| NM_015383 | NBPF14 | 3689 | 17 | 10 | 17 | 6 | 10 | 8 | 7 | 13 | 17 | 9 | 2 | 9 | 14 |
| NM_015384 | NIPBL | 10389 | 16 | 9 | 7 | 3 | 8 | 6 | 9 | 5 | 10 | 14 | 7 | 16 | 13 |
| NM_015385 | SORBS1 | 5963 | 114 | 29 | 114 | 26 | 67 | 25 | 6 | 14 | 9 | 3 | 39 | 4 | 13 |
| NM_015386 | COG4 | 2822 | 3 | 2 | 3 | 2 | 2 | 2 | 2 | 3 | 1 | 2 | 3 | 2 | 2 |
| NM_015387 | MOBKL3 | 2863 | 13 | 8 | 5 | 4 | 12 | 13 | 6 | 6 | 7 | 7 | 8 | 10 | 8 |
| NM_015388 | YIPF3 | 1550 | 4 | 3 | 3 | 2 | 2 | 4 | 4 | 4 | 2 | 1 | 1 | 3 | 4 |
| NM_015391 | ANAPC13 | 1207 | 10 | 6 | 5 | 2 | 6 | 7 | 10 | 7 | 5 | 3 | 4 | 5 | 9 |
| NM_015392 | NPDC1 | 1528 | 1 | 0 | 0 | 1 | 0 | 1 | 0 | 0 | 0 | 0 | 0 | 1 | 0 |
| NM_015393 | PARM1 | 5039 | 17 | 7 | 6 | 9 | 14 | 8 | 17 | 0 | 7 | 4 | 3 | 5 | 9 |
| NM_015394 | ZNF10 | 4403 | 6 | 2 | 1 | 0 | 1 | 3 | 3 | 1 | 1 | 4 | 1 | 2 | 6 |
| NM_015395 | TECPR1 | 4358 | 1 | 0 | 0 | 1 | 0 | 1 | 0 | 0 | 0 | 0 | 0 | 1 | 0 |
| NM_015396 | ARMC8 | 3832 | 6 | 4 | 3 | 2 | 5 | 5 | 4 | 3 | 3 | 4 | 6 | 6 | 4 |
| NM_015397 | DCAF12 | 3671 | 20 | 10 | 8 | 7 | 8 | 6 | 6 | 12 | 11 | 10 | 7 | 11 | 20 |
| NM_015398 | FAM149A | 2704 | 3 | 1 | 1 | 1 | 1 | 2 | 3 | 2 | 1 | 0 | 0 | 0 | 0 |
| NM_015399 | BRMS1 | 1437 | 7 | 4 | 3 | 4 | 4 | 3 | 7 | 5 | 4 | 2 | 3 | 7 | 5 |
| NM_015401 | HDAC7 | 4288 | 2 | 1 | 1 | 1 | 1 | 1 | 1 | 0 | 1 | 1 | 0 | 2 | 1 |
| NM_015404 | DFNB31 | 4047 | 1 | 0 | 0 | 0 | 0 | 0 | 0 | 0 | 0 | 0 | 0 | 0 | 1 |
| NM_015407 | ABHD14A | 1107 | 1 | 0 | 1 | 0 | 0 | 1 | 1 | 0 | 0 | 0 | 0 | 0 | 0 |
| NM_015409 | EP400 | 12265 | 4 | 3 | 2 | 2 | 2 | 3 | 2 | 1 | 2 | 4 | 2 | 3 | 4 |
| NM_015411 | SUMF2 | 2054 | 26 | 6 | 4 | 4 | 9 | 6 | 5 | 4 | 3 | 3 | 26 | 4 | 3 |
| NM_015412 | C3orf17 | 4856 | 4 | 2 | 2 | 1 | 2 | 3 | 3 | 2 | 2 | 3 | 3 | 4 | 3 |
| NM_015414 | RPL36 | 537 | 4 | 2 | 2 | 4 | 1 | 3 | 1 | 3 | 1 | 2 | 1 | 2 | 1 |
| NM_015415 | BRP44 | 789 | 131 | 36 | 31 | 24 | 45 | 47 | 37 | 131 | 10 | 10 | 22 | 9 | 35 |
| NM_015416 | LETMD1 | 2132 | 3 | 2 | 2 | 2 | 1 | 3 | 2 | 1 | 1 | 2 | 1 | 2 | 1 |
| NM_015417 | SPEF1 | 1587 | 1 | 0 | 0 | 0 | 0 | 1 | 0 | 0 | 0 | 0 | 0 | 0 | 0 |
| NM_015419 | MXRA5 | 9793 | 7 | 3 | 7 | 4 | 3 | 1 | 6 | 2 | 1 | 3 | 0 | 4 | 0 |
| NM_015420 | DCAF13 | 2639 | 6 | 4 | 2 | 1 | 4 | 3 | 6 | 5 | 2 | 3 | 3 | 6 | 5 |
| NM_015421 | TMEM186 | 1447 | 3 | 2 | 1 | 2 | 3 | 2 | 2 | 1 | 1 | 2 | 2 | 2 | 2 |
| NM_015423 | AASDHPPT | 2861 | 11 | 4 | 2 | 1 | 4 | 11 | 5 | 3 | 2 | 4 | 4 | 4 | 4 |
| NM_015424 | CHRDL2 | 1678 | 4 | 0 | 0 | 0 | 4 | 0 | 0 | 0 | 0 | 0 | 0 | 0 | 0 |
| NM_015425 | POLR1A | 6637 | 3 | 2 | 2 | 2 | 2 | 3 | 3 | 3 | 1 | 2 | 3 | 2 | 3 |
| NM_015426 | WDR51A | 2195 | 0 | 0 | 0 | 0 | 0 | 0 | 0 | 0 | 0 | 0 | 0 | 0 | 0 |
| NM_015428 | ZNF473 | 4690 | 7 | 2 | 1 | 1 | 1 | 1 | 1 | 1 | 1 | 1 | 1 | 3 | 7 |
| NM_015429 | ABI3BP | 4473 | 18 | 7 | 14 | 4 | 12 | 3 | 3 | 1 | 15 | 18 | 4 | 2 | 7 |
| NM_015430 | PAMR1 | 2785 | 1 | 0 | 0 | 1 | 1 | 1 | 0 | 0 | 0 | 0 | 0 | 0 | 0 |
| NM_015431 | TRIM58 | 5156 | 1 | 0 | 0 | 0 | 0 | 0 | 0 | 0 | 1 | 0 | 0 | 0 | 0 |
| NM_015432 | PLEKHG4 | 6803 | 0 | 0 | 0 | 0 | 0 | 0 | 0 | 0 | 0 | 0 | 0 | 0 | 0 |
| NM_015433 | FAM119B | 2701 | 2 | 1 | 1 | 0 | 0 | 0 | 2 | 1 | 1 | 1 | 0 | 1 | 1 |
| NM_015434 | INTS7 | 3462 | 2 | 1 | 1 | 1 | 1 | 1 | 1 | 1 | 0 | 1 | 1 | 1 | 2 |
| NM_015435 | RNF19A | 4330 | 8 | 5 | 5 | 2 | 5 | 6 | 7 | 3 | 8 | 5 | 3 | 7 | 8 |
| NM_015436 | RCHY1 | 4319 | 2 | 1 | 0 | 1 | 1 | 2 | 1 | 0 | 1 | 1 | 1 | 2 | 1 |
| NM_015439 | CCDC28A | 1496 | 5 | 2 | 2 | 1 | 1 | 5 | 2 | 2 | 2 | 1 | 1 | 2 | 2 |
| NM_015440 | MTHFD1L | 3598 | 5 | 2 | 1 | 1 | 2 | 5 | 1 | 1 | 3 | 3 | 0 | 3 | 3 |
| NM_015441 | OLFML2B | 3160 | 2 | 1 | 2 | 0 | 2 | 1 | 0 | 0 | 2 | 0 | 0 | 2 | 2 |
| NM_015442 | CNOT10 | 2741 | 5 | 2 | 2 | 2 | 2 | 2 | 2 | 2 | 2 | 3 | 1 | 4 | 5 |
| NM_015443 | KIAA1267 | 4963 | 7 | 4 | 3 | 5 | 3 | 2 | 4 | 2 | 4 | 7 | 4 | 7 | 6 |
| NM_015444 | TMEM158 | 1859 | 3 | 0 | 0 | 0 | 0 | 3 | 0 | 0 | 0 | 0 | 0 | 0 | 1 |
| NM_015446 | AHCTF1 | 8629 | 20 | 9 | 7 | 2 | 9 | 7 | 7 | 6 | 8 | 13 | 7 | 13 | 20 |
| NM_015447 | CAMSAP1 | 7631 | 4 | 2 | 1 | 1 | 1 | 4 | 1 | 1 | 1 | 2 | 3 | 1 | 2 |
| NM_015448 | RP11-529I1( | 799 | 5 | 2 | 1 | 1 | 2 | 5 | 3 | 0 | 2 | 1 | 1 | 1 | 4 |
| NM_015449 | C1orf43 | 1796 | 19 | 9 | 9 | 4 | 11 | 12 | 11 | 19 | 7 | 3 | 12 | 7 | 6 |
| NM_015450 | POT1 | 4082 | 5 | 2 | 1 | 0 | 2 | 2 | 2 | 1 | 2 | 3 | 1 | 4 | 5 |
| NM_015453 | THUMPD3 | 3980 | 8 | 4 | 4 | 1 | 4 | 4 | 5 | 4 | 3 | 3 | 3 | 4 | 8 |
| NM_015454 | LARP7 | 2158 | 15 | 8 | 6 | 1 | 15 | 7 | 11 | 5 | 6 | 8 | 5 | 11 | 9 |
| NM_015455 | CNOT6 | 6176 | 4 | 2 | 1 | 1 | 2 | 2 | 3 | 1 | 2 | 4 | 1 | 4 | 3 |
| NM_015456 | COBRA1 | 2566 | 1 | 1 | 0 | 1 | 1 | 1 | 0 | 0 | 0 | 0 | 0 | 1 | 1 |
| NM_015457 | ZDHHC5 | 4560 | 7 | 5 | 4 | 3 | 5 | 3 | 7 | 6 | 4 | 3 | 3 | 7 | 6 |
| NM_015458 | MTMR9 | 7405 | 4 | 1 | 1 | 1 | 1 | 4 | 1 | 1 | 1 | 1 | 1 | 1 | 2 |
| NM_015459 | ATL3 | 2256 | 15 | 7 | 15 | 5 | 7 | 5 | 5 | 8 | 6 | 7 | 2 | 8 | 7 |
| NM_015460 | MYRIP | 4869 | 6 | 2 | 1 | 1 | 1 | 6 | 1 | 4 | 0 | 1 | 1 | 3 | 1 |
| NM_015461 | ZNF521 | 4971 | 4 | 2 | 3 | 1 | 1 | 2 | 1 | 0 | 1 | 3 | 1 | 4 | 1 |
| NM_015462 | NOL11 | 2454 | 6 | 4 | 3 | 2 | 5 | 3 | 6 | 5 | 2 | 5 | 5 | 6 | 5 |
| NM_015463 | CNRIP1 | 1998 | 5 | 1 | 2 | 1 | 1 | 5 | 0 | 0 | 1 | 2 | 1 | 1 | 1 |
| NM_015464 | SOSTDC1 | 1869 | 4 | 1 | 0 | 2 | 0 | 4 | 4 | 0 | 3 | 0 | 0 | 0 | 1 |
| NM_015465 | GEMIN5 | 5404 | 2 | 2 | 1 | 1 | 1 | 2 | 1 | 1 | 1 | 2 | 2 | 2 | 2 |

| transcript | gene | transcript length (nt) | max | mean | adipose | colon | heart | hypothalamus | kidney | liver | lung | ovary | skeletalmuscle | spleen | testes |
|---|---|---|---|---|---|---|---|---|---|---|---|---|---|---|---|
| NM_015466 | PTPN23 | 5263 | 4 | 3 | 2 | 3 | 3 | 3 | 3 | 2 | 3 | 4 | 4 | 4 | 3 |
| NM_015469 | NIPSNAP3A | 1633 | 1 | 1 | 1 | 0 | 1 | 1 | 1 | 1 | 0 | 0 | 0 | 1 | 1 |
| NM_015470 | RAB11FIP5 | 4342 | 1 | 1 | 0 | 0 | 1 | 1 | 1 | 0 | 0 | 0 | 0 | 1 | 1 |
| NM_015471 | NSL1 | 13135 | 2 | 1 | 1 | 1 | 1 | 2 | 1 | 1 | 1 | 2 | 1 | 2 | 1 |
| NM_015472 | WWTR1 | 5113 | 29 | 15 | 12 | 7 | 19 | 11 | 23 | 5 | 23 | 29 | 5 | 17 | 17 |
| NM_015473 | HEATR5A | 6856 | 2 | 1 | 2 | 1 | 1 | 1 | 2 | 2 | 2 | 1 | 1 | 2 | 1 |
| NM_015474 | SAMHD1 | 2701 | 102 | 36 | 42 | 8 | 66 | 17 | 13 | 21 | 47 | 26 | 13 | 102 | 36 |
| NM_015475 | FAM98A | 2745 | 23 | 8 | 6 | 2 | 12 | 9 | 8 | 6 | 3 | 6 | 8 | 7 | 23 |
| NM_015476 | C18orf10 | 2065 | 12 | 5 | 6 | 3 | 4 | 10 | 5 | 2 | 4 | 6 | 2 | 3 | 12 |
| NM_015477 | SIN3A | 6679 | 9 | 5 | 3 | 3 | 4 | 5 | 4 | 3 | 5 | 6 | 4 | 7 | 9 |
| NM_015478 | L3MBTL | 3218 | 0 | 0 | 0 | 0 | 0 | 0 | 0 | 0 | 0 | 0 | 0 | 0 | 0 |
| NM_015480 | PVRL3 | 1650 | 25 | 7 | 4 | 5 | 3 | 8 | 6 | 10 | 3 | 8 | 2 | 2 | 25 |
| NM_015481 | ZNF385A | 2404 | 1 | 1 | 1 | 0 | 1 | 1 | 0 | 0 | 1 | 0 | 1 | 1 | 0 |
| NM_015482 | SLC22A23 | 6142 | 2 | 1 | 1 | 2 | 0 | 2 | 1 | 1 | 1 | 1 | 1 | 2 | 1 |
| NM_015483 | KBTBD2 | 3710 | 14 | 8 | 6 | 3 | 7 | 11 | 5 | 5 | 9 | 8 | 9 | 14 | 9 |
| NM_015484 | SYF2 | 1777 | 31 | 18 | 16 | 7 | 18 | 16 | 16 | 10 | 19 | 31 | 18 | 23 | 19 |
| NM_015485 | RWDD3 | 1235 | 5 | 3 | 3 | 2 | 3 | 5 | 4 | 3 | 2 | 3 | 2 | 3 | 5 |
| NM_015488 | PNKD | 3114 | 5 | 1 | 0 | 0 | 0 | 2 | 1 | 5 | 1 | 1 | 0 | 1 | 1 |
| NM_015490 | SEC31B | 4612 | 0 | 0 | 0 | 0 | 0 | 0 | 0 | 0 | 0 | 0 | 0 | 0 | 0 |
| NM_015491 | SFRS18 | 3141 | 32 | 20 | 21 | 10 | 16 | 17 | 25 | 5 | 24 | 25 | 17 | 32 | 25 |
| NM_015492 | C15orf39 | 4427 | 1 | 0 | 0 | 0 | 0 | 0 | 0 | 0 | 0 | 1 | 0 | 1 | 1 |
| NM_015493 | KANK2 | 4949 | 13 | 6 | 7 | 8 | 12 | 2 | 3 | 3 | 5 | 13 | 3 | 5 | 5 |
| NM_015496 | KIAA1429 | 5786 | 19 | 11 | 12 | 6 | 11 | 12 | 8 | 10 | 8 | 17 | 7 | 14 | 19 |
| NM_015497 | TMEM87A | 3007 | 13 | 5 | 6 | 4 | 4 | 5 | 4 | 3 | 8 | 4 | 3 | 5 | 13 |
| NM_015500 | C2CD2 | 6347 | 8 | 2 | 6 | 1 | 1 | 2 | 1 | 1 | 1 | 3 | 0 | 8 | 1 |
| NM_015503 | SH2B1 | 4865 | 2 | 1 | 1 | 2 | 1 | 1 | 1 | 0 | 1 | 1 | 1 | 2 | 2 |
| NM_015506 | MMACHC | 2850 | 6 | 2 | 1 | 1 | 2 | 2 | 2 | 6 | 0 | 1 | 1 | 1 | 1 |
| NM_015507 | EGFL6 | 2428 | 15 | 2 | 15 | 0 | 0 | 0 | 0 | 0 | 2 | 0 | 0 | 0 | 0 |
| NM_015508 | TIPARP | 3836 | 24 | 9 | 4 | 3 | 10 | 11 | 5 | 8 | 10 | 24 | 4 | 9 | 16 |
| NM_015509 | NECAP1 | 2593 | 25 | 5 | 2 | 3 | 2 | 25 | 4 | 4 | 3 | 4 | 2 | 4 | 6 |
| NM_015510 | DHRS7B | 1375 | 2 | 1 | 1 | 0 | 1 | 2 | 1 | 1 | 0 | 0 | 1 | 1 | 1 |
| NM_015511 | C20orf4 | 2647 | 2 | 2 | 1 | 1 | 2 | 2 | 2 | 2 | 1 | 2 | 1 | 2 | 2 |
| NM_015512 | DNAH1 | 13109 | 1 | 0 | 0 | 1 | 0 | 0 | 0 | 0 | 0 | 0 | 0 | 1 | 1 |
| NM_015513 | CRELD1 | 2707 | 2 | 1 | 1 | 1 | 1 | 2 | 1 | 1 | 1 | 1 | 1 | 1 | 1 |
| NM_015514 | NGDN | 1588 | 6 | 4 | 2 | 2 | 4 | 6 | 3 | 3 | 2 | 5 | 4 | 6 | 4 |
| NM_015515 | KRT23 | 2147 | 2 | 0 | 0 | 0 | 0 | 0 | 0 | 0 | 0 | 0 | 0 | 0 | 2 |
| NM_015516 | TSKU | 2710 | 4 | 1 | 1 | 0 | 0 | 0 | 1 | 4 | 0 | 0 | 0 | 0 | 0 |
| NM_015517 | HINFP | 2300 | 2 | 1 | 1 | 2 | 0 | 1 | 1 | 1 | 1 | 1 | 1 | 1 | 1 |
| NM_015520 | MAGI1 | 7235 | 5 | 3 | 3 | 4 | 3 | 4 | 4 | 4 | 4 | 1 | 1 | 5 | 3 |
| NM_015522 | DYNC2LI1 | 1137 | 10 | 5 | 4 | 2 | 3 | 9 | 9 | 2 | 4 | 7 | 1 | 3 | 10 |
| NM_015523 | REXO2 | 1158 | 11 | 5 | 4 | 3 | 11 | 3 | 7 | 3 | 4 | 4 | 2 | 5 | 5 |
| NM_015525 | IBTK | 5775 | 10 | 7 | 5 | 2 | 8 | 5 | 9 | 10 | 6 | 8 | 7 | 9 | 7 |
| NM_015526 | CLIP3 | 3349 | 100 | 16 | 6 | 7 | 5 | 100 | 3 | 1 | 2 | 37 | 1 | 7 | 9 |
| NM_015527 | TBC1D10B | 3288 | 3 | 2 | 1 | 1 | 2 | 2 | 2 | 1 | 1 | 1 | 1 | 2 | 3 |
| NM_015528 | RNF167 | 1708 | 5 | 2 | 2 | 3 | 2 | 4 | 2 | 5 | 1 | 1 | 2 | 2 | 2 |
| NM_015529 | MOXD1 | 3039 | 7 | 3 | 1 | 2 | 2 | 7 | 7 | 0 | 6 | 0 | 0 | 2 | 2 |
| NM_015530 | GORASP2 | 2499 | 15 | 10 | 7 | 5 | 15 | 9 | 10 | 11 | 7 | 9 | 8 | 12 | 11 |
| NM_015531 | C2CD3 | 6282 | 2 | 1 | 1 | 1 | 1 | 1 | 1 | 1 | 1 | 2 | 1 | 2 | 2 |
| NM_015532 | GRINL1A | 4123 | 1 | 0 | 1 | 0 | 0 | 0 | 0 | 0 | 0 | 0 | 0 | 0 | 0 |
| NM_015533 | DAK | 4241 | 3 | 1 | 1 | 1 | 0 | 1 | 2 | 3 | 0 | 1 | 0 | 1 | 1 |
| NM_015534 | ZZZ3 | 4323 | 12 | 7 | 6 | 3 | 6 | 8 | 9 | 7 | 5 | 12 | 7 | 8 | 9 |
| NM_015535 | SPATS2L | 6261 | 4 | 3 | 2 | 1 | 3 | 2 | 4 | 2 | 2 | 3 | 2 | 3 | 3 |
| NM_015537 | NELF | 3641 | 5 | 1 | 0 | 1 | 0 | 5 | 1 | 1 | 0 | 0 | 0 | 0 | 1 |
| NM_015540 | RPAP1 | 4671 | 1 | 1 | 0 | 1 | 1 | 1 | 1 | 1 | 0 | 0 | 1 | 1 | 1 |
| NM_015541 | LRIG1 | 4762 | 10 | 4 | 4 | 5 | 6 | 10 | 1 | 2 | 3 | 3 | 4 | 1 | 3 |
| NM_015542 | UPF2 | 5168 | 16 | 10 | 9 | 4 | 9 | 10 | 7 | 8 | 8 | 10 | 12 | 13 | 16 |
| NM_015543 | RAB1A | 2414 | 55 | 33 | 39 | 18 | 38 | 49 | 39 | 55 | 28 | 23 | 12 | 32 | 29 |
| NM_015544 | TMEM98 | 1795 | 2 | 1 | 0 | 1 | 1 | 1 | 1 | 0 | 1 | 2 | 0 | 0 | 1 |
| NM_015545 | PTCD1 | 3078 | 1 | 0 | 0 | 0 | 0 | 0 | 1 | 1 | 0 | 0 | 1 | 1 | 1 |
| NM_015547 | ACOT11 | 1921 | 2 | 1 | 1 | 2 | 0 | 0 | 1 | 0 | 0 | 1 | 0 | 0 | 0 |
| NM_015548 | DST | 16642 | 44 | 20 | 14 | 8 | 44 | 44 | 11 | 10 | 22 | 19 | 24 | 16 | 13 |
| NM_015549 | PLEKHG3 | 4400 | 2 | 1 | 1 | 1 | 0 | 1 | 2 | 0 | 0 | 1 | 0 | 1 | 0 |
| NM_015550 | OSBPL3 | 6707 | 3 | 2 | 2 | 1 | 1 | 1 | 3 | 0 | 2 | 2 | 1 | 2 | 1 |
| NM_015551 | SUSD5 | 5005 | 7 | 2 | 0 | 0 | 7 | 3 | 1 | 0 | 1 | 2 | 1 | 1 | 2 |
| NM_015553 | IPCEF1 | 6813 | 4 | 1 | 0 | 0 | 0 | 4 | 1 | 0 | 1 | 0 | 0 | 2 | 0 |
| NM_015554 | GLCE | 5043 | 7 | 4 | 3 | 2 | 4 | 5 | 7 | 4 | 4 | 6 | 2 | 3 | 4 |
| NM_015555 | ZNF451 | 5097 | 9 | 6 | 5 | 2 | 5 | 7 | 5 | 4 | 6 | 8 | 3 | 8 | 9 |
| NM_015556 | SIPA1L1 | 6005 | 15 | 5 | 2 | 4 | 2 | 15 | 5 | 4 | 4 | 2 | 1 | 10 | 6 |
| NM_015557 | CHD5 | 9646 | 8 | 1 | 0 | 0 | 0 | 8 | 0 | 0 | 0 | 0 | 0 | 0 | 1 |
| NM_015559 | SETBP1 | 9899 | 5 | 3 | 2 | 3 | 1 | 2 | 5 | 2 | 3 | 4 | 4 | 3 | 3 |
| NM_015560 | OPA1 | 6327 | 14 | 7 | 5 | 2 | 13 | 14 | 8 | 6 | 4 | 5 | 7 | 7 | 6 |
| NM_015562 | UBXN7 | 4732 | 6 | 3 | 3 | 2 | 3 | 4 | 2 | 1 | 4 | 6 | 2 | 5 | 5 |
| NM_015564 | LRRTM2 | 5628 | 6 | 1 | 0 | 1 | 0 | 6 | 0 | 0 | 1 | 1 | 0 | 1 | 0 |
| NM_015565 | RNF160 | 7756 | 4 | 3 | 2 | 1 | 3 | 3 | 3 | 3 | 2 | 3 | 3 | 4 | 4 |
| NM_015566 | FAM169A | 5942 | 8 | 2 | 0 | 0 | 1 | 8 | 1 | 1 | 0 | 1 | 0 | 1 | 6 |
| NM_015567 | SLITRK5 | 4446 | 2 | 0 | 0 | 0 | 0 | 2 | 0 | 0 | 0 | 0 | 0 | 0 | 0 |
| NM_015568 | PPP1R16B | 6251 | 5 | 1 | 0 | 1 | 0 | 5 | 3 | 0 | 1 | 0 | 0 | 4 | 0 |

| transcript | gene | transcript length (nt) | max | mean | adipose | colon | heart | hypothalamus | kidney | liver | lung | ovary | skeletalmuscle | spleen | testes |
|---|---|---|---|---|---|---|---|---|---|---|---|---|---|---|---|
| NM_015569 | DNM3 | 7643 | 23 | 3 | 0 | 0 | 0 | 23 | 1 | 0 | 1 | 1 | 0 | 1 | 1 |
| NM_015570 | AUTS2 | 6417 | 7 | 4 | 2 | 3 | 2 | 6 | 5 | 3 | 4 | 7 | 4 | 2 | 3 |
| NM_015571 | SENP6 | 6642 | 9 | 6 | 4 | 2 | 6 | 6 | 6 | 4 | 6 | 9 | 5 | 9 | 9 |
| NM_015575 | GIGYF2 | 7954 | 19 | 12 | 9 | 7 | 12 | 12 | 13 | 7 | 9 | 13 | 13 | 15 | 19 |
| NM_015576 | ERC2 | 6132 | 15 | 2 | 0 | 0 | 0 | 15 | 0 | 0 | 0 | 2 | 0 | 0 | 0 |
| NM_015577 | RAI14 | 4985 | 36 | 9 | 3 | 2 | 7 | 4 | 9 | 6 | 10 | 6 | 1 | 36 | 11 |
| NM_015578 | LSM14A | 3715 | 27 | 18 | 12 | 7 | 19 | 16 | 20 | 15 | 16 | 25 | 19 | 27 | 22 |
| NM_015584 | POLDIP2 | 2720 | 6 | 2 | 2 | 1 | 2 | 3 | 3 | 6 | 1 | 1 | 2 | 1 | 1 |
| NM_015585 | C20orf26 | 4120 | 2 | 0 | 0 | 0 | 1 | 0 | 0 | 0 | 0 | 0 | 2 | 0 | 1 |
| NM_015589 | SAMD4A | 6526 | 10 | 3 | 5 | 2 | 10 | 5 | 2 | 1 | 3 | 1 | 5 | 2 | 2 |
| NM_015590 | GPATCH4 | 1893 | 31 | 17 | 13 | 8 | 24 | 26 | 16 | 31 | 7 | 8 | 26 | 21 | 13 |
| NM_015594 | TBC1D29 | 668 | 0 | 0 | 0 | 0 | 0 | 0 | 0 | 0 | 0 | 0 | 0 | 0 | 0 |
| NM_015595 | SGEF | 5185 | 8 | 3 | 1 | 1 | 2 | 4 | 1 | 7 | 8 | 3 | 0 | 1 | 4 |
| NM_015596 | KLK13 | 1258 | 0 | 0 | 0 | 0 | 0 | 0 | 0 | 0 | 0 | 0 | 0 | 0 | 0 |
| NM_015597 | GPSM1 | 2264 | 0 | 0 | 0 | 0 | 0 | 0 | 0 | 0 | 0 | 0 | 0 | 0 | 0 |
| NM_015599 | PGM3 | 1994 | 16 | 9 | 14 | 3 | 9 | 8 | 6 | 16 | 6 | 12 | 3 | 7 | 14 |
| NM_015600 | ABHD12 | 1583 | 5 | 2 | 2 | 1 | 2 | 5 | 2 | 1 | 2 | 3 | 1 | 2 | 2 |
| NM_015601 | HERC4 | 4493 | 10 | 6 | 4 | 3 | 8 | 6 | 6 | 3 | 7 | 6 | 4 | 10 | 8 |
| NM_015602 | TOR1AIP1 | 3807 | 13 | 8 | 8 | 4 | 8 | 9 | 7 | 7 | 7 | 13 | 9 | 11 | 10 |
| NM_015603 | CCDC9 | 2076 | 8 | 6 | 5 | 7 | 5 | 7 | 4 | 4 | 7 | 5 | 5 | 7 | 8 |
| NM_015604 | DCAF4 | 2551 | 5 | 3 | 2 | 2 | 1 | 5 | 4 | 1 | 1 | 3 | 2 | 3 | 3 |
| NM_015605 | PTPN20B | 2478 | 0 | 0 | 0 | 0 | 0 | 0 | 0 | 0 | 0 | 0 | 0 | 0 | 0 |
| NM_015607 | C1orf77 | 1293 | 10 | 6 | 4 | 5 | 6 | 6 | 6 | 4 | 6 | 4 | 8 | 10 | 9 |
| NM_015608 | C10orf137 | 4579 | 6 | 2 | 1 | 1 | 2 | 2 | 2 | 1 | 1 | 4 | 2 | 4 | 6 |
| NM_015609 | C1orf144 | 3584 | 5 | 4 | 3 | 3 | 5 | 3 | 4 | 4 | 3 | 4 | 3 | 5 | 3 |
| NM_015610 | WIPI2 | 4489 | 5 | 3 | 3 | 2 | 3 | 5 | 3 | 3 | 2 | 3 | 1 | 5 | 5 |
| NM_015613 | LRIT1 | 2301 | 0 | 0 | 0 | 0 | 0 | 0 | 0 | 0 | 0 | 0 | 0 | 0 | 0 |
| NM_015617 | PYGO1 | 1260 | 13 | 3 | 0 | 0 | 1 | 2 | 1 | 0 | 1 | 8 | 13 | 0 | 2 |
| NM_015621 | CCDC69 | 3399 | 24 | 8 | 24 | 6 | 17 | 1 | 1 | 9 | 9 | 3 | 6 | 12 | 5 |
| NM_015622 | C7orf28A | 1824 | 0 | 0 | 0 | 0 | 0 | 0 | 0 | 0 | 0 | 0 | 0 | 0 | 0 |
| NM_015626 | WSB1 | 2832 | 57 | 22 | 22 | 12 | 20 | 16 | 23 | 8 | 31 | 24 | 9 | 57 | 20 |
| NM_015627 | LDLRAP1 | 2935 | 2 | 1 | 0 | 0 | 1 | 0 | 0 | 1 | 1 | 0 | 0 | 2 | 0 |
| NM_015629 | PRPF31 | 2183 | 12 | 7 | 8 | 8 | 4 | 12 | 6 | 10 | 5 | 5 | 5 | 8 | 8 |
| NM_015630 | EPC2 | 3641 | 13 | 6 | 4 | 2 | 6 | 6 | 5 | 3 | 4 | 13 | 5 | 10 | 9 |
| NM_015631 | TCTN3 | 2738 | 1 | 1 | 1 | 0 | 0 | 1 | 1 | 1 | 0 | 1 | 0 | 1 | 1 |
| NM_015633 | FGFR1OP2 | 2297 | 15 | 8 | 4 | 1 | 7 | 8 | 8 | 5 | 10 | 12 | 7 | 15 | 9 |
| NM_015634 | KIAA1279 | 2526 | 31 | 12 | 10 | 4 | 18 | 31 | 10 | 4 | 4 | 9 | 12 | 9 | 18 |
| NM_015635 | GAPVD1 | 6889 | 8 | 5 | 5 | 3 | 5 | 6 | 5 | 5 | 6 | 5 | 6 | 8 | 7 |
| NM_015636 | EIF2B4 | 1760 | 4 | 2 | 2 | 3 | 4 | 3 | 3 | 3 | 1 | 1 | 3 | 2 | 2 |
| NM_015638 | TRPC4AP | 3170 | 3 | 2 | 2 | 1 | 2 | 3 | 2 | 2 | 2 | 1 | 2 | 2 | 2 |
| NM_015640 | SERBP1 | 6701 | 25 | 19 | 14 | 9 | 22 | 15 | 19 | 25 | 15 | 21 | 25 | 24 | 18 |
| NM_015641 | TES | 2748 | 12 | 6 | 4 | 10 | 9 | 1 | 5 | 2 | 8 | 2 | 1 | 12 | 9 |
| NM_015642 | ZBTB20 | 3535 | 8 | 4 | 3 | 2 | 4 | 3 | 5 | 3 | 2 | 8 | 3 | 3 | 4 |
| NM_015645 | C1QTNF5 | 3917 | 0 | 0 | 0 | 0 | 0 | 0 | 0 | 0 | 0 | 0 | 0 | 0 | 0 |
| NM_015646 | RAP1B | 2107 | 207 | 95 | 113 | 33 | 84 | 69 | 79 | 71 | 134 | 75 | 40 | 207 | 139 |
| NM_015649 | IRF2BP1 | 2511 | 1 | 0 | 0 | 0 | 0 | 1 | 0 | 0 | 0 | 1 | 0 | 0 | 0 |
| NM_015650 | TRAF3IP1 | 4174 | 18 | 8 | 8 | 4 | 6 | 11 | 7 | 5 | 7 | 8 | 11 | 7 | 18 |
| NM_015651 | PHF19 | 4287 | 1 | 0 | 1 | 1 | 0 | 1 | 0 | 0 | 0 | 1 | 0 | 1 | 1 |
| NM_015652 | C10orf12 | 4545 | 9 | 5 | 3 | 4 | 4 | 4 | 4 | 2 | 5 | 7 | 5 | 9 | 6 |
| NM_015653 | RIBC2 | 1393 | 1 | 0 | 0 | 0 | 0 | 0 | 0 | 0 | 0 | 0 | 0 | 0 | 1 |
| NM_015654 | NAT9 | 1875 | 1 | 1 | 1 | 1 | 1 | 1 | 1 | 1 | 1 | 1 | 0 | 1 | 1 |
| NM_015655 | ZNF337 | 3197 | 10 | 5 | 4 | 4 | 4 | 6 | 5 | 1 | 4 | 10 | 5 | 6 | 7 |
| NM_015656 | KIF26A | 6757 | 0 | 0 | 0 | 0 | 0 | 0 | 0 | 0 | 0 | 0 | 0 | 0 | 0 |
| NM_015657 | ABCA12 | 8085 | 0 | 0 | 0 | 0 | 0 | 0 | 0 | 0 | 0 | 0 | 0 | 0 | 0 |
| NM_015658 | NOC2L | 2800 | 2 | 1 | 1 | 1 | 1 | 1 | 1 | 2 | 1 | 0 | 1 | 1 | 1 |
| NM_015659 | RSL1D1 | 5135 | 13 | 7 | 5 | 2 | 7 | 6 | 4 | 6 | 4 | 12 | 13 | 7 | 8 |
| NM_015660 | GIMAP2 | 1442 | 14 | 3 | 4 | 1 | 2 | 1 | 1 | 1 | 7 | 2 | 1 | 14 | 2 |
| NM_015662 | IFT172 | 5360 | 3 | 2 | 1 | 1 | 1 | 3 | 2 | 0 | 2 | 3 | 1 | 1 | 3 |
| NM_015665 | AAAS | 1837 | 1 | 1 | 1 | 1 | 1 | 1 | 1 | 1 | 1 | 0 | 1 | 1 | 1 |
| NM_015666 | GTPBP5 | 2936 | 1 | 1 | 1 | 1 | 0 | 1 | 1 | 1 | 0 | 0 | 1 | 1 | 1 |
| NM_015667 | FAM75A7 | 4223 | 0 | 0 | 0 | 0 | 0 | 0 | 0 | 0 | 0 | 0 | 0 | 0 | 0 |
| NM_015668 | RGS22 | 4184 | 17 | 3 | 1 | 0 | 0 | 2 | 0 | 0 | 2 | 13 | 0 | 0 | 17 |
| NM_015669 | PCDHB5 | 2905 | 1 | 0 | 0 | 0 | 0 | 1 | 0 | 0 | 0 | 1 | 0 | 0 | 0 |
| NM_015670 | SENP3 | 2485 | 4 | 2 | 1 | 2 | 2 | 3 | 2 | 1 | 2 | 2 | 1 | 3 | 4 |
| NM_015672 | RIMBP3 | 5793 | 0 | 0 | 0 | 0 | 0 | 0 | 0 | 0 | 0 | 0 | 0 | 0 | 0 |
| NM_015675 | GADD45B | 1363 | 74 | 36 | 28 | 13 | 52 | 63 | 28 | 74 | 60 | 17 | 18 | 34 | 11 |
| NM_015676 | C14orf109 | 909 | 8 | 5 | 6 | 3 | 5 | 5 | 8 | 4 | 7 | 4 | 4 | 6 | 4 |
| NM_015677 | SH3YL1 | 1786 | 9 | 4 | 3 | 5 | 1 | 6 | 9 | 1 | 6 | 3 | 1 | 3 | 2 |
| NM_015678 | NBEA | 10792 | 18 | 4 | 1 | 1 | 4 | 18 | 3 | 0 | 2 | 7 | 1 | 4 | 5 |
| NM_015679 | TRUB2 | 1439 | 3 | 1 | 2 | 1 | 2 | 3 | 2 | 3 | 1 | 1 | 1 | 1 | 1 |
| NM_015680 | C2orf24 | 2074 | 7 | 2 | 1 | 2 | 2 | 1 | 7 | 1 | 2 | 1 | 2 | 3 | 3 |
| NM_015681 | B9D1 | 928 | 0 | 0 | 0 | 0 | 0 | 0 | 0 | 0 | 0 | 0 | 0 | 0 | 0 |
| NM_015683 | ARRDC2 | 2503 | 8 | 3 | 3 | 2 | 1 | 3 | 1 | 1 | 4 | 0 | 8 | 3 | 1 |
| NM_015684 | ATP5S | 1359 | 2 | 1 | 0 | 0 | 1 | 1 | 2 | 0 | 1 | 1 | 1 | 1 | 1 |
| NM_015685 | SDCBP2 | 1279 | 4 | 1 | 0 | 4 | 0 | 1 | 0 | 1 | 1 | 0 | 0 | 0 | 0 |
| NM_015686 | FAM155B | 4019 | 3 | 0 | 0 | 0 | 3 | 0 | 0 | 0 | 0 | 0 | 0 | 0 | 0 |
| NM_015687 | FILIP1 | 4622 | 78 | 16 | 3 | 2 | 78 | 7 | 2 | 1 | 10 | 3 | 63 | 0 | 6 |

| | | transcript | | | | | | hypothala | | | | | skeletalm | | |
|---|---|---|---|---|---|---|---|---|---|---|---|---|---|---|---|
| transcript | gene | length (nt) | max | mean | adipose | colon | heart | mus | kidney | liver | lung | ovary | uscle | spleen | testes |
| NM_015688 | FAM184B | 3840 | 1 | 0 | 0 | 0 | 0 | 1 | 0 | 0 | 0 | 0 | 1 | 0 | 0 |
| NM_015689 | DENND2A | 3462 | 6 | 2 | 1 | 2 | 2 | 4 | 2 | 0 | 2 | 6 | 1 | 3 | 1 |
| NM_015690 | STK36 | 4921 | 3 | 1 | 0 | 1 | 0 | 0 | 1 | 0 | 1 | 1 | 0 | 1 | 3 |
| NM_015691 | WWC3 | 6454 | 6 | 3 | 1 | 2 | 6 | 2 | 2 | 1 | 5 | 5 | 1 | 4 | 3 |
| NM_015692 | CPAMD8 | 5988 | 1 | 0 | 0 | 0 | 0 | 0 | 0 | 0 | 1 | 0 | 0 | 0 | 0 |
| NM_015693 | INTU | 3238 | 4 | 1 | 1 | 1 | 1 | 4 | 2 | 0 | 1 | 3 | 0 | 1 | 3 |
| NM_015694 | ZNF777 | 3072 | 1 | 1 | 1 | 1 | 0 | 1 | 1 | 1 | 0 | 1 | 1 | 1 | 1 |
| NM_015695 | BRPF3 | 6023 | 6 | 3 | 1 | 3 | 2 | 3 | 4 | 3 | 2 | 3 | 2 | 3 | 6 |
| NM_015696 | GPX7 | 1228 | 2 | 1 | 1 | 1 | 2 | 1 | 1 | 0 | 1 | 1 | 1 | 1 | 1 |
| NM_015697 | COQ2 | 1640 | 16 | 9 | 7 | 6 | 15 | 4 | 6 | 12 | 7 | 9 | 10 | 16 | 8 |
| NM_015698 | GPKOW | 1652 | 3 | 2 | 2 | 2 | 2 | 3 | 1 | 1 | 1 | 1 | 2 | 2 | 1 |
| NM_015700 | NFU1 | 1179 | 14 | 7 | 10 | 2 | 14 | 7 | 8 | 4 | 4 | 4 | 12 | 6 | 5 |
| NM_015701 | ERLEC1 | 3121 | 9 | 6 | 4 | 2 | 8 | 9 | 5 | 5 | 6 | 5 | 3 | 6 | 8 |
| NM_015702 | MMADHC | 1434 | 8 | 3 | 3 | 2 | 3 | 3 | 4 | 8 | 3 | 2 | 2 | 4 | 3 |
| NM_015703 | RRP7A | 1205 | 2 | 1 | 2 | 2 | 1 | 2 | 1 | 1 | 1 | 1 | 1 | 2 | 1 |
| NM_015704 | PPPDE2 | 745 | 8 | 3 | 1 | 1 | 5 | 3 | 2 | 8 | 2 | 1 | 2 | 3 | 3 |
| NM_015705 | SGSM3 | 2989 | 2 | 1 | 1 | 2 | 1 | 1 | 1 | 1 | 1 | 1 | 0 | 1 | 1 |
| NM_015710 | GLTSCR2 | 1522 | 42 | 14 | 8 | 14 | 10 | 4 | 7 | 3 | 16 | 42 | 11 | 30 | 13 |
| NM_015711 | GLTSCR1 | 5739 | 0 | 0 | 0 | 0 | 0 | 0 | 0 | 0 | 0 | 0 | 0 | 0 | 0 |
| NM_015713 | RRM2B | 4930 | 19 | 9 | 8 | 2 | 8 | 12 | 8 | 7 | 10 | 6 | 19 | 9 | 10 |
| NM_015714 | G0S2 | 963 | 678 | 135 | 678 | 27 | 109 | 13 | 77 | 119 | 163 | 37 | 211 | 19 | 36 |
| NM_015715 | PLA2G3 | 2575 | 0 | 0 | 0 | 0 | 0 | 0 | 0 | 0 | 0 | 0 | 0 | 0 | 0 |
| NM_015716 | MINK1 | 4661 | 12 | 4 | 2 | 6 | 2 | 12 | 3 | 1 | 2 | 5 | 1 | 3 | 4 |
| NM_015717 | CD207 | 1868 | 0 | 0 | 0 | 0 | 0 | 0 | 0 | 0 | 0 | 0 | 0 | 0 | 0 |
| NM_015718 | NOX3 | 2042 | 0 | 0 | 0 | 0 | 0 | 0 | 0 | 0 | 0 | 0 | 0 | 0 | 0 |
| NM_015719 | COL5A3 | 6174 | 4 | 1 | 4 | 1 | 1 | 1 | 0 | 0 | 0 | 0 | 0 | 0 | 0 |
| NM_015720 | PODXL2 | 2171 | 7 | 1 | 0 | 0 | 0 | 7 | 0 | 0 | 0 | 1 | 0 | 0 | 1 |
| NM_015721 | GEMIN4 | 3741 | 1 | 0 | 0 | 1 | 0 | 0 | 0 | 0 | 0 | 0 | 0 | 0 | 1 |
| NM_015722 | CALY | 983 | 12 | 1 | 0 | 0 | 0 | 12 | 0 | 0 | 0 | 0 | 0 | 0 | 1 |
| NM_015723 | PNPLA8 | 3530 | 11 | 6 | 4 | 1 | 9 | 9 | 6 | 7 | 6 | 6 | 5 | 11 | 6 |
| NM_015725 | RDH8 | 1714 | 0 | 0 | 0 | 0 | 0 | 0 | 0 | 0 | 0 | 0 | 0 | 0 | 0 |
| NM_015726 | DCAF8 | 3883 | 17 | 10 | 9 | 7 | 12 | 10 | 11 | 11 | 6 | 7 | 17 | 9 | 10 |
| NM_015727 | TACR1 | 1731 | 0 | 0 | 0 | 0 | 0 | 0 | 0 | 0 | 0 | 0 | 0 | 0 | 0 |
| NM_015831 | ACHE | 2957 | 2 | 1 | 0 | 1 | 0 | 2 | 0 | 0 | 0 | 0 | 2 | 0 | 0 |
| NM_015832 | MBD2 | 1357 | 6 | 3 | 6 | 2 | 3 | 2 | 3 | 2 | 3 | 2 | 1 | 6 | 3 |
| NM_015833 | ADARB1 | 7027 | 3 | 1 | 1 | 1 | 1 | 2 | 1 | 0 | 3 | 1 | 1 | 2 | 1 |
| NM_015834 | ADARB1 | 3588 | 4 | 2 | 2 | 1 | 2 | 3 | 1 | 0 | 4 | 1 | 1 | 2 | 1 |
| NM_015836 | WARS2 | 2806 | 2 | 1 | 1 | 1 | 1 | 1 | 1 | 1 | 1 | 2 | 1 | 1 | 1 |
| NM_015837 | FCN2 | 943 | 20 | 2 | 0 | 0 | 0 | 0 | 0 | 20 | 0 | 0 | 0 | 0 | 0 |
| NM_015840 | ADAR | 6551 | 25 | 13 | 9 | 7 | 10 | 21 | 13 | 15 | 12 | 14 | 5 | 25 | 15 |
| NM_015841 | ADAR | 6494 | 25 | 13 | 9 | 7 | 10 | 21 | 13 | 15 | 12 | 14 | 5 | 25 | 15 |
| NM_015842 | LMO7 | 6773 | 52 | 17 | 0 | 8 | 52 | 11 | 20 | 12 | 43 | 4 | 23 | 2 | 10 |
| NM_015844 | MBD1 | 3092 | 1 | 1 | 1 | 1 | 1 | 1 | 1 | 1 | 0 | 1 | 1 | 1 | 1 |
| NM_015845 | MBD1 | 2981 | 1 | 1 | 1 | 1 | 1 | 1 | 1 | 1 | 0 | 1 | 1 | 1 | 1 |
| NM_015846 | MBD1 | 3260 | 1 | 1 | 1 | 1 | 1 | 1 | 1 | 1 | 0 | 1 | 1 | 1 | 1 |
| NM_015847 | MBD1 | 3113 | 1 | 1 | 1 | 1 | 1 | 1 | 1 | 1 | 0 | 1 | 1 | 1 | 1 |
| NM_015848 | KRT76 | 2529 | 0 | 0 | 0 | 0 | 0 | 0 | 0 | 0 | 0 | 0 | 0 | 0 | 0 |
| NM_015849 | CELA2B | 923 | 0 | 0 | 0 | 0 | 0 | 0 | 0 | 0 | 0 | 0 | 0 | 0 | 0 |
| NM_015850 | FGFR1 | 5895 | 6 | 3 | 6 | 3 | 6 | 3 | 2 | 0 | 3 | 5 | 1 | 1 | 4 |
| NM_015852 | ZNF117 | 6404 | 22 | 7 | 20 | 1 | 4 | 4 | 6 | 1 | 5 | 7 | 2 | 6 | 22 |
| NM_015853 | UBXN1 | 1289 | 23 | 15 | 14 | 11 | 14 | 13 | 14 | 10 | 14 | 18 | 23 | 23 | 16 |
| NM_015859 | GTF2A1 | 1736 | 12 | 6 | 5 | 3 | 4 | 8 | 6 | 4 | 5 | 6 | 4 | 7 | 12 |
| NM_015864 | FAM65B | 2434 | 22 | 5 | 1 | 1 | 2 | 6 | 1 | 1 | 9 | 1 | 6 | 22 | 1 |
| NM_015865 | SLC14A1 | 3970 | 2 | 1 | 0 | 0 | 1 | 2 | 1 | 0 | 1 | 0 | 0 | 0 | 0 |
| NM_015866 | PRDM2 | 7456 | 9 | 6 | 5 | 3 | 6 | 9 | 4 | 2 | 4 | 9 | 7 | 8 | 7 |
| NM_015868 | KIR2DL3 | 1596 | 0 | 0 | 0 | 0 | 0 | 0 | 0 | 0 | 0 | 0 | 0 | 0 | 0 |
| NM_015869 | PPARG | 1820 | 23 | 6 | 23 | 19 | 3 | 1 | 4 | 2 | 4 | 4 | 1 | 5 | 2 |
| NM_015871 | ZNF593 | 649 | 4 | 1 | 1 | 1 | 2 | 3 | 1 | 4 | 0 | 1 | 1 | 1 | 1 |
| NM_015872 | ZBTB7B | 3616 | 2 | 1 | 1 | 2 | 1 | 0 | 1 | 2 | 1 | 0 | 1 | 1 | 1 |
| NM_015873 | VILL | 2787 | 2 | 0 | 0 | 2 | 0 | 0 | 0 | 0 | 0 | 0 | 0 | 0 | 0 |
| NM_015874 | RBPJ | 2316 | 10 | 7 | 8 | 3 | 8 | 10 | 5 | 4 | 9 | 9 | 7 | 10 | 9 |
| NM_015878 | AZIN1 | 4348 | 15 | 10 | 6 | 4 | 10 | 15 | 9 | 9 | 11 | 13 | 6 | 14 | 11 |
| NM_015879 | ST8SIA3 | 10153 | 8 | 1 | 0 | 0 | 0 | 8 | 0 | 0 | 0 | 0 | 0 | 0 | 0 |
| NM_015881 | DKK3 | 2760 | 143 | 33 | 2 | 9 | 118 | 143 | 5 | 1 | 25 | 12 | 2 | 12 | 29 |
| NM_015884 | MBTPS2 | 4524 | 4 | 2 | 1 | 1 | 2 | 3 | 2 | 3 | 1 | 4 | 3 | 2 | 2 |
| NM_015885 | PCF11 | 5912 | 18 | 9 | 7 | 4 | 7 | 6 | 9 | 4 | 8 | 17 | 11 | 18 | 11 |
| NM_015886 | PI15 | 6732 | 3 | 1 | 0 | 1 | 1 | 0 | 1 | 0 | 0 | 1 | 0 | 3 | 1 |
| NM_015888 | HOOK1 | 5857 | 8 | 3 | 0 | 1 | 1 | 3 | 8 | 8 | 1 | 0 | 0 | 1 | 4 |
| NM_015889 | MED15 | 3255 | 1 | 1 | 1 | 1 | 1 | 1 | 1 | 0 | 1 | 1 | 1 | 1 | 1 |
| NM_015891 | CDC40 | 3852 | 8 | 4 | 3 | 1 | 4 | 6 | 5 | 4 | 3 | 6 | 3 | 8 | 5 |
| NM_015892 | CHST15 | 4680 | 9 | 3 | 2 | 2 | 1 | 3 | 1 | 2 | 3 | 6 | 1 | 9 | 1 |
| NM_015893 | PRLH | 264 | 0 | 0 | 0 | 0 | 0 | 0 | 0 | 0 | 0 | 0 | 0 | 0 | 0 |
| NM_015894 | STMN3 | 2255 | 5 | 1 | 0 | 0 | 0 | 5 | 0 | 0 | 0 | 0 | 0 | 1 | 0 |
| NM_015895 | GMNN | 1215 | 6 | 3 | 2 | 4 | 1 | 3 | 4 | 3 | 3 | 2 | 1 | 6 | 5 |
| NM_015896 | ZMYND10 | 1773 | 1 | 0 | 0 | 0 | 0 | 0 | 0 | 0 | 0 | 0 | 0 | 0 | 1 |
| NM_015897 | PIAS4 | 1737 | 2 | 1 | 1 | 1 | 1 | 2 | 1 | 1 | 0 | 1 | 1 | 1 | 2 |
| NM_015898 | ZBTB7A | 4441 | 5 | 3 | 2 | 5 | 2 | 4 | 2 | 2 | 2 | 1 | 2 | 4 | 2 |

| transcript | gene | transcript length (nt) | max | mean | adipose | colon | heart | hypothalamus | kidney | liver | lung | ovary | skeletalmuscle | spleen | testes |
|---|---|---|---|---|---|---|---|---|---|---|---|---|---|---|---|
| NM_015899 | PLEKHA9 | 1839 | 1 | 0 | 1 | 0 | 0 | 0 | 0 | 0 | 0 | 0 | 0 | 0 | 0 |
| NM_015900 | PLA1A | 1753 | 2 | 1 | 0 | 0 | 0 | 2 | 2 | 2 | 2 | 0 | 2 | 0 | 1 |
| NM_015901 | NUDT13 | 2104 | 1 | 1 | 1 | 1 | 1 | 0 | 1 | 0 | 0 | 1 | 1 | 1 | 1 |
| NM_015902 | UBR5 | 9394 | 13 | 8 | 8 | 4 | 7 | 7 | 7 | 6 | 8 | 10 | 7 | 11 | 13 |
| NM_015904 | EIF5B | 4698 | 43 | 19 | 17 | 4 | 43 | 19 | 19 | 18 | 11 | 13 | 25 | 18 | 19 |
| NM_015905 | TRIM24 | 4004 | 12 | 4 | 1 | 1 | 3 | 2 | 3 | 3 | 3 | 12 | 3 | 3 | 6 |
| NM_015906 | TRIM33 | 8339 | 11 | 7 | 5 | 4 | 5 | 9 | 8 | 6 | 7 | 10 | 6 | 10 | 11 |
| NM_015907 | LAP3 | 2100 | 39 | 25 | 22 | 10 | 25 | 36 | 36 | 38 | 20 | 9 | 19 | 39 | 18 |
| NM_015908 | SRRT | 3013 | 38 | 15 | 9 | 16 | 15 | 10 | 14 | 11 | 9 | 13 | 13 | 20 | 38 |
| NM_015909 | NBAS | 7282 | 8 | 5 | 4 | 3 | 7 | 8 | 5 | 3 | 4 | 8 | 6 | 6 | 8 |
| NM_015910 | C2orf86 | 3293 | 4 | 3 | 3 | 1 | 4 | 3 | 4 | 1 | 2 | 4 | 3 | 3 | 4 |
| NM_015911 | ZNF691 | 1575 | 2 | 1 | 1 | 1 | 1 | 1 | 2 | 1 | 1 | 2 | 1 | 1 | 1 |
| NM_015912 | FAM135B | 6962 | 2 | 0 | 0 | 0 | 0 | 2 | 0 | 0 | 0 | 0 | 0 | 0 | 1 |
| NM_015913 | TXNDC12 | 1599 | 7 | 3 | 4 | 2 | 3 | 4 | 3 | 7 | 3 | 2 | 1 | 5 | 4 |
| NM_015914 | TXNDC11 | 3047 | 5 | 3 | 2 | 2 | 3 | 2 | 3 | 5 | 3 | 2 | 3 | 4 | 1 |
| NM_015915 | ATL1 | 2645 | 15 | 2 | 1 | 0 | 1 | 15 | 1 | 0 | 0 | 2 | 0 | 1 | 1 |
| NM_015916 | CALHM2 | 1947 | 4 | 2 | 3 | 1 | 2 | 0 | 0 | 0 | 2 | 3 | 0 | 4 | 3 |
| NM_015917 | GSTK1 | 1057 | 14 | 8 | 12 | 10 | 10 | 6 | 14 | 12 | 5 | 2 | 4 | 10 | 4 |
| NM_015918 | POP5 | 797 | 3 | 2 | 2 | 0 | 2 | 3 | 3 | 2 | 1 | 1 | 1 | 3 | 0 |
| NM_015919 | ZNF226 | 825 | 2 | 1 | 1 | 1 | 1 | 2 | 2 | 1 | 0 | 1 | 1 | 1 | 2 |
| NM_015920 | RPS27L | 1084 | 31 | 17 | 18 | 16 | 24 | 12 | 23 | 31 | 17 | 8 | 14 | 18 | 12 |
| NM_015921 | CUTA | 1199 | 4 | 2 | 0 | 1 | 1 | 4 | 2 | 2 | 1 | 1 | 1 | 2 | 1 |
| NM_015922 | NSDHL | 1563 | 17 | 6 | 7 | 4 | 7 | 8 | 4 | 17 | 3 | 6 | 2 | 4 | 6 |
| NM_015925 | LSR | 2221 | 2 | 1 | 0 | 1 | 0 | 0 | 0 | 2 | 1 | 0 | 0 | 0 | 1 |
| NM_015926 | TEX264 | 1366 | 2 | 1 | 1 | 1 | 1 | 1 | 2 | 1 | 1 | 1 | 0 | 1 | 1 |
| NM_015927 | TGFB1I1 | 1803 | 1 | 0 | 0 | 1 | 1 | 0 | 0 | 0 | 0 | 1 | 0 | 0 | 0 |
| NM_015929 | LIPT1 | 1417 | 5 | 3 | 2 | 1 | 3 | 2 | 3 | 1 | 2 | 5 | 3 | 4 | 3 |
| NM_015931 | C3orf32 | 1542 | 0 | 0 | 0 | 0 | 0 | 0 | 0 | 0 | 0 | 0 | 0 | 0 | 0 |
| NM_015932 | POMP | 1359 | 18 | 10 | 11 | 3 | 14 | 18 | 8 | 16 | 7 | 5 | 8 | 11 | 7 |
| NM_015933 | CCDC72 | 463 | 37 | 21 | 25 | 20 | 18 | 37 | 18 | 26 | 17 | 10 | 16 | 24 | 19 |
| NM_015934 | NOP58 | 1965 | 21 | 12 | 8 | 5 | 14 | 12 | 13 | 12 | 10 | 13 | 12 | 21 | 16 |
| NM_015935 | METTL13 | 3407 | 3 | 2 | 1 | 1 | 1 | 2 | 2 | 2 | 1 | 1 | 1 | 2 | 3 |
| NM_015937 | PIGT | 2228 | 10 | 5 | 4 | 3 | 10 | 3 | 4 | 3 | 4 | 5 | 4 | 5 | 5 |
| NM_015938 | NMD3 | 2728 | 15 | 9 | 5 | 2 | 15 | 10 | 10 | 13 | 9 | 11 | 7 | 10 | 7 |
| NM_015939 | TRMT6 | 2311 | 6 | 3 | 2 | 1 | 4 | 4 | 3 | 3 | 2 | 2 | 3 | 4 | 6 |
| NM_015941 | ATP6V1H | 2153 | 17 | 5 | 4 | 2 | 3 | 17 | 9 | 2 | 4 | 2 | 2 | 4 | 3 |
| NM_015942 | MTERFD1 | 1406 | 4 | 2 | 2 | 1 | 4 | 2 | 2 | 3 | 1 | 2 | 2 | 2 | 3 |
| NM_015944 | AMDHD2 | 1584 | 0 | 0 | 0 | 0 | 0 | 0 | 0 | 0 | 0 | 0 | 0 | 0 | 0 |
| NM_015945 | SLC35C2 | 2266 | 1 | 1 | 0 | 0 | 0 | 0 | 1 | 1 | 1 | 0 | 0 | 1 | 1 |
| NM_015946 | PELO | 2921 | 1 | 1 | 1 | 0 | 1 | 1 | 1 | 1 | 1 | 0 | 0 | 1 | 0 |
| NM_015947 | ASCC1 | 2076 | 11 | 6 | 5 | 2 | 6 | 8 | 5 | 8 | 4 | 4 | 3 | 8 | 11 |
| NM_015948 | SLC35B3 | 2213 | 5 | 3 | 4 | 3 | 3 | 2 | 4 | 5 | 3 | 2 | 1 | 5 | 3 |
| NM_015949 | C7orf20 | 2088 | 2 | 1 | 1 | 1 | 1 | 2 | 2 | 1 | 1 | 1 | 1 | 2 | 2 |
| NM_015950 | MRPL2 | 1286 | 10 | 4 | 3 | 5 | 5 | 4 | 5 | 10 | 2 | 2 | 4 | 3 | 4 |
| NM_015952 | RWDD1 | 1067 | 31 | 18 | 19 | 4 | 31 | 20 | 16 | 17 | 14 | 19 | 25 | 19 | 14 |
| NM_015953 | NOSIP | 1098 | 3 | 2 | 2 | 2 | 1 | 3 | 1 | 2 | 1 | 1 | 3 | 2 | 2 |
| NM_015954 | DERA | 1724 | 14 | 6 | 5 | 5 | 6 | 4 | 14 | 13 | 5 | 2 | 4 | 8 | 4 |
| NM_015955 | MEMO1 | 1812 | 5 | 2 | 1 | 1 | 2 | 2 | 2 | 2 | 2 | 2 | 3 | 3 | 5 |
| NM_015956 | MRPL4 | 1458 | 1 | 1 | 1 | 0 | 0 | 1 | 0 | 1 | 0 | 0 | 0 | 0 | 0 |
| NM_015957 | APIP | 1258 | 6 | 4 | 5 | 2 | 5 | 4 | 6 | 4 | 6 | 3 | 5 | 5 | 4 |
| NM_015958 | DPH5 | 1788 | 9 | 5 | 4 | 2 | 5 | 6 | 4 | 6 | 4 | 9 | 4 | 6 | 4 |
| NM_015959 | TMX2 | 1667 | 33 | 16 | 12 | 18 | 16 | 22 | 20 | 33 | 12 | 8 | 6 | 14 | 16 |
| NM_015960 | CUTC | 1368 | 16 | 4 | 4 | 1 | 5 | 3 | 2 | 6 | 2 | 1 | 16 | 4 | 4 |
| NM_015962 | FCF1 | 2405 | 6 | 5 | 5 | 2 | 6 | 5 | 5 | 4 | 4 | 6 | 3 | 6 | 6 |
| NM_015963 | THAP4 | 2227 | 1 | 1 | 1 | 1 | 1 | 1 | 1 | 1 | 0 | 1 | 1 | 1 | 1 |
| NM_015964 | TPPP3 | 1041 | 8 | 2 | 1 | 2 | 1 | 8 | 1 | 0 | 4 | 0 | 2 | 0 | 1 |
| NM_015965 | NDUFA13 | 542 | 13 | 6 | 5 | 7 | 10 | 13 | 6 | 11 | 2 | 1 | 4 | 4 | 3 |
| NM_015966 | ERGIC3 | 1351 | 8 | 5 | 4 | 4 | 5 | 5 | 5 | 6 | 4 | 6 | 2 | 5 | 8 |
| NM_015967 | PTPN22 | 3641 | 4 | 1 | 0 | 0 | 0 | 0 | 0 | 0 | 2 | 0 | 0 | 4 | 1 |
| NM_015969 | MRPS17 | 585 | 5 | 2 | 1 | 1 | 3 | 3 | 2 | 5 | 1 | 2 | 2 | 2 | 2 |
| NM_015971 | MRPS7 | 1415 | 3 | 2 | 2 | 1 | 2 | 2 | 2 | 3 | 1 | 1 | 2 | 1 | 1 |
| NM_015972 | POLR1D | 692 | 18 | 10 | 6 | 5 | 15 | 5 | 8 | 10 | 11 | 18 | 13 | 11 | 8 |
| NM_015973 | GAL | 778 | 2 | 0 | 0 | 1 | 0 | 2 | 0 | 0 | 0 | 0 | 0 | 0 | 0 |
| NM_015974 | CRYL1 | 1501 | 8 | 2 | 1 | 1 | 1 | 2 | 8 | 3 | 1 | 0 | 0 | 1 | 0 |
| NM_015975 | TAF9B | 2689 | 3 | 2 | 2 | 0 | 3 | 3 | 3 | 1 | 2 | 3 | 2 | 3 | 2 |
| NM_015976 | SNX7 | 1777 | 8 | 5 | 3 | 5 | 7 | 8 | 7 | 4 | 3 | 4 | 1 | 3 | 8 |
| NM_015978 | TNNI3K | 3001 | 8 | 1 | 0 | 0 | 8 | 1 | 0 | 0 | 0 | 1 | 1 | 0 | 1 |
| NM_015979 | MED23 | 4599 | 5 | 2 | 2 | 2 | 2 | 2 | 3 | 1 | 2 | 3 | 1 | 5 | 3 |
| NM_015980 | HMP19 | 2335 | 127 | 12 | 0 | 1 | 0 | 127 | 0 | 0 | 0 | 0 | 0 | 0 | 1 |
| NM_015981 | CAMK2A | 4903 | 66 | 7 | 0 | 1 | 1 | 66 | 0 | 0 | 0 | 0 | 10 | 0 | 0 |
| NM_015982 | YBX2 | 1583 | 22 | 3 | 1 | 3 | 0 | 0 | 0 | 0 | 0 | 0 | 1 | 0 | 22 |
| NM_015983 | UBE2D4 | 1398 | 12 | 5 | 7 | 3 | 6 | 5 | 4 | 6 | 3 | 3 | 12 | 3 | 2 |
| NM_015984 | UCHL5 | 1703 | 15 | 10 | 6 | 3 | 14 | 15 | 10 | 11 | 7 | 7 | 10 | 13 | 9 |
| NM_015985 | ANGPT4 | 1921 | 0 | 0 | 0 | 0 | 0 | 0 | 0 | 0 | 0 | 0 | 0 | 0 | 0 |
| NM_015986 | CRLF3 | 2941 | 4 | 2 | 1 | 2 | 1 | 1 | 1 | 0 | 2 | 2 | 1 | 4 | 1 |
| NM_015987 | HEBP1 | 1186 | 24 | 12 | 7 | 3 | 15 | 5 | 13 | 10 | 15 | 24 | 6 | 20 | 11 |
| NM_015989 | CSAD | 2695 | 2 | 1 | 2 | 1 | 0 | 1 | 1 | 1 | 1 | 1 | 0 | 0 | 1 |

| transcript | gene | transcript length (nt) | max | mean | adipose | colon | heart | hypothalamus | kidney | liver | lung | ovary | skeletalmuscle | spleen | testes |
|---|---|---|---|---|---|---|---|---|---|---|---|---|---|---|---|
| NM_015990 | KLHL5 | 3486 | 9 | 5 | 9 | 3 | 3 | 8 | 5 | 7 | 4 | 5 | 1 | 5 | 2 |
| NM_015991 | C1QA | 1098 | 61 | 11 | 13 | 4 | 11 | 4 | 1 | 9 | 14 | 2 | 2 | 61 | 3 |
| NM_015993 | PLLP | 1496 | 3 | 0 | 0 | 0 | 0 | 3 | 1 | 0 | 1 | 0 | 0 | 1 | 0 |
| NM_015994 | ATP6V1D | 1669 | 15 | 5 | 4 | 2 | 8 | 15 | 8 | 3 | 3 | 2 | 2 | 3 | 4 |
| NM_015995 | KLF13 | 6800 | 5 | 2 | 1 | 2 | 2 | 3 | 2 | 3 | 3 | 1 | 2 | 5 | 1 |
| NM_015997 | C1orf66 | 2268 | 1 | 0 | 0 | 0 | 0 | 1 | 0 | 0 | 0 | 0 | 0 | 0 | 0 |
| NM_015999 | ADIPOR1 | 2135 | 12 | 7 | 4 | 3 | 9 | 7 | 7 | 12 | 8 | 4 | 6 | 11 | 8 |
| NM_016001 | UTP18 | 1884 | 3 | 2 | 2 | 1 | 1 | 3 | 2 | 2 | 1 | 2 | 1 | 3 | 3 |
| NM_016002 | SCCPDH | 2474 | 15 | 6 | 5 | 2 | 4 | 10 | 5 | 13 | 3 | 3 | 3 | 3 | 15 |
| NM_016003 | WIPI2 | 4435 | 5 | 3 | 3 | 2 | 3 | 5 | 3 | 3 | 2 | 3 | 1 | 5 | 5 |
| NM_016004 | IFT52 | 1675 | 7 | 2 | 3 | 2 | 1 | 3 | 2 | 1 | 2 | 3 | 0 | 2 | 7 |
| NM_016006 | ABHD5 | 5353 | 3 | 1 | 3 | 1 | 3 | 1 | 1 | 1 | 1 | 1 | 1 | 1 | 1 |
| NM_016008 | DYNC2LI1 | 1395 | 13 | 6 | 5 | 2 | 4 | 12 | 10 | 2 | 5 | 8 | 2 | 3 | 13 |
| NM_016009 | SH3GLB1 | 6373 | 21 | 11 | 17 | 3 | 17 | 7 | 7 | 6 | 9 | 6 | 21 | 14 | 11 |
| NM_016010 | FAM164A | 3347 | 5 | 2 | 1 | 1 | 1 | 5 | 2 | 0 | 3 | 5 | 1 | 2 | 3 |
| NM_016011 | MECR | 2307 | 1 | 0 | 1 | 1 | 0 | 1 | 1 | 0 | 0 | 0 | 0 | 0 | 1 |
| NM_016013 | NDUFAF1 | 1457 | 4 | 2 | 2 | 1 | 4 | 3 | 3 | 4 | 1 | 1 | 3 | 2 | 2 |
| NM_016014 | FAM108B1 | 1613 | 11 | 4 | 2 | 2 | 3 | 11 | 4 | 4 | 3 | 4 | 3 | 5 | 6 |
| NM_016016 | SLC25A39 | 1586 | 5 | 2 | 1 | 2 | 1 | 2 | 5 | 5 | 2 | 1 | 1 | 2 | 1 |
| NM_016018 | PHF20L1 | 6263 | 12 | 7 | 5 | 2 | 12 | 6 | 6 | 4 | 6 | 8 | 6 | 10 | 9 |
| NM_016019 | LUC7L2 | 2667 | 46 | 32 | 25 | 16 | 32 | 40 | 30 | 26 | 28 | 39 | 32 | 38 | 46 |
| NM_016020 | TFB1M | 1285 | 7 | 4 | 2 | 3 | 5 | 4 | 5 | 5 | 2 | 6 | 5 | 7 | 6 |
| NM_016021 | UBE2J1 | 4342 | 13 | 5 | 3 | 3 | 4 | 5 | 4 | 6 | 5 | 5 | 2 | 13 | 4 |
| NM_016022 | APH1A | 2189 | 7 | 4 | 4 | 4 | 3 | 3 | 6 | 7 | 4 | 3 | 2 | 7 | 5 |
| NM_016023 | OTUD6B | 3298 | 10 | 4 | 3 | 1 | 5 | 6 | 4 | 3 | 2 | 3 | 5 | 6 | 10 |
| NM_016024 | RBMX2 | 1528 | 7 | 5 | 5 | 3 | 7 | 6 | 4 | 3 | 5 | 6 | 3 | 6 | 4 |
| NM_016025 | METTL9 | 3195 | 8 | 4 | 3 | 1 | 4 | 4 | 8 | 1 | 4 | 3 | 3 | 6 | 3 |
| NM_016026 | RDH11 | 2567 | 49 | 16 | 7 | 4 | 8 | 28 | 31 | 49 | 11 | 8 | 3 | 12 | 15 |
| NM_016027 | LACTB2 | 1552 | 19 | 6 | 4 | 2 | 5 | 4 | 16 | 19 | 4 | 2 | 3 | 3 | 8 |
| NM_016028 | SUV420H1 | 2693 | 19 | 13 | 12 | 13 | 9 | 14 | 13 | 12 | 10 | 19 | 8 | 13 | 15 |
| NM_016029 | DHRS7 | 1389 | 44 | 13 | 6 | 4 | 13 | 9 | 17 | 13 | 11 | 12 | 44 | 10 | 6 |
| NM_016030 | TTC15 | 2524 | 5 | 2 | 2 | 2 | 3 | 4 | 3 | 2 | 1 | 2 | 2 | 2 | 5 |
| NM_016032 | ZDHHC9 | 2933 | 4 | 2 | 1 | 1 | 1 | 4 | 3 | 4 | 2 | 0 | 0 | 1 | 1 |
| NM_016033 | FAM82B | 3064 | 12 | 6 | 7 | 2 | 12 | 4 | 8 | 8 | 4 | 4 | 6 | 6 | 4 |
| NM_016034 | MRPS2 | 1505 | 2 | 1 | 0 | 1 | 1 | 1 | 1 | 2 | 0 | 1 | 1 | 0 | 0 |
| NM_016035 | COQ4 | 1572 | 2 | 1 | 1 | 1 | 1 | 1 | 2 | 2 | 1 | 1 | 1 | 1 | 1 |
| NM_016037 | UTP11L | 2050 | 51 | 16 | 12 | 5 | 18 | 24 | 7 | 16 | 7 | 9 | 51 | 11 | 13 |
| NM_016038 | SBDS | 1605 | 54 | 26 | 26 | 7 | 54 | 37 | 19 | 25 | 21 | 16 | 48 | 15 | 16 |
| NM_016039 | C14orf166 | 1051 | 125 | 67 | 65 | 28 | 125 | 66 | 44 | 57 | 39 | 78 | 67 | 56 | 111 |
| NM_016040 | TMED5 | 6065 | 22 | 10 | 9 | 2 | 12 | 7 | 14 | 22 | 9 | 5 | 10 | 12 | 6 |
| NM_016041 | DERL2 | 1124 | 3 | 2 | 2 | 2 | 2 | 1 | 2 | 3 | 1 | 3 | 1 | 3 | 2 |
| NM_016042 | EXOSC3 | 1220 | 10 | 5 | 5 | 2 | 5 | 5 | 5 | 6 | 4 | 10 | 2 | 8 | 7 |
| NM_016044 | FAHD2A | 1298 | 2 | 1 | 1 | 0 | 1 | 1 | 1 | 2 | 0 | 0 | 0 | 0 | 0 |
| NM_016045 | SLMO2 | 2563 | 12 | 8 | 10 | 7 | 10 | 9 | 9 | 11 | 6 | 5 | 4 | 12 | 10 |
| NM_016046 | EXOSC1 | 1150 | 2 | 1 | 1 | 1 | 1 | 1 | 1 | 1 | 1 | 1 | 1 | 2 | 1 |
| NM_016047 | SF3B14 | 771 | 2 | 1 | 1 | 1 | 1 | 1 | 1 | 1 | 1 | 1 | 1 | 2 | 1 |
| NM_016048 | ISOC1 | 1940 | 16 | 6 | 5 | 4 | 10 | 5 | 7 | 16 | 3 | 6 | 2 | 5 | 6 |
| NM_016049 | FAM158A | 835 | 1 | 0 | 0 | 0 | 0 | 0 | 0 | 0 | 0 | 0 | 0 | 1 | 0 |
| NM_016050 | MRPL11 | 1592 | 1 | 1 | 1 | 1 | 1 | 1 | 1 | 1 | 0 | 1 | 1 | 1 | 1 |
| NM_016052 | RRP15 | 7771 | 4 | 2 | 2 | 1 | 2 | 3 | 2 | 3 | 2 | 2 | 4 | 2 | 2 |
| NM_016053 | CCDC53 | 912 | 12 | 5 | 5 | 1 | 8 | 6 | 6 | 3 | 3 | 5 | 3 | 6 | 12 |
| NM_016055 | MRPL48 | 980 | 8 | 4 | 2 | 2 | 6 | 7 | 8 | 5 | 3 | 3 | 3 | 6 | 2 |
| NM_016056 | TMBIM4 | 1816 | 7 | 4 | 3 | 2 | 3 | 2 | 6 | 3 | 4 | 2 | 2 | 7 | 4 |
| NM_016057 | COPZ1 | 1897 | 10 | 7 | 6 | 5 | 6 | 8 | 10 | 9 | 6 | 6 | 4 | 8 | 5 |
| NM_016058 | TPRKB | 752 | 2 | 2 | 2 | 1 | 2 | 1 | 1 | 2 | 1 | 2 | 2 | 2 | 2 |
| NM_016059 | PPIL1 | 1734 | 3 | 1 | 1 | 0 | 3 | 2 | 1 | 2 | 0 | 1 | 0 | 1 | 1 |
| NM_016060 | MED31 | 1656 | 0 | 0 | 0 | 0 | 0 | 0 | 0 | 0 | 0 | 0 | 0 | 0 | 0 |
| NM_016061 | YPEL5 | 2259 | 65 | 41 | 30 | 13 | 42 | 59 | 39 | 25 | 48 | 47 | 26 | 59 | 65 |
| NM_016062 | FAM96B | 692 | 4 | 2 | 1 | 2 | 4 | 2 | 3 | 3 | 2 | 1 | 3 | 2 | 2 |
| NM_016063 | HDDC2 | 1615 | 16 | 6 | 6 | 3 | 8 | 16 | 8 | 2 | 4 | 4 | 7 | 3 | 10 |
| NM_016065 | MRPS16 | 2634 | 9 | 6 | 5 | 5 | 6 | 7 | 9 | 7 | 4 | 4 | 5 | 7 | 6 |
| NM_016066 | GLRX2 | 1128 | 1 | 0 | 1 | 0 | 1 | 1 | 0 | 0 | 0 | 0 | 0 | 0 | 1 |
| NM_016067 | MRPS18C | 1121 | 1 | 1 | 0 | 1 | 1 | 1 | 1 | 1 | 0 | 0 | 1 | 1 | 1 |
| NM_016068 | FIS1 | 739 | 5 | 2 | 3 | 1 | 4 | 3 | 5 | 2 | 2 | 0 | 2 | 3 | 1 |
| NM_016069 | Magmas | 511 | 1 | 0 | 0 | 0 | 1 | 0 | 0 | 1 | 0 | 0 | 1 | 0 | 0 |
| NM_016070 | MRPS23 | 903 | 24 | 12 | 9 | 4 | 24 | 21 | 18 | 10 | 6 | 5 | 10 | 8 | 18 |
| NM_016071 | MRPS33 | 746 | 12 | 6 | 6 | 5 | 11 | 11 | 7 | 12 | 2 | 4 | 3 | 3 | 6 |
| NM_016072 | GOLT1B | 3272 | 2 | 1 | 1 | 0 | 2 | 2 | 2 | 1 | 1 | 1 | 1 | 2 | 1 |
| NM_016073 | HDGFRP3 | 1973 | 46 | 15 | 9 | 4 | 17 | 46 | 8 | 1 | 11 | 14 | 3 | 10 | 38 |
| NM_016074 | BOLA1 | 856 | 4 | 2 | 3 | 2 | 2 | 4 | 2 | 3 | 2 | 2 | 1 | 2 | 1 |
| NM_016075 | VPS36 | 4423 | 8 | 4 | 3 | 2 | 5 | 5 | 7 | 3 | 4 | 4 | 5 | 8 | 3 |
| NM_016076 | PPPDE1 | 4092 | 12 | 5 | 4 | 4 | 6 | 2 | 3 | 3 | 7 | 5 | 3 | 5 | 12 |
| NM_016077 | PTRH2 | 798 | 8 | 3 | 3 | 2 | 3 | 4 | 3 | 8 | 2 | 2 | 2 | 3 | 3 |
| NM_016078 | FAM18B | 1789 | 0 | 0 | 0 | 0 | 0 | 0 | 0 | 0 | 0 | 0 | 0 | 0 | 0 |
| NM_016079 | VPS24 | 3172 | 18 | 12 | 15 | 9 | 18 | 15 | 13 | 4 | 11 | 9 | 13 | 12 | 10 |
| NM_016080 | GLOD4 | 1836 | 7 | 4 | 3 | 3 | 2 | 7 | 6 | 5 | 2 | 2 | 2 | 4 | 3 |
| NM_016081 | PALLD | 5809 | 113 | 30 | 15 | 17 | 113 | 8 | 14 | 3 | 25 | 33 | 26 | 42 | 30 |

| transcript | gene | transcript length (nt) | max | mean | adipose | colon | heart | hypothalamus | kidney | liver | lung | ovary | skeletalmuscle | spleen | testes |
|---|---|---|---|---|---|---|---|---|---|---|---|---|---|---|---|
| NM_016082 | CDK5RAP1 | 1944 | 3 | 2 | 2 | 3 | 2 | 3 | 3 | 3 | 2 | 2 | 2 | 3 | 2 |
| NM_016083 | CNR1 | 5716 | 7 | 1 | 1 | 0 | 0 | 7 | 0 | 0 | 1 | 0 | 0 | 1 | 1 |
| NM_016084 | RASD1 | 1744 | 16 | 4 | 8 | 1 | 2 | 8 | 3 | 16 | 1 | 3 | 2 | 1 | 2 |
| NM_016085 | C2orf28 | 1405 | 4 | 2 | 3 | 2 | 2 | 2 | 3 | 3 | 1 | 2 | 1 | 2 | 4 |
| NM_016086 | STYXL1 | 1416 | 1 | 1 | 0 | 1 | 0 | 1 | 1 | 1 | 1 | 1 | 0 | 1 | 0 |
| NM_016087 | WNT16 | 2876 | 1 | 0 | 0 | 0 | 0 | 0 | 0 | 0 | 0 | 0 | 0 | 1 | 0 |
| NM_016089 | ZNF589 | 3364 | 7 | 2 | 2 | 2 | 1 | 2 | 2 | 0 | 1 | 3 | 2 | 7 | 3 |
| NM_016090 | RBM7 | 1917 | 7 | 4 | 3 | 2 | 5 | 3 | 5 | 4 | 5 | 7 | 4 | 7 | 5 |
| NM_016091 | EIF3L | 1949 | 151 | 68 | 57 | 65 | 64 | 62 | 39 | 68 | 55 | 151 | 83 | 59 | 48 |
| NM_016093 | RPL26L1 | 710 | 3 | 1 | 1 | 1 | 1 | 2 | 2 | 3 | 0 | 1 | 1 | 1 | 2 |
| NM_016094 | COMMD2 | 1424 | 12 | 8 | 8 | 5 | 8 | 11 | 9 | 7 | 7 | 10 | 5 | 12 | 9 |
| NM_016095 | GINS2 | 1179 | 0 | 0 | 0 | 0 | 0 | 0 | 0 | 0 | 0 | 0 | 0 | 0 | 0 |
| NM_016096 | ZNF706 | 2745 | 9 | 5 | 3 | 2 | 5 | 9 | 4 | 2 | 4 | 6 | 6 | 8 | 4 |
| NM_016097 | IER3IP1 | 1481 | 29 | 15 | 14 | 7 | 19 | 17 | 21 | 29 | 12 | 9 | 13 | 14 | 14 |
| NM_016098 | BRP44L | 958 | 58 | 22 | 13 | 9 | 58 | 29 | 27 | 55 | 9 | 7 | 17 | 10 | 6 |
| NM_016099 | GOLGA7 | 1904 | 34 | 13 | 14 | 6 | 10 | 34 | 12 | 15 | 9 | 10 | 6 | 13 | 16 |
| NM_016100 | NAT5 | 1079 | 18 | 12 | 9 | 5 | 16 | 18 | 16 | 12 | 5 | 9 | 18 | 11 | 13 |
| NM_016101 | NIP7 | 2184 | 6 | 3 | 2 | 2 | 3 | 3 | 4 | 4 | 2 | 2 | 2 | 6 | 4 |
| NM_016102 | TRIM17 | 2083 | 1 | 0 | 0 | 0 | 0 | 1 | 0 | 0 | 0 | 0 | 0 | 0 | 1 |
| NM_016103 | SAR1B | 1246 | 195 | 54 | 42 | 17 | 46 | 58 | 38 | 195 | 21 | 17 | 101 | 29 | 30 |
| NM_016104 | RWDD1 | 1309 | 25 | 15 | 16 | 4 | 25 | 16 | 13 | 14 | 12 | 15 | 21 | 15 | 12 |
| NM_016106 | SCFD1 | 2156 | 18 | 13 | 14 | 5 | 14 | 15 | 12 | 17 | 11 | 15 | 11 | 16 | 18 |
| NM_016107 | ZFR | 4715 | 24 | 14 | 12 | 6 | 13 | 20 | 13 | 6 | 11 | 19 | 14 | 19 | 24 |
| NM_016108 | AIG1 | 1385 | 45 | 18 | 18 | 5 | 19 | 25 | 36 | 45 | 7 | 13 | 2 | 13 | 12 |
| NM_016111 | TELO2 | 3327 | 0 | 0 | 0 | 0 | 0 | 0 | 0 | 0 | 0 | 0 | 0 | 0 | 0 |
| NM_016112 | PKD2L1 | 3051 | 1 | 0 | 0 | 0 | 0 | 0 | 0 | 0 | 0 | 0 | 0 | 1 | 0 |
| NM_016113 | TRPV2 | 2776 | 2 | 1 | 1 | 0 | 1 | 1 | 0 | 0 | 1 | 0 | 0 | 2 | 0 |
| NM_016115 | ASB3 | 2214 | 0 | 0 | 0 | 0 | 0 | 0 | 0 | 0 | 0 | 0 | 0 | 0 | 0 |
| NM_016116 | ASB4 | 1281 | 1 | 0 | 0 | 0 | 0 | 0 | 0 | 0 | 0 | 0 | 1 | 0 | 0 |
| NM_016118 | NUB1 | 3104 | 12 | 9 | 11 | 4 | 8 | 10 | 7 | 8 | 8 | 8 | 9 | 12 | 12 |
| NM_016120 | RLIM | 10565 | 16 | 8 | 5 | 2 | 9 | 16 | 8 | 4 | 9 | 11 | 6 | 10 | 8 |
| NM_016121 | KCTD3 | 3931 | 6 | 3 | 3 | 2 | 2 | 4 | 4 | 6 | 2 | 3 | 1 | 2 | 4 |
| NM_016122 | CCDC41 | 3225 | 11 | 5 | 3 | 2 | 6 | 4 | 4 | 2 | 3 | 8 | 4 | 5 | 11 |
| NM_016123 | IRAK4 | 4293 | 2 | 1 | 1 | 0 | 1 | 1 | 1 | 1 | 1 | 1 | 0 | 2 | 1 |
| NM_016124 | RHD | 2833 | 1 | 0 | 0 | 0 | 0 | 0 | 0 | 0 | 1 | 1 | 0 | 1 | 0 |
| NM_016125 | RNFT1 | 2016 | 2 | 1 | 1 | 1 | 1 | 0 | 1 | 2 | 1 | 1 | 0 | 1 | 2 |
| NM_016126 | HSPB11 | 601 | 1 | 1 | 0 | 1 | 1 | 1 | 1 | 1 | 0 | 1 | 0 | 0 | 1 |
| NM_016127 | TMEM66 | 1977 | 116 | 45 | 22 | 18 | 33 | 116 | 42 | 68 | 42 | 48 | 14 | 53 | 38 |
| NM_016128 | COPG | 3074 | 27 | 13 | 17 | 15 | 11 | 21 | 15 | 27 | 8 | 8 | 6 | 12 | 8 |
| NM_016129 | COPS4 | 1748 | 5 | 3 | 2 | 1 | 3 | 5 | 2 | 2 | 1 | 2 | 3 | 2 | 4 |
| NM_016131 | RAB10 | 3784 | 15 | 9 | 8 | 5 | 10 | 13 | 11 | 7 | 8 | 6 | 10 | 15 | 9 |
| NM_016132 | MYEF2 | 5403 | 11 | 3 | 1 | 2 | 2 | 11 | 5 | 0 | 2 | 0 | 0 | 2 | 7 |
| NM_016133 | INSIG2 | 2583 | 13 | 7 | 4 | 2 | 8 | 12 | 7 | 9 | 7 | 7 | 3 | 13 | 7 |
| NM_016134 | PGCP | 1896 | 19 | 9 | 12 | 5 | 6 | 7 | 12 | 5 | 7 | 19 | 2 | 10 | 8 |
| NM_016135 | ETV7 | 1626 | 0 | 0 | 0 | 0 | 0 | 0 | 0 | 0 | 0 | 0 | 0 | 0 | 0 |
| NM_016138 | COQ7 | 2586 | 2 | 1 | 1 | 1 | 2 | 2 | 2 | 1 | 1 | 2 | 2 | 1 | 1 |
| NM_016139 | CHCHD2 | 815 | 5 | 2 | 2 | 1 | 3 | 2 | 3 | 5 | 2 | 2 | 2 | 2 | 2 |
| NM_016140 | TPPP3 | 1132 | 8 | 2 | 1 | 1 | 1 | 8 | 1 | 0 | 4 | 0 | 2 | 0 | 1 |
| NM_016141 | DYNC1LI1 | 2484 | 16 | 8 | 5 | 3 | 12 | 16 | 6 | 8 | 6 | 5 | 8 | 8 | 13 |
| NM_016142 | HSD17B12 | 2545 | 6 | 3 | 2 | 1 | 3 | 4 | 6 | 5 | 3 | 2 | 1 | 3 | 3 |
| NM_016143 | NSFL1C | 3644 | 5 | 3 | 3 | 2 | 2 | 5 | 3 | 3 | 2 | 2 | 4 | 4 | 3 |
| NM_016144 | COMMD10 | 1424 | 6 | 3 | 2 | 1 | 3 | 2 | 4 | 2 | 2 | 3 | 2 | 6 | 3 |
| NM_016145 | C19orf56 | 861 | 1 | 0 | 0 | 0 | 0 | 0 | 0 | 1 | 0 | 0 | 0 | 1 | 0 |
| NM_016146 | TRAPPC4 | 1198 | 8 | 4 | 2 | 2 | 4 | 8 | 5 | 3 | 3 | 2 | 3 | 6 | 5 |
| NM_016147 | PPME1 | 2453 | 10 | 3 | 2 | 2 | 3 | 10 | 3 | 2 | 2 | 2 | 4 | 2 | 5 |
| NM_016148 | SHANK1 | 6643 | 1 | 0 | 0 | 0 | 0 | 1 | 0 | 0 | 0 | 0 | 0 | 0 | 0 |
| NM_016150 | ASB2 | 2742 | 10 | 1 | 0 | 2 | 2 | 0 | 0 | 0 | 0 | 0 | 10 | 0 | 0 |
| NM_016151 | TAOK2 | 4909 | 3 | 2 | 1 | 2 | 1 | 3 | 1 | 1 | 1 | 1 | 0 | 2 | 2 |
| NM_016152 | RARB | 2772 | 4 | 2 | 1 | 0 | 4 | 2 | 3 | 1 | 2 | 3 | 1 | 4 | 1 |
| NM_016153 | HSFX1 | 1955 | 0 | 0 | 0 | 0 | 0 | 0 | 0 | 0 | 0 | 0 | 0 | 0 | 0 |
| NM_016154 | RAB4B | 1140 | 2 | 1 | 0 | 1 | 1 | 2 | 2 | 1 | 1 | 0 | 0 | 2 | 0 |
| NM_016155 | MMP17 | 2408 | 0 | 0 | 0 | 0 | 0 | 0 | 0 | 0 | 0 | 0 | 0 | 0 | 0 |
| NM_016156 | MTMR2 | 4593 | 8 | 4 | 4 | 2 | 4 | 5 | 5 | 1 | 3 | 5 | 1 | 5 | 8 |
| NM_016157 | TRO | 2321 | 10 | 2 | 1 | 1 | 1 | 10 | 1 | 0 | 0 | 3 | 1 | 0 | 1 |
| NM_016161 | A4GNT | 1771 | 0 | 0 | 0 | 0 | 0 | 0 | 0 | 0 | 0 | 0 | 0 | 0 | 0 |
| NM_016162 | ING4 | 1445 | 5 | 2 | 3 | 2 | 1 | 5 | 2 | 2 | 1 | 2 | 1 | 3 | 3 |
| NM_016166 | PIAS1 | 2280 | 12 | 8 | 4 | 4 | 7 | 9 | 6 | 5 | 11 | 10 | 5 | 12 | 11 |
| NM_016167 | NOL7 | 874 | 12 | 8 | 5 | 3 | 12 | 9 | 11 | 7 | 6 | 7 | 7 | 12 | 10 |
| NM_016169 | SUFU | 4898 | 1 | 1 | 1 | 1 | 1 | 1 | 1 | 0 | 1 | 1 | 0 | 1 | 1 |
| NM_016170 | TLX2 | 1752 | 0 | 0 | 0 | 0 | 0 | 0 | 0 | 0 | 0 | 0 | 0 | 0 | 0 |
| NM_016172 | UBAC1 | 1867 | 15 | 3 | 1 | 3 | 5 | 3 | 3 | 1 | 1 | 2 | 15 | 2 | 2 |
| NM_016173 | HEMK1 | 5861 | 1 | 0 | 0 | 1 | 0 | 0 | 0 | 0 | 0 | 1 | 0 | 0 | 0 |
| NM_016174 | CERCAM | 2689 | 1 | 0 | 0 | 0 | 0 | 1 | 0 | 0 | 0 | 0 | 0 | 0 | 0 |
| NM_016175 | C5orf45 | 1201 | 1 | 0 | 0 | 0 | 0 | 1 | 0 | 0 | 1 | 1 | 0 | 1 | 1 |
| NM_016176 | SDF4 | 2022 | 6 | 3 | 4 | 4 | 4 | 2 | 4 | 6 | 2 | 2 | 2 | 4 | 3 |
| NM_016178 | OAZ3 | 887 | 16 | 2 | 0 | 0 | 0 | 0 | 0 | 0 | 0 | 0 | 0 | 1 | 16 |
| NM_016179 | TRPC4 | 3434 | 3 | 1 | 0 | 0 | 0 | 3 | 0 | 0 | 1 | 1 | 0 | 0 | 0 |

| transcript | gene | transcript length (nt) | max | mean | adipose | colon | heart | hypothala mus | kidney | liver | lung | ovary | skeletalm uscle | spleen | testes |
|---|---|---|---|---|---|---|---|---|---|---|---|---|---|---|---|
| NM_016180 | SLC45A2 | 1717 | 0 | 0 | 0 | 0 | 0 | 0 | 0 | 0 | 0 | 0 | 0 | 0 | 0 |
| NM_016183 | MRTO4 | 2273 | 3 | 2 | 2 | 1 | 2 | 3 | 1 | 2 | 1 | 1 | 2 | 2 | 2 |
| NM_016184 | CLEC4A | 1281 | 1 | 0 | 0 | 0 | 0 | 0 | 0 | 0 | 1 | 1 | 0 | 1 | 0 |
| NM_016185 | HN1 | 1601 | 4 | 2 | 1 | 2 | 1 | 4 | 1 | 1 | 1 | 1 | 1 | 2 | 3 |
| NM_016186 | SERPINA10 | 2453 | 88 | 8 | 0 | 0 | 0 | 0 | 0 | 88 | 0 | 0 | 0 | 0 | 0 |
| NM_016188 | ACTL6B | 1531 | 1 | 0 | 0 | 0 | 0 | 1 | 0 | 0 | 0 | 0 | 0 | 0 | 0 |
| NM_016190 | CRNN | 1913 | 0 | 0 | 0 | 0 | 0 | 0 | 0 | 0 | 0 | 0 | 0 | 0 | 0 |
| NM_016192 | TMEFF2 | 1809 | 16 | 2 | 0 | 0 | 0 | 16 | 0 | 0 | 0 | 0 | 0 | 0 | 1 |
| NM_016194 | GNB5 | 3084 | 2 | 1 | 0 | 0 | 0 | 2 | 1 | 0 | 1 | 1 | 0 | 1 | 1 |
| NM_016195 | KIF20B | 6310 | 3 | 1 | 1 | 0 | 1 | 1 | 1 | 0 | 1 | 1 | 0 | 2 | 3 |
| NM_016196 | RBM19 | 4196 | 3 | 2 | 2 | 2 | 2 | 2 | 2 | 2 | 2 | 3 | 2 | 3 | 2 |
| NM_016199 | LSM7 | 520 | 8 | 5 | 4 | 8 | 4 | 8 | 4 | 4 | 3 | 5 | 4 | 6 | 6 |
| NM_016200 | LSM8 | 1307 | 15 | 8 | 7 | 4 | 10 | 7 | 6 | 6 | 8 | 9 | 5 | 15 | 11 |
| NM_016201 | AMOTL2 | 4984 | 6 | 2 | 6 | 1 | 2 | 2 | 3 | 1 | 3 | 2 | 1 | 3 | 2 |
| NM_016202 | ZNF580 | 1419 | 8 | 4 | 4 | 5 | 4 | 4 | 6 | 1 | 3 | 6 | 2 | 8 | 3 |
| NM_016203 | PRKAG2 | 3407 | 15 | 4 | 2 | 2 | 15 | 5 | 5 | 4 | 3 | 2 | 1 | 4 | 2 |
| NM_016204 | GDF2 | 1936 | 1 | 0 | 0 | 0 | 0 | 0 | 0 | 1 | 0 | 0 | 0 | 0 | 0 |
| NM_016205 | PDGFC | 3007 | 2 | 1 | 2 | 1 | 1 | 0 | 2 | 1 | 1 | 2 | 1 | 1 | 1 |
| NM_016206 | VGLL3 | 10396 | 8 | 3 | 8 | 0 | 4 | 1 | 1 | 0 | 8 | 5 | 1 | 2 | 4 |
| NM_016207 | CPSF3 | 2171 | 8 | 4 | 3 | 1 | 3 | 3 | 3 | 5 | 2 | 3 | 3 | 5 | 8 |
| NM_016208 | VPS28 | 944 | 2 | 1 | 1 | 1 | 1 | 1 | 1 | 1 | 1 | 1 | 1 | 2 | 0 |
| NM_016209 | TRAPPC2L | 629 | 3 | 1 | 1 | 1 | 1 | 3 | 1 | 1 | 1 | 1 | 1 | 1 | 1 |
| NM_016210 | C3orf18 | 2655 | 1 | 1 | 1 | 0 | 1 | 1 | 0 | 0 | 0 | 1 | 0 | 0 | 0 |
| NM_016211 | SEC31A | 4120 | 22 | 12 | 10 | 5 | 14 | 8 | 15 | 13 | 9 | 22 | 9 | 12 | 13 |
| NM_016212 | TP53TG3 | 2029 | 0 | 0 | 0 | 0 | 0 | 0 | 0 | 0 | 0 | 0 | 0 | 0 | 0 |
| NM_016212 | TP53TG3 | 2034 | 0 | 0 | 0 | 0 | 0 | 0 | 0 | 0 | 0 | 0 | 0 | 0 | 0 |
| NM_016213 | TRIP4 | 2028 | 12 | 6 | 6 | 6 | 7 | 5 | 12 | 5 | 6 | 6 | 2 | 10 | 6 |
| NM_016215 | EGFL7 | 1529 | 1 | 0 | 0 | 0 | 0 | 0 | 0 | 0 | 1 | 0 | 0 | 0 | 0 |
| NM_016216 | DBR1 | 2650 | 2 | 1 | 1 | 1 | 1 | 1 | 2 | 1 | 1 | 1 | 1 | 2 | 2 |
| NM_016217 | HECA | 5621 | 12 | 4 | 2 | 1 | 7 | 3 | 2 | 1 | 6 | 5 | 4 | 12 | 3 |
| NM_016218 | POLK | 4510 | 3 | 3 | 3 | 1 | 2 | 2 | 3 | 2 | 3 | 3 | 2 | 3 | 3 |
| NM_016219 | MAN1B1 | 2731 | 1 | 1 | 1 | 1 | 0 | 1 | 1 | 1 | 1 | 1 | 0 | 1 | 1 |
| NM_016220 | ZNF107 | 5505 | 18 | 4 | 1 | 1 | 2 | 2 | 4 | 0 | 4 | 9 | 1 | 18 | 6 |
| NM_016221 | DCTN4 | 3886 | 5 | 3 | 2 | 1 | 3 | 5 | 3 | 2 | 3 | 4 | 4 | 3 | 4 |
| NM_016222 | DDX41 | 2104 | 4 | 2 | 2 | 2 | 2 | 4 | 2 | 2 | 1 | 1 | 1 | 2 | 2 |
| NM_016223 | PACSIN3 | 1838 | 3 | 1 | 0 | 0 | 2 | 0 | 0 | 1 | 0 | 0 | 3 | 0 | 0 |
| NM_016224 | SNX9 | 4200 | 5 | 3 | 5 | 2 | 4 | 2 | 4 | 4 | 2 | 3 | 2 | 4 | 3 |
| NM_016226 | VPS29 | 1089 | 8 | 5 | 7 | 3 | 7 | 8 | 5 | 6 | 3 | 4 | 4 | 5 | 2 |
| NM_016227 | C1orf9 | 5466 | 21 | 7 | 4 | 2 | 7 | 8 | 7 | 3 | 6 | 9 | 2 | 8 | 21 |
| NM_016228 | AADAT | 2326 | 1 | 0 | 0 | 0 | 0 | 1 | 1 | 1 | 0 | 0 | 0 | 0 | 1 |
| NM_016229 | CYB5R2 | 1348 | 6 | 1 | 0 | 0 | 0 | 1 | 0 | 0 | 0 | 1 | 1 | 0 | 6 |
| NM_016230 | CYB5R4 | 2247 | 6 | 3 | 3 | 1 | 3 | 2 | 2 | 3 | 4 | 2 | 2 | 6 | 3 |
| NM_016231 | NLK | 3538 | 10 | 3 | 1 | 1 | 2 | 10 | 4 | 1 | 2 | 2 | 1 | 4 | 3 |
| NM_016232 | IL1RL1 | 2058 | 21 | 3 | 0 | 1 | 4 | 1 | 5 | 2 | 21 | 0 | 0 | 1 | 0 |
| NM_016233 | PADI3 | 3188 | 0 | 0 | 0 | 0 | 0 | 0 | 0 | 0 | 0 | 0 | 0 | 0 | 0 |
| NM_016234 | ACSL5 | 3354 | 31 | 6 | 4 | 10 | 2 | 2 | 1 | 31 | 4 | 2 | 1 | 5 | 3 |
| NM_016235 | GPRC5B | 2845 | 36 | 8 | 2 | 2 | 6 | 36 | 7 | 1 | 1 | 5 | 9 | 1 | 18 |
| NM_016237 | ANAPC5 | 2623 | 5 | 4 | 4 | 3 | 3 | 5 | 4 | 5 | 2 | 2 | 3 | 4 | 4 |
| NM_016238 | ANAPC7 | 3045 | 3 | 2 | 2 | 1 | 2 | 3 | 2 | 2 | 1 | 1 | 2 | 2 | 3 |
| NM_016239 | MYO15A | 11863 | 0 | 0 | 0 | 0 | 0 | 0 | 0 | 0 | 0 | 0 | 0 | 0 | 0 |
| NM_016240 | SCARA3 | 3631 | 3 | 1 | 1 | 1 | 0 | 3 | 0 | 0 | 0 | 1 | 0 | 0 | 0 |
| NM_016242 | EMCN | 4035 | 5 | 1 | 2 | 0 | 3 | 0 | 5 | 0 | 3 | 1 | 1 | 0 | 1 |
| NM_016243 | CYB5R1 | 1642 | 21 | 6 | 3 | 1 | 9 | 5 | 6 | 5 | 3 | 2 | 21 | 4 | 8 |
| NM_016245 | HSD17B11 | 1873 | 51 | 17 | 13 | 12 | 11 | 7 | 13 | 51 | 26 | 22 | 6 | 20 | 9 |
| NM_016246 | HSD17B14 | 1237 | 4 | 1 | 1 | 1 | 0 | 2 | 4 | 0 | 1 | 1 | 0 | 0 | 0 |
| NM_016247 | IMPG2 | 4454 | 0 | 0 | 0 | 0 | 0 | 0 | 0 | 0 | 0 | 0 | 0 | 0 | 0 |
| NM_016248 | AKAP11 | 9919 | 29 | 9 | 6 | 2 | 7 | 29 | 7 | 5 | 7 | 10 | 4 | 10 | 13 |
| NM_016249 | MAGEC2 | 1993 | 6 | 1 | 0 | 0 | 0 | 0 | 0 | 0 | 0 | 0 | 0 | 0 | 6 |
| NM_016250 | NDRG2 | 2080 | 67 | 19 | 12 | 9 | 14 | 67 | 10 | 18 | 7 | 16 | 39 | 11 | 6 |
| NM_016252 | BIRC6 | 15702 | 14 | 7 | 5 | 4 | 6 | 6 | 8 | 6 | 6 | 9 | 6 | 14 | 10 |
| NM_016255 | FAM8A1 | 4744 | 5 | 3 | 1 | 1 | 2 | 3 | 3 | 5 | 3 | 3 | 2 | 2 | 3 |
| NM_016256 | NAGPA | 2203 | 2 | 1 | 0 | 1 | 0 | 2 | 1 | 0 | 1 | 1 | 0 | 2 | 0 |
| NM_016257 | HPCAL4 | 4259 | 34 | 4 | 0 | 0 | 0 | 34 | 1 | 0 | 0 | 1 | 0 | 0 | 3 |
| NM_016258 | YTHDF2 | 3060 | 5 | 4 | 3 | 2 | 3 | 5 | 4 | 5 | 3 | 4 | 3 | 5 | 4 |
| NM_016260 | IKZF2 | 9423 | 2 | 1 | 0 | 0 | 0 | 1 | 1 | 0 | 1 | 1 | 1 | 2 | 1 |
| NM_016261 | TUBD1 | 2472 | 1 | 0 | 0 | 0 | 0 | 0 | 1 | 0 | 0 | 1 | 0 | 0 | 0 |
| NM_016262 | TUBE1 | 2296 | 6 | 3 | 2 | 2 | 3 | 3 | 3 | 6 | 2 | 3 | 1 | 2 | 5 |
| NM_016263 | FZR1 | 3615 | 1 | 1 | 0 | 1 | 0 | 1 | 1 | 1 | 1 | 0 | 0 | 1 | 1 |
| NM_016264 | ZNF44 | 2650 | 8 | 5 | 3 | 3 | 3 | 3 | 8 | 3 | 4 | 8 | 3 | 6 | 7 |
| NM_016265 | ZNF12 | 5063 | 8 | 4 | 3 | 2 | 4 | 3 | 3 | 1 | 3 | 8 | 2 | 7 | 5 |
| NM_016267 | VGLL1 | 1214 | 0 | 0 | 0 | 0 | 0 | 0 | 0 | 0 | 0 | 0 | 0 | 0 | 0 |
| NM_016269 | LEF1 | 3602 | 4 | 1 | 0 | 1 | 0 | 1 | 1 | 0 | 1 | 1 | 0 | 4 | 2 |
| NM_016270 | KLF2 | 1649 | 12 | 3 | 2 | 1 | 3 | 1 | 1 | 0 | 6 | 12 | 3 | 4 | 2 |
| NM_016271 | RNF138 | 3551 | 6 | 2 | 1 | 1 | 1 | 3 | 1 | 1 | 2 | 3 | 1 | 4 | 6 |
| NM_016272 | TOB2 | 4565 | 9 | 4 | 4 | 4 | 5 | 3 | 4 | 3 | 3 | 5 | 9 | 5 | 3 |
| NM_016274 | PLEKHO1 | 1615 | 22 | 9 | 8 | 9 | 15 | 10 | 2 | 1 | 13 | 4 | 4 | 22 | 10 |
| NM_016275 | SELT | 3490 | 5 | 3 | 2 | 1 | 3 | 5 | 4 | 3 | 3 | 2 | 1 | 5 | 3 |

| transcript | gene | transcript length (nt) | max | mean | adipose | colon | heart | hypothala mus | kidney | liver | lung | ovary | skeletalm uscle | spleen | testes |
|---|---|---|---|---|---|---|---|---|---|---|---|---|---|---|---|
| NM_016276 | SGK2 | 2120 | 19 | 5 | 7 | 5 | 0 | 2 | 19 | 17 | 0 | 0 | 0 | 0 | 1 |
| NM_016277 | RAB23 | 3011 | 6 | 3 | 1 | 3 | 4 | 4 | 1 | 1 | 3 | 6 | 2 | 1 | 3 |
| NM_016279 | CDH9 | 3075 | 6 | 1 | 0 | 0 | 0 | 6 | 2 | 0 | 0 | 0 | 0 | 0 | 0 |
| NM_016281 | TAOK3 | 4384 | 12 | 7 | 7 | 4 | 7 | 11 | 7 | 6 | 6 | 6 | 3 | 12 | 8 |
| NM_016282 | AK3 | 2642 | 8 | 4 | 4 | 2 | 7 | 3 | 8 | 6 | 4 | 3 | 6 | 4 | 2 |
| NM_016283 | TAF9 | 946 | 17 | 12 | 9 | 4 | 14 | 17 | 14 | 12 | 9 | 8 | 12 | 17 | 15 |
| NM_016284 | CNOT1 | 8431 | 27 | 13 | 8 | 10 | 11 | 9 | 13 | 13 | 10 | 15 | 10 | 21 | 27 |
| NM_016286 | DCXR | 829 | 60 | 11 | 5 | 4 | 7 | 8 | 14 | 60 | 3 | 3 | 8 | 3 | 3 |
| NM_016287 | HP1BP3 | 3921 | 20 | 16 | 15 | 16 | 14 | 20 | 17 | 13 | 14 | 18 | 9 | 18 | 18 |
| NM_016289 | CAB39 | 3828 | 17 | 11 | 10 | 6 | 12 | 17 | 10 | 8 | 12 | 5 | 13 | 14 | 9 |
| NM_016290 | UIMC1 | 2604 | 11 | 5 | 3 | 4 | 5 | 6 | 3 | 3 | 5 | 6 | 4 | 6 | 11 |
| NM_016291 | IP6K2 | 1798 | 11 | 6 | 6 | 8 | 4 | 8 | 5 | 3 | 4 | 8 | 3 | 7 | 11 |
| NM_016292 | TRAP1 | 2296 | 11 | 5 | 2 | 3 | 4 | 6 | 7 | 11 | 2 | 3 | 8 | 3 | 3 |
| NM_016293 | BIN2 | 2211 | 11 | 2 | 2 | 2 | 2 | 1 | 1 | 1 | 5 | 0 | 0 | 11 | 1 |
| NM_016297 | PCYOX1 | 5377 | 22 | 12 | 21 | 4 | 12 | 10 | 22 | 19 | 10 | 11 | 8 | 7 | 9 |
| NM_016298 | FBXO40 | 5722 | 20 | 3 | 0 | 0 | 20 | 0 | 0 | 0 | 0 | 0 | 13 | 0 | 0 |
| NM_016299 | HSPA14 | 1806 | 4 | 2 | 1 | 1 | 2 | 2 | 2 | 2 | 1 | 1 | 2 | 2 | 4 |
| NM_016300 | ARPP-21 | 3241 | 24 | 4 | 0 | 0 | 0 | 15 | 0 | 0 | 0 | 0 | 24 | 0 | 0 |
| NM_016301 | GPN3 | 1483 | 3 | 2 | 2 | 1 | 3 | 3 | 2 | 3 | 2 | 2 | 2 | 2 | 2 |
| NM_016302 | CRBN | 2201 | 14 | 7 | 4 | 3 | 6 | 14 | 5 | 5 | 5 | 9 | 6 | 9 | 9 |
| NM_016303 | WBP5 | 1089 | 49 | 24 | 23 | 9 | 32 | 27 | 24 | 6 | 21 | 49 | 4 | 25 | 38 |
| NM_016304 | RSL24D1 | 1473 | 8 | 5 | 3 | 2 | 5 | 6 | 4 | 3 | 8 | 7 | 4 | 6 | 6 |
| NM_016305 | SS18L2 | 815 | 5 | 3 | 2 | 1 | 3 | 5 | 2 | 3 | 3 | 2 | 1 | 4 | 2 |
| NM_016306 | DNAJB11 | 1689 | 92 | 30 | 19 | 12 | 47 | 23 | 24 | 92 | 21 | 13 | 9 | 47 | 25 |
| NM_016307 | PRRX2 | 1309 | 0 | 0 | 0 | 0 | 0 | 0 | 0 | 0 | 0 | 0 | 0 | 0 | 0 |
| NM_016308 | CMPK1 | 2929 | 65 | 35 | 33 | 16 | 42 | 24 | 37 | 65 | 44 | 22 | 25 | 45 | 26 |
| NM_016309 | LCMT1 | 1370 | 8 | 5 | 2 | 2 | 6 | 8 | 6 | 1 | 4 | 7 | 7 | 5 | 8 |
| NM_016310 | POLR3K | 796 | 3 | 2 | 1 | 1 | 2 | 3 | 2 | 2 | 1 | 1 | 1 | 2 | 0 |
| NM_016311 | ATPIF1 | 527 | 224 | 86 | 48 | 96 | 147 | 224 | 108 | 70 | 36 | 30 | 70 | 45 | 66 |
| NM_016312 | WBP11 | 2674 | 17 | 11 | 8 | 6 | 14 | 16 | 8 | 8 | 9 | 11 | 11 | 16 | 17 |
| NM_016315 | GULP1 | 3450 | 10 | 4 | 2 | 1 | 3 | 4 | 6 | 0 | 5 | 10 | 2 | 3 | 8 |
| NM_016316 | REV1 | 4734 | 4 | 2 | 1 | 1 | 2 | 2 | 2 | 1 | 2 | 4 | 2 | 3 | 4 |
| NM_016318 | P2RX2 | 1758 | 0 | 0 | 0 | 0 | 0 | 0 | 0 | 0 | 0 | 0 | 0 | 0 | 0 |
| NM_016319 | COPS7A | 1844 | 3 | 1 | 1 | 0 | 1 | 3 | 2 | 1 | 0 | 0 | 1 | 1 | 1 |
| NM_016320 | NUP98 | 6843 | 10 | 5 | 3 | 3 | 5 | 3 | 4 | 4 | 5 | 6 | 6 | 7 | 10 |
| NM_016321 | RHCG | 1935 | 2 | 0 | 0 | 0 | 0 | 0 | 2 | 0 | 0 | 0 | 0 | 0 | 0 |
| NM_016322 | RAB14 | 4362 | 17 | 10 | 8 | 4 | 8 | 17 | 11 | 16 | 10 | 8 | 6 | 13 | 11 |
| NM_016323 | HERC5 | 3513 | 14 | 3 | 1 | 0 | 1 | 1 | 1 | 0 | 2 | 4 | 1 | 2 | 14 |
| NM_016324 | ZNF274 | 2537 | 3 | 2 | 1 | 1 | 3 | 2 | 2 | 2 | 2 | 2 | 3 | 3 | 2 |
| NM_016325 | ZNF274 | 2742 | 4 | 2 | 2 | 1 | 3 | 2 | 3 | 2 | 2 | 3 | 3 | 4 | 2 |
| NM_016326 | CKLF | 408 | 9 | 3 | 3 | 4 | 2 | 1 | 2 | 1 | 3 | 2 | 1 | 9 | 4 |
| NM_016327 | UPB1 | 2067 | 5 | 1 | 0 | 0 | 0 | 0 | 3 | 5 | 0 | 0 | 0 | 0 | 0 |
| NM_016328 | GTF2IRD1 | 3425 | 2 | 0 | 0 | 0 | 1 | 0 | 1 | 0 | 0 | 1 | 2 | 0 | 0 |
| NM_016329 | SFMBT1 | 3495 | 4 | 1 | 1 | 1 | 1 | 1 | 2 | 1 | 1 | 1 | 2 | 1 | 4 |
| NM_016331 | ZNF639 | 3013 | 5 | 3 | 2 | 1 | 4 | 3 | 4 | 4 | 3 | 4 | 3 | 5 | 4 |
| NM_016332 | SEPX1 | 1367 | 1 | 0 | 0 | 0 | 0 | 0 | 1 | 1 | 0 | 0 | 0 | 0 | 0 |
| NM_016333 | SRRM2 | 9353 | 45 | 28 | 27 | 45 | 20 | 30 | 24 | 9 | 23 | 34 | 32 | 34 | 31 |
| NM_016334 | GPR89B | 2116 | 0 | 0 | 0 | 0 | 0 | 0 | 0 | 0 | 0 | 0 | 0 | 0 | 0 |
| NM_016335 | PRODH | 2211 | 3 | 1 | 0 | 0 | 1 | 3 | 2 | 0 | 2 | 0 | 3 | 0 | 0 |
| NM_016337 | EVL | 1842 | 9 | 3 | 3 | 3 | 1 | 9 | 2 | 2 | 2 | 2 | 1 | 6 | 2 |
| NM_016338 | IPO11 | 4388 | 10 | 3 | 2 | 1 | 3 | 3 | 3 | 2 | 2 | 3 | 2 | 3 | 10 |
| NM_016339 | RAPGEFL1 | 3727 | 2 | 0 | 0 | 2 | 0 | 2 | 0 | 0 | 0 | 0 | 0 | 0 | 0 |
| NM_016340 | RAPGEF6 | 8355 | 4 | 2 | 1 | 1 | 2 | 2 | 2 | 1 | 2 | 2 | 2 | 4 | 3 |
| NM_016341 | PLCE1 | 7991 | 5 | 2 | 2 | 4 | 3 | 2 | 3 | 0 | 2 | 2 | 2 | 5 | 2 |
| NM_016343 | CENPF | 10296 | 6 | 1 | 2 | 1 | 1 | 0 | 0 | 0 | 2 | 2 | 0 | 2 | 6 |
| NM_016346 | NR2E3 | 2103 | 0 | 0 | 0 | 0 | 0 | 0 | 0 | 0 | 0 | 0 | 0 | 0 | 0 |
| NM_016347 | NAT8B | 832 | 2 | 0 | 0 | 0 | 0 | 0 | 2 | 0 | 0 | 0 | 0 | 1 | 0 |
| NM_016350 | NIN | 6668 | 15 | 5 | 6 | 2 | 7 | 6 | 4 | 2 | 6 | 7 | 3 | 15 | 2 |
| NM_016351 | ADAM22 | 3335 | 11 | 2 | 2 | 0 | 0 | 11 | 1 | 0 | 0 | 1 | 0 | 1 | 1 |
| NM_016352 | CPA4 | 2816 | 1 | 0 | 1 | 0 | 0 | 0 | 0 | 0 | 0 | 0 | 0 | 0 | 0 |
| NM_016353 | ZDHHC2 | 4011 | 8 | 5 | 2 | 1 | 5 | 4 | 8 | 2 | 5 | 5 | 6 | 6 | 6 |
| NM_016354 | SLCO4A1 | 2776 | 0 | 0 | 0 | 0 | 0 | 0 | 0 | 0 | 0 | 0 | 0 | 0 | 0 |
| NM_016355 | DDX47 | 1817 | 19 | 13 | 11 | 7 | 10 | 18 | 11 | 19 | 10 | 12 | 10 | 15 | 19 |
| NM_016356 | DCDC2 | 4708 | 15 | 2 | 0 | 0 | 0 | 1 | 15 | 1 | 1 | 0 | 0 | 0 | 3 |
| NM_016357 | LIMA1 | 3738 | 35 | 11 | 35 | 13 | 7 | 4 | 12 | 4 | 11 | 7 | 4 | 13 | 8 |
| NM_016358 | IRX4 | 2221 | 0 | 0 | 0 | 0 | 0 | 0 | 0 | 0 | 0 | 0 | 0 | 0 | 0 |
| NM_016359 | NUSAP1 | 2420 | 3 | 1 | 1 | 3 | 1 | 0 | 0 | 1 | 2 | 2 | 1 | 2 | 2 |
| NM_016360 | TACO1 | 1457 | 7 | 2 | 1 | 1 | 1 | 2 | 3 | 7 | 1 | 0 | 1 | 1 | 1 |
| NM_016361 | ACP6 | 1808 | 2 | 1 | 1 | 1 | 1 | 1 | 2 | 1 | 1 | 1 | 1 | 1 | 1 |
| NM_016362 | GHRL | 528 | 0 | 0 | 0 | 0 | 0 | 0 | 0 | 0 | 0 | 0 | 0 | 0 | 0 |
| NM_016363 | GP6 | 2262 | 1 | 0 | 0 | 0 | 0 | 0 | 0 | 0 | 0 | 0 | 0 | 1 | 1 |
| NM_016364 | DUSP13 | 902 | 1 | 0 | 0 | 0 | 0 | 0 | 0 | 0 | 0 | 0 | 1 | 0 | 0 |
| NM_016366 | CABP2 | 957 | 0 | 0 | 0 | 0 | 0 | 0 | 0 | 0 | 0 | 0 | 0 | 0 | 0 |
| NM_016368 | ISYNA1 | 1825 | 1 | 0 | 0 | 0 | 0 | 0 | 0 | 0 | 0 | 0 | 0 | 0 | 1 |
| NM_016369 | CLDN18 | 3359 | 15 | 1 | 0 | 0 | 0 | 0 | 0 | 0 | 15 | 0 | 0 | 0 | 0 |
| NM_016370 | RAB9B | 1049 | 6 | 2 | 0 | 1 | 6 | 6 | 0 | 0 | 0 | 3 | 1 | 0 | 2 |
| NM_016371 | HSD17B7 | 1519 | 1 | 0 | 0 | 0 | 0 | 0 | 0 | 1 | 0 | 0 | 0 | 0 | 0 |

| transcript | gene | transcript length (nt) | max | mean | adipose | colon | heart | hypothalamus | kidney | liver | lung | ovary | skeletalmuscle | spleen | testes |
|---|---|---|---|---|---|---|---|---|---|---|---|---|---|---|---|
| NM_016372 | TPRA1 | 1869 | 1 | 0 | 0 | 0 | 0 | 0 | 0 | 0 | 0 | 0 | 0 | 1 | 0 |
| NM_016373 | WWOX | 2241 | 2 | 1 | 1 | 1 | 1 | 2 | 2 | 1 | 1 | 1 | 0 | 1 | 1 |
| NM_016374 | ARID4B | 6067 | 14 | 8 | 6 | 3 | 7 | 8 | 7 | 3 | 10 | 11 | 5 | 14 | 12 |
| NM_016376 | ANKFY1 | 7959 | 3 | 2 | 2 | 2 | 2 | 3 | 3 | 3 | 2 | 3 | 2 | 3 | 2 |
| NM_016377 | AKAP7 | 2799 | 16 | 6 | 5 | 2 | 5 | 7 | 4 | 16 | 4 | 9 | 3 | 5 | 10 |
| NM_016378 | VCX2 | 815 | 0 | 0 | 0 | 0 | 0 | 0 | 0 | 0 | 0 | 0 | 0 | 0 | 0 |
| NM_016379 | VCX3A | 994 | 0 | 0 | 0 | 0 | 0 | 0 | 0 | 0 | 0 | 0 | 0 | 0 | 0 |
| NM_016381 | TREX1 | 1816 | 0 | 0 | 0 | 0 | 0 | 0 | 0 | 0 | 0 | 0 | 0 | 0 | 0 |
| NM_016382 | CD244 | 2510 | 2 | 0 | 0 | 0 | 0 | 0 | 0 | 0 | 0 | 0 | 0 | 2 | 0 |
| NM_016383 | LUZP4 | 1728 | 2 | 0 | 0 | 0 | 0 | 0 | 0 | 0 | 0 | 0 | 0 | 0 | 2 |
| NM_016388 | TRAT1 | 1695 | 6 | 1 | 0 | 1 | 0 | 0 | 0 | 0 | 4 | 0 | 0 | 6 | 0 |
| NM_016390 | C9orf114 | 1856 | 6 | 3 | 3 | 3 | 3 | 5 | 4 | 4 | 2 | 4 | 1 | 6 | 3 |
| NM_016391 | NOP16 | 537 | 18 | 8 | 9 | 6 | 10 | 11 | 4 | 18 | 2 | 4 | 13 | 7 | 4 |
| NM_016395 | PTPLAD1 | 3208 | 24 | 11 | 6 | 6 | 4 | 24 | 19 | 23 | 7 | 11 | 2 | 9 | 11 |
| NM_016396 | CTDSPL2 | 4874 | 8 | 4 | 3 | 2 | 3 | 4 | 4 | 2 | 4 | 8 | 3 | 6 | 7 |
| NM_016399 | TRIAP1 | 1182 | 4 | 2 | 2 | 1 | 1 | 1 | 2 | 4 | 1 | 1 | 2 | 2 | 1 |
| NM_016400 | C15orf63 | 1331 | 43 | 19 | 16 | 11 | 31 | 20 | 17 | 43 | 11 | 11 | 24 | 16 | 11 |
| NM_016401 | C11orf73 | 1136 | 2 | 1 | 1 | 0 | 2 | 2 | 0 | 1 | 0 | 1 | 1 | 1 | 1 |
| NM_016403 | CWC15 | 1575 | 22 | 15 | 10 | 4 | 19 | 18 | 15 | 12 | 9 | 15 | 18 | 17 | 22 |
| NM_016404 | TRMT112 | 581 | 84 | 52 | 37 | 25 | 47 | 62 | 55 | 55 | 36 | 56 | 44 | 84 | 72 |
| NM_016406 | UFC1 | 1122 | 9 | 5 | 7 | 3 | 6 | 9 | 6 | 6 | 4 | 6 | 4 | 6 | 4 |
| NM_016407 | C20orf43 | 1649 | 22 | 14 | 14 | 6 | 13 | 22 | 13 | 12 | 9 | 12 | 12 | 18 | 21 |
| NM_016408 | CDK5RAP1 | 2092 | 3 | 2 | 2 | 2 | 2 | 3 | 3 | 3 | 2 | 2 | 2 | 3 | 2 |
| NM_016410 | CHMP5 | 1519 | 58 | 33 | 16 | 9 | 36 | 38 | 46 | 14 | 51 | 22 | 24 | 53 | 58 |
| NM_016413 | CPB2 | 1603 | 89 | 9 | 0 | 0 | 0 | 0 | 0 | 89 | 1 | 0 | 1 | 1 | 1 |
| NM_016417 | GLRX5 | 1171 | 52 | 26 | 12 | 13 | 52 | 24 | 32 | 25 | 12 | 22 | 36 | 18 | 40 |
| NM_016418 | NF2 | 6074 | 3 | 2 | 1 | 1 | 2 | 3 | 3 | 1 | 1 | 1 | 1 | 2 | 1 |
| NM_016422 | RNF141 | 4084 | 17 | 8 | 9 | 3 | 6 | 17 | 7 | 4 | 8 | 3 | 5 | 10 | 15 |
| NM_016423 | ZNF219 | 3080 | 4 | 1 | 4 | 1 | 2 | 1 | 1 | 1 | 1 | 1 | 1 | 1 | 1 |
| NM_016424 | LUC7L3 | 3515 | 33 | 21 | 18 | 9 | 18 | 23 | 24 | 9 | 25 | 28 | 16 | 30 | 33 |
| NM_016426 | GTSE1 | 3111 | 0 | 0 | 0 | 0 | 0 | 0 | 0 | 0 | 0 | 0 | 0 | 0 | 0 |
| NM_016427 | TCEB3B | 3046 | 0 | 0 | 0 | 0 | 0 | 0 | 0 | 0 | 0 | 0 | 0 | 0 | 0 |
| NM_016428 | ABI3 | 2109 | 3 | 1 | 0 | 0 | 0 | 1 | 0 | 1 | 1 | 0 | 0 | 3 | 0 |
| NM_016429 | COPZ2 | 903 | 15 | 5 | 15 | 3 | 6 | 2 | 3 | 4 | 2 | 2 | 8 | 2 | 3 |
| NM_016431 | MAPK8IP2 | 3165 | 4 | 0 | 0 | 0 | 0 | 4 | 0 | 0 | 0 | 0 | 0 | 0 | 0 |
| NM_016433 | GLTP | 2356 | 5 | 3 | 2 | 3 | 3 | 5 | 5 | 2 | 3 | 2 | 1 | 4 | 1 |
| NM_016434 | RTEL1 | 4462 | 0 | 0 | 0 | 0 | 0 | 0 | 0 | 0 | 0 | 0 | 0 | 0 | 0 |
| NM_016436 | PHF20 | 5889 | 6 | 3 | 2 | 1 | 3 | 5 | 2 | 1 | 4 | 3 | 5 | 6 | 4 |
| NM_016437 | TUBG2 | 1817 | 4 | 1 | 0 | 1 | 1 | 4 | 1 | 0 | 0 | 0 | 0 | 0 | 1 |
| NM_016438 | HIGD1B | 626 | 0 | 0 | 0 | 0 | 0 | 0 | 0 | 0 | 0 | 0 | 0 | 0 | 0 |
| NM_016440 | VRK3 | 2100 | 2 | 1 | 0 | 1 | 0 | 1 | 2 | 1 | 1 | 1 | 0 | 1 | 2 |
| NM_016441 | CRIM1 | 5584 | 27 | 17 | 27 | 6 | 20 | 17 | 24 | 2 | 20 | 14 | 22 | 25 | 13 |
| NM_016442 | ERAP1 | 5584 | 6 | 3 | 6 | 2 | 2 | 1 | 2 | 3 | 3 | 2 | 2 | 4 | 1 |
| NM_016445 | PLEK2 | 1460 | 3 | 1 | 0 | 3 | 0 | 0 | 1 | 3 | 0 | 0 | 0 | 0 | 1 |
| NM_016446 | TMEM8B | 2153 | 1 | 1 | 0 | 1 | 0 | 1 | 1 | 0 | 0 | 1 | 0 | 0 | 1 |
| NM_016447 | MPP6 | 2187 | 26 | 9 | 4 | 1 | 4 | 26 | 8 | 7 | 4 | 19 | 7 | 4 | 9 |
| NM_016448 | DTL | 4412 | 1 | 0 | 0 | 0 | 0 | 0 | 0 | 0 | 0 | 0 | 0 | 1 | 1 |
| NM_016449 | C22orf43 | 1279 | 2 | 0 | 0 | 0 | 0 | 0 | 0 | 0 | 0 | 0 | 0 | 0 | 2 |
| NM_016451 | COPB1 | 3490 | 10 | 6 | 6 | 3 | 6 | 5 | 8 | 10 | 5 | 5 | 3 | 7 | 7 |
| NM_016452 | CAPN9 | 2484 | 1 | 0 | 0 | 1 | 0 | 1 | 0 | 0 | 0 | 0 | 0 | 0 | 0 |
| NM_016453 | NCKIPSD | 2962 | 4 | 1 | 1 | 1 | 1 | 4 | 1 | 1 | 1 | 2 | 1 | 1 | 2 |
| NM_016454 | TMEM85 | 986 | 6 | 3 | 2 | 1 | 3 | 6 | 3 | 5 | 2 | 2 | 3 | 3 | 3 |
| NM_016456 | TMEM9 | 1538 | 9 | 3 | 1 | 3 | 2 | 9 | 5 | 3 | 2 | 4 | 2 | 1 | 4 |
| NM_016457 | PRKD2 | 3601 | 7 | 3 | 2 | 3 | 2 | 1 | 3 | 1 | 5 | 4 | 2 | 7 | 2 |
| NM_016458 | C8orf30A | 2405 | 1 | 0 | 0 | 0 | 0 | 0 | 1 | 1 | 0 | 0 | 1 | 0 | 0 |
| NM_016459 | MGC29506 | 827 | 2 | 0 | 0 | 1 | 0 | 0 | 0 | 0 | 0 | 0 | 0 | 2 | 0 |
| NM_016462 | TMEM14C | 1169 | 49 | 30 | 29 | 12 | 49 | 17 | 43 | 25 | 30 | 30 | 21 | 40 | 30 |
| NM_016463 | CXXC5 | 1447 | 1 | 0 | 0 | 0 | 0 | 1 | 1 | 1 | 0 | 0 | 0 | 1 | 0 |
| NM_016464 | TMEM138 | 1840 | 2 | 1 | 1 | 0 | 0 | 1 | 1 | 2 | 0 | 1 | 0 | 1 | 0 |
| NM_016466 | ANKRD39 | 898 | 1 | 0 | 0 | 0 | 0 | 1 | 0 | 1 | 0 | 0 | 1 | 0 | 0 |
| NM_016467 | ORMDL1 | 2182 | 12 | 5 | 4 | 2 | 5 | 5 | 5 | 4 | 6 | 4 | 3 | 12 | 8 |
| NM_016468 | COX16 | 1712 | 14 | 9 | 9 | 6 | 13 | 14 | 9 | 14 | 7 | 6 | 7 | 11 | 7 |
| NM_016470 | C20orf111 | 1572 | 30 | 14 | 11 | 5 | 10 | 11 | 7 | 11 | 12 | 16 | 23 | 17 | 30 |
| NM_016472 | C14orf129 | 2067 | 3 | 1 | 0 | 1 | 1 | 3 | 1 | 2 | 1 | 1 | 0 | 1 | 1 |
| NM_016474 | C3orf19 | 2960 | 3 | 2 | 2 | 1 | 2 | 2 | 3 | 1 | 2 | 3 | 2 | 2 | 2 |
| NM_016475 | JKAMP | 2374 | 5 | 3 | 3 | 1 | 3 | 5 | 4 | 3 | 3 | 2 | 2 | 4 | 5 |
| NM_016476 | ANAPC11 | 892 | 2 | 1 | 1 | 1 | 1 | 2 | 1 | 1 | 1 | 0 | 1 | 1 | 1 |
| NM_016478 | ZC3HC1 | 1914 | 1 | 1 | 1 | 0 | 1 | 1 | 1 | 1 | 0 | 1 | 1 | 1 | 1 |
| NM_016479 | SHISA5 | 2166 | 4 | 2 | 2 | 1 | 2 | 2 | 2 | 2 | 2 | 1 | 0 | 4 | 1 |
| NM_016480 | PAIP2 | 1483 | 60 | 24 | 23 | 8 | 31 | 60 | 14 | 20 | 17 | 19 | 18 | 27 | 28 |
| NM_016481 | C9orf156 | 1646 | 7 | 4 | 5 | 3 | 3 | 5 | 5 | 4 | 4 | 4 | 4 | 7 | 5 |
| NM_016483 | PHF7 | 2276 | 68 | 7 | 1 | 2 | 1 | 1 | 1 | 1 | 1 | 1 | 0 | 1 | 68 |
| NM_016484 | PDZD11 | 1246 | 7 | 4 | 4 | 2 | 6 | 7 | 6 | 3 | 2 | 3 | 2 | 5 | 3 |
| NM_016485 | VTA1 | 3244 | 3 | 2 | 1 | 0 | 2 | 2 | 2 | 1 | 1 | 2 | 3 | 2 | 2 |
| NM_016486 | TMEM69 | 1471 | 2 | 1 | 1 | 1 | 2 | 2 | 2 | 1 | 1 | 2 | 2 | 2 | 1 |
| NM_016487 | C6orf203 | 908 | 19 | 12 | 10 | 7 | 19 | 17 | 19 | 8 | 4 | 12 | 11 | 12 | 11 |
| NM_016488 | PPHLN1 | 3484 | 24 | 14 | 10 | 7 | 15 | 24 | 12 | 11 | 9 | 16 | 11 | 19 | 20 |

| transcript | gene | transcript length (nt) | max | mean | adipose | colon | heart | hypothalamus | kidney | liver | lung | ovary | skeletalmuscle | spleen | testes |
|---|---|---|---|---|---|---|---|---|---|---|---|---|---|---|---|
| NM_016489 | NT5C3 | 1552 | 5 | 3 | 2 | 1 | 2 | 3 | 3 | 2 | 2 | 3 | 5 | 4 | 2 |
| NM_016490 | FAM178B | 962 | 0 | 0 | 0 | 0 | 0 | 0 | 0 | 0 | 0 | 0 | 0 | 0 | 0 |
| NM_016491 | MRPL37 | 1483 | 6 | 3 | 2 | 2 | 3 | 4 | 3 | 6 | 1 | 2 | 3 | 2 | 2 |
| NM_016492 | RANGRF | 830 | 0 | 0 | 0 | 0 | 0 | 0 | 0 | 0 | 0 | 0 | 0 | 0 | 0 |
| NM_016494 | RNF181 | 716 | 5 | 3 | 3 | 2 | 4 | 4 | 5 | 4 | 3 | 2 | 2 | 5 | 2 |
| NM_016495 | TBC1D7 | 1280 | 0 | 0 | 0 | 0 | 0 | 0 | 0 | 0 | 0 | 0 | 0 | 0 | 0 |
| NM_016496 | 02 Mar | 1651 | 2 | 1 | 1 | 0 | 2 | 1 | 1 | 1 | 1 | 0 | 1 | 1 | 0 |
| NM_016497 | MRPL51 | 677 | 19 | 12 | 8 | 8 | 19 | 15 | 14 | 16 | 6 | 8 | 13 | 11 | 9 |
| NM_016498 | MTP18 | 1169 | 1 | 0 | 0 | 1 | 0 | 0 | 1 | 1 | 0 | 0 | 0 | 1 | 0 |
| NM_016499 | TMEM216 | 1275 | 1 | 0 | 0 | 0 | 0 | 0 | 1 | 0 | 0 | 1 | 0 | 1 | 1 |
| NM_016500 | CXorf26 | 1195 | 24 | 14 | 17 | 9 | 13 | 15 | 16 | 24 | 11 | 14 | 6 | 18 | 9 |
| NM_016504 | MRPL27 | 703 | 6 | 3 | 3 | 1 | 4 | 4 | 6 | 4 | 1 | 2 | 4 | 2 | 3 |
| NM_016505 | ZCCHC17 | 1602 | 107 | 43 | 11 | 6 | 58 | 38 | 34 | 6 | 40 | 55 | 61 | 58 | 107 |
| NM_016506 | KBTBD4 | 2460 | 2 | 1 | 1 | 1 | 1 | 2 | 1 | 1 | 1 | 1 | 1 | 1 | 1 |
| NM_016507 | CDK12 | 8290 | 7 | 4 | 3 | 3 | 3 | 2 | 4 | 4 | 4 | 4 | 2 | 6 | 7 |
| NM_016508 | CDKL3 | 1620 | 4 | 1 | 0 | 0 | 0 | 1 | 1 | 0 | 1 | 2 | 1 | 1 | 4 |
| NM_016509 | CLEC1B | 970 | 3 | 0 | 0 | 0 | 0 | 0 | 0 | 3 | 0 | 0 | 0 | 1 | 0 |
| NM_016510 | SCLY | 2460 | 4 | 1 | 1 | 0 | 1 | 1 | 4 | 4 | 1 | 0 | 1 | 1 | 1 |
| NM_016511 | CLEC1A | 1779 | 3 | 1 | 1 | 0 | 1 | 0 | 0 | 0 | 3 | 0 | 0 | 1 | 0 |
| NM_016512 | SPAG11B | 794 | 7 | 1 | 0 | 0 | 0 | 0 | 0 | 0 | 0 | 0 | 0 | 0 | 7 |
| NM_016513 | ICK | 6238 | 2 | 1 | 1 | 1 | 1 | 1 | 1 | 2 | 2 | 2 | 1 | 2 | 2 |
| NM_016516 | VPS54 | 3994 | 6 | 4 | 3 | 1 | 4 | 3 | 6 | 5 | 5 | 4 | 3 | 6 | 6 |
| NM_016518 | PIPOX | 2412 | 40 | 5 | 0 | 0 | 0 | 1 | 10 | 40 | 0 | 0 | 0 | 0 | 0 |
| NM_016519 | AMBN | 1976 | 0 | 0 | 0 | 0 | 0 | 0 | 0 | 0 | 0 | 0 | 0 | 0 | 0 |
| NM_016520 | C9orf78 | 1818 | 32 | 22 | 20 | 11 | 19 | 32 | 29 | 28 | 18 | 17 | 19 | 25 | 27 |
| NM_016521 | TFDP3 | 1680 | 1 | 0 | 0 | 1 | 0 | 0 | 0 | 0 | 0 | 0 | 0 | 0 | 1 |
| NM_016522 | NTM | 3375 | 10 | 1 | 2 | 1 | 1 | 10 | 0 | 0 | 1 | 0 | 0 | 0 | 0 |
| NM_016523 | KLRF1 | 1241 | 1 | 0 | 0 | 0 | 0 | 0 | 0 | 0 | 0 | 0 | 0 | 1 | 0 |
| NM_016524 | SYT17 | 1912 | 2 | 0 | 0 | 0 | 0 | 2 | 0 | 1 | 0 | 0 | 0 | 0 | 0 |
| NM_016525 | UBAP1 | 2735 | 9 | 6 | 6 | 3 | 6 | 9 | 5 | 8 | 6 | 5 | 7 | 6 | 9 |
| NM_016526 | BET1L | 2956 | 2 | 1 | 1 | 2 | 1 | 1 | 2 | 1 | 1 | 2 | 1 | 2 | 1 |
| NM_016527 | HAO2 | 1476 | 23 | 3 | 0 | 0 | 0 | 0 | 10 | 23 | 0 | 0 | 0 | 0 | 0 |
| NM_016529 | ATP8A2 | 5006 | 7 | 1 | 0 | 0 | 0 | 7 | 0 | 0 | 0 | 0 | 0 | 0 | 2 |
| NM_016530 | RAB8B | 4875 | 12 | 5 | 4 | 1 | 4 | 5 | 4 | 2 | 9 | 9 | 2 | 12 | 5 |
| NM_016531 | KLF3 | 5615 | 13 | 6 | 5 | 4 | 6 | 5 | 5 | 5 | 6 | 13 | 3 | 12 | 5 |
| NM_016532 | INPP5K | 2981 | 1 | 1 | 1 | 1 | 1 | 1 | 1 | 1 | 1 | 1 | 1 | 1 | 1 |
| NM_016533 | NINJ2 | 1062 | 1 | 1 | 1 | 0 | 1 | 1 | 0 | 1 | 1 | 0 | 1 | 1 | 0 |
| NM_016535 | ZNF581 | 1226 | 0 | 0 | 0 | 0 | 0 | 0 | 0 | 0 | 0 | 0 | 0 | 0 | 0 |
| NM_016536 | ZNF571 | 2276 | 2 | 1 | 1 | 0 | 1 | 1 | 1 | 0 | 1 | 2 | 0 | 1 | 2 |
| NM_016538 | SIRT7 | 1713 | 1 | 0 | 0 | 1 | 0 | 0 | 1 | 0 | 0 | 0 | 0 | 1 | 0 |
| NM_016538 | SIRT7 | 1720 | 1 | 0 | 0 | 1 | 0 | 0 | 1 | 0 | 0 | 0 | 0 | 1 | 0 |
| NM_016539 | SIRT6 | 1636 | 0 | 0 | 0 | 0 | 0 | 0 | 0 | 0 | 0 | 0 | 0 | 0 | 0 |
| NM_016540 | GPR83 | 4282 | 2 | 0 | 0 | 0 | 0 | 2 | 0 | 0 | 0 | 0 | 0 | 0 | 0 |
| NM_016541 | GNG13 | 984 | 0 | 0 | 0 | 0 | 0 | 0 | 0 | 0 | 0 | 0 | 0 | 0 | 0 |
| NM_016542 | MST4 | 3336 | 2 | 1 | 0 | 0 | 0 | 0 | 1 | 0 | 1 | 0 | 0 | 2 | 1 |
| NM_016543 | SIGLEC7 | 1475 | 7 | 1 | 1 | 0 | 0 | 1 | 0 | 2 | 1 | 0 | 0 | 7 | 0 |
| NM_016544 | DNAJC27 | 4853 | 2 | 1 | 0 | 0 | 1 | 1 | 1 | 0 | 0 | 1 | 0 | 0 | 2 |
| NM_016545 | IER5 | 2342 | 4 | 2 | 1 | 1 | 2 | 1 | 1 | 1 | 3 | 0 | 3 | 4 | 1 |
| NM_016546 | C1RL | 3319 | 15 | 4 | 3 | 1 | 3 | 1 | 3 | 15 | 3 | 4 | 1 | 5 | 3 |
| NM_016547 | SDF4 | 2138 | 6 | 3 | 4 | 3 | 4 | 2 | 4 | 6 | 2 | 1 | 2 | 4 | 3 |
| NM_016548 | GOLM1 | 3084 | 35 | 13 | 5 | 25 | 5 | 35 | 23 | 7 | 5 | 15 | 2 | 16 | 7 |
| NM_016551 | TM7SF3 | 4341 | 17 | 6 | 4 | 4 | 3 | 6 | 17 | 6 | 5 | 4 | 2 | 7 | 7 |
| NM_016552 | ANKMY1 | 3242 | 0 | 0 | 0 | 0 | 0 | 0 | 0 | 0 | 0 | 0 | 0 | 0 | 0 |
| NM_016553 | NUP62 | 3386 | 5 | 3 | 2 | 3 | 2 | 3 | 2 | 3 | 2 | 3 | 3 | 5 | 3 |
| NM_016556 | PSMC3IP | 1354 | 12 | 3 | 3 | 2 | 3 | 3 | 2 | 0 | 3 | 4 | 3 | 3 | 12 |
| NM_016557 | CCRL1 | 2224 | 1 | 1 | 1 | 0 | 1 | 0 | 1 | 1 | 1 | 1 | 0 | 1 | 1 |
| NM_016558 | SCAND1 | 883 | 1 | 0 | 0 | 0 | 0 | 0 | 0 | 1 | 0 | 0 | 0 | 0 | 0 |
| NM_016559 | PEX5L | 3577 | 27 | 3 | 0 | 0 | 0 | 27 | 0 | 0 | 0 | 0 | 0 | 0 | 1 |
| NM_016561 | BFAR | 3042 | 9 | 7 | 7 | 6 | 6 | 7 | 5 | 6 | 9 | 6 | 6 | 8 | 6 |
| NM_016562 | TLR7 | 4992 | 2 | 1 | 1 | 0 | 0 | 2 | 0 | 0 | 0 | 0 | 0 | 1 | 0 |
| NM_016563 | RASL12 | 2578 | 2 | 1 | 0 | 1 | 2 | 2 | 0 | 0 | 1 | 1 | 0 | 1 | 2 |
| NM_016564 | CEND1 | 1646 | 13 | 1 | 0 | 1 | 0 | 13 | 0 | 0 | 0 | 0 | 0 | 0 | 0 |
| NM_016565 | CHCHD8 | 816 | 6 | 2 | 1 | 1 | 1 | 1 | 2 | 6 | 1 | 1 | 1 | 2 | 1 |
| NM_016567 | BCCIP | 1444 | 46 | 22 | 21 | 16 | 23 | 22 | 22 | 46 | 14 | 13 | 19 | 20 | 29 |
| NM_016568 | RXFP3 | 1852 | 0 | 0 | 0 | 0 | 0 | 0 | 0 | 0 | 0 | 0 | 0 | 0 | 0 |
| NM_016569 | TBX3 | 4783 | 5 | 2 | 2 | 1 | 1 | 1 | 1 | 4 | 3 | 5 | 1 | 1 | 1 |
| NM_016570 | ERGIC2 | 1777 | 19 | 9 | 7 | 4 | 19 | 11 | 9 | 8 | 8 | 10 | 6 | 12 | 11 |
| NM_016571 | LGSN | 5633 | 1 | 0 | 0 | 0 | 0 | 0 | 1 | 0 | 0 | 0 | 0 | 0 | 0 |
| NM_016573 | GMIP | 3517 | 2 | 0 | 1 | 1 | 0 | 0 | 0 | 0 | 1 | 0 | 0 | 2 | 0 |
| NM_016574 | DRD2 | 2612 | 0 | 0 | 0 | 0 | 0 | 0 | 0 | 0 | 0 | 0 | 0 | 0 | 0 |
| NM_016576 | GMPR2 | 1867 | 7 | 4 | 4 | 2 | 3 | 5 | 6 | 4 | 4 | 4 | 2 | 7 | 3 |
| NM_016577 | RAB6B | 5560 | 27 | 3 | 1 | 1 | 2 | 27 | 0 | 0 | 0 | 0 | 0 | 1 | 1 |
| NM_016578 | RSF1 | 5134 | 21 | 12 | 8 | 3 | 11 | 19 | 10 | 5 | 11 | 21 | 11 | 17 | 17 |
| NM_016579 | CD320 | 1251 | 0 | 0 | 0 | 0 | 0 | 0 | 0 | 0 | 0 | 0 | 0 | 0 | 0 |
| NM_016580 | PCDH12 | 5182 | 2 | 0 | 1 | 0 | 0 | 0 | 0 | 0 | 1 | 0 | 0 | 2 | 0 |
| NM_016581 | ECSIT | 1684 | 1 | 1 | 0 | 1 | 1 | 1 | 1 | 1 | 0 | 1 | 1 | 0 | 0 |
| NM_016582 | SLC15A3 | 2116 | 7 | 2 | 5 | 1 | 2 | 1 | 1 | 1 | 4 | 1 | 1 | 7 | 1 |

| transcript | gene | transcript length (nt) | max | mean | adipose | colon | heart | hypothalamus | kidney | liver | lung | ovary | skeletalmuscle | spleen | testes |
|---|---|---|---|---|---|---|---|---|---|---|---|---|---|---|---|
| NM_016583 | PLUNC | 1023 | 7 | 1 | 0 | 0 | 0 | 0 | 0 | 0 | 7 | 0 | 0 | 0 | 0 |
| NM_016584 | IL23A | 1042 | 4 | 1 | 0 | 0 | 0 | 0 | 0 | 0 | 1 | 0 | 0 | 1 | 4 |
| NM_016585 | THEG | 1322 | 0 | 0 | 0 | 0 | 0 | 0 | 0 | 0 | 0 | 0 | 0 | 0 | 0 |
| NM_016586 | MBIP | 1648 | 15 | 8 | 5 | 2 | 5 | 11 | 8 | 8 | 15 | 11 | 3 | 8 | 9 |
| NM_016587 | CBX3 | 1851 | 10 | 7 | 6 | 3 | 7 | 8 | 9 | 9 | 7 | 5 | 4 | 10 | 8 |
| NM_016588 | NRN1 | 2056 | 27 | 5 | 10 | 1 | 3 | 27 | 1 | 1 | 3 | 1 | 3 | 4 | 1 |
| NM_016589 | C3orf1 | 1593 | 11 | 6 | 4 | 2 | 11 | 6 | 9 | 5 | 4 | 4 | 5 | 7 | 5 |
| NM_016591 | GCNT4 | 3435 | 1 | 0 | 0 | 0 | 0 | 1 | 1 | 0 | 0 | 1 | 0 | 1 | 1 |
| NM_016592 | GNAS | 2562 | 21 | 8 | 9 | 5 | 11 | 21 | 7 | 5 | 5 | 6 | 11 | 9 | 3 |
| NM_016593 | CYP39A1 | 2272 | 6 | 2 | 1 | 1 | 2 | 0 | 2 | 5 | 2 | 6 | 0 | 0 | 5 |
| NM_016594 | FKBP11 | 749 | 3 | 1 | 0 | 1 | 1 | 1 | 0 | 3 | 1 | 1 | 0 | 2 | 0 |
| NM_016598 | ZDHHC3 | 12685 | 2 | 1 | 2 | 2 | 1 | 1 | 2 | 1 | 1 | 1 | 1 | 2 | 2 |
| NM_016599 | MYOZ2 | 2590 | 128 | 15 | 0 | 0 | 128 | 0 | 0 | 0 | 0 | 0 | 34 | 0 | 1 |
| NM_016601 | KCNK9 | 1303 | 1 | 0 | 0 | 0 | 0 | 1 | 0 | 0 | 0 | 0 | 0 | 0 | 0 |
| NM_016602 | CCR10 | 1244 | 0 | 0 | 0 | 0 | 0 | 0 | 0 | 0 | 0 | 0 | 0 | 0 | 0 |
| NM_016603 | FAM13B | 5554 | 21 | 9 | 6 | 5 | 9 | 10 | 6 | 2 | 9 | 13 | 3 | 17 | 21 |
| NM_016604 | KDM3B | 6813 | 6 | 4 | 4 | 4 | 4 | 4 | 4 | 2 | 3 | 5 | 4 | 6 | 5 |
| NM_016605 | FAM53C | 4392 | 4 | 2 | 1 | 2 | 2 | 2 | 1 | 1 | 2 | 1 | 2 | 2 | 4 |
| NM_016606 | REEP2 | 2099 | 4 | 0 | 0 | 0 | 0 | 4 | 0 | 0 | 0 | 0 | 0 | 0 | 0 |
| NM_016607 | ARMCX3 | 3395 | 32 | 17 | 17 | 6 | 23 | 32 | 16 | 9 | 13 | 14 | 11 | 23 | 17 |
| NM_016608 | ARMCX1 | 2133 | 16 | 6 | 6 | 3 | 10 | 7 | 4 | 1 | 5 | 16 | 6 | 6 | 6 |
| NM_016609 | SLC22A17 | 2344 | 22 | 2 | 0 | 1 | 0 | 22 | 1 | 0 | 0 | 0 | 0 | 0 | 1 |
| NM_016612 | SLC25A37 | 1865 | 2 | 1 | 0 | 0 | 1 | 0 | 1 | 0 | 2 | 1 | 1 | 1 | 0 |
| NM_016613 | FAM198B | 4839 | 29 | 9 | 15 | 2 | 20 | 5 | 3 | 1 | 5 | 29 | 1 | 11 | 6 |
| NM_016614 | TTRAP | 1925 | 40 | 20 | 14 | 25 | 19 | 20 | 40 | 15 | 19 | 13 | 7 | 24 | 19 |
| NM_016615 | SLC6A13 | 2185 | 19 | 2 | 0 | 0 | 0 | 3 | 19 | 0 | 0 | 0 | 0 | 0 | 0 |
| NM_016616 | TXNDC3 | 2309 | 1 | 0 | 0 | 0 | 0 | 0 | 0 | 0 | 0 | 0 | 0 | 0 | 1 |
| NM_016617 | UFM1 | 2600 | 27 | 18 | 17 | 4 | 20 | 27 | 24 | 23 | 15 | 20 | 9 | 26 | 18 |
| NM_016618 | KRCC1 | 1753 | 10 | 6 | 7 | 4 | 8 | 6 | 6 | 5 | 5 | 6 | 4 | 10 | 7 |
| NM_016619 | PLAC8 | 1393 | 29 | 6 | 1 | 17 | 1 | 1 | 0 | 5 | 7 | 0 | 0 | 29 | 0 |
| NM_016620 | ZNF644 | 2056 | 12 | 7 | 5 | 2 | 7 | 8 | 5 | 5 | 5 | 11 | 8 | 11 | 12 |
| NM_016621 | PHF21A | 7178 | 5 | 2 | 2 | 2 | 3 | 3 | 2 | 1 | 3 | 3 | 1 | 5 | 4 |
| NM_016622 | MRPL35 | 3311 | 8 | 4 | 2 | 2 | 7 | 8 | 5 | 5 | 1 | 2 | 3 | 3 | 2 |
| NM_016623 | FAM49B | 2196 | 13 | 5 | 5 | 2 | 4 | 8 | 3 | 2 | 5 | 2 | 2 | 13 | 5 |
| NM_016625 | RSRC1 | 1628 | 22 | 13 | 14 | 5 | 13 | 22 | 10 | 9 | 9 | 16 | 9 | 15 | 15 |
| NM_016626 | MEX3C | 4140 | 9 | 4 | 2 | 2 | 3 | 3 | 3 | 2 | 4 | 6 | 2 | 8 | 9 |
| NM_016627 | AMZ2 | 1980 | 8 | 4 | 6 | 1 | 3 | 8 | 3 | 2 | 3 | 2 | 3 | 3 | 7 |
| NM_016628 | WAC | 3415 | 36 | 24 | 20 | 15 | 26 | 26 | 19 | 15 | 25 | 29 | 17 | 36 | 32 |
| NM_016630 | SPG21 | 1798 | 3 | 2 | 1 | 1 | 2 | 1 | 2 | 2 | 2 | 2 | 1 | 3 | 2 |
| NM_016631 | C21orf66 | 3995 | 4 | 3 | 3 | 2 | 2 | 2 | 3 | 2 | 3 | 3 | 2 | 4 | 4 |
| NM_016632 | ARL17A | 850 | 2 | 1 | 1 | 1 | 0 | 0 | 1 | 0 | 1 | 2 | 1 | 2 | 2 |
| NM_016633 | AHSP | 506 | 1 | 0 | 0 | 0 | 0 | 0 | 0 | 0 | 1 | 0 | 0 | 0 | 0 |
| NM_016638 | ARL6IP4 | 1318 | 35 | 25 | 22 | 17 | 35 | 30 | 21 | 22 | 19 | 27 | 27 | 33 | 21 |
| NM_016639 | TNFRSF12A | 1032 | 2 | 1 | 0 | 0 | 2 | 1 | 1 | 1 | 0 | 0 | 0 | 0 | 0 |
| NM_016640 | MRPS30 | 1670 | 5 | 3 | 2 | 2 | 2 | 5 | 3 | 3 | 1 | 2 | 3 | 3 | 2 |
| NM_016641 | GDE1 | 2937 | 8 | 5 | 6 | 5 | 7 | 8 | 5 | 6 | 7 | 2 | 3 | 4 | 4 |
| NM_016642 | SPTBN5 | 11723 | 0 | 0 | 0 | 0 | 0 | 0 | 0 | 0 | 0 | 0 | 0 | 0 | 0 |
| NM_016643 | ZNF771 | 1454 | 2 | 1 | 1 | 0 | 1 | 2 | 2 | 2 | 1 | 1 | 1 | 0 | 1 |
| NM_016644 | PRR16 | 1763 | 1 | 0 | 0 | 0 | 1 | 1 | 0 | 0 | 1 | 0 | 0 | 0 | 0 |
| NM_016647 | C8orf55 | 2264 | 1 | 0 | 0 | 0 | 0 | 0 | 1 | 1 | 0 | 0 | 0 | 0 | 0 |
| NM_016648 | LARP7 | 2286 | 15 | 7 | 6 | 1 | 15 | 7 | 10 | 5 | 6 | 8 | 5 | 11 | 9 |
| NM_016649 | ESF1 | 3216 | 11 | 7 | 7 | 2 | 9 | 10 | 7 | 5 | 4 | 9 | 9 | 8 | 11 |
| NM_016651 | DACT1 | 3861 | 3 | 1 | 1 | 1 | 1 | 2 | 1 | 0 | 1 | 3 | 0 | 1 | 1 |
| NM_016652 | CRNKL1 | 4406 | 15 | 9 | 5 | 3 | 8 | 10 | 9 | 7 | 6 | 15 | 13 | 12 | 12 |
| NM_016653 | ZAK | 3862 | 16 | 5 | 4 | 1 | 14 | 1 | 0 | 3 | 2 | 5 | 16 | 2 | 2 |
| NM_016654 | GABPB1 | 3016 | 5 | 2 | 1 | 1 | 2 | 2 | 2 | 2 | 2 | 3 | 2 | 5 | 3 |
| NM_016655 | GABPB1 | 1611 | 6 | 3 | 2 | 1 | 2 | 2 | 2 | 2 | 2 | 4 | 2 | 6 | 5 |
| NM_016656 | RRAGB | 2207 | 8 | 3 | 2 | 2 | 2 | 8 | 3 | 0 | 2 | 3 | 2 | 3 | 4 |
| NM_016657 | KDELR3 | 928 | 5 | 3 | 3 | 2 | 5 | 1 | 1 | 3 | 3 | 5 | 2 | 2 | 3 |
| NM_016724 | FOLR1 | 1195 | 18 | 3 | 0 | 0 | 0 | 6 | 18 | 0 | 12 | 0 | 0 | 0 | 0 |
| NM_016725 | FOLR1 | 1129 | 19 | 4 | 0 | 0 | 0 | 7 | 19 | 0 | 12 | 0 | 0 | 0 | 0 |
| NM_016729 | FOLR1 | 964 | 23 | 4 | 0 | 0 | 0 | 8 | 23 | 0 | 15 | 0 | 0 | 0 | 0 |
| NM_016732 | RALY | 1575 | 5 | 4 | 3 | 4 | 5 | 5 | 4 | 3 | 4 | 4 | 4 | 5 | 4 |
| NM_016733 | LIMK2 | 3848 | 4 | 2 | 1 | 2 | 1 | 2 | 2 | 1 | 4 | 1 | 1 | 4 | 2 |
| NM_016734 | PAX5 | 3650 | 2 | 0 | 0 | 0 | 0 | 0 | 0 | 0 | 0 | 0 | 0 | 2 | 0 |
| NM_016815 | GYPC | 1014 | 53 | 20 | 53 | 9 | 29 | 4 | 7 | 4 | 19 | 25 | 18 | 45 | 10 |
| NM_016816 | OAS1 | 1663 | 4 | 2 | 4 | 2 | 1 | 1 | 2 | 2 | 2 | 1 | 2 | 3 | 1 |
| NM_016817 | OAS2 | 3539 | 3 | 1 | 1 | 0 | 1 | 0 | 0 | 0 | 1 | 1 | 1 | 3 | 0 |
| NM_016818 | ABCG1 | 2959 | 16 | 4 | 1 | 3 | 3 | 4 | 1 | 1 | 6 | 2 | 7 | 16 | 2 |
| NM_016819 | OGG1 | 1878 | 1 | 0 | 0 | 0 | 0 | 0 | 1 | 0 | 0 | 0 | 0 | 0 | 0 |
| NM_016820 | OGG1 | 1651 | 1 | 0 | 0 | 0 | 0 | 0 | 1 | 0 | 0 | 0 | 0 | 0 | 0 |
| NM_016821 | OGG1 | 2151 | 1 | 0 | 0 | 0 | 0 | 0 | 1 | 0 | 0 | 0 | 0 | 0 | 0 |
| NM_016823 | CRK | 2397 | 10 | 6 | 7 | 4 | 5 | 6 | 5 | 10 | 5 | 4 | 3 | 6 | 6 |
| NM_016824 | ADD3 | 4444 | 28 | 12 | 9 | 6 | 16 | 28 | 20 | 2 | 10 | 11 | 3 | 10 | 17 |
| NM_016826 | OGG1 | 1950 | 1 | 0 | 0 | 0 | 0 | 0 | 1 | 0 | 0 | 0 | 0 | 0 | 0 |
| NM_016827 | OGG1 | 1768 | 1 | 0 | 0 | 0 | 0 | 0 | 1 | 0 | 0 | 0 | 0 | 0 | 0 |
| NM_016828 | OGG1 | 2251 | 1 | 0 | 0 | 0 | 0 | 0 | 1 | 0 | 0 | 0 | 0 | 0 | 0 |

| | | transcript | | | | | | hypothala | | | | | skeletalm | | |
|---|---|---|---|---|---|---|---|---|---|---|---|---|---|---|---|
| transcript | gene | length (nt) | max | mean | adipose | colon | heart | mus | kidney | liver | lung | ovary | uscle | spleen | testes |
| NM_016829 | OGG1 | 2204 | 1 | 0 | 0 | 0 | 0 | 0 | 1 | 0 | 0 | 0 | 0 | 0 | 0 |
| NM_016830 | VAMP1 | 1091 | 1 | 1 | 0 | 1 | 1 | 1 | 1 | 0 | 1 | 1 | 0 | 1 | 1 |
| NM_016831 | PER3 | 6203 | 14 | 6 | 4 | 4 | 4 | 9 | 4 | 3 | 4 | 14 | 6 | 3 | 6 |
| NM_016834 | MAPT | 5636 | 27 | 4 | 2 | 1 | 1 | 27 | 3 | 0 | 0 | 0 | 5 | 0 | 0 |
| NM_016835 | MAPT | 6761 | 23 | 3 | 2 | 1 | 1 | 23 | 3 | 0 | 0 | 0 | 4 | 0 | 0 |
| NM_016836 | RBMS1 | 4285 | 10 | 4 | 6 | 2 | 6 | 1 | 4 | 1 | 7 | 5 | 2 | 10 | 3 |
| NM_016841 | MAPT | 5543 | 27 | 4 | 2 | 1 | 1 | 27 | 4 | 0 | 0 | 0 | 5 | 0 | 0 |
| NM_016848 | SHC3 | 2407 | 6 | 1 | 0 | 0 | 0 | 6 | 0 | 0 | 0 | 0 | 0 | 0 | 0 |
| NM_016929 | CLIC5 | 5711 | 20 | 6 | 1 | 2 | 20 | 1 | 7 | 0 | 7 | 0 | 17 | 7 | 0 |
| NM_016930 | STX18 | 2157 | 7 | 4 | 4 | 3 | 3 | 5 | 7 | 4 | 2 | 4 | 2 | 4 | 7 |
| NM_016931 | NOX4 | 4378 | 21 | 3 | 2 | 0 | 3 | 0 | 21 | 0 | 1 | 1 | 1 | 0 | 1 |
| NM_016932 | SIX2 | 2153 | 1 | 0 | 0 | 0 | 0 | 0 | 0 | 0 | 0 | 0 | 1 | 0 | 0 |
| NM_016936 | UBN1 | 6477 | 3 | 2 | 2 | 2 | 2 | 2 | 2 | 2 | 1 | 1 | 1 | 3 | 2 |
| NM_016937 | POLA1 | 5447 | 4 | 2 | 2 | 2 | 2 | 2 | 2 | 1 | 2 | 4 | 3 | 4 | 3 |
| NM_016938 | EFEMP2 | 2003 | 2 | 1 | 2 | 1 | 2 | 1 | 1 | 0 | 1 | 1 | 0 | 1 | 1 |
| NM_016940 | RWDD2B | 1703 | 3 | 1 | 1 | 1 | 1 | 1 | 2 | 3 | 1 | 1 | 3 | 1 | 2 |
| NM_016941 | DLL3 | 2383 | 0 | 0 | 0 | 0 | 0 | 0 | 0 | 0 | 0 | 0 | 0 | 0 | 0 |
| NM_016943 | TAS2R3 | 1101 | 1 | 0 | 0 | 0 | 0 | 1 | 0 | 0 | 0 | 1 | 0 | 1 | 0 |
| NM_016944 | TAS2R4 | 900 | 1 | 0 | 0 | 0 | 0 | 0 | 1 | 0 | 0 | 1 | 0 | 0 | 1 |
| NM_016945 | TAS2R16 | 996 | 0 | 0 | 0 | 0 | 0 | 0 | 0 | 0 | 0 | 0 | 0 | 0 | 0 |
| NM_016946 | F11R | 4830 | 13 | 5 | 4 | 4 | 2 | 1 | 6 | 8 | 13 | 2 | 1 | 8 | 2 |
| NM_016948 | PARD6A | 1263 | 0 | 0 | 0 | 0 | 0 | 0 | 0 | 0 | 0 | 0 | 0 | 0 | 0 |
| NM_016950 | SPOCK3 | 2988 | 34 | 3 | 0 | 1 | 0 | 34 | 0 | 0 | 0 | 1 | 0 | 0 | 1 |
| NM_016951 | CKLF | 663 | 9 | 3 | 3 | 3 | 2 | 1 | 2 | 1 | 3 | 2 | 1 | 9 | 3 |
| NM_016952 | CDON | 8047 | 4 | 1 | 1 | 1 | 1 | 0 | 0 | 0 | 0 | 4 | 0 | 0 | 1 |
| NM_016953 | PDE11A | 9278 | 1 | 0 | 1 | 0 | 0 | 0 | 0 | 1 | 1 | 0 | 1 | 0 | 0 |
| NM_016954 | TBX22 | 2248 | 3 | 0 | 0 | 0 | 0 | 0 | 0 | 0 | 0 | 0 | 0 | 0 | 3 |
| NM_016955 | SEPSECS | 5644 | 4 | 1 | 1 | 0 | 1 | 1 | 2 | 4 | 1 | 2 | 1 | 1 | 2 |
| NM_017409 | HOXC10 | 1959 | 18 | 3 | 2 | 0 | 0 | 0 | 10 | 0 | 0 | 3 | 18 | 0 | 1 |
| NM_017410 | HOXC13 | 2396 | 0 | 0 | 0 | 0 | 0 | 0 | 0 | 0 | 0 | 0 | 0 | 0 | 0 |
| NM_017411 | SMN2 | 1627 | 0 | 0 | 0 | 0 | 0 | 0 | 0 | 0 | 0 | 0 | 0 | 0 | 0 |
| NM_017412 | FZD3 | 3912 | 11 | 3 | 0 | 1 | 1 | 11 | 2 | 0 | 1 | 6 | 1 | 1 | 6 |
| NM_017413 | APLN | 3116 | 0 | 0 | 0 | 0 | 0 | 0 | 0 | 0 | 0 | 0 | 0 | 0 | 0 |
| NM_017414 | USP18 | 2035 | 2 | 1 | 2 | 0 | 1 | 0 | 1 | 1 | 1 | 0 | 1 | 1 | 1 |
| NM_017415 | KLHL3 | 6806 | 1 | 1 | 0 | 0 | 1 | 1 | 1 | 0 | 0 | 1 | 0 | 0 | 1 |
| NM_017416 | IL1RAPL2 | 2985 | 0 | 0 | 0 | 0 | 0 | 0 | 0 | 0 | 0 | 0 | 0 | 0 | 0 |
| NM_017417 | GALNT8 | 2135 | 2 | 0 | 0 | 2 | 0 | 0 | 0 | 0 | 0 | 0 | 0 | 0 | 0 |
| NM_017418 | 01 Dec | 1251 | 0 | 0 | 0 | 0 | 0 | 0 | 0 | 0 | 0 | 0 | 0 | 0 | 0 |
| NM_017419 | ACCN5 | 1692 | 0 | 0 | 0 | 0 | 0 | 0 | 0 | 0 | 0 | 0 | 0 | 0 | 0 |
| NM_017420 | SIX4 | 6215 | 2 | 0 | 0 | 0 | 0 | 0 | 0 | 0 | 0 | 0 | 2 | 0 | 0 |
| NM_017421 | COQ3 | 1265 | 10 | 3 | 3 | 3 | 10 | 6 | 4 | 4 | 1 | 2 | 3 | 2 | 2 |
| NM_017422 | CALML5 | 876 | 0 | 0 | 0 | 0 | 0 | 0 | 0 | 0 | 0 | 0 | 0 | 0 | 0 |
| NM_017423 | GALNT7 | 4307 | 6 | 3 | 2 | 6 | 2 | 3 | 2 | 0 | 3 | 3 | 1 | 4 | 5 |
| NM_017424 | CECR1 | 3927 | 7 | 2 | 2 | 1 | 2 | 1 | 1 | 1 | 3 | 1 | 0 | 7 | 1 |
| NM_017425 | SPA17 | 935 | 8 | 1 | 0 | 0 | 1 | 2 | 2 | 0 | 2 | 0 | 0 | 0 | 8 |
| NM_017426 | NUP54 | 2354 | 10 | 5 | 6 | 2 | 4 | 5 | 4 | 6 | 4 | 8 | 4 | 7 | 10 |
| NM_017429 | BCMO1 | 2427 | 2 | 1 | 0 | 1 | 0 | 1 | 2 | 2 | 0 | 0 | 0 | 0 | 1 |
| NM_017431 | PRKAG3 | 2299 | 0 | 0 | 0 | 0 | 0 | 0 | 0 | 0 | 0 | 0 | 0 | 0 | 0 |
| NM_017432 | PTOV1 | 1884 | 7 | 4 | 1 | 3 | 4 | 7 | 4 | 3 | 3 | 6 | 7 | 3 | 4 |
| NM_017433 | MYO3A | 5784 | 1 | 0 | 0 | 0 | 0 | 0 | 0 | 0 | 0 | 0 | 0 | 0 | 1 |
| NM_017434 | DUOX1 | 5677 | 2 | 0 | 0 | 0 | 0 | 0 | 0 | 0 | 2 | 0 | 0 | 0 | 0 |
| NM_017435 | SLCO1C1 | 3363 | 1 | 0 | 0 | 0 | 0 | 1 | 0 | 0 | 0 | 0 | 0 | 0 | 0 |
| NM_017436 | A4GALT | 2092 | 2 | 1 | 1 | 0 | 1 | 0 | 2 | 0 | 1 | 0 | 0 | 1 | 0 |
| NM_017437 | CPSF2 | 5041 | 9 | 5 | 4 | 2 | 5 | 4 | 5 | 5 | 6 | 5 | 6 | 9 | 9 |
| NM_017438 | SETD4 | 3342 | 1 | 1 | 1 | 1 | 1 | 1 | 1 | 1 | 0 | 1 | 0 | 1 | 1 |
| NM_017439 | PION | 3251 | 4 | 1 | 1 | 1 | 1 | 1 | 2 | 2 | 3 | 1 | 0 | 4 | 0 |
| NM_017440 | MDM1 | 2969 | 3 | 1 | 1 | 1 | 1 | 2 | 1 | 0 | 1 | 1 | 0 | 2 | 3 |
| NM_017442 | TLR9 | 3868 | 1 | 0 | 0 | 0 | 0 | 0 | 0 | 0 | 0 | 0 | 0 | 1 | 0 |
| NM_017443 | POLE3 | 2262 | 15 | 8 | 7 | 5 | 8 | 9 | 5 | 9 | 5 | 6 | 9 | 12 | 15 |
| NM_017444 | CHRAC1 | 2500 | 2 | 1 | 1 | 1 | 1 | 1 | 1 | 2 | 1 | 1 | 1 | 2 | 1 |
| NM_017446 | MRPL39 | 1082 | 4 | 2 | 2 | 1 | 2 | 2 | 3 | 3 | 1 | 3 | 1 | 1 | 4 |
| NM_017447 | C21orf91 | 5430 | 5 | 2 | 1 | 0 | 1 | 3 | 1 | 1 | 2 | 2 | 1 | 2 | 5 |
| NM_017448 | LDHC | 1229 | 3 | 0 | 0 | 0 | 0 | 0 | 0 | 0 | 0 | 0 | 0 | 0 | 3 |
| NM_017449 | EPHB2 | 4866 | 3 | 1 | 1 | 3 | 0 | 1 | 0 | 0 | 0 | 1 | 0 | 0 | 1 |
| NM_017450 | BAIAP2 | 3188 | 2 | 1 | 0 | 1 | 0 | 2 | 1 | 2 | 1 | 0 | 0 | 0 | 1 |
| NM_017451 | BAIAP2 | 2894 | 2 | 1 | 0 | 1 | 0 | 2 | 1 | 2 | 1 | 0 | 0 | 0 | 1 |
| NM_017452 | STAU1 | 3395 | 14 | 9 | 7 | 6 | 11 | 11 | 12 | 14 | 8 | 7 | 9 | 10 | 9 |
| NM_017453 | STAU1 | 3684 | 13 | 9 | 6 | 5 | 10 | 10 | 11 | 13 | 8 | 7 | 8 | 9 | 9 |
| NM_017454 | STAU1 | 3320 | 14 | 10 | 7 | 6 | 11 | 11 | 12 | 14 | 9 | 7 | 9 | 10 | 10 |
| NM_017455 | NPTN | 2105 | 21 | 9 | 5 | 7 | 15 | 21 | 10 | 4 | 8 | 6 | 3 | 12 | 13 |
| NM_017456 | CYTH1 | 3308 | 16 | 6 | 5 | 7 | 5 | 6 | 3 | 4 | 10 | 3 | 6 | 16 | 4 |
| NM_017457 | CYTH2 | 4606 | 4 | 2 | 2 | 3 | 1 | 4 | 2 | 1 | 2 | 2 | 1 | 2 | 3 |
| NM_017458 | MVP | 2833 | 17 | 8 | 6 | 17 | 5 | 3 | 15 | 6 | 9 | 4 | 2 | 9 | 7 |
| NM_017459 | MFAP2 | 1082 | 3 | 1 | 1 | 1 | 1 | 0 | 0 | 0 | 1 | 3 | 0 | 0 | 1 |
| NM_017460 | CYP3A4 | 2768 | 101 | 9 | 0 | 0 | 0 | 0 | 0 | 101 | 0 | 1 | 0 | 1 | 0 |
| NM_017481 | UBQLN3 | 2345 | 6 | 1 | 0 | 0 | 0 | 0 | 0 | 0 | 0 | 0 | 0 | 0 | 6 |
| NM_017482 | ADD2 | 3763 | 5 | 0 | 0 | 0 | 0 | 5 | 0 | 0 | 0 | 0 | 0 | 0 | 0 |

| transcript | gene | transcript length (nt) | max | mean | adipose | colon | heart | hypothalamus | kidney | liver | lung | ovary | skeletalmuscle | spleen | testes |
|---|---|---|---|---|---|---|---|---|---|---|---|---|---|---|---|
| NM_017483 | ADD2 | 3022 | 29 | 3 | 0 | 1 | 0 | 29 | 0 | 0 | 0 | 0 | 0 | 0 | 0 |
| NM_017484 | ADD2 | 2845 | 5 | 1 | 0 | 0 | 0 | 5 | 0 | 0 | 0 | 0 | 0 | 0 | 0 |
| NM_017488 | ADD2 | 4026 | 22 | 2 | 0 | 0 | 0 | 22 | 0 | 0 | 0 | 0 | 0 | 0 | 0 |
| NM_017489 | TERF1 | 2960 | 6 | 3 | 3 | 1 | 3 | 6 | 5 | 3 | 3 | 5 | 2 | 4 | 4 |
| NM_017490 | MARK2 | 4515 | 5 | 3 | 2 | 3 | 2 | 5 | 2 | 2 | 2 | 2 | 2 | 5 | 2 |
| NM_017491 | WDR1 | 3143 | 12 | 8 | 9 | 9 | 10 | 9 | 12 | 6 | 7 | 6 | 4 | 12 | 7 |
| NM_017493 | OTUD4 | 938 | 4 | 3 | 2 | 1 | 3 | 3 | 2 | 3 | 2 | 2 | 3 | 4 | 4 |
| NM_017495 | RBM38 | 2378 | 8 | 2 | 0 | 3 | 3 | 1 | 1 | 0 | 1 | 2 | 8 | 4 | 2 |
| NM_017503 | SURF2 | 844 | 13 | 6 | 2 | 3 | 5 | 7 | 8 | 8 | 3 | 4 | 6 | 5 | 13 |
| NM_017504 | OR2M4 | 936 | 0 | 0 | 0 | 0 | 0 | 0 | 0 | 0 | 0 | 0 | 0 | 0 | 0 |
| NM_017506 | OR7A5 | 2138 | 0 | 0 | 0 | 0 | 0 | 0 | 0 | 0 | 0 | 0 | 0 | 0 | 0 |
| NM_017508 | SOX6 | 8978 | 2 | 1 | 0 | 0 | 1 | 1 | 2 | 0 | 0 | 0 | 1 | 0 | 1 |
| NM_017509 | KLK15 | 1309 | 0 | 0 | 0 | 0 | 0 | 0 | 0 | 0 | 0 | 0 | 0 | 0 | 0 |
| NM_017510 | TMED9 | 1393 | 117 | 34 | 32 | 36 | 32 | 33 | 22 | 117 | 26 | 12 | 8 | 37 | 23 |
| NM_017512 | ENOSF1 | 1851 | 3 | 1 | 0 | 1 | 0 | 0 | 3 | 2 | 1 | 1 | 0 | 1 | 1 |
| NM_017514 | PLXNA3 | 6745 | 0 | 0 | 0 | 0 | 0 | 0 | 0 | 0 | 0 | 0 | 0 | 0 | 0 |
| NM_017515 | SLC35F2 | 3168 | 1 | 0 | 0 | 1 | 0 | 1 | 1 | 0 | 0 | 0 | 0 | 1 | 1 |
| NM_017516 | RAB39 | 1782 | 2 | 1 | 0 | 0 | 0 | 1 | 1 | 0 | 1 | 0 | 0 | 2 | 1 |
| NM_017518 | HAUS7 | 1877 | 1 | 0 | 0 | 0 | 1 | 1 | 0 | 0 | 0 | 0 | 0 | 0 | 0 |
| NM_017519 | ARID1B | 8225 | 4 | 2 | 2 | 2 | 2 | 2 | 2 | 1 | 2 | 3 | 2 | 4 | 2 |
| NM_017520 | MPHOSPH8 | 3217 | 44 | 24 | 20 | 11 | 25 | 28 | 22 | 10 | 23 | 44 | 14 | 36 | 30 |
| NM_017521 | FEV | 1879 | 0 | 0 | 0 | 0 | 0 | 0 | 0 | 0 | 0 | 0 | 0 | 0 | 0 |
| NM_017522 | LRP8 | 3718 | 4 | 1 | 0 | 0 | 0 | 2 | 0 | 0 | 0 | 0 | 0 | 0 | 4 |
| NM_017523 | XAF1 | 3624 | 8 | 3 | 3 | 1 | 3 | 2 | 1 | 2 | 3 | 8 | 3 | 7 | 3 |
| NM_017525 | CDC42BPG | 4939 | 1 | 0 | 0 | 1 | 0 | 0 | 0 | 0 | 0 | 0 | 0 | 0 | 0 |
| NM_017526 | LEPROT | 4642 | 8 | 5 | 6 | 2 | 6 | 3 | 8 | 4 | 6 | 5 | 2 | 7 | 4 |
| NM_017527 | LY6K | 1708 | 0 | 0 | 0 | 0 | 0 | 0 | 0 | 0 | 0 | 0 | 0 | 0 | 0 |
| NM_017528 | WBSCR22 | 1230 | 10 | 5 | 3 | 2 | 6 | 5 | 4 | 4 | 2 | 3 | 5 | 4 | 10 |
| NM_017530 | ZNF821 | 1822 | 1 | 0 | 0 | 0 | 0 | 1 | 0 | 0 | 0 | 0 | 0 | 0 | 1 |
| NM_017533 | MYH4 | 6015 | 0 | 0 | 0 | 0 | 0 | 0 | 0 | 0 | 0 | 0 | 0 | 0 | 0 |
| NM_017534 | MYH2 | 6086 | 405 | 37 | 1 | 0 | 0 | 0 | 0 | 0 | 0 | 0 | 405 | 0 | 0 |
| NM_017539 | DNAH3 | 12351 | 1 | 0 | 0 | 0 | 0 | 0 | 0 | 0 | 1 | 0 | 0 | 0 | 1 |
| NM_017540 | GALNT10 | 4361 | 7 | 2 | 1 | 1 | 1 | 1 | 1 | 1 | 1 | 7 | 0 | 3 | 1 |
| NM_017541 | CRYGS | 842 | 2 | 1 | 1 | 1 | 0 | 1 | 1 | 0 | 1 | 0 | 0 | 2 | 1 |
| NM_017542 | POGK | 3852 | 5 | 3 | 2 | 2 | 2 | 5 | 3 | 2 | 3 | 3 | 1 | 4 | 3 |
| NM_017544 | NKRF | 3218 | 11 | 3 | 2 | 1 | 3 | 11 | 3 | 2 | 2 | 4 | 2 | 5 | 2 |
| NM_017545 | HAO1 | 1746 | 27 | 2 | 0 | 0 | 0 | 0 | 0 | 27 | 0 | 0 | 0 | 0 | 0 |
| NM_017546 | C2orf29 | 2518 | 9 | 6 | 4 | 5 | 8 | 6 | 7 | 8 | 4 | 5 | 6 | 8 | 9 |
| NM_017547 | FOXRED1 | 1951 | 1 | 1 | 1 | 0 | 0 | 1 | 1 | 1 | 0 | 0 | 0 | 1 | 1 |
| NM_017548 | CDV3 | 3458 | 22 | 12 | 14 | 4 | 15 | 8 | 8 | 9 | 11 | 8 | 22 | 20 | 11 |
| NM_017549 | EPDR1 | 2598 | 17 | 6 | 10 | 1 | 4 | 17 | 4 | 1 | 5 | 5 | 6 | 5 | 6 |
| NM_017550 | MIER2 | 2762 | 1 | 1 | 1 | 1 | 1 | 1 | 0 | 0 | 0 | 1 | 1 | 1 | 1 |
| NM_017551 | GRID1 | 5849 | 4 | 1 | 0 | 0 | 0 | 4 | 0 | 0 | 0 | 0 | 0 | 0 | 1 |
| NM_017552 | ATAD2B | 8053 | 6 | 3 | 2 | 2 | 3 | 2 | 3 | 2 | 4 | 4 | 3 | 6 | 5 |
| NM_017553 | INO80 | 6226 | 6 | 4 | 3 | 2 | 3 | 3 | 4 | 3 | 3 | 4 | 5 | 6 | 4 |
| NM_017554 | PARP14 | 7708 | 32 | 13 | 13 | 4 | 11 | 6 | 10 | 19 | 23 | 12 | 8 | 32 | 6 |
| NM_017556 | FBLIM1 | 3349 | 1 | 0 | 0 | 1 | 0 | 0 | 0 | 0 | 0 | 0 | 0 | 0 | 0 |
| NM_017558 | HYDIN | 3659 | 2 | 0 | 0 | 0 | 0 | 2 | 1 | 0 | 1 | 0 | 0 | 0 | 1 |
| NM_017559 | FNDC8 | 1355 | 0 | 0 | 0 | 0 | 0 | 0 | 0 | 0 | 0 | 0 | 0 | 0 | 0 |
| NM_017560 | ZNF853 | 3576 | 2 | 1 | 0 | 1 | 1 | 2 | 0 | 0 | 0 | 1 | 1 | 0 | 0 |
| NM_017561 | FAM22F | 2561 | 0 | 0 | 0 | 0 | 0 | 0 | 0 | 0 | 0 | 0 | 0 | 0 | 0 |
| NM_017563 | IL17RD | 8720 | 6 | 1 | 1 | 1 | 1 | 2 | 3 | 0 | 0 | 6 | 0 | 1 | 2 |
| NM_017564 | STAB2 | 8230 | 10 | 1 | 0 | 0 | 0 | 0 | 0 | 1 | 0 | 0 | 0 | 10 | 0 |
| NM_017565 | FAM20A | 4274 | 8 | 3 | 2 | 1 | 2 | 1 | 5 | 8 | 3 | 2 | 1 | 3 | 4 |
| NM_017566 | KLHDC4 | 1874 | 1 | 1 | 1 | 1 | 0 | 1 | 1 | 0 | 1 | 0 | 1 | 1 | 1 |
| NM_017567 | NAGK | 1574 | 8 | 3 | 4 | 3 | 3 | 4 | 3 | 2 | 3 | 2 | 1 | 8 | 2 |
| NM_017569 | FAM48A | 2924 | 7 | 3 | 1 | 2 | 2 | 1 | 3 | 1 | 2 | 3 | 2 | 4 | 7 |
| NM_017570 | OPLAH | 4011 | 0 | 0 | 0 | 0 | 0 | 0 | 0 | 0 | 0 | 0 | 0 | 0 | 0 |
| NM_017572 | MKNK2 | 1758 | 11 | 3 | 2 | 2 | 3 | 1 | 1 | 2 | 3 | 1 | 11 | 5 | 1 |
| NM_017573 | PCSK4 | 2661 | 0 | 0 | 0 | 0 | 0 | 0 | 0 | 0 | 0 | 0 | 0 | 0 | 0 |
| NM_017575 | SMG6 | 5968 | 10 | 6 | 5 | 8 | 5 | 8 | 6 | 2 | 5 | 10 | 4 | 6 | 10 |
| NM_017576 | KIF27 | 4651 | 5 | 1 | 1 | 0 | 1 | 1 | 1 | 0 | 2 | 2 | 1 | 1 | 5 |
| NM_017577 | GRAMD1C | 3761 | 4 | 1 | 0 | 1 | 0 | 3 | 4 | 1 | 1 | 0 | 0 | 1 | 4 |
| NM_017578 | ROPN1 | 1103 | 7 | 1 | 0 | 0 | 0 | 0 | 0 | 0 | 0 | 0 | 0 | 0 | 7 |
| NM_017579 | DMBT1 | 7656 | 3 | 0 | 0 | 1 | 0 | 0 | 0 | 0 | 3 | 0 | 0 | 0 | 0 |
| NM_017580 | ZRANB1 | 4942 | 15 | 8 | 4 | 3 | 8 | 6 | 5 | 7 | 6 | 11 | 15 | 7 | 12 |
| NM_017581 | CHRNA9 | 1892 | 0 | 0 | 0 | 0 | 0 | 0 | 0 | 0 | 0 | 0 | 0 | 0 | 0 |
| NM_017582 | UBE2Q1 | 3223 | 6 | 3 | 3 | 2 | 3 | 6 | 3 | 4 | 2 | 3 | 2 | 5 | 4 |
| NM_017583 | TRIM44 | 4337 | 43 | 24 | 20 | 19 | 22 | 43 | 35 | 17 | 22 | 18 | 10 | 32 | 26 |
| NM_017584 | MIOX | 1437 | 9 | 1 | 0 | 0 | 0 | 0 | 9 | 0 | 0 | 0 | 0 | 0 | 0 |
| NM_017585 | SLC2A6 | 2545 | 0 | 0 | 0 | 0 | 0 | 0 | 0 | 0 | 0 | 0 | 0 | 0 | 0 |
| NM_017586 | C9orf7 | 2755 | 1 | 0 | 0 | 1 | 0 | 0 | 1 | 1 | 0 | 0 | 0 | 1 | 0 |
| NM_017588 | WDR5 | 3155 | 3 | 2 | 2 | 2 | 2 | 2 | 2 | 3 | 1 | 2 | 2 | 2 | 3 |
| NM_017589 | BTG4 | 998 | 1 | 0 | 0 | 0 | 0 | 0 | 0 | 0 | 0 | 0 | 0 | 0 | 1 |
| NM_017590 | ZC3H7B | 5868 | 3 | 2 | 1 | 1 | 1 | 3 | 3 | 2 | 1 | 2 | 1 | 2 | 1 |
| NM_017592 | MED29 | 3567 | 6 | 3 | 2 | 1 | 2 | 6 | 4 | 3 | 2 | 4 | 2 | 4 | 3 |
| NM_017593 | BMP2K | 2666 | 6 | 3 | 3 | 1 | 3 | 5 | 3 | 2 | 2 | 2 | 1 | 6 | 5 |

| | | transcript | | | | | | hypothala | | | | | skeletalm | | |
|---|---|---|---|---|---|---|---|---|---|---|---|---|---|---|---|
| transcript | gene | length (nt) | max | mean | adipose | colon | heart | mus | kidney | liver | lung | ovary | uscle | spleen | testes |
| NM_017594 | DIRAS2 | 4107 | 25 | 2 | 0 | 0 | 0 | 25 | 0 | 0 | 0 | 0 | 0 | 0 | 0 |
| NM_017595 | NKIRAS2 | 2469 | 3 | 2 | 1 | 1 | 3 | 1 | 1 | 1 | 2 | 1 | 1 | 2 | 2 |
| NM_017596 | KIF21B | 9895 | 6 | 1 | 0 | 1 | 0 | 6 | 0 | 0 | 1 | 0 | 0 | 2 | 1 |
| NM_017599 | VEZT | 4562 | 10 | 6 | 5 | 3 | 8 | 8 | 5 | 4 | 6 | 9 | 10 | 6 | 6 |
| NM_017600 | GOLGA2L1 | 2263 | 1 | 0 | 0 | 0 | 0 | 0 | 1 | 0 | 0 | 0 | 0 | 0 | 0 |
| NM_017602 | OTUD5 | 2923 | 18 | 10 | 5 | 7 | 10 | 16 | 11 | 5 | 10 | 14 | 9 | 18 | 9 |
| NM_017607 | PPP1R12C | 2924 | 2 | 1 | 1 | 2 | 2 | 2 | 0 | 1 | 1 | 0 | 0 | 1 | 1 |
| NM_017610 | RNF111 | 5537 | 8 | 5 | 5 | 3 | 4 | 7 | 4 | 3 | 5 | 6 | 4 | 8 | 6 |
| NM_017611 | SLC43A3 | 2597 | 6 | 1 | 2 | 0 | 1 | 0 | 0 | 6 | 1 | 1 | 1 | 2 | 1 |
| NM_017612 | ZCCHC8 | 2864 | 4 | 2 | 2 | 2 | 2 | 1 | 3 | 2 | 3 | 3 | 2 | 4 | 3 |
| NM_017613 | DONSON | 2170 | 7 | 2 | 1 | 1 | 2 | 2 | 1 | 2 | 2 | 2 | 1 | 2 | 7 |
| NM_017614 | BHMT2 | 1993 | 60 | 12 | 13 | 1 | 2 | 1 | 60 | 50 | 0 | 1 | 1 | 0 | 3 |
| NM_017615 | NSMCE4A | 1399 | 7 | 5 | 7 | 5 | 5 | 4 | 5 | 5 | 3 | 4 | 4 | 7 | 2 |
| NM_017617 | NOTCH1 | 9295 | 2 | 1 | 0 | 1 | 1 | 1 | 1 | 0 | 1 | 0 | 0 | 2 | 0 |
| NM_017619 | RNPC3 | 765 | 1 | 1 | 1 | 1 | 0 | 1 | 1 | 0 | 1 | 1 | 0 | 1 | 1 |
| NM_017620 | ILF3 | 6119 | 14 | 10 | 6 | 9 | 10 | 12 | 9 | 6 | 7 | 13 | 11 | 14 | 12 |
| NM_017621 | ALKBH4 | 2065 | 0 | 0 | 0 | 0 | 0 | 0 | 0 | 0 | 0 | 0 | 0 | 0 | 0 |
| NM_017622 | C17orf59 | 1914 | 0 | 0 | 0 | 0 | 0 | 0 | 0 | 0 | 0 | 0 | 0 | 0 | 0 |
| NM_017623 | CNNM3 | 4916 | 3 | 1 | 0 | 1 | 1 | 1 | 1 | 2 | 1 | 2 | 3 | 1 | 1 |
| NM_017625 | ITLN1 | 1182 | 71 | 9 | 0 | 71 | 3 | 0 | 1 | 2 | 11 | 6 | 0 | 1 | 6 |
| NM_017626 | DNAJB12 | 3198 | 4 | 3 | 3 | 2 | 3 | 3 | 3 | 3 | 2 | 2 | 3 | 4 | 3 |
| NM_017628 | TET2 | 9215 | 4 | 2 | 2 | 1 | 2 | 1 | 2 | 1 | 3 | 3 | 3 | 4 | 2 |
| NM_017629 | EIF2C4 | 4758 | 5 | 2 | 2 | 1 | 3 | 2 | 1 | 1 | 4 | 2 | 2 | 5 | 2 |
| NM_017631 | DDX60 | 6072 | 5 | 2 | 2 | 2 | 1 | 1 | 2 | 1 | 3 | 5 | 2 | 4 | 2 |
| NM_017632 | CDKN2AIP | 2374 | 5 | 3 | 3 | 2 | 2 | 4 | 3 | 2 | 3 | 4 | 2 | 5 | 4 |
| NM_017633 | FAM46A | 5612 | 8 | 3 | 2 | 1 | 8 | 1 | 2 | 4 | 3 | 8 | 2 | 4 | 3 |
| NM_017634 | KCTD9 | 3337 | 18 | 7 | 5 | 3 | 18 | 4 | 5 | 3 | 8 | 5 | 6 | 7 | 9 |
| NM_017635 | SUV420H1 | 4562 | 12 | 8 | 7 | 7 | 6 | 9 | 8 | 8 | 5 | 12 | 6 | 9 | 8 |
| NM_017636 | TRPM4 | 4055 | 2 | 0 | 0 | 2 | 0 | 0 | 0 | 0 | 0 | 0 | 0 | 0 | 0 |
| NM_017637 | BNC2 | 12926 | 9 | 2 | 1 | 2 | 1 | 0 | 2 | 0 | 1 | 9 | 6 | 0 | 3 |
| NM_017638 | MED18 | 1869 | 1 | 0 | 0 | 0 | 0 | 0 | 0 | 1 | 0 | 0 | 0 | 1 | 0 |
| NM_017639 | DCHS2 | 8912 | 2 | 0 | 0 | 1 | 0 | 2 | 0 | 0 | 0 | 0 | 0 | 0 | 0 |
| NM_017640 | LRRC16A | 5304 | 14 | 4 | 2 | 4 | 3 | 4 | 6 | 2 | 6 | 3 | 0 | 2 | 14 |
| NM_017641 | KIF21A | 6328 | 39 | 8 | 2 | 2 | 9 | 39 | 12 | 8 | 4 | 2 | 3 | 2 | 10 |
| NM_017643 | MBTD1 | 5382 | 3 | 2 | 1 | 1 | 1 | 1 | 1 | 1 | 1 | 3 | 1 | 2 | 3 |
| NM_017644 | KLHL24 | 7328 | 16 | 7 | 5 | 2 | 9 | 7 | 5 | 3 | 10 | 9 | 16 | 7 | 4 |
| NM_017645 | HAUS6 | 6321 | 5 | 2 | 2 | 1 | 1 | 3 | 1 | 1 | 2 | 2 | 1 | 3 | 5 |
| NM_017646 | TRIT1 | 2128 | 3 | 2 | 1 | 1 | 3 | 2 | 3 | 2 | 1 | 2 | 1 | 2 | 3 |
| NM_017647 | FTSJ3 | 3559 | 6 | 4 | 3 | 3 | 5 | 3 | 6 | 5 | 2 | 4 | 3 | 6 | 4 |
| NM_017649 | CNNM2 | 4055 | 5 | 2 | 1 | 3 | 1 | 4 | 5 | 2 | 2 | 4 | 1 | 1 | 2 |
| NM_017650 | PPP1R9A | 9691 | 8 | 2 | 1 | 1 | 2 | 8 | 2 | 0 | 1 | 2 | 1 | 1 | 4 |
| NM_017651 | AHI1 | 5561 | 16 | 6 | 4 | 2 | 4 | 8 | 6 | 1 | 5 | 10 | 3 | 4 | 16 |
| NM_017652 | ZNF586 | 2215 | 11 | 6 | 3 | 6 | 5 | 3 | 6 | 2 | 6 | 11 | 5 | 11 | 6 |
| NM_017653 | DYM | 2628 | 6 | 5 | 4 | 2 | 4 | 5 | 4 | 3 | 4 | 6 | 6 | 4 | 6 |
| NM_017654 | SAMD9 | 6836 | 11 | 3 | 2 | 1 | 3 | 1 | 2 | 1 | 3 | 5 | 2 | 11 | 2 |
| NM_017655 | GIPC2 | 2823 | 8 | 3 | 1 | 4 | 1 | 0 | 8 | 2 | 1 | 1 | 0 | 8 | 1 |
| NM_017656 | ZNF562 | 5568 | 5 | 3 | 2 | 2 | 4 | 4 | 3 | 2 | 2 | 5 | 1 | 5 | 3 |
| NM_017657 | AFTPH | 4013 | 9 | 5 | 3 | 3 | 3 | 5 | 9 | 4 | 6 | 4 | 3 | 9 | 6 |
| NM_017658 | KLHL28 | 6551 | 3 | 2 | 1 | 1 | 2 | 3 | 1 | 1 | 3 | 3 | 2 | 3 | 2 |
| NM_017659 | QPCTL | 2303 | 0 | 0 | 0 | 0 | 0 | 0 | 0 | 0 | 0 | 0 | 0 | 0 | 0 |
| NM_017660 | GATAD2A | 5672 | 12 | 7 | 7 | 8 | 5 | 5 | 8 | 8 | 7 | 3 | 3 | 9 | 12 |
| NM_017661 | ZNF280D | 4479 | 14 | 7 | 5 | 2 | 5 | 7 | 5 | 3 | 5 | 14 | 10 | 8 | 8 |
| NM_017662 | TRPM6 | 8425 | 1 | 0 | 0 | 1 | 0 | 0 | 0 | 0 | 0 | 0 | 0 | 0 | 0 |
| NM_017664 | ANKRD10 | 2495 | 3 | 2 | 2 | 1 | 2 | 1 | 2 | 1 | 2 | 1 | 3 | 3 | 1 |
| NM_017665 | ZCCHC10 | 2122 | 7 | 3 | 1 | 1 | 3 | 2 | 3 | 1 | 4 | 3 | 3 | 7 | 6 |
| NM_017666 | ZNF280C | 4637 | 5 | 1 | 0 | 0 | 0 | 1 | 1 | 0 | 1 | 2 | 1 | 1 | 5 |
| NM_017667 | CCDC132 | 3613 | 6 | 3 | 2 | 1 | 3 | 6 | 4 | 2 | 3 | 4 | 3 | 5 | 4 |
| NM_017668 | NDE1 | 3218 | 9 | 3 | 2 | 9 | 2 | 3 | 1 | 0 | 2 | 2 | 1 | 4 | 4 |
| NM_017669 | ERCC6L | 4207 | 1 | 0 | 0 | 0 | 0 | 0 | 0 | 0 | 0 | 1 | 0 | 1 | 0 |
| NM_017670 | OTUB1 | 2294 | 6 | 2 | 1 | 2 | 2 | 6 | 3 | 2 | 2 | 1 | 2 | 2 | 2 |
| NM_017671 | FERMT1 | 5151 | 6 | 1 | 0 | 6 | 0 | 0 | 3 | 0 | 0 | 2 | 0 | 0 | 1 |
| NM_017672 | TRPM7 | 7247 | 17 | 10 | 8 | 5 | 12 | 4 | 17 | 13 | 9 | 11 | 6 | 15 | 11 |
| NM_017673 | C1orf26 | 3846 | 14 | 4 | 2 | 1 | 3 | 3 | 3 | 1 | 3 | 9 | 3 | 4 | 14 |
| NM_017675 | PCDH24 | 4095 | 1 | 0 | 0 | 1 | 0 | 0 | 1 | 1 | 0 | 0 | 0 | 0 | 0 |
| NM_017676 | GIN1 | 3549 | 2 | 1 | 1 | 0 | 2 | 1 | 2 | 1 | 1 | 2 | 2 | 2 | 2 |
| NM_017677 | MTMR8 | 2639 | 1 | 1 | 0 | 1 | 0 | 0 | 1 | 0 | 0 | 1 | 0 | 1 | 1 |
| NM_017678 | FAM55D | 1699 | 7 | 1 | 0 | 7 | 0 | 0 | 0 | 0 | 0 | 1 | 0 | 0 | 0 |
| NM_017679 | BCAS3 | 3564 | 6 | 3 | 2 | 2 | 2 | 6 | 3 | 1 | 3 | 3 | 3 | 2 | 4 |
| NM_017680 | ASPN | 2468 | 5 | 2 | 3 | 1 | 5 | 0 | 1 | 2 | 2 | 2 | 1 | 0 | 1 |
| NM_017681 | NUP62CL | 1761 | 8 | 1 | 0 | 1 | 0 | 0 | 1 | 0 | 0 | 0 | 0 | 0 | 8 |
| NM_017682 | BEST2 | 1910 | 1 | 0 | 0 | 1 | 0 | 0 | 0 | 0 | 0 | 0 | 0 | 0 | 0 |
| NM_017684 | VPS13C | 13273 | 19 | 10 | 9 | 3 | 9 | 9 | 10 | 4 | 14 | 12 | 9 | 19 | 11 |
| NM_017686 | GDAP2 | 8876 | 3 | 2 | 1 | 1 | 2 | 2 | 2 | 1 | 2 | 2 | 1 | 3 | 3 |
| NM_017688 | BSPRY | 2348 | 1 | 0 | 0 | 1 | 0 | 0 | 1 | 0 | 0 | 0 | 0 | 0 | 0 |
| NM_017691 | LRRC49 | 2793 | 2 | 1 | 0 | 0 | 1 | 2 | 0 | 0 | 0 | 1 | 0 | 0 | 1 |
| NM_017692 | APTX | 1999 | 7 | 3 | 3 | 3 | 2 | 7 | 4 | 3 | 3 | 3 | 2 | 3 | 5 |
| NM_017693 | BIVM | 3863 | 4 | 2 | 1 | 1 | 3 | 2 | 4 | 2 | 1 | 2 | 1 | 2 | 1 |

| | | transcript | | | | | | hypothala | | | | | skeletalm | | |
|---|---|---|---|---|---|---|---|---|---|---|---|---|---|---|---|
| transcript | gene | length (nt) | max | mean | adipose | colon | heart | mus | kidney | liver | lung | ovary | uscle | spleen | testes |
| NM_017694 | MFSD6 | 4797 | 14 | 3 | 2 | 3 | 3 | 14 | 2 | 0 | 4 | 2 | 0 | 5 | 2 |
| NM_017697 | ESRP1 | 3806 | 6 | 1 | 0 | 6 | 0 | 0 | 2 | 0 | 3 | 0 | 0 | 0 | 1 |
| NM_017698 | TMEM164 | 4977 | 2 | 1 | 1 | 1 | 2 | 1 | 1 | 0 | 1 | 1 | 1 | 1 | 0 |
| NM_017699 | SIDT1 | 5002 | 2 | 1 | 0 | 1 | 0 | 1 | 0 | 0 | 0 | 0 | 0 | 2 | 0 |
| NM_017700 | FLJ20184 | 1437 | 4 | 1 | 0 | 3 | 0 | 0 | 3 | 0 | 4 | 0 | 0 | 0 | 0 |
| NM_017702 | DEF8 | 904 | 1 | 1 | 1 | 1 | 0 | 1 | 1 | 1 | 0 | 0 | 0 | 1 | 0 |
| NM_017703 | FBXL12 | 1852 | 4 | 2 | 2 | 3 | 3 | 1 | 2 | 2 | 2 | 4 | 2 | 3 | 2 |
| NM_017704 | ANKRD49 | 1905 | 4 | 2 | 1 | 1 | 1 | 1 | 2 | 1 | 2 | 1 | 0 | 4 | 2 |
| NM_017705 | PAQR5 | 5186 | 5 | 1 | 0 | 1 | 0 | 0 | 5 | 0 | 1 | 0 | 0 | 0 | 0 |
| NM_017706 | WDR55 | 2580 | 2 | 1 | 1 | 2 | 1 | 1 | 1 | 2 | 1 | 0 | 0 | 2 | 1 |
| NM_017707 | ASAP3 | 4129 | 2 | 1 | 0 | 1 | 1 | 1 | 1 | 1 | 1 | 2 | 1 | 0 | 1 |
| NM_017708 | FAM83E | 2011 | 0 | 0 | 0 | 0 | 0 | 0 | 0 | 0 | 0 | 0 | 0 | 0 | 0 |
| NM_017709 | FAM46C | 5720 | 8 | 3 | 1 | 4 | 2 | 0 | 3 | 1 | 5 | 3 | 3 | 8 | 4 |
| NM_017711 | GDPD2 | 2166 | 8 | 1 | 0 | 0 | 0 | 1 | 0 | 0 | 0 | 0 | 0 | 8 | 0 |
| NM_017712 | PGPEP1 | 7084 | 4 | 2 | 1 | 2 | 1 | 1 | 4 | 3 | 1 | 1 | 1 | 2 | 1 |
| NM_017714 | TASP1 | 2351 | 3 | 2 | 1 | 1 | 2 | 2 | 2 | 1 | 1 | 2 | 0 | 2 | 3 |
| NM_017715 | ZNF3 | 1465 | 2 | 1 | 0 | 1 | 0 | 1 | 1 | 1 | 0 | 2 | 1 | 1 | 0 |
| NM_017716 | MS4A12 | 1159 | 5 | 0 | 0 | 5 | 0 | 0 | 0 | 0 | 0 | 0 | 0 | 0 | 0 |
| NM_017719 | SNRK | 5215 | 22 | 10 | 12 | 3 | 7 | 8 | 9 | 5 | 20 | 7 | 6 | 22 | 7 |
| NM_017720 | STAP2 | 1537 | 4 | 1 | 0 | 4 | 0 | 0 | 1 | 1 | 0 | 1 | 0 | 0 | 0 |
| NM_017721 | CC2D1A | 3640 | 3 | 1 | 1 | 3 | 1 | 1 | 1 | 1 | 1 | 1 | 1 | 1 | 1 |
| NM_017722 | TRMT1 | 2265 | 1 | 1 | 1 | 1 | 0 | 1 | 1 | 1 | 0 | 1 | 0 | 1 | 0 |
| NM_017723 | C9orf167 | 4069 | 1 | 0 | 0 | 1 | 0 | 0 | 0 | 0 | 0 | 0 | 0 | 1 | 0 |
| NM_017724 | LRRFIP2 | 2851 | 20 | 12 | 8 | 8 | 20 | 10 | 9 | 15 | 10 | 12 | 11 | 16 | 15 |
| NM_017726 | PPP1R14D | 757 | 2 | 0 | 0 | 2 | 0 | 0 | 0 | 0 | 0 | 0 | 0 | 0 | 0 |
| NM_017727 | TMEM214 | 3040 | 2 | 2 | 2 | 1 | 2 | 1 | 2 | 2 | 1 | 1 | 1 | 2 | 1 |
| NM_017728 | TMEM104 | 4749 | 1 | 0 | 0 | 0 | 0 | 0 | 0 | 1 | 0 | 0 | 0 | 0 | 0 |
| NM_017729 | EPS8L1 | 2202 | 1 | 0 | 0 | 1 | 0 | 0 | 0 | 0 | 0 | 0 | 0 | 0 | 0 |
| NM_017730 | QRICH1 | 3315 | 8 | 5 | 4 | 4 | 4 | 5 | 4 | 3 | 3 | 6 | 5 | 7 | 8 |
| NM_017733 | PIGG | 3226 | 3 | 2 | 2 | 2 | 1 | 2 | 3 | 2 | 2 | 2 | 1 | 3 | 2 |
| NM_017734 | PALMD | 2577 | 94 | 21 | 94 | 2 | 44 | 7 | 4 | 11 | 6 | 10 | 45 | 3 | 10 |
| NM_017735 | TTC27 | 2876 | 9 | 5 | 4 | 3 | 5 | 3 | 4 | 4 | 3 | 8 | 4 | 9 | 8 |
| NM_017736 | THUMPD1 | 4413 | 23 | 10 | 7 | 3 | 8 | 9 | 12 | 6 | 8 | 23 | 7 | 12 | 13 |
| NM_017737 | FNBP1L | 3889 | 21 | 9 | 6 | 4 | 10 | 9 | 21 | 4 | 11 | 9 | 6 | 4 | 11 |
| NM_017738 | CNTLN | 5514 | 12 | 4 | 3 | 1 | 3 | 1 | 3 | 5 | 3 | 12 | 2 | 5 | 7 |
| NM_017739 | POMGNT1 | 2735 | 2 | 1 | 1 | 1 | 1 | 2 | 1 | 1 | 0 | 0 | 1 | 1 | 1 |
| NM_017740 | ZDHHC7 | 3162 | 12 | 9 | 9 | 11 | 10 | 7 | 8 | 5 | 8 | 10 | 8 | 12 | 6 |
| NM_017741 | DCAF16 | 4548 | 8 | 3 | 1 | 1 | 2 | 2 | 3 | 0 | 2 | 8 | 2 | 3 | 5 |
| NM_017742 | ZCCHC2 | 5543 | 8 | 6 | 4 | 2 | 7 | 4 | 3 | 8 | 6 | 5 | 8 | 8 | 7 |
| NM_017743 | DPP8 | 3796 | 9 | 5 | 5 | 3 | 6 | 9 | 5 | 3 | 4 | 5 | 5 | 8 | 8 |
| NM_017744 | ST7L | 4527 | 5 | 2 | 1 | 2 | 1 | 2 | 1 | 1 | 1 | 5 | 1 | 1 | 5 |
| NM_017745 | BCOR | 6321 | 3 | 2 | 1 | 2 | 2 | 2 | 2 | 1 | 2 | 3 | 3 | 3 | 3 |
| NM_017746 | TEX10 | 3119 | 6 | 3 | 2 | 1 | 2 | 3 | 2 | 1 | 2 | 4 | 3 | 4 | 6 |
| NM_017747 | ANKHD1 | 8191 | 1 | 0 | 0 | 0 | 0 | 0 | 0 | 0 | 0 | 1 | 0 | 1 | 0 |
| NM_017748 | CCDC49 | 2330 | 4 | 3 | 3 | 2 | 2 | 3 | 3 | 2 | 3 | 3 | 3 | 4 | 3 |
| NM_017749 | AMBRA1 | 5023 | 2 | 2 | 1 | 2 | 1 | 2 | 2 | 1 | 1 | 1 | 1 | 2 | 2 |
| NM_017750 | RETSAT | 3310 | 28 | 6 | 28 | 11 | 2 | 2 | 6 | 7 | 3 | 2 | 2 | 5 | 2 |
| NM_017751 | SMPD4 | 4182 | 0 | 0 | 0 | 0 | 0 | 0 | 0 | 0 | 0 | 0 | 0 | 0 | 0 |
| NM_017752 | TBC1D8B | 5710 | 3 | 1 | 1 | 1 | 1 | 1 | 3 | 2 | 1 | 1 | 0 | 1 | 3 |
| NM_017753 | LPPR1 | 2322 | 3 | 1 | 0 | 0 | 0 | 3 | 3 | 0 | 0 | 0 | 0 | 0 | 0 |
| NM_017754 | UHRF1BP1 | 9633 | 3 | 2 | 2 | 1 | 2 | 3 | 2 | 2 | 2 | 2 | 2 | 3 | 3 |
| NM_017755 | NSUN2 | 3056 | 8 | 4 | 3 | 3 | 4 | 3 | 4 | 8 | 3 | 3 | 5 | 6 | 5 |
| NM_017757 | ZNF407 | 8008 | 7 | 4 | 3 | 1 | 3 | 3 | 4 | 2 | 3 | 7 | 3 | 6 | 5 |
| NM_017758 | ALKBH5 | 3433 | 37 | 14 | 4 | 5 | 26 | 9 | 12 | 8 | 10 | 12 | 37 | 14 | 18 |
| NM_017759 | INO80D | 14136 | 4 | 2 | 2 | 2 | 2 | 1 | 3 | 1 | 2 | 4 | 2 | 4 | 3 |
| NM_017760 | NCAPG2 | 3932 | 2 | 1 | 1 | 1 | 1 | 1 | 0 | 0 | 1 | 1 | 1 | 1 | 2 |
| NM_017761 | PNRC2 | 2412 | 35 | 20 | 16 | 10 | 26 | 15 | 24 | 9 | 28 | 26 | 14 | 35 | 18 |
| NM_017762 | MTMR10 | 4961 | 10 | 6 | 5 | 3 | 5 | 7 | 10 | 5 | 4 | 4 | 7 | 5 | 5 |
| NM_017763 | RNF43 | 4557 | 3 | 1 | 0 | 3 | 0 | 0 | 1 | 1 | 1 | 0 | 0 | 0 | 1 |
| NM_017765 | PQLC2 | 1885 | 0 | 0 | 0 | 0 | 0 | 0 | 0 | 0 | 0 | 0 | 0 | 0 | 0 |
| NM_017766 | CASZ1 | 4405 | 2 | 1 | 0 | 2 | 2 | 0 | 1 | 0 | 1 | 0 | 2 | 0 | 0 |
| NM_017767 | SLC39A4 | 2318 | 1 | 0 | 0 | 1 | 0 | 0 | 1 | 0 | 0 | 0 | 0 | 0 | 0 |
| NM_017768 | LRRC40 | 2847 | 7 | 4 | 3 | 1 | 3 | 7 | 5 | 7 | 2 | 4 | 3 | 5 | 6 |
| NM_017769 | G2E3 | 5581 | 3 | 1 | 1 | 0 | 1 | 2 | 1 | 1 | 1 | 2 | 1 | 2 | 3 |
| NM_017770 | ELOVL2 | 4073 | 3 | 1 | 0 | 0 | 1 | 2 | 0 | 3 | 0 | 0 | 0 | 0 | 3 |
| NM_017771 | PXK | 2027 | 6 | 3 | 3 | 1 | 2 | 6 | 3 | 1 | 3 | 2 | 2 | 5 | 2 |
| NM_017772 | TBC1D22B | 3462 | 1 | 1 | 0 | 0 | 1 | 1 | 1 | 0 | 0 | 1 | 1 | 1 | 1 |
| NM_017773 | LAX1 | 3276 | 3 | 1 | 0 | 1 | 0 | 0 | 0 | 0 | 1 | 0 | 0 | 3 | 0 |
| NM_017774 | CDKAL1 | 2408 | 6 | 3 | 2 | 2 | 3 | 4 | 3 | 2 | 3 | 6 | 3 | 5 | 5 |
| NM_017775 | TTC19 | 2784 | 10 | 6 | 2 | 4 | 6 | 10 | 9 | 4 | 4 | 8 | 3 | 7 | 4 |
| NM_017776 | ZNF673 | 2427 | 4 | 2 | 1 | 1 | 1 | 2 | 2 | 1 | 2 | 3 | 1 | 4 | 2 |
| NM_017777 | MKS1 | 2398 | 1 | 1 | 0 | 0 | 0 | 1 | 1 | 0 | 1 | 1 | 0 | 0 | 1 |
| NM_017778 | WHSC1L1 | 3995 | 18 | 10 | 7 | 4 | 8 | 12 | 9 | 4 | 12 | 18 | 6 | 16 | 10 |
| NM_017779 | DEPDC1 | 4481 | 2 | 0 | 0 | 0 | 0 | 0 | 0 | 0 | 0 | 0 | 0 | 0 | 2 |
| NM_017780 | CHD7 | 10446 | 5 | 2 | 1 | 3 | 2 | 2 | 3 | 1 | 3 | 1 | 4 | 5 | 2 |
| NM_017781 | CYP2W1 | 2304 | 0 | 0 | 0 | 0 | 0 | 0 | 0 | 0 | 0 | 0 | 0 | 0 | 0 |
| NM_017782 | C10orf18 | 9866 | 10 | 4 | 2 | 2 | 4 | 4 | 4 | 4 | 3 | 5 | 4 | 6 | 10 |

| transcript | gene | transcript length (nt) | max | mean | adipose | colon | heart | hypothalamus | kidney | liver | lung | ovary | skeletalmuscle | spleen | testes |
|---|---|---|---|---|---|---|---|---|---|---|---|---|---|---|---|
| NM_017784 | OSBPL10 | 3923 | 10 | 3 | 1 | 3 | 1 | 2 | 7 | 0 | 2 | 1 | 1 | 6 | 10 |
| NM_017785 | CCDC99 | 2667 | 16 | 4 | 2 | 1 | 3 | 5 | 2 | 1 | 2 | 4 | 1 | 4 | 16 |
| NM_017786 | GOLSYN | 3113 | 11 | 2 | 1 | 2 | 1 | 11 | 1 | 6 | 1 | 1 | 0 | 0 | 1 |
| NM_017787 | C10orf26 | 4222 | 7 | 4 | 5 | 3 | 4 | 3 | 3 | 7 | 4 | 4 | 5 | 6 | 4 |
| NM_017789 | SEMA4C | 3552 | 4 | 2 | 2 | 3 | 2 | 4 | 1 | 1 | 1 | 3 | 1 | 4 | 1 |
| NM_017790 | RGS3 | 2547 | 2 | 1 | 0 | 1 | 2 | 0 | 1 | 0 | 1 | 0 | 0 | 2 | 1 |
| NM_017791 | FLVCR2 | 3654 | 5 | 2 | 1 | 1 | 1 | 1 | 1 | 4 | 2 | 0 | 0 | 5 | 3 |
| NM_017793 | RPP25 | 2333 | 2 | 1 | 1 | 2 | 1 | 1 | 1 | 0 | 1 | 2 | 0 | 2 | 1 |
| NM_017794 | KIAA1797 | 6095 | 6 | 2 | 2 | 2 | 3 | 6 | 2 | 1 | 2 | 2 | 1 | 2 | 2 |
| NM_017797 | BTBD2 | 2618 | 2 | 1 | 0 | 1 | 0 | 2 | 1 | 0 | 0 | 1 | 1 | 1 | 0 |
| NM_017798 | YTHDF1 | 3250 | 2 | 1 | 1 | 1 | 1 | 2 | 1 | 1 | 1 | 1 | 1 | 2 | 1 |
| NM_017799 | C14orf101 | 4263 | 2 | 1 | 1 | 0 | 1 | 1 | 1 | 1 | 1 | 1 | 0 | 2 | 1 |
| NM_017801 | CMTM6 | 3366 | 12 | 5 | 3 | 2 | 3 | 3 | 4 | 11 | 7 | 3 | 1 | 12 | 11 |
| NM_017802 | HEATR2 | 3414 | 1 | 0 | 0 | 0 | 0 | 0 | 1 | 0 | 0 | 0 | 0 | 0 | 0 |
| NM_017803 | DUS2L | 1926 | 1 | 1 | 1 | 0 | 0 | 1 | 1 | 1 | 0 | 1 | 0 | 1 | 1 |
| NM_017805 | RASIP1 | 3302 | 1 | 0 | 0 | 0 | 0 | 0 | 0 | 0 | 1 | 0 | 0 | 1 | 0 |
| NM_017806 | LIME1 | 1199 | 0 | 0 | 0 | 0 | 0 | 0 | 0 | 0 | 0 | 0 | 0 | 0 | 0 |
| NM_017807 | OSGEP | 1547 | 8 | 4 | 3 | 4 | 4 | 3 | 4 | 2 | 2 | 3 | 3 | 8 | 2 |
| NM_017809 | NXF2 | 3848 | 0 | 0 | 0 | 0 | 0 | 0 | 0 | 0 | 0 | 0 | 0 | 0 | 0 |
| NM_017810 | ZNF434 | 3027 | 2 | 1 | 1 | 1 | 1 | 2 | 1 | 1 | 1 | 1 | 0 | 2 | 2 |
| NM_017811 | UBE2R2 | 4457 | 7 | 5 | 5 | 3 | 5 | 6 | 5 | 6 | 6 | 5 | 5 | 7 | 7 |
| NM_017812 | CHCHD3 | 1590 | 37 | 14 | 10 | 6 | 37 | 9 | 16 | 10 | 6 | 7 | 29 | 11 | 9 |
| NM_017813 | IMPAD1 | 7227 | 9 | 4 | 9 | 2 | 4 | 6 | 4 | 5 | 2 | 2 | 4 | 3 | 3 |
| NM_017814 | TMEM161A | 1779 | 1 | 0 | 0 | 0 | 0 | 0 | 0 | 1 | 0 | 0 | 0 | 0 | 0 |
| NM_017815 | HAUS4 | 1629 | 8 | 4 | 7 | 4 | 4 | 3 | 3 | 2 | 5 | 7 | 3 | 8 | 3 |
| NM_017816 | LYAR | 1566 | 13 | 3 | 2 | 1 | 4 | 2 | 1 | 2 | 2 | 3 | 3 | 5 | 13 |
| NM_017817 | RAB20 | 1509 | 2 | 1 | 1 | 0 | 1 | 1 | 1 | 2 | 1 | 0 | 0 | 2 | 0 |
| NM_017818 | WDR8 | 1698 | 0 | 0 | 0 | 0 | 0 | 0 | 0 | 0 | 0 | 0 | 0 | 0 | 0 |
| NM_017819 | RG9MTD1 | 1612 | 6 | 3 | 2 | 1 | 5 | 4 | 4 | 6 | 1 | 3 | 2 | 4 | 3 |
| NM_017820 | EXD3 | 2881 | 0 | 0 | 0 | 0 | 0 | 0 | 0 | 0 | 0 | 0 | 0 | 0 | 0 |
| NM_017821 | RHBDL2 | 1837 | 6 | 1 | 0 | 6 | 0 | 3 | 0 | 0 | 0 | 0 | 0 | 0 | 3 |
| NM_017822 | C12orf41 | 2386 | 4 | 2 | 1 | 2 | 2 | 3 | 3 | 1 | 2 | 2 | 2 | 4 | 4 |
| NM_017823 | DUSP23 | 685 | 11 | 3 | 2 | 1 | 3 | 1 | 11 | 5 | 2 | 1 | 1 | 2 | 1 |
| NM_017824 | 05 Mar | 3926 | 3 | 2 | 3 | 2 | 3 | 3 | 3 | 3 | 2 | 3 | 2 | 2 | 3 |
| NM_017825 | ADPRHL2 | 1690 | 1 | 1 | 0 | 0 | 0 | 1 | 1 | 0 | 0 | 0 | 0 | 0 | 1 |
| NM_017826 | SOHLH2 | 2191 | 6 | 1 | 0 | 0 | 0 | 0 | 0 | 0 | 0 | 0 | 0 | 0 | 6 |
| NM_017827 | SARS2 | 2059 | 0 | 0 | 0 | 0 | 0 | 0 | 0 | 0 | 0 | 0 | 0 | 0 | 0 |
| NM_017828 | COMMD4 | 925 | 2 | 1 | 1 | 2 | 1 | 1 | 1 | 1 | 1 | 1 | 0 | 1 | 0 |
| NM_017829 | CECR5 | 1726 | 1 | 0 | 0 | 1 | 1 | 0 | 1 | 1 | 0 | 0 | 0 | 1 | 0 |
| NM_017830 | OCIAD1 | 2004 | 11 | 6 | 3 | 2 | 7 | 11 | 8 | 6 | 3 | 5 | 5 | 6 | 5 |
| NM_017831 | RNF125 | 5888 | 2 | 1 | 1 | 1 | 1 | 1 | 0 | 1 | 1 | 0 | 0 | 2 | 1 |
| NM_017832 | C9orf6 | 1909 | 1 | 1 | 1 | 0 | 1 | 1 | 1 | 0 | 1 | 1 | 1 | 1 | 1 |
| NM_017833 | DNAJC28 | 1912 | 2 | 1 | 1 | 0 | 1 | 2 | 2 | 1 | 1 | 0 | 1 | 1 | 1 |
| NM_017836 | SLC41A3 | 2349 | 3 | 1 | 1 | 1 | 1 | 3 | 1 | 0 | 0 | 1 | 1 | 1 | 2 |
| NM_017837 | PIGV | 2365 | 4 | 2 | 1 | 1 | 1 | 2 | 3 | 2 | 2 | 2 | 1 | 2 | 4 |
| NM_017838 | NHP2 | 853 | 4 | 3 | 2 | 2 | 3 | 3 | 3 | 4 | 2 | 2 | 2 | 3 | 3 |
| NM_017839 | LPCAT2 | 5387 | 7 | 3 | 3 | 1 | 3 | 4 | 3 | 1 | 3 | 4 | 1 | 7 | 4 |
| NM_017840 | MRPL16 | 1182 | 12 | 8 | 5 | 5 | 12 | 7 | 9 | 11 | 5 | 5 | 6 | 9 | 8 |
| NM_017841 | SDHAF2 | 1209 | 9 | 6 | 5 | 6 | 7 | 7 | 6 | 9 | 4 | 6 | 6 | 8 | 6 |
| NM_017842 | SLC48A1 | 3033 | 3 | 1 | 1 | 1 | 1 | 3 | 1 | 1 | 1 | 1 | 1 | 2 | 1 |
| NM_017843 | BCAS4 | 1342 | 0 | 0 | 0 | 0 | 0 | 0 | 0 | 0 | 0 | 0 | 0 | 0 | 0 |
| NM_017844 | ANKMY1 | 2466 | 0 | 0 | 0 | 0 | 0 | 0 | 0 | 0 | 0 | 0 | 0 | 0 | 0 |
| NM_017845 | COMMD8 | 1411 | 9 | 4 | 3 | 1 | 4 | 5 | 9 | 4 | 4 | 2 | 1 | 6 | 6 |
| NM_017846 | TRNAU1AP | 1867 | 2 | 1 | 2 | 1 | 2 | 1 | 1 | 1 | 1 | 1 | 1 | 2 | 1 |
| NM_017847 | C1orf27 | 3895 | 5 | 3 | 3 | 1 | 3 | 3 | 5 | 5 | 3 | 3 | 2 | 4 | 4 |
| NM_017848 | FAM120C | 8083 | 3 | 1 | 1 | 0 | 1 | 3 | 1 | 0 | 1 | 2 | 1 | 1 | 2 |
| NM_017849 | TMEM127 | 4208 | 5 | 3 | 3 | 2 | 5 | 4 | 4 | 3 | 3 | 3 | 2 | 5 | 2 |
| NM_017850 | C1orf109 | 2355 | 3 | 2 | 2 | 1 | 1 | 3 | 2 | 1 | 1 | 1 | 1 | 2 | 2 |
| NM_017851 | PARP16 | 2534 | 3 | 2 | 1 | 2 | 1 | 1 | 2 | 2 | 1 | 3 | 1 | 2 | 2 |
| NM_017852 | NLRP2 | 3510 | 2 | 1 | 0 | 1 | 0 | 1 | 1 | 0 | 1 | 0 | 0 | 1 | 2 |
| NM_017853 | TXNL4B | 2539 | 2 | 1 | 1 | 1 | 1 | 1 | 1 | 1 | 1 | 1 | 0 | 1 | 2 |
| NM_017854 | TMEM160 | 655 | 1 | 0 | 0 | 0 | 0 | 1 | 0 | 0 | 0 | 0 | 0 | 1 | 0 |
| NM_017855 | ODAM | 1288 | 0 | 0 | 0 | 0 | 0 | 0 | 0 | 0 | 0 | 0 | 0 | 0 | 0 |
| NM_017856 | GEMIN8 | 3259 | 2 | 1 | 1 | 1 | 1 | 2 | 1 | 0 | 1 | 2 | 0 | 1 | 1 |
| NM_017857 | SSH3 | 2876 | 1 | 0 | 0 | 1 | 0 | 0 | 1 | 0 | 1 | 0 | 0 | 0 | 0 |
| NM_017858 | TIPIN | 1280 | 5 | 2 | 1 | 2 | 2 | 3 | 1 | 1 | 1 | 2 | 1 | 3 | 5 |
| NM_017859 | UCKL1 | 1830 | 2 | 1 | 1 | 2 | 2 | 1 | 1 | 0 | 1 | 1 | 2 | 2 | 1 |
| NM_017860 | C1orf56 | 2028 | 3 | 1 | 0 | 1 | 1 | 1 | 1 | 1 | 0 | 0 | 0 | 0 | 3 |
| NM_017861 | PIGX | 3037 | 5 | 2 | 2 | 2 | 2 | 5 | 3 | 1 | 2 | 2 | 1 | 2 | 2 |
| NM_017863 | CXorf48 | 2161 | 1 | 0 | 0 | 0 | 0 | 0 | 0 | 0 | 0 | 0 | 0 | 0 | 1 |
| NM_017864 | INTS8 | 3373 | 11 | 5 | 3 | 4 | 7 | 4 | 4 | 3 | 4 | 7 | 4 | 8 | 11 |
| NM_017865 | ZNF692 | 1931 | 2 | 1 | 1 | 1 | 1 | 1 | 1 | 0 | 1 | 1 | 1 | 1 | 2 |
| NM_017866 | TMEM70 | 2027 | 11 | 3 | 1 | 0 | 4 | 2 | 2 | 3 | 1 | 2 | 11 | 3 | 2 |
| NM_017867 | C4orf27 | 1216 | 7 | 3 | 3 | 1 | 3 | 7 | 3 | 2 | 2 | 4 | 2 | 3 | 3 |
| NM_017868 | TTC12 | 2315 | 5 | 2 | 1 | 1 | 1 | 1 | 2 | 2 | 1 | 2 | 1 | 2 | 5 |
| NM_017869 | BANP | 2219 | 2 | 1 | 1 | 2 | 1 | 1 | 1 | 1 | 2 | 2 | 1 | 2 | 2 |
| NM_017870 | TMEM132A | 3520 | 1 | 0 | 0 | 0 | 0 | 1 | 0 | 0 | 0 | 0 | 0 | 0 | 0 |

| transcript | gene | transcript length (nt) | max | mean | adipose | colon | heart | hypothalamus | kidney | liver | lung | ovary | skeletalmuscle | spleen | testes |
|---|---|---|---|---|---|---|---|---|---|---|---|---|---|---|---|
| NM_017871 | CPSF3L | 2135 | 1 | 1 | 1 | 1 | 1 | 1 | 1 | 1 | 0 | 1 | 1 | 1 | 1 |
| NM_017872 | THG1L | 1305 | 3 | 1 | 1 | 1 | 1 | 1 | 1 | 1 | 2 | 3 | 1 | 3 | 2 |
| NM_017873 | ASB6 | 4618 | 1 | 0 | 0 | 0 | 0 | 1 | 0 | 0 | 0 | 1 | 0 | 1 | 0 |
| NM_017875 | SLC25A38 | 2124 | 5 | 3 | 2 | 3 | 2 | 3 | 3 | 5 | 2 | 4 | 2 | 4 | 2 |
| NM_017877 | C2orf18 | 3912 | 2 | 1 | 1 | 0 | 0 | 0 | 2 | 1 | 1 | 0 | 0 | 1 | 1 |
| NM_017878 | HRASLS2 | 742 | 1 | 0 | 0 | 0 | 0 | 0 | 1 | 0 | 1 | 0 | 0 | 0 | 0 |
| NM_017879 | ZNF416 | 2509 | 1 | 1 | 1 | 0 | 0 | 1 | 1 | 0 | 0 | 1 | 1 | 1 | 1 |
| NM_017880 | C2orf42 | 2524 | 4 | 1 | 1 | 0 | 1 | 1 | 1 | 1 | 0 | 1 | 1 | 1 | 4 |
| NM_017881 | C9orf95 | 1193 | 2 | 1 | 1 | 0 | 1 | 1 | 2 | 1 | 2 | 2 | 1 | 2 | 1 |
| NM_017882 | CLN6 | 2240 | 3 | 2 | 3 | 2 | 1 | 2 | 3 | 3 | 1 | 1 | 1 | 3 | 1 |
| NM_017883 | WDR13 | 2125 | 3 | 2 | 1 | 1 | 3 | 3 | 3 | 3 | 1 | 2 | 1 | 2 | 1 |
| NM_017884 | PINX1 | 1047 | 3 | 1 | 1 | 1 | 1 | 1 | 1 | 3 | 1 | 1 | 2 | 2 | 1 |
| NM_017885 | HCFC1R1 | 1000 | 52 | 18 | 9 | 10 | 19 | 29 | 22 | 4 | 15 | 18 | 52 | 12 | 13 |
| NM_017886 | ULK4 | 4359 | 9 | 3 | 3 | 2 | 2 | 4 | 4 | 1 | 3 | 4 | 1 | 4 | 9 |
| NM_017887 | C1orf123 | 1084 | 5 | 3 | 4 | 2 | 4 | 5 | 4 | 3 | 2 | 3 | 5 | 3 | 3 |
| NM_017888 | ACSM5 | 2419 | 9 | 2 | 2 | 0 | 0 | 0 | 3 | 9 | 0 | 0 | 0 | 0 | 1 |
| NM_017890 | VPS13B | 14094 | 7 | 3 | 3 | 2 | 2 | 3 | 3 | 1 | 3 | 7 | 2 | 5 | 5 |
| NM_017891 | C1orf159 | 2104 | 0 | 0 | 0 | 0 | 0 | 0 | 0 | 0 | 0 | 0 | 0 | 0 | 0 |
| NM_017892 | PRPF40A | 7528 | 15 | 9 | 6 | 2 | 9 | 7 | 11 | 9 | 8 | 9 | 8 | 15 | 15 |
| NM_017893 | SEMA4G | 4400 | 1 | 0 | 0 | 1 | 0 | 0 | 0 | 1 | 0 | 0 | 0 | 0 | 0 |
| NM_017894 | ZSCAN2 | 988 | 1 | 0 | 0 | 0 | 0 | 0 | 1 | 0 | 0 | 0 | 0 | 0 | 0 |
| NM_017895 | DDX27 | 2693 | 12 | 7 | 6 | 5 | 7 | 7 | 7 | 6 | 5 | 8 | 7 | 12 | 9 |
| NM_017896 | C20orf11 | 4427 | 3 | 2 | 1 | 1 | 1 | 2 | 2 | 2 | 1 | 3 | 1 | 2 | 2 |
| NM_017897 | OXSM | 1526 | 3 | 1 | 0 | 1 | 1 | 1 | 1 | 3 | 0 | 0 | 1 | 1 | 1 |
| NM_017898 | MOSC2 | 1573 | 11 | 3 | 2 | 1 | 1 | 1 | 11 | 7 | 3 | 1 | 0 | 2 | 3 |
| NM_017899 | TESC | 1017 | 1 | 0 | 0 | 0 | 1 | 1 | 0 | 0 | 0 | 0 | 0 | 0 | 0 |
| NM_017900 | AURKAIP1 | 990 | 10 | 5 | 5 | 6 | 7 | 7 | 6 | 10 | 2 | 1 | 5 | 4 | 4 |
| NM_017901 | TPCN1 | 5254 | 4 | 2 | 1 | 2 | 2 | 1 | 4 | 1 | 1 | 2 | 1 | 1 | 1 |
| NM_017902 | HIF1AN | 6912 | 5 | 4 | 2 | 4 | 4 | 4 | 4 | 1 | 2 | 4 | 5 | 4 | 5 |
| NM_017904 | TTC22 | 2219 | 1 | 0 | 0 | 1 | 0 | 0 | 0 | 0 | 0 | 0 | 0 | 0 | 0 |
| NM_017905 | TMCO3 | 3086 | 8 | 6 | 7 | 4 | 6 | 6 | 7 | 8 | 6 | 4 | 2 | 8 | 6 |
| NM_017906 | PAK1IP1 | 1495 | 4 | 3 | 2 | 1 | 3 | 4 | 3 | 4 | 2 | 2 | 3 | 4 | 3 |
| NM_017907 | C11orf59 | 1068 | 5 | 3 | 3 | 2 | 3 | 4 | 4 | 2 | 2 | 2 | 3 | 5 | 2 |
| NM_017908 | ZNF446 | 1974 | 0 | 0 | 0 | 0 | 0 | 0 | 0 | 0 | 0 | 0 | 0 | 0 | 0 |
| NM_017909 | RMND1 | 1911 | 4 | 3 | 3 | 1 | 3 | 4 | 3 | 4 | 1 | 4 | 1 | 3 | 2 |
| NM_017910 | TRMT61B | 1841 | 2 | 2 | 1 | 1 | 2 | 1 | 2 | 1 | 1 | 2 | 1 | 2 | 2 |
| NM_017911 | FAM118A | 2913 | 8 | 4 | 2 | 5 | 3 | 3 | 2 | 1 | 3 | 4 | 4 | 7 | 8 |
| NM_017912 | HERC6 | 3888 | 5 | 2 | 2 | 1 | 3 | 4 | 2 | 1 | 2 | 5 | 4 | 2 | 3 |
| NM_017913 | CDC37L1 | 1698 | 18 | 10 | 7 | 2 | 18 | 12 | 8 | 18 | 5 | 7 | 15 | 6 | 10 |
| NM_017914 | C19orf24 | 895 | 2 | 1 | 0 | 0 | 0 | 0 | 1 | 2 | 1 | 0 | 0 | 0 | 0 |
| NM_017915 | C12orf48 | 3189 | 0 | 0 | 0 | 0 | 0 | 0 | 0 | 0 | 0 | 0 | 0 | 0 | 0 |
| NM_017916 | PIH1D1 | 1246 | 2 | 1 | 1 | 1 | 1 | 2 | 2 | 2 | 1 | 1 | 1 | 2 | 1 |
| NM_017917 | PPP2R3C | 1834 | 5 | 4 | 5 | 1 | 4 | 5 | 3 | 4 | 3 | 3 | 3 | 5 | 5 |
| NM_017918 | CCDC109B | 1274 | 4 | 1 | 2 | 1 | 2 | 1 | 0 | 0 | 2 | 2 | 1 | 4 | 1 |
| NM_017919 | STX17 | 6908 | 5 | 3 | 2 | 2 | 2 | 3 | 3 | 3 | 2 | 5 | 3 | 4 | 3 |
| NM_017920 | URGCP | 3591 | 7 | 4 | 3 | 6 | 4 | 7 | 7 | 4 | 2 | 3 | 3 | 4 | 3 |
| NM_017921 | NPLOC4 | 4381 | 7 | 5 | 7 | 4 | 5 | 6 | 7 | 7 | 3 | 2 | 6 | 6 | 4 |
| NM_017922 | PRPF39 | 2868 | 4 | 3 | 3 | 2 | 2 | 2 | 2 | 2 | 3 | 3 | 2 | 4 | 3 |
| NM_017923 | 01 Mar | 5389 | 7 | 1 | 1 | 0 | 1 | 2 | 0 | 0 | 2 | 1 | 0 | 7 | 1 |
| NM_017924 | C14orf119 | 2840 | 2 | 1 | 1 | 1 | 1 | 1 | 1 | 1 | 2 | 1 | 1 | 2 | 2 |
| NM_017925 | DENND4C | 7023 | 11 | 6 | 9 | 3 | 6 | 3 | 6 | 4 | 7 | 9 | 3 | 6 | 11 |
| NM_017926 | C14orf118 | 2475 | 6 | 5 | 3 | 4 | 5 | 5 | 5 | 1 | 5 | 5 | 5 | 6 | 6 |
| NM_017929 | PEX26 | 2493 | 1 | 0 | 0 | 1 | 0 | 0 | 1 | 0 | 0 | 0 | 0 | 1 | 0 |
| NM_017931 | TTC38 | 2610 | 12 | 3 | 1 | 4 | 1 | 0 | 8 | 12 | 1 | 1 | 1 | 2 | 1 |
| NM_017933 | PID1 | 2822 | 9 | 2 | 3 | 1 | 3 | 2 | 1 | 9 | 2 | 3 | 1 | 2 | 1 |
| NM_017934 | PHIP | 11965 | 9 | 5 | 3 | 2 | 5 | 4 | 5 | 1 | 7 | 8 | 6 | 9 | 7 |
| NM_017935 | BANK1 | 3443 | 29 | 4 | 3 | 1 | 0 | 0 | 1 | 0 | 2 | 0 | 0 | 29 | 2 |
| NM_017938 | FAM70A | 3497 | 3 | 1 | 0 | 0 | 0 | 3 | 0 | 0 | 0 | 3 | 0 | 0 | 0 |
| NM_017940 | NBPF1 | 4339 | 18 | 7 | 7 | 9 | 5 | 8 | 5 | 4 | 3 | 11 | 1 | 5 | 18 |
| NM_017941 | C17orf80 | 3639 | 5 | 2 | 2 | 1 | 3 | 2 | 3 | 1 | 1 | 3 | 3 | 3 | 5 |
| NM_017943 | FBXO34 | 3464 | 5 | 3 | 1 | 3 | 2 | 5 | 4 | 1 | 2 | 2 | 4 | 3 | 5 |
| NM_017944 | USP47 | 7777 | 22 | 11 | 7 | 3 | 22 | 10 | 9 | 9 | 10 | 10 | 17 | 10 | 14 |
| NM_017945 | SLC35A5 | 2940 | 5 | 3 | 2 | 1 | 2 | 4 | 4 | 3 | 3 | 4 | 1 | 5 | 5 |
| NM_017946 | FKBP14 | 2247 | 3 | 2 | 2 | 1 | 3 | 1 | 1 | 1 | 2 | 2 | 1 | 2 | 2 |
| NM_017947 | MOCOS | 2727 | 8 | 1 | 0 | 0 | 0 | 0 | 0 | 8 | 0 | 1 | 0 | 0 | 1 |
| NM_017948 | NOL8 | 4319 | 8 | 4 | 4 | 2 | 4 | 3 | 4 | 3 | 3 | 6 | 4 | 8 | 6 |
| NM_017949 | CUEDC1 | 2193 | 1 | 1 | 0 | 1 | 1 | 1 | 1 | 0 | 0 | 0 | 1 | 0 | 0 |
| NM_017950 | CCDC40 | 4294 | 3 | 1 | 1 | 1 | 1 | 1 | 2 | 0 | 1 | 0 | 0 | 1 | 3 |
| NM_017951 | SMPD4 | 4269 | 0 | 0 | 0 | 0 | 0 | 0 | 0 | 0 | 0 | 0 | 0 | 0 | 0 |
| NM_017952 | PTCD3 | 6734 | 5 | 3 | 2 | 1 | 5 | 3 | 4 | 3 | 2 | 4 | 5 | 3 | 5 |
| NM_017953 | ZNHIT6 | 6196 | 7 | 4 | 5 | 2 | 4 | 7 | 4 | 3 | 2 | 5 | 3 | 5 | 4 |
| NM_017954 | CADPS2 | 6054 | 5 | 2 | 1 | 1 | 2 | 3 | 5 | 2 | 3 | 2 | 0 | 1 | 1 |
| NM_017955 | CDCA4 | 2453 | 1 | 0 | 0 | 0 | 0 | 0 | 0 | 0 | 0 | 0 | 0 | 0 | 1 |
| NM_017956 | TRMT12 | 2219 | 6 | 3 | 3 | 2 | 3 | 6 | 4 | 3 | 3 | 3 | 2 | 4 | 4 |
| NM_017957 | EPN3 | 3927 | 0 | 0 | 0 | 0 | 0 | 0 | 0 | 0 | 0 | 0 | 0 | 0 | 0 |
| NM_017958 | PLEKHB2 | 4305 | 31 | 13 | 7 | 7 | 10 | 31 | 27 | 5 | 14 | 4 | 3 | 19 | 12 |
| NM_017964 | SLC30A6 | 2450 | 1 | 0 | 0 | 0 | 0 | 0 | 0 | 0 | 0 | 0 | 0 | 1 | 1 |

| transcript | gene | transcript length (nt) | max | mean | adipose | colon | heart | hypothalamus | kidney | liver | lung | ovary | skeletalmuscle | spleen | testes |
|---|---|---|---|---|---|---|---|---|---|---|---|---|---|---|---|
| NM_017966 | VPS37C | 2692 | 1 | 0 | 0 | 0 | 0 | 0 | 0 | 0 | 0 | 0 | 0 | 1 | 0 |
| NM_017969 | IWS1 | 3000 | 22 | 15 | 14 | 7 | 21 | 22 | 15 | 17 | 10 | 10 | 16 | 21 | 16 |
| NM_017970 | C14orf102 | 3808 | 3 | 2 | 2 | 2 | 2 | 2 | 3 | 2 | 1 | 3 | 2 | 2 | 2 |
| NM_017971 | MRPL20 | 733 | 34 | 19 | 21 | 11 | 31 | 34 | 19 | 27 | 9 | 8 | 25 | 13 | 8 |
| NM_017972 | C14orf118 | 2460 | 6 | 5 | 3 | 4 | 5 | 5 | 5 | 1 | 5 | 5 | 5 | 6 | 6 |
| NM_017974 | ATG16L1 | 3348 | 3 | 2 | 2 | 3 | 2 | 3 | 2 | 3 | 2 | 1 | 2 | 2 | 3 |
| NM_017975 | ZWILCH | 3182 | 2 | 0 | 0 | 0 | 0 | 0 | 0 | 0 | 0 | 0 | 0 | 0 | 2 |
| NM_017978 | ANKHD1 | 2112 | 0 | 0 | 0 | 0 | 0 | 0 | 0 | 0 | 0 | 0 | 0 | 0 | 0 |
| NM_017980 | LIMS2 | 2162 | 1 | 0 | 0 | 1 | 1 | 1 | 0 | 0 | 0 | 0 | 0 | 0 | 0 |
| NM_017982 | SUSD4 | 3027 | 3 | 1 | 0 | 0 | 0 | 3 | 1 | 0 | 0 | 1 | 0 | 0 | 0 |
| NM_017983 | WIPI1 | 1924 | 8 | 4 | 5 | 4 | 8 | 2 | 4 | 3 | 4 | 2 | 5 | 3 | 2 |
| NM_017984 | ZCWPW1 | 2337 | 10 | 2 | 1 | 1 | 1 | 1 | 1 | 1 | 0 | 1 | 1 | 2 | 10 |
| NM_017986 | GPR172B | 2381 | 0 | 0 | 0 | 0 | 0 | 0 | 0 | 0 | 0 | 0 | 0 | 0 | 0 |
| NM_017987 | RUFY2 | 4503 | 8 | 4 | 3 | 3 | 2 | 8 | 4 | 1 | 4 | 7 | 3 | 4 | 5 |
| NM_017988 | SCYL2 | 3804 | 7 | 5 | 3 | 2 | 6 | 5 | 7 | 6 | 5 | 4 | 3 | 6 | 6 |
| NM_017990 | PDPR | 7999 | 5 | 2 | 2 | 2 | 1 | 2 | 5 | 1 | 2 | 2 | 2 | 3 | 3 |
| NM_017991 | KIAA1310 | 5285 | 5 | 3 | 2 | 2 | 3 | 3 | 4 | 2 | 3 | 4 | 5 | 4 | 4 |
| NM_017993 | ENOX1 | 3032 | 2 | 1 | 0 | 1 | 1 | 2 | 1 | 0 | 0 | 1 | 1 | 0 | 1 |
| NM_017994 | C7orf42 | 4238 | 17 | 11 | 9 | 6 | 10 | 16 | 11 | 13 | 10 | 17 | 5 | 15 | 15 |
| NM_017995 | C9orf86 | 2050 | 1 | 1 | 1 | 1 | 0 | 1 | 1 | 0 | 0 | 0 | 1 | 1 | 0 |
| NM_017996 | DET1 | 2343 | 1 | 1 | 0 | 1 | 0 | 1 | 1 | 0 | 0 | 1 | 1 | 1 | 1 |
| NM_017998 | C9orf40 | 2325 | 4 | 2 | 2 | 3 | 1 | 4 | 1 | 0 | 1 | 1 | 0 | 2 | 4 |
| NM_017999 | RNF31 | 3556 | 1 | 1 | 0 | 1 | 1 | 1 | 1 | 1 | 1 | 1 | 1 | 1 | 1 |
| NM_018000 | MREG | 3213 | 3 | 1 | 3 | 1 | 0 | 1 | 1 | 1 | 1 | 0 | 0 | 1 | 1 |
| NM_018002 | OXR1 | 4554 | 30 | 10 | 8 | 2 | 7 | 30 | 9 | 6 | 7 | 8 | 7 | 9 | 13 |
| NM_018003 | UACA | 6857 | 37 | 12 | 10 | 2 | 13 | 4 | 13 | 3 | 10 | 14 | 37 | 14 | 11 |
| NM_018004 | TMEM45A | 1564 | 4 | 1 | 0 | 0 | 1 | 0 | 0 | 4 | 0 | 0 | 0 | 0 | 1 |
| NM_018006 | TRMU | 1964 | 1 | 1 | 0 | 1 | 1 | 1 | 1 | 1 | 0 | 0 | 0 | 1 | 0 |
| NM_018008 | FEZF2 | 2086 | 1 | 0 | 0 | 0 | 0 | 1 | 0 | 0 | 0 | 0 | 0 | 0 | 0 |
| NM_018009 | TAPBPL | 1818 | 1 | 0 | 1 | 1 | 0 | 0 | 1 | 0 | 1 | 0 | 0 | 1 | 0 |
| NM_018010 | IFT57 | 3203 | 11 | 5 | 3 | 1 | 4 | 6 | 7 | 0 | 11 | 4 | 1 | 6 | 10 |
| NM_018011 | ARGLU1 | 1751 | 15 | 9 | 7 | 9 | 8 | 12 | 8 | 6 | 13 | 6 | 5 | 13 | 15 |
| NM_018012 | KIF26B | 7287 | 1 | 0 | 0 | 0 | 0 | 1 | 0 | 0 | 0 | 0 | 0 | 0 | 0 |
| NM_018013 | SOBP | 6232 | 8 | 2 | 1 | 1 | 3 | 8 | 2 | 0 | 1 | 5 | 3 | 1 | 2 |
| NM_018014 | BCL11A | 3958 | 4 | 1 | 0 | 1 | 0 | 4 | 0 | 0 | 0 | 0 | 0 | 3 | 0 |
| NM_018015 | CXorf57 | 3904 | 2 | 1 | 0 | 0 | 0 | 2 | 1 | 0 | 0 | 1 | 0 | 0 | 1 |
| NM_018017 | C10orf118 | 6277 | 6 | 4 | 2 | 1 | 4 | 3 | 6 | 1 | 5 | 3 | 2 | 6 | 6 |
| NM_018018 | SLC38A4 | 3983 | 51 | 5 | 0 | 0 | 0 | 0 | 1 | 51 | 0 | 1 | 2 | 0 | 0 |
| NM_018019 | MED9 | 2222 | 4 | 2 | 2 | 2 | 3 | 4 | 2 | 2 | 1 | 2 | 2 | 2 | 2 |
| NM_018022 | TMEM51 | 1911 | 2 | 1 | 0 | 1 | 0 | 0 | 2 | 0 | 0 | 1 | 0 | 1 | 0 |
| NM_018023 | YEATS2 | 6506 | 6 | 4 | 2 | 3 | 3 | 6 | 3 | 1 | 4 | 6 | 2 | 5 | 5 |
| NM_018024 | WDYHV1 | 1348 | 0 | 0 | 0 | 0 | 0 | 0 | 0 | 0 | 0 | 0 | 0 | 0 | 0 |
| NM_018025 | GPATCH1 | 3362 | 3 | 2 | 2 | 2 | 1 | 3 | 3 | 1 | 1 | 3 | 2 | 3 | 3 |
| NM_018026 | PACS1 | 4488 | 6 | 3 | 3 | 1 | 6 | 5 | 3 | 1 | 3 | 4 | 1 | 5 | 5 |
| NM_018027 | FRMD4A | 6804 | 7 | 2 | 5 | 1 | 2 | 3 | 2 | 1 | 1 | 1 | 1 | 7 | 1 |
| NM_018028 | SAMD4B | 4490 | 2 | 1 | 1 | 1 | 1 | 1 | 1 | 0 | 1 | 2 | 1 | 1 | 2 |
| NM_018029 | FLJ10213 | 1662 | 1 | 1 | 0 | 0 | 1 | 0 | 0 | 0 | 1 | 1 | 0 | 1 | 1 |
| NM_018030 | OSBPL1A | 3315 | 26 | 10 | 13 | 3 | 13 | 26 | 10 | 4 | 6 | 8 | 18 | 4 | 6 |
| NM_018031 | WDR6 | 4259 | 3 | 2 | 1 | 2 | 1 | 1 | 3 | 2 | 1 | 2 | 1 | 2 | 2 |
| NM_018032 | LUC7L | 2082 | 8 | 5 | 4 | 6 | 3 | 7 | 5 | 3 | 5 | 5 | 3 | 7 | 8 |
| NM_018034 | WDR70 | 2122 | 12 | 8 | 6 | 6 | 7 | 6 | 7 | 5 | 6 | 11 | 10 | 12 | 11 |
| NM_018035 | ATP5SL | 1725 | 4 | 2 | 2 | 2 | 3 | 3 | 3 | 4 | 2 | 1 | 1 | 2 | 1 |
| NM_018036 | ATG2B | 11099 | 3 | 2 | 1 | 1 | 1 | 3 | 2 | 2 | 1 | 2 | 2 | 2 | 2 |
| NM_018039 | KDM4D | 2972 | 3 | 0 | 0 | 0 | 0 | 0 | 0 | 0 | 0 | 0 | 0 | 0 | 3 |
| NM_018040 | GPATCH2 | 2336 | 7 | 4 | 4 | 3 | 3 | 7 | 5 | 3 | 3 | 5 | 2 | 6 | 7 |
| NM_018042 | SLFN12 | 2390 | 7 | 2 | 3 | 1 | 2 | 1 | 2 | 0 | 3 | 5 | 1 | 7 | 1 |
| NM_018043 | ANO1 | 4807 | 17 | 3 | 1 | 1 | 2 | 0 | 1 | 17 | 1 | 0 | 1 | 0 | 3 |
| NM_018044 | NSUN5 | 2446 | 0 | 0 | 0 | 0 | 0 | 0 | 0 | 0 | 0 | 0 | 0 | 0 | 0 |
| NM_018045 | BSDC1 | 2856 | 3 | 2 | 2 | 2 | 1 | 3 | 1 | 2 | 1 | 2 | 1 | 2 | 2 |
| NM_018046 | AGGF1 | 4465 | 5 | 3 | 2 | 1 | 3 | 4 | 4 | 3 | 2 | 4 | 2 | 5 | 5 |
| NM_018047 | RBM22 | 2346 | 11 | 6 | 5 | 3 | 5 | 11 | 4 | 4 | 4 | 8 | 6 | 8 | 4 |
| NM_018048 | MAGOHB | 2579 | 1 | 0 | 0 | 0 | 0 | 0 | 0 | 0 | 0 | 0 | 0 | 1 | 0 |
| NM_018049 | PLEKHJ1 | 1215 | 2 | 1 | 0 | 1 | 1 | 1 | 2 | 0 | 1 | 0 | 1 | 1 | 1 |
| NM_018050 | MANSC1 | 2297 | 1 | 1 | 1 | 1 | 0 | 1 | 1 | 1 | 1 | 1 | 0 | 1 | 1 |
| NM_018051 | WDR60 | 3772 | 17 | 10 | 11 | 6 | 13 | 8 | 12 | 4 | 11 | 9 | 8 | 9 | 17 |
| NM_018052 | VAC14 | 3096 | 4 | 2 | 2 | 2 | 1 | 2 | 2 | 2 | 1 | 1 | 1 | 4 | 2 |
| NM_018053 | XKR8 | 2158 | 1 | 0 | 0 | 0 | 0 | 0 | 0 | 0 | 0 | 0 | 0 | 1 | 0 |
| NM_018054 | ARHGAP17 | 3244 | 9 | 4 | 4 | 4 | 4 | 2 | 3 | 2 | 3 | 3 | 3 | 9 | 5 |
| NM_018055 | NODAL | 2086 | 1 | 0 | 0 | 0 | 0 | 0 | 0 | 0 | 0 | 0 | 1 | 0 | 0 |
| NM_018056 | TMEM39B | 1818 | 3 | 2 | 1 | 2 | 1 | 2 | 1 | 1 | 2 | 3 | 1 | 3 | 3 |
| NM_018057 | SLC6A15 | 4256 | 3 | 0 | 0 | 0 | 0 | 3 | 0 | 0 | 0 | 0 | 0 | 0 | 0 |
| NM_018058 | CRTAC1 | 2889 | 2 | 0 | 0 | 0 | 0 | 1 | 0 | 0 | 2 | 0 | 0 | 0 | 0 |
| NM_018059 | RADIL | 3670 | 1 | 0 | 0 | 0 | 0 | 1 | 0 | 0 | 0 | 0 | 0 | 0 | 1 |
| NM_018060 | IARS2 | 3549 | 17 | 12 | 16 | 7 | 15 | 14 | 14 | 17 | 7 | 10 | 8 | 10 | 10 |
| NM_018061 | PRPF38B | 3703 | 39 | 18 | 18 | 7 | 18 | 16 | 16 | 12 | 22 | 17 | 16 | 39 | 20 |
| NM_018062 | FANCL | 1717 | 19 | 7 | 4 | 3 | 5 | 8 | 4 | 3 | 7 | 19 | 5 | 10 | 15 |
| NM_018063 | HELLS | 3099 | 5 | 2 | 1 | 2 | 1 | 0 | 1 | 1 | 1 | 2 | 0 | 3 | 5 |

| transcript | gene | transcript length (nt) | max | mean | adipose | colon | heart | hypothalamus | kidney | liver | lung | ovary | skeletalmuscle | spleen | testes |
|---|---|---|---|---|---|---|---|---|---|---|---|---|---|---|---|
| NM_018064 | AKIRIN2 | 1930 | 32 | 16 | 11 | 5 | 15 | 17 | 7 | 9 | 12 | 16 | 30 | 32 | 17 |
| NM_018066 | GPN2 | 1510 | 0 | 0 | 0 | 0 | 0 | 0 | 0 | 0 | 0 | 0 | 0 | 0 | 0 |
| NM_018067 | MAP7D1 | 3324 | 6 | 3 | 5 | 2 | 4 | 6 | 1 | 0 | 2 | 1 | 3 | 3 | 1 |
| NM_018068 | PIWIL2 | 3636 | 4 | 1 | 0 | 0 | 0 | 0 | 0 | 0 | 0 | 0 | 0 | 0 | 4 |
| NM_018070 | SSBP3 | 2014 | 11 | 4 | 2 | 3 | 3 | 8 | 2 | 2 | 2 | 11 | 3 | 3 | 5 |
| NM_018071 | FLJ10357 | 5458 | 2 | 1 | 2 | 1 | 1 | 1 | 1 | 0 | 0 | 1 | 0 | 2 | 1 |
| NM_018072 | HEATR1 | 6791 | 10 | 5 | 3 | 2 | 5 | 3 | 4 | 3 | 2 | 7 | 4 | 9 | 10 |
| NM_018073 | TRIM68 | 3300 | 2 | 1 | 1 | 1 | 1 | 1 | 2 | 1 | 1 | 1 | 1 | 1 | 1 |
| NM_018074 | CCDC94 | 1397 | 2 | 1 | 1 | 1 | 1 | 2 | 2 | 1 | 1 | 1 | 1 | 1 | 1 |
| NM_018075 | ANO10 | 2734 | 8 | 5 | 7 | 8 | 4 | 7 | 7 | 3 | 5 | 4 | 2 | 4 | 5 |
| NM_018076 | ARMC4 | 3572 | 4 | 0 | 0 | 0 | 0 | 0 | 0 | 0 | 1 | 0 | 0 | 0 | 4 |
| NM_018077 | RBM28 | 2809 | 11 | 8 | 10 | 6 | 9 | 8 | 10 | 6 | 6 | 10 | 8 | 9 | 11 |
| NM_018078 | LARP1B | 3957 | 20 | 8 | 4 | 4 | 6 | 9 | 10 | 17 | 5 | 6 | 6 | 5 | 20 |
| NM_018079 | SRBD1 | 3693 | 4 | 2 | 2 | 1 | 2 | 2 | 2 | 2 | 2 | 2 | 1 | 4 | 3 |
| NM_018080 | VPS13C | 11012 | 21 | 11 | 11 | 4 | 11 | 11 | 12 | 5 | 16 | 14 | 10 | 21 | 12 |
| NM_018081 | WRAP53 | 1962 | 0 | 0 | 0 | 0 | 0 | 0 | 0 | 0 | 0 | 0 | 0 | 0 | 0 |
| NM_018082 | POLR3B | 4271 | 4 | 2 | 2 | 1 | 2 | 2 | 3 | 4 | 1 | 3 | 3 | 2 | 3 |
| NM_018083 | ZNF358 | 1953 | 13 | 5 | 2 | 1 | 10 | 2 | 4 | 0 | 3 | 10 | 13 | 4 | 3 |
| NM_018084 | CCDC88A | 9730 | 18 | 7 | 10 | 1 | 7 | 18 | 3 | 3 | 7 | 6 | 3 | 10 | 14 |
| NM_018085 | IPO9 | 11416 | 4 | 2 | 1 | 1 | 2 | 4 | 2 | 2 | 2 | 3 | 1 | 2 | 2 |
| NM_018086 | FIGN | 4535 | 2 | 1 | 1 | 0 | 1 | 1 | 1 | 1 | 0 | 2 | 0 | 2 | 1 |
| NM_018087 | TMEM48 | 4823 | 3 | 2 | 1 | 2 | 1 | 2 | 2 | 1 | 1 | 2 | 1 | 3 | 3 |
| NM_018088 | FAM90A1 | 2516 | 0 | 0 | 0 | 0 | 0 | 0 | 0 | 0 | 0 | 0 | 0 | 0 | 0 |
| NM_018089 | ANKZF1 | 2579 | 3 | 2 | 1 | 3 | 2 | 2 | 3 | 1 | 2 | 1 | 2 | 3 | 2 |
| NM_018090 | NECAP2 | 2079 | 6 | 2 | 2 | 1 | 2 | 1 | 2 | 1 | 2 | 2 | 0 | 6 | 3 |
| NM_018091 | ELP3 | 3219 | 13 | 8 | 8 | 5 | 7 | 13 | 9 | 12 | 4 | 8 | 7 | 8 | 9 |
| NM_018092 | NETO2 | 3653 | 2 | 1 | 0 | 1 | 0 | 2 | 0 | 0 | 0 | 0 | 0 | 2 | 1 |
| NM_018093 | WDR74 | 1366 | 4 | 3 | 1 | 2 | 3 | 3 | 3 | 4 | 1 | 3 | 4 | 4 | 2 |
| NM_018094 | GSPT2 | 2533 | 6 | 3 | 2 | 1 | 3 | 6 | 3 | 3 | 1 | 4 | 1 | 3 | 6 |
| NM_018095 | KBTBD4 | 2401 | 2 | 1 | 1 | 1 | 1 | 2 | 1 | 1 | 1 | 1 | 1 | 1 | 1 |
| NM_018096 | NLE1 | 2574 | 0 | 0 | 0 | 0 | 0 | 0 | 0 | 0 | 0 | 0 | 0 | 0 | 0 |
| NM_018097 | HAUS2 | 3944 | 3 | 2 | 2 | 1 | 2 | 3 | 1 | 1 | 2 | 1 | 1 | 2 | 2 |
| NM_018098 | ECT2 | 3911 | 4 | 1 | 1 | 1 | 1 | 1 | 1 | 0 | 2 | 1 | 0 | 2 | 4 |
| NM_018099 | FAR2 | 2095 | 6 | 2 | 4 | 4 | 2 | 6 | 1 | 0 | 2 | 2 | 1 | 4 | 1 |
| NM_018100 | EFHC1 | 5582 | 3 | 1 | 1 | 1 | 1 | 1 | 1 | 0 | 1 | 2 | 0 | 1 | 3 |
| NM_018101 | CDCA8 | 2303 | 1 | 0 | 0 | 1 | 0 | 0 | 0 | 0 | 0 | 0 | 0 | 1 | 1 |
| NM_018102 | ZNF334 | 2455 | 5 | 2 | 0 | 0 | 1 | 3 | 3 | 0 | 1 | 5 | 1 | 1 | 4 |
| NM_018103 | LRRC8D | 3772 | 6 | 3 | 2 | 1 | 2 | 6 | 6 | 5 | 1 | 2 | 1 | 6 | 5 |
| NM_018105 | THAP1 | 2167 | 4 | 3 | 2 | 1 | 3 | 3 | 3 | 2 | 2 | 3 | 3 | 4 | 4 |
| NM_018106 | ZDHHC4 | 1395 | 4 | 2 | 2 | 1 | 2 | 3 | 2 | 2 | 1 | 2 | 1 | 2 | 4 |
| NM_018107 | RBM23 | 2556 | 14 | 8 | 5 | 6 | 6 | 9 | 9 | 7 | 6 | 10 | 6 | 14 | 11 |
| NM_018109 | MTPAP | 5620 | 4 | 2 | 2 | 1 | 2 | 4 | 2 | 2 | 1 | 2 | 2 | 2 | 2 |
| NM_018110 | DOK4 | 2750 | 3 | 1 | 0 | 2 | 1 | 1 | 2 | 3 | 1 | 1 | 0 | 1 | 1 |
| NM_018111 | C19orf73 | 744 | 0 | 0 | 0 | 0 | 0 | 0 | 0 | 0 | 0 | 0 | 0 | 0 | 0 |
| NM_018112 | TMEM38B | 2076 | 20 | 7 | 1 | 2 | 14 | 2 | 11 | 4 | 6 | 4 | 20 | 4 | 8 |
| NM_018113 | LMBR1L | 2344 | 5 | 1 | 1 | 2 | 1 | 1 | 1 | 1 | 1 | 1 | 0 | 2 | 5 |
| NM_018114 | DALRD3 | 2242 | 0 | 0 | 0 | 0 | 0 | 0 | 0 | 0 | 0 | 0 | 0 | 0 | 0 |
| NM_018115 | SDAD1 | 3018 | 7 | 5 | 4 | 1 | 6 | 7 | 5 | 5 | 3 | 5 | 5 | 7 | 7 |
| NM_018116 | MSTO1 | 2440 | 0 | 0 | 0 | 0 | 0 | 0 | 0 | 0 | 0 | 0 | 0 | 0 | 0 |
| NM_018117 | BRWD2 | 4724 | 7 | 4 | 5 | 2 | 3 | 4 | 6 | 3 | 3 | 4 | 1 | 7 | 4 |
| NM_018119 | POLR3E | 2605 | 5 | 3 | 2 | 3 | 3 | 4 | 3 | 4 | 2 | 2 | 5 | 3 | 2 |
| NM_018120 | ARMC1 | 2639 | 5 | 3 | 2 | 2 | 4 | 5 | 4 | 3 | 2 | 4 | 4 | 4 | 4 |
| NM_018121 | FAM178A | 7286 | 22 | 8 | 4 | 2 | 5 | 7 | 5 | 2 | 4 | 19 | 9 | 12 | 22 |
| NM_018122 | DARS2 | 3348 | 3 | 2 | 2 | 2 | 2 | 1 | 3 | 2 | 1 | 1 | 1 | 3 | 1 |
| NM_018124 | RFWD3 | 4952 | 6 | 2 | 2 | 2 | 1 | 1 | 2 | 1 | 1 | 2 | 1 | 4 | 6 |
| NM_018125 | ARHGEF10L | 4494 | 2 | 1 | 1 | 2 | 1 | 1 | 1 | 2 | 0 | 0 | 1 | 0 | 0 |
| NM_018126 | TMEM33 | 7717 | 4 | 3 | 1 | 1 | 2 | 3 | 4 | 4 | 3 | 3 | 1 | 4 | 2 |
| NM_018127 | ELAC2 | 3803 | 3 | 2 | 1 | 1 | 2 | 2 | 3 | 2 | 1 | 2 | 2 | 3 | 2 |
| NM_018128 | TSR1 | 4880 | 6 | 3 | 3 | 2 | 3 | 3 | 2 | 4 | 1 | 2 | 6 | 3 | 3 |
| NM_018129 | PNPO | 3464 | 38 | 9 | 5 | 5 | 6 | 12 | 15 | 38 | 3 | 3 | 5 | 6 | 4 |
| NM_018130 | SHQ1 | 2848 | 4 | 2 | 1 | 2 | 4 | 3 | 2 | 2 | 1 | 2 | 2 | 3 | 4 |
| NM_018131 | CEP55 | 2638 | 7 | 1 | 2 | 1 | 1 | 0 | 0 | 0 | 1 | 1 | 0 | 2 | 7 |
| NM_018132 | CENPQ | 1728 | 2 | 1 | 1 | 0 | 1 | 1 | 1 | 1 | 1 | 1 | 1 | 1 | 2 |
| NM_018133 | MSL2 | 4696 | 11 | 5 | 3 | 2 | 4 | 4 | 4 | 2 | 4 | 11 | 6 | 10 | 5 |
| NM_018134 | IQCC | 2053 | 1 | 0 | 0 | 1 | 0 | 1 | 0 | 0 | 0 | 1 | 0 | 0 | 1 |
| NM_018135 | MRPS18A | 1060 | 3 | 2 | 2 | 1 | 2 | 2 | 2 | 2 | 1 | 1 | 3 | 2 | 1 |
| NM_018136 | ASPM | 10888 | 2 | 0 | 0 | 0 | 0 | 0 | 0 | 0 | 0 | 0 | 0 | 1 | 2 |
| NM_018137 | PRMT6 | 2641 | 2 | 1 | 0 | 1 | 1 | 1 | 2 | 1 | 0 | 1 | 0 | 1 | 1 |
| NM_018138 | TBCCD1 | 2711 | 1 | 1 | 1 | 1 | 1 | 1 | 1 | 1 | 0 | 1 | 1 | 1 | 1 |
| NM_018139 | C14orf104 | 2963 | 2 | 1 | 1 | 1 | 1 | 1 | 2 | 2 | 1 | 1 | 1 | 2 | 1 |
| NM_018140 | CEP72 | 2414 | 0 | 0 | 0 | 0 | 0 | 0 | 0 | 0 | 0 | 0 | 0 | 0 | 0 |
| NM_018141 | MRPS10 | 2140 | 4 | 2 | 1 | 1 | 4 | 3 | 3 | 3 | 1 | 1 | 3 | 2 | 2 |
| NM_018142 | INTS10 | 2534 | 6 | 3 | 4 | 3 | 3 | 3 | 5 | 6 | 4 | 2 | 1 | 4 | 2 |
| NM_018143 | KLHL11 | 2326 | 15 | 6 | 2 | 2 | 3 | 15 | 5 | 3 | 3 | 11 | 2 | 7 | 13 |
| NM_018144 | SEC61A2 | 2528 | 7 | 2 | 0 | 1 | 1 | 7 | 1 | 2 | 1 | 1 | 1 | 2 | 2 |
| NM_018145 | FAM82A2 | 2251 | 5 | 3 | 5 | 2 | 2 | 5 | 4 | 5 | 3 | 1 | 1 | 2 | 2 |
| NM_018146 | RNMTL1 | 1801 | 3 | 2 | 1 | 2 | 2 | 2 | 3 | 3 | 1 | 2 | 3 | 2 | 3 |

| transcript | gene | transcript length (nt) | max | mean | adipose | colon | heart | hypothala mus | kidney | liver | lung | ovary | skeletalm uscle | spleen | testes |
|---|---|---|---|---|---|---|---|---|---|---|---|---|---|---|---|
| NM_018147 | FAIM | 924 | 10 | 4 | 10 | 2 | 3 | 8 | 6 | 5 | 3 | 4 | 2 | 3 | 3 |
| NM_018148 | LINS1 | 2577 | 3 | 1 | 1 | 2 | 1 | 1 | 1 | 1 | 1 | 2 | 1 | 3 | 2 |
| NM_018149 | C17orf71 | 3266 | 1 | 1 | 0 | 1 | 0 | 1 | 1 | 1 | 0 | 1 | 0 | 1 | 1 |
| NM_018150 | RNF220 | 3010 | 7 | 3 | 2 | 3 | 2 | 7 | 2 | 2 | 2 | 2 | 2 | 2 | 3 |
| NM_018151 | RIF1 | 8383 | 21 | 11 | 7 | 3 | 8 | 12 | 8 | 6 | 9 | 20 | 14 | 14 | 21 |
| NM_018153 | ANTXR1 | 2344 | 16 | 5 | 4 | 5 | 5 | 6 | 1 | 0 | 4 | 16 | 3 | 4 | 5 |
| NM_018154 | ASF1B | 1731 | 1 | 0 | 0 | 1 | 0 | 0 | 0 | 0 | 0 | 0 | 0 | 1 | 1 |
| NM_018155 | SLC25A36 | 4658 | 5 | 3 | 2 | 1 | 4 | 2 | 4 | 0 | 3 | 4 | 3 | 3 | 5 |
| NM_018156 | VPS13D | 16253 | 9 | 6 | 4 | 4 | 9 | 7 | 7 | 4 | 5 | 5 | 6 | 6 | 5 |
| NM_018157 | RIC8B | 4989 | 3 | 2 | 1 | 1 | 2 | 3 | 2 | 1 | 1 | 1 | 1 | 2 | 2 |
| NM_018158 | SLC4A1AP | 2970 | 12 | 6 | 5 | 2 | 6 | 12 | 5 | 3 | 4 | 4 | 5 | 5 | 10 |
| NM_018159 | NUDT11 | 2396 | 4 | 1 | 0 | 0 | 0 | 1 | 0 | 0 | 0 | 0 | 0 | 0 | 4 |
| NM_018161 | NADSYN1 | 2430 | 2 | 1 | 0 | 2 | 1 | 0 | 1 | 1 | 1 | 1 | 0 | 2 | 1 |
| NM_018163 | DNAJC17 | 993 | 10 | 6 | 5 | 6 | 4 | 9 | 6 | 10 | 4 | 7 | 3 | 8 | 3 |
| NM_018164 | C12orf11 | 2976 | 5 | 3 | 2 | 1 | 3 | 3 | 3 | 3 | 2 | 2 | 3 | 5 | 5 |
| NM_018165 | PBRM1 | 7532 | 9 | 5 | 5 | 2 | 5 | 3 | 4 | 4 | 5 | 8 | 5 | 9 | 7 |
| NM_018166 | FAM176B | 1054 | 0 | 0 | 0 | 0 | 0 | 0 | 0 | 0 | 0 | 0 | 0 | 0 | 0 |
| NM_018167 | BTBD7 | 2568 | 6 | 3 | 2 | 2 | 2 | 4 | 6 | 2 | 2 | 5 | 2 | 5 | 3 |
| NM_018168 | C14orf105 | 2512 | 16 | 2 | 0 | 0 | 0 | 0 | 16 | 3 | 0 | 0 | 0 | 0 | 1 |
| NM_018169 | C12orf35 | 6220 | 57 | 14 | 5 | 2 | 7 | 4 | 6 | 7 | 14 | 57 | 4 | 39 | 11 |
| NM_018170 | RPRD1A | 4267 | 15 | 7 | 5 | 4 | 8 | 15 | 6 | 4 | 4 | 5 | 6 | 7 | 11 |
| NM_018171 | APPL2 | 3230 | 18 | 6 | 3 | 6 | 4 | 3 | 6 | 2 | 4 | 6 | 3 | 8 | 18 |
| NM_018172 | FAM86C | 2111 | 1 | 0 | 0 | 0 | 0 | 0 | 1 | 0 | 0 | 0 | 1 | 0 | 0 |
| NM_018173 | PLEKHG6 | 2874 | 2 | 0 | 1 | 2 | 0 | 0 | 1 | 0 | 0 | 0 | 0 | 0 | 0 |
| NM_018174 | MAP1S | 3276 | 0 | 0 | 0 | 0 | 0 | 0 | 0 | 0 | 0 | 0 | 0 | 0 | 0 |
| NM_018176 | LGI2 | 6339 | 2 | 1 | 1 | 0 | 2 | 1 | 1 | 0 | 0 | 0 | 0 | 0 | 1 |
| NM_018177 | N4BP2 | 6813 | 8 | 4 | 2 | 1 | 3 | 3 | 3 | 5 | 3 | 8 | 1 | 5 | 4 |
| NM_018178 | GOLPH3L | 3171 | 14 | 8 | 5 | 9 | 6 | 8 | 9 | 5 | 6 | 14 | 7 | 9 | 8 |
| NM_018179 | ATF7IP | 4661 | 13 | 8 | 9 | 4 | 6 | 7 | 5 | 4 | 5 | 10 | 9 | 13 | 11 |
| NM_018180 | DHX32 | 3070 | 5 | 3 | 2 | 3 | 4 | 2 | 3 | 3 | 2 | 4 | 2 | 3 | 5 |
| NM_018181 | ZNF532 | 6464 | 9 | 4 | 3 | 2 | 4 | 5 | 2 | 0 | 2 | 9 | 6 | 3 | 5 |
| NM_018182 | C17orf63 | 4196 | 2 | 1 | 1 | 1 | 1 | 1 | 2 | 1 | 1 | 2 | 2 | 1 | 2 |
| NM_018183 | SBNO1 | 10978 | 22 | 7 | 4 | 3 | 6 | 9 | 6 | 5 | 4 | 7 | 5 | 7 | 22 |
| NM_018184 | ARL8B | 2985 | 34 | 20 | 15 | 5 | 27 | 34 | 21 | 12 | 20 | 15 | 25 | 25 | 22 |
| NM_018186 | C1orf112 | 3363 | 1 | 0 | 0 | 0 | 0 | 0 | 0 | 0 | 0 | 0 | 0 | 1 | 1 |
| NM_018188 | ATAD3A | 2611 | 1 | 0 | 0 | 0 | 0 | 1 | 0 | 1 | 0 | 0 | 0 | 0 | 0 |
| NM_018189 | DPPA4 | 2817 | 1 | 0 | 0 | 0 | 0 | 0 | 0 | 0 | 0 | 0 | 0 | 0 | 1 |
| NM_018190 | BBS7 | 2606 | 8 | 3 | 2 | 1 | 2 | 8 | 3 | 1 | 3 | 4 | 1 | 3 | 4 |
| NM_018191 | RCBTB1 | 4033 | 9 | 4 | 3 | 2 | 4 | 9 | 5 | 3 | 4 | 2 | 1 | 5 | 4 |
| NM_018192 | LEPREL1 | 3698 | 17 | 5 | 1 | 1 | 2 | 1 | 14 | 1 | 5 | 7 | 0 | 17 | 1 |
| NM_018193 | FANCI | 4569 | 4 | 1 | 1 | 1 | 0 | 0 | 0 | 0 | 1 | 1 | 0 | 1 | 4 |
| NM_018194 | HHAT | 3621 | 1 | 0 | 0 | 0 | 0 | 0 | 1 | 0 | 0 | 1 | 0 | 1 | 1 |
| NM_018195 | C11orf57 | 3741 | 3 | 2 | 2 | 1 | 3 | 3 | 3 | 1 | 2 | 3 | 2 | 3 | 3 |
| NM_018196 | TMLHE | 2855 | 4 | 2 | 2 | 2 | 4 | 2 | 3 | 2 | 1 | 3 | 4 | 3 | 2 |
| NM_018197 | ZFP64 | 3263 | 1 | 1 | 1 | 1 | 0 | 1 | 1 | 0 | 0 | 1 | 0 | 1 | 1 |
| NM_018198 | DNAJC11 | 3292 | 13 | 7 | 5 | 9 | 9 | 9 | 13 | 11 | 3 | 6 | 4 | 8 | 5 |
| NM_018199 | EXD2 | 2831 | 4 | 2 | 3 | 2 | 2 | 4 | 3 | 2 | 1 | 2 | 1 | 2 | 3 |
| NM_018200 | HMG20A | 3885 | 6 | 3 | 2 | 2 | 3 | 4 | 3 | 1 | 2 | 3 | 3 | 6 | 4 |
| NM_018201 | TBC1D13 | 3879 | 4 | 1 | 1 | 1 | 1 | 2 | 4 | 1 | 1 | 1 | 1 | 2 | 1 |
| NM_018202 | TMEM57 | 3936 | 13 | 5 | 2 | 1 | 3 | 4 | 4 | 13 | 3 | 7 | 9 | 5 | 5 |
| NM_018203 | KLHDC8A | 2500 | 4 | 0 | 0 | 0 | 0 | 0 | 0 | 0 | 0 | 4 | 0 | 0 | 0 |
| NM_018204 | CKAP2 | 3733 | 7 | 2 | 2 | 1 | 2 | 3 | 2 | 1 | 2 | 2 | 1 | 5 | 7 |
| NM_018205 | LRRC20 | 2909 | 2 | 1 | 0 | 0 | 0 | 1 | 0 | 1 | 0 | 0 | 2 | 0 | 0 |
| NM_018206 | VPS35 | 3285 | 20 | 12 | 9 | 4 | 16 | 20 | 13 | 5 | 10 | 15 | 7 | 18 | 20 |
| NM_018207 | TRIM62 | 3840 | 0 | 0 | 0 | 0 | 0 | 0 | 0 | 0 | 0 | 0 | 0 | 0 | 0 |
| NM_018208 | ETNK2 | 2610 | 10 | 2 | 0 | 0 | 0 | 0 | 5 | 10 | 0 | 1 | 0 | 1 | 2 |
| NM_018209 | ARFGAP1 | 3250 | 1 | 1 | 0 | 1 | 1 | 1 | 1 | 1 | 1 | 0 | 0 | 1 | 1 |
| NM_018210 | CARKD | 2661 | 2 | 1 | 1 | 1 | 1 | 2 | 2 | 1 | 1 | 2 | 1 | 1 | 2 |
| NM_018211 | RAVER2 | 4359 | 5 | 3 | 1 | 4 | 5 | 3 | 3 | 0 | 3 | 3 | 3 | 3 | 2 |
| NM_018212 | ENAH | 13109 | 19 | 9 | 6 | 5 | 14 | 13 | 4 | 2 | 7 | 13 | 9 | 8 | 19 |
| NM_018214 | LRRC1 | 3180 | 5 | 2 | 2 | 5 | 1 | 3 | 4 | 0 | 3 | 2 | 1 | 1 | 5 |
| NM_018215 | PNMAL1 | 3696 | 27 | 4 | 0 | 3 | 1 | 27 | 2 | 0 | 0 | 2 | 0 | 0 | 7 |
| NM_018216 | PANK4 | 2642 | 1 | 1 | 0 | 1 | 1 | 1 | 0 | 0 | 1 | 0 | 1 | 1 | 0 |
| NM_018217 | EDEM2 | 1913 | 9 | 3 | 2 | 2 | 2 | 2 | 3 | 9 | 2 | 1 | 1 | 4 | 2 |
| NM_018218 | USP40 | 5376 | 3 | 2 | 2 | 1 | 1 | 1 | 3 | 2 | 2 | 3 | 1 | 2 | 3 |
| NM_018219 | CCDC87 | 2915 | 1 | 0 | 0 | 0 | 0 | 0 | 0 | 0 | 0 | 0 | 0 | 0 | 1 |
| NM_018221 | MOBKL1B | 1329 | 16 | 7 | 6 | 6 | 6 | 5 | 9 | 5 | 9 | 5 | 2 | 16 | 6 |
| NM_018222 | PARVA | 3106 | 15 | 7 | 15 | 9 | 12 | 4 | 7 | 4 | 7 | 9 | 2 | 6 | 7 |
| NM_018223 | CHFR | 3158 | 2 | 1 | 1 | 1 | 1 | 1 | 1 | 1 | 1 | 1 | 1 | 2 | 1 |
| NM_018224 | C7orf44 | 857 | 4 | 3 | 1 | 2 | 3 | 3 | 4 | 1 | 4 | 2 | 2 | 4 | 3 |
| NM_018225 | SMU1 | 7091 | 6 | 3 | 3 | 2 | 3 | 6 | 3 | 4 | 2 | 4 | 3 | 4 | 3 |
| NM_018226 | RNPEPL1 | 2912 | 1 | 0 | 0 | 0 | 0 | 0 | 0 | 0 | 0 | 0 | 1 | 1 | 0 |
| NM_018227 | UBA6 | 6447 | 7 | 4 | 3 | 1 | 3 | 5 | 4 | 4 | 4 | 4 | 2 | 7 | 6 |
| NM_018228 | C14orf115 | 3416 | 0 | 0 | 0 | 0 | 0 | 0 | 0 | 0 | 0 | 0 | 0 | 0 | 0 |
| NM_018229 | MUDENG | 3094 | 8 | 4 | 2 | 2 | 5 | 6 | 8 | 3 | 3 | 3 | 3 | 7 | 5 |
| NM_018230 | NUP133 | 4170 | 6 | 4 | 3 | 2 | 5 | 4 | 4 | 3 | 3 | 6 | 4 | 5 | 5 |
| NM_018231 | SLC38A7 | 2773 | 1 | 1 | 1 | 0 | 1 | 0 | 1 | 1 | 1 | 0 | 0 | 1 | 1 |

| transcript | gene | transcript length (nt) | max | mean | adipose | colon | heart | hypothalamus | kidney | liver | lung | ovary | skeletalmuscle | spleen | testes |
|---|---|---|---|---|---|---|---|---|---|---|---|---|---|---|---|
| NM_018232 | FAM21B | 4326 | 0 | 0 | 0 | 0 | 0 | 0 | 0 | 0 | 0 | 0 | 0 | 0 | 0 |
| NM_018233 | OGFOD1 | 3020 | 8 | 4 | 3 | 2 | 4 | 6 | 4 | 5 | 3 | 3 | 8 | 4 | 4 |
| NM_018234 | STEAP3 | 3937 | 11 | 2 | 1 | 1 | 2 | 1 | 1 | 11 | 1 | 0 | 2 | 0 | 1 |
| NM_018235 | CNDP2 | 5071 | 15 | 4 | 2 | 2 | 2 | 4 | 15 | 6 | 3 | 1 | 1 | 5 | 2 |
| NM_018237 | CCAR1 | 3858 | 22 | 15 | 13 | 7 | 15 | 12 | 18 | 13 | 16 | 15 | 12 | 22 | 21 |
| NM_018238 | AGK | 2925 | 5 | 2 | 2 | 2 | 2 | 5 | 3 | 1 | 2 | 2 | 1 | 2 | 2 |
| NM_018239 | LRRC20 | 2927 | 2 | 1 | 0 | 0 | 0 | 1 | 0 | 1 | 0 | 0 | 2 | 0 | 0 |
| NM_018240 | KIRREL | 3612 | 2 | 1 | 2 | 1 | 1 | 1 | 1 | 0 | 1 | 1 | 0 | 1 | 1 |
| NM_018241 | TMEM184C | 2973 | 3 | 2 | 1 | 2 | 2 | 2 | 3 | 2 | 1 | 2 | 1 | 2 | 2 |
| NM_018242 | SLC47A1 | 3279 | 10 | 2 | 0 | 0 | 0 | 1 | 10 | 4 | 0 | 0 | 1 | 0 | 1 |
| NM_018243 | 11 Sep | 5555 | 34 | 12 | 34 | 6 | 9 | 20 | 18 | 8 | 9 | 9 | 3 | 11 | 5 |
| NM_018244 | UQCC | 2324 | 11 | 5 | 5 | 5 | 8 | 11 | 5 | 6 | 2 | 3 | 6 | 4 | 4 |
| NM_018245 | OGDHL | 3761 | 8 | 1 | 0 | 0 | 0 | 3 | 8 | 1 | 0 | 0 | 0 | 0 | 0 |
| NM_018246 | CCDC25 | 3640 | 25 | 12 | 12 | 5 | 11 | 25 | 11 | 22 | 6 | 7 | 8 | 10 | 13 |
| NM_018247 | TMEM30A | 4544 | 45 | 24 | 16 | 7 | 24 | 45 | 25 | 24 | 37 | 19 | 12 | 34 | 22 |
| NM_018248 | NEIL3 | 2402 | 0 | 0 | 0 | 0 | 0 | 0 | 0 | 0 | 0 | 0 | 0 | 0 | 0 |
| NM_018249 | CDK5RAP2 | 6230 | 16 | 8 | 6 | 4 | 9 | 6 | 4 | 9 | 5 | 11 | 16 | 8 | 11 |
| NM_018250 | INTS9 | 2759 | 1 | 1 | 0 | 0 | 1 | 1 | 1 | 0 | 0 | 0 | 0 | 1 | 1 |
| NM_018252 | TMEM206 | 1962 | 3 | 1 | 1 | 1 | 1 | 3 | 2 | 0 | 1 | 1 | 1 | 2 | 1 |
| NM_018254 | RCOR3 | 4270 | 3 | 2 | 1 | 1 | 1 | 1 | 2 | 1 | 2 | 3 | 3 | 2 | 3 |
| NM_018255 | ELP2 | 2494 | 10 | 6 | 4 | 2 | 5 | 6 | 5 | 7 | 4 | 10 | 7 | 6 | 7 |
| NM_018256 | WDR12 | 2281 | 6 | 4 | 3 | 2 | 5 | 6 | 6 | 5 | 1 | 3 | 3 | 4 | 5 |
| NM_018257 | PCMTD2 | 3912 | 12 | 6 | 3 | 3 | 7 | 6 | 7 | 3 | 5 | 12 | 6 | 8 | 8 |
| NM_018259 | TTC17 | 3481 | 19 | 12 | 10 | 8 | 11 | 8 | 12 | 14 | 12 | 19 | 8 | 17 | 16 |
| NM_018260 | ZNF701 | 2864 | 6 | 3 | 2 | 1 | 3 | 2 | 4 | 0 | 2 | 5 | 1 | 6 | 5 |
| NM_018261 | EXOC1 | 3426 | 11 | 6 | 7 | 3 | 6 | 11 | 6 | 3 | 5 | 7 | 3 | 8 | 8 |
| NM_018262 | IFT122 | 3837 | 11 | 3 | 3 | 3 | 2 | 3 | 4 | 1 | 3 | 3 | 1 | 2 | 11 |
| NM_018263 | ASXL2 | 7174 | 12 | 6 | 5 | 4 | 6 | 6 | 7 | 4 | 5 | 6 | 5 | 12 | 9 |
| NM_018264 | TYW1 | 3330 | 1 | 1 | 1 | 1 | 1 | 1 | 1 | 1 | 1 | 1 | 1 | 1 | 1 |
| NM_018265 | C1orf106 | 4128 | 1 | 0 | 0 | 1 | 0 | 0 | 1 | 0 | 0 | 0 | 0 | 0 | 0 |
| NM_018266 | TMEM39A | 2957 | 4 | 2 | 1 | 1 | 2 | 1 | 2 | 1 | 3 | 3 | 1 | 4 | 3 |
| NM_018268 | WDR41 | 2558 | 2 | 1 | 2 | 0 | 1 | 2 | 1 | 1 | 1 | 1 | 1 | 2 | 2 |
| NM_018269 | ADI1 | 1676 | 81 | 20 | 13 | 7 | 28 | 7 | 28 | 81 | 8 | 8 | 11 | 19 | 7 |
| NM_018270 | C20orf20 | 1634 | 13 | 4 | 2 | 3 | 4 | 3 | 3 | 2 | 3 | 4 | 3 | 6 | 13 |
| NM_018271 | THNSL2 | 2112 | 3 | 1 | 1 | 0 | 0 | 1 | 3 | 3 | 0 | 1 | 1 | 0 | 1 |
| NM_018272 | CASC1 | 2510 | 8 | 2 | 0 | 0 | 0 | 3 | 2 | 0 | 2 | 1 | 0 | 0 | 8 |
| NM_018273 | TMEM143 | 2249 | 1 | 0 | 0 | 0 | 1 | 0 | 0 | 0 | 0 | 0 | 1 | 0 | 0 |
| NM_018275 | C7orf43 | 2514 | 1 | 1 | 0 | 1 | 0 | 1 | 1 | 0 | 0 | 0 | 0 | 1 | 1 |
| NM_018279 | TMEM19 | 4658 | 3 | 2 | 1 | 2 | 2 | 3 | 3 | 2 | 1 | 1 | 1 | 3 | 1 |
| NM_018280 | C22orf26 | 1836 | 0 | 0 | 0 | 0 | 0 | 0 | 0 | 0 | 0 | 0 | 0 | 0 | 0 |
| NM_018281 | ECHDC2 | 1175 | 5 | 1 | 1 | 1 | 0 | 1 | 2 | 5 | 0 | 0 | 0 | 0 | 1 |
| NM_018283 | NUDT15 | 2022 | 3 | 2 | 1 | 1 | 2 | 2 | 3 | 3 | 1 | 1 | 1 | 2 | 2 |
| NM_018284 | GBP3 | 3040 | 8 | 4 | 5 | 4 | 4 | 4 | 7 | 0 | 8 | 3 | 1 | 7 | 3 |
| NM_018285 | IMP3 | 1239 | 3 | 2 | 1 | 2 | 2 | 2 | 2 | 2 | 2 | 1 | 2 | 3 | 1 |
| NM_018286 | TMEM100 | 1755 | 31 | 5 | 6 | 1 | 3 | 1 | 0 | 2 | 31 | 2 | 1 | 1 | 1 |
| NM_018287 | ARHGAP12 | 4172 | 16 | 10 | 8 | 7 | 6 | 15 | 9 | 9 | 8 | 16 | 5 | 13 | 10 |
| NM_018288 | PHF10 | 1674 | 4 | 3 | 2 | 1 | 3 | 4 | 2 | 2 | 3 | 4 | 2 | 4 | 2 |
| NM_018289 | VPS53 | 2694 | 9 | 4 | 4 | 4 | 2 | 9 | 3 | 3 | 3 | 2 | 2 | 3 | 4 |
| NM_018290 | PGM2 | 3238 | 3 | 2 | 1 | 1 | 2 | 1 | 1 | 1 | 2 | 2 | 0 | 3 | 3 |
| NM_018291 | FGGY | 2014 | 4 | 1 | 1 | 1 | 1 | 1 | 2 | 4 | 1 | 1 | 4 | 1 | 1 |
| NM_018292 | QRSL1 | 4116 | 3 | 2 | 1 | 1 | 3 | 2 | 2 | 3 | 1 | 1 | 2 | 2 | 1 |
| NM_018293 | ZNF654 | 4957 | 4 | 2 | 1 | 0 | 2 | 2 | 2 | 2 | 2 | 3 | 2 | 3 | 4 |
| NM_018294 | CWF19L1 | 2633 | 1 | 1 | 1 | 0 | 1 | 1 | 1 | 0 | 1 | 0 | 0 | 1 | 1 |
| NM_018295 | TMEM140 | 2054 | 4 | 2 | 3 | 1 | 1 | 1 | 2 | 2 | 2 | 1 | 4 | 3 | 1 |
| NM_018296 | LRRC36 | 2400 | 3 | 0 | 0 | 0 | 0 | 0 | 0 | 0 | 0 | 0 | 0 | 0 | 3 |
| NM_018297 | NGLY1 | 2588 | 6 | 3 | 2 | 2 | 3 | 3 | 3 | 4 | 2 | 2 | 4 | 4 | 6 |
| NM_018298 | MCOLN3 | 2796 | 1 | 0 | 0 | 0 | 0 | 0 | 1 | 0 | 1 | 0 | 0 | 0 | 0 |
| NM_018299 | UBE2W | 4006 | 7 | 5 | 3 | 1 | 6 | 7 | 6 | 3 | 5 | 5 | 6 | 7 | 7 |
| NM_018300 | ZNF83 | 2601 | 19 | 10 | 10 | 2 | 9 | 6 | 17 | 1 | 13 | 19 | 2 | 19 | 14 |
| NM_018301 | RBM41 | 1661 | 17 | 10 | 8 | 8 | 10 | 8 | 10 | 7 | 10 | 10 | 8 | 12 | 17 |
| NM_018302 | C4orf19 | 3633 | 1 | 0 | 0 | 0 | 0 | 0 | 1 | 1 | 0 | 0 | 0 | 0 | 0 |
| NM_018303 | EXOC2 | 4442 | 2 | 1 | 1 | 1 | 1 | 2 | 1 | 1 | 1 | 1 | 1 | 2 | 2 |
| NM_018304 | PRR11 | 1317 | 5 | 2 | 2 | 3 | 1 | 2 | 0 | 0 | 2 | 2 | 0 | 5 | 2 |
| NM_018306 | TMEM40 | 1710 | 0 | 0 | 0 | 0 | 0 | 0 | 0 | 0 | 0 | 0 | 0 | 0 | 0 |
| NM_018307 | RHOT1 | 3089 | 23 | 14 | 9 | 5 | 19 | 23 | 13 | 7 | 16 | 14 | 13 | 17 | 16 |
| NM_018309 | TBC1D23 | 3798 | 5 | 3 | 3 | 1 | 4 | 2 | 4 | 3 | 3 | 4 | 3 | 5 | 4 |
| NM_018310 | BRF2 | 1975 | 1 | 1 | 1 | 1 | 1 | 1 | 0 | 0 | 0 | 1 | 1 | 1 | 1 |
| NM_018312 | SAPS3 | 4968 | 23 | 15 | 9 | 6 | 18 | 9 | 16 | 9 | 13 | 20 | 17 | 23 | 23 |
| NM_018313 | PBRM1 | 7574 | 9 | 5 | 5 | 2 | 5 | 3 | 4 | 4 | 5 | 8 | 5 | 9 | 7 |
| NM_018314 | UEVLD | 1995 | 5 | 4 | 4 | 2 | 4 | 4 | 4 | 4 | 4 | 3 | 3 | 5 | 4 |
| NM_018315 | FBXW7 | 3735 | 22 | 5 | 2 | 5 | 7 | 22 | 1 | 2 | 2 | 2 | 6 | 5 | 4 |
| NM_018316 | KLHL26 | 3122 | 1 | 0 | 0 | 0 | 0 | 1 | 0 | 0 | 0 | 0 | 0 | 0 | 0 |
| NM_018317 | TBC1D19 | 2199 | 4 | 1 | 1 | 0 | 1 | 2 | 4 | 0 | 1 | 3 | 1 | 1 | 2 |
| NM_018318 | CCDC91 | 2335 | 10 | 6 | 5 | 2 | 8 | 10 | 6 | 3 | 5 | 5 | 5 | 8 | 9 |
| NM_018319 | TDP1 | 3747 | 2 | 1 | 1 | 1 | 0 | 1 | 1 | 0 | 0 | 1 | 0 | 2 | 1 |
| NM_018320 | RNF121 | 2604 | 3 | 2 | 1 | 2 | 2 | 2 | 3 | 2 | 1 | 2 | 2 | 2 | 2 |
| NM_018321 | BRIX1 | 1274 | 6 | 4 | 3 | 2 | 5 | 6 | 5 | 4 | 3 | 3 | 3 | 6 | 6 |

| transcript | gene | transcript length (nt) | max | mean | adipose | colon | heart | hypothalamus | kidney | liver | lung | ovary | skeletalmuscle | spleen | testes |
|---|---|---|---|---|---|---|---|---|---|---|---|---|---|---|---|
| NM_018322 | C6orf64 | 1920 | 5 | 2 | 1 | 1 | 1 | 2 | 2 | 1 | 1 | 2 | 1 | 2 | 5 |
| NM_018323 | PI4K2B | 3601 | 6 | 2 | 1 | 1 | 1 | 1 | 2 | 6 | 2 | 1 | 1 | 3 | 1 |
| NM_018324 | OLAH | 1834 | 1 | 0 | 0 | 0 | 0 | 0 | 0 | 0 | 1 | 0 | 0 | 1 | 1 |
| NM_018325 | C9orf72 | 3233 | 10 | 5 | 4 | 2 | 4 | 10 | 2 | 3 | 6 | 7 | 3 | 9 | 7 |
| NM_018326 | GIMAP4 | 1965 | 83 | 23 | 22 | 6 | 28 | 10 | 10 | 11 | 44 | 9 | 15 | 83 | 14 |
| NM_018327 | SPTLC3 | 3845 | 7 | 2 | 2 | 1 | 1 | 2 | 7 | 5 | 2 | 2 | 0 | 1 | 4 |
| NM_018328 | MBD5 | 5309 | 3 | 1 | 1 | 1 | 1 | 2 | 2 | 0 | 1 | 3 | 1 | 1 | 2 |
| NM_018330 | KIAA1598 | 3629 | 45 | 11 | 9 | 4 | 4 | 45 | 11 | 12 | 9 | 5 | 1 | 8 | 16 |
| NM_018332 | DDX19A | 2900 | 3 | 2 | 2 | 2 | 2 | 2 | 2 | 2 | 1 | 1 | 3 | 3 | 2 |
| NM_018334 | LRRN3 | 3538 | 5 | 1 | 1 | 0 | 1 | 2 | 1 | 0 | 1 | 2 | 1 | 1 | 5 |
| NM_018335 | ZNF839 | 3319 | 2 | 1 | 1 | 1 | 1 | 1 | 1 | 0 | 0 | 1 | 1 | 1 | 2 |
| NM_018337 | ZNF444 | 2043 | 1 | 0 | 0 | 1 | 0 | 0 | 0 | 1 | 0 | 0 | 0 | 0 | 1 |
| NM_018338 | WDR52 | 3281 | 3 | 1 | 1 | 2 | 1 | 2 | 2 | 0 | 2 | 2 | 1 | 2 | 3 |
| NM_018339 | RFK | 2707 | 10 | 3 | 2 | 2 | 3 | 10 | 4 | 5 | 2 | 1 | 1 | 3 | 4 |
| NM_018340 | CPPED1 | 6143 | 3 | 2 | 1 | 0 | 1 | 1 | 3 | 2 | 1 | 1 | 0 | 3 | 2 |
| NM_018341 | C6orf70 | 2078 | 5 | 2 | 2 | 1 | 1 | 4 | 2 | 3 | 2 | 2 | 2 | 3 | 5 |
| NM_018342 | TMEM144 | 3317 | 11 | 2 | 1 | 0 | 1 | 11 | 1 | 0 | 1 | 1 | 0 | 1 | 1 |
| NM_018343 | RIOK2 | 3962 | 3 | 2 | 1 | 1 | 2 | 2 | 2 | 2 | 1 | 3 | 2 | 2 | 2 |
| NM_018344 | SLC29A3 | 2267 | 1 | 0 | 1 | 0 | 0 | 0 | 0 | 0 | 0 | 1 | 0 | 1 | 0 |
| NM_018346 | RSAD1 | 2499 | 7 | 5 | 4 | 3 | 6 | 6 | 7 | 5 | 3 | 4 | 6 | 6 | 3 |
| NM_018347 | C20orf29 | 1832 | 0 | 0 | 0 | 0 | 0 | 0 | 0 | 0 | 0 | 0 | 0 | 0 | 0 |
| NM_018348 | FTSJD1 | 4171 | 4 | 2 | 1 | 1 | 1 | 1 | 3 | 2 | 1 | 2 | 1 | 4 | 3 |
| NM_018349 | MCTP2 | 7619 | 6 | 1 | 0 | 2 | 0 | 0 | 1 | 0 | 3 | 0 | 0 | 6 | 1 |
| NM_018351 | FGD6 | 9288 | 6 | 3 | 2 | 2 | 4 | 3 | 3 | 1 | 4 | 6 | 1 | 3 | 3 |
| NM_018352 | C4orf43 | 1918 | 9 | 5 | 3 | 2 | 4 | 3 | 4 | 4 | 3 | 7 | 3 | 9 | 8 |
| NM_018353 | C14orf106 | 4777 | 6 | 2 | 2 | 1 | 2 | 1 | 1 | 1 | 3 | 4 | 0 | 6 | 4 |
| NM_018354 | C20orf46 | 1720 | 0 | 0 | 0 | 0 | 0 | 0 | 0 | 0 | 0 | 0 | 0 | 0 | 0 |
| NM_018355 | ZNF415 | 2270 | 27 | 10 | 7 | 3 | 14 | 20 | 6 | 1 | 3 | 13 | 3 | 10 | 27 |
| NM_018356 | C5orf22 | 3612 | 8 | 4 | 2 | 2 | 6 | 8 | 3 | 4 | 3 | 3 | 8 | 5 | 5 |
| NM_018357 | LARP6 | 2049 | 3 | 1 | 1 | 0 | 1 | 3 | 0 | 0 | 0 | 1 | 0 | 0 | 1 |
| NM_018358 | ABCF3 | 2572 | 7 | 5 | 4 | 4 | 5 | 7 | 5 | 6 | 2 | 4 | 5 | 4 | 5 |
| NM_018359 | UFSP2 | 2379 | 4 | 2 | 1 | 1 | 2 | 2 | 3 | 1 | 2 | 4 | 2 | 2 | 4 |
| NM_018360 | CXorf15 | 4394 | 11 | 5 | 5 | 2 | 4 | 4 | 4 | 4 | 4 | 11 | 5 | 5 | 6 |
| NM_018361 | AGPAT5 | 5519 | 14 | 5 | 1 | 2 | 2 | 8 | 4 | 6 | 2 | 3 | 4 | 5 | 14 |
| NM_018362 | LIN7C | 4846 | 9 | 5 | 4 | 2 | 4 | 9 | 7 | 8 | 4 | 7 | 3 | 6 | 4 |
| NM_018363 | RNLS | 2180 | 1 | 0 | 0 | 0 | 1 | 0 | 1 | 0 | 0 | 1 | 0 | 0 | 0 |
| NM_018364 | RSBN1 | 6593 | 8 | 3 | 2 | 1 | 2 | 3 | 3 | 1 | 4 | 8 | 4 | 5 | 5 |
| NM_018365 | MNS1 | 2023 | 15 | 4 | 2 | 0 | 2 | 4 | 6 | 0 | 3 | 4 | 1 | 1 | 15 |
| NM_018366 | CNO | 1546 | 0 | 0 | 0 | 0 | 0 | 0 | 0 | 0 | 0 | 0 | 0 | 0 | 0 |
| NM_018367 | ACER3 | 4387 | 4 | 2 | 3 | 1 | 2 | 3 | 3 | 1 | 3 | 1 | 1 | 4 | 2 |
| NM_018368 | LMBRD1 | 2306 | 10 | 6 | 3 | 1 | 5 | 7 | 10 | 7 | 7 | 5 | 3 | 7 | 6 |
| NM_018369 | DEPDC1B | 2503 | 0 | 0 | 0 | 0 | 0 | 0 | 0 | 0 | 0 | 0 | 0 | 0 | 0 |
| NM_018370 | DRAM1 | 3543 | 4 | 1 | 1 | 0 | 1 | 0 | 2 | 0 | 4 | 1 | 0 | 1 | 1 |
| NM_018371 | CSGALNACT | 3872 | 7 | 3 | 2 | 1 | 7 | 4 | 2 | 1 | 3 | 5 | 1 | 4 | 1 |
| NM_018372 | C1orf103 | 3335 | 10 | 3 | 1 | 0 | 3 | 3 | 2 | 2 | 1 | 5 | 3 | 5 | 10 |
| NM_018373 | SYNJ2BP | 7060 | 13 | 6 | 6 | 3 | 10 | 5 | 13 | 7 | 5 | 5 | 6 | 6 | 5 |
| NM_018374 | TMEM106B | 6513 | 6 | 4 | 2 | 1 | 5 | 6 | 6 | 5 | 3 | 5 | 5 | 5 | 6 |
| NM_018375 | SLC39A9 | 5399 | 5 | 2 | 2 | 1 | 1 | 3 | 3 | 5 | 1 | 2 | 0 | 2 | 2 |
| NM_018376 | NIPSNAP3B | 1752 | 4 | 1 | 1 | 0 | 4 | 1 | 0 | 0 | 0 | 1 | 2 | 1 | 1 |
| NM_018378 | FBXL8 | 1599 | 0 | 0 | 0 | 0 | 0 | 0 | 0 | 0 | 0 | 0 | 0 | 0 | 0 |
| NM_018379 | FAM63A | 2839 | 3 | 1 | 1 | 1 | 1 | 2 | 3 | 1 | 1 | 1 | 0 | 1 | 2 |
| NM_018380 | DDX28 | 2592 | 1 | 1 | 0 | 0 | 0 | 1 | 1 | 0 | 0 | 1 | 1 | 1 | 1 |
| NM_018381 | C19orf66 | 2124 | 1 | 0 | 0 | 0 | 0 | 0 | 0 | 1 | 0 | 0 | 0 | 0 | 0 |
| NM_018383 | WDR33 | 6283 | 4 | 3 | 2 | 2 | 3 | 3 | 3 | 3 | 2 | 3 | 2 | 4 | 4 |
| NM_018384 | GIMAP5 | 1861 | 6 | 2 | 2 | 1 | 3 | 1 | 1 | 1 | 4 | 1 | 1 | 6 | 1 |
| NM_018385 | LSG1 | 3571 | 8 | 5 | 3 | 3 | 5 | 4 | 6 | 5 | 3 | 4 | 5 | 6 | 8 |
| NM_018386 | PCID2 | 1671 | 7 | 4 | 4 | 2 | 4 | 5 | 2 | 4 | 2 | 3 | 3 | 5 | 7 |
| NM_018387 | STRBP | 3338 | 25 | 9 | 3 | 7 | 5 | 20 | 8 | 5 | 5 | 4 | 2 | 14 | 25 |
| NM_018388 | MBNL3 | 2661 | 20 | 4 | 1 | 0 | 2 | 1 | 2 | 20 | 1 | 1 | 0 | 3 | 15 |
| NM_018389 | SLC35C1 | 3487 | 1 | 0 | 0 | 1 | 0 | 0 | 0 | 1 | 0 | 0 | 0 | 0 | 0 |
| NM_018390 | PLCXD1 | 5302 | 1 | 0 | 0 | 1 | 0 | 0 | 0 | 0 | 0 | 1 | 0 | 0 | 0 |
| NM_018392 | C4orf21 | 6739 | 2 | 1 | 0 | 0 | 0 | 1 | 0 | 0 | 1 | 1 | 1 | 1 | 2 |
| NM_018393 | TCP11L1 | 2816 | 3 | 1 | 1 | 1 | 1 | 3 | 1 | 1 | 1 | 1 | 1 | 1 | 1 |
| NM_018394 | ABHD10 | 2644 | 7 | 4 | 3 | 1 | 4 | 7 | 7 | 3 | 2 | 5 | 3 | 4 | 6 |
| NM_018396 | METTL2B | 2182 | 2 | 1 | 2 | 1 | 1 | 1 | 1 | 1 | 1 | 1 | 1 | 1 | 1 |
| NM_018397 | CHDH | 3641 | 5 | 1 | 0 | 1 | 0 | 1 | 5 | 2 | 0 | 0 | 0 | 0 | 1 |
| NM_018398 | CACNA2D3 | 3675 | 5 | 1 | 0 | 0 | 5 | 5 | 1 | 0 | 0 | 1 | 2 | 0 | 1 |
| NM_018400 | SCN3B | 6066 | 24 | 3 | 0 | 0 | 0 | 24 | 0 | 0 | 0 | 1 | 2 | 0 | 1 |
| NM_018401 | STK32B | 3224 | 6 | 1 | 1 | 0 | 1 | 1 | 6 | 0 | 0 | 1 | 0 | 0 | 2 |
| NM_018402 | IL26 | 1047 | 0 | 0 | 0 | 0 | 0 | 0 | 0 | 0 | 0 | 0 | 0 | 0 | 0 |
| NM_018403 | DCP1A | 5991 | 4 | 3 | 2 | 2 | 3 | 2 | 2 | 1 | 2 | 3 | 3 | 4 | 3 |
| NM_018404 | ADAP2 | 2749 | 3 | 1 | 3 | 1 | 1 | 1 | 1 | 1 | 1 | 0 | 0 | 2 | 0 |
| NM_018405 | C17orf79 | 936 | 14 | 6 | 7 | 2 | 7 | 14 | 6 | 6 | 3 | 5 | 4 | 4 | 12 |
| NM_018406 | MUC4 | 16722 | 33 | 4 | 0 | 33 | 0 | 0 | 1 | 0 | 6 | 0 | 0 | 0 | 1 |
| NM_018407 | LAPTM4B | 2238 | 14 | 5 | 1 | 1 | 14 | 3 | 6 | 2 | 2 | 5 | 5 | 7 | 8 |
| NM_018409 | LRP2BP | 5157 | 2 | 1 | 0 | 1 | 1 | 1 | 1 | 0 | 2 | 1 | 1 | 2 | 1 |
| NM_018410 | HJURP | 3050 | 2 | 0 | 1 | 2 | 0 | 0 | 0 | 0 | 0 | 0 | 0 | 1 | 2 |

| transcript | gene | transcript length (nt) | max | mean | adipose | colon | heart | hypothala mus | kidney | liver | lung | ovary | skeletalm uscle | spleen | testes |
|---|---|---|---|---|---|---|---|---|---|---|---|---|---|---|---|
| NM_018411 | HR | 5349 | 1 | 0 | 0 | 1 | 0 | 0 | 0 | 0 | 0 | 0 | 0 | 0 | 1 |
| NM_018412 | ST7 | 2043 | 1 | 1 | 1 | 0 | 1 | 1 | 1 | 1 | 1 | 1 | 1 | 1 | 1 |
| NM_018413 | CHST11 | 1652 | 11 | 3 | 2 | 1 | 2 | 4 | 1 | 0 | 6 | 2 | 0 | 11 | 1 |
| NM_018414 | ST6GALNAC | 2544 | 10 | 1 | 0 | 10 | 0 | 0 | 0 | 0 | 1 | 0 | 0 | 1 | 1 |
| NM_018416 | FOXJ2 | 5482 | 3 | 2 | 1 | 1 | 3 | 1 | 1 | 1 | 2 | 3 | 3 | 2 | 2 |
| NM_018417 | ADCY10 | 5421 | 1 | 0 | 0 | 0 | 0 | 0 | 0 | 0 | 0 | 0 | 0 | 0 | 1 |
| NM_018418 | SPATA7 | 1989 | 4 | 2 | 1 | 0 | 1 | 2 | 2 | 0 | 1 | 4 | 1 | 1 | 4 |
| NM_018419 | SOX18 | 1705 | 1 | 0 | 1 | 0 | 1 | 0 | 0 | 0 | 0 | 0 | 0 | 0 | 0 |
| NM_018420 | SLC22A15 | 4720 | 3 | 1 | 0 | 0 | 1 | 3 | 1 | 0 | 1 | 0 | 0 | 1 | 1 |
| NM_018421 | TBC1D2 | 3316 | 1 | 0 | 0 | 0 | 0 | 0 | 0 | 0 | 1 | 0 | 0 | 0 | 0 |
| NM_018423 | STYK1 | 2995 | 1 | 0 | 0 | 0 | 0 | 1 | 0 | 0 | 0 | 0 | 0 | 0 | 0 |
| NM_018424 | EPB41L4B | 3782 | 8 | 2 | 4 | 4 | 0 | 1 | 1 | 8 | 1 | 1 | 0 | 0 | 1 |
| NM_018425 | PI4K2A | 4185 | 4 | 1 | 2 | 0 | 1 | 2 | 1 | 1 | 1 | 4 | 1 | 2 | 1 |
| NM_018426 | TMEM63B | 3199 | 8 | 4 | 2 | 4 | 2 | 7 | 3 | 4 | 5 | 1 | 4 | 2 | 8 |
| NM_018427 | RRN3 | 3746 | 5 | 3 | 3 | 2 | 3 | 3 | 2 | 2 | 2 | 2 | 4 | 4 | 5 |
| NM_018428 | UTP6 | 2065 | 8 | 6 | 6 | 5 | 6 | 5 | 5 | 6 | 4 | 4 | 5 | 7 | 8 |
| NM_018429 | BDP1 | 11075 | 17 | 12 | 11 | 4 | 13 | 16 | 12 | 6 | 10 | 16 | 10 | 17 | 16 |
| NM_018430 | TSNAXIP1 | 2446 | 3 | 1 | 0 | 0 | 0 | 1 | 0 | 0 | 1 | 0 | 0 | 0 | 3 |
| NM_018431 | DOK5 | 1833 | 9 | 2 | 2 | 0 | 4 | 9 | 1 | 0 | 0 | 1 | 2 | 0 | 0 |
| NM_018433 | KDM3A | 4889 | 11 | 5 | 4 | 3 | 5 | 5 | 4 | 2 | 6 | 6 | 5 | 7 | 11 |
| NM_018434 | RNF130 | 1847 | 26 | 17 | 26 | 7 | 15 | 23 | 12 | 24 | 21 | 14 | 6 | 24 | 9 |
| NM_018436 | ALLC | 1444 | 3 | 0 | 0 | 0 | 0 | 0 | 1 | 0 | 0 | 0 | 0 | 0 | 3 |
| NM_018437 | HEMGN | 2188 | 12 | 2 | 0 | 0 | 0 | 0 | 0 | 0 | 2 | 0 | 0 | 1 | 12 |
| NM_018438 | FBXO6 | 1547 | 1 | 0 | 0 | 0 | 0 | 1 | 0 | 1 | 0 | 0 | 0 | 0 | 0 |
| NM_018439 | IMPACT | 3758 | 6 | 3 | 2 | 1 | 2 | 3 | 3 | 2 | 2 | 3 | 1 | 3 | 6 |
| NM_018440 | PAG1 | 10743 | 7 | 2 | 1 | 2 | 1 | 2 | 1 | 1 | 3 | 1 | 0 | 7 | 0 |
| NM_018441 | PECR | 1873 | 5 | 2 | 5 | 1 | 0 | 1 | 4 | 4 | 1 | 1 | 0 | 1 | 1 |
| NM_018442 | DCAF6 | 3293 | 125 | 34 | 18 | 9 | 58 | 24 | 15 | 29 | 14 | 20 | 125 | 16 | 41 |
| NM_018443 | ZNF302 | 2623 | 7 | 4 | 3 | 1 | 5 | 5 | 4 | 2 | 3 | 6 | 5 | 7 | 5 |
| NM_018444 | PDP1 | 4276 | 11 | 4 | 2 | 1 | 10 | 11 | 4 | 1 | 2 | 3 | 5 | 4 | 6 |
| NM_018445 | SELS | 1234 | 32 | 18 | 13 | 11 | 32 | 17 | 17 | 24 | 13 | 10 | 10 | 25 | 29 |
| NM_018446 | GLT8D1 | 2095 | 3 | 1 | 1 | 1 | 1 | 2 | 2 | 3 | 1 | 2 | 1 | 1 | 1 |
| NM_018447 | TMEM111 | 1080 | 15 | 9 | 10 | 3 | 13 | 10 | 8 | 13 | 5 | 5 | 15 | 7 | 9 |
| NM_018448 | CAND1 | 5908 | 11 | 6 | 5 | 2 | 4 | 11 | 6 | 5 | 5 | 7 | 3 | 7 | 10 |
| NM_018449 | UBAP2 | 4284 | 12 | 7 | 5 | 4 | 6 | 8 | 6 | 3 | 5 | 7 | 11 | 7 | 12 |
| NM_018451 | CENPJ | 5173 | 4 | 1 | 1 | 1 | 1 | 1 | 1 | 0 | 1 | 1 | 1 | 2 | 4 |
| NM_018452 | C6orf35 | 5025 | 2 | 1 | 1 | 1 | 2 | 2 | 1 | 0 | 1 | 1 | 1 | 1 | 1 |
| NM_018453 | EAPP | 1348 | 24 | 17 | 16 | 12 | 11 | 24 | 14 | 20 | 13 | 24 | 12 | 19 | 17 |
| NM_018454 | NUSAP1 | 2417 | 3 | 1 | 1 | 3 | 1 | 0 | 0 | 1 | 2 | 2 | 1 | 2 | 2 |
| NM_018455 | CENPN | 1449 | 1 | 0 | 0 | 0 | 1 | 1 | 0 | 1 | 0 | 0 | 0 | 1 | 0 |
| NM_018456 | EAF2 | 1020 | 6 | 2 | 1 | 2 | 1 | 2 | 6 | 1 | 2 | 1 | 1 | 6 | 2 |
| NM_018457 | PRR13 | 1195 | 43 | 15 | 11 | 16 | 9 | 14 | 43 | 15 | 14 | 6 | 5 | 28 | 6 |
| NM_018460 | ARHGAP15 | 1759 | 15 | 2 | 2 | 1 | 1 | 0 | 1 | 0 | 3 | 0 | 0 | 15 | 0 |
| NM_018461 | PPP2R2D | 1850 | 5 | 4 | 2 | 3 | 3 | 4 | 3 | 2 | 3 | 5 | 4 | 5 | 5 |
| NM_018462 | C3orf10 | 1176 | 6 | 5 | 6 | 3 | 6 | 5 | 6 | 4 | 5 | 3 | 3 | 6 | 6 |
| NM_018463 | ITFG2 | 2361 | 4 | 2 | 2 | 2 | 1 | 1 | 2 | 1 | 1 | 3 | 1 | 4 | 1 |
| NM_018464 | CISD1 | 2075 | 7 | 3 | 1 | 0 | 7 | 5 | 5 | 2 | 1 | 1 | 3 | 2 | 1 |
| NM_018465 | C9orf46 | 916 | 9 | 5 | 7 | 4 | 6 | 3 | 9 | 5 | 6 | 3 | 4 | 5 | 3 |
| NM_018466 | ALG13 | 2993 | 3 | 2 | 1 | 1 | 1 | 1 | 2 | 1 | 2 | 3 | 0 | 3 | 2 |
| NM_018467 | USE1 | 856 | 6 | 3 | 3 | 2 | 4 | 6 | 2 | 4 | 1 | 3 | 4 | 2 | 1 |
| NM_018469 | TEX2 | 5090 | 18 | 10 | 8 | 3 | 11 | 17 | 7 | 18 | 6 | 7 | 8 | 8 | 14 |
| NM_018471 | ZC3H15 | 2133 | 28 | 16 | 15 | 4 | 17 | 28 | 21 | 18 | 14 | 12 | 12 | 19 | 20 |
| NM_018473 | ACOT13 | 4102 | 5 | 3 | 3 | 2 | 3 | 2 | 5 | 5 | 1 | 2 | 2 | 2 | 2 |
| NM_018474 | PLK1S1 | 2196 | 18 | 7 | 4 | 3 | 7 | 9 | 5 | 2 | 4 | 18 | 8 | 5 | 16 |
| NM_018475 | TMEM165 | 1965 | 5 | 2 | 3 | 1 | 5 | 2 | 3 | 1 | 4 | 2 | 1 | 4 | 2 |
| NM_018476 | BEX1 | 862 | 166 | 20 | 0 | 2 | 3 | 166 | 2 | 1 | 1 | 3 | 0 | 1 | 47 |
| NM_018477 | ACTR10 | 2441 | 2 | 2 | 1 | 1 | 2 | 2 | 2 | 1 | 1 | 1 | 1 | 2 | 2 |
| NM_018479 | ECHDC1 | 2286 | 24 | 11 | 24 | 4 | 8 | 12 | 16 | 15 | 12 | 5 | 5 | 9 | 6 |
| NM_018480 | TMEM126B | 1119 | 16 | 9 | 9 | 2 | 11 | 13 | 16 | 10 | 8 | 6 | 4 | 12 | 6 |
| NM_018482 | ASAP1 | 6042 | 24 | 10 | 8 | 3 | 11 | 13 | 5 | 3 | 12 | 11 | 4 | 24 | 15 |
| NM_018484 | SLC22A11 | 2512 | 7 | 1 | 0 | 0 | 0 | 0 | 7 | 0 | 0 | 0 | 0 | 0 | 0 |
| NM_018485 | GPR77 | 1287 | 1 | 0 | 0 | 0 | 0 | 0 | 0 | 0 | 0 | 0 | 0 | 1 | 0 |
| NM_018486 | HDAC8 | 2013 | 2 | 1 | 1 | 1 | 1 | 2 | 2 | 1 | 1 | 2 | 1 | 1 | 1 |
| NM_018487 | TMEM176A | 1038 | 37 | 8 | 2 | 5 | 3 | 2 | 24 | 37 | 2 | 3 | 1 | 7 | 1 |
| NM_018488 | TBX4 | 2470 | 3 | 0 | 0 | 0 | 0 | 0 | 0 | 0 | 3 | 0 | 0 | 0 | 0 |
| NM_018489 | ASH1L | 11774 | 10 | 6 | 5 | 3 | 6 | 10 | 6 | 3 | 5 | 10 | 7 | 7 | 8 |
| NM_018490 | LGR4 | 5247 | 8 | 3 | 2 | 2 | 4 | 2 | 8 | 7 | 1 | 4 | 1 | 2 | 5 |
| NM_018491 | CBWD1 | 1723 | 2 | 1 | 1 | 0 | 1 | 1 | 2 | 1 | 1 | 1 | 0 | 1 | 1 |
| NM_018492 | PBK | 1854 | 13 | 2 | 0 | 2 | 0 | 0 | 0 | 0 | 1 | 0 | 0 | 0 | 13 |
| NM_018494 | LRDD | 3030 | 0 | 0 | 0 | 0 | 0 | 0 | 0 | 0 | 0 | 0 | 0 | 0 | 0 |
| NM_018502 | TMCO6 | 1889 | 1 | 0 | 0 | 0 | 0 | 0 | 0 | 0 | 0 | 0 | 0 | 1 | 0 |
| NM_018509 | LRRC59 | 2828 | 23 | 8 | 7 | 8 | 9 | 7 | 9 | 23 | 6 | 3 | 2 | 9 | 7 |
| NM_018518 | MCM10 | 4520 | 0 | 0 | 0 | 0 | 0 | 0 | 0 | 0 | 0 | 0 | 0 | 0 | 0 |
| NM_018527 | NARG1L | 1642 | 3 | 2 | 1 | 1 | 1 | 2 | 1 | 1 | 1 | 2 | 2 | 2 | 3 |
| NM_018530 | GSDMB | 1626 | 13 | 2 | 2 | 13 | 1 | 1 | 1 | 0 | 1 | 1 | 0 | 2 | 2 |
| NM_018534 | NRP2 | 4142 | 8 | 3 | 6 | 5 | 1 | 4 | 1 | 1 | 3 | 8 | 1 | 1 | 1 |
| NM_018538 | ERMAP | 3351 | 2 | 1 | 2 | 1 | 0 | 1 | 1 | 1 | 1 | 1 | 0 | 1 | 1 |

| transcript | gene | transcript length (nt) | max | mean | adipose | colon | heart | hypothala mus | kidney | liver | lung | ovary | skeletalm uscle | spleen | testes |
|---|---|---|---|---|---|---|---|---|---|---|---|---|---|---|---|
| NM_018553 | C17orf85 | 3797 | 4 | 3 | 2 | 3 | 3 | 3 | 3 | 2 | 3 | 4 | 2 | 4 | 4 |
| NM_018555 | ZNF331 | 5143 | 8 | 3 | 2 | 1 | 3 | 2 | 1 | 1 | 4 | 8 | 1 | 2 | 2 |
| NM_018556 | SIRPG | 1716 | 1 | 0 | 0 | 0 | 0 | 0 | 0 | 0 | 0 | 0 | 0 | 1 | 0 |
| NM_018557 | LRP1B | 16531 | 9 | 1 | 0 | 0 | 0 | 9 | 0 | 0 | 0 | 0 | 0 | 0 | 0 |
| NM_018558 | GABRQ | 2000 | 2 | 0 | 0 | 0 | 0 | 2 | 0 | 0 | 0 | 0 | 0 | 0 | 0 |
| NM_018559 | KIAA1704 | 1392 | 10 | 6 | 4 | 2 | 6 | 10 | 7 | 3 | 5 | 9 | 5 | 8 | 9 |
| NM_018561 | USP49 | 3177 | 2 | 1 | 1 | 1 | 0 | 1 | 0 | 0 | 1 | 1 | 0 | 1 | 2 |
| NM_018566 | YOD1 | 6265 | 3 | 2 | 1 | 0 | 2 | 2 | 2 | 2 | 3 | 2 | 1 | 3 | 2 |
| NM_018569 | AP1AR | 2908 | 10 | 6 | 3 | 3 | 4 | 10 | 8 | 4 | 5 | 6 | 3 | 9 | 8 |
| NM_018571 | STRADB | 2298 | 4 | 2 | 1 | 1 | 2 | 1 | 4 | 2 | 2 | 1 | 3 | 2 | 2 |
| NM_018584 | CAMK2N1 | 2351 | 33 | 6 | 0 | 5 | 1 | 33 | 9 | 5 | 2 | 2 | 0 | 1 | 2 |
| NM_018590 | CSGALNACT | 3723 | 17 | 7 | 7 | 2 | 6 | 6 | 4 | 5 | 17 | 7 | 4 | 15 | 5 |
| NM_018593 | SLC16A10 | 2889 | 2 | 1 | 1 | 0 | 1 | 0 | 2 | 0 | 1 | 0 | 2 | 1 | 1 |
| NM_018602 | DNAJA4 | 3186 | 113 | 20 | 4 | 5 | 113 | 32 | 5 | 0 | 12 | 3 | 14 | 7 | 22 |
| NM_018622 | PARL | 1412 | 13 | 8 | 10 | 7 | 8 | 13 | 7 | 11 | 6 | 6 | 5 | 7 | 8 |
| NM_018638 | ETNK1 | 7101 | 10 | 6 | 3 | 4 | 3 | 9 | 7 | 6 | 8 | 5 | 2 | 10 | 8 |
| NM_018639 | WSB2 | 2817 | 12 | 4 | 3 | 2 | 6 | 12 | 4 | 5 | 2 | 2 | 3 | 4 | 2 |
| NM_018640 | LMO3 | 3727 | 15 | 2 | 2 | 1 | 1 | 15 | 0 | 0 | 4 | 0 | 0 | 0 | 1 |
| NM_018641 | CHST12 | 2069 | 1 | 0 | 0 | 0 | 1 | 0 | 0 | 0 | 0 | 0 | 0 | 1 | 0 |
| NM_018643 | TREM1 | 920 | 22 | 4 | 2 | 0 | 5 | 1 | 0 | 1 | 22 | 1 | 0 | 9 | 1 |
| NM_018644 | B3GAT1 | 3524 | 16 | 1 | 0 | 0 | 0 | 16 | 0 | 0 | 0 | 0 | 0 | 0 | 0 |
| NM_018645 | HES6 | 1368 | 0 | 0 | 0 | 0 | 0 | 0 | 0 | 0 | 0 | 0 | 0 | 0 | 0 |
| NM_018646 | TRPV6 | 2894 | 0 | 0 | 0 | 0 | 0 | 0 | 0 | 0 | 0 | 0 | 0 | 0 | 0 |
| NM_018647 | TNFRSF19 | 1485 | 2 | 1 | 0 | 0 | 2 | 0 | 2 | 0 | 1 | 0 | 0 | 0 | 1 |
| NM_018648 | NOP10 | 535 | 19 | 11 | 12 | 8 | 14 | 12 | 13 | 15 | 13 | 4 | 6 | 19 | 5 |
| NM_018649 | H2AFY2 | 2179 | 1 | 0 | 0 | 0 | 0 | 1 | 1 | 0 | 0 | 1 | 0 | 0 | 1 |
| NM_018650 | MARK1 | 5276 | 5 | 1 | 3 | 1 | 2 | 5 | 1 | 0 | 1 | 1 | 0 | 1 | 3 |
| NM_018651 | ZNF167 | 3464 | 2 | 1 | 1 | 0 | 1 | 1 | 1 | 0 | 0 | 2 | 0 | 1 | 2 |
| NM_018652 | GOLGA6B | 3177 | 0 | 0 | 0 | 0 | 0 | 0 | 0 | 0 | 0 | 0 | 0 | 0 | 0 |
| NM_018653 | GPRC5C | 1820 | 1 | 0 | 0 | 0 | 0 | 0 | 1 | 1 | 0 | 0 | 0 | 0 | 0 |
| NM_018654 | GPRC5D | 1038 | 2 | 0 | 0 | 0 | 0 | 0 | 0 | 0 | 2 | 0 | 0 | 0 | 0 |
| NM_018655 | LENEP | 649 | 0 | 0 | 0 | 0 | 0 | 0 | 0 | 0 | 0 | 0 | 0 | 0 | 0 |
| NM_018656 | SLC35E3 | 2347 | 2 | 1 | 1 | 1 | 1 | 2 | 2 | 1 | 2 | 2 | 1 | 2 | 1 |
| NM_018657 | MYNN | 3133 | 3 | 1 | 1 | 0 | 2 | 1 | 2 | 1 | 1 | 2 | 1 | 3 | 2 |
| NM_018658 | KCNJ16 | 4004 | 35 | 4 | 0 | 0 | 0 | 3 | 35 | 0 | 0 | 0 | 0 | 0 | 1 |
| NM_018659 | CYTL1 | 1000 | 2 | 1 | 1 | 1 | 2 | 2 | 0 | 0 | 2 | 1 | 0 | 0 | 0 |
| NM_018660 | ZNF395 | 4802 | 9 | 3 | 2 | 3 | 3 | 1 | 3 | 2 | 4 | 9 | 2 | 4 | 5 |
| NM_018661 | DEFB103A | 324 | 0 | 0 | 0 | 0 | 0 | 0 | 0 | 0 | 0 | 0 | 0 | 0 | 0 |
| NM_018662 | DISC1 | 7059 | 0 | 0 | 0 | 0 | 0 | 0 | 0 | 0 | 0 | 0 | 0 | 0 | 0 |
| NM_018663 | PXMP2 | 957 | 19 | 4 | 2 | 4 | 4 | 2 | 8 | 19 | 1 | 1 | 2 | 1 | 2 |
| NM_018664 | BATF3 | 981 | 11 | 3 | 2 | 2 | 3 | 11 | 1 | 0 | 2 | 1 | 1 | 10 | 1 |
| NM_018665 | DDX43 | 2689 | 8 | 1 | 1 | 0 | 0 | 0 | 0 | 0 | 0 | 1 | 0 | 1 | 8 |
| NM_018666 | SAGE1 | 3046 | 1 | 0 | 0 | 0 | 0 | 0 | 0 | 0 | 0 | 0 | 0 | 0 | 1 |
| NM_018667 | SMPD3 | 5267 | 1 | 0 | 0 | 1 | 0 | 1 | 0 | 0 | 0 | 0 | 0 | 0 | 0 |
| NM_018668 | VPS33B | 2639 | 1 | 0 | 0 | 0 | 0 | 1 | 0 | 0 | 0 | 0 | 0 | 0 | 0 |
| NM_018669 | WDR4 | 2135 | 1 | 0 | 0 | 0 | 0 | 0 | 0 | 0 | 0 | 0 | 0 | 1 | 0 |
| NM_018670 | MESP1 | 1160 | 0 | 0 | 0 | 0 | 0 | 0 | 0 | 0 | 0 | 0 | 0 | 0 | 0 |
| NM_018671 | UNC45A | 3552 | 3 | 2 | 3 | 3 | 2 | 2 | 3 | 2 | 2 | 3 | 1 | 3 | 2 |
| NM_018672 | ABCA5 | 9249 | 8 | 2 | 1 | 1 | 1 | 1 | 2 | 2 | 1 | 1 | 8 | 1 | 2 |
| NM_018674 | ACCN4 | 2895 | 1 | 0 | 0 | 0 | 0 | 1 | 0 | 0 | 0 | 0 | 0 | 0 | 0 |
| NM_018676 | THSD1 | 3346 | 1 | 0 | 1 | 0 | 0 | 0 | 0 | 0 | 0 | 0 | 0 | 1 | 0 |
| NM_018677 | ACSS2 | 2979 | 6 | 3 | 4 | 4 | 4 | 1 | 6 | 2 | 2 | 1 | 5 | 1 | 1 |
| NM_018679 | TCP11 | 2023 | 7 | 1 | 0 | 0 | 0 | 0 | 0 | 0 | 0 | 0 | 0 | 0 | 7 |
| NM_018682 | MLL5 | 6715 | 27 | 15 | 9 | 13 | 13 | 13 | 12 | 4 | 20 | 24 | 11 | 27 | 17 |
| NM_018683 | RNF114 | 2467 | 12 | 6 | 3 | 4 | 7 | 5 | 8 | 6 | 6 | 6 | 7 | 12 | 8 |
| NM_018684 | ZC4H2 | 2156 | 4 | 2 | 1 | 2 | 2 | 4 | 2 | 2 | 1 | 3 | 1 | 1 | 1 |
| NM_018685 | ANLN | 4770 | 16 | 2 | 1 | 1 | 1 | 16 | 0 | 0 | 1 | 1 | 0 | 1 | 2 |
| NM_018686 | CMAS | 1741 | 10 | 5 | 2 | 3 | 4 | 10 | 3 | 8 | 3 | 2 | 1 | 3 | 10 |
| NM_018687 | LOC55908 | 1014 | 4 | 1 | 3 | 0 | 0 | 0 | 0 | 4 | 0 | 0 | 0 | 0 | 0 |
| NM_018688 | BIN3 | 1847 | 9 | 5 | 8 | 9 | 6 | 4 | 5 | 4 | 3 | 5 | 2 | 8 | 5 |
| NM_018689 | KIAA1199 | 7080 | 7 | 1 | 0 | 0 | 0 | 7 | 0 | 0 | 2 | 1 | 0 | 0 | 1 |
| NM_018690 | APOB48R | 3733 | 26 | 7 | 5 | 26 | 2 | 2 | 1 | 2 | 11 | 0 | 0 | 24 | 1 |
| NM_018691 | FAM114A2 | 2884 | 13 | 6 | 5 | 1 | 8 | 3 | 6 | 5 | 5 | 7 | 5 | 8 | 13 |
| NM_018693 | FBXO11 | 2567 | 23 | 14 | 11 | 4 | 11 | 18 | 11 | 7 | 12 | 23 | 13 | 19 | 22 |
| NM_018694 | ARL6IP4 | 1375 | 33 | 24 | 21 | 17 | 33 | 29 | 21 | 21 | 18 | 26 | 26 | 31 | 20 |
| NM_018695 | ERBB2IP | 6916 | 28 | 19 | 11 | 7 | 16 | 26 | 21 | 22 | 17 | 20 | 18 | 28 | 19 |
| NM_018696 | ELAC1 | 2233 | 1 | 0 | 1 | 1 | 0 | 1 | 0 | 0 | 0 | 0 | 0 | 0 | 0 |
| NM_018697 | LANCL2 | 4353 | 3 | 1 | 1 | 0 | 0 | 3 | 1 | 1 | 0 | 2 | 1 | 1 | 2 |
| NM_018698 | NXT2 | 2692 | 6 | 1 | 1 | 0 | 0 | 1 | 1 | 1 | 1 | 1 | 0 | 1 | 6 |
| NM_018699 | PRDM5 | 2480 | 14 | 4 | 2 | 2 | 5 | 4 | 3 | 0 | 4 | 14 | 2 | 2 | 8 |
| NM_018700 | TRIM36 | 4437 | 15 | 2 | 0 | 1 | 0 | 8 | 0 | 0 | 0 | 1 | 0 | 0 | 15 |
| NM_018702 | ADARB2 | 3654 | 1 | 0 | 0 | 0 | 0 | 1 | 0 | 0 | 0 | 0 | 0 | 0 | 0 |
| NM_018703 | RBBP6 | 6734 | 9 | 5 | 3 | 1 | 5 | 5 | 5 | 3 | 4 | 8 | 6 | 9 | 9 |
| NM_018704 | CTTNBP2NL | 5899 | 8 | 5 | 6 | 6 | 4 | 6 | 5 | 2 | 8 | 4 | 2 | 8 | 4 |
| NM_018706 | DHTKD1 | 5196 | 7 | 2 | 1 | 1 | 1 | 1 | 4 | 7 | 1 | 1 | 1 | 2 | 1 |
| NM_018708 | FEM1A | 3844 | 10 | 2 | 1 | 1 | 2 | 1 | 1 | 1 | 0 | 0 | 10 | 1 | 0 |
| NM_018710 | TMEM55A | 2406 | 5 | 2 | 2 | 1 | 5 | 4 | 2 | 1 | 2 | 2 | 1 | 3 | 3 |

| transcript | gene | transcript length (nt) | max | mean | adipose | colon | heart | hypothalamus | kidney | liver | lung | ovary | skeletalmuscle | spleen | testes |
|---|---|---|---|---|---|---|---|---|---|---|---|---|---|---|---|
| NM_018711 | SVOP | 2740 | 8 | 1 | 0 | 2 | 0 | 8 | 0 | 0 | 0 | 0 | 0 | 0 | 0 |
| NM_018712 | ELMOD1 | 2991 | 15 | 2 | 0 | 0 | 1 | 15 | 0 | 0 | 0 | 0 | 0 | 0 | 1 |
| NM_018713 | SLC30A10 | 2854 | 11 | 1 | 0 | 3 | 0 | 2 | 0 | 11 | 0 | 0 | 0 | 0 | 0 |
| NM_018714 | COG1 | 3034 | 3 | 2 | 1 | 2 | 1 | 3 | 2 | 1 | 1 | 2 | 2 | 2 | 2 |
| NM_018715 | RCC2 | 4091 | 5 | 2 | 2 | 3 | 2 | 2 | 2 | 1 | 3 | 2 | 1 | 5 | 3 |
| NM_018717 | MAML3 | 7088 | 2 | 1 | 0 | 1 | 0 | 0 | 0 | 1 | 1 | 2 | 0 | 1 | 1 |
| NM_018718 | TSGA14 | 3526 | 3 | 1 | 1 | 0 | 0 | 3 | 1 | 0 | 0 | 1 | 0 | 1 | 2 |
| NM_018719 | CDCA7L | 2907 | 2 | 1 | 0 | 1 | 2 | 1 | 0 | 0 | 2 | 2 | 0 | 2 | 2 |
| NM_018723 | A2BP1 | 4775 | 8 | 2 | 0 | 0 | 3 | 8 | 0 | 0 | 0 | 0 | 8 | 0 | 0 |
| NM_018724 | IL20 | 1234 | 0 | 0 | 0 | 0 | 0 | 0 | 0 | 0 | 0 | 0 | 0 | 0 | 0 |
| NM_018725 | IL17RB | 2040 | 6 | 1 | 0 | 0 | 1 | 2 | 6 | 2 | 0 | 1 | 1 | 1 | 1 |
| NM_018727 | TRPV1 | 4046 | 1 | 0 | 0 | 1 | 0 | 0 | 1 | 0 | 0 | 0 | 0 | 0 | 0 |
| NM_018728 | MYO5C | 6945 | 5 | 2 | 2 | 4 | 1 | 1 | 3 | 1 | 5 | 1 | 1 | 3 | 4 |
| NM_018833 | TAP2 | 2540 | 3 | 2 | 2 | 1 | 1 | 2 | 1 | 1 | 2 | 1 | 1 | 3 | 1 |
| NM_018834 | MATR3 | 3907 | 92 | 57 | 33 | 14 | 64 | 92 | 57 | 28 | 47 | 82 | 58 | 74 | 81 |
| NM_018835 | RC3H2 | 3565 | 13 | 7 | 5 | 4 | 9 | 13 | 6 | 5 | 8 | 7 | 6 | 12 | 8 |
| NM_018836 | AJAP1 | 2923 | 9 | 1 | 0 | 0 | 0 | 9 | 1 | 0 | 0 | 1 | 0 | 1 | 1 |
| NM_018837 | SULF2 | 3899 | 38 | 9 | 4 | 6 | 9 | 8 | 11 | 7 | 3 | 38 | 2 | 4 | 9 |
| NM_018838 | NDUFA12 | 599 | 12 | 6 | 3 | 3 | 12 | 7 | 6 | 7 | 4 | 2 | 7 | 7 | 3 |
| NM_018839 | NSFL1C | 3551 | 4 | 2 | 2 | 2 | 2 | 4 | 2 | 2 | 1 | 2 | 3 | 2 | 2 |
| NM_018840 | C20orf24 | 1084 | 20 | 5 | 4 | 4 | 3 | 4 | 4 | 20 | 3 | 1 | 3 | 6 | 2 |
| NM_018841 | GNG12 | 4396 | 8 | 4 | 6 | 2 | 4 | 2 | 8 | 2 | 4 | 5 | 1 | 6 | 4 |
| NM_018842 | BAIAP2L1 | 3664 | 3 | 1 | 0 | 3 | 0 | 0 | 1 | 2 | 2 | 0 | 0 | 0 | 1 |
| NM_018843 | SLC25A40 | 1514 | 5 | 2 | 1 | 1 | 2 | 4 | 3 | 1 | 2 | 3 | 1 | 5 | 3 |
| NM_018844 | BCAP29 | 6019 | 18 | 8 | 8 | 2 | 18 | 12 | 4 | 7 | 7 | 6 | 6 | 8 | 14 |
| NM_018845 | RAG1AP1 | 1343 | 10 | 6 | 6 | 8 | 4 | 3 | 6 | 10 | 7 | 2 | 1 | 10 | 5 |
| NM_018846 | KLHL7 | 3306 | 6 | 2 | 1 | 0 | 6 | 4 | 2 | 1 | 1 | 2 | 1 | 1 | 3 |
| NM_018847 | KLHL9 | 4412 | 5 | 3 | 3 | 2 | 3 | 4 | 4 | 2 | 2 | 5 | 1 | 4 | 4 |
| NM_018848 | MKKS | 2662 | 5 | 3 | 3 | 2 | 4 | 5 | 4 | 5 | 2 | 2 | 1 | 3 | 2 |
| NM_018849 | ABCB4 | 3988 | 23 | 3 | 0 | 0 | 1 | 0 | 0 | 23 | 0 | 0 | 1 | 3 | 0 |
| NM_018850 | ABCB4 | 3826 | 24 | 3 | 0 | 0 | 1 | 0 | 0 | 24 | 0 | 0 | 1 | 3 | 0 |
| NM_018890 | RAC1 | 2398 | 49 | 28 | 24 | 20 | 31 | 49 | 27 | 18 | 31 | 25 | 26 | 35 | 23 |
| NM_018891 | LAMC2 | 4522 | 3 | 1 | 0 | 2 | 0 | 0 | 1 | 0 | 3 | 0 | 0 | 0 | 0 |
| NM_018896 | CACNA1G | 7825 | 0 | 0 | 0 | 0 | 0 | 0 | 0 | 0 | 0 | 0 | 0 | 0 | 0 |
| NM_018897 | DNAH7 | 12394 | 3 | 1 | 0 | 0 | 0 | 1 | 2 | 0 | 1 | 0 | 0 | 0 | 3 |
| NM_018898 | PCDHAC1 | 5475 | 0 | 0 | 0 | 0 | 0 | 0 | 0 | 0 | 0 | 0 | 0 | 0 | 0 |
| NM_018899 | PCDHAC2 | 6036 | 1 | 0 | 0 | 0 | 0 | 1 | 0 | 0 | 0 | 0 | 0 | 0 | 0 |
| NM_018900 | PCDHA1 | 5260 | 0 | 0 | 0 | 0 | 0 | 0 | 0 | 0 | 0 | 0 | 0 | 0 | 0 |
| NM_018901 | PCDHA10 | 5254 | 0 | 0 | 0 | 0 | 0 | 0 | 0 | 0 | 0 | 0 | 0 | 0 | 0 |
| NM_018902 | PCDHA11 | 6115 | 0 | 0 | 0 | 0 | 0 | 0 | 0 | 0 | 0 | 0 | 0 | 0 | 0 |
| NM_018903 | PCDHA12 | 5360 | 0 | 0 | 0 | 0 | 0 | 0 | 0 | 0 | 0 | 0 | 0 | 0 | 0 |
| NM_018904 | PCDHA13 | 5260 | 0 | 0 | 0 | 0 | 0 | 0 | 0 | 0 | 0 | 0 | 0 | 0 | 0 |
| NM_018905 | PCDHA2 | 5360 | 0 | 0 | 0 | 0 | 0 | 0 | 0 | 0 | 0 | 0 | 0 | 0 | 0 |
| NM_018906 | PCDHA3 | 5260 | 0 | 0 | 0 | 0 | 0 | 0 | 0 | 0 | 0 | 0 | 0 | 0 | 0 |
| NM_018907 | PCDHA4 | 5352 | 0 | 0 | 0 | 0 | 0 | 0 | 0 | 0 | 0 | 0 | 0 | 0 | 0 |
| NM_018908 | PCDHA5 | 5218 | 0 | 0 | 0 | 0 | 0 | 0 | 0 | 0 | 0 | 0 | 0 | 0 | 0 |
| NM_018909 | PCDHA6 | 5287 | 0 | 0 | 0 | 0 | 0 | 0 | 0 | 0 | 0 | 0 | 0 | 0 | 0 |
| NM_018910 | PCDHA7 | 5221 | 0 | 0 | 0 | 0 | 0 | 0 | 0 | 0 | 0 | 0 | 0 | 0 | 0 |
| NM_018911 | PCDHA8 | 5260 | 0 | 0 | 0 | 0 | 0 | 0 | 0 | 0 | 0 | 0 | 0 | 0 | 0 |
| NM_018912 | PCDHGA1 | 4602 | 0 | 0 | 0 | 0 | 0 | 0 | 0 | 0 | 0 | 0 | 0 | 0 | 0 |
| NM_018913 | PCDHGA10 | 4617 | 1 | 1 | 0 | 0 | 1 | 1 | 0 | 0 | 1 | 1 | 1 | 0 | 1 |
| NM_018914 | PCDHGA11 | 4872 | 1 | 0 | 0 | 0 | 0 | 0 | 0 | 0 | 0 | 1 | 1 | 0 | 0 |
| NM_018915 | PCDHGA2 | 4790 | 1 | 0 | 0 | 0 | 1 | 1 | 0 | 0 | 0 | 1 | 0 | 0 | 1 |
| NM_018916 | PCDHGA3 | 4605 | 0 | 0 | 0 | 0 | 0 | 0 | 0 | 0 | 0 | 0 | 0 | 0 | 0 |
| NM_018917 | PCDHGA4 | 4602 | 1 | 0 | 0 | 0 | 0 | 1 | 0 | 0 | 0 | 1 | 0 | 0 | 0 |
| NM_018918 | PCDHGA5 | 4602 | 0 | 0 | 0 | 0 | 0 | 0 | 0 | 0 | 0 | 0 | 0 | 0 | 0 |
| NM_018919 | PCDHGA6 | 4605 | 1 | 0 | 0 | 0 | 0 | 1 | 0 | 0 | 0 | 1 | 0 | 0 | 0 |
| NM_018920 | PCDHGA7 | 4605 | 1 | 0 | 0 | 0 | 0 | 0 | 0 | 0 | 0 | 1 | 0 | 0 | 0 |
| NM_018921 | PCDHGA9 | 4605 | 1 | 0 | 0 | 0 | 0 | 1 | 0 | 0 | 0 | 1 | 0 | 0 | 0 |
| NM_018922 | PCDHGB1 | 4590 | 1 | 0 | 0 | 0 | 0 | 1 | 0 | 0 | 0 | 0 | 0 | 0 | 0 |
| NM_018923 | PCDHGB2 | 4602 | 2 | 1 | 0 | 0 | 2 | 2 | 1 | 0 | 1 | 2 | 1 | 0 | 1 |
| NM_018924 | PCDHGB3 | 4596 | 1 | 0 | 0 | 0 | 1 | 0 | 0 | 0 | 0 | 0 | 0 | 0 | 0 |
| NM_018925 | PCDHGB5 | 4578 | 1 | 0 | 0 | 0 | 1 | 1 | 0 | 0 | 0 | 1 | 1 | 0 | 1 |
| NM_018926 | PCDHGB6 | 4599 | 1 | 1 | 1 | 0 | 1 | 1 | 0 | 0 | 1 | 1 | 1 | 1 | 1 |
| NM_018927 | PCDHGB7 | 4741 | 1 | 1 | 0 | 0 | 1 | 1 | 1 | 0 | 1 | 1 | 1 | 0 | 1 |
| NM_018928 | PCDHGC4 | 4623 | 1 | 0 | 0 | 0 | 0 | 1 | 0 | 0 | 0 | 0 | 0 | 0 | 0 |
| NM_018929 | PCDHGC5 | 4641 | 4 | 0 | 0 | 0 | 0 | 4 | 0 | 0 | 0 | 0 | 0 | 0 | 0 |
| NM_018930 | PCDHB10 | 3262 | 1 | 0 | 0 | 0 | 0 | 1 | 0 | 0 | 0 | 1 | 0 | 0 | 0 |
| NM_018931 | PCDHB11 | 3271 | 2 | 1 | 0 | 0 | 0 | 1 | 0 | 0 | 0 | 2 | 1 | 0 | 1 |
| NM_018932 | PCDHB12 | 3408 | 1 | 0 | 0 | 0 | 0 | 1 | 0 | 0 | 0 | 0 | 0 | 0 | 0 |
| NM_018933 | PCDHB13 | 3485 | 1 | 0 | 0 | 0 | 1 | 1 | 0 | 0 | 0 | 1 | 0 | 0 | 1 |
| NM_018934 | PCDHB14 | 2784 | 1 | 1 | 0 | 0 | 1 | 1 | 0 | 0 | 0 | 1 | 0 | 0 | 1 |
| NM_018935 | PCDHB15 | 2655 | 1 | 0 | 0 | 0 | 0 | 0 | 0 | 0 | 0 | 1 | 0 | 1 | 1 |
| NM_018936 | PCDHB2 | 2728 | 1 | 0 | 0 | 0 | 0 | 0 | 0 | 0 | 0 | 0 | 0 | 0 | 1 |
| NM_018937 | PCDHB3 | 3173 | 0 | 0 | 0 | 0 | 0 | 0 | 0 | 0 | 0 | 0 | 0 | 0 | 0 |
| NM_018938 | PCDHB4 | 3621 | 3 | 1 | 1 | 0 | 1 | 3 | 0 | 0 | 0 | 1 | 0 | 0 | 1 |
| NM_018939 | PCDHB6 | 3030 | 1 | 0 | 0 | 0 | 0 | 1 | 0 | 0 | 0 | 0 | 0 | 0 | 0 |

| transcript | gene | transcript length (nt) | max | mean | adipose | colon | heart | hypothalamus | kidney | liver | lung | ovary | skeletalmuscle | spleen | testes |
|---|---|---|---|---|---|---|---|---|---|---|---|---|---|---|---|
| NM_018940 | PCDHB7 | 3715 | 1 | 0 | 0 | 0 | 0 | 1 | 0 | 0 | 0 | 1 | 0 | 0 | 0 |
| NM_018941 | CLN8 | 7169 | 1 | 0 | 0 | 0 | 0 | 1 | 1 | 0 | 0 | 0 | 0 | 1 | 1 |
| NM_018942 | HMX1 | 1896 | 0 | 0 | 0 | 0 | 0 | 0 | 0 | 0 | 0 | 0 | 0 | 0 | 0 |
| NM_018943 | TUBA8 | 2018 | 4 | 1 | 1 | 0 | 3 | 2 | 0 | 0 | 0 | 0 | 4 | 0 | 1 |
| NM_018944 | C21orf45 | 1571 | 2 | 1 | 0 | 1 | 0 | 1 | 1 | 0 | 0 | 1 | 0 | 1 | 2 |
| NM_018945 | PDE7B | 5387 | 7 | 2 | 1 | 1 | 2 | 3 | 2 | 1 | 0 | 7 | 1 | 1 | 1 |
| NM_018946 | NANS | 1239 | 15 | 7 | 4 | 15 | 8 | 7 | 4 | 13 | 6 | 3 | 2 | 8 | 6 |
| NM_018947 | CYCS | 5508 | 9 | 4 | 2 | 2 | 9 | 6 | 5 | 4 | 2 | 4 | 3 | 2 | 1 |
| NM_018948 | ERRFI1 | 3128 | 37 | 7 | 3 | 1 | 2 | 3 | 5 | 37 | 6 | 6 | 6 | 2 | 2 |
| NM_018949 | UTS2R | 1170 | 0 | 0 | 0 | 0 | 0 | 0 | 0 | 0 | 0 | 0 | 0 | 0 | 0 |
| NM_018950 | HLA-F | 1284 | 25 | 6 | 5 | 9 | 2 | 3 | 2 | 2 | 17 | 1 | 2 | 25 | 2 |
| NM_018951 | HOXA10 | 2571 | 1 | 0 | 0 | 1 | 0 | 0 | 1 | 0 | 0 | 0 | 1 | 0 | 0 |
| NM_018952 | HOXB6 | 1676 | 3 | 1 | 0 | 3 | 0 | 0 | 2 | 0 | 0 | 0 | 0 | 0 | 1 |
| NM_018953 | HOXC5 | 1612 | 1 | 0 | 0 | 0 | 0 | 0 | 0 | 0 | 0 | 1 | 0 | 0 | 0 |
| NM_018955 | UBB | 971 | 76 | 42 | 39 | 39 | 30 | 76 | 48 | 76 | 28 | 13 | 33 | 36 | 42 |
| NM_018956 | C9orf9 | 719 | 4 | 1 | 1 | 0 | 1 | 1 | 1 | 0 | 1 | 0 | 0 | 0 | 4 |
| NM_018957 | SH3BP1 | 2576 | 1 | 0 | 0 | 1 | 0 | 0 | 0 | 0 | 0 | 0 | 0 | 1 | 0 |
| NM_018958 | C15orf2 | 8053 | 1 | 0 | 0 | 0 | 0 | 0 | 0 | 0 | 0 | 1 | 0 | 0 | 1 |
| NM_018959 | DAZAP1 | 2184 | 3 | 2 | 2 | 2 | 3 | 1 | 2 | 3 | 2 | 1 | 2 | 3 | 3 |
| NM_018960 | GNMT | 1068 | 5 | 1 | 0 | 0 | 0 | 0 | 0 | 5 | 0 | 0 | 0 | 0 | 0 |
| NM_018961 | UBASH3A | 2492 | 0 | 0 | 0 | 0 | 0 | 0 | 0 | 0 | 0 | 0 | 0 | 0 | 0 |
| NM_018962 | DSCR6 | 2232 | 0 | 0 | 0 | 0 | 0 | 0 | 0 | 0 | 0 | 0 | 0 | 0 | 0 |
| NM_018963 | BRWD1 | 8650 | 15 | 8 | 4 | 3 | 8 | 8 | 7 | 4 | 8 | 12 | 10 | 11 | 15 |
| NM_018964 | SLC37A1 | 3094 | 2 | 1 | 0 | 2 | 0 | 1 | 1 | 0 | 1 | 0 | 1 | 1 | 1 |
| NM_018965 | TREM2 | 1051 | 1 | 0 | 1 | 0 | 0 | 1 | 0 | 0 | 0 | 0 | 0 | 0 | 0 |
| NM_018967 | SNTG1 | 1889 | 4 | 1 | 0 | 0 | 0 | 4 | 0 | 0 | 0 | 0 | 0 | 2 | 0 |
| NM_018968 | SNTG2 | 1885 | 0 | 0 | 0 | 0 | 0 | 0 | 0 | 0 | 0 | 0 | 0 | 0 | 0 |
| NM_018969 | GPR173 | 4249 | 1 | 0 | 0 | 0 | 0 | 1 | 0 | 0 | 0 | 0 | 0 | 0 | 0 |
| NM_018970 | GPR85 | 4933 | 1 | 0 | 0 | 0 | 0 | 1 | 0 | 0 | 0 | 0 | 0 | 0 | 0 |
| NM_018971 | GPR27 | 1128 | 0 | 0 | 0 | 0 | 0 | 0 | 0 | 0 | 0 | 0 | 0 | 0 | 0 |
| NM_018972 | GDAP1 | 3889 | 15 | 2 | 1 | 0 | 0 | 15 | 1 | 0 | 0 | 2 | 1 | 1 | 2 |
| NM_018973 | DPM3 | 517 | 1 | 1 | 1 | 0 | 1 | 1 | 1 | 1 | 1 | 0 | 0 | 0 | 0 |
| NM_018974 | UNC93A | 2110 | 1 | 0 | 0 | 0 | 0 | 0 | 0 | 1 | 0 | 0 | 0 | 0 | 0 |
| NM_018975 | TERF2IP | 2116 | 55 | 14 | 10 | 4 | 12 | 55 | 9 | 7 | 10 | 9 | 6 | 14 | 15 |
| NM_018976 | SLC38A2 | 4883 | 36 | 14 | 7 | 4 | 19 | 8 | 7 | 36 | 17 | 17 | 8 | 17 | 10 |
| NM_018977 | NLGN3 | 3923 | 9 | 1 | 0 | 1 | 0 | 9 | 0 | 0 | 0 | 2 | 0 | 0 | 2 |
| NM_018979 | WNK1 | 10316 | 21 | 12 | 12 | 4 | 21 | 16 | 17 | 8 | 11 | 8 | 11 | 14 | 15 |
| NM_018980 | TAS2R5 | 1150 | 1 | 0 | 0 | 0 | 0 | 0 | 1 | 0 | 0 | 1 | 0 | 0 | 1 |
| NM_018981 | DNAJC10 | 4193 | 11 | 6 | 4 | 2 | 9 | 6 | 7 | 6 | 6 | 6 | 2 | 11 | 8 |
| NM_018982 | YIPF1 | 1804 | 7 | 3 | 2 | 2 | 3 | 5 | 4 | 7 | 2 | 2 | 2 | 4 | 3 |
| NM_018983 | GAR1 | 1267 | 16 | 9 | 4 | 7 | 12 | 6 | 11 | 5 | 7 | 12 | 7 | 16 | 12 |
| NM_018984 | SSH1 | 8534 | 3 | 2 | 2 | 1 | 2 | 2 | 2 | 1 | 2 | 2 | 2 | 3 | 2 |
| NM_018986 | SH3TC1 | 4260 | 0 | 0 | 0 | 0 | 0 | 0 | 0 | 0 | 0 | 0 | 0 | 0 | 0 |
| NM_018988 | GFOD1 | 2994 | 5 | 2 | 1 | 1 | 5 | 5 | 0 | 1 | 2 | 0 | 2 | 3 | 0 |
| NM_018989 | RBM27 | 6540 | 4 | 3 | 1 | 1 | 3 | 3 | 2 | 2 | 2 | 3 | 2 | 3 | 4 |
| NM_018990 | SASH3 | 2691 | 7 | 1 | 1 | 1 | 1 | 1 | 0 | 0 | 1 | 0 | 0 | 7 | 0 |
| NM_018991 | STAG3L1 | 1826 | 6 | 3 | 2 | 4 | 2 | 2 | 5 | 1 | 3 | 4 | 2 | 6 | 5 |
| NM_018992 | KCTD5 | 2461 | 2 | 1 | 1 | 2 | 0 | 1 | 1 | 1 | 0 | 0 | 0 | 1 | 1 |
| NM_018993 | RIN2 | 4244 | 8 | 4 | 5 | 3 | 6 | 4 | 5 | 1 | 8 | 5 | 3 | 5 | 3 |
| NM_018994 | FBXO42 | 2976 | 4 | 3 | 2 | 2 | 4 | 4 | 2 | 2 | 2 | 4 | 4 | 3 | 4 |
| NM_018995 | MOV10L1 | 3956 | 4 | 0 | 0 | 0 | 0 | 0 | 0 | 0 | 0 | 1 | 0 | 0 | 4 |
| NM_018996 | TNRC6C | 9663 | 4 | 2 | 2 | 1 | 2 | 4 | 1 | 1 | 1 | 3 | 2 | 2 | 2 |
| NM_018997 | MRPS21 | 939 | 12 | 6 | 5 | 3 | 7 | 12 | 7 | 6 | 3 | 5 | 4 | 6 | 5 |
| NM_018998 | FBXW5 | 2345 | 2 | 1 | 1 | 1 | 1 | 1 | 1 | 2 | 1 | 0 | 2 | 1 | 1 |
| NM_018999 | FAM190B | 7676 | 10 | 5 | 4 | 1 | 6 | 10 | 3 | 3 | 5 | 5 | 5 | 4 | 6 |
| NM_019000 | FAM134B | 3084 | 64 | 14 | 3 | 1 | 30 | 14 | 23 | 4 | 3 | 1 | 64 | 1 | 6 |
| NM_019001 | XRN1 | 10092 | 9 | 5 | 3 | 2 | 4 | 3 | 4 | 5 | 7 | 7 | 7 | 9 | 6 |
| NM_019002 | ETAA1 | 3283 | 5 | 3 | 1 | 1 | 3 | 2 | 5 | 2 | 3 | 5 | 1 | 5 | 5 |
| NM_019003 | SPIN2A | 1319 | 1 | 0 | 0 | 1 | 0 | 0 | 0 | 0 | 0 | 1 | 0 | 1 | 0 |
| NM_019004 | ANKIB1 | 6081 | 8 | 4 | 4 | 1 | 4 | 8 | 7 | 1 | 3 | 5 | 2 | 5 | 8 |
| NM_019005 | MIOS | 3323 | 7 | 3 | 2 | 1 | 3 | 3 | 3 | 1 | 2 | 3 | 7 | 5 | 4 |
| NM_019006 | ZFAND6 | 1678 | 22 | 10 | 10 | 5 | 9 | 6 | 10 | 10 | 11 | 5 | 8 | 13 | 22 |
| NM_019007 | ARMCX6 | 1848 | 4 | 2 | 2 | 2 | 2 | 3 | 2 | 1 | 2 | 2 | 1 | 4 | 3 |
| NM_019008 | SMCR7L | 5699 | 3 | 2 | 2 | 2 | 1 | 2 | 2 | 3 | 1 | 1 | 0 | 2 | 2 |
| NM_019009 | TOLLIP | 3604 | 2 | 1 | 0 | 0 | 1 | 2 | 2 | 1 | 0 | 0 | 1 | 1 | 1 |
| NM_019010 | KRT20 | 1737 | 52 | 5 | 0 | 52 | 0 | 0 | 0 | 0 | 0 | 0 | 0 | 0 | 0 |
| NM_019012 | PLEKHA5 | 4315 | 24 | 10 | 5 | 4 | 18 | 11 | 22 | 6 | 8 | 24 | 2 | 2 | 13 |
| NM_019013 | FAM64A | 1483 | 0 | 0 | 0 | 0 | 0 | 0 | 0 | 0 | 0 | 0 | 0 | 0 | 0 |
| NM_019014 | POLR1B | 5409 | 2 | 1 | 1 | 1 | 1 | 1 | 1 | 2 | 0 | 1 | 1 | 2 | 2 |
| NM_019015 | CHPF2 | 3970 | 4 | 2 | 1 | 1 | 3 | 2 | 2 | 1 | 1 | 2 | 1 | 4 | 2 |
| NM_019016 | KRT24 | 1881 | 1 | 0 | 0 | 1 | 0 | 0 | 0 | 0 | 0 | 0 | 0 | 0 | 0 |
| NM_019018 | FAM105A | 7046 | 7 | 2 | 1 | 3 | 2 | 1 | 1 | 0 | 7 | 1 | 0 | 5 | 1 |
| NM_019020 | TBC1D16 | 3259 | 8 | 3 | 8 | 1 | 3 | 1 | 5 | 3 | 2 | 2 | 1 | 3 | 1 |
| NM_019021 | C11orf71 | 647 | 1 | 0 | 0 | 0 | 0 | 0 | 0 | 0 | 0 | 0 | 0 | 0 | 1 |
| NM_019022 | TMX3 | 4745 | 5 | 2 | 2 | 1 | 2 | 2 | 2 | 1 | 2 | 2 | 1 | 5 | 2 |
| NM_019023 | PRMT7 | 2390 | 1 | 1 | 1 | 1 | 1 | 1 | 1 | 1 | 0 | 1 | 1 | 1 | 1 |
| NM_019024 | HEATR5B | 6792 | 6 | 3 | 3 | 2 | 4 | 5 | 3 | 2 | 3 | 4 | 2 | 6 | 4 |

| transcript | gene | transcript length (nt) | max | mean | adipose | colon | heart | hypothalamus | kidney | liver | lung | ovary | skeletalmuscle | spleen | testes |
|---|---|---|---|---|---|---|---|---|---|---|---|---|---|---|---|
| NM_019026 | TMCO1 | 4446 | 22 | 12 | 9 | 5 | 11 | 13 | 16 | 22 | 9 | 11 | 5 | 14 | 18 |
| NM_019027 | RBM47 | 4485 | 11 | 3 | 2 | 5 | 1 | 0 | 11 | 4 | 3 | 0 | 0 | 4 | 2 |
| NM_019028 | ZDHHC13 | 2434 | 3 | 1 | 1 | 2 | 2 | 3 | 2 | 0 | 1 | 1 | 1 | 2 | 2 |
| NM_019029 | CPVL | 1779 | 46 | 9 | 7 | 2 | 14 | 3 | 12 | 2 | 8 | 3 | 3 | 46 | 5 |
| NM_019030 | DHX29 | 4473 | 5 | 4 | 3 | 1 | 5 | 4 | 5 | 5 | 4 | 3 | 4 | 4 | 4 |
| NM_019032 | ADAMTSL4 | 4201 | 1 | 0 | 0 | 0 | 1 | 0 | 0 | 0 | 1 | 1 | 0 | 1 | 0 |
| NM_019034 | RHOF | 2435 | 0 | 0 | 0 | 0 | 0 | 0 | 0 | 0 | 0 | 0 | 0 | 0 | 0 |
| NM_019035 | PCDH18 | 5906 | 9 | 3 | 7 | 2 | 3 | 2 | 2 | 0 | 1 | 9 | 2 | 1 | 4 |
| NM_019036 | HMGCLL1 | 2561 | 6 | 1 | 0 | 0 | 0 | 6 | 0 | 0 | 1 | 0 | 0 | 0 | 2 |
| NM_019037 | EXOSC4 | 895 | 1 | 0 | 0 | 0 | 0 | 0 | 0 | 0 | 0 | 0 | 0 | 0 | 1 |
| NM_019040 | ELP4 | 1567 | 6 | 3 | 2 | 1 | 4 | 4 | 5 | 3 | 2 | 3 | 3 | 6 | 6 |
| NM_019041 | MTRF1L | 3878 | 8 | 4 | 3 | 1 | 7 | 4 | 5 | 5 | 3 | 3 | 3 | 8 | 3 |
| NM_019042 | PUS7 | 3482 | 3 | 2 | 2 | 1 | 2 | 2 | 2 | 2 | 2 | 2 | 3 | 2 | 3 |
| NM_019043 | APBB1IP | 2637 | 38 | 9 | 19 | 2 | 4 | 9 | 3 | 16 | 8 | 2 | 1 | 38 | 1 |
| NM_019044 | CCDC93 | 6794 | 5 | 3 | 3 | 3 | 5 | 2 | 4 | 1 | 3 | 4 | 2 | 5 | 4 |
| NM_019045 | WDR44 | 4140 | 6 | 4 | 3 | 1 | 4 | 5 | 3 | 2 | 3 | 4 | 3 | 5 | 6 |
| NM_019046 | ANKRD16 | 2626 | 1 | 0 | 0 | 0 | 0 | 0 | 0 | 0 | 0 | 1 | 0 | 0 | 0 |
| NM_019048 | ASNSD1 | 2470 | 16 | 10 | 4 | 2 | 14 | 6 | 8 | 7 | 7 | 13 | 16 | 14 | 16 |
| NM_019050 | USP53 | 6595 | 21 | 11 | 8 | 3 | 21 | 5 | 11 | 4 | 19 | 7 | 17 | 14 | 11 |
| NM_019051 | MRPL50 | 1021 | 13 | 7 | 5 | 2 | 11 | 13 | 7 | 13 | 4 | 6 | 4 | 8 | 8 |
| NM_019052 | CCHCR1 | 2625 | 2 | 1 | 1 | 1 | 0 | 0 | 1 | 0 | 0 | 1 | 0 | 0 | 2 |
| NM_019053 | EXOC6 | 3619 | 9 | 5 | 2 | 2 | 3 | 7 | 3 | 5 | 4 | 3 | 7 | 7 | 9 |
| NM_019054 | FAM35A | 3404 | 1 | 1 | 0 | 0 | 0 | 0 | 1 | 1 | 1 | 0 | 0 | 1 | 0 |
| NM_019055 | ROBO4 | 3836 | 5 | 1 | 1 | 0 | 1 | 1 | 1 | 1 | 2 | 1 | 1 | 5 | 0 |
| NM_019056 | NDUFB11 | 609 | 12 | 7 | 3 | 6 | 11 | 8 | 6 | 12 | 2 | 4 | 12 | 5 | 2 |
| NM_019057 | FAM193B | 4353 | 1 | 0 | 0 | 1 | 0 | 1 | 0 | 0 | 1 | 0 | 0 | 1 | 1 |
| NM_019058 | DDIT4 | 1752 | 18 | 7 | 9 | 2 | 4 | 2 | 4 | 18 | 10 | 5 | 8 | 11 | 3 |
| NM_019059 | TOMM7 | 728 | 272 | 160 | 131 | 90 | 210 | 215 | 94 | 90 | 127 | 272 | 271 | 152 | 105 |
| NM_019060 | CRCT1 | 694 | 0 | 0 | 0 | 0 | 0 | 0 | 0 | 0 | 0 | 0 | 0 | 0 | 0 |
| NM_019062 | RNF186 | 1250 | 2 | 0 | 0 | 0 | 0 | 0 | 2 | 0 | 0 | 0 | 0 | 0 | 0 |
| NM_019063 | EML4 | 5547 | 10 | 4 | 3 | 3 | 2 | 2 | 5 | 7 | 6 | 4 | 1 | 10 | 5 |
| NM_019065 | NECAB2 | 1606 | 4 | 0 | 0 | 0 | 0 | 4 | 0 | 0 | 0 | 0 | 0 | 0 | 0 |
| NM_019066 | MAGEL2 | 4296 | 1 | 0 | 0 | 0 | 0 | 1 | 0 | 0 | 0 | 0 | 0 | 0 | 0 |
| NM_019067 | GNL3L | 6826 | 5 | 3 | 2 | 3 | 4 | 2 | 3 | 2 | 2 | 3 | 3 | 5 | 2 |
| NM_019069 | WDR5B | 4183 | 2 | 1 | 1 | 0 | 1 | 1 | 1 | 1 | 1 | 2 | 1 | 1 | 1 |
| NM_019070 | DDX49 | 1820 | 1 | 1 | 0 | 1 | 1 | 1 | 1 | 1 | 0 | 1 | 1 | 1 | 1 |
| NM_019071 | ING3 | 2204 | 4 | 1 | 1 | 1 | 1 | 2 | 2 | 0 | 1 | 1 | 1 | 4 | 1 |
| NM_019072 | SGTB | 5453 | 8 | 2 | 1 | 0 | 1 | 8 | 1 | 0 | 2 | 1 | 1 | 2 | 1 |
| NM_019073 | SPATA6 | 1739 | 13 | 4 | 2 | 2 | 3 | 4 | 3 | 1 | 4 | 6 | 1 | 4 | 13 |
| NM_019074 | DLL4 | 3383 | 4 | 1 | 2 | 1 | 1 | 0 | 1 | 0 | 4 | 1 | 1 | 2 | 1 |
| NM_019075 | UGT1A10 | 2385 | 4 | 0 | 0 | 4 | 0 | 0 | 0 | 0 | 0 | 0 | 0 | 0 | 0 |
| NM_019076 | UGT1A8 | 2396 | 0 | 0 | 0 | 0 | 0 | 0 | 0 | 0 | 0 | 0 | 0 | 0 | 0 |
| NM_019077 | UGT1A7 | 2333 | 1 | 0 | 0 | 0 | 0 | 0 | 1 | 0 | 0 | 0 | 0 | 0 | 0 |
| NM_019078 | UGT1A5 | 2345 | 0 | 0 | 0 | 0 | 0 | 0 | 0 | 0 | 0 | 0 | 0 | 0 | 0 |
| NM_019079 | L1TD1 | 3876 | 8 | 1 | 0 | 2 | 0 | 0 | 0 | 0 | 1 | 0 | 0 | 2 | 8 |
| NM_019080 | NDFIP2 | 4648 | 16 | 4 | 1 | 2 | 2 | 16 | 5 | 4 | 2 | 1 | 2 | 2 | 2 |
| NM_019082 | DDX56 | 2485 | 1 | 0 | 0 | 0 | 0 | 1 | 1 | 1 | 0 | 0 | 0 | 1 | 1 |
| NM_019083 | CCDC76 | 3142 | 3 | 2 | 2 | 1 | 2 | 1 | 2 | 1 | 1 | 2 | 2 | 3 | 3 |
| NM_019084 | CCNJ | 4103 | 3 | 1 | 1 | 1 | 1 | 1 | 1 | 1 | 1 | 2 | 1 | 3 | 2 |
| NM_019086 | VSIG10 | 5001 | 3 | 1 | 1 | 3 | 1 | 0 | 1 | 1 | 2 | 2 | 1 | 1 | 1 |
| NM_019087 | ARL15 | 2486 | 4 | 2 | 2 | 1 | 2 | 2 | 3 | 1 | 2 | 1 | 1 | 3 | 4 |
| NM_019088 | PAF1 | 2132 | 10 | 7 | 6 | 3 | 6 | 10 | 8 | 9 | 5 | 6 | 5 | 8 | 8 |
| NM_019089 | HES2 | 4258 | 0 | 0 | 0 | 0 | 0 | 0 | 0 | 0 | 0 | 0 | 0 | 0 | 0 |
| NM_019090 | KIAA1383 | 5455 | 1 | 0 | 0 | 0 | 0 | 0 | 0 | 0 | 0 | 0 | 0 | 0 | 1 |
| NM_019091 | PLEKHA3 | 2469 | 3 | 2 | 2 | 1 | 3 | 3 | 2 | 1 | 1 | 2 | 1 | 2 | 3 |
| NM_019093 | UGT1A3 | 2345 | 0 | 0 | 0 | 0 | 0 | 0 | 0 | 0 | 0 | 0 | 0 | 0 | 0 |
| NM_019094 | NUDT4 | 4810 | 0 | 0 | 0 | 0 | 0 | 0 | 0 | 0 | 0 | 0 | 0 | 0 | 0 |
| NM_019095 | CRLS1 | 3953 | 6 | 2 | 1 | 1 | 2 | 1 | 3 | 3 | 6 | 1 | 1 | 1 | 1 |
| NM_019096 | GTPBP2 | 2979 | 2 | 1 | 1 | 2 | 1 | 1 | 1 | 1 | 1 | 1 | 1 | 1 | 1 |
| NM_019098 | CNGB3 | 4347 | 0 | 0 | 0 | 0 | 0 | 0 | 0 | 0 | 0 | 0 | 0 | 0 | 0 |
| NM_019099 | C1orf183 | 5980 | 3 | 1 | 1 | 1 | 3 | 3 | 1 | 0 | 1 | 1 | 1 | 2 | 2 |
| NM_019100 | DMAP1 | 1766 | 1 | 1 | 1 | 1 | 1 | 1 | 1 | 1 | 0 | 1 | 1 | 1 | 1 |
| NM_019101 | APOM | 761 | 4 | 1 | 0 | 0 | 0 | 0 | 1 | 4 | 0 | 1 | 0 | 0 | 0 |
| NM_019102 | HOXA5 | 1332 | 1 | 1 | 1 | 1 | 0 | 0 | 1 | 0 | 1 | 0 | 0 | 0 | 0 |
| NM_019103 | ZMAT5 | 1033 | 0 | 0 | 0 | 0 | 0 | 0 | 0 | 0 | 0 | 0 | 0 | 0 | 0 |
| NM_019104 | LIN37 | 900 | 1 | 1 | 1 | 1 | 0 | 1 | 1 | 0 | 1 | 1 | 1 | 1 | 1 |
| NM_019105 | TNXB | 10147 | 3 | 2 | 3 | 3 | 2 | 0 | 0 | 0 | 2 | 3 | 1 | 1 | 1 |
| NM_019105 | TNXB | 13125 | 3 | 1 | 3 | 2 | 2 | 0 | 0 | 0 | 1 | 2 | 1 | 1 | 1 |
| NM_019106 | 03 Sep | 4656 | 17 | 2 | 0 | 1 | 0 | 17 | 0 | 0 | 0 | 0 | 0 | 0 | 0 |
| NM_019107 | C19orf10 | 1051 | 22 | 5 | 3 | 2 | 4 | 4 | 5 | 22 | 4 | 2 | 1 | 5 | 2 |
| NM_019108 | C19orf61 | 2353 | 2 | 1 | 1 | 1 | 1 | 1 | 1 | 1 | 1 | 1 | 0 | 2 | 1 |
| NM_019109 | ALG1 | 3932 | 1 | 0 | 0 | 0 | 0 | 0 | 0 | 0 | 0 | 0 | 0 | 1 | 0 |
| NM_019110 | ZKSCAN4 | 2286 | 3 | 1 | 1 | 1 | 1 | 1 | 1 | 1 | 1 | 1 | 0 | 3 | 3 |
| NM_019111 | HLA-DRA | 1253 | 286 | 82 | 76 | 60 | 30 | 38 | 62 | 18 | 286 | 20 | 12 | 271 | 26 |
| NM_019111 | HLA-DRA | 1254 | 286 | 82 | 76 | 60 | 29 | 38 | 62 | 18 | 286 | 20 | 12 | 271 | 26 |
| NM_019112 | ABCA7 | 6818 | 0 | 0 | 0 | 0 | 0 | 0 | 0 | 0 | 0 | 0 | 0 | 0 | 0 |
| NM_019113 | FGF21 | 885 | 0 | 0 | 0 | 0 | 0 | 0 | 0 | 0 | 0 | 0 | 0 | 0 | 0 |

| transcript | gene | transcript length (nt) | max | mean | adipose | colon | heart | hypothalamus | kidney | liver | lung | ovary | skeletalmuscle | spleen | testes |
|---|---|---|---|---|---|---|---|---|---|---|---|---|---|---|---|
| NM_019114 | EPB41L4B | 5578 | 2 | 1 | 2 | 2 | 1 | 2 | 0 | 2 | 0 | 0 | 1 | 0 | 0 |
| NM_019116 | UBFD1 | 4931 | 8 | 4 | 3 | 2 | 5 | 6 | 3 | 3 | 1 | 3 | 5 | 3 | 8 |
| NM_019117 | KLHL4 | 5855 | 6 | 1 | 0 | 0 | 0 | 3 | 0 | 0 | 0 | 5 | 0 | 1 | 6 |
| NM_019118 | C1orf91 | 1082 | 1 | 1 | 0 | 0 | 0 | 1 | 1 | 0 | 1 | 0 | 0 | 1 | 1 |
| NM_019119 | PCDHB9 | 4219 | 1 | 0 | 0 | 0 | 0 | 0 | 0 | 0 | 0 | 1 | 0 | 0 | 0 |
| NM_019120 | PCDHB8 | 2592 | 0 | 0 | 0 | 0 | 0 | 0 | 0 | 0 | 0 | 0 | 0 | 0 | 0 |
| NM_019554 | S100A4 | 564 | 28 | 5 | 28 | 2 | 4 | 0 | 1 | 1 | 6 | 0 | 1 | 5 | 2 |
| NM_019555 | ARHGEF3 | 3602 | 7 | 4 | 1 | 1 | 3 | 7 | 6 | 0 | 6 | 4 | 2 | 5 | 7 |
| NM_019556 | MOSPD1 | 2358 | 6 | 3 | 2 | 1 | 6 | 2 | 2 | 1 | 2 | 3 | 2 | 3 | 6 |
| NM_019557 | FAM54B | 2103 | 12 | 5 | 4 | 3 | 12 | 6 | 5 | 4 | 2 | 5 | 6 | 4 | 7 |
| NM_019558 | HOXD8 | 1879 | 7 | 1 | 1 | 1 | 0 | 0 | 7 | 0 | 0 | 2 | 1 | 2 | 2 |
| NM_019589 | YLPM1 | 8137 | 29 | 17 | 12 | 11 | 14 | 26 | 16 | 7 | 12 | 25 | 13 | 21 | 29 |
| NM_019590 | KIAA1217 | 7754 | 30 | 10 | 7 | 5 | 6 | 7 | 12 | 5 | 6 | 7 | 30 | 6 | 24 |
| NM_019591 | ZNF26 | 3059 | 3 | 2 | 2 | 1 | 1 | 2 | 2 | 1 | 1 | 2 | 1 | 3 | 2 |
| NM_019592 | RNF20 | 3940 | 16 | 10 | 12 | 5 | 13 | 16 | 10 | 7 | 6 | 11 | 11 | 12 | 12 |
| NM_019593 | RP5-1022P6 | 5459 | 12 | 5 | 4 | 2 | 4 | 3 | 3 | 1 | 9 | 5 | 3 | 12 | 5 |
| NM_019594 | LRRC8A | 4354 | 5 | 2 | 1 | 2 | 2 | 5 | 2 | 2 | 3 | 2 | 1 | 4 | 2 |
| NM_019595 | ITSN2 | 6031 | 10 | 6 | 7 | 2 | 5 | 6 | 6 | 4 | 7 | 4 | 5 | 10 | 6 |
| NM_019596 | C21orf62 | 4044 | 1 | 0 | 0 | 0 | 0 | 0 | 1 | 0 | 0 | 1 | 0 | 0 | 0 |
| NM_019597 | HNRNPH2 | 2311 | 45 | 27 | 19 | 11 | 28 | 45 | 32 | 29 | 24 | 20 | 26 | 36 | 25 |
| NM_019598 | KLK12 | 945 | 0 | 0 | 0 | 0 | 0 | 0 | 0 | 0 | 0 | 0 | 0 | 0 | 0 |
| NM_019599 | TAS2R1 | 1355 | 0 | 0 | 0 | 0 | 0 | 0 | 0 | 0 | 0 | 0 | 0 | 0 | 0 |
| NM_019600 | KIAA1370 | 4217 | 4 | 2 | 2 | 1 | 1 | 1 | 2 | 2 | 2 | 2 | 2 | 4 | 4 |
| NM_019601 | SUSD2 | 3183 | 3 | 1 | 0 | 0 | 0 | 0 | 2 | 0 | 3 | 0 | 0 | 0 | 0 |
| NM_019602 | BTNL2 | 1368 | 0 | 0 | 0 | 0 | 0 | 0 | 0 | 0 | 0 | 0 | 0 | 0 | 0 |
| NM_019604 | CRTAM | 2422 | 1 | 0 | 0 | 0 | 0 | 0 | 0 | 0 | 0 | 0 | 0 | 1 | 0 |
| NM_019605 | SERTAD4 | 2006 | 2 | 1 | 0 | 1 | 1 | 1 | 1 | 0 | 0 | 2 | 0 | 0 | 0 |
| NM_019606 | MEPCE | 2747 | 4 | 2 | 1 | 2 | 1 | 2 | 2 | 2 | 1 | 4 | 2 | 4 | 2 |
| NM_019607 | C8orf44 | 1817 | 3 | 1 | 1 | 1 | 1 | 1 | 2 | 0 | 1 | 3 | 1 | 3 | 2 |
| NM_019609 | CPXM1 | 2390 | 2 | 0 | 1 | 0 | 0 | 0 | 0 | 0 | 0 | 2 | 0 | 0 | 1 |
| NM_019610 | RBMXL1 | 4986 | 5 | 2 | 1 | 1 | 2 | 2 | 2 | 2 | 2 | 3 | 1 | 3 | 5 |
| NM_019612 | IRGC | 1604 | 2 | 0 | 0 | 0 | 0 | 0 | 0 | 0 | 0 | 0 | 0 | 0 | 2 |
| NM_019613 | WDR45L | 2587 | 4 | 2 | 2 | 1 | 2 | 4 | 3 | 2 | 1 | 2 | 1 | 2 | 4 |
| NM_019616 | F7 | 3059 | 63 | 16 | 6 | 8 | 13 | 8 | 10 | 63 | 16 | 12 | 11 | 11 | 15 |
| NM_019617 | GKN1 | 820 | 0 | 0 | 0 | 0 | 0 | 0 | 0 | 0 | 0 | 0 | 0 | 0 | 0 |
| NM_019618 | IL1F9 | 1180 | 0 | 0 | 0 | 0 | 0 | 0 | 0 | 0 | 0 | 0 | 0 | 0 | 0 |
| NM_019619 | PARD3 | 4071 | 14 | 7 | 4 | 5 | 9 | 4 | 8 | 6 | 6 | 14 | 9 | 2 | 9 |
| NM_019624 | ABCB9 | 3401 | 0 | 0 | 0 | 0 | 0 | 0 | 0 | 0 | 0 | 0 | 0 | 0 | 0 |
| NM_019625 | ABCB9 | 3530 | 0 | 0 | 0 | 0 | 0 | 0 | 0 | 0 | 0 | 0 | 0 | 0 | 0 |
| NM_019644 | ANKRD7 | 1355 | 30 | 3 | 0 | 0 | 0 | 0 | 0 | 0 | 0 | 0 | 0 | 0 | 30 |
| NM_019839 | LTB4R2 | 3099 | 1 | 0 | 0 | 0 | 0 | 0 | 0 | 0 | 0 | 0 | 0 | 1 | 0 |
| NM_019841 | TRPV5 | 2486 | 0 | 0 | 0 | 0 | 0 | 0 | 0 | 0 | 0 | 0 | 0 | 0 | 0 |
| NM_019842 | KCNQ5 | 6580 | 10 | 2 | 0 | 0 | 0 | 7 | 0 | 0 | 0 | 0 | 10 | 1 | 0 |
| NM_019843 | EIF4ENIF1 | 3700 | 11 | 6 | 4 | 5 | 4 | 9 | 6 | 2 | 4 | 6 | 5 | 6 | 11 |
| NM_019844 | SLCO1B3 | 2712 | 12 | 1 | 0 | 0 | 0 | 0 | 0 | 12 | 0 | 0 | 0 | 0 | 0 |
| NM_019845 | RPRM | 1471 | 1 | 0 | 0 | 0 | 0 | 1 | 0 | 0 | 0 | 0 | 0 | 0 | 0 |
| NM_019848 | SLC10A3 | 2128 | 2 | 1 | 0 | 1 | 1 | 0 | 1 | 0 | 1 | 2 | 1 | 1 | 1 |
| NM_019849 | SLC7A10 | 1946 | 0 | 0 | 0 | 0 | 0 | 0 | 0 | 0 | 0 | 0 | 0 | 0 | 0 |
| NM_019850 | NGEF | 3184 | 4 | 1 | 0 | 4 | 0 | 4 | 2 | 4 | 0 | 0 | 0 | 0 | 0 |
| NM_019851 | FGF20 | 1016 | 0 | 0 | 0 | 0 | 0 | 0 | 0 | 0 | 0 | 0 | 0 | 0 | 0 |
| NM_019852 | METTL3 | 1955 | 2 | 1 | 1 | 1 | 1 | 1 | 1 | 1 | 1 | 1 | 1 | 2 | 2 |
| NM_019854 | PRMT8 | 2342 | 2 | 0 | 0 | 0 | 0 | 2 | 0 | 0 | 0 | 0 | 0 | 0 | 0 |
| NM_019855 | CABP5 | 1251 | 0 | 0 | 0 | 0 | 0 | 0 | 0 | 0 | 0 | 0 | 0 | 0 | 0 |
| NM_019856 | MYOM1 | 5559 | 149 | 23 | 4 | 1 | 91 | 2 | 0 | 4 | 1 | 1 | 149 | 0 | 3 |
| NM_019857 | CTPS2 | 3833 | 3 | 1 | 1 | 1 | 1 | 1 | 1 | 1 | 1 | 3 | 1 | 1 | 1 |
| NM_019858 | GPR162 | 2516 | 1 | 0 | 0 | 0 | 0 | 1 | 0 | 0 | 0 | 0 | 0 | 0 | 0 |
| NM_019859 | HTR7 | 3344 | 1 | 0 | 0 | 0 | 0 | 1 | 0 | 0 | 0 | 0 | 0 | 0 | 1 |
| NM_019860 | HTR7 | 3251 | 1 | 0 | 0 | 0 | 0 | 1 | 0 | 0 | 0 | 0 | 0 | 0 | 1 |
| NM_019862 | ABCC1 | 6386 | 3 | 1 | 1 | 1 | 1 | 1 | 1 | 0 | 1 | 1 | 1 | 2 | 3 |
| NM_019863 | F8 | 2604 | 6 | 2 | 2 | 0 | 6 | 2 | 1 | 2 | 2 | 2 | 2 | 4 | 3 |
| NM_019884 | GSK3A | 2179 | 6 | 2 | 2 | 2 | 2 | 6 | 2 | 2 | 2 | 1 | 2 | 2 | 2 |
| NM_019885 | CYP26B1 | 4528 | 1 | 0 | 0 | 0 | 0 | 1 | 0 | 0 | 0 | 0 | 0 | 0 | 0 |
| NM_019886 | CHST7 | 2311 | 1 | 0 | 0 | 0 | 1 | 0 | 0 | 0 | 0 | 1 | 0 | 1 | 0 |
| NM_019887 | DIABLO | 2243 | 10 | 5 | 3 | 3 | 4 | 8 | 6 | 5 | 2 | 4 | 2 | 4 | 10 |
| NM_019888 | MC3R | 1084 | 0 | 0 | 0 | 0 | 0 | 0 | 0 | 0 | 0 | 0 | 0 | 0 | 0 |
| NM_019891 | ERO1LB | 5052 | 3 | 2 | 1 | 0 | 1 | 2 | 3 | 2 | 1 | 2 | 0 | 3 | 2 |
| NM_019892 | INPP5E | 3375 | 0 | 0 | 0 | 0 | 0 | 0 | 0 | 0 | 0 | 0 | 0 | 0 | 0 |
| NM_019893 | ASAH2 | 2416 | 1 | 0 | 0 | 0 | 0 | 0 | 0 | 0 | 0 | 0 | 0 | 0 | 1 |
| NM_019894 | TMPRSS4 | 3533 | 2 | 0 | 0 | 2 | 0 | 0 | 1 | 0 | 0 | 0 | 0 | 0 | 0 |
| NM_019895 | CLDND1 | 2235 | 40 | 8 | 4 | 3 | 6 | 40 | 3 | 4 | 5 | 4 | 2 | 7 | 9 |
| NM_019896 | POLE4 | 696 | 16 | 11 | 14 | 9 | 10 | 10 | 9 | 16 | 11 | 8 | 11 | 13 | 14 |
| NM_019897 | OR2S2 | 1047 | 0 | 0 | 0 | 0 | 0 | 0 | 0 | 0 | 0 | 0 | 0 | 0 | 0 |
| NM_019898 | ABCC1 | 6395 | 3 | 1 | 1 | 1 | 1 | 1 | 1 | 0 | 1 | 1 | 1 | 2 | 3 |
| NM_019899 | ABCC1 | 6218 | 3 | 1 | 1 | 1 | 1 | 1 | 1 | 0 | 1 | 1 | 1 | 2 | 3 |
| NM_019900 | ABCC1 | 6368 | 3 | 1 | 1 | 1 | 1 | 1 | 1 | 0 | 1 | 1 | 1 | 2 | 3 |
| NM_019903 | ADD3 | 4185 | 30 | 13 | 10 | 7 | 17 | 30 | 21 | 2 | 11 | 12 | 3 | 10 | 17 |
| NM_020039 | ACCN2 | 3916 | 2 | 0 | 0 | 0 | 0 | 2 | 0 | 0 | 0 | 0 | 0 | 0 | 0 |

| transcript | gene | transcript length (nt) | max | mean | adipose | colon | heart | hypothalamus | kidney | liver | lung | ovary | skeletalmuscle | spleen | testes |
|---|---|---|---|---|---|---|---|---|---|---|---|---|---|---|---|
| NM_020040 | TUBB4Q | 1303 | 0 | 0 | 0 | 0 | 0 | 0 | 0 | 0 | 0 | 0 | 0 | 0 | 0 |
| NM_020041 | SLC2A9 | 1857 | 3 | 1 | 0 | 0 | 0 | 0 | 3 | 1 | 1 | 0 | 0 | 0 | 0 |
| NM_020056 | HLA-DQA2 | 1707 | 3 | 1 | 1 | 0 | 0 | 0 | 0 | 0 | 3 | 0 | 0 | 3 | 0 |
| NM_020056 | HLA-DQA2 | 1708 | 3 | 1 | 1 | 0 | 0 | 0 | 0 | 0 | 3 | 0 | 0 | 3 | 0 |
| NM_020056 | HLA-DQA2 | 1709 | 3 | 1 | 1 | 0 | 0 | 0 | 0 | 0 | 3 | 0 | 0 | 3 | 0 |
| NM_020061 | OPN1LW | 1234 | 0 | 0 | 0 | 0 | 0 | 0 | 0 | 0 | 0 | 0 | 0 | 0 | 0 |
| NM_020062 | SLC2A4RG | 2279 | 3 | 1 | 1 | 1 | 1 | 0 | 3 | 1 | 0 | 1 | 1 | 1 | 1 |
| NM_020063 | BARHL2 | 1496 | 0 | 0 | 0 | 0 | 0 | 0 | 0 | 0 | 0 | 0 | 0 | 0 | 0 |
| NM_020064 | BARHL1 | 1907 | 0 | 0 | 0 | 0 | 0 | 0 | 0 | 0 | 0 | 0 | 0 | 0 | 0 |
| NM_020066 | FMN2 | 6429 | 7 | 1 | 0 | 0 | 0 | 7 | 0 | 0 | 0 | 0 | 0 | 0 | 0 |
| NM_020069 | ACRV1 | 1267 | 2 | 0 | 0 | 0 | 0 | 0 | 0 | 0 | 0 | 0 | 0 | 0 | 2 |
| NM_020070 | IGLL1 | 900 | 5 | 1 | 0 | 2 | 0 | 0 | 0 | 0 | 3 | 0 | 0 | 5 | 0 |
| NM_020107 | ACRV1 | 1159 | 2 | 0 | 0 | 0 | 0 | 0 | 0 | 0 | 0 | 0 | 0 | 0 | 2 |
| NM_020108 | ACRV1 | 1114 | 2 | 0 | 0 | 0 | 0 | 0 | 0 | 0 | 0 | 0 | 0 | 0 | 2 |
| NM_020109 | ACRV1 | 1057 | 2 | 0 | 0 | 0 | 0 | 0 | 0 | 0 | 0 | 0 | 0 | 0 | 2 |
| NM_020110 | ACRV1 | 892 | 2 | 0 | 0 | 0 | 0 | 0 | 0 | 0 | 0 | 0 | 0 | 0 | 2 |
| NM_020111 | ACRV1 | 1039 | 2 | 0 | 0 | 0 | 0 | 0 | 0 | 0 | 0 | 0 | 0 | 0 | 2 |
| NM_020113 | ACRV1 | 994 | 1 | 0 | 0 | 0 | 0 | 0 | 0 | 0 | 0 | 0 | 0 | 0 | 1 |
| NM_020115 | ACRV1 | 772 | 1 | 0 | 0 | 0 | 0 | 0 | 0 | 0 | 0 | 0 | 0 | 0 | 1 |
| NM_020116 | FSTL5 | 4830 | 3 | 0 | 0 | 0 | 0 | 3 | 0 | 0 | 0 | 0 | 0 | 0 | 0 |
| NM_020117 | LARS | 4849 | 16 | 10 | 11 | 5 | 13 | 16 | 10 | 6 | 6 | 10 | 13 | 13 | 11 |
| NM_020119 | ZC3HAV1 | 7171 | 12 | 5 | 3 | 3 | 3 | 2 | 6 | 4 | 6 | 6 | 1 | 12 | 6 |
| NM_020120 | UGGT1 | 10777 | 7 | 4 | 3 | 2 | 4 | 3 | 4 | 7 | 3 | 2 | 2 | 6 | 3 |
| NM_020121 | UGGT2 | 4927 | 3 | 2 | 1 | 0 | 2 | 1 | 2 | 1 | 1 | 2 | 1 | 2 | 3 |
| NM_020122 | KCMF1 | 7553 | 3 | 2 | 1 | 1 | 2 | 2 | 1 | 2 | 1 | 2 | 3 | 2 | 2 |
| NM_020123 | TM9SF3 | 6140 | 13 | 9 | 8 | 6 | 9 | 10 | 10 | 13 | 9 | 13 | 4 | 10 | 10 |
| NM_020124 | IFNK | 1140 | 1 | 0 | 0 | 0 | 0 | 0 | 0 | 0 | 0 | 0 | 0 | 1 | 0 |
| NM_020125 | SLAMF8 | 3300 | 2 | 0 | 1 | 0 | 1 | 0 | 0 | 0 | 1 | 0 | 0 | 2 | 0 |
| NM_020126 | SPHK2 | 2963 | 3 | 1 | 0 | 1 | 0 | 2 | 3 | 1 | 1 | 1 | 1 | 1 | 1 |
| NM_020127 | TUFT1 | 3111 | 7 | 2 | 2 | 3 | 1 | 2 | 7 | 1 | 2 | 2 | 1 | 1 | 2 |
| NM_020128 | MDM1 | 2286 | 2 | 1 | 1 | 1 | 1 | 2 | 1 | 1 | 1 | 2 | 0 | 2 | 1 |
| NM_020129 | LGALS14 | 778 | 0 | 0 | 0 | 0 | 0 | 0 | 0 | 0 | 0 | 0 | 0 | 0 | 0 |
| NM_020130 | C8orf4 | 1833 | 47 | 15 | 10 | 7 | 10 | 4 | 17 | 23 | 47 | 7 | 9 | 26 | 3 |
| NM_020131 | UBQLN4 | 3507 | 3 | 2 | 2 | 2 | 2 | 3 | 2 | 2 | 2 | 2 | 3 | 2 | 3 |
| NM_020132 | AGPAT3 | 6555 | 12 | 5 | 6 | 3 | 5 | 8 | 12 | 8 | 3 | 2 | 4 | 5 | 3 |
| NM_020133 | AGPAT4 | 7879 | 3 | 1 | 1 | 0 | 0 | 3 | 1 | 0 | 0 | 1 | 0 | 1 | 0 |
| NM_020134 | DPYSL5 | 5209 | 4 | 0 | 0 | 1 | 0 | 4 | 0 | 0 | 0 | 0 | 0 | 0 | 0 |
| NM_020135 | WRNIP1 | 2652 | 3 | 2 | 1 | 2 | 3 | 3 | 3 | 2 | 3 | 2 | 2 | 3 | 3 |
| NM_020137 | GRIPAP1 | 3032 | 11 | 4 | 2 | 3 | 3 | 11 | 4 | 3 | 3 | 2 | 2 | 5 | 5 |
| NM_020139 | BDH2 | 2920 | 19 | 4 | 2 | 1 | 3 | 4 | 19 | 2 | 2 | 3 | 1 | 2 | 3 |
| NM_020140 | ANKS1B | 1714 | 17 | 2 | 0 | 0 | 0 | 17 | 1 | 0 | 0 | 1 | 0 | 0 | 1 |
| NM_020141 | TMEM167B | 2750 | 15 | 11 | 9 | 4 | 13 | 13 | 10 | 7 | 14 | 10 | 7 | 15 | 15 |
| NM_020141 | TMEM167B | 2751 | 15 | 11 | 9 | 4 | 13 | 13 | 10 | 7 | 14 | 10 | 7 | 15 | 15 |
| NM_020142 | NDUFA4L2 | 1188 | 3 | 1 | 1 | 0 | 2 | 1 | 0 | 0 | 1 | 0 | 0 | 3 | 0 |
| NM_020143 | PNO1 | 1978 | 2 | 1 | 1 | 1 | 1 | 1 | 1 | 2 | 1 | 0 | 1 | 1 | 1 |
| NM_020144 | PAPOLB | 4257 | 11 | 1 | 0 | 0 | 0 | 0 | 0 | 0 | 0 | 0 | 0 | 0 | 11 |
| NM_020145 | SH3GLB2 | 1984 | 2 | 1 | 0 | 1 | 1 | 2 | 1 | 0 | 1 | 1 | 0 | 1 | 1 |
| NM_020147 | THAP10 | 2028 | 0 | 0 | 0 | 0 | 0 | 0 | 0 | 0 | 0 | 0 | 0 | 0 | 0 |
| NM_020148 | SPIRE1 | 5402 | 14 | 5 | 4 | 1 | 9 | 14 | 5 | 1 | 5 | 4 | 6 | 3 | 6 |
| NM_020150 | SAR1A | 2994 | 18 | 9 | 18 | 4 | 11 | 10 | 9 | 8 | 9 | 9 | 5 | 9 | 7 |
| NM_020151 | STARD7 | 3379 | 82 | 30 | 14 | 15 | 82 | 31 | 26 | 28 | 24 | 15 | 50 | 32 | 15 |
| NM_020152 | C21orf7 | 1995 | 12 | 2 | 1 | 0 | 3 | 0 | 1 | 1 | 3 | 1 | 12 | 2 | 1 |
| NM_020153 | C11orf60 | 1875 | 5 | 2 | 2 | 2 | 2 | 5 | 3 | 1 | 2 | 4 | 1 | 2 | 3 |
| NM_020154 | C15orf24 | 1053 | 21 | 13 | 12 | 9 | 15 | 19 | 14 | 21 | 11 | 6 | 8 | 12 | 18 |
| NM_020155 | GPR137 | 1688 | 1 | 0 | 0 | 0 | 0 | 0 | 0 | 0 | 0 | 0 | 0 | 0 | 1 |
| NM_020156 | C1GALT1 | 1938 | 10 | 6 | 6 | 2 | 5 | 10 | 9 | 5 | 5 | 3 | 3 | 10 | 4 |
| NM_020157 | OTOR | 1477 | 0 | 0 | 0 | 0 | 0 | 0 | 0 | 0 | 0 | 0 | 0 | 0 | 0 |
| NM_020158 | EXOSC5 | 993 | 1 | 0 | 0 | 0 | 0 | 1 | 0 | 1 | 0 | 0 | 0 | 0 | 0 |
| NM_020159 | SMARCAD1 | 5094 | 4 | 2 | 1 | 0 | 1 | 2 | 2 | 1 | 2 | 3 | 1 | 3 | 4 |
| NM_020160 | MEIS3 | 2158 | 1 | 0 | 0 | 0 | 0 | 1 | 0 | 0 | 0 | 0 | 0 | 0 | 0 |
| NM_020161 | C2orf83 | 1992 | 0 | 0 | 0 | 0 | 0 | 0 | 0 | 0 | 0 | 0 | 0 | 0 | 0 |
| NM_020162 | DHX33 | 4060 | 2 | 1 | 1 | 1 | 1 | 2 | 1 | 1 | 0 | 1 | 1 | 1 | 1 |
| NM_020163 | SEMA3G | 4700 | 23 | 3 | 23 | 1 | 2 | 1 | 2 | 0 | 2 | 0 | 1 | 3 | 0 |
| NM_020164 | ASPH | 3272 | 41 | 10 | 24 | 2 | 11 | 5 | 4 | 2 | 8 | 4 | 41 | 6 | 3 |
| NM_020165 | RAD18 | 5737 | 2 | 1 | 1 | 1 | 1 | 1 | 1 | 1 | 1 | 2 | 1 | 2 | 2 |
| NM_020166 | MCCC1 | 2535 | 17 | 10 | 13 | 5 | 11 | 9 | 17 | 13 | 10 | 8 | 5 | 8 | 7 |
| NM_020167 | NMUR2 | 2064 | 0 | 0 | 0 | 0 | 0 | 0 | 0 | 0 | 0 | 0 | 0 | 0 | 0 |
| NM_020168 | PAK6 | 3901 | 1 | 0 | 0 | 0 | 0 | 1 | 0 | 0 | 0 | 0 | 0 | 0 | 1 |
| NM_020169 | LXN | 1116 | 2 | 1 | 1 | 2 | 1 | 0 | 2 | 0 | 2 | 2 | 0 | 1 | 2 |
| NM_020170 | NCLN | 3733 | 4 | 2 | 2 | 3 | 1 | 2 | 1 | 4 | 1 | 1 | 1 | 2 | 1 |
| NM_020171 | GPR108 | 2214 | 1 | 1 | 1 | 1 | 1 | 1 | 1 | 1 | 1 | 1 | 0 | 1 | 1 |
| NM_020175 | DUS3L | 2109 | 1 | 0 | 0 | 0 | 0 | 0 | 0 | 1 | 0 | 1 | 0 | 1 | 0 |
| NM_020177 | FEM1C | 5813 | 15 | 7 | 5 | 3 | 15 | 9 | 4 | 5 | 9 | 6 | 8 | 7 | 11 |
| NM_020178 | CA10 | 3244 | 9 | 1 | 0 | 0 | 0 | 9 | 1 | 0 | 0 | 0 | 0 | 0 | 0 |
| NM_020179 | C11orf75 | 975 | 1 | 0 | 0 | 1 | 0 | 0 | 0 | 0 | 0 | 0 | 0 | 1 | 0 |
| NM_020180 | BRUNOL4 | 4054 | 12 | 1 | 0 | 1 | 0 | 12 | 0 | 0 | 0 | 0 | 0 | 0 | 0 |
| NM_020182 | PMEPA1 | 4914 | 7 | 2 | 7 | 1 | 3 | 2 | 1 | 0 | 1 | 3 | 0 | 1 | 1 |

| | | transcript | | | | | | hypothala | | | | | skeletalm | | |
|---|---|---|---|---|---|---|---|---|---|---|---|---|---|---|---|
| transcript | gene | length (nt) | max | mean | adipose | colon | heart | mus | kidney | liver | lung | ovary | uscle | spleen | testes |
| NM_020183 | ARNTL2 | 1930 | 1 | 0 | 0 | 0 | 1 | 1 | 0 | 0 | 1 | 0 | 0 | 0 | 0 |
| NM_020184 | CNNM4 | 4800 | 1 | 0 | 0 | 1 | 1 | 0 | 0 | 0 | 0 | 0 | 1 | 0 | 0 |
| NM_020185 | DUSP22 | 1481 | 8 | 5 | 5 | 3 | 6 | 5 | 3 | 4 | 4 | 3 | 3 | 6 | 8 |
| NM_020186 | ACN9 | 2057 | 1 | 1 | 0 | 0 | 1 | 1 | 1 | 1 | 0 | 0 | 0 | 1 | 1 |
| NM_020187 | C3orf37 | 1620 | 4 | 2 | 2 | 2 | 3 | 4 | 2 | 3 | 2 | 2 | 2 | 2 | 3 |
| NM_020188 | C16orf61 | 728 | 32 | 13 | 9 | 5 | 32 | 26 | 16 | 18 | 8 | 5 | 7 | 10 | 14 |
| NM_020189 | ENY2 | 569 | 54 | 30 | 32 | 18 | 38 | 40 | 33 | 54 | 24 | 18 | 18 | 34 | 26 |
| NM_020190 | OLFML3 | 1814 | 14 | 5 | 6 | 4 | 7 | 3 | 4 | 1 | 6 | 14 | 1 | 1 | 4 |
| NM_020191 | MRPS22 | 1122 | 9 | 4 | 3 | 2 | 6 | 5 | 4 | 9 | 1 | 3 | 4 | 3 | 4 |
| NM_020192 | C7orf36 | 856 | 4 | 2 | 2 | 2 | 2 | 4 | 2 | 2 | 1 | 2 | 1 | 1 | 2 |
| NM_020193 | C11orf30 | 5511 | 3 | 2 | 1 | 1 | 2 | 1 | 1 | 1 | 2 | 3 | 2 | 2 | 2 |
| NM_020194 | MFF | 2011 | 22 | 10 | 6 | 3 | 11 | 16 | 10 | 6 | 6 | 10 | 8 | 9 | 22 |
| NM_020195 | SDR39U1 | 1231 | 0 | 0 | 0 | 0 | 0 | 0 | 0 | 0 | 0 | 0 | 0 | 0 | 0 |
| NM_020196 | XAB2 | 2655 | 1 | 1 | 0 | 1 | 0 | 1 | 1 | 0 | 0 | 1 | 0 | 1 | 1 |
| NM_020197 | SMYD2 | 1685 | 28 | 7 | 4 | 2 | 28 | 7 | 4 | 5 | 3 | 2 | 10 | 5 | 7 |
| NM_020198 | CCDC47 | 3419 | 51 | 25 | 21 | 9 | 30 | 33 | 25 | 51 | 20 | 15 | 24 | 24 | 24 |
| NM_020199 | C5orf15 | 2280 | 9 | 4 | 2 | 1 | 5 | 2 | 9 | 3 | 4 | 4 | 2 | 5 | 4 |
| NM_020200 | PRTFDC1 | 1926 | 3 | 1 | 1 | 1 | 1 | 3 | 0 | 0 | 0 | 2 | 0 | 1 | 0 |
| NM_020201 | NT5M | 1588 | 0 | 0 | 0 | 0 | 0 | 0 | 0 | 0 | 0 | 0 | 0 | 0 | 0 |
| NM_020202 | NIT2 | 1271 | 20 | 7 | 8 | 3 | 6 | 7 | 16 | 20 | 3 | 3 | 5 | 4 | 3 |
| NM_020203 | MEPE | 1963 | 2 | 0 | 0 | 0 | 0 | 2 | 0 | 0 | 0 | 0 | 0 | 0 | 0 |
| NM_020204 | LHX9 | 2515 | 1 | 0 | 0 | 0 | 0 | 0 | 0 | 0 | 0 | 1 | 0 | 0 | 1 |
| NM_020205 | OTUD7B | 6412 | 11 | 7 | 8 | 5 | 6 | 9 | 5 | 5 | 7 | 7 | 11 | 4 | 8 |
| NM_020208 | SLC6A20 | 5432 | 1 | 0 | 0 | 0 | 0 | 1 | 0 | 0 | 0 | 0 | 0 | 0 | 0 |
| NM_020209 | SHD | 2576 | 0 | 0 | 0 | 0 | 0 | 0 | 0 | 0 | 0 | 0 | 0 | 0 | 0 |
| NM_020210 | SEMA4B | 3814 | 2 | 1 | 0 | 1 | 1 | 1 | 1 | 1 | 1 | 1 | 0 | 2 | 1 |
| NM_020211 | RGMA | 3226 | 3 | 1 | 1 | 1 | 1 | 3 | 0 | 0 | 0 | 1 | 2 | 0 | 1 |
| NM_020212 | WDR93 | 2409 | 1 | 0 | 0 | 0 | 0 | 0 | 0 | 0 | 0 | 0 | 0 | 0 | 1 |
| NM_020214 | PARP6 | 2626 | 7 | 4 | 4 | 3 | 3 | 7 | 3 | 2 | 2 | 3 | 5 | 3 | 5 |
| NM_020216 | RNPEP | 2413 | 5 | 3 | 3 | 5 | 2 | 2 | 3 | 3 | 3 | 2 | 1 | 4 | 3 |
| NM_020218 | ATXN7L3 | 3614 | 5 | 2 | 1 | 2 | 2 | 5 | 2 | 1 | 2 | 1 | 1 | 3 | 2 |
| NM_020219 | CEACAM19 | 2248 | 1 | 0 | 0 | 1 | 0 | 0 | 0 | 0 | 0 | 0 | 0 | 0 | 0 |
| NM_020223 | FAM20C | 1094 | 1 | 0 | 0 | 0 | 0 | 0 | 1 | 0 | 0 | 0 | 0 | 0 | 0 |
| NM_020225 | STOX2 | 4654 | 6 | 1 | 0 | 0 | 1 | 6 | 1 | 0 | 1 | 1 | 0 | 2 | 1 |
| NM_020226 | PRDM8 | 4097 | 2 | 0 | 0 | 0 | 0 | 2 | 0 | 0 | 0 | 0 | 0 | 0 | 0 |
| NM_020227 | PRDM9 | 3691 | 1 | 0 | 0 | 0 | 0 | 0 | 0 | 0 | 0 | 0 | 0 | 0 | 1 |
| NM_020228 | PRDM10 | 6322 | 2 | 1 | 1 | 1 | 1 | 1 | 1 | 1 | 1 | 1 | 1 | 2 | 1 |
| NM_020229 | PRDM11 | 2229 | 0 | 0 | 0 | 0 | 0 | 0 | 0 | 0 | 0 | 0 | 0 | 0 | 0 |
| NM_020230 | PPAN | 1662 | 0 | 0 | 0 | 0 | 0 | 0 | 0 | 0 | 0 | 0 | 0 | 0 | 0 |
| NM_020232 | PSMG2 | 1047 | 5 | 3 | 3 | 2 | 4 | 3 | 4 | 5 | 2 | 4 | 2 | 4 | 5 |
| NM_020233 | C17orf48 | 1518 | 1 | 1 | 1 | 0 | 1 | 1 | 1 | 1 | 1 | 1 | 1 | 1 | 1 |
| NM_020234 | DTWD1 | 2749 | 3 | 1 | 1 | 0 | 1 | 1 | 1 | 1 | 1 | 3 | 1 | 2 | 1 |
| NM_020235 | BBX | 8806 | 42 | 12 | 7 | 2 | 10 | 10 | 12 | 3 | 9 | 16 | 4 | 14 | 42 |
| NM_020236 | MRPL1 | 1359 | 10 | 6 | 3 | 2 | 10 | 7 | 9 | 8 | 3 | 6 | 7 | 7 | 5 |
| NM_020238 | INCENP | 4134 | 4 | 2 | 2 | 3 | 2 | 4 | 2 | 1 | 2 | 2 | 1 | 3 | 3 |
| NM_020239 | CDC42SE1 | 3047 | 16 | 5 | 3 | 3 | 4 | 3 | 4 | 2 | 9 | 2 | 1 | 16 | 3 |
| NM_020240 | CDC42SE2 | 3656 | 21 | 8 | 4 | 4 | 3 | 15 | 9 | 4 | 8 | 6 | 2 | 21 | 9 |
| NM_020242 | KIF15 | 4837 | 3 | 1 | 0 | 1 | 0 | 0 | 0 | 0 | 0 | 0 | 0 | 1 | 3 |
| NM_020243 | TOMM22 | 1378 | 3 | 2 | 1 | 2 | 2 | 3 | 3 | 3 | 1 | 2 | 1 | 3 | 3 |
| NM_020244 | CHPT1 | 1567 | 9 | 4 | 2 | 1 | 9 | 4 | 6 | 5 | 3 | 1 | 1 | 4 | 3 |
| NM_020245 | TULP4 | 11119 | 11 | 5 | 4 | 3 | 4 | 11 | 4 | 2 | 3 | 5 | 4 | 5 | 5 |
| NM_020246 | SLC12A9 | 3294 | 1 | 0 | 0 | 1 | 0 | 0 | 0 | 0 | 0 | 0 | 0 | 1 | 0 |
| NM_020247 | CABC1 | 2924 | 28 | 5 | 5 | 2 | 10 | 2 | 2 | 5 | 1 | 2 | 28 | 2 | 1 |
| NM_020248 | CTNNBIP1 | 2995 | 2 | 1 | 1 | 1 | 1 | 2 | 1 | 1 | 2 | 1 | 0 | 1 | 2 |
| NM_020251 | ARRB1 | 2180 | 6 | 3 | 4 | 3 | 2 | 5 | 1 | 1 | 5 | 1 | 1 | 6 | 1 |
| NM_020297 | ABCC9 | 8324 | 10 | 3 | 3 | 1 | 5 | 1 | 1 | 10 | 2 | 2 | 6 | 1 | 1 |
| NM_020298 | ABCC9 | 4562 | 15 | 4 | 4 | 1 | 7 | 2 | 2 | 15 | 2 | 2 | 8 | 1 | 2 |
| NM_020299 | AKR1B10 | 1595 | 3 | 1 | 0 | 3 | 0 | 0 | 2 | 1 | 0 | 0 | 1 | 0 | 0 |
| NM_020300 | MGST1 | 923 | 424 | 99 | 357 | 32 | 43 | 40 | 96 | 424 | 63 | 3 | 11 | 4 | 20 |
| NM_020307 | CCNL1 | 2624 | 15 | 8 | 7 | 6 | 5 | 5 | 8 | 6 | 15 | 12 | 5 | 12 | 6 |
| NM_020309 | SLC17A7 | 2975 | 11 | 1 | 0 | 0 | 0 | 11 | 0 | 0 | 0 | 0 | 0 | 0 | 0 |
| NM_020310 | MNT | 4842 | 2 | 1 | 1 | 1 | 1 | 2 | 1 | 0 | 1 | 1 | 1 | 2 | 2 |
| NM_020311 | CXCR7 | 2100 | 2 | 1 | 1 | 0 | 0 | 0 | 0 | 1 | 0 | 0 | 0 | 2 | 0 |
| NM_020312 | COQ9 | 1616 | 11 | 6 | 4 | 5 | 10 | 4 | 11 | 10 | 2 | 2 | 9 | 3 | 3 |
| NM_020313 | CIAPIN1 | 2105 | 1 | 1 | 0 | 1 | 1 | 1 | 1 | 1 | 0 | 0 | 0 | 1 | 1 |
| NM_020314 | C16orf62 | 3550 | 4 | 2 | 3 | 2 | 2 | 4 | 3 | 2 | 1 | 2 | 2 | 2 | 2 |
| NM_020315 | PDXP | 2005 | 12 | 2 | 0 | 1 | 1 | 12 | 1 | 3 | 0 | 0 | 0 | 1 | 1 |
| NM_020317 | C1orf63 | 1465 | 4 | 2 | 2 | 2 | 1 | 1 | 2 | 0 | 3 | 2 | 1 | 4 | 2 |
| NM_020318 | PAPPA2 | 6920 | 2 | 0 | 0 | 0 | 0 | 1 | 2 | 0 | 0 | 0 | 0 | 0 | 0 |
| NM_020319 | ANKMY2 | 2500 | 18 | 8 | 7 | 4 | 10 | 18 | 8 | 3 | 5 | 12 | 7 | 7 | 7 |
| NM_020320 | RARS2 | 1831 | 14 | 10 | 10 | 5 | 9 | 6 | 10 | 12 | 10 | 14 | 7 | 14 | 13 |
| NM_020321 | ACCN3 | 2088 | 0 | 0 | 0 | 0 | 0 | 0 | 0 | 0 | 0 | 0 | 0 | 0 | 0 |
| NM_020322 | ACCN3 | 2009 | 0 | 0 | 0 | 0 | 0 | 0 | 0 | 0 | 0 | 0 | 0 | 0 | 0 |
| NM_020327 | ACVR1B | 1783 | 14 | 6 | 2 | 4 | 4 | 9 | 14 | 6 | 4 | 2 | 4 | 6 | 6 |
| NM_020328 | ACVR1B | 1708 | 14 | 6 | 3 | 4 | 4 | 9 | 14 | 7 | 4 | 2 | 4 | 6 | 6 |
| NM_020335 | VANGL2 | 5345 | 1 | 0 | 0 | 1 | 0 | 1 | 0 | 0 | 1 | 1 | 0 | 0 | 0 |
| NM_020336 | RALGAPB | 8636 | 7 | 5 | 3 | 3 | 5 | 7 | 6 | 2 | 4 | 5 | 3 | 7 | 7 |

| transcript | gene | transcript length (nt) | max | mean | adipose | colon | heart | hypothalamus | kidney | liver | lung | ovary | skeletalmuscle | spleen | testes |
|---|---|---|---|---|---|---|---|---|---|---|---|---|---|---|---|
| NM_020337 | ANKRD50 | 8795 | 6 | 3 | 2 | 1 | 3 | 5 | 2 | 2 | 3 | 6 | 1 | 2 | 6 |
| NM_020338 | ZMIZ1 | 7555 | 4 | 2 | 1 | 1 | 2 | 2 | 2 | 1 | 2 | 2 | 1 | 4 | 1 |
| NM_020340 | KIAA1244 | 14877 | 8 | 1 | 0 | 1 | 0 | 8 | 1 | 0 | 2 | 0 | 0 | 0 | 0 |
| NM_020341 | PAK7 | 4777 | 2 | 0 | 0 | 0 | 0 | 2 | 0 | 0 | 0 | 0 | 0 | 0 | 0 |
| NM_020342 | SLC39A10 | 5322 | 21 | 6 | 3 | 1 | 4 | 21 | 5 | 1 | 4 | 6 | 2 | 11 | 8 |
| NM_020343 | RALGAPA2 | 6287 | 9 | 5 | 3 | 4 | 3 | 1 | 5 | 9 | 9 | 3 | 5 | 4 | 5 |
| NM_020344 | SLC24A2 | 2221 | 10 | 1 | 0 | 0 | 0 | 10 | 0 | 0 | 0 | 0 | 0 | 0 | 0 |
| NM_020345 | NKIRAS1 | 1995 | 10 | 3 | 2 | 1 | 2 | 10 | 2 | 3 | 1 | 1 | 3 | 1 | 3 |
| NM_020346 | SLC17A6 | 3920 | 3 | 0 | 0 | 0 | 0 | 3 | 0 | 0 | 0 | 0 | 0 | 0 | 0 |
| NM_020347 | LZTFL1 | 3398 | 8 | 2 | 2 | 1 | 1 | 6 | 2 | 1 | 2 | 2 | 1 | 2 | 8 |
| NM_020348 | CNNM1 | 5959 | 1 | 0 | 0 | 0 | 0 | 1 | 0 | 0 | 0 | 0 | 0 | 0 | 1 |
| NM_020349 | ANKRD2 | 1451 | 13 | 2 | 0 | 0 | 4 | 0 | 0 | 0 | 0 | 0 | 13 | 0 | 0 |
| NM_020350 | AGTRAP | 1183 | 1 | 0 | 0 | 0 | 1 | 1 | 0 | 0 | 0 | 0 | 0 | 1 | 0 |
| NM_020351 | COL8A1 | 2596 | 4 | 1 | 2 | 0 | 4 | 0 | 1 | 0 | 3 | 1 | 0 | 0 | 1 |
| NM_020353 | PLSCR4 | 3305 | 10 | 5 | 6 | 2 | 5 | 5 | 4 | 3 | 8 | 8 | 2 | 7 | 10 |
| NM_020354 | ENTPD7 | 8555 | 2 | 1 | 0 | 1 | 1 | 0 | 1 | 2 | 1 | 1 | 0 | 2 | 1 |
| NM_020356 | CASS4 | 3155 | 1 | 0 | 1 | 0 | 0 | 0 | 0 | 0 | 1 | 0 | 0 | 1 | 0 |
| NM_020357 | PCNP | 2239 | 17 | 12 | 8 | 5 | 14 | 15 | 11 | 9 | 13 | 17 | 11 | 16 | 11 |
| NM_020358 | TRIM49 | 2163 | 0 | 0 | 0 | 0 | 0 | 0 | 0 | 0 | 0 | 0 | 0 | 0 | 0 |
| NM_020359 | PLSCR2 | 1265 | 1 | 0 | 0 | 0 | 0 | 0 | 0 | 0 | 0 | 0 | 0 | 0 | 1 |
| NM_020360 | PLSCR3 | 1746 | 3 | 1 | 0 | 0 | 2 | 0 | 1 | 0 | 2 | 2 | 1 | 3 | 1 |
| NM_020361 | CPA6 | 1904 | 1 | 0 | 0 | 1 | 0 | 0 | 0 | 0 | 0 | 0 | 0 | 0 | 1 |
| NM_020362 | C1orf128 | 1620 | 32 | 9 | 7 | 4 | 5 | 32 | 5 | 9 | 5 | 5 | 7 | 7 | 9 |
| NM_020363 | DAZ2 | 3756 | 0 | 0 | 0 | 0 | 0 | 0 | 0 | 0 | 0 | 0 | 0 | 0 | 0 |
| NM_020364 | DAZ3 | 3388 | 0 | 0 | 0 | 0 | 0 | 0 | 0 | 0 | 0 | 0 | 0 | 0 | 0 |
| NM_020365 | EIF2B3 | 1994 | 3 | 1 | 3 | 1 | 1 | 2 | 1 | 1 | 1 | 1 | 2 | 1 | 2 |
| NM_020366 | RPGRIP1 | 3946 | 3 | 0 | 0 | 0 | 0 | 0 | 0 | 0 | 0 | 0 | 0 | 0 | 3 |
| NM_020367 | PARP11 | 4492 | 5 | 2 | 1 | 1 | 1 | 2 | 1 | 1 | 1 | 5 | 1 | 3 | 3 |
| NM_020368 | UTP3 | 2072 | 17 | 8 | 6 | 5 | 9 | 11 | 10 | 10 | 5 | 7 | 5 | 17 | 8 |
| NM_020369 | FSCN3 | 1883 | 5 | 0 | 0 | 0 | 0 | 0 | 0 | 0 | 0 | 0 | 0 | 0 | 5 |
| NM_020370 | GPR84 | 1496 | 0 | 0 | 0 | 0 | 0 | 0 | 0 | 0 | 0 | 0 | 0 | 0 | 0 |
| NM_020371 | AVEN | 1531 | 4 | 2 | 2 | 1 | 4 | 1 | 1 | 2 | 1 | 1 | 2 | 2 | 2 |
| NM_020372 | SLC22A17 | 2003 | 9 | 1 | 0 | 0 | 0 | 9 | 1 | 0 | 0 | 0 | 0 | 0 | 1 |
| NM_020373 | ANO2 | 3717 | 3 | 1 | 2 | 0 | 1 | 1 | 0 | 0 | 3 | 1 | 1 | 1 | 1 |
| NM_020374 | C12orf4 | 3819 | 5 | 3 | 3 | 1 | 3 | 5 | 3 | 1 | 2 | 2 | 3 | 4 | 4 |
| NM_020375 | C12orf5 | 8225 | 1 | 1 | 1 | 0 | 1 | 1 | 1 | 1 | 0 | 0 | 1 | 1 | 1 |
| NM_020376 | PNPLA2 | 2072 | 23 | 5 | 23 | 3 | 6 | 1 | 3 | 1 | 4 | 1 | 5 | 3 | 2 |
| NM_020377 | CYSLTR2 | 2548 | 2 | 1 | 0 | 0 | 2 | 1 | 0 | 0 | 1 | 1 | 0 | 1 | 1 |
| NM_020378 | NAT14 | 1313 | 1 | 0 | 0 | 0 | 0 | 1 | 0 | 0 | 0 | 0 | 0 | 0 | 0 |
| NM_020379 | MAN1C1 | 3201 | 5 | 1 | 1 | 0 | 1 | 2 | 5 | 2 | 1 | 1 | 0 | 2 | 1 |
| NM_020381 | PDSS2 | 3552 | 5 | 2 | 1 | 1 | 2 | 1 | 4 | 5 | 1 | 1 | 1 | 2 | 1 |
| NM_020382 | SETD8 | 2751 | 3 | 2 | 2 | 3 | 3 | 3 | 2 | 1 | 2 | 2 | 2 | 3 | 2 |
| NM_020383 | XPNPEP1 | 2539 | 2 | 1 | 1 | 2 | 1 | 2 | 2 | 1 | 1 | 1 | 1 | 2 | 1 |
| NM_020384 | CLDN2 | 2959 | 16 | 2 | 0 | 0 | 0 | 1 | 16 | 5 | 0 | 0 | 0 | 0 | 1 |
| NM_020385 | REXO4 | 2360 | 5 | 3 | 2 | 3 | 2 | 4 | 2 | 4 | 1 | 4 | 3 | 5 | 3 |
| NM_020386 | HRASLS | 1062 | 2 | 0 | 0 | 0 | 0 | 0 | 0 | 0 | 0 | 0 | 1 | 0 | 2 |
| NM_020387 | RAB25 | 1085 | 0 | 0 | 0 | 0 | 0 | 0 | 0 | 0 | 0 | 0 | 0 | 0 | 0 |
| NM_020388 | DST | 9354 | 30 | 12 | 8 | 4 | 30 | 19 | 5 | 4 | 13 | 14 | 20 | 10 | 5 |
| NM_020389 | TRPC7 | 2935 | 0 | 0 | 0 | 0 | 0 | 0 | 0 | 0 | 0 | 0 | 0 | 0 | 0 |
| NM_020390 | EIF5A2 | 5537 | 2 | 1 | 0 | 0 | 1 | 2 | 1 | 0 | 1 | 1 | 0 | 1 | 2 |
| NM_020393 | PGLYRP4 | 1833 | 0 | 0 | 0 | 0 | 0 | 0 | 0 | 0 | 0 | 0 | 0 | 0 | 0 |
| NM_020394 | ZNF695 | 3336 | 0 | 0 | 0 | 0 | 0 | 0 | 0 | 0 | 0 | 0 | 0 | 0 | 0 |
| NM_020395 | INTS12 | 1752 | 3 | 1 | 1 | 0 | 2 | 2 | 1 | 3 | 1 | 1 | 1 | 1 | 1 |
| NM_020396 | BCL2L10 | 887 | 0 | 0 | 0 | 0 | 0 | 0 | 0 | 0 | 0 | 0 | 0 | 0 | 0 |
| NM_020397 | CAMK1D | 1722 | 6 | 2 | 0 | 2 | 1 | 6 | 0 | 3 | 1 | 1 | 0 | 2 | 1 |
| NM_020398 | SPINLW1 | 1952 | 23 | 2 | 0 | 0 | 0 | 0 | 0 | 0 | 0 | 0 | 0 | 0 | 23 |
| NM_020399 | GOPC | 4603 | 6 | 3 | 2 | 2 | 4 | 6 | 3 | 3 | 2 | 4 | 3 | 4 | 4 |
| NM_020400 | LPAR5 | 2855 | 1 | 0 | 0 | 1 | 0 | 1 | 0 | 0 | 0 | 0 | 0 | 1 | 0 |
| NM_020401 | NUP107 | 3124 | 9 | 6 | 6 | 2 | 6 | 4 | 5 | 4 | 5 | 9 | 6 | 9 | 9 |
| NM_020402 | CHRNA10 | 1945 | 3 | 0 | 0 | 0 | 0 | 0 | 0 | 0 | 0 | 0 | 3 | 0 | 0 |
| NM_020403 | PCDH9 | 6124 | 60 | 9 | 14 | 1 | 8 | 60 | 3 | 3 | 1 | 1 | 1 | 2 | 1 |
| NM_020404 | CD248 | 2558 | 23 | 3 | 23 | 3 | 2 | 1 | 1 | 0 | 0 | 1 | 1 | 1 | 1 |
| NM_020405 | PLXDC1 | 6251 | 1 | 0 | 0 | 0 | 1 | 0 | 0 | 0 | 0 | 1 | 0 | 0 | 0 |
| NM_020406 | CD177 | 2364 | 15 | 3 | 0 | 15 | 3 | 0 | 0 | 1 | 3 | 0 | 0 | 13 | 0 |
| NM_020407 | RHBG | 1788 | 0 | 0 | 0 | 0 | 0 | 0 | 0 | 0 | 0 | 0 | 0 | 0 | 0 |
| NM_020408 | LYRM4 | 1485 | 2 | 1 | 1 | 0 | 1 | 1 | 1 | 0 | 1 | 1 | 0 | 2 | 1 |
| NM_020409 | MRPL47 | 1180 | 2 | 1 | 1 | 0 | 2 | 2 | 2 | 2 | 0 | 1 | 1 | 1 | 2 |
| NM_020410 | ATP13A1 | 3864 | 1 | 1 | 0 | 1 | 0 | 1 | 1 | 1 | 0 | 1 | 0 | 1 | 1 |
| NM_020411 | XAGE1D | 619 | 0 | 0 | 0 | 0 | 0 | 0 | 0 | 0 | 0 | 0 | 0 | 0 | 0 |
| NM_020411 | XAGE1D | 620 | 0 | 0 | 0 | 0 | 0 | 0 | 0 | 0 | 0 | 0 | 0 | 0 | 0 |
| NM_020412 | CHMP1B | 3060 | 27 | 8 | 4 | 3 | 6 | 8 | 6 | 4 | 8 | 4 | 27 | 6 | 6 |
| NM_020414 | DDX24 | 2948 | 57 | 25 | 21 | 11 | 29 | 57 | 18 | 14 | 18 | 22 | 20 | 29 | 41 |
| NM_020415 | RETN | 478 | 1 | 0 | 0 | 0 | 1 | 0 | 0 | 0 | 1 | 0 | 0 | 1 | 0 |
| NM_020416 | PPP2R2C | 4438 | 15 | 2 | 0 | 0 | 0 | 15 | 0 | 0 | 0 | 0 | 0 | 0 | 0 |
| NM_020418 | PCBP4 | 2599 | 1 | 0 | 0 | 0 | 1 | 1 | 0 | 0 | 0 | 0 | 1 | 0 | 0 |
| NM_020420 | DAZ4 | 3270 | 0 | 0 | 0 | 0 | 0 | 0 | 0 | 0 | 0 | 0 | 0 | 0 | 0 |
| NM_020421 | ADCK1 | 2233 | 1 | 0 | 0 | 0 | 0 | 1 | 0 | 0 | 0 | 0 | 0 | 0 | 0 |

| transcript | gene | transcript length (nt) | max | mean | adipose | colon | heart | hypothala mus | kidney | liver | lung | ovary | skeletalm uscle | spleen | testes |
|---|---|---|---|---|---|---|---|---|---|---|---|---|---|---|---|
| NM_020422 | TMEM159 | 1907 | 9 | 4 | 4 | 4 | 8 | 1 | 2 | 0 | 3 | 9 | 5 | 1 | 5 |
| NM_020423 | SCYL3 | 3315 | 4 | 3 | 3 | 2 | 2 | 2 | 4 | 3 | 2 | 2 | 2 | 4 | 4 |
| NM_020424 | LYRM1 | 1565 | 8 | 4 | 4 | 1 | 6 | 4 | 6 | 8 | 3 | 4 | 3 | 3 | 3 |
| NM_020425 | C6orf162 | 2413 | 2 | 1 | 1 | 1 | 1 | 2 | 2 | 1 | 1 | 2 | 1 | 1 | 2 |
| NM_020426 | LYZL6 | 872 | 1 | 0 | 0 | 0 | 0 | 0 | 0 | 0 | 0 | 0 | 0 | 0 | 1 |
| NM_020427 | SLURP1 | 537 | 0 | 0 | 0 | 0 | 0 | 0 | 0 | 0 | 0 | 0 | 0 | 0 | 0 |
| NM_020428 | SLC44A2 | 3433 | 25 | 9 | 6 | 6 | 6 | 8 | 8 | 3 | 12 | 3 | 5 | 25 | 21 |
| NM_020429 | SMURF1 | 5737 | 4 | 2 | 2 | 2 | 2 | 3 | 2 | 1 | 2 | 3 | 1 | 2 | 4 |
| NM_020431 | TMEM63C | 5402 | 1 | 0 | 0 | 0 | 0 | 1 | 0 | 0 | 0 | 0 | 0 | 0 | 0 |
| NM_020432 | PHTF2 | 4996 | 5 | 2 | 2 | 1 | 2 | 2 | 1 | 1 | 2 | 1 | 5 | 3 | 2 |
| NM_020433 | JPH2 | 4785 | 2 | 0 | 0 | 1 | 2 | 0 | 0 | 0 | 0 | 0 | 2 | 0 | 0 |
| NM_020435 | GJC2 | 2243 | 1 | 0 | 0 | 0 | 0 | 1 | 0 | 0 | 0 | 0 | 0 | 0 | 0 |
| NM_020436 | SALL4 | 3484 | 0 | 0 | 0 | 0 | 0 | 0 | 0 | 0 | 0 | 0 | 0 | 0 | 0 |
| NM_020437 | ASPHD2 | 3313 | 1 | 0 | 0 | 0 | 0 | 1 | 0 | 0 | 0 | 0 | 0 | 0 | 0 |
| NM_020438 | DOLPP1 | 2175 | 1 | 1 | 1 | 1 | 0 | 1 | 1 | 1 | 0 | 0 | 0 | 1 | 1 |
| NM_020439 | CAMK1G | 2470 | 1 | 0 | 0 | 0 | 0 | 1 | 0 | 0 | 0 | 1 | 0 | 0 | 0 |
| NM_020440 | PTGFRN | 6160 | 9 | 2 | 1 | 4 | 9 | 1 | 1 | 1 | 1 | 3 | 0 | 1 | 3 |
| NM_020441 | CORO1B | 1910 | 2 | 1 | 1 | 1 | 1 | 0 | 1 | 0 | 1 | 0 | 0 | 2 | 0 |
| NM_020442 | VARS2 | 3605 | 1 | 1 | 1 | 1 | 0 | 1 | 1 | 1 | 0 | 0 | 1 | 1 | 1 |
| NM_020443 | NAV1 | 13023 | 4 | 2 | 4 | 1 | 3 | 4 | 1 | 0 | 1 | 1 | 1 | 1 | 2 |
| NM_020444 | KIAA1191 | 2827 | 10 | 4 | 2 | 3 | 3 | 7 | 10 | 4 | 2 | 4 | 2 | 3 | 2 |
| NM_020445 | ACTR3B | 2199 | 3 | 1 | 0 | 0 | 0 | 3 | 1 | 1 | 0 | 0 | 1 | 1 | 1 |
| NM_020447 | C15orf17 | 3550 | 4 | 2 | 3 | 2 | 3 | 4 | 2 | 0 | 2 | 2 | 2 | 2 | 2 |
| NM_020448 | NIPAL3 | 5425 | 15 | 3 | 1 | 3 | 3 | 15 | 4 | 1 | 3 | 2 | 1 | 3 | 2 |
| NM_020451 | SEPN1 | 4332 | 3 | 1 | 1 | 1 | 2 | 1 | 2 | 1 | 1 | 2 | 1 | 3 | 1 |
| NM_020452 | ATP8B2 | 5857 | 3 | 1 | 1 | 1 | 1 | 1 | 1 | 0 | 1 | 2 | 0 | 2 | 3 |
| NM_020453 | ATP10D | 6550 | 7 | 3 | 1 | 1 | 2 | 2 | 6 | 2 | 3 | 4 | 0 | 7 | 2 |
| NM_020455 | GPR126 | 6961 | 7 | 2 | 1 | 1 | 1 | 0 | 2 | 7 | 3 | 0 | 0 | 3 | 1 |
| NM_020456 | C13orf1 | 3202 | 4 | 2 | 1 | 1 | 2 | 4 | 2 | 2 | 1 | 1 | 3 | 1 | 1 |
| NM_020457 | THAP11 | 1886 | 2 | 1 | 1 | 1 | 1 | 2 | 1 | 1 | 1 | 1 | 1 | 2 | 1 |
| NM_020458 | TTC7A | 5158 | 1 | 1 | 1 | 1 | 0 | 0 | 1 | 0 | 1 | 0 | 0 | 1 | 1 |
| NM_020459 | PAIP2B | 6300 | 20 | 8 | 1 | 3 | 16 | 20 | 7 | 12 | 2 | 7 | 16 | 1 | 2 |
| NM_020461 | TUBGCP6 | 6019 | 1 | 0 | 0 | 1 | 0 | 1 | 0 | 0 | 0 | 1 | 0 | 1 | 1 |
| NM_020463 | SMEK2 | 4203 | 21 | 15 | 18 | 7 | 11 | 14 | 18 | 18 | 13 | 16 | 8 | 21 | 16 |
| NM_020464 | NHSL1 | 7490 | 7 | 3 | 1 | 5 | 3 | 3 | 4 | 7 | 3 | 1 | 0 | 2 | 1 |
| NM_020465 | NDRG4 | 3335 | 41 | 7 | 0 | 1 | 22 | 41 | 0 | 0 | 1 | 0 | 0 | 2 | 6 |
| NM_020466 | LYRM2 | 5355 | 4 | 2 | 2 | 1 | 3 | 4 | 3 | 2 | 2 | 2 | 2 | 2 | 3 |
| NM_020468 | SNX14 | 3345 | 7 | 4 | 2 | 1 | 3 | 4 | 6 | 3 | 4 | 3 | 1 | 4 | 7 |
| NM_020469 | ABO | 1579 | 112 | 26 | 5 | 112 | 3 | 3 | 58 | 1 | 10 | 67 | 2 | 7 | 23 |
| NM_020470 | YIF1A | 1121 | 1 | 0 | 0 | 0 | 0 | 0 | 0 | 1 | 0 | 0 | 0 | 0 | 0 |
| NM_020473 | PIGA | 2947 | 2 | 1 | 0 | 0 | 1 | 1 | 1 | 1 | 2 | 1 | 0 | 2 | 1 |
| NM_020474 | GALNT1 | 3837 | 16 | 10 | 11 | 4 | 11 | 9 | 6 | 14 | 8 | 8 | 6 | 16 | 13 |
| NM_020475 | ANK1 | 8157 | 2 | 0 | 0 | 0 | 1 | 1 | 0 | 0 | 0 | 0 | 2 | 0 | 0 |
| NM_020476 | ANK1 | 8232 | 2 | 0 | 0 | 0 | 1 | 1 | 0 | 0 | 0 | 0 | 2 | 0 | 0 |
| NM_020477 | ANK1 | 7746 | 2 | 0 | 0 | 0 | 1 | 1 | 0 | 0 | 0 | 0 | 2 | 0 | 0 |
| NM_020478 | ANK1 | 3295 | 7 | 1 | 0 | 0 | 3 | 1 | 0 | 0 | 0 | 0 | 7 | 0 | 0 |
| NM_020480 | ANK1 | 3094 | 7 | 1 | 0 | 0 | 3 | 1 | 0 | 0 | 0 | 0 | 7 | 0 | 0 |
| NM_020482 | FHL5 | 2124 | 7 | 2 | 2 | 0 | 2 | 1 | 1 | 0 | 2 | 2 | 2 | 2 | 7 |
| NM_020485 | RHCE | 1618 | 0 | 0 | 0 | 0 | 0 | 0 | 0 | 0 | 0 | 0 | 0 | 0 | 0 |
| NM_020524 | PBXIP1 | 3199 | 25 | 14 | 13 | 13 | 10 | 25 | 10 | 3 | 11 | 21 | 16 | 14 | 11 |
| NM_020525 | IL22 | 1147 | 0 | 0 | 0 | 0 | 0 | 0 | 0 | 0 | 0 | 0 | 0 | 0 | 0 |
| NM_020526 | EPHA8 | 4996 | 0 | 0 | 0 | 0 | 0 | 0 | 0 | 0 | 0 | 0 | 0 | 0 | 0 |
| NM_020528 | PCBP3 | 1989 | 2 | 1 | 1 | 0 | 1 | 2 | 0 | 0 | 0 | 1 | 0 | 2 | 1 |
| NM_020529 | NFKBIA | 1567 | 92 | 43 | 31 | 32 | 34 | 21 | 22 | 49 | 92 | 47 | 32 | 84 | 24 |
| NM_020530 | OSM | 1864 | 0 | 0 | 0 | 0 | 0 | 0 | 0 | 0 | 0 | 0 | 0 | 0 | 0 |
| NM_020531 | C20orf3 | 2221 | 27 | 6 | 7 | 3 | 3 | 7 | 3 | 27 | 3 | 2 | 1 | 5 | 3 |
| NM_020532 | RTN4 | 4842 | 141 | 38 | 64 | 11 | 33 | 141 | 35 | 13 | 27 | 15 | 26 | 23 | 26 |
| NM_020533 | MCOLN1 | 2097 | 5 | 1 | 1 | 0 | 1 | 1 | 1 | 1 | 1 | 0 | 1 | 5 | 1 |
| NM_020535 | KIR2DL5A | 1596 | 0 | 0 | 0 | 0 | 0 | 0 | 0 | 0 | 0 | 0 | 0 | 0 | 0 |
| NM_020536 | CSRP2BP | 3712 | 2 | 1 | 1 | 0 | 1 | 2 | 1 | 1 | 0 | 1 | 1 | 1 | 1 |
| NM_020546 | ADCY2 | 6553 | 6 | 1 | 0 | 0 | 0 | 6 | 0 | 0 | 0 | 0 | 3 | 0 | 1 |
| NM_020547 | AMHR2 | 1863 | 0 | 0 | 0 | 0 | 0 | 0 | 0 | 0 | 0 | 0 | 0 | 0 | 0 |
| NM_020548 | DBI | 754 | 377 | 168 | 291 | 103 | 208 | 235 | 278 | 377 | 99 | 35 | 37 | 96 | 85 |
| NM_020549 | CHAT | 2458 | 0 | 0 | 0 | 0 | 0 | 0 | 0 | 0 | 0 | 0 | 0 | 0 | 0 |
| NM_020630 | RET | 4159 | 5 | 0 | 0 | 5 | 0 | 0 | 0 | 0 | 0 | 0 | 0 | 0 | 0 |
| NM_020631 | PLEKHG5 | 4643 | 2 | 1 | 2 | 1 | 1 | 2 | 0 | 0 | 0 | 1 | 0 | 2 | 0 |
| NM_020632 | ATP6V0A4 | 3136 | 15 | 1 | 0 | 0 | 0 | 0 | 15 | 0 | 0 | 0 | 0 | 0 | 0 |
| NM_020633 | VN1R1 | 1313 | 1 | 1 | 0 | 0 | 0 | 1 | 0 | 0 | 0 | 0 | 1 | 1 | 1 |
| NM_020634 | GDF3 | 1224 | 0 | 0 | 0 | 0 | 0 | 0 | 0 | 0 | 0 | 0 | 0 | 0 | 0 |
| NM_020637 | FGF22 | 513 | 0 | 0 | 0 | 0 | 0 | 0 | 0 | 0 | 0 | 0 | 0 | 0 | 0 |
| NM_020638 | FGF23 | 3018 | 1 | 0 | 0 | 0 | 0 | 0 | 0 | 0 | 1 | 0 | 0 | 0 | 0 |
| NM_020639 | RIPK4 | 3872 | 1 | 0 | 0 | 0 | 0 | 0 | 1 | 1 | 0 | 0 | 0 | 0 | 0 |
| NM_020640 | DCUN1D1 | 3152 | 9 | 6 | 4 | 2 | 5 | 7 | 6 | 8 | 5 | 5 | 6 | 6 | 9 |
| NM_020641 | C9orf11 | 1035 | 1 | 0 | 0 | 0 | 0 | 0 | 0 | 0 | 0 | 0 | 0 | 0 | 1 |
| NM_020642 | C11orf17 | 1308 | 4 | 2 | 2 | 2 | 2 | 4 | 2 | 2 | 2 | 2 | 2 | 3 | 3 |
| NM_020643 | C11orf16 | 1925 | 0 | 0 | 0 | 0 | 0 | 0 | 0 | 0 | 0 | 0 | 0 | 0 | 0 |
| NM_020644 | TMEM9B | 1753 | 7 | 3 | 2 | 2 | 4 | 7 | 5 | 4 | 4 | 1 | 2 | 4 | 3 |

| transcript | gene | transcript length (nt) | max | mean | adipose | colon | heart | hypothalamus | kidney | liver | lung | ovary | skeletalmuscle | spleen | testes |
|---|---|---|---|---|---|---|---|---|---|---|---|---|---|---|---|
| NM_020645 | NRIP3 | 3809 | 6 | 1 | 1 | 0 | 0 | 6 | 0 | 0 | 0 | 0 | 0 | 0 | 2 |
| NM_020646 | ASCL3 | 650 | 1 | 0 | 0 | 0 | 0 | 0 | 0 | 0 | 0 | 0 | 0 | 0 | 1 |
| NM_020647 | JPH1 | 4373 | 17 | 2 | 0 | 0 | 2 | 1 | 0 | 0 | 0 | 0 | 17 | 0 | 0 |
| NM_020648 | TWSG1 | 3755 | 10 | 4 | 3 | 2 | 5 | 3 | 7 | 1 | 5 | 10 | 2 | 5 | 7 |
| NM_020649 | CBX8 | 1521 | 2 | 1 | 0 | 1 | 1 | 0 | 1 | 0 | 1 | 0 | 0 | 1 | 2 |
| NM_020650 | RCN3 | 1854 | 1 | 0 | 1 | 1 | 1 | 0 | 0 | 0 | 0 | 0 | 0 | 0 | 0 |
| NM_020651 | PELI1 | 3767 | 5 | 2 | 2 | 2 | 2 | 2 | 1 | 1 | 5 | 1 | 1 | 5 | 3 |
| NM_020652 | ZNF286A | 5543 | 2 | 1 | 0 | 0 | 1 | 1 | 1 | 0 | 1 | 2 | 0 | 1 | 1 |
| NM_020653 | ZNF287 | 4278 | 4 | 2 | 1 | 0 | 2 | 4 | 2 | 0 | 1 | 4 | 1 | 2 | 2 |
| NM_020654 | SENP7 | 4933 | 6 | 3 | 2 | 1 | 2 | 2 | 3 | 1 | 2 | 6 | 2 | 5 | 4 |
| NM_020655 | JPH3 | 3996 | 5 | 0 | 0 | 0 | 0 | 5 | 0 | 0 | 0 | 0 | 0 | 0 | 0 |
| NM_020657 | ZNF304 | 4416 | 4 | 2 | 1 | 1 | 2 | 2 | 2 | 1 | 1 | 3 | 1 | 4 | 3 |
| NM_020659 | TTYH1 | 2029 | 2 | 0 | 0 | 0 | 0 | 2 | 0 | 0 | 0 | 0 | 0 | 0 | 0 |
| NM_020660 | GJD2 | 966 | 0 | 0 | 0 | 0 | 0 | 0 | 0 | 0 | 0 | 0 | 0 | 0 | 0 |
| NM_020661 | AICDA | 2794 | 0 | 0 | 0 | 0 | 0 | 0 | 0 | 0 | 0 | 0 | 0 | 0 | 0 |
| NM_020662 | MRS2 | 3348 | 4 | 2 | 3 | 1 | 4 | 2 | 3 | 4 | 2 | 1 | 3 | 2 | 2 |
| NM_020663 | RHOJ | 1905 | 4 | 2 | 2 | 2 | 4 | 1 | 1 | 0 | 3 | 3 | 1 | 3 | 1 |
| NM_020664 | DECR2 | 1583 | 1 | 0 | 0 | 0 | 0 | 0 | 0 | 1 | 0 | 0 | 0 | 0 | 0 |
| NM_020665 | TMEM27 | 1578 | 33 | 3 | 0 | 0 | 0 | 0 | 33 | 1 | 0 | 0 | 0 | 0 | 0 |
| NM_020666 | CLK4 | 2507 | 5 | 4 | 4 | 3 | 3 | 3 | 3 | 1 | 4 | 4 | 5 | 5 | 5 |
| NM_020672 | S100A14 | 1042 | 54 | 7 | 0 | 54 | 0 | 0 | 3 | 2 | 22 | 0 | 0 | 0 | 1 |
| NM_020673 | RAB22A | 8651 | 3 | 2 | 1 | 1 | 1 | 3 | 2 | 1 | 1 | 2 | 1 | 2 | 2 |
| NM_020675 | SPC25 | 1346 | 1 | 0 | 0 | 1 | 0 | 0 | 0 | 0 | 0 | 0 | 0 | 1 | 1 |
| NM_020676 | ABHD6 | 2393 | 5 | 2 | 1 | 1 | 1 | 3 | 3 | 5 | 1 | 0 | 0 | 4 | 1 |
| NM_020677 | NMRAL1 | 1362 | 3 | 2 | 1 | 1 | 1 | 3 | 3 | 3 | 1 | 1 | 0 | 2 | 1 |
| NM_020678 | LRTM1 | 1277 | 0 | 0 | 0 | 0 | 0 | 0 | 0 | 0 | 0 | 0 | 0 | 0 | 0 |
| NM_020679 | MIF4GD | 1410 | 1 | 1 | 1 | 1 | 1 | 1 | 1 | 1 | 1 | 1 | 1 | 1 | 1 |
| NM_020680 | SCYL1 | 2649 | 3 | 2 | 2 | 1 | 2 | 3 | 2 | 3 | 2 | 2 | 3 | 3 | 2 |
| NM_020682 | AS3MT | 2466 | 5 | 2 | 0 | 0 | 4 | 1 | 3 | 5 | 1 | 1 | 1 | 5 | 3 |
| NM_020683 | ADORA3 | 2150 | 1 | 0 | 0 | 0 | 1 | 1 | 0 | 0 | 1 | 0 | 0 | 1 | 1 |
| NM_020685 | C3orf14 | 767 | 4 | 1 | 0 | 0 | 1 | 4 | 1 | 0 | 1 | 0 | 0 | 0 | 1 |
| NM_020686 | ABAT | 4814 | 46 | 9 | 0 | 1 | 3 | 22 | 19 | 46 | 1 | 2 | 0 | 1 | 5 |
| NM_020689 | SLC24A3 | 3889 | 6 | 2 | 4 | 2 | 1 | 6 | 0 | 0 | 1 | 3 | 4 | 1 | 1 |
| NM_020690 | ANKHD1-EII | 8319 | 0 | 0 | 0 | 0 | 0 | 0 | 0 | 0 | 0 | 0 | 0 | 0 | 0 |
| NM_020692 | GALNTL1 | 3148 | 2 | 0 | 0 | 0 | 1 | 2 | 0 | 0 | 0 | 0 | 0 | 0 | 0 |
| NM_020693 | DSCAML1 | 6899 | 1 | 0 | 0 | 0 | 0 | 1 | 0 | 0 | 0 | 0 | 0 | 0 | 0 |
| NM_020695 | REXO1 | 4581 | 1 | 0 | 0 | 0 | 0 | 1 | 1 | 0 | 0 | 0 | 0 | 1 | 1 |
| NM_020696 | KIAA1143 | 5138 | 8 | 4 | 2 | 1 | 6 | 4 | 4 | 2 | 3 | 8 | 8 | 5 | 5 |
| NM_020697 | KCNS2 | 3166 | 1 | 0 | 0 | 0 | 0 | 1 | 0 | 0 | 0 | 0 | 0 | 0 | 0 |
| NM_020698 | TMCC3 | 5863 | 4 | 2 | 2 | 2 | 1 | 3 | 2 | 1 | 1 | 1 | 3 | 4 | 1 |
| NM_020699 | GATAD2B | 7476 | 10 | 6 | 5 | 5 | 5 | 9 | 4 | 4 | 7 | 9 | 8 | 10 | 7 |
| NM_020700 | PPM1H | 6199 | 5 | 1 | 0 | 0 | 0 | 5 | 2 | 1 | 0 | 2 | 0 | 0 | 0 |
| NM_020701 | ISY1 | 1758 | 19 | 11 | 12 | 9 | 14 | 9 | 11 | 8 | 9 | 14 | 9 | 19 | 12 |
| NM_020702 | KIAA1161 | 4202 | 3 | 1 | 0 | 2 | 0 | 2 | 1 | 3 | 0 | 0 | 1 | 0 | 0 |
| NM_020703 | AMIGO1 | 2471 | 5 | 1 | 1 | 1 | 1 | 5 | 1 | 0 | 1 | 1 | 2 | 1 | 1 |
| NM_020704 | FAM40B | 5112 | 4 | 1 | 0 | 0 | 2 | 2 | 3 | 0 | 1 | 0 | 4 | 0 | 2 |
| NM_020705 | TBC1D24 | 4331 | 1 | 0 | 0 | 0 | 0 | 1 | 1 | 0 | 0 | 0 | 0 | 0 | 0 |
| NM_020706 | SFRS15 | 4269 | 12 | 7 | 4 | 4 | 6 | 7 | 6 | 6 | 6 | 8 | 6 | 12 | 11 |
| NM_020707 | HHATL | 1864 | 2 | 0 | 0 | 0 | 2 | 0 | 0 | 0 | 0 | 0 | 2 | 0 | 0 |
| NM_020708 | SLC12A5 | 6059 | 17 | 2 | 0 | 0 | 0 | 17 | 0 | 0 | 0 | 0 | 0 | 0 | 0 |
| NM_020709 | PNMAL2 | 4722 | 12 | 1 | 1 | 0 | 0 | 12 | 0 | 0 | 0 | 1 | 0 | 1 | 0 |
| NM_020710 | LRRC47 | 2648 | 11 | 5 | 2 | 2 | 6 | 6 | 4 | 4 | 2 | 5 | 11 | 4 | 5 |
| NM_020711 | ERMN | 3775 | 29 | 3 | 0 | 0 | 0 | 29 | 0 | 0 | 0 | 0 | 0 | 0 | 0 |
| NM_020713 | ZNF512B | 5919 | 1 | 0 | 0 | 0 | 0 | 1 | 0 | 0 | 0 | 0 | 0 | 0 | 0 |
| NM_020714 | ZNF490 | 6098 | 4 | 2 | 2 | 1 | 1 | 4 | 2 | 1 | 1 | 4 | 1 | 4 | 2 |
| NM_020715 | PLEKHH1 | 6540 | 4 | 1 | 0 | 1 | 0 | 4 | 1 | 0 | 0 | 1 | 0 | 0 | 1 |
| NM_020716 | GRAMD1B | 2761 | 5 | 1 | 0 | 0 | 1 | 5 | 2 | 0 | 0 | 1 | 0 | 0 | 3 |
| NM_020717 | SHROOM4 | 9556 | 4 | 1 | 1 | 0 | 2 | 1 | 2 | 0 | 4 | 1 | 0 | 0 | 0 |
| NM_020718 | USP31 | 10698 | 3 | 2 | 1 | 1 | 3 | 3 | 1 | 1 | 1 | 1 | 1 | 1 | 3 |
| NM_020719 | PRR12 | 6943 | 2 | 1 | 1 | 1 | 1 | 2 | 1 | 0 | 1 | 2 | 1 | 2 | 1 |
| NM_020721 | KIAA1210 | 7824 | 28 | 3 | 0 | 0 | 0 | 0 | 0 | 0 | 0 | 0 | 0 | 0 | 28 |
| NM_020722 | KIAA1211 | 7013 | 3 | 1 | 0 | 2 | 0 | 3 | 0 | 0 | 0 | 1 | 0 | 1 | 3 |
| NM_020724 | RNF150 | 4661 | 9 | 4 | 7 | 3 | 9 | 5 | 3 | 0 | 0 | 6 | 3 | 6 | 2 |
| NM_020725 | ATXN7L1 | 5691 | 2 | 1 | 1 | 1 | 0 | 1 | 2 | 1 | 1 | 2 | 0 | 2 | 2 |
| NM_020726 | NLN | 2877 | 11 | 4 | 4 | 1 | 3 | 6 | 4 | 11 | 1 | 2 | 4 | 2 | 2 |
| NM_020727 | ZNF295 | 7414 | 2 | 1 | 1 | 0 | 1 | 1 | 1 | 1 | 1 | 2 | 1 | 2 | 2 |
| NM_020728 | ESYT2 | 5957 | 3 | 2 | 2 | 1 | 2 | 1 | 2 | 1 | 2 | 3 | 2 | 2 | 2 |
| NM_020729 | ODF2L | 2295 | 9 | 4 | 2 | 1 | 3 | 4 | 5 | 0 | 7 | 4 | 1 | 9 | 3 |
| NM_020730 | DLG3 | 5074 | 5 | 1 | 0 | 2 | 1 | 5 | 2 | 0 | 1 | 2 | 0 | 3 | 1 |
| NM_020731 | AHRR | 5660 | 1 | 0 | 0 | 0 | 0 | 0 | 0 | 0 | 0 | 0 | 0 | 1 | 0 |
| NM_020732 | ARID1B | 8105 | 4 | 2 | 2 | 2 | 2 | 2 | 2 | 1 | 2 | 3 | 2 | 4 | 3 |
| NM_020733 | HEG1 | 9156 | 10 | 4 | 4 | 2 | 10 | 4 | 4 | 1 | 5 | 4 | 1 | 6 | 6 |
| NM_020734 | RIMKLB | 5830 | 7 | 3 | 4 | 1 | 2 | 3 | 3 | 0 | 3 | 4 | 2 | 3 | 7 |
| NM_020737 | LRFN2 | 3144 | 0 | 0 | 0 | 0 | 0 | 0 | 0 | 0 | 0 | 0 | 0 | 0 | 0 |
| NM_020738 | KIDINS220 | 7361 | 23 | 12 | 9 | 5 | 13 | 23 | 12 | 7 | 10 | 20 | 10 | 13 | 12 |
| NM_020739 | CCPG1 | 6698 | 37 | 18 | 24 | 3 | 25 | 14 | 18 | 37 | 20 | 9 | 12 | 20 | 15 |
| NM_020740 | ANKFY1 | 1910 | 3 | 2 | 3 | 2 | 1 | 3 | 2 | 3 | 1 | 2 | 2 | 3 | 2 |

| transcript | gene | transcript length (nt) | max | mean | adipose | colon | heart | hypothala mus | kidney | liver | lung | ovary | skeletalm uscle | spleen | testes |
|---|---|---|---|---|---|---|---|---|---|---|---|---|---|---|---|
| NM_020741 | KIAA1257 | 1710 | 8 | 1 | 0 | 0 | 0 | 1 | 0 | 0 | 1 | 1 | 0 | 0 | 8 |
| NM_020742 | NLGN4X | 5690 | 6 | 1 | 0 | 0 | 1 | 4 | 0 | 0 | 0 | 6 | 0 | 1 | 1 |
| NM_020744 | MTA3 | 1857 | 13 | 6 | 4 | 6 | 3 | 13 | 6 | 3 | 3 | 11 | 1 | 3 | 9 |
| NM_020745 | AARS2 | 4782 | 2 | 1 | 1 | 1 | 1 | 2 | 1 | 1 | 0 | 1 | 1 | 1 | 1 |
| NM_020746 | MAVS | 11760 | 6 | 4 | 3 | 5 | 5 | 2 | 4 | 2 | 2 | 5 | 6 | 5 | 2 |
| NM_020747 | ZNF608 | 5645 | 6 | 3 | 1 | 2 | 3 | 5 | 4 | 1 | 3 | 3 | 3 | 2 | 6 |
| NM_020748 | INTS2 | 5879 | 2 | 1 | 1 | 1 | 1 | 1 | 1 | 1 | 1 | 1 | 1 | 2 | 2 |
| NM_020749 | MTUS1 | 3802 | 27 | 14 | 10 | 6 | 27 | 22 | 7 | 15 | 10 | 20 | 18 | 9 | 10 |
| NM_020750 | XPO5 | 5363 | 3 | 2 | 1 | 1 | 1 | 2 | 2 | 2 | 1 | 1 | 1 | 2 | 3 |
| NM_020751 | COG6 | 3609 | 2 | 1 | 1 | 1 | 1 | 2 | 2 | 1 | 1 | 2 | 1 | 1 | 2 |
| NM_020752 | GPR158 | 6662 | 4 | 0 | 0 | 0 | 0 | 4 | 0 | 0 | 0 | 0 | 0 | 0 | 0 |
| NM_020753 | CASKIN2 | 4977 | 1 | 0 | 0 | 0 | 1 | 0 | 1 | 0 | 1 | 0 | 0 | 1 | 0 |
| NM_020754 | CDGAP | 8079 | 3 | 2 | 3 | 1 | 3 | 2 | 1 | 1 | 3 | 2 | 0 | 2 | 1 |
| NM_020755 | SERINC1 | 3128 | 77 | 35 | 36 | 10 | 40 | 77 | 32 | 25 | 40 | 32 | 27 | 34 | 32 |
| NM_020759 | STARD9 | 15583 | 3 | 1 | 2 | 2 | 1 | 1 | 0 | 0 | 1 | 3 | 0 | 1 | 3 |
| NM_020760 | HECW2 | 6926 | 7 | 3 | 1 | 0 | 4 | 6 | 1 | 2 | 5 | 1 | 0 | 7 | 3 |
| NM_020761 | RPTOR | 6856 | 2 | 1 | 1 | 2 | 1 | 2 | 1 | 1 | 1 | 1 | 1 | 2 | 1 |
| NM_020762 | SRGAP1 | 8475 | 3 | 2 | 2 | 1 | 1 | 2 | 2 | 0 | 2 | 3 | 0 | 3 | 3 |
| NM_020764 | CASKIN1 | 5695 | 1 | 0 | 0 | 0 | 0 | 1 | 0 | 0 | 0 | 0 | 0 | 0 | 0 |
| NM_020765 | UBR4 | 15882 | 7 | 5 | 4 | 3 | 4 | 5 | 5 | 5 | 3 | 5 | 6 | 5 | 7 |
| NM_020766 | PCDH19 | 9612 | 6 | 1 | 1 | 1 | 0 | 6 | 0 | 0 | 0 | 1 | 0 | 0 | 0 |
| NM_020768 | KCTD16 | 5183 | 4 | 0 | 0 | 0 | 0 | 4 | 0 | 0 | 0 | 0 | 0 | 0 | 0 |
| NM_020769 | RGAG1 | 5425 | 5 | 1 | 0 | 0 | 0 | 0 | 0 | 0 | 0 | 0 | 0 | 0 | 5 |
| NM_020770 | CGN | 5101 | 12 | 3 | 0 | 12 | 0 | 1 | 7 | 8 | 6 | 0 | 0 | 0 | 4 |
| NM_020771 | HACE1 | 4576 | 3 | 2 | 1 | 1 | 1 | 3 | 2 | 0 | 2 | 2 | 1 | 2 | 3 |
| NM_020772 | NUFIP2 | 10880 | 12 | 7 | 5 | 3 | 8 | 7 | 6 | 5 | 7 | 7 | 6 | 12 | 8 |
| NM_020773 | TBC1D14 | 5009 | 9 | 5 | 3 | 4 | 5 | 7 | 9 | 3 | 4 | 3 | 4 | 8 | 5 |
| NM_020774 | MIB1 | 9306 | 16 | 7 | 4 | 5 | 6 | 16 | 6 | 5 | 6 | 9 | 5 | 6 | 12 |
| NM_020775 | KIAA1324 | 7030 | 3 | 1 | 0 | 3 | 0 | 2 | 0 | 0 | 1 | 0 | 0 | 0 | 1 |
| NM_020776 | KIAA1328 | 4854 | 4 | 2 | 2 | 1 | 1 | 2 | 1 | 0 | 2 | 4 | 1 | 2 | 4 |
| NM_020777 | SORCS2 | 6053 | 1 | 0 | 0 | 0 | 0 | 1 | 0 | 0 | 0 | 0 | 0 | 0 | 0 |
| NM_020778 | ALPK3 | 10915 | 22 | 4 | 1 | 1 | 10 | 1 | 2 | 0 | 1 | 0 | 22 | 0 | 1 |
| NM_020779 | WDR35 | 6911 | 4 | 2 | 1 | 1 | 1 | 3 | 3 | 1 | 1 | 3 | 1 | 1 | 4 |
| NM_020780 | PTCHD2 | 5214 | 0 | 0 | 0 | 0 | 0 | 0 | 0 | 0 | 0 | 0 | 0 | 0 | 0 |
| NM_020781 | ZNF398 | 5492 | 4 | 2 | 2 | 2 | 2 | 3 | 2 | 1 | 2 | 4 | 2 | 4 | 4 |
| NM_020782 | KLHDC5 | 6469 | 9 | 3 | 1 | 2 | 2 | 9 | 3 | 1 | 2 | 3 | 1 | 2 | 7 |
| NM_020783 | SYT4 | 4096 | 27 | 3 | 0 | 0 | 0 | 27 | 0 | 0 | 0 | 0 | 0 | 0 | 0 |
| NM_020784 | TXNDC16 | 4640 | 17 | 6 | 3 | 2 | 4 | 8 | 3 | 1 | 7 | 11 | 6 | 9 | 17 |
| NM_020785 | CC2D2A | 1664 | 2 | 1 | 0 | 0 | 0 | 1 | 2 | 0 | 1 | 1 | 0 | 0 | 1 |
| NM_020786 | PDP2 | 3830 | 4 | 2 | 4 | 1 | 1 | 3 | 4 | 2 | 1 | 3 | 1 | 1 | 3 |
| NM_020787 | ZNF624 | 4235 | 6 | 3 | 2 | 0 | 2 | 3 | 2 | 0 | 2 | 6 | 1 | 5 | 5 |
| NM_020789 | IGSF9 | 3996 | 0 | 0 | 0 | 0 | 0 | 0 | 0 | 0 | 0 | 0 | 0 | 0 | 0 |
| NM_020791 | TAOK1 | 4662 | 34 | 16 | 19 | 6 | 17 | 34 | 11 | 8 | 11 | 15 | 11 | 17 | 26 |
| NM_020792 | NCEH1 | 4290 | 4 | 2 | 3 | 0 | 2 | 4 | 2 | 1 | 1 | 0 | 0 | 2 | 0 |
| NM_020794 | LRRC7 | 6245 | 8 | 1 | 0 | 0 | 0 | 8 | 0 | 0 | 0 | 0 | 0 | 0 | 0 |
| NM_020795 | NLGN2 | 4642 | 3 | 1 | 0 | 1 | 1 | 3 | 0 | 0 | 0 | 1 | 0 | 1 | 1 |
| NM_020796 | SEMA6A | 6860 | 6 | 3 | 1 | 5 | 1 | 6 | 2 | 1 | 3 | 2 | 1 | 5 | 3 |
| NM_020798 | USP35 | 4197 | 2 | 1 | 0 | 1 | 1 | 2 | 1 | 0 | 1 | 1 | 0 | 1 | 2 |
| NM_020799 | STAMBPL1 | 1997 | 2 | 1 | 1 | 1 | 1 | 2 | 1 | 0 | 1 | 2 | 0 | 2 | 1 |
| NM_020800 | IFT80 | 4292 | 7 | 3 | 2 | 1 | 2 | 5 | 4 | 1 | 4 | 4 | 1 | 3 | 7 |
| NM_020801 | ARRDC3 | 4160 | 57 | 19 | 16 | 6 | 9 | 10 | 20 | 3 | 34 | 57 | 16 | 22 | 17 |
| NM_020802 | KIAA1377 | 7043 | 4 | 2 | 2 | 0 | 1 | 3 | 1 | 0 | 2 | 3 | 0 | 1 | 4 |
| NM_020803 | KLHL8 | 4576 | 11 | 7 | 5 | 3 | 7 | 9 | 8 | 7 | 6 | 7 | 11 | 11 | 8 |
| NM_020804 | PACSIN1 | 4234 | 25 | 2 | 0 | 0 | 1 | 25 | 0 | 0 | 0 | 0 | 0 | 0 | 0 |
| NM_020805 | KLHL14 | 4261 | 3 | 0 | 0 | 0 | 0 | 0 | 1 | 0 | 0 | 0 | 0 | 3 | 0 |
| NM_020806 | GPHN | 4302 | 7 | 3 | 1 | 2 | 2 | 5 | 6 | 7 | 1 | 2 | 2 | 1 | 2 |
| NM_020807 | ZNF319 | 4220 | 0 | 0 | 0 | 0 | 0 | 0 | 0 | 0 | 0 | 0 | 0 | 0 | 0 |
| NM_020808 | SIPA1L2 | 6488 | 12 | 4 | 2 | 4 | 12 | 7 | 2 | 3 | 4 | 5 | 2 | 5 | 2 |
| NM_020809 | ARHGAP20 | 6189 | 5 | 1 | 3 | 0 | 0 | 4 | 0 | 0 | 1 | 1 | 1 | 1 | 5 |
| NM_020810 | TRMT5 | 5281 | 1 | 1 | 1 | 0 | 1 | 1 | 1 | 1 | 0 | 1 | 1 | 1 | 1 |
| NM_020811 | ATPGD1 | 3942 | 4 | 0 | 0 | 0 | 0 | 4 | 0 | 0 | 0 | 0 | 0 | 0 | 0 |
| NM_020812 | DOCK6 | 6387 | 2 | 0 | 2 | 0 | 0 | 0 | 0 | 1 | 1 | 0 | 0 | 0 | 0 |
| NM_020813 | ZNF471 | 4966 | 5 | 2 | 1 | 0 | 2 | 2 | 2 | 0 | 1 | 5 | 1 | 1 | 3 |
| NM_020814 | 04 Mar | 4450 | 0 | 0 | 0 | 0 | 0 | 0 | 0 | 0 | 0 | 0 | 0 | 0 | 0 |
| NM_020815 | PCDH10 | 3935 | 9 | 1 | 0 | 0 | 1 | 9 | 1 | 0 | 0 | 1 | 0 | 0 | 1 |
| NM_020816 | KIF17 | 3778 | 1 | 0 | 0 | 0 | 0 | 1 | 0 | 0 | 0 | 0 | 0 | 0 | 1 |
| NM_020817 | KIAA1407 | 3976 | 4 | 2 | 1 | 1 | 1 | 2 | 3 | 0 | 2 | 2 | 0 | 3 | 4 |
| NM_020818 | KIAA1409 | 8471 | 1 | 0 | 0 | 0 | 0 | 1 | 0 | 0 | 0 | 0 | 0 | 0 | 0 |
| NM_020819 | FAM135A | 5592 | 10 | 5 | 2 | 3 | 5 | 8 | 6 | 2 | 5 | 8 | 3 | 5 | 10 |
| NM_020820 | PREX1 | 6633 | 13 | 3 | 1 | 2 | 2 | 8 | 1 | 1 | 5 | 1 | 0 | 13 | 1 |
| NM_020821 | VPS13C | 13400 | 19 | 10 | 9 | 3 | 9 | 9 | 10 | 4 | 14 | 12 | 8 | 19 | 10 |
| NM_020822 | KCNT1 | 4767 | 1 | 0 | 0 | 0 | 0 | 1 | 0 | 0 | 0 | 0 | 0 | 0 | 0 |
| NM_020823 | TMEM181 | 5397 | 8 | 4 | 3 | 2 | 3 | 6 | 2 | 4 | 3 | 2 | 2 | 5 | 8 |
| NM_020824 | ARHGAP21 | 7167 | 20 | 7 | 13 | 3 | 7 | 20 | 5 | 4 | 5 | 9 | 3 | 5 | 7 |
| NM_020825 | CRAMP1L | 7671 | 2 | 1 | 1 | 1 | 1 | 1 | 1 | 0 | 1 | 1 | 1 | 1 | 2 |
| NM_020826 | SYT13 | 5157 | 17 | 2 | 0 | 2 | 1 | 17 | 2 | 0 | 0 | 0 | 0 | 0 | 0 |
| NM_020827 | KIAA1430 | 4879 | 12 | 6 | 4 | 1 | 4 | 6 | 6 | 5 | 6 | 8 | 3 | 9 | 12 |

| transcript | gene | transcript length (nt) | max | mean | adipose | colon | heart | hypothalamus | kidney | liver | lung | ovary | skeletalmuscle | spleen | testes |
|---|---|---|---|---|---|---|---|---|---|---|---|---|---|---|---|
| NM_020828 | ZFP28 | 4087 | 6 | 2 | 1 | 0 | 2 | 3 | 2 | 0 | 1 | 6 | 1 | 2 | 4 |
| NM_020829 | KIAA1432 | 6436 | 4 | 2 | 2 | 2 | 1 | 2 | 2 | 2 | 2 | 3 | 2 | 4 | 2 |
| NM_020830 | WDFY1 | 4607 | 7 | 4 | 3 | 2 | 3 | 4 | 4 | 3 | 4 | 7 | 3 | 6 | 5 |
| NM_020831 | MKL1 | 4490 | 4 | 2 | 1 | 2 | 2 | 4 | 1 | 1 | 2 | 2 | 1 | 4 | 3 |
| NM_020832 | ZNF687 | 4508 | 1 | 1 | 0 | 0 | 1 | 1 | 1 | 0 | 0 | 1 | 0 | 1 | 1 |
| NM_020834 | HOMEZ | 3741 | 4 | 2 | 1 | 2 | 2 | 1 | 3 | 1 | 1 | 3 | 2 | 2 | 4 |
| NM_020836 | BEGAIN | 2674 | 1 | 0 | 0 | 0 | 0 | 1 | 0 | 0 | 0 | 0 | 0 | 0 | 0 |
| NM_020839 | WDR48 | 3691 | 7 | 4 | 3 | 2 | 2 | 6 | 4 | 2 | 3 | 4 | 2 | 4 | 7 |
| NM_020840 | FNIP2 | 5786 | 13 | 6 | 5 | 2 | 11 | 4 | 13 | 7 | 6 | 3 | 2 | 7 | 4 |
| NM_020841 | OSBPL8 | 7241 | 12 | 6 | 6 | 1 | 6 | 7 | 8 | 3 | 8 | 6 | 5 | 12 | 7 |
| NM_020843 | SCAPER | 4707 | 15 | 9 | 5 | 4 | 9 | 15 | 7 | 4 | 7 | 10 | 8 | 9 | 15 |
| NM_020844 | C8orf79 | 9588 | 3 | 1 | 0 | 0 | 1 | 3 | 1 | 0 | 1 | 0 | 0 | 1 | 1 |
| NM_020845 | PITPNM2 | 6736 | 2 | 1 | 0 | 1 | 1 | 2 | 1 | 1 | 1 | 1 | 0 | 1 | 1 |
| NM_020846 | SLAIN2 | 6011 | 7 | 4 | 4 | 1 | 6 | 2 | 3 | 3 | 4 | 4 | 7 | 4 | 5 |
| NM_020848 | KIAA1462 | 9300 | 16 | 6 | 6 | 6 | 5 | 6 | 3 | 0 | 10 | 6 | 2 | 1 | 16 |
| NM_020850 | RANBP10 | 5305 | 3 | 1 | 1 | 1 | 1 | 1 | 2 | 3 | 1 | 2 | 2 | 2 | 1 |
| NM_020851 | ISLR2 | 4386 | 0 | 0 | 0 | 0 | 0 | 0 | 0 | 0 | 0 | 0 | 0 | 0 | 0 |
| NM_020853 | KIAA1467 | 4711 | 7 | 1 | 0 | 0 | 0 | 7 | 1 | 0 | 0 | 0 | 0 | 1 | 1 |
| NM_020854 | KIAA1468 | 5454 | 6 | 3 | 2 | 3 | 2 | 6 | 4 | 2 | 3 | 4 | 2 | 4 | 4 |
| NM_020855 | ZNF492 | 4245 | 0 | 0 | 0 | 0 | 0 | 0 | 0 | 0 | 0 | 0 | 0 | 0 | 0 |
| NM_020856 | TSHZ3 | 4913 | 5 | 1 | 1 | 1 | 1 | 1 | 0 | 0 | 1 | 5 | 0 | 1 | 1 |
| NM_020857 | VPS18 | 3895 | 2 | 1 | 1 | 1 | 1 | 1 | 2 | 1 | 1 | 1 | 1 | 1 | 1 |
| NM_020858 | SEMA6D | 5913 | 8 | 3 | 1 | 2 | 2 | 7 | 5 | 1 | 1 | 8 | 1 | 0 | 1 |
| NM_020859 | SHROOM3 | 11017 | 3 | 1 | 0 | 3 | 1 | 1 | 2 | 3 | 2 | 0 | 0 | 1 | 0 |
| NM_020860 | STIM2 | 5154 | 6 | 3 | 2 | 1 | 2 | 4 | 2 | 2 | 3 | 6 | 1 | 6 | 2 |
| NM_020861 | ZBTB2 | 3086 | 3 | 2 | 1 | 1 | 2 | 1 | 1 | 1 | 2 | 2 | 1 | 3 | 2 |
| NM_020862 | LRFN1 | 3132 | 0 | 0 | 0 | 0 | 0 | 0 | 0 | 0 | 0 | 0 | 0 | 0 | 0 |
| NM_020863 | ZFAT | 4611 | 2 | 1 | 1 | 0 | 0 | 1 | 2 | 0 | 0 | 1 | 0 | 1 | 1 |
| NM_020864 | KIAA1486 | 4590 | 1 | 0 | 0 | 0 | 0 | 1 | 0 | 0 | 0 | 0 | 0 | 0 | 0 |
| NM_020865 | DHX36 | 3611 | 13 | 7 | 4 | 2 | 6 | 8 | 6 | 6 | 6 | 8 | 8 | 8 | 13 |
| NM_020866 | KLHL1 | 4149 | 1 | 0 | 0 | 0 | 0 | 1 | 0 | 0 | 0 | 0 | 0 | 0 | 0 |
| NM_020868 | DPP10 | 4863 | 5 | 0 | 0 | 0 | 0 | 5 | 0 | 0 | 0 | 0 | 0 | 0 | 0 |
| NM_020870 | SH3RF1 | 5265 | 8 | 4 | 2 | 4 | 3 | 8 | 5 | 2 | 4 | 4 | 3 | 2 | 5 |
| NM_020871 | LRCH2 | 4925 | 4 | 1 | 1 | 0 | 1 | 3 | 0 | 0 | 2 | 4 | 0 | 1 | 2 |
| NM_020872 | CNTN3 | 4997 | 2 | 1 | 1 | 1 | 1 | 2 | 1 | 1 | 0 | 0 | 0 | 1 | 0 |
| NM_020873 | LRRN1 | 3823 | 3 | 1 | 0 | 0 | 0 | 3 | 0 | 0 | 0 | 1 | 1 | 0 | 3 |
| NM_020876 | ARHGAP23 | 6275 | 3 | 1 | 0 | 0 | 1 | 3 | 1 | 0 | 1 | 1 | 0 | 3 | 1 |
| NM_020877 | DNAH2 | 13505 | 0 | 0 | 0 | 0 | 0 | 0 | 0 | 0 | 0 | 0 | 0 | 0 | 0 |
| NM_020879 | CCDC146 | 3333 | 7 | 2 | 1 | 1 | 2 | 2 | 2 | 0 | 3 | 2 | 1 | 2 | 7 |
| NM_020880 | ZNF530 | 2964 | 1 | 0 | 0 | 0 | 0 | 0 | 0 | 0 | 0 | 0 | 0 | 1 | 0 |
| NM_020882 | COL20A1 | 4171 | 0 | 0 | 0 | 0 | 0 | 0 | 0 | 0 | 0 | 0 | 0 | 0 | 0 |
| NM_020883 | ZSWIM5 | 5836 | 1 | 1 | 0 | 1 | 0 | 1 | 1 | 0 | 0 | 1 | 0 | 0 | 1 |
| NM_020884 | MYH7B | 6289 | 2 | 0 | 0 | 0 | 2 | 0 | 0 | 0 | 0 | 0 | 1 | 0 | 0 |
| NM_020886 | USP28 | 4632 | 23 | 7 | 3 | 4 | 23 | 3 | 4 | 3 | 4 | 6 | 16 | 7 | 6 |
| NM_020888 | KIAA1522 | 5412 | 5 | 2 | 1 | 5 | 1 | 1 | 4 | 1 | 1 | 2 | 0 | 0 | 1 |
| NM_020889 | PHF12 | 2957 | 3 | 2 | 1 | 2 | 1 | 1 | 1 | 1 | 3 | 2 | 2 | 3 | 2 |
| NM_020890 | KIAA1524 | 4265 | 1 | 0 | 0 | 0 | 0 | 0 | 0 | 0 | 0 | 0 | 0 | 1 | 1 |
| NM_020892 | DTX2 | 2819 | 0 | 0 | 0 | 0 | 0 | 0 | 0 | 0 | 0 | 0 | 0 | 0 | 0 |
| NM_020893 | KIAA1529 | 6269 | 4 | 1 | 1 | 1 | 0 | 1 | 1 | 0 | 1 | 1 | 0 | 1 | 4 |
| NM_020894 | KIAA1530 | 4665 | 2 | 1 | 1 | 2 | 0 | 0 | 1 | 0 | 1 | 0 | 0 | 1 | 2 |
| NM_020895 | GRAMD1A | 2640 | 1 | 0 | 0 | 0 | 0 | 0 | 0 | 0 | 1 | 1 | 0 | 1 | 1 |
| NM_020896 | OSBPL5 | 3887 | 1 | 0 | 0 | 1 | 0 | 0 | 0 | 0 | 0 | 0 | 0 | 1 | 0 |
| NM_020897 | HCN3 | 3717 | 0 | 0 | 0 | 0 | 0 | 0 | 0 | 0 | 0 | 0 | 0 | 0 | 0 |
| NM_020898 | CALCOCO1 | 3045 | 12 | 8 | 12 | 8 | 11 | 7 | 6 | 2 | 8 | 6 | 12 | 8 | 7 |
| NM_020899 | ZBTB4 | 5942 | 7 | 4 | 5 | 3 | 5 | 7 | 4 | 1 | 5 | 6 | 3 | 5 | 4 |
| NM_020901 | PHRF1 | 5521 | 2 | 1 | 1 | 2 | 1 | 1 | 1 | 1 | 1 | 1 | 0 | 1 | 1 |
| NM_020902 | KIAA1543 | 4104 | 0 | 0 | 0 | 0 | 0 | 0 | 0 | 0 | 0 | 0 | 0 | 0 | 0 |
| NM_020903 | USP29 | 3606 | 0 | 0 | 0 | 0 | 0 | 0 | 0 | 0 | 0 | 0 | 0 | 0 | 0 |
| NM_020904 | PLEKHA4 | 3083 | 32 | 14 | 13 | 8 | 32 | 8 | 8 | 13 | 7 | 25 | 10 | 12 | 15 |
| NM_020905 | RDH14 | 1600 | 10 | 6 | 4 | 3 | 10 | 8 | 5 | 6 | 4 | 4 | 6 | 6 | 5 |
| NM_020908 | TLE3 | 5295 | 5 | 2 | 3 | 3 | 1 | 2 | 2 | 1 | 3 | 2 | 1 | 5 | 2 |
| NM_020909 | EPB41L5 | 6540 | 13 | 6 | 2 | 2 | 2 | 5 | 12 | 10 | 7 | 7 | 3 | 2 | 13 |
| NM_020910 | KIAA1549 | 12377 | 1 | 0 | 0 | 0 | 0 | 1 | 1 | 0 | 0 | 1 | 0 | 0 | 0 |
| NM_020911 | PLXNA4 | 13061 | 5 | 1 | 5 | 0 | 2 | 3 | 0 | 0 | 0 | 1 | 0 | 2 | 0 |
| NM_020912 | FLYWCH1 | 2536 | 0 | 0 | 0 | 0 | 0 | 0 | 0 | 0 | 0 | 0 | 0 | 0 | 0 |
| NM_020914 | RNF213 | 18701 | 19 | 7 | 7 | 6 | 5 | 3 | 13 | 5 | 9 | 6 | 4 | 19 | 4 |
| NM_020917 | ZFP14 | 5645 | 3 | 1 | 1 | 1 | 1 | 2 | 2 | 0 | 1 | 3 | 0 | 2 | 1 |
| NM_020918 | GPAM | 6371 | 83 | 10 | 83 | 5 | 3 | 2 | 1 | 11 | 1 | 1 | 2 | 1 | 3 |
| NM_020919 | ALS2 | 6917 | 5 | 3 | 2 | 2 | 4 | 5 | 3 | 5 | 2 | 2 | 3 | 2 | 2 |
| NM_020920 | CHD8 | 7474 | 7 | 5 | 3 | 3 | 4 | 7 | 5 | 4 | 3 | 6 | 4 | 7 | 6 |
| NM_020921 | NIN | 10293 | 12 | 4 | 4 | 2 | 5 | 4 | 3 | 2 | 5 | 5 | 3 | 12 | 2 |
| NM_020922 | WNK3 | 7744 | 13 | 2 | 0 | 0 | 1 | 2 | 1 | 4 | 0 | 3 | 0 | 0 | 13 |
| NM_020923 | ZDBF2 | 10146 | 5 | 2 | 1 | 0 | 1 | 4 | 1 | 1 | 1 | 5 | 1 | 2 | 2 |
| NM_020924 | ZBTB26 | 1910 | 3 | 1 | 1 | 0 | 1 | 1 | 1 | 0 | 1 | 2 | 1 | 1 | 3 |
| NM_020925 | CACHD1 | 5274 | 8 | 2 | 3 | 0 | 1 | 5 | 2 | 1 | 2 | 8 | 1 | 3 | 2 |
| NM_020927 | VAT1L | 3766 | 11 | 1 | 0 | 1 | 1 | 11 | 0 | 0 | 0 | 0 | 0 | 0 | 0 |
| NM_020928 | ZSWIM6 | 5502 | 5 | 2 | 2 | 3 | 2 | 5 | 2 | 1 | 3 | 2 | 1 | 4 | 3 |

| transcript | gene | transcript length (nt) | max | mean | adipose | colon | heart | hypothalamus | kidney | liver | lung | ovary | skeletalmuscle | spleen | testes |
|---|---|---|---|---|---|---|---|---|---|---|---|---|---|---|---|
| NM_020929 | LRRC4C | 4054 | 15 | 2 | 0 | 0 | 1 | 15 | 0 | 0 | 0 | 0 | 0 | 0 | 0 |
| NM_020931 | KIAA1586 | 2894 | 5 | 2 | 1 | 0 | 2 | 3 | 1 | 1 | 2 | 5 | 1 | 4 | 3 |
| NM_020932 | MAGEE1 | 3628 | 12 | 1 | 0 | 1 | 1 | 12 | 1 | 0 | 0 | 1 | 0 | 1 | 0 |
| NM_020933 | ZNF317 | 4043 | 4 | 2 | 1 | 2 | 2 | 2 | 2 | 2 | 1 | 2 | 2 | 1 | 4 | 2 |
| NM_020935 | USP37 | 8032 | 5 | 2 | 1 | 1 | 1 | 2 | 2 | 1 | 1 | 3 | 2 | 2 | 5 |
| NM_020936 | DDX55 | 2622 | 2 | 1 | 1 | 1 | 1 | 2 | 1 | 1 | 1 | 1 | 1 | 2 | 1 |
| NM_020937 | FANCM | 7128 | 2 | 1 | 1 | 0 | 1 | 1 | 1 | 1 | 1 | 2 | 1 | 2 | 2 |
| NM_020939 | CPNE5 | 3339 | 3 | 1 | 0 | 0 | 1 | 3 | 0 | 0 | 0 | 0 | 0 | 0 | 1 |
| NM_020940 | FAM160B1 | 5806 | 2 | 2 | 2 | 1 | 2 | 1 | 1 | 2 | 2 | 2 | 1 | 2 | 2 |
| NM_020943 | CWC22 | 3279 | 10 | 6 | 7 | 2 | 6 | 7 | 7 | 5 | 5 | 8 | 5 | 10 | 8 |
| NM_020944 | GBA2 | 3611 | 4 | 2 | 1 | 3 | 2 | 4 | 4 | 2 | 1 | 1 | 1 | 2 | 1 |
| NM_020945 | WDFY4 | 10013 | 3 | 1 | 1 | 0 | 0 | 0 | 0 | 0 | 1 | 0 | 0 | 3 | 0 |
| NM_020946 | DENND1A | 5041 | 4 | 2 | 1 | 1 | 1 | 4 | 3 | 2 | 1 | 2 | 1 | 3 | 2 |
| NM_020947 | KIAA1609 | 5029 | 1 | 0 | 0 | 0 | 0 | 1 | 1 | 0 | 0 | 1 | 0 | 0 | 1 |
| NM_020948 | MIER1 | 5625 | 12 | 7 | 5 | 4 | 6 | 5 | 6 | 6 | 10 | 10 | 4 | 11 | 12 |
| NM_020949 | SLC7A14 | 10133 | 5 | 1 | 0 | 0 | 0 | 5 | 0 | 0 | 0 | 0 | 0 | 0 | 0 |
| NM_020950 | KIAA1614 | 4155 | 0 | 0 | 0 | 0 | 0 | 0 | 0 | 0 | 0 | 0 | 0 | 0 | 0 |
| NM_020951 | ZNF529 | 5144 | 1 | 1 | 0 | 0 | 1 | 1 | 1 | 0 | 1 | 1 | 1 | 1 | 1 |
| NM_020952 | TRPM3 | 5923 | 6 | 1 | 0 | 0 | 0 | 6 | 6 | 0 | 0 | 1 | 0 | 0 | 1 |
| NM_020954 | RNF213 | 5317 | 25 | 10 | 8 | 9 | 8 | 3 | 18 | 5 | 15 | 8 | 6 | 25 | 6 |
| NM_020956 | PRX | 5506 | 4 | 1 | 0 | 0 | 0 | 1 | 1 | 0 | 4 | 1 | 1 | 0 | 0 |
| NM_020957 | PCDHB16 | 4817 | 4 | 1 | 1 | 1 | 2 | 2 | 1 | 0 | 1 | 4 | 1 | 1 | 2 |
| NM_020958 | PPP4R4 | 901 | 3 | 1 | 0 | 0 | 0 | 3 | 0 | 0 | 1 | 1 | 0 | 0 | 1 |
| NM_020959 | ANO8 | 4152 | 1 | 0 | 0 | 0 | 0 | 1 | 0 | 0 | 0 | 0 | 0 | 0 | 0 |
| NM_020960 | GPR107 | 6934 | 6 | 4 | 3 | 3 | 4 | 6 | 5 | 3 | 3 | 3 | 2 | 4 | 4 |
| NM_020961 | METTL14 | 2107 | 7 | 4 | 3 | 2 | 3 | 4 | 4 | 5 | 3 | 6 | 2 | 6 | 7 |
| NM_020962 | IGDCC4 | 6485 | 1 | 0 | 0 | 0 | 0 | 1 | 0 | 0 | 0 | 1 | 0 | 0 | 0 |
| NM_020963 | MOV10 | 3636 | 3 | 1 | 1 | 1 | 1 | 1 | 2 | 1 | 1 | 1 | 0 | 2 | 3 |
| NM_020964 | KIAA1632 | 12698 | 2 | 1 | 1 | 1 | 1 | 2 | 2 | 1 | 1 | 2 | 1 | 2 | 2 |
| NM_020967 | NCOA5 | 3207 | 11 | 6 | 5 | 6 | 5 | 5 | 5 | 3 | 3 | 8 | 5 | 10 | 11 |
| NM_020971 | SPTBN4 | 8687 | 2 | 0 | 0 | 0 | 0 | 2 | 0 | 0 | 0 | 0 | 0 | 0 | 0 |
| NM_020972 | ZFYVE28 | 4110 | 1 | 0 | 0 | 0 | 0 | 1 | 0 | 0 | 0 | 0 | 0 | 1 | 1 |
| NM_020973 | GBA3 | 2147 | 14 | 2 | 0 | 1 | 0 | 0 | 14 | 4 | 0 | 0 | 0 | 2 | 0 |
| NM_020974 | SCUBE2 | 3737 | 2 | 0 | 2 | 1 | 0 | 0 | 0 | 0 | 0 | 0 | 0 | 0 | 0 |
| NM_020975 | RET | 5615 | 2 | 0 | 0 | 2 | 0 | 1 | 0 | 0 | 0 | 0 | 0 | 0 | 0 |
| NM_020977 | ANK2 | 7988 | 53 | 11 | 4 | 2 | 12 | 53 | 25 | 0 | 1 | 8 | 4 | 4 | 3 |
| NM_020978 | AMY2B | 2179 | 1 | 0 | 0 | 0 | 0 | 1 | 1 | 0 | 1 | 0 | 1 | 1 | 0 |
| NM_020979 | SH2B2 | 1893 | 0 | 0 | 0 | 0 | 0 | 0 | 0 | 0 | 0 | 0 | 0 | 0 | 0 |
| NM_020980 | AQP9 | 3021 | 29 | 5 | 2 | 0 | 1 | 0 | 0 | 29 | 7 | 0 | 0 | 10 | 1 |
| NM_020981 | B3GALT1 | 2168 | 1 | 0 | 0 | 1 | 0 | 1 | 0 | 0 | 0 | 0 | 0 | 0 | 0 |
| NM_020982 | CLDN9 | 2050 | 0 | 0 | 0 | 0 | 0 | 0 | 0 | 0 | 0 | 0 | 0 | 0 | 0 |
| NM_020983 | ADCY6 | 5877 | 3 | 1 | 3 | 2 | 2 | 1 | 2 | 0 | 1 | 1 | 0 | 1 | 1 |
| NM_020984 | CHAT | 2124 | 0 | 0 | 0 | 0 | 0 | 0 | 0 | 0 | 0 | 0 | 0 | 0 | 0 |
| NM_020985 | CHAT | 2286 | 0 | 0 | 0 | 0 | 0 | 0 | 0 | 0 | 0 | 0 | 0 | 0 | 0 |
| NM_020986 | CHAT | 2114 | 0 | 0 | 0 | 0 | 0 | 0 | 0 | 0 | 0 | 0 | 0 | 0 | 0 |
| NM_020987 | ANK3 | 14771 | 21 | 6 | 0 | 1 | 14 | 21 | 11 | 1 | 2 | 3 | 4 | 3 | 3 |
| NM_020988 | GNAO1 | 3332 | 31 | 3 | 0 | 2 | 3 | 31 | 0 | 0 | 0 | 0 | 0 | 0 | 1 |
| NM_020989 | CRYGC | 629 | 0 | 0 | 0 | 0 | 0 | 0 | 0 | 0 | 0 | 0 | 0 | 0 | 0 |
| NM_020990 | CKMT1B | 1779 | 1 | 0 | 0 | 1 | 0 | 1 | 0 | 0 | 0 | 0 | 0 | 0 | 0 |
| NM_020991 | CSH2 | 883 | 0 | 0 | 0 | 0 | 0 | 0 | 0 | 0 | 0 | 0 | 0 | 0 | 0 |
| NM_020992 | PDLIM1 | 1451 | 19 | 7 | 3 | 10 | 10 | 1 | 2 | 4 | 12 | 1 | 19 | 14 | 2 |
| NM_020993 | BCL7A | 3714 | 1 | 1 | 0 | 0 | 1 | 1 | 1 | 0 | 0 | 0 | 1 | 1 | 0 |
| NM_020994 | CTAG2 | 977 | 0 | 0 | 0 | 0 | 0 | 0 | 0 | 0 | 0 | 0 | 0 | 0 | 0 |
| NM_020995 | HPR | 1242 | 129 | 14 | 0 | 1 | 11 | 0 | 3 | 129 | 0 | 1 | 1 | 1 | 1 |
| NM_020996 | FGF6 | 744 | 0 | 0 | 0 | 0 | 0 | 0 | 0 | 0 | 0 | 0 | 0 | 0 | 0 |
| NM_020997 | LEFTY1 | 1616 | 2 | 0 | 0 | 2 | 0 | 0 | 0 | 0 | 0 | 0 | 0 | 0 | 0 |
| NM_020998 | MST1 | 2330 | 2 | 0 | 0 | 0 | 0 | 0 | 0 | 2 | 0 | 0 | 0 | 0 | 0 |
| NM_020999 | NEUROG3 | 1167 | 0 | 0 | 0 | 0 | 0 | 0 | 0 | 0 | 0 | 0 | 0 | 0 | 0 |
| NM_021002 | IFNA6 | 570 | 0 | 0 | 0 | 0 | 0 | 0 | 0 | 0 | 0 | 0 | 0 | 0 | 0 |
| NM_021003 | PPM1A | 8205 | 15 | 8 | 6 | 3 | 10 | 13 | 8 | 7 | 6 | 7 | 15 | 7 | 9 |
| NM_021004 | DHRS4 | 1278 | 5 | 2 | 1 | 1 | 1 | 1 | 5 | 5 | 1 | 1 | 0 | 1 | 1 |
| NM_021005 | NR2F2 | 5108 | 24 | 7 | 3 | 2 | 4 | 2 | 8 | 2 | 5 | 24 | 2 | 16 | 8 |
| NM_021006 | CCL3L1 | 782 | 0 | 0 | 0 | 0 | 0 | 0 | 0 | 0 | 0 | 0 | 0 | 0 | 0 |
| NM_021006 | CCL3L1 | 783 | 0 | 0 | 0 | 0 | 0 | 0 | 0 | 0 | 0 | 0 | 0 | 0 | 0 |
| NM_021007 | SCN2A | 8860 | 37 | 4 | 0 | 0 | 0 | 37 | 2 | 0 | 0 | 0 | 0 | 0 | 0 |
| NM_021008 | DEAF1 | 2716 | 1 | 0 | 0 | 0 | 0 | 1 | 0 | 0 | 0 | 0 | 0 | 0 | 0 |
| NM_021009 | UBC | 2574 | 1,120 | 385 | 307 | 185 | 340 | 313 | 204 | 361 | 392 | 229 | 1,120 | 406 | 380 |
| NM_021010 | DEFA5 | 449 | 4 | 0 | 0 | 4 | 0 | 0 | 0 | 0 | 0 | 0 | 0 | 0 | 0 |
| NM_021012 | KCNJ12 | 5230 | 1 | 0 | 0 | 0 | 0 | 0 | 0 | 0 | 0 | 0 | 1 | 0 | 0 |
| NM_021013 | KRT34 | 1713 | 0 | 0 | 0 | 0 | 0 | 0 | 0 | 0 | 0 | 0 | 0 | 0 | 0 |
| NM_021014 | SSX3 | 1273 | 1 | 0 | 0 | 0 | 0 | 0 | 0 | 0 | 0 | 0 | 0 | 0 | 1 |
| NM_021015 | SSX5 | 1384 | 0 | 0 | 0 | 0 | 0 | 0 | 0 | 0 | 0 | 0 | 0 | 0 | 0 |
| NM_021016 | PSG3 | 1908 | 0 | 0 | 0 | 0 | 0 | 0 | 0 | 0 | 0 | 0 | 0 | 0 | 0 |
| NM_021018 | HIST1H3F | 466 | 3 | 1 | 1 | 3 | 0 | 0 | 0 | 0 | 1 | 0 | 0 | 1 | 0 |
| NM_021019 | MYL6 | 754 | 115 | 42 | 52 | 115 | 35 | 21 | 45 | 22 | 38 | 24 | 8 | 69 | 29 |
| NM_021020 | LZTS1 | 5458 | 1 | 0 | 0 | 0 | 0 | 1 | 0 | 0 | 0 | 0 | 0 | 0 | 0 |
| NM_021021 | SNTB1 | 4974 | 20 | 4 | 2 | 1 | 1 | 3 | 3 | 20 | 6 | 0 | 2 | 4 | 1 |

| transcript | gene | transcript length (nt) | max | mean | adipose | colon | heart | hypothalamus | kidney | liver | lung | ovary | skeletalmuscle | spleen | testes |
|---|---|---|---|---|---|---|---|---|---|---|---|---|---|---|---|
| NM_021023 | CFHR3 | 1640 | 4 | 0 | 0 | 0 | 0 | 0 | 0 | 4 | 0 | 0 | 0 | 0 | 0 |
| NM_021025 | TLX3 | 1493 | 0 | 0 | 0 | 0 | 0 | 0 | 0 | 0 | 0 | 0 | 0 | 0 | 0 |
| NM_021026 | RFPL1 | 1496 | 0 | 0 | 0 | 0 | 0 | 0 | 0 | 0 | 0 | 0 | 0 | 0 | 0 |
| NM_021027 | UGT1A9 | 2376 | 36 | 4 | 0 | 0 | 0 | 0 | 36 | 4 | 0 | 0 | 0 | 0 | 0 |
| NM_021029 | RPL36A | 452 | 281 | 124 | 120 | 108 | 111 | 87 | 88 | 105 | 99 | 281 | 121 | 179 | 66 |
| NM_021030 | ZNF14 | 2963 | 9 | 3 | 2 | 1 | 2 | 5 | 3 | 0 | 2 | 9 | 2 | 4 | 4 |
| NM_021032 | FGF12 | 3672 | 11 | 2 | 0 | 0 | 11 | 10 | 0 | 0 | 0 | 0 | 0 | 2 | 0 |
| NM_021033 | RAP2A | 4357 | 20 | 7 | 6 | 1 | 8 | 20 | 5 | 3 | 7 | 5 | 3 | 10 | 4 |
| NM_021034 | IFITM3 | 668 | 45 | 19 | 8 | 4 | 38 | 9 | 14 | 32 | 23 | 15 | 13 | 45 | 11 |
| NM_021035 | ZNFX1 | 7369 | 9 | 6 | 7 | 4 | 7 | 6 | 5 | 3 | 8 | 6 | 6 | 9 | 5 |
| NM_021038 | MBNL1 | 6404 | 24 | 12 | 13 | 5 | 16 | 7 | 6 | 5 | 16 | 8 | 24 | 20 | 9 |
| NM_021044 | DHH | 1934 | 1 | 0 | 0 | 0 | 0 | 0 | 0 | 0 | 0 | 0 | 0 | 0 | 1 |
| NM_021045 | ZNF248 | 4883 | 4 | 2 | 1 | 1 | 1 | 4 | 2 | 0 | 1 | 4 | 1 | 2 | 2 |
| NM_021046 | KRTAP5-8 | 1183 | 0 | 0 | 0 | 0 | 0 | 0 | 0 | 0 | 0 | 0 | 0 | 0 | 0 |
| NM_021047 | ZNF253 | 2329 | 31 | 10 | 4 | 4 | 7 | 10 | 12 | 3 | 5 | 31 | 9 | 15 | 10 |
| NM_021048 | MAGEA10 | 1502 | 0 | 0 | 0 | 0 | 0 | 0 | 0 | 0 | 0 | 0 | 0 | 0 | 0 |
| NM_021049 | MAGEA5 | 1692 | 0 | 0 | 0 | 0 | 0 | 0 | 0 | 0 | 0 | 0 | 0 | 0 | 0 |
| NM_021052 | HIST1H2AE | 564 | 10 | 4 | 3 | 10 | 1 | 4 | 1 | 0 | 5 | 3 | 0 | 6 | 5 |
| NM_021057 | IFNA7 | 737 | 0 | 0 | 0 | 0 | 0 | 0 | 0 | 0 | 0 | 0 | 0 | 0 | 0 |
| NM_021058 | HIST1H2BJ | 481 | 6 | 2 | 2 | 3 | 0 | 0 | 0 | 1 | 2 | 0 | 0 | 3 | 6 |
| NM_021059 | HIST2H3C | 507 | 0 | 0 | 0 | 0 | 0 | 0 | 0 | 0 | 0 | 0 | 0 | 0 | 0 |
| NM_021061 | ZNF250 | 6364 | 2 | 1 | 0 | 1 | 1 | 1 | 1 | 0 | 1 | 2 | 1 | 1 | 1 |
| NM_021062 | HIST1H2BB | 431 | 3 | 0 | 0 | 1 | 0 | 0 | 0 | 0 | 1 | 0 | 0 | 0 | 3 |
| NM_021063 | HIST1H2BD | 523 | 65 | 25 | 16 | 65 | 9 | 23 | 23 | 15 | 49 | 17 | 6 | 31 | 18 |
| NM_021064 | HIST1H2AG | 497 | 1 | 0 | 0 | 1 | 0 | 0 | 0 | 0 | 1 | 0 | 0 | 1 | 1 |
| NM_021065 | HIST1H2AD | 460 | 0 | 0 | 0 | 0 | 0 | 0 | 0 | 0 | 0 | 0 | 0 | 0 | 0 |
| NM_021066 | HIST1H2AJ | 439 | 2 | 1 | 0 | 2 | 0 | 0 | 0 | 0 | 1 | 0 | 0 | 2 | 1 |
| NM_021067 | GINS1 | 3289 | 1 | 0 | 0 | 1 | 0 | 0 | 0 | 0 | 0 | 0 | 0 | 0 | 1 |
| NM_021068 | IFNA4 | 981 | 0 | 0 | 0 | 0 | 0 | 0 | 0 | 0 | 0 | 0 | 0 | 0 | 0 |
| NM_021069 | SORBS2 | 6344 | 36 | 6 | 1 | 3 | 36 | 5 | 3 | 5 | 1 | 2 | 2 | 3 | 1 |
| NM_021070 | LTBP3 | 4560 | 4 | 2 | 2 | 2 | 2 | 2 | 2 | 1 | 2 | 4 | 1 | 2 | 2 |
| NM_021071 | ART4 | 1371 | 12 | 2 | 0 | 0 | 4 | 0 | 1 | 12 | 1 | 0 | 0 | 8 | 1 |
| NM_021072 | HCN1 | 5369 | 6 | 1 | 0 | 0 | 0 | 6 | 0 | 0 | 0 | 0 | 0 | 0 | 0 |
| NM_021073 | BMP5 | 2171 | 2 | 1 | 0 | 1 | 1 | 1 | 0 | 0 | 2 | 0 | 0 | 0 | 0 |
| NM_021074 | NDUFV2 | 929 | 259 | 101 | 53 | 47 | 259 | 85 | 110 | 157 | 63 | 38 | 170 | 67 | 67 |
| NM_021075 | NDUFV3 | 2130 | 4 | 3 | 2 | 2 | 4 | 3 | 3 | 3 | 1 | 2 | 4 | 2 | 1 |
| NM_021076 | NEFH | 3717 | 39 | 5 | 0 | 1 | 0 | 39 | 1 | 0 | 0 | 7 | 0 | 1 | 4 |
| NM_021077 | NMB | 1030 | 1 | 0 | 1 | 0 | 0 | 0 | 0 | 0 | 0 | 0 | 0 | 0 | 0 |
| NM_021078 | KAT2A | 3112 | 2 | 1 | 1 | 2 | 1 | 2 | 1 | 0 | 1 | 1 | 1 | 2 | 2 |
| NM_021079 | NMT1 | 4884 | 7 | 4 | 4 | 3 | 4 | 7 | 5 | 5 | 3 | 2 | 4 | 5 | 3 |
| NM_021080 | DAB1 | 2580 | 2 | 0 | 0 | 0 | 0 | 2 | 0 | 1 | 0 | 0 | 0 | 0 | 0 |
| NM_021081 | GHRH | 453 | 0 | 0 | 0 | 0 | 0 | 0 | 0 | 0 | 0 | 0 | 0 | 0 | 0 |
| NM_021082 | SLC15A2 | 5523 | 3 | 1 | 0 | 0 | 0 | 2 | 3 | 0 | 2 | 0 | 0 | 1 | 2 |
| NM_021083 | XK | 5085 | 1 | 0 | 0 | 1 | 1 | 1 | 0 | 0 | 0 | 0 | 0 | 0 | 0 |
| NM_021088 | ZNF2 | 3953 | 1 | 1 | 0 | 0 | 0 | 1 | 1 | 0 | 0 | 1 | 1 | 1 | 1 |
| NM_021089 | ZNF8 | 2210 | 3 | 2 | 1 | 1 | 2 | 3 | 2 | 1 | 1 | 2 | 1 | 3 | 3 |
| NM_021090 | MTMR3 | 8992 | 3 | 2 | 2 | 2 | 2 | 3 | 2 | 2 | 2 | 2 | 2 | 3 | 3 |
| NM_021094 | SLCO1A2 | 7211 | 13 | 1 | 0 | 0 | 0 | 13 | 0 | 0 | 1 | 0 | 0 | 0 | 0 |
| NM_021095 | SLC5A6 | 3250 | 5 | 1 | 1 | 0 | 0 | 1 | 0 | 5 | 0 | 0 | 0 | 1 | 1 |
| NM_021096 | CACNA1I | 10003 | 1 | 0 | 0 | 0 | 0 | 1 | 0 | 0 | 0 | 0 | 0 | 0 | 0 |
| NM_021097 | SLC8A1 | 6053 | 79 | 13 | 3 | 5 | 79 | 30 | 11 | 1 | 4 | 2 | 1 | 10 | 2 |
| NM_021098 | CACNA1H | 8088 | 1 | 0 | 0 | 1 | 0 | 0 | 0 | 0 | 0 | 1 | 0 | 0 | 0 |
| NM_021100 | NFS1 | 2371 | 4 | 2 | 1 | 1 | 2 | 3 | 4 | 3 | 1 | 1 | 1 | 1 | 2 |
| NM_021101 | CLDN1 | 3429 | 13 | 2 | 0 | 0 | 0 | 0 | 1 | 13 | 1 | 1 | 0 | 0 | 2 |
| NM_021102 | SPINT2 | 1801 | 10 | 4 | 0 | 10 | 2 | 7 | 10 | 0 | 6 | 2 | 0 | 3 | 6 |
| NM_021103 | TMSB10 | 482 | 83 | 45 | 63 | 56 | 41 | 72 | 18 | 11 | 53 | 56 | 7 | 83 | 32 |
| NM_021104 | RPL41 | 469 | 180 | 100 | 92 | 105 | 58 | 70 | 84 | 180 | 105 | 107 | 84 | 135 | 77 |
| NM_021105 | PLSCR1 | 2210 | 18 | 7 | 5 | 4 | 6 | 6 | 7 | 5 | 11 | 6 | 3 | 18 | 6 |
| NM_021106 | RGS3 | 2862 | 2 | 1 | 2 | 1 | 2 | 1 | 2 | 0 | 2 | 0 | 0 | 1 | 2 |
| NM_021107 | MRPS12 | 1077 | 2 | 0 | 0 | 1 | 0 | 1 | 1 | 2 | 0 | 0 | 0 | 0 | 0 |
| NM_021109 | TMSB4X | 629 | 0 | 0 | 0 | 0 | 0 | 0 | 0 | 0 | 0 | 0 | 0 | 0 | 0 |
| NM_021110 | COL14A1 | 6459 | 36 | 10 | 13 | 7 | 10 | 1 | 6 | 2 | 6 | 36 | 2 | 14 | 11 |
| NM_021111 | RECK | 4411 | 5 | 3 | 5 | 1 | 4 | 2 | 2 | 1 | 4 | 5 | 1 | 4 | 3 |
| NM_021114 | SPINK2 | 591 | 17 | 2 | 0 | 0 | 0 | 0 | 0 | 0 | 0 | 0 | 0 | 0 | 17 |
| NM_021115 | SEZ6L | 3406 | 13 | 1 | 0 | 0 | 0 | 13 | 0 | 0 | 0 | 0 | 0 | 0 | 0 |
| NM_021116 | ADCY1 | 12498 | 7 | 1 | 0 | 0 | 0 | 7 | 0 | 1 | 0 | 0 | 0 | 0 | 0 |
| NM_021117 | CRY2 | 4204 | 3 | 2 | 1 | 1 | 1 | 3 | 1 | 2 | 1 | 1 | 3 | 1 | 2 |
| NM_021118 | CYLC1 | 2011 | 6 | 1 | 0 | 0 | 0 | 0 | 0 | 0 | 0 | 0 | 0 | 0 | 6 |
| NM_021120 | DLG3 | 6019 | 4 | 1 | 0 | 1 | 0 | 4 | 1 | 0 | 1 | 1 | 0 | 2 | 1 |
| NM_021121 | EEF1B2 | 836 | 115 | 66 | 39 | 32 | 70 | 44 | 56 | 82 | 61 | 85 | 96 | 115 | 44 |
| NM_021123 | GAGE7 | 497 | 0 | 0 | 0 | 0 | 0 | 0 | 0 | 0 | 0 | 0 | 0 | 0 | 0 |
| NM_021126 | MPST | 1438 | 1 | 0 | 0 | 0 | 0 | 0 | 0 | 1 | 0 | 0 | 0 | 0 | 0 |
| NM_021127 | PMAIP1 | 1936 | 1 | 0 | 0 | 0 | 0 | 0 | 0 | 0 | 1 | 0 | 0 | 1 | 0 |
| NM_021128 | POLR2L | 875 | 31 | 12 | 31 | 9 | 15 | 14 | 11 | 16 | 13 | 2 | 7 | 11 | 5 |
| NM_021129 | PPA1 | 1302 | 35 | 15 | 35 | 9 | 10 | 28 | 14 | 17 | 13 | 4 | 7 | 11 | 14 |
| NM_021130 | PPIA | 2276 | 133 | 73 | 75 | 65 | 80 | 129 | 74 | 133 | 43 | 46 | 19 | 104 | 34 |
| NM_021131 | PPP2R4 | 2649 | 2 | 1 | 0 | 1 | 1 | 2 | 1 | 1 | 0 | 0 | 0 | 1 | 1 |

| transcript | gene | transcript length (nt) | max | mean | adipose | colon | heart | hypothalamus | kidney | liver | lung | ovary | skeletalmuscle | spleen | testes |
|---|---|---|---|---|---|---|---|---|---|---|---|---|---|---|---|
| NM_021132 | PPP3CB | 3146 | 91 | 29 | 26 | 11 | 25 | 91 | 13 | 8 | 14 | 25 | 63 | 19 | 24 |
| NM_021133 | RNASEL | 4148 | 3 | 1 | 1 | 1 | 1 | 1 | 2 | 0 | 1 | 2 | 1 | 3 | 1 |
| NM_021134 | MRPL23 | 740 | 3 | 1 | 1 | 2 | 1 | 2 | 1 | 3 | 1 | 1 | 2 | 1 | 0 |
| NM_021135 | RPS6KA2 | 5817 | 7 | 4 | 2 | 1 | 7 | 7 | 2 | 1 | 6 | 4 | 1 | 7 | 1 |
| NM_021136 | RTN1 | 3309 | 40 | 4 | 1 | 1 | 1 | 40 | 0 | 0 | 1 | 0 | 0 | 4 | 1 |
| NM_021137 | TNFAIP1 | 3807 | 7 | 3 | 1 | 1 | 5 | 3 | 3 | 4 | 2 | 1 | 1 | 7 | 2 |
| NM_021138 | TRAF2 | 2277 | 0 | 0 | 0 | 0 | 0 | 0 | 0 | 0 | 0 | 0 | 0 | 0 | 0 |
| NM_021139 | UGT2B4 | 2103 | 171 | 16 | 0 | 0 | 4 | 0 | 0 | 171 | 0 | 1 | 0 | 1 | 0 |
| NM_021140 | KDM6A | 5770 | 3 | 2 | 1 | 1 | 1 | 1 | 2 | 1 | 2 | 3 | 2 | 2 | 2 |
| NM_021141 | XRCC5 | 3428 | 20 | 14 | 11 | 9 | 14 | 15 | 15 | 19 | 12 | 15 | 10 | 20 | 15 |
| NM_021143 | ZNF20 | 2341 | 4 | 2 | 1 | 1 | 1 | 2 | 3 | 1 | 2 | 3 | 0 | 3 | 4 |
| NM_021144 | PSIP1 | 1666 | 110 | 38 | 18 | 8 | 22 | 110 | 20 | 9 | 23 | 76 | 23 | 46 | 60 |
| NM_021145 | DMTF1 | 4036 | 8 | 4 | 3 | 3 | 3 | 5 | 5 | 1 | 4 | 5 | 2 | 8 | 8 |
| NM_021146 | ANGPTL7 | 2238 | 4 | 1 | 0 | 1 | 2 | 0 | 2 | 0 | 0 | 0 | 4 | 0 | 1 |
| NM_021147 | CCNO | 1432 | 0 | 0 | 0 | 0 | 0 | 0 | 0 | 0 | 0 | 0 | 0 | 0 | 0 |
| NM_021148 | ZNF273 | 4325 | 3 | 1 | 1 | 0 | 1 | 3 | 1 | 0 | 2 | 2 | 1 | 3 | 3 |
| NM_021149 | COTL1 | 1825 | 34 | 8 | 6 | 5 | 4 | 7 | 9 | 2 | 12 | 2 | 1 | 34 | 3 |
| NM_021150 | GRIP1 | 5047 | 1 | 0 | 0 | 0 | 0 | 1 | 0 | 0 | 0 | 0 | 0 | 1 | 1 |
| NM_021151 | CROT | 3236 | 2 | 1 | 1 | 1 | 1 | 1 | 2 | 1 | 0 | 1 | 1 | 1 | 1 |
| NM_021153 | CDH19 | 3185 | 29 | 4 | 0 | 1 | 29 | 2 | 0 | 2 | 1 | 0 | 1 | 2 | 2 |
| NM_021154 | PSAT1 | 2050 | 13 | 4 | 0 | 0 | 0 | 12 | 12 | 13 | 1 | 1 | 0 | 1 | 2 |
| NM_021155 | CD209 | 4327 | 5 | 2 | 5 | 1 | 4 | 1 | 1 | 1 | 1 | 1 | 0 | 1 | 1 |
| NM_021156 | TMX4 | 2365 | 88 | 25 | 14 | 8 | 33 | 36 | 17 | 24 | 15 | 18 | 12 | 14 | 88 |
| NM_021158 | TRIB3 | 2452 | 1 | 0 | 0 | 0 | 0 | 0 | 0 | 1 | 0 | 0 | 0 | 0 | 0 |
| NM_021159 | RAP1GDS1 | 3758 | 12 | 3 | 2 | 1 | 2 | 12 | 3 | 3 | 2 | 2 | 0 | 5 | 3 |
| NM_021160 | BAT5 | 1960 | 2 | 1 | 1 | 1 | 1 | 2 | 1 | 1 | 1 | 1 | 1 | 2 | 1 |
| NM_021161 | KCNK10 | 7499 | 0 | 0 | 0 | 0 | 0 | 0 | 0 | 0 | 0 | 0 | 0 | 0 | 0 |
| NM_021163 | RBAK | 6551 | 6 | 3 | 2 | 1 | 4 | 3 | 4 | 2 | 3 | 6 | 2 | 6 | 4 |
| NM_021165 | FAM5B | 3558 | 10 | 1 | 0 | 0 | 0 | 10 | 0 | 0 | 0 | 0 | 0 | 0 | 0 |
| NM_021167 | GATAD1 | 3955 | 2 | 1 | 1 | 1 | 1 | 1 | 2 | 1 | 1 | 2 | 1 | 1 | 2 |
| NM_021168 | RAB40C | 2609 | 1 | 0 | 0 | 0 | 0 | 1 | 0 | 0 | 0 | 0 | 0 | 0 | 0 |
| NM_021170 | HES4 | 961 | 0 | 0 | 0 | 0 | 0 | 0 | 0 | 0 | 0 | 0 | 0 | 0 | 0 |
| NM_021173 | POLD4 | 951 | 2 | 1 | 1 | 1 | 1 | 0 | 1 | 2 | 1 | 0 | 1 | 1 | 0 |
| NM_021174 | KIAA1967 | 3979 | 5 | 3 | 2 | 3 | 2 | 5 | 3 | 2 | 2 | 3 | 2 | 3 | 4 |
| NM_021175 | HAMP | 426 | 12 | 2 | 1 | 0 | 2 | 3 | 0 | 12 | 0 | 0 | 0 | 0 | 0 |
| NM_021176 | G6PC2 | 3081 | 0 | 0 | 0 | 0 | 0 | 0 | 0 | 0 | 0 | 0 | 0 | 0 | 0 |
| NM_021177 | LSM2 | 857 | 2 | 1 | 1 | 1 | 0 | 1 | 1 | 0 | 1 | 1 | 0 | 2 | 1 |
| NM_021178 | CCNB1IP1 | 1489 | 10 | 5 | 2 | 3 | 8 | 4 | 6 | 3 | 6 | 10 | 4 | 3 | 5 |
| NM_021179 | C1orf114 | 1866 | 9 | 2 | 0 | 0 | 1 | 6 | 3 | 0 | 2 | 3 | 0 | 0 | 9 |
| NM_021180 | GRHL3 | 2700 | 1 | 0 | 0 | 0 | 0 | 1 | 0 | 0 | 0 | 0 | 0 | 0 | 0 |
| NM_021181 | SLAMF7 | 2657 | 6 | 1 | 1 | 2 | 0 | 0 | 0 | 0 | 2 | 0 | 0 | 6 | 0 |
| NM_021182 | HMHB1 | 384 | 0 | 0 | 0 | 0 | 0 | 0 | 0 | 0 | 0 | 0 | 0 | 0 | 0 |
| NM_021183 | RAP2C | 3933 | 8 | 4 | 4 | 2 | 4 | 3 | 6 | 8 | 4 | 3 | 3 | 7 | 5 |
| NM_021184 | C6orf47 | 2475 | 4 | 2 | 1 | 3 | 2 | 2 | 3 | 2 | 2 | 2 | 1 | 4 | 1 |
| NM_021184 | C6orf47 | 2476 | 4 | 2 | 1 | 3 | 2 | 2 | 3 | 2 | 2 | 2 | 1 | 4 | 1 |
| NM_021185 | CATSPERG | 3724 | 1 | 0 | 0 | 1 | 0 | 0 | 0 | 0 | 0 | 0 | 0 | 0 | 1 |
| NM_021186 | ZP4 | 1635 | 0 | 0 | 0 | 0 | 0 | 0 | 0 | 0 | 0 | 0 | 0 | 0 | 0 |
| NM_021187 | CYP4F11 | 3395 | 15 | 2 | 0 | 1 | 0 | 1 | 1 | 15 | 0 | 0 | 0 | 0 | 0 |
| NM_021188 | ZNF410 | 2178 | 8 | 5 | 4 | 4 | 3 | 5 | 4 | 4 | 4 | 6 | 2 | 6 | 8 |
| NM_021189 | CADM3 | 3698 | 15 | 2 | 2 | 2 | 0 | 15 | 0 | 0 | 0 | 0 | 0 | 1 | 0 |
| NM_021190 | PTBP2 | 3480 | 9 | 3 | 2 | 2 | 2 | 9 | 2 | 1 | 2 | 3 | 3 | 3 | 5 |
| NM_021191 | NEUROD4 | 3956 | 0 | 0 | 0 | 0 | 0 | 0 | 0 | 0 | 0 | 0 | 0 | 0 | 0 |
| NM_021192 | HOXD11 | 1463 | 1 | 0 | 0 | 1 | 0 | 0 | 1 | 0 | 0 | 0 | 0 | 0 | 0 |
| NM_021193 | HOXD12 | 813 | 0 | 0 | 0 | 0 | 0 | 0 | 0 | 0 | 0 | 0 | 0 | 0 | 0 |
| NM_021194 | SLC30A1 | 2018 | 10 | 5 | 4 | 2 | 3 | 4 | 7 | 10 | 3 | 4 | 2 | 9 | 7 |
| NM_021195 | CLDN6 | 1370 | 0 | 0 | 0 | 0 | 0 | 0 | 0 | 0 | 0 | 0 | 0 | 0 | 0 |
| NM_021196 | SLC4A5 | 3449 | 2 | 0 | 0 | 0 | 0 | 0 | 0 | 0 | 0 | 0 | 0 | 0 | 2 |
| NM_021197 | WFDC1 | 1378 | 1 | 0 | 0 | 1 | 0 | 1 | 1 | 0 | 0 | 1 | 0 | 0 | 1 |
| NM_021198 | CTDSP1 | 2638 | 2 | 1 | 1 | 1 | 1 | 0 | 2 | 1 | 1 | 1 | 0 | 1 | 1 |
| NM_021199 | SQRDL | 1672 | 11 | 5 | 5 | 11 | 2 | 2 | 3 | 9 | 6 | 1 | 6 | 6 | 1 |
| NM_021200 | PLEKHB1 | 2380 | 50 | 5 | 0 | 1 | 1 | 50 | 1 | 0 | 1 | 1 | 0 | 1 | 2 |
| NM_021201 | MS4A7 | 3008 | 31 | 5 | 5 | 1 | 4 | 0 | 1 | 2 | 7 | 1 | 1 | 31 | 1 |
| NM_021202 | TP53INP2 | 4124 | 13 | 4 | 1 | 4 | 11 | 13 | 6 | 2 | 2 | 2 | 2 | 1 | 3 |
| NM_021203 | SRPRB | 1745 | 56 | 13 | 10 | 5 | 11 | 12 | 13 | 56 | 6 | 6 | 5 | 11 | 11 |
| NM_021204 | ENOPH1 | 2014 | 27 | 6 | 5 | 3 | 4 | 27 | 6 | 6 | 4 | 4 | 2 | 7 | 3 |
| NM_021205 | RHOU | 4322 | 11 | 5 | 2 | 1 | 4 | 11 | 2 | 7 | 5 | 6 | 3 | 8 | 9 |
| NM_021209 | NLRC4 | 3363 | 5 | 1 | 1 | 0 | 1 | 0 | 0 | 1 | 1 | 0 | 0 | 5 | 0 |
| NM_021210 | TRAPPC1 | 819 | 18 | 9 | 9 | 8 | 10 | 18 | 9 | 10 | 7 | 6 | 5 | 14 | 7 |
| NM_021211 | ZBED5 | 2738 | 10 | 6 | 4 | 2 | 8 | 7 | 8 | 2 | 4 | 10 | 7 | 10 | 7 |
| NM_021213 | PCTP | 2041 | 22 | 5 | 4 | 4 | 4 | 2 | 5 | 22 | 3 | 2 | 2 | 5 | 2 |
| NM_021214 | FAM108C1 | 2360 | 2 | 1 | 0 | 2 | 0 | 2 | 1 | 0 | 1 | 0 | 0 | 1 | 1 |
| NM_021215 | RPRD1B | 3872 | 9 | 3 | 2 | 2 | 3 | 2 | 2 | 9 | 2 | 3 | 3 | 3 | 3 |
| NM_021216 | ZNF71 | 3127 | 0 | 0 | 0 | 0 | 0 | 0 | 0 | 0 | 0 | 0 | 0 | 0 | 0 |
| NM_021217 | ZNF77 | 2040 | 2 | 1 | 1 | 1 | 1 | 2 | 1 | 0 | 1 | 2 | 1 | 1 | 1 |
| NM_021218 | C9orf80 | 1519 | 4 | 2 | 1 | 1 | 2 | 3 | 4 | 1 | 2 | 2 | 1 | 3 | 2 |
| NM_021219 | JAM2 | 1693 | 7 | 2 | 3 | 1 | 3 | 4 | 1 | 0 | 2 | 3 | 1 | 0 | 7 |
| NM_021220 | OVOL2 | 1555 | 1 | 0 | 0 | 1 | 0 | 0 | 0 | 0 | 1 | 0 | 0 | 0 | 1 |

| transcript | gene | transcript length (nt) | max | mean | adipose | colon | heart | hypothalamus | kidney | liver | lung | ovary | skeletalmuscle | spleen | testes |
|---|---|---|---|---|---|---|---|---|---|---|---|---|---|---|---|
| NM_021221 | LY6G5B | 774 | 2 | 1 | 0 | 2 | 0 | 0 | 1 | 0 | 1 | 0 | 0 | 1 | 0 |
| NM_021222 | PRUNE | 2977 | 2 | 1 | 1 | 1 | 2 | 1 | 2 | 0 | 1 | 1 | 1 | 1 | 1 |
| NM_021223 | MYL7 | 597 | 115 | 10 | 0 | 0 | 115 | 0 | 0 | 0 | 0 | 0 | 0 | 0 | 0 |
| NM_021224 | ZNF462 | 8295 | 9 | 4 | 3 | 3 | 3 | 5 | 4 | 0 | 2 | 9 | 7 | 4 | 5 |
| NM_021225 | PROL1 | 1043 | 0 | 0 | 0 | 0 | 0 | 0 | 0 | 0 | 0 | 0 | 0 | 0 | 0 |
| NM_021226 | ARHGAP22 | 2749 | 2 | 0 | 1 | 0 | 0 | 2 | 0 | 0 | 0 | 1 | 0 | 0 | 1 |
| NM_021227 | OSTC | 1080 | 15 | 6 | 2 | 2 | 15 | 2 | 14 | 7 | 11 | 2 | 0 | 3 | 9 |
| NM_021228 | SCAF1 | 4312 | 1 | 1 | 1 | 1 | 0 | 1 | 1 | 0 | 0 | 0 | 1 | 1 | 1 |
| NM_021229 | NTN4 | 3615 | 46 | 10 | 3 | 1 | 11 | 2 | 16 | 5 | 7 | 10 | 1 | 46 | 4 |
| NM_021231 | C19orf29 | 2676 | 1 | 0 | 0 | 1 | 0 | 0 | 0 | 0 | 0 | 0 | 0 | 0 | 1 |
| NM_021232 | PRODH2 | 1677 | 2 | 0 | 0 | 0 | 0 | 0 | 2 | 0 | 0 | 0 | 0 | 0 | 0 |
| NM_021233 | DNASE2B | 1259 | 1 | 0 | 1 | 0 | 0 | 0 | 0 | 0 | 0 | 0 | 0 | 0 | 0 |
| NM_021235 | EPS15L1 | 2774 | 6 | 3 | 2 | 2 | 5 | 4 | 4 | 1 | 2 | 4 | 6 | 3 | 3 |
| NM_021237 | SELK | 818 | 5 | 3 | 3 | 2 | 5 | 4 | 3 | 4 | 3 | 3 | 3 | 3 | 4 |
| NM_021238 | FAM60A | 3107 | 14 | 6 | 2 | 3 | 4 | 2 | 9 | 1 | 7 | 14 | 1 | 13 | 10 |
| NM_021239 | RBM25 | 4301 | 26 | 17 | 19 | 8 | 17 | 24 | 17 | 10 | 16 | 16 | 15 | 22 | 26 |
| NM_021240 | DMRT3 | 2184 | 0 | 0 | 0 | 0 | 0 | 0 | 0 | 0 | 0 | 0 | 0 | 0 | 0 |
| NM_021241 | WIZ | 4168 | 2 | 1 | 1 | 1 | 1 | 2 | 1 | 1 | 1 | 1 | 1 | 1 | 1 |
| NM_021242 | MID1IP1 | 2442 | 7 | 3 | 1 | 1 | 4 | 4 | 2 | 3 | 5 | 2 | 7 | 2 | 4 |
| NM_021243 | C6orf115 | 845 | 29 | 10 | 5 | 9 | 6 | 4 | 15 | 15 | 8 | 1 | 1 | 29 | 11 |
| NM_021244 | RRAGD | 4924 | 14 | 5 | 5 | 1 | 10 | 7 | 8 | 2 | 3 | 1 | 14 | 4 | 3 |
| NM_021245 | MYOZ1 | 1541 | 220 | 20 | 1 | 0 | 1 | 0 | 0 | 0 | 0 | 0 | 220 | 0 | 0 |
| NM_021246 | LY6G6D | 402 | 0 | 0 | 0 | 0 | 0 | 0 | 0 | 0 | 0 | 0 | 0 | 0 | 0 |
| NM_021247 | PRM3 | 309 | 2 | 0 | 0 | 0 | 0 | 0 | 0 | 0 | 0 | 0 | 0 | 0 | 2 |
| NM_021248 | CDH22 | 3657 | 1 | 0 | 0 | 0 | 0 | 1 | 0 | 0 | 0 | 0 | 0 | 0 | 0 |
| NM_021249 | SNX6 | 3080 | 16 | 9 | 12 | 5 | 9 | 8 | 8 | 5 | 8 | 6 | 5 | 16 | 15 |
| NM_021250 | LILRA5 | 1365 | 9 | 1 | 0 | 0 | 1 | 0 | 0 | 0 | 1 | 0 | 0 | 9 | 0 |
| NM_021251 | CAPN10 | 1024 | 1 | 0 | 0 | 0 | 1 | 0 | 0 | 0 | 0 | 0 | 0 | 0 | 0 |
| NM_021252 | RAB18 | 2790 | 8 | 4 | 4 | 2 | 5 | 8 | 6 | 6 | 4 | 2 | 2 | 5 | 5 |
| NM_021253 | TRIM39 | 3338 | 3 | 2 | 2 | 1 | 2 | 2 | 2 | 1 | 2 | 2 | 2 | 2 | 3 |
| NM_021254 | C21orf59 | 1052 | 13 | 7 | 6 | 3 | 7 | 13 | 10 | 8 | 6 | 5 | 6 | 6 | 10 |
| NM_021255 | PELI2 | 5696 | 5 | 2 | 2 | 1 | 1 | 1 | 3 | 0 | 3 | 5 | 1 | 3 | 3 |
| NM_021257 | NGB | 1876 | 0 | 0 | 0 | 0 | 0 | 0 | 0 | 0 | 0 | 0 | 0 | 0 | 0 |
| NM_021258 | IL22RA1 | 2798 | 1 | 0 | 0 | 1 | 0 | 0 | 0 | 0 | 0 | 0 | 0 | 0 | 0 |
| NM_021259 | TMEM8A | 3656 | 2 | 1 | 0 | 1 | 1 | 0 | 2 | 1 | 1 | 0 | 1 | 1 | 2 |
| NM_021260 | ZFYVE1 | 4405 | 2 | 1 | 2 | 1 | 1 | 2 | 2 | 1 | 1 | 1 | 1 | 2 | 2 |
| NM_021267 | LASS1 | 2558 | 0 | 0 | 0 | 0 | 0 | 0 | 0 | 0 | 0 | 0 | 0 | 0 | 0 |
| NM_021268 | IFNA17 | 980 | 0 | 0 | 0 | 0 | 0 | 0 | 0 | 0 | 0 | 0 | 0 | 0 | 0 |
| NM_021269 | ZNF708 | 4003 | 36 | 9 | 3 | 1 | 6 | 13 | 7 | 1 | 5 | 36 | 3 | 12 | 13 |
| NM_021270 | LAIR2 | 648 | 1 | 0 | 0 | 0 | 0 | 0 | 0 | 0 | 1 | 0 | 0 | 1 | 0 |
| NM_021569 | GRIN1 | 4265 | 6 | 1 | 0 | 0 | 0 | 6 | 0 | 0 | 0 | 0 | 0 | 0 | 0 |
| NM_021570 | BARX1 | 1482 | 0 | 0 | 0 | 0 | 0 | 0 | 0 | 0 | 0 | 0 | 0 | 0 | 0 |
| NM_021571 | CARD18 | 658 | 0 | 0 | 0 | 0 | 0 | 0 | 0 | 0 | 0 | 0 | 0 | 0 | 0 |
| NM_021572 | ENPP5 | 2943 | 9 | 2 | 0 | 0 | 1 | 3 | 3 | 0 | 1 | 1 | 1 | 1 | 9 |
| NM_021574 | BCR | 6795 | 2 | 1 | 1 | 1 | 1 | 2 | 1 | 1 | 1 | 0 | 0 | 1 | 2 |
| NM_021575 | AP2S1 | 776 | 2 | 1 | 1 | 1 | 1 | 2 | 1 | 1 | 1 | 0 | 0 | 1 | 0 |
| NM_021599 | ADAMTS2 | 1701 | 0 | 0 | 0 | 0 | 0 | 0 | 0 | 0 | 0 | 0 | 0 | 0 | 0 |
| NM_021601 | CD79A | 1144 | 44 | 4 | 0 | 3 | 0 | 0 | 0 | 0 | 2 | 0 | 0 | 44 | 0 |
| NM_021602 | CD79B | 949 | 4 | 0 | 0 | 0 | 0 | 0 | 0 | 0 | 0 | 0 | 0 | 4 | 0 |
| NM_021603 | FXYD2 | 578 | 41 | 4 | 0 | 0 | 0 | 0 | 41 | 1 | 0 | 0 | 0 | 0 | 0 |
| NM_021614 | KCNN2 | 2515 | 2 | 0 | 0 | 0 | 1 | 2 | 0 | 1 | 0 | 0 | 0 | 0 | 0 |
| NM_021615 | CHST6 | 6885 | 2 | 0 | 0 | 0 | 0 | 2 | 0 | 0 | 0 | 0 | 0 | 0 | 0 |
| NM_021616 | TRIM34 | 2286 | 0 | 0 | 0 | 0 | 0 | 0 | 0 | 0 | 0 | 0 | 0 | 0 | 0 |
| NM_021619 | PRDM12 | 2492 | 0 | 0 | 0 | 0 | 0 | 0 | 0 | 0 | 0 | 0 | 0 | 0 | 0 |
| NM_021620 | PRDM13 | 3204 | 0 | 0 | 0 | 0 | 0 | 0 | 0 | 0 | 0 | 0 | 0 | 0 | 0 |
| NM_021622 | PLEKHA1 | 3693 | 13 | 7 | 4 | 2 | 11 | 7 | 13 | 7 | 8 | 6 | 8 | 6 | 6 |
| NM_021623 | PLEKHA2 | 5641 | 15 | 5 | 3 | 3 | 7 | 5 | 5 | 1 | 7 | 5 | 2 | 15 | 4 |
| NM_021624 | HRH4 | 3667 | 0 | 0 | 0 | 0 | 0 | 0 | 0 | 0 | 0 | 0 | 0 | 0 | 0 |
| NM_021625 | TRPV4 | 3237 | 0 | 0 | 0 | 0 | 0 | 0 | 0 | 0 | 0 | 0 | 0 | 0 | 0 |
| NM_021626 | SCPEP1 | 1945 | 11 | 4 | 11 | 3 | 3 | 2 | 6 | 2 | 8 | 2 | 1 | 6 | 2 |
| NM_021627 | SENP2 | 3194 | 12 | 7 | 4 | 3 | 6 | 12 | 6 | 7 | 4 | 9 | 5 | 7 | 12 |
| NM_021628 | ALOXE3 | 3233 | 0 | 0 | 0 | 0 | 0 | 0 | 0 | 0 | 0 | 0 | 0 | 0 | 0 |
| NM_021629 | GNB4 | 6428 | 11 | 4 | 4 | 1 | 6 | 6 | 2 | 1 | 6 | 4 | 1 | 11 | 3 |
| NM_021630 | PDLIM2 | 1846 | 2 | 1 | 0 | 0 | 1 | 0 | 1 | 0 | 1 | 0 | 1 | 2 | 0 |
| NM_021632 | ZNF350 | 2341 | 5 | 2 | 1 | 0 | 2 | 2 | 2 | 1 | 1 | 5 | 2 | 4 | 3 |
| NM_021633 | KLHL12 | 3338 | 14 | 7 | 6 | 4 | 7 | 13 | 9 | 5 | 5 | 7 | 4 | 9 | 14 |
| NM_021634 | RXFP1 | 2412 | 1 | 0 | 0 | 0 | 0 | 1 | 0 | 0 | 1 | 0 | 0 | 0 | 0 |
| NM_021635 | PBOV1 | 2501 | 0 | 0 | 0 | 0 | 0 | 0 | 0 | 0 | 0 | 0 | 0 | 0 | 0 |
| NM_021636 | LGR6 | 3345 | 0 | 0 | 0 | 0 | 0 | 0 | 0 | 0 | 0 | 0 | 0 | 0 | 0 |
| NM_021637 | TMEM35 | 2068 | 7 | 1 | 0 | 2 | 0 | 7 | 0 | 0 | 0 | 1 | 0 | 0 | 1 |
| NM_021639 | GPBP1L1 | 3736 | 7 | 4 | 3 | 4 | 4 | 3 | 6 | 4 | 5 | 3 | 3 | 7 | 5 |
| NM_021640 | C12orf10 | 1215 | 7 | 2 | 2 | 1 | 2 | 7 | 3 | 3 | 1 | 3 | 1 | 2 | 2 |
| NM_021641 | ADAM12 | 3418 | 8 | 1 | 8 | 0 | 0 | 1 | 0 | 0 | 0 | 2 | 0 | 0 | 0 |
| NM_021642 | FCGR2A | 2425 | 16 | 5 | 6 | 1 | 11 | 5 | 2 | 1 | 13 | 3 | 1 | 16 | 1 |
| NM_021643 | TRIB2 | 4406 | 7 | 3 | 1 | 1 | 3 | 3 | 3 | 1 | 3 | 6 | 1 | 7 | 5 |
| NM_021644 | HNRNPH3 | 2367 | 113 | 75 | 52 | 39 | 90 | 89 | 69 | 27 | 79 | 113 | 69 | 106 | 95 |
| NM_021645 | UTP14C | 5527 | 7 | 5 | 5 | 2 | 5 | 7 | 6 | 6 | 3 | 6 | 3 | 6 | 4 |

| transcript | gene | transcript length (nt) | max | mean | adipose | colon | heart | hypothalamus | kidney | liver | lung | ovary | skeletalmuscle | spleen | testes |
|---|---|---|---|---|---|---|---|---|---|---|---|---|---|---|---|
| NM_021646 | ZNF500 | 3252 | 0 | 0 | 0 | 0 | 0 | 0 | 0 | 0 | 0 | 0 | 0 | 0 | 0 |
| NM_021647 | MFAP3L | 6189 | 7 | 2 | 0 | 0 | 0 | 4 | 6 | 7 | 0 | 0 | 0 | 0 | 1 |
| NM_021648 | TSPYL4 | 4131 | 16 | 3 | 1 | 1 | 4 | 16 | 3 | 0 | 1 | 3 | 1 | 2 | 3 |
| NM_021649 | TICAM2 | 2989 | 0 | 0 | 0 | 0 | 0 | 0 | 0 | 0 | 0 | 0 | 0 | 0 | 0 |
| NM_021705 | NFYA | 6131 | 4 | 2 | 1 | 1 | 2 | 2 | 2 | 1 | 2 | 4 | 2 | 3 | 3 |
| NM_021706 | LAIR1 | 2765 | 3 | 1 | 3 | 0 | 1 | 1 | 0 | 0 | 1 | 0 | 0 | 3 | 1 |
| NM_021709 | SIVA1 | 576 | 1 | 1 | 1 | 0 | 1 | 1 | 0 | 0 | 0 | 0 | 0 | 1 | 0 |
| NM_021721 | ADAM22 | 2636 | 9 | 1 | 1 | 0 | 0 | 9 | 1 | 0 | 0 | 1 | 0 | 0 | 1 |
| NM_021722 | ADAM22 | 3381 | 10 | 2 | 2 | 0 | 0 | 10 | 1 | 0 | 0 | 1 | 0 | 1 | 1 |
| NM_021723 | ADAM22 | 3443 | 10 | 2 | 2 | 0 | 0 | 10 | 1 | 0 | 0 | 1 | 0 | 1 | 1 |
| NM_021724 | NR1D1 | 2766 | 31 | 9 | 7 | 10 | 3 | 6 | 6 | 4 | 10 | 13 | 31 | 2 | 8 |
| NM_021727 | FADS3 | 1779 | 4 | 1 | 4 | 1 | 3 | 1 | 1 | 0 | 1 | 0 | 0 | 1 | 1 |
| NM_021728 | OTX2 | 2196 | 1 | 0 | 0 | 0 | 0 | 1 | 0 | 0 | 0 | 0 | 0 | 0 | 0 |
| NM_021729 | VPS11 | 3222 | 3 | 2 | 2 | 1 | 1 | 3 | 1 | 1 | 1 | 2 | 1 | 2 | 2 |
| NM_021731 | C19orf28 | 2284 | 2 | 0 | 1 | 0 | 0 | 0 | 2 | 0 | 0 | 0 | 0 | 0 | 0 |
| NM_021732 | AVPI1 | 1392 | 2 | 1 | 1 | 1 | 0 | 1 | 0 | 2 | 1 | 2 | 1 | 2 | 1 |
| NM_021733 | TSKS | 1812 | 1 | 0 | 0 | 0 | 0 | 0 | 0 | 0 | 0 | 0 | 0 | 0 | 1 |
| NM_021734 | SLC25A19 | 1642 | 2 | 1 | 0 | 1 | 1 | 1 | 0 | 0 | 0 | 0 | 0 | 1 | 2 |
| NM_021735 | CLCN6 | 1076 | 6 | 2 | 2 | 2 | 2 | 6 | 1 | 1 | 1 | 2 | 1 | 1 | 1 |
| NM_021736 | CLCN6 | 1175 | 6 | 2 | 2 | 2 | 2 | 6 | 1 | 1 | 1 | 2 | 1 | 1 | 1 |
| NM_021737 | CLCN6 | 1040 | 7 | 2 | 2 | 2 | 2 | 7 | 1 | 1 | 1 | 2 | 1 | 1 | 1 |
| NM_021738 | SVIL | 8297 | 212 | 35 | 7 | 34 | 50 | 5 | 9 | 2 | 9 | 36 | 212 | 5 | 20 |
| NM_021777 | ADAM28 | 2108 | 7 | 2 | 1 | 4 | 0 | 2 | 1 | 0 | 2 | 0 | 0 | 7 | 1 |
| NM_021783 | EDA2R | 3369 | 1 | 0 | 0 | 0 | 0 | 0 | 0 | 0 | 0 | 0 | 1 | 0 | 1 |
| NM_021784 | FOXA2 | 2423 | 1 | 0 | 0 | 0 | 0 | 0 | 0 | 1 | 1 | 0 | 0 | 0 | 0 |
| NM_021785 | RAI2 | 2226 | 2 | 1 | 1 | 1 | 2 | 1 | 1 | 0 | 1 | 2 | 0 | 1 | 1 |
| NM_021794 | ADAM30 | 2958 | 2 | 0 | 0 | 0 | 0 | 0 | 0 | 0 | 0 | 0 | 0 | 0 | 2 |
| NM_021795 | ELK4 | 3028 | 5 | 2 | 1 | 1 | 2 | 1 | 2 | 1 | 2 | 2 | 2 | 5 | 1 |
| NM_021796 | PLAC1 | 1126 | 0 | 0 | 0 | 0 | 0 | 0 | 0 | 0 | 0 | 0 | 0 | 0 | 0 |
| NM_021797 | CHIA | 1360 | 0 | 0 | 0 | 0 | 0 | 0 | 0 | 0 | 0 | 0 | 0 | 0 | 0 |
| NM_021798 | IL21R | 3248 | 1 | 0 | 0 | 0 | 0 | 0 | 0 | 0 | 0 | 0 | 0 | 1 | 0 |
| NM_021800 | DNAJC12 | 1212 | 16 | 5 | 0 | 1 | 4 | 16 | 4 | 15 | 0 | 2 | 2 | 0 | 4 |
| NM_021801 | MMP26 | 991 | 0 | 0 | 0 | 0 | 0 | 0 | 0 | 0 | 0 | 0 | 0 | 0 | 0 |
| NM_021803 | IL21 | 616 | 0 | 0 | 0 | 0 | 0 | 0 | 0 | 0 | 0 | 0 | 0 | 0 | 0 |
| NM_021804 | ACE2 | 3507 | 50 | 8 | 1 | 2 | 7 | 0 | 25 | 1 | 1 | 1 | 0 | 0 | 50 |
| NM_021805 | SIGIRR | 1639 | 1 | 0 | 0 | 0 | 0 | 0 | 1 | 0 | 0 | 0 | 0 | 0 | 0 |
| NM_021806 | FAM3A | 1606 | 1 | 0 | 0 | 0 | 0 | 0 | 1 | 0 | 0 | 0 | 0 | 0 | 0 |
| NM_021807 | EXOC4 | 4192 | 7 | 4 | 4 | 5 | 3 | 4 | 4 | 2 | 4 | 6 | 4 | 7 | 5 |
| NM_021808 | GALNT9 | 1727 | 1 | 0 | 0 | 0 | 0 | 1 | 0 | 0 | 0 | 0 | 0 | 0 | 0 |
| NM_021809 | TGIF2 | 3405 | 3 | 1 | 1 | 1 | 1 | 1 | 1 | 1 | 2 | 3 | 0 | 2 | 2 |
| NM_021810 | CDH26 | 1089 | 0 | 0 | 0 | 0 | 0 | 0 | 0 | 0 | 0 | 0 | 0 | 0 | 0 |
| NM_021813 | BACH2 | 9214 | 1 | 0 | 0 | 0 | 0 | 0 | 0 | 0 | 0 | 1 | 0 | 1 | 0 |
| NM_021814 | ELOVL5 | 2965 | 138 | 47 | 138 | 8 | 31 | 49 | 23 | 50 | 39 | 24 | 4 | 48 | 98 |
| NM_021815 | SLC5A7 | 5145 | 0 | 0 | 0 | 0 | 0 | 0 | 0 | 0 | 0 | 0 | 0 | 0 | 0 |
| NM_021817 | HAPLN2 | 1771 | 2 | 0 | 0 | 0 | 0 | 2 | 0 | 0 | 0 | 0 | 0 | 0 | 0 |
| NM_021818 | SAV1 | 3031 | 6 | 4 | 3 | 1 | 4 | 2 | 3 | 1 | 5 | 6 | 3 | 6 | 6 |
| NM_021819 | LMAN1L | 1736 | 0 | 0 | 0 | 0 | 0 | 0 | 0 | 0 | 0 | 0 | 0 | 0 | 0 |
| NM_021821 | MRPS35 | 1865 | 7 | 3 | 2 | 2 | 5 | 4 | 7 | 7 | 1 | 2 | 3 | 3 | 3 |
| NM_021822 | APOBEC3G | 1818 | 7 | 2 | 1 | 1 | 1 | 0 | 1 | 0 | 2 | 3 | 0 | 5 | 7 |
| NM_021823 | PPCDC | 2219 | 0 | 0 | 0 | 0 | 0 | 0 | 0 | 0 | 0 | 0 | 0 | 0 | 0 |
| NM_021824 | NIF3L1 | 1631 | 2 | 1 | 1 | 1 | 1 | 1 | 1 | 1 | 1 | 1 | 1 | 2 | 1 |
| NM_021825 | CCDC90B | 1578 | 49 | 22 | 19 | 5 | 25 | 28 | 19 | 17 | 13 | 27 | 12 | 30 | 49 |
| NM_021826 | FASTKD5 | 2863 | 8 | 4 | 2 | 2 | 6 | 3 | 6 | 8 | 2 | 3 | 5 | 5 | 4 |
| NM_021827 | CCDC81 | 2510 | 3 | 1 | 0 | 0 | 0 | 1 | 0 | 0 | 1 | 0 | 0 | 0 | 3 |
| NM_021828 | HPSE2 | 4348 | 4 | 1 | 1 | 1 | 0 | 4 | 0 | 0 | 1 | 0 | 0 | 0 | 1 |
| NM_021830 | C10orf2 | 3621 | 6 | 3 | 1 | 2 | 3 | 3 | 3 | 3 | 1 | 3 | 4 | 5 | 6 |
| NM_021831 | AGBL5 | 3237 | 8 | 1 | 1 | 1 | 0 | 1 | 2 | 1 | 0 | 1 | 0 | 1 | 8 |
| NM_021833 | UCP1 | 1000 | 0 | 0 | 0 | 0 | 0 | 0 | 0 | 0 | 0 | 0 | 0 | 0 | 0 |
| NM_021870 | FGG | 1761 | 4,140 | 385 | 0 | 7 | 9 | 0 | 4 | 4,140 | 15 | 15 | 8 | 19 | 14 |
| NM_021871 | FGA | 2210 | 14,697 | 1,362 | 0 | 30 | 39 | 0 | 15 | 14,697 | 18 | 52 | 30 | 62 | 41 |
| NM_021872 | CDC25B | 3551 | 3 | 1 | 1 | 1 | 1 | 1 | 1 | 0 | 3 | 1 | 1 | 2 | 1 |
| NM_021873 | CDC25B | 3674 | 3 | 1 | 1 | 1 | 1 | 1 | 1 | 0 | 3 | 1 | 1 | 2 | 1 |
| NM_021902 | FXYD1 | 513 | 19 | 5 | 1 | 2 | 12 | 4 | 1 | 6 | 3 | 4 | 19 | 1 | 1 |
| NM_021903 | GABBR1 | 4287 | 13 | 2 | 0 | 1 | 1 | 13 | 0 | 0 | 1 | 1 | 0 | 1 | 0 |
| NM_021904 | GABBR1 | 4351 | 13 | 2 | 0 | 1 | 1 | 13 | 0 | 0 | 1 | 1 | 0 | 1 | 1 |
| NM_021907 | DTNB | 2474 | 11 | 2 | 1 | 2 | 1 | 11 | 3 | 1 | 2 | 3 | 1 | 2 | 2 |
| NM_021908 | ST7 | 2899 | 1 | 1 | 1 | 0 | 1 | 1 | 1 | 1 | 1 | 1 | 1 | 1 | 1 |
| NM_021910 | FXYD3 | 1534 | 10 | 1 | 0 | 10 | 0 | 0 | 0 | 0 | 1 | 0 | 0 | 0 | 0 |
| NM_021911 | GABRB2 | 7409 | 20 | 2 | 1 | 0 | 0 | 20 | 1 | 0 | 0 | 0 | 0 | 3 | 0 |
| NM_021912 | GABRB3 | 5724 | 9 | 1 | 0 | 0 | 0 | 9 | 1 | 0 | 0 | 0 | 0 | 0 | 0 |
| NM_021913 | AXL | 4737 | 5 | 2 | 3 | 2 | 3 | 3 | 1 | 1 | 3 | 2 | 1 | 5 | 4 |
| NM_021914 | CFL2 | 3125 | 30 | 9 | 4 | 2 | 28 | 7 | 3 | 12 | 3 | 4 | 30 | 3 | 5 |
| NM_021915 | ZNF69 | 1659 | 1 | 0 | 0 | 0 | 0 | 0 | 1 | 0 | 0 | 1 | 0 | 1 | 1 |
| NM_021916 | ZNF70 | 4017 | 3 | 1 | 1 | 1 | 1 | 1 | 1 | 0 | 0 | 3 | 1 | 1 | 1 |
| NM_021920 | SCT | 514 | 0 | 0 | 0 | 0 | 0 | 0 | 0 | 0 | 0 | 0 | 0 | 0 | 0 |
| NM_021922 | FANCE | 2555 | 2 | 1 | 1 | 1 | 0 | 0 | 0 | 0 | 0 | 0 | 2 | 1 | 0 |
| NM_021923 | FGFRL1 | 3088 | 5 | 2 | 2 | 1 | 3 | 2 | 3 | 5 | 1 | 3 | 1 | 1 | 1 |

| transcript | gene | transcript length (nt) | max | mean | adipose | colon | heart | hypothalamus | kidney | liver | lung | ovary | skeletalmuscle | spleen | testes |
|---|---|---|---|---|---|---|---|---|---|---|---|---|---|---|---|
| NM_021924 | MUPCDH | 3419 | 5 | 1 | 0 | 5 | 0 | 0 | 5 | 3 | 0 | 0 | 0 | 0 | 0 |
| NM_021925 | C2orf43 | 2856 | 4 | 2 | 2 | 1 | 1 | 2 | 4 | 3 | 1 | 2 | 0 | 4 | 3 |
| NM_021926 | ALX4 | 5466 | 0 | 0 | 0 | 0 | 0 | 0 | 0 | 0 | 0 | 0 | 0 | 0 | 0 |
| NM_021927 | GUF1 | 4216 | 3 | 2 | 1 | 0 | 2 | 2 | 3 | 2 | 1 | 3 | 3 | 2 | 3 |
| NM_021928 | SPCS3 | 4594 | 4 | 2 | 1 | 1 | 3 | 2 | 3 | 2 | 2 | 1 | 1 | 4 | 1 |
| NM_021930 | RINT1 | 2976 | 2 | 2 | 2 | 1 | 2 | 2 | 2 | 2 | 1 | 2 | 2 | 2 | 2 |
| NM_021931 | DHX35 | 3301 | 12 | 5 | 2 | 4 | 6 | 3 | 6 | 2 | 3 | 6 | 5 | 8 | 12 |
| NM_021932 | RIC8A | 2699 | 4 | 2 | 2 | 2 | 2 | 3 | 3 | 2 | 2 | 1 | 1 | 4 | 3 |
| NM_021933 | MIIP | 1588 | 1 | 0 | 0 | 0 | 0 | 0 | 0 | 0 | 0 | 0 | 0 | 1 | 0 |
| NM_021934 | C12orf44 | 1437 | 1 | 1 | 1 | 1 | 1 | 1 | 1 | 1 | 1 | 0 | 0 | 1 | 1 |
| NM_021935 | PROK2 | 1550 | 0 | 0 | 0 | 0 | 0 | 0 | 0 | 0 | 0 | 0 | 0 | 0 | 0 |
| NM_021936 | PAPPA2 | 4370 | 1 | 0 | 0 | 0 | 0 | 1 | 1 | 0 | 0 | 0 | 0 | 0 | 0 |
| NM_021937 | EEFSEC | 2216 | 0 | 0 | 0 | 0 | 0 | 0 | 0 | 0 | 0 | 0 | 0 | 0 | 0 |
| NM_021938 | BRUNOL5 | 1853 | 2 | 0 | 0 | 0 | 0 | 2 | 0 | 0 | 0 | 0 | 0 | 0 | 0 |
| NM_021939 | FKBP10 | 2883 | 2 | 1 | 1 | 1 | 2 | 1 | 0 | 0 | 1 | 2 | 0 | 1 | 1 |
| NM_021940 | SMAP1 | 3250 | 6 | 2 | 2 | 1 | 2 | 6 | 1 | 1 | 1 | 1 | 2 | 2 | 2 |
| NM_021942 | C4orf41 | 4526 | 5 | 4 | 3 | 2 | 3 | 5 | 4 | 3 | 3 | 5 | 3 | 5 | 4 |
| NM_021943 | ZFAND3 | 3331 | 8 | 6 | 5 | 3 | 6 | 7 | 6 | 5 | 4 | 5 | 8 | 6 | 8 |
| NM_021944 | C14orf93 | 2360 | 2 | 1 | 1 | 1 | 0 | 1 | 1 | 0 | 1 | 2 | 1 | 1 | 1 |
| NM_021945 | SLC22A23 | 5899 | 2 | 1 | 1 | 2 | 0 | 2 | 1 | 1 | 1 | 1 | 1 | 2 | 1 |
| NM_021946 | BCORL1 | 6740 | 4 | 1 | 0 | 1 | 1 | 1 | 1 | 0 | 1 | 1 | 1 | 2 | 4 |
| NM_021947 | SRR | 2477 | 1 | 0 | 0 | 0 | 0 | 1 | 0 | 0 | 0 | 0 | 0 | 0 | 0 |
| NM_021948 | BCAN | 3466 | 9 | 1 | 0 | 0 | 0 | 9 | 0 | 0 | 0 | 0 | 0 | 0 | 0 |
| NM_021949 | ATP2B3 | 6574 | 3 | 0 | 0 | 0 | 0 | 3 | 0 | 0 | 0 | 0 | 0 | 0 | 0 |
| NM_021950 | MS4A1 | 3331 | 12 | 1 | 0 | 0 | 0 | 0 | 0 | 0 | 0 | 0 | 0 | 12 | 0 |
| NM_021951 | DMRT1 | 2222 | 2 | 0 | 0 | 0 | 0 | 0 | 0 | 0 | 0 | 0 | 0 | 0 | 2 |
| NM_021952 | ELAVL4 | 2101 | 8 | 1 | 0 | 0 | 0 | 8 | 0 | 0 | 0 | 0 | 0 | 0 | 1 |
| NM_021953 | FOXM1 | 3512 | 1 | 0 | 0 | 1 | 0 | 0 | 0 | 0 | 0 | 0 | 0 | 0 | 1 |
| NM_021954 | GJA3 | 5205 | 2 | 0 | 0 | 0 | 2 | 0 | 0 | 0 | 0 | 0 | 0 | 0 | 0 |
| NM_021955 | GNGT1 | 628 | 0 | 0 | 0 | 0 | 0 | 0 | 0 | 0 | 0 | 0 | 0 | 0 | 0 |
| NM_021956 | GRIK2 | 4591 | 5 | 1 | 0 | 0 | 1 | 5 | 0 | 0 | 0 | 0 | 0 | 0 | 1 |
| NM_021957 | GYS2 | 3132 | 23 | 2 | 1 | 0 | 0 | 0 | 0 | 23 | 0 | 0 | 0 | 0 | 0 |
| NM_021958 | HLX | 2278 | 3 | 2 | 2 | 3 | 1 | 1 | 0 | 2 | 2 | 1 | 1 | 2 | 1 |
| NM_021959 | PPP1R11 | 1662 | 24 | 17 | 12 | 12 | 19 | 24 | 20 | 18 | 15 | 17 | 13 | 23 | 17 |
| NM_021960 | MCL1 | 3999 | 31 | 16 | 12 | 8 | 16 | 10 | 11 | 25 | 25 | 19 | 14 | 31 | 10 |
| NM_021961 | TEAD1 | 9431 | 11 | 5 | 5 | 2 | 9 | 3 | 5 | 4 | 4 | 7 | 11 | 2 | 4 |
| NM_021962 | ABR | 5251 | 30 | 6 | 3 | 11 | 7 | 30 | 2 | 1 | 4 | 3 | 1 | 7 | 2 |
| NM_021963 | NAP1L2 | 2531 | 45 | 6 | 1 | 1 | 3 | 45 | 5 | 0 | 1 | 3 | 1 | 3 | 2 |
| NM_021964 | ZNF148 | 9543 | 8 | 5 | 3 | 2 | 4 | 6 | 4 | 4 | 4 | 8 | 4 | 6 | 6 |
| NM_021965 | PGM5 | 3338 | 10 | 3 | 1 | 6 | 10 | 0 | 1 | 1 | 1 | 2 | 3 | 1 | 2 |
| NM_021966 | TCL1A | 1396 | 1 | 0 | 0 | 0 | 0 | 0 | 0 | 0 | 0 | 0 | 0 | 1 | 0 |
| NM_021967 | SERF1A | 1912 | 0 | 0 | 0 | 0 | 0 | 0 | 0 | 0 | 0 | 0 | 0 | 0 | 0 |
| NM_021968 | HIST1H4J | 356 | 0 | 0 | 0 | 0 | 0 | 0 | 0 | 0 | 0 | 0 | 0 | 0 | 0 |
| NM_021969 | NR0B2 | 1270 | 0 | 0 | 0 | 0 | 0 | 0 | 0 | 0 | 0 | 0 | 0 | 0 | 0 |
| NM_021970 | MAPKSP1 | 4280 | 5 | 3 | 2 | 1 | 3 | 5 | 4 | 2 | 2 | 4 | 2 | 3 | 3 |
| NM_021971 | GMPPB | 1561 | 1 | 0 | 0 | 1 | 0 | 0 | 0 | 1 | 1 | 0 | 0 | 0 | 0 |
| NM_021972 | SPHK1 | 1863 | 0 | 0 | 0 | 0 | 0 | 0 | 0 | 0 | 0 | 0 | 0 | 0 | 0 |
| NM_021973 | HAND2 | 2368 | 10 | 2 | 0 | 4 | 10 | 0 | 0 | 1 | 0 | 4 | 0 | 1 | 1 |
| NM_021974 | POLR2F | 2101 | 7 | 4 | 4 | 4 | 5 | 7 | 5 | 7 | 4 | 2 | 2 | 4 | 3 |
| NM_021975 | RELA | 2577 | 2 | 1 | 1 | 1 | 1 | 1 | 1 | 2 | 1 | 2 | 2 | 2 | 1 |
| NM_021976 | RXRB | 2875 | 1 | 1 | 1 | 1 | 1 | 1 | 1 | 1 | 1 | 1 | 0 | 1 | 1 |
| NM_021976 | RXRB | 2876 | 1 | 1 | 1 | 1 | 1 | 1 | 1 | 1 | 1 | 1 | 0 | 1 | 1 |
| NM_021977 | SLC22A3 | 5624 | 3 | 1 | 1 | 0 | 0 | 0 | 0 | 2 | 1 | 1 | 3 | 0 | 1 |
| NM_021978 | ST14 | 3300 | 6 | 1 | 1 | 6 | 0 | 0 | 2 | 0 | 1 | 0 | 0 | 0 | 0 |
| NM_021979 | HSPA2 | 2769 | 63 | 15 | 1 | 9 | 7 | 63 | 14 | 1 | 3 | 4 | 23 | 14 | 27 |
| NM_021980 | OPTN | 3376 | 202 | 44 | 33 | 9 | 55 | 31 | 24 | 16 | 18 | 36 | 202 | 22 | 36 |
| NM_021982 | SEC24A | 6387 | 6 | 2 | 2 | 1 | 2 | 1 | 2 | 6 | 2 | 2 | 2 | 3 | 2 |
| NM_021988 | UBE2V1 | 2539 | 0 | 0 | 0 | 0 | 0 | 0 | 0 | 0 | 0 | 0 | 0 | 0 | 0 |
| NM_021991 | JUP | 3211 | 2 | 1 | 0 | 1 | 1 | 0 | 2 | 1 | 1 | 0 | 0 | 0 | 0 |
| NM_021992 | TMSB15A | 666 | 1 | 0 | 0 | 0 | 0 | 0 | 0 | 0 | 1 | 0 | 0 | 0 | 0 |
| NM_021994 | ZNF277 | 2687 | 3 | 2 | 1 | 1 | 2 | 2 | 3 | 2 | 3 | 3 | 2 | 3 | 2 |
| NM_021995 | UTS2 | 633 | 1 | 0 | 0 | 0 | 0 | 0 | 0 | 0 | 1 | 0 | 0 | 0 | 0 |
| NM_021996 | GBGT1 | 1949 | 1 | 0 | 0 | 1 | 0 | 0 | 0 | 0 | 0 | 0 | 0 | 1 | 0 |
| NM_021998 | ZNF711 | 4168 | 20 | 3 | 0 | 0 | 1 | 5 | 2 | 0 | 0 | 3 | 0 | 1 | 20 |
| NM_021999 | ITM2B | 1896 | 275 | 114 | 104 | 31 | 119 | 141 | 275 | 61 | 113 | 138 | 41 | 148 | 86 |
| NM_022002 | NR1I2 | 2743 | 5 | 1 | 0 | 1 | 0 | 0 | 0 | 5 | 0 | 0 | 0 | 0 | 0 |
| NM_022003 | FXYD6 | 2054 | 14 | 3 | 1 | 2 | 1 | 14 | 4 | 1 | 1 | 5 | 0 | 3 | 4 |
| NM_022006 | FXYD7 | 713 | 3 | 0 | 0 | 0 | 0 | 3 | 0 | 0 | 0 | 0 | 0 | 0 | 0 |
| NM_022034 | CUZD1 | 2276 | 0 | 0 | 0 | 0 | 0 | 0 | 0 | 0 | 0 | 0 | 0 | 0 | 0 |
| NM_022036 | GPRC5C | 2373 | 1 | 0 | 0 | 0 | 0 | 0 | 1 | 1 | 0 | 0 | 0 | 0 | 0 |
| NM_022037 | TIA1 | 4620 | 4 | 2 | 1 | 1 | 2 | 1 | 3 | 1 | 2 | 4 | 1 | 4 | 2 |
| NM_022039 | FBXW4 | 2212 | 10 | 8 | 9 | 9 | 9 | 10 | 8 | 5 | 9 | 4 | 7 | 6 | 8 |
| NM_022041 | GAN | 4542 | 6 | 2 | 1 | 2 | 2 | 6 | 1 | 1 | 1 | 2 | 1 | 1 | 1 |
| NM_022042 | SLC26A1 | 3404 | 0 | 0 | 0 | 0 | 0 | 0 | 0 | 0 | 0 | 0 | 0 | 0 | 0 |
| NM_022044 | SDF2L1 | 874 | 5 | 2 | 1 | 0 | 3 | 1 | 2 | 5 | 2 | 0 | 0 | 4 | 1 |
| NM_022045 | MTBP | 3059 | 2 | 1 | 0 | 0 | 0 | 0 | 0 | 0 | 0 | 2 | 1 | 1 | 2 |
| NM_022046 | KLK14 | 1133 | 0 | 0 | 0 | 0 | 0 | 0 | 0 | 0 | 0 | 0 | 0 | 0 | 0 |

| | | transcript | | | | | | hypothala | | | | | skeletalm | | |
|---|---|---|---|---|---|---|---|---|---|---|---|---|---|---|---|
| transcript | gene | length (nt) | max | mean | adipose | colon | heart | mus | kidney | liver | lung | ovary | uscle | spleen | testes |
| NM_022047 | DEF6 | 2296 | 3 | 1 | 0 | 1 | 0 | 0 | 0 | 0 | 1 | 0 | 0 | 3 | 0 |
| NM_022048 | CSNK1G1 | 8163 | 3 | 2 | 1 | 1 | 1 | 2 | 2 | 1 | 1 | 3 | 1 | 3 | 2 |
| NM_022049 | GPR88 | 3465 | 1 | 0 | 0 | 0 | 0 | 1 | 0 | 0 | 0 | 0 | 0 | 1 | 0 |
| NM_022051 | EGLN1 | 7096 | 26 | 6 | 2 | 1 | 9 | 3 | 4 | 3 | 2 | 4 | 26 | 4 | 4 |
| NM_022052 | NXF3 | 1895 | 4 | 0 | 0 | 0 | 0 | 0 | 0 | 0 | 0 | 0 | 0 | 0 | 4 |
| NM_022053 | NXF2 | 2322 | 0 | 0 | 0 | 0 | 0 | 0 | 0 | 0 | 0 | 0 | 0 | 0 | 0 |
| NM_022054 | KCNK13 | 2517 | 1 | 0 | 0 | 0 | 0 | 0 | 0 | 0 | 0 | 0 | 0 | 0 | 1 |
| NM_022055 | KCNK12 | 1424 | 0 | 0 | 0 | 0 | 0 | 0 | 0 | 0 | 0 | 0 | 0 | 0 | 0 |
| NM_022058 | SLC4A10 | 5575 | 24 | 2 | 0 | 1 | 0 | 24 | 0 | 0 | 0 | 0 | 0 | 0 | 0 |
| NM_022059 | CXCL16 | 2324 | 12 | 4 | 4 | 1 | 2 | 2 | 2 | 3 | 8 | 2 | 0 | 12 | 3 |
| NM_022060 | ABHD4 | 2509 | 8 | 3 | 3 | 1 | 3 | 2 | 3 | 2 | 2 | 3 | 2 | 3 | 8 |
| NM_022061 | MRPL17 | 2366 | 6 | 4 | 3 | 2 | 6 | 5 | 6 | 4 | 3 | 3 | 3 | 6 | 4 |
| NM_022062 | PKNOX2 | 3730 | 4 | 1 | 0 | 0 | 1 | 3 | 0 | 0 | 1 | 4 | 2 | 1 | 1 |
| NM_022063 | C10orf84 | 2766 | 11 | 7 | 10 | 3 | 6 | 11 | 7 | 7 | 4 | 6 | 6 | 8 | 8 |
| NM_022064 | RNF123 | 4255 | 1 | 1 | 0 | 0 | 0 | 1 | 1 | 1 | 0 | 0 | 1 | 1 | 0 |
| NM_022065 | THADA | 6114 | 3 | 2 | 2 | 1 | 2 | 1 | 2 | 3 | 2 | 3 | 1 | 3 | 3 |
| NM_022066 | UBE2O | 5355 | 3 | 1 | 0 | 1 | 1 | 3 | 1 | 0 | 0 | 1 | 1 | 1 | 1 |
| NM_022067 | C14orf133 | 2761 | 2 | 1 | 1 | 1 | 1 | 1 | 1 | 1 | 1 | 1 | 1 | 1 | 2 |
| NM_022068 | FAM38B | 9713 | 2 | 1 | 0 | 1 | 0 | 1 | 0 | 1 | 2 | 1 | 0 | 2 | 0 |
| NM_022070 | HEATR6 | 3935 | 2 | 1 | 1 | 1 | 1 | 2 | 1 | 0 | 1 | 2 | 1 | 1 | 2 |
| NM_022071 | SH2D4A | 2860 | 5 | 2 | 2 | 2 | 2 | 1 | 3 | 5 | 2 | 4 | 0 | 1 | 1 |
| NM_022072 | NSUN3 | 1435 | 3 | 1 | 0 | 0 | 1 | 1 | 1 | 1 | 1 | 1 | 0 | 3 | 2 |
| NM_022073 | EGLN3 | 2722 | 13 | 2 | 0 | 2 | 13 | 4 | 2 | 0 | 1 | 0 | 1 | 1 | 1 |
| NM_022074 | FAM111A | 3592 | 5 | 2 | 4 | 1 | 2 | 1 | 3 | 1 | 4 | 2 | 1 | 5 | 3 |
| NM_022075 | LASS2 | 2465 | 23 | 7 | 3 | 3 | 4 | 5 | 16 | 23 | 7 | 4 | 1 | 5 | 4 |
| NM_022076 | DUSP21 | 884 | 0 | 0 | 0 | 0 | 0 | 0 | 0 | 0 | 0 | 0 | 0 | 0 | 0 |
| NM_022077 | MANBAL | 1315 | 4 | 2 | 2 | 2 | 2 | 4 | 3 | 2 | 1 | 1 | 0 | 2 | 3 |
| NM_022078 | GPATCH3 | 2128 | 1 | 1 | 1 | 1 | 1 | 1 | 1 | 1 | 1 | 1 | 1 | 1 | 1 |
| NM_022079 | HERC4 | 4517 | 10 | 6 | 4 | 3 | 8 | 6 | 6 | 3 | 7 | 6 | 4 | 10 | 8 |
| NM_022080 | NAPB | 3870 | 57 | 7 | 1 | 2 | 1 | 57 | 1 | 1 | 2 | 2 | 1 | 2 | 3 |
| NM_022081 | HPS4 | 4539 | 3 | 2 | 2 | 1 | 1 | 2 | 2 | 1 | 1 | 3 | 1 | 2 | 3 |
| NM_022082 | SLC17A9 | 2592 | 1 | 0 | 0 | 0 | 0 | 0 | 0 | 0 | 0 | 0 | 0 | 1 | 0 |
| NM_022087 | GALNT11 | 2722 | 11 | 3 | 2 | 1 | 1 | 6 | 11 | 1 | 2 | 2 | 1 | 2 | 1 |
| NM_022088 | ZFP64 | 3101 | 1 | 1 | 1 | 1 | 0 | 1 | 1 | 0 | 0 | 1 | 0 | 1 | 1 |
| NM_022089 | ATP13A2 | 3996 | 2 | 0 | 0 | 0 | 0 | 2 | 0 | 0 | 0 | 0 | 0 | 1 | 0 |
| NM_022090 | C5orf54 | 2807 | 1 | 0 | 0 | 0 | 0 | 0 | 0 | 0 | 0 | 1 | 0 | 0 | 1 |
| NM_022091 | ASCC3 | 3470 | 3 | 1 | 1 | 0 | 1 | 1 | 2 | 2 | 2 | 1 | 1 | 3 | 2 |
| NM_022092 | CHTF18 | 3090 | 0 | 0 | 0 | 0 | 0 | 0 | 0 | 0 | 0 | 0 | 0 | 0 | 0 |
| NM_022093 | TNN | 5008 | 1 | 0 | 1 | 0 | 0 | 0 | 0 | 0 | 0 | 0 | 0 | 0 | 0 |
| NM_022094 | CIDEC | 1277 | 20 | 2 | 20 | 0 | 0 | 0 | 0 | 0 | 0 | 0 | 0 | 0 | 0 |
| NM_022095 | ZNF335 | 4452 | 0 | 0 | 0 | 0 | 0 | 0 | 0 | 0 | 0 | 0 | 0 | 0 | 0 |
| NM_022096 | ANKRD5 | 3823 | 5 | 1 | 1 | 0 | 0 | 1 | 1 | 1 | 0 | 1 | 0 | 1 | 5 |
| NM_022097 | CHP2 | 2366 | 13 | 1 | 0 | 13 | 0 | 0 | 0 | 0 | 0 | 0 | 0 | 0 | 2 |
| NM_022098 | XPNPEP3 | 3057 | 5 | 3 | 3 | 2 | 4 | 3 | 3 | 4 | 1 | 2 | 2 | 2 | 5 |
| NM_022100 | MRPS14 | 1081 | 3 | 2 | 2 | 1 | 2 | 3 | 2 | 2 | 1 | 1 | 1 | 2 | 1 |
| NM_022101 | CXorf56 | 904 | 10 | 5 | 6 | 3 | 3 | 10 | 4 | 3 | 3 | 5 | 5 | 5 | 5 |
| NM_022103 | ZNF667 | 3860 | 4 | 1 | 1 | 0 | 1 | 4 | 1 | 0 | 0 | 2 | 0 | 1 | 1 |
| NM_022104 | PCIF1 | 2697 | 1 | 1 | 0 | 1 | 1 | 1 | 1 | 0 | 1 | 1 | 1 | 1 | 0 |
| NM_022105 | DIDO1 | 2798 | 12 | 9 | 8 | 11 | 6 | 8 | 8 | 6 | 8 | 12 | 7 | 12 | 11 |
| NM_022106 | C20orf177 | 3800 | 3 | 1 | 0 | 0 | 1 | 3 | 1 | 0 | 1 | 1 | 1 | 1 | 1 |
| NM_022107 | GPSM3 | 1456 | 11 | 2 | 1 | 1 | 1 | 1 | 0 | 1 | 3 | 0 | 0 | 11 | 0 |
| NM_022110 | FKBPL | 1344 | 1 | 1 | 0 | 1 | 0 | 1 | 0 | 1 | 0 | 1 | 1 | 1 | 0 |
| NM_022111 | CLSPN | 4379 | 6 | 1 | 2 | 1 | 1 | 1 | 0 | 0 | 1 | 0 | 0 | 3 | 6 |
| NM_022112 | TP53AIP1 | 2654 | 0 | 0 | 0 | 0 | 0 | 0 | 0 | 0 | 0 | 0 | 0 | 0 | 0 |
| NM_022113 | KIF13A | 5940 | 16 | 8 | 10 | 6 | 13 | 5 | 5 | 4 | 7 | 9 | 16 | 4 | 5 |
| NM_022114 | PRDM16 | 8718 | 1 | 0 | 0 | 0 | 0 | 1 | 1 | 0 | 0 | 0 | 0 | 0 | 0 |
| NM_022115 | PRDM15 | 4710 | 2 | 1 | 0 | 1 | 1 | 1 | 1 | 0 | 1 | 1 | 1 | 1 | 2 |
| NM_022116 | FIGNL1 | 3524 | 2 | 1 | 1 | 1 | 1 | 1 | 1 | 1 | 1 | 2 | 1 | 1 | 1 |
| NM_022117 | TSPYL2 | 2828 | 177 | 59 | 18 | 18 | 37 | 177 | 12 | 10 | 30 | 107 | 33 | 35 | 169 |
| NM_022118 | RBM26 | 3614 | 17 | 11 | 7 | 6 | 8 | 13 | 9 | 5 | 9 | 17 | 9 | 17 | 17 |
| NM_022119 | PRSS22 | 1386 | 0 | 0 | 0 | 0 | 0 | 0 | 0 | 0 | 0 | 0 | 0 | 0 | 0 |
| NM_022120 | OXCT2 | 1824 | 1 | 0 | 0 | 0 | 0 | 0 | 0 | 0 | 0 | 0 | 0 | 0 | 1 |
| NM_022121 | PERP | 4301 | 11 | 4 | 3 | 2 | 8 | 1 | 4 | 11 | 5 | 1 | 1 | 0 | 3 |
| NM_022122 | MMP27 | 1647 | 0 | 0 | 0 | 0 | 0 | 0 | 0 | 0 | 0 | 0 | 0 | 0 | 0 |
| NM_022123 | NPAS3 | 5837 | 3 | 0 | 0 | 0 | 0 | 3 | 0 | 0 | 0 | 1 | 0 | 0 | 0 |
| NM_022124 | CDH23 | 11111 | 1 | 0 | 0 | 0 | 0 | 0 | 0 | 0 | 0 | 1 | 0 | 0 | 0 |
| NM_022126 | LHPP | 1684 | 3 | 1 | 1 | 1 | 1 | 3 | 2 | 2 | 1 | 0 | 0 | 1 | 1 |
| NM_022127 | SLC28A3 | 2209 | 0 | 0 | 0 | 0 | 0 | 0 | 0 | 0 | 0 | 0 | 0 | 0 | 0 |
| NM_022128 | RBKS | 1196 | 6 | 2 | 0 | 0 | 2 | 1 | 4 | 6 | 1 | 1 | 1 | 1 | 2 |
| NM_022129 | PBLD | 2587 | 15 | 3 | 1 | 2 | 0 | 1 | 15 | 8 | 0 | 0 | 0 | 0 | 1 |
| NM_022130 | GOLPH3 | 2676 | 43 | 17 | 14 | 12 | 15 | 13 | 15 | 43 | 18 | 14 | 5 | 20 | 14 |
| NM_022131 | CLSTN2 | 4880 | 18 | 3 | 5 | 0 | 1 | 6 | 1 | 0 | 1 | 18 | 0 | 0 | 2 |
| NM_022132 | MCCC2 | 3673 | 43 | 12 | 8 | 7 | 17 | 9 | 18 | 43 | 5 | 7 | 4 | 8 | 7 |
| NM_022133 | SNX16 | 3456 | 3 | 2 | 3 | 1 | 1 | 3 | 1 | 1 | 2 | 2 | 1 | 3 | 3 |
| NM_022134 | GAL3ST2 | 1449 | 0 | 0 | 0 | 0 | 0 | 0 | 0 | 0 | 0 | 0 | 0 | 0 | 0 |
| NM_022135 | POPDC2 | 1644 | 34 | 4 | 0 | 1 | 34 | 0 | 0 | 0 | 0 | 0 | 6 | 0 | 0 |
| NM_022136 | SAMSN1 | 1888 | 17 | 4 | 2 | 0 | 3 | 2 | 1 | 2 | 9 | 1 | 0 | 17 | 1 |

| transcript | gene | transcript length (nt) | max | mean | adipose | colon | heart | hypothalamus | kidney | liver | lung | ovary | skeletalmuscle | spleen | testes |
|---|---|---|---|---|---|---|---|---|---|---|---|---|---|---|---|
| NM_022137 | SMOC1 | 3666 | 6 | 1 | 0 | 0 | 0 | 3 | 0 | 6 | 0 | 0 | 0 | 2 | 2 |
| NM_022138 | SMOC2 | 3149 | 35 | 8 | 3 | 3 | 9 | 2 | 2 | 1 | 3 | 35 | 5 | 1 | 22 |
| NM_022139 | GFRA4 | 1427 | 0 | 0 | 0 | 0 | 0 | 0 | 0 | 0 | 0 | 0 | 0 | 0 | 0 |
| NM_022140 | EPB41L4A | 4707 | 6 | 3 | 2 | 1 | 3 | 3 | 6 | 1 | 3 | 4 | 2 | 3 | 3 |
| NM_022141 | PARVG | 2208 | 2 | 0 | 0 | 0 | 0 | 0 | 0 | 0 | 0 | 0 | 0 | 2 | 0 |
| NM_022142 | ELSPBP1 | 1031 | 10 | 1 | 0 | 0 | 0 | 0 | 0 | 0 | 0 | 0 | 0 | 0 | 10 |
| NM_022143 | LRRC4 | 3707 | 5 | 2 | 0 | 1 | 1 | 5 | 2 | 1 | 1 | 2 | 0 | 2 | 1 |
| NM_022144 | TNMD | 1348 | 41 | 4 | 41 | 0 | 0 | 0 | 1 | 0 | 0 | 2 | 2 | 0 | 1 |
| NM_022145 | CENPK | 1731 | 5 | 1 | 1 | 2 | 0 | 0 | 0 | 0 | 2 | 1 | 0 | 2 | 5 |
| NM_022146 | NPFFR1 | 1287 | 0 | 0 | 0 | 0 | 0 | 0 | 0 | 0 | 0 | 0 | 0 | 0 | 0 |
| NM_022147 | RTP4 | 1011 | 1 | 0 | 0 | 0 | 0 | 0 | 0 | 0 | 0 | 0 | 0 | 1 | 0 |
| NM_022148 | CRLF2 | 770 | 0 | 0 | 0 | 0 | 0 | 0 | 0 | 0 | 0 | 0 | 0 | 0 | 0 |
| NM_022149 | MAGEF1 | 1682 | 6 | 2 | 1 | 2 | 2 | 6 | 4 | 2 | 1 | 2 | 2 | 2 | 1 |
| NM_022150 | NPVF | 1188 | 0 | 0 | 0 | 0 | 0 | 0 | 0 | 0 | 0 | 0 | 0 | 0 | 0 |
| NM_022151 | MOAP1 | 2417 | 66 | 13 | 5 | 5 | 12 | 66 | 9 | 6 | 6 | 5 | 7 | 8 | 7 |
| NM_022152 | TMBIM1 | 2349 | 20 | 8 | 20 | 6 | 7 | 7 | 9 | 12 | 8 | 4 | 6 | 6 | 4 |
| NM_022153 | C10orf54 | 4774 | 4 | 1 | 1 | 0 | 1 | 0 | 0 | 0 | 1 | 1 | 0 | 4 | 0 |
| NM_022154 | SLC39A8 | 3292 | 13 | 3 | 2 | 1 | 3 | 1 | 2 | 3 | 13 | 1 | 0 | 2 | 1 |
| NM_022156 | DUS1L | 1864 | 3 | 1 | 1 | 1 | 1 | 1 | 1 | 3 | 1 | 1 | 1 | 3 | 1 |
| NM_022157 | RRAGC | 1428 | 7 | 4 | 7 | 3 | 3 | 4 | 5 | 2 | 4 | 2 | 1 | 4 | 5 |
| NM_022158 | FN3K | 1433 | 12 | 3 | 1 | 2 | 2 | 5 | 12 | 5 | 1 | 1 | 1 | 1 | 1 |
| NM_022159 | ELTD1 | 3619 | 16 | 4 | 5 | 0 | 5 | 1 | 2 | 2 | 5 | 2 | 3 | 16 | 2 |
| NM_022160 | DMRTA1 | 2302 | 3 | 0 | 0 | 0 | 0 | 0 | 1 | 3 | 0 | 0 | 0 | 0 | 0 |
| NM_022161 | BIRC7 | 1268 | 0 | 0 | 0 | 0 | 0 | 0 | 0 | 0 | 0 | 0 | 0 | 0 | 0 |
| NM_022162 | NOD2 | 4485 | 0 | 0 | 0 | 0 | 0 | 0 | 0 | 0 | 0 | 0 | 0 | 0 | 0 |
| NM_022163 | MRPL46 | 1000 | 6 | 3 | 2 | 3 | 5 | 6 | 4 | 6 | 1 | 2 | 4 | 2 | 2 |
| NM_022164 | TINAGL1 | 2235 | 9 | 4 | 4 | 3 | 8 | 1 | 6 | 2 | 3 | 1 | 2 | 9 | 1 |
| NM_022165 | LIN7B | 755 | 2 | 0 | 0 | 0 | 0 | 2 | 0 | 0 | 0 | 0 | 1 | 0 | 0 |
| NM_022166 | XYLT1 | 9336 | 3 | 1 | 1 | 2 | 1 | 2 | 0 | 0 | 1 | 1 | 0 | 3 | 1 |
| NM_022167 | XYLT2 | 3567 | 0 | 0 | 0 | 0 | 0 | 0 | 0 | 0 | 0 | 0 | 0 | 0 | 0 |
| NM_022168 | IFIH1 | 3421 | 8 | 3 | 3 | 1 | 3 | 2 | 4 | 3 | 4 | 3 | 3 | 8 | 3 |
| NM_022169 | ABCG4 | 3833 | 0 | 0 | 0 | 0 | 0 | 0 | 0 | 0 | 0 | 0 | 0 | 0 | 0 |
| NM_022170 | EIF4H | 2546 | 66 | 41 | 36 | 29 | 38 | 66 | 32 | 50 | 32 | 36 | 63 | 36 | 27 |
| NM_022171 | TCTA | 2147 | 12 | 6 | 7 | 3 | 5 | 8 | 12 | 9 | 4 | 5 | 3 | 7 | 3 |
| NM_022172 | PC | 3959 | 7 | 1 | 2 | 0 | 0 | 1 | 2 | 7 | 0 | 0 | 0 | 0 | 0 |
| NM_022173 | TIA1 | 4653 | 4 | 2 | 1 | 1 | 2 | 1 | 3 | 1 | 2 | 4 | 1 | 4 | 2 |
| NM_022304 | HRH2 | 3095 | 2 | 0 | 0 | 0 | 2 | 1 | 0 | 0 | 1 | 0 | 0 | 1 | 0 |
| NM_022307 | ICA1 | 2257 | 5 | 2 | 2 | 2 | 5 | 3 | 2 | 0 | 1 | 0 | 0 | 1 | 2 |
| NM_022334 | ITGB1BP1 | 1778 | 23 | 5 | 23 | 2 | 3 | 8 | 4 | 2 | 3 | 4 | 2 | 4 | 3 |
| NM_022336 | EDAR | 4214 | 0 | 0 | 0 | 0 | 0 | 0 | 0 | 0 | 0 | 0 | 0 | 0 | 0 |
| NM_022337 | RAB38 | 1408 | 0 | 0 | 0 | 0 | 0 | 0 | 0 | 0 | 0 | 0 | 0 | 0 | 0 |
| NM_022338 | C11orf24 | 2102 | 4 | 2 | 2 | 2 | 2 | 1 | 1 | 4 | 1 | 1 | 1 | 2 | 2 |
| NM_022340 | ZFYVE20 | 6678 | 8 | 4 | 3 | 2 | 4 | 8 | 2 | 3 | 2 | 5 | 3 | 3 | 4 |
| NM_022341 | PDF | 1158 | 0 | 0 | 0 | 0 | 0 | 0 | 0 | 0 | 0 | 0 | 0 | 0 | 0 |
| NM_022342 | KIF9 | 3161 | 3 | 1 | 0 | 0 | 0 | 1 | 1 | 1 | 0 | 0 | 0 | 0 | 3 |
| NM_022343 | GLIPR2 | 1883 | 5 | 1 | 5 | 2 | 1 | 2 | 0 | 0 | 2 | 1 | 0 | 2 | 1 |
| NM_022344 | C17orf75 | 1615 | 2 | 1 | 1 | 0 | 1 | 2 | 2 | 1 | 1 | 1 | 1 | 1 | 2 |
| NM_022346 | NCAPG | 4663 | 2 | 0 | 0 | 0 | 0 | 0 | 0 | 0 | 0 | 0 | 0 | 1 | 2 |
| NM_022347 | TOR1AIP2 | 1662 | 5 | 3 | 2 | 1 | 3 | 2 | 2 | 5 | 2 | 4 | 2 | 3 | 3 |
| NM_022349 | MS4A6A | 1419 | 57 | 20 | 39 | 9 | 26 | 7 | 4 | 27 | 35 | 6 | 5 | 57 | 8 |
| NM_022350 | ERAP2 | 5690 | 11 | 3 | 4 | 2 | 2 | 1 | 2 | 3 | 5 | 3 | 0 | 11 | 1 |
| NM_022351 | NECAB1 | 5139 | 8 | 2 | 0 | 0 | 5 | 8 | 1 | 0 | 2 | 2 | 0 | 1 | 6 |
| NM_022353 | OSGEPL1 | 2316 | 1 | 1 | 0 | 0 | 0 | 1 | 1 | 0 | 0 | 1 | 0 | 1 | 1 |
| NM_022355 | DPEP2 | 1707 | 1 | 0 | 0 | 0 | 0 | 0 | 0 | 0 | 0 | 0 | 0 | 1 | 0 |
| NM_022356 | LEPRE1 | 2686 | 1 | 1 | 1 | 0 | 1 | 0 | 0 | 1 | 0 | 1 | 0 | 1 | 1 |
| NM_022357 | DPEP3 | 1738 | 1 | 0 | 0 | 0 | 0 | 0 | 0 | 0 | 0 | 0 | 0 | 0 | 1 |
| NM_022358 | KCNK15 | 1246 | 0 | 0 | 0 | 0 | 0 | 0 | 0 | 0 | 0 | 0 | 0 | 0 | 0 |
| NM_022359 | PDE4DIP | 1586 | 166 | 37 | 9 | 2 | 166 | 38 | 3 | 4 | 7 | 1 | 163 | 3 | 6 |
| NM_022360 | FAM12B | 896 | 3 | 0 | 0 | 0 | 0 | 0 | 0 | 0 | 0 | 0 | 0 | 0 | 3 |
| NM_022361 | POPDC3 | 1840 | 5 | 1 | 0 | 0 | 1 | 1 | 0 | 0 | 0 | 0 | 5 | 0 | 0 |
| NM_022362 | MMS19 | 3686 | 3 | 2 | 2 | 2 | 2 | 3 | 2 | 1 | 2 | 2 | 2 | 2 | 3 |
| NM_022363 | LHX5 | 2084 | 0 | 0 | 0 | 0 | 0 | 0 | 0 | 0 | 0 | 0 | 0 | 0 | 0 |
| NM_022365 | DNAJC1 | 2091 | 5 | 2 | 2 | 1 | 2 | 1 | 3 | 5 | 2 | 1 | 1 | 2 | 2 |
| NM_022366 | TFB2M | 1781 | 2 | 1 | 0 | 0 | 1 | 0 | 1 | 2 | 0 | 0 | 0 | 0 | 1 |
| NM_022367 | SEMA4A | 3230 | 2 | 1 | 0 | 0 | 0 | 2 | 1 | 0 | 1 | 0 | 0 | 1 | 0 |
| NM_022368 | PJA1 | 2175 | 16 | 5 | 7 | 3 | 3 | 16 | 3 | 1 | 3 | 6 | 1 | 5 | 11 |
| NM_022369 | STRA6 | 2797 | 0 | 0 | 0 | 0 | 0 | 0 | 0 | 0 | 0 | 0 | 0 | 0 | 0 |
| NM_022370 | ROBO3 | 4554 | 0 | 0 | 0 | 0 | 0 | 0 | 0 | 0 | 0 | 0 | 0 | 0 | 0 |
| NM_022371 | TOR3A | 2122 | 11 | 5 | 5 | 4 | 3 | 6 | 5 | 4 | 3 | 4 | 5 | 11 | 3 |
| NM_022372 | MLST8 | 1640 | 3 | 2 | 1 | 1 | 1 | 3 | 2 | 3 | 1 | 1 | 1 | 1 | 2 |
| NM_022373 | HERPUD2 | 2910 | 5 | 3 | 3 | 1 | 2 | 3 | 2 | 3 | 3 | 5 | 1 | 3 | 3 |
| NM_022374 | ATL2 | 3364 | 9 | 5 | 2 | 3 | 4 | 5 | 6 | 6 | 4 | 9 | 9 | 7 | 5 |
| NM_022375 | OCLM | 883 | 1 | 1 | 1 | 1 | 0 | 0 | 1 | 0 | 1 | 1 | 0 | 1 | 0 |
| NM_022377 | ICAM4 | 1420 | 0 | 0 | 0 | 0 | 0 | 0 | 0 | 0 | 0 | 0 | 0 | 0 | 0 |
| NM_022405 | SLC6A20 | 5321 | 1 | 0 | 0 | 0 | 0 | 1 | 0 | 0 | 0 | 0 | 0 | 0 | 0 |
| NM_022406 | XRCC4 | 1655 | 4 | 2 | 1 | 1 | 3 | 2 | 2 | 1 | 1 | 1 | 0 | 3 | 4 |
| NM_022436 | ABCG5 | 2740 | 1 | 0 | 0 | 0 | 0 | 0 | 0 | 1 | 0 | 0 | 0 | 0 | 0 |

| transcript | gene | transcript length (nt) | max | mean | adipose | colon | heart | hypothalamus | kidney | liver | lung | ovary | skeletalmuscle | spleen | testes |
|---|---|---|---|---|---|---|---|---|---|---|---|---|---|---|---|
| NM_022437 | ABCG8 | 2665 | 11 | 1 | 0 | 0 | 0 | 0 | 0 | 11 | 0 | 0 | 0 | 0 | 0 |
| NM_022438 | MAL | 930 | 11 | 2 | 0 | 0 | 0 | 8 | 11 | 0 | 1 | 0 | 0 | 0 | 0 |
| NM_022439 | MAL | 888 | 12 | 2 | 0 | 0 | 0 | 8 | 12 | 0 | 1 | 0 | 0 | 0 | 0 |
| NM_022440 | MAL | 762 | 13 | 2 | 0 | 0 | 0 | 10 | 13 | 0 | 1 | 0 | 0 | 0 | 0 |
| NM_022442 | UBE2V1 | 2179 | 0 | 0 | 0 | 0 | 0 | 0 | 0 | 0 | 0 | 0 | 0 | 0 | 0 |
| NM_022443 | MLF1 | 2205 | 10 | 2 | 0 | 0 | 5 | 2 | 1 | 0 | 1 | 1 | 1 | 0 | 10 |
| NM_022444 | SLC13A1 | 3797 | 19 | 2 | 0 | 0 | 0 | 0 | 19 | 0 | 0 | 0 | 0 | 0 | 0 |
| NM_022445 | TPK1 | 2439 | 3 | 1 | 1 | 1 | 2 | 2 | 1 | 1 | 1 | 1 | 0 | 3 | 1 |
| NM_022449 | RAB17 | 1966 | 5 | 1 | 0 | 1 | 0 | 0 | 5 | 5 | 1 | 0 | 0 | 0 | 0 |
| NM_022450 | RHBDF1 | 2992 | 1 | 0 | 0 | 0 | 0 | 0 | 0 | 0 | 1 | 0 | 0 | 0 | 0 |
| NM_022451 | NOC3L | 3436 | 9 | 5 | 4 | 1 | 4 | 5 | 5 | 4 | 3 | 6 | 5 | 9 | 8 |
| NM_022453 | RNF25 | 1561 | 2 | 1 | 1 | 1 | 1 | 2 | 1 | 1 | 0 | 1 | 1 | 2 | 1 |
| NM_022454 | SOX17 | 2350 | 0 | 0 | 0 | 0 | 0 | 0 | 0 | 0 | 0 | 0 | 0 | 0 | 0 |
| NM_022455 | NSD1 | 12983 | 7 | 4 | 4 | 3 | 4 | 5 | 5 | 2 | 3 | 7 | 4 | 7 | 6 |
| NM_022456 | RAB3IP | 9347 | 7 | 2 | 0 | 1 | 2 | 4 | 7 | 1 | 2 | 0 | 1 | 1 | 2 |
| NM_022457 | RFWD2 | 2774 | 8 | 4 | 4 | 3 | 3 | 5 | 4 | 4 | 4 | 3 | 2 | 8 | 7 |
| NM_022458 | LMBR1 | 4890 | 7 | 2 | 1 | 1 | 2 | 3 | 3 | 2 | 2 | 2 | 1 | 2 | 7 |
| NM_022459 | XPO4 | 9885 | 7 | 2 | 1 | 1 | 2 | 1 | 2 | 1 | 2 | 2 | 7 | 3 | 3 |
| NM_022460 | HS1BP3 | 2403 | 2 | 1 | 1 | 1 | 1 | 1 | 2 | 1 | 1 | 0 | 1 | 1 | 1 |
| NM_022461 | AZI2 | 3397 | 11 | 7 | 11 | 2 | 7 | 8 | 7 | 8 | 9 | 7 | 4 | 9 | 7 |
| NM_022462 | HIF3A | 5908 | 0 | 0 | 0 | 0 | 0 | 0 | 0 | 0 | 0 | 0 | 0 | 0 | 0 |
| NM_022463 | NXN | 3004 | 2 | 0 | 0 | 1 | 0 | 0 | 0 | 0 | 1 | 1 | 2 | 0 | 0 |
| NM_022464 | SIL1 | 1900 | 18 | 7 | 8 | 4 | 6 | 4 | 7 | 18 | 5 | 4 | 6 | 5 | 11 |
| NM_022465 | IKZF4 | 5451 | 1 | 1 | 0 | 0 | 1 | 1 | 1 | 0 | 0 | 1 | 1 | 0 | 1 |
| NM_022466 | IKZF5 | 1658 | 3 | 2 | 2 | 1 | 1 | 3 | 1 | 2 | 1 | 2 | 1 | 2 | 2 |
| NM_022467 | CHST8 | 2479 | 0 | 0 | 0 | 0 | 0 | 0 | 0 | 0 | 0 | 0 | 0 | 0 | 0 |
| NM_022468 | MMP25 | 3551 | 2 | 0 | 0 | 0 | 0 | 0 | 0 | 0 | 0 | 0 | 0 | 2 | 0 |
| NM_022469 | GREM2 | 4183 | 6 | 1 | 0 | 4 | 0 | 2 | 0 | 6 | 0 | 1 | 1 | 0 | 1 |
| NM_022470 | ZMAT3 | 2409 | 4 | 2 | 1 | 1 | 2 | 4 | 2 | 1 | 1 | 1 | 1 | 2 | 2 |
| NM_022473 | ZFP106 | 10460 | 104 | 28 | 21 | 4 | 104 | 15 | 10 | 5 | 13 | 13 | 90 | 18 | 15 |
| NM_022474 | MPP5 | 5357 | 10 | 5 | 3 | 3 | 4 | 8 | 10 | 4 | 5 | 3 | 4 | 4 | 6 |
| NM_022475 | HHIP | 3530 | 8 | 2 | 0 | 2 | 0 | 5 | 1 | 2 | 8 | 1 | 0 | 0 | 4 |
| NM_022476 | AKTIP | 2193 | 11 | 6 | 5 | 1 | 10 | 11 | 6 | 5 | 3 | 4 | 7 | 4 | 7 |
| NM_022477 | NDRG3 | 2928 | 56 | 12 | 6 | 6 | 10 | 56 | 8 | 3 | 7 | 11 | 4 | 12 | 12 |
| NM_022478 | CDH24 | 3553 | 0 | 0 | 0 | 0 | 0 | 0 | 0 | 0 | 0 | 0 | 0 | 0 | 0 |
| NM_022479 | WBSCR17 | 3250 | 7 | 1 | 0 | 0 | 2 | 7 | 0 | 0 | 0 | 2 | 0 | 0 | 1 |
| NM_022480 | KLHL25 | 3681 | 0 | 0 | 0 | 0 | 0 | 0 | 0 | 0 | 0 | 0 | 0 | 0 | 0 |
| NM_022481 | ARAP3 | 5262 | 0 | 0 | 0 | 0 | 0 | 0 | 0 | 0 | 0 | 0 | 0 | 0 | 0 |
| NM_022482 | GZF1 | 4741 | 6 | 3 | 2 | 2 | 6 | 6 | 3 | 2 | 2 | 5 | 2 | 3 | 6 |
| NM_022483 | C5orf28 | 2761 | 1 | 0 | 0 | 0 | 0 | 0 | 0 | 0 | 0 | 0 | 0 | 1 | 0 |
| NM_022484 | TMEM168 | 3951 | 4 | 3 | 2 | 2 | 3 | 2 | 3 | 1 | 2 | 3 | 1 | 4 | 4 |
| NM_022485 | MTMR14 | 2161 | 14 | 8 | 7 | 5 | 12 | 5 | 8 | 8 | 7 | 5 | 8 | 14 | 9 |
| NM_022486 | SUSD1 | 2996 | 9 | 5 | 7 | 5 | 5 | 9 | 7 | 3 | 4 | 1 | 1 | 9 | 7 |
| NM_022487 | DCLRE1C | 3644 | 4 | 1 | 1 | 1 | 1 | 1 | 1 | 0 | 1 | 1 | 1 | 4 | 1 |
| NM_022488 | ATG3 | 1242 | 40 | 23 | 21 | 8 | 26 | 29 | 23 | 22 | 19 | 18 | 10 | 40 | 37 |
| NM_022489 | INF2 | 4710 | 2 | 1 | 1 | 2 | 0 | 1 | 0 | 1 | 1 | 0 | 0 | 1 | 1 |
| NM_022490 | POLR1E | 1854 | 6 | 3 | 2 | 2 | 3 | 3 | 1 | 5 | 2 | 6 | 3 | 3 | 2 |
| NM_022491 | SUDS3 | 4728 | 7 | 4 | 3 | 3 | 4 | 7 | 5 | 2 | 3 | 5 | 3 | 4 | 5 |
| NM_022492 | TTC31 | 2929 | 2 | 1 | 1 | 2 | 1 | 1 | 2 | 2 | 1 | 1 | 1 | 2 | 2 |
| NM_022493 | NARFL | 2091 | 1 | 1 | 0 | 1 | 1 | 1 | 1 | 1 | 0 | 1 | 0 | 0 | 0 |
| NM_022494 | ZDHHC6 | 2170 | 6 | 3 | 2 | 2 | 3 | 3 | 6 | 2 | 2 | 3 | 1 | 5 | 3 |
| NM_022495 | C14orf135 | 3932 | 9 | 6 | 3 | 2 | 7 | 6 | 8 | 2 | 4 | 9 | 3 | 8 | 8 |
| NM_022496 | ACTR6 | 1755 | 6 | 2 | 1 | 0 | 3 | 6 | 3 | 2 | 2 | 1 | 1 | 3 | 3 |
| NM_022497 | MRPS25 | 4566 | 5 | 3 | 2 | 3 | 4 | 4 | 3 | 5 | 2 | 1 | 2 | 2 | 2 |
| NM_022549 | FEZ1 | 1730 | 31 | 4 | 1 | 1 | 5 | 31 | 0 | 0 | 1 | 1 | 1 | 1 | 2 |
| NM_022550 | XRCC4 | 1696 | 4 | 2 | 1 | 1 | 3 | 2 | 2 | 1 | 1 | 1 | 0 | 3 | 4 |
| NM_022551 | RPS18 | 542 | 870 | 389 | 303 | 309 | 295 | 152 | 208 | 323 | 393 | 870 | 372 | 710 | 342 |
| NM_022552 | DNMT3A | 4299 | 9 | 4 | 3 | 5 | 3 | 4 | 3 | 3 | 2 | 9 | 5 | 6 | 5 |
| NM_022553 | VPS52 | 2951 | 3 | 2 | 1 | 2 | 1 | 3 | 2 | 2 | 1 | 2 | 1 | 2 | 2 |
| NM_022555 | HLA-DRB3 | 1158 | 0 | 0 | 0 | 0 | 0 | 0 | 0 | 0 | 0 | 0 | 0 | 0 | 0 |
| NM_022556 | GH2 | 776 | 0 | 0 | 0 | 0 | 0 | 0 | 0 | 0 | 0 | 0 | 0 | 0 | 0 |
| NM_022557 | GH2 | 1078 | 0 | 0 | 0 | 0 | 0 | 0 | 0 | 0 | 0 | 0 | 0 | 0 | 0 |
| NM_022558 | GH2 | 819 | 0 | 0 | 0 | 0 | 0 | 0 | 0 | 0 | 0 | 0 | 0 | 0 | 0 |
| NM_022559 | GH1 | 777 | 0 | 0 | 0 | 0 | 0 | 0 | 0 | 0 | 0 | 0 | 0 | 0 | 0 |
| NM_022560 | GH1 | 702 | 0 | 0 | 0 | 0 | 0 | 0 | 0 | 0 | 0 | 0 | 0 | 0 | 0 |
| NM_022561 | GH1 | 537 | 1 | 0 | 0 | 0 | 1 | 0 | 0 | 0 | 0 | 0 | 0 | 0 | 0 |
| NM_022562 | GH1 | 376 | 1 | 0 | 0 | 0 | 1 | 0 | 0 | 0 | 0 | 0 | 0 | 0 | 0 |
| NM_022564 | MMP16 | 1794 | 6 | 1 | 1 | 1 | 1 | 6 | 0 | 0 | 0 | 2 | 0 | 0 | 1 |
| NM_022566 | MESDC1 | 3051 | 4 | 2 | 1 | 1 | 2 | 2 | 1 | 1 | 3 | 2 | 0 | 4 | 0 |
| NM_022567 | NYX | 2629 | 0 | 0 | 0 | 0 | 0 | 0 | 0 | 0 | 0 | 0 | 0 | 0 | 0 |
| NM_022568 | ALDH8A1 | 2542 | 30 | 4 | 0 | 0 | 0 | 0 | 11 | 30 | 0 | 0 | 0 | 0 | 0 |
| NM_022569 | NDST4 | 3343 | 1 | 0 | 0 | 0 | 0 | 1 | 0 | 0 | 0 | 0 | 0 | 0 | 0 |
| NM_022570 | CLEC7A | 2470 | 11 | 3 | 2 | 1 | 4 | 2 | 1 | 2 | 8 | 1 | 0 | 11 | 1 |
| NM_022571 | GPR135 | 1820 | 0 | 0 | 0 | 0 | 0 | 0 | 0 | 0 | 0 | 0 | 0 | 0 | 0 |
| NM_022572 | PNKD | 2971 | 5 | 1 | 0 | 0 | 0 | 2 | 1 | 5 | 1 | 1 | 0 | 1 | 1 |
| NM_022573 | TSPY2 | 1160 | 1 | 0 | 0 | 0 | 0 | 0 | 0 | 0 | 0 | 0 | 0 | 0 | 1 |
| NM_022574 | GIGYF1 | 6329 | 3 | 2 | 2 | 3 | 1 | 2 | 2 | 1 | 1 | 3 | 2 | 2 | 2 |

| transcript | gene | transcript length (nt) | max | mean | adipose | colon | heart | hypothalamus | kidney | liver | lung | ovary | skeletalmuscle | spleen | testes |
|---|---|---|---|---|---|---|---|---|---|---|---|---|---|---|---|
| NM_022575 | VPS16 | 2726 | 2 | 1 | 1 | 1 | 2 | 2 | 2 | 1 | 1 | 1 | 1 | 2 | 1 |
| NM_022576 | PDC | 1173 | 0 | 0 | 0 | 0 | 0 | 0 | 0 | 0 | 0 | 0 | 0 | 0 | 0 |
| NM_022579 | CSHL1 | 837 | 0 | 0 | 0 | 0 | 0 | 0 | 0 | 0 | 0 | 0 | 0 | 0 | 0 |
| NM_022580 | CSHL1 | 588 | 1 | 0 | 0 | 0 | 0 | 1 | 0 | 0 | 0 | 0 | 0 | 0 | 0 |
| NM_022581 | CSHL1 | 768 | 0 | 0 | 0 | 0 | 0 | 0 | 0 | 0 | 0 | 0 | 0 | 0 | 0 |
| NM_022640 | CSH1 | 1132 | 0 | 0 | 0 | 0 | 0 | 0 | 0 | 0 | 0 | 0 | 0 | 0 | 0 |
| NM_022641 | CSH1 | 594 | 0 | 0 | 0 | 0 | 0 | 0 | 0 | 0 | 0 | 0 | 0 | 0 | 0 |
| NM_022644 | CSH2 | 1135 | 0 | 0 | 0 | 0 | 0 | 0 | 0 | 0 | 0 | 0 | 0 | 0 | 0 |
| NM_022645 | CSH2 | 598 | 0 | 0 | 0 | 0 | 0 | 0 | 0 | 0 | 0 | 0 | 0 | 0 | 0 |
| NM_022648 | TNS1 | 10276 | 68 | 23 | 68 | 32 | 36 | 6 | 19 | 5 | 24 | 15 | 12 | 19 | 16 |
| NM_022650 | RASA1 | 3745 | 14 | 5 | 4 | 2 | 4 | 5 | 5 | 1 | 6 | 14 | 3 | 10 | 6 |
| NM_022652 | DUSP6 | 2382 | 15 | 6 | 9 | 3 | 5 | 4 | 6 | 6 | 11 | 4 | 1 | 15 | 2 |
| NM_022658 | HOXC8 | 2288 | 2 | 0 | 2 | 0 | 0 | 0 | 1 | 0 | 0 | 0 | 1 | 0 | 1 |
| NM_022659 | EBF2 | 2297 | 2 | 1 | 2 | 0 | 2 | 0 | 0 | 0 | 0 | 0 | 1 | 0 | 0 |
| NM_022661 | SPANXC | 404 | 0 | 0 | 0 | 0 | 0 | 0 | 0 | 0 | 0 | 0 | 0 | 0 | 0 |
| NM_022662 | ANAPC1 | 6310 | 3 | 2 | 1 | 1 | 1 | 2 | 2 | 2 | 1 | 2 | 1 | 2 | 3 |
| NM_022664 | ECM1 | 1768 | 4 | 2 | 4 | 1 | 2 | 1 | 1 | 2 | 1 | 1 | 1 | 1 | 2 |
| NM_022716 | PRRX1 | 3999 | 20 | 6 | 12 | 0 | 11 | 3 | 1 | 0 | 5 | 5 | 20 | 1 | 11 |
| NM_022717 | SNRNP35 | 966 | 35 | 24 | 22 | 20 | 35 | 31 | 19 | 32 | 17 | 18 | 33 | 26 | 15 |
| NM_022719 | DGCR14 | 5385 | 1 | 0 | 0 | 0 | 0 | 0 | 0 | 0 | 0 | 0 | 0 | 0 | 1 |
| NM_022720 | DGCR8 | 4441 | 5 | 3 | 2 | 4 | 2 | 2 | 3 | 2 | 2 | 3 | 2 | 3 | 5 |
| NM_022725 | FANCF | 3309 | 1 | 0 | 0 | 0 | 0 | 0 | 1 | 0 | 0 | 1 | 0 | 1 | 0 |
| NM_022726 | ELOVL4 | 3060 | 5 | 1 | 0 | 0 | 0 | 5 | 0 | 0 | 0 | 0 | 0 | 0 | 2 |
| NM_022727 | TRMT2A | 2905 | 1 | 0 | 1 | 0 | 0 | 1 | 0 | 1 | 0 | 0 | 0 | 1 | 1 |
| NM_022728 | NEUROD6 | 2156 | 4 | 0 | 0 | 0 | 0 | 4 | 0 | 0 | 0 | 0 | 0 | 0 | 0 |
| NM_022730 | COPS7B | 1977 | 2 | 1 | 1 | 2 | 1 | 2 | 1 | 1 | 1 | 1 | 1 | 2 | 2 |
| NM_022731 | NUCKS1 | 6453 | 64 | 33 | 28 | 11 | 38 | 64 | 26 | 30 | 22 | 54 | 27 | 28 | 30 |
| NM_022733 | SMAP2 | 2576 | 94 | 21 | 12 | 6 | 11 | 31 | 5 | 23 | 28 | 4 | 7 | 94 | 8 |
| NM_022734 | METT11D1 | 1713 | 5 | 3 | 2 | 5 | 3 | 2 | 3 | 3 | 2 | 2 | 2 | 4 | 2 |
| NM_022735 | ACBD3 | 3565 | 13 | 8 | 7 | 4 | 11 | 9 | 8 | 6 | 10 | 8 | 7 | 9 | 13 |
| NM_022736 | MFSD1 | 2361 | 11 | 4 | 6 | 1 | 5 | 2 | 5 | 4 | 8 | 3 | 1 | 11 | 2 |
| NM_022737 | LPPR2 | 2680 | 2 | 0 | 0 | 0 | 0 | 2 | 0 | 0 | 0 | 0 | 0 | 0 | 0 |
| NM_022739 | SMURF2 | 3866 | 5 | 3 | 3 | 2 | 3 | 4 | 3 | 1 | 5 | 5 | 2 | 4 | 5 |
| NM_022740 | HIPK2 | 15224 | 22 | 11 | 11 | 4 | 19 | 22 | 19 | 14 | 6 | 3 | 6 | 13 | 2 |
| NM_022742 | CCDC136 | 4168 | 22 | 3 | 1 | 3 | 2 | 8 | 0 | 0 | 0 | 0 | 1 | 0 | 22 |
| NM_022743 | SMYD3 | 1463 | 2 | 1 | 0 | 1 | 1 | 2 | 1 | 1 | 1 | 1 | 1 | 1 | 1 |
| NM_022744 | C16orf58 | 2760 | 2 | 1 | 1 | 1 | 0 | 1 | 2 | 2 | 0 | 1 | 0 | 1 | 1 |
| NM_022745 | ATPAF1 | 4129 | 16 | 6 | 4 | 2 | 16 | 12 | 7 | 6 | 2 | 3 | 6 | 3 | 4 |
| NM_022746 | MOSC1 | 2242 | 10 | 2 | 10 | 1 | 1 | 1 | 1 | 5 | 1 | 0 | 0 | 1 | 0 |
| NM_022748 | TNS3 | 7627 | 6 | 4 | 5 | 2 | 2 | 3 | 5 | 5 | 3 | 4 | 1 | 6 | 2 |
| NM_022749 | FAM160B2 | 3731 | 1 | 1 | 1 | 1 | 1 | 1 | 1 | 0 | 1 | 0 | 0 | 1 | 1 |
| NM_022750 | PARP12 | 3791 | 3 | 1 | 1 | 1 | 1 | 0 | 1 | 1 | 2 | 1 | 1 | 3 | 1 |
| NM_022751 | FAM59A | 3041 | 3 | 1 | 2 | 1 | 1 | 2 | 3 | 1 | 1 | 0 | 1 | 1 | 1 |
| NM_022752 | ZNF574 | 3131 | 0 | 0 | 0 | 0 | 0 | 0 | 0 | 0 | 0 | 0 | 0 | 0 | 0 |
| NM_022753 | S100PBP | 4298 | 14 | 4 | 3 | 3 | 2 | 3 | 2 | 1 | 3 | 6 | 2 | 4 | 14 |
| NM_022754 | SFXN1 | 2991 | 8 | 3 | 1 | 1 | 2 | 6 | 5 | 8 | 1 | 2 | 1 | 2 | 2 |
| NM_022755 | IPPK | 4401 | 0 | 0 | 0 | 0 | 0 | 0 | 0 | 0 | 0 | 0 | 0 | 0 | 0 |
| NM_022756 | MEAF6 | 2147 | 40 | 13 | 11 | 9 | 9 | 40 | 8 | 9 | 8 | 13 | 7 | 12 | 20 |
| NM_022757 | CCDC14 | 4182 | 3 | 1 | 1 | 1 | 1 | 2 | 2 | 1 | 2 | 1 | 1 | 2 | 3 |
| NM_022758 | C6orf106 | 4216 | 10 | 6 | 6 | 3 | 7 | 5 | 6 | 10 | 5 | 3 | 10 | 5 | 4 |
| NM_022760 | FAM113A | 1999 | 1 | 0 | 0 | 0 | 0 | 0 | 1 | 0 | 0 | 0 | 0 | 1 | 0 |
| NM_022761 | C11orf1 | 983 | 2 | 1 | 1 | 1 | 1 | 2 | 1 | 1 | 1 | 1 | 0 | 1 | 1 |
| NM_022762 | RMND5B | 1808 | 1 | 1 | 0 | 1 | 1 | 1 | 1 | 1 | 0 | 1 | 1 | 1 | 1 |
| NM_022763 | FNDC3B | 7012 | 10 | 7 | 9 | 4 | 9 | 2 | 6 | 7 | 8 | 10 | 5 | 8 | 5 |
| NM_022764 | MTHFSD | 3035 | 3 | 2 | 1 | 1 | 1 | 1 | 2 | 1 | 1 | 2 | 2 | 2 | 3 |
| NM_022765 | MICAL1 | 3642 | 2 | 1 | 1 | 2 | 1 | 1 | 1 | 0 | 1 | 0 | 0 | 2 | 1 |
| NM_022766 | CERK | 4445 | 4 | 2 | 1 | 2 | 4 | 2 | 3 | 1 | 2 | 2 | 1 | 2 | 1 |
| NM_022767 | AEN | 3116 | 1 | 0 | 0 | 0 | 0 | 0 | 0 | 1 | 0 | 0 | 0 | 0 | 0 |
| NM_022768 | RBM15 | 3431 | 3 | 2 | 1 | 2 | 1 | 1 | 1 | 1 | 1 | 2 | 2 | 3 | 2 |
| NM_022769 | CRTC3 | 5171 | 3 | 1 | 1 | 1 | 2 | 1 | 1 | 0 | 1 | 3 | 1 | 2 | 2 |
| NM_022770 | GINS3 | 2274 | 1 | 0 | 1 | 0 | 1 | 0 | 0 | 0 | 0 | 0 | 0 | 1 | 1 |
| NM_022771 | TBC1D15 | 5794 | 6 | 4 | 4 | 1 | 3 | 4 | 3 | 4 | 4 | 4 | 4 | 6 | 4 |
| NM_022772 | EPS8L2 | 3140 | 12 | 3 | 0 | 6 | 0 | 0 | 12 | 5 | 3 | 0 | 0 | 0 | 1 |
| NM_022773 | LMF1 | 2606 | 0 | 0 | 0 | 0 | 0 | 0 | 0 | 0 | 0 | 0 | 0 | 0 | 0 |
| NM_022774 | DEM1 | 2203 | 2 | 1 | 1 | 1 | 1 | 2 | 1 | 1 | 1 | 1 | 0 | 1 | 1 |
| NM_022776 | OSBPL11 | 4632 | 7 | 4 | 4 | 1 | 2 | 3 | 2 | 6 | 3 | 2 | 6 | 7 | 5 |
| NM_022777 | RABL5 | 2427 | 5 | 2 | 1 | 1 | 2 | 4 | 4 | 1 | 1 | 2 | 1 | 1 | 5 |
| NM_022778 | CCDC21 | 3935 | 5 | 2 | 1 | 1 | 3 | 2 | 1 | 1 | 1 | 0 | 5 | 1 | 4 |
| NM_022779 | DDX31 | 3285 | 1 | 1 | 0 | 1 | 1 | 1 | 1 | 1 | 1 | 1 | 1 | 1 | 1 |
| NM_022780 | RMND5A | 6183 | 13 | 6 | 4 | 4 | 10 | 6 | 5 | 8 | 3 | 4 | 13 | 7 | 4 |
| NM_022781 | RNF38 | 5089 | 7 | 3 | 2 | 2 | 3 | 4 | 3 | 1 | 3 | 4 | 2 | 4 | 7 |
| NM_022782 | MPHOSPH9 | 3611 | 5 | 3 | 2 | 1 | 3 | 3 | 3 | 2 | 2 | 3 | 2 | 4 | 5 |
| NM_022783 | DEPDC6 | 2631 | 17 | 4 | 2 | 3 | 2 | 2 | 9 | 2 | 1 | 8 | 17 | 3 | 1 |
| NM_022784 | IQCH | 997 | 0 | 0 | 0 | 0 | 0 | 0 | 0 | 0 | 0 | 0 | 0 | 0 | 0 |
| NM_022785 | EFCAB6 | 4800 | 7 | 1 | 0 | 0 | 1 | 2 | 2 | 0 | 1 | 2 | 0 | 0 | 7 |
| NM_022786 | ARV1 | 1445 | 16 | 9 | 6 | 5 | 12 | 16 | 11 | 7 | 5 | 6 | 5 | 8 | 13 |
| NM_022787 | NMNAT1 | 3781 | 1 | 1 | 1 | 1 | 1 | 1 | 1 | 1 | 0 | 1 | 1 | 1 | 1 |

| transcript | gene | transcript length (nt) | max | mean | adipose | colon | heart | hypothalamus | kidney | liver | lung | ovary | skeletalmuscle | spleen | testes |
|---|---|---|---|---|---|---|---|---|---|---|---|---|---|---|---|
| NM_022788 | P2RY12 | 1502 | 1 | 0 | 1 | 0 | 0 | 1 | 0 | 0 | 0 | 0 | 0 | 1 | 0 |
| NM_022789 | IL25 | 1367 | 0 | 0 | 0 | 0 | 0 | 0 | 0 | 0 | 0 | 0 | 0 | 0 | 0 |
| NM_022802 | CTBP2 | 4752 | 7 | 4 | 3 | 5 | 4 | 5 | 5 | 1 | 5 | 7 | 2 | 5 | 5 |
| NM_022803 | UCP3 | 2239 | 8 | 1 | 0 | 0 | 0 | 0 | 0 | 0 | 0 | 0 | 8 | 0 | 0 |
| NM_022804 | SNURF | 422 | 0 | 0 | 0 | 0 | 0 | 0 | 0 | 0 | 0 | 0 | 0 | 0 | 0 |
| NM_022805 | SNRPN | 1605 | 1 | 0 | 0 | 0 | 0 | 1 | 0 | 0 | 0 | 0 | 0 | 0 | 0 |
| NM_022806 | SNRPN | 1616 | 1 | 0 | 0 | 0 | 0 | 1 | 0 | 0 | 0 | 0 | 0 | 0 | 0 |
| NM_022807 | SNRPN | 1751 | 2 | 0 | 0 | 0 | 0 | 2 | 0 | 0 | 0 | 0 | 0 | 0 | 0 |
| NM_022808 | SNRPN | 1643 | 1 | 0 | 0 | 0 | 0 | 1 | 0 | 0 | 0 | 0 | 0 | 0 | 0 |
| NM_022809 | CDC25C | 1896 | 4 | 0 | 0 | 1 | 0 | 0 | 0 | 0 | 0 | 0 | 0 | 0 | 4 |
| NM_022810 | SLC25A14 | 1586 | 3 | 1 | 0 | 0 | 0 | 3 | 1 | 0 | 0 | 1 | 0 | 0 | 1 |
| NM_022817 | PER2 | 6342 | 2 | 1 | 0 | 1 | 0 | 2 | 0 | 0 | 1 | 2 | 1 | 1 | 2 |
| NM_022818 | MAP1LC3B | 2287 | 10 | 5 | 4 | 2 | 10 | 9 | 4 | 4 | 6 | 2 | 7 | 5 | 4 |
| NM_022819 | PLA2G2F | 2719 | 0 | 0 | 0 | 0 | 0 | 0 | 0 | 0 | 0 | 0 | 0 | 0 | 0 |
| NM_022820 | CYP3A43 | 2167 | 1 | 0 | 0 | 0 | 0 | 0 | 0 | 1 | 0 | 0 | 0 | 0 | 0 |
| NM_022821 | ELOVL1 | 1483 | 5 | 2 | 3 | 2 | 2 | 5 | 3 | 2 | 1 | 1 | 1 | 2 | 1 |
| NM_022822 | KLC2 | 2989 | 3 | 1 | 0 | 0 | 1 | 3 | 0 | 0 | 0 | 0 | 1 | 0 | 1 |
| NM_022823 | FNDC4 | 1670 | 59 | 10 | 9 | 1 | 2 | 18 | 1 | 59 | 1 | 5 | 0 | 4 | 4 |
| NM_022825 | PORCN | 1849 | 0 | 0 | 0 | 0 | 0 | 0 | 0 | 0 | 0 | 0 | 0 | 0 | 0 |
| NM_022826 | 07 Mar | 3468 | 23 | 16 | 12 | 6 | 19 | 13 | 13 | 11 | 19 | 20 | 16 | 23 | 23 |
| NM_022827 | SPATA20 | 2607 | 3 | 1 | 2 | 1 | 1 | 3 | 2 | 1 | 0 | 2 | 1 | 1 | 2 |
| NM_022828 | YTHDC2 | 6308 | 5 | 3 | 2 | 1 | 3 | 3 | 3 | 3 | 2 | 3 | 2 | 5 | 5 |
| NM_022829 | SLC13A3 | 4045 | 63 | 6 | 0 | 0 | 0 | 2 | 63 | 0 | 0 | 0 | 0 | 0 | 0 |
| NM_022830 | TUT1 | 2826 | 4 | 3 | 3 | 2 | 2 | 3 | 4 | 4 | 2 | 3 | 3 | 3 | 4 |
| NM_022831 | AIDA | 3006 | 10 | 6 | 6 | 2 | 8 | 4 | 6 | 5 | 8 | 6 | 4 | 9 | 10 |
| NM_022832 | USP46 | 7963 | 3 | 1 | 0 | 0 | 2 | 3 | 1 | 0 | 0 | 1 | 1 | 1 | 1 |
| NM_022833 | FAM129B | 3949 | 4 | 2 | 1 | 3 | 2 | 2 | 2 | 1 | 4 | 1 | 0 | 2 | 1 |
| NM_022834 | VWA1 | 4658 | 1 | 0 | 0 | 0 | 0 | 1 | 0 | 1 | 0 | 0 | 0 | 0 | 0 |
| NM_022835 | PLEKHG2 | 7607 | 1 | 0 | 0 | 0 | 1 | 0 | 0 | 0 | 1 | 1 | 0 | 1 | 0 |
| NM_022836 | DCLRE1B | 3651 | 1 | 0 | 1 | 0 | 0 | 1 | 0 | 0 | 1 | 1 | 0 | 1 | 0 |
| NM_022838 | ARMCX5 | 2672 | 2 | 1 | 1 | 1 | 1 | 2 | 1 | 1 | 1 | 1 | 0 | 1 | 2 |
| NM_022839 | MRPS11 | 1135 | 4 | 2 | 2 | 2 | 2 | 2 | 2 | 4 | 0 | 1 | 1 | 1 | 1 |
| NM_022840 | METTL4 | 3674 | 3 | 1 | 1 | 1 | 2 | 1 | 2 | 1 | 1 | 2 | 1 | 3 | 2 |
| NM_022841 | RFX7 | 7195 | 4 | 2 | 1 | 1 | 1 | 2 | 2 | 1 | 2 | 3 | 4 | 4 | 3 |
| NM_022842 | CDCP1 | 6007 | 1 | 0 | 0 | 1 | 0 | 0 | 1 | 0 | 0 | 0 | 0 | 0 | 0 |
| NM_022843 | PCDH20 | 4777 | 6 | 1 | 0 | 1 | 3 | 6 | 0 | 2 | 1 | 0 | 1 | 0 | 1 |
| NM_022844 | MYH11 | 6921 | 399 | 85 | 15 | 399 | 59 | 20 | 20 | 1 | 46 | 86 | 26 | 29 | 231 |
| NM_022845 | CBFB | 3102 | 6 | 3 | 3 | 2 | 4 | 4 | 3 | 2 | 4 | 3 | 5 | 6 | 3 |
| NM_022872 | IFI6 | 848 | 7 | 4 | 5 | 0 | 4 | 5 | 1 | 2 | 4 | 4 | 7 | 3 | 2 |
| NM_022873 | IFI6 | 860 | 7 | 4 | 5 | 0 | 4 | 5 | 1 | 2 | 4 | 4 | 7 | 3 | 2 |
| NM_022874 | SMN1 | 1526 | 0 | 0 | 0 | 0 | 0 | 0 | 0 | 0 | 0 | 0 | 0 | 0 | 0 |
| NM_022875 | SMN2 | 1573 | 0 | 0 | 0 | 0 | 0 | 0 | 0 | 0 | 0 | 0 | 0 | 0 | 0 |
| NM_022876 | SMN2 | 1531 | 0 | 0 | 0 | 0 | 0 | 0 | 0 | 0 | 0 | 0 | 0 | 0 | 0 |
| NM_022877 | SMN2 | 1477 | 0 | 0 | 0 | 0 | 0 | 0 | 0 | 0 | 0 | 0 | 0 | 0 | 0 |
| NM_022892 | NAIP | 4476 | 20 | 5 | 2 | 2 | 4 | 5 | 3 | 2 | 4 | 4 | 1 | 20 | 3 |
| NM_022892 | NAIP | 4477 | 20 | 5 | 2 | 2 | 4 | 5 | 3 | 2 | 4 | 4 | 1 | 20 | 3 |
| NM_022892 | NAIP | 5880 | 15 | 4 | 2 | 2 | 3 | 3 | 2 | 1 | 3 | 3 | 1 | 15 | 3 |
| NM_022893 | BCL11A | 5946 | 2 | 0 | 0 | 0 | 0 | 1 | 0 | 0 | 0 | 0 | 0 | 2 | 0 |
| NM_022894 | PAPOLG | 4245 | 8 | 4 | 3 | 2 | 3 | 5 | 3 | 3 | 4 | 6 | 4 | 8 | 6 |
| NM_022895 | C12orf43 | 1920 | 1 | 0 | 0 | 0 | 0 | 1 | 1 | 0 | 0 | 1 | 0 | 0 | 0 |
| NM_022896 | LPIN3 | 4363 | 0 | 0 | 0 | 0 | 0 | 0 | 0 | 0 | 0 | 0 | 0 | 0 | 0 |
| NM_022897 | RANBP17 | 4440 | 1 | 0 | 0 | 0 | 0 | 0 | 0 | 0 | 0 | 0 | 0 | 0 | 1 |
| NM_022898 | BCL11B | 7602 | 1 | 0 | 0 | 0 | 0 | 1 | 0 | 0 | 0 | 0 | 0 | 1 | 0 |
| NM_022899 | ACTR8 | 3525 | 3 | 1 | 1 | 2 | 2 | 3 | 2 | 1 | 0 | 1 | 1 | 2 | 1 |
| NM_022900 | CASD1 | 3879 | 3 | 1 | 1 | 1 | 1 | 2 | 1 | 1 | 1 | 2 | 1 | 3 | 2 |
| NM_022901 | LRRC19 | 3600 | 4 | 0 | 0 | 1 | 0 | 0 | 4 | 0 | 0 | 0 | 0 | 0 | 0 |
| NM_022902 | SLC30A5 | 2935 | 15 | 10 | 11 | 3 | 12 | 9 | 11 | 15 | 9 | 12 | 4 | 14 | 14 |
| NM_022903 | CCDC71 | 1812 | 0 | 0 | 0 | 0 | 0 | 0 | 0 | 0 | 0 | 0 | 0 | 0 | 0 |
| NM_022904 | RASAL3 | 3290 | 1 | 0 | 0 | 0 | 0 | 0 | 0 | 0 | 0 | 0 | 0 | 1 | 0 |
| NM_022905 | TTC23 | 3562 | 2 | 1 | 1 | 2 | 1 | 1 | 2 | 1 | 1 | 2 | 1 | 1 | 2 |
| NM_022906 | STAG3L4 | 2135 | 2 | 1 | 1 | 1 | 1 | 1 | 2 | 0 | 1 | 2 | 1 | 2 | 1 |
| NM_022908 | NT5DC2 | 2088 | 5 | 2 | 1 | 2 | 5 | 2 | 1 | 1 | 1 | 3 | 1 | 1 | 3 |
| NM_022909 | CENPH | 654 | 6 | 1 | 1 | 1 | 1 | 1 | 0 | 0 | 1 | 1 | 1 | 2 | 6 |
| NM_022909 | CENPH | 1389 | 3 | 1 | 1 | 0 | 0 | 0 | 0 | 0 | 1 | 1 | 0 | 1 | 3 |
| NM_022910 | NDRG4 | 3393 | 40 | 7 | 0 | 1 | 22 | 40 | 0 | 0 | 1 | 0 | 0 | 2 | 6 |
| NM_022911 | SLC26A6 | 2643 | 1 | 0 | 0 | 1 | 0 | 0 | 1 | 0 | 0 | 0 | 0 | 0 | 0 |
| NM_022912 | REEP1 | 3853 | 24 | 4 | 0 | 1 | 2 | 10 | 1 | 0 | 1 | 0 | 1 | 1 | 24 |
| NM_022913 | GPBP1 | 3529 | 31 | 15 | 9 | 5 | 13 | 17 | 13 | 7 | 17 | 22 | 13 | 20 | 31 |
| NM_022914 | ACD | 2057 | 0 | 0 | 0 | 0 | 0 | 0 | 0 | 0 | 0 | 0 | 0 | 0 | 0 |
| NM_022915 | MRPL44 | 1748 | 2 | 1 | 0 | 0 | 1 | 0 | 1 | 2 | 0 | 0 | 1 | 1 | 1 |
| NM_022916 | VPS33A | 2603 | 6 | 3 | 3 | 2 | 2 | 6 | 3 | 4 | 2 | 3 | 2 | 3 | 5 |
| NM_022917 | NOL6 | 4853 | 2 | 1 | 1 | 0 | 1 | 1 | 1 | 2 | 1 | 1 | 1 | 1 | 1 |
| NM_022918 | TMEM135 | 9079 | 2 | 1 | 2 | 1 | 1 | 2 | 1 | 1 | 1 | 2 | 1 | 1 | 2 |
| NM_022963 | FGFR4 | 2791 | 2 | 1 | 0 | 1 | 0 | 0 | 2 | 1 | 1 | 0 | 0 | 1 | 0 |
| NM_022965 | FGFR3 | 3951 | 5 | 1 | 0 | 1 | 0 | 5 | 2 | 1 | 1 | 0 | 0 | 0 | 1 |
| NM_022968 | SERF1A | 707 | 1 | 0 | 0 | 1 | 0 | 0 | 0 | 0 | 0 | 0 | 0 | 0 | 1 |
| NM_022968 | SERF1A | 710 | 1 | 0 | 0 | 1 | 0 | 0 | 0 | 0 | 0 | 0 | 0 | 0 | 1 |

| transcript | gene | transcript length (nt) | max | mean | adipose | colon | heart | hypothalamus | kidney | liver | lung | ovary | skeletalmuscle | spleen | testes |
|---|---|---|---|---|---|---|---|---|---|---|---|---|---|---|---|
| NM_022970 | FGFR2 | 4643 | 8 | 3 | 1 | 5 | 0 | 8 | 7 | 2 | 2 | 3 | 0 | 0 | 1 |
| NM_022977 | ACSL4 | 5335 | 27 | 13 | 15 | 2 | 13 | 17 | 16 | 2 | 19 | 7 | 14 | 15 | 27 |
| NM_022978 | SERF1B | 1889 | 0 | 0 | 0 | 0 | 0 | 0 | 0 | 0 | 0 | 0 | 0 | 0 | 0 |
| NM_023000 | ARID4A | 5610 | 8 | 5 | 5 | 2 | 4 | 6 | 4 | 3 | 6 | 6 | 2 | 8 | 5 |
| NM_023001 | ARID4A | 5565 | 8 | 5 | 5 | 2 | 4 | 6 | 4 | 3 | 6 | 6 | 2 | 8 | 5 |
| NM_023002 | HAPLN4 | 3439 | 1 | 0 | 0 | 0 | 0 | 1 | 0 | 0 | 0 | 0 | 0 | 0 | 0 |
| NM_023003 | TM6SF1 | 1908 | 4 | 1 | 1 | 0 | 1 | 4 | 1 | 0 | 1 | 1 | 2 | 2 | 1 |
| NM_023004 | RTN4R | 1919 | 1 | 0 | 0 | 0 | 0 | 1 | 0 | 0 | 0 | 0 | 0 | 0 | 0 |
| NM_023007 | JMJD4 | 2594 | 0 | 0 | 0 | 0 | 0 | 0 | 0 | 0 | 0 | 0 | 0 | 0 | 0 |
| NM_023008 | KRI1 | 3005 | 5 | 3 | 2 | 3 | 2 | 3 | 2 | 2 | 2 | 3 | 4 | 5 | 4 |
| NM_023009 | MARCKSL1 | 1545 | 15 | 5 | 1 | 3 | 3 | 15 | 2 | 2 | 3 | 14 | 1 | 6 | 12 |
| NM_023010 | UPF3B | 2342 | 10 | 6 | 5 | 3 | 5 | 9 | 6 | 4 | 7 | 6 | 5 | 9 | 10 |
| NM_023011 | UPF3A | 2359 | 34 | 16 | 15 | 10 | 10 | 25 | 17 | 9 | 12 | 16 | 8 | 17 | 34 |
| NM_023012 | RSRC2 | 2252 | 40 | 29 | 25 | 13 | 32 | 40 | 28 | 19 | 31 | 30 | 30 | 35 | 37 |
| NM_023013 | PRAMEF1 | 1606 | 0 | 0 | 0 | 0 | 0 | 0 | 0 | 0 | 0 | 0 | 0 | 0 | 0 |
| NM_023014 | PRAMEF2 | 1642 | 0 | 0 | 0 | 0 | 0 | 0 | 0 | 0 | 0 | 0 | 0 | 0 | 0 |
| NM_023015 | INTS3 | 4482 | 11 | 7 | 6 | 8 | 7 | 7 | 10 | 4 | 7 | 9 | 3 | 10 | 11 |
| NM_023016 | ANKRD57 | 4654 | 4 | 2 | 1 | 1 | 1 | 1 | 2 | 4 | 1 | 3 | 0 | 2 | 2 |
| NM_023018 | NADK | 3235 | 10 | 5 | 7 | 4 | 3 | 4 | 3 | 9 | 5 | 2 | 1 | 10 | 5 |
| NM_023019 | DCTN1 | 4162 | 37 | 14 | 11 | 9 | 19 | 37 | 10 | 6 | 8 | 11 | 10 | 12 | 23 |
| NM_023033 | METTL1 | 1122 | 1 | 0 | 0 | 0 | 0 | 0 | 1 | 0 | 0 | 0 | 0 | 0 | 0 |
| NM_023034 | WHSC1L1 | 5430 | 18 | 10 | 7 | 5 | 8 | 14 | 9 | 3 | 12 | 18 | 6 | 15 | 10 |
| NM_023036 | DNAI2 | 2267 | 0 | 0 | 0 | 0 | 0 | 0 | 0 | 0 | 0 | 0 | 0 | 0 | 0 |
| NM_023037 | FRY | 10717 | 8 | 3 | 2 | 1 | 8 | 7 | 2 | 1 | 2 | 2 | 2 | 2 | 2 |
| NM_023039 | ANKRA2 | 2047 | 1 | 0 | 0 | 0 | 0 | 0 | 0 | 0 | 0 | 1 | 0 | 1 | 1 |
| NM_023067 | FOXL2 | 2916 | 1 | 0 | 0 | 0 | 0 | 0 | 0 | 0 | 0 | 1 | 0 | 0 | 0 |
| NM_023068 | SIGLEC1 | 6720 | 2 | 1 | 2 | 1 | 2 | 0 | 0 | 0 | 1 | 0 | 0 | 1 | 1 |
| NM_023070 | ZNF643 | 1976 | 8 | 2 | 1 | 1 | 1 | 8 | 1 | 0 | 1 | 3 | 0 | 3 | 4 |
| NM_023071 | SPATS2 | 3796 | 3 | 1 | 1 | 1 | 1 | 3 | 1 | 0 | 1 | 2 | 1 | 1 | 3 |
| NM_023072 | ZSWIM4 | 4339 | 1 | 0 | 0 | 0 | 0 | 0 | 0 | 0 | 1 | 0 | 0 | 0 | 0 |
| NM_023073 | C5orf42 | 11199 | 5 | 2 | 2 | 1 | 2 | 3 | 3 | 0 | 2 | 5 | 1 | 3 | 4 |
| NM_023074 | ZNF649 | 3169 | 7 | 4 | 2 | 2 | 4 | 6 | 3 | 1 | 3 | 7 | 2 | 5 | 4 |
| NM_023075 | MPPE1 | 2804 | 3 | 1 | 1 | 1 | 1 | 3 | 1 | 0 | 0 | 0 | 1 | 1 | 0 |
| NM_023076 | UNKL | 4121 | 2 | 1 | 1 | 1 | 1 | 1 | 1 | 1 | 1 | 2 | 1 | 1 | 1 |
| NM_023077 | C1orf163 | 1614 | 5 | 3 | 2 | 2 | 2 | 4 | 3 | 5 | 1 | 1 | 3 | 2 | 3 |
| NM_023078 | PYCRL | 2658 | 0 | 0 | 0 | 0 | 0 | 0 | 0 | 0 | 0 | 0 | 0 | 0 | 0 |
| NM_023079 | UBE2Z | 3090 | 11 | 6 | 4 | 4 | 6 | 10 | 7 | 4 | 5 | 6 | 4 | 11 | 9 |
| NM_023080 | C8orf33 | 2617 | 3 | 2 | 1 | 1 | 2 | 2 | 2 | 1 | 1 | 3 | 1 | 3 | 3 |
| NM_023083 | CAPN10 | 2626 | 0 | 0 | 0 | 0 | 0 | 0 | 0 | 0 | 0 | 0 | 0 | 0 | 0 |
| NM_023085 | CAPN10 | 2161 | 0 | 0 | 0 | 0 | 0 | 0 | 0 | 0 | 0 | 0 | 0 | 0 | 0 |
| NM_023089 | CAPN10 | 1304 | 0 | 0 | 0 | 0 | 0 | 0 | 0 | 0 | 0 | 0 | 0 | 0 | 0 |
| NM_023105 | FGFR1 | 5634 | 7 | 3 | 7 | 3 | 6 | 3 | 2 | 0 | 3 | 6 | 1 | 2 | 4 |
| NM_023106 | FGFR1 | 5628 | 7 | 3 | 7 | 3 | 6 | 3 | 2 | 0 | 3 | 6 | 1 | 2 | 4 |
| NM_023107 | FGFR1 | 2786 | 6 | 3 | 6 | 3 | 4 | 2 | 2 | 0 | 2 | 5 | 1 | 1 | 3 |
| NM_023108 | FGFR1 | 2780 | 6 | 3 | 6 | 3 | 4 | 2 | 2 | 0 | 2 | 5 | 1 | 1 | 3 |
| NM_023110 | FGFR1 | 5901 | 6 | 3 | 6 | 3 | 6 | 3 | 2 | 0 | 3 | 5 | 1 | 1 | 4 |
| NM_023111 | FGFR1 | 5790 | 6 | 3 | 6 | 3 | 6 | 3 | 2 | 0 | 3 | 5 | 1 | 2 | 4 |
| NM_023112 | OTUB2 | 3873 | 0 | 0 | 0 | 0 | 0 | 0 | 0 | 0 | 0 | 0 | 0 | 0 | 0 |
| NM_023915 | GPR87 | 1519 | 0 | 0 | 0 | 0 | 0 | 0 | 0 | 0 | 0 | 0 | 0 | 0 | 0 |
| NM_023917 | TAS2R9 | 1075 | 0 | 0 | 0 | 0 | 0 | 0 | 0 | 0 | 0 | 0 | 0 | 0 | 0 |
| NM_023918 | TAS2R8 | 930 | 0 | 0 | 0 | 0 | 0 | 0 | 0 | 0 | 0 | 0 | 0 | 0 | 0 |
| NM_023919 | TAS2R7 | 1096 | 0 | 0 | 0 | 0 | 0 | 0 | 0 | 0 | 0 | 0 | 0 | 0 | 0 |
| NM_023920 | TAS2R13 | 1637 | 2 | 1 | 0 | 0 | 0 | 0 | 1 | 0 | 1 | 2 | 1 | 1 | 0 |
| NM_023921 | TAS2R10 | 924 | 0 | 0 | 0 | 0 | 0 | 0 | 0 | 0 | 0 | 0 | 0 | 0 | 0 |
| NM_023922 | TAS2R14 | 954 | 1 | 0 | 0 | 0 | 0 | 0 | 1 | 0 | 0 | 0 | 0 | 1 | 0 |
| NM_023923 | PHACTR4 | 6194 | 5 | 3 | 2 | 3 | 3 | 2 | 5 | 3 | 3 | 4 | 3 | 5 | 4 |
| NM_023924 | BRD9 | 2693 | 4 | 3 | 3 | 4 | 3 | 4 | 3 | 1 | 2 | 3 | 3 | 3 | 4 |
| NM_023925 | CAPRIN2 | 4335 | 6 | 3 | 2 | 2 | 3 | 6 | 3 | 1 | 2 | 4 | 2 | 3 | 6 |
| NM_023926 | ZSCAN18 | 2928 | 7 | 2 | 1 | 1 | 2 | 7 | 2 | 0 | 1 | 2 | 1 | 2 | 2 |
| NM_023927 | GRAMD3 | 2932 | 2 | 1 | 0 | 0 | 1 | 2 | 1 | 1 | 1 | 2 | 0 | 1 | 1 |
| NM_023928 | AACS | 3321 | 4 | 2 | 4 | 3 | 2 | 4 | 2 | 1 | 1 | 1 | 0 | 1 | 3 |
| NM_023929 | ZBTB10 | 5989 | 10 | 5 | 3 | 2 | 5 | 4 | 5 | 3 | 6 | 10 | 5 | 6 | 7 |
| NM_023930 | KCTD14 | 1671 | 5 | 2 | 0 | 2 | 0 | 1 | 5 | 4 | 3 | 0 | 0 | 0 | 2 |
| NM_023931 | ZNF747 | 2371 | 0 | 0 | 0 | 0 | 0 | 0 | 0 | 0 | 0 | 0 | 0 | 0 | 0 |
| NM_023932 | DLK2 | 1548 | 0 | 0 | 0 | 0 | 0 | 0 | 0 | 0 | 0 | 0 | 0 | 0 | 0 |
| NM_023933 | FAM173A | 858 | 1 | 0 | 0 | 0 | 0 | 1 | 0 | 1 | 0 | 0 | 0 | 0 | 0 |
| NM_023934 | FUNDC2 | 2964 | 10 | 4 | 2 | 1 | 10 | 2 | 3 | 2 | 2 | 4 | 6 | 3 | 3 |
| NM_023935 | DDRGK1 | 1254 | 21 | 10 | 11 | 9 | 7 | 16 | 13 | 21 | 11 | 5 | 4 | 7 | 7 |
| NM_023936 | MRPS34 | 1003 | 7 | 4 | 3 | 4 | 4 | 5 | 4 | 7 | 2 | 2 | 4 | 4 | 2 |
| NM_023937 | MRPL34 | 968 | 9 | 4 | 3 | 1 | 8 | 3 | 7 | 9 | 2 | 2 | 3 | 4 | 4 |
| NM_023938 | C1orf116 | 5502 | 6 | 1 | 0 | 1 | 0 | 0 | 1 | 0 | 6 | 0 | 0 | 0 | 0 |
| NM_023940 | RASL11B | 1957 | 2 | 0 | 0 | 0 | 0 | 0 | 1 | 0 | 0 | 1 | 0 | 2 | 0 |
| NM_023942 | LRRC61 | 1792 | 1 | 0 | 0 | 0 | 0 | 0 | 0 | 1 | 0 | 0 | 0 | 0 | 0 |
| NM_023943 | TMEM108 | 3790 | 1 | 0 | 0 | 0 | 0 | 1 | 0 | 0 | 0 | 0 | 0 | 0 | 0 |
| NM_023944 | CYP4F12 | 1725 | 1 | 0 | 0 | 1 | 0 | 0 | 0 | 1 | 0 | 0 | 0 | 0 | 0 |
| NM_023945 | MS4A5 | 722 | 1 | 0 | 0 | 0 | 0 | 0 | 0 | 0 | 0 | 0 | 0 | 0 | 1 |
| NM_023946 | LYNX1 | 1288 | 1 | 0 | 0 | 0 | 0 | 1 | 0 | 0 | 0 | 0 | 0 | 0 | 0 |

| transcript | gene | transcript length (nt) | max | mean | adipose | colon | heart | hypothalamus | kidney | liver | lung | ovary | skeletalmuscle | spleen | testes |
|---|---|---|---|---|---|---|---|---|---|---|---|---|---|---|---|
| NM_023947 | CHID1 | 3323 | 2 | 1 | 1 | 1 | 1 | 1 | 1 | 2 | 1 | 1 | 1 | 2 | 1 |
| NM_023948 | MOSPD3 | 1203 | 1 | 1 | 1 | 1 | 1 | 1 | 1 | 1 | 1 | 1 | 0 | 1 | 1 |
| NM_024003 | L1CAM | 5014 | 4 | 1 | 0 | 1 | 0 | 4 | 1 | 0 | 0 | 0 | 0 | 0 | 0 |
| NM_024006 | VKORC1 | 998 | 13 | 3 | 3 | 3 | 2 | 1 | 4 | 13 | 2 | 3 | 1 | 3 | 3 |
| NM_024007 | EBF1 | 5274 | 11 | 3 | 11 | 1 | 2 | 0 | 1 | 0 | 1 | 4 | 2 | 2 | 4 |
| NM_024009 | GJB3 | 2217 | 0 | 0 | 0 | 0 | 0 | 0 | 0 | 0 | 0 | 0 | 0 | 0 | 0 |
| NM_024010 | MTRR | 3272 | 7 | 4 | 3 | 1 | 3 | 2 | 5 | 7 | 4 | 3 | 5 | 5 | 3 |
| NM_024011 | CDK11A | 2627 | 1 | 0 | 0 | 0 | 0 | 0 | 0 | 0 | 0 | 0 | 1 | 0 | 0 |
| NM_024012 | HTR5A | 2400 | 4 | 0 | 0 | 0 | 0 | 4 | 0 | 0 | 0 | 0 | 0 | 0 | 0 |
| NM_024013 | IFNA1 | 876 | 0 | 0 | 0 | 0 | 0 | 0 | 0 | 0 | 0 | 0 | 0 | 0 | 0 |
| NM_024014 | HOXA6 | 863 | 0 | 0 | 0 | 0 | 0 | 0 | 0 | 0 | 0 | 0 | 0 | 0 | 0 |
| NM_024015 | HOXB4 | 2033 | 2 | 1 | 1 | 1 | 0 | 0 | 2 | 0 | 1 | 0 | 0 | 1 | 1 |
| NM_024016 | HOXB8 | 1823 | 2 | 0 | 0 | 1 | 0 | 0 | 2 | 0 | 0 | 0 | 0 | 0 | 1 |
| NM_024017 | HOXB9 | 2700 | 3 | 0 | 0 | 3 | 0 | 0 | 2 | 0 | 0 | 0 | 0 | 0 | 1 |
| NM_024019 | NEUROG2 | 2287 | 0 | 0 | 0 | 0 | 0 | 0 | 0 | 0 | 0 | 0 | 0 | 0 | 0 |
| NM_024021 | MS4A4A | 1600 | 18 | 6 | 11 | 1 | 12 | 1 | 1 | 2 | 17 | 2 | 1 | 18 | 2 |
| NM_024022 | TMPRSS3 | 2441 | 0 | 0 | 0 | 0 | 0 | 0 | 0 | 0 | 0 | 0 | 0 | 0 | 0 |
| NM_024025 | DUSP26 | 1649 | 8 | 2 | 0 | 0 | 3 | 4 | 0 | 0 | 0 | 1 | 8 | 0 | 0 |
| NM_024026 | MRP63 | 2655 | 1 | 1 | 0 | 0 | 1 | 1 | 1 | 1 | 0 | 0 | 1 | 1 | 0 |
| NM_024027 | COLEC11 | 1303 | 1 | 0 | 0 | 0 | 0 | 0 | 1 | 1 | 0 | 0 | 0 | 0 | 0 |
| NM_024028 | PCYOX1L | 2551 | 4 | 1 | 1 | 1 | 1 | 4 | 1 | 0 | 1 | 2 | 0 | 2 | 3 |
| NM_024029 | YIPF2 | 1504 | 1 | 1 | 1 | 1 | 0 | 1 | 1 | 1 | 1 | 1 | 0 | 1 | 1 |
| NM_024031 | PRR14 | 2118 | 3 | 2 | 1 | 1 | 1 | 1 | 1 | 1 | 2 | 2 | 1 | 3 | 2 |
| NM_024032 | C17orf53 | 2673 | 0 | 0 | 0 | 0 | 0 | 0 | 0 | 0 | 0 | 0 | 0 | 0 | 0 |
| NM_024033 | C7orf49 | 1572 | 10 | 6 | 6 | 6 | 5 | 5 | 9 | 6 | 4 | 5 | 5 | 10 | 7 |
| NM_024034 | GDAP1L1 | 2244 | 3 | 0 | 0 | 0 | 0 | 3 | 0 | 0 | 0 | 0 | 0 | 0 | 0 |
| NM_024036 | LRFN4 | 2528 | 0 | 0 | 0 | 0 | 0 | 0 | 0 | 0 | 0 | 0 | 0 | 0 | 0 |
| NM_024037 | C1orf135 | 2061 | 1 | 0 | 0 | 0 | 0 | 0 | 0 | 0 | 0 | 0 | 0 | 0 | 1 |
| NM_024038 | C19orf43 | 910 | 144 | 61 | 22 | 46 | 90 | 26 | 59 | 39 | 64 | 55 | 52 | 144 | 73 |
| NM_024039 | MIS12 | 2501 | 7 | 4 | 2 | 2 | 3 | 4 | 4 | 5 | 3 | 4 | 2 | 7 | 5 |
| NM_024040 | CUEDC2 | 1196 | 6 | 2 | 3 | 1 | 2 | 6 | 2 | 1 | 1 | 1 | 2 | 1 | 2 |
| NM_024041 | SCNM1 | 858 | 4 | 2 | 2 | 2 | 2 | 3 | 1 | 1 | 1 | 2 | 3 | 4 | 1 |
| NM_024042 | METRN | 1092 | 2 | 0 | 0 | 0 | 0 | 2 | 1 | 0 | 0 | 0 | 0 | 1 | 0 |
| NM_024043 | DBNDD1 | 2075 | 2 | 0 | 0 | 0 | 0 | 2 | 1 | 0 | 0 | 0 | 1 | 0 | 0 |
| NM_024044 | GIYD2 | 1112 | 0 | 0 | 0 | 0 | 0 | 0 | 0 | 0 | 0 | 0 | 0 | 0 | 0 |
| NM_024045 | DDX50 | 2538 | 14 | 8 | 8 | 3 | 8 | 8 | 7 | 5 | 6 | 13 | 7 | 11 | 14 |
| NM_024046 | CAMKV | 3028 | 10 | 1 | 0 | 0 | 0 | 10 | 0 | 0 | 0 | 0 | 0 | 0 | 0 |
| NM_024047 | NUDT9 | 1702 | 22 | 10 | 7 | 4 | 10 | 15 | 14 | 22 | 6 | 7 | 6 | 7 | 11 |
| NM_024050 | DDA1 | 4075 | 3 | 1 | 1 | 1 | 2 | 3 | 1 | 1 | 1 | 1 | 1 | 2 | 2 |
| NM_024051 | GGCT | 1165 | 16 | 11 | 10 | 6 | 10 | 16 | 16 | 11 | 13 | 7 | 3 | 13 | 10 |
| NM_024052 | C17orf39 | 4232 | 3 | 1 | 1 | 1 | 1 | 1 | 1 | 1 | 1 | 1 | 2 | 1 | 3 |
| NM_024053 | CENPM | 930 | 0 | 0 | 0 | 0 | 0 | 0 | 0 | 0 | 0 | 0 | 0 | 0 | 0 |
| NM_024054 | C7orf25 | 1820 | 4 | 2 | 2 | 1 | 3 | 3 | 3 | 1 | 2 | 2 | 1 | 3 | 4 |
| NM_024055 | SLC30A5 | 1317 | 2 | 1 | 1 | 0 | 1 | 1 | 1 | 2 | 1 | 2 | 0 | 1 | 2 |
| NM_024056 | TMEM106C | 1516 | 13 | 7 | 4 | 13 | 8 | 6 | 11 | 3 | 5 | 5 | 2 | 13 | 7 |
| NM_024057 | NUP37 | 1205 | 1 | 1 | 1 | 0 | 1 | 0 | 1 | 0 | 1 | 0 | 0 | 1 | 1 |
| NM_024059 | C20orf195 | 1111 | 1 | 0 | 0 | 0 | 0 | 0 | 0 | 0 | 0 | 0 | 0 | 0 | 1 |
| NM_024060 | AHNAK | 1090 | 55 | 18 | 55 | 28 | 16 | 6 | 10 | 4 | 27 | 15 | 17 | 9 | 10 |
| NM_024061 | ZNF655 | 1585 | 2 | 1 | 1 | 1 | 1 | 0 | 1 | 1 | 1 | 0 | 0 | 2 | 1 |
| NM_024063 | SPATA5L1 | 2577 | 1 | 1 | 1 | 1 | 1 | 1 | 1 | 1 | 0 | 1 | 1 | 1 | 1 |
| NM_024065 | PDCL3 | 1075 | 3 | 2 | 2 | 1 | 2 | 3 | 3 | 3 | 1 | 2 | 3 | 3 | 3 |
| NM_024066 | ERI3 | 1743 | 2 | 1 | 1 | 1 | 0 | 2 | 2 | 0 | 0 | 1 | 0 | 1 | 1 |
| NM_024067 | C7orf26 | 1913 | 1 | 0 | 0 | 0 | 0 | 1 | 0 | 0 | 0 | 0 | 0 | 1 | 0 |
| NM_024068 | OBFC2B | 1400 | 11 | 6 | 6 | 6 | 4 | 11 | 6 | 10 | 3 | 4 | 5 | 6 | 5 |
| NM_024069 | C19orf50 | 1418 | 6 | 4 | 4 | 2 | 5 | 5 | 4 | 4 | 3 | 3 | 3 | 6 | 4 |
| NM_024070 | PVRIG | 1569 | 1 | 0 | 0 | 1 | 0 | 0 | 0 | 0 | 0 | 0 | 1 | 1 | 0 |
| NM_024071 | ZFYVE21 | 1383 | 11 | 5 | 6 | 3 | 5 | 11 | 5 | 4 | 3 | 8 | 2 | 4 | 6 |
| NM_024072 | DDX54 | 4378 | 3 | 2 | 1 | 1 | 2 | 1 | 2 | 3 | 1 | 1 | 3 | 2 | 1 |
| NM_024074 | TMEM38A | 1625 | 3 | 0 | 0 | 0 | 0 | 0 | 0 | 0 | 0 | 0 | 3 | 0 | 0 |
| NM_024075 | TSEN34 | 2297 | 1 | 1 | 1 | 0 | 0 | 0 | 1 | 1 | 1 | 1 | 0 | 1 | 1 |
| NM_024076 | KCTD15 | 4932 | 1 | 0 | 0 | 1 | 0 | 0 | 0 | 0 | 0 | 1 | 0 | 1 | 0 |
| NM_024077 | SECISBP2 | 3467 | 9 | 5 | 3 | 2 | 3 | 5 | 5 | 4 | 4 | 6 | 4 | 6 | 9 |
| NM_024078 | NOC4L | 1632 | 0 | 0 | 0 | 0 | 0 | 0 | 0 | 0 | 0 | 0 | 0 | 0 | 0 |
| NM_024079 | ALG8 | 1668 | 5 | 2 | 2 | 1 | 2 | 2 | 3 | 5 | 2 | 2 | 1 | 3 | 3 |
| NM_024080 | TRPM8 | 5621 | 5 | 0 | 0 | 0 | 0 | 0 | 0 | 5 | 0 | 0 | 0 | 0 | 0 |
| NM_024081 | PRRG4 | 1966 | 5 | 2 | 0 | 2 | 1 | 0 | 5 | 5 | 5 | 0 | 0 | 1 | 1 |
| NM_024082 | PRRG3 | 1594 | 0 | 0 | 0 | 0 | 0 | 0 | 0 | 0 | 0 | 0 | 0 | 0 | 0 |
| NM_024083 | ASPSCR1 | 1843 | 0 | 0 | 0 | 0 | 0 | 0 | 0 | 0 | 0 | 0 | 0 | 0 | 0 |
| NM_024085 | ATG9A | 3721 | 5 | 2 | 1 | 2 | 3 | 4 | 2 | 3 | 2 | 1 | 5 | 2 | 3 |
| NM_024086 | METT10D | 5753 | 4 | 2 | 2 | 1 | 2 | 2 | 2 | 1 | 2 | 4 | 2 | 3 | 3 |
| NM_024087 | ASB9 | 1760 | 2 | 0 | 0 | 0 | 0 | 0 | 1 | 1 | 0 | 0 | 0 | 1 | 2 |
| NM_024089 | KDELC1 | 2107 | 1 | 1 | 1 | 0 | 1 | 0 | 1 | 1 | 0 | 1 | 0 | 1 | 1 |
| NM_024090 | ELOVL6 | 3391 | 5 | 1 | 0 | 1 | 1 | 2 | 1 | 5 | 0 | 0 | 0 | 0 | 0 |
| NM_024091 | FASTKD3 | 2402 | 1 | 0 | 0 | 0 | 0 | 0 | 1 | 1 | 0 | 0 | 0 | 1 | 1 |
| NM_024092 | TMEM109 | 2386 | 5 | 3 | 3 | 2 | 5 | 1 | 5 | 2 | 4 | 4 | 3 | 5 | 2 |
| NM_024093 | C2orf49 | 887 | 20 | 12 | 11 | 7 | 13 | 20 | 8 | 7 | 7 | 20 | 12 | 15 | 18 |
| NM_024094 | DSCC1 | 2268 | 1 | 0 | 0 | 0 | 0 | 0 | 0 | 0 | 0 | 0 | 0 | 0 | 1 |

| transcript | gene | transcript length (nt) | max | mean | adipose | colon | heart | hypothalamus | kidney | liver | lung | ovary | skeletalmuscle | spleen | testes |
|---|---|---|---|---|---|---|---|---|---|---|---|---|---|---|---|
| NM_024095 | ASB8 | 2613 | 15 | 4 | 2 | 2 | 4 | 3 | 4 | 2 | 1 | 3 | 15 | 4 | 3 |
| NM_024096 | DCTPP1 | 1120 | 3 | 2 | 2 | 2 | 2 | 3 | 3 | 1 | 1 | 1 | 2 | 3 | 3 |
| NM_024097 | C1orf50 | 1017 | 2 | 1 | 1 | 1 | 1 | 1 | 1 | 2 | 0 | 1 | 1 | 1 | 2 |
| NM_024098 | CCDC86 | 2015 | 1 | 0 | 0 | 0 | 0 | 0 | 0 | 0 | 0 | 0 | 0 | 0 | 1 |
| NM_024099 | C11orf48 | 1320 | 6 | 3 | 2 | 2 | 3 | 5 | 3 | 6 | 1 | 2 | 2 | 4 | 3 |
| NM_024100 | WDR18 | 1551 | 0 | 0 | 0 | 0 | 0 | 0 | 0 | 0 | 0 | 0 | 0 | 0 | 0 |
| NM_024101 | MLPH | 3756 | 2 | 1 | 0 | 1 | 1 | 0 | 0 | 1 | 2 | 0 | 0 | 0 | 0 |
| NM_024102 | WDR77 | 2408 | 5 | 3 | 3 | 2 | 5 | 3 | 4 | 4 | 2 | 1 | 3 | 4 | 2 |
| NM_024103 | SLC25A23 | 3460 | 6 | 2 | 1 | 1 | 2 | 6 | 2 | 1 | 1 | 1 | 1 | 1 | 1 |
| NM_024104 | C19orf42 | 1373 | 7 | 4 | 6 | 3 | 3 | 7 | 6 | 6 | 2 | 5 | 2 | 5 | 4 |
| NM_024105 | ALG12 | 2374 | 1 | 0 | 0 | 1 | 0 | 0 | 0 | 1 | 0 | 0 | 0 | 0 | 0 |
| NM_024106 | ZNF426 | 2304 | 6 | 2 | 2 | 1 | 2 | 2 | 2 | 0 | 2 | 6 | 1 | 3 | 2 |
| NM_024107 | TMUB2 | 1922 | 1 | 1 | 1 | 1 | 1 | 1 | 1 | 0 | 1 | 1 | 1 | 1 | 1 |
| NM_024108 | TRAPPC6A | 795 | 2 | 1 | 1 | 0 | 0 | 1 | 2 | 1 | 1 | 0 | 0 | 1 | 0 |
| NM_024109 | C16orf68 | 1570 | 4 | 2 | 1 | 1 | 3 | 1 | 1 | 1 | 1 | 1 | 4 | 1 | 3 |
| NM_024110 | CARD14 | 3916 | 0 | 0 | 0 | 0 | 0 | 0 | 0 | 0 | 0 | 0 | 0 | 0 | 0 |
| NM_024111 | CHAC1 | 1560 | 1 | 0 | 0 | 0 | 0 | 0 | 0 | 0 | 0 | 0 | 1 | 0 | 0 |
| NM_024112 | C9orf16 | 713 | 0 | 0 | 0 | 0 | 0 | 0 | 0 | 0 | 0 | 0 | 0 | 0 | 0 |
| NM_024113 | C11orf49 | 1711 | 4 | 2 | 1 | 1 | 1 | 4 | 2 | 0 | 1 | 2 | 1 | 1 | 2 |
| NM_024114 | TRIM48 | 1444 | 0 | 0 | 0 | 0 | 0 | 0 | 0 | 0 | 0 | 0 | 0 | 0 | 0 |
| NM_024116 | TAF1D | 1268 | 23 | 14 | 9 | 4 | 14 | 8 | 13 | 6 | 21 | 23 | 17 | 23 | 15 |
| NM_024117 | MAPKAP1 | 3287 | 5 | 4 | 4 | 3 | 5 | 5 | 4 | 5 | 3 | 3 | 3 | 4 | 3 |
| NM_024119 | DHX58 | 2615 | 2 | 1 | 0 | 0 | 0 | 1 | 1 | 2 | 0 | 0 | 0 | 1 | 1 |
| NM_024120 | C20orf7 | 2290 | 4 | 2 | 2 | 2 | 4 | 4 | 4 | 3 | 1 | 1 | 2 | 2 | 2 |
| NM_024122 | APOO | 1128 | 14 | 6 | 3 | 2 | 12 | 14 | 10 | 6 | 2 | 2 | 3 | 3 | 5 |
| NM_024164 | TPSB2 | 1166 | 0 | 0 | 0 | 0 | 0 | 0 | 0 | 0 | 0 | 0 | 0 | 0 | 0 |
| NM_024165 | PHF1 | 2294 | 1 | 1 | 1 | 1 | 1 | 1 | 1 | 0 | 1 | 1 | 1 | 1 | 1 |
| NM_024292 | UBL5 | 519 | 33 | 14 | 7 | 6 | 33 | 15 | 18 | 11 | 16 | 8 | 15 | 18 | 7 |
| NM_024293 | FAM134A | 4614 | 7 | 4 | 4 | 3 | 3 | 7 | 4 | 3 | 3 | 4 | 2 | 6 | 5 |
| NM_024294 | C6orf106 | 4414 | 12 | 7 | 7 | 3 | 8 | 6 | 6 | 12 | 5 | 3 | 11 | 6 | 5 |
| NM_024295 | DERL1 | 3161 | 7 | 4 | 3 | 2 | 5 | 3 | 3 | 7 | 3 | 3 | 4 | 6 | 4 |
| NM_024296 | CCDC28B | 877 | 3 | 1 | 0 | 0 | 1 | 2 | 1 | 0 | 1 | 1 | 3 | 1 | 3 |
| NM_024297 | PHF23 | 2037 | 4 | 2 | 1 | 2 | 1 | 2 | 2 | 2 | 1 | 1 | 1 | 4 | 1 |
| NM_024298 | MBOAT7 | 2598 | 2 | 1 | 0 | 0 | 0 | 2 | 1 | 0 | 1 | 1 | 0 | 1 | 1 |
| NM_024299 | PPDPF | 777 | 27 | 11 | 15 | 27 | 13 | 13 | 16 | 5 | 8 | 7 | 11 | 5 | 4 |
| NM_024300 | CHCHD7 | 1666 | 26 | 13 | 9 | 9 | 16 | 26 | 13 | 8 | 11 | 9 | 8 | 22 | 8 |
| NM_024301 | FKRP | 3422 | 3 | 2 | 1 | 1 | 2 | 2 | 2 | 1 | 1 | 1 | 1 | 2 | 3 |
| NM_024302 | MMP28 | 2464 | 2 | 0 | 1 | 1 | 0 | 1 | 0 | 0 | 1 | 0 | 0 | 0 | 2 |
| NM_024303 | ZSCAN5A | 1922 | 2 | 0 | 1 | 0 | 0 | 1 | 0 | 1 | 0 | 0 | 0 | 0 | 2 |
| NM_024306 | FA2H | 2417 | 7 | 1 | 0 | 2 | 0 | 7 | 1 | 0 | 0 | 0 | 0 | 0 | 0 |
| NM_024307 | GDPD3 | 1098 | 1 | 0 | 0 | 1 | 0 | 0 | 1 | 0 | 0 | 0 | 0 | 0 | 0 |
| NM_024308 | DHRS11 | 1598 | 6 | 1 | 2 | 6 | 1 | 2 | 2 | 1 | 0 | 0 | 1 | 0 | 0 |
| NM_024309 | TNIP2 | 1974 | 7 | 5 | 7 | 7 | 5 | 4 | 5 | 5 | 3 | 3 | 3 | 7 | 6 |
| NM_024310 | PLEKHF1 | 1739 | 1 | 0 | 0 | 0 | 0 | 0 | 0 | 0 | 0 | 0 | 1 | 0 | 0 |
| NM_024311 | MFSD11 | 2508 | 2 | 1 | 1 | 1 | 1 | 1 | 1 | 1 | 1 | 1 | 1 | 1 | 2 |
| NM_024312 | GNPTAB | 5615 | 9 | 5 | 3 | 3 | 6 | 8 | 5 | 2 | 6 | 3 | 2 | 9 | 4 |
| NM_024313 | NOL12 | 2840 | 2 | 1 | 1 | 2 | 0 | 1 | 1 | 1 | 0 | 0 | 0 | 1 | 1 |
| NM_024315 | C7orf23 | 1043 | 7 | 3 | 2 | 1 | 1 | 2 | 4 | 1 | 5 | 4 | 1 | 7 | 3 |
| NM_024316 | LENG1 | 888 | 12 | 8 | 9 | 8 | 10 | 12 | 7 | 7 | 7 | 7 | 9 | 10 | 7 |
| NM_024318 | LILRA6 | 3930 | 2 | 1 | 1 | 0 | 1 | 0 | 0 | 0 | 1 | 0 | 0 | 2 | 0 |
| NM_024319 | C1orf35 | 1290 | 3 | 2 | 2 | 2 | 1 | 3 | 1 | 1 | 1 | 2 | 1 | 1 | 2 |
| NM_024320 | PRR15L | 1384 | 23 | 3 | 0 | 23 | 0 | 0 | 6 | 0 | 5 | 0 | 0 | 0 | 1 |
| NM_024321 | RBM42 | 1650 | 3 | 2 | 2 | 2 | 2 | 3 | 2 | 3 | 1 | 1 | 2 | 2 | 1 |
| NM_024322 | CENPO | 1625 | 3 | 1 | 1 | 2 | 1 | 3 | 1 | 1 | 1 | 1 | 0 | 2 | 3 |
| NM_024323 | C19orf57 | 2420 | 0 | 0 | 0 | 0 | 0 | 0 | 0 | 0 | 0 | 0 | 0 | 0 | 0 |
| NM_024324 | CRELD2 | 1428 | 13 | 2 | 1 | 2 | 2 | 1 | 1 | 13 | 1 | 0 | 0 | 2 | 1 |
| NM_024325 | ZNF343 | 3628 | 3 | 2 | 3 | 2 | 2 | 2 | 2 | 2 | 1 | 3 | 3 | 3 | 3 |
| NM_024326 | FBXL15 | 2362 | 0 | 0 | 0 | 0 | 0 | 0 | 0 | 0 | 0 | 0 | 0 | 0 | 0 |
| NM_024327 | ZNF576 | 1899 | 1 | 1 | 0 | 0 | 1 | 0 | 1 | 1 | 0 | 0 | 0 | 0 | 0 |
| NM_024328 | THTPA | 2200 | 1 | 1 | 1 | 1 | 1 | 1 | 1 | 0 | 0 | 0 | 0 | 1 | 1 |
| NM_024329 | EFHD2 | 2420 | 8 | 3 | 2 | 4 | 2 | 5 | 1 | 1 | 4 | 1 | 0 | 8 | 7 |
| NM_024330 | SLC27A3 | 2413 | 3 | 1 | 2 | 1 | 1 | 0 | 1 | 3 | 2 | 0 | 0 | 0 | 1 |
| NM_024331 | TTPAL | 6215 | 6 | 1 | 0 | 0 | 1 | 1 | 1 | 6 | 1 | 2 | 0 | 1 | 1 |
| NM_024332 | BRCC3 | 2914 | 8 | 6 | 6 | 2 | 6 | 5 | 8 | 6 | 5 | 5 | 6 | 8 | 3 |
| NM_024333 | FSD1 | 1847 | 1 | 0 | 0 | 0 | 0 | 1 | 0 | 0 | 0 | 0 | 0 | 0 | 0 |
| NM_024334 | TMEM43 | 3342 | 10 | 6 | 9 | 5 | 10 | 4 | 5 | 2 | 6 | 9 | 4 | 8 | 6 |
| NM_024335 | IRX6 | 2329 | 1 | 0 | 1 | 0 | 0 | 0 | 0 | 0 | 0 | 0 | 0 | 0 | 0 |
| NM_024336 | IRX3 | 2308 | 2 | 0 | 0 | 0 | 1 | 0 | 1 | 0 | 2 | 0 | 0 | 0 | 0 |
| NM_024337 | IRX1 | 1858 | 1 | 0 | 1 | 0 | 0 | 1 | 1 | 0 | 0 | 0 | 0 | 0 | 0 |
| NM_024339 | THOC6 | 1420 | 0 | 0 | 0 | 0 | 0 | 0 | 0 | 0 | 0 | 0 | 0 | 0 | 0 |
| NM_024341 | ZNF557 | 6001 | 4 | 2 | 1 | 1 | 1 | 2 | 2 | 1 | 1 | 4 | 1 | 3 | 2 |
| NM_024344 | CAPN3 | 3298 | 42 | 6 | 1 | 2 | 0 | 3 | 8 | 0 | 1 | 1 | 42 | 1 | 1 |
| NM_024345 | DCAF10 | 7914 | 4 | 3 | 2 | 1 | 2 | 2 | 3 | 4 | 2 | 4 | 2 | 3 | 4 |
| NM_024348 | DCTN3 | 924 | 9 | 3 | 4 | 2 | 3 | 9 | 3 | 4 | 2 | 1 | 2 | 2 | 2 |
| NM_024407 | NDUFS7 | 786 | 0 | 0 | 0 | 0 | 0 | 0 | 0 | 0 | 0 | 0 | 0 | 0 | 0 |
| NM_024408 | NOTCH2 | 11425 | 11 | 6 | 11 | 3 | 3 | 4 | 3 | 5 | 5 | 8 | 1 | 9 | 11 |
| NM_024409 | NPPC | 381 | 0 | 0 | 0 | 0 | 0 | 0 | 0 | 0 | 0 | 0 | 0 | 0 | 0 |

| transcript | gene | transcript length (nt) | max | mean | adipose | colon | heart | hypothalamus | kidney | liver | lung | ovary | skeletalmuscle | spleen | testes |
|---|---|---|---|---|---|---|---|---|---|---|---|---|---|---|---|
| NM_024410 | ODF1 | 994 | 57 | 5 | 0 | 0 | 0 | 0 | 0 | 0 | 0 | 0 | 0 | 0 | 57 |
| NM_024411 | PDYN | 2557 | 3 | 0 | 0 | 0 | 0 | 3 | 0 | 0 | 0 | 0 | 0 | 0 | 0 |
| NM_024415 | DDX4 | 2879 | 41 | 4 | 0 | 0 | 0 | 0 | 0 | 0 | 0 | 0 | 0 | 0 | 41 |
| NM_024417 | FDXR | 1883 | 5 | 1 | 1 | 1 | 0 | 1 | 1 | 2 | 0 | 2 | 1 | 2 | 5 |
| NM_024418 | BZRAP1 | 7512 | 6 | 1 | 0 | 1 | 0 | 6 | 1 | 0 | 1 | 1 | 0 | 1 | 0 |
| NM_024419 | PGS1 | 2191 | 1 | 1 | 0 | 1 | 0 | 1 | 0 | 0 | 1 | 0 | 0 | 1 | 1 |
| NM_024420 | PLA2G4A | 2928 | 4 | 1 | 4 | 1 | 1 | 1 | 2 | 0 | 2 | 1 | 0 | 1 | 2 |
| NM_024421 | DSC1 | 4208 | 3 | 0 | 0 | 0 | 3 | 0 | 0 | 0 | 0 | 0 | 0 | 0 | 0 |
| NM_024422 | DSC2 | 5199 | 10 | 4 | 3 | 10 | 9 | 2 | 5 | 4 | 4 | 1 | 1 | 7 | 1 |
| NM_024423 | DSC3 | 6978 | 1 | 0 | 0 | 0 | 0 | 0 | 0 | 0 | 0 | 0 | 0 | 0 | 1 |
| NM_024424 | WT1 | 3020 | 8 | 2 | 0 | 0 | 0 | 0 | 1 | 0 | 0 | 7 | 0 | 3 | 8 |
| NM_024425 | WT1 | 2978 | 8 | 2 | 0 | 0 | 0 | 0 | 1 | 0 | 0 | 7 | 0 | 3 | 8 |
| NM_024426 | WT1 | 3029 | 8 | 2 | 0 | 0 | 0 | 0 | 1 | 0 | 0 | 7 | 0 | 3 | 8 |
| NM_024429 | PRKAG2 | 2232 | 21 | 6 | 3 | 3 | 21 | 8 | 7 | 5 | 4 | 2 | 1 | 5 | 4 |
| NM_024430 | PSTPIP2 | 3024 | 16 | 2 | 1 | 1 | 1 | 1 | 1 | 2 | 2 | 1 | 2 | 16 | 1 |
| NM_024482 | GMEB1 | 1929 | 9 | 4 | 4 | 4 | 3 | 4 | 4 | 2 | 3 | 5 | 4 | 9 | 6 |
| NM_024490 | ATP10A | 5234 | 0 | 0 | 0 | 0 | 0 | 0 | 0 | 0 | 0 | 0 | 0 | 0 | 0 |
| NM_024491 | CEP70 | 2662 | 9 | 5 | 2 | 2 | 6 | 6 | 8 | 5 | 6 | 7 | 4 | 3 | 9 |
| NM_024493 | ZKSCAN3 | 2295 | 1 | 1 | 1 | 1 | 0 | 1 | 1 | 0 | 1 | 1 | 0 | 1 | 1 |
| NM_024494 | WNT2B | 2468 | 4 | 1 | 0 | 1 | 0 | 1 | 0 | 0 | 0 | 4 | 0 | 0 | 2 |
| NM_024496 | C14orf4 | 4149 | 3 | 1 | 1 | 1 | 1 | 1 | 1 | 1 | 3 | 2 | 1 | 2 | 2 |
| NM_024501 | HOXD1 | 1975 | 0 | 0 | 0 | 0 | 0 | 0 | 0 | 0 | 0 | 0 | 0 | 0 | 0 |
| NM_024503 | HIVEP3 | 8872 | 1 | 0 | 1 | 0 | 0 | 1 | 0 | 0 | 1 | 0 | 0 | 1 | 0 |
| NM_024504 | PRDM14 | 2207 | 0 | 0 | 0 | 0 | 0 | 0 | 0 | 0 | 0 | 0 | 0 | 0 | 0 |
| NM_024505 | NOX5 | 2582 | 0 | 0 | 0 | 0 | 0 | 0 | 0 | 0 | 0 | 0 | 0 | 0 | 0 |
| NM_024506 | GLB1L | 2495 | 1 | 0 | 0 | 0 | 0 | 0 | 1 | 0 | 0 | 0 | 0 | 0 | 1 |
| NM_024507 | KREMEN2 | 1975 | 0 | 0 | 0 | 0 | 0 | 0 | 0 | 0 | 0 | 0 | 0 | 0 | 0 |
| NM_024508 | ZBED2 | 2187 | 1 | 0 | 0 | 0 | 0 | 0 | 0 | 0 | 1 | 0 | 0 | 0 | 0 |
| NM_024509 | LRFN3 | 2515 | 0 | 0 | 0 | 0 | 0 | 0 | 0 | 0 | 0 | 0 | 0 | 0 | 0 |
| NM_024511 | HAUS3 | 5600 | 6 | 3 | 3 | 1 | 2 | 2 | 3 | 1 | 3 | 4 | 2 | 5 | 6 |
| NM_024512 | LRRC2 | 5106 | 28 | 5 | 0 | 0 | 14 | 1 | 1 | 1 | 0 | 2 | 28 | 2 | 0 |
| NM_024513 | FYCO1 | 8505 | 12 | 5 | 5 | 5 | 10 | 2 | 3 | 3 | 4 | 6 | 12 | 4 | 3 |
| NM_024514 | CYP2R1 | 1618 | 2 | 1 | 1 | 0 | 1 | 1 | 1 | 1 | 1 | 1 | 0 | 2 | 2 |
| NM_024515 | WDR25 | 2119 | 1 | 1 | 0 | 1 | 0 | 1 | 1 | 0 | 1 | 1 | 1 | 1 | 0 |
| NM_024516 | C16orf53 | 3825 | 3 | 2 | 1 | 2 | 2 | 2 | 2 | 2 | 1 | 3 | 1 | 2 | 2 |
| NM_024518 | ULBP3 | 735 | 4 | 1 | 0 | 0 | 0 | 0 | 0 | 0 | 0 | 0 | 0 | 0 | 4 |
| NM_024519 | FAM65A | 4070 | 7 | 4 | 4 | 3 | 5 | 6 | 5 | 1 | 3 | 4 | 3 | 7 | 2 |
| NM_024520 | C2orf47 | 1509 | 2 | 1 | 0 | 0 | 1 | 1 | 2 | 2 | 0 | 0 | 0 | 1 | 0 |
| NM_024522 | NKAIN1 | 2493 | 1 | 0 | 0 | 0 | 0 | 1 | 0 | 0 | 0 | 0 | 0 | 0 | 0 |
| NM_024523 | GCC1 | 4132 | 3 | 2 | 2 | 2 | 2 | 2 | 2 | 2 | 2 | 2 | 1 | 3 | 3 |
| NM_024524 | ATP13A3 | 7328 | 29 | 11 | 14 | 3 | 20 | 8 | 16 | 29 | 8 | 6 | 3 | 13 | 6 |
| NM_024525 | TTC13 | 3276 | 3 | 1 | 2 | 1 | 1 | 3 | 1 | 1 | 1 | 1 | 1 | 2 | 1 |
| NM_024526 | EPS8L3 | 2148 | 2 | 0 | 0 | 2 | 0 | 0 | 0 | 0 | 0 | 0 | 0 | 0 | 0 |
| NM_024527 | ABHD8 | 2090 | 1 | 0 | 0 | 0 | 0 | 1 | 0 | 0 | 0 | 0 | 0 | 0 | 0 |
| NM_024528 | NKAP | 1575 | 10 | 7 | 7 | 3 | 6 | 10 | 7 | 7 | 6 | 8 | 6 | 8 | 10 |
| NM_024529 | CDC73 | 5937 | 10 | 7 | 8 | 4 | 8 | 8 | 7 | 5 | 8 | 10 | 5 | 9 | 10 |
| NM_024531 | GPR172A | 1938 | 0 | 0 | 0 | 0 | 0 | 0 | 0 | 0 | 0 | 0 | 0 | 0 | 0 |
| NM_024532 | SPAG16 | 2174 | 9 | 2 | 1 | 1 | 1 | 5 | 3 | 1 | 2 | 2 | 1 | 1 | 9 |
| NM_024533 | CHST5 | 3250 | 1 | 0 | 0 | 1 | 0 | 0 | 0 | 0 | 0 | 0 | 0 | 0 | 0 |
| NM_024535 | CORO7 | 3514 | 0 | 0 | 0 | 0 | 0 | 0 | 0 | 0 | 0 | 0 | 0 | 0 | 0 |
| NM_024536 | CHPF | 2991 | 3 | 1 | 0 | 0 | 1 | 1 | 0 | 2 | 0 | 1 | 0 | 0 | 3 |
| NM_024537 | CARS2 | 1862 | 2 | 2 | 2 | 2 | 2 | 1 | 2 | 2 | 1 | 2 | 2 | 2 | 1 |
| NM_024539 | RNF128 | 2677 | 6 | 1 | 0 | 1 | 0 | 1 | 6 | 6 | 1 | 0 | 1 | 0 | 0 |
| NM_024540 | MRPL24 | 886 | 6 | 3 | 4 | 2 | 3 | 4 | 3 | 6 | 1 | 2 | 2 | 2 | 1 |
| NM_024541 | C10orf76 | 4101 | 3 | 2 | 1 | 2 | 2 | 1 | 2 | 1 | 1 | 1 | 1 | 3 | 2 |
| NM_024544 | MUL1 | 2458 | 1 | 1 | 0 | 1 | 1 | 1 | 1 | 1 | 0 | 0 | 0 | 1 | 1 |
| NM_024545 | SAP130 | 4068 | 6 | 2 | 1 | 1 | 1 | 2 | 1 | 1 | 1 | 2 | 1 | 2 | 6 |
| NM_024546 | RNF219 | 3534 | 6 | 2 | 2 | 0 | 2 | 4 | 1 | 1 | 2 | 3 | 2 | 4 | 6 |
| NM_024548 | CEP97 | 4411 | 6 | 3 | 1 | 1 | 2 | 6 | 2 | 1 | 2 | 4 | 3 | 4 | 6 |
| NM_024549 | TCTN1 | 1919 | 1 | 1 | 0 | 0 | 1 | 0 | 1 | 0 | 0 | 1 | 0 | 1 | 1 |
| NM_024551 | ADIPOR2 | 3965 | 8 | 5 | 7 | 2 | 4 | 4 | 3 | 8 | 5 | 3 | 3 | 4 | 6 |
| NM_024552 | LASS4 | 1814 | 2 | 1 | 1 | 0 | 1 | 1 | 2 | 2 | 1 | 0 | 0 | 1 | 1 |
| NM_024553 | CCDC132 | 1190 | 6 | 3 | 2 | 1 | 2 | 6 | 4 | 2 | 3 | 4 | 2 | 5 | 5 |
| NM_024554 | PGBD5 | 3444 | 2 | 0 | 0 | 0 | 0 | 2 | 0 | 0 | 0 | 0 | 0 | 0 | 0 |
| NM_024555 | FBXL6 | 1727 | 0 | 0 | 0 | 0 | 0 | 0 | 0 | 0 | 0 | 0 | 0 | 0 | 0 |
| NM_024556 | FAM118B | 1752 | 10 | 5 | 4 | 5 | 10 | 8 | 4 | 4 | 3 | 2 | 4 | 6 | 7 |
| NM_024557 | RIC3 | 5809 | 3 | 1 | 0 | 0 | 1 | 3 | 1 | 0 | 0 | 2 | 0 | 1 | 2 |
| NM_024558 | C14orf138 | 1784 | 5 | 2 | 1 | 1 | 2 | 3 | 2 | 2 | 2 | 2 | 1 | 5 | 3 |
| NM_024560 | ACSS3 | 3033 | 8 | 3 | 4 | 0 | 3 | 1 | 5 | 8 | 2 | 4 | 1 | 1 | 2 |
| NM_024561 | NARG1L | 4347 | 3 | 1 | 1 | 1 | 1 | 2 | 1 | 1 | 1 | 2 | 2 | 2 | 3 |
| NM_024562 | TMCO7 | 4818 | 2 | 1 | 1 | 1 | 1 | 2 | 1 | 2 | 1 | 1 | 1 | 1 | 2 |
| NM_024563 | C5orf23 | 2875 | 15 | 4 | 15 | 0 | 4 | 0 | 14 | 1 | 5 | 2 | 0 | 1 | 1 |
| NM_024565 | CCNJL | 3274 | 1 | 0 | 0 | 1 | 0 | 0 | 0 | 0 | 1 | 1 | 0 | 1 | 1 |
| NM_024567 | HMBOX1 | 3231 | 5 | 3 | 3 | 2 | 3 | 5 | 3 | 2 | 4 | 4 | 2 | 5 | 4 |
| NM_024569 | MPZL1 | 4906 | 9 | 4 | 4 | 3 | 5 | 3 | 3 | 2 | 4 | 5 | 1 | 4 | 9 |
| NM_024570 | RNASEH2B | 1551 | 4 | 2 | 2 | 1 | 3 | 3 | 2 | 1 | 2 | 2 | 1 | 4 | 3 |
| NM_024571 | SNRNP25 | 1086 | 7 | 3 | 1 | 2 | 5 | 7 | 2 | 3 | 2 | 1 | 5 | 2 | 2 |

| transcript | gene | transcript length (nt) | max | mean | adipose | colon | heart | hypothalamus | kidney | liver | lung | ovary | skeletalmuscle | spleen | testes |
|---|---|---|---|---|---|---|---|---|---|---|---|---|---|---|---|
| NM_024572 | GALNT14 | 2712 | 4 | 1 | 0 | 0 | 0 | 1 | 4 | 0 | 0 | 0 | 0 | 1 | 0 |
| NM_024573 | C6orf211 | 2572 | 4 | 2 | 2 | 1 | 3 | 2 | 4 | 3 | 2 | 2 | 2 | 2 | 2 |
| NM_024574 | C4orf31 | 2871 | 20 | 4 | 1 | 1 | 1 | 1 | 9 | 0 | 20 | 9 | 1 | 0 | 1 |
| NM_024575 | TNFAIP8L2 | 1178 | 2 | 0 | 0 | 0 | 0 | 1 | 0 | 0 | 1 | 0 | 0 | 2 | 0 |
| NM_024576 | OGFRL1 | 1740 | 119 | 30 | 43 | 5 | 26 | 119 | 15 | 5 | 32 | 15 | 12 | 41 | 16 |
| NM_024577 | SH3TC2 | 26580 | 1 | 0 | 0 | 0 | 0 | 1 | 0 | 0 | 0 | 0 | 0 | 0 | 1 |
| NM_024578 | OCEL1 | 1091 | 1 | 1 | 1 | 1 | 1 | 1 | 1 | 1 | 1 | 0 | 0 | 1 | 0 |
| NM_024579 | C1orf54 | 504 | 7 | 3 | 4 | 3 | 5 | 5 | 3 | 2 | 4 | 1 | 0 | 7 | 3 |
| NM_024580 | EFTUD1 | 3685 | 4 | 2 | 2 | 1 | 3 | 2 | 2 | 2 | 1 | 1 | 2 | 2 | 4 |
| NM_024581 | FAM184A | 4043 | 10 | 3 | 1 | 0 | 1 | 7 | 4 | 1 | 3 | 2 | 1 | 1 | 10 |
| NM_024582 | FAT4 | 16110 | 5 | 2 | 2 | 1 | 4 | 2 | 2 | 1 | 3 | 5 | 1 | 5 | 2 |
| NM_024583 | SCRN3 | 2468 | 14 | 8 | 8 | 3 | 11 | 14 | 9 | 9 | 4 | 5 | 6 | 8 | 7 |
| NM_024584 | CCDC121 | 2337 | 2 | 1 | 1 | 0 | 1 | 1 | 1 | 0 | 1 | 1 | 1 | 1 | 2 |
| NM_024585 | ARMC7 | 2126 | 3 | 1 | 1 | 1 | 1 | 1 | 3 | 1 | 1 | 1 | 1 | 2 | 1 |
| NM_024586 | OSBPL9 | 2907 | 35 | 16 | 10 | 4 | 13 | 7 | 20 | 35 | 13 | 15 | 19 | 14 | 24 |
| NM_024587 | TMEM53 | 1600 | 0 | 0 | 0 | 0 | 0 | 0 | 0 | 0 | 0 | 0 | 0 | 0 | 0 |
| NM_024589 | ROGDI | 1458 | 4 | 1 | 0 | 1 | 1 | 4 | 1 | 0 | 0 | 0 | 0 | 1 | 0 |
| NM_024590 | ARSJ | 4678 | 2 | 1 | 0 | 1 | 1 | 0 | 2 | 0 | 1 | 0 | 0 | 0 | 0 |
| NM_024591 | CHMP6 | 1664 | 1 | 1 | 0 | 1 | 1 | 1 | 1 | 0 | 0 | 0 | 0 | 0 | 0 |
| NM_024592 | SRD5A3 | 2660 | 1 | 0 | 0 | 0 | 0 | 0 | 0 | 1 | 0 | 0 | 0 | 0 | 0 |
| NM_024593 | EFCAB1 | 2144 | 1 | 0 | 0 | 0 | 0 | 1 | 0 | 0 | 1 | 1 | 0 | 0 | 1 |
| NM_024594 | PANK3 | 3359 | 18 | 10 | 6 | 8 | 8 | 12 | 10 | 18 | 7 | 8 | 4 | 11 | 12 |
| NM_024595 | AKIRIN1 | 2724 | 14 | 7 | 4 | 4 | 8 | 10 | 8 | 8 | 5 | 3 | 2 | 9 | 14 |
| NM_024596 | MCPH1 | 8032 | 3 | 2 | 1 | 1 | 2 | 2 | 2 | 1 | 1 | 2 | 2 | 3 | 2 |
| NM_024597 | MAP7D3 | 3027 | 23 | 9 | 18 | 1 | 13 | 2 | 5 | 3 | 4 | 12 | 8 | 9 | 23 |
| NM_024598 | C16orf57 | 2254 | 1 | 1 | 1 | 1 | 1 | 1 | 1 | 1 | 1 | 0 | 0 | 1 | 1 |
| NM_024599 | RHBDF2 | 3604 | 1 | 0 | 0 | 0 | 0 | 0 | 0 | 0 | 1 | 0 | 0 | 1 | 0 |
| NM_024600 | TMEM204 | 943 | 0 | 0 | 0 | 0 | 0 | 0 | 0 | 0 | 0 | 0 | 0 | 0 | 0 |
| NM_024602 | HECTD3 | 3628 | 3 | 2 | 3 | 3 | 1 | 1 | 2 | 3 | 1 | 1 | 2 | 2 | 2 |
| NM_024603 | BEND5 | 1328 | 3 | 1 | 1 | 1 | 0 | 3 | 2 | 0 | 0 | 3 | 1 | 2 | 2 |
| NM_024604 | RPAP3 | 3686 | 6 | 4 | 3 | 1 | 4 | 5 | 3 | 3 | 2 | 5 | 5 | 6 | 6 |
| NM_024605 | ARHGAP10 | 3056 | 10 | 6 | 8 | 3 | 9 | 2 | 4 | 2 | 4 | 10 | 7 | 4 | 8 |
| NM_024607 | PPP1R3B | 5560 | 20 | 6 | 2 | 1 | 6 | 1 | 2 | 20 | 3 | 3 | 15 | 4 | 5 |
| NM_024608 | NEIL1 | 1890 | 0 | 0 | 0 | 0 | 0 | 0 | 0 | 0 | 0 | 0 | 0 | 0 | 0 |
| NM_024610 | HSPBAP1 | 1936 | 6 | 2 | 3 | 1 | 2 | 1 | 2 | 1 | 2 | 1 | 6 | 3 | 3 |
| NM_024611 | NARG2 | 7209 | 4 | 3 | 3 | 1 | 2 | 2 | 3 | 1 | 2 | 4 | 2 | 4 | 4 |
| NM_024612 | DHX40 | 3666 | 8 | 4 | 4 | 2 | 4 | 4 | 6 | 2 | 4 | 7 | 3 | 5 | 8 |
| NM_024613 | PLEKHF2 | 2874 | 7 | 3 | 2 | 1 | 2 | 1 | 5 | 2 | 3 | 2 | 2 | 7 | 2 |
| NM_024615 | PARP8 | 2956 | 12 | 3 | 2 | 1 | 2 | 3 | 2 | 1 | 6 | 2 | 0 | 12 | 2 |
| NM_024616 | C3orf52 | 2278 | 1 | 1 | 0 | 1 | 1 | 0 | 1 | 0 | 1 | 0 | 0 | 0 | 0 |
| NM_024617 | ZCCHC6 | 5647 | 19 | 8 | 7 | 5 | 8 | 5 | 6 | 12 | 13 | 7 | 5 | 19 | 7 |
| NM_024618 | NLRX1 | 3745 | 0 | 0 | 0 | 0 | 0 | 0 | 0 | 0 | 0 | 0 | 0 | 0 | 0 |
| NM_024619 | FN3KRP | 1822 | 2 | 1 | 1 | 2 | 1 | 2 | 2 | 1 | 1 | 1 | 0 | 1 | 2 |
| NM_024620 | ZNF329 | 3413 | 6 | 3 | 2 | 2 | 3 | 5 | 4 | 1 | 2 | 4 | 1 | 4 | 6 |
| NM_024621 | VEPH1 | 4310 | 5 | 1 | 0 | 0 | 0 | 0 | 4 | 0 | 5 | 0 | 0 | 0 | 0 |
| NM_024622 | FASTKD1 | 3004 | 3 | 2 | 1 | 2 | 3 | 2 | 2 | 2 | 1 | 0 | 1 | 3 | 2 |
| NM_024623 | OGFOD2 | 2080 | 0 | 0 | 0 | 0 | 0 | 0 | 0 | 0 | 0 | 0 | 0 | 0 | 0 |
| NM_024624 | SMC6 | 5172 | 14 | 4 | 3 | 1 | 4 | 4 | 3 | 3 | 3 | 3 | 3 | 8 | 14 |
| NM_024625 | ZC3HAV1 | 3182 | 9 | 4 | 3 | 4 | 2 | 2 | 4 | 2 | 5 | 3 | 1 | 9 | 4 |
| NM_024626 | VTCN1 | 2605 | 3 | 0 | 0 | 0 | 0 | 0 | 3 | 0 | 0 | 0 | 0 | 0 | 1 |
| NM_024627 | C22orf29 | 6635 | 1 | 1 | 0 | 1 | 0 | 1 | 1 | 0 | 1 | 1 | 0 | 1 | 1 |
| NM_024628 | SLC12A8 | 3510 | 0 | 0 | 0 | 0 | 0 | 0 | 0 | 0 | 0 | 0 | 0 | 0 | 0 |
| NM_024629 | MLF1IP | 2549 | 11 | 2 | 1 | 1 | 1 | 0 | 0 | 0 | 1 | 0 | 0 | 1 | 11 |
| NM_024630 | ZDHHC14 | 2787 | 1 | 0 | 0 | 0 | 1 | 1 | 1 | 0 | 0 | 0 | 0 | 1 | 1 |
| NM_024631 | C11orf61 | 2225 | 2 | 1 | 1 | 1 | 1 | 1 | 2 | 0 | 1 | 2 | 1 | 2 | 1 |
| NM_024632 | SAP30L | 6224 | 3 | 2 | 2 | 2 | 2 | 3 | 2 | 2 | 1 | 3 | 2 | 3 | 3 |
| NM_024635 | MAK10 | 2657 | 4 | 3 | 2 | 2 | 2 | 3 | 3 | 2 | 2 | 4 | 2 | 2 | 4 |
| NM_024636 | STEAP4 | 4454 | 28 | 9 | 19 | 1 | 28 | 2 | 5 | 10 | 12 | 1 | 4 | 4 | 15 |
| NM_024637 | GAL3ST4 | 2540 | 0 | 0 | 0 | 0 | 0 | 0 | 0 | 0 | 0 | 0 | 0 | 0 | 0 |
| NM_024638 | QTRTD1 | 3995 | 5 | 2 | 1 | 1 | 1 | 1 | 3 | 1 | 2 | 2 | 1 | 5 | 3 |
| NM_024639 | ZNF322A | 4800 | 3 | 1 | 1 | 0 | 1 | 1 | 1 | 0 | 1 | 2 | 1 | 2 | 3 |
| NM_024640 | YRDC | 1836 | 1 | 0 | 0 | 0 | 0 | 1 | 0 | 0 | 0 | 0 | 0 | 0 | 0 |
| NM_024641 | MANEA | 4574 | 5 | 2 | 1 | 0 | 1 | 1 | 2 | 5 | 1 | 2 | 0 | 2 | 1 |
| NM_024642 | GALNT12 | 2731 | 6 | 1 | 1 | 6 | 0 | 0 | 1 | 0 | 2 | 0 | 0 | 0 | 1 |
| NM_024643 | FAM164C | 2076 | 6 | 1 | 0 | 0 | 1 | 1 | 1 | 1 | 0 | 0 | 0 | 0 | 6 |
| NM_024644 | C14orf169 | 2462 | 1 | 0 | 0 | 0 | 0 | 0 | 0 | 0 | 0 | 0 | 0 | 1 | 1 |
| NM_024645 | ZMAT4 | 2462 | 3 | 0 | 0 | 0 | 0 | 3 | 0 | 0 | 0 | 0 | 0 | 0 | 0 |
| NM_024646 | ZYG11B | 8140 | 11 | 5 | 2 | 1 | 6 | 6 | 4 | 5 | 2 | 4 | 11 | 3 | 5 |
| NM_024648 | C17orf101 | 1235 | 1 | 0 | 0 | 1 | 0 | 0 | 0 | 1 | 0 | 0 | 0 | 1 | 0 |
| NM_024649 | BBS1 | 3370 | 2 | 1 | 0 | 1 | 0 | 1 | 2 | 0 | 0 | 1 | 0 | 1 | 1 |
| NM_024650 | C11orf80 | 2323 | 1 | 0 | 0 | 1 | 0 | 1 | 1 | 0 | 0 | 0 | 0 | 0 | 1 |
| NM_024652 | LRRK1 | 7631 | 2 | 0 | 1 | 1 | 0 | 0 | 0 | 0 | 0 | 0 | 0 | 2 | 1 |
| NM_024653 | PRKRIP1 | 2181 | 3 | 2 | 2 | 2 | 1 | 3 | 2 | 1 | 1 | 2 | 1 | 2 | 3 |
| NM_024654 | NOL9 | 6710 | 3 | 2 | 1 | 1 | 2 | 2 | 2 | 2 | 1 | 2 | 2 | 3 | 2 |
| NM_024656 | GLT25D1 | 3593 | 4 | 2 | 3 | 1 | 3 | 1 | 1 | 1 | 2 | 1 | 1 | 4 | 1 |
| NM_024657 | MORC4 | 3802 | 5 | 2 | 2 | 1 | 1 | 1 | 1 | 4 | 2 | 2 | 1 | 1 | 5 |
| NM_024658 | IPO4 | 3600 | 3 | 1 | 1 | 1 | 1 | 1 | 3 | 3 | 1 | 0 | 1 | 1 | 3 |

| transcript | gene | transcript length (nt) | max | mean | adipose | colon | heart | hypothalamus | kidney | liver | lung | ovary | skeletalmuscle | spleen | testes |
|---|---|---|---|---|---|---|---|---|---|---|---|---|---|---|---|
| NM_024659 | GTDC1 | 2405 | 6 | 2 | 3 | 0 | 2 | 6 | 1 | 1 | 2 | 2 | 1 | 4 | 3 |
| NM_024660 | TMEM149 | 1196 | 1 | 0 | 0 | 0 | 0 | 0 | 0 | 1 | 0 | 0 | 0 | 0 | 0 |
| NM_024661 | CCDC51 | 1506 | 1 | 1 | 1 | 1 | 0 | 1 | 1 | 1 | 0 | 0 | 0 | 1 | 1 |
| NM_024662 | NAT10 | 4040 | 5 | 3 | 3 | 3 | 3 | 5 | 3 | 3 | 2 | 4 | 3 | 5 | 4 |
| NM_024663 | NPEPL1 | 2186 | 1 | 1 | 1 | 1 | 0 | 0 | 1 | 0 | 0 | 0 | 0 | 1 | 1 |
| NM_024664 | PPCS | 1489 | 10 | 5 | 7 | 4 | 5 | 6 | 10 | 7 | 3 | 4 | 3 | 6 | 4 |
| NM_024665 | TBL1XR1 | 6550 | 14 | 10 | 8 | 7 | 11 | 10 | 9 | 7 | 12 | 13 | 6 | 14 | 10 |
| NM_024666 | AAGAB | 2845 | 10 | 5 | 4 | 3 | 6 | 10 | 7 | 4 | 4 | 2 | 5 | 6 | 6 |
| NM_024667 | VPS37B | 2745 | 3 | 2 | 1 | 3 | 1 | 1 | 2 | 0 | 3 | 1 | 0 | 3 | 2 |
| NM_024668 | ANKHD1 | 2145 | 0 | 0 | 0 | 0 | 0 | 0 | 0 | 0 | 0 | 0 | 0 | 0 | 0 |
| NM_024669 | ANKRD55 | 2500 | 0 | 0 | 0 | 0 | 0 | 0 | 0 | 0 | 0 | 0 | 0 | 0 | 0 |
| NM_024670 | SUV39H2 | 3077 | 4 | 2 | 1 | 1 | 1 | 3 | 1 | 2 | 1 | 3 | 1 | 2 | 4 |
| NM_024671 | ZNF768 | 2315 | 4 | 2 | 2 | 4 | 1 | 2 | 2 | 1 | 1 | 1 | 1 | 1 | 2 |
| NM_024672 | THAP9 | 4038 | 1 | 1 | 0 | 0 | 0 | 1 | 1 | 0 | 0 | 1 | 1 | 1 | 1 |
| NM_024674 | LIN28 | 4014 | 0 | 0 | 0 | 0 | 0 | 0 | 0 | 0 | 0 | 0 | 0 | 0 | 0 |
| NM_024675 | PALB2 | 4058 | 4 | 2 | 1 | 3 | 1 | 2 | 2 | 2 | 1 | 2 | 1 | 3 | 4 |
| NM_024676 | C1orf113 | 2325 | 1 | 0 | 0 | 0 | 0 | 0 | 1 | 0 | 0 | 0 | 0 | 0 | 0 |
| NM_024677 | NSUN7 | 3698 | 1 | 0 | 0 | 0 | 0 | 0 | 0 | 0 | 0 | 0 | 0 | 0 | 1 |
| NM_024678 | NARS2 | 2509 | 2 | 1 | 1 | 1 | 1 | 2 | 1 | 1 | 1 | 1 | 1 | 1 | 1 |
| NM_024680 | E2F8 | 3721 | 1 | 0 | 0 | 1 | 1 | 0 | 0 | 0 | 0 | 0 | 1 | 1 | 0 |
| NM_024681 | KCTD17 | 1691 | 1 | 0 | 0 | 0 | 0 | 1 | 0 | 0 | 0 | 0 | 0 | 0 | 0 |
| NM_024682 | TBC1D17 | 2371 | 2 | 1 | 0 | 1 | 1 | 1 | 1 | 1 | 1 | 1 | 2 | 0 | 1 |
| NM_024683 | C17orf42 | 1339 | 1 | 1 | 1 | 1 | 1 | 1 | 1 | 0 | 0 | 1 | 1 | 1 | 0 |
| NM_024684 | C11orf67 | 504 | 29 | 15 | 27 | 9 | 29 | 9 | 11 | 7 | 12 | 12 | 29 | 9 | 9 |
| NM_024685 | BBS10 | 3583 | 4 | 3 | 3 | 1 | 2 | 4 | 3 | 1 | 2 | 4 | 2 | 4 | 3 |
| NM_024686 | TTLL7 | 3648 | 12 | 2 | 1 | 0 | 1 | 12 | 1 | 0 | 1 | 0 | 1 | 0 | 2 |
| NM_024687 | ZBBX | 3230 | 6 | 1 | 0 | 0 | 0 | 1 | 0 | 0 | 2 | 0 | 0 | 0 | 6 |
| NM_024688 | C10orf68 | 2719 | 13 | 2 | 0 | 0 | 0 | 1 | 1 | 0 | 1 | 1 | 0 | 1 | 13 |
| NM_024689 | CXorf36 | 2512 | 1 | 1 | 1 | 0 | 1 | 0 | 0 | 0 | 1 | 0 | 0 | 1 | 1 |
| NM_024690 | MUC16 | 43816 | 1 | 0 | 0 | 0 | 0 | 0 | 0 | 0 | 1 | 0 | 0 | 0 | 0 |
| NM_024691 | ZNF419 | 2363 | 2 | 1 | 1 | 1 | 1 | 2 | 1 | 0 | 1 | 2 | 1 | 2 | 1 |
| NM_024692 | CLIP4 | 4276 | 10 | 4 | 3 | 1 | 5 | 5 | 2 | 1 | 4 | 5 | 10 | 3 | 3 |
| NM_024693 | ECHDC3 | 1654 | 15 | 7 | 4 | 1 | 11 | 1 | 13 | 15 | 3 | 3 | 15 | 4 | 2 |
| NM_024694 | C6orf103 | 5289 | 1 | 0 | 0 | 0 | 0 | 0 | 0 | 0 | 0 | 0 | 0 | 0 | 1 |
| NM_024697 | ZNF385D | 1810 | 2 | 1 | 0 | 0 | 2 | 2 | 0 | 0 | 0 | 1 | 0 | 0 | 1 |
| NM_024698 | SLC25A22 | 2754 | 3 | 1 | 0 | 0 | 0 | 3 | 1 | 1 | 0 | 0 | 0 | 0 | 0 |
| NM_024699 | ZFAND1 | 1793 | 9 | 6 | 5 | 3 | 4 | 8 | 6 | 6 | 6 | 9 | 4 | 9 | 5 |
| NM_024700 | SNIP1 | 2471 | 8 | 5 | 3 | 3 | 3 | 5 | 3 | 5 | 2 | 6 | 3 | 7 | 8 |
| NM_024701 | ASB13 | 2727 | 2 | 1 | 0 | 1 | 0 | 1 | 1 | 2 | 0 | 0 | 0 | 1 | 0 |
| NM_024702 | ZNF750 | 3191 | 1 | 0 | 0 | 0 | 0 | 0 | 0 | 0 | 0 | 0 | 1 | 0 | 0 |
| NM_024704 | KIF16B | 5261 | 5 | 3 | 3 | 3 | 3 | 3 | 4 | 3 | 3 | 5 | 3 | 4 | 4 |
| NM_024705 | DHRS12 | 1970 | 1 | 1 | 1 | 0 | 0 | 0 | 1 | 1 | 0 | 1 | 0 | 1 | 0 |
| NM_024706 | ZNF668 | 2625 | 0 | 0 | 0 | 0 | 0 | 0 | 0 | 0 | 0 | 0 | 0 | 0 | 0 |
| NM_024707 | GEMIN7 | 1647 | 1 | 0 | 0 | 0 | 0 | 0 | 1 | 0 | 0 | 0 | 0 | 1 | 0 |
| NM_024708 | ASB7 | 1752 | 5 | 3 | 2 | 3 | 2 | 2 | 4 | 3 | 2 | 3 | 1 | 5 | 5 |
| NM_024709 | C1orf115 | 2972 | 21 | 9 | 9 | 7 | 3 | 18 | 12 | 21 | 6 | 5 | 1 | 12 | 3 |
| NM_024710 | ISOC2 | 1170 | 4 | 1 | 1 | 1 | 1 | 1 | 2 | 4 | 0 | 0 | 0 | 1 | 1 |
| NM_024711 | GIMAP6 | 3644 | 11 | 2 | 3 | 1 | 3 | 1 | 2 | 1 | 3 | 1 | 1 | 11 | 1 |
| NM_024712 | ELMO3 | 2512 | 0 | 0 | 0 | 0 | 0 | 0 | 0 | 0 | 0 | 0 | 0 | 0 | 0 |
| NM_024713 | C15orf29 | 2740 | 2 | 1 | 1 | 0 | 1 | 1 | 1 | 1 | 2 | 2 | 1 | 2 | 2 |
| NM_024715 | TXNDC15 | 3689 | 14 | 9 | 7 | 5 | 13 | 14 | 10 | 9 | 7 | 10 | 5 | 11 | 10 |
| NM_024717 | MCTP1 | 3910 | 12 | 3 | 6 | 1 | 3 | 12 | 1 | 1 | 5 | 1 | 1 | 4 | 3 |
| NM_024718 | C9orf86 | 3123 | 6 | 3 | 2 | 4 | 3 | 6 | 3 | 1 | 2 | 1 | 4 | 3 | 2 |
| NM_024719 | GRTP1 | 1406 | 7 | 2 | 0 | 2 | 0 | 0 | 3 | 7 | 0 | 1 | 1 | 1 | 2 |
| NM_024721 | ZFHX4 | 13823 | 3 | 1 | 3 | 1 | 0 | 2 | 0 | 1 | 0 | 1 | 2 | 0 | 1 |
| NM_024722 | ACBD4 | 2019 | 3 | 1 | 1 | 1 | 0 | 1 | 1 | 3 | 0 | 1 | 0 | 0 | 0 |
| NM_024725 | CCDC82 | 2730 | 9 | 6 | 6 | 2 | 5 | 9 | 6 | 3 | 8 | 5 | 2 | 9 | 9 |
| NM_024726 | IQCA1 | 3188 | 5 | 1 | 0 | 0 | 0 | 5 | 2 | 0 | 1 | 1 | 0 | 2 | 2 |
| NM_024727 | LRRC31 | 2465 | 4 | 0 | 0 | 4 | 0 | 0 | 0 | 0 | 0 | 0 | 0 | 0 | 0 |
| NM_024728 | C7orf10 | 1608 | 4 | 1 | 0 | 0 | 0 | 0 | 4 | 1 | 0 | 1 | 0 | 0 | 0 |
| NM_024729 | MYH14 | 6789 | 6 | 2 | 2 | 6 | 3 | 2 | 1 | 2 | 2 | 0 | 6 | 0 | 0 |
| NM_024730 | RERGL | 1134 | 4 | 1 | 4 | 1 | 1 | 1 | 1 | 0 | 0 | 3 | 2 | 0 | 1 |
| NM_024731 | KLHL36 | 2169 | 0 | 0 | 0 | 0 | 0 | 0 | 0 | 0 | 0 | 0 | 0 | 0 | 0 |
| NM_024733 | ZNF665 | 3291 | 2 | 0 | 0 | 0 | 0 | 0 | 0 | 0 | 0 | 2 | 0 | 0 | 0 |
| NM_024734 | CLMN | 12750 | 5 | 2 | 1 | 3 | 1 | 2 | 5 | 3 | 1 | 0 | 0 | 1 | 1 |
| NM_024735 | FBXO31 | 3614 | 3 | 1 | 1 | 2 | 1 | 2 | 1 | 2 | 1 | 1 | 3 | 1 | 1 |
| NM_024736 | GSDMD | 1759 | 1 | 0 | 0 | 0 | 0 | 0 | 0 | 1 | 0 | 0 | 0 | 1 | 0 |
| NM_024738 | C12orf49 | 2775 | 3 | 2 | 1 | 1 | 1 | 3 | 3 | 1 | 3 | 1 | 1 | 2 | 1 |
| NM_024740 | ALG9 | 6159 | 5 | 3 | 5 | 1 | 3 | 3 | 2 | 4 | 2 | 4 | 2 | 3 | 4 |
| NM_024741 | ZNF408 | 2441 | 0 | 0 | 0 | 0 | 0 | 0 | 0 | 0 | 0 | 0 | 0 | 0 | 0 |
| NM_024742 | ARMC5 | 4673 | 0 | 0 | 0 | 0 | 0 | 0 | 0 | 0 | 0 | 0 | 0 | 0 | 0 |
| NM_024743 | UGT2A3 | 2969 | 6 | 1 | 0 | 1 | 0 | 0 | 6 | 1 | 0 | 0 | 0 | 0 | 0 |
| NM_024744 | ALS2CR8 | 5929 | 2 | 1 | 1 | 0 | 0 | 1 | 1 | 0 | 1 | 1 | 1 | 2 | 2 |
| NM_024745 | SHCBP1 | 3232 | 1 | 0 | 1 | 0 | 0 | 0 | 1 | 0 | 0 | 0 | 0 | 1 | 1 |
| NM_024746 | HHIPL2 | 2574 | 1 | 0 | 0 | 0 | 0 | 0 | 0 | 0 | 0 | 0 | 0 | 0 | 1 |
| NM_024747 | HPS6 | 2647 | 0 | 0 | 0 | 0 | 0 | 0 | 0 | 0 | 0 | 0 | 0 | 0 | 0 |
| NM_024749 | VASH2 | 4335 | 0 | 0 | 0 | 0 | 0 | 0 | 0 | 0 | 0 | 0 | 0 | 0 | 0 |

| transcript | gene | transcript length (nt) | max | mean | adipose | colon | heart | hypothalamus | kidney | liver | lung | ovary | skeletalmuscle | spleen | testes |
|---|---|---|---|---|---|---|---|---|---|---|---|---|---|---|---|
| NM_024750 | LRRC2 | 4907 | 29 | 5 | 0 | 0 | 14 | 1 | 1 | 1 | 0 | 2 | 29 | 2 | 0 |
| NM_024751 | GSTCD | 4043 | 1 | 0 | 0 | 0 | 0 | 1 | 1 | 0 | 1 | 0 | 0 | 1 | 0 |
| NM_024753 | TTC21B | 4896 | 5 | 3 | 2 | 1 | 3 | 4 | 4 | 2 | 3 | 5 | 3 | 4 | 5 |
| NM_024754 | PTCD2 | 2103 | 1 | 1 | 1 | 0 | 1 | 1 | 1 | 1 | 0 | 1 | 1 | 1 | 1 |
| NM_024755 | SLTM | 4147 | 35 | 20 | 21 | 10 | 24 | 19 | 22 | 16 | 15 | 19 | 21 | 35 | 22 |
| NM_024756 | MMRN2 | 4177 | 2 | 1 | 2 | 1 | 2 | 1 | 1 | 1 | 1 | 1 | 1 | 2 | 1 |
| NM_024757 | EHMT1 | 5105 | 4 | 2 | 2 | 3 | 1 | 2 | 2 | 2 | 2 | 3 | 2 | 4 | 4 |
| NM_024758 | AGMAT | 2196 | 22 | 4 | 0 | 2 | 0 | 1 | 22 | 17 | 0 | 0 | 2 | 1 | 0 |
| NM_024759 | NIPAL2 | 2255 | 1 | 1 | 1 | 1 | 1 | 1 | 1 | 0 | 1 | 1 | 0 | 1 | 1 |
| NM_024760 | TLE6 | 1930 | 1 | 0 | 0 | 0 | 0 | 0 | 0 | 1 | 0 | 0 | 0 | 0 | 0 |
| NM_024761 | MOBKL2B | 6525 | 10 | 3 | 1 | 2 | 0 | 3 | 2 | 1 | 2 | 10 | 2 | 4 | 3 |
| NM_024762 | ZNF552 | 2352 | 4 | 2 | 1 | 4 | 1 | 1 | 4 | 1 | 2 | 2 | 1 | 4 | 1 |
| NM_024763 | WDR78 | 3843 | 6 | 2 | 0 | 0 | 1 | 2 | 2 | 0 | 2 | 2 | 0 | 0 | 6 |
| NM_024764 | CATSPERB | 3606 | 0 | 0 | 0 | 0 | 0 | 0 | 0 | 0 | 0 | 0 | 0 | 0 | 0 |
| NM_024766 | C2orf34 | 1553 | 1 | 0 | 0 | 0 | 0 | 0 | 1 | 0 | 0 | 0 | 0 | 1 | 1 |
| NM_024767 | DLC1 | 2452 | 11 | 5 | 11 | 1 | 10 | 3 | 2 | 0 | 3 | 10 | 5 | 1 | 7 |
| NM_024768 | CCDC48 | 2689 | 1 | 0 | 0 | 1 | 0 | 0 | 0 | 0 | 0 | 1 | 0 | 0 | 0 |
| NM_024769 | ASAM | 2630 | 30 | 5 | 30 | 4 | 5 | 1 | 1 | 0 | 2 | 4 | 0 | 4 | 2 |
| NM_024770 | METTL8 | 8134 | 3 | 2 | 2 | 1 | 2 | 2 | 2 | 2 | 1 | 2 | 1 | 3 | 3 |
| NM_024771 | NAT11 | 3336 | 2 | 1 | 0 | 1 | 1 | 1 | 1 | 0 | 0 | 0 | 0 | 2 | 1 |
| NM_024772 | ZMYM1 | 4170 | 2 | 1 | 1 | 1 | 0 | 1 | 1 | 1 | 1 | 2 | 0 | 2 | 1 |
| NM_024773 | JMJD5 | 2471 | 4 | 1 | 0 | 0 | 0 | 0 | 0 | 4 | 0 | 0 | 0 | 1 | 0 |
| NM_024775 | GEMIN6 | 692 | 9 | 4 | 4 | 3 | 2 | 4 | 4 | 9 | 2 | 3 | 2 | 5 | 6 |
| NM_024776 | SGK269 | 11576 | 27 | 8 | 8 | 4 | 10 | 6 | 3 | 1 | 9 | 6 | 3 | 27 | 10 |
| NM_024778 | LONRF3 | 2484 | 7 | 2 | 1 | 1 | 2 | 1 | 2 | 4 | 4 | 1 | 7 | 1 | 2 |
| NM_024779 | PIP4K2C | 3218 | 2 | 1 | 1 | 1 | 0 | 2 | 2 | 1 | 1 | 1 | 0 | 1 | 1 |
| NM_024780 | TMC5 | 3686 | 10 | 2 | 0 | 4 | 0 | 0 | 0 | 0 | 10 | 0 | 0 | 0 | 2 |
| NM_024781 | CCDC102B | 2711 | 12 | 3 | 3 | 2 | 2 | 3 | 2 | 0 | 12 | 1 | 0 | 2 | 3 |
| NM_024782 | NHEJ1 | 2103 | 10 | 5 | 5 | 6 | 3 | 3 | 10 | 5 | 3 | 6 | 5 | 5 | 9 |
| NM_024783 | AGBL2 | 3391 | 2 | 0 | 0 | 0 | 0 | 0 | 0 | 0 | 0 | 0 | 0 | 0 | 2 |
| NM_024784 | ZBTB3 | 2972 | 0 | 0 | 0 | 0 | 0 | 0 | 0 | 0 | 0 | 0 | 0 | 0 | 0 |
| NM_024785 | FAM124B | 2594 | 1 | 0 | 1 | 0 | 0 | 0 | 0 | 0 | 0 | 0 | 0 | 0 | 0 |
| NM_024786 | ZDHHC11 | 2606 | 1 | 0 | 0 | 0 | 1 | 1 | 1 | 0 | 0 | 0 | 0 | 0 | 1 |
| NM_024787 | RNF122 | 1868 | 2 | 1 | 0 | 0 | 2 | 0 | 1 | 0 | 1 | 2 | 1 | 0 | 0 |
| NM_024789 | TMEM180 | 2834 | 0 | 0 | 0 | 0 | 0 | 0 | 0 | 0 | 0 | 0 | 0 | 0 | 0 |
| NM_024790 | CSPP1 | 4365 | 14 | 5 | 3 | 2 | 4 | 5 | 7 | 2 | 5 | 6 | 6 | 5 | 14 |
| NM_024791 | PDZD3 | 2245 | 2 | 0 | 0 | 1 | 0 | 0 | 2 | 0 | 0 | 0 | 0 | 0 | 0 |
| NM_024792 | FAM57A | 2080 | 2 | 1 | 1 | 2 | 1 | 1 | 1 | 0 | 1 | 2 | 0 | 1 | 1 |
| NM_024793 | CLUAP1 | 2221 | 10 | 5 | 4 | 2 | 5 | 10 | 6 | 2 | 4 | 8 | 4 | 3 | 10 |
| NM_024794 | EPHX3 | 1809 | 0 | 0 | 0 | 0 | 0 | 0 | 0 | 0 | 0 | 0 | 0 | 0 | 0 |
| NM_024795 | TM4SF20 | 2294 | 0 | 0 | 0 | 0 | 0 | 0 | 0 | 0 | 0 | 0 | 0 | 0 | 0 |
| NM_024798 | SNX22 | 3614 | 1 | 0 | 0 | 0 | 0 | 1 | 0 | 0 | 0 | 0 | 0 | 0 | 0 |
| NM_024800 | NEK11 | 2952 | 5 | 2 | 1 | 1 | 1 | 5 | 2 | 0 | 2 | 3 | 0 | 1 | 5 |
| NM_024803 | TUBAL3 | 1798 | 1 | 0 | 0 | 1 | 0 | 0 | 1 | 0 | 0 | 0 | 0 | 0 | 0 |
| NM_024804 | ZNF669 | 1980 | 2 | 1 | 1 | 0 | 1 | 1 | 1 | 0 | 1 | 1 | 1 | 2 | 2 |
| NM_024805 | C18orf22 | 1404 | 12 | 3 | 5 | 2 | 3 | 2 | 3 | 12 | 2 | 2 | 2 | 3 | 2 |
| NM_024806 | C11orf63 | 2911 | 6 | 2 | 2 | 1 | 1 | 2 | 2 | 0 | 1 | 1 | 0 | 2 | 6 |
| NM_024807 | TREML2 | 3758 | 2 | 0 | 0 | 0 | 0 | 0 | 0 | 0 | 0 | 0 | 0 | 2 | 0 |
| NM_024808 | C13orf34 | 2773 | 0 | 0 | 0 | 0 | 0 | 0 | 0 | 0 | 0 | 0 | 0 | 0 | 0 |
| NM_024809 | TCTN2 | 2909 | 2 | 1 | 0 | 0 | 0 | 1 | 1 | 0 | 1 | 2 | 0 | 0 | 1 |
| NM_024811 | CPSF7 | 3687 | 5 | 4 | 3 | 3 | 3 | 5 | 4 | 3 | 3 | 5 | 5 | 5 | 5 |
| NM_024812 | BAALC | 2849 | 44 | 5 | 1 | 1 | 1 | 44 | 1 | 0 | 1 | 1 | 0 | 6 | 0 |
| NM_024813 | RPAP2 | 3112 | 3 | 2 | 2 | 1 | 2 | 3 | 3 | 2 | 2 | 3 | 3 | 3 | 3 |
| NM_024814 | CBLL1 | 4265 | 8 | 5 | 3 | 3 | 5 | 5 | 4 | 3 | 4 | 8 | 7 | 8 | 6 |
| NM_024815 | NUDT18 | 1521 | 1 | 0 | 1 | 0 | 0 | 0 | 0 | 0 | 0 | 0 | 0 | 0 | 1 |
| NM_024816 | RABEP2 | 2280 | 1 | 0 | 0 | 1 | 0 | 0 | 1 | 0 | 0 | 0 | 0 | 1 | 1 |
| NM_024817 | THSD4 | 9145 | 5 | 1 | 0 | 1 | 5 | 1 | 2 | 1 | 1 | 1 | 0 | 0 | 1 |
| NM_024818 | UBA5 | 3031 | 6 | 4 | 3 | 1 | 4 | 5 | 6 | 5 | 3 | 3 | 3 | 5 | 4 |
| NM_024819 | DCAKD | 2123 | 4 | 1 | 2 | 2 | 1 | 4 | 1 | 1 | 1 | 1 | 1 | 1 | 1 |
| NM_024820 | DENND1A | 2132 | 7 | 4 | 2 | 3 | 3 | 7 | 5 | 3 | 2 | 4 | 2 | 5 | 3 |
| NM_024821 | CCDC134 | 1275 | 1 | 1 | 0 | 1 | 0 | 1 | 1 | 1 | 1 | 1 | 1 | 1 | 1 |
| NM_024824 | ZC3H14 | 4193 | 17 | 8 | 6 | 4 | 6 | 9 | 7 | 12 | 4 | 9 | 4 | 6 | 17 |
| NM_024825 | PODNL1 | 3331 | 0 | 0 | 0 | 0 | 0 | 0 | 0 | 0 | 0 | 0 | 0 | 0 | 0 |
| NM_024827 | HDAC11 | 2917 | 1 | 0 | 0 | 1 | 0 | 1 | 1 | 0 | 0 | 0 | 0 | 0 | 0 |
| NM_024828 | C9orf82 | 2814 | 11 | 6 | 4 | 5 | 4 | 6 | 8 | 11 | 3 | 4 | 3 | 7 | 9 |
| NM_024829 | PLBD1 | 1932 | 35 | 12 | 16 | 9 | 18 | 3 | 9 | 4 | 22 | 4 | 3 | 35 | 6 |
| NM_024830 | LPCAT1 | 3946 | 26 | 6 | 2 | 1 | 3 | 5 | 2 | 1 | 19 | 2 | 0 | 26 | 2 |
| NM_024831 | TGS1 | 3692 | 6 | 3 | 2 | 1 | 4 | 3 | 4 | 2 | 2 | 5 | 3 | 6 | 5 |
| NM_024832 | RIN3 | 3847 | 4 | 1 | 1 | 1 | 1 | 0 | 0 | 1 | 2 | 1 | 0 | 4 | 1 |
| NM_024833 | ZNF671 | 2434 | 1 | 1 | 1 | 1 | 1 | 1 | 1 | 0 | 1 | 0 | 1 | 1 | 1 |
| NM_024834 | C10orf119 | 4241 | 7 | 4 | 2 | 2 | 5 | 3 | 4 | 4 | 4 | 4 | 4 | 7 | 3 |
| NM_024835 | GGNBP2 | 2778 | 14 | 10 | 8 | 5 | 11 | 11 | 10 | 7 | 9 | 11 | 9 | 14 | 14 |
| NM_024836 | ZNF672 | 2936 | 1 | 1 | 0 | 1 | 1 | 1 | 1 | 1 | 1 | 0 | 1 | 0 | 0 |
| NM_024837 | ATP8B4 | 5677 | 2 | 1 | 2 | 0 | 1 | 1 | 1 | 1 | 2 | 1 | 0 | 2 | 1 |
| NM_024838 | THNSL1 | 3770 | 2 | 1 | 0 | 0 | 0 | 2 | 2 | 2 | 0 | 2 | 0 | 1 | 1 |
| NM_024839 | RPP21 | 522 | 1 | 1 | 0 | 1 | 1 | 1 | 1 | 1 | 1 | 1 | 0 | 1 | 1 |
| NM_024840 | ZNF613 | 2225 | 3 | 2 | 1 | 1 | 2 | 2 | 2 | 1 | 1 | 2 | 1 | 3 | 2 |

| transcript | gene | transcript length (nt) | max | mean | adipose | colon | heart | hypothalamus | kidney | liver | lung | ovary | skeletalmuscle | spleen | testes |
|---|---|---|---|---|---|---|---|---|---|---|---|---|---|---|---|
| NM_024841 | PRR5L | 3949 | 7 | 1 | 1 | 2 | 0 | 1 | 0 | 0 | 1 | 2 | 0 | 7 | 0 |
| NM_024843 | CYBRD1 | 4357 | 37 | 16 | 37 | 6 | 18 | 7 | 5 | 2 | 35 | 28 | 9 | 7 | 19 |
| NM_024844 | NUP85 | 2311 | 2 | 1 | 1 | 1 | 1 | 1 | 1 | 1 | 1 | 1 | 1 | 2 | 1 |
| NM_024845 | NAT15 | 2650 | 5 | 3 | 2 | 3 | 3 | 5 | 5 | 5 | 2 | 3 | 1 | 4 | 3 |
| NM_024847 | TMC7 | 4397 | 1 | 0 | 0 | 1 | 0 | 1 | 0 | 0 | 0 | 0 | 0 | 0 | 1 |
| NM_024848 | MORN1 | 1641 | 2 | 1 | 1 | 2 | 1 | 2 | 2 | 1 | 1 | 2 | 0 | 0 | 2 |
| NM_024850 | BTNL8 | 2227 | 1 | 0 | 0 | 1 | 0 | 0 | 0 | 0 | 0 | 0 | 0 | 0 | 1 |
| NM_024852 | EIF2C3 | 3556 | 6 | 4 | 3 | 2 | 3 | 4 | 3 | 3 | 3 | 6 | 2 | 4 | 5 |
| NM_024854 | PYROXD1 | 4122 | 4 | 3 | 3 | 1 | 3 | 3 | 4 | 4 | 2 | 2 | 3 | 3 | 3 |
| NM_024855 | ACTR5 | 2479 | 1 | 0 | 0 | 1 | 0 | 1 | 0 | 1 | 0 | 0 | 0 | 1 | 1 |
| NM_024857 | ATAD5 | 6242 | 2 | 1 | 0 | 0 | 0 | 0 | 1 | 0 | 1 | 1 | 0 | 2 | 1 |
| NM_024859 | MAGIX | 2150 | 0 | 0 | 0 | 0 | 0 | 0 | 0 | 0 | 0 | 0 | 0 | 0 | 0 |
| NM_024860 | SETD6 | 2906 | 4 | 2 | 2 | 2 | 2 | 2 | 4 | 3 | 2 | 2 | 3 | 3 | 3 |
| NM_024861 | C2orf54 | 1958 | 0 | 0 | 0 | 0 | 0 | 0 | 0 | 0 | 0 | 0 | 0 | 0 | 0 |
| NM_024863 | TCEAL4 | 1345 | 331 | 113 | 58 | 21 | 146 | 160 | 79 | 59 | 74 | 331 | 34 | 92 | 184 |
| NM_024864 | MRM1 | 1833 | 1 | 0 | 0 | 1 | 0 | 0 | 1 | 1 | 0 | 0 | 0 | 0 | 0 |
| NM_024865 | NANOG | 2098 | 1 | 0 | 0 | 0 | 0 | 0 | 1 | 0 | 0 | 1 | 0 | 0 | 1 |
| NM_024866 | ADM2 | 4246 | 1 | 0 | 0 | 0 | 0 | 0 | 1 | 0 | 0 | 0 | 0 | 0 | 0 |
| NM_024867 | SPEF2 | 5638 | 2 | 1 | 0 | 0 | 0 | 1 | 2 | 0 | 1 | 1 | 0 | 1 | 2 |
| NM_024869 | GRRP1 | 1524 | 1 | 0 | 0 | 0 | 1 | 0 | 0 | 0 | 0 | 0 | 0 | 0 | 0 |
| NM_024870 | PREX2 | 5132 | 11 | 4 | 8 | 1 | 4 | 11 | 2 | 1 | 4 | 3 | 2 | 2 | 3 |
| NM_024871 | MAP6D1 | 2138 | 13 | 2 | 0 | 0 | 1 | 13 | 0 | 0 | 0 | 0 | 1 | 0 | 1 |
| NM_024872 | DOK3 | 1728 | 1 | 0 | 0 | 0 | 0 | 0 | 0 | 0 | 0 | 0 | 0 | 1 | 0 |
| NM_024873 | TNIP3 | 2396 | 3 | 0 | 0 | 0 | 0 | 0 | 0 | 0 | 3 | 0 | 0 | 1 | 0 |
| NM_024874 | KIAA0319L | 4811 | 8 | 4 | 2 | 3 | 6 | 6 | 5 | 8 | 3 | 3 | 4 | 4 | 3 |
| NM_024875 | SYNPO2L | 4116 | 30 | 5 | 0 | 0 | 25 | 0 | 0 | 0 | 0 | 0 | 30 | 0 | 0 |
| NM_024876 | ADCK4 | 2443 | 0 | 0 | 0 | 0 | 0 | 0 | 0 | 0 | 0 | 0 | 0 | 0 | 0 |
| NM_024877 | CNTD2 | 1816 | 0 | 0 | 0 | 0 | 0 | 0 | 0 | 0 | 0 | 0 | 0 | 0 | 0 |
| NM_024881 | SLC35E1 | 5093 | 5 | 4 | 4 | 3 | 4 | 5 | 4 | 4 | 4 | 4 | 4 | 5 | 3 |
| NM_024884 | L2HGDH | 6095 | 2 | 1 | 0 | 0 | 1 | 1 | 2 | 1 | 0 | 1 | 1 | 0 | 1 |
| NM_024885 | TAF7L | 2354 | 18 | 2 | 0 | 0 | 0 | 1 | 0 | 0 | 0 | 0 | 0 | 0 | 18 |
| NM_024886 | C10orf95 | 1469 | 0 | 0 | 0 | 0 | 0 | 0 | 0 | 0 | 0 | 0 | 0 | 0 | 0 |
| NM_024887 | DHDDS | 3299 | 22 | 5 | 22 | 5 | 3 | 5 | 5 | 3 | 2 | 4 | 1 | 4 | 4 |
| NM_024888 | LPPR3 | 2357 | 0 | 0 | 0 | 0 | 0 | 0 | 0 | 0 | 0 | 0 | 0 | 0 | 0 |
| NM_024889 | C10orf81 | 3249 | 1 | 0 | 0 | 0 | 0 | 0 | 0 | 0 | 1 | 0 | 0 | 0 | 0 |
| NM_024893 | TMEM90B | 2437 | 1 | 0 | 0 | 0 | 1 | 1 | 0 | 0 | 0 | 0 | 0 | 0 | 0 |
| NM_024894 | NOL10 | 3495 | 12 | 9 | 8 | 3 | 10 | 7 | 9 | 11 | 6 | 11 | 10 | 12 | 11 |
| NM_024895 | PDZD7 | 2021 | 0 | 0 | 0 | 0 | 0 | 0 | 0 | 0 | 0 | 0 | 0 | 0 | 0 |
| NM_024896 | ERMP1 | 5341 | 6 | 2 | 1 | 2 | 2 | 3 | 6 | 1 | 2 | 3 | 1 | 2 | 1 |
| NM_024897 | PAQR6 | 2117 | 24 | 2 | 0 | 0 | 0 | 24 | 0 | 0 | 0 | 0 | 0 | 0 | 1 |
| NM_024898 | DENND1C | 2795 | 6 | 2 | 0 | 3 | 0 | 0 | 6 | 5 | 3 | 0 | 0 | 2 | 0 |
| NM_024899 | CEP76 | 2869 | 6 | 2 | 1 | 1 | 1 | 2 | 1 | 2 | 1 | 2 | 1 | 2 | 6 |
| NM_024900 | PHF17 | 4190 | 8 | 6 | 7 | 3 | 7 | 5 | 8 | 3 | 5 | 8 | 5 | 7 | 7 |
| NM_024901 | DENND2D | 2042 | 13 | 3 | 2 | 2 | 1 | 1 | 4 | 1 | 3 | 1 | 0 | 13 | 2 |
| NM_024902 | DNAJC22 | 1827 | 5 | 1 | 0 | 1 | 0 | 0 | 2 | 5 | 0 | 0 | 0 | 0 | 0 |
| NM_024906 | SCD5 | 2080 | 11 | 2 | 0 | 0 | 1 | 11 | 2 | 0 | 1 | 5 | 0 | 0 | 3 |
| NM_024907 | FBXO17 | 2215 | 2 | 1 | 1 | 0 | 0 | 0 | 2 | 1 | 0 | 1 | 0 | 0 | 0 |
| NM_024908 | WDR76 | 4027 | 1 | 0 | 0 | 0 | 0 | 0 | 0 | 0 | 0 | 0 | 0 | 1 | 1 |
| NM_024909 | C6orf134 | 2161 | 4 | 1 | 0 | 1 | 0 | 4 | 1 | 0 | 0 | 1 | 0 | 1 | 1 |
| NM_024911 | GPR177 | 2765 | 10 | 6 | 4 | 7 | 4 | 10 | 10 | 2 | 4 | 6 | 1 | 4 | 8 |
| NM_024913 | C7orf58 | 5320 | 8 | 3 | 7 | 1 | 3 | 0 | 1 | 1 | 2 | 8 | 3 | 4 | 2 |
| NM_024915 | GRHL2 | 5229 | 2 | 1 | 0 | 2 | 0 | 0 | 2 | 0 | 1 | 0 | 0 | 0 | 0 |
| NM_024917 | TRMT2B | 3556 | 1 | 1 | 1 | 1 | 1 | 1 | 1 | 1 | 1 | 1 | 1 | 1 | 1 |
| NM_024918 | DSN1 | 2353 | 2 | 1 | 1 | 0 | 0 | 1 | 0 | 0 | 1 | 0 | 0 | 1 | 2 |
| NM_024919 | FRMD1 | 3028 | 1 | 0 | 0 | 1 | 0 | 0 | 0 | 0 | 0 | 0 | 0 | 0 | 0 |
| NM_024921 | POF1B | 3948 | 8 | 1 | 0 | 8 | 2 | 0 | 1 | 0 | 1 | 0 | 0 | 2 | 0 |
| NM_024922 | CES3 | 3889 | 3 | 1 | 0 | 3 | 0 | 0 | 1 | 1 | 0 | 0 | 1 | 0 | 0 |
| NM_024923 | NUP210 | 7191 | 2 | 1 | 0 | 1 | 0 | 0 | 1 | 0 | 1 | 0 | 0 | 2 | 1 |
| NM_024926 | TTC26 | 2151 | 2 | 1 | 1 | 0 | 1 | 1 | 1 | 0 | 1 | 1 | 0 | 0 | 2 |
| NM_024927 | PLEKHH3 | 3061 | 0 | 0 | 0 | 0 | 0 | 0 | 0 | 0 | 0 | 0 | 0 | 0 | 0 |
| NM_024928 | OBFC1 | 6481 | 4 | 3 | 2 | 2 | 4 | 2 | 3 | 1 | 4 | 3 | 2 | 3 | 4 |
| NM_024930 | ELOVL7 | 3881 | 4 | 1 | 1 | 0 | 1 | 3 | 4 | 0 | 1 | 0 | 0 | 1 | 0 |
| NM_024933 | ANKRD53 | 2120 | 1 | 0 | 0 | 0 | 0 | 0 | 0 | 0 | 0 | 0 | 0 | 0 | 1 |
| NM_024936 | ZCCHC4 | 2797 | 2 | 1 | 1 | 1 | 1 | 1 | 1 | 1 | 1 | 2 | 1 | 2 | 2 |
| NM_024939 | ESRP2 | 4021 | 7 | 1 | 0 | 2 | 0 | 0 | 2 | 7 | 2 | 0 | 1 | 1 | 1 |
| NM_024940 | DOCK5 | 7555 | 8 | 4 | 1 | 3 | 4 | 4 | 2 | 2 | 3 | 5 | 1 | 8 | 5 |
| NM_024941 | C5orf44 | 3100 | 6 | 3 | 2 | 1 | 3 | 6 | 3 | 3 | 2 | 2 | 2 | 3 | 4 |
| NM_024942 | C10orf88 | 3087 | 1 | 0 | 0 | 0 | 0 | 1 | 0 | 0 | 0 | 0 | 0 | 1 | 0 |
| NM_024943 | TMEM156 | 1859 | 12 | 2 | 0 | 1 | 0 | 1 | 0 | 1 | 1 | 0 | 0 | 12 | 0 |
| NM_024944 | CHODL | 2545 | 17 | 2 | 0 | 0 | 0 | 1 | 0 | 0 | 0 | 0 | 0 | 1 | 17 |
| NM_024945 | RMI1 | 3494 | 1 | 1 | 0 | 0 | 0 | 1 | 0 | 1 | 0 | 0 | 0 | 1 | 1 |
| NM_024946 | FAM192A | 2848 | 22 | 11 | 8 | 7 | 11 | 22 | 10 | 9 | 8 | 10 | 8 | 15 | 13 |
| NM_024947 | PHC3 | 12672 | 8 | 5 | 4 | 3 | 4 | 4 | 5 | 2 | 6 | 7 | 4 | 8 | 6 |
| NM_024948 | FAM188A | 2375 | 6 | 3 | 2 | 1 | 2 | 6 | 3 | 2 | 2 | 2 | 1 | 3 | 4 |
| NM_024949 | WWC2 | 8808 | 5 | 3 | 2 | 1 | 3 | 2 | 4 | 4 | 5 | 3 | 1 | 2 | 3 |
| NM_024952 | C14orf159 | 2974 | 6 | 3 | 2 | 3 | 6 | 3 | 5 | 1 | 2 | 2 | 5 | 3 | 3 |
| NM_024953 | C12orf30 | 5781 | 5 | 3 | 2 | 2 | 3 | 4 | 3 | 3 | 3 | 4 | 5 | 4 | 4 |

| transcript | gene | transcript length (nt) | max | mean | adipose | colon | heart | hypothalamus | kidney | liver | lung | ovary | skeletalmuscle | spleen | testes |
|---|---|---|---|---|---|---|---|---|---|---|---|---|---|---|---|
| NM_024954 | UBTD1 | 1557 | 0 | 0 | 0 | 0 | 0 | 0 | 0 | 0 | 0 | 0 | 0 | 0 | 0 |
| NM_024955 | FOXRED2 | 5010 | 2 | 1 | 0 | 0 | 0 | 2 | 1 | 0 | 0 | 1 | 0 | 1 | 1 |
| NM_024956 | TMEM62 | 2768 | 1 | 1 | 1 | 1 | 1 | 1 | 1 | 1 | 1 | 0 | 0 | 1 | 0 |
| NM_024958 | NRSN2 | 2385 | 6 | 1 | 0 | 0 | 1 | 6 | 1 | 0 | 0 | 1 | 0 | 0 | 1 |
| NM_024959 | SLC24A6 | 2976 | 1 | 1 | 1 | 1 | 0 | 0 | 1 | 0 | 0 | 1 | 0 | 1 | 1 |
| NM_024960 | PANK2 | 1712 | 10 | 5 | 5 | 3 | 5 | 9 | 5 | 4 | 5 | 5 | 3 | 10 | 7 |
| NM_024963 | FBXL18 | 8229 | 1 | 0 | 0 | 0 | 0 | 1 | 0 | 0 | 1 | 0 | 0 | 0 | 0 |
| NM_024966 | SEMA6D | 2290 | 6 | 2 | 2 | 2 | 1 | 6 | 4 | 1 | 1 | 5 | 1 | 0 | 1 |
| NM_024967 | ZNF556 | 1632 | 5 | 1 | 0 | 0 | 0 | 0 | 0 | 0 | 0 | 5 | 1 | 0 | 1 |
| NM_024969 | CSRNP3 | 2931 | 6 | 1 | 0 | 0 | 0 | 6 | 1 | 0 | 0 | 2 | 0 | 1 | 1 |
| NM_024971 | TRPM3 | 5959 | 6 | 1 | 0 | 0 | 0 | 6 | 6 | 0 | 0 | 1 | 0 | 0 | 1 |
| NM_024979 | MCF2L | 5406 | 2 | 1 | 1 | 1 | 1 | 2 | 1 | 0 | 1 | 0 | 0 | 0 | 0 |
| NM_024980 | GPR157 | 1153 | 0 | 0 | 0 | 0 | 0 | 0 | 0 | 0 | 0 | 0 | 0 | 0 | 0 |
| NM_024989 | PGAP1 | 11113 | 5 | 1 | 0 | 1 | 1 | 5 | 1 | 0 | 2 | 2 | 0 | 1 | 1 |
| NM_024991 | SORBS1 | 5858 | 118 | 30 | 118 | 27 | 69 | 26 | 7 | 15 | 9 | 3 | 40 | 4 | 14 |
| NM_024993 | LRRTM4 | 3623 | 3 | 0 | 0 | 0 | 0 | 3 | 0 | 0 | 0 | 0 | 0 | 0 | 0 |
| NM_024996 | GFM1 | 3467 | 14 | 7 | 5 | 3 | 7 | 8 | 9 | 14 | 3 | 3 | 7 | 6 | 8 |
| NM_024997 | ATF7IP2 | 3597 | 7 | 2 | 1 | 1 | 2 | 2 | 1 | 2 | 2 | 2 | 0 | 3 | 7 |
| NM_025000 | DCAF17 | 5826 | 3 | 2 | 1 | 1 | 2 | 2 | 2 | 1 | 1 | 2 | 1 | 3 | 3 |
| NM_025003 | ADAMTS20 | 5733 | 0 | 0 | 0 | 0 | 0 | 0 | 0 | 0 | 0 | 0 | 0 | 0 | 0 |
| NM_025004 | CCDC15 | 3893 | 4 | 1 | 1 | 0 | 0 | 0 | 0 | 0 | 0 | 1 | 0 | 1 | 4 |
| NM_025008 | ADAMTSL4 | 2893 | 1 | 0 | 0 | 0 | 1 | 0 | 0 | 0 | 0 | 0 | 0 | 0 | 0 |
| NM_025009 | CEP135 | 5670 | 3 | 1 | 1 | 0 | 1 | 1 | 2 | 0 | 1 | 3 | 1 | 3 | 3 |
| NM_025010 | KLHL18 | 4725 | 2 | 1 | 1 | 1 | 1 | 2 | 1 | 1 | 0 | 0 | 0 | 1 | 1 |
| NM_025015 | HSPA12A | 5722 | 15 | 3 | 8 | 1 | 1 | 15 | 5 | 0 | 0 | 4 | 0 | 0 | 2 |
| NM_025027 | ZNF606 | 4225 | 3 | 2 | 1 | 0 | 2 | 2 | 2 | 0 | 1 | 3 | 2 | 2 | 3 |
| NM_025029 | FAM128B | 731 | 2 | 1 | 1 | 1 | 2 | 2 | 1 | 2 | 1 | 2 | 1 | 1 | 1 |
| NM_025031 | C7orf69 | 663 | 0 | 0 | 0 | 0 | 0 | 0 | 0 | 0 | 0 | 0 | 0 | 0 | 0 |
| NM_025040 | ZNF614 | 4675 | 3 | 2 | 1 | 0 | 2 | 2 | 2 | 0 | 1 | 3 | 1 | 3 | 3 |
| NM_025041 | C3orf36 | 1667 | 1 | 0 | 0 | 0 | 0 | 0 | 1 | 0 | 1 | 0 | 0 | 1 | 0 |
| NM_025045 | BAIAP2L2 | 2145 | 1 | 0 | 0 | 1 | 0 | 0 | 1 | 0 | 0 | 0 | 0 | 0 | 0 |
| NM_025047 | ARL14 | 1286 | 0 | 0 | 0 | 0 | 0 | 0 | 0 | 0 | 0 | 0 | 0 | 0 | 0 |
| NM_025048 | GPR110 | 1422 | 1 | 0 | 0 | 0 | 0 | 0 | 1 | 0 | 0 | 0 | 0 | 0 | 0 |
| NM_025049 | PIF1 | 2655 | 0 | 0 | 0 | 0 | 0 | 0 | 0 | 0 | 0 | 0 | 0 | 0 | 0 |
| NM_025052 | YSK4 | 4165 | 1 | 0 | 0 | 0 | 0 | 0 | 0 | 0 | 1 | 0 | 0 | 0 | 1 |
| NM_025054 | VCPIP1 | 8176 | 9 | 5 | 4 | 2 | 4 | 8 | 4 | 4 | 4 | 5 | 4 | 9 | 6 |
| NM_025055 | CCDC33 | 2750 | 1 | 0 | 0 | 0 | 0 | 0 | 0 | 0 | 0 | 0 | 0 | 0 | 1 |
| NM_025057 | C14orf45 | 3106 | 9 | 2 | 2 | 1 | 2 | 2 | 3 | 0 | 3 | 2 | 0 | 1 | 9 |
| NM_025058 | TRIM46 | 3142 | 0 | 0 | 0 | 0 | 0 | 0 | 0 | 0 | 0 | 0 | 0 | 0 | 0 |
| NM_025059 | C6orf97 | 5282 | 2 | 1 | 1 | 0 | 0 | 0 | 0 | 0 | 1 | 2 | 0 | 0 | 1 |
| NM_025061 | LRRC8E | 3602 | 1 | 0 | 0 | 0 | 0 | 0 | 0 | 1 | 0 | 0 | 0 | 0 | 0 |
| NM_025063 | C1orf129 | 2257 | 0 | 0 | 0 | 0 | 0 | 0 | 0 | 0 | 0 | 0 | 0 | 0 | 0 |
| NM_025065 | RPF1 | 1975 | 5 | 4 | 3 | 1 | 5 | 4 | 3 | 5 | 2 | 3 | 3 | 5 | 4 |
| NM_025069 | ZNF703 | 2174 | 2 | 1 | 0 | 1 | 1 | 0 | 0 | 0 | 0 | 1 | 2 | 1 | 0 |
| NM_025072 | PTGES2 | 2063 | 2 | 1 | 0 | 0 | 2 | 0 | 1 | 0 | 0 | 0 | 0 | 1 | 0 |
| NM_025073 | SIKE1 | 5491 | 3 | 2 | 2 | 1 | 2 | 3 | 3 | 2 | 2 | 2 | 1 | 3 | 2 |
| NM_025074 | FRAS1 | 15624 | 3 | 1 | 0 | 1 | 0 | 1 | 3 | 0 | 1 | 0 | 0 | 0 | 0 |
| NM_025075 | THOC7 | 1008 | 21 | 11 | 10 | 8 | 17 | 21 | 11 | 11 | 9 | 7 | 10 | 9 | 7 |
| NM_025076 | UXS1 | 2084 | 18 | 8 | 4 | 3 | 9 | 8 | 18 | 4 | 13 | 7 | 3 | 11 | 11 |
| NM_025077 | TOE1 | 2392 | 1 | 1 | 0 | 1 | 0 | 0 | 1 | 0 | 0 | 1 | 1 | 1 | 1 |
| NM_025078 | PQLC1 | 2543 | 6 | 2 | 1 | 1 | 1 | 3 | 3 | 6 | 1 | 1 | 1 | 1 | 1 |
| NM_025079 | ZC3H12A | 2716 | 0 | 0 | 0 | 0 | 0 | 0 | 0 | 0 | 0 | 0 | 0 | 0 | 0 |
| NM_025080 | ASRGL1 | 2342 | 7 | 3 | 1 | 1 | 1 | 7 | 7 | 1 | 2 | 0 | 0 | 1 | 6 |
| NM_025081 | NYNRIN | 7857 | 4 | 1 | 1 | 1 | 1 | 0 | 1 | 0 | 1 | 4 | 0 | 1 | 1 |
| NM_025082 | CENPT | 2345 | 8 | 2 | 2 | 8 | 1 | 2 | 2 | 1 | 1 | 1 | 1 | 1 | 4 |
| NM_025083 | EDC3 | 3764 | 2 | 1 | 1 | 1 | 1 | 1 | 1 | 1 | 1 | 1 | 1 | 1 | 2 |
| NM_025087 | CWH43 | 2470 | 3 | 0 | 0 | 3 | 0 | 0 | 1 | 0 | 0 | 0 | 0 | 0 | 0 |
| NM_025090 | USP36 | 5234 | 3 | 2 | 1 | 2 | 1 | 1 | 2 | 0 | 2 | 3 | 2 | 2 | 2 |
| NM_025092 | ATHL1 | 3268 | 1 | 0 | 0 | 1 | 0 | 0 | 0 | 0 | 1 | 0 | 0 | 1 | 0 |
| NM_025098 | MOGAT2 | 1005 | 3 | 0 | 0 | 2 | 0 | 0 | 0 | 3 | 0 | 0 | 0 | 0 | 0 |
| NM_025099 | C17orf68 | 7072 | 2 | 1 | 0 | 1 | 0 | 0 | 1 | 0 | 1 | 1 | 0 | 2 | 0 |
| NM_025103 | IFT74 | 2163 | 13 | 5 | 3 | 1 | 4 | 6 | 7 | 4 | 3 | 8 | 2 | 4 | 13 |
| NM_025104 | DBF4B | 2543 | 1 | 0 | 0 | 1 | 0 | 0 | 0 | 0 | 0 | 0 | 0 | 0 | 1 |
| NM_025106 | SPSB1 | 3115 | 9 | 5 | 5 | 4 | 4 | 2 | 3 | 7 | 7 | 9 | 7 | 2 | 4 |
| NM_025107 | MYCT1 | 3043 | 4 | 1 | 1 | 0 | 2 | 0 | 1 | 1 | 2 | 1 | 2 | 4 | 1 |
| NM_025108 | C16orf59 | 1628 | 0 | 0 | 0 | 0 | 0 | 0 | 0 | 0 | 0 | 0 | 0 | 0 | 0 |
| NM_025109 | MYO19 | 3241 | 1 | 1 | 0 | 1 | 0 | 0 | 1 | 1 | 0 | 0 | 0 | 1 | 1 |
| NM_025112 | ZXDC | 3405 | 2 | 1 | 1 | 1 | 1 | 1 | 2 | 1 | 1 | 1 | 2 | 1 | 1 |
| NM_025113 | C13orf18 | 3977 | 2 | 0 | 0 | 0 | 0 | 0 | 0 | 0 | 0 | 0 | 0 | 2 | 1 |
| NM_025114 | CEP290 | 7955 | 5 | 3 | 2 | 1 | 2 | 3 | 3 | 0 | 2 | 5 | 1 | 4 | 4 |
| NM_025115 | C8orf41 | 2507 | 2 | 1 | 1 | 0 | 1 | 1 | 1 | 1 | 1 | 1 | 1 | 1 | 2 |
| NM_025124 | TMEM134 | 922 | 1 | 0 | 0 | 0 | 1 | 0 | 1 | 1 | 0 | 0 | 0 | 1 | 0 |
| NM_025125 | C10orf57 | 2036 | 2 | 1 | 1 | 1 | 0 | 1 | 1 | 1 | 1 | 1 | 0 | 1 | 2 |
| NM_025126 | RNF34 | 2009 | 21 | 11 | 9 | 9 | 10 | 21 | 7 | 8 | 6 | 10 | 11 | 15 | 14 |
| NM_025128 | MUS81 | 2400 | 1 | 1 | 1 | 1 | 1 | 1 | 1 | 1 | 1 | 0 | 1 | 1 | 1 |
| NM_025129 | FUZ | 1621 | 2 | 1 | 0 | 1 | 1 | 2 | 2 | 0 | 0 | 1 | 1 | 1 | 1 |
| NM_025130 | HKDC1 | 3689 | 5 | 1 | 0 | 1 | 0 | 0 | 5 | 0 | 0 | 0 | 0 | 0 | 0 |

| transcript | gene | transcript length (nt) | max | mean | adipose | colon | heart | hypothalamus | kidney | liver | lung | ovary | skeletalmuscle | spleen | testes |
|---|---|---|---|---|---|---|---|---|---|---|---|---|---|---|---|
| NM_025132 | WDR19 | 4520 | 3 | 1 | 2 | 1 | 1 | 2 | 2 | 1 | 2 | 3 | 1 | 1 | 3 |
| NM_025133 | FBXO11 | 3937 | 20 | 12 | 10 | 3 | 10 | 16 | 10 | 6 | 10 | 20 | 12 | 17 | 20 |
| NM_025134 | CHD9 | 11462 | 12 | 8 | 8 | 3 | 7 | 8 | 9 | 6 | 7 | 12 | 6 | 9 | 8 |
| NM_025135 | FHOD3 | 4942 | 30 | 5 | 0 | 1 | 30 | 6 | 7 | 0 | 0 | 3 | 6 | 1 | 3 |
| NM_025136 | OPA3 | 7871 | 1 | 1 | 0 | 1 | 1 | 1 | 1 | 1 | 0 | 0 | 0 | 1 | 0 |
| NM_025137 | SPG11 | 7788 | 8 | 5 | 4 | 3 | 5 | 4 | 6 | 4 | 5 | 4 | 4 | 8 | 6 |
| NM_025138 | C13orf23 | 5208 | 5 | 2 | 1 | 2 | 2 | 2 | 2 | 1 | 2 | 2 | 1 | 5 | 2 |
| NM_025139 | ARMC9 | 2218 | 4 | 2 | 1 | 2 | 1 | 4 | 1 | 0 | 1 | 4 | 0 | 1 | 3 |
| NM_025140 | CCDC92 | 1793 | 21 | 7 | 9 | 3 | 9 | 21 | 6 | 3 | 4 | 4 | 4 | 4 | 7 |
| NM_025141 | TM2D3 | 1329 | 7 | 3 | 2 | 1 | 3 | 7 | 2 | 3 | 2 | 2 | 2 | 2 | 2 |
| NM_025144 | ALPK1 | 5412 | 2 | 1 | 1 | 1 | 1 | 1 | 2 | 0 | 2 | 1 | 1 | 2 | 1 |
| NM_025145 | C10orf79 | 5365 | 11 | 2 | 0 | 0 | 0 | 3 | 1 | 0 | 4 | 1 | 0 | 0 | 11 |
| NM_025146 | NAT13 | 3576 | 15 | 8 | 7 | 4 | 7 | 8 | 8 | 12 | 6 | 6 | 15 | 10 | 10 |
| NM_025147 | COQ10B | 1974 | 10 | 6 | 3 | 2 | 10 | 9 | 9 | 9 | 6 | 4 | 2 | 9 | 6 |
| NM_025149 | ACSF2 | 2239 | 21 | 4 | 5 | 5 | 1 | 1 | 21 | 3 | 2 | 2 | 2 | 2 | 2 |
| NM_025150 | TARS2 | 2373 | 4 | 2 | 2 | 1 | 2 | 3 | 2 | 4 | 1 | 2 | 2 | 2 | 2 |
| NM_025151 | RAB11FIP1 | 4222 | 14 | 4 | 2 | 7 | 3 | 1 | 4 | 1 | 14 | 2 | 1 | 6 | 5 |
| NM_025152 | NUBPL | 3058 | 6 | 4 | 5 | 3 | 6 | 4 | 5 | 3 | 2 | 5 | 3 | 4 | 5 |
| NM_025153 | ATP10B | 7566 | 2 | 0 | 0 | 2 | 0 | 1 | 0 | 0 | 0 | 0 | 0 | 0 | 0 |
| NM_025154 | UNC84A | 3812 | 2 | 2 | 1 | 1 | 2 | 1 | 1 | 1 | 2 | 1 | 2 | 2 | 2 |
| NM_025155 | PAAF1 | 1585 | 4 | 2 | 1 | 2 | 1 | 4 | 2 | 1 | 1 | 2 | 0 | 2 | 2 |
| NM_025157 | PXN | 3800 | 4 | 2 | 2 | 2 | 2 | 1 | 2 | 1 | 2 | 1 | 2 | 4 | 2 |
| NM_025158 | RUFY1 | 2626 | 10 | 5 | 8 | 4 | 4 | 5 | 7 | 4 | 5 | 5 | 3 | 10 | 4 |
| NM_025159 | CXorf21 | 1927 | 5 | 1 | 3 | 1 | 1 | 1 | 1 | 0 | 2 | 1 | 0 | 5 | 0 |
| NM_025160 | WDR26 | 6872 | 14 | 10 | 10 | 5 | 10 | 8 | 8 | 7 | 14 | 8 | 10 | 14 | 11 |
| NM_025161 | C17orf70 | 3845 | 0 | 0 | 0 | 0 | 0 | 0 | 0 | 0 | 0 | 0 | 0 | 0 | 0 |
| NM_025163 | PIGZ | 2701 | 1 | 0 | 0 | 1 | 0 | 0 | 0 | 0 | 0 | 0 | 0 | 0 | 0 |
| NM_025164 | SIK3 | 6069 | 6 | 3 | 2 | 2 | 2 | 6 | 3 | 3 | 1 | 3 | 3 | 3 | 4 |
| NM_025165 | ELL3 | 2113 | 2 | 1 | 0 | 1 | 0 | 0 | 0 | 0 | 0 | 0 | 0 | 1 | 2 |
| NM_025169 | ZNF167 | 1737 | 1 | 0 | 0 | 0 | 0 | 0 | 0 | 0 | 0 | 1 | 0 | 0 | 0 |
| NM_025170 | PREX2 | 3024 | 11 | 4 | 7 | 1 | 5 | 11 | 2 | 1 | 5 | 3 | 2 | 2 | 4 |
| NM_025176 | NINL | 4972 | 8 | 2 | 1 | 2 | 1 | 2 | 8 | 1 | 1 | 4 | 0 | 1 | 3 |
| NM_025179 | PLXNA2 | 11443 | 3 | 1 | 1 | 1 | 1 | 3 | 1 | 1 | 1 | 1 | 1 | 0 | 0 |
| NM_025180 | CEP63 | 6054 | 11 | 6 | 6 | 1 | 8 | 6 | 5 | 3 | 4 | 5 | 9 | 6 | 11 |
| NM_025181 | SLC35F5 | 2848 | 16 | 7 | 3 | 2 | 9 | 3 | 16 | 6 | 6 | 7 | 5 | 8 | 8 |
| NM_025182 | KIAA1539 | 3028 | 1 | 1 | 1 | 1 | 1 | 1 | 0 | 0 | 1 | 0 | 0 | 1 | 0 |
| NM_025184 | EFHC2 | 3252 | 1 | 0 | 0 | 0 | 1 | 1 | 0 | 0 | 0 | 0 | 0 | 0 | 1 |
| NM_025185 | TANC2 | 11747 | 10 | 3 | 1 | 1 | 1 | 10 | 2 | 0 | 3 | 3 | 0 | 2 | 7 |
| NM_025187 | C16orf70 | 2841 | 2 | 1 | 1 | 0 | 1 | 1 | 1 | 2 | 1 | 1 | 1 | 1 | 1 |
| NM_025188 | TRIM45 | 3581 | 1 | 0 | 0 | 0 | 0 | 0 | 0 | 0 | 0 | 0 | 1 | 0 | 1 |
| NM_025189 | ZNF430 | 2649 | 16 | 5 | 3 | 2 | 3 | 4 | 3 | 0 | 4 | 16 | 2 | 9 | 6 |
| NM_025190 | ANKRD36B | 5986 | 8 | 2 | 1 | 1 | 1 | 1 | 1 | 0 | 2 | 6 | 1 | 4 | 8 |
| NM_025191 | EDEM3 | 6632 | 7 | 5 | 4 | 3 | 5 | 6 | 4 | 5 | 5 | 3 | 3 | 7 | 5 |
| NM_025193 | HSD3B7 | 2185 | 2 | 1 | 1 | 0 | 0 | 0 | 1 | 2 | 0 | 0 | 0 | 1 | 0 |
| NM_025194 | ITPKC | 3385 | 1 | 0 | 0 | 0 | 0 | 1 | 0 | 0 | 1 | 0 | 0 | 1 | 0 |
| NM_025195 | TRIB1 | 3632 | 18 | 6 | 2 | 2 | 6 | 2 | 6 | 18 | 8 | 1 | 5 | 8 | 3 |
| NM_025196 | GRPEL1 | 1504 | 20 | 7 | 5 | 3 | 7 | 10 | 7 | 20 | 3 | 3 | 4 | 6 | 6 |
| NM_025198 | MTERFD3 | 1765 | 6 | 4 | 5 | 2 | 5 | 5 | 6 | 5 | 2 | 3 | 2 | 3 | 5 |
| NM_025201 | PLEKHO2 | 3719 | 5 | 1 | 2 | 1 | 1 | 1 | 0 | 0 | 2 | 0 | 0 | 5 | 1 |
| NM_025202 | EFHD1 | 1989 | 39 | 12 | 3 | 2 | 10 | 24 | 39 | 3 | 3 | 19 | 8 | 17 | 6 |
| NM_025203 | C2orf44 | 3894 | 1 | 0 | 0 | 0 | 0 | 1 | 0 | 0 | 0 | 0 | 0 | 0 | 1 |
| NM_025204 | TRABD | 2315 | 1 | 0 | 0 | 0 | 0 | 0 | 1 | 1 | 1 | 0 | 0 | 1 | 0 |
| NM_025205 | MED28 | 1281 | 1 | 1 | 1 | 1 | 1 | 1 | 1 | 1 | 1 | 1 | 1 | 1 | 1 |
| NM_025207 | FLAD1 | 2192 | 2 | 1 | 2 | 1 | 1 | 1 | 1 | 2 | 1 | 0 | 1 | 1 | 1 |
| NM_025208 | PDGFD | 3993 | 6 | 1 | 2 | 0 | 4 | 0 | 1 | 0 | 0 | 6 | 0 | 1 | 1 |
| NM_025209 | EPC1 | 2913 | 4 | 3 | 2 | 2 | 2 | 2 | 3 | 2 | 2 | 4 | 3 | 4 | 3 |
| NM_025211 | GKAP1 | 1872 | 25 | 5 | 2 | 1 | 3 | 5 | 2 | 1 | 2 | 3 | 7 | 1 | 25 |
| NM_025212 | CXXC4 | 748 | 1 | 0 | 0 | 0 | 0 | 0 | 0 | 0 | 0 | 0 | 0 | 0 | 1 |
| NM_025213 | SPTBN4 | 2419 | 2 | 0 | 0 | 0 | 0 | 2 | 0 | 0 | 0 | 0 | 0 | 0 | 0 |
| NM_025214 | CCDC68 | 4130 | 4 | 2 | 0 | 1 | 2 | 1 | 1 | 2 | 3 | 4 | 0 | 1 | 4 |
| NM_025215 | PUS1 | 2014 | 2 | 1 | 1 | 2 | 1 | 2 | 1 | 2 | 1 | 1 | 1 | 2 | 1 |
| NM_025216 | WNT10A | 2375 | 0 | 0 | 0 | 0 | 0 | 0 | 0 | 0 | 0 | 0 | 0 | 0 | 0 |
| NM_025217 | ULBP2 | 1346 | 0 | 0 | 0 | 0 | 0 | 0 | 0 | 0 | 0 | 0 | 0 | 0 | 0 |
| NM_025218 | ULBP1 | 3142 | 1 | 0 | 0 | 0 | 0 | 0 | 0 | 0 | 0 | 0 | 0 | 0 | 1 |
| NM_025219 | DNAJC5 | 3241 | 15 | 4 | 3 | 5 | 3 | 15 | 3 | 3 | 2 | 1 | 1 | 4 | 3 |
| NM_025220 | ADAM33 | 3519 | 1 | 0 | 1 | 0 | 0 | 0 | 0 | 0 | 0 | 0 | 0 | 0 | 0 |
| NM_025221 | KCNIP4 | 2159 | 8 | 1 | 0 | 0 | 0 | 8 | 1 | 0 | 0 | 1 | 0 | 0 | 0 |
| NM_025222 | WDR82 | 4292 | 24 | 12 | 9 | 7 | 12 | 24 | 8 | 7 | 8 | 15 | 11 | 17 | 15 |
| NM_025224 | ZBTB46 | 4376 | 3 | 1 | 0 | 0 | 1 | 1 | 1 | 0 | 0 | 1 | 0 | 3 | 0 |
| NM_025225 | PNPLA3 | 2805 | 2 | 1 | 1 | 1 | 0 | 1 | 1 | 2 | 1 | 1 | 2 | 1 | 1 |
| NM_025227 | BPIL1 | 1897 | 0 | 0 | 0 | 0 | 0 | 0 | 0 | 0 | 0 | 0 | 0 | 0 | 0 |
| NM_025228 | TRAF3IP3 | 2058 | 8 | 1 | 0 | 1 | 0 | 1 | 0 | 0 | 2 | 0 | 0 | 8 | 0 |
| NM_025229 | SEL1L2 | 2230 | 1 | 0 | 0 | 0 | 0 | 0 | 0 | 0 | 0 | 0 | 0 | 0 | 1 |
| NM_025230 | DCAF11 | 4303 | 16 | 4 | 2 | 3 | 3 | 5 | 5 | 16 | 2 | 2 | 3 | 4 | 2 |
| NM_025231 | ZSCAN16 | 1275 | 5 | 2 | 1 | 2 | 1 | 2 | 3 | 2 | 2 | 1 | 1 | 5 | 2 |
| NM_025232 | REEP4 | 1694 | 19 | 9 | 8 | 16 | 5 | 6 | 4 | 5 | 9 | 5 | 2 | 19 | 19 |
| NM_025233 | COASY | 2553 | 4 | 2 | 1 | 2 | 1 | 2 | 4 | 4 | 2 | 1 | 1 | 3 | 1 |

| transcript | gene | transcript length (nt) | max | mean | adipose | colon | heart | hypothalamus | kidney | liver | lung | ovary | skeletalmuscle | spleen | testes |
|---|---|---|---|---|---|---|---|---|---|---|---|---|---|---|---|
| NM_025234 | WDR61 | 1190 | 5 | 3 | 4 | 2 | 2 | 5 | 4 | 4 | 2 | 2 | 2 | 3 | 4 |
| NM_025235 | TNKS2 | 6273 | 11 | 7 | 5 | 2 | 4 | 10 | 5 | 4 | 6 | 8 | 10 | 11 | 6 |
| NM_025236 | RNF39 | 2165 | 2 | 1 | 0 | 1 | 0 | 1 | 0 | 0 | 1 | 2 | 0 | 1 | 1 |
| NM_025236 | RNF39 | 2170 | 2 | 1 | 0 | 1 | 0 | 1 | 0 | 0 | 1 | 2 | 0 | 1 | 1 |
| NM_025237 | SOST | 2300 | 1 | 0 | 0 | 0 | 0 | 0 | 1 | 0 | 0 | 0 | 0 | 0 | 0 |
| NM_025238 | BTBD1 | 3290 | 58 | 22 | 15 | 6 | 40 | 31 | 15 | 13 | 11 | 10 | 58 | 19 | 23 |
| NM_025239 | PDCD1LG2 | 2418 | 2 | 1 | 1 | 0 | 0 | 0 | 0 | 0 | 1 | 0 | 1 | 2 | 1 |
| NM_025240 | CD276 | 2747 | 4 | 2 | 4 | 2 | 4 | 1 | 2 | 2 | 1 | 3 | 1 | 2 | 2 |
| NM_025241 | UBXN6 | 1656 | 4 | 2 | 2 | 2 | 1 | 4 | 2 | 2 | 1 | 1 | 2 | 1 | 2 |
| NM_025243 | SLC19A3 | 3767 | 25 | 3 | 25 | 2 | 0 | 1 | 2 | 2 | 2 | 0 | 0 | 0 | 0 |
| NM_025244 | TSGA10 | 3685 | 11 | 2 | 1 | 0 | 0 | 2 | 2 | 0 | 1 | 1 | 0 | 1 | 11 |
| NM_025245 | PBX4 | 1438 | 0 | 0 | 0 | 0 | 0 | 0 | 0 | 0 | 0 | 0 | 0 | 0 | 0 |
| NM_025246 | TMEM22 | 2060 | 2 | 1 | 1 | 0 | 1 | 2 | 0 | 0 | 1 | 1 | 0 | 1 | 1 |
| NM_025247 | ACAD10 | 4013 | 4 | 1 | 2 | 2 | 1 | 1 | 4 | 2 | 1 | 1 | 1 | 1 | 1 |
| NM_025248 | SRCIN1 | 7050 | 3 | 0 | 0 | 0 | 0 | 3 | 0 | 0 | 0 | 0 | 0 | 0 | 0 |
| NM_025249 | KIAA1683 | 3842 | 3 | 1 | 0 | 0 | 0 | 0 | 0 | 0 | 0 | 3 | 0 | 0 | 2 |
| NM_025250 | TTYH3 | 4822 | 5 | 2 | 2 | 1 | 2 | 2 | 3 | 2 | 2 | 1 | 0 | 5 | 1 |
| NM_025251 | KIAA1688 | 5143 | 1 | 0 | 0 | 0 | 0 | 1 | 0 | 0 | 0 | 0 | 0 | 0 | 1 |
| NM_025256 | EHMT2 | 3865 | 4 | 2 | 2 | 2 | 1 | 4 | 2 | 1 | 1 | 3 | 1 | 3 | 3 |
| NM_025256 | EHMT2 | 3866 | 4 | 2 | 2 | 2 | 1 | 4 | 2 | 1 | 1 | 3 | 1 | 3 | 3 |
| NM_025257 | SLC44A4 | 2633 | 3 | 1 | 0 | 3 | 0 | 0 | 2 | 0 | 0 | 0 | 0 | 0 | 0 |
| NM_025258 | C6orf27 | 2914 | 0 | 0 | 0 | 0 | 0 | 0 | 0 | 0 | 0 | 0 | 0 | 0 | 0 |
| NM_025259 | MSH5 | 2363 | 1 | 1 | 1 | 1 | 0 | 0 | 1 | 0 | 0 | 1 | 0 | 1 | 1 |
| NM_025259 | MSH5 | 2739 | 1 | 1 | 0 | 1 | 0 | 0 | 1 | 0 | 0 | 1 | 0 | 1 | 1 |
| NM_025260 | C6orf25 | 745 | 0 | 0 | 0 | 0 | 0 | 0 | 0 | 0 | 0 | 0 | 0 | 0 | 0 |
| NM_025261 | LY6G6C | 880 | 0 | 0 | 0 | 0 | 0 | 0 | 0 | 0 | 0 | 0 | 0 | 0 | 0 |
| NM_025262 | LY6G5C | 726 | 0 | 0 | 0 | 0 | 0 | 0 | 0 | 0 | 0 | 0 | 0 | 0 | 0 |
| NM_025263 | PRR3 | 2841 | 1 | 1 | 0 | 0 | 0 | 0 | 1 | 0 | 1 | 1 | 0 | 1 | 1 |
| NM_025264 | THUMPD2 | 2224 | 5 | 3 | 2 | 1 | 3 | 3 | 3 | 1 | 2 | 5 | 4 | 3 | 4 |
| NM_025265 | TSEN2 | 2384 | 3 | 2 | 1 | 3 | 2 | 2 | 2 | 1 | 1 | 2 | 2 | 1 | 2 |
| NM_025267 | AARSD1 | 1788 | 11 | 6 | 5 | 3 | 8 | 10 | 5 | 6 | 3 | 5 | 11 | 4 | 7 |
| NM_025268 | TMEM121 | 1519 | 1 | 0 | 0 | 0 | 0 | 1 | 0 | 0 | 0 | 1 | 0 | 1 | 0 |
| NM_030567 | PRR7 | 1476 | 0 | 0 | 0 | 0 | 0 | 0 | 0 | 0 | 0 | 0 | 0 | 0 | 0 |
| NM_030568 | KHDC1 | 1133 | 0 | 0 | 0 | 0 | 0 | 0 | 0 | 0 | 0 | 0 | 0 | 0 | 0 |
| NM_030569 | ITIH5 | 6319 | 84 | 11 | 84 | 9 | 2 | 4 | 5 | 0 | 4 | 6 | 2 | 1 | 6 |
| NM_030570 | UPK3B | 1609 | 1 | 0 | 0 | 0 | 0 | 0 | 0 | 0 | 1 | 0 | 0 | 0 | 0 |
| NM_030571 | NDFIP1 | 3597 | 66 | 18 | 14 | 8 | 12 | 66 | 17 | 21 | 9 | 12 | 5 | 13 | 16 |
| NM_030572 | C12orf39 | 583 | 0 | 0 | 0 | 0 | 0 | 0 | 0 | 0 | 0 | 0 | 0 | 0 | 0 |
| NM_030573 | THAP7 | 1242 | 1 | 1 | 1 | 0 | 0 | 1 | 1 | 1 | 0 | 1 | 1 | 1 | 1 |
| NM_030576 | LIMD2 | 3225 | 1 | 0 | 0 | 0 | 0 | 0 | 0 | 0 | 0 | 0 | 0 | 1 | 0 |
| NM_030577 | TMEM177 | 1397 | 1 | 0 | 0 | 0 | 0 | 0 | 1 | 1 | 0 | 0 | 0 | 0 | 1 |
| NM_030578 | B9D2 | 1031 | 0 | 0 | 0 | 0 | 0 | 0 | 0 | 0 | 0 | 0 | 0 | 0 | 0 |
| NM_030579 | CYB5B | 4286 | 5 | 2 | 2 | 2 | 2 | 5 | 3 | 2 | 2 | 1 | 1 | 3 | 3 |
| NM_030580 | ZNF34 | 1935 | 5 | 2 | 2 | 2 | 2 | 3 | 2 | 1 | 2 | 5 | 2 | 2 | 4 |
| NM_030581 | WDR59 | 3663 | 2 | 1 | 1 | 1 | 1 | 1 | 2 | 2 | 1 | 2 | 1 | 1 | 1 |
| NM_030582 | COL18A1 | 5894 | 6 | 2 | 2 | 1 | 2 | 1 | 4 | 6 | 1 | 3 | 0 | 2 | 1 |
| NM_030583 | MATN2 | 4051 | 11 | 3 | 5 | 3 | 3 | 3 | 3 | 3 | 1 | 11 | 0 | 1 | 6 |
| NM_030589 | CYP2A7 | 2128 | 6 | 1 | 0 | 0 | 0 | 0 | 0 | 6 | 0 | 0 | 0 | 0 | 0 |
| NM_030590 | MATN4 | 2130 | 0 | 0 | 0 | 0 | 0 | 0 | 0 | 0 | 0 | 0 | 0 | 0 | 0 |
| NM_030592 | MATN4 | 2007 | 0 | 0 | 0 | 0 | 0 | 0 | 0 | 0 | 0 | 0 | 0 | 0 | 0 |
| NM_030593 | SIRT2 | 1910 | 3 | 1 | 1 | 0 | 1 | 3 | 1 | 0 | 1 | 1 | 2 | 1 | 0 |
| NM_030594 | CPEB1 | 3184 | 3 | 1 | 0 | 0 | 0 | 2 | 0 | 0 | 0 | 0 | 0 | 0 | 3 |
| NM_030613 | ZFP2 | 2405 | 2 | 1 | 1 | 0 | 1 | 2 | 1 | 0 | 0 | 2 | 1 | 1 | 2 |
| NM_030615 | KIF25 | 1510 | 0 | 0 | 0 | 0 | 0 | 0 | 0 | 0 | 0 | 0 | 0 | 0 | 0 |
| NM_030621 | DICER1 | 10221 | 16 | 8 | 8 | 3 | 7 | 8 | 9 | 8 | 8 | 9 | 6 | 16 | 8 |
| NM_030622 | CYP2S1 | 2621 | 2 | 0 | 0 | 2 | 0 | 1 | 0 | 0 | 1 | 0 | 0 | 1 | 0 |
| NM_030623 | SPHKAP | 6826 | 7 | 1 | 0 | 0 | 7 | 6 | 0 | 0 | 0 | 0 | 0 | 0 | 0 |
| NM_030624 | KLHL15 | 6273 | 20 | 5 | 2 | 1 | 3 | 4 | 3 | 5 | 2 | 5 | 2 | 3 | 20 |
| NM_030625 | TET1 | 9584 | 4 | 1 | 1 | 1 | 1 | 1 | 1 | 0 | 0 | 4 | 1 | 1 | 1 |
| NM_030626 | LRRC27 | 8052 | 1 | 0 | 0 | 0 | 0 | 0 | 0 | 0 | 0 | 0 | 0 | 0 | 1 |
| NM_030627 | CPEB4 | 7769 | 14 | 8 | 4 | 1 | 14 | 13 | 11 | 7 | 6 | 3 | 12 | 8 | 5 |
| NM_030628 | INTS5 | 3285 | 2 | 1 | 0 | 1 | 1 | 1 | 1 | 1 | 0 | 1 | 1 | 2 | 1 |
| NM_030629 | CMIP | 4131 | 15 | 5 | 3 | 5 | 3 | 15 | 5 | 1 | 5 | 5 | 1 | 6 | 3 |
| NM_030630 | C17orf28 | 3342 | 1 | 0 | 0 | 0 | 0 | 1 | 0 | 0 | 0 | 0 | 0 | 0 | 0 |
| NM_030631 | SLC25A21 | 1995 | 1 | 0 | 0 | 0 | 0 | 0 | 0 | 0 | 0 | 0 | 0 | 0 | 1 |
| NM_030632 | ASXL3 | 7642 | 3 | 1 | 0 | 0 | 0 | 1 | 1 | 0 | 0 | 3 | 0 | 0 | 1 |
| NM_030634 | ZNF436 | 4315 | 7 | 3 | 4 | 1 | 3 | 7 | 2 | 1 | 2 | 7 | 1 | 4 | 4 |
| NM_030636 | EEPD1 | 4687 | 2 | 1 | 1 | 1 | 0 | 0 | 0 | 0 | 0 | 0 | 2 | 1 | 0 |
| NM_030637 | DDHD1 | 12875 | 2 | 1 | 1 | 0 | 1 | 2 | 1 | 0 | 1 | 2 | 0 | 2 | 2 |
| NM_030639 | BHLHB9 | 3985 | 5 | 1 | 0 | 0 | 1 | 5 | 1 | 0 | 0 | 1 | 0 | 1 | 1 |
| NM_030640 | DUSP16 | 3566 | 7 | 3 | 2 | 2 | 2 | 3 | 2 | 7 | 4 | 3 | 3 | 4 | 4 |
| NM_030641 | APOL6 | 10139 | 12 | 4 | 12 | 2 | 2 | 2 | 3 | 5 | 4 | 3 | 2 | 5 | 1 |
| NM_030642 | APOL5 | 1437 | 0 | 0 | 0 | 0 | 0 | 0 | 0 | 0 | 0 | 0 | 0 | 0 | 0 |
| NM_030643 | APOL4 | 3415 | 2 | 1 | 2 | 1 | 1 | 1 | 1 | 0 | 2 | 1 | 1 | 2 | 1 |
| NM_030645 | SH3BP5L | 3182 | 1 | 0 | 0 | 0 | 0 | 1 | 1 | 0 | 0 | 0 | 0 | 1 | 0 |
| NM_030647 | JHDM1D | 9178 | 6 | 2 | 2 | 1 | 2 | 1 | 1 | 1 | 3 | 1 | 6 | 3 | 2 |
| NM_030648 | SETD7 | 7012 | 25 | 16 | 25 | 6 | 25 | 11 | 6 | 23 | 9 | 14 | 23 | 16 | 17 |

| transcript | gene | transcript length (nt) | max | mean | adipose | colon | heart | hypothalamus | kidney | liver | lung | ovary | skeletalmuscle | spleen | testes |
|---|---|---|---|---|---|---|---|---|---|---|---|---|---|---|---|
| NM_030649 | ACAP3 | 3759 | 1 | 0 | 0 | 0 | 0 | 1 | 0 | 0 | 0 | 0 | 0 | 0 | 0 |
| NM_030650 | KIAA1715 | 5765 | 8 | 4 | 4 | 1 | 4 | 6 | 3 | 4 | 3 | 4 | 8 | 4 | 3 |
| NM_030651 | PRRT1 | 1896 | 6 | 1 | 0 | 0 | 0 | 6 | 0 | 0 | 0 | 2 | 0 | 0 | 0 |
| NM_030652 | EGFL8 | 1269 | 3 | 1 | 0 | 2 | 0 | 1 | 1 | 0 | 1 | 2 | 0 | 1 | 3 |
| NM_030653 | DDX11 | 3927 | 0 | 0 | 0 | 0 | 0 | 0 | 0 | 0 | 0 | 0 | 0 | 0 | 0 |
| NM_030657 | LIM2 | 983 | 0 | 0 | 0 | 0 | 0 | 0 | 0 | 0 | 0 | 0 | 0 | 0 | 0 |
| NM_030660 | ATXN3 | 6758 | 4 | 2 | 2 | 1 | 2 | 2 | 2 | 1 | 2 | 4 | 1 | 3 | 3 |
| NM_030661 | HOXA3 | 3257 | 1 | 0 | 0 | 1 | 0 | 0 | 1 | 0 | 0 | 0 | 1 | 0 | 0 |
| NM_030662 | MAP2K2 | 1733 | 9 | 6 | 4 | 6 | 7 | 7 | 4 | 9 | 5 | 4 | 8 | 6 | 5 |
| NM_030663 | SMCP | 770 | 20 | 2 | 0 | 0 | 0 | 0 | 0 | 0 | 0 | 0 | 0 | 0 | 20 |
| NM_030664 | PTER | 3738 | 4 | 1 | 1 | 0 | 1 | 3 | 4 | 2 | 1 | 0 | 1 | 2 | 1 |
| NM_030665 | RAI1 | 7660 | 1 | 0 | 0 | 1 | 0 | 1 | 0 | 0 | 0 | 0 | 0 | 1 | 0 |
| NM_030666 | SERPINB1 | 1292 | 68 | 24 | 13 | 15 | 22 | 11 | 16 | 23 | 64 | 11 | 7 | 68 | 11 |
| NM_030667 | PTPRO | 5153 | 3 | 1 | 0 | 0 | 0 | 3 | 3 | 0 | 0 | 0 | 0 | 0 | 0 |
| NM_030668 | PTPRO | 2948 | 3 | 1 | 1 | 0 | 0 | 3 | 2 | 0 | 1 | 0 | 0 | 1 | 0 |
| NM_030669 | PTPRO | 3032 | 3 | 1 | 1 | 0 | 0 | 3 | 2 | 0 | 1 | 0 | 0 | 1 | 0 |
| NM_030670 | PTPRO | 3980 | 2 | 1 | 1 | 1 | 0 | 2 | 2 | 0 | 1 | 0 | 0 | 1 | 0 |
| NM_030671 | PTPRO | 4064 | 2 | 1 | 1 | 1 | 0 | 2 | 2 | 0 | 1 | 0 | 0 | 1 | 0 |
| NM_030672 | ARHGAP28 | 2046 | 9 | 2 | 1 | 1 | 0 | 0 | 2 | 0 | 1 | 4 | 3 | 5 | 9 |
| NM_030674 | SLC38A1 | 8484 | 8 | 4 | 0 | 2 | 8 | 7 | 3 | 1 | 3 | 1 | 4 | 8 | 1 |
| NM_030751 | ZEB1 | 5370 | 26 | 14 | 19 | 4 | 26 | 25 | 5 | 4 | 18 | 10 | 24 | 10 | 14 |
| NM_030752 | TCP1 | 2453 | 39 | 27 | 22 | 12 | 32 | 39 | 27 | 34 | 17 | 30 | 25 | 26 | 35 |
| NM_030753 | WNT3 | 1495 | 1 | 0 | 0 | 0 | 0 | 1 | 0 | 0 | 0 | 0 | 0 | 0 | 0 |
| NM_030754 | SAA2 | 543 | 2,706 | 260 | 73 | 6 | 12 | 0 | 4 | 2,706 | 8 | 13 | 10 | 20 | 11 |
| NM_030755 | TMX1 | 4103 | 10 | 5 | 3 | 2 | 5 | 4 | 6 | 6 | 5 | 4 | 2 | 10 | 6 |
| NM_030756 | TCF7L2 | 4001 | 6 | 3 | 3 | 3 | 2 | 1 | 2 | 1 | 3 | 6 | 1 | 5 | 3 |
| NM_030758 | OSBP2 | 4340 | 1 | 0 | 0 | 0 | 0 | 1 | 0 | 0 | 0 | 0 | 0 | 0 | 0 |
| NM_030759 | NRBF2 | 1896 | 7 | 3 | 3 | 1 | 2 | 3 | 2 | 7 | 2 | 2 | 3 | 6 | 3 |
| NM_030760 | S1PR5 | 2340 | 4 | 1 | 0 | 0 | 0 | 4 | 0 | 0 | 1 | 0 | 0 | 3 | 0 |
| NM_030761 | WNT4 | 3905 | 1 | 0 | 0 | 0 | 0 | 0 | 0 | 0 | 0 | 1 | 0 | 0 | 0 |
| NM_030762 | BHLHE41 | 3778 | 28 | 7 | 9 | 4 | 3 | 28 | 2 | 0 | 4 | 6 | 13 | 5 | 6 |
| NM_030763 | HMGN5 | 2126 | 66 | 9 | 2 | 1 | 2 | 7 | 8 | 4 | 3 | 5 | 0 | 4 | 66 |
| NM_030764 | FCRL2 | 2588 | 3 | 0 | 0 | 0 | 0 | 0 | 0 | 0 | 0 | 0 | 0 | 3 | 0 |
| NM_030765 | B3GNT4 | 1504 | 0 | 0 | 0 | 0 | 0 | 0 | 0 | 0 | 0 | 0 | 0 | 0 | 0 |
| NM_030766 | BCL2L14 | 2037 | 2 | 0 | 0 | 2 | 0 | 0 | 0 | 0 | 0 | 0 | 0 | 0 | 2 |
| NM_030767 | AKNA | 7382 | 5 | 1 | 1 | 1 | 0 | 0 | 0 | 0 | 1 | 1 | 0 | 5 | 0 |
| NM_030768 | ILKAP | 1443 | 4 | 3 | 2 | 3 | 2 | 4 | 2 | 2 | 2 | 2 | 2 | 3 | 3 |
| NM_030769 | NPL | 1552 | 14 | 5 | 4 | 1 | 3 | 1 | 14 | 2 | 9 | 2 | 1 | 11 | 2 |
| NM_030770 | TMPRSS5 | 2210 | 0 | 0 | 0 | 0 | 0 | 0 | 0 | 0 | 0 | 0 | 0 | 0 | 0 |
| NM_030771 | CCDC34 | 1483 | 2 | 1 | 1 | 1 | 0 | 2 | 0 | 0 | 0 | 1 | 0 | 0 | 2 |
| NM_030772 | GJA9 | 2313 | 1 | 0 | 0 | 1 | 0 | 0 | 0 | 0 | 0 | 0 | 0 | 0 | 1 |
| NM_030773 | TUBB1 | 3496 | 3 | 1 | 0 | 0 | 0 | 0 | 0 | 0 | 1 | 0 | 0 | 3 | 0 |
| NM_030774 | OR51E2 | 2785 | 0 | 0 | 0 | 0 | 0 | 0 | 0 | 0 | 0 | 0 | 0 | 0 | 0 |
| NM_030775 | WNT5B | 2184 | 0 | 0 | 0 | 0 | 0 | 0 | 0 | 0 | 0 | 0 | 0 | 0 | 0 |
| NM_030776 | ZBP1 | 2298 | 1 | 0 | 0 | 1 | 0 | 0 | 0 | 0 | 0 | 0 | 0 | 0 | 0 |
| NM_030777 | SLC2A10 | 4235 | 2 | 1 | 0 | 0 | 0 | 0 | 0 | 2 | 0 | 1 | 0 | 0 | 0 |
| NM_030779 | KCNH6 | 3404 | 0 | 0 | 0 | 0 | 0 | 0 | 0 | 0 | 0 | 0 | 0 | 0 | 0 |
| NM_030780 | SLC25A32 | 3023 | 5 | 3 | 2 | 1 | 3 | 4 | 2 | 3 | 2 | 3 | 2 | 5 | 3 |
| NM_030782 | CLPTM1L | 2147 | 8 | 3 | 3 | 1 | 2 | 2 | 5 | 8 | 2 | 1 | 1 | 4 | 2 |
| NM_030783 | PTDSS2 | 2445 | 1 | 1 | 1 | 1 | 1 | 1 | 1 | 0 | 1 | 1 | 0 | 1 | 1 |
| NM_030784 | GPR63 | 2079 | 0 | 0 | 0 | 0 | 0 | 0 | 0 | 0 | 0 | 0 | 0 | 0 | 0 |
| NM_030785 | RSPH6A | 2483 | 1 | 0 | 0 | 0 | 0 | 0 | 0 | 0 | 0 | 0 | 0 | 0 | 1 |
| NM_030786 | SYNC | 3493 | 12 | 3 | 3 | 2 | 4 | 1 | 0 | 0 | 1 | 1 | 12 | 1 | 2 |
| NM_030787 | CFHR5 | 2828 | 21 | 2 | 0 | 0 | 0 | 0 | 0 | 21 | 0 | 0 | 0 | 0 | 0 |
| NM_030788 | TM7SF4 | 1952 | 0 | 0 | 0 | 0 | 0 | 0 | 0 | 0 | 0 | 0 | 0 | 0 | 0 |
| NM_030789 | HM13 | 1596 | 60 | 17 | 17 | 8 | 14 | 11 | 17 | 60 | 14 | 9 | 5 | 18 | 14 |
| NM_030790 | ITFG1 | 2230 | 16 | 6 | 4 | 3 | 5 | 16 | 6 | 5 | 4 | 5 | 4 | 6 | 10 |
| NM_030791 | SGPP1 | 3308 | 9 | 3 | 2 | 1 | 2 | 3 | 9 | 4 | 3 | 1 | 1 | 7 | 3 |
| NM_030792 | GDPD5 | 3174 | 2 | 0 | 1 | 0 | 0 | 0 | 0 | 0 | 0 | 0 | 0 | 2 | 0 |
| NM_030793 | FBXO38 | 4151 | 8 | 5 | 5 | 2 | 6 | 7 | 5 | 5 | 4 | 5 | 5 | 8 | 6 |
| NM_030794 | TDRD3 | 3016 | 6 | 4 | 3 | 2 | 5 | 4 | 4 | 2 | 2 | 6 | 4 | 3 | 4 |
| NM_030795 | STMN4 | 1314 | 62 | 6 | 0 | 2 | 0 | 62 | 0 | 0 | 0 | 0 | 0 | 0 | 1 |
| NM_030796 | VOPP1 | 2944 | 12 | 5 | 6 | 4 | 3 | 6 | 4 | 3 | 4 | 3 | 2 | 12 | 2 |
| NM_030797 | FAM49A | 1490 | 40 | 11 | 7 | 2 | 4 | 40 | 1 | 1 | 12 | 6 | 6 | 35 | 3 |
| NM_030798 | WBSCR16 | 2378 | 4 | 2 | 2 | 1 | 3 | 2 | 4 | 3 | 2 | 2 | 2 | 3 | 3 |
| NM_030799 | YIPF5 | 3198 | 2 | 2 | 1 | 1 | 2 | 2 | 2 | 2 | 1 | 2 | 1 | 2 | 2 |
| NM_030800 | C15orf44 | 2521 | 3 | 2 | 2 | 2 | 2 | 2 | 3 | 3 | 1 | 3 | 1 | 3 | 3 |
| NM_030801 | MAGED4B | 2602 | 0 | 0 | 0 | 0 | 0 | 0 | 0 | 0 | 0 | 0 | 0 | 0 | 0 |
| NM_030802 | FAM117A | 2359 | 4 | 1 | 1 | 1 | 1 | 1 | 1 | 1 | 1 | 2 | 0 | 4 | 2 |
| NM_030803 | ATG16L1 | 3405 | 3 | 2 | 2 | 3 | 2 | 3 | 2 | 3 | 2 | 1 | 2 | 2 | 3 |
| NM_030805 | LMAN2L | 2407 | 3 | 2 | 2 | 2 | 3 | 2 | 3 | 3 | 1 | 3 | 2 | 2 | 2 |
| NM_030806 | C1orf21 | 10308 | 9 | 3 | 1 | 3 | 3 | 9 | 1 | 2 | 3 | 3 | 8 | 3 | 2 |
| NM_030807 | SLC2A11 | 2390 | 1 | 0 | 0 | 0 | 0 | 0 | 1 | 0 | 0 | 0 | 0 | 0 | 0 |
| NM_030808 | NDEL1 | 2375 | 17 | 8 | 9 | 4 | 8 | 17 | 5 | 5 | 8 | 11 | 5 | 11 | 8 |
| NM_030809 | CSRNP2 | 4469 | 3 | 2 | 1 | 1 | 2 | 3 | 1 | 1 | 1 | 2 | 2 | 1 | 2 |
| NM_030810 | TXNDC5 | 2956 | 18 | 8 | 6 | 8 | 8 | 3 | 8 | 18 | 8 | 8 | 1 | 15 | 5 |
| NM_030811 | MRPS26 | 1039 | 2 | 1 | 1 | 1 | 0 | 2 | 1 | 0 | 0 | 0 | 0 | 0 | 0 |

| transcript | gene | transcript length (nt) | max | mean | adipose | colon | heart | hypothalamus | kidney | liver | lung | ovary | skeletalmuscle | spleen | testes |
|---|---|---|---|---|---|---|---|---|---|---|---|---|---|---|---|
| NM_030812 | ACTL8 | 1859 | 0 | 0 | 0 | 0 | 0 | 0 | 0 | 0 | 0 | 0 | 0 | 0 | 0 |
| NM_030813 | CLPB | 3115 | 2 | 1 | 1 | 0 | 1 | 2 | 1 | 2 | 0 | 1 | 1 | 1 | 2 |
| NM_030815 | PDRG1 | 1373 | 2 | 1 | 1 | 1 | 0 | 1 | 1 | 0 | 1 | 0 | 0 | 1 | 2 |
| NM_030816 | ANKRD13C | 5667 | 7 | 4 | 3 | 2 | 4 | 7 | 4 | 3 | 4 | 7 | 4 | 6 | 4 |
| NM_030817 | APOLD1 | 4645 | 29 | 7 | 29 | 1 | 9 | 9 | 1 | 4 | 4 | 2 | 7 | 6 | 2 |
| NM_030818 | CCDC130 | 1632 | 1 | 1 | 0 | 1 | 1 | 1 | 0 | 0 | 0 | 0 | 1 | 1 | 0 |
| NM_030819 | GFOD2 | 2074 | 3 | 1 | 1 | 1 | 1 | 2 | 1 | 2 | 1 | 1 | 1 | 3 | 1 |
| NM_030820 | COL21A1 | 4173 | 2 | 1 | 0 | 0 | 2 | 1 | 0 | 0 | 1 | 1 | 0 | 0 | 1 |
| NM_030821 | PLA2G12A | 5234 | 3 | 2 | 1 | 1 | 3 | 2 | 3 | 2 | 1 | 2 | 2 | 2 | 1 |
| NM_030824 | ZNF442 | 2825 | 6 | 2 | 0 | 0 | 1 | 1 | 3 | 1 | 1 | 6 | 2 | 2 | 2 |
| NM_030876 | OR5V1 | 1048 | 0 | 0 | 0 | 0 | 0 | 0 | 0 | 0 | 0 | 0 | 0 | 0 | 0 |
| NM_030877 | CTNNBL1 | 1888 | 51 | 23 | 17 | 17 | 21 | 20 | 16 | 29 | 16 | 21 | 14 | 51 | 26 |
| NM_030881 | DDX17 | 3661 | 54 | 31 | 25 | 21 | 25 | 24 | 37 | 12 | 40 | 38 | 31 | 54 | 37 |
| NM_030882 | APOL2 | 2545 | 21 | 11 | 14 | 6 | 13 | 21 | 11 | 19 | 12 | 5 | 6 | 14 | 4 |
| NM_030883 | OR2H1 | 2996 | 0 | 0 | 0 | 0 | 0 | 0 | 0 | 0 | 0 | 0 | 0 | 0 | 0 |
| NM_030884 | MAP4 | 5153 | 97 | 16 | 5 | 3 | 48 | 5 | 3 | 2 | 3 | 3 | 97 | 3 | 6 |
| NM_030885 | MAP4 | 3204 | 22 | 11 | 12 | 5 | 19 | 22 | 6 | 4 | 6 | 7 | 19 | 6 | 10 |
| NM_030891 | LRRC3 | 2505 | 1 | 0 | 0 | 0 | 0 | 0 | 0 | 1 | 0 | 1 | 0 | 0 | 1 |
| NM_030893 | CD1E | 2063 | 0 | 0 | 0 | 0 | 0 | 0 | 0 | 0 | 0 | 0 | 0 | 0 | 0 |
| NM_030895 | ZNF696 | 4684 | 1 | 0 | 0 | 0 | 0 | 0 | 1 | 0 | 0 | 1 | 0 | 1 | 0 |
| NM_030899 | ZNF323 | 3036 | 3 | 1 | 1 | 1 | 1 | 2 | 1 | 1 | 1 | 3 | 1 | 1 | 3 |
| NM_030900 | TBRG4 | 1917 | 2 | 1 | 0 | 1 | 1 | 1 | 1 | 2 | 1 | 1 | 1 | 1 | 1 |
| NM_030901 | OR7A17 | 930 | 0 | 0 | 0 | 0 | 0 | 0 | 0 | 0 | 0 | 0 | 0 | 0 | 0 |
| NM_030903 | OR2W1 | 963 | 0 | 0 | 0 | 0 | 0 | 0 | 0 | 0 | 0 | 0 | 0 | 0 | 0 |
| NM_030904 | OR2T1 | 1110 | 0 | 0 | 0 | 0 | 0 | 0 | 0 | 0 | 0 | 0 | 0 | 0 | 0 |
| NM_030905 | OR2J2 | 1041 | 0 | 0 | 0 | 0 | 0 | 0 | 0 | 0 | 0 | 0 | 0 | 0 | 0 |
| NM_030906 | STK33 | 2707 | 10 | 3 | 0 | 1 | 5 | 3 | 4 | 0 | 2 | 6 | 1 | 0 | 10 |
| NM_030907 | C1orf89 | 1559 | 1 | 0 | 0 | 0 | 0 | 1 | 0 | 0 | 0 | 0 | 0 | 0 | 0 |
| NM_030908 | OR2A4 | 933 | 0 | 0 | 0 | 0 | 0 | 0 | 0 | 0 | 0 | 0 | 0 | 0 | 0 |
| NM_030911 | CDADC1 | 3341 | 3 | 1 | 1 | 1 | 1 | 1 | 3 | 1 | 1 | 2 | 1 | 2 | 2 |
| NM_030912 | TRIM8 | 2744 | 3 | 2 | 3 | 2 | 3 | 3 | 3 | 1 | 3 | 2 | 2 | 2 | 2 |
| NM_030913 | SEMA6C | 3890 | 2 | 0 | 0 | 0 | 0 | 0 | 0 | 0 | 0 | 0 | 2 | 0 | 1 |
| NM_030914 | URM1 | 1370 | 1 | 0 | 1 | 0 | 1 | 0 | 1 | 1 | 0 | 0 | 0 | 1 | 0 |
| NM_030915 | LBH | 2938 | 23 | 9 | 1 | 3 | 23 | 3 | 6 | 2 | 16 | 12 | 2 | 22 | 4 |
| NM_030916 | PVRL4 | 3502 | 1 | 0 | 0 | 1 | 0 | 0 | 0 | 0 | 1 | 0 | 0 | 0 | 0 |
| NM_030917 | FIP1L1 | 2457 | 42 | 23 | 19 | 11 | 18 | 38 | 15 | 15 | 16 | 31 | 14 | 30 | 42 |
| NM_030918 | SNX27 | 7085 | 8 | 5 | 3 | 3 | 5 | 8 | 8 | 3 | 5 | 5 | 5 | 6 | 4 |
| NM_030919 | FAM83D | 2429 | 1 | 0 | 0 | 1 | 0 | 0 | 0 | 1 | 1 | 0 | 0 | 0 | 1 |
| NM_030920 | ANP32E | 3452 | 28 | 15 | 11 | 6 | 17 | 28 | 17 | 12 | 18 | 12 | 14 | 22 | 12 |
| NM_030922 | NIPA2 | 2570 | 2 | 1 | 1 | 1 | 1 | 1 | 1 | 1 | 1 | 1 | 0 | 2 | 1 |
| NM_030923 | TMEM163 | 1892 | 2 | 0 | 0 | 0 | 0 | 2 | 0 | 0 | 0 | 0 | 0 | 0 | 1 |
| NM_030924 | ACSBG2 | 2712 | 5 | 0 | 0 | 0 | 0 | 0 | 0 | 0 | 0 | 0 | 0 | 0 | 5 |
| NM_030925 | CAB39L | 3676 | 5 | 2 | 2 | 1 | 2 | 5 | 1 | 0 | 2 | 4 | 0 | 0 | 2 |
| NM_030926 | ITM2C | 2073 | 18 | 4 | 1 | 13 | 1 | 18 | 4 | 1 | 1 | 2 | 0 | 4 | 1 |
| NM_030927 | TSPAN14 | 5579 | 6 | 3 | 4 | 2 | 2 | 2 | 3 | 2 | 5 | 2 | 1 | 6 | 2 |
| NM_030928 | CDT1 | 2675 | 0 | 0 | 0 | 0 | 0 | 0 | 0 | 0 | 0 | 0 | 0 | 0 | 0 |
| NM_030929 | KAZALD1 | 2513 | 1 | 0 | 0 | 0 | 0 | 0 | 0 | 0 | 0 | 0 | 0 | 1 | 0 |
| NM_030930 | UNC93B1 | 2315 | 2 | 1 | 1 | 0 | 1 | 0 | 0 | 0 | 1 | 0 | 0 | 2 | 0 |
| NM_030931 | DEFB126 | 413 | 3 | 0 | 0 | 0 | 0 | 0 | 0 | 0 | 0 | 0 | 0 | 0 | 3 |
| NM_030932 | DIAPH3 | 2735 | 3 | 1 | 0 | 1 | 0 | 0 | 0 | 0 | 1 | 0 | 0 | 0 | 3 |
| NM_030933 | C1orf14 | 2365 | 18 | 2 | 0 | 0 | 0 | 0 | 0 | 0 | 0 | 0 | 0 | 0 | 18 |
| NM_030934 | C1orf25 | 4407 | 5 | 2 | 1 | 1 | 2 | 2 | 3 | 1 | 2 | 3 | 4 | 3 | 5 |
| NM_030935 | TSC22D4 | 2383 | 1 | 1 | 0 | 1 | 1 | 1 | 1 | 0 | 1 | 1 | 0 | 1 | 1 |
| NM_030936 | RNF32 | 1742 | 3 | 0 | 0 | 0 | 0 | 0 | 0 | 0 | 0 | 0 | 0 | 0 | 3 |
| NM_030937 | CCNL2 | 3114 | 3 | 2 | 2 | 3 | 1 | 2 | 2 | 1 | 1 | 1 | 1 | 2 | 1 |
| NM_030938 | TMEM49 | 2174 | 38 | 22 | 17 | 17 | 14 | 31 | 14 | 29 | 27 | 21 | 12 | 38 | 24 |
| NM_030939 | C6orf62 | 2471 | 53 | 19 | 16 | 6 | 14 | 11 | 18 | 9 | 18 | 28 | 11 | 53 | 24 |
| NM_030940 | ISCA1 | 2002 | 6 | 3 | 2 | 2 | 5 | 6 | 5 | 2 | 1 | 3 | 3 | 3 | 3 |
| NM_030941 | LOC81691 | 2775 | 43 | 4 | 0 | 1 | 1 | 1 | 0 | 0 | 0 | 1 | 0 | 0 | 43 |
| NM_030943 | AMN | 1557 | 0 | 0 | 0 | 0 | 0 | 0 | 0 | 0 | 0 | 0 | 0 | 0 | 0 |
| NM_030945 | C1QTNF3 | 3553 | 1 | 0 | 0 | 1 | 0 | 1 | 1 | 0 | 0 | 1 | 0 | 0 | 1 |
| NM_030946 | OR14J1 | 966 | 0 | 0 | 0 | 0 | 0 | 0 | 0 | 0 | 0 | 0 | 0 | 0 | 0 |
| NM_030948 | PHACTR1 | 2150 | 14 | 4 | 1 | 1 | 13 | 14 | 2 | 0 | 5 | 1 | 0 | 5 | 1 |
| NM_030949 | PPP1R14C | 2211 | 3 | 1 | 0 | 1 | 3 | 1 | 0 | 0 | 1 | 0 | 1 | 0 | 0 |
| NM_030952 | NUAK2 | 3401 | 3 | 1 | 0 | 1 | 0 | 0 | 3 | 0 | 2 | 0 | 0 | 2 | 1 |
| NM_030953 | TIGD6 | 3491 | 1 | 0 | 0 | 0 | 0 | 0 | 1 | 0 | 0 | 1 | 0 | 0 | 1 |
| NM_030954 | RNF170 | 3886 | 2 | 1 | 1 | 0 | 1 | 2 | 2 | 1 | 1 | 2 | 1 | 1 | 1 |
| NM_030955 | ADAMTS12 | 4954 | 3 | 1 | 3 | 0 | 0 | 0 | 0 | 0 | 1 | 1 | 0 | 0 | 1 |
| NM_030956 | TLR10 | 3576 | 3 | 0 | 0 | 0 | 0 | 1 | 0 | 0 | 0 | 0 | 0 | 3 | 0 |
| NM_030957 | ADAMTS10 | 4237 | 1 | 0 | 0 | 0 | 0 | 0 | 0 | 0 | 0 | 1 | 0 | 0 | 0 |
| NM_030958 | SLCO5A1 | 3788 | 2 | 0 | 0 | 0 | 2 | 0 | 0 | 0 | 0 | 0 | 2 | 0 | 0 |
| NM_030959 | OR12D3 | 1869 | 0 | 0 | 0 | 0 | 0 | 0 | 0 | 0 | 0 | 0 | 0 | 0 | 0 |
| NM_030960 | SPACA1 | 1499 | 4 | 0 | 0 | 0 | 0 | 0 | 0 | 0 | 0 | 0 | 0 | 0 | 4 |
| NM_030961 | TRIM56 | 3527 | 2 | 1 | 1 | 1 | 1 | 1 | 1 | 1 | 2 | 2 | 1 | 2 | 1 |
| NM_030962 | SBF2 | 7439 | 6 | 4 | 4 | 2 | 3 | 4 | 4 | 2 | 3 | 6 | 2 | 4 | 4 |
| NM_030963 | RNF146 | 2142 | 16 | 9 | 9 | 3 | 9 | 16 | 8 | 5 | 9 | 9 | 11 | 13 | 10 |
| NM_030964 | SPRY4 | 4992 | 2 | 1 | 1 | 1 | 1 | 1 | 1 | 1 | 2 | 0 | 0 | 1 | 0 |

| | | transcript | | | | | | hypothala | | | | | skeletalm | | |
|---|---|---|---|---|---|---|---|---|---|---|---|---|---|---|---|
| transcript | gene | length (nt) | max | mean | adipose | colon | heart | mus | kidney | liver | lung | ovary | uscle | spleen | testes |
| NM_030965 | ST6GALNAC | 2032 | 8 | 1 | 0 | 0 | 1 | 8 | 0 | 0 | 2 | 2 | 0 | 0 | 1 |
| NM_030966 | KRTAP1-3 | 971 | 0 | 0 | 0 | 0 | 0 | 0 | 0 | 0 | 0 | 0 | 0 | 0 | 0 |
| NM_030967 | KRTAP1-1 | 903 | 0 | 0 | 0 | 0 | 0 | 0 | 0 | 0 | 0 | 0 | 0 | 0 | 0 |
| NM_030968 | C1QTNF1 | 2886 | 4 | 1 | 1 | 0 | 4 | 0 | 0 | 0 | 0 | 0 | 0 | 1 | 0 |
| NM_030969 | TMEM14B | 972 | 118 | 61 | 60 | 18 | 118 | 48 | 77 | 48 | 47 | 56 | 75 | 70 | 59 |
| NM_030971 | SFXN3 | 3090 | 3 | 2 | 2 | 1 | 2 | 3 | 2 | 0 | 2 | 1 | 0 | 2 | 2 |
| NM_030972 | ZNF611 | 4557 | 6 | 3 | 2 | 1 | 2 | 4 | 6 | 1 | 3 | 6 | 1 | 5 | 3 |
| NM_030973 | MED25 | 2333 | 1 | 0 | 0 | 0 | 0 | 0 | 0 | 0 | 0 | 0 | 1 | 0 | 1 |
| NM_030974 | SHARPIN | 1816 | 4 | 3 | 2 | 2 | 3 | 2 | 2 | 3 | 2 | 1 | 4 | 3 | 4 |
| NM_030975 | KRTAP9-9 | 936 | 0 | 0 | 0 | 0 | 0 | 0 | 0 | 0 | 0 | 0 | 0 | 0 | 0 |
| NM_030978 | ARPC5L | 1025 | 5 | 3 | 1 | 1 | 5 | 5 | 3 | 4 | 2 | 1 | 1 | 5 | 3 |
| NM_030979 | PABPC3 | 2429 | 90 | 35 | 32 | 23 | 20 | 15 | 13 | 23 | 39 | 77 | 12 | 90 | 41 |
| NM_030980 | ISG20L2 | 2051 | 8 | 3 | 1 | 2 | 2 | 2 | 2 | 2 | 2 | 3 | 1 | 4 | 8 |
| NM_030981 | RAB1B | 1955 | 16 | 12 | 8 | 9 | 14 | 11 | 14 | 13 | 8 | 10 | 15 | 16 | 8 |
| NM_030984 | TBXAS1 | 1901 | 41 | 8 | 6 | 2 | 5 | 4 | 2 | 5 | 14 | 2 | 1 | 41 | 2 |
| NM_031157 | HNRNPA1 | 1925 | 33 | 10 | 3 | 4 | 11 | 5 | 6 | 5 | 5 | 33 | 16 | 10 | 6 |
| NM_031200 | CCR9 | 2544 | 1 | 0 | 0 | 0 | 0 | 0 | 0 | 0 | 0 | 0 | 0 | 1 | 0 |
| NM_031203 | HNRNPM | 2414 | 23 | 16 | 14 | 15 | 16 | 14 | 16 | 21 | 12 | 17 | 12 | 23 | 18 |
| NM_031204 | CABP2 | 786 | 0 | 0 | 0 | 0 | 0 | 0 | 0 | 0 | 0 | 0 | 0 | 0 | 0 |
| NM_031205 | CABP1 | 1178 | 9 | 1 | 0 | 0 | 0 | 9 | 1 | 0 | 0 | 0 | 0 | 0 | 0 |
| NM_031206 | LAS1L | 2464 | 11 | 5 | 5 | 5 | 3 | 4 | 5 | 7 | 4 | 5 | 2 | 5 | 11 |
| NM_031207 | HYI | 968 | 0 | 0 | 0 | 0 | 0 | 0 | 0 | 0 | 0 | 0 | 0 | 0 | 0 |
| NM_031208 | FAHD1 | 1684 | 8 | 3 | 1 | 3 | 2 | 3 | 8 | 7 | 1 | 1 | 1 | 1 | 2 |
| NM_031209 | QTRT1 | 1333 | 1 | 0 | 1 | 1 | 0 | 1 | 0 | 1 | 0 | 0 | 0 | 0 | 0 |
| NM_031210 | C14orf156 | 399 | 29 | 18 | 20 | 7 | 29 | 21 | 28 | 27 | 11 | 6 | 15 | 18 | 15 |
| NM_031212 | SLC25A28 | 1555 | 4 | 2 | 2 | 2 | 2 | 2 | 2 | 3 | 2 | 2 | 3 | 3 | 4 |
| NM_031213 | FAM108A1 | 1716 | 5 | 2 | 2 | 2 | 2 | 5 | 1 | 1 | 2 | 1 | 1 | 2 | 1 |
| NM_031215 | CABLES2 | 3785 | 2 | 0 | 0 | 1 | 1 | 1 | 0 | 0 | 0 | 0 | 0 | 0 | 2 |
| NM_031216 | SEH1L | 3506 | 3 | 2 | 2 | 1 | 1 | 3 | 2 | 3 | 2 | 2 | 1 | 3 | 2 |
| NM_031217 | KIF18A | 3463 | 2 | 0 | 0 | 0 | 0 | 0 | 0 | 0 | 0 | 0 | 0 | 0 | 2 |
| NM_031218 | ZNF93 | 2794 | 12 | 3 | 2 | 1 | 2 | 2 | 3 | 0 | 2 | 12 | 2 | 5 | 4 |
| NM_031219 | HDHD3 | 1721 | 2 | 0 | 0 | 1 | 0 | 0 | 2 | 1 | 0 | 0 | 0 | 0 | 0 |
| NM_031220 | PITPNM3 | 7151 | 6 | 1 | 0 | 1 | 0 | 4 | 0 | 0 | 0 | 2 | 0 | 6 | 0 |
| NM_031226 | CYP19A1 | 4514 | 3 | 0 | 0 | 0 | 0 | 1 | 0 | 0 | 0 | 3 | 0 | 0 | 0 |
| NM_031229 | RBCK1 | 2762 | 1 | 0 | 0 | 1 | 0 | 0 | 1 | 1 | 0 | 0 | 0 | 1 | 0 |
| NM_031231 | NECAB3 | 1937 | 0 | 0 | 0 | 0 | 0 | 0 | 0 | 0 | 0 | 0 | 0 | 0 | 0 |
| NM_031232 | NECAB3 | 2039 | 0 | 0 | 0 | 0 | 0 | 0 | 0 | 0 | 0 | 0 | 0 | 0 | 0 |
| NM_031243 | HNRNPA2B1 | 3702 | 393 | 209 | 102 | 83 | 291 | 137 | 246 | 188 | 227 | 194 | 208 | 393 | 227 |
| NM_031244 | SIRT5 | 2379 | 0 | 0 | 0 | 0 | 0 | 0 | 0 | 0 | 0 | 0 | 0 | 0 | 0 |
| NM_031246 | PSG2 | 1533 | 0 | 0 | 0 | 0 | 0 | 0 | 0 | 0 | 0 | 0 | 0 | 0 | 0 |
| NM_031262 | HNRNPK | 2918 | 32 | 21 | 18 | 11 | 23 | 24 | 23 | 22 | 21 | 21 | 16 | 32 | 21 |
| NM_031263 | HNRNPK | 2943 | 32 | 21 | 18 | 11 | 23 | 24 | 23 | 22 | 21 | 20 | 16 | 32 | 21 |
| NM_031264 | MUPCDH | 2837 | 5 | 1 | 0 | 5 | 0 | 0 | 5 | 3 | 0 | 0 | 0 | 0 | 0 |
| NM_031266 | HNRNPAB | 1816 | 22 | 12 | 9 | 12 | 14 | 10 | 13 | 22 | 9 | 6 | 11 | 17 | 7 |
| NM_031267 | CDK13 | 6793 | 5 | 3 | 3 | 3 | 3 | 3 | 3 | 2 | 3 | 5 | 3 | 5 | 5 |
| NM_031268 | PDPK1 | 6855 | 6 | 3 | 2 | 3 | 3 | 6 | 4 | 2 | 2 | 2 | 2 | 4 | 2 |
| NM_031271 | TEX15 | 10112 | 11 | 1 | 0 | 0 | 0 | 0 | 0 | 0 | 0 | 0 | 0 | 0 | 11 |
| NM_031272 | TEX14 | 4807 | 6 | 1 | 0 | 0 | 0 | 0 | 0 | 0 | 0 | 0 | 0 | 0 | 6 |
| NM_031273 | TEX13B | 1248 | 0 | 0 | 0 | 0 | 0 | 0 | 0 | 0 | 0 | 0 | 0 | 0 | 0 |
| NM_031274 | TEX13A | 1379 | 1 | 0 | 0 | 0 | 0 | 0 | 0 | 0 | 0 | 0 | 0 | 0 | 1 |
| NM_031275 | TEX12 | 1144 | 3 | 0 | 0 | 0 | 0 | 0 | 0 | 0 | 0 | 0 | 0 | 0 | 3 |
| NM_031276 | TEX11 | 3018 | 4 | 0 | 0 | 0 | 0 | 0 | 0 | 0 | 0 | 0 | 0 | 0 | 4 |
| NM_031277 | RNF17 | 5107 | 5 | 0 | 0 | 0 | 0 | 0 | 0 | 0 | 0 | 0 | 0 | 0 | 5 |
| NM_031279 | AGXT2L1 | 2117 | 45 | 8 | 0 | 1 | 9 | 45 | 1 | 37 | 0 | 0 | 0 | 1 | 0 |
| NM_031280 | MRPS15 | 965 | 9 | 6 | 6 | 2 | 7 | 4 | 8 | 9 | 4 | 2 | 7 | 4 | 8 |
| NM_031281 | FCRL5 | 5390 | 2 | 0 | 0 | 0 | 0 | 0 | 0 | 0 | 0 | 0 | 0 | 2 | 0 |
| NM_031282 | FCRL4 | 3458 | 0 | 0 | 0 | 0 | 0 | 0 | 0 | 0 | 0 | 0 | 0 | 0 | 0 |
| NM_031283 | TCF7L1 | 2761 | 5 | 2 | 2 | 2 | 2 | 1 | 2 | 1 | 3 | 5 | 2 | 4 | 2 |
| NM_031284 | ADPGK | 2559 | 5 | 2 | 2 | 2 | 1 | 1 | 2 | 1 | 2 | 1 | 0 | 5 | 1 |
| NM_031286 | SH3BGRL3 | 1115 | 21 | 7 | 9 | 5 | 8 | 3 | 6 | 3 | 11 | 3 | 2 | 21 | 2 |
| NM_031287 | SF3B5 | 737 | 2 | 1 | 2 | 2 | 1 | 2 | 2 | 2 | 1 | 0 | 1 | 2 | 2 |
| NM_031288 | INO80B | 1212 | 6 | 4 | 3 | 5 | 3 | 5 | 4 | 2 | 3 | 4 | 3 | 6 | 6 |
| NM_031289 | GSG1 | 2066 | 11 | 1 | 0 | 0 | 0 | 0 | 0 | 0 | 0 | 0 | 0 | 0 | 11 |
| NM_031290 | CCDC70 | 1154 | 4 | 0 | 0 | 0 | 0 | 0 | 0 | 0 | 0 | 0 | 0 | 0 | 4 |
| NM_031291 | SLC25A31 | 1811 | 1 | 0 | 0 | 0 | 0 | 0 | 0 | 0 | 0 | 0 | 0 | 0 | 1 |
| NM_031292 | PUS7L | 3953 | 3 | 2 | 1 | 0 | 2 | 2 | 2 | 1 | 1 | 3 | 1 | 3 | 2 |
| NM_031293 | PMFBP1 | 3436 | 2 | 0 | 0 | 0 | 0 | 0 | 0 | 0 | 0 | 0 | 0 | 0 | 2 |
| NM_031294 | LRRC48 | 2066 | 1 | 0 | 0 | 0 | 0 | 1 | 1 | 0 | 0 | 0 | 0 | 0 | 1 |
| NM_031296 | RAB33B | 3868 | 1 | 0 | 0 | 0 | 1 | 0 | 0 | 1 | 1 | 1 | 0 | 1 | 0 |
| NM_031297 | RNF208 | 1069 | 13 | 2 | 1 | 1 | 3 | 13 | 2 | 1 | 1 | 0 | 2 | 0 | 1 |
| NM_031298 | TMEM93 | 675 | 4 | 2 | 1 | 1 | 4 | 2 | 3 | 4 | 0 | 0 | 3 | 2 | 2 |
| NM_031299 | CDCA3 | 1170 | 0 | 0 | 0 | 0 | 0 | 0 | 0 | 0 | 0 | 0 | 0 | 0 | 0 |
| NM_031300 | MXD3 | 1465 | 0 | 0 | 0 | 0 | 0 | 0 | 0 | 0 | 0 | 0 | 0 | 0 | 0 |
| NM_031301 | APH1B | 4192 | 5 | 2 | 1 | 0 | 1 | 3 | 1 | 1 | 2 | 2 | 1 | 4 | 5 |
| NM_031302 | GLT8D2 | 1877 | 2 | 1 | 1 | 1 | 1 | 1 | 0 | 0 | 1 | 2 | 0 | 0 | 1 |
| NM_031303 | KATNAL2 | 2832 | 1 | 1 | 1 | 0 | 0 | 1 | 1 | 0 | 0 | 1 | 0 | 1 | 1 |
| NM_031304 | DOHH | 1742 | 5 | 2 | 2 | 1 | 1 | 5 | 2 | 2 | 1 | 1 | 1 | 1 | 1 |

| | | transcript | | | | | | hypothala | | | | | skeletalm | | |
|---|---|---|---|---|---|---|---|---|---|---|---|---|---|---|---|
| transcript | gene | length (nt) | max | mean | adipose | colon | heart | mus | kidney | liver | lung | ovary | uscle | spleen | testes |
| NM_031305 | ARHGAP24 | 4574 | 12 | 3 | 5 | 1 | 3 | 3 | 12 | 1 | 3 | 1 | 2 | 6 | 1 |
| NM_031307 | PUS3 | 1843 | 16 | 5 | 2 | 1 | 4 | 4 | 3 | 16 | 3 | 3 | 5 | 5 | 4 |
| NM_031308 | EPPK1 | 7523 | 2 | 1 | 0 | 2 | 0 | 0 | 1 | 0 | 1 | 0 | 0 | 1 | 0 |
| NM_031309 | SCRT1 | 3552 | 0 | 0 | 0 | 0 | 0 | 0 | 0 | 0 | 0 | 0 | 0 | 0 | 0 |
| NM_031310 | PLVAP | 2276 | 42 | 10 | 25 | 6 | 11 | 1 | 10 | 1 | 4 | 0 | 5 | 42 | 2 |
| NM_031311 | CPVL | 1691 | 48 | 10 | 7 | 2 | 14 | 3 | 13 | 2 | 8 | 3 | 3 | 48 | 5 |
| NM_031313 | ALPPL2 | 2495 | 0 | 0 | 0 | 0 | 0 | 0 | 0 | 0 | 0 | 0 | 0 | 0 | 0 |
| NM_031314 | HNRNPC | 3251 | 37 | 22 | 14 | 13 | 18 | 31 | 22 | 20 | 22 | 22 | 17 | 37 | 23 |
| NM_031361 | COL4A3BP | 5317 | 11 | 7 | 11 | 3 | 5 | 8 | 6 | 4 | 8 | 7 | 7 | 10 | 8 |
| NM_031362 | COL4A3 | 7923 | 2 | 0 | 0 | 0 | 0 | 0 | 2 | 0 | 1 | 0 | 1 | 1 | 0 |
| NM_031365 | COL4A3 | 7630 | 2 | 0 | 0 | 0 | 0 | 0 | 2 | 0 | 1 | 0 | 1 | 1 | 0 |
| NM_031366 | COL4A3 | 7420 | 2 | 1 | 0 | 0 | 0 | 0 | 2 | 0 | 1 | 0 | 1 | 1 | 0 |
| NM_031369 | HNRNPD | 2200 | 100 | 60 | 38 | 44 | 58 | 68 | 50 | 45 | 59 | 83 | 51 | 100 | 66 |
| NM_031370 | HNRNPD | 2257 | 103 | 61 | 38 | 47 | 59 | 69 | 52 | 45 | 60 | 84 | 52 | 103 | 67 |
| NM_031371 | ARID4B | 5809 | 13 | 7 | 5 | 2 | 6 | 6 | 6 | 3 | 8 | 10 | 5 | 13 | 11 |
| NM_031372 | HNRPDL | 3510 | 80 | 51 | 36 | 23 | 56 | 66 | 46 | 49 | 47 | 80 | 49 | 65 | 45 |
| NM_031407 | HUWE1 | 14725 | 18 | 13 | 10 | 8 | 15 | 17 | 13 | 11 | 8 | 17 | 17 | 14 | 18 |
| NM_031409 | CCR6 | 3479 | 1 | 0 | 0 | 0 | 0 | 0 | 0 | 0 | 0 | 0 | 0 | 1 | 0 |
| NM_031410 | PCDHA1 | 2570 | 0 | 0 | 0 | 0 | 0 | 0 | 0 | 0 | 0 | 0 | 0 | 0 | 0 |
| NM_031411 | PCDHA1 | 4468 | 0 | 0 | 0 | 0 | 0 | 0 | 0 | 0 | 0 | 0 | 0 | 0 | 0 |
| NM_031412 | GABARAPL1 | 1867 | 70 | 29 | 16 | 5 | 70 | 61 | 58 | 22 | 26 | 12 | 29 | 11 | 11 |
| NM_031413 | CECR2 | 5636 | 3 | 1 | 0 | 0 | 0 | 1 | 0 | 1 | 1 | 1 | 3 | 0 | 1 |
| NM_031414 | STK31 | 3269 | 10 | 1 | 0 | 0 | 0 | 0 | 0 | 0 | 0 | 0 | 0 | 0 | 10 |
| NM_031415 | GSDMC | 2714 | 3 | 0 | 0 | 0 | 0 | 0 | 0 | 0 | 0 | 0 | 0 | 3 | 0 |
| NM_031417 | MARK4 | 4917 | 7 | 3 | 1 | 1 | 2 | 5 | 1 | 2 | 1 | 3 | 3 | 2 | 7 |
| NM_031418 | ANO3 | 5936 | 5 | 1 | 1 | 0 | 0 | 5 | 0 | 0 | 0 | 0 | 0 | 0 | 0 |
| NM_031419 | NFKBIZ | 3923 | 12 | 5 | 3 | 5 | 4 | 2 | 4 | 5 | 12 | 9 | 2 | 11 | 2 |
| NM_031420 | MRPL9 | 1292 | 11 | 7 | 4 | 3 | 8 | 8 | 9 | 7 | 5 | 8 | 11 | 9 | 7 |
| NM_031421 | TTC25 | 2083 | 16 | 2 | 0 | 0 | 0 | 1 | 1 | 0 | 3 | 1 | 0 | 0 | 16 |
| NM_031422 | CHST9 | 2244 | 3 | 1 | 0 | 0 | 0 | 1 | 0 | 2 | 2 | 0 | 0 | 2 | 3 |
| NM_031423 | NUF2 | 1843 | 7 | 1 | 1 | 0 | 0 | 0 | 0 | 0 | 1 | 0 | 0 | 1 | 7 |
| NM_031424 | FAM110A | 1638 | 3 | 1 | 0 | 1 | 1 | 0 | 1 | 0 | 2 | 0 | 1 | 3 | 0 |
| NM_031426 | AIF1L | 3388 | 44 | 9 | 4 | 1 | 4 | 10 | 44 | 0 | 1 | 1 | 0 | 32 | 2 |
| NM_031427 | DNAL1 | 6742 | 6 | 2 | 2 | 1 | 2 | 6 | 3 | 1 | 2 | 2 | 1 | 1 | 3 |
| NM_031429 | RTBDN | 1328 | 0 | 0 | 0 | 0 | 0 | 0 | 0 | 0 | 0 | 0 | 0 | 0 | 0 |
| NM_031430 | RILP | 1791 | 1 | 1 | 1 | 1 | 1 | 0 | 1 | 1 | 1 | 0 | 1 | 0 | 0 |
| NM_031431 | COG3 | 4482 | 9 | 4 | 2 | 3 | 4 | 3 | 5 | 9 | 4 | 4 | 3 | 5 | 5 |
| NM_031432 | UCK1 | 2178 | 7 | 5 | 4 | 3 | 6 | 7 | 6 | 6 | 3 | 4 | 5 | 5 | 4 |
| NM_031433 | MFRP | 3917 | 0 | 0 | 0 | 0 | 0 | 0 | 0 | 0 | 0 | 0 | 0 | 0 | 0 |
| NM_031434 | TMUB1 | 1797 | 5 | 2 | 1 | 2 | 1 | 2 | 2 | 5 | 1 | 1 | 2 | 2 | 1 |
| NM_031435 | THAP2 | 4724 | 1 | 0 | 0 | 0 | 1 | 1 | 0 | 0 | 0 | 1 | 0 | 1 | 0 |
| NM_031438 | NUDT12 | 3488 | 4 | 2 | 1 | 1 | 1 | 3 | 4 | 4 | 1 | 1 | 1 | 1 | 1 |
| NM_031439 | SOX7 | 3216 | 7 | 2 | 1 | 0 | 3 | 1 | 1 | 0 | 7 | 1 | 2 | 1 | 1 |
| NM_031440 | RTP3 | 817 | 1 | 0 | 0 | 0 | 0 | 0 | 0 | 1 | 0 | 0 | 0 | 0 | 0 |
| NM_031442 | TMEM47 | 4054 | 5 | 3 | 2 | 1 | 5 | 5 | 3 | 2 | 5 | 3 | 2 | 1 | 3 |
| NM_031443 | CCM2 | 1894 | 4 | 2 | 2 | 1 | 1 | 3 | 1 | 4 | 1 | 0 | 1 | 3 | 2 |
| NM_031444 | C22orf13 | 3605 | 23 | 6 | 6 | 6 | 7 | 2 | 8 | 23 | 3 | 2 | 5 | 5 | 3 |
| NM_031445 | AMMECR1L | 4646 | 3 | 2 | 2 | 1 | 2 | 1 | 2 | 1 | 1 | 2 | 1 | 2 | 3 |
| NM_031446 | C18orf21 | 1039 | 6 | 3 | 2 | 1 | 2 | 3 | 2 | 2 | 1 | 3 | 3 | 4 | 6 |
| NM_031448 | C19orf12 | 4683 | 2 | 1 | 2 | 0 | 1 | 1 | 1 | 2 | 0 | 1 | 1 | 1 | 1 |
| NM_031449 | ZMIZ2 | 5143 | 2 | 1 | 1 | 2 | 1 | 2 | 2 | 0 | 1 | 1 | 1 | 2 | 1 |
| NM_031450 | C11orf68 | 1554 | 1 | 1 | 0 | 0 | 1 | 1 | 0 | 0 | 1 | 1 | 1 | 1 | 1 |
| NM_031451 | TEX101 | 1583 | 11 | 1 | 0 | 0 | 0 | 0 | 0 | 0 | 0 | 0 | 0 | 0 | 11 |
| NM_031452 | FAM103A1 | 1146 | 2 | 1 | 1 | 1 | 1 | 2 | 1 | 2 | 1 | 1 | 1 | 2 | 1 |
| NM_031453 | FAM107B | 3785 | 44 | 17 | 16 | 7 | 4 | 19 | 44 | 34 | 17 | 3 | 1 | 20 | 19 |
| NM_031454 | SELO | 2315 | 0 | 0 | 0 | 0 | 0 | 0 | 0 | 0 | 0 | 0 | 0 | 0 | 0 |
| NM_031455 | CCDC3 | 2738 | 6 | 2 | 6 | 1 | 2 | 1 | 0 | 0 | 1 | 4 | 1 | 1 | 2 |
| NM_031456 | FBXW10 | 3439 | 1 | 0 | 0 | 0 | 0 | 0 | 0 | 0 | 0 | 0 | 0 | 0 | 1 |
| NM_031457 | MS4A8B | 1354 | 1 | 0 | 0 | 0 | 0 | 0 | 0 | 0 | 1 | 0 | 0 | 0 | 0 |
| NM_031458 | PARP9 | 3341 | 13 | 6 | 3 | 2 | 5 | 3 | 5 | 13 | 7 | 4 | 6 | 11 | 4 |
| NM_031459 | SESN2 | 3485 | 1 | 1 | 0 | 0 | 0 | 1 | 1 | 1 | 0 | 1 | 0 | 1 | 1 |
| NM_031460 | KCNK17 | 1565 | 0 | 0 | 0 | 0 | 0 | 0 | 0 | 0 | 0 | 0 | 0 | 0 | 0 |
| NM_031461 | CRISPLD1 | 4192 | 2 | 0 | 0 | 0 | 1 | 2 | 0 | 0 | 0 | 1 | 0 | 0 | 0 |
| NM_031462 | CD99L2 | 3605 | 24 | 8 | 11 | 3 | 6 | 24 | 5 | 7 | 3 | 4 | 11 | 6 | 4 |
| NM_031463 | HSDL1 | 3668 | 5 | 2 | 1 | 1 | 2 | 5 | 2 | 1 | 1 | 3 | 1 | 2 | 2 |
| NM_031464 | RPS6KL1 | 3017 | 1 | 0 | 0 | 0 | 0 | 1 | 0 | 0 | 0 | 0 | 0 | 0 | 0 |
| NM_031466 | TRAPPC9 | 4474 | 5 | 3 | 1 | 3 | 2 | 5 | 2 | 1 | 2 | 3 | 3 | 4 | 2 |
| NM_031467 | SLC4A9 | 3271 | 1 | 0 | 0 | 0 | 0 | 0 | 1 | 0 | 0 | 0 | 0 | 0 | 0 |
| NM_031468 | CALN1 | 9461 | 7 | 1 | 0 | 0 | 0 | 7 | 0 | 0 | 0 | 0 | 0 | 0 | 0 |
| NM_031469 | SH3BGRL2 | 4646 | 4 | 2 | 2 | 3 | 1 | 4 | 2 | 3 | 2 | 1 | 1 | 1 | 1 |
| NM_031471 | FERMT3 | 2540 | 1 | 0 | 1 | 0 | 0 | 0 | 0 | 0 | 0 | 0 | 0 | 1 | 0 |
| NM_031472 | TRPT1 | 968 | 3 | 1 | 1 | 0 | 2 | 1 | 1 | 1 | 0 | 0 | 3 | 1 | 1 |
| NM_031473 | IFT81 | 2309 | 7 | 3 | 1 | 1 | 2 | 3 | 3 | 1 | 2 | 5 | 1 | 2 | 7 |
| NM_031474 | NRIP2 | 2776 | 2 | 1 | 0 | 1 | 1 | 2 | 2 | 0 | 0 | 2 | 1 | 2 | 1 |
| NM_031475 | ESPN | 3531 | 1 | 0 | 0 | 0 | 0 | 0 | 1 | 1 | 0 | 0 | 0 | 0 | 1 |
| NM_031476 | CRISPLD2 | 4589 | 9 | 4 | 2 | 2 | 4 | 1 | 3 | 1 | 9 | 3 | 1 | 5 | 7 |
| NM_031477 | YPEL3 | 1588 | 12 | 6 | 3 | 3 | 5 | 12 | 2 | 1 | 6 | 7 | 12 | 9 | 2 |

| transcript | gene | transcript length (nt) | max | mean | adipose | colon | heart | hypothalamus | kidney | liver | lung | ovary | skeletalmuscle | spleen | testes |
|---|---|---|---|---|---|---|---|---|---|---|---|---|---|---|---|
| NM_031478 | FAM57B | 1919 | 1 | 0 | 0 | 0 | 0 | 1 | 0 | 0 | 0 | 0 | 0 | 0 | 0 |
| NM_031479 | INHBE | 2437 | 12 | 1 | 0 | 0 | 0 | 0 | 0 | 12 | 0 | 0 | 0 | 0 | 0 |
| NM_031480 | RIOK1 | 2477 | 6 | 4 | 3 | 2 | 4 | 5 | 3 | 2 | 3 | 4 | 3 | 6 | 6 |
| NM_031481 | SLC25A18 | 2087 | 5 | 1 | 0 | 0 | 0 | 5 | 0 | 4 | 0 | 0 | 0 | 0 | 0 |
| NM_031482 | ATG10 | 2286 | 2 | 2 | 1 | 1 | 2 | 2 | 2 | 1 | 1 | 2 | 1 | 2 | 2 |
| NM_031483 | ITCH | 6379 | 6 | 4 | 3 | 2 | 3 | 6 | 4 | 5 | 3 | 4 | 2 | 4 | 5 |
| NM_031485 | GRWD1 | 2455 | 8 | 4 | 4 | 5 | 2 | 5 | 3 | 8 | 2 | 2 | 3 | 4 | 4 |
| NM_031486 | ZNF484 | 2867 | 12 | 6 | 4 | 1 | 5 | 7 | 6 | 2 | 5 | 12 | 4 | 11 | 7 |
| NM_031488 | L3MBTL2 | 3192 | 1 | 1 | 1 | 1 | 1 | 1 | 1 | 1 | 0 | 1 | 0 | 1 | 1 |
| NM_031490 | LONP2 | 4342 | 53 | 15 | 9 | 7 | 14 | 16 | 13 | 53 | 8 | 16 | 11 | 13 | 9 |
| NM_031491 | RBP5 | 940 | 80 | 14 | 2 | 3 | 1 | 0 | 80 | 35 | 2 | 1 | 0 | 28 | 3 |
| NM_031492 | RBM4B | 1722 | 3 | 2 | 1 | 2 | 1 | 2 | 2 | 1 | 2 | 3 | 1 | 3 | 2 |
| NM_031495 | PCDHA2 | 2619 | 0 | 0 | 0 | 0 | 0 | 0 | 0 | 0 | 0 | 0 | 0 | 0 | 0 |
| NM_031497 | PCDHA3 | 2475 | 0 | 0 | 0 | 0 | 0 | 0 | 0 | 0 | 0 | 0 | 0 | 0 | 0 |
| NM_031498 | GNGT2 | 1040 | 1 | 0 | 0 | 0 | 0 | 0 | 0 | 0 | 0 | 0 | 0 | 1 | 0 |
| NM_031500 | PCDHA4 | 2498 | 0 | 0 | 0 | 0 | 0 | 0 | 0 | 0 | 0 | 0 | 0 | 0 | 0 |
| NM_031501 | PCDHA5 | 2451 | 0 | 0 | 0 | 0 | 0 | 0 | 0 | 0 | 0 | 0 | 0 | 0 | 0 |
| NM_031844 | HNRNPU | 6846 | 67 | 41 | 23 | 15 | 43 | 32 | 43 | 24 | 45 | 65 | 45 | 67 | 48 |
| NM_031845 | MAP2 | 5377 | 159 | 17 | 1 | 2 | 3 | 159 | 1 | 1 | 8 | 3 | 1 | 1 | 4 |
| NM_031847 | MAP2 | 5470 | 156 | 16 | 1 | 2 | 3 | 156 | 1 | 1 | 8 | 3 | 1 | 1 | 4 |
| NM_031848 | PCDHA6 | 2439 | 0 | 0 | 0 | 0 | 0 | 0 | 0 | 0 | 0 | 0 | 0 | 0 | 0 |
| NM_031849 | PCDHA6 | 4495 | 0 | 0 | 0 | 0 | 0 | 0 | 0 | 0 | 0 | 0 | 0 | 0 | 0 |
| NM_031850 | AGTR1 | 2414 | 5 | 1 | 2 | 0 | 1 | 0 | 1 | 5 | 1 | 0 | 1 | 1 | 0 |
| NM_031852 | PCDHA7 | 2370 | 0 | 0 | 0 | 0 | 0 | 0 | 0 | 0 | 0 | 0 | 0 | 0 | 0 |
| NM_031854 | KRTAP4-12 | 1075 | 0 | 0 | 0 | 0 | 0 | 0 | 0 | 0 | 0 | 0 | 0 | 0 | 0 |
| NM_031856 | PCDHA8 | 2445 | 0 | 0 | 0 | 0 | 0 | 0 | 0 | 0 | 0 | 0 | 0 | 0 | 0 |
| NM_031857 | PCDHA9 | 5984 | 0 | 0 | 0 | 0 | 0 | 0 | 0 | 0 | 0 | 0 | 0 | 0 | 0 |
| NM_031858 | NBR1 | 4974 | 16 | 12 | 9 | 8 | 13 | 12 | 13 | 10 | 8 | 12 | 16 | 14 | 13 |
| NM_031859 | PCDHA10 | 2535 | 0 | 0 | 0 | 0 | 0 | 0 | 0 | 0 | 0 | 0 | 0 | 0 | 0 |
| NM_031860 | PCDHA10 | 4465 | 0 | 0 | 0 | 0 | 0 | 0 | 0 | 0 | 0 | 0 | 0 | 0 | 0 |
| NM_031861 | PCDHA11 | 3291 | 1 | 0 | 0 | 0 | 0 | 1 | 1 | 0 | 0 | 0 | 0 | 0 | 0 |
| NM_031862 | NBR1 | 4655 | 17 | 12 | 10 | 8 | 14 | 13 | 14 | 10 | 9 | 12 | 17 | 15 | 13 |
| NM_031864 | PCDHA12 | 2506 | 0 | 0 | 0 | 0 | 0 | 0 | 0 | 0 | 0 | 0 | 0 | 0 | 0 |
| NM_031865 | PCDHA13 | 2427 | 1 | 0 | 0 | 0 | 0 | 1 | 0 | 0 | 0 | 0 | 0 | 0 | 1 |
| NM_031866 | FZD8 | 3186 | 1 | 0 | 0 | 0 | 1 | 1 | 1 | 0 | 0 | 0 | 0 | 1 | 0 |
| NM_031882 | PCDHAC1 | 3084 | 0 | 0 | 0 | 0 | 0 | 0 | 0 | 0 | 0 | 0 | 0 | 0 | 0 |
| NM_031883 | PCDHAC2 | 2655 | 2 | 0 | 0 | 0 | 0 | 2 | 0 | 0 | 0 | 0 | 0 | 0 | 0 |
| NM_031885 | BBS2 | 2814 | 13 | 5 | 3 | 2 | 4 | 13 | 7 | 1 | 3 | 6 | 5 | 6 | 7 |
| NM_031886 | KCNA7 | 4372 | 12 | 1 | 0 | 0 | 0 | 0 | 0 | 0 | 0 | 0 | 12 | 0 | 0 |
| NM_031889 | ENAM | 5674 | 1 | 0 | 0 | 0 | 1 | 0 | 1 | 0 | 0 | 0 | 1 | 0 | 0 |
| NM_031890 | CECR6 | 5025 | 1 | 0 | 0 | 0 | 0 | 1 | 0 | 0 | 0 | 0 | 0 | 0 | 0 |
| NM_031891 | CDH20 | 2855 | 6 | 1 | 0 | 0 | 0 | 6 | 0 | 0 | 0 | 0 | 0 | 0 | 0 |
| NM_031892 | SH3KBP1 | 4766 | 8 | 3 | 8 | 3 | 3 | 5 | 2 | 2 | 4 | 2 | 1 | 7 | 2 |
| NM_031894 | FTHL17 | 813 | 0 | 0 | 0 | 0 | 0 | 0 | 0 | 0 | 0 | 0 | 0 | 0 | 0 |
| NM_031895 | CACNG8 | 1417 | 0 | 0 | 0 | 0 | 0 | 0 | 0 | 0 | 0 | 0 | 0 | 0 | 0 |
| NM_031896 | CACNG7 | 1564 | 1 | 0 | 0 | 0 | 0 | 1 | 0 | 0 | 0 | 0 | 0 | 0 | 0 |
| NM_031897 | CACNG6 | 1637 | 2 | 0 | 0 | 0 | 0 | 0 | 0 | 0 | 0 | 0 | 2 | 0 | 0 |
| NM_031898 | TEKT3 | 1782 | 8 | 1 | 0 | 0 | 0 | 0 | 0 | 0 | 0 | 0 | 0 | 0 | 8 |
| NM_031899 | GORASP1 | 3057 | 2 | 1 | 1 | 1 | 1 | 2 | 1 | 1 | 0 | 0 | 0 | 1 | 1 |
| NM_031900 | AGXT2 | 2242 | 41 | 5 | 0 | 0 | 0 | 0 | 41 | 12 | 0 | 0 | 0 | 0 | 0 |
| NM_031901 | MRPS21 | 503 | 22 | 10 | 10 | 5 | 13 | 22 | 12 | 10 | 6 | 9 | 7 | 11 | 9 |
| NM_031902 | MRPS5 | 1661 | 31 | 19 | 12 | 17 | 25 | 16 | 26 | 31 | 14 | 11 | 16 | 20 | 17 |
| NM_031903 | MRPL32 | 892 | 31 | 19 | 13 | 10 | 28 | 23 | 28 | 31 | 14 | 15 | 12 | 22 | 18 |
| NM_031904 | FRMD8 | 3751 | 0 | 0 | 0 | 0 | 0 | 0 | 0 | 0 | 0 | 0 | 0 | 0 | 0 |
| NM_031905 | ARMC10 | 2629 | 8 | 5 | 3 | 2 | 5 | 8 | 5 | 5 | 4 | 8 | 2 | 6 | 6 |
| NM_031907 | USP26 | 2794 | 2 | 0 | 0 | 0 | 0 | 0 | 0 | 0 | 0 | 0 | 0 | 0 | 2 |
| NM_031908 | C1QTNF2 | 2397 | 3 | 0 | 0 | 0 | 0 | 0 | 0 | 1 | 3 | 0 | 0 | 0 | 0 |
| NM_031909 | C1QTNF4 | 1414 | 0 | 0 | 0 | 0 | 0 | 0 | 0 | 0 | 0 | 0 | 0 | 0 | 0 |
| NM_031910 | C1QTNF6 | 2936 | 0 | 0 | 0 | 0 | 0 | 0 | 0 | 0 | 0 | 0 | 0 | 0 | 0 |
| NM_031911 | C1QTNF7 | 4347 | 6 | 2 | 3 | 1 | 1 | 0 | 2 | 0 | 2 | 6 | 1 | 3 | 2 |
| NM_031912 | SYT15 | 4862 | 1 | 0 | 1 | 0 | 0 | 0 | 1 | 0 | 1 | 0 | 0 | 0 | 1 |
| NM_031913 | ESYT3 | 4447 | 1 | 0 | 0 | 0 | 0 | 0 | 0 | 0 | 1 | 0 | 0 | 0 | 0 |
| NM_031914 | SYT16 | 3137 | 9 | 1 | 0 | 0 | 0 | 9 | 0 | 0 | 0 | 0 | 0 | 0 | 1 |
| NM_031915 | SETDB2 | 6219 | 5 | 3 | 2 | 1 | 3 | 2 | 3 | 1 | 2 | 4 | 1 | 4 | 5 |
| NM_031916 | ROPN1L | 1043 | 31 | 3 | 0 | 0 | 0 | 0 | 0 | 0 | 2 | 0 | 0 | 2 | 31 |
| NM_031917 | ANGPTL6 | 1857 | 1 | 0 | 0 | 0 | 0 | 0 | 0 | 1 | 0 | 0 | 0 | 0 | 0 |
| NM_031918 | KLF16 | 2886 | 1 | 0 | 0 | 0 | 0 | 1 | 0 | 0 | 0 | 0 | 0 | 0 | 0 |
| NM_031919 | FSD1L | 660 | 3 | 1 | 0 | 0 | 0 | 3 | 1 | 0 | 0 | 0 | 2 | 0 | 1 |
| NM_031921 | ATAD3B | 2433 | 0 | 0 | 0 | 0 | 0 | 0 | 0 | 0 | 0 | 0 | 0 | 0 | 0 |
| NM_031922 | REPS1 | 3548 | 5 | 3 | 1 | 1 | 2 | 3 | 2 | 5 | 1 | 3 | 1 | 4 | 5 |
| NM_031923 | TAF3 | 2790 | 9 | 4 | 2 | 1 | 5 | 4 | 3 | 2 | 3 | 4 | 5 | 4 | 9 |
| NM_031924 | RSPH3 | 2176 | 9 | 5 | 4 | 3 | 4 | 7 | 7 | 1 | 5 | 5 | 2 | 4 | 9 |
| NM_031925 | TMEM120A | 1470 | 2 | 2 | 1 | 1 | 2 | 1 | 2 | 2 | 1 | 2 | 2 | 2 | 2 |
| NM_031934 | RAB34 | 1766 | 2 | 1 | 1 | 1 | 1 | 1 | 1 | 0 | 1 | 2 | 0 | 1 | 1 |
| NM_031935 | HMCN1 | 18212 | 3 | 1 | 2 | 1 | 2 | 0 | 0 | 0 | 3 | 1 | 0 | 2 | 0 |
| NM_031936 | GPR61 | 2823 | 2 | 0 | 0 | 0 | 0 | 2 | 0 | 0 | 0 | 0 | 0 | 0 | 0 |
| NM_031937 | TBC1D10A | 1936 | 1 | 1 | 1 | 1 | 0 | 1 | 1 | 1 | 1 | 1 | 0 | 1 | 1 |

| transcript | gene | transcript length (nt) | max | mean | adipose | colon | heart | hypothalamus | kidney | liver | lung | ovary | skeletalmuscle | spleen | testes |
|---|---|---|---|---|---|---|---|---|---|---|---|---|---|---|---|
| NM_031938 | BCO2 | 2909 | 6 | 1 | 0 | 0 | 6 | 1 | 0 | 3 | 0 | 1 | 1 | 0 | 1 |
| NM_031939 | MRO | 5258 | 20 | 3 | 1 | 1 | 0 | 7 | 3 | 0 | 0 | 2 | 0 | 1 | 20 |
| NM_031940 | TM2D2 | 3641 | 4 | 3 | 2 | 1 | 3 | 4 | 4 | 3 | 2 | 2 | 1 | 4 | 3 |
| NM_031941 | USHBP1 | 2410 | 0 | 0 | 0 | 0 | 0 | 0 | 0 | 0 | 0 | 0 | 0 | 0 | 0 |
| NM_031942 | CDCA7 | 2810 | 1 | 0 | 0 | 1 | 0 | 0 | 0 | 0 | 0 | 0 | 0 | 0 | 1 |
| NM_031944 | MIXL1 | 699 | 0 | 0 | 0 | 0 | 0 | 0 | 0 | 0 | 0 | 0 | 0 | 0 | 0 |
| NM_031945 | TSPAN10 | 1613 | 0 | 0 | 0 | 0 | 0 | 0 | 0 | 0 | 0 | 0 | 0 | 0 | 0 |
| NM_031946 | AGAP3 | 3232 | 6 | 2 | 4 | 3 | 1 | 6 | 1 | 1 | 3 | 2 | 1 | 2 | 1 |
| NM_031947 | SLC25A2 | 1417 | 0 | 0 | 0 | 0 | 0 | 0 | 0 | 0 | 0 | 0 | 0 | 0 | 0 |
| NM_031948 | PRSS27 | 1462 | 0 | 0 | 0 | 0 | 0 | 0 | 0 | 0 | 0 | 0 | 0 | 0 | 0 |
| NM_031949 | TTLL2 | 2877 | 2 | 0 | 0 | 0 | 0 | 0 | 0 | 0 | 0 | 0 | 0 | 0 | 2 |
| NM_031950 | FGFBP2 | 1170 | 1 | 0 | 1 | 0 | 1 | 0 | 0 | 0 | 1 | 0 | 0 | 1 | 1 |
| NM_031951 | WDR87 | 8947 | 8 | 1 | 0 | 0 | 0 | 0 | 0 | 0 | 0 | 0 | 0 | 0 | 8 |
| NM_031952 | SPATA9 | 1228 | 3 | 1 | 3 | 0 | 0 | 0 | 0 | 0 | 0 | 1 | 0 | 0 | 3 |
| NM_031953 | SNX25 | 3213 | 15 | 6 | 3 | 4 | 5 | 7 | 6 | 3 | 15 | 4 | 3 | 10 | 9 |
| NM_031954 | KCTD10 | 3970 | 5 | 3 | 5 | 3 | 4 | 5 | 4 | 1 | 3 | 2 | 1 | 4 | 3 |
| NM_031955 | SPATA16 | 2081 | 11 | 1 | 0 | 0 | 0 | 0 | 0 | 0 | 0 | 0 | 0 | 0 | 11 |
| NM_031956 | TTC29 | 1786 | 11 | 1 | 0 | 0 | 0 | 1 | 0 | 0 | 1 | 0 | 0 | 0 | 11 |
| NM_031957 | KRTAP1-5 | 1176 | 0 | 0 | 0 | 0 | 0 | 0 | 0 | 0 | 0 | 0 | 0 | 0 | 0 |
| NM_031958 | KRTAP3-1 | 593 | 0 | 0 | 0 | 0 | 0 | 0 | 0 | 0 | 0 | 0 | 0 | 0 | 0 |
| NM_031959 | KRTAP3-2 | 694 | 0 | 0 | 0 | 0 | 0 | 0 | 0 | 0 | 0 | 0 | 0 | 0 | 0 |
| NM_031960 | KRTAP4-8 | 1140 | 0 | 0 | 0 | 0 | 0 | 0 | 0 | 0 | 0 | 0 | 0 | 0 | 0 |
| NM_031961 | KRTAP9-2 | 1005 | 0 | 0 | 0 | 0 | 0 | 0 | 0 | 0 | 0 | 0 | 0 | 0 | 0 |
| NM_031962 | KRTAP9-3 | 992 | 0 | 0 | 0 | 0 | 0 | 0 | 0 | 0 | 0 | 0 | 0 | 0 | 0 |
| NM_031963 | KRTAP9-8 | 987 | 0 | 0 | 0 | 0 | 0 | 0 | 0 | 0 | 0 | 0 | 0 | 0 | 0 |
| NM_031964 | KRTAP17-1 | 779 | 0 | 0 | 0 | 0 | 0 | 0 | 0 | 0 | 0 | 0 | 0 | 0 | 0 |
| NM_031965 | GSG2 | 2796 | 2 | 0 | 0 | 1 | 0 | 0 | 0 | 0 | 0 | 0 | 0 | 1 | 2 |
| NM_031966 | CCNB1 | 2091 | 4 | 1 | 1 | 2 | 0 | 1 | 1 | 0 | 1 | 1 | 0 | 2 | 4 |
| NM_031968 | NARF | 1759 | 4 | 2 | 2 | 2 | 3 | 3 | 2 | 2 | 2 | 2 | 2 | 4 | 3 |
| NM_031989 | PCBP2 | 3159 | 43 | 24 | 19 | 9 | 26 | 17 | 34 | 18 | 20 | 43 | 20 | 31 | 30 |
| NM_031990 | PTBP1 | 3260 | 21 | 11 | 7 | 12 | 8 | 5 | 11 | 16 | 13 | 12 | 4 | 21 | 12 |
| NM_031991 | PTBP1 | 3203 | 22 | 11 | 8 | 12 | 9 | 5 | 12 | 16 | 13 | 12 | 4 | 22 | 12 |
| NM_031992 | EIF4H | 2486 | 67 | 41 | 37 | 30 | 39 | 67 | 33 | 52 | 33 | 37 | 64 | 37 | 28 |
| NM_031993 | PCDHGA1 | 2472 | 1 | 0 | 0 | 0 | 1 | 0 | 0 | 0 | 0 | 0 | 0 | 0 | 0 |
| NM_032009 | PCDHGA2 | 2657 | 2 | 1 | 0 | 0 | 1 | 2 | 0 | 0 | 0 | 1 | 0 | 0 | 2 |
| NM_032011 | PCDHGA3 | 2490 | 1 | 0 | 0 | 0 | 1 | 1 | 0 | 0 | 0 | 1 | 0 | 0 | 1 |
| NM_032012 | C9orf5 | 7997 | 8 | 6 | 5 | 2 | 7 | 7 | 8 | 7 | 7 | 7 | 7 | 5 | 5 |
| NM_032013 | NDRG3 | 2964 | 55 | 12 | 6 | 6 | 10 | 55 | 8 | 3 | 7 | 11 | 4 | 12 | 12 |
| NM_032014 | MRPS24 | 696 | 3 | 1 | 1 | 0 | 3 | 1 | 2 | 2 | 0 | 1 | 1 | 1 | 0 |
| NM_032015 | RNF26 | 2786 | 1 | 0 | 0 | 1 | 0 | 0 | 1 | 0 | 0 | 0 | 0 | 0 | 1 |
| NM_032016 | STARD3NL | 1617 | 11 | 6 | 3 | 2 | 6 | 9 | 8 | 3 | 10 | 5 | 1 | 11 | 10 |
| NM_032017 | STK40 | 3846 | 5 | 3 | 4 | 2 | 4 | 2 | 2 | 3 | 3 | 2 | 4 | 4 | 5 |
| NM_032018 | C1orf124 | 2800 | 2 | 1 | 1 | 1 | 1 | 1 | 1 | 1 | 1 | 1 | 2 | 1 | 2 |
| NM_032019 | HDAC10 | 2704 | 0 | 0 | 0 | 0 | 0 | 0 | 0 | 0 | 0 | 0 | 0 | 0 | 0 |
| NM_032020 | FUCA2 | 2096 | 6 | 3 | 5 | 3 | 2 | 2 | 4 | 6 | 2 | 2 | 0 | 3 | 2 |
| NM_032021 | TMEM133 | 1856 | 1 | 1 | 1 | 0 | 1 | 0 | 1 | 1 | 1 | 1 | 0 | 1 | 0 |
| NM_032023 | RASSF4 | 2495 | 4 | 1 | 2 | 1 | 2 | 3 | 4 | 1 | 1 | 0 | 0 | 2 | 1 |
| NM_032024 | C10orf11 | 892 | 2 | 1 | 2 | 1 | 1 | 0 | 1 | 0 | 1 | 0 | 0 | 0 | 1 |
| NM_032025 | EIF2A | 3879 | 7 | 5 | 4 | 1 | 7 | 6 | 5 | 7 | 4 | 7 | 6 | 7 | 5 |
| NM_032026 | TATDN1 | 1036 | 9 | 6 | 6 | 2 | 6 | 9 | 6 | 7 | 4 | 8 | 7 | 6 | 7 |
| NM_032027 | TM2D1 | 1250 | 6 | 3 | 2 | 2 | 2 | 6 | 2 | 2 | 3 | 3 | 1 | 4 | 2 |
| NM_032028 | TSSK1B | 2478 | 1 | 0 | 0 | 0 | 0 | 0 | 0 | 0 | 0 | 0 | 0 | 0 | 1 |
| NM_032029 | FCAMR | 1980 | 13 | 1 | 0 | 0 | 0 | 0 | 13 | 0 | 0 | 0 | 0 | 0 | 0 |
| NM_032030 | FKSG83 | 1164 | 0 | 0 | 0 | 0 | 0 | 0 | 0 | 0 | 0 | 0 | 0 | 0 | 0 |
| NM_032034 | SLC4A11 | 3094 | 1 | 0 | 0 | 0 | 0 | 0 | 1 | 0 | 0 | 0 | 0 | 0 | 0 |
| NM_032036 | IFI27L2 | 467 | 3 | 1 | 1 | 1 | 1 | 3 | 1 | 0 | 2 | 1 | 0 | 1 | 3 |
| NM_032037 | TSSK6 | 1442 | 0 | 0 | 0 | 0 | 0 | 0 | 0 | 0 | 0 | 0 | 0 | 0 | 0 |
| NM_032038 | SPNS1 | 2212 | 0 | 0 | 0 | 0 | 0 | 0 | 0 | 0 | 0 | 0 | 0 | 0 | 0 |
| NM_032039 | ITFG3 | 2951 | 1 | 0 | 0 | 0 | 0 | 0 | 0 | 0 | 0 | 0 | 1 | 0 | 0 |
| NM_032040 | CCDC8 | 3333 | 10 | 3 | 2 | 1 | 5 | 2 | 3 | 0 | 1 | 10 | 0 | 1 | 4 |
| NM_032041 | NCALD | 3718 | 61 | 10 | 4 | 4 | 4 | 61 | 6 | 3 | 4 | 17 | 3 | 2 | 4 |
| NM_032042 | FAM172A | 4719 | 4 | 2 | 3 | 1 | 2 | 3 | 2 | 1 | 2 | 4 | 1 | 3 | 2 |
| NM_032043 | BRIP1 | 4563 | 2 | 0 | 0 | 2 | 0 | 0 | 0 | 0 | 0 | 1 | 0 | 0 | 1 |
| NM_032044 | REG4 | 1342 | 1 | 0 | 0 | 1 | 0 | 0 | 0 | 0 | 0 | 0 | 0 | 0 | 0 |
| NM_032045 | KREMEN1 | 2719 | 2 | 1 | 1 | 1 | 1 | 1 | 1 | 0 | 1 | 1 | 1 | 2 | 2 |
| NM_032047 | B3GNT5 | 4123 | 6 | 2 | 3 | 3 | 1 | 2 | 1 | 0 | 6 | 2 | 2 | 2 | 2 |
| NM_032048 | EMILIN2 | 4009 | 15 | 4 | 15 | 1 | 10 | 1 | 1 | 1 | 5 | 3 | 1 | 5 | 2 |
| NM_032049 | AGTR1 | 2230 | 5 | 1 | 2 | 0 | 1 | 0 | 1 | 5 | 1 | 0 | 1 | 1 | 0 |
| NM_032050 | PATZ1 | 3674 | 1 | 1 | 0 | 1 | 0 | 1 | 1 | 0 | 1 | 1 | 1 | 1 | 1 |
| NM_032051 | PATZ1 | 3001 | 1 | 0 | 0 | 0 | 0 | 0 | 1 | 0 | 0 | 1 | 0 | 1 | 0 |
| NM_032052 | PATZ1 | 3747 | 1 | 1 | 0 | 1 | 0 | 1 | 1 | 0 | 1 | 1 | 1 | 1 | 1 |
| NM_032053 | PCDHGA4 | 2529 | 1 | 1 | 1 | 0 | 1 | 1 | 0 | 0 | 0 | 1 | 1 | 0 | 1 |
| NM_032054 | PCDHGA5 | 2442 | 1 | 0 | 0 | 0 | 1 | 1 | 0 | 0 | 0 | 1 | 0 | 0 | 0 |
| NM_032086 | PCDHGA6 | 2457 | 2 | 1 | 0 | 0 | 1 | 1 | 0 | 0 | 0 | 2 | 1 | 0 | 1 |
| NM_032087 | PCDHGA7 | 2721 | 1 | 0 | 0 | 0 | 1 | 0 | 0 | 0 | 0 | 1 | 1 | 0 | 0 |
| NM_032088 | PCDHGA8 | 5503 | 0 | 0 | 0 | 0 | 0 | 0 | 0 | 0 | 0 | 0 | 0 | 0 | 0 |
| NM_032089 | PCDHGA9 | 2487 | 3 | 1 | 0 | 0 | 1 | 3 | 1 | 0 | 0 | 1 | 1 | 1 | 1 |

| transcript | gene | transcript length (nt) | max | mean | adipose | colon | heart | hypothala mus | kidney | liver | lung | ovary | skeletalm uscle | spleen | testes |
|---|---|---|---|---|---|---|---|---|---|---|---|---|---|---|---|
| NM_032090 | PCDHGA10 | 2553 | 3 | 1 | 1 | 1 | 2 | 2 | 1 | 1 | 1 | 3 | 2 | 1 | 1 |
| NM_032091 | PCDHGA11 | 3117 | 1 | 1 | 0 | 0 | 1 | 1 | 0 | 0 | 0 | 1 | 1 | 0 | 0 |
| NM_032092 | PCDHGA11 | 4317 | 1 | 0 | 0 | 0 | 0 | 1 | 0 | 0 | 0 | 1 | 1 | 0 | 0 |
| NM_032094 | PCDHGA12 | 2632 | 1 | 0 | 0 | 0 | 1 | 1 | 0 | 0 | 0 | 1 | 0 | 0 | 0 |
| NM_032095 | PCDHGB1 | 2433 | 1 | 0 | 0 | 0 | 0 | 1 | 0 | 0 | 0 | 0 | 0 | 0 | 0 |
| NM_032096 | PCDHGB2 | 2436 | 4 | 2 | 1 | 1 | 3 | 4 | 1 | 1 | 1 | 4 | 1 | 1 | 1 |
| NM_032097 | PCDHGB3 | 2445 | 1 | 0 | 0 | 0 | 1 | 1 | 0 | 0 | 0 | 1 | 1 | 0 | 0 |
| NM_032098 | PCDHGB4 | 2412 | 1 | 1 | 0 | 0 | 1 | 1 | 1 | 1 | 0 | 1 | 1 | 0 | 1 |
| NM_032099 | PCDHGB5 | 2457 | 2 | 1 | 0 | 0 | 1 | 2 | 1 | 0 | 1 | 2 | 1 | 0 | 1 |
| NM_032100 | PCDHGB6 | 2463 | 3 | 1 | 1 | 1 | 1 | 2 | 1 | 1 | 1 | 3 | 2 | 1 | 1 |
| NM_032101 | PCDHGB7 | 2715 | 2 | 1 | 1 | 1 | 1 | 1 | 1 | 0 | 1 | 2 | 1 | 1 | 1 |
| NM_032102 | SFRS2B | 4333 | 4 | 2 | 1 | 1 | 2 | 2 | 3 | 2 | 1 | 2 | 1 | 4 | 1 |
| NM_032103 | PPP1R12B | 9463 | 45 | 10 | 3 | 14 | 45 | 5 | 3 | 1 | 5 | 10 | 10 | 6 | 5 |
| NM_032104 | PPP1R12B | 9644 | 44 | 10 | 3 | 14 | 44 | 5 | 3 | 1 | 5 | 10 | 10 | 6 | 5 |
| NM_032105 | PPP1R12B | 11289 | 40 | 9 | 3 | 12 | 40 | 6 | 3 | 1 | 5 | 9 | 11 | 5 | 5 |
| NM_032107 | L3MBTL | 3336 | 0 | 0 | 0 | 0 | 0 | 0 | 0 | 0 | 0 | 0 | 0 | 0 | 0 |
| NM_032108 | SEMA6B | 3714 | 1 | 0 | 0 | 0 | 0 | 1 | 0 | 0 | 0 | 0 | 0 | 0 | 0 |
| NM_032109 | OTP | 2678 | 0 | 0 | 0 | 0 | 0 | 0 | 0 | 0 | 0 | 0 | 0 | 0 | 0 |
| NM_032110 | DMRTA2 | 3102 | 0 | 0 | 0 | 0 | 0 | 0 | 0 | 0 | 0 | 0 | 0 | 0 | 0 |
| NM_032111 | MRPL14 | 775 | 33 | 15 | 9 | 13 | 15 | 23 | 6 | 33 | 12 | 7 | 31 | 11 | 9 |
| NM_032112 | MRPL43 | 1016 | 6 | 3 | 1 | 2 | 3 | 6 | 4 | 5 | 2 | 3 | 3 | 2 | 4 |
| NM_032115 | KCNK16 | 1247 | 0 | 0 | 0 | 0 | 0 | 0 | 0 | 0 | 0 | 0 | 0 | 0 | 0 |
| NM_032116 | KATNAL1 | 7514 | 7 | 3 | 3 | 2 | 5 | 7 | 1 | 1 | 2 | 2 | 2 | 2 | 3 |
| NM_032117 | MND1 | 941 | 4 | 1 | 0 | 1 | 0 | 0 | 0 | 0 | 1 | 0 | 0 | 1 | 4 |
| NM_032118 | WDR54 | 1119 | 2 | 1 | 0 | 0 | 0 | 2 | 1 | 0 | 1 | 0 | 0 | 1 | 1 |
| NM_032119 | GPR98 | 19333 | 3 | 1 | 0 | 0 | 0 | 3 | 1 | 0 | 1 | 0 | 0 | 0 | 0 |
| NM_032120 | C7orf64 | 1717 | 15 | 9 | 6 | 4 | 8 | 10 | 8 | 4 | 7 | 14 | 7 | 15 | 13 |
| NM_032121 | MAGT1 | 4019 | 19 | 6 | 3 | 2 | 6 | 4 | 7 | 19 | 5 | 4 | 2 | 7 | 4 |
| NM_032122 | DTNBP1 | 1394 | 12 | 6 | 4 | 3 | 9 | 12 | 7 | 2 | 4 | 5 | 6 | 10 | 6 |
| NM_032123 | KIRREL2 | 2724 | 0 | 0 | 0 | 0 | 0 | 0 | 0 | 0 | 0 | 0 | 0 | 0 | 0 |
| NM_032124 | HDHD2 | 2185 | 10 | 4 | 3 | 2 | 4 | 10 | 7 | 5 | 3 | 3 | 2 | 5 | 4 |
| NM_032125 | TMEM222 | 1614 | 2 | 1 | 1 | 1 | 1 | 2 | 2 | 1 | 1 | 1 | 1 | 1 | 1 |
| NM_032126 | C1orf49 | 1023 | 6 | 1 | 0 | 0 | 0 | 0 | 0 | 0 | 0 | 0 | 0 | 0 | 6 |
| NM_032127 | FAM160A2 | 3492 | 2 | 1 | 0 | 2 | 1 | 1 | 1 | 1 | 1 | 1 | 0 | 1 | 2 |
| NM_032128 | SLC10A7 | 1803 | 2 | 1 | 1 | 1 | 1 | 1 | 1 | 2 | 1 | 1 | 0 | 2 | 1 |
| NM_032129 | PLEKHN1 | 2398 | 0 | 0 | 0 | 0 | 0 | 0 | 0 | 0 | 0 | 0 | 0 | 0 | 0 |
| NM_032130 | FAM186B | 2954 | 2 | 0 | 0 | 0 | 0 | 0 | 0 | 0 | 0 | 0 | 0 | 0 | 2 |
| NM_032131 | ARMC2 | 3413 | 5 | 1 | 0 | 0 | 2 | 2 | 1 | 0 | 1 | 1 | 1 | 1 | 5 |
| NM_032132 | HORMAD1 | 1879 | 13 | 1 | 0 | 0 | 0 | 0 | 0 | 0 | 0 | 0 | 0 | 1 | 13 |
| NM_032133 | MYCBPAP | 3189 | 8 | 1 | 0 | 0 | 0 | 0 | 0 | 0 | 0 | 0 | 0 | 0 | 8 |
| NM_032134 | QRICH2 | 5359 | 1 | 0 | 0 | 0 | 0 | 0 | 0 | 0 | 0 | 0 | 0 | 0 | 1 |
| NM_032135 | FSCB | 3148 | 3 | 0 | 0 | 0 | 0 | 0 | 0 | 0 | 0 | 0 | 0 | 0 | 3 |
| NM_032136 | TKTL2 | 2801 | 8 | 1 | 0 | 0 | 0 | 0 | 0 | 0 | 0 | 0 | 0 | 0 | 8 |
| NM_032137 | C3orf20 | 3278 | 0 | 0 | 0 | 0 | 0 | 0 | 0 | 0 | 0 | 0 | 0 | 0 | 0 |
| NM_032138 | KBTBD7 | 2992 | 2 | 1 | 0 | 1 | 0 | 2 | 1 | 1 | 1 | 1 | 1 | 2 | 2 |
| NM_032139 | ANKRD27 | 4453 | 2 | 2 | 2 | 2 | 1 | 2 | 2 | 1 | 1 | 1 | 2 | 2 | 2 |
| NM_032140 | C16orf48 | 1538 | 2 | 1 | 1 | 2 | 1 | 2 | 2 | 1 | 1 | 2 | 1 | 1 | 2 |
| NM_032141 | CCDC55 | 2499 | 34 | 22 | 26 | 5 | 34 | 27 | 22 | 15 | 16 | 25 | 17 | 25 | 26 |
| NM_032142 | CEP192 | 7974 | 5 | 3 | 2 | 2 | 3 | 3 | 2 | 1 | 3 | 5 | 3 | 4 | 5 |
| NM_032143 | ZRANB3 | 3695 | 4 | 1 | 1 | 0 | 1 | 2 | 1 | 1 | 1 | 2 | 2 | 1 | 4 |
| NM_032144 | RAB6C | 3077 | 0 | 0 | 0 | 0 | 0 | 0 | 0 | 0 | 0 | 0 | 0 | 0 | 0 |
| NM_032145 | FBXO30 | 4407 | 6 | 4 | 3 | 2 | 4 | 4 | 3 | 1 | 2 | 4 | 6 | 4 | 6 |
| NM_032146 | ARL6 | 1394 | 2 | 1 | 0 | 0 | 0 | 2 | 1 | 0 | 0 | 1 | 0 | 0 | 1 |
| NM_032147 | USP44 | 3468 | 5 | 1 | 0 | 0 | 0 | 0 | 1 | 0 | 2 | 1 | 0 | 1 | 5 |
| NM_032148 | SLC41A2 | 3544 | 21 | 4 | 3 | 3 | 2 | 3 | 3 | 21 | 3 | 2 | 0 | 1 | 3 |
| NM_032149 | C4orf17 | 1634 | 2 | 0 | 0 | 0 | 0 | 0 | 0 | 0 | 0 | 0 | 0 | 0 | 2 |
| NM_032151 | PCBD2 | 2378 | 5 | 3 | 3 | 2 | 4 | 3 | 3 | 4 | 2 | 2 | 5 | 2 | 1 |
| NM_032152 | PRAM1 | 2163 | 1 | 0 | 0 | 0 | 0 | 0 | 0 | 0 | 1 | 0 | 0 | 1 | 0 |
| NM_032153 | ZIC4 | 4089 | 1 | 0 | 0 | 0 | 0 | 1 | 0 | 0 | 0 | 0 | 0 | 0 | 0 |
| NM_032154 | PCGF6 | 2005 | 2 | 1 | 1 | 1 | 1 | 1 | 1 | 2 | 1 | 1 | 1 | 1 | 1 |
| NM_032156 | CAPRIN2 | 4399 | 5 | 3 | 2 | 2 | 3 | 5 | 3 | 1 | 2 | 4 | 2 | 3 | 5 |
| NM_032158 | NSUN5C | 1751 | 0 | 0 | 0 | 0 | 0 | 0 | 0 | 0 | 0 | 0 | 0 | 0 | 0 |
| NM_032160 | DSEL | 9281 | 6 | 3 | 5 | 1 | 3 | 6 | 3 | 2 | 2 | 3 | 1 | 2 | 2 |
| NM_032164 | ZNF394 | 2145 | 12 | 6 | 5 | 4 | 6 | 4 | 5 | 5 | 7 | 10 | 4 | 12 | 8 |
| NM_032165 | LRRIQ1 | 4457 | 2 | 0 | 0 | 0 | 0 | 1 | 1 | 0 | 1 | 0 | 0 | 0 | 2 |
| NM_032166 | ATRIP | 2428 | 2 | 1 | 0 | 0 | 1 | 1 | 1 | 1 | 0 | 1 | 1 | 1 | 2 |
| NM_032167 | RUNDC2A | 2248 | 6 | 2 | 2 | 2 | 1 | 2 | 6 | 1 | 1 | 2 | 1 | 2 | 1 |
| NM_032168 | WDR75 | 2674 | 6 | 3 | 2 | 1 | 3 | 4 | 3 | 2 | 2 | 4 | 2 | 6 | 4 |
| NM_032169 | ACAD11 | 3556 | 8 | 3 | 2 | 1 | 2 | 1 | 8 | 8 | 1 | 1 | 1 | 1 | 1 |
| NM_032171 | CEP78 | 2702 | 2 | 1 | 1 | 1 | 0 | 1 | 1 | 1 | 1 | 1 | 0 | 1 | 2 |
| NM_032172 | USP42 | 5119 | 2 | 1 | 0 | 1 | 1 | 1 | 1 | 0 | 1 | 2 | 1 | 2 | 2 |
| NM_032173 | ZNRF3 | 6541 | 2 | 1 | 0 | 0 | 0 | 1 | 2 | 1 | 0 | 1 | 0 | 0 | 1 |
| NM_032174 | TOMM40L | 2678 | 1 | 1 | 1 | 1 | 1 | 1 | 1 | 1 | 0 | 0 | 0 | 0 | 0 |
| NM_032175 | UTP15 | 3655 | 5 | 3 | 2 | 1 | 4 | 3 | 3 | 4 | 2 | 2 | 4 | 3 | 5 |
| NM_032177 | PHAX | 1751 | 11 | 5 | 5 | 2 | 4 | 11 | 5 | 5 | 2 | 6 | 4 | 7 | 5 |
| NM_032178 | SLC7A6OS | 1627 | 4 | 2 | 3 | 3 | 3 | 4 | 1 | 2 | 2 | 2 | 2 | 3 | 3 |
| NM_032180 | FAM161A | 3692 | 2 | 1 | 0 | 0 | 0 | 1 | 1 | 0 | 1 | 1 | 0 | 0 | 2 |

| transcript | gene | transcript length (nt) | max | mean | adipose | colon | heart | hypothalamus | kidney | liver | lung | ovary | skeletalmuscle | spleen | testes |
|---|---|---|---|---|---|---|---|---|---|---|---|---|---|---|---|
| NM_032181 | FAM176A | 1815 | 10 | 2 | 0 | 0 | 0 | 0 | 5 | 10 | 1 | 0 | 0 | 2 | 1 |
| NM_032182 | FAM175B | 2992 | 5 | 4 | 3 | 2 | 3 | 5 | 4 | 4 | 3 | 3 | 4 | 5 | 4 |
| NM_032184 | SYDE2 | 4711 | 3 | 2 | 1 | 1 | 3 | 1 | 3 | 2 | 3 | 3 | 1 | 1 | 2 |
| NM_032186 | ZNF644 | 1980 | 12 | 7 | 5 | 2 | 7 | 8 | 5 | 5 | 6 | 12 | 9 | 12 | 12 |
| NM_032188 | MYST1 | 1528 | 32 | 20 | 8 | 11 | 26 | 16 | 17 | 10 | 14 | 32 | 26 | 26 | 30 |
| NM_032189 | ATP11A | 8768 | 6 | 3 | 1 | 1 | 5 | 2 | 4 | 2 | 6 | 1 | 1 | 3 | 4 |
| NM_032192 | PPP1R1B | 1841 | 63 | 12 | 25 | 33 | 0 | 63 | 1 | 0 | 3 | 1 | 0 | 0 | 7 |
| NM_032193 | RNASEH2C | 2785 | 1 | 1 | 0 | 1 | 1 | 1 | 1 | 0 | 1 | 1 | 0 | 1 | 1 |
| NM_032194 | RPF2 | 949 | 18 | 12 | 10 | 5 | 18 | 10 | 13 | 14 | 7 | 11 | 17 | 15 | 12 |
| NM_032195 | SON | 7497 | 28 | 19 | 16 | 10 | 16 | 25 | 16 | 15 | 16 | 23 | 17 | 28 | 26 |
| NM_032199 | ARID5B | 7507 | 44 | 16 | 15 | 4 | 20 | 9 | 12 | 2 | 13 | 17 | 44 | 24 | 18 |
| NM_032204 | ASCC2 | 2780 | 7 | 4 | 5 | 4 | 4 | 3 | 4 | 7 | 3 | 3 | 6 | 6 | 5 |
| NM_032205 | PHF20L1 | 2269 | 23 | 13 | 9 | 5 | 23 | 13 | 12 | 7 | 12 | 17 | 12 | 20 | 19 |
| NM_032206 | NLRC5 | 6822 | 1 | 0 | 0 | 1 | 0 | 0 | 0 | 0 | 1 | 0 | 0 | 1 | 0 |
| NM_032207 | C19orf44 | 3346 | 2 | 1 | 1 | 1 | 0 | 0 | 1 | 0 | 0 | 1 | 1 | 0 | 2 |
| NM_032208 | ANTXR1 | 5891 | 26 | 7 | 4 | 4 | 7 | 7 | 2 | 0 | 7 | 26 | 4 | 6 | 6 |
| NM_032211 | LOXL4 | 3657 | 1 | 0 | 1 | 0 | 0 | 0 | 0 | 0 | 0 | 0 | 1 | 0 | 1 |
| NM_032213 | ELMOD3 | 2770 | 4 | 1 | 4 | 1 | 1 | 1 | 1 | 0 | 1 | 0 | 0 | 1 | 1 |
| NM_032214 | SLA2 | 2495 | 1 | 0 | 0 | 0 | 0 | 0 | 0 | 0 | 0 | 0 | 0 | 1 | 0 |
| NM_032217 | ANKRD17 | 9375 | 13 | 10 | 7 | 5 | 11 | 13 | 11 | 8 | 8 | 11 | 11 | 13 | 10 |
| NM_032219 | MFSD7 | 1866 | 0 | 0 | 0 | 0 | 0 | 0 | 0 | 0 | 0 | 0 | 0 | 0 | 0 |
| NM_032221 | CHD6 | 10389 | 8 | 4 | 3 | 3 | 4 | 7 | 4 | 3 | 3 | 8 | 3 | 5 | 6 |
| NM_032222 | FAM188B | 2733 | 3 | 1 | 1 | 1 | 1 | 1 | 1 | 0 | 1 | 1 | 0 | 0 | 3 |
| NM_032223 | PCNXL3 | 6566 | 1 | 0 | 0 | 0 | 0 | 0 | 1 | 0 | 0 | 0 | 0 | 1 | 1 |
| NM_032226 | ZCCHC7 | 2685 | 14 | 7 | 5 | 4 | 4 | 9 | 4 | 3 | 6 | 12 | 5 | 14 | 8 |
| NM_032227 | TMEM164 | 5568 | 2 | 1 | 1 | 1 | 2 | 1 | 1 | 0 | 1 | 1 | 1 | 1 | 0 |
| NM_032228 | FAR1 | 5273 | 5 | 3 | 2 | 1 | 2 | 4 | 3 | 0 | 3 | 2 | 2 | 5 | 3 |
| NM_032229 | SLITRK6 | 4181 | 1 | 0 | 0 | 1 | 0 | 0 | 0 | 0 | 1 | 0 | 0 | 0 | 0 |
| NM_032230 | C12orf26 | 1975 | 1 | 1 | 0 | 1 | 1 | 1 | 1 | 0 | 0 | 1 | 1 | 1 | 1 |
| NM_032231 | FAM96A | 1092 | 30 | 8 | 5 | 4 | 8 | 5 | 9 | 30 | 7 | 2 | 3 | 11 | 4 |
| NM_032233 | SETD3 | 2888 | 36 | 22 | 17 | 10 | 25 | 25 | 35 | 19 | 17 | 14 | 25 | 18 | 36 |
| NM_032236 | USP48 | 4464 | 11 | 7 | 6 | 3 | 7 | 8 | 6 | 3 | 6 | 11 | 4 | 11 | 8 |
| NM_032237 | SGK196 | 1615 | 10 | 3 | 2 | 5 | 1 | 10 | 2 | 2 | 1 | 3 | 2 | 3 | 3 |
| NM_032239 | LARP1B | 2707 | 22 | 10 | 5 | 6 | 8 | 10 | 10 | 22 | 6 | 6 | 7 | 6 | 19 |
| NM_032242 | PLXNA1 | 9066 | 1 | 1 | 1 | 0 | 1 | 1 | 0 | 0 | 1 | 1 | 0 | 1 | 1 |
| NM_032243 | TXNDC2 | 1926 | 3 | 0 | 0 | 0 | 0 | 0 | 0 | 0 | 0 | 0 | 0 | 0 | 3 |
| NM_032246 | MEX3B | 3398 | 1 | 0 | 0 | 0 | 0 | 0 | 0 | 0 | 0 | 1 | 0 | 0 | 1 |
| NM_032250 | ANKRD20A1 | 3511 | 0 | 0 | 0 | 0 | 0 | 0 | 0 | 0 | 0 | 0 | 0 | 0 | 0 |
| NM_032251 | CCDC88B | 4919 | 1 | 0 | 0 | 1 | 0 | 0 | 0 | 0 | 0 | 0 | 0 | 1 | 0 |
| NM_032256 | TMEM117 | 2697 | 1 | 1 | 0 | 1 | 1 | 1 | 1 | 1 | 0 | 0 | 0 | 0 | 1 |
| NM_032257 | ZMYND12 | 1772 | 3 | 1 | 0 | 1 | 0 | 1 | 1 | 1 | 1 | 0 | 0 | 0 | 3 |
| NM_032258 | TBC1D3F | 2121 | 0 | 0 | 0 | 0 | 0 | 0 | 0 | 0 | 0 | 0 | 0 | 0 | 0 |
| NM_032259 | WDR24 | 3165 | 0 | 0 | 0 | 0 | 0 | 0 | 0 | 0 | 0 | 0 | 0 | 0 | 0 |
| NM_032260 | RGPD5 | 3304 | 0 | 0 | 0 | 0 | 0 | 0 | 0 | 0 | 0 | 0 | 0 | 0 | 0 |
| NM_032261 | C21orf56 | 1164 | 0 | 0 | 0 | 0 | 0 | 0 | 0 | 0 | 0 | 0 | 0 | 0 | 0 |
| NM_032263 | IQCG | 2257 | 9 | 3 | 3 | 1 | 2 | 4 | 3 | 1 | 4 | 3 | 1 | 2 | 9 |
| NM_032264 | NBPF3 | 3766 | 3 | 1 | 1 | 1 | 0 | 1 | 1 | 0 | 0 | 1 | 0 | 1 | 3 |
| NM_032265 | ZMYND15 | 2273 | 2 | 0 | 1 | 0 | 0 | 0 | 0 | 0 | 0 | 0 | 0 | 1 | 2 |
| NM_032266 | C2orf16 | 6201 | 1 | 0 | 0 | 0 | 0 | 0 | 0 | 0 | 0 | 0 | 0 | 0 | 1 |
| NM_032268 | ZNRF1 | 4633 | 0 | 0 | 0 | 0 | 0 | 0 | 0 | 0 | 0 | 0 | 0 | 0 | 0 |
| NM_032269 | CCDC135 | 2910 | 4 | 0 | 0 | 0 | 0 | 0 | 0 | 0 | 0 | 0 | 0 | 0 | 4 |
| NM_032270 | LRRC8C | 7206 | 3 | 1 | 1 | 0 | 1 | 1 | 1 | 0 | 1 | 2 | 1 | 3 | 1 |
| NM_032271 | TRAF7 | 3667 | 1 | 1 | 0 | 1 | 1 | 1 | 1 | 1 | 1 | 0 | 1 | 1 | 1 |
| NM_032272 | MAF1 | 1775 | 2 | 1 | 1 | 1 | 2 | 1 | 1 | 1 | 1 | 1 | 2 | 2 | 1 |
| NM_032273 | TMEM126A | 726 | 3 | 1 | 1 | 1 | 2 | 1 | 2 | 3 | 1 | 1 | 1 | 1 | 1 |
| NM_032276 | RHBDD1 | 4854 | 5 | 3 | 2 | 2 | 3 | 3 | 4 | 3 | 2 | 5 | 2 | 4 | 3 |
| NM_032279 | ATP13A4 | 4274 | 6 | 1 | 0 | 0 | 0 | 2 | 0 | 0 | 6 | 0 | 0 | 0 | 0 |
| NM_032280 | ZCCHC9 | 1788 | 4 | 2 | 2 | 1 | 2 | 1 | 2 | 4 | 1 | 2 | 1 | 2 | 2 |
| NM_032281 | ELAVL3 | 4661 | 15 | 1 | 0 | 0 | 0 | 15 | 0 | 0 | 0 | 0 | 0 | 0 | 0 |
| NM_032283 | ZDHHC18 | 3136 | 1 | 0 | 0 | 0 | 0 | 0 | 0 | 0 | 0 | 0 | 0 | 1 | 0 |
| NM_032285 | MRI1 | 3036 | 1 | 1 | 0 | 1 | 0 | 1 | 1 | 0 | 0 | 1 | 1 | 1 | 0 |
| NM_032286 | MED10 | 1028 | 3 | 2 | 1 | 1 | 2 | 3 | 1 | 2 | 2 | 1 | 1 | 2 | 2 |
| NM_032287 | LDOC1L | 5324 | 5 | 2 | 2 | 1 | 3 | 5 | 2 | 1 | 1 | 3 | 1 | 3 | 3 |
| NM_032288 | FYTTD1 | 3713 | 11 | 6 | 5 | 2 | 9 | 5 | 5 | 5 | 5 | 5 | 9 | 7 | 11 |
| NM_032289 | PSD2 | 4510 | 8 | 1 | 0 | 0 | 0 | 8 | 0 | 0 | 0 | 0 | 0 | 0 | 0 |
| NM_032290 | ANKRD32 | 3813 | 4 | 1 | 0 | 1 | 1 | 2 | 1 | 1 | 2 | 1 | 0 | 3 | 4 |
| NM_032291 | SGIP1 | 4694 | 7 | 1 | 0 | 0 | 0 | 7 | 1 | 0 | 1 | 0 | 0 | 0 | 1 |
| NM_032292 | GON4L | 4737 | 8 | 6 | 5 | 5 | 4 | 6 | 7 | 3 | 5 | 7 | 5 | 8 | 8 |
| NM_032293 | GARNL3 | 3736 | 4 | 1 | 0 | 0 | 1 | 4 | 1 | 0 | 0 | 1 | 0 | 0 | 1 |
| NM_032294 | CAMKK1 | 3554 | 2 | 0 | 0 | 0 | 0 | 2 | 0 | 0 | 0 | 0 | 0 | 0 | 0 |
| NM_032295 | SLC37A3 | 3009 | 3 | 2 | 2 | 3 | 2 | 3 | 3 | 1 | 2 | 2 | 1 | 2 | 2 |
| NM_032296 | FLYWCH1 | 4992 | 0 | 0 | 0 | 0 | 0 | 0 | 0 | 0 | 0 | 0 | 0 | 0 | 0 |
| NM_032298 | SYT3 | 2913 | 2 | 0 | 0 | 0 | 0 | 2 | 0 | 0 | 0 | 0 | 0 | 0 | 0 |
| NM_032299 | DCUN1D5 | 12732 | 5 | 1 | 1 | 0 | 1 | 5 | 1 | 0 | 1 | 1 | 1 | 1 | 1 |
| NM_032300 | TCHP | 3156 | 5 | 3 | 2 | 3 | 2 | 1 | 3 | 1 | 2 | 2 | 2 | 5 | 4 |
| NM_032301 | FBXW9 | 1737 | 0 | 0 | 0 | 0 | 0 | 0 | 0 | 0 | 0 | 0 | 0 | 0 | 0 |
| NM_032302 | PSMG3 | 1388 | 1 | 1 | 1 | 1 | 1 | 1 | 1 | 0 | 1 | 0 | 0 | 1 | 1 |

| transcript | gene | transcript length (nt) | max | mean | adipose | colon | heart | hypothalamus | kidney | liver | lung | ovary | skeletalmuscle | spleen | testes |
|---|---|---|---|---|---|---|---|---|---|---|---|---|---|---|---|
| NM_032303 | HSDL2 | 3210 | 36 | 20 | 28 | 6 | 32 | 16 | 21 | 36 | 10 | 25 | 27 | 11 | 11 |
| NM_032304 | HAGHL | 1352 | 0 | 0 | 0 | 0 | 0 | 0 | 0 | 0 | 0 | 0 | 0 | 0 | 0 |
| NM_032305 | POLR3GL | 1165 | 49 | 29 | 43 | 18 | 49 | 32 | 15 | 26 | 22 | 19 | 41 | 32 | 23 |
| NM_032306 | ALKBH7 | 1110 | 1 | 0 | 0 | 0 | 0 | 0 | 1 | 0 | 0 | 0 | 0 | 0 | 1 |
| NM_032307 | C9orf64 | 2473 | 8 | 5 | 4 | 3 | 5 | 3 | 6 | 8 | 3 | 5 | 3 | 4 | 7 |
| NM_032309 | CHCHD5 | 673 | 2 | 1 | 1 | 1 | 2 | 1 | 1 | 2 | 1 | 0 | 1 | 0 | 2 |
| NM_032310 | C9orf89 | 906 | 0 | 0 | 0 | 0 | 0 | 0 | 0 | 0 | 0 | 0 | 0 | 0 | 0 |
| NM_032311 | POLDIP3 | 3435 | 5 | 3 | 1 | 3 | 2 | 3 | 3 | 2 | 2 | 5 | 2 | 3 | 3 |
| NM_032312 | YIPF4 | 1964 | 6 | 4 | 4 | 2 | 6 | 6 | 4 | 6 | 4 | 4 | 3 | 4 | 4 |
| NM_032313 | C4orf14 | 2272 | 4 | 3 | 2 | 2 | 2 | 3 | 2 | 2 | 2 | 4 | 4 | 4 | 2 |
| NM_032314 | COQ5 | 1509 | 5 | 2 | 3 | 2 | 2 | 3 | 3 | 5 | 1 | 2 | 2 | 2 | 2 |
| NM_032315 | SLC25A33 | 1461 | 4 | 1 | 1 | 0 | 1 | 1 | 2 | 3 | 0 | 1 | 1 | 1 | 4 |
| NM_032316 | NICN1 | 3227 | 3 | 1 | 1 | 1 | 1 | 3 | 1 | 0 | 1 | 1 | 0 | 1 | 1 |
| NM_032317 | DNAJC30 | 2534 | 2 | 1 | 1 | 1 | 1 | 2 | 2 | 1 | 1 | 2 | 1 | 2 | 2 |
| NM_032319 | C2orf7 | 1083 | 1 | 0 | 0 | 0 | 0 | 0 | 1 | 0 | 0 | 0 | 0 | 1 | 0 |
| NM_032320 | BTBD10 | 2507 | 21 | 6 | 4 | 4 | 7 | 21 | 4 | 3 | 4 | 6 | 3 | 6 | 9 |
| NM_032321 | C2orf88 | 3962 | 4 | 2 | 1 | 2 | 3 | 4 | 2 | 2 | 1 | 1 | 2 | 1 | 3 |
| NM_032322 | RNF135 | 2153 | 2 | 1 | 2 | 1 | 1 | 0 | 2 | 0 | 1 | 1 | 0 | 1 | 1 |
| NM_032323 | TMEM79 | 2201 | 1 | 0 | 0 | 1 | 0 | 0 | 0 | 0 | 0 | 0 | 0 | 0 | 0 |
| NM_032324 | C1orf57 | 902 | 9 | 4 | 3 | 3 | 5 | 9 | 5 | 5 | 3 | 3 | 3 | 4 | 5 |
| NM_032325 | EIF1AD | 2836 | 3 | 2 | 1 | 2 | 1 | 2 | 1 | 1 | 1 | 2 | 2 | 3 | 2 |
| NM_032326 | TMEM175 | 1772 | 1 | 0 | 0 | 0 | 0 | 0 | 0 | 0 | 0 | 0 | 0 | 1 | 0 |
| NM_032327 | ZDHHC16 | 1799 | 3 | 2 | 2 | 1 | 3 | 2 | 3 | 3 | 2 | 2 | 1 | 3 | 2 |
| NM_032328 | EFCAB2 | 1240 | 13 | 5 | 4 | 1 | 6 | 13 | 5 | 3 | 5 | 4 | 3 | 2 | 7 |
| NM_032329 | ING5 | 5198 | 2 | 1 | 1 | 1 | 1 | 2 | 2 | 1 | 1 | 2 | 1 | 1 | 1 |
| NM_032330 | CAPNS2 | 1009 | 1 | 0 | 0 | 0 | 0 | 0 | 0 | 0 | 0 | 0 | 0 | 1 | 0 |
| NM_032331 | ECE2 | 990 | 2 | 0 | 0 | 0 | 0 | 0 | 0 | 2 | 0 | 0 | 0 | 0 | 0 |
| NM_032332 | MORG1 | 1196 | 0 | 0 | 0 | 0 | 0 | 0 | 0 | 0 | 0 | 0 | 0 | 0 | 0 |
| NM_032333 | C10orf58 | 1748 | 55 | 13 | 55 | 9 | 10 | 21 | 16 | 8 | 2 | 4 | 3 | 4 | 10 |
| NM_032334 | UTP23 | 3689 | 2 | 1 | 1 | 0 | 1 | 1 | 1 | 1 | 1 | 2 | 1 | 2 | 2 |
| NM_032335 | PHF6 | 1210 | 12 | 3 | 1 | 0 | 2 | 2 | 3 | 1 | 2 | 12 | 1 | 5 | 4 |
| NM_032336 | GINS4 | 3839 | 1 | 0 | 0 | 1 | 0 | 0 | 0 | 0 | 0 | 0 | 0 | 1 | 1 |
| NM_032338 | LLPH | 1216 | 46 | 29 | 28 | 10 | 46 | 36 | 25 | 42 | 14 | 31 | 17 | 38 | 37 |
| NM_032339 | C17orf37 | 754 | 5 | 3 | 2 | 1 | 4 | 4 | 5 | 3 | 2 | 1 | 2 | 5 | 3 |
| NM_032340 | C6orf125 | 506 | 10 | 4 | 1 | 2 | 10 | 6 | 4 | 3 | 1 | 1 | 3 | 3 | 3 |
| NM_032341 | DDI2 | 1759 | 21 | 7 | 8 | 3 | 4 | 5 | 6 | 12 | 6 | 4 | 21 | 8 | 5 |
| NM_032342 | C9orf125 | 2089 | 5 | 1 | 0 | 2 | 2 | 5 | 2 | 1 | 1 | 2 | 0 | 0 | 1 |
| NM_032343 | CHCHD6 | 946 | 4 | 1 | 1 | 0 | 0 | 4 | 0 | 0 | 1 | 1 | 0 | 0 | 1 |
| NM_032344 | NUDT22 | 1118 | 1 | 0 | 0 | 1 | 0 | 0 | 0 | 1 | 0 | 0 | 0 | 0 | 0 |
| NM_032345 | WIBG | 1226 | 1 | 1 | 1 | 1 | 1 | 1 | 1 | 1 | 1 | 1 | 0 | 1 | 1 |
| NM_032346 | PDCD2L | 1157 | 1 | 0 | 0 | 0 | 1 | 1 | 0 | 1 | 0 | 0 | 1 | 0 | 0 |
| NM_032347 | ZNF397 | 1482 | 3 | 2 | 1 | 2 | 2 | 2 | 2 | 0 | 1 | 2 | 2 | 3 | 1 |
| NM_032348 | MXRA8 | 2273 | 3 | 1 | 3 | 1 | 1 | 1 | 1 | 0 | 1 | 2 | 0 | 1 | 1 |
| NM_032349 | NUDT16L1 | 1313 | 1 | 0 | 0 | 1 | 0 | 0 | 1 | 0 | 0 | 0 | 0 | 0 | 0 |
| NM_032350 | C7orf50 | 1293 | 8 | 4 | 3 | 4 | 6 | 8 | 7 | 3 | 4 | 5 | 4 | 3 | 2 |
| NM_032351 | MRPL45 | 1696 | 12 | 6 | 5 | 4 | 6 | 6 | 7 | 12 | 4 | 6 | 7 | 6 | 3 |
| NM_032352 | BRMS1L | 2584 | 5 | 2 | 1 | 1 | 3 | 5 | 2 | 1 | 1 | 2 | 2 | 2 | 2 |
| NM_032353 | VPS25 | 1101 | 4 | 3 | 3 | 3 | 2 | 3 | 4 | 3 | 1 | 1 | 2 | 3 | 3 |
| NM_032354 | TMEM107 | 1776 | 3 | 2 | 2 | 1 | 1 | 3 | 2 | 1 | 2 | 1 | 1 | 2 | 2 |
| NM_032355 | MON1A | 2229 | 1 | 0 | 1 | 0 | 0 | 1 | 0 | 1 | 0 | 0 | 0 | 0 | 0 |
| NM_032356 | LSMD1 | 999 | 1 | 1 | 1 | 1 | 1 | 1 | 1 | 1 | 1 | 0 | 1 | 1 | 1 |
| NM_032357 | CCDC115 | 1644 | 4 | 2 | 2 | 2 | 2 | 4 | 3 | 1 | 2 | 4 | 1 | 3 | 2 |
| NM_032358 | CCDC77 | 2367 | 3 | 2 | 1 | 1 | 1 | 2 | 1 | 1 | 1 | 2 | 1 | 2 | 3 |
| NM_032359 | C3orf26 | 1230 | 20 | 8 | 6 | 2 | 10 | 20 | 5 | 8 | 3 | 6 | 14 | 6 | 11 |
| NM_032360 | ACBD6 | 1615 | 5 | 3 | 2 | 2 | 3 | 3 | 3 | 1 | 2 | 5 | 2 | 4 | 5 |
| NM_032361 | THOC3 | 1828 | 0 | 0 | 0 | 0 | 0 | 0 | 0 | 0 | 0 | 0 | 0 | 0 | 0 |
| NM_032364 | DNAJC14 | 3330 | 5 | 3 | 2 | 2 | 2 | 4 | 3 | 3 | 2 | 2 | 1 | 5 | 4 |
| NM_032366 | C16orf13 | 840 | 5 | 2 | 1 | 1 | 2 | 5 | 3 | 2 | 1 | 1 | 3 | 2 | 1 |
| NM_032367 | ZBED3 | 1419 | 0 | 0 | 0 | 0 | 0 | 0 | 0 | 0 | 0 | 0 | 0 | 0 | 0 |
| NM_032368 | LZIC | 1407 | 15 | 10 | 8 | 7 | 10 | 15 | 9 | 8 | 6 | 11 | 5 | 9 | 15 |
| NM_032369 | HVCN1 | 1711 | 3 | 1 | 1 | 0 | 1 | 1 | 0 | 0 | 1 | 1 | 0 | 3 | 2 |
| NM_032370 | ZNF414 | 1196 | 1 | 0 | 0 | 0 | 0 | 0 | 0 | 0 | 0 | 1 | 0 | 1 | 1 |
| NM_032372 | DYDC2 | 1842 | 1 | 0 | 0 | 0 | 0 | 1 | 0 | 0 | 1 | 0 | 0 | 0 | 1 |
| NM_032373 | PCGF5 | 7127 | 13 | 8 | 3 | 3 | 13 | 11 | 8 | 5 | 9 | 8 | 8 | 9 | 6 |
| NM_032374 | C14orf153 | 1235 | 7 | 4 | 4 | 2 | 6 | 5 | 4 | 3 | 2 | 3 | 7 | 4 | 6 |
| NM_032375 | AKT1S1 | 2374 | 4 | 2 | 2 | 3 | 3 | 2 | 3 | 4 | 1 | 2 | 4 | 2 | 3 |
| NM_032376 | TMEM101 | 1539 | 10 | 5 | 2 | 3 | 4 | 5 | 10 | 4 | 5 | 4 | 3 | 5 | 7 |
| NM_032377 | ELOF1 | 1019 | 3 | 2 | 2 | 2 | 1 | 1 | 2 | 2 | 1 | 1 | 1 | 2 | 3 |
| NM_032378 | EEF1D | 2467 | 22 | 14 | 10 | 15 | 17 | 5 | 10 | 16 | 14 | 19 | 15 | 22 | 9 |
| NM_032379 | SYTL2 | 2404 | 10 | 4 | 3 | 6 | 2 | 10 | 8 | 1 | 4 | 2 | 2 | 3 | 3 |
| NM_032380 | GFM2 | 3093 | 4 | 3 | 1 | 1 | 4 | 4 | 3 | 3 | 2 | 2 | 2 | 3 | 4 |
| NM_032382 | COG8 | 2495 | 1 | 0 | 0 | 0 | 0 | 0 | 1 | 0 | 0 | 0 | 0 | 0 | 1 |
| NM_032383 | HPS3 | 4451 | 6 | 3 | 3 | 2 | 2 | 3 | 4 | 1 | 3 | 3 | 1 | 6 | 4 |
| NM_032385 | C5orf4 | 2997 | 6 | 4 | 4 | 3 | 3 | 6 | 3 | 6 | 5 | 6 | 1 | 2 | 5 |
| NM_032387 | WNK4 | 4173 | 3 | 0 | 0 | 1 | 0 | 0 | 3 | 0 | 0 | 0 | 0 | 0 | 0 |
| NM_032389 | ARFGAP2 | 2762 | 4 | 2 | 2 | 2 | 1 | 4 | 2 | 4 | 1 | 2 | 2 | 2 | 2 |
| NM_032390 | MKI67IP | 1737 | 6 | 3 | 2 | 1 | 4 | 6 | 3 | 3 | 2 | 4 | 4 | 6 | 3 |

| transcript | gene | transcript length (nt) | max | mean | adipose | colon | heart | hypothala mus | kidney | liver | lung | ovary | skeletalm uscle | spleen | testes |
|---|---|---|---|---|---|---|---|---|---|---|---|---|---|---|---|
| NM_032391 | PRAC | 383 | 0 | 0 | 0 | 0 | 0 | 0 | 0 | 0 | 0 | 0 | 0 | 0 | 0 |
| NM_032402 | PCDHGC3 | 2794 | 9 | 2 | 2 | 1 | 2 | 9 | 1 | 0 | 4 | 2 | 1 | 1 | 1 |
| NM_032403 | PCDHGC3 | 2326 | 3 | 1 | 1 | 1 | 0 | 3 | 0 | 0 | 1 | 0 | 0 | 0 | 0 |
| NM_032405 | TMPRSS3 | 1342 | 0 | 0 | 0 | 0 | 0 | 0 | 0 | 0 | 0 | 0 | 0 | 0 | 0 |
| NM_032406 | PCDHGC4 | 2616 | 1 | 0 | 0 | 0 | 0 | 1 | 0 | 0 | 0 | 0 | 0 | 0 | 0 |
| NM_032407 | PCDHGC5 | 2637 | 7 | 1 | 0 | 0 | 0 | 7 | 0 | 0 | 0 | 0 | 0 | 0 | 0 |
| NM_032408 | BAZ1B | 6115 | 17 | 12 | 13 | 6 | 13 | 14 | 10 | 10 | 10 | 17 | 11 | 16 | 14 |
| NM_032409 | PINK1 | 2660 | 49 | 20 | 12 | 8 | 39 | 49 | 19 | 15 | 8 | 10 | 31 | 10 | 14 |
| NM_032410 | HOOK3 | 14439 | 6 | 4 | 4 | 1 | 4 | 6 | 3 | 3 | 5 | 4 | 5 | 4 | 4 |
| NM_032411 | C2orf40 | 782 | 31 | 9 | 14 | 5 | 4 | 31 | 12 | 1 | 10 | 3 | 4 | 5 | 14 |
| NM_032412 | C5orf32 | 910 | 6 | 2 | 2 | 1 | 2 | 1 | 6 | 1 | 2 | 0 | 3 | 1 | 1 |
| NM_032413 | C15orf48 | 917 | 1 | 0 | 0 | 1 | 0 | 0 | 0 | 0 | 0 | 0 | 0 | 0 | 0 |
| NM_032414 | PROK1 | 1372 | 9 | 1 | 1 | 0 | 0 | 0 | 0 | 0 | 0 | 6 | 0 | 0 | 9 |
| NM_032415 | CARD11 | 4372 | 4 | 0 | 0 | 1 | 0 | 0 | 0 | 0 | 0 | 0 | 0 | 4 | 0 |
| NM_032417 | SPANXD | 384 | 0 | 0 | 0 | 0 | 0 | 0 | 0 | 0 | 0 | 0 | 0 | 0 | 0 |
| NM_032420 | PCDH1 | 4765 | 4 | 1 | 1 | 3 | 1 | 4 | 2 | 2 | 1 | 0 | 0 | 0 | 0 |
| NM_032421 | CLIP2 | 5458 | 4 | 1 | 1 | 2 | 0 | 4 | 0 | 0 | 1 | 1 | 0 | 1 | 0 |
| NM_032423 | ZNF528 | 3978 | 4 | 2 | 1 | 1 | 2 | 2 | 3 | 0 | 2 | 4 | 1 | 3 | 3 |
| NM_032424 | KIAA1826 | 4087 | 9 | 4 | 3 | 1 | 5 | 9 | 4 | 1 | 2 | 4 | 3 | 4 | 4 |
| NM_032425 | HHIPL1 | 2238 | 0 | 0 | 0 | 0 | 0 | 0 | 0 | 0 | 0 | 0 | 0 | 0 | 0 |
| NM_032427 | MAML2 | 5428 | 5 | 2 | 3 | 2 | 2 | 2 | 3 | 0 | 3 | 3 | 1 | 5 | 2 |
| NM_032429 | LZTS2 | 2741 | 1 | 0 | 0 | 0 | 1 | 0 | 0 | 0 | 0 | 0 | 0 | 0 | 0 |
| NM_032430 | BRSK1 | 3081 | 6 | 1 | 0 | 0 | 0 | 6 | 0 | 0 | 0 | 0 | 0 | 0 | 0 |
| NM_032431 | SYVN1 | 3048 | 2 | 1 | 1 | 1 | 0 | 1 | 1 | 2 | 0 | 0 | 0 | 1 | 1 |
| NM_032432 | ABLIM2 | 3389 | 21 | 3 | 0 | 0 | 1 | 3 | 0 | 0 | 0 | 0 | 21 | 3 | 0 |
| NM_032433 | ZNF333 | 3767 | 4 | 2 | 2 | 1 | 1 | 2 | 1 | 0 | 1 | 4 | 2 | 2 | 3 |
| NM_032434 | ZNF512 | 3426 | 5 | 3 | 2 | 2 | 2 | 5 | 3 | 1 | 3 | 5 | 1 | 4 | 5 |
| NM_032435 | KIAA1804 | 5809 | 3 | 1 | 0 | 1 | 0 | 0 | 2 | 0 | 1 | 0 | 0 | 0 | 3 |
| NM_032436 | ZNF828 | 3817 | 4 | 3 | 2 | 2 | 2 | 4 | 3 | 2 | 1 | 4 | 2 | 4 | 3 |
| NM_032437 | EFCAB7 | 2360 | 6 | 2 | 1 | 1 | 2 | 3 | 2 | 0 | 1 | 2 | 6 | 1 | 3 |
| NM_032438 | L3MBTL3 | 4200 | 6 | 3 | 2 | 1 | 2 | 3 | 2 | 1 | 3 | 5 | 1 | 6 | 6 |
| NM_032439 | PHYHIPL | 3578 | 16 | 2 | 0 | 0 | 0 | 16 | 2 | 0 | 0 | 0 | 0 | 0 | 3 |
| NM_032440 | LCOR | 4834 | 22 | 12 | 5 | 5 | 8 | 18 | 9 | 6 | 12 | 16 | 22 | 16 | 14 |
| NM_032441 | ZMAT1 | 6122 | 4 | 2 | 1 | 0 | 1 | 2 | 4 | 0 | 2 | 2 | 1 | 3 | 3 |
| NM_032442 | NEURL4 | 5187 | 1 | 0 | 0 | 1 | 0 | 1 | 1 | 0 | 0 | 0 | 0 | 0 | 1 |
| NM_032444 | BTBD12 | 7292 | 1 | 0 | 0 | 0 | 0 | 1 | 0 | 0 | 0 | 0 | 0 | 1 | 1 |
| NM_032445 | MEGF11 | 5914 | 1 | 0 | 0 | 0 | 0 | 1 | 0 | 0 | 0 | 0 | 0 | 0 | 0 |
| NM_032446 | MEGF10 | 7685 | 3 | 0 | 0 | 0 | 0 | 3 | 0 | 0 | 0 | 0 | 1 | 0 | 0 |
| NM_032447 | FBN3 | 8967 | 0 | 0 | 0 | 0 | 0 | 0 | 0 | 0 | 0 | 0 | 0 | 0 | 0 |
| NM_032448 | FAM120B | 3239 | 12 | 7 | 6 | 6 | 6 | 10 | 7 | 5 | 5 | 9 | 5 | 9 | 12 |
| NM_032449 | CC2D1B | 5639 | 2 | 1 | 1 | 1 | 1 | 1 | 2 | 1 | 1 | 1 | 1 | 2 | 1 |
| NM_032450 | HEATR7A | 5363 | 1 | 0 | 0 | 1 | 0 | 0 | 0 | 0 | 0 | 0 | 0 | 0 | 0 |
| NM_032451 | SPIRE2 | 3244 | 2 | 0 | 0 | 2 | 0 | 0 | 0 | 0 | 0 | 0 | 0 | 0 | 1 |
| NM_032452 | JPH4 | 4404 | 6 | 1 | 0 | 0 | 0 | 6 | 0 | 0 | 0 | 1 | 0 | 0 | 0 |
| NM_032453 | ZNF527 | 5126 | 5 | 3 | 2 | 1 | 2 | 4 | 2 | 1 | 1 | 5 | 2 | 3 | 5 |
| NM_032454 | STK19 | 444 | 5 | 2 | 1 | 1 | 1 | 4 | 5 | 2 | 1 | 2 | 0 | 3 | 1 |
| NM_032454 | STK19 | 1632 | 1 | 1 | 0 | 0 | 0 | 1 | 1 | 1 | 0 | 1 | 0 | 1 | 0 |
| NM_032456 | PCDH7 | 4921 | 6 | 2 | 0 | 1 | 3 | 6 | 0 | 0 | 1 | 1 | 0 | 4 | 1 |
| NM_032457 | PCDH7 | 8561 | 8 | 2 | 0 | 1 | 4 | 8 | 0 | 0 | 1 | 1 | 0 | 4 | 1 |
| NM_032458 | PHF6 | 4762 | 13 | 4 | 2 | 1 | 2 | 4 | 3 | 1 | 2 | 13 | 1 | 6 | 5 |
| NM_032459 | EFS | 2839 | 1 | 0 | 0 | 0 | 0 | 1 | 0 | 0 | 0 | 1 | 0 | 0 | 0 |
| NM_032461 | SPANXB1 | 469 | 0 | 0 | 0 | 0 | 0 | 0 | 0 | 0 | 0 | 0 | 0 | 0 | 0 |
| NM_032463 | LAT2 | 2121 | 7 | 1 | 0 | 1 | 1 | 3 | 0 | 1 | 2 | 0 | 0 | 7 | 0 |
| NM_032464 | LAT2 | 2191 | 7 | 1 | 0 | 1 | 1 | 3 | 0 | 1 | 2 | 0 | 0 | 7 | 0 |
| NM_032466 | ASPH | 3774 | 75 | 15 | 75 | 5 | 15 | 14 | 10 | 8 | 13 | 9 | 5 | 10 | 6 |
| NM_032467 | ASPH | 3227 | 41 | 10 | 24 | 2 | 12 | 5 | 4 | 2 | 8 | 4 | 41 | 6 | 3 |
| NM_032468 | ASPH | 3624 | 78 | 16 | 78 | 5 | 15 | 14 | 11 | 9 | 14 | 10 | 5 | 11 | 6 |
| NM_032470 | TNXB | 3125 | 0 | 0 | 0 | 0 | 0 | 0 | 0 | 0 | 0 | 0 | 0 | 0 | 0 |
| NM_032471 | PKIB | 1812 | 4 | 1 | 0 | 4 | 0 | 2 | 0 | 0 | 0 | 0 | 0 | 0 | 0 |
| NM_032472 | PPIL3 | 1178 | 4 | 2 | 2 | 1 | 2 | 4 | 2 | 3 | 1 | 3 | 1 | 3 | 3 |
| NM_032476 | MRPS6 | 990 | 46 | 19 | 16 | 12 | 17 | 22 | 46 | 10 | 12 | 15 | 13 | 12 | 35 |
| NM_032477 | MRPL41 | 604 | 1 | 1 | 0 | 0 | 1 | 0 | 1 | 1 | 0 | 0 | 1 | 1 | 0 |
| NM_032478 | MRPL38 | 1575 | 3 | 1 | 1 | 1 | 2 | 2 | 2 | 3 | 1 | 1 | 2 | 1 | 1 |
| NM_032479 | MRPL36 | 614 | 18 | 9 | 6 | 5 | 10 | 8 | 9 | 18 | 5 | 5 | 11 | 6 | 12 |
| NM_032482 | DOT1L | 7436 | 3 | 1 | 1 | 1 | 1 | 0 | 1 | 1 | 1 | 1 | 1 | 1 | 3 |
| NM_032483 | PPAPDC1B | 865 | 3 | 1 | 1 | 1 | 0 | 1 | 1 | 3 | 0 | 1 | 0 | 1 | 1 |
| NM_032484 | GHDC | 2445 | 4 | 1 | 1 | 1 | 1 | 1 | 4 | 1 | 2 | 2 | 1 | 3 | 1 |
| NM_032485 | MCM8 | 3694 | 4 | 1 | 1 | 2 | 1 | 1 | 1 | 0 | 1 | 1 | 1 | 2 | 4 |
| NM_032486 | DCTN5 | 3323 | 8 | 4 | 3 | 3 | 3 | 8 | 5 | 2 | 2 | 3 | 2 | 5 | 6 |
| NM_032487 | ARPM1 | 2001 | 4 | 1 | 0 | 0 | 1 | 0 | 0 | 1 | 0 | 0 | 0 | 0 | 4 |
| NM_032488 | CNFN | 528 | 0 | 0 | 0 | 0 | 0 | 0 | 0 | 0 | 0 | 0 | 0 | 0 | 0 |
| NM_032489 | ACRBP | 1886 | 16 | 2 | 1 | 0 | 0 | 0 | 0 | 0 | 0 | 0 | 0 | 1 | 16 |
| NM_032490 | C14orf142 | 1176 | 2 | 1 | 0 | 1 | 1 | 1 | 1 | 0 | 1 | 0 | 1 | 2 | 0 |
| NM_032491 | RFX4 | 3366 | 6 | 1 | 0 | 0 | 0 | 5 | 0 | 0 | 0 | 0 | 0 | 0 | 6 |
| NM_032492 | JAGN1 | 1658 | 1 | 1 | 1 | 1 | 1 | 1 | 1 | 1 | 1 | 1 | 1 | 1 | 1 |
| NM_032493 | AP1M1 | 2355 | 2 | 1 | 1 | 1 | 1 | 2 | 2 | 2 | 1 | 1 | 1 | 2 | 1 |
| NM_032494 | ZC3H8 | 1601 | 9 | 5 | 3 | 2 | 7 | 5 | 5 | 2 | 2 | 6 | 8 | 9 | 6 |

| transcript | gene | transcript length (nt) | max | mean | adipose | colon | heart | hypothala mus | kidney | liver | lung | ovary | skeletalm uscle | spleen | testes |
|---|---|---|---|---|---|---|---|---|---|---|---|---|---|---|---|
| NM_032495 | HOPX | 1543 | 12 | 2 | 0 | 0 | 2 | 5 | 0 | 0 | 12 | 0 | 0 | 0 | 2 |
| NM_032496 | ARHGAP9 | 2597 | 7 | 1 | 1 | 1 | 1 | 1 | 0 | 0 | 1 | 0 | 0 | 7 | 2 |
| NM_032497 | ZNF559 | 3042 | 17 | 6 | 5 | 2 | 5 | 4 | 6 | 1 | 4 | 17 | 3 | 10 | 12 |
| NM_032498 | RHOXF2 | 1297 | 0 | 0 | 0 | 0 | 0 | 0 | 0 | 0 | 0 | 0 | 0 | 0 | 0 |
| NM_032499 | C15orf41 | 2595 | 7 | 1 | 0 | 0 | 7 | 1 | 0 | 0 | 0 | 1 | 1 | 0 | 1 |
| NM_032501 | ACSS1 | 4473 | 4 | 2 | 1 | 1 | 3 | 2 | 4 | 1 | 1 | 1 | 1 | 2 | 3 |
| NM_032503 | MCHR2 | 2144 | 0 | 0 | 0 | 0 | 0 | 0 | 0 | 0 | 0 | 0 | 0 | 0 | 0 |
| NM_032504 | UNC80 | 13562 | 7 | 1 | 0 | 0 | 0 | 7 | 0 | 0 | 0 | 0 | 0 | 0 | 1 |
| NM_032505 | KBTBD8 | 4680 | 6 | 2 | 0 | 0 | 2 | 2 | 0 | 1 | 1 | 0 | 1 | 6 | 5 |
| NM_032506 | KIAA1841 | 3116 | 11 | 4 | 5 | 3 | 3 | 11 | 2 | 1 | 4 | 3 | 3 | 3 | 4 |
| NM_032507 | PGBD1 | 3065 | 1 | 1 | 1 | 0 | 1 | 1 | 1 | 0 | 0 | 1 | 0 | 1 | 1 |
| NM_032508 | TMEM185A | 2821 | 2 | 1 | 0 | 0 | 1 | 1 | 0 | 0 | 0 | 1 | 0 | 1 | 2 |
| NM_032509 | MAK16 | 3573 | 9 | 6 | 5 | 2 | 6 | 7 | 5 | 7 | 3 | 6 | 6 | 9 | 8 |
| NM_032510 | PARD6G | 3836 | 2 | 1 | 0 | 1 | 0 | 0 | 1 | 0 | 0 | 2 | 0 | 1 | 1 |
| NM_032511 | C6orf168 | 9760 | 3 | 0 | 0 | 0 | 0 | 3 | 0 | 0 | 0 | 0 | 0 | 0 | 0 |
| NM_032512 | PDZD4 | 3745 | 9 | 1 | 0 | 0 | 0 | 9 | 0 | 0 | 0 | 0 | 0 | 0 | 0 |
| NM_032513 | SLC30A2 | 2326 | 2 | 0 | 0 | 0 | 0 | 0 | 2 | 0 | 0 | 0 | 0 | 0 | 0 |
| NM_032514 | MAP1LC3A | 967 | 4 | 1 | 0 | 1 | 1 | 4 | 1 | 0 | 1 | 1 | 1 | 0 | 1 |
| NM_032515 | BOK | 2601 | 1 | 0 | 0 | 0 | 0 | 0 | 0 | 1 | 0 | 0 | 0 | 0 | 0 |
| NM_032517 | LYZL1 | 812 | 1 | 0 | 0 | 0 | 0 | 0 | 0 | 0 | 0 | 0 | 0 | 0 | 1 |
| NM_032518 | COL25A1 | 2667 | 3 | 1 | 2 | 0 | 0 | 3 | 1 | 0 | 0 | 0 | 0 | 0 | 1 |
| NM_032520 | GNPTG | 1213 | 6 | 3 | 4 | 2 | 3 | 6 | 5 | 3 | 3 | 2 | 2 | 4 | 3 |
| NM_032521 | PARD6B | 4615 | 7 | 1 | 0 | 0 | 0 | 0 | 7 | 1 | 3 | 0 | 0 | 1 | 2 |
| NM_032522 | ZBTB37 | 1370 | 5 | 3 | 3 | 2 | 1 | 3 | 4 | 2 | 2 | 4 | 1 | 5 | 5 |
| NM_032523 | OSBPL6 | 3772 | 7 | 1 | 0 | 0 | 1 | 3 | 1 | 1 | 1 | 1 | 7 | 0 | 0 |
| NM_032524 | KRTAP4-4 | 1078 | 0 | 0 | 0 | 0 | 0 | 0 | 0 | 0 | 0 | 0 | 0 | 0 | 0 |
| NM_032525 | TUBB6 | 1815 | 28 | 8 | 20 | 6 | 28 | 5 | 3 | 1 | 6 | 7 | 2 | 7 | 5 |
| NM_032526 | NT5C1A | 1107 | 0 | 0 | 0 | 0 | 0 | 0 | 0 | 0 | 0 | 0 | 0 | 0 | 0 |
| NM_032527 | ZGPAT | 1997 | 3 | 1 | 1 | 2 | 1 | 2 | 3 | 3 | 1 | 1 | 1 | 2 | 1 |
| NM_032528 | ST6GAL2 | 6855 | 2 | 0 | 0 | 0 | 1 | 2 | 0 | 0 | 0 | 1 | 0 | 0 | 2 |
| NM_032530 | ZNF594 | 4877 | 10 | 3 | 1 | 1 | 2 | 3 | 3 | 0 | 1 | 10 | 3 | 5 | 4 |
| NM_032531 | KIRREL3 | 3776 | 0 | 0 | 0 | 0 | 0 | 0 | 0 | 0 | 0 | 0 | 0 | 0 | 0 |
| NM_032532 | FNDC1 | 6551 | 1 | 0 | 1 | 1 | 0 | 0 | 0 | 0 | 0 | 0 | 0 | 0 | 0 |
| NM_032534 | KRBA1 | 4019 | 0 | 0 | 0 | 0 | 0 | 0 | 0 | 0 | 0 | 0 | 0 | 0 | 0 |
| NM_032536 | NTNG2 | 3091 | 0 | 0 | 0 | 0 | 0 | 0 | 0 | 0 | 0 | 0 | 0 | 0 | 0 |
| NM_032538 | TTBK1 | 7128 | 2 | 0 | 0 | 0 | 0 | 2 | 0 | 0 | 0 | 0 | 0 | 0 | 0 |
| NM_032539 | SLITRK2 | 4405 | 3 | 0 | 0 | 0 | 0 | 3 | 0 | 0 | 0 | 0 | 0 | 1 | 0 |
| NM_032545 | CFC1 | 1009 | 0 | 0 | 0 | 0 | 0 | 0 | 0 | 0 | 0 | 0 | 0 | 0 | 0 |
| NM_032546 | TRIM54 | 1757 | 10 | 1 | 0 | 0 | 5 | 0 | 0 | 0 | 0 | 0 | 10 | 0 | 1 |
| NM_032547 | SCOC | 1964 | 10 | 3 | 2 | 1 | 5 | 10 | 6 | 3 | 2 | 2 | 2 | 3 | 2 |
| NM_032548 | ABTB1 | 1981 | 0 | 0 | 0 | 0 | 0 | 0 | 0 | 0 | 0 | 0 | 0 | 0 | 0 |
| NM_032549 | IMMP2L | 1519 | 4 | 1 | 1 | 1 | 1 | 1 | 4 | 1 | 1 | 1 | 1 | 1 | 1 |
| NM_032550 | AFAP1L2 | 3964 | 10 | 2 | 1 | 1 | 0 | 2 | 2 | 0 | 0 | 2 | 0 | 10 | 1 |
| NM_032551 | KISS1R | 1625 | 0 | 0 | 0 | 0 | 0 | 0 | 0 | 0 | 0 | 0 | 0 | 0 | 0 |
| NM_032552 | DAB2IP | 5453 | 3 | 2 | 1 | 2 | 2 | 3 | 3 | 1 | 2 | 2 | 2 | 3 | 2 |
| NM_032553 | GPR174 | 1258 | 4 | 1 | 0 | 1 | 0 | 0 | 0 | 0 | 2 | 0 | 0 | 4 | 0 |
| NM_032554 | GPR81 | 2977 | 2 | 0 | 2 | 0 | 0 | 0 | 0 | 0 | 0 | 0 | 0 | 1 | 0 |
| NM_032556 | IL1F10 | 1366 | 0 | 0 | 0 | 0 | 0 | 0 | 0 | 0 | 0 | 0 | 0 | 0 | 0 |
| NM_032557 | USP38 | 5194 | 3 | 1 | 1 | 1 | 2 | 1 | 2 | 3 | 1 | 1 | 2 | 2 | 1 |
| NM_032558 | HIATL1 | 3298 | 3 | 2 | 2 | 1 | 2 | 2 | 3 | 1 | 3 | 2 | 1 | 3 | 2 |
| NM_032559 | KIF2B | 2335 | 9 | 1 | 0 | 0 | 0 | 0 | 0 | 0 | 0 | 0 | 0 | 0 | 9 |
| NM_032560 | SMEK1 | 3893 | 23 | 13 | 8 | 6 | 13 | 9 | 14 | 12 | 13 | 18 | 9 | 23 | 23 |
| NM_032561 | C22orf23 | 1560 | 3 | 0 | 0 | 0 | 0 | 0 | 0 | 0 | 0 | 0 | 0 | 0 | 3 |
| NM_032562 | PLA2G12B | 1092 | 11 | 1 | 0 | 0 | 0 | 0 | 3 | 11 | 0 | 0 | 0 | 0 | 0 |
| NM_032563 | LCE3D | 610 | 0 | 0 | 0 | 0 | 0 | 0 | 0 | 0 | 0 | 0 | 0 | 0 | 0 |
| NM_032564 | DGAT2 | 2452 | 14 | 2 | 14 | 0 | 0 | 0 | 0 | 5 | 0 | 0 | 0 | 1 | 0 |
| NM_032565 | EBPL | 978 | 13 | 4 | 1 | 2 | 2 | 3 | 3 | 13 | 3 | 8 | 2 | 6 | 3 |
| NM_032566 | SPINK7 | 549 | 0 | 0 | 0 | 0 | 0 | 0 | 0 | 0 | 0 | 0 | 0 | 0 | 0 |
| NM_032567 | SPZ1 | 1869 | 3 | 0 | 0 | 0 | 0 | 0 | 0 | 0 | 0 | 0 | 0 | 0 | 3 |
| NM_032569 | GLYR1 | 3732 | 13 | 8 | 5 | 7 | 8 | 13 | 12 | 9 | 5 | 5 | 5 | 11 | 7 |
| NM_032571 | EMR3 | 2301 | 1 | 0 | 0 | 0 | 0 | 0 | 0 | 0 | 0 | 0 | 0 | 1 | 0 |
| NM_032572 | RNASE7 | 1498 | 0 | 0 | 0 | 0 | 0 | 0 | 0 | 0 | 0 | 0 | 0 | 0 | 0 |
| NM_032573 | CCDC62 | 3103 | 19 | 2 | 0 | 0 | 0 | 1 | 0 | 0 | 0 | 0 | 1 | 1 | 19 |
| NM_032574 | DPY30 | 800 | 13 | 5 | 5 | 5 | 4 | 13 | 5 | 7 | 4 | 4 | 2 | 4 | 2 |
| NM_032575 | GLIS2 | 3705 | 2 | 1 | 0 | 0 | 1 | 0 | 2 | 0 | 1 | 1 | 0 | 0 | 0 |
| NM_032576 | CYorf15B | 3314 | 1 | 0 | 0 | 0 | 0 | 0 | 0 | 0 | 0 | 0 | 0 | 1 | 1 |
| NM_032578 | MYPN | 5756 | 70 | 10 | 0 | 0 | 42 | 0 | 0 | 0 | 0 | 0 | 70 | 0 | 1 |
| NM_032579 | RETNLB | 573 | 21 | 2 | 0 | 21 | 0 | 0 | 0 | 0 | 0 | 0 | 0 | 0 | 0 |
| NM_032580 | HES7 | 1667 | 2 | 0 | 0 | 0 | 1 | 0 | 1 | 0 | 0 | 0 | 0 | 2 | 0 |
| NM_032581 | FAM126A | 6151 | 6 | 4 | 4 | 1 | 4 | 5 | 2 | 2 | 4 | 2 | 6 | 6 | 4 |
| NM_032582 | USP32 | 7026 | 9 | 4 | 2 | 1 | 4 | 9 | 3 | 4 | 4 | 3 | 4 | 6 | 9 |
| NM_032583 | ABCC11 | 4576 | 1 | 0 | 0 | 0 | 0 | 0 | 0 | 1 | 0 | 0 | 0 | 0 | 0 |
| NM_032584 | ZNF347 | 3502 | 12 | 4 | 3 | 1 | 4 | 3 | 3 | 1 | 2 | 6 | 1 | 12 | 5 |
| NM_032587 | CARD6 | 4097 | 10 | 3 | 6 | 0 | 4 | 1 | 2 | 2 | 5 | 2 | 1 | 10 | 2 |
| NM_032588 | TRIM63 | 1764 | 313 | 36 | 1 | 0 | 82 | 0 | 1 | 0 | 0 | 0 | 313 | 0 | 1 |
| NM_032590 | KDM2B | 5307 | 2 | 1 | 1 | 1 | 0 | 1 | 1 | 1 | 1 | 1 | 1 | 2 | 1 |
| NM_032591 | SLC9A7 | 2200 | 1 | 0 | 0 | 0 | 0 | 1 | 0 | 1 | 0 | 1 | 0 | 0 | 0 |

| transcript | gene | transcript length (nt) | max | mean | adipose | colon | heart | hypothala mus | kidney | liver | lung | ovary | skeletalm uscle | spleen | testes |
|---|---|---|---|---|---|---|---|---|---|---|---|---|---|---|---|
| NM_032592 | ACCS | 2173 | 1 | 0 | 1 | 1 | 0 | 0 | 1 | 0 | 0 | 0 | 0 | 1 | 1 |
| NM_032593 | HINT2 | 652 | 12 | 5 | 7 | 3 | 6 | 4 | 8 | 12 | 3 | 2 | 3 | 4 | 2 |
| NM_032594 | INSM2 | 3013 | 0 | 0 | 0 | 0 | 0 | 0 | 0 | 0 | 0 | 0 | 0 | 0 | 0 |
| NM_032595 | PPP1R9B | 4048 | 36 | 11 | 9 | 7 | 7 | 36 | 6 | 2 | 13 | 7 | 3 | 19 | 13 |
| NM_032596 | C9orf24 | 1170 | 3 | 1 | 0 | 0 | 1 | 3 | 0 | 0 | 2 | 0 | 0 | 0 | 1 |
| NM_032597 | MS4A14 | 3352 | 11 | 2 | 1 | 0 | 1 | 0 | 0 | 0 | 1 | 0 | 0 | 1 | 11 |
| NM_032598 | SPATA22 | 1453 | 8 | 1 | 0 | 0 | 0 | 0 | 0 | 0 | 0 | 0 | 0 | 0 | 8 |
| NM_032599 | FAM71F1 | 1718 | 9 | 1 | 0 | 0 | 0 | 1 | 0 | 0 | 0 | 0 | 0 | 0 | 9 |
| NM_032600 | CCDC54 | 1294 | 19 | 2 | 0 | 0 | 0 | 0 | 0 | 0 | 0 | 0 | 0 | 0 | 19 |
| NM_032601 | MCEE | 870 | 12 | 4 | 4 | 2 | 8 | 4 | 4 | 12 | 2 | 2 | 2 | 2 | 2 |
| NM_032602 | GJA10 | 1632 | 0 | 0 | 0 | 0 | 0 | 0 | 0 | 0 | 0 | 0 | 0 | 0 | 0 |
| NM_032603 | LOXL3 | 3121 | 0 | 0 | 0 | 0 | 0 | 0 | 0 | 0 | 0 | 0 | 0 | 0 | 0 |
| NM_032604 | ABHD1 | 1446 | 1 | 0 | 0 | 0 | 0 | 0 | 0 | 0 | 0 | 0 | 0 | 0 | 1 |
| NM_032606 | CAPS2 | 2135 | 1 | 0 | 0 | 0 | 0 | 1 | 0 | 0 | 0 | 0 | 0 | 0 | 0 |
| NM_032607 | CREB3L3 | 2584 | 93 | 9 | 0 | 1 | 0 | 0 | 1 | 93 | 0 | 1 | 0 | 1 | 0 |
| NM_032608 | MYO18B | 8565 | 26 | 4 | 0 | 0 | 12 | 0 | 0 | 0 | 0 | 0 | 26 | 0 | 0 |
| NM_032609 | COX4I2 | 684 | 1 | 0 | 0 | 0 | 0 | 0 | 0 | 0 | 1 | 0 | 0 | 0 | 0 |
| NM_032611 | PTP4A3 | 1332 | 13 | 3 | 0 | 0 | 13 | 1 | 1 | 0 | 1 | 1 | 10 | 1 | 1 |
| NM_032620 | GTPBP3 | 2572 | 0 | 0 | 0 | 0 | 0 | 0 | 0 | 0 | 0 | 0 | 0 | 0 | 0 |
| NM_032621 | BEX2 | 891 | 109 | 16 | 0 | 3 | 6 | 109 | 11 | 0 | 4 | 15 | 5 | 6 | 20 |
| NM_032622 | LNX1 | 2840 | 3 | 1 | 0 | 0 | 1 | 3 | 2 | 1 | 0 | 0 | 0 | 0 | 1 |
| NM_032623 | C4orf49 | 1339 | 4 | 1 | 0 | 0 | 0 | 0 | 0 | 0 | 0 | 4 | 0 | 0 | 1 |
| NM_032626 | RBBP6 | 1763 | 1 | 1 | 0 | 0 | 1 | 1 | 0 | 0 | 0 | 1 | 1 | 1 | 1 |
| NM_032627 | SSBP4 | 1711 | 2 | 1 | 0 | 0 | 0 | 2 | 1 | 0 | 0 | 1 | 0 | 1 | 2 |
| NM_032630 | CINP | 979 | 4 | 2 | 2 | 1 | 2 | 4 | 2 | 3 | 1 | 2 | 1 | 2 | 2 |
| NM_032631 | HDGF2 | 2306 | 14 | 8 | 5 | 7 | 6 | 12 | 7 | 5 | 5 | 11 | 14 | 7 | 13 |
| NM_032632 | PAPOLA | 4509 | 45 | 25 | 15 | 10 | 35 | 21 | 23 | 22 | 22 | 28 | 15 | 45 | 42 |
| NM_032634 | PIGO | 4067 | 1 | 0 | 0 | 1 | 0 | 1 | 1 | 1 | 0 | 0 | 0 | 1 | 0 |
| NM_032635 | TMEM147 | 851 | 4 | 2 | 1 | 1 | 4 | 2 | 3 | 2 | 2 | 1 | 2 | 2 | 2 |
| NM_032636 | PSRC1 | 1719 | 11 | 2 | 0 | 2 | 0 | 11 | 0 | 1 | 1 | 1 | 0 | 1 | 4 |
| NM_032637 | SKP2 | 1443 | 1 | 0 | 0 | 0 | 0 | 0 | 0 | 0 | 0 | 1 | 0 | 1 | 0 |
| NM_032638 | GATA2 | 3374 | 1 | 0 | 1 | 0 | 0 | 0 | 1 | 0 | 1 | 0 | 0 | 0 | 0 |
| NM_032639 | PLEKHA8 | 1982 | 0 | 0 | 0 | 0 | 0 | 0 | 0 | 0 | 0 | 0 | 0 | 0 | 0 |
| NM_032641 | SPSB2 | 1254 | 0 | 0 | 0 | 0 | 0 | 0 | 0 | 0 | 0 | 0 | 0 | 0 | 0 |
| NM_032642 | WNT5B | 2223 | 0 | 0 | 0 | 0 | 0 | 0 | 0 | 0 | 0 | 0 | 0 | 0 | 0 |
| NM_032643 | IRF5 | 2753 | 0 | 0 | 0 | 0 | 0 | 0 | 0 | 0 | 0 | 0 | 0 | 0 | 0 |
| NM_032645 | RAPSN | 1487 | 1 | 0 | 0 | 0 | 0 | 0 | 0 | 0 | 0 | 0 | 1 | 0 | 0 |
| NM_032646 | TTYH2 | 3445 | 5 | 1 | 0 | 0 | 1 | 5 | 0 | 0 | 0 | 1 | 1 | 1 | 2 |
| NM_032647 | CBX2 | 1061 | 0 | 0 | 0 | 0 | 0 | 0 | 0 | 0 | 0 | 0 | 0 | 0 | 0 |
| NM_032648 | FAM167B | 956 | 0 | 0 | 0 | 0 | 0 | 0 | 0 | 0 | 0 | 0 | 0 | 0 | 0 |
| NM_032649 | CNDP1 | 2198 | 12 | 1 | 0 | 0 | 0 | 12 | 0 | 3 | 0 | 0 | 0 | 0 | 0 |
| NM_032656 | DHX37 | 4549 | 2 | 1 | 1 | 1 | 1 | 2 | 1 | 2 | 1 | 1 | 2 | 2 | 1 |
| NM_032663 | USP30 | 3753 | 2 | 1 | 1 | 1 | 1 | 1 | 1 | 1 | 0 | 2 | 1 | 1 | 1 |
| NM_032664 | FUT10 | 3547 | 3 | 1 | 1 | 1 | 1 | 1 | 1 | 0 | 1 | 3 | 1 | 1 | 1 |
| NM_032667 | BSCL2 | 1507 | 11 | 2 | 1 | 1 | 1 | 11 | 2 | 2 | 2 | 1 | 1 | 1 | 4 |
| NM_032673 | PCGF1 | 906 | 3 | 2 | 2 | 2 | 2 | 3 | 2 | 3 | 2 | 3 | 2 | 3 | 2 |
| NM_032679 | ZNF577 | 3084 | 15 | 7 | 4 | 4 | 5 | 8 | 6 | 1 | 4 | 15 | 6 | 7 | 14 |
| NM_032680 | EFCAB4B | 2289 | 2 | 1 | 0 | 2 | 0 | 0 | 0 | 0 | 0 | 0 | 0 | 1 | 0 |
| NM_032681 | SPRYD5 | 1625 | 0 | 0 | 0 | 0 | 0 | 0 | 0 | 0 | 0 | 0 | 0 | 0 | 0 |
| NM_032682 | FOXP1 | 6222 | 18 | 7 | 5 | 6 | 6 | 5 | 3 | 4 | 10 | 18 | 5 | 10 | 6 |
| NM_032683 | MPV17L2 | 1358 | 0 | 0 | 0 | 0 | 0 | 0 | 0 | 0 | 0 | 0 | 0 | 0 | 0 |
| NM_032687 | CYHR1 | 1198 | 1 | 0 | 1 | 1 | 0 | 1 | 1 | 0 | 0 | 0 | 0 | 0 | 1 |
| NM_032689 | ZNF607 | 4352 | 2 | 1 | 1 | 1 | 1 | 2 | 2 | 0 | 1 | 2 | 0 | 2 | 2 |
| NM_032693 | ARD1B | 1516 | 1 | 0 | 0 | 0 | 0 | 0 | 0 | 0 | 0 | 0 | 0 | 0 | 1 |
| NM_032701 | SUV420H2 | 2309 | 0 | 0 | 0 | 0 | 0 | 0 | 0 | 0 | 0 | 0 | 0 | 0 | 0 |
| NM_032704 | TUBA1C | 1553 | 24 | 11 | 10 | 15 | 10 | 4 | 24 | 23 | 12 | 3 | 1 | 11 | 8 |
| NM_032709 | PYROXD2 | 2064 | 1 | 0 | 1 | 0 | 0 | 0 | 0 | 0 | 0 | 0 | 0 | 0 | 0 |
| NM_032711 | MAFG | 5033 | 2 | 1 | 0 | 1 | 1 | 2 | 1 | 1 | 1 | 0 | 1 | 1 | 1 |
| NM_032711 | MAFG | 5060 | 2 | 1 | 0 | 1 | 1 | 2 | 1 | 1 | 1 | 0 | 1 | 1 | 1 |
| NM_032714 | INF2 | 1688 | 0 | 0 | 0 | 0 | 0 | 0 | 0 | 0 | 0 | 0 | 0 | 0 | 0 |
| NM_032717 | AGPAT9 | 2534 | 7 | 2 | 1 | 1 | 3 | 0 | 7 | 1 | 0 | 1 | 3 | 1 | 0 |
| NM_032718 | MFSD9 | 2706 | 1 | 0 | 0 | 0 | 0 | 0 | 1 | 1 | 0 | 0 | 0 | 0 | 0 |
| NM_032725 | BUD13 | 2204 | 9 | 5 | 4 | 4 | 4 | 4 | 3 | 3 | 5 | 6 | 5 | 7 | 9 |
| NM_032726 | PLCD4 | 3088 | 2 | 1 | 0 | 1 | 0 | 0 | 1 | 0 | 0 | 0 | 2 | 0 | 0 |
| NM_032727 | INA | 3229 | 12 | 1 | 0 | 0 | 0 | 12 | 0 | 0 | 0 | 0 | 0 | 0 | 0 |
| NM_032728 | PPAPDC3 | 2095 | 1 | 0 | 0 | 0 | 1 | 0 | 0 | 0 | 0 | 0 | 1 | 0 | 0 |
| NM_032730 | RTN4IP1 | 2636 | 2 | 1 | 0 | 1 | 2 | 2 | 2 | 2 | 1 | 1 | 1 | 1 | 2 |
| NM_032731 | TXNDC17 | 2075 | 1 | 0 | 0 | 1 | 0 | 0 | 1 | 1 | 0 | 0 | 0 | 1 | 0 |
| NM_032732 | IL17RC | 2390 | 0 | 0 | 0 | 0 | 0 | 0 | 0 | 0 | 0 | 0 | 0 | 0 | 0 |
| NM_032735 | BEST3 | 3532 | 8 | 1 | 0 | 0 | 0 | 0 | 0 | 0 | 0 | 0 | 8 | 0 | 0 |
| NM_032737 | LMNB2 | 4635 | 2 | 1 | 1 | 2 | 1 | 1 | 1 | 1 | 1 | 1 | 1 | 2 | 1 |
| NM_032738 | FCRLA | 2345 | 2 | 0 | 0 | 0 | 0 | 0 | 0 | 0 | 0 | 0 | 0 | 2 | 0 |
| NM_032740 | SFT2D3 | 2811 | 2 | 1 | 1 | 1 | 1 | 1 | 1 | 1 | 1 | 1 | 1 | 2 | 1 |
| NM_032741 | AGPAT1 | 1990 | 1 | 1 | 0 | 0 | 1 | 1 | 1 | 1 | 1 | 1 | 1 | 1 | 1 |
| NM_032744 | C6orf105 | 1802 | 7 | 1 | 0 | 7 | 0 | 1 | 1 | 0 | 1 | 0 | 0 | 1 | 1 |
| NM_032747 | USMG5 | 553 | 31 | 9 | 7 | 4 | 31 | 12 | 14 | 7 | 5 | 3 | 8 | 7 | 3 |
| NM_032750 | ABHD14B | 2436 | 9 | 3 | 5 | 3 | 1 | 1 | 9 | 5 | 4 | 3 | 1 | 4 | 2 |

| transcript | gene | transcript length (nt) | max | mean | adipose | colon | heart | hypothala mus | kidney | liver | lung | ovary | skeletalm uscle | spleen | testes |
|---|---|---|---|---|---|---|---|---|---|---|---|---|---|---|---|
| NM_032752 | ZNF496 | 2428 | 0 | 0 | 0 | 0 | 0 | 0 | 0 | 0 | 0 | 0 | 0 | 0 | 0 |
| NM_032753 | RAX2 | 2153 | 0 | 0 | 0 | 0 | 0 | 0 | 0 | 0 | 0 | 0 | 0 | 0 | 0 |
| NM_032756 | HPDL | 1802 | 0 | 0 | 0 | 0 | 0 | 0 | 0 | 0 | 0 | 0 | 0 | 0 | 0 |
| NM_032758 | PHF5A | 1065 | 5 | 3 | 3 | 3 | 3 | 4 | 2 | 4 | 3 | 2 | 3 | 5 | 2 |
| NM_032765 | TRIM52 | 2228 | 5 | 3 | 3 | 3 | 1 | 3 | 3 | 1 | 3 | 3 | 2 | 5 | 3 |
| NM_032772 | ZNF503 | 3179 | 2 | 1 | 0 | 0 | 0 | 0 | 1 | 0 | 1 | 2 | 0 | 0 | 0 |
| NM_032773 | LRCH3 | 2258 | 21 | 13 | 10 | 13 | 10 | 9 | 10 | 9 | 12 | 21 | 12 | 19 | 19 |
| NM_032775 | KLHL22 | 2568 | 1 | 0 | 0 | 0 | 0 | 1 | 0 | 0 | 0 | 0 | 0 | 0 | 0 |
| NM_032776 | JMJD1C | 8740 | 14 | 8 | 7 | 3 | 7 | 6 | 8 | 5 | 10 | 14 | 6 | 14 | 12 |
| NM_032777 | GPR124 | 5999 | 1 | 0 | 1 | 0 | 0 | 0 | 0 | 0 | 0 | 1 | 0 | 1 | 0 |
| NM_032778 | MINA | 5268 | 2 | 1 | 1 | 1 | 1 | 2 | 2 | 2 | 1 | 1 | 2 | 2 | 1 |
| NM_032779 | CCDC142 | 4338 | 1 | 0 | 0 | 1 | 0 | 0 | 0 | 0 | 0 | 0 | 0 | 0 | 0 |
| NM_032780 | TMEM25 | 2529 | 2 | 1 | 0 | 0 | 0 | 2 | 1 | 0 | 0 | 1 | 0 | 0 | 1 |
| NM_032781 | PTPN5 | 3090 | 6 | 1 | 0 | 0 | 0 | 6 | 0 | 0 | 0 | 0 | 0 | 0 | 0 |
| NM_032782 | HAVCR2 | 2320 | 12 | 4 | 10 | 1 | 2 | 2 | 12 | 1 | 5 | 1 | 0 | 7 | 1 |
| NM_032783 | CBR4 | 3455 | 4 | 3 | 2 | 1 | 3 | 3 | 4 | 4 | 2 | 2 | 2 | 3 | 3 |
| NM_032784 | RSPO3 | 2141 | 7 | 2 | 7 | 1 | 0 | 3 | 1 | 1 | 1 | 0 | 4 | 1 | 7 |
| NM_032785 | AGBL4 | 2988 | 3 | 0 | 0 | 0 | 0 | 3 | 0 | 0 | 0 | 0 | 0 | 0 | 0 |
| NM_032786 | ZC3H10 | 2121 | 2 | 1 | 1 | 1 | 1 | 1 | 1 | 1 | 1 | 2 | 2 | 2 | 2 |
| NM_032787 | GPR128 | 3138 | 5 | 1 | 0 | 0 | 0 | 0 | 0 | 5 | 0 | 0 | 0 | 1 | 0 |
| NM_032788 | ZNF514 | 3204 | 11 | 4 | 4 | 3 | 3 | 4 | 3 | 1 | 3 | 11 | 4 | 6 | 6 |
| NM_032789 | PARP10 | 3518 | 1 | 0 | 0 | 0 | 0 | 0 | 1 | 0 | 0 | 0 | 0 | 1 | 0 |
| NM_032790 | ORAI1 | 1502 | 0 | 0 | 0 | 0 | 0 | 0 | 0 | 0 | 0 | 0 | 0 | 0 | 0 |
| NM_032792 | ZBTB45 | 2352 | 0 | 0 | 0 | 0 | 0 | 0 | 0 | 0 | 0 | 0 | 0 | 0 | 0 |
| NM_032793 | MFSD2A | 2161 | 6 | 1 | 0 | 0 | 0 | 0 | 0 | 6 | 1 | 0 | 0 | 0 | 1 |
| NM_032795 | RPUSD4 | 2511 | 9 | 3 | 2 | 2 | 5 | 2 | 2 | 3 | 1 | 2 | 9 | 3 | 1 |
| NM_032796 | SYAP1 | 3524 | 18 | 8 | 6 | 4 | 8 | 7 | 10 | 18 | 6 | 9 | 6 | 8 | 7 |
| NM_032797 | AIFM2 | 3240 | 24 | 5 | 24 | 3 | 6 | 1 | 2 | 6 | 2 | 1 | 2 | 3 | 1 |
| NM_032799 | ZDHHC12 | 1151 | 0 | 0 | 0 | 0 | 0 | 0 | 0 | 0 | 0 | 0 | 0 | 0 | 0 |
| NM_032800 | C1orf198 | 3761 | 4 | 2 | 2 | 1 | 3 | 4 | 1 | 0 | 3 | 2 | 3 | 1 | 1 |
| NM_032801 | JAM3 | 3665 | 10 | 3 | 1 | 2 | 4 | 7 | 1 | 0 | 3 | 4 | 1 | 3 | 10 |
| NM_032802 | SPPL2A | 2003 | 5 | 3 | 2 | 3 | 2 | 2 | 4 | 5 | 2 | 1 | 4 | 2 | 1 |
| NM_032803 | SLC7A3 | 2251 | 0 | 0 | 0 | 0 | 0 | 0 | 0 | 0 | 0 | 0 | 0 | 0 | 0 |
| NM_032804 | ADO | 3724 | 5 | 2 | 1 | 1 | 2 | 3 | 2 | 1 | 1 | 2 | 1 | 3 | 5 |
| NM_032805 | ZSCAN10 | 2463 | 0 | 0 | 0 | 0 | 0 | 0 | 0 | 0 | 0 | 0 | 0 | 0 | 0 |
| NM_032806 | C3orf39 | 2538 | 1 | 0 | 0 | 0 | 0 | 1 | 0 | 0 | 0 | 1 | 0 | 0 | 0 |
| NM_032807 | FBXO18 | 3702 | 7 | 4 | 3 | 3 | 7 | 3 | 4 | 3 | 3 | 4 | 3 | 6 | 6 |
| NM_032808 | LINGO1 | 2932 | 13 | 1 | 0 | 0 | 0 | 13 | 0 | 0 | 0 | 0 | 0 | 1 | 0 |
| NM_032809 | FAM73B | 3587 | 1 | 0 | 0 | 1 | 0 | 1 | 1 | 0 | 0 | 0 | 0 | 0 | 0 |
| NM_032810 | ATAD1 | 3034 | 10 | 5 | 3 | 2 | 10 | 10 | 5 | 4 | 4 | 4 | 4 | 4 | 7 |
| NM_032811 | TBRG1 | 5162 | 7 | 3 | 3 | 2 | 4 | 7 | 4 | 2 | 3 | 3 | 2 | 5 | 3 |
| NM_032812 | PLXDC2 | 2594 | 14 | 6 | 7 | 3 | 5 | 7 | 7 | 1 | 14 | 7 | 3 | 6 | 9 |
| NM_032813 | TMTC4 | 3754 | 3 | 1 | 0 | 1 | 1 | 3 | 2 | 1 | 1 | 1 | 1 | 1 | 1 |
| NM_032814 | RNFT2 | 2415 | 3 | 0 | 0 | 0 | 0 | 3 | 0 | 0 | 0 | 0 | 0 | 0 | 0 |
| NM_032815 | NFATC2IP | 3858 | 3 | 1 | 1 | 2 | 2 | 1 | 1 | 1 | 1 | 1 | 1 | 3 | 2 |
| NM_032816 | CCDC123 | 2605 | 3 | 2 | 2 | 1 | 1 | 2 | 2 | 1 | 1 | 3 | 1 | 2 | 2 |
| NM_032817 | ITIH5 | 5659 | 47 | 6 | 47 | 5 | 1 | 2 | 3 | 0 | 2 | 3 | 1 | 0 | 3 |
| NM_032818 | C9orf100 | 3763 | 0 | 0 | 0 | 0 | 0 | 0 | 0 | 0 | 0 | 0 | 0 | 0 | 0 |
| NM_032819 | ZNF341 | 3316 | 0 | 0 | 0 | 0 | 0 | 0 | 0 | 0 | 0 | 0 | 0 | 0 | 0 |
| NM_032821 | HYDIN | 15685 | 1 | 0 | 0 | 0 | 0 | 1 | 0 | 0 | 1 | 0 | 0 | 0 | 1 |
| NM_032822 | FAM136A | 1807 | 7 | 5 | 4 | 5 | 5 | 5 | 5 | 7 | 3 | 3 | 2 | 5 | 5 |
| NM_032823 | C9orf3 | 2820 | 9 | 3 | 1 | 4 | 9 | 2 | 5 | 2 | 2 | 3 | 4 | 2 | 2 |
| NM_032824 | TMEM87B | 5212 | 4 | 2 | 1 | 2 | 1 | 1 | 1 | 1 | 2 | 1 | 0 | 4 | 1 |
| NM_032825 | ZNF382 | 2813 | 3 | 1 | 0 | 0 | 2 | 3 | 1 | 0 | 1 | 2 | 0 | 2 | 2 |
| NM_032826 | SLC35B4 | 6813 | 4 | 2 | 1 | 1 | 1 | 4 | 2 | 1 | 1 | 2 | 0 | 2 | 1 |
| NM_032827 | ATOH8 | 5862 | 0 | 0 | 0 | 0 | 0 | 0 | 0 | 0 | 0 | 0 | 0 | 0 | 0 |
| NM_032828 | ZNF587 | 6845 | 3 | 2 | 1 | 3 | 2 | 2 | 2 | 1 | 1 | 3 | 1 | 2 | 2 |
| NM_032829 | C12orf34 | 2731 | 1 | 0 | 0 | 0 | 0 | 1 | 0 | 0 | 0 | 0 | 0 | 1 | 1 |
| NM_032830 | CIRH1A | 2254 | 6 | 3 | 1 | 1 | 3 | 3 | 6 | 3 | 1 | 2 | 4 | 2 | 3 |
| NM_032831 | ORAI2 | 10660 | 1 | 0 | 0 | 0 | 0 | 0 | 0 | 0 | 0 | 0 | 0 | 1 | 0 |
| NM_032832 | LRP11 | 3618 | 14 | 4 | 5 | 3 | 3 | 14 | 5 | 1 | 5 | 5 | 1 | 2 | 5 |
| NM_032833 | PPP1R15B | 5275 | 7 | 4 | 3 | 2 | 4 | 3 | 5 | 7 | 5 | 6 | 2 | 6 | 4 |
| NM_032834 | ALG10 | 3127 | 0 | 0 | 0 | 0 | 0 | 0 | 0 | 0 | 0 | 0 | 0 | 0 | 0 |
| NM_032836 | FIZ1 | 2660 | 0 | 0 | 0 | 0 | 0 | 0 | 0 | 0 | 0 | 0 | 0 | 0 | 0 |
| NM_032837 | FAM104A | 2825 | 7 | 2 | 2 | 1 | 2 | 2 | 1 | 2 | 2 | 2 | 2 | 3 | 7 |
| NM_032838 | ZNF566 | 5196 | 3 | 1 | 1 | 1 | 1 | 1 | 1 | 1 | 1 | 3 | 1 | 2 | 1 |
| NM_032839 | DIRC2 | 3337 | 4 | 2 | 2 | 1 | 3 | 1 | 2 | 1 | 2 | 2 | 4 | 2 | 2 |
| NM_032840 | SPRYD3 | 2568 | 26 | 7 | 8 | 4 | 7 | 26 | 6 | 2 | 5 | 5 | 2 | 5 | 6 |
| NM_032842 | TMEM209 | 3545 | 2 | 1 | 1 | 1 | 1 | 1 | 1 | 1 | 1 | 1 | 0 | 1 | 2 |
| NM_032843 | FIBCD1 | 3256 | 0 | 0 | 0 | 0 | 0 | 0 | 0 | 0 | 0 | 0 | 0 | 0 | 0 |
| NM_032844 | MASTL | 3659 | 3 | 1 | 1 | 0 | 1 | 1 | 1 | 1 | 2 | 1 | 1 | 2 | 3 |
| NM_032846 | RAB2B | 2927 | 3 | 1 | 1 | 1 | 1 | 3 | 1 | 1 | 1 | 1 | 1 | 1 | 1 |
| NM_032847 | C8orf76 | 1286 | 3 | 2 | 2 | 2 | 2 | 3 | 2 | 3 | 2 | 2 | 1 | 3 | 2 |
| NM_032848 | C12orf52 | 1775 | 3 | 2 | 1 | 1 | 1 | 3 | 2 | 3 | 1 | 1 | 1 | 2 | 1 |
| NM_032849 | C13orf33 | 2390 | 8 | 2 | 8 | 1 | 3 | 2 | 0 | 0 | 2 | 1 | 1 | 0 | 3 |
| NM_032852 | ATG4C | 1807 | 9 | 4 | 3 | 1 | 3 | 7 | 4 | 4 | 3 | 4 | 2 | 9 | 3 |
| NM_032853 | MUM1 | 4101 | 2 | 1 | 1 | 1 | 0 | 2 | 1 | 0 | 0 | 2 | 0 | 1 | 2 |

| transcript | gene | transcript length (nt) | max | mean | adipose | colon | heart | hypothalamus | kidney | liver | lung | ovary | skeletalmuscle | spleen | testes |
|---|---|---|---|---|---|---|---|---|---|---|---|---|---|---|---|
| NM_032854 | CORO6 | 2397 | 3 | 0 | 0 | 0 | 0 | 0 | 0 | 0 | 0 | 0 | 3 | 0 | 0 |
| NM_032855 | HSH2D | 2363 | 3 | 0 | 0 | 1 | 0 | 0 | 0 | 0 | 1 | 0 | 0 | 3 | 0 |
| NM_032856 | WDR73 | 1843 | 2 | 1 | 1 | 1 | 1 | 2 | 2 | 1 | 1 | 2 | 1 | 2 | 2 |
| NM_032857 | LACTB | 1939 | 8 | 5 | 4 | 1 | 7 | 3 | 6 | 7 | 7 | 2 | 5 | 8 | 4 |
| NM_032858 | MAEL | 1731 | 10 | 1 | 0 | 0 | 0 | 0 | 0 | 0 | 0 | 0 | 0 | 0 | 10 |
| NM_032859 | ABHD13 | 5302 | 3 | 1 | 1 | 0 | 1 | 1 | 1 | 1 | 2 | 1 | 1 | 3 | 1 |
| NM_032860 | LTV1 | 1814 | 21 | 14 | 12 | 5 | 15 | 15 | 11 | 21 | 7 | 12 | 15 | 20 | 18 |
| NM_032861 | SERAC1 | 3969 | 3 | 2 | 1 | 1 | 1 | 3 | 2 | 1 | 1 | 1 | 1 | 2 | 2 |
| NM_032862 | TIGD5 | 2411 | 0 | 0 | 0 | 0 | 0 | 0 | 0 | 0 | 0 | 0 | 0 | 0 | 0 |
| NM_032864 | PRPF38A | 2772 | 16 | 9 | 6 | 6 | 6 | 11 | 9 | 5 | 6 | 16 | 5 | 15 | 12 |
| NM_032865 | TNS4 | 4072 | 1 | 0 | 0 | 1 | 0 | 0 | 0 | 0 | 0 | 0 | 0 | 0 | 0 |
| NM_032866 | CGNL1 | 7218 | 62 | 11 | 1 | 1 | 8 | 6 | 62 | 14 | 9 | 6 | 1 | 0 | 11 |
| NM_032867 | MICALCL | 3044 | 1 | 0 | 0 | 0 | 0 | 0 | 0 | 0 | 1 | 0 | 0 | 1 | 1 |
| NM_032868 | MPND | 1566 | 2 | 1 | 1 | 2 | 0 | 0 | 2 | 0 | 1 | 0 | 0 | 1 | 0 |
| NM_032869 | NUDCD1 | 3953 | 3 | 2 | 1 | 0 | 2 | 2 | 2 | 1 | 1 | 2 | 1 | 3 | 3 |
| NM_032870 | SFRS18 | 3221 | 31 | 19 | 20 | 10 | 15 | 17 | 25 | 5 | 23 | 24 | 17 | 31 | 24 |
| NM_032871 | RELT | 3503 | 1 | 0 | 0 | 0 | 0 | 0 | 0 | 0 | 1 | 0 | 0 | 1 | 0 |
| NM_032872 | SYTL1 | 1898 | 0 | 0 | 0 | 0 | 0 | 0 | 0 | 0 | 0 | 0 | 0 | 0 | 0 |
| NM_032873 | UBASH3B | 6903 | 9 | 1 | 0 | 0 | 1 | 2 | 0 | 0 | 1 | 0 | 0 | 9 | 1 |
| NM_032875 | FBXL20 | 2405 | 7 | 3 | 4 | 3 | 1 | 5 | 2 | 2 | 3 | 3 | 2 | 3 | 7 |
| NM_032876 | JUB | 4232 | 2 | 1 | 0 | 0 | 0 | 0 | 2 | 1 | 1 | 1 | 0 | 0 | 0 |
| NM_032878 | ALKBH6 | 977 | 1 | 0 | 0 | 0 | 0 | 1 | 0 | 0 | 0 | 0 | 0 | 0 | 0 |
| NM_032880 | IGSF21 | 1946 | 6 | 1 | 1 | 0 | 1 | 6 | 0 | 0 | 0 | 0 | 0 | 4 | 0 |
| NM_032881 | LSM10 | 850 | 1 | 1 | 1 | 0 | 0 | 1 | 0 | 1 | 0 | 0 | 1 | 1 | 0 |
| NM_032882 | PNMA6A | 2273 | 1 | 0 | 0 | 0 | 0 | 1 | 1 | 0 | 0 | 0 | 0 | 0 | 0 |
| NM_032883 | TOX2 | 2542 | 1 | 0 | 0 | 0 | 0 | 1 | 0 | 0 | 1 | 0 | 0 | 0 | 1 |
| NM_032884 | C1orf94 | 1995 | 0 | 0 | 0 | 0 | 0 | 0 | 0 | 0 | 0 | 0 | 0 | 0 | 0 |
| NM_032885 | ATG4D | 1896 | 0 | 0 | 0 | 0 | 0 | 0 | 0 | 0 | 0 | 0 | 0 | 0 | 0 |
| NM_032888 | COL27A1 | 5583 | 3 | 1 | 0 | 1 | 1 | 0 | 3 | 0 | 0 | 1 | 0 | 0 | 1 |
| NM_032889 | MFSD5 | 1760 | 1 | 0 | 0 | 0 | 1 | 0 | 1 | 0 | 0 | 0 | 0 | 1 | 0 |
| NM_032890 | DISP1 | 4743 | 3 | 1 | 1 | 1 | 1 | 0 | 2 | 1 | 2 | 1 | 0 | 1 | 3 |
| NM_032892 | FRMD5 | 2243 | 1 | 0 | 0 | 0 | 0 | 1 | 0 | 0 | 0 | 0 | 0 | 0 | 0 |
| NM_032898 | C3orf34 | 2160 | 3 | 1 | 0 | 0 | 1 | 3 | 1 | 0 | 1 | 1 | 0 | 2 | 2 |
| NM_032899 | FAM83A | 2929 | 0 | 0 | 0 | 0 | 0 | 0 | 0 | 0 | 0 | 0 | 0 | 0 | 0 |
| NM_032900 | ARHGAP19 | 5463 | 5 | 2 | 1 | 1 | 1 | 1 | 1 | 1 | 2 | 2 | 1 | 4 | 5 |
| NM_032901 | C12orf62 | 601 | 24 | 10 | 8 | 6 | 24 | 15 | 15 | 12 | 6 | 5 | 9 | 10 | 4 |
| NM_032902 | PPP1R16A | 2274 | 0 | 0 | 0 | 0 | 0 | 0 | 0 | 0 | 0 | 0 | 0 | 0 | 0 |
| NM_032905 | RBM17 | 3333 | 44 | 28 | 30 | 18 | 35 | 30 | 21 | 20 | 30 | 23 | 29 | 44 | 32 |
| NM_032906 | PIGY | 1353 | 8 | 6 | 6 | 3 | 7 | 8 | 7 | 7 | 5 | 7 | 5 | 7 | 7 |
| NM_032907 | UBL7 | 1418 | 2 | 1 | 1 | 1 | 1 | 2 | 1 | 1 | 1 | 1 | 1 | 1 | 1 |
| NM_032918 | RERG | 2217 | 6 | 2 | 3 | 1 | 1 | 1 | 4 | 0 | 2 | 3 | 0 | 6 | 5 |
| NM_032924 | ZNF3 | 2820 | 17 | 7 | 4 | 6 | 6 | 5 | 8 | 5 | 5 | 17 | 6 | 10 | 7 |
| NM_032926 | TCEAL3 | 1113 | 112 | 45 | 15 | 11 | 63 | 112 | 35 | 8 | 32 | 85 | 9 | 59 | 71 |
| NM_032927 | TMEM128 | 1732 | 1 | 0 | 0 | 0 | 0 | 1 | 1 | 0 | 0 | 0 | 0 | 0 | 1 |
| NM_032928 | TMEM141 | 858 | 4 | 2 | 1 | 3 | 2 | 1 | 4 | 2 | 1 | 0 | 0 | 1 | 1 |
| NM_032930 | C11orf70 | 1257 | 7 | 1 | 0 | 0 | 0 | 1 | 1 | 0 | 3 | 1 | 0 | 0 | 7 |
| NM_032932 | RAB11FIP4 | 8625 | 7 | 1 | 0 | 1 | 0 | 7 | 1 | 0 | 0 | 0 | 0 | 1 | 1 |
| NM_032933 | C18orf45 | 2992 | 0 | 0 | 0 | 0 | 0 | 0 | 0 | 0 | 0 | 0 | 0 | 0 | 0 |
| NM_032935 | MT4 | 294 | 0 | 0 | 0 | 0 | 0 | 0 | 0 | 0 | 0 | 0 | 0 | 0 | 0 |
| NM_032936 | TMEM60 | 872 | 4 | 2 | 1 | 0 | 1 | 1 | 2 | 2 | 1 | 3 | 2 | 4 | 3 |
| NM_032937 | C9orf37 | 1451 | 0 | 0 | 0 | 0 | 0 | 0 | 0 | 0 | 0 | 0 | 0 | 0 | 0 |
| NM_032940 | POLR2C | 1804 | 2 | 1 | 1 | 1 | 1 | 2 | 1 | 1 | 1 | 1 | 1 | 1 | 1 |
| NM_032941 | RECQL | 3581 | 8 | 4 | 5 | 2 | 4 | 2 | 3 | 4 | 5 | 3 | 2 | 8 | 3 |
| NM_032943 | SYTL2 | 4275 | 9 | 4 | 3 | 9 | 2 | 8 | 8 | 1 | 5 | 2 | 2 | 3 | 3 |
| NM_032944 | STK31 | 3513 | 9 | 1 | 0 | 0 | 0 | 0 | 0 | 0 | 0 | 0 | 0 | 0 | 9 |
| NM_032945 | TNFRSF6B | 1456 | 3 | 1 | 0 | 0 | 0 | 1 | 0 | 0 | 2 | 0 | 0 | 3 | 0 |
| NM_032946 | NXF5 | 1613 | 0 | 0 | 0 | 0 | 0 | 0 | 0 | 0 | 0 | 0 | 0 | 0 | 0 |
| NM_032947 | C5orf62 | 1975 | 3 | 1 | 3 | 1 | 2 | 0 | 1 | 0 | 2 | 1 | 1 | 2 | 1 |
| NM_032949 | PCDH8 | 3711 | 2 | 0 | 0 | 0 | 0 | 2 | 0 | 0 | 0 | 0 | 0 | 0 | 0 |
| NM_032951 | MLXIPL | 3278 | 5 | 1 | 2 | 0 | 0 | 1 | 3 | 5 | 0 | 0 | 1 | 0 | 1 |
| NM_032952 | MLXIPL | 3221 | 5 | 1 | 2 | 0 | 0 | 1 | 3 | 5 | 0 | 0 | 1 | 0 | 1 |
| NM_032953 | MLXIPL | 3272 | 5 | 1 | 2 | 0 | 0 | 1 | 3 | 5 | 0 | 0 | 1 | 0 | 1 |
| NM_032954 | MLXIPL | 3215 | 5 | 1 | 2 | 0 | 0 | 1 | 3 | 5 | 0 | 0 | 1 | 0 | 1 |
| NM_032955 | AIF1 | 503 | 110 | 22 | 23 | 3 | 22 | 38 | 8 | 7 | 26 | 5 | 2 | 110 | 4 |
| NM_032957 | RTEL1 | 4636 | 0 | 0 | 0 | 0 | 0 | 0 | 0 | 0 | 0 | 0 | 0 | 0 | 0 |
| NM_032959 | POLR2J2 | 1714 | 1 | 1 | 1 | 1 | 1 | 1 | 1 | 0 | 1 | 1 | 1 | 0 | 0 |
| NM_032960 | MAPKAPK2 | 3052 | 23 | 8 | 4 | 5 | 23 | 3 | 9 | 6 | 8 | 5 | 11 | 13 | 6 |
| NM_032961 | PCDH10 | 5366 | 9 | 1 | 0 | 0 | 1 | 9 | 1 | 0 | 0 | 1 | 0 | 0 | 1 |
| NM_032962 | CCL14 | 554 | 0 | 0 | 0 | 0 | 0 | 0 | 0 | 0 | 0 | 0 | 0 | 0 | 0 |
| NM_032963 | CCL14 | 506 | 0 | 0 | 0 | 0 | 0 | 0 | 0 | 0 | 0 | 0 | 0 | 0 | 0 |
| NM_032965 | CCL15 | 1050 | 0 | 0 | 0 | 0 | 0 | 0 | 0 | 0 | 0 | 0 | 0 | 0 | 0 |
| NM_032966 | CXCR5 | 2871 | 3 | 0 | 0 | 0 | 0 | 0 | 0 | 0 | 0 | 0 | 0 | 3 | 0 |
| NM_032967 | PCDH11X | 4767 | 0 | 0 | 0 | 0 | 0 | 0 | 0 | 0 | 0 | 0 | 0 | 0 | 0 |
| NM_032968 | PCDH11X | 9172 | 0 | 0 | 0 | 0 | 0 | 0 | 0 | 0 | 0 | 0 | 0 | 0 | 0 |
| NM_032969 | PCDH11X | 9142 | 0 | 0 | 0 | 0 | 0 | 0 | 0 | 0 | 0 | 0 | 0 | 0 | 0 |
| NM_032970 | SEC22C | 1378 | 5 | 2 | 2 | 1 | 3 | 5 | 2 | 1 | 2 | 2 | 1 | 3 | 3 |
| NM_032971 | PCDH11Y | 4710 | 2 | 0 | 0 | 0 | 0 | 2 | 0 | 0 | 0 | 0 | 0 | 0 | 0 |

| | | transcript | | | | | | hypothala | | | | | skeletalm | | |
|---|---|---|---|---|---|---|---|---|---|---|---|---|---|---|---|
| transcript | gene | length (nt) | max | mean | adipose | colon | heart | mus | kidney | liver | lung | ovary | uscle | spleen | testes |
| NM_032972 | PCDH11Y | 4964 | 2 | 0 | 0 | 0 | 0 | 2 | 0 | 0 | 0 | 0 | 0 | 0 | 0 |
| NM_032973 | PCDH11Y | 9038 | 1 | 0 | 0 | 0 | 0 | 1 | 0 | 0 | 0 | 0 | 0 | 0 | 0 |
| NM_032974 | CASP10 | 2288 | 7 | 2 | 3 | 2 | 2 | 1 | 2 | 2 | 5 | 1 | 1 | 7 | 1 |
| NM_032975 | DTNA | 6522 | 17 | 3 | 1 | 1 | 4 | 17 | 0 | 0 | 1 | 1 | 4 | 1 | 2 |
| NM_032977 | CASP10 | 5906 | 4 | 1 | 2 | 1 | 1 | 0 | 1 | 1 | 3 | 1 | 0 | 4 | 1 |
| NM_032978 | DTNA | 4570 | 19 | 3 | 1 | 1 | 7 | 19 | 0 | 0 | 1 | 1 | 4 | 1 | 2 |
| NM_032979 | DTNA | 4544 | 17 | 3 | 1 | 1 | 7 | 17 | 0 | 0 | 1 | 1 | 4 | 1 | 2 |
| NM_032980 | DTNA | 5674 | 12 | 2 | 1 | 1 | 1 | 12 | 0 | 0 | 1 | 0 | 2 | 1 | 1 |
| NM_032981 | DTNA | 3618 | 11 | 2 | 0 | 0 | 3 | 11 | 0 | 0 | 0 | 0 | 2 | 0 | 1 |
| NM_032982 | CASP2 | 4127 | 4 | 2 | 1 | 2 | 1 | 1 | 2 | 0 | 2 | 3 | 1 | 4 | 2 |
| NM_032983 | CASP2 | 3909 | 4 | 2 | 1 | 2 | 1 | 1 | 2 | 1 | 2 | 3 | 1 | 4 | 2 |
| NM_032985 | SEC23B | 3431 | 7 | 4 | 2 | 2 | 2 | 5 | 7 | 5 | 4 | 2 | 0 | 6 | 5 |
| NM_032986 | SEC23B | 3110 | 8 | 4 | 2 | 2 | 3 | 5 | 8 | 6 | 4 | 2 | 0 | 7 | 5 |
| NM_032989 | BAD | 969 | 1 | 0 | 1 | 1 | 0 | 1 | 1 | 0 | 0 | 0 | 0 | 0 | 0 |
| NM_032991 | CASP3 | 2506 | 3 | 1 | 1 | 1 | 1 | 1 | 1 | 1 | 2 | 1 | 1 | 3 | 1 |
| NM_032992 | CASP6 | 1394 | 1 | 1 | 0 | 1 | 0 | 0 | 1 | 1 | 1 | 1 | 0 | 1 | 0 |
| NM_032993 | GAR1 | 1008 | 20 | 11 | 6 | 9 | 14 | 8 | 14 | 6 | 9 | 15 | 9 | 20 | 15 |
| NM_032995 | ARHGEF4 | 4257 | 3 | 0 | 0 | 0 | 0 | 3 | 0 | 0 | 0 | 1 | 0 | 0 | 0 |
| NM_032996 | CASP9 | 1539 | 1 | 1 | 1 | 1 | 0 | 1 | 1 | 0 | 0 | 1 | 0 | 1 | 1 |
| NM_032997 | ZWINT | 1851 | 3 | 1 | 1 | 3 | 1 | 0 | 1 | 0 | 1 | 1 | 0 | 1 | 2 |
| NM_032998 | DEDD | 2361 | 2 | 2 | 1 | 1 | 1 | 1 | 2 | 1 | 1 | 2 | 2 | 2 | 2 |
| NM_032999 | GTF2I | 4529 | 8 | 5 | 5 | 3 | 5 | 5 | 8 | 5 | 4 | 7 | 6 | 7 | 5 |
| NM_033000 | GTF2I | 4469 | 9 | 6 | 5 | 3 | 5 | 5 | 9 | 5 | 4 | 7 | 6 | 7 | 5 |
| NM_033001 | GTF2I | 4466 | 9 | 6 | 5 | 3 | 5 | 5 | 9 | 5 | 4 | 7 | 6 | 7 | 5 |
| NM_033004 | NLRP1 | 5613 | 3 | 1 | 2 | 1 | 1 | 0 | 0 | 0 | 3 | 0 | 0 | 3 | 1 |
| NM_033006 | NLRP1 | 5523 | 3 | 1 | 2 | 1 | 1 | 0 | 0 | 0 | 3 | 1 | 1 | 3 | 1 |
| NM_033007 | NLRP1 | 5391 | 3 | 1 | 3 | 1 | 1 | 0 | 0 | 0 | 3 | 1 | 1 | 3 | 1 |
| NM_033008 | PCBP4 | 2007 | 1 | 0 | 0 | 0 | 1 | 1 | 0 | 0 | 0 | 0 | 1 | 0 | 0 |
| NM_033010 | PCBP4 | 1962 | 1 | 0 | 0 | 0 | 1 | 1 | 0 | 0 | 0 | 0 | 1 | 0 | 0 |
| NM_033011 | PLAT | 3035 | 8 | 3 | 1 | 3 | 3 | 1 | 8 | 0 | 3 | 5 | 1 | 0 | 5 |
| NM_033012 | TNFSF11 | 2359 | 0 | 0 | 0 | 0 | 0 | 0 | 0 | 0 | 0 | 0 | 0 | 0 | 0 |
| NM_033013 | NR1I2 | 4306 | 4 | 1 | 0 | 1 | 0 | 0 | 0 | 4 | 0 | 0 | 0 | 0 | 0 |
| NM_033014 | OGN | 3067 | 26 | 5 | 9 | 2 | 8 | 6 | 1 | 0 | 3 | 26 | 1 | 1 | 4 |
| NM_033015 | FASTK | 1429 | 1 | 0 | 0 | 0 | 0 | 0 | 1 | 0 | 0 | 0 | 0 | 0 | 0 |
| NM_033016 | PDGFB | 2378 | 2 | 1 | 1 | 1 | 1 | 1 | 1 | 0 | 2 | 0 | 0 | 0 | 0 |
| NM_033017 | TRIM4 | 3383 | 5 | 3 | 2 | 2 | 2 | 2 | 3 | 2 | 3 | 5 | 1 | 5 | 4 |
| NM_033018 | CDK16 | 3258 | 7 | 4 | 3 | 4 | 5 | 7 | 5 | 2 | 3 | 2 | 6 | 3 | 6 |
| NM_033020 | TRIM33 | 8288 | 10 | 7 | 5 | 4 | 5 | 9 | 8 | 5 | 7 | 10 | 6 | 10 | 10 |
| NM_033022 | RPS24 | 653 | 613 | 272 | 346 | 137 | 254 | 281 | 160 | 318 | 205 | 613 | 239 | 260 | 180 |
| NM_033023 | PDGFA | 2736 | 4 | 2 | 1 | 1 | 2 | 4 | 1 | 1 | 2 | 0 | 1 | 3 | 1 |
| NM_033025 | SYDE1 | 3254 | 0 | 0 | 0 | 0 | 0 | 0 | 0 | 0 | 0 | 0 | 0 | 0 | 0 |
| NM_033026 | PCLO | 20288 | 14 | 2 | 1 | 1 | 1 | 14 | 3 | 0 | 0 | 0 | 0 | 0 | 2 |
| NM_033027 | CSRNP1 | 3186 | 9 | 3 | 3 | 1 | 2 | 2 | 2 | 9 | 5 | 6 | 3 | 3 | 2 |
| NM_033028 | BBS4 | 2491 | 2 | 1 | 1 | 0 | 1 | 2 | 2 | 1 | 1 | 1 | 1 | 1 | 1 |
| NM_033029 | LMLN | 7041 | 2 | 1 | 1 | 0 | 1 | 1 | 1 | 0 | 0 | 1 | 0 | 1 | 2 |
| NM_033030 | BOLL | 2838 | 2 | 0 | 0 | 0 | 0 | 0 | 0 | 0 | 0 | 0 | 0 | 0 | 2 |
| NM_033031 | CCNB3 | 4511 | 2 | 0 | 0 | 0 | 0 | 0 | 0 | 0 | 0 | 0 | 0 | 0 | 2 |
| NM_033032 | KRTAP2-2 | 711 | 0 | 0 | 0 | 0 | 0 | 0 | 0 | 0 | 0 | 0 | 0 | 0 | 0 |
| NM_033033 | KRT82 | 2681 | 0 | 0 | 0 | 0 | 0 | 0 | 0 | 0 | 0 | 0 | 0 | 0 | 0 |
| NM_033034 | TRIM5 | 2990 | 4 | 2 | 1 | 2 | 1 | 1 | 2 | 2 | 2 | 3 | 1 | 4 | 2 |
| NM_033035 | TSLP | 2629 | 0 | 0 | 0 | 0 | 0 | 0 | 0 | 0 | 0 | 0 | 0 | 0 | 0 |
| NM_033036 | GAL3ST3 | 3320 | 1 | 0 | 0 | 0 | 0 | 1 | 0 | 0 | 0 | 0 | 0 | 0 | 0 |
| NM_033043 | CGB5 | 880 | 0 | 0 | 0 | 0 | 0 | 0 | 0 | 0 | 0 | 0 | 0 | 0 | 0 |
| NM_033044 | MACF1 | 19271 | 49 | 25 | 22 | 10 | 33 | 29 | 19 | 11 | 49 | 31 | 10 | 32 | 24 |
| NM_033045 | KRT84 | 2073 | 0 | 0 | 0 | 0 | 0 | 0 | 0 | 0 | 0 | 0 | 0 | 0 | 0 |
| NM_033046 | RTKN | 2621 | 3 | 1 | 0 | 1 | 1 | 2 | 3 | 3 | 0 | 0 | 0 | 0 | 0 |
| NM_033048 | CPXCR1 | 1629 | 0 | 0 | 0 | 0 | 0 | 0 | 0 | 0 | 0 | 0 | 0 | 0 | 0 |
| NM_033049 | MUC13 | 2861 | 18 | 2 | 0 | 18 | 0 | 0 | 2 | 0 | 0 | 0 | 0 | 0 | 0 |
| NM_033050 | SUCNR1 | 1650 | 10 | 2 | 2 | 0 | 0 | 0 | 10 | 0 | 0 | 0 | 0 | 5 | 1 |
| NM_033051 | SLC46A2 | 2501 | 1 | 0 | 0 | 0 | 0 | 0 | 0 | 0 | 1 | 0 | 0 | 0 | 0 |
| NM_033053 | DMRTC1 | 1100 | 0 | 0 | 0 | 0 | 0 | 0 | 0 | 0 | 0 | 0 | 0 | 0 | 0 |
| NM_033054 | MYO1G | 3279 | 1 | 0 | 0 | 0 | 0 | 0 | 0 | 0 | 1 | 0 | 0 | 1 | 0 |
| NM_033055 | HIAT1 | 2637 | 3 | 2 | 1 | 1 | 2 | 3 | 2 | 1 | 2 | 3 | 1 | 3 | 3 |
| NM_033056 | PCDH15 | 7021 | 1 | 0 | 0 | 0 | 0 | 1 | 0 | 0 | 0 | 0 | 0 | 0 | 0 |
| NM_033057 | OR2B2 | 1074 | 0 | 0 | 0 | 0 | 0 | 0 | 0 | 0 | 0 | 0 | 0 | 0 | 0 |
| NM_033058 | TRIM55 | 2851 | 8 | 2 | 0 | 0 | 8 | 0 | 1 | 3 | 0 | 0 | 4 | 0 | 1 |
| NM_033059 | KRTAP4-11 | 1171 | 0 | 0 | 0 | 0 | 0 | 0 | 0 | 0 | 0 | 0 | 0 | 0 | 0 |
| NM_033060 | KRTAP4-1 | 739 | 0 | 0 | 0 | 0 | 0 | 0 | 0 | 0 | 0 | 0 | 0 | 0 | 0 |
| NM_033061 | KRTAP4-7 | 936 | 0 | 0 | 0 | 0 | 0 | 0 | 0 | 0 | 0 | 0 | 0 | 0 | 0 |
| NM_033062 | KRTAP4-2 | 763 | 0 | 0 | 0 | 0 | 0 | 0 | 0 | 0 | 0 | 0 | 0 | 0 | 0 |
| NM_033063 | MAP6 | 2648 | 7 | 1 | 0 | 1 | 0 | 7 | 1 | 0 | 0 | 1 | 0 | 0 | 1 |
| NM_033064 | ATCAY | 5036 | 7 | 1 | 0 | 0 | 0 | 7 | 0 | 0 | 0 | 0 | 1 | 0 | 0 |
| NM_033066 | MPP4 | 2458 | 0 | 0 | 0 | 0 | 0 | 0 | 0 | 0 | 0 | 0 | 0 | 0 | 0 |
| NM_033067 | DMRTB1 | 1907 | 3 | 0 | 0 | 0 | 0 | 0 | 0 | 0 | 0 | 0 | 0 | 0 | 3 |
| NM_033068 | ACPT | 1347 | 0 | 0 | 0 | 0 | 0 | 0 | 0 | 0 | 0 | 0 | 0 | 0 | 0 |
| NM_033069 | C6orf114 | 1527 | 2 | 1 | 0 | 1 | 1 | 0 | 0 | 1 | 1 | 0 | 0 | 2 | 0 |
| NM_033070 | CECR5 | 1801 | 1 | 0 | 0 | 1 | 1 | 0 | 1 | 1 | 0 | 0 | 0 | 1 | 0 |

| transcript | gene | transcript length (nt) | max | mean | adipose | colon | heart | hypothalamus | kidney | liver | lung | ovary | skeletalmuscle | spleen | testes |
|---|---|---|---|---|---|---|---|---|---|---|---|---|---|---|---|
| NM_033071 | SYNE1 | 27436 | 20 | 10 | 6 | 2 | 9 | 17 | 11 | 2 | 13 | 20 | 4 | 16 | 14 |
| NM_033081 | DIDO1 | 8544 | 5 | 3 | 3 | 4 | 3 | 3 | 4 | 3 | 3 | 4 | 3 | 5 | 4 |
| NM_033082 | SARNP | 926 | 62 | 36 | 43 | 20 | 45 | 62 | 28 | 32 | 26 | 30 | 31 | 37 | 46 |
| NM_033083 | EAF1 | 4488 | 4 | 3 | 2 | 2 | 2 | 4 | 3 | 4 | 2 | 2 | 1 | 4 | 2 |
| NM_033084 | FANCD2 | 5204 | 1 | 0 | 0 | 1 | 0 | 0 | 0 | 0 | 1 | 1 | 0 | 1 | 1 |
| NM_033085 | FATE1 | 1069 | 61 | 6 | 0 | 0 | 0 | 0 | 0 | 0 | 0 | 0 | 0 | 0 | 61 |
| NM_033086 | FGD3 | 3353 | 2 | 0 | 0 | 0 | 0 | 0 | 0 | 0 | 1 | 0 | 0 | 2 | 0 |
| NM_033087 | ALG2 | 2830 | 8 | 5 | 3 | 2 | 3 | 4 | 7 | 8 | 4 | 5 | 2 | 6 | 6 |
| NM_033088 | FAM40A | 3262 | 3 | 2 | 1 | 1 | 1 | 3 | 1 | 1 | 1 | 1 | 1 | 3 | 2 |
| NM_033089 | ZCCHC3 | 2760 | 6 | 3 | 2 | 2 | 3 | 4 | 3 | 2 | 2 | 6 | 1 | 6 | 5 |
| NM_033090 | GREB1 | 2347 | 35 | 4 | 0 | 0 | 0 | 1 | 0 | 0 | 0 | 35 | 0 | 0 | 3 |
| NM_033091 | TRIM4 | 3305 | 5 | 3 | 2 | 2 | 2 | 2 | 3 | 2 | 3 | 5 | 1 | 5 | 4 |
| NM_033092 | TRIM5 | 1685 | 6 | 3 | 2 | 3 | 2 | 1 | 3 | 3 | 3 | 5 | 1 | 6 | 3 |
| NM_033093 | TRIM5 | 1763 | 6 | 3 | 2 | 3 | 2 | 1 | 3 | 3 | 3 | 5 | 1 | 6 | 3 |
| NM_033100 | PCDH21 | 5324 | 4 | 1 | 0 | 4 | 0 | 2 | 0 | 0 | 0 | 0 | 0 | 0 | 0 |
| NM_033101 | LGALS12 | 1733 | 2 | 0 | 2 | 0 | 0 | 0 | 0 | 0 | 0 | 0 | 0 | 0 | 0 |
| NM_033102 | SLC45A3 | 3382 | 1 | 0 | 0 | 0 | 0 | 1 | 0 | 0 | 0 | 0 | 0 | 1 | 0 |
| NM_033103 | RHPN2 | 3530 | 4 | 1 | 0 | 2 | 0 | 1 | 4 | 3 | 1 | 0 | 0 | 0 | 0 |
| NM_033104 | STON2 | 2919 | 2 | 1 | 1 | 0 | 0 | 2 | 2 | 0 | 0 | 0 | 0 | 2 | 0 |
| NM_033105 | DNAJC5B | 1380 | 2 | 0 | 0 | 0 | 0 | 0 | 0 | 0 | 0 | 0 | 0 | 0 | 2 |
| NM_033106 | GALP | 938 | 0 | 0 | 0 | 0 | 0 | 0 | 0 | 0 | 0 | 0 | 0 | 0 | 0 |
| NM_033107 | GTPBP10 | 3412 | 3 | 2 | 2 | 1 | 2 | 2 | 2 | 2 | 1 | 3 | 2 | 2 | 3 |
| NM_033108 | HSFY1 | 1427 | 0 | 0 | 0 | 0 | 0 | 0 | 0 | 0 | 0 | 0 | 0 | 0 | 0 |
| NM_033109 | PNPT1 | 4579 | 5 | 3 | 2 | 1 | 4 | 4 | 4 | 5 | 2 | 2 | 4 | 4 | 4 |
| NM_033110 | A2LD1 | 1227 | 1 | 0 | 0 | 0 | 0 | 0 | 1 | 0 | 0 | 0 | 0 | 0 | 0 |
| NM_033111 | N4BP2L2 | 2482 | 26 | 17 | 12 | 7 | 21 | 18 | 17 | 7 | 18 | 19 | 26 | 19 | 19 |
| NM_033112 | C6orf153 | 1159 | 10 | 6 | 6 | 3 | 6 | 6 | 7 | 10 | 3 | 2 | 5 | 6 | 5 |
| NM_033113 | ZNF628 | 3847 | 0 | 0 | 0 | 0 | 0 | 0 | 0 | 0 | 0 | 0 | 0 | 0 | 0 |
| NM_033114 | ZCRB1 | 1844 | 11 | 6 | 7 | 2 | 8 | 10 | 7 | 4 | 4 | 5 | 5 | 7 | 11 |
| NM_033115 | TBCK | 3352 | 4 | 2 | 1 | 1 | 1 | 2 | 3 | 0 | 2 | 4 | 1 | 3 | 3 |
| NM_033116 | NEK9 | 5542 | 11 | 7 | 8 | 5 | 10 | 6 | 5 | 7 | 4 | 11 | 7 | 9 | 10 |
| NM_033117 | RBM18 | 3068 | 5 | 3 | 3 | 1 | 5 | 5 | 3 | 2 | 2 | 3 | 4 | 4 | 3 |
| NM_033118 | MYLK2 | 2793 | 12 | 1 | 0 | 0 | 0 | 0 | 0 | 0 | 0 | 0 | 12 | 0 | 0 |
| NM_033119 | NKD1 | 2586 | 2 | 1 | 0 | 1 | 0 | 1 | 1 | 0 | 2 | 1 | 0 | 1 | 1 |
| NM_033120 | NKD2 | 1798 | 0 | 0 | 0 | 0 | 0 | 0 | 0 | 0 | 0 | 0 | 0 | 0 | 0 |
| NM_033121 | ANKRD13A | 3908 | 10 | 4 | 3 | 3 | 5 | 3 | 5 | 3 | 5 | 4 | 2 | 10 | 7 |
| NM_033122 | C4orf35 | 1544 | 9 | 1 | 0 | 0 | 0 | 0 | 0 | 0 | 0 | 0 | 0 | 0 | 9 |
| NM_033123 | PLCZ1 | 2075 | 9 | 1 | 0 | 0 | 0 | 0 | 1 | 0 | 0 | 0 | 0 | 0 | 9 |
| NM_033124 | CCDC65 | 1814 | 16 | 2 | 0 | 0 | 0 | 2 | 1 | 0 | 2 | 1 | 0 | 1 | 16 |
| NM_033125 | SLC22A16 | 1969 | 2 | 0 | 0 | 0 | 0 | 0 | 0 | 0 | 0 | 0 | 0 | 0 | 2 |
| NM_033126 | PSKH2 | 1158 | 0 | 0 | 0 | 0 | 0 | 0 | 0 | 0 | 0 | 0 | 0 | 0 | 0 |
| NM_033127 | SEC16B | 4024 | 12 | 2 | 0 | 1 | 0 | 0 | 1 | 12 | 0 | 1 | 1 | 0 | 0 |
| NM_033128 | SCIN | 2571 | 16 | 3 | 2 | 3 | 0 | 7 | 16 | 0 | 1 | 0 | 0 | 0 | 1 |
| NM_033129 | SCRT2 | 3576 | 0 | 0 | 0 | 0 | 0 | 0 | 0 | 0 | 0 | 0 | 0 | 0 | 0 |
| NM_033130 | SIGLEC10 | 3788 | 5 | 1 | 0 | 0 | 0 | 1 | 0 | 1 | 1 | 0 | 0 | 5 | 0 |
| NM_033131 | WNT3A | 2932 | 0 | 0 | 0 | 0 | 0 | 0 | 0 | 0 | 0 | 0 | 0 | 0 | 0 |
| NM_033132 | ZIC5 | 4597 | 1 | 0 | 0 | 0 | 0 | 0 | 0 | 0 | 0 | 0 | 0 | 0 | 1 |
| NM_033133 | CNP | 5222 | 15 | 2 | 1 | 1 | 1 | 15 | 1 | 1 | 1 | 1 | 0 | 1 | 1 |
| NM_033135 | PDGFD | 3975 | 6 | 1 | 2 | 0 | 4 | 0 | 1 | 0 | 0 | 6 | 0 | 1 | 1 |
| NM_033136 | FGF1 | 4041 | 41 | 7 | 5 | 1 | 7 | 41 | 15 | 0 | 1 | 0 | 1 | 1 | 1 |
| NM_033137 | FGF1 | 3507 | 47 | 8 | 6 | 1 | 8 | 47 | 17 | 0 | 1 | 0 | 1 | 1 | 1 |
| NM_033138 | CALD1 | 5231 | 132 | 71 | 48 | 45 | 118 | 28 | 60 | 48 | 56 | 132 | 24 | 98 | 127 |
| NM_033139 | CALD1 | 4281 | 129 | 62 | 48 | 28 | 89 | 22 | 54 | 48 | 49 | 129 | 20 | 97 | 97 |
| NM_033140 | CALD1 | 4203 | 131 | 63 | 49 | 28 | 90 | 22 | 55 | 49 | 49 | 131 | 20 | 99 | 98 |
| NM_033141 | MAP3K9 | 5600 | 2 | 0 | 0 | 0 | 0 | 2 | 0 | 0 | 0 | 0 | 0 | 0 | 0 |
| NM_033142 | CGB7 | 880 | 0 | 0 | 0 | 0 | 0 | 0 | 0 | 0 | 0 | 0 | 0 | 0 | 0 |
| NM_033143 | FGF5 | 5285 | 0 | 0 | 0 | 0 | 0 | 0 | 0 | 0 | 0 | 0 | 0 | 0 | 0 |
| NM_033147 | DTNB | 2384 | 11 | 3 | 1 | 2 | 1 | 11 | 3 | 1 | 2 | 3 | 1 | 2 | 2 |
| NM_033148 | DTNB | 2294 | 11 | 3 | 1 | 2 | 1 | 11 | 3 | 1 | 2 | 3 | 1 | 2 | 3 |
| NM_033150 | COL2A1 | 4880 | 0 | 0 | 0 | 0 | 0 | 0 | 0 | 0 | 0 | 0 | 0 | 0 | 0 |
| NM_033151 | ABCC11 | 4862 | 0 | 0 | 0 | 0 | 0 | 0 | 0 | 0 | 0 | 0 | 0 | 0 | 0 |
| NM_033157 | CALD1 | 4541 | 130 | 62 | 47 | 29 | 88 | 22 | 54 | 46 | 50 | 130 | 20 | 100 | 96 |
| NM_033158 | HYAL2 | 1974 | 4 | 1 | 1 | 0 | 1 | 0 | 1 | 1 | 1 | 0 | 0 | 4 | 1 |
| NM_033159 | HYAL1 | 2034 | 9 | 2 | 0 | 0 | 2 | 0 | 5 | 9 | 1 | 0 | 0 | 4 | 0 |
| NM_033160 | ZNF658 | 4025 | 0 | 0 | 0 | 0 | 0 | 0 | 0 | 0 | 0 | 0 | 0 | 0 | 0 |
| NM_033161 | SURF4 | 2969 | 43 | 12 | 14 | 6 | 12 | 7 | 13 | 43 | 8 | 7 | 5 | 11 | 10 |
| NM_033163 | FGF8 | 1104 | 0 | 0 | 0 | 0 | 0 | 0 | 0 | 0 | 0 | 0 | 0 | 0 | 0 |
| NM_033164 | FGF8 | 1071 | 0 | 0 | 0 | 0 | 0 | 0 | 0 | 0 | 0 | 0 | 0 | 0 | 0 |
| NM_033165 | FGF8 | 984 | 1 | 0 | 0 | 0 | 1 | 0 | 0 | 0 | 0 | 0 | 0 | 0 | 0 |
| NM_033167 | B3GALNT1 | 3437 | 6 | 2 | 0 | 0 | 5 | 6 | 3 | 0 | 1 | 2 | 0 | 1 | 2 |
| NM_033168 | B3GALNT1 | 3223 | 7 | 2 | 1 | 0 | 6 | 7 | 3 | 0 | 1 | 2 | 0 | 1 | 2 |
| NM_033169 | B3GALNT1 | 3291 | 7 | 2 | 0 | 0 | 6 | 7 | 3 | 0 | 1 | 2 | 0 | 1 | 2 |
| NM_033170 | B3GALT5 | 2629 | 1 | 0 | 0 | 1 | 0 | 0 | 0 | 0 | 0 | 0 | 0 | 0 | 0 |
| NM_033171 | B3GALT5 | 2871 | 1 | 0 | 0 | 1 | 0 | 0 | 0 | 0 | 0 | 0 | 0 | 0 | 0 |
| NM_033172 | B3GALT5 | 2711 | 1 | 0 | 0 | 1 | 0 | 0 | 0 | 0 | 0 | 0 | 0 | 0 | 0 |
| NM_033173 | B3GALT5 | 3409 | 1 | 0 | 0 | 1 | 0 | 0 | 0 | 0 | 0 | 0 | 0 | 0 | 0 |
| NM_033176 | NKX2-4 | 1619 | 0 | 0 | 0 | 0 | 0 | 0 | 0 | 0 | 0 | 0 | 0 | 0 | 0 |

| transcript | gene | transcript length (nt) | max | mean | adipose | colon | heart | hypothalamus | kidney | liver | lung | ovary | skeletalmuscle | spleen | testes |
|---|---|---|---|---|---|---|---|---|---|---|---|---|---|---|---|
| NM_033177 | BAT4 | 1897 | 3 | 2 | 2 | 2 | 2 | 2 | 2 | 1 | 2 | 3 | 3 | 3 | 3 |
| NM_033178 | DUX4 | 1603 | 0 | 0 | 0 | 0 | 0 | 0 | 0 | 0 | 0 | 0 | 0 | 0 | 0 |
| NM_033178 | DUX4 | 1604 | 0 | 0 | 0 | 0 | 0 | 0 | 0 | 0 | 0 | 0 | 0 | 0 | 0 |
| NM_033178 | DUX4 | 1617 | 0 | 0 | 0 | 0 | 0 | 0 | 0 | 0 | 0 | 0 | 0 | 0 | 0 |
| NM_033179 | OR51B4 | 933 | 0 | 0 | 0 | 0 | 0 | 0 | 0 | 0 | 0 | 0 | 0 | 0 | 0 |
| NM_033180 | OR51B2 | 1055 | 0 | 0 | 0 | 0 | 0 | 0 | 0 | 0 | 0 | 0 | 0 | 0 | 0 |
| NM_033181 | CNR1 | 5371 | 8 | 1 | 1 | 0 | 0 | 8 | 0 | 0 | 1 | 0 | 0 | 1 | 1 |
| NM_033182 | FBXO44 | 2948 | 1 | 0 | 0 | 0 | 0 | 1 | 0 | 0 | 0 | 0 | 0 | 0 | 0 |
| NM_033183 | CGB8 | 887 | 0 | 0 | 0 | 0 | 0 | 0 | 0 | 0 | 0 | 0 | 0 | 0 | 0 |
| NM_033184 | KRTAP2-4 | 763 | 0 | 0 | 0 | 0 | 0 | 0 | 0 | 0 | 0 | 0 | 0 | 0 | 0 |
| NM_033185 | KRTAP3-3 | 704 | 0 | 0 | 0 | 0 | 0 | 0 | 0 | 0 | 0 | 0 | 0 | 0 | 0 |
| NM_033187 | KRTAP4-3 | 942 | 0 | 0 | 0 | 0 | 0 | 0 | 0 | 0 | 0 | 0 | 0 | 0 | 0 |
| NM_033188 | KRTAP4-5 | 879 | 0 | 0 | 0 | 0 | 0 | 0 | 0 | 0 | 0 | 0 | 0 | 0 | 0 |
| NM_033191 | KRTAP9-4 | 967 | 0 | 0 | 0 | 0 | 0 | 0 | 0 | 0 | 0 | 0 | 0 | 0 | 0 |
| NM_033194 | HSPB9 | 616 | 0 | 0 | 0 | 0 | 0 | 0 | 0 | 0 | 0 | 0 | 0 | 0 | 0 |
| NM_033195 | LDHAL6B | 1668 | 11 | 1 | 0 | 0 | 0 | 0 | 0 | 0 | 0 | 0 | 0 | 0 | 11 |
| NM_033196 | ZNF682 | 3207 | 2 | 1 | 1 | 0 | 1 | 2 | 1 | 0 | 1 | 1 | 0 | 2 | 2 |
| NM_033197 | C20orf114 | 1727 | 8 | 1 | 0 | 0 | 0 | 0 | 0 | 0 | 8 | 0 | 0 | 0 | 0 |
| NM_033198 | PIGS | 2873 | 6 | 3 | 3 | 2 | 5 | 4 | 6 | 1 | 3 | 3 | 2 | 5 | 4 |
| NM_033199 | UCN2 | 1485 | 0 | 0 | 0 | 0 | 0 | 0 | 0 | 0 | 0 | 0 | 0 | 0 | 0 |
| NM_033200 | LMF2 | 2599 | 1 | 0 | 0 | 0 | 0 | 0 | 1 | 1 | 0 | 1 | 0 | 0 | 0 |
| NM_033201 | C16orf45 | 2230 | 17 | 4 | 3 | 2 | 5 | 17 | 3 | 1 | 1 | 2 | 3 | 1 | 4 |
| NM_033204 | ZNF101 | 1450 | 16 | 4 | 2 | 2 | 2 | 2 | 3 | 1 | 3 | 3 | 1 | 16 | 3 |
| NM_033207 | OPALIN | 3588 | 7 | 1 | 0 | 0 | 0 | 7 | 0 | 0 | 0 | 0 | 0 | 0 | 0 |
| NM_033208 | TIGD7 | 2569 | 7 | 3 | 2 | 1 | 3 | 3 | 2 | 0 | 1 | 6 | 2 | 3 | 7 |
| NM_033210 | ZNF502 | 3271 | 4 | 2 | 2 | 0 | 2 | 2 | 2 | 0 | 1 | 4 | 2 | 2 | 4 |
| NM_033211 | C5orf30 | 3010 | 5 | 2 | 2 | 1 | 1 | 5 | 1 | 0 | 1 | 1 | 1 | 1 | 2 |
| NM_033212 | CCDC102A | 2427 | 1 | 0 | 0 | 0 | 0 | 0 | 0 | 0 | 1 | 0 | 0 | 0 | 0 |
| NM_033213 | ZNF670 | 2005 | 1 | 1 | 0 | 0 | 0 | 1 | 1 | 0 | 0 | 1 | 0 | 1 | 1 |
| NM_033214 | GK2 | 1866 | 8 | 1 | 0 | 0 | 0 | 0 | 0 | 0 | 0 | 0 | 0 | 0 | 8 |
| NM_033215 | PPP1R3F | 2941 | 2 | 0 | 0 | 0 | 1 | 2 | 0 | 0 | 0 | 0 | 1 | 0 | 0 |
| NM_033219 | TRIM14 | 1479 | 5 | 2 | 1 | 2 | 0 | 1 | 2 | 3 | 1 | 0 | 0 | 5 | 1 |
| NM_033220 | TRIM14 | 1773 | 4 | 1 | 1 | 2 | 0 | 1 | 1 | 2 | 1 | 0 | 0 | 4 | 1 |
| NM_033221 | TRIM14 | 3693 | 8 | 2 | 2 | 2 | 1 | 1 | 4 | 3 | 2 | 1 | 0 | 8 | 1 |
| NM_033222 | PSIP1 | 3376 | 59 | 21 | 11 | 4 | 13 | 59 | 12 | 5 | 13 | 42 | 13 | 27 | 33 |
| NM_033223 | GABRG3 | 1677 | 2 | 0 | 0 | 0 | 0 | 2 | 0 | 0 | 0 | 0 | 0 | 0 | 0 |
| NM_033224 | PURB | 9069 | 5 | 3 | 2 | 2 | 3 | 5 | 3 | 1 | 3 | 4 | 2 | 3 | 3 |
| NM_033225 | CSMD1 | 14317 | 2 | 0 | 0 | 0 | 0 | 2 | 0 | 0 | 0 | 0 | 0 | 0 | 0 |
| NM_033226 | ABCC12 | 5168 | 0 | 0 | 0 | 0 | 0 | 0 | 0 | 0 | 0 | 0 | 0 | 0 | 0 |
| NM_033227 | TRIM23 | 2415 | 8 | 3 | 2 | 1 | 3 | 8 | 2 | 2 | 2 | 3 | 2 | 3 | 4 |
| NM_033228 | TRIM23 | 2386 | 8 | 3 | 2 | 1 | 2 | 8 | 2 | 2 | 2 | 3 | 2 | 3 | 3 |
| NM_033229 | TRIM15 | 2224 | 1 | 0 | 0 | 1 | 0 | 0 | 1 | 0 | 0 | 0 | 0 | 0 | 0 |
| NM_033238 | PML | 5595 | 2 | 1 | 1 | 1 | 1 | 0 | 1 | 0 | 1 | 1 | 1 | 2 | 1 |
| NM_033239 | PML | 3073 | 1 | 0 | 1 | 0 | 0 | 0 | 0 | 0 | 1 | 0 | 0 | 1 | 0 |
| NM_033240 | PML | 3714 | 1 | 0 | 0 | 0 | 0 | 0 | 0 | 0 | 1 | 0 | 0 | 1 | 0 |
| NM_033244 | PML | 3081 | 1 | 0 | 1 | 0 | 0 | 0 | 0 | 0 | 1 | 0 | 0 | 1 | 0 |
| NM_033246 | PML | 1835 | 4 | 2 | 1 | 1 | 2 | 0 | 1 | 0 | 3 | 2 | 1 | 4 | 2 |
| NM_033247 | PML | 1782 | 1 | 0 | 0 | 0 | 0 | 0 | 0 | 0 | 0 | 0 | 0 | 1 | 0 |
| NM_033249 | PML | 2094 | 4 | 1 | 1 | 1 | 2 | 0 | 1 | 0 | 3 | 2 | 1 | 4 | 2 |
| NM_033250 | PML | 2929 | 1 | 0 | 1 | 0 | 0 | 0 | 0 | 0 | 1 | 0 | 0 | 1 | 0 |
| NM_033251 | RPL13 | 1271 | 69 | 28 | 18 | 21 | 25 | 22 | 16 | 18 | 25 | 69 | 35 | 42 | 18 |
| NM_033253 | NT5C1B | 2682 | 2 | 0 | 0 | 0 | 0 | 0 | 0 | 0 | 0 | 0 | 0 | 0 | 2 |
| NM_033254 | BOC | 4280 | 2 | 1 | 1 | 1 | 0 | 0 | 0 | 0 | 0 | 2 | 0 | 0 | 1 |
| NM_033255 | EPSTI1 | 1508 | 17 | 7 | 9 | 3 | 8 | 3 | 6 | 2 | 8 | 7 | 5 | 17 | 7 |
| NM_033256 | PPP1R14A | 718 | 16 | 9 | 7 | 11 | 14 | 15 | 2 | 1 | 12 | 4 | 2 | 16 | 15 |
| NM_033257 | DGCR6L | 1158 | 1 | 0 | 0 | 0 | 0 | 0 | 0 | 1 | 0 | 0 | 0 | 0 | 0 |
| NM_033258 | GNG8 | 213 | 0 | 0 | 0 | 0 | 0 | 0 | 0 | 0 | 0 | 0 | 0 | 0 | 0 |
| NM_033259 | CAMK2N2 | 1360 | 1 | 0 | 0 | 0 | 0 | 1 | 0 | 0 | 0 | 0 | 0 | 0 | 0 |
| NM_033260 | FOXQ1 | 2319 | 1 | 0 | 0 | 0 | 0 | 0 | 1 | 0 | 0 | 0 | 0 | 0 | 0 |
| NM_033261 | IDI2 | 1359 | 35 | 4 | 1 | 0 | 1 | 1 | 0 | 1 | 0 | 1 | 35 | 1 | 1 |
| NM_033262 | SLC8A3 | 5262 | 10 | 1 | 0 | 0 | 0 | 5 | 0 | 0 | 0 | 0 | 10 | 0 | 0 |
| NM_033266 | ERN2 | 3620 | 1 | 0 | 0 | 1 | 0 | 0 | 0 | 0 | 0 | 0 | 0 | 0 | 0 |
| NM_033267 | IRX2 | 3055 | 2 | 1 | 1 | 0 | 0 | 0 | 2 | 0 | 2 | 0 | 0 | 0 | 1 |
| NM_033271 | BTBD6 | 1983 | 3 | 1 | 3 | 1 | 1 | 1 | 1 | 1 | 1 | 1 | 2 | 1 | 1 |
| NM_033272 | KCNH7 | 4225 | 2 | 0 | 0 | 0 | 0 | 2 | 0 | 0 | 0 | 0 | 0 | 1 | 0 |
| NM_033273 | ZNF479 | 2067 | 0 | 0 | 0 | 0 | 0 | 0 | 0 | 0 | 0 | 0 | 0 | 0 | 0 |
| NM_033274 | ADAM19 | 6466 | 5 | 1 | 0 | 2 | 4 | 0 | 0 | 1 | 1 | 1 | 0 | 5 | 1 |
| NM_033276 | XRCC6BP1 | 1160 | 4 | 1 | 1 | 1 | 1 | 1 | 1 | 0 | 1 | 1 | 2 | 1 | 4 |
| NM_033277 | LACRT | 508 | 0 | 0 | 0 | 0 | 0 | 0 | 0 | 0 | 0 | 0 | 0 | 0 | 0 |
| NM_033278 | TRIM3 | 2872 | 1 | 0 | 0 | 0 | 0 | 1 | 0 | 0 | 0 | 0 | 0 | 0 | 0 |
| NM_033280 | SEC11C | 781 | 134 | 34 | 13 | 11 | 19 | 72 | 31 | 134 | 19 | 10 | 4 | 39 | 17 |
| NM_033281 | MRPS36 | 1329 | 12 | 6 | 5 | 4 | 11 | 9 | 9 | 12 | 3 | 2 | 4 | 4 | 3 |
| NM_033282 | OPN4 | 2372 | 0 | 0 | 0 | 0 | 0 | 0 | 0 | 0 | 0 | 0 | 0 | 0 | 0 |
| NM_033284 | TBL1Y | 2407 | 0 | 0 | 0 | 0 | 0 | 0 | 0 | 0 | 0 | 0 | 0 | 0 | 0 |
| NM_033285 | TP53INP1 | 5613 | 8 | 3 | 1 | 1 | 1 | 0 | 2 | 5 | 4 | 3 | 1 | 8 | 5 |
| NM_033286 | C15orf23 | 1756 | 10 | 3 | 2 | 3 | 2 | 4 | 1 | 5 | 2 | 2 | 3 | 3 | 10 |
| NM_033288 | ZNF160 | 4219 | 29 | 10 | 7 | 3 | 8 | 8 | 7 | 2 | 6 | 16 | 4 | 29 | 18 |

| transcript | gene | transcript length (nt) | max | mean | adipose | colon | heart | hypothalamus | kidney | liver | lung | ovary | skeletalmuscle | spleen | testes |
|---|---|---|---|---|---|---|---|---|---|---|---|---|---|---|---|
| NM_033290 | MID1 | 6009 | 2 | 1 | 2 | 1 | 2 | 1 | 1 | 0 | 2 | 1 | 0 | 0 | 2 |
| NM_033292 | CASP1 | 1355 | 10 | 3 | 2 | 3 | 1 | 2 | 1 | 1 | 6 | 1 | 1 | 10 | 1 |
| NM_033293 | CASP1 | 1076 | 12 | 3 | 3 | 4 | 2 | 2 | 1 | 1 | 7 | 1 | 1 | 12 | 1 |
| NM_033294 | CASP1 | 932 | 5 | 1 | 1 | 1 | 1 | 1 | 0 | 0 | 2 | 0 | 0 | 5 | 0 |
| NM_033295 | CASP1 | 407 | 5 | 1 | 1 | 2 | 1 | 1 | 0 | 0 | 2 | 0 | 0 | 5 | 0 |
| NM_033296 | MRFAP1 | 1534 | 373 | 174 | 124 | 74 | 211 | 373 | 143 | 202 | 129 | 195 | 135 | 176 | 151 |
| NM_033297 | NLRP12 | 2621 | 1 | 0 | 0 | 0 | 0 | 0 | 0 | 0 | 0 | 0 | 0 | 1 | 0 |
| NM_033300 | LRP8 | 4097 | 6 | 1 | 0 | 0 | 0 | 2 | 0 | 0 | 0 | 0 | 0 | 0 | 6 |
| NM_033301 | RPL8 | 920 | 207 | 103 | 75 | 98 | 109 | 64 | 75 | 92 | 94 | 207 | 109 | 151 | 55 |
| NM_033302 | ADRA1A | 2089 | 3 | 1 | 1 | 0 | 1 | 1 | 0 | 3 | 0 | 0 | 0 | 1 | 0 |
| NM_033303 | ADRA1A | 2304 | 1 | 0 | 0 | 0 | 0 | 0 | 0 | 1 | 0 | 0 | 0 | 0 | 0 |
| NM_033304 | ADRA1A | 2001 | 11 | 2 | 4 | 0 | 2 | 2 | 0 | 11 | 1 | 0 | 0 | 3 | 0 |
| NM_033305 | VPS13A | 11262 | 10 | 5 | 3 | 2 | 7 | 5 | 8 | 2 | 4 | 3 | 10 | 7 | 8 |
| NM_033306 | CASP4 | 1338 | 22 | 8 | 8 | 4 | 6 | 3 | 5 | 11 | 13 | 6 | 2 | 22 | 3 |
| NM_033309 | B3GNT9 | 2705 | 1 | 0 | 0 | 0 | 0 | 0 | 0 | 0 | 0 | 1 | 0 | 0 | 0 |
| NM_033310 | KCNK4 | 1810 | 0 | 0 | 0 | 0 | 0 | 0 | 0 | 0 | 0 | 0 | 0 | 0 | 0 |
| NM_033312 | CDC14A | 2446 | 4 | 2 | 1 | 2 | 1 | 1 | 2 | 0 | 3 | 2 | 1 | 3 | 4 |
| NM_033313 | CDC14A | 2544 | 1 | 1 | 0 | 1 | 0 | 0 | 1 | 0 | 1 | 0 | 0 | 1 | 1 |
| NM_033315 | RASL10B | 3193 | 2 | 0 | 0 | 0 | 1 | 1 | 0 | 0 | 0 | 0 | 2 | 0 | 0 |
| NM_033316 | MFI2 | 1674 | 1 | 0 | 0 | 0 | 0 | 0 | 1 | 0 | 0 | 0 | 0 | 0 | 0 |
| NM_033317 | DMKN | 1933 | 1 | 0 | 0 | 0 | 0 | 1 | 1 | 0 | 1 | 0 | 0 | 0 | 0 |
| NM_033318 | C22orf32 | 1567 | 2 | 1 | 1 | 0 | 2 | 1 | 2 | 1 | 1 | 2 | 1 | 1 | 1 |
| NM_033319 | CENPL | 2477 | 1 | 0 | 0 | 0 | 0 | 0 | 0 | 0 | 0 | 0 | 0 | 1 | 0 |
| NM_033326 | SOX6 | 8864 | 2 | 1 | 0 | 0 | 1 | 1 | 2 | 0 | 0 | 0 | 1 | 0 | 1 |
| NM_033328 | CAPZA3 | 1078 | 8 | 1 | 0 | 0 | 0 | 0 | 0 | 0 | 0 | 0 | 0 | 0 | 8 |
| NM_033329 | SIGLEC12 | 1736 | 2 | 0 | 0 | 0 | 0 | 0 | 0 | 1 | 0 | 0 | 0 | 2 | 0 |
| NM_033331 | CDC14B | 5589 | 4 | 2 | 3 | 1 | 4 | 3 | 3 | 4 | 1 | 2 | 0 | 1 | 3 |
| NM_033334 | NR6A1 | 1902 | 3 | 0 | 0 | 0 | 0 | 0 | 0 | 0 | 0 | 0 | 0 | 0 | 3 |
| NM_033337 | CAV3 | 1421 | 3 | 0 | 0 | 0 | 1 | 0 | 0 | 0 | 0 | 0 | 3 | 0 | 0 |
| NM_033338 | CASP7 | 2696 | 9 | 5 | 4 | 8 | 4 | 2 | 5 | 4 | 8 | 5 | 3 | 9 | 4 |
| NM_033339 | CASP7 | 2663 | 10 | 5 | 4 | 8 | 4 | 2 | 5 | 4 | 8 | 5 | 3 | 10 | 4 |
| NM_033340 | CASP7 | 2417 | 10 | 6 | 5 | 8 | 5 | 2 | 5 | 4 | 9 | 6 | 3 | 10 | 4 |
| NM_033341 | BIRC8 | 2022 | 2 | 0 | 0 | 0 | 0 | 0 | 0 | 0 | 0 | 0 | 0 | 0 | 2 |
| NM_033342 | TRIM7 | 1107 | 1 | 0 | 0 | 0 | 0 | 0 | 0 | 0 | 0 | 0 | 1 | 0 | 0 |
| NM_033343 | LHX4 | 1870 | 0 | 0 | 0 | 0 | 0 | 0 | 0 | 0 | 0 | 0 | 0 | 0 | 0 |
| NM_033345 | RGS8 | 783 | 1 | 0 | 0 | 0 | 0 | 1 | 0 | 0 | 0 | 0 | 0 | 0 | 0 |
| NM_033347 | KCNK7 | 1298 | 0 | 0 | 0 | 0 | 0 | 0 | 0 | 0 | 0 | 0 | 0 | 0 | 0 |
| NM_033348 | KCNK7 | 1372 | 0 | 0 | 0 | 0 | 0 | 0 | 0 | 0 | 0 | 0 | 0 | 0 | 0 |
| NM_033355 | CASP8 | 2766 | 9 | 3 | 4 | 3 | 1 | 1 | 3 | 2 | 5 | 2 | 0 | 9 | 2 |
| NM_033356 | CASP8 | 2652 | 10 | 3 | 4 | 3 | 1 | 1 | 3 | 2 | 5 | 2 | 0 | 10 | 2 |
| NM_033358 | CASP8 | 1123 | 13 | 4 | 5 | 3 | 2 | 1 | 4 | 2 | 7 | 2 | 0 | 13 | 3 |
| NM_033360 | KRAS | 5421 | 13 | 7 | 3 | 4 | 6 | 13 | 6 | 6 | 7 | 5 | 4 | 11 | 8 |
| NM_033362 | MRPS12 | 1046 | 1 | 0 | 0 | 0 | 0 | 1 | 1 | 1 | 0 | 0 | 0 | 0 | 0 |
| NM_033363 | MRPS12 | 973 | 2 | 0 | 0 | 1 | 0 | 1 | 1 | 2 | 0 | 0 | 0 | 0 | 0 |
| NM_033364 | C3orf15 | 4433 | 7 | 1 | 0 | 0 | 0 | 1 | 1 | 0 | 1 | 2 | 0 | 7 | 3 |
| NM_033375 | MYO1C | 4736 | 15 | 5 | 15 | 6 | 7 | 1 | 4 | 3 | 5 | 2 | 3 | 4 | 3 |
| NM_033377 | CGB1 | 732 | 0 | 0 | 0 | 0 | 0 | 0 | 0 | 0 | 0 | 0 | 0 | 0 | 0 |
| NM_033378 | CGB2 | 732 | 0 | 0 | 0 | 0 | 0 | 0 | 0 | 0 | 0 | 0 | 0 | 0 | 0 |
| NM_033379 | CDK1 | 1736 | 0 | 0 | 0 | 0 | 0 | 0 | 0 | 0 | 0 | 0 | 0 | 0 | 0 |
| NM_033380 | COL4A5 | 6432 | 1 | 1 | 0 | 1 | 1 | 1 | 1 | 0 | 1 | 1 | 0 | 0 | 1 |
| NM_033381 | COL4A5 | 6436 | 1 | 1 | 0 | 1 | 1 | 1 | 1 | 0 | 1 | 1 | 0 | 0 | 1 |
| NM_033382 | SEC14L2 | 1824 | 6 | 1 | 0 | 1 | 0 | 1 | 1 | 6 | 0 | 1 | 0 | 1 | 1 |
| NM_033386 | MICALL1 | 3865 | 2 | 1 | 1 | 1 | 0 | 1 | 0 | 2 | 0 | 0 | 0 | 1 | 1 |
| NM_033387 | FAM78A | 3936 | 4 | 1 | 1 | 0 | 3 | 1 | 0 | 0 | 1 | 1 | 2 | 4 | 2 |
| NM_033388 | ATG16L2 | 2145 | 2 | 1 | 0 | 1 | 0 | 0 | 0 | 0 | 1 | 0 | 0 | 2 | 0 |
| NM_033389 | SSH2 | 9166 | 15 | 5 | 3 | 2 | 4 | 3 | 3 | 1 | 7 | 1 | 15 | 15 | 3 |
| NM_033390 | ZC3H12C | 8807 | 3 | 2 | 1 | 1 | 2 | 1 | 2 | 1 | 2 | 2 | 3 | 1 | 2 |
| NM_033393 | FHDC1 | 6480 | 1 | 0 | 0 | 1 | 0 | 0 | 0 | 0 | 1 | 0 | 0 | 1 | 0 |
| NM_033394 | TANC1 | 7505 | 4 | 2 | 1 | 2 | 4 | 2 | 2 | 1 | 3 | 2 | 0 | 1 | 1 |
| NM_033395 | KIAA1731 | 8044 | 6 | 3 | 2 | 1 | 3 | 2 | 3 | 1 | 3 | 5 | 2 | 6 | 6 |
| NM_033396 | TNKS1BP1 | 5820 | 12 | 7 | 12 | 6 | 6 | 3 | 4 | 7 | 9 | 6 | 6 | 6 | 6 |
| NM_033397 | ITPRIP | 4364 | 4 | 2 | 2 | 1 | 4 | 1 | 1 | 1 | 3 | 3 | 1 | 2 | 2 |
| NM_033401 | CNTNAP4 | 4866 | 17 | 2 | 0 | 0 | 0 | 17 | 0 | 0 | 0 | 0 | 0 | 0 | 0 |
| NM_033402 | LRRCC1 | 3821 | 6 | 2 | 1 | 1 | 2 | 2 | 2 | 0 | 3 | 3 | 1 | 2 | 6 |
| NM_033403 | DCLK3 | 5344 | 1 | 0 | 0 | 0 | 0 | 1 | 0 | 0 | 0 | 0 | 0 | 0 | 1 |
| NM_033405 | PRIC285 | 7507 | 0 | 0 | 0 | 0 | 0 | 0 | 0 | 0 | 0 | 0 | 0 | 0 | 0 |
| NM_033406 | FBXO3 | 1652 | 8 | 5 | 3 | 2 | 5 | 7 | 8 | 5 | 5 | 4 | 7 | 5 | 5 |
| NM_033407 | DOCK7 | 7052 | 9 | 4 | 6 | 2 | 3 | 6 | 3 | 2 | 2 | 6 | 1 | 4 | 9 |
| NM_033408 | TMEM205 | 916 | 5 | 2 | 1 | 1 | 2 | 5 | 4 | 5 | 1 | 1 | 1 | 1 | 1 |
| NM_033409 | C20orf54 | 2695 | 6 | 1 | 0 | 0 | 0 | 0 | 1 | 0 | 0 | 0 | 0 | 0 | 6 |
| NM_033410 | ZNF764 | 2738 | 1 | 0 | 0 | 0 | 0 | 0 | 0 | 0 | 0 | 0 | 0 | 1 | 1 |
| NM_033411 | RWDD2A | 1415 | 2 | 1 | 1 | 0 | 1 | 2 | 1 | 1 | 1 | 1 | 0 | 1 | 2 |
| NM_033412 | MCART1 | 1404 | 0 | 0 | 0 | 0 | 0 | 0 | 0 | 0 | 0 | 0 | 0 | 0 | 0 |
| NM_033413 | LRRC46 | 1428 | 1 | 0 | 0 | 0 | 0 | 0 | 0 | 0 | 0 | 0 | 0 | 0 | 1 |
| NM_033414 | ZNF622 | 1692 | 11 | 5 | 4 | 3 | 11 | 6 | 4 | 4 | 4 | 3 | 4 | 4 | 3 |
| NM_033415 | ARMC6 | 2289 | 1 | 0 | 0 | 0 | 0 | 1 | 0 | 0 | 0 | 0 | 0 | 1 | 0 |
| NM_033416 | IMP4 | 1049 | 22 | 11 | 11 | 10 | 10 | 18 | 8 | 22 | 5 | 5 | 12 | 9 | 6 |

| transcript | gene | transcript length (nt) | max | mean | adipose | colon | heart | hypothalamus | kidney | liver | lung | ovary | skeletalmuscle | spleen | testes |
|---|---|---|---|---|---|---|---|---|---|---|---|---|---|---|---|
| NM_033417 | HAUS8 | 1456 | 1 | 0 | 0 | 0 | 0 | 0 | 0 | 0 | 0 | 0 | 0 | 1 | 1 |
| NM_033418 | C1orf156 | 1443 | 4 | 2 | 2 | 1 | 2 | 2 | 2 | 2 | 1 | 4 | 1 | 4 | 2 |
| NM_033419 | PGAP3 | 2687 | 1 | 1 | 0 | 1 | 0 | 0 | 1 | 1 | 0 | 0 | 0 | 0 | 1 |
| NM_033420 | C19orf6 | 2608 | 1 | 1 | 1 | 1 | 1 | 1 | 1 | 1 | 1 | 0 | 1 | 1 | 1 |
| NM_033421 | SNX21 | 3173 | 1 | 1 | 1 | 1 | 1 | 1 | 1 | 0 | 1 | 1 | 1 | 1 | 1 |
| NM_033423 | GZMH | 902 | 1 | 0 | 0 | 0 | 0 | 0 | 0 | 0 | 0 | 0 | 0 | 1 | 0 |
| NM_033425 | DIXDC1 | 5429 | 5 | 3 | 4 | 2 | 4 | 5 | 3 | 1 | 2 | 2 | 2 | 2 | 4 |
| NM_033426 | KIAA1737 | 4348 | 23 | 7 | 4 | 4 | 9 | 14 | 4 | 3 | 3 | 3 | 23 | 4 | 5 |
| NM_033427 | CTTNBP2 | 5969 | 4 | 1 | 1 | 1 | 1 | 4 | 2 | 0 | 0 | 2 | 0 | 2 | 1 |
| NM_033428 | C9orf123 | 2486 | 10 | 5 | 3 | 3 | 10 | 6 | 5 | 3 | 3 | 5 | 5 | 6 | 3 |
| NM_033429 | CALML4 | 4635 | 3 | 2 | 1 | 3 | 2 | 1 | 3 | 1 | 2 | 2 | 1 | 3 | 2 |
| NM_033430 | PDE5A | 6820 | 6 | 3 | 2 | 4 | 5 | 2 | 2 | 0 | 6 | 3 | 3 | 2 | 6 |
| NM_033437 | PDE5A | 6735 | 6 | 3 | 2 | 4 | 5 | 2 | 2 | 0 | 6 | 3 | 3 | 2 | 6 |
| NM_033438 | SLAMF9 | 1154 | 1 | 0 | 1 | 0 | 0 | 0 | 0 | 0 | 0 | 0 | 0 | 0 | 0 |
| NM_033439 | IL33 | 2644 | 14 | 4 | 5 | 0 | 14 | 3 | 1 | 2 | 7 | 2 | 2 | 3 | 4 |
| NM_033440 | CELA2A | 919 | 0 | 0 | 0 | 0 | 0 | 0 | 0 | 0 | 0 | 0 | 0 | 0 | 0 |
| NM_033445 | HIST3H2A | 496 | 0 | 0 | 0 | 0 | 0 | 0 | 0 | 0 | 0 | 0 | 0 | 0 | 0 |
| NM_033446 | FAM125B | 4818 | 3 | 1 | 1 | 1 | 1 | 3 | 0 | 0 | 1 | 1 | 1 | 2 | 1 |
| NM_033448 | KRT71 | 2264 | 0 | 0 | 0 | 0 | 0 | 0 | 0 | 0 | 0 | 0 | 0 | 0 | 0 |
| NM_033449 | FCHSD1 | 4320 | 0 | 0 | 0 | 0 | 0 | 0 | 0 | 0 | 0 | 0 | 0 | 0 | 0 |
| NM_033450 | ABCC10 | 5088 | 0 | 0 | 0 | 0 | 0 | 0 | 0 | 0 | 0 | 0 | 0 | 0 | 0 |
| NM_033452 | TRIM47 | 2263 | 2 | 1 | 0 | 0 | 1 | 1 | 1 | 0 | 1 | 0 | 1 | 2 | 0 |
| NM_033453 | ITPA | 1125 | 2 | 1 | 2 | 2 | 1 | 2 | 2 | 2 | 1 | 0 | 0 | 1 | 0 |
| NM_033455 | KCNK7 | 1471 | 0 | 0 | 0 | 0 | 0 | 0 | 0 | 0 | 0 | 0 | 0 | 0 | 0 |
| NM_033467 | MMEL1 | 2902 | 0 | 0 | 0 | 0 | 0 | 0 | 0 | 0 | 0 | 0 | 0 | 0 | 0 |
| NM_033468 | ZNF257 | 3520 | 4 | 1 | 0 | 0 | 1 | 0 | 1 | 0 | 1 | 4 | 0 | 3 | 2 |
| NM_033480 | FBXO9 | 4687 | 5 | 3 | 4 | 2 | 3 | 5 | 4 | 5 | 2 | 2 | 1 | 5 | 4 |
| NM_033481 | FBXO9 | 4564 | 6 | 4 | 4 | 2 | 3 | 5 | 4 | 6 | 2 | 2 | 1 | 5 | 4 |
| NM_033482 | POM121L2 | 3170 | 0 | 0 | 0 | 0 | 0 | 0 | 0 | 0 | 0 | 0 | 0 | 0 | 0 |
| NM_033484 | FBXO4 | 1727 | 2 | 1 | 1 | 0 | 1 | 1 | 1 | 1 | 1 | 1 | 0 | 2 | 1 |
| NM_033486 | CDK11B | 2453 | 1 | 1 | 1 | 1 | 1 | 1 | 0 | 1 | 1 | 1 | 1 | 1 | 1 |
| NM_033487 | CDK11B | 2193 | 2 | 1 | 1 | 1 | 1 | 1 | 0 | 1 | 1 | 1 | 2 | 1 | 1 |
| NM_033488 | CDK11B | 2467 | 1 | 1 | 1 | 1 | 1 | 1 | 0 | 1 | 1 | 1 | 1 | 1 | 1 |
| NM_033489 | CDK11B | 2500 | 1 | 1 | 1 | 1 | 1 | 1 | 0 | 1 | 1 | 1 | 1 | 1 | 1 |
| NM_033492 | CDK11B | 2447 | 2 | 1 | 1 | 1 | 1 | 1 | 0 | 1 | 1 | 1 | 2 | 1 | 1 |
| NM_033493 | CDK11B | 2420 | 2 | 1 | 1 | 1 | 1 | 1 | 0 | 1 | 1 | 1 | 2 | 1 | 1 |
| NM_033495 | KLHL13 | 3972 | 3 | 1 | 0 | 0 | 0 | 1 | 3 | 0 | 0 | 1 | 0 | 0 | 1 |
| NM_033496 | HK1 | 3598 | 34 | 12 | 8 | 8 | 28 | 34 | 6 | 1 | 9 | 4 | 14 | 14 | 8 |
| NM_033497 | HK1 | 3816 | 32 | 12 | 8 | 7 | 27 | 32 | 5 | 1 | 9 | 4 | 13 | 13 | 8 |
| NM_033498 | HK1 | 3870 | 32 | 11 | 8 | 7 | 26 | 32 | 5 | 1 | 9 | 3 | 13 | 13 | 8 |
| NM_033500 | HK1 | 3963 | 31 | 11 | 8 | 7 | 26 | 31 | 5 | 1 | 8 | 3 | 13 | 13 | 8 |
| NM_033502 | TRERF1 | 7580 | 1 | 0 | 0 | 0 | 0 | 0 | 0 | 0 | 0 | 1 | 0 | 1 | 1 |
| NM_033503 | BMF | 4538 | 2 | 1 | 1 | 1 | 0 | 0 | 0 | 0 | 1 | 1 | 0 | 2 | 0 |
| NM_033504 | TMEM54 | 993 | 9 | 2 | 1 | 9 | 1 | 2 | 2 | 0 | 1 | 0 | 0 | 0 | 0 |
| NM_033505 | SELI | 8129 | 5 | 2 | 1 | 1 | 1 | 5 | 2 | 4 | 1 | 1 | 1 | 2 | 2 |
| NM_033506 | FBXO24 | 2254 | 10 | 1 | 0 | 0 | 0 | 0 | 0 | 0 | 0 | 0 | 0 | 0 | 10 |
| NM_033507 | GCK | 2434 | 0 | 0 | 0 | 0 | 0 | 0 | 0 | 0 | 0 | 0 | 0 | 0 | 0 |
| NM_033508 | GCK | 2558 | 0 | 0 | 0 | 0 | 0 | 0 | 0 | 0 | 0 | 0 | 0 | 0 | 0 |
| NM_033510 | DISP2 | 5032 | 0 | 0 | 0 | 0 | 0 | 0 | 0 | 0 | 0 | 0 | 0 | 0 | 0 |
| NM_033512 | TSPYL5 | 4463 | 15 | 4 | 1 | 1 | 5 | 4 | 3 | 0 | 1 | 8 | 2 | 3 | 15 |
| NM_033513 | C19orf20 | 1114 | 0 | 0 | 0 | 0 | 0 | 0 | 0 | 0 | 0 | 0 | 0 | 0 | 0 |
| NM_033514 | LIMS3 | 1182 | 1 | 0 | 0 | 0 | 0 | 0 | 0 | 0 | 0 | 0 | 0 | 0 | 1 |
| NM_033515 | ARHGAP18 | 3499 | 6 | 3 | 4 | 1 | 2 | 1 | 5 | 0 | 6 | 2 | 1 | 4 | 4 |
| NM_033516 | TPD52L3 | 2568 | 6 | 1 | 0 | 0 | 0 | 0 | 0 | 0 | 0 | 0 | 0 | 0 | 6 |
| NM_033518 | SLC38A5 | 1989 | 0 | 0 | 0 | 0 | 0 | 0 | 0 | 0 | 0 | 0 | 0 | 0 | 0 |
| NM_033520 | C19orf33 | 461 | 0 | 0 | 0 | 0 | 0 | 0 | 0 | 0 | 0 | 0 | 0 | 0 | 0 |
| NM_033529 | CDK11A | 2597 | 1 | 0 | 0 | 0 | 0 | 0 | 0 | 0 | 0 | 0 | 1 | 0 | 0 |
| NM_033535 | FBXL5 | 3475 | 25 | 16 | 17 | 5 | 14 | 17 | 25 | 13 | 15 | 13 | 13 | 20 | 21 |
| NM_033540 | MFN1 | 3513 | 4 | 2 | 2 | 1 | 4 | 2 | 3 | 2 | 2 | 2 | 2 | 4 | 3 |
| NM_033542 | SYS1 | 2823 | 6 | 4 | 3 | 2 | 4 | 4 | 5 | 5 | 3 | 6 | 3 | 5 | 3 |
| NM_033543 | CEACAM21 | 1356 | 1 | 0 | 0 | 0 | 0 | 1 | 0 | 0 | 0 | 1 | 0 | 1 | 0 |
| NM_033544 | RCCD1 | 2767 | 3 | 1 | 1 | 2 | 1 | 3 | 2 | 1 | 1 | 1 | 1 | 1 | 1 |
| NM_033546 | MYL12B | 1004 | 35 | 25 | 31 | 16 | 26 | 35 | 33 | 35 | 27 | 14 | 13 | 28 | 17 |
| NM_033547 | INTS4 | 3149 | 2 | 1 | 1 | 2 | 1 | 2 | 1 | 1 | 1 | 2 | 1 | 2 | 2 |
| NM_033549 | TRIM41 | 3620 | 5 | 3 | 3 | 3 | 2 | 5 | 3 | 2 | 2 | 2 | 2 | 3 | 4 |
| NM_033550 | TP53RK | 3373 | 3 | 2 | 1 | 1 | 2 | 2 | 2 | 1 | 1 | 2 | 1 | 3 | 3 |
| NM_033551 | LARP1 | 7227 | 16 | 9 | 9 | 7 | 9 | 16 | 10 | 10 | 5 | 7 | 7 | 10 | 8 |
| NM_033553 | GUCA2A | 575 | 9 | 1 | 0 | 9 | 0 | 0 | 0 | 0 | 0 | 0 | 0 | 0 | 0 |
| NM_033554 | HLA-DPA1 | 1157 | 132 | 38 | 37 | 26 | 21 | 25 | 19 | 16 | 99 | 18 | 11 | 132 | 14 |
| NM_033557 | YIF1B | 2905 | 1 | 1 | 0 | 1 | 1 | 1 | 0 | 1 | 1 | 0 | 0 | 1 | 0 |
| NM_033624 | FBXO21 | 4233 | 35 | 13 | 3 | 4 | 11 | 17 | 14 | 8 | 7 | 24 | 8 | 12 | 35 |
| NM_033625 | RPL34 | 987 | 144 | 74 | 86 | 42 | 74 | 68 | 47 | 72 | 75 | 144 | 49 | 110 | 47 |
| NM_033626 | CCDC120 | 3972 | 0 | 0 | 0 | 0 | 0 | 0 | 0 | 0 | 0 | 0 | 0 | 0 | 0 |
| NM_033629 | TREX1 | 1496 | 0 | 0 | 0 | 0 | 0 | 0 | 0 | 0 | 0 | 0 | 0 | 0 | 0 |
| NM_033630 | SCAND1 | 775 | 1 | 0 | 0 | 0 | 0 | 0 | 0 | 1 | 0 | 0 | 0 | 0 | 0 |
| NM_033631 | LUZP1 | 8487 | 5 | 3 | 3 | 2 | 5 | 5 | 3 | 1 | 3 | 2 | 1 | 4 | 2 |
| NM_033632 | FBXW7 | 3896 | 23 | 8 | 5 | 8 | 9 | 23 | 4 | 4 | 7 | 5 | 7 | 10 | 8 |

| transcript | gene | transcript length (nt) | max | mean | adipose | colon | heart | hypothalamus | kidney | liver | lung | ovary | skeletalmuscle | spleen | testes |
|---|---|---|---|---|---|---|---|---|---|---|---|---|---|---|---|
| NM_033637 | BTRC | 6121 | 13 | 4 | 2 | 2 | 5 | 13 | 3 | 2 | 2 | 5 | 3 | 4 | 7 |
| NM_033641 | COL4A6 | 6698 | 1 | 0 | 0 | 0 | 0 | 0 | 0 | 0 | 0 | 1 | 0 | 0 | 0 |
| NM_033642 | FGF13 | 1938 | 6 | 1 | 1 | 0 | 0 | 6 | 1 | 0 | 0 | 1 | 1 | 1 | 1 |
| NM_033643 | RPL36 | 433 | 5 | 2 | 2 | 5 | 1 | 3 | 1 | 4 | 2 | 2 | 2 | 2 | 1 |
| NM_033644 | FBXW11 | 4536 | 9 | 4 | 2 | 1 | 5 | 9 | 3 | 3 | 2 | 4 | 3 | 4 | 3 |
| NM_033645 | FBXW11 | 4473 | 9 | 4 | 2 | 1 | 5 | 9 | 4 | 3 | 3 | 4 | 3 | 4 | 3 |
| NM_033646 | CDH7 | 2728 | 2 | 0 | 0 | 0 | 0 | 2 | 0 | 0 | 0 | 0 | 0 | 0 | 0 |
| NM_033647 | HELB | 3368 | 5 | 1 | 1 | 1 | 1 | 1 | 1 | 1 | 1 | 2 | 0 | 5 | 1 |
| NM_033655 | CNTNAP3 | 5229 | 2 | 1 | 1 | 0 | 1 | 2 | 2 | 0 | 1 | 0 | 0 | 1 | 1 |
| NM_033656 | BRWD1 | 12986 | 12 | 7 | 3 | 2 | 7 | 6 | 6 | 4 | 7 | 10 | 8 | 9 | 12 |
| NM_033657 | DAP3 | 1649 | 7 | 5 | 5 | 3 | 7 | 5 | 5 | 7 | 5 | 2 | 5 | 6 | 7 |
| NM_033661 | WDR4 | 1508 | 1 | 0 | 0 | 1 | 0 | 0 | 1 | 1 | 0 | 0 | 0 | 1 | 0 |
| NM_033663 | DRD3 | 1541 | 0 | 0 | 0 | 0 | 0 | 0 | 0 | 0 | 0 | 0 | 0 | 0 | 0 |
| NM_033666 | ITGB1 | 3388 | 114 | 51 | 114 | 22 | 98 | 15 | 37 | 57 | 50 | 40 | 25 | 69 | 36 |
| NM_033667 | ITGB1 | 3765 | 124 | 59 | 124 | 24 | 115 | 16 | 44 | 63 | 61 | 47 | 28 | 83 | 41 |
| NM_033668 | ITGB1 | 3730 | 125 | 59 | 125 | 24 | 116 | 16 | 45 | 63 | 61 | 48 | 28 | 83 | 42 |
| NM_033669 | ITGB1 | 3747 | 124 | 59 | 124 | 24 | 115 | 16 | 44 | 63 | 61 | 47 | 28 | 83 | 42 |
| NM_033670 | CCNB3 | 1199 | 0 | 0 | 0 | 0 | 0 | 0 | 0 | 0 | 0 | 0 | 0 | 0 | 0 |
| NM_037370 | CCNDBP1 | 2317 | 14 | 8 | 11 | 5 | 10 | 13 | 7 | 8 | 7 | 3 | 7 | 14 | 7 |
| NM_044472 | CDC42 | 1513 | 44 | 26 | 34 | 23 | 20 | 44 | 22 | 30 | 27 | 17 | 12 | 35 | 21 |
| NM_048368 | CTDP1 | 3587 | 1 | 1 | 1 | 1 | 1 | 1 | 1 | 1 | 1 | 0 | 1 | 1 | 1 |
| NM_052811 | TRIM13 | 6840 | 5 | 3 | 2 | 1 | 3 | 2 | 3 | 1 | 3 | 4 | 3 | 5 | 4 |
| NM_052813 | CARD9 | 2122 | 0 | 0 | 0 | 0 | 0 | 0 | 0 | 0 | 0 | 0 | 0 | 0 | 0 |
| NM_052814 | CARD9 | 1813 | 0 | 0 | 0 | 0 | 0 | 0 | 0 | 0 | 0 | 0 | 0 | 0 | 0 |
| NM_052817 | MID2 | 7253 | 1 | 1 | 1 | 0 | 1 | 1 | 1 | 1 | 1 | 1 | 1 | 1 | 1 |
| NM_052818 | N4BP2L1 | 3047 | 3 | 1 | 1 | 0 | 0 | 2 | 1 | 3 | 2 | 2 | 1 | 2 | 1 |
| NM_052819 | CARD14 | 1633 | 0 | 0 | 0 | 0 | 0 | 0 | 0 | 0 | 0 | 0 | 0 | 0 | 0 |
| NM_052820 | CORO2A | 5491 | 2 | 1 | 0 | 1 | 0 | 0 | 0 | 0 | 1 | 0 | 0 | 1 | 2 |
| NM_052821 | WDR5 | 3070 | 3 | 2 | 2 | 2 | 2 | 2 | 2 | 3 | 1 | 2 | 2 | 2 | 3 |
| NM_052827 | CDK2 | 2191 | 4 | 2 | 2 | 3 | 2 | 1 | 1 | 3 | 4 | 2 | 1 | 4 | 4 |
| NM_052828 | TRIM10 | 3005 | 1 | 0 | 0 | 0 | 0 | 0 | 1 | 0 | 0 | 0 | 0 | 0 | 0 |
| NM_052831 | C6orf192 | 2425 | 1 | 1 | 1 | 0 | 0 | 1 | 1 | 0 | 1 | 1 | 0 | 1 | 0 |
| NM_052832 | SLC26A7 | 5263 | 4 | 0 | 0 | 0 | 0 | 0 | 4 | 0 | 0 | 0 | 0 | 0 | 0 |
| NM_052834 | WDR7 | 7183 | 8 | 3 | 2 | 1 | 2 | 8 | 2 | 2 | 2 | 2 | 2 | 3 | 3 |
| NM_052836 | CDH23 | 2058 | 1 | 0 | 0 | 0 | 0 | 0 | 0 | 0 | 0 | 1 | 0 | 0 | 1 |
| NM_052837 | SCAMP3 | 1511 | 3 | 2 | 3 | 2 | 3 | 3 | 3 | 3 | 2 | 2 | 2 | 3 | 2 |
| NM_052838 | 01 Sep | 1590 | 3 | 1 | 1 | 1 | 0 | 1 | 0 | 0 | 1 | 1 | 0 | 3 | 1 |
| NM_052839 | PANX2 | 3052 | 1 | 0 | 0 | 0 | 0 | 1 | 0 | 0 | 0 | 0 | 0 | 0 | 0 |
[truncated: 817,862 more chars]
