# Supplementary material for: Digital Genome-Wide ncRNA Expression, Including SnoRNAs, across 11 Human Tissues Using PolyA-Neutral Amplification
Source: PLoS One. 2010 Jul 26;5(7):e11779. doi: 10.1371/journal.pone.0011779 (PMC2909899; doi:10.1371/journal.pone.0011779)
Supplement: Table S8 — Reads per million aligned overlapped by each lincRNA. (0.15 MB PDF) [file pone.0011779.s011.pdf]

location	span (nt)	max	mean	adipose	colon	heart	hypothalamus	kidney	liver	lung	ovary	skeletal muscle	spleen	testes	Comments
chr11:84867425-84942100	74676	73,814	45,409	41,288	51,061	36,027	51,258	38,725	73,814	46,392	46,353	47,628	40,018	26,939	Locus contains a 28S rRNA-like element, line elements, and high conservation in an intron of mRNA DLG2.
chr11:65022925-65031750	8826	20,255	11,279	13,377	7,422	11,233	3,986	20,255	1,504	19,810	14,704	11,628	12,850	7,301	Locus contains ncRNA MALAT1.
chr2:202890425-202914025	23601	2,716	1,523	1,726	823	1,204	2,716	1,260	1,758	1,800	843	927	1,979	1,720	Locus contains a SRP_RNA-related
chr21:14365075-14379325	14251	2,229	842	2,229	1,512	606	845	611	492	774	368	522	440	865	Locus contains a 28S_rRNA-related
chr11:64946775-64971250	24476	2,137	868	1,155	1,044	623	342	1,295	179	2,137	458	1,128	693	493	Locus contains ncRNA NEAT1.
chr15:22809575-22919700	110126	1,816	557	114	186	430	1,816	1,006	114	287	783	516	357	520	PWS locus, including snoRNA clusters HBII-85 and HBII-52, IPW (ncRNA), and more
chr12:39871225-40111600	240376	1,446	566	464	334	1,446	587	1,274	283	199	363	454	217	606	Locus contains mRNA PDZRN4 but reads are elsewhere
chr8:67992975-68000050	7076	1,339	621	299	405	290	293	367	253	1,049	822	1,029	1,339	681	Locus contains ncRNA SNHG6, snoRNA HBII-276, and mRNA C8orf45. Reads are from HBII-276.
chr4:113827100-114091750	264651	1,197	490	436	356	452	369	223	523	342	1,197	556	609	330	Locus contains hY3_RNA-related, SRP_RNA-related, and mRNA.
chr7:130178825-130451400	272576	1,188	418	202	213	217	105	299	152	668	1,188	482	742	330	Locus contains ncRNA FLJ43663 (NR_015431.1)
chr12:126216200-126231125	14926	792	391	792	541	274	638	388	565	275	191	230	205	207	Locus contains a 28S rRNA-like gene.
chr8:125239925-125298400	58476	726	95	4	105	9	5	12	726	32	10	12	109	15	Locus contains ESTs
chrX:73336425-73429775	93351	618	253	231	239	164	84	325	68	310	618	127	406	208	Locus contains misRNA NCRNA00182, miRNAs, and ESTs.
chr6:21774653-22329825	555173	597	154	80	123	79	132	118	86	141	597	65	124	150	Locus contains no known genes but many ESTs and mRNAs.
chr2:106659950-106724850	64901	586	261	170	117	207	94	341	129	393	586	83	459	295	Locus contains ESTs
chr2:209099000-209402250	303251	567	83	22	42	22	19	567	13	41	65	19	49	53	
chr4:88997950-89088100	90151	550	249	165	84	355	550	148	138	144	477	293	207	180	
chr2:186421575-187045450	623876	543	192	104	165	97	91	272	47	162	543	120	246	270	Interesting -- locus contains no genes but high conservation and general transcription.
chrX:72949400-72989313	39914	480	127	1	235	74	1	241	0	156	480	208	1	7	Locus contains ncRNA XIST.
chr3:62910725-63091200	180476	464	96	7	464	87	5	121	209	94	24	3	21	25	
chr8:32905675-33033300	127626	456	79	16	15	38	19	456	7	20	13	236	19	26	
chrX:93434300-94126850	692551	455	107	33	49	35	25	279	25	455	86	28	60	99	
chr6:72128125-72186342	58218	392	63	21	11	34	18	392	11	92	26	29	34	30	Locus contains ncRNA C6orf155 and miRNAs
chr20:34091925-34103650	11726	386	190	141	88	229	386	172	79	202	274	191	168	162	Locus contains ESTs and ncRNA LOC647979 (NR_027451).
chr1:220524025-220686875	162851	376	174	376	200	162	97	181	41	315	55	68	51	367	
chr5:148767275-148793000	25726	368	81	40	368	52	6	27	4	34	193	13	79	70	Locus contains ncRNA LOC728264 and miRNAs.
chr14:100364750-100405275	40526	367	69	11	27	18	170	17	4	13	367	8	49	76	Locus contains ncRNA MEGS.
chrX:110643475-110767925	124451	358	196	147	107	198	121	157	107	195	358	332	230	200	
chr8:134423875-134497625	73751	358	137	137	57	95	131	140	110	84	358	151	92	156	
chr19:20755700-20823525	67826	354	73	29	40	27	45	58	11	46	354	22	94	77	
chr7:137497625-137771725	274101	338	155	86	134	124	99	123	78	276	210	78	338	162	
chr10:19970175-20140000	169826	336	78	24	105	43	34	61	336	27	149	23	21	33	
chrX:64126100-64618150	492051	334	144	74	197	80	87	188	41	136	334	58	226	163	
chr6:77666425-78134625	468201	300	89	44	58	63	25	146	19	62	300	22	109	138	Locus containsno genes or ESTs
chr4:67977175-68014325	37151	294	113	140	42	54	294	85	204	89	90	62	105	73	
chr6:106965550-107033200	67651	290	146	79	62	217	290	122	118	175	146	123	191	83	
chr4:12676250-12957000	280751	290	128	89	97	132	290	118	75	83	185	77	134	130	Locus contains ncRNA HSP90AB2P
chr14:70858200-71037050	178851	284	91	45	88	48	37	97	33	187	67	11	284	100	Locus contains ESTs
chr5:60665775-60714725	48951	277	78	33	66	47	32	78	36	102	277	18	118	55	Locus contains ESTs
chr14:100459225-100609600	150376	275	82	24	23	34	242	24	18	26	275	20	92	121	Locus contains snoRNA cluster 14q
chr3:175644725-176014550	369826	264	72	73	50	45	27	82	11	55	264	44	66	74	Locus contains ESTs and mRNAs
chr17:67895375-68098675	203301	264	35	5	264	5	21	12	4	31	10	3	8	20	
chr8:17399650-17430625	30976	231	36	4	5	9	5	13	74	9	231	29	7	13	Locus contains ESTs
chr5:88250875-88580200	329326	214	54	20	34	21	26	31	8	44	214	84	64	48	
chrX:114863475-114997900	134426	200	78	20	118	69	48	169	16	59	200	12	44	102	
chr5:33032900-33307175	274276	197	83	56	73	76	40	52	62	78	197	62	128	84	
chr10:6228325-6278100	49776	194	82	71	67	47	194	59	71	63	85	75	122	48	
chr2:134595725-134694600	98876	190	56	30	91	43	18	51	9	69	64	9	190	37	
chr22:25386775-25639800	253026	183	100	71	146	63	99	90	58	97	134	55	183	105	
chrX:55952950-56258125	305176	174	86	61	92	48	35	129	20	89	174	37	136	119	
chr1:172098475-172103825	5351	174	101	104	106	39	89	80	96	149	174	53	153	66	Locus contains ncRNA GAS5 snoRNA RNA C/D box cluster, including U44, U47, U74-81. U76 is highest expressed.
chr8:58277700-58297325	19626	172	27	1	0	0	1	118	0	0	0	0	0	172	
chrX:73081400-73149600	68201	172	60	33	41	40	29	80	20	59	172	54	77	59	
chr7:18241000-18493675	252676	168	37	32	23	27	20	16	7	31	28	8	168	49	
chr20:36470500-36515575	45076	161	73	59	149	25	51	48	60	93	51	21	83	161	Looks great!! Contains ncRNA ACA60, U71a, and U71d
chrX:135771475-135783050	11576	160	73	29	35	68	24	70	19	68	108	79	160	141	
chr2:127007450-127034900	27451	159	60	62	32	52	159	49	22	66	45	20	96	56	
chr10:67000950-67213100	212151	158	40	10	24	13	10	74	17	23	158	7	75	33	
chr5:144793925-145104175	310251	156	61	29	156	70	18	38	9	64	145	21	55	67	
chr1:141730725-141862525	131801	156	80	59	47	68	28	57	17	88	156	58	153	143	
chrX:148863050-149024650	161601	156	57	29	69	22	24	93	7	57	156	30	52	89	
chr11:86297350-86316725	19376	151	36	151	9	23	12	31	1	30	79	19	15	24	Locus contains ESTs
chr9:2442800-2542025	99226	150	23	31	7	14	7	11	4	10	150	4	7	7	
chr6:119718475-120097975	379501	144	52	54	35	30	22	85	8	44	83	18	144	53	
chrX:97432775-97543875	111101	142	84	141	80	47	62	74	87	87	142	24	104	72	
chr3:115526175-115535575	9401	141	54	41	10	64	30	78	36	35	141	51	48	62	Locus contains ESTs, conserved blocks, and a possible 3' UTR extension of a nearby mRNAs
chr10:46514400-46569425	55026	141	57	53	43	45	78	31	59	44	141	32	60	38	
chr8:130300925-130810225	509301	139	35	18	40	14	13	31	17	33	37	13	139	29	
chr11:39456550-40064650	608101	137	61	31	64	33	28	62	18	61	137	32	106	101	
chr5:60715925-60749750	33826	136	57	37	56	44	33	51	33	83	136	12	92	55	
chr8:127908350-128572000	663651	133	68	39	72	41	35	133	62	90	73	33	90	77	
chr1:206037850-206108750	70901	133	66	42	70	45	22	101	9	85	105	58	133	51	
chr3:64987550-65012925	25376	131	19	3	6	4	2	20	2	15	131	4	6	12	
chr16:83806950-84141650	334701	127	62	49	127	29	14	48	36	40	125	81	50	79	
chrX:74681500-74831575	150076	127	50	24	45	32	39	67	13	45	127	23	54	82	
chr8:135860125-135912450	52326	126	49	23	29	47	8	126	33	72	78	48	41	31	
chr12:90890975-91048525	157551	124	32	17	14	15	6	24	18	56	48	11	124	15	
chrX:62523600-62696725	173126	123	63	37	62	49	35	95	20	68	123	34	95	71	
chr18:35328475-35629325	300851	122	23	7	38	7	5	11	3	13	20	7	15	122	
chr1:245418575-245440175	21601	122	35	31	22	24	14	35	9	50	25	11	122	37	
chr21:29671250-29823325	152076	122	48	26	38	36	20	63	21	73	39	65	122	28	
chr7:80790500-80803425	12926	119	44	83	119	31	74	33	26	31	17	20	20	33	
chr3:152670000-152720900	50901	119	19	8	9	7	5	9	2	18	119	5	12	12	
chr20:53108150-53174325	66176	116	58	46	49	53	25	32	52	51	116	78	92	50	
chr7:33631025-33852800	221776	115	34	22	31	17	12	44	5	37	115	9	49	32	
chr22:44829900-44895200	65301	114	35	21	64	26	14	40	4	29	114	12	35	22	
chr7:19918100-20126725	208626	114	74	67	61	67	51	76	79	82	114	47	107	63	
chr13:23456900-23626575	169676	114	72	51	32	111	84	72	59	70	64	114	83	54	
chr7:63370950-63480450	109501	113	46	26	24	26	38	52	7	36	113	18	94	77	
chr1:121029075-121187200	158126	110	35	24	39	21	110	18	23	24	11	11	41	65	
chr4:106079150-106264700	185551	110	23	9	17	9	7	110	6	17	29	10	19	21	
chr21:28840100-29136975	296876	109	50	23	52	28	19	42	15	76	89	36	109	57	
chr1:65291650-65304400	12751	109	24	19	12	11	6	17	3	21	109	23	30	15	
chr6:29809950-29848275	38326	108	41	28	18	42	108	24	40	48	36	28	53	23	
chr15:19183225-19203975	20751	108	52	48	28	51	40	41	57	49	49	36	108	63	
chr2:54096175-54165975	69801	106	72	64	55	71	74	68	53	60	95	63	106	87	
chr10:119231725-119242550	10826	106	21	16	3	3	34	106	0	0	41	5	0	27	Locus contains ncRNA EMX2OS
chr8:92141050-92151400	10351	103	50	28	10	87	103	57	27	30	42	40	53	78	Locus contains ESTs and mRNAs. Data suggest a novel, strand-specific ncRNA.
chr14:52757725-52912850	155126	103	31	26	32	13	9	25	5	28	54	11	34	103	
chr6:3121200-3139125	17926	101	14	14	8	2	101	8	10	1	1	1	1	2	
chrX:45482950-45595350	112401	101	32	21	32	11	25	25	10	73	101	8	27	20	
chr6:34352100-34361825	9726	99	36	23	12	32	99	41	15	21	47	48	34	23	
chr8:60225475-60995275	769801	99	57	38	60	33	32	99	33	80	91	29	68	68	
chr14:85408675-85959800	551126	98	52	35	60	34	28	60	15	61	86	35	98	61	
chr17:42482352-42533565	51214	98	45	27	49	32	39	54	10	42	98	21	71	55	
chr5:102119525-102221625	102101	97	46	27	22	49	11	46	9	97	77	54	79	33	
chr13:49793725-49890200	96476	97	51	53	31	50	37	32	39	56	97	40	77	46	
chr12:45112400-45261050	148651	97	47	17	45	16	19	90	45	40	97	39	56	53	
chrX:101911200-102072375	161176	96	47	38	46	31	33	71	14	48	96	22	65	54	
chr10:49590325-49693725	103401	96	23	17	28	11	9	14	14	21	21	10	96	9	
chr12:32445125-32538825	93701	95	36	15	86	19	9	95	11	41	59	5	18	40	
chr13:49555025-49595650	40626	95	35	22	23	28	9	30	13	61	42	42	95	25	
chrX:112699025-113202875	503851	93	38	20	93	20	18	41	15	42	57	20	51	42	
chr22:29695634-29706450	10817	93	57	45	37	52	58	68	29	47	88	31	82	93	Locus contains ncRNA TUG1.
chr6:86438525-86452775	14251	93	43	29	30	26	33	25	13	63	93	72	55	30	Locus contains ncRNA U50
chr5:92890475-92942875	52401	92	20	6	7	5	25	17	2	18	92	1	21	29	
chr8:128891450-129010774	119325	92	25	12	27	12	7	9	5	22	92	15	48	31	
chr2:123438725-124477200	1038476	91	57	40	66	34	38	64	31	82	91	32	72	73	
chr14:95576925-95731700	154776	91	20	8	16	8	91	10	3	35	17	4	14	10	
chr3:64872075-64896750	24676	90	14	5	7	5	3	14	1	15	90	4	5	10	
chr6:14338050-14736875	398826	89	36	25	34	25	15	40	14	89	33	23	65	31	
chr7:66441725-66470800	29076	88	14	3	8	4	4	13	1	5	11	88	8	6	
chr18:72307325-72331925	24601	88	20	6	12	7	3	15	6	18	88	10	23	30	
chr1:93548254-93583962	35709	88	33	22	20	14	17	46	4	38	88	9	56	51	

location	span (nt)	max	mean	adipose	colon	heart	hypothalamus	kidney	liver	lung	ovary	skeletal muscle	spleen	testes	Comments
chr1:174109725-174170025	60301	86	54	58	86	38	46	25	82	47	73	38	78	24	
chr6:42833450-42867750	34301	86	34	18	46	17	13	34	4	27	86	32	69	29	
chr13:81905500-83297700	1392201	86	56	34	76	34	56	70	20	61	86	28	75	76	
chr3:13011600-13315275	303676	86	46	61	59	27	65	22	33	55	20	23	86	51	
chrX:149113300-149357500	244201	85	42	34	30	29	31	39	26	59	85	24	35	69	
chr13:99372350-99383275	10926	82	35	82	63	8	68	13	49	24	8	11	25	35	
chr1:197068975-197174200	105226	81	21	10	5	12	14	16	5	56	12	2	81	15	
chr3:196904200-196928625	24426	80	41	16	74	23	31	80	9	29	71	34	34	44	
chr10:42319800-42366925	47126	80	36	23	20	25	46	36	7	34	75	13	80	34	
chr7:135330850-135602275	271426	79	29	17	27	14	12	19	7	37	28	10	79	68	
chr7:104403400-104440275	36876	79	32	15	41	18	8	43	9	24	69	15	79	29	
chr1:173389275-173428400	39126	79	25	35	12	23	12	26	18	43	12	6	79	10	
chr5:131713500-131731950	18451	78	31	18	41	19	11	78	6	15	39	60	16	31	
chr19:32905775-33020475	114701	78	44	31	35	32	37	56	19	32	75	15	78	72	
chrX:139623150-139637100	13951	78	10	3	10	4	78	1	0	2	2	3	6	4	Locus contains ncRNA RP1-177G6.2
chr8:129045250-129106528	61279	77	20	6	24	8	3	5	2	16	77	12	39	29	
chr6:30364275-30401550	37276	77	34	21	35	25	22	49	13	28	77	13	58	30	
chr4:3542725-3692050	149326	76	10	2	7	2	2	76	3	4	4	1	3	5	
chr2:217838575-218189250	350676	76	35	44	34	45	16	30	5	34	54	13	76	37	
chr13:39764925-39866925	102001	74	29	60	17	14	9	17	74	43	27	20	16	20	
chr5:127320775-127407525	86751	73	36	24	24	29	29	42	12	25	69	25	73	38	
chr8:90781700-90817050	35351	72	26	17	20	21	16	38	5	26	72	7	40	31	
chr16:10338750-10419300	80551	72	21	8	15	12	8	16	10	27	23	3	33	72	
chr13:66853600-67679750	826151	71	38	27	38	26	24	43	15	44	71	22	49	55	
chr11:27551550-27606750	55201	71	32	23	15	33	22	28	26	26	71	42	43	26	
chr1:191490425-191504575	14151	70	31	25	9	26	36	35	6	30	70	29	41	33	
chr5:141048275-141198325	150051	69	20	18	30	17	23	25	2	69	10	12	5	12	
chr3:64827825-64839350	11526	69	10	3	3	3	2	11	0	11	69	3	3	7	
chr8:52950675-53051275	100601	68	30	16	21	20	10	33	7	34	57	33	68	31	
chr5:67121850-67148800	26951	68	8	0	1	1	0	2	68	6	2	2	1	7	
chr1:237661375-237853250	191876	67	17	5	26	6	11	18	6	19	67	5	13	17	
chr7:78921550-78942375	20826	66	38	50	9	36	41	66	16	27	65	15	51	41	
chrX:53141125-53204125	63001	66	33	24	29	23	23	41	9	31	66	33	43	37	
chr8:114557150-115165825	608676	66	36	21	42	24	21	41	15	48	66	20	56	43	
chr1:83879475-84089425	209951	66	22	4	17	10	10	66	17	14	26	6	37	37	
chr8:61960000-62228150	268151	65	33	22	51	29	25	26	15	30	35	18	65	50	
chr7:55494425-55505775	11351	65	20	22	16	18	16	23	11	16	17	10	65	11	
chr7:22570350-22697250	126901	65	15	7	12	12	6	6	6	25	6	9	65	16	
chr5:27086125-27661000	574876	65	31	17	30	23	18	65	13	55	37	11	36	39	
chr3:8312700-8451425	138726	64	22	21	17	11	21	34	4	16	64	11	19	26	
chrX:71049200-71256150	206951	64	40	33	59	36	34	27	32	64	59	22	46	33	
chr11:121514300-121586150	71851	63	16	6	7	15	12	25	4	16	63	3	3	17	
chr2:177854175-177922425	68251	63	14	11	8	7	11	63	2	8	12	5	12	13	
chr3:3963675-4110125	146451	62	38	25	37	29	27	48	19	33	54	33	55	62	
chr16:71044100-71322025	277926	62	32	17	43	18	17	43	14	28	54	15	45	62	
chr6:155200400-155254075	53676	62	30	13	29	23	13	31	8	29	62	33	59	27	
chr6:11226350-11254175	27826	62	16	7	8	14	62	9	15	9	14	10	14	11	
chr10:92917275-92951850	34576	61	21	7	9	19	13	24	9	22	61	14	44	12	
chr11:128587275-128651150	63876	60	24	11	25	13	11	37	10	26	60	6	22	46	
chr8:129111400-129188100	76701	60	15	4	14	6	3	4	2	9	60	11	34	22	
chr13:44814075-44848225	34151	60	24	15	39	12	12	35	4	26	60	14	20	26	
chr2:75354900-75436900	82001	59	11	3	6	3	3	14	2	10	12	3	59	7	
chr6:159938625-159994650	56026	59	15	6	9	11	4	13	2	26	9	59	17	8	
chr14:100425525-100457175	31651	58	13	3	7	6	11	3	4	4	58	2	24	19	
chr5:119466150-119800500	334351	58	32	18	39	21	24	33	13	41	58	19	47	43	
chrX:57186025-57301200	115176	57	31	19	37	18	17	39	8	28	57	19	53	44	
chrX:97651200-98066850	415651	56	30	15	37	18	16	38	7	31	56	18	42	47	
chr6:138942775-139061900	119126	56	19	15	29	13	10	27	11	25	12	3	56	11	
chr12:104249300-104514975	265676	56	25	11	32	12	10	41	7	29	35	13	33	56	
chr4:80197250-80386675	189426	56	26	13	18	22	30	24	4	26	51	25	19	56	
chrX:117025575-117121675	96101	56	24	14	27	16	9	54	4	21	56	14	23	29	
chr4:15848225-15898675	50451	55	23	13	20	9	18	55	3	10	55	6	33	28	
chr3:157935925-158021825	85901	55	31	21	25	28	20	45	38	24	37	22	55	25	
chr22:24179500-24251325	71826	55	21	13	18	14	8	22	3	23	55	14	24	36	
chr16:85065635-85099967	34333	54	11	0	54	0	2	1	3	54	1	0	0	2	
chr13:32786375-32821550	35176	54	22	33	19	24	7	23	9	26	54	6	23	20	
chr2:200278025-200421800	143776	54	27	26	16	13	40	41	11	14	25	54	36	25	
chr3:156439750-156538375	98626	54	14	6	11	6	4	16	3	9	17	54	11	14	
chr14:57814000-57830600	16601	54	33	35	31	28	30	39	24	38	38	18	54	34	
chr5:60766375-60837150	70776	54	24	15	23	15	13	22	10	54	35	7	43	23	
chr6:139850800-140190150	339351	54	21	14	27	10	8	20	16	54	19	6	25	28	
chr15:92584350-92637350	53001	54	12	2	21	6	1	7	5	28	4	1	54	6	
chr7:65641550-65680075	38526	53	26	27	22	17	30	24	12	22	53	12	28	34	
chr14:65797475-66001975	204501	53	28	17	24	12	9	53	18	19	49	20	39	53	
chr8:38735375-38749400	14026	53	27	37	53	19	10	32	7	29	44	8	33	28	
chrX:56439250-56581650	142401	53	30	18	33	19	15	43	8	33	53	23	42	42	
chr1:111806075-111816900	10826	53	16	13	4	53	21	11	4	7	16	35	9	9	
chr6:40724100-41036875	312776	52	18	8	17	15	10	16	4	15	52	19	18	22	
chr2:70066250-70116975	50726	52	22	15	20	10	15	28	5	18	52	22	26	32	
chrX:78326100-78453100	127001	52	15	7	16	9	5	15	3	20	18	5	52	14	
chr1:202791825-202842475	50651	52	17	10	13	9	10	17	5	23	14	16	52	13	
chr10:64906075-64942725	36651	52	20	14	16	14	9	26	5	20	52	6	36	27	
chr1:98211850-98471750	259901	52	16	4	14	5	52	8	19	9	28	6	10	20	
chr11:82461075-82481150	20076	52	21	12	14	16	11	29	4	12	52	20	40	19	
chr20:3798525-3805650	7126	51	33	27	39	45	15	38	15	20	44	51	44	22	
chr1:42162000-42267700	105701	51	25	23	17	16	12	28	7	43	42	13	51	24	
chr2:195896350-196221150	324801	51	20	11	28	11	9	19	6	18	29	11	29	51	
chr12:31876125-31998175	122051	51	16	16	20	8	9	10	16	12	12	9	51	11	
chr8:129572750-129912400	339651	51	28	17	34	22	16	32	11	32	51	18	38	39	
chr7:126777625-126793150	15526	51	22	15	13	19	10	19	11	36	28	16	51	23	
chr12:19574525-19658050	83526	51	25	8	26	14	15	51	6	35	31	18	47	23	
chr8:28968300-28978875	10576	50	31	19	21	32	40	30	16	31	46	18	50	33	
chr2:186001225-186204525	203301	50	22	11	18	13	12	36	7	23	50	13	25	35	
chr13:73697875-73923825	225951	50	19	11	23	13	11	19	4	20	30	9	50	21	
chr2:181682350-182018875	336526	50	19	9	21	8	6	17	5	46	21	7	50	18	
chr2:192264725-192294900	30176	50	12	11	5	5	1	8	2	28	7	5	50	10	
chr2:58654425-59202850	548426	50	23	10	26	10	24	31	6	21	50	8	21	42	
chr11:94263975-94302225	38251	50	19	17	7	17	15	21	11	14	50	27	15	20	
chrX:79369600-79479675	110076	50	17	9	11	11	5	26	4	13	50	7	15	37	
chr10:62084800-62177500	92701	50	21	5	16	6	4	49	3	35	50	4	40	21	
chrX:16117550-16299075	181526	49	27	16	34	17	18	33	8	29	49	16	40	33	
chr13:19153500-19172700	19201	49	24	13	17	14	9	26	7	31	49	14	49	30	
chr1:179576475-179696250	119776	49	7	1	3	3	6	49	0	3	3	2	3	6	
chr9:21869775-21928825	59051	49	21	18	15	14	8	33	9	16	49	13	39	17	
chr2:20586400-20667150	80751	48	20	14	10	25	24	17	48	17	16	14	16	16	
chr4:56611450-56711475	100026	48	17	8	20	5	11	11	10	48	24	4	20	29	
chr21:24709250-24804075	94826	48	9	6	6	5	2	6	3	9	8	3	48	9	
chr5:55603225-55802650	199426	48	21	14	15	20	15	48	16	19	20	17	22	26	
chr6:119217000-119228400	11401	47	9	7	4	3	3	4	8	47	6	4	4	8	
chr22:22581300-22604300	23001	47	17	10	47	9	9	14	19	9	23	4	23	22	
chr1:91770325-91851575	81251	47	23	20	16	21	47	15	16	15	29	18	29	24	
chrX:15600875-15661300	60426	47	18	8	12	13	10	23	10	22	47	8	22	19	
chr2:179468900-179623050	154151	47	22	17	20	47	8	23	4	35	24	12	22	30	
chr16:57438450-57663625	225176	47	22	13	27	13	14	26	8	27	34	11	26	47	
chr19:24010225-24039125	28901	47	18	9	24	10	7	24	5	22	47	4	30	15	
chr4:2526025-2584625	58601	46	21	13	36	13	10	23	5	19	46	11	33	26	
chr11:6710925-6721500	10576	46	6	1	3	1	0	3	1	9	2	1	46	2	
chr10:29739925-29773925	34001	46	22	20	17	18	17	26	9	22	46	10	32	30	
chr22:23738700-23794025	55326	46	14	5	46	8	13	26	3	12	20	2	5	12	
chr17:41006725-41017400	10676	46	19	11	40	10	6	25	4	15	25	15	46	17	
chr4:38176050-38315200	139151	46	12	6	12	5	9	8	5	16	46	4	12	13	
chr4:91269325-91343025	73701	45	18	8	15	9	5	43	1	14	16	4	45	37	
chrX:135821775-135914025	92251	45	24	16	34	12	19	25	10	30	45	10	30	31	
chr13:87831725-88426225	594501	45	27	15	29	14	13	39	35	45	36	14	27	34	

location	span (nt)	max	mean	adipose	colon	heart	hypothalamus	kidney	liver	lung	ovary	skeletal muscle	spleen	testes	Comments
chr2:46715775-46757875	42101	45	15	8	15	7	7	14	2	17	45	11	20	17	
chr15:19352325-19432225	79901	44	11	4	6	7	5	7	2	7	23	5	9	44	
chr19:56887550-56898800	11251	44	17	11	19	16	11	24	1	19	44	4	17	24	
chr13:83992850-84271550	278701	44	26	22	21	36	28	30	14	26	24	14	44	26	
chr10:131738400-131809350	70951	44	23	12	20	13	15	44	43	15	23	12	30	25	
chr12:9168650-9183575	14926	43	13	8	4	7	3	6	24	23	10	6	43	7	
chr6:80305150-80328275	23126	43	15	6	4	7	10	22	1	12	43	14	12	32	
chr8:102254625-102268925	14301	43	13	4	10	7	43	8	2	13	13	22	18	6	
chr5:87601475-87624775	23301	43	21	8	12	17	18	34	5	16	43	16	35	26	
chr12:56061700-56107050	45351	43	27	15	42	20	10	33	9	23	43	22	42	40	
chr15:33242325-33301050	58726	43	16	8	7	7	6	25	6	18	43	13	14	25	
chr6:143894500-143965675	71176	42	16	11	41	12	5	19	5	21	7	2	42	14	
chr3:113791425-113801250	9826	42	9	42	5	15	2	2	0	6	10	1	9	6	
chrX:38899925-39313125	413201	42	20	12	20	13	10	23	7	29	32	14	42	23	
chr1:223242600-223310100	67501	42	14	6	9	4	6	25	6	13	42	3	10	29	
chr21:20244475-20681850	437376	42	20	12	42	12	9	21	6	21	31	13	32	25	
chr11:128428400-128530950	102551	42	16	7	12	7	13	25	6	18	42	7	15	28	
chr12:45059775-45107700	47926	41	19	8	16	9	9	31	7	16	41	15	29	25	
chr7:93066750-93219450	152701	41	19	11	15	14	8	41	9	22	35	12	23	18	
chr12:131170325-131183625	13301	41	10	4	22	4	3	5	7	11	5	2	41	6	
chrX:131196250-131276325	80076	41	23	19	18	16	11	41	6	25	40	13	29	32	
chr13:108675775-108729525	53751	41	7	3	4	2	2	4	2	41	5	3	4	4	
chr4:120611850-120629500	17651	40	16	12	9	10	7	21	4	16	40	9	26	20	
chr18:31361475-31448725	87251	40	17	10	21	13	8	15	7	16	34	5	40	24	
chr18:33973225-34880125	906901	40	28	19	40	18	16	36	11	36	35	19	35	37	
chr10:6819700-6929500	109801	40	19	9	15	9	12	19	13	31	40	11	30	21	
chr7:108939950-109124550	184601	40	19	10	19	11	10	24	5	23	40	10	31	23	
chr12:40410875-40597275	186401	39	20	14	16	20	7	28	5	27	39	8	28	25	
chrX:133503575-133510625	7051	39	7	1	0	1	1	9	0	1	39	3	4	15	
chr16:21928100-21978250	50151	39	17	7	16	12	6	19	7	17	39	7	37	20	
chr3:64927975-64938675	10701	39	6	1	3	2	1	6	0	6	39	2	2	5	
chr2:61010650-61020850	10201	39	14	8	8	8	6	13	10	24	19	9	39	8	
chr4:80685625-80990600	304976	39	21	15	21	23	9	34	6	19	39	18	25	28	
chr11:70199775-70396875	197101	39	14	5	39	3	9	38	12	26	5	3	7	9	
chrX:63275525-63315950	40426	39	9	5	6	5	5	13	3	5	39	4	9	10	
chr13:49505400-49521157	15758	39	13	9	8	7	3	8	3	20	15	17	39	10	
chr1:176232625-176271000	38376	39	12	9	13	7	4	39	5	6	16	9	22	8	
chr4:129552425-129700850	148426	38	23	28	18	15	7	36	5	33	37	9	25	38	
chr6:152061150-152115700	54551	38	9	5	5	5	2	12	7	8	38	4	11	7	
chr7:30183275-30279675	96401	38	16	22	11	9	5	18	2	24	38	7	35	11	
chr8:122077375-122309675	232301	38	19	11	23	11	9	18	11	14	27	38	22	20	
chr5:18520075-18782025	261951	38	14	7	14	7	8	13	4	22	16	6	38	15	
chr3:108009550-108113225	103676	38	12	4	9	8	13	12	1	8	38	3	7	23	
chr14:97429125-98123725	694601	38	25	16	34	14	19	37	12	38	35	10	35	27	
chr11:121023500-121139975	116476	38	8	3	11	4	4	6	2	10	7	3	38	7	
chr8:91182275-91466575	284301	38	19	12	23	12	12	25	5	16	38	12	24	25	
chr2:239037850-239126125	88276	38	10	5	8	4	7	9	2	8	11	4	15	38	
chr3:42031400-42080525	49126	37	17	14	30	13	6	21	6	24	18	9	37	14	
chr1:217494600-217545100	50501	37	13	9	7	9	6	17	2	9	37	19	13	12	
chr3:158376800-158454175	77376	37	16	9	14	7	6	19	14	37	16	7	23	22	
chr3:44017175-44136825	119651	37	9	1	6	1	17	37	1	8	2	1	23	5	
chr3:5443625-5798075	354451	37	23	15	24	12	11	28	7	34	37	13	34	37	
chr6:98325525-98699475	373951	37	22	16	26	13	17	26	8	23	37	13	27	32	
chr2:64266275-64379225	112951	37	9	3	7	4	4	7	1	10	6	2	37	12	
chr6:20056200-20196375	140176	36	19	13	28	13	8	16	17	18	36	22	23	19	
chr8:126787400-127048475	261076	36	17	9	20	9	8	17	11	36	19	6	36	20	
chr16:72904500-72918375	13876	36	19	9	34	10	9	31	3	12	36	12	30	18	
chr2:145164825-145304400	139576	36	10	6	10	5	4	9	3	10	14	5	11	36	
chr1:172235150-172257677	22528	36	13	7	13	9	4	15	3	15	22	5	36	11	
chr5:43102225-43120425	18201	36	17	14	18	12	12	22	3	13	36	11	34	17	
chr21:9722975-9895475	172501	36	17	9	30	9	10	29	5	10	36	6	14	25	
chrX:4475750-5016575	540826	36	21	20	30	18	11	20	14	36	30	10	23	23	
chr13:40314375-40358125	43751	36	19	9	25	15	9	23	5	18	30	9	35	36	
chr12:96400875-96429925	29051	36	7	0	1	2	8	36	0	10	2	0	0	16	
chrX:53701825-53727175	25351	36	16	11	13	11	8	20	8	16	36	7	28	22	
chr5:27762975-28081775	318801	36	21	12	23	11	13	30	5	28	36	14	26	35	
chr6:72090100-72115900	25801	36	8	6	2	5	3	36	2	10	5	7	5	5	
chr7:30552675-30585400	32726	36	20	24	23	14	13	28	4	22	25	14	36	20	
chr3:190327775-190360400	32626	35	13	35	5	8	1	5	17	17	10	18	9	15	
chr14:70090650-70114250	23601	35	16	10	11	10	17	16	3	12	35	25	19	17	
chr6:158578775-158644850	66076	35	21	16	29	13	13	31	8	22	35	9	30	26	
chr6:118763025-118848700	85676	35	10	4	8	6	3	7	2	6	11	35	13	9	
chr2:70121450-70166850	45401	35	16	10	18	9	8	22	7	13	35	12	23	23	
chr10:124977275-125220900	243626	35	16	7	31	11	8	11	3	19	26	6	35	19	
chr10:79193175-79209400	16226	35	7	4	4	1	3	6	3	8	5	3	9	35	
chr12:58661275-59020350	359076	35	15	10	14	9	7	14	5	18	20	10	35	22	
chr11:111652575-111823525	170951	35	21	16	27	18	14	21	8	28	35	15	25	29	
chr3:17879250-18142350	263101	35	21	35	24	12	25	21	17	20	27	9	17	26	
chr5:173017250-173229650	212401	34	13	27	12	4	8	12	34	9	8	5	13	7	
chr3:171889875-171953950	64076	34	8	2	3	4	14	3	1	5	34	2	2	15	
chr10:53930025-54182600	252576	34	17	12	26	15	13	19	7	20	34	8	20	17	
chr1:35413250-35421400	8151	34	14	11	10	9	9	18	4	11	34	9	23	13	
chr3:161693475-161700625	7151	34	11	9	5	14	9	7	8	6	9	34	13	7	
chr6:14904575-15226225	321651	34	19	12	34	10	9	16	10	28	26	7	22	32	
chr7:64088850-64099775	10926	33	8	33	5	5	4	6	1	5	7	1	10	15	
chr7:7042600-7120400	77801	33	13	7	19	7	8	33	4	13	14	5	14	15	
chr2:233144650-233160800	16151	33	18	12	15	16	33	28	14	10	12	12	22	17	
chr14:41931100-42357500	426401	33	20	16	22	11	10	20	10	32	30	11	24	33	
chrX:108881550-109095625	214076	33	18	10	21	12	10	21	6	21	33	12	27	22	
chr1:85752775-85814125	61351	33	11	4	5	12	4	9	2	14	33	5	32	8	
chr4:78314425-78523100	208676	33	14	8	22	9	6	17	5	16	21	6	33	15	
chr13:39567725-39693050	125326	32	12	32	7	8	5	6	24	11	11	15	4	7	
chr16:70839225-70989525	150301	32	15	8	17	10	6	19	5	15	26	9	21	32	
chr13:86323275-86723250	399976	32	18	12	20	11	10	21	9	32	29	10	22	26	
chr10:33370300-33439775	69476	32	11	4	8	20	3	6	6	17	14	6	32	7	
chr1:185236575-185449925	213351	32	14	9	15	7	8	20	3	13	32	7	22	16	
chr2:213402125-213552350	150226	32	15	9	13	10	8	18	3	19	21	16	32	16	
chr4:235050-250725	15676	32	12	7	3	6	11	8	2	10	32	6	13	29	
chr15:79800050-79949450	149401	32	18	7	23	10	9	18	6	16	32	18	24	31	
chr3:108443925-108458725	14801	32	8	2	2	5	7	4	1	6	32	2	4	21	
chr14:53031875-53057875	26001	32	7	6	6	1	1	1	1	6	15	1	4	32	
chr16:14306225-14332725	26501	32	11	19	11	11	8	6	3	4	11	32	4	9	
chr11:86071725-86123800	52076	32	11	5	9	5	3	15	2	8	32	18	10	19	
chr2:114441025-114483825	42801	31	9	4	5	4	5	8	5	12	5	5	31	19	
chr2:216182125-216421975	239851	31	13	7	14	8	8	10	4	16	26	5	11	31	
chr11:97181050-97533050	352001	31	15	9	20	9	8	16	4	17	31	9	22	22	
chr1:89646850-89720000	73151	31	18	14	11	10	12	17	12	31	27	13	30	17	
chr15:48435425-48450866	15442	31	14	7	10	10	11	10	2	16	24	11	31	23	
chr21:34451725-34596450	144726	31	13	6	14	7	7	31	3	12	23	10	14	14	
chr3:195671625-195688075	16451	31	16	20	9	21	6	19	10	31	29	5	19	12	
chr7:140065900-140073425	7526	31	16	10	7	10	31	17	9	18	21	9	22	19	
chr10:9685125-9966750	281626	31	16	9	18	9	13	17	4	20	28	8	18	31	
chr6:131664050-131812075	148026	30	11	5	14	6	4	10	3	30	12	4	25	12	
chrX:109471950-109537125	65176	30	7	2	4	6	5	4	2	3	11	3	5	30	
chr2:218542550-218575300	32751	30	8	14	10	5	3	6	0	5	30	2	4	6	
chr1:199749575-199812300	62726	30	19	16	30	15	21	16	18	23	17	9	24	18	
chrX:56788625-56810725	22101	30	12	6	9	7	4	16	1	18	30	3	27	11	
chr8:104008750-104088225	79476	29	14	8	11	6	12	26	3	17	29	10	17	17	
chr2:138865825-138928025	62201	29	14	10	10	12	9	17	5	11	29	7	26	19	
chr5:38858825-38880150	21326	29	11	4	5	8	5	16	4	18	29	4	13	15	
chr9:131276675-131307550	30876	29	17	16	18	12	12	29	9	24	28	6	16	16	
chr20:48468950-48485200	16251	29	9	8	2	6	29	9	12	22	2	2	1	7	
chr13:64705550-64987675	282126	29	16	11	18	8	8	15	10	29	26	8	19	25	
chr11:22854575-22984925	130351	29	13	11	15	9	4	16	9	12	29	7	18	18	

location	span (nt)	max	mean	adipose	colon	heart	hypothalamus	kidney	liver	lung	ovary	skeletal muscle	spleen	testes	Comments
chr18:40170025-40351650	181626	29	18	10	20	11	9	26	11	21	29	10	24	26	
chr11:133854275-133909025	54751	29	8	2	29	2	2	27	3	6	6	2	5	5	
chr3:172974575-173020300	45726	29	8	6	17	4	4	3	3	5	29	1	14	5	
chr1:70914525-71011625	97101	28	8	5	5	5	3	28	3	9	10	4	7	9	
chr3:36894500-36969325	74826	28	13	8	23	7	7	6	4	18	27	2	28	12	
chr13:21470600-21702150	231551	28	13	9	15	6	3	28	7	17	6	2	23	27	
chr1:59032550-59113800	81251	28	10	6	12	3	7	12	1	10	28	9	19	8	
chr2:157897775-157942400	44626	28	10	6	8	5	4	8	3	18	13	4	28	9	
chr4:53272125-53310900	38776	28	10	3	10	6	5	10	7	17	11	4	9	28	
chr4:54914475-55161475	247001	28	15	8	23	9	7	18	7	20	28	6	19	21	
chr3:163927725-164333150	405426	28	15	9	22	10	9	17	4	15	28	9	19	21	
chr6:2198150-2222350	24201	28	15	11	19	10	6	27	7	15	28	7	22	15	
chr9:2193975-2294325	100351	28	10	5	6	4	4	7	7	28	22	4	12	9	
chrX:50921225-51011000	89776	27	10	5	9	5	6	10	2	9	27	6	20	11	
chr2:46863875-46966525	102651	27	10	7	10	4	7	7	5	27	17	7	12	9	
chr11:115542275-115819825	277551	27	10	6	15	5	11	27	10	8	7	3	8	13	
chr7:7895500-7963275	67776	27	12	7	8	6	4	15	2	16	27	14	20	16	
chrX:6755350-6907125	151776	27	17	13	22	11	8	25	6	18	26	12	27	20	
chr22:16799450-16847225	47776	27	13	5	27	11	7	21	3	12	14	7	21	14	
chr12:9371350-9397650	26301	27	4	1	2	1	1	3	1	2	2	1	2	27	
chr1:118176175-118202375	26201	27	13	10	5	16	18	27	4	10	14	13	16	12	
chr6:147231675-147541450	309776	26	15	6	22	9	14	12	4	16	26	6	25	26	
chrX:100582750-100611900	29151	26	10	7	10	5	4	14	2	9	26	7	15	13	
chr10:97629600-97656950	27351	26	10	5	7	8	4	14	2	12	15	4	26	10	
chr2:74066300-74115525	49226	26	11	10	26	8	3	10	3	17	11	2	25	6	
chr6:126421650-126451675	30026	26	10	3	5	6	4	21	11	5	26	10	14	8	
chr4:116354750-116650400	295651	26	15	10	20	11	9	17	5	18	23	7	19	26	
chr19:52838475-52873175	34701	26	10	7	26	7	5	9	2	14	9	10	13	9	
chr2:81715400-81955300	239901	26	14	10	17	9	8	18	5	13	26	9	19	20	
chr13:37514150-37567175	53026	26	13	6	8	23	6	26	4	11	22	6	12	15	
chr2:216786600-216817600	31001	26	10	10	6	6	26	9	3	11	9	10	11	7	
chr11:23093750-23240675	146926	26	14	7	21	8	14	17	5	17	26	7	19	17	
chr2:31919400-31946050	26651	25	11	4	7	8	5	9	4	9	19	16	25	12	
chr14:60598575-60663800	65226	25	14	16	13	7	8	18	2	16	25	16	14	19	
chr4:24126625-24137100	10476	25	10	2	5	7	9	10	2	10	25	8	21	7	
chr1:171471125-171555075	83951	25	13	6	9	8	7	15	6	16	25	8	23	14	
chr2:91630925-91689500	58576	25	15	19	23	16	17	17	8	7	13	12	3	25	
chr6:127722975-127746550	23576	25	11	6	7	10	5	12	8	13	25	6	15	15	
chr5:123269425-123559950	290526	25	15	10	17	12	12	15	5	18	25	10	19	20	
chr13:109079875-109141250	61376	25	8	3	6	4	5	5	2	12	7	3	25	13	
chr7:63559775-63598600	38826	25	8	4	6	5	6	9	2	9	25	3	11	12	
chr4:7154500-7226600	72101	25	13	8	9	10	23	25	9	13	11	11	16	10	
chr14:95058100-95070625	12526	25	12	9	15	5	8	10	7	11	25	7	23	13	
chr2:40800500-41088800	288301	25	14	12	19	10	9	12	5	23	25	7	12	23	
chr6:6628525-6756250	127726	25	11	11	10	7	4	25	5	13	12	13	9	18	
chr1:85705400-85746650	41251	25	10	5	3	13	4	7	5	18	13	6	25	8	
chrX:62428575-62446375	17801	24	11	8	5	9	24	8	4	7	18	8	11	14	
chr7:37575125-37663375	88251	24	13	5	12	7	19	13	7	12	18	8	24	23	
chr11:120733150-120748050	14901	24	13	10	13	19	13	10	6	10	20	11	6	24	
chr1:93931500-94066425	134926	24	16	11	16	14	8	19	4	19	24	23	20	20	
chr5:169491450-169587275	95826	24	5	2	4	1	1	24	1	5	4	1	4	3	
chr12:45330500-45384750	54251	24	10	3	8	4	5	24	7	9	19	10	11	12	
chr11:12937650-12965350	27701	24	4	0	1	1	3	24	0	2	1	2	1	6	
chr19:11961475-11976575	15101	24	7	5	7	4	3	5	7	24	5	3	6	10	
chr22:30445350-30475986	30637	24	13	12	23	9	10	13	4	10	22	4	24	15	
chr7:12729150-12829100	99951	24	9	4	10	12	3	5	4	9	10	24	6	9	
chr12:5102450-5197825	95376	24	7	6	24	3	5	6	2	11	5	3	5	6	
chr1:209631400-209656375	24976	24	9	5	8	5	4	12	5	7	13	3	13	24	
chr1:23477400-23492375	14976	24	10	4	7	5	3	11	3	8	24	10	20	14	
chr13:83475500-83726025	250526	24	9	5	9	4	5	7	5	18	10	4	11	24	
chr8:29402750-29731425	328676	24	16	13	20	10	11	16	8	17	24	23	23	14	
chr2:63825225-63857575	32351	24	6	23	2	2	3	2	3	2	5	24	3	4	
chr2:111674975-111729375	54401	23	9	6	5	9	5	9	1	14	14	3	23	6	
chr2:183740700-183793025	52326	23	9	4	9	6	3	8	3	9	23	9	17	10	
chr5:148209825-148249425	39601	23	9	10	4	11	3	3	2	23	3	20	13	2	
chr9:131132400-131148400	16001	23	6	4	4	3	4	23	1	8	3	1	10	3	
chr3:158291000-158313675	22676	23	3	1	1	23	1	1	1	3	1	1	1	1	
chr12:61283950-61301575	17626	23	9	5	5	7	4	13	4	10	15	8	23	6	
chr2:193651275-193911400	260126	23	13	10	14	8	7	15	5	14	23	7	21	15	
chr5:111819800-111931900	112101	23	13	10	14	8	6	16	4	14	21	6	23	16	
chr6:33913800-33955400	41601	23	3	1	2	1	1	23	0	1	1	0	1	4	
chr18:31720150-31782325	62176	23	8	4	7	6	3	15	2	5	10	3	7	23	
chr13:31411725-31443975	32251	23	6	5	3	23	1	7	3	6	7	3	5	4	
chr7:92358275-92489825	131551	23	14	10	16	9	8	15	8	23	21	6	16	20	
chr3:114236500-114337000	100501	23	11	5	13	9	7	12	3	9	23	7	21	14	
chr19:7320850-7339925	19076	23	5	1	11	2	2	2	0	4	3	1	23	2	
chrX:119236975-119259025	22051	23	8	12	5	4	5	8	4	7	23	4	13	7	
chr2:165216625-165235250	18626	23	7	11	2	3	1	23	5	6	8	2	11	2	
chr7:96103850-96148750	44901	23	9	3	7	9	4	12	2	7	23	9	19	7	
chr18:51712175-51765200	53026	23	4	0	1	1	1	3	1	3	23	0	11	3	
chr6:111912800-111930550	17751	23	10	11	7	5	5	10	3	14	23	7	15	12	
chrX:64926450-65001925	75476	23	15	11	13	14	11	18	9	15	22	12	23	21	
chr18:34964525-35098275	133751	23	6	3	6	2	2	9	2	6	7	3	6	23	
chr11:59416375-59538600	122226	23	11	6	12	8	7	18	4	10	23	6	16	15	
chr5:52567275-52762975	195701	22	11	7	15	7	5	11	3	11	22	7	15	15	
chr4:15266300-15304725	38426	22	15	17	7	11	19	22	9	15	16	6	22	17	
chr1:58845000-58872925	27926	22	9	9	8	7	5	22	12	8	6	5	9	7	
chr20:57146000-57188600	42601	22	5	0	4	1	2	2	0	11	2	0	22	4	
chr2:64956975-65041825	84851	22	10	7	12	4	6	9	3	10	11	6	22	14	
chr5:117175975-117309050	133076	22	14	6	13	15	9	19	4	18	21	6	22	20	
chr21:16915600-16926550	10951	22	4	3	1	2	1	4	1	2	22	3	0	5	
chr11:34675025-34851400	176376	22	14	8	19	10	12	14	4	15	20	8	21	22	
chr1:203936725-203948947	12223	22	7	2	5	4	3	5	2	4	16	6	22	6	
chr10:110110175-110275525	165351	22	11	6	14	6	6	22	3	9	18	6	17	16	
chr8:8814750-8878550	63801	22	10	7	11	5	7	22	2	19	9	3	14	8	
chr1:191151850-191212425	60576	22	9	6	5	6	4	5	5	17	6	22	11	13	
chr4:108966225-109026800	60576	22	9	5	9	12	4	16	10	22	7	3	9	6	
chr6:3944675-3963450	18776	22	10	5	9	5	3	11	3	22	16	6	18	12	
chr13:107690275-107702750	12476	22	8	5	3	7	3	9	4	12	8	5	22	5	
chrX:130649675-130814125	164451	22	11	8	12	7	8	9	5	18	18	5	22	11	
chr6:111946650-111981100	34451	21	11	12	9	7	9	10	2	13	21	10	16	11	
chr10:112658400-112668025	9626	21	6	2	3	3	3	4	1	5	21	2	12	7	
chr11:130235450-130250500	15051	21	12	15	7	10	21	14	6	8	14	7	13	12	
chr1:110606700-110664300	57601	21	11	14	6	11	7	7	8	8	21	17	12	9	
chr9:2746150-2763400	17251	21	10	5	8	6	10	21	1	5	17	11	10	10	
chr2:24208525-24239425	30901	21	9	6	7	7	6	8	1	9	21	9	10	13	
chr3:13663800-13793125	129326	21	9	9	9	5	8	8	8	21	10	3	8	12	
chr4:74354350-74381575	27226	21	9	5	6	4	5	15	4	10	21	4	11	11	
chr7:20842975-21086525	243551	21	13	9	19	7	8	14	6	14	18	8	16	21	
chr13:50001250-50161600	160351	21	11	4	21	6	4	8	3	9	12	20	14	19	
chr21:15555275-15773775	218501	21	12	21	9	4	5	13	18	10	10	18	11	12	
chr3:9403550-9411575	8026	21	11	6	9	9	6	14	5	9	17	6	21	15	
chr7:30386175-30420575	34401	21	11	8	18	4	5	19	3	13	9	12	21	8	
chr5:98293600-98305025	11426	21	9	4	3	6	6	10	2	6	21	18	21	6	
chr11:38040175-38218375	178201	21	13	7	18	7	5	20	6	19	21	7	13	21	
chr19:63509250-63520475	11226	21	13	9	11	10	16	14	9	12	21	5	20	12	
chr11:58486575-58554025	67451	21	13	8	13	7	8	18	7	13	21	6	21	17	
chr13:55006675-55166650	159976	21	11	6	11	6	6	14	3	13	20	6	21	11	
chr5:134953975-135046175	92201	21	5	3	5	2	2	21	2	5	3	1	3	7	
chr20:56260575-56317475	56901	21	11	8	21	6	4	13	2	15	19	1	19	11	
chr2:39574775-39654150	79376	21	13	18	9	12	12	21	6	14	18	12	10	7	
chr4:109632100-109739300	107201	20	11	5	11	16	6	20	5	13	15	5	15	13	
chr1:32287575-32302500	14926	20	8	5	11	4	4	6	2	14	20	5	11	8	
chr18:5222477-5236926	14450	20	14	15	9	20	14	11	11	10	20	14	20	14	

location	span (nt)	max	mean	adipose	colon	heart	hypothalamus	kidney	liver	lung	ovary	skeletal muscle	spleen	testes	Comments
chr7:9904850-10020150	115301	20	6	3	6	3	2	6	2	6	8	3	7	20	
chr17:30860150-30875450	15301	20	5	3	3	3	1	3	2	12	5	2	20	2	
chr3:71738000-71797250	59251	20	8	4	7	3	3	6	2	18	15	2	20	6	
chr5:175530325-175540750	10426	20	3	0	1	0	0	1	0	0	12	0	2	20	
chr1:22292500-22313675	21176	20	8	3	10	6	2	7	2	8	18	6	20	6	
chr13:30077400-30089300	11901	20	5	3	2	1	2	5	0	2	20	4	6	4	
chr11:17334725-17362725	28001	20	8	2	11	3	17	20	1	5	10	10	6	7	
chr9:21481050-21587675	106626	20	11	6	9	5	5	17	8	20	15	4	12	16	
chr6:129882650-129921300	38651	20	8	7	5	6	3	14	1	20	11	4	9	5	
chr18:4368300-4442750	74451	20	7	2	6	2	12	2	0	7	20	1	6	20	
chr10:71853850-71860875	7026	20	13	19	6	14	8	20	9	12	14	11	18	11	
chr2:187712425-187798175	85751	20	6	6	4	4	2	7	1	7	12	2	7	20	
chr1:101291125-101334500	43376	20	6	3	3	4	2	7	1	6	9	3	20	6	
chr18:956025-1015450	59426	20	7	4	8	4	3	7	2	7	20	3	9	8	
chr1:158198825-158225975	27151	20	3	2	20	1	1	1	1	1	2	1	1	1	
chr8:142313025-142336125	23101	20	8	4	8	7	16	12	1	8	6	3	8	20	
chr21:38511875-38532825	20951	19	5	3	1	2	1	2	5	19	1	1	12	7	
chr18:66398450-66514300	115851	19	10	5	11	4	6	17	7	15	9	4	8	19	
chr7:32919525-32950200	30676	19	11	11	16	9	10	8	4	16	19	8	12	10	
chrX:64727500-64769875	42376	19	7	4	10	4	3	7	1	11	19	3	10	7	
chr4:14744675-14820275	75601	19	11	8	11	12	9	15	3	12	19	6	16	14	
chr2:43167775-43268825	101051	19	9	5	19	7	4	6	3	14	9	5	16	9	
chr7:45950100-46055950	105851	19	10	4	10	5	5	11	4	10	19	6	18	15	
chr7:65806575-65829700	23126	19	9	6	15	5	5	8	6	15	19	3	11	9	
chr3:186480050-186516225	36176	19	8	4	5	5	6	13	4	6	19	2	10	15	
chr2:207387650-207464200	76551	19	10	7	8	5	11	19	7	13	14	11	6	10	
chr4:24416150-24422275	6126	19	7	6	4	7	19	5	3	6	12	3	5	9	
chr18:43586600-43607850	21251	19	8	5	4	4	7	8	2	9	19	5	13	7	
chr4:9776300-10014350	238051	19	8	3	9	4	4	8	3	8	15	5	11	19	
chr5:53893950-54004750	110801	19	9	6	6	8	6	11	6	19	16	4	12	11	
chr12:58277175-58299650	22476	19	5	1	2	5	1	6	1	6	7	2	19	3	
chr5:141750225-141851975	101751	19	7	7	8	4	5	7	2	19	5	4	8	5	
chr6:167309700-167331675	21976	19	7	4	8	2	3	10	2	7	10	2	14	19	
chr12:71550025-71815050	265026	19	10	7	12	7	6	14	4	10	19	6	15	16	
chr15:54850700-54891650	40951	19	6	5	4	3	3	6	1	6	19	3	8	8	
chr19:21530650-21577025	46376	19	8	7	11	4	3	7	1	7	19	7	7	14	
chr7:134416925-134469600	52676	19	12	11	7	9	10	16	4	8	16	12	17	19	
chr15:28935575-28965125	29551	19	6	4	5	4	4	3	5	19	4	1	7	13	
chr2:62348300-62566800	218501	19	12	8	19	8	6	16	7	19	16	5	18	12	
chr2:60086175-60209975	123801	19	11	7	12	9	6	14	5	11	19	6	14	14	
chr3:87221775-87276850	55076	19	6	3	9	3	4	5	1	5	19	3	7	7	
chr2:86718725-86789500	70776	19	9	5	12	5	6	10	3	8	19	5	14	11	
chr3:39600250-39734000	133751	19	12	10	14	10	8	15	6	17	19	8	13	15	
chr2:156711975-156818525	106551	19	10	4	19	4	4	14	7	17	12	3	9	12	
chr20:35569025-35576975	7951	19	8	10	11	3	2	9	2	7	10	19	8	4	
chr2:85039975-85050950	10976	19	8	4	15	4	3	6	2	7	12	6	19	7	
chr2:63710675-63736750	26076	19	6	19	4	2	2	3	3	3	5	13	5	6	
chr5:115659375-115672450	13076	18	7	4	4	6	4	11	2	4	18	5	10	7	
chr18:14043400-14092850	49451	18	9	6	10	5	8	9	3	8	18	4	11	13	
chr8:50050850-50118750	67901	18	11	7	12	9	6	12	4	13	18	6	14	15	
chr12:66683375-66716700	33326	18	4	1	4	3	0	1	1	4	1	9	18	1	
chr12:78463525-78491000	27476	18	8	1	17	3	2	14	4	10	18	1	11	8	
chr8:96031525-96053050	21526	18	7	4	7	2	3	6	2	8	18	8	10	10	
chr6:14268375-14287575	19201	18	4	5	1	0	1	3	4	18	2	0	8	2	
chr10:114579650-114681675	102026	18	11	6	17	6	8	15	3	15	18	7	16	10	
chr12:88455700-88470575	14876	18	8	2	4	3	3	11	2	8	18	6	17	10	
chr3:107171400-107322450	151051	18	9	7	10	6	3	7	3	16	13	4	10	18	
chr9:4859500-4886050	26551	18	8	7	5	3	5	9	4	6	16	18	8	5	
chr13:102930525-103035800	105276	18	8	5	8	4	3	7	6	18	10	3	7	13	
chr8:128876225-128890125	13901	18	4	1	4	2	0	1	1	3	18	2	7	4	
chr5:42999600-43035650	36051	18	5	4	3	4	3	3	3	9	4	2	18	6	
chrX:61861575-61957675	96101	18	13	15	7	17	15	15	7	11	18	9	18	16	
chr3:132569625-132581425	11801	18	7	2	4	4	4	16	2	5	18	2	11	4	
chr4:10580400-10591100	10701	18	3	1	1	1	0	1	3	18	1	0	1	2	
chr3:88233150-88258600	25451	18	9	6	5	5	3	10	3	15	18	6	15	11	
chrX:148295250-148343850	48601	18	7	4	8	4	3	8	2	6	18	4	10	9	
chr3:179056150-179146000	89851	18	10	8	10	7	5	16	8	12	18	5	13	10	
chr4:4596450-4633950	37501	18	8	5	11	4	7	12	1	4	18	3	12	9	
chr5:80216225-80255550	39326	18	6	5	4	3	5	18	3	4	6	5	7	4	
chr18:70409300-70433425	24126	18	7	4	4	5	5	10	3	5	15	2	18	8	
chr5:127413650-127433475	19826	17	7	4	6	5	4	8	1	5	17	4	17	7	
chr8:2156200-2269600	113401	17	9	5	17	6	5	13	3	14	14	5	11	12	
chr1:146173150-146194275	21126	17	8	7	4	5	7	6	4	7	15	2	10	17	
chr15:40664800-40688350	23551	17	8	6	9	4	2	4	3	17	12	2	7	17	
chr7:34182075-34270925	88851	17	8	5	10	5	3	10	2	9	17	4	11	11	
chr10:38813750-38924000	110251	17	4	2	12	1	2	2	0	1	2	1	2	17	
chr17:41052691-41073901	21211	17	8	4	13	4	8	9	1	7	17	3	14	15	
chr4:84354450-84393075	38626	17	8	3	8	4	3	9	3	6	17	7	17	12	
chr3:131096775-131170750	73976	17	8	4	10	5	8	8	2	7	17	4	12	9	
chr19:42146075-42209750	63676	17	10	4	9	7	12	10	2	9	14	11	17	17	
chr11:70540100-70637075	96976	17	7	3	16	2	3	17	10	8	5	2	5	5	
chr7:105313050-105325325	12276	17	6	7	12	1	7	17	1	2	8	1	2	5	
chr7:134656800-134676775	19976	17	4	4	2	1	1	2	4	17	2	1	1	4	
chr8:90684400-90697825	13426	17	5	3	2	4	4	9	0	4	17	1	4	6	
chr5:149846625-149861900	15276	17	7	3	8	5	2	8	5	8	11	1	17	4	
chr1:222769950-222845375	75426	17	5	3	5	3	5	7	1	5	17	3	7	5	
chr4:123291775-123305200	13426	17	7	6	6	4	3	9	1	11	17	1	15	9	
chr3:154391400-154411650	20251	17	7	17	3	3	6	2	5	11	3	2	4	17	
chr12:74301700-74547775	246076	17	11	7	12	6	6	15	5	14	17	7	13	16	
chr10:89898950-89909300	10351	17	2	0	0	0	0	1	0	1	0	17	1	1	
chr12:125726100-125824025	97926	17	7	4	8	3	4	6	5	13	5	2	17	13	
chr2:37629600-37715550	85951	17	8	5	9	5	3	7	11	12	9	7	17	8	
chr2:143157200-143262500	105301	17	9	6	10	6	6	10	4	10	14	4	10	17	
chr5:121017625-121116700	99076	17	9	6	9	6	4	11	4	11	17	6	13	15	
chr20:48695925-48741175	45251	17	5	3	6	4	2	17	2	3	5	2	3	4	
chr12:52809275-52855625	46351	17	6	8	17	3	2	9	1	4	6	4	6	9	
chr11:94940400-95122275	181876	17	8	5	11	5	6	8	2	9	13	3	17	11	
chr6:109112925-109128100	15176	16	8	4	8	8	2	7	2	9	10	16	16	4	
chr3:16120050-16171700	51651	16	4	3	2	2	3	4	1	2	6	16	3	3	
chr2:224184075-224295925	111851	16	9	6	11	6	6	7	7	16	8	7	11	9	
chr11:103977525-104180775	203251	16	8	7	8	14	3	5	5	14	10	2	7	16	
chr8:66664400-66675375	10976	16	8	7	4	6	16	8	3	4	15	8	7	6	
chr5:106317850-106369550	51701	16	8	4	7	4	16	9	1	6	9	10	9	11	
chr4:76201975-76270600	68626	16	10	5	16	5	4	13	4	14	15	5	14	12	
chr12:67055575-67125325	69751	16	6	4	11	2	1	8	1	10	5	2	16	5	
chrX:138078900-138094350	15451	16	5	6	4	2	1	7	1	2	6	16	4	7	
chr1:33641707-33668475	26769	16	8	4	6	9	6	8	2	6	13	5	16	8	
chr4:104483350-104521000	37651	16	4	3	1	2	1	2	6	16	3	1	1	5	
chr10:47057100-47109425	52326	16	4	1	5	1	0	5	1	5	1	0	16	6	
chr16:78259025-78345200	86176	16	7	4	5	3	3	10	4	16	15	2	3	13	
chrX:45782500-45808725	26226	16	9	8	16	7	8	8	8	7	9	4	12	10	
chr11:121753000-121870050	117051	16	8	5	8	4	5	7	4	16	11	4	10	14	
chr3:153786825-153905900	119076	16	9	5	6	6	12	7	5	16	9	15	7	8	
chr5:31025075-31188875	163801	16	8	6	7	4	4	9	5	14	16	5	14	9	
chr20:30902300-30911125	8826	16	10	7	6	10	14	16	3	13	10	4	14	14	
chr17:26911725-26949250	37526	16	5	16	8	5	1	4	4	3	12	1	3	2	
chr13:110206850-110287225	80376	16	4	2	3	2	2	3	3	8	3	1	4	16	
chr19:41757025-41772825	15801	16	8	5	6	5	8	10	2	9	14	2	16	9	
chr12:7153400-7167400	14001	16	6	3	9	3	2	14	3	6	4	2	16	4	
chr5:12678075-12800025	121951	16	7	6	6	4	4	7	4	11	9	3	16	13	
chr4:37873600-38109850	236251	16	9	6	13	5	9	16	4	9	12	4	11	11	
chr7:155933475-155996100	62626	16	5	2	16	2	2	7	2	4	5	3	4	6	
chr1:191667675-191731275	63601	16	6	2	4	2	2	4	8	7	16	2	8	5	
chr3:88209750-88229200	19451	16	7	4	4	4	3	7	2	10	16	3	10	9	

location	span (nt)	max	mean	adipose	colon	heart	hypothalamus	kidney	liver	lung	ovary	skeletal muscle	spleen	testes	Comments
chr9:33001575-33014725	13151	16	8	6	7	4	4	10	2	8	16	8	11	14	
chr4:26418775-26459200	40426	16	5	1	6	2	2	16	1	4	12	1	4	6	
chr4:9729950-9746600	16651	16	2	0	0	0	0	1	0	0	1	0	1	16	
chr14:38898625-38914350	15726	16	5	1	4	3	1	16	3	4	6	4	9	4	
chr8:126604125-126708875	104751	16	8	4	9	5	4	16	8	10	12	4	9	10	
chr2:183481775-183495675	13901	15	8	3	5	8	5	9	3	13	14	15	6	6	
chr7:22860450-22876975	16526	15	5	3	5	5	1	2	3	7	7	15	8	4	
chr8:37377750-37470000	92251	15	8	6	10	5	4	9	2	6	12	4	15	11	
chr5:6882550-6906100	23551	15	7	4	2	4	8	12	6	8	5	4	15	7	
chr3:6508350-6577400	69051	15	7	5	10	4	3	9	3	6	10	3	9	15	
chr6:32970575-32980325	9751	15	4	1	2	3	4	3	1	5	4	2	15	5	
chr6:5830225-5909500	79276	15	9	6	13	5	4	10	3	9	15	4	12	12	
chrX:124289750-124417275	127526	15	8	5	8	6	6	9	3	9	15	3	10	9	
chr3:22263700-22385225	121526	15	4	3	4	1	3	3	2	4	7	2	5	15	
chr6:125271300-125315975	44676	15	6	3	5	3	3	4	1	7	10	15	4	6	
chr15:38123550-38145575	22026	15	11	7	14	12	11	13	2	9	15	11	15	14	
chr6:45632625-45776550	143926	15	8	5	11	4	4	8	2	13	13	3	15	11	
chr8:134678950-134886525	207576	15	9	7	15	6	6	9	3	11	14	6	13	10	
chr8:96366700-96483000	116301	15	8	3	9	4	3	13	2	10	15	6	14	10	
chr13:50430725-50449550	18826	15	5	3	3	3	2	4	1	6	10	2	15	5	
chr10:39116700-39194725	78026	15	3	1	15	1	1	1	0	0	1	0	2	7	
chr3:109306850-109354500	47651	15	2	1	2	1	1	1	0	1	1	1	15	1	
chr9:238650-254175	15526	15	4	4	3	2	2	5	1	9	1	1	15	3	
chr9:2768700-2781525	12826	15	7	3	6	4	7	14	2	6	15	7	7	4	
chr8:20314425-20440800	126376	15	5	2	6	3	2	6	2	15	4	2	4	6	
chr1:230326450-230420025	93576	15	5	5	5	3	4	4	6	15	6	2	4	7	
chr14:47751025-47801225	50201	15	4	2	3	1	3	6	1	3	15	2	4	4	
chr6:4958950-5001875	42926	15	8	6	8	5	6	10	5	9	11	4	15	9	
chr3:111683075-111833475	150401	15	7	5	8	4	4	8	2	7	15	5	11	11	
chr2:238197525-238231375	33851	15	7	4	5	9	3	10	2	8	10	12	15	3	
chr8:96157275-96189075	31801	14	8	7	14	7	12	5	6	14	6	4	8	6	
chr12:30619850-30648175	28326	14	6	3	6	4	3	7	2	4	14	6	7	7	
chr13:90799275-90818300	19026	14	5	1	2	2	1	4	6	3	13	1	14	3	
chr2:42909150-43019900	110751	14	8	5	10	6	4	8	2	9	14	3	12	9	
chr7:37994575-38045650	51076	14	6	3	4	2	14	7	2	7	11	2	5	6	
chr2:111158925-111171375	12451	14	2	0	1	0	0	1	0	1	1	0	1	14	
chr8:135395125-135545175	150051	14	7	5	9	4	3	10	2	7	8	4	14	8	
chr2:218303850-218326725	22876	14	7	14	13	9	2	12	1	6	5	2	7	4	
chr10:122712875-122729225	16351	14	2	1	1	0	0	1	0	14	1	0	0	1	
chr7:26409925-26454600	44676	14	5	3	3	1	2	9	0	3	8	3	6	14	
chr3:179314975-179522075	207101	14	8	5	9	6	6	10	1	11	14	4	10	11	
chr18:30822250-30849925	27676	14	3	1	5	1	7	2	1	2	2	14	2	1	
chr10:28852675-28860675	8001	14	9	8	3	11	12	13	5	7	14	5	14	10	
chr6:130618000-130674625	56626	14	5	3	4	2	2	4	3	9	14	1	6	4	
chr3:18261750-18340875	79126	14	4	2	3	2	2	3	3	14	3	1	3	6	
chr2:152335500-152361525	26026	14	7	3	6	6	3	9	2	11	13	9	14	6	
chr7:17207775-17298625	90851	14	7	5	10	4	4	8	4	8	14	4	11	9	
chr7:112534500-112586075	51576	14	5	3	2	12	3	14	3	4	3	4	6	4	
chr3:31215650-31350275	134626	14	4	2	5	2	2	5	1	6	14	2	4	6	
chr7:81280875-81388725	107851	14	8	5	9	4	4	13	2	7	14	5	10	10	
chr12:122180525-122199700	19176	14	4	1	6	2	1	6	1	4	14	1	3	3	
chrX:63958875-64010675	51801	14	7	4	6	5	4	11	4	10	14	6	10	6	
chr8:132833800-132925750	91951	14	8	7	8	5	5	10	3	9	14	6	14	11	
chr12:123133225-123150050	16826	14	4	2	14	1	6	6	0	3	4	1	3	3	
chr14:58952300-58981800	29501	14	7	6	5	8	5	11	2	4	14	11	6	6	
chr5:115676975-115691975	15001	14	6	2	2	3	1	8	2	5	14	7	11	6	
chr8:142343125-142385200	42076	14	7	6	11	4	3	11	0	14	10	2	6	11	
chr2:168054925-168131600	76676	14	8	4	14	5	8	7	2	8	13	6	11	13	
chr14:55845175-55901650	56476	14	6	4	5	3	1	13	1	7	14	2	13	5	
chr11:27140600-27204675	64076	14	7	4	8	5	4	14	2	5	10	5	12	9	
chr8:96058825-96075650	16826	14	7	4	5	3	4	4	5	14	12	8	11	11	
chr5:85778600-85902825	124226	14	9	5	11	5	5	12	4	8	14	5	12	14	
chr13:40963250-41020000	56751	14	5	3	7	2	3	6	1	5	14	4	5	5	
chr11:87745400-87779800	34401	14	6	4	6	4	3	8	1	8	14	3	13	7	
chr6:82479050-82494400	15351	14	4	1	1	5	1	3	2	5	14	2	6	4	
chr15:60516650-60535375	18726	14	5	14	7	3	4	6	0	1	8	2	4	3	
chr3:4205450-4228475	23026	14	6	3	2	4	2	7	2	6	12	5	11	14	
chr2:204328625-204374375	45751	14	7	5	9	6	6	6	7	6	14	4	11	7	
chr1:223490025-223552350	62326	14	6	3	6	4	3	10	2	7	14	2	5	13	
chr19:52805475-52829480	24006	14	6	3	14	4	3	7	2	7	7	5	10	5	
chr3:130558200-130572550	14351	14	6	3	8	4	8	5	1	5	14	3	10	5	
chr3:189028725-189090525	61801	14	7	5	8	5	5	11	2	8	14	6	10	9	
chr2:237508075-237634675	126601	14	6	5	6	4	3	9	3	6	14	4	6	8	
chr14:91291375-91306825	15451	14	5	1	6	6	1	6	1	14	4	1	10	5	
chr7:41824675-41897575	72901	14	3	2	2	1	2	2	4	14	2	1	5	4	
chr2:47401150-47424250	23101	14	5	2	13	2	14	5	1	3	7	1	5	4	
chr1:141594750-141604925	10176	14	6	4	7	4	3	4	1	3	14	3	12	9	
chr12:14272950-14321775	48826	14	7	4	9	5	5	9	2	9	14	3	8	10	
chr16:13349700-13364025	14326	14	3	3	3	1	3	3	4	2	14	1	3	2	
chr1:171137475-171174300	36826	13	4	2	2	2	1	13	0	8	4	1	4	3	
chr6:3582800-3664950	82151	13	8	4	9	6	13	8	2	10	12	5	10	13	
chr1:73192025-73234050	42026	13	6	3	7	4	4	9	1	6	13	4	10	8	
chr21:18208125-18299975	91851	13	7	3	10	5	2	8	3	8	9	3	9	13	
chr5:57279250-57389025	109776	13	7	4	9	4	4	13	3	8	13	4	10	9	
chr9:23587225-23674450	87226	13	4	1	4	5	5	5	1	3	6	1	3	13	
chr3:153267725-153412750	145026	13	7	5	10	4	3	6	2	10	8	5	13	9	
chr13:29122375-29189475	67101	13	6	3	13	3	2	6	5	4	13	3	11	6	
chr13:47278600-47390025	111426	13	10	13	10	6	5	13	5	10	12	10	13	13	
chr12:88272125-88284300	12176	13	6	6	6	6	4	13	1	6	9	2	11	5	
chr1:142295250-142311025	15776	13	7	5	10	5	7	6	3	4	13	2	8	13	
chr5:29549850-29625000	75151	13	6	4	6	4	2	6	2	7	13	4	8	8	
chr21:25856650-25874075	17426	13	4	2	2	2	2	2	0	5	6	2	13	5	
chr7:77116150-77141875	25726	13	5	2	7	4	2	6	2	6	7	5	13	3	
chr14:49552850-49579050	26201	13	3	1	4	1	1	2	1	8	1	1	13	3	
chr9:127876000-127959875	83876	13	7	3	9	4	4	10	2	8	13	4	8	8	
chr15:62540775-62558475	17701	13	6	3	9	4	2	8	1	7	12	2	13	5	
chr3:4343750-4357600	13851	13	6	5	7	4	5	9	2	6	10	4	13	5	
chr13:48083800-48168625	84826	13	7	3	9	6	3	8	2	9	13	3	12	6	
chr4:68258750-68278525	19776	13	7	4	3	7	6	13	6	3	10	4	11	6	
chr3:190140525-190153400	12876	13	3	7	1	2	1	2	13	2	1	1	2	1	
chr5:10213975-10266750	52776	13	7	4	8	4	4	12	1	6	13	6	11	8	
chr6:41183175-41203475	20301	13	6	3	6	3	2	5	4	13	9	8	8	10	
chr1:87930500-88173750	243251	13	7	6	13	5	6	6	2	10	10	3	7	7	
chr1:222690675-222713925	23251	13	6	4	9	3	2	7	1	4	13	6	8	6	
chr12:64277400-64357675	80276	13	8	7	5	8	11	8	6	9	13	6	12	7	
chr13:19075800-19097525	21726	13	6	3	6	3	3	7	2	7	13	4	7	8	
chrX:127995400-128076925	81526	13	8	6	12	6	6	10	3	11	13	5	11	9	
chr5:150289375-150308950	19576	13	5	2	2	4	2	9	0	3	13	2	4	9	
chrX:75319875-75330775	10901	13	7	5	3	5	3	9	12	7	6	7	13	5	
chr3:37389200-37409350	20151	13	7	4	7	4	2	10	3	13	8	12	8	6	
chr7:125329375-125521800	192426	13	9	8	12	6	5	11	3	12	12	5	13	10	
chr14:95413500-95462925	49426	13	2	0	1	0	13	0	0	0	0	0	0	2	
chr5:25365475-25481600	116126	13	7	4	9	7	4	10	2	10	13	5	10	10	
chr14:27161250-27210900	49651	13	4	2	3	1	5	13	1	2	4	1	3	4	
chr10:118564075-118598450	34376	13	5	2	5	3	6	13	1	5	8	1	11	6	
chr2:167280525-167386875	106351	13	6	3	6	3	3	6	1	6	13	5	8	7	
chr8:118749225-118816450	67226	13	6	5	6	5	2	4	4	13	8	2	12	9	
chr3:177467675-177586425	118751	12	7	3	8	5	4	9	3	10	12	4	10	10	
chrX:67684350-67726050	41701	12	8	4	8	5	4	9	3	10	12	5	12	9	
chr19:57268625-57289533	20909	12	5	4	5	4	2	5	2	5	12	2	10	6	
chr4:55363650-55399250	35601	12	6	4	7	4	3	7	2	5	12	5	10	9	
chrX:102738075-102748350	10276	12	3	1	2	2	2	4	1	2	12	3	5	4	
chr12:123664425-123811800	147376	12	7	9	12	4	3	5	8	8	7	3	6	7	
chr12:41907575-41950125	42551	12	5	3	5	3	3	5	1	4	12	2	6	6	

location	span (nt)	max	mean	adipose	colon	heart	hypothalamus	kidney	liver	lung	ovary	skeletal muscle	spleen	testes	Comments
chr1:22450050-22467875	17826	12	3	1	4	1	1	1	12	2	2	1	1	1	
chr1:9129450-9164075	34626	12	7	10	8	5	3	4	1	12	12	4	6	11	
chr8:110241350-110293300	51951	12	5	1	3	5	2	5	1	6	9	10	12	4	
chr3:67820975-67872650	51676	12	6	4	7	4	2	12	6	5	12	3	6	8	
chr1:245000875-245012875	12001	12	5	4	8	3	5	7	3	2	12	4	3	7	
chr9:32926300-32939200	12901	12	3	2	1	1	2	12	4	1	3	1	3	2	
chr13:72807725-72937000	129276	12	6	3	12	3	3	6	2	6	10	2	8	7	
chr6:126028200-126084725	56526	12	6	3	4	3	3	10	2	7	9	4	12	6	
chr8:130092325-130220700	128376	12	6	4	8	2	3	6	5	12	8	2	12	7	
chr13:79335200-79412750	77551	12	7	6	6	4	4	7	2	6	12	6	9	9	
chr2:121545350-121619175	73826	12	6	6	10	4	7	6	2	6	12	4	6	6	
chr2:192461425-192508825	47401	12	5	2	5	6	2	3	1	9	12	1	4	5	
chr4:4962125-5022325	60201	12	7	5	10	4	4	7	4	12	10	3	9	11	
chr8:79929700-79960525	30826	12	5	2	4	2	2	9	4	12	4	1	12	5	
chr13:50544475-50665475	121001	12	6	4	7	3	4	8	2	7	9	5	10	12	
chr12:50511350-50526675	15326	12	2	0	1	1	0	12	0	2	1	0	1	1	
chr13:49455650-49466250	10601	12	5	3	4	3	2	3	0	5	5	12	11	5	
chr18:52870875-52909150	38276	12	7	4	8	5	4	9	2	8	12	4	8	9	
chr17:21373950-21390575	16626	12	4	4	3	4	12	2	3	2	4	2	4	4	
chr3:37839075-37851625	12551	12	5	2	3	7	1	5	2	2	10	2	12	4	
chr18:37312775-37462750	149976	12	6	3	12	4	5	4	4	5	11	2	5	7	
chr4:109327675-109455425	127751	12	6	4	8	2	3	7	1	5	8	3	12	12	
chr12:111269000-111299725	30726	12	6	2	12	5	3	8	2	9	10	2	9	8	
chr19:35205200-35223675	18476	12	5	2	3	2	5	6	2	3	12	5	10	5	
chr1:199858800-199883250	24451	12	5	11	11	5	7	2	1	12	2	2	4	3	
chr3:139242175-139254800	12626	12	4	3	3	2	1	6	0	4	12	1	5	2	
chr13:53710800-53826900	116101	12	5	3	4	4	3	7	2	5	12	3	7	7	
chr2:19091050-19143450	52401	12	7	5	5	4	3	12	3	8	11	6	8	8	
chr5:95329600-95376075	46476	12	5	4	4	3	2	6	3	10	12	3	6	7	
chr4:9413775-9428250	14476	12	2	1	1	1	0	12	1	1	1	0	2	1	
chr12:45538425-45587250	48826	12	5	2	3	3	3	10	1	4	12	11	4	7	
chr14:96479600-96696875	217276	12	7	5	12	4	5	7	2	10	9	2	10	8	
chr3:180146275-180207400	61126	12	6	5	5	5	5	7	5	6	4	4	12	6	
chr1:113341575-113352750	11176	12	4	2	4	3	2	2	1	2	12	4	5	3	
chr12:46324075-46343100	19026	12	5	2	3	3	5	4	2	2	12	5	8	6	
chr19:21811825-21825825	14001	12	5	2	8	3	2	5	1	5	12	1	11	5	
chr13:75944675-76053050	108376	12	6	5	6	4	3	5	4	11	10	3	6	12	
chr8:90285550-90313750	28201	12	6	3	6	4	4	9	1	4	12	4	7	7	
chr13:37380475-37450700	70226	12	5	3	5	5	2	12	2	5	11	2	7	6	
chr2:218521300-218534150	12851	12	3	5	5	2	0	2	0	2	12	1	2	2	
chr6:141206225-141265250	59026	12	7	12	8	8	3	6	2	10	9	4	10	9	
chr3:103307650-103425850	118201	12	7	6	8	5	3	9	2	7	12	5	11	9	
chr8:83217050-83316925	99876	11	6	3	9	4	3	6	2	6	9	4	8	11	
chr17:34952496-34983927	31432	11	7	5	9	4	3	10	3	8	11	5	11	8	
chr6:167232425-167255350	22926	11	3	1	2	1	3	1	0	4	3	0	9	11	
chr1:105438075-105481400	43326	11	3	1	2	1	1	2	3	11	2	1	2	3	
chr12:84499575-84625775	126201	11	5	2	6	3	3	5	3	6	6	2	5	11	
chr14:84031800-84050525	18726	11	6	3	7	4	6	11	1	9	11	3	6	8	
chr2:101190700-101222900	32201	11	5	2	4	3	1	6	1	3	11	4	11	4	
chr2:73789225-73801625	12401	11	2	0	1	0	1	5	0	1	4	0	11	2	
chr12:3767950-3783200	15251	11	4	2	2	2	3	3	1	3	11	2	10	3	
chr2:105547175-105636100	88926	11	3	2	4	2	1	3	4	11	2	1	2	6	
chr6:155257875-155278175	20301	11	6	3	7	5	2	5	1	5	11	7	11	5	
chr5:74363725-74384025	20301	11	5	2	6	2	3	9	1	5	11	1	8	7	
chrX:49575900-49588600	12701	11	3	1	3	1	1	11	1	2	7	1	3	3	
chr1:196813275-196869300	56026	11	3	1	1	1	1	4	2	11	2	0	3	5	
chr4:82382450-82416400	33951	11	3	2	11	1	1	2	6	7	1	1	1	2	
chr7:105333950-105351950	18001	11	4	6	8	1	6	11	1	1	5	1	1	4	
chr21:43576100-43643725	67626	11	4	2	6	3	3	5	0	3	5	2	11	2	
chr3:152871900-152886900	15001	11	3	2	3	2	1	2	3	11	2	1	2	4	
chr3:59325725-59437400	111676	11	7	5	9	5	4	9	2	5	11	5	9	9	
chr15:39365175-39377125	11951	11	7	5	10	4	3	10	4	10	11	5	11	5	
chr14:87558425-87570400	11976	11	3	1	1	1	1	2	11	4	2	1	6	2	
chr17:15326100-15332950	6851	11	4	2	8	2	3	4	1	3	11	1	5	2	
chr6:41475450-41577700	102251	11	5	3	11	3	4	4	2	6	7	2	5	7	
chr12:59462625-59500450	37826	11	6	4	6	4	4	7	1	6	11	3	7	10	
chr1:66644600-66679850	35251	11	5	3	5	4	2	6	3	5	11	5	7	6	
chr4:125047300-125156250	108951	11	6	3	7	11	2	7	1	5	8	7	5	6	
chr4:5953425-5987275	33851	11	2	0	1	0	11	1	0	1	1	0	2	1	
chr1:72768625-72851975	83351	11	4	2	3	2	3	3	2	8	11	1	2	4	
chr2:158279100-158295500	16401	11	4	1	3	3	1	11	1	2	6	5	3	3	
chr21:14711200-14767900	56701	11	3	2	2	1	1	2	2	11	3	0	2	3	
chr10:54913075-54953825	40751	11	6	3	7	4	4	9	1	7	11	3	9	8	
chr8:142095575-142171400	75826	11	3	2	6	2	1	3	2	11	4	1	3	3	
chr11:2404925-2415425	10501	11	2	0	11	0	0	3	0	1	0	0	2	2	
chr12:129625700-129695450	69751	11	5	1	9	2	9	9	1	3	11	1	3	5	
chr6:57029975-57042500	12526	11	4	1	3	2	4	3	1	3	11	2	7	4	
chr6:166166775-166177900	11126	11	2	0	1	1	2	8	0	0	11	0	0	3	
chr1:226324900-226335050	10151	11	3	1	3	2	1	2	0	2	11	1	6	3	
chr5:68020875-68039900	19026	11	6	4	7	4	3	6	5	5	11	4	7	7	
chr6:81204400-81225475	21076	11	4	2	4	1	2	8	2	3	11	2	3	4	
chr8:72732550-72834350	101801	11	6	4	8	4	4	8	2	7	11	3	9	9	
chr3:113609400-113639400	30001	11	4	2	3	2	3	7	1	6	7	2	11	4	
chr21:34226200-34287850	61651	11	6	2	10	4	2	11	3	9	7	6	10	5	
chr7:63682825-63758200	75376	10	6	2	8	3	4	5	2	10	10	2	7	10	
chr16:53502500-53520209	17710	10	4	5	0	6	2	4	1	8	6	2	1	10	
chr8:70066100-70122500	56401	10	6	5	7	5	3	7	3	5	10	4	9	10	
chr8:104430300-104445100	14801	10	5	5	10	5	9	4	3	6	4	4	6	3	
chr8:27472050-27491175	19126	10	3	1	7	1	1	10	1	1	4	2	1	1	
chr8:9004100-9020950	16851	10	2	1	1	1	1	1	2	10	2	2	2	2	
chr8:67007800-67084250	76451	10	7	5	9	4	3	8	2	9	10	3	10	10	
chr8:75469125-75486275	17151	10	5	2	5	3	4	8	1	5	10	3	9	5	
chr2:191398300-191442800	44501	10	3	2	4	2	1	3	1	5	3	2	10	5	
chr12:58495000-58544275	49276	10	5	3	5	3	2	6	3	10	8	2	7	8	
chrX:106618050-106664800	46751	10	3	1	4	1	5	2	0	3	10	1	3	3	
chr6:3842475-3856791	14317	10	2	1	1	10	0	0	1	1	0	3	1	1	
chr15:49996975-50010800	13826	10	4	1	5	5	1	4	1	5	10	3	10	3	
chr13:39913400-39947425	34026	10	4	3	5	3	1	4	0	5	10	5	4	3	
chr19:39973225-40013800	40576	10	3	1	4	2	1	3	1	3	4	1	5	10	
chr1:199822075-199856100	34026	10	4	5	8	4	4	2	1	10	2	2	3	3	
chr4:24772175-24788500	16326	10	5	4	4	2	2	7	2	5	6	3	10	6	
chr13:43721900-43784850	62951	10	3	2	2	1	1	6	1	1	2	10	2	2	
chr7:65959125-65978250	19126	10	4	3	4	2	1	4	1	4	6	3	7	10	
chr11:45007750-45055500	47751	10	2	1	1	1	0	1	0	2	10	1	1	1	
chr10:48090350-48146425	56076	10	2	10	2	1	1	1	0	1	2	1	1	1	
chr1:114594500-114609650	15151	10	1	0	0	0	0	10	0	0	0	0	0	0	
chr18:57387175-57433350	46176	10	3	2	3	3	1	10	0	3	3	2	2	2	
chr6:143375000-143402275	27276	10	5	3	4	2	3	10	2	3	8	3	6	6	
chr1:143238675-143262750	24076	10	3	1	2	10	3	1	0	3	1	3	2	1	
chr1:164701025-164758425	57401	10	6	4	6	4	2	6	3	10	8	3	8	8	
chr2:64438550-64482125	43576	10	3	1	4	1	1	4	0	3	3	5	10	3	
chr21:22804275-22835300	31026	10	3	2	3	1	1	2	3	10	2	1	4	5	
chr6:30889850-30922825	32976	10	3	1	4	1	2	2	1	4	4	1	10	3	
chr8:58900375-58914400	14026	10	2	2	1	1	0	1	4	10	1	0	0	2	
chr2:207788050-207840375	52326	10	2	2	2	1	1	1	0	2	2	0	3	10	
chr4:101852975-101870350	17376	10	2	2	1	1	1	1	3	10	1	1	1	3	
chr6:155282650-155328225	45576	10	5	3	10	4	1	4	2	7	7	6	7	3	
chrX:137166825-137196100	29276	10	5	3	6	4	3	8	1	5	10	4	6	6	
chr8:128640050-128779150	139101	10	5	3	7	3	2	4	4	10	5	2	5	6	
chr7:127705000-127725825	20826	10	5	4	6	4	4	7	2	5	10	4	7	6	
chr8:138400700-138502875	102176	10	5	4	6	3	3	7	1	6	10	3	5	5	
chr3:184643050-184657550	14501	10	4	2	2	3	6	5	2	3	2	2	4	10	
chr1:236697975-236712250	14276	10	2	1	1	1	0	10	0	1	2	0	1	1	
chr6:57636750-57672625	35876	10	5	4	4	3	3	5	4	10	8	3	7	7	

location	span (nt)	max	mean	adipose	colon	heart	hypothalamus	kidney	liver	lung	ovary	skeletal muscle	spleen	testes	Comments
chr6:126461350-126482675	21326	10	4	1	2	2	1	9	4	2	10	5	4	4	
chr7:155386625-155451325	64701	10	3	1	4	1	1	10	1	2	3	1	2	2	
chr14:22834350-22839975	5626	10	4	2	1	2	4	3	2	10	4	5	5	4	
chr7:20477725-20548875	71151	10	5	3	3	3	2	4	4	10	7	3	6	8	
chr8:124540800-124568875	28076	10	5	1	5	4	3	6	3	7	10	4	9	4	
chr16:25178750-25189800	11051	10	4	1	6	2	1	3	0	1	10	2	8	4	
chr3:37861975-37876925	14951	10	3	1	6	1	2	7	0	2	10	0	3	3	
chr4:53376500-53401675	25176	10	5	2	5	3	3	5	1	4	10	4	7	6	
chr6:84732350-84748000	15651	10	3	1	1	2	1	2	2	4	2	3	10	3	
chr1:35058475-35091575	33101	10	7	5	10	5	4	7	4	8	9	5	9	7	
chr5:94014600-94029225	14626	10	4	2	2	3	2	3	2	6	6	1	8	10	
chr5:127435825-127445650	9826	10	4	2	3	2	2	5	1	3	10	1	7	4	
chr2:177947475-177965575	18101	10	4	4	2	4	2	7	1	3	6	2	10	8	
chr1:191884000-191929100	45101	10	3	1	3	1	0	2	10	3	6	1	5	2	
chr20:971000-1032775	61776	10	5	4	5	2	2	10	2	4	9	1	6	8	
chr4:26657100-26713550	56451	9	5	4	9	3	3	5	1	7	7	2	9	5	
chr2:122483375-122493675	10301	9	4	2	5	2	1	4	0	4	9	2	6	7	
chr13:30254825-30309675	54851	9	3	2	3	2	1	1	0	6	2	1	9	2	
chr3:160049375-160087675	38301	9	7	4	6	9	3	6	1	7	9	9	9	9	
chr8:551925-588750	36826	9	4	3	4	2	1	6	1	4	5	1	9	6	
chr8:110304925-110316200	11276	9	4	1	1	4	2	4	2	3	7	6	9	3	
chr1:227077300-227144400	67101	9	3	2	3	2	2	2	1	9	4	1	5	3	
chr2:191748300-191787275	38976	9	4	3	3	2	1	3	3	9	7	1	6	4	
chr2:23485150-23511825	26676	9	5	5	9	3	2	6	1	6	8	4	8	3	
chr11:117173700-117185525	11826	9	2	1	2	0	0	6	0	1	9	0	2	2	
chr11:129074200-129114425	40226	9	5	3	7	2	2	6	1	4	8	3	7	9	
chr5:1759400-1776250	16851	9	5	3	7	3	1	5	1	4	9	3	6	9	
chr18:40517050-40532650	15601	9	4	2	5	2	2	9	1	4	7	2	9	3	
chr1:48285375-48309925	24551	9	1	0	1	0	0	9	0	0	0	0	0	1	
chr8:20871025-20973775	102751	9	4	3	3	2	3	2	5	9	3	1	3	5	
chr14:45824500-45861375	36876	9	2	1	2	1	1	9	0	1	2	1	2	1	
chr2:155952550-156008475	55926	9	4	2	4	2	1	3	2	5	5	1	5	9	
chr3:88191225-88208250	17026	9	5	3	3	3	1	5	1	9	9	2	9	7	
chr6:1395175-1499250	104076	9	5	5	5	4	4	4	3	6	9	4	6	9	
chr15:23112925-23128125	15201	9	5	2	4	3	7	5	1	3	7	9	6	3	
chr15:46800350-46810550	10201	9	3	3	1	3	9	4	2	3	3	3	4	4	
chr4:6714950-6738350	23401	9	5	3	6	3	9	4	1	4	6	3	5	6	
chrX:42677625-42779075	101451	9	4	2	5	2	2	4	1	3	9	2	6	4	
chr2:19395750-19410525	14776	9	3	3	2	2	2	4	1	2	9	2	5	5	
chr2:145589275-145631750	42476	9	3	1	0	1	0	9	0	2	5	1	1	7	
chr5:54056975-54069450	12476	9	1	0	0	1	1	0	0	0	0	0	9	1	
chr6:154759925-154767025	7101	9	3	0	2	2	1	9	5	2	3	0	4	3	
chrX:138783749-138795925	12177	9	4	2	6	2	1	4	1	4	7	1	9	4	
chrX:68001200-68090950	89751	9	3	2	9	3	2	3	1	4	4	2	4	3	
chr14:86260825-86341250	80426	9	5	3	6	3	3	7	2	6	9	5	6	7	
chr9:124209250-124254675	45426	9	5	3	8	3	2	6	1	5	9	4	8	8	
chr15:52749925-52822450	72526	9	5	3	6	3	3	9	1	4	8	4	7	7	
chr5:39280900-39292500	11601	9	2	1	2	2	1	1	1	1	1	1	9	1	
chr7:11275675-11302400	26726	9	4	7	2	2	1	2	6	8	9	1	2	4	
chrX:13885025-13926975	41951	9	4	2	4	3	2	5	1	4	9	2	7	6	
chr7:63143400-63159850	16451	9	2	1	2	1	1	2	0	1	9	1	3	2	
chr13:56129650-56140775	11126	9	2	2	1	1	1	2	3	9	2	1	1	3	
chr8:76283650-76351975	68326	9	4	2	4	2	2	9	2	4	5	2	4	4	
chr7:115726625-115762575	35951	9	3	9	1	2	1	1	0	3	5	1	3	3	
chr12:18869375-18895800	26426	9	2	1	1	1	1	9	1	2	2	1	2	2	
chr1:113301525-113321425	19901	9	4	2	5	4	2	2	1	2	9	6	8	5	
chr10:116533050-116566275	33226	9	3	3	4	1	2	3	2	3	9	1	4	5	
chr5:153455300-153511350	56051	9	5	4	5	4	4	6	3	5	9	4	8	6	
chr11:129695825-129745775	49951	9	4	2	5	2	1	7	1	3	5	1	5	9	
chr3:3878525-3899175	20651	9	5	3	4	3	3	6	1	4	8	4	7	9	
chr9:101603550-101614575	11026	9	3	1	1	1	1	4	0	1	9	7	2	5	
chr8:21389625-21523225	133601	9	5	6	9	2	3	4	2	4	4	7	8	4	
chr2:107594300-107750025	155726	9	5	4	6	4	2	5	2	5	8	3	7	9	
chr13:70096550-70376150	279601	9	6	3	8	3	3	6	3	9	8	3	7	8	
chr6:114828600-114909700	81101	9	6	3	9	3	7	6	2	7	8	4	7	7	
chr17:49425-59050	9626	9	3	1	3	1	0	3	1	3	6	1	9	1	
chr5:1183775-1239275	55501	9	2	0	3	1	1	3	1	1	1	1	2	9	
chr18:33065350-33076375	11026	9	2	1	1	0	1	1	0	2	9	2	2	2	
chr4:1162925-1175425	12501	9	4	2	3	1	2	8	1	4	9	1	4	4	
chr5:77321525-77331525	10001	9	2	1	1	2	0	3	1	2	3	2	9	2	
chr8:122409075-122455775	46701	9	5	4	6	3	2	6	2	5	9	6	7	6	
chr1:194117800-194158050	40251	9	2	2	2	1	1	1	3	9	1	1	1	3	
chr2:207931600-208000325	68726	9	4	5	5	2	3	4	1	4	6	2	9	7	
chr6:30109600-30121550	11951	9	4	3	2	2	3	4	1	4	6	2	5	9	
chr18:65797050-65814575	17526	9	3	0	1	1	0	8	0	1	9	5	5	2	
chr15:40538400-40556025	17626	9	5	2	5	6	3	4	2	5	6	4	9	5	
chr3:8816950-8886100	69151	9	4	2	8	2	4	3	1	4	7	3	9	7	
chr3:27841100-27880125	39026	9	4	3	4	2	1	4	4	9	3	2	4	6	
chr7:93425600-93443100	17501	9	3	3	1	3	1	3	1	5	3	2	9	2	
chr14:60832450-60851000	18551	9	4	2	5	2	1	4	1	4	9	4	8	5	
chr1:51794250-51821775	27526	9	3	1	9	1	1	3	2	2	2	3	2	3	
chr15:72566300-72592700	26401	9	4	3	9	2	2	4	1	3	7	3	8	6	
chr16:63817525-63848450	30926	9	4	2	4	3	3	6	1	4	9	2	6	5	
chr12:95534400-95556825	22426	9	2	1	2	1	2	1	2	9	2	0	2	4	
chr15:77428250-77465075	36826	9	3	2	4	2	2	3	2	9	4	1	7	3	
chr10:129903575-129998050	94476	9	4	2	5	2	2	5	1	5	9	2	6	5	
chr2:239460050-239536125	76076	9	2	3	4	1	0	1	0	1	9	1	1	1	
chr5:82197325-82250800	53476	8	5	3	5	3	2	8	1	5	8	3	6	5	
chr14:20977125-20993325	16201	8	4	2	8	3	2	6	1	3	7	1	8	4	
chr15:86833575-86843600	10026	8	2	1	2	1	0	2	0	0	8	1	1	2	
chr18:11646850-11658050	11201	8	1	0	0	0	0	0	0	0	0	0	0	8	
chr14:75762425-75799950	37526	8	3	2	3	3	1	8	1	2	2	3	4	4	
chr14:98529150-98680525	151376	8	4	2	7	2	2	4	1	5	5	1	8	4	
chr12:92829775-92882150	52376	8	5	2	4	3	3	8	2	3	8	8	5	5	
chr3:154164450-154202725	38276	8	4	3	6	3	2	4	1	6	8	3	6	7	
chr5:170105175-170132700	27526	8	4	2	4	2	3	7	1	5	8	2	6	4	
chr1:179329700-179339750	10051	8	2	1	1	1	1	1	2	8	1	1	1	2	
chr1:94958600-94969225	10626	8	2	0	8	1	1	6	0	2	1	0	1	1	
chr8:2545200-2594500	49301	8	3	3	2	1	1	8	0	1	4	1	4	7	
chr9:2673000-2701050	28051	8	5	3	5	2	3	8	1	4	8	4	6	6	
chr13:75491350-75542100	50751	8	4	2	2	2	2	3	3	8	6	2	3	5	
chr18:51549225-51625400	76176	8	5	3	6	5	4	5	2	6	8	3	7	8	
chr5:88659975-88751600	91626	8	4	2	4	2	2	3	2	6	8	4	5	4	
chr15:67738650-67775700	37051	8	2	1	8	1	0	8	0	3	2	0	1	3	
chr1:184733575-184761100	27526	8	4	3	3	3	3	5	2	7	6	3	8	5	
chr15:70415750-70422125	6376	8	4	3	3	2	2	8	0	4	8	2	6	3	
chr2:75001325-75029025	27701	8	3	0	8	0	3	4	4	3	1	3	1	4	
chr4:52952325-52974425	22101	8	5	3	7	7	3	4	2	8	6	3	8	5	
chr10:119256775-119270300	13526	8	2	1	1	0	1	7	0	1	8	0	0	2	
chr12:75176700-75230575	53876	8	4	4	4	4	3	4	1	4	4	8	6	4	
chr12:96435000-96447575	12576	8	2	0	0	0	1	8	0	3	1	0	0	4	
chrX:119347600-119374125	26526	8	4	3	6	3	3	5	2	4	8	3	6	7	
chr18:18732600-18754800	22201	8	2	1	2	1	1	4	1	2	2	0	2	8	
chr3:72239600-72471675	232076	8	5	3	8	2	8	8	3	5	8	2	5	5	
chr6:36223550-36259875	36326	8	4	2	4	2	4	3	1	3	8	4	8	7	
chr2:90959050-90986900	27851	8	1	0	8	0	0	0	0	1	0	1	1	1	
chr2:113075625-113090775	15151	8	4	2	4	2	2	4	1	4	6	3	8	5	
chr18:70941800-71033250	91451	8	4	2	7	4	3	3	1	4	8	2	6	5	
chrX:47769775-47794250	24476	8	3	2	6	2	2	3	0	3	8	1	4	3	
chr3:84058675-84073875	15201	8	2	1	1	0	0	1	3	8	1	1	0	2	
chr2:114283350-114336075	52726	8	3	2	4	2	2	2	1	3	5	2	8	5	
chr7:115859275-115880850	21576	8	3	8	1	4	0	1	0	5	8	1	3	2	
chr19:15279000-15289200	10201	8	5	3	8	3	2	6	2	7	7	3	8	5	
chr3:12995350-13009850	14501	8	2	1	3	2	1	1	0	4	2	1	8	1	

location	span (nt)	max	mean	adipose	colon	heart	hypothalamus	kidney	liver	lung	ovary	skeletal muscle	spleen	testes	Comments
chr11:14889900-14935000	45101	8	3	3	2	1	1	7	2	4	4	1	8	3	
chr14:96996650-97122525	125876	8	3	1	4	2	1	3	1	5	3	1	8	3	
chr18:46799075-46810100	11026	8	4	3	3	3	2	4	2	3	8	4	5	4	
chrX:57912775-57943025	30251	8	3	1	3	1	3	3	1	3	8	1	4	2	
chr11:121425850-121455950	30101	8	3	1	3	5	8	2	1	3	7	2	2	3	
chr1:210367025-210384325	17301	8	2	2	2	1	1	2	1	2	8	2	3	2	
chr10:31608475-31625250	16776	8	4	2	4	2	2	5	1	5	8	3	5	4	
chr4:111456725-111486750	30026	8	4	4	6	3	2	5	1	4	8	2	6	6	
chr3:195446400-195513525	67126	8	3	2	3	1	1	3	8	8	4	1	1	4	
chr2:77168475-77216350	47876	8	3	2	3	2	8	3	1	4	4	1	3	3	
chr9:36409075-36421725	12651	8	4	2	5	1	1	5	0	4	6	1	6	8	
chr5:147308325-147389600	81276	8	4	3	5	3	2	7	0	4	8	3	6	6	
chr8:125541150-125555450	14301	8	3	1	2	2	2	3	1	2	8	1	7	2	
chr15:75573800-75655225	81426	8	4	2	6	4	8	3	0	3	4	3	7	3	
chr12:69889725-69989850	100126	8	5	3	6	3	2	5	3	8	6	4	4	8	
chr10:120422300-120428500	6201	8	3	2	1	2	4	2	0	3	8	5	5	4	
chr4:77361150-77383100	21951	8	3	1	3	1	2	3	8	2	3	3	2	8	
chr7:5433575-5444700	11126	8	2	1	5	1	2	2	0	2	8	1	2	1	
chr3:167355350-167366325	10976	8	2	1	2	1	1	8	0	2	3	1	3	1	
chrX:113528900-113613550	84651	8	4	3	6	3	4	6	1	5	8	3	5	5	
chr6:28189525-28199325	9801	8	4	4	3	3	4	5	2	3	8	3	8	4	
chr1:179196475-179208700	12226	8	3	3	3	2	3	4	1	3	8	2	5	2	
chr14:68133150-68148025	14876	8	2	2	2	0	1	1	0	2	8	1	4	1	
chr8:108150775-108322000	171226	8	5	4	6	3	4	4	2	6	8	5	6	7	
chr11:96952375-97086200	133826	8	5	3	6	4	2	7	2	4	8	3	8	6	
chr22:27033450-27045625	12176	8	3	2	3	2	1	3	1	3	8	2	4	4	
chr1:212579525-212590244	10720	8	4	4	3	5	2	7	1	3	6	8	4	4	
chr2:30968700-30980475	11776	8	1	0	0	0	0	8	0	0	0	0	0	0	
chr16:47237250-47265300	28051	8	5	3	8	3	2	6	2	6	8	3	8	7	
chr2:16335875-16523025	187151	8	4	3	8	2	2	3	1	3	5	2	4	6	
chr20:58089800-58106500	16701	8	1	0	1	0	1	0	0	0	0	0	1	8	
chr12:29121750-29172275	50526	8	5	4	5	5	4	7	2	5	8	3	8	6	
chr10:4934575-4967950	33376	8	3	2	2	3	2	2	6	2	4	1	3	8	
chr2:217088775-217129475	40701	8	3	1	2	1	2	1	1	8	2	3	3	5	
chr11:106680375-106691525	11151	8	3	1	1	2	3	6	1	3	4	4	8	3	
chr5:133796150-133810875	14726	8	2	0	1	3	1	1	0	1	0	8	1	1	
chr13:96654250-96665325	11076	8	2	1	3	1	1	1	3	8	2	0	0	5	
chr14:33522725-33553200	30476	8	3	1	4	3	2	2	1	8	4	2	6	3	
chr5:100411875-100464525	52651	8	4	3	7	3	3	5	2	5	8	2	5	5	
chrX:39654075-39718125	64051	8	2	1	8	1	1	1	0	1	3	1	2	2	
chr6:29346025-29356300	10276	8	4	4	5	3	2	4	1	3	8	3	5	6	
chr7:125011450-125108750	97301	8	3	2	4	2	1	2	2	8	2	1	2	3	
chr14:34498300-34508825	10526	8	4	1	3	2	1	8	1	6	6	1	7	4	
chr14:97192075-97236700	44626	8	4	3	4	3	3	6	8	5	5	3	4	5	
chr4:75129050-75167025	37976	8	3	2	1	1	1	2	3	8	2	1	2	4	
chr7:129031750-129038650	6901	8	4	3	7	2	3	7	1	3	7	1	8	6	
chr9:5129600-5146200	16601	8	3	2	2	3	1	4	1	4	5	4	8	5	
chr6:98783950-98895400	111451	8	4	2	6	2	5	5	1	5	6	2	5	8	
chr1:221252075-221314050	61976	8	5	3	5	4	4	6	2	8	7	5	6	5	
chr7:80594925-80732275	137351	8	4	2	6	3	3	5	2	4	8	2	6	6	
chr2:157739200-157801400	62201	8	3	2	3	2	3	3	1	2	3	2	4	8	
chr22:33904725-33953150	48426	8	4	2	4	7	4	4	3	4	8	3	5	6	
chr2:105915600-105995825	80226	8	4	2	5	2	3	5	5	8	5	2	6	6	
chr7:30517800-30529075	11276	8	4	4	3	3	2	7	1	4	6	4	8	4	
chr1:119352575-119365400	12826	8	3	2	1	2	1	4	1	5	8	1	3	3	
chr10:6663500-6677950	14451	8	2	1	2	0	3	3	0	3	2	8	4	1	
chr14:50799425-50830400	30976	7	4	2	2	2	2	4	1	7	4	1	7	6	
chr6:132319775-132332075	12301	7	2	1	2	1	1	1	0	1	7	0	1	2	
chr7:77147550-77161525	13976	7	4	2	7	3	3	6	4	5	5	2	3	4	
chr11:72124275-72140250	15976	7	2	1	3	1	1	1	1	3	2	0	7	0	
chr5:49667825-49697900	30076	7	2	1	2	2	1	2	1	4	2	1	7	2	
chr10:80162000-80341225	179226	7	3	4	6	2	7	3	1	3	3	2	2	3	
chrX:125218650-125243775	25126	7	5	3	7	2	4	5	2	6	6	2	6	5	
chr2:167180050-167221900	41851	7	3	2	2	2	1	3	2	7	4	1	4	5	
chr15:47173125-47199050	25926	7	4	3	3	3	5	4	2	5	4	6	7	2	
chr5:1597500-1620100	22601	7	2	1	2	0	1	2	0	7	2	0	3	1	
chr8:50277200-50391700	114501	7	5	4	5	4	3	6	1	6	7	5	5	7	
chr7:7252400-7267000	14601	7	2	0	1	2	2	4	1	3	1	2	7	1	
chrX:138752200-138762250	10051	7	4	2	6	2	1	4	1	4	6	2	7	5	
chrX:6577525-6619425	41901	7	3	1	2	2	1	4	1	3	7	2	3	3	
chr2:7917975-7969300	51326	7	4	2	4	2	2	5	2	7	6	2	4	5	
chr14:80950175-80970975	20801	7	3	1	2	1	2	7	0	1	3	1	7	2	
chr5:92788525-92800450	11926	7	2	0	0	0	1	3	1	1	7	0	2	2	
chrX:115823450-115840525	17076	7	3	1	3	2	2	6	0	4	7	1	4	4	
chr6:53704600-53744825	40226	7	3	1	4	1	2	7	0	4	5	1	5	5	
chr12:130395250-130407325	12076	7	2	2	1	1	1	1	3	7	1	0	0	2	
chr2:46288525-46300250	11726	7	2	1	1	1	1	2	3	7	2	1	2	3	
chr1:81414200-81494775	80576	7	4	3	5	3	2	4	3	5	7	2	5	4	
chrX:44194775-44217950	23176	7	2	1	7	1	1	2	0	2	2	0	1	1	
chr1:229087100-229097900	10801	7	4	3	5	2	4	2	2	4	4	4	5	7	
chr6:2737475-2768400	30926	7	3	3	2	3	2	3	1	3	7	3	6	3	
chr8:108779875-108813100	33226	7	4	3	6	2	2	5	1	4	7	2	6	4	
chrX:38601375-38677625	76251	7	4	2	5	2	2	4	1	5	7	3	5	5	
chr8:105676400-105757350	80951	7	3	2	4	2	2	3	1	3	5	1	4	7	
chr6:12007000-12022300	15301	7	3	2	1	4	7	6	3	6	1	0	1	5	
chr16:11464875-11492800	27926	7	3	2	3	1	1	2	1	4	2	3	7	3	
chr8:6541450-6552025	10576	7	3	1	5	1	1	5	0	2	4	1	7	4	
chr3:47182200-47192800	10601	7	4	2	4	2	3	7	1	5	6	3	4	6	
chr4:368275-378300	10026	7	3	2	2	2	2	2	1	1	7	3	4	4	
chrX:10963875-11001100	37226	7	4	3	6	3	2	5	1	3	7	2	5	6	
chr2:239994925-240122575	127651	7	3	3	7	2	2	3	1	5	5	1	4	4	
chr5:56013475-56103075	89601	7	4	2	5	3	2	4	1	4	7	2	7	5	
chrX:102885925-102902750	16826	7	2	2	1	1	1	1	2	7	2	0	1	3	
chr17:7716675-7728025	11351	7	4	2	3	5	5	4	0	3	7	4	7	6	
chr5:95945275-96020850	75576	7	4	3	3	4	4	5	1	4	7	2	6	4	
chr9:15989575-16042100	52526	7	2	1	2	1	2	4	0	2	7	1	2	4	
chr20:5134450-5152500	18051	7	5	2	5	4	7	4	1	4	7	6	7	4	
chr2:101422625-101454925	32301	7	4	3	4	2	2	5	1	4	7	2	6	5	
chr5:44862425-44872625	10201	7	3	1	1	2	3	5	1	2	5	7	4	2	
chr12:45939700-45986400	46701	7	4	2	4	2	3	3	1	4	7	1	6	5	
chr2:42416350-42427850	11501	7	3	2	4	2	1	6	1	3	7	1	7	3	
chr4:125320325-125379400	59076	7	3	2	3	2	1	3	1	4	4	1	3	7	
chr2:167967500-168003750	36251	7	4	2	7	2	4	3	1	3	6	3	5	5	
chr21:19714950-19740475	25526	7	3	2	2	2	2	3	1	2	6	1	5	7	
chr17:71097925-71111875	13951	7	3	2	7	1	1	3	0	4	6	1	6	2	
chr5:36729300-36761900	32601	7	1	1	2	1	1	1	0	1	1	1	7	1	
chr5:53692150-53755875	63726	7	4	3	4	2	3	6	2	5	7	3	5	5	
chr1:236138375-236165900	27526	7	4	3	4	3	3	4	2	4	7	2	5	4	
chr6:169521225-169539250	18026	7	1	0	0	0	1	2	0	2	7	0	1	2	
chr9:460700-475275	14576	7	2	1	2	1	0	7	0	1	1	1	2	6	
chr3:115417750-115427925	10176	7	1	0	1	0	0	0	0	3	0	0	7	0	
chr5:74941275-74952975	11701	7	3	1	7	1	1	7	1	4	4	1	2	3	
chr17:10598925-10629325	30401	7	3	2	5	1	2	4	3	2	7	1	3	4	
chr1:161532100-161545575	13476	7	3	1	1	1	1	4	1	2	7	2	4	7	
chr11:119530475-119566750	36276	7	2	1	7	1	1	5	2	1	4	0	2	1	
chr5:153788925-153803825	14901	7	3	1	4	2	1	3	1	2	5	1	7	2	
chr3:24611450-24674150	62701	7	3	1	2	1	3	7	2	4	4	2	3	3	
chr5:93465825-93476900	11076	7	3	2	2	2	1	3	1	2	7	0	6	3	
chr13:79861975-79897575	35601	7	3	2	3	2	2	3	2	6	3	2	7	4	
chr8:94562350-94588700	26351	7	3	2	4	2	1	3	2	3	6	2	6	7	
chr3:136560550-136594175	33626	7	2	2	1	1	2	2	3	7	2	1	4	2	
chrX:74466525-74477225	10701	7	2	2	2	1	1	2	2	7	2	1	2	2	
chr8:103749000-103836675	87676	7	4	3	7	2	3	4	1	5	4	2	5	4	
chr4:12579600-12608800	29201	7	2	2	1	2	3	2	7	2	2	0	2	2	

location	span (nt)	max	mean	adipose	colon	heart	hypothalamus	kidney	liver	lung	ovary	skeletal muscle	spleen	testes	Comments
chr10:1085825-1091600	5776	7	2	1	2	1	1	4	1	2	3	1	7	4	
chr2:57699575-57740425	40851	7	3	2	1	1	1	2	1	2	6	2	7	4	
chr8:28538200-28593425	55226	7	4	2	6	3	3	3	2	5	7	2	4	4	
chr11:68926100-69048700	122601	7	3	2	7	2	2	3	2	3	3	1	3	2	
chr7:99948500-99966925	18426	7	4	4	7	2	2	4	2	6	3	2	4	6	
chr6:2414150-2484475	70326	7	4	3	4	2	5	7	4	4	4	1	3	3	
chr11:7855925-7880950	25026	7	2	1	1	1	1	2	1	7	1	0	2	4	
chr6:48152775-48178175	25401	7	2	1	1	3	0	2	1	7	2	1	1	2	
chr16:85445825-85562825	117001	7	4	3	7	2	5	4	1	4	4	2	4	6	
chr2:181636500-181651275	14776	7	4	1	4	2	3	6	1	2	7	3	6	7	
chr3:130058000-130074800	16801	7	2	4	2	1	1	1	0	1	2	0	1	7	
chrX:79626350-79664125	37776	7	4	2	4	4	2	6	2	4	6	2	5	7	
chr11:61150700-61162450	11751	7	4	4	4	4	1	4	7	4	4	3	6	3	
chr10:113880000-113890875	10876	7	2	7	6	1	0	0	1	1	1	0	0	0	
chr8:104323575-104358050	34476	7	3	2	2	3	5	7	1	5	4	1	5	4	
chr9:114007475-114015775	8301	7	3	1	2	3	1	4	1	4	3	1	7	4	
chr6:56917375-56926575	9201	7	2	1	0	1	4	1	1	7	1	0	1	1	
chr7:63000650-63023550	22901	7	4	2	3	4	3	3	3	7	6	1	5	5	
chr6:37180500-37212625	32126	6	2	2	5	2	2	2	1	6	2	1	2	2	
chr22:26646350-26652975	6626	6	3	2	3	2	2	3	2	3	6	2	5	5	
chr7:29552375-29568225	15851	6	1	0	6	0	0	2	0	1	2	0	0	1	
chr12:64452900-64492900	40001	6	2	1	2	1	0	2	0	1	6	1	1	1	
chr21:39415875-39444300	28426	6	3	2	3	2	3	2	3	3	6	4	5	4	
chr10:116460425-116473650	13226	6	3	4	4	3	2	6	0	2	3	2	2	2	
chr12:2733775-2756550	22776	6	3	2	4	2	1	2	1	3	6	2	4	4	
chr12:5248100-5298825	50726	6	3	2	6	2	2	4	1	3	6	2	4	5	
chr5:139072325-139135900	63576	6	4	3	6	2	3	3	2	6	3	2	5	4	
chr2:237685400-237732100	46701	6	3	1	2	2	0	2	0	1	6	3	3	6	
chr1:85836625-85880575	43951	6	4	1	5	2	2	5	2	3	6	4	6	3	
chr1:91132500-91146825	14326	6	3	1	2	2	1	3	1	3	6	2	6	2	
chr10:76728025-76749800	21776	6	3	2	4	2	2	4	2	6	4	3	6	3	
chr6:11758275-11776200	17926	6	2	1	1	1	1	1	2	6	2	0	1	2	
chr6:74040350-74054750	14401	6	2	1	3	1	2	2	1	2	6	0	3	3	
chr6:18142500-18217725	75226	6	4	2	6	2	3	4	3	6	5	1	5	6	
chr1:120940875-120953875	13001	6	4	3	2	5	4	5	6	3	5	3	6	3	
chr4:72215925-72232750	16826	6	4	3	4	4	4	5	3	5	6	2	4	5	
chr4:125759125-125791075	31951	6	3	1	3	3	1	2	1	4	6	1	4	2	
chr8:106235775-106344550	108776	6	4	3	4	3	2	4	1	5	6	3	5	5	
chr5:134493025-134619900	126876	6	3	2	5	1	1	3	0	2	5	6	3	4	
chr9:127207725-127218875	11151	6	1	1	2	0	1	1	1	2	6	0	1	1	
chr14:99267075-99297500	30426	6	2	1	6	2	1	4	0	5	2	1	2	2	
chr13:19287250-19304250	17001	6	3	2	3	3	2	3	1	3	5	3	6	4	
chr5:8490875-8526025	35151	6	4	5	2	6	3	6	1	3	3	3	4	6	
chr13:38747350-38802275	54926	6	3	2	3	3	1	2	1	4	6	2	5	4	
chr15:79036700-79049525	12826	6	3	2	4	2	2	4	1	3	6	2	4	4	
chr12:95477300-95516900	39601	6	2	0	1	1	2	1	0	5	3	0	2	6	
chr17:39456275-39467525	11251	6	4	3	6	3	2	5	2	3	6	2	6	5	
chr13:47534400-47540050	5651	6	2	1	1	3	0	4	1	3	6	2	5	2	
chr4:8541575-8566850	25276	6	3	2	3	1	3	4	1	3	6	2	2	4	
chr7:36737750-36788825	51076	6	2	1	1	1	2	4	0	5	3	0	6	3	
chr15:79524225-79548025	23801	6	2	0	1	1	1	1	2	1	6	1	3	4	
chr8:111817150-111883075	65926	6	3	2	4	4	2	3	1	3	6	1	5	4	
chr6:139674475-139684850	10376	6	1	0	1	0	0	0	0	1	3	0	6	2	
chr3:43162175-43282975	120801	6	3	2	6	2	2	3	2	3	6	2	3	4	
chr1:39776600-39792825	16226	6	3	2	3	1	2	3	0	2	6	5	3	3	
chr4:54483050-54499200	16151	6	2	1	1	1	1	2	2	6	2	2	2	2	
chr9:137244375-137371000	126626	6	2	2	6	2	2	2	1	4	3	1	3	2	
chr16:3113750-3124850	11101	6	3	2	5	2	2	4	1	2	6	2	5	2	
chr5:57860400-57875950	15551	6	3	1	4	2	2	4	1	4	6	2	5	4	
chr5:90759300-90789150	29851	6	3	1	4	1	1	3	1	3	3	3	5	6	
chr21:43712475-43723950	11476	6	2	1	2	1	0	0	6	2	3	1	1	2	
chr10:62726900-62746900	20001	6	1	1	1	1	0	1	3	6	0	0	0	2	
chr2:146013750-146223325	209576	6	4	2	5	2	3	4	2	6	5	2	4	4	
chr12:7324400-7339725	15326	6	3	2	2	2	2	3	2	3	6	2	4	4	
chr13:74376150-74419100	42951	6	3	2	4	2	2	3	1	3	6	2	4	4	
chr2:42002850-42053225	50376	6	2	2	3	1	1	2	0	4	6	1	3	3	
chr9:14575325-14587525	12201	6	2	1	2	1	2	3	0	2	3	1	6	3	
chr13:84444275-84578075	133801	6	4	2	6	2	2	3	2	5	5	2	4	6	
chr2:41924000-41937475	13476	6	3	4	3	3	2	2	3	3	6	2	4	4	
chr7:37800325-37812150	11826	6	2	1	1	1	0	1	2	6	1	0	3	2	
chr12:30814550-30854925	40376	6	3	3	2	2	3	4	1	2	6	3	3	4	
chr15:55822375-55857650	35276	6	1	1	1	1	1	1	1	6	1	0	1	1	
chr4:99326125-99342425	16301	6	1	1	1	0	1	1	0	6	1	0	2	2	
chr6:83161900-83211150	49251	6	3	3	3	4	2	5	0	5	4	1	6	4	
chr4:124718875-124749675	30801	6	2	1	1	1	1	1	1	6	2	1	2	4	
chr6:3926525-3939100	12576	6	3	2	3	1	2	4	1	6	5	2	6	4	
chr8:37109300-37205775	96476	6	3	1	5	2	1	3	1	3	6	2	4	4	
chr11:30371700-30382775	11076	6	2	1	1	1	3	6	1	1	1	2	1	1	
chr2:145689775-145757350	67576	6	2	1	2	1	1	6	2	6	2	1	1	3	
chr7:35750875-35764150	13276	6	3	2	2	1	6	5	1	1	3	2	2	5	
chr6:44114325-44170550	56226	6	2	1	2	1	1	1	0	5	4	6	3	2	
chr19:47466700-47476275	9576	6	4	2	5	3	3	4	2	5	4	3	6	5	
chr5:79329500-79349525	20026	6	2	0	1	1	3	6	0	1	5	2	3	6	
chrX:58337500-58371350	33851	6	2	5	1	1	3	2	0	1	2	1	2	6	
chr13:42278100-42340125	62026	6	4	4	6	4	2	4	2	5	5	3	4	5	
chr22:22567775-22575550	7776	6	2	1	6	1	1	1	2	1	3	1	2	4	
chr7:8311175-8366500	55326	6	3	2	4	3	2	4	1	4	6	2	4	5	
chr15:77191800-77204850	13051	6	1	1	0	1	1	1	2	6	0	0	0	2	
chr8:90958775-90977600	18826	6	3	1	3	1	2	4	2	3	5	1	6	2	
chr10:27288300-27300050	11751	6	2	2	2	1	1	6	1	2	4	1	3	2	
chr8:80224875-80250800	25926	6	3	2	4	2	2	3	2	5	6	2	3	5	
chr4:90216125-90233175	17051	6	4	3	6	2	2	6	1	4	5	2	5	5	
chr13:49658575-49701625	43051	6	2	0	5	1	1	3	2	2	2	1	6	3	
chr8:26350000-26365750	15751	6	3	2	2	2	1	5	0	5	6	1	3	2	
chrX:48460225-48477975	17751	6	3	1	6	1	2	3	0	2	6	1	5	6	
chr14:80937850-80948425	10576	6	2	1	1	1	1	6	0	1	3	0	6	1	
chr13:29410325-29519200	108876	6	4	2	5	3	3	3	2	5	6	2	4	5	
chr4:79780850-79806375	25526	6	2	2	1	1	6	1	0	1	1	1	3	1	
chr16:11375775-11386450	10676	6	1	0	1	1	0	0	0	1	1	1	6	1	
chr8:123572275-123648225	75951	6	2	1	2	6	1	2	1	2	3	1	2	2	
chr2:59545675-59619200	73526	6	4	2	6	2	2	5	3	5	5	2	4	4	
chr1:179407300-179428500	21201	6	2	1	1	1	1	1	2	6	1	0	1	2	
chr4:90584375-90650325	65951	6	2	1	2	1	1	2	1	2	4	1	3	6	
chr4:93394950-93417950	23001	6	3	1	4	2	2	4	1	3	6	2	2	3	
chr5:99862475-99884050	21576	6	2	1	3	1	1	3	1	2	4	1	3	6	
chr11:37603350-37656150	52801	6	3	1	4	2	2	4	1	4	5	3	5	6	
chr8:47941875-47969750	27876	6	1	0	0	0	0	0	0	0	0	0	6	1	
chr16:87351225-87378000	26776	6	2	1	6	1	0	3	0	2	2	0	3	1	
chr12:118517650-118534025	16376	6	2	1	1	1	1	2	2	6	3	4	2	2	
chr4:107142375-107160000	17626	6	3	2	3	1	1	3	2	6	5	1	3	3	
chrX:36682025-36716325	34301	6	2	2	1	0	1	1	2	6	4	1	5	2	
chr11:128956550-128999900	43351	6	3	1	2	3	2	3	1	2	4	2	4	6	
chr11:72539200-72584250	45051	6	2	1	2	4	0	1	0	2	5	0	2	6	
chr3:72727375-72817150	89776	6	3	2	6	2	2	3	1	4	4	2	4	5	
chr13:47479700-47493175	13476	6	4	3	5	2	2	4	4	5	6	2	3	4	
chr4:6046775-6060425	13651	6	2	1	3	1	1	1	0	2	1	0	6	1	
chr7:39733400-39765800	32401	6	3	2	5	2	2	4	1	3	5	1	6	3	
chr16:49755350-49769950	14601	6	1	0	1	0	1	6	1	0	5	0	0	1	
chr11:64825000-64835850	10851	6	2	3	6	1	1	1	0	2	3	0	4	1	
chr16:66082250-66096075	13826	5	3	2	5	1	2	4	0	2	5	1	5	2	
chr2:91040800-91059550	18751	5	3	2	5	2	2	2	1	3	3	1	5	3	
chr1:235143375-235157150	13776	5	2	0	1	2	1	5	0	1	2	4	1	2	
chr21:35616700-35632175	15476	5	1	0	0	0	0	2	0	0	0	0	0	5	
chr4:106466500-106490525	24026	5	3	2	4	3	2	5	1	3	5	5	5	2	

location	span (nt)	max	mean	adipose	colon	heart	hypothalamus	kidney	liver	lung	ovary	skeletal muscle	spleen	testes	Comments
chr14:49172775-49179275	6501	5	2	1	4	2	1	4	0	1	4	1	5	2	
chr2:48342900-48376875	33976	5	2	1	2	1	1	2	1	3	2	1	5	4	
chr5:120635200-120699075	63876	5	3	2	5	2	2	4	5	3	5	2	3	4	
chr10:90729225-90740200	10976	5	2	3	1	2	5	1	1	4	4	1	3	2	
chr4:2757175-2787100	29926	5	2	1	5	2	1	5	1	4	1	0	5	1	
chr4:85418625-85432325	13701	5	3	2	3	2	1	3	1	2	5	2	4	3	
chr8:2683850-2700525	16676	5	2	2	1	0	1	5	0	2	3	0	3	2	
chr2:85975525-86007075	31551	5	3	3	3	2	5	2	0	4	3	1	4	2	
chr5:92830575-92842275	11701	5	1	0	1	1	1	2	1	1	5	0	2	2	
chr13:100010550-100034475	23926	5	3	3	3	2	1	4	0	2	3	1	5	3	
chr5:55919625-55956375	36751	5	2	1	1	1	0	5	0	1	2	1	3	2	
chr2:136680450-136726125	45676	5	1	1	2	1	0	1	0	2	2	1	5	2	
chr4:45434200-45475850	41651	5	3	2	3	2	1	2	1	2	5	2	4	3	
chr10:5146300-5161275	14976	5	2	1	1	1	0	2	5	1	3	0	1	5	
chr1:59255200-59301300	46101	5	2	2	2	1	1	2	0	2	5	4	4	1	
chrX:56824125-56848925	24801	5	2	1	4	2	1	3	0	2	5	1	5	3	
chr8:24112325-24140875	28551	5	1	0	1	0	0	5	0	1	1	0	2	2	
chr3:131414550-131449200	34651	5	2	0	2	1	1	3	0	2	5	1	2	3	
chr1:89521625-89559825	38201	5	2	1	2	1	1	2	1	2	4	1	5	3	
chr8:96278825-96304600	25776	5	1	0	2	1	0	5	0	1	1	0	1	1	
chr5:42794450-42825750	31301	5	3	1	2	2	4	2	1	1	4	2	4	5	
chr12:68755950-68789750	33801	5	3	3	4	2	4	3	1	4	5	3	3	5	
chr2:37838875-37869900	31026	5	2	1	2	0	1	1	1	5	1	3	1	1	
chr8:117182000-117203125	21126	5	1	1	1	0	0	0	1	5	0	0	0	1	
chr1:201273200-201284425	11226	5	1	0	1	1	5	1	0	0	0	4	0	2	
chr13:111594475-111683250	88776	5	2	2	3	1	2	2	1	3	3	1	3	5	
chr6:27771225-27791475	20251	5	2	1	3	1	1	1	0	2	5	1	4	2	
chr2:134424550-134510625	86076	5	2	2	2	5	2	1	1	1	2	1	2	4	
chr5:142064125-142106675	42551	5	2	2	3	2	1	2	1	4	2	1	5	2	
chr12:92933675-92975575	41901	5	3	2	5	2	3	4	1	3	5	1	4	3	
chr2:53906400-53920900	14501	5	3	2	2	2	3	4	1	4	5	4	4	3	
chr6:155702975-155724025	21051	5	2	1	3	1	1	4	0	2	5	1	3	3	
chrX:38748625-38829075	80451	5	2	1	2	1	1	3	0	2	5	1	2	4	
chr7:15034875-15075100	40226	5	3	3	4	2	2	5	2	2	4	1	3	3	
chr8:67466625-67481800	15176	5	2	1	1	1	1	5	2	1	2	1	1	5	
chr5:133864950-133875950	11001	5	1	1	5	1	0	1	0	1	0	1	1	1	
chr1:91072125-91085975	13851	5	1	0	1	1	2	1	1	1	5	0	1	1	
chr4:35648950-35702775	53826	5	2	1	4	1	2	2	1	4	1	1	5	2	
chr5:138990975-139005450	14476	5	2	0	4	1	0	2	0	1	3	1	5	1	
chr6:4111525-4130600	19076	5	1	1	2	1	1	1	0	1	1	1	5	1	
chr10:126423300-126430700	7401	5	2	1	3	1	3	2	0	2	5	1	3	4	
chr2:84550150-84567200	17051	5	2	1	3	1	1	5	1	1	2	1	2	3	
chr2:47589325-47600350	11026	5	2	0	1	1	5	2	0	1	5	1	1	3	
chr2:120176200-120227875	51676	5	3	2	3	1	2	4	0	2	5	1	3	5	
chr5:149961075-149975750	14676	5	1	1	5	1	0	1	0	1	1	1	1	0	
chr13:29782300-29797600	15301	5	2	2	2	1	1	2	0	3	4	1	5	1	
chr1:191511500-191522500	11001	5	2	3	1	2	2	3	1	3	3	5	4	1	
chr13:26522125-26538050	15926	5	2	1	2	1	0	2	0	5	2	1	4	1	
chr5:141603100-141658125	55026	5	3	2	5	2	2	4	2	4	4	1	4	2	
chr10:44150000-44171575	21576	5	2	3	3	2	1	2	0	1	1	1	5	1	
chr6:27484175-27507175	23001	5	2	3	2	1	3	2	0	5	3	2	2	3	
chr11:37861200-37931225	70026	5	2	1	2	1	1	1	0	1	5	1	2	2	
chr14:76534800-76551800	17001	5	1	1	1	1	1	1	0	2	0	1	5	0	
chr5:114562825-114570725	7901	5	2	2	2	2	3	2	1	2	3	3	5	3	
chr8:55512850-55528575	15726	5	1	1	1	0	0	1	0	1	1	0	5	1	
chr6:43923350-44037900	114551	5	2	2	3	1	2	3	1	3	5	1	4	3	
chr11:84662925-84675450	12526	5	1	1	0	1	0	2	0	1	5	1	1	1	
chr12:124248075-124327650	79576	5	2	1	3	2	5	1	0	2	3	1	2	4	
chr2:174393650-174464925	71276	5	3	1	5	2	2	4	1	3	4	2	4	3	
chr6:74607850-74640200	32351	5	3	2	4	2	1	3	1	5	3	1	3	3	
chr14:33403725-33434925	31201	5	2	1	5	2	1	3	1	3	3	2	3	2	
chr12:129125675-129208050	82376	5	3	2	5	2	2	2	1	3	5	2	3	3	
chr18:33500650-33550675	50026	5	2	1	3	1	2	1	0	2	5	1	3	2	
chr7:50198525-50260725	62201	5	2	1	3	1	1	3	1	2	5	1	3	3	
chr8:86963950-87054175	90226	5	3	2	4	2	2	3	1	3	5	2	3	3	
chr4:128977825-128988500	10676	5	2	0	2	1	5	4	1	1	3	1	1	1	
chr10:17290425-17301825	11401	5	1	1	1	0	0	1	0	2	2	0	5	1	
chr9:36392125-36402600	10476	5	2	1	2	1	1	3	1	2	3	1	5	3	
chr8:16431250-16489400	58151	5	2	5	2	1	0	4	0	5	1	1	2	4	
chrX:40136950-40206350	69401	5	2	1	4	1	1	2	1	3	5	2	3	2	
chr7:124655325-124692375	37051	5	3	1	4	2	2	4	1	3	5	1	4	2	
chr22:26399650-26467825	68176	5	2	1	4	2	1	2	1	5	2	3	2	2	
chr2:235183250-235262900	79651	5	2	1	3	1	1	2	5	3	3	1	2	3	
chr4:76679425-76695050	15626	5	3	3	1	2	2	4	1	2	5	2	4	3	
chr8:125829300-125904725	75426	5	3	2	4	2	1	3	1	3	5	2	4	3	
chr11:76132025-76162975	30951	5	1	2	1	0	0	5	1	0	2	0	1	1	
chr13:37779325-37804375	25051	5	2	1	2	1	0	5	2	2	3	0	2	2	
chr1:227445225-227455500	10276	5	1	1	2	1	1	1	0	5	1	1	3	2	
chr14:103403900-103436425	32526	5	1	1	5	1	1	2	1	2	1	0	2	1	
chr4:10302925-10319675	16751	5	1	0	1	0	0	5	0	0	0	0	1	0	
chrX:55818100-55829600	11501	5	2	1	1	1	1	3	0	1	5	1	4	2	
chr7:61383725-61394675	10951	5	1	0	5	0	0	0	0	0	0	0	0	0	
chrX:21818800-21841025	22226	5	2	2	2	2	2	3	1	2	3	5	4	2	
chr2:48237675-48250500	12826	5	2	1	2	1	1	2	1	2	5	2	3	3	
chr3:111003125-111018900	15776	5	1	0	1	1	0	5	0	0	0	0	0	1	
chr5:54198250-54243900	45651	5	2	1	1	1	1	5	0	1	3	1	2	2	
chr12:4829050-4840850	11801	5	1	0	1	1	5	0	0	1	1	0	1	0	
chr14:104347300-104362400	15101	5	2	1	5	1	1	2	1	4	3	1	3	2	
chr7:149967450-150001750	34301	4	2	1	2	1	1	1	0	2	3	1	4	2	
chr13:89971500-90052625	81126	4	2	1	2	1	1	2	0	2	3	1	2	4	
chr1:100787025-100809750	22726	4	2	1	2	1	1	4	0	1	3	2	4	1	
chr10:112068400-112115800	47401	4	2	1	4	1	1	2	0	1	2	1	3	2	
chr6:20445050-20482600	37551	4	2	1	4	1	1	1	0	1	4	1	3	3	
chr2:10614350-10628300	13951	4	3	2	2	3	3	3	2	2	4	4	4	3	
chr9:117281075-117332525	51451	4	2	1	3	1	1	1	0	1	2	1	2	4	
chr1:118021050-118060375	39326	4	2	1	3	1	1	4	0	3	3	2	3	2	
chr2:224419425-224431400	11976	4	2	2	4	2	1	2	1	3	3	2	4	3	
chrX:51150500-51160675	10176	4	2	0	1	1	4	1	0	1	4	0	4	3	
chr14:69792625-69829600	36976	4	2	1	2	1	0	2	0	1	2	1	2	4	
chr4:76853725-76868150	14426	4	2	0	3	1	1	2	1	1	2	4	2	2	
chr2:175621975-175641350	19376	4	2	1	1	1	0	3	1	2	2	0	4	1	
chr2:38224625-38236425	11801	4	2	2	3	1	1	3	0	1	2	2	4	2	
chr5:626650-642950	16301	4	1	0	1	0	0	1	0	0	1	0	1	4	
chr10:74428350-74435575	7226	4	1	0	0	1	0	1	0	2	3	4	2	0	
chr4:106491125-106505350	14226	4	2	1	2	2	2	4	1	2	4	4	3	1	
chr10:31098800-31139450	40651	4	3	3	4	2	3	2	4	4	2	1	2	2	
chr13:79054625-79103425	48801	4	3	2	3	2	1	4	1	4	4	2	4	4	
chr5:159600-182725	23126	4	2	1	2	1	1	3	0	1	4	1	2	2	
chr18:51434050-51464400	30351	4	1	1	2	0	0	1	0	1	1	1	1	4	
chr6:14808225-14856150	47926	4	2	2	3	1	1	2	1	4	2	1	2	4	
chr1:89153625-89168975	15351	4	2	1	2	1	2	2	1	3	4	1	4	2	
chr17:66488275-66507650	19376	4	1	0	1	0	1	1	0	1	4	1	1	1	
chr1:38655800-38689600	33801	4	2	1	1	1	1	4	0	2	3	1	4	2	
chr2:3151200-3164600	13401	4	2	1	4	1	1	4	0	0	2	1	4	2	
chr5:148435300-148452150	16851	4	2	1	3	2	2	3	1	2	4	1	3	4	
chr8:95986900-96001825	14926	4	2	1	3	1	0	1	0	2	4	2	3	1	
chr2:3671525-3682050	10526	4	2	2	3	2	1	4	0	4	2	0	2	2	
chr1:185637825-185716750	78926	4	2	1	2	0	1	2	1	2	4	1	3	2	
chr9:123192750-123210375	17626	4	2	1	2	1	1	2	1	2	4	1	4	4	
chr1:94851700-94869225	17526	4	2	2	3	2	2	4	1	2	4	1	2	2	
chr4:5594075-5607075	13001	4	2	1	2	1	1	4	1	1	4	1	2	2	
chr20:48336525-48346625	10101	4	1	1	0	0	0	0	1	1	0	0	4	0	
chr8:75160600-75178150	17551	4	1	1	1	2	1	0	0	1	1	1	4	1	
chr15:65581525-65598300	16776	4	2	2	2	1	1	4	1	2	4	2	3	3	

location	span (nt)	max	mean	adipose	colon	heart	hypothalamus	kidney	liver	lung	ovary	skeletal muscle	spleen	testes	Comments
chr11:113096775-113107650	10876	4	2	2	1	1	1	2	1	2	4	3	3	3	
chr11:28328725-28349150	20426	4	2	2	1	1	1	3	1	3	4	2	3	4	
chr17:37055675-37077700	22026	4	1	0	4	0	1	1	0	1	1	0	1	1	
chr5:5410650-5437825	27176	4	2	1	2	1	2	3	1	2	4	1	3	4	
chr4:11108100-11128250	20151	4	2	1	2	1	1	3	0	2	2	1	3	4	
chr1:154326000-154341900	15901	4	1	1	3	1	1	1	0	1	4	1	1	1	
chr21:20864700-20924525	59826	4	2	2	3	1	1	3	0	2	4	2	2	2	
chr3:135322675-135340275	17601	4	2	1	2	1	1	2	0	1	4	3	4	2	
chr15:64997350-65018875	21526	4	2	4	1	2	0	3	0	2	2	1	1	2	
chr13:29572725-29629450	56726	4	2	2	4	1	1	1	1	2	3	1	2	2	
chr2:120936925-121024600	87676	4	2	2	4	1	1	2	1	3	3	2	2	3	
chr13:98845000-98872975	27976	4	3	2	4	1	2	2	1	3	4	2	4	3	
chr4:41790675-41800725	10051	4	2	1	1	1	1	3	1	3	3	2	4	2	
chr20:56582075-56648350	66276	4	2	1	3	1	2	4	0	1	2	0	1	2	
chr4:24423850-24436150	12301	4	2	2	2	4	4	2	1	2	3	1	2	2	
chr4:25707575-25774725	67151	4	2	3	4	2	2	2	0	4	3	2	2	3	
chr9:123914275-123933950	19676	4	1	1	2	0	0	4	0	1	1	1	1	1	
chr10:89817350-89828825	11476	4	1	0	0	0	0	0	0	1	0	0	4	0	
chr5:171206800-171220925	14126	4	2	2	2	2	0	2	0	2	3	1	4	1	
chr10:17544300-17574475	30176	4	2	1	2	1	1	2	1	4	3	1	2	2	
chr5:141712700-141725675	12976	4	1	1	2	1	1	1	0	4	1	0	1	2	
chr12:1511350-1531175	19826	4	2	1	4	1	1	1	0	2	4	1	1	2	
chr2:8625475-8656675	31201	4	1	0	4	0	0	2	1	1	1	0	2	1	
chr1:33624900-33640125	15226	4	2	1	2	2	1	2	0	2	3	2	4	1	
chr2:68184950-68197450	12501	4	2	1	1	1	2	1	0	1	4	2	2	3	
chr2:238241425-238260675	19251	4	2	1	2	3	1	4	1	4	4	3	4	1	
chr11:109591300-109599000	7701	4	2	1	1	1	1	1	0	2	4	3	2	1	
chr5:67711025-67754775	43751	4	1	1	1	1	1	2	4	1	2	0	1	2	
chr8:94942275-94989225	46951	4	1	1	2	1	0	1	0	1	2	1	2	4	
chr1:27717600-27729175	11576	4	2	0	3	1	0	1	0	1	3	4	2	1	
chr6:44054725-44073196	18472	4	1	1	2	1	1	1	0	1	1	4	1	0	
chr1:161547975-161558200	10226	4	2	2	1	1	1	3	0	1	4	1	3	3	
chr1:197692000-197775950	83951	4	2	3	3	2	1	3	1	3	4	1	3	3	
chr12:55531725-55548275	16551	4	1	1	1	1	0	1	1	1	4	1	1	1	
chr13:35836025-35848350	12326	4	1	0	1	1	1	1	0	1	4	0	1	1	
chr12:108546875-108559575	12701	4	1	0	4	1	1	1	0	1	1	1	2	1	
chr6:15803725-15840100	36376	4	2	1	4	1	1	3	1	2	3	1	2	4	
chr1:237448850-237518300	69451	4	2	1	3	0	0	2	1	2	2	1	2	4	
chr4:65264625-65308600	43976	4	2	2	4	1	1	3	1	2	4	2	3	3	
chr5:167625475-167637500	12026	4	1	1	2	1	1	4	1	4	1	0	1	1	
chr8:121151225-121164350	13126	4	1	1	1	1	0	1	0	0	1	4	1	1	
chr12:4015975-4071825	55851	4	2	1	3	1	1	3	1	2	4	1	3	3	
chr12:59079125-59174275	95151	4	2	1	3	2	1	3	1	3	4	2	3	3	
chr2:127375700-127418650	42951	4	2	1	1	1	2	2	1	2	4	1	4	2	
chr8:37300175-37362850	62676	4	2	2	3	1	1	2	4	1	3	1	1	3	
chr14:95273700-95291125	17426	4	1	0	1	0	1	1	1	1	1	0	4	1	
chr16:83714775-83742000	27226	4	2	1	4	1	1	3	0	1	2	1	4	1	
chr5:81999675-82050175	50501	4	2	1	2	2	1	3	0	4	2	1	1	2	
chr6:132493825-132536700	42876	4	2	2	2	1	2	2	1	2	4	0	3	4	
chr12:46439750-46452350	12601	4	1	1	1	1	1	4	0	1	4	0	1	1	
chr5:40478375-40500725	22351	4	2	1	3	2	1	2	1	2	4	1	4	3	
chr10:102320175-102331900	11726	4	1	0	1	1	4	0	0	2	1	1	1	1	
chr4:285700-301175	15476	4	2	1	3	2	2	4	1	1	4	1	4	4	
chrX:111827950-111846700	18751	4	2	1	2	1	1	2	0	1	4	2	2	3	
chr13:104871700-104904225	32526	4	1	1	4	1	0	1	0	1	3	0	0	1	
chr10:44057525-44069225	11701	4	2	1	2	1	1	2	0	2	3	1	2	4	
chr2:28505600-28531825	26226	4	1	1	2	0	0	1	0	1	1	4	1	1	
chr6:131076150-131124300	48151	4	2	1	2	1	1	3	1	3	4	1	4	3	
chrX:57018625-57030000	11376	4	2	1	2	1	1	3	1	1	4	1	3	1	
chr5:158747225-158807525	60301	4	2	1	3	1	1	2	0	1	2	1	4	3	
chr3:195235225-195254025	18801	4	1	1	1	1	1	1	1	3	1	1	1	4	
chr14:98364250-98474750	110501	4	2	1	4	1	2	3	2	2	4	1	3	2	
chr12:12775375-12786225	10851	4	1	1	2	0	1	1	0	1	4	0	3	1	
chr2:62229225-62239350	10126	4	2	1	2	1	1	3	1	1	4	2	4	2	
chr2:202531900-202562225	30326	4	1	1	2	1	1	1	2	4	1	0	1	1	
chr18:69451350-69507600	56251	4	2	3	4	3	1	3	1	3	3	2	2	3	
chr9:5564750-5571550	6801	4	2	1	2	1	2	1	0	1	1	4	3	2	
chr10:114927875-114959800	31926	4	2	1	2	1	1	2	1	1	2	1	4	2	
chr11:10696225-10707675	11451	4	2	1	2	2	1	2	0	2	1	1	4	2	
chr7:86800800-86810825	10026	4	2	1	1	2	2	3	1	2	2	1	4	2	
chr21:43744000-43763000	19001	4	1	1	1	0	1	1	0	1	2	1	0	4	
chr3:31131575-31154225	22651	4	1	0	1	0	1	1	0	1	4	0	0	1	
chr4:119994100-120020550	26451	4	2	1	2	1	1	2	1	2	4	1	4	3	
chrX:42991675-43019825	28151	4	2	0	2	1	1	2	0	1	4	1	2	2	
chr2:57876000-57907450	31451	4	1	1	1	1	0	0	0	1	3	0	4	3	
chr10:54673950-54691500	17551	4	1	1	1	1	0	1	1	4	1	1	0	1	
chr21:18370050-18390050	20001	4	2	1	1	1	1	2	0	2	2	1	3	4	
chr10:44839900-44867575	27676	4	2	3	2	1	1	2	2	2	4	0	2	3	
chr14:40496250-40534675	38426	4	2	1	2	1	1	2	1	2	4	1	3	2	
chr6:31363000-31416375	53376	4	2	1	3	1	2	2	1	3	4	2	2	2	
chr9:5300525-5316625	16101	4	2	2	3	2	3	3	1	2	4	1	3	2	
chrX:124133150-124148375	15226	4	1	0	1	0	1	4	0	1	0	0	1	1	
chr1:198597975-198611175	13201	4	1	1	1	0	1	2	1	3	1	1	4	1	
chr1:203671525-203684250	12726	4	1	0	0	0	0	1	0	1	1	0	0	4	
chr11:9738200-9748525	10326	4	2	2	3	1	1	2	0	2	4	1	2	2	
chr14:55229950-55253225	23276	4	2	1	1	1	1	2	0	1	1	4	3	2	
chr10:3964425-3977400	12976	4	1	0	1	1	0	0	0	0	1	1	1	4	
chr12:28607450-28619725	12276	4	2	3	1	2	1	2	1	1	3	3	4	1	
chr10:80394300-80443425	49126	4	2	3	4	0	2	1	0	2	2	1	2	1	
chr4:55462350-55525325	62976	4	2	1	3	2	1	2	1	2	4	1	2	2	
chr14:76222450-76238625	16176	4	1	1	1	1	1	1	1	2	2	0	2	4	
chr2:200692850-200728325	35476	4	1	1	2	1	1	1	0	1	2	1	2	4	
chr8:21766900-21799225	32326	4	2	1	4	1	1	1	0	1	1	2	2	1	
chr5:32567225-32584425	17201	4	1	1	2	1	1	1	1	4	2	0	3	2	
chr11:94007350-94057525	50176	4	2	2	2	1	3	2	2	3	2	1	3	4	
chr11:127925850-127944925	19076	4	1	1	1	1	0	1	0	3	1	0	4	0	
chr5:66581100-66618700	37601	4	2	1	2	1	1	2	1	2	3	1	4	2	
chr1:226142500-226163525	21026	4	1	0	4	0	1	0	0	1	1	2	1	1	
chr2:76662200-76684675	22476	4	2	1	4	1	1	2	1	3	3	1	3	3	
chr13:43541700-43575500	33801	4	2	1	2	1	1	2	1	4	1	1	2	3	
chr17:14447875-14462575	14701	4	1	1	1	1	0	1	2	4	1	0	1	1	
chr2:193983125-194058900	75776	4	2	1	3	1	1	2	1	4	3	1	2	3	
chr15:81191300-81205175	13876	4	2	1	2	1	1	2	1	3	2	1	4	3	
chr3:144386625-144409775	23151	4	1	1	1	0	1	1	0	1	1	1	4	1	
chr17:50603800-50615775	11976	4	1	0	0	1	1	4	0	1	4	1	1	2	
chr1:192286275-192304050	17776	4	1	0	1	0	0	1	4	1	2	0	1	1	
chr2:161805425-161819450	14026	4	1	0	1	0	0	1	4	3	0	0	3	2	
chr11:47579600-47590300	10701	4	2	2	3	1	4	2	3	1	3	2	3	2	
chr2:177202562-177210548	7987	4	1	1	1	0	0	3	0	0	3	0	2	4	
chr12:32083800-32099625	15826	4	2	1	3	1	0	1	0	2	3	1	4	1	
chrX:112428775-112448300	19526	4	2	1	2	1	1	2	0	2	4	1	2	2	
chr18:43736200-43765550	29351	4	2	1	4	1	1	2	1	2	3	1	2	2	
chr4:6554025-6576400	22376	4	1	0	4	1	3	1	0	1	1	0	2	1	
chr1:38952425-38967525	15101	4	1	0	1	0	0	4	0	0	1	0	1	1	
chr11:2316375-2338500	22126	4	2	1	2	1	2	2	0	2	4	1	2	2	
chr5:61966575-61986950	20376	4	2	1	1	2	1	2	1	4	4	3	2	2	
chr11:68711325-68782100	70776	4	2	1	4	1	1	2	1	3	3	1	2	3	
chrX:7876450-7886700	10251	4	2	0	2	1	1	4	2	1	3	1	2	3	
chrX:119142325-119155025	12701	4	1	1	1	0	1	1	0	1	4	0	1	1	
chr8:118825700-118840650	14951	4	1	1	1	1	0	1	0	2	3	1	4	2	
chr1:43203850-43224600	20751	3	2	1	3	1	2	2	0	2	3	0	2	2	
chr3:32802975-32815650	12676	3	1	1	3	1	1	2	0	1	2	1	2	2	
chr7:64833500-64843700	10201	3	2	0	3	1	2	2	0	1	3	0	3	1	
chr2:155447775-155502825	55051	3	2	1	2	1	0	1	0	1	3	1	2	3	

location	span (nt)	max	mean	adipose	colon	heart	hypothalamus	kidney	liver	lung	ovary	skeletal muscle	spleen	testes	Comments
chr16:83762925-83776250	13326	3	1	0	2	0	0	2	0	0	3	2	1	1	
chr2:158472500-158484150	11651	3	1	1	1	0	1	2	0	1	3	0	1	1	
chr10:77993900-78008175	14276	3	1	1	0	1	0	2	0	1	3	0	3	2	
chr6:143711975-143724400	12426	3	2	2	1	1	1	3	1	1	3	0	2	1	
chr12:109859650-109871275	11626	3	1	0	0	0	3	0	0	1	0	3	0	1	
chr2:128803850-128856725	52876	3	1	1	2	1	1	2	1	1	2	0	3	1	
chr17:60528775-60538900	10126	3	2	1	3	1	1	1	2	3	2	2	1	2	
chr4:37438825-37458300	19476	3	1	1	2	0	0	0	0	1	1	0	3	0	
chr4:56497950-56508950	11001	3	1	1	0	1	1	1	1	1	1	0	3	1	
chr16:83112375-83139300	26926	3	1	1	3	1	1	1	1	3	1	0	2	2	
chr2:188216850-188242950	26101	3	2	1	3	1	1	1	0	3	2	1	2	3	
chr2:231927600-231939325	11726	3	1	1	2	0	1	1	0	1	3	0	0	1	
chr1:82690075-82738225	48151	3	1	1	1	0	0	1	1	3	0	0	0	2	
chr14:72115775-72140300	24526	3	1	1	1	1	1	1	2	3	2	1	0	1	
chr20:29267675-29298475	30801	3	1	1	3	0	0	1	0	1	0	0	0	1	
chr2:104872525-104884550	12026	3	1	0	2	0	1	3	0	1	0	0	0	0	
chr6:91443800-91462575	18776	3	1	0	0	0	0	0	0	0	0	1	3	0	
chr2:128912250-128946725	34476	3	2	1	1	1	0	3	1	2	3	0	3	2	
chr21:10142425-10168325	25901	3	2	1	3	1	2	2	1	1	2	1	1	3	
chr5:142858650-142910425	51776	3	2	1	3	2	1	3	0	2	3	1	2	2	
chr20:1594825-1615675	20851	3	1	0	1	0	1	1	0	1	1	0	3	0	
chr5:86440850-86461100	20251	3	1	0	1	0	3	0	0	0	0	0	1	0	
chr6:164108650-164125500	16851	3	1	0	0	0	0	0	0	1	0	0	1	3	
chr6:126536000-126549075	13076	3	2	1	1	1	1	3	2	1	3	1	2	2	
chr5:78088475-78102000	13526	3	1	0	1	0	0	3	0	0	1	0	1	1	
chr12:91259775-91314700	54926	3	1	2	1	1	1	2	0	1	2	1	3	1	
chr4:57290325-57305175	14851	3	2	1	2	1	2	2	1	2	2	1	3	2	
chr4:14873975-14887950	13976	3	2	1	2	2	1	3	0	3	3	1	3	2	
chr6:56857225-56873850	16626	3	2	3	3	1	1	2	1	3	3	1	2	2	
chr4:84754000-84780025	26026	3	2	2	3	2	2	3	2	2	3	2	3	2	
chr21:39131050-39141200	10151	3	1	0	1	0	0	0	3	1	1	1	1	1	
chr1:231084925-231131050	46126	3	1	1	3	1	1	1	0	1	2	1	1	2	
chr4:24273450-24285525	12076	3	1	1	1	0	0	0	0	0	0	0	0	3	
chr1:157219375-157229975	10601	3	1	0	1	1	0	1	0	1	0	0	3	0	
chrX:43070225-43119975	49751	3	2	1	2	1	1	2	1	3	3	1	3	3	
chr13:72717475-72744200	26726	3	1	0	3	0	1	1	0	1	2	0	1	1	
chr6:135461825-135493175	31351	3	2	1	3	1	1	1	1	3	3	0	1	2	
chr15:89814100-89836700	22601	3	1	1	1	0	0	3	0	1	2	1	1	2	
chr21:32528625-32540925	12301	3	1	1	1	1	1	1	0	1	3	2	3	1	
chr14:50080825-50091175	10351	3	1	1	2	1	1	3	0	1	2	0	3	2	
chrX:70452900-70467450	14551	3	1	0	3	1	1	2	0	2	3	1	2	2	
chr9:117609800-117640825	31026	3	1	2	2	1	0	1	0	1	1	0	1	3	
chr1:110140000-110161150	21151	3	1	1	1	1	1	1	1	2	3	1	1	1	
chr2:67455100-67475300	20201	3	1	1	1	1	0	2	0	1	3	0	1	2	
chr2:145391450-145406075	14626	3	1	0	0	0	0	0	0	0	0	0	0	3	
chr3:120042175-120054600	12426	3	1	0	1	1	1	3	1	1	1	1	1	3	
chr2:47707900-47740825	32926	3	1	1	3	1	1	2	0	1	2	1	1	1	
chr7:115705750-115719100	13351	3	1	3	0	1	0	0	0	1	1	0	1	1	
chr5:120503325-120556975	53651	3	1	1	1	1	0	1	0	2	2	0	1	3	
chr17:13902350-13912750	10401	3	1	0	1	1	1	1	1	1	3	1	3	0	
chr3:156892700-156920200	27501	3	1	0	3	1	1	2	0	3	1	0	1	3	
chr1:180367800-180398625	30826	3	2	1	2	1	1	1	3	2	2	1	2	3	
chr6:140930925-140944025	13101	3	1	1	0	0	0	0	0	0	3	0	0	1	
chr5:126209425-126222425	13001	3	1	1	2	1	0	1	0	2	1	0	3	1	
chr1:38349575-38436300	86726	3	2	1	3	1	3	2	1	2	2	1	2	3	
chr10:63244600-63290375	45776	3	2	1	3	1	1	2	0	1	2	0	2	3	
chr6:163707275-163722675	15401	3	1	2	3	1	1	1	0	1	2	1	3	1	
chr11:68866050-68876750	10701	3	1	3	1	0	0	0	1	0	0	0	0	1	
chr8:17033050-17043750	10701	3	1	1	1	1	1	1	1	3	1	1	3	2	
chr14:65329850-65390700	60851	3	1	1	3	1	1	3	1	1	2	0	2	2	
chr2:129013175-129078275	65101	3	2	1	3	1	1	3	3	2	1	0	1	2	
chr2:19934400-19954800	20401	3	1	0	1	1	1	3	1	1	2	1	3	1	
chr10:11477175-11516650	39476	3	1	1	2	1	1	2	0	1	3	1	2	2	
chrX:16349775-16384450	34676	3	2	2	3	1	1	1	1	2	2	1	3	2	
chr11:125559275-125576425	17151	3	2	0	2	2	1	2	0	1	2	3	2	2	
chr16:85173525-85183625	10101	3	1	1	3	2	1	3	0	2	1	0	1	2	
chr18:71155200-71236250	81051	3	2	1	3	1	1	2	0	2	3	1	2	3	
chr6:48613250-48635850	22601	3	1	0	1	1	0	1	0	1	3	1	1	1	
chr14:95159375-95182150	22776	3	1	0	2	0	0	1	1	1	1	0	2	3	
chr7:135091600-135161700	70101	3	2	1	3	2	3	2	0	3	2	1	2	2	
chr12:3887700-3912800	25101	3	1	0	1	0	0	0	3	1	0	0	1	1	
chrX:121189550-121211000	21451	3	2	1	1	1	1	2	1	2	3	2	3	2	
chr4:75405950-75439100	33151	3	1	1	1	1	1	1	1	2	3	1	3	2	
chr5:5376800-5390800	14001	3	1	0	1	0	0	1	0	0	3	0	0	1	
chr7:140858550-140880350	21801	3	1	1	1	1	1	1	0	0	3	0	1	2	
chr5:6309175-6335000	25826	3	1	0	1	0	2	2	0	1	3	1	2	2	
chr7:152227150-152252575	25426	3	2	1	3	1	1	3	1	2	2	2	2	1	
chrX:47499275-47526100	26826	3	2	1	2	1	1	2	0	1	3	1	2	2	
chr12:92102025-92112725	10701	3	2	3	2	1	1	1	0	2	2	1	1	2	
chr14:61092950-61102975	10026	3	1	1	1	1	0	1	1	3	1	0	2	1	
chr10:79477000-79490450	13451	3	1	1	2	1	0	1	0	2	1	0	2	3	
chr9:109835775-109863175	27401	3	1	0	0	1	0	0	0	1	1	0	1	3	
chr5:171169700-171196400	26701	3	2	1	3	1	0	2	0	2	2	2	3	2	
chr5:36449325-36493325	44001	3	2	1	3	1	2	3	1	3	2	1	2	2	
chr8:117452225-117463525	11301	3	1	1	2	1	1	1	0	1	3	1	2	1	
chrX:128656625-128669100	12476	3	1	0	1	0	1	1	3	1	0	1	1	1	
chr2:856250-875200	18951	3	1	0	1	0	1	2	0	0	3	0	1	3	
chr10:90790650-90810150	19501	3	1	1	1	1	1	1	0	3	3	1	2	1	
chr2:59760925-59778075	17151	3	1	1	2	1	1	2	0	1	3	1	2	2	
chr6:37852300-37874075	21776	3	1	0	1	0	1	2	0	1	3	0	1	1	
chr14:70218275-70241100	22826	3	1	0	3	1	1	1	0	1	1	1	3	1	
chr1:83290500-83321125	30626	3	1	1	1	0	0	3	1	1	1	1	2	2	
chrX:45204175-45226275	22101	3	1	1	1	1	1	2	1	2	3	1	1	2	
chrX:106809250-106830375	21126	3	1	1	1	1	0	1	1	3	2	1	1	1	
chr14:20357225-20368875	11651	3	2	1	3	1	1	2	1	3	2	1	3	3	
chr9:132216250-132228100	11851	3	1	1	3	0	0	0	0	0	0	1	0	0	
chr1:157733100-157744550	11451	3	1	0	0	1	0	1	3	0	1	0	0	0	
chr12:28147950-28179550	31601	3	1	0	1	1	2	3	1	1	1	0	1	1	
chr8:101561650-101582050	20401	3	1	1	3	1	0	3	1	1	1	1	2	1	
chr8:96764575-96799350	34776	3	2	1	3	1	1	2	0	2	2	1	2	2	
chr1:219344150-219386650	42501	3	1	1	1	1	1	2	0	2	1	1	2	3	
chr4:129807075-129835750	28676	3	2	1	2	2	1	3	1	2	1	1	2	1	
chr21:25773375-25797750	24376	3	1	1	2	1	1	2	0	1	3	1	2	2	
chrX:12985100-12999000	13901	3	1	1	1	1	1	1	1	2	3	1	1	1	
chr20:30614850-30628550	13701	3	1	0	2	0	0	1	0	1	0	0	3	0	
chr2:74047225-74063900	16676	3	1	0	3	1	0	1	0	1	1	1	1	1	
chr6:44551950-44649875	97926	3	1	1	3	1	1	1	1	1	2	0	1	2	
chr10:43321675-43337750	16076	3	1	1	2	1	1	1	0	1	2	0	3	1	
chr2:18650725-18664975	14251	3	2	1	2	1	1	3	1	1	2	1	3	2	
chr18:10967400-10990075	22676	3	2	1	3	1	1	2	0	2	3	1	2	3	
chr13:85112300-85176950	64651	3	2	1	3	1	1	1	0	2	3	0	3	2	
chr18:24926225-24960300	34076	3	1	1	3	0	1	1	0	2	1	1	1	1	
chr3:154613700-154630125	16426	3	1	1	1	1	0	1	0	3	2	1	1	2	
chr3:9316650-9327225	10576	3	1	1	1	1	1	3	0	2	2	1	1	2	
chr4:21970900-21980925	10026	3	1	0	3	0	1	2	0	0	1	3	0	0	
chr13:93951875-93969425	17551	3	2	2	3	2	2	2	3	3	2	1	2	2	
chr3:128335025-128366725	31701	3	2	2	2	2	2	2	3	2	2	1	1	2	
chr6:47497200-47512025	14826	3	1	1	2	0	1	2	0	1	3	1	1	1	
chr10:119334750-119346225	11476	3	1	0	0	0	0	3	0	0	1	0	0	1	
chr12:109163775-109180275	16501	3	1	1	3	1	1	2	0	1	2	1	1	1	
chr3:67392950-67407275	14326	3	1	1	1	1	1	3	1	2	2	1	2	1	
chr12:27251975-27262525	10551	3	1	0	1	0	0	1	0	1	1	1	1	3	
chr5:25161275-25192725	31451	3	1	0	1	1	1	1	0	1	2	0	1	3	
chr14:68342850-68361800	18951	3	1	1	1	0	0	1	1	1	1	0	3	1	

location	span (nt)	max	mean	adipose	colon	heart	hypothalamus	kidney	liver	lung	ovary	skeletal muscle	spleen	testes	Comments
chr2:235007225-235027750	20526	3	1	1	2	1	1	1	0	2	1	0	3	2	
chr2:235436300-235461250	24951	3	1	0	2	0	1	2	1	1	2	0	3	2	
chr5:72582075-72621975	39901	3	1	0	1	1	3	2	1	1	1	0	1	1	
chr7:17050950-17064125	13176	3	2	1	2	1	1	1	1	3	3	1	2	2	
chr21:43431675-43445875	14201	3	1	1	2	1	1	3	0	1	2	1	2	2	
chr3:138221350-138270375	49026	3	2	1	2	1	0	1	1	3	2	1	1	3	
chr4:108912750-108928175	15426	3	1	0	1	1	0	2	1	2	2	1	3	1	
chr18:51844825-51856000	11176	3	1	0	0	0	0	2	0	0	3	0	2	1	
chr1:55603825-55618950	15126	3	1	0	0	0	0	3	0	0	0	0	1	0	
chr7:44985750-44993250	7501	3	1	0	1	0	1	0	0	2	2	0	3	1	
chr1:170043625-170063325	19701	3	1	0	1	0	0	1	0	1	3	1	2	2	
chrX:112520800-112559300	38501	3	1	1	1	1	1	2	1	2	3	1	1	2	
chr6:113779025-113797250	18226	3	1	0	0	0	0	1	0	3	0	0	0	1	
chrX:7521975-7555900	33926	3	2	1	2	1	1	2	1	3	2	1	2	2	
chr10:82403500-82422325	18826	3	1	0	3	0	0	1	0	1	1	0	1	1	
chr12:84376050-84390000	13951	3	1	1	3	1	1	1	1	1	2	1	2	2	
chr8:23401500-23433375	31876	3	1	1	1	0	1	1	0	1	2	1	3	1	
chr4:60197375-60231625	34251	3	2	1	2	1	1	2	1	2	3	1	3	2	
chr7:132000925-132019600	18676	3	1	1	2	1	1	1	0	1	3	1	2	1	
chr1:54700800-54715125	14326	3	1	1	2	1	0	1	1	3	1	0	1	1	
chr17:35920625-35944325	23701	3	1	1	2	1	1	1	0	3	1	1	3	1	
chr14:82180425-82193800	13376	3	1	1	3	0	0	1	0	1	2	0	2	2	
chr15:68264425-68364650	100226	3	2	2	3	1	1	2	1	2	2	1	2	2	
chr6:24848150-24866900	18751	3	1	1	3	1	1	1	0	1	2	0	2	1	
chr20:38277400-38346375	68976	3	2	1	2	1	1	2	1	2	3	1	2	2	
chr7:3112050-3122375	10326	3	1	0	0	0	0	0	0	0	3	0	2	1	
chr13:54046725-54067800	21076	3	1	1	1	1	1	1	1	1	3	0	0	1	
chr11:123446150-123461375	15226	3	1	0	1	0	0	1	0	0	0	0	0	3	
chr1:110967750-110987075	19326	3	1	1	3	1	0	1	0	2	1	0	1	1	
chr2:145044975-145101650	56676	3	1	1	2	1	0	1	0	2	3	1	2	1	
chrX:113383525-113403350	19826	3	1	1	3	1	1	1	0	1	2	1	1	1	
chr12:112666800-112695075	28276	3	1	0	1	0	2	0	0	1	1	1	0	3	
chr6:107039875-107055450	15576	3	1	2	1	1	0	3	0	1	1	0	1	1	
chrX:39545025-39558350	13326	3	1	1	1	0	1	3	0	1	1	0	1	1	
chr2:209877875-209898250	20376	3	1	1	1	1	1	1	0	2	2	1	3	1	
chr8:98281700-98293000	11301	3	1	0	2	1	2	2	0	2	3	1	1	2	
chr13:20795375-20806850	11476	3	1	1	1	1	1	2	0	1	1	1	3	2	
chr16:64354175-64369575	15401	3	1	0	1	0	0	1	0	3	1	0	1	1	
chr4:97862925-97919950	57026	3	1	1	2	1	1	2	0	3	1	1	2	2	
chr6:45930950-45960325	29376	3	0	0	1	1	0	0	0	3	0	1	0	0	
chr2:113338800-113367300	28501	3	1	1	1	0	0	0	1	2	0	0	0	3	
chr5:100029775-100049925	20151	2	1	1	2	1	1	1	0	2	2	1	2	2	
chr6:40364375-40431825	67451	2	1	0	2	1	1	1	2	1	1	1	2	1	
chr12:66382725-66396775	14051	2	1	0	1	1	0	1	0	1	1	2	2	1	
chr8:8448600-8464875	16276	2	1	1	2	1	0	1	1	1	1	0	2	2	
chr10:5323425-5346050	22626	2	1	1	1	1	1	2	2	2	1	1	2	1	
chr11:61497150-61510050	12901	2	1	0	1	0	0	2	1	1	1	0	1	1	
chr16:82518800-82529525	10726	2	1	1	2	1	0	2	0	0	2	1	1	1	
chr17:52638425-52662275	23851	2	1	1	2	1	1	2	0	1	2	1	2	2	
chr18:19102650-19116400	13751	2	1	1	2	1	1	1	0	1	2	0	2	2	
chr2:15837300-15865800	28501	2	1	1	2	1	1	1	1	2	2	0	1	1	
chr2:208068275-208086800	18526	2	1	1	1	0	0	1	0	1	1	0	2	1	
chr5:130418650-130430500	11851	2	1	0	2	0	0	1	0	1	2	0	2	1	
chr6:23772575-23786650	14076	2	1	1	1	1	0	1	0	2	2	1	1	1	
chr2:129171850-129204875	33026	2	1	1	2	0	1	1	0	1	2	1	2	1	
chr2:207882050-207916600	34551	2	1	2	2	0	0	1	0	2	1	0	1	2	
chr10:31640150-31647375	7226	2	1	1	0	1	1	1	0	1	2	1	1	1	
chr13:32944875-32958750	13876	2	1	1	1	0	0	1	0	1	2	0	1	1	
chr10:8185475-8199525	14051	2	1	1	1	1	1	2	0	2	1	0	2	1	
chr1:37631125-37664925	33801	2	1	1	2	1	1	1	0	2	1	2	1	2	
chr13:37652175-37671200	19026	2	1	1	1	1	0	2	0	1	1	1	1	1	
chr13:30514900-30551075	36176	2	1	1	1	0	2	0	0	1	1	0	1	1	
chr3:52030875-52042150	11276	2	0	0	0	0	0	1	0	0	0	2	0	0	
chr4:13433500-13445875	12376	2	1	0	1	2	0	1	0	0	1	0	1	2	
chr12:74122750-74141150	18401	2	1	0	1	1	0	1	0	1	2	2	1	2	
chr3:191350675-191373100	22426	2	1	1	1	0	1	2	0	1	2	0	2	2	
chr4:13280500-13304225	23726	2	1	0	0	0	0	2	0	0	2	0	0	2	
chr9:99670975-99681450	10476	2	1	1	1	0	1	1	1	2	1	1	2	1	
chr12:99650125-99682025	31901	2	1	0	1	0	0	0	0	0	1	0	0	2	
chr11:118872950-118890300	17351	2	1	1	2	1	1	2	0	1	2	1	1	1	
chr14:63186050-63201475	15426	2	1	1	2	1	0	2	1	2	2	1	2	2	
chr14:37627700-37638050	10351	2	1	0	1	1	1	1	0	1	2	1	2	2	
chr3:174419600-174446075	26476	2	1	1	2	1	0	2	0	2	2	1	2	1	
chr3:15547200-15558900	11701	2	1	1	1	0	1	2	1	1	2	1	2	2	
chr9:101349675-101362675	13001	2	1	0	0	1	0	2	0	1	2	1	1	2	
chr2:197532200-197543775	11576	2	1	0	0	1	0	0	0	1	0	0	2	1	
chr13:23020375-23031325	10951	2	1	0	1	2	1	0	0	1	1	0	1	1	
chr12:46293050-46303950	10901	2	1	0	0	0	1	1	0	0	2	1	1	1	
chr1:226983650-227005375	21726	2	1	1	2	1	1	1	0	2	1	1	1	1	
chr4:24088325-24111350	23026	2	1	0	2	0	0	1	0	1	2	1	1	1	
chr5:126425050-126439050	14001	2	1	1	1	0	0	1	0	0	1	0	2	2	
chr12:28059800-28074275	14476	2	1	1	0	0	0	1	0	2	0	0	0	1	
chr6:114419675-114434275	14601	2	1	2	1	1	2	1	1	2	2	1	2	1	
chr10:46439775-46457625	17851	2	1	0	2	0	0	0	0	1	1	0	0	1	
chr10:72470575-72517275	46701	2	1	1	2	1	1	1	0	1	1	0	1	1	
chr17:21173800-21193125	19326	2	1	0	2	1	0	1	0	1	1	0	1	1	
chr5:151402350-151417950	15601	2	1	2	1	1	1	2	1	2	1	1	1	2	
chr5:67021350-67033475	12126	2	1	0	1	0	0	1	1	2	0	2	0	1	
chr2:46610375-46622325	11951	2	1	2	1	2	1	2	0	2	2	1	2	1	
chr2:68699850-68713300	13451	2	1	1	1	1	1	1	0	1	2	1	1	2	
chr2:1582100-1606250	24151	2	1	1	2	1	1	1	0	2	1	0	1	1	
chr3:15903550-15915000	11451	2	1	0	1	1	0	1	0	0	2	1	1	1	
chr2:48144125-48183025	38901	2	1	1	1	0	1	1	0	1	1	1	2	2	
chr8:125928050-125946450	18401	2	1	0	1	1	1	1	1	1	1	0	2	2	
chr11:35030825-35051075	20251	2	1	1	2	1	0	1	0	2	2	1	2	1	
chr14:99762875-99774250	11376	2	1	0	1	0	0	1	0	1	2	0	1	0	
chr4:11175800-11186900	11101	2	1	0	0	0	0	1	0	1	2	0	1	1	
chrX:44865675-44877100	11426	2	1	0	1	1	1	1	1	1	2	1	1	1	
chr15:68593550-68621775	28226	2	1	0	2	0	1	1	0	1	1	1	2	2	
chr6:158103850-158116625	12776	2	1	2	1	0	0	1	0	0	0	1	1	0	
chr1:115446650-115482075	35426	2	1	1	2	1	1	2	0	1	2	0	1	1	
chr1:5284450-5323200	38751	2	1	0	2	1	0	0	0	1	1	0	1	1	
chr6:28803400-28817200	13801	2	1	1	2	1	1	1	0	1	2	1	2	2	
chr2:143529050-143548825	19776	2	1	0	1	0	0	1	1	1	1	0	2	1	
chr6:83144225-83155775	11551	2	1	0	1	1	1	1	0	1	1	0	2	0	
chr5:95612950-95629950	17001	2	1	1	1	1	0	2	0	2	2	1	1	1	
chr6:132270225-132303825	33601	2	1	1	2	1	0	1	0	1	2	1	1	1	
chr8:80982575-80993550	10976	2	1	0	1	1	1	2	1	1	2	2	2	1	
chr4:97596350-97637675	41326	2	1	1	2	1	1	2	1	2	2	0	2	2	
chr15:77908600-77919075	10476	2	1	1	1	1	1	2	1	1	2	1	2	1	
chr1:219188650-219213500	24851	2	1	0	2	0	0	1	0	1	1	1	2	2	
chr5:108581425-108615525	34101	2	1	1	1	0	0	2	1	1	1	1	2	1	
chr8:8935475-8948675	13201	2	1	1	1	1	0	1	1	1	2	0	2	1	
chr1:58946300-58965675	19376	2	1	1	1	1	1	1	0	2	1	0	0	1	
chr7:12309550-12324625	15076	2	1	1	1	0	0	1	0	1	2	0	2	2	
chr8:99470550-99481825	11276	2	1	1	0	0	0	1	1	1	2	0	1	2	
chr14:21939225-21987475	48251	2	1	0	2	0	1	2	0	1	1	0	2	1	
chr1:79669875-79726875	57001	2	1	1	1	0	1	1	0	1	2	1	2	2	
chr15:27756650-27770700	14051	2	1	0	1	0	1	2	0	0	1	0	0	2	
chr13:44167650-44182450	14801	2	1	1	2	1	1	1	1	1	2	1	1	1	
chrX:39865200-39876225	11026	2	1	1	2	1	0	1	1	1	1	0	1	1	
chr8:28599675-28613975	14301	2	1	0	1	1	1	1	0	1	2	1	1	2	
chr4:109840575-109856475	15901	2	1	1	1	1	1	2	0	1	1	1	1	1	
chr1:110890825-110912275	21451	2	0	2	0	0	0	0	0	1	0	0	0	0	

location	span (nt)	max	mean	adipose	colon	heart	hypothalamus	kidney	liver	lung	ovary	skeletal muscle	spleen	testes	Comments
chr5:65651750-65665975	14226	2	1	0	1	2	0	1	0	0	1	1	0	2	
chr7:134059975-134078575	18601	2	1	1	2	1	1	1	0	1	2	2	1	2	
chr10:14583425-14595550	12126	2	1	0	0	0	0	2	1	1	0	0	2	1	
chr14:59839275-59854025	14751	2	1	0	1	1	0	1	1	1	1	1	2	1	
chr1:227938125-227966450	28326	2	1	1	2	1	1	1	0	1	2	0	1	2	
chr1:192052425-192067650	15226	2	1	0	1	1	0	1	2	1	2	1	1	1	
chr14:103819375-103843275	23901	2	0	0	1	0	1	1	0	0	2	0	0	0	
chr16:86886600-86897900	11301	2	0	0	0	0	0	0	0	0	2	0	0	0	
chr7:129874900-129897725	22826	2	1	1	2	1	0	1	0	1	1	2	1	1	
chr7:36085575-36119300	33726	2	1	0	1	0	0	1	0	1	1	2	1	0	
chr1:229493875-229512225	18351	2	1	1	2	0	1	1	0	1	2	1	1	1	
chr17:45292550-45303650	11101	2	1	1	2	0	1	1	0	2	1	2	1	1	
chr7:156071100-156081325	10226	2	1	1	2	0	1	2	1	0	1	0	1	1	
chrX:16485950-16503000	17051	2	1	0	2	1	0	1	0	1	2	1	1	1	
chr12:1905425-1919900	14476	2	1	0	1	1	1	1	0	1	2	1	2	2	
chr2:173862575-173875725	13151	2	1	1	1	1	1	1	2	1	1	1	1	1	
chr5:7315850-7326875	11026	2	1	0	1	1	0	2	0	2	1	1	1	2	
chr14:102278900-102297625	18726	2	1	0	2	1	0	1	1	1	1	0	1	1	
chr2:102247075-102260750	13676	2	1	0	1	0	0	0	0	2	0	0	2	0	
chr9:110220050-110234450	14401	2	1	0	1	1	0	1	0	1	2	1	1	1	
chr2:112100575-112126675	26101	2	1	1	2	1	0	2	0	2	1	2	1	1	
chr1:108668675-108685775	17101	2	1	0	0	0	0	0	0	0	1	0	1	2	
chr14:96861925-96885100	23176	2	1	0	1	0	0	1	0	1	2	0	0	1	
chr2:68414075-68436975	22901	2	1	0	2	1	1	1	0	1	2	1	2	1	
chr8:59599400-59611475	12076	2	1	1	1	1	1	1	0	1	2	1	1	1	
chr1:111258200-111271650	13451	2	1	0	1	0	0	1	1	2	1	0	2	1	
chr3:28780450-28795425	14976	2	1	1	2	0	0	1	0	1	1	0	1	1	
chr8:107124300-107141200	16901	2	1	1	2	0	0	1	0	0	2	0	0	1	
chr7:154880875-154899050	18176	2	0	0	0	0	2	0	0	0	0	0	0	0	
chr4:119116800-119129925	13126	2	1	0	1	0	0	1	0	1	1	0	2	1	
chr4:109537600-109559575	21976	2	1	1	2	1	1	2	0	1	1	0	1	1	
chr7:63974350-63986200	11851	2	1	0	2	0	1	0	0	1	0	0	0	2	
chr9:110453000-110477975	24976	2	1	1	1	1	1	2	0	1	1	0	1	2	
chr2:177413225-177449975	36751	2	1	0	1	0	1	1	0	0	2	0	0	1	
chr6:3720750-3734450	13701	2	0	0	0	0	0	0	2	0	0	0	0	0	
chr3:127457650-127470750	13101	2	0	2	0	0	0	1	0	1	0	0	0	0	
chr16:1370125-1384875	14751	2	1	1	2	0	1	1	0	2	2	0	2	1	
chr7:154956450-154968800	12351	2	1	1	2	0	1	1	1	1	1	0	1	1	
chr8:86661875-86704300	42426	2	1	1	2	1	1	1	0	1	2	0	1	1	
chr15:35840950-35861750	20801	2	1	1	1	1	0	2	1	1	2	1	1	1	
chr12:27470200-27503925	33726	2	1	1	2	1	2	1	1	2	1	1	1	2	
chr16:85209025-85242650	33626	2	1	0	1	2	1	2	0	1	1	1	1	1	
chr1:230946300-230968050	21751	2	1	1	1	0	0	1	0	0	1	0	1	2	
chr5:153310925-153336875	25951	2	1	1	1	1	0	2	0	1	1	1	2	2	
chr16:64844625-64859475	14851	2	1	0	0	0	0	1	0	1	2	0	0	1	
chr2:75102425-75113500	11076	2	1	0	0	0	0	1	0	0	2	0	1	1	
chr3:140070825-140082475	11651	2	1	0	2	1	0	1	0	1	2	1	1	1	
chr3:72052025-72073225	21201	2	1	0	2	0	0	1	0	1	1	0	1	1	
chr21:26566775-26598525	31751	2	1	0	1	1	1	1	1	2	1	1	1	1	
chr4:6803950-6814375	10426	2	1	0	1	0	0	1	1	2	1	0	0	1	
chrX:46061075-46074800	13726	2	1	2	1	1	0	2	0	1	1	0	1	1	
chr1:198069025-198095775	26751	2	1	0	1	0	0	1	0	1	1	1	1	2	
chr6:28257900-28284400	26501	2	1	0	1	1	0	1	0	1	2	0	1	1	
chr8:133765050-133775675	10626	2	1	1	1	1	0	0	0	1	1	0	2	0	
chrX:68155600-68165750	10151	2	0	0	2	0	0	1	0	1	0	0	1	0	
chr16:85714350-85726850	12501	2	1	0	1	0	1	1	0	0	1	0	0	2	
chr14:58685825-58705500	19676	2	1	0	2	0	0	1	0	1	1	0	1	1	
chr12:37070650-37099175	28526	2	1	1	1	0	1	1	0	1	2	1	1	1	
chr14:50957850-50968625	10776	2	0	0	0	0	0	0	0	0	2	0	0	0	
chr2:208088275-208101600	13326	2	1	1	1	1	0	0	0	1	2	0	2	1	
chr16:24382475-24394475	12001	2	1	0	1	1	1	1	0	2	1	0	1	2	
chr12:91114125-91124300	10176	2	1	1	0	0	0	1	0	2	1	0	1	1	
chr3:14365525-14403925	38401	2	1	1	1	1	0	1	0	2	1	2	1	1	
chr3:18718325-18755600	37276	2	1	1	2	1	0	1	0	2	2	1	2	1	
chrX:9188975-9205300	16326	2	1	0	1	0	1	0	0	2	0	0	0	1	
chr17:31380950-31404075	23126	2	1	0	2	1	1	2	1	1	2	0	1	2	
chrX:8034225-8044325	10101	2	1	0	1	0	0	1	0	0	2	1	1	1	
chr1:89972700-89985275	12576	2	1	1	1	0	0	1	0	1	1	0	2	1	
chr14:75090375-75100625	10251	2	1	1	1	1	1	1	0	1	0	0	2	1	
chr7:17693175-17708075	14901	2	1	0	1	0	0	0	0	0	0	1	2	0	
chr1:186622825-186667625	44801	2	1	1	2	0	1	1	0	1	1	0	1	1	
chr14:54626650-54641500	14851	2	1	0	2	0	0	0	0	0	1	0	1	1	
chr2:195714425-195743825	29401	2	1	0	1	1	0	1	0	1	1	2	1	2	
chr10:120025775-120037250	11476	2	1	1	2	1	0	2	0	1	1	1	1	1	
chrX:108755875-108765750	9876	2	0	0	0	0	0	0	0	1	0	1	2	0	
chr5:170925150-170938100	12951	2	1	0	2	0	1	0	0	0	0	0	1	1	
chr8:123180400-123245250	64851	2	1	1	2	2	1	1	0	1	2	0	1	1	
chrX:45935775-45952650	16876	2	1	1	2	1	0	1	0	1	1	0	1	1	
chr9:108063575-108084000	20426	2	1	0	1	0	0	1	0	0	1	2	1	1	
chr10:21588375-21603075	14701	2	1	0	1	0	0	1	0	1	2	0	0	1	
chr8:116879400-116915750	36351	2	1	1	1	1	1	2	0	1	2	1	1	2	
chr13:90287700-90298800	11101	2	1	0	1	1	1	2	0	1	1	1	1	1	
chr5:172802300-172835075	32776	2	1	1	1	1	1	1	0	1	1	1	0	2	
chr6:319725-331650	11926	2	1	0	0	0	0	0	1	1	1	1	2	1	
chr1:190336425-190386300	49876	2	1	0	2	1	1	1	0	1	2	0	1	1	
chr1:32953825-32968200	14376	2	1	1	2	0	2	2	1	1	1	0	1	1	
chr10:46461825-46483150	21326	2	1	0	2	0	1	1	0	1	1	0	1	1	
chr18:69428125-69440375	12251	2	1	1	2	1	0	0	0	1	0	1	0	0	
chr8:135270400-135285950	15551	2	1	1	2	1	1	1	0	1	2	0	1	1	
chr11:12061300-12075325	14026	2	1	0	1	0	0	0	0	1	0	0	1	2	
chr7:148684475-148711675	27201	2	1	1	1	1	1	1	0	1	2	1	1	2	
chr7:37491050-37501225	10176	2	1	1	1	0	0	1	0	1	1	0	1	2	
chr20:21494900-21542475	47576	2	1	1	2	0	1	2	1	1	1	1	1	1	
chr4:12494675-12506850	12176	2	1	1	1	1	0	2	0	0	1	0	1	1	
chr1:209780925-209794325	13401	2	1	1	1	1	2	1	1	1	2	1	1	1	
chr11:44514100-44530500	16401	2	1	0	1	1	1	1	0	1	2	0	1	1	
chr7:19668825-19689525	20701	2	1	0	0	1	0	1	0	0	2	1	1	1	
chr21:23019950-23043675	23726	2	0	0	1	0	0	0	0	1	0	0	0	2	
chr1:116599350-116640475	41126	2	1	0	2	0	1	1	0	1	1	0	0	1	
chr12:12899550-12917075	17526	2	1	1	2	1	1	1	0	1	1	1	2	2	
chr4:84501725-84520225	18501	2	1	0	2	1	1	1	0	1	1	2	1	1	
chr2:19843425-19853775	10351	2	1	1	1	1	0	2	1	0	1	1	1	1	
chr6:88777300-88787350	10051	2	0	0	0	0	0	2	0	0	0	0	0	0	
chr7:45237050-45257100	20051	2	1	0	1	2	0	1	0	1	1	0	1	0	
chr3:162490600-162502750	12151	2	1	1	1	1	0	1	0	0	1	1	1	2	
chr6:95297000-95320325	23326	2	1	0	1	0	2	0	0	0	1	0	0	1	
chr3:67211075-67230650	19576	2	1	1	1	1	1	2	0	1	1	0	1	1	
chr8:90569375-90582100	12726	2	1	0	1	1	1	2	0	0	2	1	0	1	
chr15:52877675-52913225	35551	2	1	1	1	1	0	1	1	2	1	1	1	2	
chr11:127666200-127694325	28126	2	1	1	1	0	0	1	0	1	0	0	2	0	
chr16:65001425-65016550	15126	2	1	1	1	0	0	0	0	0	0	0	2	1	
chr7:107281875-107305375	23501	2	1	0	2	0	0	1	0	0	1	0	1	1	
chr16:72642800-72653775	10976	2	1	1	1	1	0	1	1	1	2	1	1	1	
chrX:47435100-47446900	11801	2	0	0	0	0	0	1	0	0	2	0	0	0	
chr17:6706675-6717550	10876	2	1	0	1	0	1	2	1	1	1	0	0	1	
chr7:139125175-139135400	10226	2	1	1	1	0	0	2	0	1	1	0	1	1	
chr3:178961675-178979250	17576	2	1	0	2	0	0	0	0	2	0	0	1	0	
chr4:74767950-74796275	28326	2	1	1	1	0	0	2	0	1	2	0	1	0	
chr21:39892800-39905250	12451	2	1	0	1	0	2	0	0	0	1	0	0	0	
chr4:105886425-105897000	10576	2	0	0	0	0	0	2	0	0	0	0	0	1	
chr8:20714075-20728800	14726	2	0	0	0	0	0	0	2	0	0	0	0	1	
chr1:211527625-211546900	19276	2	1	0	1	1	2	1	0	1	2	2	1	1	
chr7:127535725-127551125	15401	2	1	1	1	0	1	1	0	1	1	2	1	1	
chr14:56622350-56632675	10326	2	0	0	0	0	0	0	0	0	2	0	0	1	

location	span (nt)	max	mean	adipose	colon	heart	hypothalamus	kidney	liver	lung	ovary	skeletal muscle	spleen	testes	Comments
chr11:11610275-11623250	12976	2	1	1	2	1	1	1	0	2	1	1	1	1	
chr2:20228225-20252075	23851	2	1	1	2	0	0	1	0	2	1	0	1	1	
chr13:94099200-94126650	27451	2	1	1	2	1	1	1	0	1	1	1	1	1	
chr12:60293850-60330425	36576	2	1	0	1	1	1	1	0	1	2	0	1	1	
chr15:86016750-86031775	15026	2	0	0	0	0	0	0	0	0	0	0	0	2	
chr12:130862425-130873450	11026	2	1	1	2	1	0	1	0	1	1	1	1	1	
chr8:49497300-49508350	11051	2	0	0	2	0	0	0	0	0	0	0	0	0	
chrX:13179325-13195650	16326	2	0	0	0	0	0	0	0	2	0	0	0	0	
chr2:60308225-60325275	17051	2	1	0	1	1	1	1	0	1	2	0	1	2	
chr10:33295350-33306125	10776	2	1	1	1	0	0	0	0	1	2	1	1	1	
chr4:28047250-28057975	10726	2	0	0	0	0	0	0	0	0	2	0	0	0	
chr2:118070075-118082125	12051	2	1	1	1	1	1	2	1	1	2	1	2	2	
chr7:126966450-126978000	11551	2	1	0	1	0	0	1	1	1	1	0	1	2	
chr2:191182625-191194800	12176	2	1	1	1	1	1	1	0	1	1	1	2	2	
chr12:71402075-71451500	49426	2	1	1	2	1	1	1	0	1	2	2	2	2	
chr20:41540650-41550875	10226	2	1	0	1	1	0	1	0	1	2	1	1	0	
chr7:25945000-25956500	11501	2	1	0	1	0	0	1	1	1	2	0	1	0	
chr2:140320750-140345675	24926	2	1	1	2	0	0	1	0	1	2	0	1	1	
chr5:123989500-124000000	10501	2	1	0	2	1	0	1	0	2	1	1	1	0	
chr2:100743825-100764825	21001	2	0	0	1	0	0	2	0	0	1	0	0	0	
chr4:123259225-123275150	15926	2	1	1	0	0	1	0	0	1	1	0	2	0	
chrX:97117325-97138975	21651	2	1	1	0	1	1	1	0	1	1	1	2	1	
chr13:62527475-62547500	20026	2	0	0	1	0	0	0	0	0	0	0	0	2	
chr2:3018400-3029600	11201	2	1	0	2	0	1	0	0	0	0	0	1	1	
chr5:67639050-67653625	14576	2	1	0	2	0	0	1	1	1	0	0	1	1	
chr8:126534450-126548675	14226	2	1	0	2	1	0	1	0	1	1	0	1	1	
chr9:136478450-136494350	15901	2	1	0	2	0	0	0	0	0	0	2	1	0	
chr3:140886175-140908425	22251	2	1	0	0	0	0	1	0	0	2	1	1	1	
chr5:71698825-71709925	11101	2	1	0	0	1	1	1	0	0	2	0	2	1	
chrX:17692075-17705000	12926	2	0	0	0	0	1	0	0	0	2	0	0	1	
chr18:52269550-52287850	18301	2	1	1	1	0	0	0	0	0	1	0	2	1	
chr2:239386225-239398275	12051	2	1	0	0	0	0	0	2	1	1	0	1	1	
chr11:75618975-75681950	62976	2	1	0	2	0	1	1	0	1	1	0	1	1	
chr12:12035425-12048075	12651	2	1	0	2	1	0	1	0	1	1	0	1	0	
chr13:76119825-76133875	14051	2	1	1	2	1	0	1	1	1	1	0	2	2	
chr4:75749750-75816175	66426	2	1	1	2	1	1	1	0	1	1	0	1	1	
chr6:105257475-105276750	19276	2	1	0	1	0	0	1	0	1	1	1	1	2	
chr4:82927700-82939425	11726	2	1	1	1	1	1	2	0	1	1	0	1	1	
chr13:73143700-73156425	12726	2	0	0	0	0	0	1	0	0	1	1	2	0	
chr4:45663225-45675625	12401	2	1	0	0	1	0	1	0	1	1	1	2	1	
chrX:42589900-42605225	15326	2	0	0	1	0	0	0	0	0	0	0	1	2	
chr1:117139575-117159550	19976	2	1	0	2	0	0	0	0	1	1	0	2	1	
chr10:124096325-124117225	20901	2	1	0	2	0	0	1	0	1	1	0	1	1	
chr16:73375750-73388725	12976	2	1	1	2	0	0	0	0	1	1	0	1	1	
chr8:49339350-49364275	24926	2	1	0	2	1	1	1	0	1	1	1	1	1	
chr10:116737575-116751575	14001	2	1	0	1	0	0	1	0	0	1	0	1	2	
chr6:167563325-167591625	28301	2	1	0	0	0	1	1	0	1	1	0	1	2	
chr13:26929500-26940825	11326	2	0	0	1	0	0	0	1	2	0	0	0	0	
chr8:101497275-101508975	11701	2	1	0	1	0	0	1	0	2	0	0	0	1	
chr8:55859150-55874925	15776	2	1	0	0	0	0	2	0	2	1	0	1	1	
chr12:66122475-66133100	10626	2	1	0	0	0	0	0	0	1	1	2	0	1	
chr2:102818025-102837000	18976	2	1	0	1	2	0	1	0	1	1	2	1	1	
chr2:60939000-60949925	10926	2	0	0	0	0	0	1	0	0	2	0	0	0	
chr21:40002450-40025400	22951	2	1	1	1	1	1	1	0	1	2	0	1	1	
chr6:168840750-168858250	17501	2	0	0	0	0	0	0	0	0	2	0	0	1	
chrX:33839175-33855650	16476	2	1	0	0	1	0	0	0	0	2	1	0	1	
chr4:122340200-122357975	17776	2	1	0	1	0	0	1	0	1	1	0	2	1	
chr8:134223000-134240175	17176	2	1	0	1	0	0	1	0	1	0	0	2	0	
chr1:233083800-233131200	47401	2	1	0	2	1	1	1	1	1	1	1	1	1	
chr10:79868075-79885175	17101	2	1	0	2	0	1	0	0	1	1	0	1	1	
chr12:22424300-22444300	20001	2	1	0	2	0	1	1	0	1	1	1	1	1	
chr10:74794275-74801925	7651	2	1	0	1	1	1	1	0	1	2	1	1	1	
chr3:129629700-129653875	24176	2	1	1	1	1	1	1	0	1	2	1	1	1	
chr6:168228500-168281475	52976	2	1	0	1	0	0	1	0	2	0	0	1	1	
chr3:40633100-40647875	14776	2	1	0	1	0	0	1	0	0	1	0	2	1	
chr6:56831150-56842350	11201	2	1	1	2	0	1	0	0	0	1	0	1	0	
chr1:233176125-233186700	10576	2	1	0	1	1	0	0	2	1	1	0	1	0	
chr2:83365875-83377675	11801	2	1	0	1	1	0	0	0	1	2	0	1	1	
chr3:39253550-39266600	13051	2	1	0	1	0	0	0	0	1	2	0	1	1	
chr4:103259950-103278600	18651	2	1	0	1	1	0	1	0	1	2	1	1	1	
chr15:95199150-95215225	16076	2	0	0	1	0	0	0	1	2	0	0	0	1	
chr2:33927975-33955425	27451	2	1	0	1	1	0	1	0	1	1	1	1	2	
chr3:165381975-165394700	12726	2	0	0	0	0	0	2	0	1	0	0	0	0	
chr12:129742550-129761400	18851	2	1	0	1	1	1	1	0	1	2	0	1	1	
chr11:93342525-93370800	28276	2	1	0	2	0	0	1	0	1	1	1	1	1	
chr13:111038175-111051075	12901	2	1	0	2	0	0	0	0	1	1	0	1	1	
chr18:10217025-10229050	12026	2	1	0	2	1	0	0	0	1	0	0	1	1	
chr7:64405850-64428525	22676	2	1	1	2	0	1	1	0	1	1	1	1	1	
chr11:113400350-113410875	10526	2	0	0	0	0	0	0	0	0	0	2	0	0	
chr13:77485575-77499475	13901	2	0	0	0	0	1	2	0	0	0	0	0	1	
chr8:87388450-87400450	12001	1	1	0	1	0	0	1	0	0	1	0	1	1	
chr15:72297100-72307675	10576	1	1	0	1	0	0	0	0	1	1	0	1	1	
chr18:31270875-31282100	11226	1	1	0	1	0	0	1	0	1	1	0	1	1	
chr7:1314975-1333125	18151	1	1	0	1	0	0	0	0	1	1	0	1	1	
chr12:62078650-62106025	27376	1	1	1	1	1	1	1	0	1	1	0	1	1	
chr7:38128125-38146550	18426	1	1	0	1	0	0	1	0	1	1	0	1	0	
chr18:45525275-45537075	11801	1	1	0	0	0	0	1	0	0	1	0	1	1	
chr5:123734825-123757500	22676	1	1	1	1	1	1	1	0	1	1	1	1	1	
chr2:131299475-131330850	31376	1	1	0	1	0	1	0	0	0	0	0	1	1	
chr15:34482575-34514925	32351	1	1	0	1	0	0	1	0	0	1	0	1	1	
chr4:100514375-100532350	17976	1	1	1	0	1	0	1	1	1	1	0	1	0	
chr3:59513350-59536300	22951	1	1	0	1	0	0	1	0	1	1	0	0	1	
chr5:172551525-172566225	14701	1	1	0	1	0	1	1	0	1	1	0	1	1	
chr6:106317650-106342450	24801	1	0	0	1	0	0	0	0	1	1	0	1	1	
chr8:143050700-143089375	38676	1	1	0	1	0	1	1	0	1	0	0	1	0	
chr7:10108575-10127025	18451	1	1	0	1	1	1	1	0	1	1	0	1	1	
chr1:94192050-94206150	14101	1	1	0	0	0	0	1	0	1	1	0	1	1	
chr10:72048325-72060125	11801	1	1	0	1	0	1	1	0	1	1	0	1	0	
chr13:65073950-65093750	19801	1	1	1	1	1	0	1	0	1	1	1	1	1	
chrX:42410125-42420750	10626	1	0	0	0	0	0	1	0	0	0	0	0	0	
chr10:9125775-9138650	12876	1	0	1	0	0	1	1	0	0	0	0	0	1	
chr6:137747450-137784475	37026	1	1	1	1	0	1	1	1	1	1	0	1	1	
chr8:58422575-58434950	12376	1	1	1	1	1	0	1	1	1	1	0	1	1	
chr8:6504100-6520650	16551	1	1	0	1	1	1	1	0	0	1	1	0	1	
chrX:47681075-47693375	12301	1	1	1	1	0	1	1	1	1	1	0	1	1	
chr12:4208850-4236425	27576	1	1	1	1	1	1	1	0	1	1	0	1	1	
chr14:65527250-65542300	15051	1	1	1	1	0	0	1	1	1	1	0	0	1	
chr7:12555150-12568375	13226	1	1	0	1	0	0	1	0	1	1	0	0	1	
chr13:32328175-32365200	37026	1	1	0	1	0	0	1	0	1	1	1	1	1	
chr5:131205050-131217175	12126	1	1	0	0	0	0	0	0	1	1	0	1	1	
chr8:127174725-127195150	20426	1	1	0	1	0	0	1	0	1	1	0	1	1	
chr1:41622050-41638350	16301	1	1	0	1	0	1	1	0	1	1	1	0	1	
chr7:139990575-140001525	10951	1	1	0	1	0	0	0	0	0	1	0	1	1	
chr5:147208200-147229425	21226	1	1	1	1	1	1	1	0	1	1	0	1	1	
chr10:58632875-58649825	16951	1	1	0	1	1	0	1	0	0	1	0	0	1	
chr7:26058825-26077900	19076	1	1	0	1	0	1	1	0	1	1	0	0	1	
chr1:47977525-47990050	12526	1	1	0	1	0	0	1	0	1	1	0	1	1	
chr9:136969250-136980175	10926	1	0	0	0	0	0	0	0	0	1	0	0	0	
chr12:103278000-103288275	10276	1	0	0	0	0	0	1	0	0	1	1	1	0	
chr4:92760700-92774625	13926	1	1	0	1	0	0	1	0	1	1	0	1	1	
chr18:71295125-71308850	13726	1	1	0	0	1	1	0	0	0	1	0	0	1	
chr1:36804800-36856000	51201	1	1	0	1	0	0	1	0	1	1	0	1	0	
chr13:102433875-102457175	23301	1	1	1	1	1	0	1	0	1	1	1	1	1	
chr15:32723275-32748900	25626	1	1	1	1	0	1	1	0	1	1	1	0	1	

location	span (nt)	max	mean	adipose	colon	heart	hypothalamus	kidney	liver	lung	ovary	skeletal muscle	spleen	testes	Comments
chr9:110587325-110599600	12276	1	1	1	1	0	0	0	0	0	1	0	1	1	
chr4:126684025-126702050	18026	1	1	0	1	0	0	1	0	1	1	0	1	1	
chr1:227336900-227362675	25776	1	1	1	1	1	1	1	0	1	1	0	0	1	
chr9:20275050-20313050	38001	1	1	0	0	0	0	0	0	1	1	0	1	1	
chr10:43662450-43681050	18601	1	1	1	0	1	1	0	0	0	0	0	1	1	
chr10:95690050-95706050	16001	1	1	0	1	0	0	0	0	0	1	0	1	1	
chr8:107241625-107255900	14276	1	1	1	1	0	0	1	0	1	1	0	1	1	
chr5:172935725-172946925	11201	1	1	1	0	1	0	1	0	0	1	1	1	1	
chr1:61790650-61807975	17326	1	0	0	0	0	0	0	0	0	1	0	0	1	
chr18:12755775-12765850	10076	1	0	0	1	0	0	0	0	1	1	0	1	1	
chr3:34601800-34619400	17601	1	1	0	0	1	1	1	0	1	1	0	1	1	
chr12:123289800-123300925	11126	1	0	0	0	1	0	0	1	0	1	0	0	1	
chr1:210766975-210778775	11801	1	1	0	1	0	0	1	0	1	1	0	1	1	
chr4:3267300-3280150	12851	1	1	0	1	0	0	0	0	1	1	0	0	1	
chr13:25892925-25912325	19401	1	1	0	0	1	0	1	0	1	1	1	1	1	
chr16:11202325-11213275	10951	1	1	0	1	0	0	1	0	1	1	1	1	1	
chr16:85636250-85652025	15776	1	0	0	1	0	1	0	0	0	0	0	0	1	
chr18:65815775-65821875	6101	1	1	0	0	0	0	1	0	0	1	1	1	1	
chr7:131200300-131215700	15401	1	1	0	1	1	0	1	0	1	1	0	1	1	
chr18:62495250-62511675	16426	1	1	0	0	1	0	1	0	1	1	0	1	1	
chr11:3318675-3329000	10326	1	0	0	0	0	0	1	0	0	0	0	1	0	
chr18:50499900-50530325	30426	1	1	1	1	1	0	1	0	1	1	0	1	1	
chr21:36918225-36929075	10851	1	0	0	0	0	0	1	0	0	0	0	0	0	
chr1:184977425-184989700	12276	1	0	0	1	0	0	1	0	0	1	0	1	1	
chr10:20704225-20728925	24701	1	1	1	1	1	0	1	0	1	1	0	1	1	
chr8:102116975-102146725	29751	1	1	0	1	0	0	0	0	1	1	0	1	1	
chr8:62947050-62968950	21901	1	0	0	1	0	0	1	0	0	0	0	0	1	
chr6:160265000-160304725	39726	1	1	0	1	0	1	1	0	1	1	1	1	1	
chr7:106011900-106044800	32901	1	1	1	1	0	0	1	0	0	0	1	1	1	
chr3:1054400-1066350	11951	1	1	1	1	1	0	1	0	0	1	1	0	1	
chr13:96567750-96587100	19351	1	0	0	0	0	1	0	0	0	1	1	0	1	
chr15:75776925-75798350	21426	1	1	0	1	0	1	1	0	1	1	0	0	1	
chr5:138004150-138033350	29201	1	1	0	1	0	0	0	0	1	1	0	1	1	
chr6:169980975-170009950	28976	1	0	0	1	0	0	1	0	0	1	0	0	1	
chr2:65553800-65571600	17801	1	1	0	1	0	0	1	0	1	1	0	1	1	
chr3:14234200-14253875	19676	1	1	0	1	1	0	1	0	1	1	1	1	1	
chr3:151675700-151689700	14001	1	1	0	1	1	0	1	0	1	1	1	1	1	
chr13:36774375-36786575	12201	1	0	0	1	0	0	0	0	0	0	0	1	1	
chr4:60023500-60045050	21551	1	1	0	1	1	1	1	0	1	1	0	1	1	
chr5:82720450-82740300	19851	1	1	0	1	0	0	1	0	1	1	1	1	1	
chr11:70708525-70763300	54776	1	1	1	1	1	1	1	0	1	1	0	1	1	
chr8:129364550-129425050	60501	1	1	1	1	0	1	1	0	1	1	0	1	1	
chr2:174618925-174633575	14651	1	0	0	1	0	0	0	0	0	1	0	0	1	
chr3:162956025-162984275	28251	1	1	0	1	1	0	1	0	1	1	0	1	1	
chr4:80131600-80143150	11551	1	1	0	1	1	1	0	0	1	1	0	1	1	
chr5:82352125-82366025	13901	1	1	0	0	1	1	1	0	0	1	0	1	1	
chr1:232846750-232871450	24701	1	1	0	1	0	0	1	0	1	1	1	1	1	
chr18:3275925-3287175	11251	1	1	0	1	1	0	1	0	1	1	0	1	1	
chr18:73132350-73145125	12776	1	1	1	1	0	0	1	0	0	1	0	0	1	
chr5:171039675-171075250	35576	1	0	0	1	0	0	1	0	0	1	0	0	1	
chr7:133743150-133761500	18351	1	1	1	1	0	1	0	0	0	0	0	0	1	
chr1:157866600-157887050	20451	1	1	0	1	0	0	1	0	1	1	0	0	1	
chr14:100893675-100910100	16426	1	0	0	0	0	0	0	0	0	1	0	0	1	
chr8:67374100-67384800	10701	1	0	0	0	0	0	1	0	1	1	0	0	1	
chr11:76004575-76019350	14776	1	1	0	1	0	0	1	0	1	1	1	1	1	
chr5:150980275-150993600	13326	1	1	1	1	1	1	1	0	1	1	1	1	1	
chr1:242144675-242161200	16526	1	0	0	0	0	1	0	0	0	1	0	1	0	
chr13:109450900-109474050	23151	1	0	0	0	0	1	0	0	0	0	0	0	1	
chr14:88668550-88683450	14901	1	0	0	0	0	0	0	0	1	1	1	1	0	
chr1:242224475-242238875	14401	1	0	0	1	0	1	0	0	0	1	0	0	1	
chr1:196602575-196613925	11351	1	0	0	0	0	0	0	0	0	0	0	0	1	
chr5:65527325-65546300	18976	1	1	1	1	0	0	1	0	1	0	1	0	1	
chr11:60195475-60207650	12176	1	0	0	1	0	0	0	0	0	0	0	1	1	
chr17:13668000-13679475	11476	1	1	0	1	0	0	1	0	0	1	0	1	0	
chr1:232737950-232749825	11876	1	0	0	1	0	0	0	0	0	0	0	0	0	
chr16:80725025-80730300	5276	1	0	0	1	0	1	0	0	0	0	0	0	0	
chr4:11720650-11746750	26101	1	1	0	1	1	0	1	0	1	1	0	1	1	
chr5:172072175-172091825	19651	1	0	0	1	0	0	1	1	1	0	0	1	0	
chr11:64016000-64031350	15351	1	1	1	1	0	1	0	0	1	1	0	1	1	
chr5:93854775-93865400	10626	1	0	0	0	0	0	1	0	0	1	0	1	1	
chr8:99411775-99424650	12876	1	1	0	1	1	0	1	0	1	1	0	1	1	
chr11:86907850-86918625	10776	1	0	0	0	0	0	0	0	1	0	0	1	1	
chr7:142857900-142879425	21526	1	1	0	1	0	0	0	0	1	1	0	1	1	
chr18:66573100-66583300	10201	1	0	0	1	0	0	1	0	0	0	0	0	1	
chr11:118079725-118089900	10176	1	1	0	1	1	0	1	1	1	1	0	1	1	
chr2:127221550-127268625	47076	1	1	1	1	0	0	1	0	1	0	0	0	0	
chr6:12422400-12439200	16801	1	1	0	1	0	0	0	0	1	1	0	1	1	
chr8:29955875-29977325	21451	1	1	0	1	0	0	1	0	1	1	0	1	1	
chr13:57331500-57346525	15026	1	0	0	0	0	0	1	0	0	1	0	1	1	
chr3:107753000-107766100	13101	1	0	0	0	0	1	1	0	0	1	0	0	0	
chr3:190099900-190110100	10201	1	1	0	1	1	0	1	0	1	1	1	1	0	
chr3:53080350-53090725	10376	1	1	1	1	0	1	1	0	1	1	1	1	0	
chr12:53675775-53689275	13501	1	1	0	1	1	0	0	0	0	1	0	1	1	
chr6:164379775-164403250	23476	1	1	0	1	0	1	1	0	1	1	0	1	1	
chr10:79685025-79696100	11076	1	0	0	0	0	1	0	0	0	0	0	0	0	
chr4:103556475-103569975	13501	1	1	0	1	0	1	1	0	1	1	0	1	0	
chr2:173956250-173969375	13126	1	0	0	1	1	0	0	0	0	1	1	0	1	
chr4:24699025-24711050	12026	1	1	1	1	0	0	1	0	1	1	1	1	0	
chr10:63773225-63790825	17601	1	1	0	1	0	1	1	0	1	1	0	1	0	
chr13:70805100-70838950	33851	1	1	1	1	0	0	1	0	1	1	0	1	1	
chr20:47658050-47669650	11601	1	1	0	1	0	0	1	0	1	0	0	1	1	
chr1:232962350-232985575	23226	1	1	0	1	0	1	1	0	1	0	0	0	1	
chr13:80149275-80161275	12001	1	0	0	1	0	0	0	0	0	0	0	1	1	
chr5:15074475-15098700	24226	1	0	0	1	0	0	1	0	1	1	0	1	1	
chr12:88147075-88171675	24601	1	1	0	0	1	0	1	0	1	1	1	1	1	
chr20:12132900-12146175	13276	1	0	0	0	0	0	1	0	1	1	0	0	1	
chr3:151267675-151279475	11801	1	0	0	0	0	1	0	0	0	1	1	0	0	
chr5:153865150-153884225	19076	1	1	1	1	0	0	1	0	1	1	0	1	1	
chr8:131665675-131689950	24276	1	1	1	1	1	1	1	0	1	1	0	1	1	
chr10:58524700-58561000	36301	1	1	1	1	0	0	1	1	1	1	0	1	1	
chr3:188285650-188297650	12001	1	1	0	1	1	0	1	0	1	1	0	1	1	
chr6:94572500-94596150	23651	1	0	0	1	0	0	1	0	0	0	0	0	0	
chr11:8260275-8275625	15351	1	0	0	1	0	0	1	0	0	0	0	0	0	
chr2:172916050-172928325	12276	1	0	0	1	0	0	0	0	0	0	0	1	0	
chr3:67036325-67048375	12051	1	0	0	1	0	0	0	0	0	1	0	0	0	
chr8:143175675-143202700	27026	1	1	1	1	0	1	1	0	0	1	0	1	1	
chrX:13390150-13402350	12201	1	0	0	1	0	0	0	0	1	1	0	0	0	
chrX:149445825-149463350	17526	1	0	0	1	0	0	0	0	0	1	0	1	1	
chr11:129971550-129983400	11851	1	1	1	0	0	0	1	0	0	1	0	1	1	
chr2:10067850-10080200	12351	1	0	1	1	0	0	0	1	0	1	1	0	1	
chr6:27356350-27374325	17976	1	1	1	1	0	0	1	0	1	1	0	1	1	
chr13:70523025-70535900	12876	1	1	1	1	0	0	1	0	1	1	0	1	1	
chr5:174407925-174419375	11451	1	1	0	1	0	0	1	0	1	1	0	1	1	
chrX:22623000-22636625	13626	1	0	0	0	0	0	0	0	0	0	1	1	1	
chr10:83047075-83063275	16201	1	0	0	1	0	0	1	0	0	1	0	0	1	
chr16:32487800-32498825	11026	1	1	0	1	0	0	1	0	1	1	1	1	1	
chrX:96977850-96994250	16401	1	0	0	0	0	0	0	0	0	0	0	0	1	
chr10:120357125-120373825	16701	1	1	0	1	0	0	1	0	0	1	0	1	1	
chr12:95328975-95344075	15101	1	0	0	1	1	0	1	0	1	1	0	1	0	
chr13:26444150-26456575	12426	1	0	0	1	0	1	0	0	0	0	0	1	0	
chr18:75510350-75521775	11426	1	0	0	1	0	0	0	0	0	0	1	1	0	
chr2:234448625-234460150	11526	1	1	0	1	0	0	1	0	1	1	0	1	1	
chr9:30285875-30296925	11051	1	0	0	1	0	0	0	0	0	1	0	1	1	
chr7:93318600-93332975	14376	1	0	0	1	1	0	1	0	0	1	0	0	0	

location	span (nt)	max	mean	adipose	colon	heart	hypothalamus	kidney	liver	lung	ovary	skeletal muscle	spleen	testes	Comments
chr10:119185750-119206425	20676	1	1	0	1	1	0	1	0	1	1	0	1	1	
chr1:206192400-206213400	21001	1	1	1	1	0	0	1	0	1	1	0	1	1	
chr3:129747975-129758550	10576	1	0	0	0	0	0	0	0	1	0	0	1	0	
chr5:20567625-20582975	15351	1	1	0	0	0	1	1	0	1	1	1	1	1	
chr5:58269850-58284050	14201	1	0	0	0	0	0	0	0	1	0	0	0	1	
chr7:107961175-107971925	10751	1	1	0	0	1	0	1	0	1	1	1	1	1	
chr10:79649925-79660825	10901	1	0	0	0	0	1	0	0	0	0	0	0	0	
chr12:131342275-131366850	24576	1	0	0	0	0	1	1	0	0	0	0	1	0	
chr6:141828150-141851300	23151	1	1	0	1	1	1	1	0	1	1	0	1	1	
chr9:115624525-115638900	14376	1	0	0	0	0	0	0	0	0	1	0	1	1	
chrX:130474825-130486100	11276	1	0	0	1	0	0	1	0	0	1	0	0	1	
chr15:54368275-54379425	11151	1	0	0	0	0	0	0	0	0	1	0	0	1	
chr20:23933300-23963300	30001	1	0	0	0	0	0	1	0	0	0	0	0	1	
chr20:23069750-23081475	11726	1	0	0	1	0	0	0	0	1	0	1	0	0	
chr14:100655175-100665700	10526	1	0	0	0	0	0	0	0	0	0	0	1	0	
chr11:125478700-125489900	11201	1	0	0	0	0	1	0	0	1	1	0	0	1	
chr8:143698500-143708725	10226	1	1	0	1	0	0	1	0	1	1	0	1	1	
chr1:211844700-211858375	13676	1	0	0	0	0	0	0	0	0	0	0	0	1	
chr7:132089250-132105750	16501	1	0	0	0	0	0	0	0	0	0	0	0	1	
chr1:142366925-142378900	11976	1	0	0	1	0	0	0	0	0	1	0	0	1	
chr10:125967200-125988150	20951	1	0	0	1	0	0	0	0	1	0	0	0	0	
chr12:125949425-125960625	11201	1	1	0	1	0	1	1	0	0	1	0	1	1	
chr5:158952700-158963825	11126	1	0	0	1	0	1	0	0	0	1	0	1	0	
chr10:5063025-5074225	11201	1	0	1	0	0	0	0	0	0	0	0	1	1	
chr2:130348325-130363275	14951	1	1	0	1	1	1	0	0	1	1	0	1	1	
chr7:85086825-85104450	17626	1	0	0	1	0	1	0	0	0	0	0	1	1	
chr12:83580475-83606200	25726	1	1	0	1	0	0	1	0	1	1	0	0	1	
chr13:21926150-21946050	19901	1	1	0	1	1	0	1	0	1	1	0	1	0	
chr4:55732800-55750175	17376	1	0	0	1	0	1	0	0	1	1	0	1	1	
chr6:137544875-137554925	10051	1	1	0	1	0	1	1	1	1	1	0	1	1	
chr1:84998125-85009475	11351	1	0	0	1	0	0	0	0	1	1	0	0	1	
chrX:13066500-13084525	18026	1	0	0	0	0	0	1	0	0	1	0	1	1	
chr6:137463000-137483475	20476	1	0	0	0	0	0	0	0	0	1	0	1	0	
chr6:40057500-40082950	25451	1	0	1	1	0	1	0	0	1	0	0	1	0	
chrX:107150775-107167475	16701	1	0	0	0	0	0	0	0	1	0	0	1	0	
chr18:18297450-18310625	13176	1	0	0	0	0	0	1	1	0	0	0	0	1	
chr8:115369100-115387225	18126	1	0	0	0	0	0	0	0	0	0	0	0	1	
chr1:18263675-18285975	22301	1	0	0	1	0	0	1	0	1	1	0	0	0	
chr1:56367475-56378825	11351	1	1	0	1	0	0	1	0	1	1	0	1	0	
chr13:110967175-110979375	12201	1	0	0	1	0	0	0	0	0	0	0	0	1	
chr2:112179825-112193875	14051	1	0	0	1	0	0	1	0	0	1	1	0	0	
chr7:86596600-86613925	17326	1	0	0	0	0	0	1	0	0	1	0	1	1	
chr8:124686250-124701500	15251	1	0	0	0	0	0	0	0	1	1	0	1	1	
chr2:206825950-206841100	15151	1	0	0	1	0	0	0	0	1	1	0	1	1	
chr2:148061475-148073025	11551	1	0	0	0	0	0	0	0	0	0	0	0	1	
chr5:68249800-68267150	17351	1	0	0	0	0	1	1	0	1	1	0	1	1	
chr1:212355225-212367225	12001	1	0	0	0	0	1	0	0	0	0	0	0	0	
chr2:100672675-100683975	11301	1	0	0	0	0	0	1	0	0	0	0	0	0	
chr2:177566750-177577650	10901	1	0	0	1	0	0	0	0	0	1	0	1	1	
chr8:135113725-135131100	17376	1	0	0	0	0	0	0	0	0	1	0	1	0	
chr1:245015075-245029300	14226	1	1	0	1	0	0	1	0	0	1	1	1	0	
chr10:102392025-102402450	10426	1	0	0	1	0	0	0	0	0	0	0	1	0	
chr11:39334375-39350600	16226	1	0	0	1	0	0	0	0	1	0	0	1	0	
chr15:97788575-97799125	10551	1	0	0	1	0	0	0	0	0	0	0	0	0	
chr18:74488625-74501875	13251	1	1	0	1	0	1	1	0	1	1	0	1	0	
chr13:44374400-44385800	11401	1	0	0	0	0	0	0	0	0	0	0	0	1	
chr2:72003825-72014850	11026	1	0	0	0	0	0	0	0	0	0	0	0	1	
chr21:44053175-44063175	10001	1	0	1	0	0	0	0	0	0	1	0	1	1	
chr12:113054050-113092400	38351	1	0	0	0	0	0	0	0	1	0	0	0	1	
chr1:4325150-4335375	10226	1	0	0	0	0	0	0	0	0	0	0	0	1	
chrX:45440850-45465775	24926	1	1	0	1	0	0	1	0	1	1	0	1	1	
chr11:44677300-44698225	20926	1	1	0	1	1	0	0	0	1	1	1	1	0	
chr16:26291625-26301925	10301	1	0	0	1	0	0	0	0	0	0	0	0	0	
chr15:77299925-77310325	10401	1	0	0	0	0	1	0	0	0	0	0	0	0	
chr5:122085725-122100025	14301	1	0	0	1	0	0	0	0	1	1	0	0	1	
chr8:143507200-143521850	14651	1	1	0	1	0	0	1	0	1	1	1	1	0	
chr8:8654675-8664850	10176	1	1	0	1	0	0	1	1	0	1	1	1	1	
chr2:68059800-68077075	17276	1	0	0	0	0	0	0	0	0	1	0	1	1	
chr5:32651700-32662150	10451	1	0	0	0	0	0	1	0	1	0	0	1	0	
chr4:25052450-25069275	16826	1	0	0	1	0	0	1	0	1	1	0	0	0	
chr14:70669500-70682000	12501	1	0	0	1	0	0	1	0	1	1	0	1	0	
chr13:90193525-90216950	23426	1	0	0	0	0	0	1	0	1	0	0	0	1	
chr4:108726075-108736900	10826	1	0	0	0	0	0	0	0	1	1	0	1	0	
chr6:112406400-112459300	52901	1	1	1	1	0	0	0	0	1	1	0	1	1	
chr11:127550150-127566775	16626	1	0	0	0	0	0	1	0	1	1	0	1	1	
chr18:45392375-45412075	19701	1	1	0	1	1	0	0	0	1	1	0	1	1	
chr8:24784000-24803475	19476	1	1	0	1	0	1	0	0	1	0	0	1	1	
chr9:125140075-125157500	17426	1	1	0	1	0	1	1	0	1	0	0	1	0	
chr4:102845400-102859100	13701	1	0	0	1	0	0	1	0	1	1	1	1	0	
chr2:176938125-176952700	14576	1	0	0	0	0	0	0	0	1	0	0	0	1	
chr1:190448450-190465925	17476	1	0	0	1	0	0	0	0	1	0	0	0	1	
chr12:115292100-115302600	10501	1	0	0	1	0	0	0	0	1	0	0	0	0	
chr15:47839050-47854350	15301	1	0	0	1	0	0	1	0	1	0	0	1	1	
chr16:82854175-82865325	11151	1	0	0	1	0	1	0	0	0	1	0	0	1	
chr2:223543900-223554575	10676	1	0	0	1	0	0	0	0	0	1	0	0	0	
chr2:231458400-231474450	16051	1	0	0	1	0	1	0	0	0	0	0	0	1	
chr2:237208375-237219525	11151	1	0	0	1	0	0	0	0	0	0	0	0	0	
chr2:71085625-71099625	14001	1	0	0	1	0	0	0	0	1	1	0	1	1	
chr20:2090500-2103375	12876	1	0	0	1	0	0	0	0	1	1	0	0	1	
chr9:110109500-110126925	17426	1	0	0	0	1	0	0	0	0	0	0	0	1	
chr12:97320125-97330750	10626	1	0	0	0	0	0	0	0	0	1	0	1	1	
chr7:79757325-79769550	12226	1	1	1	0	1	0	1	1	1	1	0	0	1	
chr18:37965050-37976600	11551	1	1	0	1	0	0	1	0	1	1	0	1	1	
chr2:206943350-206958650	15301	1	0	0	1	0	0	0	0	1	1	0	1	1	
chr8:79243350-79253700	10351	1	0	1	1	0	0	0	0	1	0	0	1	0	
chr5:81125650-81136150	10501	1	0	0	1	0	0	0	1	0	0	0	0	0	
chr7:155540650-155553700	13051	1	0	0	1	0	0	0	0	0	0	0	0	1	
chr6:150647775-150661325	13551	1	0	0	0	0	0	0	0	1	1	1	0	1	
chr3:31017300-31040525	23226	1	0	0	1	0	0	0	0	0	1	0	0	1	
chr4:4855350-4865400	10051	1	0	0	0	0	1	1	0	0	1	0	0	0	
chr2:192023525-192035025	11501	1	1	0	1	0	0	1	0	1	1	0	1	1	
chr20:56824850-56837175	12326	1	0	0	1	0	0	1	0	0	0	1	0	1	
chr6:140260300-140278725	18426	1	0	0	0	0	0	1	0	0	1	0	1	1	
chr6:78243750-78269775	26026	1	0	0	1	0	1	0	0	0	0	0	1	1	
chr14:70369075-70380850	11776	1	0	0	1	0	0	1	0	1	0	0	0	0	
chr18:22231500-22244975	13476	1	0	0	1	0	0	0	0	0	1	0	1	1	
chr21:26974675-27001175	26501	1	0	0	1	0	0	1	0	1	1	0	0	1	
chr3:140313275-140326250	12976	1	0	0	1	0	0	0	0	0	1	0	0	1	
chr3:158712275-158734225	21951	1	1	1	1	0	0	1	0	1	0	0	1	1	
chrX:102123350-102139900	16551	1	0	0	1	0	0	1	0	0	1	0	0	0	
chr1:225586950-225598475	11526	1	1	0	1	0	0	1	0	1	1	0	1	0	
chr7:25285175-25303700	18526	1	0	0	0	0	0	1	0	1	0	0	0	0	
chr20:56126025-56136400	10376	1	0	0	0	0	0	0	0	1	1	0	0	1	
chr1:201504525-201520825	16301	1	0	0	1	0	0	0	0	0	1	0	1	0	
chr1:86455725-86478575	22851	1	0	0	1	0	0	1	0	1	1	0	1	1	
chr4:67908675-67922125	13451	1	0	0	1	0	0	0	0	0	0	1	1	1	
chr14:93405000-93415700	10701	1	0	0	0	0	0	0	0	0	0	1	0	1	
chr11:8171725-8184025	12301	1	0	0	0	0	0	1	0	0	0	0	0	1	
chr9:99536025-99552150	16126	1	0	0	0	0	0	0	0	0	0	0	1	1	
chr2:31234450-31256675	22226	1	0	0	1	0	0	0	0	0	1	0	1	1	
chr2:31769200-31779600	10401	1	0	0	0	0	0	0	1	0	0	0	0	1	
chr5:124845325-124857875	12551	1	0	0	1	0	0	0	0	0	1	0	0	0	
chr2:238009325-238034900	25576	1	0	0	1	0	0	0	0	0	0	1	0	0	
chr2:88735550-88749975	14426	1	0	0	0	0	0	1	0	1	1	1	1	1	

location	span (nt)	max	mean	adipose	colon	heart	hypothalamus	kidney	liver	lung	ovary	skeletal muscle	spleen	testes	Comments
chrX:148733000-148752575	19576	1	0	0	0	0	0	1	0	0	0	0	1	0	
chr11:127986200-127997625	11426	1	0	0	1	1	0	0	0	0	0	0	0	0	
chr8:140183025-140239350	56326	1	0	0	1	0	0	1	0	0	0	0	0	1	
chr1:233220100-233231000	10901	1	0	0	0	0	0	0	0	0	1	0	0	0	
chr14:71447525-71461400	13876	1	0	0	1	0	0	0	0	1	0	0	1	1	
chr3:40986025-41002750	16726	1	0	0	0	0	0	0	0	0	0	0	0	1	
chr5:102017350-102033375	16026	1	0	0	0	0	0	0	0	0	0	0	0	1	
chr1:38888750-38900850	12101	1	0	0	1	0	0	1	0	0	0	0	0	0	
chr10:7584675-7594825	10151	1	0	0	1	0	0	0	0	0	0	0	0	0	
chr18:44239350-44253025	13676	1	0	0	1	0	0	0	0	0	0	0	0	0	
chr2:187440075-187467625	27551	1	0	0	1	0	0	0	0	1	0	0	1	0	
chr2:45358400-45376050	17651	1	0	0	1	0	0	0	0	0	0	0	0	0	
chr4:102619950-102632850	12901	1	0	0	1	0	0	1	0	0	0	0	1	0	
chr5:109275850-109296200	20351	1	0	1	1	0	0	1	0	0	1	0	1	1	
chr2:66891375-66902150	10776	1	0	0	0	0	0	0	0	0	1	0	1	1	
chr1:61193225-61207625	14401	1	0	0	0	0	1	0	0	0	1	0	1	1	
chr2:151418900-151431450	12551	1	0	0	0	0	0	1	0	1	1	0	1	1	
chr10:112221425-112232275	10851	1	0	0	0	0	0	0	0	1	1	0	1	1	
chr13:76807675-76829275	21601	1	0	0	1	0	0	1	0	1	0	0	1	0	
chr21:35050150-35061650	11501	1	0	0	1	0	0	0	0	0	0	0	1	0	
chr8:18025325-18043650	18326	1	0	0	0	0	0	1	0	1	1	0	1	0	
chr6:167428450-167439825	11376	1	0	0	0	0	0	1	0	0	0	0	1	1	
chr1:108865050-108879725	14676	1	0	0	0	0	0	1	0	0	1	1	0	1	
chr7:46184850-46198900	14051	1	0	0	0	0	0	1	0	0	1	0	0	0	
chr18:42451550-42482700	31151	1	0	0	1	0	0	0	0	0	0	0	0	0	
chr3:138272725-138291175	18451	1	0	0	1	0	0	0	0	0	0	0	0	0	
chr4:1743950-1756700	12751	1	0	0	1	0	0	0	0	0	0	0	0	0	
chr8:123825700-123843275	17576	1	0	0	1	0	0	1	0	0	0	0	0	1	
chr2:136867875-136878800	10926	1	0	0	1	1	0	0	0	1	1	0	1	0	
chr1:87457500-87472250	14751	1	0	0	1	0	0	1	0	1	0	0	0	0	
chrX:115315750-115327875	12126	1	0	0	0	0	0	1	0	0	0	0	0	0	
chr8:21182750-21199450	16701	1	0	0	1	0	0	0	0	0	0	0	0	1	
chr8:43637850-43650650	12801	1	0	0	0	0	0	0	0	1	0	0	1	0	
chr20:55589775-55605050	15276	1	0	0	0	0	0	1	0	0	0	0	0	0	
chr8:127449850-127469100	19251	1	0	0	0	0	0	0	0	0	0	0	0	1	
chr1:157472725-157487250	14526	1	0	0	1	0	0	0	0	0	0	0	0	0	
chr10:115138875-115151400	12526	1	0	0	1	0	0	0	0	0	0	0	0	0	
chr14:94916400-94934950	18551	1	0	0	1	0	0	0	0	0	0	0	1	0	
chr5:126931200-126948250	17051	1	0	0	0	0	0	0	0	1	0	0	1	0	
chr18:60131975-60143950	11976	1	0	0	0	1	0	1	0	0	1	0	1	0	
chr4:114929225-114940000	10776	1	0	0	0	1	0	1	0	0	1	0	0	0	
chr1:242310200-242327800	17601	1	0	0	1	0	0	0	0	1	0	0	0	0	
chr1:221930725-221941025	10301	1	0	0	0	0	0	0	0	0	1	0	0	1	
chr1:246337475-246347500	10026	1	0	0	0	0	0	0	0	1	0	0	0	1	
chr13:80059725-80075900	16176	1	0	0	0	0	0	0	0	0	0	0	0	1	
chr4:13730175-13740250	10076	1	0	0	0	0	0	1	0	0	0	0	0	0	
chr8:66160600-66171525	10926	1	0	0	0	0	0	1	0	0	1	0	0	1	
chr15:68065575-68082800	17226	1	0	1	1	0	1	0	0	0	0	0	0	1	
chr1:10834200-10846175	11976	1	0	0	1	0	0	1	0	0	0	0	0	0	
chr18:57739625-57750700	11076	1	0	0	0	0	0	0	0	0	1	0	0	1	
chr2:142652025-142668175	16151	1	0	0	1	0	0	0	0	0	1	0	0	0	
chr2:237859350-237869800	10451	1	0	0	0	0	0	0	0	0	1	0	0	0	
chr4:1021700-1040275	18576	1	0	0	1	0	0	1	0	0	1	0	0	1	
chr4:1589400-1605200	15801	1	0	0	0	0	0	0	0	0	1	1	0	1	
chr6:44847950-44860775	12826	1	0	0	0	0	0	1	0	0	0	0	0	1	
chr18:8443475-8460025	16551	1	0	0	0	0	0	0	0	0	1	0	1	0	
chr5:3790400-3802175	11776	1	0	0	1	0	0	1	0	1	1	0	1	0	
chrX:112619875-112636550	16676	1	0	0	0	0	0	0	0	1	0	0	1	0	
chr4:35430750-35443150	12401	1	0	0	0	0	0	0	0	0	0	0	0	1	
chr5:10822825-10832850	10026	1	0	0	0	0	0	0	0	0	0	0	0	1	
chr1:34594000-34611425	17426	1	0	0	1	0	0	0	0	0	0	0	0	0	
chr11:27837225-27857975	20751	1	0	0	1	0	0	0	0	0	0	0	0	0	
chr4:84893350-84907125	13776	1	0	0	1	0	0	0	0	0	0	0	0	0	
chr5:134674675-134685525	10851	1	0	0	1	0	0	0	0	0	0	0	0	0	
chr2:2821100-2843950	22851	1	0	1	0	0	1	0	0	0	0	0	0	0	
chr1:197930275-197948150	17876	1	0	0	0	0	0	0	0	0	1	0	1	0	
chr21:24182150-24197250	15101	1	0	0	0	0	0	0	0	0	0	0	0	1	
chr6:125885000-125895925	10926	1	0	0	0	0	0	0	0	0	0	0	0	1	
chr3:154809400-154830550	21151	1	0	0	0	0	0	0	0	1	1	0	0	1	
chr13:24548975-24561125	12151	1	0	0	1	1	0	1	0	1	1	0	0	0	
chr13:46522450-46536325	13876	1	0	0	0	0	0	1	0	0	0	0	0	1	
chr6:168331950-168343050	11101	1	0	0	0	0	0	0	1	0	0	0	0	0	
chr3:113997225-114007525	10301	1	0	0	0	0	1	0	0	1	0	0	0	1	
chr2:85812350-85823000	10651	1	0	0	1	0	0	0	0	0	1	0	0	1	
chr13:21203525-21215225	11701	1	0	0	1	0	0	0	0	0	0	0	0	0	
chr4:23939275-23950400	11126	1	0	0	1	0	1	0	0	0	0	0	0	0	
chr8:26092850-26111900	19051	1	0	0	1	0	1	0	0	0	0	0	0	0	
chrX:30716225-30736150	19926	1	0	0	1	0	0	0	0	0	0	0	0	0	
chrX:5192350-5206925	14576	1	0	0	1	0	0	0	0	0	1	0	0	1	
chr3:188363175-188375850	12676	1	0	0	1	0	0	0	0	1	0	0	1	0	
chr7:106276275-106286550	10276	1	0	0	0	0	0	0	0	0	1	0	1	0	
chr1:29914900-29928125	13226	1	0	0	0	0	0	0	0	0	1	0	0	0	
chr2:134177450-134192075	14626	1	0	0	0	1	0	0	0	1	0	0	0	0	
chr12:115504200-115514350	10151	1	0	0	1	0	0	0	0	1	0	0	0	0	
chr13:94306375-94318750	12376	1	0	0	1	0	0	0	0	0	0	0	0	0	
chr5:54380675-54392025	11351	1	0	0	1	0	0	0	0	0	0	0	0	0	
chr6:130952300-130967925	15626	1	0	0	1	0	0	0	0	0	0	0	0	0	
chr3:177853550-177864125	10576	1	0	0	0	0	0	0	0	0	0	0	0	1	
chr7:175450-189200	13751	1	0	0	0	0	0	0	0	0	0	0	1	0	
chr2:201342075-201354925	12851	1	0	0	1	0	1	0	0	0	0	0	0	0	
chr2:151669825-151682775	12951	1	0	0	0	0	0	0	0	0	0	0	0	1	
chr8:23664975-23675150	10176	1	0	0	0	0	0	0	0	0	1	0	0	0	
chrX:20329325-20341425	12101	1	0	0	0	0	0	0	0	0	1	0	0	0	
chr2:145864000-145902775	38776	1	0	0	1	0	0	1	0	0	1	0	1	0	
chr6:34632150-34647850	15701	1	0	0	0	0	0	1	0	1	1	0	0	1	
chr5:176041950-176075125	33176	1	0	0	1	0	0	0	0	0	1	0	1	0	
chr2:235906000-235923850	17851	1	0	0	1	0	0	0	0	0	0	0	0	1	
chr14:24222400-24234725	12326	1	0	0	0	0	0	0	0	0	1	0	0	0	
chr6:47425050-47444950	19901	1	0	0	1	0	0	0	0	0	1	0	0	0	
chr10:72360000-72376125	16126	1	0	0	1	0	0	0	0	0	0	0	0	0	
chr16:86135375-86147125	11751	1	0	0	1	0	0	0	0	0	0	0	0	0	
chr18:73358625-73372325	13701	1	0	0	1	0	0	0	0	0	0	0	0	0	
chr18:75427775-75446650	18876	1	0	0	1	0	0	0	0	0	0	0	0	0	
chr4:79842650-79853700	11051	1	0	0	1	0	0	0	0	0	0	0	0	1	
chr9:136571925-136590625	18701	1	0	0	1	0	0	0	0	0	0	0	0	0	
chr8:38628300-38642625	14326	1	0	0	0	0	0	0	0	0	1	0	0	1	
chr12:66812300-66822625	10326	1	0	0	0	0	0	0	0	0	0	0	1	0	
chr3:188521875-188539200	17326	1	0	0	0	0	0	0	0	0	1	0	0	0	
chr8:113083525-113098000	14476	1	0	0	0	0	0	0	0	0	1	0	0	1	
chr13:54457650-54471175	13526	1	0	0	0	0	0	0	0	0	0	0	0	1	
chr1:41728875-41743675	14801	1	0	0	0	0	0	0	0	1	1	0	0	0	
chr2:144839850-144851925	12076	1	0	0	0	0	0	0	0	0	1	0	0	0	
chr5:169663425-169690075	26651	1	0	0	0	0	0	0	0	0	1	0	0	0	
chr1:155641250-155657075	15826	1	0	0	0	0	0	0	0	1	0	0	0	0	
chr18:10498025-10508725	10701	1	0	0	0	0	0	0	0	1	0	0	0	0	
chr10:120265025-120275550	10526	1	0	0	0	0	0	0	0	0	0	0	0	1	
chr13:42662375-42676425	14051	1	0	0	0	0	0	0	0	0	0	0	0	1	
chr21:28521775-28537600	15826	1	0	0	0	0	0	0	0	0	0	0	0	1	
chr3:45182150-45196400	14251	1	0	0	1	0	0	0	0	0	0	0	0	1	
chrX:45353850-45364375	10526	1	0	0	0	0	0	0	0	0	0	0	0	1	
chr2:204255125-204267050	11926	1	0	0	0	0	1	0	0	0	0	0	0	0	
chr16:31588925-31601750	12826	1	0	0	0	0	0	1	0	1	0	0	0	0	
chr13:102352250-102366025	13776	1	0	0	0	0	0	0	0	0	0	0	0	1	
chr5:72743475-72759550	16076	1	0	0	0	0	0	0	0	0	0	0	0	1	

location	span (nt)	max	mean	adipose	colon	heart	hypothalamus	kidney	liver	lung	ovary	skeletal muscle	spleen	testes	Comments
chr11:2185000-2195750	10751	1	0	0	1	0	0	0	0	0	0	0	0	0	
chr13:103304175-103316475	12301	1	0	0	1	0	0	0	0	0	0	0	0	0	
chr2:118641200-118655550	14351	1	0	0	1	0	0	0	0	0	0	0	0	0	
chr4:117477050-117499075	22026	1	0	0	1	0	0	0	0	0	0	0	0	0	
chr5:3618525-3632100	13576	1	0	0	1	0	0	0	0	0	0	0	0	0	
chr9:12944125-12955625	11501	1	0	0	0	0	0	0	0	1	0	0	0	0	
chr18:58292100-58302600	10501	1	0	0	0	0	0	0	0	0	0	0	1	0	
chr5:65876625-65888825	12201	1	0	0	0	0	0	0	0	0	0	0	1	0	
chr8:102348200-102365375	17176	1	0	0	0	0	0	0	0	0	0	0	1	0	
chr10:64272675-64282950	10276	1	0	0	0	0	0	0	0	0	0	0	0	1	
chr18:74372500-74382950	10451	1	0	0	0	0	0	0	0	0	0	0	0	1	
chr2:59937350-59959625	22276	1	0	0	0	0	0	1	0	0	0	0	0	0	
chr13:68163750-68188075	24326	0	0	0	0	0	0	0	0	0	0	0	0	0	
chr14:60768300-60778400	10101	0	0	0	0	0	0	0	0	0	0	0	0	0	
chr16:77894925-77907775	12851	0	0	0	0	0	0	0	0	0	0	0	0	0	
chr12:36728900-36740300	11401	0	0	0	0	0	0	0	0	0	0	0	0	0	
chr16:49113275-49131950	18676	0	0	0	0	0	0	0	0	0	0	0	0	0	
chr3:16732325-16743650	11326	0	0	0	0	0	0	0	0	0	0	0	0	0	
chr4:97235825-97246525	10701	0	0	0	0	0	0	0	0	0	0	0	0	0	
chr5:171923650-171936650	13001	0	0	0	0	0	0	0	0	0	0	0	0	0	
chr2:235101975-235113800	11826	0	0	0	0	0	0	0	0	0	0	0	0	0	
chr11:101930825-101946125	15301	0	0	0	0	0	0	0	0	0	0	0	0	0	
chr5:1006500-1016800	10301	0	0	0	0	0	0	0	0	0	0	0	0	0	
chr10:33691650-33706325	14676	0	0	0	0	0	0	0	0	0	0	0	0	0	
chr5:2915250-2928275	13026	0	0	0	0	0	0	0	0	0	0	0	0	0	
chr8:37650675-37661525	10851	0	0	0	0	0	0	0	0	0	0	0	0	0	
chr18:35727600-35746750	19151	0	0	0	0	0	0	0	0	0	0	0	0	0	
chr13:19779350-19799600	20251	0	0	0	0	0	0	0	0	0	0	0	0	0	
chr13:21343525-21353675	10151	0	0	0	0	0	0	0	0	0	0	0	0	0	
chr2:107085125-107104250	19126	0	0	0	0	0	0	0	0	0	0	0	0	0	
chr8:49778575-49789975	11401	0	0	0	0	0	0	0	0	0	0	0	0	0	
chr8:97516400-97526500	10101	0	0	0	0	0	0	0	0	0	0	0	0	0	
chr4:59170525-59185400	14876	0	0	0	0	0	0	0	0	0	0	0	0	0	
chr7:25523625-25539425	15801	0	0	0	0	0	0	0	0	0	0	0	0	0	
chr1:46758275-46770275	12001	0	0	0	0	0	0	0	0	0	0	0	0	0	
chr4:99827550-99842325	14776	0	0	0	0	0	0	0	0	0	0	0	0	0	
chr7:92334500-92344850	10351	0	0	0	0	0	0	0	0	0	0	0	0	0	
chr6:89328975-89341875	12901	0	0	0	0	0	0	0	0	0	0	0	0	0	
chr10:62230700-62240900	10201	0	0	0	0	0	0	0	0	0	0	0	0	0	
chr8:20493075-20506350	13276	0	0	0	0	0	0	0	0	0	0	0	0	0	
chr7:124822950-124833175	10226	0	0	0	0	0	0	0	0	0	0	0	0	0	
chr4:3799050-3822500	23451	0	0	0	0	0	0	0	0	0	0	0	0	0	
chr14:100197725-100219000	21276	0	0	0	0	0	0	0	0	0	0	0	0	0	
chr3:41141450-41165950	24501	0	0	0	0	0	0	0	0	0	0	0	0	0	
chr1:155711350-155725225	13876	0	0	0	0	0	0	0	0	0	0	0	0	0	
chr3:169303700-169322700	19001	0	0	0	0	0	0	0	0	0	0	0	0	0	
chr1:100547525-100561800	14276	0	0	0	0	0	0	0	0	0	0	0	0	0	
chr12:66912600-66922875	10276	0	0	0	0	0	0	0	0	0	0	0	0	0	
chr6:3426225-3440625	14401	0	0	0	0	0	0	0	0	0	0	0	0	0	
chr7:154606775-154624950	18176	0	0	0	0	0	0	0	0	0	0	0	0	0	
chr21:22930950-22941850	10901	0	0	0	0	0	0	0	0	0	0	0	0	0	
chr18:10070425-10083725	13301	0	0	0	0	0	0	0	0	0	0	0	0	0	
chr5:41915550-41927950	12401	0	0	0	0	0	0	0	0	0	0	0	0	0	
chr4:6608650-6619000	10351	0	0	0	0	0	0	0	0	0	0	0	0	0	
chr9:6054675-6064900	10226	0	0	0	0	0	0	0	0	0	0	0	0	0	
chr10:4568200-4583225	15026	0	0	0	0	0	0	0	0	0	0	0	0	0	
chr1:226789375-226804525	15151	0	0	0	0	0	0	0	0	0	0	0	0	0	
chrX:68323375-68348925	25551	0	0	0	0	0	0	0	0	0	0	0	0	0	
chr11:29287925-29298875	10951	0	0	0	0	0	0	0	0	0	0	0	0	0	
chr12:90728475-90738500	10026	0	0	0	0	0	0	0	0	0	0	0	0	0	
chr5:9972325-9986625	14301	0	0	0	0	0	0	0	0	0	0	0	0	0	
chr8:109813675-109824100	10426	0	0	0	0	0	0	0	0	0	0	0	0	0	
chr1:230573375-230588800	15426	0	0	0	0	0	0	0	0	0	0	0	0	0	
chr13:27285150-27300025	14876	0	0	0	0	0	0	0	0	0	0	0	0	0	
chr16:84629500-84646475	16976	0	0	0	0	0	0	0	0	0	0	0	0	0	
chr18:71985450-71997700	12251	0	0	0	0	0	0	0	0	0	0	0	0	0	
chr8:47056500-47068675	12176	0	0	0	0	0	0	0	0	0	0	0	0	0	
chr2:151317425-151330125	12701	0	0	0	0	0	0	0	0	0	0	0	0	0	
chr20:24726575-24738550	11976	0	0	0	0	0	0	0	0	0	0	0	0	0	
chr2:129258125-129268825	10701	0	0	0	0	0	0	0	0	0	0	0	0	0	
chr4:115617300-115634500	17201	0	0	0	0	0	0	0	0	0	0	0	0	0	
chr8:117684425-117694800	10376	0	0	0	0	0	0	0	0	0	0	0	0	0	
chr15:75899850-75913050	13201	0	0	0	0	0	0	0	0	0	0	0	0	0	
chr3:980375-1000225	19851	0	0	0	0	0	0	0	0	0	0	0	0	0	
chrX:136212300-136224525	12226	0	0	0	0	0	0	0	0	0	0	0	0	0	
chr5:124766050-124776075	10026	0	0	0	0	0	0	0	0	0	0	0	0	0	
chr1:54356225-54366425	10201	0	0	0	0	0	0	0	0	0	0	0	0	0	
chr10:79782425-79793050	10626	0	0	0	0	0	0	0	0	0	0	0	0	0	
chr10:80083325-80095925	12601	0	0	0	0	0	0	0	0	0	0	0	0	0	
chr17:44887200-44904100	16901	0	0	0	0	0	0	0	0	0	0	0	0	0	
chr18:20526500-20537775	11276	0	0	0	0	0	0	0	0	0	0	0	0	0	
chr3:53148200-53163075	14876	0	0	0	0	0	0	0	0	0	0	0	0	0	
chr13:75360325-75373025	12701	0	0	0	0	0	0	0	0	0	0	0	0	0	
chr11:13877025-13889575	12551	0	0	0	0	0	0	0	0	0	0	0	0	0	
chr8:29804225-29817650	13426	0	0	0	0	0	0	0	0	0	0	0	0	0	
chr13:104555125-104567000	11876	0	0	0	0	0	0	0	0	0	0	0	0	0	
chr13:31280650-31291700	11051	0	0	0	0	0	0	0	0	0	0	0	0	0	
chr1:242433950-242449875	15926	0	0	0	0	0	0	0	0	0	0	0	0	0	
chr14:98295825-98307425	11601	0	0	0	0	0	0	0	0	0	0	0	0	0	
chr3:71002675-71016650	13976	0	0	0	0	0	0	0	0	0	0	0	0	0	
chr6:91184500-91207375	22876	0	0	0	0	0	0	0	0	0	0	0	0	0	
chr7:129188850-129205475	16626	0	0	0	0	0	0	0	0	0	0	0	0	0	
chr6:63131625-63144250	12626	0	0	0	0	0	0	0	0	0	0	0	0	0	
chr1:84487175-84498675	11501	0	0	0	0	0	0	0	0	0	0	0	0	0	
chr1:150676175-150689200	13026	0	0	0	0	0	0	0	0	0	0	0	0	0	
chr2:234704700-234716175	11476	0	0	0	0	0	0	0	0	0	0	0	0	0	
chr11:12043925-12055650	11726	0	0	0	0	0	0	0	0	0	0	0	0	0	
chr18:69751425-69764700	13276	0	0	0	0	0	0	0	0	0	0	0	0	0	
chr3:185828975-185840500	11526	0	0	0	0	0	0	0	0	0	0	0	0	0	
chr12:33624000-33638850	14851	0	0	0	0	0	0	0	0	0	0	0	0	0	
chr1:2350825-2395175	44351	0	0	0	0	0	0	0	0	0	0	0	0	0	
chr13:71468300-71482450	14151	0	0	0	0	0	0	0	0	0	0	0	0	0	
chr5:3881075-3894525	13451	0	0	0	0	0	0	0	0	0	0	0	0	0	
chr1:112343150-112361000	17851	0	0	0	0	0	0	0	0	0	0	0	0	0	
chr1:201431425-201441525	10101	0	0	0	0	0	0	0	0	0	0	0	0	0	
chr3:153975125-153989575	14451	0	0	0	0	0	0	0	0	0	0	0	0	0	
chr5:102800100-102810350	10251	0	0	0	0	0	0	0	0	0	0	0	0	0	
chr7:41596600-41607700	11101	0	0	0	0	0	0	0	0	0	0	0	0	0	
chr13:48021375-48033500	12126	0	0	0	0	0	0	0	0	0	0	0	0	0	
chr1:163349950-163361375	11426	0	0	0	0	0	0	0	0	0	0	0	0	0	
chr8:20622500-20634450	11951	0	0	0	0	0	0	0	0	0	0	0	0	0	
chr2:100217575-100229800	12226	0	0	0	0	0	0	0	0	0	0	0	0	0	
chr8:133140825-133161650	20826	0	0	0	0	0	0	0	0	0	0	0	0	0	
chr8:142857525-142870300	12776	0	0	0	0	0	0	0	0	0	0	0	0	0	
chrX:45030125-45040475	10351	0	0	0	0	0	0	0	0	0	0	0	0	0	
chr2:120864150-120877825	13676	0	0	0	0	0	0	0	0	0	0	0	0	0	
chr6:130131950-130145525	13576	0	0	0	0	0	0	0	0	0	0	0	0	0	
chr12:59599600-59621750	22151	0	0	0	0	0	0	0	0	0	0	0	0	0	
chr8:140419225-140429850	10626	0	0	0	0	0	0	0	0	0	0	0	0	0	
chr13:52409200-52419600	10401	0	0	0	0	0	0	0	0	0	0	0	0	0	
chr10:32000350-32014075	13726	0	0	0	0	0	0	0	0	0	0	0	0	0	
chr2:231247700-231258825	11126	0	0	0	0	0	0	0	0	0	0	0	0	0	
chr21:41969525-41979600	10076	0	0	0	0	0	0	0	0	0	0	0	0	0	
chr5:158012750-158032025	19276	0	0	0	0	0	0	0	0	0	0	0	0	0	

location	span (nt)	max	mean	adipose	colon	heart	hypothalamus	kidney	liver	lung	ovary	skeletal muscle	spleen	testes	Comments
chr7:139158975-139169025	10051	0	0	0	0	0	0	0	0	0	0	0	0	0	
chr8:66380600-66391625	11026	0	0	0	0	0	0	0	0	0	0	0	0	0	
chr6:168412975-168429225	16251	0	0	0	0	0	0	0	0	0	0	0	0	0	
chr8:143272275-143284725	12451	0	0	0	0	0	0	0	0	0	0	0	0	0	
chr6:113800850-113811850	11001	0	0	0	0	0	0	0	0	0	0	0	0	0	
chr1:190265225-190278725	13501	0	0	0	0	0	0	0	0	0	0	0	0	0	
chr6:44743250-44755800	12551	0	0	0	0	0	0	0	0	0	0	0	0	0	
chr18:70288175-70298975	10801	0	0	0	0	0	0	0	0	0	0	0	0	0	
chr9:31332775-31343225	10451	0	0	0	0	0	0	0	0	0	0	0	0	0	
chr2:159281300-159295375	14076	0	0	0	0	0	0	0	0	0	0	0	0	0	
chr2:84045950-84056500	10551	0	0	0	0	0	0	0	0	0	0	0	0	0	
chr2:122289675-122301200	11526	0	0	0	0	0	0	0	0	0	0	0	0	0	
chr8:143487875-143498025	10151	0	0	0	0	0	0	0	0	0	0	0	0	0	
chrX:68211200-68232250	21051	0	0	0	0	0	0	0	0	0	0	0	0	0	
chr10:42792000-42802200	10201	0	0	0	0	0	0	0	0	0	0	0	0	0	
chr11:38356650-38373850	17201	0	0	0	0	0	0	0	0	0	0	0	0	0	
chr4:15520650-15531025	10376	0	0	0	0	0	0	0	0	0	0	0	0	0	
chr14:92422900-92433575	10676	0	0	0	0	0	0	0	0	0	0	0	0	0	
chr10:71159500-71173350	13851	0	0	0	0	0	0	0	0	0	0	0	0	0	
chr2:149073750-149085000	11251	0	0	0	0	0	0	0	0	0	0	0	0	0	
chr2:60376250-60386500	10251	0	0	0	0	0	0	0	0	0	0	0	0	0	
chr4:127914425-127929275	14851	0	0	0	0	0	0	0	0	0	0	0	0	0	
chr9:136300550-136313775	13226	0	0	0	0	0	0	0	0	0	0	0	0	0	
chr12:44764175-44775050	10876	0	0	0	0	0	0	0	0	0	0	0	0	0	
chr18:37147675-37159675	12001	0	0	0	0	0	0	0	0	0	0	0	0	0	
chr5:3276050-3292025	15976	0	0	0	0	0	0	0	0	0	0	0	0	0	
chr2:239599675-239614825	15151	0	0	0	0	0	0	0	0	0	0	0	0	0	
chr8:140577700-140593825	16126	0	0	0	0	0	0	0	0	0	0	0	0	0	
chr14:101121575-101132150	10576	0	0	0	0	0	0	0	0	0	0	0	0	0	
chr7:54998325-55008375	10051	0	0	0	0	0	0	0	0	0	0	0	0	0	
chr4:81264400-81281800	17401	0	0	0	0	0	0	0	0	0	0	0	0	0	
chr4:121140275-121151000	10726	0	0	0	0	0	0	0	0	0	0	0	0	0	
chr5:107200075-107210175	10101	0	0	0	0	0	0	0	0	0	0	0	0	0	
chr10:1842775-1853600	10826	0	0	0	0	0	0	0	0	0	0	0	0	0	
